# A study of transposable element-associated structural variations (TASVs) using a de novo-assembled Korean genome

**DOI:** 10.1038/s12276-021-00586-y

**Published:** 2021-04-08

**Authors:** Seyoung Mun, Songmi Kim, Wooseok Lee, Keunsoo Kang, Thomas J. Meyer, Bok-Ghee Han, Kyudong Han, Heui-Soo Kim

**Affiliations:** 1 Department of Nanobiomedical Science, Dankook University, Cheonan, 31116 Republic of Korea; 2 DKU-Theragen Institute for NGS analysis (DTiNa), Cheonan, 31116 Republic of Korea; 3 Center for Bio‑Medical Engineering Core Facility, Dankook University, Cheonan, 31116 Republic of Korea; 4 Department of Microbiology, Dankook University, Cheonan, 31116 Republic of Korea; 5 CCR Collaborative Bioinformatics Resource, National Cancer Institute, National Institutes of Health, Bethesda, MD USA; 6 Advanced Biomedical Computational Science, Frederick National Laboratory for Cancer Research, Frederick, MD USA; 7 Center for Genome Science, National Institutes of Health, Korea Centers for Disease Control and Prevention, Osong, 28160 Republic of Korea; 8 Department of Biological Sciences, Pusan National University, Busan, 46283 Republic of Korea

**Keywords:** Comparative genomics, Mobile elements, Next-generation sequencing

## Abstract

Advances in next-generation sequencing (NGS) technology have made personal genome sequencing possible, and indeed, many individual human genomes have now been sequenced. Comparisons of these individual genomes have revealed substantial genomic differences between human populations as well as between individuals from closely related ethnic groups. Transposable elements (TEs) are known to be one of the major sources of these variations and act through various mechanisms, including de novo insertion, insertion-mediated deletion, and TE–TE recombination-mediated deletion. In this study, we carried out de novo whole-genome sequencing of one Korean individual (KPGP9) via multiple insert-size libraries. The de novo whole-genome assembly resulted in 31,305 scaffolds with a scaffold N50 size of 13.23 Mb. Furthermore, through computational data analysis and experimental verification, we revealed that 182 TE-associated structural variation (TASV) insertions and 89 TASV deletions contributed 64,232 bp in sequence gain and 82,772 bp in sequence loss, respectively, in the KPGP9 genome relative to the hg19 reference genome. We also verified structural differences associated with TASVs by comparative analysis with TASVs in recent genomes (AK1 and TCGA genomes) and reported their details. Here, we constructed a new Korean de novo whole-genome assembly and provide the first study, to our knowledge, focused on the identification of TASVs in an individual Korean genome. Our findings again highlight the role of TEs as a major driver of structural variations in human individual genomes.

## Genomics: Following the footprints of ‘jumping genes’

A novel strategy for genome analysis offers insights into the distribution and impact on genome variation of transposable elements, DNA sequences that can replicate and relocate themselves at different chromosomal regions. These sequences, also known as ‘jumping genes’, comprise up to 50% of the genome, but it has proven challenging to map them with existing techniques. Seyoung Mun of Dankook University, Cheonan, South Korea, and coworkers have developed a sequencing and computational analysis strategy that allowed them to accurately map transposable elements across the genome of a Korean individual. These data revealed hundreds of insertion and deletion events relative to an existing reference map of the genome, showing significant alterations in the chromosomal structure. The authors speculate that such widespread transposition events could potentially contribute to individual differences in gene expression and risk of disease.

## Introduction

As next-generation sequencing (NGS) technologies developed by Illumina^1^, Pacific Biosciences^2^, and Ion Torrent^3^ have been commercialized, genomic research on a variety of species, including humans, has accelerated^4^. Studies related to various genetic diseases, including cancers and phenotypic traits, have yielded many accurate human individual genome datasets^5–8^. The integration of these genomic datasets, which are mostly comprised of whole-genome resequencing and whole-exome sequencing data, has allowed the exploration of a wide range of complex variation types, such as single-nucleotide polymorphisms (SNPs), insertions and deletions (INDELs), inversions, rearrangements, copy number variations (CNVs), and structural variations mediated by transposable elements (TEs)^9–11^.

It is well established that TEs account for at least 50% of the human genome^12^. They are among the key factors contributing to structural variations and are classified as transposons (cut-and-paste) or retrotransposons (copy-and-paste) on the basis of their mechanisms of mobilization^13–16^. Among TEs, non-long terminal repeat (non-LTR) retrotransposons, such as *Alu* elements, long interspersed elements (LINEs), SINE-R/VNTR/*Alu*-like (SVA) elements, and LTR retrotransposons, including human endogenous retroviruses (HERVs), are still retrotranspositionally competent in the human genome^17–20^. Comparative genomic studies of primate genomes show that TEs have played a significant role in shaping primate genomic architecture^21–24^. The fact that some TEs are currently active in the human genome indicates their potential to structurally alter the human genome. Examples are known whereby TEs have influenced the expression and structure of gene products by supplying alternative splice donor/acceptor and transcription binding sites^25,26^. As recognition of the importance of TEs has grown, many researchers have studied the relationship between TE-associated genetic variations and the risks of carcinogenesis^27–29^ and other genetic disorders^30,31^. Furthermore, new methods using high-throughput NGS methods, such as TE capture sequencing and bioinformatic techniques, have allowed more detailed determination of TE contributions to the human genome^32,33^. Despite considerable progress in our understanding of the biology and distribution of TEs and their impact on the structure, function, and evolution of the human genome, a great deal of uncertainty still remains concerning differences between individuals. As many previous studies used only short sequence reads for sequence mapping and repeat assembly, their ability to interrogate certain types of variations was limited. These challenging tasks include the identification of private structural variations, high copy number gene families, INDELs of intermediate size, and especially TEs, whose high copy number and sequence identity can cause problems for both mapping and assembly^34,35^. To overcome these limitations in genome analysis based on short reads, a practical method for the de novo assembly of large genomes from short sequence reads using SOAPdenovo2 was developed. This method employs short-read sequencing in parallel with multiple insert-size libraries, cost-effectively enabling not only the construction of de novo genomes but also the accurate detection of structural variations. The SOAPdenovo2 assembler consists of six systematic steps, as follows: read-error correction, *de Bruijn graph* (DBG) construction, contig assembly, paired-end (PE) read mapping, scaffold construction, and gap closure. In the DBG-based sequence assembly method, it is very important to choose a proper *k*-mer size for the determination of optimized contigs. The use of a large *k*-mer size can solve the problem of short repetitive sequences when the sequencing depth is sufficient and can improve the quality of the resulting contig assembly. Complementarily, the use of small-sized *k*-mers can reduce some negative effects on assembly related to sequencing error and heterozygosity. Utilizing the advantages of both large and small *k*-mers during assembly, the SOAPdenovo2 assembler provides an effective solution for large genome assembly and identification of TE-associated structural variations (TASVs)^36,37^.

Here, we report the de novo-assembled draft genome and genetic information for a Korean individual (KPGP9) generated using the SOAPdenovo2 assembler with multiple insert-size libraries (Supplementary Fig. S1). Using this pipeline, the draft genome for KPGP9 was constructed from raw sequencing data, yielding a genome of ~2.86 Gb in length, with 105.16X coverage and a scaffold N50 size of 13.23 Mb. Here, we provide a detailed description of the TASVs identified from this draft genome and show a total of 271 TASVs that have contributed to the generation of an appreciable proportion of genomic variation between human individuals.

## Materials and methods

### DNA sample preparation and genome sequencing

The blood sample was donated from a Korean male (KPGP9) following the Korean ethical guidelines for human genome research. We extracted genomic DNA using the QIAamp DNA Blood Mini Kit (Qiagen, Germany) according to the manufacturer’s instructions. Libraries of various insert sizes were constructed using paired-end (insert size: ~170, 500, and 800 bp) and mate-pair (insert size: 2, 5, 10, 20, and 40 kb) methods. Size selection for the intended libraries was conducted using a Covaris S2 Ultrasonicator system (Covaris, USA). The final libraries were sequenced on an Illumina Hi-Seq 2500 platform using the TruSeq Paired-End Cluster Kit v3 (Illumina, USA). A total of 315.47 Gb of sequence data were produced using 9 lanes. The read lengths of paired-end and mate-pair libraries were 90–100 bp and 49 bp, respectively (Supplementary Fig. S1 and Supplementary Table S1).

### Raw data filtering

Prior to genome assembly, we carried out sequence filtering to reduce assembly error due to low-quality reads. The reads with low quality were filtered out as follows: (i) reads with multiple poly-A sequences or ambiguous bases (represented by the letter N); (ii) reads with ≥40% low-quality bases (base quality ≤7) in paired-end libraries (~170, 500, and 800 bp) or mate-pair libraries (2, 5, 10, 20, and 40 kb); (iii) reads containing adaptor sequences; (iv) cases in which read1 and read2 overlapped by ≥10 bp; (v) low-quality bases trimmed using SICKLE (https://github.com/najoshi/sickle); and (vi) PCR duplications that were identified using PICARD (http://picard.sourceforge.net/index.shtml). Thus, we used only high-quality sequence reads for further analysis.

### De novo assembly of the KPGP9 genome

The corrected reads were used for de novo assembly using the *De Bruijn* graph assembly algorithm implemented in SOAPdenovo ver. 2.04 with default parameters. The resulting assembled genome had a total length of 2.86 Gb and an N50 scaffold length of 13.23 Mb^38^ (Table 1). First, sequence reads from short-insert libraries were used for the construction of a *De Bruijn* graph. In the contig step of SOAPdenovo2 assembly, we excluded erroneous connections such as clip tips, low-coverage links, small repeats, and merged bubbles with the following parameters: “contig -g inputGraph -R -M 1 -D”. Second, we realigned the qualified reads to contig sequences to measure the amount of shared paired-end relationships between contigs, followed by the construction of the scaffolds stepwise from the short-insert size to long-insert-size paired-ends. Finally, we used paired-end reads to close the gaps within the constructed scaffolds^36^. This application of de novo genome assembly combined with stringent filtering and conditioning of the data allowed us to construct a high-quality KPGP9 draft genome. Alignment of large-scale scaffold blocks to the human reference genome was carried out using algorithms in SyMAP software (http://www.agcol.arizona.edu/software/symap/, v4.2), which is one of the tools for detecting orthologous segments of the genome^39,40^. The large-scale scaffold regions were finally visualized using the webtool idiographica (http://www.ncrna.org/idiographica/)^41^. The de novo-assembled genome data for KPGP9 and the coordinates of scaffolds aligned to the human reference genome are available to download at ftp://210.102.196.176.

**Table 1:** Summary of KPGP9 de novo assembly statistics.

Step	Size	Sequences	Total Size (bp)	N50	N90	Longest	Scaffold genome coverage (%)
Contig	–	1,439,891	2,860,663,260	49,974	8719	604,770	
Scaffolds (filtering steps)	2 K	1,344,398	2,895,066,623	300,579	54,077	2,055,172	
	5 K	1,327,495	2,921,071,090	1,027,730	172,103	6,646,083	
	10 K	1,321,040	2,956,383,059	8,116,997	977,239	45,450,559	
	20 K	1,320,902	2,961,961,259	11,947,405	1,320,382	77,015,144	
	40 K	1,182,591	2,963,912,249	13,083,805	1,336,383	75,957,870	
Gap filled	>500	31,505	2,862,402,237	13,235,598	1,787,215	75,412,104	92.30

### Repeat annotation

Tandem repeats in the KPGP9-assembled scaffolds were identified using Tandem Repeats Finder13 (http://tandem.bu.edu/trf/trf.html, version 4.04), with parameters set to “2 7 7 80 10 50 2000 -f -m -h –d”^42^. To screen TEs in the genome, a homology-based repeat element search was implemented using RepeatMasker (http://www.repeatmasker.org/cgi-bin/WEBRepeatMasker, v4.0.6) and Repbase, a database of known repeats (http://www.girinst.org/repbase)^43,44^. All repeats were sequentially categorized into their relevant types.

### Data mining and manual inspection of TASVs

We sorted events by the ages of repeat subfamilies, reasoning that the youngest TE subfamilies are most likely to be still active in the human genome. We extracted sequences for LINE-1 (L1HS), *Alu* (10 *Alu*Y subfamilies), HERV (HERV-K), and SVA (SVA A to F) elements, along with approximately 2–4 kb of flanking region from the KPGP9 scaffolds on either side of the TE. Sequence extraction was performed by a custom Perl script. The extracted candidate sequences were mapped to the human reference genome. Initially, a total of 19,224 putative TASVs (14,548 insertion and 4676 deletion candidates) were identified by comparison of KPGP9 to the human reference genome. Candidate insertions less than 100 bp in size were filtered out, but candidate deletions were included in the downstream analysis without size selection.

To isolate precise TASVs, we first filtered out uncertain candidates containing either ambiguous sequences (represented by the letter N) or small insertion sizes (<100 bp) or lacking target site duplications (TSDs), which are the universal hallmark of retrotransposons generated at both ends of most TE insertions^45^. In this way, we were able to avoid assembly bias and systematic errors to effectively screen putative TASV insertions^34,35^. The flanking sequences were utilized as queries for BLAST-Like Alignment Tool (BLAT) (http://genome.ucsc.edu/cgi-bin/hgBlat?command=start) searches against the human genome assembly (hg19; February 2009 freeze)^46^. We examined the alignment of the flanking sequences with the corresponding regions in other nonhuman primate genomes, including chimpanzee (panTro5; May 2016), gorilla (gorGor5; May 2016), and orangutan (ponAbe2; July 2007 freeze), to ensure that the TASV candidates were unique to the KPGP9 genome. Repeat elements in the flanking sequences of TASV candidates from the KPGP9 genome and its counterparts from the human reference genome were annotated with RepeatMasker (http://www.repeatmasker.org/cgi-bin/WEBRepeatMasker)^43,47^. In this way, we filtered out the TASV insertions previously reported and shared with the KPGP9 genome. To refine the set of TASV deletion candidates, we initially identified deletion regions in scaffolds by comparing them with the human reference genome (hg19) (Supplementary Fig. S1). Next, the flanking sequences on either side of these deletion breakpoint loci were subjected to RepeatMasker analysis (http://www.repeatmasker.org/cgi-bin/WEBRepeatMasker)^47^. Next, we selected and analyzed only those candidates anchoring repeat elements in both flanking sequences of the breakpoint. Candidates that were unrelated to the TE-mediated deletion were filtered out according to internal sequence variations in the element, incompletely assembled regions (i.e., N stretches), or variations caused by simple repeats. Manual inspection was performed to identify the mechanism of each using alignments with their flanking sequences using BioEdit software v.7.0.5.3^48^.

### Experimental validation of precise TASVs

To validate all TASVs, we conducted PCR amplification and DNA sequencing as described below. Oligonucleotide primers for PCR amplification of each locus were designed using Primer3 software^49^. PCR amplification of each locus was performed with initial denaturation at 95 °C for 10 min; 35 cycles of 30 s of denaturation at 95 °C, 30 s at the annealing temperature, and 1 to 4 min of extension at 72 °C (depending on the expected size of the PCR product); and a 5 min final extension at 72 °C. Each PCR was performed in a 25 μL reaction containing 10 μL of 2X Lamp *Pfu* DNA Polymerase (BioFact, South Korea), forward and reverse oligonucleotide primers (10 pmol/μL each), and more than 20 ng of target template DNA. PCR confirmation analyses were conducted in two different human genomic DNA panels. One DNA panel purchased from the Coriell Institute for Medical Research was composed of 80 human individuals (20 individuals from each of four different populations: Asian, South American, European, and African American). The other panel was composed of 38 genomic DNA samples that were extracted from whole blood of healthy Korean donors. KPGP9 DNA was used as a control in every round of experiments. Each DNA sample was qualified and quantified by using a spectrometer (TITERTEK, Germany). The PCR products were loaded on a 2% agarose gel for electrophoresis, stained with EcoDye™ Staining Solution (Biofact, South Korea), and visualized using UV fluorescence. For several candidates involving stretches of ambiguous sequence (Ns) or otherwise identified as uncertain amplicons, the PCR products were confirmed with Sanger DNA sequencing using an ABI 3500 Genetic Analyzer (Applied Biosystems, USA). We gratefully acknowledge the Center for Bio-Medical Engineering Core Facility at Dankook University for providing equipment, including a Sanger sequencer.

### Comparison analysis with the public genomic variant database

In comparing the TASVs with large-scale genomic variants, we used public data for the AK1 genome^50^, The Cancer Genome Atlas (TCGA)^51^, and the 1000 Genomes SV dataset^8^, downloaded from dbVar (estd219)^52^. BEDTools (v2.27.0) software^53^ was used to identify the overlap between TASVs and genomic features such as CNVs, TE insertions, and deletions from the public database. To verify the precision of the TASV position from the KPGP9 genome, the predicted insertion and deletion sites in other data were compared, and the margin of error that results from comparing the corresponding TASV sites with AK1 and TCGA was calculated.

### Inferring clinical impacts and visualizing the identified TASVs from the KPGP9 genome

To observe risk genes associated with phenotypic abnormalities and disease, all genes associated with TASV insertion or deletion events were searched in the DisGeNET^54^ and OMIM databases^55^. We analyzed sequence homology for TASV insertions harbored in the UTR regions of the genes through a search of the miRBase database^56^. The ideogram figure indicating the positions along chromosomes of common SNPs in three Koreans and our TASV insertions and deletions were created using the webtool idiographica (http://www.ncrna.org/idiographica/)^41^.

### *Alu* and L1 densities in genic region

To measure the *Alu* and L1 densities within the human genes, we used RefSeq gene data from the UCSC human genome (hg19). We extracted all repeat information from the RepeatMasker track using the UCSC genome table browser. *Alu* and L1 insertions in genic regions were extracted by comparison with the locations of human reference genes. The composition ratio of *Alu* and L1 elements in genes was calculated based on length (*Alu* and L1/gene length).

### Ethical approval

This study was approved by the Central Ethics Committee on Research involving human subjects of the Genome Research Foundation (GRF), an Institutional Review Board (IRB) certified by the Korean office of Human Research Protection (IRB number 20101202-001).

## Results

### Sequencing and de novo assembly of a Korean individual genome

We generated sequencing data from total of nine libraries, including three paired-end (PE) libraries and six mate-paired (MP) libraries, with the Illumina HiSeq 2500 platform. The raw sequence data yielded by these PE and MP libraries consisted of 201.27 Gb and 114.2 Gb, respectively (Supplementary Table S1). A total of 284.24 Gb of high-quality reads (94.7X coverage) covered approximately 99.23% of the human reference genome (hg19; http://genome.ucsc.edu/), as measured by mapping-based methods (Supplementary Table S2). De novo assembly of the KPGP9 genome was performed using the *De Bruijn* graph assembly algorithm with SOAPdenovo ver. 2.04^36,38^. This highly accurate de novo assembly resulted from a total of 1,439,891 contigs and 31,505 scaffolds, including the longest scaffold of length 75,412,104 bp (Table 1). A total of 555 large-scale scaffold blocks were used for genome alignments (Supplementary Table S3 and Supplementary Fig. S2). As with this result, we constructed the de novo draft genome with sufficiently long scaffolds for the detection of TASVs from the Korean KPGP9 genome. We investigated the composition of the repeat elements using RepeatModeler 1.0.8 (Supplementary Table S4).

### Computational data mining of TE-associated structural variations (TASVs)

To investigate TASVs in the KPGP9 genome, we focused on two different structural variations derived from TE insertions and TE-mediated genomic deletions. We initially extracted all TE-containing sequences within the scaffolds corresponding to four retrotransposon families (LINE-1, *Alu*, LTR retrotransposon, and SVA elements), along with their 5’ and 3’ flanking sequences. A total of 14,548 TASV insertions and 4676 TASV deletion candidates were initially selected from all TE-containing genomic regions. As described in the Materials and Methods, uncertain insertion candidates were excluded. After BLAT analysis, we found 352 nonreference TE insertions, 97 of which were previously reported in the Database of Retrotransposon Insertion Polymorphisms (dbRIP; http://falcon.roswellpark.org:9090)^57^. Thus, our analysis of TASV insertion candidates identified a total of 255 novel, nonreference TE insertions in the KPGP9 genome (Table 2).

**Table 2:** Summary of TASV insertions in the KPGP9 individual genome.

Class	Subfamily	Total candidates	Filtered out	dbRIP	Validation by PCR	False positive	Not working	Total confirmed	Contributions to KPGP9 (loss)
*Alu*	*Alu*Ya5 (NCAI^a^)	2823	2669	50	104	11	2	84 (7)	26,333 bp (−35 bp)
	*Alu*Ya8	541	528	3	10	2	–	8	2580 bp
	*Alu*Yb8 (NCAI^a^)	1991	1923	19	49	5	–	40 (4)	13,568 bp (−54 bp)
	*Alu*Yb9 (NCAI^a^)	351	332	5	14	–	–	12 (2)	3946 bp (−3270 bp)
	*Alu*Yc3	556	528	5	23	17	2	4	987 bp
	*Alu*Yd8 (NCAI^a^)	192	183	–	9	6	–	2 (1)	798 bp (−9 bp)
	*Alu*Yg6 (NCAI^a^)	895	880	5	10	3	3	3 (1)	1150 bp (−1 bp)
	*Alu*Yh9	354	339	2	13	11	1	1	135 bp
	*Alu*Yk11	168	167	0	1	1	–	0	–
	*Alu*Yk12	127	125	1	1	–	–	1	111 bp
LINE-1	L1HS	3124	3117	3	4	1	–	4	6399 bp
LTR	HERV-K	224	222	–	2	1	–	1	684 bp
	HERVK14C	87	85	–	2	1	–	1	2725 bp
	LTR5_HS	653	649	–	4	–	–	4	2541 bp
SVA	SVA_A	1,050	1,048	–	2	2	–	0	–
	SVA_E	615	613	–	2	2	–	0	–
	SVA_F	797	788	4	5	3	–	2	2275 bp
		**14,548**	**14,196**	**97**	**255**	**65**	**8**	**182**	**64,232** **bp (−3369** **bp)**

^a^Non-Classical *Alu* Insertion.

For genomic deletions by retrotransposons, we focused on two mechanisms known to produce human genomic rearrangements: nonallelic homologous recombination (NAHR) and nonhomologous end joining (NHEJ), both of which can be mediated by retrotransposons^24,58,59^. NAHR between LINE-1 or *Alu* repeats has also emerged as a major driver of copy number variation (CNV) in primate genomes^23,24^. DNA double-strand breaks (DSBs) are typically one of the most dangerous types of DNA damage in the human genome and can be repaired by TEs, especially LINE-1 and *Alu* elements, using either NHEJ or homologous recombination (HR)^60^. In previous studies, it has been reported that retrotransposon-mediated genomic deletions may be linked to certain cancers and genetic disorders and have contributed to the evolution of genetic differences among and between human populations^61^. After a comparative analysis of the KPGP draft genome and the human reference genome, 1355 and 3321 retrotransposon-mediated deletion candidates associated with LINE-1 and *Alu* elements, respectively, were detected. After manual inspection using BLAT and multiple sequence alignment analysis, the numbers of deletions associated with L1 and *Alu* were 40 and 240, respectively. Thus, we categorized each event as belonging to one of four categories: 131 NAHR-*Alu* recombination-mediated deletions (ARMDs), 109 NHEJ-ARMDs, seven NAHR-L1 recombination-associated deletions (L1RADs), and 33 NHEJ-L1RADs (Table 3).

**Table 3:** Summary of TASV deletions in the KPGP9 individual genome.

Class	Total candidates	Filtered out	Subclass	Validation by PCR	False positive	Not working	Total confirmed	Contributions to KPGP9
ARMD	3321	3081	NAHR	131	73	7	51	42,909 bp
			NHEJ	109	32	11	26	7313 bp
L1RMD	1355	1315	NAHR	7	5	0	2	3177 bp
			NHEJ	33	18	5	10	29,373 bp
**Total**	**4676**	**4396**		**280**	**128**	**23**	**89**	**82,772** **bp**

### Experimental validation for TASVs

To experimentally validate our computationally detected TASV insertions and deletions, we designed target-specific primers for a total of 255 insertion and 280 deletion candidates. PCR amplification was performed in a panel consisting of material from 80 non-Korean individuals (20 individuals from each of four populations) and 39 Korean individuals, including KPGP9 DNA (Supplementary Table S5). Several loci containing poly(N) stretches or displaying unexpected amplicon sizes were resequenced *via* Sanger sequencing. By applying the processes mentioned above, a total of 65 insertion (25.39%) and 128 deletion (45.71%) candidates were eliminated as false positives. A further eight insertion (3.12%) and 23 deletion candidates (8.12%) failed to produce any amplicons. Of the original 255 TASV insertion candidates, we were able to experimentally confirm 182 (71.37%) as authentic (Table 2 and Supplementary Data S1). In addition, 89 (31.78%) out of 280 TASV deletion candidates were confirmed as authentic (Table 3 and Supplementary Data S2). All TASV loci inspected through this approach are listed in Supplementary Table S6 and Supplementary Table S7. In summary, 271 TASVs accounting for 54.6% of the 496 TASV candidates identified through manual inspection were authentic (Supplementary Fig. S3). Our results once again indicated the importance of experimental validation in NGS-based human TASV analysis (Supplementary Fig. S4).

Among our set of 182 authentic TASV insertions, we experimentally validated 155 *Alu*, four L1 and six LTR retrotransposons, and two SVA insertions that exhibit the hallmarks of retrotransposition expected of classic insertions^62,63^. Meanwhile, the remaining 15 TASV insertions were found to be non-classical *Alu* insertions (NCAIs), which were inserted into the genome via an alternative pathway involved in DSB repair. These NCAIs were characterized by the absence of TSDs and several other structural features (e.g., truncated *Alu* and absence of the poly-A tail) and were accompanied by deletions of the preinsertion sequence^64,65^. Of the six LTR retrotransposon insertions, four were found to be solitary LTRs caused by unequal homologous recombination between the two LTRs, but the site was designated as empty in the human reference genome (the Human DNA panel we analyzed showed the empty and solitary LTR insertion alleles; with more experimental evidence, we would expect three different types of genotypes (i.e., trimorphism): absence of the HERV element, presence of the HERV element, and presence of a solitary LTR)^66^. The remaining two LTR retrotransposon insertions were HERV-K insertions with internal sequence deletions^67^. The homologous loci of these insertion sites in the human reference genome contained only solitary LTRs (Supplementary Data S1 and Supplementary Table S6). We investigated the associations of TASV insertion with disease and found that 11 TASV insertions were located in the intragenic regions of 10 genes closely related to 16 hereditary diseases identified in recent studies (Table 4).

**Table 4:** Clinical implication of genes-associated with novel TASVs in the KPGP9 genome.

Gene symbol	Genes	Types of TASV (density of the given TE)	Disease (# OMIM. Phenotype)	Inheritance	UMLS ID	Source	No. of SNPs	No. of publications
*Insertion*								
*PRPH2*^*a*^	Peripherin 2	*Alu*Ya5 insertion (33.54%)	Retinitis Pigmentosa 7 (#608133)	Autosomal dominant inheritance	umls:C1842475	CLINVAR, CTD_human, MGD, UNIPROT	9	6
			Patterned dystrophy of retinal pigment epithelium (#169150, #608161)	Autosomal dominant inheritance	umls:C1868569	CLINVAR, CTD_human, UNIPROT	7	4
			CHOROIDAL DYSTROPHY, CENTRAL AREOLAR 2 (#613105)	Autosomal dominant inheritance	umls:C2751290	BeFree, CLINVAR, CTD_human, UNIPROT	4	2
*DMD*^*a*^	Dystrophin	*Alu*Ya5 insertion (6.24%)	Muscular Dystrophy, Duchenne (# 310200)	X-linked recessive inheritance	umls:C0013264	BeFree, CLINVAR, CTD_human, GAD, LHGDN, MGD, ORPHANET, UNIPROT	175	535
			Becker Muscular Dystrophy (# 300376)	X-linked recessive inheritance	umls:C0917713	BeFree, CLINVAR, GAD, MGD, ORPHANET, UNIPROT	136	205
			DMD-associated dilated cardiomyopathy	X-linked recessive inheritance	umls:C3668940	BeFree, CLINVAR, CTD_human, GAD, UNIPROT	213	35
*EPB41*	Erythrocyte membrane protein band 4.1	Two *Alu*Ya5 insertion (31.54%)	Hereditary Elliptocytosis 1	Autosomal dominant inheritance	umls:C2678497	CLINVAR, CTD_human, MGD	1	29
*MTPAP*^*a*^	Mitochondrial poly(A) polymerase	*Alu*Ya5 insertion (31.51%)	SPASTIC ATAXIA 4, AUTOSOMAL RECESSIVE (#613672)	Autosomal recessive inheritance	umls:C3150925	CLINVAR, CTD_human, ORPHANET, UNIPROT	1	1
*XDH*	Xanthine dehydrogenase	*Alu*Ya5 insertion (4.74%)	Xanthinuria, Type I	Autosomal recessive inheritance	umls:C0268118	BeFree, CLINVAR, CTD_human, ORPHANET, UNIPROT	2	8
*PDE8B*^*a*^	Phosphodiesterase 8B	*Alu*Ya5 insertion (7.07%)	Striatal Degeneration, Autosomal Dominant (#609161)	Autosomal dominant inheritance	umls:C1836694	BeFree, CLINVAR, CTD_human, ORPHANET	0	1
*CASK*^*a*^	Calcium/calmodulin-dependent serine protein kinase	*Alu*Yb8 insertion (13.84%)	Mental Retardation and Microcephaly With Pontine And Cerebellar Hypoplasia (#300749)	X-linked dominant inheritance	umls:C2677903	CLINVAR, CTD_human, ORPHANET, UNIPROT	20	1
*FTO*^*a*^	Fat mass and obesity associated	*Alu*Yb8 insertion (10.15%)	Diabetes Mellitus, Non-Insulin-Dependent	Autosomal dominant inheritance	umls:C0011860	BeFree, CTD_human, GAD, GWASCAT	61	116
			Obesity/BODY MASS INDEX QUANTITATIVE TRAIT (# 612460)	Polygenic inheritance	umls:C0028754	BeFree, CTD_human, GAD, GWASCAT	36	345
			Growth Retardation, Developmental Delay, Coarse Facies, And Early Death (#612938)	Autosomal recessive multiple congenital	umls:C2752001	CLINVAR, CTD_human, ORPHANET, UNIPROT	2	1
*KCNJ6*^*a*^	Potassium channel, inwardly rectifying subfamily J, member 6	*Alu*Yb8 insertion (4.7%)	KEPPEN-LUBINSKY SYNDROME (#614098)	Undefined	umls:C3279800	BeFree, CLINVAR, ORPHANET, UNIPROT	2	1
*RAF1*^*a*^	Raf-1 proto-oncogene, serine/threonine kinase	SVA_F insertion (0%)	Noonan Syndrome/CARDIOMYOPATHY, DILATED (#615916)	Autosomal dominant inheritance	umls:C0028326	BeFree, CLINVAR, CTD_human, GAD, LHGDN, ORPHANET	5	25
*Deletion*								
*FA2H*^*a*^	Fatty acid 2-hydroxylase	NAHR-ARMDs (22.04%)	Leukodystrophy, Dysmyelinating, And Spastic Paraparesis With Or Without Dystonia (#612319)	Autosomal recessive inheritance	umls:C3496228	CTD_human, MGD, ORPHANET, UNIPROT	0	3
*EHMT1*^*a*^	Euchromatic histone-lysine N-methyltransferase 1	NAHR-ARMDs (20.66%)	Kleefstra Syndrome (#610253)	Autosomal dominant inheritance	umls:C0795833	BeFree, CLINVAR, CTD_human, MGD, UNIPROT	16	8
*INSR*^*a*^	Insulin receptor	NAHR-ARMDs (40.47%)	Diabetes Mellitus, Non-Insulin-Dependent	Autosomal dominant inheritance	umls:C0011860	BeFree, CLINVAR, GAD, RGD, UNIPROT	11	114
			Insulin Resistance (#262190)	Autosomal dominant inheritance	umls:C0021655	CLINVAR, CTD_human, GAD, RGD	3	8
			Donohue Syndrome (#246200)	Autosomal dominant inheritance	umls:C0265344	BeFree, CLINVAR, CTD_human, GAD, ORPHANET, UNIPROT	17	46
			Rabson-Mendenhall Syndrome	Autosomal recessive inheritance	umls:C0271695	BeFree, CLINVAR, ORPHANET, UNIPROT	4	19
			Hyperinsulinemic Hypoglycemia, Familial, 5 (#609968)	Autosomal dominant inheritance	umls:C1864952	CLINVAR, CTD_human, ORPHANET, UNIPROT	3	1
*SERPINF2*	Serpin peptidase inhibitor, clade F (alpha-2 antiplasmin, pigment epithelium derived factor), member 2	NHEJ-ARMDs (38.39%)	ALPHA-2-PLASMIN INHIBITOR DEFICIENCY	Autosomal recessive inheritance	umls:C2752081	CLINVAR, MGD, ORPHANET, UNIPROT	2	1
*PCCB*^*a*^	Propionyl CoA carboxylase, beta polypeptide	NHEJ-L1RMDs (40.53%)	Propionic acidemia (#606054)	Autosomal recessive inheritance	umls:C0268579	BeFree, CLINVAR, CTD_human, ORPHANET, UNIPROT	24	23

^a^The genes annotated in OMIM phenotype data.

In addition, we categorized 89 TASV deletions based on their deletion mechanisms. A total of 51 NAHR-ARMDs, 26 NHEJ-ARMDs, two NAHR-L1RADs, and 10 NHEJ-L1RADs were validated as new genomic deletions (Supplementary Data S2 and Supplementary Table S7). We experimentally confirmed that all TASV deletions were polymorphic in our panel of five different human populations, including 38 Korean individuals (e.g., Supplementary Fig. S5). We believe these results support the concept that data mining of TASVs within subpopulation DNA panels can be an effective strategy for pursuing population-specific genomic research^68^. In addition, our analysis of TASVs has demonstrated that these events have contributed to the genetic polymorphism observed in human populations.

### Contribution of TASVs in genomic variation

As previously suggested, TEs can cause genetic diversity and instability through their insertions into genic regions or through homologous recombination between two TE copies with high sequence similarity in the human genome. To characterize these processes in our de novo genome, we ascertained the contributions of identified TASVs to genomic variations and structural alterations, which are associated with genes containing TASVs^69^. A total of 182 TASV insertions by *Alu*, L1, SVA elements, and LTR retrotransposons are responsible for a gain of 64,232 bp of sequence within the KPGP9 genome relative to the reference genome. A total of 170 of these consisted of *Alu* insertions, contributing 49,608 bp of sequence, with an average length of 292 bp (range: 111 to 598 bp). A majority of the *Alu* insertions are members of the *Alu*Ya5 subfamily, which accounted for 26,333 bp of the sequence gain. We also found 15 NCAIs, which include insertion of the TE sequence and also genomic deletions at each insertion site. These insertions contain truncated *Alu* sequences with no TSDs. In total, these NCAI events resulted in the deletion of 3369 bp of genomic sequences. However, these are not simple deletions, as TE sequence is also inserted during such events. For example, one of our NCAI insertions resulted in the addition of 139 bp of sequence in the form of a truncated *Alu*Yb9 element, but also the deletion of 3270 bp of pre-integration sequence, for a net sequence loss of 3131 bp. The remaining events, consisting of four L1HS, two SVA elements, and six LTR retrotransposon insertions, contributed sequence gains of 6399, 2275, and 5950 bp, respectively, in the KPGP9 genome (Table 2).

**Fig. 1:**
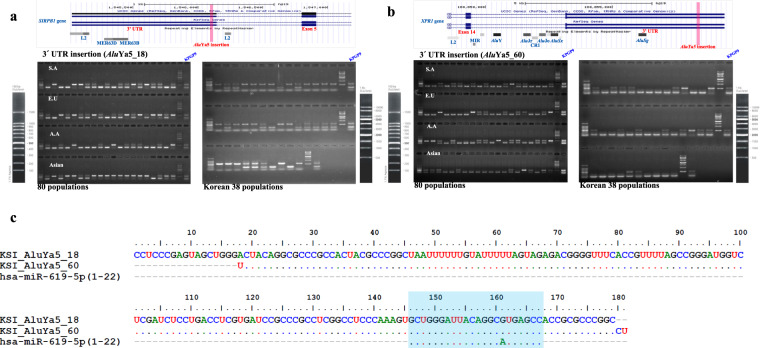
Novel *Alu*Ya5 insertion events in the 3’UTRs of the *SIRPB1* and *XPR1* genes. Polymorphic insertion testing was performed for two AluYa5 insertions located in the 3’UTR regions of the **a** SIRPB1 and **b** XPR1 genes. A screenshot of the UCSC human genome browser (hg19) shows the location of each AluYa5 insertion in the 3’UTR of each gene; adjacent repeats are displayed in gray boxes. The results of PCR amplification from a panel of 80 geographically diverse individuals and 38 Korean samples are shown in this figure (Supplementary Table S4). The upper and lower bands denote the presence of an AluYa5 insertion and its absence, respectively. **c** The blue box shows the identical sequences of miRNA (hsa-miR-619) binding to the AluYa5 elements inserted in the 3’UTR regions of the SIRPB1 and XPR1 genes.

To investigate the genetic impact of TASV insertions, we identified 80 of 182 TASV insertions (43.95%) as existing in intragenic regions by comparing the insertion positions with the RefSeq Gene database^70^. Of these 80 TASV insertions, 78 are located in the intronic regions of 78 genes. Interestingly, the remaining two intragenic TASV insertions are *Alu* elements that have inserted into the 3′ untranslated regions (UTRs) of two genes. One inserted into the signal-regulatory protein beta 1 (*SIRPB1*) gene, which is a member of the immunoglobulin superfamily^71^. The other inserted into the xenotropic and polytropic retrovirus receptor 1 (*XPR1*) gene, which is known to be a phosphate exporter molecule^72^. We confirmed that these two *Alu* insertions into the *SIRPB1* and *XPR1* genes are polymorphic on our panel of five human populations, including 38 Korean individuals (Fig. 1). It was previously found that TEs, especially *Alu* insertions in gene regions, may be considered a potential resource for some microRNAs (miRNAs) as a rare genetic event that provides miRNA target sites for transcriptional regulation. We therefore also investigated the influence of these insertions on miRNA binding sites. Both *Alu*Ya5 insertions in the *SIRPB1* and *XPR1* genes were found to contain a potential binding site for the miRNA hsa-miR-619 (MIMAT0026622) in a search for homologous miRNA sequences. In recent studies, it has been demonstrated that the majority of mature hsa-miR-619-5p binding sites are located in the 3’UTRs (214 sites) of 201 human genes^73^ and derived from the *Alu* element^74^. This indicates that the embedded *Alu* insertions at the 3′ UTR in both genes may act as a translation inhibitor interacting with miRNA, a function that is highly conserved among *Alu* sequences^75^.

**Fig. 2:**
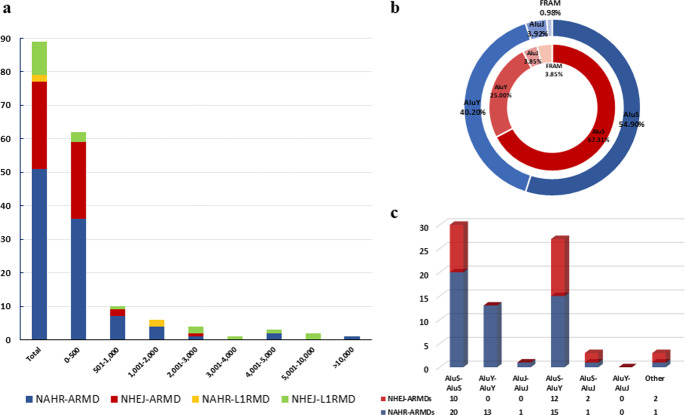
Size distribution of TASV deletion events and *Alu* subfamily composition involved in ARMD events. **a** Size distribution of genomic deletions by insertion mechanism. The number of TASV deletions in 500 bp bins is shown on the y-axis. **b** The composition of *Alu* subfamilies involved in NAHR-ARMD (navy) and NHEJ-ARMD (red) events. **c** The x-axis indicates *Alu* subfamily contributions to the ARMDs observed in this study. The total number of events by mechanism is shown on the *y*-axis.

We also identified 89 TASV deletions that removed a total of 82,772 bp of sequence in the KPGP9 genome. Among those occurring between *Alu* elements and L1s, the 51 NAHR-ARMD events accounted for a large portion of the total genomic sequence deleted (42,909 bp) from the KPGP9 genome (Table 3). Most (75%) TASV deletions were less than 1 kb in length, and only four deletion events were >5 kb (Fig. 2a). The largest genomic deletion we observed was 13,698 bp in size and was caused by an NAHR-ARMD event between two *Alu*Sz6 insertions in the intronic region of the ankyrin repeat domain 30B (*ANKRD30B*) gene. In addition, we investigated homologous reference sequences in the recombination breakpoints of NAHR deletions and in the microhomology sequences of NHEJ deletions. The length of homologous sequence stretches between *Alu*-*Alu* recombinants ranged from 3 to 283 bp, with 43 bp being the average. Three positions along the *Alu* elements participating in these events, including the *Alu* recombination “hotspots” previously identified based on *Alu* consensus sequences (*Alu*S, *Alu*J, and *Alu*Y families), were found to have slightly higher recombination frequencies (Supplementary Fig. S6)^21^. We also found microhomologies ranging from 1 to 18 bp in size, which implied a microhomology-mediated end joining mechanism in 15 out of 26 *Alu* NHEJ-ARMD events^76^. Among a total of 10 NHEJ-L1RAD events, we verified homologous sequences associated with deletions in only seven events. All details of our homologous sequence and microhomology investigations at TASV deletion loci are detailed in Supplementary Data S2 and Supplementary Table S7.

To verify which subfamilies are most commonly involved in TASV deletions, we examined the distribution of *Alu* and L1 subfamilies among these events. A total of 29 *Alu* subfamilies were associated with our ARMD events (14 *Alu*S, 11 *Alu*Y, 2 *Alu*J, FLAM, and FRAM). We further found that 91 *Alu*S insertions were among the 154 *Alu* insertions associated with deletions, including ~54% NAHR-ARMD and ~67% NHEJ-ARMD events. This observation of an overrepresentation of *Alu*S insertions in ARMDs is in accord with the results of previous studies. It is perhaps unsurprising that the most successful *Alu* subfamily in terms of copy number (*Alu*S) has also been involved with the most ARMD events^21^. Previous studies also found that the *Alu*J subfamily contributed less to the detected ARMD events, an observation thought likely to be driven by the fact that the older *Alu*J copies had accumulated more point mutations, thereby lowering their chances of being involved in recombination events^23^. Consistent with these expectations, we also observed substantially fewer contributions of *Alu*J insertions to our detected deletion events (Fig. 2b). We investigated all *Alu* copy interaction combinations located in the homologous upstream and downstream regions of each ARMD breakpoint and found that 20 NAHR-ARMDs and 10 NHEJ-ARMDs appeared to have occurred between *Alu*S copies. The *Alu*S*-Alu*Y combinations were also found at high frequency, while we identified only a small number of ARMD events (4 of 77) associated with *Alu*J copies (Fig. 2c). For L1RADs, members of 16 different L1 subfamilies, including L1HS, were involved in L1RAD events in various combinations, as shown in Supplementary Table S7. Of our 89 authentic TASV deletion events, 40 occurred in the intronic regions of 40 genes. These included 24 NAHR-ARMDs, 11 NHEJ-ARMDs, one NAHR-L1RAD, and four NHEJ-L1RADs. The ~51% of authentic TASV deletions occurring in intronic regions are responsible for the loss of 42,538 bp of sequence.

### Comparison with the published genomic databases

**Fig. 3:**
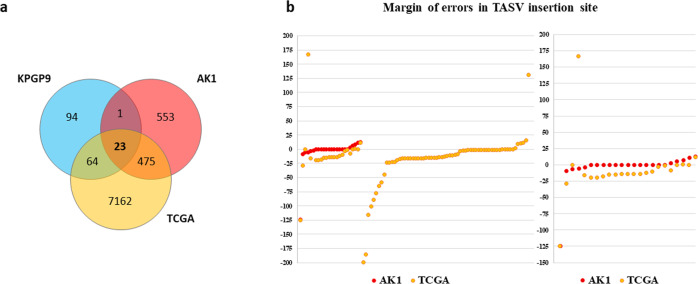
Comparisons of TASV insertions among three genome datasets. **a** The numbers of TASV insertions were compared between KPGP9, another Korean genome (AK1), and TCGA genome data. Only 23 loci were shared among the three genome datasets, and 94 were unique to KPGP9. **b** The red and orange dots represent the margin of error in the TASV insertion points of AK1 and TCGA data, respectively, based on the TASV insertion points detected in the KPGP genome. The left side shows the margin of error for 88 common TASV insertions to KPGP, and the right side shows the margin of error for 23 TASV insertions shared in both genome datasets.

With careful bioinformatics identification of TASVs followed by rigorous experimental validation, we confirmed that TASVs contribute to genetic differences within different ethnic groups. The majority of TASVs in this study are polymorphic among human populations. We first compared and analyzed all TASVs in a thorough search of the Database of Genomic Structural Variation (dbVar) in NCBI^77^. This database provides detailed information on human SVs from multiple studies, including the 1000 Genomes Project (1000 GP). The results showed that 26 out of a total of 182 TASV insertions and 24 out of 89 TASV deletions were previously observed but not experimentally confirmed (Supplementary Table S6 and Supplementary Table S7). Additionally, we compared and analyzed the population allele frequencies of 26 confirmed insertions and 24 deletions, which averaged 0.279 and 0.496, respectively. In particular, AluYc3_4 (ALU_umary_ALU_7707) had an average allele frequency of 0.17 in five superpopulations (African, American, East Asian, European, South Asian) and was observed only in East Asians (0.04), Africans (0.047), and South Asians (0.011). NHEJ-L1RAD_6 (UW_VH_1123) and NHEJ-L1RAD_7 (YL_CN_LWK_752) were confirmed to be dominant in all but the African population (Supplementary Table S8). Next, a comparative analysis was conducted to evaluate their distribution in recently published studies focusing on TASV insertions, such as the Korean AK1 genome^50^ and TCGA^51^, and how accurate the identification was. Of the 10,077 insertion events found in the AK1 genome, we selected 1097 TE-associated insertion events, which are newly identified cases (707 *Alu*, 177 L1, 18 HERV, and 195 SVA) and compared with TASV insertions in the KPGP9 genome. Only 24 events are shared with AK1. Furthermore, among a total of 7724 TE-associated insertion events from TCGA data, 87 TASV events were shared in both. Compared to previous studies, we have confirmed that a total of 94 TASV insertions (approximately 51 %) were found uniquely in the KPGP9 genome (Fig. 3 and Supplementary Table S9). To analyze the exact insertion site between the given data, we investigated the error of the insertion sites based on the manually investigated TSD. Observing the error of the expected location of the TASV insertions, which that were shared among the three datasets, AK1 had an average margin of error of only 4 bp, but in TCGA, AK1 had a margin of error of more than 15 bp in both upstream and downstream sequences (Fig. 3). NGS analysis with short-read sequencing data is straightforward to explore for a wide range of genome variants because they generate sufficient query bulk data in a short period and have a guaranteed price advantage. However, there are still difficulties in determining exact INDEL sites, identifying the full-length sequences of insertion variations, and providing base-level resolution from NGS data. Therefore, validation tests with PCR and Sanger sequencing are needed. AK1, on the other hand, was highly efficient in identifying convoluted and repetitive TEs because it used a newly assembled genome generated using single-molecule real-time sequencing, physical mapping, microfluidics-based linked reads, and bacterial artificial chromosome (BAC) sequencing approaches.

## Discussion

In summary, the aim of this study was to establish a de novo-assembled Korean genome and to identify new TASVs through comparative analysis with the human reference genome. The use of the SOAPdenovo2 assembler based on the parallel assembly method with various insert-size libraries has enabled de novo construction of this Korean genome, which was then suitable for the identification of precise TASVs. Through this, we verified that many genomic variations associated with TEs contribute to polymorphisms in an individual Korean genome relative to the reference genome and a population panel, and we report the detailed genomic information of these TASVs.

**Fig. 4:**
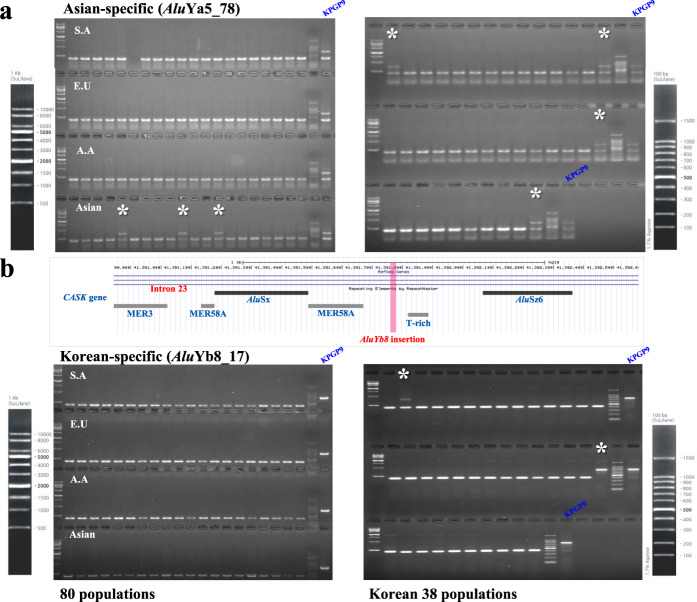
Polymorphic *Alu* insertions in Asian populations. The PCR results for two *Alu* insertions, **a** *Alu*Ya5_78 and **b** *Alu*Yb8_17, are shown. A screenshot of the UCSC human genome browser (hg19) shows the polymorphic *Alu*Yb8 insertion at intron 23 of the *CASK* gene; adjacent repeats are displayed in gray boxes. PCR amplification was conducted from 80 DNA samples representing four different populations (South American, European, African American, and Asian) and 38 Korean DNA samples (Supplementary Table S5). The upper bands (marked with asterisks) indicate the presence of an *Alu* insertion, and the lower bands indicate its absence at the corresponding genomic locus.

Recent studies into the mechanisms of genetic diseases and cancer have underlined the clinical relevance of TEs, which can alter sequences through insertional mutagenesis and recombination-mediated deletion and modify the epigenetic regulation of nearby loci^69,78^. To study the potential impact of TEs in our de novo genome on the genic loci associated with disease, we were able to identify 10 genes bearing 10 *Alu* insertions and one SVA_F insertion. These loci are associated with the 16 diseases listed in Table 4. Of note, two *Alu*Ya5 insertions are observed in the intronic region of the *EPB41* gene, which encodes a red blood cell cytoskeleton protein (protein 4.1) and is associated with the autosomal dominant hereditary disease elliptocytosis^79^. From previous studies, it is well known that approximately one million *Alu* insertions represent more than 10.6% of the human genome and that the density of *Alu* insertions in the genome is 306 per megabase (Mb)^80,81^. Here, we estimated the exact composition of *Alu* and L1 elements in genic regions based on UCSC human RefSeq Gene data (hg19)^80^. A total of 27,767 gene locations were involved in this calculation, and we found that 20,972 and 17,340 genes were associated with at least one *Alu* or L1 element, respectively. The densities of *Alu* and L1 elements in genic regions were 14.91% and 11.81%, respectively (Supplementary Fig. S7). The *EPB41* gene contains a markedly higher density (31%) of *Alu* insertions. We speculate that this high density of *Alu* insertions could lead to genomic instability by providing many sites of high sequence similarity between which ARMD may occur in the *EPB41* gene. In addition, the *PRPH2* and *MTPAP* genes were shown to have high densities of *Alu* insertions (also over 30%). *PRPH2* is associated with retinitis pigmentosa (RP)^82^, while *MTPAP* is associated with spastic ataxia-4 (SPAX4)^83^. Interestingly, one *Alu*Yb8 insertion (chrX: 41391772-41391772) that is only commonly polymorphic in the Korean population (Fig. 4) was inserted into the *CASK* gene, which is closely linked to mental retardation and microcephaly with pontine and cerebellar hypoplasia (MICPCH)^84,85^. A previous study found that an NAHR event between an *Alu*Jb insertion in intron 6 and an *Alu*Sq2 insertion in intron 8 was responsible for an 11,218 bp deletion in a MICPCH patient^86^. Based on this association, we wondered whether the novel *Alu*Yb8 insertion could be a potential risk factor for *CASK* gene mutation, at least in the Korean population, because it contributes one possible site to participate in an ARMD event. Additionally, we investigated disease-associated genes that were also associated with our TASV deletions. As before, this was accomplished by using DisGeNET and OMIM, and we found three NAHR-ARMDs, one NHEJ-ARMD, and one NHEJ-L1RAD to be associated with five disease-associated genes (*FA2H*^87^, *EHMT1*^88^, *INSR*^89^, *SERPINF2*^90^, and *PCCB*^91^). Analysis of the TE density in these genes showed that the five TASV deletions occurred in regions that contained higher densities of the involved TE families than expected from average *Alu* and L1 densities (*Alu*: 14.91%; L1: 11.81%) in genic regions, based on UCSC human RefSeq gene data (hg19) (Table 4). All details of TASV insertion loci are annotated in Supplementary Data S1 and Supplementary Table S6.

Despite retrotransposition and TE-mediated deletion having been noted as one of the major drivers of genomic structural variation in humans and other nonhuman primates, analyzing the TEs underlying these polymorphisms has proven challenging with high-throughput resequencing methods. As TEs are highly repetitive and abundant, detecting novel TASVs with usual strategies for mapping to the reference genome is difficult. In attempts to overcome this difficulty, various software programs for detecting TEs in WGRS data have been developed, including Tangram^92^, Mobile Element Locator Tool (MELT)^93^, and RetroSeq^94^. These tools detect novel TE insertions, including *Alu*, L1, SVA, and LTR transposons, by searching first for discordant read pairs and split reads in WGRS data at the genomic locations of TEs in the reference. The ability of these broadly applicable software programs has been proven by benchmarking on 1000 GP^95^ and TCGA data^96^.

The advent of these methods has enabled meaningful analyses of TASVs using high-throughput sequencing data in studies involving population genetics, human diseases, and clinical genomics. However, limitations remain. It can be expensive to generate the appropriately deep coverage of the whole genome required to identify these events with confidence. Furthermore, it is difficult to detect the actual breakpoint in the nonreference TE insertion region due to the interval between two aligned mate reads. The complete and interior sequences of repetitive TE insertions cannot be determined by methods that use only short-read mapping. To overcome these difficulties, it is necessary to include sequencing validation with traditional Sanger technology or synthetic long read sequencing technologies that allow the construction of synthetic long reads from short sequencing reads such as the Moleculo method^97^. In the studies using AK1^50^ and TCGA data^51^, it was possible to identify even more events because these datasets did not focus only on the most recently inserted youngest TEs but all events associated with structural variants caused by TEs. In particular, analysis of TCGA genome data of 200 normal subjects revealed approximately 800 TASV insertions per person on average. It is true that the computational framework using the short-read mapping approach, as mentioned in the results, has a wide margin of error in finding insertion locations, and it is challenging to search for TSDs and their alternative mechanisms accurately. Given these differences in analysis, our study also identified new TASV insert events in the KPGP9 individual genomes and provided more detail for each event by checking the potential mechanisms in sequence.

Although our analysis of a de novo Korean-assembled genome created from a combination of PE and MP libraries with a variety of insert sizes does not provide a large number of TASVs compared to previous studies, we have fully validated and characterized each TASV with respect to features such as TSDs, mechanism, exact size, and complete sequence. Our meticulous analysis of TASVs based on their nucleotide sequence enabled us to determine the likely mechanisms responsible for these structural variations in the KPGP9 genome. These included events derived from classical insertion, NCAI, NAHR, and NHEJ mechanisms. We speculate that these TASVs may influence the regulation of several genes. The mechanisms by which such influence could occur include direct mutation or epigenetic alteration of the enhancer region of these genes^26^, as well as the introduction of novel splice sites by insertions into the intronic regions_ENREF_100^98^. We believe that as future studies produce larger numbers of high-quality phased genomes, the genetic and phenotypic relationships of TASV events between ethnic groups or between normal and patient groups will be further validated as an important source of variation.

## Supplementary information

Supplementary Information

## Data Availability

The data can also be accessed through BioProject accession number PRJNA408178 for whole-genome sequence data. All raw sequence data are available at the NCBI Sequence Read Archive (SRA) (http://www.ncbi.nlm.nih.gov/sra) with BioSample accession number SAMN07680091 and accessible through SRA accession number SRR6059039-6059046. The detailed sequence data for the TASVs are available in Supplementary Data S1 and S2.
