# Supplementary material for: A study of transposable element-associated structural variations (TASVs) using a de novo-assembled Korean genome
Source: Exp Mol Med. 2021 Apr 8;53(4):615–30. doi: 10.1038/s12276-021-00586-y (PMC8102501; doi:10.1038/s12276-021-00586-y)
Supplement: Supplementary file 1 — Supplementary Information [file 12276_2021_586_MOESM1_ESM.pdf]

A study of transposable element-associated structural variations (TASVs) using a *de novo* assembled Korean genome

Seyoung Mun[1, 2, 3], Songmi Kim[1, 2], Wooseok Lee[3], Keunsoo Kang[4], Thomas J. Meyer[5], Bok-Ghee Han[6, $], Kyudong Han[2, 3, 4, $], and Heui-Soo Kim[7, $]

[1]Department of Nanobiomedical Science, Dankook University, Cheonan 31116, Republic of Korea

[2]DKU-Theragen institute for NGS analysis (DTiNa), Cheonan 31116, Republic of Korea

[3]Center for Bio-Medical Engineering Core Facility, Dankook University, Cheonan 31116, Republic of Korea

[4]Department of Microbiology, Dankook University, Cheonan 31116, Republic of Korea

[5]Collaborative Bioinformatics Resource, Center for Cancer Research, Frederick National Laboratory for Cancer Research sponsored by the National Cancer Institute, National Institutes of Health, Bethesda, MD 20892, USA

[6]Center for Genome Science, National Institutes of Health, Korea Centers for Disease Control and Prevention, Osong 28160, Republic of Korea

[7]Department of Biological Sciences, Pusan National University, Busan 46283, Republic of Korea

First-author
Seyoung Mun
Mailing address: Department of Nanobiomedical Science & Center for Bio-Medical Engineering Core Facility, Dankook University, Cheonan 31116, Republic of Korea
Phone: +82 -41-550-3567
Fax: +82 -41-559-7937
E-mail: munseyoung@gmail.com

Co-authors
Songmi Kim E-mail: songmik66@gmail.com
Wooseok Lee E-mail: wooseoklee87@gmail.com
Keunsoo Kang E-mail: kangk1204@gmail.com
Thomas J. Meyer E-mail: artificial.selection@gmail.com

[$] Co-corresponding authors
Bok-Ghee Han
Mailing address: Center for Genome Science, National Institutes of Health, Korea Centers for Disease Control and Prevention, Osong 28160, Republic of Korea
Phone: +82 -43-719-8800
Fax: +82-43-719-8869
E-mail: bokghee@korea.kr
Kyudong Han
Mailing address: Department of Microbiology, College of Science & Technology & Center for Bio-Medical Engineering Core Facility, Dankook University, Cheonan 31116, Republic of Korea
Phone: +82 -41-550-3567
Fax: +82 -41-559-7937
E-mail: kyudong.han@gmail.com or jim97@dankook.ac.kr

Heui-Soo Kim
Mailing address: Department of Biological Sciences, Pusan National University, Busan 46283, Republic of Korea
Phone: +82-51-510-2259
Fax: +82-51-581-2962
E-mail: khs307@pusan.ac.kr

Supplementary Information

Supplementary Figures

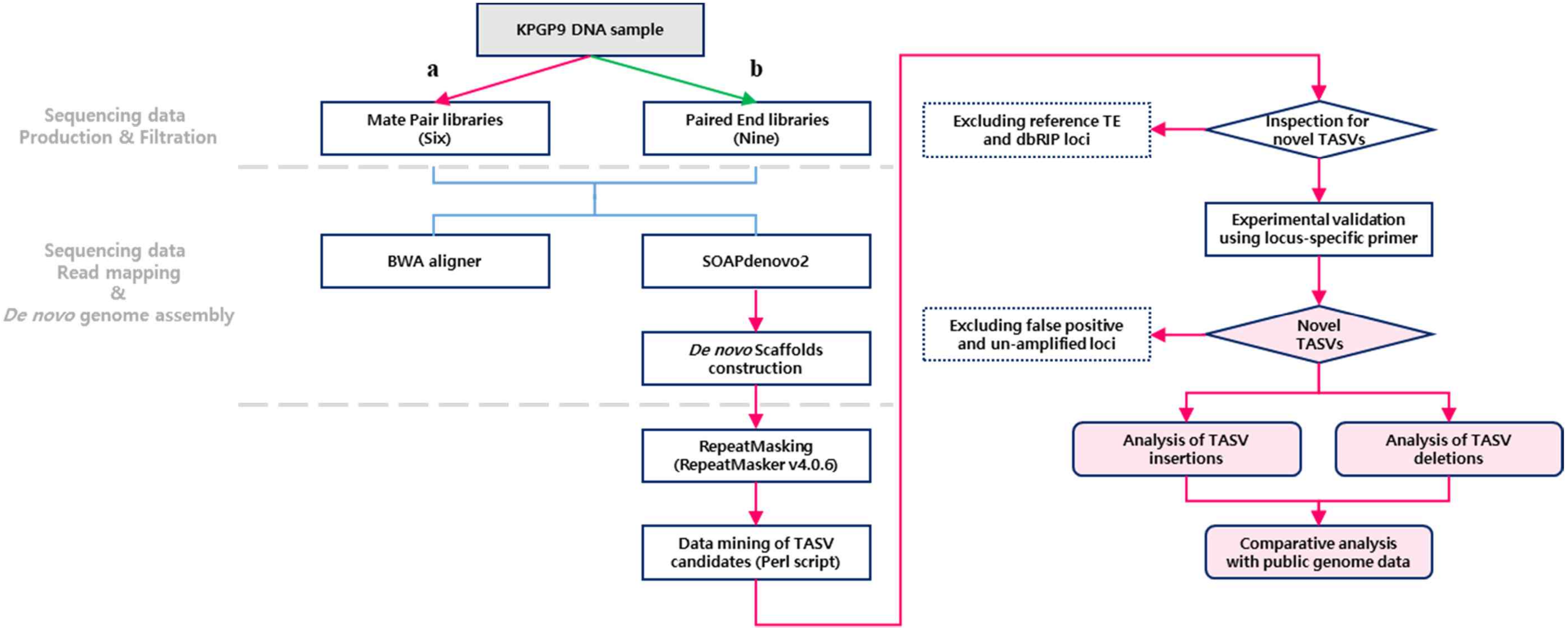

Supplementary Figure S1. Study design and workflow for the KPGP9 genome assay

De novo genome assembly, variant analysis, and genome phasing was enabled via the parallel whole-genome sequencing approach. The genomic data from libraries with multiple insert sizes (both PE and MP) were used for the construction of genome scaffolds using the SOAPdenovo2 assembler. **a** Sequence mapping for the KPGP9 genome was performed against the human reference genome (hg19). **b** TE-

containing regions from the assembled scaffolds were extracted by using a Perl script and manually inspected to identify the precise TASVs through sequence comparison with the human reference genome (hg19). All TASV candidates were experimentally validated. The genes associated with these TASVs were used for the further investigation, such as GO term enrichment and gene-disease association analyses.

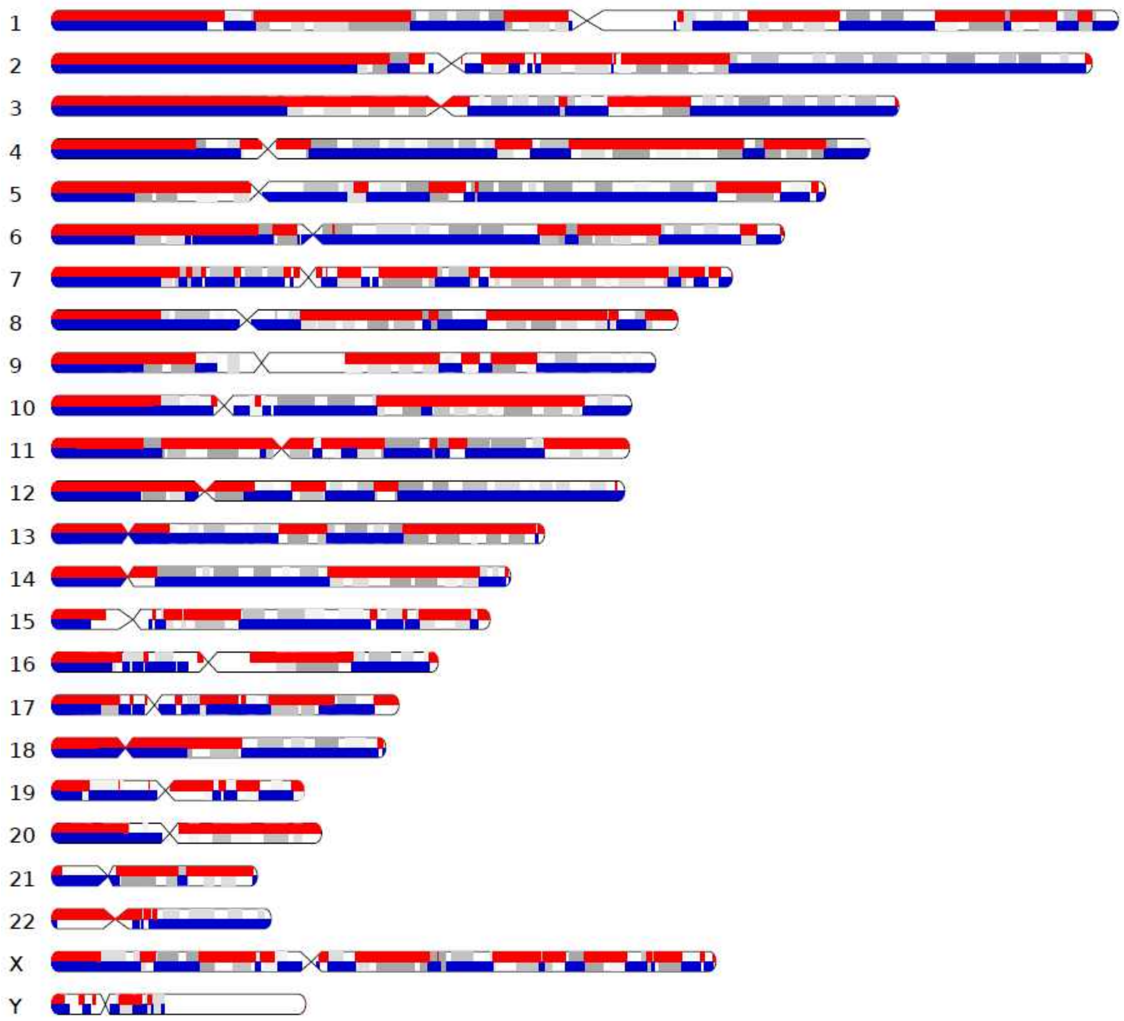

Supplementary Figure S2. Chromosomal mapping of the large-scale scaffold blocks

A total of 555 Large-scale scaffold selected through scaffold alignment using SyMAP software were plotted on the human idiogram.

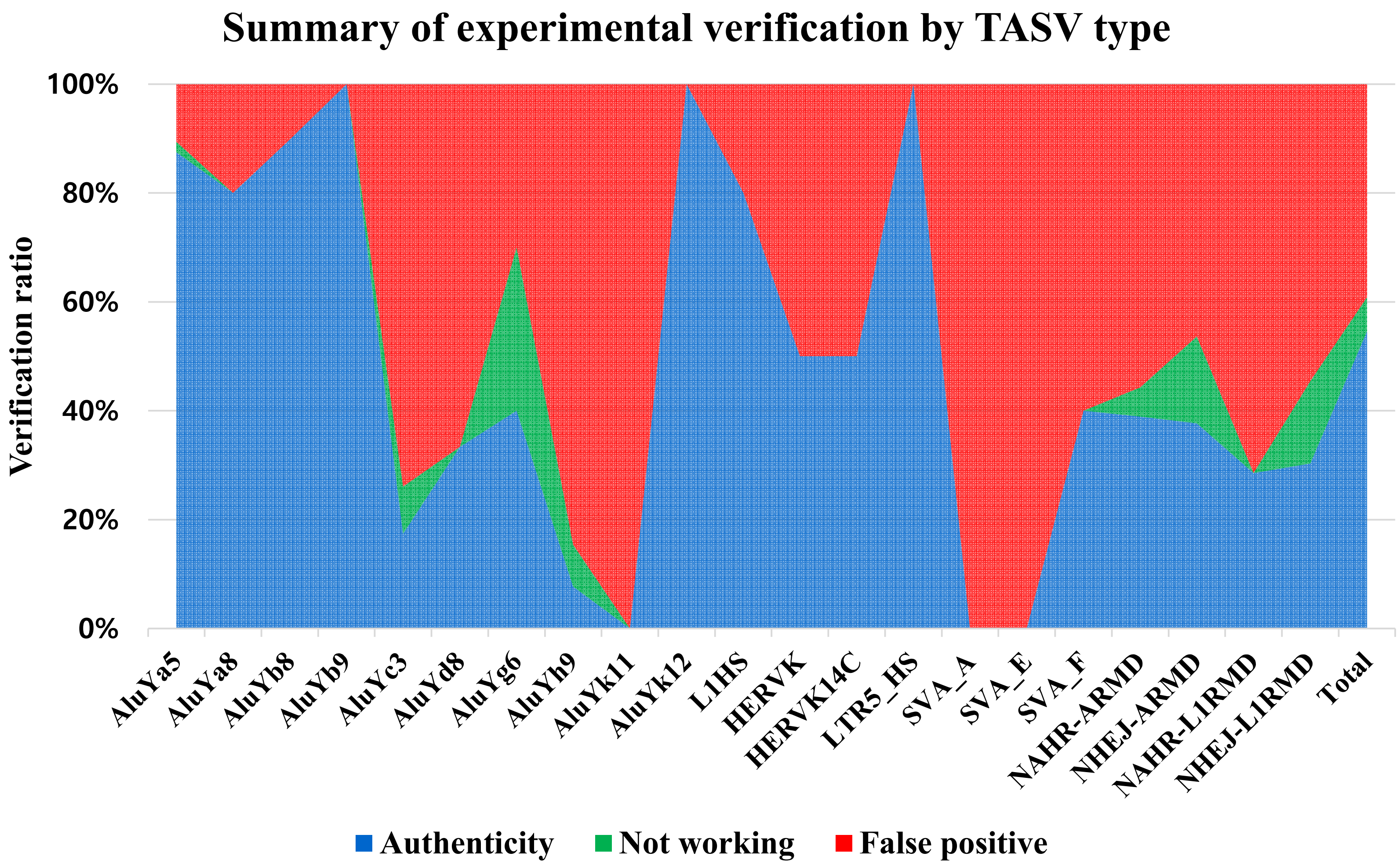

Supplementary Figure S3. Area chart showing the ratio of experimental verification by TASV types

The 21 TASV types are listed on the x-axis and the experimental verification rates for each type are shown on the y-axis. The verification ratio of the 496 TASV candidates is located at the end of the area chart. Blue, green, and red zones indicate for authenticity, not working, and false positives, respectively. Through the experimental verification process, 182 TASV insertions and 89 TASV deletions, accounting for 54.6%, out of 496 TASVs were finally confirmed.

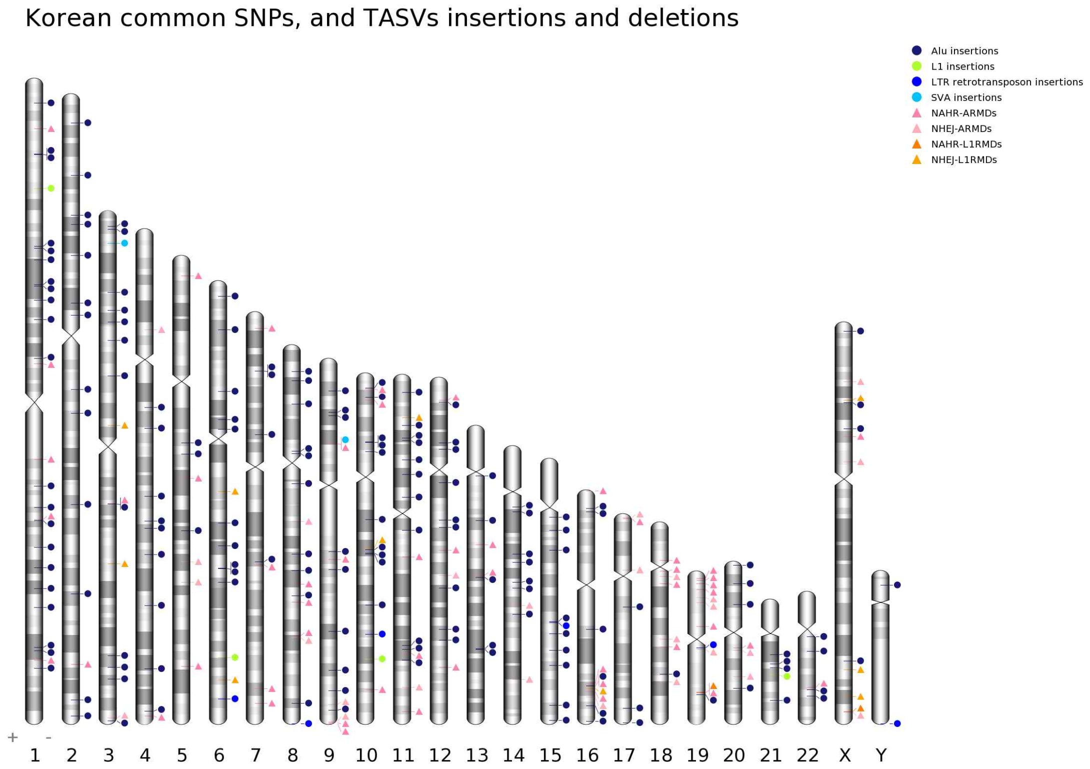

Supplementary Figure S4. Chromosomal overview of the genomic variations investigated in our study

All TASV events identified in the KPGP9 genome are projected onto a human idiogram. Each event is represented in different colors following the legend on top of the idiogram.

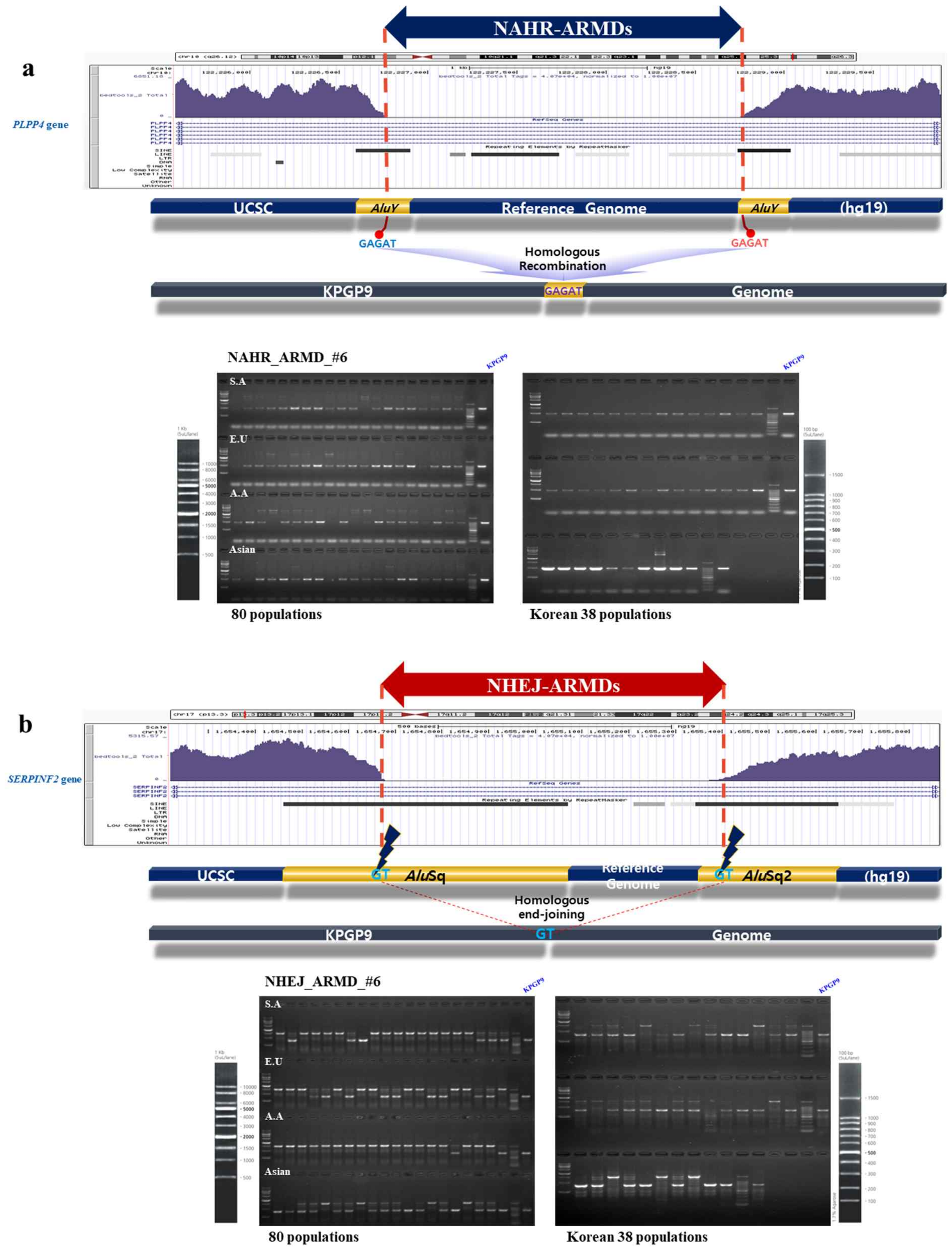
a
NAHR-ARMDs
PLPP4 gene
UCSC
AluY
Reference Genome
AluY
(hg19)
GAGAT
Homologous Recombination
GAGAT
KPGP9
GAGAT
Genome
NAHR_ARMD_#6
S.A
E.U
A.A
Asian
KPGP9
80 populations
Korean 38 populations
b
NHEJ-ARMDs
SERPINF2 gene
UCSC
GT
AluSq
Reference Genome
GT
AluSq2
(hg19)
Homologous end-joining
KPGP9
GT
Genome
NHEJ_ARMD_#6
S.A
E.U
A.A
Asian
KPGP9
80 populations
Korean 38 populations

c

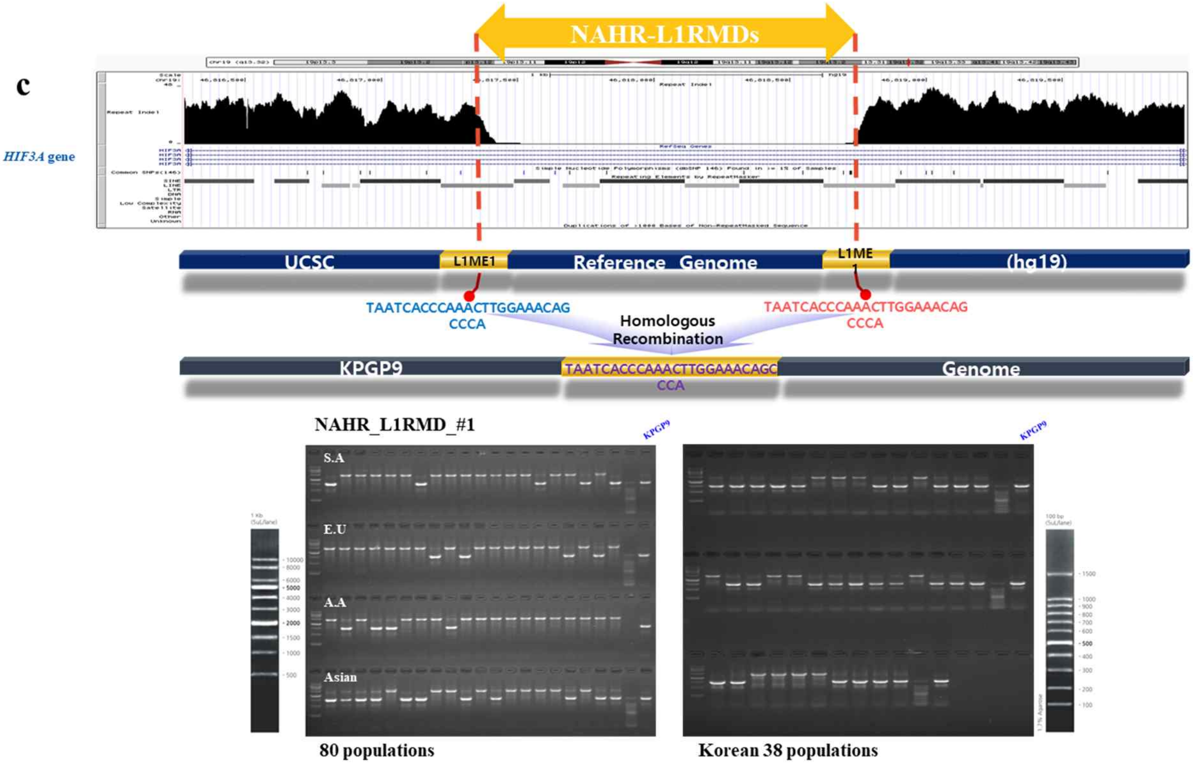
NAHR-L1RMDs
HIF3A gene
UCSC
L1ME1
Reference Genome
L1ME1
(hg19)
TAATCACCCAAACTTGGAAACAG CCCA
TAATCACCCAAACTTGGAAACAG CCCA
Homologous Recombination
KPGP9
TAATCACCCAAACTTGGAAACAGCCCA
Genome
NAHR_L1RMD_#1
KPGP9
S.A
E.U
A.A
Asian
80 populations
Korean 38 populations

d

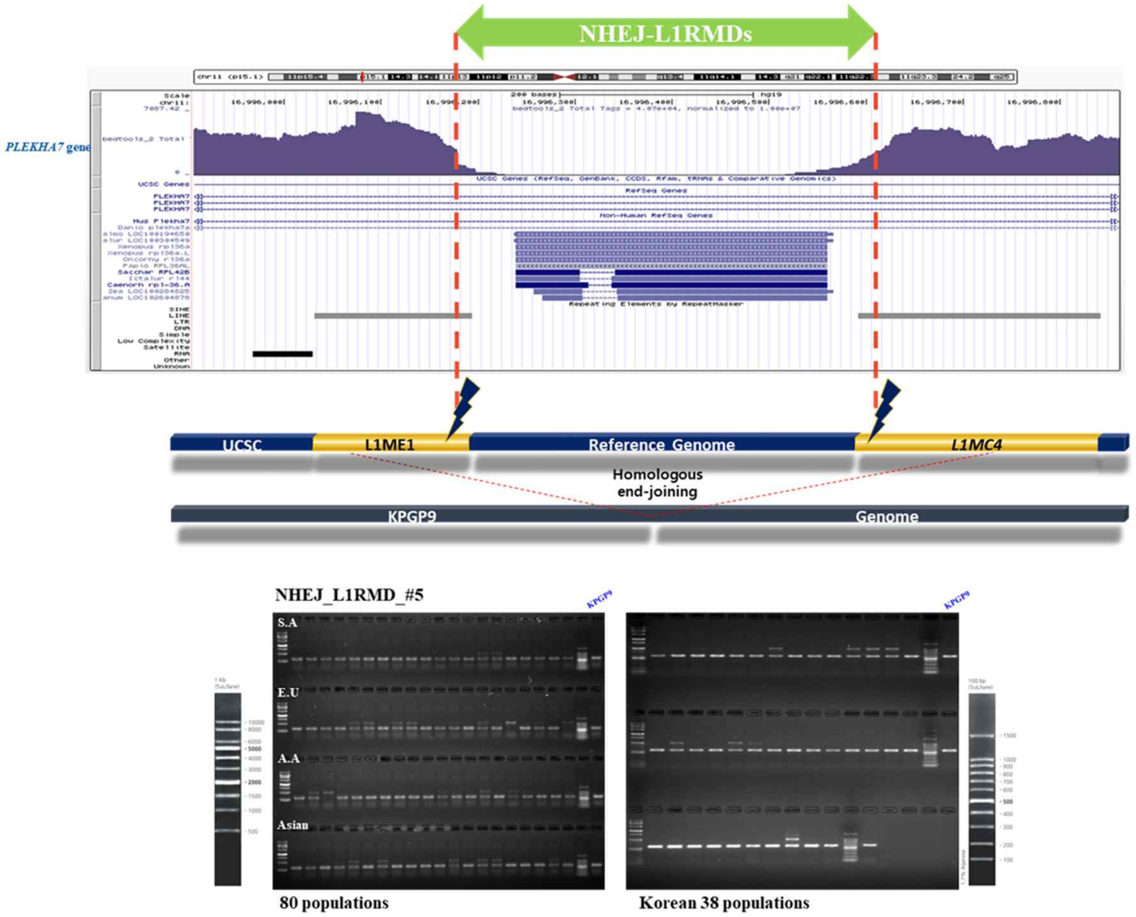
NHEJ-L1RMDs
PLEKHA7 gene
UCSC
L1ME1
Reference Genome
L1MC4
Homologous end-joining
KPGP9
Genome
NHEJ_L1RMD_#5
KPGP9
S.A
E.U
A.A
Asian
80 populations
Korean 38 populations

Supplementary Figure S5. Representative examples and PCR confirmation of TASV deletions in the KPGP9 genome

The expected read depth at the position of **a** NAHR-ARMD, **b** NHEJ-ARMD, **c** NAHR-L1RMD, and **d** NHEJ-L1RMD events are visualized in the UCSC genome browser. Diagrams representing the structure of each TASV deletion in the KPGP9 genome are shown under the screen shot of the UCSC genome browser (hg19). The gel chromatographs of each locus are shown. The lower-size bands indicate the presence of TASV deletions and the upper-size bands indicate the non-deletion at each counterpart.

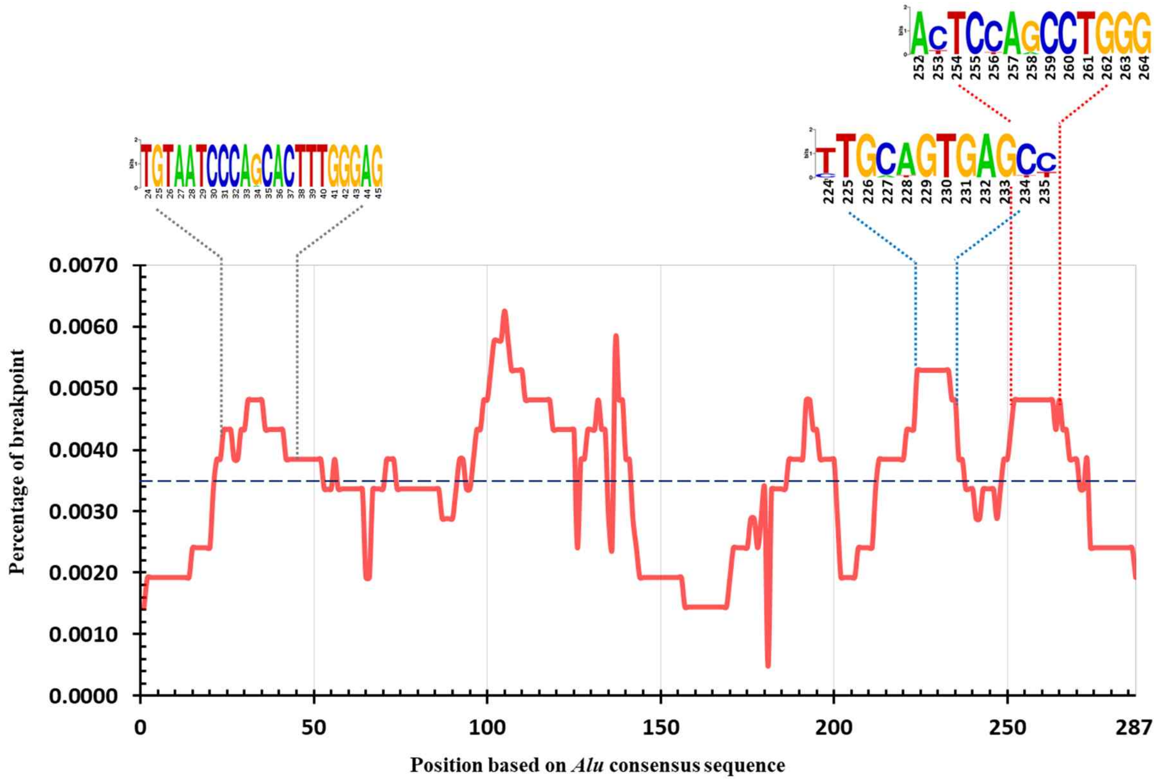

Supplementary Figure S6. Recombination breakpoints of *Alu* elements involved in NAHR-ARMD events

Three spots within the *Alu* element, represented by their conserved sequences, were found to have higher frequencies of recombination as visualized in this WebLogo analysis (http://weblogo.berkeley.edu/logo.cgi). The dashed line indicates the average percentage (0.0035%) of NAHR-ARMD breakpoints across the full-length *Alu* consensus sequence (*Alu*S, *Alu*J, and *Alu*Y families).

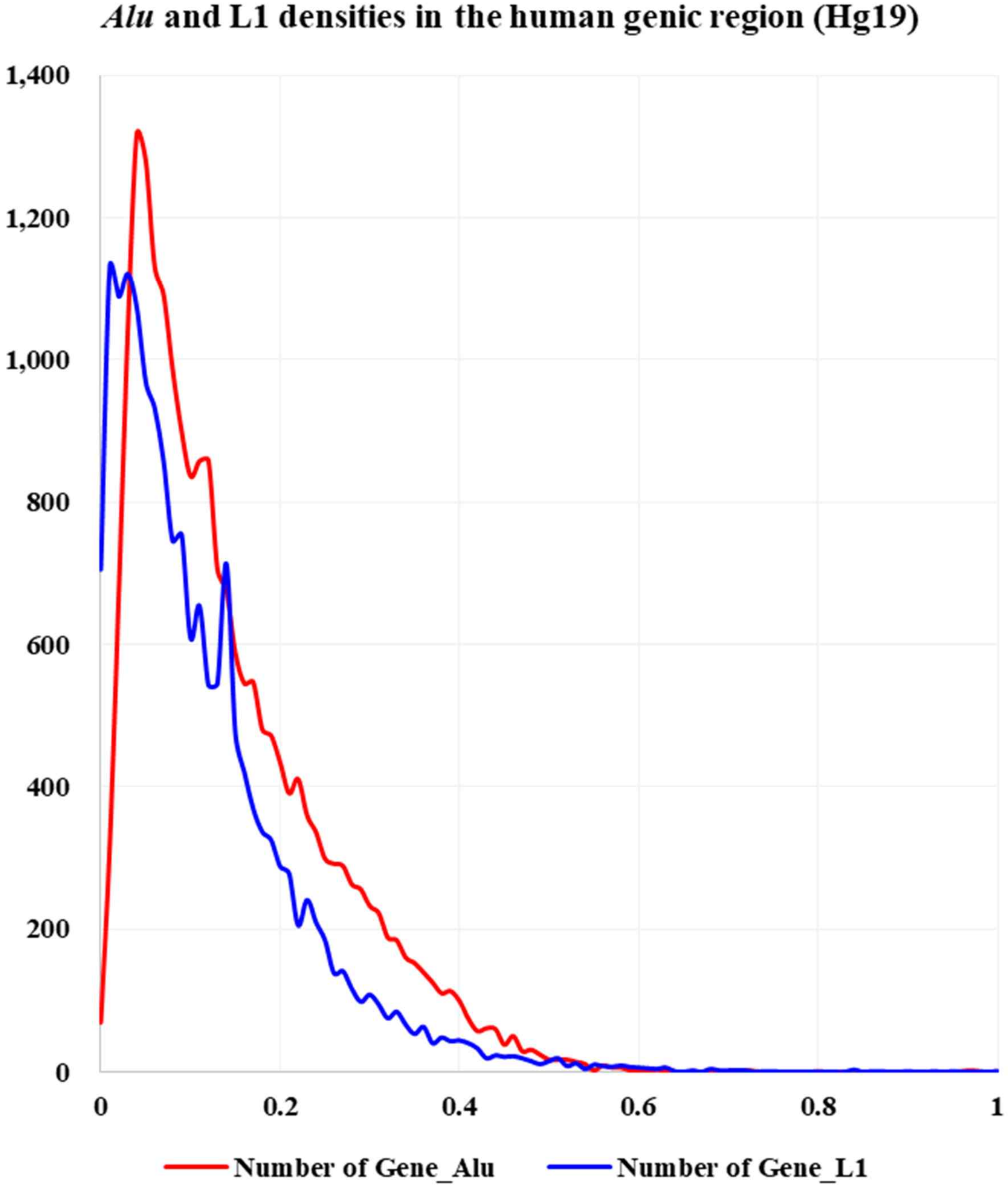

Supplementary Figure S7. ***Alu*** **and L1 densities in the human genic regions (hg19)**

The x-axis shows the density of *Alu* (red) and L1 (blue) insertions in genic regions, and the y-axis indicates the number of genes corresponding to their densities.

Supplementary Table list

The following are the supplementary data related to this article:

Supplementary Table S1. Multiple insert-sizes of sequencing libraries and genome coverage

Supplementary Table S2. Genome coverage against the human reference genome (hg19) by mapping-based methods

Supplementary Table S3. Scaffold blocks of the Korean assembled genome conserved with the human reference genome (hg19)

Supplementary Table S4. Repeat composition of the KPGP9 individual genome

Supplementary Table S5. List of DNA samples used

Supplementary Table S6. List of validated novel TASV insertions in the KPGP9 genome

Supplementary Table S7. List of validated novel TASV deletions in the KPGP9 genome

Supplementary Table S8. Population allele frequency of 50 TASVs identified from 1000GP

Supplementary Table S9. TASV comparison with AK1 and TCGA data

Supplementary Table S1. Multiple insert-sizes of sequencing libraries and genome coverage

Libraries	Insert Size	Read length (bp)	Raw data (Gb)	Sequence depth (X)
Paired-End	170 bp	100	35.8 Gb	11.93
	170 bp	100	38.69 Gb	12.9
	500 bp	90	29.66 Gb	9.89
	500 bp	90	13.89 Gb	4.63
	500 bp	90	14.43 Gb	4.81
	500 bp	90	13.21 Gb	4.4
	500 bp	90	12.39 Gb	4.13
	500 bp	90	10.36 Gb	3.45
	800 bp	100	32.84 Gb	10.95
Mate-Pair	2 Kb	49	19.62 Gb	6.54
	2 Kb	49	19.84 Gb	6.61
	5 Kb	49	18.87 Gb	6.29
	10 Kb	49	19.08 Gb	6.36
	20 Kb	49	18.08 Gb	6.03
	40 Kb	49	18.71 Gb	6.24
Total	-	-	315.47 Gb	105.16

Supplementary Table S2. Genome coverage against the human reference genome (hg19) by mapping-based methods

Chromosome	Length	Aligned Length	Coverage (%)
chr1	225,280,621	222,417,188	98.73%
chr2	238,204,518	237,091,262	99.53%
chr3	194,797,135	194,436,078	99.81%
chr4	187,661,676	187,130,587	99.72%
chr5	177,695,260	176,332,341	99.23%
chr6	167,395,066	166,980,884	99.75%
chr7	155,353,663	154,013,116	99.14%
chr8	142,888,922	142,169,143	99.50%
chr9	120,143,431	116,578,808	97.03%
chr10	131,314,738	130,220,572	99.17%
chr11	131,129,516	130,846,246	99.78%
chr12	130,481,393	130,211,413	99.79%
chr13	95,589,878	95,378,256	99.78%
chr14	88,289,540	88,058,180	99.74%
chr15	81,694,766	80,141,852	98.10%
chr16	78,884,753	77,215,048	97.88%
chr17	77,795,210	76,928,004	98.89%
chr18	74,657,229	74,505,935	99.80%
chr19	55,808,983	55,257,111	99.01%
chr20	59,505,520	59,372,939	99.78%
chr21	35,106,642	34,979,024	99.64%
chr22	34,894,545	34,518,269	98.92%
chrX	151,100,560	150,002,731	99.27%
chrY	25,653,566	24,568,143	95.77%
Total	2,861,327,131	2,839,353,130	99.23%

Supplementary Table S3. Scaffold blocks of the Korean assembled genome conserved with the human reference genome (hg19)

Group1	Group2	Block	Start1	End1	Start2	End2	Hits
chr1	Scaffold66	1	13,485,610	16,477,520	18,519	2,800,558	110
chr1	Scaffold80	1	222,716,562	224,065,570	26,781	1,314,794	47
chr1	Scaffold87	1	146,491,883	147,382,186	26,838	896,932	34
chr1	Scaffold94	1	83,950,680	85,964,196	20,953	2,056,124	85
chr1	Scaffold102	1	239,810,623	242,497,420	10,191	2,703,022	103
chr1	Scaffold105	1	121,159,224	121,406,185	6,289	201,545	8
chr1	Scaffold112	1	219,790,536	222,625,308	22,295	2,879,821	115
chr1	Scaffold122	1	234,946,144	239,752,624	33,821	4,836,710	185
chr1	Scaffold144	1	228,768,511	234,904,148	9,686	6,156,654	239
chr1	Scaffold157	1	8,880,954	12,876,103	6,995	4,042,844	152
chr1	Scaffold173	1	105,801,135	108,755,156	9,679	2,973,942	117
chr1	Scaffold224	1	243,373,994	248,580,922	13,507	5,258,508	204
chr1	Scaffold272	1	38,474,604	40,224,278	18,345	1,778,911	61
chr1	Scaffold285	1	2,689,560	8,861,222	33,413	6,149,584	212
chr1	Scaffold306	1	828,505	1,571,084	22,720	786,403	39
chr1	Scaffold327	1	40,277,454	47,571,050	40,316	7,133,190	263
chr1	Scaffold388	1	109,016,898	111,873,063	5,000	2,868,066	113
chr1	Scaffold453	1	1,696,202	2,538,822	37,118	890,505	29
chr1	Scaffold485	1	16,502,314	17,221,809	11,918	357,500	16
chr1	Scaffold540	1	184,064,136	184,271,275	27,141	235,631	9
chr1	Scaffold673	1	206,605,398	219,756,570	21,411	13,213,527	517
chr1	Scaffold875	1	248,593,870	249,204,591	23,873	271,716	12
chr1	Scaffold988	1	19,901,368	25,734,348	22,324	5,888,797	225
chr1	Scaffold1568	1	145,408,040	145,606,815	31,547	230,545	9
chr1	Scaffold2018	1	25,755,670	38,431,264	23,815	12,666,594	496
chr1	Scaffold3177	1	161,663,662	162,767,245	22,713	1,130,280	43
chr1	Scaffold3237	1	111,911,211	120,508,049	33,831	8,649,624	307
chr1	Scaffold4620	1	17,226,186	19,872,440	8,972	2,699,999	107
chr1	Scaffold4637	1	224,235,228	228,762,084	1,241	4,513,336	179

chr1	Scaffold5515	1	149,789,345	161,421,302	5,307	11,613,643	438
chr1	Scaffold31459	1	242,531,202	243,062,702	35,101	569,855	22
chr1	Scaffold31463	1	184,314,281	206,315,628	111	21,818,983	868
chr1	Scaffold31477	1	162,813,392	184,013,348	25,068	21,325,590	783
chr1	Scaffold31478	1	86,000,725	105,774,474	3,815	19,660,311	694
chr1	Scaffold31486	1	47,391,665	83,609,837	23,429	36,150,391	1260
chr2	Scaffold37	1	88,316,534	89,066,559	14,241	762,969	33
chr2	Scaffold79	1	241,648,951	243,035,290	10,000	1,408,390	53
chr2	Scaffold158	1	98,266,253	100,548,369	22,220	2,298,136	79
chr2	Scaffold172	1	109,145,068	110,487,855	22,055	1,216,318	46
chr2	Scaffold229	1	57,134,407	57,403,979	19,242	287,614	13
chr2	Scaffold251	1	148,664,711	152,426,510	15,047	3,690,040	148
chr2	Scaffold328	1	78,708,683	83,546,923	10,865	4,856,731	178
chr2	Scaffold347	1	71,272,722	74,020,167	10,000	2,753,618	98
chr2	Scaffold413	1	113,216,683	114,159,878	7,877	955,168	35
chr2	Scaffold494	1	100,576,343	110,429,145	25,517	6,432,590	254
chr2	Scaffold497	1	158,438,855	161,585,425	27,146	3,198,244	115
chr2	Scaffold508	1	74,028,915	78,683,987	6,875	4,645,530	167
chr2	Scaffold539	1	111,404,543	111,993,273	15,637	605,249	25
chr2	Scaffold612	1	152,446,176	158,390,497	21,110	5,978,170	237
chr2	Scaffold913	1	108,557,094	109,101,854	18,108	563,845	25
chr2	Scaffold945	1	95,557,809	95,763,111	40,320	245,808	8
chr2	Scaffold961	1	161,646,305	170,801,342	8,394	9,187,151	335
chr2	Scaffold972	1	112,640,386	113,104,843	7,909	480,222	19
chr2	Scaffold986	1	130,982,706	131,139,582	20,030	176,926	7
chr2	Scaffold1652	1	83,567,579	86,931,337	17,538	3,389,385	135
chr2	Scaffold1824	1	57,437,802	71,266,298	17,973	13,880,839	511
chr2	Scaffold2219	1	131,504,143	131,918,658	17,066	432,017	17
chr2	Scaffold2475	1	107,131,818	108,435,170	17,365	1,312,906	53
chr2	Scaffold4721	1	114,441,189	130,775,400	20,842	16,445,471	595
chr2	Scaffold7296	1	96,749,672	98,062,630	20,206	1,017,573	35

chr2	Scaffold31452	1	133,129,320	148,636,263	31,155	15,615,843	617
chr2	Scaffold31456	1	38,072,886	57,108,368	21,014	19,097,560	761
chr2	Scaffold31481	1	180,957	38,052,125	4,020	37,876,543	1504
chr2	Scaffold31494	1	170,852,239	241,623,541	9,305	70,913,421	2820
chr3	Scaffold64	1	197,410,495	197,857,962	22,554	461,127	15
chr3	Scaffold121	1	11,969,827	15,156,257	13,589	3,236,775	122
chr3	Scaffold641	1	118,786,032	120,285,637	24,322	1,525,579	54
chr3	Scaffold903	1	19,651,483	19,886,994	6,665	241,755	10
chr3	Scaffold993	1	80,707	634,750	10,181	564,389	23
chr3	Scaffold1402	1	195,541,655	195,659,376	34,813	155,573	8
chr3	Scaffold2701	1	90,276,030	97,374,023	99,409	3,988,920	132
chr3	Scaffold31422	1	120,309,600	129,728,658	25,346	9,507,534	362
chr3	Scaffold31442	1	682,376	11,917,849	34,881	11,316,912	403
chr3	Scaffold31453	1	195,389,036	197,343,295	23,944	1,644,250	62
chr3	Scaffold31460	1	74,519,473	93,582,129	330,695	16,320,605	610
chr3	Scaffold31464	1	97,414,823	118,753,783	30,549	21,454,344	758
chr3	Scaffold31470	1	129,943,486	149,372,882	29,275	19,499,932	691
chr3	Scaffold31489	1	149,400,562	195,169,236	30,267	45,951,174	1777
chr3	Scaffold31492	1	19,912,278	74,487,963	14,495	54,766,693	2193
chr3	Scaffold31494	1	15,183,029	19,599,684	70,961,334	75,397,542	180
chr4	Scaffold55	1	132,831,348	133,532,917	4,949	711,799	23
chr4	Scaffold83	1	83,508	3,533,730	15,000	3,479,160	121
chr4	Scaffold101	1	8,644,299	9,064,452	94,266	502,829	14
chr4	Scaffold114	1	116,808,421	119,325,977	21,630	2,544,724	91
chr4	Scaffold296	1	128,409,427	132,583,434	14,609	4,196,359	156
chr4	Scaffold299	1	161,598,789	166,160,974	36,702	4,599,235	180
chr4	Scaffold303	1	4,183,298	8,053,223	11,639	3,903,669	159
chr4	Scaffold513	1	93,896,952	95,009,854	36,255	1,160,583	36
chr4	Scaffold522	1	3,585,758	3,901,352	30,847	358,833	10
chr4	Scaffold564	1	33,964,078	44,091,856	29,631	10,167,795	372
chr4	Scaffold592	1	121,132,185	128,385,651	12,267	7,254,875	278

chr4	Scaffold810	1	180,582,539	190,565,716	22,619	10,041,217	353
chr4	Scaffold887	1	59,738,569	60,270,045	754	489,896	20
chr4	Scaffold893	1	119,605,737	120,261,332	20,000	674,158	27
chr4	Scaffold1236	1	166,541,275	180,558,937	12,434	14,066,466	568
chr4	Scaffold1437	1	166,206,416	166,502,870	28,475	325,104	13
chr4	Scaffold1507	1	8,077,243	8,616,319	12,263	559,023	16
chr4	Scaffold1745	1	95,035,970	103,799,828	10,000	8,817,276	319
chr4	Scaffold2213	1	52,680,272	59,733,771	4,624	7,069,635	276
chr4	Scaffold2350	1	190,689,045	190,928,054	16,361	259,658	9
chr4	Scaffold2519	1	103,887,481	112,081,035	52,604	8,244,233	297
chr4	Scaffold2953	1	120,306,777	121,108,648	13,361	814,336	33
chr4	Scaffold5781	1	44,119,569	49,086,024	20,000	4,996,302	180
chr4	Scaffold31426	1	112,126,745	116,782,731	34,367	4,707,062	155
chr4	Scaffold31431	1	145,087,102	161,572,812	26,330	16,576,073	599
chr4	Scaffold31444	1	9,699,172	33,905,382	45,105	24,355,197	853
chr4	Scaffold31446	1	133,582,146	145,061,608	46,022	11,491,975	412
chr4	Scaffold31490	1	60,311,717	93,845,790	22,304	33,440,526	1169
chr5	Scaffold28	1	20,935,520	21,270,362	16,640	354,808	13
chr5	Scaffold33	1	68,445,806	68,874,166	24,075	457,186	12
chr5	Scaffold60	1	19,172,353	20,690,762	13,542	1,531,630	62
chr5	Scaffold76	1	179,092,057	180,501,172	13,722	1,394,974	51
chr5	Scaffold95	1	70,751,927	73,331,211	24,438	2,607,648	96
chr5	Scaffold100	1	49,471,457	49,872,609	23,092	432,599	15
chr5	Scaffold138	1	177,492,737	179,071,537	12,579	1,597,141	63
chr5	Scaffold145	1	140,563,111	142,335,763	33,272	1,809,569	54
chr5	Scaffold287	1	94,567,390	96,547,810	33,495	2,016,063	75
chr5	Scaffold325	1	170,376,148	175,317,153	32,697	5,000,201	165
chr5	Scaffold484	1	88,238,403	94,541,374	25,000	6,338,129	211
chr5	Scaffold575	1	17,605,157	19,144,300	17,252	1,551,233	62
chr5	Scaffold643	1	73,372,469	73,815,689	16,304	461,837	16
chr5	Scaffold735	1	42,709	8,721,817	29,997	8,662,050	313

chr5	Scaffold897	1	22,222,401	34,080,766	34,251	11,975,773	462
chr5	Scaffold1011	1	175,722,530	177,074,320	12,937	1,370,163	48
chr5	Scaffold1017	1	180,526,698	180,677,935	73,533	228,116	7
chr5	Scaffold1071	1	8,761,670	17,507,298	32,357	8,782,421	318
chr5	Scaffold1519	1	98,921,068	99,398,419	43,066	526,678	19
chr5	Scaffold6183	1	96,579,254	98,735,095	17,537	2,172,163	71
chr5	Scaffold11509	1	21,539,252	21,984,320	17,212	464,625	19
chr5	Scaffold31436	1	142,357,735	155,473,283	25,004	13,106,472	521
chr5	Scaffold31447	1	155,510,063	170,340,925	2,197	14,901,541	593
chr5	Scaffold31450	1	73,846,827	88,203,045	7,229	14,469,890	560
chr5	Scaffold31461	1	34,379,255	46,350,231	15,772	12,043,796	440
chr5	Scaffold31473	1	49,923,507	68,410,799	27,443	18,536,635	668
chr5	Scaffold31488	1	99,452,146	99,687,509	4,556	240,460	10
chr5	Scaffold31491	1	99,740,291	140,557,664	19,266	40,949,823	1475
chr6	Scaffold84	1	26,969,733	29,123,725	22,862	2,201,587	78
chr6	Scaffold86	1	31,370,249	32,425,129	13,816	1,037,066	41
chr6	Scaffold98	1	160,948,868	164,876,300	16,739	3,843,508	143
chr6	Scaffold332	1	120,062,504	122,949,107	15,898	2,902,274	125
chr6	Scaffold358	1	29,134,758	29,676,910	9,990	554,357	22
chr6	Scaffold425	1	164,901,322	170,343,024	13,308	5,552,448	220
chr6	Scaffold620	1	113,816,437	120,035,351	10,623	6,245,381	236
chr6	Scaffold630	1	3,650,936	11,872,176	15,853	8,275,203	294
chr6	Scaffold651	1	170,361,933	170,899,382	19,413	572,375	22
chr6	Scaffold718	1	58,446,653	58,749,460	32,460	338,027	8
chr6	Scaffold833	1	65,829,480	66,000,134	11,915	184,610	9
chr6	Scaffold941	1	157,017,750	161,029,125	14,349	4,004,809	160
chr6	Scaffold962	1	29,862,670	31,321,331	21,535	1,415,658	51
chr6	Scaffold1237	1	176,507	303,113	28,449	155,495	7
chr6	Scaffold1348	1	32,736,119	33,011,186	19,420	294,655	13
chr6	Scaffold1459	1	57,610,604	57,896,885	49,568	335,123	9
chr6	Scaffold3024	1	402,791	3,626,625	63,922	3,306,544	113

chr6	Scaffold3674	1	142,028,977	156,981,376	9,972	15,042,477	597
chr6	Scaffold3944	1	51,645,834	57,187,449	14,425	5,571,634	192
chr6	Scaffold6259	1	58,742,325	65,782,197	19,572	3,920,841	145
chr6	Scaffold21962	1	11,895,210	26,657,646	18,508	14,874,533	539
chr6	Scaffold31410	1	33,037,316	51,620,164	11,283	18,777,045	736
chr6	Scaffold31423	1	122,975,654	141,992,837	6,938	19,082,392	759
chr6	Scaffold31493	1	66,171,387	113,778,527	27,408	48,122,909	1917
chr7	Scaffold31	1	57,195,468	57,896,250	33,724	706,380	18
chr7	Scaffold49	1	64,733,315	64,919,085	6,669	194,355	8
chr7	Scaffold50	1	153,857,382	156,129,850	6,721	2,201,507	91
chr7	Scaffold51	1	63,306,566	64,237,186	13,262	944,763	40
chr7	Scaffold88	1	146,712,729	152,484,240	776	2,903,813	121
chr7	Scaffold123	1	150,319,211	152,460,832	5,428	2,152,770	73
chr7	Scaffold124	1	42,765,019	44,005,056	19,156	1,266,402	46
chr7	Scaffold155	1	64,487,248	66,539,769	42,645	1,291,360	44
chr7	Scaffold159	1	35,250,742	35,944,150	73,729	768,856	26
chr7	Scaffold186	1	144,080,755	146,681,016	9,658	2,609,730	96
chr7	Scaffold190	1	153,496,442	153,682,971	11,305	199,055	8
chr7	Scaffold199	1	36,030,662	41,402,237	25,000	5,407,498	186
chr7	Scaffold239	1	99,318,315	99,883,030	256,027	794,202	20
chr7	Scaffold277	1	2,907,185	5,899,208	36,385	3,049,209	108
chr7	Scaffold286	1	61,991,192	62,974,663	26,714	761,716	20
chr7	Scaffold291	1	99,950,876	100,612,720	24,638	649,378	27
chr7	Scaffold309	1	31,609,957	32,729,342	32,254	1,133,853	48
chr7	Scaffold315	1	29,754,262	31,584,219	30,266	1,866,528	71
chr7	Scaffold316	1	128,328,208	129,110,610	25,838	815,236	28
chr7	Scaffold321	1	73,360,698	74,136,525	5,000	840,324	31
chr7	Scaffold353	1	68,249,124	71,985,088	16,958	3,750,843	130
chr7	Scaffold412	1	6,045,090	6,825,174	13,542	818,532	28
chr7	Scaffold489	1	64,261,005	64,471,805	9,270	199,432	8
chr7	Scaffold517	1	158,162,499	158,902,129	25,968	780,593	18

chr7	Scaffold533	1	97,575,131	99,312,051	21,160	1,811,739	66
chr7	Scaffold567	1	55,848,344	56,400,917	20,170	576,557	19
chr7	Scaffold580	1	65,026	2,882,713	23,805	2,819,803	98
chr7	Scaffold676	1	152,545,947	153,411,994	31,502	908,657	33
chr7	Scaffold722	1	66,822,352	68,222,870	11,010	1,421,167	52
chr7	Scaffold738	1	100,660,087	101,960,107	18,022	1,326,468	50
chr7	Scaffold823	1	7,069,981	10,998,054	3,901,597	7,843,212	160
chr7	Scaffold843	1	32,714,529	35,107,858	23,769	2,090,769	74
chr7	Scaffold904	1	143,291,564	144,037,974	4,166	343,769	16
chr7	Scaffold983	1	44,067,176	45,790,573	112,936	1,848,211	59
chr7	Scaffold1130	1	158,930,107	159,111,112	16,076	186,531	8
chr7	Scaffold1210	1	72,453,282	73,336,398	27,639	639,320	26
chr7	Scaffold1347	1	56,478,753	56,803,682	14,048	338,357	13
chr7	Scaffold1481	1	41,426,476	42,715,101	20,485	1,322,138	50
chr7	Scaffold1526	1	102,463,987	102,805,073	24,862	367,897	12
chr7	Scaffold1529	1	32,791,095	33,135,947	16,573	218,417	8
chr7	Scaffold1626	1	149,994,056	150,283,911	25,196	318,854	12
chr7	Scaffold1641	1	157,941,903	158,110,223	4,342	175,308	8
chr7	Scaffold1658	1	54,394,940	55,717,629	23,912	1,346,794	54
chr7	Scaffold2160	1	75,139,088	76,063,092	39,474	945,882	38
chr7	Scaffold3410	1	129,134,272	143,212,504	17,949	14,167,548	498
chr7	Scaffold3994	1	45,854,441	54,274,467	12,412	8,415,433	306
chr7	Scaffold31413	1	11,020,372	29,694,548	14,671	18,762,103	634
chr7	Scaffold31429	1	89,786,968	97,498,027	10,000	7,723,705	290
chr7	Scaffold31433	1	76,801,717	89,736,942	15,654	12,982,145	539
chr7	Scaffold31438	1	156,153,992	157,926,289	14,352	1,796,911	74
chr7	Scaffold31483	1	102,929,627	128,206,873	10,629	25,347,774	909
chr8	Scaffold65	1	86,860,181	88,325,427	22,501	1,493,800	52
chr8	Scaffold75	1	415,584	2,169,398	29,999	1,777,762	68
chr8	Scaffold126	1	132,296,335	133,777,456	16,227	1,504,859	58
chr8	Scaffold259	1	139,094,787	146,294,584	28,805	7,104,687	262

chr8	Scaffold265	1	46,904,218	47,915,472	32,473	1,041,471	36
chr8	Scaffold283	1	101,717,674	108,572,731	35,357	6,902,383	269
chr8	Scaffold357	1	37,772,681	43,752,371	11,883	6,054,203	207
chr8	Scaffold399	1	138,734,440	139,042,529	8,612	316,258	10
chr8	Scaffold654	1	8,114,124	11,852,618	24,821	3,791,056	145
chr8	Scaffold899	1	130,211,736	132,222,557	38,056	2,072,037	84
chr8	Scaffold1211	1	129,939,098	130,183,485	29,001	273,993	8
chr8	Scaffold2040	1	187,193	362,953	5,612	189,386	7
chr8	Scaffold2417	1	133,818,745	138,716,014	9,297	4,932,751	192
chr8	Scaffold2763	1	88,363,477	90,189,099	19,091	1,845,982	69
chr8	Scaffold4417	1	90,215,900	101,692,335	6,644	11,504,325	472
chr8	Scaffold31430	1	2,210,226	6,815,705	13,499	4,541,435	180
chr8	Scaffold31459	1	47,948,912	58,102,392	607,358	10,785,885	400
chr8	Scaffold31465	1	108,618,936	129,716,662	27,458	21,195,694	837
chr8	Scaffold31467	1	12,556,934	37,726,247	93,355	25,368,795	894
chr8	Scaffold31485	1	58,118,648	86,456,658	17,605	28,430,905	1112
chr9	Scaffold21	1	90,568,476	90,728,862	14,062	175,076	8
chr9	Scaffold103	1	86,445,829	90,499,476	12,804	2,114,489	84
chr9	Scaffold147	1	90,768,174	95,646,475	12,017	4,778,619	156
chr9	Scaffold292	1	97,205,576	99,733,927	1,038	2,480,750	90
chr9	Scaffold396	1	33,599,587	38,525,350	33,120	4,986,297	190
chr9	Scaffold452	1	95,695,929	97,048,887	25,666	1,381,732	52
chr9	Scaffold3674	1	100,006,219	102,841,616	15,093,078	17,931,910	114
chr9	Scaffold5467	1	102,868,261	113,423,084	18,915	10,610,839	424
chr9	Scaffold31437	1	15,626,534	21,188,845	15,925	5,601,716	206
chr9	Scaffold31440	1	68,752,726	88,364,787	30,477	17,404,684	686
chr9	Scaffold31443	1	217,839	15,577,975	19,007	15,428,039	564
chr9	Scaffold31449	1	21,188,555	33,542,655	12,037	12,387,793	443
chr9	Scaffold31455	1	113,446,692	141,009,665	4,545	27,783,418	969
chr10	Scaffold70	1	37,475,516	38,541,905	30,511	1,085,130	41
chr10	Scaffold92	1	86,560,897	88,752,799	51,504	2,246,343	74

chr10	Scaffold131	1	76,007,742	81,267,055	25,000	5,310,399	194
chr10	Scaffold187	1	42,865,794	46,149,420	23,990	3,370,002	126
chr10	Scaffold194	1	82,024,961	86,499,482	14,001	4,502,933	164
chr10	Scaffold240	1	33,214,502	37,470,784	10,005	4,261,862	160
chr10	Scaffold293	1	10,021,981	11,666,447	25,497	1,681,118	63
chr10	Scaffold298	1	49,409,649	51,066,567	13,677	1,677,509	69
chr10	Scaffold311	1	11,697,668	11,947,682	11,071	259,651	11
chr10	Scaffold443	1	131,721	9,994,871	45,546	9,951,437	341
chr10	Scaffold800	1	81,789,674	81,974,930	20,243	201,693	8
chr10	Scaffold801	1	51,961,894	52,432,562	19,609	492,027	20
chr10	Scaffold1182	1	12,036,715	25,176,920	20,000	13,011,986	456
chr10	Scaffold1367	1	124,351,018	135,368,642	20,668	11,109,807	385
chr10	Scaffold1448	1	118,379,091	124,322,162	12,568,679	18,539,793	214
chr10	Scaffold1702	1	25,201,586	26,443,835	11,759	1,263,830	43
chr10	Scaffold1803	1	47,776,692	48,741,917	8,105	468,511	18
chr10	Scaffold31469	1	26,509,726	33,170,148	42,242	6,791,809	228
chr10	Scaffold31474	1	81,479,227	118,372,250	21,095	29,213,649	1139
chr10	Scaffold31475	1	52,568,691	75,958,465	28,637	23,534,794	850
chr11	Scaffold44	1	3,689,679	4,223,732	14,643	552,114	22
chr11	Scaffold125	1	60,998,241	62,860,803	7,022	1,843,443	70
chr11	Scaffold129	1	130,309,596	134,926,424	19,829	4,672,760	191
chr11	Scaffold182	1	86,970,159	88,782,910	10,359	1,788,469	57
chr11	Scaffold228	1	67,598,941	77,610,789	23,914	6,263,352	236
chr11	Scaffold258	1	48,915,579	49,843,758	14,693	906,390	23
chr11	Scaffold258	2	88,580,693	89,810,055	34,496	827,139	13
chr11	Scaffold266	1	69,802,737	71,313,047	21,920	1,575,468	58
chr11	Scaffold280	1	63,209,200	67,451,815	40,626	4,316,470	163
chr11	Scaffold333	1	205,333	3,420,235	89,027	3,309,604	140
chr11	Scaffold343	1	92,802,528	96,898,473	25,083	3,975,658	145
chr11	Scaffold375	1	96,934,508	103,261,789	23,713	6,400,167	247
chr11	Scaffold430	1	88,911,407	89,453,032	27,985	571,121	15

chr11	Scaffold535	1	103,309,743	114,485,488	20,799	11,233,502	406
chr11	Scaffold581	1	50,344,144	51,580,693	5,511	466,106	15
chr11	Scaffold634	1	77,642,811	86,948,693	10,817	9,337,917	377
chr11	Scaffold823	1	45,055,941	51,516,868	13,553	3,876,369	146
chr11	Scaffold1001	1	114,531,860	115,064,093	22,458	561,731	22
chr11	Scaffold1190	1	50,103,084	50,293,974	15,173	204,960	7
chr11	Scaffold2444	1	89,837,773	92,779,704	19,794	2,983,272	115
chr11	Scaffold6294	1	48,463,695	55,618,465	14,563	1,315,984	46
chr11	Scaffold7192	1	62,860,196	63,158,908	28,776	317,582	13
chr11	Scaffold10188	1	115,089,926	130,282,474	12,468	15,338,098	511
chr11	Scaffold31435	1	67,776,623	69,778,263	3,568	1,985,466	75
chr11	Scaffold31466	1	25,790,366	45,020,482	25,115	19,339,006	712
chr11	Scaffold31471	1	4,406,881	25,503,251	22,634	21,231,385	784
chr11	Scaffold31480	1	49,891,163	60,986,764	13,534	5,373,507	190
chr12	Scaffold47	1	51,965,960	53,516,524	31,122	1,558,315	65
chr12	Scaffold73	1	9,707,397	10,642,841	3,559	944,147	36
chr12	Scaffold116	1	31,351,582	34,108,206	11,718	2,774,590	103
chr12	Scaffold207	1	61,226,397	63,575,678	20,000	2,370,990	74
chr12	Scaffold260	1	8,526,219	9,421,025	19,052	899,947	35
chr12	Scaffold294	1	42,086,927	45,043,978	36,026	3,004,095	106
chr12	Scaffold355	1	131,775,718	133,815,922	25,612	1,595,388	65
chr12	Scaffold356	1	37,937,994	42,043,100	108,308	4,282,216	144
chr12	Scaffold429	1	45,084,179	51,953,578	22,229	6,906,009	264
chr12	Scaffold471	1	75,372,616	80,851,977	4,010	5,496,162	220
chr12	Scaffold476	1	53,554,994	55,707,005	20,426	2,193,501	82
chr12	Scaffold507	1	63,584,780	63,960,005	11,319	379,841	14
chr12	Scaffold524	1	188,884	8,312,596	10,034	8,208,708	313
chr12	Scaffold585	1	56,001,300	61,179,538	22,088	5,213,832	205
chr12	Scaffold712	1	55,748,895	55,946,264	10,145	206,762	9
chr12	Scaffold1075	1	131,810,923	132,124,491	13,352	331,467	13
chr12	Scaffold1144	1	63,998,234	75,334,225	15,413	11,377,756	392

chr12	Scaffold1890	1	34,158,798	34,837,801	9,898	689,952	23
chr12	Scaffold2171	1	80,892,146	84,760,990	34,734	3,904,945	129
chr12	Scaffold31472	1	10,677,975	31,192,973	30,640	20,645,490	787
chr12	Scaffold31487	1	84,778,788	131,777,260	12,704	47,239,291	1684
chr13	Scaffold77	1	110,496,745	112,663,690	7,064	2,195,204	86
chr13	Scaffold81	1	25,056,031	25,499,485	11,998	461,881	19
chr13	Scaffold195	1	82,005,710	83,735,341	9,934	1,743,047	69
chr13	Scaffold250	1	20,102,386	25,028,641	19,505	4,961,171	168
chr13	Scaffold322	1	112,690,338	112,962,524	20,000	263,072	12
chr13	Scaffold600	1	19,463,988	19,989,506	38,971	564,398	20
chr13	Scaffold632	1	113,638,518	115,101,902	15,335	1,326,392	55
chr13	Scaffold990	1	112,996,059	113,613,731	30,843	649,372	23
chr13	Scaffold2045	1	57,744,385	64,317,287	1,755	6,594,798	223
chr13	Scaffold2103	1	64,426,694	81,956,310	15,000	17,574,226	656
chr13	Scaffold7024	1	53,167,012	57,701,203	14,955	4,609,260	176
chr13	Scaffold31462	1	25,586,165	52,770,281	29,168	27,346,707	975
chr13	Scaffold31479	1	83,762,451	110,452,625	29,258	26,706,581	1055
chr14	Scaffold146	1	52,249,702	52,983,043	19,116	759,091	28
chr14	Scaffold184	1	91,676,013	100,002,899	25,000	8,399,526	287
chr14	Scaffold313	1	24,445,360	28,215,421	45,729	3,793,339	138
chr14	Scaffold352	1	20,303,508	24,443,178	5,612	4,168,102	159
chr14	Scaffold474	1	100,903,297	106,153,355	30,714	5,223,354	208
chr14	Scaffold776	1	106,586,199	106,795,175	56,916	265,889	7
chr14	Scaffold958	1	100,029,210	100,882,523	15,734	871,073	31
chr14	Scaffold1085	1	106,055,826	106,514,285	11,051	357,057	15
chr14	Scaffold1213	1	106,901,691	107,274,490	56,176	430,258	15
chr14	Scaffold1580	1	64,789,815	81,889,253	8,621	17,161,016	662
chr14	Scaffold2687	1	53,023,593	64,758,067	32,350	11,799,831	427
chr14	Scaffold31445	1	81,906,307	91,625,866	23,602	9,787,357	383
chr14	Scaffold31482	1	28,256,443	52,198,558	22,045	24,036,411	860
chr15	Scaffold36	1	32,033,169	32,466,273	22,842	453,780	18

chr15	Scaffold54	1	43,921,278	45,103,868	33,867	1,120,381	43
chr15	Scaffold63	1	32,934,765	34,573,068	14,660	1,664,109	65
chr15	Scaffold67	1	24,425,703	26,343,869	18,079	1,777,600	71
chr15	Scaffold71	1	26,372,387	28,915,509	10,000	2,243,567	70
chr15	Scaffold137	1	55,756,986	59,748,474	18,185	4,024,990	132
chr15	Scaffold154	1	89,101,080	91,904,911	8,813	2,823,220	117
chr15	Scaffold163	1	72,987,280	74,349,618	8,442	1,381,797	57
chr15	Scaffold168	1	29,097,188	30,350,773	22,344	1,245,574	49
chr15	Scaffold214	1	22,761,899	23,094,578	19,996	357,726	12
chr15	Scaffold319	1	99,575,023	102,297,727	16,876	2,731,773	101
chr15	Scaffold365	1	76,043,424	81,983,365	8,795	5,965,010	232
chr15	Scaffold409	1	74,424,824	75,994,567	34,806	1,598,913	64
chr15	Scaffold418	1	82,820,336	84,856,350	13,990	1,661,002	62
chr15	Scaffold504	1	91,931,474	97,897,512	19,784	6,012,881	197
chr15	Scaffold609	1	97,926,994	99,546,787	20,525	1,638,335	65
chr15	Scaffold748	1	23,639,728	24,359,376	27,118	748,651	27
chr15	Scaffold842	1	72,256,841	72,922,144	25,000	704,257	25
chr15	Scaffold1045	1	31,062,244	31,886,671	7,435	836,618	33
chr15	Scaffold1448	1	59,778,502	72,206,289	25,515	12,551,885	485
chr15	Scaffold1819	1	82,012,210	82,894,465	20,730	628,474	27
chr15	Scaffold2584	1	85,103,112	85,706,067	33,741	636,124	25
chr15	Scaffold8029	1	85,879,327	89,085,696	6,671	3,213,070	114
chr15	Scaffold9112	1	45,166,781	55,731,535	37,606	10,677,042	349
chr15	Scaffold31448	1	34,889,102	43,955,249	25,506	9,048,530	332
chr16	Scaffold35	1	29,668,476	30,196,844	5,297	535,293	23
chr16	Scaffold42	1	15,488,918	16,304,575	11,663	836,515	29
chr16	Scaffold52	1	16,875,208	18,162,020	28,718	1,315,751	52
chr16	Scaffold235	1	88,229,510	90,166,021	21,494	1,968,497	63
chr16	Scaffold244	1	18,957,192	20,610,802	60,543	1,561,250	51
chr16	Scaffold336	1	5,371,667	14,838,870	48,164	9,487,634	333
chr16	Scaffold379	1	2,719,423	5,285,068	38,419	2,669,790	100

chr16	Scaffold444	1	69,711,518	70,202,734	15,908	283,722	12
chr16	Scaffold450	1	70,301,978	70,860,965	32,824	592,410	23
chr16	Scaffold461	1	98,900	2,586,167	15,420	2,575,204	91
chr16	Scaffold613	1	28,836,433	29,032,647	14,728	211,436	9
chr16	Scaffold700	1	21,957,746	22,439,948	12,977	498,694	17
chr16	Scaffold743	1	20,608,659	21,344,394	17,092	756,490	27
chr16	Scaffold1110	1	46,507,518	55,798,253	31,890	9,338,648	324
chr16	Scaffold1122	1	30,360,643	31,933,368	19,298	1,595,565	60
chr16	Scaffold1510	1	21,574,753	21,737,273	22,546	186,314	7
chr16	Scaffold1768	1	21,788,879	28,340,879	37,443	5,806,624	225
chr16	Scaffold2188	1	34,201,338	35,231,953	18,660	1,048,480	35
chr16	Scaffold15645	1	71,191,985	74,352,114	24,004	3,203,599	104
chr16	Scaffold31441	1	55,855,253	69,683,301	12,975	13,893,256	506
chr16	Scaffold31457	1	74,611,458	88,173,887	28,645	13,715,302	468
chr17	Scaffold38	1	14,369,709	15,431,502	11,705	1,079,688	45
chr17	Scaffold45	1	16,781,518	18,310,525	9,251	1,560,722	65
chr17	Scaffold62	1	41,411,615	43,503,004	10,952	2,129,079	74
chr17	Scaffold91	1	72,485,931	75,400,909	23,882	3,008,570	107
chr17	Scaffold99	1	75,436,773	81,159,617	24,637	5,803,496	196
chr17	Scaffold113	1	30,423,335	34,480,066	7,907	4,081,158	158
chr17	Scaffold120	1	36,704,654	41,377,118	22,427	4,728,390	168
chr17	Scaffold142	1	1,176,582	3,152,400	5,035	1,963,209	71
chr17	Scaffold149	1	21,686,608	21,885,668	23,701	221,570	8
chr17	Scaffold209	1	25,324,508	28,825,990	492,630	3,916,564	129
chr17	Scaffold209	2	44,666,978	45,084,681	19,314	437,515	18
chr17	Scaffold245	1	36,356,772	50,915,516	6,324	5,295,790	192
chr17	Scaffold256	1	50,964,063	58,038,360	24,011	7,140,098	238
chr17	Scaffold264	1	19,155,251	20,205,808	20,398	1,072,524	44
chr17	Scaffold462	1	21,940,177	22,223,898	20,459	303,739	12
chr17	Scaffold552	1	45,137,568	45,537,450	36,556	438,006	10
chr17	Scaffold553	1	58,303,006	60,370,395	11,523	1,931,611	70

chr17	Scaffold647	1	62,658,475	72,438,756	54,798	9,596,326	329
chr17	Scaffold662	1	86,951	710,130	5,220	535,499	23
chr17	Scaffold689	1	36,433,659	36,626,213	56,542	249,994	7
chr17	Scaffold848	1	34,821,975	36,217,147	18,548	1,418,643	57
chr17	Scaffold885	1	21,249,435	21,482,454	25,562	263,512	11
chr17	Scaffold968	1	29,032,499	30,366,062	14,598	1,353,323	52
chr17	Scaffold1188	1	15,787,120	16,575,352	15,294	803,804	30
chr17	Scaffold1509	1	18,502,086	18,899,997	29,896	187,968	8
chr17	Scaffold1571	1	20,773,728	21,208,237	119,355	559,320	16
chr17	Scaffold1677	1	3,160,188	15,492,311	10,317	11,257,202	433
chr17	Scaffold1743	1	723,402	1,018,122	8,758	304,957	13
chr17	Scaffold2016	1	58,116,467	58,272,326	13,967	170,137	7
chr17	Scaffold31417	1	43,731,984	44,278,599	14,981	564,579	24
chr17	Scaffold31484	1	60,604,565	66,058,324	21,020	2,288,231	80
chr18	Scaffold32	1	11,656,172	12,155,045	20,657	515,489	21
chr18	Scaffold41	1	10,622,037	11,591,054	33,685	1,008,571	36
chr18	Scaffold185	1	44,549,817	47,902,698	26,460	3,389,415	136
chr18	Scaffold196	1	12,228,622	14,305,479	111,002	2,194,228	80
chr18	Scaffold253	1	77,412,184	78,006,613	17,031	625,298	23
chr18	Scaffold406	1	76,210,505	77,375,158	9,313	1,179,632	46
chr18	Scaffold3667	1	74,053,178	76,183,914	10,000	2,099,890	73
chr18	Scaffold31454	1	39,461,422	44,525,100	10,000	5,091,189	172
chr18	Scaffold31468	1	47,940,940	74,020,970	10,839	26,030,175	1040
chr18	Scaffold31476	1	18,540,051	39,436,488	10,535,309	31,490,782	737
chr18	Scaffold31476	2	143,827	10,591,713	13,922	10,503,502	422
chr19	Scaffold53	1	36,834,592	37,735,184	31,448	974,427	35
chr19	Scaffold78	1	48,448,561	50,566,073	27,794	2,160,103	77
chr19	Scaffold93	1	43,288,293	43,713,532	57,909	493,337	14
chr19	Scaffold97	1	37,781,032	39,274,127	9,837	1,493,838	60
chr19	Scaffold166	1	56,304,975	59,073,011	24,819	2,807,046	111
chr19	Scaffold216	1	22,134,371	24,558,590	12,905	1,727,091	65

chr19	Scaffold268	1	29,877,139	36,780,000	38,595	6,972,112	255
chr19	Scaffold330	1	15,964,270	22,415,150	13,014	6,568,725	237
chr19	Scaffold387	1	39,301,257	40,381,083	21,518	1,095,490	37
chr19	Scaffold401	1	631,987	7,048,901	10,304	6,501,748	230
chr19	Scaffold556	1	22,608,551	22,817,857	32,695	241,865	9
chr19	Scaffold687	1	265,576	605,768	15,212	362,792	17
chr19	Scaffold714	1	43,840,265	48,301,430	10,335	4,500,745	163
chr19	Scaffold811	1	50,665,707	56,254,064	18,876	5,748,498	228
chr19	Scaffold1104	1	7,080,204	8,827,089	23,788	1,680,096	61
chr19	Scaffold1168	1	8,902,286	15,672,120	4,611	6,814,022	247
chr19	Scaffold1254	1	15,696,396	15,941,522	8,981	254,905	9
chr19	Scaffold3127	1	40,389,788	43,218,310	43,872	2,835,053	108
chr19	Scaffold7309	1	27,906,421	29,817,049	22,316	1,945,555	76
chr20	Scaffold211	1	85,000	1,569,733	9,933	1,485,216	56
chr20	Scaffold1049	1	4,041,909	8,032,453	6,369	4,022,064	157
chr20	Scaffold1274	1	1,568,606	3,988,668	23,980	2,433,397	92
chr20	Scaffold6874	1	29,827,990	46,490,382	102,107	16,807,475	570
chr20	Scaffold13626	1	8,178,634	25,727,894	19,904	17,649,333	620
chr20	Scaffold31495	1	46,530,289	62,898,955	15,000	16,458,364	608
chr21	Scaffold284	1	47,098,464	48,097,154	20,443	1,027,961	36
chr21	Scaffold529	1	31,562,741	47,073,584	19,951	15,617,566	528
chr21	Scaffold1310	1	29,385,960	31,521,583	21,326	2,168,820	87
chr21	Scaffold1815	1	15,419,935	29,325,262	27,302	14,059,596	478
chr22	Scaffold29	1	21,096,952	21,444,121	22,751	375,429	14
chr22	Scaffold39	1	22,669,725	22,962,678	14,827	327,931	15
chr22	Scaffold40	1	18,893,177	20,289,893	23,810	1,430,953	55
chr22	Scaffold58	1	17,515,732	18,647,316	7,553	1,162,972	46
chr22	Scaffold200	1	23,682,088	28,619,331	9,596	3,590,284	142
chr22	Scaffold201	1	23,699,183	24,327,798	8,432	624,385	25
chr22	Scaffold486	1	48,761,115	51,177,718	16,817	2,450,322	89
chr22	Scaffold646	1	21,752,386	22,603,282	10,000	813,189	33

chr22	Scaffold839	1	24,694,651	24,986,561	30,014	321,127	12
chr22	Scaffold1047	1	24,291,972	24,630,971	12,942	257,319	11
chr22	Scaffold1138	1	23,022,095	23,630,951	42,986	650,698	28
chr22	Scaffold1381	1	20,736,881	21,016,881	23,401	324,059	11
chr22	Scaffold2197	1	28,640,083	48,736,945	10,355	20,263,942	721
chrX	Scaffold27	1	90,266,128	90,460,590	23,647	218,300	7
chrX	Scaffold30	1	89,561,422	89,866,563	35,217	344,351	8
chrX	Scaffold34	1	8,523,975	9,360,438	10,945	851,809	31
chrX	Scaffold108	1	165,230	2,658,757	31,260	2,402,966	53
chrX	Scaffold140	1	154,567,777	155,216,932	35,075	610,071	21
chrX	Scaffold141	1	89,038,219	89,505,403	29,309	507,306	15
chrX	Scaffold160	1	21,273,329	24,138,179	16,564	2,942,393	112
chrX	Scaffold161	1	61,905,113	62,441,401	43,527	533,702	17
chrX	Scaffold210	1	119,337,278	120,000,362	14,679	674,802	28
chrX	Scaffold212	1	148,628,518	151,525,098	23,339	2,466,609	103
chrX	Scaffold220	1	116,320,966	120,088,565	22,013	2,901,365	115
chrX	Scaffold226	1	154,137,633	154,549,801	14,991	433,322	17
chrX	Scaffold227	1	51,483,259	52,008,234	15,505	517,901	18
chrX	Scaffold239	1	47,653,179	47,866,908	11,739	221,545	10
chrX	Scaffold278	1	37,388,519	41,257,168	10,000	3,888,711	146
chrX	Scaffold326	1	140,361,754	140,623,595	19,248	288,936	7
chrX	Scaffold335	1	140,855,872	146,246,872	27,130	5,379,849	196
chrX	Scaffold337	1	114,172,905	114,946,965	12,632	789,399	35
chrX	Scaffold345	1	115,001,042	116,267,145	17,101	1,237,439	51
chrX	Scaffold354	1	98,268,441	101,428,939	24,812	3,183,873	126
chrX	Scaffold366	1	41,279,998	47,619,567	6,090	6,355,976	228
chrX	Scaffold382	1	152,574,243	153,870,224	14,919	1,203,040	39
chrX	Scaffold457	1	49,318,163	51,313,415	19,583	1,906,175	71
chrX	Scaffold458	1	52,937,480	53,856,555	25,575	912,000	33
chrX	Scaffold502	1	139,092,147	140,075,624	4,984	968,841	39
chrX	Scaffold511	1	53,893,109	55,520,625	12,394	1,611,188	65

chrX	Scaffold557	1	34,396,396	35,604,607	13,970	1,219,496	52
chrX	Scaffold589	1	90,508,692	90,851,062	37,066	378,439	14
chrX	Scaffold598	1	71,032,132	71,957,274	17,338	942,573	35
chrX	Scaffold621	1	134,370,713	134,549,601	8,679	186,998	8
chrX	Scaffold693	1	76,993,932	80,304,820	11,494	3,331,157	123
chrX	Scaffold787	1	88,072,374	88,590,060	22,348	561,350	19
chrX	Scaffold924	1	20,810,383	21,239,070	10,069	438,498	11
chrX	Scaffold932	1	147,448,158	148,612,064	20,769	1,187,513	43
chrX	Scaffold953	1	72,018,700	72,273,588	5,822	169,305	9
chrX	Scaffold984	1	120,131,451	124,387,913	18,072	4,290,416	163
chrX	Scaffold997	1	103,362,821	103,829,868	17,343	482,435	19
chrX	Scaffold1014	1	48,312,972	48,683,234	30,983	407,180	16
chrX	Scaffold1113	1	37,079,770	37,356,379	18,414	258,970	10
chrX	Scaffold1329	1	56,944,323	57,428,324	20,639	513,652	18
chrX	Scaffold1432	1	48,740,492	49,317,054	9,541	436,807	17
chrX	Scaffold1501	1	134,982,342	139,065,886	13,899	4,104,179	143
chrX	Scaffold1704	1	146,271,311	147,180,318	16,665	910,628	38
chrX	Scaffold2059	1	134,224,675	134,849,851	14,235	292,808	9
chrX	Scaffold2395	1	35,630,005	37,451,019	10,425	1,370,255	45
chrX	Scaffold2668	1	24,157,508	25,920,392	3,348	1,803,606	59
chrX	Scaffold2771	1	6,471,225	6,931,305	15,889	476,213	20
chrX	Scaffold2807	1	153,890,120	154,104,933	11,005	227,307	10
chrX	Scaffold2816	1	151,963,373	152,340,860	5,000	284,761	11
chrX	Scaffold3020	1	147,202,377	147,392,677	12,786	212,627	8
chrX	Scaffold3048	1	151,548,115	151,956,067	5,992	342,186	14
chrX	Scaffold3622	1	57,463,708	58,554,854	10,820	1,104,470	42
chrX	Scaffold3873	1	126,952,272	134,240,942	8,051	7,335,972	294
chrX	Scaffold4065	1	69,985,210	70,965,634	19,827	984,313	36
chrX	Scaffold4256	1	124,412,708	126,904,202	24,988	2,509,709	99
chrX	Scaffold6080	1	101,090,828	103,115,735	23,678	1,529,209	63
chrX	Scaffold8046	1	80,339,924	88,016,053	18,923	7,684,166	313

chrX	Scaffold8314	1	25,972,889	34,377,878	9,104	8,453,987	332
chrX	Scaffold11543	1	2,702,973	6,442,510	12,962	3,690,214	121
chrX	Scaffold24429	1	92,385,442	98,243,404	27,016	5,899,247	197
chrX	Scaffold31427	1	62,498,071	64,570,008	12,120	2,081,337	74
chrX	Scaffold31428	1	55,551,013	56,795,862	10,797	1,211,392	48
chrX	Scaffold31432	1	6,967,198	8,471,075	20,000	1,487,733	52
chrX	Scaffold31434	1	9,381,240	20,755,101	5,446	11,361,023	412
chrX	Scaffold31439	1	72,351,874	76,962,126	34,582	4,594,150	165
chrX	Scaffold31451	1	64,614,863	69,923,364	58,121	5,347,876	214
chrX	Scaffold31458	1	103,854,169	114,126,777	6,762	10,278,720	366
chrY	Scaffold30	1	3,751,212	4,057,378	35,166	344,351	8
chrY	Scaffold89	1	6,571,674	7,433,260	50,074	914,317	32
chrY	Scaffold108	1	115,230	2,608,757	31,260	2,402,966	53
chrY	Scaffold140	1	59,084,405	59,319,938	35,075	277,841	8
chrY	Scaffold141	1	3,307,754	3,688,713	28,283	502,076	11
chrY	Scaffold206	1	9,539,355	10,080,010	43,196	573,985	12
chrY	Scaffold233	1	23,238,050	23,659,368	14,641	417,852	16
chrY	Scaffold261	1	2,663,463	3,037,595	38,251	414,807	13
chrY	Scaffold549	1	13,810,633	15,787,070	8,425	2,024,062	72
chrY	Scaffold602	1	22,495,459	23,160,916	9,530	659,167	25
chrY	Scaffold707	1	19,462,302	20,256,636	26,556	489,640	11
chrY	Scaffold707	2	20,310,480	20,598,538	148,054	435,983	7
chrY	Scaffold720	1	18,903,078	19,411,408	23,330	535,388	19
chrY	Scaffold1301	1	16,168,537	17,994,779	34,996	1,845,744	67
chrY	Scaffold1847	1	20,622,098	22,161,130	77,853	1,399,690	55
chrY	Scaffold2025	1	18,554,815	18,866,717	25,456	338,866	14
chrY	Scaffold3501	1	7,516,858	8,966,560	1,605	1,436,360	56
chrY	Scaffold11543	1	16,525,861	21,080,325	1,383,743	3,643,840	8
chrY	Scaffold31408	1	25,866,133	26,164,716	21,044	321,334	9
chrY	Scaffold31432	1	15,823,794	17,599,659	176,210	1,714,530	12

Supplementary Table S4. Repeat composition of the KPGP9 individual genome

Type		Number of Elements	Length(bp)	(%) in the genome
Retrotransposons	SINEs	2,864,361	402,930,137	14.08%
	LINEs	3,003,055	690,651,505	24.13%
	LTR elements	1,256,037	265,066,144	9.26%
	Retroposon	9,177	2,468,947	0.09%
	Total	7,132,630	1,361,116,733	47.55%
DNA transposons	DNA	831,769	109,925,653	3.84%
	RC	2,052	428,502	0.01%
	Total	833,821	110,354,155	3.86%
Small RNA		13,970	1,148,909	0.04%
Satellites		15,362	18,996,845	0.66%
Simple repeats		819,164	44,100,603	1.54%
Low complexity		118,053	6,544,669	0.23%
Unclassified		22,077	5,012,923	0.18%
Total			1,547,274,837	54.06%

Supplementary Table S5. List of DNA samples used

Population 80									
South American	1	NA17301	Andes	African American	1	NA17031	African Americans
	2	NA17302			2	NA17032	
	3	NA17303			3	NA17033	
	4	NA17304			4	NA17034	
	5	NA17305			5	NA17035	
	6	NA17306			6	NA17036	
	7	NA17307			7	NA17037	
	8	NA17308			8	NA17038	
	9	NA17309			9	NA17039	
	10	NA17310			10	NA17040	
	11	NA17311	Brazil, Guyana, and Venezuela		11	NA17105	African Americans
	12	NA17312			12	NA17106	
	13	NA17313			13	NA17107	
	14	NA17314			14	NA17108	
	15	NA17315			15	NA17133	
	16	NA17316			16	NA17134	
	17	NA17317			17	NA17135	
	18	NA17318			18	NA17136	
	19	NA17319			19	NA17109	
	20	NA17320			20	NA17110	
European	1	NA17001	Northern Europeans	Asian	1	NA17051	Japeneses
	2	NA17002			2	NA17052	
	3	NA17003			3	NA17053	
	4	NA17004			4	NA17054	
	5	NA17005			5	NA17055	
	6	NA17006			6	NA17056	
	7	NA17007			7	NA17057	
	8	NA17008			8	NA17058	
	9	NA17009			9	NA17059	
	10	NA17010			10	NA17060	
	11	NA13820	Russians (Zversky district Northeast of Moscow)		11	NA17081	Southeast Asians (Excluding Japanese and Chineses)
	12	NA13838			12	NA17082	
	13	NA13849			13	NA17083	
	14	NA13852			14	NA17084	
	15	NA13876			15	NA17085	
	16	NA13877			16	NA17086	
	17	NA13911			17	NA17087	
	18	NA13912			18	NA17088	
	19	NA13913			19	NA17089	
	20	NA13914			20	NA17090	

Supplementary Table S6. List of validated novel TASV insertions in the KPGP9 genome

NO	repClass	Family	Subfamily	Scaffold#	Orientation	Position (Hg19)	TSD	Insertion Size	TSD size	Deletion size by insertion-mediated deletion	Adjacent gene	Gene symbol	RefSeq	OMIM	Forward Primer	Reverse Primer	Variation region accession (dbVar)	Variation region ID	NOTE
											***Alu* Ya5**								
1	SINE	*Alu*	*Alu* Ya5	Scaffold2638-605135-605416	+	chr1:165659304-165659304	AATATACATATAT	290	13		Homo sapiens aldehyde dehydrogenase 9 family, member A1 (ALDH9A1)	ALDH9A1	NM_000696	602733	GTGCTAAGGTATTGCTATGG	GTGGTAGGTCTGTGGTGAC	esv3821086	ALU_umary_ALU_568	
2	SINE	*Alu*	*Alu* Ya5	Scaffold4551-4391627-4391889	C	chr3:50063025-50063025	TTTTTTTTTTTTTT	224	14		Homo sapiens RNA binding motif protein 6 (RBM6)	RBM6	NM_005777	606886	GGCGAATAGGTAGATGCAC	CTGCTGTAACCCTGATACC			High GC
3	SINE	*Alu*	*Alu* Ya5	Scaffold10222-341979-342230	C	chr21:21324518-21324518	-	244	0	6	-		-	-	CCACCCAATTCTCAGACC	CACATGATTAGGAAGAGCAGG			
4	SINE	*Alu*	*Alu* Ya5	Scaffold4348-551877-552175	+	chr17:75092428-75092428	AAGAGTTCGAGACCA	228	15		Homo sapiens SEC14-like lipid binding 1 (SEC14L1)	SEC14L1	NM_001204410	601504	GCTGTCTCCTGCTGTCATTC	CAGTCTGGTGTTGGATTACATC			
5	SINE	*Alu*	*Alu* Ya5	Scaffold16601-735477-735706	+	chr9:105450619-105450619	-	228	0	20	-		-	-	GAGAGAGGGCAGAAGATGG	GAAGCAGAGCCTCACCAATC	esv3858405	ALU_umary_ALU_7615	
6	SINE	*Alu*	*Alu* Ya5	Scaffold1254-496783-496969	+	chr6:42670319-42670319	-	186	0	0	Homo sapiens peripherin 2 (retinal degeneration, slow) (PRPH2)	PRPH2	NM_000322	179605	GCTTCCTCACCAAATCTTCC	GCAATGTAAGCAAATCCTCG			
7	SINE	*Alu*	*Alu* Ya5	Scaffold16064-858104-858251	+	chr8:53791031-53791031	-	144	0	9	-		-	-	GCTGTTGCCTGGACACTC	GCAGCAGAAGAAACTTGG			
8	SINE	*Alu*	*Alu* Ya5	Scaffold498-2422037-2422344	C	chr15:35473614-35473614	CCCTGTGTTCACTATTCTT	320	19		-		-	-	GTGTTAGTGCCAGCAAGC	GTCAGGGAACTAAAGTCACAT			
9	SINE	*Alu*	*Alu* Ya5	Scaffold1051-342768-343037	C	chr1:65573259-65573259	CCTTTCTCCTTTTC	279	14		-		-	-	CTGCCATTCCTTCACAGAC	GCTTGGGAAGTATGGAGATAG			
10	SINE	*Alu*	*Alu* Ya5	Scaffold1073-160543-160841	C	chr17:36169329-36169329	GGATATCTTCTTTT	306	14		-		-	-	GCTCCAAACACTGGCTGGC	GCAGAAGGCAGACAATGAAG			
11	SINE	*Alu*	*Alu* Ya5	Scaffold1337-1252784-1252994	+	chr12:40832707-40832707	:TTCCTGTAGAGTAAAGAGAAT	300	89		Homo sapiens mucin 19, oligomeric (MUC19)	MUC19	NM_173600	612170	GGCATATGAATTGTGCTAGGG	CATAGCCTCCTCAGCCATTAC			
12	SINE	*Alu*	*Alu* Ya5	Scaffold1464-150754-151054	+	chr11:110277916-110277916	AAAAATACCGGGCCCG	312	16		-		-	-	CCTTGGTAGTTGTTAGTTGAGC	CCATTCCTTCACTGGAGC			
13	SINE	*Alu*	*Alu* Ya5	Scaffold1755-1394897-1395205	+	chr9:81596118-81596118	AAGAAATACAATGATG	322	16		-		-	-	GAGTATCTGAACAATGGCAGG	GCCCTACTTGATGGGAATC			
14	SINE	*Alu*	*Alu* Ya5	Scaffold1780-4824802-4824987	+	chr16:7227223-7227223	AAGAACCAAG	222	10		Homo sapiens RNA binding protein, fox-1 homolog (C. elegans) 1 (RBFOX	RBFOX1	NM_018723	605104	CCAGGCATTCTTCCCAGTG	CCTCAGCCAATAGCAGTCTCC			
15	SINE	*Alu*	*Alu* Ya5	Scaffold2658-3501402-3501582	C	chr6:53649677-53649677	TTTATATGCCTTTT	262	14		-		-	-	GCTCAGCATTGGTTTGG	CAAACAGGCTAACAACAAG			
16	SINE	*Alu*	*Alu* Ya5	Scaffold3713-484678-484813	C	chr15:73983319-73983319	TATGCTTTT	214	9		Homo sapiens CD276 molecule (CD276)	CD276	NM_001024736	605715	GCTGCGATCTTATCACAAGG	GGACTCCTCAAATATGTCTGC	esv3875583	ALU_umary_ALU_10812	
17	SINE	*Alu*	*Alu* Ya5	Scaffold5650-2672938-2673178	C	chr1:9573320-9573320	TCTACTTTACTTTTT	348	15		-		-	-	CCCGTGTTAGGAATGCCTG	CGAACCTCCACCCACTGG			
18	SINE	*Alu*	*Alu* Ya5	Scaffold5938-1540413-1540593	+	chr20:1546089-1546089	:ATGGCAGGGAAGGAAGGAAG	319	140		Homo sapiens signal-regulatory protein beta 1 (SIRPB1)	SIRPB1	NM_006065	603889	CAGATGACCTGGGAGAGGAG	GTCCACCCATAACAGGCATC			
19	SINE	*Alu*	*Alu* Ya5	Scaffold6216-273930-274113	C	chr20:16831730-16831730	TACAAGATATTTTTT	264	15		-		-	-	GCACACCTTCTTTTGAGG	CACTGGGTTTCTGTATTTGAG			
20	SINE	*Alu*	*Alu* Ya5	Scaffold6778-1173547-1173827	+	chrX:31275305-31275305	AGAAGTGGGCGTCTC	261	15		Homo sapiens dystrophin (DMD)	DMD	NM_004020	300377	CCTATTGTGTTCAAACCCTG	GCATGGTAGGTTACACATTCC			High GC
21	SINE	*Alu*	*Alu* Ya5	Scaffold6842-893826-893990	C	chr4:145396177-145396177	TATGGTAATGTGTTTTT	296	17		-		-	-	GGTCTTGCACATGTTCG	CCCCAGCAGTATGTTAGC			
22	SINE	*Alu*	*Alu* Ya5	Scaffold7277-770410-770651	C	chr3:196602023-196602023	AAGCAGACACATTTT	383	15		Homo sapiens SUMO1/sentrin specific peptidase 5 (SENP5)	SENP5	NM_152699	612845	GTTCAAGAAGCCTGGGTAAAGC	GGATTACTTGATTGAGCCTGG			
23	SINE	*Alu*	*Alu* Ya5	Scaffold7847-1253437-1253603	+	chr12:101451672-101451672	AAGAAACCTGCACGTTCC	266	18		Homo sapiens anoctamin 4 (ANO4)	ANO4	NM_001286615	610111	GTAGATGACGATTTGAAGGG	GGACCTCCAGAATAATGCTG			High GC
24	SINE	*Alu*	*Alu* Ya5	Scaffold7857-1475280-1475433	C	chr8:13975418-13975418	GAACTCATCCTTTTT	202	15		Homo sapiens sarcoglycan, zeta (SGCZ)	SGCZ	NM_139167	608113	GCAGAGTATAGCATCTCACTGG	CCTCAATCCAGTTAGAATGGC			
25	SINE	*Alu*	*Alu* Ya5	Scaffold8353-1406912-1407152	C	chr2:85541281-85541281	TATTTTTAGTTTT	283	13		-		-	-	GAGGCTGGCATTGGTGG	GAGTTTGAGACCATGACCTC			
26	SINE	*Alu*	*Alu* Ya5	Scaffold9154-1740349-1740570	+	chr1:220955345-220955345	AAAAGAGAAATCCT	329	14		Homo sapiens mitochondrial amidoxime reducing component 2 (MARC2)	MARC2	NM_017898	614127	GCTCCAAAGAATGTTAAGCC	CCAAGGAGCTTCTTTTATGC			
27	SINE	*Alu*	*Alu* Ya5	Scaffold9449-191631-191907	+	chr1:219575677-219575677	AAAAAAAGAAAAGGC	280	15		-		-	-	CATGGAGGTCACTGGTCAC	GCCCCATGCTAGGTAGTAG			
28	SINE	*Alu*	*Alu* Ya5	Scaffold9661-2389101-2389391	+	chr5:106181896-106181896	AAGATCCAGTTTGTA	320	15		Homo sapiens uncharacterized LOC102467213 (LOC102467213)	LOC102467213	NR_104671		GCCTCATTGTTAGCCTCAGC	CCAAAATGCAGACAATGAATG			
29	SINE	*Alu*	*Alu* Ya5	Scaffold9676-68882-69153	+	chr14:23673669-23673669	AAAAAGGGGGACTAAAAGTA(	294	35		-		-	-	GAGTTATTTTGCACGGCA	GTCTCCAATCTCATCTACC			
30	SINE	*Alu*	*Alu* Ya5	Scaffold9692-874427-874643	C	chr16:89292322-89292322	CAGAATGCATCTT	285	13		-		-	-	CAGGAAAGAGCTGGACAAAGG	GTCGAGTTTAGCGGCTGTG			
31	SINE	*Alu*	*Alu* Ya5	Scaffold9708-1139681-1139969	+	chr2:62502896-62502896	AAGAATGTATAGCT	298	14		-		-	-	GGACCAAGAGGGGAAACAG	CAGTCTGCTGATCCCGATG			
32	SINE	*Alu*	*Alu* Ya5	Scaffold10546-619886-620026	+	chr13:86167443-86167443	-	142	0	0	-		-	-	GCAACCTTTCCACTCTGG	GCAAATGTTATTATGCCTATGG			
33	SINE	*Alu*	*Alu* Ya5	Scaffold10986-833152-833306	+	chr4:125660008-125660008	-	155	0	0	-		-	-	GACAGTGTATGCGAGTAAGC	CTTGAGGCTAACCATTGTC			
34	SINE	*Alu*	*Alu* Ya5	Scaffold11190-97324-97550	C	chr15:67645787-67645787	GATAACTTTC	365	10		Homo sapiens IQ motif containing H (IQCH)	IQCH	NM_001031715	612523	CCCAAAGTACTGGAATGTTC	CCTGTTTCTGCTGGTGG			
35	SINE	*Alu*	*Alu* Ya5	Scaffold11731-977646-977787	C	chr8:96784471-96784471	TCTGTTATTCTATTTT	219	16		Homo sapiens C8orf37 antisense RNA 1 (C8orf37-AS1)	C8orf37-AS1	NR_038207		CAGAGTCGGGTGTGCTGC	CTTAACCAGGGCTCCATAACC			
36	SINE	*Alu*	*Alu* Ya5	Scaffold12188-3828034-3828155	C	chr2:80769010-80769010	TCTAAATGTTTTTAATATGGA	280	144		Homo sapiens catenin (cadherin-associated protein), alpha 2 (CTNNA2)	CTNNA2	NM_001282600	114025	CAACTCCAAACCACACTCAG	GCTTCACAATTATCTGGGG			
37	SINE	*Alu*	*Alu* Ya5	Scaffold12428-30637-30768	+	chr4:169756756-169756756	GCTCTGTTATCAAATTTTAAGA(	186	51		Homo sapiens palladin, cytoskeletal associated protein (PALLD)	PALLD	NM_001166108	608092	CAGTCTCCCAGGTGTTTAGCC	CTCTTTGAGCCAGGCACAGTG			
38	SINE	*Alu*	*Alu* Ya5	Scaffold12887-1479719-1479867	C	chr9:21038486-21038486	CCCTGCAACATCTTTT	214	16		-		-	-	CTGGTCATTATTAAAGGCAGTG	CCATTATTCAAGGAAGAAAGG			
39	SINE	*Alu*	*Alu* Ya5	Scaffold13200-212724-212942	+	chr9:22289707-22289707	AGAAAGGAAACACAGGC	270	17		-		-	-	GAATACATCAGAGCAGAACAG	GATGAGATGGTGTGCAAG			
40	SINE	*Alu*	*Alu* Ya5	Scaffold14286-4814423-4814652	C	chr1:29383135-29383135	CATAGTTATTTTCT	316	14		Homo sapiens erythrocyte membrane protein band 4.1 (EPB41)	EPB41	NM_203342	130500	CCAGGCTGTGATCCACCC	GAGACTGAAGGCCACCTCTG			
41	SINE	*Alu*	*Alu* Ya5	Scaffold14286-4875436-4875745	+	chr1:29322463-29322463	AAGAAATAAGGCTGGGTG	325	18		Homo sapiens erythrocyte membrane protein band 4.1 (EPB41)	EPB41	NM_203342	130500	CTGGATCTTGGTCTGAATGG	CCATGTTGCCCAGGTTACTC			
42	SINE	*Alu*	*Alu* Ya5	Scaffold14795-3905856-3906083	C	chr1:70091056-70091056	CAGTCTTAGGATCTTT	323	16		-		-	-	GTGGAACCTATAGTGATGGC	GGAAATGCTACATGCCAC	esv3819552	ALU_umary_ALU_226	
43	SINE	*Alu*	*Alu* Ya5	Scaffold16040-1100576-1100886	C	chr16:75655154-75655154	GGCATTTTATTTTTCT	322	16		Homo sapiens adenosine deaminase, tRNA-specific 1 (ADAT1)	ADAT1	NM_012091	604230	GGGTTGCAGACTCACTTGC	GGGATTTACTAGAGGGCGG			
44	SINE	*Alu*	*Alu* Ya5	Scaffold16041-196443-196589	+	chrY:5719834-5719834	:TTGTGTTTCTTTTTGAGATTAC	327	180		-		-	-	GTCTCACCTCTTCAATTTAGG	GATTGCAAACATGGTCCAC			
45	SINE	*Alu*	*Alu* Ya5	Scaffold16053-1713177-1713512	C	chr10:69631054-69631054	TTTGTTTGTTT	328	11		-		-	-	GATCCACCCACCTCACCTC	GGTTGAGGCTGTAGCAGTAAGC			
46	SINE	*Alu*	*Alu* Ya5	Scaffold16436-2508175-2508476	C	chr10:30614762-30614762	CCCACCCCACTTTTTT	312	16		Homo sapiens mitochondrial poly(A) polymerase (MTPAP)	MTPAP	NM_018109	613669	CTTGTGGGTGAAGATTCGAG	GACCTTTGAATTGCCTCCAG			
47	SINE	*Alu*	*Alu* Ya5	Scaffold16914-1578785-1579093	C	chr6:102220563-102220563	CTCAACTTTTGATTCTTT	367	18		Homo sapiens glutamate receptor, ionotropic, kainate 2 (GRIK2)	GRIK2	NM_175768	138244	GCCTGAAAGCAGATGTTGG	GAGTGGGACAAACAAGTAAACC			
48	SINE	*Alu*	*Alu* Ya5	Scaffold16962-506310-506610	C	chr7:22854479-22854479	TGGGCTACATTT	310	12		Homo sapiens translocase of outer mitochondrial membrane 7 homolog (yeast) (T(	TOMM7	NM_019059	607980	GCTGGTTTCGAACTCGTG	CTCCAATAGAGTGTAGGAAG			
49	SINE	*Alu*	*Alu* Ya5	Scaffold16962-560182-560398	+	chr7:22800807-22800807	AAAACAATCAG	230	11		-		-	-	GAGGCCAGAGGGAATGAC	GCACTGAGAGCCACATTAGAC			
50	SINE	*Alu*	*Alu* Ya5	Scaffold63205-462802-463112	+	chr8:10327346-10327346	AAGAAAATTGTTTTT	333	15		-		-	-	GCCGACTTCACTGTTATTCCTC	GAAAGACACAGGGGACGGAC			
51	SINE	*Alu*	*Alu* Ya5	Scaffold84228-3153261-3153579	C	chr11:33235105-33235105	GATGAAGAGGAATTTTT	331	17		-		-	-	CGGAAAGAGGGATTTGAAGG	CCACAGACTTTCTTGACTGGG	esv3863454	ALU_umary_ALU_8493	
52	SINE	*Alu*	*Alu* Ya5	Scaffold2142-424628-424808	+	chr17:80590463-80590463	ITTTTTTAGCCAGTCACCTTTCT(	305	118		Homo sapiens WD repeat domain 45B (WDR45B)	WDR45B	NM_019613	609226	GGGTGTTGTGTGCTCAGC	CAGTACCTTTGTTGGGAGGG			
53	SINE	*Alu*	*Alu* Ya5	Scaffold2151-1635568-1635707	C	chr2:46928950-46928950	TGTGGCGGTTTTGTTAAAATGG	300	210		Homo sapiens suppressor of cytokine signaling 5 (SOCS5)	SOCS5	NM_014011	607094	GCATTGTTAGCGTTAGTGC	GAGGAAGGAGAGCAATACC	esv3824113	ALU_umary_ALU_1171	
54	SINE	*Alu*	*Alu* Ya5	Scaffold2633-5607493-5607803	C	chr2:31560685-31560685	GGGGCTTcCTCTTTTT	322	16		Homo sapiens xanthine dehydrogenase (XDH)	XDH	NM_000379	607633	GGACTGGGTTCTTCCTGATACTC	GTTGAGGTCTGCGGATGG			
55	SINE	*Alu*	*Alu* Ya5	Scaffold3523-1824351-1824656	C	chr11:38802069-38802069	AATAAACCACTTT	316	13		-		-	-	GAGTTGGCGTGTTCCCAT	CCACTAGCTCACCAGCAGC			
56	SINE	*Alu*	*Alu* Ya5	Scaffold3703-210432-210676	+	chr2:11353525-11353525	.GTTTCACTGGAACGTAGCCAC.	416	183		Homo sapiens Rho-associated, coiled-coil containing protein kinase 2 (ROCK	ROCK2	NM_004850	604002	GCTCTGTAGCCTCATTCTG	GAGCAAAGACATAGAAGATGG			
57	SINE	*Alu*	*Alu* Ya5	Scaffold3709-260020-260277	C	chr6:6035740-6035740	GTGCTTTCTCTTTTCT	325	16		-		-	-	GTGGCAAGAGAGAGGAGAG	CACGAGATAGGATAAAGTGTGG			
58	SINE	*Alu*	*Alu* Ya5	Scaffold4423-1481504-1481814	C	chr11:24792942-24792942	CTATTCATGTGATTTTT	322	17		Homo sapiens leucine zipper protein 2 (LUZP2)	LUZP2	NM_001009909	608178	CCAGCTTTAGTAGACAGTAGGTC	GATGTGCTATGGAAGGTAGG			
59	SINE	*Alu*	*Alu* Ya5	Scaffold9442-6066823-6067025	+	chr8:23042318-23042318	ACCACGAGCAATGTCAATTAC	304	101		-		-	-	CCTACTCAGTTGTCTTGGAGGC	CGTGGTGGCTCACTGTGG			
60	SINE	*Alu*	*Alu* Ya5	Scaffold13190-391314-391475	C	chr1:180858548-180858548	GCTATCTCAATTTTA	179	15		Homo sapiens xenotropic and polytropic retrovirus receptor 1 (XPR1)	XPR1	NM_004736	605237	GGTGATTTTGTAGTTGAGAGAGC	CTGGTAAGCAGATTTCAGGTC			
61	SINE	*Alu*	*Alu* Ya5	Scaffold13294-3398577-3398707	+	chr1:227602198-227602198	ATTGCAGAGCATTCCCAATCA(	410	95		-		-	-	GTGGGAAGAGCCGGTTG	GCACCTACTGAACTTGGAAGTC			
62	SINE	*Alu*	*Alu* Ya5	Scaffold14550-2906989-2907299	C	chr6:18899149-18899149	TCATTCTTTTATTTT	314	15		-		-	-	GGAGTGGTTTCATTGGCTG	GCAACCAAGCATCCAATTCT			
63	SINE	*Alu*	*Alu* Ya5	Scaffold15038-105959-106272	C	chr1:93167519-93167519	TGTATGTTATTTT	326	13		Homo sapiens ecotropic viral integration site 5 (EVI5)	EVI5	NM_005665	602942	CATACAGCATGTGCAGAACTG	GGATTGTTGCTTATGCAGG			
64	SINE	*Alu*	*Alu* Ya5	Scaffold15563-8994633-8994940	C	chr3:176144029-176144029	AATTTCTTTTTTTTTT	314	16		-		-	-	CATGTCAAGGCCAGTTCTTC	CAGGACACTCACAGAATAAGG			
65	SINE	*Alu*	*Alu* Ya5	Scaffold16034-7683297-7683428	+	chr11:106170467-106170467	GAAATAACTTTGCC	352	14		-		-	-	CAGTAGTGAGGAGGAGCC	CATGACTGTTGGCATCCC			
66	SINE	*Alu*	*Alu* Ya5	Scaffold16052-128293-128440	+	chr13:36716723-36716723	GAGGcGGAGCTTGCAGTGAGC(	300	30		-		-	-	CCAATGAACTAAGCATCTAAC	GCTATGATAAGGCATGAGC			
67	SINE	*Alu*	*Alu* Ya5	Scaffold16369-703225-703335	C	chr12:55156566-55156566	CATTAGGTATATCTcCTAATGC	283	25		-		-	-	GCCATCCACAAATAACGAC	GAGAACATTTGGACACAGGA			
68	SINE	*Alu*	*Alu* Ya5	Scaffold14800-161410-161709	C	chr14:45014830-45014830	TGAG	550	4		-		-	-	GCATTCTCTGAGGGTACAAGC	CATCATAATGGGTGTGAGGTG			
69	SINE	*Alu*	*Alu* Ya5	Scaffold1490-60189-60423	C	chr2:114106432-114106432	AAGAAATCAGAAAA	313	14		-		-	-	GCCGCACAAAACTCCTGCT	GGATGGTGCATAAGGCAGGG			
70	SINE	*Alu*	*Alu* Ya5	Scaffold7861-518867-519193	+	chr2:123330645-123330645	AAGAAAAATCCACTGA	370	16		-		-	-	TTGTGATGGTTGTACTGCCC	GGTCAGACAGACTTCCTGGC			
71	SINE	*Alu*	*Alu* Ya5	Scaffold3707-5995421-5995618	+	chr2:233845893-233845893	-	196	0	0	Homo sapiens neuronal guanine nucleotide exchange factor (NGEF)	NGEF	NM_019850	605991	AATCACCAGCTACTGCAGGG	AAGGACCAGTGAAGCTCCC	esv3828594	ALU_umary_ALU_2051	
72	SINE	*Alu*	*Alu* Ya5	Scaffold6436-4283357-4283607	C	chr3:171626373-171626373	TCCTGTTAATTTCTT	307	15		-		-	-	CCAAAAGTGGTGGTGTATGCC	GCTAGGGACAAAGACACTGCCC			
73	SINE	*Alu*	*Alu* Ya5	Scaffold15791-6062824-6063019	+	chr5:76716195-76716195	AAAACCTACTCAT	310	13		Homo sapiens phosphodiesterase 8B (PDE8B)	PDE8B	NM_001029851	603390	GTGAACAGCATCAACAAGCC	AGACTGGTCATGGGATGGG			
74	SINE	*Alu*	*Alu* Ya5	Scaffold14134-5602316-5602459	+	chr6:93576134-93576134	AAAAAGACTGTATG	322	14		-		-	-	CACTCCTAAGAGATCATCCCT	CTCCTCTTCCACTGACACCT			
75	SINE	*Alu*	*Alu* Ya5	Scaffold13910-1194632-1194724	C	chr6:116307096-116307096	AGAATGCTGAGTGCTT	140	16		Homo sapiens fyn-related Src family tyrosine kinase (FRK)	FRK	NM_002031	606573	AGGATAGAGACAGCGGTGAGGG	GCTGGTTTGGTCATGGCTCG	esv3846361	ALU_umary_ALU_5500	
76	SINE	*Alu*	*Alu* Ya5	Scaffold13717-765201-765505	+	chr8:138296182-138296182	CAATAtCTTCAG	316	12		-		-	-	GTCACCTCAGGGAAGGATGG	CTCTTACTTCAGTGCTGGTGC			
77	SINE	*Alu*	*Alu* Ya5	Scaffold10516-436226-436378	C	chr9:12601791-12601791	AGTAAATAT	215	9		-		-	-	CAAGGAGCCAGATAACGCG	AGTTAGAGTGGGAATGGTGGG	esv3856661	ALU_umary_ALU_7291	
78	SINE	*Alu*	*Alu* Ya5	Scaffold14588-526215-526528	+	chr10:5897299-5897299	:TGGGTGACAGAGGGAGACTA	303	77		-		-	-	ATGCCACTGCACTTCCATC	CCCAAAGATATGAGCCACC			Specific to Asian
79	SINE	*Alu*	*Alu* Ya5	Scaffold9158-641473-641486	+	chr10:56664643-56664643	AAAAAAAACAAAAAG	363	15		-		-	-	GGTAAAGTGTGGAGAAAAGGG	AAGAATCTGGACTTTGAGGCC			
80	SINE	*Alu*	*Alu* Ya5	Scaffold15236-101934-102075	+	chr11:104786590-104786590	AAGAATTTAAATATTGA	222	17		Homo sapiens caspase 4, apoptosis-related cysteine peptidase pseudogene (LOC6	LOC643733	NR_034078	643733	TTCTAAATCACCAGTCAAGCG	CCTTGACAGGAGACCAGCC			
81	SINE	*Alu*	*Alu* Ya5	Scaffold6480-595471-595673	C	chr12:27865795-27865795	AAAAATAGACAATTAT	241	16		Homo sapiens mitochondrial ribosomal protein S35 (MRPS35), transcript vari	MRPS35	NM_001190864	611995	CTTTCCTGACCATCCTGTGG	CGCTGTGATAGAAGTGTGGC			
82	SINE	*Alu*	*Alu* Ya5	Scaffold13173-2102376-2102680	+	chr12:58083259-58083259	AAAAAAaATTATAGTTTG	315	18		-		-	-	AGGATGATGGAGTTTGAGGG	TTATCAGAACAGCATTGGGC			
83	SINE	*Alu*	*Alu* Ya5	Scaffold4038-1998288-1998508	+	chr12:81316218-81316218	AGAATCTGCTGGCACT	273	16		Homo sapiens lin-7 homolog A (C. elegans) (LIN7A)	LIN7A	NM_004664	603380	CCCTGATGGGATTATTAGCC	AAGACAGAGGATTGCTGAGG			
84	SINE	*Alu*	*Alu* Ya5	Scaffold9496-671468-671764	+	chr14:25582749-25582749	ATATATATTTTA	319	12		-		-	-	GTGTCCACTTAGACCTCAGG	GGTGGTGTAAAATGTAGCCC			
85	SINE	*Alu*	*Alu* Ya5	Scaffold1807-892485-892795	+	chr15:63086025-63086025	AGAAACTTGGCA	320	12		Homo sapiens talin 2 (TLN2)	TLN2	NM_015059	607349	CTATTCTCCCCCAGTATGCG	CAGGTGTCCTAAATCAGGGA			
86	SINE	*Alu*	*Alu* Ya5	Scaffold7085-892715-892987	C	chr15:79796871-79796871	AAGAATGTATGTG	318	13		-		-	-	GAATCACTGGTGGAGTCTGC	CTCCATTCACTCCAGCATCT			
87	SINE	*Alu*	*Alu* Ya5	Scaffold6443-3148378-3148596	C	chr21:24976488-24976488	AGAGAGAAAt	375	10		-		-	-	TTGAGTCAGTCCAGGGAAGG	GTTGGCAAAACAGCCTCTTC			
88	SINE	*Alu*	*Alu* Ya5	Scaffold6443-4682118-4682300	+	chr21:26497431-26497431	AGGATG	183	6		-		-	-	GGGAGCCCACAAAATAGTCA	GGGCTTACTTTCACCAATGA	esv3886444	ALU_umary_ALU_12334	
89	SINE	*Alu*	*Alu* Ya5	Scaffold3772-201563-201847		chrX:130922942-130922942	AAAATGTGAC	291	10		Homo sapiens firre intergenic repeating RNA element (FIRRE)	FIRRE	NR_026975	-	GCGTACAACAACACTGGG	TCTCCACCTCTCCACTGATA			
90	SINE	*Alu*	*Alu* Ya5	Scaffold14588-4143009-4143175	C	chr10:9511985-9511985	AAAAAAATTTGCA	211	13		-		-	-	GGTTCACACTTTGCCTTAGGG	CATGTGTGAATGTGAATGGC			
91	SINE	*Alu*	*Alu* Ya5	Scaffold5099-734647-734945	+	chr4:185978799-185978799	AAAATTCAG	308	9		-		-	-	GTGAACCGTGCTTAAAAGAGGC	GCAATGTAGTGATATATGCCTGC			

***Alu* Ya8**																			
1	SINE	*Alu*	*Alu* Ya8	Scaffold1761-1335113-1335220	C	chr11:47580062-47580062	AGCGAGAC	516	8		-		-	-	CCACTGATGAGTCTGTGTCA	GGAAATGATGATAGCCAGG			
2	SINE	*Alu*	*Alu* Ya8	Scaffold2353-2617237-2617542	+	chr8:42040896-42040896	AGAACACTAC	312	10		Homo sapiens plasminogen activator, tissue (PLAT)	PLAT	NM_033011	173370	ACAGGACAGCTCACAGTTCT	CTCATTCCCCATTACTCTCA			
3	SINE	*Alu*	*Alu* Ya8	Scaffold2628-2327996-2328307	+	chr3:31401627-31401627	AAAAAGTTAATTACA	333	15		-		-	-	CTTGTTGGAGTTGAGGCATC	TGAGTTACACTGTGAAAGGCC			
4	SINE	*Alu*	*Alu* Ya8	Scaffold5807-117396-117488	+	chr4:112699426-112699426	AAACATATCTTTT	348	13		-		-	-	AGGATATGCTGCTACTGGAA	CCCACACTTAGCAATCTTTC			
5	SINE	*Alu*	*Alu* Ya8	Scaffold12158-2008551-2008651	+	chr14:41840257-41840257	AAAACAGGGTGATTA	314	15		-		-	-	GCAGATGTTGGTCAAAGAAGAC	GAACACATTGACACAGGAAGG			
6	SINE	*Alu*	*Alu* Ya8	Scaffold14281-1358920-1359021	C	chr12:25463893-25463893	AGTTCTTGATGGTGGCACTAA	157	55		-		-	-	GACCAGTGTGATATGTGGA	AGGCAAGGTGTAGTTACCG			
7	SINE	*Alu*	*Alu* Ya8	Scaffold14564-7020-7099	C	chr15:100899777-100899777	ATCGTTCTTGTTTCTCCAGTTCC	274	156		-		-	-	GTGTGTGGGCATTCATAGTA	TGAGAAGGGATTGGTGTCC			
8	SINE	*Alu*	*Alu* Ya8	Scaffold15820-1508418-1508538	C	chr8:80751704-80751704	CAGAGATTTCTT	326	12		Homo sapiens uncharacterized LOC101927040 (LOC101927040)	LOC101927040	NR_110954	-	ACCTGTAGATCATTAGGGGAG	GATGAATGGGCAGGACACA			
***Alu* Yb8**																			
1	SINE	*Alu*	*Alu* Yb8	Scaffold4423-2317559-2317843	+	chr11:25605138-25605138	-	285	0	14	-		-	-	CTCAAGATAACCCTCACCC	GGTAACTCATCAGCAGCC	esv3863278	ALU_umary_ALU_8456	
2	SINE	*Alu*	*Alu* Yb8	Scaffold6439-7922999-7923207	C	chr1:188832839-188832839	-	208	0	0	-		-	-	ACACTATGCCCCAGAGAATG	GAAAGACCACAAGGGAGGAAG			
3	SINE	*Alu*	*Alu* Yb8	Scaffold5076-4464479-4464808	C	chr20:48943826-48943826	TTGTATTTTTAGTAGAGAcGGG	194	30		-		-	-	CAGGCAAGACTTCTATGATAC	CAGTGTCCAGTTGTTTGGTC			
4	SINE	*Alu*	*Alu* Yb8	Scaffold6756-4571276-4571588	C	chr10:26961080-26961080	ATTTTTTTTT	184	10		-		-	-	AAAATCAACAATGCCTGTCC	CTACACAGTGCCCATCCC			
5	SINE	*Alu*	*Alu* Yb8	Scaffold12434-4104190-4104493	C	chr19:47809223-47809223	TTTTT	174	5		-		-	-	GTGACCTGGCTTCCTGAACC	CTTGGAGCCTACGGCACA			
6	SINE	*Alu*	*Alu* Yb8	Scaffold9148-1211806-1211948	+	chr2:192840053-192840053	-	143	0	10	ens transmembrane protein with EGF-like and two follistatin-like domains 2	TMEFF2	NM_001305134	605734	GCCAGTGGTTAATGGTGC	TAGCCCATTCCTGGTATTGC	esv3827562	ALU_umary_ALU_1860	
7	SINE	*Alu*	*Alu* Yb8	Scaffold49-334993-335301	C	chr15:22770068-22770068	TAAATATTTT	315	10		-		-	-	GCTTCACATACTCACCATT	GACAAGCCACATACTCAGAG			
8	SINE	*Alu*	*Alu* Yb8	Scaffold1230-602330-602676	+	chr1:64959061-64959061	AAGAAAACAATAGGGGG	354	17		Homo sapiens cache domain containing 1 (CACHD1)	CACHD1	NM_020925	-	TGCTGTCTTTTACACTGACG	GTTGCTTGAAGAATAACGGC			
9	SINE	*Alu*	*Alu* Yb8	Scaffold1231-2979587-2979903	C	chr3:42898418-42898418	GAGCCTAATTCTT	328	13		Homo sapiens atypical chemokine receptor 2 (ACKR2),	ACKR2	NM_001296	602648	CTTCACGATTGCCTGCTTGG	CCTCACCACCACCACCCTG	esv3830096	ALU_umary_ALU_2319	
10	SINE	*Alu*	*Alu* Yb8	Scaffold1254-3006087-3006398	+	chr4:68944769-68944769	AAAAAAaGGC	316	10		Homo sapiens transmembrane protease, serine 11F (TMPRSS11F)	TMPRSS11F	NM_207407	-	AACCATAGTCCCCCAATGC	CCTACCTGCCTGTCTCCC	esv3835555	ALU_umary_ALU_3368	
11	SINE	*Alu*	*Alu* Yb8	Scaffold1774-3449494-3449811	C	chr2:50602629-50602629	AATTCTGAAGCTC	331	13		Homo sapiens neurexin 1 (NRXN1)	NRXN1	NM_001135659	600565	TATTCATACCAGGCTTAGC	CTTGTATCATAGAGCCCTC			
12	SINE	*Alu*	*Alu* Yb8	Scaffold1780-3512926-3513209	C	chr16:8536494-8536494	TTCTCAGGATGTTTCT	297	16		-		-	-	ATGGAAGCAGAGAGAGTGC	GCAGTTTTGAAGAGGGAC			
13	SINE	*Alu*	*Alu* Yb8	Scaffold1913-81388-81700	+	chr2:158510355-158510355	AAGAAAGTAAGAGAGAG	324	17		-		-	-	GTCTCACCAGGTCAAAGGC	GCTTGTCTGTTGTTCAATGC			
14	SINE	*Alu*	*Alu* Yb8	Scaffold2140-4721399-4721696	C	chr3:63540950-63540950	AGAAAAAcACTTCC	312	14		Homo sapiens synaptoporin (SYNPR)	SYNPR	NM_144642	-	GGTTGCTCTAGCCTTGTGG	TGATGATAAAGCCATTCACG			
15	SINE	*Alu*	*Alu* Yb8	Scaffold2353-3390563-3390792	+	chr8:41271458-41271458	TGTGTC	236	6		-		-	-	GAGAACAAGCCCCACTGC	GCTCATTGCCTTCTCCCTC			
16	SINE	*Alu*	*Alu* Yb8	Scaffold3195-74833-75066	C	chr11:7093736-7093736	ACCTGCATTCTT	298	12		-		-	-	TGCCAGAGATAGCAGGGC	CCAGACTTGGGTGCTTAGC			
17	SINE	*Alu*	*Alu* Yb8	Scaffold3317-2875896-2876183	C	chrX:41391772-41391772	TCCATTTTTTT	357	11		Homo sapiens calcium/calmodulin-dependent serine protein kinase (MAGUK family	CASK	NM_001126055	300172	CCAGTTTGTCACCTCCTGA	ATGAACCACCACAGGAGGA			Specific to Asian
18	SINE	*Alu*	*Alu* Yb8	Scaffold3524-466832-467142	+	chr22:23170871-23170871	AAGAAGTAGAGGAATG	336	16		-		-	-	CTTCCCTCCAGCCCTACA	CTTGTCTATCTCACCCAGTGT			
19	SINE	*Alu*	*Alu* Yb8	Scaffold3717-1643730-1644047	+	chr2:239883393-239883393	gaaaaaggactaata	329	15		-		-	-	GGATCCTCATCTGTTACCCT	CCTTTACATCACATCCATGC			
20	SINE	*Alu*	*Alu* Yb8	Scaffold4019-3293963-3294277	C	chr4:115550925-115550925	GCTTTCTT	321	8		Homo sapiens UDP glycosyltransferase 8 (UGT8)	UGT8	NM_001128174	601291	TTCTCTGAGGGAGTTCCAGC	TATAAACAGGGGCAGTGCTG			
21	SINE	*Alu*	*Alu* Yb8	Scaffold4319-2904259-2904575	+	chr22:37925124-37925124	GTGAGCCGAGATTGCGCCACT	598	31		-		-	-	CAGAGAGCAGGATAGGACCC	CCCAGCGACTCAGTTCCCTA			
22	SINE	*Alu*	*Alu* Yb8	Scaffold4319-5407141-5407448	C	chr22:40403459-40403459	cctcctccttcttt	315	14		Homo sapiens family with sequence similarity 83, member F (FAM83F)	FAM83F	NM_138435	-	AGAGACCAGGGATGGAGG	ATGCCAGTAGAGTGGGAACC			
23	SINE	*Alu*	*Alu* Yb8	Scaffold6231-154264-154590	C	chr12:9871146-9871146	CCAAAATTCATATTTT	338	16		Homo sapiens C-type lectin-like 1 (CLECL1)	CLECL1	NM_001253750	607467	GCTACATTTGTGAGTTTGTCC	AGAACAAGGAGGGACATTC			
24	SINE	*Alu*	*Alu* Yb8	Scaffold7584-171256-171536	C	chr14:65015035-65015035	GAGTTTCT	319	8		-		-	-	CCCTCACAGGACACTTGGAG	ATTTCGCATGTGATAGAGGG	esv3873283	ALU_umary_ALU_10368	
25	SINE	*Alu*	*Alu* Yb8	Scaffold7847-5239238-5239518	C	chr12:97496294-97496294	CTACATTTGTTTTT	344	14		-		-	-	CAGTCCTTTATCAGGTATGCC	CAGTAGATGATTTTGGTGCC			
26	SINE	*Alu*	*Alu* Yb8	Scaffold8110-616558-616804	+	chr3:7133324-7133324	CCTTTTCAACAAATATATTTGG	327	77		Homo sapiens glutamate receptor, metabotropic 7 (GRM7)	GRM7	NM_000844	604101	TTGGATTTTCACTCCCTTCC	CTGTCAAAAACGCTCATTCC			
27	SINE	*Alu*	*Alu* Yb8	Scaffold8133-1077171-1077499	+	chr18:58774980-58774980	AAAAAGACAACTATA	341	15		-		-	-	AACACTAACAAAGCACCAAGC	GAGAATTTCCCCCTCAGGAA			
28	SINE	*Alu*	*Alu* Yb8	Scaffold8587-799131-799434	+	chr11:19962993-19962993	AATAAATAGCATGAGGTT	347	18		Homo sapiens neuron navigator 2 (NAV2)	NAV2	NM_001111018	607026	ATCAAATGGGGTTGGTCGC	CAGAGGCTGAAAGATGAGTCC			
29	SINE	*Alu*	*Alu* Yb8	Scaffold8739-1323469-1323719	+	chr14:54816727-54816727	CCTTCCCGAATAAATTTTGCGT	311	55		-		-	-	AGAACGGCAGGAAGGAATCC	CCAAAAAAGTGGGGAGATGG			
30	SINE	*Alu*	*Alu* Yb8	Scaffold9165-103041-103345	+	chr16:53874969-53874969	AAGAAGTGCCT	311	11		Homo sapiens fat mass and obesity associated (FTO)	FTO	NM_001080432	610966	CGTGAGTTCTTTTCTGGTCC	GACATGACTGATTGGCTGG			
31	SINE	*Alu*	*Alu* Yb8	Scaffold9672-2052388-2052664	+	chr3:38399700-38399700	AAAGTTTGGT	285	10		Homo sapiens xylulokinase homolog (H. influenzae) (XYLB)	XYLB	NM_005108	604049	GCTATTTGTGCTTTGGTGC	TCTAGTGCTGGTGCGTTG	esv3829993	ALU_umary_ALU_2298	
32	SINE	*Alu*	*Alu* Yb8	Scaffold11674-6584416-6584733	+	chr6:111107853-111107853	AGAAATGTGTT	335	11		Homo sapiens cyclin-dependent kinase 19 (CDK19)	CDK19	NM_001300964	614720	GCTTGATGGCTTCTGTGGC	TAATGAAGCAAGCCCCAGG			
33	SINE	*Alu*	*Alu* Yb8	Scaffold12391-2396714-2396998	C	chr21:39005523-39005523	ACGAGGTAATTTTT	324	14		Homo sapiens potassium channel, inwardly rectifying subfamily J, member 6 (K	KCNJ6	NM_002240	600877	GCCTATTTCCCGTGAATT	ACCAGAGTTTGTGGCTGC			
34	SINE	*Alu*	*Alu* Yb8	Scaffold13646-191471-191790	C	chr22:17705290-17705290	AACAAGTGCTAATAA	335	15		-		-	-	CCAACAGTACCAGTGAGAGC	GGAATGTCTGTGGCTCTAGG			
35	SINE	*Alu*	*Alu* Yb8	Scaffold14277-2749392-2749507	C	chr4:103134338-103134338	GTAAATTTGAACAATTTATAAT	317	203		-		-	-	GCTTCTCTTCCAAATTGCCC	GCTTCCTCCTTCCTTGGTGC			
36	SINE	*Alu*	*Alu* Yb8	Scaffold15242-961087-961414	C	chr16:83133229-83133229	CTACATATTACTTCT	341	15		Homo sapiens cadherin 13 (CDH13)	CDH13	NM_001220489	601364	AGTGGAGAATCAAGGGAAGG	GGTGCTTTGAATGAACCAAG			
37	SINE	*Alu*	*Alu* Yb8	Scaffold15563-4652654-4652952	+	chr3:180485096-180485096	AAAACTGTACTCAC	309	14		Homo sapiens uncharacterized LOC101928882 (LOC101928882)	LOC101928882	NR_109986	-	AATCCAGAGTGCAGAGTGGG	GTGAAGGTGGCGATCAGC			
38	SINE	*Alu*	*Alu* Yb8	Scaffold15791-1653676-1653968	+	chr5:72333142-72333142	AAAAGTATATGTGT	303	14		Homo sapiens FCH domain only 2 (FCHO2)	FCHO2	NM_001146032	613438	TAGCATCAGCAGTTATGAGC	CCTATGTTGAGGTTTCATGC			
39	SINE	*Alu*	*Alu* Yb8	Scaffold16598-6754296-6754623	C	chr9:74545779-74545779	AGATATCTTTAAGTTT	341	16		Homo sapiens chromosome 9 open reading frame 85 (C9orf85)	C9orf85	NM_182505	-	CTGTACTTTGGTCAGGTGTCC	TAATATGACCAGAGCGAGGC			
40	SINE	*Alu*	*Alu* Yb8	Scaffold15239-333019-333335	C	chr1:157364488-157364488	CTCATGCATGG	327	11		-		-	-	GAGCTACCACAGCACTTGGC	CCAGAGAGGCAGATATGTGACC			
41	SINE	*Alu*	*Alu* Yb8	Scaffold8772-3828102-3828352	+	chr10:68217408-68217408	-	228	0	30	Homo sapiens catenin (cadherin-associated protein), alpha 3 (CTNNA3)	CTNNA3	NM_013266	607667	GATTGTACCACCCCACTCC	GGAGAATGGAAAGTTGTTGC	esv3860969	ALU_umary_ALU_8046	
42	SINE	*Alu*	*Alu* Yb8	Scaffold16904-571241-571523	+	chr1:108143467-108143467	AATAGTTCAG	290	10		Homo sapiens vav 3 guanine nucleotide exchange factor (VAV3)	VAV3	NM_001079874	605541	CAATGCCACTATTAGAGACACG	CCTTATCTCACACTGACACAGC			
43	SINE	*Alu*	*Alu* Yb8	Scaffold12674-678892-679193	C	chr11:60294387-60294387	tgccccactaatattttat	315	19		Homo sapiens membrane-spanning 4-domains, subfamily A, member 13 (MS4	MS4A13	NM_001100909	-	ATCCTTCACCTGTAATGACG	TGGACAAGCAGTAAGTGACC			
44	SINE	*Alu*	*Alu* Yb8	Scaffold23-142365-142683	+	chr15:27766781-27766781	TTTTGGG	325	7		Homo sapiens gamma-aminobutyric acid (GABA) A receptor, gamma 3 (GABI	GABRG3	NM_033223	600233	TCTTGGCATCTGGTCTACC	ACCATTCAGACATACATTCCC			
***Alu* Yb9**																			
1	SINE	*Alu*	*Alu* Yb9	Scaffold2988-5009447-5009660	C	chr9:120391684-120391684	AGGAGACTACTTTC	263	14		-		-	-	CACTGGGTGAGCTTGATCTG	GAGACTTCACCTTGGGGTCC			
2	SINE	*Alu*	*Alu* Yb9	Scaffold3569-403792-403941	+	chr1:79583075-79583075	TAAAGGACCATCACATTCTAC	327	177		-		-	-	GATCTTATCAAGTCCTGTAGCC	GGTTCATGAGTGCTTGTGATG			
3	SINE	*Alu*	*Alu* Yb9	Scaffold4525-352704-352782	C	chr7:96543282-96543282	-	139	0	3270	-		-	-	GAACTATCTCCTTTCAGGGCT	GCATCTGGTGAGCACTTTCT	esv3850524	ALU_umary_ALU_6225	
4	SINE	*Alu*	*Alu* Yb9	Scaffold4578-103530-103671	C	chr8:87080544-87080544	CAGTGAATGATTCTTTT	233	17		Homo sapiens protein serine kinase H2 (PSKH2)	PSKH2	NM_033126	-	GAGAGTGGAGGGGTAAAAGTG	ACAGCCTTGCTCGTGAGTCC			
5	SINE	*Alu*	*Alu* Yb9	Scaffold5649-873123-873336	C	chr13:58621051-58621051	AAAATGAATAATATT	235	15		-		-	-	ACTCCTACTATGCACCAGGC	GGCTGACAGGGATTAAGTGTG			
6	SINE	*Alu*	*Alu* Yb9	Scaffold5671-281592-281743	+	chr3:6104036-6104036	aaagattccatcct	170	14		-		-	-	TTCCAAATGGGACTGGTGTC	CGAGGGGGAAGTTCAGTTG			
7	SINE	*Alu*	*Alu* Yb9	Scaffold6246-1108733-1108981	+	chr1:80300084-80300084	CATTCCATGTGCTTATAAAACA	280	29		-		-	-	GCAGCAAAACCAAGCACAGG	CTCCACCATCTGGAGTTCTG			
8	SINE	*Alu*	*Alu* Yb9	Scaffold6756-4463130-4463466	+	chr10:26852965-26852965	AAAAAgAAAAAATA	338	14		Homo sapiens amyloid beta (A4) precursor protein-binding, family B, member 1 interacting	APBB1IP	NM_019043	609036	TTCCCAAGCTGCCTGTTCAG	TCTGCTTAGTGGCCTCTTACC			
9	SINE	*Alu*	*Alu* Yb9	Scaffold8313-376550-376872	C	chr14:52643753-52643753	CCTGGAGTTTCTTT	332	14		-		-	-	CATGGAGCAGTCCTGTAGGC	GCTAGTCATGAACAAGAACCC			
10	SINE	*Alu*	*Alu* Yb9	Scaffold10990-1181919-1182079	C	chr3:113047144-113047144	CAATCTCTTTCT	388	12		-		-	-	GCCCTTCTTGCTTTACTGTG	CCAGGACTTGGGATACTTCG			
11	SINE	*Alu*	*Alu* Yb9	Scaffold11674-6424086-6424404	+	chr6:110948424-110948424	AAAAATACTTTTCAG	332	15		Homo sapiens cyclin-dependent kinase 19 (CDK19)	CDK19	NM_001300964	614720	CAGAAAGCAACCTTCATGGG	GCACACACCCATAGTCTGAAC			
12	SINE	*Alu*	*Alu* Yb9	Scaffold15026-183472-183802	C	chr13:19606159-19606159	TGAACATTTC	341	10		-		-	-	CCACTGAGCAAGTTGAGA	ATGGCCTAAACTACGAATG	esv3869558	ALU_umary_ALU_9593	
13	SINE	*Alu*	*Alu* Yb9	Scaffold16053-1348011-1348325	C	chr10:69995891-69995891	CAAAATTGCTAATTCT	326	16		-		-	-	GGCCCGCATCTATCATTTACC	ACCAGTCACTTCCCAGTCAG			
14	SINE	*Alu*	*Alu* Yb9	Scaffold5495-121380-121627	C	chrX:3599704-3599704	-	242	0	0	Homo sapiens protein kinase, X-linked (PRKX)	PRKX	NM_005044	300083	AATGTCTTGAACCAGGGAGC	GTGATGATAAACACGGTGGC	esv3888111	ALU_umary_ALU_12549	
***Alu* Yc3**																			
1	SINE	*Alu*	*Alu* Yc3	Scaffold22-929540-929740	C	chr1:85620124-85620124	CCAGACAGTTATT	514	13		-		-	-	CTCAACCTTGTTTCTTTAGGG	TCTGTACTTGGTGATGGTGC	esv3819907	ALU_umary_ALU_319	*Alu* recombinant in hg19
2	SINE	*Alu*	*Alu* Yc3	Scaffold2647-2656613-2656726	C	chr15:95166018-95166018	CTTGAAATAACATCTCATGATC	148	36		-		-	-	CCAACCTCTTTTCTTTCCC	ATAGTGGATTGGCTGAATGC			
3	SINE	*Alu*	*Alu* Yc3	Scaffold6431-6940126-6940296	C	chr10:89815963-89815963	AGTATCAAGTCATTCT	185	16		-		-	-	ACATCAGGAGACATGAGGTTG	GCTCACAGTAAGAAGTCGGG			
4	SINE	*Alu*	*Alu* Yc3	Scaffold9444-548694-548826	+	chr9:128319942-128319942	ccacacaagg	140	10		Homo sapiens mitogen-activated protein kinase associated protein 1 (MAPKA	MAPKAP1	NM_001006621	610558	TCTGCCTTCCCCCTCCTT	ATGGGTTGTAGTGGAAGCCG	esv3858923	ALU_umary_ALU_7707	
***Alu* Yd8**																			
1	SINE	*Alu*	*Alu* Yd8	Scaffold490-519820-520051	+	chr1:170603447-170603447	AATA	237	4		-		-	-	CGTGGTATGGCTCTTTCAC	TGCTTCATTGAGAGTCTGCT	esv3821175	ALU_umary_ALU_585	
2	SINE	*Alu*	*Alu* Yd8	Scaffold3702-1178759-1179083	+	chr7:47552077-47552077	TAAAATATTTAAG	337	13		Homo sapiens tensin 3 (TNS3)	TNS3	NM_022748	606825	CAACCCAAGTTCTCCAGGT	CTGTCACTTGAGTTCTGGTG			
3	SINE	*Alu*	*Alu* Yd8	Scaffold16936-3927859-3928082	C	chr20:8670594-8670594	-	224	0	9	Homo sapiens phospholipase C, beta 1 (phosphoinositide-specific) (PLCB1	PLCB1	NM_182734	607120	ATGAAGAAGGGTGGTGAGAG	AACCCCCTGCTGCCTAAC			
***Alu* Yg6**																			
1	SINE	*Alu*	*Alu* Yg6	Scaffold577-15815-16111	+	chr6:57510291-57510291	GATATAATATTTAAGG	312	16		Homo sapiens primase, DNA, polypeptide 2 (58kDa) (PRIM2)	PRIM2	NM_000947	176636	ACCTCGCTTCAAGTTGTCAG	CTGGAAACCATCATTCTCAG			
2	SINE	*Alu*	*Alu* Yg6	Scaffold4172-702091-702405	C	chr4:77023808-77023808	AATATTAACTTTT	383	13		Homo sapiens ADP-ribosyltransferase 3 (ART3), transcript variant 3	ART3	NM_001130017	603086	GCTGAGCACAGAAGTTTGGC	GGCTAGTGACCCAGAATCTCTC			
3	SINE	*Alu*	*Alu* Yg6	Scaffold11661-935338-935394	+	chr9:135759089-135759089	-	138	0	1	Homo sapiens chromosome 9 open reading frame 9 (C9orf9)	C9orf9	NM_018956	-	AGCACCTAAATCACAGCAGG	CTTTTGTGAGAGGGGACTGG			
4	SINE	*Alu*	*Alu* Yg6	Scaffold14554-625319-625621	+	chr1:204238455-204238455	AGAAACCTCATCTATTGTC	317	19		Homo sapiens pleckstrin homology domain containing, family A member 6 (PLEI	PLEKHA6	NM_014935	607771	ACATTCCACCCCTTTGGGC	CTGTGGCAACCTCATCTGTA			
***Alu* Yh9**																			
1	SINE	*Alu*	*Alu* Yh9	Scaffold9875-2429-2475	C	chr1:196781796-196781796	AAGTATTTACATTTTTT	135	17		-		-	-	TGTCAACCACTCTCAAGG	TGAGAATACAAAAATGGGC			
***Alu* Yk12**																			
1	SINE	*Alu*	*Alu* Yk12	Scaffold10546	C	chr13:86362565-86362565	CATTGTTGTCTT	111	12		-		-	-	CCGTCTACTAAAGTGGCTG	GCAAAGAAGGTATATGTATGGC			
**L1HS**																			
1	LINE	L1	L1HS	Scaffold8776-3149934-3150755	C	chr10:110458460-110458460	CTTT	838	4		-		-	-	GAAGTGATTCCCTCCTTTAGTTC	CCTGGAGAAAGAGGAGAATCTG			
2	LINE	L1	L1HS	Scaffold10609-167777-169006	C,C,+	chr21:29074161-29074161	GATTTACATTTCTT	2745	14		-		-	-	CCCATTATCGAACTTACTTGC	CTGAAGAGCCTATACTCCG			
3	LINE	L1	L1HS	Scaffold12895-1876184-1877460	C	chr6:145500927-145500927	ATTCAACATTCTT	1310	13		-		-	-	CAATAGCCGAATCAAGCAACC	CATAGTGTCTCTGGTCTCTAC			
4	LINE	L1	L1HS	Scaffold13660-1094413-1095879	C	chr1:42488951-42488951	AACATTTCTT	1506	10		Homo sapiens human immunodeficiency virus type I enhancer binding protein 3 (H	HIVEP3	NR_038261	606649	GAAGGTAGGATAGGGTGAGC	CTTCTCCTACTCTGGGCTTG			
**LTR5HS**																			
1	LTR	ERVK	LTR5HS	Scaffold1239-174695-175663	+	chr6:161270904-161270904	CCCAGAGAAAAGA	998	13		-		-	-	ACCCAGATGAAAGGTGACC	CTGGTTTCACCATTGTCTT	esv3847467	SVA_umary_SVA_320	
2	LTR	ERVK	LTR5HS	Scaffold1807-1181863-1182787	C	chr15:63374600-63374600	AGAAAAAAGG	930	10		-		-	-	CCTGGGTGACAGTTGCTCTG	TGAGATGGTGGTGGTTGTGG	esv3875382	SVA_umary_SVA_637	
3	LTR	ERVK	LTR5HS	Scaffold7830-16787-17000	C	chr19:29855780-29855780	TTGTCACATCTCCATCTCTCTC	383	169		Homo sapiens uncharacterized LOC284395 (LOC284395)	LOC284395	NR_040029	-	CACACCACATTTCACCTGGC	GACATTCAGCTTATCTTGGG	esv3883553	SVA_umary_SVA_748	

4	LTR	ERVK	LTR5HS	Scaffold13397-1849507-1849652	C,+	chr10:101016124-101016124	TGTGTAGGGG	230	10	-		-	-	CAAAGTTGGGCTTATCCAGG	TCTTGCTGATTGCACTGTCC	
										**HERVK**						
1	LTR	ERVK	HERVK	Scaffold6057-11637-11951	+	chr8:146252361-146252361	GTCAA	684	5	-		-	-	AGAGATCAGACTGTTACTG	GCAGTGCATTATGCCTGTG	
2	LTR	ERVK	HERVK14C	Scaffold13916	+	chrY:59139339-59139339	GTTTT	2725	5	-		-	-	GCTTCCTCAAAGAATCCAGC	GCATCGAAGGCTACCCAG	LTR recombinant in hg19
										**SVA_F**						
1	NE Retropos	SVA	SVA_F	SVA_F-Scaffold8597-396226-396311	C	chr9:33130548-33130548	TCAATATCCTT	1675	11	Homo sapiens UDP-Gal:betaGlcNAc beta 1,4- galactosyltransferase, polypeptide 1 (I	B4GALT1	NM_001497	137060	GCCGTTGAAGTTGGAATGC	CCATTTGGCTTATGCCTCTG	High GC
2	NE Retropos	SVA	SVA_F	SVA_F-Scaffold15806-1280400-128086	C	chr3:12701175-12701175	AGAACACTTTCTTTC	600	15	Homo sapiens Raf-1 proto-oncogene, serine/threonine kinase (RAF1)	RAF1	NM_002880	164760	GTAATGGGCAAACCAGAACC	CCTCTCTCCTCAGCCTACC	High GC

	Element	Gene
**Alu genic**	76	74
**L1 genic**	1	1
**LTR genic**	1	1
**SVA genic**	2	2
**Total**	80	78

Table S7. List of validated novel TASV deletions in the KPGP9 genome

NAHR-ARMDs

NO	Class	Scaffold#	Orientation	Position range (Hg19)	Subfamily		Homologous Sequences	(bp)	Deletion Size	Gene symbol	Refseq	OMIM	Forward Primer	Reverse Primer	Variation region accession (dbVar)	Variation region ID
1	NAHR	Scaffold809 654223-656521	+	chr8:99465143-99465470	AluSg	AluSx1	CGTGGT	6	328		–	–	TCTATACTGGAGTCTGTTGG	TAGCTTCAGTTACATTCACG		
2	NAHR	Scaffold3189 441568-443877	+	chr11:130189307-130189484	AluY	AluSc8	GGAGGCTGAGGCAGG	15	178		–	–	TCAATCAATTCTGCCTGTGC	GCCCTTTCACTGTCTTGAGC		
3	NAHR	Scaffold16047 983136-985437	+	chr11:70445554-70445740	AluYe5	AluSg4	–	-	184	SHANK2	NM_012309	603290	GACATCAGTGAAACTACCTAG	GCATTGACCCAGAATAGACC		
4	NAHR	Scaffold15799 363849-364156	+	chr1:147124959-147125264	AluSx4	AluY	CATTCCAACCTGGGTGACA	74	306	ACP6	NM_016361	611471	GACTGTGTACCCTAAAGGC	CTATGTCTCTAAAGCTCTTCC		
5	NAHR	Scaffold1019 1951464-1953753	+	chr5:158420019-158420136	AluJo	AluSz	CCATCTCTACTAAAATACA	36	118	EBF1	NM_001290360	164343	CCAGATACCAACCAGAGACC	TCTGGGAACAGCATCATGAG		
6	NAHR	Scaffold10740 3485204-3487498	+	chr10:122226657-122228805	AluY	AluY	GAGAT	5	2149	PLPP4	NM_001030059	–	AGCAATACTTGAACAGGTGC	CAAAGATGGCATGTGTTAGG	esv3862204	SI_BD_10957
7	NAHR	Scaffold1016 4856679-4858976	+	chr9:140974168-140974299	AluSx1	AluSx3	CTCCAGCCTGGGCAACAGA	23	132	CACNA1B	NM_000718	601012	CAAGGGCATTGAGGAGGCAG	CTTGGAGTCAGGGCCACAGG		
8	NAHR	Scaffold4058 474463-476587	+	chr8:92400441-92400556	AluYa8	AluYc	CGGGAGGCGGAGCTTGCAG	27	116	SLC26A7	NM_001282357	608479	GAGTGATGTGGCTTTCAGAGC	CCATTGCTATCCTTGACACTC		
9	NAHR	Scaffold14588 5465171-5467475	+	chr10:10809809-10809978	AluY	AluYc	TTAGCC	6	170		–	–	CACTTCATCTTCAGACAACCC	TGCTTGTGATGCTGAGATGC		
10	NAHR	Scaffold528 3222812-3223115	+	chr20:33477174-33477306	AluSx3	AluYj4	ATCCCAGCACTTTGGGAGG	31	133	ACSS2	NM_001242393	605832	CTCTTTCACTCCCCAGACCC	ATGTGCTGCTGCCTGGTGTG		
11	NAHR	Scaffold16040 198931-199238	+	chr16:74762703-74762930	AluSc8	AluSc	GTCTCAAAAAAAAAAAAAA	33	228	FA2H	NM_024306	611026	TGGAGCTGAAACCTGCAAAC	TTGTCTCCAAGGCAAGGATG		
12	NAHR	Scaffold16040 1171017-1171314	+	chr16:75723509-75723816	AluY	AluY	CGTCTCTACTAAAAATACA	39	308		–	–	TGAAGCACAGCAGGGAGTTG	GCCTTGCTAGAGCCTCCCAC		
13	NAHR	Scaffold4044 579296-579606	+	chr7:151057986-151058129	FLAM_C	AluJb	CCAGCACTTTG	11	144	NUB1	NM_016118	607981	TTGGAGGGTGATAGTGAAGC	CATGTGCTATGTTGCTAGTCG		
14	NAHR	Scaffold10901 250430-250739	+	chrX:44437439-44437717	AluSc	AluSg	GGCC	4	279		–	–	AGGCTGGTCTCGGAACTCC	CCACTGTTGAGACCTACTGC		
15	NAHR	Scaffold2051 3593-3886	+	chr18:14807619-14821544	AluSz6	AluSz6	CCGGAGCTGTGAGAATCAC	48	13698	ANKRD30B	NM_001145029	616565	CCAAGCTAATGGTAGAATGC	CACATACCTGCAACACGG		
16	NAHR	Scaffold8735 415612-415919	+	chr19:21514122-21514302	AluYa5	AluSx	–	-	181		–	–	CATCTCTCTTCGGTTCTGAGG	GGAAGGAGGGCAATAGGTGG		
17	NAHR	Scaffold12434 3240591-3240887	c	chr19:46942052-46943472	AluSx1	AluSx1	TAGCTGGGACTACAAGCGC	283	1421		–	–	GCTGTCACCTCCTCTCTTGC	GCAGAAGGAACAGTCAGTGC		
18	NAHR	Scaffold6455 96896-97177	+	chr12:9016842-9017868	AluSq2	AluSp	GCTCATGCCTGT	12	1027	A2ML1	NM_144670	610627	GGGCTTCTCTTCTACTCAGG	TCTCACACACCAAGTGGCTG	esv3866389	SI_BD_11943
19	NAHR	Scaffold1060 496749-497073	+	chr9:77524156-77524342	AluYb8	AluSz6	TTAGC	5	187		–	–	CACCCAGGACAGAGGAATGG	GAGAAGTTCCTCCTGATTCC	esv3857764	DEL_pindel_28601
20	NAHR	Scaffold14588 879917-880191	+	chr10:6251980-6252941	AluY	AluYc	GCCTGTAATCCCAGC	15	962	PFKFB3	NM_001145443	605319	GGGTAGAGCATTTGACTG	TGCCCAGCCAATACTTACC		
21	NAHR	Scaffold12649 2345765-2346044	+	chr1:19562891-19563201	AluSp	AluSz	CACCTGTAATCCCAGCACT	55	311	EMC1	NM_015047	–	GTATCCCACCGAGAATGCC	ATGACTCAGTGGGCTACCG		
22	NAHR	Scaffold8597 478573-478869	+	chr9:33213176-33214036	AluSx1	AluSx3	TGAGGCAGGAGAATC	15	861		–	–	CGAACAGCCTGGACAGAGC	CTGGAGACAGTCACCCATCC	esv3857349	EM_DL_DEL101120420
23	NAHR	Scaffold10228 4171017-4171314	+	chr2:220232686-220233190	AluSx	AluSc	ACTCCAGCCTGGGTGACAG	25	505		–	–	GTATCAGCTTGTCAACTCCC	TACACAGATCCTTGTCTCCG	esv3828244	BI_GS_DEL1_B5_P0471_256
24	NAHR	Scaffold5430 3029171-3029452	+	chr13:46005385-46005721	AluSq2	AluSp	CAACATGGTGAAACCC	16	337		–	–	GTAAGTAGCCATTTAGGTTGC	TAGGGCTGTAAACACCACC		
25	NAHR	Scaffold5954 2043283-2043584	+	chr19:2909503-2910825	AluY	AluY	GCGGATCACGACGTCA	16	1323	ZNF57	NM_173480	–	CAATACAGGCTGAGAAGTGG	CCCTTCAGTTATCACTCTCC	esv3882817	EM_DL_DEL10483187
26	NAHR	Scaffold5954 2874436-2874742	+	chr19:3711053-3711347	AluSx1	AluY	GTGGTGG	7	295	TJP3	NM_001267560	612689	ACCATCTACGCTAAGGTGC	GTATGGGTACTTGTTCTTCC	esv3882842	SI_BD_16171
27	NAHR	Scaffold5954 4053811-4054119	+	chr19:4885651-4886214	AluSc	AluY	AGGCGGAGGTTGCAGTGAG	26	564		–	–	GTTGGTCCCACCCTATGG	GTGGGCAGATGTCTTTGAGG		
28	NAHR	Scaffold1016 4524114-4524424	+	chr9:140663165-140663300	AluSp	AluYa5	CGTCTCTACTAAAAATACA	34	136	EHMT1	NM_024757	607001	CAAGATACAAGACAGGACC	CTGTGATGTTCAGACCAGC		
29	NAHR	Scaffold9291 345963-346276	+	chr18:48215371-48215507	AluSz6	AluSp	GGGCGGATCACATGAAGTCA	80	137	MAPK4	NM_001292040	176949	GATACAACGGACATCAGGG	ACGCCTGGCTGGACAACC		
30	NAHR	Scaffold4319 2114943-2115248	+	chr22:37143175-37147900	AluSx1	AluSx1	AGCTACT	7	4726		–	–	TGAGGGCAAGAGACCAGG	GTCAGACAGCACAGGTTCC	esv3887464	YL_CN_GIH_3320
31	NAHR	Scaffold7862 241399-241694	+	chr7:145501456-145501763	AluSx4	AluSg	AGGTCAGGAGTTCGAGACC	153	308		–	–	CTTTCTTGTGTCAACTGG	TCTGGTTCATGGACTTGC		
32	NAHR	Scaffold1058 790080-790212	+	chr17:1912703-1913566	AluSg	AluSx1	ACATGGTGAA	10	864	RTN4RL1	NM_178568	610461	GCTTCTCTCTCCTAGCAGG	ACAACGATGAACTGCCTGCC	esv3878676	BI_GS_DEL1_B2_P2513_505
33	NAHR	Scaffold10990 1044469-1044767	+	chr3:112924192-112924328	AluY	AluSx	TTGCAGTGAGCC	12	137		–	–	CTCAGCCTAACAAGTGAAGG	CAACTCAGGCATTCCTACTGG		
34	NAHR	Scaffold15785 392507-392805	+	chr7:6489397-6490113	AluSc	AluSx	CACTCTAGCCTGGCAACAG	79	717		–	–	GCTCATCACCCCACTGTGC	CCTGACCAACTCACAGCC		
35	NAHR	Scaffold259 1176504-1176805	+	chr1:110187169-110191400	AluY	AluY	CCATCCTGGCTAACACGGT	48	4232		–	–	CCTCTTGAATAGCAACTCTCC	GATTGAACTCATTGCCTCCC	esv3820394	EM_DL_DEL04008
36	NAHR	Scaffold13926 246583-246875	+	chr12:66710707-66710884	AluY	AluSx1	CAGGAGAAT	9	178	HELB	NM_033647	614539	GGAGTTATCCTCTGTCAGC	AGCACCTTCTATCTCCAGG		
37	NAHR	Scaffold9881 149226-149541	+	chr19:7203976-7204135	AluSx1	AluSq2	GAGGCAGAGATTGCAGTGA	24	160	INSR	NM_000208	147670	CAGCAGCCAGCCTGATAGG	ATGGCGGTCTTGAATGTGC		
38	NAHR	Scaffold13294 428732-429020	+	chr1:224640308-224640648	AluY	AluSp	CCCGTCTCTACTAAAAATA	29	341		–	–	ATGGTTGGCGTTCATAGTGGC	TGTAGTCCCAGCCATGAAGG		
39	NAHR	Scaffold5081 840701-841027	+	chr5:8027067-8027359	AluYk3	AluY	GCCACTGCACTCCAGCCTG	24	293		–	–	GCTAATGGAAATACATGAC	ATAACGCATGAACCAGGGG		
40	NAHR	Scaffold4023 421910-422195	+	chr18:18960854-18960995	AluSg4	AluYk3	–	-	142	GREB1L	NM_001142966	–	GGAGTCTTAGGAGCCTGGC	GTCATTCTGCTCCATCACCC		
41	NAHR	Scaffold4023 5375949-5376243	c	chr18:23827927-23828575	AluJb	AluJb	CTCACTATGCCCAGCTAAT	282	649	TAF4B	NM_001293725	601689	ATCTCAGTGCCTACCTGTCC	GTTTCAGCACTCAATTCACCG		
42	NAHR	Scaffold9447 740344-740637	+	chr4:187850751-187850884	AluY	AluYm1	CCGAGGCGGGCGGATCACG	56	134		–	–	CTCCCTGTCTGACTGAGCC	GCAAAGCCGTAACTGGAAGG		
43	NAHR	Scaffold16034 9256984-9257264	+	chr11:107733068-107733263	AluSg	AluSx3	TCGTGCCATTGCACTCCAG	74	196		–	–	GGAGAGATTATCCTGTGGGC	GGAGCCTCTGAGCTTCTGG		
44	NAHR	Scaffold16034 2009358-2009671	+	chr16:77859769-77859951	AluY	AluYa5	TGCACTCCAGCCTGGGCGA	31	183	VAT1L	NM_020927	–	CACATGGACAGCAAAGCCC	CACACCTGACAGATGACGC		
45	NAHR	Scaffold7842 100452-100732	+	chr7:97655798-97657598	AluSx3	AluY	AAA	3	1801		–	–	GTTGGAGACTGTAGAGAGCTATG	CTGTGGCAACTCACAATGTAG	esv3850555	SI_BD_8024
46	NAHR	Scaffold5150 9152828-9153128	+	chr8:112406330-112406498	AluYc3	AluSx3	CAGGAGAAT	9	169		–	–	CTAACCGCAGTCACACACAC	GACTGACAGATATGGCAACCC		
47	NAHR	Scaffold2632 4512664-4512809	+	chr13:57669140-57669284	AluSx3	AluSq2	TGGCCAA	7	145		–	–	GGAATTATGGGTTGGGTGCG	AACCTCCTTCCACAGTTCC		
48	NAHR	Scaffold490 141155-141455	+	chr1:170224174-170224403	AluY	AluY	AAAAATTAGCCG	12	230		–	–	GGTGGAAGGATTGCCTGACC	CTCCACCTACAGCACCACC		
49	NAHR	Scaffold11012 428385-428560	c	chr16:502549-502868	AluY	AluY	TTCACCATGTTAGCCAGGA	188	320	RAB11FIP3	NM_014700	608738	GAAGGACTTAGAATTGGGTGC	CCTGGTTTAGAGGGGAAGG		
50	NAHR	Scaffold12159 1028338-1028449	+	chr5:85988501-85988638	AluYc	AluYc	CTCGGGAGGCGGAGCTTGC	78	138		–	–	GGCAACAGAGCGATACTGC	GTCATCCATGTAGCCAGCC		
51	NAHR	Scaffold1763 2657355-2657665	+	chr12:112063548-112063675	AluSq10	AluSx3	cgggaggctgaggcagGAG	26	128		–	–	CAACAGAGTGAGACCCCG	GAAGTCATTGGAAGGAGAGC		

NHEJ-ARMDs

NO	Class	Scaffold#	Orientation	Position (Hg19)			Microhomology	(bp)	Deletion Size		Refseq	OMIM	Forward Primer	Reverse Primer		
1	NHEJ	Scaffold15803 87485-89866	+	chrX:150409556-150409654	AluYm1	AluSp	–	0	99		–	–	TGAGGTCCACTGAGATGTGC	TCTGCTCATGTCCTTTGTCC		
2	NHEJ	Scaffold16357 164440-166710	+	chr20:44465124-44465202	AluY	FRAM	–	0	79	SNX21	NM_152897	–	ACTACTCCAATTTCAGCAGG	CTTCCAGTGTATGAACCCAG		
3	NHEJ	Scaffold11358 1342234-1342528	+	chr4:38883946-38884049	AluYb8	AluSp	A	1	104	FAM114A1	NR_033290	–	GAGCTGGTTGGTAACAGG	CCTCAAAGTCTATTCTGCC		
4	NHEJ	Scaffold16618 102066-102379	+	chr18:45295091-45295228	AluY	AluSx	–	0	138		–	–	CACATACCTATCAGAATGGC	ATGAGACCTGAGCAGAGG		
5	NHEJ	Scaffold1016 4101021-4101251	c	chr9:140253510-140255974	AluSc8	AluY	–	0	2465	EXD3	NM_017820	–	GTCTGTAGTGATGTCAATGC	GCAAAGAGGCAATAGATGC	esv3859224	UW_VH_816
6	NHEJ	Scaffold1058 516096-516304	+	chr17:1654676-1655407	AluSq	AluSq2	GT	2	732	SERPINF2	NM_001165921	613168	GCGTACTCTTCCTTCATCC	CACTGCTCCTCCACTTCC	esv3878669	BI_GS_DEL1_B4_P2513_123
7	NHEJ	Scaffold10752 157469-157754	+	chr20:34514051-34514186	AluSc8	AluYi6_4	–	0	136	PHF20	NM_016436	610335	GCTTATGGTTATTCTTAGG	ACTGGGAGTCACATAGGTCC		
8	NHEJ	Scaffold9444 5609992-5610293	c	chr9:133452241-133452366	AluSq2	AluSx1	gTTG	4	126		–	–	CTTGCTGTGTTGTTGAAGTGC	ACGTAGTGGAGGCAACACC		
9	NHEJ	Scaffold14085 161832-162050	+	chr17:21756105-21756225	AluSp	FRAM	–	0	121		–	–	GGAGGGTGAATCAACTGAGG	CTCAGGCAGGAGACAGTGG		
10	NHEJ	Scaffold752 343330-343639	c	chrX:54124445-54124556	AluSq	AluY	GG	2	112	FAM120C	NM_001300788	300741	GAAGACATCCAGCAAGTG	GTCATTTACCCATCTGTGC		
11	NHEJ	Scaffold9667 6902633-6902936	+	chr5:118221122-118221245	AluSc8	AluSp	A	1	124	DTWD2	NM_001308081	–	CTCACCCACAGAACCTGAAC	GGTTTCTGTATGGGTCTGGC		
12	NHEJ	Scaffold16538 3186318-3186584	c	chr8:68310583-68310701	AluSq	AluSc	–	0	119		–	–	GTAGGAGGAATTGTGGTAGGC	AGGACCTCACACACTCACC		
13	NHEJ	Scaffold4023 2512543-2512833	+	chr18:21012662-21012873	AluSg	AluJb	G	1	212	TMEM241	NM_032933	615430	CTGAAGCTCTTGCCTCACC	CTGGCTGATGACGTGTTCC		
14	NHEJ	Scaffold16034 4091341-4091647	+	chr16:79922816-79923014	AluSp	AluY	CATGCCTGTAATCCCAGC	18	199		–	–	TGTCACAGGGGTTGAGCCC	CAAGCGTGATACACTCTCAGG		
15	NHEJ	Scaffold5150 9912351-9912634	+	chr8:113166420-113166543	AluSx1	AluSz	–	0	124		–	–	GTACAAATAACAGTGGCAGGC	ACTAGGGAGTAGAACGGTGG		

Total Deletion size	
	82,772
NAHR-ARMDs	
	42,909
NHEJ-ARMDs	
	7,313
NAHR-L1RMDs	
	3,177
NHEJ-L1RMDs	
	29,373
Total	
	82,772

16	NHEJ	Scaffold1772 874007-874289	+	chr14:90124229-90124344	AluSc8	AluSx1	A	1	116		–	–	ACGCCAGAGAGGAAGCAGG	CCTGACCTATGGAAACTACG		
17	NHEJ	Scaffold7830 453697-453797	c	chr19:30289452-30289557	AluSc	AluSz	CC	2	106		–	–	GACAGATTCCAAACATGATG	GTTGGGATTGTGACTCAGGC		
18	NHEJ	Scaffold6763 6877483-6877613	c	chr5:125974104-125974307	AluY	AluSc8	GT	2	204		–	–	GTGAGGATATGCCAAGAACC	GATGGATGGTTGCCTAAGG		
19	NHEJ	Scaffold11648 1901931-1902071	c	chrX:23146535-23146715	AluSq2	AluSq2	–	0	181	PTCHD1-AS	NR_073010	–	CACTGGTTATGGTTTGGTAGG	GTGCAGGTGAGAACTAAGC		
20	NHEJ	Scaffold8133 3994540-3994749	+	chr18:61657106-61657428	AluYa5	AluSx	–	0	323		–	–	CTGGTTTTTCTCTCTTCTCACC	GATTTGGTAGCCATATCACG		
21	NHEJ	Scaffold15812 6432609-6432774	+	chr11:120855362-120855543	AluSg	AluSz	–	0	182	GRIK4	NM_014619	600282	GCAAGTAGTTAGAGCAGTCC	CTGTAGCAACATCTCACACC		
22	NHEJ	Scaffold11677 279275-279504	c	chr14:61806272-61806395	AluSx	AluY	TC	2	124	PRKCH	NM_006255	605437	GCCAACAGAGGACTGAAAGG	CATCCTCTCCTGTCCTCC		
23	NHEJ	Scaffold1249 1478929-1479225	+	chr19:10352149-10352319	AluY	AluSp	aCTCC	5	171		–	–	TAGCCTGATGTGGCTGGG	CTCGACCTCCTGGGCTGACG		
24	NHEJ	Scaffold8858 53661-53821	+	chr3:195569217-195569932	AluSq2	AluSx1	G	1	716		–	–	CTAGAAATGTATGAAAGTGCC	CAGGTCAGCCTCCAAACCC	esv3833732	BI_GS_DEL1_B3_P0690_30
25	NHEJ	Scaffold1249 3562544-3562841	c	chr19:12423760-12423882	AluSg	AluY	GG	2	123		–	–	GTGGTATGCTAACACCTTCC	TTCTTCAACCTCTAACACAGG		
26	NHEJ	Scaffold12145 953342-953476	c	chr12:76482998-76483174	AluJb	AluSp	AA	2	177		–	–	GGATCTCTCTGTGTTATTGC	GAGAAATGTATGTAAGTTCCC		
							**NAHR-L1RMDs**									
**NO**	**Class**	**Scaffold#**	**Orientation**	**Position (Hg19)**			**Homologous Sequences**	**(bp)**	**Deletion Size**		**Refseq**	**OMIM**	**Forward Primer**	**Reverse Primer**		
1	NAHR	Scaffold12434-3117278-3117546	c	chr19:46817339-46818739	L1ME1	L1ME1	CACCCAAACTTGGAAACAG	27	1401	HIF3A	NM_152795	609976	CCCATCGTGATTTTCCCAC	CACGCAAAGATAGATGGTTT	esv3883991	SI_BD_16603
2	NAHR	Scaffold2335-1054021-1054119	+	chrX:150293941-150295716	L1PA8	L1PB4	GAA	3	1776		–	–	CAACATACCCAAGTCTGTGG	TAGGACACATACTGAAGGC	esv3890388	BI_GS_DEL1_B4_P3048_39
							**NHEJ-L1RMDs**									
**NO**	**Class**	**Scaffold#**	**Orientation**	**Position (Hg19)**			**Microhomology**	**(bp)**	**Deletion Size**		**Refseq**	**OMIM**	**Forward Primer**	**Reverse Primer**		
1	NHEJ	Scaffold11650-3508863-3509085	c	chr6:153958368-153961524	L1MEd	L1MA1	AAAT	4	3157		–	–	GAAGATGTATGAGGCTGTG	TTTTGACATGCGTATTAGCTTT	esv3847294	P_gs_CNV_6_153958312_153961510
2	NHEJ	Scaffold14809-2015734-2016063	+	chrX:134161923-134162057	L1HS	L1PB2	–	0	135		–	–	AATTTGGGGAAAGGACACC	TCGTACTCAAAGGTGAAAGCC	esv3890142	DEL_pindel_54641
3	NHEJ	Scaffold8772-3125693-3126283	c	chr10:67515124-67517406	L1MB5	L1MB5	AA	2	2283	LINC01515	NR_120647	–	TGGAGTCAGGACCACAGAG	GGGAATAACCTAAGTGAGC		
4	NHEJ	Scaffold11663-1227744-1228852	+	chrX:144422075-144424505	L1M1	L1M1	CA	2	2431		–	–	GAAGTAGAAGAAAGTCACAGAG	TCCCCTTCACCTTTTGCC	esv3890308	BI_GS_DEL1_B1_P3042_253
5	NHEJ	Scaffold8727-9925320-9925703	c	chr11:16996173-16996596	L1ME1	L1MC4a	AAAAACCTG	9	424	PLEKHA7	NM_175058	612686	TTTTTGGAGTTGTCTTCC	ATTTCGTCATTGGGTGCC	esv3863009	BI_GS_DEL1_B2_P1840_657
6	NHEJ	Scaffold16034-689412-690672	+	chr16:76539134-76544025	L1ME1	L1MB8	T	1	4892	CNTNAP4	NM_033401	610518	CATGTAATAAGTCAAGGAGAGG	GTAGAAGACGAGACAACTGC	esv3878049	UW_VH_1123
7	NHEJ	Scaffold9879-2588357-2588815	c	chr3:136021014-136026199	L1MC4	L1M4c	–	0	5186	PCCB	NM_000532	232050	GTTATTGTTTCCATTCTCCC	GAGAGTACACAGGGCATCC	esv3832215	YL_CN_LWK_752
8	NHEJ	Scaffold6778-231728-232541	c	chrX:30347766-30348598	L1MB7	L1MB7	–	0	833		–	–	CATCATCCAACAAAAAGCC	TCAAAGATTATTCCAAGAGG	esv3888549	BI_GS_DEL1_B4_P2928_61
9	NHEJ	Scaffold3188-533045-533766	+	chr3:82916665-82916837	L1PA2	L1M4c	AT	2	173				ATTTTAGGTGAGGACAGCC	CCTCTTTTTTTGTCTTGCTGG		
10	NHEJ	Scaffold8589-2772864-2772919	c	chr6:81283720-81293578	L2a	L1MB8	T	1	9859				GTCACTTCCCTTTGTTCCC	ACCCCTTTCTTTGACTGGG	esv3845520	BI_GS_DEL1_B4_P1148_45

Supplementary Table S8. Population allele frequency of 50 TASVs identified from 1000GP

NO	Type	Subfamily	Variation region accession (dbVar)	Variation region ID	AF	EAS_AF	EUR_AF	AFR_AF	AMR_AF	SAS_AF	EAS_HWE_chisq	EUR_HWE_chisq	AFR_HWE_chisq	AMR_HWE_chisq	SAS_HWE_chisq
1	*Alu* Insertion	*Alu* Ya5	esv3821086	ALU_umary_ALU_568	0.4301	0.5218	0.3698	0.3941	0.4107	0.4601	0.0000	0.0000	0.0000	0.0001	0.0000
5		*Alu* Ya5	esv3858405	ALU_umary_ALU_7615	0.2338	0.2976	0.1879	0.1233	0.1945	0.3926	0.0000	0.0172	0.7621	0.0506	0.0000
16		*Alu* Ya5	esv3875583	ALU_umary_ALU_10812	0.2322	0.2738	0.3171	0.1210	0.2464	0.2423	0.0019	0.0000	0.1155	0.0023	0.0571
42		*Alu* Ya5	esv3819552	ALU_umary_ALU_226	0.2804	0.4187	0.2565	0.2020	0.2608	0.2822	0.0000	0.0348	0.6330	0.0567	0.0003
51		*Alu* Ya5	esv3863454	ALU_umary_ALU_8493	0.0651	0.1190	0.0666	0.0106	0.0692	0.0787	0.0100	0.2779	0.9628	0.3837	0.1677
53		*Alu* Ya5	esv3824113	ALU_umary_ALU_1171	0.4539	0.3829	0.3966	0.5348	0.4683	0.4663	0.0000	0.0062	0.0000	0.0050	0.0538
71		*Alu* Ya5	esv3828594	ALU_umary_ALU_2051	0.3433	0.2361	0.3569	0.4410	0.2853	0.3487	0.0000	0.0000	0.0000	0.0000	0.0000
75		*Alu* Ya5	esv3846361	ALU_umary_ALU_5500	0.2921	0.4623	0.2515	0.1551	0.2608	0.3661	0.0000	0.0000	0.0134	0.0054	0.0000
77		*Alu* Ya5	esv3856661	ALU_umary_ALU_7291	0.2402	0.5258	0.1064	0.1687	0.3141	0.1278	0.0000	0.4510	0.9986	0.9982	0.9228
88		*Alu* Ya5	esv3886444	ALU_umary_ALU_12334	0.3792	0.5357	0.3787	0.2458	0.3876	0.3926	0.0000	0.0000	0.0250	0.0420	0.0000
1		*Alu* Yb8	esv3863278	ALU_umary_ALU_8456	0.3908	0.5496	0.4920	0.0809	0.4928	0.4693	0.0000	0.0000	0.4760	0.0000	0.0000
6		*Alu* Yb8	esv3827562	ALU_umary_ALU_1860	0.2817	0.4821	0.3787	0.0204	0.2637	0.3415	0.0000	0.0000	0.8662	0.0801	0.0001
9		*Alu* Yb8	esv3830096	ALU_umary_ALU_2319	0.2536	0.4683	0.2674	0.0242	0.3862	0.2342	0.0000	0.0030	0.8160	0.3112	0.9989
10		*Alu* Yb8	esv3835555	ALU_umary_ALU_3368	0.2570	0.3125	0.3519	0.1112	0.2594	0.2975	0.3263	0.0203	0.4586	0.7591	0.2880
24		*Alu* Yb8	esv3873283	ALU_umary_ALU_10368	0.3215	0.4315	0.3519	0.1914	0.3314	0.3456	0.0000	0.0000	0.0000	0.0000	0.0000
31		*Alu* Yb8	esv3829993	ALU_umary_ALU_2298	0.3341	0.3790	0.3559	0.2474	0.3386	0.3793	0.0000	0.0000	0.0000	0.0000	0.0000
41		*Alu* Yb8	esv3860969	ALU_umary_ALU_8046	0.0743	0.0794	0.0427	0.1271	0.0360	0.0573	0.1537	0.9961	0.0098	0.7848	0.8801
3		*Alu* Yb9	esv3850524	ALU_umary_ALU_6225	-	-	-	-	-	-	-	-	-	-	-
12		*Alu* Yb9	esv3869558	ALU_umary_ALU_9593	0.2208	0.2470	0.2087	0.2231	0.2839	0.1585	0.0000	0.0000	0.0000	0.0004	0.3484
14		*Alu* Yb9	esv3888111	ALU_umary_ALU_12549	-	-	-	-	-	-	-	-	-	-	-
1		*Alu* Yc3	esv3819907	ALU_umary_ALU_319	0.4832	0.5060	0.5040	0.4652	0.4611	0.4785	0.0000	0.0000	0.0000	0.0000	0.0000
4		*Alu* Yc3	esv3858923	ALU_umary_ALU_7707	0.0170	0.0407	0.0000	0.0000	0.0476	0.0112	0.6357	0.0000	0.0000	0.6489	0.9689
1		*Alu* Yd8	esv3821175	ALU_umary_ALU_585	0.2005	0.2143	0.2028	0.1460	0.1513	0.2924	0.0003	0.0022	0.0414	0.0457	0.0000
1	HERV Insertion	LTR5HS	esv3847467	SVA_umary_SVA_320	0.3421	0.4613	0.2992	0.3041	0.3069	0.3395	0.0000	0.0000	0.0000	0.0000	0.0000
2		LTR5HS	esv3875382	SVA_umary_SVA_637	0.3512	0.4633	0.3449	0.3200	0.2565	0.3517	0.0000	0.0000	0.0000	0.0000	0.0000
3		LTR5HS	esv3883553	SVA_umary_SVA_748	0.2147	0.3165	0.2147	0.2156	0.1614	0.1462	0.0000	0.0000	0.0000	0.0062	0.0008
6	NAHR-ARMD	AluY AluY	esv3862204	SI_BD_10957	0.7326	0.9117	0.8250	0.4228	0.8444	0.7924	0.8390	0.7605	0.8791	0.8084	0.1627
18		AluSq2 AluSp	esv3866389	SI_BD_11943	0.9261	1.0000	1.0000	0.7368	0.9683	1.0000	0.0000	0.0000	0.0013	0.0000	0.0000
19		AluYb8 AluSz6	esv3857764	DEL_pindel_28601	0.5004	0.5804	0.4920	0.4410	0.4683	0.5297	0.0000	0.0000	0.0000	0.0000	0.0000
22		AluSx1 AluSx3	esv3857349	EM_DL_DEL101120420	0.4736	0.6786	0.5318	0.2360	0.4928	0.5102	0.1798	0.8434	0.9261	0.5273	0.9727
23		AluSx AluSc	esv3828244	BI_GS_DEL1_B5_P0471_256	0.1160	0.2004	0.1064	0.0121	0.1499	0.1554	0.7450	0.9489	0.0133	0.8786	0.8230
25		AluY AluY	esv3882817	EM_DL_DEL10483187	0.7636	0.7173	0.8807	0.6467	0.8415	0.7935	0.3850	0.7365	0.9962	0.3265	0.0416
26		AluSx1 AluY	esv3882842	SI_BD_16171	0.7746	0.9931	0.6899	0.6399	0.7896	0.8078	0.9878	0.0005	0.0004	0.5001	0.0008
30		AluSx1 AluSx1	esv3887464	YL_CN_GIH_3320	0.5964	0.8562	0.7237	0.2315	0.7277	0.5982	0.9789	0.5925	0.9533	0.8285	1.0000
32		AluSg AluSx1	esv3878676	BI_GS_DEL1_B2_P2513_505	0.4237	0.5367	0.3151	0.3949	0.4063	0.4703	0.9266	0.0737	0.9455	0.1531	0.5723
35		AluY AluY	esv3820394	EM_DL_DEL04008	0.6042	0.6716	0.5895	0.5424	0.7161	0.5542	0.7616	0.0791	0.1090	0.9999	0.9231
45		AluSx3 AluY	esv3850555	SI_BD_8024	0.1206	0.1567	0.1302	0.0401	0.1470	0.1636	0.3359	0.2125	0.1470	0.3262	0.9996
5	NHEJ-ARMD	AluSc8 AluY	esv3859224	UW_VH_816	0.6847	0.8532	0.6561	0.4561	0.7075	0.8333	0.5372	0.5539	0.9273	0.8356	0.9908
6		AluSq AluSq2	esv3878669	BI_GS_DEL1_B4_P2513_123	0.2380	0.4812	0.2823	0.0310	0.2118	0.2403	0.2508	0.5343	0.8948	0.1075	0.8581
24		AluSq2 AluSx1	esv3833732	BI_GS_DEL1_B3_P0690_30	0.4439	0.7728	0.2753	0.2837	0.5086	0.4489	0.7467	0.6524	0.8303	0.0897	0.3015
1	NAHR-L1RAD	L1ME1 L1ME1	esv3883991	SI_BD_16603	0.1773	0.3194	0.1044	0.1021	0.2450	0.1595	0.7653	0.7795	0.9990	0.9432	0.9824
2		L1PA8 L1PB4	esv3890388	BI_GS_DEL1_B4_P3048_39	-	-	-	-	-	-	-	-	-	-	-
1	NHEJ-L1RAD	L1MEd L1MA1	esv3847294	DUP_gs_CNV_6_153958312_153961510	-	-	-	-	-	-	-	-	-	-	-
2		L1HS L1PB2	esv3890142	DEL_pindel_54641	-	-	-	-	-	-	-	-	-	-	-
4		L1M1 L1M1	esv3890308	BI_GS_DEL1_B1_P3042_253	-	-	-	-	-	-	-	-	-	-	-
5		L1ME1 L1MC4a	esv3863009	BI_GS_DEL1_B2_P1840_657	0.7927	0.8710	0.6491	0.7693	0.8213	0.8712	0.0320	0.2874	0.1546	0.7810	0.1443
6		L1ME1 L1MB8	esv3878049	UW_VH_1123	0.5236	0.7728	0.7237	0.1051	0.5231	0.6268	0.8806	0.2390	0.8641	0.8045	0.9355
7		L1MC4 L1M4c	esv3832215	YL_CN_LWK_752	0.4509	0.7857	0.4483	0.0537	0.6037	0.5368	0.1677	0.6560	0.7046	0.1023	0.9305
8		L1MB7 L1MB7	esv3888549	BI_GS_DEL1_B4_P2928_61	-	-	-	-	-	-	-	-	-	-	-
10		L2a L1MB8	esv3845520	BI_GS_DEL1_B4_P1148_45	0.0847	0.1815	0.0547	0.0008	0.0994	0.1186	0.4209	0.4346	0.9998	0.9433	0.8891

Supplementary Table S9. TASV comparison with AK1 and TCGA data

	KPGP9_Korean												
**Numbers**	**Chromosome**	**Start**	**End**	**epeat candida**	**Insertion Size**	**TSD size**	**insertion-m**	**Gene symbol**	**egion accessi**	**iation region**	**Chromosome**	**Start**	**End**
659	1	204238455	204238455	AluYg6_4	317	19	NA	PLEKHA6	NA	NA	1	204238455	204238455
708	1	170603447	170603447	AluYd8_1	237	4	NA	NA	esv3821175	_umary_ALU	1	170603434	170603434
1241	2	46928950	46928950	AluYa5_53	300	210	NA	SOCS5	esv3824113	_umary_ALU_	2	46928950	46928950
1260	2	80769010	80769010	AluYa5_36	280	144	NA	CTNNA2	NA	NA	2	80769010	80769010
1364	2	239883393	239883393	AluYb8_19	329	15	NA	NA	NA	NA	2	239883393	239883393
1829	3	38399700	38399700	AluYb8_31	285	10	NA	XYLB	esv3829993	_umary_ALU_	3	38399697	38399697
1870	3	63540950	63540950	AluYb8_14	312	14	NA	SYNPR	NA	NA	3	63540950	63540950
1880	3	50063025	50063025	AluYa5_2	224	14	NA	RBM6	NA	NA	3	50063025	50063025
3002	5	76716195	76716195	AluYa5_73	310	13	NA	PDE8B	NA	NA	5	76716195	76716195
3411	6	161270904	161270904	LTR5HS_1	998	13	NA	NA	esv3847467	_umary_SVA	6	161270898	161270898
4495	8	53791031	53791031	AluYa5_7	144	0	9	NA	NA	NA	8	53791040	53791040
4877	9	21038486	21038486	AluYa5_38	214	16	NA	NA	NA	NA	9	21038489	21038489
5226	10	26961080	26961080	AluYb8_4	184	10	NA	NA	NA	NA	10	26961086	26961086
5591	11	19962993	19962993	AluYb8_28	347	18	NA	NAV2	NA	NA	11	19962993	19962993
6358	13	19606159	19606159	AluYb9_12	341	10	NA	NA	esv3869558	_umary_ALU_	13	19606159	19606159
6642	14	52643753	52643753	AluYb9_9	332	14	NA	NA	NA	NA	14	52643753	52643753
6853	15	63374600	63374600	LTR5HS_2	930	10	NA	NA	esv3875382	_umary_SVA	15	63374592	63374592
6874	15	63086025	63086025	AluYa5_85	320	12	NA	TLN2	NA	NA	15	63086025	63086025
7074	16	53874969	53874969	AluYb8_30	311	11	NA	FTO	NA	NA	16	53874969	53874969
7665	19	47809223	47809223	AluYb8_5	174	5	NA	NA	NA	NA	19	47809228	47809228
7799	20	1546089	1546089	AluYa5_18	319	140	NA	SIRPB1	NA	NA	20	1546213	1546213
7939	21	26497431	26497431	AluYa5_88	183	6	NA	NA	esv3886444	umary_ALU_	21	26497420	26497420
8049	22	17705290	17705290	AluYb8_34	335	15	NA	NA	NA	NA	22	17705290	17705290

AK1_Korean													
Repeat type	Insertion Size	Gene	ene descripti	Gene type	Position	Type	Repeat (%)	Novelty	Chrom	Start	End	nsposon Elen	sposon Subfa
SINE/Alu	318	PLEKHA6	main contain	rotein_codin	intron	INS	0.937	NA	1	204238474	204238474	ALU	AluYa5
SINE/Alu	315	RP1-79C4.1	. lincRNA	lincRNA	promoter	INS	0.943	NA	1	170603435	170603435	ALU	AluYa5
SINE/Alu	310	SOCS5	r of cytokine	rotein_codin	intron	INS	0.997	NA	2	46928953	46928953	ALU	AluYa5
SINE/Alu	270	CTNNA2	n-associated	protein_codin	intron	INS	0.974	NA	2	80769024	80769024	ALU	AluYa5
SINE/Alu	328	NA	NA	NA	intergenic	INS	0.951	NA	2	239883408	239883408	ALU	AluYb8
SINE/Alu	293	XYLB	e homolog (H	rotein_codin	intron	INS	0.963	NA	3	38399708	38399708	ALU	AluYb8
SINE/Alu	312	SYNPR	synaptoporin	rotein_codin	intron	INS	0.952	NA	3	63540965	63540965	ALU	AluYb8
SINE/Alu	263	RBM6	nding motif p	protein_codin	intron	INS	0.996	NA	3	50063039	50063039	ALU	AluYa5
SINE/Alu	325	PDE8B	sphodiesteras	rotein_codin	intron	INS	0.957	NA	5	76716209	76716209	ALU	AluYa5
LTR/ERVK	975	NA	NA	NA	intergenic	INS	99%	NA	6	161270904	161270904	SVA	SVA_B
SINE/Alu	249	NA	NA	NA	intergenic	INS	0.952	NA	8	53791060	53791060	ALU	AluYa5
SINE/Alu	342	NA	NA	NA	intergenic	INS	0.968	NA	9	21038502	21038502	ALU	AluYa5
SINE/Alu	198	RP13-16H11.	. lincRNA	lincRNA	intron	INS	0.985	NA	10	26961080	26961080	ALU	AluYb8
SINE/Alu	348	NAV2	uron navigato	rotein_codin	intron	INS	0.911	NA	11	19963012	19963012	ALU	AluYb8
SINE/Alu	339	GTF2IP3	ption factor II	pseudogene	intron	INS	0.968	NA	13	19606169	19606169	ALU	AluYb9
SINE/Alu	328	NA	NA	NA	intergenic	INS	0.93	NA	14	52643767	52643767	ALU	AluYb8
LTR/ERVK	974	TPM1	omyosin 1 (al	rotein_codin	enhancer	INS	99%	NA	15	63374599	63374599	SVA	SVA_B
SINE/Alu	323	TLN2	talin 2	rotein_codin	intron	INS	0.957	NA	15	63086026	63086026	ALU	AluYa5
SINE/Alu	242	FTO	and obesity a	rotein_codin	intron	INS	0.848	NA	16	53874981	53874981	ALU	AluYb8
SINE/Alu	174	C5AR1	t component 5	rotein_codin	promoter	INS	0.994	NA	19	47809056	47809056	ALU	AluYb8
SINE/Alu	308	RP4-576H24.	aracterized p	rotein_codin	intron	INS	0.951	NA	20	1546214	1546214	ALU	AluYa5
SINE/Alu	337	NA	NA	NA	intergenic	INS	0.964	NA	21	26497431	26497431	ALU	AluYa5
SINE/Alu	332	NA	NA	NA	intergenic	INS	0.952	NA	22	17705308	17705308	ALU	AluYb8

							TCGA_Normal						
ınsposon Len	sitions on Re	verted Eleme	TSD Length	ΓSD Sequenc	Insert Motif	Gene	Gene Region	Nearby CNV	Nearby linc	R'erence Retro	viously annot	supportind	ıer of forward
281	1-282	TRUE	17	CTCATCTA'	GTCTTCC	PLEKHA6	9bp after exo:	.	.	.	Known	92	60
281	1-282	FALSE	17	ıAAAGTATG.	GGAAAAA	.	.	.	'ONS_000003	ıuJr#SINE#Al	Known	54	28
268	14-282	TRUE	3	TTT	TTTATGC	SOCS5	56bp after exc	.	.	E#MIR, MIR#	Known	64	34
250	32-282	TRUE	14	CTAAATGTtı	tttaata	CTNNA2	3bp before exc	.	.	T-rich#Low_	Known	85	39
288	1-289	TRUE	14	ıaaaaggactaatı	atatcca	.	.	.	.	'B1#LINE#L	Known	60	35
260	29-289	TRUE	11	aaaagtttggt	ggtataa	XYLB	5bp before ex	.	.	., (TG)n#Simp	Known	66	47
288	1-289	TRUE	15	AAAATACT'	CCTAAGT	SYNPR	5bp before ex	.	.	A16#LINE#L	Known	88	49
281	1-282	FALSE	14	ttttttttttttt	tttaaga	RBM6	51bp before ex	.	.	AluS, FLAM_	Known	49	8
281	1-282	TRUE	14	ACCTACTC	ATGTACC	PDE8B	0bp after exon	.	.	.	Known	83	53
436	936-1372	FALSE	6	TACGCC	GCCCAGA	.	.	.	.	.	Novel	76	50
208	74-282	TRUE	20	;AATTTTTA	TTTGTGA	.	.	.	.	.	Known	83	44
281	1-282	TRUE	49	ctgtgactggcC(	TTTGTAA	.	.	.	.	E#FLAM, Alı	Known	65	26
240	49-289	TRUE	14	attttttattgtt	taatttt	.	.	.	.	#AluS, AluSq	Known	97	41
288	1-289	TRUE	22	AATAGCAT	TTGAATT	NAV2	63bp after exc	.	.	.3#LINE#CR	Known	81	45
271	1-272	TRUE	10	GAACATTT	TTCAAAA	.	.	G_FILT2800	.	uSz#SINE#Al	Known	53	43
278	11-289	TRUE	14	cctggagtttcttt	tttaaga	.	.	.	.	T1N2#LTR#E	Known	54	26
436	936-1372	TRUE	7	aGAAAAA	AAAAggc	.	.	.	.	AluJ, AluJb#S	Novel	66	15
281	1-282	TRUE	12	AAACTTGG	TTAGAAA	TLN2	50bp after exo	.	.	IR#SINE#MI	Known	76	55
288	1-289	FALSE	12	GAAGTGCC	CTGTAGA	FTO	8bp before ex	.	.	Charlie, MER	Known	122	68
220	69-289	TRUE	20	Atttttttttttttttt	AGAtttt	.	.	.	.	E#L1ME, Alu	Known	39	16
281	1-282	FALSE	15	ıagaaatgtttcctı	ttaagaa	SIRPB1	9bp after exon	.	.	L2c#LINE#L2	Known	59	23
281	1-282	TRUE	11	AGGAAGGA'	ATGTATT	.	.	.	.	.	Known	76	43
288	1-289	TRUE	18	ıcaagtgctaataa	tttagaa	.	.	.	.	, (CAAA)n#S	Known	76	34

Supplementary Data

Supplementary Data S1. Sequence information of the manually inspected TASV insertion regions

Supplementary Data S2. Sequence information of the manually inspected TASV deletion regions

ber of reverse	ward breakp	verse breakp	orward conti	Reverse conti	Allele Fractio	f samples wit	ples with inse
32	204238474	204238458	ACTCCATT	GGAGGCTG	0.81818182	116	X-A05S;TCG
26	170603451	170603435	TCAGAGTG	ATTTATTT	0.8	65	64-1680;LUA
30	46928953	46928951	CAGAGCG	AAAATTCA	0.67241379	127	-A05D;TCG
46	80769024	80769011	CAAGTCT	CCCAAAGT	0.65671642	106	5S;TCGA-A
25	239883408	239883395	GGCTCACT	GAGTAGC	0.76923077	39	A-06-0686;G
19	38399708	38399698	CTAGAGCA	GGCGCAA	0.71641791	153	CV-7255;TC
39	63540965	63540951	TAGGACAT	GAACCTC	0.68181818	81	AD-TCGA-55
41	50063039	50063026	.	GGCAACA	0.55172414	77	LUAD-TCG
30	76716209	76716196	AATGTGA	GGTTTCA	0.90384615	32	22;LUAD-TC
26	161270904	161270899	TGCTTTTC	CCCCTCTT	0.63513514	103	A-D1-A16G;
39	53791060	53791041	TTTGTATT	AAAAATA	0.62831858	49	UAD-TCGA-
39	21038502	21038454	ACACACA	TTAATTT	0.58571429	82	666;LUAD-T
56	26961093	26961080	CTCAGCC	CCCGGGG	0.57142857	29	44-2666;LUA
36	19963012	19962991	TTCAAGC	AATTAGCC	0.79365079	28	A-06-5415;G
10	19606169	19606160	CTTTTGAA	TTTGAAAT	0.64285714	41	JAD-TCGA-4
28	52643767	52643754	TCCATCA	CCCAAAGT	0.75714286	42	GA-06-5415;
51	63374599	63374593	GTGACCCT	CTGGGATT	0.56603774	91	UAD-TCGA-
21	63086037	63086026	GACAGAGC	TTAGCCG	0.63043478	52	LUAD-TCGA
54	53874981	53874970	ACTGTTAC	GGTTCACG	0.78181818	104	1CI;TCGA-E
23	47809075	47809056	CCCGGGAA	CCAGCTAC	0.39344262	34	AD-TCGA-6
36	1546228	1546214	TCATTCAT	CAGAGTCC	0.72222222	86	A-67-6215;L
33	26497431	26497421	TTTATGAT	CGCTCTGT	0.87301587	100	-2665;LUAD-
42	17705308	17705291	ACCTTCCA	GCTCACGC	0.55555556	136	-AP-A05A;T

Data Note S1. Sequence information of the manually inspected TASV insertion regions

AluYa5

Instruction
Green box: Primer information
Red letter: Target site duplication
Black letter: Insertion sequences
Green letter: Deleted sequenced by insertion-mediated deletion

>KSI_AluYa5_1

>Scaffold2638-605135-605416
AGTCCCAGCACTTTGGGAAGCTGAGGCAGGAGGATGACTTGAGCCCAGGAGATCAAGACCATCCTGGGCAACATGAATAAACCCGCTCTCTACAAAAAATACAAAAATTAGCCAAGTGTGGTGACA TG
AGTCTGTAGTCCCAGCTACTCAGGACGCTGAGGTGGGAGGATCGTTTGAGCCCAGAAGGTCAAGGCCATAGTGAGCTATGATTCCACTACTGTGCTTCAGCTGGGGAGACAGAGCGAGACCTTGT CTC
AAAAAACAAAACAAAACAGAAAAGAAATGCTGAAGAAATTCCTTCTAGCAGAAGAGAAAATGACAGCAGACAGATATCTGGGTCTACACAAATGACTTATGAGCACCAGAAATGGTAAATATGTGG AC
AAATGTGAAATATTTTACCTTATTCTTAAAATATTTTAAAAAATAATTGTTTACAGCAATACTTTTTTTTAAATTCCAGCCTGGGAAACATGGCGTAA aCCtATCTCTACAAAAAATACAAACAAAAA
gTAGCCAGGTATGGTGGTGCACATATACGGGAAGCTGAGGCAAGAGGATCCCTTGAGCCCAGGAGGTTGAGGCTGTAGTGAGCCATGATTGTGCCACTGCACTCCAGCCTGGGTGACAGAGCAAGA CC
CTGTCAAAAAAGAACAAAGAACTGCAGATCTTGATTAACTAGAAAAATCACTTAAAAGAAAA tTGAGGGCTAATATGATTGTAGGTGTGGTCACAGAGTGGGATCTATTCAGTTATGGAATAAATTGG
GGTGAAGGCAGGTTCAGAGCACGATaATCAGACAAGTGTTTTTGATTAATAAACACTTAATAATAAGCAGTGGGTTTTTGATTAATAAAACACCACACAGGTATCAAAGGAAAAGTAAAGGGGAGGGT
AAAGTAAAAAGAAATGAAATTTAGTATCAGACAACAGATTTCAGAGCAAAAAATATTATTAGATATTACCCAGGATCAATAAAAGTCATTTCCTAACAATAAAGGAGCCAATTCTTAACAGCCCT ACA
ATGCTGCTCATTCACAGGAAAAAAAAATGGGCAAATGAAAATAATAAATTATCTGCTTTGAGTTATCTTTGGTTTATCGAATTAAAAATAAATTTTTAATAACGGGGAAAATACTTCGTTGGGAGAA AA
TTCTATATGAATATTTTGATATTTCTGCATGGTCAGGGCTTTCCAAGCAAAGGACACTGACGAGTTGATTGAATAGCAAAGGCCTTGAAAGGCAGAGACAGTATCTCCCTAAAGATAAA cAAATCTGG
AGAAATTTAGAAATGTAAACCTCTTTACTTCCTGTTCCTGAAACATGTGCTTATACTCCAGGATAATAAACTTAGTGATACCTTCCCTCTCAACCCTGGAGATTTGCTTAATAAGTAATCTCAATC TC
TCTCACTCTCTCCCCAGAGAAGAGGATGGGCAGATGTGCCAGTAgTTTCTATTTAAATGAATCTCAGAAGTTTAGGGTTCTTCTCCTTTGGTGCAACTTCCTGCATGCACAGGGATCTCACCTGGTTC
TCATCCCATCCCCTCACTCACCGTGGTGACCGGTGCTTTGAAAACAAAAAAACTAGGCCGGATGCAGTGGCTCATGCCTGTAATCCCAGCACTTTGGAAGGCCAAGGCAGGCGGATCACCTAAGGT CA
GGAGTTcGAGACGAGCCTGGCCAACATGGGGAgACTCTGTCTCTACTAAAAATACAAAAAATAGCCAGGCGTGGTGTCATGTGCCTGTAATCCCAGCTATTCAGGAGTCTGAGGCAGGAGAATCGCTT
GAACCCAGGAGGCGGAGGTTGCAGTGAGCCAAGATTGTGCCATTGCACTCCAGCCTGGGTGACAGAGTGAGACTCCATCACAAAAAAAAAAATAAT AATAAACAGAAAcTAGTGCTAAGGTATTGCTAT
GGCTGCTGCATTTTGCTGTAATAAACCATCCTTCTCTGTGATCCAAAAAGCCTCATGTCTCTTGCTAG AATATATATATGTATaatcccagcactttgggaggccgaggcgggcggatcacgaggtca
ggagatcaagaccatcccggctaaaacggtgaaaccccgtctctactaaaaatacaaaaaattagccgggcgtagtggcgggcgcctgtagtcccagctacttgggaggctgaggcgggagaat ggt
gtgaacccgggaggcggagcttgcagtgagccgagatcccgccactgcactccagcctgggcgacagagcgagactccgtctc aaaaaaaaaaaaaaaaaaaAAAATATACATATATTACACATAATT
GTGTAATTTACAACTATAATATAGAATATATGTATATTATATAGATTATATATTATTCTATGTGTATAACATAGCTTATATGTGTATAATTATGTATATGTATATTATATGTGTTTTATATGTAT ATG
TATGTAATATATACACATATACACACACACTCTTGAAAGCAGGGAAACTCTCAGACCCGTCACAGTTTCTGACGTAACATACT TATGACAAAAAGTCACCACAGACCTACCACTCAGCTATCCATGAT
TCTTATTTTGATGTATTCTCTTCTGAATTTTCTTTCTATAATGAACATCTGGTACTTCTGATCTTTAAGACTATAAAAAGTTTAACTATCAGAGTCCAGAATCAAGATGGCCGAATAGGAGCAGC TCt
GATCTGCATCTCCCAGCGTGATCAATGCAGAAGACGGTGATTTCTGCATTTCCAACAGAGGTACTGGGTTCATCTCATTAGGA CTGGTTCACCTGGGAAGCACAAGGGGTCAGGGAATTCCCTTTCCT
AGCCAAGGGAAGCCGTGACAGATGGgACCTGGGAAATCGGGACACTCCCACCCTAATACTGCGCTTTTCCAACAGTCTTAGCAAACGGCACACCAGGAGATTATATCCTGCAGCTGGCTCGGCGGGTC
CCCCACCCATGGAGCTTTGCTCACTGCTATAACAGCAGTCCAAGATGGAACTGCGAAG tTGGCAGTGAAGCTGGGGGAGGGGCGTCTGCCATTCCTGAGGCTTGACTAGGTAAACAAAGCAGCCAGGA
AGCTCGAACCGAGTGGAGCCCACCACAGCTCAAGGAGGCCTGCCTGCCTCTGTAAACTGCACCTCTGGCGGCAGGGCATAGCTGAACAAAAGGCAGCGGAAACTTCTGCAGACTTAAACGTCCCT GTC
TGACAGCTTTGAAAAGAGTAGTGGTTCTTCCAGCATGGAGTTTGAGATCTGAGAATGGACAGACTGCCTCCTCAAGTGGGTCC CTGACCCCCAAGTAGCCTAACTGGGAGACACCTCCCAGTCGAGGC
CGACTGACACCTCATACAGGTGGGTGCCCTTCTGGGACAAAGCTTCTAGAGGAAGGATCAGGCAGCAACATTTGCTGTTCTGCAATATTTGCTGTTCTACAGCCTCCGCTGGTGATACCCAGGCA AAC
AGGGTCTGGAGTGGACCTCCAGCAAACTCCAACAGACCTGCAGCTGAGGGTCCTGACTATTAGAAGGAAAACTAACAGAAAGG AATAGCATCAACATCAACAAAAAGGACATCCACACCAAAACCCCA
TATGTAGGTCAGCATCATCAAAAACCAAAGGTAGATAAAACCACAAAGATGGGGAGAAACCAGAACAGAAAAGCTGAAAATTCTCAAAACCAGAGTGCCTCTTCTCCTCCAAAGGATCGCAGCTC CTC
ACCAGCAATGGAACAAAGCTGCACGGAGAATGACTTAGACAAGTGACAGAAGTAAGCTTCAGAAGATCGGTAATAACAAACTT CTCTGAGGTAAAGGATGATGTTTGAACCCATCACAAAGAAGCTAA
AAACCCTGAATTCTACCAGAGATACAAAGATGAGCTGGTACTATTCCTTCTGAAACTATTCCAATCATTAGAAAAAGAGGGAACCTCCCTAACTCATTTTATGAGGCCAGCATCAGCCTGATACC AAG
ACATAACAAAAAAAGAGAATTTTAGACCAATATCCCTGATGAACATCGATGCAAAAATCCTCAATAAAATACTGGCAAACAGA ATCCAGTAGCACATCAAAAAGCTTATCCACCATGATCAAGTTGGC
TTCATCCCTGGGATGCAAGGCTGGTTCAACATACACAAATCAATAAACGTAATCCATCATATAAACAGAACCAAAGACAAAAACCACGACTATCTCAACAGACGCAGAAAAGGCCTTCGACAAAT TTC
AACAGCCCTTCATGCTAAAAACTCTCAATATACTAGGTATTGATGGGACATATCTCA A

1784	17.4	0.0	0.0	UnnamedSequence	3	283	(3999)	+	AluJb	SINE/Alu	29	309	(3)	1	
571	20.7	5.6	0.6	UnnamedSequence	284	443	(3839)	+	L1MEc	LINE/L1	1774	1941	(4459)	2	
1191	13.9	7.9	1.9	UnnamedSequence	460	661	(3621)	+	AluJb	SINE/Alu	85	298	(14)	3	
242	24.1	2.1	12.8	UnnamedSequence	917	1011	(3271)	+	L1MEc	LINE/L1	2513	2598	(3723)	2	
1235	24.1	6.8	8.9	UnnamedSequence	1141	1551	(2731)	+	LTR40A1	LTR/ERVL	2	404	(180)	4	
310	30.1	2.4	10.9	UnnamedSequence	1476	1591	(2691)	+	LTR40b	LTR/ERVL	196	297	(165)	5	*
2295	11.7	0.0	0.0	UnnamedSequence	1592	1899	(2383)	+	AluSx	SINE/Alu	1	308	(4)	6	
310	30.1	2.4	10.9	UnnamedSequence	1900	1991	(2291)	+	LTR40b	LTR/ERVL	298	387	(75)	5	
2593	1.4	0.0	0.4	UnnamedSequence	2001	2282	(2000)	+	AluYa5	SINE/Alu	24	304	(7)	7	
36	21.3	8.5	1.3	UnnamedSequence	2283	2394	(1888)	+	(AT)n	Simple_repeat	1	123	(0)	8	
26	5.0	4.9	0.0	UnnamedSequence	2395	2435	(1847)	+	(TATGTA)n	Simple_repeat	1	43	(0)	9	
36	21.3	8.5	1.3	UnnamedSequence	2436	2453	(1829)	+	(AT)n	Simple_repeat	2	20	(0)	10	
276	12.5	2.1	0.0	UnnamedSequence	2464	2511	(1771)	+	LTR40A1	LTR/ERVL	536	584	(0)	4	
7948	9.5	5.2	0.2	UnnamedSequence	2663	3850	(432)	+	L1P2	LINE/L1	8	1255	(4929)	11	
3221	7.3	3.2	0.0	UnnamedSequence	3846	4282	(0)	+	L1P2	LINE/L1	3558	4008	(2154)	11	*

>HG19Seq chr1:165657302-165661308
AGTCCCAGCACTTTGGGAAGCTGAGGCAGGAGGATGACTTGAGCCCAGGAGATCAAGACCATCCTGGGCAACATGAATAAACCCGCTCTCTACAAAAAATACAAAAATTAGCCAAGTGTGGTGACA TG
AGTCTGTAGTCCCAGCTACTCAGGACGCTGAGGTGGGAGGATCGTTTGAGCCCAGAAGGTCAAGGCCATAGTGAGCTATGATTCCACTACTGTGCTTCAGCTGGGGAGACAGAGCGAGACCTTGT CTC
AAAAAACAAAACAAAACAGAAAAGAAATGCTGAAGAAATTCCTTCTAGCAGAAGAGAAAATGACAGCAGACAGATATCTGGGTCTACACAAATGACTTATGAGCACCAGAAATGGTAAATATGTGG AC
AAATGTGAAATATTTTACCTTATTCTTAAAATATTTTAAAAAATAATTGTTTACAGCAATACTTTTTTTTAAATTCCAGCCTGGGAAACATGGCGTAA cCCcATCTCTACAAAAAATACAAACAAAAA
tTAGCCAGGTATGGTGGTGCACATATACGGGAAGCTGAGGCAAGAGGATCCCTTGAGCCCAGGAGGTTGAGGCTGTAGTGAGCCATGATTGTGCCACTGCACTCCAGCCTGGGTGACAGAGCAAGA CC
CTGTCAAAAAAGAACAAAGAACTGCAGATCTTGATTAACTAGAAAAATCACTTAAAAGAAAA cTGAGGGCTAATATGATTGTAGGTGTGGTCACAGAGTGGGATCTATTCAGTTATGGAATAAATTGG
GGTGAAGGCAGGTTCAGAGCACGATcATCAGACAAGTGTTTTTGATTAATAAACACTTAATAATAAGCAGTGGGTTTTTGATTAATAAAACACCACACAGGTATCAAAGGAAAAGTAAAGGGGAGGGT
AAAGTAAAAAGAAATGAAATTTAGTATCAGACAACAGATTTCAGAGCAAAAAATATTATTAGATATTACCCAGGATCAATAAAAGTCATTTCCTAACAATAAAGGAGCCAATTCTTAACAGCCCT ACA
ATGCTGCTCATTCACAGGAAAAAAAATGGGCAAATGAAAATAATAAATTATCTGCTTTGAGTTATCTTTGGTTTATCGAATTAAAAATAAATTTTTAATAACGGGGAAAATACTTCGTTGGGAGAA AA
TTCTATATGAATATTTTGATATTTCTGCATGGTCAGGGCTTTCCAAGCAAAGGACACTGACGAGTTGATTGAATAGCAAAGGCCTTGAAAGGCAGAGACAGTATCTCCCTAAAGATAAA gAAATCTGG
AGAAATTTAGAAATGTAAACCTCTTTACTTCCTGTTCCTGAAACATGTGCTTATACTCCAGGATAATAAACTTAGTGATACCTTCCCTCTCAACCCTGGAGATTTGCTTAATAAGTAATCTCAATC TC
TCTCACTCTCTCCCCAGAGAAGAGGATGGGCAGATGTGCCAGTAaTTTCTATTTAAATGAATCTCAGAAGTTTAGGGTTCTTCTCCTTTGGTGCAACTTCCTGCATGCACAGGGATCTCACCTGGTTC
TCATCCCATCCCCTCACTCACCGTGGTGACCGGTGCTTTGAAAACAAAAAAACTAGGCCGGATGCAGTGGCTCATGCCTGTAATCCCAGCACTTTGGAAGGCCAAGGCAGGCGGATCACCTAAGGT CA
GGAGTTtGAGACGAGCCTGGCCAACATGGGGAaACTCTGTCTCTACTAAAAATACAAAAAATAGCCAGGCGTGGTGTCATGTGCCTGTAATCCCAGCTATTCAGGAGTCTGAGGCAGGAGAATCGCTT
GAACCCAGGAGGCGGAGGTTGCAGTGAGCCAAGATTGTGCCATTGCACTCCAGCCTGGGTGACAGAGTGAGACTCCATCACAAAAAAAAAAATAAT AataaATAAACAGAAAtTAGTGCTAAGGTATTG
CTATGGCTGCTGCATTTTGCTGTAATAAACCATCCTTCTCTGTGATCCAAAAAGCCTCATGTCTCTTGCTAG AATATATATATGTATAAAATATACATATATTACACATAATTGTGTAATTTACAACT
ATAATATAGAATATATGTATATTATATAGATTATATATTATTCTATGTGTATAACATAGCTTATATGTGTATAATTATGTATATGTATATTATATGTGTTTATATGTATATGTATGTAATATATA CA
CATATACACACACACTCTTGAAAGCAGGGAAACTCTCAGACCCGTCACAGTTTCTGACGTAACATACTTATGACAAAAA GTCACCACAGACCTACCACTCAGCTATCCATGATTCTTATTTTGATGTA
TTCTCTTCTGAATTTTCTTTCTATAATGAACATCTGGTACTTCTGATCTTTAAGACTATAAAAAGTTTAACTATCAGAGTCCAGAATCAAGATGGCCGAATAGGAGCAGCTC cGATCTGCATCTCCCA
GCGTGATCAATGCAGAAGACGGTGATTTCTGCATTTCCAACAGAGGTACTGGGTTCATCTCATTAGGACTGGTTCACCTGGGAAGCACAAGGGGTCAGGGAATTCCCTTTCCTAGCCAAGGGAAG CCG
TGACAGATGGtACCTGGGAAATCGGGACACTCCCACCCTAATACTGCGCTTTTCCAACAGTCTTAGCAAACGGCACACCAGGAGATTATATCCTGCAGCTGGCTCGGCGGGTC CCCCACCCATGGAGC
TTTGCTCACTGCTATAACAGCAGTCCAAGATGGAACTGCGAAGgTGGCAGTGAAGCTGGGGGAGGGGCGTCTGCCATTCCTGAGGCTTGACTAGGTAAACAAAGCAGCCAGGAAGCTCGAACCGAGTG
GAGCCCACCACAGCTCAAGGAGGCCTGCCTGCCTCTGTAAACTGCACCTCTGGCGGCAGGGCATAGCTGAACAAAAGGCAGCGGAAACTTCTGCAGACTTAAACGTCCCTGTC TGACAGCTTTGAAAA
GAGTAGTGGTTCTTCCAGCATGGAGTTTGAGATCTGAGAATGGACAGACTGCCTCCTCAAGTGGGTCCCTGACCCCCAAGTAGCCTAACTGGGAGACACCTCCCAGTCGAGGCCGACTGACACCT CAT
ACAGGTGGGTGCCCTTCTGGGACAAAGCTTCTAGAGGAAGGATCAGGCAGCAACATTTGCTGTTCTGCAATATTTGCTGTTCTACAGCCTCCGCTGGTGATACCCAGGCAAAC AGGGTCTGGAGTGGA
CCTCCAGCAAACTCCAACAGACCTGCAGCTGAGGGTCCTGACTATTAGAAGGAAAACTAACAGAAAGGAATAGCATCAACATCAACAAAAAGGACATCCACACCAAAACCCCATATGTAGGTCAG CAT
CATCAAAAACCAAAGGTAGATAAAACCACAAAGATGGGGAGAAACCAGAACAGAAAAGCTGAAAATTCTCAAAACCAGAGTGCCTCTTCTCCTCCAAAGGATCGCAGCTCCTC ACCAGCAATGGAACA
AAGCTGCACGGAGAATGACTTAGACAAGTGACAGAAGTAAGCTTCAGAAGATCGGTAATAACAAACTTCTCTGAGGTAAAGGATGATGTTTGAACCCATCACAAAGAAGCTAAAAACCCTGAATT CTA
CCAGAGATACAAAGATGAGCTGGTACTATTCCTTCTGAAACTATTCCAATCATTAGAAAAAGAGGGAACCTCCCTAACTCATTTTATGAGGCCAGCATCAGCCTGATACCAAG ACATAACAAAAAAAG

AGAATTTTAGACCAATATCCCTGATGAACATCGATGCAAAAATCCTCAATAAAATACTGGCAAACAGAATCCAGTAGCACATCAAAAAGCTTATCCACCATGATCAAGTTGGCTTCATCCCTGGG ATG
CAAGGCTGGTTCAACATACACAAATCAATAAACGTAATCCATCATATAAACAGAACCAAAGACAAAAACCACGACTATCTCAACAGACGCAGAAAAGGCCTTCGACAAATTTC AACAGCCCTTCATGC
TAAAAACTCTCAATATACTAGGTATTGATGGGACATATCTCAA

1784	17.4	0.0	0.0	UnnamedSequence	3	283	(3728)	+	AluJb	SINE/Alu	29	309	(3)	1	
571	20.7	5.6	0.6	UnnamedSequence	284	443	(3568)	+	L1MEc	LINE/L1	1774	1941	(4459)	2	
1256	12.4	7.9	1.9	UnnamedSequence	460	661	(3350)	+	AluJb	SINE/Alu	85	298	(14)	3	
242	24.1	2.1	12.8	UnnamedSequence	917	1011	(3000)	+	L1MEc	LINE/L1	2513	2598	(3723)	2	
1221	24.3	6.8	8.9	UnnamedSequence	1141	1551	(2460)	+	LTR40A1	LTR/ERVL	2	404	(180)	4	
303	29.5	3.8	12.4	UnnamedSequence	1476	1591	(2420)	+	LTR40b	LTR/ERVL	196	302	(160)	5	*
2306	11.9	0.0	0.0	UnnamedSequence	1592	1903	(2108)	+	AluSx	SINE/Alu	1	312	(0)	6	
303	29.5	3.8	12.4	UnnamedSequence	1904	1995	(2016)	+	LTR40b	LTR/ERVL	303	388	(74)	5	
40	21.7	8.8	0.6	UnnamedSequence	1996	2123	(1888)	+	(AT)n	Simple_repeat	1	140	(0)	7	
26	5.0	4.9	0.0	UnnamedSequence	2124	2164	(1847)	+	(TATGTA)n	Simple_repeat	1	43	(0)	8	
40	21.7	8.8	0.6	UnnamedSequence	2165	2182	(1829)	+	(AT)n	Simple_repeat	1	19	(0)	9	
276	12.5	2.1	0.0	UnnamedSequence	2193	2240	(1771)	+	LTR40A1	LTR/ERVL	536	584	(0)	4	
7986	9.3	5.2	0.2	UnnamedSequence	2392	3579	(432)	+	L1P2	LINE/L1	8	1255	(4929)	10	
3221	7.3	3.2	0.0	UnnamedSequence	3575	4011	(0)	+	L1P2	LINE/L1	3558	4008	(2154)	10	*

>KSI_AluYa5_2 (We have tried to verify that there is insertion in this region, but we couldn't confirmed the exact sequences by high GC-content concentrated in this insertion region.)

>Scaffold4551-4391627-4391889
TGAATTTAATAATTGCTGTGATTACTCTGGCATTATACGCTCACAAATAAAATGTTTGGTGAttttttTTTTTTTTTTTTTTTTTTTTGGAGACAGATTCTTGCTCTGTCACCCAGGCTGTGCAATGA
TGTGATCTCAGCTTACTGCAACCTCCGAGTTCAAGTGATTCTCGTGCCTCAGCCTCTCGAGTAGCTGGGATTACAGGCACCCGCCATCATGCCTGGCTAATTTTTGTATTTTTGTAGAGATGGGGTTT
CACCATGTTGGCCAGGCTGGTCTTGAACTCCTGACTTCAGGTGATCCACCCATCTCAGCCTCCCAAAGTGCTGGGATTACAGGTGTGAGCCACTGCTCCCAGCCGGGTGTGATATTTTTAATAAA ACA
AGTATTCAAATTCACTTACAGGACCAATGAAAGAATCGTTTGTCGTAATTTTATGCCAAAGGGTACTTGTGGCTTAAGATAAACTTCCCATAATGACATTATCCACAGATTCAAAAAGTAGTTTATCT
TAAACAACTTCTGTGACATTTTAAAATGATGTGGCTTAGAAAATTGCTAGGTTATCTAAAATGGCTCTATTGATGATGTAAATGTAGCACATGAAGAGCTTGAATAAAATAGACTTTTGAAGTGT GCA
AATGGAAAGAACAGTCCTTCTAAATAATTATTTCCCCTCCCTTTTATTGACGTATACATACAGAAAAGATATCATGTCGTAAGTGTATTGCTTAGTGAATTACTCCAAAGTTGGATATACCTGGTTAA
CCACCACCTGAATGAAAAAAACAGAACACTGCTTCATATGGAGAAGCCCCTCCTGCCCCTCCTGGTCATTGTCCTTTTCATCCCTCCCACAGGTAGTCACTGAGTTCTAATACCACAGAGTCTTT TGA
CTTTCTTTTGAGCCTTATGTAATTAGAATCACAAAAGATGTATTCTTTTGCCTGACTTTTATACTTAGTATTGTTTTTGAAATTCATCTTGTGTGTAACTGCGATTTGTTCATTTTCATTGCTTAGTG
AATTATTCCAAAGTTGGATATACCTGGTTAACCACCACCCGAATGAAAAAAACAGTTTTTGGCCGGGCACGATGGCTCACGCCTGTTATCCCAGCACTTTGGGAGGCTGAAGCGTGCAGATTACG AGG
TCAGGAGATCAAGACCATCCTGGCTAACACGGTGAAACCCCGTCTCTACTAAAAATACAAAAAATTAGCTGGGCGTGGTGACGGGCCCCTGTAGTCCCAGCTACTCAGGAGGCTGAGGCAGGACACCT
GTAATCCCAGCTACTTGAGATGCTGAAACAGGAGAGTGGCGTGAACTTGGGAGATGGAGCTTGCAGTGAGCCGAGATTGCGCCACTGCACTCCAGCCTGGGCGACAGAGCAAGACTCCGTCTCAA AAA
ACAAAAAAaAAAAAACAAGAAAACAGTTTTCCAGTCTAAGAATGTATTACAATTTATTCAAATTCCACTCTAGATGGACTGTGGGTTTTTTTTTTTCCCCCATTTGGAGCTATGGCAAATGATGTTTT
TTCAAAGTTGTTATTTCTCAGCCAGGCGCGGTGGCTCACGCCTGTAATCCCAGTACTTTGGGAGACTGAGGTGGGCAGATCACCTGAGGTCAGAAGCAAGACCAGCCTGGCTAACATGGCGAAAC CCC
GTCTTTTCTAAAAATACAAAAATTAGCCAGGTGTGGTGATGGGCACCTGTAATCCCAGCTACACAGGAGGCTGAGGCAGGATAATCACTTGAACCCAGGAGGTAGAGGTTGCAGTGAGCTGAGATCAC
ACCACTGCACTCCAGCCTGGGTGACAGAGCGAGACTCTATCTCAAAAAAGAAAACAAAACACCACGGAATTGTTATTTCTCTTGGCGAATAGGTAGATGCACTTATTCCTGTTAATATATACCTACCT
GTGAATGTGCTTGTTGGATTTTCTATGTATCTTCTGTCTGCCACCTAGAAATTTAACCTTTTATATATATACAACTTTAA TTTTTtttttttttttttttttttttggggggcaggctgctcccggc
gcccnnnnnnnnnnnnnnnnnnnnnnnnnnnnnnnnnnnnngggttcacgccattctcctgcctcagcctcccaagtagctgggactacaggcgcccgccactacgcccggctaatttttttgtatttt
tagtagagacgaggtttcaccgttttagccgggatggtctcgatctcctgacctcgtgatccgcccgcctcggcctcccaaagtg TTTTTTTTTTTTTAAGAGACAGGGTGTCACTATGTTGCCCAG
GCTGGTTGGGAACTCCTGGCCTTAAGCCGTCCTCCTGCTTCAGTCTCCCAAAGTGTTGGGAATATAGGCGTGAGCCACTGTGCCCCACTGT TCAAGTTTTCATTGATTGCTGCCTACATATAGTTGTT
CAACAGCTATTGATTCCCCCTGCTCTGTATATATGTCTCCTAGTGTAGGTATCAGGGTTACAGCAGTAATTAAGACCACATTATTTCATTTTATCATTTAAATATATAAGACTAATTGATAAATTAAG
TATAGAACTTTGACCAACATGGTGAAACCCCATCTCTACTAGAAATACAAAAATTAGCTGGGTGTGGTGGCAGACGCCTGTAATCCCAGCTACTCAGGAGGC CGAGGCAGAACTGCTTGGAGATGGAG
GTTGCAGTGAACCAATATCAGACCACTATACTCCAGCTTGGATGACAGAGGGAGACTTTGTCTCTTTTTTTTTTTCTTTTTTTTTGAGACGGAATCTCGCCGTCTTCCAGGCTGGAGTGCAGTGG CAC
GATCTCGGCTCACTGCAGCCTCCGCCTCCCGGGTTCAAGCGATTCTTCTACCTCAGCCTTCCGAGTAGCTGGGATTACAGGCACCCACCACCATGCCCGGCT AATTTTTGTATTTTTAGTAGACAGGG
TTTCACCATGTTGGCCAGGCTGGTCTCAAACCCCTGACCTCAAGtGATCAACCTGCTTTGGTCTCCCAAAGTGCTAGGATTATAGGCGTGAGCCACTGTGCCCGGCCCTTTTTTTTTTTTTGGAGAC
AGAATTTCGCCCAGTTGCCAGACTGGAGTGCAGTGGCACGATCTCAGCTCACTGCAACCTCTGCTTCATGGGTTCAAGCCATTTTCCTGCCTCAGCCTCCCA AATAGCTGGGACTACAGGCATGCACC
ACCACGTCTGGCTAATTTTTTGTATTTTTAGTAAAGCCAGAGTCCCAAAGTGCTGGGACTAGGCAGGCGTGAACCACCACGCCTGGCCAAGACTCTGTCTCTCAAAAAAAAAAAAAAGAAAAAAA AAT
ATAGGACTTTGGGAGGCCGAGGCAGGCAGATCACCTGAGGTCAAAAGTTTGAGACCAGCCTGACTAACATGGTGAATCCCCATATCTACCAAAAAATACAAA AATTAGGCAGGTGTGGTGGCGTGCAC
CTGTAGTCCCAGCTATTGGGGAAGCCGAGGTGGGAGATTGTACCTGGGAGGCAGTGAGCAGAGATCGCACCACTGCACTCCAGCCTGGGTGACAGAGTGAGACCTTGTCTCAC CAAAAAAAAAAATAG
CATAGGTAGGCATTTGATGATTTGATGATTTCATTCGCATCCCTAAAAGTTTATTTGTTCCTGGaTCGTCAGATAGCTTTTGGCCATCTTCCTGTTGAGAAAATTGATGTACCCTTCTGGAGTCCTC
CAATTTTCCATTATAATATGGTAAGTGGGAGCTAGAGCTTTGGGTAAGAATTGGATGTGATAAGGAGGATGAGTTTTGCAGTGGTGTGCATGGTTAGGAGGAGAAAAAGCTGGAGGCAGAGTGT TCA
CTTAGAGGCTTGGGGTAGGAGGGGTAGGTTTAAGTGGTGCTCATCTGGGCCAGAATAGGGCAAAAAGGGAAGAATGAAATAACCAGATGTCTTTGCTTTGTC AGTAGTCTTGCAGCCCTGAAAGCTTT
TTTTGTTGTGTTATATTTGTTGTAATTGAGGTATAATCCACATAACATAAAACTTACCTCTTTCAAGTGTACAATTTAGTAGTTTTTAGTATATTCATAAAATTGTGCAACTATCACCACTGA cACCA
GAACATTTCTGGGAACAAAAAGAAACTATATATCCATTAAGAGTCACTCTCCATTTTCTCCTACTTCCTTCTCTACCCCCAGTCATCTGCTAGTCGGCTTTC TGTCTCTATAGATTTGCCTGCTCTGG
ATATTTCATATAAATGGAATCATATACCATATGGTCTTT

2003	10.7	4.1	0.0	TeRefseq	63	360	(3903)	C	AluSx1	SINE/Alu	(0)	312	1	1	
903	24.4	7.2	0.3	TeRefseq	394	672	(3591)	+	Kanga2_a	DNA/TcMar-Tc2	386	683	(204)	2	
913	27.1	7.9	3.9	TeRefseq	680	1048	(3215)	C	L1ME3A	LINE/L1	(11)	6162	5780	3	
370	23.5	12.9	1.2	TeRefseq	911	1065	(3198)	C	L1MD2	LINE/L1	(431)	5928	5756	4	*
2306	6.6	0.0	12.3	TeRefseq	1085	1431	(2832)	+	AluY	SINE/Alu	1	309	(2)	5	
243	26.4	2.9	7.1	TeRefseq	1433	1534	(2729)	C	L1ME3A	LINE/L1	(439)	5803	5706	3	
2268	10.8	0.7	0.0	TeRefseq	1557	1851	(2412)	+	AluSx1	SINE/Alu	2	298	(14)	6	

28	0.0	0.0	0.0	TeRefseq	2001	2027	(2236)	+	(T)n	Simple_repeat	1	27	(0)	7
1637	1.1	0.0	0.0	TeRefseq	2090	2263	(2000)	C	AluYa5	SINE/Alu	(105)	206	33	8
829	15.9	0.0	0.0	TeRefseq	2264	2389	(1874)	C	FLAM_C	SINE/Alu	(13)	130	5	9
1148	14.4	5.8	0.0	TeRefseq	2571	2744	(1519)	+	AluSx1	SINE/Alu	91	274	(38)	10
2374	9.8	1.3	0.0	TeRefseq	2745	3051	(1212)	C	AluSx1	SINE/Alu	(1)	311	1	11
1338	14.1	0.5	0.0	TeRefseq	3053	3243	(1020)	C	AluSx3	SINE/Alu	(15)	296	105	12
279	9.3	0.0	4.7	TeRefseq	3244	3288	(975)	C	Alu	SINE/Alu	(90)	43	1	13
20	4.3	0.0	0.0	TeRefseq	3304	3327	(936)	+	(A)n	Simple_repeat	1	24	(0)	14
1601	13.9	5.6	0.4	TeRefseq	3331	3581	(682)	+	AluSx	SINE/Alu	33	296	(16)	15
284	22.9	3.5	3.5	TeRefseq	3604	3689	(574)	+	Kanga2_a	DNA/TcMar-Tc2	754	839	(48)	16
1295	22.0	2.6	3.0	TeRefseq	3953	4263	(0)	C	L1MB7	LINE/L1	(137)	6197	5895	17

>Hg19Seq chr3:50061021-50065027
TGAATTTAATAATTGCTGTGATTACTCTGGCATTATACGCTCACAAATAAAATGTTTGGTG ATTTTTTTTTTTTTTTTTTTTGGAGACAGATTCTTGCTCTGTCACCCAGGCTGTGCAATGATGTGAT
CTCAGCTTACTGCAACCTCCGAGTTCAAGTGATTCTCGTGCCTCAGCCTCTCGAGTAGCTGGGATTACAGGCACCCGCCATCA TGCCTGGCTAATTTTTGTATTTTTGTAGAGATGGGGTTTCACCAT
GTTGGCCAGGCTGGTCTTGAACTCCTGACTTCAGGTGATCCACCCATCTCAGCCTCCCAAAGTGCTGGGATTACAGGTGTGAGCCACTGCTCCCAGCCGGGTGTGATATTTTTAATAAAACAAGT ATT
CAAATTCACTTACAGGACCAATGAAAGAATCGTTTGTCGTAATTTTATGCCAAAGGGTACTTGTGGCTTAAGATAAACTTCCC ATAATGACATTATCCACAGATTCAAAAAGTAGTTTATCTTAAACA
ACTTCTGTGACATTTTAAAATGATGTGGCTTAGAAAATTGCTAGGTTATCTAAAATGGCTCTATTGATGATGTAAATGTAGCACATGAAGAGCTTGAATAAAATAGACTTTTGAAGTGTGCAAAT GGA
AAGAACAGTCCTTCTAAATAATTATTTCCCCTCCCTTTTATTGACGTATACATACAGAAAAGATATCATGTCGTAAGTGTATT GCTTAGTGAATTACTCCAAAGTTGGATATACCTGGTTAACCACCA
CCTGAATGAAAAAAACAGAACACTGCTTCATATGGAGAAGCCCCTCCTGCCCCTCCTGGTCATTGTCCTTTTCATCCCTCCCACAGGTAGTCACTGAGTTCTAATACCACAGAGTCTTTTGACTT TCT
TTTGAGCCTTATGTAATTAGAATCACAAAAGATGTATTCTTTTGCCTGACTTTTATACTTAGTATTGTTTTTGAAATTCATCT TGTGTGTAACTGCGATTTGTTCATTTTCATTGCTTAGTGAATTAT
TCCAAAGTTGGATATACCTGGTTAACCACCACCCGAATGAAAAAAACAGTTTTTGGCCGGGCACGATGGCTCACGCCTGTTATCCCAGCACTTTGGGAGGCTGAAGCGTGCAGATTACGAGGTCA GGA
GATCAAGACCATCCTGGCTAACACGGTGAAACCCCGTCTCTACTAAAAATACAAAAAATTAGCTGGGCGTGGTGACGGCCCC TGTAGTCCCAGCTACTCAGGAGGCTGAGGCAGGACACCTGTAATC
CCAGCTACTTGAGATGCTGAAACAGGAGAGTGGCGTGAACTTGGGAGATGGAGCTTGCAGTGAGCCGAGATTGCGCCACTGCACTCCAGCCTGGGCGACAGAGCAAGACTCCGTCTCAAAAAACA AAA
AAcAAAAAACAAGAAAACAGTTTTCCAGTCTAAGAATGTATTACAATTTATTCAAATTCCACTCTAGATGGACTGTGGGTTTTTTTTTTTCCCCCATTTGGAGCTATGGCAAATGATGTTTTTTCAAA
GTTGTTATTTCTCAGCCAGGCGCGGTGGCTCACGCCTGTAATCCCAGTACTTTGGGAGACTGAGGTGGGCAGATCACCTGAGGTCAGAAGCAAGACCAGCCTGGCTAACATGGCGAAACCCCGTC TTT
TCTAAAAATACAAAAATTAGCCAGGTGTGGTGATGGGCACCTGTAATCCCAGCTACACAGGAGGCTGAGGCAGGATAATCACTTGAACCCAGGAGGTAGAGGTTGCAGTGAGCTGAGATCACACCACT
GCACTCCAGCCTGGGTGACAGAGCGAGACTCTATCTCAAAAAAGAAAACAAAACACCACGGAATTGTTATTTCTCTT GGCGAATAGGTAGATGCACTTATTCCTGTTAATATATACCTACCTGTGAAT
GTGCTTGTTGGATTTTCTATGTATCTTCTGTCTGCCACCTAGAAATTTAACCTTTTATATATATACAACTTTAATTTT TTTTTTTTTTTTTTTAAGAGACAGGGTGTCACTATGTTGCCCAGGCTGGT
TGGGAACTCCTGGCCTTAAGCCGTCCTCCTGCTTCAGTCTCCCAAAGTGTTGGGAATATAGGCGTGAGCCACTGTGCCCCACTGTTCAAGTTTTCATTGATTGCTGCCTACATATAGTTGTTCAA CAG
CTATTGATTCCCCCTGCTCTGTATATATGTCTCCTAGTGTAGGTATCAGGGTTACAGCAGTAATTAAGACCACATTATTTCATTTTATCATTTAAATATATAAGACTAATTGATAAATTAAGTATAGA
ACTTTGACCAACATGGTGAAACCCCATCTCTACTAGAAATACAAAAATTAGCTGGGTGTGGTGGCAGACGCCTGTAATCCCAGCTACTCAGGAGGCCGAGGCAGAACTGCTTGGAGATGGAGGTT GCA
GTGAACCAATATCAGACCACTATACTCCAGCTTGGATGACAGAGGGAGACTTTGTCTCTTTTTTTTTTTCTTTTTTTTTGAGACGGAATCTCGCCGTCTTCCAGGCTGGAGTGCAGTGGCACGATCTC
GGCTCACTGCAGCCTCCGCCTCCCGGGTTCAAGCGATTCTTCTACCTCAGCCTTCCGAGTAGCTGGGATTACAGGCACCCACCACCATGCCCGGCTAATTTTTGTATTTTTAGTAGACAGGGTTT CAC
CATGTTGGCCAGGCTGGTCTCAAACCCCTGACCTCAAGgGATCAACCTGCTTTGGTCTCCCAAAGTGCTAGGATTATAGGCGTGAGCCACTGTGCCCGGCCCTTTTTTTTTTTTTTGGAGACAGAATT
TCGCCCAGTTGCCAGACTGGAGTGCAGTGGCACGATCTCAGCTCACTGCAACCTCTGCTTCATGGGTTCAAGCCATTTTCCTGCCTCAGCCTCCCAAATAGCTGGGACTACAGGCATGCACCACC ACG
TCTGGCTAATTTTTTGTATTTTTAGTAAAGCCAGAGTCCCAAAGTGCTGGGACTAGGCAGGCGTGAACCACCACGCCTGGCCAAGACTCTGTCTCTCAAAAAAAAAAAAAAGAAAAAAAAATATAGGA
CTTTGGAGGCCGAGGCAGGCAGATCACCTGAGGTCAAAAGTTTGAGACCAGCCTGACTAACATGGTGAATCCCCATATCTACCAAAAAATACAAAAATTAGGCAGGTGTGGTGGCGTGCACCTG TAG
TCCCAGCTATTGGGGAAGCCGAGGTGGGAGATTGTACCTGGGAGGCAGTGAGCAGAGATCGCACCACTGCACTCCAGCCTGGGTGACAGAGTGAGACCTTGTCTCAC CaaaaaaaaaaaAAAAAAAAA
AATAGCATAGGTAGGCATTTGATGATTTGATGATTTCATTCGCATCCCTAAAAGTTTATTTGTTCCTGG gTCGTCAGATAGCTTTTTGGCCATCTTCCTGTTGAGAAAATTGATGTACCCTTCTGGAG
TCCTCCAATTTTCCATTATAATATGGTAAGTGGGAGCTAGAGCTTTGGGTAAGAATTGGGATGTGATAAGGAGGATGAGTTTTGCAGTGGTGTGCATGGTTAGGAGGAGAAAAAGCTGGAGGCAGAGT
GTTCACTTAGAGGCTTGGGGTAGGAGGGGTAGGTTTAAGTGGTGCTCATCTGGGCCAGAATAGGGCAAAAAGGGAAGAATGAAATAACCAGATGTCTTTGCTTTGTCAGTAGTCTTGCAGCCCTG AAA
GCTTTTTTTGTTGTGTTATATTTGTTGTAATTGAGGTATAATCCACATAACATAAAACTTACCTCTTTCAAGTGTACAATTTAGTAGTTTTTAGTATATTCATAAAATTGTGCAACTATCACCACTGA
tACCAGAACATTTCTGGGAACAAAAAGAAACTATATATCCATTAAGAGTCACTCTCCATTTTCTCCTACTTCCTTCTCTACCCCCAGTCATCTGCTAGTCGGCTTTCTGTCTCTATAGATTTGCCT GC
TCTGGATATTTCATATAAATGGAATCATATACCATATGGTCTTT

2168	10.3	3.8	0.0	Hg19Seq	63	354	(3658)	C	AluSx1	SINE/Alu	(9)	303	1	1	
903	24.4	7.2	0.3	Hg19Seq	388	666	(3346)	+	Kanga2_a	DNA/TcMar-Tc2	386	683	(204)	2	
913	27.1	7.9	3.9	Hg19Seq	674	1042	(2970)	C	L1ME3A	LINE/L1	(11)	6162	5780	3	
370	23.5	12.9	1.2	Hg19Seq	905	1059	(2953)	C	L1MD2	LINE/L1	(431)	5928	5756	4	*
2282	6.9	0.0	12.3	Hg19Seq	1079	1425	(2587)	+	AluY	SINE/Alu	1	309	(2)	5	
243	26.4	2.9	7.1	Hg19Seq	1427	1528	(2484)	C	L1ME3A	LINE/L1	(439)	5803	5706	3	
2268	10.8	0.7	0.0	Hg19Seq	1551	1845	(2167)	+	AluSx1	SINE/Alu	2	298	(14)	6	
856	15.5	0.0	0.0	Hg19Seq	1999	2127	(1885)	C	FLAM_C	SINE/Alu	(10)	133	5	7	
1148	14.4	5.8	0.0	Hg19Seq	2309	2482	(1530)	+	AluSx1	SINE/Alu	91	274	(38)	8	
2350	10.1	1.3	0.0	Hg19Seq	2483	2789	(1223)	C	AluSx1	SINE/Alu	(1)	311	1	9	
1338	14.1	0.5	0.0	Hg19Seq	2791	2981	(1031)	C	AluSx3	SINE/Alu	(15)	296	105	10	
279	9.3	0.0	4.7	Hg19Seq	2982	3026	(986)	C	Alu	SINE/Alu	(90)	43	1	11	
20	4.3	0.0	0.0	Hg19Seq	3042	3065	(947)	+	(A)n	Simple_repeat	1	24	(0)	12	
1700	13.4	5.3	0.4	Hg19Seq	3069	3330	(682)	+	AluSx	SINE/Alu	33	307	(5)	13	
286	22.9	3.5	3.5	Hg19Seq	3353	3438	(574)	+	Kanga2_a	DNA/TcMar-Tc2	754	839	(48)	14	
1310	21.6	2.6	3.0	Hg19Seq	3702	4012	(0)	C	L1MB7	LINE/L1	(137)	6197	5895	15	

>KSI_AluYa5_3

>Scaffold10222-341979-342230
GAAGGAATATCAACGAAAAATCGTGTATATCAATGGAAAAAATCAAACATTTAGTTCATGTTTCACAACAATTTTAACAATGTAGTGATTCTAATTGCCCTATTTCAAGTTTAAATAAATTTGTAA TA
TATAATTTGCATTTAAATGTTTCCCCTAAATTTTGATTTATAAACAACATTTCAATGGCTTTGTTGAGTCAACCATAGAACTCAAAAATTCTTATTCAGATCTTTCCCTGTTCCCACTTATGTTG CCT
ATGCTAGAATTGTCAGTGTTGTGGGACAGAGACAAgTAGGTGAGTCCCAGTAAAAGTTTTCACTTCCTCTATTGTAAGAACCCTTTACCTGTGAACATAACCACCAAAGTCTAAAAATACAGTATTTA
GGCTGTCTTGCATACAGTCTGGCCTGTGACTAAGTTCTGGCATAGAAGTGTTACAAGGAATCTTCTAAGGATCCTTTGTCAAAGACAATTCGAACACTTTATTCTTCTCTCTTATATCGCCTGGA ATA
AGGATGGGGTACCTGGAGCTCTGGATCATGTATTTCATAAGGGATAAATTGTTTAGCACAAGGGAGTATCAATCTCTAAAAACTTCATGGGCTAAGAAGTTTTATCATCATTAGACCACCTGGCTC TT
GACTATTATGAGGGAAAAATAACTAACTTATTGAAGTCACTGATATATGGGTTATGTGCAACTCAGAAGGAACTTAACATTTGCTGCTACAAATTTGTGCTAACAGCTTCTGTTTATGGTTTGCT ATA
ATACATCTTACAAATATAAGGTTAAACTTCGTTTCTATTTTATACAAATAAACACAGCGATTAAAAAGATAATGCTTCTTACATGAAACAATTGTGGTAGATACTAGCTAGTGCATCTTGGATATT TG
TGGATAACAGTTCTGCTCTACCACTAGCCCCAGGGTACCTCACCATCCCTGCCTATACCCTTATAAATGTGTTGCTTTATTAAGGCATTTGAAATGACTCCCTCTGAGTGTGTTACCTGTGCAAA TTG
AACACTGAAAATGGCATAATTAATACTAAGTTTTCCACTAATTGGCATAGGATGAAATGTGGGTGTAGAGGCAAGCAGGTGACAGATTAAGTCGTTTGGCAAGTATAAATACTACACATAATTTAG CT
TCATTTAACTGCTCTAGAGGGAAACTAGTCAGGCCAAATGTGTTGATATGGATAAACTAACTCAGAATTAGGAAATACTTTAAGTACCTGGGAACTGTCAGCTAGCTTAGTTAATTAAATTCTCA ACT
CAGATGGGACTTTAGTCAGTGAGATACAAATTTCGGAATATCCCTGGAATATAGAAGGGGAAGAAACCCAGTGACTCAGGGAGAATAGCAATTTTTCAGCGGCCTTTGATGAGTAGCCTATGGGTA TC
TAGGTGTCCTATTCCCCAGGCACCCTAAGTATTACTTTGTGTCTCCATGGTTATTCAG TTCAACACAAGCAACTTGTTTCCAGGTGGCTGGAATTACCTCTGAAAAGTGGTATTTTATATGGGCTGTA
TGTTGGTAGGTTAGGCACTTAGCAATGAGGGTAAACAGATTATCCATGGTGAGCAAGGGTCTATTTTTAGTGTCCATGGAGAGCCTCAATATTTATCACATGGTCACTTTGTATATGGACCAACAA TT
CATGCTTGGTTGTTAGAATTATTCACCTCGGCCAGTTAGAAAACTGACCTCTTTTTCTGCCTATTGGTTCAGTGGCATAAATCTCAAAACTGTTGGCAGGTGGGTAAGAAAAGTCAGCCTCAACT TCC
AAACCACTGAAGATGTGAATACTTTTCTAGGGAGAAATATTTATCCATTGAGTACCAAAACTTCCAGAT GTCCTAATCTGATTCAAGATTTGGAAGAAACAGTATCTTACATATATCTTACAATATCT
TCTATATCTTTAGTTGTAATTGGGAGAGAGCTCCTGCCCCTGTCTTCCACCCAATTCTCAGACCAAGATATTCTCACTAAGGAGTAGcagtggcggggtctcggctcactgcaagctccgcctcccgg
gttcacgccattctcccgcctcagcctcccaagtagctgggactacaggcgcccgccactacgcccggctaatttttttgtatttttagtagagacggggtttcaccgttttagccgggatggtct cga
tctcctgacctcgtgatccgcccgcctcggcctcccaaagtgctgggattacaggcgtgagccaccgcgcccggc CCACATCCGATTTCTTAAAATCTCTGGCATATCATCCCATCATTCTACTTGAG
CCTGCTCTTCCTAATCATGTGTTCAAACCAATAAATCCTGTGCATGTAATCATATTGTTATATTTCCTTTACCGTAAGTTAAGGAAAGTGGTCCTATGAGATTACAAGTTGATACATAGGCATTCTGT
AAGACCATGAGAGGTAATAAGGTATAAGGAAGGGGTCCAGTTTCCATTTTCTGCATATGGCTGGTCAATTCTGCCAGCATCATTTAT TAAATATGGAATCATTTCCTTATTGCTTGTTTTTGTCAGGC
TTGTCAAAAATCAGATGGTTGATGTGTGGTTTTATTTCTGAGTTCTCTATTCTGTTCCATTGATCTGTGTACAAGTACCATGTTGTTTTGGTTACTGTAGCCTTGTAGCATAGTTTGAAGTCAGG TAG
CGTGATGCCTCCAGCTTTGTTCTTTTTGCTTAGGATTGTCTTGGCTATATGGGCCCTTTTTTGGTTCCATATGAAATTTAAAGTAGT TTTTTTCTAATTCTATGAAGAAAATCAATGGTAGTTGGGGA
TATAGCACTGAATCTATAAATTACTTTAGCAGTATGGCCATTTTCATGATATTGATTCTTCCTATCCATGAGCATGGAACGTTTTTCTGTCTGTTTGTGTTGTCTCTGATTTCCTTGAGCAGTGG TTT
GTAGTTCTCCTTGAAGAGGTCCTTGACCTCAATTGTTAGCCGTATTCCTAGGTATTTTATTCTCTTGTGGCAAAAATTAACTCAAGG TGGATTAGACACTTAAATGTAAAACCCAAAACTATAAAAAC
CCTAAAAGAAAACCTAAGCCATACTATTCAGGACCTAGGCATGGGCAAAGATTTTATGATGAGATCATCAAAAGCAATTGCTACAAAAGCAACAATTGACAAATGGGATCTAATTGCATTAAAGA GCT
TCTGCACAGCAAAATAAACTATCATCAGAGTGAACAGGCAACCTACAGAATGGGAGAACATTTCTGCAATCTGTCTTTCTGACAAAG GTCTAATATCCTGTATCTACAAGGAACTTAAGCAAATTTAC
AAGGATAAAAATGAACAATCCCATTAAAAAGTGGGCAAAGGATAGGAATACACACCTCTCAAGAGAAGGCATACATGCAGACAACAAACATATGAAAAAAAGCTCAACATCACTGATGATCAGAG AAA
TGCAAATCAAAACCACACAGTGAAATATCATCTTGTGCCAATCAGAAAGGCAATTATTAAAAAGTCAATAAACAGATGCTGCAAGGT TGAGGAGAAATAGGAACATTTTTATGGTGTTGGTGGGAATG
GAAATTAGTTTAACCATTGTGGAAGATAGTGTAGTGATTCCTCGAGAATCTAGAACCAGAAATACCATTTGACACAGCAATCCCTTTACTGGGTATACACACCCAAAGGAATATAAAGCATTCTA TTA
TAAAGATACATGCACACATATGTTCATTGCATCACCATTTACAATAGCAAAGATGTGGAATTAACCTAAATCCCCATCAATGATAAA CTGGGTAAAGAAAATATAGTACATATACACCATGGAATACT
ATGCCACCATAAAAGGAAGATCATGTCCTTTGCAGGGACATGGATGAAGCTGGAAGCTATTATTCTCAGTAAACTAATGCAGGAATAAAATAACCAAACACTGCATGTTTTCACTTACAGTGGGA GCT
GAACACATGGACACAGGGAGCGGAACAATAAACTCTGGGG CCTCTTGGGGAGGTGAGGGAGCATCAGGAAAAATAGCTAATGCATGC CAGGCTTAATACCTAGGTGATGGGTTAATAGGTGCAGCAAA
CCATCATGGCACACATTTACCTACGTAACAAACCTGCACATGCTGCGCATGCACTCTGAAACTTAAAATGAAATAAAATTTAAAAAAAGAAAGGGAATAATGCCAGAAGCATTGCAATAAAGAAA GGA
CATTTAATACTCAGAATATATCGATACT

319	24.6	8.7	2.5	TeRefseq	276	425	(3827)	+	MLT1J	LTR/ERVL-MaLR	1	159	(353)	1	
307	28.5	17.4	1.4	TeRefseq	896	1016	(3236)	+	LTR33	LTR/ERVL	350	489	(26)	2	
466	32.5	9.4	0.5	TeRefseq	1466	1657	(2595)	C	ERVL-E-int	LTR/ERVL	(1416)	4251	4043	3	
379	27.1	5.5	14.8	TeRefseq	1660	1858	(2394)	C	ERVL-E-int	LTR/ERVL	(699)	4968	4786	4	
2414	0.8	0.0	0.4	TeRefseq	2001	2252	(2000)	C	AluYa5	SINE/Alu	(60)	251	1	5	
3974	7.9	3.5	0.3	TeRefseq	2449	3017	(1235)	C	L1PA10	LINE/L1	(1169)	4977	4391	6	*
5729	11.0	2.1	0.8	TeRefseq	3015	4184	(68)	+	L1PA10	LINE/L1	4979	6167	(1)	6	

```
>Hg19seq chr21:21322512-21326525
tagacgaagttagcttttacttgcacacaatgtggaattttggtgaagcagaaaatcacctcattatgaaaaatatattgatggtttcaatatcaatga GAAGGAATATCAACGAAAAATCGTGTAT
ATCAATGGAAAAAATCAAACATTTAGTTCATGTTTCACAACAATTTTAACAATGTAGTGATTCTAATTGCCCTATTTCAAGTTTAAATAAATTTGTAATATATAATTTGCATTTAAATGTTTCCC CTA
AATTTTGATTTATAAACAACATTTCAATGGCTTTGTTGAGTCAACCATAGAACTCAAAAATTCTTATTCAGATCTTTCCCTGTTCCCACTTATGTTGCCTATGCTAGAATTGTCAGTGTTGTGGG ACA
GAGACAAcTAGGTGAGTCCCAGTAAAAGTTTTCACTTCCTCTATTGTAAGAACCCTTTACCTGTGAACATAACCACCAAAGTCTAAAAATACAGTATTTAGGCTGTCTTGCATACAGTCTGGCCTGTG
ACTAAGTTCTGGCATAGAAGTGTTACAAGGAATCTTCTAAGGATCCTTTGTCAAAGACAATTCGAACACTTTATTCTTCTCTCTTATATCGCCTGGAATAAGGATGGGGTACCTGGAGCTCTGGA TCA
TGTATTTCATAAGGGATAAATTGTTTAGCACAAGGGAGTATCAATCTCTAAAAACTTCATGGGCTAAGAAGTTTTATCATCATTAGACCACCTGGCTCTTGACTATTATGAGGGAAAAATAACTA ACT
TATTGAAGTCACTGATATATGGGTTATGTGCAACTCAGAAGGAACTTAACATTTGCTGCTACAAATTTGTGCTAACAGCTTCTGTTTATGGTTTGCTATAATACATCTTACAAATATAAGGTTAA ACT
TCGTTTCTATTTTATACAAATAAACACAGCGATTAAAAAGATAATGCTTCTTACATGAAACAATTGTGGTAGATACTAGCTAGTGCATCTTGGATATTTGTGGATAACAGTTCTGCTCTACCACT AGC
CCCAGGGTACCTCACCATCCCTGCCTATACCCTTATAAATGTGTTGCTTTATTAAGGCATTTGAAATGACTCCCTCTGAGTGTGTTACCTGTGCAAATTGAACACTGAAAATGGCATAATTAATA CTA
AGTTTTCCACTAATTGGCATAGGATGAAATGTGGGTGTAGAGGCAAGCAGGTGACAGATTAAGTCGTTTGGCAAGTATAAATACTACACATAATTTAGCTTCATTTAACTGCTCTAGAGGGAAAC TAG
TCAGGCCAAATGTGTTGATATGGATAAACTAACTCAGAATTAGGAAATACTTTAAGTACCTGGGAACTGTCAGCTAGCTTAGTTAATTAAATTCTCAACTCAGATGGGACTTTAGTCAGTGAGAT ACA
AATTTCGGAATATCCCTGGAATATAGAAGGGGAAGAAACCCAGTGACTCAGGGAGAATAGCAATTTTTCAGCGGCCTTTGATGAGTAGCCTATGGGTATCTAGGTGTCCTATTCCCCAGGCACCC TAA
GTATTACTTTGTGTCTCCATGGTTATTCAGTTCAACACAAGCAACTTGTTTCCAGGTGGCTGGAATTACCTCTGAAAAGTGGTATTTTATATGGGCTGTATGTTGGTAGGTTAGGCACTTAGCAA TGA
GGGTAAACAGATTATCCATGGTGAGCAAGGGTCTATTTTTAGTGTCCATGGAGAGCCTCAATATTTATCACATGGTCACTTTGTATATGGACCAACAATTCATGCTTGGTTGTTAGAATTATTCA CCT
CGGCCAGTTAGAAAACTGACCTCTTTTTCTGCCTATTGGTTCAGTGGCATAAATCTCAAAACTGTTGGCAGGTGGGTAAGAAAAGTCAGCCTCAACTTCCAAACCACTGAAGATGTGAATACTTT TCT
AGGGAGAAATATTTATCCATTGAGTACCAAAACTTCCAGATGTCCTAATCTGATTCAAGATTTGGAAGAAACAGTATCTTACATATATCTTACAATATCTTCTATATCTTTAGTTGTAATTGGGA GAG
AGCTCCTGCCCCTGTCTTCCACCCAATTCTCAGACCAAGATATTCTCACTAAGGAGTAGttgtcaCCACATCCGATTTCTTAAAATCTCTGGCATATCATCCCATCATTCTACTTGAGCCTGCTCTTC
CTAATCATGTGTTCAAACCAATAAATCCTGTGCATGTAATCATATTGTTATATTTCCTTTACCGTAAGTTAAGGAAAGTGGTCCTATGAGATTACAAGTTGATACATAGGCATTCTGTAAGACCATGA
GAGGTAATAAGGTATAAGGAAGGGGTCCAGTTTCCATTTTCTGCATATGGCTGGTCAATTCTGCCAGCATCATTTATTAAATATGGAATCATTTCCTTATTGCTTGTTTTTGTCAGGCTTGTCAA AAA
TCAGATGGTTGATGTGTGGTTTTATTTCTGAGTTCTCTATTCTGTTCCATTGATCTGTGTACAAGTACCATGTTGTTTTGGTTACTGTAGCCTTGTAGCATAGTTTGAAGTCAGGTAGCGTGATG CCT
CCAGCTTTGTTCTTTTTGCTTAGGATTGTCTTGGCTATATGGGCCCTTTTTTGGTTCCATATGAAATTTAAAGTAGTTTTTTTCTAATTCTATGAAGAAAATCAATGGTAGTTGGGGATATAGCA CTG
AATCTATAAATTACTTTAGCAGTATGGCCATTTTCATGATATTGATTCTTCCTATCCATGAGCATGGAACGTTTTTCTGTCTGTTTGTGTTGTCTCTGATTTCCTTGAGCAGTGGTTTGTAGTTC TCC
TTGAAGAGGTCCTTGACCTCAATTGTTAGCCGTATTCCTAGGTATTTTATTCTCTTGTGGCAAAAATTAACTCAAGGTGGATTAGACACTTAAATGTAAAACCCAAAACTATAAAAACCCTAAAA GAA
AACCTAAGCCATACTATTCAGGACCTAGGCATGGGCAAAGATTTTATGATGAGATCATCAAAAGCAATTGCTACAAAAGCAACAATTGACAAATGGGATCTAATTGCATTAAAGAGCTTCTGCAC AGC
AAAATAAACTATCATCAGAGTGAACAGGCAACCTACAGAATGGGAGAACATTTCTGCAATCTGTCTTTCTGACAAAGGTCTAATATCCTGTATCTACAAGGAACTTAAGCAAATTTACAAGGATA AAA
ATGAACAATCCCATTAAAAAGTGGGCAAAGGATAGGAATACACACCTCTCAAGAGAAGGCATACATGCAGACAACAAACATATGAAAAAAAGCTCAACATCACTGATGATCAGAGAAATGCAAAT CAA
AACCACACAGTGAAATATCATCTTGTGCCAATCAGAAAGGCAATTATTAAAAAGTCAATAAACAGATGCTGCAAGGTTGAGGAGAAATAGGAACATTTTTATGGTGTTGGTGGGAATGGAAATTA GTT
TAACCATTGTGGAAGATAGTGTAGTGATTCCTCGAGAATCTAGAACCAGAAATACCATTTGACACAGCAATCCCTTTACTGGGTATACACACCCAAAGGAATATAAAGCATTCTATTATAAAGAT ACA
TGCACACATATGTTCATTGCATCACCATTTACAATAGCAAAGATGTGGAATTAACCTAAATCCCCATCAATGATAAACTGGGTAAAGAAAATATAGTACATATACACCATGGAATACTATGCCAC CAT
AAAAGGAAGATCATGTCCTTTGCAGGGACATGGATGAAGCTGGAAGCTATTATTCTCAGTAAACTAATGCAGGAATAAAATAACCAAACACTGCATGTTTTCACTTACAGTGGGAGCTGAACACA TGG
ACACAGGGAGCGGAACAATAAACTCTGGGGCCTCTTGGGGAGGTGAGGGAGCATCAGGAAAAATAGCTAATGCATGCCAGGCTTAATACCTAGGTGATGGGTTAATAGGTGCAGCAAACCATCAT GGC
ACACATTTACCTACGTAACAAACCTGCACATGCTGCGCATGCACTCTGAAACTTAAAATGAAATAAAATTTAAAAAAAGAAAGGGAATAATGCCAGAAGCATTGCAATAAAGAAGGACATTTAA TAC
TCAGAATATATCGATACTtatgagaataaactcctgactccgcattcacccacggacatttttaaaatctcatgtcaaagaaccaacaaactgaaaagggtgaccaaagtgaataaat
```

343	23.9	8.7	2.5	Hg19seq	376	525	(3689)	+	MLT1J	LTR/ERVL-MaLR	1	159	(353)	1	
307	28.5	17.4	1.4	Hg19seq	996	1116	(3098)	+	LTR33	LTR/ERVL	350	489	(26)	2	
466	32.5	9.4	0.5	Hg19seq	1566	1757	(2457)	C	ERVL-E-int	LTR/ERVL	(1416)	4251	4043	3	
379	27.1	5.5	14.8	Hg19seq	1760	1958	(2256)	C	ERVL-E-int	LTR/ERVL	(699)	4968	4786	4	
3974	7.9	3.5	0.3	Hg19seq	2311	2879	(1335)	C	L1PA10	LINE/L1	(1169)	4977	4391	5	*
5729	11.0	2.1	0.8	Hg19seq	2877	4046	(168)	+	L1PA10	LINE/L1	4979	6167	(1)	5	

>KSI_AluYa5_4

```
> Scaffold4348-551877-552175
CAAAGTGCTGGGATTACAGGTATGAGCTACCGCGCCCGGCACAAAGCATTTTCAGCTGTGAAATTCCCTGGGGTGCTGCAGTAGAGGAAGGTTTGGGGTCTTGACGGGTGCTATTTGCCACGGAAA GA
TGCCTTCCTGCTCCCAGGGCAGGAGAGCCGAGGTAAGACTTACTGTAGGCTGTCGTTTTTTTTGTTTGTTTTTTGTCTTTGCGATGGAGTCTCACTCTGTCGCCAGGCTGGAGTGCAGTGGCATG ATC
TTGGCTCACTGCAGAACCTCgcCCTCCCAGGTTCAAGCGATTCTCCTGCCTCAGCCTCCCAAGTAACTGGGATTACAGGCACATGCCCCCACAACCAGCTAATTTTTTATTTTTAGTAGAGACAGGGT
TTCACATGTTGGCCAGGCTGGTCTTGAACTCCTGACCTCAGGTGATCCGCCCGCCTCGGCCTCCCAAAGTGCTGGGATTACAG gCATGAGCCACTGCGCCCAGCCAGGCTGTTGTTTTTTTACCTCCT
TGTTTGCACAATTTGGGCCACTCACAAGAGTGTATACCCTGTGATAAACAGTTACCTACATTCTCCTCTGCATGCTTGTCTTTAGAGGAAGGAAATGTATTAATTGCCCAAAGTAATATATTGTGT TA
AGATGTGATATATACTGGGGAAAAAAAAAGTGTATATTGACATTTCTGGAATAAACCACTTTGATTCCCAGTTACTGACTCGTTCTCCTGTGTGGTGTCTTTCCTGTATGCAGAAGGCCGGCTTC CCC
ACAGGGGCTGGGGGTGACTTGTCTGTCCTTGGAATATGAGTCTTTGCAAGCAGCTGCTGCCCATGTGCACAGTTCCTCGAGGAGTTGGTTCAATCTTGGTTTGGGAGTTTGATTGGAAGGATTAGA TC
CTAGGCTCCCCTGAGCAGTAACATCTTATTTATAGCGTGGAAAATGGACAGAGCTGTTGATACTTAGGAGAGCTTTCCTCATTATCACCCCGAGGAGAGTGCCTGAGTCTCCAAGAGGTCATTGT TGG
TCTGCAAGCTCTTAAGGCAGAAGAGTCTGCCAGTAACAGGGAAATCAGGACAGGATGGGAATGAGTGTCCCAAGGCAGTGACAGAAGCTGAGGCTGAACCTTCCCAGGAATCCTCCACCTTCAGGG TG
CCACCTTGTCTCTGCATGAAGTGTCTGCAGACACACTGCCTGAAAGTGGCTCCTTAACAATGAACCTGCTTGCCTTTTCTCTGTCACTAGACCCCAAGACAATGAAGAGCCTTCATTGGCTGGGC ATG
GTGGCTTGCGCCTGTAATCCCAGCACTTTAGGAGGCCGAGGCGGGTGGTCAGGAGTTCGAAAGCAGCCTGACCAACATGGTGAAACCCCATCTCTACTAAAAATACAAAATTCAACCAGGTGTCGT GG
CAGGCACCTGTAATCCCAGCTGCTCGGGAGGCTGAGGCATGAGAATCAGTTGAACCCAGGAGACAGAGGTTTCGGTGAGCCAAGGTCACACCACTGCACTCCAGCCTAGAAGACAGAGCAAGACT TTG
TCTCAAAAAAAAAAATTAACTAATTACTtAAATAAATAAACAGCCTTCACTAATTCTCCTACACAGTAATGGGCGCGCTCCTCCTACACAGGACTGGCATGCTGGGCATCTTTCTGGGTGATGGGAGG
GAGGCCTAGACTCCATGGGTGGAGGCCTAGGAAGGTTTTGTGTGGCTGTCAGGAACATTCCCTGGCCTTGGCTGTCTCCTGCTGTCATTCAGTTCCAGCTCCTTGCAGTCAGTCTTTTTTTTTTTTTT
TTTTTTAAATAGAGATGAGGTCTCACTGTCTTGCCCAGACTGGTCTTGAACTCCTGGGCTCAGGTGATCCTCCCACCTCAGCCTCCCAAAGTGTTGGAATCACAGGCATGAGCCATCACGCCTGG CTA
CAGATGGTGCTTTCAATATTAGCAATGGATAATTAAATATGTGGTTAAGAAGTTGTATTTAAGGTTAAAAgTCACTATGAGGCTGGGTGTGGTGGCTCAAACCTGTAAT CCCAGCACTTTGGGCCAAG
GCAGGCGGATCACCTGAGGTCaagagatcgagaccatcccggctaaaacggtgaaaccccgtctctactaaaaatacaaaaaaattagccgggcgtagtggcgggcgcctgtagtcccagctacttgg
gaggctgaggcaggagaatggcgtgaacccgggaggcggagcttgcagtgagccgagatcccgccactgcactccagcctgggcgacagagcgagactccgtctcaaaa aaaaaaaaaaaaAAGAGTT
CGAGACCAGCCTGGCCAACATGCTGAAACCCTGTCTCTACTAAAAATACAAAAATTAGCCAGGCGTTATGTTGCGTACCTATAATCCCAGCTACTCGGGAGGCTGAGGCAGGAGAACCGCATGAACCT
GGGAGGCAGCGGTTGCAGTGAGCCGAGATAGCGCCACTACACTCCAGCCTGGGTGACAGTGAGACTCCATCTCAAAAAAAAAAAAaGTCACCGTGGGACACA gGCCCTGTATCAGAAAAGGTATAGAAA
TTAAAGAACAGTTAAGTCAAGATTTTGAATTTCTCTCTTCGTCCACTAGAGGATGTAATCCAACACCAGACTGTGTTCTCTGATGTAACTTGGTATAGAGTTTGGGTTTTGGGGCTTTTTTGTTCTGT
TTTGATTTGTGTAATTTAGCCAAGACTTTCTGTGACCAAAAAGTTATGTCTGTTGTAACTTGGTACAGA GTTTGGATTTTGGGGTTTTTTTGTTTTGTTTTGATTTGTG TAATTTAGCCAAGACTTTC
TGTCACCAAAAAGTTATGCAGAAGATTCATCTGCGGGGCTGGGCTCTTAGTTACGAATCTGTCCGGTTGTCCCAGTGGAATCAGTCCAGGTGTCTTGGTTCGTGATAAGTGCTTTCCTTGGAAGC GTT
TATCATGTCAGGAACCAAGCACAGGCACTGCACGTGGAGATAAGAGCGTGTTCTAATACATTTCTCCGTTCCAATGTACTCCCAGTGCCAGCTGGTGAGAACATCGGGA AAGATTTCTTTGTAAGGA
ATCCAGTGTTAACACGTACCAGTATTTGAAGTCCCAGACAGTGTGCATTAAGGGTTTAGAAGTGTCTCTGGCATGAACATTGCTTATAACTTGAAATTGACAGGTACTTATCAAGACCAAACAAA GTT
TGCACAAAGCCAGCACAGTTAAGAGTGCTGATTGTGGCCGGGCACGGTGGCTCACGCCTGTAATCCCAGCACTTTGGGAGGCCGAGGCGGGCGGATCACAAGGTCAAGA GATCAAGACCATCCTGGCC
AACATGGTGAAACCCCGTCTCTATTAAAAGTACAAAAATTAGCCGGGCGTAGTGGCGCACGCCTGTAATCCCAACTACTCGGGAGGCTGAGGCAGGAGAATCTCTTAAACCTGGGAGGCAGAGGT TGC
AGTGAGCCGGGATTGTGCCACTGCACTCCAGCCTGGCGACAGAGCAAGACTCCATCTCAAGAAAAAAAAATTTAAAAAAAAAGAGTGCTGATTGTTAGCTGGTGGGAAAGT GGACTTGCTGGGTGGGGGC
GGGGGAGTGGGGAGAATGACCATTCTGAAAGTGTAATCTTCAACAGAGTACTGTACGTTCTCTGGCTTATTAGCCCGGTGAACAGCTTTCTGATGGTTTTAGTTGAACAAGTATTGCTGGATATG TTA
AAATACAGCCAGAGGCAAAACATACTCCCTCCTCTCAGAGAACTTATATCCCAGGATAGGGGAGACTGGAAACAGGTCATTAGAATAT GCAGAATAACAGATGAGTGCTATGGAGAGAAAGACAGCAG
CCCCACTACGGAGGAGCACGGGATGGGGTTTGtAATTTTAGATGGGTGGCCTTTAACCAAATCCTTCAGTGAGAGTAAGTCACATGATGACCAGGAAGAGCACCCCAGCAGGGGAGGCAGCCAGTGCC
CTGGCACACAAGCAAAAGGAGTTGATGAGAGGGCTGGGGGGCTGAGGCCAGATCTCATGGGGCCTCACATGGAGGAAAGTGTGGGACTTCTGCCTTTTCTCTGAATAGT GTCCGTAGCTTACATATTC
TATCTACTCTTGCCTTTTAGGAACCATTTTTCCCTCTAATGGTAGTTTACGTTAGAAGTATTTCCTGTTCTAGGCCCGGTGCAGTGGCTCATGTCTGTAATCCCAGCACTTTGGGAGGCCAAGGC AGG
TGGATCACCTGAGGTCAGGAGTTCGAAACCAGCCTGGCCAACATGGCAAAACCCCATCTCTACTAAAAATACAA A
```

309	7.5	0.0	0.0	TeRefseq	1	40	(4259)	C	Alu	SINE/Alu	(92)	41	2	1
2318	8.8	1.0	0.7	TeRefseq	184	489	(3810)	C	AluSx	SINE/Alu	(5)	307	1	2
1989	13.5	3.9	0.0	TeRefseq	1270	1551	(2748)	+	AluSx1	SINE/Alu	1	293	(19)	3
950	13.3	0.0	2.3	TeRefseq	1784	1918	(2381)	C	FLAM_C	SINE/Alu	(10)	133	2	4
2606	2.7	1.0	1.0	TeRefseq	2001	2299	(2000)	+	AluYa5	SINE/Alu	1	299	(12)	5
1698	9.2	0.9	0.0	TeRefseq	2300	2517	(1782)	+	AluSx	SINE/Alu	75	294	(18)	6
14	16.0	0.0	5.4	TeRefseq	2758	2796	(1503)	+	(GTTTT)n	Simple_repeat	1	37	(0)	7
2528	6.3	0.0	1.0	TeRefseq	3236	3536	(763)	+	AluSc	SINE/Alu	1	298	(11)	8

383	31.8	10.8	4.6	TeRefseq	3692	3992	(307)	C	L2a	LINE/L2	(43)	3383	3018	9
1075	9.2	0.0	0.0	TeRefseq	4169	4299	(0)	+	AluSx	SINE/Alu	1	131	(181)	10

>Hg19seq chr17:75090360-75094430

CAAAGTGCTGGGATTACAGGTATGAGCTACCGCGCCCGGCACAAAGCATTTTCAGCTGTGAAATTCCCTGGGGTGCTGCAGTAGAGGAAGGTTTGGGGTCTTGACGGGTGCTATTTGCCACGGAAA GA
TGCCTTCCTGCTCCCAGGGCAGGAGAGCCGAGGTAAGACTTACTGTAGGCTGTCGTTTTTTTTGTTTGTTTTTTGTCTTTGCGATGGAGTCTCACTCTGTCGCCAGGCTGGAGTGCAGTGGCATG ATC
TTGGCTCACTGCAGAACCTCcaCCTCCCAGGTTCAAGCGATTCTCCTGCCTCAGCCTCCCAAGTAACTGGGATTACAGGCACATGCCCCCACAACCAGCTAATTTTTTATTTTTAGTAGAGACAGGGT
TTCACATGTTGGCCAGGCTGGTCTTGAACTCCTGACCTCAGGTGATCCGCCCGCCTCGGCCTCCCAAAGTGCTGGGATTACAG aCATGAGCCACTGCGCCCAGCCAGGCTGTTGTTTTTTTACCTCCT
TGTTTGCACAATTTGGGCCACTCACAAGAGTGTATACCCTGTGATAAACAGTTACCTACATTCTCCTCTGCATGCTTGTCTTTAGAGGAAGGAAATGTATTAATTGCCCAAAGTAATATATTGTGT TA
AGATGTGATATATACTGGGAAAAAAAAAGTGTATATTGACATTTCTGGAATAAACCACTTTGATTCCCAGTTACTGACTCGTTCTCCTGTGTGGTGTCTTTCCTGTATGCAGAAGGCCGGCTTC CCC
ACAGGGGCTGGGGGTGACTTGTCTGTCCTTGGAATATGAGTCTTTGCAAGCAGCTGCTGCCCATGTGCACAGTTCCTCGAGGAGTTGGTTCAATCTTGGTTTGGGAGTTTGATTGGAAGGATTAGA TC
CTAGGCTCCCCTGAGCAGTAACATCTTATTTATAGCGTGGAAAATGGACAGAGCTGTTGATACTTAGGAGAGCTTTCCTCATTATCACCCCGAGGAGAGTGCCTGAGTCTCCAAGAGGTCATTGT TGG
TCTGCAAGCTCTTAAGGCAGAAGAGTCTGCCAGTAACAGGGAAATCAGGACAGGATGGGAATGAGTGTCCCAAGGCAGTGACAGAAGCTGAGGCTGAACCTTCCCAGGAATCCTCCACCTTCAGGG TG
CCACCTTGTCTCTGCATGAAGTGTCTGCAGACACACTGCCTGAAAGTGGCTCCTTAACAATGAACCTGCTTGCCTTTTCTCTGTCACTAGACCCCAAGACAATGAAGAGCCTTCATTGGCTGGGC ATG
GTGGCTTGCGCCTGTAATCCCAGCACTTTAGGAGGCCGAGGCGGGTGGTCAGGAGTTCGAAAGCAGCCTGACCAACATGGTGAAACCCCATCTCTACTAAAAATACAAAATTCAACCAGGTGTCGT GG
CAGGCACCTGTAATCCCAGCTGCTCGGGAGGCTGAGGCATGAGAATCAGTTGAACCCAGGAGACAGAGGTTTCGGTGAGCCAAGGTCACACCACTGCACTCCAGCCTAGAAGACAGAGCAAGACT TTG
TCTCAAAAAAAAAAAaTTAACTAATTACTgAAATAAATAAACAGCCTTCACTAATTCTCCTACACAGTAATGGGCGCGCTCCTCCTACACAGGACTGGCATGCTGGGCATCTTTCTGGGTGATGGGAG
GGAGGCCTAGACTCCATGGGTGGAGGCCTAGGAAGGTTTTGTGTGGCTGTCAGGAACATTCCCTGGCCTTG GCTGTCTCCTGCTGTCATTCAGTTCCAGCTCCTTGCAGTCAGTCTTTTTTTTTTTTT
TTTTTTAAATAGAGATGAGGTCTCACTGTCTTGCCCAGACTGGTCTTGAACTCCTGGGCTCAGGTGATCCTCCCACCTCAGCCTCCCAAAGTGTTGGAATCACAGGCATGAGCCATCACGCCTG GCT
ACAGATGGTGCTTTCAATATTAGCAATGGATAATTAAATATGTGGTTAAGAAGTTGTATTTAAGGT TAAAAaTCACTATGAGGCTGGGTGTGGTGGCTCAAACCTGTAATCCCAGCACTTTGGGCCAA
GGCAGGCGGATCACCTGAGGTCAAGAGTTCGAGACCAGCCTGGCCAACATGCTGAAACCCTGTCTCTACTAAAAATACAAAAATTAGCCAGGCGTTATGTTGCGTACCTATAATCCCAGCTACTCGGG
AGGCTGAGGCAGGAGAACCGCATGAACCTGGGAGGCAGCGGTTGCAGTGAGCCGAGATAGCGCCACTACACTCCAGCCTGGGTGACAGTGA GACTCCATCTCAAAAAAAAAAAAGTCACCGTGGGACAC
AaGCCCTGTATCAGAAAAGGTATAGAAATTAAAGAACAGTTAAGTCAAGATTTTGAATTTCTCTCTTCGTCCACTAGAG GATGTAATCCAACACCAGACTGTGTTCTCTGATGTAACTTGGTATAGAG
TTTGGGTTTTGGGGCTTTTTTGTTCTGTTTTGATTTGTGTAATTTAGCCAAGACTTTCTGTGACCAAAAAGTTATGTCTGTTGTAACTTGG TACAGAGTTTGGATTTTGGGGTTTTTTTGTTTTGTTT
TGATTTGTGTAATTTAGCCAAGACTTTCTGTCACCAAAAAGTTATGCAGAAGATTCATCTGCGGGGCTGGGCTCTTAGTTACGAATCTGTCCGGTTGTCCCAGTGGAATCAGTCCAGGTGTCTTG GTT
CGTGATAAGTGCTTTCCTTGGAAGCGTTTATCATGTCAGGAACCAAGCACAGGCACTGCACGTGGAGATAAGAGCGTGTTCTAATACATTT CTCCGTTCCAATGTACTCCCAGTGCCAGCTGGTGAGA
ACATCGGGAAAGATTTTCTTTGTAAGGAATCCAGTGTTAACACGTACCAGTATTTGAAGTCCCAGACAGTGTGCATTAAGGGTTTAGAAGTGTCTCTGGCATGAACATTGCTTATAACTTGAAAT TGA
CAGGTACTTATCAAGACCAAACAAAGTTTGCACAAAGCCAGCACAGTTAAGAGTGCTGATTGTGGCCGGGCACGGTGGCTCACGCCTGTAA TCCCAGCACTTTGGGAGGCCGAGGCGGGCGGATCACA
AGGTCAAGAGATCAAGACCATCCTGGCCAACATGGTGAAACCCCGTCTCTATTAAAAGTACAAAAATTAGCCGGGCGTAGTGGCGCACGCCTGTAATCCCAACTACTCGGGAGGCTGAGGCAGGA GAA
TCTCTTAAACCTGGGAGGCAGAGGTTGCAGTGAGCCGGGATTGTGCCACTGCACTCCAGCCTGGCGACAGAGCAAGACTCCATCTCAAGAA AAAAAATTTAAAAAAAAGAGTGCTGATTGTTAGCTGG
TGGGAAAGTGGACTTGCTGGGTGGGGGCGGGGGAGTGGGGAGAATGACCATTCTGAAAGTGTAATCTTCAACAGAGTACTGTACGTTCTCTGGCTTATTAGCCCGGTGAACAGCTTTCTGATGGT TTT
AGTTGAACAAGTATTGCTGGATATGTTAAAATACAGCCAGAGGCAAAACATACTCCCTCCTCTCAGAGAACTTATATCCCAGGATAGGGGA GACTGGAAACAGGTCATTAGAATATGCAGAATAACAG
ATGAGTGCTATGGAGAGAAAGACAGCAGCCCCACTACGGAGGAGCACGGGATGGGGTTTG cAATTTTAGATGGGTGGCCTTTAACCAAATCCTTCAGTGAGAGTAAGTCACATGATGACCAGGAAGAG
CACCCCAGCAGGGGAGGCAGCCAGTGCCCTGGCACACAAGCAAAAGGAGTTGATGAGAGGGCTGGGGGGCTGAGGCCAGATCTCATGGGGC CTCACATGGAGGAAAGTGTGGACTTCTGCCTTTTCT
CTGAATAGTGTCCGTAGCTTACATATTCTATCTACTCTTGCCTTTTAGGAACCATTTTTCCCTCTAATGGTAGTTTACGTTAGAAGTATTTCCTGTTCTAGGCCCGGTGCAGTGGCTCATGTCTG TAA
TCCCAGCACTTTGGGAGGCCAAGGCAGGTGGATCACCTGAGGTCAGGAGTTCGAAACCAGCCTGGCCAACATGGCAAAACCCCATCTCTAC TAAAAATACAAA

1191	5.7	0.0	0.7	Hg19seq	1	140	(4131)	C	AluSx	SINE/Alu	(172)	140	2	1
2342	8.8	1.0	0.7	Hg19seq	284	589	(3682)	C	AluSx	SINE/Alu	(5)	307	1	2
1998	13.4	3.9	0.0	Hg19seq	1370	1652	(2619)	+	AluSx1	SINE/Alu	1	294	(18)	3
950	13.3	0.0	2.3	Hg19seq	1885	2019	(2252)	C	FLAM_C	SINE/Alu	(10)	133	2	4
2212	9.7	1.7	0.0	Hg19seq	2102	2389	(1882)	+	AluSx	SINE/Alu	1	293	(19)	5
14	16.0	0.0	5.4	Hg19seq	2630	2668	(1603)	+	(GTTTT)n	Simple_repeat	1	37	(0)	6
2528	6.3	0.0	1.0	Hg19seq	3108	3408	(863)	+	AluSc	SINE/Alu	1	298	(11)	7
392	31.4	10.8	4.6	Hg19seq	3564	3864	(407)	C	L2a	LINE/L2	(43)	3383	3018	8
1817	11.3	0.0	0.0	Hg19seq	4041	4271	(0)	+	AluSz6	SINE/Alu	1	231	(81)	9

>KSI_AluYa5_5

> Scaffold16601-735477-735706

TCAGGTTCCCCTATTTCAGTAAACAAAAAGAACTACGAATCTTCAGGTGAAACTTTTGCAGCAAAAAAATGAATAGAAAGAAAAAACAAAAGAAATAGAATATTGTGCTTGATTTTTGTAGAATCAA T
ACTTCCTTTTTGACAAGTCTTAAATCAAATTAGCAGAAACTTGAGGGAAATGCATATTGTATTTTTAAAAGTACACAGTAAAGTTGGAAATATTATGCATATGATGTATGTAAAGCAATAGTCCC TCA
CTTAGGCACAATTTTATTTTCCATAGTTTCAGTTATGTGTGGTCAACTGTGGTCCAAAAATACTAACAGGAAAATTTCAGAAACAAACAATTTGTAAGTTTTAAATTGTGCATCCTTCTGAATATCA T
GAAATCTCACATTGTCTTGCACTCTCCCACTGAGAACATGcATTATCCCTTCATCTAGCATATCGACGCTGTATTAACCACCCACTAGTCACTTAGTAGCTCCCTTGGCTATCACATAGACTGTTGTG
ATATCACAACTGTGTGTTCAAGTTACTCTTATTTTACTTAATAAGGGCCCCAAGTGCAAGAGTAGTGATGCTGGCAATTTGAATTTGCAAAAGTTTCTGTAAAATTGCCAAGTTTCTGTAAAAAG CTG
CTGGCTTTTTATTTATTTATTTATTTATTTATTTATTTATTTATTTatttatttGAGACCAGGTCTTGCTCTGACTCCTAGGCTGGAGTGCAGTGGTGTGGTCACCACCTTAAGTGAAAAGTGAAACT
TTATCATAGATATGTACGGTTGATGAAAAAATATAGTATATATAGAATTTGGTGCTATATGCAAGTTCAGGCATTCACTGGGGGGCTGGAACATTTTCTCCATAGATAAGGGGAAACAACTATAC TAT
TATTTCTTATGAGAAGACAATAAAATCTGAAAATTGAAGCGAAGTGTTCTGAGGGTTACTCTTGGTTGATTGAAGAAGACAGCTTTTAAAAATCCAACTATGGGCCAGGCGCGGTGGCTCACAACTAT
AATCCCAGCACTTTGGGAGGCCAAGGCCGGCGGATCACGAGGTCAGGAGATCGAGACCATCCTGgCTAACACGGTCAAAACCCGTCTCTACTAAAAATACAAAAAATTAGCCAGGCACGGTGGCAGGC
GCCTGTAGTCCCAGCTACTCGGGAGGCTGAGGCAGGAAAATGGCGTGAACCCAGGAGGCAGAGCTTGCAGTGAGCCGAGACAGCACCACTGCAGTCCAGCCTGGGAGAAAAAGCGAGACTCTGTCTCA
AAAAAAAAAAAAAAAAAAAAAAATTCAACTATAAGAAGTGATAAATACTTATTATAAACACTATCATCACTGTGATTTGTCTCTATTTGGGTTCGGTGAGTTTTAAAATTGCTCAATGTTGACCCCAA
GGAGAAAACAACATAGTGGAAACAATGTTGGGTTGATTTATATGTCATTCTACTGTAGAAATCCCTCTGTGCAAGCCTGGAATTGTGGTTTCGGATAATGTGGTAGGCAGGAGCACATTAAGTACTCA
ATGGCTAACACTCATAAGATTGTGATTTCCTTTCCTTTGAAACAACAAAGCAGAACTCTAATACGATGTGCAGTATCAGGCATGGCAGAGGAAGCATGCACAGTGTAAAGAGGGGAACGCTGCTA TCT
CTAGGATGTGCTTATGATGCGACAGTTGAGAGAGGGCAGAAGATGGAATGAATAAAGACTTTATCCTGTCAACTAGAACCACGTTAAACTTAACGATATGTATGGACGGTTTACATTTTGAACCTGGC
CTGTTTCAATCCAATCCCATACTTGCTGTATGGTTATGGTTAATGACAAAAACAGTATGGACACATACATGCGCATGCACACACACACACACACacTCTCTCTCTCTCTCTCTCCCCCCCAAATGTCTTTT
GGATTTAAAAGAAAGTATCTGAATATTGAACATTCAGCCATTATATACAGTCATAAAACCTCTTTTAAAAAAAAACAAGCAGgccgggcgcggtggctcacgcctgtaatcccagcactttgggaggcc
gaggcgggcggatcacgaggtcaggagatcgagaccatcccggctaaaacggtgaaaccccgtctctactaaaaatacaaaaaattagccgggcgtagtggcgggcgcctgtagtcccagctact tgg
gaggctgaggcaggagaatggcgtgaacccgggaggcggagcttgcagtgagcCTCTTCTTAGTAGACTTTTAAAAAGTAATATACATATAAAAAGATGCAAAGCATAAAGTTTCAGCTCATCAAATT
TATACCTAGTAAACAAACCTATGTAACTCCCATCCACATGAAGAAAGAGAATATGATAGTCACCATATAGATAGAAGGAGGACTTCATGTTCCTGTCCACTTATTACCTCACCTCTAAATGCAAC CAT
GATCTTTCCTTCCATCATTACAGATTAATCTGGGCTGGTTTTAAATACATGTAAATAGAATCATCATATGGGCTCTCTTAGGTTAGGCTTCTTTTATTCAACATTATATTGAAGATTTATTTTATTAC
ATGCCACATTAGGTACTTTATTTTTATTTTTGGAGATTTTCTCATTGAATAAATATACCATGATTTCTTTATCCATTTTACTATTACTGAACATCTAGTTTGTTTTCAGGCTTTGTCTATTAGAA AGG
TATTATTGTTTAACCTCTTAGTCCTATCTTTTAGTGCACATATGGGCATATTTTTGTTAGGTTTCTTACCAGAAATTCATTATTTTGAATTTTAGGgTCTGTGTATGTATGTATGCATGTATTTATCT
ATCTATCTACCTATgTATGTATGTATGAATGTATCTATCTATCAATCTGTCAGCAAATAGTTTCTAAAGTTGCTAGAACAATTTCGTCTCCCACCAGTAATGAATTAGAGTTCCTAATGTTCTTCAGC
ATCACCAATTCTCATAATGCAGATATTGCTTCATATTTTCAGTGGTTTATAAGACAAACATTTGCTCCTCTTACAAATCTGCaTATCGATTGGTGAGGCTCTGCTTCAGGCTGAAGGTTTGGTTCAGG
TTTGCTTTGTATGTCTTCATTTTCCTTGGTCCAGGGCATAGTTaTCTTATAAAAAATGACAAGAGCAGCAGGTAAGAACTTTGCAATATCCTTTAAGGCTTCTGCCCAGATTGTCACTTTTGCCCACA
TTCTGTTGGCAAAAGCAAGTCATATGCACAGGTCAAAAATCAATTGTCTAAAGTTTgTAATACTCTGCCTACTCTAATGTTAAGGTATGGCAACAATCGTATGGCAAGGACCATAGATATATATAATT
CTAATACAGAGGACAGAATAATCAGGATTTTTTTaaATTTcATTTTAGTCAGCATGGTGGGAATATATTGATACCCCATTGTTGATTTAAGTTGCATTTTACTAAGTATGAATGCTGtTTATCATTTT
AAATGCTTATTTGATATCCAGATATCTTTTGTGAAGTGACAAAGTTTTCTTTTTTTACCATTTTCTGATTATATTGTCTATTATTTATTCTTTGATATATGAACACTATATATTCTGAATATCAG TCC
TTTGTAAATTATATGTTGCAAATATTTTATCCCAGTATGTAGTTTATATATGTGCCCTATTAATGGTATATTTTGATGTTCTTAACTTTAATAGGGTACACATTATTTTT TCTAATTTTTCAACTACT
TCgCATCTTCTTGAAGATATGTTTCCTACTCTTAGTCAAAAATGTACTTGTTGTTATAAtTCTCACATGTAGATCTGCAATCCATTTGAAATTGATTTTTCTATATCCTGTTACTTAGAGAATCAAAT
TCATTATTTTCAtGATAAAATCCAAATTGAGCTAGCACTACTTATGAAAGAAAAGTCCATTTTATTTCCCATTGAATTGCATTGGCAAGTCTCTCAAAAGTCAAGGATCCATGTATTA GTAGCTATCT
GTCCAGATTCTCTATTCTGCgTCATCACTCCATTTGTCTATATAGTTAATGCAAATTTCACACTGCCCTAATTTTACTTGCTTGATAATAAATCTTGATATGTGTTAGTATAAATCCTAAAG tTTTCA
TTCCTTTTTATGGTTACCTCGACTCTTTATAGTATTTTGCTTTTTGACAGAAAgTCAACAAACAGCTTGCCAATATCCACAAAAACCTACTTACAGCTTTTGATTACAATGAAACTATAAATCACCTA
GGAGGA

11	11.3	6.9	0.0	TeRefseq	72	100	(4130)	+	(AATAGA)n	Simple_repeat	1	31	(0)	1
1871	23.3	1.8	11.3	TeRefseq	244	647	(3583)	+	MER44B	DNA/TcMar-Tigger	1	397	(153)	2
52	0.0	0.0	0.0	TeRefseq	648	694	(3536)	+	(TTTA)n	Simple_repeat	1	47	(0)	3
1871	23.3	1.8	11.3	TeRefseq	695	889	(3341)	+	MER44B	DNA/TcMar-Tigger	398	549	(1)	2
2606	6.6	0.0	0.0	TeRefseq	999	1303	(2927)	+	AluYk4	SINE/Alu	1	305	(6)	4
17	21.8	0.0	0.0	TeRefseq	1853	1886	(2344)	+	(AC)n	Simple_repeat	1	34	(0)	5
17	0.0	0.0	0.0	TeRefseq	1886	1904	(2326)	+	(CT)n	Simple_repeat	1	19	(0)	6
2266	0.0	0.0	0.0	TeRefseq	2001	2230	(2000)	+	AluYa5	SINE/Alu	1	230	(81)	7

1109	31.9	3.2	1.9	TeRefseq	2240	2796	(1434)	C	L1ME3A	LINE/L1	(7)	6166	5603	8
31	18.2	0.0	0.0	TeRefseq	2797	2858	(1372)	+	(ATGT)n	Simple_repeat	1	62	(0)	9
30	22.9	0.0	0.0	TeRefseq	2859	2867	(1363)	+	(ATCT)n	Simple_repeat	1	71	(0)	10
302	23.2	2.4	1.2	TeRefseq	2870	2952	(1278)	C	L1M5	LINE/L1	(2413)	5582	5499	11
304	23.1	11.5	1.1	TeRefseq	3155	3244	(986)	C	MLT1I	LTR/ERVL-MaLR	(230)	181	79	12
1438	27.2	7.7	2.3	TeRefseq	3366	4220	(10)	C	L1M5	LINE/L1	(966)	5478	4571	11

>Hg19seq chr9:105448619-105452640
TCAGGTTCCCCTATTTCAGTAAACAAAAAGAACTACGAATCTTCAGGTGAAACTTTTGCAGCAAAAAAATGAATAGAAAGAAAAAACAAAAGAAATAGAATATTGTGCTTGATTTTTGTAGAATCA AT
ACTTCCTTTTTGACAAGTCTTAAATCAAATTAGCAGAAACTTGAGGGAAATGCATATTGTATTTTTAAAAGTACACAGTAAAGTTGGAAATATTATGCATATGATGTATGTAAAGCAATAGTCCCTCA
CTTAGGCACAATTTTATTTTCCATAGTTTCAGTTATGTGTGGTCAACTGTGGTCCAAAAATACTAACAGGAAAATTTCAGAAACAAACAATTTGTAAGTTTTAAATTGTGCATCCTTCTGAATAT CAT
GAAATCTCACATTGTCTTGCACTCTCCCACTGAGAACATGaATTATCCCTTCATCTAGCATATCGACGCTGTATTAACCACCCACTAGTCACTTAGTAGCTCCCTTGGCTATCACATAGACTGTTGTG
ATATCACAACTGTGTGTTCAAGTTACTCTTATTTTACTTAATAAGGGCCCCAAGTGCAAGAGTAGTGATGCTGGCAATTTGAATTTGCAAAAGTTTCTGTAAAATTGCCAAGTTTCTGTAAAAAG CTG
CTGGCTTTTTATTTATTTATTTATTTATTTATTTATTTATTTATTTGAGACCAGGTCTTGCTCTGACTCCTAGGCTGGAGTGCAGTGGTGTGGTCACCACCTTAAGTGAAAAGTGAAACTTTATCATA
GATATGTACGGTTGATGAAAAAATATAGTATATATAGAATTTGGTGCTATATGCAAGTTCAGGCATTCACTGGGGGGCTGGAACATTTTCTCCATAGATAAGGGGAAACAACTATACTATTATTT CTT
ATGAGAAGACAATAAAATCTGAAAATTGAAGCGAAGTGTTCTGAGGGTTACTCTTGGTTGATTGAAGAAGACAG CTTTTAAAAATCCAACTATGGGCCAGGCGCGGTGGCTCACAACTATAATCCCAG
CACTTTGGGAGGCCAAGGCCGGCGGATCACGAGGTCAGGAGATCGAGACCATCCTG aCTAACACGGTCAAAACCCGTCTCTACTAAAAATACAAAAAATTAGCCAGGCACGGTGGCAGGCGCCTGTAG
TCCCAGCTACTCGGGAGGCTGAGGCAGGAAAATGGCGTGAACCCAGGAGGCAGAGCTTGCAGTGAGCCGAGACAGCACCACTGCAGTCCAGCCTGGGAGAAAAAGCGAGACTCTGTCTCAAAAAA AAA
AAAAAAAAAAAAAAAaTTCAACTATAAGAAGTGATAAATACTTATTATAAACACTAT CATCACTGTGATTTGTCTCTATTTGGGTTCGGTGAGTTTTAAAATTGCTCAATGTTGACCCCAAGGAGAAA
ACAACATAGTGGAAACAATGTTGGGTTGATTTATATGTCATTCTACTGTAGAAATCCCTCTGTGCAAGCCTGGAATTGTGGTTTCGGATAATGTGGTAGGCAGGAGCACATTAAGTACTCAATGG CTA
ACACTCATAAGATTGTGATTTCCTTTCCTTTGAAACAACAAAGCAGAACTCTAATAC GATGTGCAGTATCAGGCATGGCAGAGGAAGCATGCACAGTGTAAAGAGGGGAACGCTGCTATCTCTAGGAT
GTGCTTATGATGCGACAGTTGAGAGAGGGCAGAAGATGGAATGAATAAAGACTTTATCCTGTCAACTAGAACCACGTTAAACTTAACGATATGTATGGACGGTTTACATTTTGAACCTGGCCTGTTTC
AATCCAATCCCATACTTGCTGTATGGTTATGGTTAATGACAAAAACAGTATGGACAC ATACATGCGCATGCACACACACACACACTCTCTCTCTCTCTCTCTCtcCCCCCAAATGTCTTTTGGATTTA
AAAGAAAGTATCTGAATATTGAACATTCAGCCATTATATACAGTCATAAAAC CTCTTTTAAAAAAAACAAGCAGaatgactctaaaggctgaatCTCTTCTTAGTAGACTTTTAAAAAGTAATATACA
TATAAAAAGATGCAAAGCATAAAGTTTCAGCTCATCAAATTTATACCTAGTAAACAA ACCTATGTAACTCCCATCCACATGAAGAAAGAGAATATGATAGTCACCATATAGATAGAAGGAGGACTTCA
TGTTCCTGTCCACTTATTACCTCACCTCTAAATGCAACCATGATCTTTCCTTCCATCATTACAGATTAATCTGGGCTGGTTTTAAATACATGTAAATAGAATCATCATATGGGCTCTCTTAGGTT AGG
CTTCTTTTATTCAACATTATATTGAAGATTTATTTTATTACATGCCACATTAGGTAC TTTATTTTTATTTTTGGAGATTTTCTCATTGAATAAATATACCATGATTTCTTTATCCATTTTACTATTAC
TGAACATCTAGTTTGTTTTCAGGCTTTGTCTATTAGAAAGGTATTATTGTTTAACCTCTTAGTCCTATCTTTTAGTGCACATATGGGCATATTTTTGTTAGGTTTCTTACCAGAAATTCATTATT TTG
AATTTTAGGaTCTGTGTATGTATGTATGCATGTATTTATCTATCTATCTACCTAT aTATGTATGTATGAATGTATCTATCTATCAATCTGTCAGCAAATAGTTTCTAAAGTTGCTAGAACAATTTCGT
CTCCCACCAGTAATGAATTAGAGTTCCTAATGTTCTTCAGCATCACCAATTCTCATAATGCAGATATTGCTTCATATTTTCAGTGGTTTATAAGACAAACATTTGCTCCTCTTACAAATCTGC gTATC
GATTGGTGAGGCTCTGCTTCAGGCTGAAGGTTTGGTTCAGGTTTGCTTTGTATGTCT TCATTTTCCTTGGTCCAGGGCATAGTTtTCTTATAAAAAATGACAAGAGCAGCAGGTAAGAACTTTGCAAT
ATCCTTTAAGGCTTCTGCCCAGATTGTCACTTTTGCCCACATTCTGTTGGCAAAAGCAAGTCATATGCACAGGTCAAAAATCAATTGTCTAAAGTTT aTAATACTCTGCCTACTCTAATGTTAAGGTA
TGGCAACAATCGTATGGCAAGGACCATAGATATATATAATTCTAATACAGAGGACAG AATAATCAGGATTTTTTTttATTTtATTTTAGTCAGCATGGTGGGAATATATTGATACCCCATTGTTGATT
TAAGTTGCATTTTACTAAGTATGAATGCTGaTTATCATTTTAAATGCTTATTTGATATCCAGATATCTTTTGTGAAGTGACAAAGTTTTCTTTTTTTACCATTTTCTGATTATATTGTCTATTATTTA
TTCTTTGATATATGAACACTATATATTCTGAATATCAGTCCTTTGTAAATTATATGTTGCAAATATTTTATCCCAGTATGTAGTTTATATATGTGCCCTATTAATGGTATATTTTGATGTTCTTA ACT
TTAATAGGGTACACATTATTTTTTtCTAATTTTTCAACTACTTCtCATCTTCTTGAAGATATGTTTCCTACTCTTAGTCAAAAATGTACTTGTTGTTATAA cTCTCACATGTAGATCTGCAATCCATT
TGAAATTGATTTTTCTATATCCTGTTACTTAGAGAATCAAATTCATTATTTTCA cGATAAAATCCAAATTGAGCTAGCACTACTTATGAAAGAAAAGTCCATTTTATTTCCCATTGAATTGCATTGGC
AAGTCTCTCAAAAGTCAAGGATCCATGTATTAGTAGCTATCTGTCCAGATTCTCTATTCTGCaTCATCACTCCATTTGTCTATATAGTTAATGCAAATTTCACACTGCCCTAATTTTACTTGCTTGAT
AATAAATCTTGATATGTGTTAGTATAAATCCTAAAGcTTTCATTCCTTTTTATGGTTACCTCGACTCTTTATAGTATTTTGCTTTTTGACAGAAA aTCAACAAACAGCTTGCCAATATCCACAAAAAC
CTACTTACAGCTTTTGATTACAATGAAACTATAAATCACCTAGGAGGAaattgatatctttacagtattatgttttccactcagtaaaactagaataacttaaagaatgtataattaaactatgaata
tttaagaaattatcaatgac

11	11.3	6.9	0.0	Hg19seq	172	200	(4016)	+	(AATAGA)n	Simple_repeat	1	31	(0)	1
1891	23.1	1.8	11.3	Hg19seq	344	747	(3469)	+	MER44B	DNA/TcMar-Tigger	1	397	(153)	2
43	0.0	0.0	0.0	Hg19seq	748	786	(3430)	+	(TTTA)n	Simple_repeat	1	39	(0)	3
1891	23.1	1.8	11.3	Hg19seq	787	981	(3235)	+	MER44B	DNA/TcMar-Tigger	398	549	(1)	2

2598	6.9	0.0	0.0	Hg19seq	1091	1396	(2820)	+	AluYk4	SINE/Alu	1	306	(5)	4	
15	23.5	0.0	0.0	Hg19seq	1946	1977	(2239)	+	(AC)n	Simple_repeat	1	32	(0)	5	*
19	0.0	0.0	0.0	Hg19seq	1977	1997	(2219)	+	(CT)n	Simple_repeat	1	21	(0)	6	
1147	31.3	3.3	1.7	Hg19seq	2125	2635	(1581)	C	L1ME3A	LINE/L1	(7)	6166	5648	7	
35	17.9	0.0	0.0	Hg19seq	2675	2743	(1473)	+	(TGTA)n	Simple_repeat	1	69	(0)	8	
34	23.9	0.0	0.0	Hg19seq	2744	2752	(1464)	+	(ATCT)n	Simple_repeat	6	83	(0)	9	
301	23.2	2.4	1.2	Hg19seq	2755	2837	(1379)	C	L1M5	LINE/L1	(2413)	5582	5499	10	
302	23.2	11.5	1.1	Hg19seq	3040	3129	(1087)	C	MLT1I	LTR/ERVL-MaLR	(230)	181	79	11	
1727	28.0	7.6	1.7	Hg19seq	3251	4150	(66)	C	L1M5	LINE/L1	(684)	5478	4527	10	

>KSI_AluYa5_6

> Scaffold1254-496783-496969
AATAAATATTTTTACAGCAGAAAAAGTCACCTCCTCCATGAAGTCCTCTTGATGTGAACGCGTCCACTCTGATCCCCTCCTGC gTTCCTGAAGGAGGCTCTCTGCACTGTGAGTTTTGCACTTGGTAG
TGATCCTCCCTTTGGCTCATAAATTAGACATCATCTCCTGACTGGATTGTGAAGCCTAGGCAGGCACAGCCAAGATCAGACTC tTAACATTATGCTCAGCCACCCTTGGCCCATCAGACCTCTGTCCA
TTAAAGGCCCCAAGTAAAAGGGGCATTTGAATGCCCTCTTCCCAATAAGCTGAT cCTTGTCTAATGCAGTCCACTAGATTCCTGTAAAGGTGGGGCCACTAAGAGGCTGCCAATGGAACCCCATTCAT
TTTCCCACTGACTTTTCTTTCTTTTTTTTTTTTTTGGAGATAGAGTCTGGCTCTGTTAGAGTGCAGTGTTGAGATCATGGCTTACAGCAGCCTTGACCTCCAGGGCTCAGGTGATCCTCCTGCCTGAG
CCAGGAGGATCCAGGACTACATGTGTGCACCACCACACAAAGCTAATTTTTATGTTTTTGTTTTTGTTGCTCAAGCTGGAGTGC AATGGCGTGATCTCaGCTCACCACAACCTCCACCTCCCAGGTTC
AAGCAATTCTCCTGCCTCAGCCCCCAAGTAGCTGGGATTACAGGCATGCACCATTAGGCCCGGCTAATCTTGTATTTTTAGGTAGAGACAGGGTTTCTCCATGTTGGTCAGGCTGGTTGCAAACT CCC
AACCTCAGGTGATCCCCCGCCTCGGCCTCCCAAAGTGCTGAGATTACAGGCATAAGCCACCGCGCCTGGCCGTCATTTTTGTGT TTTTTGTAGAGATGAGATCTTGCTATGTTGCC cGGGCTGGTCTA
AACTCCTGGGCTCAATTGATCCTCCTGCCTTAGCCTCCCAAATCCTAGCTAATGCCTGCTAGGATTACAGGTGTGAGCCACCGCTCCTGGCTCCGCTGACTTCTTTTATGCCTCCATCTCTGGGG CTC
ATGAGATCCTCCCATCTTGGCCATCCGAGTACCTGAGTAGCTG gGACTATAGGCATGCGCCACCACGTCCAGCAATTTTTTTTT TTTTTTTTTTTTTTTTTTtttttttttttttGCTCACTATGT
TGCCCAGGCTGGTCTTGAACTCCTGAGCTCAATGCTAGTGAACTTTTATGACTTCTTTTATATTCAGTCTTCTTTTTGTGTGTTTTCAGGGAATGTGGCCATCTAAATTTCCAGCAGTATGTTCC TCT
CCTCATTCTACCCACCCAAGACTCTGTGTCAAGGAGTCAGCGACCAATAAATAGTCATGAATATGCTGTGTTTGCCTCAGCATT AAAAGAAGGCTTCGGCTGGGCGCAGTGGCTCACGCCTGTAATCC
CAGCACTTTAGGAGGCCGAGGTTGGGGGATCACCTGTGGTCAGGAATTCAAGACCAGCCCAGCCAACATGGTCAAACCTCGTCTCTACTAAAAATACAAAATCAGCCAGGCGTGGTGGTGGGCGC CTG
TAATCCCAGCTATTTGGAAGGGGCTGAGGCAGAAGAATCACTTGAACCCAGGAGTCTGAGGTTGCCGTGAGCTGAGATCGCGCC ACTGCACTCCAGCCTGGGCAACAAGAGTGAAACTCTGTCTTAAA
AAAAAAAAAAGGCGGCTTCCTGGACATACAGTCACCCTTTAGTTTCATGTGGTGAGTTAGAGGAGGTGTTTATCAGCCACAGGCAGGATGCACTGGCTGATAGCATTAGGACAGTGACTCATTTAAAG
GAATCTTTGGGAATCTCCTCAATGGAAAAAGCTTCCTCACCAAATCTTCCTGGCTTTGTACATTGGGATTTTGAAGTTTGGATTTTCTGCTCTCAAGGTAGCTCAAAGCAGCTACCCTGAGTCACCAT
GCCACCTCATTTACTAACAGGCTCTCATTCAACACCTGAGCTGTGCCTGGCATATGGGTACATGTTTAAGAAATATCTC Tggccgggcgcggtggctcacgcctgtaatcccagcactttgggaggcc
gaggcgggcggatcacgaggtcaggagatcgagaccatcccggctaaaacggtgaaaccccgtctctactaaaaatacaaaaa attagccgggcgtagtggcgggcgcctgtagtcccagctactcg
ggaggctgagGACCACGTGTGGTGGCTCACGCCTGTAATCCCAGCACTTTGGGAGGCCGAGGCCGAGGCTGGTGGATTATTTGAGGTCAGGAGTTCAAGACCAGCTTGGCCAACATGGTAAAACCCCA
TCTCTACTAAAAATACAAAAACTGGCTAGGCGTGGTGGTGCACGCCTGTAATCACAGCTACTTGGGAGGCTGAGACAGGAGAAT TGCTTGAACCTGGGAGGTGGAGCTTGCAGTGAGCTGAGATCGTG
CCATTGCACTCCAGCCTGGGTGACAGAGCAAGACTTCATCTCAAAAAAAAAGAAAGAAATATCTCTTGGCCAGATGCAGTGGCACACACCTGTAATCCCAGCACTTTGGGGGACCAAGGTAAGAG GAT
TGCTTGAAGCCAGGAGTTAGAGAGCAGCCTGGGCAACATAGCAAGACCCCATTTCTACAGAAGACTCTACGGAGTATGACTCAG GGCACCTGTTTTCAGCTACTCTGCATGTGCCTATAGTCCTAGCT
ACTCAGAAGGCTGAGGTGGGAAGAGCCCTGAGCCCAGGAGTTCAAGGCTGCAGTGAGCTACGATCATGCCCCTGCACTCCAGCCTGGG cGACAGGTGAATTTTTCTTCTTGTCTCTAAAAAAAAATAAT
AATAAACAATTTAAAATAATAAAAATAAAAAATGAATATCTCTTTAACCAAAGAGCGAATTCAGC ACACTCACCGAGGATTTGCTTACATTGCAAGCCCTATAGCTACAGAGAAACTGAGCGTTGCAG
ATAAGATGAACCCACACATAAtTAGACTAACAGGTAAATGAGGACCAGCATCgCAAGAAGCACAAATCCAAACAGTACCCATTTCCTGAACACCTGCACATGCTGTTCATGTTGCCTCCCAATAGTCC
CCAAAGCTAAGTGCTGTCACCCCATATAACAGAGAGGTTAAATGACTAAGACCTCGGTACCAAAAAGTGGGGA CACAGGATGAACCTCCAAAACATGCTAACTAAAGAAGCCAAACGTAATATGGGT
CCATTTATATGAAATGTCCAAAATACATAAATCCAGAGAACAGTTGCCAGGGGCTGGAGGGCAGGTTGGGAGGTGGTGACTGCTTCATGGGTGTGAGATTTTCTT aGGGGCTGATGGAAGTCTTTTGG
AACTAGGTAGAGGTGCTTGCACAACATTGTAAATCAACTAGATGACACCAAATTGTTTACCTTTTATAAATCT CACCTCAATTAAAAAAACAATTGTCGGTCGGGTGCAGTGCTCACACCTGTAAACC
CAGACTTCGGGAGGCCGAGGCAGGCAGATCACGAGGTCAGGAGATCGAGACCATCCTGGCTAATGCAGTGAAAACCTGTCTCTACTACAAATATAAAAAATTAGCCGGGCATGGTGGCACATGCC TGT
AGTCCCAGCTATCGGGAGGCTGAGGCAGAAGGATCCCTTGAACCCAGGAGAGGGAGGTTGCAGTGAGCTGAGA TCATGCCACTGCACTCCAGCCTGGGCAACAgagagactcagcctcaaaataaaaa
aaaataaaaaaaaaaatttaagtggtgggaaagaaaccaaaaaagaaataaatgaataaataaataaatCAGAGAGACTCAGCCTCAAAATAAAAAAAAAATAAAAAAAAATAAATTTAAGTGGTGGGA
AAGAAACCAAAAAAGAAATAAATGAATAAATAAATAAATAAATAAATACAAAGGTGGAGGCCCTGGGTGGGG TTCAAGCCCAGACTGATCTGACCCACAGCTCCACTGAAGGCTG TTTCCAAAGAGG
GAGGCATGCTCTCCAAGCCTGGCTCCGCCCCCATTAGACCCAAATGGGACCGGAGGCTCTCCTTACCCTCTACCCCCAGCTGGCCCAGGGCCTACCTCGAAGAGCCAAATGAGGAGCGTGACGAC ACC
CATGGAGTTCATGAGGCTGCTGTAGTAGCTCAGCAGGGCAGCCCTGCAGCCACGCACCCACAGGTTGAGCTCC TCCGTCTGGTGGTCGTAA

1457	14.9	3.7	11.0	TeRefseq	402	574	(3613)	C	AluJo	SINE/Alu	(11)	301	129	1
2037	9.5	1.1	0.4	TeRefseq	576	839	(3348)	C	AluSp	SINE/Alu	(47)	266	1	2
1457	14.9	3.7	11.0	TeRefseq	840	987	(3200)	C	AluJo	SINE/Alu	(184)	128	2	1
551	15.2	18.8	5.1	TeRefseq	1006	1099	(3088)	C	AluJr	SINE/Alu	(92)	220	116	3
46	0.0	0.0	0.0	TeRefseq	1100	1141	(3046)	+	(T)n	Simple_repeat	1	42	(0)	4
551	15.2	18.8	5.1	TeRefseq	1142	1184	(3003)	C	AluJr	SINE/Alu	(198)	114	65	3
2271	9.8	0.3	0.7	TeRefseq	1378	1674	(2513)	+	AluSq	SINE/Alu	1	296	(17)	5
188	27.1	2.0	2.0	TeRefseq	1950	1998	(2189)	+	L2a	LINE/L2	3363	3411	(15)	6
1740	0.5	0.0	0.5	TeRefseq	2001	2187	(2000)	+	AluYa5	SINE/Alu	1	186	(125)	7

2254	10.4	0.0	2.0	TeRefseq	2193	2491	(1696)	+	AluSz	SINE/Alu	7	299	(13)	8
1553	16.9	0.6	7.9	TeRefseq	2500	2825	(1362)	+	AluJr	SINE/Alu	1	304	(8)	9
1002	17.6	21.9	0.6	TeRefseq	3150	3418	(769)	+	L1MB5	LINE/L1	5849	6174	(0)	10
2209	10.6	1.7	0.0	TeRefseq	3427	3728	(459)	+	AluSc8	SINE/Alu	1	307	(4)	11
23	26.6	1.2	3.6	TeRefseq	3742	3798	(389)	+	A-rich	Low_complexity	1	83	(0)	12
19	11.7	0.0	0.0	TeRefseq	3799	3826	(361)	+	(A)n	Simple_repeat	1	28	(0)	13
30	3.1	0.0	0.0	TeRefseq	3856	3888	(299)	+	(AAAT)n	Simple_repeat	1	33	(0)	14

>hg19seq chr6:42668320-42672320
AATAAATATTTTTACAGCAGAAAAAGTCACCTCCTCCATGAAGTCCTCTTGATGTGAACGCGTCCACTCTGATCCCCTCCTGC tTTCCTGAAGGAGGCTCTCTGCACTGTGAGTTTTGCACTTGGTAG
TGATCCTCCCTTTGGCTCATAAATTAGACATCATCTCCTGACTGGATTGTGAAGCCTAGGCAGGCACAGCCAAGATCAG ACTCcTAACATTATGCTCAGCCACCCTTGGCCCATCAGACCTCTGTCCA
TTAAAGGCCCCAAGTAAAAGGGGCATTTGAATGCCCTCTTCCCAATAAGCTGAT tCTTGTCTAATGCAGTCCACTAGATTCCTGTAAAGGTGGGGCCACTAAGAGGCTGCCAATGGAACCCCATTCAT
TTTCCCACTGACTTTTCTTTCTTTTTTTTTTTTTTtGGAGATAGAGTCTGGCTCTGTTAGAGTGCAGTGTTGAGATCAT GGCTTACAGCAGCCTTGACCTCCAGGGCTCAGGTGATCCTCCTGCCTGA
GCCAGGAGGATCCAGGACTACATGTGTGCACCACCACACAAAGCTAATTTTTATGTTTTTGTTTTTGTTGCTCAAGCTGGAGTGCAATGGCGTGATCTC gGCTCACCACAACCTCCACCTCCCAGGTT
CAAGCAATTCTCCTGCCTCAGCCCCCAAGTAGCTGGGATTACAGGCATGCACCATTAGGCCCGGCTAATCTTGTATTTT TAGGTAGAGACAGGGTTTCTCCATGTTGGTCAGGCTGGTTGCAAACTCC
CAACCTCAGGTGATCCCCCGCCTCGGCCTCCCAAAGTGCTGAGATTACAGGCATAAGCCACCGCGCCTGGCCGTCATTTTTGTGTTTTTTGTAGAGATGAGATCTTGCTATGTTGCC tGGGCTGGTCT
AAACTCCTGGGCTCAATTGATCCTCCTGCCTTAGCCTCCCAAATCCTAGCTAATGCCTGCTAGGATTACAGGTGTGAGC CACCGCTCCTGGCTCCGCTGACTTCTTTTATGCCTCCATCTCTGGGGCT
CATGAGATCCTCCCATCTTGGCCATCCGAGTACCTGAGTAGCTGaGACTATAGGCATGCGCCACCACGTCCAGCAATTTTTTTTTTTTTTTTTTTTTTTTTagtagagatgaggGCTCACTATGTT
GCCCAGGCTGGTCTTGAACTCCTGAGCTCAATGCTAGTGAACTTTTATGACTTCTTTTATATTCAGTCTTCTTTTTGTGTGTTTTCAGGGAATGTGGCCATCTAAATTTCCAGCAGTATGTTCCT CTC
CTCATTCTACCCACCCAAGACTCTGTGTCAAGGAGTCAGCGACCAATAAATAGTCATGAATATGCTGTGTTTGCCTCAGCATTAAAAGAAGGCTTCGGCTGGGCGCAGTGGCTCACG CCTGTAATCCC
AGCACTTTAGGAGGCCGAGGTTGGGGGATCACCTGTGGTCAGGAATTCAAGACCAGCCCAGCCAACATGGTCAAACCTCGTCTCTACTAAAAATACAAAATCAGCCAGGCGTGGTGGTGGGCGCC TGT
AATCCCAGCTATTTGGAAGGGGCTGAGGCAGAAGAATCACTTGAACCCAGGAGTCTGAGGTTGCCGTGAGCTGAGATCGCGCCACTGCACTCCAGCCTGGGCAACAAGAGTGAAACT CTGTCTTAAAA
AAAAAAAAAGGCGGCTTCCTGGACATACAGTCACCCTTTAGTTTCATGTGGTGAGTTAGAGGAGGTGTTTATCAGCCACAGGCAGGATGCACTGGCTGATAGCATTAGGACAGTGACTCATTTAA AGG
AATCTTTGGGAATCTCCTCAATGGAAAAAGCTTCCTCACCAAATCTTCCTGGCTTTGTACATTGGGATTTTGAAGTTTGGATTTTCTGCTCTCAAGGTAGCTCAAAGCAGCTACCCT GAGTCACCATG
CCACCTCATTTACTAACAGGCTCTCATTCAACACCTGAGCTGTGCCTGGCATATGGGTACATGTTTAAGAAATATCTC TGACCACGTGTGGTGGCTCACGCCTGTAATCCCAGCACTTTGGGAGGCCG
AGGCCGAGGCTGGTGGATTATTTGAGGTCAGGAGTTCAAGACCAGCTTGGCCAACATGGTAAAACCCCATCTCTACTAAAAATACAAAAACTGGCTAGGCGTGGTGGTGCACGCCTG TAATCACAGCT
ACTTGGGAGGCTGAGACAGGAGAATTGCTTGAACCTGGGAGGTGGAGCTTGCA GTGAGCTGAGATCGTGCCATTGCACTCCAGCCTGGGTGACAGAGCAAGACTTCATCTCAAAAAAAAAGAAAGAAA
TATCTCTTGGCCAGATGCAGTGGCACACACCTGTAATCCCAGCACTTTGGGGGACCAAGGTAAGAGGATTGCTTGAAGCCAGGAGTTAGAGAGCAGCCTGGGCAACATAGCAAGACC CCATTTCTACA
GAAGACTCTACGGAGTATGACTCAGGGCACCTGTTTTCAGCTACTCTGCATGTGCCTATAGTCCTAGCTACTCAGAAGGCTGAGGTGGGAAGAGCCCTGAGCCCAGGAGTTCAAGGCTGCAGTGA GCT
ACGATCATGCCCCTGCACTCCAGCCTGGGtGACAGGTGAATTTTTCTTCTTGTCTCTAAAAAAAATAATAATAAACAATTTAAAATAATAAAAATAAAAAATGAATATCTCTTTAA CCAAAGAGCGAA
TTCAGCACACTCACCGAGGATTTGCTTACATTGCAAGCCCTATAGCTACAGAGAAACTGAGCGTTGCAGATAAGATGAACCCACACATAA cTAGACTAACAGGTAAATGAGGACCAGCATCcCAAGAA
GCACAAATCCAAACAGTACCCATTTCCTGAACACCTGCACATGCTGTTCATGTTGCCTCCCAATAGTCCCCAAAGCTAAGTGCTGTCACCCCATATAACAGAGAGGTTAAATGACTA AGACCTCGGTA
CCAAAAAGTGGGGACACAGGATGAACCTCCAAAACATGCTAACTAAAAGAAGCCAAACGTAATATGGGTCCATTTATATGAAATGTCCAAAATACATAAATCCAGAGAACAGTTGCCAGGGGCTG GAG
GGCAGGTTGGGAGGTGGTGACTGCTTCATGGGTGTGAGATTTTCTTgGGGGCTGATGGAAGTCTTTTGGAACTAGGTAGAGGTGCTTGCACAACATTGTAAATCAACTAGATGACAC CAAATTGTTTA
CCTTTTATAAATCTCACCTCAATTAAAAAAACAATTGTCGGTCGGGTGCAGTGCTCACACCTGTAAACCCAGACTTCGGGAGGCCGAGGCAGGCAGATCACGAGGTCAGGAGATCGAGACCATCC TGG
CTAATGCAGTGAAAACCTGTCTCTACTACAAATATAAAAAATTAGCCGGGCATGGTGGCACATGCCTGTAGTCCCAGCTATCGGGAGGCTGAGGCAGAAGGATCCCTTGAACCCAGG AGAGGGAGGTT
GCAGTGAGCTGAGATCATGCCACTGCACTCCAGCCTGGGCAACACAGAGAGACTCAGCCTCAAAATAAAAAAAAAATAAAAAAAAATAAATTTAAGTGGTGGGAAAGAAACCAAAAAAGAAATAAATGA
ATAAATAAATAAATAAATAAATACAAAAGGTGGAGGCCCTGGGTGGGGTTCAAGCCCAGACTGATCTGACCCACAGCTCCACTGAAGGCTGTTTCCAAAGAGGGAGGCATGCTCTCC AAGCCTGGCTC
CGCCCCCATTAGACCCAAATGGGACCGGAGGCTCTCCTTACCCTCTACCCCCAGCTGGCCCAGGGCCTACCTCGAAGAGCCAAATGAGGAGCGTGACGACACCCATGGAGTTCATGAGGCTGCTG TAG
TAGCTCAGCAGGGCAGCCCTGCAGCCACGCACCCACAGGTTGAGCTCCTCCGTCTGGTGGTCGTA A

1452	15.2	3.7	11.0	hg19seq	502	675	(3431)	C	AluJo	SINE/Alu	(10)	302	129	1
2051	9.1	1.1	0.4	hg19seq	677	940	(3166)	C	AluSp	SINE/Alu	(47)	266	1	2
1452	15.2	3.7	11.0	hg19seq	941	1088	(3018)	C	AluJo	SINE/Alu	(184)	128	2	1
692	16.6	9.3	5.8	hg19seq	1107	1200	(2906)	C	AluJr	SINE/Alu	(92)	220	135	3
28	0.0	0.0	0.0	hg19seq	1201	1227	(2879)	+	(T)n	Simple_repeat	1	27	(0)	4

692	16.6	9.3	5.8	hg19seq	1228	1283	(2823)	C	AluJr	SINE/Alu	(178)	134	65	3
2271	9.8	0.3	0.7	hg19seq	1477	1773	(2333)	+	AluSq	SINE/Alu	1	296	(17)	5
188	27.1	2.0	2.0	hg19seq	2049	2097	(2009)	+	L2a	LINE/L2	3363	3411	(15)	6
2254	10.4	0.0	2.0	hg19seq	2106	2404	(1702)	+	AluSz	SINE/Alu	7	299	(13)	7
1539	17.2	0.6	7.9	hg19seq	2413	2738	(1368)	+	AluJr	SINE/Alu	1	304	(8)	8
1006	17.6	21.9	0.6	hg19seq	3063	3331	(775)	+	L1MB5	LINE/L1	5849	6174	(0)	9
2205	11.4	1.0	0.0	hg19seq	3340	3645	(461)	+	AluSc8	SINE/Alu	1	309	(2)	10
30	3.1	0.0	0.0	hg19seq	3675	3707	(399)	+	(AAAT)n	Simple_repeat	1	33	(0)	11

>KSI_AluYa5_7

> Scaffold16064-858104-858251

GTATGCATACGTTGGTTGGGCCTGGAATGAAGGGACATCTCAAAGTGTGGGGGTGCTTACAGGTAACAGGTGGATTTAAAGATTTTTTGGTTGGCAATTGATTGAAAGAGTTGAGCTTTGCCAAAA GA
GTTCAAGTCACTAAAAAGAAATGCTTAAGTTAAGATAAGGGGAGTTGTGGAAGCCATGGTTCTTGTTATTTGGATGAAGCCTCTAAGTAGTAGGTTTGTATTAGTCCATTCTCACGCTGCTATGA AGG
CATACCTGAGACTGGATAACTTATAAAGGAGAGGGGTTTAATTGACTCACAGTTCCACATGCTGGGGAGGCCTCAGAAAACTTAGAATCCCGGCGGAAAGGGAAGCAAACAC gTCCTTTTTCACATGA
CAGCAGGAAAGAGAAGAATGAGAGCCAAGTGAAGGAGAAGCCCCTTATAAAACCATCAGATCTCGTAAGAACTTATTATCAGGAGAATAGCATGTGGAAAACCACCCCCATGATTCAATTACCTC CCA
CTGGGAACCTCCCACTGGGTCCCTCCCACCACATATGGGGATTATGGGAACTACAATTCAAG aTAAGATTTGGGTGGAAACACAGCCAAACCATATTAAGGCTTCAGAGGGAA TAGATGGTAAATGTC
TCTTTTTGGACCTTAAATGGTGTCAGACCCTTAGTTAAATCTCTCCTGAATTTGGAAAAGACCTGGAAAGGGAGGGAGATTCTCTACAGATGCAAGTATCCCTTGCAACAGACAGCATTTGCAGG GTC
ATTTCAAAATACTTCAATGAAATACATTTTGGGGTAAAATACTTTGATTTTCTTGAGACCTTGCTATTGATCAGGTGATGCTATACCAGAGATGGAATTTGATGTCTTCTTGT CACAAAGAGTCTGTC
TGGTGAGTCTTTAATCTCgGTTTTAATGTTAATTCTGGTCTCGTGCCTAAACTCCAAAAGGCAGGGAGTTTATAATGAAGCATGTCAGACCTCGCCTCCCATCATGGTCTGGAATTCAGTTTTTTAGC
TTTCCCTTGGCCCAGAGAGGGTCCATGCAGTTTGTTAAGGGG cTTACAATTTTATTTTGGTTTACACTATGGATCATATCCATGGAGTTCTTGCGATTTTGTGGGCCTCCAGT TAAAAACTCTGGTCT
GTATGTGATTTGGTTTGGCATGTTGTGGCTCATGAAGCCTGTAAAACAGTGCAGATATGACCACTGTCAATGTTTGCC ATGCAGCAATCTCAGCAGCTACCTCGTTGCCTCACAAATGGAAGAACTTA
AAAAATTTGGAAAGTAAAGAGAGCATGATGCCATCTCTGATTTGGATGCAAGCAGGCAGTAAGGCACAAATAACAAACAAATGTGAAAGTCTTTATGTAATATTTTTGATCGT TTCATTAGGCATTTG
TTTTCTTAATTTTTTAAACACATCAAATTTAAAGATAGAGAACAAAGGGATAAAACTATAGGACTCAAGAAGCAGCACTAAAGCATATAATCTGTTTATACTAGAACTAAAATAATCTCATCAAT AAT
TGGCTGTTAACTCCATGCTTGTCACTTAGAGAGGAGGAAAAACAAAAGGGTATATTCTGAAACACAGATCGAGCAAGTAAATTATTGAGTGATTCACAGTAAGTTGACATCCC TAGCTGTTGCCTGGA
CACTCAGATTCTAAATTGATGGTGACCATTCCCTGGTGACTCAGAAAACTTAAATAAAAACAGAATCAAGCAAATCTAGCCAAGTTGAGGGTCCACCTTCTCCTAAAAGATTGTTTACTTGTTTATTT
AAGACATCAATTCTCAGGCTCTATCTCTCCCTTCCATGGCACTAACTCTCACTTGAACCAACAGTAGCTAAGTACACAGAACCAAATAAAGGTGGCAAAGTTATTTAGAAGCC AGGGGAATAAAATTG
TCTAATGCAATAGTCTGTACATATATGTATTTGCTATTTACATAAGCTTATAATTTTCATCATCATCATCAGTATTGTTG AAGctccgcctcccgggttcacgccattctcctgcctcagcctcccaa
gtagctgggactacaggcgcccgccactacgcccggctaatttttttgtatttttagtagagacggggtttcaccgttttagccgggatggtctcgatc TAGCTGAATTTTTAATTTTTGTGAATCTG
CTAGGTAAAATCACGTTTTATGATTAGCAGCAACGTTGAAGCCATCCAAGTTTCTTCTGCTGCAAATTATCTTTTATTCTTTGTTGAGATTTCAATTGTGTTCTAAAATATTTTTTCTTTTATTTGCA
GAAAGACATTTTTATATATTTGCTTTTTACATTTTAAGATAGCTGTTTATTAGTATTTGTGGTCTTTTTACATTTAATTCCTGGGAACATGTTTTACTGCTTTGCATAACATA TACTTTTTGAAATAT
AGTTATTTTTTAACTGATTTTATTCCTGATATCCCCAATTAATTATAGACATAATATTTATTCCTCTGATAGTCAGTTTTCCAAGAGACACTGGTTTCTAAGAATGACTAAGTTTTAAAACTTTT GAT
TAGAGAATCACAAATGAATAAACATAATTTGAAATCTCTATTATCACTAAATCTTTTTCTAGATGTAAAGTATATAGTTAGTAAAAAGATAATACATAGTTTAAACTAATATT TAAAATAAATTGTAT
GTGTGTATGCAAATATGAAGAGAAAATATATTAGATTTTTAATATAACAGCTTTACTGAGATAAAATTTA cAAACCATACAATTCACACATTTAAGGTGTATAATTAAATAGTTTATAGTATATTCAC
AGAGcTGTGCAACCATTGCTCACTCAATTTTGAAACATTTTTACCATCCCTAGACAGGAATTTCTTAATAATGTGATCCAGTAGTAGTCACAATCTATTCTTGACAATTACTA ATTCACTTTCTGTCT
CTATAGATTTGCCTATTCTGGAAATTTCATAAATGGAATCGTATAATATATGGTCTTTTGTAACTGACTTCCGTCACTTAGCAAAGTTTTTTCAAGGTCTATCTATGTTTTAACATGTATCAGTG TTT
CATTCCTTTTTTATTGCTAAATAATCATCAATCAGTTGATGGACATTTGAGCCTTTTCTACCTGACTATCAAAATTAATGCTATAACATTGCTCTAAAAGTTTTTATGTGGAC ATAGTAATTTTCCTT
GGGGAGATAGCTAAAATTGCTTAACCTTTTAAGTAACTATCAGACTGTTTTCTAAAGCAGCTGTACCATTTTATGTCCCCACCAGCAGTGTGTGAGGGTTTCACTTTATATCCTCAACAGTGTTT GTT
ATCATCTTTTTACCAATAGCCATCATAGTGGGTATGAAGTGGTACCTCACTGTGGTTTTTATTTGTATTACCCTGATGACTAATGATGTCGAGCATCTCTTCATGTGCTTGTG AGCCTTATTATATTT
GGACAAACGTTTATTCAAACCTTTTGCCCATTTTTAAATTGGGTTATCTTATTGAATCAAAGAGTTCTTCGTATATTCTAAATTAAAGTCTCTTATCAGATATATGGTTTGCAAAATATCTTCTT GTG
TGTATTGTTTTCACTTTCTTAATGATATTCATTGAAGCATAGGAGTTTTAAATTTTGATGAAGTTAAATTTATTTATTTTTTCTTTTGTTGCTTCTGCTTTTGGTATCATAGC TATGAAACCACTGCT
TAACCCAAGTTCTCCAAGATTTATATCTACATTTTCTCCAAGGAGTTTTTAAAATTTTAATACTAATATTTAGGTCTTTGATCCATTTTAAGTCAGTTTCTGATATGT TATGATGTAGGTTACACAGC
TGTCCTAACCAAAACTATTTGATTATAGAACCAACAGGTTAGTATGCTTGCTGCACAGTAACAGACCAATTACACGGAGACAGCAGGGCTTGCAGCAGAGAAAGAGTTTAATG ATTGCAGGGTACCAA
GCAAGGGGATTGGAAGAGACCTTCAAATCCATCTCCCTGAGGAGTTCTGGGCTGGGAATTTTAAGGGGATTGTAGTGGGTGAGGGGCTAGAAAATTGGGATTGTTGATTAGTTGGGTTAAGGGGG ATA
AAATCATCAGGACATGGAAATTGCATTCTTTGGTGAGTCAGCCCCTTGTAGG

3878	14.7	3.1	2.1	TeRefseq	5	224	(3924)	C	MER4D	LTR/ERV1	(195)	708	495	1
2344	11.2	1.8	4.8	TeRefseq	225	610	(3538)	C	THE1C	LTR/ERVL-MaLR	(0)	375	1	2
3878	14.7	3.1	2.1	TeRefseq	611	1090	(3058)	C	MER4D	LTR/ERV1	(409)	494	1	1
1395	0.0	0.0	0.7	TeRefseq	2001	2148	(2000)	C	AluYa5	SINE/Alu	(91)	220	74	3
2270	19.6	11.6	0.3	TeRefseq	2733	3831	(317)	C	L1MB8	LINE/L1	(5)	6173	4961	4
2000	13.3	0.0	0.0	TeRefseq	3863	4148	(0)	C	LTR26	LTR/ERV1	(2)	601	316	5

>chr8:53789029-53793041

GTATGCATACGTTGGTTGGGCCTGGAATGAAGGGACATCTCAAAGTGTGGGGGTGCTTACAGGTAACAGGTGGATTTAAAGATTTTTTGGTTGGCAATTGATTGAAAGAGTTGAG CTTTGCCAAAAGA

GTTCAAGTCACTAAAAAGAAATGCTTAAGTTAAGATAAGGGGAGTTGTGGAAGCCATGGTTCTTGTTATTTGGATGAAGCCTCTAAGTAGTAGGTTTGTATTAGTCCATTCTCACGCTGCTATGA AGG
CATACCTGAGACTGGATAACTTATAAAGGAGAGGGGTTTAATTGACTCACAGTTCCACATGCTGGGGAGGCCTCAGAAAACTTAGAATCCCGGCGGAAAGGGAAGCAAACAC aTCCTTTTTCACATGA
CAGCAGGAAAGAGAAGAATGAGAGCCAAGTGAAGGAGAAGCCCCTTATAAAACCATCAGATCTCGTAAGAACTTATTATCAGGAGAATAGCATGTGGAAAACCACCCCCATGATTCAATTACCTC CCA
CTGGGAACCTCCCACTGGGTCCCTCCCACCACATATGGGGATTATGGGAACTACAATTCAAG gTAAGATTTGGGTGGAAACACAGCCAAACCATATTAAGGCTTCAGAGGGAATA GATGGTAAATGTC
TCTTTTTGGACCTTAAATGGTGTCAGACCCTTAGTTAAATCTCTCCTGAATTTGGAAAAGACCTGGAAAGGGAGGGAGATTCTCTACAGATGCAAGTATCCCTTGCAACAGACAGCATTTGCAGG GTC
ATTTCAAAATACTTCAATGAAATACATTTTGGGGTAAAATACTTTGATTTTCTTGAGACCTTGCTATTGATCAGGTGATGCTATACCAGAGATGGAATTTGATGTCTTCTTGTCA CAAAGAGTCTGTC
TGGTGAGTCTTTAATCTCaGTTTTAATGTTAATTCTGGTCTCGTGCCTAAACTCCAAAAGGCAGGGAGTTTATAATGAAGCATGTCAGACCTCGCCTCCCATCATGGTCTGGAATTCAGTTTTTTAGC
TTTCCCTTGGCCCAGAGAGGGTCCATGCAGTTTGTTAAGGGG tTTACAATTTTATTTTGGTTTACACTATGGATCATATCCATGGAGTTCTTGCGATTTTGTGGGCCTCCAGTTA AAAACTCTGGTCT
GTATGTGATTTGGTTTGGCATGTTGTGGCTCATGAAGCCTGTAAAACAGTGCAGATATGACCACTGTCAATGTTTGCCATGCAGCAATCTCAGCAGCTACCTCGTTGCCTCACAAATGGAAGAAC TTA
AAAAATTTGGAAAGTAAAGAGAGCATGATGCCATCTCTGATTTGGATGCAAGCAGGCAGTAAGGCACAAATAACAAACAAATGTGAAAGTCTTTATGTAATATTTTTGATCGTTT CATTAGGCATTTG
TTTTCTTAATTTTTTAAACACATCAAATTTAAAGATAGAGAACAAAGGGATAAAACTATAGGACTCAAGAAGCAGCACTAAAGCATATAATCTGTTTATACTAGAACTAAAATAATCTCATCAAT AAT
TGGCTGTTAACTCCATGCTTGTCACTTAGAGAGGAGGAAAAACAAAAGGGTATATTCTGAAACACAGATCGAGCAAGTAAATTATTGAGTGATTCACAGTAAGTTGACATCCCTA GCTGTTGCCTGGA
CACTCAGATTCTAAATTGATGGTGACCATTCCCTGGTGACTCAGAAAACTTAAATAAAAACAGAATCAAGCAAATCTAGCCAAGTTGAGGGTCCACCTTCTCCTAAAAGATTGTTTACTTGTTTATTT
AAGACATCAATTCTCAGGCTCTATCTCTCCCTTCCATGGCACTAACTCTCACTTGAACCAACAGTAGCTAAGTACACAGAACCAAATAAAGGTGGCAAAGTTATTTAGAAGCCAG GGGAATAAAATTG
TCTAATGCAATAGTCTGTACATATATGTATTTGCTATTTACATAAGCTTATAATTTTCATCATCATCATCAGTATTGTTGAA GggctattttTAGCTGAATTTTTAATTTTTGTGAATCTGCTAGGTA
AAATCACGTTTTATGATTAGCAGCAACGTTGAAGCCATCCAAGTTTCTTCTGCTGCAAATTATCTTTTATTCTTTGTTGAGATTTCAATTGTGTTCTAAAATATTTTTTCTTTTATTTGCAGAAAGAC
ATTTTTATATATTTGCTTTTTACATTTTAAGATAGCTGTTTATTAGTATTTGTGGTCTTTTTACATTTAATTCCTGGGAACATGTTTTACTGCTTTGCATAACATATACTTTTTGAAATATAGTT ATT
TTTTAACTGATTTTATTCCTGATATCCCCAATTAATTATAGACATAATATTTATTC CTCTGATAGTCAGTTTTCCAAGAGACACTGGTTTCTAAGAATGACTAAGTTTTAAAACTTTTGATTAGAGAA
TCACAAATGAATAAACATAATTTGAAATCTCTATTATCACTAAATCTTTTTCTAGATGTAAAGTATATAGTTAGTAAAAAGATAATACATAGTTTAAACTAATATTTAAAATAAATTGTATGTGT GTA
TGCAAATATGAAGAGAAAATATATTAGATTTTTAATATAACAGCTTTACTGAGATA AAATTTAtAAACCATACAATTCACACATTTAAGGTGTATAATTAAATAGTTTATAGTATATTCACAGAG aTG
TGCAACCATTGCTCACTCAATTTTGAAACATTTTTACCATCCCTAGACAGGAATTTCTTAATAATGTGATCCAGTAGTAGTCACAATCTATTCTTGACAATTACTAATTCACTTTCTGTCTCTAT AGA
TTTGCCTATTCTGGAAATTTCATAAATGGAATCGTATAATATATGGTCTTTTGTAA CTGACTTCCGTCACTTAGCAAAGTTTTTTCAAGGTCTATCTATGTTTTAACATGTATCAGTGTTTCATTCCT
TTTTTATTGCTAAATAATCATCAATCAGTTGATGGACATTTGAGCCTTTTCTACCTGACTATCAAAATTAATGCTATAACATTGCTCTAAAAGTTTTTATGTGGACATAGTAATTTTCCTTGGGG AGA
TAGCTAAAATTGCTTAACCTTTTAAGTAACTATCAGACTGTTTTCTAAAGCAGCTG TACCATTTTATGTCCCCACCAGCAGTGTGTGAGGGTTTCACTTTATATCCTCAACAGTGTTTGTTATCATCT
TTTTACCAATAGCCATCATAGTGGGTATGAAGTGGTACCTCACTGTGGTTTTTATTTGTATTACCCTGATGACTAATGATGTCGAGCATCTCTTCATGTGCTTGTGAGCCTTATTATATTTGGAC AAA
CGTTTATTCAAACCTTTTGCCCATTTTTAAATTGGGTTATCTTATTGAATCAAAGA GTTCTTCGTATATTCTAAATTAAAGTCTCTTATCAGATATATGGTTTGCAAAATATCTTCTTGTGTGTATTG
TTTTCACTTTCTTAATGATATTCATTGAAGCATAGGAGTTTTAAATTTTGATGAAGTTAAATTTATTTATTTTTTCTTTTGTTGCTTCTGCTTTTGGTATCATAGCTATGAAACCACTGCTTAAC CCA
AGTTCTCCAAGATTTATATCTACATTTTCTCCAAGGAGTTTTTAAAATTTTAATAC TAATATTTAGGTCTTTGATCCATTTTAAGTCAGTTTCTGATATGTTATGATGTAGGTTACACAGCTGTCCTA
ACCAAAACTATTTGATTATAGAACCAACAGGTTAGTATGCTTGCTGCACAGTAACAGACCAATTACACGGAGACAGCAGGGCTTGCAGCAGAGAAAGAGTTTAATGATTGCAGGGTACCAAGCAA GGG
GATTGGAAGAGACCTTCAAATCCATCTCCCTGAGGAGTTCTGGGCTGGGAATTTTA AGGGGATTGTAGTGGGTGAGGGGCTAGAAAATTGGGATTGTTGATTAGTTGGGTTAAGGGGGATAAAATCAT
CAGGACATGGAAATTGCATTCTTTGGTGAGTCAGCCCCTTGTAGG

4392	14.8	2.7	3.2	Hg19seq	1	324	(3889)	C	MER4D	LTR/ERV1	(102)	801	495	1
2290	11.7	1.8	4.8	Hg19seq	325	710	(3503)	C	THE1C	LTR/ERVL-MaLR	(0)	375	1	2
4392	14.8	2.7	3.2	Hg19seq	711	1190	(3023)	C	MER4D	LTR/ERV1	(409)	494	1	1
1945	19.4	11.5	0.8	Hg19seq	2698	3796	(417)	C	L1MB8	LINE/L1	(5)	6173	4961	3
2141	14.2	0.0	0.0	Hg19seq	3828	4138	(75)	C	LTR26	LTR/ERV1	(2)	601	291	4

>KSI_AluYa5_8

>Scaffold498-2422037-2422344
CACACACACACACACACTTGCCCgGTGGGTTCCACAATGCCTAATTAATTAAAAGATGTATTTGCCTACTTTGCTTACTTGCCCAGGCTGGCACACGATCATGGCTCACTGCAGCCTTGACCTCCTAG
GCCCAAGCGATCCTCCCACGTCAGCCTCCCAAGTGGCTGGAACTACAAGTGTGTGCCACCATGCCCAGATAAGTTTTTAACTTTCTTTTTTCTTTCTTTCTTTTGTAGAGACAGGGATCTTATC ATG
TTGCCCAGGCTGTGGACTGATCTTGAACTCCTGGACTCAAGAGATCCTCCCATCTCAGCCTCCCAAAGTGCTGGGATTATAGGCATAAGCCACCATGCCTGCCCTACTTTACTTTCTTGAAAAAAATG
TGATATCTTGTCCTTTTAAATCAATACTTTAGTAAACAATTTTAATATATATTTCTTATGGCCAAAGTGAAATAGGCATTGAAAGTAATTTTTAAAATTCTGTTTTTATCTATTTTCCATTAAAA AAG
GCAATATAAAAATACAAAAATATGCATTTAAGCAAATAATGAATGTAGCTGTGTTGACATTAGTGTTCTCTCAGTTACTACTCCCACTGATTAAGGGCACTGATACTAGCCAGACAACTGAGGCTTAA
AATATGGTTCCACCACCAATTGGCTTTGAGCTCTTAGGGCAGTCACTTAACTTTTCAATTTTCTCATTTGTAAAATGGGGATAGGACTATTTCCCATCTCAGAGTTAAA ATAATAAcAACATTTTGTA
GTATTTACAGATGTGTCTCGATGATTGCCTTATATTTGGGCATTGTCACATTACTCATGCCTGTGTTTACATTTGTGTTAGTGCCAGCAAGCACCTAAACATTCTTTCTCCTTTGCCTCCCTCTGGAA
GCTTCAGCAAACTTTTCACTTTGTTTTAGTTAAGGTATTCATTTGGTGCCAACATATGTAAACGCAGGCACTAAATATAGCAGCCATCC Tccctgtgttcactattctttttttttttttttttttt
ttgagacggagtctcgctctgtcgcccaggctggagtgcagtggcgggatctcggttcactgcaagctccgcctcccgggttcacgccattctcctgcctcagcctcccaagtagctgggactac agg
cgcccgccactacgcccggctaatttttttgtatttttagtagagacggggtttcaccgttttagccgggatggtctcgatctcctgacctcgtgatccgcccgcctcg gcctcccaaagtgctggga
ttacaggcgtgagccaccgcgcccggCCCTGTGTTCACTATTCTTGCCCTCAGATTCTCTTGTCAGTTATAAAAAcAAAACTCTGCTTTTTAGTAAAAAAAAAAAATTATCTTTTAATAGTTTAATAG
AATGTGACTTTAGTTCCCTGACTTAATCAATTGAGATTATGATTGACATCAGGAAATAATATTGCAG TGAGTAATGTACTACTTTAACTGTCTTACATGATCAGAAATA GAGCTTAGGTGTACTTAAT
ACATAAACAATGCCCTTGTCCCAAGTACAGGCCAAATGTTAAGA cGACATGCTGAGCAAAAGGTTTTACAATAGTTCACATAGAGAGAAATAGTGGAAGAAATGAGTCATTTCATGACATATTGTAGC
TTAGCTATACCAACCAGAAAAGAAGTCACACTATAGGAGTCAGCACTTACTTGGTAAGGAGAATTATCATGAGACCTATTTTTGTAAGTATTAATTAGCAGTAGCTCAT TAGCAGTGTGTAGAAGTAA
ATGTATAATATACCTTTGTTTAATGATATTTCTTTATTAATCAATGTTGTAAAG ATTAGTAAAATGATTGTCTTTCTAAATAAAGATGTATGTAtTTgtgACTAAAAATAATTACAGCATGTGTCACA
ATAAAGTAATACTTTTAATTATAGTTGTATTGGTTTTGTAGGTTTGTTTTTATTTAGTCTTTACATTTGTTTTGGTTGTATAGTTGTATAAAACCTATAGATAAAGGAATTCATGGCTAATTTTA CAG
TTTTACATGTATAAGTATAATAACATAAGAAAATAATGCTGATCAAAACTGGACA tCTCTAAGAATTTTTTTTTTTTTTTTTTTTTTTTTTTACTTTTAAGGCCAAAA ACCCTGAGAAATGCTATACTAG
AGTAAACTGCTTTCTTTGCAGAGTGTTTCAGACTATATTATTACCAAAAGTAATGTGCAATTCTGGCTGAAAATTTAATACGTTTAGTTTCTATTGAAGAACTGGGATAGGTTTGTGAGCTTAAT TAT
TATT

15	0.0	0.0	0.0	AluYa5-Scaffold498-2422037-2422344	1	17	(2291)	+	(CA)n	Simple_repeat	1	17	(0)	1
1438	14.5	2.8	10.3	AluYa5-Scaffold498-2422037-2422344	79	360	(1948)	C	AluJb	SINE/Alu	(49)	263	1	2
428	31.0	6.9	0.0	AluYa5-Scaffold498-2422037-2422344	600	744	(1564)	+	MIR	SINE/MIR	15	169	(93)	3
2877	0.7	0.0	0.3	AluYa5-Scaffold498-2422037-2422344	1001	1308	(1000)	C	AluYa5	SINE/Alu	(4)	307	1	4
945	30.5	4.0	0.9	AluYa5-Scaffold498-2422037-2422344	1478	1801	(507)	+	Charlie29b	DNA/hAT-Charlie	216	549	(645)	5
659	25.2	1.0	1.0	AluYa5-Scaffold498-2422037-2422344	1906	2113	(195)	+	Charlie29b	DNA/hAT-Charlie	937	1144	(50)	6
26	0.0	0.0	0.0	AluYa5-Scaffold498-2422037-2422344	2114	2138	(170)	+	(T)n	Simple_repeat	1	25	(0)	7

>hg19 chr15:35472629-35474627
CACACACACACACACACTTGCCCaGTGGGTTCCACAATGCCTAATTAATTAAAAGATGTATTTGCCTACTTTGCTTACTTGCCCAGGCTGGCACACGATCATGGCTCACTGCAGCCTTGACCTCCTAG
GCCCAAGCGATCCTCCCACGTCAGCCTCCCAAGTGGCTGGAACTACAAGTGTGTGCCACCATGCCCAGATAAGTTTTTAACTTTCTTTTTTCTTTCTTTCTTTTGTAGAGACAGGGATCTTATC ATG
TTGCCCAGGCTGTGGACTGATCTTGAACTCCTGGACTCAAGAGATCCTCCCATCTCAGCCTCCCAAAGTGCTGGGATTATAGGCATAAGCCACCATGCCTGCCCTACTTTACTTTCTTGAAAAAAATG
TGATATCTTGTCCTTTTAAATCAATACTTTAGTAAACAATTTTAATATATATTTCTTATGGCCAAAGTGAAATAGGCATTGAAAGTAATTTTTAAAATTCTGTTTTTATCTATTTTCCATTAAAA AAG
GCAATATAAAAATACAAAAATATGCATTTAAGCAAATAATGAATGTAGCTGTGTTGACATTAGTGTTCTCTCAGTTACTACTCCCACTGATTAAGGGCACTGATACTAGCCAGACAACTGAGGCTTAA
AATATGGTTCCACCACCAATTGGCTTTGAGCTCTTAGGGCAGTCACTTAACTTTTCAATTTTCTCATTTGTAAAATGGGGATAGGACTATTTCCCATCTCAGAGTTAAAATAATAA tAACATTTTGTA
GTATTTACAGATGTGTCTCGATGATTGCCTTATATTTGGGCATTGTCACATTACTCATGCCTGTGTTTACATTTGTGTTAGTGCCAGCAAGCACCTAAACATTCTTTCTCCTTTGCCTCCCTCTGGAA
GCTTCAGCAAACTTTTCACTTTGTTTTAGTTAAGGTATTCATTTGGTGCCAACATATGTAAACGCAGGCACTAAATATAGCAGCCATCC TCCCTGTGTTCACTATTCTTGCCCTCAGATTCTCTTGTC
AGTTATAAAAAAAAACTCTGCTTTTTAGTAAAAAAAAAAAAaTTATCTTTTAATAGTTTAATAGAATGTGACTTTAGTTCCCTGACTTAATCAATTGAGATTATGATTGACATCAGGAAATAATATTG
CAGTGAGTAATGTACTACTTTAACTGTCTTACATGATCAGAAATAGAGCTTAGGTGTACTTAATACATAAACAATGCCCTTGTCCCAAGTACAGGCCAAATGTTAAGA tGACATGCTGAGCAAAAGGT
TTTACAATAGTTCACATAGAGAGAAATAGTGGAAGAAATGAGTCATTTCATGACATATTGTAGCTTAGCTATACCAACCAGAAAAGAAGTCACACTATAGGAGTCAGCACTTACTTGGTAAGGAGAAT
TATCATGAGACCTATTTTTGTAAGTATTAATTAGCAGTAGCTCATTAGCAGTGTGTAGAAGTAAATGTATAATATACCTTTGTTTAATGATATTTCTTTATTAATCAATGTTGTAAAG AagaTTAGTA
AAATGATTGTCTTTCTAAATAAAGATGTATGTAaTTACTAAAAATAATTACAGCATGTGTCACAATAAAGTAATACTTTTAATTATAGTTGTATTGGTTTTGTAGGTTTGTTTTTATTTAGTCTTTAC

ATTTGTTTTGGTTGTATAGTTGTATAAAACCTATAGATAAAGGAATTCATGGCTAATTTTACAGTTTTACATGTATAAGTATAATAACATAAGAAAATAATGCTGATCAAAACTGGACA aCTCTAAGA
AtttttttttttTTTTTTTTTTTTTTTTTTTTTTTTTACTTTTAAGGCCAAAAACCCTGAGAAATGCTATACTAGAGTAAACTGCTTTCTTTGCAGAGTGTTTCAGACTATATTATTACCAAAAGTAA
TGTGCAATTCTGGCTGAAAATTTAATACGTTTAGTTTCTATTGAAGAACTGGGATAGGTTTGTGAGCTTAATTATTAT T

15	0.0	0.0	0.0	hg19	1	17	(1982)	+	(CA)n	Simple_repeat	1	17	(0)	8
1438	14.5	2.8	10.3	hg19	79	360	(1639)	C	AluJb	SINE/Alu	(49)	263	1	9
428	31.0	6.9	0.0	hg19	600	744	(1255)	+	MIR	SINE/MIR	15	169	(93)	10
928	30.8	4.0	0.9	hg19	1158	1481	(518)	+	Charlie29b	DNA/hAT-Charlie	216	549	(645)	11
647	23.3	1.0	1.0	hg19	1586	1780	(219)	+	Charlie29b	DNA/hAT-Charlie	937	1131	(63)	12
39	0.0	0.0	0.0	hg19	1794	1829	(170)	+	(T)n	Simple_repeat	1	36	(0)	13

>KSI_AluYa5_9

>Scaffold1051-342768-343037
TGGCTTCAACAGCCCTTCCATTTACCATCGTGGAATTCTTCTCCAGGGTTTCACCATGGAGTCATCTAGCCATGTTGACAGGGACAGCCCCTCTAACTTCAGCTTGAGTTTTGCTGGAGTCTGGGA GT
CTGGGATTGGGCTGCTCCCTGAATCTGTCAGGAGCCCTAGTCTCTTGCCATAGCCACCGTCAGATTCCAGCCTTCCAAAATCCTGATCTACTGACCCAGGC ACTGCAACTATACTAGGTCTCCAAGTT
TCCATTTATATTTGAATTGCCCACTGGACTAAAAGCTCTTCTCTGAATCTCCATCTACTTATCTGAGTCCTGACACCCTACTTAGTTCTTTTGTTCTTTTTTTGTAAGAAACGGGGTCTCACTCT GCC
TCCCAGGCTGGAGTGCAGTAGCATGAGCACCGCTTACTATATCTTTGAACTCCCAGGCTTAAGCAATTCTCCCGCCTTAGCCTCTGGAGTAGCTGAGACTA CAGGCACACACCATCATGCCTGGTTAA
TTTAAAAACAAATTTTCTTTTCttTTTTTTTTTTTTTTAGAGATAGGGTTTCCTTATGTTGCCAAGGCTGATCTCGAACTCCTGGCCTCAAGAAATCCTCTCTTCTTAGCCTCCCAAAGTGCTGAGAT
TATAGGCCTATTGAGCCCAGCTCCTACTTAGTTCTTGCTGGAATCCACCTCTTCCTATTTG TGTGACTTGCAGCAACTAACCTAATCTTTCTGAATCTCCG TTTTCTACTTCTTAGGCTGTTGCAAGG
ACAAAATGAAGTGCCAAAGACAGACAGACATTGGTATAGATACAGATAAGGATATAGATAGAGGTATGC AGATATTCAAGAAACATTAGTTTTTCATCCCTATATTTACAAATCTCAGCTTCTGGTGC
TATGTCCATTACTTCAGGCCTCCTTCACTTGCCATGAAAGCCTGAGTCTGCCATTCCTTCACAGACATTCTATGTGTCCACTGTTCCCATGCCATCCTTTCTCCTTTTCtttttctttttttttttt
tttgagatggagtctcgctctgtcgcccaggctggagtgcagtggcgggatctcggctcactgcaagctccgcctcccgggttcacgccattctcctgcctgagcctcccaagtagctgggacta cag
gcgcccgccactacgcccggctaatttttgtatttttagtagagacggggtttcaccgttttagccgggatggtctcgatctcctgacctcgtg atccacccgcctcggcctcccaacctttctcct
tttcATTCCTAGCCTGCACCGCTGAGGTCCCTGCAGGCACTATGGTTTAGCCA GCACAGCTCACAGCAGACTCATCTGCTTGGATCATCTTTCTTTCTCCCCTTCTTCATCCCTATCTCCATACTTCC
CAAGCATCCTTGATACTCAAAGTTTGGTGCAGCAGCAGTATCACCTGGGTGCGTGTTAGAAATGCAGTATGTGATGCCCCACTCCAGACCTGGAA TTCTGCATTTTGACAGGGTTAACATTCCTCCTC
CAGGAATCTTTCTCTGATGTCCCTGTGGTAAGTTAAGGGCTCCTATGCTATGCGCCATCC CACAGGAATCAGCCTGCCATAGAACCAAGGAAACTCATTCCTGGCCTCGGGACCCAC gTGTGCAAAAG
TCTACAGGCTAAAAATATCCTAGAGCATGTGGTGTATGGATTAATTTATCCCTTCCATTGCAACGATTTTTTTTTTTTTTTGAGACGGAGTCTCACTCTGTCACTCAGGCTGGAGTGCAGTGGCGTGA
TCTCAGCTCACTGCAATCTCTGCCTCCCAGGTTCAAATGATTCTCCTGCCTCAGCCTCCCGAGTAGCTGCAATTACAGGCATGCACCAG cTGTGTCTGGCTAATTTTTGTATTTTTAGTAGAGATGGA
GTTTCACCATGTTGGCCAGGCTAATCTCGAACTCTTGACCTCAGGCGATCCGCCTGCCTCAGCCTCC CAAAGTGCTGGGGTTACCGGTGTGAGGCACCACACCCAGCCTCCACTGCAATGATTTATGT
ACATATCTGTCTCCCTTAGTCATCCATGAGTTCCTTAAGAGCAGGAACTGAGTCCTGTTGATCTATTTCTAATAACTGGCATAGTGTCAGGCATAGAAGTACACAGATAGGACAGCAACAAGAAG ATA
GGTTTGGAGAGGGATTTCTTCTGGGCTTAGTTCATTGTCCATCAAACTGGTATCCTGAGTATCTTGA GCTTTCTTCTAGAGCAGTAGGATAAGA

1507	18.3	0.0	8.5	AluYa5-Scaffold1051-342768-343037	342	647	(1623)	C	AluJr	SINE/Alu	(10)	302	21	1
201	29.7	0.0	0.0	AluYa5-Scaffold1051-342768-343037	684	747	(1523)	+	MIRb	SINE/MIR	73	136	(132)	2
12	21.7	0.0	0.0	AluYa5-Scaffold1051-342768-343037	803	834	(1436)	+	A-rich	Low_complexity	1	32	(0)	3
2489	1.9	0.0	0.0	AluYa5-Scaffold1051-342768-343037	1001	1270	(1000)	C	AluYa5	SINE/Alu	(3)	308	39	4
525	14.0	15.9	0.0	AluYa5-Scaffold1051-342768-343037	1416	1522	(748)	+	MER5A1	DNA/hAT-Charlie	1	124	(42)	5
2259	11.7	0.0	0.3	AluYa5-Scaffold1051-342768-343037	1731	2028	(242)	C	AluSx	SINE/Alu	(15)	297	1	6
321	34.1	4.5	0.0	AluYa5-Scaffold1051-342768-343037	2032	2143	(127)	+	L2c	LINE/L2	3234	3349	(38)	7

>hg19 chr1:65571270-65573259
TGGCTTCAACAGCCCTTCCATTTACCATCGTGGAATTCTTCT CCAGGGTTTCACCATGGAGTCATCTAGCCATGTTGACAGGGACAGCCCCTCTAACTTCAGCTTGAGTTTTGCTGGAGTCTGGGAGT
CTGGGATTGGGCTGCTCCCTGAATCTGTCAGGAGCCCTAGTCTCTTGCCATAGCCACCGTCAGATTCCAGCCTTCCAAAATCCTGATCTACTGACCCAGGCACTGCAACTATACTAGGTCTCCAA GTT
TCCATTTATATTTGAATTGCCCACTGGACTAAAAGCTCTTCT CTGAATCTCCATCTACTTATCTGAGTCCTGACACCCTACTTAGTTCTTTTGTTCTTTTTTTGTAAGAAACGGGGTCTCACTCTGCC
TCCCAGGCTGGAGTGCAGTAGCATGAGCACCGCTTACTATATCTTTGAACTCCCAGGCTTAAGCAATTCTCCCGCCTTAGCCTCTGGAGTAGCTGAGACTACAGGCACACACCATCATGCCTGGT TAA
TTTAAAAACAAATTTTCTTTTCTTTTTTTTTTTTTTAGAGATAGGGTTTCCTTATGTTGCCAAGGCTGATCTCGAACTCCTGGCCTCAAGAAATCCTCTCTTCTTAGCCTCCCAAAGTGCTGAGATTA
TAGGCCTATTGAGCCCAGCTCCTACTTAGTTCTTGCTGGAATCCACCTCTTCCTATTTGTGTGACTTGCAGCAACTAACCTAATCTTTCTGAATCTCCGTTTTCTACTTCTTAGGCTGTTGCAAG GAC
AAAATGAAGTGCCAAAGACAGACAGACATTGGTATAGATACA GATAAGGATATAGATAGAGGTATGCAGATATTCAAGAAACATTAGTTTTTCATCCCTATATTTACAAATCTCAGCTTCTGGTGCTA
TGTCCATTACTTCAGGCCTCCTTCACTTGCCATGAAAGCCTGAGTCTGCCATTCCTTCACAGACATTCTATGTGTCCACTGTTCCCATGCCATCCTTTCTCCTTTTCATTCCTAGCCTGCACCGCTGA
GGTCCCTGCAGGCACTATGGTTTAGCCAGCACAGCTCACAGCAGACTCATCTGCTTGGATCATCTTTCTTTCTCCCCTTCTTCATCC CTATCTCCATACTTCCCAAGCATCCTTGATACTCAAAGTTT
GGTGCAGCAGCAGTATCACCTGGGTGCGTGTTAGAAATGCAGTATGTGATGCCCCACTCCAGACCTGGAATTCTGCATTTTGACAGGGTTAACATTCCTCCTCCAGGA ATCTTTCTCTGATGTCCCTG
TGGTAAGTTAAGGGCTCCTATGCTATGCGCCATCCCACAGGAATCAGCCTGCCATAGAACCAAGGAAACTCATTCCTGGCCTCGGGACCCAC aTGTGCAAAAGTCTACAGGCTAAAAATATCCTAGAG
CATGTGGTGTATGGATTAATTTATCCCTTCCATTGCAACGAtTTTTTTTTTTTTTTTGAGACGGAGTCTCACTCTGTCACTCAGGCTGGAGTGCAGTGGCGTGATCTC AGCTCACTGCAATCTCTGCC
TCCCAGGTTCAAATGATTCTCCTGCCTCAGCCTCCCGAGTAGCTGCAATTACAGGCATGCACCAG tTGTGTCTGGCTAATTTTTGTATTTTTAGTAGAGATGGAGTTTCACCATGTTGGCCAGGCTAA
TCTCGAACTCTTGACCTCAGGCGATCCGCCTGCCTCAGCCTCCCAAAGTGCTGGGGTTACCGGTGTGAGGCACCACACCCAGCCTCCACTGCAATGATTTATGTACAT ATCTGTCTCCCTTAGTCATC
CATGAGTTCCTTAAGAGCAGGAACTGAGTCCTGTTGATCTATTTCTAATAACTGGCATAGTGTCAGGCATAGAAGTACACAGATAGGACAGCAACAAGAAGATAGGTTTGGAGAGGGATTTCTTC TGG

GCTTAGTTCATTGTCCATCAAACTGGTATCCTGAGTATCTTGAGCTTTCTTCTAGAGCAGTAGGATAAGA

1519	18.4	0.0	7.8	hg19	342	645	(1345)	C	AluJr	SINE/Alu	(10)	302	21	8
201	29.7	0.0	0.0	hg19	682	745	(1245)	+	MIRb	SINE/MIR	73	136	(132)	9
12	21.7	0.0	0.0	hg19	801	832	(1158)	+	A-rich	Low_complexity	1	32	(0)	10
525	14.0	15.9	0.0	hg19	1135	1241	(749)	+	MER5A1	DNA/hAT-Charlie	1	124	(42)	11
2253	12.0	0.0	0.3	hg19	1450	1748	(242)	C	AluSx	SINE/Alu	(14)	298	1	12
321	34.1	4.5	0.0	hg19	1752	1863	(127)	+	L2c	LINE/L2	3234	3349	(38)	13

>KSI_AluYa5_10

>Scaffold1073-160543-160841
CAGTGCCCCAGAAATCTCACTGGTGCCCTCTCTCAGTTCTGTTCAGCCCCCACTACCCACCTACTCCAAGAGAAAGCAAAGATAATAACTTTAAACCCATAGATTGTTTTTGCCTATTTTTGAATT TT
ATATAAATGGAACAGAATATAAAATCTTTGGTATATTGCTTCTCTTACTATGTATGTTTATGAGGT gTATTCATGTTGTTGCATGTAATAGTTCAaTTATTCTCATACTAAATAGTATTCCATTTTAT
GACTACAGCACAATTTATCCATTCTACTTTTGAGAGATATTTGGGTAGTTTCCTGGTT TtttttttttttTTTTTTTTTTTTTTTTTTTTTTTTTGAGACAGAGTTTTGCTCTTGTTGCCCAGGCT
GGAGTGCAATGGCATGATCTTGGCTCACTGAAACCTCTGCCTCCTGGGTTCAAGTGATTCTCCTGCC TCAGCCTCCCGAGTAGCTGGGATTACAGGCATGTGCCACCACGCCCAGCTAATTTTGTATT
TGCAGTAGAGACAAGGTTTCTCCATGTTGGTCAGGCTGGTCTCAAACTCCTGACCTCAGGTGATCCACCCGCCTTGGCCTCCCAAAGTGCTGGGATTATAGGCGTGAGCCACCGCGCCTGGCCTC CTG
TTTTGaGCTATTACTAATgGTTCTGCTGTGAGCATTCTTTTGGTAAATACCTTTTGATGAAAATACG TGAGCAATTCTATCGGGAATTTTTCCAGGAGTGGAATTGCCAAGTCACAGTGCATGGATAT
GTTTAGCTTTAAAGATGATTTCCAAAGGAAATTGATAAGTATACACATCCAGCACTGAATATGAGTCCTAGAA GCTCCAAACACTGGCTGGCATTTGATATCATTTGTCTTTTTCATTTTAGCTACTC
TGGTGAATGTAcAGTGATATTATGTTATGCTTTTAAACTGCATTTCTTTGAGGAGTAGTGAAGTTGA GCACTATGTTCATATCTTTATCAGCCACTTggatatcttcttttttttttttttgagacg
gagtcttgctctgtcacccaggctggagtgcaggggcgcgatctcggctcactgcaagctccgcctcccgggttcacgccattctcctgcctcagcctcccaagtagctgggactacaggcgccc gcc
actacgcccggctaatttttgtatttttagtagagacggggtttcaccgttttagccgggatggtctcgatctcctgacctcgtgatccgcccgcctcggcctcccaaag tgctgggattacaggcg
tgagccaccgcgcccggccGGATATCTTCTTTTGAATAGTATTTGTTCAAaTATTTTGCTCATTTTTCTGTTGGTACATCTTCATTGTCTGCCTTCTGCTTATTAATGATCAATGTTCTTTATATATT
CTAAAAATGGGTTCTTTGTGCATTGTGTATTGCAAATATCTATTCTCATTTGTCTTTTTACTCACTTCATGATGTCTTTTGATGAACAGAAGCTTTAAATTTTAATCTAGT CCAATTTATTAAGTTTT
GGTTAAGGCTTTTTACGTCCTGCTTAAGAATCTGCCATCCTACAAAGATCATGAAGATATTTTATGTCTTATTCTAGAATATTTATTAGTTTTTCTTCTACATTTAGATCTATAAGGCACCTAAA TTA
TTTTTTATGTATGGTGTGAGGTAGGCATTTGGGATTCATTTGTTTCCCACATGCATAGTCATGAAAATGAATAAAATTGTGAATTTGGCA cAGTTCAAATAATACTAACTCCTTTTTGAAATATTCTG
CAGAACCTCAAtttttttTTTTTTTTTTTTTTTTTGAGATGTGGGTCTTAATATGTTGCCCAGGCTGGTTTCAAACTCCTGGGCTCAAGCAATCCTCCCACCTCAGCCTCCTGAGGATCTGGGATTA
CATGCTACTGTGCCTGGCCTAATTAGATTTGATGAATGAATTAATTATTTTAAATTAATTCAGTATTTTTACACATCTAGTGTTTGCCAGTTTAGCTAGGTTGGTAAAGAA AGTCCTCTCAGAGCCAA
CATTTGAGCTCAGACCTGATTGATGAAATGAAGCCAGCCACAAGGGGATTGGGGGAAGAACATTTCAGGAAGAGGCAAGAGGTAGGTAAGTAAAGTTTGATATGTTCAAGGAACAAGAGGAAGGC CAG
CACCGCTGAAGTGTAATGCCCAAGATGATGTGAAATACAAGACAATTCTTATGAAT gGGAGAGCAAAGGAAAGAGAACCAGACGGGGATCACTAGAAGATAATGGAGCAAT GCTTTCAAAGTC

2532	24.1	4.1	6.1	AluYa5-Scaffold1073-160543-160841	1	312	(1987)	C	L1ME3A	LINE/L1	(135)	6038	5730	1
2302	8.5	0.3	0.0	AluYa5-Scaffold1073-160543-160841	313	635	(1664)	C	AluSp	SINE/Alu	(0)	313	1	2
2532	24.1	4.1	6.1	AluYa5-Scaffold1073-160543-160841	636	1000	(1299)	C	L1ME3A	LINE/L1	(444)	5729	5360	1
2770	1.7	0.0	0.0	AluYa5-Scaffold1073-160543-160841	1001	1299	(1000)	C	AluYa5	SINE/Alu	(12)	299	1	3
2532	21.3	5.8	4.9	AluYa5-Scaffold1073-160543-160841	1300	1721	(578)	C	L1ME3A	LINE/L1	(814)	5359	4931	1
679	15.4	5.5	0.9	AluYa5-Scaffold1073-160543-160841	1804	1938	(361)	C	FLAM_A	SINE/Alu	(0)	142	1	4
13	19.2	0.0	8.9	AluYa5-Scaffold1073-160543-160841	1940	1988	(311)	+	(TAAT)n	Simple_repeat	1	45	(0)	5
420	29.2	10.7	0.5	AluYa5-Scaffold1073-160543-160841	1999	2194	(105)	C	L2a	LINE/L2	(240)	3186	2971	6
235	11.1	0.0	2.8	AluYa5-Scaffold1073-160543-160841	2262	2298	(1)	+	HAL1	LINE/L1	1579	1614	(851)	7

>hg19 chr17:36168344-36170322
CAGTGCCCCAGAAATCTCACTGGTGCCCTCTCTCAGTTCTGTTCAGCCCCCACTACCCACCTACTCCAAGAGAAAGCAAAGATAATA ACTTTAAACCCATAGATTGTTTTTGCCTATTTTTGAATTTT
ATATAAATGGAACAGAATATAAAATCTTTGGTATATTGCTTCTCTTACTATGTATGTTTATGAGGT aTATTCATGTTGTTGCATGTAATAGTTCAtTTATTCTCATACTAAATAGTATTCCATTTTAT
GACTACAGCACAATTTATCCATTCTACTTTTGAGAGATATTTGGGTAGTTTCCTGGTT TgtttgTTTTTTTTTTTTTTTTTTTTTTTTTTGAGACAGAGTTTTGCTCTTGTTGCCCAGGCTGGAGTGC
AATGGCATGATCTTGGCTCACTGAAACCTCTGCCTCCTGGGTTCAAGTGATTCTCCTGCCTCAGCCTCCCGAGTAGCTGGGATTACAGGCATGTGCCACCACGCCCAGCTAATTTTGTATTTGCA GTA
GAGACAAGGTTTCTCCATGTTGGTCAGGCTGGTCTCAAACTCCTGACCTCAGGTGATCCACCCGCCTTGGCCTCCCAAAGTGCTGGG ATTATAGGCGTGAGCCACCGCGCCTGGCCTCCTGTTTTGgG
CTATTACTAATaGTTCTGCTGTGAGCATTCTTTTGGTAAATACCTTTTGATGAAAATACGTGAGCAATTCTATCGGGAATTTTTCCAGGAGTGGAATTGCCAAGTCACAGTGCATGGATATGTTTAGC
TTTAAAGATGATTTCCAAAGGAAATTGATAAGTATACACATCCAGCACTGAATATGAGTCCTAGAA GCTCCAAACACTGGCTGGCATTTGATATCATTTGTCTTTTTCATTTTAGCTACTCTGGTGAA
TGTAtAGTGATATTATGTTATGCTTTTAAACTGCATTTCTTTGAGGAGTAGTGAAGTTGAGCACTATGTTCATATCTTTATCAGCCACT TGGATATCTTCTTTTGAATAGTATTTGTTCAAtTATTTT
GCTCATTTTTCTGTTGGTACATCTTCATTGTCTGCCTTCTGCTTATTAATGATCAATGTTCTTTATATATTCTAAAAATGGGTTCTTTGTGCATTGTGTATTGCAAATATCTATTCTCATTTGTCTTT
TTACTCACTTCATGATGTCTTTTGATGAACAGAAGCTTTAAATTTTAATCTAGTCCAATTTATTAAGTTTTGGTTAAGGCTTTTTACGTCCTGCTTAAGAATCTGCCATCCTACAAAGATCATGA AGA
TATTTTATGTCTTATTCTAGAATATTTATTAGTTTTTCTTCTACATTTAGATCTATAAGGCACCTAAATTATTTTTTATGTATGGTGTGAGGTAGGCATTTGGGATTCATTTGTTTCCCACATGCATA
GTCATGAAAATGAATAAAATTGTGAATTTGGCAaAGTTCAAATAATACTAACTCCTTTTTGAAATATTCTGCAGAACCTCA ATTTTTTTTTTTTTTTTTTGAGATGTGGGTCTTAATATGTTGCCCAG

GCTGGTTTCAAACTCCTGGGCTCAAGCAATCCTCCCACCTCA GCCTCCTGAGGATCTGGGATTACATGCTACTGTGCCTGGCCTAATTAGATTTGATGAATGAATTAATTATTTTAAATTAATTCAGT
ATTTTTACACATCTAGTGTTTGCCAGTTTAGCTAGGTTGGTAAAGAAAGTCCTCTCAGAGCCAACATTTGAGCTCAGACCTGATTGATGAAATGAAGCCAGCCACAAGGGGATTGGGGGAAGAAC ATT
TCAGGAAGAGGCAAGAGGTAGGTAAGTAAAGTTTGATATGTT CAAGGAACAAGAGGAAGGCCAGCACCGCTGAAGTGTAATGCCCAAGATGATGTGAAATACAAGACAATTCTTATGAAT aGGAGAGC
AAAGGAAAGAGAACCAGACGGGGATCACTAGAAGATAATGGAGCAATGCTTTCAAAGT C

2605	23.6	4.1	6.1	hg19	1	320	(1659)	C	L1ME3A	LINE/L1	(135)	6038	5730	8
2302	8.5	0.3	0.0	hg19	321	628	(1351)	C	AluSp	SINE/Alu	(0)	313	1	9
2605	22.2	5.2	4.4	hg19	629	1408	(571)	C	L1ME3A	LINE/L1	(4 44)	5729	4931	8
805	14.2	4.7	0.8	hg19	1492	1618	(361)	C	FLAM_A	SINE/Alu	(10)	132	1	10
13	18.2	0.0	10.4	hg19	1620	1668	(311)	+	(AATT)n	Simple_repeat	4	48	(0)	11
420	29.2	10.7	0.5	hg19	1679	1874	(105)	C	L2a	LINE/L2	(240)	3186	2971	12
235	11.1	0.0	2.8	hg19	1942	1978	(1)	+	HAL1	LINE/L1	1579	1614	(851)	13

>KSI_AluYa5_11

>Scaffold1337-1252784-1252994

CAAGGACTTAAAAAATTCATAATCTCTATTATGAAAACTCATGATAATATCTATAAACATCAAGCTGAAGTAAAATTGTTTGTTTCCCTGGATATGTACATATTCTTTCTTTCTTTTGTCTCTGTA CC
AACAGAACATTCTTGCCCAAGTGGCCTAGTGTTCAAGTACAATGTAAAAGCCTGTAATAGTTCTTGCCGATCCTTGTCAGAAAGAGATAGGAGCTGTGATGTAGAAGATGTTCCAGTTGATGGAT GCA
CCTGCCCTGATGCAATGTACCAGAATAATGAAGGAAACTGTGTACTGAAATCTCAGTGTGACTGTTACATAAACGACGAGGTCATGCAACCAGGCAAACTCATCCATATTGATGACAATAAATGGT AT
GGAAATTATACGTATACTTGCTATGACACTGAAATCAAGAGACTGCGTTCTTTTCAGATAAATGATCTTTCGTCAGTATGAAGTCTTTTTAGACCTACCATACTTATGATTCTTATTTATTAACA AAA
ATCTATTTTATTCTGGAAGGCTCATTGCATCCTAATGGATATAGATGACTTATCCCACTCTGGTTTTGTTTTAACACCAGTAGAAAAATATTCTGGTAGCTTCTATATACCAGATGTGAAATGATT TC
ATATTCAGAAGATGCTCTCTAGCTGTCTCTCAGTAGTCATCATTCAACAAACACTCATTAAGTGTCTGCAGTGTGCCAGGCCTTGTGCTGAGTGCTGGAGTTGCAAAGCTGAACGAACATTGTCCTGG
ACTCCGAGCAAGCAGGGCACAAACCACTAGAAGGAGGTTTATATACTAGAACATGGTAGCAGAAGGGGCCAGGCACTACCTGAGGAGATTAAGGAAAACTTCTCAGAGAAGACA GGCATATGAATTGT
GCTAGGGAATGGATCtttttccagacaaaattgaaatgtagaggatgacttcctgtagagtaaagagaatgcacagatgtaggaaatgtttgaaaaaattttac ggccgggcgcggtggctcacgcct
gtaatcccagcactttgggaggccgaggcgggcggatcacgaggtcaggagatcgagaccatcccggctaaaacggtgaaaccccgtctctactaaaaatacaaaaaattagccgggcgtagtggc gg
gcgcctgtagtcccagctacttgggaggctgaggcaggagaatggcgtgaacccgggag TTTTTCCAGACAAAATTGAAATGTAGAGGATGACTTCCTGT AGAGTAAAGAGAATGCACAGATGTAGGA
AATGTTTGAAAAAATTTTACACATGCGGGTTGTTTTGTATAGGATTGGGCTAGAAACATTGACAGTGGTCAGATCATAACCAAGAATTTGGACTTAGTTTCTGAAGAATTTAAGAATTTTGACTATTT
TGTAATGGCTGAGGAGGCTATGATTTCTACAGTTTGGAGCAAATTCATTTTTTTCTTTTTTTTATTTTTATTTTTTTTGAGATGGGGTCTCACTTCAATTGCCCAGGCTGGAGTTCAGTGGCACAGTT
TTGGCTCACTGCAGCTCCAACCTCCCTGGGCTCAAGTGATTCTCCCACCGCAGCCTCC TGAGTAGCTGGGACTACAGGTGCATGCCACCATGCCCAGCTAATATTTTGTATTTTTAGTAGATACGGTA
TTTCACCATGTTGCCCAGGCTGGTCTTGAACTCCTGGACTTAAGCAAT CCACCTGCCTTGGCTTCCCAAAGTGCTGAGATTACAGGCATGAGCCACTGTGCTCAGCCTGGAGCAAAGTAATTTAAGCA
TGTTTCTAATTCCATAGAGAGGTGTAGGAATTCTTGCCAAAATGAAGTAGCCACTCAAAAATATTTTGAATATGCTTCCTTTCTTTTCTTTGGTTTCATGAGGCTATCATTAACAGTAAGTAATT TTT
CTTGGTCATTCAAAAGGAAAGCCTAGGTAACCCCTTTATATCAAAGCC CATGAGCCTTTTGCTGGGGTTTGATAATGATAATTTCTTTTTCTCTGTAGTTTTTGGGGAATCAATAGATTAACTTTTTA
ATGGTGCTTAATAAAGAATTACACTATTTTATCTGTATTGGATATTAGCAAATATGCAGTCTGCTCTTTTTTTCATGGGAATCAATAAATTAACTTTTCAATGAATCAGGCTAGGACCTCCAAAT TAC
AGTTCCCTTCACCACTACCCTATTTGAGACTCTCC

371	26.1	0.2	2.9	AluYa5-Scaffold1337-1252784-1252994	680	776	(1435)	C	L2b	LINE/L2	(6)	3381	3293	1
2028	0.0	0.0	0.0	AluYa5-Scaffold1337-1252784-1252994	1001	1211	(1000)	+	AluYa5	SINE/Alu	1	211	(100)	2
2037	15.6	0.0	1.0	AluYa5-Scaffold1337-1252784-1252994	1457	1771	(440)	C	AluJb	SINE/Alu	(0)	312	1	3

>hg19 chr12:40831797-40833707

CAAGGACTTAAAAAATTCATAATCTCTATTATGAAAACTCATGATAATATCTATAAACATCAAGCTGAAGTAAAATTGTTTGTTTCCCTGGATATGTACATATTC TTTCTTTCTTTTGTCTCTGTACC
AACAGAACATTCTTGCCCAAGTGGCCTAGTGTTCAAGTACAATGTAAAAGCCTGTAATAGTTCTTGCCGATCCTTGTCAGAAAGAGATAGGAGCTGTGATGTAGAAGATGTTCCAGTTGATGGAT GCA
CCTGCCCTGATGCAATGTACCAGAATAATGAAGGAAACTGTGTACTGAAATCTCAGTGTGACTGTTACATAAACGACGAGGTCATGCAACCAGGCAAACTCATCC ATATTGATGACAATAAATGGTAT
GGAAATTATACGTATACTTGCTATGACACTGAAATCAAGAGACTGCGTTCTTTTCAGATAAATGATCTTTCGTCAGTATGAAGTCTTTTTAGACCTACCATACTTATGATTCTTATTTATTAACA AAA
ATCTATTTTATTCTGGAAGGCTCATTGCATCCTAATGGATATAGATGACTTATCCCACTCTGGTTTTGTTTTAACACCAGTAGAAAAATATTCTGGTAGCTTCTA TATACCAGATGTGAAATGATTTC
ATATTCAGAAGATGCTCTCTAGCTGTCTCTCAGTAGTCATCATTCAACAAACACTCATTAAGTGTCTGCAGTGTGCCAGGCCTTGTGCTGAGTGCTGGAGTTGCAAAGCTGAACGAACATTGTCC TGG
ACTCCGAGCAAGCAGGGCACAAACCACTAGAAGGAGGTTTATATACTAGAACATGGTAGCAGAAGGGGCCAGGCACTACCTGAGGAGATTAAGGAAAACTTCTCA GAGAAGACAGGCATATGAATTGT
GCTAGGGAATGGATCTTTTTCCAGACAAAATTGAAATGTAGAGGATGACTTCCTGTAGAGTAAAGAGAATGCACAGATGTAGGAAATGTTTGAAAAAATTTTAC ACATGCGGGTTGTTTTGTATAGGA
TTGGGCTAGAAACATTGACAGTGGTCAGATCATAACCAAGAATTTGGACTTAGTTTCTGAAGAATTTAAGAATTTTGACTATTTT GTAATGGCTGAGGAGGCTATGATTTCTACAGTTTGGAGCAAAT
TCATTTTTTTCTTTTTTTTATTTTTATTTTTTTTGAGATGGGGTCTCACTTCAATTGCCCAGGCTGGAGTTCAGTGGCACAGTTTTGGCTCACTGCAGCTCCAACCTCCCTGGGCTCAAGTGATT CTC
CCACCGCAGCCTCCTGAGTAGCTGGGACTACAGGTGCATGCCACCATGCCCAGCTAATATTTTGTATTTTTAGTAGATACGGTAT TTCACCATGTTGCCCAGGCTGGTCTTGAACTCCTGGACTTAAG
CAATCCACCTGCCTTGGCTTCCCAAAGTGCTGAGATTACAGGCATGAGCCACTGTGCTCAGCCTGGAGCAAAGTAATTTAAGCATGTTTCTAATTCCATAGAGAGGTGTAGGAATTCTTGCCAAA ATG
AAGTAGCCACTCAAAAATATTTTGAATATGCTTCCTTTCTTTTCTTTGGTTTCATGAGGCTATCATTAACAGTAAGTAATTTTTC TTGGTCATTCAAAAGGAAAGCCTAGGTAACCCCTTTATATCAA
AGCCCATGAGCCTTTTGCTGGGGTTTGATAATGATAATTTCTTTTTCTCTGTAGTTTTTGGGGAATCAATAGATTAACTTTTTAATGGTGCTTAATAAAGAATTACACTATTTTATCTGTATTGG ATA
TTAGCAAATATGCAGTCTGCTCTTTTTTTCATGGGAATCAATAAATTAACTTTTCAATGAATCAGGCTAGGACCTCCAAATTACA GTTCCCTTCACCACTACCCTATTTGAGACTCTCC

371	26.1	0.2	2.9	hg19	680	776	(1135)	C	L2b	LINE/L2	(6)	3381	3293	4
2037	15.6	0.0	1.0	hg19	1157	1471	(440)	C	AluJb	SI NE/Alu	(0)	312	1	5

>KSI_AluYa5_12

>Scaffold1464-150754-151054
gagtccGAGGCAGGAGAATCACTTGAATCCAGGAGGTGGAGGTTGCAGTGAGTCTGTGTCCGGAATTGGTTCCTTCTGGTGGGTTCTTGGTCTTGCTGACTTCAAGAATGAACCCATGAACCCTCACA
GTGAGTGTTACAGTTCTTAAAGATGGCGTGTCCAGAGTTTGTTCATTCAGATGTTCAGATGTGTCCGAAATTTCTTCCTTCCAGTGGGTTCGTGGTCTTGCTGACTTCAGGAGTGAAGCCACAGA CCT
TCGCAGTGAGTGTTACAGCTCTTAAAGGTGGCATGTCTGAAGTCGATTGTTCCTCCCCATGGGTTCGTGGTCTCGCTGACTTCAGGAGTGAAGCTGAAGACCTTCGCAGTGAGTGTTGCAGCTCATAA
AGGTGGTGTGGACCCAAAGAGTGAGCAGCAGCAAGATTTATTGTGAAGAGTGAAAGAACAAAGCTTCCACAGCATGGAAGGGGACCCGAGTGGGTTGCCACTGCTGACTCAGGTGGCCAGCTTTT ATT
CCCTTATTTGGCCCTGCCCGCATCCTACTGATTGGTCCATTTTACAGAGTGCTGATTGGTCCGTTTTTACAGAGTGCTGATTGGTGCATTTACAAACCTCTAGCTAGACACATAGCACTGATTGGTGT
GTTTACAATCCTCTAGCTAGACAGAAgAGTTCTCCAAGTCCCCACCCAACCCAGAAGCCCAGCTGGCTTCACCTCTCAATCTCCCCACTTAACAAGACACCCCAACTGCTGTTGGGAATTGGGTGATG
ACCACTCTAGCTACTTCCTGCTGGATAGGGGCAAAGAAAGGACCCTGCAGTTGTAGTGTCCTCCAGAGGGGAACTCTTTAGGCCATTGAAAGGGCCAGCGGGTTGGTCCAGGGGT CCTTGGTAGTTGT
TAGTTGAGCTCATTTGGGGTTCCATTTGTAAGATCATCTGTAGCTTGATAGCCTCGATCCTAGAGGAAACAAATTTGACAAGGAGGTT AAAAATACCGGGCCCGggccgggcgcggtggctcacgcct
gtaatcccagcactttgggaggccgaggcgggcggatcacgaggtcaggagatcgagaccatcctggctaaaacggtgaaaccccgtctctactaaaaatacaaaaaattagccgggcgtagtggcgg
gcgcctgtagtcccagctacttgggaggctgaggcaggagaatggcgtgaacccgggaggcggagcttgcagtgagccgagatcccgccactgcactccagcctgggcgacagagcgagactccg tct
caaaaaaaaaaaaaaaaaaaatcccgggcccgAAGGTGAGTAATAGCAAGAgGGCTGCCATGGGACCTAGAAAGGGGAGAAGCCATGTTGCCAACTCCAGAGGCTGGTATAAGAGCTTGAAAGATGTT
GTCTGATTTTGGAAGCCTTTTCCTGTAAATGCCGGGTAGCATCTCGTACTATCCCTGACTGGTTAGTGTAAA AACAACACTCTTCCCCTAAGAAGGTGCAGAGTCCTCCTTTCTCAGCAGTGAGGAGG
TCTAGCCTCAGCAGTTTTGGAGAGTCACTGCTGCGAAAGAGTCTATTTGGGATTCTGGAGTAAGGATAGATTTCGTTATCTCTTGCAAACTGTCAGAGAAATCCTTTGAGAGTGTGTGGCAGTAG GAT
AATGAAGTAGATAAACCGGCTATTCCGGTTCCTTTAGCAGTAGCCATTCCTAACCCTATAAGTAGGGGTATT AGTTGTATGGCTCTGTGCTGACAGACTTGAGCTTTGAGGGATACTGATAGGGTCTG
ATTTCCTGGGGCAATGTTAATGTTAGGACTTAGAAAGACTAAGGTACAGATGCCTGTCCAGTTAGTGGAGAGGCAGATATGTTGATGTTCCACATAAGAAGAATATACATTGGCTGGGTAGACAG AAC
TGGTTGTGTATGTTAAAAAGGTGTGTGAGTTTGTTATTTTCATTTTCCCATACTTCTAGAGTAATTGCCAAG GTAGCTCCAGTGAAGGAATGGAAAGGGGTGTTGGGAGCAAACTGAGTGGCTCCCTG
TGTTCTATTTTCCCATTGGAGAAAAAGCCTTTTTGTATCTACTAGGAACCATTTGAGAGAGTGATTGAAAGAAGGGATGAGAAGGCATTCACTAGTGGTGGGGGCACTGATGCAGGGGGTCCAGG GAT
GAATGGTCATGGAGGGAGTATGTTTGTCATTACAAAACTTGGACTGTTTGTTAAGCAGGGAGGAGGTGATGA TTTTTGGGGACCCAGAGAAGTGGACAAACTGTCTGAATGGAGCTGTTTGAGT

408	10.0	0.0	0.0	AluYa5-Scaffold1464-150754-151054	5	54	(2246)	+	AluSq10	SINE/Alu	182	231	(82)	1
5314	7.5	3.6	0.0	AluYa5-Scaffold1464-150754-151054	55	718	(1582)	C	LTR12_	LTR/ERV1	(0)	688	1	2
10339	6.1	0.6	0.9	AluYa5-Scaffold1464-150754-151054	719	1000	(1300)	C	HERV9-int	LTR/ERV1	(0)	8436	8152	2
2870	0.3	0.0	0.0	AluYa5-Scaffold1464-150754-151054	1001	1301	(999)	+	AluYa5	SINE/Alu	1	301	(10)	3
10339	6.1	0.6	0.9	AluYa5-Scaffold1464-150754-151054	1302	2300	(0)	C	HERV9-int	LTR/ERV1	(285)	8151	7160	2

>chr11:110275934-110277916
GAGGCAGGAGAATCACTTGAATCCAGGAGGTGGAGGTTGCAGTGAGTCTGTGTCCGGAATTGGTTCCTTCTGGTGGGTTCTTGGTCTTGCTGACTTCAAGAATGAACCCATGAACCCTCACAGTGA GT
GTTACAGTTCTTAAAGATGGCGTGTCCAGAGTTTGTTCATTCAGATGTTCAGATGTGTCCGAAATTTCTTCCTTCCAGTGGGTTCGTGGTCTTGCTGACT TCAGGAGTGAAGCCACAGACCTTCGCAG
TGAGTGTTACAGCTCTTAAAGGTGGCATGTCTGAAGTCGATTGTTCCTCCCCATGGGTTCGTGGTCTCGCTGACTTCAGGAGTGAAGCTGAAGACCTTCGCAGTGAGTGTTGCAGCTCATAAAGG TGG
TGTGGACCCAAAGAGTGAGCAGCAGCAAGATTTATTGTGAAGAGTGAAAGAACAAAGCTTCCACAGCATGGAAGGGGACCCGAGTGGGTTGCCACTGCTG ACTCAGGTGGCCAGCTTTTATTCCCTTA
TTTGGCCCTGCCCGCATCCTACTGATTGGTCCATTTTACAGAGTGCTGATTGGTCCGTTTTTACAGAGTGCTGATTGGTGCATTTACAAACCTCTAGCTAGACACATAGCACTGATTGGTGTGTT TAC
AATCCTCTAGCTAGACAGAAaAGTTCTCCAAGTCCCCACCCAACCCAGAAGCCCAGCTGGCTTCACCTCTCAATCTCCCCACTTAACAAGACACCCCAAC TGCTGTTGGGAATTGGGTGATGACCACT
CTAGCTACTTCCTGCTGGATAGGGGCAAAGAAAGGACCCTGCAGTTGTAGTGTCCTCCAGAGGGGAACTCTTTAGGCCATTGAAAGGGCCAGCGGGTTGGTCCAGGGGT CCTTGGTAGTTGTTAGTTG
AGCTCATTTGGGGTTCCATTTGTAAGATCATCTGTAGCTTGATAGCCTCGATCCTAGAGGAAACAAATTTGACAAGGAGGTT AAAAATACCGGGCCCGAAGGTGAGTAATAGCAAGAtGGCTGCCATG
GGACCTAGAAAGGGGAGAAGCCATGTTGCCAACTCCAGAGGCTGGTATAAGAGCTTGAAAGATGTTGTCTGATTTTGGAAGCCTTTTCCTGTAAATGCCGGGTAGCATCTCGTACTATCCCTGAC TGG
TTAGTGTAAAAACAACACTCTTCCCCTAAGAAGGTGCAGAGTCCTCCTTTCTCAGCAGTGAGGAGGTCTAGCCTCAGCAGTTTTGGAGAGTCACTGCTG CGAAAGAGTCTATTTGGGATTCTGGAGTA
AGGATAGATTTCGTTATCTCTTGCAAACTGTCAGAGAAATCCTTTGAGAGTGTGTGGCAGTAGGATAATGAAGTAGATAAACCGGCTATTCCGGTTCCTTTAGCAGTAGCCATTCCTAACCCTAT AAG
TAGGGGTATTAGTTGTATGGCTCTGTGCTGACAGACTTGAGCTTTGAGGGATACTGATAGGGTCTGATTTCCTGGGGCAATGTTAATGTTAGGACTTAG AAAGACTAAGGTACAGATGCCTGTCCAGT
TAGTGGAGAGGCAGATATGTTGATGTTCCACATAAGAAGAATATACATTGGCTGGGTAGACAGAACTGGTTGTGTATGTTAAAAAGGTGTGTGAGTTTGTTATTTTCATTTTCCCATACTTCTAG AGT
AATTGCCAAGGTAGCTCCAGTGAAGGAATGGAAAGGGGTGTTGGGAGCAAACTGAGTGGCTCCCTGTGTTCTATTTTCCCATTGGAGAAAAAGCCTTTT TGTATCTACTAGGAACCATTTGAGAGAGT
GATTGAAAGAAGGGATGAGAAGGCATTCACTAGTGGTGGGGGCACTGATGCAGGGGGTCCAGGGATGAATGGTCATGGAGGGAGTATGTTTGTCATTACAAAACTTGGACTGTTTGTTAAGCAGG GAG
GAGGTGATGATTTTTGGGGACCCAGAGAAGTGGACAAACTGTCTGAATGGAGCTGTTTGAGT G

374	12.5	0.0	0.0	hg19	1	48	(1935)	+	AluSp	SINE/Alu	184	231	(82)	4
5334	7.4	3.6	0.0	hg19	49	712	(1271)	C	LTR12_	LTR/ERV1	(0)	688	1	5
10469	6.0	0.6	0.0	hg19	713	1983	(0)	C	HERV9-int	LTR/ERV1	(0)	8436	7159	5

>KSI_AluYa5_13

>Scaffold1755-1394897-1395205
GGTGAAACTCCGTCTCTACTAAAAATACAAAAAATTAGCTGGGCGTGGTGGCGGGTGCCTGTAGTCCCAGCTACTTGGGAGGCTGAGGTAGGAGAATGATGTGAACCCGGGAGGC gGAGCTTGCAATG
AGCCGAGATTGCGTCACTGCACTCCAGCCTGGGCAACAGAGTGAGACTCCGTCTCAAAAAAAAAAAAAACCAAAAAAAATCAAACCCAGATGGGTCCCACCCTCATAATTTCTGCTCCAGTAAAT CTG
GGGTGAACCCAAGAATTTGTATGTCTATAAACTTCCCAGGCCATATTGCTGCTGTCACAGGAACTACCCTTTGAGAACCCCTGACCTATGACACCGTGGCTGCCTCCTTGCCTCCC TTTCATTGAACT
AGGCGAAATGAGTATCTGAACAATGGCAGGATTTACATTATTAAAAGATGTTGTGAACGTTGCTTATCCTAAATTCCCTCTTCTGTAAATCTTATTTAAT ATGTTAGATTAAAAGTAAATTAGAGGGG
AAGGTTATATAGTGAAAGAAGAAAAGGGAAAGAAGGGAAGGGGGTTTAGTGAGGCAAATAATGTCTGTTTATATTAATTTACATTCCAGTGATATGAAGAACATCTGAGAATGTTA AATTATGATCAC
TACTGCCTACTGGTAGAATTCTAGATCAATTGGAGAGAATGTAAGACATTTCTACTCTTCCAACTTTACAGATGCAAAAAGGGGTTCTGAGAGCTGAACTGTGTTTTAGACTCAGAAACATGCTT TTT
AATGTCATATTGAGTAATTGAACATTTTTTTCTTCCAAGGACAAGTTTTCTGACTTTTAATCTTAACTTTGTCACAAAGGGAATAGAAAGAGAAGCCTCTGGAGAATTTCTCAAAT GGAAGCACATTT
TAATTTTGGGATGATGTTAATGGAAACACTATAATACAAATTCTCTTTTCCTTTGAAAAATTTTCTGAGAGGCAAAATAGTACAACAT Taagaaatacaatgatggccgggcgcggtggctcacgcct
gtaatcccagcactttgggaggccgaggcgggcggatcacgaggtcaggagatcgagaccatcccggctaaaatggtgaaaccccgtctctactaaaaatacaaaaaattagccgggcgtagtgg cgg
gcgcctgtagtcccagctactcgggaggctgaggcgggagaatggcgtgaacccgggaggcggagcttgcagtgagccgagatcccgccactgcactccagcctg ggcgacagagcgagactccgtct
caaaaaaaaaaaaaaaaaaaaaaaaaAAGAAATACAATGATGTGGCAAACAGCCCTTCTTTGGGGCTTTtGGGATTGAAAATAAAGTTTCTCAAAGGTGATTCCCATCAAGTAGGGCAAAGGTTCAG
CAACATGGATCTTTTCGAGATTTATTTGTATTTTCCCAGAGGTTTCTGAGAGGTGGCCTGTTCCTGCT gTGTGACTATCTTCAGAGATGGTGTTTCCTGGTGTGT GACCCTCAGCATATTAAAATTCT
GAGATAGTCAGTTTAAAAAAAACTAAAGCTCTGCAATTTAGTGGCTGTCATTAAAAGACACAGGGTGGTAAACAAGATGATTATTTGAAAATAGAAATAGAAAAAAAAGAAAACATTCTTAGAGG ATA
TTCTTCAGCTCCTCATTATCTCCAGCCTGCGTTACTGTCCAGTTTACTTAGCAGCCTCCAGTCTTAGTGACCCTTCAATTCGCTCCACTCCCATCCAAGTGCTCT TTCTAATACGATTTTATTCCTGC
CCTGCTTAGAATTCCTCAATAATTCACCATTTTCTTCAGGAAAATAATAGACTACCTTTTGTGTTGTATCCCAAACAGGGTTGCACGTGATTCATTTCACTGTCCACTATGTCTTAAGTTGCAGT AAC
GCTAAGCTCCTACTCTTCCTTCAAACCTGATTTTTTTTTTTTTTTTTTtGAGATTCACTTTTGTTGCCCAGGCTGcGGTGCGTTGGCACGATCACGGCTCACTGCAGCCTCCACCTCCCAGGTTCAAGC
AATTCTCCTGCCTCAGCTTCCCGAGAAGCTAGGATTACAGGCACCCGCCACCATGCCTGGCTAAGTTTTTATATTTTTAGTAGAGACGGGGTTTCACCATGTTGGCCAGGCTGGTCTCAAACTCC TGA
CCTCAGGTGATCCACCTGACTCGGCTTCCCAAAGTGCTGGGATTACAGGCGTGAGCCACCACACCTGGCCAAACCTAATATTCTTTCTCATGACTCACTATGTTT CCCTCAGCTTTCCTCAGTCTGGA
AAAAC

1734	7.2	0.0	0.0	AluYa5-Scaffold1755-1394897-1395205	1	207	(2102)	+	AluYc	SINE/Alu	87	293	(6)	1
569	22.3	6.2	0.0	AluYa5-Scaffold1755-1394897-1395205	210	339	(1970)	C	MER5A	DNA/hAT-Charlie	(51)	138	1	2
2908	1.0	0.0	0.0	AluYa5-Scaffold1755-1394897-1395205	1001	1309	(1000)	+	AluYa5	SINE/Alu	1	309	(2)	3
2253	9.5	2.0	0.3	AluYa5-Scaffold1755-1394897-1395205	1951	2246	(63)	C	AluSq2	SINE/Alu	(12)	301	1	4

>hg19 chr9:81595134-81597119
GGTGAAACTCCGTCTCTACTAAAAATACAAAAAATTAGCTGGGCGTGGTGGCGGGTGCCTGTAGTCCCAGCTACTTGGGAGGCTGAGGTAGGAGAATGATGTGAACCCGGGAGGC aGAGCTTGCAATG
AGCCGAGATTGCGTCACTGCACTCCAGCCTGGGCAACAGAGTGAGACTCCGTCTCAAAAAAAAAAAAAACCAAAAAAAATCAAACCCAGATGGGTCCCACCCTCATAATTTCTGCTCCAGTAAAT CTG
GGGTGAACCCAAGAATTTGTATGTCTATAAACTTCCCAGGCCATATTGCTGCTGTCACAGGAACTACCCTTTGAGAACCCCTGACCTATGACACCGTGGCTGCCTCCTTGCCTCCC TTTCATTGAACT
AGGCGAAATGAGTATCTGAACAATGGCAGGATTTACATTATTAAAAGATGTTGTGAACGTTGCTTATCCTAAATTCCCTCTTCTGTAAATCTTATTTAATATGTTAGATTAAAAGTAAATTAGAGGGG
AAGGTTATATAGTGAAAGAAGAAAAGGGAAAGAAGGGAAGGGGGTTTAGTGAGGCAAATAATGTCTGTTTATATTAATTTACATTCCAGTGATATGAAGAACATCTGAGAATGTTA AATTATGATCAC
TACTGCCTACTGGTAGAATTCTAGATCAATTGGAGAGAATGTAAGACATTTCTACTCTTCCAACTTTACAGATGCAAAAAGGGGTTCTGAGAGCTGAACTGTGTTTTAGACTCAGAAACATGCTT TTT
AATGTCATATTGAGTAATTGAACATTTTTTTCTTCCAAGGACAAGTTTTCTGACTTTTAATCTTAACTTTGTCACAAAGGGAATAGAAAGAGAAGCCTCTGGAGAATTTCTCAAAT GGAAGCACATTT
TAATTTTGGGATGATGTTAATGGAAACACTATAATACAAATTCTCTTTTCCTTTGAAAAATTTTCTGAGAGGCAAAATAGTACAACAT TAAGAAATACAATGATGTGGCAAACAGCCCTTCTTTGGGG
CTTTcGGGATTGAAAATAAAGTTTCTCAAAGGTGATTCCCATCAAGTAGGGCAAAGGTTCAGCAACATGGATCTTTTCGAGATTTATTTGTATTTTCCCAGAGGTTTCTGAGAGGT GGCCTGTTCCTG
CTcTGTGACTATCTTCAGAGATGGTGTTTCCTGGTGTGTGACCCTCAGCATATTAAAATTCTGAGATAGTCAGTTTAAAAAAAACTAAAGCTCTGCAATTTAGTGGCTGTCATTAAAAGACACAGGGT
GGTAAACAAGATGATTATTTGAAAATAGAAATAGAAAAAAAAGAAAACATTCTTAGAGGATATTCTTCAGCTCCTCATTATCTCCAGCCTGCGTTACTGTCCAGTTTACTTAGCAG CCTCCAGTCTTA
GTGACCCTTCAATTCGCTCCACTCCCATCCAAGTGCTCTTTCTAATACGATTTTATTCCTGCCCTGCTTAGAATTCCTCAATAATTCACCATTTTCTTCAGGAAAATAATAGACTACCTTTTGTG TTG
TATCCCAAACAGGGTTGCACGTGATTCATTTCACTGTCCACTATGTCTTAAGTTGCAGTAACGCTAAGCTCCTACTCTTCCTTCAAACCTGATTTTTTTTTTTTTTTT TGAGATTCACTTTTGTTGCC
CAGGCTGtGGTGCGTTGGCACGATCACGGCTCACTGCAGCCTCCACCTCCCAGGTTCAAGCAATTCTCCTGCCTCAGCTTCCCGAGAAGCTAGGATTACAGGCACCCGCCACCATGCCTGGCTAAGTT
TTTATATTTTTAGTAGAGACGGGGTTTCACCATGTTGGCCAGGCTGGTCTCAAACTCCTGACCTCAGGTGATCCACCTGACTCGGCTTCCCAAAGTGCTGGGATTACAGGCGTGAG CCACCACACCTG
GCCAAACCTAATATTCTTTCTCATGACTCACTATGTTTCCCTCAGCTTTCCTCAGTCTGGAAAAAC

1719	7.7	0.0	0.0	hg19	1	207	(1779)	+	AluYc	SINE/Alu	87	293	(6)	5
569	22.3	6.2	0.0	hg19	210	339	(1647)	C	MER5A	DNA/hAT -Charlie	(51)	138	1	6
2244	9.5	2.0	0.3	hg19	1629	1923	(63)	C	AluSq2	SINE/Alu	(13)	300	1	7

>KSI_AluYa5_14

>Scaffold1780-4824802-4824987
CTTTGATTGGAAGCGGTTGTATTGTCGCTATTCTCCATGTGGGGAAAACTACTCCTGGACAAAAATGCATGCCAGTCAGCAAAGTGTCGTGTTCTTTGCACAGTTCTACAATACTAAATGGAGTTG TC
TCCGAACCAGCTTTTTATGACAGGCAATGTCTGAATTTATTTGCAACCTGGGGTGGTTCCTGAATTTTGTGCTCAATGAGACTTCATGGGGTAGTGAGTTTTTATTTTTCTATTGAAAACTACCT TGT
AAATGAATTACTAAAGCGATACTATAAAATTCTATTCTGAGATTAGAGGCATTTAAATGGGAAATATCTAAAAAATTTTCCTCTCCCCCCATTCCTGCTTTCTCTCTGTCTTACA cTTCTGTATTTTT
TTCTTTACATTTATTACCACAGATGTTTTGTGCATATGTGTATAGGTAAGTACATATTTATTTATATGTGTCGTGTCTGTCTTACCTTGACTATAATAAAAGTAGGAATATCACCTTATTGTGTT CAT
TGTTTCATTCTCAATACCTAGAACAAGGATCGGAAACCgTTTCCAGTAAAGGGGCAAACATTAAATATTTCAGGCTTTGCCACCCATATAGTTTCTGTCACTAATCATCTGTGTCGTTGTAGCACAAA
CACAGCCATAATCGATATGCAAATGAAGGGGTGTGGCTGTGTTCCACTATAACTGTATTTACAAAAACAAGAGGAGAGCTGGATTTTTCCCGAGAGGTATAGTTTACTGACATAATAG ATACCTCGTA
CACATAATAGATACCTCGTACATATCTATTATATAAATGAATATCTTAACTGTGTGTGGTCAAATTATAGTCACAAAGTACACTCTATATG CCAGGCATTCTTCCCAGTGCCAAAGATGTAAAATCAA
ATATAACATAAAAACGATAAAAGCTAAAGAAATGCAAAATGGTGCTAAGCACTTACATGCATTATCTCATGTAATCCTATCCATGATTTT AAGAACCAAGtattggccgggcgcggtggctcacgcct
gtaatcccagcactttgggaggccgaggcgggcggatcacgagatcaggagatcgagaccatcccggctaaaacggtgaaaccccgtctctactaaaaatacaaaaaattagccgggcgtagtgg cgg
gcgcctgtagtcccagctacttgggaggctgaggaaaaaaaaaaaaaaaaaaaaa aagaaccaagtATTATTATTAATCTCATTTTACAGTTTCATTAACCAAAGCTAACACAGTTAAGGAGCCTGTC
CAAAATCACACACCTAGCATGTAGCACCaGGATTCCAATAAAAAAAATTTGATTAAAAGTACATGCTCCTAACCACAC CAGCGGTCTGTGAGTGGAGACTGCTATTGGCTGAGGTCCATCTTGGTTAC
ATGTCAAAATGAACTGACACTCACATCGGATGGCTGCTAAGCACGCACCTCCCAGATACTTAAGTTTCTGCATTTACAGCTTTCCAGGTTTAAAAGTCTG TGCAGCAGAGGAATTGGTGTCTGTTTCT
TTGGGCCTTTATATGCTCAGAGGGCTAATATAAGAACCTGGAGTCCTTTCAACTCCTCTAAAGAGTGACCTTTTCCTAGATTACTGCAACCCAGCTGAGAAACATGTGCAGAGACTTGCTTTACT CTG
AGAAGGAGAGAGGAAGACATTTTATCAAAAAAATGAAAGAAATAATACCAACTACTGCGTGGCTGGAAGAGGTAACCTCGGGAAGCTTGAACTGACAGGG TAACACGTGGCTTTCAAGCTGCACACTG
GAGGCACACAGCTGTTGGGGTGgGATAATGGATCCAAACCCTACCTTTCAAAGTGTGCCTTATGCACAAATGGGATCCTTTGCCTATGAGCCCTATGTGCCTACCTGTGGCATGTTCACACAACCAGC
CCATAGGCTCAATGAGTATaACCAGGCAGgGAAAGTCTCAGCTCCCTGTCACACATTTCTGAATCCACaTTAAAGGGGGAGGAGCTCTTTCTCCCACTTCAGGCCCCTGCCCATGCCTTTGCTGACTT
ATAAGGGAAAGGAAATTATAGGTAGTAATGTAAGATTCCTCAAGATTCACTGAGACCCATTTCACAGGaGAAGAAGGTTATTTCATTGAAAGATCATGCACTTTGTTCTCACTTATAAGTGGGAGCTG
AAAAATGAAA

194	29.9	6.5	11.5	AluYa5-Scaffold1780-4824802-4824987	336	535	(1651)	+	L2a	LINE/L2	3184	3374	(52)		1
1030	23.6	1.4	0.0	AluYa5-Scaffold1780-4824802-4824987	536	751	(1435)	C	MER58A	DNA/hAT-Charlie	(0)	224	6		2
13	27.2	0.0	0.0	AluYa5-Scaffold1780-4824802-4824987	881	924	(1262)	+	A-rich	Low_complexity	1	44	(0)		3
243	25.4	1.6	0.0	AluYa5-Scaffold1780-4824802-4824987	938	1000	(1186)	C	MIRb	SINE/MIR	(47)	221	158		4
1772	0.5	0.0	0.0	AluYa5-Scaffold1780-4824802-4824987	1001	1186	(1000)	+	AluYa5	SINE/Alu	1	186	(125)		5
22	4.0	0.0	0.0	AluYa5-Scaffold1780-4824802-4824987	1187	1212	(974)	+	(A)n	Simple_repeat	1	26	(0)		6
349	30.0	4.9	1.4	AluYa5-Scaffold1780-4824802-4824987	1215	1356	(830)	C	MIR	SINE/MIR	(102)	160	14		4
279	5.7	0.0	0.0	AluYa5-Scaffold1780-4824802-4824987	2152	2186	(0)	+	L1PA10	LINE/L1	5912	5946	(222)		7

>hg19 chr16:7225259-7227223
CTTTGATTGGAAGCGGTTGTATTGTCGCTATTCTCCATGTGGGGAAAACTACTCCTGGACAAAAATGCATGCCAGTCAGCAAAGTGTCGTGTTCT TTGCACAGTTCTACAATACTAAATGGAGTTGTC
TCCGAACCAGCTTTTTATGACAGGCAATGTCTGAATTTATTTGCAACCTGGGGTGGTTCCTGAATTTTGTGCTCAATGAGACTTCATGGGGTAGTGAGTTTTTATTTTTCTATTGAAAACTACCT TGT
AAATGAATTACTAAAGCGATACTATAAAATTCTATTCTGAGATTAGAGGCATTTAAATGGGAAATATCTAAAAAATTTTCCTCTCCCCCCATTCC TGCTTTCTCTCTGTCTTACAtTTCTGTATTTTT
TTCTTTACATTTATTACCACAGATGTTTTGTGCATATGTGTATAGGTAAGTACATATTTATTTATATGTGTCGTGTCTGTCTTACCTTGACTATAATAAAAGTAGGAATATCACCTTATTGTGTT CAT
TGTTTCATTCTCAATACCTAGAACAAGGATCGGAAACCtTTTCCAGTAAAGGGGCAAACATTAAATATTTCAGGCTTTGCCACCCATATAGTTTC TGTCACTAATCATCTGTGTCGTTGTAGCACAAA
CACAGCCATAATCGATATGCAAATGAAGGGGTGTGGCTGTGTTCCACTATAACTGTATTTACAAAAACAAGAGGAGAGCTGGATTTTTCCCGAGAGGTATAGTTTACTGACATAATAGATACCTC GTA
CACATAATAGATACCTCGTACATATCTATTATATAAATGAATATCTTAACTGTGTGTGGTCAAATTATAGTCACAAAGTACACTCTATATG CCAGGCATTCTTCCCAGTGCCAAAGATGTAAAATCAA
ATATAACATAAAAACGATAAAAGCTAAAGAAATGCAAAATGGTGCTAAGCACTTACATGCATTATCTCATGTAATCCTATCCATGATTTT AAGAACCAAGaATTATTATTAATCTCATTTTACAGTTT
CATTAACCAAAGCTAACACAGTTAAGGAGCCTGTCCAAAATCACACACCTAGCATGTAGCACC gGGATTCCAATAAAAAAAATTTGATTAAAAGTACATGCTCCTAACCACACCAGCGGTCTGTGAGT
GGAGACTGCTATTGGCTGAGGTCCATCTTGGTTACATGTCAAAATGAACTGACACTCACATCGGATGGCTGCTAAGCACGCACCTCCCAGATACTTAAGTTTCTGCATTTACAGCTTTCCAGGTTTAA
AAGTCTGTGCAGCAGAGGAATTGGTGTCTGTTTCTTTGGGCCTTTATATGCTCAGAGGGCTAATATAAGAACCTGGAGTCCTTTCAACTCCTCTA AAGAGTGACCTTTTCCTAGATTACTGCAACCCA
GCTGAGAAACATGTGCAGAGACTTGCTTTACTCTGAGAAGGAGAGAGGAAGACATTTTATCAAAAAAATGAAAGAAATAATACCAACTACTGCGTGGCTGGAAGAGGTAACCTCGGGAAGCTTGA ACT
GACAGGGTAACACGTGGCTTTCAAGCTGCACACTGGAGGCACACAGCTGTTGGGGTG aGATAATGGATCCAAACCCTACCTTTCAAAGTGTGCCT TATGCACAAATGGGATCCTTTGCCTATGAGCCC
TATGTGCCTACCTGTGGCATGTTCACACAACCAGCCCATAGGCTCAATGAGTAT gACCAGGCAGcGAAAGTCTCAGCTCCCTGTCACACATTTCTGAATCCAC gTTAAAGGGGGAGGAGCTCTTTCTC

CCACTTCAGGCCCCTGCCCATGCCTTTGCTGACTTATAAGGGAAAGGAAATTATAGGTAGTAATGTAAGATTCCTCAAGATTCACTGAGACCCATTTCACAGG cGAAGAAGGTTATTTCATTGAAAGA
TCATGCACTTTGTTCTCACTTATAAGTGGGAGCTGAAAAATGAAA

1054	23.1	1.4	0.0	hg19	536	751	(1214)	C	MER58A	DNA/hAT-Charlie	(0)	224	6	8
13	27.2	0.0	0.0	hg19	881	924	(1041)	+	A-rich	Low_complexity	1	44	(0)	9
494	30.1	4.0	1.0	hg19	938	1135	(830)	C	MIR	SINE/MIR	(45)	217	14	10
279	5.7	0.0	0.0	hg19	1931	1965	(0)	+	L1PA10	LINE/L1	5912	5946	(222)	11

>KSI_AluYa5_15

>Scaffold2658-3501402-3501582
TTCCTAAACTAATGTCCTGAAGCATTTCTCTTATGTTTTCTTCTAGAAGTTTTATTGTTTTGGATCTTACTTTTAGGTCTTTTGTCTATTTTGAGTTGACTTTTTATAGGTTGAGATGTAGGAATC TA
GTTTTAATCTTCTGCATATGGATATTCAGTTTTTCCAGCACCATTTATTGAAGAGACTGTCCTTTTCCCAATGTGCCTTATTGGCACCTTCATCAATAATGAGTTAGCCATAGATACATTAATTA ATT
TCTGAGTTATCTATTCTGTTTCATTGGTCTGTGTGTCTGTTTTTATGCCAGTATCATGCTGTTTTGGTTACTACCGCTTTGTAAGATATTTTGAAGGCTGGTAGTGTGATTCCTCTAACTTGGCTG GC
TGGCTTGCTTGTTTATTTGTTTCATTGTTTTGCTCAGCATTGGTTTGGCTACTTGGAGTCTTTTGTGGTTTCATACAAATTTTAGGATTTCTTTTTGTATTTCTGTGAAGAATATCATGGCATTTTG
GTAAGGATAGCATTGAATCTGTAGTTTACTTTGGGTAGTATTGTCATTGTAACAATATTAATTCTTATAATCCATGAGCTTGAGATGTCTTTCCATTTGTTTGTATCTTCATTATTTCATTCTTCA GT
GTTTTGCAGTTTCTTGTAAAAGTCTTTCACCTCCTTGATTACATTTATTCCTAGATATTTTATTTTTTGTAGCTATTACAAATGGGATTGCATTCTTGATTTTATTTTCAGCTAGTTCATTGTT CAT
GTATGGAAATGCTGCTTTTTTATATTGATTTCATATCCTGCAACTTTACTGAATTTGTTTTATTCTGTTTTTTGATAGAGTCTTTAGGTTTTTCTCTACATAAGATCATGGAATTTGCAAACAAGA AC
AAATTGATTTCCTGCTATTCCAATTTTATATGCCTTTTtttttttttttttttttttttttgaaggggtcttggtgttttgcccgcggctggggggggggggggggcctcccaagtagctgggactaca
ggcgcccgccactacgcccggctaattttttgtatttttagtagagacggggtttcaccgttttagccgggatggtctcgatctcctgacctcgtgatccgcccgcctcggcctcccaaagtgctg gg
attacaggcgtgagccaccgcgcccggcctttatatgccttttATGTCCTTCTCTTGACTAATTGTTCTGGCTAATACTTCTAATACTATGTAGAATAAGTGTGGTGAAAGTGGACATACTTGTCTTC
TTCCAGTTCTTaGAAAAAAATCTTTTAGCTTTTCCCTATTCAGTATGATGTTAGCTGTGAGTTTGTCATATATGGCCTTTATTATTTTGAGGTATGTTCCTTCTGTGCCTAGTTGGTTGAAAGTTTTT
ATTATAAACAGATGTAGAATTTTATCAAGTGCTTTCTCGGCATCTATTGAGATGATCATATGGTTTTTAGTAGTCCTTCATTCTATTGATGAGATGTATGACATTTAATGATTTGCATATATTGA ACC
ATCCTTGTATTCGTGAGATAAATAACACTTGACCATGGTGTATTATCTTTTTGATGTGTTCTTGGATTGGATTTACTAGTATTTTGTTGAGGATTTTTGTGTCTGTGTTTATCAGGAATACTGGCC TA
TAGTTTCCTTTTTTTGTTGTGTCCTTGTCTGCTTTTGGTACCAGGGTTaTGCTGGCCTCATAAAATAAGTTAAGAAAAATTTTCTCTGCTTCATTTTGTGAAATAGTTCGACAAGAATTGATACTAAT
TCTTTAAAGATTCAGGAGAATTCAGCAGTGAAGCCATCCAGTCTTTGACATTTATTTATTGGGAGATTTTCTGTTACTGACTCAATCTTGTTA CTTGTTGTTAGCCTGTTTGAGTTTTCTGTTTTTTC
TTGGTTGAATTTTGGTAGATTGTTTATGTCCAGGAAATTATCTATTTTCTTTTGGTTTTTTAATTTATTGTCATGTATTTGTTCATAGTAGTCTCTAATAACTCTTTGTATTTCTGTGGTATCT GTT
GTGACATCTCCTTTTAATTTATTTTATTTATTTGGATCATCTCTCTTTTTTCTTACTCTAGCTAATAGTTTGTTGCTTTTGGTTATCTTCTCCAAAAAAAAACGCAACTTTTTGTTTCAGTGATTT TT
GTACT

2186	16.5	0.3	0.0	AluYa5-Scaffold2658-3501402-3501582	3	377	(1804)	C	L1M2	LINE/L1	(1065)	5078	4703	1
2699	15.9	2.9	0.2	AluYa5-Scaffold2658-3501402-3501582	412	929	(1252)	C	L1M2	LINE/L1	(1447)	4696	4165	1
28	0.0	0.0	0.0	AluYa5-Scaffold2658-3501402-3501582	930	956	(1225)	+	(T)n	Simple_repeat	1	27	(0)	2
1739	0.0	0.0	0.0	AluYa5-Scaffold2658-3501402-3501582	1001	1181	(1000)	C	AluYa5	SINE/Alu	(130)	181	1	3
5203	17.4	1.1	1.0	AluYa5-Scaffold2658-3501402-3501582	1182	2181	(0)	C	L1M2	LINE/L1	(1969)	4174	3174	1

>chr6:53647759-53649677
TTCCTAAACTAATGTCCTGAAGCATTTCTCTTATGTTTTCTTCTAGAAGTTTTATTGTTTTGGATCTTACTTTTAGGTCTTTTGTCTATTTTGAGTTGACTTTTTATAGGTTGAGATGTAGGAATC TA
GTTTTAATCTTCTGCATATGGATATTCAGTTTTTCCAGCACCATTTATTGAAGAGACTGTCCTTTTCCCAATGTGCCTTATTGGCACCTTCATCAATAATGAGTTAGCCATAGATACATTAATTA ATT
TCTGAGTTATCTATTCTGTTTCATTGGTCTGTGTGTCTGTTTTTATGCCAGTATCATGCTGTTTTGGTTACTACCGCTTTGTAAGATATTTTGAAGGCTGGTAGTGTGATTCCTCTAACTTGGCTG GC
TGGCTTGCTTGTTTATTTGTTTCATTGTTTTGCTCAGCATTGGTTTGGCTACTTGGAGTCTTTTGTGGTTTCATACAAATTTTAGGATTTCTTTTTGTATTTCTGTGAAGAATATCATGGCATTTTG
GTAAGGATAGCATTGAATCTGTAGTTTACTTTGGGTAGTATTGTCATTGTAACAATATTAATTCTTATAATCCATGAGCTTGAGATGTCTTTCCATTTGTTTGTATCTTCATTATTTCATTCTTCA GT
GTTTTGCAGTTTCTTGTAAAAGTCTTTCACCTCCTTGATTACATTTATTCCTAGATATTTTATTTTTTGTAGCTATTACAAATGGGATTGCATTCTTGATTTTATTTTCAGCTAGTTCATTGTT CAT
GTATGGAAATGCTGCTTTTTTATATTGATTTCATATCCTGCAACTTTACTGAATTTGTTTTATTCTGTTTTTTGATAGAGTCTTTAGGTTTTTCTCTACATAAGATCATGGAATTTGCAAACAAGA AC
AAATTGATTTCCTGCTATTCCAATTTTATATGCCTTTTATGTCCTTCTCTTGACTAATTGTTCTGGCTAATACTTCTAATACTATGTAGAATAAGTGTGGTGAAAGTGGACATACTTGTCTTCTTCCAG
TTCTTgGAAAAAAATCTTTTAGCTTTTCCCTATTCAGTATGATGTTAGCTGTGAGTTTGTCATATATGGCCTTTATTATTTTGAGGTATGTTCCTTCTGTGCCTAGTTGGTTGAAAGTTTTTATTATA
AACAGATGTAGAATTTTATCAAGTGCTTTCTCGGCATCTATTGAGATGATCATATGGTTTTTAGTAGTCCTTCATTCTATTGATGAGATGTATGACATTTAATGATTTGCATATATTGAACCATC CTT
GTATTCGTGAGATAAATAACACTTGACCATGGTGTATTATCTTTTTGATGTGTTCTTGGATTGGATTTACTAGTATTTTGTTGAGGATTTTTGTGTCTGTGTTTATCAGGAATACTGGCCTATAGT TT
CCTTTTTTTGTTGTGTCCTTGTCTGCTTTTGGTACCAGGGTTtTGCTGGCCTCATAAAATAAGTTAAGAAAAATTTTCTCTGCTTCATTTTGTGAAATAGTTCGACAAGAATTGATACTAATTCTTTA
AAGATTCAGGAGAATTCAGCAGTGAAGCCATCCAGTCTTTGACATTTATTTATTGGGAGATTTTCTGTTACTGACTCAATCTTGTTA CTTGTTGTTAGCCTGTTTGAGTTTTCTGTTTTTTCTTGGTT
GAATTTTGGTAGATTGTTTATGTCCAGGAAATTATCTATTTTCTTTTGGTTTTTTAATTTATTGTCATGTATTTGTTCATAGTAGTCTCTAATAACTCTTTGTATTTCTGTGGTATCTGTTGTG ACA
TCTCCTTTTAATTTATTTTATTTATTTGGATCATCTCTCTTTTTTCTTACTCTAGCTAATAGTTTGTTGCTTTTGGTTATCTTCTCCAAAAAAAAACGCAACTTTTTGTTTCAGTGATTTTTGTAC T

9795	16.9	1.4	2.0	hg19	3	1919	(0)	C	L1M2	LINE/L1	(1065)	5078	3174	4

>KSI_AluYa5_16

>Scaffold3713-484678-484813
TCTCACCCTTATTCTGACACTCCAGAGTTGCATTCACTCTCAGGCATCCAGATCCTTTCCCCATATACAAACATCCATCCATCCATATATCTATCCATCTCTCTATCTAAACACACATGCATTCAT TC
GTTCACTCATTGAATCGTGCATTGAGAACCCATTATGTGtCAAGCCATTGCTAGAATTAAGGATGCAATAGTTAAATAAAGCAGATGTGGTTCCTTCTCAGGGAGGTTCTCATATATCATGAAGAAAA
CAGACTGGGTGCAATGAATGACAGGGTCCTTCTTAGAGTGGCAGATCTCTTACCTGGCTTTTCTGTCAAGGTGACCCTCAGAATATGGAGGTGTTACCTTTTATCTTAGTCCATGTTTTACTGCT ATA
ACAGAATACCACAGCCTGGGTAATATATAAAGAAAAAAAG GTTATTTGGCTTGTAGTTCTGGAGACTGGGAAGTCCAAGGGCATGGTGCTGGCATCTGGCGAGGGCCTTCTTGCTGCATTATCCTATT
GTGGAAGGGCATGTGAGCGTGCAGTACAGACAGTATGGGGGCAGCACTTACGCTTTTATCAGGA aCCCACTTTTCCTGTGATAACAGCATTAATCTCTTCACTTCTTACAGGCCCCACCTCTTAATAC
TGTTACAGTGGCAATTAAATTTCAACATGAGTCTTAAAGC ACCCATGTCATCTAGGAAGAAGAAACTGTATTTGCAGCAACACTGTGAGGTAGGAAAGGGAGTCCTATGTTGTAGGAACTGGAAGCAG
CCCACTGTGGTGAGGGGCAAGTAGGAAGAGGTGAGGCTGGGCAGGTGGGCAGGGCCAGGGCAGATGGGGCTCTGTAGGCTA TGGAGGAGCTGCGATCTTATCACAAGGAAAGTGAACAGCCCTATGAT
TAACTATTGAAGGCAGTGATATAATTTATGCTTTTtttttttttttaattttttttttttttgagcggagatctctctgtctcccccagttgggggagcggtttttgtatttttttttagtaga
gacggggtttcaccgttttagccaggatggtctcgatctcctgacctcgtgatccgcccgcctcggcctcccaaagtgctgggattacaggcgtgagccaccgcgcccggcc tatgcttttATATTAA
TAGCAATTATTCTGGCTGTTGTATAGAGTATAGAT TGGAGAACAGAATTGGGTGGGGGAACTAGAGAAGGTGAACAGACTGGTGAAGAGGCTTTTATAGTTATCCAGGCAAGAGATGATGGTGACTTG
GAAGACCAGGAAGAGAAGCAGGGGTAGAGAGAAATGGAGATAGTGAAGATATAGCAGACATATTTGAGGAGTCCTTCAGTTACAGATAATAGAAAACTAGCTCAAGCACATTTAAGAAAAAAAAAAAAA
GACTTGTTTAAGTACCTAGGCAGTCTATGTGTGCA GCTTGCTTCAGGAATAGGGGCTTAAATGATGGTTTTAGGGCTCTATATCCTTCTAGCTTCCATCTCTGCCTCTGTTCATTTGTTTGGATCAAT
TCTTTAGATTGTTGGTAGCAGACACATTTATTGCTAACAGTCCCAGACCGTATCCTTCTTAGTAATCTCAACAAGAAAGAGTCTTTTTTGATGGCTGCAGCAGCAAGGCTCTGGGGAAGTCTCTG ATT
GGGTGTGGCTTGGCTTACAGCTCATTCTCGAACTAATCACTGAGTGGGGGCAGGGCAGGTTTCTCCATGAGCCAGGTCTTGTAACATGCCCAGGCCCTAGGCTGGGAGGACAGCTCCACCAGTACAGC
ATGGACTGGGCTCCCTGCAGGAGTGCGGAGCCTTGCTACCAGAAGGGGCCAGCATAGACCAAGAGAAGGGCACTGTAGAATAAAGGACATGGA GCAAAATAACTGTGTTAAGACTAGCTACCAAAGAC
GGAGACGATCTGCTCGGCATTGAATTTGCCTCTTT GTCAAGGAAAGAAGGAGCAGAAGATGTTAAGAAGGAATTGAAGTTTAGGCCATACGGGGAGGTGCTCATCTGTTTTAACTGTATTTGTCTTCT
GGCCCCTGGGATTGCCTGTCCTGGTGTGGGGTGAACTTGGAGACAGAATCTCTGAGAAGCTGTCAGGGATATTTCAGGCCAGTCTAG G

17	18.6	0.0	0.0	AluYa5-Scaffold3713-484678-484813	73	109	(2027)	+	(ATCT)n	Simple_repeat	1	37	(0)	1
308	34.1	3.6	3.6	AluYa5-Scaffold3713-484678-484813	125	261	(1875)	C	L2b	LINE/L2	(0)	3375	3239	2
1150	19.7	4.5	3.0	AluYa5-Scaffold3713-484678-484813	358	679	(1457)	C	MSTD	LTR/ERVL-MaLR	(0)	396	25	3
212	26.4	13.2	2.7	AluYa5-Scaffold3713-484678-484813	691	826	(1310)	C	L2b	LINE/L2	(319)	3068	2919	2
26	6.4	0.0	0.0	AluYa5-Scaffold3713-484678-484813	928	960	(1176)	+	(T)n	Simple_repeat	1	33	(0)	4
1202	0.7	0.0	3.0	AluYa5-Scaffold3713-484678-484813	1001	1136	(1000)	C	AluYa5	SINE/Alu	(179)	132	1	5
381	26.1	2.6	10.1	AluYa5-Scaffold3713-484678-484813	1141	1332	(804)	C	L2b	LINE/L2	(603)	2816	2638	2
475	31.6	9.6	4.4	AluYa5-Scaffold3713-484678-484813	1459	1802	(334)	C	MLT1K	LTR/ERVL-MaLR	(134)	461	101	6

>hg19 chr15:73982389-73984310
TCTCACCCTTATTCTGACACTCCAGAGTTGCATTCACTCTCAGGCATCCAGATCCTTTCCCCATATACAAACATCCATCCATCCATATATCTATCCATCTCTCTATCTAAACACACATGCATTCAT TC
GTTCACTCATTGAATCGTGCATTGAGAACCCATTATGTG cCAAGCCATTGCTAGAATTAAGGATGCAATAGTTAAATAAAGCAGATGTGGTTCCTTCTCAGGGAGGTTCTCATATATCATGAAGAAAA
CAGACTGGGTGCAATGAATGACAGGGTCCTTCTTAGAGTGGCAGATCTCTTACCTGGCTTTTCTGTCAAGGTGACCCTCAGAATATGGAGGTGTTACCTTTTATCTTAGTCCATGTTTTACTGCTA TA
ACAGAATACCACAGCCTGGGTAATATATAAAGAAAAAAAGGTTATTTGGCTTGTAGTTCTGGAGACTGGGAAGTCCAAGGGCATGGTGCTGGCATCTGGCGAGGGCCTTCTTGCTGCATTATCCT ATT
GTGGAAGGGCATGTGAGCGTGCAGTACAGACAGTATGGGGGCAGCACTTACGCTTTTATCAGGA gCCCACTTTTCCTGTGATAACAGCATTAATCTCTTCACTTCTTACAGGCCCCACCTCTTAATAC
TGTTACAGTGGCAATTAAATTTCAACATGAGTCTTAAAGCACCCATGTCATCTAGGAAGAAGAAACTGTATTTGCAGCAACACTGTGAGGTAGGAAAGGGAGTCCTATGTTGTAGGAACTGGAAG CAG
CCCACTGTGGTGAGGGGCAAGTAGGAAGAGGTGAGGCTGGGCAGGTGGGCAGGGCCAGGGCAGATGGGGCTCTGTAGGCTATGGAGGA GCTGCGATCTTATCACAAGGAAAGTGAACAGCCCTATGAT
TAACTATTGAAGGCAGTGATATAATTTATGCTTTTATATTAATAGCAATTATTCTGGCTGTTGTATAGAGTATAGATTGGAGAACAGAATTGGGTGGGGGAACTAGAGAAGGTGAACAGACTGGTGAA
GAGGCTTTTATAGTTATCCAGGCAAGAGATGATGGTGACTTGGAAGACCAGGAAGAGAAGCAGGGGTAGAGAGAAATGGAGATAGTGAAGATATA GCAGACATATTTGAGGAGTCCTTCAGTTACAGA
TAATAGAAAACTAGCTCAAGCACATTTAAGAAAAAAAAAAAAAGACTTGTTTAAGTACCTAGGCAGTCTATGTGTGCAGCTTGCTTCAGGAATAGGGGCTTAAATGATGGTTTTAGGGCTCTATAT CCT
TCTAGCTTCCATCTCTGCCTCTGTTCATTTGTTTGGATCAATTCTTTAGATTGTTGGTAGCAGACACATTTATTGCTAACAGTCCCAGACCGTATCCTTCTTAGTAATCTCAACAAGAAAGAGTCT TT
TTTGATGGCTGCAGCAGCAAGGCTCTGGGGAAGTCTCTGATTGGGTGTGGCTTGGCTTACAGCTCATTCTCGAACTAATCACTGAGTGGGGGCAGGGCAGGTTTCTCCATGAGCCAGGTCTTGTA ACA
TGCCCAGGCCCTAGGCTGGGAGGACAGCTCCACCAGTACAGCATGGACTGGGCTCCCTGCAGGAGTGCGGAGCCTTGCTACCAGAAGGGGCCAGCATAGACCAAGAGAAGGGCACTGTAGAATAAA GG
ACATGGAGCAAAATAACTGTGTTAAGACTAGCTACCAAAGACGGAGACGATCTGCTCGGCATTGAATTTGCCTCTTTGTCAAGGAAAGAAGGAGCAGAAGATGTTAAGAAGGAATTGAAGTTTAG GCC

ATACGGGGAGGTGCTCATCTGTTTTAACTGTATTTGTCTTCTGGCCCCTGGGATTGCCTGTCCTGGTGTGGGGTGAACTTGGAGACAGAATCTCTGAGAAGCTGTCAGGGATATTTCAGGCCAGTC TA
GG

17	18.6	0.0	0.0	hg19	73	109	(1813)	+	(ATCT)n	Simple_repeat	1	37	(0)	7
320	33.3	3.6	3.6	hg19	125	261	(1661)	C	L2b	LINE/L2	(0)	3375	3239	8
1131	20.0	4.5	3.0	hg19	358	679	(1243)	C	MSTD	LTR/ERVL-MaLR	(0)	396	25	9
517	31.2	7.2	6.2	hg19	691	1118	(804)	C	L2b	LINE/L2	(350)	3069	2638	8
475	31.6	9.6	4.4	hg19	1245	1588	(334)	C	MLT1K	LTR/ERVL-MaLR	(134)	461	101	10

>KSI_AluYa5_17

>Scaffold5650-2672938-2673178
CGGTTTGGGGAGGGTCAGAATCTTGTAGTTTGCAGCTGCATGACTCCTAAACCATCATTCCTAATCTTTTGGCTAATCTGTTAGTCCTACAAAGGCAGTCTAGTTCCTAGGCAAGAATAATGTTTG TT
TGGGGAAAGGGCTGTTATTATCTTTGCTTTAAACTATTAAAAAAAAAGGGTGCAGTGGCTCACGCCTGTAATCTCAGCACTTTGGGAGGCCAAGGCAGGCAGATCACTTGAGACTAGCCTGGCTA ACA
GCGAAAGTCTGTCTCTACTAAAAATACAAAAAATTAGCTTGGCCATGGTGGCACAAACCTGTAATCCCAGCTACTCAGGTGGCTGAGGTGCAAGAATCACTTGAA CCCGTGTTAGGAATGCCTGTTCC
CTGGTGCCATAAAGAAATAGCACTTGAACGTAAATTTAATTTCCTTAGCAAGGCCATTTTTTAACTTTCTGCAGAAAGGGTACACTCGCCAGCAGTTTTGCCATGAGAGTACACTGAACAAAGGAGAC
AGGGTCATTTATAACCTGATGCGTCCACCCTACTGCTGTGTCTGGTTTCCATTGGCTGGAACGGAACCTCGTATTCTGTATTTGTCCTGATTTGCTAGCAACTTAAAACTTCTAAAAAAGGCAAAA AC
AGAGGAGAACAAAGGAAGGGGGAAGTAACTCGTGGAATGCTGAGAAAGGTAAAAACACCTTCAAATAAGGAAGAGGAACAGGCTATGAGCTAATGCTTGCTTGGACCAGTATAAGCATGCCAGAG CAA
ATATTTAGGCTAAATTGTGGGAGCTAAGAACATAAAGTACATTGATTTCTTTATTATGGCTAGCAGATATTCAAGAATATTAGCACAGGTCTTTGAATAAATTTTGCTTCTAAGAGAGGTTACTAT TT
ATTCCTAATTAGACAGGGAAGAACATCTTTTAAGAGGAACTTCTACTTTACTTTTTttttttttttttttttttttcacaTTTTTTTTTTTTTTTTTTTTTTTTTGAGACGGAGTCTCGCTCTGTCGC
CCAGGCTGGAGTGCAGTGGCggcgggatctcggctcactgcaagctccgcctcccgggttcacgccattctcctgcctcagcctcccaagtagctgggactacaggcgcccgccactacgcccggcta
atttttgtatttttagtagagacggggtttcaccgttttagccgggatggtctcgatctcctgacctcgtgatccgcccg cctcggcctcccaaagtgctgggattacaggcgtgagccaccgcgcc
cggcctctactttactttttACACCCAGTGGGTGGAGGTTtGATCATGGCACAATCTCGGCTCACTGTACTCCAGCCTGGGTGACACAGCAAGACCCTGTCTCCAAAAAAATAAATAAATAAAAATAA
AAATGGCCAAGCGTGGTGGCTCACGCCTGTAATCCCAGCACTTTGGGAGGCCGAGGCGGGCGGATCACGAGGTCAGGAGA gCGAGACCATCgTGGCTAACAGGGTGAGACCCTGTCTcTACTAAAAAT
ACAAAAAATTAGCCAGGCacGGTGGnnnnnnnnnnnAGTCCCAGCTGCTCGGGAGGCTGAGGCAGGAGAATGGCGTGAACCCGGGAGGCGGAGCTTGCAG gGAGCTGAGATCGCGCCACTGCACTGCA
GCCTGGGGGACAGAGCGAGACTCCGTCTCaaaaaAAAAAAAAAAAATAAATAAATAAATAAAaAAATAAAAAATAAACTGTAAACTAAGTTCTCCCCAAAGTTAGTTCAGCCTACACCCCGGAGTGAACC
AGGACAGCTTGGAGATTAGAAGCAAGATGGAGTTGGTTAGGTCAGCTCTCTTTCAAATTTTGTCATGGCAGTTTCAGCAGGGCAGAAAGAACCCATCATGGGATTCCATGGCTTCCAGGTGATGA AAG
CTCTCTTTCCCCTGGCGTTGCCTCTGCCGCTTTCCCTCCGCCCTCCCTGCTTGCCTGGCGTGGCCTCCTCTGTGGCTCCTT CTCTTCTGCCCCCTGTGCTGGGTCCCAGGCCCACCCTTCTGAAGCGG
CTACGTTGTCTGGGGTAAATACCCTGGGGTTCATCGTCTTGCCCCAGAAAAACTTAGGACACGGACACACATGAGGCGTTTAGGAGTGGAGGTTCAACAAGCAAGAGAAAGAGAAAGAAAGAGGA ACA
CAGCTCTCTCTCTAGTGAGAGAGAGGGGACTTCTGAGAGGAAAAGGCTGGCCAGCGGTGGATGCGCC aAATTTGTTTTTGTTTTTGTTTTTGAGACAGAGTCTCACTTT

1075	13.3	0.0	1.8	AluYa5-Scaffold5650-2672938-2673178	1	168	(2073)	C	LTR8	LTR/ERV1	(384)	307	143	1
1178	13.2	8.4	1.0	AluYa5-Scaffold5650-2672938-2673178	176	365	(1876)	+	AluSz6	SINE/Alu	5	208	(104)	2
4771	6.5	0.2	0.0	AluYa5-Scaffold5650-2672938-2673178	366	947	(1294)	C	LTR10C	LTR/ERV1	(0)	591	9	3
27	0.0	0.0	0.0	AluYa5-Scaffold5650-2672938-2673178	948	973	(1268)	+	(T)n	Simple_repeat	1	26	(0)	4
2319	0.0	0.0	0.0	AluYa5-Scaffold5650-2672938-2673178	1001	1241	(1000)	C	AluYa5	SINE/Alu	(70)	241	1	5
372	19.8	4.0	6.1	AluYa5-Scaffold5650-2672938-2673178	1259	1359	(882)	+	AluSg7	SINE/Alu	201	299	(10)	6
2451	9.7	0.0	0.0	AluYa5-Scaffold5650-2672938-2673178	1369	1677	(564)	+	AluY	SINE/Alu	1	309	(2)	7
826	14.2	9.7	0.0	AluYa5-Scaffold5650-2672938-2673178	1691	1824	(417)	C	LTR8	LTR/ERV1	(544)	147	1	1
1187	16.0	0.0	4.5	AluYa5-Scaffold5650-2672938-2673178	1997	2205	(36)	C	LTR9	LTR/ERV1	(0)	612	413	8

>chr1:9571381-9573320
CGGTTTGGGGAGGGTCAGAATCTTGTAGTTTGCAGCTGCATGACTCCTAAACCATCATTCCTAATCTTTTGGCTAATCTGTTAGTCCTACAAAGGCAGTCTAGTTCCTAGGCAAGAATAATGTTTG TT
TGGGGAAAGGGCTGTTATTATCTTTGCTTTAAACTATTAAAAAAAAAGGGTGCAGTGGCTCACGCCTGTAATCTCAGCACTTTGGGAGG CCAAGGCAGGCAGATCACTTGAGACTAGCCTGGCTAACA
GCGAAAGTCTGTCTCTACTAAAAATACAAAAAATTAGCTTGGCCATGGTGGCACAAACCTGTAATCCCAGCTACTCAGGTGGCTGAGGTGCAAGAATCACTTGAA CCCGTGTTAGGAATGCCTGTTCC
CTGGTGCCATAAAGAAATAGCACTTGAACGTAAATTTAATTTCCTTAGCAAGGCCATTTTTTAACTTTCTGCAGAAAGGGTACACTCGC CAGCAGTTTTGCCATGAGAGTACACTGAACAAAGGAGAC
AGGGTCATTTATAACCTGATGCGTCCACCCTACTGCTGTGTCTGGTTTCCATTGGCTGGAACGGAACCTCGTATTCTGTATTTGTCCTGATTTGCTAGCAACTTAAAACTTCTAAAAAAGGCAAA AAC
AGAGGAGAACAAAGGAAGGGGGAAGTAACTCGTGGAATGCTGAGAAAGGTAAAAACACCTTCAAATAAGGAAGAGGAACAGGCTATGAG CTAATGCTTGCTTGGACCAGTATAAGCATGCCAGAGCAA
ATATTTAGGCTAAATTGTGGGAGCTAAGAACATAAAGTACATTGATTTCTTTATTATGGCTAGCAGATATTCAAGAATATTAGCACAGGTCTTTGAATAAATTTTGCTTCTAAGAGAGGTTACTA TTT
ATTCCTAATTAGACAGGGAAGAACATCTTTTAAGAGGAACTTCTACTTTACTTTTTACACCCAGTGGGTGGAGGTTcGATCATGGCACAATCTCGGCTCACTGTACTCCAGCCTGGGTGACACAGCAA
GACCCTGTCTCCAAAAAAATAAATAAATAAAAATAAAAATGGCCAAGCGTGGTGGCTCACGCCTGTAATCCCAGCACTTTGGGAGGCCGAGGCGGGCGGATCACGAGGTCAGGAGA tCGAGACCATCc
TGGCTAACAGGGTGAGACCCTGTCTtTACTAAAAATACAAAAAATTAGCCAGGCgtGGTGGcgggtgcctgtAGTCCCAGCTGCTCGGGAGGCTGAGGCAGGAGAATGGCGTGAACCCGGGAGGCGGA
GCTTGCAGtGAGCTGAGATCGCGCCACTGCACTGCAGCCTGGGGGACAGAGCGAGACTCCGTCT CAAAAAAAAAAAATAAATAAATAAATAAAtaaataaatAAATAAAAAATAAACTGTAAACTAAGTT
CTCCCCAAAGTTAGTTCAGCCTACACCCCGGAGTGAACCAGGACAGCTTGGAGATTAGAAGCAAGATGGAGTTGGTTAGGTCAGCTCTCTTTCAAATTTTGTCATGGCAGTTTCAGCAGGGCAGA AAG

AACCCATCATGGGATTCCATGGCTTCCAGGTGATGAAAGCTCTCTTTCCCCTGGCGTTGCCTCTGCCGCTTTCCCTCCGCCCTCCCTGCTTGCCTGGCGTGG CCTCCTCTGTGGCTCCTTCTCTTCTG
CCCCCTGTGCTGGGTCCCAGGCCCACCCTTCTGAAGCGGCTACGTTGTCTGGGGTAAATACCCTGGGGTTCATCGTCTTGCCCCAGAAAAACTTAGGACACGGACACACATGAGGCGTTTAGGAG TGG
AGGTTCAACAAGCAAGAGAAAGAGAAAGAAAGAGGAACACAGCTCTCTCTCTAGTGAGAGAGAGGGGACTTCTGAGAGGAAAAGGCTGGCCAGCGGTGGATG CGCCgAATTTGTTTTTGTTTTTGTTT
TTGAGACAGAGTCTCACTTT

1075	13.3	0.0	1.8	hg19	1	168	(1772)	C	LTR8	LTR/ERV1	(384)	307	143	9
1517	17.1	5.0	2.6	hg19	176	365	(1575)	+	AluSx3	SINE/Alu	5	207	(104)	10
4815	6.4	0.2	0.0	hg19	366	955	(985)	C	LTR10C	LTR/ERV1	(0)	591	1	11
1517	17.1	5.0	2.6	hg19	956	1063	(877)	+	AluSx3	SINE/Alu	208	309	(2)	10
2547	4.5	0.0	0.0	hg19	1065	1353	(587)	+	AluY	SINE/Alu	1	289	(22)	12
38	0.0	0.0	0.0	hg19	1354	1388	(552)	+	(AAAT)n	Simple_repeat	1	35	(0)	13
826	14.2	9.7	0.0	hg19	1390	1523	(417)	C	LTR8	LTR/ERV1	(544)	147	1	9
1199	15.5	0.0	4.5	hg19	1696	1904	(36)	C	LTR9	LTR/ERV1	(0)	612	413	14

>KSI_AluYa5_18

>Scaffold5938-1540413-1540593

GTGGAGGTGAGAGGTGTGGAGAATAGGGATTTAACTCTGAGGTTTCCAGCAGCAGGCTTGGACTGGGCCCTGAAAACCAATTGTTAAATTTCAGGAATCTTGCAATCTGGTTGTTTAACTGTTGGC AG
TGTGAAATTGGTCAGGTTGGGAGTTATTACACTAGAGAAATTGGCAAGCACTGCAAAACAGCTTTTGGTTTTTCCCTTTCAGAAAGTCCATTGTTAAACATTTACCATCTCATGTAAGTGGACAC ATG
CATTTTGGAGGCAGAACTGAATATGAGTTGTAAAGGGCAGTCATCGAGGGCTCCTCAATGTTGGGCTGTGGCAGCATTTCTACCACAAGCAGCAGGACTGGGAAGGCTGCAGGATACTAGAAGAA ATA
CTGTCTCCATCACAGAGAGAGAAGGGAGAGCTTCTTTCTTTTTGGGTTTGAGGGATAGGATGCTTGGACAAAGAGGAAAGAGAATCATTTTTTTCTTAAAATTGGTATAGGGGTTCATGTGTATT CAG
GAGGCAGTAGAGAACCTCAACAAGGCTGGGGGAGTTGGGGTAGGCAGGAATCCAGAAGACAGGATAACTCTTGAGAGCCTGGAGAGAGTCTGTGGTTTATGGCCAGTCCCAAGGCGACGGATGGGA GA
AGTCCTGGTGTGTTTAGATTTGGAGTGTTTCTCACCTTCTAGGGAGGTGCCATGAGTCAGTGCCCAGAGCCCAATCCCATGGCCCCTGCTCAGGACCGTGAATAGAGACACTGCTTTGGGGCATC ATG
GTCTTGTGAAGGTGGAACAGGAGCGTGTGGGCAGGCAGATGACCTGGGAGAGGAGGCGTTTGGAGCTGAGTGTGGGGAAGGTTCTCGCCAGCTCcttctctcaggctgagcttctcaagaaaggagga
ggcagcagagactgagggtcctacatggcagggaaggaaggaaggagctgtgggaaaacacatccacattcattcatctgctcatcatttaagaaatgtttcctg gccgggcgcggtggctcacgcct
gtaatcccagcactttgggaggccgaggcgggcggatcacgaggtcaggagatcgagaccatcccggctaaaacggtgaaaccccgtctctactaaaaatacaaaaaattagccgggcgtagtggc gg
gcgcctgtagtcccagctactcgggaggCCTTCTCTCAGGCTGAGCTTCTCAAGAAAGGAGGAGGCAGCAGAGACTGAGGGTCCTACATGGCAGGGAAGGAAGGAAGGAGCTGTTGGAAAACACATCC
ACATTCATTCATCTGCTCATCATTTAAGAAATGTTTCCTGAGAGCCTGATATGTCCCTAGTGCTGGTCTCCTCTTGGGATGCCTGTTATGGGTGGACAGGCAGACCTGAGATAGGACTCTGCCAGCTC
AGCACTTCTTCCAAGTCCTGATGCTCCCTCTTCCCCGCTTTCCACTCAAGCACACAGAAGGTCAGAGCAGGAACAGCCCTCAGAGCAGCCTATCTGGGAATTCTCAACACTACGGTGGGGATTAC TTT
GAACCCATGGAGTCCCAGCAGTGGCCTCCAAGGGACCACCCACTCAGTTGGCCCTCACTTAGACCCCTCTGTCCACCTTTTCCATGGGACTCCTAGGCCACTGGGGACAACTTTGAACCCCCTTGA AG
CATCCCACCTCCTGTAATACCCTTGACCATCCCAACTCCTGCACACTCCCTGGGGGTTGGGAGCCCCATACAGGGTTCCCTGAGACTCCTGAGTATCCTGAGGGAGCCCTGTCACCTTCTGTAAA CTC
CACTGACCCTGAAGCTGCATGGCATGTGAGCTGGGCCCTCCTGCTGGCCTTGCCTTTCTGGAGGAGCCCTTGGTCAGTCCTCCCTCTCCTAAACTTACAGTCAGGCCTTCTGTTTCCAGCAGATGT AG
ATGGCAGAGACACCAACCACCAGTAGCAGCTTGGGGCCCAGGAGGAGAGCTACGAGGAGTGGAGCAGTAGGAGCCAGCGCTGCTTCTGGAAATCAGGGAAGAGGAGGAGCCATGAGAGGGGCCAC CTG
GGAGCTCATCCCAGGGGCCTCCACTTACCCCATCTGAGGCTACCTGGGCCCATCAATCCTCTGAAGCTTTTGAACTTGCATCCTGCTCTGGCTTTCCTGACTCCAGCCGAAAGCAAAGGCCAGGGA AA
GTGGC

312	24.6	1.4	0.0	AluYa5-Scaffold5938-1540413-1540593	51	119	(2062)	+	MER63B	DNA/hAT-Blackjack	15	84	(352)	1
534	20.5	1.7	2.6	AluYa5-Scaffold5938-1540413-1540593	121	235	(1946)	+	MER63B	DNA/hAT-Blackjack	312	425	(11)	2
1722	0.6	0.0	0.0	AluYa5-Scaffold5938-1540413-1540593	1001	1181	(1000)	+	AluYa5	SINE/Alu	1	181	(130)	3
186	25.5	0.0	0.0	AluYa5-Scaffold5938-1540413-1540593	1300	1346	(835)	C	L2c	LINE/L2	(6)	3381	3335	4

>chr20:1545228-1547089

GTGGAGGTGAGAGGTGTGGAGAATAGGGATTTAACTCTGAGGTTTCCAGCAGCAGGCTTGGACTGGGCCCTGAAAACCAATTGTTAAATTTCAGGAA TCTTGCAATCTGGTTGTTTAACTGTTGGCAG
TGTGAAATTGGTCAGGTTGGGAGTTATTACACTAGAGAAATTGGCAAGCACTGCAAAACAGCTTTTGGTTTTTCCCTTTCAGAAAGTCCATTGTTAAACATTTACCATCTCATGTAAGTGGACAC ATG
CATTTTGGAGGCAGAACTGAATATGAGTTGTAAAGGGCAGTCATCGAGGGCTCCTCAATGTTGGGCTGTGGCAGCATTTCTACCACAAGCAGCAGGA CTGGGAAGGCTGCAGGATACTAGAAGAAATA
CTGTCTCCATCACAGAGAGAGAAGGGAGAGCTTCTTTCTTTTTGGGTTTGAGGGATAGGATGCTTGGACAAAGAGGAAAGAGAATCATTTTTTTCTTAAAATTGGTATAGGGGTTCATGTGTATT CAG
GAGGCAGTAGAGAACCTCAACAAGGCTGGGGGAGTTGGGGTAGGCAGGAATCCAGAAGACAGGATAACTCTTGAGAGCCTGGAGAGAGTCTGTGGTT TATGGCCAGTCCCAAGGCGACGGATGGGAGA
AGTCCTGGTGTGTTTAGATTTGGAGTGTTTCTCACCTTCTAGGGAGGTGCCATGAGTCAGTGCCCAGAGCCCAATCCCATGGCCCCTGCTCAGGACCGTGAATAGAGACACTGCTTTGGGGCATC ATG
GTCTTGTGAAGGTGGAACAGGAGCGTGTGGGCAGGCAGATGACCTGGGAGAGGAGGCGTTTGGAGCTGAGTGTGGGGAAGGTTCTCGCCAGCTCCTTCTCTCAGGCTGAGCTTCTCAAGAAAGGAGGA
GGCAGCAGAGACTGAGGGTCCTACATGGCAGGGAAGGAAGGAAGGAGCTGTTGGAAAACACATCCACATTCATTCATCTGCTCATCATTTAAGAAATGTTTCCTG AGAGCCTGATATGTCCCTAGTGC
TGGTCTCCTCTTGGGATGCCTGTTATGGGTGGACAGGCAGACCTGAGATAGGACTCTGCCAGCTCAGCACTTCTTCCAAGTCCTGATGCTCCCTCTT CCCCGCTTTCCACTCAAGCACACAGAAGGTC
AGAGCAGGAACAGCCCTCAGAGCAGCCTATCTGGGAATTCTCAACACTACGGTGGGGATTACTTTGAACCCATGGAGTCCCAGCAGTGGCCTCCAAGGGACCACCCACTCAGTTGGCCCTCACTT AGA
CCCCTCTGTCCACCTTTTCCATGGGACTCCTAGGCCACTGGGGACAACTTTGAACCCCCTTGAAGCATCCCACCTCCTGTAATACCCTTGACCATCC CAACTCCTGCACACTCCCTGGGGGTTGGGAG
CCCCATACAGGGTTCCCTGAGACTCCTGAGTATCCTGAGGGAGCCCTGTCACCTTCTGTAAACTCCACTGACCCTGAAGCTGCATGGCATGTGAGCTGGGCCCTCCTGCTGGCCTTGCCTTTCTG GAG
GAGCCCTTGGTCAGTCCTCCCTCTCCTAAACTTACAGTCAGGCCTTCTGTTTCCAGCAGATGTAGATGGCAGAGACACCAACCACCAGTAGCAGCTT GGGGCCCAGGAGGAGAGCTACGAGGAGTGGA
GCAGTAGGAGCCAGCGCTGCTTCTGGAAATCAGGGAAGAGGAGGAGCCATGAGAGGGGCCACCTGGGAGCTCATCCCAGGGGCCTCCACTTACCCCATCTGAGGCTACCTGGGCCCATCAATCCT CTG
AAGCTTTTGAACTTGCATCCTGCTCTGGCTTTCCTGACTCCAGCCGAAAGCAAAGGCCAGGGAAAGTGG C

312	24.6	1.4	0.0	hg19	51	119	(1743)	+	MER63B	DNA/hAT-Blackjack	15	84	(352)	5

534	20.5	1.7	2.6	hg19	121	235	(1627)	+	MER63B	DNA/hAT-Blackjack	312	425	(11)	6
186	25.5	0.0	0.0	hg19	981	1027	(835)	C	L2c	LINE/L2	(6)	3381	3335	7

>KSI_AluYa5_19

>Scaffold6216-273930-274113
AATTCAAGGTTGTTTCAATATTAGGAGATTTATCATTACGTTCCAACATATTAA cAGAAATGACTAGAAAATATGACTGTCTCTGACAGTGAAAAGCATTTAATAAAGTTCAATATTTATTCTTATTA
ACAAGCAAACAGCCTCCTAATTAACTAAGATTAGAAGACTACCTTCTTAATATGATTATCTATCTCATACAAACATAGAAACCTCCTGATTGATGGGAAAGTAAGAAATGTGTGACAAGGCAAAG ATG
TCCACAACCACTGCTGTTATTTAACCCTGTTTTGGGGGGCCTAGCTATTGCATTT AGGCAAGAGGAAGaAATAAATATTTTTAAATTGGAGAGAAGGTGGTGAAATTATTATGGATTGTATATGATAT
GATCTGTATACTTGGAAAACCCAAGAGAATAAACTGGAAAACTATTAGAACCAGAATTAAGAAACTGCCTGGGGACAAAATATGCAAAAATACTCTACTCCTACATAAATAACACCAAAACAGAA GTT
ATGAGGAAGAAATGATCCCAGTTAAAGCAGTAATATATGTTGAGTATAGAAGAAA TAGTGGATAGAAATAAACAACAAACGTACAACATGTATATGAAGAGAATATTAAAATGTTACTTAAGAATATG
AAAAAAATGCACACCTTCTTTTGAGGTAGAAAGGATAAGcAACATTTTTTAATTCATTGAAATAGGAATACTAATACTAAGGAAAT ATGAAGGAATTGAATGAATAAAGTTAAAATAAATTAATTCAA
AAATATAAGAAATAAATTTTCAAAGCAAATTTTATATATAATAGAATTTCTTTAA CAGAATGCTTAATTATTTAAAACATTCTAAATTTTATGAAAAAATAAACAAGCAAAAATTACTAGCATATGAA
TGAAGAACTGTTTAAACCAGGTATTACAAGATATTTTTTtttttttttttttttttttttttttttttttttttgagggggtccccctttccccccgggggggcagcctcccaagtagctgggacta
caggcgcccgccactacgcccggctaatttttttgtatttttagtagagacggggtttcaccgttttagccgggatggtctcgatctcttgacctcgtgatccgcccgcctcggcctcccaaagt gct
gggattacaggcgtgagccaccgcgcccggcctacaagatattttttAGGTATAATAAAAGCCCTCAAATACAGAAACCCAGTGAAACAGAAAAAAAGCGTTACAAAGTAAACCCAAATTTTACTGGC
AGCTGGTATATGACAAAGACAATTCTGCATATCAGCGGGGGAGAT GAATTATCCATTGAGTGATGCAGAAACAACTAAGTGGCTTCTGGAGAAAGATATATATCTGCGTCTCTGCCTGTGCTAAATAA
ATTTTGGAAAAATAAAAAATATAAATGTAGAAACAAAGC CCCAAAAGCACTAAACTAAAACATGATTTTTCATAATCTTGAAATGAAGGAAGTCTTTTTAAGCACGAAGACATAAAGAAGAGAAGGAA
TGGAGAGAGGAAGAGAGTGTTAGGGTAAGAAGGGAAGGATAGAGGCAGGAAAAAGAGAGACGGAAGATAAAAGATCACAAAAAAAAATCATAATTTCAGCAACACAAAAATATTCTTTAAAAAGTC CCA
ATGAGGCTGGGTTGAGTGGTCATGCCTGTAATCCCAGCA CTTAGGGAGGCCAAGGGAGATGGATTGCTTGAGTCCTGGAGTCTGAGACAAGCTTGGGCAACGTGGCAAAACCCCATCTCTACAAAAAA
CAAAACaAAAACAAAAATTAGCCAGGCATGGTGGTGTGCACCTGTAGCCCcGCTACTTAGGAGGTCGAGGTGGtAGGATCACTTGAGCCCAGGAGTTGAGGCTGCTGTGAGCCAAGATGGTGCCAtCA
CACTCCAGCCTGGGTAACAGAGTGAGACCCTATTTCAAA AAAGAaaaaaaaaaaagagAAAAAAAAAGTCAAAATGCAAATTATCATCTATGAATAAAATGGCAGCACATATTACAGATAGAACTCATT
TTCTTGATTACTACATATCAAGTTGATAAAGGAGTAATAACATATAAAAATGGAGAAAGAATGTGAGCAAAAAGGGTAGGAAATCTTAAGCATATGAATAATGTTCAATCTCATTCTCAATAACA AAA
AAAATTAA

852	28.8	9.7	3.5	AluYa5-Scaffold6216-273930-274113	1	646	(1538)	+	L1M5	LINE/L1	3816	4491	(1655)	1
14	23.4	2.1	2.1	AluYa5-Scaffold6216-273930-274113	739	785	(1399)	+	(AATA)n	Simple_repeat	1	47	(0)	2
44	0.0	0.0	0.0	AluYa5-Scaffold6216-273930-274113	930	969	(1215)	+	(T)n	Simple_repeat	1	40	(0)	3
1713	0.5	0.0	0.6	AluYa5-Scaffold6216-273930-274113	1001	1184	(1000)	C	AluYa5	SINE/Alu	(128)	183	1	4
1803	17.3	0.9	2.9	AluYa5-Scaffold6216-273930-274113	1669	1986	(198)	+	AluJr	SINE/Alu	1	312	(0)	5

>hg19 chr20:16829819-16831730
AATTCAAGGTTGTTTCAATATTAGGAGATTTATCATTACGTTCCAACATATTAA tAGAAATGACTAGAAAATATGACTGTCTCTGACAGTGAAAAGCATTTAATAAAGTTCAATATTTATTCTTATTA
ACAAGCAAACAGCCTCCTAATTAACTAAGATTAGAAGACTACCTTCTTAATATGATTATCTATCTCATACAAACATAGAAACCTCCTGATTGATGGGAAAGTAAGAAATGTGTGACAAGGCAAAG ATG
TCCACAACCACTGCTGTTATTTAACCCTGTTTTGGGGGGCCTAGCTATTGCATTT AGGCAAGAGGAAGtAATAAATATTTTTAAATTGGAGAGAAGGTGGTGAAATTATTATGGATTGTATATGATAT
GATCTGTATACTTGGAAAACCCAAGAGAATAAACTGGAAAACTATTAGAACCAGAATTAAGAAACTGCCTGGGGACAAAATATGCAAAAATACTCTACTCCTACATAAATAACACCAAAACAGAA GTT
ATGAGGAAGAAATGATCCCAGTTAAAGCAGTAATATATGTTGAGTATAGAAGAAA TAGTGGATAGAAATAAACAACAAACGTACAACATGTATATGAAGAGAATATTAAAATGTTACTTAAGAATATG
AAAAAAATGCACACCTTCTTTTGAGGTAGAAAGGATAAGtAACATTTTTTAATTCATTGAAATAGGAATACTAATACTAAGGAAATATGAAGGAATTGAATGAATAAAGTTAAAATAAATTAATTCAA
AAATATAAGAAATAAATTTTCAAAGCAAATTTTATATATAATAGAATTTCTTTAA CAGAATGaattCTTAATTATTTAAAACATTCTAAATTTTATGAAAAAATAAACAAGCAAAAATTACTAGCATA
TGAATGAAGAACTGTTTAAACCAGGTATTACAAGATATTTTTTAGGTATAATAAAAGCCCTCAAATACAGAAACCCAGTGAAACAGAAAAAAAGCGTTACAAAGTAAACCCAAATTTTACTGGCAGCT
GGTATATGACAAAGACAATTCTGCATATCAGCGGGGGAGATGAATTATCCATTGA GTGATGCAGAAACAACTAAGTGGCTTCTGGAGAAAGATATATATCTGCGTCTCTGCCTGTGCTAAATAAATTT
TGGAAAAATAAAAAATATAAATGTAGAAACAAAGCCCCAAAAGCACTAAACTAAAACATGATTTTTCATAATCTTGAAATGAAGGAAGTCTTTTTAAGCACGAAGACATAAAGAAGAGAAGGAAT GGA
GAGAGGAAGAGAGTGTTAGGGTAAGAAGGGAAGGATAGAGGCAGGAAAAAGAGAG ACGGAAGATAAAAGATCACAAAAAAAAATCATAATTTCAGCAACACAAAAATATTCTTTAAAAAGTCCCAATGA
GGCTGGGTTGAGTGGTCATGCCTGTAATCCCAGCACTTAGGGAGGCCAAGGGAGATGGATTGCTTGAGTCCTGGAGTCTGAGACAAGCTTGGGCAACGTGGCAAAACCCCATCTCTACAAAAAAC AAA
ACgAAAACAAAAATTAGCCAGGCATGGTGGTGTGCACCTGTAGtCCCaGCTACTTAGGAGGTCGAGGTGGaAGGATCACTTGAGCCCAGGAGTTGAGGCTGCTGTGAGCCAAGATGGTGCCA gCACAC
TCCAGCCTGGGTAACAGAGTGAGACCCTATTTCAAAAAAGAAAAAAAAAAAGTCAAAATGCAAATTATCATCTATGAATAAAATGGCAGCACATATTACAGATAGAACTCATTTTCTTGATTACTACAT
ATCAAGTTGATAAAGGAGTAATAACATATAAAAATGGAGAAAGAATGT GAGCAAAAAGGGTAGGAAATCTTAAGCATATGAATAATGTTCAATCTCATTCTCAATAACAAAAAAAAATTA A

812	29.1	9.7	3.5	hg19	1	646	(1266)	+	L1M5	LINE/L1	3816	4491	(1655)	6
14	23.4	2.1	2.1	hg19	739	785	(1127)	+	(AATA)n	Simple_repeat	1	47	(0)	7
1776	17.0	0.7	3.0	hg19	1409	1714	(198)	+	AluJr	SINE/Alu	1	299	(13)	8

>KSI_AluYa5_20 (We have tried to verify that there is insertion in this region, but we couldn't confirmed the exact sequences by high GC-content concentrated in this insertion region.)

>Scaffold6778-1173547-1173827
CACAGACAAATGCTGAAAGAAAACTGATTTCCAGTTGCTTTTAGTGATTTATTCCC TGTGAAAATCCAAGACAATGCTTTTGTCAAACCATTAATGTGATGTAAAAAATTTTCTGGGGACTCAAATTC
CTAATGACTGTTCAGTCATCATAGTGAACCTTTAAATTATTTCTTTACTATGTCTTGCTTTGTTTGTGTAGGACTCTGGCAATATTTTCTCTCAATTTTTTGTGTAGGTACACAGCCGATAAATG AGG
GAAACACAATATTTCTAACACTTGATGGAAACCTCCTCTGCAAAGCCATTTTAGAT CTGTTGGAAATAATCTAAAATTAATTATCTTATATTGTTTTTTtATCTATTTTTTAATCAAAAAAGTTCTAT
TTTCTCTACCACTAAAATGTCTACATCTGCATTTTTCACAACTGGACATACAGACTAAAAATACATGAGATGATCTCTTTGAGTTTAAAGTTACCTGACAAAGTCCATGAGAATCAGGGTCCTTA AGT
ATAAATTATCTACATATATGTGGGGCGTGTGTGTGTGTGTGTGTGTGTGTGTGTGT GGCTACTGCTGCTTTTCTGTCATAAGTTCTCATTAGTTAATGAATTGTAGCTATAGAGACACTAAGTAAGAA
ATGGAATGGGGGTATCGACGAAGAAACTTTTTTACCAAACATCTAACTGTAAATAATATAAATTCATTGTAGAAAATCAGTGGAAAAGGAATGAAAAAGAAAACCACCCAGAATGTATTAGAGAG CTC
CAATGGTAATATCTCAAGCAATATTATAGCTTTTCATCAGTAAATACTTGCTACTT TTTCAGTTCTAACAAAATGTTTTCACCTTCTAATCCTATTGTGTTCAAACCCTGGGTGCAGGGGAACACCTG
AATGCTTTTGAGGGCAAGGCAGGTAACATAAACATGTGTTGAACAGGCTGCTTGGGAACTTTGGCAAATTAGAGAATGGAAATCTCCT Tagaagtgggcgtctcgctcacgcctgtaatcccagcact
ttgggaggccgaggcgggcggatcacgaggtcaggagatcgagaccatcccggctaaaacggtgaaaccccgtctctactaaaaatacaaaaaattagccgggcgtagtggcgggcgcctgtagt ccc
agctacttgggaggctgaggcaggagaatggcgtgaacccgggannnnnnnnnnnnnnnnnnnnnnnnnnnnnnnnnnnngggaaaaaggcgagcccccaaaaaaaaaaaaaaaaaaaaaaaaaa
aAGAAGTGGGCGTCTCTAGGAATGTGTAACCTACCATGCATACTGTGAACCACTGTAGCTTCCACGGTAGAGTATGCCTGGAGTGGGGGTACAGAGGGAAAAAGCAACATCTTGATATTTTCAGCACC
TCACCAGAGACATGAGGTTCAGGTTCGATTAGGAGAGGGGGCTAGATGTGGTCTTAAACATTTGACACGCCAGACCACAACACCATTTCTGCTTTCAAGATACC TTTCACCAACTGACTGcAGTTGAA
GGCTTCCGACCATTTCATCCAGCTCAAAGAGCAATTTGGACCTAAAGGGATACCTCTTGAAGAAGAAAAATATAAACTAATAAGTAAAAACTAAAATCAATGAAAAGTTTCTTTTCAGTATTTTA TGC
TTATGTTTCGACTCTGAAACACTGTAATTTTGGAGAGGGGGAAAGGCAGTGCTGGTAGTTAATCTTATATGGGATCTCAA GGATGTAGGCAGGCTGGGCGCTGTGGCTTACGCTTGTAATCCTAACAA
TTTGGGAGGCCAAGGTGGGAGGATCACCTGAGGTCAGGAGTTCGAGACCAGCCTAGCCAACATGGCAAAACCCCGTCCCTAGTAAAAATACAAAAATTAGCTGGGTATGGTGGCGGGTGCCTAGT AAT
CCCAGCTACTCGGGAGGCTGAAGCAGGAGAATCACTTCATCCTGGGGAGGCGGAGGTTGCCGTGAGCCAAGATCGTGCCA CTGCATTCCAGCCTGGGTGACAGAGTGAGAATCTGTCTCAAAAAACAA
ACAAACAAAAAAGGATGTAGCCATATAACAATCATTCTATATGTGCCAGGCAACAGTGAAAGGAGTGCCCATTGTTTAGGACCCACCATACCATTTCCAGAAATACCACTCAAGTAACTTACTAT TCA
TAGGTAAAACTATCAATTCCTATATACGTGTAGCAGGTATGTAGTAACCAACATAAATATACTTTACCTAGTAAATAAAA TCAATGAGAACAGTGAAAGTACTAA

36	0.0	0.0	0.0	AluYa5-Scaffold6778-1173547-1173827	539	569	(1712)	+	(GT)n	Simple_repeat	1	31	(0)	1
278	33.0	0.0	2.1	AluYa5-Scaffold6778-1173547-1173827	897	995	(1286)	C	MER102b	DNA/hAT-Charlie	(17)	324	228	2
2033	14.2	5.7	0.0	AluYa5-Scaffold6778-1173547-1173827	1001	1281	(1000)	+	AluYa5	SINE/Alu	15	311	(0)	3
2202	11.5	0.0	0.7	AluYa5-Scaffold6778-1173547-1173827	1756	2060	(221)	+	AluSx1	SINE/Alu	1	303	(9)	4

>chrX:31274322-31276305
CACAGACAAATGCTGAAAGAAAACTGATTTCCAGTTGCTTTTAGTGATTTATTCCCTGTGAAAATCCAAGACAATGCTTTTGTCAAACCATTAATGTGATGTAAAAAATTTTCTGGGGACTCAAAT TC
CTAATGACTGTTCAGTCATCATAGTGAACCTTTAAATTATTTCTTTACTATGTCTTGCTTTGTTTGTGTAGGACTCTGGCAATATTTTCTCTCAATTTTTTGTGTAGGTACACAGCCGATAAATG AGG
GAAACACAATATTTCTAACACTTGATGGAAACCTCCTCTGCAAAGCCATTTTAGATCTGTTGGAAATAATCTAAAATTAATTATCTTATATTGTTTTT TATCTATTTTTTAATCAAAAAAGTTCTATT
TTCTCTACCACTAAAATGTCTACATCTGCATTTTTCACAACTGGACATACAGACTAAAAATACATGAGATGATCTCTTTGAGTTTAAAGTTACCTGACAAAGTCCATGAGAATCAGGGTCCTTAA GTA
TAAATTATCTACATATATGTGGGGCGTGTGTGTGTGTGTGTGTGTGTGTGTGTGTGGCTACTGCTGCTTTTCTGTCATAAGTTCTCATTAGTTAATGAAT TGTAGCTATAGAGACACTAAGTAAGAAA
TGGAATGGGGGTATCGACGAAGAAACTTTTTTACCAAACATCTAACTGTAAATAATATAAATTCATTGTAGAAAATCAGTGGAAAAGGAATGAAAAAGAAAACCACCCAGAATGTATTAGAGAGC TCC
AATGGTAATATCTCAAGCAATATTATAGCTTTTCATCAGTAAATACTTGCTACTTTTTCAGTTCTAACAAAATGTTTTCACCTTCTAAT CCTATTGTGTTCAAACCCTGGGTGCAGGGGAACACCTGA
ATGCTTTTGAGGGCAAGGCAGGTAACATAAACATGTGTTGAACAGGCTGCTTGGGAACTTTGGCAAATTAGAGAATGGAAATCTCCT TAGAAGTGGGCGTCTCTAGGAATGTGTAACCTACCATGCAT
ACTGTGAACCACTGTAGCTTCCACGGTAGAGTATGCCTGGAGTGGGGGTACAGAGGGAAAAAGCAACATCTTGATATTTTCAGCACCTCACCAGAGACAT GAGGTTCAGGTTCGATTAGGAGAGGGGG
CTAGATGTGGTCTTAAACATTTGACACGCCAGACCACAACACCATTTCTGCTTTCAAGATACCTTTCACCAACTGACTG tAGTTGAAGGCTTCCGACCATTTCATCCAGCTCAAAGAGCAATTTGGAC
CTAAAGGGATACCTCTTGAAGAAGAAAAATATAAACTAATAAGTAAAAACTAAAATCAATGAAAAGTTTCTTTTCAGTATTTTATGCTTATGTTTCGACT CTGAAACACTGTAATTTTGGAGAGGGGG
AAAGGCAGTGCTGGTAGTTAATCTTATATGGGATCTCAAGGATGTAGGCAGGCTGGGCGCTGTGGCTTACGCTTGTAATCCTAACAATTTGGGAGGCCAAGGTGGGAGGATCACCTGAGGTCAGG AGT
TCGAGACCAGCCTAGCCAACATGGCAAAACCCCGTCCCTAGTAAAAATACAAAAATTAGCTGGGTATGGTGGCGGGTGCCTAGTAATCCCAGCTACTCGG GAGGCTGAAGCAGGAGAATCACTTCATC
CTGGGGAGGCGGAGGTTGCCGTGAGCCAAGATCGTGCCACTGCATTCCAGCCTGGGTGACAGAGTGAGAATCTGTCTCAAAAAACAAACAAACAAAAAAGGATGTAGCCATATAACAATCATTCT ATA
TGTGCCAGGCAACAGTGAAAGGAGTGCCCATTGTTTAGGACCCACCATACCATTTCCAGAAATACCACTCAAGTAACTTACTATTCATAGGTAAAACTAT CAATTCCTATATACGTGTAGCAGGTATG
TAGTAACCAACATAAATATACTTTACCTAGTAAATAAAATCAATGAGAACAGTGAAAGTACTA A

36	0.0	0.0	0.0	hg19	538	568	(1416)	+	(GT)n	Simple_repeat	1	31	(0)	5
278	33.0	0.0	2.1	hg19	896	994	(990)	C	MER102b	DNA/hAT-Charlie	(17)	324	228	6
2202	11.5	0.0	0.7	hg19	1459	1763	(221)	+	AluSx1	SINE/Alu	1	303	(9)	7

>KSI_AluYa5_21

>Scaffold6842-893826-893990
TTTTCTCCCAGATTGTTGCTTATCTTCATTTTCTTTAGAGTGGCTTTTAAAGAGCAGAAGTTTAAAATTTTGATAAAGTCCAATTTATAAGTGTTTTCTTTTGTGATTTGTGCTTTATGTGCCAAT CT
AGGAAAACTTTACCAAACTCAAGTTGATGTGAATTTTCTCCTAAGTTTTCTCCTATACAATTTTTAATTTTAACCTTTGTGTTTAGGTCTATACTAACTAA TAGCTAATTTTTATAAAGAGCATGAGG
CAATGAATTGAGGTTTTTTTTGGATATAAATATTTAGGTATTCAACACTATTTTCTAA gAAGACTTTTCTCTCCTCACCAAATTACTTTAATATTATTGTCAAATATCAATTTGTCATTTACAAGTAG
GTCTGCTTCTAAGCTCTCTGCTCTGTTATTTTGATCTATAGGTCTATTCTTATGTCAATATCACACCATCTTGATTGGTCCAGCTTTATAGTAAATCTTGA AATCAGGTAGTATGAGTGACTCAACTT
TCTTCTACTGTTTCAAAATTGTTCTTTTACTAGAAGAGTGTTTGCATTTTCATATAGATTTTAGATTCAAGTTATTTATTTTTAGAAATAGAACAGGTGGGATTTTAATTGGGGTGGTATAAAAT TTA
TATATTAATATGAGAAAAGATGACATCTCAATATTGAATCTTCTGATCTATTAAAGTGATTTATCTCCTCATTTACTTAGGTATCCTTCTCTGAGCAAAGC TTTATAGTTTTCAGTGTAcGGGTCTTG
CACATGTTCGTTAAGTTTATCACTAGGTACTTTGTgTTTTTTAAATTTTTTAAATTTTAGCTTCCAATTGTGCATTGCTATAAACATTTACATATGGTTTT TATGGTAATGTGTTTTTtttttttttt
tttttttttttttttttttttttttaaaggggggttcgggtttttttttttttttttccaggctggtctcgaacccctgggctcaagtgatcctcctgcct tatggactacaggcgcccgccactacg
cccggctaatttttgtatttttagtagagacggggtttcaccgttttagccgggatggtctcgatctcctgacctcgtgatccgcccgcctcggcctcccaaagtgctgggattacaggcgtga gcc
accgcgcccggcctatggtaatgtgtttttATCCTCCTCAGATAAATAGCTAACATACTGCTGGGGCAAAATGCAGATATGTATGTTTAACATGTTAAGATTTTTTTACATATTGATCTTTTATCCTG
CCATCAGGCTAAACTCTTCTATAAATTCCTGAAGCATTTTTTTCTGAATTTCCTAGGATTTTCTACATAAACAGTTATGTCACTGGCAATTAAAGACAGTTTTCTCTCTTCCTTTCTGATCTGTA TAT
CTTTTATTTCTTTCTTTCGCTTCACTGTACAATGTTGAATATAATTTGTAAAAGTAGAAATCTTTGCCTTGTTCTCAATCTTAGGGGGAAACATCAAATTT CTATTAATATGTAAGATATTAGCTGTA
GTGTTTTTTTTGCCGATGCCCTTTATTGGGTTAAGGGAGTTCCTTTATATTCCCTAGTTTGCTGAGCATTTTTATCATGAATGGATGCTGAATTTTGTCAAATAGTTTTCCTGTACCTGTTGAGA TGA
TTATATACTTTCTGTTTGTCTATCTATATAGTAAATATTGATTGATTTTAAAATGTTGAACTATCTTTGAATTTCTGGGTTAAACACCACTTAGTCATGAT ATATTATTTTTACATACTGGTGGATTT
TTTTTTGCCAATATTTTGTTAAACAGTTTTGCATCTATATCTATGAGGATTATTGGCCTTCCGTTTTATTTTTTCATGATATGTTTATCTGGTTTTGGTGTCAAGGTATGCTGGCCTCACACAAT GAG
TTGGAGAGTGTATCTACTTCttTTTTTTTTTTTTTTTGAAAGAGTTTGTGTAGAATTTTCATTATTGTTTTCTTAATTATTTGGCAGAATTGTCCAGTGAA GCCATCTGTGGCTTGAGTTTTCTTTGA
GGGTGACTTAAAAAAAAAATTCAATTTCTTTTGTTCATGTTTTTACATTGCTAAAATTATTATCAATAAATTATACTATTCTTTTTAACTTCTGTAGAACCTAGAGAGCTGCTCCAT C

2340	28.8	4.8	0.6	AluYa5-Scaffold6842-893826-893990	1	848	(1317)	C	L1M5	LINE/L1	(922)	5224	4341	1
46	0.0	0.0	0.0	AluYa5-Scaffold6842-893826-893990	882	923	(1242)	+	(T)n	Simple_repeat	1	42	(0)	2
378	6.8	0.0	0.0	AluYa5-Scaffold6842-893826-893990	954	997	(1168)	C	FLAM_A	SINE/Alu	(50)	92	49	3
1585	0.0	0.0	0.0	AluYa5-Scaffold6842-893826-893990	1001	1165	(1000)	C	AluYa5	SINE/Alu	(146)	165	1	4
2840	23.7	4.5	1.8	AluYa5-Scaffold6842-893826-893990	1255	2078	(87)	C	L1M5	LINE/L1	(1826)	4320	3475	1

>chr4:145394311-145396177
TTTTCTCCCAGATTGTTGCTTATCTTCATTTTCTTTAGAGTGGCTTTTAAAGAGCAGAAGTTTAAAATTTTGATAAAGTCCAATTTATAAGTGTTTTCT TTTGTGATTTGTGCTTTATGTGCCAATCT
AGGAAAACTTTACCAAACTCAAGTTGATGTGAATTTTCTCCTAAGTTTTCTCCTATACAATTTTTAATTTTAACCTTTGTGTTTAGGTCTATACTAACTAATAGCTAATTTTTATAAAGAGCATG AGG
CAATGAATTGAGGTTTTTTTTGGATATAAATATTTAGGTATTCAACACTATTTTCTAA aAAGACTTTTCTCTCCTCACCAAATTACTTTAATATTATTG TCAAATATCAATTTGTCATTTACAAGTAG
GTCTGCTTCTAAGCTCTCTGCTCTGTTATTTTGATCTATAGGTCTATTCTTATGTCAATATCACACCATCTTGATTGGTCCAGCTTTATAGTAAATCTTGAAATCAGGTAGTATGAGTGACTCAA CTT
TCTTCTACTGTTTCAAAATTGTTCTTTTACTAGAAGAGTGTTTGCATTTTCATATAGATTTTAGATTCAAGTTATTTATTTTTAGAAATAGAACAGGTG GGATTTTAATTGGGGTGGTATAAAATTTA
TATATTAATATGAGAAAAGATGACATCTCAATATTGAATCTTCTGATCTATTAAAGTGATTTATCTCCTCATTTACTTAGGTATCCTTCTCTGAGCAAAGCTTTATAGTTTTCAGTGTA tGGGTCTTG
CACATGTTCGTTAAGTTTATCACTAGGTACTTTGTaTTTTTTAAATTTTTTAAATTTTAGCTTCCAATTGTGCATTGCTATAAACATTTACATATGGTT TTTATGGTAATGTGTTTTTATCCTCCTCA
GATAAATAGCTAACATACTGCTGGGGCAAAATGCAGATATGTATGTTTAACATGTTAAGATTTTTTTACATATTGATCTTTTATCCTGCCATCAGGCTAAACTCTTCTATAAATTCCTGAAGCATTTT
TTTCTGAATTTCCTAGGATTTTCTACATAAACAGTTATGTCACTGGCAATTAAAGACAGTTTTCTCTCTTCCTTTCTGATCTGTATATCTTTTATTTCT TTCTTTCGCTTCACTGTACAATGTTGAAT
ATAATTTGTAAAAGTAGAAATCTTTGCCTTGTTCTCAATCTTAGGGGGAAACATCAAATTTCTATTAATATGTAAGATATTAGCTGTAGTGTTTTTTTTGCCGATGCCCTTTATTGGGTTAAGGG AGT
TCCTTTATATTCCCTAGTTTGCTGAGCATTTTTATCATGAATGGATGCTGAATTTTGTCAAATAGTTTTCCTGTACCTGTTGAGATGATTATATACTTT CTGTTTGTCTATCTATATAGTAAATATTG
ATTGATTTTAAAATGTTGAACTATCTTTGAATTTCTGGGTTAAACACCACTTAGTCATGATATATTATTTTTACATACTGGTGGATTTTTTTTTGCCAATATTTTGTTAAACAGTTTTGCATCTA TAT
CTATGAGGATTATTGGCCTTCCGTTTTATTTTTTCATGATATGTTTATCTGGTTTTGGTGTCAAGGTATGCTGGCCTCACACAATGAGTTGGAGAGTGT ATCTACTTCTTTTTTTTTTTTTTTTGAAAG
AGTTTGTGTAGAATTTTCATTATTGTTTTCTTAATTATTTGGCAGAATTGTCCAGTGAAGCCATCTGTGGCTTGAGTTTTCTTTGAGGGTGACTTAAAAAAAAAATTCAATTTCTTTTGTTCATGT TTT
TACATTGCTAAAATTATTATCAATAAATTATACTATTCTTTTTAACTTCTGTAGAACCTAGAGAGCTGCTCCAT C

2331	29.0	4.9	0.7	hg19	1	890	(977)	C	L1M5	LINE/L1	(922)	5224	4297	5	
254	27.2	7.1	2.5	hg19	844	956	(911)	C	L1M5	LINE/L1	(458)	5706	5589	6	*

2336	23.8	6.1	3.1	hg19		959	1780	(87)	C	L1M5	LINE/L1	(1826)	4320	3475	5

>KSI_AluYa5_22

>Scaffold7277-770410-770651
GACGGAGATCGATTAGGGGAAGGTTATACTGACTATGCCTTTAGTCTTTCACATGTTCAATTTGAGATGACCATTAGAAGTCCAAGTGGATATATACAGCTAGATATACAGTTCAAGAATTCTGAA AA
GAGGACATCATGTGAATTCCAGTTTTCAGCTTAGAGTAACCAAAGCTATAGAAAATGATAAGATCACCTAGAAAGAATTTGTAAAAGGTAGAAGAGAATGAGAGAATGTACCCTAGGGCAATAGC CCA
AGGAACTCTAGGAATTTTTTTTTTTTTCCCACTCTTGTTGCCCAGGCTGGAGTACAATGGCACGATCTCAGCTCACTGCAACCTCCGCCTCCCGGGTTCAAGCAATTCTCCTGCCTCAGGCTCCCA AG
TGGCTGGGgTTACAaGCATGCGCCACCACGCCCAGCCAATTTTGTATTTTTAGTAGAGACCGGGTTTCTCCATGTTGGTCAGGCTGGTCTCGACCTCTGACCTCAGGTGATCCACCCAACTCAGCCTC
CCAAAGTGCTGGGATTACAGGTGTGCCCAGCTGGAACTCCAGGATTTAAGAAATAAAGAAAGACTAAGCAAAGGAAAGTGAAAAAAAATGACCAGGGAGATAAGGTCAAAAATCAGGTAAAGGTG GTA
TCAGCAAAGCTAGAACAGGTTTCTAAAAAGAGGGAAATGGTCAGTTAAAGGTTGTTGAGAAGTCGAGCAAGATAAGGATTAAAAGTGTTCACTGAATTCTCTTGACATTTGCAGGAGAAATTTTAGTA
CAATGGAAAGGACAAAAACCAAGGTGGAACAAAAAAGGCGATATATAAAAATTGTTCAAGAAGCCTGGGTAAAGCTCAAACATTTTTTTAGTGGAGATTCTTATACATGTTTAAATTCTGATGAAGCA
CTTTACTTCTGGATAAAGACTGCAGATTAAGCAGACACATTTTttttttttttttttttttttttttttttttgtagggggcgctccctcatctccccggg ggTTTTTTTTTTTTTTTTTTTTT
TGAGACGGAGTCTCGCTCTGTCGCCCAGGCTGGAGTGCAGTGGCGGGA tctcggctcactgcaagctccgcctcccgggttcacgccattctcctgcctcagcctcccaagtagctgggactacaggc
gcccgccactacgcccggctaatttttgtatttttagtagagacggggtttca ctgtttttagccgggatggtctcgatctcctgacctcgtgatccgcccgcctcggcctcccaaagtgctgggat
tacaggcgtgagccaccgcgcccggccaagcagacacattttACCATCATTCCCTCTTAAAACTTCATTAAGAATAAAAAAATTGTCCT TTATCTTTTTTTTTTTTTTAAAAAAAAAAGGTCTCATTCT
GTCAGCCATGGCTCACTGAAGCCCCAAACTCCCAGGCTCAATCAAGTAATCCTCCTGACTTAGCCTTCTGAGTACCTAAGATTACAGGTGTGAGCCAATAAATTAATTTTTTAAAAGAAAAAACCAGC
CAGGCACCATGGTGCATGTCTATAATCCCAGCTACTCAAGAGGCTGAGGCAGAAGGATCCTTTGAGGTCAAGAATTTGAGACCAACCTGGGCAACACAGCAAGACCCATTCCTAAATA ACTAATTAAA
ATTAAATTATGTTTTTTGTTTTGTTTTGTTTTTTGAGATGGAGTCTCACTATGTCACCCAGGCTATAGTGCAGTGGCACATCT tAGCTCACTGCAAGCTCCGCCTCCCAGGTTCAAGGATTCTCCTGC
CTCAGCCTCCCGAGTAGCTGGGACTTCAGGCGCCCGCCACCACACCCGGCTAATTTTTTGTATTTTTAGTAGAGATGGGGTTTCACCGTGTTAGCCAGGATGGTCTTGATCTCCTGACCTCGCAA TCC
cCCCGCCTCAGACTCCCAAAGTGCTGGGATTACAGGCGTGAGCCACCACGCCCGGCTGCCAAGAATTTTTTAACTTAATTTTTTAAAGATATAATTTACCTACAGTGAAATACATAAACCTGAAGG GC
TTTTGTTTTTTTAAAGCATATGTACACAAAATGGAACACCGAACAACAGCAAAAACATTTTGAAAGTTGGAATGTGGATTGACTTAAAGATCTAAAGACGTTAAGTCTCGGCTGGGGCCGGGCGC GGT
GGCTCAtGCCTGTAATCCCAGCACTTTGGGAGGCCGAGGCAGGCGAATCACCTGAGGTCAGGAGTTCAAGACCAGCCTGACCAACATGGAGAAACCCCGTCTCTACTAAAATACAAACATTAGCCGGG
CAT

591	30.0	10.8	5.5	AluYa5-Scaffold7277-770410-770651	40	270	(1972)	C	L2	LINE/L2	(957)	2462	2224	1
1992	9.4	4.1	0.0	AluYa5-Scaffold7277-770410-770651	271	537	(1705)	C	AluSp	SINE/Alu	(18)	295	18	2
591	30.0	10.8	5.5	AluYa5-Scaffold7277-770410-770651	538	788	(1454)	C	L2	LINE/L2	(1196)	2223	1956	1
39	0.0	0.0	0.0	AluYa5-Scaffold7277-770410-770651	936	971	(1271)	+	(T)n	Simple_repeat	1	36	(0)	3
2209	2.9	0.0	0.4	AluYa5-Scaffold7277-770410-770651	1001	1242	(1000)	C	AluYa5	SINE/Alu	(70)	241	1	4
462	20.4	17.5	4.5	AluYa5-Scaffold7277-770410-770651	1304	1440	(802)	C	AluJb	SINE/Alu	(11)	301	148	5
669	22.9	0.8	0.0	AluYa5-Scaffold7277-770410-770651	1470	1587	(655)	+	FLAM_A	SINE/Alu	1	119	(23)	6
2342	8.3	0.7	0.0	AluYa5-Scaffold7277-770410-770651	1611	1911	(331)	C	AluY	SINE/Alu	(7)	304	2	7
1239	4.9	0.7	0.0	AluYa5-Scaffold7277-770410-770651	2099	2242	(0)	+	AluSp	SINE/Alu	1	145	(168)	8

>chr3:196600100-196602023
GACGGAGATCGATTAGGGGAAGGTTATACTGACTATGCCTTTAGTCTTTCACATGTTCAATTTGAGATGACCATTAGAAGTCCAAGTGGATATATACAGCTAGATATACAGTTCAAGAATTCTGAA AA
GAGGACATCATGTGAATTCCAGTTTTCAGCTTAGAGTAACCAAAGCTATAGAAAATGATAAGATCACCTAGAAAGAATTTGTAAAAGGTAGAAGAGAATGAGAGAATGTACCCTAGGGCAATAGC CCA
AGGAACTCTAGGAATTTTTTTTTTTTTCCCACTCTTGTTGCCCAGGCTGGAGTACAATGGCACGATCTCAGCTCACTGCAACCTCCGCCTCCCGGGTTCAAGCAATTCTCCTGCCTCAGGCTCCCA AG
TGGCTGGGaTTACAgGCATGCGCCACCACGCCCAGCCAATTTTGTATTTTTAGTAGAGACCGGGTTTCTCCATGTTGGTCAGGCTGGTCTCGACCTCTGACCTCAGGTGATCCACCCAACTCAGCCTC
CCAAAGTGCTGGGATTACAGGTGTGCCCAGCTGGAACTCCAGGATTTAAGAAATAAAGAAAGACTAAGCAAAGGAAAGTGAAAAAAAATGACCAGGGAGATAAGGTCAAAAATCAGGTAAAGGTGG TA
TCAGCAAAGCTAGAACAGGTTTCTAAAAAGAGGGAAATGGTCAGTTAAAGGTTGTTGAGAAGTCGAGCAAGATAAGGATTAAAAGTGTTCACTGAATTCTCTTGACATTTGCAGGAGAAATTTTA GTA
CAATGGAAAGGACAAAAACCAAGGTGGAACAAAAAAGGCGATATATAAAAATTGTTCAAGAAGCCTGGGTAAAGCTCAAACATTTTTTTAGTGGAGATTCTTATACATGTTTAAATTCTGATGAAGCA
CTTTACTTCTGGATAAAGACTGCAGATTAAGCAGACACATTTTACCATCATTCCCTCTTAAAACTTCATTAAGAATAAAAAAATTGTCCTTTATCTTTTTTTTTTTTTTAAAAAAAAAAGGTCTCATTC
TGTCAGCCATGGCTCACTGAAGCCCCAAACTCCCAGGCTCAATCAAGTAATCCTCCTGACTTAGCCTTCTGAGTACCTAAGATTACAGGTGTGAGCCAATAAATTAATTTTTTAAAAGAAAAAACCAG
CCAGGCACCATGGTGCATGTCTATAATCCCAGCTACTCAAGAGGCTGAGGCAGAAGGATCCTTTGAGGTCAAGAATTTGAGACCAACCTGGGCAACACAGCAAGACCCATTCCTAAATAACTAAT TAA
AATTAAATTATGTTTTTTGTTTTGTTTTGTTTTTTGAGATGGAGTCTCACTATGTCACCCAGGCTATAGTGCAGTGGCACATCT cAGCTCACTGCAAGCTCCGCCTCCCAGGTTCAAGGATTCTCCTG
CCTCAGCCTCCCGAGTAGCTGGGACTTCAGGCGCCCGCCACCACACCCGGCTAATTTTTTGTATTTTTAGTAGAGATGGGGTTTCACCGTGTTAGCCAGGATGGTCTTGATCTCCTGACCTCGCA ATC

CaCCCGCCTCAGACTCCCAAAGTGCTGGGATTACAGGCGTGAGCCACCACGCCCGGCTGCCAAGAATTTTTTAACTTAATTTTTTAAAGATATAATTTACCTACAGTGAAATACATAAACCTGAAGG G
CTTTTGTTTTTTTAAAGCATATGTACACAAAATGGAACACCGAACAACAGCAAAAACATTTTGAAAGTTGGAATGTGGATTGACTTAAAGATCTAAAGACGTTAAGTCTCGGCTGGGGCCGGGCG CGG
TGGCTCAcGCCTGTAATCCCAGCACTTTGGGAGGCCGAGGCAGGCGAATCACCTGAGGTCAGGAGTTCAAGACCAGCCTGACCAACATGGAGAAACCCCGTCTCTACTAAAATACAAACATTAGCCGG
GCAT

591	30.0	10.8	5.5	chr3:196600100-196602023	40	270	(1654)	C	L2	LINE/L2	(957)	2462	2224	9
2024	8.6	4.1	0.0	chr3:196600100-196602023	271	537	(1387)	C	AluSp	SINE/Alu	(18)	295	18	10
591	30.0	10.8	5.5	chr3:196600100-196602023	538	788	(1136)	C	L2	LINE/L2	(1196)	2223	1956	9
462	20.4	17.5	4.5	chr3:196600100-196602023	986	1122	(802)	C	AluJb	SINE/Alu	(11)	301	148	11
669	22.9	0.8	0.0	chr3:196600100-196602023	1152	1269	(655)	+	FLAM_A	SINE/Alu	1	119	(23)	12
2368	8.0	0.7	0.0	chr3:196600100-196602023	1293	1593	(331)	C	AluY	SINE/Alu	(7)	304	2	13
1254	4.2	0.7	0.0	chr3:196600100-196602023	1781	1924	(0)	+	AluSp	SINE/Alu	1	145	(168)	14

>KSI_AluYa5_23 (We have tried to verify that there is insertion in this region, but we couldn't confirmed the exact sequences by high GC-content concentrated in this insertion region.)

>Scaffold7847-1253437-1253603
AAAGCCAAAATAGACAAATGGGATCTAATTAAACTAAAGAGCTTCTGCACAGCAATGGAAACTATCATCAGAGTGAACAGGCAACCTATAGAATGGGAGAAAATTTTTGC cATCTATCtATCaGACAA
AGAGCTAATATCCAGAATCTACAAGCAACTTAAACAACTTTACAGGAAAATACAAACAACCCCATAAAAAGTGGGCAAAGGATATGAACAGAAACTTCTCAAAAGAAGACATTTACACAGGCAAC AAA
CATATGAAAAAAAGCTCATCATCACTGGTCATTAGAGAAATGCAAATCAAAACCACAATGAGATACCATCTCATGCCAGTTAGAATGGCAATCACTAAAAAGTCAGGAAACAACAGATGCTGG AGAGG
ATGTGGAGAAATAGGAACGCTTTTACACTAATTACACTTTTGGTGGGAGTGTAAATTAGTTCAATCATTGTGGAAACCAATGTGGTGACTCCTCAAGGATCTAGAACCAGAAATACCATTTGACC CAG
CAATCCCATTACTGAGTATACACCCAAAGGATTATAAATCATTCTACTATAAAGACACATGCACACGTATATTTATTGTGGCACTATTCACAATAGCAAAGACTTCGAACCAACCCAAATGCC CATCA
ATGATAGACTGAATAAAGAAAATGTGGCACATACACAcCATGGAATACTATGCAGCCATAAAAAAGAATGAGTTCATGTCCTTTgCAGGGACATGGATGAAGCTGGAAACCATCATTCTCAGCAAACT
AACATAGGAAGAGAAAACCAAACACTGCATGTTCTCACTCCTAAGTGGGAGTTGAACAATGAGAACACATGGACACAGGGCAGGGACCATCACACACTGGGGCCTGTTGGGGGATAGGGGGAT CGGGG
AGGGAAAGCATTAGGAGAAATACCTAATGTAGATGACGATTTGAAGGGTGCAGCAAACCACcATGGCACATGTATACCTATGTAAGAAACCTGCACGTTCCgtcggccgggcgcggtggctcacgcct
gtaatcccagcactttgggaggccgaggcgggcggatcacgaggtcaggagatcgagaccatcccggctaaaacggtgaaaccccgtctctactaaaaatacaaaaaattagccgggcgtagtgg cgg
gggcccgtcgtgccacgtcctttggtgggggggggggggnnnnnnnnnnnggggggggggcgggcgacagcgcccccgaaaaaaaaaaaaaaaaaaaaaaaaaaagaaacctgcacgttccACACAT
GTATCCCAGAACTTGAAGTATAAAAAAATAAAAGAAAGAAAGAAACAGGATTGTCTTAGTTAATACAGGAAAAGCTTTTGGCAAAATTCTATACCCATTCATGATTTTTAAAACCCTCAGTTCCT AAT
AATAGAAGGAATGTCCTTCAACTTGATAACAGACATATATGAAAACCTGTGGTTTCTATCATACTTAATGTTGAAAGACTAAACACATTCCCCTAAGACTG GGCATAAGGCAAGGATGTCTAGTACAT
AACTTGTATCCAGCATTATTCTGGAGGTCCTAACCAGTATAAGGCAAGAAAAAGAAATAAAACTCATATAGATTAGAAAGAAAGAAAACTTATTTATTTTATAAATTTCTTAATCATCTATGTTGAAA
ATATTAAGGAATCTATAAAAAAGCTAGAAGCACTAATAACTGAGTTTAGTAAGTCACAAGATATATGGTCAATATACAAAAGTTAATTGTATTTCTATGTA GTAGCAATGAATAACTGGCAATTGAAA
TTACAGAAAAATACTAtTTTGTAAAAAACTCAAAAAATCCAATgTATAGGCATAAATTTAACAAAATATCTGGAAGCCTTGTACATTGAAAATAATAAGACATTGCTTAGAGAAATTACAGAATATGT
AAATAAACAAAAAGCTAAATGACACTCAATCTTTCCAAACTGACATTGATTCAACTTAATCCCAATCAAAATATCAACACTTTTTTTGAAGAAATTTAAGT GCTGATTCTACAATTTTTTTGGAAATG
CAAAGAACCTAAAACAGCAAAACAATTTTTGAAAAGAAAAATATGATTTTAACAGTTATTATGCCATAATAATCAAGACAGTGGATTATTGCCATGTGTACACATACACATCAAACAT A

6831	6.1	0.3	0.9	AluYa5-Scaffold7847-1253437-1253603	1	998	(1169)	+	L1PA7	LINE/L1	5122	6112	(42)	1
1511	2.4	0.0	0.0	AluYa5-Scaffold7847-1253437-1253603	1001	1167	(1000)	+	AluYa5	SINE/Alu	1	167	(144)	2
23	0.0	0.0	0.0	AluYa5-Scaffold7847-1253437-1253603	1232	1256	(911)	+	(A)n	Simple_repeat	1	25	(0)	3
350	10.1	0.0	8.3	AluYa5-Scaffold7847-1253437-1253603	1257	1321	(846)	+	L1PA8	LINE/L1	6112	6171	(1)	4
1952	25.6	6.2	0.7	AluYa5-Scaffold7847-1253437-1253603	1325	2127	(40)	+	L1M4	LINE/L1	3890	4739	(1407)	5

>chr12:101449784-101451672
AAAGCCAAAATAGACAAATGGGATCTAATTAAACTAAAGAGCTTCTGCACAGCAATGGAAACTATCATCAGAGTGAACAGGCAACCTATAGAATGGGAGAAAATTTTTGC aATCTATCcATCtGACAA
AGAGCTAATATCCAGAATCTACAAGCAACTTAAACAACTTTACAGGAAAATACAAACAACCCCATAAAAAGTGGGCAAAGGATATGAACAGAAACTTCTCAAAAGAAGACATTTACACAGGCAAC AAA
CATATGAAAAAAAGCTCATCATCACTGGTCATTAGAGAAATGCAAATCAAAACCACAATGAGATACCATCTCATGCCAGTTAGAATGGCAATCACTAAAAAGTCAGGAAACAACAGATGCTGG AGAGG

ATGTGGAGAAATAGGAACGCTTTTACACTAATTACACTTTTGGTGGGAGTGTAAATTAGTTCAATCATTGTGGAAACCAATGTGGTGACTCCTCAAGGATCTAGAACCAGAAATACCATTTGACC CAG
CAATCCCATTACTGAGTATACACCCAAAGGATTATAAATCATTCTACTATAAAGACACATGCACACGTATATTTATTGTGGCACTATTCACAATAGCAAAGACTTCGAACCAACCCAAATGCC CATCA
ATGATAGACTGAATAAAGAAAATGTGGCACATACACAaCATGGAATACTATGCAGCCATAAAAAAGAATGAGTTCATGTCCTTTtCAGGGACATGGATGAAGCTGGAAACCATCATTCTCAGCAAACT
AACATAGGAAGAGAAAACCAAACACTGCATGTTCTCACTCCTAAGTGGGAGTTGAACAATGAGAACACATGGACACAGGGCAGGGACCATCACACACTGGGGCCTGTTGGGGGATAGGGGGAT CGGGG
AGGGAAAGCATTAGGAGAAATACCTAATGTAGATGACGATTTGAAGGGTGCAGCAAACCACgATGGCACATGTATACCTATGTAAGAAACCTGCACGTTCCACACATGTATCCCAGAACTTGAAGTAT
AAAAAAATAAAAGAAAGAAAGAAACAGGATTGTCTTAGTTAATACAGGAAAAGCTTTTGGCAAAATTCTATACCCATTCATGATTTTTAAAACCCTCAGTTCCTAATAATAGAAGGAATGTCC TTCAA
CTTGATAACAGACATATATGAAAACCTGTGGTTTCTATCATACTTAATGTTGAAAGACTAAACACATTCCCCTAAGACTGGGCATAAGGCAAGGATGTCTAGTACATAACTTGTATC CAGCATTATTC
TGGAGGTCCTAACCAGTATAAGGCAAGAAAAAGAAATAAAACTCATATAGATTAGAAAGAAAGAAAACTTATTTATTTTATAAATTTCTTAATCATCTATGTTGAAAATATTAAGGAATCTAT AAAAA
AGCTAGAAGCACTAATAACTGAGTTTAGTAAGTCACAAGATATATGGTCAATATACAAAAGTTAATTGTATTTCTATGTAGTAGCAATGAATAACTGGCAATTGAAATTACAGAAAAATACT ATTTGT
AAAAAACTCAAAAAATCCAATaTATAGGCATAAATTTAACAAAATATCTGGAAGCCTTGTACATTGAAAATAATAAGACATTGCTTAGAGAAATTACAGAATATGTAAATAAACAAAAAGCTA AATGA
CACTCAATCTTTCCAAACTGACATTGATTCAACTTAATCCCAATCAAAATATCAACACTTTTTTTGAAGAAATTTAAGTGCTGATTCTACAATTTTTTTGGAAATGCAAAGAACCTAAAACAGCA AAA
CAATTTTTGAAAAGAAAAATATGATTTTAACAGTTATTATGCCATAATAATCAAGACAGTGGATTATTGCCATGTGTACACATACACATCAAACAT A

6980	6.6	0.3	0.9	hg19	1	1040	(849)	+	L1PA7	LINE/L1	5122	6154	(0)	6	
1978	25.7	6.0	0.7	hg19	1024	1849	(40)	+	L1M4	LINE/L1	3867	4739	(1407)	7	*

>KSI_AluYa5_24

>Scaffold7857-1475280-1475433
CCATTACTGCTTTTTGCAATTTATAACGATTAAAATTATCCATAGTTTACAGGGAAGTCCAAGCATCTTCAAAATTATTGGGCTATTATGGTAACCTGTGTTTTAAAGGTTGCCATAATATCTTTA AA
GATGGCATGTATCTTTAAAGATGGTCGTCCTATGACCTACAAAATGTATCTTTAAAGATGTATCTTTAAAGGTGTTTGTCCTCTGACCTACAAAAAT AATGCATTAGAGATGAGCATTAGACCTGCTG
ATATAAATGGGCCTCTTCTGATAAGTCAGAGGTAAAATACACAACTTTTATAATCCAGATATGTGCTTTTACATTTTGGGAATCCCTTGATCCACTTAGCAAATATCTCACCATGAACAGGGTAA ACA
GATATTCAATATTTTCAATAGGTtgtggTTTTTTTTTTTTGAGAAAGCTTAAATTGTGACACTGAGATGCACACA TTGTGTGTGTGTGTGTGTGTGTGTGTGAATGCAGAGTATAGCATCTCACTGGG
GAGTACTTGACCCGTGTGTGTGTGTGTGTATGTGTACTGACACATTTACAAAATAAAATTCCAATCTTGTAAATACCTTTCCAGAGAATTGAAAAAAAAGCTGATGCCCTAATATATGAAGTTTTTAT
TATCATACTTTAAGTTCTGGAGTACCTGTGCAGAATGTGCAGGTTTGTTACATA aGTATACGTGTGCCATAGTGGTTTGCTGCACCCATCAACCCATCATCTACATTAGGTATTTCTCCTAATGTTAT
CCCTCCCTTAGTCCCACATCCCCTAATAGGCCCCgGTGTGTGATGTTCCTCTCCCTGTGTCCATGTGTTCTCATTGTTCAA cTCCCACTTATGAGTGAGAACATGCAGTTTTGATTTTCTCTTCTTG
TGTTCGTTTGCTGAGAATGATGTTATCTAGCTTCATCCATGTCCCTGCAAAGGACA Tgaactcatcctttttttttttttttttttttttttttttttttttttttacccgccactacgcccggctaatt
ttttgtatttttagtagagacggggtttcaccgttttagccgggatggtctcgatctcctgacctcgtgatccgcccgcctcggcctcccaaagtgctgggattacaggcgtgagccaccgcgcc cgg
cccGAACTCATCCTTTTTATGGTTGCTTAGTATTCCATGGTTTATATGTGCCACATTTTCTTTATGCAGTCTATCAT TGATTGACATTTGGGTTGGTTCCAAGTCTTTGCTATTGTGAACAATGCTGC
AATAAACATAGCTGTGCATGTGTCTTTATAGTAGAATGATTTATAATCCTCTAGGTATATAACCAGTAATGGGATTTCTAGGTCAAATGGTATTTCTAGTTCTAGATCCTTGAGGAATCGCCACA TTG
TCTTCCACAATGGTTGAACTAATTTGCACTCCTACCAACAGTGTAAAAGTGTTCCTATTTCTCCACATTCTCTCCAG CATCTGTTGTTTCCTGACTTTTTAATGATCGCCATTCTAACTGGATTGAGG
TGGTTTTGATTTGCATTTCTCTAATGACCAGTGATGGTGAGGTGTTTTTCATAATTGTTGGCCACATAAATGTCTTCTTTTGAGAAGTGTCTGTTCATATCCTTCACCCACTTTTTGATGGGGTT GTT
TTTTTCTTGTAAATTTAAGTTCTTTGTAGATTCTGGATATTAGCCCTTTGTCAGATAAAGAGATTGCAAAAACTTTC TCCCATTCTGTAGGTTGCCTGTTCACTCTGATGATAGTTTCTTTTGCTATG
CAGAAGCTCTTTAGTTTAATTAGATCCCATTTGTCTATTTTGGCTTTTGTTGCCATTGCTTTTGGTGTTTTAGTCATGAGGTCTTTGCCCACACCTATGTCCTGAGTGGTATTGCCTAGGTTTTC TTC
CAGGGTTTTTATGGTTTTAGGTGTTACATTTAGGTCTGTAATCCATCTTGAGTTAATTTTTGTATAAGGTGCTAGGA AAGGGTCCAGTGTCAGTTTTCTGCATATGGCTAGCCAGTTTTCCCAACAGC
ATTTATTAAATAGGGAATCCTTTTCCAATTGCTTGTTTTTGTCAGATTTGTCAAAGATCAGATGGTTGTGGATGTGTGGTGTTATTTCCGAAGTTTCTGTTC tGTT

30	0.0	0.0	0.0	AluYa5-Scaffold7857-1475280-1475433	461	486	(1668)	+	(TG)n	Simple_repeat	1	26	(0)	1
18	4.8	0.0	0.0	AluYa5-Scaffold7857-1475280-1475433	526	547	(1607)	+	(GT)n	Simple_repeat	1	22	(0)	2
2555	8.5	0.0	0.0	AluYa5-Scaffold7857-1475280-1475433	634	963	(1191)	C	L1PA7	LINE/L1	(5)	6149	5820	3
40	0.0	0.0	0.0	AluYa5-Scaffold7857-1475280-1475433	964	1000	(1154)	+	(T)n	Simple_repeat	1	37	(0)	4
1469	0.0	0.0	0.0	AluYa5-Scaffold7857-1475280-1475433	1002	1154	(1000)	C	AluYa5	SINE/Alu	(158)	153	1	5
4096	7.0	3.2	0.0	AluYa5-Scaffold7857-1475280-1475433	1156	2154	(0)	C	L1PA7	LINE/L1	(325)	5829	4807	3

>chr8:13974455-13976417
CCATTACTGCTTTTTGCAATTTATAACGATTAAAATTATCCATAGTTTACAGGGAAGTCCAAGCATCTTCAAAATTATTGGGCTATTATGGTAACCTGT GTTTTAAAGGTTGCCATAATATCTTTAAA
GATGGCATGTATCTTTAAAGATGGTCGTCCTATGACCTACAAAATGTATCTTTAAAGATGTATCTTTAAAGGTGTTTGTCCTCTGACCTACAAAAATAATGCATTAGAGATGAGCATTAGACCTG CTG
ATATAAATGGGCCTCTTCTGATAAGTCAGAGGTAAAATACACAACTTTTATAATCCAGATATGTGCTTTTACATTTTGGGAATCCCTTGATCCACTTAG CAAATATCTCACCATGAACAGGGTAAACA
GATATTCAATATTTTCAATAGGTagtgtttTTTTTTTTTTTTGAGAAAGCTTAAATTGTGACACTGAGATGCACACATctgtgtgtgTGTGTGTGTGTGTGTGTGTGTGTGAATGCAGAGTATAGC
ATCTCACTGGGGAGTACTTGACCCGTGTGTGTGTGTGTGTATGTGTACTGACACATTTACAAAATAAAATTCCAATCTTGTAAATACCTTTCCAGAGAA TTGAAAAAAAAGCTGATGCCCTAATATAT
GAAGTTTTTATTATCATACTTTAAGTTCTGGAGTACCTGTGCAGAATGTGCAGGTTTGTTACATA gGTATACGTGTGCCATAGTGGTTTGCTGCACCCATCAACCCATCATCTACATTAGGTATTTCT
CCTAATGTTATCCCTCCCTTAGTCCCACATCCCCTAATAGGCCCCaGTGTGTGATGTTCCTCTCCCTGTGTCCATGTGTTCTCATTGTTCAA tTCCCACTTATGAGTGAGAACATGCAGTTTTGATT
TTCTCTTCTTGTGTTCGTTTGCTGAGAATGATGTTATCTAGCTTCATCCATGTCCCTGCAAAGGACA TGAACTCATCCTTTTTATGGTTGCTTAGTATTCCATGGTTTATATGTGCCACATTTTCTTT
ATGCAGTCTATCATTGATTGACATTTGGGTTGGTTCCAAGTCTTTGCTATTGTGAACAATGCTGCAATAAACATAGCTGTGCATGTGTCTTTATAGTAG AATGATTTATAATCCTCTAGGTATATAAC
CAGTAATGGGATTTCTAGGTCAAATGGTATTTCTAGTTCTAGATCCTTGAGGAATCGCCACATTGTCTTCCACAATGGTTGAACTAATTTGCACTCCTACCAACAGTGTAAAAGTGTTCCTATTT CTC
CACATTCTCTCCAGCATCTGTTGTTTCCTGACTTTTTAATGATCGCCATTCTAACTGGATTGAGGTGGTTTTGATTTGCATTTCTCTAATGACCAGTGATGGTGAGGTGTTTTTCATAATTGTTGGCC
ACATAAATGTCTTCTTTTGAGAAGTGTCTGTTCATATCCTTCACCCACTTTTTGATGGGGTTGTTTTTTTCTTGTAAATTTAAGTTCTTTGTAGATTCTGGATATTAGCCCTTTGTCAGATAAAG AGA
TTGCAAAAACTTTCTCCCATTCTGTAGGTTGCCTGTTCACTCTGATGATAGTTTCTTTTGCTATGCAGAAGCTCTTTAGTTTAATTAGATCCCATTTGT CTATTTTGGCTTTTGTTGCCATTGCTTTT
GGTGTTTTAGTCATGAGGTCTTTGCCCACACCTATGTCCTGAGTGGTATTGCCTAGGTTTTCTTCCAGGGTTTTTATGGTTTTAGGTGTTACATTTAGGTCTGTAATCCATCTTGAGTTAATTTT TGT
ATAAGGTGCTAGGAAAGGGTCCAGTGTCAGTTTTCTGCATATGGCTAGCCAGTTTTCCCAACAGCATTTATTAAATAGGGAATCCTTTTCCAATTGCTT GTTTTTGTCAGATTTGTCAAAGATCAGAT
GGTTGTGGATGTGTGGTGTTATTTCCGAAGTTTCTGTTCcGTT

40	0.0	0.0	0.0	hg19	464	497	(1466)	+	(TG)n	Simple_repeat	1	34	(0)	6		
18	4.8	0.0	0.0	hg19	537	558	(1405)	+	(GT)n	Simple_repeat	1	22	(0)	7		
6524	7.4	2.6	0.0	hg19	645	1963	(0)	C	L1PA7	LINE/L1	(5)	6149	480	7	8	

>KSI_AluYa5_25

>Scaffold8353-1406912-1407152
TCCCAAGTAGCTGGGATTACAGGCATGTACCACCACACCCACTAATGTTTGTATTTTCGGTAGAGATGGGGTTTCACCACGTTGGCCAGGCTATTTTCAAACTCCTGACCTCAAGTGATCTGCCTG CC
TCGTCCTCCCAAAGTGCTGGGATTGAAGGCGTGAGTCACCGTGCCCGGCCTGCTTCGTTCATGTAACTAA gTATTTGTGAGGCCCTCAGGTTGTGTTCAGGAGGAAGAGACACAGGAACACAGATGGA
GGGCAAGAGGGACAGATGGCTCTAGCTGGGCTGCCTGGGTGAGATGCTGGGGCCTGGCCAG GACAGTGTCAGTTCCCAGAGGTCCTGGGACAGGGGAGAAGGGAGGTCAGGGTCACAGAGAGAGAGGC
AGGAAGTAGCAAAGGCAGAGTCTGCCTGTGCACCAAGCCCGACCCCCCTGCTCTGCCCCTGGGCCGTCTCT ACCTCACCCCCTCAGCTCCCCCGGTCTGAGAGAGGGGAGGCCGAGC cGGCTGCAGAC
CCAaCCACATGGGGCAGGGGAAGGAGAGGCTGGCATTGGTGGAGCCACCCACTGCACTGCCAGTCATACAAAGAAGTCTATCACACAGAATTATGTGCAGTATTTTTTTTTTTTTGAGA CAGTTTTGAT
CTTGTCTCTCAGGCTGGGTTGCAATGGCACAATCTCGGCTCACTGCAACCTCCACCTCCTGGCTTCAAGCG ATTCTCTGGCCTCAGCCTCCCGAGTAGCTGGGACTACAGGCGGCCGCCACCATGCCC
GGGTAAGTTTTGTATTTTTAGTAGAGAGGTTTCACCATGTTGGCCAGGCTGGTCTCGAACTCCTGACCTCAGGTGATCTGCCCGTCTCGGCCTCCCAATGTGTTGGGATTACAGGCGTGAACCAC CGC
GCCTGGCCTATAGTACTTTTTAATGAACGAAATGACTATGTTACTGGTTTATGTATTTGCTA TATTTTTAgttttttttttttttttttttttttttttgggggggggtctccctctgtcgcccaggc
tggagtgcagtggcgggatctcggctcactgcaagctccgcctcccgggttcacgccattctcctgcctcagcctcctgggctaatttttttgtatttttagtagagacggggtttcaccgtttta gcc
gggatggtctcgatctcctgacctcgtgatccgcccgcctcggcctcccaaagtgctgggattacaggcgt gagccaccgcgcccggcctatttttaGTTTTAAACAAAAAAGTTTAAGAAGTAAAAA
AAAAAATGTTTTTGTTTTTGTTTTCTTTGAGAGGGAGTCTCGCATTGCTGCCCAGGCTGGAGTACAGTGGCATGATCTCAGCTCACTGCAACCTC CGCCTCCCGGGTTCAAGAGATTCTCCTGCCTCA
ACCTCCCTAGTAGCTGGGATTACAGGCGCCCGCCACCGTGTCCGGCTAATTTTTATATTTTTAGTAGAGTT GGGGTTTTGAGGTCATGGTCTCAAACTCTTGATCTCAAGTGATCTGCCCACCTTGGC
CTCCCAAAATGCTGAGATTAGCAGGCATAAGCTACTGCACCCAGCCCCAAAAAAGACTTTAATTAGAAAAAAGCTCATAGAATAAGGATATAAAGAAAATATCTTTGTGCAGCTGTACAGTGTAT TTG
CGTTTTAAGCTAAGTGTTATTACAAGAGTCAAAAAGTGTTTAAGTTGATAAAGTAAAAAAGTTAAACTAAGGTTAATTTATTCTTGAAGAAAGAAGAAT ATTTCTTTATAAACCTAGTGTTGCCTAAG
TGTACAGCGTCATGGTTACTCACCACTCACTGACGCACCCAGAGCAGCTGCCAGTCCTGCAAGCTCCATGCCTGGGAAATGCCCTATACAGCTATGCCATCTTTTATCTTGTTTTTTGTTTTTTT TTT
CCCAAGACAGAATTTTGATCTTGTTGCCCAGGCTGGAGTGCAACGGTGCGATCTCGGCTCACCGCA tCCTCTGCCTCCTGGGTTCAAGCAATTCTCCTGCCTCAGCCACCTGAGTAGCTGGGATTACA
GGCGCCCACCACCACGCCCAGTTACTTTTTGTATTTTTAGTAGAGACGGGGTTTCATCATGTTGGCCAGGCTGGTCGCTAACTCCTGACCTCAGGTGATCCACCCTGCTCGGCCTCCCAAAGTGC TGG
GATTACAGGCTTGAGCCACTGCACCCGGCCCATCTTTTATCTTTTACACCAGATTTTACTGCAC C

1284	12.9	0.6	0.0	UnnamedSequence	1	178	(2063)	C	AluSz	SINE/Alu	(133)	179	1	1
556	15.2	0.9	2.6	UnnamedSequence	559	614	(1627)	+	Tigger3	DNA/TcMar-Tigger	175	228	(2801)	2
2233	8.3	1.7	0.0	UnnamedSequence	615	904	(1337)	C	AluSq2	SINE/Alu	(18)	295	1	3
556	15.2	0.9	2.6	UnnamedSequence	905	962	(1279)	+	Tigger3	DNA/TcMar-Tigger	229	287	(2742)	2
30	0.0	0.0	0.0	UnnamedSequence	968	995	(1246)	+	(T)n	Simple_repeat	1	28	(0)	4
1962	0.8	14.5	0.0	UnnamedSequence	1001	1241	(1000)	C	AluYa5	SINE/Alu	(35)	276	1	5
1142	9.5	8.3	4.4	UnnamedSequence	1245	1288	(953)	+	Tigger3b	DNA/TcMar-Tigger	535	575	(656)	6
2007	12.9	3.1	0.3	UnnamedSequence	1289	1582	(659)	C	AluSx1	SINE/Alu	(10)	302	1	7
1142	11.7	11.2	3.9	UnnamedSequence	1583	1893	(348)	+	Tigger3b	DNA/TcMar-Tigger	576	809	(422)	6
2343	11.2	0.0	0.0	UnnamedSequence	1894	2206	(35)	C	AluSq2	SINE/Alu	(0)	313	1	8
821	17.2	18.3	2.8	UnnamedSequence	2207	2241	(0)	+	Tigger3	DNA/TcMar-Tigger	2744	2775	(254)	6

>chr2:85539319-85541281
TCCCAAGTAGCTGGGATTACAGGCATGTACCACCACACCCACTAATGTTTGTATTTTCGGTAGAGATGGGGTTTCACCACGTTGGCCAGGCTATTTTCAAACTCCTGACCTCAAGTGATCTGCCTG CC
TCGTCCTCCCAAAGTGCTGGGATTGAAGGCGTGAGTCACCGTGCCCGGCCTGCTTCGTTCATGTAACTAA aTATTTGTGAGGCCCTCAGGTTGTGTTCAGGAGGAAGAGACACAGGAACAC AGATGGA
GGGCAAGAGGGACAGATGGCTCTAGCTGGGCTGCCTGGGTGAGATGCTGGGGCCTGGCCAGGACAGTGTCAGTTCCCAGAGGTCCTGGGACAGGGGAGAAGGGAGGTCAGGGTCACAGAGAGAGA GGC
AGGAAGTAGCAAAGGCAGAGTCTGCCTGTGCACCAAGCCCGACCCCCCTGCTCTGCCCCTGGGCCGTCTCTACCTCACCCCCTCAGCTCCCCCGGTCTGAGAGAGGGGAGGCCGAGC aGGCTGCAGAC
CCAgCCACATGGGGCAGGGGAAGGAGAGGCTGGCATTGGTGGAGCCACCCACTGCACTGCCAGTCATACAAAGAAGTCTATCACACAGAATTATGTGCAGTATTTTTTTTTTTTTGAGA CagAGTTTTG
ATCTTGTCTCTCAGGCTGGGTTGCAATGGCACAATCTCGGCTCACTGCAACCTCCACCTCCTGGCTTCAAGCGATTCTCTGGCCTCAGCCTCCCGAGTAGCTGGGACTACAGGCGGCCGCCACCA TGC
CCGGGTAAGTTTTGTATTTTTAGTAGAGAGGTTTCACCATGTTGGCCAGGCTGGTCTCGAACTCCTGACCTCAGGTGATCTGCCCGTCTCGGCCTCCCAATGTGTTGGGATTACAGGCGTGA ACCACC
GCGCCTGGCCTATAGTACTTTTTAATGAACGAAATGACTATGTTACTGGTTTATGTATTTGCTA TATTTTTAttGTTTTAAACAAAAAAGTTTAAGAAGTAAAAAAAAAAATGTTTTTGTTTTTGTTT
TCTTTGAGAGGGAGTCTCGCATTGCTGCCCAGGCTGGAGTACAGTGGCATGATCTCAGCTCACTGCAACCTCCGCCTCCCGGGTTCAAGAGATTCTCCTGCCTCAACCTCCCTAGTAGCTGG GATTAC
AGGCGCCCGCCACCGTGTCCGGCTAATTTTTATATTTTTAGTAGAGTTGGGGTTTT GAGGTCATGGTCTCAAACTCTTGATCTCAAGTGATCTGCCCACCTTGGCCTCCCAAAATGCTGAGATTAGCA
GGCATAAGCTACTGCACCCAGCCCCAAAAAAGACTTTAATTAGAAAAAAGCTCATAGAATAAGGATATAAAGAAAATATCTTTGTGCAGCTGTACAGTGTATTTGCGTTTTAAGCTAAGTGT TATTAC

AAGAGTCAAAAAGTGTTTAAGTTGATAAAGTAAAAAAGTTAAACTAAGGTTAATTTATTCTTGAAGAAAGAAGAATATTTCTTTATAAACCTAGTGTTGCCTAAGTGTACAGCGTCATGGTTACT CAC
CACTCACTGACGCACCCAGAGCAGCTGCCAGTCCTGCAAGCTCCATGCCTGGGAAATGCCCTATACAGCTATGCCATCTTTTATCTTGTTTTTTGTTTTTTTTT TcCCCAAGACAGAATTTTGATCTT
GTTGCCCAGGCTGGAGTGCAACGGTGCGATCTCGGCTCACCGCA aCCTCTGCCTCCTGGGTTCAAGCAATTCTCCTGCCTCAGCCACCTGAGTAGCTGGGATTACAGGCGCCCACCACCACGCCCAGT
TACTTTTTGTATTTTTAGTAGAGACGGGGTTTCATCATGTTGGCCAGGCTGGTCGCTAACTCCTGACCTCAGGTGATCCACCCTGCTCGGCCTCCCAAAGTGCTGGGATTACAGGCTTGAGC CACTGC
ACCCGGCCCATCTTTTATCTTTTACACCAGATTTTACTGCACC

1285	12.9	0.6	0.0	hg19	1	178	(1785)	C	AluSz	SINE/Alu	(133)	179	1	10	
610	14.0	5.7	2.3	hg19	559	614	(1349)	+	Tigger3	DNA/TcMar-Tigger	175	228	(2801)	11	
2284	8.6	1.0	0.0	hg19	615	906	(1057)	C	AluSq2	SINE/Alu	(18)	295	1	12	
610	14.0	5.7	2.3	hg19	907	973	(990)	+	Tigger3	DNA/TcMar-Tigger	229	302	(2727)	11	*
1252	11.0	8.0	5.1	hg19	944	1009	(954)	+	Tigger3b	DNA/TcMar-Tigger	515	575	(656)	13	
1990	12.9	3.1	0.3	hg19	1010	1303	(660)	C	AluSx1	SINE/Alu	(10)	302	1	14	
1252	11.0	8.0	5.1	hg19	1304	1523	(440)	+	Tigger3b	DNA/TcMar-Tigger	576	809	(422)	13	
621	15.4	3.9	4.7	hg19	1523	1615	(348)	+	Tigger3b	DNA/TcMar-Tigger	850	946	(285)	15	*
2339	11.2	0.0	0.0	hg19	1616	1928	(35)	C	AluSq2	SINE/Alu	(0)	313	1	16	
621	15.4	3.9	4.7	hg19	1929	1963	(0)	+	Tigger3b	DNA/TcMar-Tigger	947	977	(254)	15	

>KSI_AluYa5_26

>Scaffold9154-1740349-1740570
AAAAAATTAACACAAGAAAATGCAGCAATGGCTTCAAGACAGCGTATTCAGAAGCAAAGCTGACCCTTTACCATATCACCCTAGTGGATCCATCACTCATCACTACACCATCCGATACACAGGGTC AC
CAACTCTCAGGCTTCCAATTTTTTCCACTGAATAATAGATCCCAAAAAGTGGAGACAACTTGTACAATTCCCTCTCAGAAGGATCACACAGGCGGTAGCTGAAACAAGCAAAAATTCAATCATCA GAC
ACAAAAAGGAAATAATAATCGAAGTATTCTCAATAGAACTCAAGTTTGCCTTCCAAGTTATTTATAAATACACAGCAAGAATTTTCTAAAATGTTGCGAGAGAAGTCTGTGGACAATTAGTCAAAC TG
CAAATAATTCTAGTTTTTCTAGAAGTATCCTGAAAGTCTACGCAATTTCTCCATGGCTTCAGTTTAAGTCCAAAAAGAAAAAAACTTATGTGTAAGTATAAAATAAATACTTTGTCTATATGGGT ACC
TTCAACTTCACAACTCATTGAAACAAATGCCCCTAAAACTCAAAATGGAATGGCCCTGAACTCTACAAATCTTCTGTTTAGTGCATTTAAGCACCATGCTTTCAACCCCCTCCAGGTTTATGAGCC CA
AAGCTGGGGTTCACTGGTGAACAAAAATTCAGACAGACATGAAGTTGGCAGATGGGTCTGGACTACTGCAGATGTTAATCTTTTTCCAGTGTCCTGTCTAAAGAAAACAATAACTTTCACAGCAT CTT
AAATAATTATCACTACATCTTAAAAATCAAAAACAAAATAGAATTGTAAATCTGAAGTCATCATGAAATAACCTCTTTTCTAAGCTAAACCCATTGGAAACAATCCACAAAACAGAGGAAAGAGAA AC
AAAATAGAATACAGCTCCAAAGAATGTTAAGCCTAAATCACTATCTTCGTTGGGAACTAAAGTCAGCCAAACTTCTTTAAATAACAAAACAAAAGAGAAATCCTggccgggcgcggtggctcacgcct
gtaatcccagcactttgggaggccgaggcgggcggatcacgaggtcaggagatcgagaccatcccggctaaaacggtgaaaccccgtctctactaaaaatacaaaaaattagccgggcgtagtggc gg
gcgcctgtagtcccagctactcgggaggctgaggcaggagaatggcgtgaacccgggaggcgGAGCTTGCAGTGAGCCGAGATCCCGCCACTGCACTCCAGCCTGGGCGACAGAGCGAGACTCCG TCT
CAAAAAAAAAAAaaaaaaaaaaaaaaaaaaaaaaaaaagagaaatcctTAGCATAAAAGAAGCTCCTTGGTAAAAAAGCTCTGACAACTCATTACATTCATTTTTTTCTAATATTTCATTATGCAGT
TTCAATTATTCTCATATTCCTTGATATTGATTCTAATAAAGGAATTTGAGTTGCAATAAACATGATTAAATAAAGTGTTTAAGGCCTACATTTGGAAAGACATATATAAATAGTATTAAAAATTG ATA
TGAGAGATACTAAAATTTCAAAATGTAGTATTATAaAATTAAAAGTTTATCAAAGCTGGGTATGGTGGCTCATGCCTGTAATCCCAGCATGTTGGGAGGCAGGCGAATCACTGAGGCAAGGAGTTTGA
GACCAGCCTGGCCAACATGGTGAAACCCTGTCTCTGCTAAAAATAACAATAATCACCCAGGCATGGTGGCACACACCTGTAGTCCCAGCTACTCAGGAGGCTGAGGCAGGATCACTTGAACCCAG AAA
GTGGAGGTTGCAGTGAGCTAAGATCACGCCACTGCACTCCAGCCTGGGGGACAGAGTGAGGCTCCTCT CAAAAAAAAAAAAAAAAAAGTTTATCCAAAGCACTAGAGGATCCATCCAGAAAAAGAGAGG
AAGCAACAGTGCTCTCATTAACCAATAGACGCGAATCAGTCAGAAGGTAGACTGGAAGGTTCTCCGGAGAGAAAGGTCATATCTTCCCTAAAGCAGACATAGCACCTAAACCCATAGGTAGTTCT GGG
GTGGAGATAATATTTAAAATACACTGGCTGTGGTGGTATTCTACCTCTTCAGGGTGTC CAGTGGCTGTTTCCTGTCTATGACTCCAGTGTCTGGGTCCACCGTTGTCAAAATACACCTGTTATGCAGT
AACCAAGGGTTAGGTGATGCTTAGGAACCAATGAATGGGCCACACCACAGTCCTGGGGTCTAGCTAGGCTCCTTACCTGGGGCATGCCATTACCTTTTTCACTTCTACA C

2044	3.6	0.0	0.0	AluYa5-Scaffold9154-1740349-1740570	1001	1222	(1000)	+	AluYa5	SINE/Alu	1	222	(89)	1
28	6.1	0.0	0.0	AluYa5-Scaffold9154-1740349-1740570	1227	1261	(961)	+	(A)n	Simple_repeat	1	35	(0)	2
1971	12.2	3.8	0.3	AluYa5-Scaffold9154-1740349-1740570	1527	1814	(408)	+	AluSx	SINE/Alu	2	299	(13)	3

>chr1:220953388-220955345
AAAAAATTAACACAAGAAAATGCAGCAATGGCTTCAAGACAGCGTATTCAGAAGCAAAGCTGACCCTTTACCATATCACCCTAGTGGATCCATCACTCATCACTACACCATCCGATACACAGGGTC AC
CAACTCTCAGGCTTCCAATTTTTTCCACTGAATAATAGATCCC AAAAAGTGGAGACAACTTGTACAATTCCCTCTCAGAAGGATCACACAGGCGGTAGCTGAAACAAGCAAAAATTCAATCATCAGAC
ACAAAAAGGAAATAATAATCGAAGTATTCTCAATAGAACTCAAGTTTGCCTTCCAAGTTATTTATAAATACACAGCAAGAATTTTCTAAAATGTTGCGAGAGAAGTCTGTGGACAATTAGTCAAA CTG
CAAATAATTCTAGTTTTTCTAGAAGTATCCTGAAAGTCTACGC AATTTCTCCATGGCTTCAGTTTAAGTCCAAAAAGAAAAAAACTTATGTGTAAGTATAAAATAAATACTTTGTCTATATGGGTACC
TTCAACTTCACAACTCATTGAAACAAATGCCCCTAAAACTCAAAATGGAATGGCCCTGAACTCTACAAATCTTCTGTTTAGTGCATTTAAGCACCATGCTTTCAACCCCCTCCAGGTTTATGAGC CCA
AAGCTGGGGTTCACTGGTGAACAAAAATTCAGACAGACATGAA GTTGGCAGATGGGTCTGGACTACTGCAGATGTTAATCTTTTTCCAGTGTCCTGTCTAAAGAAAACAATAACTTTCACAGCATCTT
AAATAATTATCACTACATCTTAAAAATCAAAAACAAAATAGAATTGTAAATCTGAAGTCATCATGAAATAACCTCTTTTCTAAGCTAAACCCATTGGAAACAATCCACAAAACAGAGGAAAGAGA AAC
AAAATAGAATACAGCTCCAAAGAATGTTAAGCCTAAATCACTATCTTCGTTGGGAACTAAAGTCAGCCAAACTTCTTTAAATAACAAAACAAAAGAGAAATCCTTAGCATAAAAGAAGCTCCTTGGTA
AAAAAGCTCTGACAACTCATTACATTCATTTTTTTCTAATATTTCATTATGCAGTTTCAATTATTCTCATATTCCTTGATATTGATTCTAATAAAGGAATTTGAGTTGCAATAAACATGATTAAA TAA
AGTGTTTAAGGCCTACATTTGGAAAGACATATATAAATAGTATTAAAAATTGATATGAGAGATACTAAAATTTCAAAATGTAGTATTATA gAATTAAAAGTTTATCAAAGCTGGGTATGGTGGCTCAT
GCCTGTAATCCCAGCATGTTGGGAGGCAGGCGAATCACTGAGGCAAGGAGTTTGAGACCAGCCTGGCCAACATGGTGAAACCCTGTCTCTGCTAAAAATAACAATAATCACCCAGGCATGGTGGC ACA
CACCTGTAGTCCCAGCTACTCAGGAGGCTGAGGCAGGATCACTTGAACCCAGAAAGTGGAGGTTGCAGTGAGCTAAGATCACGCCACTGCACTCCAGCCTGGGGGACAGAGTGAGGCTCCTCT CaAAA
AAAAAAAAAAAAAAGTTTATCCAAAGCACTAGAGGATCCATCCAGAAAAAGAGAGGAAGCAACAGTGCTCTCATTAACCAATAGACGCGAATCAGTCAGAAGGTAGACTGGAAGGTTCTCCGGAG AGA
AAGGTCATATCTTCCCTAAAGCAGACATAGCACCTAAACCCATAGGTAGTTCTGGGGTGGAGATAATATTTAAAATACACTGGCTGTGGTGGTATTCTACCTCTTCAGGGTGTCCAGTGGCTGTT TCC
TGTCTATGACTCCAGTGTCTGGGTCCACCGTTGTCAAAATACACCTGTTATGCAGTAACCAAGGGTTAGGTGATGCTTAGGAACCAATGAATGGGCCACACCACAGTCCTGGGGTCTAGCTAGGC TCC
TTACCTGGGGCATGCCATTACCTTTTTCACTTCTACAC

1980	12.1	3.8	0.3	hg19	1262	1550	(408)	+	AluSx	SINE/Alu	2	300	(12)	4

>KSI_AluYa5_27

>Scaffold9449-191631-191907
TCTTTTTTGTTTGGGATCTGATTCAAGAGCCTCAATTCAGCTTATTGCAGAACTAACTAAACTAAACCTCAAAGGAAAAATTTTAAAAATGATTTTTATAGCAATGACATCATTATATAAAATGTT GG
CATTTCATATTTTCCAAACAGAAACTTGGAAATAACAGAATACAAGTTAAATAAAACAAATTGAAACTAATCCAATTCTCTGTTTTTCTTTCCCTTTTTAAAAACCAAGCTTTTTATTGCCAGCT TTT
ACTTTGCATAATAATATATGATGAATTGTCCAAGAAAGACCCAGTGGGTACATTTCTCTTAGAAATAAGCCTCTCTATGCAATAGGAGGCCAAGTTCTAGGGCAACATACTTATATAATCATGTAA GA
TTTTCCTTATTGCTAACAATTGACCAACCTGGGCATTTGTCAAATATGTAAACACATGGAGGTCACTGGTCACCAGAGGATTGAAAACACAAATCACTCTATTCATATCCATCCCTAGTTCTAGAAAA
ACAATACCAAATTAAAGCATATATCCTACTAGCAATAGGACTAGCAAAATTATTTTGGTATACCAGAGCTACATGTTATACATTTTGCTACTTATGGATTTAATTACTCAGGATAATTATACACAT GG
CTACAACTGTAAACTTTATAATATTTTGCTGGCATACCTTCCAAAAGAGTTTGAAAAGCTATGAATTTAATTCTATATTTGCATTTGACATATAAAGTACTTCATTGTAATTTGAAATAGTAGTC AAA
GATATAATTTTCATTATAAATATTACTATTTTATAATTTTATAAAATTTATCACAAAAATTGAAACACTTTTATAAATAGATATCAAATAGAAAAATAAAAATATTATTGAAAATGTATCTCTTGA cA
GATGTATGGGATTTCCTTTCCCCAATTATTCACTAACACATGAATGAATATGTTTTATTGCAAGAAGGAGGCAGGGTTAAGTGGCAATT AAAAAAAGAAAAGGCgcactttgggaggccgaggcgggc
ggatcacgaggtcaggagatcgagaccatcccggctaaaacggtgaaaccccgtctctactaaaaatacaaaatattagccgggcgtagtggcgggcgcctgtagtcccagctacttgggaggctg ag
gcaggagaatggcgtgaacccgggaggcggagcttgcagtgagccgagatcccgccactgcactccagcctgggcgacagagcgagactccgtctcaaaaa aaaaaaaaaaaaaaaaaaagaaaaggc
ATTAGGCTACTACCTAGCATGGGGCTTATAAACATTATTAAGAATTTTTGTTAAGAGCATTACTTACAGGAGAATTTATCATTTATTTGTAAGTTTTGCCAAATAAATAAACAGTATTAATCAACATA
CAGTAGTGATAAATTAGCAGATCAAAAATTGATGAGAGCAATTTGTGAAAAAATTATTGAAAATGTTTTTGGAGCCTGAATAAATAATTAGAAAAAACAAAATGTTAACTAACCCCACTATATATTTC
TTTTATCTAAAAATGTAATATTGATTTATAAGAAATAATAATAAAACTTCTAATCTCTTCTTAAAATCATGTAAAATATACAACTGAGATATAGACTGAGATACAAGGTAATTGAGAATATTCATC AA
ACCCTTTTTTATTGGATATGGGAAAAATAAAGAGATGGTGTGTTTTGCAGTAGAGTGAAATTTTCCAGCATGCTTTTTAATAAACTTTGAATATTCCAATCATTAGATAGATAGCTAGATAGGCA GAC
AGATAGATAGATAGATGATAGACAGGCCGGGTGCAGTGGCTCATGCCTATAATCCCGGCACTCTAGGAGGTTGAGGCAGGTGGATAACTTGAGGTCAGGAGTTCAAGACCAGCCTGGCCAAAATGG TG
AAACCCCACCCTGTCTGTACTAATAATACAAAAATTAGCTGGGTGTGGTGGTGACC tCCTGTAATCCCAGCTACTCGAGAGGCTAGATACAAGAATCATTTGAACCTGGGAGACAGAGGTTGCAGTGA
GCTGAGATCGTTCCACTGCATTCCAGCCTGGGCAACACAGCAAGACTCTGTCT CAAAAAAAAAAAAAAGATAGATAGACAGATAGATAGATAGATAGGGCACACAGCTTTGCCATGAGTTTACTGCCTG
GGTGAAATCAGGCATTGGAGGCTACAGAATTTCTTATTGATGATATTTGCTTTGCTTCCATAGATTTATCACTCTGGGTGACACATTCTTTAACAATAGT T

231	29.9	0.0	8.6	AluYa5-Scaffold9449-191631-191907	488	575	(1702)	+	MamSINE1	SINE/tRNA-RTE	197	277	(13)	1
496	25.3	3.6	6.5	AluYa5-Scaffold9449-191631-191907	664	985	(1292)	C	Charlie2b	DNA/hAT-Charlie	(503)	2279	2071	2
2601	0.7	0.0	0.0	AluYa5-Scaffold9449-191631-191907	1001	1277	(1000)	+	AluYa5	SINE/Alu	34	310	(1)	3
1390	29.2	3.9	1.1	AluYa5-Scaffold9449-191631-191907	1445	1767	(510)	C	Charlie2b	DNA/hAT-Charlie	(1765)	1017	683	4
27	9.2	2.1	0.0	AluYa5-Scaffold9449-191631-191907	1768	1814	(463)	+	(TAGA)n	Simple_repeat	1	48	(0)	5
2014	13.4	0.3	1.7	AluYa5-Scaffold9449-191631-191907	1817	2114	(163)	+	AluSz	SINE/Alu	1	294	(18)	6
27	3.5	0.0	0.0	AluYa5-Scaffold9449-191631-191907	2115	2144	(133)	+	(AGAT)n	Simple_repeat	1	30	(0)	7
1390	29.2	3.9	1.1	AluYa5-Scaffold9449-191631-191907	2145	2276	(1)	C	Charlie2b	DNA/hAT-Charlie	(2102)	680	546	4

>chr1:219573680-219575677
TCTTTTTTGTTTGGGATCTGATTCAAGAGCCTCAATTCAGCTTATTGCAGAACTAACTAAACTAAACCTCAAAGGAAAAATTTTAAAAATGATTTTTATAGCAATGACATCATTATATAAAATGTT GG
CATTTCATATTTTCCAAACAGAAACTTGGAAATAACAGAATACAAGTTAAATAAAACAAATTGAAACTAATCCAATTCTCTGTTTTTCTTTCCCTTTTTAAAAACCAAGCTTTTTATTGCCAGCT TTT
ACTTTGCATAATAATATATGATGAATTGTCCAAGAAAGACCCAGTGGGTACATTTCTCTTAGAAATAAGCCTCTCTATGCAATAGGAGGCCAAGTTCTAGGGCAACATACTTATATAATCATGTAA GA
TTTTCCTTATTGCTAACAATTGACCAACCTGGGCATTTGTCAAATATGTAAACACATGGAGGTCACTGGTCACCAGAGGATTGAAAACACAAATCACTCTATTCATATCCATCCCTAGTTCTAGAAAA
ACAATACCAAATTAAAGCATATATCCTACTAGCAATAGGACTAGCAAAATTATTTTGGTATACCAGAGCTACATGTTATACATTTTGCTACTTATGGATTTAATTACTCAGGATAATTATACACAT GG
CTACAACTGTAAACTTTATAATATTTTGCTGGCATACCTTCCAAAAGAGTTTGAAAAGCTATGAATTTAATTCTATATTTGCATTTGACATATAAAGTACTTCATTGTAATTTGAAATAGTAGTC AAA
GATATAATTTTCATTATAAATATTACTATTTTATAATTTTATAAAATTTATCACAAAAATTGAAACACTTTTATAAATAGATATCAAATAGAAAAATAAAAATATTATTGAAAATGTATCTCTTGA tA
GATGTATGGGATTTCCTTTCCCCAATTATTCACTAACACATGAATGAATATGTTTTATTGCAAGAAGGAGGCAGGGTTAAGTGGCAATT AAAAAAAGAAAAGGCATTAGGCTACTACCTAGCATGGGG
CTTATAAACATTATTAAGAATTTTTGTTAAGAGCATTACTTACAGGAGAATTTATCATTTATTTGTAAGTTTTGCCAAATAAATAAACAGTATTAATCAACATACAGTAGTGATAAATTAGCAGAT CA
AAAATTGATGAGAGCAATTTGTGAAAAAATTATTGAAAATGTTTTTGGAGCCTGAATAAATAATTAGAAAAAACAAAATGTTAACTAACCCCACTATATATTTCTTTTATCTAAAAATGTAATAT TGA
TTTATAAGAAATAATAATAAAACTTCTAATCTCTTCTTAAAATCATGTAAAATATACAACTGAGATATAGACTGAGATACAAGGTAATTGAGAATATTCATCAAACCCTTTTTTATTGGATATGGG AA
AAATAAAGAGATGGTGTGTTTTGCAGTAGAGTGAAATTTTCCAGCATGCTTTTTAATAAACTTTGAATATTCCAATCATTAGATAGATAGCTAGATAGGCAGACAGATAGATAGATAGATGATAG ACA
GGCCGGGTGCAGTGGCTCATGCCTATAATCCCGGCACTCTAGGAGGTTGAGGCAGGTGGATAACTTGAGGTCAGGAGTTCAAGACCAGCCTGGCCAAAATGGTGAAACCCCACCCTGTCTGTACTA AT
AATACAAAAATTAGCTGGGTGTGGTGGTGACCaCCTGTAATCCCAGCTACTCGAGAGGCTAGATACAAGAATCATTTGAACCTGGGAGACAGAGGTTGCAGTGAGCTGAGATCGTTCCACTGCATTCC

AGCCTGGGCAACACAGCAAGACTCTGTCTCaAAAAAAAAAAAAAGATAGATAGACAGATAGATAGATAGATAGGGCACACAGCTTTGCCATGAGTTTACTGCCTGGGTGAAATCAGGCATTGGAGGCT
ACAGAATTTCTTATTGATGATATTTGCTTTGCTTCCATAGATTTATCACTCTGGGTGACACATTCTTTAACAATAGT T

231	29.9	0.0	8.6	hg19	488	575	(1423)	+	MamSINE1	SINE/tRNA-RTE	197	277	(13)	8
506	25.1	3.6	6.5	hg19	664	985	(1013)	C	Charlie2b	DNA/hAT-Charlie	(503)	2279	2071	9
1390	29.2	3.9	1.1	hg19	1165	1487	(511)	C	Charlie2b	DNA/hAT-Charlie	(1765)	1017	683	10
27	9.2	2.1	0.0	hg19	1488	1534	(464)	+	(TAGA)n	Simple_repeat	1	48	(0)	11
2034	13.4	0.3	1.7	hg19	1537	1835	(163)	+	AluSz	SINE/Alu	1	295	(17)	12
27	3.5	0.0	0.0	hg19	1836	1865	(133)	+	(AGAT)n	Simple_repeat	1	30	(0)	13
1390	29.2	3.9	1.1	hg19	1866	1997	(1)	C	Charlie2b	DNA/hAT-Charlie	(2102)	680	546	10

>KSI_AluYa5_28

>Scaffold9661-2389101-2389391
CAGATTTAAATGGCCCGTTAACCATATTTCAACATAATATTTTACTTATATAGATGGGGAGAACTTTAAAAATCTAGATTTATCTGTAGTGGTTTTTAAATGAATTTTTCTTTAATATGTGCACCA TC
CTTAAATATAGCTATCCTTAGAAATATACATGCGGCCGGGCGCAGTGGCTCACGCCTGTAATCCCAGCACTTTGGGAGGCCAAGGCGGGCGGATCACAAGGTCAGGAGAT tGAGACCATCCTGGCTAA
CACAGTGAAACCCCaTCTCTACTAAAAATACAAAAAATTAGCCGGGCgTGGTGGCAGGCGCCTGTAGTCCCAGCTACTCGGGAGGCTGAGGCAGGAGAATGGCGTGAACCCGGGAGGCGGAGCTTGCA
GTGAGCTGAGATCGTGCCACTGCACTCCAGCCTGGGTGACAGCGTGAGACTCTGTCTCAAAAAAAAAAGAAAAAAAAAAATATATATGTAT GTATATATATATATATTTATACATGCTCAAATATAACA
ATACTCAGTGACTTACCACTATATTATAATGTAAACAAAGTAAAATTATTAAGCTGATGTAGTGAAAATCTACACATGAAGAGTCATTATTCTTAAAAAATTCAAAGTATTTAAGGAGGAGGCACA GT
TTTTCCATAGTTTAAATGTTTAGTTTTGCTTGAAAACCATAAAACATTTTGTTTTGTAATAAATTATTAATTAGAAGATTATGTTAGCTTTCCAGGCAAAAGTTGAA GCCTCATTGTTAGCCTCAGC
ATCTTGATTTGGTAAAAATTGAAAATGAAGAGTCAAAACAATATTAGTGGATTGAGGTCATAAAAAATTAGCAAAAGCTTTAATTTGTTGATGGAAATCATGCCATTGTTATCCAAATGCGAAAA GC
TCCTAATAGCAAATGTAGTATCATTAGTGTTTTTTTTTTTGGCCTTTTTTTTGGTTTGTTTTTCATCTCTTGGATTCTTGCTGTTCAGTAAGATCCAGTTTGTAggccgggcgcggtggctcacgcct
gtaatcccagcactttgggaggccgaggcgggcggatcacgaggtcaagagatcgagaccatcccggctaaaacggtgaaccccgtctctactaaaaatacaaaaaaat taGCCGGGCGTAGTGGCG
GGCGCCTGTAGTCCCAGCTACTTGGGAGGCTGAGGCAGGAAAATGGCGTGAACCCGGGAGGCGGAGCTTGCAGTGAGCCGAGATCCCGCCACTGCACTCCAGCC TGGGCGACAGAGCGAGACTCCGTC
TCAAAAAAAAAAAAAAAAAAAAAAAaagatccagtttgtaTTCATTCATTGTCTGCATTTTGGTTTATAGATATACAATTGTTCAGAAAAAGATGTGCTCAAAGTGCCTATAATTCATATCTTTTGCT
AAATAGTCAAGAGAAAATCTTTCAATGTTGTTATTTCATTTTATGGTAAACAACAGAGATATCCCCCTTTTTATAATGATCTTACTGTGGATTATTTATATTAT TTTGCTTTCTTTTGATAGTAAAAT
GTAGAACTATTATAAAATAAAGTTTTAATGTTTTTGATCATGCCTATTTGGCAGCAGTTTATAATAGCAAATTGAATATACTTAAAAGTATACTTACATGTACTACATATGGGTAATTTAAAGTA TTT
TCATGGCAATAGTATCAATGGTGAATTTATAGTTTCCTAATCTATGTAAATAATATTAGTAAATTACCGGAGATAATTACTTGTAAGTGGCTTAAAATATGTTT TTTATGTCAATAATCTATAAATTA
AGAACCATAGATTTATTATATTTCTACTAACCTTCCATGAAGATTAATATATGTGACCAGTAGGTTGAATAAACCTGAGTCATGACATGAAATTTTTAATTGGAAGAAAATGTCCCATACCTTTT TAT
AAAAGCTCTACAGTTTTTCCATGGATTTTCTGCTTATGCTTAGCTAATAGTCTTTGAATTACTTGAGTTGTTTGAACATAGTTATTCAACTCACCAAATAGCAT ATGCAACCTATAATAACAATGTAA
ATTTCAATTTGCCCTCTCCTCTCTTATTTCCACCTGGAAGCCTCCAGTAAGATAAAGCAACTACGATTTGACAGTTCTATACATTTTTGTTAGCTCACTTCTTCTCCCACTCCATACTGATGTTA TTT
CTCCAGGTTTCTGGAGAGAAGAGAGGCTAGAACAACAGGTAACGAAATATAACTACTCTTGCTGAGCTGGCCACAGATGTTTTAAGTGCTGCTTTCATTTTTGG TATACCTTCCTTTCACAGACTCT

22	27.2	0.0	0.0	AluYa5-Scaffold9661-2389101-2389391	131	161	(2130)	+	(TATA)n	Simple_repeat	1	31	(0)	1
2675	4.6	0.0	0.0	AluYa5-Scaffold9661-2389101-2389391	162	463	(1828)	+	AluY	SINE/Alu	1	302	(9)	2
22	27.2	0.0	0.0	AluYa5-Scaffold9661-2389101-2389391	464	498	(1793)	+	(TATA)n	Simple_repeat	4	38	(0)	3
1248	42.3	3.8	0.3	AluYa5-Scaffold9661-2389101-2389391	1001	1291	(1000)	+	AluYa5	SINE/Alu	1	301	(10)	4

>chr5:106179911-106181896
CAGATTTAAATGGCCCGTTAACCATATTTCAACATAATATTTTACTTATATAGATGGGGAGAACTTTAAAAATCTAGATTTATCTGTAGTGGTTTTTAAATGAATTTTTCTTTAATATGTGCACCA TC
CTTAAATATAGCTATCCTTAGAAATATACATGCGGCCGGGCGCAGTGGCTCACGCCTGTAA TCCCAGCACTTTGGGAGGCCAAGGCGGGCGGATCACAAGGTCAGGAGAT cGAGACCATCCTGGCTAA
CACAGTGAAACCCCgTCTCTACTAAAAATACAAAAAATTAGCCGGGCaTGGTGGCAGGCGCCTGTAGTCCCAGCTACTCGGGAGGCTGAGGCAGGAGAATGGCGTGAACCCGGGAGGCGGAGCTTGCA
GTGAGCTGAGATCGTGCCACTGCACTCCAGCCTGGGTGACAGCGTGAGACTCTGTCTCAAAAAAAAAAGAAAAAAAAAAATATATATGTAT GtaTATATATATATATATTTATACATGCTCAAATATAA
CAATACTCAGTGACTTACCACTATATTATAATGTAAACAAAGTAAAAT TATTAAGCTGATGTAGTGAAAATCTACACATGAAGAGTCATTATTCTTAAAAAATTCAAAGTATTTAAGGAGGAGGCACA
GTTTTTCCATAGTTTAAATGTTTAGTTTTGCTTGAAAACCATAAAACATTTTGTTTTGTAATAAATTATTAATTAGAAGATTATGTTAGCTTTCCAGGCAAAAGTTGAA GCCTCATTGTTAGCCTCA
GCATCTTGATTTGGTAAAAATTGAAAATGAAGAGTCAAAACAATATTA GTGGATTGAGGTCATAAAAAATTAGCAAAAGCTTTAATTTGTTGATGGAAATCATGCCATTGTTATCCAAATGCGAAAA
GCTCCTAATAGCAAATGTAGTATCATTAGTGtTTTTTTTTTTTGGCCTTTTTTTTGGTTTGTTTTTCATCTCTTGGATTCTTGCTGTTCAGTAAGATCCAGTTTGTATTCATTCATTGTCTGCATTTT
GGTTTATAGATATACAATTGTTCAGAAAAAGATGTGCTCAAAGTGCCT ATAATTCATATCTTTTGCTAAATAGTCAAGAGAAAATCTTTCAATGTTGTTATTTCATTTTATGGTAAACAACAGAGATA
TCCCCCTTTTTATAATGATCTTACTGTGGATTATTTATATTATTTTGCTTTCTTTTGATAGTAAAATGTAGAACTATTATAAAATAAAGTTTTAATGTTTTTGATCATGCCTATTTGGCAGCAGT TTA
TAATAGCAAATTGAATATACTTAAAAGTATACTTACATGTACTACATA TGGGTAATTTAAAGTATTTTCATGGCAATAGTATCAATGGTGAATTTATAGTTTCCTAATCTATGTAAATAATATTAGTA
AATTACCGGAGATAATTACTTGTAAGTGGCTTAAAATATGTTTTTTATGTCAATAATCTATAAATTAAGAACCATAGATTTATTATATTTCTACTAACCTTCCATGAAGATTAATATATGTGACC AGT
AGGTTGAATAAACCTGAGTCATGACATGAAATTTTTAATTGGAAGAAA ATGTCCCATACCTTTTTATAAAAGCTCTACAGTTTTTCCATGGATTTTCTGCTTATGCTTAGCTAATAGTCTTTGAATTA
CTTGAGTTGTTTGAACATAGTTATTCAACTCACCAAATAGCATATGCAACCTATAATAACAATGTAAATTTCAATTTGCCCTCTCCTCTCTTATTTCCACCTGGAAGCCTCCAGTAAGATAAAGC AAC
TACGATTTGACAGTTCTATACATTTTTGTTAGCTCACTTCTTCTCCCA CTCCATACTGATGTTATTTCTCCAGGTTTCTGGAGAGAAGAGAGGCTAGAACAACAGGTAACGAAATATAACTACTCTTG
CTGAGCTGGCCACAGATGTTTTAAGTGCTGCTTTCATTTTTGGTATACCTTCCTTTCACAGACTC T

23	26.2	0.0	0.0	hg19	131	161	(1825)	+	(TATA)n	Simple_repeat	1	31	(0)	5

2690	4.3	0.0	0.0	hg19	162	463	(1523)	+	AluY	SINE/Alu	1	302	(9)	6
23	26.2	0.0	0.0	hg19	464	500	(1486)	+	(TATA)n	Simple_repeat	4	40	(0)	7
16	23.3	0.0	0.0	hg19	928	962	(1024)	+	(T)n	Simple_repeat	1	35	(0)	8

>KSI_AluYa5_29

>Scaffold9676-68882-69153
GGGATAATTGGCTAGCCACAGGTAGGAGAATGAAACTAGATCCTCATCTCTCACCTTATACAAAAATCAACTCAAGATGGATTAAGGACTTAAATCTAAGACCTGAAACTATAAAAACTCTAGAAG AT
AATTTTGGAAAAATGCTTCTAGACATTGGCTTAGGCAAGGATTTCATGACCAAGAACCCAAAAGCAAATATAATAAAAACAAAGATAAATAGTTGGGACTTAATTAAACTAAA GAGTTATTTTGCACG
GCAAAAGGAACAGTCAGCAGAGTAAACAGACAACCCACAGAGTGGGAGAAAATCTTCACAATCTATCCATCTGACAAAGGACTAATATCCGGAATCTACAACGAACTCAAACAAATCAGTAAGAAAAA
AACAAACAATCCCATCAAAAAGTGGGCTAAGGACATGAATAGACAATTCTCAAG gGAAGATATACAAATGGCAAACAAACGTATGCTCAGCATCACTAGTGATCAGGGAAATGCAAATCAAAACCACA
ATGCAACACCACCTTACTCCTGCAAGAATGGCTATAATCAAAAAATCAAAAAACTGTAGATGTTGGCATGGATACGGTGATCAGAGAACACTTCTACACTACTGGTGGTAATGTAAACTAGTACAG CC
ACTAGGGAAAACAGTGTGGAGATTCCTTAAATAACTAAAGTAAGGCCTACCATGGTGGCTCATGCCTGTAATCCCAGCACTTTGGGAGGCC gAGGCAGGTGGATCACATGAGGTCAGGAGTTCAACAC
CAGCCTGACCAACATGGTGAAACCCCATCTCTACTAAAAATACAAAATTAGCCAGGTGTGGTGGCACACACTTATAATCTCAGCTACTTGAGAGGCTGAGGCATGAGAATTGCTTGAACCCGGAAG GT
GGAcATTGCAGTGAGCCAAGATCGTGCCATTGCACTCCAGCGTGGGTGACAGAGCAAGGCTCCATCT Caaaaaaaaaaaagggggactaaaagtagaactaccgggccgggcgcggtggctcacgccc
gtaatcccagcactttgggaggccgaggcgggcggatcacgaggtcaggagatcgagaccatcccggctaaaacggtgaaaccccgtctctactaaaaatacaaaaaattagccgggcgtggtggc ga
gcgcctgtagtcccagctacttgggaggctgaggcaggagaatggggtgaacccgggagggggggctgccgggcagcgtgggtgacagagcaaggctccatctca AAAAAAAAAAAAAAGGGGGACT
AAAAGTAGAACTACCATTCGATCCAGCAATACCACTACTGGGTATAGACCCAGAGGAAAAGAAGTCATTATACAAAAAAGATATTTGCACGCACATGTTTATAGCAGCACAATTCACAATTGCAAAAT
tGTGGAACCAACCCAAATGCCCATCAATCAACAAGTGGATAAAGAAATTGT GGTAGATGAGATTGGAGAC TATTATTGTAAGTGAAGTAGCCCAGGAATGGAAAACCAAACATCATATGTTCTCACTG
ATCTGTGGAAGCTAAGCTATAAAGATACACAGGCATAAGAATGATATAATGGACTTTGGGGACTTTGGGATCTTGGGGGTGAGGGTTGGGGGGATGAGAGATAAAAGACTACAAATAGGGTGCAGT GT
ATACTGCTTGGGTGATGGGTACACCAAAATCTCACAAATCACCACTAAAGAACTCAGTTATGTAACCAAATACTCCCTGTTCCCCAAAAAACCTATGGAAAAATTAAAAAACAAAATAAAATGAA ATA
AAATAAAGTCCTTGTAACATTGAGTTAGAGGGCTTTAACTCCTGGGTCTAAAATGGACACCAAGTCCTGTTAAATTTTAAACACTCACAGCA ATTTTTTTTTTTTTTTTTTGAGACAGGGTCTCGCTC
TGTCaCCCAGGCTgGAGTGCAGTGcCCAATCTcGGCTCACTGCcACCTCCcCCTCCCAGGTTCACGCCATTCTCCTGCCTCAGCCTCTCGAGTAGCTGGGACTACAGGTGCCCACCACCATGCCCGGC
TAATTTTTTGTATTTTTAGTAGAGACAGGGTTTCACCGTGTAAGCCAGGGTG tTCTCGATCTCCTGACCTCGTGATCCACCCGCCTCGGCCTCCCGAAGTGCTGGGATTACAGGCGTGAGCCATCACG
CCCAGCCTAAACACTGACAGCAATTAAAGCCTCATCTTTAGGCCCTGTAGA AGATGCCAATCAAAATAAAATGCATTCCTGAGACACAGGGCAAG A

3763	6.4	1.9	0.3	AluYa5-Scaffold9676-68882-69153	1	693	(1579)	+	L1PB1	LINE/L1	4919	5617	(534)	1
2148	11.3	0.3	0.0	AluYa5-Scaffold9676-68882-69153	694	976	(1296)	+	AluSx	SINE/Alu	11	294	(18)	2
2151	5.2	8.8	0.0	AluYa5-Scaffold9676-68882-69153	1001	1272	(1000)	+	AluYa5	SINE/Alu	1	296	(15)	3
1917	11.2	13.6	2.6	AluYa5-Scaffold9676-68882-69153	1276	1775	(497)	+	L1PB1	LINE/L1	5596	6150	(1)	1
1164	5.2	0.0	11.5	AluYa5-Scaffold9676-68882-69153	1796	1885	(387)	+	MER4-int	LTR/ERV1	4012	4093	(2505)	4
2428	7.4	0.3	0.0	AluYa5-Scaffold9676-68882-69153	1886	2183	(89)	C	AluY	SINE/Alu	(12)	299	1	5
1164	5.2	0.0	11.5	AluYa5-Scaffold9676-68882-69153	2184	2266	(6)	+	MER4-int	LTR/ERV1	4094	4167	(2431)	4

>chr14:23671690-23673669
GGGATAATTGGCTAGCCACAGGTAGGAGAATGAAACTAGATCCTCATCTCTCACCTTATACAAAAATCAACTCAAGATGGATTAAGGACTTAAATCTAAGACCTGAAACTATAAAAACTCTAGAAG AT
AATTTTGGAAAAATGCTTCTAGACATTGGCTTAGGCAAGGATTTCATGACCAAGAACCCAAAAGCAAATATAATAAAAACAAAGATAAATAGTTGGGACTTAATTAAACTAAA GAGTTATTTTGCACG
GCAAAAGGAACAGTCAGCAGAGTAAACAGACAACCCACAGAGTGGGAGAAAATCTTCACAATCTATCCATCTGACAAAGGACTAATATCCGGAATCTACAACGAACTCAAACAAATCAGTAAGAAAAA
AACAAACAATCCCATCAAAAAGTGGGCTAAGGACATGAATAGACAATTCTCAAG aGAAGATATACAAATGGCAAACAAACGTATGCTCAGCATCACTAGTGATCAGGGAAATGCAAATCA AAACCACA
ATGCAACACCACCTTACTCCTGCAAGAATGGCTATAATCAAAAAATCAAAAAACTGTAGATGTTGGCATGGATACGGTGATCAGAGAACACTTCTACACTACTGGTGGTAATGTAAACTAGTACA GCC
ACTAGGGAAAACAGTGTGGAGATTCCTTAAATAACTAAAGTAAGGCCTACCATGGTGGCTCATGCCTGTAATCCCAGCACTTTGGGAGGCC aAGGCAGGTGGATCACATGAGGTCAGGAGTTCAACAC
CAGCCTGACCAACATGGTGAAACCCCATCTCTACTAAAAATACAAAATTAGCCAGGTGTGGTGGCACACACTTATAATCTCAGCTACTTGAGAGGCTGAGGCATGAGAATTGCTTGAACCCGGAA GGT
GGAgATTGCAGTGAGCCAAGATCGTGCCATTGCACTCCAGCGTGGGTGACAGAGCAAGGCTCCATCT CAAAAAAAAAAAAAAGGGGGACTAAAAGTAGAACTACCATTCGATCCAGCAATACCACTAC
TGGGTATAGACCCAGAGGAAAAGAAGTCATTATACAAAAAAGATATTTGCACGCACATGTTTATAGCAGCACAATTCACAATTGCAAAAT cGTGGAACCAACCCAAATGCCCATCAATCAACAAGTGG
ATAAAGAAATTGTGGTAGATGAGATTGGAGACTATTATTGTAAGTGAAGTAGCCCAGGAATGGAAAACCAAACATCATATGTTCTCACTGATCTGTGGAAGCTAAGCTATAAAGATACACAGGCATAA

GAATGATATAATGGACTTTGGGGACTTTGGGATCTTGGGGGTGAGGGTTGGGGGGATGAGAGATAAAAGACTACAAATAGGGTGCAGTGTATACTGCTTGGGTGATGGGTACACCAAAATCTCAC AAA
TCACCACTAAAGAACTCAGTTATGTAACCAAATACTCCCTGTTCCCCAAAAAACCTATGGAAAAATTAAAAAACAAAATAAAATGAAATAAAATAAAGTCCTTGTAACATTGAGTTAGAGGGCTTTAA
CTCCTGGGTCTAAAATGGACACCAAGTCCTGTTAAATTTTAAACACTCACAGCAAttTTTTTTTTTTTTTTTTTTGAGACAGGGTCTCGCTCTGTCgCCCAGGCTaGAGTGCAGTGaCCAATCTTtGGC
TCACTGCaACCTCCaCCTCCCAGGTTCACGCCATTCTCCTGCCTCAGCCTCTCGAGTAGCTGGGACTACAGGTGCCCACCACCATGCCCGGCTAATTTTTTGTATTTTTAGTAGAGACAGGGTTTCAC
CGTGTAAGCCAGGGTGgTCTCGATCTCCTGACCTCGTGATCCACCCGCCTCGGCCTCCCGAAGTGCTGGGATTACAGGCGTGAGCCATCACGCCCAGCCTAAACACTGACAGCAATTAAAGCCTCATC
TTTAGGCCCTGTAGAAGATGCCAATCAAAATAAAATGCATTCCTGAGACACAGGGCAAGA

3775	6.2	1.9	0.3	hg19	1	693	(1287)	+	L1PB	LINE/L1	4919	5617	(534)	6	
2177	11.2	0.3	0.0	hg19	694	978	(1002)	+	AluSx	SINE/Alu	11	296	(16)	7	
1466	25.6	2.5	6.2	hg19	979	1172	(808)	+	L1M5	LINE/L1	5608	5785	(457)	8	*
1917	12.3	0.6	4.3	hg19	1168	1481	(499)	+	L1PB1	LINE/L1	5848	6150	(1)	9	
1164	5.2	0.0	11.5	hg19	1502	1591	(389)	+	MER4-int	LTR/ERV1	4012	4093	(2505)	10	
2503	7.0	0.3	0.0	hg19	1592	1891	(89)	C	AluY	SINE/Alu	(10)	301	1	11	
1164	5.2	0.0	11.5	hg19	1892	1974	(6)	+	MER4-int	LTR/ERV1	4094	4167	(2431)	10	

>KSI_AluYa5_30

>Scaffold9692-874427-874643
ATCCTGCACCATCTGCTCTAACTTCGAAAAGTCTGTGCGCACAAAACAGCTCTGCGATCTCAAGACCTCGTCATGTGTGCGCTGATGTGTGTCTGAAGCACCAAGTTTCAAGACAGGATGAGGTCC TC
CCAAATTCCGCACATTCAGAGTGTTCTCTACAGACAACCCCTGCCTGAGTGAATCCTGCTTCTCTAAATTCTCTCAATTTTGCTCATTTTCTATAGTGGAATTCCCAATCTTTTATGAGTGTGAT AAA
TAAAAAAATCTCTCTTGTTAATCTTACCGTCTGTGTCTCATTTGATGCTCTGAACCAAGATCTATCCTGCCGAAGTGCTGGCCCTTTGGTTTTAAGTCGCCATTCTGAAAAGAA AaaaaaAATGACCT
AAAAACGATCAAACTCATTGGTATTTGTTTTCATATAAGACATGGCAGAAAAAAAAACAAAGCAAAGATAGATTTAAGGAGTTTGGCTATGTGTAAAATTTGGAATCATAGGAACTGGGAGTCAT AAT
TTAGAAGATTTTAAAATACATTTGGTCCATAAAGAAGAAATGTGAGGTGAACAGAAACAATATTATCAATGAAAGAAAG CAGGAAAGAGCTGGACAAAGGGCATACATTCAAAACACAAACTTGGCCA
GGCGTGGCAGCACGCACCTGTAATCCCAGCTACTCAGGAGGCTGAGGCAGAAGAACTGAGTGAACCCAGGAGGCAGAGTTTGCAGTGAGCCAAGATTGTGCCACTGCACTCCAGCCTGGGCAACA GAG
aAAGACTCCGTCTCAAAAAAAAAAAAAAAAAAAACCCACAAACTGAAAGAACAAAACAGCAGGTTTTTTATGAGATCACAAGAAAGGGAAAATCCAGATCAGATGGAAAATAAGGAATTACGGGAAGCT
CAGGATACCCAGAGCCAACAGCCTCATGTTCTGTGCCAGAATGCATCTTttttttttttttttttttttttttttttttgggggggttctcccttttgcccccnncctcccgggttcacgccat
tctcctgcctcagcctcccaagtagctgggactacaggcgcccacgcccggctaattttttttttttgtatttttagtagagacggggt ttcaccgttttagccgggatggtctcgatctcctgacct
cgtgatccgcccgcctcggcctcccaaagtgctgggattacaggcgtga gccaccgcgcccggcccagaatgcatcttAAAACGTGCACTCAGGATcCTTCCACAGCCGCTAAACTCGACACTCAGGA
TCCTCCCACAGCCGCTaAACTTGACACAATCCTTCCATAGCCGCTAAACTTGACACTCAGGATCCTCCCACAGCTGCTAAACTTGACATCGACCAGGCGTGCTCTTCACACT TactcACCTTGGAGAA
CTGCTCTCCGCCCTGCCCTCAACTCCTCTTCCTGCTCCAGCCAATAGAT CACACTGGGTTGGAACAGGTGATGTCCTGTACACAGGGAAAGAGACATCGATGGAAGAGGCTCAGGCAGGGGATGACAA
ACCTACCCGTGAACAGGAACTACATTTCCTTTTTTTTATTATTTTTGAGACAGCGTCTCGCGCTGTTGCCCAGGCTGGAGTGCAGTGGTGCAATCTCGGCTCACTGCAACGTCTGCCTCCTGGGTTC
AAGTGATTCTCTTGCCTCAGCCTCCTAAGTAGCTGGGACTATAGGTGCC CGCCACCAtGCCTGGCTAGTTTTTTGTATTTTTAGTAGAGATGGGGTTTCACCGTGTTGGTCAGAATGGTCTCAATCTC
CTGACCTTGTGATCCGCCCACCCCGGCCTCTCAAAAGTGCTAGGATTACAGGCATGAGCCACTGCGTCTGGCGTCCCAGAGTGCT gGGATTACAGGCGTGAGCCACTGCGCCCAGCCTCCCAAAGTGC
TGGGATTACAGGCATGAGCCACTGCGTCCAGCATCCCAGAGTGCTGGGA TTACAGGCGTGGCCACCACACCCTGCCTCCCAGAGTGCTGGGATTACAGCCCTGAGCCACTGCGCCTGGCCTCCTAGAG
TGCTGGGATTACAGCCCTGAGCCACTGCACCCAGCCTCCCAGAGTGCTCGGATTACAGCCCT aAGCCACTGCgCCtgGCCTCCCAGAGTGCTgGGATTACAGCCCTgAGCCACcGCGCCcGGCCTCCC
AGAGTGCTGGGATTACAGCCCTGAGCCACTGCACCCAGCCT

1277	12.8	0.6	0.0	AluYa5-Scaffold9692-874427-874643	623	801	(1416)	+	AluSx	SINE/Alu	122	301	(11)	1
39	0.0	0.0	0.0	AluYa5-Scaffold9692-874427-874643	944	979	(1238)	+	(T)n	Simple_repeat	1	36	(0)	2
1870	1.4	2.8	2.8	AluYa5-Scaffold9692-874427-874643	1001	1217	(1000)	C	AluYa5	SINE/Alu	(94)	217	1	3
2226	12.5	0.0	0.3	AluYa5-Scaffold9692-874427-874643	1561	1864	(353)	C	AluSc8	SINE/Alu	(7)	304	2	4
343	6.8	0.0	0.0	AluYa5-Scaffold9692-874427-874643	1866	1909	(308)	C	Alu	SINE/Alu	(257)	44	1	5
383	17.5	7.1	5.5	AluYa5-Scaffold9692-874427-874643	1910	2172	(45)	C	AluY	SINE/Alu	(133)	169	1	6
398	12.7	1.6	1.6	AluYa5-Scaffold9692-874427-874643	2173	2216	(1)	C	AluYm1	SINE/Alu	(267)	44	1	7

>chr16:89290349-89292322
ATCCTGCACCATCTGCTCTAACTTCGAAAAGTCTGTGCGCACAAAACAGCTCTGCGATCTCAAGACCTCGTCATGTGTGCGCTGATGTGTGTCTGAAGCACCAAGTTTCAAGACAGGATGAGGTCC TC
CCAAATTCCGCACATTCAGAGTGTTCTCTACAGACAACCCCTGCCTGAGTGAATCCTGCTTCTCTAAATTCTCTCAATTTTGCTCATTTTCT ATAGTGGAATTCCCAATCTTTTATGAGTGTGATAAA
TAAAAAAATCTCTCTTGTTAATCTTACCGTCTGTGTCTCATTTGATGCTCTGAACCAAGATCTATCCTGCCGAAGTGCTGGCCCTTTGGTTTTAAGTCGCCATTCTGAAAAGAA AgaagAATGACCTA
AAAACGATCAAACTCATTGGTATTTGTTTTCATATAAGACATGGCAGAAAAAAAAACAAAGCAAAGATAGATTTAAGGAGTTTGGCTATGTG TAAAATTTGGAATCATAGGAACTGGGAGTCATAATT
TAGAAGATTTTAAAATACATTTGGTCCATAAAGAAGAAATGTGAGGTGAACAGAAACAATATTATCAATGAAAGAAAG CAGGAAAGAGCTGGACAAAGGGCATACATTCAAAACACAAACTTGGCCAG
GCGTGGCAGCACGCACCTGTAATCCCAGCTACTCAGGAGGCTGAGGCAGAAGAACTGAGTGAACCCAGGAGGCAGAGTTTGCAGTGAGCCAAGATTGTGCCACTGCACTCCAGCCTGGGCAACAG AGc
AAGACTCCGTCTCaaaAAAAAAAAAAAAAAAAAAAACCCACAAACTGAAAGAACAAAACAGCAGGTTTTTTATGAGATCACAAGAAAGGGAAAATCCAGA TCAGATGGAAAATAAGGAATTACGGGAAG
CTCAGGATACCCAGAGCCAACAGCCTCATGTTCTGTGCCAGAATGCATCTTAAAACGTGCACTCAGGATtCTTCCACAGCCGCTAAACTCGACACTCAGGATCCTCCCACAGCCGCTtAACTTGACAC
AATCCTTCCATAGCCGCTAAACTTGACACTCAGGATCCTCCCACAGCTGCTAAACTTGACATCGACCAGGCGTGCTCTTCACACT TACCTTGGAGAACTGCTCTCCGCCCTGCCCTCAACTCCTCTTC
CTGCTCCAGCCAATAGATCACACTGGGTTGGAACAGGTGATGTCCTGTACACAGGGAAAGAGACATCGATGGAAGAGGCTCAGGCAGGGGATGACAAACCTACCCGTGAACAGGAACTACATTTC CTT
TTTTTTATTATTTTTGAGACAGCGTCTCGCGCTGTTGCCCAGGCTGGAGTGCAGTGGTGCAATCTCGGCTCACTGCAACGTCTGCCTCCTGGGTTCAA GTGATTCTCTTGCCTCAGCCTCCTAAGTA
GCTGGGACTATAGGTGCCCGCCACCAcGCCTGGCTAGTTTTTTGTATTTTTAGTAGAGATGGGGTTTCACCGTGTTGGTCAGAATGGTCTCAATCTCCTGACCTTGTGATCCGCCCACCCCGGCCTCT
CAAAAGTGCTAGGATTACAGGCATGAGCCACTGCGTCTGGCGTCCCAGAGTGCT cGGATTACAGGCGTGAGCCACTGCGCCCAGCCTCCCAAAGTGCTG GGATTACAGGCATGAGCCACTGCGTCCAG
CATCCCAGAGTGCTGGGATTACAGGCGTGGCCACCACACCCTGCCTCCCAGAGTGCTGGGATTACAGCCCTGAGCCACTGCGCCTGGCCTCCTAGAGTGCTGGGATTACAGCCCTGAGCCACTGC ACC

CAGCCTCCCAGAGTGCTCGGATTACAGCCCTgAGCCACTGCaCCcaGCCTCCCAGAGTGCTcGGATTACAGCCCTaAGCCACtGCGCCtGGCCTCCCAGAGTGCTGGGATTACAGCCCTGAGCCACcg
cgcccggcctcccagagtgctgggattacagccctgagccacTGCACCCAGCCT

1330	12.1	0.6	0.0	chr16:89290349-89292322	622	803	(1171)	+	AluSx	SINE/Alu	122	304	(8)	8
2241	12.2	0.0	0.3	chr16:89290349-89292322	1274	1577	(397)	C	AluSc8	SINE/Alu	(7)	304	2	9
383	20.6	4.6	3.3	chr16:89290349-89292322	1580	1885	(89)	C	AluY	SINE/Alu	(112)	190	1	10
424	5.7	1.9	0.0	chr16:89290349-89292322	1886	1929	(45)	C	AluYm1	SINE/Alu	(266)	45	1	11
398	12.7	1.6	1.6	chr16:89290349-89292322	1930	1973	(1)	C	AluYm1	SINE/Alu	(267)	44	1	12

>KSI_AluYa5_31

>Scaffold9708-1139681-1139969

AAGACAAAGATTAACCCCTCTCACCAACACTGAGCATGTGTATACTTTATATAACAGTTGAAGCTATCAGCTTGTTTGGAGATACAGGTGTTTATTAGCAGAATTTTCATGCTGCTAATAAAGATATA
CTCAAGACTGGGCAATTTACGAAAGAAAGAGGTTTAATGTACTTACAGTTGCACATGGCTGGGGAGGCCTCACAATCATGGCAGAAGGCAAGGAGGAGCAAGTCACATCTTACGTAGATGGCGGCAGG
CAAAGAGAGAGAACCTGTGCAGGGAAACTCCTCTTTATAAAACCATCAGATCTCATGAGACTTATTCACTATCACGAGAACAGCATGAGAAAGAACCGCCCCCATGATTCAATTACCTCCCACTGGGT
CCCTCCTACAACACATAGAGATTGTAGGAGTTACCATTCAAGCTGAGATTTAGGTACGACACAACTAAACTATGTCAGGCCTGCAAGGATGAGGCCAAGATTCTCCATGAAGGTGTGGTCAGTCCaAA
TGTCAAGCTCTAGACAGCCTGGAGAGGAGTATGTTCCCTCATTCTCACATTGCACCCAGGTGTTGAAATGTCCTCAGAACCCACAAGGGTGCTTTTCAGTGGACAGTGTTAAGATGGCATGCCCATGT
GGAGACCAGTCACGAGCGACGAAACAAGGACATTTCCAAATCAAATGTGACTTTGATCTAATTAATCACTTCACTGAGACTTATGATGTATTCTGTGCTTCTTTTCCTTATTTTGGACATGTGTGAAG
AGGGGGCTGGGACCAAGAGGGGAAACAGCAAGTTCATCATGAAATGTTAAGTGAAAATAGaTAGGATATTAAACTAGATCACAGACACGCACAGAGATATTAAACTAGATCATCATACACACACACAC
ACACACACACACACACAtatATATATATATATATGCACATACACATACATTCTGTGATGGTTAATTTTATGTCTCAGTTTGACTGGGCTTAAGAATGTATAGCTggccgggcgcggtggctcacgcct
gtaatcccagcactttgggaggccgaggcgggcggatcacgaggtcaggaccatcccggctaaaacggtgaaaccccgtctctactaaaaatacaaaacattagccgggcgtagtggcgggcgcctgt
agtcccagctacttgggaggctgaggcaggagaatggcgtgaacccgggaggcggagcttgcagtgagccgagattgtgccactgcactccagcctgggcgacagagcgagactccgtctcaaaaaaa
aaaaaagaatgtatagctAACTGGTAAAACATTCTCTCTGGGTGTGTCTGTGAGGATGTTTCTGGAAGAGATTAGCATCGGGATCAGCAGACTGAGTAAAGAAGATACCCTCACCAATGTGGGTGGGC
ATGATCCAATCTGTTGAGAACCCAAATAGAATGAAAAGGCGGAGGAAGGGCGAATTCATTCTCTCAGCTAAAGTTTGGATATCCATCTTCTCAGGCCCTCAGACATCAGTGCTTCCAGTTCTTAGGCC
TTCGGACTTAGACTGGGACTTGAACCATCAGCCTCCCACTTCTTAGGCCTTCAGACTCTGACCAGGATACATGCCATCTGCtACACCCCACCCTCTACCCTGTTCTTAGGTCTTTGGATTAGAACTGG
GACTTATACCATTGACTTCCCTGGTTCTCAGGCCTTTGGGCTTGGACTGGAACTACATCACCAACTCTCCTGGTGTCTGAGATAGGAAAAACCAAATCGCTTTTCCTACCCTCACACACTCACCACTC
AATACAATACTTCACCTCTAGTCACCAAAATGTGTGTCATCAGCCAATTCTCCATCAGGTGCCAACTGGGTATATACAATTTAACTCAATTCTGACATTATCTACCTGGAGTTAGCATCAGACACCAC
AGGTTAAGGGCTCAGTCCCACAAGAAGTCCCAGGAGAGTGCCCCCACTTTAGATGCCAGTCACAAGTTCTCCAGGTTGTGACTGTATATCTGACCAACTGGCCATAAATTGGGAGTTTCCATGAATCC
CTCCTCAGGTTCAATTAATTTGTTAGCATGGCTTACAAAACTCAGGGAAACTATTTATGTTTACCCATTTAATATAAAGGACCTTAAAAAGAACACAGAAGAACAACCAAAGAGAAGAGATGCATAGG
GTGAGATATGCAGGAAGGGGCATGGAGCTTCTGTGCCCTTGTCAGCACGCAACCCTCTGGTACCTCCATGTGTTCAGCAACaGGGAAGCTCTCTGAATCCCATAGCTCAGGGA

2431	12.0	0.8	2.6	AluYa5-Scaffold9708-1139681-1139969	104	461	(1828)	C	THE1B	LTR/ERVL-MaLR	(12)	352	1	1
34	0.0	0.0	0.0	AluYa5-Scaffold9708-1139681-1139969	885	913	(1376)	+	(AC)n	Simple_repeat	1	29	(0)	2
17	21.0	0.0	0.0	AluYa5-Scaffold9708-1139681-1139969	914	946	(1343)	+	(AT)n	Simple_repeat	1	33	(0)	3
2168	17.6	0.8	3.2	AluYa5-Scaffold9708-1139681-1139969	949	1000	(1289)	+	MLT2B1	LTR/ERVL	1	50	(470)	4
2604	1.7	2.8	0.0	AluYa5-Scaffold9708-1139681-1139969	1001	1289	(1000)	+	AluYa5	SINE/Alu	1	297	(14)	5
2168	18.5	0.6	3.8	AluYa5-Scaffold9708-1139681-1139969	1290	1742	(547)	+	MLT2B1	LTR/ERVL	49	431	(123)	4
2478	17.9	4.1	7.8	AluYa5-Scaffold9708-1139681-1139969	1747	2279	(10)	C	MER21C	LTR/ERVL	(4)	934	420	6

>chr2:62500908-62502896

AAGACAAAGATTAACCCCTCTCACCAACACTGAGCATGTGTATACTTTATATAACAGTTGAAGCTATCAGCTTGTTTGGAGATACAGGTGTTTATTAGCAGAATTTTCATGCTGCTAATAAAGATATA
CTCAAGACTGGGCAATTTACGAAAGAAAGAGGTTTAATGTACTTACAGTTGCACATGGCTGGGGAGGCCTCACAATCATGGCAGAAGGCAAGGAGGAGCAAGTCACATCTTACGTAGATGGCGGCAGG
CAAAGAGAGAGAACCTGTGCAGGGAAACTCCTCTTTATAAAACCATCAGATCTCATGAGACTTATTCACTATCACGAGAACAGCATGAGAAAGAACCGCCCCCATGATTCAATTACCTCCCACTGGGT
CCCTCCTACAACACATAGAGATTGTAGGAGTTACCATTCAAGCTGAGATTTAGGTACGACACAACTAAACTATGTCAGGCCTGCAAGGATGAGGCCAAGATTCTCCATGAAGGTGTGGTCAGTCCcAA
TGTCAAGCTCTAGACAGCCTGGAGAGGAGTATGTTCCCTCATTCTCACATTGCACCCAGGTGTTGAAATGTCCTCAGAACCCACAAGGGTGCTTTTCAGTGGACAGTGTTAAGATGGCATGCCCATGT
GGAGACCAGTCACGAGCGACGAAACAAGGACATTTCCAAATCAAATGTGACTTTGATCTAATTAATCACTTCACTGAGACTTATGATGTATTCTGTGCTTCTTTTCCTTATTTTGGACATGTGTGAAG
AGGGGGCTGGGACCAAGAGGGGAAACAGCAAGTTCATCATGAAATGTTAAGTGAAAATAGgTAGGATATTAAACTAGATCACAGACACGCACAGAGATATTAAACTAGATCATCATACACACACACAC
ACACACACACACACACAcATATATATATATATATGCACATACACATACATTCTGTGATGGTTAATTTTATGTCTCAGTTTGACTGGGCTTAAGAATGTATAGCTAACTGGTAAAACATTCTCTCTGGGTG
TGTCTGTGAGGATGTTTCTGGAAGAGATTAGCATCGGGATCAGCAGACTGAGTAAAGAAGATACCCTCACCAATGTGGGTGGGCATGATCCAATCTGTTGAGAACCCAAATAGAATGAAAAGGCGGAG
GAAGGGCGAATTCATTCTCTCAGCTAAAGTTTGGATATCCATCTTCTCAGGCCCTCAGACATCAGTGCTTCCAGTTCTTAGGCCTTCGGACTTAGACTGGGACTTGAACCATCAGCCTCCCACTTCTT
AGGCCTTCAGACTCTGACCAGGATACATGCCATCTGCcACACCCCACCCTCTACCCTGTTCTTAGGTCTTTGGATTAGAACTGGGACTTATACCATTGACTTCCCTGGTTCTCAGGCCTTTGGGCTTG
GACTGGAACTACATCACCAACTCTCCTGGTGTCTGAGATAGGAAAAACCAAATCGCTTTTCCTACCCTCACACACTCACCACTCAATACAATACTTCACCTCTAGTCACCAAAATGTGTGTCATCAGC
CAATTCTCCATCAGGTGCCAACTGGGTATATACAATTTAACTCAATTCTGACATTATCTACCTGGAGTTAGCATCAGACACCACAGGTTAAGGGCTCAGTCCCACAAGAAGTCCCAGGAGAGTGCCCC
CACTTTAGATGCCAGTCACAAGTTCTCCAGGTTGTGACTGTATATCTGACCAACTGGCCATAAATTGGGAGTTTCCATGAATCCCTCCTCAGGTTCAATTAATTTGTTAGCATGGCTTACAAAACTCA

GGGAAACTATTTATGTTTACCCATTTAATATAAAGGACCTTAAAAAGAACACAGAAGAACAACCAAAGAGAAGAGATGCATAG GGTGAGATATGCAGGAAGGGGCATGGAGCTTCTGTGCCCTTGTCA
GCACGCAACCCTCTGGTACCTCCATGTGTTCAGCAACgGGGAAGCTCTCTGAATCCCATAGCTCAGGGA

2431	12.0	0.8	2.6	chr2:62500908-62502896	104	461	(1528)	C	THE1B	LTR/ERVL-MaLR	(12)	352	1	7
36	0.0	0.0	0.0	chr2:62500908-62502896	885	915	(1074)	+	(AC)n	Simple_repeat	1	31	(0)	8
2230	19.0	0.7	1.9	chr2:62500908-62502896	947	1442	(547)	+	MLT2B1	LTR/ERVL	1	431	(123)	9
2479	17.9	4.1	7.8	chr2:62500908-62502896	1447	1979	(10)	C	MER21C	LTR/ERVL	(4)	934	420	10

>KSI_AluYa5_32

>Scaffold10546-619886-620026
CTATGGGTAGATTGAGACAAAGACCAACACCAATAAAATTTTACTTCTTGTTAGAACTTTGGCTGCTTTACTAGAGAAATATTCTAGGAAAGAAAAATCTGTACCAACAGAAAAGAGTCTTATGAG AA
TCCTGGAAGCTCCTCTCGTAGGTGGTTTGCCCTTAGGCATTAGAGTTGCTGAGTGATTTGCAGATGGCTCCTCAACTTTTAATTCAAAGGTAATATTTTGTTTTCAGTCTTCCTTTCTTGGTCTC AAG
TGAGTCTTTATAAAAGAGAATTAAAGGACCTAGTCTCACTGTGTAAAAACACATTTTTGGCATGTATCAGGGGTCCAGCACTATAAACAGTCATGATCCTTATTTATACGGATATTGGTCTTGCCA CT
GGTATTCTAATGATTAGGGCCTCATGTCAAGGTTTGTACAGGACAATTTTCTGTGTATAACAAATACCTAACAAACTAAAATCGGAATTGTAATAACCCATAAAAATACATATTATAGCATAGGA AAG
TATTCACAATGAAAAATGTGTATGTCCCCTCAAAGCAGATGGTATAATCCCAGGAGGCAGACAAACGGGTGCATAAACAAGATAGTGATTGTGAAGGAAGCATGCACTAAAGTGAGTATGGTGCTT CG
AGATCCTGTGAGATTCCCAAACCAAAATGATTGGGATTTTGGCGTTTACCTGCTATGCCTAAAGACATGAAACCTTCCCATGATAGGAAGGTGGATGACACCGAGCCACTCCCTAGTCATCAGGG TAA
AAGTATGCTCTAACAGAAATTTATGTTTGCATAAAGTTTGCCTGAATTTCTTGGAGGCCTTCCAAACATTAATAAGACTATATTATAGAGGCAAACATTGAAATGGATTGGATGGTGGAAGCCTGA CA
AGTGTTCTTGGGAGATAGACAACTCTGCCCCAGGGTGGGCCAGGCATTCTAAGAATCTAATC GCAACCTTTCCACTCTGGCTTAAGATAACTAAATGAAaCaaatagtggcgggcgcctgtagtccca
gctacttgggaggctgaggcaggagaatggcgtgaccccgggaggcggagcttgcagtgagccgagatcccgccactgcactccagcctggaaaaaaaaaaaaaaaaaaaaaaa AAGAGTTTGTTTT
CTACTCAGTCTGACTTTCCCATAATCTGCCCAAAGAACTGCTGTTTTATTTTAGGTAAAAATAA CCATAGGCATAATAACATTTGCCTTTACGAGTTTAGGAAAGTGAAAAGTATATTTAAAATTGAG
TCATAATATATGTCACATGAATATACAGAAAAAAACTGGTAGTAGTTGGTGAATTAATTAGAGATTCAAGACCTACATAGTATCTGTAGCTCTCAGGTAATATTTTACAGAGTGAAACCGTAACA TAC
CAACTTATTAAAATTATGACTACCTTCATCCATTGTGGGTTTGTTTTATCAACTCATATTTAAAATCTGGAAATTCTTTTAATTATATAAACTATTAAAAT ACCTGGCTTTAATCCAGGAGGTCAGTA
GGTCTAATACTGGGCTACTATGTTGAGAGTGGGAAATTATTGGTAAATTCTAATATTGAGTATAAAAAATCATGTGCACCAATTTAACTAAGCCCTCAGTTTACCACTGTGAGGGCCAAAATCTA CAA
AGCTGTGTACCTCCTAGCCATTGGCTGAAGTCAAATGGTCCACCTCAGAAAATATATAGAAATAGGAGTAAAACCTTAAAGGTCATGGTTTTCATTTTGAT GCCATTTGGAGATTATTTTAATATGGG
TCAAATTAAAAGAAAGCATTTTTTTCCTCTGGGCATGGTGGTTCATGTCTGTAATTCCAGCACTTAAAGGAGGCTGAGGCAGGCGGATCACTTGGGGTCAGGAGTTTGAGACCACCCTCAACATG GTG
AAACCCTGTCTCTACTAAAAATATAAACATTAGCTGGGCATGGTGGTACACATCTGTAATTCTAGCTAGTGGGGAGGCTGAGGCAGGCAAATCGCTTGAAC CCAGGAGGTGGAGGTTGCAGTGAGCTG
AGATTGCACCACCGCACTTGAGCCTGGGTGGGAGGGCAAGAGTCAATCTCAAACAAAACAAACAAACAAACAAAAAATAAATAAATAAGAAA G

1159	0.7	16.3	0.0	AluYa5-Scaffold10546-619886-620026	1001	1141	(1000)	+	AluYa5	SINE/Alu	144	307	(4)	1
1911	16.7	1.0	0.3	AluYa5-Scaffold10546-619886-620026	1821	2125	(16)	+	AluSz	SINE/Alu	3	309	(3)	2

>chr13:86165445-86167443
CTATGGGTAGATTGAGACAAAGACCAACACCAATAAAATTTTACTTCTTGTTAGAACTTTGGCTGCTTTACTAGAGAAATATTCTAGGAAAGAAAAATCTGTACCAA CAGAAAAGAGTCTTATGAGAA
TCCTGGAAGCTCCTCTCGTAGGTGGTTTGCCCTTAGGCATTAGAGTTGCTGAGTGATTTGCAGATGGCTCCTCAACTTTTAATTCAAAGGTAATATTTTGTTTTCAGTCTTCCTTTCTTGGTCTC AAG
TGAGTCTTTATAAAAGAGAATTAAAGGACCTAGTCTCACTGTGTAAAAACACATTTTTGGCATGTATCAGGGGTCCAGCACTATAAACAGTCATGATCCTTATTTAT ACGGATATTGGTCTTGCCACT
GGTATTCTAATGATTAGGGCCTCATGTCAAGGTTTGTACAGGACAATTTTCTGTGTATAACAAATACCTAACAAACTAAAATCGGAATTGTAATAACCCATAAAAATACATATTATAGCATAGGA AAG
TATTCACAATGAAAAATGTGTATGTCCCCTCAAAGCAGATGGTATAATCCCAGGAGGCAGACAAACGGGTGCATAAACAAGATAGTGATTGTGAAGGAAGCATGCAC TAAAGTGAGTATGGTGCTTCG
AGATCCTGTGAGATTCCCAAACCAAAATGATTGGGATTTTGGCGTTTACCTGCTATGCCTAAAGACATGAAACCTTCCCATGATAGGAAGGTGGATGACACCGAGCCACTCCCTAGTCATCAGGG TAA
AAGTATGCTCTAACAGAAATTTATGTTTGCATAAAGTTTGCCTGAATTTCTTGGAGGCCTTCCAAACATTAATAAGACTATATTATAGAGGCAAACATTGAAATGGA TTGGATGGTGGAAGCCTGACA
AGTGTTCTTGGGAGATAGACAACTCTGCCCCAGGGTGGGCCAGGCATTCTAAGAATCTAATC GCAACCTTTCCACTCTGGCTTAAGATAACTAAATGAAtCAAGAGTTTGTTTTCTACTCAGTCTGAC
TTTCCCATAATCTGCCCAAAGAACTGCTGTTTTATTTTAGGTAAAAATAA CCATAGGCATAATAACATTTGCCTTTACGAGTTTAGGAAAGTGAAAAGTATATTTAAAATTGAGTCATAATATATGTC
ACATGAATATACAGAAAAAAACTGGTAGTAGTTGGTGAATTAATTAGAGATTCAAGACCTACATAGTATCTGTAGCTCTCAGGTAATATTTTACAGAGTGAAACCGTAACATACCAACTTATTAA AAT
TATGACTACCTTCATCCATTGTGGGTTTGTTTTATCAACTCATATTTAAAATCTGGAAATTCTTTTAATTAT ATAAACTATTAAAATACCTGGCTTTAATCCAGGAGGTCAGTAGGTCTAATACTGGG
CTACTATGTTGAGAGTGGGAAATTATTGGTAAATTCTAATATTGAGTATAAAAAATCATGTGCACCAATTTAACTAAGCCCTCAGTTTACCACTGTGAGGGCCAAAATCTACAAAGCTGTGTACC TCC
TAGCCATTGGCTGAAGTCAAATGGTCCACCTCAGAAAATATATAGAAATAGGAGTAAAACCTTAAAGGTCAT GGTTTTCATTTTGATGCCATTTGGAGATTATTTTAATATGGGTCAAATTAAAAGAA
AGCATTTTTTTCCTCTGGGCATGGTGGTTCATGTCTGTAATTCCAGCACTTAAAGGAGGCTGAGGCAGGCGGATCACTTGGGGTCAGGAGTTTGAGACCACCCTCAACATGGTGAAACCCTGTCT CTA
CTAAAAATATAAACATTAGCTGGGCATGGTGGTACACATCTGTAATTCTAGCTAGTGGGGAGGCTGAGGCAG GCAAATCGCTTGAACCCAGGAGGTGGAGGTTGCAGTGAGCTGAGATTGCACCACCG
CACTTGAGCCTGGGTGGGAGGGCAAGAGTCAATCTCAAACAAAACAAACAAACAAACAAAAAATAAATAAATAAGAAA G

1911	16.7	1.0	0.3	chr13:86165445-86167443	1679	1983	(16)	+	AluSz	SINE/Alu	3	309	(3)	3

>KSI_AluYa5_33

>Scaffold10986-833152-833306
TTGTAAGTGATGTAATTAAGCCATTTGCAAGGCAGATCTTCTGGCTTCAATCAAGCTTCAGACTACAGCCCAGGCCAACATT TTGACAACAACCTCATTGGAGACGCTGAGCTAGAGCCATCGACTCC
TGAATTCCTCATCAGGAGACATTGTGTGATATTATATAATGAATGTTTATTGTTGTTTGAAGCTAAATCTTGAGATCATTTATATAGAGTGATAGAGAATTAATACAAGAGGTTAAGTGCTGCCA AAT
GTTATATCTGTTAGCAGACATAAGTTACATTCTCCAATATTAGCTTGAAATACCAAAGGTGAAATTTTACCTTCCAAATGTT GCATCTTTTGTGTTTTTGTTATTACTTCAGATCTTGAGTGAGGAAG
AGGGGTTCCAGTTATTGGGTCCCCTAAAACACTAACATTATGCTTTGCATTTTATCATTCATTCCCTATTCAGCTAGCATTCTGTTGAACATTTACTAATTCTAAGCACGGTGCAAAAGTTTTGA ATG
CCAAAAGAGTAAGAAGACCTTTTCCACTGCCATTAAAGTTTGCATAGACTTATGAAAAATGGACATAATTATTAATAACTAC TAAATTATATCATAGTCAAATCTCATAAACTGGTTAATTGGCTATG
GGGGCAGAAGGAGAAGAGTTCAGCTAGTATAAAATTGATTTGATATATTTAATTAAAATTATCTCTCATATGTA GACAGTGTATGCGAGTAAGCCCAGCCCAATGGGTTATCACACTTATGATAATCC
TTTTTGAGGAGAGAAACATAGTGTTGAGAGCATAAACTCTGGAGCGAGGCTGCCTCAATCTCTCTCCTGGCTCTGCCTCTTGGGACTGGTGTGATTTTGGAAAATTAACTTTTGGTGCCTTTTTC TGC
ATCTGTCAAATGAGGGTTAAATGGGTGAATACATGTGCAGTATTTAGAGTAGTGCCTAGCACCATATTATTGTTTGGTATTACTATTAGAATTTACA AAATCTTggccgggcgcggtggctcacgcct
gtaatcccagcactttgggaggccgaggcgggcggatcacgaggtcaggagatcgagaccatcccggctaaaaacggtgaaaccccgtctctactaaaaatacaaaaaattagccgggcgtagtg gcg
ggcTTCATTATGTTGACAATGGTTAGCCTCAAGATCCTGAAACTCTGATTCATGCATATAAGAGCCAAGTCATACGTTGTTGTTTTCATAAATTAGT TTGCCAAAACATGTATTGAAGATGTCTACTA
GTGTTCCAAGAGCTTCGAGAGAAATCTTTGCTCTTTCTACAGCAGATAGGCTCATGTGCTGGAATTGCTTGTGTCCATTTTTTGATGCACTACCCACAATTACCTTATTAATGTTATCCTATGAG AAA
CAATTCAAGATTTTTTATTTCCTGAAGCTCTTCACATAACCAGACAACAATGCAAAATAAATATTCTTTATCTTGTTCAAATTCATACTTTTTCAAA TAAAAAATGTTTTATTCCATATTCTAGTTCA
AGTTGTTCAGTAGTCCCCTCTTTTCATAATTGTTCTAAGTTATTAAATTTTTATGGAATGTAAAGCACTTAATAGTTTTTGTTAAATAAAATGTTTTACTTACTAAGCTATATATCTAAGGTGCA AAG
GAGACAGGCTTTCCCACATTGTATTAACTACATTATGGCATCTTTTGTTCACTATCATTCAAAATGATAGAACTTTAAAATAAAAAAAAAAAAAACAG GAGACAGTGGAGAGGGAGTCACAAAGAATGT
CTTGTGCAAAAAGAAAAATGAGTTAATGAGCATGTTCCAATACAATGGACTAATGTTTGTGTCCTCTCAAAATTAATATGTTGATATTCTAACCCCCAATATGATTGTATTAAGAGATGGAGCCT TGG
TAAGGTAATTAGGTCATGAGGGTGGAGCCTCAAGAAATTTCATCAGCTTCATGTTAAATCCATCTGGTACTTACACTTCTTGTGAGTTCAAATAAAA ATCCCATAAAGTTATATCCTGTGAAATTTAA
TAGAATCCACCTAATTCAGAGTTTTATTTACAATGCATTTATGGCCCCATTTATTGTAAAATGAGAATAATTTCAAATAATAATTTTAAGTTCCATGTTATTTGCA A

876	24.8	7.2	2.0	AluYa5-Scaffold10986-833152-833306	1	235	(1920)	+	MLT1F	LTR/ERVL-MaLR	302	548	(0)	1
467	27.1	3.3	0.6	AluYa5-Scaffold10986-833152-833306	270	421	(1734)	+	LTR79	LTR/ERVL	372	527	(0)	2
402	25.6	25.9	0.0	AluYa5-Scaffold10986-833152-833306	783	958	(1197)	+	MIR	SINE/MIR	8	229	(33)	3
1449	0.0	0.0	0.7	AluYa5-Scaffold10986-833152-833306	1001	1155	(1000)	+	AluYa5	SINE/Alu	1	154	(157)	4
687	15.1	1.7	0.8	AluYa5-Scaffold10986-833152-833306	1837	1956	(199)	+	MLT1A0	LTR/ERVL-MaLR	5	125	(240)	5

>chr4:125658009-125660008
TTGTAAGTGATGTAATTAAGCCATTTGCAAGGCAGATCTTCTGGCTTCAATCAAGCTTCAGACTACAGCCCAGGCCAACATTTTGACAACAACCTCATTGGAGACGCTGAGCTAGAGCCATCGACT CC
TGAATTCCTCATCAGGAGACATTGTGTGATATTATATAATGAATGTTTATTGTTGTTTGAAGCTAAATCTTGAGAT CATTTATATAGAGTGATAGAGAATTAATACAAGAGGTTAAGTGCTGCCAAAT
GTTATATCTGTTAGCAGACATAAGTTACATTCTCCAATATTAGCTTGAAATACCAAAGGTGAAATTTTACCTTCCAAATGTTGCATCTTTTGTGTTTTTGTTATTACTTCAGATCTTGAGTGAGG AAG
AGGGGTTCCAGTTATTGGGTCCCCTAAAACACTAACATTATGCTTTGCATTTTATCATTCATTCCCTATTCAGCTA GCATTCTGTTGAACATTTACTAATTCTAAGCACGGTGCAAAAGTTTTGAATG
CCAAAAGAGTAAGAAGACCTTTTCCACTGCCATTAAAGTTTGCATAGACTTATGAAAAATGGACATAATTATTAATAACTACTAAATTATATCATAGTCAAATCTCATAAACTGGTTAATTGGCT ATG
GGGGCAGAAGGAGAAGAGTTCAGCTAGTATAAAATTGATTTGATATATTTAATTAAAATTATCTCTCATATGTA GACAGTGTATGCGAGTAAGCCCAGCCCAATGGGTTATCACACTTATGATAATCC
TTTTTGAGGAGAGAAACATAGTGTTGAGAGCATAAACTCTGGAGCGAGGCTGCCTCAATCTCTCTCCTGGCTCTGCCTCTTGGGACTGGTGTGATTTTGGAAAATTAACTTTTGGTGCCTTTTTC TGC
ATCTGTCAAATGAGGGTTAAATGGGTGAATACATGTGCAGTATTTAGAGTAGTGCCTAGCACCATATTATTGTTTG GTATTACTATTAGAATTTACAAAATCTTTTCATTATGTTGACAATGGTTAGC
CTCAAGATCCTGAAACTCTGATTCATGCATATAAGAGCCAAGTCATACGTTGTTGTTTTCATAAATTAGTTTGCCAAAACATGTATTGAAGATGTCTACTAGTGTTCCAAGAGCTTCGAGAGAAATCT
TTGCTCTTTCTACAGCAGATAGGCTCATGTGCTGGAATTGCTTGTGTCCATTTTTTGATGCACTACCCACAATTAC CTTATTAATGTTATCCTATGAGAAACAATTCAAGATTTTTTATTTCCTGAAG
CTCTTCACATAACCAGACAACAATGCAAAATAAATATTCTTTATCTTGTTCAAATTCATACTTTTTCAAATAAAAAATGTTTTATTCCATATTCTAGTTCAAGTTGTTCAGTAGTCCCCTCTTTT CAT
AATTGTTCTAAGTTATTAAATTTTTATGGAATGTAAAGCACTTAATAGTTTTTGTTAAATAAAATGTTTTACTTAC TAAGCTATATATCTAAGGTGCAAAGGAGACAGGCTTTCCCACATTGTATTAA
CTACATTATGGCATCTTTTGTTCACTATCATTCAAAATGATAGAACTTTAAAATAAAAAAAAAAAAAACAGGAGACAGTGGAGAGGGAGTCACAAAGAATGTCTTGTGCAAAAAGAAAAATGAGTT AAT
GAGCATGTTCCAATACAATGGACTAATGTTTGTGTCCTCTCAAAATTAATATGTTGATATTCTAACCCCCAATATG ATTGTATTAAGAGATGGAGCCTTGGTAAGGTAATTAGGTCATGAGGGTGGAG
CCTCAAGAAATTTCATCAGCTTCATGTTAAATCCATCTGGTACTTACACTTCTTGTGAGTTCAAATAAAAATCCCATAAAGTTATATCCTGTGAAATTTAATAGAATCCACCTAATTCAGAGTTT TAT
TTACAATGCATTTATGGCCCCATTTATTGTAAAATGAGAATAATTTCAAATAATAATTTTAAGTTCCATGTTATTT GCAA

876	24.8	7.2	2.0	chr4:125658009-125660008	1	235	(1765)	+	MLT1F	LTR/ERVL-MaLR	302	548	(0)	6

467	27.1	3.3	0.6	chr4:125658009-125660008	270	421	(1579)	+	LTR79	LTR/ERVL	372	527	(0)	7
402	25.6	25.9	0.0	chr4:125658009-125660008	783	958	(1042)	+	MIR	SINE/MIR	8	229	(33)	8
687	15.1	1.7	0.8	chr4:125658009-125660008	1682	1801	(199)	+	MLT1A0	LTR/ERVL-MaLR	5	125	(240)	9

>KSI_AluYa5_34

>Scaffold11190-97324-97550
AGGCAGAGGTTGCAGTGAGCTGAGATCACGCCATTGCACTCCAGCTTGTGCTACAGAGTGAGACTCTGTCTCAAAAAAAAA AtAAATAAATAAATATAAAATAAAAAAATAAAGAAGCCAATTTTTAA
TTATTTTGGGTCATTTTTGTTTTTACTTTCTTAATGTCTGCTTTTATTTTCATTATTTCCTTTCATTTTTTTTCCCAGTTTACTGCTTTTCTTTTTCTGTCTTCTTGTGTTTAATTAGGCTTACG TCA
CTTATTATCAATTGTTCTTGTTTTCTTTTTTCTTTATTTTTTTTTTTGAGACTGAGTCTCGCTCTGTCACTCAGGCTGGATTGCAGTGGAGCAATCTTGGCTCACTGAAACCTCTGCCTCCTGGGT TC
AAGCAATTCTCCTGCCTCAGCCTCCTGAGTAGCTGAGATTACAGGTGCGCACCACCACGCCTGGCTAATTTTTGTATTTTTAGTAAAGACGGGGTTTCACCATTTTAGTCAGGCTGGTCTTGAAC ACT
TGACCTCGTGATCCACCCACCTTAGCCTCCCAAAGTACTGGAATGTTCCTGTTTTCTACTAAATGCATATAAACAATGGATGAGGTTGCTCAGAAGTGATGAAAGACATTGGTCTACAGATTCAAGAA
TCCCAATAAATACAAAGCAGGATAAATAAAAAGACCTATATGCCTAGATGCATCACAATGAACCTGTAGAACACAAAAGACAAAAAGAACTTATTTCAACTAGAGAGGAAACAAAAGACAAATTG TTT
TCATCgACAATTTTAGCCACTATCTACCACTGACTCCTCAATAGTCCA CAGGCTCCTCAATAGCAGATATAATTGCTAGAAAACAATGAAATAATATATTGAATGTGCTGACAA GATAACTTTCtttt
tttttttcttttttttttttttttttttttttttttttt ttttttTTTTTTTCTTTTTTTTTTTTTTTTGAGACGGAGTCTCGCTCTGTCGCCCAGGCTGGAGAGCAGTGGCGGGATCTCGGC tcac
tgcaagctccgcctcccgggttcacgccattctcctgcctcagcctcc caagtagctgggactacaggcgcccgccactacgcccggctaatttttttgtatttttagtagagacggggtttcaccgtt
ttagccgggatggtctcgatctcctgacctcgtgatccgcccgcctcggcctcccaaagtgctgggattacaggcgtgagccaccgcgcccggcc gataactttcAAAAGTCAGGACATAAACACATT
TTGAGGCAAATAAAAGTTGAAAGAGTTTACCACCAGCAGAAACAGGCTTGTACTAGTGGAAATTGAAAGAATATAATTTAGGGAGATGGAAGTGAATGCAGTATTGAAAGTCAGAGATGCAACAAGGA
ATGAAGAGCAAAGACAGTGACAAATCTGTTGGCTAATATAACAAATTCTGATTACATAAAAAACATTAACAATAATGTCTTGTGGGTTTTTTTAAAAAGATACAACTAAAATACATAAGAAAAAT AGA
TGTTACATCAGGAGGGAGATGGAGTTAAAATGTTCTGAGGTTTTTATA CTGTTAAAGGGAAAAGTACAGATATTTATTAACTTTAGACTTTGATAAGTAATATATGCATGTGAAAATAAGAAGCTAAT
CACAATAAGAATATAAAAGGAATGTATCAATTCCAAAATAATAGAGGAATAAAAGTATAATGAGGAAAAATAATCTATTCAAAAGAAGACAATAAAGGAAAGAAAATGAAATAGCAACAAAACAG GGC
AAGGCACAAACAGAAACAGTCTAGATGGAAGAAATATTATCAAAATAT ATTAGTAATTAGAATAACTGTAAATACAGTAAATGCTAAATGGATAAC aATGGTCAGAATGATTTTTTTTTAAATTCAGC
TATATACTATTTATAAAAGACAGACTTAAAACATATTGCTACAGAAAGATTAAAAGTAAAAAAATGAGAAAAGATATGCCAGGCAAACAATAACCAAAAGGAAGCTGATGTGGCTATATTAACAG ACA
AAATGCTATTTATGCTAAAAAGCAATTATAGAGATAATGAAGGACAGT GTGCAATGATAAAAGTTCCCATTTACTAGTAAGATATAATAACTATAGTAAGAGTTGGCAAGGATGTGAATCCACAGAGT
ATCTTACACACTGAAGGTAGAAATGTAAATTGATGCAATGACTTTAAAAGACAGTTTGGCATTACTTTCT A

665	13.9	0.0	0.0	AluYa5-Scaffold11190-97324-97550	1	101	(2126)	+	AluSx3	SINE/Alu	210	310	(1)	1	
227	25.6	3.0	7.0	AluYa5-Scaffold11190-97324-97550	142	276	(1951)	C	L1M5	LINE/L1	(2976)	3170	3035	2	
2113	11.4	0.0	0.0	AluYa5-Scaffold11190-97324-97550	277	556	(1671)	C	AluSg	SINE/Alu	(3)	307	28	3	
227	25.6	3.0	7.0	AluYa5-Scaffold11190-97324-97550	557	584	(1643)	C	L1M5	LINE/L1	(3112)	3034	3013	2	*
637	24.0	4.6	10.7	AluYa5-Scaffold11190-97324-97550	583	892	(1335)	+	L1MEd	LINE/L1	1233	1523	(5598)	4	
51	2.0	0.0	0.0	AluYa5-Scaffold11190-97324-97550	893	943	(1284)	+	(T)n	Simple_repeat	1	51	(0)	5	
2182	0.0	0.0	0.0	AluYa5-Scaffold11190-97324-97550	1001	1227	(1000)	C	AluYa5	SINE/Alu	(84)	227	1	6	
1585	25.5	5.1	4.4	AluYa5-Scaffold11190-97324-97550	1235	2220	(7)	+	L1MEd	LINE/L1	1556	2445	(3701)	4	

>chr15:67643905-67645787
AGGCAGAGGTTGCAGTGAGCTGAGATCACGCCATTGCACTCCAGCTTGTGCTACAGAGTGAGACTCTGTCTCAAAAAAAAA AaaAAATAAATAAATATAAAATAAAAAAATAAAGAAGCCAATTTTTA
ATTATTTTGGGTCATTTTTGTTTTTACTTTCTTAATGTCTGCTTTTATTTTCATTATTTCCTTTCATTTTTTTTCCCAGTTTACTGCTTTTCTTTTTCTGTCTTCTTGTGTTTAATTAGGCTTAC GTC
ACTTATTATCAATTGTTCTTGTTTTCTTTTTTCTTTATTTTTTTTTTTGAGACTGAGTCTCGCTCTGTCACTCAGGCTGGATTGCAGTGGAGCAATCTTGGCTCACTGAAACCTCTGCCTCCTGGG TT
CAAGCAATTCTCCTGCCTCAGCCTCCTGAGTAGCTGAGATTACAGGTGCGCACCACCACGCCTGGCTAATTTTTGTATTTTTAGTAAAGACGGGGTTTCACCATTTTAGTCAGGCTGGTCTTGAA CAC
TTGACCTCGTGATCCACCCACCTTAGCCTCCCAAAGTACTGGAATGTTCCTGTTTTCTACTAAATGCATATAAACAATGGATGAGGTTGCTCAGAAGTGATGAAAGACATTGGTCTACAGATTCAAGA
ATCCCAATAAATACAAAGCAGGATAAATAAAAAGACCTATATGCCTAGATGCATCACAATGAACCTGTAGAACACAAAAGACAAAAAGAACTTATTTCAACTAGAGAGGAAACAAAAGACAAATT GTT
TTCATCcACAATTTTAGCCACTATCTACCACTGACTCCTCAATAGTCCACAGGCTCCTCAATAGCAGATATAATTGCTAGAAAACAATGAAATAATATATTGAATGTGCTGACAA GATAACTTTCAAA
AGTCAGGACATAAACACATTTTGAGGCAAATAAAAGTTGAAAGAGTTTACCACCAGCAGAAACAGGCTTGTACTAGTGGAAATTGAAAGAATATAATTTAGGGAGATGGAAGTGAATGCAGTATTGAA
AGTCAGAGATGCAACAAGGAATGAAGAGCAAAGACAGTGACAAATCTGTTGGCTAATATAACAAATTCTGATTACATAAAAAACATTAACAATAATGTCTTGTGGGTTTTTTTAAAAAGATACAAC TA
AAATACATAAGAAAAATAGATGTTACATCAGGAGGGAGATGGAGTTAAAATGTTCTGAGGTTTTTATACTGTTAAAGGGAAAAGTACAGATATTTATTAACTTTAGACTTTGATAAGTAATATAT GCA
TGTGAAAATAAGAAGCTAATCACAATAAGAATATAAAAGGAATGTATCAATTCCAAAATAATAGAGGAATAAAAGTATAATGAGGAAAAATAATCTATTCAAAAGAAGACAATAAAGGAAAGAAAA TG
AAATAGCAACAAAACAGGGCAAGGCACAAACAGAAACAGTCTAGATGGAAGAAATATTATCAAAATATATTAGTAATTAGAATAACTGTAAATACAGTAAATGCTAAATGGATAAC gATGGTCAGAAT
GATTTTTTTTTAAATTCAGCTATATACTATTTATAAAAGACAGACTTAAAACATATTGCTACAGAAAGATTAAAAGTAAAAAAATGAGAAAAGATATGCCAGGCAAACAATAACCAAAAGGAAGCT GA

TGTGGCTATATTAACAGACAAAATGCTATTTATGCTAAAAAGCAATTATAGAGATAATGAAGGACAGTGTGCAATGATAAAAGTTCCCATTTACTAGTAAGATATAATAACTATAGTAAGAGTTG GCA
AGGATGTGAATCCACAGAGTATCTTACACACTGAAGGTAGAAATGTAAATTGATGCAATGACTTTAAAAGACAGTTTGGCATTACTTTCT A

701	12.8	0.0	0.0	chr15:67643905-67645787	1	102	(1781)	+	AluSx3	SINE/Alu	210	311	(0)	7	
227	25.6	3.0	7.0	chr15:67643905-67645787	143	277	(1606)	C	L1M5	LINE/L1	(2976)	3170	3035	8	
2113	11.4	0.0	0.0	chr15:67643905-67645787	278	557	(1326)	C	AluSg	SINE/Alu	(3)	307	28	9	
227	25.6	3.0	7.0	chr15:67643905-67645787	558	585	(1298)	C	L1M5	LINE/L1	(3112)	3034	3013	8	*
2010	26.8	6.0	4.2	chr15:67643905-67645787	584	1876	(7)	+	L1MEd	LINE/L1	1408	2620	(3701)	10	

>KSI_AluYa5_35

```
>Scaffold11731-977646-977787
AAGGAGGTTACCTGGTTTAATTGTTGTTGTTGTTTGAAGGGGGAACAAAGAGATAAAGAAATGTTTTTATTAACTTTAATGAAACAAAAATGGAGCCTGAGTTTTTGTTCTTATTTTGG tTTGCT
GGATCGCTTGCATAAGTTGTAACTAACTGTTCTCTTCATGTTGTAAAAGTAAAATTCCCAAAGCCTTGGGATTATAGTCAACAGGATAGAAAAAGCTATTTTATGTACCTAGCTATGGCCAAAAA GAC
TTTTTGAAAAGAGTATTATTACAGAGCTTGGGGCTAATCTACTGGAATCCCCTGAGCCTTTCTTTCACTACACAGGTATCTTAGTAGATCTAGAGGGTACAATCACTCGTGACAAGCAATGAA TATAC
AACAGGCTTCTAGAACATTCATTTCATCAGCACAAGAAACTTGGGAATATCTATATTCAAATACATTGAAATGGGGATATTTAGAGCCAAAAGCTATGTAAATCCCATTACCCCTTGAAAATGGA TGT
CTACATTCCTATGAATGTAGCTTTCCTCAGCTCCTATTCCCCATGATTCTCAGAAGTATGAAATTGATAAATATCCAATTACACCTTGTAGAGGAGACTTAGCAACCAGCGTTCATACTGGCC TCACT
TATTCTTTTTTTTAAAAATGTGACTATTTTAAGTATTATTTGGATAACAAAATCTCAACCTGAAACACCTAGAGCCAGGGGTCCAGGATGCTCCAGCTGATGATCACGTATGCTTACTAACGCTC CCT
GCTGTGATGCTGGCACACAAGGTGAAAAAGCAGAGTCGGGTGTGCTGCTTTTTGGAATGAAAACTTAGTATGAATTTGTTCACTTGGCATTTGCCATTACTTCCAAAAATGAAGACCAAAAATTCTTG
GTAATTAAAGTTTCTGCATATTTAGATTCTGTTATTCTATTTTttttttttttttttttttttttttttttaggggggttttctctctccccccagccgggggggcccggctaatttttgtattttt
agtagagacggggtttcaccattttagccgggatggtctcgatctcct gacctcgtgatccgcccgcctcggcctcccaaagtgctgggattacaggcgtgagccaccgcgcccggcc tctgttattc
tattttACAATGTATGTGATTTTTTTTTTTttttGAgTTAACATGGCATTTGAAAACCTATTGCAAGCAATAGAACTATCCTCCCCAGAAATCT aAGTTTCAGGGGATTCACCTGGGGTTATGGAGCC
CTGGTTAAGAGCTCACAAGATAATGAAAACAGCATGATACAAAAACCTACAGCTAACATCACACTGAATGACAAGAAATTCGAAGTTTTCTCACTGAAATCAG TAACAAGGCACGGATTTTTCTCTCA
CCATTCCTTTTCAACATTGTACTGGAATTTCTAGCTAATGCAATAATATAAGAAAAGGAAATGAAAGGTATACAGATAGGGAAGGAAAAATAAAACTGTCTTTATTCACAGATGGCATGATTGTC TAT
GTAAAAACCCAAAAGGACTGACAAAAGACTCTTAGAACTAAAGTCAAA TATGGTGGTGTGTGTCTGTAGTCCCAGCTACCCAAGAGGGTGAGGTGGAAAGATCACTTGAGGCCAGGAGTTCAAGGCTG
TAGTGCAGTATGATTGCATCTGGAAAAAGCCGCTGCACATCAGCCTGGGCAACATTGTGAAATCCCATATCTTAAAAAT GAAAAAAAAAAAATCCTGGAACTAATAAGCAATTATTGCAGAGCTGCAGA
ATACAAAGTTAACATACATGCAAAAGTCAATTCCTTTCTTATATGCCA GCAAGAAACAAGTGGAATTTGAAATGAAAAACACAATATCATTTACATTAGCACCCTACAAAATGAAATACTT gGGTATA
AATCTAACAAAATATATTAAGATATATATGAAAAAACCTACATAACTCCAATGAACAAAATTAAAGAACTAAGCAAATGAAAAGATATTC cATGTTCATGGACAGGAAGACTCAATATTGTCAAGATG
TGAGGCCTTCCCAATTTGATCTGCAGATCCA aTGCAATCCCAATCAAAATCCAAGGAAGTTATTTTGTGAATATTTACAAACTGATTCTAAAGT
```

13	0.0	0.0	0.0	AluYa5-Scaffold11731-977646-977787	21	37	(2105)	+	(TTG)n	Simple_repeat	1	17	(0)	1
34	0.0	0.0	0.0	AluYa5-Scaffold11731-977646-977787	936	967	(1175)	+	(T)n	Simple_repeat	1	32	(0)	2
1347	0.7	0.0	0.0	AluYa5-Scaffold11731-977646-977787	1001	1142	(1000)	C	AluYa5	SINE/Alu	(169)	142	1	3
1621	14.0	1.6	0.0	AluYa5-Scaffold11731-977646-977787	1321	1577	(565)	+	L1MC	LINE/L1	4013	4273	(1873)	4
987	20.2	0.0	0.0	AluYa5-Scaffold11731-977646-977787	1588	1755	(387)	+	FAM	SINE/Alu	10	177	(8)	5
2397	13.1	1.0	1.0	AluYa5-Scaffold11731-977646-977787	1756	2142	(0)	+	L1MC	LINE/L1	4262	4648	(1498)	4

```
>chr8:96782551-96784471
AAGGAGGTTACCTGGTTTAATTGTTGTTGTTGTTTGAAGGGGGAACAAAGAGATAAAGAAATGTTTTTATTAACTTTAATGAAACAAAAATGGAGCCTGAGTTTTTGTTCTTATTTTGG cTTGCT
GGATCGCTTGCATAAGTTGTAACTAACTGTTCTCTTCATGTTGTAAAAGTAAAATTCCCAAAGCCTTGGGATTATAGTCAACAGGATAGAAAAAGCTATTTTATGTACCTAGCTATGGCCAAAAA GAC
TTTTTGAAAAGAGTATTATTACAGAGCTTGGGGCTAATCTACTGGAATCCCCTGAGCCTTTCTTTCACTACACAGGTATCTTAGTAGATCTAGAGGGTACAATCACTCGTGACAAGCAATGAA TATAC
AACAGGCTTCTAGAACATTCATTTCATCAGCACAAGAAACTTGGGAATATCTATATTCAAATACATTGAAATGGGGATATTTAGAGCCAAAAGCTATGTAAATCCCATTACCCCTTGAAAATGGA TGT
CTACATTCCTATGAATGTAGCTTTCCTCAGCTCCTATTCCCCATGATTCTCAGAAGTATGAAATTGATAAATATCCAATTACACCTTGTAGAGGAGACTTAGCAACCAGCGTTCATACTGGCC TCACT
TATTCTTTTTTTTAAAAATGTGACTATTTTAAGTATTATTTGGATAACAAAATCTCAACCTGAAACACCTAGAGCCAGGGGTCCAGGATGCTCCAGCTGATGATCACGTATGCTTACTAACGCTC CCT
GCTGTGATGCTGGCACACAAGGTGAAAAAGCAGAGTCGGGTGTGCTGCTTTTTGGAATGAAAACTTAGTATGAATTTGTTCACTTGGCATTTGCCATTACTTCCAAAAATGAAGACCAAAAAT TCTTG
GTAATTAAAGTTTCTGCATATTTAGATTCTGTTATTCTATTTTACAATGTATGTGATTTTTTTTTTTGAaTTAACATGGCATTTGAAAACCTATTGCAAGCAATAGAACTATCCTCCCCAGAAATCT g
AGTTTCAGGGGATTCACCTGGGGTTATGGAGCCCTGGTTAAGAGCTCACAAGATAATGAAAACAGCATGATACAAAAACCTACAGCTAACATCACACTGAATGACAAGAAATTCGAAGTTTTC TCACT
GAAATCAGTAACAAGGCACGGATTTTTCTCTCACCATTCCTTTTCAACATTGTACTGGAATTTCTAGCTAATGCAATAATATAAGAAAAGGAAATGAAAGGTATACAGATAGGGAAGGAAAAATA AAA
CTGTCTTTATTCACAGATGGCATGATTGTCTATGTAAAAACCCAAAAGGACTGACAAAAGACTCTTAGAACTAAAGTCAAATATGGTGGTGTGTGTCTGTAGTCCCAGCTACCCAAGAGGGTG AGGTG
GAAAGATCACTTGAGGCCAGGAGTTCAAGGCTGTAGTGCAGTATGATTGCATCTGGAAAAAGCCGCTGCACATCAGCCTGGGCAACATTGTGAAATCCCATATCTTAAAAAT GaaAAAAAAAAAAATC
CTGGAACTAATAAGCAATTATTGCAGAGCTGCAGAATACAAAGTTAACATACATGCAAAAGTCAATTCCTTTCTTATATGCCAGCAAGAAACAAGTGGAATTTGAAATGAAAAACACAATATC ATTTA
CATTAGCACCCTACAAAATGAAATACTTaGGTATAAATCTAACAAAATATATTAAGATATATATGAAAAAACCTACATAACTCCAATGAACAAAATTAAAGAACTAAGCAAATGAAAAGATATTC tAT
GTTCATGGACAGGAAGACTCAATATTGTCAAGATGTGAGGCCTTCCCAATTTGATCTGCAGATCCA gTGCAATCCCAATCAAAATCCAAGGAAGTTATTTTGTGAATATTTACAAACTGATTC TAAAG
T
```

13	0.0	0.0	0.0	chr8:96782551-96784471	21	37	(1884)	+	(TTG)n	Simple_repeat	1	17	(0)	6
1621	14.0	1.6	0.0	chr8:96782551-96784471	1098	1354	(567)	+	L1MC	LINE/L1	4013	4273	(1873)	7
1005	20.0	0.0	0.0	chr8:96782551-96784471	1365	1534	(387)	+	FAM	SINE/Alu	10	179	(6)	8
2381	13.3	1.0	1.0	chr8:96782551-96784471	1535	1921	(0)	+	L1MC	LINE/L1	4262	4648	(1498)	7

>KSI_AluYa5_36

>Scaffold12188-3828034-3828155
CTGATGAAATTCTAGAAATCAAAATATATAAAACCAAGTTGATAGTCACCACTGGGCCATTGGGTCCAGTGAGAAGCAGAGAAAGACAAGCCAAAAGAAAAAAGGGAAGGTAGGGATTTGGATAGA GC
CCATGACCAACTTAAAATATGTAAGCCTGCCTCTGAAGATACTGAAATCCTTAGCATCATATTGGAAATATTTTCAGAATGTTTCCAAAGAAAATACAAGGTGAATGAAATTCTGGGTTTTAAGT GGA
ATTAAATAGCAGGAGTACGTTGCAGAGTATAGGCTGCCAGACTTTAGGATTTATGGATCAGTACAATTAAAGAGAATAAATAACTGGAGGACTATCAAGAGGTGCCAGCTCTTTATTTTTGCCAAG CA
TTGCTAGCTTGGTTTCCCCCTACTAAGATTCTGTTGGGTGAGGAGTTAGGCCTAAGGAATAATAGAGTTTAATTTAAACAAAAGATGAGAAAACAGTTCAAAAGATACACAAAGAAAAGTTTATT TTC
ACTATGTGGAAGTTACTAGTTAGCGTTTCCTTCTGTTAGAGTCCAGCTCTTCTGAGCTCTAAGTCCCACAGGGTCGACTTCTGCCAGGTGACTCGCAGTCAGCATAAGTGGGAAGCTCTGCGATCT GG
GGAGCTATGCTGTGACTGACAGAGTCCAGTTACAGCACTCTTCATTTGTGCTCAGGTAGGTACCCTCTACTGTCTGCAAGTCT CAACTCCAAACCACACTCAGGTTTCCCTAGCTTACACTGTGCTCC
CTGGAGACACGCACAGATGCAAGAAAGTGTTTTCCAGAGCTGTTGATATAATACTTTGTGGCTGCTACTTTTT Ttctaaatgtttttaatatggaaaatgctaagcatatcccaaagtaaagaatgt
gtaatgaaccatcatacactgtcactcagcttcaacaattataaactaatggcgaatcttgtttttctacatttctacccatttcccca tggcccccccccccccgccactacgcccggctaattt
tttgtatttttagtagagacggggtttcaccgttttagccgggatggtctcgatctcctgacctcgtgatccgcccgcctcggcctcccaaagtgctg TCTAAATGTTTTTAATATGGAAAATGCTA
AGCATATCCCAAAGTgAAGAATGTGTAATGAACCATCATACACTGTCACTCAGCTTCAACAATTATAAACTAATGGCGAATCTTGTTTTTCTACAcTTCTACCCATTTCCCCA ACCCCAGATAATTG
TGAAGCAAATTCCAAACATCAGTTCATCAATAAACATCTTGATTATATAGCTCTAAAAGCTATAGATTCTTT TAAAAAAAAAGTACCATTAGCATGCTTTAAAATAATAATAACTCCTCAGTATCATC
AAATGTCTAGTTAATATTTAAATTTCCTTGATGGTTTCATAAATGTAGTCTTAT gTACTTATTTATTTACATATTTATGTaTATATAtgTATATTATAGTTTGTTTTTCTATTCAGGACACAAATGA
GATCTATGTCTTTTTTCCAaCAGTTTTAACCATCAGGATTTTTATGATTGCATGTATGTACTTTTATTTTACATGTTTCTTTTCCTCTTGAATTTTCTGTAAATTGAAAATTGATCCTAAATGGCTG
ACCTTTTCTTTTTTTGGCAAGAATATTTCATGGACAGTGGTATGTCACTCAGTCAAGAAACACATGATCTCTGGTTGTCACTCTTTCTTTTTTTAATGTTAACCACTGATGACTT GGTCTGGATCCAT
TATTTCATTAAGCGTGTTGGTATTTTCTTATGCTTTTTACAGAAATGCAAATAATTACCTGATTCTTGGGGCATTTTTAAGTTTTCATATTGTTGTGCTAAACTCTAGCAATTATTCACTGTTTG CAA
AAGTGTGATCTCAAGTCTTTTGCACAGCAAGCAGGGATGGTACGGGGCAAAAACAGCCATCATCTAAAATTATCACTATTTCATAGAATGAGAGGTTGTTCAAAAGCAGATATAG GAAAAATTTAATT
GAATATTAAAAGGTAGAACTTTAAATACATATATCTTTCTAGAAGATTCTCCACTATCCTGATTTTTTCCCCC C

320	20.7	2.1	5.3	AluYa5-Scaffold12188-3828034-3828155	288	384	(1738)	C	Charlie18a	DNA/hAT-Charlie	(4)	338	245	1
625	20.5	2.7	2.0	AluYa5-Scaffold12188-3828034-3828155	837	985	(1137)	C	L1MC5	LINE/L1	(0)	7947	7798	2
1167	0.0	0.0	0.0	AluYa5-Scaffold12188-3828034-3828155	1001	1122	(1000)	C	AluYa5	SINE/Alu	(158)	153	32	3
1333	21.3	5.5	0.8	AluYa5-Scaffold12188-3828034-3828155	1132	1476	(646)	C	L1MC5	LINE/L1	(12)	7935	7575	4
16	11.7	3.6	0.0	AluYa5-Scaffold12188-3828034-3828155	1479	1506	(616)	+	(AT)n	Simple_repeat	1	29	(0)	5
12	13.6	6.8	4.4	AluYa5-Scaffold12188-3828034-3828155	1507	1522	(600)	+	(ATATT)n	Simple_repeat	1	45	(0)	6
698	26.1	5.6	1.9	AluYa5-Scaffold12188-3828034-3828155	1558	1807	(315)	C	L1MC5	LINE/L1	(511)	7436	7178	4

>chr2:80768169-80770009
CTGATGAAATTCTAGAAATCAAAATATATAAAACCAAGTTGATAGTCACCACTGGGCCATTGGGTCCAGTGAGAAGCAGAGAAAGACAAGCCAAAAGAAAAAAGGGAAGGTAGGGATTTGGATAGA GC
CCATGACCAACTTAAAATATGTAAGCCTGCCTCTGAAGATACTGAAATCCTTAGCATCATATTGGAAATATTTTCAGAATGTTTCCAAAGAAAATACAAGGTGAATGAAATTCTGGGTTTTAAGT GGA
ATTAAATAGCAGGAGTACGTTGCAGAGTATAGGCTGCCAGACTTTAGGATTTATGGATCAGTACAATTAAAGAGAATAAATAACTGGAGGACTATCAAGAGGTGCCAGCTCTTTATTTTTGCCAAG CA
TTGCTAGCTTGGTTTCCCCCTACTAAGATTCTGTTGGGTGAGGAGTTAGGCCTAAGGAATAATAGAGTTTAATTTAAACAAAAGATGAGAAAACAGTTCAAAAGATACACAAAGAAAAGTTTATT TTC
ACTATGTGGAAGTTACTAGTTAGCGTTTCCTTCTGTTAGAGTCCAGCTCTTCTGAGCTCTAAGTCCCACAGGGTCGACTTCTGCCAGGTGACTCGCAGTCAGCATAAGTGGGAAGCTCTGCGATCT GG
GGAGCTATGCTGTGACTGACAGAGTCCAGTTACAGCACTCTTCATTTGTGCTCAGGTAGGTACCCTCTACTGTCTGCAAGTCT CAACTCCAAACCACACTCAGGTTTCCCTAGCTTACACTGTGCTCC
CTGGAGACACGCACAGATGCAAGAAAGTGTTTTCCAGAGCTGTTGATATAATACTTTGTGGCTGCTACTTTTT TTCTAAATGTTTTTAATATGGAAAATGCTAAGCATATCCCAAAGTaAAGAATGT
GTAATGAACCATCATACACTGTCACTCAGCTTCAACAATTATAAACTAATGGCGAATCTTGTTTTTCTACAtTTCTACCCATTTCCCCA ACCCCAGATAATTGTGAAGCAAATTCCAAACATCAGTT
CATCAATAAACATCTTGATTATATAGCTCTAAAAGCTATAGATTCTTT TtAAAAAAAAAGTACCATTAGCATGCTTTAAAATAATAATAACTCCTCAGTATCATCAAATGTCTAGTTAATATTTAAAT
TTCCTTGATGGTTTCATAAATGTAGTCTTATtTACTTATTTATTTACATATTTATGTgTATATATATATTATAGTTTGTTTTTCTATTCAGGACACAAATGAGATCTATGTCTTTTTTCCA gCAGTT
TTTAACCATCAGGATTTTTATGATTGCATGTATGTACTTTTATTTTACATGTTTCTTTTCCTCTTGAATTTTCTGTAAATTGAAAATTGATCCTAAATGGCTGACCTTTTCTTTTTTTGGCAAGAA TA
TTTCATGGACAGTGGTATGTCACTCAGTCAAGAAACACATGATCTCTGGTTGTCACTCTTTCTTTTTTTAATGTTAACCACTGATGACTTGGTCTGGATCCATTATTTCATTAAGCGTGTTGGTA TTT
TCTTATGCTTTTTACAGAAATGCAAATAATTACCTGATTCTTGGGGCATTTTTAAGTTTTCATATTGTTGTGCTAAACTCTAGCAATTATTCACTGTTTGCAAAAGTGTGATCTCAAGTCTTTTGC AC
AGCAAGCAGGGATGGTACGGGGCAAAAACAGCCATCATCTAAAATTATCACTATTTCATAGAATGAGAGGTTGTTCAAAAGCAGATATAGGAAAAATTTAATTGAATATTAAAAGGTAGAACTTT AAA
TACATATATCTTTCTAGAAGATTCTCCACTATCCTGATTTTTTCCCCC C

320	20.7	2.1	5.3	chr2:80768169-80770009	288	384	(1457)	C	Charlie18a	DNA/hAT-Charlie	(4)	338	245	7
1312	22.1	5.6	1.1	chr2:80768169-80770009	837	1193	(648)	C	L1MC5	LINE/L1	(0)	7947	7575	8
15	17.7	3.1	0.0	chr2:80768169-80770009	1194	1225	(616)	+	(AT)n	Simple_repeat	1	33	(0)	9
695	26.2	6.1	1.8	chr2:80768169-80770009	1263	1526	(315)	C	L1MC5	LINE/L1	(495)	7452	7178	8

>KSI_AluYa5_37

>Scaffold12428-30637-30768
CAGGTGGAAGTTGCAGTGAGCCAAGATTACACCACTGCACTCCAGCCTGGGTGTCAGAGCGAGACTCCATCTAAAAATAAAAAAAAAAATTAAATGTCACCACCTTGCATTGTACTTGATCTTCAGA AT
TTTACTCTATTGTCTTAAGCCTTTAAAACTTTGCTCATGAACTATATTATTTGGACTACCTTTGAGGGTAGTTGCATTTTTGCATTTTTGCAAAATATTATTTTCCCTAGGTTACAGTAAACCTT TGA
CTCTGTTATATTTGAAGCAACTAATTATTCCTGAAACATATAAAGCATTTCCTGGCAATGTTGGTGGAGTTCATACTGCTCAAGGTGCTGAAGTCATTATTTCAAGGGGAAATTATTAGCGTGTTC CA
ACAGTTCTTAATTGTCCAAACAATTGTTGATTTCCCCCCTTTCCAGATTTCTTTCACAATCATTGGGAACTATAAAAAGGTGTATTCGACATTTTCTTTTGTGTATATTTTCCACTTCAACAATT TTG
TATCAAAACGTATGGCACTTTTTCAGCACTTATTTCAAGAGATCC aGATAAACTCTGAGTAATCACAATTCACTATACTTCAAAAATTGCCTCCCATTCAGTATTTTACAAATAATCTTCTACAAGTA
AATGGGGAAAATAGACACTAACCATATACTTTTTAAAAATAATACATTAAAAGAGCACTAAAGCGTTCTTTGAAAGTGCTCCTGGTACTATATACGTATTGGCTCATTCTAGCAATGTTCTTTTG AGA
CACCATACAAGAAATGGAACTCGACCAGATTT cAGAAAAAGCTGCAGAGAAGGTGAGGATTTGTTCCAGGGCCATACAGAGAGGAAATTGAGACACTCATTTCTTCTTAGTT CAGTCTCCCAGGTGTT
TAGCCCTAGATCTCTTTGAGATTGCACGAATTTAAGGGATTTTTT Taaaaaaacagggaagctctgttatcaaattttaagagaggataaagaatta gggatggggccgggcgcggtggctcacgcct
gtaatcccagcactttgggaggccgaggtgggcggatcatgaggtcaggagatcgagaccatcccggctaaaacggtgaaaccccgtctctactaaaaatacaa TAAAAAACAGGGAAGCTCTGTTAT
CAAATTTTAAGAGAGGATAAAGAATTA GAGATGGTAGATGAGAAATGTTTCATCCAAAGAGTTTTTTGTTTGTTTTGTTGTTTTTTTGAGACATAGTCTCGCTCTGTCACCCAGGCTGGAGTGCAGTG
GCATGATCTGGGCTCACTGCAGCCTCTGCCTCCCAGGTTCAAGCGATTCTCGTGCCTCAGCCTCCTGAGTAGCTTGGATTATAGGCGTGAGC CACTGTGCCTGGCTCAAAGAGTTTTAATGAATAGAA
AACAGTGACCCATTCTTGTAGAAAAGACATGGAGATAAAATGAAATAAGGAGAGCCTGAGGTGTCAGGGAAGGAGAAAGAGCATTTTGAAACTGGTGGCAGGTACAATTCTCTGCCTAACTCCGT GGA
CTACTTAGGGCACAGATTTCTTCCTGCTCAGTCAGTGTTAGCTGCCTTCCTGGTTATGTGCTTTAGGGTTGACC aTAATTGGTAACATAAGCCATTCCAGTTAGGTGAGCCTC TCTTCCTTGTGTATT
TTGGATAATGACTACGAGGAAACAAGATGGAAATGTGGGATGGTTAGCTAATGTCAGTGCCGAGTCTCCCTGCTCAATGCGCGTTAGCCCAACTGCCAAAGCTCTGCCACTATGGTAAGTGAAGA TGG
TGATTTTCAGTTTGGCAGAGACTGTTGGAAGGCGTAATGGCTTATTTGATGGAAAAGATTTCCTGCTGCGGGAGAAGCTGCCATTTGCAGACAAGGTGTGTCAGCCAGAAGAA ATGAGCAATAACCGT
TTTCTGGTTTCTACTGGCAAAAGCTTTGTATTTTTTTCATATAGAAGTGTTAAAAATAGATTCATAAGAAAATGCATGAAACCAAGTAAAAAGTACAGACACTGAAATTTCACCAATGAATGGGA GAG
AAGAGAGCTAACCTTTGTTGAATAGTTATTGCTAACCAGGTGCTTCAGTTCTCACAAAATTGTGTGAGGTAGGCCCGCCACAT T

631	12.6	0.0	0.0	AluYa5-Scaffold12428-30637-30768	2	88	(2044)	+	AluSx3	SINE/Alu	210	296	(15)	1
1208	2.3	0.0	0.0	AluYa5-Scaffold12428-30637-30768	1001	1132	(1000)	+	AluYa5	SINE/Alu	1	132	(179)	2
1256	12.1	0.0	0.0	AluYa5-Scaffold12428-30637-30768	1214	1386	(746)	C	AluSz6	SINE/Alu	(4)	308	136	3

>chr4:169755815-169757760
CAGGTGGAAGTTGCAGTGAGCCAAGATTACACCACTGCACTCCAGCCTGGGTGTCAGAGCGAGACTCCATCTAAAAATAAAAAAAAAAATTAAATGTCACCACCTTGCATTGTACTTGATCTTCAGA AT
TTTACTCTATTGTCTTAAGCCTTTAAAACTTTGCTCATGAACTATATTATTTGGACTACCTTTGAGGGTAGTTGCATTTTTGCATTTTTGCAAAATATTATTTTCCCTAGGTTACAGTAAACCTTTGA
CTCTGTTATATTTGAAGCAACTAATTATTCCTGAAACATATAAAGCATTTCCTGGCAATGTTGGTGGAGTTCATACTGCTCAAGGTGCTGAAGTCATTATTTCAAGGGGAAATTATTAGCGTGTT CCA
ACAGTTCTTAATTGTCCAAACAATTGTTGATTTCCCCCCTTTCCAGATTTCTTTCACAATCATTGGGAACTATAAAAAGGTGTATTCGACATTTTCTTTTGTGTATATTTTCCACTTCAACAATTTTG
TATCAAAACGTATGGCACTTTTTCAGCACTTATTTCAAGAGATCC gGATAAACTCTGAGTAATCACAATTCACTATACTTCAAAAATTGCCTCCCATTCAGTATTTTACAAATAATCTTCTACAAGTA
AATGGGGAAAATAGACACTAACCATATACTTTTTAAAAATAATACATTAAAAGAGCACTAAAGCGTTCTTTGAAAGTGCTCCTGGTACTATATACGTATTGGCTCATTCTAGCAATGTTCTTTTGAGA
CACCATACAAGAAATGGAACTCGACCAGATTT gAGAAAAAGCTGCAGAGAAGGTGAGGATTTGTTCCAGGGCCATACAGAGAGGAAATTGAGACACTCATTTCTTCTTAGTT CAGTCTCCCAGGTGTT
TAGCCCTAGATCTCTTTGAGATTGCACGAATTTAAGGGATTTTTT TTAAAAAACAGGGAAGCTCTGTTATCAAATTTTAAGAGAGGATAAAGAATTA GAGATGGTAGATGAGAAATGTTTCATCCAAA
GAGTTTTTTGTTTGTTTTGTTGTTTTTTTGAGACATAGTCTCGCTCTGTCACCCAGGCTGGAGTGCAGTGGCATGATCTGGGCTCACTGCAGCCTCTGCCTCCCAGGTTCAAGCGATTCTCGTGC CTC
AGCCTCCTGAGTAGCTTGGATTATAGGCGTGAGC CACTGTGCCTGGCTCAAAGAGTTTTAATGAATAGAAAACAGTGACCCATTCTTGTAGAAAAGA CATGGAGATAAAATGAAATAAGGAGAGCCTG
AGGTGTCAGGGAAGGAGAAAGAGCATTTTGAAACTGGTGGCAGGTACAATTCTCTGCCTAACTCCGTGGACTACTTAGGGCACAGATTTCTTCCTGCTCAGTCAGTGTTAGCTGCCTTCCTGGTT ATG
TGCTTTAGGGTTGACCgTAATTGGTAACATAAGCCATTCCAGTTAGGTGAGCCTCTCTTCCTTGTGTATTTTGGATAATGACTACGAGGAAACAAGA TGGAAATGTGGGATGGTTAGCTAATGTCAGT
GCCGAGTCTCCCTGCTCAATGCGCGTTAGCCCAACTGCCAAAGCTCTGCCACTATGGTAAGTGAAGATGGTGATTTTCAGTTTGGCAGAGACTGTTGGAAGGCGTAATGGCTTATTTGATGGAAA AGA
TTTCCTGCTGCGGGAGAAGCTGCCATTTGCAGACAAGGTGTGTCAGCCAGAAGAAATGAGCAATAACCGTTTTCTGGTTTCTACTGGCAAAAGCTTT GTATTTTTTTCATATAGAAGTGTTAAAAATA
GATTCATAAGAAAATGCATGAAACCAAGTAAAAAGTACAGACACTGAAATTTCACCAATGAATGGGAGAGAAGAGAGCTAACCTTTGTTGAATAGTTATTGCTAACCAGGTGCTTCAGTTCTCAC AAA
ATTGTGTGAGGTAGGCCCGCCACATT

631	12.6	0.0	0.0	chr4:169755815-169757760	2	88	(1858)	+	AluSx3	SINE/Alu	210	296	(15)	4
1256	12.1	0.0	0.0	chr4:169755815-169757760	1028	1200	(746)	C	AluSz6	SINE/Alu	(4)	308	136	5

>KSI_AluYa5_38

>Scaffold12887-1479719-1479867
CCCGTATTTTCATATCCTAGGTTTTTGTTCCCTGTGGCTTATTGTAAAACTCAATCCACAGCTCTCTAAGCTTTGCTATCCTAAAAAGAGTCTTTGGGGGAACTGAGAAATTCCTTTTATTCAAAG CC
AGTGCTTCACTGGGGACTGCAAGAGCCCACTTTGATAGGTAAAAGCCCAGCATGAAACCATTTCATTCCTTTACATTCTTCCTACCACAAGCCAAATTTCCTGAGGCAAAAAGGATGATTCTGGC AGG
CTAGGAAGGTCATGCAAAGAGATGCAAAAAGAAAATCATATTACGGTCATCCCTTGGTATCCATGGGGGATTGGTTCCAGGACAGGCTACCTGCCCCACTCCAAGATACCACAGTCCAGAATTCTC AA
GCCCGTTATATAAGAGGACATAATTGCATATAACCTAtACACATTCTCCCATATGCTTTCAATCTAGATTACTTGTAATATTTAATACCATGTACACATTATATAAATAGTTGTGATATATTTTTTAA
TTTTTTTCCCAAGTATTTTCCATCTGCAGTTACAGAACTCTCAGATATGGAGAGCCAACTGTAATATGTTTCAAAACTGTTATTCCTGCTATATATAGTTAAGGTTTTATTTAAATTCACAATTTTCA
TTTCTATATATTCTTGTTCAACATCATTGCCTACGATTACTGAAAATGACTTTTCTTAGGTTAACTCACCTTATAAACTGCTTAGAAGAATCACTCAGAGAAAAGCAAGTTATATGCTAATTTTT TAG
AGTTTAGTTTTTTGGTGATGGGAACATTATTTGGGTTGCAGATATAAGTAGTTTCAAAAGAAGATAACTTTTATAGTATCTCAGATAAAATCATTTGTGTTATATT CTGGTCATTATTAAAGGCAGTG
TTGCTTTTTGGTAGTCTAAGGTAAGAGGTTTTCTAATGAGCCTGTAAGAATTTAAGAGTTTGTAATTGTATGATATACACACAGTCAAGAAGCAAAACGGCAACTTTTTTTTTTTTTTTTCTAGT AGA
GACTGAGTCTTGCTATGTTGCTTAGGCTGGTCTTGAACTCCTGGGCTCAAGTGATCCTCCCACCTCAGCCTCCTGAGTAACTGGGATTACAGGCATGAGTCACTGTGACTGG Cccctgcaacatcttt
tttttttttttttttttttttttttttttttatgggaccacaggcgcccgccactacgcccggctaatttttgtatttttagtagagacggggtttcaccgttttagccgggatggtctcgat ct
cctgacctcgtgatccgcctgcctcggcctcccaaagtgctgggattacaggcgtgagccaccgcgcccggCCCTGCAACATCTTTTGTAATCTATTTTTGAATAAGGAAAGTTAAAAAACAAAGAGG
TAAAATTAAAAGACATGATCTAAGCATTTAAAATTAATTTGTCTAAAAGTTATTGAA AGCCAGGCATTGTTGCTTATGCCGGTAATCCCAGCAACTGTGGAGGCTGAGGTGGGAGGATTGCTTGAGTC
CTGGAGTTCAAGACCAGCCTCTTTAAAAAGAAAAGTTATTGACTCACTGGATGTTTAAATAGATT AACACAGTTCATATTATATACTTATATAAACTTAACAATGCTAGGAAATGCTTGTCCCCAGAA
AAATATGACCAGAAACCTTTCTTCCTTGAATAATGGGTTTCTGGGATCAAGAAAAAAGATTTACTCTCCAGATTCTTTTATTCTGTATTGCCTCCTAAAGGTTGGCTATTCTCTCAATTCAGAGGGAT
TAGATAAGAAGATCATAAAGATTTTTATGATCTGGTTCTAATACTCTAAAATGCCATGATTAATTCAATTTCTCAAATATATGCTTTCTATAAAAGCTGTTCCTAGAAAAGCCATCAGCTGTAAA TGT
TGTTAATGTTTGTACTTCTTCATTCATGCTTTGGTTTTATATTTTtACTCTGCTGTTTGTGTATACTTTCCACAGAATTGTTTATCAAAAATATTGTGTAAGACCTGGGCCTTTATTGGCCCTAATTG
TTTATCAAAATTATTGTGTAACACCTGGGCCCTTATTATCTCCTTTGGGAGATAATAAATATAAAGGCAAAAAGACCTGGCCTCCATACTCAAGAAGATT A

475	27.8	5.7	0.5	AluYa5-Scaffold12887-1479719-1479867	120	296	(1853)	C	MamGypLTR4	LTR/Gypsy	(719)	219	34	1
934	17.6	5.7	6.2	AluYa5-Scaffold12887-1479719-1479867	300	511	(1638)	C	MER2	DNA/TcMar-Tigger	(0)	345	135	2
1621	13.2	0.6	5.6	AluYa5-Scaffold12887-1479719-1479867	1001	1149	(1000)	C	AluYa5	SINE/Alu	(8)	303	168	3
38	0.0	0.0	0.0	AluYa5-Scaffold12887-1479719-1479867	1150	1184	(965)	+	(T)n	Simple_repeat	1	35	(0)	4
1621	13.2	0.6	5.6	AluYa5-Scaffold12887-1479719-1479867	1185	1353	(796)	C	AluYa5	SINE/Alu	(144)	167	1	3
525	20.0	0.0	0.0	AluYa5-Scaffold12887-1479719-1479867	1467	1556	(593)	+	AluJo	SINE/Alu	2	91	(221)	5

>chr9:21037350-21039284
CCCGTATTTTCATATCCTAGGTTTTTGTTCCCTGTGGCTTATTGTAAAACTCAATCCACAGCTCTCTAAGCTTTGCTATCCTAAAAAGAGTCTTTGGGGGAACTGAGAAATTCCTTTTATTCAAAG CC
AGTGCTTCACTGGGGACTGCAAGAGCCCACTTTGATAGGTAAAAGCCCAGCATGAAACCATTTCATTCCTTTACATTCTTCCTACCACAAGCCAAATTTCCTGAGGCAAAAAGGATGATTCTGGC AGG
CTAGGAAGGTCATGCAAAGAGATGCAAAAAGAAAATCATATTACGGTCATCCCTTGGTATCCATGGGGGATTGGTTCCAGGACAGGCTACCTGCCCCACTCCAAGATACCACAGTCCAGAATTCT CAA
GCCCGTTATATAAGAGGACATAATTGCATATAACCTAcACACATTCTCCCATATGCTTTCAATCTAGATTACTTGTAATATTTAATACCATGTACACATTATATAAATAGTTGTGATATATTTTTTAA
TTTTTTTCCCAAGTATTTTCCATCTGCAGTTACAGAACTCTCAGATATGGAGAGCCAACTGTAATATGTTTCAAAACTGTTATTCCTGCTATATATAGTTAAGGTTTTATTTAAATTCACAATTT TCA
TTTCTATATATTCTTGTTCAACATCATTGCCTACGATTACTGAAAATGACTTTTCTTAGGTTAACTCACCTTATAAACTGCTTAGAAGAATCACTCAGAGAAAAGCAAGTTATATGCTAATTTTT TAG
AGTTTAGTTTTTTGGTGATGGGAACATTATTTGGGTTGCAGATATAAGTAGTTTCAAAAGAAGATAACTTTTATAGTATCTCAGATAAAATCATTTGTGTTATATT CTGGTCATTATTAAAGGCAGTG
TTGCTTTTTGGTAGTCTAAGGTAAGAGGTTTTCTAATGAGCCTGTAAGAATTTAAGAGTTTGTAATTGTATGATATACACACAGTCAAGAAGCAAAACGGCAACTTTTTTTTTTTTTTTTCTAGT AGA
GACTGAGTCTTGCTATGTTGCTTAGGCTGGTCTTGAACTCCTGGGCTCAAGTGATCCTCCCACCTCAGCCTCCTGAGTAACTGGGATTACAGGCATGAGTCACTGTGACTGG CCCCTGCAACATCTTT
TGTAATCTATTTTTGAATAAGGAAAGTTAAAAAACAAAGAGGTAAAATTAAAAGACATGATCTAAGCATTTAAAATTAATTTGTCTAAAAGTTATTGAAAGCCAGGCATTGTTGCTTATGCCGGTA AT
CCCAGCAACTGTGGAGGCTGAGGTGGGAGGATTGCTTGAGTCCTGGAGTTCAAGACCAGCCTCTTTAAAAAGAAAAGTTATTGACTCACTGGATGTTTAAATAGATTAACACAGTTCATATTATA TAC
TTATATAAACTTAACAATGCTAGGAAATGCTTGTCCCCAGAAAAATATGACCAGAAACCTTTCTTCCTTGAATAATGGGTTTCTGGGATCAAGAAAAAAGATTTACTCTCCAGATTCTTTTATTCTGT
ATTGCCTCCTAAAGGTTGGCTATTCTCTCAATTCAGAGGGATTAGATAAGAAGATCATAAAGATTTTTATGATCTGGTTCTAATACTCTAAAATGCCATGATTAATTCAATTTCTCAAATATATG CTT
TCTATAAAAGCTGTTCCTAGAAAAGCCATCAGCTGTAAATGTTGTTAATGTTTGTACTTCTTCATTCATGCTTTGGTTTTATATTTT cACTCTGCTGTTTGTGTATACTTTCCACAGAATTGTTTATC
AAAAATATTGTGTAAGACCTGGGCCTTTATTGGCCCTAATTGTTTATCAAAATTATTGTGTAACACCTGGGCCCTTATTATCTCCTTTGGGAGATAATAAATATAAAGGCAAAAAGACCTGGCCT CCA
TACTCAAGAAGATTA

475	27.8	5.7	0.5	chr9:21037350-21039284	120	296	(1639)	C	MamGypLTR4	LTR/Gypsy	(719)	219	34	6
949	17.1	5.7	6.2	chr9:21037350-21039284	300	511	(1424)	C	MER2	DNA/TcMar-Tigger	(0)	345	135	7
918	16.2	0.0	0.0	chr9:21037350-21039284	1002	1137	(798)	C	FLAM_A	SINE/Alu	(6)	136	1	8
525	20.0	0.0	0.0	chr9:21037350-21039284	1253	1342	(593)	+	AluJo	SINE/Alu	2	91	(221)	9

>KSI_AluYa5_39

>Scaffold13200-212724-212942

ATATTTATTAGAATCTCATCTTCTCTAGGCTTTACTCTTTTGCTGAAGGACCTTTAAATATCTTTACCTAACTTCCTTTTATTCATATACAGTATTGCCAAAAAATGTGTTCCAACAACTATTAGT TT
GGGGTGTTGATCATACAAAATGTAAAAGATGAGAGGCTCTATGATCATATAAATGTGGAAGTTACTACTTCCACAAATTTAAACCAGTTTCTTTACTGCAGGAGTTCTCAGAGCCTTTAACTTGT GTT
TTACAAGCTAAACCTCCAGGAAATAGTGATAGTATGCCTAACTGTCCCAATTTGTTTGGCTTTGAAAATCTTGTTTTTGTCGAGCAACTGTGGGACAATATTATATTAAGTACATTTCAGAAAACT GA
GATTTAGTTGGAATTCTAGACGTTCACTATTTCAAATTGTTTAGGACGTAGGGGCCTTTTGACTCTACCACATGGGACTTTCGGCCATTCCATCTAGTGAACTGCCGAAGAGCTCTATGGGAGAC CAG
GGTGAAGGAAAATGTTAAACACAGGAGAGGTACAAGTAATTGTGCTTTCCCCTGTGCCAGCATGGAAAAAGGTTTCATCATTTTTGGACATATTGTAGGTCTCCATTGTATCATGGTCATTTTTAT TT
TTATCACTGCATGTGGTGTCCATTTGTTAAATTTAAGGTAATATATCTATGAGAACACCTGAAACACATTTATCAACTTAAAAAAAACATACTCAGAACATGAAAAGTACTATGCCAATTAATTA TCT
TGAATACATCAGAGCAGAACAGATTTTAGAAAGTCATTTGTAAGGTTTTGGCTGTATTTCCTGAAATGTTTTTGATGGGATTTTAATGTTTTAAATCTGACAGCTGCTCCTG cAAATGGAATACTGTT
TGTCAGCAAGCTACAGTTAACTCATTTCGATAAATAGGTAATTTGTTCCCTTAGAAAACAGTTGAAAGCATTCACTGACATTTGAGT AGAAAGGAAACACAGGCggccgggcgcggtggctcacgcct
gtaatcccagcactttgggaggccgaggcgggcggatcacgaggtcaggagatcgagaccatcccggctaaaacggtgaaaccccgtctctactaaaaatacaaaaaattagccgggcgtagtggc gg
gcgcctgtagtcccagctacttgggaggctgaggcaggagaatggcgtgaacccgggaggcggagctaaaaaaaaaaaaaaaaaaaaaaaaaaaaaaaa agaaaggaaacacaggcAATCTGTGGT
GGGGGGAAGATCAAATCTTGCACACCATCTCATCCCAAACTCTAGGAAGGTGAAAAAGACATTGGACACATAAGGGAAGATTGCTCTTGTGTCTCTAGTGAAAGGGATTAAATTGCAAGTAATTCTCT
TTTACCACCTGAGATAGTAAAAATATGCTATAATATGGAAATCTGGTTCCTGT GTGCCATGATTTAAATGTTTTTGACTCCTTTAAAATTCATGTTGAAACTTAATTTCCAATGCAACATTATTAAAA
GGTGTGACCTTTAGGGGTGATTGAGTCATGAGGGCTACGCCTTCATGAATGAGATTAGGTAGCCTTATAAAAGGGCTTGATGGAGGAAATTCACCCCTTTTTGCCTTTCCATCCCTTCTGCCATGT GA
TGATACAGTGTTCTCCCGCTCCAGAGGATGCAGCATCAAGGTGCCATCCTAGAAGCAGAGAGCAGCCCTCACCAGACATGATGCTGGTGGCTTGATCTTGGACTTCCCAGGCTCCAGAACTGGGA AAA
AATAACTTTCTCTGCTTTATAAATTACCCAGTCTAAGGTATTTTGACATACCTGCATAAATGGACTAAGACACTGTGTGTGACTTTTTGCATTTACTTTAAAGAGACATAATATTCAGAGCCTCTT TC
TAGATCTCATGCCTTCATGCATTAAGAAACGCTCTTACTGTGGATGACATGAAATTCAGACATTTTCTTCCAAAGGCCTCTCGGTCAAGACAACTCCTCTTCTAACAATCCATTGATGCAGCTAG CAG
TTTGCTGGGCCAAATTAAAGACTTTTATTTTTCTCAAGCCTTTGGTACTCCCAATTGTCTATCTACTCTCCATGTTAGCCATACAAACACACACCATCAATTTCTGGTCAGGAAAACAAAAACCAT CA
TAACATTTCAAAAACAGAGGTTTTCATACAAGGAATTCGGTGC

514	32.0	4.6	3.9	AluYa5-Scaffold13200-212724-212942	102	381	(1838)	+	MER103C	DNA/hAT-Charlie	12	293	(4)	1
2106	0.0	0.0	0.0	AluYa5-Scaffold13200-212724-212942	1001	1219	(1000)	+	AluYa5	SINE/Alu	1	219	(92)	2
34	2.6	0.0	0.0	AluYa5-Scaffold13200-212724-212942	1220	1258	(961)	+	(A)n	Simple_repeat	1	39	(0)	3
2272	16.0	2.2	0.7	AluYa5-Scaffold13200-212724-212942	1463	1864	(355)	+	MLT1A1	LTR/ERVL-MaLR	1	408	(0)	4
253	23.8	1.6	0.0	AluYa5-Scaffold13200-212724-212942	2151	2213	(6)	C	LTR33A	LTR/ERVL	(14)	506	443	5

>chr9:22287759-22289707

ATATTTATTAGAATCTCATCTTCTCTAGGCTTTACTCTTTTGCTGAAGGACCTTTAAATATCTTTACCTAACTTCCTTTTATTCATATACAGTATTGCCAAAAAATGTGTTCCAACAACTATTAGT TT
GGGGTGTTGATCATACAAAATGTAAAAGATGAGAGGCTCTATGATCATATAAATGTGGAAGTTACTACTTCCACAAATTTAAACCAGTTTCTTTACTGCAGGAGTTCTCAGAGCCTTTAACTTGT GTT
TTACAAGCTAAACCTCCAGGAAATAGTGATAGTATGCCTAACTGTCCCAATTTGTTTGGCTTTGAAAATCTTGTTTTTGTCGAGCAACTGTGGGACAATATTATATTAAGTACATTTCAGAAAACT GA
GATTTAGTTGGAATTCTAGACGTTCACTATTTCAAATTGTTTAGGACGTAGGGGCCTTTTGACTCTACCACATGGGACTTTCGGCCATTCCATCTAGTGAACTGCCGAAGAGCTCTATGGGAGAC CAG
GGTGAAGGAAAATGTTAAACACAGGAGAGGTACAAGTAATTGTGCTTTCCCCTGTGCCAGCATGGAAAAAGGTTTCATCATTTTTGGACATATTGTAGGTCTCCATTGTATCATGGTCATTTTTAT TT
TTATCACTGCATGTGGTGTCCATTTGTTAAATTTAAGGTAATATATCTATGAGAACACCTGAAACACATTTATCAACTTAAAAAAAACATACTCAGAACATGAAAAGTACTATGCCAATTAATTA TCT
TGAATACATCAGAGCAGAACAGATTTTAGAAAGTCATTTGTAAGGTTTTGGCTGTATTTCCTGAAATGTTTTTGATGGGATTTTAATGTTTTAAATCTGACAGCTGCTCCTG aAAATGGAATACTGTT
TGTCAGCAAGCTACAGTTAACTCATTTCGATAAATAGGTAATTTGTTCCCTTAGAAAACAGTTGAAAGCATTCACTGACATTTGAGT AGAAAGGAAACACAGGCAATCTGTGGTGGGGGGAAGATCAA
ATCTTGCACACCATCTCATCCCAAACTCTAGGAAGGTGAAAAAGACATTGGACACATAAGGGAAGATTGCTCTTGTGTCTCTAGTGAAAGGGATTAAATTGCAAGTAATTCTCTTTTACCACCTGAGA
TAGTAAAAATATGCTATAATATGGAAATCTGGTTCCTGTGTGCCATGATTTAAATGTTTTTGACTCCTTTAAAATTCATGTTGAAACTTAATTTCCAATGCAACATTATTAAAAGGTGTGACCTT TAG
GGGTGATTGAGTCATGAGGGCTACGCCTTCATGAATGAGATTAGGTAGCCTTATAAAAGGGCTTGATGGAGGAAATTCACCCCTTTTTGCCTTTCCATCCCTTCTGCCATGTGATGATACAGTGTT CT
CCCGCTCCAGAGGATGCAGCATCAAGGTGCCATCCTAGAAGCAGAGAGCAGCCCTCACCAGACATGATGCTGGTGGCTTGATCTTGGACTTCCCAGGCTCCAGAACTGGGAAAAAATAACTTTCT CTG
CTTTATAAATTACCCAGTCTAAGGTATTTTGACATACCTGCATAAATGGACTAAGACACTGTGTGTGACTTTTTGCATTTACTTTAAAGAGACATAATATTCAGAGCCTCTTTCTAGATCTCATGC CT
TCATGCATTAAGAAACGCTCTTACTGTGGATGACATGAAATTCAGACATTTTCTTCCAAAGGCCTCTCGGTCAAGACAACTCCTCTTCTAACAATCCATTGATGCAGCTAGCAGTTTGCTGGGCC AAA
TTAAAGACTTTTATTTTTCTCAAGCCTTTGGTACTCCCAATTGTCTATCTACTCTCCATGTTAGCCATACAAACACACACCATCAATTTCTGGTCAGGAAAACAAAAACCATCATAACATTTCAAA AA
CAGAGGTTTTCATACAAGGAATTCGGTGC

514	32.0	4.6	3.9	chr9:22287759-22289707	102	381	(1568)	+	MER103C	DNA/hAT-Charlie	12	293	(4)	6
2272	16.0	2.2	0.7	chr9:22287759-22289707	1193	1594	(355)	+	MLT1A1	LTR/ERVL-MaLR	1	408	(0)	7
253	23.8	1.6	0.0	chr9:22287759-22289707	1881	1943	(6)	C	LTR33A	LTR/ERVL	(14)	506	443	8

>KSI_AluYa5_40

>Scaffold14286-4814423-4814652
AACCTGAGAGGCGGAGGTTGCAGTGAGCTGAGATCGCACCATTGCACTCCAGCCTGGGTGACAGAGCAAGACTCCGTCTCAAAAAAAAGAATTGAGGCCAAGTAGCAACAGTAGTTCTGTGTATGC CC
CTACACAGAACATTAAATGAGATAATATACATGGGGGAGACAGtACCTACAGGTATACTGGCAGAATATATCTCATTAAAATGCCAACAAAAAGCTATCTCTATCTGGCACCAAACTAATGTCAGATA
ATGAAATTTACAGTTACAAGGCTACTTTATCACTACTATTGCTTTATATAATAAATATGGCTTCAAGTAAATGCCATAGTTTTTTTTTTGTTTTGTTTTGTTTTGTTTTGTTGTTTTTTGTTTTTT TT
TTTTTTGAGACGGAGTCTCGTTCTGTCACCCAGGCTGGAGTGCAGCGGCGCGATC Tnnnnnnnnnnnnnnnnnnnnnnnnnnnnnnnnnnnnnnnnnnnnnnnnnnnnnnnnnnnnnnnn
nnnnnnnnnnnnnnnnnnnnnnnnnnnnnnnnnnnnnnnnnnnnnnnnnnnnnnnnnnnnnnnnnnnnnnnnnnnnnnnnnnnnnnnnnnnnnnnnnnnnnnnnnnnnnnnnnn nn
nnnnnnnnnnnnnnnnnnnnnnnnnnnnnnnnnnnnnnnnnnnnnnnnnnnnnnnnnnnnnnnnnnnnnnnnnnnnnnnnnnnnnnnnnnnnnnnnnnnnnnnnnnnnnnnn nnn
nnnnnnnnnnnnnnnnnnnnnnnGCCCAGCTAATTTTTGTATTTTTAGTAAAGACGGGGTTTGCCATGTTGGCCAGGCTGTGATCCACCCGTCCTGGCCTCCCAAATTGCTGGGATTACGGGCGTG
AGCCACCGTGCCTGCCGCCATAGTTATTTTCTttttttttttttttttttttttttttttttttttttttggaggggcctccccttccccccccgcgggggctcactgcaagctccgcctccc
gggttcacgccattctcctgcctcagcctcccaagtagctgggactacaggcgcccgccactacgcccggctaattttttgcatttttagtagagacggggtttcaccgttttagccaggatggtc tc
gatctcctgacctcgtgatccgcccgcctcggcctcccaaagtgctgggattacaggcgtgagccaccgcgcccggcc catagttattttctAACTGCTATGTATAAAATCAGCTCAGAGTCACCCTT
AATTCTGCCACCACTATTACAATAATAAGTTATCAGAGGTGGCCTTCAGTCTCATTTCAACACAAGTATCTCGAATCATATATTTATGTAGAATTAATTGGCAACAAAGACCCCCTATTGGCACTTAA
AATGGAAAGCATATACCAGGTAGTGATCTCTTCACTCTGCCAAGTTCCCAAGAGCAAAGGGAGCCTATTGTTGAAGGTATTTATGTCTGTGTTATAGGCAAAAAGGAGAAAAAGTGAGTCAGCAG AAA
GCCAGAGGAATCTTAGCCACTAGGAAGATTCCCCTGACAATTCCAGTAATCCAACACTTTTTAGTTAGTATAACAAATAGAGGGATAGTCAAAAGTGCCTGGTGAGATATTCTAACATTAAGAACT GA
AGAGGTAGTCTAACATTAAACAGGCATTGTTGACAGTGAACCTGATGACCAATGTCATAAACATTTGGCTAGGTACAAAAATGTAAACACCTACCATTCACTCAATCTGTTTTTCTCTCTGGACT CAA
GGTAGTCCCAAATGGACAGTAAcACTGTATGTCATATTTAACAGTTTATTTATTATTATTATTTTTTGAGAC aGAGTCTTGCTCTGTCACTCAGGCTGGAGTGCAGTGGTGTGATCTGGGCTCACTGC
AACCTCTGCCTCCCAGGTTCAAGCAATTCTCCTGCCT cAgCCTCCCaAGTAGCTGGGATTACAGGCACCTGCCATCATGCCCAGCTAATTTTTGTATTTTTAGTAAAGACGGGGCTTCACTATTTGAG
ACAGCGTCTCACTTTGTCACCCAGGCTGAAGTGCAGTGGGCGATCTCGGTGCACTGAAGCCTCTTCAACCTCTCAGGTTCAAGCTATCCTTCTGTCTCAGTCCCCCAAGGAGCTGGGACTACAGAT GT
GCGTCACCATACCCAGCTAGTTTTTTTATTTTTTGTAGACATGGGGTTTCACC A

751	8.8	0.0	0.0	AluYa5-Scaffold14286-4814423-4814652	1	91	(2139)	+	AluSx3	SINE/Alu	202	292	(19)	1
543	7.6	0.0	0.0	AluYa5-Scaffold14286-4814423-4814652	336	440	(1790)	C	AluY	SINE/Alu	(0)	311	232	2
685	11.9	17.8	0.0	AluYa5-Scaffold14286-4814423-4814652	793	910	(1320)	C	AluY	SINE/Alu	(169)	142	4	2
46	2.2	0.0	0.0	AluYa5-Scaffold14286-4814423-4814652	923	968	(1262)	+	(T)n	Simple_repeat	1	46	(0)	3
2180	0.9	0.0	0.0	AluYa5-Scaffold14286-4814423-4814652	1001	1230	(1000)	C	AluYa5	SINE/Alu	(81)	230	1	4
1550	13.3	0.0	17.7	AluYa5-Scaffold14286-4814423-4814652	1841	2193	(37)	C	AluSx1	SINE/Alu	(11)	301	2	5
261	10.8	0.0	0.0	AluYa5-Scaffold14286-4814423-4814652	2194	2230	(0)	C	AluJb	SINE/Alu	(176)	136	10 0	6

>chr1:29381179-29383135
AACCTGAGAGGCGGAGGTTGCAGTGAGCTGAGATCGCACCATTGCACTCCAGCCTGGGTGACAGAGCAAGACTCCGTCTCAAAAAAAAGAATTGAGGCCAAGTAGCAACAGTAGTTCTGTGTATGC CC
CTACACAGAACATTAAATGAGATAATATACATGGGGGAGACAG cACCTACAGGTATACTGGCAGAATATATCTCATTAAAATGCCAACAAAAAGC TATCTCTATCTGGCACCAAACTAATGTCAGATA
ATGAAATTTACAGTTACAAGGCTACTTTATCACTACTATTGCTTTATATAATAAATATGGCTTCAAGTAAATGCCATAGTTTTTTTTTTGTTTTGTTTTGTTTTGTTTTGTTGTTTTTTGTTTTT TTT
TTTTTTGAGACGGAGTCTCGTTCTGTCACCCAGGCTGGAGTGCAGCGGCGCGATC Tcggctcactgcaagctccgcctcccgggttcacgccatt ctcctgcctcggcctcccgagtagctgagacta
cacgcgcctgccaccacgcctggctaattttttgtatttttagtagacggggtttcaccatgttggccaggatggtctcaatctcctgaccttgtgatccgcctgcctcggcctcccaaagtgct ggg
attacaggcgtgagccaccgcgcccggccctttttttttttttttttttttgag acagtcttgctctgttgcccaggctggcgtggcgtgcagtggcgcaatctcagctcactgcaacctctgcct
cccaggttcaagtgattctcctgcctcagcctcccaaatagctgggattacaggtgtctgccagcag GCCCAGCTAATTTTTGTATTTTTAGTAAAGACGGGGTTTGCCATGTTGGCCAGGCTGTGA
TCCACCCGTCCTGGCCTCCCAAATTGCTGGGATTACGGGCGTGAGCCACCGTGCCTGCCGCCATAGTTATTTTCTAACTGCTATGTATAAAATCAGCTCAGAGTCACCCTTAATTCTGCCACCACTAT
TACAATAATAAGTTATCAGAGGTGGCCTTCAGTCTCATTTCAACACAAGTATCTCGAATCATATATTTATGTAGAATTAATTGGCAACAAAGACCCCCTATTGGCACTTAAAATGGAAAGCATATACC
AGGTAGTGATCTCTTCACTCTGCCAAGTTCCCAAGAGCAAAGGGAGCCTATTGTTGAAGGTATTTATGTCTGTGTTATAGGCAAAAAGGAGAAAA AGTGAGTCAGCAGAAAGCCAGAGGAATCTTAGC
CACTAGGAAGATTCCCCTGACAATTCCAGTAATCCAACACTTTTTAGTTAGTATAACAAATAGAGGGATAGTCAAAAGTGCCTGGTGAGATATTCTAACATTAAGAACTGAAGAGGTAGTCTAAC ATT
AAACAGGCATTGTTGACAGTGAACCTGATGACCAATGTCATAAACATTTGGCTAGGTACAAAAATGTAAACACCTACCATTCACTCAATCTGTTT TTCTCTCTGGACTCAAGGTAGTCCCAAATGGAC
AGTAAaACTGTATGTCATATTTAACAGTTTATTTATTATTATTATTTTTTGAGAC gGAGTCTTGCTCTGTCACTCAGGCTGGAGTGCAGTGGTGTGATCTGGGCTCACTGCAACCTCTGCCTCCCAGG
TTCAAGCAATTCTCCTGCCTtAaCCTCCCcAGTAGCTGGGATTACAGGCACCTGCCATCATGCCCAGCTAATTTTTGTATTTTTAGTAAAGACGGGGCTTCACTATTTGAGACAGCGTCTCACTTTGT

CACCCAGGCTGAAGTGCAGTGGGCGATCTCGGTGCACTGAAGCCTCTTCAACCTCTCAGGTTCAAGCTATCCTTCTGTCTCAGTCCCCCAAGGAGCTGGGACTACAGATGTGCGTCACCATACCC AGC
TAGTTTTTTTATTTTTTGTAGACATGGGGTTTCACCA

```
751    8.8  0.0  0.0  chr1:29381179-29383135                  1    91 (1866) + AluSx3   SINE/Alu        202    292   (19)   7
 2629    5.2  0.7  0.0  chr1:29381179-29383135                  336   669 (1288) C AluY     SINE/Alu        (0)    311     1   8
 1949    9.9  7.8  1.7  chr1:29381179-29383135                  671   953 (1004) C AluSg    SINE/Alu        (7)    303     4   9
 1524   13.6  0.0 17.7  chr1:29381179-29383135                 1568  1920   (37) C AluSx1   SINE/Alu       (11)    301     2  10
  261   10.8  0.0  0.0  chr1:29381179-29383135                 1921  1957    (0) C AluJb    SINE/Alu      (176)    136   100  11
```

>KSI_AluYa5_41

>Scaffold14286-4875436-4875745
TTAATGACTATTAATAATTCACATAAATGTTCATTCATTGTCAGTCTTAACTCATCCATATCAAATAATTTTCTTTTTTTCTCCCATGTTTTCATTCTAAATGACAGAAGATGATTTGAAAATGCT TC
ATGTATTTTCCCTAATCCCTTATTCATAATTCTAAAATCCAAATGGCAAATTTTGACTT GAACTGTAAAGAGACTATTTATAATCTTAATTTATCCACCTCAGAATGAATCTTCATATCTTTTACCAT
AGAAATACTATGTCTGATCATGTGGGTGCTGCTACAGACACTGCTGGGTTTATTATAAAATATGCAGAATATGTACTTATTCCCTTGCTAAACTCCAAAATATTCTGAATTCTAGTTTTTATTTT GTT
TTGTTTTGAGACGGAGTCTCACTCTGTCGCCAGGCTGGAGTGCAGTGGTGTGATCTCAG CTCACTGCAACCTCTACCTCCTGGGTTCAAGCGATTCTCCTGCCTCAGCCTCCCAAGCAGCTGGGACTA
CAGGTGCGTGCCACCACACCCAGCTAATTTTTGTATTTTTAGTAGAGACGGGGTTTCACCATGTTGGTCAGTATGGTCTCGATTTCTTGACCTCGTGATCCACCTGCCTCGGCCTCCCAAAGTGC TGG
GATTACAGGCATGAGCCACCGTGCCCGGCCTCAAAATATT CTGAATTCTATGACACATCTGTCCCCAAGGGCTTCATATATAAGAGAT CCATATCTGGATCTTGGTCTGAATGGGAAGAATAGGTGTA
AAATAGCTTTCCCCATCTCTAAATTAGTTAGTGCAAGAAGCAGAAAAAGCAGACCCTAAGGCTCTAGTTAGGTGTATAGTACCTGTTTCAAAGTACAATTACATCTTTCTTGATTTCCTTTTTAA ACT
TAGAGAATACCTATTAGTAAATATAGCATAATATTTTAGATGATTTGTGATTGAACATATTTACATTTGCAAATAAATAATGTGATT AAGAAATAAGGCTGGGTGgccgggcgcggtggctcacgcct
gtaatcccagcactttgggaggccgaggcgggcggatcacgaggtcaggagatcgagaccatcccggctaaaacggtgaaaccccgtctctactaaaaatacaaaaaattagccgggcgtagtgg cgg
gcgcctgtagtcccagctacttgggaggctgaggcaggagaatggcgtgaacccgggaggcggagcttgcagtgagccgagatctcgccactgcactccagcctgggcgacaga gcgagactccgtct
caaaaaaaaaaaaaaaaaaaaaaaaaaaagaaataaggctgggtgtGGTGACTCATGCCTATAATCTTAGTACTTTGGGAGGCTGAGGCAGGCAGATCGCTTGAGCCGGGGAGCTCAA GAGTAACCT
GGGCAACATGGTGAGAACCTGTCTCTACAAAAAATATAAAAAATTAGCCAGGTGTGATGGTATGTGCCTGTAGTCCCAGCTACTCAGGAGGCTGAGGTGGGAGGATCACCTGAG CCCAGGAGGTTGAG
GCTGCAGTGAGCTGTCACTGCGCCACTGCACTTCAACCTGGGCAACAGAGCAAGACCCTGTCTCAAATAAATAAATAAATAAATAAATAAATAAATAAATAAATAAAATAGTTTGTTCCACATAT ATG
TGAATTACACACTTTGTCAATAAAAGCTGTTACTAGTAACAATTGCAATTGCTTATATATCAAGCATATCATAATAAACAACTACTGTTCCAGTTTGAAAAATATATAGTTAGT AGATTTTTTTAAAA
TTTCAACTTGCTCTGGCCGGGCGCGGTGGCTCAAGCCTGTAAACCCAGCACTTTGGGAGGCCGAGGCGGGCAGATCACGAGGTCAGGAGATCGAGACCAGCCTGGCTAACACGGTGAAACCCCGT CTC
TACTTAAAACACAAAAAACTAGCCAGGCGTGGTGGCGGGTGCCTGTAGTCCCAGCTACTCTGGAGGCTGAGGCAGGAGAATGGCGTGAACCCAGGAGGCAGAGCTTGCAGTGAG CCGAGATTGTACCA
CTGCACTCCAGCCTGGACGACAGAGAGAGACTCTGTCTCAAAAAACAAACAAACAAAAAAAAAAATTGTCAACTTGTCCTGAAGGAGCAAAAAGAGTAACTGAATAACTCTGATATTTTTCTTCAA TCC
TGATTCAATCACTTATTCTTTCAGAAAACAGAAAATTTTAAGAAATGGCATTTACTGGACTTAAAGACTTACTCATCATCTTCCCATAATCCCACCTCCAGAAAATCACCATTA ATACTTCCATCTAC
AGAAAT

898	20.8	1.6	10.6	AluYa5-Scaffold14286-4875436-4875745	173	371	(1939)	+	MER46C	DNA/TcMar-Tigger	92	291	(47)	1
2478	7.7	0.0	0.0	AluYa5-Scaffold14286-4875436-4875745	372	670	(1640)	C	AluSc	SINE/Alu	(10)	299	1	2
898	20.8	1.6	10.6	AluYa5-Scaffold14286-4875436-4875745	671	728	(1582)	+	MER46C	DNA/TcMar-Tigger	292	328	(10)	1
2951	0.3	0.0	0.0	AluYa5-Scaffold14286-4875436-4875745	1001	1310	(1000)	+	AluYa5	SINE/Alu	1	310	(1)	3
1916	16.6	0.0	0.3	AluYa5-Scaffold14286-4875436-4875745	1318	1600	(710)	+	AluJb	SINE/Alu	1	282	(30)	4
47	0.0	0.0	0.0	AluYa5-Scaffold14286-4875436-4875745	1601	1643	(667)	+	(AAAT)n	Simple_repeat	1	43	(0)	5
2545	6.5	0.0	0.0	AluYa5-Scaffold14286-4875436-4875745	1807	2112	(198)	+	AluYm1	SINE/Alu	1	306	(5)	6

>chr1:29320479-29322463
TTAATGACTATTAATAATTCACATAAATGTTCATTCATTGTCAGTCTTAACTCATCCATATCAAATAATTTTCTTTTTTTCTCCCATGTTTTCATTCTAAATGACAGAAGATGATTTGAAAATGCT TC
ATGTATTTTCCCTAATCCCTTATTCATAATTCTAAAATCCAAATGGCAAATTTTGACTTGAACTGTAAAGAGACTATTTATAATCTTAATTTATCCACCTCAGAATGAATCTTCATATCTTTTAC CAT
AGAAATACTATGTCTGATCATGTGGGTGCTGCTACAGACACTGCTGGGTTTATTATAAAATATGCAGAATATGTACTTATTCCCTTGCTAAACTCCAAAATATTCTGAATTCTAGTTTTTATTTTG TT
TTGTTTTGAGACGGAGTCTCACTCTGTCGCCAGGCTGGAGTGCAGTGGTGTGATCTCAGCTCACTGCAACCTCTACCTCCTGGGTTCAAGCGATTCTCCTGCCTCAGCCTCCCAAGCAGCTGGGA CTA
CAGGTGCGTGCCACCACACCCAGCTAATTTTTGTATTTTTAGTAGAGACGGGGTTTCACCATGTTGGTCAGTATGGTCTCGATTTCTTGACCTCGTGATCCACCTGCCTCGGCCTCCCAAAGTGCT GG
GATTACAGGCATGAGCCACCGTGCCCGGCCTCAAAATATTCTGAATTCTATGACACATCTGTCCCCAAGGGCTTCATATATAAGAGATCCATAT CTGGATCTTGGTCTGAATGGGAAGAATAGGTGTA
AAATAGCTTTCCCCATCTCTAAATTAGTTAGTGCAAGAAGCAGAAAAAGCAGACCCTAAGGCTCTAGTTAGGTGTATAGTACCTGTTTCAAAGTACAATTACATCTTTCTTGATTTCCTTTTTAA ACT
TAGAGAATACCTATTAGTAAATATAGCATAATATTTTAGATGATTTGTGATTGAACATATTTACATTTGCAAATAAATAATGTGATT AAGAAATAAGGCTGGGTGcGGTGACTCATGCCTATAATCTT
AGTACTTTGGGAGGCTGAGGCAGGCAGATCGCTTGAGCCGGGGAGCTCAAGAGTAACCTGGGCAACATGGTGAGAACCTGTCTCTACAAAAAATATAAAAAATTAGCCAGGTGTGATGGTATGTGCCT
GTAGTCCCAGCTACTCAGGAGGCTGAGGTGGGAGGATCACCTGAGCCCAGGAGGTTGAGGCTGCAGTGAGCTGTCACTGCGCCACTGCACTTCAACCTGGGCAACAGAGCAAGA CCCTGTCTCAAATA
AATAAATAAATAAATAAATAAATAAATAAATAAATAAAATAGTTTGTTCCACATATATGTGAATTACACACTTTGTCAATAAAAGCTGTTACTAGTAACAATTGCAATTGCTTATATATCAAGCA TAT
CATAATAAACAACTACTGTTCCAGTTTGAAAAATATATAGTTAGTAGATTTTTTTAAAATTTCAACTTGCTCTGGCCGGGCGCGGTGGCTCAAGCCTGTAAACCCAGCACTTTG GGAGGCCGAGGCGG
GCAGATCACGAGGTCAGGAGATCGAGACCAGCCTGGCTAACACGGTGAAACCCCGTCTCTACTTAAAACACAAAAAACTAGCCAGGCGTGGTGGCGGGTGCCTGTAGTCCCAGCTACTCTGGAGG CTG

AGGCAGGAGAATGGCGTGAACCCAGGAGGCAGAGCTTGCAGTGAGCCGAGATTGTACCACTGCACTCCAGCCTGGACGACAGAGAGAGACTCTGTCTCAAAAAACAAACAAACA AAAAAAAAATTGTC
AACTTGTCCTGAAGGAGCAAAAAGAGTAACTGAATAACTCTGATATTTTTCTTCAATCCTGATTCAATCACTTATTCTTTCAGAAAACAGAAAATTTTAAGAAATGGCATTTACTGGACTTAAAG ACT
TACTCATCATCTTCCCATAATCCCACCTCCAGAAAATCACCATTAATACTTCCATCTACAGAAA T

898	20.8	1.6	10.6	chr1:29320479-29322463	173	371	(1614)	+	MER46C	DNA/TcMar-Tigger	92	291	(47)	7
2478	7.7	0.0	0.0	chr1:29320479-29322463	372	670	(1315)	C	AluSc	SINE/Alu	(10)	299	1	8
898	20.8	1.6	10.6	chr1:29320479-29322463	671	728	(1257)	+	MER46C	DNA/TcMar-Tigger	292	328	(10)	7
1931	16.2	0.0	0.3	chr1:29320479-29322463	993	1275	(710)	+	AluJb	SINE/Alu	1	282	(30)	9
47	0.0	0.0	0.0	chr1:29320479-29322463	1276	1318	(667)	+	(AAAT)n	Simple_repeat	1	43	(0)	10
2545	6.5	0.0	0.0	chr1:29320479-29322463	1482	1787	(198)	+	AluYm1	SINE/Alu	1	306	(5)	11

>KSI_AluYa5_42

>Scaffold14795-3905856-3906083
CTTTTTGTATCCTGCTTAAGAAATATTTAGATATCCAAAGGCATGAAAATATTTTCCTGCATTAAATTCCAAAAGTTCTTTGATTTTTACATTTTAATCTACACTCATCTAAATTTTACTTTTGTA TA
TGTCATGATGTGGATGTCAAGTTTATTTCATTTTGTCCAATATGACTATCCAATTCACCTAGCTGCATTTACTGAAAAGCTCATCCTTTTCTGAATGTTTAGCAGTGTCATATTTGTTGCTAAT CAA
AGTCCACATATGCCTAATCACCTTCTGGACTGTTGTTTTTCATTGATCTATTTCTCAGTCCTTAAGCCATTACCATATATCTTTATTACTGTAACCTTATATTAATTTATACCATTTGTGAGAATA TT
CTCTTTCCTTATTCTTCAATACTGTCATAAAACTAAGCTATACCATCCCCATTTTCATATACATTTTAGAAACAATTCGTCAAAGACCTCAAAAAATTGTCATTTTTATTGAGAAAATTTGTATA AGG
CTGGCATTATTTTTTCTTTAAATGATTAGCAAAATTCATTTCTAAAGCCGTCTGAGCAAGGAGTTTTCTTTGTGGGGAGATTTTAATTACAGATTAAATATCTTTAATAGTTAAAAGGAGTATTTT GG
TTTTTCTCCTCTTATGTCAATTTTATTAAGCTGTATTTTATGGGAATTTGTCTTTTGATTTCAAATATGTGAGCATAAACTTGGTTATAATATTCTTTAACAATTTTTTATATT GTGGAACCTATAGT
GATGGCCTCATTTTAATTCCTGATATTTATACCTCCACTATTTTTTTTTCTGAA TcagtcttaggatctttTTTTTTTTTTTTTTTTTTTTTTTTTGAGACGGAGTCTCGCTCTGTCGCCCAGGCTGG
AGTGCAGTGGCGGGATCTCGGCtcactgcaagctccgcctcccgggttcacgccattctcctgcttcagcctcccaagtagctggaactacaggcgcccgccactacgcccggctaatttttttgtat
ttttagtagagacggggtttcaccgttttagccgggatggtctcgatctcctgacctcgtgatccgcccgcctcggcctcccaaagtgctgggattacaggcgtgagccaccgcgcccggcc CAGTCT
TAGGATCTTTATCAATTTTGTCAGTCTTTCCaAAGAACCAAGTTTGGGTTTTATTAATTATCTGCACTCTATGTTTTCTTGATATTTTATTAATTTATTCTATTAA cTTTACTATTTCCCACCTTCCT
ATATTTTTAGATGTTTAATTTACTGTTATTCTTAAGAACTGTAAATGAATGCTTGATTACTTATATCCAGACTTTATTTTTAATTAATATATTCATTTATAGCTATAAACACCTCACAAGTTTGGC TA
TAAATAGATTTTAGCTATAAACAAATATAGCTACAAAATTCCCACAAATGTGGCATGTAGCATTTCCATTATTGTTGTTTCAAAAATATGTTTTAATTTGTATTTTGAGTTCTTCTTTGATCCGGGGG
CTGTTTAGAATACTTTTTTCTGAATTTCTAAACATCTGGAATTTTCTAGTTACCTTTATGCCAATTATTTCTTGCTAAATTCCAAATATTTGAAATTTGAAGAAACTCGTATGTTCCATGTGTATT TG
AATACAATGTGTGTTTTGCCATTGGTCTGGTGTTCTATAGATGTAAATAAGATAATTTTTAAAAATCGTATTGTTCAAATGTCCTATATTCTTACTAATTTTGTTA cTGCTTGTTCAATGAAAATTT
CTCACTATGattgTAAATGATCTATTTTTGCTTTTAATTCTATAAACTTTTACTTAACTTGTATGAGGCTCCATTATTAGTTATGTAACATCTGAGATTATTCTGAGATGGATTTCAGTCCATCTTGG
GACCCGTTAATATCTGATTCATTCTTATTTCTAGGGTGTAACCCTCAGGAACATCTTGCTTGAGGACCTTGATCTTATTTTGGCCCCAAAAGATTGTCAGAATCTATGGTATGTTTTGAGAACTG GCA
GATGCCCCTGAGGAAAAGGCTAGACTCAGCTTTCTGGGTATTTATTGCCCTGGATCTTGACCCCATATTTCTTTACTGCCTTGTTAACTCTCTGATG C

1097	28.9	5.6	1.4	AluYa5-Scaffold14795-3905856-3906083	1	498	(1730)	C	L1MEg	LINE/L1	(1036)	5110	4592	1	*
1110	23.6	7.3	0.3	AluYa5-Scaffold14795-3905856-3906083	487	830	(1398)	C	L1MEg	LINE/L1	(2536)	3610	3243	1	
43	0.0	0.0	0.0	AluYa5-Scaffold14795-3905856-3906083	837	875	(1353)	+	(T)n	Simple_repeat	1	39	(0)	2	
2122	0.9	0.0	0.4	AluYa5-Scaffold14795-3905856-3906083	1001	1228	(1000)	C	AluYa5	SINE/Alu	(84)	227	1	3	
537	28.6	2.1	0.8	AluYa5-Scaffold14795-3905856-3906083	1229	1471	(757)	C	L1MEg	LINE/L1	(2897)	3249	3004	1	
613	21.1	3.2	6.2	AluYa5-Scaffold14795-3905856-3906083	1486	1702	(526)	C	L1MEg	LINE/L1	(3134)	3012	2802	1	
540	21.1	16.5	1.6	AluYa5-Scaffold14795-3905856-3906083	1729	1892	(336)	C	L1MEg	LINE/L1	(3436)	2710	2523	1	
279	32.1	0.0	0.0	AluYa5-Scaffold14795-3905856-3906083	1967	2047	(181)	C	L1MEg	LINE/L1	(5643)	599	519	1	
315	27.4	14.0	0.8	AluYa5-Scaffold14795-3905856-3906083	2115	2228	(0)	C	L1MEg	LINE/L1	(5856)	386	258	1	

>chr1:70090234-70092052
CTTTTTGTATCCTGCTTAAGAAATATTTAGATATCCAAAGGCATGAAAATATTTTCCTGCATTAAATTCCAAAAGTTCTTTGATTTTTACATTTTAATCTACACTCATCTAAATTTTACTTTTGTA TA
TGTCATGATGTGGATGTCAAGTTTATTTCATTTTGTCCAATATGACTATCCAATTCACCTAGCTGCATTTACTGAAAAGCTCAT CCTTTTCTGAATGTTTAGCAGTGTCATATTTGTTGCTAATCAA
AGTCCACATATGCCTAATCACCTTCTGGACTGTTGTTTTTCATTGATCTATTTCTCAGTCCTTAAGCCATTACCATATATCTTTATTACTGTAACCTTATATTAATTTATACCATTTGTGAGAAT ATT
CTCTTTCCTTATTCTTCAATACTGTCATAAAACTAAGCTATACCATCCCCATTTTCATATACATTTTAGAAACAATTCGTCAAAG ACCTCAAAAAATTGTCATTTTTATTGAGAAAATTTGTATAAGG
CTGGCATTATTTTTTCTTTAAATGATTAGCAAAATTCATTTCTAAAGCCGTCTGAGCAAGGAGTTTTCTTTGTGGGGAGATTTTAATTACAGATTAAATATCTTTAATAGTTAAAAGGAGTATTT TGG
TTTTTCTCCTCTTATGTCAATTTTATTAAGCTGTATTTTATGGGAATTTGTCTTTTGATTTCAAATATGTGAGCATAAACTTGGT TATAATATTCTTTAACAATTTTTTATATTGTGGAACCTATAGT
GATGGCCTCATTTTAATTCCTGATATTTATACCTCCACTATTTTTTTTTCTGAA TCAGTCTTAGGATCTTTATCAATTTTGTCAGTCTTTCCgAAGAACCAAGTTTGGGTTTTATTAATTATCTGCAC
TCTATGTTTTCTTGATATTTTATTAATTTATTCTATTAAgTTTACTATTTCCCACCTTCCTATATTTTTAGATGTTTAATTTACT GTTATTCTTAAGAACTGTAAATGAATGCTTGATTACTTATATC
CAGACTTTATTTTTAATTAATATATTCATTTATAGCTATAAACACCTCACAAGTTTGGCTATAAATAGATTTTAGCTATAAACAAATATAGCTACAAAATTCCCACAAAT GTGGCATGTAGCATTTCC
ATTATTGTTGTTTCAAAAATATGTTTTAATTTGTATTTTGAGTTCTTCTTTGATCCGGGGGCTGTTTAGAATACTTTTTTCTGAA TTTCTAAACATCTGGAATTTTCTAGTTACCTTTATGCCAATTA
TTTCTTGCTAAATTCCAAATATTTGAAATTTGAAGAAACTCGTATGTTCCATGTGTATTTGAATACAATGTGTGTTTTGCCATTGGTCTGGTGTTCTATAGATGTAAATAAGATAATTTTTTAAA AAT
CGTATTGTTCAAATGTCCTATATTCTTACTAATTTTGTTAtTGCTTGTTCAATGAAAATTTCTCACTAT GTAAATGATCTATTTTTGCTTTTAATTCTATAAACTTTTACTTAACTTGTATGAGGCTC
CATTATTAGTTATGTAACATCTGAGATTATTCTGAGATGGATTTCAGTCCATCTTGGGACCCGTTAATATCTGATTCATTCTTATTTCTAGGGTGTAACCCTCAGGAACATCTTGCTTGAGGACC TTG

ATCTTATTTTGGCCCCAAAAGATTGTCAGAATCTATGGTATGTTTTGAGAACTGGCAGATGCCCCTGAGGAAAAGGCTAGACTCA GCTTTCTGGGTATTTATTGCCCTGGATCTTGACCCCATATTTC
TTTACTGCCTTGTTAACTCTCTGATGC

1097	28.9	5.6	1.4	chr1:70090234-70092052	1	498	(1321)	C	L1MEg	LINE/L1	(1036)	5110	4592	4	*
1261	26.7	5.9	6.2	chr1:70090234-70092052	487	1297	(522)	C	L1MEg	LINE/L1	(2536)	3610	2802	4	
494	20.1	5.1	2.8	chr1:70090234-70092052	1324	1461	(358)	C	L1MEg	LINE/L1	(3436)	2710	2570	4	
279	32.1	0.0	0.0	chr1:70090234-70092052	1558	1638	(181)	C	L1MEg	LINE/L1	(5643)	599	519	4	
315	27.4	14.0	0.8	chr1:70090234-70092052	1706	1819	(0)	C	L1MEg	LINE/L1	(5856)	386	258	4	

>KSI_AluYa5_43

>Scaffold16040-1100576-1100886
TTCTTCCTCATTTTGGACTGTCCTATGCATTTTGTTCCTGTTCCCATTGACACAACTTCCTTTGTCACTGTGGGAAAAAAACAAATCGTGTGAGTTCTGAGAAACGGAATGTTCCCCGGTGTTCAG GT
GTGATCAGCCTGCACACTCCTCTAGGGGATTCCCAGAGGCCCACTCAACCAAGGTCTGCGGTTCCAGCATGGCGGTCTCCCGCCATCAGAGGTACTGCGTCACCTAGCAGAGGACAAGCACATAT GCC
TAACATCGTAGTTCACACCCTACCCACGACTGTACCCTCACAGTACAGCAATGCATAATTTTACCATCTCTCACAGTGAGGAGCATGCCAAGAAGAAAAAGTCTCACCTTGCACCGGCTTATCAGG GG
TGTCGCAGGCCTTGTCAGCTGGAGATTGTATCTTCACCACCGCTGCCAATAATGTCCACTCATGGTTTGGCTCAGGCTTCCCCTTCTTGGGCAGCCTGATCCCATAGTGTTCATAGCATAGCTGA GCA
ATCTCATCCGCGGTCCACATGGTCTGAGCTGGTATTGAGACCTTGATCAAAAACCACAACCATCAGAAGTACGGAAAGGAGAACCAGTGCACGATGCTACAGCCTCTCATATCCATGCTTCGAGTC TT
TCAAATGGAGCAGACCGAGGTTTCCCTATCTGGAACTCTATGGATCATTTTCTTCTACTATCTCCCTGCCCCCTTTTCATCTTTAATGAAAGATCAAAGAAACCCCAAATTCTACCAATCCCCAA AAT
TATTGTTTCAAGTTTTTCTTAATGTTATATATCTGATACATTTCTAAGGAGTCTGCATCCTCTACTCAACATTAAAGGACACGAGAAGGAAATGTCCTCACTACTCCAGTTTTCA tTGGAAACGGCAT
GTAAAGGGTTGCAGACTCACTTGCTTTGGGCAGACCCTCACTGTATAAGGTTTATCTTTCTGGACAGATGTTCTTATCTGTCTTCTTTTTCCAggcattttatttttcttttttcttttttttttttt
ttttttgagacggagtctcgctctgtcgcccaggctggagtgcagtggcgggatctcggctcactgcaagctccgcctcccgggttcacgccattctcctgcctcagcctcccaagtagctggga cta
caggcgcccgccactacgcccggctaatttttgtatttttagtagagacggggtttcaccgttttagccgggatggtctcgatctcctgacctcgtgatccgcccgcc tcggcctcccaaagtgctg
ggattacaggcgtgagccaccgcgcccggccGGCATTTTATTTTTCTCCTTTCCCCTACAGCAGAGTGTTTCTCTACCGCCCTCTAGTAAATCCCCACTGAAATGTTTCTCAGCAGTTACGTGTTCTC
AcAGAGCTCTACTTAAGGGTGTGGACTTTGGATTCACCCTTTCTGGGTTCTAATTTTGGCTTT GCCACAAATTAACTGTGATGCTGAATAGACTACTTAACCTTGCTGG GTCTGTTTCCTCATCTGTC
AAAATAGTGATAAtAATAGTATCTAAaCTATTTAGATTATTGTGAAGATTAAATAATACTTAGGGTTACTGTGAGGGTTAAATAAGTAATACTTATAAAAAGCAATTGCTACTATGTTGAGCATAATT
AAGGTCAACTGTGATTACTATTACAAAAAATAACATTTGATGATTTTCCTGGTTAATTTGCCTTGAGCTACCTGGTGGTGGGGGTGAAAGGGGGAGGGCTGTAATGGGC ATAGAAAGCCATGAATTAA
AAAACCATCAAGCAGATCCCTAGGATACTTTGCAGGGAAATCTCTCACTCTTGGCCCTATCAATGCTCACTAGGCACATCCTGATCTCATTTGGACTCAATTATACAAAAAGCTTCCCAGTAGAA GAT
GAGCTTGTGATAGACATAGCTAAGCACTGTAAAACCAAACTGAAGGCCGGGCGCGGTGGCTCACCTGAGGTCAGGAGTTCGAGGCCAGCCTGGCCAACGTGGTGAAACC CTGTCTCTACTGAAAATAC
AAAAAAATTAGCCGGGCGTGATGGCGCGTGCCTGTAGTCCCAGCTACTCGGGAGGCTGAGGCAGGAGAATCGCTTGAACCCGGGAGG tGGAGGTCGCAATGAGCCGAGATTGTGCCACTGCACTCCAG
GCTGGGTGACAGAGCGAGACTCCCTCTCAAAAAAACAAAAAAGGCTTTCCTGAGCTCCAGTGCATGCAGCTTTTGAAAGATGGTATTGTTATGAATCCCCTGCAGCGCA ACGTGCAGGCTCGCTACTG
TAGTGAA

2941	0.6	0.0	0.0	AluYa5-Scaffold16040-1100576-1100886	1001	1311	(1000)	C	AluYa5	SINE/Alu	(0)	311	1	1
609	26.5	2.3	1.1	AluYa5-Scaffold16040-1100576-1100886	1421	1592	(719)	+	MIR	SINE/MIR	18	191	(71)	2
188	18.6	2.3	0.0	AluYa5-Scaffold16040-1100576-1100886	1597	1639	(672)	+	MIR	SINE/MIR	167	210	(52)	2
2005	7.4	0.4	1.2	AluYa5-Scaffold16040-1100576-1100886	1962	2218	(93)	+	AluSx	SINE/Alu	42	296	(16)	3

>chr16:75654166-75656154
TTCTTCCTCATTTTGGACTGTCCTATGCATTTTGTTCCTGTTCCCATTGACACAACTTCCTTTGTCACTGTGGGAAAAAAACAAATCGTGTGAGTTCTGAGAAACGGAATGTTCCCCGGTGTTCAG GT
GTGATCAGCCTGCACACTCCTCTAGGGGATTCCCAGAGGCCCACTCAACCAAGGTCTGCGGTTCCA GCATGGCGGTCTCCCGCCATCAGAGGTACTGCGTCACCTAGCAGAGGACAAGCACATATGCC
TAACATCGTAGTTCACACCCTACCCACGACTGTACCCTCACAGTACAGCAATGCATAATTTTACCATCTCTCACAGTGAGGAGCATGCCAAGAAGAAAAAGTCTCACCTTGCACCGGCTTATCAG GGG
TGTCGCAGGCCTTGTCAGCTGGAGATTGTATCTTCACCACCGCTGCCAATAATGTCCACTCATGGT TTGGCTCAGGCTTCCCCTTCTTGGGCAGCCTGATCCCATAGTGTTCATAGCATAGCTGAGCA
ATCTCATCCGCGGTCCACATGGTCTGAGCTGGTATTGAGACCTTGATCAAAAACCACAACCATCAGAAGTACGGAAAGGAGAACCAGTGCACGATGCTACAGCCTCTCATATCCATGCTTCGAGT CTT
TCAAATGGAGCAGACCGAGGTTTCCCTATCTGGAACTCTATGGATCATTTTCTTCTACTATCTCCC TGCCCCCTTTTCATCTTTAATGAAAGATCAAAGAAACCCCAAATTCTACCAATCCCCAAAAT
TATTGTTTCAAGTTTTTCTTAATGTTATATATCTGATACATTTCTAAGGAGTCTGCATCCTCTACTCAACATTAAAGGACACGAGAAGGAAATGTCCTCACTACTCCAGTTTTCA cTGGAAACGGCAT
GTAAAGGGTTGCAGACTCACTTGCTTTGGGCAGACCCTCACTGTATAAGGTTTATCTTTCTGGACAGATGTTCTTATCTGTCTTCTTTTTCCAGGCATTTTATTTTTCTCCTTTCCCCTACAGCAGAG

TGTTTCTCTACCGCCCTCTAGTAAATCCCCACTGAAATGTTTCTCAGCAGTTACGTGTTCTCAtAGAGCTCTACTTAAGGGTGTGGACTTTGGATTCACCCTTTCTGGGTTCTAATTTTGGCTTTGCC
ACAAATTAACTGTGATGCTGAATAGACTACTTAACCTTGCTGGGTCTGTTTCCTCATCTGTCAAAATAGTGATAA cAATAGTATCTAAcCTATTTAGATTATTGTGAAGATTAAATAATACTTAGGGT
TACTGTGAGGGTTAAATAAGTAATACTTATAAAAAGCAATTGCTACTATGTTGAGCATAATTAAGGTCAACTGTGATTACTATTACAAAAAATAACATTTGATGATTTTCCTGGTTAATTTGCCT TGA
GCTACCTGGTGGTGGGGGTGAAAGGGGGAGGGCTGTAATGGGCATAGAAAGCCATGAATTAAAAAACCATCAAGCAGATCCCTAGGATACTTTGCAGGGAAATCTCTCACTCTTGGCCCTATCAATGC
TCACTAGGCACATCCTGATCTCATTTGGACTCAATTATACAAAAAGCTTCCCAGTAGAAGATGAGCTTGTGATAGACATAGCTAAGCACTGTAAAACCAAACTGAAGGCCGGGCGCGGTGGCTCA CCT
GAGGTCAGGAGTTCGAGGCCAGCCTGGCCAACGTGGTGAAACCCTGTCTCTACTGAAAATACAAAAAAATTAGCCGGGCGTGATGGCGCGTGCCTGTAGTCCCAGCTACTCGGGAGGCTGAGGCAGGA
GAATCGCTTGAACCCGGGAGGcGGAGGTCGCAATGAGCCGAGATTGTGCCACTGCACTCCAGGCTGGGTGACAGAGCGAGACTCCCTCTCAAAAAAACAAAAAAGGCTTTCCTGAGCTCCAGTGCATG
CAGCTTTTGAAAGATGGTATTGTTATGAATCCCCTGCAGCGCAACGTGCAGGCTCGCTACTGTAGTGAA

624	26.5	2.3	1.1	chr16:75654166-75656154	1099	1270	(719)	+	MIR	SINE/MIR	18	191	(71)	4
188	18.6	2.3	0.0	chr16:75654166-75656154	1275	1317	(672)	+	MIR	SINE/MIR	167	210	(52)	4
2020	7.0	0.4	1.2	chr16:75654166-75656154	1640	1896	(93)	+	AluSx	SINE/Alu	42	296	(16)	5

>KSI_AluYa5_44

>Scaffold16041-196443-196589
CTATCAGTTTGAGTCCCTCATGGTAATTCTACATTTTAACTTTTTGAGGAACTCTCAAACTCTGCTTTTGATGGAGGCTGCAACATTTTGCATTTGCACAAGTAATGCAAAAAGTTCCAATTTCCC CA
CATCCgTTACAATTGATGTTATTTTTGAATATTTTTTAGTAGCCATTCTAGTGGGTGGG aAGTGGTATCTCATTGTAGTTTTGATTTGCATTTTCATAAGACTAATGATG tTAAGAATATTTTCATAT
GCTTATTAGCCATTTATATATCTTCTGTGGAACAATGTCTGTTGAAGTACTTTGACCATTTTTAATTGAGTTACCTTTTTGTAAGAGTTCTTTATATATTCTAGATGTTAAATCTTAAGATATTA TCT
GCAAATATATTCTTCAATTCTGTGGGTTGTGTTTTCATTATCTTGATAGTGTCCTTTGAT ATACAAAAGTTAATTTTTATGAAGTCGAATTTACCTTTTTTTTCTCTTAATTGTAGTTTTGGTATGAT
ATTTAAGTAACTATTGCCAAATAAAGGGTCATGAAAATTTGTTCCTATGATTTCTACTAAGAATTTTGGA GTCTCACCTCTTCAATTTAGGTCTTTCATTCATTTTGAGCTATTTTCAGATACGCTGT
AAAGTAAgGATTTCACaTCATTCAATACATTTGCATATCCAGTTTTATCAGCTCCATTTG TTGAACAACTTTCCTTCCACATTGAATAGCCTTGGCACTTTGTTATAAATCAATTAGTGATATATGCG
AAGGTTTACTTCCGAGCTCTCATTTCTATTCTCTTGGCCTTT aTGTATATCCCTATGTGAGAACCATACTATGTTTTGTGTACTATAGTAAGTTTTGAAATCAGGACATGTGAGTCCTCCACTTTGTT
TCTTGTGTTTCTTTTTGAGATTACTTTTTGCTATTCAGGGTATCgTGAAAATCCATGTGA ATTTTaGAATAGATAATTCTATTTTTAAAATCATGCATTTTTTT ggccgggcgcggtggctcacgcct
gtaatcccagcactttgggaggccgaggcgggcggatcacgaggtcaggagatcgagaccatcccggctaaaacggtgaaaccctgtctctactaaaaatacaaaaaattagccgggcgtagt ctatg
tgagaaccatactatgttttgtgtactatagtaagttttgaaatcaggacatgtgagtcc tccactttgtttcttgtgtttctttttgagattactttttgctattcagggtatc atgaaaatccatg
tgaattttggaatagataattctatttttaaaatcatgcattttttt AAAAGGCCATTAAAAAATTGTGGGCTGGGCATT GTGGACCATGTTTGCAATCTTAGCACTTTGGGAGGTGAAGGCAGGAAG
ATTGCTTGAGCCCAGGAGTTCAGGACAGCCTGGGCAACATAGACCACATCTCTATTAAAAAAAATTATATATTAAAGAAAAATCAAATTGTATTGAACCTGTATGTCGCTTTGGGGACTATTGTC CTC
TTAACAACACAGATGTCTTTTCATTTACTTTTTGTGTATCTATTAGGGTTCTCTAGAGGGACAGACCTAATAGGATAGATGTATATGTAAAGGGGAATT TATTAGGGAATACTGATTCACACAATCAC
AAGGTGAGGTCTCACAATAAGAAGTCTGCAAGCTGAGGAGCAAGGAAGCTAGTCCGGTTCCCAAAGCTGAAGAACTTGGAGTCTGA tGTTCAAGGGCAGGAAGCTTCCAGCATGGGAGAAAGATGTAG
GCCAGAAGACTAAACTAGTCTAGTCTTTTCATTTTCTTTTGCCTGCTTTTATTCTGGC cGCACTGGCAGCTGATTAGATTGTGCCCACCCATATTGAGG GTGGATCTGCCTTTCCCAATCCAATTATT
CAAATGTTAATCTCCTTTGGCAACACCCTCACAGACACACTCAGGAGCAATACTTTGCATCCTCGATCCAATCAAGTTGACCCTCAACATTAACCATCACAAGTCCACCCCTTGTCAACTTGAAC TCA
TACACATCTGAAATCATACATAATCTTAAGTAAAGACAATAATAAGGTCATAATTACACCTAACATAATACAACTGTCTTTTGTACAGTAGGAAATGC A

1588	17.5	6.4	1.7	AluYa5-Scaffold16041-196443-196589	20	362	(1785)	C	L1M4	LINE/L1	(563)	5617	5259	1	*
2379	21.6	4.5	2.6	AluYa5-Scaffold16041-196443-196589	234	966	(1181)	C	L1M4	LINE/L1	(743)	5403	4639	2	
1394	0.7	0.0	0.0	AluYa5-Scaffold16041-196443-196589	1001	1147	(1000)	+	AluYa5	SINE/Alu	1	147	(164)	3	
444	20.6	6.2	6.2	AluYa5-Scaffold16041-196443-196589	1149	1293	(854)	C	L1M4	LINE/L1	(1363)	4783	4639	1	
669	17.9	4.1	1.6	AluYa5-Scaffold16041-196443-196589	1350	1472	(675)	+	FLAM_C	SINE/Alu	1	126	(17)	4	
262	21.9	0.0	0.3	AluYa5-Scaffold16041-196443-196589	1495	1555	(592)	C	L1M4	LINE/L1	(1554)	4592	4533	1	
3404	10.0	0.4	0.0	AluYa5-Scaffold16041-196443-196589	1571	2021	(126)	C	MLT2A2	LTR/ERVL	(0)	453	1	5	
898	7.9	3.2	0.0	AluYa5-Scaffold16041-196443-196589	2022	2147	(0)	C	HERVL-int	LTR/ERVL	(0)	5654	5525	5	

>chrY:5718015-5719834
CTATCAGTTTGAGTCCCTCATGGTAATTCTACATTTTAACTTTTTGAGGAACTCTCAAACTCTGCTTTTGATGGAGGCTGCAACATTTTGCATTTGCACAAGTAATGCAAAAAGTTCCAATTTCCC CA
CATCCaTTACAATTGATGTTATTTTTGAATATTTTTTAGTAGCCATTCTAGTGGGTGGG gAGTGGTATCTCATTGTAGTTTTGATTTGCATTTTCA TAAGACTAATGATGcTAAGAATATTTTCATAT
GCTTATTAGCCATTTATATATCTTCTGTGGAACAATGTCTGTTGAAGTACTTTGACCATTTTTAATTGAGTTACCTTTTTGTAAGAGTTCTTTATATATTCTAGATGTTAAATCTTAAGATATTA TCT
GCAAATATATTCTTCAATTCTGTGGGTTGTGTTTTCATTATCTTGATAGTGTCCTTTGATATACAAAAGTTAATTTTTATGAAGTCGAATTTACCT TTTTTTTCTCTTAATTGTAGTTTTGGTATGAT
ATTTAAGTAACTATTGCCAAATAAAGGGTCATGAAAATTTGTTCCTATGATTTCTACTAAGAATTTTGGA GTCTCACCTCTTCAATTTAGGTCTTTCATTCATTTTGAGCTATTTTCAGATACGCTGT
AAAGTAAaGATTTCACgTCATTCAATACATTTGCATATCCAGTTTTATCAGCTCCATTTGTTGAACAACTTTCCTTCCACATTGAATAGCCTTGGCACTTTGTTATAAATCAATTAGTGATATATGCG
AAGGTTTACTTCCGAGCTCTCATTTCTATTCTCTTGGCCTTT tTGTATATCCCTATGTGAGAACCATACTATGTTTTGTGTACTATAGTAAGTTTTGAAATCAGGACATGTGAGTCCTCCACTTTGTT
TCTTGTGTTTCTTTTTGAGATTACTTTTTGCTATTCAGGGTATCaTGAAAATCCATGTGAATTTTgGAATAGATAATTCTATTTTTAAAATCATGCATTTTTTT AAAAGGCCATTAAAAAATTGTGGG
CTGGGCATTGTGGACCATGTTTGCAATCTTAGCACTTTGGGAGGTGAAGGCAGGAAGATTGCTTGAGCCCAGGAGTTCAGGACAGCCTGGGCAACATAGACCACATCTCTATTAAAAAAAATTATATA
TTAAAGAAAAATCAAATTGTATTGAACCTGTATGTCGCTTTGGGGACTATTGTCCTCTTAACAACACAGATGTCTTTTCATTTACTTTTTGTGTATCTATTAGGGTTCTCTAGAGGGACAGACCTAAT
AGGATAGATGTATATGTAAAGGGGAATTTATTAGGGAATACTGATTCACACAATCACAAGGTGAGGTCTCACAATAAGAAGTCTGCAAGCTGAGGAGCAAGGAAGCTAGTCCGGTTCCCAAAGCT GAA
GAACTTGGAGTCTGAcGTTCAAGGGCAGGAAGCTTCCAGCATGGGAGAAAGATGTAGGCCAGAAGACTAAACTAGTCTAGTCTTTTCATTTTCTTTTGCCTGCTTTTATTCTGGC tGCACTGGCAGCT
GATTAGATTGTGCCCACCCATATTGAGGGTGGATCTGCCTTTCCCAATCCAATTATTCAAATGTTAATCTCCTTTGGCAACACCCTCACAGACACACTCAGGAGCAATACTTTGCATCCTCGATC CAA

TCAAGTTGACCCTCAACATTAACCATCACAAGTCCACCCCTTGTCAACTTGAACTCATACACATCTGAAATCATACATAATCTTAAGTAAAGACAATAATAAGGTCATAATTACACCTAACATAATAC
AACTGTCTTTTGTACAGTAGGAAATGCA

1558	18.1	6.4	1.7	chrY:5718015-5719834	20	362	(1458)	C	L1M4	LINE/L1	(563)	5617	5259	6	*
2318	19.1	6.0	2.0	chrY:5718015-5719834	234	966	(854)	C	L1M4	LINE/L1	(743)	5403	4639	7	
669	17.9	4.1	1.6	chrY:5718015-5719834	1023	1145	(675)	+	FLAM_C	SINE/Alu	1	126	(17)	8	
262	21.9	0.0	0.3	chrY:5718015-5719834	1168	1228	(592)	C	L1M4	LINE/L1	(1554)	4592	4533	6	
3372	10.4	0.4	0.0	chrY:5718015-5719834	1244	1694	(126)	C	MLT2A2	LTR/ERVL	(0)	453	1	9	
898	7.9	3.2	0.0	chrY:5718015-5719834	1695	1820	(0)	C	HERVL-int	LTR/ERVL	(0)	5654	5525	9	

>KSI_AluYa5_45

>Scaffold16053-1713177-1713512
ACATTTTCATTTCTTTTTTTTCAAATCTGCATGCCTTTTATTTAGTTGTTCTTGCCTTATTGTACTGGTTATGATTTCATACAATGTTGAAGGGGAGTAAAGACAACAGATATCCTTATCTTGTTC CA
GATATTAGGAGGAAACATTAGTTCTTTCACTATCTTTTTTAAGACAAAGTGTCTCACTCTCTTTTCCAGGCTGGAGTGCAGTTGTGCTTACTGTAACCTTGAACTCCTTGGCTCAAGCAGTCCTC CTG
CCTCAGCCTCCCAAGTAGCTGGAACCATAGGTGCATGCCACCATGCCCGGCTAATTTTTTAATCTTTGTAGAGACAGGGTCTCACTGTGTTGCCCAGGCTAGTTTTGAACTCCTGGGCTCAAAGGA TG
CTCCCACCTCGGCCTCCCAAAGTGTTAGAATTATAGGCATGAGCCACTGTGCCTGGTCCCAGGCTTTCACTATTTAGTATGACGTTAGCTGTAAGTCTTTTAGTTTTAGTTTTTTTTTTTTTAACA GAT
GTTCTTTTTCAGGTGAAGAAAGTTTCTTTTATTACTAGTTTGCTAAGAGTTTTTATTATAAATAGGTGTTAAATGTTGCTAA AtTTTTTTTTTGCATCTATTGAGATGATCATATGGTTTTTCTTCTT
GGGCTGTTGAAATGGTGAATTACCTTCTTTTTTTTTTTTTTTTTGGTGTAGGGGGACAGAGTCTTCCTCTTGTCACCCAGGCTGGAGTGCAATGGCACAATCTTGGCTCACTGCAACCTCTGCCTCCC
AGGTTCAAGCAATTCTCCTGCCTCAGCCTCCCAAGTAGCTGGAATTACAGGCACCCACCACCATACCTGGCTAATTTTTGTATTTTTAGTAGAGACAGGGTTTTGCCATGTTGGCCAGGCTGGTCT CG
AACTCCTGACCTCATGATCCACCCACCTCACCTCATGATCCACCCACCTCAGCCTTTCCCAAATTGCTGGAATTACAGGCATGAGCCACCGCGTCCGGCCTGGTTTT GTTTTGTTTGTTTGTTTgttt
gtttgtttgtttttttttttttttttttttgagacggagtctcgctctgtcgcccaggctggagtgcagtggcgtgatctcggctcactgcaagttccgcctcccgggttcacgccattctcctg cc
tcagcctcccaagtagctgggactacaggcgcccgccactacgcccggctaattttttgtatttttagtagagacggggtttcaccgttttagccgggatggtctcgatctcctgacctcgtgat cca
cccgcctcggcctcccaaagtgctgggattacaggcgtgagccaccgcgcccggcctttgtttgtttTTTTAAGACAGGGTCTTACTCTGTCACCCAGGCCAGAGTGCAGTGGCGTGACCATAGCTT
ACTGCTACAGCCTCAACCTCCTGGGCTCAAGCGATCCTCTGGCCTCAGCCTCCTGAATAGCTGTACAAGGAATGTGCCACCATGCCTGACTAATTTTTAAATATTTTGGCCAGGTGCAATAGCTCATA
CTTATAATCCAGAAATTTGTGAGGCCAGGGCAGGCAAATCGCTTGAGCCCAGGAGTTTGAGAATAGCCTGGGCAACATAGCAAAACCCTGTATCTACAAAAAGAAAACCAAACCAAACCAAACCA AAT
ATTAGCCAGGTGTGGTGGTGTGGGCCTGTAGTCCCAGCTACTGAGGAGGCCGAGGTGGGAGAACGATCTGAACCCAGGAAAGATGGTTGAGGCTGCAGTGAGGTGTAATCATGCCACTGCACTCCAGC
CTGGGTGACAGAGTGAGACCCTGTTTCAATAATAATAATAATGATAATAATAATTTTTATATTTGTAGGGACAGGGTTTCGCTTTGTCACCCAGGCTGGTCTCAAACTCCTGGGCTCAACCAGTC CTC
CTGCCTTGGCCTCCAAAAGTGCTGGGATTATAGGCGTGAGCCATCATGCCTAGCCCACTAATTGATTTTCGAATGTTGAGTTAGCCTTGCATTTCCAAAATAACTCCCATCAGATTCAATTTGATAAT
ATTTTGTGGAAGATTGTTGTATCTATGATCATCGGTGACATTGATCTGTAGTTTTAATTTCTTATATCTTTCTCTTGTTTTGGTATCAGAGTAACACAGACTAAAAAGTATAAGGAGTTGGAAAG TGT
TTCCTTCTCTTCTGTTTTCTGGTATTATTTCTTCCTTAAATGTTTGATAGACTGGAGTTGTCTACCTTGGATAATTTTTTAACTGCAGATACAAGATAGCTTGTATCCTTCAAGAAATTGATCCAGCC
AGGCTCAGGGGCTCATGCCTGTAATCCCAGCA

730	21.0	1.5	10.0	AluYa5-Scaffold16053-1713177-1713512	1	162	(2174)	C	L1MD	LINE/L1	(1945)	4201	4054	1
1759	16.1	3.6	0.7	AluYa5-Scaffold16053-1713177-1713512	163	442	(1894)	C	AluJo	SINE/Alu	(24)	288	1	2
822	19.7	0.9	10.4	AluYa5-Scaffold16053-1713177-1713512	443	663	(1673)	C	L1MC	LINE/L1	(2092)	4054	3853	3
2154	10.2	0.0	7.8	AluYa5-Scaffold16053-1713177-1713512	665	996	(1340)	C	AluSq2	SINE/Alu	(5)	308	1	4
2820	1.0	0.0	0.0	AluYa5-Scaffold16053-1713177-1713512	1001	1336	(1000)	C	AluYa5	SINE/Alu	(0)	311	1	5
969	16.1	2.5	2.5	AluYa5-Scaffold16053-1713177-1713512	1337	1498	(838)	C	AluJb	SINE/Alu	(14)	298	137	6
1528	18.0	0.3	8.1	AluYa5-Scaffold16053-1713177-1713512	1516	1821	(515)	+	AluJo	SINE/Alu	1	284	(28)	7
14	5.0	0.0	0.0	AluYa5-Scaffold16053-1713177-1713512	1822	1842	(494)	+	(TAA)n	Simple_repeat	1	21	(0)	8
895	15.0	2.3	0.0	AluYa5-Scaffold16053-1713177-1713512	1843	1975	(361)	C	AluJo	SINE/Alu	(176)	136	1	9
310	20.4	0.0	0.0	AluYa5-Scaffold16053-1713177-1713512	1976	2029	(307)	C	L1MC	LINE/L1	(2293)	3853	3800	3
716	20.8	11.8	1.4	AluYa5-Scaffold16053-1713177-1713512	2033	2227	(109)	C	L1M4	LINE/L1	(2384)	3762	3548	10
226	14.3	0.0	0.0	AluYa5-Scaffold16053-1713177-1713512	2302	2336	(0)	+	Alu	SINE/Alu	2	36	(97)	11

>chr10:69629047-69631054
ACATTTTCATTTCTTTTTTTTCAAATCTGCATGCCTTTTATTTAGTTGTTCTTGCCTTATTGTACTGGTTATGATTTCATACAATGTTGAAGGGGAGTAAAGACAACAGATATCCTTATCTTGTTC CA
GATATTAGGAGGAAACATTAGTTCTTTCACTATCTTTTTTAAGACAAAGTGTCTCACTCTCTTTTCCAGGCTGGAGTGCAGTTGTGCTTACTGTAACCTTGAACTCCTTGGCTCAAGCAGTCCTC CTG
CCTCAGCCTCCCAAGTAGCTGGAACCATAGGTGCATGCCACCATGCCCGGCTAATTTTTTAATCTTTGTAGAGACAGGGTCTCACTGTGTTGCCCAGGCTAGTTTTGAACTCCTGGGCTCAAAGGA TG
CTCCCACCTCGGCCTCCCAAAGTGTTAGAATTATAGGCATGAGCCACTGTGCCTGGTCCCAGGCTTTCACTATTTAGTATGACGTTAGCTGTAAGTCTTTTAGTTTTAGTTTTTTTTTTTTTAACA GAT
GTTCTTTTTCAGGTGAAGAAAGTTTCTTTTATTACTAGTTTGCTAAGAGTTTTTATTATAAATAGGTGTTAAATGTTGCTAA ATTTTTTTTTGCATCTATTGAGATGATCATATGGTTTTTCTTCTTG
GGCTGTTGAAATGGTGAATTACCTTCtTTTTTTTTTTTTTTTTTGGTGTAGGGGGACAGAGTCTTCCTCTTGTCACCCAGGCTGGAGTGCAATGGCACAATCTTGGCTCACTGCAACCTCTGCCTCCC
AGGTTCAAGCAATTCTCCTGCCTCAGCCTCCCAAGTAGCTGGAATTACAGGCACCCACCACCATACCTGGCTAATTTTTGTATTTTTAGTAGAGACAGGGTTTTGCCATGTTGGCCAGGCTGGTC TCG
AACTCCTGACCTCATGATCCACCCACCTCACCTCATGATCCACCCACCTCAGCCTTTCCCAAATTGCTGGAATTACAGGCATGAGCCACCGCGTCCGGCCTGGTTTTGTTTTG TTTGTTTGTTTTTTT
AAGACAGGGTCTTACTCTGTCACCCAGGCCAGAGTGCAGTGGCGTGACCATA GCTTACTGCTACAGCCTCAACCTCCTGGGCTCAAGCGATCCTCTGGCCTCAGCCTCCTGAATAGCTGTACAAGGAA

TGTGCCACCATGCCTGACTAATTTTTAAATATTTTGGCCAGGTGCAATAGCTCATACTTATAATCCAGAAATTTGTGAGGCCAGGGCAGGCAAATCGCTTGAGCCCAGGAGTTTGAGAATAGCCTGGG
CAACATAGCAAAACCCTGTATCTACAAAAAGAAAACCAAACCAAACCAAACCAAATATTAGCCAGGTGTGGTGGTGTGGGCCTGTAGTCCCAGCTACTGAGGAGGCCGAGGTGGGAGAACGATCT GAA
CCCAGGAAAGATGGTTGAGGCTGCAGTGAGGTGTAATCATGCCACTGCACTCCAGCCTGGGTGACAGAGTGAGACCCTGTTTCAATAATAATAATAATGATAATAATAATTTTTATATTTGTAGGGAC
AGGGTTTCGCTTTGTCACCCAGGCTGGTCTCAAACTCCTGGGCTCAACCAGTCCTCCTGCCTTGGCCTCCAAAAGTGCTGGGATTATAGGCGTGAGCCATCATGCCTAGCCCACTAATTGATTTT CGA
ATGTTGAGTTAGCCTTGCATTTCCAAAATAACTCCCATCAGATTCAATTTGATAATATTTTGTGGAAGATTGTTGTATCTATGATCATCGGTGACATTGATCTGTAGTTTTAATTTCTTATATCTTTC
TCTTGTTTTGGTATCAGAGTAACACAGACTAAAAAGTATAAGGAGTTGGAAAGTGTTTCCTTCTCTTCTGTTTTCTGGTATTATTTCTTCCTTAAATGTTTGATAGACTGGAGTTGTCTACCTTG GAT
AATTTTTTAACTGCAGATACAAGATAGCTTGTATCCTTCAAGAAATTGATCCAGCCAGGCTCAGGGGCTCATGCCTGTAATCCCAGC A

730	21.0	1.5	10.0	chr10:69629047-69631054	1	162	(1705)	C	L1MD	LINE/L1	(1945)	4201	4054	12	
1759	16.1	3.6	0.7	chr10:69629047-69631054	163	442	(1425)	C	AluJo	SINE/Alu	(24)	288	1	13	
811	19.3	1.4	10.4	chr10:69629047-69631054	443	662	(1205)	C	L1MC	LINE/L1	(2092)	4054	3853	14	
775	18.0	1.2	12.0	chr10:69629047-69631054	449	663	(1204)	C	L1M5	LINE/L1	(2098)	4048	3852	15	*
2163	10.2	0.0	7.8	chr10:69629047-69631054	664	996	(871)	C	AluSq2	SINE/Alu	(4)	309	1	16	
981	16.3	2.4	2.4	chr10:69629047-69631054	1005	1170	(697)	C	AluJb	SINE/Alu	(10)	302	137	17	
775	18.0	1.2	12.0	chr10:69629047-69631054	1171	1185	(682)	C	L1M5	LINE/L1	(2300)	3846	3833	15	
1528	18.0	0.3	8.1	chr10:69629047-69631054	1188	1493	(374)	+	AluJo	SINE/Alu	1	284	(28)	18	
14	5.0	0.0	0.0	chr10:69629047-69631054	1494	1514	(353)	+	(TAA)n	Simple_repeat	1	21	(0)	19	
895	15.0	2.3	0.0	chr10:69629047-69631054	1515	1647	(220)	C	AluJo	SINE/Alu	(176)	136	1	20	
310	20.4	0.0	0.0	chr10:69629047-69631054	1648	1701	(166)	C	L1MC	LINE/L1	(2293)	3853	3800	14	
658	21.4	1.9	1.9	chr10:69629047-69631054	1705	1866	(1)	C	L1M4	LINE/L1	(2384)	3762	3601	21	

>KSI_AluYa5_46

>Scaffold16436-2508175-2508476
TTAGCCAGCATGATCACATGTGCCTGTCCCAGCTCGGAAGGCTGAGGCAGGAGAATTGATTGAAGCCAGGAGGCGGAGGTTGCATTGCAGTGAGCCAAGATTGCGGCACTGCACTCTAGCCTACGC GA
CAGAGCAAGACTATGTCTCAAAAAAAAAAAAAAGAAAAAGAAAAAGAAGAAACAGCAACTTAGAAATTCATTGTGATTAAGTGTTTTATGTATGAAAATGAAGCTAAATTAAGCTAAAAATAAATA TCA
AAAAGTATAAGGTATCTTGATATAAAGAAAAAAACATAATTACAAAGATAAATGTAATTTTGTCCTATTTTACATACTACAAAGAAAATATAATAATGATAGAAAGTACAGTATTATATTTTTATA CT
AAAATTTGTCAAGAAATCACAGACTTTACTCCATGAGTTTTACTTGAGAGAAAGTCGCAAGTACTTATGATTGG CTTGTGGGTGAAGATTCGAG TTTAATCAAAACTGCTCTAGCCTTGATTTTCTGA
ATCTATTTTCAGTAATGATTATTTAGCTAACAAATAACATAGTGAAATGAACTCATGCAATGAGAAGTTAAGAAATAAACTTCTCAATTAAGAAATTTTCCCTACT GTGGGCCAGGGTCAGTGGCTCA
CGCCTGTAATCCCAGCACTCTAGGAGGCCAAAGCAGGCAGATCACTTCAGGTCAGGAGTTCGAGACCAGCCTGGCCAACAGGGTGAAACCCCGTCTCCACTAAAAAAACAAAAATTAGCCAGCGT GAT
GGCATGCATCTACAATCCCAGCTACTTGGGAGGCTGAGGCACAAGAATCGCTTGAACCCAGGAGGTGGAGGTTGCAGTGAGCCGAGATCGTGCAGTGTGCTGCACTCCAGCCTAGGCAACAGAGTA AG
ACTCCGTCTCAAAAGTAATAATGATAAAAAAATGAAATAAAGAAATTTTCCCTGTTGTGAAACCAGGAAAAGTCATCAGTACAAAAGAACTTT Tcccaccccactttttttttttttttttttttgaga
cggagtctcgctctgtcgcccaggctggagtgcagtggcgggatctcggctcactgcaagctccgcctcccgggttcacgccattctcctgcctcagcctcccaagtagctgggac tacaggcgcccg
ccactacgcccggctaattttttgtatttttagtagagacggggtttcaccgttttagccgggatggtctcgatctcttgacctcgtgatccgcccgcctcggcctcccaaacttgagggatta cag
gcgtgagccaccgcgcccggccCCCACCCCACTTTTTTAAGCAAACAAATCACTAGGATAATACAATGCACATAGGCTGAACAAAAGCCATCCGGTCTTAAGCTCTTCCAACCACTTTTGGACCTAAG
TAAAGGGGACATTTGTCTAGTACACAAAGTTGACTATATAT CTGGAGGCAATTCAAAGGTC AACTTCAAGTTGGTATTTTATTTTCTAAGTATTCTTAGAAGGCTTGTACTATAAAT AGGCATCTTTC
TTTCTTATTTTACTTTTTTCTTTTTTTGCCATCAGAAATTTCTCCTAAAAAATAGGCATCTTTCACACTACACACTACCAGGCTGCACAAGTAAGAATAATGAAATACATTTCTTCTCAGAGGTT AGA
TATGTGAACATTTTAACACAATATCCTAACACTGTTTAGGAACACCAAATTATCAACAGGTATAA tGTAAGAGAAAAGATTACTTACTTTTCAATCCAAGTGATAAATTTTTAAAAA ATTATTATTCA
AGCATGTTTAAGATTACAAAGCCACTGTTTCTGTGTGAGAAACACTAAGCTAATATATCAGAATATTCATATTTAAAATAATAAATGTAAGCGGTCATACCTATTGTTCGTAGTCAAATCACACT GAA
ATCCGGAGGCCTGGTGTGAGAACCTCACGAGCGGACACCGGGCATTTAATATTTTTTGCACACCCACACAGCCAGGGCCAAAGTGGTCAAGGCACTCTCCTAACACAGACAGGATCT TCTGAGTTGCA
ATTCTTTCTGAAGGAACATTTTTCACTTGAAATTCCATCAGAAAATTTCCTGAGATCTGAAAAATAAACACATTTCTAAATAGGAGCTTTTGGTAAAAAAAACAATTTTTTAATAAGCTTTTGTA AAA
GgATGCTCTAAATCACTGCTGGAAAGCAAAACAAAATAAGAAAAAAACAACCCACAAAATAAAAACTCTTCAAGAATCACAGCCAAAAGCAAACTTAAAAGATCCCCAGTTGCCTT CTGTTTCTC

1012	14.3	4.0	2.8	AluYa5-Scaffold16436-2508175-2508476	1	175	(2127)	+	AluSx3	SINE/Alu	133	309	(2)	1
13	22.6	3.6	3.6	AluYa5-Scaffold16436-2508175-2508476	258	312	(1990)	+	A-rich	Low_complexity	1	55	(0)	2
2100	13.0	0.3	1.3	AluYa5-Scaffold16436-2508175-2508476	622	928	(1374)	+	AluSz	SINE/Alu	1	304	(8)	3
2735	1.7	0.0	0.3	AluYa5-Scaffold16436-2508175-2508476	1001	1302	(1000)	C	AluYa5	SINE/Alu	(10)	301	1	4
15	10.4	3.0	3.0	AluYa5-Scaffold16436-2508175-2508476	1531	1563	(739)	+	(TCTT)n	Simple_repeat	1	33	(0)	5
14	15.4	0.0	7.7	AluYa5-Scaffold16436-2508175-2508476	2199	2240	(62)	+	A-rich	Low_complexity	1	39	(0)	6

>chr10:30613773-30615762
TTAGCCAGCATGATCACATGTGCCTGTCCCAGCTCGGAAGGCTGAGGCAGGAGAATTGATTGAAGCCAGGAGGCGGAGGTTGCATTGCAGTGAGCCAAGATTGCGGCACTGCACTCTAGCCTACGC GA
CAGAGCAAGACTATGTCTCAAAAAAAAAAAAAAGAAAAAGAAAAAGAAGAAACAGC AACTTAGAAATTCATTGTGATTAAGTGTTTTATGTATGAAAATGAAGCTAAATTAAGCTAAAAATAAATATCA
AAAAGTATAAGGTATCTTGATATAAAGAAAAAAACATAATTACAAAGATAAATGTAATTTTGTCCTATTTTACATACTACAAAGAAAATATAATAATGATAGAAAGTACAGTATTATATTTTTAT ACT
AAAATTTGTCAAGAAATCACAGACTTTACTCCATGAGTTTTACTTGAGAGAAAGT CGCAAGTACTTATGATTGG CTTGTGGGTGAAGATTCGAG TTTAATCAAAACTGCTCTAGCCTTGATTTTCTGA
ATCTATTTTCAGTAATGATTATTTAGCTAACAAATAACATAGTGAAATGAACTCATGCAATGAGAAGTTAAGAAATAAACTTCTCAATTAAGAAATTTTCCCTACTGTGGGCCAGGGTCAGTGGC TCA
CGCCTGTAATCCCAGCACTCTAGGAGGCCAAAGCAGGCAGATCACTTCAGGTCAGGAGTTCGAGACCAGCCTGGCCAACAGGGTGAAACCCCGT CTCCACTAAAAAAACAAAAATTAGCCAGCGTGAT
GGCATGCATCTACAATCCCAGCTACTTGGGAGGCTGAGGCACAAGAATCGCTTGAACCCAGGAGGTGGAGGTTGCAGTGAGCCGAGATCGTGCAGTGTGCTGCACTCCAGCCTAGGCAACAGAGT AAG
ACTCCGTCTCAAAAGTAATAATGATAAAAAAATGAAATAAAGAAATTTTCCCTGTTGTGAAACCAGGAAAAGTCATCAGTACAAAAGAACTTT TCCCACCCCACTTTTTTAAGCAAACAAATCACTAG
GATAATACAATGCACATAGGCTGAACAAAAGCCATCCGGTCTTAAGCTCTTCCAACCACTTTTGGACCTAAGTAAAGGGGACATTTGTCTAGTACACAAAGTTGACTATATAT CTGGAGGCAATTCAA
AGGTCAACTTCAAGTTGGTATTTTATTTTCTAAGTATTCTTAGAAGGCTTGTACTATAAATAGGCATCTTTCTTTCTTATTTTACTTTTTTCTT TTTTTGCCATCAGAAATTTCTCCTAAAAAATAGG
CATCTTTCACACTACACACTACCAGGCTGCACAAGTAAGAATAATGAAATACATTTCTTCTCAGAGGTTAGATATGTGAACATTTTAACACAATATCCTAACACTGTTTAGGAACACCAAATTAT CAA
CAGGTATAAcGTAAGAGAAAAGATTACTTACTTTTCAATCCAAGTGATAAATTTTTAAAAAATTATTATTCAAGCATGTTTAAGATTACAAAGC CACTGTTTCTGTGTGAGAAACACTAAGCTAATAT
ATCAGAATATTCATATTTAAAATAATAAATGTAAGCGGTCATACCTATTGTTCGTAGTCAAATCACACTGAAATCCGGAGGCCTGGTGTGAGAACCTCACGAGCGGACACCGGGCATTTAATATT TTT
TGCACACCCACACAGCCAGGGCCAAAGTGGTCAAGGCACTCTCCTAACACAGACAGGATCTTCTGAGTTGCAATTCTTTCTGAAGGAACATTTT TCACTTGAAATTCCATCAGAAAATTTCCTGAGAT
CTGAAAAATAAACACATTTCTAAATAGGAGCTTTTGGTAAAAAAAACAATTTTTTAATAAGCTTTTGTAAAAG aATGCTCTAAATCACTGCTGGAAAGCAAAACAAAATAAGAAAAAAACAACCCACA
AAATAAAAACTCTTCAAGAATCACAGCCAAAAGCAAACTTAAAAGATCCCCAGTTGCCTTCTGTTTCT C

1012	14.3	4.0	2.8	chr10:30613773-30615762	1	175	(1815)	+	AluSx3	SINE/Alu	133	309	(2)	7
13	22.6	3.6	3.6	chr10:30613773-30615762	258	312	(1678)	+	A-rich	Low_complexity	1	55	(0)	8
2100	13.0	0.3	1.3	chr10:30613773-30615762	622	928	(1062)	+	AluSz	SINE/Alu	1	304	(8)	9
15	10.4	3.0	3.0	chr10:30613773-30615762	1219	1251	(739)	+	(TCTT)n	Simple_repeat	1	33	(0)	10
14	15.4	0.0	7.7	chr10:30613773-30615762	1887	1928	(62)	+	A-rich	Low_complexity	1	39	(0)	11

>KSI_AluYa5_47

>Scaffold16914-1578785-1579093
TTATTATTATACTTTAAGTTTTAGGGTACATGTGCACAATATGCAGGTTTGTTACATATGTATACATGTGCCATGTTGGTGTGCTGCATCCATTAACTTGTCATTTACATTAGGTATATCTCCTA AAG
CTTCATCCATGTCCCTACAAAGGACATGAACTCATCATTTTTTATGGCTGCATAGTATTCCATGATGTATATGTGCCACATTTTCTTAATCCCGTCTATCATTGTTGGACATTTGGGTTGGTTCC AAG
TCTTTGCTATTGTGAATAGTGTCGCAATAAACATACGTGTGCATGTGTGTTTATAGTTATCTTTCTTTTAAAGTACAGGATAATTTGTCTTCAAACATAGCTGGATCCAGGTGCCCAAAGAAAGTC CT
TATCAACAGGTTCCTCTTCTCCCACTTCCTCTCTTTTCTCTACTGGCTTCATTCTCAGAGGGAGATATAAAGGTGGCATCAAGGTTTATAATCCACCAGTCTGGGACCCCACAAGAAAGAAAGTA CCT
CTTTCCTAATGGCTTCCTCAAAAAGCTTGGGTTTAACTAATGAGCTGAGCTTGTTTCACTTGGCCATGCCTGAAGCAATAGCTGTGACAAGGCCT GAAATATATGTTTATACCTGGAGCTAGGAGATT
AAAGCAGGAAATGGACTGAAGTGGAAGAGGATTCTGCTGCAGTAAGAAGAGATGTTGAGAAGGCAGAACAACAGTTCCCACTGAAGATGCCACTGAGTTACTAGGACCATACTCCTGGTCTTCTG AGT
TCCATATTCTACTCTTTGAGTCTTTCACATGGATCAGTTCTAAGATGAAGTAAGAATAAACTTAAATAAATCCTTTAATGTTTATCCTCTAAAAGGACAAGAATATTTTAATA GCCTGAAAGCAGATG
TTGGGAATCTCAGAAAATGAATAAAGTATAATCTTGAAAAAAACTAGTGAAACTTCTGGTTTGATCTCTTCCCTAATGCCACCATTCC Tctcaacttttgattcttttttttttttttttttttttt
ttttttttgacggggtctcccccctgtcccccgcggggTTTTTTTTTGAGACGGAGTCTCGGTCTGTCCCCCAGGCTGGAGTGCAGTGGCGGGATCTCGGCTCACTGCAAGCTCCGCCTCCCGGGTTC
ACGCCATTCTCCTGCCTCAGCCTCCCAagtagctgggacgccgggcccccgccactacgcccggctaattttttttgtatttttagtagagacggggtttcaccgttttagccgggatggtctcgatct
cttgacctcgtgatccgcccgcctcggcctcccaaagtgctgggattacaggcgtgagccaccgcgcccggcCTCAACTTTTGATTCTTTAGCTTTTTTCCACTCCTTTTATTTTTTGAAGCTATTGA
TGTTGCTAGAAAAATCTTTGAAGCAAAGgCTACTCTCTAACATTAATAGATAGGAACTACTCCAGATGAAATTAAGTACCCTTCTATTACCCTGCATATGGCATTGCATTAAAATTTT GGTTTACTTG
TTTGTCCCACTCACATACTGTAAGTACTCCAAAGAACTAAATCATCTTTGATTTCCAAGTTTTATTGCCCAACCACATAATTGTATTTGTTGTCAGTAACACATTAATAACATAAGTGTTGTTGAATG
ACCAAATCAAACTGTAATGCACTTACAGACACAGGAAACAACACAATGAGAAAGTGTATGTTCATATGGGGCAGGGATATTCAAGAGAACTAAGAATGAGCAAGCAGAGGCATTTCCTCATGGCAATA
AAACAATAATTAGTTAAAGCAATATATTTTCAAAATAGGAAACATTTATGTTAATTAAAATAGTGAAGAGGGCCAGGTGCTGTGGCTCATGCCTGTAATCCCAGCACTTTGGGAGGCCGAGGCGG GCG
GATCACGAGGTGAGGAGATCGAGACCATCCTGGCTAACATGGTGAAACCCCATCTCTACTAAAAACACAAAAAATTAGCCAGGCAAGGTGGCACGCGCCTATAGTCTCAGCTACTCGGGAGGCTCAGG
CAGGAGAATCGCTTGAACCCGGGAGGAGGAGGTTGCAGTGAGCCGAGATCGTGCCATTGCACTCCAGCCTGGCAACAGAGCAAGACTTTGTCTCAAAAAAAAAAAAAA AaaaGGAAAAAAAAAACTAG
TGAAGAGATGGAAGGTCTTCAAGTTAAGGATCTGAGGTACACTATAAATATGAGTGTAGAAATCAATTCTTATTTAAAAAACAAAACCAAAAACTCTGTCCCATCTAAATTGATATTATCAGTTGTTA
ACATACTTGAGATTTTCAGTTAAGTTGTTTTATAATTATGACATATTT A

1066	5.7	0.7	1.4	AluYa5-Scaffold16914-1578785-1579093	1	143	(2166)	C	L1PA4	LINE/L1	(8)	6147	6006	1
1473	2.4	0.0	0.0	AluYa5-Scaffold16914-1578785-1579093	144	312	(1997)	C	L1PA3	LINE/L1	(314)	5841	5 673	2
376	28.2	4.3	0.0	AluYa5-Scaffold16914-1578785-1579093	326	442	(1867)	C	MLT1K	LTR/ERVL-MaLR	(93)	502	381	3
403	33.1	2.1	0.0	AluYa5-Scaffold16914-1578785-1579093	463	604	(1705)	C	MLT1K	LTR/ERVL-MaLR	(256)	339	195	3
1535	30.1	1.4	0.4	AluYa5-Scaffold16914-1578785-1579093	1001	1309	(1000)	C	AluYa5	SINE/Alu	(0)	311	1	4
2528	7.4	0.3	0.0	AluYa5-Scaffold16914-1578785-1579093	1819	2128	(181)	+	AluSc8	SINE/Alu	1	311	(0)	5

>chr6:102219416-102221561
TTATTATTATACTTTAAGTTTTAGGGTACATGTGCACAATATGCAGGTTTGTTACATATGTATACATGTGCCATGTTGGTGTGCTGCATCCATTAACTTGTCATTTACATTAGGTATATCTCCTA Atg
ctatccctccccccctcccccaccccacaacagtccctgttgtgtgatgttccccttcctgtgtccatgtg ttctcattgttcaattcccacctatgattgagaacatgcggtgtttggttttttgt
ccttgcgatagtttgctgagaatgatggtttccAGCTTCATCCATGTCCCTACAAAGGACATGAACTCATCATTTTTTATGGCTGCATAGTATTCCATGATGTATATGTGCCACATTTTCTTAATCCC
GTCTATCATTGTTGGACATTTGGGTTGGTTCCAAGTCTTTGCTATTGTGAATAGTGTCGCAATAAACATAC GTGTGCATGTGTGTTTATAGTTATCTTTCTTTTAAAGTACAGGATAATTTGTCTTCA
AACATAGCTGGATCCAGGTGCCCAAAGAAAGTCCTTATCAACAGGTTCCTCTTCTCCCACTTCCTCTCTTTTCTCTACTGGCTTCATTCTCAGAGGGAGATATAAAGGTGGCATCAAGGTTTATA ATC
CACCAGTCTGGGACCCCACAAGAAAGAAAGTACCTCTTTCCTAATGGCTTCCTCAAAAAGCTTGGGTTTAA CTAATGAGCTGAGCTTGTTTCACTTGGCCATGCCTGAAGCAATAGCTGTGACAAGGC
CTGAAATATATGTTTATACCTGGAGCTAGGAGATTAAAGCAGGAAATGGACTGAAGTGGAAGAGGATTCTGCTGCAGTAAGAAGAGATGTTGAGAAGGCAGAACAACAGTTCCCACTGAAGATGC CAC
TGAGTTACTAGGACCATACTCCTGGTCTTCTGAGTTCCATATTCTACTCTTTGAGTCTTTCACATGGATCA GTTCTAAGATGAAGTAAGAATAAACTTAAATAAATCCTTTAATGTTTATCCTCTAAA
AGGACAAGAATATTTTAATAGCCTGAAAGCAGATGTTGGGAATCTCAGAAAATGAATAAAGTATAATCTTGAAAAAAACTAGTGAAACTTCTGGTTTGATCTCTTCCCTAATGCCACCATTCC TCTCA
ACTTTTGATTCTTTAGCTTTTTTCCACTCCTTTTATTTTTTGAAGCTATTGATGTTGCTAGAAAAATCTTTGAAGCAAAG aCTACTCTCTAACATTAATAGATAGGAACTACTCCAGATGAAATTAAG
TACCCTTCTATTACCCTGCATATGGCATTGCATTAAAATTTTGGTTTACTTGTTTGTCCCACTCACATACTGTAAGTACTCCAAAGAACTAAATCATCTTTGATTTCCAAGTTTTATTGCCCAACCAC
ATAATTGTATTTGTTGTCAGTAACACATTAATAACATAAGTGTTGTTGAATGACCAAATCAAACTGTAATGCACTTACAGACACAGGAAACAACACAATGAGAAAGTGTATGTTCATATGGGGCAGGG
ATATTCAAGAGAACTAAGAATGAGCAAGCAGAGGCATTTCCTCATGGCAATAAAACAATAATTAGTTAAAGCAATATATTTTCAAAATAGGAAACATTTATGTTAATTAAAATAGTGAAGAGGGC CAG
GTGCTGTGGCTCATGCCTGTAATCCCAGCACTTTGGGAGGCCGAGGCGGGCGGATCACGAGGTGAGGAGATCGAGACCATCCTGGCTAACATGGTGAAACCCCATCTCTACTAAAAACACAAAAAATT
AGCCAGGCAAGGTGGCACGCGCCTATAGTCTCAGCTACTCGGGAGGCTCAGGCAGGAGAATCGCTTGAACCCGGGAGGAGGAGGTTGCAGTGAGCCGAGATCGTGCCATTGCACTCCAGCCTGGC AAC

AGAGCAAGACTTTGTCTCAAAAAAAAAAAAAAAAGGAAAAAAAAAACTAGTGAAGAGATGGAAGGTCTTCAAGTTAAGGATCTGAGGTACACTATAAATATGAGTGTAGAAATCAATTCTTATTTAAAA
AACAAAACCAAAAACTCTGTCCCATCTAAATTGATATTATCAGTTGTTAACATACTTGAGATTTTCAGTTAAGTTGTTTTATAATTATGACATATTT A

4072	2.5	0.2	0.2	chr6:102219416-102221561	1	475	(1671)	C	L1PA4	LINE/L1	(8)	6147	5673	6
376	28.2	4.3	0.0	chr6:102219416-102221561	489	605	(1541)	C	MLT1K	LTR/ERVL-MaLR	(93)	502	381	7
403	33.1	2.1	0.0	chr6:102219416-102221561	626	767	(1379)	C	MLT1K	LTR/ERVL-MaLR	(256)	339	195	7
2501	7.5	0.3	0.0	chr6:102219416-102221561	1659	1965	(181)	+	AluSc8	SINE/Alu	1	308	(3)	8

>KSI_AluYa5_48

>Scaffold16962-506310-506610
GTTTATATAATACTTATGTAAATAATACAGTTAATTATCTACTAGTTTGTTTCCCTTGCCAGGTTGTTGGGTTGTTTGAGATTAGGACCACATTCATCTTAGTATCCTCTTTCACCCAGGATGACT CA
TTGTATGTGGTCAGCAAATATTGGTTGACATGAATGCCTTATTCAAAAGTACTTTAATATATAAGGGGCAGTGTCTTTGTAAAACATTTAGGCAAAATAT GTGACTATCAAGGTTAAGAGAATCATTT
CCATTATCTGCATAAGGCTGAGCAAGATTTCCTTTTTATATTTTTGCTGCCCATTACCATTGAAGAAGAGACCTTCATAGATCTTTTTTTGTGTGTTTTTTTCTGTCACCCAGGCTGGAGTGCAG TGG
CGCAGTCATGGCTCACTGCAGCCTTGACCTCCTGAGCTCAGGTGGTCCTCCCACCTCAGCCTCCCGAGTAGCCAGGACCACAGGCACGC aTCACCACACCTGGCTAAttTTTTTTTTTTTTTTTTTTT
TGAGTCGGAGTCTTGCcCTGTCcCCCAGGCTGGAGTGCAATGGTACAATCTCGGCTCACTGCAACCTCCGCCTCCCAGGTTCAAGCGATTCTCCTGCCTCAGCCTCCTGAGTAGCTGGTATTACAGGC
ATGCACCACCAAGTCCGGCTAATTTTTGTATTTTTGTAtTTTTTTTTTTTTTTAAGAGACAGGGTTTCACCATGTTTCCCAGGCTGGTTTCGAACTCGTGGGCTCAGGCGATCCACCCACCTCAGCCT
CCAAAGTACTGGGATTACAGGCATGAACTATCATGCCTGGCCAATAATTTATTTTCATATCTTAACTGTGTGCAAAAATACTTAGGGTTTAAGAGGCGATTTTAATACACATTTGAAAATTGGAAATA
GATGTGTGTTTTCTTCTTAGGCACAGGGAAAATATTTTTTAAAAATAAACTTTACAAAACTGTCAGTGTCATTTAATAATGCAGTTTTATTCAAC TGGGCTACATTTctttttttttttttttttgaga
cggagtctcgctctgtcgcccaggctggagtgcagtggcgggatctcggctcactgcaagctccgcctcccgggttcacgccattctcccgcctcagcctcccaagtagctgggactacaggcgc ccg
ccactacgcccggctaatttttgtatttttagtagagacggggtttcaccattttagccgggatggtctcgatctcctgacctcgtgatccgcccgcct cggcctcccaaagtgctgggattacagg
cgtgagccaccgcgcccggcctgggctacatttTTAAGCAGCAGGATTTAGAAATGAAACCATCTCTTTTCTCCTTCCTACACTCTATTGGAGTCAGCAAATGATTTGGGAGTTTGGGCTTCTTAGTG
GCTGGAATGTTTGGGGTAAGAGTTAGTTGAGGTGGAAGTGGAGGCTAATTGCTCCCCAGGAACTTAATGGTTGTAAGGGCACTTAGGCCACATCGCACTCTACCCACTTAAATGATAT ATAACTATCT
CAGAGCCTCAATTCCCACATTAAAATAGGGATCGTAGTTGGGAGTTGTGAACTTTCCTCAGTGTCTAATGCACAGAAACCATTCAATAAATGTTATTTTAGTATTACTGATACTG CATCTAaTAATGA
GATAGCTTTGTTTTCTGGGTTTATCAGGCCTACCTGTTAATGGAAATCAGTAATCGTTTTCCTTTTGATTGACTTCATTTTATGTCTGCTCACATTTTTGTTTTTGTTTATGATAGGTAACTGTG CTC
AAAAGAAAAGTGTATTTGAATACAGTTCTGTATATTTAATTTGTTTGTTCAGTGGGATTGATCCTTGTCTTTCACAACTGAATTTGATAGATGAGGAAACAAACATAAATGTATA AAATTTTCTAGGT
CTTAGGTTATGTCTAGAAGATTTTTATTTCGGAATTTAATTTCTTTCTTCTCTTCACAAACATTTTAGCCTACTTTGGGGATAAAGGATTATTTGGTCTTCTGGATTTGGAGGCAATCAGCGGAC AGC
ATGaAAGATGTGTGCTCTGGCTCGGATAAGAGATGGGACATCATTCAGTCACTAGTTGGATGGCACAAGGCTCTTCACAGACGCATCTGTAGCAGAGTGGA tCTTGTACTAACTTATGATAGAATGTA
TCAGAATAAATGTTTTTAACAGTGTTATGCTTGAGTCTTTTTTGAATTGAACATGCTAGGATTATCAGGTAAAAATTAAGCCTTTTGGTACTTTCTCAAAACAG aGAATTTATGTGTaTACCTAG

257	31.1	6.9	4.9	AluYa5-Scaffold16962-506310-506610	6	164	(2137)	+	L2c	LINE/L2	3223	3384	(3)	1
986	14.9	0.0	0.0	AluYa5-Scaffold16962-506310-506610	358	491	(1810)	C	AluJb	SINE/Alu	(45)	267	134	2
2089	11.0	0.6	5.6	AluYa5-Scaffold16962-506310-506610	492	810	(1491)	C	AluSx	SINE/Alu	(8)	304	1	3
2836	1.0	0.0	0.0	AluYa5-Scaffold16962-506310-506610	1001	1301	(1000)	C	AluYa5	SINE/Alu	(10)	301	1	4

>chr7:22852492-22854479
GTTTATATAATACTTATGTAAATAATACAGTTAATTATCTACTAGTTTGTTTCCCTTGCCAGGTTGTTGGGTTGTTTGAGATTAGGACCACATTCATCTTAGTATCCTCTTTCACCCAGGATGACT CA
TTGTATGTGGTCAGCAAATATTGGTTGACATGAATGCCTTATTCAAAAGTACTTTAATATATAAGGGGCAGTGTCTTTGTAAAACATTTAGGCAAAATATGTGACTATCAAGGTTAAGAGAATCA TTT
CCATTATCTGCATAAGGCTGAGCAAGATTTCCTTTTTATATTTTTGCTGCCCATTACCATTGAAGAAGAGACCTTCATAGATCTTTTTTTGTGTGTTTTTTTCTGTCACCCAGGCTGGAGTGCAGT GG
CGCAGTCATGGCTCACTGCAGCCTTGACCTCCTGAGCTCAGGTGGTCCTCCCACCTCAGCCTCCCGAGTAGCCAGGACCACAGGCACGC gTCACCACACCTGGCTAATTTTTTTTTTTTTTTTTTTTG
AGTCGGAGTCTTGCtCTGTCaCCCAGGCTGGAGTGCAATGGTACAATCTCGGCTCACTGCAACCTCCGCCTCCCAGGTTCAAGCGATTCTCCTGCCTCAGCCTCCTGAGTAGCTGGTATTACAGGCAT
GCACCACCAAGTCCGGCTAATTTTTGTATTTTTGTATTTTTTTTTTTTTTAAGAGACAGGGTTTCACCATGTTTCCCAGGCTGGTTTCGAACTCGTGGGCTCAGGCGATCCACCCACCTCAGCCTCCA
AAGTACTGGGATTACAGGCATGAACTATCATGCCTGGCCAATAATTTATTTTCATATCTTAACTGTGTGCAAAAATACTTAGGGTTTAAGAGGCGATTTTAATACACATTTGAAAATTGGAAATAG AT
GTGTGTTTTCTTCTTAGGCACAGGGAAAATATTTTTTAAAAATAAACTTTACAAAACTGTCAGTGTCATTTAATAATGCAGTTTTATTCAAC TGGGCTACATTTTTAAGCAGCAGGATTTAGAAATGA
AACCATCTCTTTTCTCCTTCCTACACTCTATTGGAGTCAGCAAATGATTTGGGAGTTTGGGCTTCTTAGTGGCTGGAATGTTTGGGGTAAGAGTTAGTTGAGGTGGAAGTGGAGGCTAATTGCTCCCC
AGGAACTTAATGGTTGTAAGGGCACTTAGGCCACATCGCACTCTACCCACTTAAATGATATATAACTATCTCAGAGCCTCAATTCCCACATTAAAATAGGGATCG TAGTTGGGAGTTGTGAACTTTCC

TCAGTGTCTAATGCACAGAAACCATTCAATAAATGTTATTTTAGTATTACTGATACTGCATCTA gTAATGAGATAGCTTTGTTTTCTGGGTTTATCAGGCCTACCTGTTAATGGAAATCAGTAATCGT
TTTCCTTTTGATTGACTTCATTTTATGTCTGCTCACATTTTTGTTTTTGTTTATGATAGGTAACTGTGCTCAAAAGAAAAGTGTATTTGAATACAGTTCTGTATA TTTAATTTGTTTGTTCAGTGGGA
TTGATCCTTGTCTTTCACAACTGAATTTGATAGATGAGGAAACAAACATAAATGTATAAAATTTTCTAGGTCTTAGGTTATGTCTAGAAGATTTTTATTTCGGAATTTAATTTCTTTCTTCTCTT CAC
AAACATTTTAGCCTACTTTGGGGATAAAGGATTATTTGGTCTTCTGGATTTGGAGGCAATCAGCGGACAGCATG gAAGATGTGTGCTCTGGCTCGGATAAGAGAT GGGACATCATTCAGTCACTAGTT
GGATGGCACAAGGCTCTTCACAGACGCATCTGTAGCAGAGTGGA aCTTGTACTAACTTATGATAGAATGTATCAGAATAAATGTTTTTAACAGTGTTATGCTTGAGTCTTTTTTGAATTGAACATGCT
AGGATTATCAGGTAAAAATTAAGCCTTTTGGTACTTTCTCAAAACAG gGAATTTATGTGTgTACCTAG

257	31.1	6.9	4.9	chr7:22852492-22854479	6	164	(1824)	+	L2c	LINE/L2	3223	3384	(3)	5	
1001	14.2	0.0	0.0	chr7:22852492-22854479	358	491	(1497)	C	AluJb	SINE/Alu	(45)	267	134	6	
2105	10.8	0.6	5.3	chr7:22852492-22854479	492	807	(1181)	C	AluSx	SINE/Alu	(10)	302	1	7	

>KSI_AluYa5_49

>Scaffold16962-560182-560398
ATTTCTTtAGCCAAAGGACCAGAGAAGGGATGGCCTAGAAAAATAGAAAAGCTTGAGATAATAACCACCAGACTCCAGCCAAATATACAGAAAAAACTATGGCCCCACTACCTCCCCCAACATGGGAG
GCCAAGTGAGAAGCCCAGCCTTCTACCCCCATGAGGCTATAAGGAGGCACCCAACACCCCACTGGGGTAGTGTCAGGGAATGCTTAGTGAAGAGCTGGGACTTTTACCTCTGCCAGGAAGCAATG AGT
CCCCTTCCTTCTTTTCCACTGTGGTGCTAGGAGAGACCATGTGGAGAGCCTGGGCTTCCACTCCCTCCATCCTTAAGGAGGTGGTCTTTCCCCTCCCCAATAGGATGTCAGTTAAGGCCTAGTGGAGA
GTCAGTACCTTCACCAACACTCAGCAgCAACAAGGCCACCACCTACCACCTCCCCAGCTATGTCAGATGCATCCAGATGGGGAGCAGGCACTGCTCTCCAGGCAAGGTG gTATCACTGGAGGCCCAGT
GGGGAATCTGAATTCCCACCCCTGCCTAGTAGTGACTAGAGGCCCCTCTCACCCTGGGTGTCAACAGGGCAAGTAGAGAATCTAGACTATCTTTTCTATCTGAAGACTAAAGACAAAGAAAA gTTCTT
AAAAGCAGCTAGAGAAAAATAACACATTACCTATTACCCATAGCAAAAAAAAAAGTTTGAATGACAAGCAGATTTCTCATCAGAAACCATG GAGGCCAGAGGGAATGACATAACACTCTTCAAGTGCT
GAGAGAAAAGAACTGTCAACCCAGAATTCTATGTCCAGTGAAAATATCCTTCAAGAATGAAGGGGAAATAAAGACATTCTCAGATGATGGAAAACTAAAAAAAATTGTTGCCAGCAGACCTATTCTAA
AAGAATGGCTAAAGGAAGTTCTCTAGACAAAAAAGAAATAGTAAAATAAAAAGTCTTGGAACAGTAGGAAGGAAGAAAGAACATGGGATGAGT AAAACAATCAGggccgggcgcggtggctcacgcct
gtaatcccagcactttgggaggccgaggcgggcggatcacgaggtcaggagatcgagaccatcctggctaaaacggtgaaaccccgtctctactaaaaatacaaaaaattagccgggcgtagtggcgg
gcgcctgtagtcccagctacttgggaggctgaggcaggagaatggcgtgaacccgggaggcggagaa aaaacaatcagTAAAAACAAGACTTTCCTTTTCTTGAGTTGTTAAATTATGTTTGATTATT
GAAGCAAAAGTCTAACTGTCTAATGTGGCTCTCAGTGCATGGAGAGGAAATAAGACAATTATATTCTAAAGAGAGGGAATAAAGTGATAT aAAGAGACATAAGGTCTCTATACTTCATTCTAACTGGT
AAAATGTTGACACCAGTGGACTCAGATAAATTAAATGTCTGTAATGTAATACTTAGAGAAACCACTAAAAGAGATCTACAAAGAAAATCACTCAAAAGCACTA cAGGCATATCAAAATGGCATTTAAA
AAATGTTTGAGTCATCTACTAGAAGGCAGAAAAATAAAAGCAGAGAAACAGAAATACAAAGATCAAAGATAAAGTAAAAAATAAAATGGCATATTTAAGCCTTAACAGATCAGCAATTACATTAAACA
TAAATAGTTTAAACATACCAATTAAAAGACAAATTAGCATAGTGGACTTAAAAAAATGACTCAATTATATGTTGTATAAGAAATTCACTTCAAGTAGTTCAAATTATACATGTAGCTTAAAAATA AAG
GATGGAAAATACACACCATGCAAACTTTAATCAAAATAAAGCAGATGTGGCTCTGTTATTATCAGATACAGTAGAGTTTAGATAAAAGAAACATATCAAAAACAGAGAAGGACATTACACAATGATAA
AAGGtTCAATTCACCAAGAAAACATAGCAATCCCTAGTGTGTGTTCACCAAACAACAGAACTGCAAAATATATGAAGCAAAAACGGATAGAAAGAAAAAGTTATGGATAAATCCACAGTTCTCATTGG
TGACTTCAACACTCCTCTCTCAACTAGAACTAGCTGTTGATAGAACAACTAGAGAGAAACAGCATCAAAGATGTAGAGAACTCAACATGACAATCAACCAACAGGATCTAATTGACAATAAAACACTT
CATTTAACAATAGTAGAATTCATGTTTTTTTCAAGTACCC T

1652	28.2	4.2	1.9	AluYa5-Scaffold16962-560182-560398	2	567	(1650)	+	L1MDa	LINE/L1	302	972	(5661)	1	
568	22.4	1.9	0.0	AluYa5-Scaffold16962-560182-560398	494	600	(1617)	+	L1MDa	LINE/L1	823	931	(5702)	2	*
4598	16.1	2.4	3.0	AluYa5-Scaffold16962-560182-560398	611	1000	(1217)	+	L1MDa	LINE/L1	1716	2092	(4541)	1	
2072	0.5	0.0	0.0	AluYa5-Scaffold16962-560182-560398	1001	1217	(1000)	+	AluYa5	SINE/Alu	1	217	(94)	3	
4598	16.9	2.2	2.8	AluYa5-Scaffold16962-560182-560398	1218	2216	(1)	+	L1MDa	LINE/L1	2093	3092	(3445)	1	

>chr7:22798821-22800807
ATTTCTTcAGCCAAAGGACCAGAGAAGGGATGGCCTAGAAAAATAGAAAAGCTTGAGATAATAACCACCAGACTCCAGCCAAATATACAGAAAAAACTATGGC CCCACTACCTCCCCCAACATGGGAG
GCCAAGTGAGAAGCCCAGCCTTCTACCCCCATGAGGCTATAAGGAGGCACCCAACACCCCACTGGGGTAGTGTCAGGGAATGCTTAGTGAAGAGCTGGGACTTTTACCTCTGCCAGGAAGCAATG AGT
CCCCTTCCTTCTTTTCCACTGTGGTGCTAGGAGAGACCATGTGGAGAGCCTGGGCTTCCACTCCCTCCATCCTTAAGGAGGTGGTCTTTCCCCTCCCCAATAG GATGTCAGTTAAGGCCTAGTGGAGA
GTCAGTACCTTCACCAACACTCAGCAaCAACAAGGCCACCACCTACCACCTCCCCAGCTATGTCAGATGCATCCAGATGGGGAGCAGGCACTGCTCTCCAGGCAAGGTG tTATCACTGGAGGCCCAGT
GGGGAATCTGAATTCCCACCCCTGCCTAGTAGTGACTAGAGGCCCCTCTCACCCTGGGTGTCAACAGGGCAAGTAGAGAATCTAGACTATCTTTTCTATCTGA AGACTAAAGACAAAGAAAAaTTCTT
AAAAGCAGCTAGAGAAAAATAACACATTACCTATTACCCATAGCAAAAAAAAAAGTTTGAATGACAAGCAGATTTCTCATCAGAAACCATG GAGGCCAGAGGGAATGACATAACACTCTTCAAGTGCT
GAGAGAAAAGAACTGTCAACCCAGAATTCTATGTCCAGTGAAAATATCCTTCAAGAATGAAGGGGAAATAAAGACATTCTCAGATGATGGAAAACTAAAAAAA ATTGTTGCCAGCAGACCTATTCTAA
AAGAATGGCTAAAGGAAGTTCTCTAGACAAAAAAGAAATAGTAAAATAAAAAGTCTTGGAACAGTAGGAAGGAAGAAAGAACATGGGATGAGT AAAACAATCAGTAAAAACAAGACTTTCCTTTTCTT
GAGTTGTTAAATTATGTTTGATTATTGAAGCAAAAGTCTAACTGTCTAATGTGGCTCTCAGTGCATGGAGAGGAAATAAGACAATTATATTCTAAAGAGAGGG AATAAAGTGATATgAAGAGACATAA
GGTCTCTATACTTCATTCTAACTGGTAAAATGTTGACACCAGTGGACTCAGATAAATTAAATGTCTGTAATGTAATACTTAGAGAAACCACTAAAAGAGATCTACAAAGAAAATCACTCAAAAGC ACT
AtAGGCATATCAAAATGGCATTTAAAAAATGTTTGAGTCATCTACTAGAAGGCAGAAAAATAAAAGCAGAGAAACAGAAATACAAAGATCAAAGATAAAGTAA AAAATAAAATGGCATATTTAAGCCT
TAACAGATCAGCAATTACATTAAACATAAATAGTTTAAACATACCAATTAAAAGACAAATTAGCATAGTGGACTTAAAAAAATGACTCAATTATATGTTGTATAAGAAATTCACTTCAAGTAGTT CAA
ATTATACATGTAGCTTAAAAATAAAGGATGGAAAATACACACCATGCAAACTTTAATCAAAATAAAGCAGATGTGGCTCTGTTATTATCAGATACAGTAGAGT TTAGATAAAAGAAACATATCAAAAA
CAGAGAAGGACATTACACAATGATAAAAGGgTCAATTCACCAAGAAAACATAGCAATCCCTAGTGTGTGTTCACCAAACAACAGAACTGCAAAATATATGAAGCAAAAACGGATAGAAAGAAAAAGTT
ATGGATAAATCCACAGTTCTCATTGGTGACTTCAACACTCCTCTCTCAACTAGAACTAGCTGTTGATAGAACAACTAGAGAGAAACAGCATCAAAGATGTAGA GAACTCAACATGACAATCAACCAAC
AGGATCTAATTGACAATAAAACACTTCATTTAACAATAGTAGAATTCATGTTTTTTTCAAGTACCC T

1616	28.6	4.2	1.9	chr7:22798821-22800807	2	567	(1420)	+	L1MDa	LINE/L1	302	972	(5661)	4	
560	22.6	1.9	0.0	chr7:22798821-22800807	495	600	(1387)	+	L1MDa	LINE/L1	824	931	(5702)	5	*
4697	17.1	2.3	1.9	chr7:22798821-22800807	611	1986	(1)	+	L1MDa	LINE/L1	1716	3092	(3445)	4	

>KSI_AluYa5_50

>Scaffold63205-462802-463112
CCTTTCCATGTTGTAGAAGGACATTAGAAAGTGTCTCTGCCCAGCTGGGCGCAGTGGCTCACGCCTGTAATCCCAGCACTTTGGGAGACCGAGGCGGGCGGATCACAAGGTCAAGAGATTGAGACC AT
CCTGGCCAACACGGTGAAACCCTGTGTCTACTAAAAATACAAAAATTAGCTGGACATGGTGGCATGCGCCTGTAATCCCAGC TACTTGGGAGACTGAGGCAGGAGAATTGCCTGAACCTGGGAGGTGG
AGGTTGCACTGAGtGGAGATTGCGCCACTGCACTCCAGCCTGGTGATGGAGCGAGACTACGTCAAGGAAGGAAGGAAGGAAGGAAGGAAG GgAGGGAGGGAGGGAGGGAGGGAnnnnnnnnnnnnnn
nnnnnnnnnnnnnnnGGAGGGAGGGGAGGGGAAGGGAGGGGAAGGAAGAAAGGAAGGGAGTCTCTGCTCATCCAGACATTG CATTTCATAATGTCCCCTTTGCATCTAGGCAAGACCACGGGACTAA
TTTTCAACACTGGAATTTAAGCATAAGTTAAAAAGTCTGCCGACTTCACTGTTATTCCTCTGATGATAGTATGCATTGTTTTGCTGTTTTTGAGATTTTTTTTCCCTTATACTTAGTTTTCAGCAGTT
TGAACATGGTATTCCTAGGTGTGGTTCTCTTGTATTTACCTTGCTGGTGACACACAAAATTATTTCAGTTGATGAG gTGATGTCTTTCATCAATTCTTGGAAGTTCTTGCCTGTCGTATCTCCAAATA
CTGACTCGGCCTCAATTTCTGCTTTCTTCTTCTTTTAAAATTCCTAGGCTTGCTTTAGCAGGTCTTTATGCCCTTAAACATAAGGTTTCTGCATTTTATCTGAGTTTTCTAGTTATTTTTGGCAA GTG
CTTTAATTTCCTGCAAAGTTTTCCATCCTGCCTGGAAGAGTAAGTTCTTATATAAAATTCTTTTTCTCTGTAGTTTA AATCTTTGTTTCAAGAAAATTGTTTTTggccgggcgcggtggctcacgcct
gtaatcccagcactttgggaggccgaggcgggcggatcacgaggtcaggagatcgagaccatcccggctaaaacggtgaaaccccgtctctactaaaaatacaaaaaattagccgggcgtagtgg cgg
gcgcctgtagtcccagctgcttgggaggctgaggcaggagaatggcgtgaacccgggaggcagagcttgcagtgagc cgagatcccgccactgcactccagcctgggcgacagagcgagactccgtct
caaaaaaaaaaaaaaaaaaaaaaaaaaaaaaaaaaaaaaagaaaattgtttttATTCCTTCTGTTATGGATTTCAAAGGCTTTTCAGTTCCAATTTTTTCCCTTTTCTTTACTTCAGGTATACTGGTT
ACTCACATGCCGGGTCTAAGTTGTCCGTCCCCTGTGTCTTTCATCTTATTCCTGCAATTCCGCATTGAATCGTGCATGAATGTCTGCTGCGTTCTTCTTCTTTTTTGTTGGTTTGTTTGTTTGAGATG
GAGTCTTACTCTGTGTCCCAGGCTGGAGTGCAGTGGCATGATCTCAGCTCACTGAAACCTCCATGTCCTGGGTTCAGAATTGCTTGAACCTCCACTTCCGGGGTTCAAGCAATTCT GGTGCTTGAACT
AGAGTAGCTGAGACTACAGGTGTGCACCACCACGCTCGGCTAATTTTTGTATTTGTAGTAGAGACGGGGTTTCACCA TGTTGGCCAGGCTGGTCTTGAACCCCCGAGCTCAGGTTATCTGCCCGCCTC
AGCCTCCCAAAGTGCTGGGATTAAAGGCATGAGTCACCATTCCTGGCCCATGTCTTCTGCATTCTGCATGAATAGCTTACATCAGCCCACCTTGTCACTTACAAGGGATTGGGTTCTTGTTTTGC TGC
TGCTTGTTTGGTTTTAACTTCACCAATTCTATTTTCTTACTTTTTATGTCTCTATCCATTTCATTTCTTTTTTTTTC TTTTATAAACTTATGTATTACAGGGGCAACTTTAATTTATTTGGGTAAAGA
TGATAAAGCAGCTAAAATTTTCTATTTCCTGGAGTACATCCAACAAAATTTTATTTATTTTTATATCTATATATGTGTATTTTATCTATCTATATCTGTATCTACCTACTCATATGTAGAGAGAT TTA
TAAATATTAATATATATAACCTAATGCTAAAGTGTATTCATGCTTTTTTGTTGTTTTCATACATTTTTGTTTTTGCG GTTGATTTATGACCTCAGGTTAGATCTAACCAGCTCAGCCTTTAACTCCAA
AATACAA

2187	10.1	0.0	0.0	AluYa5-Scaffold63205-462802-463112	44	319	(1992)	+	AluSc	SINE/Alu	2	277	(32)	1
30	0.0	0.0	0.0	AluYa5-Scaffold63205-462802-463112	320	347	(1964)	+	(AAGG)n	Simple_repeat	1	28	(0)	2
17	0.0	0.0	0.0	AluYa5-Scaffold63205-462802-463112	348	369	(1942)	+	(GAGG)n	Simple_repeat	1	22	(0)	3
19	11.8	0.0	5.0	AluYa5-Scaffold63205-462802-463112	403	444	(1867)	+	G-rich	Low_complexity	1	40	(0)	4
697	32.6	2.9	1.4	AluYa5-Scaffold63205-462802-463112	539	812	(1499)	C	L1MEg	LINE/L1	(4790)	2543	2266	5
510	27.3	0.8	0.2	AluYa5-Scaffold63205-462802-463112	806	943	(1368)	C	L1MEg	LINE/L1	(6108)	127	1	5 *
2943	0.6	0.0	0.0	AluYa5-Scaffold63205-462802-463112	1001	1311	(1000)	+	AluYa5	SINE/Alu	1	311	(0)	6
1777	13.5	0.9	10.6	AluYa5-Scaffold63205-462802-463112	1500	1840	(471)	C	AluSx	SINE/Alu	(1)	311	1	7
28	29.1	1.1	2.1	AluYa5-Scaffold63205-462802-463112	2100	2194	(117)	+	(TA)n	Simple_repeat	1	94	(0)	8

>chr8:10325291-10327346
CCTTTCCATGTTGTAGAAGGACATTAGAAAGTGTCTCTGCCCAGCTGGGCGCAGTGGCTCACGCCTGTAATCCCAGCACTTTGGGAGACCGAGGCGGGCGGATCACAAGGTCAAGAGATTGAGACCAT
CCTGGCCAACACGGTGAAACCCTGTGTCTACTAAAAATACAAAAATTAGCTGGACATGGTGGCATGCGCCTGTAATCCCAGCTACTTGGGAGACTGAGGCAGGAGAATTGCCTGAACCTGGGAGG TGG
AGGTTGCACTGAGcGGAGATTGCGCCACTGCACTCCAGCCTGGTGATGGAGCGAGACTACGTCAAGGAAGGAAGGAAGGAAGGAAGGAAG GaaggaAGGGAGGGAGGGAGGGAGGGAgggagggagga
agggagggagggggcgagggagggagggaaggagggggcgagggagggagggaaggagggggcgagggagggagggaaggagggggcgagggag GGAGGGAGGGGAGGGGAAGGGAGGGGAAGGAAGA
AAGGAAGGGAGTCTCTGCTCATCCAGACATTGCATTTCATAATGTCCCCTTTGCATCTAGGCAAGACCACGGGACTAATTTTCAACACTGGAATTTAAGCATAAGTTAAAAAGTCT GCCGACTTCACT
GTTATTCCTCTGATGATAGTATGCATTGTTTTGCTGTTTTTGAGATTTTTTTTCCCTTATACTTAGTTTTCAGCAGTTTGAACATGGTATTCCTA GGTGTGGTTCTCTTGTATTTACCTTGCTGGTGA
CACACAAAATTATTTCAGTTGATGAGtTGATGTCTTTCATCAATTCTTGGAAGTTCTTGCCTGTCGTATCTCCAAATACTGACTCGGCCTCAATTTCTGCTTTCTTCTTCTTTTAAAATTCCTAGGCT
TGCTTTAGCAGGTCTTTATGCCCTTAAACATAAGGTTTCTGCATTTTATCTGAGTTTTCTAGTTATTTTTGGCAAGTGCTTTAATTTCCTGCAAA GTTTTCCATCCTGCCTGGAAGAGTAAGTTCTTA
TATAAAATTCTTTTTCTCTGTAGTTTAAATCTTTGTTTCAAGAAAATTGTTTTTATTCCTTCTGTTATGGATTTCAAAGGCTTTTCAGTTCCAATTTTTTCCCTTTTCTTTACTTCAGGTATACTGGT
TACTCACATGCCGGGTCTAAGTTGTCCGTCCCCTGTGTCTTTCATCTTATTCCTGCAATTCCGCATTGAATCGTGCATGAATGTCTGCTGCGTTC TTCTTCTTTTTTGTTGGTTTGTTTGTTTGAGAT
GGAGTCTTACTCTGTGTCCCAGGCTGGAGTGCAGTGGCATGATCTCAGCTCACTGAAACCTCCATGTCCTGGGTTCAGAATTGCTTGAACCTCCACTTCCGGGGTTCAAGCAATTCTGGTGCTTG AAC

TAGAGTAGCTGAGACTACAGGTGTGCACCACCACGCTCGGCTAATTTTTGTATTTGTAGTAGAGACGGGGTTTCACCATGTTGGCCAGGCTGGTC TTGAACCCCCGAGCTCAGGTTATCTGCCCGCCT
CAGCCTCCCAAAGTGCTGGGATTAAAGGCATGAGTCACCATTCCTGGCCCATGTCTTCTGCATTCTGCATGAATAGCTTACATCAGCCCACCTTGTCACTTACAAGGGATTGGGTTCTTGTTTTG CTG
CTGCTTGTTTGGTTTTAACTTCACCAATTCTATTTTCTTACTTTTTATGTCTCTATCCATTTCATTTCTTTTTTTTTCTTTTATAAACTTATGTA TTACAGGGGCAACTTTAATTTATTTGGGTAAAG
ATGATAAAGCAGCTAAAATTTTCTATTTCCTGGAGTACATCCAACAAAATTTTATTTATTTTTATATCTATATATGTGTATTTTATCTATCTATATCTGTATCTACCTACTCATATGTAGAGAGA TTT
ATAAATATTAATATATATAACCTAATGCTAAAGTGTATTCATGCTTTTTTGTTGTTTTCATACATTTTTGTTTTTGCGGTTGATTTATGACCTCA GGTTAGATCTAACCAGCTCAGCCTTTAACTCCA
AAATACAA

2202	9.8	0.0	0.0	chr8:10325291-10327346	44	319	(1737)	+	AluSc	SINE/Alu	2	277	(32)	9	
80	24.7	2.0	0.5	chr8:10325291-10327346	320	523	(1533)	+	GA-rich	Low_complexity	1	207	(0)	10	
716	32.2	2.9	1.4	chr8:10325291-10327346	617	890	(1166)	C	L1MEg	LINE/L1	(4790)	2543	2266	11	
510	27.3	0.8	0.2	chr8:10325291-10327346	884	1021	(1035)	C	L1MEg	LINE/L1	(6108)	127	1	11	*
1777	13.5	0.9	10.6	chr8:10325291-10327346	1245	1585	(471)	C	AluSx	SINE/Alu	(1)	311	1	12	
28	29.1	1.1	2.1	chr8:10325291-10327346	1845	1939	(117)	+	(TA)n	Simple_repeat	1	94	(0)	13	

>KSI_AluYa5_51

>Scaffold84228-3153261-3153579
TTTTTAGTAGAGACAGGGTTTCACCGTATTAGCCAGCATGGTCTCCATCTCCTGACCTTGTGATCCGCTCGCCTCGGCCTCCCAAAGTGCTGGGATTACAGGCGTGAGCCACCATGCCTGGCCTAT TC
CAGCACTTTTGGAGGCTGAGGCGAGAGAATCACCTGAACCCAGGAGTTCAAGACCAGCCTGGGCAACATGATGAAACCCCATCTCTACAAAACATACAAAAATAAGCTGAGTGTGGTGTCGTAAG CCT
GTGGTCCCAGCTACTCACAGCTACTCAGGAGGCTGAAGTAGAAGGATTTATTGAGCCAGGAAGGTCGAGGCTATAGTGAGCTGTGATTATGCCACTGCACTCCAGCCTGGGTGACAGAACGAATCT CT
GTCCCAAAAATAAATAAATGAGTAAATAAGAAAAGTTTAAAATAAGAATAGGCTGGGTGGCTCACGCCCGTAATCCCAGCACTTTAGGAGGCTAAGGAGGGCGGATGACTGAGGTCAGGAGTTCG AGA
CCACTTTGGGCAACATGGTGAAACCCCGTCTCTAGTAAAACTACAAAATTTAACTAGGTGTGGTGGCACATGCCTGTAATCCCACCTACTCAGGAAGCTGAGGCAGGAGAGTCACTTGAACCCGGG AG
GCGGAGGGTGCAGTGAGCCGAGATCGTGCACTCCAGCCTGGGTGACAGAGCGAGACTTTGTCTCAAAAAAATAAATAAATAAAAATAAAAATAATTTAAATGTCTATCAATAGATAAATAAATTA TGG
TAATCCATACCACAGAATAATTTATTGTAGTTACAAATGAATCTATAAGATTTCTATGTATTAACCTGCAGAGAATCTTTAAGGCACAGTAAGGGAAAGACATTTATTACAAGAGAAAAAATTTTG GA
TATATATATGCCGATATATACATATATGTAGAATTCACACCAAACCTTTGAGAAAGGTTATTCTCTGGAA CGGAAAGAGGGATTTGAAGGTAgatgaagaggaattttttttttttttttttttttt
tttttttttttttgagacggagtctcgctctgtcgcccaggctggagtgcagtggcgggatctcggctcactgcaagctccgcctcccgggttcacgccattctcctgcctcagcctcccaagta gc
tgggactacaggcgcccgccactacgcccagctaatttttgtatttttagtagagacggggtttcaccatgttagccgggatggtctcgatctcctgacctcgtgatccgcccgcctcggcctc cca
aagtgctgggattacaggcgtgagccaccgcgcccggccGATGAAGAGGAATTTTTATATTTTGCTATATATTTTTTTCTGTTGTTTGCATTTGCATTTTTACAATAAGAATGTGTGTATATAATAGT
TGTCAAAAGATATCATTTATTTATTTATTTGAGACAGGGTCTCACTCCGTTACCTAGGCTGGTGTGTGATGGCATGATCACAGCGCACTGCAGCTTTGACCTCCCAGGCTCAAGTGATCCTCCCACCT
CAGCTTCCCGGGTAGCTGGGACTACAGGCATGTGCCACCACACCTGGCTAATTTTTTTGTATTTTTTGTAGGGATGAGGTCTCACCATGTTGCCCAGGCTGGTCTTGAACTAGGCTCAAGCGATCC AC
CCACCCCGGCCACTCAAAAGTTTTGGGATTACAGGTGTGAGCCGCTGCTCCCAACAAAATATATTTTTATAAATAAATCAAGATGAAAACATTTTAGACAATAAAGATGATTTTTTTTAAACTGA AGA
AATCAATTGAATGGCTTTAGGAAAGGAGATTAGATTTTTGTTTAAGCAATAACTTTTCTTTTCTTTCCTTTTCTTTTCTTCTTCTTTTTTTTTTTTTTTTTTTT TGAGACAGAGTCTCGCTTTGTTGC
CCAGGCTGGAGTGCAATGGTGCAATCTCAGCTCACTGCAGCCTTCACCTCACCCACCTCCTAACACTGTCACTTTGGGAATCACATTTATACAAGAGTTTTGGTGAGGACAAATTATATTGAAAT CAT
GGAATTAGTCATAATGAATTGCTGTTGGTATTCCAATGTCTTCAGACCTCCTGACTGAAGAGGAAGACAGAACTGTAGT CCCAGTCAAGAAAGTCTGTGGTCTGGAAACAACCAAAGTTCCAGTGACT
TACTGTTTCATGTTCTCTTACTTCATCATATCCTGTTTCCATTTTTTAAGATCAACTGGGTTTGGACTTCTGAAGAACACAGCATGGAGGGTAAATGACTGGGGCTCCAGGAGGCCACATTCTGG GCA
GGGAGGATTAATGTC

1012	8.1	0.0	0.0	AluYa5-Scaffold84228-3153261-3153579	1	123	(2196)	C	AluYj4	SINE/Alu	(188)	123	1	1
1713	15.5	0.0	3.7	AluYa5-Scaffold84228-3153261-3153579	125	402	(1917)	+	AluJb	SINE/Alu	28	295	(17)	2
2057	11.3	2.4	0.0	AluYa5-Scaffold84228-3153261-3153579	440	731	(1588)	+	AluSx	SINE/Alu	11	309	(3)	3
343	24.2	7.1	1.5	AluYa5-Scaffold84228-3153261-3153579	733	858	(1461)	+	L1ME3Cz	LINE/L1	5734	5866	(384)	4
2651	1.1	0.0	0.0	AluYa5-Scaffold84228-3153261-3153579	1001	1319	(1000)	C	AluYa5	SINE/Alu	(0)	311	1	5
1850	16.0	1.0	1.0	AluYa5-Scaffold84228-3153261-3153579	1424	1716	(603)	C	AluJb	SINE/Alu	(15)	297	5	6
773	12.6	0.0	0.0	AluYa5-Scaffold84228-3153261-3153579	1868	1970	(349)	C	AluSx3	SINE/Alu	(0)	311	209	7
419	19.0	5.1	0.0	AluYa5-Scaffold84228-3153261-3153579	1972	2050	(269)	C	MSTB2	LTR/ERVL-MaLR	(372)	85	3	8

>chr11:33234118-33236106
TTTTTAGTAGAGACAGGGTTTCACCGTATTAGCCAGCATGGTCTCCATCTCCTGACCTTGTGATCCGCTCGCCTCGGCCTCCCAAAGT GCTGGGATTACAGGCGTGAGCCACCATGCCTGGCCTATTC
CAGCACTTTTGGAGGCTGAGGCGAGAGAATCACCTGAACCCAGGAGTTCAAGACCAGCCTGGGCAACATGATGAAACCCCATCTCTACAAAACATACAAAAATAAGCTGAGTGTGGTGTCGTAAG CCT
GTGGTCCCAGCTACTCACAGCTACTCAGGAGGCTGAAGTAGAAGGATTTATTGAGCCAGGAAGGTCGAGGCTATAGTGAGCTGTGATT ATGCCACTGCACTCCAGCCTGGGTGACAGAACGAATCTCT
GTCCCAAAAATAAATAAATGAGTAAATAAGAAAAGTTTAAAATAAGAATAGGCTGGGTGGCTCACGCCCGTAATCCCAGCACTTTAGGAGGCTAAGGAGGGCGGATGACTGAGGTCAGGAGTTCG AGA
CCACTTTGGGCAACATGGTGAAACCCCGTCTCTAGTAAAACTACAAAATTTAACTAGGTGTGGTGGCACATGCCTGTAATCCCACCTA CTCAGGAAGCTGAGGCAGGAGAGTCACTTGAACCCGGGAG
GCGGAGGGTGCAGTGAGCCGAGATCGTGCACTCCAGCCTGGGTGACAGAGCGAGACTTTGTCTCAAAAAAATAAATAAATAAAAATAAAAATAATTTAAATGTCTATCAATAGATAAATAAATTA TGG
TAATCCATACCACAGAATAATTTATTGTAGTTACAAATGAATCTATAAGATTTCTATGTATTAACCTGCAGAGAATCTTTAAGGCACA GTAAGGGAAAGACATTTATTACAAGAGAAAAAATTTTGGA
TATATATATGCCGATATATACATATATGTAGAATTCACACCAAACCTTTGAGAAAGGTTATTCTCTGGAA CGGAAAGAGGGATTTGAAGGTAGATGAAGAGGAATTTTTATATTTTGCTATATATTTT
TTTCTGTTGTTTGCATTTGCATTTTTACAATAAGAATGTGTGTATATAATAGTTGTCAAAAGATATCATTTATTTATTTATTTGAGAC AGGGTCTCACTCCGTTACCTAGGCTGGTGTGTGATGGCAT
GATCACAGCGCACTGCAGCTTTGACCTCCCAGGCTCAAGTGATCCTCCCACCTCAGCTTCCCGGGTAGCTGGGACTACAGGCATGTGCCACCACACCTGGCTAATTTTTTTGTATTTTTTGTAGG GAT
GAGGTCTCACCATGTTGCCCAGGCTGGTCTTGAACTAGGCTCAAGCGATCCACCCACCCCGGCCACTCAAAAGTTTTGGGATTACAGG TGTGAGCCGCTGCTCCCAACAAAATATATTTTTATAAATA
AATCAAGATGAAAACATTTTAGACAATAAAGATGATTTTTTTTAAACTGAAGAAATCAATTGAATGGCTTTAGGAAAGGAGATTAGATTTTTGTTTAAGCAATAACTTTTCTTTTCTTTCCTTTT CTT

TTCTTCTTCTTTTTTTTTTTTTTTTTTTTTtGAGACAGAGTCTCGCTTTGTTGCCCAGGCTGGAGTGCAATGGTGCAATCTCAGCTCA CTGCAGCCTTCACCTCACCCACCTCCTAACACTGTCACTT
TGGGAATCACATTTATACAAGAGTTTTGGTGAGGACAAATTATATTGAAATCATGGAATTAGTCATAATGAATTGCTGTTGGTATTCCAATGTCTTCAGACCTCCTGACTGAAGAGGAAGACAGA ACT
GTAGTCCCAGTCAAGAAAGTCTGTGGTCTGGAAACAACCAAAGTTCCAGTGACTTACTGTTTCATGTTCTCTTACTTCATCATATCCT GTTTCCATTTTTTAAGATCAACTGGGTTTGGACTTCTGAA
GAACACAGCATGGAGGGTAAATGACTGGGGCTCCAGGAGGCCACATTCTGGGCAGGGAGGATTAATGT C

1012	8.1	0.0	0.0	chr11:33234118-33236106	1	123	(1866)	C	AluYj4	SINE/Alu	(188)	123	1	9
1713	15.5	0.0	3.7	chr11:33234118-33236106	125	402	(1587)	+	AluJb	SINE/Alu	28	295	(17)	10
2057	11.3	2.4	0.0	chr11:33234118-33236106	440	731	(1258)	+	AluSx	SINE/Alu	11	309	(3)	11
343	24.2	7.1	1.5	chr11:33234118-33236106	733	858	(1131)	+	L1ME3Cz	LINE/L1	5734	5866	(384)	12
1850	16.0	1.0	1.0	chr11:33234118-33236106	1093	1385	(604)	C	AluJb	SINE/Alu	(15)	297	5	13
773	12.6	0.0	0.0	chr11:33234118-33236106	1538	1640	(349)	C	AluSx3	SINE/Alu	(0)	311	209	14
419	19.0	5.1	0.0	chr11:33234118-33236106	1642	1720	(269)	C	MSTB2	LTR/ERVL-MaLR	(372)	85	3	15

>KSI_AluYa5_52

>Scaffold2142-424628-424808
TTCTCATCAGTGTGCCCCTGTTGCTCCCCTCAGAGAGCATTGGTCATCCTTTTGTCATGGAAGACCTCTGAAAATTTGTGGACATTTTCCTCTTACGTAATTCATGAACAGTTGGGAGCATTTCAG AC
TTCGGTGGCTCACACTTACAATCCCAGCATTTTGGGAGGCTGAGATGGCAGGATTGTTTGAGACCAGGAGTTCACGACCAGCCTGGGCAATACAGTATGACCTCGCCTCTACAAAAACTTTTCTG AAA
AAATTAACCAGGCGCGGTGATGTGTGCCTGTAGTCCCAGCTACTCGGGAGGCTGAAGCAGGAGGAGTCCTTGAGCCCGGGAGTTTGAGGCTGTAGTGAGCTGTGATCACTTCACTGCACTCAAGCC TG
AGCAACACAGCAAGACCCTGTTTCTTAAAAAAAAAGGAAATGATAAAAGTCACTCAGCTTTTGCATTAAAGTTTTGTGGGGTTTTTTTGGTTTGTTTTTATTTTTGTTTTTATTTATTTATCTTG AGA
TAGAGTCTCGCTCTGTCACCAGGCTGGAGTGCAGTGGTGTGATCTCGGCTCACTGCAGCCTCAGCCTCCGGGGTTCAAGTGATTCTCCTGCCTCAGCTCCTGAGTGGCTGGGACTACAGGTGCCTG CC
ACCACGCCGGCTAATTTTTGTATTTGTAGTAAAGACAGC aTTTCACCATGTTGGCCCAGATGGTCTCAGTCTCTTGACCTCATCATCTGCCCGCCTCAGCCTCCCAAGCATTAAAGCTTTTAGTATGC
TGTCTTGGAGTTGACAGGTTTTGCCACCTCTTGAAATGTGAATTCAAGTATTTCATACATTTTGCTGAGTTAGCTTCCTA GGGTGTTGTGTGCTCAGCCTTTATGAAACAGTGAACATGATGACAGTA
AACTGAGAGAATTTGGAAACCTAGCACTCACTATTTTTTTAGCCAGTCACCTTTCTCATTTCTGA tGATAGTTAGATATAAATTGCTGATTAAAAATGAGTCCTGgccgggcgcggtggctcacgcct
gtaatcccagcactttgggaggccgaggcgggcggatcacgaggtcaggagatcgagaccatcccggctaaaacggtgaaaccccgtctctactaaaaatacaaaaaattagccgggcgtagtgg cgg
gggcccggggtgcccgcccacttggggggggggggg catgatgacagtaaactgagagaatttggaaacctagcactcactatttttttagccagtcacctttct catttctgacgatagttagatat
aaattgctgattaaaaatgagtcctgTAGGCCGGGCACAGTGGCTCATGTCTGTAGTCCCAGCATTTTGGGAGGCCGAGGCAGACTGATCGCCTGAGTTCAGGAGTTTGAGATCAGCCTGGCCAGCAT
AGTGAAACCCCATCTCTACTAAAAATACAAAAATTAGCTGGGCATGGTGGCACATGCTTGTAATCCCAACTACCTGGGGA aGCTGAGGCAGGAGAGTGGCTTGAACCCAGGAGGTGGAGGTTGCAGTA
AGCtGAGATCGCGCGACTGCACTCTAGCCTGGGTGACAGAGCAAGACTCC aTCTCAAAAAAAAAAAAAAAAAAAAATGAGTCCTGTATTCCCAGCTACTTGGGAAGCTGAGGCAGGAGGATCATGTGA
GCCCAGGAGTTCAAGGCTGCAGTGAGCTGTGATTGTACCTGTGAATAGCTACTGCATTCCAGCCTGGGCAACACAGTGAGATCTTGTCTCTTACAAAAAATAGAA AATTGCTGCGTTACCTGTCTTGA
CTTTCTCTTTTCTTTTTTATTGTAAAATATACATATTAACTTGTAACATTGGTCTTTTTAAAACTATCATTTCAGAATTTCTAGAAGGAGGAGTTGGCCATGTTGAAATGTTATTTCGCTGCAAC TAT
TTAGCTTTAGTTGGTGGTGGAAAAAAGCCGAAATA CCCTCCCAACAAAGGTACTGTAATTGTGTATTGAAGAATCATGCCTCACGTGTCCCACCCAGTCTTTTTT TGAATGCGTTTATGTTTGTGGCC
ATGTGTGATTTTCCCTAGTAGTTCTGTGAAATATCCAGGACTGGTCACTGGGGAGGAAGCAGGCGAAGAGGGCACCAGGTCTCTATTCCCAGATGGAGCACCTAAAGAGCATCACTCGCTTGCAT TGT
CATAG

1790	17.0	0.0	2.4	AluYa5	131	424	(1757)	+	AluJr	SINE/Alu	10	296	(16)	1
1874	12.3	0.7	0.0	AluYa5	479	747	(1434)	C	AluSc	SINE/Alu	(0)	309	39	2
1538	4.4	0.0	0.6	AluYa5	1001	1181	(1000)	+	AluYa5	SINE/Alu	1	180	(131)	3
2272	11.8	0.0	0.3	AluYa5	1309	1612	(569)	+	AluSx	SINE/Alu	1	303	(9)	4
1033	16.2	0.0	0.0	AluYa5	1618	1771	(410)	+	FAM	SINE/Alu	21	174	(11)	5

>hg19 chr17:80588585-80590463
TTCTCATCAGTGTGCCCCTGTTGCTCCCCTCAGAGAGCATTGGTCATCCTTTTGTCATGGAAGACCTCTGAAAATTTGTGGACATTTTCCTCTTACGTAATTCATGAACAGTTGGGAGCATTTCAG AC
TTCGGTGGCTCACACTTACAATCCCAGCATTTTGGGAGGCTGAGATGGCAGGATTGTTTGAGACCAGGAGTTCACGACCAGCCTGGGCAATACAGTATGACCTCGCCTCTACAAAAACTTTTCTG AAA
AAATTAACCAGGCGCGGTGATGTGTGCCTGTAGTCCCAGCTACTCGGGAGGCTGAAGCAGGAGGAGTCCTTGAGCCCGGGAGTTTGAGGCTGTAGTGAGCTGTGATCACTTCACTGCACTCAAGCC TG
AGCAACACAGCAAGACCCTGTTTCTTAAAAAAAAAGGAAATGATAAAAGTCACTCAGCTTTTGCATTAAAGTTTTGTGGGGTTTTTTTGGTTTGTTTTTATTTTTGTTTTTATTTATTTATCTTG AGA
TAGAGTCTCGCTCTGTCACCAGGCTGGAGTGCAGTGGTGTGATCTCGGCTCACTGCAGCCTCAGCCTCCGGGGTTCAAGTGATTCTCCTGCCTCAGCTCCTGAGTGGCTGGGACTACAGGTGCCTG CC
ACCACGCCGGCTAATTTTTGTATTTGTAGTAAAGACAGC gTTTCACCATGTTGGCCCAGATGGTCTCAGTCTCTTGACCTCATCATCTGCCCGCCTCAGCCTCCCAAGCATTAAAGCTTTTAGTATGC
TGTCTTGGAGTTGACAGGTTTTGCCACCTCTTGAAATGTGAATTCAAGTATTTCATACATTTTGCTGAGTTAGCTTCCTA GGGTGTTGTGTGCTCAGCCTTTATGAAACAGTGAACATGATGACAGTA
AACTGAGAGAATTTGGAAACCTAGCACTCACTATTTTTTTAGCCAGTCACCTTTCTCATTTCTGAcGATAGTTAGATATAAATTGCTGATTAAAAATGAGTCCTG TAGGCCGGGCACAGTGGCTCATG
TCTGTAGTCCCAGCATTTTGGGAGGCCGAGGCAGACTGATCGCCTGAGTTCAGGAGTTTGAGATCAGCCTGGCCAGCATAGTGAAACCCCATCTCTACTAAAAATACAAAAATTAGCTGGGCATGG TG
GCACATGCTTGTAATCCCAACTACCTGGGGAgGCTGAGGCAGGAGAGTGGCTTGAACCCAGGAGGTGGAGGTTGCAGTAAGC cGAGATCGCGCGACTGCACTCTAGCCTGGGTGACAGAGCAAGACTC
CtTCTCaaaAAAAAAAAAAAAAAAAAAAAAATGAGTCCTGTATTCCCAGCTACTTGGGAAGCTGAGGCAGGAGGATCATGTGAGCCCAGGAGTTCAAGGCTGCAGTGAGCTGTGATTGTACCTGTGAAT
AGCTACTGCATTCCAGCCTGGGCAACACAGTGAGATCTTGTCTCTTACAAAAAATAGAAAATTGCTGCGTTACCTGTCTTGACTTTCTCTTTTCTTTTTTATTGTAAAATATACATATTAACTTG TAA
CATTGGTCTTTTTAAAACTATCATTTCAGAATTTCTAGAAGGAGGAGTTGGCCATGTTGAAATGTTATTTCGCTGCAACTATTTAGCTTTAGTTGGTGGTGGAAAAAAGCCGAAATA CCCTCCCAACA
AAGGTACTGTAATTGTGTATTGAAGAATCATGCCTCACGTGTCCCACCCAGTCTTTTTTTGAATGCGTTTATGTTTGTGGCCATGTGTGATTTTCCCTAGTAGTTCTGTGAAATATCCAGGACTGGTC
ACTGGGGAGGAAGCAGGCGAAGAGGGCACCAGGTCTCTATTCCCAGATGGAGCACCTAAAGAGCATCACTCGCTTGCATTGTCATA G

1790	17.0	0.0	2.4	hg19	131	424	(1455)	+	AluJr	SINE/Alu	10	296	(16)	6
1889	11.9	0.7	0.0	hg19	479	747	(1132)	C	AluSc	SINE/Alu	(0)	309	39	7
2318	11.1	0.0	0.3	hg19	1004	1310	(569)	+	AluSx	SINE/Alu	1	306	(6)	8
1033	16.2	0.0	0.0	hg19	1316	1469	(410)	+	FAM	SINE/Alu	21	174	(11)	9

>KSI_AluYa5_53

>Scaffold2151-1635568-1635707
TCCATCAGAGAAAACATTTCTGCAAAAAGGTGTTAATATTTTCAGTTGGCACCTTCTTAGTTCTTGAATAACTAATGGATAATTATATATATTTTCTCCTTATTGT CCTTGAATTTTACTATTCGTCC
TCAGGGAGAATGTACTGGCTTACAATACAGTAGCCTAAAATATTGAAACCTAACACCTTTTAACATTATAATAGCTTCTACATTATTCACCTTGAGAATCATCTATGGCAAACCAGTGATTAGGG AAG
CTTTCCCCTATCACCTTTGTTTAGATTGGGCTGTTTTCTCCTTTTGTAGAACTAAGGAAAAGTAAGACTCACTGGCTGTGAAGTAGTAGTAATAGCTAAACAGAAT GAGAATGGGAGGCTTGGAAAAA
AGATGTATGTGTATGAGTCACGTTTCTTCTAGAGCTGGAAGGGTAGACCATGGAGATGATATTTTGCAGATTAGGAAACCAAGGCCCAGAGCAGTTGAAGGGATTAAATTGCTATTTAATGGTGG GGC
CAGGATTGGTGGGCAGGTCTGTGGACTCCAGTCCAGAGATTGCTTTATCACTTTCACACTGTTTATTTCTAAAGCATTCTCGTGCCAACTGTTAATGGTTTTTGGA TTATTTGTGATTTGTTCCCTTC
TAAGGATGAAGAAGTTTGAGTTGACTACTTTTATCCATGAAATTTTGTAAAACTGGGTGAGCTTTTATTTAAAAGTTTAAAACAATATGCT GCATTGTTAGCGTTAGTGCTGATAGTTCCCTGTCATT
GGAAAGAGACAAACTGTCATTTAGGTGAAAATATTAAATGAATGCCCTAGAGTAGTTTAGCATTCTATAAT Ttttatgctaaaattaattgcttaaaagattttcaaaattcagaaggcttctatatt
ttgaaagtaaattatgtgaacattattattttcagttagtgtggcggttttgttaaaatgggtttagtacctttaaaaagagagtagaattacatgatcattat ccggctaatttttgtatttttag
tagagacggggtttcaccgttttagccgggatggtctcgatctcctgacctcgtgatccgcccgcctcggcc tcccaaagtgctgggattacaggcgtgagccaccgcgcccggcc TTTATGCTAAAA
TTAATTGCTTAAAAGATTTTCAAAATTCAGAAGGCTTCTgTATTTTGAAAGTAAATTATGTGAACATTATTATTTTCAGTTAGTGTGGCGGTTTTGTTAAAATGGGTTTAGTACCTTTAAAAAGA GAG
TAGAATTACATGATCATTATGAATACATCATTCCTATCATAGTTATAATAGAGAAAGACAGGATTTGTAT CCCAGCCCCTTCACATGATCTTAGGCAAGTGATTTAACCACTGTGTACCTGAGTTTAC
TCGTTTGTAAAGGGAGGGTGTTAAAAGTACCTACCTCACAGCATAGTGGTGAGGTTTAACTGTGCCTACCTAC TACATAGAAAGGGTACAGCAAATATTAGACATTATTCTCTGTGGTTCGTTAGATG
TAAGCAGGCAGTTGTGTGCCCATTTATTTCCTTGCTGTACACGCACTTTCAAGTTACAAAAATTCCTAGAAA AATGCACGATCAAATCATACTCTTTGGTAAGGGGGTACTTGGTACAAATGATGCTC
TGATTTTTGGTATTGCTCTCCTTCCTCTGCTTACCTTGGACCTTTAATTAGGTCTGCATATCAAATTAGAGTGAATGTGTTACTGTTTCTGAATGTCATTGGGAGATCCTTGTTGCAGTTTCATTATT
CTAGAACAAAAATATTAATAATTGATTTGCAGATATTCCAGTGAAAATACAGTACTAGAGTACTAGAGACCT GCTGGGTACACTTAGGTGAGGAAGTCAGGAATTTATAAGGTCTATTATTGATCTCA
GCTCTTCTCTGCCAGTGGAACCATGGGAAAATCATTGAACCCTGTTTTCTATTAAACGAATATTTTGATGACTTCATTCATTAAGCTGTGAGATTTTAGTGATAAAATGTGTGTGACTCCAATTT TAT
AACATATGGTTCTTTTTAATTTGCTGTAACCCAGGTCCAACTAGTGTCATGACCATGTGTTTTAGCAAATGT TTTTGACGGTGACAGTTTGA

275	24.2	19.4	2.6	AluYa5-Scaffold2151-1635568-1635707	415	576	(1564)	C	MIR3	SINE/MIR	(23)	185	3	1
1342	0.0	0.0	0.0	AluYa5-Scaffold2151-1635568-1635707	1001	1140	(1000)	C	AluYa5	SINE/Alu	(171)	140	1	2
457	26.1	8.5	0.0	AluYa5-Scaffold2151-1635568-1635707	1341	1470	(670)	+	MIR	SINE/MIR	50	190	(72)	3

>hg19 chr2:46928111-46929950
TCCATCAGAGAAAACATTTCTGCAAAAAGGTGTTAATATTTTCAGTTGGCACCTTCTTAGTTCTTGAATAACTAATGGATAATTATATATATTTTCTCCTTATTGTCCTTGAATTTTACTATTCGT CC
TCAGGGAGAATGTACTGGCTTACAATACAGTAGCCTAAAATATTGAAACCTAACACCTTTTAACATTATAATAGCTTCTACATTATTCACCTTGAGAATCATCTATGGCAAACCAGTGATTAGGG AAG
CTTTCCCCTATCACCTTTGTTTAGATTGGGCTGTTTTCTCCTTTTGTAGAACTAAGGAAAAGTAAGACTCACTGGCTGTGAAGTAGTAGTAATAGCTAAACAGAATGAGAATGGGAGGCTTGGAAA AA
AGATGTATGTGTATGAGTCACGTTTCTTCTAGAGCTGGAAGGGTAGACCATGGAGATGATATTTTGCAGATTAGGAAACCAAGGCCCAGAGCAGTTGAAGGGATTAAATTGCTATTTAATGGTGG GGC
CAGGATTGGTGGGCAGGTCTGTGGACTCCAGTCCAGAGATTGCTTTATCACTTTCACACTGTTTATTTCTAAAGCATTCTCGTGCCAACTGTTAATGGTTTTTGGATTATTTGTGATTTGTTCCCT TC
TAAGGATGAAGAAGTTTGAGTTGACTACTTTTATCCATGAAATTTTGTAAAACTGGGTGAGCTTTTATTTAAAAGTTTAAAACAATATGCT GCATTGTTAGCGTTAGTGCTGATAGTTCCCTGTCATT
GGAAAGAGACAAACTGTCATTTAGGTGAAAATATTAAATGAATGCCCTAGAGTAGTTTAGCATTCTATAAT TTTTATGCTAAAATTAATTGCTTAAAAGATTTTCAAAATTCAGAAGGCTTCTaTATT
TTGAAAGTAAATTATGTGAACATTATTATTTTCAGTTAGTGTGGCGGTTTTGTTAAAATGGGTTTAGTACCTTTAAAAAGAGAGTAGAATTACATGATCATTATGAATACATCATTCCTATCATA GTT
ATAATAGAGAAAGACAGGATTTGTATCCCAGCCCCTTCACATGATCTTAGGCAAGTGATTTAACCACTGTGTACCTGAGTTTACTCGTTTGTAAAGGGAGGGTGTTAAAAGTACCTACCTCACAGCAT
AGTGGTGAGGTTTAACTGTGCCTACCTACTACATAGAAAGGGTACAGCAAATATTAGACATTATTCTCTGTGGTTCGTTAGATGTAAGCAGGCAGTTGTGTGCCCATTTATTTCCTTGCTGTACA CGC
ACTTTCAAGTTACAAAAATTCCTAGAAAAATGCACGATCAAATCATACTCTTTGGTAAGGGGGTACTTGGTACAAATGATGCTCTGATTTTT GGTATTGCTCTCCTTCCTCTGCTTACCTTGGACCTT
TAATTAGGTCTGCATATCAAATTAGAGTGAATGTGTTACTGTTTCTGAATGTCATTGGGAGATCCTTGTTGCAGTTTCATTATTCTAGAACAAAAATATTAATAATTGATTTGCAGATATTCCAG TGA
AAATACAGTACTAGAGTACTAGAGACCTGCTGGGTACACTTAGGTGAGGAAGTCAGGAATTTATAAGGTCTATTATTGATCTCAGCTCTTCTCTGCCAGTGGAACCATGGGAAAATCATTGAACCC TG
TTTTCTATTAAACGAATATTTTGATGACTTCATTCATTAAGCTGTGAGATTTTAGTGATAAAATGTGTGTGACTCCAATTTTATAACATATGGTTCTTTTTAATTTGCTGTAACCCAGGTCCAAC TAG
TGTCATGACCATGTGTTTTAGCAAATGTTTTTGACGGTGACAGTTTGA

275	24.2	19.4	2.6	hg19	415	576	(1264)	C	MIR3	SINE/MIR	(23)	185	3	4
457	26.1	8.5	0.0	hg19	1041	1170	(670)	+	MIR	SINE/MIR	50	190	(72)	5

>KSI_AluYa5_54

>Scaffold2633-5607493-5607803
AGGCTGTTGGAGAGCCGCCCCTCTTCCTGGCTGCTTCTATCTTCTTTGCCATCAAAGATGCCATCCGTGCAGCTCGAGCTCAGCACACAGGTAATAACGTGAAGGAACTCTTCCGGCTAGACAGCC CT
GCCACCCCGGAGAAGATCCGCAATGCCTGCGTGGACAAGTTCACCACCCTGGTATGATGCCCCTCAAGCATCCACTCAGTCCAGGGGAGGCCAGGGATAGGCTGGCAAACTGCAGGGAAGGTTCT GCA
CAGAGGAGAGGTGTTGGGCTCTCTCCTAACCGGGCAGCCTTGCATCAATGGCCACCCTTCATTGTGCATGGTATGAAGCACACACTCGAACTTGCAAGAGAATAATGAGAAACCTCCTTAAAGCTA TA
GCTTGTAGCTGGGCCTCCTTAGAGATGGGAGGAATGTGTGGTGCACAGTCATAACTCACAGATTTTATTATTTTCCATCTAGATAAGGATGATGGATGAAACTCCAGAGAGCCTAATCTTTAGTA TGA
GTCAGTCGCTAGCATTTTTTTTTTTAATTTATGTTAAGGACACTGTATGAATATTGGGGGGATGGAGGGAAAAAGAGGAAATAATTAATTAGATCAACCACATTTGTCCTGTGATGATCATGTGCC AT
TCTGATACTGGGGACACATAGATGTCCTTGAGGACTTTGCAACCTTGGGAAACCAGATGATTCCATTATAGGGTGATTGATTACTGCCTTGGAGGTGTATGTGGCAGAAAGTAGTAGAAGAATTC CGT
AGTCATTGGAATCACTTTAAGAAGCACCTATGTGATACTTGTAGTTGGCAATGCCTAACAAAAGAAGACTTTGCTAGGACGGTTGCTTCCGCTTTGGGGATCTCAGAACCAGCCCAGCCTCTGAGT GG
TGCTGAAGTGGGCTCTGTACAGTGGCTTTAATTGCCTTTGCAACTGCAACCTGTGTGGAGTTAGCA GGACTGGGTTCTTCCTGATACTCAGGTGGGGCTTcCTCTTTTTttttttttttttttttt
ttttttgagacggagtctcgctctgtcgcccaggctggagtgcagtggcgggatctcggctcactgcaagctccgcctcccgggttcacgccattctcctgcctcagcctcccaagtagctgggac ta
caggcgcccgccactacgcccggctaattttttgtatttttagtagagacggggtttcaccgttttagccgggatggtctcgatctcctgacctcgtgatccgcccgcctcggcctcccgaagtg ctg
ggattacaggcgtgagccaccgcgcccggccggggcttcctctttttAAAATTCGATATGCTTTTGGATTTATAGGTAAAGTGGGTACATGTGGTCCCAAGTGGGGAGGGCACAGAGGAACCCCAGG
GGCAGACAAATCCACCATCCGCAGACCTCAACACAGTGGGTGAAGGAGGCATATGGGTCCCCATTCAGGTCTTCCTTGGGTCTACCAAGAGCTTTAGCTATGGAGTGAGAACTACACAGACCGTGTGG
CACTCCCAGGCAGTAAAGATGCCCACATCTGTGCAGCAACCTACCCACAGCCCAGAACCTTCCCACTATGGCTCCCAATGCTGACAATCACCCAAACCCATTTCCCTGAGAGGAGTGGGTGGCATG GC
TCCCAATAAGCTGCCACAGTTTGCAAGGCGAGCTCTTTGCCAACAGTCTGATGCAGAACTCAGTCATTTTCGTAAGCTCTCTGTGGCCCCAGACCTGAAACAAAGATCCGTTTCTATGTTACAAC ATA
CCAGCGATTTAAAAATTCTTGCCATGGATTTGCATGAGCAGCTGATTCTATGAACTTTTTAAGGACTGTGAGTTTCAAAAGGGATCACATCTGTATTGTTTGTCACCATATCTCCAAAAC gTAATAGA
ACGCCTGCCATGTGGAAAGTGCTCAGTGAAAATAAATGAATGGCTTAGGAAATTAAGGAGTCCTGCCCTGAGGCATTCTCAACAGCTAGGCCTGCAGAGTGGTGCTTGGCCTTGGAATGATGGTT GGC
AGTGGTGGGAGGTGAGGTATGCAAGAGAGTGGGAGGAGCATGATCAGCAGACCCTGGCAATGAGAACACTATCATTCCTTCCTAGTGTGTCACTGGTGTCCCAGAAAACTGCAAACCCTGGTCTGT GA
GGGTCTAAAGAGAGAGTCCTCAGCAGAGTCTTCTTGTGCTGCCTTTGGGCTTCCATGGAGCAGGAGGAACATACCACAGAACATGGATCTATTAAAGTCACAGAATGACAGACCTGTGATTTGTC AAG
ATGGGAT

2688	0.4	0.0	0.0	AluYa5	1001	1311	(1000)	C	AluYa5	SINE/Alu	(0)	311	1	1
276	33.6	13.1	0.0	AluYa5	1855	1961	(350)	+	L2b	LINE/L2	3255	3375	(0)	2

>hg19 chr2:31558697-31560685
AGGCTGTTGGAGAGCCGCCCCTCTTCCTGGCTGCTTCTATCTTCTTTGCCATCAAAGATGCCATCCGTGCAGCTCGAGCTCAGCACACAGGTAATAACGTGAAGGAACTCTTCCGGCTAGACAGCC CT
GCCACCCCGGAGAAGATCCGCAATGCCTGCGTGGACAAGTTCACCACCCTGGTATGATGCCCCTCAAGCATCCACTCAGTCCAGGGGAGGCCAGGGATAGGCTGGCAAACTGCAGGGAAGGTTCT GCA
CAGAGGAGAGGTGTTGGGCTCTCTCCTAACCGGGCAGCCTTGCATCAATGGCCACCCTTCATTGTGCATGGTATGAAGCACACACTCGAACTTGCAAGAGAATAATGAGAAACCTCCTTAAAGCTA TA
GCTTGTAGCTGGGCCTCCTTAGAGATGGGAGGAATGTGTGGTGCACAGTCATAACTCACAGATTTTATTATTTTCCATCTAGATAAGGATGATGGATGAAACTCCAGAGAGCCTAATCTTTAGTA TGA
GTCAGTCGCTAGCATTTTTTTTTTTAATTTATGTTAAGGACACTGTATGAATATTGGGGGGATGGAGGGAAAAAGAGGAAATAATTAATTAGATCAACCACATTTGTCCTGTGATGATCATGTGCC AT
TCTGATACTGGGGACACATAGATGTCCTTGAGGACTTTGCAACCTTGGGAAACCAGATGATTCCATTATAGGGTGATTGATTACTGCCTTGGAGGTGTATGTGGCAGAAAGTAGTAGAAGAATTC CGT
AGTCATTGGAATCACTTTAAGAAGCACCTATGTGATACTTGTAGTTGGCAATGCCTAACAAAAGAAGACTTTGCTAGGACGGTTGCTTCCGCTTTGGGGATCTCAGAACCAGCCCAGCCTCTGAGT GG
TGCTGAAGTGGGCTCTGTACAGTGGCTTTAATTGCCTTTGCAACTGCAACCTGTGTGGAGTTAGCA GGACTGGGTTCTTCCTGATACTCAGGTGGGGCTTtCTCTTTTTAAAATTCGATATGCTTTTT
GGATTTATAGGTAAAGTGGGTACATGTGGTCCCAAGTGGGGAGGGCACAGAGGAACCCCAGGGGCAGACAAATCCA CCATCCGCAGACCTCAACACAGTGGGTGAAGGAGGCATATGGGTCCCCATTC
AGGTCTTCCTTGGGTCTACCAAGAGCTTTAGCTATGGAGTGAGAACTACACAGACCGTGTGGCACTCCCAGGCAGTAAAGATGCCCACATCTGTGCAGCAACCTACCCACAGCCCAGAACCTTCC CAC
TATGGCTCCCAATGCTGACAATCACCCAAACCCATTTCCCTGAGAGGAGTGGGTGGCATGGCTCCCAATAAGCTGCCACAGTTTGCAAGGCGAGCTCTTTGCCAACAGTCTGATGCAGAACTCAGT CA
TTTTCGTAAGCTCTCTGTGGCCCCAGACCTGAAACAAAGATCCGTTTCTATGTTACAACATACCAGCGATTTAAAAATTCTTGCCATGGATTTGCATGAGCAGCTGATTCTATGAACTTTTTAAG GAC
TGTGAGTTTCAAAAGGGATCACATCTGTATTGTTTGTCACCATATCTCCAAAAC cTAATAGAACGCCTGCCATGTGGAAAGTGCTCAGTGAAAATAAATGAATGGCTTAGGAAATTAAGGAGTCCTGC
CCTGAGGCATTCTCAACAGCTAGGCCTGCAGAGTGGTGCTTGGCCTTGGAATGATGGTTGGCAGTGGTGGGAGGTGAGGTATGCAAGAGAGTGGGAGGAGCATGATCAGCAGACCCTGGCAATGA GAA
CACTATCATTCCTTCCTAGTGTGTCACTGGTGTCCCAGAAAACTGCAAACCCTGGTCTGTGAGGGTCTAAAGAGAGAGTCCTCAGCAGAGTCTTCTTGTGCTGCCTTTGGGCTTCCATGGAGCAGG AG
GAACATACCACAGAACATGGATCTATTAAAGTCACAGAATGACAGACCTGTGATTTGTCAAGATGGGA T

299	32.7	13.1	0.0	hg19	1533	1639	(350)	+	L2b	LINE/L2	3255	3375	(0)	3

>KSI_AluYa5_55

>Scaffold3523-1824351-1824656
GCAGATCACAAGGTGAAGAGATTGAGACCATCCTGGCCAAAGTGGTGAAACCCCGTCTCTACTAAAAATACAAAAATTAGCTGGTGTGGT gGTGAGCGCCTGTAGTCCCAGCTACTAGGGAGGCTGAG
GCAGAAGAATCACTTGAACCCAGAAGGCAGAGGTTGCAGTGAGCCAAGATTGTGCCACTGCACTCCAGCCTGGCAACAGAGC gAGACTCTGTCCCAGAAAATAAATAAATAAATAAATAAATAAATAA
ATAAATAAATAAATAAAATATaATAAAGATATTTGCCAGCCATTTATCTGATAAAGGATTAATAATCCAACACATATAAGAAA cCTTTCTTATTTTTATCATTACTTTTATTGAATTTTTTTAAACTT
CATGAGTACATGAATATCCAGATGGTTAATTTTCAGCTTCTCTGCCTTATTTTAATATGCTTATTGGCTATTTTAATTTTCTTCCCTACCTATTCTTCATTAAATCATTTTTGCCTTTTTCATAA aTC
CTTATCCATCTTGGTTATAAGTAGATAAGGCTTAAAATTTCCAGTAGGTAAAGACAATAAACACACTGTTTTGTCACTCCCTTAAATTGTT TTTTTTTTTTTAGAAGCCATGAGCTTCTAATGTTTCT
GCCCCATCTCCAATTTTATCCACCCATCCTTTTTTCTGGTTTTCAAATTATCTTTGCTTTGAGTC gGTGTTATTCACTGGAAAAGCAAAGTGACATACTCCTACAGAAGGACTGTCGACTCTCCAGTC
ACATTATGCTTCCCATCACTACCACATTATAGAAGTATCAATTAGAGGTGATGACAAGATGTTCTACCAAATACATATTTTCTCAAAATGA AGCAGGCATATTAGAGAAAATCTTTGACAAAATGCCA
TTCATATTCCCGTAACTCCTGTCCTTGATTTTGCATGCTAGTCAAAAAATGTTAACACATAGTCAACACTTT GAGTTGGCGTGTTCCCATGTTAAATAAACCACTTTtttttttttttttttttttt
tgagacggagtctcgctctgtcgcccaggctggagtgcagtggcgggatctcggctcactgcaagctccgcctcccgggttcacgccattctcctgcctcagcctcccaagtagctgggactaca ggc
gcccgccactacgcccggctaatttttgtatttttagtagagacggggtttcaccgttttagccgggatggtctcgatctcctgacctcgtgatccgcccgcctcg gcctcccaaagtgctgggatt
acaggcgtgagccaccgcgcccggccaataaaccactttCTAAGTCAAAATTATTTTGGTTATAATTAACAGAAAATCATTTGAAAACAACTAAACAAAAACCAGGAATACAGGG ATATCTTATAGAT
TTATATTGCAGGAAGCTCTCCTGAGGGTCTGCAGTCAATATTTGGAAACTGCCAGTAATGAAGACCAACGACTCTTCCTCTTCCTCTTTCTCTCATGGCC aCTTATTACTTACATCACTGATTTATTT
TTGCATATGCTTCATTTTTCTTTCTGAAGAATCATTTTCTCTATTATCTCCATGCCAAATAATAACCA tTCGGATCCTGTGTGTATAAGTGCTCTATTCACTTGTAAGCGTTTCGAAGAAAATTGCA
TTGGACAAAGTTAAACAGGCAAGGAATATCTTATTCAAAACCATTGTAACAGGAAATAGAGATTGAACTCAACTCCAGTGAAAGAAAAGGAGGTTTGTA GCTGCTGGTGAGCTAGTGGAAAAGCACTG
GAGGATGTTAGCAGGGAGATAGTTCTAtGGGATTAAATTAAGCACAATGCCTCATTCTTAAGTTTACAAATGTTTTGTTCTCTGTAATTAGGCTATTCGGATATGCTAATTGGTGTCCATTAAAGTTA
GGTTCCTACTCCTACTCAACAGCAACTGGGGTATGGAATACTATTTTTCCTGATGACTACATTTTGAACGGATGGCTCTCAGGTCTTCAGAGAGGAACGTTTCTGAA TTATTAAACTGGTGAGAGCTA
GTAGAAGATATATACACATTTCATAGAGGCAGAGAGCATTTGGACAAGTGTTCTGAAGTAAATCCTCAAAGAACAGGAAGATGGGGCCTATAGTATGGATTAAACCAGTCTAAACTTTAGTCAAA CTG
AAGGAAACTTTAAGCCATCTTAGTAAAGCCCAAAGTTATAATCAGTTTTCATTTGGATTCCAAATTCAAAGTGAGTAAAATTGATTGCCTCAGGGAAGGTTAAATGT CTAATCCAGGTCCAATCAGAA
TT

1742	9.8	0.5	0.0	AluYa5-Scaffold3523-1824351-1824656	1	224	(2082)	+	AluSc	SINE/Alu	56	280	(29)	1
53	0.0	0.0	3.6	AluYa5-Scaffold3523-1824351-1824656	227	283	(2023)	+	(AAAT)n	Simple_repeat	1	55	(0)	2
303	14.6	0.0	1.8	AluYa5-Scaffold3523-1824351-1824656	285	340	(1966)	+	L1M3	LINE/L1	5218	5272	(868)	3
11	20.2	2.9	0.0	AluYa5-Scaffold3523-1824351-1824656	343	376	(1930)	+	(TTTTTA)n	Simple_repeat	1	35	(0)	4
2705	0.0	0.0	0.0	AluYa5-Scaffold3523-1824351-1824656	1001	1306	(1000)	C	AluYa5	SINE/Alu	(0)	311	1	5
260	29.6	6.6	14.6	AluYa5-Scaffold3523-1824351-1824656	1319	1591	(715)	C	MLT1O	LTR/ERVL-MaLR	(2)	540	287	6
572	21.2	4.3	0.0	AluYa5-Scaffold3523-1824351-1824656	1652	1827	(479)	C	MER67D	LTR/ERV1	(5)	509	304	7
984	23.4	3.7	9.4	AluYa5-Scaffold3523-1824351-1824656	1875	2200	(106)	C	MER67D	LTR/ERV1	(203)	311	3	7

>chr11:38800076-38802069
GCAGATCACAAGGTGAAGAGATTGAGACCATCCTGGCCAAAGTGGTGAAACCCCGTCTCTACTAAAAATACAAAAATTAGCTGGTGTGGT cGTGAGCGCCTGTAGTCCCAGCTACTAGGGAGGCTGAG
GCAGAAGAATCACTTGAACCCAGAAGGCAGAGGTTGCAGTGAGCCAAGATTGTGCCACTGCACTCCAGCCTGGCAACAGAGC aAGACTCTGTCCCAGAAAATAAATAAATAAATAAATAAATAAATAA
ATAAATAAATAAATAAataaAATATcATAAAGATATTTGCCAGCCATTTATCTGATAAAGGATTAATAATCCAACACATATAAGAAA aCTTTCTTATTTTTATCATTACTTTTATTGAATTTTTTTAA
ACTTCATGAGTACATGAATATCCAGATGGTTAATTTTCAGCTTCTCTGCCTTATTTTAATATGCTTATTGGCTATTTTAATTTTCTTCCCTACCT ATTCTTCATTAAATCATTTTTGCCTTTTTCATA
AgTCCTTATCCATCTTGGTTATAAGTAGATAAGGCTTAAAATTTCCAGTAGGTAAAGACAATAAACACACTGTTTTGTCACTCCCTTAAATTGTTTTTTTTTTTTTAGAAGCCATGAGCTTCTAATG T
TTCTGCCCCATCTCCAATTTTATCCACCCATCCTTTTTTCTGGTTTTCAAATTATCTTTGCTTTGAGTC aGTGTTATTCACTGGAAAAGCAAAGTGACATACTCCTACAGAAGGACTGTCGACTCTCC
AGTCACATTATGCTTCCCATCACTACCACATTATAGAAGTATCAATTAGAGGTGATGACAAGATGTTCTACCAAATACATATTTTCTCAAAATGAAGCAGGCATATTAGAGAAAATCTTTGACAA AAT
GCCATTCATATTCCCGTAACTCCTGTCCTTGATTTTGCATGCTAGTCAAAAAATGTTAACACATAGTCAACACTTT GAGTTGGCGTGTTCCCATGTTAAATAAACCACTTTCTAAGTCAAAATTATTT
TGGTTATAATTAACAGAAAATCATTTGAAAACAACTAAACAAAAACCAGGAATACAGGGATATCTTATAGATTTATATTGCAGGAAGCTCTCCTGAGGGTCTGCAGTCAATATTTGGAAACTGCC AGT
AATGAAGACCAACGACTCTTCCTCTTCCTCTTTCTCTCATGGCCgCTTATTACTTACATCACTGATTTATTTTTGCATATGCTTCATTTTTCTTT CTGAAGAATCATTTTCTCTATTATCTCCATGCC
AAATAATAACCAaTCGGATCCTGTGTGTATAAGTGCTCTATTCACTTGTAAGCGTTTCGAAGAAAAATTGCATTGGACAAAGTTAAACAGGCAAGGAATATCTTATTCAAAACCATTGTAACAGGAAA
TAGAGATTGAACTCAACTCCAGTGAAAGAAAAGGAGGTTTGTAGCTGCTGGTGAGCTAGTGGAAAAGCACTGGAGGATGTTAGCAGGGAGATAGTTCTA cGGGATTAAATTAAGCACAATGCCTCATT
CTTAAGTTTACAAATGTTTTGTTCTCTGTAATTAGGCTATTCGGATATGCTAATTGGTGTCCATTAAAGTTAGGTTCCTACTCCTACTCAACAGCAACTGGGGTATGGAATACTATTTTTCCTGA TGA

CTACATTTTGAACGGATGGCTCTCAGGTCTTCAGAGAGGAACGTTTCTGAATTATTAAACTG GTGAGAGCTAGTAGAAGATATATACACATTTCATAGAGGCAGAGAGCATTTGGACAAGTGTTCTGA
AGTAAATCCTCAAAGAACAGGAAGATGGGGCCTATAGTATGGATTAAACCAGTCTAAACTTTAGTCAAACTGAAGGAAACTTTAAGCCATCTTAGTAAAGCCCAAAGTTATAATCAGTTTTCATT TGG
ATTCCAAATTCAAAGTGAGTAAAATTGATTGCCTCAGGGAAGGTTAAATGTCTAATCCAGGT CCAATCAGAATT

1701	10.7	0.5	0.0	hg19	1	224	(1770)	+	AluSc	SINE/Alu	56	280	(29)	8
57	0.0	0.0	0.0	hg19	227	277	(1717)	+	(AAAT)n	Simple_repeat	1	51	(0)	9
299	15.8	0.0	1.8	hg19	286	343	(1651)	+	L1M3	LINE/L1	5215	5271	(869)	10
11	20.2	2.9	0.0	hg19	347	380	(1614)	+	(TTTTTA)n	Simple_repeat	1	35	(0)	11
247	30.0	6.6	14.6	hg19	1007	1279	(715)	C	MLT1O	LTR/ERVL-MaLR	(2)	540	287	12
557	21.8	4.3	0.0	hg19	1340	1515	(479)	C	MER67D	LTR/ERV1	(5)	509	304	13
984	23.4	3.7	9.4	hg19	1563	1888	(106)	C	MER67D	LTR/ERV1	(203)	311	3	13

>KSI_AluYa5_56

>Scaffold3703-210432-210676
AAGCCCCAATgCTCCTCCTATTTTCTACATAAACCCCAAAGGTTTTCTAATTCTTTGATTTTTATAGTTCATATCATAAATATGATAAAGGCTTTTTCCAAATAAAGCCTTTCATACAAAAAATGAT
TTCTCAAACCACATAATCCACCAACAGAGTAATCTACTCTGTCCTAAAAAAGAAAAAAAAAAATCACTACTTCACACAGTCTTTTCCCAGATGGGCTATTCCACTCACATGATTTCAGCTACTTA CAT
ACCAATGACTCAAATATTTCTAACTTCTACTACTATAGCCTACTGCAGTGCAGACATGCTAATTTGGCTGTCCCATAGGTACCCTCAAACTTAAGAGGACTAAAGTGGGTTTATCACTGTCTCATGAG
CTATTTTTCCTTTATTCTTTATTAACAGCATCACTATATATCTTGTCACAACCTACAAATCTGGGACTCATCTTTGACTGCTACTCATATGCCATATCTAATCAACTA cTAAGTGCTGCCAAATATTC
CTCTTCCAAATATTTCTGAGTCTTCCAATTTTTCCAACCTTTTAATGGCTGTACTAAGTCTCCCATTATTCCTTATCAGCATTACTGAAAAAGTCTACTGACCAGTCTCTCTACCTCTAGTCTTGTCC
TCCTCAAATCTTTTCTCCACACAGTATACAGCGATCTATCTTTAATGTACCTCTAATAAGATCATCCAATTTAAAGCCCTTAAGTGGCTAGCCCATTACTACATTCAAAATTTCTTAATAGAGTA TTC
GCTATCTTGCATGATCTACCTCATGCCGAGCTCTGTAGCCTCATTCTGACcactgaattcctcatgtccttacataacaaccacattgtgatcttcaggccaatctaacctgatgccttttttcttttt
taagtttcactggaacgtagccacatccattccttcatgaatcacagcagggctgaacagttgcaagaccatataacccacagaa gataaaatatttattaccaggccgggcgcggtggctcacgcct
gtaatcccagcactttgggaggccgaggcgggcggatcacgaggtcaggagatcgagaccatcctggctaaaacggtgaaaccccgtctctactaaaaatacaaaaaattagccgggcgtagtggcgg
gcgcctgtagtcccagctacttgggaggctgaggcaggagaatggcgtgaacccgggaggcggagcttgcagtgagccgag CCACTGAATTCCTCATGTCCTTACATAACAACCACATTGTGATCTTC
AGGCCAATCTAACCTGATGCCTTTTTCTTTTTTAAGTTTCACTGGAACGTAGCCACATCCATTCCTTCATGAATCACAGCAGGGCTGAACAGTTcCAAGACCATATAACCCACAGAAGATAAAATATT
TATTACCATGCCTTTAAACGATTAAAATTTGCTGACTCCCACTCTATACTCCAGAAAACAAACAATGCCTTTTTTATTTT CCATCTTCTATGTCTTTGCTCATGCTGTTCTCCCTAACTTAGAATGTG
CCTTTGCCCTACGGACCTATCCTGCTGTCAGGTTAGGGATGCATCCCATACACCCAATACATATACCCTGTCCATAAGTCTTATCATTCTATTTGCCATACTGTCATTAGAATTATCTTTGTGCTTTA
TTATTACTTTTAAACTTAACTATTCAACTGTGACATTATTCAAGGCAAGGACTGCATCCTGATATTTCTACCACATATAACACACTGAATATTTAGCTGAGTAAATAAATAAGTGAATGAAAATA AAA
TGCTAACTGCATAGACTCTAGAAATCATCCCTTTCTTAGAGTATTTTAAAAGAAAAACTTCTAAATGCCAAAAGTCATGGTGACTTTATATATGGCATTTTTAAGACATGTTATGAGTCGAAATTTGA
GTTTTCCTTGGGTCCTAGAGAGATCCTCATTATCTACACTCCAGGATGGGTCCCCTTACTTCATGAGCAACCAGCATGCTGAGGGCTGCTTGTACAGTGTTATGGTAAGCCTGCAGATACATCTC TTA
TCTACACATCAAGTCCAAATCAAAAGGAATCCATGAGCATGTGGAAATATTCCAGTTAGTGACTTCCCTTAGTTTAGAATAACTTTTCTAACCTGAAAGTCTACTGTCAATTTTCTTATGCAAAAATA
TAAAACATGTTTTACCTTTTCCAAATCAGTAAATCTCTCCTGAAGTTGTCTCTTCTCCAGTTCTACTT T

382	34.9	6.5	2.2	AluYa5-Scaffold3703-210432-210676	400	751	(1494)	+	L2	LINE/L2	2509	2875	(544)	1
240	26.7	6.7	0.0	AluYa5-Scaffold3703-210432-210676	940	999	(1246)	+	MER58A	DNA/hAT-Charlie	128	191	(33)	2
2241	1.2	2.5	0.0	AluYa5-Scaffold3703-210432-210676	1001	1245	(1000)	+	AluYa5	SINE/Alu	1	251	(60)	3
279	25.8	6.5	3.2	AluYa5-Scaffold3703-210432-210676	1356	1447	(798)	+	MER58A	DNA/hAT-Charlie	128	222	(2)	4

>hg19 chr2:11352708-11354536
AAGCCCCAATaCTCCTCCTATTTTCTACATAAACCCCAAAGGTTTTCTAATTCTTTGATTTTTATAGTTCATATCATAAATATGATAAAGGCTTTTTCCAAATAAAGCCTTTCATACAAAAAATGAT
TTCTCAAACCACATAATCCACCAACAGAGTAATCTACTCTGTCCTAAAAAAGAAAAAAAAAAATCACTACTTCACACAGTCTTTTCCCAGATGGGCTATTCCACTCACATGATTTCAGCTACTTA CAT
ACCAATGACTCAAATATTTCTAACTTCTACTACTATAGCCTACTGCAGTGCAGACATGCTAATTTGGCTGTCCCATAGGTACCCTCAAACTTAAGAGGACTAAAGTGGGTTTATCACTGTCTCATGAG
CTATTTTTCCTTTATTCTTTATTAACAGCATCACTATATATCTTGTCACAACCTACAAATCTGGGACTCATCTTTGACTGCTACTCATATGCCATATCTAATCAACTA tTAAGTGCTGCCAAATATTC
CTCTTCCAAATATTTCTGAGTCTTCCAATTTTTCCAACCTTTTAATGGCTGTACTAAGTCTCCCATTATTCCTTATCAGCATTACTGAAAAAGTCTACTGACCAGTCTCTCTACCTCTAGTCTTGTCC
TCCTCAAATCTTTTCTCCACACAGTATACAGCGATCTATCTTTAATGTACCTCTAATAAGATCATCCAATTTAAAGCCCTTAAGTGGCTAGCCCATTACTACATTCAAAATTTCTTAATAGAGTA TTC
GCTATCTTGCATGATCTACCTCATGCCGAGCTCTGTAGCCTCATTCTGACCACTGAATTCCTCATGTCCTTACATAACAACCACATTGTGATCTTCAGGCCAATCTAACCTGATGCCTTTTTCTTTTT
TAAGTTTCACTGGAACGTAGCCACATCCATTCCTTCATGAATCACAGCAGGGCTGAACAGTTgCAAGACCATATAACCCACAGAAGATAAAATATTTATTACCA TGCCTTTAAACGATTAAAATTTGC
TGACTCCCACTCTATACTCCAGAAAACAAACAATGCCTTTTTTATTTTCCATCTTCTATGTCTTTGCTCATGCTGTTCTCCCTAACTTAGAATGTGCCTTTGCCCTACGGACCTATCCTGCTGTCAGG
TTAGGGATGCATCCCATACACCCAATACATATACCCTGTCCATAAGTCTTATCATTCTATTTGCCATACTGTCATTAGAATTATCTTTGTGCTTTATTATTACTTTTAAACTTAACTATTCAACT GTG
ACATTATTCAAGGCAAGGACTGCATCCTGATATTTCTACCACATATAACACACTGAATATTTAGCTGAGTAAATAAATAAGTGAATGAAAATAAAATGCTAACTGCATAGACTCTAGAAATCATCCCT
TTCTTAGAGTATTTTAAAAGAAAAACTTCTAAATGCCAAAAGTCATGGTGACTTTATATATGGCATTTTTAAGACATGTTATGAGTCGAAATTTGAGTTTTCCTTGGGTCCTAGAGAGATCCTCA TTA
TCTACACTCCAGGATGGGTCCCCTTACTTCATGAGCAACCAGCATGCTGAGGGCTGCTTGTACAGTGTTATGGTAAGCCTGCAGATACATCTCTTATCTACACATCAAGTCCAAATCAAAAGGAATCC
ATGAGCATGTGGAAATATTCCAGTTAGTGACTTCCCTTAGTTTAGAATAACTTTTCTAACCTGAAAGTCTACTGTCAATTTTCTTATGCAAAAATATAAAACATGTTTTACCTTTTCCAAATCAG TAA
ATCTCTCCTGAAGTTGTCTCTTCTCCAGTTCTACTTT

382	34.9	6.5	2.2	hg19	400	751	(1078)	+	L2	LINE/L2	2509	2875	(544)	5
307	24.7	6.5	3.2	hg19	940	1031	(798)	+	MER58A	DNA/hAT-Charlie	128	222	(2)	6

>KSI_AluYa5_57

>Scaffold3709-260020-260277
TGGAATGCCCTCCAACTGCCTCTACACCCACTCAAATCCTCCTCTTCTAAGCTAGGTCGAGGGCCTCCCTAATTTGCCTCCTTTACTCAGATTTTCAAGTTCCTCTTTGTGCCAAGCACTGTGCTG TA
CACTGAGATGTAGGACTTGTAGCTCCCTTCTCAGATAACTTGTGACAAAGATTTACTCATCAATGAGAGTACCCAGCCTCCCTCCTCCCACTTCCAGCCCACTCAGAACAACTTTGT cTGTCAGTTTG
GATGCTTCACCTTCGCCACCTTCCAAAGAGAGTCTTTGCCAGGCTGGCTTCAGTGGCTTTGTAATGCAGAATTAAGAGAATGTATAGAGGAAAGGTAAGAGACAACAGAAGACTCATGATTTAAAA AA
AAGAGAGAGAGTTAAGAGAGGCAAAAcAGACAAAATAAAACATATGGGTTGGGTATCAGTAAAGTTAGGGGTGTTACGTTTGAACAGCTTTAGAGCAGCCACAGCTATCAGG GAAGAGAGAAATAAAC
TTCTGAAGAGTGTTGTTGTAGGTTAAAAATTAGATCCCTTTTCATGTTCTTGAGCCTCAAGAAACATTTCTAATTACTTTTGGTTACTATATTGTGCTCTTTTGAATGCAACAGCATAAGAAGCCT GG
GCTATATGGTCTTCAGTTTATGTTATAATTATATTTAACATCTAAACTGAGTTACTCTTGAGAGTAAAAGGGGAAACTATTAATGATTATTCTGGGATAACAGGCATAAACTGGGACTGTGCTGGGCA
AACCAGGATGTCCAGCCACCCAGCTTGTAGTATAATAAAATGTGGCAAGTAAAGGAGTGgATGTAcCCAGGACATCTCTGAGGCTCAAAGAATGAGCATCTCACTCAGCGGGTGGCAAGAGAGAGGAG
AGGATTTTTCTTCTGGAGAAGAAAAATCTTTCCACTTCTATGGGCCTACAGTGTGCTTTCTCTTTTCTttttttttttttttttttttttttttttGAGACGGAGTCTCGCTCTGTCRCccaggctgg
agtgcagtggcgggatctcggctcactgcaagctccgcctccctggttcacgccattctcctgcctcagcctcccaagtagctgggactacaggcgcccgccactacgcccggctaattttttgt att
tttagtagagacggggtttcaccgttttagccgggatggtctcgatctcctgacctcgtgatccgcccgcctcggcctcccaaagtgctgggattacaggcgtgagccaccgcgcccgg ccgtgcttt
ctcttttctAAATTAAACAACCTATATCACCTGTTAAAAACCACACTTTATCCTATCTCGTGTTAACTGTTTTTTTTTCTGGAGTGTCAACAAAGAGTAAAAAAAGGAAAACATCAATCTTTTATAGC
ATTGAAAGGAAAGAATATCATTCTTTTATATCTCTCACAATGTTGGGCAGTGTGGGAGA TAAAAAAAAAGCGCAATaACTACATGTTGCTTGAGTAATTTGTTCCCCTAAACCAACCAA AATACGCAT
CCATTTTCCAGTTATTTTGTAATGGCCTTTGAGCAAATGCTCAGTACTGGGGCAATAGTATAATGTCAATACTTTCAGGCAAGCAGGAAAGCATTTCTATTTTTGATTAATGGAGAGGAGAGTGA ACT
AGAGTCTCATTTACCAGGCTTTGCCAACATCCCAAATCATCACTTAACCGTCCAACACATTTATACCTACGTTGCCAAGTGGAGGTAAACACTATTTATCTTCATCTGCGGAAAAGG AAAATGCTC
ATTAGGCTCACCATCACTTTCCTCCCTCCACCCCCAATGTGCTTCtCCAACCTCAGTTGGCTTGAGTAAAGAATTTCCAACACGGTTCTGTAATCAGTGGGGAGGGCCCAAGAGAAAATCCACAATAT
CCCTTGTTCAGTTACATTCACTGTGATGTGGAAGCTTAGAAAACAGGAAATCCACCTTTAGCAGCTCATTTGAGGCAGCAAATTTCTGGCTGGGGTGGGGAGAACTTTGCTGCAAAATC ACATCTTTG
AACCCTCCAGAGTACAGATGGAGTGCTGAAAGTTGGGAAAGACCAGTGAAGTGGGCTGTAGTAAACGCCTAGACTTCAGACAGGAATGCTGCAGGCAGGGAAAGGAACTAACCTATGATGACCCA AAA
TGAGTCTTTTGATTTAGATATCAAGTCAATCCTGGCTTCCAGGCCAAGCCATCGCATCCCCTGTGACTTGCATGTATATGCCCAGATGGTCTGAAG T

493	28.7	4.6	6.2	AluYa5-Scaffold3709-260020-260277	172	368	(1890)	+	LTR103_Mam	LTR/ERV1?	9	202	(273)	1
583	26.5	0.0	0.0	AluYa5-Scaffold3709-260020-260277	673	789	(1469)	C	MER94	DNA/hAT-Blackjack	(15)	119	3	2
31	3.0	0.0	0.0	AluYa5-Scaffold3709-260020-260277	959	992	(1266)	+	(T)n	Simple_repeat	1	34	(0)	3
2457	0.4	0.0	0.0	AluYa5-Scaffold3709-260020-260277	1001	1258	(1000)	C	AluYa5	SINE/Alu	(53)	258	1	4
461	10.2	0.0	0.0	AluYa5-Scaffold3709-260020-260277	2200	2258	(0)	+	LTR7Y	LTR/ERV1	17	75	(397)	5

>hg19 chr6:6033775-6035740
TGGAATGCCCTCCAACTGCCTCTACACCCACTCAAATCCTCCTCTTCTAAGCTAGGTCGAGGGCCTCCCTAATTTGCCTCCTTTACTCAGATTTTCAAGTTCCTCTTTGTGCCAAGCACTGTGCTG TA
CACTGAGATGTAGGACTTGTAGCTCCCTTCTCAGATAACTTGTGACAAAGATTTACTCATCAATGAGAGTACCCAGCCTCCCTCCTCCCACTTCCAGCCCACTCAGAACAACTTTGT tTGTCAGTTTG
GATGCTTCACCTTCGCCACCTTCCAAAGAGAGTCTTTGCCAGGCTGGCTTCAGTGGCTTTGTAATGCAGAATTAAGAGAATGTATAGAGGAAAGGTAAGAGACAACAGAAGACTCATGATTTAAAA AA
AAGAGAGAGAGTTAAGAGAGGCAAAAgAGACAAAATAAAACATATGGGTTGGGTATCAGTAAAGTTAGGGGTGTTACGTTTGAACAGCTTTAGAGCAGCCACAGCTATCAGG GatacttggggAAGAG
AGAAATAAACTTCTGAAGAGTGTTGTTGTAGGTTAAAAATTAGATCCCTTTTCATGTTCTTGAGCCTCAAGAAACATTTCTAATTACTTTTGGTTACTATATTGTGCTCTTTTGAATGCAACAGCA TA
AGAAGCCTGGGCTATATGGTCTTCAGTTTATgtttatGTTATAATTATATTTAACATCTAAACTGAGTTACTCTTGAGAGTAAAAGGGGAAACTATTAATGATTATTCTGGGATAACAGGCATAAACT
GGGACTGTGCTGGGCAAACCAGGATGTCCAGCCACCCAGCTTGTAGTATAATAAAATGTGGCAAGTAAAGGAGTG aATGTAtCCAGGACATCTCTGAGGCTCAAAGAATGAGCATCTCACTCAGCGGG
TGGCAAGAGAGAGGAGAGGATTTTTCTTCTGGAGAAGAAAAATCTTTCCACTTCTATGGGCCTACAGTGTGCTTTCTCTTTTCTAAATTAAACAACCTATATCACCTGTTAAAAACCACACTTTATCC
TATCTCGTGTTAACTGTTTTTTTTTCTGGAGTGTCAACAAAGAGTAAAAAAAGGAAAACATCAATCTTTTATAGCATTGAAAGGAAAGAATATCATTCTTTTATATCTCTCACAATGTTGGGCAGTGT
GGGAGATaaAAAAAAAAAGCGCAATtACTACATGTTGCTTGAGTAATTTGTTCCCCTAAACCAACCAAAATACGCATCCATTTTCCAGTTATTTTGTAATGGCCTTTGAGCAAATGCTCAGTACTGGG
GCAATAGTATAATGTCAATACTTTCAGGCAAGCAGGAAAGCATTTCTATTTTTGATTAATGGAGAGGAGAGTGAACTAGAGTCTCATTTACCAGGCTTTGCCAACATCCCAAATCATCACTTAACC GT
CCAACACATTTATACCTACGTTGCCAAGTGGAGGTAAACACTATTTATCTTCATCTGCGGAAAAGGAAAATGCTCATTAGGCTCACCATCACTTTCCTCCCTCCACCCCCAATGTGCTTC cCCAAC
CTCAGTTGGCTTGAGTAAAGAATTTCCAACACGGTTCTGTAATCAGTGGGGAGGGCCCAAGAGAAAATCCACAATATCCCTTGTTCAGTTACATTCACTGTGATGTGGAAGCTTAGAAAACAGGAA AT
CCACCTTTAGCAGCTCATTTGAGGCAGCAAATTTCTGGCTGGGGTGGGGAGAACTTTGCTGCAAAATCACATCTTTGAACCCTCCAGAGTACAGATGGAGTGCTGAAAGTTGGGAAAGACCAGTG AAG
TGGGCTGTAGTAAACGCCTAGACTTCAGACAGGAATGCTGCAGGCAGGGAAAGGAACTAACCTATGATGACCCAAAATGAGTCTTTTGATTTAGATATCAAGTCAATCCTGGCTTCCAGGCCAAGC CA
TCGCATCCCCTGTGACTTGCATGTATATGCCCAGATGGTCTGAAGT

513	28.1	4.6	6.2	hg19	172	368	(1598)	+	LTR103_Mam	LTR/ERV1?	9	202	(27 3)	6
243	32.7	9.8	4.7	hg19	477	659	(1307)	+	LTR103_Mam	LTR/ERV1?	261	452	(23)	6
583	26.5	0.0	0.0	hg19	689	805	(1161)	C	MER94	DNA/hAT -Blackjack	(15)	119	3	7
461	10.2	0.0	0.0	hg19	1908	1966	(0)	+	LTR7Y	LTR/ERV1	17	75	(397)	8

>KSI_AluYa5_58

>Scaffold4423-1481504-1481814
TTCCTTTTATTATTTCATGTCTCTATTTTCCTTTCTTTCAAGTCTCTCAGTTATCTTTCTCATCTGTTTGTTATATGGACCATATAGTTTACTACAATTCTGGCTTATTAATATGTCTTCTTCAGC TC
CTGTTATCAACTGATTTAATGTCCTTTTATATTTTcTTTTAATGTCATTGCTCTAAGTGCCTGAGAGAAAAATCTTGCACAATTCAAGTCACTGGCCAATATCGGATTCCCAAGTAGTTCCAAATAGC
CATTGACACATATCCAGGAATATGATGAATAAAACATGATTTTTAGGGGCAATAAAGCAGTTACTATTCTTGGTTATGGCAAAATTTGTTCTTTTAAGAAAGGAATTTATTAATTCCTATAGATA TCT
TACAAAATCGTTTGAAGAGCTAAAAATACAGCTTATAGGTTGTACCTTAGAAAAACTCTACAAACACACCACTGATTTGGCTGTCTTGAGAAAGCACTACCTCCGCCTGAATCAGGAAGTTGCCAGC
CTGCCTGCTGCCCTGCCACTGCAGGCTCCAGCAaCCACACACCCTGGGCCTTCTGCAACAGAGGATGGATAGCATTTAGCCTATTTTCTCAAGTAACAAATTCCTGTATCCACATATTGCACAGATCA
TGTCTGAATAGTAAAGCCTAAGTCATATCTGACCTAGAGCCGCAATGAATTTTCAGATTTTGTTTATTTTTATTTGTCTGCTTTAATTTTTCATGGGAAATATAGATTTGATACTATAGAAAGCTTTC
ACATTGCAGAAcCTAGTCCTCATAAATTAGTAACCTGTGTATTAAAGGCAACCTTTCTTAATAAAGAGTTATAGTCCATGATAA CCAGCTTTAGTAGACAGTAGGTCATCATTGAAGGCTATTGAATA
AGTGAGAAACAAGTTGTGAGTACCAATGATGGAGTACTCAATAGTGTGTGCATGTATGTTATAGTGTCTTATGTATATACATGGCATCGTA Tctattcatgtgatttttttttttttttttttttt
tttttgagacggagtctcgctctgtcgcccaggctggagtgcagtggcggggtctcggctcactgcaagctccgcctcccgggttcacgccattctcctgcctcagc ctcccaagtagctgggaccac
aggcgcccgccactacgcccggctaatttttgtatttttagtagagacggggtttcaccgttttagccgggatggtctcgatctcctgacctcgtgatccgcccgcctcggcctcccaaagtg ctg
ggattacaggcgtgagccaccgcgcccggcCTATTCATGTGATTTTTAACTTTGCATTTCTCAGAATTATTTTCTTAACATGATGCAAATaCCTTGAAGTTAGAAACCTACCTTCCATAGCACATCAT
AGTTCACATTTTTCACAAAGACAACACAGGGCCAGGACTTTGCATATATTGAGAATATATTAAGACCATATTCAAGTCTGTTCTTACTAGTACTAATGAATTACATGAATTAAGAAAAA tACACTACC
TCCAAATTTGGAGATAAAAGCTTAATTACTGACATGGTTTGGCTGTGTCCCTACCCAAATCTCACCTTAATTTGTAATAATCCCCACGTGTCAAAGGGTGGGGGCCA GGTGGAGATAATTGAATCATG
GGGGCAGTTTTCCCCGTACTGTTCTCCCAGTAGTGAATAAGTCTCATGAGATCTGATGGTTTTATAAATTAGAGTTACCCTGCACAAGCTCTTCTTGCCTGACCTGACACCGTGTAAGACATGAC TTT
GCTCCTCGTTCACCTACCTCCATGATTGTCAGACCTCCCCAGCCATGTGGAACTGTGAGTCAATTAAGCCTCTTTCCTTTATAAATTACCCAGTCTCAGTTATGTCT TTATTAGCAGCATGAGAAGAG
GACAATACAATTCCTAATTAGCAATATTCCTTCATAGGAATGCTTAAAGACCCCACAATATACAAAGACTGATATCTGAGAATCACTGGCCATCCTGAAATGATGCAGAAAAACAGTATATGTGT TGT
GTTTTTCAAGGAAGAGGGCCTAGATTCTCAGAAGAAGTCCTTGACTAAAATAAAAAAAACAATAAAGGTCAATAACCAATGATCTAGGGAAAACTCTAAATTTTAAG AAGAAAACTCACTTTGGTTCA
ACTCTTGTGGCTGTAAATCTTTCTAAAATAAATTATTCTACCTTCTTGCCTCAATAAACACACTGAAGTGAAGATGATGGGATATTTCTTAACATGATAGAGTGCTTCAGGGTCACTATTCTGAA AAT
GAATTCT

349	29.8	6.9	3.6	AluYa5-Scaffold4423-1481504-1481814	348	507	(1804)	C	LTR67B	LTR/ERVL	(47)	573	409	1
2641	0.7	0.0	0.4	AluYa5-Scaffold4423-1481504-1481814	1001	1311	(1000)	C	AluYa5	SINE/Alu	(0)	311	1	2
2220	13.2	2.2	2.2	AluYa5-Scaffold4423-1481504-1481814	1566	1929	(382)	+	THE1B	LTR/ERVL-MaLR	1	364	(0)	3
255	30.1	0.0	7.9	AluYa5-Scaffold4423-1481504-1481814	2173	2309	(2)	C	Tigger15a	DNA/TcMar-Tigger	(107)	608	482	4

>chr11:24791956-24793943
TTCCTTTTATTATTTCATGTCTCTATTTTCCTTTCTTTCAAGTCTCTCAGTTATCTTTCTCATCTGTTTGTTATATGGACCATATAGTTTACTACAATTCTGGCTTATTAATATGTCTTCTTCAGC TC
CTGTTATCAACTGATTTAATGTCCTTTTATATTTTgTTTTAATGTCATTGCTCTAAGTGCCTGAGAGAAAAATCTTGCACAATTCAAGTCACTGGCCAATATCGGATTCCCAAGTAGTTCCAAATAGC
CATTGACACATATCCAGGAATATGATGAATAAAACATGATTTTTAGGGGCAATAAAGCAGTTACTATTCTTGGTTATGGCAAAATTTGTTCTTTTAAGAAAGGAATTTATTAATTCCTATAGATA TCT
TACAAAATCGTTTGAAGAGCTAAAAATACAGCTTATAGGTTGTACCTTAGAAAAACTCTACAAACACACCAC TGATTTGGCTGTCTTGAGAAAGCACTACCTCCGCCTGAATCAGGAAGTTGCCAGC
CTGCCTGCTGCCCTGCCACTGCAGGCTCCAGCACCACACACCCTGGGCCTTCTGCAACAGAGGATGGATAGCATTTAGCCTATTTTCTCAAGTAACAAATTCCTGTATCCACATATTGCACAGATCAT
GTCTGAATAGTAAAGCCTAAGTCATATCTGACCTAGAGCCGCAATGAATTTTCAGATTTTGTTTATTTTTATT TGTCTGCTTTAATTTTTCATGGGAAATATAGATTTGATACTATAGAAAGCTTTCA
CATTGCAGAAtCTAGTCCTCATAAATTAGTAACCTGTGTATTAAAGGCAACCTTTCTTAATAAAGAGTTATAGTCCATGATAA CCAGCTTTAGTAGACAGTAGGTCATCATTGAAGGCTATTGAATAA
GTGAGAAACAAGTTGTGAGTACCAATGATGGAGTACTCAATAGTGTGTGCATGTATGTTATAGTGTCTTATGTATATACATGGCATCGTA TCTATTCATGTGATTTTTAACTTTGCATTTCTCAGAAT
TATTTTCTTAACATGATGCAAATgCCTTGAAGTTAGAAACCTACCTTCCATAGCACATCATAGTTCACATTTTTCACAAAGACAACACAGGGCCAGGACTTTGCAT ATATTGAGAATATATTAAGACC
ATATTCAAGTCTGTTCTTACTAGTACTAATGAATTACATGAATTAAGAAAAA gACACTACCTCCAAATTTGGAGATAAAAGCTTAATTACTGACATGGTTTGGCTGTGTCCCTACCCAAATCTCACCT
TAATTTGTAATAATCCCCACGTGTCAAAGGGTGGGGGCCAGGTGGAGATAATTGAATCATGGGGGCAGTTTTCCCCGTACTGTTCTCCCAGTAGTGAATAAGTCTC ATGAGATCTGATGGTTTTATAA
ATTAGAGTTACCCTGCACAAGCTCTTCTTGCCTGACCTGACACCGTGTAAGACATGACTTTGCTCCTCGTTCACCTACCTCCATGATTGTCAGACCTCCCCAGCCATGTGGAACTGTGAGTCAAT TAA
GCCTCTTTCCTTTATAAATTACCCAGTCTCAGTTATGTCTTTATTAGCAGCATGAGAAGAGGACAATACAATTCCTAATTAGCAATATTCCTTCATAGGAATGCTT AAAGACCCCACAATATACAAAG
ACTGATATCTGAGAATCACTGGCCATCCTGAAATGATGCAGAAAAACAGTATATGTGTTGTGTTTTTCAAGGAAGAGGGCCTAGATTCTCAGAAGAAGTCCTTGACTAAAATAAAAAAAACAATA AAG
GTCAATAACCAATGATCTAGGGAAAACTCTAAATTTTAAGAAGAAAACTCACTTTGGTTCAACTCTTGTGGCTGTAAATCTTTCTAAAATAAATTATTCTACCTTC TTGCCTCAATAAACACACTGAA
GTGAAGATGATGGGATATTTCTTAACATGATAGAGTGCTTCAGGGTCACTATTCTGAAAATGAATTCT

349	29.8	6.9	3.6	hg19	348	507	(1481)	C	LTR67B	LTR/ERVL	(47)	573	4 09	5

2220	13.2	2.2	2.2	hg19		1243	1606	(382)	+	THE1B	LTR/ERVL-MaLR	1	364	(0)	6
255	30.1	0.0	7.9	hg19		1850	1986	(2)	C	Tigger15a	DNA/TcMar-Tigger	(107)	608	482	7

>KSI_AluYa5_59

>Scaffold9442-6066823-6067025
ATGGCTGGTGTGCTCTCTCGATTTTGCAGGTTTGACCAAGGTAGCTTAGGAAGTTTAGGTAAATAGAGCAAATGATAAATGGTTGGAAATGCATAGGAAACAAAATGGCTGTTCATAGAACCAAAT AC
AAGCCTTCCATTAGAAACTAAAACACGGCCGGGCACGGTGGCTTATGCCTGTAATCCCAGCACTTTGGGAGGCTGAGGCGAGTGGATCATGAGGTCAAGAGAACGAG tCCACACTGcCCAACATGGaG
AAACCCCGTCTCTACTAcAAATACAAAAATTAGCTGGGCATGGTGGCGCACACCTGTAGTCCCAGCTGTTCGGGAGGCTGAGGCAGGGGAATCACTTTAACCCAGGAGGTGGAGGTTGCATTGAGCCA
AGATCACGCCACTGCACTCCAGCCTGGTGACAGAGCGAGACTCCATCTCAAAAAAAAAAAAAAAAAAGAAAAGAAACTAAAACACGACAATGGACTTACAATTGTATGCATAAGCCAAACCCCAAG GAA
AACAAACAGCAGCTAAATGCAAATTAAAAGCAAAAACAAATGAACAGGACACCAACCCCCAATTTTTTTT CCTACTCAGTTGTCTTGGAGGCTACCATGTTACCCAGAGCCTAAAAAAAAAAAAAAA
AAAGAATATTTTATTCCTGGTTTACGAATTAATGTCTTTAAGTCCACCAATCCCACTGTATATTTTGTGCAATTAAGAAATTCACTTTAGGCATGTGACCAGTAAGTACTTTATTGCTAGTACTATCT
CTGCAAAAGAGCAGTACAGTGTGAAACAAAGCAATGCAAGCGTGGGTGTGAAATGTGGCCCCTTGTTAAATAGGACTTCATGATGAACTATATTAAAGAAGAATTGGCAAACTGCCAGTGTATTTC TT
TACaatacttcttactttaccttcatcaagactaagcgctttaaccacgagcaatgtcaattagccaaacctttcaattttctatcagattttaaagaatattt ggccgggcgcagtggctcacgcct
gtaatcccagcactttgggaggccgaggcgggcggatcacgaggtcaggagatcgagaccatcctggctaaaacggtgaaaccccgtctctactaaaaatacaaaaaattagccgggcgtagtggc gg
gggggcggggcggtgcgtacttgggaggctgaggcaggagaatggcgtgaaAATACTTCTTACTTTACCTTCATCAAGACTAAGCGCTTTAACCACGAGCAATGTCAATTAGCCAAACTTTTCAATTT
TCTATCAGATTTTAAAGAATATTTTACAATGTACTTGCATGGATCATTGATGACATACTAGGACTTTCTATTTTGTCCTAAATTTTTTTCTGTCTTGTTTTAATAATCATTCAATTTATATTAGAACA
TATTCATATATTCATCATACAAGACTTTTTCTCATACAAAGTTATTCTCTTTTCTTTCTAACCTTCATTACCAAAAATATATCTTCACCATAATTTTTTTCTTTTCCTTTTTTTTTTTTTTT TtGTTA
GAAATGAGCTTTTTGGCAGGTCACCCAGGCTGATCTTGAACTCCTAAGGTCAAGCGATCCACTTGCTTCAGCCTC CCACAGTGAGCCACCACGCCTCCCATAAATCCATAACTTACTTCACATCTCTC
TCCCCTACTTACTGGTTCCTTTCTACCTTGTTTAATAAATAACCTTTGCAAGTCCATAATTAGAATTAACCTTTAGATAACTTCTGAATTAGACTAATTTATTCTTTTTCTTAATAAGAACACAT CTT
TGGCAAATCTTATGTACAGAATTATACACTAACTACAATTGTTATCCTTAGTAACTTTAAATTTTAGTGAAAACTAGGAAGCAAAAAGTCCTGAACTGTCTATCAATAGAAAACACTAGCATTTTC TA
GATGGGTTTTCCATAAGGAGGAACAGTCAGACTGCAGGTTTTTCTTTCCCTTTCTCGTAACCAGCCTAAGAAACAATATTTTACATTTTATCAAAATAATTCCCAGCCAGGTGCAGTGGCTCATG CCT
GTAATCCCAGCACTTTGGGAGGCCAAGGTGGGTGGATTACTTGAGCCCAAGAGTTCAAGACCAGCCTGGGCAATATGGCAAAACCTTACAGCTACCAAAAATACCAAAAAGAAAAAATTAGGCCGG GC
ACCTTGGCTTACCCTGTAATCCCAGCA

4413	11.7	0.6	2.0	AluYa5-Scaffold9442-6066823-6067025	1	154	(2049)	C	LOR1-int	LTR/ERV1	(5311)	2809	2658	1	
2341	10.5	0.0	0.0	AluYa5-Scaffold9442-6066823-6067025	155	459	(1744)	+	AluSc	SINE/Alu	1	305	(4)	2	
4413	11.7	0.6	2.0	AluYa5-Scaffold9442-6066823-6067025	460	1000	(1203)	C	LOR1-int	LTR/ERV1	(5463)	2657	2124	1	
1638	4.9	1.5	1.5	AluYa5-Scaffold9442-6066823-6067025	1001	1203	(1000)	+	AluYa5	SINE/Alu	1	203	(108)	3	
727	10.0	0.9	0.0	AluYa5-Scaffold9442-6066823-6067025	1204	1313	(890)	C	LOR1-int	LTR/ERV1	(5895)	2225	2115	1	
641	19.7	2.6	8.4	AluYa5-Scaffold9442-6066823-6067025	1301	1519	(684)	C	LOR1-int	LTR/ERV1	(6080)	2040	1858	1	*
493	23.0	0.0	1.0	AluYa5-Scaffold9442-6066823-6067025	1520	1619	(584)	C	FLAM_C	SINE/Alu	(10)	133	35	4	
1890	9.3	2.0	2.0	AluYa5-Scaffold9442-6066823-6067025	1640	1936	(267)	C	LOR1-int	LTR/ERV1	(6248)	1872	1576	1	
552	17.0	2.8	1.8	AluYa5-Scaffold9442-6066823-6067025	1914	2021	(182)	+	MER4-int	LTR/ERV1	3802	3910	(2688)	5	*
997	15.2	0.6	5.3	AluYa5-Scaffold9442-6066823-6067025	2026	2202	(1)	+	AluSz6	SINE/Alu	2	170	(142)	6	

>hg19 chr8:23041419-23043317
ATGGCTGGTGTGCTCTCTCGATTTTGCAGGTTTGACCAAGGTAGCTTAGGAAGTTTAGGTAAATAGAGCAAATGATAAATGGTTGGAAATGCATAGGAAACAAAATGGCTGTTCATAGAACCAAAT AC
AAGCCTTCCATTAGAAACTAAAACACGGCCGGGCACGGTGGCTTATGCCTGTAATCCCAGCACTTTGGGAGGCTGAGGCGAGTGGATCATGAGGTCAAGAGAACGAG aCCACACTGgCCAACATGGtG
AAACCCCGTCTCTACTAaAAATACAAAAATTAGCTGGGCATGGTGGCGCACACCTGTAGTCCCAGCTGTTCGGGAGGCTGAGGCAGGGGAATCACTTTAACCCAGGAGGTGGAGGTTGCATTGAGCCA
AGATCACGCCACTGCACTCCAGCCTGGTGACAGAGCGAGACTCCATCTCAAAAAAAAAAAAAAAAAAGAAAAGAAACTAAAACACGACAATGGACTTACAATTGTATGCATAAGCCAAACCCCAAG GAA
AACAAACAGCAGCTAAATGCAAATTAAAAGCAAAAACAAATGAACAGGACACCAACCCCCAATTTTTTTT CCTACTCAGTTGTCTTGGAGGCTACCATGTTACCCAGAGCCTAAAAAAAAAAAAAAA
AAAaGAATATTTTATTCCTGGTTTACGAATTAATGTCTTTAAGTCCACCAATCCCACTGTATATTTTGTGCAATTAAGAAATTCACTTTAGGCATGTGACCAGTAAGTACTTTATTGCTAGTACTATC
TCTGCAAAAGAGCAGTACAGTGTGAAACAAAGCAATGCAAGCGTGGGTGTGAAATGTGGCCCCTTGTTAAATAGGACTTCATGATGAACTATATTAAAGAAGAATTGGCAAACTGCCAGTGTATTT CT
TTACAATACTTCTTACTTTACCTTCATCAAGACTAAGCGCTTTAACCACGAGCAATGTCAATTAGCCAAACTTTTCAATTTTCTATCAGATTTTAAAGAATATTT TACAATGTACTTGCATGGATCAT
TGATGACATACTAGGACTTTCTATTTTGTCCTAAATTTTTTTCTGTCTTGTTTTAATAATCATTCAATTTATATTAGAACATATTCATATATTCATCATACAAGACTTTTTCTCATACAAAGTTAT TC
TCTTTTCTTTCTAACCTTCATTACCAAAAATATATCTTCACCATAATTTTTTTCTTTTCCTTTTTTTTTTTTTTT TGTTAGAAATGAGCTTTTTGGCAGGTCACCCAGGCTGATCTTGAACTCCTAAG
GTCAAGCGATCCACTTGCTTCAGCCTCCCACAGTGAGCCACCACGCCTCCCATAAATCCATAACTTACTTCACATCTCTCTCCCCTACTTACTGGTTCCTTTCTACCTTGTTTAATAAATAACCTTTG
CAAGTCCATAATTAGAATTAACCTTTAGATAACTTCTGAATTAGACTAATTTATTCTTTTTCTTAATAAGAACACATCTTTGGCAAATCTTATGTACAGAATTATACACTAACTACAATTGTTAT CCT

TAGTAACTTTAAATTTTAGTGAAAACTAGGAAGCAAAAAGTCCTGAACTGTCTATCAATAGAAAACACTAGCATTTTCTAGATGGGTTTTCCATAAGGAGGAACAGTCAGACTGCAGGTTTTTCTT TC
CCTTTCTCGTAACCAGCCTAAGAAACAATATTTTACATTTTATCAAAATAATTCCCAGCCAGGTGCAGTGGCTCATGCCTGTAATCCCAGCACTTTGGGAGGCCAAGGTGGGTGGATTACTTGAG CCC
AAGAGTTCAAGACCAGCCTGGGCAATATGGCAAAACCTTACAGCTACCAAAAATACCAAAAAGAAAAAATTAGGCCGGGCACCTTGGCTTACCCTGTAATCCCAGC A

4450	11.7	0.6	2.2	hg19	1	154	(1745)	C	LOR1-int	LTR/ERV1	(5311)	2809	2658	7	
2445	9.2	0.0	0.0	hg19	155	459	(1440)	+	AluSc	SINE/Alu	1	305	(4)	8	
4450	11.7	0.6	2.2	hg19	460	1010	(889)	C	LOR1-int	LTR/ERV1	(5463)	2657	2115	7	
641	20.1	1.4	9.0	hg19	987	1205	(694)	C	LOR1-int	LTR/ERV1	(6080)	2040	1858	7	*
493	23.0	0.0	1.0	hg19	1216	1315	(584)	C	FLAM_C	SINE/Alu	(10)	133	35	9	
1890	9.3	2.0	2.0	hg19	1336	1632	(267)	C	LOR1-int	LTR/ERV1	(6248)	1872	1576	7	
552	17.0	2.8	1.8	hg19	1610	1717	(182)	+	MER4-int	LTR/ERV1	3802	3910	(2688)	10	*
997	15.2	0.6	5.3	hg19	1722	1898	(1)	+	AluSz6	SINE/Alu	2	170	(142)	11	

>KSI_AluYa5_60

>Scaffold13190-391314-391475
TTTCCCTCTGATTTGTGGTGGCACAGAGGAAAGGGCATAGGAAGAACTAAGGTAATATGGTGGAAGTTTAGTCAAGTAACTGGTTTGAAGGTATACGAGACTGTGTACTTAGTAGATATACTGAAT CC
AAGGCAAACAATAGGTTATTTGATTGTCCTCCTTAGGTTGATGGCACCATACAAAAAACTGAGGGTGGCTTTGATTTGACGATAAAATAAACCAAAGGTTTGCTACAAGGAAACATACTTCAGTT GCA
AAGTTGTATAATGATTCTCCTCACTTTAAGGGAAACTCTCATCTTGTTCTCACTTCCTCTTCCACCATAAATAAGCAAACATATTCTGGAAGGCTGTTTTCTAGGGGCAACTCAGTATCAGTTTTG AT
AATTCATACAAGAAAGGCATCATGAGCCATGCCAATCAACAGGCTCCTCTCTGTTCTCAGGCAAACTTCCTGTATGGACTACCTTAGGCTCTTCCAAACCTAATGAGATGTTTTTACTTTTTCAT ATT
TGGAGAGTATGCTGGTTAGATCATTGGTTTGTAACACCAACCAGTGCAAATTTCTCATGCTATCATATAATCAACTCTGTCCCACATTACCATGTGCTCTAACATTGTATAGACTTAAAATACAAT GC
AGAAAGTTGAGAGTTTGTAGGATTTACTATCAAGACTGACTTCTGAAATGCAGAAACGTATGTGTTTGCATTTAGAAAACCTTTAACACTCCTTGTCTTAGCTAATGATCACAAGCAAGAAAAAT TTA
GATCACTCAGAAAAAGAATATGAACAAAGCTGATAAATTGGATTTATTCTTCTTTACCTAACTTTTGCAGAAGTTCTACCAAAGCTTCAATTGTCTTCACCTGGTCACTAGAGACCGTATCTGTAA TG
ACACTGAATTCCTATTAGATTTGAGAAAAGTGTGGTGATTTTGTAGTTGAGAGAGCTACAAATTCAACTTTCTTTTTTTCTCTCAGAAGCTATCTCAATTTTAtctacaggcgcccgccactacgccc
ggctaatttttgtatttttagtagagacggggtttcaccgttttagccgggatggtctcgatctcctgacctcgtgatccgcccgcctcggcctcccaaagtgctgggattacaggcgtgagcca cc
gcgcccggcctgctatctcaattttaAAAACTCAAATGTTATACAGTAAGAGGTACTTGTTTTTTGAGAGGAAAATTTCATGTAAACCATTAAAAGAAAACAATAAT GACCTGAAATCTGCTTACCAG
ATTTGTATCCCACATTTTAAAACAATGCCACAGATAGGGTTATTTTCTTAATTATTTTCCTTAGAATAAGAAATTTTTGTGGTCACTGCTTAGAAAACTGCATTTGGGAATAAATAATTATTTCCT GA
ATAATGCAAAGTAATGGGGAAAAGATGATAGGAAAATAAAGGCAGGTCTTTCCCAGCAATTTAATAACCACTAAGCGGACAGATGGAGGCATCACCTTCGCGTCATCAGGCTCACGTACTTTCCT AAC
TGCCTTGCTGCGGCTTACTGCCAACGTCCAACATCTTCTTGCTCTTCTCTGCTATCTCCAAAGTTATTTCACTTATCTTCAATGAATGTCTGTTAATCTGATACCTGCATGACTCAATACTACTTT AC
TTTTTTCTGTTCTTACCTTGCCGTAGTTCACCTATCCAGAAATGGATGATCTGCTTTCTCTAGTTACTGTGAATGTACTTTGGTCAAAGCATGGTGGAAAGATAATATACATCAACACGTTACTT CTG
GTAAAGAGCTCAGTTAAAGTCACAGAACTCTCATGTTTAGAATATCAGGAAGTCAGGGATCCTAGGGGCTGGGGCCCATTTTCCTTCTTTAAACGGTTTTTATAAGTTACTACTGAGGTGAATGGT GT
TCTCCCACACCTTTGCTTCTGACGGAAGTTAAGCAGCTTGCCTAAGGAGCCTAATGGTAGAGTGCTGAAAAAAAGTTGCACATGGGTTTTTGACTACTTTCCAATCTTACAAATGACCATCATAA CCT
AATGATTTAATCTTTGATTTCATTGTTTCTCTGTATGCTATGAAATCACCAATTACGCCACTGTAATGGTATAGGCAGCCTTAAATATAGGCACTCATTTGTGCATAGACAGT A

1556 0.0 0.0 0.0 AluYa5-Scaffold13190-391314-391475 1001 1162 (1000) C AluYa5 SINE/Alu (149) 162 1 1

>hg19 chr1:180856566-180858548
TTTCCCTCTGATTTGTGGTGGCACAGAGGAAAGGGCATAGGAAGAACTAAGGTAATATGGTGGAAGTTTAGTCAAGTAACTGGTTTGAAGGTATACGAGACTGTGTACTTAGTAGATATACTGAAT CC
AAGGCAAACAATAGGTTATTTGATTGTCCTCCTTAGGTTGATGGCACCATACAAAAAACTGAGGGTGGCTTTGATTTGACGATAAAATAAACCAAAGGTTTGC TACAAGGAAACATACTTCAGTTGCA
AAGTTGTATAATGATTCTCCTCACTTTAAGGGAAACTCTCATCTTGTTCTCACTTCCTCTTCCACCATAAATAAGCAAACATATTCTGGAAGGCTGTTTTCTAGGGGCAACTCAGTATCAGTTTT GAT
AATTCATACAAGAAAGGCATCATGAGCCATGCCAATCAACAGGCTCCTCTCTGTTCTCAGGCAAACTTCCTGTATGGACTACCTTAGGCTCTTCCAAACCTAA TGAGATGTTTTTACTTTTTCATATT
TGGAGAGTATGCTGGTTAGATCATTGGTTTGTAACACCAACCAGTGCAAATTTCTCATGCTATCATATAATCAACTCTGTCCCACATTACCATGTGCTCTAACATTGTATAGACTTAAAATACAA TGC
AGAAAGTTGAGAGTTTGTAGGATTTACTATCAAGACTGACTTCTGAAATGCAGAAACGTATGTGTTTGCATTTAGAAAACCTTTAACACTCCTTGTCTTAGCT AATGATCACAAGCAAGAAAAATTTA
GATCACTCAGAAAAAGAATATGAACAAAGCTGATAAATTGGATTTATTCTTCTTTACCTAACTTTTGCAGAAGTTCTACCAAAGCTTCAATTGTCTTCACCTGGTCACTAGAGACCGTATCTGTA ATG
ACACTGAATTCCTATTAGATTTGAGAAAAGTGTGGTGATTTTGTAGTTGAGAGAGCTACAAATTCAACTTTCTTTTTTTCTCTCAGAAGCTATCTCAATTTTAAAAACTCAAATGTTATACAGTAAGA
GGTACTTGTTTTTTGAGAGGAAAATTTCATGTAAACCATTAAAAGAAAACAATAAT GACCTGAAATCTGCTTACCAGATTTGTATCCCACATTTTAAAACAATGCCACAGATAGGGTTATTTTCTTAA
TTATTTTCCTTAGAATAAGAAATTTTTGTGGTCACTGCTTAGAAAACTGCATTTGGGAATAAATAATTATTTCCTGAATAATGCAAAGTAATGGGGAAAAGAT GATAGGAAAATAAAGGCAGGTCTTT
CCCAGCAATTTAATAACCACTAAGCGGACAGATGGAGGCATCACCTTCGCGTCATCAGGCTCACGTACTTTCCTAACTGCCTTGCTGCGGCTTACTGCCAACGTCCAACATCTTCTTGCTCTTCT CTG
CTATCTCCAAAGTTATTTCACTTATCTTCAATGAATGTCTGTTAATCTGATACCTGCATGACTCAATACTACTTTACTTTTTTCTGTTCTTACCTTGCCGTAG TTCACCTATCCAGAAATGGATGATC
TGCTTTCTCTAGTTACTGTGAATGTACTTTGGTCAAAGCATGGTGGAAAGATAATATACATCAACACGTTACTTCTGGTAAAGAGCTCAGTTAAAGTCACAGAACTCTCATGTTTAGAATATCAG GAA
GTCAGGGATCCTAGGGGCTGGGGCCCATTTTCCTTCTTTAAACGGTTTTTATAAGTTACTACTGAGGTGAATGGTGTTCTCCCACACCTTTGCTTCTGACGGA AGTTAAGCAGCTTGCCTAAGGAGCC
TAATGGTAGAGTGCTGAAAAAAAGTTGCACATGGGTTTTTGACTACTTTCCAATCTTACAAATGACCATCATAACCTAATGATTTAATCTTTGATTTCATTGTTTCTCTGTATGCTATGAAATCA CCA
ATTACGCCACTGTAATGGTATAGGCAGCCTTAAATATAGGCACTCATTTGTGCATAGACAGT A

>KSI_AluYa5_61

1FR

>Scaffold13294-3398577-3398707
GAGGCAGAGGTTGCAGTGAGCTGAGATGGCACCACTGTACTCTAGCCTAGGTGATAGAGCAAGACCCTGGCTCAAAAAATAAATTAAATAAAGTGAGCAGGGCGTGGTGACTCACGCTTGTAATCC CA
GTACTTTGGGAGGCTGAGGCAGGTGGATCACCTGAGGTCAGGAGTTCAAGACCAGCCTGGCCAACATGGTGAAACCCTGTCTCTACTAAAAATACAAA AATTAGGCAGGTGTGGTGGCTCATGCCTGT
AATCCCAGCTAcTCAGGAGGCTGAGGCAGGAGAATCACTTGAACCAGGAGGCAGAAGTTGCAGGGAGCCAAGATGGTGC cACTGCACTCCACCCTGGGCAACAGAGTGAGGCTCCGTCTCAAAAGAAA
TTAAATAAATAAATAAAGTGAGAGATGTGCAACTCTTTTACTTGAACACTTAAGAGGCCACTGTAGGGTTATTAATTGACCTAATTTTAATACTGTTG TATCTCATGGAATAGGGAGGACCCTGAGGA
GAGGGAGAGAAATGGGGGAACAGCCAGTCAGTGGGAAGAGCCGGTTGGTGGAGCAATCAGAGCACACACATTTATTGATCAAGTTCATCTTCTTATGTGGGTGCTGCCCATGACTCCCCAAAACAATT
ACAACAGTAACATTGGAGATCACTGATCACAGATCAGTATAACAGAAATAATAATGATGAAAAAGTTTGAAATATTGTGAGAATTACAGAAACGTGACACAGAGACCATGAAATGAGCACATGCT GTT
AGAAAAATGTCAAAGATAGGCTGGGGCATGGTGGCTCATACCTGTCA CCCCAGCACTTTGGGAGGCCAAGGTGAGAGAATCACTTGAGCCCAGGAGTTCAAGACAGTCTGGGCAACATAGTGACAGCC
TCATCTCTTAAAAAAAAAAAAAAAAgttaaaccaggtatgcctttaattgcagagcattcccaatcactcagcccaccactgctcctttaagaaggctctttgt ggccgggcgcggtggctcacgcct
gtaatcccagcactttgggaggccgaggcgggcggatcacgaggtca ggagatcgagaccatcccggctaaaatggtgaaaccccgtctctactaaaaatacaaaaa ATTAGCCGGGCGTAGTGGCGG
GCGCCTGTAGTCCCAGCTACTTGGGAGGCTGAGGCAGGAGAATGGCGTGAACCCGGGAGGCGGAGCTTGCAGTGAGCCGAGATCCCGCCACTGCACTCCAGCCTGGGCGACAGAGCGAGACTCCG TCT
CAAAAAAAAAAAAAAAAAAAAAAgcctcatcttttaaaaaaaaaaaaaaGTTAAACCAGGTATGCCTTTAATTGCAGAGCATTCCCAATCACTCAGCCCACCACTGCTCCTTTAAGAAGGCTCTTTG
TGATGTTCTGTGGTTCTAGCCCTGCACATTCCCAGTTTCCTCTTGCATGATTCTACCTTCCACTGAAACAGAGCAAGATCTGCTTAAGATCCTATAAAAATGACTTTGGCATGTGCCCTTTGCCTGG G
AACAGCTTTTTCAGTTGCTGCTGCTGCTGTGTAATATGTTAGG TTACATGGACTACCATTTTTTGTTAAAGAGAATGACTTCCAAGTTCAGTAGGTGCTTCTTGAAAATATAGTTCCCCGTGATGCAA
TAATAATTGATTCTGTTATTTCCTATTCTTGTTCTTATGTATGTGACATATGACATGTAACTGAGACACAGTTCCTATATTTAATATTTGCTGTTTGTTCCACAATCTTTTCATAGATGGGATTA GGT
TGAACATTACCCTGTTCTATCTGAATTATATGTAATATGCTATAAAT TTGAATAATCCACGGTAAGGTGATTTTAAGCTTAACTCTGCCCCAGCAGGTGATAATTATTTTATTAGAGCATTCTGTATC
TAGTCACTCCGCATAGTGTTGACTCTTGGTTTGAAGTCACACCTCATGCCTGTGGTTCAGCATGCATCTTTGGGCCTCAATGTCTTAGAAAAACTTAGCTTAGTGGACTGGATTTACAGACCTGG GGA
GGACATGGAAATTTCAAGTTTCTCATCCAGAATCTTCTTCCAACTTG CTGTGTGGCCTTCCACGCGTCATTTCATACTCGCTCGATCATTTCTTCATTTCAGCATGGAAACAGAGCCCATTAGCTTCT
CAAGGGCATTGTAAGAAAGGGGAGAGCAGTGGTCAAAAAGGAAACAAACTGAAAAATATCAAGATCCCTTCAATAGTTCTGACCTCTAGTGAAAAAAGGAACAACGTGGTACCGGA A

552	16.9	0.0	0.0	AluYa5-Scaffold13294-3398577-3398707	1	83	(2048)	+	AluSx3	SINE/Alu	209	291	(20)	1
2238	10.8	0.3	0.7	AluYa5-Scaffold13294-3398577-3398707	97	401	(1730)	+	AluSx	SINE/Alu	2	305	(7)	2
2290	13.1	1.8	5.6	AluYa5-Scaffold13294-3398577-3398707	402	790	(1341)	C	Tigger1	DNA/TcMar-Tigger	(1947)	471	97	3
861	13.9	0.8	0.8	AluYa5-Scaffold13294-3398577-3398707	792	920	(1211)	+	FLAM_C	SINE/Alu	5	133	(10)	4
1241	0.8	0.0	0.0	AluYa5-Scaffold13294-3398577-3398707	1001	1131	(1000)	+	AluYa5	SINE/Alu	1	131	(180)	5

>hg19 chr1:227601278-227603161
GAGGCAGAGGTTGCAGTGAGCTGAGATGGCACCACTGTACTCTAGCCTAGGTGATAGAGCAAGACCCTGGCTCAAAAAATAAATTAAATAAAGTGAGCAGGGCGTGGTGACTCACGCTTGTAATCC CA
GTACTTTGGGAGGCTGAGGCAGGTGGATCACCTGAGGTCAGGAGTTCAAGACCAGCCTGGCCAACATGGTGAAACCCTGTCTCTACTAAAAATACAAAAATTAGGCAGGTGTGGTGGCTCATGCC TGT
AATCCCAGCTAtTCAGGAGGCTGAGGCAGGAGAATCACTTGAACCAGGAGGCAGAAGTTGCAGGGAGCCAAGATGGTGC tACTGCACTCCACCCTGGGCAACAGAGTGAGGCTCCGTCTCAAAAGAAA
TTAAATAAATAAATAAAGTGAGAGATGTGCAACTCTTTTACTTGAACACTTAAGAGGCCACTGTAGGGTTATTAATTGACCTAATTTTAATACTGTTGTATCTCATGGAATAGGGAGGACCCTGA GGA
GAGGGAGAGAAATGGGGGAACAGCCAGTCAGTGGGAAGAGCCGGTTGGTGGAGCAATCAGAGCACACACATTTATTGATCAAGTTCATCTTCTTATGTGGGTGCTGCCCATGACTCCCCAAAACAATT
ACAACAGTAACATTGGAGATCACTGATCACAGATCAGTATAACAGAAATAATAATGATGAAAAAGTTTGAAATATTGTGAGAATTACAGAAACGTGACACAGAGACCATGAAATGAGCACATGCT GTT
AGAAAAATGTCAAAGATAGGCTGGGGCATGGTGGCTCATACCTGTCACCCCAGCACTTTGGGAGGCCAAGGTGAGAGAATCACTTGAGCCCAGGAGTTCAAGACAGTCTGGGCAACATAGTGACAG CC
TCATCTCTTAAAAAAAAAAAAAAAAGTTAAACCAGGTATGCCTTTAATTGCAGAGCATTCCCAATCACTCAGCCCACCACTGCTCCTTTAAGAAGGCTCTTTGTG ATGTTCTGTGGTTCTAGCCCTGC
ACATTCCCAGTTTCCTCTTGCATGATTCTACCTTCCACTGAAACAGAGCAAGATCTGCTTAAGATCCTATAAAAATGACTTTGGCATGTGCCCTTTGCCTGGGAACAGCTTTTTCAGTTGCTGCTG CT
GCTGTGTAATATGTTAGGTTACATGGACTACCATTTTTTGTTAAAGAGAATGACTTCCAAGTTCAGTAGGTGCTTCTTGAAAATATAGTTCCCCGTGATGCAATAATAATTGATTCTGTTATTTCCTA
TTCTTGTTCTTATGTATGTGACATATGACATGTAACTGAGACACAGTTCCTATATTTAATATTTGCTGTTTGTTCCACAATCTTTTCATAGATGGGATTAGGTTGAACATTACCCTGTTCTATCTG AA
TTATATGTAATATGCTATAAATTTGAATAATCCACGGTAAGGTGATTTTAAGCTTAACTCTGCCCCAGCAGGTGATAATTATTTTATTAGAGCATTCTGTATCTAGTCACTCCGCATAGTGTTGA CTC
TTGGTTTGAAGTCACACCTCATGCCTGTGGTTCAGCATGCATCTTTGGGCCTCAATGTCTTAGAAAAACTTAGCTTAGTGGACTGGATTTACAGACCTGGGGAGGACATGGAAATTTCAAGTTTCT CA
TCCAGAATCTTCTTCCAACTTGCTGTGTGGCCTTCCACGCGTCATTTCATACTCGCTCGATCATTTCTTCATTTCAGCATGGAAACAGAGCCCATTAGCTTCTCAAGGGCATTGTAAGAAAGGGG AGA
GCAGTGGTCAAAAAGGAAACAAACTGAAAAATATCAAGATCCCTTCAATAGTTCTGACCTCTAGTGAAAAAAGGAACAACGTGGTACCGGA A

552	16.9	0.0	0.0	chr1:227601278-227603161	1	83	(1801)	+	AluSx3	SINE/Alu	209	291	(20)	6
2208	11.5	0.3	0.7	chr1:227601278-227603161	97	401	(1483)	+	AluSx	SINE/Alu	2	305	(7)	7
2290	13.1	1.8	5.6	chr1:227601278-227603161	402	790	(1094)	C	Tigger1	DNA/TcMar-Tigger	(1947)	471	97	8
861	13.9	0.8	0.8	chr1:227601278-227603161	792	920	(964)	+	FLAM_C	SINE/Alu	5	133	(10)	9

>KSI_AluYa5_62

>Scaffold14550-2906989-2907299
CTTTCTCTCTCTCTCTCTCTTTTTAATACAGAGTTTCACTCTTGTcGCCCAGGCTGGAGTGCAGTGGTGTGATCTTGGCTCACTGCAACCTCCGCCTCCTGGGTTCAAGCGATTCTCCTGCCTCAGTC
TCCAGAGTAGCTGGGATTACAGGCACCCACCAGCATGCCTGGCTAATTTTTGTATTTTTAGTAGAGACTGAGTTTCACCACATTGATCATGCTGGTCTCGAACTCCTGACCTCAGGTGATCCACC CGC
CTTGGCTGGGGATTACAGGGGTGAGCCACTGTGCCCAGCCAAGAGT CTACATTTCTGTTCAGTAGCCCTCCACAAACCATTTCAAGTGTCACTCTGATGTAGACTACAC aACGTGTGTGCCTGAAATA
TTTGCAGGGCCTGAGTGTGCAAGTAAAGATGGAGCCGATGCGATGCTGCTACCGCCGTCCTGAACTGCTGTTATGTGTGGTTCTCTTCCCTGCTCCTGGGACACCTCTGACTCTGCTTACCCTCC TCC
CAGAGTGGTGACAGGCCATGGGGAATGAAGGTTTAATGTGCCTTTG TCAGTTCACCATATTTTCAAACACCAAGGTTTTAAAGAGCAAGGAAAGTAGAAATAGAAGGAAAATAATCAACACATACATA
AAGTAATAGATATGAGGAAAAATAATTACTATGTATGAACACAAATGACATGGAAAAATCCAGATGCTTTCTGGTCCTGTATCATCAAAAAGTCATCATATACACAAAATAAATGTACTTATTTT TTA
TTTGTGGATTTTTTCTTTTGCAAGATGTTGGCAATAAAGACCTCAT TTCAACATTACTGTTACCATTATTACTATTGCTATGAGTTATTAAACAGTACTACAGGTTATTTCCTGTTTGAAAGATGAAA
CCTAGAAATGGGAGAATGGAGTGGTTTCATTGGCTGAAAAGGGCAAGAAAACAAAAGAACTTCATGTTTGCTACTTTCAATCCCAACTCTTCCTCTAACT Atcattcttttatttattttttttttt
tgagacggagtctcgctctgtcgcccaggctggagtgcagtggcgggatctcggctcactgcaagctccgcctcccgggttcacgccattctcctgcctcagcctcccaagtagctgggactaca ggc
gcccgccactacgcccggctaattttttttttgtatttttagtagagacggggtttcaccatttagccgggatggtctcgatctcctgacctcgtgatccgcccgcctcggcc tcccaaagtgctg
ggattacaggcgtgagccaccgcgcccggccTCATTCTTTTATTTTTATTTTTTATTTATTTTTTATTTTTTTGAGACAGGGTCTAACTCTGTCACCTAG GCTGGACGGCTCGATCTCAGCTCACTGC
AACCTCCACCTCCCgGGTTCAAGCAATTCTCCTGCCTCAGCCTCCCAAGTAGCTGAGATTACAGGTGTGTGCCACCACACCTGGCTAATTTTTGTATTTTTAGTAGAGACAGGGT TTCACCACGTTGG
CCAGGCTGTTCTCAAACCCCTGACCTCAAATGATTTGCCTGCCTCGGCTAAGATTGCAGGTGTGAGCCACTGCACCCAGCCACTGTCATTCTTTTAATAGTATGCAGCTATGAATTATTTAAATT AGA
ATTGGATGCTTGGTTGCAAGAAGACATTTGCAACATTTCTGTTGAAGATTAGTATAAAATATATAAGAAATAATTCTAAAATATGCAAGAGAAAGATAAAACATTCTATAGATAA TGTGGCAAAAgAA
ATAATCAATTTACAAAACAGAAATCTGAATGGCTGAGAAATACATGAAGAGACATTCATTCTTACTGATAAAGAAGTGAAAATAAAGATAAAACACCAACTTGTGTCTATGCGATTGGCAAAAAA CAA
CTCAGACAATATCAAGTGCTGACAAGCATGAGGAGAAACCAGTATCATCCATCACTGCTGATTGTCACGAGGGCTGGTCCAGCCCTGTTGAGGTGCAATATAACTGTGAAATTAA GTTAAAAATGCTC
TGTGCTTCAACAATTCTTTTGTAGGTTTTTCTACAGGGAAAACCTTTACAAATATTTTTCTGTAAGAAAACCTGTACAAAGATGAAGTACTTTGTTGAACTACTACAGATTGTAGTGTAGAATTG AAG
ACAACCCAAGCTACCATCTCATGAGTAACAGGGAAGTTAAGTGTAGAAAATGCACAAAAAGTGATTGTATACAGGAGGAATGACTGTATACAGAAGACTTCTTTAGGAAGAAAGT GAGGCTCCCACTT
GTCACAA

2070	12.5	3.7	0.0	AluYa5-Scaffold14550-2906989-2907299	2	296	(2015)	C	AluSq2	SINE/Alu	(7)	306	1	1
2790	1.3	0.0	1.6	AluYa5-Scaffold14550-2906989-2907299	1001	1311	(1000)	C	AluYa5	SINE/Alu	(5)	306	1	2
1995	12.9	6.1	0.0	AluYa5-Scaffold14550-2906989-2907299	1324	1617	(694)	C	AluSz	SINE/Alu	(0)	312	1	3
620	30.8	7.6	3.8	AluYa5-Scaffold14550-2906989-2907299	1683	2253	(58)	+	L1ME3Cz	LINE/L1	5219	5808	(442)	4

>hg19 chr6:18898149-18900149
CTTTCTCTCTCTCTCTCTCTctTTTTttAATACAGAGTTTCACTCTTGTtGCCCAGGCTGGAGTGCAGTGGTGTGATCTTGGCTCACTGCAACCTCCGCCTCCTGGGTTCAAGCGATTCTCCTGCCTC
AGTCTCCAGAGTAGCTGGGATTACAGGCACCCACCAGCATGCCTGGCTAATTTTTGTATTTTTAGTAGAGACTGAGTTTCACCACATTGATCATGCTGGTCTCGAACTCCTGACCTCAGGTGATC CAC
CCGCCTTGGCTGGGGATTACAGGGGTGAGCCACTGTGCCCAGCCAAGAGT CTACATTTCTGTTCAGTAGCCCTCCACAAACCATTTCAAGTGTCACTCTGATGTAGACTACAC cACGTGTGTGCCTGA
AATATTTGCAGGGCCTGAGTGTGCAAGTAAAGATGGAGCCGATGCGATGCTGCTACCGCCGTCCTGAACTGCTGTTATGTGTGGTTCTCTTCCCTGCTCCTGGGACACCTCTGACTCTGCTTACC CTC
CTCCCAGAGTGGTGACAGGCCATGGGGAATGAAGGTTTAATGTGCCTTTG TCAGTTCACCATATTTTCAAACACCAAGGTTTTAAAGAGCAAGGAAAGTAGAAATAGAAGGAAAATAATCAACACATA
CATAAAGTAATAGATATGAGGAAAAATAATTACTATGTATGAACACAAATGACATGGAAAAATCCAGATGCTTTCTGGTCCTGTATCATCAAAAAGTCATCATATACACAAAATAAATGTACTTA TTT
TTTATTTGTGGATTTTTTCTTTTGCAAGATGTTGGCAATAAAGACCTCAT TTCAACATTACTGTTACCATTATTACTATTGCTATGAGTTATTAAACAGTACTACAGGTTATTTCCTGTTTGAAAGAT
GAAACCTAGAAATGGGAGAATGGAGTGGTTTCATTGGCTGAAAAGGGCAAGAAAACAAAAGAACTTCATGTTTGCTACTTTCAATCCCAACTCTTCCTCTAACT ATCATTCTTTTATTTTTATTTTTT
ATTTATTTTTTATTTTTTTGAGACAGGGTCTAACTCTGTCACCTAGGCTG GACGGCTCGATCTCAGCTCACTGCAACCTCCACCTCCC aGGTTCAAGCAATTCTCCTGCCTCAGCCTCCCAAGTAGCT
GAGATTACAGGTGTGTGCCACCACACCTGGCTAATTTTTGTATTTTTAGTAGAGACAGGGTTTCACCACGTTGGCCAGGCTGTTCTCAAACCCCTGACCTCAAATGATTTGCCTGCCTCGGCTAA GAT
TGCAGGTGTGAGCCACTGCACCCAGCCACTGTCATTCTTTTAATAGTATG CAGCTATGAATTATTTAAATTAGAATTGGATGCTTGGTTGCAAGAAGACATTTGCAACATTTCTGTTGAAGATTAGTA

TAAAATATATAAGAAATAATTCTAAAATATGCAAGAGAAAGATAAAACATTCTATAGATAATGTGGCAAAA tAAATAATCAATTTACAAAACAGAAATCTGAATGGCTGAGAAATACATGAAGAGACA
TTCATTCTTACTGATAAAGAAGTGAAAATAAAGATAAAACACCAACTTGT GTCTATGCGATTGGCAAAAAACAACTCAGACAATATCAAGTGCTGACAAGCATGAGGAGAAACCAGTATCATCCATCA
CTGCTGATTGTCACGAGGGCTGGTCCAGCCCTGTTGAGGTGCAATATAACTGTGAAATTAAGTTAAAAATGCTCTGTGCTTCAACAATTCTTTTGTAGGTTTTTCTACAGGGAAAACCTTTACAA ATA
TTTTTCTGTAAGAAAACCTGTACAAAGATGAAGTACTTTGTTGAACTACT ACAGATTGTAGTGTAGAATTGAAGACAACCCAAGCTACCATCTCATGAGTAACAGGGAAGTTAAGTGTAGAAAATGCA
CAAAAAGTGATTGTATACAGGAGGAATGACTGTATACAGAAGACTTCTTTAGGAAGAAAGTGAGGCTCCCACTTGTCACA A

2106	12.4	3.7	0.0	chr6:18898149-18900149	2	300	(1701)	C	AluSq	SINE/Alu	(3)	310	1	5
1980	13.3	6.1	0.0	chr6:18898149-18900149	1014	1307	(694)	C	AluSz	SINE/Alu	(0)	312	1	6
619	31.0	7.6	3.8	chr6:18898149-18900149	1373	1943	(58)	+	L1ME3Cz	LINE/L1	5219	5808	(442)	7

>KSI_AluYa5_63

>Scaffold15038-105959-106272
ATTAACAAATCTAGTTTTGCATTGTATTATTTATAACCTAATTCCACTTAGAAATCAATAACCACTGGGAGCACTTCTTATGTGTTCTTATATATGCTGTAACACAAAGTAAAAATTATTTTTATT AT
AAATCAGTGTCTCTGACATCAGGTCTCTTCTACTTAGAAAAGTGTATACTCCTGACAGCAACAAGAGCTTTAAAACTCAAAGCAGTTGGGAAGCTGAAGCAAAGCATCAGGAAACGGTTTCTGAT TCC
TACTTTGCTTTTATCTACCAGGATGTGTGATCTTGGGTAAGCCACTGAACTTTCACTGCCTATCTCCTATGAGCCAAACACCATGCTAGGCTTCTACATACATTACCTCAAATAACCCTCACAACT AA
TCTGAAAGTAGATAGTATTATCTTACTAAGGCTCAGAGAGAGGTTAAATAATCTAGCCTAAATT CATACAGCATGTGCAGAACTGAGTTGCAAAACCACCCTATATGACTCAAACCAAGTATCTCCAT
GATTCCTTTTCAGGACTAAAATCTGAGACTAAATAAAGAGTAAGATGGACTTAACATTTCAACAAAGCTGAAGAGATAATACTATATGCCATTTCCCTTCAAAGCTATGATTATC tTACCCCCGAATT
CTGAAAACATTCCACCCAAACCCACTGCACACTCAATAACAAAGTTATATCCCTAGGGGGTAAAAGTGGCTGAGTACCACTATTTTAGGGCATGCAGTGAGGTATGCATAACAAGCTTTTAGCTC CAT
GATAAATTAAATTCTCTATAAATCTCTAAAGATGTTGTACAATATAAATAAGATTTTCTTACCAACACTTTAATTATTTCCTCCTTGGTTAGAAAAAGATTACTCTTCATTAAATGTTAACAAATA AG
ATTTTAAACTACTGTAGATaCAAGCTTTAGTAGAATATTACAGTAAAAATGTATCTATCATGAAGTTGAAATTTCATTTGGTGGTAAGATATTA Ctgtatgttattttttttttttttttttttttt
tttttttgagacggagtctcgctctgtcgcccaggctggagtgcagtggcgggatctcggctcactgcaagctccgcctcccgggttcacgccattctcctgcctcagcctcccaagtagctggg ac
cacaggcgcccgccactacgcccggctaatttttgtatttttagtagagacggggtttcaccgttttagccgggatggtctcgatctcctgacctcgtgatccgcccgcctcggcctcccaaa gtg
ctgggattacaggcgtgagccaccgcgcccggcctactgTATGTTATTTTAACATGTTTAATCTTTTAAAATGCAAACTAAATATTTGTATTCAAATTATGCCTAGGAAAAAATACCCATATCCACTT
AtATTTAATAAAACAAAGATGCTTTTTAAACTCTCCAATCCAACTTAATAAAAAATTTCATTGGACATTTAATACTTATATTAACTTA CCTGCATAAGCAACAATCCAACTATAAAAGCACTTCCTTG
ACAGTAACCAACCTCACGATCTACTAAAGAGTAAGCCTAAAAAGTAAAAAAAAGTTGTTCAAAATACATGGTAAGTTATACCTTCCTTCTCCAAAAAACCTAAAATTGGCAAGACAG gCAGATTAAAA
AACATTTATTTTTGAGAAAAGCTCTTGCTTGCTTGTTATAGATGTTATCCTATACATATTTAAAAAGCAAATGTTTTATTACATGAAATTTTATTTTGACAATTTCCCAATCCAAACTTGCTAGC TCT
AAAGACATAAAATAAAATAAAATAAAATAAAATAAAATAAAATAAAATAA AataaaGGAATTTGTAACAGGGCTTAAAAATCAAGTAAAATTGAGCTGGGCACAGTGGCTCATGCCTATAATCCCAGC
ACTTTGGGAGGCAGAGGCAGGCGGATCACCTGAGGtCAGGAGTTCGAGATCAGCCTGGCCAACATAGTGAAACCCTGTCTCTACTAAAAATACAAAAATTAGCTGGGCGTAGTGGCGGG gGCgTGTAA
TCCCAGCTACTAGGGAGGCTGAGGCAGGAGAATTGCTTGAACCTGGGAGGTGGAGGTTGCAGTGAGCTGAGATCACACCGCTGCACTCCAGCCTGGGCAAAAAGAGTGCAACTCCATCTCAAAAA AgA
AAAAAAAAAATTCAAGTAAAATTTATATTGATGTATATAAAACTAGACTAGGTTTAAGGCAAAGGATTGGCGAAATTGAAATGATTTAACAAGAAGTCAGTATTTCAAAGAAGTAGCTAAAAG AAAAA
GTAACTGTGG

203	26.6	2.9	6.1	AluYa5-Scaffold15038-105959-106272	235	302	(2012)	+	MIR3	SINE/MIR	40	105	(103)	1
397	24.9	10.2	9.6	AluYa5-Scaffold15038-105959-106272	323	518	(1796)	C	MIRb	SINE/MIR	(31)	237	41	2
2658	0.3	0.0	0.4	AluYa5-Scaffold15038-105959-106272	1001	1314	(1000)	C	AluYa5	SINE/Alu	(0)	311	1	3
55	0.0	0.0	0.0	AluYa5-Scaffold15038-105959-106272	1799	1848	(466)	+	(ATAAA)n	Simple_repeat	1	50	(0)	4
2402	9.0	0.0	0.0	AluYa5-Scaffold15038-105959-106272	1887	2186	(128)	+	AluSq	SINE/Alu	2	301	(12)	5

>hg19 chr1:93166529-93168508
ATTAACAAATCTAGTTTTGCATTGTATTATTTATAACCTAATTCCACTTAGAAATCAATAACCACTGGGAGCACTTCTTATGTGTTCTTATATATGCTGTAACACAAAGTAAAAATTATTTTTATT AT
AAATCAGTGTCTCTGACATCAGGTCTCTTCTACTTAGAAAAGTGTATACTCCTGACAGCAACAAGAGCTTTAAAACTCAAAGCAGTTGGGAAGCTGAAGCAAAGCATCAGGAAACGGTTTCTGAT TCC
TACTTTGCTTTTATCTACCAGGATGTGTGATCTTGGGTAAGCCACTGAACTTTCACTGCCTATCTCCTATGAGCCAAACACCATGCTAGGCTTCTACATACATTACCTCAAATAACCCTCACAACT AA
TCTGAAAGTAGATAGTATTATCTTACTAAGGCTCAGAGAGAGGTTAAATAATCTAGCCTAAATT CATACAGCATGTGCAGAACTGAGTTGCAAAACCACCCTATATGACTCAAACCAAGTATCTCCAT
GATTCCTTTTCAGGACTAAAATCTGAGACTAAATAAAGAGTAAGATGGACTTAACATTTCAACAAAGCTGAAGAGATAATACTATATGCCATTTCCCTTCAAAGCTATGATTATC cTACCCCCGAATT
CTGAAAACATTCCACCCAAACCCACTGCACACTCAATAACAAAGTTATATCCCTAGGGGGTAAAAGTGGCTGAGTACCACTATTTTAGGGCATGCAGTGAGGTATGCATAACAAGCTTTTAGCTC CAT
GATAAATTAAATTCTCTATAAATCTCTAAAGATGTTGTACAATATAAATAAGATTTTCTTACCAACACTTTAATTATTTCCTCCTTGGTTAGAAAAAGATTACTCTTCATTAAATGTTAACAAATA AG
ATTTTAAACTACTGTAGATtCAAGCTTTAGTAGAATATTACAGTAAAAATGTATCTATCATGAAGTTGAAATTTCATTTGGTGGTAAGATATTA CTATGTTATTTTAACATGTTTAATCTTTTAAAAT
GCAAACTAAATATTTGTATTCAAATTATGCCTAGGAAAAAATACCCATATCCACTTA cATTTAATAAAACAAAGATGCTTTTTAAACTCTCCAATCCAACTTAATAAAAAATTTCATTGGACATTTAA
TACTTATATTAACTTACCTGCATAAGCAACAATCCAACTATAAAAGCACTTCCTTGACAGTAACCAACCTCACGATCTACTAAAGAGTAAGCCTAAAAAGTAAAAA AAAGTTGTTCAAAATACATGGT
AAGTTATACCTTCCTTCTCCAAAAAACCTAAAATTGGCAAGACAG aCAGATTAAAAAACATTTATTTTTGAGAAAAGCTCTTGCTTGCTTGTTATAGATGTTATCCTATACATATTTAAAAAGCAAAT
GTTTTATTACATGAAATTTTATTTTGACAATTTCCCAATCCAAACTTGCTAGCTCTAAAGACATAAAATAAAATAAAATAAAATAAAATAAAATAAAATAAAATAA AGGAATTTGTAACAGGGCTTAA
AAATCAAGTAAAATTGAGCTGGGCACAGTGGCTCATGCCTATAATCCCAGCACTTTGGGAGGCAGAGGCAGGCGGATCACCTGAGG cCAGGAGTTCGAGATCAGCCTGGCCAACATAGTGAAACCCTG
TCTCTACTAAAAATACAAAAATTAGCTGGGCGTAGTGGCGGG cGCaTGTAATCCCAGCTACTAGGGAGGCTGAGGCAGGAGAATTGCTTGAACCTGGGAGGTGGAG GTTGCAGTGAGCTGAGATCACA
CCGCTGCACTCCAGCCTGGGCAAAAAGAGTGCAACTCCATCTCAAAAA AAAAAAAAAAAATTCAAGTAAAATTTATATTGATGTATATAAAACTAGACTAGGTTTAAGGCAAAGGATTGGCGAAATTG
AAATGATTTAACAAGAAGTCAGTATTTCAAAGAAGTAGCTAAAAGAAAAAGTAACTGTG G

203	26.6	2.9	6.1	chr1:93166529-93168508	235	302	(1678)	+	MIR3	SINE/MIR	40	105	(103)	6
397	24.9	10.2	9.6	chr1:93166529-93168508	323	518	(1462)	C	MIRb	SINE/MIR	(31)	237	41	7
49	0.0	0.0	0.0	chr1:93166529-93168508	1471	1515	(465)	+	(ATAAA)n	Simple_repeat	1	45	(0)	8
2419	8.7	0.0	0.0	chr1:93166529-93168508	1554	1852	(128)	+	AluSq	SINE/Alu	2	300	(13)	9

>KSI_AluYa5_64

>Scaffold15563-8994633-8994940
TGCgCATAAGACAAGGCATAAAAGTCCCCCATTGCTGCTAGTCCAACTCTGACTTCTCTTCCCAGGCAGCCTCTGTGGACAGTGTATGTTCCTTGGAACCTCCTTTCTCTGTATTTATATGTATATAC
GTGTACGTATATGTTATACATACAGATCTGTATGTATATGTTATACAAACATCTGTATGTATATA GATAAGCAGCatATGTTTAAAAATATAAATGTAAAATATTAATATAAATGATATAAAATATAA
ATTAACAACGTAAATACATTGCTTTTGGTTAGTTTTATTTATGATACATATGTTTCGGAAATCTTTCCATGTTATTGCATATGGGTGTATCTGAATTTCTGGTCTGCCATTATATGGATATATGG CAA
ATTATTTAATCTTTCCTCCACTAAACAGTACATTTGGGGAGGGGGTTTGCTATTACAAGCAATGTTCCAGAGAATATCTTGTAATTATAAATATTTTC ATAAATGTAGAAGTAAGTTGTATTTAAAAA
TAATAGATACCTAGAAGCAGAAGTGTTGCTTAAAGTACATGAACtTTTTTTTTTTTTTTTTTTTTTTTTTGAGATGGAGTCTTGCTCTGTCTCCCAGGCTAGAGTGTAATGGTGCGATCTCAGCTCA
CTGCAACCTCCGACTCCCAGGTTCAAGTGACTCTCCTGCCTTAGCCTCCCAAGTAGCTCGGATCACAGGCACCCACCACCACACCCGGCTAGTTTTTG TATTTTTAGTAGAGACAGGGTTTCACCATG
TTGGTCGGGCTGGTCTTTAACTCCTGACCTCAAGCAATCCACCCGCCTTGGCGTCCCAAAATGCTGGGATTACAGG cGTGAGCGACCAcTCCCGGCGATGCATTTTtGAATGTAGTAAAAACTGCTAG
ACTGTCTCCATTTAGACCCCATGGACCACATTCACTCTAACAATGCATGTCAAGGCCAGTTCTTCTGAATCCTTCCCTGCATTCATTCTTTAGTCTTTAATTTCTTTTTTTTTTTttttttttttttt
ttttagacggggtctcgctctgtcgcccaggctggagtgcagtggcgggatctcggctcactgcaagctccgcctcccgggttcacgccattctcctgcctcagcctcccaagtagctgagacta aag
gcgcccgccactacgcccggctaatttttgtatttttagtagagacggggtttcaccgttttagccgggatggtctcgatctcctgacctcgtgatc cgcccgcctcggcctcccaaagtgctggga
ttacaggcgtgagccaccgcgcccggccaatttctttttttttttAATCAGGGGGAAAAATCTCTCTTTTTTGACATTTCTCATAATTGCTTGATACATTTAGTAGCTTTTCATT CCTTATTCTGTGA
GTGTCCTGTTTATATTTCTTTACCCTTTTTCCTACTAGGCATTTTTTTTTTCTTTTCAAGTTAATTTATATAAGTTCTTTCTCATAGAAATCAACCTT TTGAGTAAGATACATATATTTTCACCCAAA
CTGTCATTTGTTTTTAACTTCATTAATTATTTCTTTCTAAAGGAAGATTTTAATTTTATAAGGTAAATTAATTATCATTTTCCTTTCCTGTTTTACAGTTTTGTGTCTGACTTATCTCTTTAATC CAC
GAGATTAATTTTTATTCATTATGTGTAATAGATTGTGTTAGGGAACACAGCTGCTGA GATATATATAATGAGTCCAACAGAATAGAATAGAAGTGCTT CACTCAAGAAATACCCCGGGTGGTTCAGGC
CAATGGTGACTAGGGGTGGAAGGTGGGCTGCTTGGCTTCAGAGTGCTTTGGGGGTCCAGGCAACTCTAGCTCTGCCATTTGCCATTTTAGGTTTGCAAAGCTTGCCAGAGGGTCGTTTTTTATTG CAG
TTCACGTGAGAGAAAAGATGAGGGGGAAGCACGTGTGTGAGTTTTTCTGGGTTGTGTCTGGAAGTGGTACCCATTACCTCTCATGCAGGATTCCCTAA AATCAGCCACAGGTCACATGGCATTCCCCA
GAAGAAATGGATTTAGAAACAGCTGGCACCCTCCGCCTCAGACTATGGATTTATTTTGCTTCTGTAAATGGCTATCTCATCTTCCTGTGATCACGTATTAAGCGGGCCAGCTTTTGACTGGGAAT ATC
ACCTTTACCGTAAGTTACACTGACATATACAAGATCTGTTAGGAACTCTCCTTCTGTCAACC gATTTGTTTATTCTGGCATGAATTCCATGaGGTGTTACTTCAGGTACTTTGTGAGAGTGACACACC
TTAT

20	23.6	3.7	3.7	AluYa5-Scaffold15563-8994633-8994940	112	192	(2116)	+	(TATGTA)n	Simple_repeat	1	81	(0)	1
22	7.1	4.2	4.2	AluYa5-Scaffold15563-8994633-8994940	213	260	(2048)	+	A-rich	Low_complexity	1	48	(0)	2
386	29.8	8.8	7.3	AluYa5-Scaffold15563-8994633-8994940	304	556	(1752)	C	L1ME3G	LINE/L1	(312)	5865	5606	3
2094	12.5	0.0	0.0	AluYa5-Scaffold15563-8994633-8994940	557	864	(1444)	C	AluSx1	SINE/Alu	(0)	312	2	4
305	30.4	11.9	7.9	AluYa5-Scaffold15563-8994633-8994940	865	1000	(1308)	C	L1ME3G	LINE/L1	(645)	5605	5472	3
2637	1.1	0.0	0.0	AluYa5-Scaffold15563-8994633-8994940	1001	1308	(1000)	C	AluYa5	SINE/Alu	(0)	311	1	5
305	30.4	11.9	7.9	AluYa5-Scaffold15563-8994633-8994940	1309	1488	(820)	C	L1ME3G	LINE/L1	(779)	5471	5274	3
242	32.4	5.6	4.3	AluYa5-Scaffold15563-8994633-8994940	1805	2036	(272)	C	MLT1J1	LTR/ERVL-MaLR	(114)	330	96	6

>hg19 chr3:176141978-176144029
TGCaCATAAGACAAGGCATAAAAGTCCCCCATTGCTGCTAGTCCAACTCTGACTTCTCTTCCCAGGCAGCCTCTGTGGACAGTGTATGTTCCTTGGAACCT CCTTTCTCTGTATTTATATGTATATAC
GTGTACGTATATGTTATACATACAGATCTGTATGTATATGTTATACAAACATCTGTATGTATATA GatagATAAGCAGCATGTTTAAAAATATAAATGTAAAATATTAATATAAATGATATAAAATAT
AAATTAACAACGTAAATACATTGCTTTTGGTTAGTTTTATTTATGATACATATGTTTCGGAAATCTTTCCATGTTATTGCATATGGGTGTATCTGAATTTC TGGTCTGCCATTATATGGATATATGGC
AAATTATTTAATCTTTCCTCCACTAAACAGTACATTTGGGGAGGGGGTTTGCTATTACAAGCAATGTTCCAGAGAATATCTTGTAATTATAAATATTTTCATAAATGTAGAAGTAAGTTGTATTT AAA
AATAATAGATACCTAGAAGCAGAAGTGTTGCTTAAAGTACATGAACTTTTTTTTTTTTTTTTTTTTTTTTTGAGATGGAGTCTTGCTCTGTCTCCCAGGC TAGAGTGTAATGGTGCGATCTCAGCTC
ACTGCAACCTCCGACTCCCAGGTTCAAGTGACTCTCCTGCCTTAGCCTCCCAAGTAGCTCGGATCACAGGCACCCACCACCACACCCGGCTAGTTTTTGTATTTTTAGTAGAGACAGGGTTTCAC CAT
GTTGGTCGGGCTGGTCTTTAACTCCTGACCTCAAGCAATCCACCCGCCTTGGCGTCCCAAAATGCTGGGATTACAGG aGTGAGCGACCAaTCCCGGCGATGCATTTTaGAATGTAGTAAAAACTGCTA
GACTGTCTCCATTTAGACCCCATGGACCACATTCACTCTAACAATGCATGTCAAGGCCAGTTCTTCTGAATCCTTCCCTGCATTCATTCTTTAGTCTTTAATTTCTTTTTTTTTTTAATCAGGGGGAAA
AAATCTCTCTTTTTTGACATTTCTCATAATTGCTTGATACATTTAGTAGCTTTTCATT CCTTATTCTGTGAGTGTCCTGTTTATATTTCTTTACCCTTTTTCCTACTAGGCATTTTTTTTTTCTTTTC
AAGTTAATTTATATAAGTTCTTTCTCATAGAAATCAACCTTTTGAGTAAGATACATATATTTTCACCCAAACTGTCATTTGTTTTTAACTTCATTAATTATTTCTTTCTAAAGGAAGATTTTAAT TTT
ATAAGGTAAATTAATTATCATTTTCCTTTCCTGTTTTACAGTTTTGTGTCTGACTTATCTCTTTAATCCACGAGATTAA TTTTTATTCATTATGTGTAATAGATTGTGTTAGGGAACACAGCTGCTGA
GgcaattagattaaatatatatatatatatatatatatatatatatatatatatATATATATAATGAGTCCAACAGAATAGAATAGAAGTGCTTCACTCAAGAAATACCCCGGGTGGTTCAGGC
CAATGGTGACTAGGGGTGGAAGGTGGGCTGCTTGGCTTCAGAGTGCTTTGGGGGTCCAGGCAACTCTAGCTCTGCCATT TGCCATTTTAGGTTTGCAAAGCTTGCCAGAGGGTCGTTTTTTATTGCAG

TTCACGTGAGAGAAAAGATGAGGGGGAAGCACGTGTGTGAGTTTTTCTGGGTTGTGTCTGGAAGTGGTACCCATTACCTCTCATGCAGGATTCCCTAAAATCAGCCACAGGTCACATGGCATTCC CCA
GAAGAAATGGATTTAGAAACAGCTGGCACCCTCCGCCTCAGACTATGGATTTATTTTGCTTCTGTAAATGGCTATCTCA TCTTCCTGTGATCACGTATTAAGCGGGCCAGCTTTTGACTGGGAATATC
ACCTTTACCGTAAGTTACACTGACATATACAAGATCTGTTAGGAACTCTCCTTCTGTCAACC cATTTGTTTATTCTGGCATGAATTCCATGgGGTGTTACTTCAGGTACTTTGTGAGAGTGACACACC
TTAT

20	23.6	3.7	3.7	chr3:176141978-176144029	112	192	(1860)	+	(TATGTA)n	Simple_repeat	1	81	(0)	7
22	7.1	4.2	4.2	chr3:176141978-176144029	215	262	(1790)	+	A-rich	Low_complexity	1	48	(0)	8
372	30.5	11.2	7.1	chr3:176141978-176144029	274	558	(1494)	C	L1ME3G	LINE/L1	(254)	5891	5606	9
2042	13.2	0.0	0.0	chr3:176141978-176144029	559	865	(1187)	C	AluSx1	SINE/Alu	(0)	312	2	10
372	30.5	11.2	7.1	chr3:176141978-176144029	866	1175	(877)	C	L1ME3G	LINE/L1	(536)	5609	5273	9
62	0.0	0.0	0.0	chr3:176141978-176144029	1423	1475	(577)	+	(AT)n	Simple_repeat	1	53	(0)	11
242	32.4	5.6	4.3	chr3:176141978-176144029	1549	1780	(272)	C	MLT1J1	LTR/ERVL-MaLR	(114)	330	96	12

>KSI_AluYa5_65

>Scaffold16034-7683297-7683428
TAAGCATTTAGATCTCCTTCCAAATGGATTGGTTATAGATGTCTGTGAGGCACACACATTCAAGTCTTTCTGTTACTTCAATTTGTGTAGTCATAGGTGATGGCTTTTCAACATAGTCAGAGGTAA GA
TTTAATGTCAAATTGGTCTTAGTGGGGACAAATTGGAGCTTAGTTCCTCCCACCTGTAGGTGTGGTGGCAAATCTAACAGTTAGGTTGTTCCTTGAGGTGATAATCCTGGAAAAATTGATGAGTG AGT
TTTCTTCCCATTGGGCCCTAGTAGCAACGAGTATGTGGAGAAGGAGGAAGAGGAAGTTATTTCTCATTGTGATCCTGCCTTAAACCTAGCAAGGACAGAAAGAAAAGGTCCTTACCAG cGGGAGGGGC
TGTCCAGATGGTAGAACAAGAAAACGGTAATTAGGGTGAAGAGAAAAATTAGTTTTCCTATGCTTACTCACAGCATTATGTGTTTGCATCTGGCTATCGGTTCTTGTAAACCTGTAGTGGAGGCC TTC
CACTCATTCACGGGAGGAAAGGGGGCTTTCTGGATGAGGCTAGAAACATTCTGGTGAAAGCCTTTTAATTGGGAGAGGCTGGAGATCCCTGGTAGCTTGACTGCAGTTGGAGTAGCCAGGATCACC CA
GTCCTAGGGCCCTTTCACTTAGGCAACCATTGATCCTCTGGGGAGCCTTCTTTCCAAATCTTAAGAAGGACTTGGTCTCCTG GTTGACCTTTAGAGGAGATTGTCCCTCTTTTATAGAAGTTGATGGT
ACTTTATTCCCAAAATGTAATATTGCTTTCTGAATCTGATCCACATTGATAATACCATATGTTTTGACTCATTTCTCAGTCCAGTAAAATGT CAGTAGTGAGGAGGAGCCTCCTGTAATCCATTTCAA
ATGGACTGGGTTTTAAGTTGCTCTTTGGGGCAGTGTGAATCTGAAAGAGTGCAATAGGAAATAGCCTTATCCAGGTTTGCTATGTTTCT Tgaaataactttgccggccgggcgcggtggctcacgcct
gtaatcccagcactttgggaggccgaggcgggcggatcacgaggtcaggagatcgagaccatcccggctaaaacggtgaaaccccgtc tctactaaaaatacaaaaaaATTAGCCGGGCGTAGTGGCG
GGCGCCTGTAGTCCCAGCTACTTGGGAGGCTGAGGCAGGAGAATGGCGTGAACCCGGGAGGCGGAGCTTGCAGTGAGCCGAGATCCCGCCACTGCACTCCAGCCTGGGCGACAGAGCGAGACTCC GTC
TCAAAAAAAAAAAAAAAAAAAAAAAAAAAAAAAAAAAAAAAAAAAAaaaaaaaaaa GAAATAACTTTGCCAAAGTTCTTTTAAGGGTATGATTCATCCTTTCTACC TTTCCTCAGGATTGGGGATG
CCAACAGTCATGTATTTATAATCTATTCCCAGGACTTCCAAAAAGCTTTGAGTGATTTTAGCTATGAAACAGGGTCTATTGTTTCCCTGGAGTGACTTTGGAAGACCGAATTGAGGAATTACTTCTTT
TAGACTTTCGCAAGTATCAGTGGCCTTTTCAGACTGGGTTGGATATGCTTTGACCCAGCCAGTAAAAGTGTCCATGAAAATGAGAAGGTATTTCTATCCCAAAAGAGA GGGCACCTGGGTAATATTTC
TTTGCTAGTCTTCCACAGGGTAGGTTCCCTgGTGTTGGACTGGCTTGACTAGGGCAAGAGGAACCAGTTGAGTCTGGGGATTGTTTTGAATACGTAATTTGCAGGCCATGGTTACTTGACTTACAGTC
TTTCTTGGCCCCTTTCCAGAAGAGGTATTTTGTACTAATTCCCAAAAGATATCCCTCCTATAGTGAGTAACTTCATGTAGGTACTAAATAAAT aTTCACTGCCAAGTGCGGGGATAAGGACTTTACTG
TCCTTAACCAACCACCTGGCACTCCTCTTTTCCTATTCCCACTTCTCAGTGTCATCCTGTTCTTGTTGGGAGTACTGAGGTTAGCTGGGAAGTCCCAAAGGGTTGATTATTAGTACCATTACCTG GTC
ATCTTTCTGTGCTTAAGCTGCTTATTTTGTGGTCCTATCAGCTAATTTCCTTGGCTAATAAGCAATCTGTCCCTCCGATGGCCTTTGCAGTGAATGACTTCCACCTGG CCAGGGAATTGTACAGCTTC
CAGAAGAGCTAAGtCTAGATCCCTATGCTTTATTGGGAAATTTTTAGCTGTTAGCATACCC C

625	32.1	5.2	3.9	AluYa5-Scaffold16034-7683297-7683428	333	999	(1133)	C	PRIMA41-int	LTR/ERV1	(1673)	5003	4608	1
1265	0.0	0.0	0.0	AluYa5-Scaffold16034-7683297-7683428	1001	1132	(1000)	+	AluYa5	SINE/Alu	1	132	(179)	2
41	2.2	0.0	0.0	AluYa5-Scaffold16034-7683297-7683428	1189	1235	(897)	+	(A)n	Simple_repeat	1	47	(0)	3
925	33.9	2.6	1.8	AluYa5-Scaffold16034-7683297-7683428	1236	1688	(444)	C	PRIMA41-int	LTR/ERV1	(2027)	5729	5273	1
276	26.6	0.0	0.0	AluYa5-Scaffold16034-7683297-7683428	2019	2082	(50)	C	HERVS71-int	LTR/ERV1	(3238)	5740	5677	4

>hg19 chr11:106169481-106171367
TAAGCATTTAGATCTCCTTCCAAATGGATTGGTTATAGATGTCTGTGAGGCACACACATTCAAGTCTTTCTGTTACTTCAATTTGTGTAGTCATAGGTGATGGCTTTTCAACATAGTCAGAGGTAA GA
TTTAATGTCAAATTGGTCTTAGTGGGGACAAATTGGAGCTTAGTTCCTCCCACCTGTAGGTGTGGTGGCAAATCTAACAGTTAGGTTGTTCCTTGAGGTGATAATCCTGGAAAAATTGATGAGTG AGT
TTTCTTCCCATTGGGCCCTAGTAGCAACGAGTATGTGGAGAAGGAGGAAGAGGAAGTTATTTCTCATTGTGATCCTGCCTTAAACCTAGCAAGGACAGAAAGAAAAGGTCCTTACCAG tGGGAGGGGC
TGTCCAGATGGTAGAACAAGAAAACGGTAATTAGGGTGAAGAGAAAAATTAGTTTTCCTATGCTTACTCACAGCATTATGTGTTTGCATCTGGCTATCGGTTCTTGTAAACCTGTAGTGGAGGCC TTC
CACTCATTCACGGGAGGAAAGGGGGCTTTCTGGATGAGGCTAGAAACATTCTGGTGAAAGCCTTTTAATTGGGAGAGGCTGGAGATCCCTGGTAGCTTGACTGCAGTTGGAGTAGCCAGGATCACC CA
GTCCTAGGGCCCTTTCACTTAGGCAACCATTGATCCTCTGGGGAGCCTTCTTTCCAAATCTTAAGAAGGACTTGGTCTCCTG GgTTGACCTTTAGAGGAGATTGTCCCTCTTTTATAGAAGTTGATGG
TACTTTATTCCCAAAATGTAATATTGCTTTCTGAATCTGATCCACATTGATAATACCATATGTTTTGACTCATTTCTCAGTCCAGTAAAATGT CAGTAGTGAGGAGGAGCCTCCTGTAATCCATTTCA
AATGGACTGGGTTTTAAGTTGCTCTTTGGGGCAGTGTGAATCTGAAAGAGTGCAATAGGAAATAGCCTTATCCAGGTTTGCTATGTTTCT TGAAATAACTTTGCCAAAGTTCTTTTAAGGGTATGATT
CATCCTTTCTACCTTTCCTCAGGATTGGGGATGCCAACAGTCATGTATTTATAATCTATTCCCAGGACTTCCAAAAAGCTTTGAGTGATTTTAGCTATGAAACAGGGTCTATTGTTTCCCTGGAGTGA
CTTTGGAAGACCGAATTGAGGAATTACTTCTTTTAGACTTTCGCAAGTATCAGTGGCCTTTTCAGACTGGGTTGGATATGCTTTGACCCAGCCAGTAAAAGTGTCCATGAAAATGAGAAGGTATT TCT
ATCCCAAAAGAGAGGGCACCTGGGTAATATTTCTTTGCTAGTCTTCCACAGGGTAGGTTCCCT aGTGTTGGACTGGCTTGACTAGGGCAAGAGGAACCAGTTGAGTCTGGGGATTGTTTTGAATACGT
AATTTGCAGGCCATGGTTACTTGACTTACAGTCTTTCTTGGCCCCTTTCCAGAAGAGGTATTTTGTACTAATTCCCAAAAGATATCCCTCCTATAGTGAGTAACTTCATGTAGGTACTAAATAAA TtT
TCACTGCCAAGTGCGGGGATAAGGACTTTACTGTCCTTAACCAACCACCTGGCACTCCTCTTTTCCTATTCCCACTTCTCAGTGTCATCCTGTTCTTGTTGGGAGTACTGAGGTTAGCTGGGAAGT CC
CAAAGGGTTGATTATTAGTACCATTACCTGGTCATCTTTCTGTGCTTAAGCTGCTTATTTTGTGGTCCTATCAGCTAATTTCCTTGGCTAATAAGCAATCTGTCCCTCCGATGGCCTTTGCAGTG AAT
GACTTCCACCTGGCCAGGGAATTGTACAGCTTCCAGAAGAGCTAAG aCTAGATCCCTATGCTTTATTGGGAAATTTTTAGCTGTTAGCATACCC C

1438	33.0	4.2	3.1	chr11:106169481-106171367	333	1443	(444)	C	PRIMA41-int	LTR/ERV1	(1636)	6120	4608	5

278	27.9	0.0	0.0	chr11:106169481-106171367	1774	1841	(46)	C	HERVS71-int	LTR/ERV1	(3238)	5740	5673	6

>KSI_AluYa5_66

>Scaffold16052-128293-128440

AATTAATGTAAAATAAGATATGAAAGGAAAGTGAAAGAAACATAGAACAAGAGGAACAATTATAAAGCAGAAAATAAGATGCTGAAAATAACCCAAATATTTTAGTAATTACAAAAATATAACTAT GC
TAAAAACTCCATTTAAGAGGCCAAATTGCTAGACTGGAATTTTTAGATCTAGCTCTATGCTGTTGATAAGAGACACATCTAAAATATAACAAGACAATAACATTTAAAATATAAAGAAGGAAAAA AAT
AACATACAAATACTAACAAAGTTCAAATAAGTATATTAAGGTAAAAATTTCTAGGCTTACATAATGATAAAAAAGTCAATTGATCAAGAACATACAATCTATTTCTACCCACCAGCACAAAATAAT AT
GACCTTAATAATATGAAGCAAGAATTGACAGAATTATAAAAAGAAAATAGAAAAAAATCAACAAATTTCCAAATATTGATTCATACAGTCAATATTCTTCCCTACAATGCAATTAAGTAGGAAAT GAG
TAATTTAAGGAAAAAAAATTAAATGTATATATTTAAAGTATTTGAAATATTCTTTTAGTTGTACATTGTTCAAAGGAGAAATCATAATGGCCTTTAGAAAATACTTTAGAAAA CCAATGAACTAAGCA
TCTAACTTCAGAAGTTAGAAAGAAAACAACAGGCTGGGCACGGTAGCTCACATCTGTAATCCCAGCACTTTGGGAGGCTGAAGCAGGCGGATCATGAGGTCAGGAGATCGAGACCATCCTGGTTAACA
CGGTGGAACATCATCTCTGCTAAAAATACAAAAAAATTAGCCGGACGTGGTGGTGGACGCCTGTAGTCCCAGCTACTCGGCAGGCTGAGGCAGGATAATGGAGTGA Accgggaggtggagcttgcag
tgagccaagactgtgccactgcactccagcctgggcgacagagtgagaccctgtctcaaa AAAAAAAAAAAAAAAAAGGCCGGGCGCGGTGGCTCACGCCTGTAATCCCAGCACTTTGGGAGGCCGAGG
CGGGCGGATCACGAGGTCAGGAGATCGAGACCATCCCAGCTAAAACGGTGAAACCCCGTCTCTACTAAAAATACAAAAAATTAGCCGGGCGTAGTGGCGGG cgcctgtagtcccagctacttgggagg
ctgaggcaggagaatggcgtgaacCCGGGAGGcGGAGCTTGCAGTGAGCCgAGAtcccGCCACTGCACTCCAGCCTGGGCGACAGAGcGAGACtCcGTCTCAAAAAAAAAAAAAAAAAAAAACCAGAAT
AAAGCCATAATAAGATTTATAAAAAGACAAATATAAGGGACTAC TGGCAGAAAACATGCTATGATAGCTCTCAAGATTTCTGCATCCTTGAGCACACAACCAGCATAATCTCTTTCTGTTGACTGTGG
ATGCACCTGACTTAATTAGGTGAGCCCTTTAAAAGAGGGTTTAGAGGTCAGAGACAGAGG TCTGAGAAATTCAAAGGTTCAGCAGGTGCTCTTCTTCTGTCCTGGAGGTATGCAAATAGCCATGTTGT
AAACTGCCTGCTGGGGCCACATGGCAAAGAATTGTGTTTAGCTGTAGAAGTTAAGGAGCCAACAAGCAAATGGGGAATTCAGCCATATAACTATAAGGAAATGAATTCTGCCAAAACCAGTGAGC TTG
GAAGAAGAGCTCATGCCTTATCATAGCCCCAGCCCCACCTTGAGAAGAGCATACAGCTAA CCATACCCAGACATAAAACAATAAATCAGTTTTGTTTCAGATAGTCAGTTTGCAGCCATTTGTCATAC
AGCAATAAAAAATTAATAGAGTTGATTTAATGTTTTAGAAAACAAATACACAATATAGAGAATCATCAAAGACAAAAGCTGGTTCCTTAAAAACTCAAATAAAATAGATCAAACTTTGGAAAGAT AAA
GTAAGAATAAAATAAAGAAGGTGAAAGTGAATAATATTAAGAATAAAAAAGTAGATCTAA CTGTGCATGCTTCAGTTATTTGAAAGATAATAAaAGTTTATGACAACACATTTTAAAACTTAGATGAA
ATAGTCTTCAAAAATATAACTTACCAAAACTGACTTGAAAAGAAATATAGGATCAGAATTTAAATCTTCCCATAATGAAAACAACAGGCCCACAGAAAAAAAAAA AaCAGGTGAGTTCTGCCAAATAT
TGAAGGAACAAATAATTTCCATCCTGCAAAAATTATTCTTAAGTATAAGTAAAGAGAACA TTCCCCTCAATTTATTTTAAGAGACTAGCAAAAGCAT

640	24.5	4.8	4.5	AluYa5-Scaffold16052-128293-128440	1	295	(1853)	+	L1MEd	LINE/L1	2210	2516	(3805)	1	
298	21.4	5.9	7.5	AluYa5-Scaffold16052-128293-128440	313	448	(1700)	+	L1MEd	LINE/L1	2572	2705	(3616)	1	
272	26.7	5.6	3.3	AluYa5-Scaffold16052-128293-128440	442	619	(1529)	+	L1MEd	LINE/L1	2925	3106	(3215)	1	*
2147	10.2	0.3	0.7	AluYa5-Scaffold16052-128293-128440	672	956	(1192)	+	AluY	SINE/Alu	1	284	(27)	2	
1416	0.0	0.0	0.0	AluYa5-Scaffold16052-128293-128440	1001	1148	(1000)	+	AluYa5	SINE/Alu	154	301	(10)	3	
699	24.4	13.5	7.2	AluYa5-Scaffold16052-128293-128440	1281	1685	(463)	+	MLT1E1A	LTR/ERVL-MaLR	221	651	(2)	4	
588	25.0	5.2	1.9	AluYa5-Scaffold16052-128293-128440	1703	2144	(4)	+	L1MEd	LINE/L1	3333	3827	(2494)	1	

>chr13:36715848-36717824

AATTAATGTAAAATAAGATATGAAAGGAAAGTGAAAGAAACATAGAACAAGAGGAACAATTATAAAGCAGAAAATAAGATGCTGAAAATAACCCAAATATTTTAGTAATTACAAAAATATAACTAT GC
TAAAAACTCCATTTAAGAGGCCAAATTGCTAGACTGGAATTTTTAGATCTAGCTCTATGCTGTTGATAAGAGACACATCTAAAATA TAACAAGACAATAACATTTAAAATATAAAGAAGGAAAAAAAT
AACATACAAATACTAACAAAGTTCAAATAAGTATATTAAGGTAAAAATTTCTAGGCTTACATAATGATAAAAAAGTCAATTGATCAAGAACATACAATCTATTTCTACCCACCAGCACAAAATAA TAT
GACCTTAATAATATGAAGCAAGAATTGACAGAATTATAAAAAGAAAATAGAAAAAAATCAACAAATTTCCAAATATTGATTCATAC AGTCAATATTCTTCCCTACAATGCAATTAAGTAGGAAATGAG
TAATTTAAGGAAAAAAAATTAAATGTATATATTTAAAGTATTTGAAATATTCTTTTAGTTGTACATTGTTCAAAGGAGAAATCATAATGGCCTTTAGAAAATACTTTAGAAAA CCAATGAACTAAGCA
TCTAACTTCAGAAGTTAGAAAGAAAACAACAGGCTGGGCACGGTAGCTCACATCTGTAATCCCAGCACTTTGGGAGGCTGAAGCAG GCGGATCATGAGGTCAGGAGATCGAGACCATCCTGGTTAACA
CGGTGGAACATCATCTCTGCTAAAAATACAAAAAAATTAGCCGGACGTGGTGGTGGACGCCTGTAGTCCCAGCTACTCGGCAGGCTGAGGCAGGATAATGGAGTGA ACCGGGAGGtGGAGCTTGCAG
TGAGCCaAGActgtGCCACTGCACTCCAGCCTGGGCGACAGAGtGAGACcCtGTCTCAAAAAAAAAAAAAAAAAAAAAaaaacCCAGAATAAAGCCATAATAAGATTTATAAAAAGACAAATATAAGGG
ACTACTGGCAGAAAACATGCTATGATAGCTCTCAAGATTTCTGCATCCTTGAGCACACAACCAGCATAATCTCTTTCTGTTGACTGTGGATGCACCTGACTTAATTAGGTGAGCCCTTTAAAAGA GGG
TTTAGAGGTCAGAGACAGAGGTCTGAGAAATTCAAAGGTTCAGCAGGTGCTCTTCTTCTGTCCTGGAGGTATGCAAATAGCCATGT TGTAAACTGCCTGCTGGGGCCACATGGCAAAGAATTGTGTTT
AGCTGTAGAAGTTAAGGAGCCAACAAGCAAATGGGGAATTCAGCCATATAACTATAAGGAAATGAATTCTGCCAAAACCAGTGAGCTTGGAAGAAGA GCTCATGCCTTATCATAGCCCCAGCCCCACC
TTGAGAAGAGCATACAGCTAACCATACCCAGACATAAAACAATAAATCAGTTTTGTTTCAGATAGTCAGTTTGCAGCCATTTGTCA TACAGCAATAAAAAATTAATAGAGTTGATTTAATGTTTTAGA
AAACAAATACACAATATAGAGAATCATCAAAGACAAAAGCTGGTTCCTTAAAAACTCAAATAAAATAGATCAAACTTTGGAAAGATAAAGTAAGAATAAAATAAAGAAGGTGAAAGTGAATAATA TTA
AGAATAAAAAAGTAGATCTAACTGTGCATGCTTCAGTTATTTGAAAGATAATAA gAGTTTATGACAACACATTTTAAAACTTAGAT GAAATAGTCTTCAAAAATATAACTTACCAAAACTGACTTGAA
AAGAAATATAGGATCAGAATTTAAATCTTCCCATAATGAAAACAACAGGCCCACAGAAAAAAAAAA ACAGGTGAGTTCTGCCAAATATTGAAGGAACAAATAATTTCCATCCTGCAAAAATTATTCTT

AAGTATAAGTAAAGAGAACATTCCCCTCAATTTATTTTAAGAGACTAGCAAAAGCAT

640	24.5	4.8	4.5	chr13:36715848-36717824	1	295	(1682)	+	L1MEd	LINE/L1	2210	2516	(3805)	5
298	21.4	5.9	7.5	chr13:36715848-36717824	313	448	(1529)	+	L1MEd	LINE/L1	2572	2705	(3616)	5
272	26.7	5.6	3.3	chr13:36715848-36717824	442	619	(1358)	+	L1MEd	LINE/L1	2925	3106	(3215)	5 *
2336	9.5	0.3	0.7	chr13:36715848-36717824	672	977	(1000)	+	AluY	SINE/Alu	1	305	(6)	6
699	24.4	13.5	7.2	chr13:36715848-36717824	1111	1515	(462)	+	MLT1E1A	LTR/ERVL-MaLR	221	651	(2)	7
610	24.8	5.2	1.6	chr13:36715848-36717824	1533	1973	(4)	+	L1MEd	LINE/L1	3333	3827	(2494)	5

>KSI_AluYa5_67

>Scaffold16369-703225-703335
TAATAAAGACTATCAGTTCCTGAAATTATTAGAAAgGCTGGAGAAAAAGATGTCCAAGCTTCTGGTAAAAATGCTAACAACTTTGCCACAGGACTGGTTTGATGAGAAAACCTCCTGCATGGCCATGG
GTGCTGCCAAGTACTAAATGCCAAGAATTTGATTAAAACAACAGTAAAACGTATTTCCCTATCAGAAATCAGTCCTGCAATCATTGCTGTCCTTTCAACCTGTATGTTTTGGCAGGCAATATATG CCA
GCAAAATGAAGGCTGTTTTCACACACTGCTCATCTCCAGGCTAAGGTCTTGTGAAAGTTCCTAAAATTAGAAGAGCTAGTTCACAAGCCTGCATTCTGGTTG CAAGAGAAGCTGAGAAACTGCAGTAT
AATTTCAATGTCGTAGGACTATACACAGCATTTTATTTAATTCTTTTAAACAGAATGGACATTATTATCTATATTTTATAAATGAAGATATTGAAGTATATTAATTTTGATAAATTATAGGTTAT TTT
GATTTTTGATTTTATATAGATAATCAGGCCATCCACAAATAACGACATTTTTTTCTTTCTTCCTTTCCAACTATTTGACCCTTTATTTCCTTTTCTGATTTTAATAATCAGGCTAGAAACTCTAGTAT
AATGAGAATAAATGTAGTAATAGCAGCACTCTCTGAATTATTTCTGATTTTTTTTCATGTTCCAAAGCTTTCTTTTTTACTATTCTTATTATACTTTAAGTTCTAGGGTACATGTGCACAACGTG CAG
GTTTGTTACATAGGTATACATGTGCCATGTTGATGTGCTGCACCCGTTAACTCGTCATTTACATTAGGTATATCTcCTAATGCTAtccctccccacccctcccctcaccctacagcagtccccagtg
tgtgatgttccgcttcctatgtccaagtgttctcattgttcagttcccacctatgagtgagaacatgcggtgtttggtttttttgtccttgaaagaggtattttcgactacaggcgcccgccacta cgc
ccggctaatttttttgtatttttagtagagacggggtttcaccgttttagccgggatggtctcgatctcctgacctcgtgatccgcacattaggtatatcttctaatgctaCCCCTCCCCTCTCCCC
CAACCCCATGACAGGCCCTGGTGTGTGATGTTCCCCATCCTGTGTCCAAATGTTCTCATTGTTCAATTCCCACCTATGAGTGAGAACATGCGGTGTTTGTTTTTCTGTCCTTGCAATAGTTTGCTCAG
AATGATGGTTTCCAGCTTCATCCATGTCCCTACAAAGGAGATGAACTCATCCTTTTTTATGGCTGCATAGTATTCCATGGTGTATATGTGCCACATTTTCTTAATCCAGTCTATCATTGATGGACATT
TGGGTTAGTTCCAAGTCTTTGCTATTGTGAATTTTTTTTTTTTTTGAAACGGTGTTTTGCTTTTGTTGCCCAGGCTGGAGTGTAATGTCATGAACTCGGCTCACTGCAAACTCCACCTCCTGGGTTCA
AGCAATTCTTCTGCTTCAGCCTCCTGAGTAGCTGGGATTACAGGCACGCACCACCACTCCCGGCTAATTTTGTATTTTTAGTAAAGATGGGGTTTCTGCATGTTGGTCAGGCTGGTCTCGAACTCCCA
ACCTCAGGTGATCcGCCTGCCTCAGCCACAGAAGtGCTCGGATTACAGGCATGAGCCACCGCACCCGGCCTATTTCTGATTGTAAGATAAGTGCATTCAGTAATTTACAATTGAATA tGATGTTTCCT
GTAAATTATTTGTAAATAACCTTAATTTTTCATGTTAAGAAAGTTTCCTCTATTCCTTGTTTTCTAAGACTTTTAACAATGAATGGATGTTGAATGCTACCAATGCTATTTCTACATTTAATTAGATA
ATCATATATGTTTTATTTTAATATCTAAATATGATGAGTAATAATAACACACTTCTTTTTAAAAAATTCTTTTCAGAAAGAAGTCCAGTTTTCCTGAAGTAGTGTCTTGTCTTTAAGATAGTTAG GCT
CACTTTGCTGCTAATTTGATCATATTTTTGCATCTTTATATAAACAAGATTAGCCTGTGATAC

547	26.4	2.9	3.4	AluYa5-Scaffold16369-703225-703335	483	690	(1421)	C	L1M5	LINE/L1	(1871)	4275	4069	1
2130	4.8	0.0	2.2	AluYa5-Scaffold16369-703225-703335	713	986	(1125)	C	L1PA5	LINE/L1	(0)	6154	5887	2
1004	0.0	0.0	1.8	AluYa5-Scaffold16369-703225-703335	1001	1111	(1000)	C	AluYa5	SINE/Alu	(147)	164	56	3
2745	4.9	0.0	0.0	AluYa5-Scaffold16369-703225-703335	1112	1439	(672)	C	L1PA5	LINE/L1	(114)	6040	5713	4
2188	10.8	0.7	0.0	AluYa5-Scaffold16369-703225-703335	1440	1734	(377)	C	AluSp	SINE/Alu	(16)	297	1	5
2207	14.1	0.8	1.3	AluYa5-Scaffold16369-703225-703335	1735	1780	(331)	C	L1PA10	LINE/L1	(456)	5712	5667	6
723	27.7	4.8	1.6	AluYa5-Scaffold16369-703225-703335	1757	2109	(2)	C	L1M5	LINE/L1	(2088)	4058	3695	1 *

>chr12:55154738-55156566
TAATAAAGACTATCAGTTCCTGAAATTATTAGAAAaGCTGGAGAAAAAGATGTCCAAGCTTCTGGTAAAAATGCTAACAACTTTGCCACAGGACTGGTTTGATGAGAAAACCTCCTGCATGGCCATGG
GTGCTGCCAAGTACTAAATGCCAAGAATTTGATTAAAACAACAGTAAAACGTATTTCCCTATCAGAAATCAGTCCTGCAATCATTGCTGTCCTTTCAACCTGTATGTTTTGGCAGGCAATATATG CCA
GCAAAATGAAGGCTGTTTTCACACACTGCTCATCTCCAGGCTAAGGTCTTGTGAAAGTTCCTAAAATTAGAAGAGCTAGTTCACAAGCCTGCATTCTGGTTGCAAGAGAAGCTGAGAAACTGCAGTAT
AATTTCAATGTCGTAGGACTATACACAGCATTTTATTTAATTCTTTTAAACAGAATGGACATTATTATCTATATTTTATAAATGAAGATATTGAAGTATATTAATTTTGATAAATTATAGGTTAT TTT
GATTTTTGATTTTATATAGATAATCAGGCCATCCACAAATAACGACATTTTTTTCTTTCTTCCTTTCCAACTATTTGACCCTTTATTTCCTTTTCTGATTTTAATAATCAGGCTAGAAACTCTAGTAT
AATGAGAATAAATGTAGTAATAGCAGCACTCTCTGAATTATTTCTGATTTTTTTTCATGTTCCAAAGCTTTCTTTTTTACTATTCTTATTATACTTTAAGTTCTAGGGTACATGTGCACAACGTG CAG
GTTTGTTACATAGGTATACATGTGCCATGTTGATGTGCTGCACCCGTTAACTCGTCATTTACATTAGGTATATCTtCTAATGCTACCCCTCCCCTCTCCCCCAACCCCATGACAGGCCCTGGTGTGTG

ATGTTCCCCATCCTGTGTCCAAATGTTCTCATTGTTCAATTCCCACCTATGAGTGAGAACATGCGGTGTTTGTTTTTCTGTCCTTGCAATAGTTTGCTCAGAATGATGGTTTCCAGCTTCATCCATGT
CCCTACAAAGGAGATGAACTCATCCTTTTTTATGGC TGCATAGTATTCCATGGTGTATATGTGCCACATTTTCTTAATCCAGTCTATCATTGATGGACATTTGGGTTAGTTCCAAGTCTTTGCTATTG
TGAAtTTTTTTTTTTTTTTGAAACGGTGTTTTGCTTTTGTTGCCCAGGCTGGAGTGTAATGTCATGAACTCGGCTCACTGCAAACTCCACCTCCTGGGTTCAAGCAATTCTTCTGCTTCAGCCTCCTG
AGTAGCTGGGATTACAGGCACGCACCACCACTCCCG GCTAATTTTGTATTTTTAGTAAAGATGGGGTTTCTGCATGTTGGTCAGGCTGGTCTCGAACTCCCAACCTCAGGTGATC tGCCTGCCTCAGC
CACAGAAGaGCTCGGATTACAGGCATGAGCCACCGCACCCGGCCTATTTCTGATTGTAAGATAAGTGCATTCAGTAATTTACAATTGAATA cGATGTTTCCTGTAAATTATTTGTAAATAACCTTAAT
TTTTCATGTTAAGAAAGTTTCCTCTATTCCTTGTTT TCTAAGACTTTTAACAATGAATGGATGTTGAATGCTACCAATGCTATTTCTACATTTAATTAGATAATCATATATGTTTTATTTTAATATCT
AAATATGATGAGTAATAATAACACACTTCTTTTTAAAAAATTCTTTTCAGAAAGAAGTCCAGTTTTCCTGAAGTAGTGTCTTGTCTTTAAGATAGTTAGGCTCACTTTGCTGCTAATTTGATCAT ATT
TTTGCATCTTTATATAAACAAGATTAGCCTGTGATAC

547	26.4	2.9	3.4	chr12:55154738-55156566	483	690	(1139)	C	L1M5	LINE/L1	(1871)	4275	4069	7
3643	4.8	0.0	0.5	chr12:55154738-55156566	713	1156	(673)	C	L1PA5	LINE/L1	(0)	6154	5713	8
2155	11.5	0.7	0.0	chr12:55154738-55156566	1157	1452	(377)	C	AluSp	SINE/Alu	(15)	298	1	9
713	27.9	4.8	1.6	chr12:55154738-55156566	1475	1827	(2)	C	L1M5	LINE/L1	(2088)	4058	3695	7

>KSI_AluYa5_68

>Scaffold14800-161410-161709
TAGTTCTTCCCCTGCCCCTGAATGAAAACTTGGGCAGACCCCATAACATCTCTGGTCTCTTTTAGGTAACATAAAAGTTTCAATAGTCACTAGAGATCACAGCATCTCAATATAGCAATTGATGTG AT
CTTTTCTTTGTAATTATCAAATATCCAAACTTGCTTTAAAATTGAGGCTTCTAATTAGCATTCCTAAATCCTTTTCTTTCTCATCTCATAAACCCTTTTTGTGGCCCTTCAGGTAGAGAGGAGGT AGG
AAAAGAGTTCTCTTTCAATTGGCATTGTCATCCTTGTCTGGCACCTGTGCTCTCTTCTGGGTTCAATAGGTGTTTAAAAACAAGTCTATTTCTTGTAAATGCTGGTGATGTTTCACCTAAGACAAG TT
TCCTTATAGAGGACAGCATATCTAATTTATACTgAGTCATTTACTTATTTTTTTTAGCACCGTGTAACCCAGCAGTACCTTCAGATTGTTTTTACTGAGATCTATTTTGTACCTCTAGTTAGTTTTTT
GGACAGGGCCTAAGATCAATAAAGCCAGCTTTTTCCTCCTGGTTTTTGAAGGGAAATTCAT GCATTCTCTGAGGGTACAAGCCATGTGCAACTCAGGAAATTCTTCCTTGCTTTGCCATTAGAACAGC
TAGCCAGAGTAAATCATGATGTAGATTGCACAGGTATAAATAGACAACAGACCACTAGGTCCTCCAATTGCAAATGACACGTTAAGTACTC TGAGgtacctcactgaaatccttctgccaagatgaaa
gcccattttctccccagagcagtagagcgtcagttctctttagagaaatgctttctttcatatccttcaaattcaaatccttttgtaacctcaatatgaatgaggggtttgaactgtgaaattct ttg
taaatcatgcatatgttctagcatacccttttgggatgtacctgtggtcttttgtac agacaaaaattcttttctttgcatcctgttacatttatt tttataacattcttttttttttttttttgagac
ggagtctcgctctgtcgcccaggctggagtgcagtggcaggatctcggctcactgcaagctccgcctcccgggttcacgccattctcctgcctcagcctcccaagtagctgggactacaggcgcc cgc
cactacgcccggctaatttttttgtatttttagtagagacggggtttcaccgttttagccgggatggtctcgatctcctgacctcgtgatccgccc gcctcggcctcccaaagtgctgggattacaggc
gtgagCCACCGCGCCCGGCCAACATTCTTAATAAATCATAAAAGGCACTTAACACAAAGATGAAAACAACATATTCTATGACATAGGTTTTATCGAAAGTTGGTGGGACTACAACAAACTAAACAGTG
TCTGTACAGAAAAAGAAACaATCAATAAAAAGAAAACAGAATGGGGGGAAATATTTGCAAAACCATGTACTGAGTAAAAGCTTAATGTCCAAATA TATAAAGAACTCATGCAACTCAGTAGCAATAAA
GCCAAATAATCCTATTAAAAATGGGCAAAGAACCTGAATAGATATGTTTTTTCTAAAAAGTCTTACAAATGGCCAACAAGTATAGGAAGAAATGTTCAAAATCATGGATCATCAGAGAAATATAA ATA
TAAATCAGAAATATAAATATAAATCAAAGCCACAATGAGATCT CACCTCACACCCATTATGATGATTATTATAAAAAAGACAAGAGATAACAAGT GATGGCAAGAATGTGGAGAAATGACCTTTAATA
CTGTTGGTGGGAAGGTAAATTGTTAACAACTGTTTTGGAAAAAAGTATGGAAGTTCCTCCAAAAAATTAAAAATAGAACTACCATATGATCGAGCAATCCTACTTCAGGGTATATATCCAAAAGA AAT
GAAATTAGTGTCTCTAAAGGATATCTGTACCCCCAAGTTCACAATAGCCAAGATACAAAAATAACCTATGTATCCATCAATAGGTGAATGGATAA AGAAACTGTTGTGTGTTTGTGTGTGTATATGTG
TATGTGTGTGTGTATATACATATATTTGTATATGTAATATACACACACACATACATATATACA TATATATTATATACATACATATATAATATATACACATACATATATGTACATATACTATACACATA
TATAATGAAATATTATTCATATAAAAGAAGGAAATCCTGCCATTTTTGACAACATTGATGTAA tTGGAGGCAATTATGCTAAGTGAAATACACCA GACATAGAAAGACAAATACTGTATGATaT

744	22.5	2.8	1.1	AluYa5-Scaffold14800-161410-161709	1	180	(2120)	C	LTR78	LTR/ERV1	(98)	1206	1024	1
374	32.1	7.9	3.8	AluYa5-Scaffold14800-161410-161709	357	583	(1717)	C	LTR78	LTR/ERV1	(577)	727	492	2
287	26.6	21.3	0.6	AluYa5-Scaffold14800-161410-161709	799	934	(1366)	C	LTR78	LTR/ERV1	(1083)	221	58	2
2844	0.7	0.0	0.0	AluYa5-Scaffold14800-161410-161709	1001	1300	(1000)	C	AluYa5	SINE/Alu	(11)	300	1	3
2607	15.8	5.5	1.4	AluYa5-Scaffold14800-161410-161709	1383	1653	(647)	+	L1MA9	LINE/L1	5133	5416	(896)	4
27	3.1	0.0	2.9	AluYa5-Scaffold14800-161410-161709	1654	1688	(612)	+	(AAATAT)n	Simple_repeat	1	34	(0)	5
2607	15.3	6.1	1.1	AluYa5-Scaffold14800-161410-161709	1689	2031	(269)	+	L1MA9	LINE/L1	5417	5785	(527)	4
50	25.3	2.4	0.0	AluYa5-Scaffold14800-161410-161709	2041	2165	(135)	+	(TA)n	Simple_repeat	1	128	(0)	6
760	13.8	3.1	0.0	AluYa5-Scaffold14800-161410-161709	2169	2298	(2)	+	L1MA9	LINE/L1	5782	5915	(397)	4

>hg19 chr14:45013076-45014830
TAGTTCTTCCCCTGCCCCTGAATGAAAACTTGGGCAGACCCCATAACATCTCTGGTCTCTTTTAGGTAACATAAAAGTTTCAATAGTCACTAGAGATCACAGCATCTCAATATAGCAATTGATGTG AT
CTTTTCTTTGTAATTATCAAATATCCAAACTTGCTTTAAAATTGAGGCTTCTAATTAGCATTCCTAAATCCTTTTCTTTCTCATCTCATAAACCCTTTTTGTGGCCCTTCAGGTAGAGAGGAGGT AGG
AAAAGAGTTCTCTTTCAATTGGCATTGTCATCCTTGTCTGGCACCTGTGCTCTCTTCTGGGTTCAATAGGTGTTTAAAAACAAGTCTATTTCTTGTAAATGCTGGTGATGTTTCACCTAAGACAAG TT
TCCTTATAGAGGACAGCATATCTAATTTATACTaAGTCATTTACTTATTTTTTTTAGCACCGTGTAACCCAGCAGTACCTTCAGATTGTTTTTACTGAGATCTATTTTGTACCTCTAGTTAGTTTTTT
GGACAGGGCCTAAGATCAATAAAGCCAGCTTTTTCCTCCTGGTTTTTGAAGGGAAATTCATGCATTCTCTGAGGGTACAAGCCATGTGCAACTCAGGAAATTCTTCCTTGCTTTGCCATTAGAACA GC
TAGCCAGAGTAAATCATGATGTAGATTGCACAGGTATAAATAGACAACAGACCACTAGGTCCTCCAATTGCAAATGACACGTTAAGTACTCTGA GCCACCGCGCCCGGCCAACATTCTTAATAAATCA
TAAAAGGCACTTAACACAAAGATGAAAACAACATATTCTATGACATAGGTTTTATCGAAAGTTGGTGGGACTACAACAAACTAAACAGTGTCTGTACAGAAAAAGAAAC cATCAATAAAAAGAAAACA
GAATGGGGGGAAATATTTGCAAAACCATGTACTGAGTAAAAGCTTAATGTCCAAATATATAAAGAACTCATGCAACTCAGTAGCAATAAAGCCAAATAATCCTATTAAAAATGGGCAAAGAACCT GAA
TAGATATGTTTTTTCTAAAAAGTCTTACAAATGGCCAACAAGTATAGGAAGAAATGTTCAAAATCATGGATCATCAGAGAAATATAAATATAAATCAGAAATATAAATATAAATCAAAGCCACAAT GA
GATCTCACCTCACACCCATTATGATGATTATTATAAAAAAGACAAGAGATAACAAGTGATGGCAAGAATGTGGAGAAATGACCTTTAATACTGTTGGTGGGAAGGTAAATTGTTAACAACTGTTT TGG
AAAAAAGTATGGAAGTTCCTCCAAAAAATTAAAAATAGAACTACCATATGATCGAGCAATCCTACTTCAGGGTATATATCCAAAAGAAATGAAATTAGTGTCTCTAAAGGATATCTGTACCCCCAA GT
TCACAATAGCCAAGATACAAAAATAACCTATGTATCCATCAATAGGTGAATGGATAAAGAAACTGTTGTGTGTTTGTGTGTGTATATGTGTATGTGTGTGTGTATATACATATATTTGTATATGT AAT
ATACACACACACATACATATATACATataatATATATTATATACATACATATATAATATATACACATACATATATGTACATATACTATACACATATATAATGAAATATTATTCATATAAAAGAAGGAA

ATCCTGCCATTTTTGACAACATTGATGTAAcTGGAGGCAATTATGCTAAGTGAAATACACCAGACATAGAAAGACAAATACTGTATGATcT

744	22.5	2.8	1.1	chr14:45013076-45014830	1	180	(1575)	C	LTR78	LTR/ERV1	(98)	1206	1024	7
360	32.5	7.9	3.8	chr14:45013076-45014830	357	583	(1172)	C	LTR78	LTR/ERV1	(577)	727	492	8
2607	16.0	5.5	1.4	chr14:45013076-45014830	833	1103	(652)	+	L1MA9	LINE/L1	5133	5416	(896)	9
27	3.1	0.0	2.9	chr14:45013076-45014830	1104	1138	(617)	+	(AAATAT)n	Simple_repeat	1	34	(0)	10
2607	15.3	6.1	1.1	chr14:45013076-45014830	1139	1481	(274)	+	L1MA9	LINE/L1	5417	5785	(527)	9
53	24.0	3.1	0.0	chr14:45013076-45014830	1491	1620	(135)	+	(TA)n	Simple_repeat	1	134	(0)	11
800	12.9	3.0	0.0	chr14:45013076-45014830	1624	1755	(0)	+	L1MA9	LINE/L1	5782	5917	(395)	9

>KSI_AluYa5_69

>Scaffold1490-60189-60423
TCCTCTCCTCATTAACTATGACTCTTCTAATTTTTATTTATCCATCAACATTGCTATGATTTCATTGATGATCAAGACTCATTCTTGACTTCTGTCTTCTCCTTCATTCTCTATGTCTTATTCAGT AA
CCAAATCAATTTATTcTCCCTTTCATAACATTTTTCCTATCATTATTGCCAGAACCCTAGTTTAGGCCTTCACACCGTGTTTCATTAGCCTCCTAACCTGATTTCTCATGGACAGTTTCAATGCCAT
GTTGACTCATCATGCCCCTTCTCCACACCAGATTTTACAAAGGCTTCCTTTGCCAACCCTTATGGGAGAGCTTTACAGCCCTTTTAGTAAAAGGACTTGTCTTCAGCCAAACACATTCACTAACAT TA
AGGTTTCTTAATTTTTGTAGCTACTCTCACCACCATGCTTATACATGTGTGAAATTCTGGGCTCAGCAGTCCCCAGAAACCTTCTCAGATCACCCCCTTCCACCGAGTCCTCTTTCTTTCCTATG GGT
CtCTgTGCAGGCACATCAATCTTTGGTAACATCCTGCCCGGCATGACCATCATCCTGATTCATGTACATCTTATACCTTGTACCCTGTGTCTG GCCGCACAAAACTCCTGCTCCTGCTCTGAGTCACC
TACTGTTTCTCTGATACACTGTGGCATTCATTAAATTCAGTGGGATTAATGGGTGGAGTATTGTTTCTCAGGACACAGGAGTAGGTTGGGGTTGGCTTCCTTTAAATTAGTGCAGACTTAGGCTA TCT
TCCTTGATGCTCTTGATGTTGACAGACCAACTCTCTATTTCTCATTGACCCCTTTTCTAAGCAATGTGCTCAATTTTCCCAGCTCTCCTTAGTTCCTAATTTCTCTCAACA gTTCACTTCtCTTGGTT
CCCTTTTGGGACATGGTCTTGATTCACATCTTACTGAGCTCTCTGGGACACAGTGTAATAGGCTGTAGTCATAGACTCAGAAAAAGGGA Caagaaatcagaaaaggccgggcgcggtggctcacgcct
gtaatcccagcactttgggaggccgaggcgggcggatcacgaggtcaggagatcgagaccatcccggctaaaacggtgaaaccccgtctctactaaaaatacaaaaaattag ccgggcgtagtggcgg
gggggggtggggctgcctacttgggaggctgaggcaggagaatggcgtgaacccgggaggcggagcttgcagtgagccgagattgccccccgggggcacaaggggaggcccccccaaaaaaaa aaa
aaaaaaaaaaaaaaaaaaAAGAAATCAGAAAATTGTATTCCTTTCATAGCACTGGGGAAGTCACTTACCTTCTTTGACTCTCCATTGTCTTCCATGGTAAACTACAAATAG TAGTATTTCCCCTCTC
AGGACTGGTGTGTGATCCCATGAGATAATGAACATGGAAAACCTTTGCACAACATGAAGCAGTATGTGAGAGGTGTTATCAGCCC TCCTTCTCTGCACCTTCAGATATTGCTGTCTCTACCCACAAAG
TGCTTCCTTTACCTTTCATCTCACCTGCTGTCTCCATCTGACTCTGGCTGCAGCAACTTCAGTCTTTCCCTCTTCGTGCATTCTTTCCTCTCTATTGACAATCCTTT CCCTGCCTTATGCACCATCCT
CACTTTCCTAGCATGTAAACAACCTCTTACTTGATCCTTCTGTCTGTTCCACCTCCCACCAGCCACTGTCCTTTTCCCTTTCCTTTTGTAACCAGAGAGGACAACACGTAGTCCCTCCTGCTGGC TCA
TTACCTACTATTTCCATAGCTCCTGCATTTCTTCCCAATGACTCTCCCTAAGCCAGCAGATCTGTTTAAGTTCTCAGTTCTTATC CTCCTTAAACTCATTGTTTCGTCTTCTTTTCTGATTCAGTATC
ACCCCATCCTTTTACAGTGGGCTTTTCTCCAGGCCTAGTCTTCTTGCCATCTGCTCTACCCCCACACAGGGAACCATAATGCACTACATTCTCTTATTTTA CTCCATTCAGATTGAATCATACTGGGA
GTACTGTGTTTACTTCTAGCTTTCACCTTCAGTGAGAATTAT gACCAACTCAGGGATATTCAATAAGGAGCACCAAGATGGTAAGGGCACTAGAATGATCTCCTCTTATGGA ACACTTGATAGAATGG
ATGTATTTAACATACAGAAGAGAAAATCCAGAGAAAATGTACACTTTGCTGTCTTAA C

13	15.0	2.5	2.5	UnnamedSequence	975	1000	(1234)	+	A-rich	Low_complexity	1	26	(0)	1
2190	8.4	4.0	0.0	UnnamedSequence	1001	1312	(922)	+	AluYk2	SINE/Alu	1	311	(0)	2
318	28.7	2.9	2.7	UnnamedSequence	1334	1489	(745)	+	MIRb	SINE/MIR	96	263	(5)	3
351	39.5	2.3	2.3	UnnamedSequence	2012	2231	(3)	+	L3	LINE/CR1	3555	3774	(325)	4

>hg19 chr2:114105447-114107367
TCCTCTCCTCATTAACTATGACTCTTCTAATTTTTATTTATCCATCAACATTGCTATGATTTCATTGATGATCAAGACTCATTCTTGACTTCTGTCTTCTCCTTCATTCTCTATGTCTTATTCAGT AA
CCAAATCAATTTATTaTCCCTTTCATAACATTTTTCCTATCATTATTGCCAGAACCCTAGTTTAGGCCTTCACACCGTGTTTCATTAGCCTCCTAACCTGATTTCTCATGGACAGTTTCAATGCCAT
GTTGACTCATCATGCCCCTTCTCCACACCAGATTTTACAAAGGCTTCCTTTGCCAACCCTTATGGGAGAGCTTTACAGCCCTTTTAGTAAAAGGACTTGTCTTCAGCCAAACACATTCACTAACA TTA
AGGTTTCTTAATTTTTGTAGCTACTCTCACCACCATGCTTATACATGTGTGAAATTCTGGGCTCAGCAGTCCCCAGAAACCTTCTCAGATCACCCCCTTCCACCGAGTCCTCTTTCTTTCCTATGGGT
CcCTtTGCAGGCACATCAATCTTTGGTAACATCCTGCCCGGCATGACCATCATCCTGATTCATGTACATCTTATACCTTGTACCCTGTGTCTG GCCGCACAAAACTCCTGCTCCTGCTCTGAGTCACC
TACTGTTTCTCTGATACACTGTGGCATTCATTAAATTCAGTGGGATTAATGGGTGGAGTATTGTTTCTCAGGACACAGGAGTAGGTTGGGGTTGGCTTCCTTTAAATTAGTGCAGACTTAGGCTATCT
TCCTTGATGCTCTTGATGTTGACAGACCAACTCTCTATTTCTCATTGACCCCTTTTCTAAGCAATGTGCTCAATTTTCCCAGCTCTCCTTAGTTCCTAATTTCTCTCAACA aTTCACTTCcCTTGGTT
CCCTTTTGGGACATGGTCTTGATTCACATCTTACTGAGCTCTCTGGGACACAGTGTAATAGGCTGTAGTCATAGACTCAGAAAAAGGGA CAAGAAATCAGAAAATTGTATTCCTTTCATAGCACTGGG
GAAGTCACTTACCTTCTTTGACTCTCCATTGTCTTCCATGGTAAACTACAAATAGTAGTATTTCCCCTCTCAGGACTGGTGTGTGATCCCATGAGATAATGAACATGGAAAACCTTTGCACAACA TGA
AGCAGTATGTGAGAGGTGTTATCAGCCCTCCTTCTCTGCACCTTCAGATATTGCTGTCTCTACCCACAAAGTGCTTCCTTTACCTTTCATCTCACCTGCTGTCTCCATCTGACTCTGGCTGCAGCAAC
TTCAGTCTTTCCCTCTTCGTGCATTCTTTCCTCTCTATTGACAATCCTTT CCCTGCCTTATGCACCATCCTCACTTTCCTAGCATGTAAACAACCTCTTACTTGATCCTTCTGTCTGTTCCACCTCCC
ACCAGCCACTGTCCTTTTCCCTTTCCTTTTGTAACCAGAGAGGACAACACGTAGTCCCTCCTGCTGGCTCATTACCTACTATTTCCATAGCTCCTGCATTTCTTCCCAATGACTCTCCCTAAGCCAGC
AGATCTGTTTAAGTTCTCAGTTCTTATCCTCCTTAAACTCATTGTTTCGTCTTCTTTTCTGATTCAGTATCACCCCATCCTTTTACAGTGGGCTTTTCTCCAGGCCTAGTCTTCTTGCCATCTGC TCT
ACCCCCACACAGGGAACCATAATGCACTACATTCTCTTATTTTACTCCATTCAGATTGAATCATACTGGGAGTACTGTGTTTACTTCTAGCTTTCACCTTCAGTGAGAATTAT tACCAACTCAGGGAT
ATTCAATAAGGAGCACCAAGATGGTAAGGGCACTAGAATGATCTCCTCTTATGGAACACTTGATAGAATGGATGTATTTAACATACAGAAGAGAAAATCCAGAGAAAATGTACACTTTGCTGTCT TAA
C

>KSI_AluYa5_70

>Scaffold7861-518867-519193
TACATGTATTAACATTGATATAAACATTACACTGAGTGAAAAGTTGTAGAAAGATACATTT tATCAGTTACATATATTTGAGAAACACAGGAAATACTATATATTCCCTCAAATGTTTTTGAGCAATG
CCTTCTCCTTTTTCTCCTTGATTGAGTTCTGAGTTAGGTGAAACAGAGATAAGCCATCACTTCTTTTTTCA cCCTAGGGTGAAATTTGATATCACCCTATGATATTTGTTATTTGAACAATAAGAATA
TTTTTTCATAAGTATTTTCTTTAGTTGTCCCCAAATAAGTTAATATTACAAATATAGAATAGGCTTGTTTACATTAAAAACACCCTTCTCCATCAAAGAAA TTGTGATGGTTGTACTGCCCTTTCACC
CCTTAGGTGGTTGCACTGCCCTTTCACCCCTCCTTCCTACTCCCACCCTGGATAAGCCACAGCTCCGGGGCACGTGAGAGTAATTTCTTATAAGATTTCTCTGTGATAGACTTCAGAAGACAAGTTAT
ATACTCATGAGATTCTGAAATTGTAAAATATTAGTGCCAAAAATCCCTCAAGAACTGAATTATCTTTTACAACACCTCAATGAAAAGACTAAGAACTGAAGGTTAGTGGGGTGTATTCACCTTTCTAG
GGTCACATAATGAAGCAGGGCATGTAATACACAGCAATAAAGGAGAAGAAAGGGATTAAATGCTGAAGTAAGTGGTGAGGTTAACAATTCTAAAAAAGGCAGAAACAAAATTGATTTGAGTTGTTTAA
AGGTTACTTAACATTTCATGTAAACTTTCATAGTACAGAAAACTATAGGAAAGCATTCCAAATTTGCGGACCACAGTCTGATAAAAGAAATGAATAGCTTATTCTTAAACAAGATCTGTTTGGCC CAT
CTAGGAAGTTATGCTGGGAAAGACATGAAATTAACACGCATTAAGAGATAGACCCTCTATCTGTGCAATGTTTCTTCTCTCATAAT Taagaaaaatccactgaggccgggcgcggtggctcacgcctg
taatcccagcactttgggaggccgaggcgggcggatcacgaggtcaggagatcgagaccatcccggctaaaacggtgaaaccccgtctctactaaaaatacaaaaaattagccgggcgtagtggc ggg
cgcctgtagtcccagctacttgggaggctgaggcaggagaatggcgtgaacccgggaggcggagcttgcagtgagccgagatcccgccactgcactccag CCTGGGCGACAGAGCGAGACTCCGTCTC
AAAAAAAAAAAAccgcccccgcccggggcgaaaggggaagccccttcaaaaaaaaaaaaaaaaaaaaaaaa AAGAAAAATCCACTGAATTCATGCCAGGAAGTCTGTCTGACCCTGATGCCTACCT
TACTGTCACCATAAAACACTGTCTTCCAGGATGTTGGGGAATGGAGAGATTGAATGGGTGTACATTTTGGGAAAAAAAAACTTCAAAGGCAAATATTTTGTGTGCGGCTTTAATGCTAAGACCAGC AAT
TAGCTATTGAGGATAATTATACCATGGGGCTAACTTCTATGACATTTAAATTCGTTTT CATTGTGAATCATTTCAGCCATATAAAAAAGAATAGCAATATAAAAAATAATCCATG TGTTTACTAATAT
GATTTATGAAATGTTGACATTTTGCTGTATTTGTTTCATGTTGTTTTTGTTTATCTTAGAAGTTTTAGATAAGATAAAAGCTCCTCCTCTTTTTGTTCCCCATCTTTCAGTCCCAGAAGTAACCA TTT
CCTGAAGCAACTATTTTGGTGTGTGGCCTCCTGAAGCTACTTCATTCTTTACAACATGTCTACCCTATATATTCTTATGTGTATAAAGCCTATATAATATTTCCTGTGTTTCTCA AATATATGTAACT
GATAAAATATATCCTTTTGCAACTTTTCAATGTAATGGTTATATCAATGTTGGTACATGTAGCTCTCATTCATTTATCTATGCTTATTAATTTGATGTATTATTTTAAACTAGTTGATTGAAGGA ATA
GCAAGCATTTAATTTTTTTCTCTAATTGTAATAATTTTGTTTTTCTATGATTTTTTTATTATAGAATTCTGAAAAGAACATTGTGTTACATGTGTCCTTGTGACAAATTTTTCTA AGGTAGCTACCTA
ATTGTGGACATTTCTAGATAGTAATGTATGAACATCTTAAAATTAATGAGATACTGCCTCAAGGCTCTCCAAAATGGTAGTTGTTTTGTGTGCCATATTGG ATAAATATTTGTGTGTACTTTTCAAAT
TGCCAACTAGTTGAGTTCCAGATTATCATTCAGAGTCTGACAGGTAACAAGTCAC

484	12.8	1.3	0.0	UnnamedSequence	104	181	(2145)	C	L1MB4	LINE/L1	(6127)	480	402	1
188	40.0	2.2	2.9	UnnamedSequence	524	662	(1664)	C	MIR3	SINE/MIR	(2)	206	2	2
2650	2.8	0.6	5.8	UnnamedSequence	1000	1326	(1000)	+	AluYa5	SINE/Alu	1	311	(0)	3
524	26.6	10.7	8.3	UnnamedSequence	1563	2224	(102)	C	L1ME3C	LINE/L1	(8)	6242	5556	4

>hg19 chr2:123329663-123331651
TACATGTATTAACATTGATATAAACATTACACTGAGTGAAAAGTTGTAGAAAGATACATTT cATCAGTTACATATATTTGAGAAACACAGGAAATACTATATATTCCCTCAAATGTTTTTGAGCAATG
CCTTCTCCTTTTTCTCCTTGATTGAGTTCTGAGTTAGGTGAAACAGAGATAAGCCATCACTTCTTTTTTCA tCCTAGGGTGAAATTTGATATCACCCTATGATATTTGTTATTTGAACAATAAGAATA
TTTTTTCATAAGTATTTTCTTTAGTTGTCCCCAAATAAGTTAATATTACAAATATAGAATAG GCTTGTTTACATTAAAAACACCCTTCTCCATCAAAGAAATTGTGATGGTTGTACTGCCCTTTCACC
CCTTAGGTGGTTGCACTGCCCTTTCACCCCTCCTTCCTACTCCCACCCTGGATAAGCCACAGCTCCGGGGCACGTGAGAGTAATTTCTTATAAGATTTCTCTGTGATAGACTTCAGAAGACAAGT TAT
ATACTCATGAGATTCTGAAATTGTAAAATATTAGTGCCAAAAATCCCTCAAGAACTGAATTA TCTTTTACAACACCTCAATGAAAAGACTAAGAACTGAAGGTTAGTGGGGTGTATTCACCTTTCTAG
GGTCACATAATGAAGCAGGGCATGTAATACACAGCAATAAAGGAGAAGAAAGGGATTAAATGCTGAAGTAAGTGGTGAGGTTAACAATTCTAAAAAAGGCAGAAACAAAATTGATTTGAGTTGTT TAA
AGGTTACTTAACATTTCATGTAAACTTTCATAGTACAGAAAACTATAGGAAAGCATTCCAAA TTTGCGGACCACAGTCTGATAAAAGAAATGAATAGCTTATTCTTAAACAAGATCTGTTTGGCCCAT
CTAGGAAGTTATGCTGGGAAAGACATGAAATTAACACGCATTAAGAGATAGACCCTCTATCTGTGCAATGTTTCTTCTCTCATAAT TAAGAAAAATCCACTGAATTCATGCCAGGAAGTCTGTCTGAC
CCTGATGCCTACCTTACTGTCACCATAAAACACTGTCTTCCAGGATGTTGGGGAATGGAGAG ATTGAATGGGTGTACATTTTGGGAAAAAAAAACTTCAAAGGCAAATATTTTGTGTGCGGCTTTAATG
CTAAGACCAGCAATTAGCTATTGAGGATAATTATACCATGGGGCTAACTTCTATGACATTTAAATTCGTTTTCATTGTGAATCATTTCAGCCATATAAAAAAGAATAGCAATATAAAAAATAATC CAT
GTGTTTACTAATATGATTTATGAAATGTTGACATTTTGCTGTATTTGTTTCATGTTGTTTTT GTTTATCTTAGAAGTTTTAGATAAGATAAAAGCTCCTCCTCTTTTTGTTCCCCATCTTTCAGTCCC
AGAAGTAACCATTTCCTGAAGCAACTATTTTGGTGTGTGGCCTCCTGAAGCTACTTCATTCTTTACAACATGTCTACCCTATATATTCTTATGTGTATAAAGCCTATATAATATTTCCTGTGTTT CTC
AAATATATGTAACTGATAAAATATATCCTTTTGCAACTTTTCAATGTAATGGTTATATCAAT GTTGGTACATGTAGCTCTCATTCATTTATCTATGCTTATTAATTTGATGTATTATTTTAAACTAGT
TGATTGAAGGAATAGCAAGCATTTAATTTTTTTCTCTAATTGTAATAATTTTGTTTTTCTATGATTTTTTTATTATAGAATTCTGAAAAGAACATTGTGTTACATGTGTCCTTGTGACAAATTTT TCT
AAGGTAGCTACCTAATTGTGGACATTTCTAGATAGTAATGTATGAACATCTTAAAATTAATG AGATACTGCCTCAAGGCTCTCCAAAATGGTAGTTGTTTTGTGTGCCATATTGGATAAATATTTGTG
TGTACTTTTCAAATTGCCAACTAGTTGAGTTCCAGATTATCATTCAGAGTCTGACAGGTAACAAGTCAC

```
250   19.1 21.9  0.0  hg19_2       1   105 (1884) + L1ME3Cz  LINE/L1     5830   5957  (293)   1 *
  493   12.8  1.3  0.0  hg19_2      104   181 (1808) C L1MB4    LINE/L1    (6127)    480    402   2
  735   26.8 11.3  7.1  hg19_2     1226  1887  (102) C L1ME3Cz  LINE/L1       (8)   6242   5555   3
```

>KSI_AluYa5_71

```
>Scaffold3707-5995421-5995618
CAGGAGCTGAGGTGGCTGTTACACCTCCCAGCACTGGGCTGCATGAATCAGTGTGTGAATCAGTGTCCTGGGAGGTCAGGACACAAGGGGACAAGGAGGTGGGGAGTTGTGGGGAGCTGGCTGCAG GC
TCAGCTGTAGCAATCACCAGCTACTGCAGGGGAGGAAGGCACAGGGCATGTGGGGAGGAGCAAGGTGGGTGTGGAGAGGTGAAGTTTCTAGACTGGATCTCATCCAAGTGCAGACATCATCCGGGGCT
CTtGTTAAATCACAGATTCTGACTCAGGAGGTCCTGGGTGGGGGCTGAGACTGCATTTCTTTTTTCTTTTCTTTTTTTTTGACAGAGTCTTGCTCTGTCGCCTAGGCTGGCGTGCAGTGGCGCGATC
TCAGCTCACTGCAACCTCTGCCTCCTGGGTTCAAATGATTCTTCTGCCTCAGTTTCCCAAGTAGCTGGGATTATAGGTGCCTATCACCGTGCCTGGCTACGTTTTTCTTTTTAAATAGAGGCAGG GTT
TCACCATGTTGGCCAAGCTGGTCTCAAACTCCTGACCTCAAGTGATCCACCCACCTCAGCCTCCCAGAGTGTTGGGATTACAGGCATGAGTCAGACAGCTCGCAGAGCATTCTCATTTGCTAGTCC CT
AGAGCCCCCTCAGGCAAAGTCCTAGGGGAACACCGCCACCAGGGCATCCTCAGCAGGTGCATGAGGGAGAAGCTGCCTGGGCGAGAGCAGCGGCCCTTGCTTTGGCCAGGTCTAGGGCACATGGA GTG
CCTGCGCAGGTCAGAGGCAAGCAGGAGTCCTGATCTGGAGAAGGACTGGCACCAGTAGGGAAGGGGCAGCTGGAGCCCTGCCATCTCTCGCTATC aTCTTCTGCCTCTCAACTAGGGGAATGGAGGGG
TAAGGGTAGGAGAAGGTGGAAAACAAGAGAAAGGAGGATGTCTCCTCCCCAGGGCCGAGGGCCTCCCAAGCACCTGGAATTGCCTTTGCAAAAAGTATAACTGAG Gccgggcgcggtggctcacgcct
gtaatcccagcactttgggaggccgaggcgggcggatcacgaggtcaggagatcgagaccatcccggctaaaacggtgaaaccccgtctctactaaaaatacaaaaaattagccgggcgtagtgg cgg
gcgcctgtagtcccagctacttgggaggctgaggcaggagaatggc AAATTGTGACAGTGAAAGAAATCAGACCTAACCAACTCCACCTTGCTTCTAACCTTTAAG CTGTCCTTGTTCATTCCTGGGC
ATAGGCCAAACTATAGGAAGGAATTCAGTTCACGGTTTGACTCTGAAACAAAATTGCTAATAGCCCTTTCCCGAAAAGACCCCTTTCTTGCCTGGGGACCAGTCTGCCTTT GCAGGACTAACAAATTA
GCTACACGAGTAGAAATTACGGTTTAGGGGTCCTGCAGCCTCTGACTCCAAGAGTCTGACCCTCCCCACGTTGCTCCTGGGGATAACATCACTATTGTAAAACCTA AGATCAGtGCTTGAGATGTTTT
GCAGGCCCTGCACTGGATGGATCAGCTGACACCACCCAGACCGTGGTAATCTGGCTCAACCAGTTCTGCCACCCCACCCAGGAACAGAAGACAGCAAGCAAACCTCACTTCAATCCCTATGATTC CAT
CTCCAACCTGATCAATCAGTACTCCCCACTTCCCGAGACCCTACCTGCCAAATTATCCTTAAAAACTCCAATCCCCGAATGCTTGGGGAGACTGATTTGAGTAATA ATAAAACTCCCATCTCCCGCAC
AGCTGGCTCTGCGTGAATTACTATTTCTCCATTGCAATTCCCCTGTCTTAATAAATTGGCTCTGTCTAGGCAGTAGGCAAGGTGAACCCAC gGGGTGTTTATACACCTGCTCGGGAACAGGCCCCTCT
AGGTGCTGGGGACAGCTCAGTGAGACAGGCCTGGCCTCCTTGGAGCTCGCAGGGGCCACCAGAGGGAAAAAAGGACTACAAGGGAGAGGGAAGTGGCAGAGCCCAT TGCAGGGGAGGTACTGGTGAGG
AGCAGGTCACAGGGGAGGCCACAGCCCTGGGTGACTGGGACTGGGGGAGCTTCACTGGTCCTTGGAGCTGGGACCTTGGATTTCAGCTTCAGCCCAGCAGGAGGCATGGGCAGCCCCATGCCTGGAGC
ACAAGGGCAGCTGCTGCCCAGC
```

383	15.8	5.3	0.0	AluYa5	236	311	(1887)	C	MER5A1	DNA/hAT-Charlie	(21)	145	66	1
2060	13.6	1.0	0.3	AluYa5	312	605	(1593)	C	AluSx1	SINE/Alu	(4)	308	13	2
3361	17.9	2.2	7.7	AluYa5	971	1000	(1198)	+	LTR8	LTR/ERV1	1	29	(662)	3
1902	0.0	0.0	0.0	AluYa5	1001	1198	(1000)	+	AluYa5	SINE/Alu	1	198	(113)	4
3361	17.9	2.2	7.7	AluYa5	1199	1895	(303)	+	LTR8	LTR/ERV1	30	691	(0)	3

```
>hg19 chr2:233844893-233846893
CAGGAGCTGAGGTGGCTGTTACACCTCCCAGCACTGGGCTGCATGAATCAGTGTGTGAATCAGTGTCCTGGGAGGTCAGGACACAAGGGGACAAGGAGGTGGGGAGTTGTGGGGAGCTGGCTGCAG GC
TCAGCTGTAGCAATCACCAGCTACTGCAGGGGAGGAAGGCACAGGGCATGTGGGGAGGAGCAAGGTGGGTGTGGAGAGGTGAAGTTTCTAGACTGGATCTCATCCAAGTGCAGACATCATCCGGGGCT
CTcGTTAAATCACAGATTCTGACTCAGGAGGTCCTGGGTGGGGGCTGAGACTGCATTTCTTTTTTCTTTTCTTTTTTTTTGACAGAGTCTTGCTCTGTCGCCTAGGCTGGCGTGCAGTGGCGCGATC
TCAGCTCACTGCAACCTCTGCCTCCTGGGTTCAAATGATTCTTCTGCCTCAGTTTCCCAAGTAGCTGGGATTATAGGTGCCTATCACCGTGCCTGGCTACGTTTTTCTTTTTAAATAGAGGCAGG GTT
TCACCATGTTGGCCAAGCTGGTCTCAAACTCCTGACCTCAAGTGATCCACCCACCTCAGCCTCCCAGAGTGTTGGGATTACAGGCATGAGTCAGACAGCTCGCAGAGCATTCTCATTTGCTAGTCC CT
AGAGCCCCCTCAGGCAAAGTCCTAGGGGAACACCGCCACCAGGGCATCCTCAGCAGGTGCATGAGGGAGAAGCTGCCTGGGCGAGAGCAGCGGCCCTTGCTTTGGCCAGGTCTAGGGCACATGGA GTG
CCTGCGCAGGTCAGAGGCAAGCAGGAGTCCTGATCTGGAGAAGGACTGGCACCAGTAGGGAAGGGGCAGCTGGAGCCCTGCCATCTCTCGCTATC gTCTTCTGCCTCTCAACTAGGGGAATGGAGGGG
TAAGGGTAGGAGAAGGTGGAAAACAAGAGAAAGGAGGATGTCTCCTCCCCAGGGCCGAGGGCCTCCCAAGCACCTGGAATTGCCTTTGCAAAAAGTATAACTGAG GAAATTGTGACAGTGAAAGAAAT
CAGACCTAACCAACTCCACCTTGCTTCTAACCTTTAAGCTGTCCTTGTTCATTCCTGGGCATAGGCCAAACTATAGGAAGGAATTCAGTTCACGGTTTGACTCTGAAACAAAATTGCTAATAGCCC TT
TCCCGAAAAGACCCCTTTCTTGCCTGGGGACCAGTCTGCCTTTGCAGGACTAACAAATTAGCTACACGAGTAGAAATTACGGTTTAGGGGTCCTGCAGCCTCTGACTCCAAGAGTCTGACCCTCC CCA
CGTTGCTCCTGGGGATAACATCACTATTGTAAAACCTAAGATCAGcGCTTGAGATGTTTTGCAGGCCCTGCACTGGATGGATCAGCTGACACCACCCAGACCGTGGTAATCTGGCTCAACCAGTTCTG
CCACCCCACCCAGGAACAGAAGACAGCAAGCAAACCTCACTTCAATCCCTATGATTCCATCTCCAACCTGATCAATCAGTACTCCCCACTTCCCGAGACCCTACCTGCCAAATTATCCTTAAAAA CTC
CAATCCCCGAATGCTTGGGGAGACTGATTTGAGTAATAATAAAACTCCCATCTCCCGCACAGCTGGCTCTGCGTGAATTACTATTTCTCCATTGCAATTCCCCTGTCTTAATAAATTGGCTCTGTC TA
GGCAGTAGGCAAGGTGAACCCACtGGGTGTTTATACACCTGCTCGGGAACAGGCCCCTCTAGGTGCTGGGGACAGCTCAGTGAGACAGGCCTGGCCTCCTTGGAGCTCGCAGGGGCCACCAGAGGGAA
AAAAGGACTACAAGGGAGAGGGAAGTGGCAGAGCCCATTGCAGGGGAGGTACTGGTGAGGAGCAGGTCACAGGGGAGGCCACAGCCCTGGGTGACTGGGACTGG GGGAGCTTCACTGGTCCTTGGAGC
TGGGACCTTGGATTTCAGCTTCAGCCCAGCAGGAGGCATGGGCAGCCCCATGCCTGGAGCACAAGGGCAGCTGCTGCCCAG C
```

364	17.1	5.3	0.0	hg19	236	311	(1691)	C	MER5A1	DNA/hAT-Charlie	(21)	145	66	5
2060	13.6	1.0	0.3	hg19	312	605	(1397)	C	AluSx1	SINE/Alu	(4)	308	13	6
3360	17.9	2.2	7.8	hg19	971	1699	(303)	+	LTR8	LTR/ERV1	1	691	(0)	7

>KSI_AluYa5_72

>Scaffold6436-4283357-4283607
GACTCCAGTACCCTGTGGGTAAAgCACACACCCACATACTTCCGAAAGACAAATAGTTCgTAAAGATCATACTGTCTAAACTAAATCACATTAAATGGAAAAATCTTCCTAACAGCTCTGACAATCTA
CAAAGACTTATCCTAAAATAAACCCTGAAAGTAGACCTTTACATGATTACCTAATGCCATTCTTCTTTTCCTTTCTTCAATCTCCAACTTCCAAACTATGGCCGAAGGTTTT cCTAGGGACAAAGACA
CTGCCCATCACAAGTCCTGCTCAAACAGAAAACCCATTCACCCTTGATCTTTACCCTTAACTACACAAAAATACTGGCAGACACTTTGAATGTACAGGTTTAGACTTAGGCCAACTTGAATTTTTAGG
TAGGGATTTCTATTGCACCATTTGTCTCTAAGTGGAATAAGGTCTGTGAAACATTTACTTACCACTGCCCTTCCCACGAGCTAGCAAAAGCTCCTAGCAATGACATTTAGGAATTGTTTTGTTTT TAT
TTTTATTTTCCTATTTCTTTACATGACGTGTAATCTAAGCTTGACCTATTTAAAGAATAAATGTAAAAAAGGACAAATGAAACGATTAAT aGTCTAGAGGTGAGCATTGTTTACTATATCCTAGTTTT
AATTTTTCCTATGCCATTTTTAAATAAAATTGGGATTCTATTGGATAGAGCATTACAAGACTGCTTCTTGTGGAAAGAATGTTCCTGAATGCCTTTCACAACACCTAGATGCTAGATGCTTGCTT TTA
CAATACTCCCTCCTTATCCAGTTTCCCTTTCTGTGTTTTAGTTACCCACATTCAACCACAGTCCAAAAATATTACATACAATATTTTGAGAGAGAAAAACTACATTCACATAACTTTTATTACAGTAT
ATTGTTATTATTCTATTTTCATTATTAAGTTATtcctgttaatttctttttttttttttttttttttttttttttttgggggggcttccctttttcccccccccagtgcagtggcgggatctcggctc
actgcaagctctgcctcccgggttcacgccattctcctgcctcagcctcccaagtagctgggactacaggtgcccgccactacgcccggctaatttttttgtatttttagtagagacggggtttcac
cgttttagccgggatggtctcgatctcctgacctcgtgatccgcccgcctcggcctcccaaagtgctgggattacaggcgtgagccaccgcgcccggcc TCCTGTTAATTTCTTACTATGTCTAATTT
ATAAATTAAACTTTCTCATAGGTGTGTATGTATGCATAGGAAAAAGCATAGCATATATAGGGTTCAGTACTATCTGCAGTTTCAGGCATGCACTGGGGGTCTTGAAACAT CTCCTGAGGATAAAGGGA
GTCTACTATGTCTGAATTCCGATATAATTCACATTCCATAAGATTTATCTTTTTAAAATGTAAAATTCAGTGGTTTTTAGCATATTCCCAAAGTTATGCAACCATCATTATTATCTGATTCCAGA ACA
GTTTCATGATCCAAAAAGAAACTCCATACCCCTAAAAGCAGTCACTCTCCACCCCTTCTACCGTAAGCCCCTGGCAAACACTAAGCTACTTTCAATCTCTAGGGATTTGCCTACTTTAGATATATTAT
ACAAATGAAATCACACAACATGTGGTCTTTTATGACTGGCTTCTTTCACTTACCATAATGTTTTCAAAGCTCAGCCATTTATGAGTACTTCATTCCTTTTTATGACTGAGTGATATTCCACTGTA TGA
ATATATCAATTTTGTTTATCCATTAATCAGTTGGTGGACATTTGTGTTGCTTCCAATTTGGGGCTGTTATGAATAATACTGCTGTAAACATTTGTGTACAAGTTTCTGGGTGAACATATGTTTCATTT
CTCTTGGGTATTTACCTAGAAGTAGAATTACTGGATCATATTTTAACTCTATGTTTAACCATTTAAGGAATTTTCAAACTATTTTCCAAAGCAGCTGCACCATTTTAAAGTCGCACCAGCAGGGT ATG
AGGGTTCCAGTTTCCCCACATTCTTACCAAGGTTTACTGGTATCTTTCTTTTTTATTATAGCTATCAAATAGCTTTTGTAAAGTGGTATATCATTAAGGGTTTGATTTACATTTCTCTGATAGGCTAA
TGAGCATCTTCACATTTTATTGGCCATTTACATATCTTCTGTGGAGAAGTATCCATTCAGATGTTGTGCTTATT TTTAAATTGGGCTATTTGTCTTATTGTTGAGTTGTAAGAGTTCTGTATATATTC
TGGACACTGGACTCTTATCAGATATATGATTTGCAAATATTTTTTCCATTCTGTATGTTGTCTTCTCATTTTCTTTTTTGAAACTTTTATTGTTATTATTATTATTTTAGAGACAGGTTCTTGCTCTG
TCACCCAGGTTGGAGTACAGTGGTGCCATCGTAGCTCATTGCAGCCTTGAATTCCTGGGTTCAAGCAATCCTCCTGCCTCAGTCTCCTGAGTAGCTAGGATGACAGGTGTGCCACCATGCCTGGT TAA
TTTTTTTTGTTTTCTTTCAGAGACAGGGTCTCACTATATTGCCCAGGCTGGACTTGAACTCCTGGACTTAAGCTATCTTCCCACCTCCATCTCCCAGAGCACTGGGATTACAGGTGTGAGCCACCACA
CCCAACCTTACTTTCTTAATAATGTCCTTTAGAGCACAAGAGTTTTTATTTTGATTAAGTTCAACTTGCCTTTTGGGGGTAGGGGGCAGGGTTGTTCATGCTTATGGTATTATATCTAAGAAACA TTG
CCTGATCAAAGGTCACAAAGATTTACACCTATGTCTTCTTATTTTTTTCTTTCTTCTTTTCTTTCTTTCTTTCTTTCCTTCTTTCTTTCTTTTTTGACAAGGTCTTGCTATTTTGCTCAGATTGGTGT
ACAGTAGCTGTTCACAGGTGTAAGCATCAAAGTGTCCTGCAGCCTCAAACTCTTGGCCTCAAGTGATCCTCCTGGCTTATCTCATGAGTAGCTGGGACTACA GGCATACACCACCACTTTTGGCTTTA
TATCTATGTTTTTAAAGAGTTTTGGCTTTTACATTTAGATCTTTAATTTATTTTTAGTTAATTTTTATATGGTGTAAGGTTAGGAATCCAACTTTATTCTTTTGCGTATGAATTTCTAGTTGCACCAG
CACGATTTATTGAAAAGACGTATCTTTCCCCCATTGAATAATCTTGACACTCTAGCCAAAAATCAATTGACCATAAATATGAGTTTATTTCTAAATCTTCAGCCCTATACCATTGATCTATATGT TTA
CCCTAATGCCAGTACCACACAGCTTTTCCTTAAAAAGAGAGATAGAGTGCTGAGAATTTGAAAGACATTGCTCTGAATTTGTACATCAATTTGGAGATATTACTATAACAATGTTAAGTTTTCAATCT
AGGAACAGGGATATCTTTCTATTTATTTAGATTTTTTAAAGTTTTTTTTTTCAGAATGTTCTGTAGTTTTCAGTATGTAACTCTTGCAGTTTTTTGTTAACTTTGTTTCTGAGTTATTTCCGTTT CAT
TTCATTTTGTCCATTGCCAGTGTGTAAAAATACAGTTGATTTTTGTATATTGATCCTCTGTCTACAACATTGCTTGTTTATTACTACTAATATTAATACTAATAGGTTTTTGGGGATCCTGTAGAATT
TTCTACATACAAGATCATCTGCAATTAGAAATGGATTTACTTCTTTTCTAATCTGAATGCCTTCTTTTCCTTCTTTTTCTTGCTTAATTGCTCTGGTTACAACCTCAAGTACCATGCTGAATAGA AAT
GGCAAAAACAGATATCTTTATCTTCTTCTTGATCTTAGGAAGAAAGCTTTTAGTCTTTCATCATTAAATATAATGTTAGCTTTGGATATTTTGTAGAAGTCCTTTGTAAGGTTGAAGAAGTTCCCATC
CTTTCCTGGTTTTTATTATGAAATGTGTGGTATTTTTATTAAACACTTTTATGCGATATTGTGATGATTATCTGAATTTTTCTATTAATATTGTATATTATAGTAATTGCTTTTCACAGGTTGAG CCA
AACTTGGACCCGTAGGATAAAACCTACTTAGTCATGATGTATCATCTTTTCAAATGTTGCTGGGTTTTGTTTGTTAGTATATCGTTGAGGATGTTTGCATCTGTATACATAAAAGATATGTATGTCCT
TTTCTCCTTGTGATGTCTTTGCCTAGT

14	17.5	0.0	0.0	AluYa5	501	532	(3719)	+	(TTTTAT)n	Simple_repeat	1	32	(0)	1
1610	11.8	5.2	14.2	AluYa5	769	942	(3309)	+	MER44A	DNA/TcMar-Tigger	1	180	(159)	2
33	0.0	0.0	0.0	AluYa5	943	973	(3278)	+	(T)n	Simple_repeat	1	31	(0)	3
1610	11.8	5.2	14.2	AluYa5	974	1000	(3251)	+	MER44A	DNA/TcMar-Tigger	181	181	(158)	2
2329	0.8	0.0	0.8	AluYa5	1001	1251	(3000)	C	AluYa5	SINE/Alu	(62)	249	1	4
1610	11.8	5.2	14.2	AluYa5	1252	1415	(2836)	+	MER44A	DNA/TcMar-Tigger	181	338	(1)	2
4867	16.6	3.0	2.3	AluYa5	1429	2388	(1863)	C	L1MB7	LINE/L1	(24)	6160	5195	5
1827	17.6	1.0	1.0	AluYa5	2389	2695	(1556)	C	AluJo	SINE/Alu	(5)	307	1	6

1596	16.1	3.3	8.6	AluYa5	2696	2849	(1402)	C	L1MB7	LINE/L1	(952)	5194	5049	5
623	22.1	1.6	9.0	AluYa5	2850	3085	(1166)	C	AluJr	SINE/Alu	(0)	312	121	7
1445	17.5	1.5	0.8	AluYa5	3090	3348	(903)	C	L1MB7	LINE/L1	(1118)	5028	4768	5
3399	19.2	9.9	1.8	AluYa5	3377	4251	(0)	C	L1MB7	LINE/L1	(1536)	4610	3666	5

>hg19 chr3:171625446-171627373
GACTCCAGTACCCTGTGGGTAAAaCACACACCCACATACTTCCGAAAGACAAATAGTTCtTAAAGATCATACTGTCTAAACTAAATCACATTAAATGGAAAAATCTTCCTAACAGCTCTGACAATCTA
CAAAGACTTATCCTAAAATAAACCCTGAAAGTAGACCTTTACATGATTACCTAATGCCATTCTTCTTT TCCTTTCTTCAATCTCCAACTTCCAAACTATGGCCGAAGGTTTTgCTAGGGACAAAGACA
CTGCCCATCACAAGTCCTGCTCAAACAGAAAACCCATTCACCCTTGATCTTTACCCTTAACTACACAAAAATACTGGCAGACACTTTGAATGTACAGGTTTAGACTTAGGCCAACTTGAATTTTTAGG
TAGGGATTTCTATTGCACCATTTGTCTCTAAGTGGAATAAGGTCTGTGAAACATTTACTTACCACTGCCCTTCCCACGAGCTAGCAAAAGCTCCTAGCAATGACATTTAGGAATTGTTTTGTTTT TAT
TTTTATTTTCCTATTTCTTTACATGACGTGTAATCTAAGCTTGACCTATTTAAAGAATAAATGTAAAAAAGGACAAATGAAACGATTAAT tGTCTAGAGGTGAGCATTGTTTACTATATCCTAGTTTT
AATTTTTCCTATGCCATTTTTAAATAAAATTGGGATTCTATTGGATAGAGCATTACAAGACTGCTTCTTGTGGAAAGAATGTTCCTGAATGCCTTTCACAACACCTAGATGCTAGATGCTTGCTT TTA
CAATACTCCCTCCTTATCCAGTTTCCCTTTCTGTGTTTTAGTTACCCACATTCAACCACAGTCCAAAAATATTACATACAATATTTTGAGAGAGAAAAACTACATTCACATAACTTTTATTACAGTAT
ATTGTTATTATTCTATTTTCATTATTAAGTTATTCCTGTTAATTTCTTACTATGTCTAATTTATAAATTAAACTTTCTCATAGGTGTGTATGTATGCATAGGAAAAAGCATAGCATATATAGGGTTCA
GTACTATCTGCAGTTTCAGGCATGCACTGGGGGTCTTGAAACATCTCCTGAGGATAAAGGGAGTCTACTATGTCTGAATTCCGATATAATTCACATTCCATAAGATTTATCTTTTTAAAATGTAAAAT
TCAGTGGTTTTTAGCATATTCCCAAAGTTATGCAACCATCATTATTATCTGATTCCAGAACAGTTTCATGATCCAAAAAGAAACTCCATACCCCTAAAAGCAGTCACTCTCCACCCCTTCTACCG TAA
GCCCCTGGCAAACACTAAGCTACTTTCAATCTCTAGGGATTTGCCTACTTTAGATATATTATACAAATGAAATCACACAACATGTGGTCTTTTATGACTGGCTTCTTTCACTTACCATAATGTTTTCA
AAGCTCAGCCATTTATGAGTACTTCATTCCTTTTTATGACTGAGTGATATTCCACTGTATGAATATATCAATTTTGTTTATCCATTAATCAGTTGGTGGACATTTGTGTTGCTTCCAATTTGGGG CTG
TTATGAATAATACTGCTGTAAACATTTGTGTACAAGTTTCTGGGTGAACATATGTTTCATTTCTCTTGGGTATTTACCTAGAAGTAGAATTACTGGATCATATTTTAACTCTATGTTTAACCATTTAA
GGAATTTTCAAACTATTTTCCAAAGCAGCTGCACCATTTTAAAGTCGCACCAGCAGGGTATGAGGGTTCCAGTTTCCCCACATTCTTACCAAGGTTTACTGGTATCTTTCTTTTTTATTATAGCT ATC
AAATAGCTTTTGTAAAGTGGTATATCATTAAGGGTTTGATTTACATTTCTCTGATAGGCTAATGAGCATCTTCACATTTTATTGGCCATTTACATATCTTCTGTGGAGAAGTATCCATTCAGATGTTG
TGCTTATTTTTAAATTGGGCTATTTGTCTTATTGTTGAGTTGTAAGAGTTCTGTATATATTCTGGACACTGGACTCTTATCAGATATATGATTTGCAAATATTTTTTCCATTCTGTATGTTGTCTTCT
CATTTTCTTTTTTGAAACTTTTATTGTTATTATTATTATTTTAGAGACAGGTTCTTGCTCTGTCACCCAGGTTGGAGTACAGTGGTGCCATCGTAGCTCATTGCAGCCTTGAATTCCTGGGTTCAAGC
AATCCTCCTGCCTCAGTCTCCTGAGTAGCTAGGATGACAGGTGTGCCACCATGCCTGGTTAATTTTTTTTGTTTTCTTTCAGAGACAGGGTCTCACTATATTGCCCAGGCTGGACTTGAACTCCT GGA
CTTAAGCTATCTTCCCACCTCCATCTCCCAGAGCACTGGGATTACAGGTGTGAGCCACCACACCCAACCTTACTTTCTTAATAATGTCCTTTAGAGCACAAGAGTTTTTATTTTGATTAAGTTCAACT
TGCCTTTTGGGGGTAGGGGGCAGGGTTGTTCATGCTTATGGTATTATATCTAAGAAACATTGCCTGATCAAAGGTCACAAAGATTTACACCTATGTCTTCTTATTTTTTTCTTTCTTCTTTTCTT TCT
TTCTTTCTTTCCTTCTTTCTTTCTTTTTTGACAAGGTCTTGCTATTTTGCTCAGATTGGTGTACAGTAGCTGTTCACAGGTGTAAGCATCAAAGTGTCCTGCAGCCTCAAACTCTTGGCCTCAAGTGA
TCCTCCTGGCTTATCTCATGAGTAGCTGGGACTACAGGCATACACCACCACTTTTGGCTTTATATCTATGTTTTTAAAGAGTTTTGGCTTTTACATTTAGATCTTTAATTTATTTTTAGTTAATTTTT
ATATGGTGTAAGGTTAGGAATCCAACTTTATTCTTTTGCGTATGAATTTCTAGTTGCACCAGCACGATTTATTGAAAAGACGTATCTTTCCCCCATTGAATAATCTTGACACTCTAGCCAAAAATCAA
TTGACCATAAATATGAGTTTATTTCTAAATCTTCAGCCCTATACCATTGATCTATATGTTTACCCTAATGCCAGTACCACACAGCTTTTCCTTAAAAAGAGAGATAGAGTGCTGAGAATTTGAAA GAC
ATTGCTCTGAATTTGTACATCAATTTGGAGATATTACTATAACAATGTTAAGTTTTCAATCTAGGAACAGGGATATCTTTCTATTTATTTAGATTTTTAAAGTTTTTTTTTCAGAATGTTCTGTAG
TTTTCAGTATGTAACTCTTGCAGTTTTTTGTTAACTTTGTTTCTGAGTTATTTCCGTTTCATTTCATTTTGTCCATTGCCAGTGTGTAAAAATACAGTTGATTTTTGTATATTGATCCTCTGTCT ACA
ACATTGCTTGTTTATTACTACTAATATTAATACTAATAGGTTTTTGGGGATCCTGTAGAATTTTCTACATACAAGATCATCTGCAATTAGAAATGGATTTACTTCTTTTCTAATCTGAATGCCTTCTT
TTCCTTCTTTTTCTTGCTTAATTGCTCTGGTTACAACCTCAAGTACCATGCTGAATAGAAATGGCAAAAACAGATATCTTTATCTTCTTCTTGATCTTAGGAAGAAAGCTTTTAGTCTTTCATCA TTA
AATATAATGTTAGCTTTGGATATTTTGTAGAAGTCCTTTGTAAGGTTGAAGAAGTTCCCATCCTTTCCTGGTTTTTATTATGAAATGTGTGGTATTTTTATTAAACACTTTTATGCGATATTGTGATG
ATTATCTGAATTTTTCTATTAATATTGTATATTATAGTAATTGCTTTTCACAGGTTGAGCCAAACTTGGACCCGTAGGATAAAACCTACTTAGTCATGATGTATCATCTTTTCAAATGTTGCTGG GTT
TTGTTTGTTAGTATATCGTTGAGGATGTTTGCATCTGTATACATAAAAGATATGTATGTCCTTTTCTCCTTGTGATGTCTTTGCCTAG T

14	17.5	0.0	0.0	hg19	501	532	(3397)	+	(TTTTAT)n	Simple_repeat	1	32	(0)	8
1836	12.2	5.8	1.8	hg19	769	1093	(2836)	+	MER44A	DNA/TcMar-Tigger	1	338	(1)	9
4867	16.6	3.0	2.3	hg19	1107	2066	(1863)	C	L1MB7	LINE/L1	(24)	6160	5195	10
1827	17.6	1.0	1.0	hg19	2067	2373	(1556)	C	AluJo	SINE/Alu	(5)	307	1	11
1596	16.1	3.3	8.6	hg19	2374	2527	(1402)	C	L1MB7	LINE/L1	(952)	5194	5049	10
623	22.1	1.6	9.0	hg19	2528	2763	(1166)	C	AluJr	SINE/Alu	(0)	312	121	12
1445	17.5	1.5	0.8	hg19	2768	3026	(903)	C	L1MB7	LINE/L1	(1118)	5028	4768	10
3399	19.2	9.9	1.8	hg19	3055	3929	(0)	C	L1MB7	LINE/L1	(1536)	4610	3666	10

>KSI_AluYa5_73

>Scaffold15791-6062824-6063019
GTTTGCACAGAGTGCAGTGGGCCAGCTTATGTGGCTGCCGTGCCAGCCTGCCATTTCCTGCCACCTTACTGTTTTGATGTTCTCAAGGGAAGGGAGAGGGGAATCACCTTGTCAGATAAACGTCCA CC
ATGTGACAGGTACCTTCTCTCTTTTTTAAAATACTCATAAATCACATAAGGGTCAGCTATCACTGGGGAAAATGGAATAGCA TCTGCAAGTCTTGCTTACTGGGAGGACGAGCTCTTCTTGTGATCTG
CACACATTTCTTAACTCTGTGCCCCAGCTTCCTCCTAGGTTGTGAGGAGTGTAGTGAAATGGTGGCAGAATAGTTATAATTTGAGACACGCTGTGAACAAGCCCCTTTGTCTTGTGGTTATTTTA GGA
ACCATTATCGAACGCTGCGCCAGGCTATTATTGACATGGTTTTGGCAACAGAGATGACAAAACACTTTGAACATGTGAATAA GTTTGTGAACAGCATCAACAAGCCAATGGCAGCTGAGGTGAGTACT
GCTTTCCATGCCATAAGGCACATCCAAGTACATTTCCATAAGAGCCTGCTCTGGCCCTGAAGCATTTTCTCTCCCACTCAAATGTAAGGTGGTTATTTACTTGGAGTTGATAAAGGGCTGAAAGG ACT
GGCCACTTGTTTTCCTTGGGGATGAAAATAAACTGTGTCCACAGCAACTCTGAGCTCCTTGAAAGAAAGGCCCTGAGAAAAA GCCAGTACTGACCAGAAAAGCTTGGCCCAAAGAATATGGAAGGAGA
CGCAGACGAACAGAGACTGGCATAAATGGTGGTATTTTAGAGAATCCAAAGCCTTTACTTCTCTGTTCACTGAGCTCTTGGAAAACCTCAAAGTTGGAAGGTGTTTTAAAAAACAAAGAATGTTC CTG
ATTCACATTAAAAGCATACCAGTACCTGAAGACTATCATAAAACTGGGAGTGACTGGAAAGCAGAAGCATTTATTGTGTTTT CCTTTTTTaaaacctactcatggccgggcgcggtggctcacgcctg
taatcccagcactttgggaggccgaggcgggcggatcacgaggtcaggagatcgagaccatcccggctaaaacggtgaaaccccgtctctactaaaaatacaaaaaattagccgggcgtagtggc ggg
cgcctgtagtcccagctacttgggaggctgaggcaggagaatg GCGTGAACCCGGGAGGCGGAGCTTGCAGTGAGCCGAGATCCCGCCACTGCACTCCAGCCTGGGCGACAGAGCGAGACTCCGTCTC
AAAAAAAAAAAAAAAAAAAACCTACTCATGTACCTTGGACCTGCCTTAAGGTCCTTACCAATTTGGCTTTTAGGAACATGAAACAGAAGGTTCACACCTAATAAGATCCACTGTTCTCTGAAGCCATG
AAGCTGTTCATAACCCCATAAAGGTAGAAAAGCTTTCC GCTCATTCCTGTTGAGCGGCAGAGGTTGGCAAACTGGATGGATGTGAATGATCATCATGAGTAACTTGCTCAGTAAGGAGATGAACTGTG
TAGGACCCATCCCATGACCAGTCTGGATGGAGTGACAGTGTCTACCTCACCTATTTAAGCTCCTTCCCCCAGGGATTCCACAGCACAACACCAGAGCCCTCTCCTTCCACAGAAGACTTACAGGAGCA
AGGATCTGGGGATTTAGAGAAGGAAAAGTATAGAGATTCATAG AACTAGGAAGGTCATCCACTCATGTAAGCCCCCACCTCTGCACTGCAACTTACTATTAACTGCAGTATTTATAGTAAACAGTATT
ACTTTGATAAGTAATCAAAAACCTCCAGAACATATTCTGCCCATTGATAGTCTAATAAATAGGTCCAGTCTTAAACCCCCCAAATGTCATGCAGCCTTTTGGCTTGGCAGAGCTCTGGAGACCTA ATT
GTTTTTACACAATCAATTTGATGGTGTCTTTAAAAGTGGAGAA TAACAATCTTTCAGGTTCTATGTAAATGGCCTGTGATGTGTGTACCAACT tGTGTCCTGGCCACATGGCTCAGGACACTGACATC
ACCACTAGGTGTGGACCAGTCTTTCTAAACATGGCTGAACACACCTGCTCCTGACTACTTCCTACTCCAAGGACCCCACGCCTACCTGGCTCACTTACCAGCCAGTCAGAAGAACAGCCAAGAAA ACA
TGAGTGGACACATGCCTTAGGAGAACCAAAGTGCGAGAGAGTT GGGTGAGTTGGGGGTGGGACAGGTCATA

1882	0.0	0.0	0.0	UnnamedSequence	1000	1195	(1000)	+	AluYa5	SINE/Alu	1	196	(115)	1
29	3.0	0.0	0.0	UnnamedSequence	1215	1248	(947)	+	(A)n	Simple_repeat	1	34	(0)	2

>hg19 chr5:76715210-76717146
GTTTGCACAGAGTGCAGTGGGCCAGCTTATGTGGCTGCCGTGCCAGCCTGCCATTTCCTGCCACCTTACTGTTTTGATGTTCTCAAGGGAAGGGAGAGGGGAATCACCTTGTCAGATAAACGTCCA CC
ATGTGACAGGTACCTTCTCTCTTTTTTAAAATACTCATAAATCACATAAGGGTCAGCTATCACTGGGGAAAATGGAATAGCATCTGCAAGTCTTGCTTAC TGGGAGGACGAGCTCTTCTTGTGATCTG
CACACATTTCTTAACTCTGTGCCCCAGCTTCCTCCTAGGTTGTGAGGAGTGTAGTGAAATGGTGGCAGAATAGTTATAATTTGAGACACGCTGTGAACAAGCCCCTTTGTCTTGTGGTTATTTTA GGA
ACCATTATCGAACGCTGCGCCAGGCTATTATTGACATGGTTTTGGCAACAGAGATGACAAAACACTTTGAACATGTGAATAAGTTT GTGAACAGCATCAACAAGCCAATGGCAGCTGAGGTGAGTACT
GCTTTCCATGCCATAAGGCACATCCAAGTACATTTCCATAAGAGCCTGCTCTGGCCCTGAAGCATTTTCTCTCCCACTCAAATGTAAGGTGGTTATTTACTTGGAGTTGATAAAGGGCTGAAAGG ACT
GGCCACTTGTTTTCCTTGGGGATGAAAATAAACTGTGTCCACAGCAACTCTGAGCTCCTTGAAAGAAAGGCCCTGAGAAAAAGCCAGTACTGACCAGAAA AGCTTGGCCCAAAGAATATGGAAGGAGA
CGCAGACGAACAGAGACTGGCATAAATGGTGGTATTTTAGAGAATCCAAAGCCTTTACTTCTCTGTTCACTGAGCTCTTGGAAAACCTCAAAGTTGGAAGGTGTTTTAAAAAACAAAGAATGTTC CTG
ATTCACATTAAAAGCATACCAGTACCTGAAGACTATCATAAAACTGGGAGTGACTGGAAAGCAGAAGCATTTATTGTGTTTTCCTTTTT TAAAACCTACTCATGTACCTTGGACCTGCCTTAAGGTCC
TTACCAATTTGGCTTTTAGGAACATGAAACAGAAGGTTCACACCTAATAAGATCCACTGTTCTCTGAAGCCATGAAGCTGTTCATAACCCCATAAAGGTAGAAAAGCTTTCCGCTCATTCCTGTT GAG
CGGCAGAGGTTGGCAAACTGGATGGATGTGAATGATCATCATGAGTAACTTGCTCAGTAAGGAGATGAACTGTGTAGGA CCCATCCCATGACCAGTCTGGATGGAGTGACAGTGTCTACCTCACCTAT
TTAAGCTCCTTCCCCCAGGGATTCCACAGCACAACACCAGAGCCCTCTCCTTCCACAGAAGACTTACAGGAGCAAGGATCTGGGGATTTAGAGAAGGAAAAGTATAGAGATTCATAGAACTAGGA AGG
TCATCCACTCATGTAAGCCCCCACCTCTGCACTGCAACTTACTATTAACTGCAGTATTTATAGTAAACAGTATTACTTTGATAAGTAATCAAAAACCTCC AGAACATATTCTGCCCATTGATAGTCTA
ATAAATAGGTCCAGTCTTAAACCCCCCAAATGTCATGCAGCCTTTTGGCTTGGCAGAGCTCTGGAGACCTAATTGTTTTTACACAATCAATTTGATGGTGTCTTTAAAAGTGGAGAATAACAATC TTT
CAGGTTCTATGTAAATGGCCTGTGATGTGTGTACCAACT cGTGTCCTGGCCACATGGCTCAGGACACTGACATCACCACTAGGTGTGGACCAGTCTTTCT AAACATGGCTGAACACACCTGCTCCTGA
CTACTTCCTACTCCAAGGACCCCACGCCTACCTGGCTCACTTACCAGCCAGTCAGAAGAACAGCCAAGAAAACATGAGTGGACACATGCCTTAGGAGAACCAAAGTGCGAGAGAGTTGGGTGAGT TGG
GGGTGGGACAGGTCATA

>KSI_AluYa5_74

>Scaffold14134-5602316-5602459

```
GTATTTTCATTTCACCATTTGTAAAATAAAGCAGCTGAACTTGATATCACACTAGAACTTGATCAAAAGCCCAAGAAGCAACAATGTTTTGCCAATCCCTATGTTATCTTCCCTTTTTAAAATTCT GT
AATGCTGTGATTACAAATAGCTTCCTAGTTCCTAAAAATTTGATGTTTTAAGtGTCTTACTATTTAAGGGTTTAAGAAAGGACATTTGCACAATAATATACGATATCCAAATTTAAATATAAAATATG
CCCAAGAAAAGCAGATAAGGTCTGCAGTACCTCAGTGTCCATATAATGGCATTGAACAAATGGAAACAGATGGTCTGAGAGAATTAGACATATTCTTGTCTATTAAC CACTCCTAAGAGATCATCCCT
CAAATAAGAGTTTGTCTAATAACAGTTTTAGGTCTATGCATTATAGATGAAGACATTTGAAGACCAAAAGCATTCACATCTGTAGTTTTTAAATTTTAGGCATACTGAGAGTTGTCAGGAAAAAT ATT
TCTGCTAGGGCCCTTTCACACCATATAGGTGATAACCTTATCTGCTTACATCCATGTGATGGTTTGGCCAAAGGCCACAGGTCCTTTTACAAAGCAGGGCCTACTTTTATTGTAACCAG tAAGAGCAG
ACAAAATATAATTTTCTGATTGGCAACTGTTCTTCTTTGGTTGGAAAAA GACCAGCATGTTCCAGAGGCATCAACTCTATCTTCAATGATCAGGTTAGCTAACATTTCTTTTTGGGATATTCTAGTTG
TATCAGATGCAGCCAGAAAGACCAGTCTTTCTATGATTTATATTCCTTCACCCAATTGCCTCCTTTTAACCAGTTTTCATGATATAAATTAAGGATATGCCAGACAATAAATTACTAATTTGATC AAT
TAATCCATACCAGATAATTTGATTAGCCAAAtGAATACTTGTTGAGTTCCTGCAATATCCTAGGTTCTGTGCATGCTAAACACCAGCATaaaaagactgtatgggccgggcgcggtggctcacgcctg
taatcccagcactttgggaggccgaggcgggcggatcacgaggtcaggagattgagaccatcccggctaaaacggtgaaaccccgtctctactaaaaaaatacaaaaaattagccggg CGTAGTGGC
GGGCGCCTGTGGTCCCAGCTACTTGGGAGGCTGAGGCAGGAGAATGGCGTGAACCCGGGAGGCGGAGCT TGCAGTGAGCCGAGATCCCGCCACTGCACTCCAGCCTGGGCGACAGAGCGAGACTCCGT
CTCAAAAAAAAAAAAAAAAAAAAAAAAAAAAAGACTGTATGACACAATCCTTGTCTGACTCTTGCTCCAAGATTCATAAACACTGTTAGACCAGATAGATCTGTCTTCCCTATAGATCTGTCTTCCCT
GTTACACCAGCTGGATTTGGACAACATGAGTTGCTGTGAGACCTAGACTTAGAGTAGAGGCCTAAGGAA GAATCTCTTCTGTTCTTCACTTGCTCCTCTGGTCCTCACCATTCTTCTTACATGGATAA
AAGGAAAGAGACATTATTCCTTAAAACTTAAAGCAAAGGTGACCTATGCCATCCCCTATCTTTACATTGTTTTATTCTTCCAATGGAGGGGCACCTCTATGCAGGAAGTAGCTCTAGTTCCATTA TAC
TACTTCATTGTCATGAGACAGAAACAAGTAGAGAAGAAGCAGCTGAGTATAAGAAGGAATATCATTTTT ACATTTTATATTTTACaTAATTTTCTTTCTTCCACCAAACTTTGACTGCTATTATTAAT
GTCTTTAGTTTGTTCCTGGTACATGTCAGGTGTCAGTGGAAGAGGAGAGAGGAGAATAAAATATGAAATAAGGAGAAAGAGCAGAAGGAACTGTGGCC ACTCAGAGGCACCTGGAACCTTCCTCCAGC
TAAGACTAGTCTTTGTAGACCACAGTGTAGCATGCTCTGGAGGCAGCCTGAAAAAATCATGTAGCCAGCTTAAGGAAAAATGCCAGAAAAGTGAT gAAGTTGATTCCTTCTTCCCCTTATATTCTAGA
CAGCACATTATTCATGCCGTTTCTGGCACCAAAGGATAAATTAGGCTGTCTGAATATTCTCGGCAGAAAAGAAGCTATAATAGCAAACTGAGTTATACAACATTCTGAACAAAATGCA ATAAGGCATG
TAATAGGCCAAATCAACTCCAAAGTTAAGCTTAAGAAAAAGTCCTCTGCTCTCAATTGTGTGTTT G
```

238	30.8	16.5	0.7	UnnamedSequence	441	561	(1582)	C	MamTip2	DNA/hAT-Tip100	(148)	157	18	1
1319	0.7	0.0	2.1	UnnamedSequence	1000	1143	(1000)	+	AluYa5	SINE/Alu	1	141	(170)	2
22	0.0	0.0	0.0	UnnamedSequence	1190	1213	(930)	+	(A)n	Simple_repeat	1	24	(0)	3
13	18.8	5.8	3.8	UnnamedSequence	1732	1783	(360)	+	GA-rich	Low_complexity	1	53	(0)	4

>hg19 chr6:93575150-93577069

```
GTATTTTCATTTCACCATTTGTAAAATAAAGCAGCTGAACTTGATATCACACTAGAACTTGATCAAAAGCCCAAGAAGCAACAATGTTTTGCCAATCCCTATGTTATCTTCCCTTTTTAAAATTCT GT
AATGCTGTGATTACAAATAGCTTCCTAGTTCCTAAAAATTTGATGTTTTAAGcGTCTTACTATTTAAGGGTTTAAGAAAGGACATTTGCACAATAATATACGATATCCAAATTTAAATATAAAATATG
CCCAAGAAAAGCAGATAAGGTCTGCAGTACCTCAGTGTCCATATAATGGCATTGAACAAATGGAAACAGATGGTCTGAGAGAATTAGACATATTCTTGTCTATTAAC CACTCCTAAGAGATCATCCCT
CAAATAAGAGTTTGTCTAATAACAGTTTTAGGTCTATGCATTATAGATGAAGA CATTTGAAGACCAAAAGCATTCACATCTGTAGTTTTTAAATTTTAGGCATACTGAGAGTTGTCAGGAAAAATATT
TCTGCTAGGGCCCTTTCACACCATATAGGTGATAACCTTATCTGCTTACATCCATGTGATGGTTTGGCCAAAGGCCACAGGTCCTTTTACAAAGCAGGGCCTACTTTTATTGTAACCAG cAAGAGCAG
ACAAAATATAATTTTCTGATTGGCAACTGTTCTTCTTTGGTTGGAAAA AGACCAGCATGTTCCAGAGGCATCAACTCTATCTTCAATGATCAGGTTAGCTAACATTTCTTTTTGGGATATTCTAGTTG
TATCAGATGCAGCCAGAAAGACCAGTCTTTCTATGATTTATATTCCTTCACCCAATTGCCTCCTTTTAACCAGTTTTCATGATATAAATTAAGGATATGCCAGACAATAAATTACTAATTTGATC AAT
TAATCCATACCAGATAATTTGATTAGCCAAAcGAATACTTGTTGAGTTCCTGCAATATCCTAGGTTCTGTGCATGCTAAACACCAGCATAAAAAGACTGTATGACACAATCCTTGTCTGACTCTTGCT
CCAAGATTCATAAACACTGTTAGACCAGATAGATCTGTCTTCCCTATAGATCTGTCTTCCCTGTTACACCAGCTGGATTTGGACAACATGAGTTGCTGTGAGACCTAGACTTAGAGTAGAGGCCT AAG
GAAGAATCTCTTCTGTTCTTCACTTGCTCCTCTGGTCCTCACCATTCTTCTTA CATGGATAAAAGGAAAGAGACATTATTCCTTAAAACTTAAAGCAAAGGTGACCTATGCCATCCCCTATCTTTACA
TTGTTTTATTCTTCCAATGGAGGGGCACCTCTATGCAGGAAGTAGCTCTAGTTCCATTATACTACTTCATTGTCATGAGACAGAAACAAGTAGAGAAGAAGCAGCTGAGTATAAGAAGGAATATC ATT
TTTACATTTTATATTTTACtTAATTTTCTTTCTTCCACCAAACTTTGACTGCTATTATTAATGTCTTTAGTTTGTTCCTGGTACATGTCAGGTGTCAGTGGAAGAGGAGAGAGGAGAATAAAATATGA
AATAAGGAGAAAGAGCAGAAGGAACTGTGGCCACTCAGAGGCACCTGGAACCTTCCTCCAGCTAAGACTAGTCTTTGTAGACCACAGTGTAGCATGCTCTGGAGGCAGCCTGAAAAAATCATGTAGCC
AGCTTAAGGAAAAATGCCAGAAAAGTGATtAAGTTGATTCCTTCTTCCCCTTATATTCTAGACAGCACATTATTCATGCCGTTTCTGGCACCAAAGGATAAATTAGGCTGTCTGAATATTCTCGGCAG
AAAAGAAGCTATAATAGCAAACTGAGTTATACAACATTCTGAACAAAATGCAATAAGGCATGTAATAGGCCAAATCAACTCCAAAGTTAAGCTTAAGAAAAAGTCCTCTGCTCTCAATTGTGTGT TTG
```

13	18.8	5.8	3.8	hg19_7	1509	1560	(360)	+	GA-rich	Low_complexity	1	53	(0)	1

>KSI_AluYa5_75

>Scaffold13910-1194632-1194724
ATTCAAGAAAGCTTCAATAAGCACATCCTTGTGGGTGGAATACCATCTGACAATTACAATGACATCATCCTGAGCCTTGTACTTGAAAGGCAGCATGGTGGGGAAGAGCCTGAGCATGGTCTCACA GA
GAGCTGGATTGGAAATGAGATCTGCTACTTACATGCAAATCAATTAATTCATCTTCTCTAAGCCTCAGTTTCCTCACTGTAAAGCCAAAAAGAACACTCCCTGCAGGATCATGGTGAAGATTATAATA
AATACACCTAAATCAGGGTGAGCGTTCCATGCATAGTAACTACTATTACTTGTTTCCAAAACCCCACACAAATAGCATCACTAAGCCCGGCCCTTTCATGAGCTCTCTTTGGGTGGTTACTTTTC CTT
TCAGGGACTAAGATATAAGACAAGGATAGAGACAGCGGTGAGGGCAAAACACTGCAACTACAATTTTTAGTAATGTGTTTAAATATTTAAATGCTACAATGTTTATTTTTAAAAGGAATGATGAATGA
GATCTCAATCTTATATTTCTGTACTAGAGTTTGCAAAGAATTTTCACATATTCTTTTTTATTGTACAATGGGGTGGATGTCCA CATAAGTTCTGAGTCAGATAGCCTGGCTATAAACCTCAGATTCTT
TACTTTCTAGCTGTGTGACCTTGGACAGCTACTCCATGACGCAGTTACTTCCACTGTAAATTGGTGGTAATAATAGTACCTACTTCAAATAAATATGCTAAAACATGTAGAAATGTTCAGGACAGTGT
CTAGCACAAGGGAAGCACTCTGTAGATATTATTCTCATCAACCAATAACGTAGACAGGACTGATGCTACAACTATTTTATAGATGATGAAACTGAGGCTCACGCAGTGGATGCCCAGAGCTTCTG TTC
CAATCTGGAGTGATTTTGAGATAAAGAAAAAAGATAAAGAGGAAAGTATCAATACTTTCTCCCAACAAGCAGCAAAATCATTATGATT AGAATGCTGAGTGCTTgcactttgggaggccgaggcgggc
ggatcacgaggtcaggagatcgagaccatcccggctaaaacggtgaaaccctgtctctactaaaaatncaaaaaaaaaaaaaaaaaaaaaaaaaaaaaaa agaatgctgagtgcttATATTATACATA
TATTTTATAGTAGACAACTTTTCAATACAGTGATGTTGCCTTTTGGTTGGGGGGGAACAGTTATTTGAGGCTATTTTTATACCATTAATATGTGTACAAATTTATGTCTGGGACTATTTATGTGAAAC
ATGGGGTTAATTATTCATTCTCTCCTAGCCTTTGCCATGACTCTCAATTAGATGCAACATTCTTCTCCATCCCACTTACTCAGGATGTGGCCAAATGACTTGCTTTGGTGAATTGAGTAGGGGCA ACC
ATGACAAACAGCTGTCTTCACAGAAGCTTTAAATAATTTGGTTTGCTTCTTCTTAGTCTTCTGCTCTCCGCCATGAGAAACCCATGACTCACATAGCAGCAGCTCCTTTAGCCTGAGTTCCAGAATGA
AAGGTACATGGAGATTACCCTAGGCCTGTCTACCTTAGACAGAGCCCAGTCGAGCCATGACCAAACCAGCAGCCATTATATAGCTTGATTAAACCACTGAGATTTATGGGTAAAAGTGATTAATACAG
GACATTTCAAAGAAAGCACTGTGACTTCTGTATAATGTTTAAAAAATATCAACAGATCCTGTTTTAGAGCTATCTGCTCACATCTAGTCATGTTAGCACGGATGAGTAACACTCTATCTAGCCTTTAA
TTCCTTCAGTTATGAAAACAGTGTTTTAAAGAGGTGATCTCTTCAGTTCAAGTTCTCTACTTCTAAGATTCTAGCAAGAAGGT TTACAGAAATAAAGTTATCATAACATTCAAACGTGGTGGAGAAAA
ATTATGATCTCTACTTTCTGAAAGAGAAAACTGAACCACAAACATTTAGATGAAATCTGAAAATTCTAGAGACTCATTATTGTGACAAGGCACATCCAGGGAAATGTGGTGGGTTATACATCGAGTAA
TGTGTGTTGACACTGGGATTCTGCTCTGTGGGCATCCCTTCTAC

436	26.9	14.1	1.0	UnnamedSequence	85	268	(1824)	+	MIRb	SINE/MIR	3	210	(58)	1	
314	22.4	1.3	0.0	UnnamedSequence	431	506	(1586)	+	X26_DNA	DNA/TcMar-Tigger	241	317	(574)	2	
487	28.7	9.4	0.5	UnnamedSequence	596	799	(1293)	+	MIR	SINE/MIR	25	257	(11)	3	
273	23.9	8.4	0.0	UnnamedSequence	799	869	(1223)	C	MIRc	SINE/MIR	(74)	194	118	4	*
926	2.0	0.0	0.0	UnnamedSequence	1001	1099	(993)	+	AluYa5	SINE/Alu	34	132	(179)	5	
24	3.6	0.0	0.0	UnnamedSequence	1100	1128	(964)	+	(A)n	Simple_repeat	1	29	(0)	6	
300	35.3	5.3	3.3	UnnamedSequence	1342	1549	(543)	+	MLT1I	LTR/ERVL-MaLR	72	283	(128)	7	
214	30.8	9.2	2.5	UnnamedSequence	1713	1865	(227)	+	MIR3	SINE/MIR	40	202	(6)	8	

>hg19 chr6:116306097-116308048
ATTCAAGAAAGCTTCAATAAGCACATCCTTGTGGGTGGAATACCATCTGACAATTACAATGACATCATCCTGAGCCTTGTACTTGAAAGGCAGCATGGTGGGGAAGAGCCTGAGCATGGTCTCACA GA
GAGCTGGATTGGAAATGAGATCTGCTACTTACATGCAAATCAATTAATTCATCTTCTCTAAGCCTCAGTTTCCTCACTGTAAAGCCAAAAAGAACACTCCCTGCAGGATCATGGTGAAGATTATAATA
AATACACCTAAATCAGGGTGAGCGTTCCATGCATAGTAACTACTATTACTTGTTTCCAAAACCCCACACAAATAGCATCACTAAGCCCGGCCCTTTCATGAGCTCTCTTTGGGTGGTTACTTTTC CTT
TCAGGGACTAAGATATAAGACAAGGATAGAGACAGCGGTGAGGGCAAAACACTGCAACTACAATTTTTAGTAATGTGTTTAAATATTTAAATGCTACAATGTTTATTTTTAAAAGGAATGATGAATGA
GATCTCAATCTTATATTTCTGTACTAGAGTTTGCAAAGAATTTTCACATATTCTTTTTTATTGTACAATGGGGTGGATGTCCACATAAGTTCTGAGTCAGATAGCCTGGCTATAAACCTCAGATT CTT
TACTTTCTAGCTGTGTGACCTTGGACAGCTACTCCATGACGCAGTTACTTCCACTGTAAATTGGTGGTAATAATAGTACCTACTTCAAATAAATATGCTAAAACATGTAGAAATGTTCAGGACAGTGT
CTAGCACAAGGGAAGCACTCTGTAGATATTATTCTCATCAACCAATAACGTAGACAGGACTGATGCTACAACTATTTTATAGATGATGAAACTGAGGCTCACGCAGTGGATGCCCAGAGCTTCTG TTC
CAATCTGGAGTGATTTTGAGATAAAGAAAAAAGATAAAGAGGAAAGTATCAATACTTTCTCCCAACAAGCAGCAAAATCATTATGATT AGAATGCTGAGTGCTTATATTATACATATATTTTATAGTA
GACAACTTTTCAATACAGTGATGTTGCCTTTTGGTTGGGGGGGAACAGTTATTTGAGGCTATTTTTATACCATTAATATGTGTACAAATTTATGTCTGGGACTATTTATGTGAAACATGGGGTTA ATT
ATTCATTCTCTCCTAGCCTTTGCCATGACTCTCAATTAGATGCAACATTCTTCTCCATCCCACTTACTCAGGATGTGGCCAAATGACTTGCTTTGGTGAATTGAGTAGGGGCAACCATGACAAACAGC
TGTCTTCACAGAAGCTTTAAATAATTTGGTTTGCTTCTTCTTAGTCTTCTGCTCTCCGCCATGAGAAACCCATGACTCACATAGCAGCAGCTCCTTTAGCCTGAGTTCCAGAATGAAAGGTACAT GGA
GATTACCCTAGGCCTGTCTACCTTAGACAGAGCCCAGTCGAGCCATGACCAAACCAGCAGCCATTATATAGCTTGATTAAACCACTGAGATTTATGGGTAAAAGTGATTAATACAGGACATTTCAAAG
AAAGCACTGTGACTTCTGTATAATGTTTAAAAAATATCAACAGATCCTGTTTTAGAGCTATCTGCTCACATCTAGTCATGTTAGCACGGATGAGTAACACTCTATCTAGCCTTTAATTCCTTCAG TTA
TGAAAACAGTGTTTTAAAGAGGTGATCTCTTCAGTTCAAGTTCTCTACTTCTAAGATTCTAGCAAGAAGGTTTACAGAAATAAAGTTATCATAACATTCAAACGTGGTGGAGAAAAATTATGATCTCT
ACTTTCTGAAAGAGAAAACTGAACCACAAACATTTAGATGAAATCTGAAAATTCTAGAGACTCATTATTGTGACAAGGCACATCCAGGGAAATGTGGTGGGTTATACATCGAGTAATGTGTGTTG ACA

CTGGGATTCTGCTCTGTGGGCATCCCTTCTAC

>KSI_AluYa5_76

>Scaffold13717-765201-765505
GTTGCTGGGGCATCCATTCCAGGGCACCATCAGTCTTCAGCGGCAAGGCGGAAGAGATCCAAGTAGGAGGTTTTG GTCAgCTCAGGGAAGGATGGGGGCAGTCCTTGCAGGAGCTGCTCACAGTCCGA
CTTCCAGTGGGGTCCTCCACAGAAGGGGCACAGGCTGGTGGGCTTACCTGGGTTTGGGCATTGTTTGGACCAGTGGCCTTCATTGCTGCACTTGAAACAGGCACCAGGTGGAGGTGGATTGCTAG GAG
GCTTCTTTGTGGAGCTGCGGCCTCAGGGACCTGCAGGGCCTCTAACGGTGGAGGCAA gCATTTGAAACTCTGCCTGTTTTTGCCTTTTACTTCCT CATCACAATTGTTAAAGACTTTGAAGGCTAAAT
TAAGAAGGTCTCGTTGTGGGGTTTGAGGGCCGTC gTCAAGCTTCTGAAGCTTaCACCAAATATCAGGGGTGGAcTGGGAGATGAATCGAAGGTTTAAAACAGTGGTTCTTTCTGGGCTG gCTGGGTCT
aGGTTGGTaTACTTTCTCATGCCTTCAGTTACATGAGAGAGAAAAAGGGCTGGGTTTTCGTCAGGACCTTAGGTGATTTCTGAAAGTTTTTCATA GTTTACCACTTTATGGgCAGCTTTTTTGAGTCC
TGCAAGGAGACACACAATCCTGTGGTCTGGACGGTGGCGTCTAGAGGCCCCGTCTTGATAATCCTAG TGGGGTCCTGGTTGGGGACTGCCTCTGTGCCAGTAGGCTGGGCAGGAGCTTGGTGATGAAT
TGTATCAGCATGCTCCTGAACTaGGGTCCAGATACGGTTTTGGTCTTCTGAGGTGAGGGTGGAAGAGAGGATAATGTAGAGGTCATGCCAGGTTA GTTCATAAGACTGGGTAAGGTACTGAAACTCCT
CAATATAAGAGaTAGAGTTTTCTGGAAATGAACTGCGACTGTTGTTAATTTGAGAGAGATCAGTGAGGGAGAAGGGAACATGAACTCTA aCAATAtCTTCAGggccgggcgcggtggctcacgcctgt
aatcccagcactttgggaggccgaggcgggcggatcacgaggtcaggagatcgagaccatcccggctaaaacggtgaaaccccgtctctactaaaaatacaaaaaaaaaaaattagccgggcgta gtg
gcgggcgcctgtagtcccagctacttgggaggctgaggcaggagaatggcgtgaacccgggaggcggagcttgcagtgagccgagatcccgcccctgcactc cagcctgggcgacagagcgaaactcc
gtctcaaaaaaaaaaaaaaaaaacaataccttcagTTcCTGCTACTTCCTGAAGGGAGCACTCTAGCACCAGCACTGAAGTAAGAGTagggcatgggcCAAgGATGGTGCCTcAGCGAGTATAGGCGG
TAGaGAAAGAAGAAGCTGGAAgTGGTTCCTTCTGAGGGTTTGAAGGGGGAAGGGGGGTTGAGTTGATAGGTAATGGAGGATAGATAGGGGTGTAAGGTGGCA TGATGGGTTTACAGGCTTCAGGAGAG
GGTGGTGGGGGAGGAGAATGGGTACAGGCAATACTAGAATTGTCTTGAGGAGGGGACGGTGTAGGAAAAGGAGTGGGTACTTTTGGAAGTGATGCCGGCTGGGAAGAT GGCGGGGAAGATGGCAGCTG
AGAAGATAACAAGGAAGATTGGGGAGTTAAAGAAGACAGTTGAGAGGGAGAGGTAGGGGCTGGGAGTGGTGGACAGCAGTCAGCTGAATTGAATGAGGAAAA AGAGTTAGGGTCGGAAGGAGAAAGGC
AATCAGGGCGGTGAGAATGGAGGAGAAGGATTTGAACAGGTAAGCCAGAATTGCAGAGGTCGGGTTGTGATCTGAGTGCAAAACGGCCTGGACATAAGGAATTTCTGCCCATTTTTCCAGTAGTC GGC
AATAATTGCTTAAGTCAGTTAAAACTGTAAAGTCGAATGTTCTATTTGCAGGCCATTTGGACCTGTTATCTAATTC gGACTGCAGTCAGGCTGTATTGCAAAAAAGATAAGGCGCTTAGGaTGGATAT
CTTGCCTGAGGCCTAAGGTTTACAGGCTTTTTTATGGGGCAGCCTAGAGGGCTGTTTTTGGAATGGAGGACTGGGAGTTTCCGATAACCGAGGGTCAGCTTGGGAGAACAGGGAAAAGGAGAC CGT
CCTGGACGGCTGGAGGGAGACGATAAAAGGAGCAATCGTCACCGCTGCTTTTCTCGTTCCCTGAATGGGATCAAATGGCTTAGAGGCGTCCCCCTAAGACCA GATGATCAGCGAGTgCCTGGCACAC

8976	12.7	1.1	2.2	UnnamedSequence	1	998	(1305)	C	HERVH48-int	LTR/ERV1	(4367)	2287	1313	1
2723	1.0	0.0	2.0	UnnamedSequence	999	1306	(997)	+	AluYa5	SINE/Alu	1	302	(9)	2
8976	15.4	1.7	1.7	UnnamedSequence	1307	2303	(0)	C	HERVH48-int	LTR/ERV1	(5342)	1312	256	1

>hg19 chr8:138294195-138296183
AGTTGCTGGGGCATCCATTCCAGGGCACCATCAGTCTTCAGCGGCAAGGCGGAAGAGATCCAAGTAGGAGGTTTTG GTCAcCTCAGGGAAGGATGGGGGCAGTCCTTGCAGGAGCTGCTCACAGTCCG
ACTTCCAGTGGGGTCCTCCACAGAAGGGGCACAGGCTGGTGGGCTTACCTGGGTTTGGGCATTGTTTGGACCAGTGGCCTTCATTGCTGCACTTGAAACAGGCACCAGGTGGAGGTGGATTGCTA GGA
GGCTTCTTTGTGGAGCTGCGGCCTCAGGGACCTGCAGGGCCTCTAACGGTGGAGGCAA aCATTTGAAACTCTGCCTGTTTTTGCCTTTTACTTCCTCATCACAATTGTTAAAGACTTTGAAGGCTAAA
TTAAGAAGGTCTCGTTGTGGGGTTTGAGGGCCGTC aTCAAGCTTCTGAAGCTTgCACCAAATATCAGGGGTGGAtTGGGAGATGAATCGAAGGTTTAAAACAGTGGTTCTTTCTGGGCTG aCTGGGTC
TgGGTTGGTgTACTTTCTCATGCCTTCAGTTACATGAGAGAGAAAAAGGGCTGGGTTTT CGTCAGGACCTTAGGTGATTTCTGAAAGTTTTTCATAGTTTACCACTTTATGG aCAGCTTTTTTGAGTC
CTGCAAGGAGACACACAATCCTGTGGTCTGGACGGTGGCGTCTAGAGGCCCCGTCTTGATAATCCTAG TgGGGGTCCTGGTTGGGGACTGCCTCTGTGCCAGTAGGCTGGGCAGGAGCTTGGTGATGA
ATTGTATCAGCATGCTCCTGAACTgGGGTCCAGATACGGTTTTGGTCTTCTGAGGTGAGGGTGGAAGAGAGGATAATGTAGAGGTCATGCCAGGTTAGTTCATAAGACTGGGTAAGGTACTGAAACTC
CTCAATATAAGAGgTAGAGTTTTCTGGAAATGAACTGCGACTGTTGTTAATTTGAGAGAGATCAGTGAGGGAGAAGGGAACATGAACTCTA tCAATAcCTTCAGTTtCTGCTACTTCCTGAAGGGAGC
ACTCTAGCACCAGCACTGAAGTAAGAGTCAAcGATGGTGCCTgAGCGAGTATAGGCGGTAGtGAAAGAAGAAGCTGGAAaTGGTTCCTTCTGAGGGTTTGAAGGGGGAAGGGGGGTTGAGTTGATAGG
TAATGGAGGATAGATAGGGGTGTAAGGTGGCATGATGGGTTTACAGGCTTCAGGAGAGGGTGGTGGGGGAGGAGAATGGGTACAGGCAATACTAGAATTGTCTTGAGGAGGGGACGGTGTAGGAA AAG
GAGTGGGTACTTTTGGAAGTGATGCCGGCTGGGAAGAT GgtggggaagatgGCGGGGAAGATGGCAGCTGAGAAGATAACAAGGAAGATTGGGGAGTTAAAGAAGACAGTTGAGAGGGAGAGGTAGGG
GCTGGGAGTGGTGGACAGCAGTCAGCTGAATTGAATGAGGAAAAAGAGTTAGGGTCGGAAGGAGAAAGGCAATCAGGGCGGTGAGAATGGAGGAGAAGGATTTGAACAGGTAAGCCAGAATTGCA GAG
GTCGGGTTGTGATCTGAGTGCAAAACGGCCTGGACATAAGGAATTTCTGCCCATTTTTC CAGTAGTCGGCAATAATTGCTTAAGTCAGTTAAAACTGTAAAGTCGAATGTTCTATTTGCAGGCCATTT
GGACCTGTTATCTAATTCaGACTGCAGTCAGGCTGTATTGCAAAAAAGATAAGGCGCTTAGG gTGGATATCTTGCCTGAGGCCTAAGGTTTACAGGCTTTTTTATGGGGCAGCCTAGAGGGCTGTTTT
TTGGAATGGAGGACTGGGAGTTTCCGATAACCGAGGGTCAGCTTGGGAGAACAGGGAAA AAGGAGACCGTCCTGGACGGCTGGAGGGAGACGATAAAAGGAGCAATCGTCACCGCTGCTTTTCTCGTT
CCCTGAATGGGATCAAATGGCTTAGAGGCGTCCCCCTAAGACCAGATGATCAGCGAGT tCCTGGCACAC

>KSI_AluYa5_77

>Scaffold10516-436226-436378
ATTTTTAATTCTGCTACAGCAAGGAGCCAGATAACGCGTTAGTTTCTTTCAAAGTCTGATATGATTTTATGATTCTATAAAATATACAAAATTATCAACAATTAGGCCACCTGAAAAATGTTTGAATC
TAATCATCTAGATATGCACTAAAATACTAAAATAATATGCATAATATCAGATAACCTTGATAATTACTTCTTCCATTAAATAAATTTAATCTTTCAAGAAGTCTCCTGTGCTATCTACTTGACTA TCG
GAAAGGATAGGAGAGTTGGGTAACAATTAATGAAGCAGTGCCCACCTAGTGGAAAGTTCTAGTCATATTTCATAGGGCTTTCTGTTTTAACTTAT cAGCTATGACTATTTCCACCCCTTTAATCAATG
ACTTTGCAGTATATATAAAAACATAAGTGAATGGCATTCACCTGGATGCTACAAATAACATGTAAATAATAAACTCAAGATTCAAAACTAAATAGGCCAAAATATTGGAGAATAATAGGTAAATT AAA
ACTTTATTAGGAAAAATTAAAATTCATGTGGAAAGAAGCATGAGGAAACTTTCTATGGTAATAATAATGGATTTAAATCTTGATAGGAGTTTGAAT AATATTTTGAACTCAAGTTCCTGGTACACATG
CTGAGGTATTTATGAGTGTTTGTGAGAGTTTTATGCTGCCATTTTATTTAAAATACATCATAAAATAAGACAATTT gGTGAATGAATAGATGGATGTATAGAAAGATATGTAATAAAGTAAACACAAG
CAAATGTTAATTGTAGAATCTAGGTTGTGTACATGTGGTTATTCACTATATAATCTTTTCAATTTTTCATTACGTTTCAAAAGTTTCCTAATATA gGTTTGTGAAAAATTTATATGTTTGGTCTTAAA
CAATATTGAAGAATTCCTGGAACTTAATAGTATTCTAATAAGTTTGTATGGAATAAATGAATAAATAATTACATGAATAATTGAATGGTTTAAA Aagtaaatatggccgggcgcggtggctcacgcct
gtaatcccagcactttgggaggccgaggcgggcggatcacgaggtcaggagatcgagaccatcccggctaaaacggtgaaaccccgtctctactaa aaatacaaaaaattagccgggcgtagtggcgg
ggggcgggggcgccgcccccccgggggcaaaaaaaaaaaaaaaaaaaaaaagAGTAAATATTAAAAATGGAAAAAGTCTTAATGGCAGCTTGTGTGTAAAATATTCTGGGGTTTTAGGTAACCTAT
TACTTATTTTCCAAGTAATGTACCGAGCCACCAAGGAATCAAACACAATTCCAGATGATAATACAATAAACACAGTGTTCAAATTAAGGGGATAAT AGAGCCATTCTGTTCTGGTCAGAGCATATCTA
AATTATTGCATCCTCTTCCAGACACTCACCTTTACAAAATTATAACAGAGAAATGACTACCAAGAGGAGAATGACTGGGGAACAGTGAGCTCAAAAATTCAAGCCAGATAGGAAGAAGTTAAATG ATG
TAGAGATTTTCAAACTGGAGAAGAAAATCCCAGTGAAGAACCTTATAGT GGATACATTCCCACCATTCCCACTCTAACTCTTTTCACGGTTCTCATTTCAGCTCAGTTTTTAACTATTTCAGAATGCA
TTATATTACAGTCTTAATTTAATTATTTTTAAATAAATATATAATTTATTATTTTGTTTATTTTGCATGTAGTGCCAGGAACCTTATACCAATTGTTGATCTTGGTCTTACTCCTGCATGTGCACATA
CACACATAGACGCACATGCACACACACACAGAGTCTCAATTTTCAGCAAGAATCTAAGCCTCTT GAGGGAATGAACTGTGTTTTATATATATCTTT GTATTCCTCAAACTCAGCACAATTGTGCCCAT
TTATTTTATAAACATAGTGAAAAATATTTTTGTGCTAATTACTGCACTGAGATTGGAGCTTAAAAATTTATTTTAACACCTCCCCCTGCTCATAAACAGCTCATAGTCTAAAATTGGAAATGAAC ATA
AAACCCAATAATACAAATCATTGTAATCAGTACAATGACAGGAACATACATAAAATACTATGGAAACCAATATGACAGTTGCATCTAACATCCCGG AGACGA

338	37.9	7.2	3.7	UnnamedSequence	275	537	(1613)	+	L3	LINE/CR1	3079	3350	(749)	1
298	21.9	0.0	1.6	UnnamedSequence	541	605	(1545)	+	L1ME2z	LINE/L1	6011	6074	(370)	2
873	24.6	1.9	1.9	UnnamedSequence	615	875	(1275)	+	L1ME2z	LINE/L1	6181	6441	(3)	2
1500	0.0	0.0	0.0	UnnamedSequence	1001	1153	(997)	+	AluYa5	SINE/Alu	1	153	(158)	3
22	0.0	0.0	0.0	UnnamedSequence	1182	1205	(945)	+	(A)n	Simple_repeat	1	24	(0)	4
590	37.9	6.1	1.7	UnnamedSequence	1218	1563	(587)	+	L3	LINE/CR1	3386	3746	(353)	1
13	29.6	2.0	0.0	UnnamedSequence	1678	1727	(423)	+	(TTTA)n	Simple_repeat	1	51	(0)	5
19	19.7	0.0	0.0	UnnamedSequence	1787	1822	(328)	+	(CA)n	Simple_repeat	1	36	(0)	6
271	26.0	3.5	6.0	UnnamedSequence	1832	1917	(233)	+	L2d2	LINE/L2	3336	3419	(45)	7
246	33.6	5.4	1.7	UnnamedSequence	2004	2115	(35)	C	L2c	LINE/L2	(79)	3308	3193	8

>hg19 chr9:12600801-12602737
ATTTTTAATTCTGCTACAGCAAGGAGCCAGATAACGCGTTAGTTTCTTTCAAAGTCTGATATGATTTTATGATTCTATAAAATATACAAAATTATCAACAATTAGGCCACCTGAAAAATGTTTGAATC
TAATCATCTAGATATGCACTAAAATACTAAAATAATATGCATAATATCAGATAACCTTGATAATTACTTCTTCCATTAAATAAATTTAATCTTTCAAGAAGTCTCCTGTGCTATCTACTTGACTA TCG
GAAAGGATAGGAGAGTTGGGTAACAATTAATGAAGCAG TGCCCACCTAGTGGAAAGTTCTAGTCATATTTCATAGGGCTTTCTGTTTTAACTTAT tAGCTATGACTATTTCCACCCCTTTAATCAATG
ACTTTGCAGTATATATAAAAACATAAGTGAATGGCATTCACCTGGATGCTACAAATAACATGTAAATAATAAACTCAAGATTCAAAACTAAATAGGCCAAAATATTGGAGAATAATAGGTAAATT AAA
ACTTTATTAGGAAAAATTAAAATTCATGTGGAAAGAAG CATGAGGAAACTTTCTATGGTAATAATAATGGATTTAAATCTTGATAGGAGTTTGAATAATATTTTGAACTCAAGTTCCTGGTACACATG
CTGAGGTATTTATGAGTGTTTGTGAGAGTTTTATGCTGCCATTTTATTTAAAATACATCATAAAATAAGACAATTT tGTGAATGAATAGATGGATGTATAGAAAGATATGTAATAAAGTAAACACAAG
CAAATGTTAATTGTAGAATCTAGGTTGTGTACATGTGG TTATTCACTATATAATCTTTTCAATTTTTCATTACGTTTCAAAAGTTTCCTAATATA aGTTTGTGAAAAATTTATATGTTTGGTCTTAAA
CAATATTGAAGAATTCCTGGAACTTAATAGTATTCTAATAAGTTTGTATGGAATAAATGAATAAATAATTACATGAATAATTGAATGGTTTAAA AAGTAAATATTAAAAATGGAAAAAGTCTTAATGG
CAGCTTGTGTGTAAAATATTCTGGGGTTTTAGGTAACC TATTACTTATTTTCCAAGTAATGTACCGAGCCACCAAGGAATCAAACACAATTCCAGATGATAATACAATAAACACAGTGTTCAAATTAA
GGGGATAATAGAGCCATTCTGTTCTGGTCAGAGCATATCTAAATTATTGCATCCTCTTCCAGACACTCACCTTTACAAAATTATAACAGAGAAATGACTACCAAGAGGAGAATGACTGGGGAACA GTG
AGCTCAAAAATTCAAGCCAGATAGGAAGAAGTTAAATGATGTAGAGATTTTCAAACTGGAGAAGAAAATCCCAGTGAAGAACCTTATAGTGGATACATT CCCACCATTCCCACTCTAACTCTTTTCAC
GGTTCTCATTTCAGCTCAGTTTTTAACTATTTCAGAATGCATTATATTACAGTCTTAATTTAATTATTTTTAAATAAATATATAATTTATTATTTTGTTTATTTTGCATGTAGTGCCAGGAACCT TAT
ACCAATTGTTGATCTTGGTCTTACTCCTGCATGTGCAC ATACACACATAGACGCACATGCACACACACACAGAGTCTCAATTTTCAGCAAGAATCTAAGCCTCTTGAGGGAATGAACTGTGTTTTATA

TATATCTTTGTATTCCTCAAACTCAGCACAATTGTGCCCATTTATTTTATAAACATAGTGAAAAATATTTTTGTGCTAATTACTGCACTGAGATTGGAGCTTAAAAATTTATTTTAACACCTCCC CCT
GCTCATAAACAGCTCATAGTCTAAAATTGGAAATGAACATAAAACCCAATAATACAAATCATTGTAATCAGTACAATGACAGGAACATACATAAAATACTATGGAAACCAATATGACAGTTGCATCTA
ACATCCCGGAGACGAGT

>KSI_AluYa5_78

>Scaffold14588-526215-526528

TGCTTTACACGGCTAGATTTGGGGGTAATTTGCTGTGCAGCCACAGTACCTGGAACAAGAGGACAATGGCTGGAGTTGCCTAGTGGCTGTCGGTGGGCTTGTTGAAAGTGGACCTATTTGAGATGC TG
AATAGAAATTGAATTAACAAGATGTAGTCATGAAAGGGACAGAAAATGTCTTAATTATAAAAAAATTATAAAGACCCAGCCAGGCGTGATGGCTCATGCCTGTAATGCACTTTGGGAGGCGGAAG CGG
GCGGATCACCTGAGGTAGGGAGTTTGAGATCAGCCTGACCAACATGGTGAAACCCCGTCTCTACTGAAAATACAAAATCAGCTGTGCGTGGTGGCGGGC gCCTGTAATCCCAGCTACTCAGGAGGCTG
AGGCACGAGAATTGCTTGAACCCAGGAGGCAGAGGTTGCAGTGAGCCGAGATTGTGTCATTGCACTCCAACCTGGGCAACAAGAGTGAAACTCCGTCTCAAAAAAAA AtAAATAAATAAACAAATATA
AAGATACAAACCAGGCAtGGTGGCTTACGCCTGTAATTCCAGCACTTTGGAAGCTGAGGTGGGTGGATTGCTTGAAGCCAGGAGTTTGAGACCAGCCTGTCCAACATGGTGAAACCCCGTCTCTACTA
AAAATACAAAAATTAGCCAGGTGTGGTGGCACACACCTGAGTGTGTGTGTACCAGCTTCTCAGGAGGCGGAGGCATGAGAATGGCTTGAACCCAGGAGGCGGAGGTTGCAGTGAACCGAGGTC ATGCC
ACTGCACTTCCATCCCCAGGGAGGGAAGGAGCAAAAGAAAACTAGGAAAAGGGGCTAAAAATGGTGCTTGTCTCACTGCTTTCTTCAGTTGAGCTCTTCTTGGTCTTGTGCTGGTAACTTGATGATTT
TCATTCGCCTTTGACGACTCAGCCCATTAAGCcaaagaggctcacctggtgctactcagcctgggtgacagggagactatgtctcaaaaaaaaacaaaaaac aaggccgggcgcggtggctcacgcct
gtaatcccagcactttgggaggccgaggcgggcggatcacgaggtcaggagatcgagaccatcctggctaaaacggtgaaaccccgtctctactaaaaatacaaaaaattagccgggcgtagtggc gg
ggggggcagtcccagctacttgggaggctgaggcaggagaatggcgtgaacccgggaggcggagcttgcagtgagccg CAAAGAGGCTCACCTGGTGCTACTCAGCCTGGGTGACAGAGGGAGACTA
TGTCTCAAAAAAAACAAAAAACAAAAAATACAAATAAAGATATGAAGGAAGGCCCAGTTAGAATACATTTTTGAAAATACTTCTTTACATTTTTAATGACGCATGCATTTCATATTGAGAGCTTCATT
CTTTCGGTTTAATAATTTGATCCTTGTTAAAATGCAGACAATTGGCTGGGCATGGTGGCTCATATCTTTGGGAGGCTGAGGTGAGCAGATCACTTGAGCCCAGGAGTTTGAGACCAGCCT GGGCAACA
TGGTGAAACCCTGTCTCTACTAAAAAAAATACAAAAAAATAATTAGCTAGGTGTGGTGGTGCCTGCCTGTGGTCCCAGCTACTTGGGAGCCTGAGGTAGGAAGATCGCTTGAACTCAGGAGTCAG AGG
CTGCAGTAAGCCGGGATCACACCCCTGCACTCCAGCCTGAgggaCAGTGAAATCCTGTCTCAAAAAAAAAAAAAATGCAGACAACTCAAGGATTGATAAAATTTGAAATTTTTTCATTAAC ACCTTAGC
ATGAATGAAGACTGAAACCTAGTTTTCTTTTTGATAAAGACAATGGAGGCGTCCAAGGGCTAAGGACATACATCCCCAGGGAGGGAAAGAGAAAGAGAAAAAGTAGGAAAAGGGGCTAAAAATGG TGT
TTGTCTCACTGCTTTCTTCAGTTGAGCTCTTCTTGGTCTTGTGCTGCTAACTTGATGATTTTCAT TCGCCTTTGACGACTCAGCCCATTAAGC CAAAGAGGCTCACCTGGTGCGACTGTTACTCACAA
TGGCTCACACGTGTTGGGTGCACGTGCTGGGCCAAGCATCATGCACGCTGTAAGGTATATAGATGTGATTTGGTCAAGGTAGAGACCGAGGCGGATATACAGGCCTGCACGGCTCAGTGAGTTTG GTG
CGCAGGCGCACACCTCCGCTTGTTACATAACCTGTTTGTGTAAGTTCATACTTGGCTCTGAGCCACTATTGTCTGTAAAAGGTATAACTGCCCTGCTGACGCCGTACA aGTGCTTTTGAGGCTCAGCT
TGGCTCGACA

2267	9.9	1.7	0.0	AluYa5_87	207	508	(1806)	+	AluSq2	SINE/Alu	2	308	(5)	1
1687	13.7	0.4	2.8	AluYa5_87	523	778	(1536)	+	AluSz6	SINE/Alu	3	252	(60)	2
322	12.5	0.0	0.0	AluYa5_87	953	1000	(1314)	+	AluYe5	SINE/Alu	252	299	(11)	3
2370	5.5	0.3	2.0	AluYa5_87	1001	1308	(1006)	+	AluYa5	SINE/Alu	1	303	(8)	4
1761	13.2	5.6	2.7	AluYa5_87	1452	1738	(576)	+	AluJb	SINE/Alu	1	295	(17)	5
13	14.5	3.2	0.0	AluYa5_87	1874	1904	(410)	+	GA-rich	Low_complexity	1	32	(0)	6
1673	10.1	0.0	0.0	AluYa5_87	2097	2314	(0)	+	LTR18B	LTR/ERVL	1	218	(396)	7

>hg19 chr10:5896371-5898378

TGCTTTACACGGCTAGATTTGGGGGTAATTTGCTGTGCAGCCACAGTACCTGGAACAAGAGGACAATGGCTGGAGTTGCCTAGTGGCTGTCGGTGGGCTTGTTGAAAGTGGACCTATTTGAGATGC TG
AATAGAAATTGAATTAACAAGATGTAGTCATGAAAGGGACAGAAAATGTCTTAATTATAAAAAAATTATAAAGACCCAGCCAGGCGTGATGGCTCATGCCTGTAATGCACTTTGGGAGGCGGAAG CGG
GCGGATCACCTGAGGTAGGGAGTTTGAGATCAGCCTGACCAACATGGTGAAACCCCGTCTCTACTGAAAATACAAAATCAGCTGTGCGTGGTGGCGGGC aCCTGTAATCCCAGCTACTCAGGAGGCTG
AGGCACGAGAATTGCTTGAACCCAGGAGGCAGAGGTTGCAGTGAGCCGAGATTGTGTCATTGCACTCCAACCTGGGCAACAAGAGTGAAACTCCGTCTCAAAAAAAA AaaAAATAAATAAACAAATAT
AAAGATACAAACCAGGCAcGGTGGCTTACGCCTGTAATTCCAGCACTTTGGAAGCTGAGGTGGGTGGATTGCTTGAAGCCAGGAGTTTGAGACCAGCCTGTCCAACATGGTGAAACCCCGTCTCTACT
AAAAATACAAAAATTAGCCAGGTGTGGTGGCACACACCTGAGTGTGTGTGTACCAGCTTCTCAGGAGGCGGAGGCATGAGAATGGCTTGAACCCAGGAGGCGGAGGTTGCAGTGAACCGAGGTC ATGC
CACTGCACTTCCATCCCCAGGGAGGGAAGGAGCAAAAGAAAACTAGGAAAAGGGGCTAAAAATGGTGCTTGTCTCACTGCTTTCTTCAGTTGAGCTCTTCTTGGTCTTGTGCTGGTAACTTGATGATT
TTCATTCGCCTTTGACGACTCAGCCCATTAAGCCAAAGAGGCTCACCTGGTGCTACTCAGCCTGGGTGACAGAGGGAGACTATGTCTCAAAAAAAACAAAAAACAAAAAA TACAAATAAAGATATGAA
GGAAGGCCCAGTTAGAATACATTTTTGAAAATACTTCTTTACATTTTTAATGACGCATGCATTTCATATTGAGAGCTTCATTCTTTCGGTTTAATAATTTGATCCTTGTTAAAATGCAGACAATTG GC
TGGGCATGGTGGCTCATATCTTTGGGAGGCTGAGGTGAGCAGATCACTTGAGCCCAGGAGTTTGAGACCAGCCTGGGCAACATGGTGAAACCCTGTCTCTACTAAAAAAAATACAAAAAAATAATTAG
CTAGGTGTGGTGGTGCCTGCCTGTGGTCCCAGCTACTTGGGAGCCTGAGGTAGGAAGATCGCTTGAACTCAGGAGTCAGAGGCTGCAGTAAGCCGGGATCACACCCCTGCACTCCAGCCTG ACAGTGA
AATCCTGTCTCAAAAAAAAAAAAAATGCAGACAACTCAAGGATTGATAAAATTTGAAATTTTTTCATTAACACCTTAGCATGAATGAAGACTGAAACCTAGTTTTCTTTTTGATAAAGACAATGGA GGC
GTCCAAGGGCTAAGGACATACATCCCCAGGGAGGGAAAGAGAAAGAGAAAAAGTAGGAAAAGGGGCTAAAAATGGTGTTTGTCTCACTGCTTTCTTCAGTTGAGCTCTTCTTGGTCTTGTGCTGCT AA
CTTGATGATTTTCATTCGCCTTTGACGACTCAGCCCATTAAGCCAAAGAGGCTCACCTGGTGCGACTGTTACTCACAATGGCTCACACGTGTTGGGTGCACGTGCTGGGCCAAGCATCATGCACG CTG

TAAGGTATATAGATGTGATTTGGTCAAGGTAGAGACCGAGGCGGATATACAGGCCTGCACGGCTCAGTGAGTTTGGTGCGCAGGCGCACACCTCCGCTTGTTACATAACCTGTTTGTGTAAGTTCA TA
CTTGGCTCTGAGCCACTATTGTCTGTAAAAGGTATAACTGCCCTGCTGACGCCGTACA gGTGCTTTTGAGGCTCAGCTTGGCTCGACA

2288	9.9	1.6	0.0	hg19	207	509	(1499)	+	AluSq2	SINE/Alu	2	309	(4)	8
1702	13.3	0.4	2.8	hg19	524	779	(1229)	+	AluSz6	SINE/Alu	3	252	(60)	9
301	11.8	0.0	0.0	hg19	954	1004	(1004)	+	Alu	SINE/Alu	251	301	(0)	10
1714	12.7	7.1	2.7	hg19	1150	1432	(576)	+	AluJb	SINE/Alu	1	295	(17)	11
13	14.5	3.2	0.0	hg19	1568	1598	(410)	+	GA-rich	Low_complexity	1	32	(0)	12
1683	9.6	0.0	0.0	hg19	1791	2008	(0)	+	LTR18B	LTR/ERVL	1	218	(396)	13

>KSI_AluYa5_79

>Scaffold9158-641473-641486
AGGCTGCCAAGTCAAGATGTATAATTGGTAATGCTGAGCCATTTATTCTGAGTGCACTCTAGACAATGACATATTATTACTATTATGGCACAAATAACTGCCTTTTTGAAGCAAATGTTTTCACTG GC
TTTAGTTAGGATGCTGTCCTATTCCTTTTAACATgATACTGATGCCCTCAGTATTATTTTATATATGTCAATGAAACAATATCATGCTGTGAAATTTATGATAAAAGCAAAACTAAAAATCATTGGTT
GATTTTTGTTTCCCCCTCTCTTCCTTTTAACTTCAGCTTTGCAAAATCACTATGTATTCTGCTGTGGAAGTATAATTAATGTTATTTTCTTCTTATTACTATATTTTAACTTTGCATCTTAAGCCT AA
ATTTTAGCCTATTCATGTAAGGTTGTACTTTAAACAATACCTCCAATAGATTTTCATACTCCATCTAACTATACAAAGAGAAGAGACTATCTAGTTACAATTTATGTATTTGATTGTGGTAGCTT ATG
CCTACCCTAAATATTTTCCCTCCTAGGTCAACCTGGTTTGGGTTCAATTATATGATGATTAAAAAAAAAATCTCAGGTTGGAACAAAATCTATATAATAAGATGACAAAATACTTTCATAACTATT TC
AGTTATAGCTTCTACTAAGTTGCATTTACAATGATATATTTACATAACAGCATAATGGATAGAAATATAAAGAGAGAGACCTTTTGTCTCTCTGTGCCATCAGAATGAACCATGACATTTAGGTG CCT
TGAGGAAAGACTAGAAATAAACTACCAATCAGGCAAATGAACAAACAAAAACAGAATAATTTCTTTCCTAGCTGCCCCACACTTTATCACATCACTTCAGTATGCCCCACTTCCTTAACTCTAATC AA
TTTCCATGTGCCATTTGAGGTACCACAGCTCACTCTCAGAAGGTCAAGAGGTAAAGTGTGGAGAAAAGGGAAGAAGGTGGGTATAAATTaaaaaaaacaaaaagggccgggcgcggtggctcacgcct
gtaatcccagcactttgggaggccgaggcgggcggatcacgaggtcaggagatcgagaccatcccggctaaaacggtgaaaccccgtctctactaaaaatacaaaaaaaaaattagccgggcgta gtg
gcgggcgcctgtagtcccagctacttgggaggctgaggcaggagaatggcgtgaacccgggaggcggagcttgcagtgagccgagatcccgccactgcactcca gcctgggcgacagagcgagactcc
gtctcaaaaaaaaaaaaaaaaaaaaaaaaaaaaaaaaaaaaaaaaaaaaaaaaaaaaaaaaaaa AAAAAAAACAAAAAGGACAAAGATAAAATACAAGATAAATTAAAATTATATTTTTAAATT
AGCTTTTGTTCCTTGAGCATCTGCTATGTATCAGGCATTCTCTATTACCTATTTTCGTTCTGGCCACACAACTACACTATGAAGCAGATATGACTGTACCACTT TCAAAcTCATATAAGAAAACCAAT
GTTCAGAGAAGTTGTCTGAGGTCCCACACCTAATAAATAAATGACAGAGCAAGGATTTGGCCTCAAAGTCCAGATTCTTTCCTCAGTTGCACAGTGCCACATTATAAATGTGTTGATACACTGAAATA
GAAGAATAATATCAAAAGTATTTCTTTTTATTTTCTCTCAACAGGGATTAATAATAATAATTATGTCAGAACTTGGGCACAGGCTTTAATCCTTAAAATTCTTT TAATCATTATTTTATCATCATAAG
CACATACAAGCAACAtTGTATTTATTTCGCTGAAATGTATAAAATCCTGGCATAGCCAATGAATTTGTTTGATAAATTATGAGATAAATTCAGCTAATGGAGTTTCCAAGCATGCTGTCAAAGAAATA
GAATCATGCATTTATGAATTTTTCCATATTTTTCTTCATTTCTAATGCAACTAGTTAATTATTTGATCTCTTTATCACTTTAAAAGCTTTGTAATAGTTCAGAT GATGTGAGATTATTTGCAAACAAG
ATAAGAAATGCCAGTTCCTGGGACAGTTATAGGAAGCCTGAGAAGG cAAGGCTTTTCAAGGAAACTAGGTCGTGAAAGAGAATATTGCTATAATCCTTAGAAAGCTGGATAGAGTTGTCTTTTCCAAT
ATTAATATGAGGCTCAATAGTTCCATATGGACAAGAACAGGGGTTGAGATCTCTGAGTCATGCAGAACTCTTCTGTCAAAACAGAACAGTTGCAGATGTGTCAT GAAT

52	16.8	0.0	0.0	UnnamedSequence	986	1000	(1284)	+	A-rich	Low_complexity	1	15	(0)	1
2968	0.0	0.0	1.3	UnnamedSequence	1001	1315	(969)	+	AluYa5	SINE/Alu	1	311	(0)	2
52	16.8	0.0	0.0	UnnamedSequence	1316	1393	(891)	+	A-rich	Low_complexity	16	93	(0)	3
442	31.5	15.9	3.1	UnnamedSequence	1409	1634	(650)	C	MIRb	SINE/MIR	(8)	260	7	4

>hg19 chr10:56663659-56665579
AGGCTGCCAAGTCAAGATGTATAATTGGTAATGCTGAGCCATTTATTCTGAGTGCACTCTAGACAATGACATATTATTACTATTATGGCACAAATAACTGCCTTTTTGAAGCAAATGTTTTCACTG GC
TTTAGTTAGGATGCTGTCCTATTCCTTTTAACATaATACTGATGCCCTCAGTATTATTTTATATATGTCAATGAAACAATATCATGCTGTGAAATTTATGATAAAAGCAAAACTAAAAATCATTGGTT
GATTTTTGTTTCCCCCTCTCTTCCTTTTAACTTCAGCTTTGCAAAATCACTATGTATTCTGCTGTGGAAGTATAATTAATGTTATTTTCTTCTTATTACTATATTTTAACTTTGCATCTTAAGCCT AA
ATTTTAGCCTATTCATGTAAGGTTGTACTTTAAACAATACCTCCAATAGATTTTCATACTCCATCTAACTATACAAAGAGAAGAGACTATCTAGTTACAATTTATGTATTTGATTGTGGTAGCTT ATG
CCTACCCTAAATATTTTCCCTCCTAGGTCAACCTGGTTTGGGTTCAATTATATGATGATTAAAAAAAAAATCTCAGGTTGGAACAAAATCTATATAATAAGATGACAAAATACTTTCATAACTATT TC
AGTTATAGCTTCTACTAAGTTGCATTTACAATGATATATTTACATAACAGCATAATGGATAGAAATATAAAGAGAGAGACCTTTTGTCTCTCTGTGCCATCAGAATGAACCATGACATTTAGGTG CCT
TGAGGAAAGACTAGAAATAAACTACCAATCAGGCAAATGAACAAACAAAAACAGAATAATTTCTTTCCTAGCTGCCCCACACTTTATCACATCACTTCAGTATGCCCCACTTCCTTAACTCTAATC AA
TTTCCATGTGCCATTTGAGGTACCACAGCTCACTCTCAGAAGGTCAAGAGGTAAAGTGTGGAGAAAAGGGAAGAAGGTGGGTATAAATTAAAAAAAACAAAAAGGACAAAGATAAAATACAAGATAAA
TTAAAATTATATTTTTAAATTAGCTTTTGTTCCTTGAGCATCTGCTATGTATCAGGCATTCTCTATTACCTATTTTCGTTCTGGCCACACAACTACACTATGAAGCAGATATGACTGTACCACTTT CA
AAtTCATATAAGAAAACCAATGTTCAGAGAAGTTGTCTGAGGTCCCACACCTAATAAATAAATGACAGAGCAAGGATTT GGCCTCAAAGTCCAGATTCTTTCCTCAGTTGCACAGTGCCACATTATAA
ATGTGTTGATACACTGAAATAGAAGAATAATATCAAAAGTATTTCTTTTTATTTTCTCTCAACAGGGATTAATAATAATAATTATGTCAGAACTTGGGCACAGGCTTTAATCCTTAAAATTCTTTT AA
TCATTATTTTATCATCATAAGCACATACAAGCAACA cTGTATTTATTTCGCTGAAATGTATAAAATCCTGGCATAGCCAATGAATTTGTTTGATAAATTATGAGATAAATTCAGCTAATGGAGTTTCC
AAGCATGCTGTCAAAGAAATAGAATCATGCATTTATGAATTTTTCCATATTTTTCTTCATTTCTAATGCAACTAGTTAATTATTTGATCTCTTTATCACTTTAAAAGCTTTGTAATAGTTCAGATG AT
GTGAGATTATTTGCAAACAAGATAAGAAATGCCAGTTCCTGGGACAGTTATAGGAAGCCTGAGAAGG tAAGGCTTTTCAAGGAAACTAGGTCGTGAAAGAGAATATTGCTATAATCCTTAGAAAGCTG
GATAGAGTTGTCTTTTCCAATATTAATATGAGGCTCAATAGTTCCATATGGACAAGAACAGGGGTTGAGATCTCTGAGTCATGCAGAACTCTTCTGTCAAAACAGAACAGTTGCAGATGTGTCATG AA
T

>KSI_AluYa5_80

>Scaffold15236-101934-102075
AACCTCTTCAGCCTTCCTTATGTCTTATTTCCAGTCTCAATTCACATTGCATTGATTTTATTTGCTAAACATTTTCAGATTCTGACCACTTCCCTCAATTCTAATTGTTCATTACTTCTATCCAGG CC
AACATCCTTTCTTTCTTAATAAATAgGTTGATAAGAACCTTGTAAACTtATTTTCCTTTCTATTCTATTCTTCACAATGAAGAGTGTTCCAATATCTATATTTGACCATGAAACCTCTCAGCTCATTC
TAATCTAGGCCTACTCATTCACCCTCATCTTCTGATACTGTTCTGACCTTGTGCCTTATATTCAAAACACTCTGCCCTTCATTTAATTTCTAGAAATCCTCTACATTGGGATCTCTGTATTTCTAG AC
CCCACCTTCTCATTCCTTCTTGTTTGTCTACTgCTGTCTGTGTATCCTTCAAGTCTCAGATTAGACAGCACTTTTTTCATGgAATCCTGTCTGATTTTCTGAGGAGAGACAGTGTCAGAGTCACATGC
CCACAGGGCATGTTGCTTCTCCTTAGGATAGCATATCTTTTTCCCTTTATTTGTGATTCCCAGCCTACTTTTCAGTATTCACTACTAGATTTTATGCTCTGTGAGGACAGGGACCATGATCTATGC CT
GTGCTAATTTTGGCATTCGAGGGATATTTATTAATTATATTCTAAATCACCAGTCAAGCGAATGTAGGAAGAAATGAAAAAAAAAATTAGGTGTGGCTCAACCCTTGTGGAATACTGTTTTCTATTAA
CAGCAAACCAGGTAAGTTCAATTACATTTAACAGACAAAAAGTGCACATTGGAAGTTTCAAGCAGAGGGAAGTCATATTCATTCTCCTATTCATTCAGGATTATTTAAGGAGCAGCAGCTCTTGAA AC
TGCTAGTATTGGGAGTACCATTTTGGAAACATGCTTACAGTCAAGAGGAAAGATTGACATGCAAATAAGTATAATGAAACACCTTT Taagaatttaaatattgaggccgggcgcggtggctcacgcct
gtaatcccagcactttgggaggccgaggcgggcggatcacgaggtcaggagatcgagaccatcccggctaaaacggtgaaaccccgtctctactaaaaatacaaaaaattagccgggccgcccca cct
gggtggggtacggggcgtagccccgtcacaaaaaaaaaaaaaaaaaaaaaaaaAAGAATTTAAATATTGAGCAGATGCTCAAGAAAAGCATAAGCAAAGTGTGTCTCAGGTAGACGATAGCTGTCCAG
ATGAGGGATGGCTTGGCACTGAGACCTGAAGAACTGGTAGGAATTTGCCCCTTGGACAACTGG gGGCTGGTCTCCTGTCAAGGAGCAGTGTGAGAAGAAGCAAAAAGGCTTGAGGGTGTCTGATGTAA
CCAGAAAGTTAGGAGAGCTTGGCTTTAGAAGGAAAAGAAGTGTGCATCCGAGGCTGAAGTGG gAGTGATCAGAGACAGGGCAGGTATTTGAAGATATTTTCACA ACCTCAGCATCACCCCACAACCTC
AGCGGATATTTTCAGAAAATTAGAGTAGTGGCAGAGTTTGAGAGTCATGCC aACCTGAGATCACTCCTCTCAACTGACTTTTGACCTAAAGCATGCTCTGAAGGCATTCTCTCAGAATCCCCTTCAAC
TATAATATGGCAATAATAGCACAACTGTATTGCTTGGTTTAAAATATTAAATCAGAGAGACTGCATGAAATAGCTGTCATATGGTAAGTATTTTTTAAAGAAAC CATTATTATATGTATTTTTTTGTT
ATTGTAATAATCACTGAATGAAAGCTGCAGTGATAGTAAAAATGGAGAGAATCAATTAGAGGAAACAACTCACCAGCACCACAGTTTTGATTCCAGGCAGTGATGTAATGCTTAAACACTTTAAA GGA
TACTCTTCCTCAGAAGTTTTAGTTAACAATAGCTGACTAAAGTTGGTTGTTTATAATTATCAAACCACAACTTGTTTTTGACTACTGGCTTTCAATTTCTACTG TCTATTTTTTAATGCCTTGATTCA
AATCCAATGACCTGTAACATCAGCCTGCTTGGCTGAAGAGGTAGCCATGTTTATGAATTTGACTAACTGATGCCTAGCCAAATGCTTTATTA G

314	38.6	5.8	3.9	UnnamedSequence	4	636	(1505)	+	L2c	LINE/L2	2573	3320	(67)	1
1388	0.0	0.0	0.0	UnnamedSequence	1001	1142	(999)	+	AluYa5	SINE/Alu	1	142	(169)	2
24	3.6	0.0	0.0	UnnamedSequence	1182	1210	(931)	+	(A)n	Simple_repeat	1	29	(0)	3
218	27.7	12.2	5.5	UnnamedSequence	1567	1738	(403)	+	MIRb	SINE/MIR	30	212	(56)	4

>hg19 chr11:104785607-104787526
AACCTCTTCAGCCTTCCTTATGTCTTATTTCCAGTCTCAATTCACATTGCATTGATTTTATTTGCTAAACATTTTCAGATTCTGACCACTTCCCTCAATTCTAATTGTTCATTACTTCTATCCAGG CC
AACATCCTTTCTTTCTTAATAAATAaGTTGATAAGAACCTTGTAAACTgATTTTCCTTTCTATTCTATTCTTCACAATGAAGAGTGTTCCAATATCTATATTTGACCATGAAACCTCTCAGCTCATTC
TAATCTAGGCCTACTCATTCACCCTCATCTTCTGATACTGTTCTGACCTTGTGCCTTATATTCAAAACACTCTGCCCTTCATTTAATTTCTAGAAATCCTCTACATTGGGATCTCTGTATTTCTA GAC
CCCACCTTCTCATTCCTTCTTGTTTGTCTACTaCTGTCTGTGTATCCTTCAAGTCTCAGATTAGACAGCACTTTTTTCATGcAATCCTGTCTGATTTTCTGAGGAGAGACAGTGTCAGAGTCACATGC
CCACAGGGCATGTTGCTTCTCCTTAGGATAGCATATCTTTTTCCCTTTATTTGTGATTCCCAGCCTACTTTTCAGTATTCACTACTAGATTTTATGCTCTGTGAGGACAGGGACCATGATCTATG CCT
GTGCTAATTTTGGCATTCGAGGGATATTTATTAATTATATTCTAAATCACCAGTCAAGCGAATGTAGGAAGAAATGAAAAAAAAAAaTTAGGTGTGGCTCAACCCTTGTGGAATACTGTTTTCTATTA
ACAGCAAACCAGGTAAGTTCAATTACATTTAACAGACAAAAAGTGCACATTGGAAGTTTCAAGCAGAGGGAAGTCATATTCATTCTCCTATTCATTCAGGATTATTTAAGGAGCAGCAGCTCTTG AAA
CTGCTAGTATTGGGAGTACCATTTTGGAAACATGCTTACAGTCAAGAGGAAAGATTGACATGCAAATAAGTATAATGAAACACCTTT TAAGAATTTAAATATTGAGCAGATGCTCAAGAAAAGCATAA
GCAAAGTGTGTCTCAGGTAGACGATAGCTGTCCAGATGAGGGATGGCTTGGCACTGAGACCTGAAGAACTGGTAGGAATTTGCCCCTTGGACAACTGG cGGCTGGTCTCCTGTCAAGGAGCAGTGTGA
GAAGAAGCAAAAAGGCTTGAGGGTGTCTGATGTAACCAGAAAGTTAGGAGAGCTTGGCTTTAGAAGGAAAAGAAGTGTGCATCCGAGG CTGAAGTGGcAGTGATCAGAGACAGGGCAGGTATTTGAAG
ATATTTTCACAACCTCAGCATCACCCCACAACCTCAGCGGATATTTTCAGAAAATTAGAGTAGTGGCAGAGTTTGAGAGTCATGCC gACCTGAGATCACTCCTCTCAACTGACTTTTGACCTAAAGCA
TGCTCTGAAGGCATTCTCTCAGAATCCCCTTCAACTATAATATGGCAATAATAGCACAACTGTATTGCTTGGTTTAAAATATTAAATC AGAGAGACTGCATGAAATAGCTGTCATATGGTAAGTATTT
TTTAAAGAAACCATTATTATATGTATTTTTTTGTTATTGTAATAATCACTGAATGAAAGCTGCAGTGATAGTAAAAATGGAGAGAATCAATTAGAGGAAACAACTCACCAGCACCACAGTTTTGA TTC
CAGGCAGTGATGTAATGCTTAAACACTTTAAAGGATACTCTTCCTCAGAAGTTTTAGTTAACAATAGCTGACTAAAGTTGGTTGTTTA TAATTATCAAACCACAACTTGTTTTTGACTACTGGCTTTC
AATTTCTACTGTCTATTTTTTAATGCCTTGATTCAAATCCAATGACCTGTAACATCAGCCTGCTTGGCTGAAGAGGTAGCCATGTTTATGAATTTGACTAACTGATGCCTAGCCAAATGCTTTAT TAG

>KSI_AluYa5_81

>Scaffold6480-595471-595673
GTATTTTCAATAGAGACGAGGTTCCGCCATGTTGCCCAGGCTGGTCTCAAACTCCTGGGCTCAAGGGATCCGCAGGCCTCGACCTCCCACAGTGCTGGGATTACAGGAATGAGCCCCCACACTCAG CC
CCTTGGCAGGGCTTTGATTTTCTTCTTCCAGTCTTCCCAATTCGTGTACATGGAATCATCATTCATCCAATTGCTCAGAACAAAAATGTAACAGGATTTTTTTGAGACTTGTCCTCAAGCCCCA CAT
CAAATCCATCAGTAAATCCTATGAACTTTACCTTCAAAATATATCGCAAATACAACCACACCTCCTTCTCCACTATTACTTTAATCCAAGTCACCATCACCTCATTTGGATTACTGCAACAGCCTC CA
AACAGATCTTTGGCTTCCACTCTTACCCCAGTAAAGCAGCCAGTGATCTTTTAAAAACGTAAATCACATTATATCAGTTCCCTGTTCCAAGCCTTTCAATATAAAACCCAAGCCAAAAATCCAAA TGA
AATGTCTACAAACTCTTGAGAGATCAGATTATTACTTAACTAACTCTCTGACGTCTTATACTACCACTCTCTCCTCTACTCACACTTAGCTCCTGCCACATTGTCCTTCTTCCCGTTCCTTGATCA CA
CCCCTCAGATTGTTCTCAACTTTTGGCCTTTGCACTTATAGTTCCCCATACCCCAAAGGCTCTTTCCCCATATCTCTGCATGGCTTGGGGCCCTGTTCAAATGTCACTTCCTCAGAAAGAT CTTTCCT
GACCATCCTGTGGCCACTACTCCCCCCAAATCCATCATTCTCAATACTCTACTTTATTTTTCTTCAAAGCATTAAACCCTATCTGACATCTTATTTAGTTGTTTGGTTGTTTATACTATTGACTTCCC
CACCTTGCCGTATCTCCGGTGAGTGTCTAGAACACTGCATGGTATACAATAGGAGTTAAGTATTCCAAGAATTTAAGAATGAAAATTT AAAAATAGACAATTATggccgggcgcggtggctcacgcct
gtaatcccagcactttgggaggccgaggcgggcggatcacgaggtcaggagatcgagaccatcccggctaaaatggtgaaaccccgtctctactaaaaatacaaaaaattagccgggcgtagtgg cg
ggcgcctgtagtcccagctacttgggaggctgaggcaggagaatggcgtgacaaaaaaaaaaaaaaaaaaaaa aaaaatagacaattatTAGACCTATGAAGAGCCAGTATGAAGGATTAATGTATTT
ATAGTTTATCTGAAAACAATGGGAAACCATTTGTAGATC cTGAACATGAGAGCAGCAAGACAAGATCTAAATTTTGAAAACCTCATTTGGCTGTTTTGAGAATTCAAT GTAGGAGAGCAAGAGGGGAA
ACAGGAAACGGAAAAAAAACTTGTAGAAATGAGATGGTCACCTGAACTAGATAGTTGGTAGTATGAGGAGAGTAAATTAGAGATATAACAGGAAGTAGCACAGGTAGGATCCGCCTATTGATTAG ACT
CACGCCAGCTCCTCCTCAACGCTCCCGTCATTCTAGCCACACTTCTATCACAGCGCCTCTCACCCTACATAGCACTTCTCCGCTAATAAATCTGTATTTTCCACTAGAGTCCTCCGGGGTAAGCACGC
TATCAAGCATCCCTACAATCTCTGTTAAATGaAGGTCATCAACGCCTGTTGAATGAAGGCCGCCCATTTGGTGGCCTGGAATTCAGAGCAGCAATTTGTCCTAGCACAGCCGTATGACTCTCTTAATG
TTTGGGGAAATCCATGGTTCAACAGCGCCGATGCCCGATAAACAACACAGCACCAGTAACCTGTGCCAGGGGCTGTGGACGGGGGCAACACAGGAGCAGGTCACGGGGAAGGGGGATGTAAGCTT TCT
GAGCAAACTCCCAGAGCACCCAGGGCAGAAACAGGCGGCTAGAAGCCGGGCAACGCGGGTGCAGAGCACAGACCCACGTCCATGGTTTTCCTACCAGACGGGCCTGGCAGCGGACGCACTGGCCTCCC
CCGGGCGGCGCTGGCTCAGCCACCGCGGCACCCTTCATGAGGCCGGTATTCCGCTCTTGCACCTCCCCCAAAGCCCAGAGAAGACGAGACAGACACTCACGCAGGCTAGGTGTCGGGACCGGAGT GGC
CGAGTAGACGGCAGTGGAGAATGCAC

852	15.6	0.0	0.0	UnnamedSequence	1	128	(2074)	C	AluSz6	SINE/Alu	(184)	128	1	1	
963	28.4	7.3	1.7	UnnamedSequence	150	709	(1493)	+	L2c	LINE/L2	2489	3082	(337)	2	
674	27.6	18.9	5.0	UnnamedSequence	553	979	(1223)	+	L2a	LINE/L2	2931	3425	(1)	3	*
1896	0.5	0.0	0.5	UnnamedSequence	1001	1203	(999)	+	AluYa5	SINE/Alu	1	202	(109)	4	
24	0.0	0.0	0.0	UnnamedSequence	1205	1230	(972)	+	(A)n	Simple_repeat	1	26	(0)	5	
343	34.0	10.4	0.5	UnnamedSequence	1285	1476	(726)	C	L2	LINE/L2	(541)	2878	2668	6	
191	35.7	5.1	0.0	UnnamedSequence	1547	1644	(558)	+	L2c	LINE/L2	3183	3285	(102)	2	

>hg19 chr12:27863835-27865795
GTATTTTCAATAGAGACGAGGTTCCGCCATGTTGCCCAGGCTGGTCTCAAACTCCTGGGCTCAAGGGATCCGCAGGCCTCGACCTCCCACAGTGCTGGGATTACAGGAATGAGCCCCCACACTCAG CC
CCTTGGCAGGGCTTTGATTTTCTTCTTCCAGTCTTCCCAATTCGTGTACATGGAATCATCATTCATCCAATTGCTCAGAACAAAAA TGTAACAGGATTTTTTTGAGACTTGTCCTCAAGCCCCACAT
CAAATCCATCAGTAAATCCTATGAACTTTACCTTCAAAATATATCGCAAATACAACCACACCTCCTTCTCCACTATTACTTTAATCCAAGTCACCATCACCTCATTTGGATTACTGCAACAGCCT CCA
AACAGATCTTTGGCTTCCACTCTTACCCCAGTAAAGCAGCCAGTGATCTTTTAAAAACGTAAATCACATTATATCAGTTCCCTGTTC CAAGCCTTTCAATATAAAACCCAAGCCAAAAATCCAAATGA
AATGTCTACAAACTCTTGAGAGATCAGATTATTACTTAACTAACTCTCTGACGTCTTATACTACCACTCTCTCCTCTACTCACACTTAGCTCCTGCCACATTGTCCTTCTTCCCGTTCCTTGATC ACA
CCCCTCAGATTGTTCTCAACTTTTGGCCTTTGCACTTATAGTTCCCCATACCCCAAAGGCTCTTTCCCCATATCTCTGCATGGCTTG GGGCCCTGTTCAAATGTCACTTCCTCAGAAAGATCTTTCCT
GACCATCCTGTGGCCACTACTCCCCCCAAATCCATCATTCTCAATACTCTACTTTATTTTTCTTCAAAGCATTAAACCCTATCTGACATCTTATTTAGTTGTTTGGTTGTTTATACTATTGACTTCCC
CACCTTGCCGTATCTCCGGTGAGTGTCTAGAACACTGCATGGTATACAATAGGAGTTAAGTATTCCAAGAATTTAAGAATGAAAATT TAAAAATAGACAATTATTAGACCTATGAAGAGCCAGTATGA
AGGATTAATGTATTTATAGTTTATCTGAAAACAATGGGAAACCATTTGTAGATC tTGAACATGAGAGCAGCAAGACAAGATCTAAATTTTGAAAACCTCATTTGGCTGTTTTGAGAATTCAATGTAGG
AGAGCAAGAGGGGAAACAGGAAACGGAAAAAAAACTTGTAGAAATGAGATGGTCACCTGAACTAGATAGTTGGTAGTATGAGGAGAGTAAATTAGAGATATAACAGGAAGTAGCACAGGTAGGAT CCG
CCTATTGATTAGACTCACGCCAGCTCCTCCTCAACGCTCCCGTCATTCTAGCCACACTTCTATCACAGCGCCTCTCACCCTACATAGCACTTCTCCGCTAATAAATCTGTATTTTCCACTAGAGTCCT
CCGGGGTAAGCACGCTATCAAGCATCCCTACAATCTCTGTTAAATG gAGGTCATCAACGCCTGTTGAATGAAGGCCGCCCATTTGGTGGCCTGGAATTCAGAGCAGCAATTTGTCCTAGCACAGCCGT
ATGACTCTCTTAATGTTTGGGGAAATCCATGGTTCAACAGCGCCGATGCCCGATA AACAACACAGCACCAGTAACCTGTGCCAGGGGCTGTGGACGGGGGCAACACAGGAGCAGGTCACGGGGAAGGG
GGATGTAAGCTTTCTGAGCAAACTCCCAGAGCACCCAGGGCAGAAACAGGCGGCTAGAAGCCGGGCAACGCGGGTGCAGAGCACAGACCCACGTCCATGGTTTTCCTACCAGACGGGCCTGGCAG CGG
ACGCACTGGCCTCCCCCGGGCGGCGCTGGCTCAGCCACCGCGGCACCCTTCATGA GGCCGGTATTCCGCTCTTGCACCTCCCCCAAAGCCCAGAGAAGACGAGACAGACACTCACGCAGGCTAGGTGT

CGGGACCGGAGTGGCCGAGTAGACGGCAGTGGAGAATGCAC

>KSI_AluYa5_82

>Scaffold13173-2102376-2102680
AAAAGAAATAGTCAACAGAATGAACAGACAACGTGCAGAATGGGAGAAACTATTTGCAAAATACACATCCAACAAGGGTCTAATATCAGAA TATAAAAGGAACTAAAATTCGACAAAAATCTCAGATA
ACCCCATTAAAAGTGAGCAAAGGATATGAATAGACATTTTTTTCCAAAAATACATACAAATGGCCAAGAAACATATGAAAAAATGCTCAACATCACTAATCATCAGAGAAATGCAAATTAAAACC ACA
ATGAGACACCGTCTTACAACAGTCAGAATGGCTATTACTGAAAAGTCAAAAAATAACAGATGTTGGTGAGGACATGAAGAAAAGAGAACAC TTAAACACTGTTGGTGGGAATGTAAATTAGTACAACT
TCTATGGAAAACAGTATGGAGATTTCTCAAAGAACTAAAAAATAAAACTACCACTTGATTCCACAATCCCATTACTGGGTATCTACCCAAGGAAAAGAAATTACTACATCAAAAAGATACTGGCA TTT
GTACGTTTACTGCAATACTATTCACAATAGCAAAGATATGGAATCAACCTAAGTGTATATCAATGGACGACTGGTTAAAGAAAATGTGGTC TATATATACAATGAAATACTATTCAGCCATAAAAAG
AATGAAATCTGTCTTTTGCATAAACATGGATGGAATGGGAGACCATTATCTTAAGTGAAACAAGTCAGAAACAGAAAGACAAATGTTGCATGTTCTCACTTATAAGTGGGAGCTAAACAATGTGT ACA
CATGGACATAGAGTGTGGAATAATAGACAAcGGAGACTCAGAAGGATGATGGAGTTTGAGGGGCTGGATGATGAGAAATTACTTAATGGGTACAATGCATATTGTTTGGGTAATGGATACCCTAAAA
GCCCTGACTTCACCACTGCACAATCTATGCATCTAACAAAATTGCACTTGCATTCCACAAATTTATACAAAAATAATGTTGAAT Caaaaaacattatagtttgggccgggcgcggtggctcacgcctg
taatcccagcactttgggaggccgaggcgggcggatcacgaggtcaggagatcgagaccatcccggctaaaacggtgaaaccccgtctctactaaaaatacaaaaaattagccgggcgtagtggc ggg
cgcctgtagtcccagctacttgggaggctgaggcaggagaatggcgtgaacccgggaggcggagcttgcagtgagccgagatcccgccactgcactccagcct gggcgacagagcgagactccgtctc
aaaaaaaaaaaaaaaaAAAAAAaATTATAGTTTGGTGGAACATAGAATTATAGGTTGGAAATAATTTCCCTAAAAAATTTGAAGATATTTTATATTATTTTCT AGCTCCCAATGTATTTTTTCTTTTT
TAAGAGACAGGGTCTCACTCTGTCACCCAGGCTGGTGTGCAGTGGCGTGATCATGGCTGACTGCAGCCTTGACCTCCTGGGCTCAAGCAATACTCCCACCTCA GCCTCCCAAGAAGCTGGGATCACAG
GCACTCGCTACCATGCCCTACTATTTTTTTAAATTTTTTTTGTAAAGATGCAGTCTCCCTATGTTGCCCTGGCTGGTCTCAAACTCTTGGGCTCAAGTGATCCTCCCATCTTGGCCTCCCAAA GTG
CTGGGATTAGAGGAGTGAGCCACCACACTCATTCCTCCCAATGTTGATGAGTTTGTAA GCCCAATGCTGTTCTGATAAGGAATCAATTAAATACAATTAAAAATTTTATTTTTGTGTGGGCACACAGT
GGTGTATATATGTATATATTTATGGGGTACATGAGATACTCTGATACAGACATGCAATAAGTAATAATCACATCATGAAAAATGGAGTATCCATCCCCTCAAGAATTTATCCTTTGTGTTACAAA CAA
TCCGATTATACCCTTTTAGTTATTTTAAAATGTACAATTAAATTATTATTGACTATAGTCACCCTGTTATGCTATCACATACTAGGTCTTTTTCATTATTTCT ATGTTTTATACCCATTAGCCATCCC
CACTTCTCCTCAACCCCACACTACTCTTCCCAGCCTTTGGTAACCATCCTTCTACTCTCTATCTCCGTGAGTTCAACTGCTTTGGTTTTTAGCTCTCACAAATAAGTGAGAACATGCTAAGTTTG TCT
TTCTGTGCCTGGCTTATTTCATATGACATAATGACCTCCAGTTTCATCCACGTTGTTGCGAGTTACAGGATCTCATTCTTTTTCATGGCTGAATAGTAAGTAC TCCTATGTGTATACGTACTACATTT

4782	12.4	1.6	0.6	UnnamedSequence	1	877	(1427)	+	L1PB4	LINE/L1	5175	6058	(98)	1
434	20.0	0.9	0.0	UnnamedSequence	878	987	(1317)	+	L1MA4A	LINE/L1	6189	6299	(4)	2
2968	0.0	0.0	0.0	UnnamedSequence	1000	1304	(1000)	+	AluYa5	SINE/Alu	1	305	(6)	3
382	21.4	1.5	14.9	UnnamedSequence	1307	1396	(908)	C	HAL1	LINE/L1	(900)	1607	1517	4
1946	14.4	0.0	2.4	UnnamedSequence	1397	1694	(610)	C	AluJb	SINE/Alu	(17)	295	5	5
382	21.4	1.5	14.9	UnnamedSequence	1695	1740	(564)	C	HAL1	LINE/L1	(991)	1516	1487	4
3367	9.7	1.1	2.3	UnnamedSequence	1768	2304	(0)	C	L1MA2	LINE/L1	(0)	6304	5774	6

>hg19 chr12:58082279-58084267
AAAAGAAATAGTCAACAGAATGAACAGACAACGTGCAGAATGGGAGAAACTATTTGCAAAATACACATCCAACAAGGGTCTAATATCAGAATATAAAAGGAACTAAAATTCGACAAAAATCTCAGA TA
ACCCCATTAAAAGTGAGCAAAGGATATGAATAGACATTTTTTTCCAAAAATACATACAAATGGCCAAGAAACATATGAAAAAATGCTCAACATCACTAATCATCAGAGAAATGCAAATTAAAACC ACA
ATGAGACACCGTCTTACAACAGTCAGAATGGCTATTACTGAAAAGTCAAAAAATAACAGATGTTGGTGAGGACATGAAGAAAAGAGAACACTTAAACACTGTTGGTGGGAATGTAAATTAGTACAA CT
TCTATGGAAAACAGTATGGAGATTTCTCAAAGAACTAAAAAATAAAACTACCACTTGATTCCACAATCCCATTACTGGGTATCTACCCAAGGAAAAGAAATTACTACATCAAAAAGATACTGGCA TTT
GTACGTTTACTGCAATACTATTCACAATAGCAAAGATATGGAATCAACCTAAGTGTATATCAATGGACGACTGGTTAAAGAAAATGTGGTCTATATATACAATGAAATACTATTCAGCCATAAAAA AG
AATGAAATCTGTCTTTTGCATAAACATGGATGGAATGGGAGACCATTATCTTAAGTGAAACAAGTCAGAAACAGAAAGACAAATGTTGCATGTTCTCACTTATAAGTGGGAGCTAAACAATGTGT ACA
CATGGACATAGAGTGTGGAATAATAGACAAtGGAGACTCAGAAGGATGATGGAGTTTGAGGGGCTGGATGATGAGAAATTACTTAATGGGTACAATGCATATTGTTTGGGTAATGGATACCCTAAAA
GCCCTGACTTCACCACTGCACAATCTATGCATCTAACAAAATTGCACTTGCATTCCACAAATTTATACAAAAATAATGTTGAAT CAAAAAAcATTATAGTTTGGTGGAACATAGAATTATAGGTTGGA
AATAATTTCCCTAAAAAATTTGAAGATATTTTATATTATTTTCTAGCTCCCAATGTATTTTTTCTTTTTTAAGAGACAGGGTCTCACTCTGTCACCCAGGCTGGTGTGCAGTGGCGTGATCATGGC TG
ACTGCAGCCTTGACCTCCTGGGCTCAAGCAATACTCCCACCTCAGCCTCCCAAGAAGCTGGGATCACAGGCACTCGCTACCATGCCCTACTATTTTTTTAAATTTTTTTTGTAAAGATGCAGT CTC
CCTATGTTGCCCTGGCTGGTCTCAAACTCTTGGGCTCAAGTGATCCTCCCATCTTGGCCTCCCAAAGTGCTGGGATTAGAGGAGTGAGCCACCACACTCATTCCTCCCAATGTTGATGAGTTTGTA AG
CCCAATGCTGTTCTGATAAGGAATCAATTAAATACAATTAAAAATTTTATTTTTGTGTGGGCACACAGTGGTGTATATATGTATATATTTATGGGGTACATGAGATACTCTGATACAGACATGCAATA
AGTAATAATCACATCATGAAAAATGGAGTATCCATCCCCTCAAGAATTTATCCTTTGTGTTACAAACAATCCGATTATACCCTTTTAGTTATTTTAAAATGTACAATTAAATTATTATTGACTATA GT
CACCCTGTTATGCTATCACATACTAGGTCTTTTTCATTATTTCTATGTTTTATACCCATTAGCCATCCCCACTTCTCCTCAACCCCACACTACTCTTCCCAGCCTTTGGTAACCATCCTTCTACT CTC
TATCTCCGTGAGTTCAACTGCTTTGGTTTTTAGCTCTCACAAATAAGTGAGAACATGCTAAGTTTGTCTTTCTGTGCCTGGCTTATTTCATATGACATAATGACCTCCAGTTTCATCCACGTTGTT GC
GAGTTACAGGATCTCATTCTTTTTCATGGCTGAATAGTAAGTACTCCTATGTGTATACGTACTACATT T

>KSI_AluYa5_83

>Scaffold4038-1998288-1998508
AAAAGTGGTAATAGTGCCCCAAGTCTTCTTTCCTGATAGTCTTTGCCTCCTAGTGAAAGGAGAGAAAGGCATTTGGGAATGTAGCAGGCCCTGAATTTCAGTCATGAAGAGAGAATCTTGACATCC CT
TTAATGTTACTTGTGAATGTCCTAAAAGACATAGACTGTTCTTATCCAACATCATTAAAATACATGCCTTGAGGTTTTAATA tGGATTTTAAGTGGACCAAGTAATGCTATATTTTTCTAGCACTCTA
TCTGGAAAAGATGTTCTACCTAAGTAAACTTACAGATAACAGATGTTTACTCCTGTAGGCCAAAATTTTATTTTTAACCATTTTTAACCTAAGCATAGGATTTATTTCCTTTCCTATTTCATTACA GC
GtATGTTTTCTAGCAAATGGGCTTTTTGTAAACAGTGGAAGATA gAACAAATAAGGCAGTAGAATTTAATGCACATTACTGAAGGAAT gCTGAGATACTTATTTTGTGAACTCAAAGGGAATATTTGC
ATCCTTCTGGCTTCTCAGACCTATCCTACTTAGCTGAGTTTGACAGATACATGCCTCTGGATGAATGAAATATTCTGTGACCAGGCAGACATTAATTTCTTCTGATGTCACACATTTTTCAAATAT TG
CCAGTTTGGCATCACATGGTAACCACCAATAGGAAAATGTTAAGATGGGTGTATGTTATAGGCTGAATATTTCTGTCCCCCCAAAATGCATATGTTGAAGCCCTAGCCCCTAATGTGATGGTACT TGA
GGTGGGCTTTTAGGAGGTAATTAGGTTTCCATGAAGTCAGAAGGGTGGGGCCT CCCTGATGGGATTATTAGCC TTCTAAGAAGAGGAGAGCAGAGCATGCTCCCTCCCACCCTCCCCAACGCCTT cGT
GCTCACTTGATCTCCCTCTCACTTCTGACTTGTGAAGACAGATCCAGAAGGCTGCTGTTCAGAACCAGAAGAAG gCCCTCGTCAGGTAGAATCTGCTGGCACTgggccgggcgcggtggctcacgcct
gtaatcccagcactttgggaggccgaggcgggcggatcacgaggtcaggagatcgagaccatcccggctaaaaacggtgaaaccccgtctctactaaaaaatacaaaaaattagccgggcgtag tg
gcgggcgcctgtagtcccagctacttgggaggctgaggcaggagaatggcgtgaacccgggaggcggaggagaaccccgtccaaaaaaaaaaaaaaaaaaaaaa agaatctgctggcactCTGATCTT
GGAAGTGCCAGCCTCCAAAACTATGAGAAATAAACATCAGTTGCTTAAGCCACTCAGTGTATGGCATTTTGTTACAGCAGTCTGCTTTAGTCTATTTGGGCTGCTATAATAAAATACCATAGACT AGG
TCACTTATAAACTACAGATATATATTTCTTATGGTTCTGGAGTCTGGGAATTCCAAAATCAAGGTACCAGCATATTCCATGTCTGTTTAGGGCCTGTTTTTCCTGGTTCTTA GAGGACACCTTCTTGC
TGTGTCCTCACGTGGTAGAAGGGGCAAGGCAGCTCTCTGAGGCCTAATCTCCAGCGCCCTCTTGACTTAATCATATCTCAAAGTCCTTACCTTCTAACACCATCACATTGGTGATTAGGCTTCAG CAT
AGGCATTTTGGAGGGACACATTCAGGCCATACCACAGCTGGAGCTAAGAGAGCGTAGCAATCATTACATGATCTTGG CCTCAGCAATCCTCTGTCTT AAGTAGCTGAACAGTTTAGCCATAGGCCAGA
TAGATGTACATGGATATTAGAACTAGATAGCATTATCTGGCCAATATTACAACTTAATATGGTACATATTAAGGTATTCCTGTTTTTTGCTTAGTTTTATTTTTCTTCTCTGCCTTAAGAAATCT CTG
TCTTTCATGTACTAACAAGTTTTCAAAACTTCTCTTGGATCACTCTCCATTATTTTCAGAGCTTTAAAGAATATAATTTAATTATGCATATTTTTAAAGTTTCTCCTGAATATGTTTAAAG ATGAGAT
TTTCTCTTTGTAAACTGTAAATTTTCCAAATGTGATGGAGCTCTAATCAATTGGCTTATATTCAAATAATACTTTAATGATCTAACGTTGATGTGAGCCTTTGCCTATTTTGCTCTGTCTTTTTTT GTA
TTGTTTGTAATGTTGGCCCTAGGGTTTAGAGTATATATCCTTAA

297	21.8	7.1	1.7	UnnamedSequence	229	340	(1880)	C	Tigger15a	DNA/TcMar-Tigger	(1)	714	597	1
788	19.9	1.1	2.1	UnnamedSequence	694	883	(1337)	+	MLT1A	LTR/ERVL-MaLR	1	188	(186)	2
2066	0.0	0.0	1.8	UnnamedSequence	1001	1221	(999)	+	AluYa5	SINE/Alu	1	217	(94)	3
21	0.0	0.0	0.0	UnnamedSequence	1235	1257	(963)	+	(A)n	Simple_repeat	1	23	(0)	4
591	18.1	0.0	0.0	UnnamedSequence	1258	1362	(858)	+	MLT1A	LTR/ERVL-MaLR	254	358	(16)	2
1730	18.0	9.2	0.6	UnnamedSequence	1363	1698	(522)	C	MLT1A0	LTR/ERVL-MaLR	(0)	365	1	5
445	22.7	1.6	1.6	UnnamedSequence	1699	1713	(507)	+	MLT1A	LTR/ERVL-MaLR	393	406	(2)	2
183	23.1	1.9	1.9	UnnamedSequence	1735	1787	(433)	C	Penelope1_Vert	LINE/Penelope	(262)	825	773	6

>hg19 chr12:81314272-81316218
AAAAGTGGTAATAGTGCCCCAAGTCTTCTTTCCTGATAGTCTTTGCCTCCTAGTGAAAGGAGAGAAAGGCATTTGGGAATGTAGCAGGCCCTGAATTTCAGTCATGAAGAGAGAATCTTGACATCC CT
TTAATGTTACTTGTGAATGTCCTAAAAGACATAGACTGTTCTTATCCAACATCATTAAAATACATGCCTTGAGGTTTTAATA cGGATTTTAAGTGGACCAAGTAATGCTATATTTTTCTAGCACTCTA
TCTGGAAAAGATGTTCTACCTAAGTAAACTTACAGATAACAGATGTTTACTCCTGTAGGCCAAAATTTTATTTTTAACCATTTTTAACCTAAGCATAGGATTTATTTCCTTTCCTATTTCATTACA GC
GcATGTTTTCTAGCAAATGGGCTTTTTGTAAACAGTGGAAGATA aAACAAATAAGGCAGTAGAATTTAATGCACATTACTGAAGGAAT tCTGAGATACTTATTTTGTGAACTCAAAGGGAATATTTGC
ATCCTTCTGGCTTCTCAGACCTATCCTACTTAGCTGAGTTTGACAGATACATGCCTCTGGATGAATGAAATATTCTGTGACCAGGCAGACATTAATTTCTTCTGATGTCACACATTTTTCAAATAT TG
CCAGTTTGGCATCACATGGTAACCACCAATAGGAAAATGTTAAGATGGGTGTATGTTATAGGCTGAATATTTCTGTCCCCCCAAAATGCATATGTTGAAGCCCTAGCCCCTAATGTGATGGTACT TGA
GGTGGGCTTTTAGGAGGTAATTAGGTTTCCATGAAGTCAGAAGGGTGGGGCCT CCCTGATGGGATTATTAGCC TTCTAAGAAGAGGAGAGCAGAGCATGCTCCCTCCCACCCTCCCCAACGCCTT tGT
GCTCACTTGATCTCCCTCTCACTTCTGACTTGTGAAGACAGATCCAGAAGGCTGCTGTTCAGAACCAGAAGAAG aCCCTCGTCAGGTAGAATCTGCTGGCACTCTGATCTTGGAAGTGCCAGCCTCCA
AAACTATGAGAAATAAACATCAGTTGCTTAAGCCACTCAGTGTATGGCATTTTGTTACAGCAGTCTGCTTTAGTCTATTTGGGCTGCTATAATAAAATACCATAGACTAGGTCACTTATAAACTAC AG
ATATATATTTCTTATGGTTCTGGAGTCTGGGAATTCCAAAATCAAGGTACCAGCATATTCCATGTCTGTTTAGGGCCTGTTTTTCCTGGTTCTTAGAGGACACCTTCTTGCTGTGTCCTCACGTG GTA
GAAGGGGCAAGGCAGCTCTCTGAGGCCTAATCTCCAGCGCCCTCTTGACTTAATCATATCTCAAAGTCCTTACCTTCTAACACCATCACATTGGTGATTAGGCTTCAGCATAGGCATTTTGGAGGG AC
ACATTCAGGCCATACCACAGCTGGAGCTAAGAGAGCGTAGCAATCATTACATGATCTTGG CCTCAGCAATCCTCTGTCTT AAGTAGCTGAACAGTTTAGCCATAGGCCAGATAGATGTACATGGATAT
TAGAACTAGATAGCATTATCTGGCCAATATTACAACTTAATATGGTACATATTAAGGTATTCCTGTTTTTTGCTTAGTTTTATTTTTCTTCTCTGCCTTAAGAAATCTCTGTCTTTCATGTACTAA CA
AGTTTTCAAAACTTCTCTTGGATCACTCTCCATTATTTTCAGAGCTTTAAAGAATATAATTTAATTATGCATATTTTTAAAGTTTCTCCTGAATATGTTTAAAGATGAGATTTTCTCTTTGTAAA CTG

TAAATTTTCCAAATGTGATGGAGCTCTAATCAATTGGCTTATATTCAAATAATACTTTAATGATCTAACGTTGATGTGAGCCTTTGCCTATTTTGCTCTGTCTTTTTTTGTATTGTTTGTAATGTTG GC
CCTAGGGTTTAGAGTATATATCCTTAA

>KSI_AluYa5_84

>Scaffold9496-671468-671764
AGAAAGGTATGTTTCTCTCTCCTCTCTGTCCCTCTTTCTTGCCTATTAGTCACTCATGGTACAGGAAGCTCCCCTTTCTCCCAAATTGGGAAAATTTATCAGTCTATGGAGATAAATCTCTGACAA AG
TCAAATAAATCAATGAGCATACAATTGCTTATTATGGGAGCAATGGGCAAGAGCAGAAAACAAAAATAGCCATTAATATTCCTCAGGTCTGAGATCAAGTTAAAACCTTATAGAAACTAATCAAA GCT
TTCACCTTTTTATATTTGTACCTGGCTCTGTTATTATTAGAGTTATATATAAGTGCTTTTCTGAAACATATGCAAATATGATTGCCCGAATGGCAGAGGCATAATGTCAAGCATTCTGCTTTCATT AA
CAAATCTATCAGAACTTGCCCCTTAGCTTTATCACTAAGACCGTTAGTGTCTTTAAGTATTAAATAATCATAATAATAAAGAAGGAAAATGTTGTATATAATTTGCAGAGGTGCTATCTGTATTC ATT
AGGATATTATTTCAGCTGTTTAAACAGAGACCAAATCACAATAGCTGAAAACTAGATAAAAGGTTATTTCTCTCATTAAAGTTCAATCGATAGGTTCAGCAGGTACACACTGTACTCCACAAGATC AC
CCAGGGATCAAAGCTCCTTCTATCTTGTTTTGTCATTTCTTACAGTGTTGTTCTTGTCATGGTCTAAACCAGCTTACCCCACTGCCACATTTATCCATCAGCCTATGGGAAAAGGGAAAAAGGAT GTG
GAAGGTAAGCAGCTTCCTTTTGGGAACATGATCTAGAATTTTGGACGTTATTTCTGCTCACATGCCATTGGCCAGAACTTGATTGTGTGGCCACAC aTTGCTAAGAGAAACTGTGAAATGTAATTTCT
AATTGAACAGTCATGTGTCCACTTAGACCTCAGGTGGTTCTGTTACCAAACTTCACCGTGCCACATATACAACTTCTATCCTACTTAAAAATATATATTTTAgggccgggcgcggtggctcacgcctg
taatcccagcactttgggaggccgaggcgggcggatcacgaggtcaggagatcgagaccatcccggctaaaacggtgaaaccccgtctctactaaaaatacaaaaaattagccgggcgtagtggc ggg
cgcctgtagtcccagctacttgggaggctgaggcaggagaatggcgtgaacccgggaggcggagcttgcagtgagccgagatcccgccactgcactccagcc tggcgacagagcgagactccgtctc
aaaaaaaaaaaaaaaatatatatatatatatatttaATAATCATTTCCCCATGCTGTTAATAAATCTTCATGAACATCATCTTTAATGACTATGTAATGGTTGTACCTA TGAAAGTATTTCCCTGCT
GTCAAACATGTAGGTAGTTTCCAGTTGGGGATTTTATACATAGTACTGCAGTTAATATCTGCCATATGAATCTTTATTTGCTTTTCTGGTTATTTCCTTGGT ATTTATTCCTACAGGTAAGACTGTTT
ACTGAAAAGTTATACCAATTAACCTTCTCCTCAGAAGCAAATGAGGGGGCTACATTTTACACCACCATCACTTGCAATAATTTGGTCTTATTTTTTATCTTTGCAAATTTGAtCAATTTAAAATTATA
TCTAATTATAATTTTAAATTTCATTCCTTTAATTATTAGTGCATTTATTACTCATGAATTTTAACATGTTTCTGGTATTTATTAATTATTTGTATTATTTCT CTTAAGAATTCATCTTTGCCCATTTA
TTATCTTAGCTTTTTTCCAACTGTAGAACAAGATAGTAATAGTACCTGCATAATAGGGTATGGTGAGAATCAAACAAGAAAAGGAATGACAGAAACTTT tTTTCTTCTGGTTTCTTCTGAACAAGTTC
TTCAGACTTGGGCTCAGTCTATGTTGTAGCTACAATGGAGTGGTCTGAAGAGCCCTCATCACTCTGCCGCACAACCCCATCTTATTTTCTTCATCGTACTGA TGATGATCAAAGATTCTATTTTTCAA
TGGTTTACTTATTTTTGTCCCTCCCCTTCCCCACTAGAACCAGGCCCTGGTCTCTCTTCTCTACTCATATTTCTTTATCACCAAAACAGCATCTGCCACACAGTAGATGCTCAATTAATATTTTC TAG
ATAAATAAATAAACCTCCTTCCCTCACTGCCAGAATCTCTTGACCTTGTTAAAAACTTAGGCATAGTCTTCAATTACCTCTTTGACAAAGACAAGATGTGAT GCCCCCAACAAACCCTCC

826	30.9	7.2	2.8	UnnamedSequence	503	945	(1351)	C	MLT1J	LTR/ERVL-MaLR	(0)	512	51	1
19	7.0	3.2	0.0	UnnamedSequence	987	999	(1297)	+	(AT)n	Simple_repeat	1	14	(0)	2
2904	0.0	0.0	0.0	UnnamedSequence	1000	1296	(1000)	+	AluYa5	SINE/Alu	1	297	(14)	3
19	7.0	3.2	0.0	UnnamedSequence	1297	1313	(983)	+	(AT)n	Simple_repeat	1	17	(0)	4
459	29.3	10.1	0.4	UnnamedSequence	1318	1526	(770)	C	L1ME4a	LINE/L1	(259)	5865	5637	5
382	29.2	3.3	8.3	UnnamedSequence	1540	1809	(487)	C	L1ME4a	LINE/L1	(556)	5568	5295	5
221	24.6	3.3	0.0	UnnamedSequence	1810	1870	(426)	+	MIR	SINE/MIR	130	192	(70)	6
385	29.8	10.9	2.2	UnnamedSequence	1961	2186	(110)	+	L2a	LINE/L2	3171	3426	(0)	7

>hg19 chr14:25580773-25582749
AGAAAGGTATGTTTCTCTCTCCTCTCTGTCCCTCTTTCTTGCCTATTAGTCACTCATGGTACAGGAAGCTCCCCTTTCTCCCA AATTGGGAAAATTTATCAGTCTATGGAGATAAATCTCTGACAAAG
TCAAATAAATCAATGAGCATACAATTGCTTATTATGGGAGCAATGGGCAAGAGCAGAAAACAAAAATAGCCATTAATATTCCTCAGGTCTGAGATCAAGTTAAAACCTTATAGAAACTAATCAAA GCT
TTCACCTTTTTATATTTGTACCTGGCTCTGTTATTATTAGAGTTATATATAAGTGCTTTTCTGAAACATATGCAAATATGATT GCCCGAATGGCAGAGGCATAATGTCAAGCATTCTGCTTTCATTAA
CAAATCTATCAGAACTTGCCCCTTAGCTTTATCACTAAGACCGTTAGTGTCTTTAAGTATTAAATAATCATAATAATAAAGAAGGAAAATGTTGTATATAATTTGCAGAGGTGCTATCTGTATTC ATT
AGGATATTATTTCAGCTGTTTAAACAGAGACCAAATCACAATAGCTGAAAACTAGATAAAAGGTTATTTCTCTCATTAAAGTT CAATCGATAGGTTCAGCAGGTACACACTGTACTCCACAAGATCAC
CCAGGGATCAAAGCTCCTTCTATCTTGTTTTGTCATTTCTTACAGTGTTGTTCTTGTCATGGTCTAAACCAGCTTACCCCACTGCCACATTTATCCATCAGCCTATGGGAAAAGGGAAAAAGGAT GTG
GAAGGTAAGCAGCTTCCTTTTGGGAACATGATCTAGAATTTTGGACGTTATTTCTGCTCACATGCCATTGGCCAGAACTTGAT TGTGTGGCCACACcTTGCTAAGAGAAACTGTGAAATGTAATTTCT
AATTGAACAGTCATGTGTCCACTTAGACCTCAGGTGGTTCTGTTACCAAACTTCACCGTGCCACATATACAACTTCTATCCTACTTAAAAATATATATTTTAATAATCATTTCCCCATGCTGTTAATA
AATCTTCATGAACATCATCTTTAATGACTATGTAATGGTTGTACCTATGAAAGTATTTCCCTGCTGTCAAACATGTAGGTAGTTTCCAGTTGGGGATTTTATACATAGTACTGCAGTTAATATCT GCC
ATATGAATCTTTATTTGCTTTTCTGGTTATTTCCTTGGTATTTATTCCTACAGGTAAGACTGTTTACTGAAAAGTTATACCAATTAACCTTCTCCTCAGAAGC AAATGAGGGGGCTACATTTTACACC
ACCATCACTTGCAATAATTTGGTCTTATTTTTTATCTTTGCAAATTTGAaCAATTTAAAATTATATCTAATTATAATTTTAAATTTCATTCCTTTAATTATTAGTGCATTTATTACTCATGAATTTTA
ACATGTTTCTGGTATTTATTAATTATTTGTATTATTTCTCTTAAGAATTCATCTTTGCCCATTTATTATCTTAGCTTTTTTCCAACTGTAGAACAAGATAGTA ATAGTACCTGCATAATAGGGTATGG
TGAGAATCAAACAAGAAAAGGAATGACAGAAACTTTgTTTCTTCTGGTTTCTTCTGAACAAGTTCTTCAGACTTGGGCTCAGTCTATGTTGTAGCTACAATGGAGTGGTCTGAAGAGCCCTCATCACT
CTGCCGCACAACCCCATCTTATTTTCTTCATCGTACTGATGATGATCAAAGATTCTATTTTTCAATGGTTTACTTATTTTTGTCCCTCCCCTTCCCCACTAGA ACCAGGCCCTGGTCTCTCTTCTCTA

CTCATATTTCTTTATCACCAAAACAGCATCTGCCACACAGTAGATGCTCAATTAATATTTTCTAGATAAATAAATAAACCTCCTTCCCTCACTGCCAGAATCTCTTGACCTTGTTAAAAACTTAG GCA
TAGTCTTCAATTACCTCTTTGACAAAGACAAGATGTGATGCCCCCAACAAACCCTC C

>KSI_AluYa5_85

>Scaffold1807-892485-892795
ATCCGCTGTAATCAAGGACAAAAGCAGGAAACTCAGCCAAAACTAACCCAGCTGACTCAGCTGTCTCATCCCGATCTTCCCCACTGCCCAGGTTCTCCCAAAAATGGGGTGCATCAGGGAGCTGGG GT
GATCATAGGCCCAGAGAATTTGAGGGCTTAGAGGGACTATCCCATGAAGGTCAGGCCCTGGCACTCCCCCTTCTTGCAGGGCTTCCCCGTAGGCAGATTGTATCAAAGTCCCCATCAGCAGCCAG GAG
CCCCTGGTCAGGGGAGGCCTGGAGCCAGAGCCCTTTCCTGCAACCTCTGTTGACACCATCTTTCTTCTCAAGGAGGCTGCTGAGGGCTGAGGGCTGAGTCAGGAGGAGTGAAAATCCCTTTGGTGT AA
CCTCTAATTTACAGATGAGACAGTGAAGTGGGGGTGGGTTAGGCCACATGGGGCAGTGGCGTGTGCCTTTGTGGAGAAGCAGAAACTTGTCCTCGGTTTTCTGATTCTAAAGACAGTTGTTTGTA GAG
AGATTCCCATGGGGTAGTGAAGAAGGCTCTAAGATCTTAGCTAAGGTACTTCAGCTTTCCACAACTTGATTTCCTCATCTGTAAAATGAGGATAAAAATATATAATATCTGACTGATAGAACTGCA AA
GATTTACTGTGCTGATATGTGTAAAAGTGCCTGGTACAGTGCCAGACACACAGTTACACATGTAATAAATCTAGCTACTGTTTTCTCTAACTTTCCTCCAAACTTCTTTTTCCCCAGGCAGTTTT CCC
CAGTTAACCACGCCATACTCTGAATGTTTCTTCATTCCCAAGTAGCTCTTCTATTCTCCCCCAGTATGCGAGTCTTTTGCTTTGCTTTTTGTCTCGGAATCATTTTTCAGGTCAGCTGTTTTGCAGGC
TGCTTGAGGTACAGGATAAAGCATCCAGGCTTGGTGGCTTTTCCCCTTCCCTTAACTCTGGTTCATAGGACACAGAGCTCTGTCCTGTT Tagaaacttggcagggccgggcgcggtggctcacgcctg
taatcccagcactttgggaggccgaggcgggcggatcacgaggtcaggagatcgagaccatcccggctaaaacggtgaaaccccgtctctactaaaaatacaaaaaattagccgggcgtagtggcg gg
cgcctgtagtcccagctacttgggaggctgaggcaggagaatggcgtgaacccgggaggcggagcttgcagtgagccgagatcccgccactgcactccagcctgggcgacagagcgagactccgt ctc
aaaaaaaaaaaaaaaaaaaaaaaaaAGAAACTTGGCAACAGACTTGAGGGGCGGGCAGGGGGGACCCTCAACTGGAAGCTGCTGCCACAGAGGCTGCTCTGTCCGTGGCC CTCCCTGAGGCCAAGCT
ACAGTCTGGCCTTCTCCCATAGCCCACAGGGCTGTCCAGCCTCCTGGGACTCCACAGACGAGGAAATAAATCTCTCAGCTGGAGGAAGGCATTTGCCTTCCTTTCAAAGCCCTTTCTTGTTATTG AAG
TTATTCTTGCCAAGTTCCCCCATAAAAGGCCTTATAGCGGGAGCACCAGGAAGGGAGGAGAGGGGTCCGAGCTACATAGTGTTTGTAGGCATGGCCAAGCAGCTTGGTCTTCCTGAGCTGGCCTCG GC
CGCTACCTGTCCCGGCTTTGGGTCCCTGATTTAGGACACCTGGAGGGAtCACCAAAGGAGAGGAGGTGGCTCGTTCCTAGCAACTGGCCTTTTGACTGTTTCTTGCTGTGCGTCCCTGTCCTCTATGC
CTTGTGTCTCGTTATATGCCTTGATGGTGTGTATAGTGTACCCACACACAGCCATGACTTGTCTGCCTTTGTGTCCTCAGACCCTAGTGACTAGCAGATACTCAGTAAACACCTGCTGGCTGGCAC AC
CCTTCACAGAGGGTGGGATGCTATTTCTGGACTCTTTTTCTTTCACCTTGGCTAAGAATGTCTCTCTCCCAGACACATATACAACCCTGCAGGCTTCCCGTGATACTTGTCTTGGCCTTTGTCAC CCA
TCCTCTGAGGGGCTTTCCAGAACCTGGATACCTCATTTCCCGGCCAGCTCTATGCCAGTCTGACTTGTCTTTTCCTGGCAGGACCTAGTAGCATTTGAAAGAAAGGCAGATGAGGAAGAGAAACAA TT
CCTTGTGGGCTGGTCTTGGAACGTGGTAGGTACCCAGGAAATGGTCCCTGTGCCTCTCCTGCCAGACTGTGTCCACAGCCTCTGCTGAGATCTCAGCAGCACGGGGGACAGAGTGTCTCATACTC CAT
GCCTCT

508	31.9	2.2	3.3	UnnamedSequence	538	722	(1588)	+	MIR	SINE/MIR	80	262	(0)	1
2917	0.3	0.0	0.0	UnnamedSequence	1000	1310	(1000)	+	AluYa5	SINE/Alu	1	311	(0)	2
209	39.4	0.0	0.0	UnnamedSequence	1844	1914	(396)	+	L2c	LINE/L2	3310	3380	(7)	3

>hg19 chr15:63085040-63087029
ATCCGCTGTAATCAAGGACAAAAGCAGGAAACTCAGCCAAAACTAACCCAGCTGACTCAGCTGTCTCATCCCGATCTTCCCCACTGCCCAGGTTCTCCCAAAAATGGGGTGCATCAGGGAGCTGGG GT
GATCATAGGCCCAGAGAATTTGAGGGCTTAGAGGGACTATCCCATGAAGGTCAGGCCCTGGCACTCCCCCTTCTTGCAGGGCTTCCCCGTAGGCAGATTGTATCAAAGTCCCCATCAGCAGCCAGGAG
CCCCTGGTCAGGGGAGGCCTGGAGCCAGAGCCCTTTCCTGCAACCTCTGTTGACACCATCTTTCTTCTCAAGGAGGCTGCTGAGGGCTGAGGGCTGAGTCAGGAGGAGTGAAAATCCCTTTGGTG TAA
CCTCTAATTTACAGATGAGACAGTGAAGTGGGGGTGGGTTAGGCCACATGGGGCAGTGGCGTGTGCCTTTGTGGAGAAGCAGAAACTTGTCCTCGGTTTTCTGATTCTAAAGACAGTTGTTTGTAGAG
AGATTCCCATGGGGTAGTGAAGAAGGCTCTAAGATCTTAGCTAAGGTACTTCAGCTTTCCACAACTTGATTTCCTCATCTGTAAAATGAGGATAAAAATATATAATATCTGACTGATAGAACTGC AAA
GATTTACTGTGCTGATATGTGTAAAAGTGCCTGGTACAGTGCCAGACACACAGTTACACATGTAATAAATCTAGCTACTGTTTTCTCTAACTTTCCTCCAAACTTCTTTTTCCCCAGGCAGTTTTCCC
CAGTTAACCACGCCATACTCTGAATGTTTCTTCATTCCCAAGTAGCTCTTCTATTCTCCCCCAGTATGCGAGTCTTTTGCTTTGCTTTTTGTCTCGGAATCATTTTTCAGGTCAGCTGTTTTGCAGGC
TGCTTGAGGTACAGGATAAAGCATCCAGGCTTGGTGGCTTTTCCCCTTCCCTTAACTCTGGTTCATAGGACACAGAGCTCTGTCCTGTT TAGAAACTTGGCAACAGACTTGAGGGGCGGGCAGGGGGG
ACCCTCAACTGGAAGCTGCTGCCACAGAGGCTGCTCTGTCCGTGGCCCTCCCTGAGGCCAAGCTACAGTCTGGCCTTCTCCCATAGCCCACAGGGCTGTCCAGCCTCCTGGGACTCCACAGACGA GGA
AATAAATCTCTCAGCTGGAGGAAGGCATTTGCCTTCCTTTCAAAGCCCTTTCTTGTTATTGAAGTTATTCTTGCCAAGTTCCCCCATAAAAGGCCTTATAGCGGGAGCACCAGGAAGGGAGGAGAGGG
GTCCGAGCTACATAGTGTTTGTAGGCATGGCCAAGCAGCTTGGTCTTCCTGAGCTGGCCTCGGCCGCTACCTGTCCCGGCTTTGGG TCCCTGATTTAGGACACCTGGAGGGAcCACCAAAGGAGAGGA
GGTGGCTCGTTCCTAGCAACTGGCCTTTTGACTGTTTCTTGCTGTGCGTCCCTGTCCTCTATGCCTTGTGTCTCGTTATATGCCTTGATGGTGTGTATAGTGTACCCACACACAGCCATGACTTGTCT
GCCTTTGTGTCCTCAGACCCTAGTGACTAGCAGATACTCAGTAAACACCTGCTGGCTGGCACACCCTTCACAGAGGGTGGGATGCTATTTCTGGACTCTTTTTCTTTCACCTTGGCTAAGAATGT CTC
TCTCCCAGACACATATACAACCCTGCAGGCTTCCCGTGATACTTGTCTTGGCCTTTGTCACCCATCCTCTGAGGGGCTTTCCAGAACCTGGATACCTCATTTCCCGGCCAGCTCTATGCCAGTCTGAC
TTGTCTTTTCCTGGCAGGACCTAGTAGCATTTGAAAGAAAGGCAGATGAGGAAGAGAAACAATTCCTTGTGGGCTGGTCTTGGAACGTGGTAGGTACCCAGGAAATGGTCCCTGTGCCTCTCCTG CCA
GACTGTGTCCACAGCCTCTGCTGAGATCTCAGCAGCACGGGGGACAGAGTGTCTCATACTCCATGCCTC T

>KSI_AluYa5_86

>Scaffold7085-892715-892987
AGGAGGTCAGGAGTTCAAGACCAGCCTGGCCAACATAGTGAAACCCTGTCTCTACTAAAAATAGAAAAATTA gCCAAGCGTGGTGGTGGGCACCTATAGTCCCAGCTACTTGAGAGGCTGAGGGAGGA
GAATCGCTTGAACCTGGGAGGTGGATGTTGCAGTGAGCCGAGGTCATGCCACTGCACTCCAGCTTGGGCAACAGAGTGAGACTTAGTCT CaaaAAAAAAAAAAAAACAGTCAATAACAATAAATATAC
TATCAAACAACACAGTATGATTTTATGGCTCTATATATCTGTATCTAAAATTATACAAGAGCAAGGAAGATGA CATTAAAGGATATGCATCAAGTAAGAACTCAGGGTGGGA cTGTTCAGAGGTGACT
TCAGCTTTATCTCTAGTGTTTTTTTTTTTTGGTAAAGGGAAATTTATTCGTATATTGCTTGCATAATTAAGGATTACATGTGTAATTAAGGATTTCATATATAATTAAACATTAATTTAAAAATGTGG
GCAGGAGTCTTTACCTTCCCAAGACCATTTATAAATCAATTTTCTTTTCTGTCTTCTAATAAATCAGCTACTG TAAGCATCGTGAATGAGGGCCACACACTGCATAATGGTTCCCTCTGCCACTCTGG
CTTCTATTTTTACTCTTCCTTGGAACCTCCGGGCATTTGATTTCAATCTGTAATGTCGAGATTTGGAAATTAAATTTAGGTGAGTTCAAAAGAAGAGTGTCTCTAAATGTAAGCAGCTTGGGTAT TTA
CCTTTTCCCTGTTCAAACGTATGCACAAGAACCTCTCTTCTGTCTTCACGTCTGTGAGAAAATTAGTGAGAAA CAACATCAGGGAATCACTGGTGGAGTCTGCATAATTATATATGTCGGCAGCTCTG
TTCTTGGGGGATATTGTGAAATTAGGATATTTCTCTTCCCAAGCCTGATGA tTAGAAAATTTGAATCTCACATACAACCACCAAGTCTCTTTAAGAATGTATGTGgccgggcgcggtggctcacgcct
gtaatcccagcactttgggaggccgaggcgggcggatcacgaggtcaggagatcgagaccatcccagctaaaacggtgaaaccccgtctctactaaaaatacaaaaaattagccgggcgtagtgg cgg
gcgcctgtagtcccagctacttgggaggctgaggcaggagaatggcgtgaacccgggaggcggagcttgcagtgagccgaga TCCCGCCACTGCACTCCAGCCTGGCGACAGAGCGAGACTCTGTCTC
AAAAAAAAAAAAAAAAAAAAAAAAAaagaatgtatgtgATGCCCTGTATAATTCTTAATGGACTTAGAATATGCCTAAAAATTCTATTATAGCACTGTGGAGTATGAAAATTTAAAGAGAGCATACT
CATtctcaaTCTCAAGTTCAGTCACTTTGAAGAGTTTGTTGTTGATGTTGTTTTTAATGGAGAAAATGAGGTCCACAGAATGGAAGTGAATTCTCAACATCACAG AACCAcTGGGCATGGTTGTGGGC
AGATTCCAAGCCTTGATCACCCCCAACAACACAACCATTGTATTCATGTGATATTATATTTGTGCAAATGTAAATT AGATGCTGGAGTGAATGGAGAAGAGGAAACTTCCCATCTTCTGTTACACCTG
CCTGCACAAGTCCTAACAGAATGATACGGCCCAGTACAATGTGCATTTCTCCCATGGACGTTGCCTGCTTAGGAGCATATGGTCTAGGAGAGAGTTGAGGTCACC AGTATTGGTGATCTGCATGAGGA
TGAAGTGATagTGAGAGACCCTAAAGAGAATGGGCCACATAAGGCATTACCTTCAT tGTCAAGAATAAAAATAAAAGTTGTGTTTTTCTGGTCCTCAGTGATAGATACAATTTTGCTCAGTCGTCAAG
AAACTCGTTCCTTCCCCTCACCAAGGGGCAGAGGAAACACAAAGGGATGAAGCTAACACTTCTCGGCTCTCACGTGCTAGTGTTTTGCTGCTGTCCTCCTAATTC TGAATGGCAAGTGTCTTAATGCC
AAAACTGTAAAAGCTGGTCCAGGTCTGATGTGTTCAATGTCATGATTAAGAAAAAA AaaaAAAAAaAAAAACAAAAAACTGAAGTCCTACAGCCAGGTTTGAAAACGTAGCTGAACAGGTGTAGGTGA
GGAGAATGCAGCTGAGAGGTAGCACTGTTCACACATTTAACGAGTGGACATTGTGACTGGGCTTCTGAAGACATTCATATGGTGGAACATGCAGTGTA G

1922	10.3	0.0	0.0	UnnamedSequence	3	234	(2038)	+	AluSx1	SINE/Alu	67	298	(14)	1
354	21.1	0.0	5.3	UnnamedSequence	361	460	(1812)	+	L1ME3G	LINE/L1	6073	6167	(10)	2
2665	8.2	1.3	0.0	UnnamedSequence	1001	1305	(967)	+	AluYa5	SINE/Alu	1	309	(2)	3
215	30.7	6.9	0.9	UnnamedSequence	1457	1558	(714)	C	MIR3	SINE/MIR	(67)	141	34	4
24	6.8	0.0	0.0	UnnamedSequence	2093	2123	(149)	+	(A)n	Simple_repeat	1	31	(0)	5
197	30.4	1.2	6.5	UnnamedSequence	2125	2205	(67)	+	L3	LINE/CR1	3343	3419	(680)	6

>hg19 chr15:79794925-79796871
AGGAGGTCAGGAGTTCAAGACCAGCCTGGCCAACATAGTGAAACCCTGTCTCTACTAAAAATAGAAAAATTA tCCAAGCGTGGTGGTGGGCACCTATAGTCCCAGCTACTTGAGAGGCTGAGGGAGGA
GAATCGCTTGAACCTGGGAGGTGGATGTTGCAGTGAGCCGAGGTCATGCCACTGCACTCCAGCTTGGGCAACAGAGTGAGACTTAGTCT CAAAAAAAAAAAAAACAGTCAATAACAATAAATATACTAT
CAAACAACACAGTATGATTTTATGGCTCTATATATCTGTATCTAAAATTATACAAGAGCAAGGAAGATGACATTAAAGGATATGCATCAAGTAAGAACTCAGGGTGGGA tTGTTCAGAGGTGACTTCA
GCTTTATCTCTAGTGtTTTTTTTTTTTGGTAAAGGGAAATTTATTCGTATATTGCTTGCATAATTAAGGATTACATGTGTAATTAAGGATTTCATATATAATTAAACATTAATTTAAAAATGTGGGC
AGGAGTCTTTACCTTCCCAAGACCATTTATAAATCAATTTTCTTTTCTGTCTTCTAATAAATCAGCTACTGTAAGCATCGTGAATGAGGGCCACACACTGCATAATGGTTCCCTCTGCCACTCTGG CT
TCTATTTTTACTCTTCCTTGGAACCTCCGGGCATTTGATTTCAATCTGTAATGTCGAGATTTGGAAATTAAATTTAGGTGAGTTCAAAAGAAGAGTGTCTCTAAATGTAAGCAGCTTGGGTATTT ACC
TTTTCCCTGTTCAAACGTATGCACAAGAACCTCTCTTCTGTCTTCACGTCTGTGAGAAAATTAGTGAGAAACAACATCAGG GAATCACTGGTGGAGTCTGCATAATTATATATGTCGGCAGCTCTGTT
CTTGGGGGATATTGTGAAATTAGGATATTTCTCTTCCCAAGCCTGATGA cTAGAAAATTTGAATCTCACATACAACCACCAAGTCTCTTTAAGAATGTATGTGATGCCCTGTATAATTCTTAATGGAC
TTAGAATATGCCTAAAAATTCTATTATAGCACTGTGGAGTATGAAAATTTAAAGAGAGCATACTCA TTCTCAAGTTCAGTCACTTTGAAGAGTTTGTTGTTGATGTTGTTTTTAATGGAGAAAATGAG
GTCCACAGAATGGAAGTGAATTCTCAACATCACAGAACCA tTGGGCATGGTTGTGGGCAGATTCCAAGCCTTGATCACCCCCAACAACACAACCATTGTATTCA TGTGATATTATATTTGTGCAAATG
TAAATTAGATGCTGGAGTGAATGGAGAAGAGGAAACTTCCCATCTTCTGTTACACCTGCCTGCACAAGTCCTAACAGAATGATACGGCCCAGTACAATGTGCATTTCTCCCATGGACGTTGCCTGCTT
AGGAGCATATGGTCTAGGAGAGAGTTGAGGTCACCAGTATTGGTGATCTGCATGAGGATGAAGTGAT gtTGAGAGACCCTAAAGAGAATGGGCCACATAAGGCATTACCTTCATcGTCAAGAATAAAA
ATAAAAGTTGTGTTTTTCTGGTCCTCAGTGATAGATACAATTTTGCTCAGTCGTCAAGAAACTCGTTCCTTCCCCTCACCAAGGGGCAGAGGAAACACAAAGGGATGAAGCTAACACTTCTCGGC TCT
CACGTGCTAGTGTTTTGCTGCTGTCCTCCTAATTCTGAATGGCAAGTGTCTTAATGCCAAAACTGTAAAAGCTGGTCCAGGTCTGATGTGTTCAATGTCATGAT TAAGAAAAAAAcAAAAAcAAAAAC

AAAAAACTGAAGTCCTACAGCCAGGTTTGAAAACGTAGCTGAACAGGTGTAGGTGAGGAGAATGCAGCTGAGAGGTAGCACTGTTCACACATTTAACGAGTGGACATTGTGACTGGGCTTCTGAA GAC
ATTCATATGGTGGAACATGCAGTGTAG

1758	10.9	0.0	0.0	hg19_26	3	231	(1716)	+	AluSx1	SINE/Alu	67	295	(17)	1
341	19.2	1.0	7.4	hg19_26	358	458	(1489)	+	L1ME3G	LINE/L1	6073	6167	(10)	2
192	30.0	7.8	0.0	hg19_26	1134	1223	(724)	C	MIR3	SINE/MIR	(67)	141	45	3
17	15.3	0.0	0.0	hg19_26	1770	1798	(149)	+	A-rich	Low_complexity	1	29	(0)	4

>KSI_AluYa5_87

1FR

>Scaffold6443-3148378-3148596
CAATATGTGAGGCAATGGTTGCCTGTATCAGTGTGGTATTAGaGGaAATGATAGTCAGAACTTAAACTTAAAaTATATTATTAAATATCTGcCAAAGGTTTGGAGTTCTTTGTTGGAAATTTTATTGT
GcGGATATTGTAATCTTGTGAAAATTGTTTTGCTTTCTATTGAGTCAGTCCAGGGAAGGATCCAGAAGGAGAAGACGACCTTCATTAGGAAGGTTATTGAGCCATCTGTC cGTAAGCACTGCTATTTC
CTGTTATTCTAACTGATGGCAAGGATCTTTGTTTTAGTTCCTAAAAAGTTGAGAGTGGAAAAATGATTGTGTTTTATGATACAGAATGTTCA AGAcAATTTTGCAGTTGTCAcTTTTAGCACCTTCTT
TGCTAGTGAAGCAATAGCAATATATTTTGAAAAAAATGTTAAGCTCTTTTTCTGAGAAACATCTAAACAATGATTGTTTTCATCAGATCCCCTTCTTCCATTTAGGGTTACAATGTGTTTGTATT TTA
AGTGCTTTATGTGTCATACCATTAGGGGCTATGATCAGCTTGGCATGGGAAAAACACACAAAAATATGCTAGAGCATCTGTTTCCAAAAAT TAGGCTGGAAATCCAAGGCTAAATCTATTATTATCT
TGCTCTATTGAGCTGTTTTCTTATTTCTTCTACCTCAATGTgTTAGACTGAGTGTTACTAGGTTATGGtCTGAGTTATCTATCAGAGAAATACCTGAAATTGCATAGGATCATTCCTAGCATGTATTT
TCTAAATATCAGCCATCTCAGAAGTGTTAAATGAAAAACaAAAAAAAAATGTCTAATTGTTTATAATTATTAAAAGCTACTTAAGTATTTCA ACATACTGGAACAATGAACAACGTAGTGCTTCATTA
CATTTCCAAAAGGTATAAtTCTCTTTTATTTTTGATCTCCTATGAATGGAATAAAATGCTTTCAAATCTCTTCTAGAACTTCAGTGTGTTTAAGAGAGAAATcaggccaggcgcggtggctcacgcct
gtaatcccagcactttgggaggccgaggcgggcggatcacgaggtcaggagatcgagaccatcccggctaaaacggtgaaaccccgtctcta ctaaaaatacaaaaaattagccgggcgtagtggcgg
gcgcctgtagtcccagctacttgggaggctgaggcaggagaatggcgtgaacccgggaggcggagct AGGCAGGAGAATGGCGTGAACCCGGGAGGCGGAGCTTGCAGTGAGCCGAGATCCCGCCACT
GCACTCCAGCCTGGGCGACAGAGCGAGACTCCGTCTCAAAAAAAAAAAAAAAAAAAAAAAAAAAAA aaaaaaaaaaaaaaaaagagagagaaatCAAGGCAAGATATTTGCTTTGCaAAAAAAAAAAAT
CAGTGTATTAGCTGTATTTTCTGTATTTTTTTTtctcttCTtAAGATGTGTGCACTTAACCTCAACTATAAAATGAAGCTATAACACTGAATGTTAAACTAAATGAGTATCAGAACAACGGTTATTGC
TAGTTATAAGCTAACTGGATATTGTGAAAAACTGAAAACTACATTCCTCATTAAGATTTTAAAAAAATATTGCTGAGAAATCT GTGTTGCTATTTTCCCCACATTCTTCCAGAAATTTTTTATGTTCT
TTAGGATTTGCAATGACCAAGAAAATATTAAGATGATGCCTTTATATATTATAGCACAACAACAACAAAACTATATAAAATGTTCTTCTTGTGAAAAGTAGTTTTTTAACCTTTATTTGAGCCAT TCT
TGAAACAAAATTTTCCTAAGCGAAATCACAATTTGAATCCATTTAAAACTGAGTCACATTTTTCCAGGCCAGTTTTCTAAATC TACTAAAAATATTTCCTGTTAAATTAATAATAGGTCTTAGTCTCA
TAACTTCTTTAAAATTCTTGCCTTGACTCTTGACTGAACCTTAAAATtACTTAAGAGATCGGGCAACACATATGGATAaTGAAGAGGCTGTTTTGCCAACTACCGTGTCATTTCTGATCATACTCTGG
CCAACTGGTAGGTAGTTTTACAGAGGAAAACCCTGTATTTAGAGAAGCTGACTATGATTCCTCTGCACTGGCACTGCAGTGAT AAAGGTGTTCAGTCCCTCGTACTTAAAATAGTTAAGAAAATTAAG
TTCTAAAAAAATTACCCATTTCCCCTGTTATAAATCATCCATTTCCTCAGTCATAAGCAGGATGAGAGAGCATCAAAAGTTAGCCAAGCTCAGGCCGGGCGCGGTTGCTCACAC C

2141	0.5	0.0	0.0	UnnamedSequence	1001	1219	(999)	+	AluYa5	SINE/Alu	1	219	(92)	1
36	0.0	0.0	0.0	UnnamedSequence	1251	1289	(929)	+	(A)n	Simple_repeat	1	39	(0)	2

>hg19 chr21:24974582-24976488
CAATATGTGAGGCAATGGTTGCCTGTATCAGTGTGGTATTAGtGGAATGATAGTCAGAACTTAAACTTAAAcTATATTATTAAATATCTGtCAAAGGTTTGGAGTTCTTTGTTGGAAATTTTATTGTG
tGGATATTGTAATCTTGTGAAAATTGTTTTGCTTTCTATTGAGTCAGTCCAGGGAAGGATCCAGAAGGAGAAGACGACCTTCATTAGGAAGGTTATTGAGCCATCTGTC tGTAAGCACTGCTATTTCC
TGTTATTCTAACTGATGGCAAGGATCTTTGTTTTAGTTCCTAAAAAGTTGAGAGTGGAAAAATGATTGTGTTTTATGATACAGAATGTTCA AGAaAATTTTGCAGTTGTCAtTTTTAGCACCTTCTTT
GCTAGTGAAGCAATAGCAATATATTTTGAAAAAAATGTTAAGCTCTTTTTCTGAGAAACATCTAAACAATGATTGTTTTCATCAGATCCCCTTCTTCCATTTAGGGTTACAATGTGTTTGTATTT TAA
GTGCTTTATGTGTCATACCATTAGGGGCTATGATCAGCTTGGCATGGGAAAAACACACAAAAATATGCTAGAGCATCTGTTTCCAAAAAT TAGGCTGGAAATCCAAGGCTAAATCTATTATTATCTT
GCTCTATTGAGCTGTTTTCTTATTTCTTCTACCTCAATGTaTTAGACTGAGTGTTACTAGGTTATGGaCTGAGTTATCTATCAGAGAAATACCTGAAATTGCATAGGATCATTCCTAGCATGTATTTT
CTAAATATCAGCCATCTCAGAAGTGTTAAATGAAAAACAAAAAAAAAATGTCTAATTGTTTATAATTATTAAAAGCTACTTAAGTATTTCAACATACTGGAACAATGAACAACGTAGTGCTTCATTACA
TTTCCAAAAGGTATAAcTCTCTTTTATTTTTGATCTCCTATGAATGGAATAAAATGCTTTCAAATCTCTTCTAGAACTTCAGTGTGTTTA AGAGAGAAAgCAAGGCAAGATATTTGCTTTGCAAAAAA
AAAAATCAGTGTATTAGCTGTATTTTCTGTATTTTTTTTTCTcAAGATGTGTGCACTTAACCTCAACTATAAAATGAAGCTATAACACTGAATGTTAAACTAAATGAGTATCAGAACAACGGTTATTGC
TAGTTATAAGCTAACTGGATATTGTGAAAAACTGAAAACTACATTCCTCATTAAGATTTTAAAAAAATATTGCTGAGAAATCTGTGTTGCTATTTTCCCCACATTCTTCCAGAAATTTTTTATGT TCT
TTAGGATTTGCAATGACCAAGAAAATATTAAGATGATGC CTTTATATATTATAGCACAACAACAACAAAACTATATAAAATGTTCTTCTTGTGAAAAGTAGTTTTTTAACCTTTATTTGAGCCATTCT
TGAAACAAAATTTTCCTAAGCGAAATCACAATTTGAATCCATTTAAAACTGAGTCACATTTTTCCAGGCCAGTTTTCTAAATCTACTAAAAATATTTCCTGTTAAATTAATAATAGGTCTTAGTC TCA
TAACTTCTTTAAAATTCTTGCCTTGACTCTTGACTGAAC CTTAAAATgACTTAAGAGATCGGGCAACACATATGGATAgTGAAGAGGCTGTTTTGCCAACTACCGTGTCATTTCTGATCATACTCTGG
CCAACTGGTAGGTAGTTTTACAGAGGAAAACCCTGTATTTAGAGAAGCTGACTATGATTCCTCTGCACTGGCACTGCAGTGATAAAGGTGTTCAGTCCCTCGTACTTAAAATAGTTAAGAAAATT AAG
TTCTAAAAAAATTACCCATTTCCCCTGTTATAAATCATC CATTTCCTCAGTCATAAGCAGGATGAGAGAGCATCAAAAGTTAGCCAAGCTCAGGCCGGGCGCGGTTGCTCACAC C

>KSI_AluYa5_88

>Scaffold6443-4682118-4682300
TCAAAGTAATCAAATCATACATTATAAATATGTGCATCTTATAGTATGTCCATTATATCTTGTATTATTGTACAACATAAATTATACATATAAAATACCAAAAGTATGTAGACAAACTGATAATTA TT
TTTACTAAGGTTAAGTAATTATTTGTGAACTACTTTTAAAGTGAAGCTGGTGCCATTTGGGGATCAGATGTTTTAATTCCTAATTTTAATCCTTGA cGATAGAGCGTAAGTGCCCATGTGGACTCTTC
CCGGGGGGAGCCCACAAAATAGTCAGAAAAGTACTGGCTCTCTTACATAGTGCCTGAGTAGTGTACTGCCTTTATAGTTTGCTCAGCTCCTTTCATCTGTAATAGTCCACATGGAAGACAACATAGAA
CCACTGGAATCCCTAAATGATGACTATACAGATGACTCTCAGTAACTCTCTTTGTGCTATAAATACAGTATAAAAATAACTACACTGTGCTTCAATATATTCAGTTGTTGAGTAAAAGCAGGATA AGG
CCTTAAGAGAAATGGATTTCTTGACAGTTAGGGTCCATATTATCAAACTAAAATTAAA tAGCAATTTCCCCACAAGCCAATCATGCATTAGGGAGACCATCACTGTTATAAATTATGTAACAAGGACA
GAAAACATGGTTCCAAGAAGGTTCTAAATGCTTCATACTAAATGAAAGTTTAAAATCACTTGAATTTTAAGCAGAGTGATATAGACCTGGGGGGTAACAAAGTCGTTTCCAAGTTCTGATCATAC ACA
ACTGTTGATAACCAAATGTTTAACATGGGAAATAGAAGAGTGAAATAAAGGTCTATAGTTGCTTTTATTTACATTGGCCATATTTATCTCACAATAGTT ACTTGACTGCCATTGTTTGCATTTGTTTC
TACTTTAAAATTTTAAAGGAGCAGCAATTGTCTCTTTTTATGATTAGCATCAACCTCTGCATGGGTTTATGAAGAAGATGCATTGCTTTGTTAAGGA AGGATGggccgggcgcggtggctcacgcctg
taatcccagcactttgggaggccgaggcgggcggatcacgaggtcaggagatcgagaccatcctggctaaaacggtgaaaccccgtctctactaaaaatacaaaaaattagccgggcgtagtggcg gg
cgcctgtagtcccagctacttgggaggctgTAGTATTTTACCAGGAAAAAAAATAGGGTAAAGAGCCATGTAAGATGTGCTAAACATATGACCTTAAGATGCTA TATTAATTTAAAGAATGGAGACATG
GAGTAAATAATCATTGGTGAAAGTAAGCCCTTTAACTTATTTTAAACTCTGTTATTTATAATTCAGCATTTTTCAATAAGCCTTTAGGAAGCTTTCACTATTTTGACTATGTCTCTGTTATAATATTA
AGAATGGAGGTTCATAGAGTTTTACATAACATTTTTGCAACTATAAGAAATTAAACATTAATTATTGATCTGTGAGGTTTAGTTTGACATATCTGTCAAACAT ATATAATTTGCTTCTTAATAGAATC
CAGTTTAATCTCTAATATTTAAATTACAATTTACTTTGCCTTTATCGTTGGCTTTTACCTTTCCTATTATTCACATTGTCTCTTTGCTATGTTTGCTCCTCATGTGATGAATTTCTGACCTTTTA AAA
AAAGAGCATACACATTTGCAAATTTCATGATCCAAATAGTCTTTCTGAGATGAGTATTTCACCAGTTTTATAATTTCTCAATTGTAACTGTTAAGCAAATTAC AATTAACCATCTCCCAGTTTGCTCA
ACCATGTAAATGCTTGTTATTTTTGTTGATTATATAAAGCAGACTTTTTATTCTTTTGGCAAACAAGATAGAGAGGCTATAAACTAAACATCAGAATGGGCCATAAAATTTTTTCTGTTAAATCA ATT
TCCATTGCTGGCCTCAGAATATACATACAATGTAGCTTAGATATATTTCTTGAGAACATTATACCAATTTCAATACATTTGCTTTTCTTTAATAAAAAATATG TGACAGTTGTACTGAATTCAACATT
CAGCTGGCAGGGATTACCAATCAGATTCATTCAAAGTGCTCTTGGGGTCAACTTACTTTTCAGGAGCAAATAAATCTAGCAGTGTGTAGCAAGTCTTCATGAAATAAAATTATCATTTTTTATCG AGA
AACAGT

1781 0.6 0.0 0.0 UnnamedSequence 1000 1182 (1000) + AluYa5 SINE/Alu 1 183 (128) 1

>hg19 chr21:26496433-26498449
TCAAAGTAATCAAATCATACATTATAAATATGTGCATCTTATAGTATGTCCATTATATCTTGTATTATTGTACAACATAAATTATACATATAAAATACCAAAAGTATGTAGACAAACTGATAATTA TT
TTTACTAAGGTTAAGTAATTATTTGTGAACTACTTTTAAAGTGAAGCTGGTGCCATTTGGGGATCAGATGTTTTAATTCCTAATTTTAATCCTTGA tGATAGAGCGTAAGTGCCCATGTGGACTCTTC
CCGGGGGGAGCCCACAAAATAGTCAGAAAAGTACTGGCTCTCTTACATAGTGCCTGAGTAGTGTACTGCCTTTATAGTTTGCTCAGCTCCTTTCATCTGTAATAGTCCACATGGAAGACAACATAGAA
CCACTGGAATCCCTAAATGATGACTATACAGATGACTCTCAGTAACTCTCTTTGTGCTATAAATACAGTATAAAAATAACTACACTGTGCTTCAATATATTCAGTTGTTGAGTAAAAGCAGGATA AGG
CCTTAAGAGAAATGGATTTCTTGACAGTTAGGGTCCATATTATCAAACTAAAATTAAA cAGCAATTTCCCCACAAGCCAATCATGCATTAGGGAGACCATCACTGTTATAAATTATGTAACAAGGACA
GAAAACATGGTTCCAAGAAGGTTCTAAATGCTTCATACTAAATGAAAGTTTAAAATCACTTGAATTTTAAGCAGAGTGATATAGACCTGGGGGGTAACAAAGTCGTTTCCAAGTTCTGATCATAC ACA
ACTGTTGATAACCAAATGTTTAACATGGGAAATAGAAGAGTGAAATAAAGGTCTATAGTTGCTTTTATTTACATTGGCCATATTTATCTCACAATAGTTACTTGACTGCCATTGTTTGCATTTGTT TC
TACTTTAAAATTTTAAAGGAGCAGCAATTGTCTCTTTTTATGATTAGCATCAACCTCTGCATGGGTTTATGAAGAAGATGCATTGCTTTGTTAAGGA AGGATGtattacatactgtaagctTAGTATT
TTACCAGGAAAAAAAATAGGGTAAAGAGCCATGTAAGATGTGCTAAACATATGACCTTAAGATGCTATATTAATTTAAAGAATGGAGACATGGAGTAAATAA TCATTGGTGAAAGTAAGCCCTTTAACT
TATTTTAAACTCTGTTATTTATAATTCAGCATTTTTCAATAAGCCTTTAGGAAGCTTTCACTATTTTGACTATGTCTCTGTTATAATATTAAGAATGGAGGTTCATAGAGTTTTACATAACATTT TTG
CAACTATAAGAAATTAAACATTAATTATTGATCTGTGAGGTTTAGTTTGACATATCTGTCAAACATATATAATTTGCTTCTTAATAGAATCCAGTTTAATCTCTAATATTTAAATTACAATTTACT TT
GCCTTTATCGTTGGCTTTTACCTTTCCTATTATTCACATTGTCTCTTTGCTATGTTTGCTCCTCATGTGATGAATTTCTGACCTTTTAAAAAAAGAGCATACACATTTGCAAATTTCATGATCCA AAT
AGTCTTTCTGAGATGAGTATTTCACCAGTTTTATAATTTCTCAATTGTAACTGTTAAGCAAATTACAATTAACCATCTCCCAGTTTGCTCAACCATGTAAATGCTTGTTATTTTTGTTGATTATAT AA
AGCAGACTTTTTATTCTTTTGGCAAACAAGATAGAGAGGCTATAAACTAAACATCAGAATGGGCCATAAAATTTTTTCTGTTAAATCAATTTCCATTGCTGGCCTCAGAATATACATACAATGTA GCT
TAGATATATTTCTTGAGAACATTATACCAATTTCAATACATTTGCTTTTCTTTAATAAAAAATATGTGACAGTTGTACTGAATTCAACATTCAGCTGGCAGGGATTACCAATCAGATTCATTCAAA GT
GCTCTTGGGGTCAACTTACTTTTCAGGAGCAAATAAATCTAGCAGTGTGTAGCAAGTCTTCATGAAATAAAATTATCATTTTTTATCGAGAAACAG T

>KSI_AluYa5_89

>Scaffold3772-201563-201847
CAGAGTGATAGAATGGCGTACAACAACACTGGGAGACTTCAGTACTCCACTTTCAGTAATGGACAGCTTAATTAGACAAAGAAACATTGAAATTAAACTGTTCTCTAGACCAAATGAACTTAACTGAC
CTTTACAAAATATTTCATCCAATTGCTATAGAATATACATTCTTGTCATCAGCATATGGAACATTCTCCAGTATAGAGCATATGTTAGGCCACAGAACAAG GCTAAACAAATTTAAGAAAAGACCCGA
AAATATGAAATTACTAGAATAAGAGCCTGGGGAAATGCTTTAAGACATTGTTCTGGGGAAAGATTTTTTGTATAAGATCTGAAAAGCACTGGCAATCAAAGCGAAAATAGACAAATGAGAGTAAA TCA
AGCTAGAAAGCTTCTGCACAGCAAAGAAAACAACAAAGTGAGGAGACAACCTACAGAAAGAGAGAATATCTTAGCAAACTATTGATCTAACAATGGAATAA CAAATCACAATGTAAAACAAACTCAGC
TCAATAACCAAAAACCAAATTATCTGATTTACAAATGGGCAAATGATCTGAATAGGCATTTCTGAAACAAAGACACAAATGGCCAACAGATATATGAAAATACGTTAAACATCACTAACCGTCAG GGA
AACGCAAATCAAAACCATGTGAGATACCATATTACCCCAGTTCAAATGGCTCTTACTAAAAAGACAGAGAATAACAGTTGCTGGCTAGGAAGCAAAGAGGA ATGCTTGTATACTGTTGCTGTAAATGT
ATATTAATACAGCCACTATGGGACATAGGATGGAGTTTCCCCAAAAAGCTAAAAATAGAGTTACCGTATGATGCAGCAATCCCGCTGCTGAGTATATATTCA AAAGAAAGGAAACCAGTACAGCAAAG
AGATATATGCATTGCCATGTTTATTGCATCATTATTCACAATAGCAAAGATATAGAATCAACCTAAGTGTTCACTGACGGATGAATGGATTAAG AAAATGTGACggccgggcgcggtggctcacgcct
gtaatcccagcactttgggaggccgaggcgggcggatcacgaggtcaggagatcgagaccatcccggctaaaacggtgaaaccccgtctctactaaaaatacaaaaaaattagccgggcgtagtg gcg
ggcgcctgtagtcccagctacttgggaggctgaggcaggagaatggcgtgaacccgggaggcggagcttgcagtgaccccacccgggggaaaaagggagc cccccccaaaaaaaaaaaaaaaaaa
aaaaatgtgacATACATATAAAAAGGAAGATTATTGAGCCAGAAAAAGGAATAAAATCCTGTCATCTGCAGCAACATGAATGGAACTGTAGGACACTACGT gAAGTGAAATAAGCCAGATGCAGAAAG
ACAAATTATCACATGTCCTTACTCATGTGCAGGAGCTGAAAGAATCCATCTCATGGAACTATAGAGTAGAATGGTGGATACCAGAAGTTAGGAAAGGAGGGGAATGAAGAGAGGTTGGTT AATGGCTA
CAAAAATACAGTTGGACAGAAGGAGTAAGTTCTAGTGTTCAATAGCATACTTAGGGCGACCATAGTTAGCAATAATTTAATGCTTATTTCCAAATACCTAAA AGAACATTTGAAATGTTTCCAACACA
AACAAATAATAAATGCATTAGGTGATGAGTATCCCAGTTATCCTTTTTTGGTTATCACACATTTTGTGGATGCATGAAAATATATGTACTACATAAATATGTACAATTATATGCAAATTTTAAAA ATA
GGCAAAGCTCTTAAATATACAATTCTCCAAAGAAGATATACAAATAGCCAACAGTTCTATGAAAGATGCTTCCATTATTAGTCTTTTCAGAAACGTAAATCA AAACCACCTGATGGTACCAATTAACA
CCCACTAGAATGGGAATTACTTAAAAAGAACAAATATCAGTGGAGAGGTGGAGAAGTTAGAACTCTTCTAGAGTGTTGGTGGAAATGCTAAATTGTGCACCTGCTACAGAAAGCAGTTTATCAGTACT
TCAAAAATTTAAACATAGAAGGAACCATATGAGCTAGCAACTCTACTCCTATGTATACACCACATATGATTGAAAACAAATACTCAAACAAGTACTTTTATA TACTCACTGTATATATTTATTAACAA
TACCCAAAAGGTGAACATAAGCAAACATCCATCACTTGATGAATGGATAAACAAAATGTGATATATACATATGGATATTATTCAGCTACAAAATGAAATGAAGTACT G

1317	18.2	4.5	0.0	UnnamedSequence	3	249	(2035)	+	L1MA4	LINE/L1	2510	2767	(3376)	1	*
5310	17.0	2.6	0.7	UnnamedSequence	248	1000	(1284)	+	L1MA4	LINE/L1	5013	5780	(520)	1	
2324	3.9	7.4	0.3	UnnamedSequence	1001	1285	(999)	+	AluYa5	SINE/Alu	1	305	(6)	2	
5310	16.6	2.6	0.9	UnnamedSequence	1286	1774	(510)	+	L1MA4	LINE/L1	5781	6278	(22)	1	
1901	22.0	4.6	1.4	UnnamedSequence	1787	2284	(0)	+	L1MB5	LINE/L1	5319	5832	(342)	3	

>hg19 chrX:130920950-130922942
CAGAGTGATAGAATGGCGTACAACAACACTGGGAGACTTCAGTACTCCACTTTCAGTAATGGACAGCTTAATTAGACAAAGAAACATTGAAATTAAACTGTTCTCTAGACCAAATGAACTTAACTGAC
CTTTACAAAATATTTCATCCAATTGCTATAGAATATACATTCTTGTCATCAGCATATGGAACATTCTCCAGTATAGAGCATATGTTAGGCCACAGAACAAGGCTAAACAAATTTAAGAAAAGACC CGA
AAATATGAAATTACTAGAATAAGAGCCTGGGGAAATGCTTTAAGACATTGTTCTGGGGAAAGATTTTTTGTATAAGATCTGAAAAGCACTGGCAATCAAAGCGAAAATAGACAAATGAGAGTAAAT CA
AGCTAGAAAGCTTCTGCACAGCAAAGAAAACAACAAAGTGAGGAGACAACCTACAGAAAGAGAGAATATCTTAGCAAACTATTGATCTAACAATGGAATAACAAATCACAATGTAAAACAAACTC AGC
TCAATAACCAAAAACCAAATTATCTGATTTACAAATGGGCAAATGATCTGAATAGGCATTTCTGAAACAAAGACACAAATGGCCAACAGATATATGAAAATACGTTAAACATCACTAACCGTCAGG GA
AACGCAAATCAAAACCATGTGAGATACCATATTACCCCAGTTCAAATGGCTCTTACTAAAAAGACAGAGAATAACAGTTGCTGGCTAGGAAGCAAAGAGGAATGCTTGTATACTGTTGCTGTAAA TGT
ATATTAATACAGCCACTATGGGACATAGGATGGAGTTTCCCCAAAAAGCTAAAAATAGAGTTACCGTATGATGCAGCAATCCCGCTGCTGAGTATATATTCAAAAGAAAGGAAACCAGTACAGCAA AG
AGATATATGCATTGCCATGTTTATTGCATCATTATTCACAATAGCAAAGATATAGAATCAACCTAAGTGTTCACTGACGGATGAATGGATTAAG AAAATGTGACATACATATAAAAAGGAAGATTATT
GAGCCAGAAAAAGGAATAAAATCCTGTCATCTGCAGCAACATGAATGGAACTGTAGGACACTACGT tAAGTGAAATAAGCCAGATGCAGAAAGACAAATTATCACATGTCCTTACTCATGTGCAGGAG
CTGAAAGAATCCATCTCATGGAACTATAGAGTAGAATGGTGGATACCAGAAGTTAGGAAAGGAGGGGAATGAAGAGAGGTTGGTTAATGGCTACAAAAATACAGTTGGACAGAAGGAGTAAGTTC TAG
TGTTCAATAGCATACTTAGGGCGACCATAGTTAGCAATAATTTAATGCTTATTTCCAAATACCTAAAAGAACATTTGAAATGTTTCCAACACAAACAAATAATAAATGCATTAGGTGATGAGTATC CC
AGTTATCCTTTTTTGGTTATCACACATTTTGTGGATGCATGAAAATATATGTACTACATAAATATGTACAATTATATGCAAATTTTAAAAATAGGCAAAGCTCTTAAATATACAATTCTCCAAAG AAG
ATATACAAATAGCCAACAGTTCTATGAAAGATGCTTCCATTATTAGTCTTTTCAGAAACGTAAATCAAAACCACCTGATGGTACCAATTAACACCCACTAGAATGGGAATTACTTAAAAAGAACAA AT
ATCAGTGGAGAGGTGGAGAAGTTAGAACTCTTCTAGAGTGTTGGTGGAAATGCTAAATTGTGCACCTGCTACAGAAAGCAGTTTATCAGTACTTCAAAAATTTAAACATAGAAGGAACCATATGAGCT
AGCAACTCTACTCCTATGTATACACCACATATGATTGAAAACAAATACTCAAACAAGTACTTTTATATACTCACTGTATATATTTATTAACAATACCCAAAAGGTGAACATAAGCAAACATCCATC AC
TTGATGAATGGATAAACAAAATGTGATATATACATATGGATATTATTCAGCTACAAAATGAAATGAAGTACT G

>KSI_AluYa5_90

>Scaffold14588-4143009-4143175

TCCTGTTTAGCAGAGCCATTTCCATGATTTGTTGGCTCATTTTTATGTTAAA cATTTTCACAAGAATGGCTTCTTGACATCAGGGAGTCAGTCAGATATAATGGAGTTCTGCTAACAAGCCTCATAAA
AATGTAGGAAGCAGATAATAAATCAGACTTATATACTTCATAGATAATAACTTGAAATTTGGAATAATTAAGTATCTAGCACACTACAGCACTTGTGAAACATTAAGAGCTTGGTGCAAACAGAA AAA
AATGTCCACCCATATTTAAAGCTGATTTCCTTTATTAAATGAAATCACCAGGTCTCTATGCTGACACATTGGCCATCCTAACTTAGAAATATAGTATAGAGTCCTAAGGGTAACTGCTGGAATGAT GA
CTATTTCTTGGTGTTCTTTCGGAAGCTGGCTAGGTAAATAGAAGCGATGTCAAGTAATTGAGCCACAGCTGGTCAGGACATTTCATTAGCAGCATTACTATCAATCCTTGGTTAAAAGAGTAAAT GGA
AGTACTATGAATTAGTAATAGCTCTTAGAGCTGAAAGTGTGGTTCACACTTTGCCTTAGGGAAAGAAGTTTGAGAAAGGCAAAGAATGACTGAGAATACTCATCCTTTTTTTTTTTTT TAATTTTTCC
ACAGGAGTTGATTTCTCTACTAAAAGCAATACCCACAACATTGAACCCTTGATATAAAACCTGATACTGAATTTTCCACAAGGTCACCTATATTTAAGTAACATATTCAGGATTATATAATATGA GAG
TTAAAGCAGAATAATGAGAGTAAACAAAAATTGCAGTGCTAATGATACTTCGATTTGCATGGCATTTTTCATCAGCTAATTCAAAAACAATTGAATCTCTTAAAAACTGAAATGTTTTTAAAGAAA AT
GGCTGACTTTCAGTTAAAATTTTACTTTTTTAAAAAACTAACCCTGAAACTTCTACTTTAGTTCTATATTCAAACATAAAATACTCCACTT AAAAAAATTTGCAggccgggcgcggtggctcacgcct
gtaatcccagcactttgggaggccgaggcgggcggatcacgaggtcaggagatcgagaccatcccggctaaaacggtgaaaccccgtctctactaaaaatacaaaaaaattagccgggcgtagtg gcg
ggcgcctgtagtccctagacaacagagcataaaaaaaaaaaaaaaaaaaaaaaaaaaaa aaaaaaatttgcaCCTCGTATGCcATCAAGGGCCTTCATATTTTATTTTATATGTATATATTCAAATAG
GAATTCACTTATATTTGTTCAAAATGTATTAGGAAAAATCAATGTGTGGTCAGTACACACTGCATGTGAATTGTAAACAGATATTTAGTGTGTGAATATTGTCA TTGATCCACTCATTTTTACCAAGT
GATTTCTAGTTGCTAATACTAAGCTATACTGCATATTGCCAATGTTTTACCTGGTTTTAGAGCAAAGCCACATGACCAAAACCAAAAGGGAGAAAGAGGCCTTC TACTTCTATATTTATAGCCATTCA
CATTCACACATGTATATACATAAACAGTCAAGTCTACCATCATGCAAATGCTTAGAGCATGTCAAATTAAATCCCATCAGGGTACAAATCAATTTTCCTATTTTAGCTTTTTTCATTTTATTTTTCTA
CCATAAGGAGTACAGAATCCTAATCAATTCTAACTCATCAAAT TTTAGCAGAGTAGTTAAAAGAACAGACTGAAGTATGAGACAACTGAGATTTAAATCTTGAC TGCCACTTAAaAGACTCTGTGAAT
GTGGAAAGCTACTTTAAGTTTTAGATTTCATTTCTCTCATGTCTAAAATGGACATAGAAG CTCAGAAAGCTTTGGAAAGAAGAATTGCTCAAAAACATAAGCTACTGTTAATGAAATGATTATTATTT
CACAAATTTTAGCTTTCAATTATTATTTCTTACAGAGACTTGAGGAAGCAAAACTAAAGCAAAATTAAATAAACAACCTCAATGAGTTTATAGCTTAACCGAAT ATTAAAATAAAAGAAAATTGGAAC
ATATTAAGTTAATTTGTAATTCAAATAAATAGGCTAACCACACTGAA aGTGTACTTAGTGTTTGATGAACAGGCGTTCTTACCTTTATTTAGTCCATTTCACTGTTCTTTACCTGTC T

1601	0.0	0.0	0.6	UnnamedSequence	1001	1167	(999)	+	AluYa5	SINE/Alu	1	166	(145)	1
33	0.0	0.0	0.0	UnnamedSequence	1183	1218	(948)	+	(A)n	Simple_repeat	1	36	(0)	2
352	34.0	2.9	0.7	UnnamedSequence	1710	1848	(318)	+	MIR	SINE/MIR	7	148	(114)	3

>hg19 chr10:9510039-9511985

TCCTGTTTAGCAGAGCCATTTCCATGATTTGTTGGCTCATTTTTATGTTAAA tATTTTCACAAGAATGGCTTCTTGACATCAGGGAGTCAGTCAGATATAATGGAGTTCTGCTAACAAGCCTCATAAA
AATGTAGGAAGCAGATAATAAATCAGACTTATATACTTCATAGATAATAACTTGAAATTTGGAATAATTAAGTATCTAGCACACTACAGCACTTGTGAAACATTAAGAGCTTGGTGCAAACAGAA AAA
AATGTCCACCCATATTTAAAGCTGATTTCCTTTATTAAATGAAATCACCAGGTCTCTATGCTGACACATTGGCCATCCTAACTTAGAAATATAGTATAGAGTCCTAAGGGTAACTGCTGGAATGATGA
CTATTTCTTGGTGTTCTTTCGGAAGCTGGCTAGGTAAATAGAAGCGATGTCAAGTAATTGAGCCACAGCTGGTCAGGACATTTCATTAGCAGCATTACTATCAATCCTTGGTTAAAAGAGTAAAT GGA
AGTACTATGAATTAGTAATAGCTCTTAGAGCTGAAAGTGTGGTTCACACTTTGCCTTAGGGAAAGAAGTTTGAGAAAGGCAAAGAATGACTGAGAATACTCATCCTTTTTTTTTTTTT TtttaAATTT
TTCCACAGGAGTTGATTTCTCTACTAAAAGCAATACCCACAACATTGAACCCTTGATATAAAACCTGATACTGAATTTTCCACAAGGTCACCTATATTTAAGTAACATATTCAGGATTATATAAT ATG
AGAGTTAAAGCAGAATAATGAGAGTAAACAAAAATTGCAGTGCTAATGATACTTCGATTTGCATGGCATTTTTCATCAGCTAATTCAAAAACAATTGAATCTCTTAAAAACTGAAATGTTTTTAAAGA
AAATGGCTGACTTTCAGTTAAAATTTTACTTTTTTAAAAAACTAACCCTGAAACTTCTACTTTAGTTCTATATTCAAACATAAAATACTCCACTT AAAAAAATTTGCACCTCGTATGCtATCAAGGGC
CTTCATATTTTATTTTATATGTATATATTCAAATAGGAATTCACTTATATTTGTTCAAAATGTATTAGGAAAAATCAATGTGTGGTCAGTACACACTGCATGTGAATTGTAAACAGATATTTAGT GTG
TGAATATTGTCATTGATCCACTCATTTTTACCAAGTGATTTCTAGTTGCTAATACTAAGCTATACTGCATATTGCCAATGTTTTACCTGGTTTTAGAGCAAAGCCACATGACCAAAACC AAAAGGGAG
AAAGAGGCCTTCTACTTCTATATTTATAGCCATTCACATTCACACATGTATATACATAAACAGTCAAGTCTACCATCATGCAAATGCTTAGAGCATGTCAAATTAAATCCCATCAGGGTACAAATCAA
TTTTCCTATTTTAGCTTTTTTCATTTTATTTTTCTACCATAAGGAGTACAGAATCCTAATCAATTCTAACTCATCAAATTTTAGCAGAGTAGTTAAAAGAACAGACTGAAGTATGAGAC AACTGAGAT
TTAAATCTTGACTGCCACTTAAcAGACTCTGTGAATGTGGAAAGCTACTTTAAGTTTTAGATTTCATTTCTCTCATGTCTAAAATGGACATAGAAGCTCAGAAAGCTTTGGAAAGAAGAATTGCTCAA
AAACATAAGCTACTGTTAATGAAATGATTATTATTTCACAAATTTTAGCTTTCAATTATTATTTCTTACAGAGACTTGAGGAAGCAAAACTAAAGCAAAATTAAATAAACAACCTCAAT GAGTTTATA
GCTTAACCGAATATTAAAATAAAAGAAAATTGGAACATATTAAGTTAATTTGTAATTCAAATAAATAGGCTAACCACACTGAA cGTGTACTTAGTGTTTGATGAACAGGCGTTCTTACCTTTATTTAG
TCCATTTCACTGTTCTTTACCTGTCT

>KSI_AluYa5_91

>Scaffold5099-734647-734945
GTAGGGTTGTTATGAGGTTATAGCTACTTAGTACATATAAATCCTTTAGAACAGTGCTGAGCACATATAGAAATGTTTGTTATTCTTATTAGCAGTCAGTATTAAGTACTAGGTGAGAGAAGATCT AA
TAAAGCAGAAAAAGGCAGAGAAAAGAAAATGGTGGGTCTGGGCATGATGGGAGCCATGAGAGAGGAAGCTGGGGGCTGGTGAGCAGA aTTGCCATTAGGAAAGGGCCAGAGCTTCTAGAGGAAAGGAG
GAAGTAGCCACCCAAACCTTCACAGAATCCTGTCAACATCTCCAGGGACAAATCAAGCCACCCTACAGAAAG TACACTTGTATGGCCTGTTAATGCTGAGGTCGAATTCATTAGAAGAAATTGTGCAA
TGTAGAATCGAATCATTTTTAAGCATTTAAAAGCAAGAAGCAGAAGTGAACCGTGCTTAAAAGAGGCAGGCACATAGTATCTAAGAGCTTGGGCTCTGCAATTAGACTGGCACAAGTTCAAATCCCAG
CTCTGCCaTTCACTATTATCTGACCTGGGAAGGGTTACTTAGCCTTGTTGATGACTAGTTTCCTCTTGTGGAAAATGGAAATAATCATAGCTATCTCACAGGTTATTCTGAGTCTGAAAGGAAGTATT
CTATGATAATTGCTTTGCACAATGCCTGGAATATTCTAAGGCATCAATACATGTTGCTGTTGCTTACTGCTATCATAATAAAAAGAGAGAATGAACACCTGTGCATGACTTGGAAAGGGTGAGCA TGG
TAAGATATGGATGCATTTTGCTTTAGTTGCTTTGTATGATGTTCGGGCTTATAAAAGATAACTTTTGAATTTCTTTGCCAGCTCTGAATCTCCCACCACTAAGGAGCAGCATCCTATCTATGATGC CC
TCACAAAACCAGAACATCTGCTCGTGTTCTGCATGAGCAGTTATCAACAAAAAGATGTCCCAACACTCAATGGAAAACAAAGTATTTTAATGAT AAAATTCAGggccgggcgcggtggctcacgcctg
taatcccagcactttgggaggccgaggcgggcggatcacgaggtcaggagatcgagaccatcccggctaaaacggtgaaaccccgtctctactaaaaatacaaaaaattagccgggcgtagtggcg gg
cgcctgtagtcccagctgctcgggaggctgaggcaggagaatggcgtgaacccgggaggcggagcttgcagtgagccgagatcgcgccactgcactccagcctgggcgacagagcgagactccgt ctc
aaaaaaaaaaaaaaaaaaaaaaattcagTACGTGAGCTATGTTTATTTTTGATTATTTTGACATTTCAAGCTCAGAAACAGCATTCGTGAAATGGGGATTGCTGGTGAAAATTAAATGGAAAATTGGCA
CTTAAAAATTTTGCATTTTTTTCCAGCTAATTCCTTTCTAAATTCCATCCATTCCTTAAAGAGTCTTGCTGTGATGATTTCTGCTAATTTCTTTCTTGGTA GTTTCCAATAAATCAAACTTCACAGAT
AACAAGAGAATAACAAAATGaTAAAGGTGCTTCTTTCCTTCCCTTCtGAAAATAcTCATTTGCGGATTTTTTACAGAGTACAAATGAAGGCCTGAATGTCAAAGTTTTGTTTTTGTTGTTAAATGACA
AGTTGTTCCGGGGTACATATACTTTGACTTTCAGGCATTCATTCGTACTCTGTAAAAAAATTTGCAAGTGAGTAGGAACTTAAAATAAAaGCTGAAGAAATAACAAAGAAACACTAGAAAAGAAAAAA
AGTAAAAACTCAGTATCTGTGAAGCGCAATAAAATGAAGCACAACAGAAAGGGGAAAAA AATGCCAAGTTGTGTCGCCAGGAATAATAAgCCACGGTATTTCCAAGTAGATGTTTTTaTAAAATACAT
TGGGGTAGATCTTTAtGTTTACTCCTTTTCTTCACTCTGTCACAGTtTTAAAATAAAATGCTCATTATTTGGAATCATGGGAAAGAAGGC TTATCTATCCATTACGGGGAAAACTTAAAaGTGGAGTG
TTACTACTTCAAGCAGCACTATCCATATTACAAATAAAAACCTAACTAGGAATAAGTGATTTAAAAATTCCTATTCAAA GCAGGCATATATCACTACATTGCAACGTGCCACAAACAAAATAAAACTt
TAACACAGGAAAATTAATTATGGGATTTAAAAGTTGAAAACACACCTAAGGAATTCATAATTTTATGAAGGTTTGGCTTATAACTGTTAT TGATTTTAGACTGACTATCCCTTTACTCTTAGGTAA

305	18.8	12.8	1.0	UnnamedSequence	2	87	(2215)	+	MIR	SINE/MIR	167	262	(0)	1
821	26.1	2.9	1.2	UnnamedSequence	457	696	(1606)	+	MIR	SINE/MIR	10	253	(9)	2
570	31.9	6.2	2.6	UnnamedSequence	721	999	(1303)	+	Tigger10	DNA/TcMar-Tigger	172	478	(1620)	3
2892	1.0	0.0	0.0	UnnamedSequence	1000	1302	(1000)	+	AluYa5	SINE/Alu	1	303	(8)	4
421	34.5	9.3	3.3	UnnamedSequence	1303	1486	(816)	+	Tigger10	DNA/TcMar-Tigger	479	667	(1431)	3
244	19.6	0.0	0.0	UnnamedSequence	1796	1846	(456)	C	Tigger1	DNA/TcMar-Tigger	(2359)	59	9	5
450	26.5	9.0	3.7	UnnamedSequence	2037	2291	(11)	C	X11_DNA	DNA/TcMar-Tigger	(84)	403	136	6

>hg19 chr4:185976806-185978799
GTAGGGTTGTTATGAGGTTATAGCTACTTAGTACATATAAATCCTTTAGAACAGTGCTGAGCACATATAGAAATGTTTGTTATTCTTATTAGCAGTCAGTATTAAGTACTAGGTGAGAGAAGATCT AA
TAAAGCAGAAAAAGGCAGAGAAAAGAAAATGGTGGGTCTGGGCATGATGGGAGCCATGAGAGAGGAAGCTGGGGGCTGGTGAGCAGA cTTGCCATTAGGAAAGGGCCAGAGCTTCTAGAGGAAAGGAG
GAAGTAGCCACCCAAACCTTCACAGAATCCTGTCAACATCTCCAGGGACAAATCAAGCCACCCTACAGAAAGTACACTTGTATGGCCTGTTAATGCTGAGGTCGAATTCATTAGAAGAAATTGTGC AA
TGTAGAATCGAATCATTTTTAAGCATTTAAAAGCAAGAAGCAGAAGTGAACCGTGCTTAAAAGAGGCAGGCACATAGTATCTAAGAGCTTGGGCTCTGCAATTAGACTGGCACAAGTTCAAATCCCAG
CTCTGCCcTTCACTATTATCTGACCTGGGAAGGGTTACTTAGCCTTGTTGATGACTAGTTTCCTCTTGTGGAAAATGGAAATAATCATAGCTATCTCACAGGTTATTCTGAGTCTGAAAGGAAGTATT
CTATGATAATTGCTTTGCACAATGCCTGGAATATTCTAAGGCATCAATACATGTTGCTGTTGCTTACTGCTATCATAATAAAAAGAGAGAATGAACACCTGTGCATGACTTGGAAAGGGTGAGCA TGG
TAAGATATGGATGCATTTTGCTTTAGTTGCTTTGTATGATGTTCGGGCTTATAAAAGATAACTTTTGAATTTCTTTGCCAGCTCTGAATCTCCCACCACTAAGGAGCAGCATCCTATCTATGATGC CC
TCACAAAACCAGAACATCTGCTCGTGTTCTGCATGAGCAGTTATCAACAAAAAGATGTCCCAACACTCAATGGAAAACAAAGTATTTTAATGAT AAAATTCAGTACGTGAGCTATGTTTATTTTTGAT
TATTTTGACATTTCAAGCTCAGAAACAGCATTCGTGAAATGGGGATTGCTGGTGAAAATTAAATGGAAAATTGGCACTTAAAAATTTTGCATTTTTTTCCAGCTAATTCCTTTCTAAATTCCATCC AT
TCCTTAAAGAGTCTTGCTGTGATGATTTCTGCTAATTTCTTTCTTGGTAGTTTCCAATAAATCAAACTTCACAGATAACAAGAGAATAACAAAATG gTAAAGGTGCTTCTTTCCTTCCCTTCaGAAAA
TAtTCATTTGCGGATTTTTTACAGAGTACAAATGAAGGCCTGAATGTCAAAGTTTTGTTTTTGTTGTTAAATGACAAGTTGTTCCGGGGTACATATACTTTGACTTTCAGGCATTCATTCGTACTCTG
TAAAAAAATTTGCAAGTGAGTAGGAACTTAAAATAAAgGCTGAAGAAATAACAAAGAAACACTAGAAAAGAAAAAAAGTAAAAACTCAGTATCTGTGAAGCGCAATAAAATGAAGCACAACAG AAAGG
GGAAAAAAATGCCAAGTTGTGTCGCCAGGAATAATAAaCCACGGTATTTCCAAGTAGATGTTTTTgTAAAATACATTGGGGTAGATCTTTAcGTTTACTCCTTTTCTTCACTCTGTCACAGTaTTAAA
ATAAAATGCTCATTATTTGGAATCATGGGAAAGAAGGCTTATCTATCCATTACGGGGAAAACTTAAA cGTGGAGTGTTACTACTTCAAGCAGCACTATCCATATTACAAATAAAAACCTAACT AGGAA
TAAGTGATTTAAAAATTCCTATTCAAAGCAGGCATATATCACTACATTGCAACGTGCCACAAACAAAATAAAACTcTAACACAGGAAAATTAATTATGGGATTTAAAAGTTGAAAACACACCTAAGGA

ATTCATAATTTTATGAAGGTTTGGCTTATAACTGTTATTGATTTTAGACTGACTATCCCTTTACTCTTAGGTA A

AluYa8

Instruction

Green box: Primer information

Red letter: Target site duplication

Black letter: Insertion sequences

Green letter: Deleted sequenced by insertion-mediated deletion

>KSI_AluYa8_1

>Scaffold1761-1335113-1335220
TATATAATATAATATATTAAATATATAATATATATATGAGGGTTTCATCTAATATATTATATATATATATAATATATATATGAGGGTTTCATCTAATGCTTTTATCCATTGATGGCTATTGACTAG AT
CCATTATTTTACCAAAATGCAGATTTTCTGATTATATATATAAAACACTCATAGGAAATTTGACCATAGAGGGATCAGGCTCGGGCCACCTCAA CCACTGATGAGTCTGTGTCAGTAAAAGCAGGACA
GACAACTTGACATCATAGGCTATAGGCCTCCAGATGTGATGCAGTGTGACGCTCGCAGCACCGCCTGAATGTAACCAGGCCTTAAGAGTTTTCTTTCCGGCCATGCGCGGTGGCTCACGCCTATA ATC
CCAACACTTTCGGAGGCTGAGGCGGGCAGATCACCTGAGGTTAGGAGTTCAAGACCAGTCTGGCCAACATGGTGAAACCCTGTCTCTACTAAAAATACAAAAAATTAGTTGGGC ATGCTGGCAGGTAC
AGGTAATTCCAGTTACTCGGGAGGATGAGGCAGGAGAATCTCTTGAACCCAGGAGGCGGAGGTTGTAGTGAGCCGATATTGCATCATTGCACTCCAGCCTGGGCAACAAGAGCAAAACTCCATCT CAA
AAAAAAAGAGTTTTCTTTTTTTCTTTTAGAGACAAGGGCAGGGCGAGGTGGCATGTGCCTGTAAGTCCCAACTA CTCTGGAGGCTGAGGCAGGGGGATCACTTGAGTCCAGAAG TTCTGGGCTATAGT
GCAGTATGTCAATCAGGTGTCTGCACTAAGTTCTGCATCAACATCGTGACCTCCAGAGAGTGGGGGACCACCAGGTTGCCTAAGGAGTGGTGAACCGGCTCAGGCTGGAAAAGAAACAGGTCAAA ACT
CCCATGCTGATCTGTAGTGGAATCATGCCTGTGAAAAGTCACTGCAC aCCAGCCTGGGCAAtatAGCGAGACcctgtctctttatttatttatttattttgagacggagtctcgctccgtcgcccag
gctggggtgcagtggccggatctcagctcactgcaagctccgcctcccaggttcacgccattctggagatgatgaactggagatctagttaaaagtcctggccccataaatgacctgtataacct tag
ataagtaacatggccttttaagccccagtttcccactgtctctttaaaaagaaaaatagggccgggcgcggtggctcacgcctgtaatcccagcactttgggaggccgaggcgg gcggatcacgaggt
caggagatcgagaccatcccggctaaaacggtgaaacctcgtctctactaaaaatacaaaaaattagccgggcgtggtggcgggcgcctgtagtcccagctactcgggaggctgaggcaggagaa tgg
cgtgaacccgggaggcggagcttgcagtgagccgagatcccgccactgcactccagcctgggcgacgg agcgagacTCCGTCTCAAAAAAAAAAGAAAAATAGGCCGGGCGT GATGGCTCACCCCTGT
AATTGAAGCACTTTGGGAGGCCAAGGCGGGTGGATCATGAGGTCAGGAGATCGAGAGTACCCTGGCTAACACGGTGAAACCCGTCTCTACTAAAAATACAAAAATTAGCCGGATGTGGTGGCAT GTG
CCTGTAGTCCCAGCTACCCGGGAGGCTGAGGCAGGAGAATCGCTTGAACCCGGGAGGGGGAGGTTGCAGTGAGCTGAGATTGTGCCACTGGACTCCAGCCTGGGCCACAGAGCC AGACTCTGCCTCAA
AAAAAAAACAAAACAAAACAAACAAAACCAAGAAAGAAAAGTAAATAGGCCGGGCGTGGTGGCTCCCGGCACTTTGCGAGGCTGAGGCGGGCGGATCACTTGAGGTAAGGAGTTCAAGGCCAACC TGA
CCAACATGGGGAAACCCCGACTCTACTAAAAATACAAAAATTAGCTGGGCGTGGTGGTGTGCACCTGTAATCCCAGCTACTCAGGAGGCTGAGGCAGCAGAATCGCTTGAATCT GAGAGGCAGACATT
GCAGTGAGCCAAGATGGTGCCACTGTACTCCAGCATGGGCCACTGTACTCCAGCATGGGCAACAGAGCAAGACTCCGTCTCAAAATAAATAATAAATAAATAAATAAATAACCAAATAGAGACAGGGT
CTTACTCTGATGCCTAGGCTGGAGTGCAGTGTCGTGATCATAGCTCACTGCAACCTGGAACTCCTGGGCTGAAGGGATCCTCCTACCTCAGCTTCTTAAGTAGTTGGAACTGCA GATGTACATGCCAC
CATGCCAGGCTAACATTTTTAAATTTTTTATTTATATGGAGATGGTGTCTTGTTATGTTGCCTGGGCTGGTTTTGAATTCCTGGCCTCAAACAGTCCTCCTGCCTTGGCCTCCAGGGTAGCTGGG ATT
ATGGGAGTATGCCATCATGCCTGGCTATCATCATTTCCTCTATATTTATCTATTATTATATGGCTATGAATGAACCATAATTTATCAATCTCATTTTGTATCAAATTTTATGCTGTTTAAACAATGTT
ATGGTCAGGTGTCATGGCTCGCACCTGTAATCCCAAAACTTCGGAAGGCCGAGTGGGGAGGGTTGCTTGAGCTCAGGAGTTTGAGATCAGCCTGGGCAACATAGTGAGACCCCTGTCTCTACAAAAG A
AAAAAAATTAGTTGGACATGGTGGCACACACCTGTGAT CTCAGCTACTCACAAGGTTCTGGTGGAAGGATCTCATGAACCCAGGAGTTCAAGGTTGCTGTGAACTATGATAGCACTACTACACTCCAG
GCTGGGTGACAGAGCAAGACCCTGTGTCTAGAAAAGTAAAATTAAAAAAATAAAAAA TGTTACAATCTCTGCCATTATCCATGAATATTCCCTTAGGATAAATTCCTAGAAAGGTATGTAAAAGTTTA
GATTCCTCTAGGCAGATTATCTGAACTTTACTCCCATCACCACTTGGAGAAGGCTATTTCTCTGCACTCTTACTAACACTGGC TTTTTTTTTTTTTTTTTTTTGAGACAGAGTCTTGCTGTGTCGCCC
AGGCTGGAGTGCAGTGGTTCGATCTCGACTCACTGCAACCACCGTCTCCCAAGTTCAAGCTATTCTGCCATCTCAGCCTCCCGAGTAGCTGGGATTACAGGCACCCGCCATCATGCCTGGCTAAC GTT
TGTATTTTTTAGTAGAAACAGGGTTTCACCAGGTTGGT CTTGAACTCCTGACCTCAGGTGATCCATCTGCCTCGGCCTCCCAAAGTGCTAGGATTACAGGCATGAGCTACCGCGCCTGGCC GACATTG
GCTATTTTTTAAAAAAAATAAATCTTAATCTATTTGTAAGGCAAAACATGAAACAGGAAGCTTTACATACTCTTAAATCTCACACTAGGCTTTTTTTGTATTTGGACTGTTTAGCATTTTGTCTATGT
TGCTGAAACCCTTTGAAGTTACAAGATCTATTTGTGTT CTTCATGTAATTGTTCTTCCCCCCAACCTTGGGCTAACAGTCTGTGGCCTTCCTTGGTAACTAAATGCAAGAGTGATATTAATACCTTAC
AAGTTCCTCCCTTGCAAATGGGAGCCCTAAGTCAAGGCACAGATAGAATAGGAAAAAGCTTCTGGTTGCTCTTCAAGAAGTTTATCGAGTTAGATA

37	7.0	8.6	3.5	AluYa8-Scaffold1761-1335113-1335220	1	81	(2007)	+	(TA)n	Simple_repeat	1	85	(0)	1
305	17.7	15.2	0.0	AluYa8-Scaffold1761-1335113-1335220	87	165	(1923)	C	L1MC5a	LINE/L1	(797)	7233	7143	2
392	24.9	3.2	8.9	AluYa8-Scaffold1761-1335113-1335220	169	322	(1766)	+	L1MC5a	LINE/L1	7229	7374	(656)	2
2193	11.3	0.0	0.3	AluYa8-Scaffold1761-1335113-1335220	355	647	(1441)	+	AluSq2	SINE/Alu	1	292	(21)	3
2082	13.9	0.0	0.3	AluYa8-Scaffold1761-1335113-1335220	678	978	(1110)	+	7SLRNA	srpRNA	1	300	(20)	4
861	7.4	0.0	0.0	AluYa8-Scaffold1761-1335113-1335220	981	1088	(1000)	C	AluYa8	SINE/Alu	(13)	297	190	5

249	31.3	4.8	0.0	AluYa8-Scaffold1761-1335113-1335220	1103	1185	(903)	+	MIRc	SINE/MIR	46	132	(136)	6
2766	1.7	0.0	0.0	AluYa8-Scaffold1761-1335113-1335220	1212	1508	(580)	+	AluYa5	SINE/Alu	1	297	(14)	7
2346	9.7	0.3	0.0	AluYa8-Scaffold1761-1335113-1335220	1511	1819	(269)	+	AluSc8	SINE/Alu	1	310	(1)	8
1792	11.2	4.8	0.0	AluYa8-Scaffold1761-1335113-1335220	1840	2088	(0)	+	AluSz	SINE/Alu	1	261	(51)	9

>hg19 chr11:47578491-47580062

TATATAATATAATATATTAAATATATAATATATATATGAGGGTTTCATCTAATATATTATATATATATATAATATATATATGAGGGTTTCATCTAATGCTTTTATCCATTGATGGCTATTGACTAG AT
CCATTATTTTACCAAAATGCAGATTTTCTGATTATATATATAAAACACTCATAGGAAATTTGACCATAGAGGGATCAGGCTCGGGCCACCTCAA CCACTGATGAGTCTGTGTCAGTAAAAGCAGGACA
GACAACTTGACATCATAGGCTATAGGCCTCCAGATGTGATGCAGTGTGACGCTCGCAGCACCGCCTGAATGTAACCAGGCCTTAAGAGTTTTCTTTCCGGCCATGCGCGGTGGCTCACGCCTATA ATC
CCAACACTTTCGGAGGCTGAGGCGGGCAGATCACCTGAGGTTAGGAGTTCAAGACCAGTCTGGCCAACATGGTGAAACCCTGTCTCTACTAAAAATACAAAAAATTAGTTGGGC ATGCTGGCAGGTAC
AGGTAATTCCAGTTACTCGGGAGGATGAGGCAGGAGAATCTCTTGAACCCAGGAGGCGGAGGTTGTAGTGAGCCGATATTGCATCATTGCACTCCAGCCTGGGCAACAAGAGCAAAACTCCATCT CAA
AAAAAAAGAGTTTTCTTTTTTTCTTTTAGAGACAAGGGCAGGGCGAGGTGGCATGTGCCTGTAAGTCCCAACTACTCTGGAGGCTGAGGCAGGGGGATCACTTGAGTCCAGAAG TTCTGGGCTATAGT
GCAGTATGTCAATCAGGTGTCTGCACTAAGTTCTGCATCAACATCGTGACCTCCAGAGAGTGGGGGACCACCAGGTTGCCTAAGGAGTGGTGAACCGGCTCAGGCTGGAAAAGAAACAGGTCAAA ACT
CCCATGCTGATCTGTAGTGGAATCATGCCTGTGAAAAGTCACTGCAC tCCAGCCTGGGCAAcggAGCGAGACTCCGTCTCAAAAAAAAAAAGAAAAATAGGCCGGGCGTGATGGC TCACCCCTGTAATT
GAAGCACTTTGGGAGGCCAAGGCGGGTGGATCATGAGGTCAGGAGATCGAGAGTACCCTGGCTAACACGGTGAAACCCGTCTCTACTAAAAATACAAAAAATTAGCCGGATGTGGTGGCATGTGC CTG
TAGTCCCAGCTACCCGGGAGGCTGAGGCAGGAGAATCGCTTGAACCCGGGAGGGGGAGGTTGCAGTGAGCTGAGATTGTGCCACTGGACTCCAGCCTGGGCCACAGAGCCAGAC TCTGCCTCAAAAAA
AAAACAAAACAAAACAAACAAAACCAAGAAAGAAAAGTAAATAGGCCGGGCGTGGTGGCTCCCGGCACTTTGCGAGGCTGAGGCGGGCGGATCACTTGAGGTAAGGAGTTCAAGGCCAACCTGAC CAA
CATGGGGAAACCCCGACTCTACTAAAAATACAAAAATTAGCTGGGCGTGGTGGTGTGCACCTGTAATCCCAGCTACTCAGGAGGCTGAGGCAGCAGAATCGCTTGAATCTGAGA GGCAGACATTGCAG
TGAGCCAAGATGGTGCCACTGTACTCCAGCATGGGCAACAGAGCAAGACTCCGTCTCAAAATAAATAATAAATAAATAAATAAATAACCAAATAGAGACAGGGTCTTACTCTGATGCCTAGGCTGGAG
TGCAGTGTCGTGATCATAGCTCACTGCAACCTGGAACTCCTGGGCTGAAGGGATCCTCCTACCTCAGCTTCTTAAGTAGTTGGAACTGCAGATGTACATGCCACCATGCCAGGC TAACATTTTTAAAT
TTTTTATTTATATGGAGATGGTGTCTTGTTATGTTGCCTGGGCTGGTTTTGAATTCCTGGCCTCAAACAGTCCTCCTGCCTTGGCCTCCAGGGTAGCTGGGATTATGGGAGTATGCCATCAT GCCTGG
CTATCATCATTTCCTCTATATTTATCTATTATTATATGGCTATGAATGAACCATAATTTATCAATCTCATTTTGTATCAAATTTTATGCTGTTTAAACAATGTTAT GGTCAGGTGTCATGGCTCGCAC
CTGTAATCCCAAAACTTCGGAAGGCCGAGTGGGGAGGGTTGCTTGAGCTCAGGAGTTTGAGATCAGCCTGGGCAACATAGTGAGACCCCTGTCTCTACAAAAGAAAAAAAATTAGTTGGACATGG TGG
CACACACCTGTGATCTCAGCTACTCACAAGGTTCTGGTGGAAGGATCTCATGAACCCAGGAGTTCAAGGTTGCTGTGAACTATGATAGCACTACTACACTCCAGGCTGGGTGAC AGAGCAAGACCCTG
TGTCTAGAAAAGTAAAATTAAAAAAATAAAAAATGTTACAATCTCTGCCATTATCCATGAATATTCCCTTAGGATAAATTCCTAGAAAGGTATGTAAAAGTTTAGATTCCTCTAGGCAGATTATCTGA
ACTTTACTCCCATCACCACTTGGAGAAGGCTATTTCTCTGCACTCTTACTAACACTGGC TTTTTTTTTTTTTTTTTTTTGAGACAGAGTCTTGCTGTGTCGCCCAGGCTGGAGT GCAGTGGTTCGATC
TCGACTCACTGCAACCACCGTCTCCCAAGTTCAAGCTATTCTGCCATCTCAGCCTCCCGAGTAGCTGGGATTACAGGCACCCGCCATCATGCCTGGCTAACGTTTGTATTTTTTAGTAGAAACAG GGT
TTCACCAGGTTGGTCTTGAACTCCTGACCTCAGGTGATCCATCTGCCTCGGCCTCCCAAAGTGCTAGGATTACAGGCATGAGCTACCGCGCCTGGCC GACATTGGCTATTTTTTAAAAAAAATAAATC
TTAATCTATTTGTAAGGCAAAACATGAAACAGGAAGCTTTACATACTCTTAAATCTCACACTAGGCTTTTTTTGTATTTGGACTGTTTAGCATTTTGTCTATGTTGCTGAAACCCTTTGAAGTTA CAA
GATCTATTTGTGTTCTTCATGTAATTGTTCTTCCCCCCAACCTTGGGCTAACAGTCTGTGGCCTTCCTTGGTAACTAAATGCAAGAGTGATATTAATACCTTACAA GTTCCTCCCTTGCAAATGGGAG
CCCTAAGTCAAGGCACAGATAGAATAGGAAAAAGCTTCTGGTTGCTCTTCAAGAAGTTTATCGAGTTAGATA

37	7.0	8.6	3.5	hg19	1	81	(1491)	+	(TA)n	Simple_repeat	1	85	(0)	1 0
305	17.7	15.2	0.0	hg19	87	165	(1407)	C	L1MC5a	LINE/L1	(797)	7233	7143	11
392	24.9	3.2	8.9	hg19	169	322	(1250)	+	L1MC5a	LINE/L1	7229	7374	(656)	11
2193	11.3	0.0	0.3	hg19	355	647	(925)	+	AluSq2	SINE/Alu	1	292	(21)	12
2156	13.7	0.6	0.3	hg19	678	992	(580)	+	7SLRNA	srpRNA	1	3 16	(4)	13
2346	9.7	0.3	0.0	hg19	995	1303	(269)	+	AluSc8	SINE/Alu	1	310	(1)	14
1792	11.2	4.8	0.0	hg19	1324	1572	(0)	+	AluSz	SINE/Alu	1	261	(51)	15

>KSI_AluYa8_2

>Scaffold2353-2617237-2617542
GGAGCCACTTCCGGTGGAGATGAAACCCCCTTGCCATCTCTAAGTAAAAGGACTCAAAGAGGGGGAAAAAGGCTCAGGCCGGGCGTGTTGGCTCACACCTGTACTCCCAGCACTTTGGGAGGCTGA GG
CGGGCAGATCACCTGATGTTAGGAGTTCAAGACCAGCCTGGCCAACATGGTGAAACCCTGTCTCAACTAAAAATACAAAATTAGCGTAGCATGGTGGCGCATGCCTGTAACCCCACCTACTCGGG AGG
CTGAGGTAGAAGAATCGCTTGAACCCAGGAGGTGGAGATTGCAGTGAGCCAAGATCGCGCATTGCACTCCAGCCTGGGCAACAAAAGGGGAAGTGTGAGGTGTTACACTCTTCTCAGTGCCGGGCT AG
CGGCCTCCAGCTTCCTCGACCGGCAGCTCCGCTTCAAGCCCCAGCCCTGACTTGCGTGCTTCAGAATTTGTTTTTGTTTCAGTTTGAGAATTCTTTTCTTCTAGGAAACAGTGACTGCTACTTTG GGA
ATGGGTCAGCCTACCGTGGCACGCACAGCCTCACCGAGTCGGGTGCCTCCTGCCTCCCGTGGAATTCCATGATCCTGATAGGCAAGGTTTACACAGCACAGAACCCCAGTGCCCAGGCACTGGGCC TG
GGCAAACATAATTACTGCCGGTAGGTAGCACAGGGGTGGGGGGTTCAGGTCTTGGCAGAACGTGGGATTAGGGTGTGAGACGGGGGAAGATCCAATGTCTCAAGTTGCATGACAGACCCAGTGCG TGG
GAAGCACCCATGGATATTATCTAATCCAACCTCTTCACTTGCTAGAATAACACATATTGTGAAAAGCAAGGTCTACCAGTTTTCCAACCTAAATCCCAAGTTAAGGGTCCTGGCCTGTAACCATTT AG
TCCTCAGCTGTTCTCCTGACATCTTTATTGCAATGATTTGTAAGAGTTCCGTAACAGGACAGCTCACAGTTCTGTCTGACAACCCTATGAGATT**AGAACACTAC**ggccgggcgcggtggctcacgcct
gtaatcccagcactttgggaggccgaggcgggcggatcacgaggtcaggagatcgagaccatcccggctaaaacggtgaaaccccgtctctactaaaactacaaaaaatagccgggcgtagtggcg gg
cgcctgtagtcctagctacttgggaggctgaggcaggagaatggcgtgaacccgggaggcggagcttgcagtgagccgagatcccgccactgcactccagcctgggcaacagagcgagactccgt ctc
aaaaaaaaaaaaaaaaaaaaa**agaacactac**ATTACTGACTGGGTAACAAAGTTAAAGAGAAGTTCTCCTAGGGTGGGGGTGTGCTGCAAGGTCAAGATGAACTCGGTGTCCTCCCTCCCAGCTCAG
TGGTTTTCATTGGTTGACTGAGTCTCCTTCTACTCTTACATGGCCTGTGATGTGGCTGAAAATGGGATTGAAAATCTTAAACTCCTGGCCTGGTGTGGTGGTGCATGCCTGTAATCCCAGCACTT TGG
GAGGCTGAGGCAGGAGGATTGCTTGAGCCCAGGAGTTCAAGACCAGCCTGGGCAACATGGCAAGACCCCATCTCTACAAAAATTAAAAAATAAGAAGATAAAAAGGAAACCTTAAACTCTCTTATC AT
TTAGGAATCCTGATGGGGATGCCAAGCCCTGGTGCCACGTGCTGAAGAACCGCAGGCTGACGTGGGAGTACTGTGATGTGCCCTCCTGCTGTAAGGGCTGGGCCCCGGCTGCCTCCCTGCACCTG GTC
TCCCTTCTTCACCATCTCCTCGCCACTTTCTAGGCCTTCATGGCTGTCTGGTGCAGACTGTGTGCCTACCAGACTTCCAGGCTGGGTGGGGAGGGGGCTTCCATGACTGAAGCCACGGTGGGTGGG CG
GGTGTCCATGACCCATGCATGGGGGTGGTGGGGAGGGGCAAGAAGAAAGAAAAGAACACAGCAACCTCTCTGAGAAGTCAGTAGGCTCTACCCGGCTCTCTCTAGACGCCTCTATTGGTGTTGC TGAG
AGTAATGGGGAATGAGGAGGAGGCCTCGCATGCATGGCTCCTTAGATAATAAAGTGCCTTCACCACATTTGTCCAACAGCGTGGTGGAGGTGGTCTTCCTACCTCCAGTTTACTGAGTAGGAAGCAGT
CTCCCAGACGCCAGGTGGCCTGCCCAAGCTCGCTGGGCACCGGGAGGTAAAGCTAGGGCCTGCCCTCATGCCCTCTGACCCCAATGGCATGTACTTCCACTGTGAGATCTTCCACGTTTCCTGTT GAC
CT

1994	10.6	0.8	0.0	AluYa8-Scaffold2353-2617237-2617542	77	341	(1965)	+	AluSx	SINE/Alu	1	267	(45)	1
2898	0.7	0.0	0.0	AluYa8-Scaffold2353-2617237-2617542	1001	1306	(1000)	+	AluYa8	SINE/Alu	1	306	(4)	2
998	11.4	0.0	0.0	AluYa8-Scaffold2353-2617237-2617542	1495	1626	(680)	+	AluJo	SINE/Alu	1	132	(180)	3

>hg19 chr8:42038903-42040896
GGAGCCACTTCCGGTGGAGATGAAACCCCCTTGCCATCTCTAAGTAAAAGGACTCAAAGAGGGGGAAAAAGGCTCAGGCCGGGCGTGTTGGCTCACACCTGTACTCCCAGCACTTTGGGAGGCTGA GG
CGGGCAGATCACCTGATGTTAGGAGTTCAAGACCAGCCTGGCCAACATGGTGAAACCCTGTCTCAACTAAAAATACAAAATTAGCGTAGCATGGTGGCGCATGCCTGTAACCCCACCTACTCGGG AGG
CTGAGGTAGAAGAATCGCTTGAACCCAGGAGGTGGAGATTGCAGTGAGCCAAGATCGCGCATTGCACTCCAGCCTGGGCAACAAAAGGGGAAGTGTGAGGTGTTACACTCTTCTCAGTGCCGGGCT AG
CGGCCTCCAGCTTCCTCGACCGGCAGCTCCGCTTCAAGCCCCAGCCCTGACTTGCGTGCTTCAGAATTTGTTTTTGTTTCAGTTTGAGAATTCTTTTCTTCTAGGAAACAGTGACTGCTACTTTG GGA
ATGGGTCAGCCTACCGTGGCACGCACAGCCTCACCGAGTCGGGTGCCTCCTGCCTCCCGTGGAATTCCATGATCCTGATAGGCAAGGTTTACACAGCACAGAACCCCAGTGCCCAGGCACTGGGCC TG
GGCAAACATAATTACTGCCGGTAGGTAGCACAGGGGTGGGGGGTTCAGGTCTTGGCAGAACGTGGGATTAGGGTGTGAGACGGGGGAAGATCCAATGTCTCAAGTTGCATGACAGACCCAGTGCG TGG
GAAGCACCCATGGATATTATCTAATCCAACCTCTTCACTTGCTAGAATAACACATATTGTGAAAAGCAAGGTCTACCAGTTTTCCAACCTAAATCCCAAGTTAAGGGTCCTGGCCTGTAACCATTT AG
TCCTCAGCTGTTCTCCTGACATCTTTATTGCAATGATTTGTAAGAGTTCCGTAACAGGACAGCTCACAGTTCTGTCTGACAACCCTATGAGATTAGAACACTACATTACTGACTGGGTAACAAAGTTA
AAGAGAAGTTCTCCTAGGGTGGGGGTGTGCTGCAAGGTCAAGATGAACTCGGTGTCCTCCCTCCCAGCTCAGTGGTTTTCATTGGTTGACTGAGTCTCCTTCTACTCTTACATGGCCTGTGATGTG GC
TGAAAATGGGATTGAAAATCTTAAACTCCTGGCCTGGTGTGGTGGTGCATGCCTGTAATCCCAGCACTTTGGGAGGCTGAGGCAGGAGGATTGCTTGAGCCCAGGAGTTCAAGACCAGCCTGGGC AAC
ATGGCAAGACCCCATCTCTACAAAAATTAAAAAATAAGAAGATAAAAAGGAAACCTTAAACTCTCTTATCATTTAGGAATCCTGATGGGGATGCCAAGCCCTGGTGCCACGTGCTGAAGAACCGCA GG
CTGACGTGGGAGTACTGTGATGTGCCCTCCTGCTGTAAGGGCTGGGCCCCGGCTGCCTCCCTGCACCTGGTCTCCCTTCTTCACCATCTCCTCGCCACTTTCTAGGCCTTCATGGCTGTCTGGTG CAG
ACTGTGTGCCTACCAGACTTCCAGGCTGGGTGGGGAGGGGGCTTCCATGACTGAAGCCACGGTGGGTGGGCGGGTGTCCATGACCCATGCATGGGGGTGGTGGGGAGGGGCAAGAAGAAAGAAAAG AA
CACAGCAACCTCTCTGAGAAGTCAGTAGGCTCTACCCGGCTCTCTCTAGACGCCTCTATTGGTGTTGCTGAGAGTAATGGGGAATGAGGAGGAGGCCTCGCATGCATGGCTCCTTAGATAATAAAGTG
CCTTCACCACATTTGTCCAACAGCGTGGTGGAGGTGGTCTTCCTACCTCCAGTTTACTGAGTAGGAAGCAGTCTCCCAGACGCCAGGTGGCCTGCCCAAGCTCGCTGGGCACCGGGAGGTAAAGCT AG
GGCCTGCCCTCATGCCCTCTGACCCCAATGGCATGTACTTCCACTGTGAGATCTTCCACGTTTCCTGTTGACC T

1994	10.6	0.8	0.0	hg19	77	341	(1653)	+	AluSx	SINE/Alu	1	267	(45)	4
998	11.4	0.0	0.0	hg19	1183	1314	(680)	+	AluJo	SINE/Alu	1	132	(180)	5

>KSI_AluYa8_3

>Scaffold2628-2327996-2328307
TATTTAAAGTATATATATTTATATACTTTATAtatatatataaagtataaatatatataatTATATATATAAAGTATAAATATATATAATTATATATATAAAGTATATATACACTTATATATGTGTAT
ATATACTTATATATAAGTATATATATACTTTTATACAATATATATATGTATATACTTATATATGTTTCAGTGTTAAATTTATTGTTTTCAGGCCAGATTTTCTCTGAGAACATAATATACTCTTA CTC
TTTGTTGGAGGAAAAAATAGTATAAACATATACTATATAGAGAGTATATATATAACTTATGGTAAATATATATAAACTTATATATATTATATAAAAGTATATATACTTATATATGTGTATATATAC AC
TTTTATATAACATATATAATACATATATGCTTATATATGTATATGTATACCTTTATATATATATAGCTATCTTAGGATCAGCAGATATATAACATTATATAGGTTATGATCTTAATCATAACATA CAT
GCACACGTATATGTGCACACACACACACAAGCACATGGTATGATCCCAAGTTGGTGAGAAGCAAATAGACCAAAATGCCAACGATGACTTTGCTAGGATTATATTCTTGTCTTCACTTCGCTTGAC CT
GGGGAGGTGAGGATTTGGGCCATCTATAGTGAAAACCATATGGCTTTCATGATGAATAAAACTCATGAAGGAGCGTGGCCCCATGGACTCTATGGCCTTTTACCAAGCCGAATCTGTTTAAACAT CAA
TCCTTATCTGTTTGTGTAAACCTTCTTGACCTTGGAATGGGAAGAAGCAATTAGGAATTATTGGCCTTGTTGTTATATTGGTACCTTCCTCAAAACCAA CTTGTTGGAGTTGAGGCATCTTAAAAACA
ACAGGATAAAATCATCCTCTCTGGGAAATACATATACACATATGTCCACATTTTTTTTCCTAACAACTTTTGCAATAACAAGATGAAGT **AAAAAGTTAATTACA**ggccgggcgcggtggctcacgcct
gtaatcccagcactttgggaggccgaggcgggcggatcacaaggtcaggagatcgagaccatcccggctaaaacggtgaaaccccgtctctactaaaaaactacaaaaaatagccgggcgtagtgg cg
ggcgcctgtagtcctagctacttgggaggctgaggcaggagaatggcgtgaacccgggaggcggagcttgcagtgagccgagatcccgccactgcactccagcctgggcgacagagcgagactcc gtc
tcaaaaaaaaaaaaaaaaaaaaaaaaaaaaaaaaaa**aaaaagttaattaca**AATATATGCCACAAGGTCTACATTTGGGCATATATGATGTTTTTCTAAGAGATTTGTAATAGTAGCACTAGTATT
CAGCTAGTGAATGCTTTTTCTTGATATTTATTTACAGAAAGGTACTTCAGATTGGACAGATGTTAACAATGCAGTATCTTTTTTCCACGATAACTTTGTTTTGTAATAAGAGCCTTTTGCTATG ATG
TCTTTTTGACTTTTATCATGTGGGGTATAGCAAGATGCTGTTGGTTTTATTTCCTCCCCATTTATCTGTTATCTCAGTCATGCTACATAGTCACACCCATTTGGAGTTAGGCATATACCAGATAAG AT
AAGAGAAAGGGGCCTTTCACAGTGTAACTCAAGGATGAAAAGAGAGAAAGTTTCTCATAGCATAATAATGCACAAACATTGATTGTCTTAAAGAATAGTTTCCAAACCACTGTTTGTGTACACCAAAA
CCACTGGACAACTTGTGAAATCAGATTGCTATGCTTTATGTCC cGACTTTCTGAGTATTCAGTAGGTCTGAGTTGGTGGTCAtGATTTTGCATTTCTCATTTCTCAGGTGATGCTGATGCTCCTGCCC
TTGAGCAGGATGATTCAAGACCAGTGGTTCTGAATGAAATAAAATCTTTTCTTTTTTTTTTTGAGACAGAGCCTCACTCTGCTGCCCAGGCTGGAGTGCAGTGGCGTGATCTCAGCTCACTGCAA CCT
CTGCCCCCACTGGGCTCAAGCGATTCTCCTGCCTCAGCCTCCCAAGTAGCTGGGACTACAGGTGTGTGCCACCATGCCCAGCTAATTTTTGTATTTTTAGTAGAGTGGGTGTTTTGCCATGTTGGC CA
GGCTGGTCTCAAACTCCTAATCTCAAGTGATCCACTCTCCTCTGCCTCCCAAAGTGCTGGGATTACAGGCATGAGCCACCACGTCTGGCCTGAAGTAAAATCTTAATTACAAATATATGCCATAA GGT
GCACAT

131	25.3	3.1	3.7	AluYa8-Scaffold2628-2327996-2328307	9	191	(2121)	+	(TA)n	Simple_repeat	1	179	(0)	1
131	25.3	3.1	3.7	AluYa8-Scaffold2628-2327996-2328307	276	449	(1863)	+	(TA)n	Simple_repeat	1	174	(0)	2
11	29.9	0.0	0.0	AluYa8-Scaffold2628-2327996-2328307	509	547	(1765)	+	(ACACGC)n	Simple_repeat	1	39	(0)	3
2462	11.2	0.0	0.7	AluYa8-Scaffold2628-2327996-2328307	1001	1312	(1000)	+	AluYa8	SINE/Alu	1	310	(0)	4
520	25.5	3.8	4.4	AluYa8-Scaffold2628-2327996-2328307	1760	1919	(393)	C	MER5A	DNA/hAT-Charlie	(1)	188	30	5
2228	11.7	0.0	0.7	AluYa8-Scaffold2628-2327996-2328307	1969	2268	(44)	C	AluSz	SINE/Alu	(14)	298	1	6

>hg19 chr3:31399680-31401627
TATTTAAAGTATATATATTTATATACTTTATATATATATATATAAAGTATAAATATATATAATTATATATATAAAGTATATATACACTTATATATGTGTATATATACTTATATATAAGTATATATATACT
TTTATACAATATATATATGTATATACTTATATATGTTTCAGTGTTAAATTTATTGTTTTCAGGCCAGATTTTCTCTGAGAACATAATATACTCTTACTCTTTGTTGGAGGAAAAAATAGTATAAA CAT
ATACTATATAGAGAGTATATATATAACTTATGGTAAATATATATAAACTTATATATATTATA TAAAAGTATATATACTTATATATGTGTATATATACACTTTTATATAACATATATAATACATATATG
CTTATATATGTATATGTATACCTTTATATATATATAGCTATCTTAGGATCAGCAGATATATAACATTATATAGGTTATGATCTTAATCATAACATACATGCACACGTATATGTGCACACACACAC ACA
AGCACATGGTATGATCCCAAGTTGGTGAGAAGCAAATAGACCAAAATGCCAACGATGACTTT GCTAGGATTATATTCTTGTCTTCACTTCGCTTGACCTGGGGAGGTGAGGATTTGGGCCATCTATAG
TGAAAACCATATGGCTTTCATGATGAATAAAACTCATGAAGGAGCGTGGCCCCATGGACTCTATGGCCTTTTACCAAGCCGAATCTGTTTAAACATCAATCCTTATCTGTTTGTGTAAACCTTCT TGA
CCTTGGAATGGGAAGAAGCAATTAGGAATTATTGGCCTTGTTGTTATATTGGTACCTTCCTC AAAACCAACTTGTTGGAGTTGAGGCATCTTAAAAACAACAGGATAAAATCATCCTCTCTGGGAAAT
ACATATACACATATGTCCACATTTTTTTTCCTAACAACTTTTGCAATAACAAGATGAAGT AAAAAGTTAATTACAAATATATGCCACAAGGTCTACATTTGGGCATATATGATGTTTTTCTAAGAGAT
TTGTAATAGTAGCACTAGTATTCAGCTAGTGAATGCTTTTTCTTGATATTTATTTACAGAAA AGGTACTTCAGATTGGACAGATGTTAACAATGCAGTATCTTTTTTCCACGATAACTTTGTTTTGTA
ATAAGAGCCTTTTGCTATGATGTCTTTTTGACTTTTATCATGTGGGGTATAGCAAGATGCTGTTGGTTTTATTTCCTCCCCATTTATCTGTTATCTCAGTCATGCTACATAGTCACACCCATTTG GAG
TTAGGCATATACCAGATAAGATAAGAGAAAGGGGCCTTTCACAGTGTAACTCAAGGATGAAAAGAGAGAAAGTTTCTCATAGCATAATAATGCACAAACATTGATTGTCTTAAAGAATAGTTTCCAAA
CCACTGTTTGTGTACACCAAAACCACTGGACAACTTGTGAAATCAGATTGCTATGCTTTATGTCC aGACTTTCTGAGTATTCAGTAGGTCTGAGTTGGTGGTCA aGATTTTGCATTTCTCATTTCTCA
GGTGATGCTGATGCTCCTGCCCTTGAGCAGGATGATTCAAGACCAGTGGTTCTGAATGAAATAAAATCTTTTCTTTTTTTTTTTGAGACAGAGCCTCACTCTGCTGCCCAGGCTGGAGTGCAGTG GCG
TGATCTCAGCTCACTGCAACCTCTGCCCCCACTGGGCTCAAGCGATTCTCCTGCCTCAGCCTCCCAAGTAGCTGGGACTACAGGTGTGTGCCACCATGCCCAGCT AATTTTTGTATTTTTAGTAGAGT

GGGTGTTTTGCCATGTTGGCCAGGCTGGTCTCAAACTCCTAATCTCAAGTGATCCACTCTCCTCTGCCTCCCAAAGTGCTGGGATTACAGGCATGAGCCACCACGTCTGGCCTGAAGTAAAATCT TAA
TTACAAATATATGCCATAAGGTGCACAT

116	25.9	3.3	3.7	hg19	9	162	(1786)	+	(TA)n	Simple_repeat	1	151	(0)	7
116	25.9	3.3	3.7	hg19	247	420	(1528)	+	(TA)n	Simple_repeat	1	174	(0)	8
11	29.9	0.0	0.0	hg19	480	518	(1430)	+	(ACACGC)n	Simple_repeat	1	39	(0)	9
575	24.2	3.8	4.4	hg19	1396	1555	(393)	C	MER5A	DNA/hAT-Charlie	(1)	188	30	10
2228	11.7	0.0	0.7	hg19	1605	1904	(44)	C	AluSz	SINE/Alu	(14)	298	1	11

>KSI_AluYa8_4

```
>Scaffold5807-117396-117488
GCATCCCTAGATAGATTAGTGGCAGGAAATGTGGTTACAGAGTGTAGGATTTAGAAGGAGCTGAAATATGCTCCCATCAGGGCCACTTTCCACAGGCGTCTGACCTGAGCTGCTACACAAGACTC TGC
ACTCAGCTCTAGTGCTTGGTTTAATGCTCTGTTATAACTGTCTTGAAATTCTTAATAACTTTTGAATAAGAGGCTCCTATTTTTATTTAGCTCTGGGTCCCACAAATTAGCCATTCTTGCTACAT TTT
TACTAAGGGTAAGTATCTCAAATAGACAATACCAACATAAAGAATAACAGATCATTCCTTTTCAGTCAATGAAATGTGAAGTAGATCAAACGTATGATAATGTATGATAAGGTATAATAGAAAAC ACT
TTTACAGGTTTCTTGAATATTTAAATTACAATTTTCCAAAATTAGCAGCAGAATTTTCTAAATTAATGGCAATTGAGAAAAAAATGATTTTTAAACTGATTTTTCAGCACATTTGCTACTGTGGT TCC
AACTGGAATGCAGAAATCCAGTAGCACACATAAAAGAAGGAAGATGGAACTGAATGTATTTCATTTATGTTCTTCTGAATAAAGCATCTTGTAAACTGTTATTATGGCTGGAAAAATGCCGTA AGGAT
ATGCTGCTACTGGAAAAACAGACAACAGACAATCAGTTTTTAACTGAGAGAGAGAGAGAGAGAGAGAGAGAGAGAGAGAGAGTGAAAGAGATGACATAAATCAGGATGCTGAGACTATTTTTTTCCAT
AAGCTTTTAAATAAAAAAACCCCACGAATTTATGGCTATGCTGGAAAAAATAAATTTCGTACTCATTTTCGTCTTGAAGTAGTAATGTAAGACAGAACATGTACTAACATTACCGCCTGAACAAA ATA
GTTGTCTAAACCATTTAAGAAACCTAAGGTTAGAGTTGCCAATTTA AATTTATGGCTGCATGAATTCAGCTTACTTCACTGACATCTAATTAAACATATCTTTTggccgggcgcggtggctcacgcct
gtaatcccagcactttgggaggccgaggcgggggggtcacgaggcaggggatcgagaccatcccggct AAAACGGTGAAACCCCGTCTCTACTAAAAATACAAAAAAATTAGCCGGGCGTAGTGGCG
GGCGCCTGTAGTCCCAGCTACTTGGGAGGCTGAGGCAGGAGAATGG CGTGAACCCGGGAGGCGGAGCTTGCAGTGAGCCGAGATCCCGCCACTGCACTCCAGCCTGGGCGACAGAGCGAGACTCCGTC
TCAAAAAAAAAAAAAAAAAAAAAAAAAAAAAAAAAAAAAAA aaaaaaaaaaaaaaaaaaaaatatcttttAATAGTGGTTGACTTTTTGGATGTTAAATAAGTAAAGAGCATGCGAGCAATCCAATAACC
CCCATCAATCAAGGGAGAACGCAAAGAATGTCCAGCCTCCTGTTTA TCCCATCACTACCTTCCTAGATGCTGGGGCGTAGTCAGAATGGTCCTAGAAAGTTCAGTGGTGATCTCTAGCACAAGAACTT
CATGACAATCAGAAGAGAGGAAGGAAAGAATGAAGGCAGGACAGAAGAAAGGAGGGAAGGAAAGATGGAAGGAAGGAAAGGTAAAAGGAAATTTTCTGTATGTACATAAACTGTGGAATTAGGAG TAA
GGAAGAATTAGATTTACAGTGTTATAGAAAAGGCTTTTAAATCTTA CTCTTGATTTCTAGAGCTGCACATGGTCTCCTTCAAACTTGCAGACTATGTGTTTAAATATAGTGGGAAGTTCCATACATA G
AAAGATTGCTAAGTGTGGGGGGCTGGGGAGGTTTAAGACTCACACAGTCCTCCTCTGGTCTTAAACCTAGAGACAAACCACAGTCCCCTGAGTCAGAGGCAAGAGTACAATCTTCTCATTTTATATTA
TTGCATGTCTTTGGCTCTGTCTTCAAAGGTAAAGTTTTCATTCAGC AAGTCAGTGATAAACCCTGAACATATATCTAAAAATAGAAGCAAAATTAGTGCTTTCTCACATATGGTAAGGTCATACGTGA
TTTACTCATTGAAAAAAAATATTATATCCCAATGCTTTTAAATGCTTTCAAAATATGCCACAACTATAAAATTAGGAGTTTGTGAAGAAAATGCCAAAGGAAGAAGATAATATTTTATGCTAATA AAA
CAACCAAAAAGAT
```

776	22.1	3.8	1.2	AluYa8-Scaffold5807-117396-117488	78	237	(1856)	+	MER45A	DNA/hAT-Tip100	1	164	(14)	1
43	0.0	0.0	0.0	AluYa8-Scaffold5807-117396-117488	686	722	(1371)	+	(GA)n	Simple_repeat	1	37	(0)	2
817	4.3	0.0	0.0	AluYa8-Scaffold5807-117396-117488	1001	1093	(1000)	+	AluYa8	SINE/Alu	1	93	(217)	3
52	0.0	0.0	0.0	AluYa8-Scaffold5807-117396-117488	1189	1244	(849)	+	(A)n	Simple_repeat	1	56	(0)	4

```
>hg19 chr4:112697584-112699426
GCATCCCTAGATAGATTAGTGGCAGGAAATGTGGTTACAGAGTGTAGGATTTAGAAGGAGCTGAAATATGCTCCCATCAGGGCCACTTTCCACAGGCGTCTGACCTGAGCTGCTACACAAGACTCT GC
ACTCAGCTCTAGTGCTTGGTTTAATGCTCTGTTATAACTGTCTTGAAATTCTTAATAACTTTTGAATAAGAGGCTCCTATTTTTATTTAGCTCTGGGTCCCACAAATTAGCCATTCTTGCTACAT TTT
TACTAAGGGTAAGTATCTCAAATAGACAATACCAACATAAAGAATAACAGATCATTCCTTTTCAGTCAATGAAATGTGAAGTAGATCAAACGTATGATAATGTATGATAAGGTATAATAGAAAACA CT
TTTACAGGTTTCTTGAATATTTAAATTACAATTTTCCAAAATTAGCAGCAGAATTTTCTAAATTAATGGCAATTGAGAAAAAAATGATTTTTAAACTGATTTTTCAGCACATTTGCTACTGTGGT TCC
AACTGGAATGCAGAAATCCAGTAGCACACATAAAAGAAGGAAGATGGAACTGAATGTATTTCATTTATGTTCTTCTGAATAAAGCATCTTGTAAACTGTTATTATGGCTGGAAAAATGCCGTA AGGAT
ATGCTGCTACTGGAAAAACAGACAACAGACAATCAGTTTTTAACTgaGAGAGAGAGAGAGAGAGAGAGAGAGAGAGAGAGAGAGTGAAAGAGATGACATAAATCAGGATGCTGAGACTATTTTTTTCC
ATAAGCTTTTAAATAAAAAAACCCCACGAATTTATGGCTATGCTGGAAAAAATAAATTTCGTACTCATTTTCGTCTTGAAGTAGTAATGTAAGACAGAACATGTACTAACATTACCGCCTGAACAA AA
TAGTTGTCTAAACCATTTAAGAAACCTAAGGTTAGAGTTGCCAATTTAAATTTATGGCTGCATGAATTCAGCTTACTTCACTGACATCTAATT AAACATATCTTTTAATAGTGGTTGACTTTTTGGAT
GTTAAATAAGTAAAGAGCATGCGAGCAATCCAATAACCCCCATCAATCAAGGGAGAACGCAAAGAATGTCCAGCCTCCTGTTTATCCCATCACTACCTTCCTAGATGCTGGGGCGTAGTCAGAATG GT
CCTAGAAAGTTCAGTGGTGATCTCTAGCACAAGAACTTCATGACAATCAGAAGAGAGGAAGGAAAGAATGAAGGCAGGACAGAAGAAAGGAGGGAAGGAAAGATGGAAGGAAGGAAAGGTAAAAG GAA
ATTTTCTGTATGTACATAAACTGTGGAATTAGGAGTAAGGAAGAATTAGATTTACAGTGTTATAGAAAAGGCTTTTAAATCTTACTCTTGATTTCTAGAGCTGCACATGGTCTCCTTCAAACTTGC AG
ACTATGTGTTTAAATATAGTGGGAAGTTCCATACATAGAAAGATTGCTAAGTGTGGGGGGCTGGGGAGGTTTAAGACTCACACAGTCCTCCTCTGGTCTTAAACCTAGAGACAAACCACAGTCCCCTG
AGTCAGAGGCAAGAGTACAATCTTCTCATTTTATATTATTGCATGTCTTTGGCTCTGTCTTCAAAGGTAAAGTTTTCATTCAGCAAGTCAGTGATAAACCCTGAACATATATCTAAAAATAGAAGC AA
AATTAGTGCTTTCTCACATATGGTAAGGTCATACGTGATTTACTCATTGAAAAAAAATATTATATCCCAATGCTTTTAAATGCTTTCAAAATATGCCACAACTATAAAATTAGGAGTTTGTGAAG AAA
ATGCCAAAGGAAGAAGATAATATTTTATGCTAATAAAACAACCAAAAAGAT
```

776	22.1	3.8	1.2	hg19	78	237	(1606)	+	MER45A	DNA/hAT-Tip100	1	164	(14)	5
46	0.0	0.0	0.0	hg19	686	724	(1119)	+	(GA)n	Simple_repeat	1	39	(0)	6

>KSI_AluYa8_5 (sequencing file 없음. 정수형에게 있는지 확인해야함.)

```
>Scaffold12158-2008551-2008651
TTGATAAAATTTTTAAAGGACTTTTGCATTCCAACATTCAAA gATTAGATTATATTAGGTAGAATTATTGCCACTCCCCTCCCACCACCAAAGATATCCATGACTTAATC aTCAGAACCTGTTAATAT
GTTGCTTTCATGCCAAGGGAACTTTGCAGATGTGATTTGGTTAAGGACCTTGAAATTGGGAGGTTA gTCTGGATTACCCAGGTGGGCCCAATCAAATTACATATGTCCTTAACAGGGAACAACTTTGT
CCAGCTCTTGTCAGAAGGAAATGTTACTGTGGGATAATAGAAAGAAAATGTGCTGGCTTTATAAACAAAAGAATGGGGCTACCACCCAAGGAATGTGAATGATTTCTAGAA TCAAGAAAAGACAAGGA
GATAAATTCTCTCCTGGAGGCTCCAGAAAGGAGAGCAGCCCTGAAACACCTTGAGTTTAGCCCAGTGAGACCCATGGCAACTTTGTGACTTCCAGAACTGTAGTTTATACTTATATTGTATATAC TTA
TTCTGACTTAAGTATTGTGTTAGATAACCTTTCTTCTTAAGCCAGTGTCTGAAAAAGTTTGCATTTCATTGAGGTTATTTTTTCCTTAACAGTTTTGTAA ACCCATCTGTAACACCAGGTGGCAGGAG
TAATTGGATTTATTTTATAATTTAATTTATTCTATGATTGCTAATTTATTCAGAAATTATATTTTCTTATGATTGATTTTTCTTAAGACCCACCCATGATCAATCATACCATGTTTTAAAATTTA ATA
TTCTTTGGAGATCCAGGCACAGAAAGACCTATATCACACAATCCCACTCATAGGCCAAATCTAAAAATGTTGACCTCCTAGAAGTAGAGAGTAGAATGGAGTAGAATGGTG ATTACCACAGCTGGAGT
GATTGAGGGGTGGAGATTGGGGCAGATGTTGGTCAAAGAAGACAACATTTCTGTTGAATAGCAAAAGAGTAAGTTAAGGAGATCTATATaaaacagggtgattaggccgggcgcggtggctcacgcct
gtaatcccagcactttgggaggccgaggcgggcggatcacgaggtcaggagatcgagaccatcccggctaaaacggtgaaaccccgtctctactaaaaatacaaaaaattagccgggcgtggtgg cgg
gcgcctgtagtcccagctacttgggaggctgaggcaggagaatggcgtgaacccgggaggcggagcttgcagtgagccgagatcccgccactgcactccagcct gggcgacagagcgagactccgtct
caaaaaaaaaaaaaaaaaAAAACAGGGTGATTATAGTTAATACTATATTAAATTCTTAGAATATGCTTAGAGAGTGAATGTGACATGTTCTCACCGCAAAAATGATCACTACGTGAGAT aATGCACA
TGGTAACTGATTTAGTTATTCCACAATGTATATATACTTCAAAACATTACACTATATAGAGTAAATACATACAATTTTATCTGTCAATTTTTTTTAATTTTTTT ATTATACTTTAAGTTCTAGGGTAC
ATGTACACAATGTGCGGGTTTGTTACATATGTATACAT GTGCCTTGTTGATGTGCTGCACCCATTAACTCGTCATTTACATTAGGTATATCTCCTAATGCTATCCCTCCCCCTTCCCCCCACCCCACA
ACAGGCCCTGGTGTGTGATGTTCCCCTTCCTGTGTCAATGTGTTCTCGTTGTTCAATTCCCACCTATGAGTGACAACATGCGGTGTCTGGTTTTTTGTCCTTGC AATAGTTTGCTGAGAATGATGGTT
TTCAGCTTCATCCATGTCCCTACAAAGGACATGAACTCATCTTTTTTATGGCTGCATAGTATTCCATGGTGTATATGTGCCACATTTTCTTAATCCAGTCTATCATTGTTGGATATTTGGGTTGG TTC
CAAGTCTTTGCTATTGTGAATAGTGCTGCAATAAACATACTTGTGCATGTGTCTTTATATCAGCATGATTTATAATCCTTTGGGTACATACCCAGTAATGGGAT GGCTGGGTCAAATGGTATTTCTAG
TTCTAGATCCCTGAGGAATCGCCACACTGTCTTCnnnnnnnnnnnnnnnnnnnnnnnnn
```

1573	23.8	3.5	1.7	AluYa8-Scaffold12158-2008551-2008651	85	486	(1615)	+	MLT1D	LTR/ERVL-MaLR	22	430	(75)	1
813	19.3	1.4	10.4	AluYa8-Scaffold12158-2008551-2008651	781	1000	(1101)	+	L1MA8	LINE/L1	5886	6087	(204)	2
1007	51.2	4.4	0.0	AluYa8-Scaffold12158-2008551-2008651	1001	1295	(806)	+	AluYa5	SINE/Alu	1	308	(3)	3
789	21.1	5.7	0.0	AluYa8-Scaffold12158-2008551-2008651	1296	1489	(612)	+	L1MA8	LINE/L1	6077	6281	(10)	4
4912	3.8	0.2	0.3	AluYa8-Scaffold12158-2008551-2008651	1490	2074	(27)	C	L1PA5	LINE/L1	(0)	6154	5571	5

```
>hg19 chr14:41839216-41841040
TTGATAAAATTTTTAAAGGACTTTTGCATTCCAACATTCAAA aATTAGATTATATTAGGTAGAATTATTGCCACTCCCCTCCCACCACCAAAGATATCCATGACTTAATC gTCAGAACCTGTTAATAT
GTTGCTTTCATGCCAAGGGAACTTTGCAGATGTGATTTGGTTAAGGACCTTGAAATTGGGAGGTTA tTCTGGATTACCCAGGTGGGCCCAATCAAATTACATAT GTCCTTAACAGGGAACAACTTTGT
CCAGCTCTTGTCAGAAGGAAATGTTACTGTGGGATAATAGAAAGAAAATGTGCTGGCTTTATAAACAAAAGAATGGGGCTACCACCCAAGGAATGTGAATGATTTCTAGAATCAAGAAAAGACAA GGA
GATAAATTCTCTCCTGGAGGCTCCAGAAAGGAGAGCAGCCCTGAAACACCTTGAGTTTAGCCCAGTGAGACCCATGGCAACTTTGTGACTTCCAGAACTGTAGT TTATACTTATATTGTATATACTTA
TTCTGACTTAAGTATTGTGTTAGATAACCTTTCTTCTTAAGCCAGTGTCTGAAAAAGTTTGCATTTCATTGAGGTTATTTTTTCCTTAACAGTTTTGTAA ACCCATCTGTAACACCAGGTGGCAGGAG
TAATTGGATTTATTTTATAATTTAATTTATTCTATGATTGCTAATTTATTCAGAAATTATATTTTCTTATGATTGATTTTTCTTAAGACCCACCCATGATCAAT CATACCATGTTTTAAAATTTAATA
TTCTTTGGAGATatatatatatatatatatatatatatatatatatatatatgttaaatgaaataagCCAGGCACAGAAAGACCTATATCACACAATCCCACTCATAGGCCAAATCTAAAAATGTT
GACCTCCTAGAAGTAGAGAGTAGAATGGAGTAGAATGGTGATTACCACAGCTGGAGTGATTGAGGGGTGGAGATTGGG GCAGATGTTGGTCAAAGAAGACAACATTTCTGTTGAATAGCAAAAGAGTA
AGTTAAGGAGATCTATATAAAACAGGGTGATTATAGTTAATACTATATTAAATTCTTAGAATATGCTTAGAGAGTGAATGTGACATGTTCTCACCGCAAAAATGATCACTACGTGAGAT gATGCACAT
GGTAACTGATTTAGTTATTCCACAATGTATATATACTTCAAAACATTACACTATATAGAGTAAATACATACAATTTTATCTGTCAATTTTTTTTAATTTTTTTATTATACTTTAAGTTCTAGGGT ACA
TGTACACAATGTGCGGGTTTGTTACATATGTATACAT GTGCCTTGTTGATGTGCTGCACCCATTAACTCGTCATTTACATTAGGTATATCTCCTAATGCTATCCCTCCCCCTTCCCCCCA CCCCACAA
CAGGCCCTGGTGTGTGATGTTCCCCTTCCTGTGTCAATGTGTTCTCGTTGTTCAATTCCCACCTATGAGTGACAACATGCGGTGTCTGGTTTTTTGTCCTTGCAATAGTTTGCTGAGAATGATGGTTT
TCAGCTTCATCCATGTCCCTACAAAGGACATGAACTCATCTTTTTTATGGCTGCATAGTATTCCATGGTGTATATGTGCCACATTTTCTTAATCCAGTCTATCATTGTTGGATATTTGGG TTGGTTCC
AAGTCTTTGCTATTGTGAATAGTGCTGCAATAAACATACTTGTGCATGTGTCTTTATATCAGCATGATTTATAATCCTTTGGGTACATACCCAGTAATGGGATGGCTGGGTCAAATGGTATTTCT AGT
TCTAGATCCCTGAGGAATCGCCACACTGTCTTC
```

1599	23.5	3.5	1.7	hg19	85	486	(1339)	+	MLT1D	LTR/ERVL-MaLR	22	430	(75)	6

51	0.0	0.0	0.0	hg19	779	822	(1003)	+	(AT)n	Simple_repeat	1	44	(0)	7
1675	19.9	3.4	5.1	hg19	823	1240	(585)	+	L1MA8	LINE/L1	5871	6281	(10)	8
4912	3.8	0.2	0.3	hg19	1241	1825	(0)	C	L1PA5	LINE/L1	(0)	6154	5571	9

>KSI_AluYa8_6

>Scaffold14281-1358920-1359021

AGGAGGGTAGTTATCTGCAGAAGACGGCAGGGCCTTGCTCCAAAATCCTAGAGGCCTCCACTGTGATTCACCTATGGGGGCCTGTCAAAGGCTCCACACAGCACCTCTATCTGCCACTGACACCTC GA
GCACCATTGGATCTGCTAGGTCATATGGCCCAAGTGAAAGAGAAGATTGCACAGCAGACTGGACCTGTTGCAGAGCCGTCTGTTCTGGGCCCCACTCAAAACTGTCAGCCTTTCAGGTCACTTGA TAA
ATGAACCAGAGTAACACACCCAAATGAGGAATGTGTTGCCTCCAAAAACCAAATAGATCCGCTAGATGTTGTGCCTCTTTCTTGGTTGTAGGAGGGGCCAAATGCAACAATTTATCCTTCACCTTA GA
AGGAATATCTTGACAGGCCCCACACCACTGAACCACTAGAAATTTTTCTGAGGTAGAAGATCCCTGAATTTTAGTCAGATTGTTTTCCCATCCTTTGGCATGCAAATGTCTCACCAATAAGTCCA GTG
TGTTTGCTACTTTCTCGCTCACTGGATCCAATCAGTATAATATCATCAATGTAATGGACCAGTGTGATATGTGGAAAGGAAAAGTGATCAAGATCTCTGAGAACAAGATTATGACACAAAGCTGGAGA
GTTGATACACCCCTGCGGTAGGACAGTAAAGGTATATTGCTGGCCTTGCCAGCTGAAGGCAAATTGCTTCTGGTGGGCTTTATGGATAGGAATAGAGACAAAGGCATTTGCCAAGTCAATGGCTG CAT
AACCCATACCAGAAGATGTATTAATTTGTTTCAAGCTATGAACACATCTGGTACAGCAGCTGCAACTGGAGTCACCACTTGGTTAAGCTTACAATAATCTGCTGTCATCCTCTGAGATCCATCTGT CT
TTTGCACAGACCAAATAGGCGAGTTGAACAGGGATGgGGTGGGAATCACCATCCCTGCATCTTTCAAGTTCTTGATGGTGGCACTAATCTCCACAATCCCTC CAcaccgttttagccgggatggtctc
gatctcctgacctcgtgatccgcccgcctcggcctcccaaagtgctgggattacaggcgtgagccaccgcgcccggcc catccctgcatctttcaagttcttgatggtggcactaatctccacaatcc
ctccaGGGATGAGATATTGTTTTTCATTTAGTATTTTTCTAGGTAGAGGCAGCTCTAATGGCTTCCATTTGGCTTTTCTCACCATAGTAGCCCTTCACCCTACAAGTCA GGGAGCCAATATGGGGGTT
CTGCCAGCTGCTAAGTATGTCTATCCCAATTATGCATTCTGGCACTGGGGAAATGACCAAAGGATGAGTCCAGGGACCCACTGGACACACTATAAGTCAGACCCGAGCTAAAACTCTACTAATTA CCT
AACCTCCATAAGCCTCTACTTTAACTGGAGGACCACAATGACATTTGGGTCCCCTGGAATCAACATCAGCTCAGAGCCAGTGTCCAGTAGTCCCCAAAAGGTCTGATCA TTCCTCTTTCCCCAGTGCA
CAGTTACTCTGGTAAAAGGCTGGAGGTTTCCTTGGGGGAGGATGGGAGAAAGATTAACAGCATAAATTGTTGGTAGTTTAGTGTGGTCGTTCCTCAAGGGGAGTCTGGCCTCCCCTTCATTCAAG GGA
TTCTGGGTCTGTAAATTTGTTCATGTCTGGAATTTGATTGAGGGGACATAATTCTTTATTTTTATAATTAAAATTAGTTTTTGTCCACTCGGCCTAGAAGCTTTTGCTT ATACAAATTACTATAGTGA
GAATGCAGTGGGCTTCTTATCAATTTCACTTCTAGGAACATCATGATTAGCCAGTGCCACAGCTCTACATGAGTCAGACTATTCTGATTGCTGCTTTGCCTCTGCTATCTCTTA CGGTAACTACACCT
TGCCTTTGAAGGTTGAATGCCATCACTTGGCCCCTGCCACCTTGACTTTCAGTTATTCCCATTGCATTTAAATTTTCTTACTAAGTGACTGTAGTTCCCACTGTAAGAC CTGGCCTACAGAGAAGGGC
AATCACAGACCTCTTTAAGGATGCAGGTGCTGCCCTCACAAATCTATTTCGCC A

SW score	perc div.	perc del.	perc ins.	query sequence	position in query begin	end	(left)		matching repeat	repeat class/family	position in repeat begin	end	(left)	ID
7824	8.3	0.8	0.2	AluYa8-Scaffold14281-1358920-1359021	1	1000	(1102)	C	HERVL-int	LTR/ERVL	(2364)	3290	2285	1
986	0.0	0.0	0.0	AluYa8-Scaffold14281-1358920-1359021	1001	1102	(1000)	C	AluYa8	SINE/Alu	(208)	102	1	2
7325	10.0	1.1	0.6	AluYa8-Scaffold14281-1358920-1359021	1103	2100	(2)	C	HERVL-int	LTR/ERVL	(3315)	2339	1337	1
14740	9.2	1.0	0.4	hg19	1	1943	(2)	C	HERVL-int	LTR/ERVL	(2364)	3290	1337	3

>hg19 chr12:25461949-25463893

AGGAGGGTAGTTATCTGCAGAAGACGGCAGGGCCTTGCTCCAAAATCCTAGAGGCCTCCACTGTGATTCACCTATGGGGGCCTGTCAAAGGCTCCACACAGCACCTCTATCTGCCACTGACACCTC GA
GCACCATTGGATCTGCTAGGTCATATGGCCCAAGTGAAAGAGAAGATTGCACAGCAGACTGGACCTGTTGCAGAGCCGTCTGTTCTGGGCCCCACTCAAAACTGTCAGCCTTTCAGGTCACTTGATAA
ATGAACCAGAGTAACACACCCAAATGAGGAATGTGTTGCCTCCAAAAACCAAATAGATCCGCTAGATGTTGTGCCTCTTTCTTGGTTGTAGGAGGGGCCAAATGCAACAATTTATCCTTCACCTT AGA
AGGAATATCTTGACAGGCCCCACACCACTGAACCACTAGAAATTTTTCTGAGGTAGAAGATCCCTGAATTTTAGTCAGATTGTTTTCCCATCCTTTGGCATGCAAATGTCTCACCAATAAGTCCAGTG
TGTTTGCTACTTTCTCGCTCACTGGATCCAATCAGTATAATATCATCAATGTAATGGACCAGTGTGATATGTGGAAAGGAAAAGTGATCAAGATCTCTGAGAACAAGATTATGACACAAAGCTGGAGA
GTTGATACACCCCTGCGGTAGGACAGTAAAGGTATATTGCTGGCCTTGCCAGCTGAAGGCAAATTGCTTCTGGTGGGCTTTATGGATAGGAATAGAGACAAAGGCATTTGCCAAGTCAATGGCTGCAT
AACCCATACCAGAAGATGTATTAATTTGTTTCAAGCTATGAACACATCTGGTACAGCAGCTGCAACTGGAGTCACCACTTGGTTAAGCTTACAATAATCTGCTGTCATCCTCTGAGATCCATCTG TCT
TTTGCACAGACCAAATAGGCGAGTTGAACAGGGATGtGGTGGGAATCACCATCCCTGCATCTTTCAAGTTCTTGATGGTGGCACTAATCTCCACAATCCCTCCAGGGATGAGATATTGTTTTTCATTT
AGTATTTTTCTAGGTAGAGGCAGCTCTAATGGCTTCCATTTGGCTTTTCTCACCATAGTAGCCCTTCACCCTACAAGTCAGGGAGCCAATATGGGGGTTCTGCCAGCTGCTAAGTATGTCTATCC CAA
TTATGCATTCTGGCACTGGGGAAATGACCAAAGGATGAGTCCAGGGACCCACTGGACACACTATAAGTCAGACCCGAGCTAAAACTCTACTAATTACCTAACCTC CATAAGCCTCTACTTTAACTGGA
GGACCACAATGACATTTGGGTCCCCTGGAATCAACATCAGCTCAGAGCCAGTGTCCAGTAGTCCCCAAAAGGTCTGATCATTCCTCTTTCCCCAGTGCACAGTTACTCTGGTAAAAGGCTGGAGG TTT
CCTTGGGGGAGGATGGGAGAAAGATTAACAGCATAAATTGTTGGTAGTTTAGTGTGGTCGTTCCTCAAGGGGAGTCTGGCCTCCCCTTCATTCAAGGGATTCTGG GTCTGTAAATTTGTTCATGTCTG
GAATTTGATTGAGGGGACATAATTCTTTATTTTTATAATTAAAATTAGTTTTTGTCCACTCGGCCTAGAAGCTTTTGCTTATACAAATTACTATAGTGAGAATGCAGTGGGCTTCTTATCAATTT CAC
TTCTAGGAACATCATGATTAGCCAGTGCCACAGCTCTACATGAGTCAGACTATTCTGATTGCTGCTTTGCCTCTGCTATCTCTTA CGGTAACTACACCTTGCCTTTGAAGGTTGAATGCCATCACTTG
GCCCCTGCCACCTTGACTTTCAGTTATTCCCATTGCATTTAAATTTTCTTACTAAGTGACTGTAGTTCCCACTGTAAGACCTGGCCTACAGAGAAGGGCAATCACAGACCTCTTTAAGGATGCAG GTG

CTGCCCTCACAAATCTATTTCGCCA

>KSI_AluYa8_7

>Scaffold14564-7020-7099
TGTGTCCCTTCTATGCCAATTTTGCTGAGAGTTTTAACCATAAAGAGATGCTGGATCTTGTAAAATGCTTTTTCTGCATCTATTGAGATGATCATTTGATTTTTGTTTTTAATTCTGTTTATGTGGTG
TATCACACTTATTGACCTGCAGATGTTAAGCCACCCTTGCATCTCTGGTGTGAAACCCACTTGATCATGGTGGATTATCTTTTTGATATGCTGTTGTATTTGGTTAGCTAGTATTTTATTGAGGA TTT
TTGCATCTGTGTTCATCAGGGATGTTGGTATATAGT TTTCTTTTTTTGTTATGTCCTTCTCTGGTTTTGATATTAGGGTTATAATGGTATCATAGAATGATTTAGTGAGGATTCTCTCTCTCTCTC
TctctGTCTCTCTCTcTCTCTCTCTCCCCTGTGGAATAGTGTCAACAGGATTGGCATCAGTTCTTCTTTGAATGTCTGATAGAATTCAGCTGTGAATCTGTCTGGTCCTGGACTTTTTTGTTGTTGGC
ATTTTTTTATTACTATTTCAATCTCGCTGCTTGTTATTGGTCTGTTCAGGGTTTCTATATCTTCCTGGTTTAATCTAGGAGGGTTGTATATTTCCAGGAATTTATCCATGTCCTCTGGGTTTTTTAGT
TTATGTGTGTGGGCATTCATAGTAGCCTTGAGTAATCTTTTGTATTTCTGTAGTATCAGTAGTAATATTTAACATTTTGTTTCTAATTAAGCTATTTGGATCTTCTCTCTTCTTTTCTTGGTTAATC
TCACTACTGGTCTATCAATTTTATTTATCTTTTTGAAGAACCGGCTTTTTGTTTCAATTATCTTTTGTATTTTTTTT tGTTTTAATTTCATTTAGTTCTGCTCTGATTGTCATTATTTCTTTTCTTCTG
CTGGGTTTGGTTTTGGATCGTTCTTGTTTCTCCAGTTCCATGAGGTGTGACCATAGATTGTCTATTTGTGCTCTTTCAGACTTTTTTATATAAGCATTTAATG TTagagacggggtttcaccgtttta
gctgggatggtctcgatctcctgacctcgtgatccgcccgcctcggcctcccgaagaaccggctttttgtttcaattatcttttgtattttttttg gttttaatttcatttagttctgctctgattgtc
attatttcttttcttctgctgggtttggttttggatcgttcttgtttctccagttccatgaggtgtgaccatagattgtctatttgtgctctttcagactttttttatataagcatttaatgtt GTGAA
CTTTCTTCTTAGCACTGCTTTTGCTGTATCCCAGAAATTTTGATAGGTTGTGCAACTATTATCATTCAGTTCAAAAAATTTTTAAATTTCCATCTTGATTTCATTGTTGACCCAACAATCATTCA GGA
GCAGGTTATTTAATTTCCATGTATTTGCCTGGTTTTGATGGTTCCTTTTGGAGCTGATTTCTAATTTTATTCCACTGTGGTGTGAGAGAGTACTTGTTATAATTTCAATTTTCTTAAATTTACTGAGA
CTTATTTTGTGGCCTATCATATGGTCTATCTTGGAGAATGTTCCATGTGCTGATAATAGAATGTATATTCTGCAGTTGTTGGGTAGAATGTTCTGTAAATATCTGTTAAGTCCATTTGTTGTAGG GTA
TAGTTTAAGTCCATTGTTCTTTGTTGACTTTCTGTCTTGATGACCTGTCTAGTGCTGTCAGTGGAGTACTGAAATCTCCCACTATTATTGTGTTGCTCTCTATCTCATTTCTTAGGTCTAGTAGTAAT
TGCTTTATAAATTTGGGAGCTCCAATATTAGGTGCATATATATTTAGGATTGTTATATTTTTCTGTTGGACTAGTCCTGTTATCATTACATAATGTCTCTCTTTGTCTTTTTTTTTAAAGTTTTT CCT
TTTTTTATTGTTATACTTTAAGTTCTGGGGTACATGTGCACAACGTGCAGGTTTGTTACATAGGTATACATGTGCCATGTTGGTTTGTTGCACCCATCAACTCGTCATTTACATTAGGTATTTCTCCT
AATGCTATTCCTCCCCCAGCCCTCCACCCACTGACAGGCCCTGGTGTGTGATGTTCCCCTCCCTGTGTCTATGTGTTCTCATTGTTCAGCTCCCACTTATGGGTGAGAACATGCGGTGTTTGGTT TCC
TCTTCTTGTGTTACTTTGCTGAGAATGACGGTTTCCAGTTTCATCCATGTCCCTGCAAACAACATGAACTCATCCTTTTTTAAAATGGCTGCATAGTATCCCATGGTGCATGTGTGCCACATTTTCTT
TATCCAGTCTATCATTGATGGGCATTTGGGTTGGTTCCAAGACTTAACTGCTTTTGCTTTAAAGTTTGTTTTGTCTGATATAAGAATAGCTACTTCTGCTCACTTTTGGTGTCCTTTGCATGGAC TAT
CTTTTTCCACCCTTTTACCTTAAGTTTATGTGAGTCCTTATGTGTTAGGTGAGTCTCCTGAGGATAGCAGAAACTTGGTTGGTGAATTCTTATCCATTCTGCCATTCTGTATATTTTAAGTGGAGCAT
TTAAGCCATTTACATTCAACATTAGTTTTGAGATGTGACACACTATTCTATTCATTGTGCTACTTATTGCCTGAATACCTTGTTTTTTTTTTTTCATTGTGTTATTGTTTGATAGGTCTTGTGAG ACA
TATGCTTTAAGGAGGGTCTATTTTGGTGTATTTTGAGGATTTGTTTCAAGATTTAGAGCTCCTTTTAGCAGTTCTTGAAGTGCTGGCTTGGTAGTGGTGAGTTCTCTCAGCATTTGTTTGTCTGGAAA
ATACTGCATCTTTCTTTCATTTATGAAGCTTAGTTTCACTGGATACAAAATTCTTGATTGATAATTGTTTTGTTTAAGGAGGCTAAAAATA GGACACCAATCCCTTCTCACTTGTAGGGTTTCTGCTG
AGAAGTTTGCTGTTACTCTGTTTTCCTTTATAGTTACCTGATACTTTCGCCTCACAGCTCTTAAGATTTGTTCCTTCATCTTGACTTTAGATAACCGGATGCATATGTGCCTAGGCGATGATCTTTTT
GCGATGAA

6361	10.2	2.0	0.1	AluYa8	1	368	(2712)	C	L1PBa	LINE/L1	(2160)	4702	4335	1
44	2.4	0.0	0.0	AluYa8	369	410	(2670)	+	(TC)n	Simple_repeat	1	42	(0)	2
6361	10.2	2.0	0.1	AluYa8	411	996	(2084)	C	L1PB	LINE/L1	(2528)	3618	3015	3
737	2.5	0.0	0.0	AluYa8	1001	1080	(2000)	C	AluYa8	SINE/Alu	(194)	116	37	4
7727	8.7	0.7	0.6	AluYa8	1081	1919	(1161)	C	L1PBa	LINE/L1	(2940)	3922	3081	1
3336	6.9	0.0	1.4	AluYa8	1920	2360	(720)	C	L1PA7	LINE/L1	(3)	6151	5717	5
7727	12.5	2.8	1.1	AluYa8	2361	3080	(0)	C	L1PBa	LINE/L1	(3782)	3080	2297	1

12898	9.4	1.3	0.4	hg19	1	368	(2434)	C	L1PBa	LINE/L1	(2160)	4702	4335	6
34	5.5	0.0	0.0	hg19	369	406	(2396)	+	(TC)n	Simple_repeat	1	38	(0)	7
12898	9.4	1.3	0.4	hg19	407	1641	(1161)	C	L1PBa	LINE/L1	(2528)	4334	3082	6
3336	6.9	0.0	1.4	hg19	1642	2082	(720)	C	L1PA7	LINE/L1	(3)	6151	5717	8
12898	12.6	2.9	1.1	hg19	2083	2802	(0)	C	L1PBa	LINE/L1	(3781)	3081	2297	6

>hg19 chr15:100897976-100899777
TGTGTCCCTTCTATGCCAATTTTGCTGAGAGTTTTAACCATAAAGAGATGCTGGATCTTGTAAAATGCTTTTTCTGCATCTATTGAGATGATCATTTGATTTTTGTTTTTAATTCTGTTTATGTGG TG
TATCACACTTATTGACCTGCAGATGTTAAGCCACCCTTGCATCTCTGGTGTGAAACCCACTTGATCATGGTGGATTATCTTTTTGATATGCTGTTGTATTTGGTTAGCTAGTATTTTATTGAGGA TTT
TTGCATCTGTGTTCATCAGGGATGTTGGTATATAGTTTTCTTTTTTTGTTATGTCCTTCTCTGGTTTTGATATTAGGGTTATAATGGTATCATAGAATGATTTAGTGAGGATTCTCTCTCTCTCTC TC

TGTCTCTCTCTgTCTCTCTCTCCCCTGTGGAATAGTGTCAACAGGATTGGCATCAGTTCTTCTTTGAATGTCTGATAGAATTCAGCTGTGAATCTGTCTGGTCCTGGACTTTTTTGTTGTTGGCATTT
TTTTATTACTATTTCAATCTCGCTGCTTGTTATTGGTCTGTTCAGGGTTTCTATATCTTCCTGGTTTAATCTAGGAGGGTTGTATATTTCCAGGAATTTATCCATGTCCTCTGGGTTTTTTAGTTT AT
GTGTGTGGGCATTCATAGTAGCCTTGAGTAATCTTTTGTATTTCTGTAGTATCAGTAGTAATATTTAACATTTTGTTTCTAATTAAGCTTATTTGGATCTTCTCTCTTCTTTTCTTGGTTAATCTCAC
TACTGGTCTATCAATTTTATTTATCTTTTTGAAGAACCGGCTTTTTGTTTCAATTATCTTTTGTATTTTTTT gGTTTTAATTTCATTTAGTTCTGCTCTGATTGTCATTATTTCTTTTCTTCTGCTGG
GTTTGGTTTTGGATCGTTCTTGTTTCTCCAGTTCCATGAGGTGTGACCATAGATTGTCTATTTGTGCTCTTTCAGACTTTTTTATATAAGCATTTAATGTT GTGAACTTTCTTCTTAGCACTGCTTTT
GCTGTATCCCAGAAATTTTGATAGGTTGTGCAACTATTATCATTCAGTTCAAAAAATTTTTAAATTTCCATCTTGATTTCATTGTTGACCCAACAATCATTCAGGAGCAGGTTATTTAATTTCCAT GT
ATTTGCCTGGTTTTGATGGTTCCTTTTGGAGCTGATTTCTAATTTTATTCCACTGTGGTGTGAGAGAGTACTTGTTATAATTTCAATTTTCTTAAATTTACTGAGACTTATTTTGTGGCCTATCA TAT
GGTCTATCTTGGAGAATGTTCCATGTGCTGATAATAGAATGTATATTCTGCAGTTGTTGGGTAGAATGTTCTGTAAATATCTGTTAAGTCCATTTGTTGTAGGGTATAGTTTAAGTCCATTGTTCT TT
GTTGACTTTCTGTCTTGATGACCTGTCTAGTGCTGTCAGTGGAGTACTGAAATCTCCCACTATTATTGTGTTGCTCTCTATCTCATTTCTTAGGTCTAGTAGTAATTGCTTTATAAATTTGGGAG CTC
CAATATTAGGTGCATATATATTTAGGATTGTTATATTTTTCTGTTGGACTAGTCCTGTTATCATTACATAATGTCTCTCTTTGTCTTTTTTTTTAAAGTTTTTCCTTTTTTTTATTGTTATACTTTA AG
TTCTGGGGTACATGTGCACAACGTGCAGGTTTGTTACATAGGTATACATGTGCCATGTTGGTTTGTTGCACCCATCAACTCGTCATTTACATTAGGTATTTCTCCTAATGCTATTCCTCCCCCAG CCC
TCCACCCACTGACAGGCCCTGGTGTGTGATGTTCCCCTCCCTGTGTCTATGTGTTCTCATTGTTCAGCTCCCACTTATGGGTGAGAACATGCGGTGTTTGGTTTCCTCTTCTTGTGTTACTTTGCT GA
GAATGACGGTTTCCAGTTTCATCCATGTCCCTGCAAACAACATGAACTCATCCTTTTTTAAAATGGCTGCATAGTATCCCATGGTGCATGTGTGCCACATTTTCTTTATCCAGTCTATCATTGAT GGG
CATTTGGGTTGGTTCCAAGACTTAACTGCTTTTGCTTTAAAGTTTGTTTTGTCTGATATAAGAATAGCTACTTCTGCTCACTTTTGGTGTCCTTTGCATGGACTATCTTTTTCCACCCTTTTACCT TA
AGTTTATGTGAGTCCTTATGTGTTAGGTGAGTCTCCTGAGGATAGCAGAAACTTGGTTGGTGAATTCTTATCCATTCTGCCATTCTGTATATTTTAAGTGGAGCATTTAAGCCATTTACATTCAA CAT
TAGTTTTGAGATGTGACACACTATTCTATTCATTGTGCTACTTATTGCCTGAATACCTTGTTTTTTTTTTTCATTGTGTTATTGTTTGATAGGTCTTGTGAGACATATGCTTTAAGGAGGGTCTA TT
TTGGTGTATTTTGAGGATTTGTTTCAAGATTTAGAGCTCCTTTTAGCAGTTCTTGAAGTGCTGGCTTGGTAGTGGTGAGTTCTCTCAGCATTTGTTTGTCTGGAAAATACTGCATCTTTCTTTCA TTT
ATGAAGCTTAGTTTCACTGGATACAAAATTCTTGATTGATAATTGTTTTGTTTAAGGAGGCTAAAAATA GGACACCAATCCCTTCTCACTTGTAGGGTTTCTGCTGAGAAGTTTGCTGTTACTCTGTT
TTCCTTTATAGTTACCTGATACTTTCGCCTCACAGCTCTTAAGATTTGTTCCTTCATCTTGACTTTAGATAACCGGATGCATATGTGCCTAGGCGATGATCTTTTTGCGATGA A

>KSI_AluYa8_8

>Scaffold15820-1508418-1508538
TTTTCCAAGCATCTATATATCTGTTCTTACACCAGCACCATACTGTCTTGATTACTGTAGATTTATGATAAGTCTTGAAATCAAGTAGTATAAGTCCGCTAACTTTGCTCTTCTTTTTAAATATTG TT
TTGGCCATCCTAGGACCTTTCCATTTCCATATAAATTTAGAATCTATTTGTCAGTTTTTACCAAAAACCTGCCAATATTTTACCTGGAGTAACTGATCTGA ACCTGTAGATCATTAGGGGAGAATTGA
TGCTTTAATAATACTGATCATTCAGTTAATAAACAGGGGATATACCTTGATTAATTTAGCTCTTTACTTTCT CTTTTTTTTTTTTTTTTTGAGATGGAATCTCACTCTGTCACCCAGGCTGGAGTGCAG
TGGCACAATCTCAGCTCACTGCAACCTCCGCCTCCCAGGTTCAAGCGATTCTCCTGCCTCAGCCCCACGAGTAGCTGGGAGTACAGGCATGTGCCACCATACCTGGCTAAATTTTGTATTTTTAG TAG
AGATGGGGTTTCATCATGTTGGTCAGGCTGGTCTCGAACTCCCAACTTCAAGTGGTCTGCCTGCCTCAGCCTCCCAAAGTGCTGGACTTACAGGCGTGAGCCATGGTGTACCAGGCCTTTACTTTC TC
TTGGGCATGTTTTATAGTTTTCGGAATACAGATATTGCACTTATTATATTTATCCCTAAATATTTCATTTTTACACAATTATAAATGGTAAGAAAACATTTTCACTTTGTGATTTTTTAATAGAC ATT
ATGTTTTAGCACAGTTTTACATTGACAGCAAAATTAAGCAGAACGTA CAGAGATTTCTTTTTTTTTTTTTTTTTTTTTTTTTTTTTTTTTTTTTTTGAGACGGAGTCTCGCTCTGTCGCCCAGGCTGGAGTG
CAGTGGCGGGATCTCGGCTCACTGCAAGCTCCGCCTCCCGGGTTCACGCCATTCTCCTGCCTCAGCCTCCCAAGTAGCTGGGACTACAGGCGCCCGCCACTACGCCCGGCTAATTTTTTGTATTt tta
gtagagacggggtttcaccgttttagccaggatggtctcgatctcctgacctcgtgatccgcccgcctcggcctcccaaagtgctgggattacaggcgtgagccaccgcgcccggcc cagagatttct
tATACATCCCCTGCTCCCACATACACACGGCCACACTACTTTCAAATTCCCACACCAGAATGGTAATGATAAAATCAATGAGCCTACAGTGACACATTACCACCACACAAAATCCATAGTTTATTAG G
ATTCACTCTTGGTGTTGTACATTCTGTGGGTTTTGACACATGTATAATGATATGTATTCACCACTATGGAATTATACAAAACAGTTTCACTGCCCTAAAGATCCTC TGTGTCCTGCCCATTCATCCTT
CCCTCCTCCCTAATTCCTGGCAACCACTGATTGTTTTAC cGTCTCCATAGTTTTGCTTTTTCCAGAATATCTTATAGTCAGAATCATACAACTTGTAGCATTTTTGACTGGCTTTCACTTGGTAACAT
GCATTTAAGTTTCCTCCATATCTTTTCATGGTTTAATAGCTCATTTCTTTTTAGTATTTCATAATACTTCATGGTCAGAATATACCA tAGTCCACCCACTTACCTACTGAAGAACATCTTGGTTACTT
TCAAGTTTGGGCAATTATAAATAAATCTGTTATAAACATTCATATGTAAGTTTTGGCTTAGACATAGGTTTTCAACTCATTTGGAAGCACAATTGCCAAAATCATATGGTAAGATTTTGTAAGTT TTG
TAAGAAACTGCCAAACTATCTTTTAAATTAGCTGTACCATTTTGTATTCCTACCAACAGTGAATGAGAGTTCCTGTTGCTCCACGTCCTCACCAGCATTTGATGTCGTCAATGTTTTGGATTTTGG CC
ATTCTAATAAGTGTGTGATGGTATCCTGTTGTTTTAATTTGTAATTTCCTAATGATATAGGATGTTGTGCATTTTTTTCATATGCTTATTTGCCACCTGTTTATATCTTCTTTGGTGAGGTGTCC AGG
TATTTTGTCTATTTTAAATCAGGTTTTTAAAATCATT cAATTTTAAGAGTTCTTTGTATGTTTTAGATAACAGTGTTTTATTAGTGTTTCAT A

1517	22.8	3.0	4.8	AluYa8-Scaffold15820-1508418-1508538	12	323	(1798)	C	L1M5	LINE/L1	(1347)	4799	4490	1
2262	11.0	0.0	0.0	AluYa8-Scaffold15820-1508418-1508538	324	615	(1506)	C	AluSz	SINE/Alu	(8)	304	13	2
1517	22.8	3.0	4.8	AluYa8-Scaffold15820-1508418-1508538	616	730	(1391)	C	L1M5	LINE/L1	(1657)	4489	4379	1
415	15.1	0.0	0.0	AluYa8-Scaffold15820-1508418-1508538	753	825	(1296)	C	L1MC1	LINE/L1	(0)	6333	6261	3
35	0.0	0.0	0.0	AluYa8-Scaffold15820-1508418-1508538	826	858	(1263)	+	(T)n	Simple_repeat	1	33	(0)	4
1150	0.8	0.0	0.0	AluYa8-Scaffold15820-1508418-1508538	1001	1121	(1000)	C	AluYa8	SINE/Alu	(189)	121	1	5
5554	16.4	4.7	0.6	AluYa8-Scaffold15820-1508418-1508538	1122	2118	(3)	C	L1MC1	LINE/L1	(63)	6270	5236	3

>hg19 chr8:80749888-80751704
TTTTCCAAGCATCTATATATCTGTTCTTACACCAGCACCATACTGTCTTGATTACTGTAGATTTATGATAAGTCTTGAAATCAAGTAGTATAAGTCCGCTAACTTTGCTCTTCTTTTTAAATATTG TT
TTGGCCATCCTAGGACCTTTCCATTTCCATATAAATTTAGAATCTATTTGTCAGTTTTTACCAAAAACCTGCCAATATTTTACCTGGAGTAACTGATCTGA ACCTGTAGATCATTAGGGGAGAATTGA
TGCTTTAATAATACTGATCATTCAGTTAATAAACAGGGGATATACCTTGATTAATTTAGCTCTTTACTTTCT CttTTTTTTTTTTTTTTTGAGATGGAATCTCACTCTGTCACCCAGGCTGGAGTGC
AGTGGCACAATCTCAGCTCACTGCAACCTCCGCCTCCCAGGTTCAAGCGATTCTCCTGCCTCAGCCCCACGAGTAGCTGGGAGTACAGGCATGTGCCACCATACCTGGCTAAATTTTGTATTTTT AGT
AGAGATGGGGTTTCATCATGTTGGTCAGGCTGGTCTCGAACTCCCAACTTCAAGTGGTCTGCCTGCCTCAGCCTCCCAAAGTGCTGGACTTACAGGCGTGAGCCATGGTGTACCAGGCCTTTACTT TC
TCTTGGGCATGTTTTATAGTTTTCGGAATACAGATATTGCACTTATTATATTTATCCCTAAATATTTCATTTTTACACAATTATAAATGGTAAGAAAACATTTTCACTTTGTGATTTTTTAATAG ACA
TTATGTTTTAGCACAGTTTTACATTGACAGCAAAATTAAGCAGAACGTA CAGAGATTTCTTATACATCCCCTGCTCCCACATACACACGGCCACACTACTTTCAAATTCCCACACCAGAATGGTAATG
ATAAAATCAATGAGCCTACAGTGACACATTACCACCACACAAAATCCATAGTTTATTAGGATTCACTCTTGGTGTTGTACATTCTGTGGGTTTTGACACATGTATAATGATATGTATTCACCACT ATG
GAATTATACAAAACAGTTTCACTGCCCTAAAGATCCTC TGTGTCCTGCCCATTCATCCTTCCCTCCTCCCTAATTCCTGGCAACCACTGATTGTTTTAC tGTCTCCATAGTTTTGCTTTTTCCAGAAT
ATCTTATAGTCAGAATCATACAACTTGTAGCATTTTTGACTGGCTTTCACTTGGTAACATGCATTTAAGTTTCCTCCATATCTTTTCATGGTTTAATAGCTCATTTCTTTTTAGTATTTCATAAT ACT
TCATGGTCAGAATATACCAcAGTCCACCCACTTACCTACTGAAGAACATCTTGGTTACTTTCAAGTTTGGGCAATTATAAATAAATCTGTTATAAACATTCATATGTAAGTTTTGGCTTAGACATAGG
TTTTCAACTCATTTGGAAGCACAATTGCCAAAATCATATGGTAAGATTTTGTAAGTTTTGTAAGAAACTGCCAAACTATCTTTTAAATTAGCTGTACCATTTTGTATTCCTACCAACAGTGAATG AGA
GTTCCTGTTGCTCCACGTCCTCACCAGCATTTGATGTCGTCAATGTTTTGGATTTTGGCCATTCTAATAAGTGTGTGATGGTATCCTGTTGTTTTAATTTGTAATTTCCTAATGATATAGGATGTT GT
GCATTTTTTTCATATGCTTATTTGCCACCTGTTTATATCTTCTTTGGTGAGGTGTCCAGGTATTTTGTCTATTTTAAATCAGGTTTTTAAAATCATT gAATTTTAAGAGTTCTTTGTATGTTTTAGAT
AACAGTGTTTTATTAGTGTTTCAT A

1517	22.8	3.0	4.8	hg19	12	323	(1494)	C	L1M5	LINE/L1	(1347)	4799	4490	6
2280	10.9	0.0	0.0	hg19	324	617	(1200)	C	AluSz	SINE/Alu	(6)	306	13	7
1517	22.8	3.0	4.8	hg19	618	732	(1085)	C	L1M5	LINE/L1	(1657)	4489	4379	6
5954	16.1	4.5	0.6	hg19	755	1814	(3)	C	L1MC1	LINE/L1	(0)	6333	5236	8

***Alu*Yb8**

Instruction

Green box: Primer information

Red letter: Target site duplication

Black letter: Insertion sequences

Green letter: Deleted sequenced by insertion-mediated deletion

>KSI_AluYb8_1

>Scaffold4423-2317559-2317843

CCAAAATGGGAGAGGTTGGGAGGTTGGTGAGGGTTGAAAAATTACCTGTTGGGTACAATGTTCAATATTTGGATGATGGGTACACTAGAAACTCAAACCTCATCACTATGCAATACACTCATGTAC AA
TACCTGCACATGTACCCCGTGGGTCTATAATTTTATGTTATTCTAGTTTATTTTTGAGACAGGGTCTCACTCTGTCACCCAGTCTAGAGTGCAATGTCC CTATCTTGGCTCAACCTCCTGGACTCAAG
TGATCCTCCTGCCTCAGCCTTCTAAGCAACTGGGGCCACAGGTGCACTCCACAATGCCTGGCAATTTTTTTGTATCTTTCGTAGAGATGGGGTTTCACTATGTTGCCCAGGCTGGTGTCAAACTC CTG
AGCTCAAGCAATCCGACTTGAAATGAATGCATCTTCAAACAAGGAGTATTTTTTAAAGTAATCATAATACCAAGAGATTATAAAGATTAAATTTTTAAA AAAGATTTAAAAGGAGCTTAGTAAGTTAA
ATTATGAGCACATTTGGGAGCGTTGACCTCCCAAAGTCTGAGATCACAGGTGTGAGCCACCACATTGGCCATGGATCCATAATTTAGAAAAATAAAAAAAAAATACACAGACACATGGATGTACT GTT
TTTCTCAGCGGAAAAAAAAAAAAaTGCATTCCTATGGACTGATGGAAGCCTTTAGATGTTGAGAAAAAAACATTGAAGAT CTCAAGATAACCCTCACCCATGACACTCTAACTCTGCAATTAAAATTCA
CCCTATAGCATTTTTCTTTTTTTCAAGTAGTTATTCACCTTCTTTGTCCTCAAATTCTTAAAAAAATAAGTATGAGAAAGCACAATTTTTCCACAAGAGAGTGAATGGCAGGATAGATGAGAAGC GAT
GTAATGAAATGCGGCTTCAAACAAGGAGTATTTTTAAAGTAATCATAATACCAAGAGATTATAAAGATTACATTTCAAAAATAGATTTAAAAGGAACTT AGTTggccgggcgcggtggctcacgcctg
taatcccagcactttgggaggccgaggcaggtggatcatgaggtcaggagatcgagaccatcctggctaacaaggtgaaaccccgtctctactaaaaatacaaaaaattagccgggcgcggtggc ggg
cgcctgtagtcccagctactcgggaggctgaggcaggagaatggcgtgaacccgggaagcggagcttgcagtgagccgagattgcgccactgcagtccg cagtccggcctgggcgacagagcgagact
ccgtAATTCTGAAGAGCATACATTATGAAATTAAACTCACCTGAAATTCATTACTTTCTTGAGTCCTCAAATGTCATCTTCCTAAAGACAGGGTTTGTTTCTTAT TAGATCTATGTAAATAACGTGGT
ATGTAGGCAGAGAGAAACTAAAACTGCCTTTTCATTTCTGGCTGCTGATGAGTTACCGCTGTGCTTGCATGACATTTACAAGCCACGTTAGTGAGGTCA CCCTCACTATACTAGATTCTCTTTTATAC
ATTATTGCCCTGGATAATTGTGTTGGAAATTGCCATCAAATGCCTTTTCTGCTATGACACTAAATAATTTGACATTATTAAAACAATGCTGGTGCCAAGCAACATGCATCAGAGTGTAGATTTTG GAG
TATGACAGACTGTATTCGCATACTGTTTTTTTGCTATTTACTCCTTGGTATGTAAGACTTTGGAGAATTTACCTAAAACTGATGCCTTATCTATGAAA TGCAGGTGACAATACAAACACATTGCACA
TAATAGAACAGGATGTATATGTTACATTTCTAACACAGTTTCTGGCAGGTAGCTCGGCTAGTGGTTAGGAGCAGACTCGGAGCTATGATACCTGGATTGTAATCTCAGTTCTGCCAGTTACTAGC TGT
ATGCAAAAGAAAACAGATACAATTTCTGCTTTAGATTTCTCATTTGTAAAGTAAGGAGGACAGTGTGACTTTTTTATGGTCATTATGAAGATTGCATGA GTAAATGTGTGTAAAATGCTTAGAACAGA
CAAATATTGATTAATTAATAAAAATTGTTTAGAAATTTAGTGACATATTTGGAGAGCCAAAAACAATTAAGAAGAAAGATTTAATTCTGTCGATTCATAATACAAAAATCAT gTATTAATATATGTTA
TATATTAATACATGCGAATTTATATAATTAGTATACATTCATATATATTCAATCTATATATACTCAGATATATATAGTATATGAGTATATATGAGTATA TGTATATGA

877	17.7	0.6	0.6	UnnamedSequence	1	159	(2125)	+	L1PA17	LINE/L1	5988	6146	(20)	1
1349	16.1	5.4	0.8	UnnamedSequence	175	398	(1886)	C	AluJb	SINE/Alu	(21)	291	58	2
261	19.6	3.9	0.0	UnnamedSequence	532	582	(1702)	C	Alu	SINE/Alu	(80)	53	1	3
2779	0.7	0.0	0.0	UnnamedSequence	1000	1284	(1000)	+	AluYb8	SINE/Alu	1	285	(33)	4
240	30.6	9.1	5.9	UnnamedSequence	1647	1778	(506)	+	MIRb	SINE/MIR	27	162	(106)	5
652	28.5	6.6	2.3	UnnamedSequence	1834	2079	(205)	+	MIR	SINE/MIR	1	257	(5)	6
33	24.1	2.2	6.8	UnnamedSequence	2145	2282	(2)	+	(AT)n	Simple_repeat	1	132	(0)	7

>hg19 chr11 25604140 25606152

CCAAAATGGGAGAGGTTGGGAGGTTGGTGAGGGTTGAAAAATTACCTGTTGGGTACAATGTTCAATATTTGGATGATGGGTACACTAGAAACTCAAACCTCATCACTATGCAATACACTCATGTAC AA
TACCTGCACATGTACCCCGTGGGTCTATAATTTTATGTTATTCTAGTTTATTTTTGAGACAGGGTCTCACTCTGTCACCCAGTCTAGAGTGCAATGTCCCTATCTTGGCTCAACCTCCTGGACTC AAG
TGATCCTCCTGCCTCAGCCTTCTAAGCAACTGGGGCCACAGGTGCACTCCACAATGCCTGGCAATTTTTTTGTATCTTTCGTAGAGATGGGGTTTCACTATGTTGCCCAGGCTGGTGTCAAACTCC TG
AGCTCAAGCAATCCGACTTGAAATGAATGCATCTTCAAACAAGGAGTATTTTTTAAAGTAATCATAATACCAAGAGATTATAAAGATTAAATTTTTAAAAAAGATTTAAAAGGAGCTTAGTAAGT TAA
ATTATGAGCACATTTGGGAGCGTTGACCTCCCAAAGTCTGAGATCACAGGTGTGAGCCACCACATTGGCCATGGATCCATAATTTAGAAAAATAAAAAAAAAATACACAGACACATGGATGTACTG TT
TTTCTCAGCGGAAAAAAAAAAAATGCATTCCTATGGACTGATGGAAGCCTTTAGATGTTGAGAAAAAAACATTGAAGAT CTCAAGATAACCCTCACCCATGACACTCTAACTCTGCAATTAAAATTCAC
CCTATAGCATTTTTCTTTTTTTCAAGTAGTTATTCACCTTCTTTGTCCTCAAATTCTTAAAAAAATAAGTATGAGAAAGCACAATTTTTCCACAAGAGAGTGAATGGCAGGATAGATGAGAAGCGA TG
TAATGAAATGCGGCTTCAAACAAGGAGTATTTTTAAAGTAATCATAATACCAAGAGATTATAAAGATTACATTTCAAAAATAGATTTAAAAGGAACTTAGT TaattatgagcacaaAATTCTGAAGAG

CATACATTATGAAATTAAACTCACCTGAAATTCATTACTTTCTTGAGTCCTCAAATGTCATCTTCCTAAAGACAGGGTTTGTTTCTTATTAGATCTATGTAAATAACGTGGTATGTAGGCAGAGAG AA
ACTAAAACTGCCTTTTCATTTCTGGCTGCTGATGAGTTACCGCTGTGCTTGCATGACATTTACAAGCCACGTTAGTGAGGTCACCCTCACTATACTAGATTCTCTTTTATACATTATTGCCCTGGATA
ATTGTGTTGGAAATTGCCATCAAATGCCTTTTCTGCTATGACACTAAATAATTTGACATTATTAAAACAATGCTGGTGCCAAGCAACATGCATCAGAGTGTAGATTTTGGAGTATGACAGACTGTA TT
CGCATACTGTTTTTTTGCTATTTTACTCCTTGGTATGTAAGACTTTGGAGAATTTACCTAAAACTGATGCCTTATCTATGAAATGCAGGTGACAATACAAACACATTGCACATAATAGAACAGGA TGT
ATATGTTACATTTCTAACACAGTTTCTGGCAGGTAGCTCGGCTAGTGGTTAGGAGCAGACTCGGAGCTATGATACCTGGATTGTAATCTCAGTTCTGCCAGTTACTAGCTGTATGCAAAAGAAAAC AG
ATACAATTTCTGCTTTAGATTTCTCATTTGTAAAGTAAGGAGGACAGTGTGACTTTTTTATGGTCATTATGAAGATTGCATGAGTAAATGTGTGTAAAATGCTTAGAACAGACAAATATTGATTA ATT
AATAAAAATTGTTTAGAAATTTAGTGACATATTTGGAGAGCCAAAAACAATTAAGAAGAAAGATTTAATTCTGTCGATTCATAATACAAAAATCAT aTATTAATATATGTTATATATTAATACATGCG
AATTTATATAATTAGTATACATTCATATATATTCAATCTATATATACTCAGATATATATAGTATATGAGTATATATGAGTATATGTATATG A

874	17.7	0.6	0.6	UnnamedSequence	1	159	(1853)	+	L1PA17	LINE/L1	5988	6146	(20)	1
1349	16.1	5.4	0.8	UnnamedSequence	175	398	(1614)	C	AluJb	SINE/Alu	(21)	291	58	2
255	19.6	3.9	0.0	UnnamedSequence	532	582	(1430)	C	Alu	SINE/Alu	(80)	53	1	3
242	30.6	9.1	5.9	UnnamedSequence	1375	1506	(506)	+	MIRb	SINE/MIR	27	162	(106)	4
659	28.5	6.6	2.3	UnnamedSequence	1562	1807	(205)	+	MIR	SINE/MIR	1	257	(5)	5
36	23.0	2.2	6.8	UnnamedSequence	1873	2010	(2)	+	(AT)n	Simple_repeat	1	132	(0)	6

>KSI_AluYb8_2

>Scaffold6439-7922999-7923207
TATTTTGTTGAGAATGTTTGCATCAATATTCATCAGAAATCTTGACCTGTAATTTTATTTTCTTATAATGTCCCTGGCTTTTATATCAGGGACTGGTCTCATAAAATGAGTTTGGAAGTGTTCCTC AC
TTTTTTGGGGGGAAAAGGTGATTAGAATTGGCATTAATTCTTATTTAAATATTTATATAATTTATCTTTAATTCATCTTAGCCTGAAATAATCTGTATTTTAGAGGTTTTTTTAAATTGCATATT CAA
TTTCTTTACTTATAAAAGGTCTGTTCTGATTTTCTTTTTCTTTATGGTTCAAACATAGTACATTTTATGTTTCTAGAAGTTATTTTATTTTTTGAGTTCTTAAACTTTTTGGTGCGTAATTGTCCA TA
GGATTCTTTTATGATCCTTTGTATTTCAGTGATATCAATTTTGACGTCTTCTCTTTCATTTGTAATTTTCTTGATTTGATTCCTGCTCATTAATTTCTTGACTATTCTAGCTAAAGTTTTGTCA ATT
TTATCTTTTTAAAAAACACTAATTTTGTTAATTTTTATGTTGTTTTCTGTTATTTACTTCATTAATCTCTCCTCTGTTTATTATTTCCTTTCTTCTCTTAACTGGGGACTCATTTTATTCTCTTAT TC
TAGTTCCTGTTGATGTGAAGTTAAGTTTTGGAGATCTTTTCATGCTCCTTAATGCAGGAATTTATCATTATAAACTTGTTGGCTGGGCGCTCACGCCTGTGAATACCAGCACTTTGGGAGGCTGA GGC
AGGCAGATCACGAGGTCAGGAGATTGAGACCGTACTAGCTAACATGGTGAAACCCCGTCTCTACTAAAAATACAAGAAATTAGCCAGGCGTGGTGGCAGGCTCCTGTAGTCCTAGCTACTCCAGAG AC
TGAGGCAGGAGAATGGCATGAACCCAGGAGGTGGAGCTCGCCGTGAGCTGAGATTGTGCCACTGCAATCCAGCCTGGGTGACAGAGAGAGACTCCATCTAAAAAAATAAAAATAAAAAAAATTAC AAA
CTTATCTCTTAGAATTACTTTAGCTGCATTTTGTGAGTTTTGATATGTTACATTTTCATTTTAGTTTGTCTCAAGACATAATTTGATTTCTCTTTTAATTTTTTTTGACCAATTGATTGTCCAAAA AT
GTGTTAATTTCCACATTTGGAATTTTTAAATTTTTCTACTATTATTGAGTTCTAATTTTCTATCTCTGCAATTAGAAAAAAAAAAATGCTTGATGTTATTTTAATCTTCTTAAGTTGTTATGTCTT GTT
TTGTGACCAAGTATACACTATGCCCCAGAGAATGTTCTTTTTACTCTACATAAGAATGTGGTATACTCTGCTGCATTTAGATAGAACGTTCTGTATGTCTGCTAGGCCCAGTTGGTCTACAGTGTTTT
CAAGTGTAATGTCTTTTTATTGATATTCTGTCTGAATAATCTATTGATTATTAGGGTAATGAAGTATCCTACTATTGCTGTATTATTGTCTATTACTCTCTTCATTTCTGTTCATATTTGCTTTA TTT
ATTATTTATTTATTTATTTTTGAGACAGGGTCTTGCTCTATTACCTAGGCTGGAGTGCAGTGCACGATCTAGGCTCACTGCAACCTCCGCCTCCTGGGTTCAAGTGATTCTCATGCCTCAGCCTCC CA
AGTAGGTGGGATTACAGGTACATGCCACCACGCTGGCTCATTTTTTTGTAATTTTAGTGGAGATGGGTTTTTACCATGTTGACCAGGCTGGTCTTGAACTCCTGACCTCAAATGACCCATCTGCC TTG
GCACCCCAAAATGCTGGGATTACAGGTGTGAGCCTCTGTGCCTGGCCCAATTTGCTTTATTTTTTTGAGTGCTATAATAT TGGATGCATATATATTTATAGTTGTTTATACTGTTGATCAATTAACCT
CCTTTTTATTTTATAATCTTCCTCATCTCTTCTGATAGTTTTTGACTACAGTCTATTTCATCTTCTGTAAATATAGACCA Cactgcaagctccgcttcccgggttcacgccattctcctgcctcagcc
tcccaagtatctgggactacaggcgcccgccaccgcgcccggctaatttttgtatttttagtagagacggggtttcaccttgttagccaggatggtctcgatctcctgacctcatgatccacccg cc
tcggcctcccaaagtgctgggattacaggcgtgTCTTCTTTCTTGATTACCATTGGCATGGGATATCTTTTTGATATCCCTTCAATTTCAGCCTATGTCTGTCTTTAAATCTAAATGAGTCTCCTGTA
GGGAGTATAGAGACATATCTTATTTTATTATTCATTGAGGCACTTTCTGTTTTCTAACTAATTTATTTCATTTATATTTAAAGTAA TTATTGATAGGTAAGAACTTACTATTGTCAATTTACTAAATG
TTTTCTAAGTGTTTTGTAAATCATTTGCTCCTTTCTTCCTCCCTTGTGGTCTTTCTTTGTGATTTGATGATTTTCTGTATGCTTTAATTCATTTATCTTTATCTTTTGTGTATATACTTTAAGTTTAT
GCTTTTTGATTACTATGAGCCTACATAAAATACCTTGTAACAGTCTAATTTAAATTAATAATTTAACTTAATTGCATACAAAAACTCTGCCCTTTTACTTCTCCCTCAACACATTTTACATTATTG AT
ATGACAATTTACATCTTTATTATAGTGTGTATCCATTAACAAATTTTTGTAGTTATACTTACTTTAAATGTTTATCTTTTTATTTTTTATTTATGCTAGAGTTAAAAGTGATTTATGTACTACCA TTA
CAGTATTGCAATATTTTAAATTTGACTACATATGTACCTTTATTAGTGAGTTTGATAAATTAACAATTTTTATATTCTAATTAGATTCCTTTCGTTTCAACTTAAAGGACTTGTAGAATTTCTTGT AA
GGATGTCTAGAGGTGATGAATTTCAGTATTTGTTTGTCTGGGAAAGTATCTTTCCTTCATTCTGAAGAACAGTTTTGGATTGTATATTATTCTTTGTTGGCAGCTTTTTTAAATGTTGGCATTTT TAA
TGTAATGTCTCACTTTCTCCTGACCTACAAGGTTTCTGCTGATAAATCTTCTGATAACATTATGGAGGTTCCCTTTTATGTGATCAGTCTCTTTTGTCTTATGGCTTTTAATATTCTCTGTTTGTA TT
TGACTTTTGACATATTGATTACAACATGGCTTATAAGCACATTGAACTTTGATTATAACTTGCCTCATAATAACAATGAAAGAAGGTTGAACTTTTTTATGTTCAACATTATATGGAATTCTTTG GGC
TTAAGGTATCTGGATGTTTATTTCCCTCTATATTGGTAAATTTTATGTAATTATTTCATTATAAGCGTCTGCCTTTTTCTCTTTCTCTGATGCTACTGAGGTTCCCTTTATGTGTATGTTCGTCTG TT
TGATGATGTCCCATAAAGCCTTTAGGTGTTCTTCACTGTTTTTTATTCTTTCTCTTGTTTTTGTTTCTCTGACTGGATAGCTTCAAATAATCTACCTTCAACTTTTTCTGCTTGGTCAAGTCTGC TGT
TAAAGGTTTTCCTTGATTTTTTTAAAAAATCAGGCACTCTTATTCTTCAATTCTAAAATTCGTTTTGTTTTTCTTGCTCTATGTTGAACATTTTAATATGTTCATTTCTTTTAAAAATTTTATTTT AA
TAGATTATCGGTCTTCCTTAGAAGTTCACTGATCCCCTTGAAGACAATTATTTTGAGTTTTTTGTCAGGAAATTTACAAATTTTTCTTTGGCGTCAGACACTGGAGCTTTTTTTTTTTGTCTTTT TTT
TAACTTTTACTTAAGTTCAGGGGTACAAGTGCAGGTTTGTTACATAAAGGTAAACGTACCATGGGGGTTTCTTGTACAAGATTATTTAATCTCCCAGGTATTAAGCCTAGTACCCATTAGTTATTT TT
CCTAATCCTCTCCGTTCTCCCATCCTCCACACTCCAGAAGGCCCCATGTGTTGTTGCCCTCTATGTGTTCATGTGTTCTCATCATTCAGCTCCCACTTATAAGTAAGAACATGCAATAAACCATG CAC
TCACTGCTGTCTTTAGAACTGATGAAACAGTCACCATCAGTCTTTACTGACAGGCACAGGTAGAGATTCTGAGGTTCTGTTAGACCTTTTATATGAATTTATCCACTTTACT C

5431	21.9	4.5	2.9	TeRefseq	1	720	(3489)	C	L1M3c	LINE/L1	(2399)	3828	3086	1	
2172	11.1	2.0	0.3	TeRefseq	721	1017	(3192)	+	AluY	SINE/Alu	1	302	(9)	2	
5431	21.9	4.5	2.9	TeRefseq	1018	1529	(2680)	C	L1M3c	LINE/L1	(3142)	3085	2574	1	
2014	15.2	0.7	0.7	TeRefseq	1530	1839	(2370)	C	AluSz6	SINE/Alu	(2)	310	1	3	
5431	21.9	4.5	2.9	TeRefseq	1840	2000	(2209)	C	L1M3c	LINE/L1	(3654)	2573	2417	1	
1980	1.4	0.0	0.0	TeRefseq	2001	2209	(2000)	C	AluYb8	SINE/Alu	(92)	226	18	4	
5431	21.9	4.5	2.9	TeRefseq	2210	2420	(1789)	C	L1M3c	LINE/L1	(3811)	2416	2199	1	
1948	30.4	6.0	5.8	TeRefseq	2229	3826	(383)	C	L1M1	LINE/L1	(3919)	3725	2126	5	*
1689	8.0	2.4	1.2	TeRefseq	3834	4086	(123)	C	L1PA13	LINE/L1	(3)	6160	5905	6	

350	23.2	29.5	0.0	TeRefseq	4098	4209	(0)	C	L1M3c	LINE/L1	(5103)	1038	894	1

>Hg19 chr1:188830839-188834839

```
TATTTTGTTGAGAATGTTTGCATCAATATTCATCAGAAATCTTGACCTGTAATTTTATTTTCTTATAATGTCCCTGGCTTTTATATCAGGGACTGGTCTCATAAAATGAGTTTGGAAGTGTTCCTC AC
TTTTTTGGGGGGAAAAGGTGATTAGAATTGGCATTAATTCTTATTTAAATATTTATATAATTTATCTTTAATTCATCTTAGCCTGAAATAATCTGTATTTTAGAGGTTTTTTTAAATTGCATATT CAA
TTTCTTTACTTATAAAAGGTCTGTTCTGATTTTCTTTTTCTTTATGGTTCAAACATAGTACATTTTATGTTTCTAGAAGTTATTTTATTTTTTGAGTTCTTAAACTTTTTGGTGCGTAATTGTCCA TA
GGATTCTTTTATGATCCTTTGTATTTCAGTGATATCAATTTTGACGTCTTCTCTTTCATTTGTAATTTTCTTGATTTGATTCCTGCTCATTAATTTTCTTGACTATTCTAGCTAAAGTTTTGTCA ATT
TTATCTTTTTAAAAAACACTAATTTTGTTAATTTTTATGTTGTTTTCTGTTATTTACTTCATTAATCTCTCCTCTGTTTATTATTTCCTTTCTTCTCTTAACTGGGGACTCATTTTATTCTCTTAT TC
TAGTTCCTGTTGATGTGAAGTTAAGTTTTGGAGATCTTTTCATGCTCCTTAATGCAGGAATTTATCATTATAAACTTGTTGGCTGGGCGCTCACGCCTGTGAATACCAGCACTTTGGGAGGCTGA GGC
AGGCAGATCACGAGGTCAGGAGATTGAGACCGTACTAGCTAACATGGTGAAACCCCGTCTCTACTAAAAATACAAGAAATTAGCCAGGCGTGGTGGCAGGCTCCTGTAGTCCTAGCTACTCCAGAG AC
TGAGGCAGGAGAATGGCATGAACCCAGGAGGTGGAGCTCGCCGTGAGCTGAGATTGTGCCACTGCAATCCAGCCTGGGTGACAGAGAGAGACTCCATCTAAAAAAATAAAAATAAAAAAAATTAC AAA
CTTATCTCTTAGAATTACTTTAGCTGCATTTTGTGAGTTTTGATATGTTACATTTTCATTTTAGTTTGTCTCAAGACATAATTTGATTTCTCTTTTAATTTTTTTTGACCAATTGATTGTCCAAAA AT
GTGTTAATTTCCACATTTGGAATTTTTAAATTTTTCTACTATTATTGAGTTCTAATTTTCTATCTCTGCAATTAGAAAAAAAAAAATGCTTGATGTTATTTTAATCTTCTTAAGTTGTTATGTCTT GTT
TTGTGACCAAGTATACACTATGCCCCAGAGAATGTTCTTTTTACTCTACATAAGAATGTGGTATACTCTGCTGCATTTAGATAGAACGTTCTGTATGTCTGCTAGGCCCAGTTGGTCTACAGTGTTTT
CAAGTGTAATGTCTTTTTATTGATATTCTGTCTGAATAATCTATTGATTATTAGGGTAATGAAGTATCCTACTATTGCTGTATTATTGTCTATTACTCTCTTCATTTCTGTTCATATTTGCTTTA TTT
ATTATTTATTTATTTATTTTTGAGACAGGGTCTTGCTCTATTACCTAGGCTGGAGTGCAGTGCACGATCTAGGCTCACTGCAACCTCCGCCTCCTGGGTTCAAGTGATTCTCATGCCTCAGCCTCC CA
AGTAGGTGGGATTACAGGTACATGCCACCACGCTGGCTCATTTTTTTGTAATTTTAGTGGAGATGGGTTTTTACCATGTTGACCAGGCTGGTCTTGAACTCCTGACCTCAAATGACCCATCTGCC TTG
GCACCCCAAAATGCTGGGATTACAGGTGTGAGCCTCTGTGCCTGGCCCAATTTGCTTTATTTTTTTGAGTGCTATAATATTGGATGCATATATATTTATAGTTGTTTATACTGTTGATCAATTAAC CT
CCTTTTTATTTTATAATCTTCCTCATCTCTTCTGATAGTTTTTGACTACAGTCTATTTCATCTTCTGTAAATATAGACCA CTCTTCTTTCTTGATTACCATTGGCATGGGATATCTTTTTGATATCCC
TTCAATTTCAGCCTATGTCTGTCTTTAAATCTAAATGAGTCTCCTGTAGGGAGTATAGAGACATATCTTATTTTATTATTCATTGAGGCACTTTCTGTTTTCTAACTAATTTATTTCATTTATATT TA
AAGTAATTATTGATAGGTAAGAACTTACTATTGTCAATTTACTAAATGTTTTCTAAGTGTTTTGTAAATCATTTGCTCCTTT CTTCCTCCCTTGTGGTCTTTCTTTGTGATTTGATGATTTTCTGTAT
GCTTTAATTCATTTATCTTTATCTTTTGTGTATATACTTTAAGTTTATGCTTTTTGATTACTATGAGCCTACATAAAATACCTTGTAACAGTCTAATTTAAATTAATAATTTAACTTAATTGCATA CA
AAAACTCTGCCCTTTTACTTCTCCCTCAACACATTTTACATTATTGATATGACAATTTACATCTTTATTATAGTGTGTATCCATTAACAAATTTTTGTAGTTATACTTACTTTAAATGTTTATCT TTT
TATTTTTTATTTATGCTAGAGTTAAAAGTGATTTATGTACTACCATTACAGTATTGCAATATTTTAAATTTGACTACATATGTACCTTTATTAGTGAGTTTGATAAATTAACAATTTTTATATTCT AA
TTAGATTCCTTTCGTTTCAACTTAAAGGACTTGTAGAATTTCTTGTAAGGATGTCTAGAGGTGATGAATTTCAGTATTTGTTTGTCTGGGAAAGTATCTTTCCTTCATTCTGAAGAACAGTTTTG GAT
TGTATATTATTCTTTGTTGGCAGCTTTTTTAAATGTTGGCATTTTTAATGTAATGTCTCACTTTCTCCTGACCTACAAGGTTTCTGCTGATAAATCTTCTGATAACATTATGGAGGTTCCCTTTTA TG
TGATCAGTCTCTTTTGTCTTATGGCTTTTAATATTCTCTGTTTGTATTTGACTTTTGACATATTGATTACAACATGGCTTATAAGCACATTGAACTTTGATTATAACTTGCCTCATAATAACAAT GAA
AGAAGGTTGAACTTTTTTATGTTCAACATTATATGGAATTCTTTGGGCTTAAGGTATCTGGATGTTTATTTCCCTCTATATTGGTAAATTTTATGTAATTATTTCATTATAAGCGTCTGCCTTTTT CT
CTTTCTCTGATGCTACTGAGGTTCCCTTTATGTGTATGTTCGTCTGTTTGATGATGTCCCATAAAGCCTTTAGGTGTTCTTCACTGTTTTTTATTCTTTCTCTTGTTTTTGTTTCTCTGACTGGA TAG
CTTCAAATAATCTACCTTCAACTTTTTCTGCTTGGTCAAGTCTGCTGTTAAAGGTTTTCCTTGATTTTTTTAAAAAATCAGGCACTCTTATTCTTCAATTCTAAAATTCGTTTTGTTTTTCTTGCT CT
ATGTTGAACATTTTAATATGTTCATTTCTTTTAAAAATTTTATTTTAATAGATTATCGGTCTTCCTTAGAAGTTCACTGATCCCCTTGAAGACAATTATTTTGAGTTTTTTGTCAGGAAATTTAC AAA
TTTTTCTTTGGCGTCAGACACTGGAGCTTTTTTTTTTTGTCTTTTTTTTAACTTTTACTTAAGTTCAGGGGTACAAGTGCAGGTTTGTTACATAAAGGTAAACGTACCATGGGGGTTTCTTGTACA AG
ATTATTTAATCTCCCAGGTATTAAGCCTAGTACCCATTAGTTATTTTTCCTAATCCTCTCCGTTCTCCCATCCTCCACACTCCAGAAGGCCCCATGTGTTGTTGCCCTCTATGTGTTCATGTGTT CTC
ATCATTCAGCTCCCACTTATAAGTAAGAACATGCAATAAACCATGCACTCACTGCTGTCTTTAGAACTGATGAAACAGTCACCATCAGTCTTTACTGACAGGCACAGGTAGAGATTCTGAGGTTCT GT
TAGACCTTTTATATGAATTTATCCACTTTACTC
```

5980	21.8	4.2	2.8	Hg19seq	1	820	(3381)	C	L1M3c	LINE/L1	(2299)	3928	308 6	1	
2172	11.1	2.0	0.3	Hg19seq	821	1117	(3084)	+	AluY	SINE/Alu	1	302	(9)	2	
5980	21.8	4.2	2.8	Hg19seq	1118	1629	(2572)	C	L1M3c	LINE/L1	(3142)	3085	2574	1	
2014	15.2	0.7	0.7	Hg19seq	1630	1939	(2262)	C	AluSz6	SINE/Alu	(2)	310	1	3	
5980	21.8	4.2	2.8	Hg19seq	1940	2312	(1889)	C	L1M3c	LINE/L1	(3654)	2573	2198	1	
1948	30.4	6.0	5.8	Hg19seq	2121	3718	(483)	C	L1M1	LINE/L1	(3919)	3725	2126	4	*
1689	8.0	2.4	1.2	Hg19seq	3726	3978	(223)	C	L1PA13	LINE/L1	(3)	6160	5905	5	
545	24.1	21.1	1.8	Hg19seq	3990	4179	(22)	C	L1M3c	LINE/L1	(5103)	1038	813	1	

>KSI_AluYb8_3

>Scaffold5076-4464479-4464808
TAAATATCCATCAGTTGAGGAAGGGTTAAACACCCTGCAGTGGTCTGAGGCTCCCCATCTTTTGAAGCACAGTCTGCACACCTCCATCACTGATGTTGAACCTGCTAGGTGCTCACTCTTCCAAGC AT
CCCTTGCAGCTAGGGGGTGGTCACGTGACCCAGGTGCCACCGATCAGACGTGCTCACTGAGACCTGGAAGCAGGGATAGTTCAGGGGCAGCAGAGAGGGCCCACCGATCCAGTGTTGTGTGTGAT TTG
GGGTACTGTCCCTGCTGACCTTAAGCCTGGTTCTGCAATCCTCCCAGGCTCCTCAAATCCCTTATTTTATTTTGAACTTTCACTGAAGTATAGCACACATAGAGAAAAATGCATAGGTCGTAAGCG AT
AACTTCATGAGCCTTCATAAGTGAGTACACTTGTGTAACCAGCCCTTCATCAAGAAACAGAATATTCCAGTTCCCTTAAGCTTCCCTTAATGCCCTCTTCCAGTCACGACTTCCCCGAGGCTAAC CAA
GGCGAGTTGTGTCTATTTCTAGTGCTTTATATAAATGGAATCAAATGATGTGTGCTTGTTTGTGTCTGGCCTCTTTCCCTTAATGTTCAAGTCTTTGAGATTCACCCATGCTGTTGAGTGTGGTTG CA
GATCcTTCATTCTCATTGTTAGGCAAAATTCCATAGTTGGAGCAGTGTCTGAGTGTGTCTCTGTTCTTTCATTCATTCTTCTACTGATGGACATTTGGGTACTTTCTAGTTAGGGGAGATTTATGAAT
AGaGCTGCTATGAATATTTATATGTGTCCTTCTTGGTGAAAATCTGTCTGCACTACTATTG AGTGTAGTCCTCTCATTGGGTCTTAcGGCAAGTGTATGTTTCACCTTATTAAGTTGCCAGTGTCTTC
TGAAGTGCTTGCACCATTTTGCATTTCTACCAGTCATGTATGAGGGTTTTTTCTAAATGCTCCGTATTTTCATCCACTCTTGGTATTTTCCATATTTTTCATTTTAGCCATTCTAGTGGGTGTAC AGT
GGTTATCTCATCAAAGTTTTCATTTGCATTTCCCTGCTGAGCAAGGATGTTGATCACCTCTGTGTGTTTATTGATCCTTTGGGTATCCCCTTTTGTGAAATGCCTACTCGAGTCTTGGCCATTTTT GT
TTATTGGATTGATTGTTCTTCCCCTCATAAAGTACTTTGAATAGACT GCTTCTCTGTTTAAATTACTGTATCAGCCAGGGCTTTCCAGAGAAACAAAACCTAACAGGATGCGTGTGTGTATATCTATT
TATATTTAATTCAATTTTTAAAGGGATTGACTCATGCAATTGTAGAAGCTGGCAAATGTGAAATTTGTAGGGCAAGCCGGCAGACTGGGGACT CAGGCAAGACTTCTATGATACACACTTAAGGCAGA
ATTCCTTCTACCAAAAACTTGGATTTTGCTCTCAAGGCCTTGAACTGATTGAATGAGGCCCACACACATTATCGAGCCCATCTACAAAATATCTTTTATTTTTATTTGTATTTATTTATTTATTT TTT
TGAGATGGAGTCTCACTCTGTTGCCCAGGTTGGAATGCAGTGGTGCAATCTCAGCTCCCTGTAACCCCCACTTCCCGGGTTCAAGTGATTCTCATGCCTCAGCCTCCCGAGTAG CTGGGATTACAGGC
GTCCCCCACCATGCCGGGCTAATTTTTTGGTGTTTTTAGTAGAGATGGGGTTTCGCCATGTTGGCCAGGCTGGTCTCGACTTCCTGACTCAAGTGATCTGCCTTCCTTGACCTCCCAAAGTGTTG AGA
TTACAGGTGTGAGCCACCATGCGTGGCTCAGAATATCTTTTTTTTTTTTTTTTTTTTT TGAGACAGAGTCTTGCTGGGTTGCCCAGGTTGGAA TGCAGTGGTGCAATCTCAGCTCACTGCAACCTCC
GCCCCTCAGGTTCAAGTGATTCTCCTGCCTCAGCCTCCCAAGTAGCTGGGACTACAGGTGCCTGCCACCATGCCTGGCAAATTT Ttttttgtatttttagtagagatggggtttttttttttgagac
ggagtctcgctctgtcgcccaggccggactgcggactgcagtggcgcaatctcggctcactgcaagctccgcttcccgggttcacgccattctcctgcctcagcctcccgagta gctgggactacagg
cgcccgccaccgcgcccggctaaTTTTTTGTATTTTTAGTAGAGACGGGGTTTCACctTGTTaGCCAGGaTGGTCTCGAtCTCCTGACCTCATGATCCaCCCGCCTcGGCCTCcCAAAGTGCTGGGAT
TACAGGcGTgAGCCACCgcgcccggccCACAGAATATCTTTATAGCAACATCTAGACTCATGTTTGACCAAACAACTGGACACTGTAACCTGGTCAAGTTGACACGTAAAATTTAACTACCACAATTA
CCCAGAGCTGTTTATGTTGCTCTCAACTCTTAGCTCTATCTAATCTACTGTGAACATACATGCACACTCACACACATGCACACACTACCAACAACAGTGGTAGACATTTCATTGTAAGTGCACAGAAG
TAAATATGGAAGGATGCTAAACTCATTACATGACCCCCTTGGGGAATGTCCAGGATGGACATCAAAGGGGATTTTAACTACAAGCTGTAACTGTAAAAAATATTTTTAAGAATATAATAATGGGT TAA
TTGTATAATTAAAATTAATATTTAAGTGGCAAAACTTTT AAAAGAAAACCAAAGGAGTCGTTAAGTCCCCATTATTCATTCAGCAAATATTTACTGAGCACCTACTTTGCAATAGTTGTTGGAGACAC
TGGATAAGACAGATCCAGTCTCTATCTTGGTGGAGCTGACATCATAGTCGAGAAGACAGGCAATAAAGAAGCAAACAGGGAAATAGCAGATGATTTCAGAAAGTGTTCAGGAGTATTCTGAAAGC AAG
CAAGCCATGTGCAGGGGCACAGTATGAATGGGGACAGGGTTATGTTGGTGTGGGAAGTCAGGGAAGGCCTCCCTGCAGTGATATTTGAGCTGAGCCCTGAAAGAGGAGAAGGAGCTGGAAGAGCACAC
TGAAAGAGGACAAGGAGCTGGAAGAGTGGGGACCAACTCGGTACATGCAAAGGTCCTGTATTAGTCCATTCTCACACTGCTAATAAAGACATACCCAAGATTTGGTAATTTACAAAGAAAAAGAA CTT
TAATGGACTTACAGTTCCACTTATAGGTCTGGGGAGGCC TCACAATCATGGCAGAAGGTGAAGGAGGAGAAAAGGCACGTCTTACATGGTGGCAGGCAAGAGATCATTGCAGGGGAACTGCCCTTTAT
AAAACCATCAGCTCTCTTGAGACTTATTCACTATCATGAGAACCGAATGGGAAAGACCCAACCCCATGATTCAATTACCTCCCACGGAATCCCTCCCACTACACATGGGGATTATGGGAGCTACA AAT
CAAGATGAGATATGGATGGGGACACAGCCAAATCATATC AGGTCCTGAGGCAGGAATGGACCAGATGAGCTGGGATGAGAACCCAAGCAAGCTTCTGACTTGGAAACCGAGGCTCAGAGAGGGATACA
CACAACTCAAGGCCACAGCACTGGACGTGGTATAACTGAACGAGAATCCAGCTGTGTGCTGGTGCAGAGCTGCCTGTACAGGAAGGAGGCCTCTGAGCTGCTCAAAGGGCTCTATCTGCACTCCC CCA
CAGAAATCCCCTTATGATCAGAGCCAAACATTGGAGACC CAGCCCCCAGGCTTGTGCCAGCCTTGCCCTGCCAGCCAAGGCCAGTATCCCCACTGAGGGGCATCTGAGGGCTCTGTACACTTTGCCTC
TGAGTTGAGGATCAATAGAAGCTACCACTGCTGTTTTACCTAATTATATAACAGTGGCCAGGGAGGAAGGTGTCAGGGATAGACGCTTTACCGGCACCATCCCATCAAATCCTGCTAAGAACTCC ATG
AAGGGGTATTATTATTCCCATTTTGCAGTTGGGAACACT GAGACTCAGAGAGATCAAATGCCTTGACTTCAGAATCTGTATTTTTTTTTTTTTTT TGAGACAGGGTCTGGCTCTGTTGCCCAGGCTGGAG
TGCAGTGACATGATCTCAGCTCACTGCAATCTCTACCTCCTGGGCTCAAGCCATCCTCCCACCTCAGCCTCCTGAGTAGCTGGGATTACAGATACATACCACCTAACCAGGCTAATTTTTGCTTT TTT
TTTTTTTTTTTTTTTTTTttGTGGAGAGGGTGTTTCTCCATGTTGCCCAGGCTGGTCTCAAACTCCTGAGCTCAAGAGATCTGCCTGCCTC gGCCTCCCAAAGTGCTA

401	26.8	1.7	10.6	TeRefseq	27	201	(4130)	+	MLT1N2	LTR/ERVL-MaLR	41	201	(361)	1
282	25.5	8.5	0.0	TeRefseq	210	303	(4028)	+	MLT1N2	LTR/ERVL-MaLR	358	459	(103)	1
2739	25.6	5.7	2.7	TeRefseq	322	1192	(3139)	C	L1ME3A	LINE/L1	(0)	6173	5279	2
1205	18.5	16.4	4.8	TeRefseq	1219	1503	(2828)	C	MLT2C1	LTR/ERVL	(0)	397	84	3
2095	13.8	0.3	0.6	TeRefseq	1508	1819	(2512)	C	AluSz	SINE/Alu	(0)	312	2	4
1477	10.8	0.0	2.0	TeRefseq	1830	2032	(2299)	C	AluSx3	SINE/Alu	(7)	304	106	5
2853	0.3	0.0	0.0	TeRefseq	2033	2331	(2000)	C	AluYb8	SINE/Alu	(19)	299	1	6
583	16.1	0.0	1.1	TeRefseq	2335	2428	(1903)	C	MLT2C1	LTR/ERVL	(371)	93	1	3

12	22.7	0.0	0.0	TeRefseq	2486	2517	(1814)	+	(ACAC)n	Simple_repeat	1	32	(0)	7
243	22.2	19.5	4.1	TeRefseq	2545	2713	(1618)	+	L1ME3G	LINE/L1	5982	6175	(2)	8
696	30.8	8.8	6.7	TeRefseq	2760	3128	(1203)	C	L2a	LINE/L2	(2)	3424	3037	9
2541	10.5	0.3	1.4	TeRefseq	3129	3496	(835)	C	THE1B	LTR/ERVL-MaLR	(0)	364	1	10
696	30.8	8.8	6.7	TeRefseq	3497	3579	(752)	C	L2a	LINE/L2	(390)	3036	2963	9
529	26.3	0.9	0.0	TeRefseq	3920	4033	(298)	C	MIRb	SINE/MIR	(51)	217	103	11
1679	15.2	2.7	0.0	TeRefseq	4049	4218	(113)	C	AluSz	SINE/Alu	(16)	296	127	12
26	0.0	0.0	0.0	TeRefseq	4219	4243	(88)	+	(T)n	Simple_repeat	1	25	(0)	13
1679	15.2	2.7	0.0	TeRefseq	4244	4330	(1)	C	AluSz	SINE/Alu	(193)	119	33	12

>Hg19 chr20:48941796-48945928
TAAATATCCATCAGTTGAGGAAGGGTTAAACACCCTGCAGTGGTCTGAGGCTCCCCATCTTTTGAAGCACAGTCTGCACACCTCCATCACTGATGTTGAACCTGCTAGGTGCTCACTCTTCCAAGC AT
CCCTTGCAGCTAGGGGGTGGTCACGTGACCCAGGT GCCACCGATCAGACGTGCTCACTGAGACCTGGAAGCAGGGATAGTTCAGGGGCAGCAGAGAGGGCCCACCGATCCAGTGTTGTGTGTGATTTG
GGGTACTGTCCCTGCTGACCTTAAGCCTGGTTCTGCAATCCTCCCAGGCTCCTCAAATCCCTTATTTTATTTTGAACTTTCACTGAAGTATAGCACACATAGAGAAAAATGCATAGGTCGTAAGC GAT
AACTTCATGAGCCTTCATAAGTGAGTACACTTGTG TAACCAGCCCTTCATCAAGAAACAGAATATTCCAGTTCCCTTAAGCTTCCCTTAATGCCCTCTTCCAGTCACGACTTCCCCGAGGCTAACCAA
GGCGAGTTGTGTCTATTTCTAGTGCTTTATATAAATGGAATCAAATGATGTGTGCTTGTTTGTGTCTGGCCTCTTTCCCTTAATGTTCAAGTCTTTGAGATTCACCCATGCTGTTGAGTGTGGTT GCA
GATCtTTCATTCTCATTGTTAGGCAAAATTCCATAGTTGGAGCAGTGTCTGAGTGTGTCTCTGTTCTTTCATTCATTCTTCTACTGATGGACATTTGGGTACTTTCTAGTTAGGGGAGATTTATGAAT
AGgGCTGCTATGAATATTTATATGTGTCCTTCTTGGTGAAAATCTGTCTGCACTACTATTGAGTGTAGTCCTCTCATTGGGTCTTA tGGCAAGTGTATGTTTCACCTTATTAAGTTGCCAGTGTCTTC
TGAAGTGCTTGCACCATTTTGCATTTCTACCAGTCATGTATGAGGGTTTTTTCTAAATGCTCCGTATTTTCATCCACTCTTGGTATTTTCCATATTTTTCATTTTAGCCATTCTAGTGGGTGTACAGT
GGTTATCTCATCAAAGTTTTCATTTGCATTTCCCTGCTGAGCAAGGATGTTGATCACCTCTGTGTGTTTATTGATCCTTTGGGTATCCCCTTTTGTGAAATGCCTACTCGAGTCTTGGCCATTTT TGT
TTATTGGATTGATTGTTCTTCCCCTCATAAAGTAC TTTGAATAGACTGCTTCTCTGTTTAAATTACTGTATCAGCCAGGGCTTTCCAGAGAAACAAAACCTAACAGGATGCGTGTGTGTATATCTATT
TATATTTAATTCAATTTTTAAAGGGATTGACTCATGCAATTGTAGAAGCTGGCAAATGTGAAATTTGTAGGGCAAGCCGGCAGACTGGGGACT CAGGCAAGACTTCTATGATACACACTTAAGGCAGA
ATTCCTTCTACCAAAAACTTGGATTTTGCTCTCAAGGCCTTGAACTGATTGAATGAGGCCCACACACATTATCGAGCCCATCTACAAAATATCTTTTATTTTTATTTGTATTTATTTATTTATTT TTT
TGAGATGGAGTCTCACTCTGTTGCCCAGGTTGGAATGCAGTGGTGCAATCTCAGCTCCCTGTAACCCCCACTTCCCGGGTTCAAGTGATTCTCATGCCTCAGCCTCCCGAGTAG CTGGGATTACAGGC
GTCCCCCACCATGCCGGGCTAATTTTTTGGTGTTTTTAGTAGAGATGGGGTTTCGCCATGTTGGCCAGGCTGGTCTCGACTTCCTGACTCAAGTGATCTGCCTTCCTTGACCTCCCAAAGTGTTG AGA
TTACAGGTGTGAGCCACCATGCGTGGCTCAGAATATCTTTTTTTTTTTTTTTTTTTTT TtGAGACAGAGTCTTGCTGGGTTGCCCAGGTTGGAATGCAGTGGTGCAATCTCAG CTCACTGCAACCTC
CGCCCCTCAGGTTCAAGTGATTCTCCTGCCTCAGCCTCCCAAGTAGCTGGGACTACAGGTGCCTGCCACCATGCCTGGCAAATTT TTTTTTTGTATTTTTAGTAGAGAtGGGGTTTCACtaTGTTgGC
CAGGtTGGTCTCGAaCTCCTGACCTCATGATCCgCCCGCCTtGGCCTCtCAAAGTGCTGGGATTACAGGtGTaAGCCACCagcccagCACAGAATATCTTTATAGCAACATCTAGACTCATGTTTGAC
CAAACAACTGGACACTGTAACCTGGTCAAGTTGACACGTAAAATTTAACTACCACAATTACCCAGAGCTGTTTATGTTGCTCTCAACTCTTAGCTCTATCTAATCTACTGTGAACATACATGCACACT
CACACACATGCACACACTACCAACAACAGTGGTAGACATTTCATTGTAAGTGCACAGAAGTAAATATGGAAGGATGCTAAACTCATTACATGACCCCCTTGGGGAATGTCCAGG ATGGACATCAAAGG
GGATTTTAACTACAAGCTGTAACTGTAAAAAATATTTTTAAGAATATAATAATGGGTTAATTGTATAATTAAAATTAATATTTAAGTGGCAAAACTTTTAAAAGAAAACCAAAGGAGTCGTTAAG TCC
CCATTATTCATTCAGCAAATATTTACTGAGCACCTACTTTGCAATAGTTGTTGGAGACACTGGATAAGACAGATCCAGTCTCTATCTTGGTGGAGCTGACATCATAGTCGAGAA GACAGGCAATAAAG
AAGCAAACAGGGAAATAGCAGATGATTTCAGAAAGTGTTCAGGAGTATTCTGAAAGCAAGCAAGCCATGTGCAGGGGCACAGTATGAATGGGGACAGGGTTATGTTGGTGTGGGAAGTCAGGGAA GGC
CTCCCTGCAGTGATATTTGAGCTGAGCCCTGAAAGAGGAGAAGGAGCTGGAAGAGCACACTGAAAGAGGACAAGGAGCTGGAAGAGTGGGGACCAACTCGGTACATGCAAAGGT CCTGTATTAGTCCA
TTCTCACACTGCTAATAAAGACATACCCAAGATTTGGTAATTTACAAAGAAAAAGAACTTTAATGGACTTACAGTTCCACTTATAGGTCTGGGGAGGCCTCACAATCATGGCAGAAGGTGAAGGA GGA
GAAAAGGCACGTCTTACATGGTGGCAGGCAAGAGATCATTGCAGGGGAACTGCCCTTTATAAAACCATCAGCTCTCTTGAGACTTATTCACTATCATGAGAACCGAATGGGAAA GACCCAACCCCATG
ATTCAATTACCTCCCACGGAATCCCTCCCACTACACATGGGGATTATGGGAGCTACAAATCAAGATGAGATATGGATGGGGACACAGCCAAATCATATCAGGTCCTGAGGCAGGAATGGACCAGA TGA
GCTGGGATGAGAACCCAAGCAAGCTTCTGACTTGGAAACCGAGGCTCAGAGAGGGATACACACAACTCAAGGCCACAGCACTGGACGTGGTATAACTGAACGAGAATCCAGCTG TGTGCTGGTGCAGA
GCTGCCTGTACAGGAAGGAGGCCTCTGAGCTGCTCAAAGGGCTCTATCTGCACTCCCCCACAGAAATCCCCTTATGATCAGAGCCAAACATTGGAGACCCAGCCCCCAGGCTTGTGCCAGCCTTG CCC
TGCCAGCCAAGGCCAGTATCCCCACTGAGGGGCATCTGAGGGCTCTGTACACTTTGCCTCTGAGTTGAGGATCAATAGAAGCTACCACTGCTGTTTTACCTAATTATATAACAG TGGCCAGGGAGGAA
GGTGTCAGGGATAGACGCTTTACCGGCACCATCCCATCAAATCCTGCTAAGAACTCCATGAAGGGGTATTATTATTCCCATTTTGCAGTTGGGAACACTGAGACTCAGAGAGATCAAATGCCTTG ACT
TCAGAATCTGTATTTTTTTTTTTTTTTtGAGACAGGGTCTGGCTCTGTTGCCCAGGCTGGAGTGCAGTGACATGATCTCAGCTCACTGCAATCTCTACCTCCTGGGCTCAAGCCA TCCTCCCACCTCAG
CCTCCTGAGTAGCTGGGATTACAGATACATACCACCTAACCAGGCTAATTTTGCTTTTTTTTTTTTTTTTTTTTTT TGTGGAGAGGGTGTTTCTCCATGTTGCCCAGGCTGGTCTCAAACTCCTGAG
CTCAAGAGATCTGCCTGCCTCaGCCTCCCAAAGTGCTA

237	24.0	31.2	0.0	Hg19seq	30	125	(4209)	+	L1ME3G	LINE/L1	5637	5762	(415)	1

401	26.8	1.7	10.6	Hg19seq	127	301	(4033)	+	MLT1N2	LTR/ERVL-MaLR	41	201	(361)	2
282	25.5	8.5	0.0	Hg19seq	310	403	(3931)	+	MLT1N2	LTR/ERVL-MaLR	358	459	(103)	2
2734	25.6	5.7	2.7	Hg19seq	422	1292	(3042)	C	L1ME3A	LINE/L1	(0)	6173	5279	3
1520	20.2	5.0	5.0	Hg19seq	1319	1607	(2727)	C	MLT2C1	LTR/ERVL	(0)	397	107	4
2095	13.8	0.3	0.6	Hg19seq	1608	1919	(2415)	C	AluSz	SINE/Alu	(0)	312	2	5
2315	10.1	0.3	1.3	Hg19seq	1930	2236	(2098)	C	AluSx3	SINE/Alu	(6)	305	2	6
1520	20.2	5.0	5.0	Hg19seq	2237	2332	(2002)	C	MLT2C1	LTR/ERVL	(301)	96	2	4
12	22.7	0.0	0.0	Hg19seq	2390	2421	(1913)	+	(ACAC)n	Simple_repeat	1	32	(0)	7
243	22.2	19.5	4.1	Hg19seq	2449	2617	(1717)	+	L1ME3G	LINE/L1	5982	6175	(2)	1
696	30.8	8.8	6.7	Hg19seq	2664	3032	(1302)	C	L2a	LINE/L2	(2)	3424	3037	8
2541	10.5	0.3	1.4	Hg19seq	3033	3400	(934)	C	THE1B	LTR/ERVL-MaLR	(0)	364	1	9
696	30.8	8.8	6.7	Hg19seq	3401	3483	(851)	C	L2a	LINE/L2	(390)	3036	2963	8
529	26.3	0.9	0.0	Hg19seq	3824	3937	(397)	C	MIRb	SINE/MIR	(51)	217	103	10
1841	15.3	0.0	5.4	Hg19seq	3953	4265	(69)	C	AluSz	SINE/Alu	(15)	297	1	11

>KSI_AluYb8_4

>Scaffold6756-4571276-4571588
AAAAATATAAAGAGTCTTCATACAAATGGGTGACAGACATGCAAAGGAAACTGTGGATGGACTCAACTTAGGCAGAGAAGAACAGAGTCATCCAGTTAAAAATTGTGGCAAATTTGACAAAC aCTATA
TAAAAACTTGGCAATAAAATACTGTTGAAATACATTAGGAATTGCAGGTGTAAGTCTAAGCACTTTGAATCAAATTGTAAAAATATAAACTACTTGGAAATCAGCACATTCTAGGTCTGTATATA AGA
TTCTTTGAGGCATTATCTAATTCTGTACATTTTTATCCAGTTCGAAATTTTCTGTTTGTATGTCTTTTGGCAATAAAATTTTAAGCAGTGGTTAAGGAAGACTATGTCCAAACAAAAGTTCAA CCAAA
GTTCCACATAATATGTTAATCACTGATGCACTAGGTCATAGGCTTTATTAATGAAAAAAAAATTTTTTTTGAGACAGAGTCTCACTCCCTCAGTCAGATTGAATTGCAATGGCATGACCTCGGCT CAT
TGCAACCTCCACCTCCTGGGTTCAAGAGATTCTtGTGCCTCAGCCTCCCTAGTAGCTGGGATTACAGGTGTGTGCCACCATGACCAGCTAATTTTTTTGTATTTTTAGTAGAGATGGAATTTC GGCAT
GTTGGCCAGGCTGGTCTCAAACTCCTGGCCTCATGTGATCCGCCTGCCTCGGCtTCCCAAAGTGCTGAAATTACAGGCATGAGCCACCTTGCCCAGTTGATCATAGGCTTTTTTTTTTTTTTTTTGAG
ACTGAGtcTCTCTCTCTCTTGCCCAGGCTaGAGTGCAGTGGCGTAATCTTGGCTCACTGCAACCTCCGCCTTCTGGGTTCAAGTGATTCTTCTGCCTCgGCCTCCTAAGTAGCTGGGACTACAGGTGC
GCGCTACCAtGCTCAGCTAATTTTTGTATTTTCAGTAGATGCgGGGTTTCGCCATGTTGGTAAGGCTGGTCTCCAGCTGCTGACCTCAGGTGTCCTCCCGCCTTGGCCTCCCAAAGTGCTGGGATTAC
AGGCATGAGCCACTaCACCTGGCCTGATCACAGGCTTTTTAATGGTGGGAATCAAATTCATCTTTGTGGCTTTAGCTTTAACAATTTGACTATGCAAAGATACACATAATAGGGACTTGGTAT GTGTT
TGCTAAATAACTCAATTTAGTGCTGGGAAAAGAATTACAGCCAACCTAATTTTATTGCAGATACAGATATTTCAGGGCTTAAAAAATTCTCCTTTGCAATATGAACCCAATCACACAATGCTAGA GCT
ACCTAGGGTAACATACTTAAATACATGGAAATATTTCATTTTGGGACATCAGCTGTTTTCTCATAGGAGCCTGATGATCTTATTCTTTCCAAAGAAACTATAGAAATGTAGGCCAGGCACAGT GGCTC
ATGCCTGTAATCCCAGCACTTTGGGAGGCCAAGGCGGGCAGATCACGAGATCGGGAGTTCCAGACCAGCCTGGCCAACATG aAGAAACCCCGTCTCTACTAAAAATACAAAAAATAGCTGGGCGTGGT
GATGGGTGCCTGTAATCCcAGCTACTTGGGAGGCTGAGGCAGGAGAATCATTTGAACCCACCAGGTGGAGGTTGCAGTGA GCCAAGATCCCACCATTGCACTCCAACCTGGGAAACAGGGCGAGGCTC
CATCTCAAAAAAAAAAAAAaGAAGAAGAAAAGGAAACGATAGAAATGACTACTTCCCTATCAATCTTTATAGTTACAGAGCTAAACACCATACAAAACATCATCTTCTTGAAATAC AAAATCAACAAT
GCCTGTCCCGAGTATCATAGTAAATCAAATGCATTACTTCTTCTTCTTCTTTTTTTTTTGAGATGAAGTTTC ACTCTTGTGGCCCAGGCTGGGTaCAATGACGTGATCTCGGCTCACTGAAACCTCCA
CCTCCCGGGTTCAAGCAATTCTCCTGCCTCAGCCTCCCAAGTAGCCGGGATTACAGACACCTGCCACCAAGCCCAGCTA ATTTTTTTTTttttttttttgagacggagtctcgctctgtcgcccaggc
cggactgcggactgcagtggcgcaatctcggctcactgcaagctccgcttcccgggttcacgccattctcctgcctcagcctcccgagtagctgg gactacaggcgcccgccaccgcgcccggctaat
tttttgtattttttttttAGTAGAGATGGGGTTTCACCATGTTGACCAGGCTGGTCTCAAACTCCTGACCTCAGCTGATCCGCCTGCCTCAGCCTCCCAAACTGCTGGGATTACAGGCGTGAGCCACCA
TGCCCGGCCGTGATGCATTTCTTAAATGTAGTTTTAACTATTCTGGGATGGGCACTGTGTAGCATGAAAAAGAAATGACCAATGCCTACTGGTGGTTTATGATCTTCCTTAGAGTTATTTGGAGGTGT
AAGAATAATAAATATTGAGCATTTAAAAAATATTTCTAAGACCTGTAAAACACTCATCTTTGATGTTCCCATTGCTTGCAACTAGAGCATTTCTAAAAA TTTTTAATAGCAAATCTTCCCCAATCTAC
TCCTGAGTTTTTAAAACATGTTTTGGAACTATGTAAAACATATATATAGTTTTATGTAAATAAATATAAATATGGTAT ATATATGTATGTTGTATGTATATAAATATATATATATATTTTAATCTTCT
GGTTTCTAAAAAACATATCCATCCCAGTGTTTGCAATATGACAAGGATATTTAATGCACTCCATAATAATACAATAGCTGGTATTTTATAGAGTACTTACTATGTGCCAACCACTATCTTTTTTTA TTT
TTTTATTTTTTTGAGATGGAGTCTCGCTCTGTTGCCCAGGCTGGAGTGCAGTGGCACAGTCTCAGCTCACTGCAACCT CCACCTCCctGGTTCAAGTGATTCTCCTGCCTCAACCTCCTGAGTAGCTG
GGATTACAGGCGCGCACCACCACGCCCAGCTAATTTTTGTATTTTTAGTAGAGACAGGGTTTCACCAC gTTGGTCAGGCTTGTCTCAAACTCCTGACCTGGTGATCCACTCCCCTC gGCCTCCCAAAG
TGCTGGAATTACAGGCGCGAGCCAGCCAACCACTATCTTATTGAGTCTGTGCAACAACCCATTATACATATGCACTGT ATAGTATTAATGAGGGCATAAAAGTTAGGACAGGTTAAGTGGATAAAAAT
GTTTTAAAATTGGACTTCtGTGATTGCACAATTTCTTTAGTAAAAAAAAATTaTATACTTGAATGAACTTTATGGTaTGTAAATTATATGTCAATAAAGCTGTTAAAAATTACACACCAGAGAGAAAG
AAAGAAGTGGGGCATCAAGTGACAGGCTTACTGTCATCTAGGAAATGTTTGGTCAACCAGAACTCA gGCATAGCTGGTCTAACTCTGGACCAGCACAGCcagcTGGTTaCTCCAGAGAAAGGAGCTAC
AtGGAAATAAAAAGATTCCATGAGGGAAACGAGGATCAGACTTTACTCAGCTGTCTCCTGCTTAGATGTTTATTTCTTCATGTCTGTATAATTCTTTAACTCCACATCCCGGCTATACATTGTTACA T
AAACATGTGCAAcCAATATTTTATATATTTCCATTTACAGATCAATTAAATATATAAGAATATATGGAAGTGTACGTT TAAAAAGTAAATGAACTATCATTATAAACTCTTATTATACCATTGTTCTT
CATGGAAATTGATTTCTTCCCTTCTTTGAGAGCACATAGTTTAGCATTTACTATTTCTCTAAAATCACAGAAGCAAAGCTGCAGCCATGAACCCACTTCATTTCTTTCATTTTGCCATTTCATCC AAT
CTATCCTAACCAAGTAAATCCAAATACTAAGGACTGCCACCACATGGCATTTAAAAATATTTTAGACTTCTTTCTCTC AaCTGCTGGGACTATTCACATTCTGAGCTGGCCTGGAAGTTCTGTATGAT
TGCCCAAACGGaCATAAATGGCTGGCAAGGAAATGAACGCCAAAGAGCTCACCCACTTCACTGTAAGAACTAAACTTGCTTTTATGTCTACAATAATGTCCCGAGTTCAAGAAGCAATTTCATTCCCT
AACCTAATGTGGAATGTTAGTGTCATCCTTCTTTCATGGACAGAAACCAGCCACCCCGATACACACACATACTCTCTA TACAAGTCCTAAGGCAGAAGCACTCAAAGGCTCAGATCCTTCTGCCTTAA
CTTCTTTGATCTTGCTAGAACCTGAACTGCAAAAAGAATTACCTTCCTTTTTTGTTGTTTTTGTTTGTTTGTTTCTTTGTTTGGAGAC A

2044	14.1	0.0	0.7	TeRefseq	447	736	(3577)	C	AluSz6	SINE/Alu	(22)	290	3	1
2190	12.0	0.3	0.7	TeRefseq	749	1048	(3265)	C	AluSx	SINE/Alu	(13)	299	1	2
2299	12.6	0.0	0.0	TeRefseq	1391	1700	(2613)	+	AluSg	SINE/Alu	1	310	(0)	3
1258	12.3	0.6	0.0	TeRefseq	1830	2000	(2313)	C	AluSq2	SINE/Alu	(8)	305	134	4
2644	4.5	0.0	1.9	TeRefseq	2001	2313	(2000)	C	AluYb8	SINE/Alu	(11)	307	1	5
36	19.4	1.3	1.3	TeRefseq	2600	2677	(1636)	+	(AT)n	Simple_repeat	1	78	(0)	6
299	27.4	15.4	6.8	TeRefseq	2753	2805	(1508)	C	MIRb	SINE/MIR	(0)	268	220	7
2344	8.9	0.0	0.0	TeRefseq	2806	3096	(1217)	C	AluSg	SINE/Alu	(7)	303	13	8

299	27.4	15.4	6.8	TeRefseq	3097	3191	(1122)	C	MIRb	SINE/MIR	(49)	219	108	7
340	19.3	14.9	0.0	TeRefseq	3192	3305	(1008)	+	L1MB7	LINE/L1	6045	6175	(9)	9
20	9.4	0.0	2.9	TeRefseq	4272	4307	(6)	+	(TTGT)n	Simple_repeat	1	35	(0)	10

```
>Hg19 chr10:26959081-26963213
ATACAAATGGGTGACAGACATGCAAAGGAAACTGTGGATGGACTCAACTTAGGCAGAGAAGAACAGAGTCATCCAGTTAAAAATTGTGGCAAATTTGACAAAC gCTATATAAAAACTTGGCAATAAAA
TACTGTTGAAATACATTAGGAATTGCAGGTGTAAGTCTAAGCACTTTGAATCAAATTGTAAAAATATAAACTACTTGGAAATCAGCACATTCTAGGTCTGTATATAAGATTCTTTGAGGCATTAT CTA
ATTCTGTACATTTTTATCCAGTTCGAAATTTTCTGTTTGTATGTCTTTTGGCAATAAAATTTTAAGCAGTGGTTAAGGAAGACTATGTCCAAACAAAAGTTCAA CCAAAGTTCCACATAATATGTTAA
TCACTGATGCACTAGGTCATAGGCTTTATTAATGAAAAAAAAATTTTTTTTGAGACAGAGTCTCACTCCCTCAGTCAGATTGAATTGCAATGGCATGACCTCGGCTCATTGCAACCTCCACCTCC TGG
GTTCAAGAGATTCTcGTGCCTCAGCCTCCCTAGTAGCTGGGATTACAGGTGTGTGCCACCATGACCAGCTAATTTTTTTGTATTTTTAGTAGAGATGGAATTTC GGCATGTTGGCCAGGCTGGTCTCA
AACTCCTGGCCTCATGTGATCCGCCTGCCTCGGCcTCCCAAAGTGCTGAAATTACAGGCATGAGCCACCTTGCCCAGTTGATCATAGGCttttTTTTTTTTTTTTTTTTTGAGACTGAGTCTCTCTCT
CTTGCCCAGGCTgGAGTGCAGTGGCGTAATCTTGGCTCACTGCAACCTCCGCCTTCTGGGTTCAAGTGATTCTTCTGCCTC aGCCTCCTAAGTAGCTGGGACTACAGGTGCGCGCTACCAaGCTCAGC
TAATTTTTGTATTTTCAGTAGATGCaGGGTTTCGCCATGTTGGTAAGGCTGGTCTCCAGCTGCTGACCTCAGGTGTCCTCCCGCCTTGGCCTCCCAAAGTGCTGGGATTACAGGCATGAGCCACT gCA
CCTGGCCTGATCACAGGCTTTTTAATGGTGGGAATCAAATTCATCTTTGTGGCTTTAGCTTTAACAATTTGACTATGCAAAGATACACATAATAGGGACTTGGT ATGTGTTTGCTAAATAACTCAATT
TAGTGCTGGGAAAAGAATTACAGCCAACCTAATTTTATTGCAGATACAGATATTTCAGGGCTTAAAAAATTCTCCTTTGCAATATGAACCCAATCACACAATGCTAGAGCTACCTAGGGTAACAT ACT
TAAATACATGGAAATATTTCATTTTGGGACATCAGCTGTTTTCTCATAGGAGCCTGATGATCTTATTCTTTCCAAAGAAACTATAGAAATGTAGGCCAGGCACA GTGGCTCATGCCTGTAATCCCAGC
ACTTTGGGAGGCCAAGGCGGGCAGATCACGAGATCGGGAGTTCCAGACCAGCCTGGCCAACATG gAGAAACCCCGTCTCTACTAAAAATACAAAAAATAGCTGGGCGTGGTGATGGGTGCCTGTAATC
CaAGCTACTTGGGAGGCTGAGGCAGGAGAATCATTTGAACCCACCAGGTGGAGGTTGCAGTGAGCCAAGATCCCACCATTGCACTCCAACCTGGGAAACAGGGC GAGGCTCCATCTCAAAAAAAAAAA
AAGAAGAAGAAAAGGAAACGATAGAAATGACTACTTCCCTATCAATCTTTATAGTTACAGAGCTAAACACCATACAAAACATCATCTTCTTGAAATAC AAAATCAACAATGCCTGTCCCGAGTATCAT
AGTAAATCAAATGCATTACTTCTTCTTCTTCTTTTTTTTTTGAGATGAAGTTTCACTCTTGTGGCCCAGGCTGGGT gCAATGACGTGATCTCGGCTCACTGAAACCTCCACCTCCCGGGTTCAAGCAA
TTCTCCTGCCTCAGCCTCCCAAGTAGCCGGGATTACAGACACCTGCCACCAAGCCCAGCTA ATTTTTTTattgTTAGTAGAGATGGGGTTTCACCATGTTGACCAGGCTGGTCTCAAACTCCTGACCT
CAGCTGATCCGCCTGCCTCAGCCTCCCAAACTGCTGGGATTACAGGCGTGAGCCACCATGCCCGGCCGTGATGCATTTCTTAAATGTAGTTTTAACTATTCT GGGATGGGCACTGTGTAGCATGAAAA
AGAAATGACCAATGCCTACTGGTGGTTTATGATCTTCCTTAGAGTTATTTGGAGGTGTAAGAATAATAAATATTGAGCATTTAAAAAATATTTCTAAGACCTGTAAAACACTCATCTTTGATGTT CCC
ATTGCTTGCAACTAGAGCATTTCTAAAAATTTTTAATAGCAAATCTTCCCCAATCTACTCCTGAGTTTTTAAAACATGTTTTGGAACTATGTAAAACATATATA TAGTTTTATGTAAATAAATATAAA
TATGGTATATATATGTATGTTGTATGTATATAAATATATATATATATATatTTTAATCTTCTGGTTTCTAAAAAACATATCCATCCCAGTGTTTGCAATATGACAAGGATATTTAATGCACTCCATAATA
ATACAATAGCTGGTATTTATAGAGTACTTACTATGTGCCAACCACTATCTTTTTTTATTTTTTTATTTTTTTGAGATGGAGTCTCGCTCTGTTGCCCAGGCTGGAGTGCAGTGGCACAGTCTCAG CTC
ACTGCAACCTCCACCTCCtgGGTTCAAGTGATTCTCCTGCCTCAACCTCCTGAGTAGCTGGGATTACAGGCGCGCACCACCACGCCCAGCTAATTTTTGTATTTTTAGTAGAGACAGGGTTTCACCAC
aTTGGTCAGGCTTGTCTCAAACTCCTGACCTGGTGATCCACTCCCCTC aGCCTCCCAAAGTGCTGGAATTACAGGCGCGAGCCAGCCAACCACTATCTTATTGAGTCTGTGCAACAACCCATTATACA
TATGCACTGTATAGTATTAATGAGGGCATAAAAGTTAGGACAGGTTAAGT GGATAAAAATGTTTTAAAATTGGACTTCcGTGATTGCACAATTTCTTTAGTAAAAAAAAATTcTATACTTGAATGAAC
TTTATGGTgTGTAAATTATATGTCAATAAAGCTGTTAAAAATTACACACCAGAGAGAAAGAAAGAAGTGGGGCATCAAGTGACAGGCTTACTGTCATCTAGGAAATGTTTGGTCAACCAGAACTCA aG
CATAGCTGGTCTAACTCTGGACCAGCACAGCTGGTTgCTCCAGAGAAAGGAGCTACAcGGAAATAAAAAGATTCCATGAGGGAAACGAGGATCAGACTTTACTCAGCTGTCTCCTGCTTAGATGTTTA
TTTCTTCATGTCTGTATAATTCTTTAACTCCACATCCCGGCTATACATTGTTACATAAACATGTGCAA tCAATATTTTATATATTTCCATTTACAGATCAATTAAATATATAAGAATATATGGAAGTG
TACGTTTAAAAGTAAATGAACTATCATTATAAACTCTTATTATACCATT GTTCTTCATGGAAATTGATTTCTTCCCTTCTTTGAGAGCACATAGTTTAGCATTTACTATTTCTCTAAAATCACAGAA
GCAAAGCTGCAGCCATGAACCCACTTCATTTCTTTCATTTTGCCATTTCATCCAATCTATCCTAACCAAGTAAATCCAAATACTAAGGACTGCCACCACATGGCATTTAAAAATATTTTAGACTT CTT
TCTCTCAgCTGCTGGGACTATTCACATTCTGAGCTGGCCTGGAAGTTCTG TATGATTGCCCAAACGGgCATAAATGGCTGGCAAGGAAATGAACGCCAAAGAGCTCACCCACTTCACTGTAAGAACTA
AACTTGCTTTTATGTCTACAATAATGTCCCGAGTTCAAGAAGCAATTTCATTCCCTAACCTAATGTGGAATGTTAGTGTCATCCTTCTTTCATGGACAGAAACCAGCCACCCCGATACACACACA TAC
TCTCTATACAAGTCCTAAGGCAGAAGCACTCAAAGGCTCAGATCCTTCTG CCTTAACTTCTTTGATCTTGCTAGAACCTGAACTGCAAAAAGAATTACCTTCCTTTTTTGTTGTTTTTGTTTGTTTGT
TTCTTTGTTTGGAGACA
```

373	11.9	0.0	0.0	Hg19seq	1	59	(4283)	+	Alu	SINE/Alu	242	300	(1)	1
2076	13.4	0.0	0.7	Hg19seq	557	846	(3496)	C	AluSz6	SINE/Alu	(22)	290	3	2
2265	11.6	0.3	0.0	Hg19seq	859	1160	(3182)	C	AluSx	SINE/Alu	(9)	303	1	3
2281	12.6	0.0	0.0	Hg19seq	1503	1811	(2531)	+	AluSg	SINE/Alu	1	309	(1)	4
2361	10.2	0.3	0.0	Hg19seq	1941	2244	(2098)	C	AluSq2	SINE/Alu	(8)	305	1	5
38	18.9	1.2	1.2	Hg19seq	2531	2610	(1732)	+	(AT)n	Simple_repeat	1	80	(0)	6
299	27.4	15.4	6.8	Hg19seq	2686	2738	(1604)	C	MIRb	SINE/MIR	(0)	268	220	7

2322	9.6	0.0	0.0	Hg19seq	2739	3029	(1313)	C	AluSg	SINE/Alu	(7)	303	13	8
299	27.4	15.4	6.8	Hg19seq	3030	3124	(1218)	C	MIRb	SINE/MIR	(49)	219	108	7
329	21.1	14.9	0.0	Hg19seq	3125	3238	(1104)	+	L1MB7	LINE/L1	6045	6175	(9)	9
889	15.2	0.0	0.0	Hg19seq	4211	4342	(0)	C	AluJb	SINE/Alu	(4)	308	177	10

>KSI_AluYb8_5

>Scaffold12434-4104190-4104493
GGCGACAAATTGAGACTCTGTCTCAGAAAAAAAAAAAAAAAGTTATTGGAACAACTGGTGAAATTTCAGTGAGGGCTGAGTATAGTGTGGTATCAGTGGCATATTTCCTTATTTTGATAAATATATT GT
GGTTATGTCAGAGAATATCCTGTCATAACCGCAATATAAGAGATACACAGTAACAAATTTATGGTAAAGGAGCATGATGTTTCCAAAGTACTCTCAAATGATTCCAGGAAAAACATAAGTAGACATAG
ATATATACGCCTGTATTATTTTTCTAGGGTTTACCAGCCCCCACAATTGCATGAGCCAATTTCTTTTTTTCTGTGTGTGTGTGACAAAGTTTCGCCCTTGTCACTCAAGGCTGGAGTGCAATGGC ACG
ATCTCGGCTGACTGCAACCTCCACCTCCCGAGTTCAAGTGATTCTTCTCCCTCAGCCTCCCAAGTAGCTGGGATTACAGGTGCCCACCACCACACCTGGCTAATTTTTTGTATTCTTAGTAGAGATGG
GGTTTCACCATGTTGGCCAGGCTGGTCTTGAACTCCTGACTTTCAGGTGATCCACCCGCCTTAGCCTCCCAAAGTGCTGGGATTACAGGCGTCAGCCACCGTGCCCGGCCTCTGTCTTTTTTTTT TTT
TTTTTTCGAGACAGGGTCTTGCTCTTTTGTCCAGGTTGCAGTGCAGTGGCACAATCATAGGTCACTGCAGCCTCGACCTCCTGGGCTCAGGTGATCCTCCTGCCTCTGCCTCCTAAGTAGCTAGGACT
ATAGGTGTGCTCCACCACACCTGGATAATTTTTTGTGTTTTGTAGAGATGGGGGTTTCACTATGTTACTTAGGCTGGTCTGGAACTCCTGGACTCCAGTGATCTTCCCACCATGGCCTCCCAAAA TGC
TAGGATTACAGGCATGAGCCATTGCTTCTGGCCCCAATTGCTTTCTTTTTCTTTCTTTCTTTTTTTTTTTTTTTTGAGGCAGAGTCTCACTTTGTCGCCCAGGCTGGAGTGCAGTGGTATGATCTCGGT
TCACTGCAACCTCTTCCTCCTGAGTTCAAGTGATTCTCCTGCCTCAGCCTCCCAAGTAGCTGGGACTACAGGTGCCTGCCACCATTCCCAGCTACTTTTTTGTATTTTTATTAAAGACGGGGTTT CAC
AATGTTGGTCAGCCTGGTCTCGAACTCTTTACCTCAAGTGATCTTCCCTCCTC aGCTTCCCAAAGTGCTGGGATTACAGGCGGGAGCCAACGCACCCGGCCGCTTTTACTATTCTTGCAACTTTTTTC
ATTTGACATTCCATCGAAATAAAAAGTTAAAAAAATTCTTTTTCTTCTGAGGGGGTGTTTTATCAAGGACACCTTTATCAAGGGTCCCGGGCCTAAAAATGTTAAGAACCCATGATTTACGGCTG AGG
AGGCAACTTCCATCCAAAATCCTAAAAGTCAAGTTCCTGTGACCTGGCTTCCTGAACCTCCCCGCTCCTGCGCCCCCTGGAGGTCAGGGCTAGAACAACACGGCATTCCTATAATGCTCCAAATCGCC
gCAAATCGACCTTGGAAATCCTGGGTGAGTAACGCAcACTGCGCACATGGCTTCAATTAGCGGAACgAAAACTTGCTGAACATCGTGTACCCGCGTGTCCATGGCCAAACACAAACTCCACCTTTGTA
AAAGTCTAGGTGAATTCCCAGCTGAATTCAGAAGCGTGACAGGAATGG GACTTTTCATTGGTGTCTTTTTCTTTTTTCTATTTTTAGAGTGGGGAGGAGTGTATTTCTGGATTGTTTAAATATTTACA
ATGAGCATGCATTTCCTTTGCAATTAAAAGAAAACCAGATTTTTTTTTTTTTTTTTT TttGAGGCAGAATGTTGCTCTGTCCCCAGGCTGGAGTGCAGTGGCGCGATCTCGGCTCACTGCAACCTCTG
CCTCCCGTGTTCAAGCAATTCTCCTGCCTCAGCCTCCTGAGTAGCTGGGACTACAAGTGCCCGCCACCACGCCCAGC TAAttttttttttttttttttgagacggagtctcgctctgtcgcccaggtcg
gactgcggactgcagtggcgcaatctcggctcactgcaagctccgcttcccgggttcacgccattctcctgcctcagcctcccgagtagctgggactacaggcgcccgccaccgcgcccggctaa tTT
TTTGTATTTTTAGTAGAGACGGGGTTTCACCATGTTAGCCAGGATAGTCTCAATCTCTTGAACTGGTGATCTGCCTGCCTCGGCTTCCCAAAGTGCTGGGATTACAGGCATGAGGACCGCACCCGGCC
GAAAACCAGATTTTTAAAAATTGAAGTTTGCTGTAGAAAAATACTGTG GGAGAAAGTTGTGGGTGGTGTTTTGTAGAGTCGCAGAAGGACATGGGAAAAAAAAATTGAGTGTGTTTCTGTAATCATCA
TAACAATCACACTCATTGTGCCGTAGGCTCCAAGGGAGGGCTCTtGAGCTGTGGTTACCAGATAAAATACAAGATTCTCAGGTAAAATGTGAAAAAAAAA AaTATTTTTGTGATCTTAACTCACTGCA
GTCTTGATCTCCTGGGCTGAAGTGATCCTCCTGCCTCAGCCTCCCAGGTAGCTGGGACCACAG aCACGTGCCACCATGCCCtGCTAATTTTTAATTTATTTTGTTGtGATGGGATCTCACTATGTTGC
CCAGGCTGCTCTCAAACTCCTGGCCTCAAGCGATCTTTCCTCGGTGTCCCCATGCCCTGGGATTACAGGTATGAAGCACCAGGCCCAACCCTGAAATTTAAAGTTAACTGGGCAGTCTG TAATGTGTT
TTTGTTTGTGTAGGTGTGTGTGCTAAATCAGGCAACCCCCTATTCTGAGCAGAGGAGTGCTTTGATCTGACTTAGATTTTAATAGGCTCCCTCTGGTTGCTGGTGGA GATGAGACTACGGAGGGGGG
CAGTGAAGATCCCAGAGAGGAGGCTGCTGTAATGGTCCAAGCAGAAGGTGATAGTGATTTGGATCAGGGTAACAGAGCCGAGGTGGTGAGAGG cTGACAGGTCATGCATCTATTTCGAAGGAAAGGCC
ATGTCCAGGCTCCTTAGCTCGGCATTCAAGGCTTCTCTCCACCACTGGACACACTTACTCTCTAGCTTCTCAGACCCTGTTGCAGATAGAATCATGATCCCCAATGA TGTCCACATCTTAATCCCCTG
AACCTGTGAACAGGTGACCTTCCATGGCAAAAGGGACTTTGCAGATAGGATGAAGTTAAAGATCTTTAGATGGGGGAGCGATTATCCTGGATTGGGTCTGGGTGGGCTCA aTGGAATCACAAGAGTCC
TTATATGAGGAAGATAGAAATTTGAGACATACGGTAGAGGAGGAGGTGATGAGACCACAGAGGCAGGGACGGGCGTGGTGCAACCACAAGCCAAGGAATTTCAGGAG CCACCTGAAGGTGGGAGAGGC
AAGGAACAGATTCTCCCCTGGAACCTCTGGAGGGAGTGGTGACATCTTGATTTTGGACTTCTGGCCTGCAGAACCATGCCAAAATAATTGTCTGGCTTTTTTTTTCTTTTTTTTTCCTTGAGGTG GAG
TCTTGCTTTGTCGCCCAGGCTGGAGTGCAGTGGTGCCATCTTGGCTCACTGCAACCTCCACCTCCCGGGTTCAAGTAATTCTTGTGCCTCAGCCTCCCGAGTAGCTG GGACTACAGGCATGCACCACC
ACACCCAGGTAATTTATGTATTTTTAGTAGAGATGGGGTTTTGCCATGTTGGCCAGGCTGGTCTTGAACTCTTGACCTCAATGATCTGCCCGCCTCAGCCTCCCAAAGTGCTGGGATTGCAGGCA TGA
GCCACTGCGCCCGGTCCTGGTGTTAAGCCCGCAGTTTGTGCTAATTGGTTACAGCAGCCTCGGGAAGGTAATCTAGGCTCCTTCCTTGCCCCAGGCTCTAGGAAGTA TCTTAGCCCCCAGCCCTGGGC
TGCAGACCTGTACCAGTCCATGGCCTGCTAGGAACCATGCAGCATCGCAGGAGGTGAGTGGCAGGTGAGTAAGTGAAGCTTCATCTGTATTTATAGCCACGCCCCTTCGCTCATATTACCGCCTG ATC
TGTCAGATCAGGGGCGGAATTACATTCTCATAAGAGCGTGAACCCAGGCCGGGCGCAGTGGCTCAAGCCTGTAATCCCAGCATTTTGGGAGGCTGAGGCGGGTGGAT CACGAGGTCAGGAGTTCGAGA
TCAGCCTGGCCAAGATGGTGAAACCACGTCTCTACTAAAAATACAAAAATTAGCCGGG cGCAGTGGTAGGCACCTGTAGT

234	12.5	0.0	0.0	TeRefseq	1	40	(4264)	+	Alu	SINE/Alu	258	297	(4)	1
625	23.7	4.6	5.1	TeRefseq	42	259	(4045)	+	L1MC4	LINE/L1	7702	7918	(124)	2
2260	10.8	0.0	1.0	TeRefseq	316	622	(3682)	C	AluSq2	SINE/Alu	(9)	304	1	3
2010	15.9	0.3	0.3	TeRefseq	629	929	(3375)	C	AluJb	SINE/Alu	(11)	301	1	4
2244	12.8	0.0	0.3	TeRefseq	942	1253	(3051)	C	AluSx1	SINE/Alu	(1)	311	1	5
230	20.6	3.2	0.0	TeRefseq	1764	1826	(2478)	+	L1ME4a	LINE/L1	6058	6122	(2)	6
1364	8.3	0.6	0.0	TeRefseq	1832	2000	(2304)	C	AluSg4	SINE/Alu	(9)	301	132	7
2603	5.3	0.3	0.0	TeRefseq	2001	2304	(2000)	C	AluYb8	SINE/Alu	(13)	305	1	8

1430	16.9	1.7	0.8	TeRefseq	2542	2778	(1526)	C	AluJr	SINE/Alu	(73)	239	1	9
772	28.4	3.7	0.0	TeRefseq	2859	3073	(1231)	C	L2	LINE/L2	(575)	2844	2622	10
1579	21.8	12.8	3.4	TeRefseq	3150	3552	(752)	+	MLT1D	LTR/ERVL-MaLR	1	446	(59)	11
2363	10.9	0.3	0.0	TeRefseq	3553	3856	(448)	C	AluSz6	SINE/Alu	(7)	305	1	12
1579	21.8	12.8	3.4	TeRefseq	3857	3912	(392)	+	MLT1D	LTR/ERVL-MaLR	447	502	(3)	11
1050	17.6	4.8	0.0	TeRefseq	3954	4141	(163)	+	MER1A	DNA/hAT-Charlie	8	204	(325)	13
1375	6.2	0.0	0.0	TeRefseq	4143	4302	(2)	+	AluSg4	SINE/Alu	1	160	(150)	14

```
>Hg19 chr19:47807224-47811353
GGCGACAAATTGAGACTCTGTCTCAGAAAAAAAAAAAAAAAGTTATTGGAACAACTGGTGAAATTTCAGTGAGGGCTGAGTATAGTGTGGTATCAGTGGCATATTTCCTTATTTTGATAAATATATT GT
GGTTATGTCAGAGAATATCCTGTCATAACCGCAATATAAGAGATACACAGTAACAAATTTATGGTAAAGGAGCATGATGTTTCCAAAGTACTCTCAAATGATTCCAGGAAAAACATAAGTAGACA TAG
ATATATACGCCTGTATTATTTTTCTAGGGTTTACCAGCCCCCACAATTGCATGAGCCAATTTCTTTTTTTCTGTGTGTGTGTGACAAAGTTTCGCCCTTGTCACTCAAGGCTGGAGTGCAATGGCA CG
ATCTCGGCTGACTGCAACCTCCACCTCCCGAGTTCAAGTGATTCTTCTCCCTCAGCCTCCCAAGTAGCTGGGATTACAGGTGCCCACCACCACACCTGGCTAATTTTTTGTATTCTTAGTAGAGA TGG
GGTTTCACCATGTTGGCCAGGCTGGTCTTGAACTCCTGACTTTCAGGTGATCCACCCGCCTTAGCCTCCCAAAGTGCTGGGATTACAGGCGTCAGCCACCGTGCCCGGCCTCTGTCTTTTTTTTTT TT
TTTTTTtCGAGACAGGGTCTTGCTCTTTTGTCCAGGTTGCAGTGCAGTGGCACAATCATAGGTCACTGCAGCCTCGACCTCCTGGGCTCAGGTGATCCTCCTGCCTCTGCCTCCTAAGTAGCTAGGAC
TATAGGTGTGCTCCACCACACCTGGATAATTTTTTGTGTTTTGTAGAGATGGGGGTTTCACTATGTTACTTAGGCTGGTCTGGAACTCCTGGACTCCAGTGATCTTCCCACCATGGCCTCCCAAAA TG
CTAGGATTACAGGCATGAGCCATTGCTTCTGGCCCCAATTGCTTTCTTTTTCTTTCTTTCTTTTTTTTTTTTTTTGAGGCAGAGTCTCACTTTGTCGCCCAGGCTGGAGTGCAGTGGTATGATCT CGG
TTCACTGCAACCTCTTCCTCCTGAGTTCAAGTGATTCTCCTGCCTCAGCCTCCCAAGTAGCTGGGACTACAGGTGCCTGCCACCATTCCCAGCTACTTTTTTGTATTTTTATTAAAGACGGGGTTT CA
CAATGTTGGTCAGCCTGGTCTCGAACTCTTTACCTCAAGTGATCTTCCCTCCTC gGCTTCCCAAAGTGCTGGGATTACAGGCGGGAGCCAACGCACCCGGCCGCTTTTACTATTCTTGCAACTTTTTT
CATTTGACATTCCATCGAAATAAAAAGTTAAAAAAATTCTTTTTCTTCTGAGGGGGTGTTTTATCAAGGACACCTTTATCAAGGGTCCCGGGCCTAAAAATGTTAAGAACCCATGATTTACGGCTG AG
GAGGCAACTTCCATCCAAAATCCTAAAAGTCAAGTTCCTGTGACCTGGCTTCCTGAACCTCCCCGCTCCTGCGCCCCCTGGAGGTCAGGGCTAGAACAACACGGCATTCCTATAATGCTCCAAATCGC
CaCAAATCGACCTTGGAAATCCTGGGTGAGTAACGCAgACTGCGCACATGGCTTCAATTAGCGGAACtAAAACTTGCTGAACATCGTGTACCCGCGTGTCCATGGCCAAACACAAACTCCACCTTTGT
AAAAGTCTAGGTGAATTCCCAGCTGAATTCAGAAGCGTGACAGGAATGGGACTTTTCATTGGTGTCTTTTTCTTTTTTCTATTTTTAGAGTGGGGAGGAGTGTATTTCTGGATTGTTTAAATATTTAC
AATGAGCATGCATTTCCTTTGCAATTAAAAGAAAACCAGATTTTTTTTTTTTTTTTTTTGAGGCAGAATGTTGCTCTGTCCCCAGGCTGGAGTGCAGTGGCGCGATCTCGGCTCACTGCAACCTCTGC
CTCCCGTGTTCAAGCAATTCTCCTGCCTCAGCCTCCTGAGTAGCTGGGACTACAAGTGCCCGCCACCACGCCCAGCTA ATTTTTGTATTTTTAGTAGAGACGGGGTTTCACCATGTTAGCCAGGATAG
TCTCAATCTCTTGAACTGGTGATCTGCCTGCCTCGGCTTCCCAAAGTGCTGGGATTACAGGCATGAGGACCGCACCCGGCCGAAAACCAGATTTTTAAAAATTGAAGTTTGCTGTAGAAAAATACT GT
GGGAGAAAGTTGTGGGTGGTGTTTTGTAGAGTCGCAGAAGGACATGGGAAAAAAAAAATTGAGTGTGTTTCTGTAATCATCATAACAATCACACTCATTGTGCCGTAGGCTCCAAGGGAGGGCTCTGAG
CTGTGGTTACCAGATAAAATACAAGATTCTCAGGTAAAATGTGAAAAAAAAA ATATTTTTGTGATCTTAACTCACTGCAGTCTTGATCTCCTGGGCTGAAGTGATCCTCCTGCCTCAGCCTCCCAGGT
AGCTGGGACCACAGgCACGTGCCACCATGCCCaGCTAATTTTTAATTTATTTTGTTGaGATGGGATCTCACTATGTTGCCCAGGCTGCTCTCAAACTCCTGGCCTCAAGCGATCTTTCCTCGGTGT CC
CCATGCCCTGGGATTACAGGTATGAAGCACCAGGCCCAACCCTGAAATTTAAAGTTAACTGGGCAGTCTGTAATGTGTTTTTGTTTGTGTAGGTGTGTGTGCTAAATCAGGCAACCCCCTATTCT GAG
CAGAGGAGTGCTTTGATCTGACTTAGATTTTAATAGGCTCCCTCTGGTTGCTGGTGGAGATGAGACTACGGAGGGGGGGCAGTGAAGATCCCAGAGAGGAGGCTGCTGTAATGGTCCAAGCAGAA GGT
GATAGTGATTTGGATCAGGGTAACAGAGCCGAGGTGGTGAGAGGtTGACAGGTCATGCATCTATTTCGAAGGAAAGGCCATGTCCAGGCTCCTTAGCTCGGCATTCAAGGCTTCTCTCCACCACTGGA
CACACTTACTCTCTAGCTTCTCAGACCCTGTTGCAGATAGAATCATGATCCCCAATGATGTCCACATCTTAATCCCCTGAACCTGTGAACAGGTGACCTTCCATGGCAAAAGGGACTTTGCAGAT AGG
ATGAAGTTAAAGATCTTTAGATGGGGGAGCGATTATCCTGGATTGGGTCTGGGTGGGCTCA gTGGAATCACAAGAGTCCTTATATGAGGAAGATAGAAATTTGAGACATACGGTAGAGGAGGAGGTGA
TGAGACCACAGAGGCAGGGACGGGCGTGGTGCAACCACAAGCCAAGGAATTTCAGGAGCCACCTGAAGGTGGGAGAGGCAAGGAACAGATTCTCCCCTGGAACCTCTGGAGGGAGTGGTGACATC TTG
ATTTTGGACTTCTGGCCTGCAGAACCATGCCAAAATAATTGTCTGGCTTTTTTTTTCTTTTTTTTTCCTTGAGGTGGAGTCTTGCTTTGTCGCCCAGGCTGGAGTGCAGTGGTGCCATCTTGGCT CAC
TGCAACCTCCACCTCCCGGGTTCAAGTAATTCTTGTGCCTCAGCCTCCCGAGTAGCTGGGACTACAGGCATGCACCACCACACCCAGGTAATTTATGTATTTTTAGTAGAGATGGGGTTTTGCCA TGT
TGGCCAGGCTGGTCTTGAACTCTTGACCTCAATGATCTGCCCGCCTCAGCCTCCCAAAGTGCTGGGATTGCAGGCATGAGCCACTGCGCCCGGTCCTGGTGTTAAGCCCGCAGTTTGTGCTAATT GGT
TACAGCAGCCTCGGGAAGGTAATCTAGGCTCCTTCCTTGCCCCAGGCTCTAGGAAGTATCTTAGCCCCCAGCCCTGGGCTGCAGACCTGTACCAGTCCATGGCCTGCTAGGAACCATGCAGCATC GCA
GGAGGTGAGTGGCAGGTGAGTAAGTGAAGCTTCATCTGTATTTATAGCCACGCCCCTTCGCTCATATTACCGCCTGATCTGTCAGATCAGGGGCGGAATTACATTCTCATAAGAGCGTGAACCCA GGC
CGGGCGCAGTGGCTCAAGCCTGTAATCCCAGCATTTTGGGAGGCTGAGGCGGGTGGATCACGAGGTCAGGAGTTCGAGATCAGCCTGGCCAAGATGGTGAAACCACGTCTCTACTAAAAATACAA AAA
TTAGCCGGGtGCAGTGGTAGGCACCTGTAGT
```

1038	12.1	0.0	0.0	Hg19seq	1	140	(4187)	+	AluSx	SINE/Alu	159	298	(14)	1
625	23.7	4.6	5.1	Hg19seq	142	359	(3968)	+	L1MC4	LINE/L1	7702	7918	(124)	2

2260	10.8	0.0	1.0	Hg19seq	416	722	(3605)	C	AluSq2	SINE/Alu	(9)	304	1	3
2019	15.9	0.3	0.3	Hg19seq	729	1030	(3297)	C	AluJb	SINE/Alu	(10)	302	1	4
2258	12.5	0.0	0.3	Hg19seq	1043	1354	(2973)	C	AluSx1	SINE/Alu	(1)	311	1	5
230	20.6	3.2	0.0	Hg19seq	1865	1927	(2400)	+	L1ME4a	LINE/L1	6058	6122	(2)	6
2380	8.4	0.3	0.0	Hg19seq	1933	2229	(2098)	C	AluSc	SINE/Alu	(11)	2 98	1	7
1482	16.0	1.7	0.8	Hg19seq	2465	2701	(1626)	C	AluJr	SINE/Alu	(73)	239	1	8
785	27.9	3.7	0.0	Hg19seq	2782	2996	(1331)	C	L2	LINE/L2	(575)	2844	2622	9
1560	22.1	12.8	3.4	Hg19seq	3073	3475	(852)	+	MLT1D	LTR/ERVL-MaLR	1	446	(59)	10
2363	10.9	0.3	0.0	Hg19seq	3476	3779	(548)	C	AluSz6	SINE/Alu	(7)	305	1	11
1560	22.1	12.8	3.4	Hg19seq	3780	3835	(492)	+	MLT1D	LTR/ERVL-MaLR	447	502	(3)	10
1050	17.6	4.8	0.0	Hg19seq	3877	4064	(263)	+	MER1A	DNA/hAT-Charlie	8	204	(325)	12
2237	6.5	0.0	0.0	Hg19seq	4066	4326	(1)	+	AluSg4	SINE/Alu	1	261	(49)	13

>KSI_AluYb8_6

>Scaffold9148-1211806-1211948
TAGGTACAGTCATAACTATAGGTGTTTCGCATACTACTGTAATAGGATACTGTATATCATTCAATATGAAGAATGTGGTTTTTTTTTGGAGGACTAAATTGCTTGGTTCTATATATCTTTTTCTTA AA
AATAAAGTATATTGGCTTTTTTGCTATAAAAGCAATTCACATTGATTTGAGAATAGTAGAAAATATAGATAGGAAAAGAACAAAAGTACTGAAAGTTCACAACCTAAAGAAGGCCAAAATCAAAA TTT
TATTTATTTACATCTGGTCTTTTTACTATGTATATATTTTGGATCTGTTTGCTTAGTTTATGCAAATGGCATTCTTTTTTATGTTTCTTTATTTTTTTTAATTGTAAGCTTAATTTCCAGCCATGT CT
ATAAAAGCTGCTCTAAAATATCAGGGAAGTTAATGTTCAGTGTGGGTCATTGTAAGTAGACCATTTAGTCTATCTGGAGAAGAATTGTAGTTTTTGGAAGTCCACTTATTATGTTAGTTG GCCAGTGG
TTAATGGTGCAGAGAAAATTTAAGATACCATCAATTGGAAAGAATATGGTAGAGACTTTCAAAGAGGAATGTAGTAGCTCATATATCAAGAGCCTCAAGCACAAAGACTCAGGTTACAAAGAGATTAA
TTAATTATAACATCATCCAAAATGGTAAAAAATGGGAAATTACCTAAATTTTCCACAACAGTGTAATGGTTAAATAATGTTACAACAAAATGATAAAATGTTATTTAAATAGAAAAAAATCATGA AGA
ATTTTGATGACATAGAAAATGAGAAGGATGCTAATTAAAAAAATATATAGGAAATATAAAAAACTAGAAGGAAATTCACCAAAATTTGACAGTGATTATTTCT AGGTAGTAGAGATAACTCTAATAAT
TGAAAAATGAAGGTCATCAGGTGATTTTTTTCTTTTTCTTCTTTTTTTTTTTTTTTTGCCTAAAAAGGGATGGTCAAGTTTTTGAATTTAAGATTTTATTCT Cggccgggcgcggtggctcacgcctg
tagtcccagcactttgggaggccgaggcgggtggatcatgaggtcaggagatcgagaccatcctggctaacaaggtgaaaccccgtctctactaaaaaatacaaaaaattagccgggc TTTAGATTTG
TTATATCAGAGCCTTTCTTTACTTATTAACAATCTCTTGTAGTAAACATGTGTAATCTGATTTCCCTCAACTTTCTCCAAACTCAAATATCTACAAATCACCTTCATGTCTTCATACTATTT TGGAAA
ATAGTTTCTCCAGTTCTCAAATGTTACTGAGTGTATTTCCATGTCCCAGGAGCTTGGCAATTTTTTTCTATCATAATTG GCAATACCAGGAATGGGCTAATTGCCTTTGGAGGTCAGAACTTCTTTAG
TAtTTTTATTTTTTTATTGGTCTTGGGTCACTAGAAGGAACTGTTACTGTGTCCTGGCTGTGGCTAGCAGTGACAAGACTGTCTTTTGAGGGCGCTGCCAGGCTTAATTGCATCCAATATAGGGATCT
TCATCTGAGATTTGGAACAATGAATCTTCCATGCCAATTAAAGATGAGTTTAATATAGAGAATATGAAGTATGCCTAGCAGAGGAACAGTTTTACAGAGCCTCAGGATCCCATCTGCCTTTCTACA AC
ACAGAATTGGACCCATACAAGCCCTTTATCATCTCTTCAAGCTAGGACATTCCAAAGAAACCAGGAGCAAAATATGAGCATGGAGAGCTTTAGGCATATTCATTTCTCTCTTATTCTCTAAATAT TTC
TCAGTGTTTTCTAAAGGATTCTGAACATAAGGTATGTTTGCTGTTTGTCTCACAACACATAATTGATATGTTATCTAAGTCAAATAGTTTGGTAATTGACAGTGAATTTTTGCAATACTGAAAATA AT
CTCACTTCTTATACAAATAAAGAGTAATTGTGTCAAATTGAGTTTAAAAGCCAGTGCCTCTGCCTATTAGTTATGGGGCCCAAATCAAGTCAAATACTCCTCTGTAGATATTTCCTTATCTGTGA AAT
GGGGATAATCATAGTATTTGCCTTACAGGTTTATTATGAGGAATACATGAGAGGATCCATATAAAACCCTCAGCAAAAGAGCTGGCAAATAAT C

260	25.7	10.7	0.6	TErefseq_16	145	293	(1849)	C	L1ME4a	LINE/L1	(5)	6119	5956	1
327	27.7	6.7	3.2	TErefseq_16	611	789	(1353)	+	L1ME4a	LINE/L1	5676	5860	(264)	2
315	16.4	1.8	0.0	TErefseq_16	826	880	(1262)	+	L1ME4a	LINE/L1	5970	6025	(99)	2
23	10.0	0.0	0.0	TErefseq_16	921	953	(1189)	+	(T)n	Simple_repeat	1	33	(0)	3
1333	0.7	0.0	0.7	TErefseq_16	1000	1142	(1000)	+	AluYb8	SINE/Alu	1	142	(176)	4
582	30.0	3.3	1.6	TErefseq_16	1959	2141	(1)	+	MIR	SINE/MIR	49	234	(28)	5

>hg19 chr2 192839054 192841063
TAGGTACAGTCATAACTATAGGTGTTTCGCATACTACTGTAATAGGATACTGTATATCATTCAATATGAAGAATGTGGTTTTTTTTTGGAGGACTAAATTGCTTGGTTCTATATATCTTTTTCTTA AA
AATAAAGTATATTGGCTTTTTTGCTATAAAAGCAATTCACATTGATTTGAGAATAGTAGAAAATATAGATAGGAAAAGAACAAAAGTACTGAAAGTTCACAACCTAAAGAAGGCCAAAATCAAAA TTT
TATTTATTTACATCTGGTCTTTTTACTATGTATATATTTTGGATCTGTTTGCTTAGTTTATGCAAATGGCATTCTTTTTTATGTTTCTTTATTTTTTTTAATTGTAAGCTTAATTTCCAGCCATGT CT
ATAAAAGCTGCTCTAAAATATCAGGGAAGTTAATGTTCAGTGTGGGTCATTGTAAGTAGACCATTTAGTCTATCTGGAGAAGAATTGTAGTTTTTGGAAGTCCACTTATTATGTTAGTTG GCCAGTGG
TTAATGGTGCAGAGAAAATTTAAGATACCATCAATTGGAAAGAATATGGTAGAGACTTTCAAAGAGGAATGTAGTAGCTCATATATCAAGAGCCTCAAGCACAAAGACTCAGGTTACAAAGAGATTAA
TTAATTATAACATCATCCAAAATGGTAAAAAATGGGAAATTACCTAAATTTTCCACAACAGTGTAATGGTTAAATAATGTTACAACAAAATGATAAAATGTTATTTAAATAGAAAAAAATCATGA AGA
ATTTTGATGACATAGAAAATGAGAAGGATGCTAATTAAAAAAATATATAGGAAATATAAAAAACTAGAAGGAAATTCACCAAAATTTGACAGTGATTATTTCTAGGTAGTAGAGATAACTCTAATA AT
TGAAAAATGAAGGTCATCAGGTGATTTTTTTCTTTTTCTTCTTTTTTTTTTTTTTTTGCCTAAAAAGGGATGGTCAAGTTTTTGAATTTAAGATTTTATTCT CacttcgtctgTTTAGATTTGTTATA
TCAGAGCCTTTCTTTACTTATTAACAATCTCTTGTAGTAAACATGTGTAATCTGATTTCCCTCAACTTTCTCCAAACTCAAATATCTACAAATCACCTTCATGTCTTCATACTATTTTGGAAAATA GT
TTCTCCAGTTCTCAAATGTTACTGAGTGTATTTCCATGTCCCAGGAGCTTGGCAATTTTTTTCTATCATAATTG GCAATACCAGGAATGGGCTAATTGCCTTTGGAGGTCAGAACTTCTTTAGTAgTT
TTATTTTTTTATTGGTCTTGGGTCACTAGAAGGAACTGTTACTGTGTCCTGGCTGTGGCTAGCAGTGACAAGACTGTCTTTTGAGGGCGCTGCCAGGCTTAATTGCATCCAATATAGGGATCTTCA TC
TGAGATTTGGAACAATGAATCTTCCATGCCAATTAAAGATGAGTTTAATATAGAGAATATGAAGTATGCCTAGCAGAGGAACAGTTTTACAGAGCCTCAGGATCCCATCTGCCTTTCTACAACAC AGA
ATTGGACCCATACAAGCCCTTTATCATCTCTTCAAGCTAGGACATTCCAAAGAAACCAGGAGCAAAATATGAGCATGGAGAGCTTTAGGCATATTCATTTCTCTCTTATTCTCTAAATATTTCTCA GT
GTTTTCTAAAGGATTCTGAACATAAGGTATGTTTGCTGTTTGTCTCACAACACATAATTGATATGTTATCTAAGTCAAATAGTTTGGTAATTGACAGTGAATTTTTGCAATACTGAAAATAATCT CAC
TTCTTATACAAATAAAGAGTAATTGTGTCAAATTGAGTTTAAAAGCCAGTGCCTCTGCCTATTAGTTATGGGGCCCAAATCAAGTCAAATACTCCTCTGTAGATATTTCCTTATCTGTGAAATGGG GA

TAATCATAGTATTTGCCTTACAGGTTTATTATGAGGAATACATGAGAGGATCCATATAAAACCCTCAGCAAAAGAGCTGGCAAATAAT C

>KSI_AluYb8_7

>Scaffold49-334993-335301
TGTGCTGCTTTTTTCTTAGGCAAACCACCCTCCATCCCAGTCCCTGGCAATCACTGATGTCTTCTCTGCCCCTATAGCTTTAGCTTTTCCAGAATGTCATATAAATGTGATCATACTGCATATAAT CT
TTGCAGCTGGTTTCTTTCACTTAATACAGTGCAGTTGAGGTTCATCCATGTCACTGCATGTATCAGTAGTTCATTACTGC gcAGTACTGTTTCATTATGTAGATGTACCACAGTTTCTTTATTCATGT
ACACATTGAAGGACATGTGAACTGTTTTCGGTTTTTGACTATAGGTTAAATATCCCTTATCCAAGATGCTTGAGACCAGAAGTGTTTCAGATTTCAGTGTTTTAGGATCTTGCAATATTTGTATA TAC
ATAATGAGATAGCTTGGGGATGGACCCAAGTCTAAGCACAAAATTTATTTATGTTTCATATACACCTTGTATAAAATAAGGCGAAGGTAATTTCATACAATA TTTTAAATATTTTCAGTGCATGAAA
CAAAGTTTGTGTTAAGTACTTAtGTGTGGAATTTTCCACTTGCATCATGTCAGTGCTCAAAAAGTTTTGGATTTGGGAGCATTACAGATTTCAGATTTTTGGATTTGGGATACTCAATCTGTATTATA
ACTAAATATTGCTATCAATTTTTGTGTACAAGATTCTCTGTGAACTTAAGTTTTTATTTCATTAGGATACTTAGAAGTAGGATGGTTGAGGCATATATGATAG GTATGTATTGAACATTTTAATGAAA
CTGCCAAACTGTTTTCCAGGATTCCTGTGCCATTTCTGCATTTCCACTAGCAATGTATGAAAAACTTCAATT GCTTCACATACTCACCATT TTTCATGCTAGTCATTCTAATAAGTTCATAGTTATAG
TCAGTATGATTTTAATTTGCACTACTGTAATGATTATGATATTGAGCATCCTTGCATTTATTTATTTGACAGATACTTTTTTTGGTGAAATGTCTGTT TAAATATTTTttttttttttttttgagac
ggagtctcgctctgtcgcccaggtgggactgcggactgcagtggcgcaatctcggctcactgcaagctccgcttcccgggttcacgccattctcctgcctcagcctcccgagtagctgggactac agg
cgcccgccaccgcgcccggctaatttttttgtatttttagtagagacggggtttcaccttgttagccaggatggtctcgatctcctgacctcatgatccacc cgcctcggcctcccaaagtgctggg
attacaggcgtgagccaccgcgcccggcctaaatattttGTTCATTAAAATTTTTTTCTTAATTTTGAGTTTTGAGAGTTCTTTATATATTCTGGGTACAAATATTTT CTCTGAGTATGTGGCTTGT
CTTTTCTTACAGTGTCCTTCACAGAGGAGAAATTTTAATTTTGATGAAGTTTACTGATATTCAGTTGTATAATACATTTTGGGTTAA TTTTTGCATATGGAGTGAGGAATGAGTGTAAGTTAACATTT
TTGCATGCGGATATTCAGTTGTTCCAGCATCATTTGGTGATATGATTATCCTTTTTCCAATGTTGCCTTTGCACCTTTATCGTAAACTAATTGACTATATTTCTGAACTCTAGTTTGTTTCCTCA TTC
TGTGTGTCTCATTTTGTCAATACTACATTGTTTTGATCACTGTAGTTTTTGTATGTCTTAAAATTATTGTGTGATTCTTGCATATCC CCCCAAAATTGTTTTGCATTTTCTAGTTTTTTTTTTTTTAC
CTTTCCATAGAAATTTTAAACTTAGTTTATATCTACAAAATATCCTGTTGGGATTTTGATTGAAGTTGCATTGAGTCTATAGATCAATTTGGGGAGAATTGATGACTAACTTGAGTCTTACTACA TGA
ACATGGAATGTCTCTTCATTATATGTTTAAAAAGTTTTATTTTATCACTATTTTAGAGTGTTAATGTACAGATTCTGTAGATGTTTT GTTAGGGTGAAAGCAATACTTCTTTGTTGTTGTTATGGTTG
GAGCTATTGAAAAGTAGTACTTTAAAAAGGATTTATTTTCCATTTGTTCATTATTGGTATATTGAAATATACTAGATCTTGCTAAACTCACTTATAAGTTATAGAAA CtTTTTTTTTGTAAATTCCTT
GGGATTTTCTACATAGATCATCATGCCATCTGCAAATATTTCTCTCATTCAAATCTAAATGTCATTTATTTATTTTTCTTATACTGG CTACAGCTTTCAGTAAAATATTGAATCATGGGTGATAAGAG
TGGAC

2670	23.1	5.9	1.5	AluYb8-Scaffold49-334993-335301	25	298	(2011)	C	L1ME1	LINE/L1	(178)	6001	5719	1
1898	13.2	8.1	0.6	AluYb8-Scaffold49-334993-335301	299	633	(1676)	C	Tigger4b	DNA/TcMar-Tigger	(1)	360	1	2
2670	23.1	5.9	1.5	AluYb8-Scaffold49-334993-335301	634	1000	(1309)	C	L1ME1	LINE/L1	(461)	5718	5332	1
2848	1.0	0.0	0.7	AluYb8-Scaffold49-334993-335301	1001	1309	(1000)	C	AluYb8	SINE/Alu	(11)	307	1	3
2670	18.1	22.2	2.7	AluYb8-Scaffold49-334993-335301	1310	1461	(848)	C	L1ME1	LINE/L1	(810)	5336	5141	1
1937	23.8	12.8	3.1	AluYb8-Scaffold49-334993-335301	1478	2309	(0)	C	L1MD	LINE/L1	(1144)	5002	4091	4

>Hg19 chr15:22767976-22770168
TGTGCTGCTTTTTTCTTAGGCAAACCACCCTCCATCCCAGTCCCTGGCAATCACTGATGTCTTCTCTGCCCCTATAGCTTTAGCTTTTCCAGAATGTCATATAAATGTGATCATACTGCATATAAT CT
TTGCAGCTGGTTTCTTTCACTTAATACAGTGCAGTTGAGGTTCATCCATGTCACTGCATGTATCAGTAGTTCATTACTGC agAGTACTGTTTCATTATGTAGATGTACCACAGTTTCTTTATTCATGT
ACACATTGAAGGACATGTGAACTGTTTTCGGTTTTTGACTATAGGTTAAATATCCCTTATCCAAGATGCTTGAGACCAGAAGTGTTTCAGATTTCAGTGTTTTAGGATCTTGCAATATTTGTATAT AC
ATAATGAGATAGCTTGGGGATGGACCCAAGTCTAAGCACAAAATTTATTTATGTTTCATATACACCTTGTATAAAATAAGGCGAAGGTAATTTCATACAATATTTTAAATATTTTCAGTGCATG AAA
CAAAGTTTGTGTTAAGTACTTAcGTGTGGAATTTTCCACTTGCATCATGTCAGTGCTCAAAAAGTTTTGGATTTGGGAGCATTACAGATTTCAGATTTTTGGATTTGGGATACTCAATCTGTATTATA
ACTAAATATTGCTATCAATTTTTGTGTACAAGATTCTCTGTGAACTTAAGTTTTTATTTCATTAGGATACTTAGAAGTAGGATGGTTGAGGCATATATGATAGGTATGTATTGAACATTTTAATG AAA
CTGCCAAACTGTTTTCCAGGATTCCTGTGCCATTTCTGCATTTCCACTAGCAATGTATGAAAAACTTCAATT GCTTCACATACTCACCATT TTTCATGCTAGTCATTCTAATAAGTTCATAGTTATAG
TCAGTATGATTTTAATTTGCACTACTGTAATGATTATGATATTGAGCATCCTTGCATTTATTTATTTGACAGATACTTTTTTTGGTGAAATGTCTGTT TAAATATTTTGTTCATTAAAATTTTTTTC
TTAATTTTGAGTTTTGAGAGTTCTTTATATATTCTGGGTACAAATATTTT CTCTGAGTATGTGGCTTGTC TTTTCTTACAGTGTCCTTCACAGAGGAGAAATTTTAATTTTGATGAAGTTTACTGATA
TTCAGTTGTATAATACATTTTGGGTTAATTTTTGCATATGGAGTGAGGAATGAGTGTAAGTTAACATTTTTGCATGCGGATATTCAGTTGTTCCAGCATCATTTGGTGATATGATTATCCTTTTT CCA
ATGTTGCCTTTGCACCTTTATCGTAAACTAATTGACTATATTTCTGAACTCTAGTTTGTTTCCTCATTCTGTGTGTCTCATTTTGTCAATACTACATTGTTTTGATCACTGTAGTTTTTGTATGTC TT
AAAATTATTGTGTGATTCTTGCATATCCCCCCAAAATTGTTTTGCATTTTCTAGTTTTTTTTTTTTTACCTTTCCATAGAAATTTTAAACTTAGTTTATATCTACAAAATATCCTGTTGGGATTT TGA
TTGAAGTTGCATTGAGTCTATAGATCAATTTGGGGAGAATTGATGACTAACTTGAGTCTTACTACATGAACATGGAATGTCTCTTCATTATATGTTTAAAAAGTTTTATTTTATCACTATTTTAGA GT
GTTAATGTACAGATTCTGTAGATGTTTTGTTAGGGTGAAAGCAATACTTCTTTGTTGTTGTTATGGTTGGAGCTATTGAAAAGTAGTACTTTAAAAAGGATTTATTTTCCATTTGTTCATTATTG GTA
TATTGAAATATACTAGATCTTGCTAAACTCACTTATAAGTTATAGAAA CTTTTTTTTGTAAATTCCTTGGGATTTTCTACATAGATCATCATGCCATCTGCAAATATTTCTCTCATTCAAATCTAAAT

GTCATTTATTTATTTTTCTTATACTGGCTACAGCTTTCAGTAAAATATTGAATCATGGGTGATAAGAGTGGAC

400	16.7	0.0	0.0	Hg19	1	60	(2133)	C	AluSx	SINE/Alu	(127)	185	126	5	
2890	23.0	6.5	1.7	Hg19	63	398	(1795)	C	L1ME1	LINE/L1	(118)	6061	5719	6	
1904	13.2	8.1	0.6	Hg19	399	733	(1460)	C	Tigger4b	DNA/TcMar-Tigger	(1)	360	1	7	
2890	23.0	6.5	1.7	Hg19	734	1167	(1026)	C	L1ME1	LINE/L1	(461)	5718	5255	6	
655	18.7	22.9	0.0	Hg19	1033	1246	(947)	C	L1MC	LINE/L1	(743)	5403	5141	8	*
1943	23.7	12.7	3.0	Hg19	1263	2170	(23)	C	L1ME1	LINE/L1	(1144)	5002	4012	6	

>KSI_AluYb8_8

```
>Scaffold1230-602330-602676
TTATTAAGCCCCTCATGGGTCATCACACTGGCTTCATGAAGGATTCAGCTTTTCACCTCATTATTTCTTCATGATCACCTCCTCCTGGAGAAGAATTAAAGTTACACAAAAAGCAAGTTTACCCAT AG
TAATAAGTGGAATGTCCTTTTATTTACCTAGCTATACATAAGATAAAATAAAAAATCTTTCACTCAAAAAGCATTTCCAAACATTTATAAGATATGCAAATGATTTCCCAGTTCACAAACGGATT TTA
GAGACATTTCGTTTGATGTGACAACAGCTCTGTGACACAGATATTAACAACTCCCGTGTTACAAAGTCCAGAGAGATTAAGTGGAGCCCCAAGTCTTTCCTTCCAAATTCATTCATTCATTCCTTC AG
CACTTACTGAGGGCCTAGTTTTGGCCTGGGCACTATTGAAGGTAGGGATTCAGGGAAAGCAGATGTAGTCTCTACTGCTAAGTATCTCACAAGGGCAACCCAAGCTTAGGTGTAAGAAAACAGGC ATC
TTTCTAGAGAAGGCTATGTCAGAGCTGAATTTTCAAGGATAAATTTCCTGTTCTTTTCACATCAACATGTCCTCCATGAATCACCAGTATTAAACTAGGCTAAGAAAAAATGAACCTGATAATCAC CT
TTGTGCCAGAAGACAAAAATAACTGGACAGGAAGCCCAGGCAGTCCCAGTGGCGGTGCTGTCTTTTACACTGACGTTAACTCTAACGATCAGCGGAGTAACCGCACAGCGCTGCTGGGCACCTGGAGC
TTATTGTTTCCGTGTGGCATATGATGAAGTATCAGAACGGGGAGGAGTATATCTCCAAGAAGGAATGTGATGAGCAGAAAAACTCATCCTGTGAGATTGGCCTAGAGAGATCTGAAGCCAGGAGCC AT
TTGGTCTTGTCCAATGGCATTAATGTACCCACAGACAAAGGCCAAAGGGGACATGGAGGCCAGAGCTGCTGGTCAACGGCTTGTTTT AAGAAAACAATAGGGGGggccgggcgcggtggctcacgcct
gtaatcccagcactttgggaggccgaggcgggggatcatgagggcaggagatcgagaccatcctggctaacaaggtgaaaccccgtctctactaaaaaaaaaaatacaaaaaattagccgggcg cgg
tggcgggcgcctgtagtcccagctactcgggaggctgaggcaggagaatggcgtgaacccgggaagcggagcttgcagtgagccgagattgcgccactgcagtc cgcagtccggcctgggcgacagag
cgagactccgtctcaaaaaaaaaaaaaaaaaaaaaaaaaaaaaaaaaaaaaaaaa aagaaaacaatagggggACCCAACTCCATGCTATCAGAATAAAGGCCATCCTTCCCCGTAT TTTTCTCAAG
TTATACAAGTGCCGTTATTCTTCAAGCAACAAAGAATTCCACTAGCCTTTTGCTAAGGATAAAACATTTCCAAAATATAAGTTGTTTAAACTGCAGGTATAGAA TAATTTCTTCAACTGTCTTCTTTT
TCCCAAATGAATAATGCTACCTAGATGAAAACAATTTTTTTCTTCACCTTTTATTCCAAATTTCTTTTTTTAAAAAAAGCTTCCATCTTTTAAAATTCAAACAGAAATACTGAGGGAAGTTCCAG TTT
CAAACCATCTTTTGTCAAGAAAAAAAACTAGTGAAAGGTACTTTCAGGAAATCAAGTACAGTATATGATTTCAACACAGCAAAGCTAGTGTCTCACTGTTAATT TTTATTAATAAAAAAAAAACAAAA
AACAAACAAACAAAAAAAACCGCTGGGTGCAGTGGCTCACACTTGTAATCCCAGTACTTTGGGAGGCTGAGGTAGGAGGACTGCTTGAGTCCAGGAGTCTGAGACTAGCCTGGGCAAGACAGTAA GAC
CCTGGCTCTACAAAAAACAAAATTTTTAATTAGCTGGACATGGTGGTGCATGCCTGTAGTCCCAGCTACTCAGGAGGCTGAGGCAGGAGGATCACTTATGCATG AGAGGTCAAGGCTGCAGTGAGCCA
AGCTGGGCAACAGAGGGTGATATGGTTTGAGTCTGTGTCCCCACCCAAGTCTCACATCAAATTGTAATTCTCATGTGTCAGGGGAGGGTCCTGGAGGGAGGTGACTGAATCATGGGGGTGGATTT CCC
CCCTGCTTTTCTTGTGATAGTGAGTTCTTGAGTTCTCATGAGATCTGATGGTATAAAAGTGTGGCACTTCCCCCCTTGCTCTTCTTTCTCTCTCCTGCCACCAT GTATAAGGCATGCCTTGCTTCCCC
TTTGCCTTCCACCATGATTGTAAGTTTCCTGAGGCCTCCCCAG
```

223	24.1	23.1	0.7	AluYb8-Scaffold1230-602330-602676	231	351	(1996)	C	MIRc	SINE/MIR	(30)	238	91	1
14	4.9	0.0	0.0	AluYb8-Scaffold1230-602330-602676	363	383	(1964)	+	(ATTC)n	Simple_repeat	1	21	(0)	2
186	35.5	2.5	2.5	AluYb8-Scaffold1230-602330-602676	476	556	(1791)	C	L2c	LINE/L2	(202)	3185	3105	3
2916	0.9	0.0	1.9	AluYb8-Scaffold1230-602330-602676	1001	1347	(1000)	+	AluYb8	SINE/Alu	1	318	(0)	4
22	15.1	0.0	0.0	AluYb8-Scaffold1230-602330-602676	1775	1811	(536)	+	A-rich	Low_complexity	1	37	(0)	5
1479	16.3	0.4	2.2	AluYb8-Scaffold1230-602330-602676	1814	2046	(301)	+	AluJo	SINE/Alu	2	230	(82)	6
1422	13.7	10.3	4.4	AluYb8-Scaffold1230-602330-602676	2066	2347	(0)	+	MSTA	LTR/ERVL-MaLR	1	298	(130)	7

```
>Hg19 chr1:64957069-64959061
TTATTAAGCCCCTCATGGGTCATCACACTGGCTTCATGAAGGATTCAGCTTTTCACCTCATTATTTCTTCATGATCACCTCCTCCTGGAGAAGAATTAAAGTTACACAAAAAGCAAGTTTACCCAT AG
TAATAAGTGGAATGTCCTTTTATTTACCTAGCTATACATAAGATAAAATAAAAAATCTTTCACTCAAAAAGCATTTCCAAACATTTATAAGATATGCAAATGATTTCCCAGTTCACAAACGGATT TTA
GAGACATTTCGTTTGATGTGACAACAGCTCTGTGACACAGATATTAACAACTCCCGTGTTACAAAGTCCAGAGAGATTAAGTGGAGCCCCAAGTCTTTCCTTCCAAATTCATTCATTCATTCCTTC AG
CACTTACTGAGGGCCTAGTTTTGGCCTGGGCACTATTGAAGGTAGGGATTCAGGGAAAGCAGATGTAGTCTCTACTGCTAAGTATCTCACAAGGGCAACCCAAGCTTAGGTGTAAGAAAACAGGC ATC
TTTCTAGAGAAGGCTATGTCAGAGCTGAATTTTCAAGGATAAATTTCCTGTTCTTTTCACATCAACATGTCCTCCATGAATCACCAGTATTAAACTAGGCTAAGAAAAAATGAACCTGATAATCAC CT
TTGTGCCAGAAGACAAAAATAACTGGACAGGAAGCCCAGGCAGTCCCAGTGGCGGTGCTGTCTTTTACACTGACGTTAACTCTAACGATCAGCGGAGTAACCGCACAGCGCTGCTGGGCACCTGGAGC
TTATTGTTTCCGTGTGGCATATGATGAAGTATCAGAACGGGGAGGAGTATATCTCCAAGAAGGAATGTGATGAGCAGAAAAACTCATCCTGTGAGATTGGCCTAGAGAGATCTGAAGCCAGGAGCC AT
TTGGTCTTGTCCAATGGCATTAATGTACCCACAGACAAAGGCCAAAGGGGACATGGAGGCCAGAGCTGCTGGTCAACGGCTTGTTTT AAGAAAACAATAGGGGGACCCAACTCCATGCTATCAGAATA
AAGGCCATCCTTCCCCGTATTTTTCTCAAGTTATACAAGTGCCGTTATTCTTCAAGCAACAAAGAATTCCACTAGCCTTTTGCTAAGGATAAAACATTTCCAAAATATAAGTTGTTTAAACTGCAGGT
ATAGAATAATTTCTTCAACTGTCTTCTTTTTCCCAAATGAATAATGCTACCTAGATGAAAACAATTTTTTTCTTCACCTTTTATTCCAAATTTCTTTTTTTAAAAAAAGCTTCCATCTTTTAAAA TTC
AAACAGAAATACTGAGGGAAGTTCCAGTTTCAAACCATCTTTTGTCAAGAAAAAAAACTAGTGAAAGGTACTTTCAGGAAATCAAGTACAGTATATGATTTCAACACAGCAAAGCTAGTGTCTCAC TG
TTAATTTTTATTAATAAAAAAAAAACAAAAAACAAACAAACAAAAAAAACCGCTGGGTGCAGTGGCTCACACTTGTAATCCCAGTACTTTGGGAGGCTGAGGTAGGAGGACTGCTTGAGTCCAGG AGT
CTGAGACTAGCCTGGGCAAGACAGTAAGACCCTGGCTCTACAAAAAACAAAATTTTTAATTAGCTGGACATGGTGGTGCATGCCTGTAGTCCCAGCTACTCAGGAGGCTGAGGCAGGAGGATCACT TA
```

TGCATGAGAGGTCAAGGCTGCAGTGAGCCAAGCTGGGCAACAGAGGGTGATATGGTTTGAGTCTGTGTCCCCACCCAAGTCTCACATCAAATTGTAATTCTCATGTGTCAGGGGAGGGTCCTGGA GGG
AGGTGACTGAATCATGGGGGTGGATTTCCCCCCTGCTTTTCTTGTGATAGTGAGTTCTTGAGTTCTCATGAGATCTGATGGTATAAAAGTGTGGCACTTCCCCCCTTGCTCTTCTTTCTCTCTCCT GC
CACCATGTATAAGGCATGCCTTGCTTCCCCTTTGCCTTCCACCATGATTGTAAGTTTCCTGAGGCCTCCCCA G

223	24.1	23.1	0.7	Hg19	331	451	(1742)	C	MIRc	SINE/MIR	(30)	238	91	8
14	4.9	0.0	0.0	Hg19	463	483	(1710)	+	(ATTC)n	Simple_repeat	1	21	(0)	9
186	35.5	2.5	2.5	Hg19	576	656	(1537)	C	L2c	LINE/L2	(202)	3185	3105	10
22	15.1	0.0	0.0	Hg19	1521	1557	(636)	+	A-rich	Low_complexity	1	37	(0)	11
1479	16.3	0.4	2.2	Hg19	1560	1792	(401)	+	AluJo	SINE/Alu	2	230	(82)	12
1997	13.3	5.0	3.7	Hg19	1812	2187	(6)	+	THE1D	LTR/ERVL-MaLR	1	381	(0)	13

>KSI_AluYb8_9

>Scaffold1231-2979587-2979903
CAAGTCAAGATCAAGGTCCATGTCAGCAGCTCAGAAGAGCTGGTGTTTAATCAATGTCAACTCCTGAGTAATAAGCCCTGATAAGACCCGAGTATACTCCTCACATCCTCCTTCTGCAGCAGCTCA CA
GGCTCCCAGCACCAGCCATGTGCTAGGAGTGTCCTTACTGCTGCCCAGAAAAGGGAGGAGTTGAAGGCCAGGAGGCCCCTGGTGCAGAGGAGAGGACAG GAAGACCAGCAGAAAAAGGCACTGGGTAA
AGTTCCTAAGGTTGATCAGGAACAGAGTTGGGCCTTAGATAAAGCCACTCAGGCTGAGCTCCTGGGGTGGGGGCAGGTCATGTGTGGTTTCCACAGCATCTGGTCCAGATGCCATCGGATAGGAG CCC
AAGGGTGGGGCAGAAGCTCACAGAGTGGGAGGACTCCAGGGCTCCCTCAGGAGAGGTTCTGTTTCAGCAGCACTGAATAGTTCCATCAGCCATGAGATC ATGGTATCATAGATACTTTGGGTCAACTG
AGGACCCGAGTCAACATTTCTTTCGAAGTCTGCCTGAAAAAAGGGGATAAAACTTTTATCAGAAAGGGCTAAGGAAAATGTTTGCAATACATATGAAAAAAGCTAACATCCTCACTATTATAAGA CCT
CATAAAATGAGCATCCCACCCATTAAAAGAATAAATAATTTACAGAAGAAATACAAGGGGGTATTGAACATATTAAAAAGTTCAAGCTCATGCAAGTAA AATATTAAAAAGTAAAATTCAGAAGTACG
GGGTAGAGCTGATTTGCATTTCTCAGTTTGTGGCCCGTGACCATCAGCAGTCTACAGGCCTCTCTTCAGTGGCT CTTCACGATTGCCTGCTTGGACCACAAATTCCTTTTTATCTCCTTCCTCTCTCC
TCCTCAAGCCCCCTTCCCCATTGGTGAATGCTCTTTGTTCAATATGAAGTCTCTCTCTCTCCCTGTCTTTCTCCTGGAACACACTACAGGTT Tgagcctaattctttttttttttttttttttttt
tttttgagacggagtctcgctctgtcgcccaggccggactgcggactgcagtggcgcaatctcggctcactgcaagctccgcttcccgggttcacgccattctcctgcctcagcctcccgagtag ctg
ggactacaggcgcccgccaccgcgcccggctaattttttgtatttttagtagagacggggtttcaccttgttagccaggatggtctcgatctcctgacc tcatgatccacccgcctcggcctcccaaa
gtgctgggattacaggcgtgagccaccgcgcccggccGAGCCTAATTCTTAGTTCATGACATACTTGTATCACTGGTTTATGTGAAGCTCTCATCAAAAAATT ATTTTAAATAATGCAATAAAATCCT
GGGATGTCACTGCCTTAGAGGACACTTAACCGTGGACAGTGACTTGTCCCATCTCCATGGTCCACCCCAATCCCATGCTGCTGGCTTCTGCCCAGGCACCTGCCGTCTTGAATTTGGTGCCTCTC ATT
CCTATGCCTTCCTGTCATCTATTTGTACTCCTTCAAAGTATATATGTTTTGTTTCATTTGTCTTAATTTTATTAAAAGAGTATCTTGTACATATTTTTATGGG ACTTAGAGATAAGTGGGCTGATTCA
AGAGGAGAGACTCACAGGGTGGTGGTGGTGAGGgTGATGGGGAAAGAATCTGCAAGGTATCCCATATTTCAGTCTTAGGTGACTGAGTAGTTGGTGGAGTCATTCAGTTAGATAGGCCTAGGAAAGAA
GAAAGGGGCTGGTGGGGAGATGATGAACTCAGTTCTGGGCAAAGCAATAATTTAGGCTCGCATTTATTGAATGTCTGCTGTGTTCCAGTCAGTTTTGTAGAAC TTAAAGTGCTCTGCATGTATTATTA
CATTTAAGCATAACAAGAACCTCACAAGACAGGCACTGTTATTATTTTCATTTTAAAGATAAGGAAACTAAGAGGGAGAAAAGTTTAGTAGGTTCTGAGCCCATCAACATGATGGATCACACCTT TCT
GCTGCGAACAGAAATATTGGATAAAATGTTTAAAATATTAAATATTTGTCATTAAAAATATTGATATGAAACTATTTACAAATAAAATATTTAAAAATTCTCA AAAAGCAAGGGAAATCCTCAGGTGA
CAGGAGTAAAGAAAACCCAAAGCCAGGGTGGATGCATTGGACATTTATGGATAAAACAAGCCCCACTGGCATCTGCCATTACTGAGGCTTGAGTAGGCGGTTTTCCCCTCGCAGTGTAAACAAAG CCA
CTGGGAAGTTCGA

15	13.9	0.0	0.0	AluYb8-Scaffold1231-2979587-2979903	946	969	(1348)	+	(TC)n	Simple_repeat	1	24	(0)	1
2754	0.3	0.0	0.0	AluYb8-Scaffold1231-2979587-2979903	1001	1317	(1000)	C	AluYb8	SINE/Alu	(0)	318	1	2
271	28.4	11.4	1.6	AluYb8-Scaffold1231-2979587-2979903	1469	1643	(674)	C	L1ME3C	LINE/L1	(48)	6075	5884	3
502	27.4	0.0	5.3	AluYb8-Scaffold1231-2979587-2979903	1853	2010	(307)	C	MIR	SINE/MIR	(12)	250	101	4
631	5.4	0.0	0.0	AluYb8-Scaffold1231-2979587-2979903	2244	2317	(0)	+	L1P3	LINE/L1	625	698	(5763)	5

>Hg19 chr3:42897419-42899407
CAAGTCAAGATCAAGGTCCATGTCAGCAGCTCAGAAGAGCTGGTGTTTAATCAATGTCAACTCCTGAGTAATAAGCCCTGATAAGACCCGAGTATACTCCTCACATCCTCCTTCTGCAGCAGCTCA CA
GGCTCCCAGCACCAGCCATGTGCTAGGAGTGTCCTTACTGCTGCCCAGAAAAGGGAGGAGTTGAAGGCCAGGAGGCCCCTGGTGCAGAGGAGAGGACAGGAAGACCAGCAGAAAAAGGCACTGGG TAA
AGTTCCTAAGGTTGATCAGGAACAGAGTTGGGCCTTAGATAAAGCCACTCAGGCTGAGCTCCTGGGGTGGGGGCAGGTCATGTGTGGTTTCCACAGCATCTGGTCCAGATGCCATCGGATAGGAGC CC
AAGGGTGGGGCAGAAGCTCACAGAGTGGGAGGACTCCAGGGCTCCCTCAGGAGAGGTTCTGTTTCAGCAGCACTGAATAGTTCCATCAGCCATGAGATCATGGTATCATAGATACTTTGGGTCAA CTG
AGGACCCGAGTCAACATTTCTTTCGAAGTCTGCCTGAAAAAAGGGGATAAAACTTTTATCAGAAAGGGCTAAGGAAAATGTTTGCAATACATATGAAAAAAGCTAACATCCTCACTATTATAAGAC CT
CATAAAATGAGCATCCCACCCATTAAAAGAATAAATAATTTACAGAAGAAATACAAGGGGGTATTGAACATATTAAAAAGTTCAAGCTCATGCAAGTAAAATATTAAAAAGTAAAATTCAGAAGT ACG
GGGTAGAGCTGATTTGCATTTCTCAGTTTGTGGCCCGTGACCATCAGCAGTCTACAGGCCTCTCTTCAGTGGCT CTTCACGATTGCCTGCTTGGACCACAAATTCCTTTTTATCTCCTTCCTCTCTCC
TCCTCAAGCCCCCTTCCCCATTGGTGAATGCTCTTTGTTCAATATGAAGTCTCTCTCTCTCCCTGTCTTTCTCCTGGAACACACTACAGGTT TGAGCCTAATTCTTAGTTCATGACATACTTGTATCA
CTGGTTTATGTGAAGCTCTCATCAAAAAATTATTTTAAATAATGCAATAAAATCCTGGGATGTCACTGCCTTAGAGGACACTTAACCGTGGACAGTGACTTGTCCCATCTCCATGGTCCACCCCAA TC
CCATGCTGCTGGCTTCTGCCCAGGCACCTGCCGTCTTGAATTTGGTGCCTCTCATTCCTATGCCTTCCTGTCATCTATTTGTACTCCTTCAAAGTATATATGTTTTGTTTCATTTGTCTTAATTT TAT
TAAAAGAGTATCTTGTACATATTTTTATGGGACTTAGAGATAAGTGGGCTGATTCAAGAGGAGAGACTCA CAGGGTGGTGGTGGTGAGGaTGATGGGGAAAGAATCTGCAAGGTATCCCATATTTCAG
TCTTAGGTGACTGAGTAGTTGGTGGAGTCATTCAGTTAGATAGGCCTAGGAAAGAAGAAAGGGGCTGGTGGGGAGATGATGAACTCAGTTCTGGGCAAAGCAATAATTTAGGCTCGCATTTATTG AAT
GTCTGCTGTGTTCCAGTCAGTTTTGTAGAACTTAAAGTGCTCTGCATGTATTATTACATTTAAGCATAACAAGAACCTCACAAGACAGGCACTGTTATTATTTTCATTTTAAAGATAAGGAAACTA AG
AGGGAGAAAAGTTTAGTAGGTTCTGAGCCCATCAACATGATGGATCACACCTTTCTGCTGCGAACAGAAATATTGGATAAAATGTTTAAAATATTAAATATTTGTCATTAAAAATATTGATATGA AAC
TATTTACAAATAAAATATTTAAAAATTCTCAAAAAGCAAGGGAAATCCTCAGGTGACAGGAGTAAAGAAAACCCAAAGCCAGGGTGGATGCATTGGACATTTATGGATAAAACAAGCCCCACTGGC AT
CTGCCATTACTGAGGCTTGAGTAGGCGGTTTTCCCCTCGCAGTGTAAACAAAGCCACTGGGAAGTTCG A

15	13.9	0.0	0.0	Hg19	1046	1069	(1120)	+	(TC)n	Simple_repeat	1	24	(0)	6
271	28.4	11.4	1.6	Hg19	1241	1415	(774)	C	L1ME3C	LINE/L1	(48)	6075	5884	7
502	27.4	0.0	5.3	Hg19	1625	1782	(407)	C	MIR	SINE/MIR	(12)	250	101	8
1404	7.5	0.0	0.0	Hg19	2016	2189	(0)	+	L1P3	LINE/L1	625	798	(5663)	9

>KSI_AluYb8_10

>Scaffold1254-3006087-3006398

CACAGCCCTCCCAGGAAGCGGTGGATGGTCCCCTGGCCTTTGGCAACCCCCATCCCACCCTTACTCCCACCTGCTTAACTCTTTTGTAACCCCTCCCCTGATCCTGGTCCAGCTGGAGAGGGGCCA AA
ACCTCTTATCTACTGATTTAATTGCCTTTCCTAATCACAGTAGGCTGGTTCACACCTCTGTGACTGTGTACATTCATTTTCCTCTTCCTACAACTTTTTTCCCCCCTGGCAAATTTCTTCAATTA CAA
CCTTACCTTACTTGAGAAACTCTAATGACAATTAAGTTAGCTCTAACTTGCCCTCCTCAAAGTTGAACACTCCTTTATTTGGGCCACCTTGTCATCTTGAATATGATTTTATTACATTAATAGCAA TT
ATCACACTGGTTTACAATAGTGTCTCTCTCACTCTACACTATGAACTGTGGTAAGGCAGGGAGTGTGATTTATGTTTATATATCCAATG cTCCACACATTGTCTGGCCCACAGTAAACACACAAGCCA
TGTTTGACAAATAAATGAGTGAATGAATAACTGAATTAATGAATTAAGAATCAACAATATGGTAAAGGGAAGAAGATGGTAACACATTGTTTTATCTGCAAATTGTGTTGCCAAGTGAAAGTAGAA AT
CTGTGGTTATCTGCAGAAAGGTGGGACCCAGGAGGCCCCAGTAGTGACCTCACATATTTGGGTCATTCCTCAGGTTTGGATCAGTTCTGAGTCATGACACTTACAATTTCACTAAGCTTCTCTGA GCC
CATTTTATTTCCATCACTCAGGGAACAGGGAGAAAAGGTGATTTTTGTATCTCTAGCACATCTGATTAATGTCCTTTGAAATTCTGAACTGTGTACATATGACACTTTGGGGAAGGATTAGGAGTC TT
ATTATATTAGAGTATAAGAAAATATCAAGATTCAATTCAAACTAAAATGTGATATTAACA AACCATAGTCCCCCAATGCATCAACCTCTATTTaaaaaatggcgggccgggcgcggtggctcacgcct
gtaatcccagcactttgggaggccgaggcgggtggatcatgaggtcaggagatcgagaccatcctggctaacaaggtgaaaccccgtctctactaagaatacaaaaaattagccgggcgcggtggc gg
gcgcctgtagtcccagctactcgggaggctgaggcaggagaatggcgtgaacccgggaagcggagcttgcagtgagccgagattgcgccactgcagtccgcagtccggcctgggcgacagagcga gac
tccgtctcaaaaaaaaaaaaaaaaaAAAAAAaGGCATATTCTGGTATAGTgCAATACTGGTGTGACCTTTACTAAAAGCTTACAAGTATTAATTCCATACTGCATGGCAGGCATTGGACTGATTCTTT
TGTTTAAAACTCACACTACCTTAGGAGGAAAAGTATCATTCTCATCAGCTCCATTTGATAGATGAAGAAACTGAGACTCAGGATATATAATCTTG TCTAAGGTGGCACAGGAAGTGGTGGCTCTGTAA
TATGAACCCAGGGAAGCCATCTTTAGATCTTGTGTTAAGCGCATTAAGTGACTTTGACAACCTTGAATTAATTACATATTTGAAGCAGATGTTAAAGTTGGT GGGAGACAGGCAGGTAGGAAGGTCAT
GGTGAAAGAAACCCTTAAAGAAGCCCTCAAAGGCATCTTTTATTTCCTGAATATAAATCTCCTAAGAGATAATCTCTTCAGAGATTGTTTACTGCTTCTAAGTTTAACAGGGGTAGGGGAACTAT TCC
TCCTCAGATACCTAATGATTGTTATAACTAGTTTCTGGAAAGTTTAACTCATATTTACTTTTTTCTGTTGGAAACTCAGTAATATAATGCTTTTAATGTGATCCAAACTACAAAAACTAA TTGTATCC
TTTCCCTTTTTACAGTTCAAACCTAAGAATAGATCATTTTACTAGAGATTATTCTATATGAAACAAGATAGTGTGCTCATGGTAAAATGGGGGAAAAAACTTCACCCTAAAATCAGATACCAATT GTA
GTCTTTGGTTTGAACTCCAGCATTAAAAAACAAAATAACATATTTGAAACAATACTTATGGCTCTAACTAATTAGCTTCATACCACCCAGGTACTGTTTTTGGCAAGT tTGAGCTCgCATCTTTCCTT
TGGTTGTGAACTAAATGCAGATTAAGTCCTCAAGGAAGATCAGTAGTTACTGTATGCCAGGTAGGGAATCCCTTTTGAGGTTCACCCATGAAAATAATCAGGCAGGTTATGCAAATTTCCTGTTA CTG
TCAAGGGA

248	37.5	6.4	6.9	AluYb8-Scaffold1254-3006087-3006398	140	532	(1780)	+	L2b	LINE/L2	2985	3375	(0)	1
14	16.4	2.9	0.0	AluYb8-Scaffold1254-3006087-3006398	533	566	(1746)	+	(GAAT)n	Simple_repeat	1	35	(0)	2
2953	0.6	0.0	0.0	AluYb8-Scaffold1254-3006087-3006398	1001	1312	(1000)	+	AluYb8	SINE/Alu	1	312	(6)	3
368	28.4	11.2	3.7	AluYb8-Scaffold1254-3006087-3006398	1355	1572	(740)	C	MIR	SINE/MIR	(6)	262	23	4

>Hg19 chr4:68943781-68945776

CACAGCCCTCCCAGGAAGCGGTGGATGGTCCCCTGGCCTTTGGCAACCCCCATCCCACCCTTACTCCC ACCTGCTTAACTCTTTTGTAACCCCTCCCCTGATCCTGGTCCAGCTGGAGAGGGGCCAAA
ACCTCTTATCTACTGATTTAATTGCCTTTCCTAATCACAGTAGGCTGGTTCACACCTCTGTGACTGTGTACATTCATTTTCCTCTTCCTACAACTTTTTTCCCCCCTGGCAAATTTCTTCAATTA CAA
CCTTACCTTACTTGAGAAACTCTAATGACAATTAAGTTAGCTCTAACTTGCCCTCCTCAAAGTTGAAC ACTCCTTTATTTGGGCCACCTTGTCATCTTGAATATGATTTTATTACATTAATAGCAATT
ATCACACTGGTTTACAATAGTGTCTCTCTCACTCTACACTATGAACTGTGGTAAGGCAGGGAGTGTGATTTATGTTTATATATCCAATG tTCCACACATTGTCTGGCCCACAGTAAACACACAAGCCA
TGTTTGACAAATAAATGAGTGAATGAATAACTGAATTAATGAATTAAGAATCAACAATATGGTAAAGG GAAGAAGATGGTAACACATTGTTTTATCTGCAAATTGTGTTGCCAAGTGAAAGTAGAAAT
CTGTGGTTATCTGCAGAAAGGTGGGACCCAGGAGGCCCCAGTAGTGACCTCACATATTTGGGTCATTCCTCAGGTTTGGATCAGTTCTGAGTCATGACACTTACAATTTCACTAAGCTTCTCTGA GCC
CATTTTATTTCCATCACTCAGGGAACAGGGAGAAAAGGTGATTTTTGTATCTCTAGCACATCTGATTA ATGTCCTTTGAAATTCTGAACTGTGTACATATGACACTTTGGGGAAGGATTAGGAGTCTT
ATTATATTAGAGTATAAGAAAATATCAAGATTCAATTCAAACTAAAATGTGATATTAACA AACCATAGTCCCCCAATGCATCAACCTCTATTTAAAAAAtGGCATATTCTGGTATAGTaCAATACTGG
TGTGACCTTTACTAAAAGCTTACAAGTATTAATTCCATACTGCATGGCAGGCATTGGACTGATTCTTTTGTTTAAAACTCACACTACCTTAGGAGGAAAAGTATCATTCTCATCAGCTCCATTTG ATA
GATGAAGAAACTGAGACTCAGGATATATAATCTTGTCTAAGGTGGCACAGGAAGTGGTGGCTCTGTAATATGAACCCAGGGAAGCCATCTTTAGATCTTGTGTTAAGCGCATTAAGTGA CTTTGACAA
CCTTGAATTAATTACATATTTGAAGCAGATGTTAAAGTTGGT GGGAGACAGGCAGGTAGGAAGGTCATGGTGAAAGAAACCCTTAAAGAAGCCCTCAAAGGCATCTTTTATTTCCTGAATATAAATCT
CCTAAGAGATAATCTCTTCAGAGATTGTTTACTGCTTCTAAGTTTAACAGGGGTAGGGGAACTATTCCTCCTCAGATACCTAATGATTGTTATAACTAGTTTCTGGAAAGTTTAACTCA TATTTACTT
TTTTCTGTTGGAAACTCAGTAATATAATGCTTTTAATGTGATCCAAACTACAAAAACTAATTGTATCCTTTCCCTTTTTACAGTTCAAACCTAAGAATAGATCATTTTACTAGAGATTATTCTAT ATG
AAACAAGATAGTGTGCTCATGGTAAAATGGGGGAAAAAACTTCACCCTAAAATCAGATACCAATTGTAGTCTTTGGTTTGAACTCCAGCATTAAAAAACAAAATAACATATTTGAAACA ATACTTATG
GCTCTAACTAATTAGCTTCATACCACCCAGGTACTGTTTTTGGCAAGT cTGAGCTCaCATCTTTCCTTTGGTTGTGAACTAAATGCAGATTAAGTCCTCAAGGAAGATCAGTAGTTACTGTATGCCAG
GTAGGGAATCCCTTTTGAGGTTCACCCATGAAAATAATCAGGCAGGTTATGCAAATTTCCTGTTACTGTCAAGGG A

18	4.0	0.0	0.0	Hg19	55	80	(2116)	+	(CTGCC)n	Simple_repeat	1	26	(0)	5
236	37.7	6.4	6.9	Hg19	240	632	(1564)	+	L2b	LINE/L2	2985	3375	(0)	6
14	16.4	2.9	0.0	Hg19	633	666	(1530)	+	(GAAT)n	Simple_repeat	1	35	(0)	7
368	28.4	11.2	3.7	Hg19	1139	1356	(840)	C	MIR	SINE/MIR	(6)	262	23	8

>KSI_AluYb8_11

>Scaffold1774-3449494-3449811
AGTTTTTGCCAAGTGGTCAAGATGTTCACTGCTGTAACTAAGAGCTTCCACTGAAG cTACTATTATCAACCACAGCACTCATAAACACATAGCACTCCTGGGCACATTTTCTATTTATTTAAGTGACT
TACCTTATTTGATCAAAAAGAAAATATTAAAATCACCATAAAACTATGTAATTAGTGATTGCATGGTGATCTTTATGTGATGAAATTTTTAACAGTTAATATCTAATATAGAATTAAGAATCCTA CAC
AATATTTTAAAGTTATATTTTCAAATCCATATTTTGTGGATTTCAAATAATTCCAGT TTGAATGCTTGCAACTGCAACAGGTTTCTGTAAAAGCTTTTGAGTTTCACAGCCTTCCTTTTGACAGATTT
CTTAAGTTATGGAATGCAGCCATCAGCAGTCTGTAGAAAAATGAAATGCTGCAAGTCTATTTTTATGCAGCTGGGATCAAGTTACTAAATGGATCAAAAGAAATAATTTCTTTGAAGAGGTGGCT GTT
GACACCAGTGCCCTAAAAATAAGTGCAATTTTGTGGGCATAAAAATTTGTTTATATC TTAGTGAAAGTAACTATAGATATATTTTCTATATATACACTTGCATTCATGATATATTGGATGCCATTCTA
AACATCTTTTAATATACCTTAGTTTAACATTGCTTTTAAGAGGAAAGTTGCATGCTTTCTCTTGAATAATTGATGTAGCTAAAG tCAATTATTCATACCAGGCTTAGCTTACTGGGTTTATAATAAAA
ACAATGATAGTAATTTACCTAACTCTTACTTTGTGCCAAATACCAGCCAAATACTTC CCAACATTATTTCATTTGATTTTAGAATTACATTTTATTTTAATAACTAGCTATTATTTTCACAATTGTAT
AGATGAAGAAGCTGATTCTTAGAGAGTGTAACTTGCCAAAGATTACAC aGCTAGCATGCCTCTGAGTTAAAATTTGAAAGCTAGTCTGTT Taattctgaagctcttttttttttttttttttttt
ttttttgagacggagtctcgctctgtcgcccaggccggactgcggactgcagtggcg caatctcggctcactgcaagctccgcttcccgggttcacgccattctcctgcctcagcctcccgagtagct
gggactacaggcgcccgccaccgcgcccggctaattttttgtatttttagtagagacggggtttcaccttgttagccaggatggtctcgatctcctgacctcctgatccacccgcctcggcctcc caa
agtgctgggattacaggcgtgagccaccgcgcccggccAATTCTGAAGCTCTTAATCACTAAATTTTATACTACCTCTTTAGGTTGGATGGCATATCAACTGATTTGAAACTATAGAAAAAAATTAGG
AAACAGAATAATCTTTTAAAATATCTTTGTATGTGAAAGTAAGCTTGCTTATAGAAAATGTAGTAAATTGAAAGACTATTACTATTGCAATAGCAATAGTCAAACTGAAGCCTATAG AACTCAAATGC
ATGTAAGTGTCAAAATAGCCAAGTAATAATGCGATATGATATAATTTTTTA TCATAATAAAAAGTCAGAAAACAAAATTCATAAAGAGGGCTCTATGATACAAGAGGAAAGGTTGATCTAAGGGTACA
AACTAAAACATTTTAAATAAAGTTTTTGGAAAAAACAATTTTAGGCTCCGAAATAAGAAATAAATACAACCCCTTCA tAGATAATAATAAAGAAATAAGCCAATAAGCCCTTAAAATAGAACGATATA
GTCTTAGCTCTGGAGGACAAAATATTCTCTACTTGGACCCTGACTCATTTA GAACTCCTGCACATAAATACATATATATATAtatataCACACACACACACACACACACATATTGACTATATTGCTGA
AGGGGAGAGTAAAGGAAATGGTGAAAGGATCATCATTTTTATTATTTCATTAATTCCTGAAGAAAACACA cACACAAATTGCAATTGAAGCTTCTAAAAAACCTAGAAAACAGTATAAAGGACTTAGA
CACAGTACAAGTATTTCCTACCATGTAAGTCTGAAAGAGAGAAAATCAATT TTTATGAAAACATAAAACAGTGATTTGGGTTGTGAACAAAATTATTTGTCTCCATTTGTGTTCTATTGGCTTACTGC
TCTGCCAAATTTTGAAGCAAAATGAAAAAAAAAAAAAAAGAGAGGGAAATTATAGCAAACCTCCCAAAACATATCCCTTAAACTCCGCAATGTATTTAAACTACCCAGGTTCTGTTCATGGATATA CTC
AAACATTGAGAAAT

499	33.2	2.7	2.7	AluYb8	781	1000	(1318)	C	MIRb	SINE/MIR	(15)	253	34	1
2730	0.7	0.0	0.0	AluYb8	1001	1318	(1000)	C	AluYb8	SINE/Alu	(0)	318	1	2
356	33.0	2.7	6.5	AluYb8	1319	1352	(966)	C	MIR	SINE/MIR	(232)	30	4	1
18	8.5	0.0	0.0	AluYb8	1856	1880	(438)	+	(AT)n	Simple_repeat	1	25	(0)	3 *
21	0.0	0.0	0.0	AluYb8	1880	1902	(416)	+	(AC)n	Simple_repeat	1	23	(0)	4

>hg19 chr2:50601643-50603623
AGTTTTTGCCAAGTGGTCAAGATGTTCACTGCTGTAACTAAGAGCTTCCACTGAAG tTACTATTATCAACCACAGCACTCATAAACACATAGCACTCCTGGGCACATTTTCTATTTATTTAAGTGACT
TACCTTATTTGATCAAAAAGAAAATATTAAAATCACCATAAAACTATGTAATTAGTGATTGCATGGTGATCTTTATGTGATGAAATTTTTAACAGTTAATATCTA ATATAGAATTAAGAATCCTACAC
AATATTTTAAAGTTATATTTTCAAATCCATATTTTGTGGATTTCAAATAATTCCAGTTTGAATGCTTGCAACTGCAACAGGTTTCTGTAAAAGCTTTTGAGTTTCACAGCCTTCCTTTTGACAGA TTT
CTTAAGTTATGGAATGCAGCCATCAGCAGTCTGTAGAAAAATGAAATGCTGCAAGTCTATTTTTATGCAGCTGGGATCAAGTTACTAAATGGATCAAAAGAAATA ATTTCTTTGAAGAGGTGGCTGTT
GACACCAGTGCCCTAAAAATAAGTGCAATTTTGTGGGCATAAAAATTTGTTTATATCTTAGTGAAAGTAACTATAGATATATTTTCTATATATACACTTGCATTCATGATATATTGGATGCCATT CTA
AACATCTTTTAATATACCTTAGTTTAACATTGCTTTTAAGAGGAAAGTTGCATGCTTTCTCTTGAATAATTGATGTAGCTAAAG aCAATTATTCATACCAGGCTTAGCTTACTGGGTTTATAATAAAA
ACAATGATAGTAATTTACCTAACTCTTACTTTGTGCCAAATACCAGCCAAATACTTCCCAACATTATTTCATTTGATTTTAGAATTACATTTTATTTTAATAACTAGCTATTATTTTCACAATTG TAT
AGATGAAGAAGCTGATTCTTAGAGAGTGTAACTTGCCAAAGATTACAC gGCTAGCATGCCTCTGAGTTAAAATTTGAAAGCTAGTCTGTT TAATTCTGAAGCTCTTAATCACTAAATTTTATACTACC
TCTTTAGGTTGGATGGCATATCAACTGATTTGAAACTATAGAAAAAAATTAGGAAACAGAATAATCTTTTAAAATATCTTTGTATGTGAAAGTAAGCTTGCTTATAGAAAATGTAGTAAATTGAA AGA
CTATTACTATTGCAATAGCAATAGTCAAACTGAAGCCTATAGAACTCAAATGCATGTAAGTGTCAAAATAGCCAAGTAATAATGCGATATGATATAATTTTTTAT CATAATAAAAAGTCAGAAAACAA
AATTCATAAAGAGGGCTCTATGATACAAGAGGAAAGGTTGATCTAAGGGTACAAACTAAAACATTTTAAATAAAGTTTTTGGAAAAAACAATTTTAGGCTCCGAAATAAGAAATAAATACAACCCCTT
CAcAGATAATAATAAAGAAATAAGCCAATAAGCCCTTAAAATAGAACGATATAGTCTTAGCTCTGGAGGACAAAATATTCTCTACTTGGACCCTGACTCATTTAG AACTCCTGCACATAAATACATAT
ATATATACACACACACACACACACACACATATTGACTATATTGCTGAAGGGGAGAGTAAAGGAAATGGTGAAAGGATCATCATTTTTATTATTTCATTAATTCCTGAAGAAAACACA tACACAAATTG
CAATTGAAGCTTCTAAAAAACCTAGAAAACAGTATAAAGGACTTAGACACAGTACAAGTATTTCCTACCATGTAAGTCTGAAAGAGAGAAAATCAATTTTTATGA AAACATAAAACAGTGATTTGGGT

TGTGAACAAAATTATTTGTCTCCATTTGTGTTCTATTGGCTTACTGCTCTGCCAAATTTTGAAGCAAAATGAAAAAAAAAAAAAAGAGAGGGAAATTATAGCAAACCTCCCAAAACATATCCCTT AAA
CTCCGCAATGTATTTAAACTACCCAGGTTCTGTTCATGGATATACTCAAACATTGAGAAA T

490	33.1	6.6	2.0	hg19	781	1026	(955)	C	MIRb	SINE/MIR	(15)	253	2	5
22	22.0	0.0	0.0	hg19	1522	1565	(416)	+	(CA)n	Simple_repeat	1	44	(0)	6

>KSI_AluYb8_12

>Scaffold1780-3512926-3513209
TTGAAATTGTCCACAAAATCTTTAGCATCCTCTGAGCAGCCATCTCTTCCTCACCTTAAGAAGCCGACTACACTGGCTGGTTGCAAATATTGACAAATTTCAGGATCACCTTGGAAGGTGGTTTTT TA
GGTTCTTTGATTTAAACTCTTCAATCATGCTCCATCAACCCACCAGTCCCAAGTCAGGCTGGTATTTGAGGCTCCTC tTGACCTGGTTTCTGGTCTTGGCTTCAACACCTCTACTCTCCCTTGTTGaA
TCTTGTGTGACAGTCTCCCTGTGGAGGTCACGATGAAGGTGAAAGCGATATGACCAGATGAAGGGACTGAGAAGCGCCAAGGAAGATGTCAGGGTAAGACTCGTTCTGGGTTCGATGTAAATATAG CC
ACAGAGATTTCCACCTCTAAGCTCTGGCCCTCCTTTCTCTACGATATTCCTATCTCCTTTCCTGCCTTCTGTCTTAGGTCAAAGAACCACAGGCAGCCACCTGAAAGACTGTGCACCATAATATT CAA
GAACATGAACTTTGGAAATCTATGTGGTCTGGATTTAGATCCTGGCTCCGCCACATACTAGCTGTGTGACTTTGGGAAAGTTAGGCTACCTGTCTGGGCCTTAATGTTGTCATCACAGCTCTTTAG CT
CAACATCAGTTCTAGTCCCCATCATCTGCAATAGCAATCATTCTCCAGGAAAAGTTTTCACCTCTTCATTTAAGGCTGCTCCAACTCTCAAGCAGTCTGGGTACCCTTTTCCCCAACTCCCACCC CAG
TTGTATCTGTTACCTGTGACCTTCCCTTCTGAATATTGCCTCAATAGCATGAACCCTTTTATACTTTGAAAGACAGCATGGAAAT ATGGAAGCAGAGAGAGTGCATAGAATGTGGAATGTGAGTCCGG
GCTCTGCTACCAGCTGTATGACCTTGGGCAGTTTATGTAACGTGGTTGAACTTGTTCCCTATTTTCAATAAAAAATCAATAATTC CCTTTTCTCAGGATGTTTCTtttttttttttttttttttttt
ttttgagacggaatctcgctctgtcgcccaggtcggactgcggactgcagtggcgcaatctcggctcactgcaagctccgcttcccgggttcacgccattctcctgcctcagcctcccgagtagc tgg
gactacaggcgcccgccaccgcgcccggctaatttttgtatttttagtagagacggggtttcactttgttagccaggacggtctcgatctcctgacctcatgat ccacccgcctcggcctcccaaag
tgttctcaggatgtttctAAGTATTAAGTGGGATGAGATGCGTATGCTTTGGGCTTCACTCATCTGTGAGTACAAATAAAACTATATTACAGTAATAACCTG cGTTCTTGACAGTAGGGAGAGCTCCT
TACCCCACTGTTTTCAAGAATCCTTATGGTGCCCATGAAT aTCCAAAAATCAGACTTGTTTCTTCTCTATGCAAGCCTAGGTAAAGTGTG GCAGCATGACCTATGTTCTCAGTTATAgCtTTCTACTG
CCTAGGTTTCAGAGAGTTGAAACTCTGTCCCTCTTCAAAACTGCAGAAACTGGAGAAGCCTTTTTTTCTTTTTCCTTTTTTTTTTTTTTTTTTTtttttttGAGACAGGGTCTTGCTCTTTTGCCCAGG
CTGGAATTCAGTGGAGTGATCATAGCTCACTGCAGCCTCAATCTCCTGGGCTCAAGCAATCCTCCCACCTCAGCCTCCAGAGGGGCAGGGAACATAGGTGCTTGC CACCATGTCCGGCTAATTAAATT
AAATTACATTTATTTGTTTGTAGAGATGGAGTTTTTCTATGTTGCCCAGGCTGGTATTGAACTCCC gCCCTCAAGTGATTCTCCCACCTCAGCCTCCCAAAGTGCTGGGACTACAGACATGAGCCACC
ACACCTGGCCAGGAAATGCTTTTTTTGAATTCTGGTACTAAAGATTTAAGT aGTAATGCTTGAAAAgTGAGAAGATAATGCTGCTTCTGTGCTATGTCCAGTGAG GGAGCCCCACAGTTTGATTCGTT
GAGGTTTTATAATAgAAGTCTCACACTCTTTTCTGTTTCTTGATGGGTTACAAATCCCTTGCTTTGGGCCTGCCAATCCCATGGGGGTGACACAGT aAGGCTGGGGTTCTGTTCCCTTACCATCACCG
GTGTTTCAGTGCAGGGGCAAGAGAATGCAGATACATCCAATGTCAGTAGGCAGGAAATACCAGCATCTGAGACTGAGGTGATATCTCACGTGAAACATGACAAATAC A

491	29.3	0.0	1.5	AluYb8-Scaffold1780-3512926-3513209	492	626	(1658)	+	MIR	SINE/MIR	1	133	(129)	1
258	31.8	6.5	0.0	AluYb8-Scaffold1780-3512926-3513209	889	995	(1289)	+	MIRb	SINE/MIR	63	176	(92)	2
2365	2.3	0.0	0.0	AluYb8-Scaffold1780-3512926-3513209	1001	1284	(1000)	C	AluYb8	SINE/Alu	(0)	318	33	3
225	26.5	2.0	0.0	AluYb8-Scaffold1780-3512926-3513209	1332	1380	(904)	C	LTR88a	LTR/Gypsy?	(19)	797	748	4
1785	14.6	0.0	4.3	AluYb8-Scaffold1780-3512926-3513209	1597	1930	(354)	C	AluJr	SINE/Alu	(0)	312	1	5

>Hg19 chr16:8534516-8536494
TTGAAATTGTCCACAAAATCTTTAGCATCCTCTGAGCAGCCATCTCTTCCTCACCTTAAGAAGCCGACTACACTGGCTGGTTGCAAATATTGACAAATTTCAGGATCACCTTGGAAGGTGGTTTTT TA
GGTTCTTTGATTTAAACTCTTCAATCATGCTCCATCAACCCACCAGTCCCAAGTCAGGCTGGTATTTGAGGCTCCTC aTGACCTGGTTTCTGGTCTTGGCTTCAACACCTCTACTCTCCCTTGTTGtA
TCTTGTGTGACAGTCTCCCTGTGGAGGTCACGATGAAGGTGAAAGCGATATGACCAGATGAAGGGACTGAGAAGCGCCAAGGAAGATGTCAGGGTAAGACTCGTTCTGGGTTCGATGTAAATATA GCC
ACAGAGATTTCCACCTCTAAGCTCTGGCCCTCCTTTCTCTACGATATTCCTATCTCCTTTCCTGCCTTCTGTCTTAGGTCAA AGAACCACAGGCAGCCACCTGAAAGACTGTGCACCATAATATTCAA
GAACATGAACTTTGGAAATCTATGTGGTCTGGATTTAGATCCTGGCTCCGCCACATACTAGCTGTGTGACTTTGGGAAAGTTAGGCTACCTGTCTGGGCCTTAATGTTGTCATCACAGCTCTTTA GCT
CAACATCAGTTCTAGTCCCCATCATCTGCAATAGCAATCATTCTCCAGGAAAAGTTTTCACCTCTTCATTTAAGGCTGCTCC AACTCTCAAGCAGTCTGGGTACCCTTTTCCCCAACTCCCACCCCAG
TTGTATCTGTTACCTGTGACCTTCCCTTCTGAATATTGCCTCAATAGCATGAACCCTTTTATACTTTGAAAGACAGCATGGAAAT ATGGAAGCAGAGAGAGTGCATAGAATGTGGAATGTGAGTCCGG
GCTCTGCTACCAGCTGTATGACCTTGGGCAGTTTATGTAACGTGGTTGAACTTGTTCCCTATTTTCAATAAAAAATCAATAA TTCCCTTTTCTCAGGATGTTTCTAAGTATTAAGTGGGATGAGATGC
GTATGCTTTGGGCTTCACTCATCTGTGAGTACAAATAAAACTATATTACAGTAATAACCTG aGTTCTTGACAGTAGGGAGAGCTCCTTACCCCACTGTTTTCAAGAATCCTTATGGTGCCCATGAAT g
TCCAAAAATCAGACTTGTTTCTTCTCTATGCAAGCCTAGGTAAAGTGTGGCAGCATGACCTATGTTCTCAGTTATA aCTTCTACTGCCTAGGTTTCAGAGAGTTGAAACTCTGTCCCTCTTCAAAACT
GCAGAAACTGGAGAAGCCTTTTTTTCTTTTTCCTTTTTTTTTTTTTTTTTTT TGAGACAGGGTCTTGCTCTTTTGCCCAGGCTGGAATTCAGTGGAGTGATCATAGCTCACTGCAGCCTCAATCTCCTGG
GCTCAAGCAATCCTCCCACCTCAGCCTCCAGAGGGGCAGGGAACATAGGTGCTTGCCACCATGTCCGGCTAATTAAATT AAATTACATTTATTTGTTTGTAGAGATGGAGTTTTTCTATGTTGCCCAG
GCTGGTATTGAACTCCCtCCCTCAAGTGATTCTCCCACCTCAGCCTCCCAAAGTGCTGGGACTACAGACATGAGCCACCACACCTGGCCAGGAAATGCTTTTTTTGAATTCTGGTACTAAAGATTTAA
GTcGTAATGCTTGAAAAcTGAGAAGATAATGCTGCTTCTGTGCTATGTCCAGTGAGGGAGCCCCACAGTTTGATTCGTT GAGGTTTTATAATAaAAGTCTCACACTCTTTTCTGTTTCTTGATGGGTT
ACAAATCCCTTGCTTTGGGCCTGCCAATCCCATGGGGGTGACACAGT gAGGCTGGGGTTCTGTTCCCTTACCATCACCGGTGTTTCAGTGCAGGGGCAAGAGAATGCAGATACATCCAATGTCAGTAG
GCAGGAAATACCAGCATCTGAGACTGAGGTGATATCTCACGTGAAACATGACAAATAC A

491	29.3	0.0	1.5	Hg19	592	726	(1453)	+	MIR	SINE/MIR	1	133	(129)	6
312	31.9	5.0	1.4	Hg19	989	1128	(1051)	+	MIRb	SINE/MIR	63	207	(61)	7
237	29.1	1.8	0.0	Hg19	1135	1189	(990)	C	LTR88a	LTR/Gypsy?	(19)	797	742	8
1978	14.5	0.0	3.9	Hg19	1402	1725	(454)	C	AluJr	SINE/Alu	(0)	312	1	9

>KSI_AluYb8_13

>Scaffold1913-81388-81700
GTCTCGTTCAAAGTTTCTCAAGCACAGTTCAACTTTGTTCTAGGACTGTGAATATTTAAATTTATGGTTATTACATATTAGAAAGGTACACTATGATTTTGTGATATAACTCAATATTACTCAGTT AT
GAATGATATGAAGGCAATAGATATAAAAATACTGGACTACAACCCTCCAGAATTAAGGATGCTGGAGATAAAATGGCTTCCCTAAAAGGCTAAAAATGAATCATTATTCTATGCTATTTATGAAA TAT
CTTGGTTAACAGGGCCTCACAGGTAGTAATGAATATAAAAATATCCCTAGATTCCAATTTCATCAATAGGCTGAGTCCCCACTCCTACCAAACTTTGTATTTGTGAAAGAGTGTAAAGCTTTGGTT TC
TACCTTTTTTCCCTTATGAAGTGGACATTCTGTTGGAGATTATATTACCTAGAAAACAGGGGACAAACCATAATACTAGATCACTGGACTAAAAGTCAAAAAATGTGAATTCTAATCAAAATTCT ATC
AAGAATTTTGAATGTCAAAGGAAAGCCAGTTCCTTTCAAAGGGCCTTAGTTTGCTCATTTTGCAAAACCAAGGACTAAAATTAAAATTGCCTAAG GTCTCACCAGGTCAAAGGCAATATAGAATAATG
TTTAAGAGACTGTCCTCAGTTATATATACTAGGTACATACATTTGGGTAAATTATCAAACTTTTTGCCTCAGTTTCACAAAAATAATCAGGATAATAATATCTACCTCATTGATTTATTGAAAGC CAC
CAATGAGTTGATATGTGTTAAGTTGTTAACACATGCTCAATAACAATAATGGTGTCTTTGTCTGTCTTGTGCTGCTATAACAGAATATCTAAGGCTGGGTAATTTAAAATGAACACAAATTTGTTG GC
TTAAAGTTCTAGAGGCTAGAAAGTCCAGTTTCAAGGTGCTGGCATCTGGCAAGGGCCTTCTTGCTGCATTATAACACAGCAGAAG ATaagaaagtaagagagagggccgggcgcggtggctcacgcct
gtaatcccagcactttgggaggccgaggcgggtggatcatgaggtcaggagatcgagaccatcctggctaacaaggtgaaaccccgtctctactaaaaatacaaaaaattagccgggtgcggtgg cgg
gcgcctgtagtcccagctactcgggaggctgaggcaggagaatggcgtgaacccgggaagcggagcttgcagtgagccgagattgcgccactgcagtctgcagt ctggcctgggcgacagagcgagac
tccgtctcaaaaaaaaaaaaaaaaaaAAGAAAGTAAGAGAGAGCAAGAGGGTCCAAATTCATTCTTTTATAAGGGTATGAACCTCACCCATGGGGTGGAGCCCTCATGGCCTAATGTCTTCTTAGAG
GTCCCACTTCTTAATATTGTTACAATGGCAATTACATTTCAACATGAGTTTTGGAGGGGACAAACATTCAAACCATAGCAGATGATAATGATGGAGAAGGTGAT GATGGTGGTGATGAAGATAATTTT
ATGTACACAATGTTATAATAGAAGACTGTAAGGTAAAGAAAATATTTAGATAATTTACAAACTACGTAACATTCTATAAAAGTACAAAGTGTGATTAAATACAGTGGT CAAGAAAGAAACTGAGTAAA
TAAACTTTGTTATATATGTCTAATGTATGTTTATCATTTTTAAGGCAACTGTTGCCTTAAGCACTTAATCACTGGGTAAATAAGTTTCCAATTCTATGAATTTC TTTACATATAAAATTACATCAAAT
TTAAGCATTTGACTGCATGTATAGGCATTGAACAACAGACAAGCTTTTTATTAGAAATAGAGATTTGAGAGTGAAACCAAATATATTATCTGTGATGACTAATACTGAGTGTCAACTTGATTGGATGG
AAGGATGCAAAGTATTGATCCTGGGTGTGTCTGTGAGGGTATTGtCAAAAAAGATTAACATTTGAGTCAGATGTTAGGTGAGGGCAGACCCACCCTTAATCTGG TTGGGCACCATTTAATCAGCTGCC
AGTGCAGCTAGAATATAAAGCAGGCAGAAAAATGTGAAAAGACAAGACTGGCCTAGCCTCCCAGCCTGCATCTTTCTCCCGTGCTGGATGCTTCCTGCCCTGGAACATCAGACTCCAAGTCCTTC AGT
TCTGGGACTCAGACTGGCTCTCCTTGCTCCTCAGCTTGCAGACAGCCTACTGTGGGACCCCTGTGATCGTGTGAGTTAATACTTAGTAAATACTAATATAATATA CTACTACTAATATAATACTTAGTT
AATACTAAT

239	36.5	3.3	2.0	AluYb8-Scaffold1913-81388-81700	462	612	(1701)	+	MIR3	SINE/MIR	23	175	(33)	1
272	32.4	7.3	1.9	AluYb8-Scaffold1913-81388-81700	667	817	(1496)	+	MIR	SINE/MIR	75	240	(28)	2
1305	22.3	13.2	1.8	AluYb8-Scaffold1913-81388-81700	821	1000	(1313)	C	MSTD	LTR/ERVL-MaLR	(0)	396	216	3
2917	1.6	0.0	0.0	AluYb8-Scaffold1913-81388-81700	1001	1313	(1000)	+	AluYb8	SINE/Alu	1	313	(5)	4
1305	22.3	13.2	1.8	AluYb8-Scaffold1913-81388-81700	1314	1488	(825)	C	MSTD	LTR/ERVL-MaLR	(181)	215	2	3
18	23.9	0.0	0.0	AluYb8-Scaffold1913-81388-81700	1489	1533	(780)	+	(GAT)n	Simple_repeat	1	45	(0)	5
2943	8.4	0.3	0.8	AluYb8-Scaffold1913-81388-81700	1883	2265	(48)	+	MLT2A1	LTR/ERVL	1	381	(63)	6
12	13.0	8.8	0.0	AluYb8-Scaffold1913-81388-81700	2266	2299	(14)	+	(TAC)n	Simple_repeat	1	37	(0)	7

>Hg19 chr2:158509273-158511461
GTCTCGTTCAAAGTTTCTCAAGCACAGTTCAACTTTGTTCTAGGACTGTGAATATTTAAATTTATGGTTATTACATATTAGAAAGGTACACTATGATTTTGTGATATAACTCAATATTACTCAGTT AT
GAATGATATGAAGGCAATAGATATAAAAATACTGGACTACAACCCTCCAGAATTAAGGATGCTGGAGATAAAATGGCTTCCCTAAAAGGCTAAAAATGAATCATTATTCTATGCTATTTATGAAA TAT
CTTGGTTAACAGGGCCTCACAGGTAGTAATGAATATAAAAATATCCCTAGATTCCAATTTCATCAATAGGCTGAGTCCCCACTCCTACCAAACTTTGTATTTGTGAAAGAGTGTAAAGCTTTGGTT TC
TACCTTTTTTCCCTTATGAAGTGGACATTCTGTTGGAGATTATATTACCTAGAAAACAGGGGACAAACCATAATACTAGATCACTGGACTAAAAGTCAAAAAATGTGAATTCTAATCAAAATTCT ATC
AAGAATTTTGAATGTCAAAGGAAAGCCAGTTCCTTTCAAAGGGCCTTAGTTTGCTCATTTTGCAAAACCAAGGACTAAAATTAAAATTGCCTAAG GTCTCACCAGGTCAAAGGCAATATAGAATAATG
TTTAAGAGACTGTCCTCAGTTATATATACTAGGTACATACATTTGGGTAAATTATCAAACTTTTTGCCTCAGTTTCACAAAAATAATCAGGATAATAATATCTACCTCATTGATTTATTGAAAGC CAC
CAATGAGTTGATATGTGTTAAGTTGTTAACACATGCTCAATAACAATAATGGTGTCTTTGTCTGTCTTGTGCTGCTATAACAGAATATCTAAGGCTGGGTAATTTAAAATGAACACAAATTTGTTG GC
TTAAAGTTCTAGAGGCTAGAAAGTCCAGTTTCAAGGTGCTGGCATCTGGCAAGGGCCTTCTTGCTGCATTATAACACAGCAGAAGA TAAGAAAGTAAGAGAGAGCAAGAGGGTCCAAATTCATTCTTT
TATAAGGGTATGAACCTCACCCATGGGGTGGAGCCCTCATGGCCTAATGTCTTCTTAGAGGTCCCACTTCTTAATATTGTTACAATGGCAATTACATTTCAACATGAGTTTTGGAGGGGACAAACA TT
CAAACCATAGCAGATGATAATGATGGAGAAGGTGATGATGGTGGTGATGAAGATAATTTTATGTACACAATGTTATAATAGAAGACTGTAAGGTAAAGAAAATATTTAGATAATTTACAAACTAC GTA
ACATTCTATAAAAGTACAAAGTGTGATTAAATACAGTGGTCAAGAAAGAAACTGAGTAAATAAACTTTGTTATATATGTCTAATGTATGTTTATCATTTTTAAGGCAACTGTTGCCTTAAGCACTT AA
TCACTGGGTAAATAAGTTTCCAATTCTATGAATTTCTTTACATATAAAATTACATCAAATTTAAGCATTTGACTGCATGTATAG GCATTGAACAACAGACAAGCTTTTTATTAGAAATAGAGATTTGA
GAGTGAAACCAAATATATTATCTGTGATGACTAATACTGAGTGTCAACTTGATTGGATGGAAGGATGCAAAGTATTGATCCTGGGTGTGTCTGTGAGGGTATTG cCAAAAAAGATTAACATTTGAGTC

AGATGTTAGGTGAGGGCAGACCCACCCTTAATCTGGTTGGGCACCATTTAATCAGCTGCCAGTGCAGCTAGAATATAAAGCAGGCAGAAAAATGTGAAAAGACAAGACTGGCCTAGCCTCCCAGC CTG
CATCTTTCTCCCGTGCTGGATGCTTCCTGCCCTGGAACATCAGACTCCAAGTCCTTCAGTTCTGGGACTCAGACTGGCTCTCCTTGCTCCTCAGCTTGCAGACAGCCTACTGTGGGACCCTGTGAT CG
TGTGAGTTAATACTTAGTAAATACTAATATAATATACTACTACTAATATAATACTTAGTTAATACTAA T

239	36.5	3.3	2.0	Hg19	562	712	(1477)	+	MIR3	SINE/MIR	23	175	(33)	8
272	32.4	7.3	1.9	Hg19	767	917	(1272)	+	MIR	SINE/MIR	75	240	(28)	9
1292	20.8	19.5	0.7	Hg19	921	1264	(925)	C	MLT1A1	LTR/ERVL-MaLR	(0)	408	1	10
18	23.9	0.0	0.0	Hg19	1265	1309	(880)	+	(GAT)n	Simple_repeat	1	45	(0)	11
3124	8.2	0.2	10.6	Hg19	1659	2148	(41)	+	MLT2A1	LTR/ERVL	1	444	(0)	12

>KSI_AluYb8_14

>Scaffold2140-4721399-4721696
GCCAAGGCCAATGTTGAGAAGGGCATTTCCTAGGTTTTCTTCCAGGAGTTTGAGGTCTTACATTTATATCTGTAATCCGTCTTGAGTTAATTTTTTTATATGGTGAAAGGTAAGGGTCCAGTTTCA TT
CTTCTTCATATGGCTAGCCATTTATCCCAGCACCATGTACTGAGTAGGGAGTTCTTTTTCTATTGCTTGTTTTTGTCAGCCTTGTCAAAGATCAGACGGTTATAAGTGTATGGTTTTATTTCTGA GTT
TTTTATCCTGTTCCATTGGTCTATGTGTCTGTTTTGTACTAGTACCATGCTGTTTT GGTTGCTCTAGCCTTGTGGTATAGTTTGAAATTGGGTATTGTGATGTCTCCAGCTTTGTTCCTTTTGCTTAA
GACTGTTTTGGCTATTCAGGCTCTTTTTTTGGGTTCCATATGAATTTTAGAATAGTTTTTTTTTTAATTCTGTGAAGAATGACATTGGTAGTTTGATAGGAATAGAATTTAATCTGTAAGTTGCT CTG
GGCAATAGGGCTATTTTAGTGATACTGATTCTTCCAgTCCATGAACATGGAAAGTATTTCCATTTATTTGTGTCATCTCTGATTTCTTTCAGCAGAGTTTTATAGTTCTCCTTGTAGAGATCTTTCAC
CTCCTTGGTTAGCTGCATTCCTAGGTATTTTATTTTCTTTGTGGCTATGGTAAATGAGATTGTGCTCTTGATTTGACTCTCAGCCTGGACATTATTGGTGTATAGAAATGCTACTGATTTTTGTA CCC
TGATTTTATATCAAAAACTTACTAAAATTGTTTATCAGTTTTAGTAGGCTTTTAGCAGATTATTTAGAATTTTCTAGACAGAAAATTATATCATCAGTGAAGAGAGATAGTTTGACTTCTTTTCCT AT
TTGGATGCCTTTTATTTTTTTCACTTGCCTGAGCGCTCTGGCTAGGACATCTAACAGATCCGTTTTAAAGGGATTGAGTTTTGTTTAGT Aagaaaaacacttccttttttttttgagacggagtctcg
ctctgtcgcccaggccggactgcggactgcagtggcgcaatctcggctcactgcaagctccgcttcccgggttcacgccattctcctgcctcagcctcccgagtagctgggactacaggcgcccg cca
ccgcgcccggctaatttttgtatttttagtagagacggggtttcaccttgttagccaggatggtctcgatctcctgacctcatgatccacccgcctcggcctc ccaaagtgctgggattacaggcgt
gagccaccgcgcccggccAGAAAAAcACTTCCTAAGTATAAGTTGGAATTTATGAGGTTCGTGAATGGCTTTATCATCACTGTTACTTTTATAGATAATTTTCCAAGTGGCACTTTATGGTCATAAAT
AAGCCTCAGTCATATCTTTTTGCAAGTGCCTCCCTAATTATCATTTCTGAAGAAAAATTTTGGCCTCTATATTAAATTATAGTTCACGACTTAGTTCCAGTTCA CACCCTGAGCTACTATATTGGATA
TAACACATCTTCCTTAGGTGTTACTTACTGAGCTGAAAGTATTGCCCAAAGCATCTTATTTTGTCTGTATCCAGTAATTTATTGAGCCTGGATGCTATCCTGATAGAGTTTACTCACTAAAATAG AAG
TTATAAAAGTTCAATCAAATTATGATCTATCAGTCATCTTACAGAACCAAGAATGTGCATTCATTTATCCAAGTACTATTTACTCAGTGCCTAAAAAATGTCAG GTGTTTGGGATCACAGAAAAAAAG
AAAACCAAGATTCCCACTATCATGGAGAAATGATATTCACAGTTGAGTGGTCAGTGGGACTGAAAAAAAAAGTAATCAAATTCATTACCAAGACAGCTTTATCTGCTAATGAGTGCTACGAATGA CAT
TAAATAGAGTGATGTAATAGCAAATGCCTAAAGGAAAGGAGCTCCTTTTGATTAATGGCAGGAAAAGGCTCTCATCTCTCAGGAGATGTTGAGCTGATGTCTGA ATGCCAAGGAGGCAGCCATGGGAA
ATGTCAGGGGGCAGAGTGATTTAGGAAAAGAGAACAGTCAACAACAAGACCTGGAGACAGGACCATGTTTGTCATAACTGAGAAACAGAAAGCAGGCCAATGTAGTTGAAACAGGGTGATGGAGG ACA
GAGCAGCAGGGGAGGAGTCTGGAGATACAGTTAGGGGCACCAAGGTAAGGCCTGGCGAGCCCTGTTAAAGAGAGGAAGCACTGAAGAATTTCAGCAGGGGGATG TCGTTGTCTAATTTATCA

6225	12.3	1.5	0.4	AluYb8-Scaffold2140-4721399-4721696	2	949	(1349)	C	L1P4	LINE/L1	(1065)	5081	4124	1
2844	0.3	0.0	0.0	AluYb8-Scaffold2140-4721399-4721696	1001	1298	(1000)	C	AluYb8	SINE/Alu	(20)	298	1	2
568	34.4	9.4	2.7	AluYb8-Scaffold2140-4721399-4721696	1723	2296	(2)	C	L2a	LINE/L2	(0)	3426	2812	3

>Hg19 chr3:63539965-63541950
GCCAAGGCCAATGTTGAGAAGGGCATTTCCTAGGTTTTCTTCCAGGAGTTTGAGGTCTTACATTTATATCTGTAATCCGTCTTGAGTTAATTTTTTTATATGGTGAAAGGTAAGGGTCCAGTTTCA TT
CTTCTTCATATGGCTAGCCATTTATCCCAGCACCATGTACTGAGTAGGGAGTTCTTTTTCTATTGCTTGTTTTTGTCAGCCTTGTCAAAGATCAGACGGTTATAAGTGTATGGTTTTATTTCTGA GTT
TTTTATCCTGTTCCATTGGTCTATGTGTCTGTTTTGTACTAGTACCATGCTGTTTT GGTTGCTCTAGCCTTGTGGTATAGTTTGAAATTGGGTATTGTGATGTCTCCAGCTTTGTTCCTTTTGCTTAA
GACTGTTTTGGCTATTCAGGCTCTTTTTTTGGGTTCCATATGAATTTTAGAATAGTTTTTTTTTTAATTCTGTGAAGAATGACATTGGTAGTTTGATAGGAATAGAATTTAATCTGTAAGTTGCT CTG
GGCAATAGGGCTATTTTAGTGATACTGATTCTTCCAaTCCATGAACATGGAAAGTATTTCCATTTATTTGTGTCATCTCTGATTTCTTTCAGCAGAGTTTTATAGTTCTCCTTGTAGAGATCTTTCAC
CTCCTTGGTTAGCTGCATTCCTAGGTATTTTATTTTCTTTGTGGCTATGGTAAATGAGATTGTGCTCTTGATTTGACTCTCAGCCTGGACATTATTGGTGTATAGAAATGCTACTGATTTTTGTA CCC
TGATTTTATATCAAAAACTTACTAAAATTGTTTATCAGTTTTAGTAGGCTTTTAGCAGATTATTTAGAATTTTCTAGACAGAAAATTATATCATCAGTGAAGAGAGATAGTTTGACTTCTTTTCCT AT
TTGGATGCCTTTTATTTTTTTCACTTGCCTGAGCGCTCTGGCTAGGACATCTAACAGATCCGTTTTAAAGGGATTGAGTTTTGTTTAGT AAGAAAAAtACTTCCTAAGTATAAGTTGGAATTTATGAG
GTTCGTGAATGGCTTTATCATCACTGTTACTTTTATAGATAATTTTCCAAGTGGCACTTTATGGTCATAAATAAGCCTCAGTCATATCTTTTTGCAAGTGCCTCCCTAATTATCATTTCTGAAGAAAA
ATTTTGGCCTCTATATTAAATTATAGTTCACGACTTAGTTCCAGTTCACACCCTGAGCTACTATATTGGATATAACACATCTTCCTTAGGTGTTACTTACTGAGCTGAAAGTATTGCCCAAAGCA TCT
TATTTTGTCTGTATCCAGTAATTTATTGAGCCTGGATGCTATCCTGATAGAGTTTACTCACTAAAATAGAAGTTATAAAAGTTCAATCAAATTATGATCTATCAGTCATCTTACAGAACCAAGAAT GT
GCATTCATTTATCCAAGTACTATTTACTCAGTGCCTAAAAAATGTCAGGTGTTTGGGATCACAGAAAAAAAGAAAACCAAGATTCCCACTATCATGGAGAAATGATATTCACAGTTGAGTGGTCA GTG
GGACTGAAAAAAAAAGTAATCAAATTCATTACCAAGACAGCTTTATCTGCTAATGAGTGCTACGAATGACATTAAATAGAGTGATGTAATAGCAAATGCCTAAAGGAAAGGAGCTCCTTTTGATTA AT
GGCAGGAAAAGGCTCTCATCTCTCAGGAGATGTTGAGCTGATGTCTGAATGCCAAGGAGGCAGCCATGGGAAATGTCAGGGGGCAGAGTGATTTAGGAAAAGAGAACAGTCAACAACAAGACCTG GAG
ACAGGACCATGTTTGTCATAACTGAGAAACAGAAAGCAGGCCAATGTAGTTGAAACAGGGTGATGGAGGACAGAGCAGCAGGGGAGGAGTCTGGAGATACAGTTAGGGGCACCAAGGTAAGGCCTG GC
GAGCCCTGTTAAAGAGAGGAAGCACTGAAGAATTTCAGCAGGGGGATGTCGTTGTCTAATTTATC A

6877	12.2	1.3	0.4	Hg19	1	1049	(1137)	C	L1P4	LINE/L1	(964)	5182	4124	4
568	34.4	9.4	2.7	Hg19	1511	2084	(102)	C	L2a	LINE/L2	(0)	3426	2812	5

>KSI_AluYb8_15

>Scaffold2353-3390563-3390792
AAATATAATAATTTGAAATCATCTATTAAGAAATTGGCTAGGTGTGGTGGCTCACATCTGTAATCCCAGTGCTTTGGGAGGCCGAGGCAGGAGAATTGCTAGAGGCCAGGAGTTTGAGACCAGCCC TA
GGCAACATAGCAAGATCTCATCTCTACAACAAGTTTTTAAAAATTAGCCAGGTGTGGTGGTTACGCCTGTAGTCCCAGCTACTTGGAAGGCTAAGGTGGGAGCATAGCTTGAGCCCAGGAGTTTG AGG
GTACAGTGGGCTATGACTGTGCCACTCTAGCCTGGGCAACAGAGCAAGACCCTGTCTTTAAAACAAACAAATAAATAAATATTACTAATCATTTTGGCATATAAGGAAAAACTCAAATATATGTTA TT
TAGAAGAAATACAATTACAAGATAACAATAGGAATGATAAAAGGAATGGACAAATGCAACAAATGGAAACCAAAATCACCCAGGAAGAGTAATATTAGTATCAAATAAAGTGGAATTTAGGACTA AAA
CACTCAGTGGAATAAAGGGAGACACTCTATAATGATTAACTACACAATTGATGAAAAAAGTCATAAACATTTATGCACCAAACTGTATGCCATCTGTGGCAATAAATCCAAAACAAAATAAAAAGA GA
ACTTGATTAAAAAAAACCCAAAATTTTAGTGGGAGGCTTAATTATCCCTCTAAAGAGGGGTAGACTTGTGTCATGCCAGGGCCGTGAGCACACATCACTAATGCCCTGTGCTGCGGTGACATTGG GTT
TGTTGGGAGAAAACTTATAAGCAGAGAGCAGCAGAGCACTGCCCTGAAACGGTTGTTATCCCACAGAATAGTCTGGTTCTAAACATCCTAGGTGCAGCATGGCCAGAGGACTAATGTTCCACCCAG GG
CACTCCACAGAGAACAAGCCCCACTGCCCATCACATTACATATTTATGGGGTAAGTCCAACAGAGGCTAGGGAACCTGGTTTTTCTGAGTAACTAAAA TGTGTCggccgggcgcggtggctcacgcct
gtaatcccagcactttgggaggccgaggcgggtggatcatgaggtcaggagatcgagaccatcctggctaacaaggtgaaaccccgtctctactaaaaatacaaaaaattagccgggcgcggtggc gg
gcgcctgtagtcccagctactcgggaggctgaggcaggagaatggcgtgaacccgggaagcggagcttgcagtgagcc tgtgtcTTGAGATCTTCCAGGTCCAAGATCAGTGCTAGCAGTCTGCATGG
GGAGGGCCCTGGGAACAGCTTTGCTTGCTGGGAATGATGACAACTGCCTGTCTCTTGCCAGGCTGGCTCAGTCCATTGGAGTCAAATCTCTACTAG GCTTCTAAGAGGGAGAAGGCAATGAGCCTGCA
GGATGTCCAGATTTGTCTCTCATAAATTCAAATCTATGTAGTACATTTTCAAAGTCAGAACATAGAGGAACTAAATAATAATATC AATAGCCTTGATACAATCAATACATAATGTTAAATCCTTTAAA
TAGAGAACATACATTATTTTTAAAATATGCATAAAACTTTATAAAAAATGAGCATGATAAAAACAATAAAGCTTTGATAGGCTACATTCTGCAGCCATGAGCCAATAAAATTAGAAAGAAACTGT AAA
AAGGGAACTAATAAATCTGAACTACTTATAAATAAGAACCACAGTCCTAGATAACACCAGGATCAAAGAAGAAATTTAAAAAGAA ATTACTATCTAGAAACAAAGAAAAGGAGAATCACTGAAATAAG
AAACCAACAAAAAAGTAGAAAAGAAAATAAACATATCAATAAATTAAGAGAGAAAAACCATATGATCACAATAGTCAATGTCAAAAAACATCAGACAATAAGTTTGAATTTATCCATCATTCTTA ATA
AAAATAGGAATAAAAAGAAGGAAACTCTTGAATATAAAGACTATCAAAACCAACAGTAAATATTATCCTAAAATGAAAGACACTA AGGTAACTTCAATTACAGAAAACCCTAGAAAGAGGAAAGCCTA
CAATCACCATTATTACTTAACATTACTTGGGCGGTTTTAGCAATGTAATAAGGCAAGAAAATTAAATAACTGGCATAAGCATTGGAAGACAAATAGATAAAACCATCTCTTTTTTCTACTAATAA GAT
AACATAGTAGAATTCCTATATATCAAGGAATCTTGGGATAAAACTCTACTGGA T

1849	16.0	2.0	1.0	AluYb8-Scaffold2353-3390563-3390792	36	335	(1895)	+	AluJr	SINE/Alu	1	303	(9)	1
345	30.1	9.7	2.8	AluYb8-Scaffold2353-3390563-3390792	345	691	(1539)	+	L1M5	LINE/L1	2259	2621	(3588)	2
2193	0.4	0.0	0.0	AluYb8-Scaffold2353-3390563-3390792	1001	1230	(1000)	+	AluYb8	SINE/Alu	1	230	(88)	3
457	25.1	9.0	6.4	AluYb8-Scaffold2353-3390563-3390792	1824	2209	(21)	+	L1M5	LINE/L1	3925	4314	(1895)	2

>Hg19 chr8:41269465-41271458
AAATATAATAATTTGAAATCATCTATTAAGAAATTGGCTAGGTGTGGTGGCTCACATCTGTAATCCCAGTGCTTTGGGAGGCCGAGGCAGGAGAATTGCTAGAGGCCAGGAGTTTGAGACCAGCCC TA
GGCAACATAGCAAGATCTCATCTCTACAACAAGTTTTTAAAAATTAGCCAGGTGTGGTGGTTACGCCTGTAGTCCCAGCTACTTGGAAGGCTAAGGTGGGAGCATAGCTTGAGCCCAGGAGTTTG AGG
GTACAGTGGGCTATGACTGTGCCACTCTAGCCTGGGCAACAGAGCAAGACCCTGTCTTTAAAACAAACAAATAAATAAATATTACTAATCATTTTGGCATATAAGGAAAAACTCAAATATATGTTA TT
TAGAAGAAATACAATTACAAGATAACAATAGGAATGATAAAAGGAATGGACAAATGCAACAAATGGAAACCAAAATCACCCAGGAAGAGTAATATTAGTATCAAATAAAGTGGAATTTAGGACTA AAA
CACTCAGTGGAATAAAGGGAGACACTCTATAATGATTAACTACACAATTGATGAAAAAAGTCATAAACATTTATGCACCAAACTGTATGCCATCTGTGGCAATAAATCCAAAACAAAATAAAAAGA GA
ACTTGATTAAAAAAAACCCAAAATTTTAGTGGGAGGCTTAATTATCCCTCTAAAGAGGGGTAGACTTGTGTCATGCCAGGGCCGTGAGCACACATCACTAATGCCCTGTGCTGCGGTGACATTGG GTT
TGTTGGGAGAAAACTTATAAGCAGAGAGCAGCAGAGCACTGCCCTGAAACGGTTGTTATCCCACAGAATAGTCTGGTTCTAAACATCCTAGGTGCAGCATGGCCAGAGGACTAATGTTCCACCCAG GG
CACTCCACAGAGAACAAGCCCCACTGCCCATCACATTACATATTTATGGGGTAAGTCCAACAGAGGCTAGGGAACCTGGTTTTTCTGAGTAACTAAAA TGTGTCTTGAGATCTTCCAGGTCCAAGATC
AGTGCTAGCAGTCTGCATGGGGAGGGCCCTGGGAACAGCTTTGCTTGCTGGGAATGATGACAACTGCCTGTCTCTTGCCAGGCTGGCTCAGTCCATTGGAGTCAAATCTCTACTAGGCTTCTAA GAGG
GAGAAGGCAATGAGCCTGCAGGATGTCCAGATTTGTCTCTCATAAATTCAAATCTATGTAGTACATTTTCAAAGTCAGAACATAGAGGAACTAAATAATAATATCAATAGCCTTGATACAATCAATAC
ATAATGTTAAATCCTTTAAATAGAGAACATACATTATTTTTAAAATATGCATAAAACTTTATAAAAAATGAGCATGATAAAAACAATAAAGCTTTGATAGGCTACATTCTGCAGCCATGAGCCAAT AA
AATTAGAAAGAAACTGTAAAAAGGGAACTAATAAATCTGAACTACTTATAAATAAGAACCACAGTCCTAGATAACACCAGGATCAAAGAAGAAATTTAAAAAGAAATTACTATCTAGAAACAAAG AAA
AGGAGAATCACTGAAATAAGAAACCAACAAAAAAGTAGAAAAGAAAATAAACATATCAATAAATTAAGAGAGAAAAACCATATGATCACAATAGTCAATGTCAAAAAACATCAGACAATAAGTTTG AA
TTTATCCATCATTCTTAATAAAAATAGGAATAAAAAGAAGGAAACTCTTGAATATAAAGACTATCAAAACCAACAGTAAATATTATCCTAAAATGAAAGACACTAAGGTAACTTCAATTACAGAA AAC
CCTAGAAAGAGGAAAGCCTACAATCACCATTATTACTTAACATTACTTGGGCGGTTTTAGCAATGTAATAAGGCAAGAAAATTAAATAACTGGCATAAGCATTGGAAGACAAATAGATAAAACCAT CT
CTTTTTTCTACTAATAAGATAACATAGTAGAATTCCTATATATCAAGGAATCTTGGGATAAAACTCTACTGGA T

316	30.1	8.7	3.7	Hg19	86	135	(1959)	+	L1M5	LINE/L1	2201	2251	(3964)	4

1849	16.0	2.0	1.0	Hg19	136	435	(1659)	+	AluJr	SINE/Alu	1	303	(9)	5
320	30.1	9.0	3.5	Hg19	436	791	(1303)	+	L1M5	LINE/L1	2252	2621	(3588)	4
457	25.1	9.0	6.4	Hg19	1688	2073	(21)	+	L1M5	LINE/L1	3925	4314	(1895)	4

>KSI_AluYb8_16

>Scaffold3195-74833-75066
CAAGAGAGACACTTGAATCTCTCAGGTGGCCTGCTTGATTTCTTCCAAGTGTACTTTGCTTTCTTTAATAAACTTTGCCTCTGCTTTAAACCTACTTCTGCCTCTCAGTTGAATTCTTTCCTCTGA GG
GGGCAAGGATCAAAATTGCTGAGAATCACTAGACTTCCGGCTGATAACAGGAAAAGGGGTCTGGCTCATCCCAGCCTTTCAGCCATCCCTGCCAAGGCAGCAGACGTGTCCAAGTTATCTGGGGC CTC
CAGAGCAATCTGGCTGCCAGGTGAATACCACCAAGGGACCCCAAGTAATACCATGTGGAGTATAAGAAATGCTCAGCTGGGCCCTTTCTGAATTCCAATCCAAGTTATGAGATTTAATAAAATCAT TA
TTGTTTTAAGCCACCAAGTTTTGGGATAGTTTGTTACACGATCAATAAATAACCAGAACAGTCTTTCACACACACACACAAAATTTATTGTGTCACGCTCTTGCCAAATGAAAGAATCTGTTTCT AGC
TCTAGCCCAAATAACCTTATATTCCCTTAAAGGAAATTAATCCTCCTTATCTTTACAATAAGCTATAGAAAGTATTCATTGTAGAAGTAGACTCCCATTTCTATTGAAACTAGTGACTCTCAACTA TT
AGTATACTTAAGAATTTGCCTGGGAAATTCTCAAAAATGCTGGTTGCTAGCTGGTAGCAGACATTCTGATTTGGTAAGTCTAGTGTGGGGTTCAGGAATCTCCATTTTAAGA tGGATACCCAAGTGAT
CTTGACACTGGTGATCCCAAGAACACATATGGAGACACATTTTAATAAAACGTGCCAGAGATAGCAGGGCTGTGAgCTAAGCCATGTCAGTGGAAGTAAGGAAAAAACAAGGCATTTTAACTGATCTT
CCCTGTCcGCATCCTCCAGCCTCATATCCAGGCTTCCCTTACCTGCATTCTTctttttttttttttttttttttttttttttttttttttttttttgagcggggtctcggctcactgcaagctccgct
tcccgggttcacgccattctcctgcctcagcctcccgagtagctgggactacaggcgcccgccaccgcgcccggctaatttttgtatttttagtagagacggggtttcaccttgttagccaggat gg
tctcgatctcctgacctcatgatccacccgcctcggcctcccaaagtgctgggattacaggcgtgagccaccgcgcccggcc acctgcattcttAAGCTCTGTATCCAATTAAAAACATACTTTTCAG
AGAGTTTTATGCTTTTCATTTTATTTTAGCCTCTCGACCACTGTGTGAAGTAGAACAGACATTTTACAGAGAAGTAGATGAATGGTATCTGACAGCTGCCAAGTGCACTATCTCTGGGCATAGAG TAT
CTGAAGCTAAGCACCCAAGTCTGGGTATATATCTATGTATGTATGTATTTATATTTATATTTTTAAAGGAATGTCAGTTTCTTCAAATCAGAAACACCACCACCAAG ACACATCTTCTTCATTATGAT
AGTTACAATCTGGCTTAATGATTAAGTGTGGATTGCTAACTCCCACAGCTTCCAGCAGCTTCTGGGCTTCCTCATCAAATGCCTCTTTGCACAACCTGAGAAAACAAGGGAACATCTCTTTTCCT GCA
TTCAATTTCCCTACAGCCCTCCACTTTCTCTGTAAAGGATTTATTTCAGGAGAAAAAAAACTGTTCTGTTAAAATAGAGAGATAATATTATAACATGGGTGAGGAGT GGGGAAGCGAACTGAGAGAGA
TACAGTGAGGGAGATAAAGTAGTGGAAGGAAGACAGAATAAATTAACAGAAGAGAAAGAGAAACCAATTAAAATTAAAAAGGATCCTCCAAGAGCCTTTTGCTATCCAAGCAAAATTAATATTAC TAC
TGATAATAATAAAAACAGAGAACATATTTATTGTTTATTATCATGCAAGGCACCATTCTAAGTACTTAGCCTATATTGACTCAGATCCTCATGATAACCTTATGGGT AGATTTTATCATTTCACCCAT
TTTACTCATAAGCAAATCAAAACAAAGAGTGATGGGGGACTAGCCTTAATTAAACATTTAGTAAGTGGTAGGGTCAGAATTCAAGCTCATCTAGGCTGGTTCCAGGGTGCTCAATATTTAACCAC TAT
GCTATATTGTATCTCTCAATATTCAGTGTTCTATGACAGAAAAACAAATAAGTGTCT A

687	19.5	16.1	2.1	AluYb8-Scaffold3195-74833-75066	1	168	(2066)	+	MER50C	LTR/ERV1	554	744	(38)	1
1196	23.4	3.2	0.4	AluYb8-Scaffold3195-74833-75066	192	444	(1790)	+	MLT1H1	LTR/ERVL-MaLR	296	555	(0)	2
366	35.6	2.2	1.7	AluYb8-Scaffold3195-74833-75066	624	803	(1431)	C	MER5A	DNA/hAT-Charlie	(1)	188	8	3
47	0.0	0.0	0.0	AluYb8-Scaffold3195-74833-75066	950	992	(1242)	+	(T)n	Simple_repeat	1	43	(0)	4
2231	0.4	0.0	0.0	AluYb8-Scaffold3195-74833-75066	1001	1234	(1000)	C	AluYb8	SINE/Alu	(84)	234	1	5
18	19.6	0.0	0.0	AluYb8-Scaffold3195-74833-75066	1434	1468	(766)	+	(TA)n	Simple_repeat	1	35	(0)	6
689	27.4	5.8	2.4	AluYb8-Scaffold3195-74833-75066	1944	2186	(48)	C	MIR	SINE/MIR	(11)	251	1	7

>Hg19 chr11:7091801-7093736
CAAGAGAGACACTTGAATCTCTCAGGTGGCCTGCTTGATTTCTTCCAAGTGTACTTTGCTTTCTTTAATAAACTTTGCCTCTGCTTTAAACCTACTTCTGCCTCTCAGTTGAATTCTTTCCTCTGA GG
GGGCAAGGATCAAAATTGCTGAGAATCACTAGACTTCCGGCTGATAACAGGAAAAGGGGTCTGGCTCATCCCAGCCTTTCAGCCATCCCTGCCAAGGCAGCAGACGTGTCCAAGTTATCTGGGGC CTC
CAGAGCAATCTGGCTGCCAGGTGAATACCACCAAGGGACCCCAAGTAATACCATGTGGAGTATAAGAAATGCTCAGCTGGGCCCTTTCTGAATTCCAATCCAAGTTATGAGATTTAATAAAATCAT TA
TTGTTTTAAGCCACCAAGTTTTGGGATAGTTTGTTACACGATCAATAAATAACCAGAACAGTCTTTCACACACACACACAAAATTTATTGTGTCACGCTCTTGCCAAATGAAAGAATCTGTTTCT AGC
TCTAGCCCAAATAACCTTATATTCCCTTAAAGGAAATTAATCCTCCTTATCTTTACAATAAGCTATAGAAAGTATTCATTGTAGAAGTAGACTCCCATTTCTATTGAAACTAGTGACTCTCAACTA TT
AGTATACTTAAGAATTTGCCTGGGAAATTCTCAAAAATGCTGGTTGCTAGCTGGTAGCAGACATTCTGATTTGGTAAGTCTAGTGTGGGGTTCAGGAATCTCCATTTTAAGA cGGATACCCAAGTGAT
CTTGACACTGGTGATCCCAAGAACACATATGGAGACACATTTTAATAAAACGTGCCAGAGATAGCAGGGCTGTGAaCTAAGCCATGTCAGTGGAAGTAAGGAAAAAACAAGGCATTTTAACTGATCTT
CCCTGTCtGCATCCTCCAGCCTCATATCCAGGCTTCCCTTACCTGCATTCTTAAGCTCTGTATCCAATTAAAAACATACTTTTCAGAGAGTTTTATGCTTTTCATTTTATTTTAGCCTCTCGACCACT
GTGTGAAGTAGAACAGACATTTTACAGAGAAGTAGATGAATGGTATCTGACAGCTGCCAAGTGCACTATCTCTGGGCATAGAGTATCTGAA GCTAAGCACCCAAGTCTGGGTATATATCTATGTATGT
ATGTATTTATATTTATATTTTTAAAGGAATGTCAGTTTCTTCAAATCAGAAACACCACCACCAAGACACATCTTCTTCATTATGATAGTTACAATCTGGCTTAATGATTAAGTGTGGATTGCTAA CTC
CCACAGCTTCCAGCAGCTTCTGGGCTTCCTCATCAAATGCCTCTTTGCACAACCTGAGAAAACAAGGGAACATCTCTTTTCCTGCATTCAATTTCCCTACAGCCCTCCACTTTCTCTGTAAAGGAT TT
ATTTCAGGAGAAAAAAAACTGTTCTGTTAAAATAGAGAGATAATATTATAACATGGGTGAGGAGTGGGGAAGCGAACTGAGAGAGATACAGTGAGGGAGATAAAGTAGTGGAAGGAAGACAGAAT AAA
TTAACAGAAGAGAAAGAGAAACCAATTAAAATTAAAAAGGATCCTCCAAGAGCCTTTTGCTATCCAAGCAAAATTAATATTACTACTGATAATAATAAAAACAGAGAACATATTTATTGTTTATTA TC
ATGCAAGGCACCATTCTAAGTACTTAGCCTATATTGACTCAGATCCTCATGATAACCTTATGGGTAGATTTTATCATTTCACCCATTTTACTCATAAGCAAATCAAAACAAAGAGTGATGGGGGA CTA

GCCTTAATTAAACATTTAGTAAGTGGTAGGGTCAGAATTCAAGCTCATCTAGGCTGGTTCCAGGGTGCTCAATATTTAACCACTATGCTATATTGTATCTCTCAATATTCAGTGTTCTATGACAGA AA
AACAAATAAGTGTCTA

1091	22.3	11.9	1.4	Hg19	1	268	(1868)	+	MER50C	LTR/ERV1	449	744	(38)	8
1196	23.4	3.2	0.4	Hg19	292	544	(1592)	+	MLT1H1	LTR/ERVL-MaLR	296	555	(0)	9
377	37.1	1.7	1.1	Hg19	724	903	(1233)	C	MER5A	DNA/hAT-Charlie	(1)	188	8	10
18	19.6	0.0	0.0	Hg19	1236	1270	(866)	+	(TA)n	Simple_repeat	1	35	(0)	11
689	27.4	5.8	2.4	Hg19	1746	1988	(148)	C	MIR	SINE/MIR	(11)	251	1	12

>KSI_AluYb8_17

>Scaffold3317-2875896-2876183
GAAAAACATTTCCTAAAGTGTGTTTTGCAGGATACTGGTCCTATTAGTATCCACAGAACTAGAGCTTATTGGCATATACTTCAGTTACACTCACTTAGGTGAGGGGTGGGCAAGCCTTTTCTGTAA AG
GACCAGATAGTACACATTTGAGGCTTTAAGGCCATACAGTCTATCACAAGCACTTAGCTCTGCTGTTGTCTCATGAAAACAACCATAGGCAATATGTAAACAAAAGCACATGGCTGTGTTCCAGT AAA
ACTTTGTTTACAAAAACAGACAGTAGGTGGCCAGGTGCAGTGGCTCACACCTGTAATCCCAGCACTTTGGGAGACTGAGGCGGGCAGATCACCTGAGGTCAGGAGTTTGAGACCAGTCTGGCCAAC AT
GGTGAAACCCCGTCTCTGTTGGAAATACAAAAAATAGCTGGGCATGGTGGTACACGCCTATAATCCCAGCTGCTCAGGAGGCTGAGGGCACAAGAATCTCTTGAACCCAGGAGGTGGAAGTTGCA GTG
AGCTGAGATCGCACCACTGCACTCCAGCCTGGGCTACAGAGTGGGACTCTGTCTCCAAAATAAAAAAATAAAAATAAAAAAAGACAGTAGACCATGTTTGGCCCATAA GCCAGTTTGTCACCTCCTGA
TTTAGGTCTTCCATAATAATTATGGGTATGTCTGGCCACAGTCATTGTGTTTTCAGTGTAACGTGTCTTTTGCTGTATCCTAACATTCTCCTTTTGCAAGATGTTATAGAACATCACTTTTCAAT AGA
ACTTTCTGTGACAATGACAGTACTTGATGTCTGTGCAGTCCAGTACAGTGGCCACTAACCATACGTGGTTATTGAATCCTTGAAATGCCACTAGTGAGACTGAGAAACTAAAATTTGAATTTTATTTA
ATTTTGGCTTATTTAAATTTAACCATATGCAGTTTTCCATTTTTTTttttttttttttttttttttttttttttttttttttttttttttttttttttttttttttgagacggagtctcgctctgtcgcc
caggccggactgcggactgcagtggcgcaatctcggctcactgcaagctctgcttcccgggttcacgccattctcctgcctcagcctcccgagtagctgggactacaggcgcccgccaccgcgcc cgg
ctaatttttgtatttttagtagagacggggtttcaccttgttagccaggatggtctcgatctcctgacctcatgatccacccgcctcggcctcccaaagtgctgggattacaggcgtgagccaccgc
gcccggcctccatttttttATTCTCCTTTTTTCCTTCTACACAAGACCCAACTGCATTAAGGCATACAGTGAGTTATATTAATCTTGGGTCATTTCACGTCTTCCTCCTGTGGTGGTTCATGAAGGGA
TAAAATACcTCTCACCTGAACACTGAAAGTTTCACCAAACCAGACAATTCACTGGCTACTATGGTGTGATGCACACAGATAAATATGGTTTCTAGAATTTAGTTCTGCAAAGCCACTGGGGGTGAGAA
CAGGGGATATGAGATATACGCTAAAGAATCCACAGCTTTTAGGAAGAGACTGGGTTTGAGTAAATCTGTTGGGAGTGTAGTAAGCTCATCAGCTCAGCCTTTTAAACCTCTTGTGCCATGCGACT TTG
CAGATACAACCAGACCTCCAAAGAAAGACGAAGAAAATGGAAAGAATTATTACTTTGTATCTCATGACCAAATGATGCAAGACATCTCTAATAACGAGTACTTGGAGTACGGCAGCCACGAGGATGCG
ATGTATGGGACAAAACTGGAGACCATCCGGAAGATCCACGAGCAGGGGCTGATTGCAATACTGGACGTGGAGCCTCAGGTAATGCCCAGCCTCCCCGAATGTCTGACATCCTGCCTGCATGCCAA TAA
GCACAGATTCTGAAATCAAAAATGCCATCAGAAAGTCACAGGTCACATAATAATTTCATAAACCACAGAAAAGTATCAGTAATGGAGGCGTTGGGGGTTCACAGTCTAGAATGCCAGCTGACTAATCG
TTGGACTCAGTGCACACACCATGTATTTAAGAAGCAGAGATTATTCTCTTAAGAGGTTTATTATATGCAGAAGCTCAGTAAGGTTCCTGATTGTGTCTACGCCTGCCAGAAATCCTACGATCATC TGA
CCTCAGGATGAGGCTCTGGCACTTTGTTATCACAACAGCCACCTTCCCTCTCTGGGTACAGCTCACGCTGGACCATCAGAGCCTCTACCAGCACATTATGTTAATCTCCAGT

1054	21.6	2.2	4.9	AluYb8-Scaffold3317-2875896-2876183	102	284	(2004)	C	MER58A	DNA/hAT-Charlie	(1)	223	39	1
2259	12.3	0.0	0.3	AluYb8-Scaffold3317-2875896-2876183	285	594	(1694)	+	AluSx	SINE/Alu	1	309	(3)	2
1054	21.6	2.2	4.9	AluYb8-Scaffold3317-2875896-2876183	595	639	(1649)	C	MER58A	DNA/hAT-Charlie	(186)	38	1	1
854	24.0	2.2	0.0	AluYb8-Scaffold3317-2875896-2876183	752	930	(1358)	+	MER3	DNA/hAT-Charlie	1	183	(26)	3
73	0.0	0.0	0.0	AluYb8-Scaffold3317-2875896-2876183	936	1000	(1288)	+	(T)n	Simple_repeat	1	65	(0)	4
2739	0.7	0.0	0.0	AluYb8-Scaffold3317-2875896-2876183	1001	1288	(1000)	C	AluYb8	SINE/Alu	(30)	288	1	5

>Hg19 chrX:41389842-41391772
GAAAAACATTTCCTAAAGTGTGTTTTGCAGGATACTGGTCCTATTAGTATCCACAGAACTAGAGCTTATTGGCATATACTTCAGTTACACTCACTTAGGTGAGGGGTGGGCAAGCCTTTTCTGTAA AG
GACCAGATAGTACACATTTGAGGCTTTAAGGCCATACAGTCTATCACAAGCACTTAGCTCTGCTGTTGTCTCATGAAAACAACCATAGGCAATATGTAAACAAAAGCACATGGCTGTGTTCCAGT AAA
ACTTTGTTTACAAAAACAGACAGTAGGTGGCCAGGTGCAGTGGCTCACACCTGTAATCCCAGCACTTTGGGAGACTGAGGCGGGCAGATCACCTGAGGTCAGGAGTTTGAGACCAGTCTGGCCAAC AT
GGTGAAACCCCGTCTCTGTTGGAAATACAAAAAATAGCTGGGCATGGTGGTACACGCCTATAATCCCAGCTGCTCAGGAGGCTGAGGGCACAAGAATCTCTTGAACCCAGGAGGTGGAAGTTGCA GTG
AGCTGAGATCGCACCACTGCACTCCAGCCTGGGCTACAGAGTGGGACTCTGTCTCCAAAATAAAAAAATAAAAATAAAAAAAGACAGTAGACCATGTTTGGCCCATAAG CCAGTTTGTCACCTCCTGA
TTTAGGTCTTCCATAATAATTATGGGTATGTCTGGCCACAGTCATTGTGTTTTCAGTGTAACGTGTCTTTTGCTGTATCCTAACATTCTCCTTTTGCAAGATGTTATAGAACATCACTTTTCAAT AGA
ACTTTCTGTGACAATGACAGTACTTGATGTCTGTGCAGTCCAGTACAGTGGCCACTAACCATACGTGGTTATTGAATCCTTGAAATGCCACTAGTGAGACTGAGAAACTAAAATTTGAATTTTATT TA
ATTTTGGCTTATTTAAATTTAACCATATGCAGTTTTCCATTTTTTTATTCTCCTTTTTTCCTTCTACACAAGACCCAACTGCATTAAGGCATACAGTGAGTTATATTAATCTTGGGTCATTTCACGTC
TTCCTCCTGTGGTGGTTCATGAAGGGATAAAATACaTCTCACCTGAACACTGAAAGTTTCACCAAACCAGACAATTCACTGGCTACTATGGTGTGATGCACACAGATAAATATGGTTTCTAGAATTTA
GTTCTGCAAAGCCACTGGGGGTGAGAACAGGGGATATGAGATATACGCTAAAGAATCCACAGCTTTTAGGAAGAGACTGGGTTTGAGTAAATCTGTTGGGAGTGTAGTAAGCTCATCAGCTCAGC CTT
TTAAACCTCTTGTGCCATGCGACTTTGCAGATACAACCAGACCTCCAAAGAAAGACGAAGAAAATGGAAAGAATTATTACTTTGTATCTCATGACCAAATGATGCAAGACATCTCTAATAACGAGTAC
TTGGAGTACGGCAGCCACGAGGATGCGATGTATGGGACAAAACTGGAGACCATCCGGAAGATCCACGAGCAGGGGCTGATTGCAATACTGGACGTGGAGCCTCAGGTAATGCCCAGCCTCCCCGA ATG
TCTGACATCCTGCCTGCATGCCAATAAGCACAGATTCTGAAATCAAAAATGCCATCAGAAAGTCACAGGTCACATAATAATTTCATAAACCACAGAAAAGTATCAGTAATGGAGGCGTTGGGGGTTCA
CAGTCTAGAATGCCAGCTGACTAATCGTTGGACTCAGTGCACACACCATGTATTTAAGAAGCAGAGATTATTCTCTTAAGAGGTTTATTATATGCAGAAGCTCAGTAAGGTTCCTGATTGTGTCT ACG
CCTGCCAGAAATCCTACGATCATCTGACCTCAGGATGAGGCTCTGGCACTTTGTTATCACAACAGCCACCTTCCCTCTCTGGGTACAGCTCACGCTGGACCATCAGAGCCTCTACCAGCACATTATGT
TAATCTCCAGT

16	33.9	3.3	0.0	Hg19	11	70	(2061)	+	GA-rich	Low_complexity	1	62	(0)	6
1054	21.6	2.2	4.9	Hg19	202	384	(1747)	C	MER58A	DNA/hAT-Charlie	(1)	223	39	7
2259	12.3	0.0	0.3	Hg19	385	694	(1437)	+	AluSx	SINE/Alu	1	309	(3)	8
1054	21.6	2.2	4.9	Hg19	695	739	(1392)	C	MER58A	DNA/hAT-Charlie	(186)	38	1	7
854	24.0	2.2	0.0	Hg19	852	1030	(1101)	+	MER3	DNA/hAT-Charlie	1	183	(26)	9

>KSI_AluYb8_18

>Scaffold3524-466832-467142
TGTTTCACAGGTGAGAAAGACATGAGTTTTAGAGTCCAtAGTGTGGAGGGTTATTGGCTGAAGTGTGCCCcGAGAATTCATACAATGAAGCTCTAATCACAGTGCATGAATGAATTGGAACATAGGgT
CTTTAATGGGGTGATTAAGTGAAAATGAAGCCAGTAGGATGGGTTCTTTTCtATCTGACAGGTGTCCTCTGAAGATGAAGAAATTTGAGCACACAGAAATGAGACACCATAGATGAGCATGCAGAGGA
AAGATCACGTGAGGACACAGAAGAAGGGAGTGATTTGCAAGCCAAGGAGATGTTACAGTAGATAACTAGGCAGACGTGACAGGGCAGGAGACCCCCTGCCCCAGGAATGTCACGCAAGCATCAGA TGA
TGGTCAGGTAGTTGTTAAAaCTGTCTCTGTGAATTAATAATTAGTCACAGCC GGCACCAAGGAAAGGCGGTCTCCCAATAGATAGAAAATACCTGAAGCTGGTGATCAGCAGCTTTCTGATAAGATCT
CAGGCCTTGGGTGAGTGGGCTCAAGCAGGCAAACTAAAAGGCAAAACAGAGGCCAGGCATGGTGGCTCACCCCTGTAATCCCAGCACTTTGGGAGGCCGAGGTGGGCCAATCACTTGAGGTCAGG AGC
gCTAGACCAGCCTGACCAACATGGTGAAACCCTGTCTCTACTAAAAATGCAA AAATTAGCCAGGTGTGGTGGTGCATGCCTATAATCCCAGCTACTCAGAAGGCTGAGCCAGGAGAATCACTTGAACC
TGGGAGATGGAGGCTGCAGTGAGCCGAGATTGCATCACTGCACCCCAGCCCAGGTGACAGAGCAAGGGTCCTTCAAAAAAAAAAAATGCAAAATAGCAGAGTTTAACCTGGATCTGAGACTCCCAT GCT
GCCCTTCCCTCCAGCCCTACAGGCAGGCTCTGCAGAGAGTGAATCACCAGTAAATACACAAACAAATATGATTACGTTCCTTTTGTTTaagaagtagaggaatgggccgggcgcggtggctcacgcct
gtaatcccagcactttgggaggccgaggcgggtggatcatgaggtcaggagatcgagaccatcctggctaacaaggtgaaaccccgtctctactaaaaatacaaaaaattagccgggcgcggtgg cgg
gcgcctgtagtcccagctactcgggaggctgaggcaggagaatggcgtgaac ccgggaagcggagcttgcagtgagccgagattgcgccactgcagtccgcagtggggcaaaagggagcccccgcca
aaaaaaaaaaaaaaaaaaaaaaaaaaaaaaaaaaaaaaaaAAGAAGTAGAGGAATGATTGAATATATTAAGTGAAAATAAGGAATACCAACACAAAGAAAAATT TAACTGTAAGTATGGTTAAAAATA
CACTGGGTGAGATAGACAAGTTAGATGTCGCAAAACAAGAGATTAGCAAACTGTATAGTGTAACACTAGAAACTATTCAAAATGAAACACATAGCAAAAAATAACGATAAAAATAAACAGCATCAATG
AGATGTAGGAGAACTTCAGACAGGCTAATATACGTGTAATTGAAGTCCTCAGAATAGAAGATGGGGTAAGAATGAAAAGAAAAAATATTTGAATGAATAATGACAAAATATGTTCCACATTTGGT GAA
AACCATAAACTGATATAACCCAGAAGCTCGATAAAACTTAAGCATAGGAAACACAAAGA aAACAACTCCAAAACACATCATAATCAAATTAATAAAATCTAGTGATGAAAAGTAGTCATTTAGGACAA
AACTACAAAGAGCAGATTTCTCATTAGGAGCAATGCAAGCAAGAAGATAGTGAAGCAATGTCTTGAAAATAATAACATGCAAAATCCAACAACCTTGAATTCTTTATCCACCAGAAACATCTTTC AAA
ACTGGAGGCAAAATAAGACTTTTTCAGACATGTGAAATCCTACAGAATTGATCACCATTAGACTCTCGCTATTAGAAATGATAAAGGACACCCCTAAACAGAAAGAAAATAATCCTACATTGAAATGT
GGTTTGACACAAAGAAATGAAAAATCAGATATGGTAGCTACCTGGATCAATTAGAAACCTCTGTATTATGTAATGTCTATAAAAAGAACAGTAATGTCTATAAAGAGAACTGTTCAGAGAAAAAT TAA
TAAGAATACAtTATGATTTGTAATAAAAATAAAAGCAAACTATATGACAAAGCAGAAAAGCGAAGGTGGGTGAAGAACATGTAATAATGGTTTTGTACTATATGTGAATATATAATAGCACCTGAAGG
TAGATTT

762	22.8	5.1	8.0	AluYb8-Scaffold3524-466832-467142	50	306	(2005)	+	MLT1B	LTR/ERVL-MaLR	2	251	(139)	1
1544	16.0	3.5	4.6	AluYb8-Scaffold3524-466832-467142	307	562	(1749)	+	MER50	LTR/ERV1	1	261	(473)	2
2113	13.1	0.7	0.0	AluYb8-Scaffold3524-466832-467142	563	853	(1458)	+	AluSz	SINE/Alu	1	293	(19)	3
1544	16.0	3.5	4.6	AluYb8-Scaffold3524-466832-467142	854	884	(1427)	+	MER50	LTR/ERV1	262	285	(449)	2
2674	2.9	2.2	0.0	AluYb8-Scaffold3524-466832-467142	1001	1311	(1000)	+	AluYb8	SINE/Alu	1	318	(0)	4
1411	25.3	9.1	2.2	AluYb8-Scaffold3524-466832-467142	1400	2310	(1)	+	L1MEc	LINE/L1	1069	2094	(4306)	5

>Hg19 chr22:23169891-23171862
ggagtcacctgcacttcatttggatgataaataaatgagatggatcttagagttggtgctgatctagtcaatgatttgggatgttgggctgaggtgaatg TGTTTCACAGGTGAGAAAGACATGAGTT
TTAGAGTCCAcAGTGTGGAGGGTTATTGGCTGAAGTGTGCCCGAGAATTCATACAATGAAGCTCTAATCACAGTGCATGAATGAATTGGAACATAG GTCTTTAATGGGGTGATTAAGTGAAAATGAAG
CCAGTAGGATGGGTTCTTTTCcATCTGACAGGTGTCCTCTGAAGATGAAGAAATTTGAGCACACAGAAATGAGACACCATAGATGAGCATGCAGAGGAAAGATCACGTGAGGACACAGAAGAAGGGAG
TGATTTGCAAGCCAAGGAGATGTTACAGTAGATAACTAGGCAGACGTGACAGGGCAGGAGACCCCCTGCCCCAGGAATGTCACGCAAGCATCAGATGATGGTCAGGTAGTTGTTAA ACTGTCTCTGTG
AATTAATAATTAGTCACAGCCGGCACCAAGGAAAGGCGGTCTCCCAATAGATAGAAAATACCTGAAGCTGGTGATCAGCAGCTTTCTGATAAGATCTCAGGCCTTGGGTGAGTGGGCTCAAGCAG GCA
AACTAAAAGGCAAAACAGAGGCCAGGCATGGTGGCTCACCCCTGTAATCCCAGCACTTTGGGAGGCCGAGGTGGGCCAATCACTTGAGGTCAGGAGC tCTAGACCAGCCTGACCAACATGGTGAAACC
CTGTCTCTACTAAAAATGCAAAAATTAGCCAGGTGTGGTGGTGCATGCCTATAATCCCAGCTACTCAGAAGGCTGAGCCAGGAGAATCACTTGAACCTGGGAGATGGAGGCTGCAGTGAGCCGAG ATT
GCATCACTGCACCCCAGCCCAGGTGACAGAGCAAGGGTCCTTCAAAAAAAAAAAATGCAAAATAGCAGAGTTTAACCTGGATCTGAGACTCCCATGCTGCC CTTCCCTCCAGCCCTACAGGCAGGCTCT
GCAGAGAGTGAATCACCAGTAAATACACAAACAAATATGATTACGTTCCTTTTGTT TAAGAAGTAGAGGAATGATTGAATATATTAAGTGAAAATAAGGAATACCAACACAAAGAAAAATTTAACTGT
AAGTATGGTTAAAAATACACTGGGTGAGATAGACAAGTTAGATGTCGCAAAACAAGAGATTAGCAAACTGTATAGTGTAACACTAGAAACTATTCAAAATGAAACACATAGCAAAAAATAACGATAAA
AATAAACAGCATCAATGAGATGTAGGAGAACTTCAGACAGGCTAATATACGTGTAATTGAAGTCCTCAGAATAGAAGATGGGGTAAGAATGAAAAGAAAAAATATTTGAATGAATAATGACAAAA TAT
GTTCCACATTTGGTGAAAACCATAAACTGATATAACCCAGAAGCTCGATAAAACTTAAGCATAGGAAACACAAAGA cAACAACTCCAAAACACATCATAATCAAATTAATAAAATCTAGTGATGAAAA
GTAGTCATTTAGGACAAAACTACAAAGAGCAGATTTCTCATTAGGAGCAATGCAAGCAAGAAGATAGTGAAGCAATGTCTTGAAAATAATAACATGCAAAATCCAACAACCTTGAATTCTTTATC CAC
CAGAAACATCTTTCAAAACTGGAGGCAAAATAAGACTTTTTCAGACATGTGAAATCCTACAGAATTGATCACCATTAGACTCTCGCTATTAGAAATGATAAAGGACACCCCTAAACAGAAAGAAA ATA
ATCCTACATTGAAATGTGGTTTGACACAAAGAAATGAAAAATCAGATATGGTAGCTACCTGGATCAATTAGAAACCTCTGTATTATGTAATGTCTATAAAAAGAACAGTAATGTCTATAAAGAGA ACT

GTTCAGAGAAAAATTAATAAGAATACAcTATGATTTGTAATAAAAATAAAAGCAAACTATATGACAAAGCAGAAAAGCGAAGGTGGGTGAAGAACATGTAATAATGGTTTTGTACTATATGTGAATAT
ATAATAGCACCTGAAGGTAGATTTtggtaagttatgtatttactgtataccctaaaacaaccattaaaataaaaccctcaacaaaaaagaaatatggctagtacgctatattagacttgtccac

432	23.4	7.7	1.4	Hg19	5	134	(2038)	+	MLT1B-int	LTR/ERVL-MaLR	1219	1356	(15)	6
721	22.6	5.9	8.0	Hg19	150	404	(1768)	+	MLT1B	LTR/ERVL-MaLR	2	251	(139)	6
1574	16.0	3.5	4.2	Hg19	405	659	(1513)	+	MER50	LTR/ERV1	1	261	(473)	7
2137	12.7	0.7	0.0	Hg19	660	950	(1222)	+	AluSz	SINE/Alu	1	293	(19)	8
1574	16.0	3.5	4.2	Hg19	951	981	(1191)	+	MER50	LTR/ERV1	262	285	(449)	7
1395	25.9	7.9	3.5	Hg19	1161	2156	(16)	+	L1MEc	LINE/L1	1069	2174	(4226)	9

>KSI_AluYb8_19

>Scaffold3717-1643730-1644047
TCCTAGAACTGGTAAATGAATTCAGCAGGGTTTCAGGATACAAAATTAATGTACACAAATCAGTAGTTCCGCTATACACCAACAGCGACCAAGCTGAGAATCAAATCAAGAACTCAATCCCTTTTA CA
ATAGCTGCAAAAAAATAATGAAATACTTAGGAATATACCTAACCGAGGAAGTGAAATACCTCTACAAGGAAAACTACAAAACACTGCTGAATGTAATCATAGACAACATAAACAAATGGAAACAC ATC
CCATGCTCACGGATGGGTAGAACCAATATTGTGAAAATTACCACACTGCCAAAAATAATCTACAAATTCAATGCAATTCCCATCAAAATACCACCATCATTCTTCACGAACTAGAAAAAAAATCCT AA
ATTTTATATCAAATGAAAAAAAAGAGTCCACATAGTCAAAGCAAGACTAACCAAAAAGAACAAATGTGAAGGCATCACATTACCTGACTTCAAACTATAGTATAGGGTCATAGTCACCAAAACAG CAT
GGTACTAGTATAAAAATAGGCACATACCAATGGAACAGAATAGAGAACCCAGAAATAAAGCCAAACTGATCTTTGACAACTAATCTT tGACAAAGCAAACAAAAACATAAAGTGGGGAAAGGACACCC
TATTCAACAAATGGTGCTGGGATAATTGGCAAGCCACGTGTAGAAGAATGAAACTGGATCCTCATCTGTTACCCTATACAAAAATCAACTCAAGATTGATCAAAGACTTAAATCTGAGACCTGAAACA
ATAAAAATTCTGGAAGGTAATATCAGAAAAACTCTTCTAGACATTGGTTTAAGCAAAGACGTCATGACCAAGAACACCAAAGCAAATGCAGCAAAAACAAAGATAAATAGATGGGACTTCATTAAA TG
AAAAAGCTTCTGCACAGGAAAATAAATAATCAGCAGAGGTAACAGACAACCCACAGAGTGGGAAAAAAATCTTCACAATCTATACATC Tgaaaaaggactaataggccgggcgcggtggctcacgcct
gtaatcccagcactttgggaggccgaggcgggtggatcatgaggtcaggagatcgagaccatcctggctaacaaggtgaaaccccgtctctactaaaaatacaaaaaattagccgggcgcggtgg cgg
gcgcctgtagtcccagctactcgggaggctgaggcaggagaatggcgtgaacccgggaagcggagcttgcagtgagccgagattgcgccactgcagtccgcagt ccggcctgggcgacagagcgagac
tccgtctcaaaaaaaaaaaaaaaaaaaaaaaaaGAAAAAGGACTAATATCCAGAATCTATAAATAACTCAAACAAATTAGCAAGAAAAAAACAAACAATCCCATCAAAAAGTGGGCAAAGGAAATGA
ATAGACAATTCTCAAAAGAAGATATACAAATGGCCAACAAGCATATGGAAAAATGCTCAGCATCACTAA cGACCAGGGAAATGCAAATCAAAACCACAATGCGATACCACCTTACTCCTGCAAGAATG
GCCATAATCAAAAAATCAAAAAATAATAGATATTGGCATGGATGTGATGTAAAGGGAACACTTTTGCACGTTGGTAGGAATGTAAACTAATACAACCACTATGGAAAACATTGTGGAGATTCTTTGAA
GAACTAAAAGTAGATGTACCATTTGATTCAGCAATCCCACTACTAGATATATATACAGGGGAAAAGAAGTCACTATACAAAATGATACTTGCATATGCATGTGT ATAGCAGCACATTTTGCAACTGCA
AAAATGTGGAATCAGCCCAAATGCCCATCAATCAACAAGTGGATAAAGAAATACATACATCTCACACTTTATCATCTATTTTTATCTATCTGTCTATCTATCTATCTATCTATCTATCTATCTAT CTA
TCTATCTATGCAATGGAATACTACTCAGCCATAAAAAGGAATAAAATATTGGCATTCACAGCAACCTGGATGGAACTGGAGATCATTATTCTCAAGTAACTCAG GAATGGAAAACCAAACATCGTATG
TTCTCACTCATAAGTGGGAGTTAATTATGAGGACGAAAAGGCAAAAGAATGATACAGTGGACTTTGGGGACTCAGGCAGAAGGGTGGGCGGGCGGTGTGAGATAAAGGACTAACACTGGGTATGG CGT
ATACTGCTCGGGTGATGGGTGCACCAAAATCTCAGAAATCACCACTGAAGAACTGATTCACATAACCAAACACCACCTGTTCCCAAAAACCTATTGAAATAAAA AATAAATTAAAAAAAACACTCTTG
AGGGAAATTCTGAT

7943	8.7	0.3	1.6	AluYb8-Scaffold3717-1643730-1644047	1	1000	(1318)	+	L1PB1	LINE/L1	4262	5259	(887)	1
3007	0.6	0.0	0.0	AluYb8-Scaffold3717-1643730-1644047	1001	1318	(1000)	+	AluYb8	SINE/Alu	1	318	(0)	2
7943	9.0	0.8	1.8	AluYb8-Scaffold3717-1643730-1644047	1319	1874	(444)	+	L1PB1	LINE/L1	5254	5791	(360)	1
56	1.9	0.0	0.0	AluYb8-Scaffold3717-1643730-1644047	1875	1929	(389)	+	(TATC)n	Simple_repeat	1	55	(0)	3
6208	9.1	1.0	1.9	AluYb8-Scaffold3717-1643730-1644047	1930	2282	(36)	+	L1PB1	LINE/L1	5792	6150	(1)	1

>Hg19 chr2:239882409-239884397
TCCTAGAACTGGTAAATGAATTCAGCAGGGTTTCAGGATACAAAATTAATGTACACAAATCAGTAGTTCCGCTATACACCAACAGCGACCAAGCTGAGAATCAAATCAAGAACTCAATCCCTTTTA CA
ATAGCTGCAAAAAAATAATGAAATACTTAGGAATATACCTAACCGAGGAAGTGAAATACCTCTACAAGGAAAACTACAAAACACTGCTGAATGTAATCATAGACAACATAAACAAATGGAAACAC ATC
CCATGCTCACGGATGGGTAGAACCAATATTGTGAAAATTACCACACTGCCAAAAATAATCTACAAATTCAATGCAATTCCCATCAAAATACCACCATCATTCTTCACGAACTAGAAAAAAAATCCT AA
ATTTTATATCAAATGAAAAAAAAGAGTCCACATAGTCAAAGCAAGACTAACCAAAAAGAACAAATGTGAAGGCATCACATTACCTGACTTCAAACTATAGTATAGGGTCATAGTCACCAAAACAG CAT
GGTACTAGTATAAAAATAGGCACATACCAATGGAACAGAATAGAGAACCCAGAAATAAAGCCAAACTGATCTTTGACAACTAATCTT cGACAAAGCAAACAAAAACATAAAGTGGGGAAAGGACACCC
TATTCAACAAATGGTGCTGGGATAATTGGCAAGCCACGTGTAGAAGAATGAAACTGGATCCTCATCTGTTACCCTATACAAAAATCAACTCAAGATTGATCAAAGACTTAAATCTGAGACCTGAAACA
ATAAAAATTCTGGAAGGTAATATCAGAAAAACTCTTCTAGACATTGGTTTAAGCAAAGACGTCATGACCAAGAACACCAAAGCAAATGCAGCAAAAACAAAGATAAATAGATGGGACTTCATTAAA TG
AAAAAGCTTCTGCACAGGAAAATAAATAATCAGCAGAGGTAACAGACAACCCACAGAGTGGGAAAAAAATCTTCACAATCTATACATC TGAAAAAGGACTAATATCCAGAATCTATAAATAACTCAAA
CAAATTAGCAAGAAAAAAACAAACAATCCCATCAAAAAGTGGGCAAAGGAAATGAATAGACAATTCTCAAAAGAAGATATACAAATGGCCAACAAGCATATGGAAAAATGCTCAGCATCACTAA tGAC
CAGGGAAATGCAAATCAAAACCACAATGCGATACCACCTTACTCCTGCAAGAATGGCCATAATCAAAAAATCAAAAAATAATAGATATTG GCATGGATGTGATGTAAAGGGAACACTTTTGCACGTTG
GTAGGAATGTAAACTAATACAACCACTATGGAAAACATTGTGGAGATTCTTTGAAGAACTAAAAGTAGATGTACCATTTGATTCAGCAATCCCACTACTAGATATATATACAGGGGAAAAGAAGTC AC
TATACAAAATGATACTTGCATATGCATGTGTATAGCAGCACATTTTGCAACTGCAAAAATGTGGAATCAGCCCAAATGCCCATCAATCAACAAGTGGATAAAGAAATACATACATCTCACACTTT ATC
ATCTATTTTTATCTATCTGTCTATCTATCTATCTATCTATCTATCTATCTATCTATCTATCTATGCAATGGAATACTACTCAGCCATAAAAAGGAATAAAATATTGGCATTCACAGCAACCTGGAT GG
AACTGGAGATCATTATTCTCAAGTAACTCAGGAATGGAAAACCAAACATCGTATGTTCTCACTCATAAGTGGGAGTTAATTATGAGGACGAAAAGGCAAAAGAATGATACAGTGGACTTTGGGGA CTC
AGGCAGAAGGGTGGGCGGGCGGTGTGAGATAAAGGACTAACACTGGGTATGGCGTATACTGCTCGGGTGATGGGTGCACCAAAATCTCAGAAATCACCACTGAAGAACTGATTCACATAACCAAAC AC

CACCTGTTCCCAAAAACCTATTGAAATAAAAAATAAATTAAAAAAAACACTCTTGAGGGAAATTCTGA T

8771	8.8	0.6	0.8	Hg19	1	1645	(444)	+	L1PB1	LINE/L1	4160	5791	(360)	4
56	1.9	0.0	0.0	Hg19	1646	1700	(389)	+	(TATC)n	Simple_repeat	1	55	(0)	5
6226	9.0	1.0	1.9	Hg19	1701	2053	(36)	+	L1PB1	LINE/L1	5792	6150	(1)	4

>KSI_AluYb8_20

>Scaffold4019-3293963-3294277
AGGAAATCCTTGTAAGCCCATAATGCTTACAGAAGCAGATGACAAGCTATTAAAAACTACAATGTTGCTTAGAAAATTTTT cTATCAGTGAACTGAACAGTTAAGTCATTGATAGTTTCAACAGTGCT
TGATTTACTTACTTCCTGGATAGATTTCTTTAACAAGAAATTTGGTTACTTACTATAAATTTGGTTTCTTACTACAAAATAATAAGAAAATACATGATTAACCTATAAAGCATCTGCAAAAACAT TTT
ACATTAATGAGTTCCCTTAGAGAATTTATTTTTTCTGCCAAACTATCATTTAACAGTTCATTAAATATTAAACTTTTATTGA GTGCCAACCACTTTTCAGGCATTCAGGGTATAGAGTACCAACCTTT
TGAGGTACTCACATTCTAGTTAAGAGAGCCATCCAACAAACAAACAAACAGTAGTTAAAGTACTAACATTTCTGAACTCAGAATTAGTAACTGGAATGAAATTATTAGTCTCTTCTAAACATTGT CAT
CCTCAATTTTCTGACCCCCACATAGCTAACAACATAGCATAGTACTACTATATAGATGAAGCTTTACATCTCCAGCTTTAAA TTTCATAAGATAACAAATTGCATATGTAATTATTAATAAAAATGGC
CTTAATTTTCCACATCTGCTGCAACCCTGGATCAAGTCTTTTACCCCCAAGGACCCTGGTCTCATATCCAAGCCTAGAGACTTCATTCATTAATGGTGAAACCAGCTTTCAATCACCATATGTAA TAG
GAAGTAATGGCTTCTATGTTAATCAGGAATACCAAGTATGGAAAG cCACTATTTTGTTTCTTCATTTCATTTTTCTTCTCATAAACTTACCTGCTCACAAGTGGCTAATCTAATAACTCTGTTGCTCA
ATCTCTCATTGTCCCCAAGATCATTCTCTGAGGGAGTTCCAGCAAATAACAAAATGACAAACAGTGAATATCTCCTGGTCTAGTCCCTGGTCCCATGTGCTTTCTTtttttttttttttttttttttt
tttgagacggagtctcgctctgtcgcccaggtcggactgcggactgcagtggcgcaatctcggctcactgcaagctccgcttcccgggttcacgccattctcctgcctcagcctcccgagtagct ggg
actacaggcgcccgccaccgcgcccggctaattttttgtatttttagtagagacggggtttcaccttgttagccaggatggtctcgatctcctgacctcatgatcc acccgcctcggcctcccaaagt
gctgggattacaggcgtgagccaccgcgcccggccactttcttATCTCTGTTTATCTCTTATCTCTCCCCAAGACTATGCTTCCTTAAATAAAAATGTCTCCACAACTGGTGAACTTAAGATGCCATA
AATACTCCTAATGTGGAAATAcAGTATCTCTGAAGAGCACCAAATGAGTGCCATGCTGCCTCAAATCATCTTTCAAGACAGCAATAGCTGGCCTGACTTTCAGCACTGCCCCTGTTTATATGCATATT
TGGAAGAGAGAAAAGACTATTTTAGTTCACAATTGGGTAATGGTATGGTTATCTTCTTTCCTCACTATCAGTAACATTAGAATAGTTATTCTAATTCATTAAAATAACTTAGTACCAGCTGGGGC AGT
GGCTCATGCCTGTAATCCCAGCACTTTGAGAAGCCAAGGTGGC ctAATCACTTGAGGTCAGGAGTTTGAGACCATCCTGGCCAACATATCGAAACCCTGTCTCTAC TGAAAATATAAAAATTAACTAG
GCGTGGTGACATACACCTCTAATCCCAGCTACTTGGGAGGGTGAGGCAGGAGAATTGCTTGAACCCAAGAGGCAGAGGCTACAGTGAGCCAAGATCATGCCACTGCACTTCAGCCTGGGTGACAG AGT
GAGACTCCATCTCAAAAAATAAAAATTAAAAAAAAAACTCAACAACTTAGCATGGATTTTTGGGTTGCTTAATGGCTAGGACAGTATACAGACACAAATTTAGAAC AAACAGATGATAGTTATAATAT
TAACATTGTTGCACTAACTGAATCACCTATTTTTGGCAACTATTTCTATTTGATACAGTTCATTAAATATTAAAGTTTTATTGAGTACCAACCATTTTTCAGGCACTCAGGTTATAGAGTATCAA CCT
TTTGAGGTACTCACATTCTAGTTAAGAGAGCCATGTAAAACAAACAAACAACAACAAAAACAGTACAACAACAGCAAACAGTAGTTAAAGTGCTATTAGGTGCTAT AAGAGTTCAAGGCTGTAGAAGT
TGAAAAGTGGC

200	27.8	14.1	3.9	AluYb8-Scaffold4019-3293963-3294277	302	443	(1872)	C	L2a	LINE/L2	(6)	3420	3265	1
2739	0.7	0.0	0.0	AluYb8-Scaffold4019-3293963-3294277	1001	1315	(1000)	C	AluYb8	SINE/Alu	(0)	318	1	2
2098	14.1	0.3	0.7	AluYb8-Scaffold4019-3293963-3294277	1654	1957	(358)	+	AluSz	SINE/Alu	2	304	(8)	3
16	6.7	0.0	11.4	AluYb8-Scaffold4019-3293963-3294277	2215	2253	(62)	+	(AAC)n	Simple_repeat	1	35	(0)	4

>Hg19 chr4:115548932-115550925
AGGAAATCCTTGTAAGCCCATAATGCTTACAGAAGCAGATGACAAGCTATTAAAAACTACAATGTTGCTTAGAAAATTTTT gTATCAGTGAACTGAACAGTTAAGTCATTGATAGTTTCAACAGTGCT
TGATTTACTTACTTCCTGGATAGATTTCTTTAACAAGAAATTTGGTT ACTTACTATAAATTTGGTTTCTTACTACAAAATAATAAGAAAATACATGATTAACCTATAAAGCATCTGCAAAAACATTTT
ACATTAATGAGTTCCCTTAGAGAATTTATTTTTTCTGCCAAACTATCATTTAACAGTTCATTAAATATTAAACTTTTATTGAGTGCCAACCACTTTTCAGGCATTCAGGGTATAGAGTACCAACC TTT
TGAGGTACTCACATTCTAGTTAAGAGAGCCATCCAACAAACAAACAA ACAGTAGTTAAAGTACTAACATTTCTGAACTCAGAATTAGTAACTGGAATGAAATTATTAGTCTCTTCTAAACATTGTCAT
CCTCAATTTTCTGACCCCCACATAGCTAACAACATAGCATAGTACTACTATATAGATGAAGCTTTACATCTCCAGCTTTAAATTTCATAAGATAACAAATTGCATATGTAATTATTAATAAAAAT GGC
CTTAATTTTCCACATCTGCTGCAACCCTGGATCAAGTCTTTTACCCC CAAGGACCCTGGTCTCATATCCAAGCCTAGAGACTTCATTCATTAATGGTGAAACCAGCTTTCAATCACCATATGTAATAG
GAAGTAATGGCTTCTATGTTAATCAGGAATACCAAGTATGGAAAG tCACTATTTTGTTTCTTCATTTCATTTTTCTTCTCATAAACTTACCTGCTCACAAGTGGCTAATCTAATAACTCTGTTGCTCA
ATCTCTCATTGTCCCCAAGATCATTCTCTGAGGGAGTTCCAGCAAATAACAAAATGACAAACAGTGAATATCTCCTGGTCTAGTCCCTGGTCCCATGTGCTTTCTTATCTCTGTTTATCTCTTATCTC
TCCCCAAGACTATGCTTCCTTAAATAAAAATGTCTCCACAACTGGTGAACTTAAGATGCCATAAATACTCCTAATGTGGAAATA tAGTATCTCTGAAGAGCACCAAATGAGTGCCATGCTGCCTCAAA
TCATCTTTCAAGACAGCAATAGCTGGCCTGACTTTCAGCACTGCCCCTGTTTATATGCATATTTGGAAGAGAGAAAAGACTATTTTAGTTCACAATTGGGTAATGGTATGGTTATCTTCTTTCCTCAC
TATCAGTAACATTAGAATAGTTATTCTAATTCATTAAAATAACTTAGTACCAGCTGGGGCAGTGGCTCATGCCTGTAATCCCAGCACTTTGAGAAGCCAAGGTGGC tgAATCACTTGAGGTCAGGAGT
TTGAGACCATCCTGGCCAACATATCGAAACCCTGTCTCTACTGAAAATATAAAAATTAACTAGGCGTGGTGACATACACCTCTAATCCCAGCTACTTGGGAGGGTGAGGCAGGAGAATTGCTTGAACC
CAAGAGGCAGAGGCTACAGTGAGCCAAGATCATGCCACTGCACTTCAGCCTGGGTGACAGAGTGAGACTCCATCTCAAAAAATAAAAATTAAAAAAAAAACTCAACAACTTAGCATGGATTTTTG GGT
TGCTTAATGGCTAGGACAGTATACAGACACAAATT TAGAACAAACAGATGATAGTTATAATATTAACATTGTTGCACTAACTGAATCACCTATTTTTGGCAACTATTTCTATTTGATACAGTTCATTA
AATATTAAAGTTTTATTGAGTACCAACCATTTTTCAGGCACTCAGGTTATAGAGTATCAACCTTTTGAGGTACTCACATTCTAGTTAAGAGAGCCATGTAAAACAAACAAACAACAACAAAAACA GTA
CAACAACAGCAAACAGTAGTTAAAGTGCTATTAGG TGCTATAAGAGTTCAAGGCTGTAGAAGTTGAAAAGTGGC

200	27.8	14.1	3.9	Hg19	402	543	(1651)	C	L2a	LINE/L2	(6)	3420	3265	5
2109	14.1	0.3	0.7	Hg19	1433	1736	(458)	+	AluSz	SINE/Alu	2	304	(8)	6
193	30.3	3.4	4.8	Hg19	1954	2102	(92)	C	L2d	LINE/L2	(102)	3323	317 7	7

>KSI_AluYb8_21

> Scaffold4319-2904259-2904575

TGTCTCCAGCCCCTATTTTTCACTTTGGTACTCTCATtAAAATCTGTCAACTCTTTTATGTATTTGTCCACTTCCGTCTCTATAGAATGTAAACCCCTGAGAGCAGAGGTGGTGTTCGCACCTCCATC
GCCGCATCCCTAGAGCCTAGCTCAGTGCCTGGCGCGCAGCTGGTACTCCAgAGATATAGGTACAATCAATGAATGAATAGTACGTAGTAGGTTCCTAGTAAATCCCACACACTAAGCCTAGCTCATCA
GTGACTGGCATACAGGTGCTCTCAAGAGATATGTGTAAAATGAATTAATTAAT AGTATGAATTAGGTTCCCAATGAGCGGGCTCCCAGAGAGCAGGATAGGACCCTAGCGCTCGGGGCAATATAATAA
TTGCTAACATTATGGCTGGGTGAGGTGGCTCGTGGCTGTAATCCCAGCACTTTGAGAGGCCAAGGCAGGTAAATCACTCAAGCCCAGGAGTCTG AGACCAGCCTGGGCAACATGATGAAATCCTGTAT
CTACAAGAAAAGTACAAAAATTAGCTGGGTGTGGTGGCACATGCCCATAGTCCCAGCTACTCGGGGGGTGCTGAGGTGGAAGGATGGCCTGACCCTCCGAAGCGGAG Gttgcagtgagccgagatcgc
gccactgcacttggggctgggtgacacagcaagaccctgtctcataaaataataataataggccaggctcggtggctcatgcctgtaatcccagcactttgagaggccaaggtgggcagaacacctgg
ggtcaggagttccagaccagcctgtccaacatagtgaaaccccgtctccaccaaaaatacaaaaattagtcaggcatggtggtacatgcctgtaattccagctacttaggaggctgaagcaggag agt
ccctggaacccgggaggcaaaggttccagtgagccgagatcgcaccattgctctccagcctggacaacgagagtgaaactctgtctcaaaaaaataataataataggccgggcgcggtggctcacgcct
gtaatcccagcactttgggaggccgaggcgggtggatcatgaggtcaggagatcgagaccatcctggctaacaaggtgaaaccccgtctctactaaaaatacaaaaaattagccgggcgcggtgg cgg
gcgcctgtagtcccagctactcaggaggctgaggcaggagaatggcgtgaacccgggaagcagagcTTGCAGTGAGCCGAGATTGCGCCACTGCAGTCCGCAGTCCGGCCTGGGCGACAGAGcGaGAC
TCCGTCTCAAAAAAAAAAAAAAGAAAAAATAATAATAATAATAATTGCCAACATTATGGAATGCTCGTTATATGCTAAGCAGCGTTCTGAGCACCTTTCCTCCCCACGACGAACCTGTGAAGCAGT CAC
TATTTATATATATATATATTTTTTTGTTTGTTTTTTTTGAGACTGAGCCTCACTTTCGTCACCCAGGCTGGAGTGCAGTGGCGCGATCTCGGCTTACTGCAACCTGCACCTCCTGGATTCAAGCGATT
CTCCTGCCTCAGCCTCCTGAGTAGCTGGGATTACAGGTGCACACCACCACTCCTGGCTAATTTTTGTATTTTTAATAGAGACGGGGTTTCACCATGTTGGCCAAGCTGGTCTCAAACTCCTGACC TCA
GGTAATCCACCCGCCTCGGCCTCTGAAAGTGCTGGGATTACAGGTATGAGCCACCGTGCCCGGCTAG CAGCCACAATTTCTATCCAGATTTCATGGATGAGGAAACTGAGGCTCAGAGGCAAGTAACT
TGCTCCAGGGCACCCAGCTATGTGTCCTGGAATTTGAACCTAGACACACTCTGGTTCATAAAGTTGGCTTGCTTTTCAACACTGCCCA CTACTGCAAGCAGGAAGACTTCCTGGAGGAGATGTGGCAG
AAGCTGGGCCTGGGAGAGTGGAACAAATTCCTACAATAGGGAACTGAGTCGCTGGGTGCAGCAGCTGGCAGGAGTGGAGCAGAGAGGGCACTGGGGAATAAAGTTGGCAGGTGTTTCAGTTATCTATT
GCTGATAGGAATCCACCCAAAAACATGGCAGCATTGACTTAGCAACAACCATATACTTAACTCAGGAATCTGCAACTGGCAGGACTCAGCAGACACAGCTCATCTCTGCTCACATGACATCAGTC CTC
TGTGGTCTCACTACCTGGCCTCTACAACATGGTGGCAGCAGGGCAGCCAGCTTCCTACATGGTGGTTTCAAAGACACATGTCAAGAGACAGCCAGAGGAAGCCATCGCCTTTTATGATCTGGCCTTGG
AAGTCAGCCAGTG

351	29.9	6.1	0.0	AluYb8-Scaffold4319-2904259-2904575	56	202	(2115)	+	L2a	LINE/L2	3271	3426	(0)	1
183	22.1	1.6	5.0	AluYb8-Scaffold4319-2904259-2904575	244	305	(2012)	+	L2a	LINE/L2	3367	3426	(0)	2
26	8.3	1.7	3.5	AluYb8-Scaffold4319-2904259-2904575	375	397	(1920)	+	(AAT)n	Simple_repeat	1	22	(0)	3
1758	17.5	0.0	1.4	AluYb8-Scaffold4319-2904259-2904575	398	689	(1628)	+	AluJb	SINE/Alu	1	288	(24)	4
26	8.3	1.7	3.5	AluYb8-Scaffold4319-2904259-2904575	690	700	(1617)	+	(AAT)n	Simple_repeat	2	12	(0)	5
2066	13.8	0.0	0.0	AluYb8-Scaffold4319-2904259-2904575	701	989	(1328)	+	AluSp	SINE/Alu	1	289	(24)	6
26	8.3	1.7	3.5	AluYb8-Scaffold4319-2904259-2904575	990	1000	(1317)	+	(AAT)n	Simple_repeat	1	11	(0)	7
2887	1.3	0.0	0.0	AluYb8-Scaffold4319-2904259-2904575	1001	1308	(1009)	+	AluYb8	SINE/Alu	1	308	(10)	8
26	8.3	1.7	3.5	AluYb8-Scaffold4319-2904259-2904575	1309	1318	(999)	+	(AAT)n	Simple_repeat	3	12	(0)	9
453	28.9	5.5	8.0	AluYb8-Scaffold4319-2904259-2904575	1319	1426	(891)	C	MIR	SINE/MIR	(0)	262	165	10
2372	9.3	0.0	0.3	AluYb8-Scaffold4319-2904259-2904575	1427	1728	(589)	C	AluSx	SINE/Alu	(10)	302	2	11
453	28.9	5.5	8.0	AluYb8-Scaffold4319-2904259-2904575	1729	1875	(442)	C	MIR	SINE/MIR	(98)	164	13	10
594	23.0	2.9	0.7	AluYb8-Scaffold4319-2904259-2904575	2032	2171	(146)	C	MLT1G1	LTR/ERVL-MaLR	(0)	579	437	12
393	23.8	10.8	2.1	AluYb8-Scaffold4319-2904259-2904575	2189	2317	(0)	C	MLT1G1	LTR/ERVL-MaLR	(285)	270	131	12

>Hg19 chr22:37924505-37926223

TGTCTCCAGCCCCTATTTTTCACTTTGGTACTCTCATcAAAATCTGTCAACTCTTTTATGTATTTGTCCACTTCCGTCTCTATAGAATGTAAAC CCCTGAGAGCAGAGGTGGTGTTCGCACCTCCATC
GCCGCATCCCTAGAGCCTAGCTCAGTGCCTGGCGCGCAGCTGGTACTCCAcAGATATAGGTACAATCAATGAATGAATAGTACGTAGTAGGTTCCTAGTAAATCCCACACACTAAGCCTAGCTCATCA
GTGACTGGCATACAGGTGCTCTCAAGAGATATGTGTAAAATGAATTAATTAATAGTATGAATTAGGTTCCCAATGAGCGGGCTCC CAGAGAGCAGGATAGGACCCTAGCGCTCGGGGCAATATAATAA
TTGCTAACATTATGGCTGGGTGAGGTGGCTCGTGGCTGTAATCCCAGCACTTTGAGAGGCCAAGGCAGGTAAATCACTCAAGCCCAGGAGTCTGAGACCAGCCTGGGCAACATGATGAAATCCTG TAT
CTACAAGAAAAGTACAAAAATTAGCTGGGTGTGGTGGCACATGCCCATAGTCCCAGCTACTCGGGGGGTGCTGAGGTGGAAGGATGGCCTGACCCTCCGAAGCGG AGGTTGCAGTGAGCCGAGATTGC
GCCACTGCAGTCCGCAGTCCGGCCTGGGCGACAGAGtGtGACTCCGTCTCAAAAAAAAAAAAAAGAAAAAATAATAATAATAATAATTGCCAACATTATGGAATGCTCGTTATATGCTAAGCAGCGTTC
TGAGCACCTTTCCTCCCCACGACGAACCTGTGAAGCAGTCACTATTTATATATATATATATATTTTTTTGTTTGTTTTTTTTGAGACTGAGCCTCACTTTCGTCACC CAGGCTGGAGTGCAGTGGCGCGA

TCTCGGCTTACTGCAACCTGCACCTCCTGGATTCAAGCGATTCTCCTGCCTCAGCCTCCTGAGTAGCTGGGATTACAGGTGCACACCACCACTCCTGGCTAATTTTTGTATTTTTAATAGAGACG GGG
TTTCACCATGTTGGCCAAGCTGGTCTCAAACTCCTGACCTCAGGTAATCCACCCGCCTCGGCCTCTGAAAGTGCTGGGATTACAGGTATGAGCCACCGTGCCCGG CTAGCAGCCACAATTTCTATCCA
GATTTCATGGATGAGGAAACTGAGGCTCAGAGGCAAGTAACTTGCTCCAGGGCACCCAGCTATGTGTCCTGGAATTTGAACCTAGACACACTCTGGTTCATAAAGTTGGCTTGCTTTTCAACACT GCC
CACTACTGCAAGCAGGAAGACTTCCTGGAGGAGATGTGGCAGAAGCTGGGCCTGGGAGAGTGGAACAAATTCCTACAA TAGGGAACTGAGTCGCTGGGTGCAGCAGCTGGCAGGAGTGGAGCAGAGAG
GGCACTGGGGAATAAAGTTGGCAGGTGTTTCAGTTATCTATTGCTGATAGGAATCCACCCAAAAACATGGCAGCATTGACTTAGCAACAACCATATACTTAACTCAGGAATCTGCAACTGGCAGG ACT
CAGCAGACACAGCTCATCTCTGCTCACATGACATCAGTCCTCTGTGGTCTCACTACCTGGCCTCTACAACATGGTGGCAGCAGGGCAGCCAGCTTCCTACATGGT GGTTTCAAAGACACATGTCAAGA
GACAGCCAGAGGAAGCCATCGCCTTTTATGATCTGGCCTTGGAAGTCAGCCAGTG

561	16.7	0.0	0.0	Hg19	1	84	(1835)	C	AluSp	SINE/Alu	(229)	84	1	13
374	29.4	9.5	1.0	Hg19	114	302	(1617)	+	L2a	LINE/L2	3222	3426	(0)	14
183	22.1	1.6	5.0	Hg19	344	405	(1514)	+	L2a	LINE/L2	3367	3426	(0)	15
1839	16.0	0.0	3.6	Hg19	498	810	(1109)	+	AluSz	SINE/Alu	1	302	(10)	16
453	28.9	5.5	8.0	Hg19	821	928	(991)	C	MIR	SINE/MIR	(0)	262	165	17
2372	9.3	0.0	0.3	Hg19	929	1230	(689)	C	AluSx	SINE/Alu	(10)	302	2	18
453	28.9	5.5	8.0	Hg19	1231	1377	(542)	C	MIR	SINE/MIR	(98)	164	13	17
594	23.0	2.9	0.7	Hg19	1534	1673	(246)	C	MLT1H1	LTR/ERVL-MaLR	(0)	579	437	19
665	23.8	10.6	1.5	Hg19	1691	1878	(41)	C	MLT1H1	LTR/ERVL-MaLR	(285)	270	66	19

>KSI_AluYb8_22

>Scaffold4319-5407141-5407448
TGCCTAGACAGACACAGCACTGGGCAGACATGTGTCCTAGGGAAGGCACACACTACACTGGGGACTGAGGACAGAGGACAATCCTGGGACAGCCCTGGGAAGAGTGAAACAAGGACCTCAACAAAG AG
CATTCCCATCACTACAGGCTTAGAGAGTGACTGTGTGAGTTAAGGCACAAGCCGGGGCCTGCTGGGTGAGTTTGGAGAACATCAAATAATCTTGTG GGGTATGAGCTGATTATCTGGGGAAAGTGGA
TAAATGCCTCTTTGGGTTGGACGCACATGAAGGGTCTGCAGCCAAGCTGAGGGCCTTGGACTTGGCTGTAGGCAGAGTCCGGAGCCGCTGAGTGGGCAGGGAAACGGGGAAACAGGGCACTTGGG TTG
TGTTACGGGGTCCGTCTGCCAAGAGGGGAACAGTCAGGGCAGCGTGAACTAGCATTGCTGCACGGTGACCCTGAGCACCTCCTGTGTGTCAGGCCC TGTCCCAGAGGAAGGGCAGAGCTCAGCCAGCT
GGGGCAGGCACCAGGCAAGATTTTATCTAGGCTTGATCACATTTCTTTTCATCTTCATAGAACCATGAAGTAGCCTCATTTTACAGGTGGAATTCAGAGGGCTTAAGTAACTTGCACAAGGCCTC ACA
GCCTGCGTGGAATCTGTGGCACTTTCCCCACTGCAGTACACTTCCGTACTGTTGGATCGATGTGAATCT AGAGACCAGGGATGGAGGAAGTTAAAGGGGTGCTCTGTGGGGGTCTTGCAGCAGGAGCC
CCTCCTGCGGGCAGCTGTGCAGCCTCCTCTCCTCTCCTCCCCTCCCCTCCTCCTCTCCTCCTCCTTTCCTCCTCCTCTCTTCCTCCTTTCCTCCTCTCCTCCTCCTCTTGTCCTCTCCTCTCCTC CCC
TGTCCTCTCCTCCTCCTCTCCTCTCCTCcTCCTCTCCTCCTCCTgcttgcctcctcctctcttcctcctctcctcctcttctgctcctttcctcctcctcctccttctttttttttttttttgaga
cggagtctcgctctgtcgcccaggccagactgcggactgcagtggcgcaatctcggctcactgcaagctccgcttcccgggttcacgccattctcctgcctcagcctcccgagtagctgggacta cag
gcgcctgccaccgcgcctggctaatttttttgtatttttagtagagacggggtttcaccttgttagccaggatggtctcgatctcttgacctcatga tccacccgccttggcctcccaaagtgctggga
ttacaggcgtaagccaccgcgcccggccctcctccttctttcctcctcctctcttcCTCCTCTcCTCCTCCTCCTCTCCTcTCCTCCTCCTCTCCTCTCCTCCTCTCCTcctctccctttcttcttcc
tctcctcctcttctcctcctctcctcttcctgtcCTCCTCCTCCTCTCCTCTtCCTCCTCctcttctcctcctccttctctcttctTCCTtTCCTCCTCCTCTCCTCTCCTCTTCCTCtcctcctcct
ctcctcttcctctcctcctccTCtTCCTCCTGTCCTCCTCCTCTCCTCTCCTCCTCTCCTCTCCTCCTCCTCTCCTCCTCCTCTCCTCTCCTCCTCCTCTCTTCTCCTCCTCCTCTCTTCTCCTCTCT
TCCTCCTCTCCTCTCCTCTCCTCTTCTCTCCTCCTCGTTTCCTCTCCTCTCCTCCTCATTTCCTCTCCTCT CCTCCTCATTTCCTCTCCTCTCTTCCTCCTGTCCTCCTCCTCTCCTCCTCTCTCCCC
AGGGTATGTACATGGGGAACTTAAGAGAAAGTTTGTTGTTTAGT GGTTCCCACTCTACTGGCATAAACATTGGTGATAATTCAGATGGGGTCTGTTGCAGTCTTGGTGACAGTCTTTGCAGTGTACCC
AGAGTACTTAACGACACCCTCTCCTGGATGCATCGTCTCCACAGAGCACCTTTCTGTGCTCTGGTGGCAGCTGTGCCAGTTAGAAGTTGCACTGGTTCCCCGCAGCCTGCCTTGAGATACTTTAC TCT
GGCAGCGGTTTTCTTAGCCGTGCATTGCGACATTCAGCATATGGGTGTAGACACCCGGTATGGC TGGGTGAACGGTTCATCTTGTGGGTTCTTCCTTAATTGGGCTGCCCTTCCCCTCTATTAGTTCA
AGTTTTGACCTTTCTGGAAGTGTCGTGTTCCCTGAGCTTTAATCAGCACCGTTGGCAGGGCCTGCTGGGCACGGCATTGAGCAGAGGAACGACATCCCCACCTTGATGTCTGTGCCGCGGGAGGC GCG
GGCT

192	6.9	0.0	0.0	AluYb8-Scaffold4319-5407141-5407448	455	483	(1825)	C	L2b	LINE/L2	(24)	3351	3323	1
275	20.0	24.4	0.0	AluYb8-Scaffold4319-5407141-5407448	553	642	(1666)	C	MIRb	SINE/MIR	(70)	198	87	2
357	6.8	9.8	0.8	AluYb8-Scaffold4319-5407141-5407448	791	1000	(1308)	+	(CCT)n	Simple_repeat	1	231	(0)	3
2827	2.6	0.0	0.0	AluYb8-Scaffold4319-5407141-5407448	1001	1308	(1000)	C	AluYb8	SINE/Alu	(10)	308	1	4
357	6.8	9.8	0.8	AluYb8-Scaffold4319-5407141-5407448	1309	1790	(518)	+	(CCT)n	Simple_repeat	1	523	(0)	5

>Hg19 chr22:40402430-40404267
TGCCTAGACAGACACAGCACTGGGCAGACATGTGTCCTAGGGAAGGCACACACTACACTGGGGACTGAGGACAGAGGACAATCCTGGGACAGCCCTGGGAAGAGTGAAACAAGGACCTCAACAAAG AG
CATTCCCATCACTACAGGCTTAGAGAGTGACTGTGTGAGTTAAGGCACAAGCCGGGGCCTGCTGGGTGAGTTTGGAGAACATCAAATAATCTTGTGGGGTATGAGCTGATTATCTGGGGAAAGTGGA
TAAATGCCTCTTTGGGTTGGACGCACATGAAGGGTCTGCAGCCAAGCTGAGGGCCTTGGACTTGGCTGTAGGCAGAGTCCGGAGCCGCTGAGTGGGCAGGGAAACGGGGAAACAGGGCACTTGGG TTG
TGTTACGGGGTCCGTCTGCCAAGAGGGGAACAGTCAGGGCAGCGTGAACTAGCATTGCTGCACGGTGACCCTGAGCACCTCCTGTGTGTCAGGCCCTGTCCCAGAGGAAGGGCAGAGCTCAGCCAGCT
GGGGCAGGCACCAGGCAAGATTTTATCTAGGCTTGATCACATTTCTTTTCATCTTCATAGAACCATGAAGTAGCCTCATTTTACAGGTGGAATTCAGAGGGCTTAAGTAACTTGCACAAGGCCTC ACA
GCCTGCGTGGAATCTGTGGCACTTTCCCCACTGCAGTACACTTCCGTACTGTTGGATCGATGTGAATCT AGAGACCAGGGATGGAGGAAGTTAAAGGGGTGCTCTGTGGGGGTCTTGCAGCAGGAGCC
CCTCCTGCGGGCAGCTGTGCAGCCTCCTCTCCTCTCCTCCCCTCCCCTCCTCCTCTCCTCCTCCTTTCCTCCTCCTCTCTTCCTCCTTTCCTCCTCTCCTCCTCCTCTTGTCCTCTCCTCTCCTC CCC
TGTCCTCTCcttctctcttcttcctttcCTCCTCCTCTCCTCTCCTCtTCCTCTCCTCCTCCTCTCCTCTtcctCTCCTCCTCCTcttcCTCCTgTCCTCCTCCTCTCCTCTCCTCCTCTCCTCTCCT
CCTCCTCTCCTCTCCTCCTCTCCTcTCCTCCTCCTCTCCTCTCCTCTTCCTCTCcTCCTCCTGTCCTCCTCCTCTCCTCTCCTCCTCTCCTCcTCCTCtcctcctcctCTCCTCTCCTCCTCCTCTCC
TCTCCTCCTCCTCTCTTCTCCTCCTCCTCTCTTCTCCTCTCTTCCTCCTCTCCTCTCCTCTCCTCTCCTCTTCTCTCCTCCTCGTTTCCTCTCCTCTCC TCCTCATTTCCTCTCCTCTCCTCCTCATTTCCTC
TCCTCTCTTCCTCCTGTCCTCCTCCTCTCCTCCTCTCTCCCCAGGGTATGTACATGGGGAACTTAAGAGAAAGTTTGTTGTTTAGT GGTTCCCACTCTACTGGCATAAACATTGGTGATAATTCAGAT
GGGGTCTGTTGCAGTCTTGGTGACAGTCTTTGCAGTGTACCCAGAGTACTTAACGACACCCTCTCCTGGATGCATCGTCTCCACAGAGCACCTTTCTGTGCTCTGGTGGCAGCTGTGCCAGTTAGAAG
TTGCACTGGTTCCCCGCAGCCTGCCTTGAGATACTTTACTCTGGCAGCGGTTTTCTTAGCCGTGCATTGCGACATTCAGCATATGGGTGTAGACACCCGGTATGGCTGGGTGAACGGTTCATCTT GTG
GGTTCTTCCTTAATTGGGCTGCCCTTCCCCTCTATTAGTTCAAGTTTTGACCTTTCTGGAAGTGTCGTGTTCCCTGAGCTTTAATCAGCACCGTTGGCAGGGCCTGCTGGGCACGGCATTGAGCAGAG
GAACGACATCCCCACCTTGATGTCTGTGCCGCGGGAGGCGCGGGC T

192	6.9	0.0	0.0	Hg19	555	583	(1455)	C	L2b	LINE/L2	(24)	3351	3323	6
275	20.0	24.4	0.0	Hg19	653	742	(1296)	C	MIRb	SINE/MIR	(70)	198	87	7
275	5.6	10.9	0.7	Hg19	891	1420	(618)	+	(CCT)n	Simple_repeat	1	584	(0)	8

>KSI_AluYb8_23

>Scaffold6231-154264-154590
ACCTACAGCTGAATTCACTTAGCATATTAAGTTATATAGCTGTTTGCTGGAAAATAGTAATTAGGCATACAAGTGAGATAATAGCTTTCTTGCATGAATTACCTTTGTACTATGTTAAAAACAA cCAG
TTGACTATATTTGCCTGGGTCTATTTTGAGCCTTCTTTTCAGTTTCACTCATCTATATATCTCTCC cTTGACAATTCTACCCAATCTTAACGAACTTAGCTTTACAGTAAGACCTGAAATTGAATAGT
GTAAGTCTTCCATTCTGCTTGCTTTGGGTTTAGCTTGCTCTTCTTTTTCTGTTTTTTTTTCTTTAAGTAGATGCTTGCATTATAAATTCTACATTATCTCCTTTTCAAATATAAGCATTCAATATT AT
ACATTTCCCTCTAAACTCTGCTTAAGCTAAAATCCATTAGTTTTGATGCTTTATATTGTTATTTTTATTCAACTCAGAATACGTTTTAAAAATTTCCCCTGATTCTTCCTCTTTGGCCTATACAT TAT
TTAAAAGTCTGTTGTTTAACTTCTAAATATTTAGGCATTTTCCAAGTATCTTTCTTTTATTAATTTCTAGTTTAATTTTATTATGATTTGAGATATACATTTTGTATGATATTTATATTTTCCACA TT
TGTAAATATTTTCAGATTTTTATGTCCCCAAATATGATCCACATGAACATTCATGTGCACTTGGAAAGAATGTACATTCCCTGTCGTATAGAGTTTCCAATAATGTCAATTAAATCTAGTTGGTT GTT
ATTGTTGTTTAGTTCTTCTGTTTCCTTACTATTTTTTTTTGTCTATTAGTTTAATTAGTTACTTAAAGAGGTGTTTACATCTCCAGCTACATTTGTGAGTTTGTCCATTTCCCCTTTCAGTTCTGCAG
TCTTTCCCTTGGTGCATGCACGTTTATAATTGACCCTTGTTATGGGCTGTGGGGCCCAGAGAGTGGAATATTATGGGCTGAATTGTACTAC Tccaaaattcatattttttttttttttttttttttt
tttttgagacggagtctcgctctgtcgcccaggccggactgcggactgcagtggcgcaatctcggctcactgcaagctccgcctcccgggttcacgccattctcctgcctcagcctcccgagtag ctg
ggactacaggcgcccgccaccgcgcccggctaattttttttgtatttttttttttttagtagagacggggtttcaccttgttagccaggatggtctcgatctcctgac ctcatgatccacccgcctcg
gcctcccaaagtgctgggattacaggcgtgagccaccgcgcccggcCCAAAATTCATATTTTGAAGTCCTAATCGCCACTATCTCAGAATGTGACTGTATATGGAGACACAACCTTTAAAGATGTAAT
TAACTTACAGTGAGGCCATTGCGGTGGGCCCTAATCCAATCTGATAATGTCCTTACAAGAAGAAATTTGGATACACAGAGACATCAGAGATGTACACACATTGA AGAAATGCCATTTGAAGGTACAGC
AAAAAGGTGATCAGCTGAAAACCAAGGAGAGATGCCTCAGAAGAAACCAAACCTGCCAACAACCTTGATCTTGGACTTCTAGGCTTTAGCTGTGAAAAATAAGTTTCTGTTGCTTTAGCTACCTA GTC
TGTGATACTTTATTGAAACAGCCCTAGCATATTAATACAACTCCTTTATTATTATGGAATGTCCCTCCTTGTTCTCAAGAGTGTCTTGGGTTTTTTTGTGTGTGTCTTTTCATATACCTCTTACATGT
TTGTTGAAAGTTGTACATCTTCATCTTGTGTAGGACAGTGGGAATTGAGTTAAAATGCTTTTGTACTTGGAAATGTGCACACAGGTCCTTCTCCTAGGTCTTTAATGTGGGCTTTGAGTTAATCA TTA
AGAGTTAGAGCCAGGTGCGGTGGCTCATGCCTGTAATAGCAACTGTTCATGAGGGTGCCGTGGGAGGAACCCT cGAGACCAGGAGTTTGAGAACAGCCTAGGCTGGGC GTGTTGGCTCACGCTTGTAA
TCCCAGTACTTTGGGAGGCTGAGGCGGGCAGATCACAAGGTCAGGAGATCGAGACCATCCTGGCTAACATGGAGAAACCCCGTCTCTACTAAAAATACAAAAAATTAGCTGGGCGTGGTGGCGGG CGC
CTGTAGTCCCAGCTACTGGGGAGGCTGAGGCAGGAGAATGGCGTGAACCCGGGAAGTGGAGCTTGCAGTGAGCCGAGATTGCATCACTGCACTCCAGCCTGGGCGAAA GAGCGAGACTTTGTCTCAAA
AACAAAAACAAAACAAAAAGAAA

570	26.1	2.1	1.6	AluYb8-Scaffold6231-154264-154590	82	268	(2059)	C	L1MC	LINE/L1	(1249)	4897	4710	1	*
1932	24.5	5.2	4.6	AluYb8-Scaffold6231-154264-154590	263	964	(1363)	C	L1MCc	LINE/L1	(3023)	3918	3212	2	
1834	18.1	1.7	8.0	AluYb8-Scaffold6231-154264-154590	965	1000	(1327)	+	MLT1B	LTR/ERVL-MaLR	1	25	(365)	3	
2629	0.7	0.0	3.5	AluYb8-Scaffold6231-154264-154590	1001	1327	(1000)	C	AluYb8	SINE/Alu	(0)	318	1	4	
1834	18.1	1.7	8.0	AluYb8-Scaffold6231-154264-154590	1328	1703	(624)	+	MLT1B	LTR/ERVL-MaLR	26	389	(1)	3	
1932	24.5	5.2	4.6	AluYb8-Scaffold6231-154264-154590	1704	1736	(591)	C	L1MCc	LINE/L1	(3730)	3211	3179	2	
336	31.8	2.8	3.4	AluYb8-Scaffold6231-154264-154590	1751	1926	(401)	C	L1MCc	LINE/L1	(5060)	1363	1189	2	
405	25.6	0.0	0.0	AluYb8-Scaffold6231-154264-154590	1930	2019	(308)	+	FLAM_A	SINE/Alu	1	90	(52)	5	
2504	8.1	0.0	0.0	AluYb8-Scaffold6231-154264-154590	2021	2327	(0)	+	AluYk2	SINE/Alu	1	307	(4)	6	

>Hg19 chr12:9870158-9872147
ACCTACAGCTGAATTCACTTAGCATATTAAGTTATATAGCTGTTTGCTGGAAAATAGTAATTAGGCATACAAGTGAGATAATAGCTTTCTTGCATGAATTACCTTTGTACTATGTTAAAAACAA tCAG
TTGACTATATTTGCCTGGGTCTATTTTGAGCCTTCTTTTCAGTTTCACTCATCTATATATCTCTCC tTTGACAATTCTACCCAATCTTAACGAACTTAGCTTTACAGTAAGACCTGAAATTGAATAGT
GTAAGTCTTCCATTCTGCTTGCTTTGGGTTTAGCTTGCTCTTCTTTTTCTGTTTTTTTTTCTTTAAGTAGATGCTTGCATTATAAATTCTACATTATCTCCTTTTCAAATATAAGCATTCAATAT TAT
ACATTTCCCTCTAAACTCTGCTTAAGCTAAAATCCATTAGTTTTGATGCTTTATATTGTTATTTTTATTCAACTCAGAATACGTTTTAAA AATTTCCCCTGATTCTTCCTCTTTGGCCTATACATTAT
TTAAAAGTCTGTTGTTTAACTTCTAAATATTTAGGCATTTTCCAAGTATCTTTCTTTTATTAATTTCTAGTTTAATTTTATTATGATTTGAGATATACATTTTGTATGATATTTATATTTTCCAC ATT
TGTAAATATTTTCAGATTTTTATGTCCCCAAATATGATCCACATGAACATTCATGTGCACTTGGAAAGAATGTACATTCCCTGTCGTATA GAGTTTCCAATAATGTCAATTAAATCTAGTTGGTTGTT
ATTGTTGTTTAGTTCTTCTGTTTCCTTACTATTTTTTTTTtGTCTATTAGTTTAATTAGTTACTTAAAGAGGTGTTTACATCTCCAGCTACATTTGTGAGTTTGTCCATTTCCCCTTTCAGTTCTGCA
GTCTTTCCCTTGGTGCATGCACGTTTATAATTGACCCTTGTTATGGGCTGTGGGGCCCAGAGAGTGGAATATTATGGGCTGAATTGTACTAC TCCAAAATTCATATTTTGAAGTCCTAATCGCCACTA
TCTCAGAATGTGACTGTATATGGAGACACAACCTTTAAAGATGTAATTAACTTACAGTGAGGCCATTGCGGTGGGCCCTAATCCAATCTGATAATGTCCTTACAAGA AGAAATTTGGATACACAGAGA
CATCAGAGATGTACACACATTGAAGAAATGCCATTTGAAGGTACAGCAAAAAGGTGATCAGCTGAAAACCAAGGAGAGATGCCTCAGAAGAAACCAAACCTGCCAACAACCTTGATCTTGGACTT CTA
GGCTTTAGCTGTGAAAAATAAGTTTCTGTTGCTTTAGCTACCTAGTCTGTGATACTTTATTGAAACAGCCCTAGCATATTAATACAACTCCTTTATTATTATG GAATGTCCCTCCTTGTTCTCAAGAG

TGTCTTGGGTTTTTTTGTGTGTGTCTTTTCATATACCTCTTACATGTTTGTTGAAAGTTGTACATCTTCATCTTGTGTAGGACAGTGGGAATTGAGTTAAAATGCTTTTGTACTTGGAAATGTGC ACA
CAGGTCCTTCTCCTAGGTCTTTAATGTGGGCTTTGAGTTAATCATTAAGAGTTAGAGCCAGGTGCGGTGGCTCATGCCTGTAATAGCAACTGTTCATGAGGGTGCCG TGGGAGGAACCCTtGAGACCA
GGAGTTTGAGAACAGCCTAGGCTGGGCGTGTTGGCTCACGCTTGTAATCCCAGTACTTTGGGAGGCTGAGGCGGGCAGATCACAAGGTCAGGAGATCGAGACCATCCTGGCTAACATGGAGAAAC CCC
GTCTCTACTAAAAATACAAAAAATTAGCTGGGCGTGGTGGCGGGCGCCTGTAGTCCCAGCTACTGGGGAGGCTGAGGCAGGAGAATGGCGTGAACCCGGGAAGTGGA GCTTGCAGTGAGCCGAGATTG
CATCACTGCACTCCAGCCTGGGCGAAAGAGCGAGACTTTGTCTCAAAAACAAAAACAAAACAAAAAGAA A

232	23.3	10.0	0.0	Hg19	54	113	(2077)	+	MLT1J	LTR/ERVL-MaLR	428	493	(19)	7
1926	24.9	4.5	4.1	Hg19	182	1065	(1125)	C	L1MCc	LINE/L1	(3023)	3918	3212	8
1923	18.6	1.8	4.6	Hg19	1066	1466	(724)	+	MLT1B	LTR/ERVL-MaLR	1	390	(0)	9
1926	24.5	5.2	4.7	Hg19	1467	1499	(691)	C	L1MCc	LINE/L1	(3730)	3211	3179	8
336	31.8	2.8	3.4	Hg19	1514	1689	(501)	C	L1MCc	LINE/L1	(5060)	1363	118 9	8
614	17.4	3.1	9.2	Hg19	1693	1783	(407)	+	AluJr	SINE/Alu	2	85	(227)	10
2504	8.1	0.0	0.0	Hg19	1784	2090	(100)	+	AluYk2	SINE/Alu	1	307	(4)	11
614	17.4	3.1	9.2	Hg19	2091	2159	(31)	+	AluJr	SINE/Alu	86	153	(159)	10

>KSI_AluYb8_24

>Scaffold7584-171256-171536
GACCAGCCTGGCCAACATGGTGAAACCCTGTCTCTACTAAAAATACAAAAATCAGCCAGGCATGGTGGCGTGCCCCTGTAGTCCCAGCTACTCAAGAGGCTGAGGTGGGAGAATCGCTTGAGCCCG GG
AGGCGGAGGTTGCAGTGAGCGGAGATCGCACTACCGTATTCCAGCCCGGGCGACAGAGTGAGACCCTGTCTCAAAACAAAACAAATGAACAAACAAAAAAACAGGAGCTGCCATCTGCAGATGTA TAG
TGTCAGAAAAAAAAAATAATTTTTCAAAAATGaaaaaaataatttttcaaaaattTTGTTAATTCAAAATTTTGCATTGCTGTTTTCTTCTGTGGAAAGTTAATTTTTTAAGGTAGAATTACAACACTA
TAAAAACCTGTCCATTGGCCGGGCGCGGTGGCTCACGCCTGTAATCCCAGCACTTTGGGAGGCCGAGGCGGGCGGATCATGAGGTCAGTAGATCGAGACCATCCTGGCTAACACGGTGAAACCCC GTC
TCTACTAAAAATAAGCCGGGCGTGGTGGGGGCTCCTGTAGTCCCAGCTACTCAGGAGACTGAGGCAGAATGGCGTGAACCCGGGAGGCGGAGTTTGCAGTGAGCCGAGATCATGCCACTGCACTC CA
GCCGGGGCGACAGAGCGAGACTCCGTCTCAAAACAAACAAACAAAAAAACAAACCTGTCCATTATCTTTTTTATAGAATTAGCATGAATTATATTCAGTTAATGTCTTGGACCTAAAATCTG aGACCA
GCAATTTAGTTATATATTATATGCTCTTAGATAGTTATATGACCTTAAGTTTCTCTTAATTAGGAAAAAAAAAAAATT CCCTCACAGGACACTTGGAGTTATCAAATATTTAGAAGTTACCTTCATCCC
TACTAAATATCATCAATTGTGTTTATTCTTCTTTACAGGGACAATCATATTTCCA GTTTTGGTTGTgagtttctttttttttttttttttttttttttttttaggctcgctctgtcgcccaggccg
gactgcggactgcagtggcgcaatctcggctcactgcaagctccgcttcccgggttcacgccattctcctgcctcagcctcccgagtagctgggactacaggcgcccgccaccgcgcctggctaat tt
tttgtatttttagtagagacggggtttcaccttgttagccaggatggtctcgatctcctgacctcatgatccacccgcctcggcctcccaaagtgctgggattacaggcgtgagccaccgcgccc ggc
cGAGTTTCTAATGTCATTTAAAAAGAAACCTAACTGGTGAAAAATGTACCTTAGG AAATCGGTGGGTCACTTTTTAAAATAAGTACTGTAATGATTAAGAGATGTAAAAACAATCTTTTCTTCCTCCT
TTTGTTTGGGGTAAAGTGATAGAGAAGTATAAGATTGTCCCTTCAGAGTTACTGAAACTTTCCTTTAAATATTATGT CCCTCTATCACATGCGAAATAACTTCTCCTTTAATTTTTAAGTAGTTATTG
TTTTGGGCTCTCAACTTTTATTTTTACCTAAGTTTTGTACATTAGACAGCCTAAGTTTATTCTGTGATAAGATATATAGATCTTAAGTTTTCTATCAGCACTTCTCCACACATTATTGGGATGAAC TG
AAGAGGTTTACTGACCACTAGTGTGGTCTTCCCGGTAATGATTATGTAGTGATTGGATGCCATCCAGATTCCTTGGTCTGTCTGCATCTTTTCTTGAGCTTTACCTCATTATTTCCCTGTCCTGT CCA
CCCCAAGTTATGTTCGCTACTTGGTGAAAAGCTCTTGGCATATGGGATAGTCTCCTTTTCAAGCGTGTCATCTTGTAGTAAGAAAGCCTTGCCTGCGGCCAGTATATA gGTGGGTTTCCATATACCTG
GACCAACTGAACAGCTGCCAGAATGGACTCAAGTGAGCAGAACATGA AatTCTAGATGATTACTATTTAATCACTGTAGCCAGTGATTGTGTATTACCAAGTAGTGAGACTGGGAGGGTGGGAATAGA
AGAAGCAGTGGGATGACTACTGACATAACTTAAAAGTTAATGGGAGTATATATCCACTACTTATGAGCCTCTCATACCAATTAAGCTGTGGTGATACAAAAGAATATACTAGGCATTCCCAATCAT GT
TGCATCATCTAGCATTGCTCACTTAGAGCTGGGAGGAAAAAAATCAGCCAGAATCAGCATTATCAACTCCGTTGCAAGAAGGAATTACAAGACACATGATTTGA A

1677	12.2	0.0	0.0	AluYb8-Scaffold7584-171256-171536	1	229	(2052)	+	AluSx	SINE/Alu	83	311	(1)	1
2378	4.8	4.1	0.0	AluYb8-Scaffold7584-171256-171536	401	693	(1588)	+	AluY	SINE/Alu	1	305	(6)	2
33	0.0	0.0	0.0	AluYb8-Scaffold7584-171256-171536	970	1000	(1281)	+	(T)n	Simple_repeat	1	31	(0)	3
2647	1.1	0.0	0.0	AluYb8-Scaffold7584-171256-171536	1001	1281	(1000)	C	AluYb8	SINE/Alu	(37)	281	1	4

>Hg19 chr14:65014094-65016033
GACCAGCCTGGCCAACATGGTGAAACCCTGTCTCTACTAAAAATACAAAAATCAGCCAGGCATGGTGGCGTGCCCCTGTAGTCCCAGCTACTCAAGAGGCTGAGGTGGGAGAATCGCTTGAGCCCG GG
AGGCGGAGGTTGCAGTGAGCGGAGATCGCACTACCGTATTCCAGCCCGGGCGACAGAGTGAGACCCTGTCTCAAAACAAAACAAATGAACAAACAAAAAAACAGGAGCTGCCATCTGCAGATGTA TAG
TGTCAGAAAAAAAAAATAATTTTTCAAAAATGgagTTGTTAATTCAAAATTTTGCATTGCTGTTTTCTTCTGTGGAAAGTTAATTTTTTAAGGTAGAATTACAACACTATAAAAACCTGTCCATTGGCC
GGGCGCGGTGGCTCACGCCTGTAATCCCAGCACTTTGGGAGGCCGAGGCGGGCGGATCATGAGGTCAGTAGATCGAGACCATCCTGGCTAACACGGTGAAACCCCGTCTCTACTAAAAATAAGC CGG
GCGTGGTGGGGGCTCCTGTAGTCCCAGCTACTCAGGAGACTGAGGCAGAATGGCGTGAACCCGGGAGGCGGAGTTTGCAGTGAGCCGAGATCATGCCACTGCACTCCAGCCGGGGCGACAGAGCGA GA
CTCCGTCTCAAAACAAACAAACAAAAAAACAAACCTGTCCATTATCTTTTTTATAGAATTAGCATGAATTATATTCAGTTAATGTCTTGGACCTAAAATCTG gGACCAGCAATTTAGTTATATATTAT
ATGCTCTTAGATAGTTATATGACCTTAAGTTTCTCTTAATTAGGAAAAAAAAAAAATT CCCTCACAGGACACTTGGAGTTATCAAATATTTAGAAGTTACCTTCATCCCTACTAAATATCATCAATTGT
GTTTATTCTTCTTTACAGGGACAATCATATTTCCAGTTTTGGTTG TGAGTTTCTAATGTCATTTAAAAAGAAACCTAACTGGTGAAAAATGTACCTTAGGAAATCGGTGGGTCACTTTTTAAAATAAG
TACTGTAATGATTAAGAGATGTAAAAACAATCTTTTCTTCCTCCTTTTGTTTGGGGTAAAGTGATAGAGAAGTATAAGATTGTCCCTTCAGAGTTACTGAAACTTTCCTTTAAATATTATGT CCCTCT
ATCACATGCGAAATAACTTCTCCTTTAATTTTTAAGTAGTTATTGTTTTGGGCT CTCAACTTTTATTTTTACCTAAGTTTTGTACATTAGACAGCCTAAGTTTATTCTGTGATAAGATATATAGATCT
TAAGTTTTCTATCAGCACTTCTCCACACATTATTGGGATGAACTGAAGAGGTTTACTGACCACTAGTGTGGTCTTCCCGGTAATGATTATGTAGTGATTGGATGCCATCCAGATTCCTTGGTCTG TCT
GCATCTTTTCTTGAGCTTTACCTCATTATTTCCCTGTCCTGTCCACCCCAAGTT ATGTTCGCTACTTGGTGAAAAGCTCTTGGCATATGGGATAGTCTCCTTTTCAAGCGTGTCATCTTGTAGTAAGA
AAGCCTTGCCTGCGGCCAGTATATAaGTGGGTTTCCATATACCTGGACCAACTGAACAGCTGCCAGAATGGACTCAAGTGAGCAGAACATGA ATCTAGATGATTACTATTTAATCACTGTAGCCAGTG
ATTGTGTATTACCAAGTAGTGAGACTGGGAGGGTGGGAATAGAAGAAGCAGTGG GATGACTACTGACATAACTTAAAAGTTAATGGGAGTATATATCCACTACTTATGAGCCTCTCATACCAATTAAG
CTGTGGTGATACAAAAGAATATACTAGGCATTCCCAATCATGTTGCATCATCTAGCATTGCTCACTTAGAGCTGGGAGGAAAAAAATCAGCCAGAATCAGCATTATCAACTCCGTTGCAAGAAGG AAT
TACAAGACACATGATTTGAA

2345	10.8	0.0	0.0	Hg19	24	329	(1811)	+	AluSz	SINE/Alu	6	311	(1)	5
2378	4.8	4.1	0.0	Hg19	481	773	(1367)	+	AluY	SINE/Alu	1	305	(6)	6

>KSI_AluYb8_25

>Scaffold7847-5239238-5239518
CGTTGTATGTTCTCACTCATAAGTGGGAGCTAAGTTATGAGGATGCAAAGGCATAAGAATGACACAATGGACTTTGGGGATTCAGGGGGAAAAGGTCAGAAGTGGGTGAGAGATAAAAGACACAAA TA
GCTGTGTTACTGCTCCAGTGATGGGTGTACCAAAATCTCACAAATCACTACTAAAGAACTTACTCATGCAACCAAACACCACCTGTTCCCCAGTAACCT ATAGAAATAAAAAATAAAATAAAATAAAT
TCCCGGTATCAATGAATGAGAGTTCCTGTGGCTCCACATCCTCACCAGCATTTGACATTGTCAGTGTTCTGAATTTGTGCCATTATAATGAGTGTGTAGTGGCTTCTCATA tTGTTTTCATTTGCATT
TCCCTGATGACAAATGTTATGGGTCATCTTTTCATATGCTTATTTGTCACCTGTATATCTTCTTTAGTGAGGTATTTGCTAAGGTCTTTGGCTCATTTT TAAATTGGGTTGTTTGTTTTCTTATTGTT
CAGTTTTAAGAGTTTTTTtTATATTTTGAATAACAGTCCTTTATCAGGTATGCCTTTTGCAAATATTTTCTCCCAGTCTATGGCTTATCTTTTTTTCTCTTGACAAAGTCTTTCACAAAGGAGAAACT
TTTAATTTCAATGATGTCCAGCTTATCAACTATTTCTTTCATGGATTGTGCCTTTAAGTTATACCTAAAAAGTCATTATCGAACTCAAAGTTATCTAGA TTTTCTCCTATGTTATCTTCTAGGAGTTT
CATAGTTTTGCATTTTACATTTAGGTCTGTGATCCACTGTGAGTTAATTTTTCTGAAAAGAATAAGGTGGTCTGTGTCTAGATTCCTTTTATTTTCTTTTTGCTTGTGGATGTTCAGTTTTTCCA GCA
CCATTCTTGAAAAAACTATCTTTTATCCATTGTATTGCCTCTACATTTGTTTTTttttttttttttttttttttttttttttttttttttagggggggt ctctcagtctcgctctgtcgcccaggccg
gactgcggactgcagtggcgcaatctcggctcactgcaagctccgcttcccgggttcacgccattctcctgcctcagcctcccgagtagctgggactacaggcgcccgccaccgcgcccggctaa ttt
tttgtatttttagtagagacggggtttcaccttgttagccaggatggtctcgatctcctgacctcatgatccacccgcctcggcctcccaaagtgctgg gattacaggcgtgagccaccgcgcccggc
ctacatttgtttttAAAAAGTTAGAATTTTTATCAATTTAGATTCACCTACCTTCAGA TACAGTAGGTGGTATAGACTGAAGGCTTGTGTCTCCCCAAAATTCATATGTTGAAATTCTATCCCCTAAT
ATGATGTTAGTAGAAGGTGGGGCCTTTGGTAAATGACTGGGTCATGAGGGCATTGTCCTCATAAATGGGATTAGTGCCCTTATGAAAGAGGCCCTGGAGAGCTCCTTCAACCCTTCTTCCAGAAT gGG
AGTTCTCACCAGACATTGAATCTACTGGCACCAGACCCTGAATCTACTGGCACCAAAATCATCTACTGATCTTGGACTACCCAGCCTCCAaaACTGTGAGAAATAAGTTTTTGTTGTCTATAACCCAC
TCAGTCTATGGTATTTTGTTATAGCAGGCCAAATGGACTAAGACAGAGGGAAATAATATGGACCAGACACCTTCTATGTATTAGGAACTGTATAGCCACTTTCACATATGCTAAGCATGTTTACA GTA
TAGATCTACTCTATTGAGAAAAAAAATGGATTTTGCACACTGCTAATTTTATGTTAAAA GAGGTTATAATTATTTTAAAGTCACTTAACTTAAAATATGTATCGAAGACCATGGTTATGATCACAGTGG
TCAACATGCCTCTCTTCAtTGAATATTGGGTACTGCATTGAGTGCATTGGCCCAGGCATCCTCTAGGGAAGATGCTATTACGATTCCCATTTTACAGATGCACAAAGTAAGCCTTGGAGAGGTCAGGC
AACTTGCCCAAGGTGACTCAGCCAGCATATGCAGGAGTCAGGACTTGAACTCGAGTCA TTCTAAATCTAAAGTTATGGGCAAACCATTTTAAATGTGGACTCAAAAAAGAAGTTGTGCACTTCCCACT
GCCCTCCaatcattAAAGAGGTCAGAGAAAAATGAATATATAGTAAACACCCTGTATCTAATTTCTCCTACCGAATTCATGAAGAATAAGTAAGTGATTCAGCC A

1628	10.4	2.5	0.4	AluYb8-Scaffold7847-5239238-5239518	1	241	(2040)	+	L1PB1	LINE/L1	5905	6150	(1)	1
3378	12.4	0.5	1.6	AluYb8-Scaffold7847-5239238-5239518	255	940	(1341)	C	L1MC	LINE/L1	(785)	5548	4870	2
45	0.0	0.0	0.0	AluYb8-Scaffold7847-5239238-5239518	946	986	(1295)	+	(T)n	Simple_repeat	1	41	(0)	3
2686	0.4	0.0	0.0	AluYb8-Scaffold7847-5239238-5239518	1001	1281	(1000)	C	AluYb8	SINE/Alu	(37)	281	1	4
1742	18.8	5.2	4.4	AluYb8-Scaffold7847-5239238-5239518	1348	1709	(572)	+	MLT1A0	LTR/ERVL-MaLR	1	365	(0)	5
508	27.5	0.0	2.3	AluYb8-Scaffold7847-5239238-5239518	1985	2118	(163)	C	MIRc	SINE/MIR	(102)	166	36	6

>Hg19 chr12:97494366-97496294
CGTTGTATGTTCTCACTCATAAGTGGGAGCTAAGTTATGAGGATGCAAAGGCATAAGAATGACACAATGGACTTTGGGGATTCAGGGGGAAAAGGTCAGAAGTGGGTGAGAGATAAAAGACACAAA TA
GCTGTGTTACTGCTCCAGTGATGGGTGTACCAAAATCTCACAAATCACTACTAAAGAACTTACTCATGCAACCAAACACCACCTGTTCCCCAGTAACCTATAGAAATAAAAAATAAAATAAAATA AAT
TCCCGGTATCAATGAATGAGAGTTCCTGTGGCTCCACATCCTCACCAGCATTTGACATTGTCAGTGTTCTGAATTTGTGCCATTATAATGAGTGTGTAGTGGCTTCTCATA cTGTTTTCATTTGCATT
TCCCTGATGACAAATGTTATGGGTCATCTTTTCATATGCTTATTTGTCACCTGTATATCTTCTTTAGTGAGGTATTTGCTAAGGTCTTTGGCTCATTTTTAAATTGGGTTGTTTGTTTTCTTATT GTT
CAGTTTTAAGAGTTTTTTgTATATTTTGAATAACAGTCCTTTATCAGGTATGCCTTTTGCAAATATTTTCTCCCAGTCTATGGCTTATCTTTTTTTCTCTTGACAAAGTCTTTCACAAAGGAGAAACT
TTTAATTTCAATGATGTCCAGCTTATCAACTATTTCTTTCATGGATTGTGCCTTTAAGTTATACCTAAAAAGTCATTATCGAACTCAAAGTTATCTAGATTTTCTCCTATGTTATCTTCTAGGAG TTT
CATAGTTTTGCATTTTACATTTAGGTCTGTGATCCACTGTGAGTTAATTTTTCTGAAAAGAATAAGGTGGTCTGTGTCTAGATTCCTTTTATTTTCTTTTTGCTTGTGGATGTTCAGTTTTTCCAG CA
CCATTCTTGAAAAAACTATCTTTTATCCATTGTATTGCCTCTACATTTGTTTTTAAAAAGTTAGAATTTTTATCAATTTAGATTCACCTACCTTCAGATACAGTAGGTGGTATAGACTGAAGGCTTGT
GTCTCCCCAAAATTCATATGTTGAAATTCTATCCCCTAATATGATGTTAGTAGAAGGTGGGGCCTTTGGTAAATGACTGGGTCATGAGGGCATTGTCCTCATAAATGGGATTAGTGCCCTTATGA AAG
AGGCCCTGGAGAGCTCCTTCAACCCTTCTTCCAGAATaGGAGTTCTCACCAGACATTGAATCTACTGGCACCAGACCCTGAATCTACTGGCACCAAAATCATCTACTGATCTTGGACTACCCAGCCTC
CAcACTGTGAGAAATAAGTTTTTGTTGTCTATAACCCACTCAGTCTATGGTATTTTGTTATAGCAGGCCAAATGGACTAAGACAGAGGGAAATAATATGGACCAGACACCTTCTATGTATTAGGAACT
GTATAGCCACTTTCACATATGCTAAGCATGTTTACAGTATAGATCTACTCTATTG AGAAAAAAAATGGATTTTGCACACTGCTAATTTTATGTTAAAAGAGGTTATAATTATTTTAAAGTCACTTAACT
TAAAATATGTATCGAAGACCATGGTTATGATCACAGTGGTCAACATGCCTCTCTTCA cTGAATATTGGGTACTGCATTGAGTGCATTGGCCCAGGCATCCTCTAGGGAAGATGCTATTACGATTCCCA
TTTTACAGATGCACAAAGTAAGCCTTGGAGAGGTCAGGCAACTTGCCCAAGGTGA CTCAGCCAGCATATGCAGGAGTCAGGACTTGAACTCGAGTCATTCTAAATCTAAAGTTATGGGCAAACCATTT

TAAATGTGGACTCAAAAAAGAAGTTGTGCACTTCCCACTGCCCTCCAAAGAGGTCAGAGAAAAATGAATATATAGTAAACACCCTGTATCTAATTTCTCCTACCGAATTCATGAAGAATAAGTAAGTG
ATTCAGCCA

2244	10.2	1.8	2.1	Hg19	1	341	(1788)	+	L1PB1	LINE/L1	5811	6150	(1)	7
3410	12.2	0.5	1.6	Hg19	355	1040	(1089)	C	L1MC	LINE/L1	(785)	5548	4870	8
1706	18.5	5.5	4.4	Hg19	1104	1464	(665)	+	MLT1A0	LTR/ERVL-MaLR	1	365	(0)	9
508	27.5	0.0	2.3	Hg19	1740	1873	(256)	C	MIRc	SINE/MIR	(102)	166	36	10

>KSI_AluYb8_26

>Scaffold8110-616558-616804
AGGAACTCAAACTAGAAACCAATGATAGATAAGATAcAGAAGAGAGAGCAGAAACTCGAAAATTTAGAAAGGATCCTCTATCATAtaCATGGGTTGTTAACTCAAGGTCAGCCATGAGTCTTTGAAG
TGCTTAAGAAAATGAATTTAGCTTCTTAATAAAATATCTTAAACTATTAGGGAATTCTATTGCAGAGCTTTTTAAAAACAGCCATTTAAGAAAATTACATA AAAAGAAAGAAATAGAATAGAGAAGAC
ATGCTGTGATGAACATTAAAAATTTGTTTCCAAATAATTAACATATTAAATGTATCTGTCATGATTTATTTGTCCCAACAAACTGATATCCTATACTTTCTACTTTGGAAAATTATTTCAAATTT GCA
TTTTGTACAAGTTGAGTGATATAAATTCTTTAGCTCTGCAGAAGAATAGAGGTATTTTATAGGTTAATAGGTGTCGAAAGGATTGATTTGTAGGCCTGGCA GTGAAGTACCAAAAAAAAAAAAaGAA
AAAAGAAAAATTATATGCTAGAGCAACCCATGAACTTGCTAAATGTTGATAACTAAAGGCCTTCTTCTGGGACTTAAATATCCTCTAGGTTTCTCCAGTGACTTAAAGTGATGCCACAGCAGGTA GCA
GAACTATAAGACATCATTTCTACCATTTGATATAAGATGTTTATTGTTTCCATCAAACACAACACATAAAATACCTTGTAAATGGCTGCAAAGCACATTAT GAAGTGCCTCCTGGTGTGTTTATGCTA
GTCCATTTTGAACATTTCCCTTTGCTACAGGCATCAAGAAATGACCTTTTTGGCAGTGTCTACTCCCTT TTGGATTTTCACTCCCTTCCAGAGTCCCATTATCTCATTAAATCATTTATGTGCAGGAG
GAGAGATGCTGTAGTGAATAGCAAAAtcccatgatttgtttttgatatgctctacgttttcaacaaatatatttggagtaaagtctatataaat aacttaattgggccgggcgcggtggctcacgcct
gtaatcccagcactttggaaggccgaggcgggtggatcatgaggtcaggagatcgagaccatcctggctaacaaggtgaaaccccgtctctactaaaaatacaaaaaattagccgggcgcggtgg cgg
gcgcctgtagtcccagctactcgggaggctgaggcaggagaatggcgtgaacccgggaagcggagcttgcagtgagccgagattgcgccactgcagt TCCCATGATTTGTTTTTGATATGCTCTACCT
TTTCAACAAATATATTTGGAGTAAAGTCTATATAAATAACTTAATT AAAGCCAGACATTGAATGTCAGGAATGAGCGTTTTTGACAGTATGGTGCGGACTGAGTTTTAAAACTCAGGATAATTTTAGA
AACCCTGAATTATACAGTAGCTACCTTATGCTACATGGTACTTTATGTGAAGTTTTTGCCTTAACACAATGTAGGACAGTTCTTTCCTGAGTGACATTGC AATGATCACACTGTAAGGAATCACATGG
TATAACTTTGCTGCTTGCAACCTTAATTTTCTCATTTGAAAAATGGAAGGATGTATTCAGTTTTTATTTTATTTATTTATTATTTATTTATTTTAATATTTTGAGATGGAATCTCACTCTGTCAC CCA
GGCTGGAGTGAAGTGGtGTGATCTCAGCTTTCTGCAACCTCCACTTTCAGGGTTCAAGCCATTCTCCCTCCTCAACCTCCGGAGAAGCTGGGACTACAGG CATTCACCATTTTTAAAGTTTCTTTTAA
TCCGTGAAGTAAAGAAATTAATGATGTGTCAGAACAGAATCTAGTTAGAAGCCCCTGAATTAACTTTTTCCATGCCTGATTAGCTATCTTCATGACAGAACTTCTAAATAGAGATAGTTCCTTAT TTG
ATCATAAATCTGACACATTTCTTATTTTATCCATTATGTTtAAGAGCAAACATTTTAGTTACCACTTCTTGGTTTAATCCAGGATCCTGTACTTGGTACT TTAATGAGTCTCGGGCAATTGACAATCA
TTTTGTACCTCAGTTTGCTTTTCTACAAAATGGTTTATTGGAAGGTTTAAATAAGCTAATCAATGTTAAATAGTCAAATGCTTACTGACAGTGAGTATTCAGAGTTAGGTCTTATTGTTTGTTCT TTA
TGTTGATGTTTTCAGCTGTAAAAATGTTCTTTGAATTGGGGGGGGCAATTTAGATTAAAAGTCACTTGAG

12	24.6	0.0	2.6	AluYb8-Scaffold8110-616558-616804	216	255	(1992)	+	GA-rich	Low_complexity	1	39	(0)	1
20	8.0	0.0	0.0	AluYb8-Scaffold8110-616558-616804	496	522	(1725)	+	(A)n	Simple_repeat	1	27	(0)	2
2341	0.8	0.0	0.0	AluYb8-Scaffold8110-616558-616804	1001	1247	(1000)	+	AluYb8	SINE/Alu	1	247	(71)	3
916	18.1	0.0	1.2	AluYb8-Scaffold8110-616558-616804	1608	1773	(474)	C	AluSx3	SINE/Alu	(1)	310	147	4
230	32.0	4.6	1.5	AluYb8-Scaffold8110-616558-616804	1952	2081	(166)	+	MIR	SINE/MIR	10	143	(119)	5

>Hg19 chr3:7132406-7134322
AGGAACTCAAACTAGAAACCAATGATAGATAAGATAgAGAAGAGAGAGCAGAAACTCGAAAATTTAGAAAGGATCCTCTATCATACATGGGTTGTTAACTCAAGGTCAGCCATGAGTCTTTGAAGTG
CTTAAGAAAATGAATTTAGCTTCTTAATAAAATATCTTAAACTATTAGGGAATTCTATTGCAGAGCTTTTTAAAAACAGCCATTTAAGAAAATTACATAAAAAGAAAGAAATAGAATAGAGAAGA CAT
GCTGTGATGAACATTAAAAATTTGTTTCCAAATAATTAACATATTAAATGTATCTGTCATGATTTATTTGTCCCAACAAACTGATAT CCTATACTTTCTACTTTGGAAAATTATTTCAAATTTGCATT
TTGTACAAGTTGAGTGATATAAATTCTTTAGCTCTGCAGAAGAATAGAGGTATTTTATAGGTTAATAGGTGTCGAAAGGATTGATTTGTAGGCCTGGCAGTGAAGTACCAAAAAAAAAAAA AGAAAAA
AGAAAAATTATATGCTAGAGCAACCCATGAACTTGCTAAATGTTGATAACTAAAGGCCTTCTTCTGGGACTTAAATATCCTCTAGGT TTCTCCAGTGACTTAAAGTGATGCCACAGCAGGTAGCAGAA
CTATAAGACATCATTTCTACCATTTGATATAAGATGTTTATTGTTTCCATCAAACACAACACATAAAATACCTTGTAAATGGCTGCAAAGCACATTATGAAGTGCCTCCTGGTGTGTTTATGCTA GTC
CATTTTGAACATTTCCCTTTGCTACAGGCATCAAGAAATGACCTTTTTGGCAGTGTCTACTCCCTT TTGGATTTTCACTCCCTTCCAGAGTCCCATTATCTCATTAAATCATTTATGTGCAGGAGGAG
AGATGCTGTAGTGAATAGCAAAATCCCATGATTTGTTTTTGATATGCTCTACCTTTTCAACAAATATATTTGGAGTAAAGTCTATATAAATAACTTAATT AAAGCCAGACATTGAATGTCAGGAATGA
GCGTTTTTGACAGTATGGTGCGGACTGAGTTTTAAAACTCAGGATAATTTTAGAAACCCTGAATTATACAGTAGCTACCTTATGCTACATGGTACTTTATGTGAAGTTTTTGCCTTAACACAATGTAG
GACAGTTCTTTCCTGAGTGACATTGCAATGATCACACTGTAAGGAATCACATGGTATAACTTTGCTGCTTGCAACCTTAATTTTCTCATTTGAAAAATGGAAGGATGTATTCAGTTTTTATTTTA TTT
ATTTATTATTTATTTATTTTAATATTTTGAGATGGAATCTCACTCTGTCACCCAGGCTGGAGTGAAGTGG cGTGATCTCAGCTTTCTGCAACCTCCACTTTCAGGGTTCAAGCCATTCTCCCTCCTCA
ACCTCCGGAGAAGCTGGGACTACAGGCATTCACCATTTTTAAAGTTTCTTTTAATCCGTGAAGTAAAGAAATTAATGATGTGTCAGAACAGAATCTAGTTAGAAGCCCCTGAATTAACTTTTTCC ATG
CCTGATTAGCTATCTTCATGACAGAACTTCTAAATAGAGATAGTTCCTTATTTGATCATAAATCTGACACATTTCTTATTTTATCCATTATGT TAAGAGCAAACATTTTAGTTACCACTTCTTGGTTT
AATCCAGGATCCTGTACTTGGTACTTTAATGAGTCTCGGGCAATTGACAATCATTTTGTACCTCAGTTTGCTTTTCTACAAAATGGTTTATTGGAAGGTTTAAATAAGCTAATCAATGTTAAATA GTC
AAATGCTTACTGACAGTGAGTATTCAGAGTTAGGTCTTATTGTTTGTTCTTTATGTTGATGTTTTCAGCTGTAAAAATGTTCTTTGAATTGGGGGGGG GgCAATTTAGATTAAAAGTCACTTGAG

12	24.6	0.0	2.6	Hg19	314	353	(1764)	+	GA-rich	Low_complexity	1	39	(0)	6
19	8.3	0.0	0.0	Hg19	594	619	(1498)	+	(A)n	Simple_repeat	1	26	(0)	7
931	17.5	0.0	1.2	Hg19	1378	1543	(574)	C	AluSx3	SINE/Alu	(1)	310	147	8
233	31.4	4.9	1.6	Hg19	1728	1850	(267)	+	MIR	SINE/MIR	17	143	(119)	9

>KSI_AluYb8_27

>Scaffold8133-1077171-1077499
AGCAATTACAGTTGATACTGTGAGAATTAGAAATACCTTTTTAAAAGAACACTTTACTTCTCTAATCAAGACAGATCAAGAAGACAGAAATATATATATAT AtatAATATTTAAAtTACATGTTATAA
TCAACCAGATGATTTTTATGTAGCAAACTTTATGCTTTCAAGTTATAGAAAACACTTCTTTTCAAGTGTACATACAAAAGAAGAAAATTGACAATATACTAAGCCAGAAAGGAAAAGGAAACCCAC AAA
CAAACAAAAACTAACAAATATATATATATTTTAAAAAGCAAACCTTTTAAAAAGTCTCAAGTCAGGAGAAAATACCAACTGAAGTTACAGAAACAACTGCAATAAAAGCAATATATATCAGAACTTAC GA
AATATAACTAAAGCAAGTCCTACAGGAAACATTTTTAAAGAAGAAATGAAAATAAATGCAGTAAGCATCTGGCTCAGAGTCAGAAAATAGAAACATAATACAAACACACCAAAAAACAAAAGGCA AAT
GAAGAAATTAGGGTACATTCATAAATTGATGACATAGCATAAATGAATGAATGAATGAATGGTGTTTTTGAAAATGATCCATAGATTTGTCACTACCTAATTTATTCAAGAGAAAAAATAAAAGAG CA
AGAGAAAGTAATCATAAGAACAGAAATGTTTAAAATTATAAGACTACTTCAGATGACTCTAGGCAACTTAATGTAAAGGCCTATATAAATTGAATAGTTTTCTAGGAGAATA cGATTCAGTGAAACTT
AGTGTGTTATACTTTCTTTAGCACAGAAGAAATTAAGAGCTAACACTAACAAAGCACCAAGCTAGGAGATCTCACTGGGTAGTTTCAATAGAAATTAGACAGTAAATAATTCCAGTGCTCCATAACTG
GGTTCAGGACTCAGAAAGAAAGAAAACATCCCAATTGTTTTTTAAAATGAATATAATATTGATAATAAAAGCCAACAAATATTTCATT Taaaaagacaactataggccgggcgcggtggctcacgcct
gtaatcccagcactttgggaggccgaggcgggtggatcatgaggtcaggagatcgagaccatcctggctaacaaggtgaaaccccgtctctactaaaaatacaaaaaagtagccgggcgcggtggc gg
gcgcctgtagtcccagctactcgggaggctgaggcaggagaatggcgggcacccgggaagcggagcttgcagtgagccgagattgcgccactgcagtccgcagtccggcctgggcgacagagcga gac
tccgtctcaaaaaaacacacacacacaaaaaaaaaaaaaaaaaaaaAAAAAGACAACTATAGATTAACCTCACTTACGAATATTAACATTAAAAAAATCCTGAGTATATCAAGCCAAAGTCAACATCA
ACTTAACAGGTAACAACTCACTGGAGTTTATTCTAAAAATATGTGAATAAACTTGGTATCAGGCAGTTATAGTCAATTATATTAATAGATCAGAGAAACT TTCCTGAGGGGGAAATTCTCATGATCAT
CTCCCTCTAGATACAAAAAAAAAAGACATTTGACAAAATTTGGCAGATTATTTATTAACAGGATAAATACACATATCACTTTTTCATCACAACTATTTAACATTCTACTGGAGATATTTACCAAT GCT
TTTAACAAGAAAATTAATTTTACTCAAGAATTTTTCTTATTCCCCAACCCTTCCAATACACAATCAATCCTATCATCTTTGTAGAAAGATGAAATCCCACACAGTGAGTAAAAGTAGTAA GATAGATA
ATAAAGTACAAAATTAATTTAAAGAACAAAATAAGATATAATTAAAAAGGAAGATAAAATAGAAGATCTCATTACAATAAAAATTTTAACTCCAGTAAATATACTTAACAAGGAAGATTCAAGAC AAT
ATTTTTGAAAATAGCTCAAATTCTCATGATGGATACGGAAGTGAAATTAAACAACTAGAAGTAAAATTACTATTTATTTAAGACTCAATATCTTACATAAGTCAATTTAAGTTCAGTGTG AACCCAGT
GAAAATACCAAAAGGTTTTTGTCCATCTGCAACTACATAATTCAATTCAAAGTTTACATGCACCACCTAAAAAGCAAGAATAGCCAGAAAATCTTTAAAAAAATAAGAGATATAAGAGTCACTGG CAT
TGCTAGATATTAAAATACAAATTTTGATAGTAAAAACTATGTTACTGTTACATGAATAGACTGACCAATGGAAAAGAATGAAAAGTTGAGAAATAAACCCAAATATATTTATATAACAAT TATCAAAT
TAGTACATAAGGATATATATATTTA

18	11.4	7.9	0.0	AluYb8-Scaffold8133-1077171-1077499	90	127	(2202)	+	(AT)n	Simple_repeat	1	41	(0)	1
17	14.9	0.0	5.1	AluYb8-Scaffold8133-1077171-1077499	533	573	(1756)	+	(ATGA)n	Simple_repeat	1	39	(0)	2
2862	1.2	0.0	3.5	AluYb8-Scaffold8133-1077171-1077499	1001	1329	(1000)	+	AluYb8	SINE/Alu	1	318	(0)	3
371	27.8	12.3	2.9	AluYb8-Scaffold8133-1077171-1077499	2000	2284	(45)	+	L1M5	LINE/L1	4533	4843	(1303)	4

>HG19 chr18:58773998-58775983
AGCAATTACAGTTGATACTGTGAGAATTAGAAATACCTTTTTAAAAGAACACTTTACTTCTCTAATCAAGACAGATCAAGAAGACAGAAATATATATATAT AaAATATTTAAAcTACATGTTATAATC
AACCAGATGATTTTTATGTAGCAAACTTTATGCTTTCAAGTTATAGAAAACACTTCTTTTCAAGTGTACATACAAAAGAAGAAAATTGACAATATACTAAGCCAGAAAGGAAAAGGAAACCCACAA ACA
AACAAAAACTAACAAATATATATATATTTTAAAAAGCAAACCTTTTAAAAAGTCTCAAGTCAGGAGAAAATACCAACTGAAGTTACAGAAACAACTGCAATAAAAGCAATATATAT CAGAACTTACGAAA
TATAACTAAAGCAAGTCCTACAGGAAACATTTTTAAAGAAGAAATGAAAATAAATGCAGTAAGCATCTGGCTCAGAGTCAGAAAATAGAAACATAATACAAACACACCAAAAAACAAAAGGCAAA TGA
AGAAATTAGGGTACATTCATAAATTGATGACATAGCATAAATGAATGAATGAATGAATGGTGTTTTTGAAAATGATCCATAGATTTGTCACTACCTAATTTATTCAAGAGAAAA AATAAAAGAGCAAG
AGAAAGTAATCATAAGAACAGAAATGTTTAAAATTATAAGACTACTTCAGATGACTCTAGGCAACTTAATGTAAAGGCCTATATAAATTGAATAGTTTTCTAGGAGAATA tGATTCAGTGAAACTTAG
TGTGTTATACTTTCTTTAGCACAGAAGAAATTAAGAGCTAACACTAACAAAGCACCAAGCTAGGAGATCTCACTGGGTAGTTTCAATAGAAATTAGACAGTAAATAATTCCAGT GCTCCATAACTGGG
TTCAGGACTCAGAAAGAAAGAAAACATCCCAATTGTTTTTTAAAATGAATATAATATTGATAATAAAAGCCAACAAATATTTCATT TAAAAAGACAACTATAGATTAACCTCACTTACGAATATTAAC
ATTAAAAAAATCCTGAGTATATCAAGCCAAAGTCAACATCAACTTAACAGGTAACAACTCACTGGAGTTTATTCTAAAAATATGTGAATAAACTTGGTATCAGGCAGTTATAGT CAATTATATTAATA
GATCAGAGAAACTTTCCTGAGGGGGAAATTCTCATGATCATCTCCCTCTAGATACAAAAAAAAAAGACATTTGACAAAATTTGGCAGATTATTTATTAACAGGATAAATACACATATCACTTTTTCAT
CACAACTATTTAACATTCTACTGGAGATATTTACCAATGCTTTTAACAAGAAAATTAATTTTACTCAAGAATTTTTCTTATTCCCCAACCCTTCCAATACACAATCAATCCTAT CATCTTTGTAGAAA
GATGAAATCCCACACAGTGAGTAAAAGTAGTAAGATAGATAATAAAGTACAAAATTAATTTAAAGAACAAAATAAGATATAATTAAAAAGGAAGATAAAATAGAAGATCTCATTACAATAAAAAT TTT
AACTCCAGTAAATATACTTAACAAGGAAGATTCAAGACAATATTTTTGAAAATAGCTCAAATTCTCATGATGGATACGGAAGTGAAATTAAACAACTAGAAGTAAAATTACTAT TTATTTAAGACTCA
ATATCTTACATAAGTCAATTTAAGTTCAGTGTGAACCCAGTGAAAATACCAAAAGGTTTTTGTCCATCTGCAACTACATAATTCAATTCAAAGTTTACATGCACCACCTAAAAAGCAAGAATAGC CAG
AAAATCTTTAAAAAAATAAGAGATATAAGAGTCACTGGCATTGCTAGATATTAAAATACAAATTTTGATAGTAAAAACTATGTTACTGTTACATGAATAGACTGACCAATGGAA AAGAATGAAAAGTT

GAGAAATAAACCCAAATATATTTATATAACAATTATCAAATTAGTACATAAGGATATATATATTTA

```
17   14.9  0.0  5.1  HG19                              631   671 (1515) + (ATGA)n  Simple_repeat     1     39    (0)    5
  371   29.0 11.4  3.2  HG19                             1757  2179    (7) + L1M5    LINE/L1        4533   4994 (1152)   6
```

>KSI_AluYb8_28

```
>Scaffold8587-799131-799434
CTGTCTCTTTTCCCCATTCCAAAACTCTGTGTCCCAAGCTCCAGCAGCTTCTAGTCAGGGAAATGAGTGGTAAGCCATGGATTTATTAGTGGCTGTGTCATTCTTCTCATATCTTACCACTTCCAC AG
TCGGGTTAAAAACTGAATCCAGGCTTAAAATCCATTTATTGAACTGAATATCCCTGACTACAGCATTTTAATAGCCACATTACAGCTGTGGAATTGGTCTGCAGCCCAGTGATTGAACCTTACCC ATC
TAGCAATTAGCCCCAaTGATTAATTTCAGAGGATTCTGGTTCCACTGCCTGGGCACGGTGATGGTGCAGAGATTCTTCAAGCCATTTGTAAGCAGTCCATCTTAGAATGATTTGGGCCCCACATTTGG
GATTGATTTTGTTTCCTTCTCTTCAAGGTTTGTAgCTTTCTTTCATATGAGTACATGGATAGCACGGGAAAAGGACCAGCAATGAGAAGGGTGTGGAGTTCAGATGGCTGCTCGAGTCTCCATGCACA
TTCCAGATTGTGTTCTGATGTGCAGACATCAAAAGAGCGAGGCCAACGCTTCAGATCCCAGCATGGTTCAGATGTGTGGCCCGTGGAGCAGTGTGGATTCCTGTCAGATGCTGAGTCTCAGCAAAA TC
CACATTTGAAAAGCAAAAGCAGGAGCCGGAGAATATTCCAAATATTACTTAGATTTCAGAGTGACAGATTGCAGTGTGTGATCTTCCCCTAATTTTTCTTTTTATCTCCATGTCTCCCCTGTACC AGG
CCTTAAGAGCTCAGAAACCCTGAAATGCTGTCAATCGGCTGCGGGGAAGAGACTGTGCAGCCAGTAGTGCTTGACTGTGCACTCTTTGGTGTCAA ATCAAATGGGGTTGGTCGCTAACAAAAATTCCT
TATCAGGGTGACATGTCTTTATATTCAAGCAGTAGAAGTCACACATGTGACTATGTTGAATTTTTTTT TttATGGAGACACTGAATaataaatagcatgaggttggccgggcgcggtggctcacgcct
gtaatcccagcactttgggaggccgaggcgggtggatcatgaggtcaggagatcgagaccatcctggctaacaaggtgaaaccctgtctctactaaaaatacaaaaaattagccgggcgcggtgg cgg
gcgcctgtagtcccagctactcgggaggctgaggcaggagaatggcgtgaacccgggaagcggagcttgcagtgagccgagattgcgccactgcagtccgcagt ccggcctgggcgacagagcgagac
tccgtctcaaaaaaaaaaaaaaaaaaaataaataaataaataaataaataAATAAATAGCATGAGGTTGAATTTAACTTACCCACAGCCAATCCTTTGAAATGACAGTGCATGGTAGGACTATTTAGAA
ATGGACATTTACATAACTATTTGATCAATTGCTTAGTTCTTCCTCTTACTTATGCAAATGGGTGGGTTTTCCTATGGTGTGTGGTCATGTACAGAGCCTTACAG TTGAGAAATGCATCTTGCATGATA
GAATTGAAGGAACCACACACACCTGCTTATCATACTGCTCATGAATTCCTTCTATAGCTTACTGA GGACTCATCTTTCAGCCTCTGCTTGAATATTTCTTGGACAAAATGTTGTTCCTTCATAAGGCA
GCCCTTTACTTTTTTGCTGTTGTTGACGGCTTCAGCAGTTAATGGAGTTCTTTATTCTTACATTTCCAATCACCGGATATAATTCAGCCT cCTGAAGCCCCAGTTTTCATTCTTTTTCATGTTGTAAC
ATATCCCTTAAGGTTTCTTTATTTCCAAGTAAAAATCCTGGATTTTTTCCATGGTCCTTTTGTAACACTTTGATATCCTCCAAGCTCAGTCATATTTGGTCACAATGCGATGCTCTCCCAATCAT GAC
TAGTGGCTTGAGGTAGAGCCCTCCTTGGCAAGGTCCAGATCGCTACAGGTAGACACT gAAGTTTTAAGACCTCTCCATATCTGTGAGCTGAAAGCTCCCTGCTG TTTCCACTGGAGCTCGATTGTGTT
TTTATTGTGTTCAGGCTGTGAGTTAGTGGGTATATCCTACATTCAGGTCATTGTCAGAGGAGGCATAATTGGCAGTTTAGAACGTGATCACCTAAGCATGTCAGGTGGGTTACTGTTTTGTGTTC CTA
CATTGCCCTTGGGATCTGATGTAGAGCCCTGGAGGAAGGCAAATATTTCCCAGCACGACCCACAAGAGGGAAACAGGACACTCATATTTACTGAGCCCTCATTT TGTGCTATGTGCCATATCATCTGA
```

2883	0.7	0.0	0.0	AluYb8-Scaffold8587-799131-799434	1001	1304	(1000)	+	AluYb8	SINE/Alu	1	304	(14)	1
35	0.0	0.0	0.0	AluYb8-Scaffold8587-799131-799434	1305	1337	(967)	+	(AAAT)n	Simple_repeat	1	33	(0)	2
256	32.4	2.9	0.0	AluYb8-Scaffold8587-799131-799434	1576	1680	(624)	C	L3	LINE/CR1	(69)	4030	3923	3

```
>Hg19 chr11:19962014-19963968
CTGTCTCTTTTCCCCATTCCAAAACTCTGTGTCCCAAGCTCCAGCAGCTTCTAGTCAGGGAAATGAGTGGTAAGCCATGGATTTATTAGTGGCTGTGTCATTCTTCTCATATCTTACCACTTCCAC AG
TCGGGTTAAAAACTGAATCCAGGCTTAAAATCCATTTATTGAACTGAATATCCCTGACTACAGCATTTTAATAGCCACATTACAGCTGTGGAATTGGTCTGCAGCCCAGTGATTGAACCTTACCC ATC
TAGCAATTAGCCCCAgTGATTAATTTCAGAGGATTCTGGTTCCACTGCCTGGGCACGGTGATGGTGCAGAGATTCTTCAAGCCATTTGTAAGCAGTCCATCTTAGAATGATTTGGGCCCCACATTTGG
GATTGATTTTGTTTCCTTCTCTTCAAGGTTTGTAtCTTTCTTTCATATGAGTACATGGATAGCACGGGAAAAGGACCAGCAATGAGAAGGGTGTGGAGTTCAGATGGCTGCTCGAGTCTCCATGCACA
TTCCAGATTGTGTTCTGATGTGCAGACATCAAAAGAGCGAGGCCAACGCTTCAGATCCCAGCATGGTTCAGATGTGTGGCCCGTGGAGCAGTGTGGATTCCTGTCAGATGCTGAGTCTCAGCAAAA TC
CACATTTGAAAAGCAAAAGCAGGAGCCGGAGAATATTCCAAATATTACTTAGATTTCAGAGTGACAGATTGCAGTGTGTGATCTTCCCCTAATTTTTCTTTTTATCTCCATGTCTCCCCTGTACC AGG
CCTTAAGAGCTCAGAAACCCTGAAATGCTGTCAATCGGCTGCGGGGAAGAGACTGTGCAGCCAGTAGTGCTTGACTGTGCACTCTTTGGTGTCAA ATCAAATGGGGTTGGTCGCTAACAAAAATTCCT
TATCAGGGTGACATGTCTTTATATTCAAGCAGTAGAAGTCACACATGTGACTATGTTGAATTTTTTTT TATGGAGACACTGAATAATAAATAGCATGAGGTTGAATTTAACTTACCCACAGCCAATCC
TTTGAAATGACAGTGCATGGTAGGACTATTTAGAAATGGACATTTACATAACTATTTGATCAATTGCTTAGTTCTTCCTCTTACTTATGCAAATGGGTGGGTTTTCCTATGGTGTGTGGTCATGTA CA
GAGCCTTACAGTTGAGAAATGCATCTTGCATGATAGAATTGAAGGAACCACACACACCTGCTTATCATACTGCTCATGAATTCCTTCTATAGCTTACTGA GGACTCATCTTTCAGCCTCTGCTTGAAT
ATTTCTTGGACAAAATGTTGTTCCTTCATAAGGCAGCCCTTTACTTTTTTGCTGTTGTTGACGGCTTCAGCAGTTAATGGAGTTCTTTATTCTTACATTTCCAATCACCGGATATAATTCAGCCT tCT
GAAGCCCCAGTTTTCATTCTTTTTCATGTTGTAACATATCCCTTAAGGTTTCTTTATTTCCAAGTAAAAATCCTGGATTTTTTCCATGGTCCTTTTGTAACACTTTGATATCCTCCAAGCTCAGT CAT
ATTTGGTCACAATGCGATGCTCTCCCAATCATGACTAGTGGCTTGAGGTAGAGCCCTCCTTGGCAAGGTCCAGATCGCTACAGGTAGACACT aAAGTTTTAAGACCTCTCCATATCTGTGAGCTGAAA
GCTCCCTGCTGTTTCCACTGGAGCTCGATTGTGTTTTTATTGTGTTCAGGCTGTGAGTTAGTGGGTATATCCTACATTCAGGTCATTGTCAGAGGAGGCATAATTGGCAGTTTAGAACGTGATCA CCT
AAGCATGTCAGGTGGGTTACTGTTTTGTGTTCCTACATTGCCCTTGGGATCTGATGTAGAGCCCTGGAGGAAGGCAAATATTTCCCAGCACGA CCCACAAGAGGGAAACAGGACACTCATATTTACTG
AGCCCTCATTTTGTGCTATGTGCCATATCATCTGA
```

259	34.0	8.9	0.0	Hg19	1327	1517	(638)	C	L3	LINE/CR1	(69)	4030	3823	4

368	29.9	7.7	0.0	Hg19	2038	2154	(1)	C	MIRb	SINE/MIR	(63)	205	80	5

>KSI_AluYb8_29

>Scaffold8739-1323469-1323719
TAAAATTCATTTGTGACTAAGTCATTATAGTGAAGTACTTGGAAACCTTTCTATTCTAGAGAGTTCTGGGCACAAACACATTTACAGTGTGCACTAATGCCAGGCTCATGTCCTCACGTCCTCCAA GG
GACAGGGATGAGCTGGGTCATAAACCAAACAGTGGGACTCTGGCAATCCCTTTTAGCTCAGGCAGATGCAAAATTGAAGACCCAAAAGAAGCCTTCCAAAGGTCACAGCAGGTTGCTCTCAGGAAAAG
AGAATCAGTAATTTATTTTTAAGCAATCTCTTAGCCTCTGTGACAGTTATGTTTCCTTGTAAGCTGCTTTGGAGCCACTCACCACTCTGAAATTGCAGAGTAGGCTACGAACAATCCGGCTTCGT AAG
AACATGCAAGTGAGGACCCACGAGGAGATATTCTTGTGCTTTTTAAAGTGCTTCTCTCACTTTGCTTCCTAAACTGCCTTAATTGAGGCAAGGGAGCCAACATGAGTGAGTTCCTTCCCGCGGCTTGC
TCTGCCGTGGAGTTCAAGGATGCCTGAGCCCTCAAAACACGGCGCGGTGCAGTTGGGCTGATGGGAAGTCCTGAACAGCTTGCTGAATTATTACTTCATTCTCGGCTGAAGCAGCAGGAGGCGAG ATG
ATGAGTGAAGGAAAAAACCCATGGACTCCAGCGTGGGCTGAAGGAACCAAAACAAACACTGAGAAGACAGGCCAATACTGAAATCTGTTTCAAGGATGGAGCGCGCAGGAATGTTTCACCATATTTTG
TCTAAGGAACAGATTTCATTTTTTAATGGCTGTATTAATCAGTGGAAGCAGAACGGCAGGAAGGAATCCGTTTTCCCCTCTCCCCAACGCCTCCCGTGGCTC GCCCGGACATTCCCCCCATTCTTTGT
ACTGAAGGTGGCCTGGAAGTTTTAGAGGCGTGAGACAGGTTCTGAACT tGCATGCCCTTTCTTTCTCCTTCCCGAATAAATTTTGCGTAAGAATATTTGGGACA ggccgggcgcggtggctcacgcct
gtaatcccagcactttgggaggccgaggcgggtggatcatgaggtcaggagatcgagaccatcctggctaacaaggtgaaaccccgtctctactaaaaatac aaaaaattagccgggcgcggtggcgg
gcgcctgtagtcccagctactcgggaggctgaggcaggagaatggcgtgaacccgggaagcggagcttgcagtgagccgagattgcgccactgcagtccgcagc gcatgccctttctttctccttccc
gaataaattttgcgtaagaatatttgggacaAGGAATCCACCATGGCGGGGTGCAATAAAAGCGTTTCACAAATTATGATTTCCCATTCTCTAGGCAGTTCA GCTCTGCCTAACAGCTCTAATTTCCT
AGACAAATAGTCCTCCCTGTCCTCTCCAGCTGCTCACAGGCGCCTCTCTCTGGGTATTCTTTTTAAAATCCCTCTCCTGGGGAATTTTTAAAAAAGCTTCTCGCACTTTTTCATGCGATTTTCAT GGT
TCCATCCATCTCCCCACTTTTTTGGTCAAAGCCTTCCTCCCAAATATCAATTGCTTTTTCAACCCGTCCATACTACTGCTCAGCTGTTGGTTGAGCTTCTCC TAGCCTCCTGCTGCTGTCACTGGGCC
AATGGAAGCCAGTCCCTTGCCTCTTTGTTCTATTATTTTATGGCAAGATAATAAACTTCTACATTTTTGAAGTACTTTTCACCGACTTGATATCAAGTATTTTGATAATCACAAAGTGATCAAAC ACA
TGTTCTCTTTTTCCAGTTACATAATGAGATCAAATATTGATTAGCAGTGAGTAGATAGATGTATATTGTACACACACACATACACCCGACCATCCTCCTTTT GGCCTATGGAGACTGTTACTCCAAAG
AGCCGACACATTTGTGTTGACATTTCAGTCATCCAAGAAAGCTGCCAATGTCACTTCCTTTGAATATTTTCCTTCAGTTTTCAGGAAAGAGTTGGTGGGACTAGCTTATCATGGATGGCATGAGC TGA
GGGGATGGTAATTTTTCACAAAAGTAGACATTGTTCGAGGCTAATAGTAAGCTGTAATCTAGACTCATAACTGTAGAGGGCGCTCTTGACTTAATAATGACA GCATTGCCATTGTCACTCCAGGAAGC
TGGGAGGTGACAGAGTATCCTTTTATTACCAAGTATTTTATGGGGAAAGAGAGAAGGTGCCTTTTGGAATAGAC A

2395 0.4 0.0 0.0 AluYb8-Scaffold8739-1323469-1323719 1001 1251 (1000) + AluYb8 SINE/Alu 1 251 (67) 1

>Hg19 chr14:54814788-54816727
TAAAATTCATTTGTGACTAAGTCATTATAGTGAAGTACTTGGAAACCTTTCTATTCTAGAGAGTTCTGGGCACAAACACATTTACAGTGTGCACTAATGCCAGGCTCATGTCCTCACGTCCTCCAA GG
GACAGGGATGAGCTGGGTCATAAACCAAACAGTGGGACTCTGGCAATCCCTTTTAGCTCAGGCAGATGCAAAATTGAAGACCCAAAAGAAGCCTTCCAAAGGTC ACAGCAGGTTGCTCTCAGGAAAAG
AGAATCAGTAATTTATTTTTAAGCAATCTCTTAGCCTCTGTGACAGTTATGTTTCCTTGTAAGCTGCTTTGGAGCCACTCACCACTCTGAAATTGCAGAGTAGGCTACGAACAATCCGGCTTCGT AAG
AACATGCAAGTGAGGACCCACGAGGAGATATTCTTGTGCTTTTTAAAGTGCTTCTCTCACTTTGCTTCCTAAACTGCCTTAATTGAGGCAAGGGAGCCAACATG AGTGAGTTCCTTCCCGCGGCTTGC
TCTGCCGTGGAGTTCAAGGATGCCTGAGCCCTCAAAACACGGCGCGGTGCAGTTGGGCTGATGGGAAGTCCTGAACAGCTTGCTGAATTATTACTTCATTCTCGGCTGAAGCAGCAGGAGGCGAG ATG
ATGAGTGAAGGAAAAAACCCATGGACTCCAGCGTGGGCTGAAGGAACCAAAACAAACACTGAGAAGACAGGCCAATACTGAAATCTGTTTCAAGGATGGAGCGC GCAGGAATGTTTCACCATATTTTG
TCTAAGGAACAGATTTCATTTTTTAATGGCTGTATTAATCAGTGGAAGCAGAACGGCAGGAAGGAATCCGTTTTCCCCTCTCCCCAACGCCTCCCGTGGCTCGCCCGGACATTCCCCCCATTCTTTGT
ACTGAAGGTGGCCTGGAAGTTTTAGAGGCGTGAGACAGGTTCTGAACT cGCATGCCCTTTCTTTCTCCTTCCCGAATAAATTTTGCGTAAGAATATTTGGGACAAGGAATCCACCATGGCGGGGTGCA
ATAAAAGCGTTTCACAAATTATGATTTCCCATTCTCTAGGCAGTTCAGCTCTGCCTAACAGCTCTAATTTCCTAGACAAATAGTCCTCCCTGTCCTCTCCAGCTGCTCACAGGCGCCTCTCTCTG GGT
ATTCTTTTTAAAATCCCTCTCCTGGGGAATTTTTAAAAAAGCTTCTCGCACTTTTTCATGCGATTTTCATGGTTCCAT CCATCTCCCCACTTTTTTGGTCAAAGCCTTCCTCCCAAATATCAATTGCT
TTTTCAACCCGTCCATACTACTGCTCAGCTGTTGGTTGAGCTTCTCCTAGCCTCCTGCTGCTGTCACTGGGCCAATGGAAGCCAGTCCCTTGCCTCTTTGTTCTATTATTTTATGGCAAGATAAT AAA
CTTCTACATTTTTGAAGTACTTTTCACCGACTTGATATCAAGTATTTTGATAATCACAAAGTGATCAAACACATGTTCTCTTTTTCCAGTTACATAATGAGATC AAATATTGATTAGCAGTGAGTAGA
TAGATGTATATTGTACACACACACATACACCCGACCATCCTCCTTTTGGCCTATGGAGACTGTTACTCCAAAGAGCCGACACATTTGTGTTGACATTTCAGTCATCCAAGAAAGCTGCCAATGTC ACT
TCCTTTGAATATTTTCCTTCAGTTTTCAGGAAAGAGTTGGTGGGACTAGCTTATCATGGATGGCATGAGCTGAGGGGATGGTAATTTTTCACAAAAGTAGACAT TGTTCGAGGCTAATAGTAAGCTGT
AATCTAGACTCATAACTGTAGAGGGCGCTCTTGACTTAATAATGACAGCATTGCCATTGTCACTCCAGGAAGCTGGGAGGTGACAGAGTATCCTTTTATTACCAAGTATTTTATGGGGAAAGAGA GAA
GGTGCCTTTTGGAATAGACA

>KSI_AluYb8_30

>Scaffold9165-103041-103345
CTGGGATTACAGGTGTGAGCCACCACACCTGGGCCTTTTTATTTCTTTTTAATTTTGTGTGGACTTCAATGGTAGAAGTTATAGTTGATTTGACCAGAAAGGGACATGTGAAAAACCTTCCTAAAA TA
TTTCCTTTTTTTTTCTTGTGCTGTTTGCTCTATCTGTAATCATTAGTGCCCCCCTAGAATGAAGCAGCCAAGTCTTGGGTTATCACAAATATAAACATAATTTTTATATCATATTCTTTTGCTAA GGT
GATCTGGATATCAATTAATTTAGCATTAATTATTATACTGTATGCTGTCATTAGTCATGTTACATTATTAGGTATTAGTCACATTACATCATAATTATTTTATGCAGCCTCAACTTCTTTACTAAA AT
GTGCAGTAGACATCAGATCACAAATATCAAGACTTTtGAACAGGTGTTGGCAAATTTTTTTTGTAAAGGGCCAGAAACAAGTACTTTTGGCTTTGTGGGTTTTATAGTCTGTGTCTTATCTACTCAAC
CTTGCTGTCTGTGTGAAAGCAGCCATTTATGTAAATGAATGGGCATGACTGTGTTCTAGTTAAACTTTATTTTGCAAAAGCAGGTCA tGGGACTGATTTGCCTGTGATCTCTGCAACCCAGCTCTTGA
GTAAGGGTCACAGACTTTGCTGTGGTTAGGCAGGTACCTCAAATGAGTAAAGTGAAGTGGCTGTGGGGACTGCGACTAGCAAAGGTCACGTCCTTGTCTAAAATGGGCAGCTACTTCTTCCCTAT AGC
CACTGTGACTGGCTCAGTGATACCAGATCTTGTAATTCAAGGGTAGCTGGGAATTTGGATTTTTAGGCAAAGTCCTTTTATCAGTTGGCAACTCATTTAGATTTGTTAAGGAAATATGGTTGAGGT CA
AGCTACACACCTTCGTGAGTTCTTTTCTGGTCCATGAACTCtAGTTTAGCAAATTCTGGTATTTTCAAGTGTAACAGTgTATTATGTATGAATaagaagtgcctggccgggcgcggtggctcacgcct
gtaatcccagcactttgggaggccgaggcgggtggatcatgaggtcaggagatcgagaccatcctggctaacaaggtgaaaccccgtctctactaaaaatacaaaaaaaattagccgggcgcggtg gc
gggcgcctgtagtcccagctactcgggaggctgaggcaggagaatggcgtgaacccgggaagcggagcttgcagtgagccgagattgcgccactgcagtccgcagtcccgcctgggagacagagc gag
actccgtctcaaaaaaaaaaAAGAAGTGCCTGTAGAATGTCATCAGACATCGATAGCAACTATCTAgTGCTGGTGCTGCCACTCACTAACCCTTGTAGTGCCCATGACAGACATCAGTAATTGATCAT
AACACTCTTTCCTGCTGAGCTAGGAGAAAGTTTCAGATTTCTTCTCCACTAGGGCTCTAAAAAGCCGCAA CCAGCCAATCAGTCATGTCCCCTGATATAAAGGTATTTATCATTCTCATTCTAGAAGG
TTTATAAAAAATGTTTTTTTAATTAACCTAGGAAATGGCTTTGGACTTTTATTGTTATTCTGTTAAAAAAACCTAATTAAAAGATGTTCTTAATTAAAAAGTACTGGAGAAAACATGTATTAGAA TGG
CAGGTATGAAAAAAGGTAAGATACTGTTAATATGGCAGTGTTAGATTTCAGACCAAACTTAGGGGAAAGAGTATCCAAAGTCAGATGTA TCCTTAAAAGTCACTTAGCAGCATTCCTTCCTTGCAGAG
ACCATGAACTTGGCTTTCTGATGTGTTCATTTTCACCATGCCTAGTGCAAATTAATGTGAATGAATAGTTACAAAGGGGAGTTTCTTTTTTTCCTTCCTTTCACTGCCTTTGACCCTTCTGCACA TTT
GGCGGTAGAGTACGcCATTCAGAATTTAGGGATTCTGTGTGTGCCATCTGGGATGCATGTATTTTTTAAATTGTTTATGTGATATATAC ATGATACAGCCCAGGGAAAACATGCTTTATTGTGCATTT
TGAAAGCTTTGCATCTTTTTGTCTTCCTGCATTACTTCTCATTTTACCCTCTGCCAGTCTAAACACTGTTGTCTTTCACGTTTGACCTTCTCTAAGAGTTCTGCTCTAGTTCTGTCGTTGAACCT CTC
TCCTCTCTAAACTTCTTTAGTACTGAGGACTGTACTAGTTTAGCAGCAATGCCATGCTTTCTTATGTCCTTTGGTTGTAAATTTGAGGC ATTTTTGTTTTTTTCTTCCAAATTAACATTTTCTTTCCC
T

241	12.5	0.0	0.0	AluYb8-Scaffold9165-103041-103345	1	32	(2273)	C	Alu	SINE/Alu	(99)	34	3	1
846	21.3	5.2	1.0	AluYb8-Scaffold9165-103041-103345	425	618	(1687)	C	MER58A	DNA/hAT-Charlie	(0)	224	23	2
588	31.9	10.6	1.5	AluYb8-Scaffold9165-103041-103345	643	954	(1351)	C	MER102b	DNA/hAT-Charlie	(1)	340	1	3
2784	1.3	0.0	0.7	AluYb8-Scaffold9165-103041-103345	1001	1305	(1000)	+	AluYb8	SINE/Alu	1	303	(15)	4
559	31.4	1.0	2.1	AluYb8-Scaffold9165-103041-103345	1332	1523	(782)	C	MER117	DNA/hAT-Charlie	(4)	193	4	5

>Hg19 chr16:53873981-53875974
CTGGGATTACAGGTGTGAGCCACCACACCTGGGCCTTTTTATTTCTTTTTAATTTTGTGTGGACTTCAATGGTAGAAGTTATAGTTGATTTGACCAGAAAGGGACATGTGAAAAACCTTCCTAAAA TA
TTTCCTTTTTTTTTCTTGTGCTGTTTGCTCTATCTGTAATCATTAGTGCCCCCCTAGAATGAAGCAGCCAAGTCTTGGGTTATCACAAATATAAACATAATTTTTATATCATATTCTTTTGCTAA GGT
GATCTGGATATCAATTAATTTAGCATTAATTATTATACTGTATGCTGTCATTAGTCATGTTACATTATTAGGTATTAGTCACATTACATCATAATTATTTTATGCAGCCTCAACTTCTTTACTAAA AT
GTGCAGTAGACATCAGATCACAAATATCAAGACTTTcGAACAGGTGTTGGCAAATTTTTTTTGTAAAGGGCCAGAAACAAGTACTTTTGGCTTTGTGGGTTTTATAGTCTGTGTCTTATCTACTCAAC
CTTGCTGTCTGTGTGAAAGCAGCCATTTATGTAAATGAATGGGCATGACTGTGTTCTAGTTAAACTTTATTTTGCAAAAGCAGGTCA cGGGACTGATTTGCCTGTGATCTCTGCAACCCAGCTCTTGA
GTAAGGGTCACAGACTTTGCTGTGGTTAGGCAGGTACCTCAAATGAGTAAAGTGAAGTGGCTGTGGGGACTGCGACTAGCAAAGGTCACGTCCTTGTCTAAAATGGGCAGCTACTTCTTCCCTAT AGC
CACTGTGACTGGCTCAGTGATACCAGATCTTGTAATTCAAGGGTAGCTGGGAATTTGGATTTTTAGGCAAAGTCCTTTTATCAGTTGGCAACTCATTTAGATTTGTTAAGGAAATATGGTTGAGGT CA
AGCTACACACCTTCGTGAGTTCTTTTCTGGTCCATGAACTCcAGTTTAGCAAATTCTGGTATTTTCAAGTGTAACAGTaTATTATGTATGAATAAGAAGTGCCTGTAGAATGTCATCAGACATCGATA
GCAACTATCTAaTGCTGGTGCTGCCACTCACTAACCCTTGTAGTGCCCATGACAGACATCAGTAATTGATCATAACACTCTTTCCTGCTGAGCTAGGAGAAAGTTTCAGATTTCTTCTCCACTAGGGC
TCTAAAAAGCCGCAACCAGCCAATCAGTCATGTCCCCTGATATAAAGGTATTTATCATTCTCATTCTAGAAGGTTTATAAAAAATGTTTTTTTAATTAACCTAGGAAATGGCTTTGGACTTTTATTGT
TATTCTGTTAAAAAAACCTAATTAAAAGATGTTCTTAATTAAAAAGTACTGGAGAAAACATGTATTAGAATGGCAGGTATGAAAAAAGGTAAGATACTGTTAATATGGCAGTGTTAGATTTCAGAC CA
AACTTAGGGGAAAGAGTATCCAAAGTCAGATGTATCCTTAAAAGTCACTTAGCAGCATTCCTTCCTTGCAGAGACCATGAACTTGGCTTTCTGATGTGTTCATTTTCACCATGCCTAGTGCAAAT TAA
TGTGAATGAATAGTTACAAAGGGGAGTTTCTTTTTTTCCTTCCTTTCACTGCCTTTGACCCTTCTGCACATTTGGCGGTAGAGTACG aCATTCAGAATTTAGGGATTCTGTGTGTGCCATCTGGGATG
CATGTATTTTTTAAATTGTTTATGTGATATATACATGATACAGCCCAGGGAAAACATGCTTTATTGTGCATTTTGAAAGCTTTGCATCTTTTTGTCTTCCTGCATTACTTCTCATTTTACCCTCT GCC
AGTCTAAACACTGTTGTCTTTCACGTTTGACCTTCTCTAAGAGTTCTGCTCTAGTTCTGTCGTTGAACCTCTCTCCTCTCTAAACTTCTTTAGTACTGAGGACTGTACTAGTTTAGCAGCAATGCC AT

GCTTTCTTATGTCCTTTGGTTGTAAATTTGAGGCATTTTTGTTTTTTTCTTCCAAATTAACATTTTCTTTCCC T

1029	10.1	0.0	0.0	Hg19	4	132	(2062)	C	AluYj4	SINE/Alu	(180)	131	3	6
865	20.8	5.2	1.0	Hg19	525	718	(1476)	C	MER58A	DNA/hAT-Charlie	(0)	224	23	7
604	31.6	10.6	1.5	Hg19	743	1054	(1140)	C	MER102b	DNA/hAT-Charlie	(1)	340	1	8
544	31.9	1.0	2.1	Hg19	1121	1312	(882)	C	MER117	DNA/hAT-Charlie	(4)	193	4	9

>KSI_AluYb8_31

>Scaffold9672-2052388-2052664
TTTTGTGTGGACATATATTTTCATTTCTATTGGGTATATTACCTAAAAgTGGAATTGCGGGGTCACATGGTAACTCTGTGTTCAACATTTTGAAGAATTGCCAAACTGTTTTCCAAAGTGGCTGTACT
ATTTTCCATTCCCACCAGTGGTAGATGGTGCCTCTAGTTTCTCTACATCCTTTTTTTTTTTTTtGAGATGGAGTCTTGCTCTGTCACCCAGGCTGGAGTGCAGTGGCGTGATCTCGGCTCACTGCAAGC
TCCACCTCCTGGGTTCATGCCATTCTCCTGTCTCAGCCTCCCTAGTAGCTGGAACTACAGGCACCCGCCACCACGCCTGGCTAATTTTTTGTATTTTTAGTAGAGATGGGGTTTCACCGTGTTAG CCA
GGATGGTCTCGATCTCCTGACCTTGTGATCCGCCCGCCTCGGCCTCCCAAAGTGCTGGGATTACAGGCGTGAGCCACCGCATCCTTGCTTAACATTTAT TATTGACTGTCTTTTTAATTACAGACGGC
AGCCATCCTAGTGGATGTGAAATGCAATCTCACTGTGCTTTTGATTTGCATTTATCTAATGATAA TGATGTTGAGTATCTTTGCCATTTGTGTATCTTCTTTAGAGAAATGTCTATTAAGATCTTTTG
CCCTTTTTTAAAAAAAACTGTGTTATTTCTCTTTTATTATTGAGTTGTAAAAGTTTTTTTTtAATGTATATTCTGGAAACAAGTCCCTTGTTAGATATGTGATCTCAAAATTTTTGTCCCATTCTGTG
GGTTGTCTTTTCACTTTCTTGATGATCCTTTGAAGCAATGTTTTTAgTTTTGATAAAGTCTAATCTTCCTACTTTTTTTCCTTTTGCTATTTGTGCTTTGGTGCAGTATTGGTTAGGTTTTGGTATCAA
TTTTATATTTACTTCACTAAAATAATTGGAAAAATTTTCCTTCTTTTCAGGTGCTCTAGAACAATTTAAATAGTATTAGAAATATTTGCTATTT AAAGTTTGGTcccagcactttgggaggccgaggc
gggtggatcatgaggttaggagatcgagaccatcctggctaacaaggtgaaaccccgtctctactaaaaatacaaaaaattagctgggcgcggtggcgggcgcctgtagtcccagctactcgga ggc
tgaggcaggagaatggcgtgaacccgggaagcggagcttgcagtgagccgagattgcgccactgcagtccgcagaccgacctgggcgaagaccc cgcccaaaaaaaaaaaaaaaaaaaaaaaaggt
ttggtATAATTTTTCTGTCAAACCATTTGGGCCTGGTGCTTTTGGGGCATGGGGTGGCTCTGGTCTTCCTTCTTAGCTGGAAATGCCAGTTTTGTTTTGTGTGTGTGTGTGTGTGTGTGTGTGTGTGT
GTGTGTGTGTGTGTGTGTGTgtgtttTTGAGACAGAGCCTTGCTCTGTTGCgCCCAGGCTGGAGTGCAGTGGCAAGATCTCAGCTCATTGCATCCT CCGCCTCCTGGGTTCAAACAATTCTCCCACCTTA
GTCTCCCAAGTAGCTGGGATTACAGGTGCCCTCCACCATGCCTGGCTAATTTTTGTATTTTTGTAGAGATGGGGTTTCACCATATTGGCCAGGTTGGTCTT tAACTCCCGACTTCAGGTGATCTGCCT
GCCTTGGCCTCCCAAAGCACTGGGATTATAGGTATGAGCCACCGTGCCCAGC tGAAATGCCAGTTTTAAGTTGTTAAATACATGGCAGAATGTC TGGTTACAGTTTTTATCTGTTTTGTGAAGGCAAC
ATTTTTTGGTGAGCTTTTATTGTCTGTAGTAAAATTTTGTTGCTCTCTTCCTCTTTTTCCCTAGTAGTATCTTTTTAAAAAATCCATATTATTGAGTATAATTTATGTACAAAAAATGCCATCCA TTA
AAAATGTATAATTAAAGGTACTTTGCCAGATATAGACCTATAGCTGTGAAACTACCACCACAATCAAGATACAGAATGTTTATATCACCC tCCAAAAGATCGCTTTTTTCCCTGCTTACCGAGACAAG
CTCGGTTGTGGAGACTCCAACGCACCAGCACTAGAGGAATGAAGACAAAGACCCAGAAATAAAGTGCAGTGTGGGAATCGGCTAAAAGCCTTTAGAGCTGAGAGCCACAAACAGAGTTTTGCCCACAT
ATTTATTGACAGTAAGCCTGTGATAAACATTGTTTCTAAAGATTATAGATTAGCTGAAAGCATT cCTTATGGGAAACAAAGCATTCTTTTCAAGAAGCAGA

2221	14.3	3.9	5.1	AluYb8-Scaffold9672-2052388-2052664	1	178	(2099)	C	L1M4	LINE/L1	(496)	5682	5506	1	
2533	5.5	0.0	0.0	AluYb8-Scaffold9672-2052388-2052664	179	468	(1809)	C	AluY	SINE/Alu	(17)	294	5	2	
2221	13.0	6.5	4.5	AluYb8-Scaffold9672-2052388-2052664	469	870	(1407)	C	L1M4	LINE/L1	(673)	5505	5102	1	
372	28.1	3.2	5.4	AluYb8-Scaffold9672-2052388-2052664	864	1000	(1277)	C	L1M5	LINE/L1	(2459)	3687	3552	3	*
2427	2.5	2.9	0.0	AluYb8-Scaffold9672-2052388-2052664	1001	1277	(1000)	+	AluYb8	SINE/Alu	29	313	(5)	4	
372	28.1	3.2	5.4	AluYb8-Scaffold9672-2052388-2052664	1278	1327	(950)	C	L1M5	LINE/L1	(2595)	3551	3504	3	
62	0.0	0.0	0.0	AluYb8-Scaffold9672-2052388-2052664	1378	1430	(847)	+	(TG)n	Simple_repeat	1	53	(0)	5	
2107	12.9	0.3	0.7	AluYb8-Scaffold9672-2052388-2052664	1431	1716	(561)	C	AluSx1	SINE/Alu	(26)	286	2	6	
383	27.0	2.1	2.1	AluYb8-Scaffold9672-2052388-2052664	1730	1869	(408)	C	L1M7	LINE/L1	(5380)	838	699	7	*
471	26.0	1.2	4.9	AluYb8-Scaffold9672-2052388-2052664	1864	2033	(244)	C	L1ME1	LINE/L1	(1)	6178	6015	8	
1641	11.6	2.1	0.0	AluYb8-Scaffold9672-2052388-2052664	2037	2277	(0)	C	MER9B	LTR/ERVK	(0)	499	254	9	

>Hg19 chr3:38398706-38400694
TTTTGTGTGGACATATATTTTCATTTCTATTGGGTATATTACCTAAAAtTGGAATTGCGGGGTCACATGGTAACTCTGTGTTCAACATTTTGAAGAATTGCCAAACTGTTTTCCAAAGTGGCTGTACT
ATTTTCCATTCCCACCAGTGGTAGATGGTGCCTCTAGTTTCTCTACATCCTTTTTTTTTTTTT TGAGATGGAGTCTTGCTCTGTCACCCAGGCTGGAGTGCAGTGGCGTGATCTCGGCTCACTGCAAGCT
CCACCTCCTGGGTTCATGCCATTCTCCTGTCTCAGCCTCCCTAGTAGCTGGAACTACAGGCACCCGCCACCACGCCTGGCTAATTTTTTGTATTTTTAGTAGAGATGGGGTTTCACCGTGTTAGC CAG
GATGGTCTCGATCTCCTGACCTTGTGATCCGCCCGCCTCGGCCTCCCAAAGTGCTGGGATTACAGGCGTGAGC CACCGCATCCTTGCTTAACATTTATTATTGACTGTCTTTTTAATTACAGACGGCA
GCCATCCTAGTGGATGTGAAATGCAATCTCACTGTGCTTTTGATTTGCATTTATCTAATGATAA TaatGATGTTGAGTATCTTTGCCATTTGTGTATCTTCTTTAGAGAAATGTCTATTAAGATCTTT
TGCCCTTTTTTAAAAAAAAaCTGTGTTATTTCTCTTTTATTATTGAGTTGTAAAAGTTTTTTTT TAATGTATATTCTGGAAACAAGTCCCTTGTTAGATATGTGATCTCAAAATTTTTGTCCCATTCTG
TGGGTTGTCTTTTCACTTTCTTGATGATCCTTTGAAGCAATGTTTTTAaTTTTGATAAAGTCTAATCTTCCTACTTTTTTTCCTTTTGCTATTTGTGCTTTGGTGCAGTATTGGTTAGGTTTTGGTATC
AATTTTATATTTACTTCACTAAAATAATTGGAAAAATTTTCCTTCTTTTCAGGTGCTCTAGAACAATTTAAAT AGTATTAGAAATATTTGCTATTTAAAAGTTTGGTATAATTTTTCTGTCAAACCAT
TTGGGCCTGGTGCTTTTGGGGCATGGGGTGGCTCTGGTCTTCCTTCTTAGCTGGAAATGCCAGTTTTGTTTTGTGTGTGTGTGTGTGTGTGTGTGTGTGTGTGTGTGTGTGTGTGTGTGTG TTTGAGACA
GAGCCTTGCTCTGTTGCaCCCAGGCTGGAGTGCAGTGGCAAGATCTCAGCTCATTGCATCCTCCGCCTCCTGGGTTCAAACAATTCTCCCACCTTAGTCTCCCAAGTAGCTGGGATTACAGGTGCCCT

CCACCATGCCTGGCTAATTTTTGTATTTTTGTAGAGATGGGGTTTCACCATATTGGCCAGGTTGGTCTT gAACTCCCGACTTCAGGTGATCTGCCTGCCTTGGCCTCCCAAAGCACTGGGATTATAGG
TATGAGCCACCGTGCCCAGCcGAAATGCCAGTTTTAAGTTGTTAAATACATGGCAGAATGTCTGGTTACAGTTTTTATCTGTTTTGTGAAGGCAACATTTTTTGGTGAGCTTTTATTGTCTGTAGTAA
AATTTTGTTGCTCTCTTCCTCTTTTTCCCTAGTAGTATCTTTTTAAAAAATCCATATTATTGAGTATAATTTATGTACAAAAAATGCCATCCATTAAAAATGTATAATTAAAGGTACTTTGCCAG ATA
TAGACCTATAGCTGTGAAACTACCACCACAATCAAGATACAGAATGTTTATATCACCC cCCAAAAGATCGCTTTTTTCCCTGCTTACCGAGACAAGCTCGGTTGTGGAGACTC CAACGCACCAGCACT
AGAGGAATGAAGACAAAGACCCAGAAATAAAGTGCAGTGTGGGAATCGGCTAAAAGCCTTTAGAGCTGAGAGCCACAAACAGAGTTTTGCCCACATATTTATTGACAGTAAGCCTGTGATAAACATTG
TTTCTAAAGATTATAGATTAGCTGAAAGCATTtCTTATGGGAAACAAAGCATTCTTTTCAAGAAGCAGA

645	13.3	0.0	0.0	Hg19	1	90	(2099)	+	MLT1B	LTR/ERVL-MaLR	301	390	(0)	10	
2219	14.9	3.6	5.5	Hg19	92	278	(1911)	C	L1M4	LINE/L1	(487)	5691	5506	11	
2524	5.5	0.0	0.0	Hg19	279	567	(1622)	C	AluY	SINE/Alu	(18)	293	5	12	
2219	12.9	6.4	4.6	Hg19	568	972	(1217)	C	L1M4	LINE/L1	(673)	5505	5102	11	
439	28.1	3.3	1.1	Hg19	966	1145	(1044)	C	L1M5	LINE/L1	(2459)	3687	3504	13	*
57	0.0	0.0	0.0	Hg19	1196	1244	(945)	+	(TG)n	Simple_repeat	1	49	(0)	14	
2110	13.0	0.3	0.7	Hg19	1245	1529	(660)	C	AluSx1	SINE/Alu	(28)	284	1	15	
383	27.0	2.1	2.1	Hg19	1542	1681	(508)	C	L1M7	LINE/L1	(5380)	838	699	16	*
471	26.0	1.2	4.9	Hg19	1676	1845	(344)	C	L1ME1	LINE/L1	(1)	6178	6015	17	
2166	12.5	1.6	0.0	Hg19	1849	2167	(22)	C	MER9B	LTR/ERVK	(0)	499	176	18	

>KSI_AluYb8_32

>Scaffold11674-6584416-6584733
AGGCACACTTAGTGTAACTTTATAGAAATACATCATAGGCCGGGCGCGGTGGCTCACACCTGTAATCCCAGTACTTTGGGAGGCCAAGGCAGGCAGATCGCCTGAGTTTGGGAGTTGGCGACCAGC CT
GACCAACATAGAGAAAACTCGTCTCTACTAAAAATACAAAATCAGCCAGGCGTGCTGGCACATGCCTGTAATCCCAACTACTCGGGAGCCTGAGGCAGAAGAATCGCTTGAACCGGGGAGGCAGA GAT
TGTCGTGAGTCGAGATGAAGCCATTGCACCCCACCCTGGGCAACAAGAGC aAAACTCCATCTCAAAAAAAAAAAAAGAAAGGAAAATAAAAAGAAATACATCATAATTTCTGCCTGACTTTTTTGCTT
TTTCATTTCTCTAGCAATGTTTTGGTATGGTACCATTCCTTCTTCCGTCTGCTTTAGTTTCAGTGTCTGTATCATAGAAGAGGAGATGTTGTAAAAGAAGTCCAAAGCAGCCTAAATAATCAGAA CCA
TTTTGCCAGCTTGTCTAACATCAATTTGTTTCCTGACACTGCTTCTTGTGTGTCTTTTTCCTCATCATCTGGTACTGGTTCGGAAGCACTCATCTCCATCAAGTCATTTATTAATTCCTCTGGTGT GC
CATCTATTAGGTCTTTAATTCTCTCGGATCTGTATTTTAAAACCCTTCACTCCATCTTTTTTTCCATACCCCCAATTTCCTTCATGATTTCCTTAACTGGCTCTGTCATAAACCCTGTGAAGTCA TGC
ACAACATCTGGGCACTGTTTTCTCCAGCAGGAATGTATTGTTTTGAGCTTGATGGCTTCTGTGGCTGTAACAACAGCATCTTAAATGGTGCAATCCTTCTAGAC tTTCATGATGTTCTCCCTATTGGG
TTTCTCTTTCATAATGTAGACAATCCTTTCCATAGGGTACCGCGTGTAATGAGCCTTAAAGGTCTTATGACCCACTGATCTACAGGCTAAAT Tagaaatgtgttggccgggcgcggtggctcacgcct
gtaatcccagcactttgggaggccgaggtgggtggatcatgaggtcaggagatcgagaccatcctggctaacaaggtgaaaccccgtctctactaaaaatacaaaaaattagccgggcgcggtgg cgg
gcgcctgtagtcccagctactcgggaggctgaggcaggagaatggcgtgaacccgggaggcggagcttgcagtgagccgagattgcgccactgcagtccgcagt ccggcctgggcgacagagcgagac
tccgtctcaaaaaaaaaaaaaaaaaaaaaaaaaaaaaaaaaaAGAAATGTGTTTGGGGGCAAATAGATCACTTCAACACCTTTAGTGTTGAACTCATTGGG CTATGGGGgATCACGGCATTGTCTA
ATACCAAAAGAACTTTGAGAGGCAGTCCCTTATTGGCAAGGTACTTCCTTAGGGAACAAAGCATTGATGGAACCAATCAAGATAAAGGGTTCTCATTGTCCAGA CTTTCTTTTGTAAAATGAAAAGAC
TGGCAGCTGGTGTTTATCTTTTCCTTCCAAGGCTCAGGGGTTAGCAGCTTTATAGATAAGGGCAGTCCTAATCATAAACCCAAACTGCATTTGCACACAATGGTACAATTCTATCCTTCTATCCC TTC
TTGCCTTAAATCCTGGGGCTTGCTTCATTACTAATTAATGTTCCTTGTGGCATTTTTTCCAGAATAAGGCCCTTTTGTCCACATTAAAAACCTATTCAGGGCCA GGCGCGGTGGCTCACACCTGTAAT
CCCCAGCACTTTGGGAGGTCCAGACGGGCGGATCACTTGAAGTCAGGAGTTCGAGACCAGCCTTGGCCAACATGGTGAAACCCTGTCTCTACTAAAAATATAAAAATTAGCTGGGCATGGTGGTG CAT
GTTTGTAATCCCAGCTACTTGGGAGGCTGAGGCAGGAGAATCGTTTGAACCTGGGAGGCAGAGTTTGCAGTGAGTGGAGATTGTGCCACTGCATTCCAGCTTGG GTGACAGAGCAAGGCTCTGTCTAA
AAaaaaCAAACAAACAAACAAAGACCTATTCAGGCAGATATCCTTTCTCCTCAATGTTTTTTTTGTTTTGTTTTGTTTTTTTGTTTTGGAGAGGGGAACAAGGTCTCACTCTGTCACCCAGGCTGGAG
TGCAGTGGAACAATCACAGCTCACTGTAGCCTCTACCTCCTGGGCTTAAGGGATTCTCCAACGTCAGCCTCACAAGTAGCTGACACTCTAGGTAACGTGCCACC ATATTTTATTTTATTTTTTTGTAG
CAACAGGGTCTGAC

7039	12.8	3.0	4.1	AluYb8-Scaffold11674-6584416-6584733	1	37	(2281)	C	Tigger2	DNA/TcMar-Tigger	(418)	2300	2264	1
2217	12.5	0.3	0.0	AluYb8-Scaffold11674-6584416-6584733	38	348	(1970)	+	AluSp	SINE/Alu	1	312	(1)	2
7039	12.8	3.0	4.1	AluYb8-Scaffold11674-6584416-6584733	349	1000	(1318)	C	Tigger2	DNA/TcMar-Tigger	(455)	2263	1608	1
2992	0.9	0.0	0.0	AluYb8-Scaffold11674-6584416-6584733	1001	1318	(1000)	+	AluYb8	SINE/Alu	1	318	(0)	3
7039	12.8	3.0	4.1	AluYb8-Scaffold11674-6584416-6584733	1319	1763	(555)	C	Tigger2	DNA/TcMar-Tigger	(1111)	1607	1165	1
2255	11.4	0.0	0.7	AluYb8-Scaffold11674-6584416-6584733	1764	2070	(248)	+	AluSz	SINE/Alu	1	305	(7)	4
7039	12.8	3.0	4.1	AluYb8-Scaffold11674-6584416-6584733	2071	2104	(214)	C	Tigger2	DNA/TcMar-Tigger	(1554)	1164	1142	1
988	19.3	2.8	3.3	AluYb8-Scaffold11674-6584416-6584733	2107	2318	(0)	C	AluJb	SINE/Alu	(0)	312	102	5

>Hg19 chr6:111106865-111108843
ttgtcttcacattgagtcggctgagaagaaagaggggagttggtcttgctgtctcaaggatggcagaggcggagg aggtagaaggggagacagaagaggcAGGCACACTTAGTGTAACTTTATAGAAA
TACATCATAGGCCGGGCGCGGTGGCTCACACCTGTAATCCCAGTACTTTGGGAGGCCAAGGCAGGCAGATCGCCTGAGTTTGGGAGTTGGCGACCAGCCTGACCAACATAGAGAAAACTCGTCTC TAC
TAAAAATACAAAATCAGCCAGGCGTGCTGGCACATGCCTGTAATCCCAACTACTCGGGAGCCTGAGGCAGAAGAA TCGCTTGAACCGGGGAGGCAGAGATTGTCGTGAGTCGAGATGAAGCCATTGCA
CCCCACCCTGGGCAACAAGAGCgAAACTCCATCTCAAAAAAAAAAAAAGAAAGGAAAATAAAAAGAAATACATCATAATTTCTGCCTGACTTTTTTGCTTTTTCATTTCTCTAGCAATGTTTTGGTAT
GGTACCATTCCTTCTTCCGTCTGCTTTAGTTTCAGTGTCTGTATCATAGAAGAGGAGATGTTGTAAAAGAAGTCC AAAGCAGCCTAAATAATCAGAACCATTTTGCCAGCTTGTCTAACATCAATTTG
TTTCCTGACACTGCTTCTTGTGTGTCTTTTTCCTCATCATCTGGTACTGGTTCGGAAGCACTCATCTCCATCAAGTCATTTATTAATTCCTCTGGTGTGCCATCTATTAGGTCTTTAATTCTCTC GGA
TCTGTATTTTAAAACCCTTCACTCCATCTTTTTTTCCATACCCCCAATTTCCTTCATGATTTCCTTAACTGGCTC TGTCATAAACCCTGTGAAGTCATGCACAACATCTGGGCACTGTTTTCTCCAGC
AGGAATGTATTGTTTTGAGCTTGATGGCTTCTGTGGCTGTAACAACAGCATCTTAAATGGTGCAATCCTTCTAGAC cTTCATGATGTTCTCCCTATTGGGTTTCTCTTTCATAATGTAGACAATCCTT
TCCATAGGGTACCGCGTGTAATGAGCCTTAAAGGTCTTATGACCCACTGATCTACAGGCTAAAT TAGAAATGTGTTTGGGGGCAAATAGATCACTTCAACACCTTTAGTGTTGAACTCATTGGGCTAT
GGGGaATCACGGCATTGTCTAATACCAAAAGAACTTTGAGAGGCAGTCCCTTATTGGCAAGGTACTTCCTTAGGGAACAAAGCATTGATGGAACCAATCAAGATAAAGGGTTCTCATTGTCCAGACTT
TCTTTTGTAAAATGAAAAGACTGGCAGCTGGTGTTTATCTTTTCCTTCCAAGGCTCAGGGGTTAGCAGCTTTATAGATAAGGGCAGTCCTAATCATAAACCCAAACTGCATTTGCACACAATGGT ACA
ATTCTATCCTTCTATCCCTTCTTGCCTTAAATCCTGGGGCTTGCTTCATTACTAATTAATGTTCCTTGTGGCATTTTTTCCAGAATAAGGCCCTTTTGTCCACATTAAAAACCTATTCAGGGCCAGGC
GCGGTGGCTCACACCTGTAATCCCCAGCACTTTGGGAGGTCCAGACGGGCGGATCACTTGAAGTCAGGAGTTCGAGACCAGCCTTGGCCAACATGGTGAAACCCTGTCTCTACTAAAAATATAAA AAT

TAGCTGGGCATGGTGGTGCATGTTTGTAATCCCAGCTACTTGGGAGGCTGAGGCAGGAGAATCGTTTGAACCTGGGAGGCAGAGTTTGCAGTGAGTGGAGATTGTGCCACTGCATTCCAGCTTGGGTG
ACAGAGCAAGGCTCTGTCTAAAACAAACAAACAAACAAAGACCTATTCAGGCAGATATCCTTTCTCCTCAATGTTTTTTTTGTTTTGTTTTGTTTTTTTGTTTTGGAGAGGGGAACAAGGTCTCACTC
TGTCACCCAGGCTGGAGTGCAGTGGAACAATCACAGCTCACTGTAGCCTCTACCTCCTGGGCTTAAGGGATTCTCCAACGTCAGCCTCACAAGTAGCTGACACTCTAGGTAACGTGCCACCATATTTT
ATTTTATTTTTTTGTAGCAACAGGGTCTGACtatgttgcccaagctggtctcaaactcctgggctcaagtgatcctcctgccttggcctgccaaagtgctgggatttataggcgtaagccatgacacc
tgg

7742	12.8	4.3	2.6	Hg19	1	137	(2042)	C	Tigger2	DNA/TcMar-Tigger	(303)	2415	2264	6
2232	12.2	0.3	0.0	Hg19	138	448	(1731)	+	AluSp	SINE/Alu	1	31 2	(1)	7
7742	12.8	4.3	2.6	Hg19	449	1528	(651)	C	Tigger2	DNA/TcMar-Tigger	(455)	2263	1165	6
2219	11.6	0.0	0.7	Hg19	1529	1831	(348)	+	AluSz	SINE/Alu	1	301	(11)	8
7742	12.8	4.3	2.6	Hg19	1832	1865	(314)	C	Tigger2	DNA/TcMar-Tigger	(1554)	1164	1142	6
1697	17.3	1.9	2.6	Hg19	1868	2179	(0)	C	AluJb	SINE/Alu	(0)	312	3	9

>KSI_AluYb8_33

>Scaffold12391-2396714-2396998
CTTTTAACATGATAAATTATATGATCAGTATTCTAAGGTTAAACCACCCTTGCATTCCTTGGAATAAACCCAACTTGATCATGGTGTCTTTTTTTCTAAACTGTTAGATTTGGTTTGCTAATATTT CT
TCTAGGATTCTTGTAGCTACACAAGTGAATAATATGGCTTGTGTTCTCCCTTTTTTTCTCTCTTTCTTGTACTATATTTATGACTTTATTATCAAGATTATTTGCCTTATAGAATGAGTTAGGA AAT
GATTCTTCTATTTCCTAGGAGAGTTTATGTAAAATTATCATGATTTCTTCCTAGAATATTTCATTGAACTCATCTTTAAGATTGTCTGGGCTTTGTAATTTCTTTATGGCAAGTTTTTAATCACTG GC
TCAATTTCATAATTGGTTATAAGGTATCTCTGCTTTTTATTTTTACTTAAATAAGTATTGGTAATTTATATGATTTAGAAAATTGTCCATTTCGTCTATATTTTTAAGTTATTATAATCTCCTTT TGT
GTTTTCAGTCTGCTGTTTCTAAAGTTATGTATACCTTTGAGTTCCCAATGCCATTTATTTTTGCCTTCTACACCTTTTACTTGGCCAGTCTGATCAGAGATTTGTCTATTTTACTAGACTTTTCAAAA
AACAACTTTTGCCTTTATTGATTCTCACTGTGGTTGACTTGTTTTATATTTTATTAATTTATACAATGATCTTTGTT GCCTATTTCCCGTGAATTAATTCTGTTGTTCATTTCCTAATGTCTTCAGTT
GAATATTCTAAACATTAATGTTCAGACTTTCTTGTTTTCTAATATATGCATTTAGGCATATAAATTTTTCTTACTTTCTATATGCCCTTTAATTTTTATATTTAGAATTTCAATATCACTCAGTCC TA
AGTACTATCAAATTTCCATTATGGTTTCTTCTTTGACATTTAGAAGTGTGGTCATATATTCCAAA ACGAGGTAATTTTTttttttttttttttttttttttgaacggagtctcgctctgtcgcccag
gccggactgcggactgcagtggcgcaatctcggctcactgcaagctccgcttcccgggttcacgccattctcctgcctcagcctcccgagtagctgggactacaggcgcccgccactgcgcccggc ta
atttttgtatttttagtagagacggggtttcaccttgttagccaggatggtctcgatctcctgacctcatgatccacccgcctcggcctcccaaagtgctgggattacaggcgtgagccaccgc gcc
cggccacgaggtaattttt AAGTTCATCTTTTCTTATTGTTTTCCGTCCTGTGCATGCAAAGCT tACGATTATATCAATTCATACACAGAATCTGGAAATCCTCTTCTCCAGT TCTCTCCTCTGTGAG
ATCCACCCCTCCCCCCGGTCGGCCCCCACTTTCAGCTCCCGGGCCTCCTTTTCTGATACTTCTTTCTCTAAAGCCAAGACTTGCTCAGCATTTGAGCCACCTGGGCTGCTGCATTGTTTTACACA ATC
GGGGCCACTCTTAGGGTAAAGCAGTCAGAGGAAAGAGGAAAAT GCAGCCACAAACTCTGGT CCCTGGAATTCCCCTTTCCCAATACTCGGTCCAAAAAGACAGGCTCTCTCTC AGTTTTGGCACCTG
TGTCACCGCTGCACTGCAGAGCAGCTGAGGACAAAGGCTGCACTCAGGGCTGGTGGGGAAGTCCTCACACTCAATTAACGGGGATTCCCACTCCCGTCTCTGACTCACAGCAGCACCTTTTCCTG GTC
CTCCGGTTAGAAAGGCAGGGTTTCTCTCTGAGATTTTCTTGCCAGCACTTGTTCTGCATTGGGGCCCCAGTTCTACACTGGGGCCACTCTCAGGTTAAAGCCAGAAAATAAAA GAGGAAGGCAGAAAA
ATTTATCCTCAGACGATTCATTCCTCAAGTTTTGACTCCCTTCTCCAATCGTCCTGCTATTGTGAACTTTCTAGAGTCTTCAGGTGGTTTCATTTTGTACATTGTCCAGAGGCCTCAACTATACT TGG
TGGGAAGGAATAGGCGTGTTATTCCCTCTTCACCAGCCCCAGAAGGCTCATCTCTTGCTTTTATTGAACTCAGGTTTAATTCAAATTATGAATTTTGTACTTTTCCAGCATTG TATTATATTTTAGCT
GAGGGTCCTTCATCTGCAGACAGACATGACGCTGGAAGCAGTAATCTCCCACTTTCTTTTCTAATGACTCACAAGCAATCTTTTTATTTAACATTAATAATACATTTC A

2181	27.9	7.3	1.3	AluYb8-Scaffold12391-2396714-2396998	1	945	(1340)	C	L1MEf	LINE/L1	(2274)	3872	2872	1
30	0.0	0.0	0.0	AluYb8-Scaffold12391-2396714-2396998	971	998	(1287)	+	(T)n	Simple_repeat	1	28	(0)	2
2742	0.0	0.0	0.0	AluYb8-Scaffold12391-2396714-2396998	1001	1285	(1000)	C	AluYb8	SINE/Alu	(33)	285	1	3
509	32.2	4.2	8.8	AluYb8-Scaffold12391-2396714-2396998	1341	1578	(707)	C	L1MEf	LINE/L1	(5778)	464	237	1
257	32.0	3.0	4.7	AluYb8-Scaffold12391-2396714-2396998	1584	1714	(571)	C	L1MEf	LINE/L1	(5854)	346	218	4
1009	30.0	3.5	2.3	AluYb8-Scaffold12391-2396714-2396998	1745	2092	(193)	C	L1MEf	LINE/L1	(5844)	398	47	1

>Hg19 chr21:39003561-39005523
CTTTTAACATGATAAATTATATGATCAGTATTCTAAGGTTAAACCACCCTTGCATTCCTTGGAATAAACCCAACTTGATCATGGTGTCTTTTTTTCTAAACTGTTAGATTTGGTTTGCTAATATTT CT
TCTAGGATTCTTGTAGCTACACAAGTGAATAATATGGCTTGTGTTCTCCCTTTTTTTCTCTCTTTCTTGTACTATATTTATGACTTTATTATCAAGATTATTTGCCTTATAGAATGAGTTAGGA AAT
GATTCTTCTATTTCCTAGGAGAGTTTATGTAAAATTATCATGATTTCTTCCTAGAATATTTCATTGAACTCATCTTTAAGATTGTCTGGGCTTTGTAATTTCTTTATGGCAAGTTTTTAATCACTG GC
TCAATTTCATAATTGGTTATAAGGTATCTCTGCTTTTTATTTTTACTTAAATAAGTATTGGTAATTTATATGATTTAGAAAATTGTCCATTTCGTCTATATTTTTAAGTTATTATAATCTCCTTT TGT
GTTTTCAGtcTCTGCTGTTTCTAAAGTTATGTATACCTTTGAGTTCCCAATGCCATTTATTTTTGCCTTCTACACCTTTTACTTGGCCAGTCTGATCAGAGATTTGTCTATTTTACTAGACTTTTCAA
AAAACAACTTTTGCCTTTATTGATTCTCACTGTGGTTGACTTGTTTTATATTTTATTAATTTATACAATGATCTTTGTT GCCTATTTCCCGTGAATTAATTCTGTTGTTCATTTCCTAATGTCTTCAG
TTGAATATTCTAAACATTAATGTTCAGACTTTCTTGTTTTCTAATATATGCATTTAGGCATATAAATTTTTCTTACTTTCTATATGCCCTTTAATTTTTATATTTAGAATTTCAATATCACTCAGT CC
TAAGTACTATCAAATTTCCATTATGGTTTCTTCTTTGACATTTAGAAGTGTGGTCATATATTCCAAA ACGAGGTAATTTTTAAGTTCATCTTTTCTTATTGTTTTCCGTCCTGTGCATGCAAAGCT cA
CGATTATATCAATTCATACACAGAATCTGGAAATCCTCTTCTCCAGTTCTCTCCTCTGTGAGATCCACCCCTCCCCCCGGTCGGCCCCCACTTTCAGCTCCCGGGCCTCCTTTTCTGATACTTCTT TC
TCTAAAGCCAAGACTTGCTCAGCATTTGAGCCACCTGGGCTGCTGCATTGTTTTACACAATCGGGGCCACTCTTAGGGTAAAGCAGTCAGAGGAAAGAGGAAAAT GCAGCCACAAACTCTGGT CCCTG
GAATTCCCCTTTCCCAATACTCGGTCCAAAAAGACAGGCTCTCTCTCAGTTTTGGCACCTGTGTCACCGCTGCACTGCAGAGCAGCTGAGGACAAAGGCTGCACTCAGGGCTGGTGGGGAAGTCC TC
ACACTCAATTAACGGGGATTCCCACTCCCGTCTCTGACTCACAGCAGCACCTTTTCCTGGTCCTCCGGTTAGAAAGGCAGGGTTTCTCTCTGAGATTTTCTTGCCAGCACTTGTTCTGCATTGGG GCC
CCAGTTCTACACTGGGGCCACTCTCAGGTTAAAGCCAGAAAATAAAAGAGGAAGGCAGAAAAATTTATCCTCAGACGATTCATTCCTCAAGTTTTGACTCCCTTCTCCAATCGTCCTGCTATTGTG AA
CTTTCTAGAGTCTTCAGGTGGTTTCATTTTGTACATTGTCCAGAGGCCTCAACTATACTTGGTGGGAAGGAATAGGCGTGTTATTCCCTCTTCACCAGCCCCAGAAGGCTCATCTCTTGCTTTTA TTG

AACTCAGGTTTAATTCAAATTATGAATTTTGTACTTTTCCAGCATTGTATTATATTTTAGCTGAGGGTCCTTCATCTGCAGACAGACATGACGCTGGAAGCAGTAATCTCCCACTTTCTTTTCTAA TG
ACTCACAAGCAATCTTTTTATTTAACATTAATAATACATTTCA

2282	27.8	7.1	1.5	Hg19	1	1102	(1061)	C	L1MEf	LINE/L1	(2161)	3985	2815	5	
510	32.2	8.6	7.8	Hg19	1101	1356	(807)	C	L1MEf	LINE/L1	(5748)	494	2 37	5	*
257	32.0	3.0	4.7	Hg19	1362	1492	(671)	C	L1MEf	LINE/L1	(5854)	346	218	6	
1009	30.0	3.5	2.3	Hg19	1523	1870	(293)	C	L1MEf	LINE/L1	(5844)	398	47	5	
185	29.1	3.7	10.3	Hg19	1994	2127	(36)	C	L3	LINE/CR1	(1257)	2842	2717	7	

>KSI_AluYb8_34

>Scaffold13646-191471-191790
CACTGTGCCTGGCTGAAAATATTTTTAAAACTATAAAGCAACTATACAATTACACAGCATAAATGATTTTCATTTTTCTTTTTTTTTGAGAAGGAGTTTCACTCTTATTGCCCAGGCTGGAGTGCA GT
GGCATGATCTCGGCTCACCACCACCTCCaCCTCCCGGGTTCAAGTGATTCTCCTGCCTCAGCCTCCCGAGTACCTGGGATTACAGGCATGTGCCACCACGCCTGGCTAATTTTTGTTTGTTTGTTTGT
TTTGTTTTGTTTTGTTTAGTAGAGACGGGGTTTCTCCATGTTGGTCAGGCTGGTCTCCAACTCCCAACCTTAGGTGATCTGCCCGCCTCGGCCTCCCAAAGTGCTGGGATTACAGGTGTAAGCCAC CG
TGCCCAGCCCAGCATAAGTAATTTTCCTTGCACAAATCTTTCTAGTATTTTTTCTGGCTTCTTAGGGTGCAGTGAACATAATTTGATCTTTTTTGAGGACTTGGTATCATTATTATGAGCATTTA TCA
TAATACTTTCTATTGCTATTGCATTATCACTCTATTTCCAATGCCAGTGAGGTGTGGAGCTTTGTTTGCCCCTGTCCTAAATGGCTTCAGACTGCTCTGCTTGTATGTATTTTTATATAATTTTTC TG
TCCCTTCCTATGCAGTTCACTTGTCATTCTGGGTAGGTCTAAATTCCAGCAATCCTCTCCCTCTAATGCTTTACTTCTTGTGGTCTA gGGACTGAAGAACAAGATACTCCAACAGTACCAGTGAGAGC
CCCTTCAATTGCTCTATGAGTTGGGGCATATTCACAAGTGCATGGATGGATTCTCAATTAGAGATTGCAGGCTGAACACATGAATTAACCTTAGTTCCTCTCCAAAACCTGCACTAAAATGA gAGTGG
GGAAAAAAATTAAGCATATATTCACAAGGTTGAAGAAAGCAAGAAAGGAGAAAATAGCAAAAAAAAACTTGGAAGATGGAAGGTACAT Aaacaagtgctaataatttttttttttttttttttttt
tttttttgagacggagtctcgctctgtcgcccaggccggactgcggactgcagtggcgcaatctcggctcactgcaagctccgcttcccgggttcacgccattctcctgcctcagcctcccgagt agc
tgggactacaggcgccggccaccgcgcccggctaattttttgtatttttagtagagacggggtttcaccttgttagccaggatggtctcgatctcctgacctc atgatccacccgcctcggcctccc
aaagtgctgggattacaggcgtgagccaccgcgcccggccAACAAGTGCTAATAATTTAGAAGACAAAAAAACAAACAAACAAACAAAAAAAACAAACACTGAATCTGAAACCAGCAGTGACGAAAGA
AACCTCGGATACCTGTGAAAGTGAGGCTGAAGGTAAGTCCAAAGCAGGAGAAATGGAGGAATGGCTGGAAAG CCTTTTTAACAAAAACCTAGAGCCACAGACATTCCTCCCACTCCCTCTTCAGCAGA
GCAGTGAAGATTTGTTTTCAGGGAAAGGTAAAACAGAGTTTCTGGCCTAGTTAAGGATGGGGTTATCATACTAAAAATGAAGACTTTTTTTAAATTTTGCATATTGAACACTGAG cCTTCCAAGCCTT
TCTCCCCTATTAGTCTCCCAAACATCAGCAACTTATATCCACAGTCAAGAAGAGGGAAAAGCCTTCTCTAGGGCTTTATCCAGCCTCACTGGAAAGACCTAATG ATATTTAAAGGGATGTACCAGGTC
ACGATCTAGTAAAACTCATGGCAACATAGCTTCCTCCCTCCCTGC tCTTCCCACATACAAAGAGCTTATAACATCCTTTTAGTACCCTACTCTTAAATATAAGTAGACAGCCAAGAGTTACTACCTTT
GAGAAATACTATTTTATGACAGAGAAAGAGCATCTCAGAAGAAAAAGAATAAGAGGGAGAAGAAAACTTTATCTAAGATTATTAGCCTTAGAGATATAAAAGAT GTTATGCCCACAAAACAACAACAG
GATTCTATAGAAAAGGAATAGGCAAAGAATGAAAAAGATGCTCTGGGGCCAGGCGCGGTGGCTCACACCTGTAATCCCAGCACTTTGGGAGGCTGAGGCGGGTGGATCACAGGGTCAGGAGATCA AGA
CCATCCTGGCTAACACGGTGAAACCCCGTCTCTACTAAAAACACAAAAAATTAGCCAGGCGTGGTGGCGGGCGCCTGTAGTCCCAGCTACCTGGGAGGCTGAGG CAGGAGAATGGCATGAACCCGGGA
GGTGGAGCTTGCAGTG

2259	8.3	0.0	7.6	AluYb8-Scaffold13646-191471-191790	67	393	(1927)	C	AluSp	SINE/Alu	(9)	304	1	1
626	26.9	14.9	3.0	AluYb8-Scaffold13646-191471-191790	418	746	(1574)	C	Tigger15a	DNA/TcMar-Tigger	(125)	590	224	2
610	27.3	15.7	6.7	AluYb8-Scaffold13646-191471-191790	817	1000	(1320)	+	HAL1	LINE/L1	1	185	(2322)	3
2698	0.7	0.0	0.3	AluYb8-Scaffold13646-191471-191790	1001	1320	(1000)	C	AluYb8	SINE/Alu	(0)	318	1	4
1115	27.2	15.9	4.7	AluYb8-Scaffold13646-191471-191790	1321	2092	(228)	+	HAL1	LINE/L1	186	1065	(1442)	3
1960	6.2	0.0	0.0	AluYb8-Scaffold13646-191471-191790	2095	2320	(0)	+	AluYk3	SINE/Alu	1	226	(85)	5

>Hg19 chr22:17704306-17706290
CACTGTGCCTGGCTGAAAATATTTTTAAAACTATAAAGCAACTATACAATTACACAGCATAAATGATTTTCATTTTTCTTTTTTTTTGAGAAGGAGTTTCACTCTTATTGCCCAGGCTGGAGTGCA GT
GGCATGATCTCGGCTCACCACCACCTCCgCCTCCCGGGTTCAAGTGATTCTCCTGCCTCAGCCTCCCGAGTACCTGGGATTACAGGCATGTGCCACCACGCCTGGCTAATTTTTGTTTGTTTGTTTGT
TTTGTTTTGTTTTGTTTAGTAGAGACGGGGTTTCTCCATGTTGGTCAGGCTGGTCTCCAACTCCCAACCTTAGGTGATCTGCCCGCCTCGGCCTCCCAAAGTGCTGGGATTACAGGTGTAAGCCAC CG
TGCCCAGCCCAGCATAAGTAATTTTCCTTGCACAAATCTTTCTAGTATTTTTTCTGGCTTCTTAGGGTGCAGTGAACATAATTTGATCTTTTTTGAGGACTTGGTATCATTATTATGAGCATTTA TCA
TAATACTTTCTATTGCTATTGCATTATCACTCTATTTCCAATGCCAGTGAGGTGTGGAGCTTTGTTTGCCCCTGTCCTAAATGGCTTCAGACTGCTCTGCTTGTATGTATTTTTATATAATTTTTC TG
TCCCTTCCTATGCAGTTCACTTGTCATTCTGGGTAGGTCTAAATTCCAGCAATCCTCTCCCTCTAATGCTTTACTTCTTGTGGTCTA cGGACTGAAGAACAAGATACTCCAACAGTACCAGTGAGAGC
CCCTTCAATTGCTCTATGAGTTGGGGCATATTCACAAGTGCATGGATGGATTCTCAATTAGAGATTGCAGGCTGAACACATGAATTAACCTTAGTTCCTCTCCAAAACCTGCACTAAAATGA cAGTGG
GGAAAAAAATTAAGCATATATTCACAAGGTTGAAGAAAGCAAGAAAGGAGAAAATAGCAAAAAAAAACTTGGAAGATGGAAGGTACAT AAACAAGTGCTAATAATTTAGAAGACAAAAAAACAAACAA
ACAAACAAAAAAAACAAACACTGAATCTGAAACCAGCAGTGACGAAAGAAACCTCGGATACCTGTGAAAGTGAGGCTGAAGGTAAGTCCAAAGCAGGAGAAATGGAGGAATGGCTGGAAAGCCTTT TT
AACAAAAACCTAGAGCCACAGACATTCCTCCCACTCCCTCTTCAGCAGAGCAGTGAAGATTTGTTTTCAGGGAAAGGTAAAACAGAGTTTCTGGCCTAGTTAAGGATGGGGTTATCATACTAAAAATG
AAGACTTTTTTTAAATTTTGCATATTGAACACTGAGtCTTCCAAGCCTTTCTCCCCTATTAGTCTCCCAAACATCAGCAACTTATATCCACAGTCAAGAAGAGGGAAAAGCCTTCTCTAGGGCTTTAT
CCAGCCTCACTGGAAAGACCTAATGATATTTAAAGGGATGTACCAGGTCACGATCTAGTAAAACTCATGGCAACATAGCTTCCTCCCTCCCTGC cCTTCCCACATACAAAGAGCTTATAACATCCTTT
TAGTACCCTACTCTTAAATATAAGTAGACAGCCAAGAGTTACTACCTTTGAGAAATACTATTTTATGACAGAGAAAGAGCATCTCAGAAGAAAAAGAATAAGAGGGAGAAGAAAACTTTATCTAA GAT
TATTAGCCTTAGAGATATAAAAGATGTTATGCCCACAAAACAACAACAGGATTCTATAGAAAAGGAATAGGCAAAGAATGAAAAAGATGCTCTGG GGCCAGGCGCGGTGGCTCACACCTGTAATCCCA
GCACTTTGGGAGGCTGAGGCGGGTGGATCACAGGGTCAGGAGATCAAGACCATCCTGGCTAACACGGTGAAACCCCGTCTCTACTAAAAACACAAAAAATTAGCCAGGCGTGGTGGCGGGCGCCT GTA

GTCCCAGCTACCTGGGAGGCTGAGGCAGGAGAATGGCATGAACCCGGGAGGTGGAGCTTGCAGT G

915	8.0	0.9	0.0	Hg19	1	113	(2072)	C	AluSg4	SINE/Alu	(195)	115	2	6
2274	8.0	0.0	7.6	Hg19	167	493	(1692)	C	AluSp	SINE/Alu	(9)	304	1	7
645	27.4	14.3	2.5	Hg19	518	846	(1339)	C	Tigger15a	DNA/TcMar-Tigger	(125)	590	224	8
1120	27.7	13.9	4.2	Hg19	917	1857	(328)	+	HAL1	LINE/L1	1	1065	(1442)	9
2541	7.0	0.0	0.0	Hg19	1860	2159	(26)	+	AluY	SINE/Alu	1	300	(11)	10

>KSI_AluYb8_35

>Scaffold14277-2749392-2749507

TGATGTCCTGACTGGAGCCTTACACATAAGGGTACTGTAGACAGCTGTCAAAATGAGATCATATGCCTTCCTTTA GGTTGAATATGACCATTTTTTAGCTTTGGCTTTTAATAGAGAAAGTTCTATCC
TGAATTATAGGTTTTGCTTCAAAATAAAAGCGATATATAAGGAAAGAACTTGATTGATTAGATAATTTAATCCTCTCAGACTTTTCCAAATTTGTCCCAAAATTTGTGTTATTGACTATTTTTTT GCT
TTAATAATAGATTGGACCAAATATTTACTAAAATATTTAAGTTCCAGCATTTGTGTTTCATTCATGTAGAAGATA CAGGCATGCAGTGTTACTACAAGAGTGTATAAACAATGAGGAAAATATTGATC
ATCACAATACTAGTGGGCTAAAAAATTGTTTTAAAAACTTCTTTACTTTGTACTAGAATTGAAATTAATATTTCTTTGTTCACATATTCATTACAATTTTTTCTTTATGTATTCCCTGCCTTTTT GTT
TTAAGGGAAATTTGAATTTCTATGGTAGCTTCTCTTCCAAATTGCCCAGAATCAACTCAACTCAGTGTTTTAAGAAACAATTGATTTCTATTATATAGATCTTTTGGTTAATCATTTCTCTGAAGCCT
TTGTTTTGAAAGGAGAACTGCAATGATATTCCCTCCATTTAAAGAAAACATACTCTTTGTTGTAATTGCAATGAAGTATACTATCTATAAAATAGTTTATATTTTATAAAACTTTTACTTATAAA CTA
TTTGGTTTTCTGTTTCTGAGTAATTCACTTAGGATAATGGCCTCCAGCCACATCTATGTTGCTGCAAAACTACAG CTATGTGTCAGGCTCAAGTTATCACATGTTAACAGAATGGTTGTAAATTTGAA
CAATTTATAATAAtGCTAATATTTATTATTTTGTAATTAAAACTGATGATAATgTGGTATGTTTTCTAAGAAAAAGTGCTTGAGTTCATCTTGTAGGACCTTT atttatttttatttatttttttt
tttgagacggagtctcgctctgtcgcccaggccggactgcggactgcagtggcgcaatctcggctcactgcaagctccgcttcccggg ttcaggataatggcctccagccacatctatgttgctgcaa
aactacagctatgtgtcaggctcaagttatcacatgttaacagaatggttgtaaatttgaacaatttataataa cgctaatatttattattttgtaattaaaactgatgataatatggtatgttttct
aagaaaaagtgcttgagttcatcttgtaggaccttt GAGTCCTCTTAAGTTTTTACTATTGTAAGGTGTCTCCTACATTTCAAATGCAAGGCTTTGTTCTATTACCCATCAATCGATCATCCTCATTA
TTGGCATTCTCACAGAATTAGAGCTGAACTGTGATAACCCAGTGTGTTTTGTGTTGCACTCTGAAAA GCACCAAGGAAGGAGGAAGCATAAAGTAGATCAAAGTAACTTCGATAGACGCACCCAGCAG
GGCAAATGGGGACAAGACAGAACCTCATTTTACCAGTAAGTCCGTACAACTTCAATGATTTATTGAAAATGACACGTCTTGGCCTGGCGCGATGGCTCAAGCCTGTAATCCCAGTACTTTGGGAG GCT
GAGGCAGGCAAATCACGATGTGAGGAGTTTAAGACCAGCCTGGCCAACATGATGAAACCCTGTCTCTACTAAAAATACAAAAAATTAGCTCACCATGGTGATG GGTGTCTGTAATTCCAGCTACTCAG
AAGGCTGAGGCAGGAGAATTGCTTGAACCCAGGAGGTGGAGGTTGCAGTGAGCCTAGATCGTGCCACTGCACTCCAGCCTGGGCAACTGCGAGACTCTGTCT CaaAAAAAAAAAAAAAAAAATAGAAAA
AGAAAATTACACATCCTACACATAAGTTCTTATGAGAGAGTGTGTCCAAACAGGTTTTTAACAGTGGGTTAAAGAGAAAAAGAAATAATTTTATGGAGCCCAT GGGAGAACTAAGTGGAAAGGAGTAC
AAAATCATTAAATATCATTTAATCACATATATACTTAAACCTGAAAACAGTGTCTCAGCTCAGAATG A

532	9.1	1.4	0.0	AluYb8-Scaffold14277-2749392-2749507	759	836	(1280)	C	L1PA17	LINE/L1	(257)	5914	5836	1
1022	2.6	0.0	0.0	AluYb8-Scaffold14277-2749392-2749507	1001	1116	(1000)	C	AluYb8	SINE/Alu	(3)	315	200	2
304	8.1	0.0	0.0	AluYb8-Scaffold14277-2749392-2749507	1117	1153	(963)	C	L1PA17	LINE/L1	(294)	5872	5836	3
2221	12.6	0.7	0.0	AluYb8-Scaffold14277-2749392-2749507	1617	1925	(191)	+	AluSx3	SINE/Alu	1	311	(0)	4

>Hg19 chr4:103132542-103134338

TGATGTCCTGACTGGAGCCTTACACATAAGGGTACTGTAGACAGCTGTCAAAATGAGATCATATGCCTTCCTTTAGGTTGAATATGACCATTTTTTAGCTTTGGCTTTTAATAGAGAAAGTTCTAT CC
TGAATTATAGGTTTTGCTTCAAAATAAAAGCGATATATAAGGAAAGAACTTGATTGATTAGATAATTTAATCCTCTCAGACTTTTCCAAATTTGTCCCAAAATTTGTGTTATTGACTATTTTTTT GCT
TTAATAATAGATTGGACCAAATATTTACTAAAATATTTAAGTTCCAGCATTTGTGTTTCATTCATGTAGAAGATACAGGCATGCAGTGTTACTACAAGAGTGTATAAACAATGAGGAAAATATTGA TC
ATCACAATACTAGTGGGCTAAAAAATTGTTTTAAAAACTTCTTTACTTTGTACTAGAATTGAAATTAATATTTCTTTGTTCACATATTCATTACAATTTTTTCTTTATGTATTCCCTGCCTTTTT GTT
TTAAGGGAAATTTGAATTTCTATGGTAGCTTCTCTTCCAAATTGCCCAGAATCAACTCAACTCAGTGTTTTAAGAAACAATTGATTTCTATTATATAGATCTTTTGGTTAATCATTTCTCTGAAGCCT
TTGTTTTGAAAGGAGAACTGCAATGATATTCCCTCCATTTAAAGAAAACATACTCTTTGTTGTAATTGCAATGAAGTATACTATCTATAAAATAGTTTATATTTTATAAAACTTTTACTTATAAA CTA
TTTGGTTTTCTGTTTCTGAGTAATTCACTTAGGATAATGGCCTCCAGCCACATCTATGTTGCTGCAAAACTACAGCTATGTGTCAGGCTCAAGTTATCACATGTTAACAGAATGGTTGTAAATTTGAA
CAATTTATAATAAcGCTAATATTTATTATTTTGTAATTAAAACTGATGATAATaTGGTATGTTTTCTAAGAAAAAGTGCTTGAGTTCATCTTGTAGGACCTTT GAGTCCTCTTAAGTTTTTACTATTG
TAAGGTGTCTCCTACATTTCAAATGCAAGGCTTTGTTCTATTACCCATCAATCGATCATCCTCATTATTGGCATTCTCACAGAATTAGAGCTGAACTGTGATAACCCAGTGTGTTTTGTGTTGCAC TC
TGAAAAGCACCAAGGAAGGAGGAAGCATAAAGTAGATCAAAGTAACTTCGATAGACGCACCCAGCAGGGCAAATGGGGACAAGACAGAACCTCATTTTACCAGTAAGTCCGTACAACTTCAATGATTT
ATTGAAAATGACACGTCTTGGCCTGGCGCGATGGCTCAAGCCTGTAATCCCAGTACTTTGGGAGGCTGAGGCAGGCAAATCACGATGTGAGGAGTTTAAGACCAGCCTGGCCAACATGATGAAACC CT
GTCTCTACTAAAAATACAAAAAATTAGCTCACCATGGTGATGGGTGTCTGTAATTCCAGCTACTCAGAAGGCTGAGGCAGGAGAATTGCTTGAACCCAGGAGGTGGAGGTTGCAGTGAGCCTAGA TCG
TGCCACTGCACTCCAGCCTGGGCAACTGCGAGACTCTGTCT CAAAAAAAAAAAAAAAAATAGAAAAAGAAAATTACACATCCTACACATAAGTTCTTATGAGAGAGTGTGTCCAAACAGGTTTTTAACA
GTGGGTTAAAGAGAAAAAGAAATAATTTTATGGAGCCCATGGGAGAACTAAGTGGAAAGGAGTACAAAATCATTAAATATCATTTAATCACATATATACTTAAACCTGAAAACAGTGTCTCAGCT CAG
AATGA

532	9.1	1.4	0.0	Hg19	859	936	(1061)	C	L1PA17	LINE/L1	(257)	5914	5836	5
2212	12.7	0.7	0.0	Hg19	1400	1707	(290)	+	AluSx3	SINE/Alu	1	310	(1)	6

>KSI_AluYb8_36

>Scaffold15242-961087-961414

GACAAATTTTATTTTTCTTTGAGCATTTCAACGGTTCACTCCATTATCTTCTGACTTGCATTGTTGCTAACAAGACATCT GCTGTCATTCTTCTCTTTTTCCTCTGTGTGCCATGTGTCTTCTTCCCT
CTAGCTGCTCTCAAGAATTTATTTTTATTAGTGGTTTTGAGCAATTTGATTATGATAAGAAACATTTTTG AGTGGAGAATCAAGGGAAGGTTGAAACAGATAAGACGTCAATGCTGGGAAAAAAGGTT
AATTGTGAGACTGGTTGTTCCTTATTTGAGCCTGATAATAACAATCTCTTCAGGTACAGGACAAATGTCATGACAGAATG CACTTTAAAAGAAAAATAATAGCTAACACTTGAAGATTGCTATATATA
CCTATGTGTGTGGGTGTGTATGTATACATACACATATACATATATATGCACATACACACCCACACACACATACATAAAGATAGTGTTCATACATATAAGATCTAACTTCTAAGATATATAAGATC CTA
TTCTAACATATTTAATATaTTGCATATATTAGAATATATATTTGAACAGTATCTCATACAAAGTATGTTTACACAAAGTA GGTGCTCTTTAAATGTTAACTATTACATAAATGTACACACACACATTT
TACACTAAAAACAATTCTATGATGTAGGTAGTATGATAATCTTCATTTTACACATTTTTTTAAGATGGAGTTTCACTCTTGTCACCCAGGCTGGAGTGCAACGGTGCAATCTCAGCTCATTGCA GCC
TCCGCCTCCTGGGTTCAAGTGATTCTCCTGCCTCAGCCTCCAGAGTAGCTGGGATTACAGGCACCTGGCACCATGCCTGG CTAATTTTTGCATTTTTAGTAGAGACAGGGTTTTGCCATGTTGGCCAG
GCTGGTCTCGAAGTCCTAACCTCATGTGATCTACCTAACTCAGCCTCCCAAAGTGCTGGGATTATAGGTGTGAGCCACCGTGCCGGGCC Tctacatattacttctttttttttttttttttttttt
ttttttttttttttttgagacggagtctcgctctgtcgcccaggtcggactgcggactgcagtggcgcaatctcggctcactgcaagctccgcttcccgggttcacgccattctcctgcctcagc ctc
ccgagtagctgggactacaggcgcccgccaccgcgcccggctaatttttgtatttttagtagagacggggtttcaccttgttagccaggatggtctcgatctcc tgacctcatgatccacccgcctc
ggcctcccaaagtgctgggattacaggcgtgagccaccgcgcccggcCTACATATTACTTCTAATCCTTAACATAACAGCCCATGATTCCACTCTTTCAATCTCGTT TCTTTTAACTTTGAACCATTC
TTTGCCAATTACTTTACCTACCTGTGTCTCAGTTTCTTCATTTGCATCATGAAGCTAATAATAAAACCAAAAGGCTGTTTTGAGTATTAAATGAGATAATTCATT TGAAAAGTGCTCAGCATAGCGCC
AGACGCGGGAGAGCgCTCAGTAAGAGTGAGGAGGTGGTACTGTTATCGCTGCTGTTTTATGCTTTCTTCTGCCTGCGAGTTCCCA CTTGGTTCATTCAAAGCACCCACACTGTCCTACAAAGCCAAGT
TAGCATGCAGCCTACTGCCCTGATCATGCAGTCACAATCTCTGTTTCTGCTAACAGTCAGTAAACACAATTTAGCATCTCGATGGGTTTCATTGCTCCACATAGG CTCATTTAGACTTTTCAACTAGA
TTAACTCATCACTCATTCAATTCGTGTGCTGTCTGAGGACACACCAGGTGCCAAGGACTGTTCTGGTTTACTAGGGTTAGAAAGGTAAATAAGAAAGATAAAAATTAAATCCCTGCCTCATGGAT CTT
ATGTATGGGGGTGGGGGAGACAAACAAAAATAATATAAATAAGTAAAATATGTGGTATGATAGATGAAAACAAGGGACATAGATACAAATAAAGCACAGAAGGGA AATAGGAAGTGTTGGGGATACCA
TATATTTTGGATACCGAGGTCAAATTTCACTGTGTGGGCAGGATTATCTCACCCATTTCAGAAGAGAGGAAACTGATGCTCAGAGAAGGGTGGTGAATTTTTTCATTTCATGCAGCATTGGTTTA GAG
ACAAGATAGAAATCAAGATTCTCCAACTTACAGTCCAAGACATATTTCTGTAGCGGGCAGAAGCTGAACTCTATTTGTGCTCCTAGGTGGAAAACAATGGTCCTG TAGTCCTCATCACCATTTCCCAT
GTCTGAAACTGTCTTTGTCTACCT

842	19.3	1.7	0.0	AluYb8-Scaffold15242-961087-961414	4	184	(2144)	C	L1MEc	LINE/L1	(4795)	1605	1422	1
26	18.0	0.0	0.0	AluYb8-Scaffold15242-961087-961414	407	458	(1870)	+	(TACACA)n	Simple_repeat	1	52	(0)	2
23	26.9	6.1	3.3	AluYb8-Scaffold15242-961087-961414	459	553	(1775)	+	(TATACA)n	Simple_repeat	1	151	(0)	3
209	23.3	0.0	0.0	AluYb8-Scaffold15242-961087-961414	633	692	(1636)	C	MIR	SINE/MIR	(69)	193	134	4
2196	12.0	0.0	0.0	AluYb8-Scaffold15242-961087-961414	695	985	(1343)	C	AluSq2	SINE/Alu	(22)	291	1	5
2739	0.7	0.0	0.0	AluYb8-Scaffold15242-961087-961414	1001	1328	(1000)	C	AluYb8	SINE/Alu	(0)	318	1	6
493	25.3	4.7	1.3	AluYb8-Scaffold15242-961087-961414	1412	1559	(769)	+	MIR	SINE/MIR	95	247	(15)	7
629	26.8	1.2	4.3	AluYb8-Scaffold15242-961087-961414	1801	2042	(286)	C	L2a	LINE/L2	(2)	3424	3190	8
246	32.8	5.4	1.5	AluYb8-Scaffold15242-961087-961414	2084	2213	(115)	C	MIRc	SINE/MIR	(103)	165	31	9

>Hg19 chr16:83132244-83134230

GACAAATTTTATTTTTCTTTGAGCATTTCAACGGTTCACTCCATTATCTTCTGACTTGCATTGTTGCTAACAAGACATCTGCTGTCATTCTTCTC TTTTTCCTCTGTGTGCCATGTGTCTTCTTCCCT
CTAGCTGCTCTCAAGAATTTATTTTTATTAGTGGTTTTGAGCAATTTGATTATGATAAGAAACATTTTTG AGTGGAGAATCAAGGGAAGGTTGAAACAGATAAGACGTCAATGCTGGGAAAAAAGGTT
AATTGTGAGACTGGTTGTTCCTTATTTGAGCCTGATAATAACAATCTCTTCAGGTACAGGACAAATGTCATGACAGAATGCACTTTAAAAGAAAA ATAATAGCTAACACTTGAAGATTGCTATATATA
CCTATGTGTGTGGGTGTGTATGTATACATACACATATACATATATATGCACATACACACCCACACACACATACATAAAGATAGTGTTCATACATATAAGATCTAACTTCTAAGATATATAAGATC CTA
TTCTAACATATTTAATATgTTGCATATATTAGAATATATATTTGAACAGTATCTCATACAAAGTATGTTTACACAAAGTAGGTGCTCTTTAAATG TTAACTATTACATAAATGTACACACACACATTT
TACACTAAAAACAATTCTATGATGTAGGTAGTATGATAATCTTCATTTTACACATTTTTTTAAGATGGAGTTTCACTCTTGTCACCCAGGCTGGAGTGCAACGGTGCAATCTCAGCTCATTGCA GCC
TCCGCCTCCTGGGTTCAAGTGATTCTCCTGCCTCAGCCTCCAGAGTAGCTGGGATTACAGGCACCTGGCACCATGCCTGGCTAATTTTTGCATTT TTAGTAGAGACAGGGTTTTGCCATGTTGGCCAG
GCTGGTCTCGAAGTCCTAACCTCATGTGATCTACCTAACTCAGCCTCCCAAAGTGCTGGGATTATAGGTGTGAGCCACCGTGCCGGGCC TCTACATATTACTTCTAATCCTTAACATAACAGCCCATG
ATTCCACTCTTTCAATCTCGTTTCTTTTAACTTTGAACCATTCTTTGCCAATTACTTTACCTACCTGTGTCTCAGTTTCTTCATTTGCATCATGA AGCTAATAATAAAACCAAAAGGCTGTTTTGAGT
ATTAAATGAGATAATTCATTTGAAAAGTGCTCAGCATAGCGCCAGACGCGGGAGAGC aCTCAGTAAGAGTGAGGAGGTGGTACTGTTATCGCTGCTGTTTTATGCTTTCTTCTGCCTGCGAGTTCCCA
CTTGGTTCATTCAAAGCACCCACACTGTCCTACAAAGCCAAGTTAGCATGCAGCCTACTGCCCTGATCATGCAGTCACAATCTCTGTTTCTGCTA ACAGTCAGTAAACACAATTTAGCATCTCGATGG
GTTTCATTGCTCCACATAGGCTCATTTAGACTTTTCAACTAGATTAACTCATCACTCATTCAATTCGTGTGCTGTCTGAGGACACACCAGGTGCCAAGGACTGTTCTGGTTTACTAGGGTTAGAA AGG

TAAATAAGAAAGATAAAAATTAAATCCCTGCCTCATGGATCTTATGTATGGGGGTGGGGGAGACAAACAAAAATAATATAAATAAGTAAAATATG TGGTATGATAGATGAAAACAAGGGACATAGATA
CAAATAAAGCACAGAAGGGAAATAGGAAGTGTTGGGGATACCATATATTTTGGATACCGAGGTCAAATTTCACTGTGTGGGCAGGATTATCTCACCCATTTCAGAAGAGAGGAAACTGATGCTCA GAG
AAGGGTGGTGAATTTTTTCATTTCATGCAGCATTGGTTTAGAGACAAGATAGAAATCAAGATTCTCCAACTTACAGTCCAAGACATATTTCTGTA GCGGGCAGAAGCTGAACTCTATTTGTGCTCCTA
GGTGGAAAACAATGGTCCTGTAGTCCTCATCACCATTTCCCATGTCTGAAACTGTCTTTGTCTACC T

941	25.8	0.7	3.2	Hg19	1	284	(1903)	C	L1MEf	LINE/L1	(4663)	1537	1261	10
26	18.0	0.0	0.0	Hg19	507	558	(1629)	+	(TACACA)n	Simple_repeat	1	52	(0)	11
23	24.8	2.7	1.3	Hg19	559	581	(1606)	+	(TATACA)n	Simple_repeat	1	76	(0)	12
209	23.3	0.0	0.0	Hg19	733	792	(1395)	C	MIR	SINE/MIR	(69)	193	134	13
2196	12.0	0.0	0.0	Hg19	795	1085	(1102)	C	AluSq2	SINE/Alu	(22)	291	1	14
481	26.0	4.7	1.3	Hg19	1171	1318	(869)	+	MIR	SINE/MIR	95	247	(15)	15
629	26.8	1.2	4.3	Hg19	1560	1801	(386)	C	L2a	LINE/L2	(2)	3424	3190	16
246	32.8	5.4	1.5	Hg19	1843	1972	(215)	C	MIRc	SINE/MIR	(103)	165	31	17

>KSI_AluYb8_37

>Scaffold15563-4652654-4652952

TGCAGTAATGTTGCAAGTATTTTTTCCTAGTTTGTCAGTTGTCTTTTTTAAATTAAATTAA ATTTTTTTTTCTGGGAAGAGAAAGTCTCCAGTGTGGACAGAAGGTGCTCTTTCTTCCCCTAGTTTAT
TGTTTGTCTTTTGATGTTACTAATGGTATTTCTTGCAATAAAAAGGTATCTATCTATCTATCT gTCTgTCTgTCTGTCTGTCTGTCTATCTATTTATTTAGAGACGGAGCCTTGCTCTGTCACCCAGG
CTGGAGTGCAGTGACACAATCTCAGCTCACTGCAACCTCCACCTCCCAGGCTCAAGCAATTCTCCTGCCTCAGCCTCTGAGTATTTGGGATTACAGGTGCCCACCACCATGCCCAGCTAATTTTT GTA
TTTTTAGTAGAGAAGGGGTTTTACCATGTTGGACCAGGCTGGTCTCAAACTCCTGACCTCAAGTGATCTGGC cGCCTTTGCTGAGACCAGCTCAATCGATACCcTAACCCAGCAGcGCTAGAGGAATT
AAAGACACACACACACAGAAATCCAGAGTGCAGAGTGGGAATCAGGGGGCTGACAGCCTTCAGAGCTGAGAGCCACGAACAGAGTTTTACCCACATATTTATTGACAGCAAGCCAGTGATAAGCAGTGTT
TCTATAGATTATAGATTAACTAAAATGGGAAACAAAAGGATGGGGTCTGGCTAGTTATCTGCAGCAGGAATG TGTCCTTAAGGCACAGATCACTCATGCTATTGTTGTGGTCTGCCCTGGGTGGGCCA
GGTGTTTCCTTGCCCTCATTCCAGTAAACCAACAACCTTCAGTGTGGGCATCATAGTCATTGTGAGCATGTCACAGTGCTGCAGAGATTCTGCTTATGGCCAGTTTTGGGGCCTGTTTATGGCCA GAT
TTGGGGGCCTGTCCCTAACATGCCTTGgCCTCCCAAAGTGCTGGGATTATAGGCATGAGCCACTGTGCCTGG CAAAAAATGTTTATTTTTAAAACTGTACTCACggccgggcgcggtggctcacgcct
gtaatcccagcactttgggaggccgaggtgggtggatcatgaggtcaggagatcgagaccatcctggctaacaaggtgaaaccccgtctctactaaaaatacaaaaaattagccgggcgcggtgg cgg
gtgcctgtagtcccagctactcgggaggctgaggcaggagaatggcgtgaacccgggaagcggagcttgcag tgagccgagattgcgccactgcagtccgcagtccggcctgggcgacagagcgagac
tccgtctcaaaaaaaaaaactgtactcacATTTCTCAATTTAAAAAATTGCCACCGGATTTTGAGTCATAGGAAGCCTCTCTTAACAATGAGGTTTAAAGAGGAATTCCCTCATGCTTTCTTCTAGTA
AATTCCATTTCTTTTGTCTAATTACATTTTAAAATATTT cTATTTTATAATTTGTGAATTTTATTCTTGTATTTGATGTGAAGTCTGCATCTAATTTTGTTTTTTTCCATAT GATTCATCAATTGACC
CAATGGGAGACAGGACTAGCTGGATTTCCTAGGCTGACTAAGAATTCCTAAGCCTAGCTGGGGAAGGTGACTGCAACCCACCTTTAAACACAGGGCTTGTAACTCAGCTCACACCTGACCAATCA GGT
AGTAAAGAGGGCTCACTAAAATACAAATTAGGCTAAAAGC AGGAGGTAAAGAAGTAGTCAAATCATATATTGCCTGAGAGCACACAGGGAGGGGCAATGATTGGGATATAAACCCCAGGACTTTGAGC
TGGGAGTGGGCAACCCCCTTTAGGTCCCCTCCCATTGTATGGGAGCTCTGTTTTCACTCTATTAAATCTTGCAACTGCACACTCTTCTGGTCC aTGTTTGTTCTGGCTTGAGCTGAACTGCTCTCTGT
CCACCACTGCTGATCGCCACCTTCACAGACCCACCATTGACTTTCACCCCTCCGGATCCGGCAGGGTGTCTGCTGCgCTTCTGATCCAGCAAGGTGCCCATTGCCCCTCCCGTTTGGGCTAGAAGCTC
GTCATTGTTCCTGTGCAGCTAAGTGCCCGGGTTTGTCCTAATCAAGCTGAACACTAGCCGCTGGGTTCCACGGTTCTCTTCCATGACCCATGGCTTCTAATAGAGCTATAACACTCACTGCATGG CCC
AAGGTTCCATTCCTTGGAATCTGTGAGGCCAAGAACCCCA GGTCAGAGAACAAAAGGCTTGCTGCCATCTTGGGAGTGGCTGCCACTATCTTGGGAGCAGCCCATCACCATCTTGGGAGCTC T

255	21.7	0.0	0.0	AluYb8-Scaffold15563-4652654-4652952	72	117	(2182)	C	LTR51	LTR/ERV1	(49)	622	577	1
25	15.1	0.0	0.0	AluYb8-Scaffold15563-4652654-4652952	175	220	(2079)	+	(TATC)n	Simple_repeat	1	46	(0)	2
1791	10.8	0.4	0.4	AluYb8-Scaffold15563-4652654-4652952	221	461	(1838)	C	AluSx1	SINE/Alu	(22)	290	50	3
3098	8.0	13.0	0.2	AluYb8-Scaffold15563-4652654-4652952	463	916	(1383)	C	MER9a3	LTR/ERVK	(0)	512	1	4
396	14.8	0.8	1.1	AluYb8-Scaffold15563-4652654-4652952	917	987	(1312)	C	FLAM_C	SINE/Alu	(80)	53	2	5
2823	1.0	0.0	0.0	AluYb8-Scaffold15563-4652654-4652952	1001	1299	(1000)	+	AluYb8	SINE/Alu	1	299	(19)	6
5309	11.7	0.5	1.1	AluYb8-Scaffold15563-4652654-4652952	1540	2299	(0)	+	LTR17	LTR/ERV1	1	756	(24)	7

>Hg19 chr3:180483104-180485096

TGCAGTAATGTTGCAAGTATTTTTTCCTAGTTTGTCAGTTGTCTTTTTTAAATTAAATTAA AtTTTTTTTTTCTGGGAAGAGAAAGTCTCCAGTGTGGACAGAAGGTGCTCTTTCTTCCCCTAGTTTA
TTGTTTGTCTTTTGATGTTACTAATGGTATTTCTTGCAATAAAAAGGTATCTATCTATCTATCT aTCTaTCTaTCTGTCTGTCTGTCTATCTATTTATTTAGAGACGGAGCCTTGCTCTGTCACCCAG
GCTGGAGTGCAGTGACACAATCTCAGCTCACTGCAACCTCCACCTCCCAGGCTCAAGCAATTCTCCTGCCTCAGCCTCTGAGTATTTGGGATTACAGGTGCCCACCACCATGCCCAGCTAATTTT TGT
ATTTTTAGTAGAGAAGGGGTTTTACCATGTTGGACCAGGCTGGTCTCAAACTCCTGACCTCAAGTGATCTGGC tGCCTTTGCTGAGACCAGCTCAATCGATACCTAACCCAGCAGtGCTAGAGGAATT
AAAGACACACACACACAGAAATCCAGAGTGCAGAGTGGGAATCAGGGGGCTGACAGCCTTCAGAGCTGAGAGCCACGAACAGAGTTTTACCCACATATTTATTGACAGCAAGCCAGTGATAAGCAGTGTT
TCTATAGATTATAGATTAACTAAAATGGGAAACAAAAGGATGGGGTCTGGCTAGTTATCTGCAGCAGGAATGTGTCCTTAAGGCACAGATCACTCATGCTATT GTTGTGGTCTGCCCTGGGTGGGCCA
GGTGTTTCCTTGCCCTCATTCCAGTAAACCAACAACCTTCAGTGTGGGCATCATAGTCATTGTGAGCATGTCACAGTGCTGCAGAGATTCTGCTTATGGCCAGTTTTGGGGCCTGTTTATGGCCA GAT
TTGGGGGCCTGTCCCTAACATGCCTTGcCCTCCCAAAGTGCTGGGATTATAGGCATGAGCCACTGTGCCTGGCAAAAAATGTTTATTTTT AAAACTGTACTCACATTTCTCAATTTAAAAAATTGCCA
CCGGATTTTGAGTCATAGGAAGCCTCTCTTAACAATGAGGTTTAAAGAGGAATTCCCTCATGCTTTCTTCTAGTAAATTCCATTTCTTTTGTCTAATTACATTTTAAAATATTT aTATTTTATAATTT
GTGAATTTTATTCTTGTATTTGATGTGAAGTCTGCATCTAATTTTGTTTTTTTCCATATGATTCATCAATTGACCCAATGGGAGACAGGACTAGCTGGATTTC CTAGGCTGACTAAGAATTCCTAAGC
CTAGCTGGGGAAGGTGACTGCAACCCACCTTTAAACACAGGGCTTGTAACTCAGCTCACACCTGACCAATCAGGTAGTAAAGAGGGCTCACTAAAATACAAATTAGGCTAAAAGCAGGAGGTAAA GAA
GTAGTCAAATCATATATTGCCTGAGAGCACACAGGGAGGGGCAATGATTGGGATATAAACCCCAGGACTTTGAGCTGGGAGTGGGCAACCCCCTTTAGGTCCC CTCCCATTGTATGGGAGCTCTGTTT
TCACTCTATTAAATCTTGCAACTGCACACTCTTCTGGTCCgTGTTTGTTCTGGCTTGAGCTGAActtCTGCTCTCTGTCCACCACTGCTGATCGCCACCTTCACAGACCCACCATTGACTTTCACCCC
TCCGGATCCGGCAGGGTGTCTGCTGCaCTTCTGATCCAGCAAGGTGCCCATTGCCCCTCCCGTTTGGGCTAGAAGCTCGTCATTGTTCCTGTGCAGCTAAGTG CCCGGGTTTGTCCTAATCAAGCTGA

ACACTAGCCGCTGGGTTCCACGGTTCTCTTCCATGACCCATGGCTTCTAATAGAGCTATAACACTCACTGCATGGCCCAAGGTTCCATTCCTTGGAATCTGTGAGGCCAAGAACCCCAGGTCAGA GAA
CAAAAGGCTTGCTGCCATCTTGGGAGTGGCTGCCACTATCTTGGGAGCAGCCCATCACCATCTTGGGAGCTC T

246	12.2	6.1	0.0	Hg19	1	49	(2144)	C	L1M	LINE/L1	(869)	5309	5258	8
255	21.7	0.0	0.0	Hg19	173	218	(1975)	C	LTR51	LTR/ERV1	(49)	622	577	9
36	6.8	0.0	0.0	Hg19	276	322	(1871)	+	(TATC)n	Simple_repeat	1	47	(0)	10
1767	11.0	0.4	0.4	Hg19	326	562	(1631)	C	AluSx1	SINE/Alu	(26)	286	50	11
3046	8.6	13.2	0.2	Hg19	564	1016	(1177)	C	MER9a3	LTR/ERVK	(0)	512	1	12
371	16.6	0.8	1.1	Hg19	1017	1087	(1106)	C	FLAM_C	SINE/Alu	(80)	53	2	13
248	31.0	3.2	8.1	Hg19	1162	1330	(863)	C	L1M5	LINE/L1	(1074)	5072	4918	14
5329	12.6	0.1	1.0	Hg19	1331	2117	(76)	+	LTR17	LTR/ERV1	1	780	(0)	15
248	31.0	3.2	8.1	Hg19	2118	2193	(0)	C	L1M5	LINE/L1	(1229)	4917	4838	14

>KSI_AluYb8_38

>Scaffold15791-1653676-1653968
AAACTTACTTCTTTTTTGTTTTTTTGTTTTTTTCTGTTTCTGTTTTTTTCTTTTTAATTTTTGAGGTAGGGTCTCACTCTGTCACTCAGGGTGGAGTGCAGTGGTGTGATCACAGCTCACTGCAGC CT
CCATCTCCTGGACTCAAGCAATCCTCTCACCCCAGCCTCCCGAGTAGCTAGGACCACAGGCGCGCACCACCACACCCAGCTAATCTTTTGTATTTTTTTGTAGAGACAGGGTTTCACCATGTTGC CTA
GGCTGGTCTTGAACTTCTGAGTTCAAGTGATCTGTCCGCTTTGGCCTTCCAAAGTGCTGGGATTATAGGCATGCATCACTGCACCTGGCCCCATATTATACTTCTAATGGGTCTGCTGTATCATCT TT
TTATCTAAAACAATAAAATTATATTTTGTGGCCCTCTAAAAAGCAAACATGATTTGACCTTTATAACATTATTTGACGTTTATAACAAAATTTAGTATAATGAATAATGTACTTTCTTTGAAGTT TTA
AGATATCTCCATATGCTTATTATGTGTAAGGTGACTTAAGGGAGGCTTAGGAAAAGAAAAGAAAATTTTATCTATAAAGTAAATGTTCTATGATCTTTTGATAAACAATAGACTATGTTTTATTAA CA
TAAGAAAAGTCTAACCAATAGGGTTGAGTGGTTGCTAACTTTATTTCTGTCATCCACATT TAGCATCAGCAGTTATGAGCAAATTATGTGTGAGGATGCAAGATGTATCCATAAGTTTAATATGTTTA
TCAGGAATGTTTATGTTTCTGTAACAATATATATTTATTTTAAAGGTATAAAACCAAGGAAAAGAAAGACCTTTGCTTTGCCAGGAATCATTAAAAAGGAAAAAGATGCAGAATCTGTGTAAGTAT TT
TAAATATCGTCAAAATCCTTTTTGTTTTTATAAGTGATTAAGAATAAGACTTTTGAATTTGGAAAGAAAAAAGTATTTTGGAAATAACT Caaaagtatatgtgtcctgtaatcccagcactttgggag
gccgaggcgggtggatcatgaggtcaggagatcgagaccatcctggctaacaaggtgaaaccccgtctctactaaaaatacaaaaaattagccgggcgcggtggcgggcgcctgtagtcccagct act
cgggaggctgaggcaggagaatggcgtgaacccgggaagcggagcttgcagtgagccgagattgcgccactgcagtccgcagtccggcctgggcgacagagcga gactccgtctcaaaaaaaaaaaa
aaaaaaaaaAAAAGTATATGTGTAAGGTGTGAAAAACAAGCCTTTCTAAATATTAATAAATACATTTGCCAATTCCTCTGGTTTTAGATCAAAGTATTATTAATTATTTGAAAATGTTAAGACATTAT
AGACAAAAGCTTTAATGTCTAGAAATGGAAAGTTTTGAAATATCCTTATTTTTGTAAAATGATAAATGCATATTTTAAAAAATTAAAAGTGCAAAGAAAACTAA AAATTgATCAAAGTCTTAATGCCT
AAGAGAAAATCTCTGGTCAACTATTTGATGAAAATCCTTCTAGACAATTCTGAGTGTACAGGCACATGGTGATACCTATACATACATTGATGGAAAATGTTAAATAGGGGAGA GAAAAGCCCTAACAT
TTTACCATTTATACTAGGTCATCATTTTTAGATGGAATATTTGGTCCACTTATAAGTCTGATTTACCTTCTTTTTTTTTTTTTGAGATGGAGTTTCACTGTTGT TGCCCAGGTTGGAATGCAATGGCG
CGATCTTGGCTTACTGCATTCTCCACCTCCCAGGTTCAAGCGATTCTCCTCCTACCTCAGCCTCCCAAGTAGCTGGAATTACGGCGCCCACCACCACATCTGGCTAATTTTTTGTATTTTTAGTA GAG
ACAGGGTTTCACCATATTGGCCAGGCTGGTCGCGAACTCCTGACCTCAGGTGAACCACCCGCCTCAGCTTCCCAAAGTGCTGGGATTACAGGCCTGAGCCACGC GCCCGGCCTGATTTACCTTCTTTT
AAAAATAAAAAATTAATATACAAAGAATATGTAATAAACATGTAAGAAATTGAGTTTAGATTAGCTAACTAAAGTTTTTGTTTGTTTGTTTGTTTTTTACTTTGGCTACACATTAGAACTACCTG GGA
AATTAAAATAAATACACCTATGCCAGGTCCCACCTCAGATCAATTAAATCAGATTCTCTAA GCATGAAACCTCAACATAGGTTTTGCTCTTTTTTGTTTTAAGCAGCCCCTTTGATT

2100	15.2	0.0	1.3	AluYb8-Scaffold15791-1653676-1653968	31	346	(1947)	C	AluJb	SINE/Alu	(0)	312	1	1
2781	0.3	0.0	0.0	AluYb8-Scaffold15791-1653676-1653968	1001	1293	(1000)	+	AluYb8	SINE/Alu	22	314	(4)	2
245	24.7	13.2	2.8	AluYb8-Scaffold15791-1653676-1653968	1454	1582	(711)	C	HAL1b	LINE/L1	(23)	2398	2257	3
2239	9.3	0.7	1.3	AluYb8-Scaffold15791-1653676-1653968	1732	2032	(261)	C	AluSq2	SINE/Alu	(14)	299	1	4
436	24.6	3.5	4.4	AluYb8-Scaffold15791-1653676-1653968	2140	2254	(39)	+	MER5B	DNA/hAT-Charlie	6	119	(59)	5

>Hg19 chr5:72332157-72334146
AAACTTACTTCTTTTTTGTTTTTTTGTTTTTTTCTGTTTCTGTTTTTTTCTTTTTAATTTTTGAGGTAGGGTCTCACTCTGTCACTCAGGGTGGAGTGCAGTGGTGTGATCACAGCTCACTGCAGC CT
CCATCTCCTGGACTCAAGCAATCCTCTCACCCCAGCCTCCCGAGTAGCTAGGACCACAGGCGCGCACCACCACACCCAGCTAATCTTTTGTATTTTTTTGTAGAGACAGGGTTTCACCATGTTGC CTA
GGCTGGTCTTGAACTTCTGAGTTCAAGTGATCTGTCCGCTTTGGCCTTCCAAAGTGCTGGGATTATAGGCATGCATCACTGCACCTGGCCCCATATTATACTTCTAATGGGTCTGCTGTATCATCT TT
TTATCTAAAACAATAAAATTATATTTTGTGGCCCTCTAAAAAGCAAACATGATTTGACCTTTATAACATTATTTGACGTTTATAACAAAATTTAGTATAATGAATAATGTACTTTCTTTGAAGTT TTA
AGATATCTCCATATGCTTATTATGTGTAAGGTGACTTAAGGGAGGCTTAGGAAAAGAAAAGAAAATTTTATCTATAAAGTAAATGTTCTATGATCTTTTGATAAACAATAGACTATGTTTTATTAA CA
TAAGAAAAGTCTAACCAATAGGGTTGAGTGGTTGCTAACTTTATTTCTGTCATCCACATT TAGCATCAGCAGTTATGAGCAAATTATGTGTGAGGATGCAAGATGTATCCATAAGTTTAATATGTTTA
TCAGGAATGTTTATGTTTCTGTAACAATATATATTTATTTTAAAGGTATAAAACCAAGGAAAAGAAAGACCTTTGCTTTGCCAGGAATCATTAAAAAGGAAAAAGATGCAGAATCTGTGTAAGTAT TT
TAAATATCGTCAAAATCCTTTTTGTTTTTATAAGTGATTAAGAATAAGACTTTTGAATTTGGAAAGAAAAAAGTATTTTGGAAATAACT CAAAAGTATATGTGTAAGGTGTGAAAAACAAGCCTTTCT
AAATATTAATAAATACATTTGCCAATTCCTCTGGTTTTAGATCAAAGTATTATTAATTATTTGAAAATGTTAAGACATTATAGACAAAAGCTTTAATGTCTAGAAATGGAAAGTTTTGAAATATCC TT
ATTTTTGTAAAATGATAAATGCATATTTTAAAAAATTAAAAGTGCAAAGAAAACTAAAAATT cATCAAAGTCTTAATGCCTAAGAGAAAATCTCTGGTCAACTATTTGATGAAAATCCTTCTAGACAA
TTCTGAGTGTACAGGCACATGGTGATACCTATACATACATTGATGGAAAATGTTAAATAGGGGAGAGAAAAGCCCTAACATTTTACCATTTATACTAGGTCATCATTTTTAGATGGAATATTTGGT CC
ACTTATAAGTCTGATTTACCTTCTTTTTTTTTTTTTGAGATGGAGTTTCACTGTTGTTGCCCAGGTTGGAATGCAATGGCGCGATCTTGGCTTACTGCATTCTCCACCTCCCAGGTTCAAGCGAT TCT
CCTCCTACCTCAGCCTCCCAAGTAGCTGGAATTACGGCGCCCACCACCACATCTGGCTAATTTTTTGTATTTTTAGTAGAGACAGGGTTTCACCATATTGGCCAGGCTGGTCGCGAACTCCTGACC TC
AGGTGAACCACCCGCCTCAGCTTCCCAAAGTGCTGGGATTACAGGCCTGAGCCACGCGCCCGGCCTGATTTACCTTCTTTTAAAAATAAAAAATTAATATACAAAGAATATGTAATAAACATGTA AGA
AATTGAGTTTAGATTAGCTAACTAAAGTTTTTGTTTGTTTGTTTGTTTTTTACTTTGGCTACACATTAGAACTACCTGGGAAATTAAAATAAATACACCTATGCCAGGTCCCACCTCAGATCAATT AA
ATCAGATTCTCTAAGCATGAAACCTCAACATAGGTTTTGCTCTTTTTTGTTTTAAGCAGCCCCTTTGATT

2100	15.2	0.0	1.3	Hg19	131	446	(1744)	C	AluJb	SINE/Alu	(0)	312	1	6
249	23.1	13.2	2.8	Hg19	1251	1379	(811)	C	HAL1b	LINE/L1	(23)	2398	225 7	7
2239	9.3	0.7	1.3	Hg19	1529	1829	(361)	C	AluSq2	SINE/Alu	(14)	299	1	8
496	25.5	3.9	11.2	Hg19	1937	2117	(73)	+	MER5B	DNA/hAT -Charlie	6	174	(4)	9

>KSI_AluYb8_39

>Scaffold16598-6754296-6754623
TTTCTAGATCAGTGATTCTCAACCTcTGTTTGTGTCTTTTTTTTTTTTTTTTTTTTTTTTGAGACAGGGTCTTGCTCTGTCACCCAGATAGAGTGCAGTGGTGGGATCACGGCTCACTGTAGCCTCCA
CCTCCCAGGCTCAAGTGATCCTTTCACTTCAGCCTCCCTAGTAGCTGGGACCACAGGCATGTGCCACCATGCCTGGCTATTTTTTTTTTTTTTTTGGTA GAGACAGAGTCTCACTATGTCACTATGTTG
CCCAGGCTGGCCTCCAACCCCTGGGGCTCAAGCAATCCTCCCACCTCAGTCTCCCAAAGTGTTGGGATTACAGGTGTGAGCCAACATGCCTGGCCTGTTTCTGTCTTTACTGTCCACATAGCCAT ACC
TTCATCATCATATACAGAAAGTGGCAAATCATAATTAACGGGAGAGTAATAAGTATTTTGGGGAAAAACAGGGAATAGAGAACCTGGAACATTATCTT CCAACAGAGAATATCCACATGAAAATTAAA
GGAAATATAGCTATATGCATGAGTTTCTATCTTCTCAGATCTTCTAGTATCCTTATCATTTAGCTCCCTGCTTGGTTGTTTATGTTTCTGGTCAATGTTCTTCTGCTTACTCAAGTCTGAGTGAT TTT
ATGTGCATGGAACATGGCTGTACTTTGGTCAGGTGTCCAGACAAAAGCATTCTTAGGGAAGAATTATTTATCTACAAAGTTTATTATAAAATAAATCT TTTGGTTTTTATTATAAAAATAATAGCAAA
CTAAATAATTTTTATTCAAAAGACTGCTACCATAAATCTACAAACCACATGTCTAATTCTGTATAAAAATTCTCCCTGCTATTTTTACAACATTCTTGTGGCTACGAGTAGGTATAATTATCCTC GTT
TTGCAGATGAGAAcCCTAAGGCTCAAAGAATTcAGAGATTTGCTTGAGGTCACAAAATTAATCAGAAGCAGCAGAGGCAGAATCCATAAAAAGATATCTTTAAGTTTttttttttttttttttttttt
ttttttttttttttttgagacggagtctcgctctgtcgcccaggccggactgcggactgcagtggcgcaatctcggctcactgcaagctccgcttcccgggttcacgccattcccctgcctcagc ctc
ccgagtagctgggactacaggcgcccgccaccgcgcccggctaattttttgtatttttagtagagacggggtttcaccttgttagccaggatggtctc gatctcctgacctcatgatccacccgcctc
ggcctcccaaagtgctgggattacaggcgtgagccaccgcgcccggccagatatctttaagtttAAGGCATGCTATTAAGTTACAAGTGGAACATGTGTTCCTATAGCT TTTTAGATCTTCTAAATAT
TAAAATAAAACACATATACTACTTGGAAGAGCACACATTTTCATTGGTACAGTGTTTTGACTTATTCTAACACTGGTATATTAACTGCTTTGTAAATAAGGCCAAACATGCTGGCTTGTTCCTAG AAT
AACATTAGAGTATTATACATTATTTAAAAGTCACAAAATTTCGAAGTTGGAAAAGTTCATTTAGAGAACAAAACATAATTTTTGCTAAGCAATGTTCTAAGAGAGGAGG ATATAGTTTTCTATGATGC
TGTTCCCAGATTTTCAACATACTGAGATCTAAACAATGAAGATCTAACTAAATGGTGAGCAATATGGGCCAACCTATGTACAATTCAACTGGAATGCAAAAAGCACCACTAAAGCAGCAATTGCA TAG
TGTCATGAGGCAACACACATGTTTTTATAAGCACTCATTAAAAGTTAAGTCACCATTTGAGATCTTGTCCAACCGGTACGGTATGCCCCAAGTTAGTGATCTTAAAAGA GACAAACCACTGATGGACT
ATTGTTTAACATGTATTAAACAAAAAAGAAGAATGAGATTTCCTAATAGGGATGTATGGAGTTAAGTCTTACAAACATAATAATTGGTAATGTCCACAAACCAAATGATTTTGTCTTTAAAAAAA ATC
TGCTTCGTAAGTCATTTACTTGGGGCAGGTGTAAGAGGACAATGACCATCTTAAAACTAACTTCCTCAAAGATAGGT GCCTCGCTCTGGTCATATTAATATGTCACAAGAATACCCTATTTATGGTAA
CAATGAATTCAAGAGTCAACTCACATCAGGAAGAAATTAGATTGTACCATGATTCATTTGGTTTAAAGCTCAGTATTCACATGTGACCTGCCTAACTTCAGTTGAGAGCATTTTCTCACTCTTAG AAA
CAGCAGAAAGAGGATTTTGATGAC

2015	13.7	0.3	3.3	AluYb8-Scaffold16598-6754296-6754623	37	351	(1977)	C	AluJb	SINE/Alu	(6)	306	1	1
453	27.3	0.8	6.3	AluYb8-Scaffold16598-6754296-6754623	849	981	(1347)	C	MIRb	SINE/MIR	(80)	188	63	2
2737	0.7	0.0	0.0	AluYb8-Scaffold16598-6754296-6754623	1001	1328	(1000)	C	AluYb8	SINE/Alu	(0)	318	1	3

>Hg19 chr9:74543793-74545779
TTTCTAGATCAGTGATTCTCAACCTtTGTTTGTGTCTTTTTTTTTTTTTTTTTTTTTTTTGAGACAGGGTCTTGCTCTGTCACCCAGATAGAGTGCAGTGGTGGGATCACGGCTCACTGTAGCCTCCA
CCTCCCAGGCTCAAGTGATCCTTTCACTTCAGCCTCCCTAGTAGCTGGGACCACAGGCATGTGCCACCATGCCTGGCTATTTTTTTTTTTTTTTTGGTAGAGACAGAGTCTCACTATGTCACTATG TTG
CCCAGGCTGGCCTCCAACCCCTGGGGCTCAAGCAATCCTCCCACCTCAGTCTCCCAAAGTGTTGGGATTACAGGTGTGAGCCAACATGCCTGGCCTGTTTCTGTCTTTACTGTCCACATAGCCATACC
TTCATCATCATATACAGAAAGTGGCAAATCATAATTAACGGGAGAGTAATAAGTATTTTGGGGAAAAACAGGGAATAGAGAACCTGGAACATTATCTTCCAACAGAGAATATCCACATGAAAATT AAA
GGAAATATAGCTATATGCATGAGTTTCTATCTTCTCAGATCTTCTAGTATCCTTATCATTTAGCTCCCTGCTTGGTTGTTTATGTTTCTGGTCAATGTTCTTCTGCTTACTCAAGTCTGAGTGATTTT
ATGTGCATGGAACATGGCTGTACTTTGGTCAGGTGTCCAGACAAAAGCATTCTTAGGGAAGAATTATTTATCTACAAAGTTTATTATAAAATAAATCTTTTGGTTTTTATTATAAAAATAATAGCAAA
CTAAATAATTTTTATTCAAAAGACTGCTACCATAAATCTACAAACCACATGTCTAATTCTGTATAAAAATTCTCCCTGCTATTTTTACAACATTCTTGTGGCTACGAGTAGGTATAATTATCCTCGTT
TTGCAGATGAGAAaCCTAAGGCTCAAAGAATTaAGAGATTTGCTTGAGGTCACAAAATTAATCAGAAGCAGCAGAGGCAGAATCCATAAAAAGATATCTTTAAGTTTAAGGCATGCTATTAAGTTACA
AGTGGAACATGTGTTCCTATAGCTTTTTAGATCTTCTAAATATTAAAATAAAACACATATACTACTTGGAAGAGCACACATTTTCATTGGTACAGTGTTTTGACTTATTCTAACACTGGTATATTAAC
TGCTTTGTAAATAAGGCCAAACATGCTGGCTTGTTCCTAGAATAACATTAGAGTATTATACATTATTTAAAAGTCACAAAATTTCGAAGTTGGAAAAGTTCATTTAGAGAACAAAACATAATTTT TGC
TAAGCAATGTTCTAAGAGAGGAGGATATAGTTTTCTATGATGCTGTTCCCAGATTTTCAACATACTGAGATCTAAACAATGAAGATCTAACTAAATGGTGAGCAATATGGGCCAACCTATGTACAATT
CAACTGGAATGCAAAAAGCACCACTAAAGCAGCAATTGCATAGTGTCATGAGGCAACACACATGTTTTTATAAGCACTCATTAAAAGTTAAGTCACCATTTGAGATCTTGTCCAACCGGTACGGT ATG
CCCCAAGTTAGTGATCTTAAAAGAGACAAACCACTGATGGACTATTGTTTAACATGTATTAAACAAAAAAGAAGAATGAGATTTCCTAATAGGGATGTATGGAGTTAAGTCTTACAAACATAATAATT
GGTAATGTCCACAAACCAAATGATTTTGTCTTTAAAAAAAATCTGCTTCGTAAGTCATTTACTTGGGGCAGGTGTAAGAGGACAATGACCATCTTAAAACTAACTTCCTCAAAGATAGGT GCCTCGCT
CTGGTCATATTAATATGTCACAAGAATACCCTATTTATGGTAACAATGAATTCAAGAGTCAACTCACATCAGGAAGAAATTAGATTGTACCATGATTCATTTGGTTTAAAGCTCAGTATTCACATGTG
ACCTGCCTAACTTCAGTTGAGAGCATTTTCTCACTCTTAGAAACAGCAGAAAGAGGATTTTGATGAC

2015	13.7	0.3	3.3	Hg19	137	451	(1736)	C	Alu Jb	SINE/Alu	(6)	306	1	4

488	25.7	0.8	6.3	Hg19	949	1081	(1106)	C	MIRb	SINE/MIR	(80)	188	63	5

>KSI_AluYb8_40

>Scaffold15239-333019-333335
ATCACTAAGGCAGTACTTTTACAAACTACAGGGTTACTAAGCTTGGGGTGTCCCCTAATGTAGATAAAGTTGCAGTAACCAAAAATTTAGATCGCAACACCCATGTCTTCTTGAATACCTTAAAAT CC
TTCCCAAGAAGAATGGGTATAAACAAGCCCAGACTGTGAAGACCACAATAAATACCTAACTCTACAATGCCCAAACACTGATGAACATCCACAAGCATCAAGACCATTCAGGAAAACATGACCTC ACC
AAATGAACTAAATAATGCACCAAGGGCAAATCCCAGAGAGGCAGATATGTGACCTGACAGAGAATTCAAAAATAGTTCTTTTGAGGAAACTGAAAGAAACTCAAGATAACACAGAGGGAATTTAGATT
CCTATGGATAAATTTAACAAAGAAATTGAAATAATTATAAAGAATCAAGTATAAATTCTAGAGTTGAAAAATGCAATTGGCATAC cGAAGAATGCATCAAAGACTCTTAATAGCAGAATCGATTAAAC
AAAAGAAAGAAGTAGGAAGTAGTGAGCTCAATGGTAGGCTATTTAAAAACACGCAGTCAGAGGAGACAAAAGAAAAAAGAATAAAAAAGAATGAAGCATGCCTAAAAGATCTAGAAAACAGCCTCA AA
AGGGCAAATCTAGGAGTTATTGGCCTTAAAGAGTAGGTAGAGACAGAGATAGGGGTAGAAAATATTTTCAAAGGGATAATAACAGAGAATTTCTGAAACCTAGAAAAAGGTATCAATATTCAAGT ACA
AGAAAGTTAAAGAACACCAAGCAGATTTTACACACAAAAAAAGAAGTCCTTCAAAAAGAACTTCTTCAAACATGAAGAAGATATAATGATTTTCCCAGAGG AACAAAAGCTGAAGGATTTCACCAACA
CCAGACCAGTCTTACAAGAAATGCTAAAGGGAATTCTTCAATCTGAAAGAAAAGGACATTAGTGAGCAATGAGAAATCATCTACAGGTACAAAA CTCATGCATGGgccgggcgcggtggctcacgcct
gtaatcccagcactttgggaggccgaggcgggtggatcatgaggtcaggagatcgagaccatcctggctaacaaggtgaaaccccgtctctactaaaaatacaaaaaattagccgggcgcggtggc gg
gcgcctgtagtcccagctactcgggaggctgaggcaggagaatggcgtgaacccgggaagcggagcttgcagtgagccgagattgcgccactgcagtccgcagtccagcctgggcgacagagcga gac
tccgtctcaaaaaaaaaaaaaaaaaaaaaaaaaaaaactcatgcatggTAGTAAGTTCCTAGAAAAACACAGAATATTATAATGCTGTAATTGTGGTATGTAAACTACTCATATCTTAAGTTAAAAGA
CAAAAAGATGAACTGATCAAAAATAATAACTATAACATTTCAAGACATAGACATTACAATAAGATATAAATAGAAACAACAAAAAGTTAAAAAGCAAGGAGATGAAGTTAAAA TACAGAGTTTTCTTA
GTTTTCTTTTTGCTTGTTTTTTTGTTTATGCAATTAGTGTTATTGGTTTAAAATAATGGGCTATAAAATATTATTTAGTACCTCATAGTACCTTAAATCTAAAAACATAAAATGGCTACACACAA AAA
AAAGCCAGAAACAAAAATATACTGCTAGAGAAGATCACTTTTACTAAAAGAAGGAGAGAAAGGAGAAAACAAGAACAAGAAGACCAGAAAACAAATAACAAAAAGGCAGCAGT AAGCCCTTACTTACC
AAATAATGGCATTGAATGTAAATGAACTAAACTCTCCAATCAAAAGACATAGAGTGGCTGAATGGGTATTAAAAAAAAAAAAAGACCCAATGATCTGTTGCCTACAAGAAAAATGCTTCAGCTGTA AAG
ACACATAGACTGAAAATAAAGAGATGGGCCAAGTGCTGTGGTAGCTCACACCTGTAATTCCAGCAGTTTAGGAGGCCAAGGCAGACAGATAACTTGAGGCCAGGAGTTGGAGA CCAACCTGGCCAACA
TGGAAAAACCCCATCTCTATTAAAAATATAAAAATTAGCCAGGAGTGGTGGCACATGCCTATAATTCCAGATACTCTGAAGGATGAGGCATGAGAAGTGATTGAACCCAGGAGGTAGAGGTTTCA ATG
AGCTGAGATGCTGTGACTGCACTCTAGCCTGGAACACAGAGAGAGACTCTGTGTCAAAAACAAAACAAAACAAAACAAAAGACGAAGAGGGTCATTATATAATGATAAAGGAA TCAATTCAAAAAACA
GCACACAACAAT

5119	11.7	2.4	1.0	UnnamedSequence	1	809	(1507)	+	L1M1	LINE/L1	1851	2670	(4895)	1
4300	11.8	3.8	2.8	UnnamedSequence	830	1000	(1316)	+	L1M1	LINE/L1	2895	3060	(4505)	1
3041	0.6	0.0	0.0	UnnamedSequence	1001	1317	(999)	+	AluYb8	SINE/Alu	1	317	(1)	2
4300	11.9	3.0	3.1	UnnamedSequence	1318	1946	(370)	+	L1M1	LINE/L1	3061	3704	(3940)	1
1862	17.7	0.0	1.0	UnnamedSequence	1947	2256	(60)	+	AluSz6	SINE/Alu	1	307	(5)	3
1062	15.7	0.1	252.3	UnnamedSequence	2257	2316	(0)	+	L1M1	LINE/L1	3705	3712	(3853)	1

>hg19 chr1 157362499 157364488
ATCACTAAGGCAGTACTTTTACAAACTACAGGGTTACTAAGCTTGGGGTGTCCCCTAATGTAGATAAAGTTGCAGTAACCAAAAATTTAGATCGCAACACCCATGTCTTCTTGAATACCTTAAAATCC
TTCCCAAGAAGAATGGGTATAAACAAGCCCAGACTGTGAAGACCACAATAAATACCTAACTCTACAATGCCCAAACACTGATGAACATCCACAAGCATCAAGACCATTCAGGAAAACATGACCTC ACC
AAATGAACTAAATAATGCACCAAGGGCAAATCCCAGAGAGGCAGATATGTGACCTGACAGAGAATTCAAAAATAGTTCTTTTGAGGAAACTGAAAGAAACTCAAGATAACACAGAGGGAATTTAGATT
CCTATGGATAAATTTAACAAAGAAATTGAAATAATTATAAAGAATCAAGTATAAATTCTAGAGTTGAAAAATGCAATTGGCATAC tGAAGAATGCATCAAAGACTCTTAATAGCAGAATCGATTAAAC
AAAAGAAAGAAGTAGGAAGTAGTGAGCTCAATGGTAGGCTATTTAAAAACACGCAGTCAGAGGAGACAAAAGAAAAAAGAATAAAAAAGAATGAAGCATGCCTAAAAGATCTAGAAAACAGCCTC AAA
AGGGCAAATCTAGGAGTTATTGGCCTTAAAGAGTAGGTAGAGACAGAGATAGGGGTAGAAAATATTTTCAAAGGGATAATAACAGA GAATTTCTGAAACCTAGAAAAAGGTATCAATATTCAAGTACA
AGAAAGTTAAAGAACACCAAGCAGATTTTACACACAAAAAAAGAAGTCCTTCAAAAAGAACTTCTTCAAACATGAAGAAGATATAATGATTTTCCCAGAGGAACAAAAGCTGAAGGATTTCACCA ACA
CCAGACCAGTCTTACAAGAAATGCTAAAGGGAATTCTTCAATCTGAAAGAAAAGGACATTAGTGAGCAATGAGAAATCATCTACAG GTACAAAACTCATGCATGGTAGTAAGTTCCTAGAAAAACACA
GAATATTATAATGCTGTAATTGTGGTATGTAAACTACTCATATCTTAAGTTAAAAGACAAAAAGATGAACTGATCAAAAATAATAACTATAACATTTCAAGACATAGACATTACAATAAGATATA AAT
AGAAACAACAAAAAGTTAAAAAGCAAGGAGATGAAGTTAAAATACAGAGTTTTCTTAGTTTTCTTTTTGCTTGTTTTTTTGTTTAT GCAATTAGTGTTATTGGTTTAAAATAATGGGCTATAAAATAT
TATTTAGTACCTCATAGTACCTTAAATCTAAAAACATAAAATGGCTACACACAAAAAAAAAGCCAGAAACAAAAATATACTGCTAGAGAAGATCACTTTTACTAAAAGAAGGAGAGAAAGGAGAAA ACA
AGAACAAGAAGACCAGAAAACAAATAACAAAAAGGCAGCAGTAAGCCCTTACTTACCAAATAATGGCATTGAATGTAAATGAACTA AACTCTCCAATCAAAAGACATAGAGTGGCTGAATGGGTATTA
AAAAAAAAAAAGACCCAATGATCTGTTGCCTACAAGAAAAATGCTTCAGCTGTAAAGACACATAGACTGAAAATAAAGAGATGG GCCAAGTGCTGTGGTAGCTCACACCTGTAATTCCAGCAGTTTAG
GAGGCCAAGGCAGACAGATAACTTGAGGCCAGGAGTTGGAGACCAACCTGGCCAACATGGAAAAACCCCATCTCTATTAAAAATAT AAAAATTAGCCAGGAGTGGTGGCACATGCCTATAATTCCAGA
TACTCTGAAGGATGAGGCATGAGAAGTGATTGAACCCAGGAGGTAGAGGTTTCAATGAGCTGAGATGCTGTGACTGCACTCTAGCCTGGAACACAGAGAGAGACTCTGTGTCAAAAACAAAACAA AAC

AAAACAAAAGACGAAGAGGGTCATTATATAATGATAAAGGAATCAATTCAAAAAACAGCACACAACAA T

>KSI_AluYb8_41

>Scaffold8772-3828102-3828352
ACAGAGCAAGACTCCATCTCAAAAAACAAACAAAAAACACAAACAAAACAACAACAACAACAACCTGACCTAAACCTCACTTTATCCAAAATTTAACTCAAAAAACATTATGATGTAAATATGAAA TG
AAAAGCCATGGAACTTTTTGAAAAAATCAGCCAGGGGTGGTGGCTCATGTCTGTAATCCTAGCACTTTGGCAGGCCAATGTGTGAGGACTGCTTGAGCCCAGAAGTTCAAGACCAGCCTGGGTAA CAT
AGTGAAAACCCATCTCTACAAATTTTTTAAAAATTAGCTGGGTGTGGTGGCATGCACTGGTAGTCCCAGCTACTTGGGAGGCTGAGGTGGGAGGATCTCTTGAGCCCAAGACGTCGTGTCTGGAAT GA
GCTTTGATTGTACCACCCCACTCCAGCCTAGGCATCAAATTGAGACCCTGTCTCAAAAAATGGAAAAAACAAAACAAAACAAAAAAACCTTTACAACTTGGTGTTAGTCTAAGAGTTCTTAGAGATAA
TAGCAAAAGCACAATCAATAATTTAAAAACAGATCAGTTAGATTTTGTCAAAATGTAAAACTTTTGCTTTACAAAAGACACTATTAGGAAAATGAAAAGCTAAACTTCAAAATTGGAGAAAATATT TT
CAAACCACATGTCTAACACAGAACTTGTATCCAGAATATATAAAGCACTCTC aAAACTCAATAAAAAaAAAACAAACAACTCAATTTAAAAATTCATGATAATATCAAAAGGCTGGTGAGAATGTGCA
GTGACTAGAACACTCCTACATTGCTGTTGGGCACTCTCTTGTTTACACTTCCCATATGCCTCAGCAATACTATTCCTGGGAATTTACTTTAGAAAAATAAAATTTATGTTCACAGAAAGATATGTA CA
TGAATATAAACACAGCTCCATTCATAATTGCTGAAAAACGAAAACAAGCCAAAGTTCTAGCA gTCAAAGCCTGACACATTTGACTGGTTAAAAAAAAAAAagttggccgggcgcggtggctcacgcctg
taatcccagcactttgggaggccgaggcgggtggatcacgaggtcaggagatcgagaccatcctggctaacaaggtgaaaccccgtctctactaaaaatacaaaaaattagccgggcgcggtggcg gg
cgcctgtagtcccagctactcgggaggctgaggcaggagaatggcgtgaacccaggaagcggagcttgcagtgagccgagattgcgccattgcagtccgta TAGCAAAATAAAATGAGCAAAAATGCC
AGATACCCAAGCCCCTTATCTCAAAGGATGACACTGAAACAAAAATAGCGAGTAATGTGATGATGTATCATCAGTAGTGGGAGCAACCTGCTGCTGCAGATCCAATTGCATCTTGCAGATAAGCAG CT
TTATACTTTGCAGTTGTGCCCAAATAACAGAGAGGCAGAGAGCCTCAGGCCTCTTCAAAACCCATGAAGTGATGAGTAACTGTCTCATGGCAGCCTCCTTGGAAGATAGGTAGAGGGGGTAGGCT CTT
CAAAGCCTCCCACACTCTCATGTTCCAGGAATATCTCAGAGCATTTTACCAAAACTTAGATCAGCTGTAGTTAAGCCTTGGCCAAATAGCCTCTCTAGAGACATGCAGGGAAGATGTTTATCATAA GC
CCAGAAACCCCTCTCCTGGGATTAGAGAGACCTTAGGGAGTGAAAAA gGTTGATCACACAAAACAATTGTATGTCAATGTTTGTAATAGCTCTATTATAATCACACAAACTGTAAACAATCCAAATGT
CTTTCAACAGGTGTATGAACAAATTTTGGTACATTCATATCATGGCATACTACTCAGCTCTAAAAAGGAAAGAATTATTGATATACTCAACGCCATAAATGAAATCACTGTGTTGTAAATTCTAGA TT
TGTATTTCATTTAATATAATTTCTTCACTTTTAAAAAGTGTTAAACTTATATTTTCTTTTGACTATTGAGGTAAAATATACGTAACACAAAATTTATCATGCAAGTCATTTGGATGTGTACAATT CAG
TGGTATTAAGTGCATTCACATTGTTTCACAACCATCATTTCTATTCCTCTTCAGAACTTTTTCATCATCCTAAACTAAAACTCTGTACCCATTAA GCAACAACTTTCCATTCTCCCCTCCCCAGATCC
TGGTGACCACTATTCTATTTTCTGCCTCTAtGGATTTAACTATTCTAGGTGTGTCTTATTAGTAGAATCAGACA

19	10.8	5.0	0.0	TErefseq_17	22	61	(2189)	+	A-rich	Low_complexity	1	42	(0)	1	
1145	23.7	3.9	0.0	TErefseq_17	62	157	(2093)	+	L1MD	LINE/L1	4951	5050	(1096)	2	
1833	18.1	0.0	1.0	TErefseq_17	158	471	(1779)	+	AluJr	SINE/Alu	2	312	(0)	3	
1145	23.7	3.9	0.0	TErefseq_17	472	733	(1517)	+	L1MD	LINE/L1	5051	5322	(824)	2	
521	24.6	22.4	0.4	TErefseq_17	727	954	(1296)	+	L1MD	LINE/L1	5474	5751	(608)	2	*
2401	1.6	0.0	0.0	TErefseq_17	1000	1250	(1000)	+	AluYb8	SINE/Alu	1	251	(67)	4	
1086	26.6	8.0	2.0	TErefseq_17	1271	1646	(604)	C	LTR75	LTR/ERVL	(129)	433	36	5	
751	23.0	3.7	2.5	TErefseq_17	1655	1895	(355)	+	L1MD1	LINE/L1	5612	5855	(387)	6	
1372	18.7	0.8	0.0	TErefseq_17	1984	2250	(0)	C	L1MB4	LINE/L1	(5)	6175	5907	7	

>hg19 chr10 68216413 68218435
ACAGAGCAAGACTCCATCTCAAAAAACAAACAAAAAACACAAACAAAACAACAACAACAACAACCTGACCTAAACCTCACTTTATCCAAAATTTAACTCAAAAAACATTATGATGTAAATATGAAA TG
AAAAGCCATGGAACTTTTTGAAAAAATCAGCCAGGGGTGGTGGCTCATGTCTGTAATCCTAGCACTTTGGCAGGCCAATGTGTGAGGACTGCTTGAGCCCAGAAGTTCAAGACCAGCCTGGGTAA CAT
AGTGAAAACCCATCTCTACAAATTTTTTAAAAATTAGCTGGGTGTGGTGGCATGCACTGGTAGTCCCAGCTACTTGGGAGGCTGAGGTGGGAGGATCTCTTGAGCCCAAGACGTCGTGTCTGGAAT GA
GCTTTGATTGTACCACCCCACTCCAGCCTAGGCATCAAATTGAGACCCTGTCTCAAAAAATGGAAAAAACAAAACAAAACAAAAAAACCTTTACAACTTGGTGTTAGTCTAAGAGTTCTTAGAGATAA
TAGCAAAAGCACAATCAATAATTTAAAAACAGATCAGTTAGATTTTGTCAAAATGTAAAACTTTTGCTTTACAAAAGACACTATTAGGAAAATGAAAAGCTAAACTTCAAAATTGGAGAAAATATT TT
CAAACCACATGTCTAACACAGAACTTGTATCCAGAATATATAAAGCACTCTC gAAACTCAATAAAAAgAAAACAAACAACTCAATTTAAAAATTCATGATAATATCAAAAGGCTGGTGAGAATGTGCA
GTGACTAGAACACTCCTACATTGCTGTTGGGCACTCTCTTGTTTACACTTCCCATATGCCTCAGCAATACTATTCCTGGGAATTTACTTTAGAAAAATAAAATTTATGTTCACAGAAAGATATGTA CA
TGAATATAAACACAGCTCCATTCATAATTGCTGAAAAACGAAAACAAGCCAAAGTTCTAGCA aTCAAAGCCTGACACATTTGACTGGTTAAAAAAAAAAAgtttatgttatatttagacagtttattac
tTAGCAAAATAAAATGAGCAAAAATGCCAGATACCCAAGCCCCTTATCTCAAAGGATGACACTGAAACAAAAATAGCGAGTAATGTGATGATGTATCATCAGTAGTGGGAGCAACCTGCTGCTGCAG A
TCCAATTGCATCTTGCAGATAAGCAGCTTTATACTTTGCAGTTGTGCCCAAATAACAGAGAGGCAGAGAGCCTCAGGCCTCTTCAAAACCCATGAAGTGATGAGTAACTGTCTCATGGCAGCCTC CTT
GGAAGATAGGTAGAGGGGGTAGGCTCTTCAAAGCCTCCCACACTCTCATGTTCCAGGAATATCTCAGAGCATTTTACCAAAACTTAGATCAGCTGTAGTTAAGCCTTGGCCAAATAGCCTCTCTAG AG
ACATGCAGGGAAGATGTTTATCATAAGCCCAGAAACCCCTCTCCTGGGATTAGAGAGACCTTAGGGAGTGAAAAA tGTTGATCACACAAAACAATTGTATGTCAATGTTTGTAATAGCTCTATTATAA
TCACACAAACTGTAAACAATCCAAATGTCTTTCAACAGGTGTATGAACAAATTTTGGTACATTCATATCATGGCATACTACTCAGCTCTAAAAAGGAAAGAATTATTGATATACTCAACGCCATAA AT

GAAATCACTGTGTTGTAAATTCTAGATTTGTATTTCATTTAATATAATTTCTTCACTTTTAAAAAGTGTTAAACTTATATTTTCTTTTGACTATTGAGGTAAAATATACGTAACACAAAATTTAT CAT
GCAAGTCATTTGGATGTGTACAATTCAGTGGTATTAAGTGCATTCACATTGTTTCACAACCATCATTTCTATTCCTCTTCAGAACTTTTTCATCATCCTAAACTAAAACTCTGTACCCATTAA GCAAC
AACTTTCCATTCTCCCCTCCCCAGATCCTGGTGACCACTATTCTATTTTCTGCCTCTA cGGATTTAACTATTCTAGGTGTGTCTTATTAGTAGAATCAGACA

>KSI_AluYb8_42

>Scaffold16904-571241-571523
GATGAAAGGAATCGTCACTGAAAAGGGCCAATcAATTTAGCTTTGTGTCACCTGGGTTCAGCTCAAGGCAGTTCTTCAGAAGCCAAGGTTAGATGTGAAAAGCCAGTAATACCACAGTGACCTGGATT
GGTAGACATAGATACATGAGTTCCTATCTGCATGTAAAAGCTGTGATTATAGTGAAAGCAATAGAGACCCAGCATTAAAAAAAAATTGTGCCAGAGAGCC TTAAAAGTTTCTACAATGATATTCTCTG
GAAAAATAACTAAAAGTACTTGTCACAGGACTAAAGTAAGCTTTTCTTATATGTTATGTATAAAATCTCAGCAATGCCACTATTAGAGACACGTGCATATGTGTTTTttTCTCCATATCTGGGCATCT
TTTGTAAGGAAGCACGTATCACAGCGTGTTAAAGGGAAGGGACTAAGAATTTTAGCAAAGCAAAATGAAACTAACAAACATTAGCAAAGGATGTAATTAT GAAAAGAAAAACAAGGAGCTGGAAAAAA
AATCTCAATATCAGAAAAGTGCTTTTATCAAGTTTAGTAAATAACTATAAAAATTGCTGAAGAGGCAGCTGAACTAATCTGAAGCTACCCCTCCATTTTGTTTCTGTTCATTTGTTGAGCAAATT TAT
ATTGAGTCCTTACAATGTGAAATCACTTTCTGGAGAACTCAAAAGGACTTAACCATTCTAAAAAGAAGCTTAAGAATCAGTAAATACGTCTACACCAAGA ACCTCACCATAGAGCAGTCCTTGGAACC
TAGGCTGGGAAATGCCACATGGATACTTGGTCAGAAGGAATGGCTCATGTTTTAGCAACCTGAACTTCTGGAAGGAGGTAGAAGATGATTAATTGGTGTGAAGGTTTCTCAAAATCAAGATCATA CAa
ACTTCAaGGAGTTACCTTTTATCATGTTGGAGTCAAATAAaACACTTGTCTATTATAAGTAAGGAGTGATCTGTGGATTTGAGTTGTTTCTTAAGAATAGTTCAGgctcacgcctgtaatcccagcac
tttgggaggccgaggcgggtggatcatgaggtcaggagatcgagaccatcctggctaacaaggtgaaaccccgtctctactaaaaatacaaaaaattagccgggcgcggtggcgggcgcctgtag tcc
cagctactcgggaggctgaggcaggagaatggcgtgaacccgggaagcggagcttgcagtgagccgagattgcgccactgcagtccgcagtccggcctgg gcgacagagcgagactccgtctcaaaaa
aaatagttcagTATCCTCTTaTTATTGaTATTAGTTTACTCTTCTATCTGTGAAACACAATAGGGCCTGGAATTAAATTTAAATCTGGAAAGTGTGTAAAACATAAGCAAACTCCAGAGCTTCTTTTA
AATTAACTGGTTTCTCAGTGAGAAGATTGGCCTTAGGTGGTTTTGACCTAACCTTTTTCCCTTGAGTTTCAATAGCTTGGTTAATCAGAATCTTTGTCTTTCTTAGTCATTAAATTCAAATCCTA AAT
GAATTTCTGTATCCAGATTACAAGGCATTAATTTTGTTCTAATTTTCATGTATGCGAGACAGTCCATGTCCAAATGCTGTTTATAAACCATCTCCATTGCCTGTTTCCTTGGTAATTGGAATGACTGG
CTCTCACTCATTATTTTTaAGAGACTTGAATTGTTGACACTGCTGTGTCAGTGTGAGATAAGGAAAGCTTAGCGATTTCTTTTGTTTAGAGTCTTCTTATTTACTTGTTGTACCTGAAGGTCAATGAC
TGTTAAGGCAGGGGTGGATTAGACTAAAATACAGAAAGCATAAAGGCAAATTGGAAAAGATCCATGGTGACACTATTTAGTTCTACCCAGTTAGAAACATGTAGGAAGATTTACCCTCGTAAGACTGT
AAAAATGGAACTAAAATATCTTCCATGCTTAGTGAAGTGATAAAGAGTGTTTTGCAGCTTACTAAGTAAGCCGCATGACCCTAGGCCTTGGAAAACTCATTTTTTCTTCCATAAAATTTGGGGGT GGG
CGAGTATTAAATAATCACCAAATCATTTTTCAGTATGATAATTCTATGGTTTCTAGCCAAATTCACATAAATTGTAATTCATAGCAAAAATCATAAAATTCATATTACCTATAACAAAATTACGTTGA
AGTTCAAAGCTATGGCTTATAATTCCTTTCAAGTTTGTTTTCACATTTGCTAAAGAAAATAACACATTTTACTCAATTTAAGTAATAAGACTAGGAGAAACTT tGGT

2749	0.3	0.0	0.0	UnnamedSequence	1001	1283	(1000)	+	AluYb8	SINE/Alu	14	296	(22)	1
191	32.5	3.4	13.1	UnnamedSequence	1983	2099	(184)	+	MIR3	SINE/MIR	93	199	(9)	2

>Hg19 chr1:108141474-108143466
GATGAAAGGAATCGTCACTGAAAAGGGCCAATgAATTTAGCTTTGTGTCACCTGGGTTCAGCTCAAGGCAGTTCTTCAGAAGCCAAGGTTAGATGTGAAAAGCCAGTAATACCACAGTGACCTGGATT
GGTAGACATAGATACATGAGTTCCTATCTGCATGTAAAAGCTGTGATTATAGTGAAAGCAATAGAGACCCAGCATTAAAAAAAAATTGTGCCAGAGAGCCTTAAAAGTTTCTACAATGATATTCT CTG
GAAAAATAACTAAAAGTACTTGTCACAGGACTAAAGTAAGCTTTTCTTATATGTTATGTATAAAATCTCAGCAATGCCACTATTAGAGACACGTGCATATGTGTTTTcgTCTCCATATCTGGGCATCT
TTTGTAAGGAAGCACGTATCACAGCGTGTTAAAGGGAAGGGACTAAGAATTTTAGCAAAGCAAAATGAAACTAACAAACATTAGCAAAGGATGTAATTATGAAAAGAAAAACAAGGAGCTGGAAA AAA
AATCTCAATATCAGAAAAGTGCTTTTATCAAGTTTAGTAAATAACTATAAAAATTGCTGAAGAGGCAGCTGAACTAATCTGAAGCTACCCCTCCATTTTGTTTCTGTTCATTTGTTGAGCAAATTTAT
ATTGAGTCCTTACAATGTGAAATCACTTTCTGGAGAACTCAAAAGGACTTAACCATTCTAAAAAGAAGCTTAAGAATCAGTAAATACGTCTACACCAAGAACCTCACCATAGAGCAGTCCTTGGA ACC
TAGGCTGGGAAATGCCACATGGATACTTGGTCAGAAGGAATGGCTCATGTTTTAGCAACCTGAACTTCTGGAAGGAGGTAGAAGATGATTAATTGGTGTGAAGGTTTCTCAAAATCAAGATCATACA g
ACTTCAtGGAGTTACCTTTTATCATGTTGGAGTCAAATAAcACACTTGTCTATTATAAGTAAGGAGTGATCTGTGGATTTGAGTTGTTTCTTAAGAATAGTTCA GTATCCTCTTgTTATTGgTATTAG
TTTACTCTTCTATCTGTGAAACACAATAGGGCCTGGAATTAAATTTAAATCTGGAAAGTGTGTAAAACATAAGCAAACTCCAGAGCTTCTTTTAAATTAACTGGTTTCTCAGTGAGAAGATTGGCCTT
AGGTGGTTTTGACCTAACCTTTTTCCCTTGAGTTTCAATAGCTTGGTTAATCAGAATCTTTGTCTTTCTTAGTCATTAAATTCAAATCCTAAATGAATTTCTGTATCCAGATTACAAGGCATTAA TTT
TGTTCTAATTTTCATGTATGCGAGACAGTCCATGTCCAAATGCTGTTTATAAACCATCTCCATTGCCTGTTTCCTTGGTAATTGGAATGACTGGCTCTCACTCATTATTTTT gAGAGACTTGAATTGT
TGACACTGCTGTGTCAGTGTGAGATAAGGAAAGCTTAGCGATTTCTTTTGTTTAGAGTCTTCTTATTTACTTGTTGTACCTGAAGGTCAATGACTGTTAAGGCAGGGGTGGATTAGACTAAAATACAG
AAAGCATAAAGGCAAATTGGAAAAGATCCATGGTGACACTATTTAGTTCTACCCAGTTAGAAACATGTAGGAAGATTTACCCTCGTAAGACTGTAAAAATGGAACTAAAATATCTTCCATGCTTAGTG
AAGTGATAAAGAGTGTTTTGCAGCTTACTAAGTAAGCCGCATGACCCTAGGCCTTGGAAAACTCATTTTTTCTTCCATAAAATTTGGGGGTGGGCGAGTATTAAATAATCACCAAATCATTTTTC AGT
ATGATAATTCTATGGTTTCTAGCCAAATTCACATAAATTGTAATTCATAGCAAAAATCATAAAATTCATATTACCTATAACAAAATTACGTTGAAGTTCAAAGCTATGGCTTATAATTCCTTTCAAGT
TTGTTTTCACATTTGCTAAAGAAAATAACACATTTTACTCAATTTAAGTAATAAGACTAGGAGAAACTT cGGT

>KSI_AluYb8_43

>Scaffold12674-678892-679193
AGTTAAGAAACTTTTCCAGGGGTTTACACACAGTGAGTACTGAGGATAGTATTTAAATAAGTGCACTCTGATCCTGGAATCTATACTATTAACCATTTTGCTATATGAGGAGTATATTTCAGATGC AT
GCACTTTTCTGACCCTGCCTGGGAACACTGAAAACTTGATGACTTTGTAGATTTAGCCAAGCCCCTTGCTTATGTGAGAAAAGTTGGCATGTAAAAGTCCTGTAAAATGTTGGCTGCCTTAGCAA GTG
TAAGTATATAATTCTGTAATGACACTAGAAAAAGATGGTGGCTTTGAGATATAAGTCTAATATACAAGCTCATTTTGTGTAACTAATCACAGCAAACTTGTGCTGAACTTGTGATTTTGAGAGACT AT
AAAACTGGTCGTTATCATTGAGTTCAGATCAGATAAATACAGACTACAATAGTTTGTCACACTGACTGCAAAAGGCAATCCTTGGCAATTATGCCATATTAAACTGATAATTAAAAGTTTATGAA TAA
GAATGGTGACATATAACTTGAAACAATTATGCACTTAATAACTTAAGTCCTCCTTTCCTATTCTCTGCCCTCAAAATTAGAACTAAAGATTTCTAGATTGAATGTTCAGGGCAACTGGACAATTTTCT
TATAATTTATAGGGAAAATCTGGTCCATGTTTGTTTACATAATCACTTGTTACAGATAATATATTTTGG GGTATTAGTGCATTTTTCTATTATTGTATCACTGTCCTCCAGGGTTTTGTTCTCCAGGT
TTGGGTTCTTTCCTCTGATGTCCCCCTTAATAATATGTCACAGGTATGGAGTGAAAGTGTGTAGGGGAGGAGGAGGAAGTTT ATCCTTCACCTGTAATGACGCTTCTACTCATTACTGAAATTGTTAT
TTGTTTTGTTCTGTTGGTCAGACAAGGTGTTTCACTCCAATTTGTCCTCACTTTAAATGTGAGATTTTGTATTAACTTTGCTAAGGTCTTT TGCCCCACTAATATTTTATttttttttgagacggagt
ctcgctctgtcgcccaggccggactgcggactgcagtggcgcaatctcggctcactgcaagctccgcttcccgggttcacgccattctcctgcctcagcctcccaagtatctgggactacaggcac cc
gccaccgcgcccggctaattttttgtatttttagtagagacggggtttcaccttgttagccaggatggtctcgatctcctgacctcatgatccacccgcttcggcctcccaaagtgctgggatta cag
gcgtgagccaccgcgcccggcctgccccactaatattttatGATTCTGAATTTAAAGCCAATATAATTAAAATAAAACTATTGTGTTATCAGTAAAAATCTTCACATTTACTGGGCAACTAAATGGAA
ACAAGTTAGGTTTATTTATATTTAGCATTTTTGGCCTTAAAGCAAAATTTATGTAAGATGCAAAGATAGGCTATGTATGAAAAATTCTATGTTAAGCCAGAAATTACTAGGTGTCTACAATTTGA ACC
ATATGTGTCAGTAGCAGAATTCTTTTGTTTATTTGCAAAATGGTTTTATTGTCCAGGATTATACTTTTCTTGTATTAAGAGAATCATTAAGTGGCTCTGTGCCTAATACATAG ACACTTGCCACTGAA
GCTTTCAAATTTTTACTCACTTTAATTCAGCAAGTCAAGAATTCAAACATTCTCTTAATATTTACTAAAACTCACAGCCTAGAATTATTTTTGATTTAAAATTTTCAATCAAGAGCTCACAACTA AAT
CAAAGAATTTCATGAGGACGAATAAAAGCCTCATCTTACCTC CTAAGATTTCAAAATTATTAAAAAGAATAATTTTAAAATTAATATGTTCCCATAGAATT GGTCACTTACTGCTTGTCCATAATTCC
TGTATGACACAGAATTAAAATGAGACAACTCTATTATTGTTAGAGTTACTGCAGTAATTGTAGTAATTATGCAGATGATGTTTATGATTAAAGTACTTATAATCTGAAAAGGAGACAGAGGGCAA ATT
TATTATATTCAGAATAAACATTTTGGTGTAATAGTTTGCATT GCTGAAAAAACTCATCTAGTTAAAACTTTGATCTAGTTTCTGAAGTCTTAGAAGTATTAAATATTAAGAAAATATTTTTCTGATTT
ATATGAAGCCCAGTTAATCTGGAGGAAACATTAACACCATCAGTCCATTAATCATTAATCTTCCATTTAGGCTAAAACATATTGTATTTTTTG GGGCCGGGCGCAGTGGCTCACGCCTGTAATCCC

285	36.3	1.9	1.9	UnnamedSequence	2	105	(2197)	C	MIR	SINE/MIR	(154)	108	5	1
2837	2.0	0.0	0.0	UnnamedSequence	1001	1302	(1000)	C	AluYb8	SINE/Alu	(16)	302	1	2
240	3.1	0.0	0.0	UnnamedSequence	2271	2302	(0)	+	Alu	SINE/Alu	1	32	(101)	3

>Hg19 chr11:60292400-60294386
AGTTAAGAAACTTTTCCAGGGGTTTACACACAGTGAGTACTGAGGATAGTATTTAAATAAGTGCACTCTGATCCTGGAATCTATACTATTAACCATTTTGCTATATGAGGAGTATATTTCAGATGC AT
GCACTTTTCTGACCCTGCCTGGGAACACTGAAAACTTGATGACTTTGTAGATTTAGCCAAGCCCCTTGCTTATGTGAGAAAAGTTGGCATGTAAAAGTCCTGTAAAATGTTGGCTGCCTTAGCAAGTG
TAAGTATATAATTCTGTAATGACACTAGAAAAAGATGGTGGCTTTGAGATATAAGTCTAATATACAAGCTCATTTTGTGTAACTAATCACAGCAAACTTGTGCTGAACTTGTGATTTTGAGAGAC TAT
AAAACTGGTCGTTATCATTGAGTTCAGATCAGATAAATACAGACTACAATAGTTTGTCACACTGACTGCAAAAGGCAATCCTTGGCAATTATGCCATATTAAACTGATAATTAAAAGTTTATGAATAA
GAATGGTGACATATAACTTGAAACAATTATGCACTTAATAACTTAAGT CCTCCTTTCCTATTCTCTGCCCTCAAAATTAGAACTAAAGATTTCTAGATTGAATGTTCAGGGCAACTGGACAATTTTCT
TATAATTTATAGGGAAAATCTGGTCCATGTTTGTTTACATAATCACTTGTTACAGATAATATATTTTGGGGTATTAGTGCATTTTTCTATTATTGTATCACTGTCCTCCAGGGTTTTGTTCTCCAGGT
TTGGGTTCTTTCCTCTGATGTCCCCCTTAATAATATGTCACAGGTATGGAGTGAAAGTGTGTAGGGGAGGAGGAGGAAGTTT ATCCTTCACCTGTAATGACGCTTCTACTCATTACTGAAATTGTTAT
TTGTTTTGTTCTGTTGGTCAGACAAGGTGTTTCACTCCAATTTGTCCTCACTTTAAATGTGAGATTTTGTATTAACTTTGCTAAGGTCTTT TGCCCCACTAATATTTTATGATTCTGAATTTAAAGCC
AATATAATTAAAATAAAACTATTGTGTTATCAGTAAAAATCTTCACATTTACTGGGCAACTAAATGGAAACAAGTTAGGTTTATTTATATTTAGCATTTTTG GCCTTAAAGCAAAATTTATGTAAGAT
GCAAAGATAGGCTATGTATGAAAAATTCTATGT TAAGCCAGAAATTACTAGGTGTCTACAATTTGAACCATATGTGTCAGTAGCAGAATTCTTTTGTTTATTTGCAAAATGGTTTTATTGTCCAGGAT
TATACTTTTCTTGTATTAAGAGAATCATTAAGTGGCTCTGTGCCTAATACATAG ACACTTGCCACTGAAGCTTTCAAATTTTTACTCACTTTAATTCAGCAA GTCAAGAATTCAAACATTCTCTTAAT
ATTTACTAAAACTCACAGCCTAGAATTATTTTTGATTTAAAATTTTCAATCAAGAGCTCACAACTAAATCAAAGAATTTCATGAGGACGAATAAAAGCCTCATCTTACCTCCTAAGATTTCAAAA TTA
TTAAAAAGAATAATTTTAAAATTAATATGTTCCCATAGAATT GGTCACTTACTGCTTGTCCATAATTCCTGTATGACACAGAATTAAAATGAGACAACTCTA TTATTGTTAGAGTTACTGCAGTAATT
GTAGTAATTATGCAGATGATGTTTATGATTAAAGTACTTATAATCTGAAAAGGAGACAGAGGGCAAATTTATTATATTCAGAATAAACATTTTGGTGTAATAGTTTGCATTGCTGAAAAAACTCA TCT
AGTTAAAACTTTGATCTAGTTTCTGAAGTCTTAGAAGTATTAAATATTAAGAAAATATTTTTCTGATTTATATGAAGCCCAGTTAATCTGGAGGAAACATTA ACACCATCAGTCCATTAATCATTAAT
CTTCCATTTAGGCTAAAACATATTGTATTTTTTGG GGCCGGGCGCAGTGGCTCACGCCTGTAATCCC

375 36.8 1.4 1.4 Hg19 60 205 (1982) C MIR SINE/MIR (112) 150 5 1

1211	3.0	0.0	0.0	Hg19	2056	2187	(0)	+	AluYg6	SINE/Alu	1	132	(179)	2

>KSI_AluYb8_44

>Scaffold23-142365-142683
AAAATCATATTATCCTTTAAGTCTTCATTGTTATAATAGCTGTTGTTAAAGTCCATGCCTACTGAGTCAGCAATAGACTTCTCTTTGCTTTTTATTAATCCTTCTTTCTTTGTAATCATGGGTAAC AT
TTTTCTACATCTTAGAATGTCTAATAAGTTCTTATTATAACTCCTTACAATGTAAAGAGTCTGCACTATATTATCTTTTTAAGAAGCTCTTTGAATTCTTCTGACAAACAGTTAATTTACTGATG GTT
CTCCTTGATCTTGTTATTGCTTGGTTCAAGCTTCATTAAAGGACGTCTAGGGTACCCCCTTTATTTCTAGGCACAGACTTTTTGGTGTCTAAACTAAACATCTTATTGTTAATAAAGTTCCTCAAT TC
TTCTGGCTATAAATTTATTATCTGACTGCACCGTGAAATTCTAGCTTCTGTGTCTTGAGCCCAGAACAACTATTCTCTGTAGGATTTTCATAGTCTCAATTTGTACAAACATAGTTATATATTAT ATC
ACTTTGAAGCTTATCTTTAGATGTCTTGTGTACAGTGACATCTAATATATGTAACTATGAAGCCATAACCCCAAGGGTTAGTCACGAAATCTGTGTTGATTTGCCTACAATTCTACCACGTATTTT CT
ATAAGCATGCAAATCTAGTATAGGTAGAGGATATTACAGGCTAATTAATCTCTTGGCATCTGGTCTACCCAGGCCCAGTGCTTTGTTCTTGAACAAACAAATAAAAAAAAACACAGAGAAATAACgAT
GCAAATATGAGAaATGTTGCAGAAATTTGAAATTGAGACAGCTTCCTCTTTTCTATAGGATTTTTTTAGGGGAAAACAATCTCTATATTCAGTCTTATATATTACCTGCCTTCAAAAAATCAAAACA
TTGAAAGTTAAGCAAAATTCCTGTCAGAAAGGAAACCTGACATAATACA tGTTGCACAGTTCTAAAACTACTGAAAGTTAGTGTTTTATTAAGAAGA TTTTGGGgccgggcgcggtggctcacgcctg
taatcccagcactttgggaggccgaggcgggtggatcatgaggtcaggagatcgagaccatcctggctaacaaggtgaaaccccgtctctactaaaaaatacaaaaaattagccgggcgcggtg gcg
ggcgcctgtagtcccagctactcgggaggctgaggcaggagaatggcgtgaacccgggaagcggagcttgcagtgagccgagattgcgccactgcagtccgcag tccggcctgggcgacagagcgaga
ctccgtctcaaaaaaaaaaaaaaaaaaaaaaaaaaaattttgggAACCAGAGAAAAATTcCATGAAAAGACTGAGTTTTAGTGTGTGCTCACAGCTGAAAAGAATGATCATTTTCTCGTTTGCTGAT
AAAGTTGCTATATACAAGCAGAGCTTCAATTATTGATCAACTATTTTTTACCAATACTATGTCTTCAGTCCTCAGAGGTGTGTGGCAAATTCTGTTTCCTGACA TTAACGTAcGGGGACTTTTGAGTT
GACAGGTTCTGGGATGTGCCTGTGTTTTTGTGATCATATCAGCCTGTGATACAGGATACTATCACAATGGATTTACTGTGAATCTCCTACGCCGTCTTGATTTAAGTGGCATACATAGAATCCCA TTG
TGAGAAAGAACAGCCTTGCTTGGCTTCTTAAGAGAAAATTCTAAGCATGGTTCAAGTCACCCTCAAATGAGATCATTCACATTTAAATAGTCTTTTACATTGAA GGTATTTTTTATGAATGCTTCTAT
ATTACAATTACGCAAATTTGTGGAAGAGATTTTTTGAAACTCACCTAATGCTGTTCTTGCTGGCGTAGCATCTTTTTTGATCCAAAATGACACCCAGGATAAAACCACAGTCAGTATACA GGGAATGT
ATGTCTGAATGGTGAAGTATCCCATTCTTCTACTCAATTCAAAATATATAGTCATGACAACATAATCACCTGTAATAAAATATTGTCAAAATTAAGACAACTTT GATGGGTCAAAAATAATTCAGGAA
AATAATTTACAATTTATTTTGTTAAGTGAATGACTTGGATAAAAATGCACAACTACAACAATATCAAATCAGGAAATGGATCAAAATGCT tGTTTTCTTAAACTTAATTTTCATAGTAtCCATGGCTG
TACCAGGCATTTAATCGGTCAGTGTAAAGGGCACAAAACGAACATATTCACGGATAGAGTGAGAACACATGCAGATCTCTTCCACGACAGGCCATGAGTTGATG AGCTATTATCgCTTGAGTCCTTCC
ATAGGTTGTACAAT

247	27.2	8.6	3.1	TErefseq_13	13	164	(2154)	C	L1M4a2	LINE/L1	(1451)	1205	1046	1
716	28.4	5.1	8.9	TErefseq_13	461	869	(1449)	C	Kanga2_a	DNA/TcMar-Tc2	(490)	397	3	2
3021	0.3	0.0	0.6	TErefseq_13	1000	1318	(1000)	+	AluYb8	SINE/Alu	1	317	(1)	3

>hg19 chr15 27764788 27766781
AAAATCATATTATCCTTTAAGTCTTCATTGTTATAATAGCTGTTGTTAAAGTCCATGCCTACTGAGTCAGCAATAGACTTCTCTTTGCTTTTTATTAATCCTTCTTTCTTTGTAATCATGGGTAAC AT
TTTTCTACATCTTAGAATGTCTAATAAGTTCTTATTATAACTCCTTACAATGTAAAGAGTCTGCACTATATTATCTTTTTAAGAAGCTCTTTGAATTCTTCTGACAAACAGTTAATTTACTGATG GTT
CTCCTTGATCTTGTTATTGCTTGGTTCAAGCTTCATTAAAGGACGTCTAGGGTACCCCCTTTATTTCTAGGCACAGACTTTTTGGTGTCTAAACTAAACATCTTATTGTTAATAAAGTTCCTCAAT TC
TTCTGGCTATAAATTTATTATCTGACTGCACCGTGAAATTCTAGCTTCTGTGTCTTGAGCCCAGAACAACTATTCTCTGTAGGATTTTCATAGTCTCAATTTGTACAAACATAGTTATATATTAT ATC
ACTTTGAAGCTTATCTTTAGATGTCTTGTGTACAGTGACATCTAATATATGTAACTATGAAGCCATAACCCCAAGGGTTAGTCACGAAATCTGTGTTGATTTGCCTACAATTCTACCACGTATTTT CT
ATAAGCATGCAAATCTAGTATAGGTAGAGGATATTACAGGCTAATTAATCTCTTGGCATCTGGTCTACCCAGGCCCAGTGCTTTGTTCTTGAACAAACAAATaAAAAAAAAACACAGAGAAATAACaA
TGCAAATATGAGAgATGTTGCAGAAATTTGAAATTGAGACAGCTTCCTCTTTTCTATAGGATTTTTTTAGGGGAAAACAATCTCTATATTCAGTCTTATATATTACCTGCCTTCAAAAAATCAAAAC
ATTGAAAGTTAAGCAAAATTCCTGTCAGAAAGGAAACCTGACATAATACA cGTTGCACAGTTCTAAAACTACTGAAAGTTAGTGTTTTATTAAGAAGA TTTTGGGAACCAGAGAAAAATTCATGAAAA
GACTGAGTTTTAGTGTGTGCTCACAGCTGAAAAGAATGATCATTTTCTCGTTTGCTGATAAAGTTGCTATATACAAGCAGAGCTTCAATTATTGATCAACTATTTTTTACCAATACTATGTCTTC AGT
CCTCAGAGGTGTGTGGCAAATTCTGTTTCCTGACATTAACGTA tGGGGACTTTTGAGTTGACAGGTTCTGGGATGTGCCTGTGTTTTTGTGATCATATCAGCCTGTGATACAGGATACTAT CACAATG
GATTTACTGTGAATCTCCTACGCCGTCTTGATTTAAGTGGCATACATAGAATCCCATTGTGAGAAAGAACAGCCTTGCTTGGCTTCTTAAGAGAAAATTCTAAGCATGGTTCAAGTCACCCTCAA ATG
AGATCATTCACATTTAAATAGTCTTTTACATTGAAGGTATTTTTTATGAATGCTTCTATATTACAATTACGCAAATTTGTGGAAGAGATTTTTTGAAACTCACCTAATGCTGTTCTTGCTG GCGTAGC
ATCTTTTTTGATCCAAAATGACACCCAGGATAAAACCACAGTCAGTATACAGGGAATGTATGTCTGAATGGTGAAGTATCCCATTCTTCTACTCAATTCAAAATATATAGTCATGACAACATAATCAC
CTGTAATAAAATATTGTCAAAATTAAGACAACTTTGATGGGTCAAAAATAATTCAGGAAAATAATTTACAATTTATTTTGTTAAGTGAATGACTTGGATAAAAATGCACAACTACAACAAT ATCAAAT
CAGGAAATGGATCAAAATGCTgGTTTTCTTAAACTTAATTTTCATAGTA aCCATGGCTGTACCAGGCATTTAATCGGTCAGTGTAAAGGGCACAAAACGAACATATTCACGGATAGAGTGAGAACACA
TGCAGATCTCTTCCACGACAGGCCATGAGTTGATGAGCTATTATC aCTTGAGTCCTTCCATAGGTTGTACAAT

***A*l*u*Yb9**

Instruction

Green box: Primer information

Red letter: Target site duplication

Black letter: Insertion sequences

Green letter: Deleted sequenced by insertion-mediated deletion

>KSI_AluYb9_1

>Scaffold2988-5009447-5009660

TGATATATATATATAGAAGGCATACTATGGGTCAGTACTAAGTATGCAACTATTTTATATCATTTAATCTTCAAGATAACTATGTCATTAGATGCATTTTTTTATCATTGTTATATCAGGGACTAT GT
AGCTAATATTACCCTCTTTTTTTTAGGTGAGGAAATACAGAAGCGCTCAGAGTGGTAGATTTTGCTTACACTGATATAACTAGTAAATGGCAGATCTAGCATATGGACCCAGATATATTTCTTTC TTA
AACCATTTCTGTTTTGTGACACATTATAAATGCCTTCAATTTAGTCATTGTCAAACTTTTATTTTATTTTCAGATGTTTAAAAAATTCTTCAAATCTTCCCTAAAACTCTTATAAAATGTGAAATT TT
TAAAAGCCAAGGAGTTATCAGTAGGCTCATATTTCTGGGAGATGAGAGAGGGCAAGCCTGTGTTACCTGAACCTGAGATAGAGATGAAGAATCTCTTTCATTCCATTTGGAGGGAACACACTTAG ATC
CCAGGGAAGAAAGATGAGTATAATTCTATGAACTGGGCCCAAGTGTGATTTCAGCTTCATGTGGAATATTGAGAAAGCTGAGGCTAGAGAACTGTAAACATTTAATCTTAATTTTAGTAGGTGAAT AC
ATCAGTTTATCTTTGTTTCTACTCTGCTTTTTGAATGGCATGGTGAGGGGCAGATTGGGCATCACATAAGATTTGCATCTCTACTTAAT CACTGGGTGAGCTTGATCTGATCATGCTAGGTTTCTGAG
TCTGTTTTTTTCTTTCATTTGTAAAGTAAGACAAAGTCTAATCAAAGTATGTCTAATCATACTATAATAATGCAACTAAAGGAGTAAGCACACCAGTGAATTCATACCATCTCCTTATTATTTGAT TG
TTCTTTGCCTTCTCAAAAGGCTATTTCTTCAGGATGCAGCTGTTTTGACTCTCTT AGGAGACTACTTTCttttttttttttttttttttttttttttttttgagcgcttcccgggttcacgccattct
cctgcctcagcctccccagtagctgggactacaggcgcccgccaccgcgcccggctaatttttgtatttttagtagagacggggtttcaccttgttagccaggatggtctcgatctcctgacctc at
gatccacccgcctcggcctcccaaagtgctgggattacaggcgtgagccaccaggcccggcc aggagactactttcAAGTAAGCTCACTATGGCACATGCTATGGTTTCCAAGGAAGGGTTTATGTGG
CAGAGGCAATGGTTAGCAGTAATAAAACAGGAAGACCCCTTAGTTTTCAGCACATTATTTCACAGATAGTGTTAAAAATCATTCTGAGCTTATAATTGAAACACTAGTTCCAGGGATGTAATTTC AAT
CCTTATATTTACTTAAGGACCCCAAGGTGAAGTCTCTTTGGATACAGATGATTTTATTCTTAAACAGAGAAAATAAT AATATCTGTATAACGGGAACATTATGGACATCAAAGGAGATATTAGTAGGC
AACTGCAGAGTTAACAGATCTTGGTTTTAATCCTGATTTTACCAATCTACAGTTTTGACTTAGCCCCTTTGTCTCTGTGAACCAACACCCACACCATCAAAAATAAGAGGCTTGCAACTTATGGT TAA
ATTTCCTTCTAGTTGATATTCTGTAATTCTGTGACTTGAACAAGGTCTCACCAAAGTAGATAAAGTCCAGGTTGTCG CCCTGCTAGTCTGGTGCTTTCTACTCTACATGTTATTTCAGCTCAGAGATT
TGTACCTTGTGGCTTTAAGGAAAGTCTTCTTTTGAACAAAAGCTGCCTGCAATGGTATCTCCAAATTGCAACCCCTCTTAGATCTTGTGAAGATCCCTGGTTGATCTGTGTCTAAGAAAATTCAC CTA
CCTGCCCAATCTGTGGTATTCTTTTGTCATGAAGTTTATCAGCTTTTGATTCATACTATACTGATAAAGAAAACATA TTACTTATGTGTCAAAGGAGCTAGTTTCAAGTACCAGATATTCTGTTGTGT
GAACTGTGGCTAGACTTCCACTCAAAAATCAACAACAGCAACAATAACAGATGCTAAGAGAGCATACATTAAAAATTCTTAGGCCTACCATGCAGGCAAAGTTAAAGGGGTAATGACTTGCTGAT GGC
ACAGAAAATAGTTCAAGCCCCAAAACCCAGACATTAAG

292	27.9	2.6	4.4	KS2	127	242	(1972)	C	MIRb	SINE/MIR	(102)	166	53	3
34	0.0	0.0	0.0	KS2	966	997	(1217)	+	(T)n	Simple_repeat	1	32	(0)	4
2016	0.9	0.0	0.0	KS2	1001	1214	(1000)	C	AluYb9	SINE/Alu	(104)	214	1	5
233	32.0	10.4	2.3	KS2	1538	1700	(514)	+	MIR3	SINE/MIR	32	207	(1)	6

>Hg19 chr9:120389734-120391684

TGATATATATATATAGAAGGCATACTATGGGTCAGTACTAAGTATGCAACTATTTTATATCATTTAATCTTCAAGATAACTATGTCATTAGATGCATTT TTTTATCATTGTTATATCAGGGACTATGT
AGCTAATATTACCCTCTTTTTTTTAGGTGAGGAAATACAGAAGCGCTCAGAGTGGTAGATTTTGCTTACACTGATATAACTAGTAAATGGCAGATCTAGCATATGGACCCAGATATATTTCTTTC TTA
AACCATTTCTGTTTTGTGACACATTATAAATGCCTTCAATTTAGTCATTGTCAAACTTTTATTTTATTTTCAGATGTTTAAAAAATTCTTCAAATCTTC CCTAAAACTCTTATAAAATGTGAAATTTT
TAAAAGCCAAGGAGTTATCAGTAGGCTCATATTTCTGGGAGATGAGAGAGGGCAAGCCTGTGTTACCTGAACCTGAGATAGAGATGAAGAATCTCTTTCATTCCATTTGGAGGGAACACACTTAG ATC
CCAGGGAAGAAAGATGAGTATAATTCTATGAACTGGGCCCAAGTGTGATTTCAGCTTCATGTGGAATATTGAGAAAGCTGAGGCTAGAGAACTGTAAAC ATTTAATCTTAATTTTAGTAGGTGAATAC
ATCAGTTTATCTTTGTTTCTACTCTGCTTTTTGAATGGCATGGTGAGGGGCAGATTGGGCATCACATAAGATTTGCATCTCTACTTAAT CACTGGGTGAGCTTGATCTGATCATGCTAGGTTTCTGAG
TCTGTTTTTTTCTTTCATTTGTAAAGTAAGACAAAGTCTAATCAAAGTATGTCTAATCATACTATAATAATGCAACTAAAGGAGTAAGCACACCAGTGAATTCATACCATCTCCTTATTATTTGA TTG
TTCTTTGCCTTCTCAAAAGGCTATTTCTTCAGGATGCAGCTGTTTTGACTCTCTT AGGAGACTACTTTCAAGTAAGCTCACTATGGCACATGCTATGGTTTCCAAGGAA GGGTTTATGTGGCAGAGGC
AATGGTTAGCAGTAATAAAACAGGAAGACCCCTTAGTTTTCAGCACATTATTTCACAGATAGTGTTAAAAATCATTCTGAGCTTATAATTGAAACACTAGTTCCAGGGATGTAATTTCAATCCTT ATA

TTTACTTAAGGACCCCAAGGTGAAGTCTCTTTGGATACAGATGATTTTATTCTTAAACAGAGAAAATAATAATATCTGTATAACGGGAACATTATGGACATCAAAGGAG ATATTAGTAGGCAACTGCA
GAGTTAACAGATCTTGGTTTTAATCCTGATTTTACCAATCTACAGTTTTGACTTAGCCCCTTTGTCTCTGTGAACCAACACCCACACCATCAAAAATAAGAGGCTTGCAACTTATGGTTAAATTT CCT
TCTAGTTGATATTCTGTAATTCTGTGACTTGAACAAGGTCTCACCAAAGTAGATAAAGTCCAGGTTGTCGCCCTGCTAGTCTGGTGCTTTCTACTCTACATGTTATTTC AGCTCAGAGATTTGTACCT
TGTGGCTTTAAGGAAAGTCTTCTTTTGAACAAAAGCTGCCTGCAATGGTATCTCCAAATTGCAACCCCTCTTAGATCTTGTGAAGATCCCTGGTTGATCTGTGTCTAAGAAAATTCACCTACCTG CCC
AATCTGTGGTATTCTTTTGTCATGAAGTTTATCAGCTTTTGATTCATACTATACTGATAAAGAAAACATATTACTTATGTGTCAAAGGAGCTAGTTTCAAGTACCAGAT ATTCTGTTGTGTGAACTGT
GGCTAGACTTCCACTCAAAAATCAACAACAGCAACAATAACAGATGCTAAGAGAGCATACATTAAAAATTCTTAGGCCTACCATGCAGGCAAAGTTAAAGGGGTAATGACTTGCTGATGGCACAG AAA
ATAGTTCAAGCCCCAAAACCCAGACATTAAG

292	27.9	2.6	4.4	Hg19	227	342	(1809)	C	MIRb	SINE/MIR	(102)	166	53	1
233	32.0	10.4	2.3	Hg19	1375	1537	(614)	+	MIR3	SINE/MIR	32	207	(1)	2

>KSI_AluYb9_2

>Scaffold3569-403792-403941

AAATTAGAATTAAAAGCTAGCTGATTTTTTTTTCACTGGCATATA aATAACCCTGAATTAAGATTTGGAAAATTTCAATTTGGTTCTGGCGATCTCATTAACTCATCAGCTTTGTTTTTATGTATTAT
AACTAGTATTaCCTTTCCAGATCTCAAGTATTTCGTGTGTACAATTATAGAATTGGAGTAAGCCATCACTGGGGCACTTTCTGGATTTTAAATTCAGTAGGTCTGAAATTCTGACACCGAAGCTGACT
GTCTCAGTTAAGGAAAGTGTTTCAGAACACTAACCTATTTCCACCTGGACACACATCCAGCAATTAGATGTTTCTATACTTTCTTCTGCTCCCACTCTTTCTTGGACCACCCCACCACTTCACTAAAA
CTGCTGATTTAAGGACATCAAATGACTTCCATGTTGATAATCCAGTGGTCAGTTCTTTTTCCTCTAGACTTTTACACGGCATTT gATACAACAAATTACTTGCTTTTTCTTGAAACATTTCTTCTTCT
GGTTCCTGGAACATTATTAACATCGTTCTCTCTTATATCTACCTCAGTATGTGACTCCTCCCACACTTTTGCTTAATCCCTTTTTCTTGACCTTTAATGACTGAAGTGTCTCAAGCGTCATTTCT CAG
ATCTCATCACTTTTTCATTTACATTCATTAGCTAAGTGATCTTATCAAGTCCTGTAGCCTTCAAAACATAGTAGCAACTCATAATATTATCTCTGGCCAGGAATTAACCCTTTAACTTCAATATTAAA
ATCCAATTGCACACAATATGTCTTTATTTGGGGACCAAATAGGCATTTCAGACTT AACATGTCCAAAACCATTATCTTGATTCCCACTATCTACCTGCTTCCAAATCTTTCCTTCTAGTCTTCCCCAT
CTAGGTAAAGGACCATCACATTCTACCAGTAGTCAAGGCAAATATTTTAAGATCATCTCTAACTCATCTCTGGAATAGAATTCCT ACTCtATAATTTAAAAATCggccgggcgcggtggctcacgcct
gtaatcccagcactttgggaggccgaggcgggtggatcatgaggtcaggagatcgagaccatcctggctaacaaggtgaaaccccgtctctactaaaaatacaaaaaattagccgggcgcggtgg caa
catgtccaaaaccattatcttgattcccactatctacctgcttccaaatctttccttctagtcttccccatctaggtaaaggacc atcacattctaccagtagtcaaggcaaatattttaagatcatc
tctaactcatctctggaatagaattcctactccataatttaaaaatcTTCTTTAAAAAATATAAGTATACAGAATTTTCTTAATTCTACTTATTTTTCATTACA tACTCATTTATCCACACCAAAACT
TATTTATTTAATCTTCAATTCATTTTTAAAAATAACATCACAAGCACTCATGAACCTATCATTCAATACAAAAAGGGTACTCAATGGATAATCTATGCATCCCCCAGCCTTCCACAACATCCGCCTGC
CACTCCTAACCCAAGTAATAACCATCAAGATGCTTCATTCACTATTTCCTTGCTGTAGGTTTATATATTTTTAACTCATCTTTATGTATTCCTCTACATTTTATTAGCTTCTTAAAAACACTTTC ATG
GTGTATGTACATTTATACTCAATATTATATTGCTGAAATTCATCTATGTTGTTGCATGTGGATGAGGTTAATTCACTCTTACTGC TTACTGATATGATTTATTAATATTAATATGTTAATATTAATTC
TATTACGTGACTATCCAACACGAGATAGATTCACTCTTGTGTCAATGAACTAAAATGATTGTTTCTAGGTTTTGGTCACTGTAAGCC aTGCATCTGTAAACAATCTTGTACATGTAAAAAGTTTCTCC
CAGATGGTAGCAGATGCTTCCAGTATCTTGCCCAGATTCTCAGGGACTCCTTTACTGGGTTCTGTTGGCACTGCTGCTAAACGCT TCCACCTGCACCTTTCCCTGAGTGACAGCACTTGCAAAATTGA
TCCAACCTTATCCGGGTTGCTGGGCTTGTGCTCCTCCCACCCATTCTCCTCACTGGAGGCAGTTGGGAGTCAATGTAGGTGTACAAAAGCCCTACTACCCA G

1192	31.2	8.1	2.6	KS	337	967	(1183)	+	L2	LINE/L2	1917	2581	(838)	1
1438	0.0	0.0	0.0	KS	1001	1150	(1000)	+	AluYb9	SINE/Alu	1	150	(168)	2
290	27.9	2.8	8.0	KS	1151	1294	(856)	+	L2	LINE/L2	2445	2581	(838)	3
425	27.0	12.0	11.3	KS	1574	1907	(243)	C	L1ME3F	LINE/L1	(225)	6018	5683	4
1177	31.4	8.1	2.6	hg19	437	1067	(956)	+	L2	LINE/L2	1917	2581	(838)	5
411	27.3	12.0	11.3	hg19	1347	1680	(343)	C	L1ME3F	LINE/L1	(225)	6018	5683	6
347	26.3	2.0	0.0	hg19	1923	2021	(2)	+	LTR16A1	LTR/ERVL	322	422	(34)	7

>hg19 chr1:79581253-79583075

AAATTAGAATTAAAAGCTAGCTGATTTTTTTTTCACTGGCATATA cATAACCCTGAATTAAGATTTGGAAAATTTCAATTTGGTTCTGGCGATCTCATTAACTCATCAGCTTTGTTTTTATGTATTAT
AACTAGTATTgCCTTTCCAGATCTCAAGTATTTCGTGTGTACAATTATAGAATTGGAGTAAGCCATCACTGGGGCACTTTCTGGATTTTAAATTCAGTAGGTCTGAAATTCTGACACCGAAGCTGACT
GTCTCAGTTAAGGAAAGTGTTTCAGAACACTAACCTATTTCCACCT GGACACACATCCAGCAATTAGATGTTTCTATACTTTCTTCTGCTCCCACTCTTTCTTGGACCACCCCACCACTTCACTAAAA
CTGCTGATTTAAGGACATCAAATGACTTCCATGTTGATAATCCAGTGGTCAGTTCTTTTTCCTCTAGACTTTTACACGGCATTT aATACAACAAATTACTTGCTTTTTCTTGAAACATTTCTTCTTCT
GGTTCCTGGAACATTATTAACATCGTTCTCTCTTATATCTACCTCA GTATGTGACTCCTCCCACACTTTTGCTTAATCCCTTTTTCTTGACCTTTAATGACTGAAGTGTCTCAAGCGTCATTTCTCAG
ATCTCATCACTTTTTCATTTACATTCATTAGCTAAGTGATCTTATCAAGTCCTGTAGCCTTCAAAACATAGTAGCAACTCATAATATTATCTCTGGCCAGGAATTAACCCTTTAACTTCAATATTAAA
ATCCAATTGCACACAATATGTCTTTATTTGGGGACCAAATAGGCAT TTCAGACTTAACATGTCCAAAACCATTATCTTGATTCCCACTATCTACCTGCTTCCAAATCTTTCCTTCTAGTCTTCCCCAT
CTAGGTAAAGGACCATCACATTCTACCAGTAGTCAAGGCAAATATTTTAAGATCATCTCTAACTCATCTCTGGAATAGAATTCCTACTCcATAATTTAAAAATC TTCTTTAAAAAATATAAGTATACA
GAATTTTCTTAATTCTACTTATTTTTCATTACA cACTCATTTATCCACACCAAAACTTATTTATTTAATCTTCAATTCATTTTTAAAAATAACATCACAAGCACTCATGAACCTATCATTCAATACAA
AAAGGGTACTCAATGGATAATCTATGCATCCCCCAGCCTTCCACAACATCCGCCTGCCACTCCTAACCCAAGTAATAACCATCAAGATGCTTCATTCACTATTTCCTTGCTGTAGGTTTATATAT TTT
TAACTCATCTTTATGTATTCCTCTACATTTTATTAGCTTCTTAAAA ACACTTTCATGGTGTATGTACATTTATACTCAATATTATATTGCTGAAATTCATCTATGTTGTTGCATGTGGATGAGGTTAA
TTCACTCTTACTGCTTACTGATATGATTTATTAATATTAATATGTTAATATTAATTCTATTACGTGACTATCCAACACGAGATAGATTCACTCTTGTGTCAATGAACTAAAATGATTGTTTCTAG GTT

TTGGTCACTGTAAGCCgTGCATCTGTAAACAATCTTGTACATGTAAAAAGTTTCTCCCAGATGGTAGCAGATGCTTCCAGTATCTTGCCCAGATTCTCAGGGACTCCTTTACTGGGTTCTGTTGGCAC
TGCTGCTAAACGCTTCCACCTGCACCTTTCCCTGAGTGACAGCACTTGCAAAATTGATCCAACCTTATCCGGGTTGCTGGGCTTGTGCTCCTCCCACCCATTCTCCTCACTGGAGGCAGTTGGGA GTC
AATGTAGGTGTACAAAAGCCCTACTACCCAG

>KSI_AluYb9_3

>Scaffold4525-352704-352782
TTTACATAACTGCCTTTGCTGAATCTTGGAAATGTGTTGATTTGTTGGATTAAAATACACAGATAAAAATCATAACACTAACTATGTGTGGTTAACTTTATATTACAAGGCCATTATGATTTCTCT TT
TGGTTCCAACTTTTGTGCTCTTTATTTTGCTTAGTGGTGAAAAAATACTAACCTTATAGAATTGGGGGCTTTCTATCTCGATTTTATAATGAGATGGCCATGTTTGTCAGCCCGTTTACCACATG ATA
CCCCACTCTTCATGGCCGCCCATGCTAGTCTCAGCTTTATACTAGGGTAGCATTCTAAGCTCTTGTTCTCCCACTACCCCTGTTAGAGCCAATTCCTCCAGGACACCAACATAAACAAGGTAGACC CT
TTCTTTCAAGTATTCTCCCTGGCTGATCGGTACTTTCTCCATTGTTAGATACAATGGTCCTGCTCCTGCTCGAGTTTTCTGAAGTTCCTGATGGCTGACCATCTGTTTATTCCTTTGGATTCTCT TAC
AAAGCCTTCATCTGGTTCTCCTGTTTTATTTCTCATAAGCTGAACTATCTCCTTTCAGGGCTCCTGTCCTCTTTCCATCCCTTTGTGTGAGCATGCCACAAGGCTCCATCCTGTCTTCTCCTCTCTT c
TAGTTGAAATCTCTTTCATTCACAGAACTTCGGCAATCACCACTCTATCCCCACACCTGCCTCTTTCATGCTTCTGCTATAAAGCAACAGATTCCTGCTATACTTACCTGAAAAGTCCCATTGATATC
CCAAGGTCAATATATTTCATCCCAAAATAAGTCCTCCTGCTTCTCTCATCTTATTATAAGTAGCACCATTTCTACAATCTCTTAAGCTCAAAACCTTTCTACAGTCTTCTACCGCCATCTAATTAG TT
ACAGTATCCTAAGTTTTCATTTAAAATGCTCCTTACTTTCAACTgaggcaggagttcagatttcatcattaacaaatatcatcataactaaaattctgcctcaactcctgcctcaacctcaacactcc
tgggaccacagctcccgccaccacacctggctaatttttttgtatttttagtagagGGCCTCCCCGGTTCACGCCATTCTTCTGCCTCAACCTCCTGAGTAGCTGGGACCACAGCTCCCGCCACCACAC
CTGGCTAATTTTTTGTATTTTTAGTAGAGACGGGGTTTCACTGTGTTAGCCAGGATGGTCTCG ATCTCCTGACCTTGTGATCCGCCCGCCTCGGCCTCCCAAAGTGCTGGGATTACAGGCATGAGCC
ACAGCACCTGGCCCAATCTATAGCTATGATTTCAATGTTTGTTTTCTCTCAAACTTATGTTAAAATTTAATTACCATTGTAATAGTATTAAGAGGTGAAACCTTGAAGAGGTGATTAAGCTCTACCTT
CATGAATGAATTAATGCCGCCATTATACAGGATTGGGTTTCTTAAGAGAGAATTCCTATAAAAGGATGAGTTTGGCCCCCTCTTGCTTTCTCTCACCCTCTCTTTGCCCATAGAATGATGCAGCA AGA
AAGTGCTCACCAGATGCTAGCCCCTTGAACCTGGATTTCCCAAACTTCAGAATCATAAGCCAATAAATTTCTGTCCATTAAAAATTACCCAGTCTGTGGTATTCTGTAGCACAAAACAAACTAAGCCA
TACAAAATAATATTTgAAGAATATGAAGAGGGTTGGAAGTATTTTAAAGACAAAAACAGCTCATGATTTAAGAAATTATTGAGATTTCCTGGGAATATGGCTCTCTTACTTTCATATCCTATAGATTA
TGATCAGATAGAAATTTAGAAATACTGGGCTTAGGTCATGAGTGGGACATAGTCCCAACCAGCATGGATTATTGTTCTAATTTAGAAACTTTCATAAAGAGAAAAAAGTAAAAGTTTTTCTTGTAT TA
AGTGAACTACTTAATACTGATTTGAAATTTTGCATAAAATATTTTGAAGGACAAAAAGCAGAATTCACTAAGAATTCAACCAAATATTCTGCAGAAAAATAGCTGGTTAAGTGTTACCTCCCCCC AAA
TCATTCCATGCATTTTCTCTCTCTGTTCATT

491	12.7	2.5	0.0	KS	1001	1079	(1000)	C	AluYb9	SINE/Alu	(126)	192	112	9
1721	7.0	0.5	0.5	KS	1081	1293	(786)	C	AluYk3	SINE/Alu	(98)	213	1	10
1411	20.1	11.9	2.5	KS	1303	1664	(415)	+	MSTD	LTR/ERVL-MaLR	2	396	(0)	11
416	30.2	0.0	4.1	KS	1667	1818	(261)	C	MER99	DNA/hAT?	(684)	147	2	12

1941	19.8	1.4	3.6	Hg19	1922	2341	(3069)	C	L1MA10	LINE/L1	(7)	6327	5917	1
3682	12.0	2.2	2.9	Hg19	2343	2507	(2903)	+	L1M3	LINE/L1	3857	4010	(2130)	2
2158	10.9	0.3	0.0	Hg19	2508	2791	(2619)	+	AluSp	SINE/Alu	1	285	(28)	3
48	2.1	0.0	0.0	Hg19	2794	2841	(2569)	+	(CATA)n	Simple_repeat	1	48	(0)	4
3682	12.0	2.2	2.9	Hg19	2842	3299	(2111)	+	L1M3	LINE/L1	4011	4479	(1661)	2
255	34.2	0.8	2.6	Hg19	3345	3461	(1949)	C	MIR3	SINE/MIR	(91)	117	3	5
2955	24.1	2.5	6.6	Hg19	3524	4221	(1189)	C	MER99	DNA/hAT?	(0)	831	145	6
2487	6.6	0.3	0.3	Hg19	4222	4524	(886)	C	AluY	SINE/Alu	(8)	303	1	7
1411	20.1	11.9	2.5	Hg19	4534	4895	(515)	+	MSTD	LTR/ERVL-MaLR	2	396	(0)	8
2955	24.1	2.5	6.6	Hg19	4896	5049	(361)	C	MER99	DNA/hAT?	(687)	144	2	6

>Hg19 chr7:96542343-96547552
TTTACATAACTGCCTTTGCTGAATCTTGGAAATGTGTTGATTTGTTGGATTAAAATACACAGATAAAAATCATAACACTAACTATGTGTGGTTAACTTTATATTACAAGGCCATTATGATTTCTCT TT
TGGTTCCAACTTTTGTGCTCTTTATTTTGCTTAGTGGTGAAAAAATACTAACCTTATAGAATTGGGGGCTTTCTATCTCGATTTTATAATGAGATGGCCATGTTTGTCAGCCCGTTT ACCACATGATA
CCCCACTCTTCATGGCCGCCCATGCTAGTCTCAGCTTTATACTAGGGTAGCATTCTAAGCTCTTGTTCTCCCACTACCCCTGTTAGAGCCAATTCCTCCAGGACACCAACATAAACAAGGTAGAC CCT
TTCTTTCAAGTATTCTCCCTGGCTGATCGGTACTTTCTCCATTGTTAGATACAATGGTCCTGCTCCTGCTCGAGTTTTCTGAAGTTCCTGATGGCTGACCATCTGTTTATTCCTTTG GATTCTCTTAC
AAAGCCTTCATCTGGTTCTCCTGTTTTATTTCTCATAAGCTGAACTATCTCCTTTCAGGGCTCCTGTCCTCTTTCCATCCCTTTGTGTGAGCATGCCACAAGGCTCCATCCTGTCTTCTCCTCTCTT t
TAGTTGAAATCTCTTTCATTCACAGAACTTCGGCAATCACCACTCTATCCCCACACCTGCCTCTTTCATGCTTCTGCTATAAAGCAACAGATTCCTGCTATACTTACCTGAAAAGTC CCATTGATATC

CCAAGGTCAATATATTTCATCCCAAAATAAGTCCTCCTGCTTCTCTCATCTTATTATAAGTAGCACCATTTCTACAATCTCTTAAGCTCAAAACCTTTCTACAGTCTTCTACCGCCATCTAATTA GTT
ACAGTATCCTAAGTTTTCATTTAAAATGCTCCTTACTTTCAACTttttctttccattttagatgctgacagccctcttcagacctgcactgccccatcatgatcaacatcatagaat gaaatatctaa
cagcatgtctgctgcaagacagtagaggaaatgtctaacatagaaaaggcctggatcgacggcttgaagtcaaacgtgtgagaaaatgtggcataactaaatactctttcccaaagtgtttttc tag
tttagctcctcccacattttccagggctcctctgactcaaaaaaattaatttgaccaatctagaattagaaaatagtaccttatttcagtaaagaacacgttgatcccagaagtaaa tgaacgtattt
attattaatttacaatgaaatatgctgacagccaacccctttccctctaaaagaaaacatattgctccgcaacctgccccagacctgtccaacttatagaaatatctgaagtgataataattgt tac
tggttttaagttataaacttagccctaatgagagagattttattattttattaacatttcatatgacaaacaataaagttccttttgatttggatttagcacaaagtatcttaaaa cgtctgaatgc
aaaggcaagtttgaaattcgccagttgggtgtgctggcgtcccccaactagctgcagcccccaattcccatcagcacaggtggctggagccaccatcgagttaaaaaaagcaaacatcagccact ggc
ctctcctctggtgactgtcagaacccactctcccacccaagggcaacttcctggcagacctttgtttttctgcccctcacagtgaccttgaaacctctcagaaataaaatgttattt cttctcatagc
ctttcagcttatcaaaaaacaacaaaaaaaaacattttattgaggtacaagcgacatacaaaaggctgtacatattcaatatatacaacttgctgagtttggagacaagtatatatccatgaaac tct
taacacaatctatgccataaacatatccatcacttcagaaagtttcctcccaccctcttcatttgttatcagtaatttttttgtgataggaacagaatgtaagatctaccctcttag caaaatttaa
gtacacaatgcaatattgttaattatgggcattatgctgtacagtagatctccagcacttatttatttgtataactgaaactttgtaccatttgactaatacttccatttccttctcaccccagc acc
tggcaaccactattctactctctgtctctatgagtttgaatttttttagattcctcacatacgtggtacattagcagaatgaaggatacaaatcacatgattatctcaatagattgga gaaaaagcatt
tgacaaacttcaacaccctttttatggtaaaaactctcaacaaattaggtacaaaaggaacttacctcaacataataaaagccaagtaaataaaaataaagctcggccgggcacagtggctcatgt ctg
taatcccagcactttgggaggctgaggctggtggatcacctgaggtcgggagttcgagaccagcctgacaaacatggagaaacccgatctctactaaaaatacaaaattagcagggc atggtggcgca
tacctgtaatcccagctactcaggaggctgaggcagtagaatcacttcaacccaggaggcagaggttgcagtgagccaaaatcatgccattgcactccagcctgggtgacaagagcaaaagtcca tct
caatgcatacatatatacatacatacatacatacatacatacatacatacataaagctcacagctaacatcacactcaatggtgaaaaactgaaaacttttcctttaagatctggaa cactgcaagga
tgcccactctcaccccttctattcaacataggactctaagtcttagcattaggtaagaaaaagaaacaaaaggcatctaaatcagaaaaagggaagtaaaatgatctctgtttttagatgacttg atc
ttacatgcagaaaatcctaaagacaccaccaaaaaattgttagaactaataaatgaattcagtcaagttgcagaatacaaaatcaacatataaaaatcagttgtatttttgtacact aataacaaaca
acccgaaaataaagagaacaatcgcatttacaacagcatcaaaataataaaatacttacgaacaaacttaactaggaggtgaaagacctgtacactgaacactataaaacattgattaaagaac ttt
tagcttcccttaaactcattatgtacaaactcattatgtaacttcctgagaagttaggaaaccgacttcaggttatacaactatagttagtggggcagtcaggattaaaacccatgg ttcctgaagtc
tggtcctgggtgactctgtcatgtcacactgccttccacccagcttgagtctacaaggagcagccctggtcagtcccaaaggggtcgggcttggatagtgattccatgtgggaatgccttcctag tat
ttcttgggaaattgttgtgtttttcaaaatcccaagaaaaaaactctcaagaaattttaaagactggaaaagtctttacaatttgcctgcaagaattaaaacattgattgctaaaac acagcaatcaa
tgtagagatgttgctgatttgaagggctccactgacatcaggtggaaagcaaccgaaggaaatatgaacacacaaagtggaggttaaggttgaccagcatgtctaggaattgagacaggagcaca gga
aagagaagtggtcactacattatgggtcagtcttgtctataactgttgcaatgttataagtgagcattcatagccatagctggtactaacagtaagtcaagcttacaaatgaaataa tgattagtatg
tctatacaaatagaatattttaaacaaatcctatgagtggtattccaagaaataacaagttttagaagcaatacaagtttttttaattggcaattttgagacatgattctgcatagtgccaagaga tct
gaactgtggtattaatcaaatactacccaatagtctgggctgacattttacagcatttgaatatttgctatgggaaaaaaaattataaatactactaaaagtaaaatttatttgtta aaaaataaaac
tctttttagatatctatggctatgatttcttttttttttttttttttgagatggagtctcgctctgtcacccaggctggagtgcagtggtgcgatcttggctcactgcaagctc GGCCTCCCCGGTTC
ACGCCATTCTTCTGCCTCAACCTCCTGAGTAGCTGGGACCACAGCTCCCGCCACCACACCTGGCTAATTTTTTGTATTTTTAGTAGAGACGGGGTTTCACTGTGTTAGCCAGGATGGTCTC GATCTCC
TGACCTTGTGATCCGCCCGCCTCGGCCTCCCAAAGTGCTGGGATTACAAGGCATGAGCCACAGCACCTGGCCCAATCTATAGCTATGATTTCAATGTTTGTTT TCTCTCAAACTTATGTTAAAATTTA
ATTACCATTGTAATAGTATTAAGAGGTGAAACCTTGAAGAGGTGATTAAGCTCTACCTTCATGAATGAATTAATGCCGCCATTATACAGGATTGGGTTTCTTAAGAGAGAATTCCTATAAAAGGA TGA
GTTTGGCCCCCTCTTGCTTTCTCTCACCCTCTCTTTGCCCATAGAATGATGCAGCAAGAAAGTGCTCACCAGATGCTAGCCCCTTGAACCTGGATTTCCCAAACTTCAGAATCATAAGCCAATAAATT
TCTGTCCATTAAAAATTACCCAGTCTGTGGTATTCTGTAGCACAAAACAAACTAAGCCATACAAAATAATATTT aAAGAATATGAAGAGGGTTGGAAGTATTTTAAAGACAAAAACAGCTCATGATTT
AAGAAATTATTGAGATTTCCTGGGAATATGGCTCTCTTACTTTCATATCCTATAGATTATGATCAGATAGAAATTTAGAAATACTGGGCTTAGGTCATGAGTGGGACATAGTCCC AACCAGCATGGAT
TATTGTTCTAATTTAGAAACTTTCATAAAGAGAAAAAAGTAAAAGTTTTTCTTGTATTAAGTGAACTACTTAATACTGATTTGAAATTTTGCATAAAATATTTTGAAGGACAAAAAGCAGAATTC ACT
AAGAATTCAACCAAATATTCTGCAGAAAAATAGCTGGTTAAGTGTTACCTCCCCCCAAATCATTCCATGCATTTTCTCTCTCTGTTCAT T

>KSI_AluYb9_4

>Scaffold4578-103530-103671

TAATGGGTGGCTCAGCTGGCTTCCCAAGGCACTGCTGGTCCTAGAATTGCATGCTCACTTCGTCACAGTGCTCTGTCCAGAATCTATGCAGAGCCATTTCCTGGCCTCCTTGAGAACTTTTAGTAC AG
TGACTCCTGGAGGATTAGTTCGGTGGCTGCTAAGCTTACAGAAGAACAAGGCTGGCTAATATGACTGAACTGAATCTGAGTAGGTACAGGGTCTTGTCAAG ACTGAATTGGTTAAATGGCATCTCTGG
TCATTTGTAGTCAGAGAGATGATGCTAGTCTGGTCTAGTTTCATCCACCCTACTAATTCCCAATCCAGGGGTAGACCCAGCCCCATAGAGTGTAAAGGCATCACAGAAGTAGAGGATCTCAACA TTA
TAAGGGCTTCAGAGATTTTAACTCAAGGCTCATCTTTAACCAATGAGTAGTTGTGTCCCCAAAGGTTAAGTGACTTGTCCAAGTTA aTGGTAGAATTGGGACTAGAACACAGTCGTCTTCCTTCTCCG
TTGTGATTCCATCTATAACTTTTGCAGAAAAGAGATGATAGAATTTAGATATGAGTATATTTACTTTCTTGTTGGGAGTTTATTATAATTGTTTCTCTCAAGCAGATTGTAACACTATTATTATT TCC
TTGTTTCAAATCTTTAATCTATGTTCAAAAATTCCCAGTTTGTATCATAGCAGCTGAGGTTAAAGTAATATTTCCAATTTACGTAGTCTCTGGGATGATAC AAAATTGTCCAACCTACTAAACTGACT
CTGAGGCTACTCTTTAAGAGAGTGGAGGGGTAAAAGTGAAGGAAATGAAAAGACCTTATAAAGGCATTCTTCCCAGCTTGATGTGATCTTTACTTAATGTTTGCAAAATATATATGATGATTCAGGAA
TAATATGTATTCAGTGAATGATTCTTTTttttttttttttttttttttttttttttttttttttgtgggggtttctcttttcccccgcgggggccggcgggcccgccaccgcgcccggctaatt
ttttgtatttttagtagagacggggtttcaccttgttagccaggatggtctcgatctcctgacctcatgatccacccgcctcggcctcccaaagtgctgggattacaggcgtgagc Cagtgaatgatt
cttttGTTCAGTGTTTATTGCTACTCCTCTGTGCTAGGCACTTTACAAGGTATAGCCACGCAAATCATAAAGTTGGTCACCATTCATGGCAG TTGAGGACTCACGAGCAAGGCTGTAGCTATAAGAAT
TTGTTATGGGACAAATTGAATAACAATCTGAGGCATTTATTTCCACCACTCTAAATCTTGGCTTCAAGTGTACTGTTTCCTCCAATGCCAAATTACTAGCATTTTTTCAAATATTGGAAATATGA AGC
CCCCTTAACAGTATTGAATAAAGCTTCTTTTATTTGAGCAAAAGCGTGTAGTTCTCAGAAATGGTAGTTTGTTTGATATGATTAATTCTGTGAACAAATGAGTTTGATGGCAGCTTTGAGGGCATAGC
GATAAATTAGCATTTACAAAGTGCTTTCATCCACAATAGAGGTAATATAAAACTTGGTTGGTCTGCAAAGCAATAGAATTTGAAATGCCTGTGATTATCTTCAAAATCACACTGAGAATGCATGT GAA
TAGAAAAGCAGAGTTGGAAAGGGAAAAGGGGAAAGAATCTATTTAAGATTACTAAATTGCTATCTGTTTTGATAAGGTAATTGGCCAGAAAATTAAATGCTCTGTGGAGAACAAAGCTGTTTTCTGTA
ATCTGGTACAGCCAAATGTCATTTTTATTCAATACACACAGGAATATAAAGAACAAACAAGAAGGCAAAAAAAAAAAGTTACAGGAAAAATAAGCTCAATAAAAGAAGTAAGACTATTTAAAGCAT AGT
TTAGTATTTGCATTTGAATCTTTGGTATTGAATATGTGATATAAAAACGTGCCTTCTCCCTGGGTACTCCCTGAGTACATTCCATTCTCTGTCATCCTTAGCTAGGAGAGGTTTTATTAAAATGAAGT
CCAGGCACAGTGGCTCATACCTGTAATCCCAACACTTTGGGAGGCTGAGGTGGGCAGATCACTTCAGGTCAGGAGTTCAAGACCAGCCTGGCA A

345	29.4	11.6	1.2	KS	357	511	(1631)	C	MIR3	SINE/MIR	(2)	206	36	4
45	0.0	0.0	0.0	KS	921	961	(1181)	+	(T)n	Simple_repeat	1	41	(0)	5
1358	0.0	0.0	0.0	KS	1001	1142	(1000)	C	AluYb9	SINE/Alu	(164)	154	13	6
12	21.6	2.1	4.3	KS	1916	1962	(180)	+	(ATATTTG)n	Simple_repeat	1	46	(0)	7
714	12.0	0.0	0.0	KS	2049	2140	(2)	+	AluSz	SINE/Alu	3	94	(218)	8

>Hg19 chr8:87078636-87080544

TAATGGGTGGCTCAGCTGGCTTCCCAAGGCACTGCTGGTCCTAGAATTGCATGCTCACTTCGTCACAGTGCTCTGTCCAGAATCTATGCAGAGCCATTTCCTGGCCTCCTTGAGAACTTTTAGTAC AG
TGACTCCTGGAGGATTAGTTCGGTGGCTGCTAAGCTTACAGAAGAACAAGGCTGGCTAATATGACTGAACTGAATCTGAGTAGGTACAGGGTCTTGTCAAGACTGAATTGGTTAAATGGCATCTC TGG
TCATTTGTAGTCAGAGAGATGATGCTAGTCTGGTCTAGTTTCATCCACCCTACTAATTCCCAATCCAGGGGTAGACCCAGCCCCATAGAGTGTAAAGGCATCACAGAAGTAGAGGATCTCAACAT TA
TAAGGGCTTCAGAGATTTTAACTCAAGGCTCATCTTTAACCAATGAGTAGTTGTGTCCCCAAAGGTTAAGTGACTTGTCCAAGTTA gTGGTAGAATTGGGACTAGAACACAGTCGTCTTCCTTCTCCG
TTGTGATTCCATCTATAACTTTTGCAGAAAAGAGATGATAGAATTTAGATATGAGTATATTTACTTTCTTGTTGGGAGTTTATTATAATTGTTTCTCTCAAGCAGATTGTAACACTATTATTATTT CC
TTGTTTCAAATCTTTAATCTATGTTCAAAAATTCCCAGTTTGTATCATAGCAGCTGAGGTTAAAGTAATATTTCCAATTTACGTAGTCTCTGGGATGATACAAAATTGTCCAACCTACTAAACTG ACT
CTGAGGCTACTCTTTAAGAGAGTGGAGGGGTAAAAGTGAAGGAAATGAAAAGACCTTATAAAGGCATTCTTCCCAGCTTGATGTGATCTTTACTTAATGTTTGCAAAATATATATGATGATTCAGGAA
TAATATGTATTCAGTGAATGATTCTTTTGTTCAGTGTTTATTGCTACTCCTCTGTGCTAGGCACTTTACAAGGTATAGCCACGCAAATCATAAAGTTGGTCACCATTCATGGCAGTTGA GGACTCACG
AGCAAGGCTGTAGCTATAAGAATTTGTTATGGGACAAATTGAATAACAATCTGAGGCATTTATTTCCACCACTCTAAATCTTGGCTTCAAGTGTACTGTTTCCTCCAATGCCAAATTACTAGCATTTT
TTCAAATATTGGAAATATGAAGCCCCCTTAACAGTATTGAATAAAGCTTCTTTTATTTGAGCAAAAGCGTGTAGTTCTCAGAAATGGTAGTTTGTTTGATATGATTAATTCTGTGAACAAATGAG TTT
GATGGCAGCTTTGAGGGCATAGCGATAAATTAGCATTTACAAAGTGCTTTCATCCACAATAGAGGTAATATAAAACTTGGTTGGTCTGCAAAGCAATAGAATTTGAAATGCCTGTGATTATCTTCA AA
ATCACACTGAGAATGCATGTGAATAGAAAAGCAGAGTTGGAAAGGGAAAAGGGGAAAGAATCTATTTAAGATTACTAAATTGCTATCTGTTTTGATAAGGTAATTGGCCAGAAAATTAAATGCTC TGT
GGAGAACAAAGCTGTTTTCTGTAATCTGGTACAGCCAAATGTCATTTTTATTCAATACACACAGGAATATAAAGAACAAACAAGAAGGCAAAAAAAAAAAGTTACAGGAAAAATAAGCTCAATAAAA GA
AGTAAGACTATTTAAAGCATAGTTTAGTATTTGCATTTGAATCTTTGGTATTGAATATGTGATATAAAAACGTGCCTTCTCCCTGGGTACTCCCTGAGTACATTCCATTCTCTGTCATCCTTAGC TAG
GAGAGGTTTTATTAAAATGAAGTCCAGGCACAGTGGCTCATACCTGTAATCCCAACACTTTGGGAGGCTGAGGTGGGCAGATCACTTCAGGTCAGGAGTTCAAGACCAGCCTGGCA A

357	28.7	11.6	1.2	Hg19	457	611	(1498)	C	MIR3	SINE/MIR	(2)	206	36	1
12	21.6	2.1	4.3	Hg19	1783	1829	(280)	+	(ATATTTG)n	Simple_repeat	1	46	(0)	2
1472	10.3	0.0	1.6	Hg19	1916	2109	(0)	+	AluSz	SINE/Alu	3	193	(119)	3

>KSI_AluYb9_5

>Scaffold5649-873123-873336
ATTATGAATAATGATACACAGTGGCATTTTAGtTATCCTTTTTGTTGGAAAGAAAAAACAAGAAAGTCACTAGCCCTTCAGAGATTTGGAAAACCAAATTTATTAAGATAAGGAAATAGAAAATGATT
GATGATTCCAAACTAGATGGGTTAGGGGAACCTTTTTAGATTCACAAACACTAACTTTTTAAACAAGAAAATCGAATAGTCAACAAATATTTGTTGAGCAGTTACTGTGAATGAGCCACTCTGCT ATA
TGCTGCAAAGCATAGACAAAAAGTAAGACACGATCTCACTCTATAAATTGTATTGCAATCTAGGAGGCAGTGAGAAACCCATATAAATGAATAATTATTTATTTAAATGGCAAAACATGATGCACA CC
ACAGAAAAGACATAAGTAAGGTACTATAGGTTTCAGACGCTACCTTCTTACCTCTCTGGGGATGACTTTAATAAATTTAAATCTTTGTTTCTGCTGTGTGACAGCTGCTGACCCTTGAGTGACTG TGG
TCTGGTGTGGAGTGTCATAGGTGGAGGCGTTAAAGAGATGGTAGCTGAAAGTACAAGGAGGAGGAGGAACAAGGTGAGTGAGAGAGCTGAGTATTAGGAATGTGGAGGGGTGGAAATTAATGGGTC AT
GGACATAAATAGGGGACTGAGGAACCCAAGAAACAAAGAAGGAGGGATTGC gTTAACAGCAGATTAGGAAAATGAAAGATATACATAGTAAGGTCATAGAATATGATGGTCAACATCTAGTTGGACAA
TTTTGCGCAAATCACTCTACCTCTTTAAGCTTTTGGTTTCATCAAAGGTAAAAAAAAATTCAGTAAATATTTATTGA ACTCCTACTATGCACCAGGCTTTTTTTCCAGGACTCGAAGATCCAAATTTA
ATAAGTCATAGTCTCTGCCTTTAAGGAGGCTGAAATGACTGTATAATACAAAAATAAAT tTTTTGTTTTAAAATTAAAAACACAaaatgaataatatttttttctgcagtggcacaatctcggctcac
tgcaagctccgcttcccgggttcacgccattctcctgcctcagcctccccagtagctgggactacaggcgcccgccaccgcgcccggctaatttttttgtatttttagtagagacggggtttcacc tt
gttagccaggatggtctcgatctcctgacctcatgatccacccgcctcggcctcccaaagtgAAAATGAATAATATTAATAAAATCAATCAAAATTAGTTGAATTTCTATGGTACACTAGATTTACAT
GGTGAGGAAGTAGTAAGGTGCAAGAGCTTTCAAGTCACCATAGATCTTGATTCAAATGGTTTCAGTCACTAATTCCCTGGAA CACACTTAATCCCTGTCAGCCTGTTGTTTCTTGCTCTGGAGAATGT
GAATAATCACATTCCgATGTAACAAATAAACTATATGAGTTATTTATTATTTTTTGTTTGATTGTTTATCCATCATTGCACACCCTAGTGCCCAGGACAGTACCTTGTCTAGTAGTCAAATAAAGAAT
ATTTGTTGAATAGAGCTTTTGGAAAGATTAAATAATGATTTGCTTAAAGTGCTTATGCACAGGAAACAGCAAATATAAATGCCAACA aGTTTATTAATTTAAAAATATTATTGGCTTTCAGCTATAAT
ATCTGTGATTTGTTGAGGAAGAAATAAATGTTTGAAGGACATATAGATGGGGGCATCATTCAGTGAGAAAGTCTTAAAATTAATTTTACTCTTTAAATATTTAAATTACTAATTGGAAAATATAACTT
ACCCATATATTGGGAATGATTCTTTCTACCAGTTTAGAAAGGCTGGCCACATATAAGTTCTTAGGGAATAATAACAAAATTTACTTTAAAAGAACATTTTAACGGCCTCTGAATAAATAAAACAC CTA
CCTTTTTCATTATGCCTTTCCCCTTAGAATCAATAGACCTGGGGCAACATTTATTATTCTAGGGACCAATACATTTTTCAGATACTTGAGGATTCCTTTCTTGGTACCCTTTCCTTTTCAGGGAAATT
GTCTAACCAATCCAAAGAATGGAGAACTCACATAAAGCCCATAAACTTTGCATGTTGCACTGTCACATATAAAGGAGAAAGACCAAACGTGAAATAAGGCAGACTGTCTTCCTCAGAAGCAGCAA CAG
TAAGCATATTTCTTTTAAGCAGATAGAGAAGGTTGAAT

300	30.6	6.5	4.5	KS	197	412	(1802)	C	L2d2	LINE/L2	(2)	3462	3243	7
202	31.3	0.0	1.6	KS	757	821	(1393)	+	MIRb	SINE/MIR	84	147	(121)	8
293	25.8	0.0	13.8	KS	826	924	(1290)	C	L2c	LINE/L2	(8)	3379	3293	9
1998	0.5	0.0	0.5	KS	1001	1214	(1000)	C	AluYb9	SINE/Alu	(71)	247	35	10
242	29.7	10.4	2.1	KS	1282	1415	(799)	+	MIRb	SINE/MIR	13	157	(111)	11
223	27.4	2.3	4.7	KS	1460	1547	(667)	+	L2a	LINE/L2	3333	3418	(8)	12

>Hg19 chr13:58620073-58622051
ATTATGAATAATGATACACAGTGGCATTTTAGcTATCCTTTTTGTTGGAAAGAAAAAACAAGAAAGTCACTA GCCCTTCAGAGATTTGGAAAACCAAATTTATTAAGATAAGGAAATAGAAAATGATT
GATGATTCCAAACTAGATGGGTTAGGGGAACCTTTTTAGATTCACAAACACTAACTTTTTAAACAAGAAAATCGAATAGTCAACAAATATTTGTTGAGCAGTTACTGTGAATGAGCCACTCTGCT ATA
TGCTGCAAAGCATAGACAAAAAGTAAGACACGATCTCACTCTATAAATTGTATTGCAATCTAGGAGGCAGTG AGAAACCCATATAAATGAATAATTATTTATTTAAATGGCAAAACATGATGCACACC
ACAGAAAAGACATAAGTAAGGTACTATAGGTTTCAGACGCTACCTTCTTACCTCTCTGGGGATGACTTTAATAAATTTAAATCTTTGTTTCTGCTGTGTGACAGCTGCTGACCCTTGAGTGACTG TGG
TCTGGTGTGGAGTGTCATAGGTGGAGGCGTTAAAGAGATGGTAGCTGAAAGTACAAGGAGGAGGAGGAACAA GGTGAGTGAGAGAGCTGAGTATTAGGAATGTGGAGGGGTGGAAATTAATGGGTCAT
GGACATAAATAGGGGACTGAGGAACCCAAGAAACAAAGAAGGAGGGATTGC aTTAACAGCAGATTAGGAAAATGAAAGATATACATAGTAAGGTCATAGAATATGATGGTCAACATCTAGTTGGACAA
TTTTGCGCAAATCACTCTACCTCTTTAAGCTTTTGGTTTCATCAAAGGTAAAAAAAAATTCAGTAAATATTT ATTGAACTCCTACTATGCACCAGGCTTTTTTTCCAGGACTCGAAGATCCAAATTTA
ATAAGTCATAGTCTCTGCCTTTAAGGAGGCTGAAATGACTGTATAATACAAAAATAAAT gTTTTGTTTTAAAATTAAAAACACAAAATGAATAATATTAATAAAATCAATCAAAATTAGTTGAATTTC
TATGGTACACTAGATTTACATGGTGAGGAAGTAGTAAGGTGCAAGAGCTTTCAAGTCACCATAGATCTTGATTCAAATGGTTTCAGTCACTAATTCCCTGGAA CACACTTAATCCCTGTCAGCCTGTT
GTTTCTTGCTCTGGAGAATGTGAATAATCACATTCC cATGTAACAAATAAACTATATGAGTTATTTATTATTTTTTGTTTGATTGTTTATCCATCATT GCACACCCTAGTGCCCAGGACAGTACCTTG
TCTAGTAGTCAAATAAAGAATATTTGTTGAATAGAGCTTTTGGAAAGATTAAATAATGATTTGCTTAAAGTGCTTATGCACAGGAAACAGCAAATATAAATGCCAACA cGTTTATTAATTTAAAAATA
TTATTGGCTTTCAGCTATAATATCTGTGATTTGTTGAGGAAGAAATAAATGTTTGAAGGACATATAGATGGGGGCATCATTCAGTGAGAAAGTCTTAA AATTAATTTTACTCTTTAAATATTTAAATT

ACTAATTGGAAAATATAACTTACCCATATATTGGGAATGATTCTTTCTACCAGTTTAGAAAGGCTGGCCACATATAAGTTCTTAGGGAATAATAACAAAATTTACTTTAAAAGAACATTTTAACG GCC
TCTGAATAAATAAAACACCTACCTTTTTCATTATGCCTTTCCCCTTAGAATCAATAGACCTGGGGCAACATTTATTATTCTAGGGACCAATACATTTT TCAGATACTTGAGGATTCCTTTCTTGGTAC
CCTTTCCTTTTCAGGGAAATTGTCTAACCAATCCAAAGAATGGAGAACTCACATAAAGCCCATAAACTTTGCATGTTGCACTGTCACATATAAAGGAGAAAGACCAAACGTGAAATAAGGCAGAC TGT
CTTCCTCAGAAGCAGCAACAGTAAGCATATTTCTTTTAAGCAGATAGAGAAGGTTGAA T

300	30.6	6.5	4.5	Hg19	297	512	(1667)	C	L2d2	LINE/L2	(2)	3462	3243	1
202	31.3	0.0	1.6	Hg19	857	921	(1258)	+	MIRb	SINE/MIR	84	147	(121)	2
293	25.8	0.0	13.8	Hg19	926	1024	(1155)	C	L2c	LINE/L2	(8)	3379	3293	3
242	29.7	10.4	2.1	Hg19	1147	1280	(899)	+	MIRb	SINE/MIR	13	157	(111)	4
223	27.4	2.3	4.7	Hg19	1325	1412	(767)	+	L2a	LINE/L2	3333	3418	(8)	5
571	14.5	0.0	1.2	Hg19	2097	2179	(0)	+	AluSp	SINE/Alu	2	83	(230)	6

>KSI_AluYb9_6

>Scaffold5671-281592-281743
TCTCCAAAGAACAATTGCCTAAAGAGTCCCTATTGAAGTATTTTTCATTTATAACAAGATCTTATGATAGTTAAAAATAAATACAGCACTGAAAACTAATTCTCTGAATATCTAATGCTGTGAACT GT
CATGTAGATCAACACACACAGTATGTTTTGGGAAATTTTCCAGCAGATCTAACATTTCTTAATTCTATTTCTTAGTTTTCTATTTTTTCAGAAGAAAAAAGTGTATTATTCTACTAATTAATGGA CTT
GTACATCTTAAAACTTAATCTTTATTTGTAATAATAGTAATACACAGAATTTTTA aAAGTCTGTAAGAAATAATAACAACCTGAATCTAAAATAAAATAAATTTTTAAAAAGGAAATAATAGCAAGAG
ACATATCGGAAGAGAGCATTTTCATTCGACTGCTCTTCGTCACCCTCCTCACCTACCTAGTTAACCACGTTTATCTCTTATGGTATTTATTGCCATAAATAATCGTAATATGCTCATATTGCTAT TTC
TAAGTTTACTAATTTAAACATTATGCATTGACTTTATATTATAGCAGTTAAGGCTTTGTTTTCCTTAGATGCATTCTTTTCATCCCTTATGAAATCTGCTTGCTTTAGGCCCCTTCTTCTAATTG AT
TTACATCATAATTATCAATGAAATAAATTATCAGAATTTGGTTATTATCTATTTTAATGCTGTTCATTTCTCAAAAAAATTATGCAGTATTATTACTATTATTTTCATACAATGTTTTGATTTCC CGT
GGTTCATAACTATATTTAAAAATTATTTTTATTTTTTTATACAGCTATGCTAATTTTTCCTAAAACATTCAAAATACATGCAACATACAAAAGCCTCTCCATATGGTTTTCCGTTATGCCATTTA TA
TCATCTCAATTGTTGTCCTTGGAGAACTTCCTCCTGGAGCCTTTTGTCCTTCTGTTCCAAATGGGACTGGTGTCTCCGTGGGCCTGCTATaaagattccatcctggccgggcgcggtggctcacgcct
gtaatcccagcactttgggaggccgaggcgggtggatcatgaggtcaggagatcgagaccatcctggctaacaaggtgaaaccccgtctctactaaaaatacaaaacattagccgggagcggtggc gg
aaaaAAAGATTCCATCCTGGGTCTTTCTGTCACCATCAACTGAACTTCCCCCTCGAGAATGCTCTGTTTCTTGAATAGGATGTTTTCCTTTGTCTTGATTTGTTCCCCCATTTTAGTAAAGCACATAT
CCACAGCTTTCCGAGAAAGTTTCCGAAAGTAAAAATGTCTGAGATTTTTCTTGCCTTAAAATGTCTTGATTAAACATAC gCTCTTAAgacGACAGAGTTTAATATTcTCCTTCTTATAGTGAGAACAT
TGCTCTGTATACTTTTTAGAGTTTCCTGACAGTAGTTCTAATACCTAATCTTTGGTACATAAGCTGTTGCATAGTCCTCCTTTCTTCTTAGTTAAAAAAACTAACATCTTCTTAATCTTATTAGT AGG
TCAGAGAGATTTTCTTGCATtATTTTTTTAAATTTCACCACTGTTTTCTTCATTCTTTTTTTCTTCAACATTATTAAATAGATGCTGGAGTTTCTTGATTCACCCTATAATTGTTTCATCTTTTCTCT
GCTGCAGTCTTTTTGTACACTTTTTGACATTATTTTTTCAAACTTTTTACTATTTTAAATTTCAGTTACTAAAGTTATCATTACCAAGAGGGTTTTTTGTTGTTGTTGTTCTCAGAAGGTCCTCT TAT
TATTTCATGAATGGAAAATCTCTATTTCTCCAAAGATCTCAATTTAAACTGCAAATGCCTTTGTTATCTTTATTCTTCCAAGTTTACCATTTTTCCCTGTTTCTTTGTGTGTTTTGATCACTCTTT CA
TGTTGAAGATTTACCAGCAATCTTTGCTAGGGTTTTTTCCCTGTCTATTTAATTTTGATGAAAAGCTACTAAAGCTATTGTAACCCCACTGAATGTTAGCCAAGCTGTGGACTGTAAATCACTTG AAG
AGTGATCTACTGGAGCAGGCTCTGGATTTCTGTGTTTATACCCCCATCTCTAGGGAATACGCCTCTGATCATGGTATTTGAGAAAAGAGGGATAGGAAATCTT A

674	31.9	5.9	4.8	AluYb9_8	360	1000	(1152)	C	HAL1	LINE/L1	(33)	2474	1827	1
1408	1.3	0.0	0.0	AluYb9_8	1001	1152	(1000)	+	AluYb9	SINE/Alu	1	152	(166)	2
524	25.8	10.7	0.5	AluYb9_8	1165	1351	(801)	C	HAL1	LINE/L1	(675)	1832	1627	1
474	28.0	13.7	1.3	AluYb9_8	1534	1935	(217)	C	HAL1	LINE/L1	(1193)	1314	864	1

>hg19 chr3:6102058-6104036
TCTCCAAAGAACAATTGCCTAAAGAGTCCCTATTGAAGTATTTTTCATTTATAACAAGATCTTATGATAGTTAAAAATAAATACAGCACTGAAAACTAATTCTCTGAATATCTAATGCTGTGAACT GT
CATGTAGATCAACACACACAGTATGTTTTGGGAAATTTTCCAGCAGATCTAACATTTCTTAATTCTATTTCTTAGTTTTCTATTTTTTCAGAAGAAAAAAGTGTATTATTCTACTAATTAATGGA CTT
GTACATCTTAAAACTTAATCTTTATTTGTAATAATAGTAATACACAGAATTTTTA cAAGTCTGTAAGAAATAATAACAACCTGAATCTAAAATAAAATAAATTTTTAAAAAGGAAATAATAGCAAG AG
ACATATCGGAAGAGAGCATTTTCATTCGACTGCTCTTCGTCACCCTCCTCACCTACCTAGTTAACCACGTTTATCTCTTATGGTATTTATTGCCATAAATAATCGTAATATGCTCATATTGCTAT TTC
TAAGTTTACTAATTTAAACATTATGCATTGACTTTATATTATAGCAGTTAAGGCTTTGTTTTCCTTAGATGCATTCTTTTCATCCCTTATGAAATCTGCTTGCTTTAGGCCCCTTCTTCTAATT GAT
TTACATCATAATTATCAATGAAATAAATTATCAGAATTTGGTTATTATCTATTTTAATGCTGTTCATTTCTCAAAAAAATTATGCAGTATTATTACTATTATTTTCATACAATGTTTTGATTTCC CGT
GGTTCATAACTATATTTAAAAATTATTTTTATTTTTTTATACAGCTATGCTAATTTTTCCTAAAACATTCAAAATACATGCAACATACAAAAGCCTCTCCATATGGTTTTCCGTTATGCCATTT ATA
TCATCTCAATTGTTGTCCTTGGAGAACTTCCTCCTGGAGCCTTTTGTCCTTCTGTTCCAAATGGGACTGGTGTCTCCGTGGGCCTGCTATAAAGATTCCATCCTGGGTCTTTCTGTCACCATCAACTG
AACTTCCCCCTCGAGAATGCTCTGTTTCTTGAATAGGATGTTTTCCTTTGTCTTGATTTGTTCCCCCATTTTAGTAAAGCACATATCCACAGCTTTCCGAGAAAGTTTCCGAAAGTAAAAATGTCT GA
GATTTTTCTTGCCTTAAAATGTCTTGATTAAACATACtCTCTTAAGACAGAGTTTAATATTtTCCTTCTTATAGTGAGAACATTGCTCTGTATACTTTTTAGAGTTTCCTGACAGTAGTTCTAATACC
TAATCTTTGGTACATAAGCTGTTGCATAGTCCTCCTTTCTTCTTAGTTAAAAAAACTAACATCTTCTTAATCTTATTAGTAGGTCAGAGAGATTTTCTTGCAT aATTTTTTTAAATTTCACCACTGTT
TTCTTCATTCTTTTTTTCTTCAACATTATTAAATAGATGCTGGAGTTTCTTGATTCACCCTATAATTGTTTCATCTTTTCTCTGCTGCAGTCTTTTTGTACACTTTTTGACATTATTTTTTCAAA CTT
TTTACTATTTTAAATTTCAGTTACTAAAGTTATCATTACCAAGAGGGTTTTTTGTTGTTGTTGTTCTCAGAAGGTCCTCTTATTATTTCATGAATGGAAAATCTCTATTTCTCCAAAGATCTCAA TTT
AAACTGCAAATGCCTTTGTTATCTTTATTCTTCCAAGTTTACCATTTTTCCCTGTTTCTTTGTGTGTTTTGATCACTCTTTCATGTTGAAGATTTACCAGCAATCTTTGCTAGGGTTTTTTCCCT GTC
TATTTAATTTTGATGAAAAGCTACTAAAGCTATTGTAACCCCACTGAATGTTAGCCAAGCTGTGGACTGTAAATCACTTGAAGAGTGATCTACTGGAGCAGGCTCTGGATTTCTGTGTTTATACC CCC
ATCTCTAGGGAATACGCCTCTGATCATGGTATTTGAGAAAAGAGGGATAGGAAATCTT A

1160	30.7	7.1	3.8	hg19	360	1181	(798)	C	HAL1	LINE/L1	(33)	2474	1627	3
454	28.2	13.7	1.3	hg19	1361	1762	(217)	C	HAL1	LINE/L1	(1193)	1314	864	3

>KSI_AluYb9_7

>Scaffold6246-1108733-1108981

TGCAGTTGAAAAACATCAAACTAATCGCTATGGTTAAtATTGGGAAATGATCTGGCTTAGTAGCAGAAGGTAGGTTTCGAGAAGAGGTGAGGAAACATTTAT gCTTTAGATTTAGGCTTGTttagaat
aTTTGAATATTTTTAAAGGAGAATTCATTCTTGTATGAATGAAGCATTCAAAAATGTGAAATTTGGGGCAGCTTGGGTGGAAATAATGCATGAAATGATCAAAGTGTAATTTCAAAGGGCTAAAACA T
GACTCATACAAATAGGGAAAGGATATATGTCAGATATTTTATTGAACACATTAATGAATGAAAGGAGAAAAATGATAAAACTGGTTTCAAAAGACTTTGAGAACTGAAG TTCATGTAAGTATGAATTT
TAGAATAGGGTCATACAAACAAAACTGATTAGTATGCTTTAAGACATTATTGAAAGAAAAACTTTAGCTGAATTAAGTTTAACAGAGTTTCACTGAGCAGAGAACTATTCACGAATCACGCAGCC TCC
CAAGCCAGAGTAGGCTCAGAGACTCCAGCGCAGCCACATAGTGGGAGAAGATTTATGGGCAGAA gAAGGAAAGTGATCTACAGAAAATGAAAGTGAGGTATAGAAACAG CCAGATTGGTTACAGCTTG
GCGTTTGCCTTATTTGAACATAATTTGAACAGTTGGCCACTTTGATTGGCCCAAACTCTGTGATCAGCACAAGAGTAGCTACAGCCTGCTTACATGTCCATTTAGGTTATAGTTCATGATCTACA GAG
AAACCTTTAGGTCAAACTTAAAATATGTAAGAAGGCAGCTTCAGACTAAACAATTTAACAGCTTCAAAGCCATAACTTTTGGAATTATATTTTCTACTAGATAGAAATC TAAAAATTAACATGCAACT
TCACAATATCTGGCTTCTAATCTATTAGATAAGCAGCAAAACCAAGCACAGGTACATTCTCCTAGAGAATTaaacAatcattccatgtgcttataaaacaatgcggccgaggcgggtggatcatgagg
tcaggagatcgagaccatcctggctaacaaggtgaaaccccgtctctactaaaaatacagaaaattagccgggcgcggtggcgggcgcctgtagtcccagctactggggaggctgaggcaggaga atg
gcgtgaacccgggaagcggagcttgcagtgagccgagattgcgccactgcagtccgcagtccagcctgggcgacagagcgagactccgtctcaaa AATCATTCCATGTGCTTATAAAACAATGCATCA
GTATGAAATTTAAAGTATATGCCATTCCTTTTTGCTTATTTCTAAGAATTGTATAATTTTTTATGAAA tAAAgAATAACAGTAAAAAAGTAGACACCCCACTATTAAGAACAATGGTTGAAACAGACA
TTATTTAATAATGTTTTAGGGGAATGTGATTCACTTCTGGAAAAACTATAACAGAACTCCAGATGGTGGAGATAGAATAACACGAAAAATATTATTAATTGCAGAAAGAGGCAAAATTCCTTGAAATT
TATACATAAATCCTGATACTAGAAAATATTCATTCATATACTAACAGGAGTATATATAGAAAGAATAAAAGTTGCTAAATGAGACCAGGACAGTGATTATCATTAGCTACATGGGGCTGAGTATT CCT
AGGGAATGAAGATTGTTGTAATTCTTGAACTTGTTCTGCATATTATTTTTAGTTTCTATGTTTTCATATTGAAACTATATAGAAAATTCCTATAGGCCCATACC ATGCTTTGTGTGTGTGTGGGGGGG
GGGGgGCACGTGCGCTTGTGTGCATGTGTGTGTATTTTTAACTCATGTTTTCTACCATAATCAGAATGTTACTAATACCTCCAGTTATAGTCATAGATAAGACTGGATAAACTCTAATTTACCCATTA
CAACCAAGATTCAGGTGACCATCATGAGTATGGAGTAAGATTCTATTCTGTGTGCCATATGTTATATAAATATAAAACATTTGTTAATAAAGAGTTTTCCCATA ACACACATAAACCACTATTTTCAA
ATATTGCTTTAGACCCATGTCAGGATTTACCTAGTCAGGTGCTTCCTTGAACATTTTTATTAAACTAATTTTGTCTTAATTACTTTAGGACATTTTGTTTAACAGACTATACAACAAATAAAGAT GGA
TCAACTTTGTGAAAAGAAATAAAAGAGCCCCAGAGGTCTGTGATAATAAGAAAATCTAAGTGTCTTTACACT T

1324	23.3	4.9	7.9	KS	227	431	(1818)	C	LTR37B	LTR/ERV1	(2)	466	242	1
2833	11.3	1.0	0.0	KS	432	828	(1421)	C	MER57B1	LTR/ERV1	(0)	401	1	2
1324	23.3	4.9	7.9	KS	829	1000	(1249)	C	LTR37B	LTR/ERV1	(227)	241	76	1
2359	0.8	0.0	0.0	KS	1001	1249	(1000)	+	AluYb9	SINE/Alu	45	293	(25)	3
1324	23.3	4.9	7.9	KS	1250	1341	(908)	C	LTR37B	LTR/ERV1	(393)	75	9	1
254	23.9	9.7	1.3	KS	1870	1941	(308)	C	LTR91A	LTR	(819)	164	87	4

>hg19 chr1:80298124-80300084

TGCAGTTGAAAAACATCAAACTAATCGCTATGGTTAAaATTGGGAAATGATCTGGCTTAGTAGCAGAAGGTAGGTTTCGAGAAGAGGTGAGGAAACATTTAT cCTTTAGATTTAGGCTTGTTTTGAAT
ATTTTTAAAGGAGAATTCATTCTTGTATGAATGAAGCATTCAAAAATGTGAAATTTGGGGCAGCTTGGGTGGAAATAATGCATGAAATGATCAAAGTGTAATTTCAAAGGGCTAAAACATGACTC ATA
CAAATAGGGAAAGGATATATGTCAGATATTTTATTGAACACATTAATGAATGAAAGGAGAAAAATGATAAAACTGGTTTCAAAAGACTTTGAGAACTGAAGTTCATGTAAGTATGAATTT TAGAATAG
GGTCATACAAACAAAACTGATTAGTATGCTTTAAGACATTATTGAAAGAAAAACTTTAGCTGAATTAAGTTTAACAGAGTTTCACTGAGCAGAGAACTATTCACGAATCACGCAGCCTCCCAAGC CAG
AGTAGGCTCAGAGACTCCAGCGCAGCCACATAGTGGGAGAAGATTTATGGGCAGAA aAAGGAAAGTGATCTACAGAAAATGAAAGTGAGGTATAGAAACAGCCAGATTGGTTACAGCTTG GCGTTTGC
CTTATTTGAACATAATTTGAACAGTTGGCCACTTTGATTGGCCCAAACTCTGTGATCAGCACAAGAGTAGCTACAGCCTGCTTACATGTCCATTTAGGTTATAGTTCATGATCTACAGAGAAACC TTT
AGGTCAAACTTAAAATATGTAAGAAGGCAGCTTCAGACTAAACAATTTAACAGCTTCAAAGCCATAACTTTTGGAATTATATTTTCTACTAGATAGAAATCTAAAAATTAACATGCAACT TCACAATA
TCTGGCTTCTAATCTATTAGATAAGCAGCAAAACCAAGCACAGGTACATTCTCCTAGAGAATTAATCATTCCATGTGCTTATAAAACAATGCATCAGTATGAAATTTAAAGTATATGCCATTCCTTTT
TGCTTATTTCTAAGAATTGTATAATTTTTTATGAAA cAAAtAATAACAGTAAAAAAGTAGACACCCCACTATTAAGAACAATGGTTGAAACAGACATTATTTAATAATGTTTTAGGGGAA TGTGATTC
ACTTCTGGAAAAACTATAACAGAACTCCAGATGGTGGAGATAGAATAACACGAAAAATATTATTAATTGCAGAAAGAGGCAAAATTCCTTGAAATTTATACATAAATCCTGATACTAGAAAATATTCA
TTCATATACTAACAGGAGTATATATAGAAAGAATAAAAGTTGCTAAATGAGACCAGGACAGTGATTATCATTAGCTACATGGGGCTGAGTATTCCTAGGGAATGAAGATTGTTGTAATTC TTGAACTT
GTTCTGCATATTATTTTTAGTTTCTATGTTTTCATATTGAAACTATATAGAAAATTCCTATAGGCCCATACCATGCTTTGTGTGTGTGTGGGGGGGGGGG tGCACGTGCGCTTGTGTGCATGTGTGTG
TATTTTTAACTCATGTTTTCTACCATAATCAGAATGTTACTAATACCTCCAGTTATAGTCATAGATAAGACTGGATAAACTCTAATTTACCCATTACAACCAAGATTCAGGTGACCATCA TGAGTATG
GAGTAAGATTCTATTCTGTGTGCCATATGTTATATAAATATAAAACATTTGTTAATAAAGAGTTTTCCCATAACACACATAAACCACTATTTTCAAATATTGCTTTAGACCCATGTCAGGATTTA CCT
AGTCAGGTGCTTCCTTGAACATTTTTATTAAACTAATTTTGTCTTAATTACTTTAGGACATTTTGTTTAACAGACTATACAACAAATAAAGATGGATCAACTTTGTGAAAAGAAATAAAA GAGCCCCA
GAGGTCTGTGATAATAAGAAAATCTAAGTGTCTTTACACT T

1438	23.6	6.2	1.8	hg19	319	523	(1638)	C	LTR37B	LTR/ERV1	(2)	466	242	5
2849	11.1	1.0	0.0	hg19	524	920	(1241)	C	MER57B1	LTR/ERV1	(0)	401	1	6
1438	23.6	6.2	1.8	hg19	921	1153	(1008)	C	LTR37B	LTR/ERV1	(227)	241	9	5
17	31.6	0.0	0.0	hg19	1587	1637	(524)	+	(TG)n	Simple_repeat	1	51	(0)	7
254	23.9	9.7	1.3	hg19	1682	1753	(408)	C	LTR91A	LTR	(819)	164	87	8

>KSI_AluYb9_8

>Scaffold6756-4463130-4463466
CTGATAGTAGCACATCCAAGTTTTAAACCCATGGGATTTTACTTGCCTTTTTTGAATAAAATAGGGCAACTAGTAGTCTGAAGCCCTATATGAAGTGCTAACTGGCCCCCAAAATCAGATTAGAAG AC
TGCTAAAAGAGtTACATTATAAATAGGGCTGGGCACAGTGGTGAGCACCTGTAGTCCCAACTACCTGGGATGCTGAGGCAGGAGGATCCCTTGAGCCCAGGAGTTCAAGGCTGCAGTGAGCTCTGGTT
GCACCACTACACTCCAGCCTGGGTGACAGATAAAGACCCTGTCTTAAAAAAAAGAAAAAAGATAAAGAAGGGTTACACTAAAAATGTGTATTTTCTATTTTCCCCATTAATGTCCTTCCTACATA CAT
CCCCAGGTGATGGAGGAGAAAATACATTGTAAAATAATAGCCAAATTGGTATAATTCTCCATAAAATTTTTTCAACCGAATAGATCAGTGAAGATTCCTTTAAATCTATCTTAAAATCATAACTAATA
ATTATTATTTAGTTACTAATTTAGTTCAAAGCTTCTCAGCTGACTGTATATATTCTGTTGAATATTTCTCTTGTGTGATTCAAAACAACCTTAAATAACTGTGACAATAGCATATCACCCAGCTA GGA
CAGGCAGGGTGCTTCACTTCAACAATTTTTCACTTCTTTCACCCCTTCATTTTGTCCATCAGCCAGTGGTGAAAACAACTAAATATTGGTATTTATCGAACTTATAATTTTGACAACCACTGCAGACC
TTTAAACGTTGTATTTCTTTAATGTTCTCATTATCTTCTCAAGCCTGACAATCTCCTCATGCGATGTGCTTTCAGCTACTATTATTTTCATTATTTCTAAATGCCAATTGAAGGACAGCATTTAA AAA
TGCTGAAAGAAATAAAAGAATCTCAGAATAGAAATCACTTAACTATATGTGAACACCAATTCCCAAGCTGCCTGTTCAGAAATTTAAATAaaaagaaaaaatatggccgggtgcggtggctcacgcct
gtaatcccagcactttgggaggccgaggcgggtggatcatgaggtcaggagatcgagaccatcctggctaacaaggtgaaaccccgtctctactaaaaatacaaaaaattagccgggcgcggtgg cgg
gcgcctgtagtcccagctactggggaggctgaggcaggagaatggcgtgaacccgggaagcggagcttgcagtgagccgagattgcgccactgcagtccgcagtccggcctgggcgacagagcgagac
tccgtctcaaaaaaaaaaaaaaaaaaaaaaaaaaaaaaaaaaaAAAAAaAAAAAAATACATCACCAGTTAAAGAGCTTATCATGTGAAGTAAATAAACTAAATAAAATAGAAACTAAATCAGAAGGCAC
TTTATTAGAAAAAAATTCCAAATTTAATCTTCAATGGTAAGAGGCCACTAAGCAGAGAATGAAATGAACTGGAACCCTGATAAACTGTAAATTGAAATGATAATTATCACTAAAACAGAGGGATTCAA
TCCAGCTGCCTGGGGATGTTTCATTATATAAATTATTATTATCTATTCCAGTATTGG CAGAGGAAGGGCTATGCGTCTATTAGACTTAGGCCAAATTTGGCATGCTGGTTTCTACCAATAGCACATGC
TGGTGATTTTACTTTTCAGAACTCACATATTTATCTCATTTGATCTCATCACAACCTGAGACACGAGTCATAATGAGGACTATGGGGCTAATTTTATATCCAGTGAACCAGCCAAGTTTCATGCA GTT
GAGAAATGGTAAACTGTCCAGACgTAGTGGCTCACGCCTGTAATTCCAAcactttgggaggccgaggcaggcggatcacctgagtccaggagttccagaccagcctggccaacatggtgaaacccgtc
tctactaaaaatacaaaaattagctgggtgtggtggtgcgggcctgtaatcccagCACTTTGGGAGGCCGAGGCAGGCGGATCACCTGAGTCCAGGAGTTGGAGACCAGAGTGGCCAATATGTAGAAA
CCCTGTCTCTACAAAAAGTACAAAAATTAGCTGGGGGTGTTGGCACACACTTGTAAT TCCAGCTAATCCGGAGGCTGAGGCATGAGGATTGCTTGAGCTCGGGAGGTGGAGTTTGCAGTGAGGGGAGA
TGCTGCCACTGCACTCCAGCCtTGGTGACAGAACGAGACTCTGCCTCAAAAAAATAAAAATAAAAATAAAAATAAAATAAAATAAAGAAAAGAAAAAGTAAACTTATTAGAGACCAGATGTCCTGTCC
TGATTCCTGGCCCACCACACTGCCTTCCCTGAA

1106	15.9	0.0	0.0	KS10	156	318	(2019)	+	FRAM	SINE/Alu	1	163	(13)	3
22	12.0	0.0	0.0	KS10	976	1000	(1337)	+	(A)n	Simple_repeat	1	25	(0)	4
3027	0.3	0.0	0.0	KS10	1001	1337	(1000)	+	AluYb9	SINE/Alu	1	318	(0)	5
1069	7.5	0.8	0.0	KS10	1810	1942	(395)	+	AluSx	SINE/Alu	3	136	(176)	6
1955	16.1	0.0	0.0	KS10	1943	2253	(84)	+	AluSz6	SINE/Alu	2	312	(0)	7

>Hg19 chr10:26851981-26853845
CTGATAGTAGCACATCCAAGTTTTAAACCCATGGGATTTTACTTGCCTTTTTTGAATAAAATAGGGCAACTAGTAGTCTGAAGCCCTATATGAAGTGCTAACTGGCCCCCAAAATCAGATTAGAAG AC
TGCTAAAAGAGcTACATTATAAATAGGGCTGGGCACAGTGGTGAGCACCTGTAGTCCCAACTACCTGGGATGCTGAGGCAGGAGGATCCCTTGAGCCCAGGAGTTCAAGGCTGCAGTGAGCTCTGGTT
GCACCACTACACTCCAGCCTGGGTGACAGATAAAGACCCTGTCTTAAAAAAAAGAAAAAAGATAAAGAAGGGTTACACTAAAAATGTGTATTTTCTATTTTCCCCATTAATGTCCTTCCTACATA CAT
CCCCAGGTGATGGAGGAGAAAATACATTGTAAAATAATAGCCAAATTGGTATAATTCTCCATAAAATTTTTTCAACCGAATAGATCAGTGAAGATTCCTTTAAATCTATCTTAAAATCATAACTAATA
ATTATTATTTAGTTACTAATTTAGTTCAAAGCTTCTCAGCTGACTGTATATATTCTGTTGAATATTTCTCTTGTGTGATTCAAAACAACCTTAAATAACTGTGACAATAGCATATCACCCAGCTA GGA
CAGGCAGGGTGCTTCACTTCAACAATTTTTCACTTCTTTCACCCCTTCATTTTGTCCATCAGCCAGTGGTGAAAACAACTAAATATTGGTATTTATCGAACTTATAATTTTGACAACCACTGCAGACC
TTTAAACGTTGTATTTCTTTAATGTTCTCATTATCTTCTCAAGCCTGACAATCTCCTCATGCGATGTGCTTTCAGCTACTATTATTTTCATTATTTCTAAATGCCAATTGAAGGACAGCATTTAA AAA
TGCTGAAAGAAATAAAAGAATCTCAGAATAGAAATCACTTAACTATATGTGAACACCAATTCCCAAGCTGCCTGTTCAGAAATTTAAATAAAAAgAAAAAATACATCACCAGTTAAAGAGCTTATCAT
GTGAAGTAAATAAACTAAATAAAATAGAAACTAAATCAGAAGGCACTTTATTAGAAAAAAATTCCAAATTTAATCTTCAAT GGTAAGAGGCCACTAAGCAGAGAATGAAATGAACTGGAACCCTGATA
AACTGTAAATTGAAATGATAATTATCACTAAAACAGAGGGATTCAATCCAGCTGCCTGGGGATGTTTCATTATATAAATTATTATTATCTATTCCAGTATTGGCAGAGGAAGGGCTATGCGTCTATTA
GACTTAGGCCAAATTTGGCATGCTGGTTTCTACCAATAGCACATGCTGGTGATTTTACTTTTCAGAACTCACATATTTATCTCATTTGATCTCATCACAACCTGAGACACGAGTCATAATGAGGA CTA
TGGGGCTAATTTTATATCCAGTGAACCAGCCAAGTTTCATGCAGTTGAGAAATGGTAAACTGTCCAGAC aTAGTGGCTCACGCCTGTAATTCCAACACTTTGGGAGGCCGAGGCAGGCGGATCACCTG
AGTCCAGGAGTTGGAGACCAGAGTGGCCAATATGTAGAAACCCTGTCTCTACAAAAAGTACAAAAATTAGCTGGGGGTGTTGGCACACACTTGTAATTCCAGCTAATCCGGAGGCTGAGGCATGA GGA

TTGCTTGAGCTCGGGAGGTGGAGTTTGCAGTGAGGGGAGATGCTGCCACTGCACTCCAGCCgTGGTGACAGAACGAGACTCTGCCTCAAAAAAATAAAAATAAAAATAAAAATAAAATAAAATAAAGA
AAAGAAAAAGTAAACTTATTAGAGACCAGATGTCCTGTCCTGATTCCTGGCCCACCACACTGCCTTCCCTGAA

1106	15.9	0.0	0.0	Hg19	256	418	(1647)	+	FRAM	SINE/Alu	1	163	(13)	1
1943	16.4	0.0	0.0	Hg19	1572	1881	(184)	+	AluSz6	SINE/Alu	3	312	(0)	2

>KSI_AluYb9_9

>Scaffold8313-376550-376872
AACAAGtCTGTAGCTCTGTCTTGCTTGGACAATTCTCTACTGGtACCTACCAAGCTGCGAAGCTTCCTTCCTGTCTTGAGGTTTCACAGCCAGTTGCTTTAAATTCCCC aCAGTGTTCCTTGCCTTGC
AGTGCTCACTACTAAGCAACAAACACATgCACACAGAGACCACTTGTCTTAGTTCCACAAACTTCTCATATAGCAACGAGAGATGCTGGAGGTAATAAACTAGGTAACTTACAGGTTCAAAGCCTCAC
ATTACTAACTGATGCTGGCTTCAAGCATTCTCGCCTTTCCTGAGCACTTCCTTATTCATCTCTTTCAGATGCTTTTCCATTTCCTGTGCTTTAACTGACACTCTTTTCTATTAGGTTAAACC gCAGGA
AACTGCTGATACTCAGCCACTTTTAACCTACAAAAATTAAAATTtCACCTGGTTCAACCTAACGAGGTTGAGATGTTTATTTACCCATTGGGGAACATGGTAACAGTTTGTGATACTGTAAAGCTGTT
CAAAACCTGTGAAAACCCTTCCTATCCTTGAGGAATTATTTTAAATCTTCATCTCTATGaAGTAAATGCCAGAAACTCACCTTCCCAGGCTGCTCCAATCAGATACAAGATTTTCTACTCAGAAAAGA
ATGACAGGAAGAGCTATTCTGCCATTGAGGAGAGACAGGAATAGGGGTGGGAACTGGGCATGGGAGGGTTCTTGACCTGGGTGGTGGTTATATGGAAGCGTGGTCTGTGATAATTCTTTTCTATT CTG
TGTTATTCTGGAACTTTAAAAAAGTTTTGGAAACTCAGTTGTGGCAAGGGTAGGGGTGAGACATTGAGCTACCAGTCTTGGTGGTGTCCGGATTCCTGTGTCAGCAGTGTGAGCTGCAGGATCTGTAT
CCAATGCTGCCCTCTGGCCTCCATGGAGCAGTCCTGTAGGCAATTTTGGCATAGTTCTTGGTGTACAGTCTCCATCACTGTTTCTCTGGCTCTcctggagtttctttTttttttttttttttttttt
ttttttttttttttttttttgaggggagtctcgctctgtcgcccaggccggactgcggactgcagtggcgcaatctcggctcactgcaagctccgcttcccgggttcacgccattctcctgcc tca
gcctccccagtagctgggactacaggcgcccgccaccgcgcccggctaattttttgtatttttagtagagacggggtttcaccttgttagccaggatggtctcgatc tcctgacctcatgatccaccc
gcctcggcctcccaaagtgctgggattacaggcgtgagccaCCTGGAGTTTCTTTAAGATgCCAAATATCCTTTAGTAAACATTCTTTCTGCTCTAATTAGCTATAGAAGGTTCTTATTTTGTTGTTG
TTGTTTTGTTTTGTTTTAAGAAATAGGATCTCACTGTGTCACTCAGGCTGGAGA GCAGAGGTGCAATCAGAGCTCACTATAGgCTTGAACTCCTGGGCTCAAGTGATTCTCCCACTTCAGCCTCCTAA
AGTGCTGGGATTACAGGCATGAGCCACTGCACCCTGCCGGGTTCTTGTTCATGACTAGCATCTAAGAGCCCTAGCTGATAAAAGGGAATTCAGGTTCTGTTCAGGCTAGACCTTCACCTGACACTGAA
AATGCTGTCCTCAAGGCCACCTGAATGCTGGCTCTGTGGAGTACTCTATGGTACTCCAGACTCTAGTGCTCCCTCTGTTCTACAATCAGAGCATGCAGGGCTGAAG TATATACAGCATGCCCTGCTG
CTTTCAGCTGACTATTATTAGCTTCTCTAAAAGTCAATTTTTTACATTAATCTCCACTACTTTCTCCTCTATAAACCCCTGTTCCCCAAGAAATACAAGCACACACACACAACCACCAGCAGCAG CAG
CAGCAGCACCCCCATCACCACCACCACAACCTGGAATACCCAGGACTATGAAAAAGTCATGACACACCACGTCTCACTAGAATGTAATCTTCATGGGCAGACTTGTC TATTTTGTTCAGTTCTCTATC
ACAGCATGTAGAATGGTGCCAGGCAGATATTAGGTACCCAGTAAATATCTGTTAAATGAATGAATATCTTGCCCTAAAATACATCTATCCATTTGTTTATCAAACGAAGAACAAATGCTTATGGA GAC
ATGGGCTCAGGCACTGGTGTGTGTATTAGGAAAGTCATGGCTCTGCCCATGAG tAGTTCGAAATGAGTTAACGTTACTAATGACCCCCACAATATATGTTTTGTCAC TTCTGTGAGCCCAgTTGGAGC
TTCTTTTTAGTGAAGCTCT

428	20.0	0.0	0.0	KS	367	446	(1877)	+	MADE2	DNA/TcMar-Mariner	1	80	(0)	6
317	29.1	5.3	2.6	KS	491	603	(1720)	+	MLT1N2	LTR/ERVL-MaLR	12	127	(435)	7
481	28.1	4.4	6.8	KS	848	1000	(1323)	+	MLT1N2	LTR/ERVL-MaLR	308	461	(101)	7
2629	0.7	0.0	0.0	KS	1001	1323	(1000)	C	AluYb9	SINE/Alu	(0)	318	11	8
481	28.1	4.4	6.8	KS	1324	1393	(930)	+	MLT1N2	LTR/ERVL-MaLR	462	526	(36)	7
894	22.2	0.0	1.1	KS	1398	1573	(750)	C	FRAM	SINE/Alu	(2)	174	1	9
23	20.5	0.0	3.5	KS	1892	1951	(372)	+	(CAC)n	Simple_repeat	1	58	(0)	10
538	18.2	5.8	0.0	KS	1993	2113	(210)	+	L2a	LINE/L2	3299	3426	(0)	11

>Hg19 chr14:52642765-52644755
AACAAGgCTGTAGCTCTGTCTTGCTTGGACAATTCTCTACTGGcACCTACCAAGCTGCGAAGCTTCCTTCCTGTCTTGAGGTTTCACAGCCAGTTGCTTTAAATTCCCC cCAGTGTTCCTTGCCTTGC
AGTGCTCACTACTAAGCAACAAACACATaCACACAGAGACCACTTGTCTTAGTTCCACAAACTTCTCATATAGCAACGAGAGATGCTGGAGGTAATAAACTAGG TAACTTACAGGTTCAAAGCCTCAC
ATTACTAACTGATGCTGGCTTCAAGCATTCTCGCCTTTCCTGAGCACTTCCTTATTCATCTCTTTCAGATGCTTTTCCATTTCCTGTGCTTTAACTGACACTCTTTTCTATTAGGTTAAACC aCAGGA
AACTGCTGATACTCAGCCACTTTTAACCTACAAAAATTAAAATT aCACCTGGTTCAACCTAACGAGGTTGAGATGTTTATTTACCCATTGGGGAACATGGTAAC AGTTTGTGATACTGTAAAGCTGTT
CAAAACCTGTGAAAACCCTTCCTATCCTTGAGGAATTATTTTAAATCTTCATCTCTATG gAGTAAATGCCAGAAACTCACCTTCCCAGGCTGCTCCAATCAGATACAAGATTTTCTACTCAGAAAAGA
ATGACAGGAAGAGCTATTCTGCCATTGAGGAGAGACAGGAATAGGGGTGGGAACTGGGCATGGGAGGGTTCTTGACCTGGGTGGTGGTTATATGGAAGCGTGGT CTGTGATAATTCTTTTCTATTCTG
TGTTATTCTGGAACTTTAAAAAAGTTTTGGAAACTCAGTTGTGGCAAGGGTAGGGGTGAGACATTGAGCTACCAGTCTTGGTGGTGTCCGGATTCCTGTGTCAGCAGTGTGAGCTGCAGGATCTG TAT
CCAATGCTGCCCTCTGGCCTCCATGGAGCAGTCCTGTAGGCAATTTTGGCATAGTTCTTGGTGTACAGTCTCCATCACTGTTTCTCTGGCTCTCCTGGAGTTTCTTTAAGATaCCAAATATCCTTTAG
TAAACATTCTTTCTGCTCTAATTAGCTATAGAAGGTTCTTATTTTGTTGTTGTTGTTTTGTTTTGTTTTAAGAAATAGGATCTCACTGTGTCACTCAGGCTGGAGAGCAGAGGTGCAATCAGAGC TCA
CTATAGcCTTGAACTCCTGGGCTCAAGTGATTCTCCCACTTCAGCCTCCTAAAGTGCTGGGATTACAGGCATGAGCCACTGCACCCTGCC GGGTTCTTGTTCATGACTAGCATCTAAGAGCCCTAGCT

GATAAAAGGGAATTCAGGTTCTGTTCAGGCTAGACCTTCACCTGACACTGAAAATGCTGTCCTCAAGGCCACCTGAATGCTGGCTCTGTGGAGTACTCTATGGTACTCCAGACTCTAGTGCTCCC TCT
GTTCTACAATCAGAGCATGCAGGGCTGAAAGTATATACAGCATGCCCTGCTGCTTTCAGCTGACTATTATTAGCTTCTCTAAAAGTCAATTTT TTACATTAATCTCCACTACTTTCTCCTCTATAAAC
CCCTGTTCCCCAAGAAATACAAGCACACACACACAACCACCAGCAGCAGCAGCAGCAGCACCCCCATCACCACCACCACAACCTGGAATACCCAGGACTATGAAAAAGTCATGACACACCACGTC TCA
CTAGAATGTAATCTTCATGGGCAGACTTGTCTATTTTGTTCAGTTCTCTATCACAGCATGTAGAATGGTGCCAGGCAGATATTAGGTACCCAG TAAATATCTGTTAAATGAATGAATATCTTGCCCTA
AAATACATCTATCCATTTGTTTATCAAACGAAGAACAAATGCTTATGGAGACATGGGCTCAGGCACTGGTGTGTGTATTAGGAAAGTCATGGCTCTGCCCATGAG cAGTTCGAAATGAGTTAACGTTA
CTAATGACCCCCACAATATATGTTTTGTCACTTCTGTGAGCCCA aTTGGAGCTTCTTTTTAGTGAAGCTCT

409	20.0	0.0	0.0	Hg19	467	546	(1645)	+	MADE2	DNA/TcMar-Mariner	1	80	(0)	1
304	30.0	5.3	2.6	Hg19	591	703	(1488)	+	MLT1N2	LTR/ERVL-MaLR	12	127	(435)	2
585	28.1	4.7	1.8	Hg19	948	1161	(1030)	+	MLT1N2	LTR/ERVL-MaLR	308	527	(35)	2
920	21.6	0.0	1.1	Hg19	1166	1341	(850)	C	FRAM	SINE/Alu	(2)	174	1	3
23	20.5	0.0	3.5	Hg19	1660	1719	(472)	+	(CAC)n	Simple_repeat	1	58	(0)	4
538	18.2	5.8	0.0	Hg19	1761	1881	(310)	+	L2a	LINE/L2	3299	3426	(0)	5

>KSI_AluYb9_10

>Scaffold10990-1181919-1182079
ATACTAAAGATGGAGACTAGGTAGGAATAACAGGAAAAAGGAGGAGGGGGAAAGAAGACAAGAAAGAGATAAGGGCTAGAACAAGTGGGAGATTTGAGTTAAGAAGAAAAAGAAATCAAGTTTCTC CA
TAACTAAGGGACCAGCCAGCTAGGATTCTTCTGCCCACTCATACTTGCATGTACTGTTCCTCTTTGTCTAAGGAATTAACACGTTCTTGAAGTATGTTCTCCTGTTTTTCAAAATTCTTCAGGAG AAG
TATTTCTTGAAATAAGGTGACATGGTGCAGGTCAGATAATTTCATCTGAGTATCTAGTTTTAGTTTCTGATGTCTAAGGAGACGGAGTTCTGCATCGAAAGTGACAACCAGTTCTTTGATCTGAGA TG
GAAAAAATATTTGCGGATACGTTAGAAGACTAGATAAATGCATTTTTAAACTATCTAACAGTACTAATTTGTATGAAGTCATTTTTTATTATGCCCTTATGGATGGAGAATTGTGTGTGTGTGTA TAG
TCACAAAAATAAATTCATTAATGGCATTAAGTTTACATAAATTAATTTATTAAGTGGTAGGAATTGTGTAACTAGAAGTCCTTCCACCTATTTCTTCACCATTAACTAAGCCATCAATCAAGAAT GCC
CTTCTTGCTTTACTGTGTAGCTTATGAGCTCTCTGAGTGGGTAGTGTTTCTATCTGTCTCCTCTTTATTCTCCATAGTATCTCTACAATCCCTTTTATATAGTAAGCACAAATAAATACGTTTCCCAA
CTTCTTATGTTGAAAATTTTCAAAACCACAGAATGGTAAATATTTTTAAATAGATAAAATAATCATGGAGCCACAAAAAAACCTGT TCaatctctttctTtttttttttttttttttttttttttttttt
ttttttttttttTTTTTTTTTTTTTTTTTTTTTTTTTTTTTTTTTTTTTTTTTTTTTTTGAGACGGAGTCTCGCTCTGTCGCCCAGGCCGGACTGCGGACTGCAGTGGCGCAATCTCGGCTCACT GCA
AGCTCCGCTTCCCGGGTTCACGCCATTCTCCTGCCTCAGCCTCCCGAGTAGCTGGGACtacaggcgcccgccaccgcgcccggctaatttttgtatttttagtagagacggggtttcaccttgtt ag
ccaggatggtctcgatctcttgacctcatgatccacccgcctcggcctcccaaagtgctgggattacaggcgtgagctaccgcgcccggc CAATCTCTTTCTAAGATTGAAGAAATTAAGGTCAAAGT
TACTTTTCCTGATAAGAGCCGAAGTATCCCAAGTCCTGGTCCAGACTTTAATCCATTATGGTCCTACTAATTTTAAGTCCTCAGTTTTGTTCTGATTTTGGTACAGCTTTCCCCCAGCTTTCCCCCCC
AGTATCACAAAGGAGATGAAGAGGTCAAGAGAGAGCCCCAAGGGCTGATTCTCCTGAGGAAACTGGAACAATAGGAAGAGACAAACCATTCTATGTTTTTAATTAGTTTGTACATGATAGGTGGC CTC
AACTGTCAAAAGCCTATAGTAAGTGAGGACCCAGACTGGGGCTGAGGAGAGAAAGAGATCTGTGGGTTTGGAGCAGTGAAACTACCAGAGAAATTTAGAGAGCT aAAAAAAAAAAAAAAAAAAAAAAA
gAGAGAGAGAAGAAAAAATTAAACTTTTAAGTAACCATCTCTATAGACTATATGTTTGGGCTCCCCTTTAAAGCTTGTTTAACAAACTTGAATTATTCCTGCATTTATTTGGAGGACCACATAGGTC T
CCAAGTCTGTAAGGATTACACTTGTTTTATTCTCTCCAATCCCAATAGCCTATAAGCTTTGTGAAGATAGAGAACATGTCTTTCTTAAGAATTGTTGTATCCCTAATCCTATCTATCAGTGTCCC TGC
ATACAGAACATCCAATAAATATTTGCTAAATTAATAAATTAGTTACAATTAAATCAATCAAAACTTTTGACTGAATATCTATTATATGTTTGACATTGTCCTAGACTATGTGGAGGAATCAGAAGTA T
ATGTGGCCCTTTGCTCAAGAATTGTGTAATTTAATTTTCAAGACAATACTAATACCCATGAAACAGCAAGGAAAATTAGTGTAAAACATGAGATGCTTATTACAGGTGCAACTGGAGTTTAGAAA CAA
GGACAGCATAAGTAATGTGGGCGGAAAATGCTGGAGAGGGTTTGGAACTGAGCCATGCTTTGAATG A

12	17.8	0.0	9.5	KS	520	565	(1596)	+	(AATT)n	Simple_repeat	1	42	(0)	6
197	33.3	4.0	1.9	KS	658	758	(1403)	+	L2d2	LINE/L2	3344	3446	(18)	7
52	0.0	0.0	0.0	KS	866	912	(1249)	+	(T)n	Simple_repeat	1	47	(0)	8
1515	1.2	0.0	0.0	KS	1001	1161	(1000)	C	AluYb9	SINE/Alu	(157)	161	1	9
25	16.8	0.0	0.0	KS	1559	1600	(561)	+	A-rich	Low_complexity	1	42	(0)	10
276	31.6	3.0	3.7	KS	1743	1877	(284)	+	L2a	LINE/L2	3293	3426	(0)	11
368	31.4	14.8	1.6	KS	1884	2161	(0)	C	L2d	LINE/L2	(1)	3424	3111	12

>Hg19 chr3:113046291-113048154
ATACTAAAGATGGAGACTAGGTAGGAATAACAGGAAAAAGGAGGAGGGGGAAAGAAGACAAGAAAGAGATAAGGGCTAGAACAAGTGGGAGATTTGAGTTAAGAAGAAAAAGAAATCAAGTTTCTC CA
TAACTAAGGGACCAGCCAGCTAGGATTCTTCTGCCCACTCATACTTGCATGTACTGTTCC TCTTTGTCTAAGGAATTAACACGTTCTTGAAGTATGTTCTCCTGTTTTTCAAAATTCTTCAGGAGAAG
TATTTCTTGAAATAAGGTGACATGGTGCAGGTCAGATAATTTCATCTGAGTATCTAGTTTTAGTTTCTGATGTCTAAGGAGACGGAGTTCTGCATCGAAAGTGACAACCAGTTCTTTGATCTGAG ATG
GAAAAAATATTTGCGGATACGTTAGAAGACTAGATAAATGCATTTTTAAACTATCTAACA GTACTAATTTGTATGAAGTCATTTTTTATTATGCCCTTATGGATGGAGAATTGTGTGTGTGTGTATAG
TCACAAAAATAAATTCATTAATGGCATTAAGTTTACATAAATTAATTTATTAAGTGGTAGGAATTGTGTAACTAGAAGTCCTTCCACCTATTTCTTCACCATTAACTAAGCCATCAATCAAGAAT GCC
CTTCTTGCTTTACTGTGTAGCTTATGAGCTCTCTGAGTGGGTAGTGTTTCTATCTGTCTC CTCTTTATTCTCCATAGTATCTCTACAATCCCTTTTATATAGTAAGCACAAATAAATACGTTTCCCAA
CTTCTTATGTTGAAAATTTTCAAAACCACAGAATGGTAAATATTTTTAAATAGATAAAATAATCATGGAGCCACAAAAAAACCTGT TCAATCTCTTTCTAAGATTGAAGAAATTAAGGTCAAAGTTACT
TTTCCTGATAAGAGCCGAAGTATCCCAAGTCCTGGTCCAGACTTTAATCCATTATGGTCCTACTAATTTTAAGTCCTCAGTTTTGTTCTGATTTTGGTACAGCTTTCCCCCAGCTTTCCCCCCCAGTA
TCACAAAGGAGATGAAGAGGTCAAGAGAGAGCCCCAAGGGCTGATTCTCCTGAGGAAACTGGAACAATAGGAAGAGACAAACCATTCTATGTTTTTAATTAGTTTGTACATGATAGGTGGCCTCA ACT
GTCAAAAGCCTATAGTAAGTGAGGACCCAGACTGG GGCTGAGGAGAGAAAGAGATCTGTGGGTTTGGAGCAGTGAAACTACCAGAGAAATTTAGAGAGCT tAAAAAAAAAAAAAAAAAAAAAAAAaaaa
aaaaacAGAGAGAGAAGAAAAAATTAAACTTTTAAGTAACCATCTCTATAGACTATATGTTTGGGCTCCCCTTTAAAGCTTGTTTAACAAACTTGAATTATTCCTGCATTTATTTGGAGGACCACATA
GGTCTCCAAGTCTGTAAGGATTACACTTGTTTTAT TCTCTCCAATCCCAATAGCCTATAAGCTTTGTGAAGATAGAGAACATGTCTTTCTTAAGAATTGTTGTATCCCTAATCCTATCTATCAGTGTC

CCTGCATACAGAACATCCAATAAATATTTGCTAAATTAATAAATTAGTTACAATTAAATCAATCAAAACTTTTGACTGAATATCTATTATATGTTTGACATTGTCCTAGACTATGTGGAGGAATC AGA
AGTATATGTGGCCCTTTGCTCAAGAATTGTGTAAT TTAATTTTCAAGACAATACTAATACCCATGAAACAGCAAGGAAAATTAGTGTAAAACATGAGATGCTTATTACAGGTGCAACTGGAGTTTAGA
AACAAGGACAGCATAAGTAATGTGGGCGGAAAATGCTGGAGAGGGTTTGGAACTGAGCCATGCTTTGAATG A

12	17.8	0.0	9.5	Hg19	620	665	(1399)	+	(AATT)n	Simple_repeat	1	42	(0)	1
197	33.3	4.0	1.9	Hg19	758	858	(1206)	+	L2d2	LINE/L2	3344	3446	(18)	2
32	13.4	0.0	0.0	Hg19	1354	1403	(661)	+	(A)n	Simple_repeat	1	50	(0)	3
276	31.6	3.0	3.7	Hg19	1546	1680	(384)	+	L2a	LINE/L2	3293	3426	(0)	4
390	34.5	11.4	2.8	Hg19	1687	2053	(11)	C	L2d	LINE/L2	(1)	3424	3027	5

>KSI_AluYb9_11

>Scaffold11674-6424086-6424404
TATATATGTTAACAGTATCTGGAAtATCTTCTTTAAATCACAGGCATAATTAATATGCTAAAATCTCAGTTTCTAGTTTAAATAACAATCTACATCTGTTTTTTAAAAAGATTAATTTAACTATTCTC
ATTGAAATTACTACCTATAATTAATGTACACTATGGTTTGAATGTTTATGTCCCCTCCAAAATTCATGTTGAAACTTAATTCTCAATGCAACAGTATTAGAAGATAAGGCCTTCCAGAATTGAGG GCT
TTGCTTTGAGGAGTGGGATTAATGCCTTGTAAAAGAGTAAAAGAGGCATCACACAGCATCTGCCCCTTTTTGCCCTTCTACCTATGTGAGGACACAGCAACAAGGTGTCACCCTGGAAG CAGAAAGCA
ACCTTCATGGGATTACAAACATGCCAGTGCCTTGACCTTGGACTTTCCAGCCTCAAGGACTGGGAGAAAACAAATTCCTGTTGTTTATAAATTACCCAGTCTAAGGTATTTTTTTATAGCAGCACAAA
AAGACTAAGACTGTGTGGAATTACTTACGTTGTTCTTCTAAAGTCTTTTTGAAGTGTGGGATATTCTGGCATCTTTCTAATATCTTCCCAGTCTTTATCTTACAAAAAAATTAAATATTGTTATA GAA
AATAATGTGTTAATTTAAAATCTCCTAATTATGTTAGTTTATAAATATACATTAAAATATGCTTTTGAGAAGAAATGATTTTGAAATATGACTCAAAATATGACTTTAAAAAGGAAAATAAATTACCT
GCAGGAAACCCCATGACACTAAATATCCGATCCAGTTGATCATGATGAAAGGGATTGCTTGTTTTTATATCTTCCTGACGACAGTGAAAAATAGGTTCCGAAGTCAACAATTCAGCAAATATACA ACC
TATTGCCCATATATCTGGGAAGGAAGAAACATAAGTTTTTGTATATATTTCCTTGGTTATAATTCCTAGATATAATCCCAGCCTACTTA TAaaaatacttttcagGccgggcgcggtggctcacgcct
gtaatcccagcactttgggaggccgaggcgggtggatcatgaggtcaggagatcgagaccatcctggctaacaaggtgaaaccccgtctctactaaaaatacaaaaaattagccgggcgcggtgg cgg
gcgcctgtagtcccagctactggggaggctgaggcaggagaatggcgttgaacccgggaagcggagtttgcagtgagccgagattgcgccactgcagtccgcagtccagcctgggcgacagagcgaga
ctccgtctcaaaaaaaaaaaaaaaaaaaaaaaaaaaaAAAAATACTTTTCAGTAAATCAATATACAAAGTACAATAAGTACATTAAGAGAACATTATTGTACTTTGTATTTTGACATATGTGTGGGT
ATGTGTGTATATATAGGTATGTATATATATGTGTGTGTATGTGTATATATATATACATGACATGAAATAGCTATATCATCATGTAACAAATTGAAAGCCAAGTAATAAAAAACATTTACTTGTAAATT
TTCTGATGACAATAAATGGGGACAGTCAATTTAGCAAGCAACAAATTCTTTAGATTTAGGCAAAGGTCTTTCTAACCACTTTTTTTGTTGTTGTTTTTAGGGTCTCTGTCACCCAGGCTGGAGTA CAG
TGGTGTAACCATGGCTCCCTGAAGCCTTGACCTCCTGGGCTCAAGGAATCCTCCTGCCTCAGCCTCCAGAGTA GTTCAGACTATGGGTGTGTGCTACTTGTACCTGGCTAATTTTTTAAAAAATTGTT
TTGTAGAGACAAGGTCTTACCATGTCTAACCACTGTTAATAATTTAATCATTAAAGTACTCAATTGATAATCTAATACCAGGCGTAATACACAGATCTGATATACTGAAACAATATCATCATCAG ACA
TGGTGACGTAATTCTTTTAAAGCCATCCAATGTATAATGATAGAGTAGAATTTATAAGTTTTGAGTCTAGACAGACAGTATAGACCATATAAAAGAATTGGGGAG aCTGGGTGCAGTGGTTCATGCCT
GTATTCCAAGTACTTTGGGAGGCTGAGGTGGGCGGATCACTTGAGGTCAGGAGTTCGAGACCATCCTGACCAACATGGTGAAACCCTGTCTCTATTAAAAATACAAAAAATTAGCCGGGTGTGGT GGC
ACACGCCTGTAGTCCCAGCTACTGAGGAGGCAGATGCAGAAGAATCTCTTGAACCCAGGGGACAGAGGTTGCAGTGAGCCAAGATCATGCCACCGTACTCCAGCTTGGGCGACAGAGTGAAACACCAT
CTTGGAAAAAAACAAA

1649	18.7	13.2	2.0	KS	159	523	(1796)	+	MLT1A1	LTR/ERVL-MaLR	3	407	(1)	5
2982	0.6	0.0	0.3	KS	1001	1319	(1000)	+	AluYb9	SINE/Alu	1	318	(0)	6
25	29.1	0.0	2.7	KS	1400	1484	(835)	+	(TA)n	Simple_repeat	1	82	(0)	7
1008	18.3	4.5	2.9	KS	1616	1817	(502)	C	AluJb	SINE/Alu	(11)	301	97	8
2038	14.6	0.0	0.3	KS	2025	2319	(0)	+	AluSz	SINE/Alu	1	294	(18)	9

>Hg19 chr6:110947439-110949425
TATATATGTTAACAGTATCTGGAAaATCTTCTTTAAATCACAGGCATAATTAATATGCTAAAATCTCAGTTTCTAGTTTAAATAACAATCTACATCTGTTTTTTAAAAAGATTAATTTAACTATTCTC
ATTGAAATTACTACCTATAATTAATGTACACTATGGTTTGAATGTTTATGTCCCCTCCAAAATTCATGTTGAAACTTAATTCTCAATGCAACAGTATTAGAAGATAAGGCCTTCCAGAATTGAGG GCT
TTGCTTTGAGGAGTGGGATTAATGCCTTGTAAAAGAGTAAAAGAGGCATCACACAGCATCTGCCCCTTTTTGCCCTTCTACCTATGTGAGGACACAGCAACAAGGTGTCACCCTGGAAG CAGAAAGCA
ACCTTCATGGGATTACAAACATGCCAGTGCCTTGACCTTGGACTTTCCAGCCTCAAGGACTGGGAGAAAACAAATTCCTGTTGTTTATAAATTACCCAGTCTAAGGTATTTTTTTATAGCAGCACAAA
AAGACTAAGACTGTGTGGAATTACTTACGTTGTTCTTCTAAAGTCTTTTTGAAGTGTGGGATATTCTGGCATCTTTCTAATATCTTCCCAGTCTTTATCTTACAAAAAAATTAAATATTGTTATAGAA
AATAATGTGTTAATTTAAAATCTCCTAATTATGTTAGTTTATAAATATACATTAAAATATGCTTTTGAGAAGAAATGATTTTGAAATATGACTCAAAATATGACTTTAAAAAGGAAAATAAATTA CCT
GCAGGAAACCCCATGACACTAAATATCCGATCCAGTTGATCATGATGAAAGGGATTGCTTGTTTTTATATCTTCCTGACGACAGTGAAAAATAGGTTCCGAAGTCAACAATTCAGCAAATATACAACC
TATTGCCCATATATCTGGGAAGGAAGAAACATAAGTTTTTGTATATATTTCCTTGGTTATAATTCCTAGATATAATCCCAGCCTACTTA TAAAAATACTTTTCAGTAAATCAATATACAAAGTACAAT
AAGTACATTAAGAGAACATTATTGTACTTTGTATTTTGACATATGTGTGGGTATGTGTGTATATATAGGTATGTATATATATGTGTGTGTATGTGTATATATATATACATGACATGAAATAGCTATAT
CATCATGTAACAAATTGAAAGCCAAGTAATAAAAAACATTTACTTGTAAATTTTCTGATGACAATAAATGGGGACAGTCAATTTAGCAAGCAACAAATTCTTTAGATTTAGGCAAAGGTCTTTCT AAC
CACTTTTTTTGTTGTTGTTTTTAGGGTCTCTGTCACCCAGGCTGGAGTACAGTGGTGTAACCATGGCTCCCTGAAGCCTTGACCTCCTGGGCTCAAGGAATCCTCCTGCCTCAGCCTCCAGAGTA GTT
CAGACTATGGGTGTGTGCTACTTGTACCTGGCTAATTTTTTAAAAAATTGTTTTGTAGAGACAAGGTCTTACCATGTCTAACCACTGTTAATAATTTAATCATTAAAGTACTCAATTGATAATCTAAT
ACCAGGCGTAATACACAGATCTGATATACTGAAACAATATCATCATCAGACATGGTGACGTAATTCTTTTAAAGCCATCCAATGTATAATGATAGAGTAGAATTTATAAGTTTTGAGTCTAGACAGAC

AGTATAGACCATATAAAAGAATTGGGGAGgCTGGGTGCAGTGGTTCATGCCTGTATTCCAAGTACTTTGGGAGGCTGAGGTGGGCGGATCACTTGAGGTCAGGAGTTCGAGACCATCCTGACCAACAT
GGTGAAACCCTGTCTCTATTAAAAATACAAAAAATTAGCCGGGTGTGGTGGCACACGCCTGTAGTCCCAGCTACTGAGGAGGCAGATGCAGAAGAATCTCTTGAACCCAGGGGACAGAGGTTGCAGTG
AGCCAAGATCATGCCACCGTACTCCAGCTTGGGCGACAGAGTGAAACACCATCTTGGAAAAAACAAA

1649	18.7	13.2	2.0	Hg19	259	623	(1564)	+	MLT1A1	LTR/ERVL-MaLR	3	407	(1)	1
25	29.1	0.0	2.7	Hg19	1168	1252	(935)	+	(TA)n	Simple_repeat	1	82	(0)	2
1008	18.3	4.5	2.9	Hg19	1384	1585	(602)	C	AluJb	SINE/Alu	(11)	301	97	3
2092	14.2	0.0	0.3	Hg19	1793	2094	(93)	+	AluSz	SINE/Alu	1	301	(11)	4

>KSI_AluYb9_12

>Scaffold15026-183472-183802
TTCCTCACTAAGTTTCATCATTTTTAACTTTTGATTTAAAGTGAGCAGGCTGCAACTCTTTCTTTTACATGAACACTTAGAGGCCACTGTAGGACTATTAACTGGCCTCATTTCACTATTATGTCT CA
GGGACTAGGGAGGCCTTAGGAGAGGAAGCGATGGGAAACCACCTGATTGGTGGATCAGTCAGAGCACACAGCATTTCCCAATAAGGTTTGCCATCTTATACTGGTGCGTTCATGGAGTTCAAAAC AAT
TACAGCAGTAACATCAAAGCTCACTGATCTCAGGTCACCACAGCACATACAACAATCATGAAAAAGTTTGAAATATTCTAAGAGTAATCAAATGTGACACAGAGAACCAAAGAGAGCACATGCTGT TG
GAAAAATGGCAcAACAGACTTGCCTGACACAAGATTGCCACAAATCTTCAATTTGTAAAAACTGCAGTATCTGCAGGGTTCAAAAAAAAAGAGTTATGCCTGTATGTATTCTGTGCCTATAGATGCCC
TGCCTTTTTTTGTTCCCTTTCAGTGTTTCACTCAGTTAACATGAAACCTATCGATAGTGTTAGGTTACGCACAGATATGAAAATTGCCATACTGTGAAAGGCCATACGTATTAACGTCCTATAGTA AA
TTTTAATACTGTGTTCCAATGTCAAATAACCTCACTACTCTAAATGAGGATTGCAAAACCCACTGAAGATTCAGATAAGCTGATT CCACTGAGCAAGTTGAGAACTGTTTAATTCTGCATCATGCTTA
AACAAACTCCTTTCTTGTTTTCAGTTCAACATCTTTATCTAGAACTCCTTATCTGAGTAAGAACTTTTCATCTATTCAAAATTTCTCTCCATTACCTTATCTCTAATGTGTCTCATTAATCCAGAG TG
AAGCCAAGCAAGGAGCTCCGTACAATAAACACTTGCCATTTTTCTTAACTCTTCGGTATCTATCCCAACAT gTTATCACTTCGGTGATTCCTTTTgaacatttcttttttttttttttttttttttt
tttttttttttttttttttgagacggagtctcgctctgtcgcccaggccggactgcggactgcagtggcgcaatctctgctcactgcaagctccgcttcccgggttcacgccattctcctgcctc agc
ctccccagtagctgggactacaggcgcccgccaccgcgcccggctaatttttgtatttttagtagagacggggtttcaccttgttagccaggatgatctcgat ctcctgacctcatgatccacccgc
ctcggcctcccaaagtgctgggattacaggcgtgagccaccgcgcccagccTGAACATTTCAAAATGTCTTCATAAACATTTCAGAAAATCATTCGTAGTTTAGGCCATGCACAGTGGCTCACACCTG
TAATCCTAGCACTTTGGGAGGCCGAGGTGAGTGGATCATTTGAAGTCAGGAGTTCAACACCAGCTTGACCAACATGGTGAAACCCCAGCTCTACTAAAAAATAC AAAAAAAAAAAaaTTAGCTAGGTG
TGGTGCCACGTGCCTGTAATCCCAGATACTCAAGAGGCTGAGGCAG GGGATTTGCTTGAACTCGGGAAGCAGAGGTTGCAGTGAGCcAAGACCTCACCACTGCACTCtAGCCTAGACAACAGAATGAG
ACTCCAACTCAAAAAtGAAAATTTTTTTTATACTTTAAACCTCATTCTGCTTTAGAAGAAAATATGACTAGTTTTATCTATAAATATTTAGGTTTAAAATCAAT GAAATAAAAATGATAATATGATGC
TTACAATATTTTACAAAATCTGTTTGAAAATCCTTTCTGGTACTGAAAAAATTGTGTCCTCTGTCCTTCAGTTCTAAAGGCAAAGAGATCTGCTTTATACTCTGTGATGCAGGCCACTTTGGCCT TAA
ACTTGGCCAGTTCTTTAAACTATAATAAGAACAGCAAATATTATGTTAAAGGCAGACATTTAAAAGCACCATTTCTGCTAGATGAATTAAAAAATCTAACACAA GGCTGGGCACAGCACCTCACGCCT
GTAGTTCCAGCACTTTGGGAGGCCGAGGCAGGCAGATCACGAGGTCAGGAGTTTGAGACCAGGCTGGCCAACATAGTGAAACCCCGTCTCTACTAAAAATGCAACTA aTAATAATAATAATGATAATA
AATAAATAAAAAGCTGGGTGTGGTGGCTCACGCCTGTAATCCCAGCTACTTGGGAGGCTGAGGCAGGAGAATCGCTTGAACCTG gGAGGCAGAGGTTGCAGTGAGTCGAGATCATGCCAATGCACTCC
AGACTGGGCAACAGCGCAAGACTCTGT

2578	18.1	2.5	0.2	KS	1	471	(1860)	C	Tigger1	DNA/TcMar-Tigger	(1907)	511	30	4
2716	1.0	0.0	0.0	KS	1001	1331	(1000)	C	AluYb9	SINE/Alu	(0)	318	1	5
1916	15.2	0.0	3.1	KS	1384	1685	(646)	+	AluSz	SINE/Alu	1	293	(19)	6
1896	9.8	0.0	10.8	KS	2025	2331	(0)	+	AluSg	SINE/Alu	1	277	(33)	7

>Hg19 chr13:19605170-19607157
TTCCTCACTAAGTTTCATCATTTTTAACTTTTGATTTAAAGTGAGCAGGCTGCAACTCTTTCTTTTACATGAACACTTAGAGGCCACTGTAGGACTATTAACTGGCCTCATTTCACTATTATGTCT CA
GGGACTAGGGAGGCCTTAGGAGAGGAAGCGATGGGAAACCACCTGATTGGTGGATCAGTCAGAGCACACAGCATTTCCCAATAAGGTTTGCCATCTTATACTGGTGCGTTCATGGAGTTCAAAAC AAT
TACAGCAGTAACATCAAAGCTCACTGATCTCAGGTCACCACAGCACATACAACAATCATGAAAAAGTTTGAAATATTCTAAGAGTAATCAAATGTGACACAGAGAACCAAAGAGAGCACATGCTG TTG
GAAAAATGGCAtAACAGACTTGCCTGACACAAGATTGCCACAAATCTTCAATTTGTAAAAACTGCAGTATCTGCAGGGTTCAAAAAAAAAGAGTTATGCCTGTATGTATTCTGTGCCTATAGATGCC C
TGCCTTTTTTTGTTCCCTTTCAGTGTTTCACTCAGTTAACATGAAACCTATCGATAGTGTTAGGTTACGCACAGATATGAAAATTGCCATACTGTGAAAGGCCATACGTATTAACGTCCTATAGT AAA
TTTTAATACTGTGTTCCAATGTCAAATAACCTCACTACTCTAAATGAGGATTGCAAAACCCACTGAAGATTCAGATAAGCTGATT CCACTGAGCAAGTTGAGAACTGTTTAATTCTGCATCATGCTTA
AACAAACTCCTTTCTTGTTTTCAGTTCAACATCTTTATCTAGAACTCCTTATCTGAGTAAGAACTTTTCATCTATTCAAAATTTCTCTCCATTACCTTATCTCTAATGTGTCTCATTAATCCAGA GTG
AAGCCAAGCAAGGAGCTCCGTACAATAAACACTTGCCATTTTTCTTAACTCTTCGGTATCTATCCCAACAT aTTATCACTTCGGTGATTCCTTTTGAACATTTCAAAATGTCTTCATAAACATTTCAG
AAAATCATTCGTAGTTTAGGCCATGCACAGTGGCTCACACCTGTAATCCTAGCACTTTGGGAGGCCGAGGTGAGTGGATCATTTGAAGTCAGGAGTTCAACACCAGCTTGACCAACATGGTGAAACCC
CAGCTCTACTAAAAAATACAAAAAAAAAAATTAGCTAGGTGTGGTGCCACGTGCCTGTAATCCCAGATACTCAAGAGGCTGAGGCAGGGGATTTGCTTGAACTCGGGAAGCAGAGGTTGCAGTGAGC g
AAGACCTCACCACTGCACTCcAGCCTAGACAACAGAATGAGACTCCAACTCAAAAAcGAAAATTTTTTTTATACTTTAAACCTCATTCTGCTTTAGAAGAAAATATGACTAGTTTTATCTATAAATAT
TTAGGTTTAAAATCAATGAAATAAAAATGATAATATGATGCTTACAATATTTTACAAAATCTGTTTGAAAATCCTTTCTGGTACTGAAAAAATTGTGTCCTCTGTCCTTCAGTTCTAAAGGCAAA GAG
ATCTGCTTTATACTCTGTGATGCAGGCCACTTTGGCCTTAAACTTGGCCAGTTCTTTAAACTATAATAAGAACAGCAAATATTATGTTAAAGGCAGACATTTAAAAGCACCATTTCTGCTAGATG AAT
TAAAAAATCTAACACAAGGCTGGGCACAGCACCTCACGCCTGTAGTTCCAGCACTTTGGGAGGCCGAGGCAGGCAGATCACGAGGTCAGGAGTTTGAGACCAGGCTGGCCAACATAGTGAAACCC CGT

CTCTACTAAAAATGCAACTAtTAATAATAATAATGATAATAAATAAATAAAAAGCTGGGTGTGGTGGCTCACGCCTGTAATCCCAGCTACTTGGGAGGCTGAGGCAGGAGAATCGCTTGAACCTG cGA
GGCAGAGGTTGCAGTGAGTCGAGATCATGCCAATGCACTCCAGACTGGGCAACAGCGCAAGACTCTG T

3086	17.4	5.5	0.2	Hg19	3	571	(1617)	C	Tigger1	DNA/TcMar-Tigger	(1790)	628	30	1
1920	15.3	0.0	2.4	Hg19	1143	1442	(746)	+	AluSz	SINE/Alu	1	293	(19)	2
2027	10.0	0.0	10.0	Hg19	1782	2110	(78)	+	AluSg	SINE/Alu	1	299	(11)	3

>KSI_AluYb9_13

>Scaffold16053-1348011-1348325
ATGAAGGCTCAGTCTTCAATTTCCATAGTTTTCTCTGCAAATTTCAGAATGAATATTTTTTTCCAAGTGTTTTCCTTTCTCTCTTTTAATTTTGTGTGTTTGTGTGTGTGTGTGTGTGTGTGTT TG
AGAGCTTTCATCTTAAAAATTAAACACAGTTTTGGATAATAAAGGAATGTTTACCCTTAAACCTATTCTACACATTTTATGTAATAATTTCTAGCC gTGGATCTTTTACCTTTAAAATAATATTTTCT
ATTTTTCTATTCCTTCTGCATGTGCAAACTCCTTCTTCCCTGGGTCCACCCCCAATTCCTGCCATCTCCTTATCTCCATCTGCCAGTTTTGATTTCTGGCCAACCACCAGATGTATGGTAACCACC AA
TTCAACTAGTAAGATCATACATTCAGTATGGAAACACTGTGGAAATTAGTCTGACATACATGTTCTTCTGGCCCAAAAGGCTATGG AtttTTTTTTTTTTTTTTTTTTTTTTGAGACAGGGTCTTGCT
CTGTCACCCAGGCTGGAGTCAGTGGTGCAATCATAGCTCACTGCAGCCTTGACCTCCTATACTCAAGCTATCCTCCCTTACTTAGCCTCTCAAGTAACTGGTACTACAGGCATTCTCCATTACTCC CA
GCTAATTAATTTTATTTCTGTAGAGACAGGGTCTTACTATGCTGCATAGGCTGGTCTGAAATTCCTGTGCTGAAATGATGGTGTGAGCCACTGCACCCAGCCTGTTTTTAAGAAGTCATTCTGC ATT
CAAAATGTGCAGCTAGCTCCCGTTCCCTACAGGTGAGGGAACATGAATCATTGTCTGGGTTATTCACTAGGAATGTGTTCATTCT GGCCCGCATCTATCATTTACCTAGTTTATTGTTCCAGTCACTC
ATTCCAGGTTATTTACTGACTACTTATTATTTTCTAGTGTCTAGTGTGCTAGACACTA GGGTTATAAAATAACTGATGCTGTCCTTTATGCACAAAATTGCTAATTCTtttttttttttttttttt
tttgagacggagtctcgctctgtcgcccaggtcggactgcggactgcagtggcgcaatctcggctcactgcaagctccgcttcccgggttcacgccattctcctgcctcagcctcccgagtagctg gg
actacaggcgcccgccaccacgcccggctaatttttgtatttttagtagagacggggtttcaccttgttagccaggatggtctcgatctcctgacctcatgatccccccgcctcggcctcccaa agt
gctgggattacaggcgtgagccaccgcgcccggccaaaattgctaattctATTCCTGAAAATTTTATTAGTGACTTGTCTGACTGGGAAGTGACTGGTAGATATCAGTTGGCCCATTGCAAAATGAGG
GGCTTGTATTAACTGATCTTCTAATTTTAAAGCCTCTAAAATGAAGGGATGCAGGAGATTTGACTTCTTGCCTTAATCTAATCATTAAATTGCTCAGTGCCTTTTAGTACAATGTTCAGTCTCTA GAT
CTCAGTCTCCTTCTCCTCTGTAAAATAAATGAAGGCTGAGCGCGGTGGCTCACGCCTGTAATTCCAGCACTTTGGGAGGCTGAGGCGGG gGGgTCACCTGAGGACAAGAGTTCGAGACCAGCCTGGCC
AACATGGTGAAACCCCGTGTCTACTAAAAATGCAAAATTAGCCGGTGTGGTGGCACATGCCTGTAATCCCAGCTATTTGGGAGGCTGAGGCAGGAGAATCACTTGAATTTGGCAGGTGGAGGTTG CAG
TGAGCTGAGATTGCACCACTGCACTCCAGCGTGGGCAAAAAGAGTGAAACTTTGTCTCAAAAAAATAAACAAACAAATAAATAAAATAAATGAAATGAA ATGTACTAGAGCAGGTTAGAAAAAGTGTCC
AAGGAACTTTGTTTGTTTTGAGACAGGGTCATACTTTGTTGCCCAGGCTGGAGTGTAGTGGTGATCACTGCTCACTGCAGCCTCCACCTCCCAGGCTCAAGCAAACCTCCCATTTCAGCCTCTCA AGT
AGCTGGGACTACAGGTGCATGCCACCACACCCAACTAATATTTTTAAAACATTTTTCATAGAGATAGGATCTCACTATATTGCCTAGGCTGGTCTCAA GCAATCCTCCCGCCTCGGCCTCCCAAAGTG
CTAGGATTATAGGCATAAGCCACTGCACCCAGCCAAGGAACTTTGTCAATAGATCTTTCTAGGATAAGAGGGTTCTGTGTTCAAGGAGATCTCAGAAATGCTGGAGTCAATAGGTTTCTTTACTC CAG
GATTTCTCAGA

33	3.1	0.0	0.0	KS	93	125	(2190)	+	(TG)n	Simple_repeat	1	33	(0)	8
1227	17.5	1.4	1.4	KS	472	719	(1596)	C	AluJo	SINE/Alu	(0)	312	60	9
2918	1.6	0.0	0.0	KS	1001	1315	(1000)	C	AluYb9	SINE/Alu	(3)	315	1	10
190	18.8	2.0	2.0	KS	1384	1432	(883)	+	MIR3	SINE/MIR	121	169	(39)	11
220	33.7	2.7	3.6	KS	1454	1564	(751)	+	MIR3	SINE/MIR	35	144	(64)	12
2211	11.7	0.7	0.0	KS	1570	1877	(438)	+	AluSq	SINE/Alu	1	310	(3)	13
1690	15.9	5.7	1.7	KS	1928	2210	(105)	C	AluJr	SINE/Alu	(18)	294	1	14
359	22.1	10.4	0.9	KS	2220	2315	(0)	+	MER103C	DNA/hAT-Charlie	46	150	(147)	15

>Hg19 chr10:69993906-69995891
ATGAAGGCTCAGTCTTCAATTTCCATAGTTTTCTCTGCAAATTTCAGAATGAATATTTTTTTCCAAGTGTTTTCCTTTCTCTCTTTTAATTTTGTGTGTTTGTGTGTGTGTGTGTGTGTGTGTT TG
AGAGCTTTCATCTTAAAAATTAAACACAGTTTTGGATAATAAAGGAATGTTTACCCTTAAACCTATTCTACACATTTTATGTAATAATTTCTAGCC aTGGATCTTTTACCTTTAAAATAATATTTTCT
ATTTTTCTATTCCTTCTGCATGTGCAAACTCCTTCTTCCCTGGGTCCACCCCCAATTCCTGCCATCTCCTTATCTCCATCTGCCAGTTTTGATTTCTGGCCAACCACCAGATGTATGGTAACCAC CAA
TTCAACTAGTAAGATCATACATTCAGTATGGAAACACTGTGGAAATTAGTCTGACATACATGTTCTTCTGGCCCAAAAGGCTATGG ATTTTTTTTTTTTTTTTTTTTTTTTGAGACAGGGTCTTGCTCTG
TCACCCAGGCTGGAGTCAGTGGTGCAATCATAGCTCACTGCAGCCTTGACCTCCTATACTCAAGCTATCCTCCCTTACTTAGCCTCTCAAGTAACTGGTACTACAGGCATTCTCCATTACTCCCA GCT
AATTAATTTTATTTCTGTAGAGACAGGGTCTTACTATGCTGCATAGGCTGGTCTGAAATTCCTGTGCTGAAATGATGGTGTGAGCCACTGCACCCAGCC TGTTTTTAAGAAGTCATTCTGCATTCAA
AATGTGCAGCTAGCTCCCGTTCCCTACAGGTGAGGGAACATGAATCATTGTCTGGGTTATTCACTAGGAATGTGTTCATTCT GGCCCGCATCTATCATTTACCTAGTTTATTGTTCCAGTCACTCATT
CCAGGTTATTTACTGACTACTTATTATTTTCTAGTGTCTAGTGTGCTAGACACTAGGGTTATAAAATAACTGATGCTGTCCTTTATGCA CAAAATTGCTAATTCTATTCCTGAAAATTTTATTAGTGA
CTTGTCTGACTGGGAAGTGACTGGTAGATATCAGTTGGCCCATTGCAAAATGAGGGGCTTGTATTAACTGATCTTCTAATTTTAAAGCCTCTAAAATGAAGGGATGCAGGAGATTTGACTTCTTGCCT
TAATCTAATCATTAAATTGCTCAGTGCCTTTTAGTACAATGTTCAGTCTCTAGATCTCAGTCTCCTTCTCCTCTGTAAAATAAATGAAGGCTGAGCGCG GTGGCTCACGCCTGTAATTCCAGCACTTT

GGGAGGCTGAGGCGGGtGGaTCACCTGAGGACAAGAGTTCGAGACCAGCCTGGCCAACATGGTGAAACCCCGTGTCTACTAAAAATGCAAAATTAGCCGGTGTGGTGGCACATGCCTGTAATCCCAGC
TATTTGGGAGGCTGAGGCAGGAGAATCACTTGAATTTGGCAGGTGGAGGTTGCAGTGAGCTGAGATTGCACCACTGCACTCCAGCGTGGGCAAAAAGAG TGAAACTTTGTCTCAAAAAATAAACAAAC
AAATAAATAAAATAAATGAAATGAAATGTACTAGAGCAGGTTAGAAAAAGTGTCCAAGGAACTTTGTTTGTTTTGAGACAGGGTCATACTTTGTTGCCCAGGCTGGAGTGTAGTGGTGATCACTG CTC
ACTGCAGCCTCCACCTCCCAGGCTCAAGCAAACCTCCCATTTCAGCCTCTCAAGTAGCTGGGACTACAGGTGCATGCCACCACACCCAACTAATATTTT TAAAACATTTTTCATAGAGATAGGATCTC
ACTATATTGCCTAGGCTGGTCTCAAGCAATCCTCCCGCCTCGGCCTCCCAAAGTGCTAGGATTATAGGCATAAGCCACTGCACCCAGCCAAGGAACTTTGTCAATAGATCTTTCTAGGATAAGAG GGT
TCTGTGTTCAAGGAGATCTCAGAAATGCTGGAGTCAATAGGTTTCTTTACTCCAGGATTTCTCAGA

33	3.1	0.0	0.0	Hg19	193	225	(1961)	+	(TG)n	Simple_repeat	1	33	(0)	1
1425	15.9	1.2	1.2	Hg19	572	816	(1370)	C	AluJo	SINE/Alu	(8)	304	60	2
190	18.8	2.0	2.0	Hg19	1155	1203	(983)	+	MIR3	SINE/MIR	121	169	(39)	3
220	33.7	2.7	3.6	Hg19	1225	1335	(851)	+	MIR3	SINE/MIR	35	144	(64)	4
2239	11.4	0.7	0.0	Hg19	1341	1648	(538)	+	AluSq	SINE/Alu	1	310	(3)	5
1690	15.9	5.7	1.7	Hg19	1699	1981	(205)	C	AluJr	SINE/Alu	(18)	294	1	6
692	24.0	6.5	1.0	Hg19	1991	2175	(11)	+	MER103C	DNA/hAT-Charlie	46	240	(57)	7

>KSI_AluYb9_14

>Scaffold5495-121380-121627
TCTGCTGAGGGTGTTGTATAATTAAAAAGGACATGCACCCATTCTCATCCCAAATTTAGAAAAATCCCCAAATTCCCAAATATACCTGATCTATACATTTCAGAGACTTAGAAGGACCATGGCAAT CT
CGGGAAGGTGTGGCTACTTTTAAGGTGGGCACGCCCTTTTTATAGCAGGTGCAATACAGTGGGCTTTTCCTCCAACATTCAAAGAGTTGTTTCTCCAGCCAAGTGCCAGACGAGGAAGGTGGATC AGA
TTTATGAGCCATTCTATGTGTGCAATTCTGATCTGTAAACATTAATGCGGGGTGGACTCAGGTCTCCTCTCTCCCACTGGAGAACCTCCTGCTTTTTGAAAGCCTCTGACAGTGTGGCCACTCTCT CT
GCATTCCCTCATCCATCTATCCCTCCTCCCCTACCCTATCCATCCTTCCCAAGCCCTTGGGTCCAACTCACACACAAACACATCCTGGCCTGTTGTTGTTGTTGTTGTTGTTGTTGTTGTTA CAG
AGTCTCACTCTGTCGCCCAGGCTGGAGTGCAATGGCGCAATCTCAGTTCACTGCAACCTCTGCCTCCTGGGTTCAAGCGATTCTCCTGCCTCAGCCTCCAAAGTAGCTGGGATTACCGGTGCCCGC CA
CCCCGCCTGGCTAATTTTTGTATTTTTAGTAGTGACAGGGTTTCACCATGTTGGCCAGGCTGGTCTCAAACTCCTGACCTCAAGTGATCCACCCACCTCGGCCTCCCAAAGTGCTAGGAGTACAG GCA
TGAGCCACTGCACCTGGCCACATCCCAGCTTTCTGTC TGGAGGGATGGGAAGAAGGAGAAAGACGACCTCCACACTCCCAATAAAGAGCAAACATCATAAACTTCAACTTTCCCAGAAAAATGCCATG
GGCATCTTCTGAGCTCCACCCAAATACAGGCATGTGTAACT tTAAACATACACATACACATTACGGTCACCAAAAAATGACAGCCTTTATCCCGAATGTTTAGACAACTCAGCAGAACTAAAATATTA
GGTGAATCACCATGACCATGGAAAATGCTGAAAGGTTCCCTTGTTAGTGAAGACTCACAACTGGCGGCTTGGGGGCTGGGGGGAATCTACAGCCAGGCACCGTAGCTCGCAACTGTAATCCCAGCA CT
TTGGGAGGCCAAGGCAAGAGGATGGCTTCAGGCCAGGAGTTCTCGACCAGCTTGGGCAACACAGCGAGAGCCCCTTGTCTACAAAAACATAGAGATCTCATCCCTATTAAAAGAAATTAGCCAGG AGT
CATGATGCACTCCTGCAGTCCCAGCTACTGGGGAGGCTGAAGTGGGAGGATCACTTGAGCCCAGGAGGTGGAGGCTGCAGTGAGCTATGGTTGCCACCGCACTGCAGTCTGGGCAACAGAATGAAA CC
CTGTCTCCAAAAATAAATAAATAAAAAATAAATAAGGCCAGGCACAGTGGTGCACACCTGTAATCCCAGCACACTGGAAGGCTGAGGTCAGAGGACTGCTTGAGCCCAGGAATTCCCGACCAGCC TGG
GCAACATGGTGAGATCCCTATCTCTACGAAAAAATACAAAAATTAGCCAGGCATGGTTGTGTGCACCTGGAGTCCCAACTGCTCAAGAGGCCGA gGCAGGAGGATTGCTTGAGCCCAGGAGGTTGAGA
CCAGCCAGGGCAACATAGTGAGACCCTGCCTCTATTTA GAAAAGAAATAGGCCGGGCACAGTAGCTCACACCTGTAATCCCAGTGCTTTGGGAGGCCAACATGGGAGGATCGCTTGAGCCCAGGAGTT
TGAGATCAGCCTGGACAACACAGTGAGACCCCAGCCTCTACTAAAAATACAAAAAAATTAGCCAGGCATGGTGGCGGGTACCTGTAATCCCAGCTACTCCAAAGGCATGAG AATGTCTTGAACCAGGG
AGCAGGAGGTTGCAGAGAGCTGAGATTGCACCACTGCACTGCAGCCTGGGCAACAGAGGGAGATTTTGTCTCAAAAAAA ATTTTTTttttttttttttgagacggagtctcgctctgtcgcccaggcc
ggactgcggactgcagtggcgcaatctcggctcactgcaagctccgcttcccgggttcacgccattctcctgcctcagcctccccagtaactgggactacaggcgcccgccaccgcgcccggcta att
ttttgtatttttttttttagtagagacggggtttcaccttgttagccaggatggtctcgatctcctgacctc TTAAATAATAAATATATCTCTCTTGGTTCAAAAATCTAAAGAGAAATAATTTTTTT
AAATGTACAAAGCGAGGTATGAGAAAGAACAGAAAACACATTTCTCACTGTGAAAAGAGATGGCCTTTTCCTCTCTTCCTCAAGCCCAGCAAACCCTAAATCCCTCTTGCCTGTGACTTTGGAGG GAA
GGAAGGAGGCGGCCAGCAGAGAcAGAAGCAAAGACAGCAGGCCACCGTGTTTATCATCACGATGCCCATTCATACTAAAGAAGTGTCCAGAAAGAGGAGAAACACGGATTTAAGTTTCTTTGGTTATA
TTTTTAAATTTTGCTAGAACTCAAAACACTTCAGGAGAGCTGTATGGAATAAACACAGGGA aCGTGACATGGAATTTCACGCGGTCACAAAACGGAAAAGAAACCAACCAACCCTGAGTACTTTCCCT
TGGGGTCCGTGGGTATATGGATGGGGTgAGGAAGGTGACGGGAGAAGGACCGTGTCTCACTGCAG CACATTCCCGGGAGTAGATTCACGGATGATCTCAGACACCAACAGCTGACACCATCTGAGCC
CAGTTCTTCCTCTGTGCTGCAAGAGCAACGCCGCACAGCTAGCTCGGCCTTTCACAGGTCACCTAAACACAGCCAATCTCCCCTCTCCCCACTTTGTGTGCATTGAATGGGGTGCCCTGGAAAAG ATA
CGcTGACgTCCTAATCCTTGGTACCTGTcAATGGGACCTTAGTTGgAAAtAGGGTCTTTGCAGATGTGATTAAGATATACACAAAG GCTGGGCACAGTGGCTCACCCCTGTAATCCCAGCACAGGAGT
TTGAGACCAGCCTGGGCAACACAGCAAGACCTCAGCTCCACTTTTTTAAAAAATGAAATAAAAAAAAAGGTATATATTAAGGTGAGGTCATCCTGAAGGTGGT cCAATATGAGTGGTGAACTAATAAG
GAGACACAGACGCAGAGGGGAAGGCCACGTGGAGAAAGAGGCAGAGAGTACAGTGATGCGGCCACAAGCCCAGGGACACTTGGAGC CCCCAGCAGCTGGGAGAGGCAGGAAGGAGCTTCCCCTGGAGC
CTCCAGAAACAACTGGATACAACTGTAGTGGACTGAACTATGGTCTCCCAGAAAGATATGTCCATGTCCTGACCCACAGAACCTGTAAATGAGACCTCATTTGGGAAAAGGGTCTTTGCAGATGT AAT
TAAGTTAAAGATCTCAAGGTGAGGTCATCCAAAATTGGAGTGGACCCTAAACACAATGACGGGTGTTCCTGTAAGAGAAGAAGAGA AGACACAGACACAGAGGAGAAGGCCAcGTGGAGACGGAGGCA
GAGACCGCAGTGATGCGGCCACAAGCTCAGGGACGCCTGGAGCCCCCAGGAGCTGGGAGAGGCAGGAAGGAGCCTCCCCTAGAGCCCGCAGAGGGAGCACAGCCCTGC tGCATCTGCACTTCAGAGTT
GTGGTCTCCAGGACTGTGAAACAATTAACTTCTGTTATTTTAAGGCACCCAGTCTGTGGTATGTTACCAAAGCAAATATGCCCAAG GGGTACAAATTCAACCAAATAGTTCCAAATTTAGCCAAGTAC
ATTCCCTAGCAATGCTGCGAGCTCACAGTCAAAGGTATAACTCCACCATCTCTGAGAACCTGAGAGAGGCTTCCAGGGTGGTCTGAACTGTGCAGACCTCAGCAAACAGCAACATCCCAGGAAAG AGT
GGGGCTAAGAAGCTACTAATCCCTATTCCTACAAAGGCAGCCTAGACATGTCTGAGCCTTCCCAGGCCGATCACTAAGGAATGTCC ACAACGCAcAGCtGCCCCAAACATGGGAGAAGACAGAAGTCT
TCAAAGGAGCCGAGATGAAAAACCAAGTTCAAATTGTAAACTAGGCTGGGCAGGGTGGCTCACGCTTGTAAGTCCAGCACTTTGGGAGGCCTAGATGGTGGGATCACTTGAGGCCGGGAGTTCAA GAC
CTGCCTGGGCAACATAGCGAGATC

357	36.5	2.3	2.3	TeRefseq	127	300	(3948)	C	MamRep137	DNA/TcMar-Tigger	(90)	420	247	1
12	18.0	5.0	2.4	TeRefseq	394	433	(3815)	+	(CATC)n	Simple_repeat	1	41	(0)	2
37	0.0	0.0	0.0	TeRefseq	475	508	(3740)	+	(TGT)n	Simple_repeat	1	34	(0)	3
2120	9.3	0.0	0.0	TeRefseq	509	787	(3461)	C	AluSx1	SINE/Alu	(33)	279	1	4
1620	14.6	0.6	6.8	TeRefseq	1116	1443	(2805)	+	AluJr	SINE/Alu	2	310	(2)	5
1319	17.1	0.0	0.9	TeRefseq	1444	1665	(2583)	+	AluJo	SINE/Alu	1	220	(92)	6
1672	15.3	2.1	1.0	TeRefseq	1714	2000	(2248)	+	AluSx1	SINE/Alu	1	290	(22)	7
2105	0.4	0.0	2.5	TeRefseq	2001	2248	(2000)	C	AluYb9	SINE/Alu	(12)	306	65	8

958	18.8	11.4	7.3	TeRefseq	2913	3029	(1219)	+	MLT1C2	LTR/ERVL-MaLR	4	108	(353)	9
371	12.0	32.4	5.9	TeRefseq	3030	3137	(1111)	+	FLAM_C	SINE/Alu	1	135	(8)	10
958	18.8	11.4	7.3	TeRefseq	3138	3335	(913)	+	MLT1C2	LTR/ERVL-MaLR	109	331	(130)	9
1841	19.6	4.9	2.5	TeRefseq	3352	3779	(469)	+	MLT1C2	LTR/ERVL-MaLR	1	438	(23)	11
709	14.7	0.0	0.0	TeRefseq	4140	4248	(0)	+	AluJr	SINE/Alu	1	109	(203)	12

>Hg19seq chrX:3598698-3600704
TCTGCTGAGGGTGTTGTATAATTAAAAAGGACATGCACCCATTCTCATCCCAAATTTAGAAAAATCCCCAAATTCCCAAATATACCTGATCTATACATTTCAGAGACTTAGAAGGACCATGGCAATCT
CGGGAAGGTGTGGCTACTTTTAAGGTGGGCACGCCCTTTTTATAGCAGGTGCAATACAGTGGGCTTTTCCTCCAACATTCAAAGAGTTGTTTCTCCAGCCAAGTGCCAGACGAGGAAGGTGGATC AGA
TTTATGAGCCATTCTATGTGTGCAATTCTGATCTGTAAACATTAATGCGGGGTGGACTCAGGTCTCCTCTCTCCCACTGGAGAACCTCCTGCTTTTTGAAAGCCTCTGACAGTGTGGCCACTCTCTCT
GCATTCCCTCATCCATCTATCCCTCCTCCCCTACCCTATCCATCCTTCCCAAGCCCTTGGGTCCAACTCACACACAAACACATCCTGGCCTGTTGTTGTTGTTGTTGTTGTTGTTGTTGTTGTTA CAG
AGTCTCACTCTGTCGCCCAGGCTGGAGTGCAATGGCGCAATCTCAGTTCACTGCAACCTCTGCCTCCTGGGTTCAAGCGATTCTCCTGCCTCAGCCTCCAAAGTAGCTGGGATTACCGGTGCCCGCCA
CCCCGCCTGGCTAATTTTTGTATTTTTAGTAGTGACAGGGTTTCACCATGTTGGCCAGGCTGGTCTCAAACTCCTGACCTCAAGTGATCCACCCACCTCGGCCTCCCAAAGTGCTAGGAGTACAG GCA
TGAGCCACTGCACCTGGCCACATCCCAGCTTTCTGTCTGGAGGGATGGAAGAAGGAGAAAGACGACCTCCACACTCCCAATAAAGAGCAAACATCATAAACTTCAACTTTCCCAGAAAAATGCCATG
GGCATCTTCTGAGCTCCACCCAAATACAGGCATGTGTAACTgTAAACATACACATACACATTACGGTCACCAAAAAATGACAGCCTTTATCCCGAATGTTTAGACAACTCAGCAGAACTAAAATATTA
GGTGAATCACCATGACCATGGAAAATGCTGAAAGGTTCCCTTGTTAGTGAAGACTCACAACTGGCGGCTTGGGGGCTGGGGGGAATCTACAGCCAGGCACCGTAGCTCGCAACTGTAATCCCAGC ACT
TTGGGAGGCCAAGGCAAGAGGATGGCTTCAGGCCAGGAGTTC TCGACCAGCTTGGGCAACACAGCGAGAGCCCCTTGTCTACAAAAACATAGAGATCTCATCCCTATTAAAGAAATTAGCCAGGAGT
CATGATGCACTCCTGCAGTCCCAGCTACTGGGGAGGCTGAAGTGGGAGGATCACTTGAGCCCAGGAGGTGGAGGCTGCAGTGAGCTATGGTTGCCACCGCACTGCAGTCTGGGCAACAGAATGAA ACC
CTGTCTCCAAAAATAAATAAATAAAAAATAAATAAGGCCAGG CACAGTGGTGCACACCTGTAATCCCAGCACACTGGAAGGCTGAGGTCAGAGGACTGCTTGAGCCCAGGAATTCCCGACCAGCCTGG
GCAACATGGTGAGATCCCTATCTCTACGAAAAAATACAAAAATTAGCCAGGCATGGTTGTGTGCACCTGGAGTCCCAACTGCTCAAGAGGCCGA aGCAGGAGGATTGCTTGAGCCCAGGAGGTTGAGA
CCAGCCAGGGCAACATAGTGAGACCCTGCCTCTATTTAGAAA AGAAATAGGCCGGGCACAGTAGCTCACACCTGTAATCCCAGTGCTTTGGGAGGCCAACATGGGAGGATCGCTTGAGCCCAGGAGTT
TGAGATCAGCCTGGACAACACAGTGAGACCCCAGCCTCTACTAAAAATACAAAAAAATTAGCCAGGCATGGTGGCGGGTACCTGTAATCCCAGCTACTCCAAAGGCATGAG AATGTCTTGAACCAGGG
AGCAGGAGGTTGCAGAGAGCTGAGATTGCACCACTGCACTGC AGCCTGGGCAACAGAGGGAGATTTTGTCTCAAAAAAAAaTTTTTTTTAAATAATAAATATATCTCTCTTGGTTCAAAAATCTAAAG
AGAAATAATTTTTTTAAATGTACAAAGCGAGGTATGAGAAAGAACAGAAAACACATTTCTCACTGTGAAAAGAGATGGCCTTTTCCTCTCTTCCTCAAGCCCAGCAAACCCTAAATCCCTCTTGC CTG
TGACTTTGGAGGGAAGGAAGGAGGCGGCCAGCAGAGAgAGAAGCAAAGACAGCAGGCCACCGTGTTTATCATCACGATGCCCATTCATACTAAAGAAGTGTCCAGAAAGAGGAGAAACACGGATTTAA
GTTTCTTTGGTTATATTTTTAAATTTTGCTAGAACTCAAAACACTTCAGGAGAGCTGTATGGAATAAACACAGGGA cCGTGACATGGAATTTCACGCGGTCACAAAACGGAAAAGAAACCAACCAACC
CTGAGTACTTTCCCTTGGGGTCCGTGGGTATATGGGATGGGGTcAGGAAGGTGACGGGAGAAGGACCGTGTCTCACTGCAGCACATTCCCGGGAGTAGATTCACGGATGATCTCAGACACCAACAGCT
GACACCATCTGAGCCCAGTTCTTCCTCTGTGCTGCAAGAGCAACGCCGCACAGCTAGCTCGGCCTTTCACAGGTCACCTAAACACAGCCAATCTCCCCTCTCCCCACTTTGTGTGCATTGAATGG GGT
GCCCTGGAAAAGATACGtTGACaTCCTAATCCTTGGTACCTGTgAATGGGACCTTAGTTGcAAAcAGGGTCTTTGCAGATGTGATTAAGATATACACAAAGGCTGGGCACAGTGGCTCACCCCTGTAA
TCCCAGCACAGGAGTTTGAGACCAGCCTGGGCAACACAGCAAGACCTCAGCTCCACTTTTTTAAAAAATGAAATAAAAAAAAAGGTATATATTAAGGTGAGGTCATCCTGAAGGTGGT gCAATATGAG
TGGTGAACTAATAAGGAGACACAGACGCAGAGGGGAAGGCCA CGTGGAGAAAGAGGCAGAGAGTACAGTGATGCGGCCACAAGCCCAGGGACACTTGGAGCCCCCAGCAGCTGGGAGAGGCAGGAAGG
AGCTTCCCCTGGAGCCTCCAGAAACAACTGGATACAACTGTAGTGGACTGAACTATGGTCTCCCAGAAAGATATGTCCATGTCCTGACCCACAGAACCTGTAAATGAGACCTCATTTGGGAAAAG GGT
CTTTGCAGATGTAATTAAGTTAAAGATCTCAAGGTGAGGTCA TCCAAAATTGGAGTGGACCCTAAACACAATGACGGGTGTTCCTGTAAGAGAAGAAGAGAAGACACAGACACAGAGGAGAAGGCCA t
GTGGAGACGGAGGCAGAGACCGCAGTGATGCGGCCACAAGCTCAGGGACGCCTGGAGCCCCCAGGAGCTGGGAGAGGCAGGAAGGAGCCTCCCCTAGAGCCCGCAGAGGGAGCACAGCCCTGC cGCAT
CTGCACTTCAGAGTTGTGGTCTCCAGGACTGTGAAACAATTAACTTCTGTTATTTTAAGGCACCCAGTCTGTGGTATGTTACCAAAGCAAATATGCCCAAGGGGTACAAATTCAACCAAATAGTTCCA
AATTTAGCCAAGTACATTCCCTAGCAATGCTGCGAGCTCACAGTCAAAGGTATAACTCCACCATCTCTGAGAACCTGAGAGAGGCTTCCAGGGTGGTCTGAACTGTGCAGACCTCAGCAAACAGC AAC
ATCCCAGGAAAGAGTGGGGCTAAGAAGCTACTAATCCCTATT CCTACAAAGGCAGCCTAGACATGTCTGAGCCTTCCCAGGCCGATCACTAAGGAATGTCCACAACGCA gAGCcGCCCCAAACATGGG
AGAAGACAGAAGTCTTCAAAGGAGCCGAGATGAAAAACCAAGTTCAAATTGTAAACTAGGCTGGGCAGGGTGGCTCACGCTTGTAAGTCCAGCACTTTGGGAGGCCTAGATGGTGGGATCACTTG AGG
CCGGGAGTTCAAGACCTGCCTGGGCAACATAGCGAGATC

446	29.9	1.8	1.2	Hg19seq	23	191	(4016)	+	MER46C	DNA/TcMar-Tigger	137	306	(32)	1
357	36.5	2.3	2.3	Hg19seq	227	400	(3807)	C	MamRep137	DNA/TcMar-Tigger	(90)	420	247	2
12	18.0	5.0	2.4	Hg19seq	494	533	(3674)	+	(CATC)n	Simple_repeat	1	41	(0)	3
37	0.0	0.0	0.0	Hg19seq	575	608	(3599)	+	(TGT)n	Simple_repeat	1	34	(0)	4
2120	9.3	0.0	0.0	Hg19seq	609	887	(3320)	C	AluSx1	SINE/Alu	(33)	279	1	5
1620	14.6	0.6	6.8	Hg19seq	1216	1543	(2664)	+	AluJr	SINE/Alu	2	310	(2)	6

1306	17.6	0.0	0.9	Hg19seq	1544	1765	(2442)	+	AluJo	SINE/Alu	1	220	(92)	7
1681	15.3	2.1	1.0	Hg19seq	1814	2101	(2106)	+	AluSx1	SINE/Alu	1	291	(21)	8
900	23.4	6.7	2.7	Hg19seq	2772	2888	(1319)	+	MLT1C2	LTR/ERVL-MaLR	4	114	(347)	9
371	12.0	32.4	5.9	Hg19seq	2889	2996	(1211)	+	FLAM_C	SINE/Alu	1	135	(8)	10
900	23.4	6.7	2.7	Hg19seq	2997	3194	(1013)	+	MLT1C2	LTR/ERVL-MaLR	115	330	(131)	9
1869	19.2	4.9	2.5	Hg19seq	3211	3638	(569)	+	MLT1C2	LTR/ERVL-MaLR	1	438	(23)	11
1176	16.8	0.5	1.5	Hg19seq	3999	4206	(1)	+	AluJr	SINE/Alu	1	206	(106)	12

AluYc3

Instruction
Green box: Primer information
Red letter: Target site duplication
Black letter: Insertion sequences
Green letter: Deleted sequenced by insertion-mediated deletion

>KSI_AluYc3_1

>Scaffold22-929540-929740
GACACACAGCACTTAGACCTGTTGATGGCTGGGATATACAGGGGGGACAGGGTAAGAAAGACTCCAATTTTGGAGCAACAGGGTTCAGATGATTAGGAAAGTGTTGTGTATATTTGTCACTGTTTT GC
CTGCTCaGCATCCAAATCTCCTTCTGCGATCATAGAATCTACTTACTATGTAAATTCTAAGAAAGGATGCAAGCTAATTCCTCTCTGCCTCCTGGAAGCTGGAGTACAATCTCATGCTTCTGTCTGGG
ACTGTGAATCAGGAGTAATGGAGAATGAGGATGTTTTCTAACTAACTCCAGTGGGGAGTACATGAGGCTGCTTAGTGACAGTGACAGCAACAGCAGTGTCATAACCAGGTATGGCCTTGATTGCTG TC
ATTCTGACGACCTAGCCTCTCTTAGTTTCTGTTTCTTTTCCAAGTCTATTTCTCAAGCTTGCCCATTAAAAAAAATTTAAAAAGAGGTATGACATATAAAGGGCACAAATCTTAAGTATGCAACC TGA
TATGTAAACACCTATGAAACCACCACCTAGACCAAAATATAAAAGACTTTCAGCACCCTCAAAGTCTTCCCTTTGATACACTATTTTCTCAACAAATT CAATTTCTGCCTGAGTTAGCAAGTCCATTT
CAGGCTGAGGCAGAATGCAATCAAGAAATCTGACTTGTGGCACTTCAGTGTTTATCACATATGACCCTTTTGCATTCAGAGTGAAAATTGGTCAGGAAAAACAGCAAAAGAGCATTTTTGTACCA AAG
TCCTCAAGTGAGTAAGCCTCAAATTAAGACTTGTTATTATTAATCATAATACAACTTTATCATACCAAAGTACCTAAAAATTGATATATTTAATTTATAAAATCTTAACCATCAAACTCAATCTGT GG
TCTGGAAACTAAATTCTTTTCAGGGACCCTACCAGTACTCAACCTTGTTTCTTTAGGGTCCCTAACATGTAAAGCTACATAATCTggAATTTTCCAGACAGTTATTTATTTTTCATTTTTTATTTTTT
GAGACGGAGTCTCGCTCTGTCGCCCAGGCTGGAGTGCAGTGGCGCAAT aTCGGCTCACTGCAACCtgtgccgcccgggttcacgccattctcctgcctcagtctcccgagtagctgggactacaggcg
cccgccatcacacctggctaatttttttgtatttttagtagagacggggt ccagacagttattatgtgggactttaaatatatatgaagtcttgctgggcgcggtggctcacgcctgtaatcccagcac
ttagggggccaaggctggcagatcacgaggtcaggagatcgagaccatcctggctaacacagtgaaaccccgtctctactaaaaaacaaaaaaccaaacaaacaaagaaacaaaaaaaaaaca caa
aaaatgagcctggcatggtggcaggcgcctgtagtcccagctactcgggaggctgaggcaggcgaatggcgtgaacccgggaggcggagcttgcaggagccgggat cgcgccactgcactccagcctg
ggcgacagagcaaggctctgtctcaaaaaaaaaaaaaaataataataaatcaaaagatatagatgaagTCTTCTGGCCTTCTTATTCTAGTAGTAACTCTATGAGCACCATCACCAAGTACAGAGGCAT
TAGTACTAATCGTTAACAGGACATTATAAGACATTTTAATAACTACTATATTAACAACACCAACGTCCAATAGAAGGAACTCACTGAAAAAACATGGAGTCATTTCAATCTCATTTGATGGCGCT CCA
TCATTCAGCTGCTCAGGCCCTACTCCTTTCATAGTCCACT GGCAGATTCTTTTAGCTCCTGCTTCAAAATATATCCAGAATCCAACCATATCTCACCATCTCTATTGTTTCTATCCTTGTCCG AATCT
TTCTAGCCTGGACTACTGCAAAACTGGATGCTTGAAGGTAATAGTGAAATGTCTTCAAAATTCTAAAGAAAAATGAGTTTGAACATAGAACTGCAGGCCCAATAAAAATAAAGTGTGAGGAAAAA ATA
AAGGCCTTGTCAAACACACAAGGACTCCTAGTTTCTCTCCCATGCACCCTTTCTGGGGGAAAAAAAGGCACCTGTAAATATACTTCAGCAAACAGAAAAGACAAATTCAAGAAAGAATGCAGC AAAAA
AAAAAAAAAAAGAAAAGAAAAAGAA

482	19.1	16.5	0.0	AluYc_1	468	582	(1619)	C	L1ME3	LINE/L1	(10)	6152	6019	1
1615	7.5	0.0	0.0	AluYc_1	1001	1201	(1000)	C	AluYc	SINE/Alu	(6)	293	93	2
2199	5.2	0.3	12.6	AluYc_1	1245	1574	(627)	+	AluY	SINE/Alu	2	295	(16)	3
440	25.7	6.2	0.0	AluYc_1	1825	1937	(264)	+	L2	LINE/L2	2602	2721	(698)	4
724	25.9	3.5	3.1	AluYc_1	1938	2164	(37)	+	HAL1ME	LINE/L1	1571	1798	(667)	5
21	11.2	0.0	0.0	AluYc_1	2172	2201	(0)	+	(A)n	Simple_repeat	1	30	(0)	6

>hg19 chr1:85619036-85620721
GACACACAGCACTTAGACCTGTTGATGGCTGGGATATACAGGGGGGACAGGGTAAGAAAGACTCCAATTTTGG AGCAACAGGGTTCAGATGATTAGGAAAGTGTTGTGTATATTTGTCACTGTTTTGC
CTGCTCgGCATCCAAATCTCCTTCTGCGATCATAGAATCTACTTACTATGTAAATTCTAAGAAAGGATGCAAGCTAATTCCTCTCTGCCTCCTGGAAGCTGGAGTACAATCTCATGCTTCTGTCTGGG
ACTGTGAATCAGGAGTAATGGAGAATGAGGATGTTTTCTAACTAACTCCAGTGGGGAGTACATGAGGCTGCTTAGTGACAGTGACAGCAACAGCAGTGTCATAACCAGGTATGGCCTTGATTGCT GTC
ATTCTGACGACCTAGCCTCTCTTAGTTTCTGTTTCTTTTCCAAGTCTATTTCTCAAGCTTGCCCATTAAAAAAAATTTAAAAAGAGGTATGACATATAAAGGGCACAAATCTTAAGTATGCAACCTGA
TATGTAAACACCTATGAAACCACCACCTAGACCAAAATATAAAAGACTTTCAGCACCCTCAAAGTCTTCCCTTTGATACACTATTTTCTCAACAAATTCAATTTCTGCCTGAGTTAGCAAGTCCA TTT
CAGGCTGAGGCAGAATGCAATCAAGAAATCTGACTTGTGGCACTTCAGTGTTTATCACATATGACCCTTTTGCATTCAGAGTGAAAATTGGTCAGGAAAAACAGCAAAAGAGCATTTTTGTACCAAAG
TCCTCAAGTGAGTAAGCCTCAAATTAAGACTTGTTATTATTAATCATAATACAACTTTATCATACCAAAGTACCTAAAAATTGATATATTTAATTTATAAAATCTTAA CCATCAAACTCAATCTGTGG
TCTGGAAACTAAATTCTTTTCAGGGACCCTACCAGTACTCAACCTTGTTTCTTTAGGGTCCCTAACATGTAAAGCTACATAATCTaaAATTTTCCAGACAGTTATTTATTTTTCATTTTTTATTTTTT

GAGACGGAGTCTCGCTCTGTCGCCCAGGCTGGAGTGCAGTGGCGCAAT cTCGGCTCACTGCAACCTCTTCTGGCCTTCTTATTCTAGTAGTAACTCTATGAGCACCATCACCAAGTACAGAGGCATTA
GTACTAATCGTTAACAGGACATTATAAGACATTTTAATAACTACTATATTAACAACACCAACGTCCAATAGAAGGAACTCACTGAAAAAACATGGAGTCATTTCAATCTCATTTGATGGCGCTCCATC
ATTCAGCTGCTCAGGCCCTACTCCTTTCATAGTCCACT GGCAGATTCTTTTAGCTCCTGCTTCAAAATATATCCAGAATCCAACCATATCTCACCATCTCTATTGTTTCTATCCTTGTCCGAATCTTT
CTAGCCTGGACTACTGCAAAACTGGATGCTTGAAGGTAATAGTGAAATGTCTTCAAAATTCTAAAGAAAAATGAGTTTGAACATAGAACTGCAGGCCCAATAAAAATAAAGTGTGAGGAAAAAATAAA
GGCCTTGTCAAACACACAAGGACTCCTAGTTTCTCTCCCATGCACCCTTTCTGGGGGAAAAAAAGGCACCTGTAAATATACTTCAGCAAACAGAAAAGACAAATTCAAGAAAGAATGCAGCAAAA AAA
AAAAAAAAAGAAAAGAAAAAAGAA

211	13.2	0.0	0.0	TeRef	42	79	(1708)	C	L2	LINE/L2	(710)	2709	2672	7
482	19.1	16.5	0.0	TeRef	568	682	(1105)	C	L1ME3	LINE/L1	(10)	6152	6019	8
750	5.5	0.0	0.0	TeRef	1101	1191	(596)	C	AluSx3	SINE/Alu	(6)	305	215	9
440	25.7	6.2	0.0	TeRef	1411	1523	(264)	+	L2	LINE/L2	2602	2721	(698)	10
724	25.9	3.5	3.1	TeRef	1524	1750	(37)	+	HAL1ME	LINE/L1	1571	1798	(667)	11
21	11.2	0.0	0.0	TeRef	1758	1787	(0)	+	(A)n	Simple_repeat	1	30	(0)	12

>KSI_AluYc3_2

>Scaffold2647-2656613-2656726
TGCAACCCCCCCTTTTACTTGAGCAAATATTAAGCTAACAGATGTTAATATGAGGCAGAGCTCCTAGTCACACACTCTAGAGTTGTGGGTATTTTCATTGTTGTTTGTAATCTATTACATCAATTA TT
TCTGCACTTGCTTTGTTGATTGCTAAATCTCACGTGTTGTAGTATGGGTTTTTTGTCCTCAAAAATAATTATTACTTTGGAATAATTCATTCCAAGTGCACATGCATTTGTTATGTTTTTTCATC ATT
TTCAATTTTTAAAATAATCTCTACTAATTATCATTATAATTGTGCCTTTCCTCAAATTGGTTCTGATCAGACTTGATGGAGTTAATGTATTTGGTTTTTAAAACATCTCAAGCCTGTGCCAAGCAT AA
CAAATATTCCATTCATGGCCTAGACATACATGACCTTCTATTATTTCTAGAATCATCTCCAGTTGCTGGCAACTTTATTGTGTTTTCACTCCAAAATATAGCTTTGTTTGTTTCTACAAAAATAT GTA
AGGTGAAAGCTAGTAAATAGACTCTGAATTCAGGTTTATCCAATTAACTAGAAAGGAATCTTTTTCCTAGTTAATTACATATTATTGGAAATGTCAGAGAGATTTAATGAACCTTCACAGAGGTCT GA
CTTTGACTCAATGAGGAGCTGATCTTTATGCAAGTTGAAGTCAGCAATTGTCTTTTTCTATTTCCCCTCTTTTGGTCTTCTATTTTGAGTCTTCTTTCCTCCTTC CCAACCTCTTTTCTTTCCCACTC
TGTAACTGCACCAGAGACATGCTCATCTCCATTCCTTCCTTTGCCCCTCCTCTCTCTGCCTTGCCTCTCATATTCTCCCTGCCTTGCCCCCTGCATTCCAACATTGTTTCTGGCTTACTTGTTCTACA
TGGTTAACCTGCCTATCCTTGATATTAGCTCATCATTATAATGCTTGGTTGCTCAAATATGTCATTAAAAAgCATCTTTCTTGAAATAACATCTCATGATGTTATTTTttttttttgagacggagtc
tcgctctgtcgcccaggctggagtgcagtggcgcgatctcggctcactgcaagccccgcctcccaggttcacgccattctcctgcctcagaa catctttcttgaaataacatctcatgatgttatttt
ATATTTTTCTATAAAATTCAATGTTTTAGTAGTTTACCAAATATGTTTTAGTATATTTCCGTGTTAGTTAGCTGTTTGGCATAAAAAAGACATAGAATTTTAAT GCATTCAGCCAATCCACTATAGTT
TTTTGTATTAAAgaTAATTTTAAACAATTATAATATATTATAGTATATATACATCCAGTGATAATATAATAAAATTAAAATTTATTATATTATAATAATGTAATTTATTGAAATCATGTGAAATTATA
ACCTCATAAGTATTTTTGTTTTGTTTATTCCTTTGGGTTTACCCTTTCGTTTCTTGTGTGCTGGAGGCTGGGTTCTGTGTCC TTGCTTGTTTTCCTGAAATCAACATCATAAGAATGATACAGTATTT
CTGAAAATACTTGTTTATTACTTTTTGCCTTTTACAATGCAAGTCTTTGAAGGTGGAAAATCTTTACGTTCTTTAGTAACCATAAAATATGATGGCGTTATTTTCAAAATAATATATACTAGTGC ATA
GCTTACAGGAAATATTCATTAATATTTGTTAACCAAATGTTTGATAAAAGAATAGAAGTGAAGGCCAGGCATGGTGGCTTACACCTGTAATCCCAGCCCTTTGGGAGG CCAAGGCAGGCAGATCACTT
GAGGCCAGGAGTTCAAGACCAGCCTGGCCAAAATGGTGAAAGTCCCATCTCTACTAAAAATATAAAAATTAGTCAGGCATGGTGGCGGGTGCCTGTAATCGCAGCTACTTGGGAGTCTGAGGCAC AAG
AAGTGCTTGAATCCTGGAGGTGGAGGTTGCAGTGAGCTAAGATTGTGCCACTGCACTCCGGCCTGGGCAACAGAGAAGTGAGATTCTGTTCAGGAATTAAAGGTCAAA TAGCAATGGCAGATGGGATG
GGATGTGTGAGCAAGCAATTTAGGTAGAACCTGTACAAAAGGCGTGGAAGAATGCATGAGCAAGT C

1061	1.8	0.0	0.0	AluYc3	1001	1114	(1000)	C	AluYc	SINE/Alu	(17)	282	169	1
25	24.8	2.9	4.0	AluYc3	1307	1408	(706)	+	(ATTAT)n	Simple_repeat	1	101	(0)	2
1967	13.4	0.0	1.4	AluYc3	1727	2009	(105)	+	AluSz6	SINE/Alu	1	279	(33)	3

>hg19 chr15:95164053-95166018
TGCAACCCCCCCTTTTACTTGAGCAAATATTAAGCTAACAGATGTTAATATGAGGCAGAGCTCCTAGTCACACACTCTAGAGTTGTGGGTATTTTCATTGTTGTTTGTAATCTATTACATCAATTA TT
TCTGCACTTGCTTTGTTGATTGCTAAATCTCACGTGTTGTAGTATGGGTTTTTTGTCCTCAAAAATAATTATTACTTTGGAATAATTCATTCCAAGTGCACATGCATTTGTTATGTTTTTTCATC ATT
TTCAATTTTTAAAATAATCTCTACTAATTATCATTATAATTGTGCCTTTCCTCAAATTGGTTCTGATCAGACTTGATGGAGTTAATGTATTTGGTTTTTAAAACATCTCAAGCCTGTGCCAAGCAT AA
CAAATATTCCATTCATGGCCTAGACATACATGACCTTCTATTATTTCTAGAATCATCTCCAGTTGCTGGCAACTTTATTGTGTTTTCACTCCAAAATATAGCTTTGTTTGTTTCTACAAAAATAT GTA
AGGTGAAAGCTAGTAAATAGACTCTGAATTCAGGTTTATCCAATTAACTAGAAAGGAATCTTTTTCCTAGTTAATTACATATTATTGGAAATGTCAGAGAGATTTAATGAACCTTCACAGAGGTCT GA
CTTTGACTCAATGAGGAGCTGATCTTTATGCAAGTTGAAGTCAGCAATTGTCTTTTTCTATTTCCCCTCTTTTGGTCTTCTATTTTGAGTCTTCTTTCCTCCTTC CCAACCTCTTTTCTTTCCCACTC
TGTAACTGCACCAGAGACATGCTCATCTCCATTCCTTCCTTTGCCCCTCCTCTCTCTGCCTTGCCTCTCATATTCTCCCTGCCTTGCCCCCTGCATTCCAACATTGTTTCTGGCTTACTTGTTCTACA
TGGTTAACCTGCCTATCCTTGATATTAGCTCATCATTATAATGCTTGGTTGCTCAAATATGTCATTAAAAA aCATCTTTCTTGAAATAACATCTCATGATGTTATTTTATATTTTTCTATAAAATTCA
ATGTTTTAGTAGTTTACCAAATATGTTTTAGTATATTTCCGTGTTAGTTAGCTGTTTGGCATAAAAAAGACATAGAATTTTAAT GCATTCAGCCAATCCACTATAGTTTTTGTATTAAAatTAATTT
TAAACAATTATAATATATTATAGTATATATACATCCAGTGATAATATAATAAAATTAAAATTTATTATATTATAATAATGTAATTTATTGAAATCATGTGAAATTATAACCTCATAAGTATTTTT GTT
TTGTTTATTCCTTTGGGTTTACCCTTTCGTTTCTTGTGTGCTGGAGGCTGGGTTCTGTGTCCTTGCTTGTTTTCCTGAAATCAACATCATAAGAATGATACAGTATTTCTGAAAATACTTGTTTATTA
CTTTTTGCCTTTTACAATGCAAGTCTTTGAAGGTGGAAAATCTTTACGTTCTTTAGTAACCATAAAATATGATGGCGTTATTTTCAAAATAATATATACTAGTGCATAGCTTACAGGAAATATTC ATT
AATATTTGTTAACCAAATGTTTGATAAAAGAATAGAAGTGAAGGCCAGGCATGGTGGCTTACACCTGTAATCCCAGCCCTTTGGGAGGCCAAGGCAGGCAGATCACTTGAGGCCAGGAGTTCAAGA CC
AGCCTGGCCAAAATGGTGAAAGTCCCATCTCTACTAAAAATATAAAAATTAGTCAGGCATGGTGGCGGGTGCCTGTAATCGCAGCTACTTGGGAGTCTGAGGCACAAGAAGTGCTTGAATCCTGG AGG
TGGAGGTTGCAGTGAGCTAAGATTGTGCCACTGCACTCCGGCCTGGGCAACAGAGAAGTGAGATTCTGTTCAGGAATTAAAGGTCAAATAGCAATGGCAGATGGGATGGGATGTGTGAGCAAGCAA TT
TAGGTAGAACCTGTACAAAAGGCGTGGAAGAATGCATGAGCAAGTC

1967	13.4	0.0	1.4	hg19	1579	1861	(105)	+	AluSz6	SINE/Alu	1	279	(33)	4

>KSI_AluYc3_3

>Scaffold6431-6940126-6940296

GCACTTTGGGAGACTGAGGCAGGATGATCACTTGAGGCCAGGAGTTAGAGATCAGTCTGGGCAACACAGGGAGACCCCATCCCTACAAAATAAAATAAAATA AAATATTAGCTGGGCATGGTGGTGCG
CACCTGTAGTCCTAGCTACTTGGCAGACTAAGGTGGGAGGATTGCTTGAGCCCAGGAGTTGGAGGATGCAGTGAGCTATGATTACACAACTACACTCCAGCCTGGAGGACAGAGCAAGACTCTAT CTC
TTAAAAAAAATTAATTAATTAAAAACCAAAAGTTATCTCTTCTGATTATAGAAGCAATACATATTTATTATAGAAAATTTAAAAAGCA CaAAAAAAAATAAAAAAATAAATTAAGTATTAGTACTGTG
AACATTAATACAGGCTTTTGTCCAAAAGGGAGCATCCTTCCTCAACAGTCTGCATAGCACATGGAAGGGGTGGCAACACCAGTGACTGTACAGCTGGGCCAACCAAAAGAGATGAATTGCTAACC TTG
ACTCCAATGAGTCCCTGGTGTGTGACATTGGAAAGTCACTTTCTGCCTCTACCCTCAGTTTCCTCATTAGTCAAACACGGATTTTTCTGATTTGTGATTTTT AAAGTGTGCCTTGGGGCTTTGTGACA
CCCTTTTAGAACAGGTGGGGGAAAGGGCTCAAACTGTCCTGCCTGTCCTCCAGCCAGCATAGAACCACATTACCTGTTTTCCATTTGGG aTTTTGGCATAAATTTGGTAGTATGTTGGATTGTTGTTG
TTGTTGTTGTTTGTTGTTGCTTTGTTaTGTTTTAAACTACACATCAGGAGACATGAGGTTGCAATTCATTTTTCCAAATACTCAATAAAGTTACTTCCAACT CTAACTTTTGATGATGATTCTAATTC
CCTTCTGACATAAATTAATATAGTCATGAACTAGGTGTAACTATAAACTC CTTTTCCTATGGGCCTACAGGAACTTTCCTAAAGATTCTAA Aagtatcaggtcattctttttttttttttttttttt
tgagacggagtctcgctctgtcgcccaggctggagtgcagtggcgggatctcggctcactgcaagctccgcctcccgggttcacgccattctcctgcctcagcctcccaaagtgctgggattaca ggc
gtgagccaccgtgcccggcctAGTATCAAGTCATTCTAAATGGAATAACTTATACCAGATCATCTATGGCTGtGGACCTTGAACAAATTtGCTTAACCTCCCCATGCTTCAATCTCCTCATCTATAAA
ATGGGGATCATATAACCCACTCTATTGGATGCAGGGGAAATGAAATTGTATCTCATACACAAAAGTGCCTAGCACACAACAAGGAATAGAGCCGTAGCTTACATTT CCCGACTTCTTACTGTGAGCCA
GGATCTGAGCTACATGTTTGGTATGCATTCCTTCATCCCGTCTACACACAAGCCTATGCAATAGGCCCTTTTTTTTTTTATTATTGTCTATTATTTCCCCATTTTACA GATGAGAAACTGAGGCTTAA
TGACATTAGGTAACTTTTTTCCATTTCACCCAGAGCTTGGGTTTGAACC cAGGTAATCAGATTCATGAGGCTATGCTATAAAATGAATAACAGTAAAAAGCATAATACTAACTGTAACTCAACTATTT
TGAGGGTTTGCTCTACAGGAGATACTATTATAAAAGCTTTGTCTTATTTAGTTCTCACATGGATCCAATGGAGC tAGCATTGACTTTACAGATTAGAAAACTGAGACA CAGAGACACTAAATGCTGTA
TCTCCACTGCCTTTTAAGTTGACTGCTTAGAACTGACTCCAACTCCTTAGGTAGCTTCCCTACTGAACATTAGGAG tCTGTGCAACTAATTGTCTAACTGATCCTTCTGAAAATATAGAATAAGAAGC
ATCCTCTAAAATGAAAACTCCCAGGGAAGGGGTACATTAACTCAGGATTTACAACAGCAAAAGGTAATCATCAAAGACTCCTTTCCTTCCCGCTCCTGCAAATCCAAT CAATTATGGGTCCTGCAGTC
TCACCTCCTAAATATTTCTCCACTCATTCCTTTCGCTCATTCCCACCTTGGCTGCTCCGGTTTGTGCCATTCTGTCTGTCAAAAGCCTCCTGCCTGCTCTCCCTGCTCCAGTCTTGACCTCC A

1700	13.2	0.0	3.5	AluYc_2	1	266	(1905)	+	AluJr	SINE/Alu	34	290	(22)	1
246	21.9	3.7	0.0	AluYc_2	289	370	(1801)	C	L1ME4a	LINE/L1	(0)	6124	6040	2
231	23.5	3.2	0.6	AluYc_2	531	619	(1552)	+	MIRb	SINE/MIR	83	172	(36)	3
22	3.1	2.9	2.9	AluYc_2	760	794	(1377)	+	(TTG)n	Simple_repeat	1	35	(0)	4
1420	6.4	0.0	0.0	AluYc_2	1001	1171	(1000)	C	AluYc	SINE/Alu	(5)	294	124	5
419	25.7	11.0	0.6	AluYc_2	1219	1363	(808)	+	MIR	SINE/MIR	78	237	(25)	6
413	29.3	6.7	8.5	AluYc_2	1375	1613	(558)	C	MIR	SINE/MIR	(5)	257	23	7
283	26.8	16.2	1.3	AluYc_2	1651	1786	(385)	C	MIR	SINE/MIR	(5)	257	102	8
291	31.6	21.7	0.5	AluYc_2	1810	1984	(187)	C	MamGypsy2-I	LTR/Gypsy	(1222)	2944	2733	9

>hg19 chr10:89813980-89815963

GCACTTTGGGAGACTGAGGCAGGATGATCACTTGAGGCCAGGAGTTAGAGATCAGTCTGGGCAACACAGGGAGACCCCATCCCTACAAAATAAAATAAAATAAAAT
ATTAGCTGGGCATGGTGGTGCGCACCTGTAGTCCTAGCTACTTGGCAGACTAAGGTGGGAGGATTGCTTGAGCCCAGGAGTTGGAGGATGCAGTGAGCTATGATTA
CACAACTACACTCCAGCCTGGAGGACAGAGCAAGACTCTATCTCTTAAAAAAAATTAATTAATTAAAAACCAAAAGTTATCTCTTCTGATTATAGAAGCAATACAT
ATTTATTATAGAAAATTTAAAAAGCACAAAAAAAATAAAAAAATAAATTAAGTATTAGTACTGTGAACATTAATACAGGCTTTTGTCCAAAAGGGAGCATCCTTCC
TCAACAGTCTGCATAGCACATGGAAGGGGTGGCAACACCAGTGACTGTACAGCTGGGCCAACCAAAAGAGATGAATTGCTAACCTTGACTCCAATGAGTCCCTGGT
GTGTGACATTGGAAAGTCACTTTCTGCCTCTACCCTCAGTTTCCTCATTAGTCAAACACGGATTTTTCTGATTTGTGATTTTTAAAGTGTGCCTTGGGGCTTTGTG
ACACCCTTTTAGAACAGGTGGGGGAAAGGGCTCAAACTGTCCTGCCTGTCCTCCAGCCAGCATAGAACCACATTACCTGTTTTCCATTTGGGgTTTTGGCATAAAT
TTGGTAGTATGTTGGATTGTTGTTGTTGTTGTTGTTTGTTGTTGCTTTGTTtTGTTTTAAACTACACATCAGGAGACATGAGGTTGCAATTCATTTTTCCAAATAC
TCAATAAAGTTACTTCCAACTCTAACTTTTGATGATGATTCTAATTCCCTTCTGACATAAATTAATATAGTCATGAACTAGGTGTAACTATAAACTCCTTTTCCTA

TGGGCCTACAGGAACTTTCCTAAAGATTCTAAAAGTATCAAGTCATTCTAAATGGAATAACTTATACCAGATCATCTATGGCTGcGGACCTTGAACAAATTGCTTA
ACCTCCCCATGCTTCAATCTCCTCATCTATAAAATGGGGATCATATAACCCACTCTATTGGATGCAGGGGAAATGAAATTGTATCTCATACACAAAAGTGCCTAGC
ACACAACAAGGAATAGAGCCGTAGCTTACATTTCCCGACTTCTTACTGTGAGCCAGGATCTGAGCTACATGTTTGGTATGCATTCCTTCATCCCGTCTACACACAA
GCCTATGCAATAGGCCCTTTTTTTTTTTATTATTGTCTATTATTTCCCCATTTTACAGATGAGAAACTGAGGCTTAATGACATTAGGTAACTTTTTTCCATTTCAC
CCAGAGCTTGGGTTTGAACCtAGGTAATCAGATTCATGAGGCTATGCTATAAAATGAATAACAGTAAAAAGCATAATACTAACTGTAACTCAACTATTTTGAGGGT
TTGCTCTACAGGAGATACTATTATAAAAGCTTTGTCTTATTTAGTTCTCACATGGATCCAATGGAGCgAGCATTGACTTTACAGATTAGAAAACTGAGACACAGAG
ACACTAAATGCTGTATCTCCACTGCCTTTTAAGTTGACTGCTTAGAACTGACTCCAACTCCTTAGGTAGCTTCCCTACTGAACATTAGGAGcCTGTGCAACTAATT
GTCTAACTGATCCTTCTGAAAATATAGAATAAGAAGCATCCTCTAAAATGAAAACTCCCAGGGAAGGGGTACATTAACTCAGGATTTACAACAGCAAAAGGTAATC
ATCAAAGACTCCTTTCCTTCCCGCTCCTGCAAATCCAATCAATTATGGGTCCTGCAGTCTCACCTCCTAAATATTTCTCCACTCATTCCTTTCGCTCATTCCCACC
TTGGCTGCTCCGGTTTGTGCCATTCTGTCTGTCAAAAGCCTCCTGCCTGCTCTCCCTGCTCCAGTCTTGACCTCCA

1700	13.2	0.0	3.5	Teref	1	266	(1718)	+	AluJr	SINE/Alu	34	290	(22)	10
266	23.7	0.0	0.0	Teref	289	364	(1620)	C	L1ME4a	LINE/L1	(0)	6124	6049	11
231	23.5	3.2	0.6	Teref	530	618	(1366)	+	MIRb	SINE/MIR	83	172	(36)	12
24	2.7	5.0	2.4	Teref	759	798	(1186)	+	(TTG)n	Simple_repeat	1	41	(0)	13
430	26.4	11.1	0.0	Teref	1033	1176	(808)	+	MIR	SINE/MIR	78	237	(25)	14
402	29.7	6.7	8.5	Teref	1188	1426	(558)	C	MIR	SINE/MIR	(5)	257	23	15
280	29.1	16.2	1.3	Teref	1464	1599	(385)	C	MIR	SINE/MIR	(5)	257	102	16
257	34.7	3.3	0.8	Teref	1676	1797	(187)	C	MamGypsy2-I	LTR/Gypsy	(1309)	2857	2733	17

>KSI_AluYc3_4

>Scaffold9444-548694-548826
AAGGAGGAAAGGGGGGAAATGACGAGGAGGAGTAGTTACGGCTTTATTTATTTTGCTGGGATTTCCTTCTCTGAAACTTTAGACACATCCCTACAGACTCCTGAAGGTCTGCTCTGTTCTGCATCT GT
AGGAGAAGCTTTACTAACTTAGAATATAGACCAGAAAGGCGAGTAAGAGGTTGCTGAACCAAGAAAATACACACAGGGCAAACGGTCAGTAATCTGTACTATTTCATCAGACGACTGAGAAAAAG AAA
ATTTATTGACACTTTTGTTCTTAATGCTCACATATAAAGATTTCACTTTACCTCGTGTTTTCAGAAACACTCCAGACACATCAAATAAACTCTCTTGAATTTTAAAATAGAAAAGGATTGACTGG GC
TATGTGTGTGTCTGGGAGTGGGAACACAGGGATGGGAGTAATTCAGGATCAAAAAACAGAGTATTAAGTTTAAAGCAGTTGTTACTC TCTGCCTTCCCCCTCCTTCCTATTCTCCAGAAGGCAGGGTT
CATAGTGAAACACTGTGTAGTCTTAGATGCCCCTCCTGGAAAGGCATATGAATCCTAGCCCCACTCCACTCTACTGAAAACTCAGTGATTTAAAGCCTCTTGCTTTAAAAAAGGACTTCTGGTTCA AA
AGACTGCTATGATCATGTGTTAACATTCATGTAGAAAAAGTTCTAGTCATATTAATAATCAATAAAAATGCAAGTAAAAACTATCGAAATGCCACTGTGTATATCTTTTAGAGTAGAAAAAAAAA CTT
CTAATACCTTGTAATGGTAAAACTATAAAAATGATTTTAGGAATTCTGTTGGTAACATTGTAAATTAGGACATCTTTCAGAGGGCTATTGTCAATAAAAAGTGAGTCTAAAGATGTTTTTACTCTG AC
CAATAATCCTACTTACAAGGCATGATCCTAAGGAAGTTGTTCAACAGAATAAAAGGTATATAGATAATGACCATCATCATCAAATTACTTAT TAAAACCACACAAGGctgaggcaggagaatggcgtg
aacccaggaggcggagcttgcagtgagccgagatcgcgccactgcactccagcctgggcgacagagccagacgctgtctcaaaaaaaaaaaaaaaaaaaaaaaaaaaaa ccacacaaggAAGCAATCT
AAATATTCTGTGATCGGCTTCCACTACAACCCATACACTTTTCTTTTATACTGCTTATCACATTTTAATTATTTGACAACTGTCTCACTACTAGATAGTAAGCTCCA TGAGGGTAGTGCTGTCTCTGC
CTAGATCTCTGGCACATAAATACAGACTGTAGGAATTAAGAACAAATACTGAAACGTTTATAATTTATTATTTTTAGATGGAGTCTCACTTTGTCGCCCAGGCTGGAGTGCAATGGCGCGATCTC AGC
TAACTGCTACCTCCGCCTCCCGGGTTCCAGCGATTCTCCTGCCTCAGACTCCCGAGTAGCTGGGATTACAGGTGCCCACCACTGCATCCGGTTAATTTTTGTATTTT TAGTAGAGACGGGGTTTCACC
ATGTTGGCCAAGCTGGTTTCGAACTCTTGACCCTCCTCGGCCTCCCAAATCCTGGCCTGCCTCGGTCTCCCAAAGTGCTGGGATTACAGGCGTGAGCCACTGCGCCTGACCTAATTTAATGTCAA GTA
AAAATAACACAAAAATATGTGTGCTACAAGTGAAACTGAGTAACATATACACACGATTATTATACAAGATTAAAAATAGTTAAGATGGAGGCATCATTGGCCAACTT TTCAATTATTTTAAATATCCT
TTAAAGTTATTTTAGTACCTCTGAAATGAAAAAGCAGGAAAAAAATTTGCTGTTATTTACACCAAACTTGACCTTGTAGTAGGGATGAAACGGCCACCATGAAAGCCTGAGGGCCCACTCCCCAGC TTA
CCCCCAGGTACTCAGTTATTCTTTCTTTGCTCAAGGGTAGAAAGAGCCAAAATACTCATATATCATATGTAGCATAACCCTATTTTCTGTAACAATCTCAAACAAAG GCAGTATGTTGAGAGAGACAG
GGAAATGTCTTCTAAAGCAATCCCTTTTGAGCAGTGAATTTGCTGGTTACTAACAGCAGGGGGTGCCAAAGGGATCGCTATTGT T

368	30.4	9.0	2.2	Scaffold9444-548694-548826	659	957	(1176)	+	L1ME4c	LINE/L1	5371	5689	(394)	4
1186	3.0	0.0	0.0	Scaffold9444-548694-548826	1001	1133	(1000)	+	AluYc	SINE/Alu	166	298	(1)	5
201	22.6	3.8	0.0	Scaffold9444-548694-548826	1216	1268	(865)	+	L2a	LINE/L2	3274	3328	(98)	6
2096	9.9	0.0	4.5	Scaffold9444-548694-548826	1344	1647	(486)	C	AluSg4	SINE/Alu	(19)	291	1	7

>Hg19 chr9:128318940-128320932
AAGGAGGAAAGGGGGGAAATGACGAGGAGGAGTAGTTACGGCTTTATTTATTTTGCTGGGATTTCCTTCTCTGAAACTTTAGACACATCCCTACAGACTCCTGAAGGTCTGCTCTGTTCTGCATCT GT
AGGAGAAGCTTTACTAACTTAGAATATAGACCAGAAAGGCGAGTAAGAGGTTGCTGAACCAAGAAAATACACACAGGGCAAACGGTCAGTAATCTGTACTATTTCATCAGACGACTGAGAAAAAG AAA
ATTTATTGACACTTTTGTTCTTAATGCTCACATATAAAGATTTCACTTTACCTCGTGTTTTCAGAAACACTCCAGACACATCAAATAAACTCTCTTGAATTTTAAAATAGAAAAGGATTGACTGG GC
TATGTGTGTGTCTGGGAGTGGGAACACAGGGATGGGAGTAATTCAGGATCAAAAAACAGAGTATTAAGTTTAAAGCAGTTGTTACT CTCTGCCTTCCCCCTCCTTCCTATTCTCCAGAAGGCAGGGTT
CATAGTGAAACACTGTGTAGTCTTAGATGCCCCTCCTGGAAAGGCATATGAATCCTAGCCCCACTCCACTCTACTGAAAACTCAGTGATTTAAAGCCTCTTGCTTTAAAAAAGGACTTCTGGTTCA AA
AGACTGCTATGATCATGTGTTAACATTCATGTAGAAAAAGTTCTAGTCATATTAATAATCAATAAAAATGCAAGTAAAAACTATCGAAATGCCACTGTGTATATCTTTTAGAGTAGAAAAAAAAA CTT
CTAATACCTTGTAATGGTAAAACTATAAAAATGATTTTAGGAATTCTGTTGGTAACATTGTAAATTAGGACATCTTTCAGAGGGCTATTGTCAATAAAAAGTGAGTCTAAAGATGTTTTTACTCTG AC
CAATAATCCTACTTACAAGGCATGATCCTAAGGAAGTTGTTCAACAGAATAAAAGGTATATAGATAATGACCATCATCATCAAATTACTTATTAAAA CCACACAAGGAAGCAATCTAAATATTCTGTG
ATCGGCTTCCACTACAACCCATACACTTTTCTTTTATACTGCTTATCACATTTTAATTATTTGACAACTGTCTCACTACTAGATAGTAAGCTCCATGAGGGTAGTGCTGTCTCTGCCTAGATCTCTGG
CACATAAATACAGACTGTAGGAATTAAGAACAAATACTGAAACGTTTATAATTTATTATTTTTAGATGGAGTCTCACTTTGTCGCCCAGGCTGGAGTGCAATGGCGCGATCTCAGCTAACTGCTA CCT
CCGCCTCCCGGGTTCCAGCGATTCTCCTGCCTCAGACTCCCGAGTAGCTGGGATTACAGGTGCCCACCACTGCATCCGGTTAATTTTTGTATTTTTAGTAGAGACGGGGTTTCACCATGTTGGCCA AG
CTGGTTTCGAACTCTTGACCCTCCTCGGCCTCCCAAATCCTGGCCTGCCTCGGTCTCCCAAAGTGCTGGGATTACAGGCGTGAGCCACTGCGCCTGACCTAATTTAATGTCAAGTAAAAATAACA CAA
AAATATGTGTGCTACAAGTGAAACTGAGTAACATATACACACGATTATTATACAAGATTAAAAATAGTTAAGATGGAGGCATCATTGGCCAACTTTTCAATTATTTTAAATATCCTTTAAAGTTAT TT
TAGTACCTCTGAAATGAAAAAGCAGGAAAAAAATTTGCTGTTATTTACACCAAACTTGACCTTGTAGTAGGGATGAAACGGCCACCATGAAAGCCTGAGGGCCCACTCCCCAGCTTACCCCCAGGT ACT
CAGTTATTCTTTCTTTGCTCAAGGGTAGAAAGAGCCAAAATACTCATATATCATATGTAGCATAACCCTATTTTCTGTAACAATCTCAAACAAAGGCAGTATGTTGAGAGAGACAGGGAAATGTCT TC
TAAAGCAATCCCTTTTGAGCAGTGAATTTGCTGGTTACTAACAGCAGGGGGTGCCAAAGGGATCGCTATTGT T

382	32.2	8.0	2.1	Hg19	659	1020	(973)	+	L1ME4c	LINE/L1	5371	5753	(330)	1
201	22.6	3.8	0.0	Hg19	1076	1128	(865)	+	L2a	LINE/L2	3274	3328	(98)	2

2096	9.9	0.0	4.5	Hg19	1204	1507	(486)	C	AluSg4	SINE/Alu	(19)	291	1	3

AluYd8

Instruction

Green box: Primer information

Red letter: Target site duplication

Black letter: Insertion sequences

Green letter: Deleted sequenced by insertion-mediated deletion

>KSI_AluYd8_1

>Scaffold490-519820-520051
TTTGTCCACACTGAGCTGCCAACAACTTGTCAATTACAGTTGAAATCTTCATATCTTGATACTGGTTCTTGCAGAAGATTTTTGCTTCTGGTCTGGTAATTGTGATTTTCTGTATTAACCTATTTC TC
TATTTTGAGTTTGCCCTGTGACCTCAGTTTTCTGAAGGATATAAGAAGAGTGTTTATTTTCAGTTTGTTCAGCTTTTTTTGTTGTTGCTTTGTTTTGAGGATGAGAATGACAATTTCCAAGTAGC TTA
CATACTGGAACAGAGACTAGAAGTCTAGTTTGTGATATAAAAATTTATAAATACATGCTATTGCATTTGGCTAATTGCTCATCCCCTAAGAAAAGAGTGAGCATTCATTTGTCTATCTGTGCTTAG AT
GACATTGATTTTAGTTTCTTTATTTTTTTCTTTCTTTCTTTCTTTTTTATTTTTTGAGA CGTGGTATGGCTCTTTCACCCAGGCTGGAGTACAGTAGTATTCTCATGGCTCACTGCAACCTCAAACTT
CTGGGCTCAAGTAATCCTCTTGCCTCAGCCTCTTGAGTAATTGGAACTACATGCATGTGCCACCATGCTCAGCTAATTTGTTTTTATTTTGATTGTAGAGATGGGGTTTTGCTGTGTTGCCCAGGC TG
GTCTCAAACTCCTGGCCTTAAGAGATCCTCCTATTTCAGCCTCCCAAAGTGTGGGGATTACAGGAATTAGCCATGCCATCTGCCTAGATGACACTGTTTCTGAGAAGTGAATAATAATATTCTGA TGT
TTTCTATAATTTTGTGCTTAATTTCAATTTCACTATGTCAGATGAGTATTGGCTTTTAGCAATACTGCTTATATTCAGTGTAATGTGAGAACTGTTAAGTAGTCAGTAGCTGCTTAACAATCTAAA AA
GAATTCTCATTCTGGTAAGCCAAATGCCATCAGATAACTTTTTCTGGTAGATGATAATGCCACTCTGAAATGTCTACTTTTTGTGGAAAAAAAGTATG Aaataggccgggcgcggtggctcacgcctg
taatcccagcactttgggaggccgaggcgggcggatcacgaggtcaggagatcgagaccacggtgaaaccccgtctctactaaaaatacaaaaaaattagccgggcgcagtggcgggcgcctgtag tc
ccagctactcgggaggctgaggcaggagaatggcgtgaacccggaaggcggagcttgcagtgagcggagatcgcgccact AATAAATTGAGTGGTCCTTAGAAAGCTGGCATTTGTTTCTAAAAACAA
TTATTTTTGATTTGGCACATCTTACATGCGTGACTACAATGTAGACTAAGGCATGCAGGCCCTGGGAAGGCAACTGCAACTTCCAATTTACTTTTTCTCTTTTTACCACAAAATCTACTGCTTTG ATA
GAATGGAGAGTCTTTCATTGAGTCATCCTTTACTAATGCTATTAATGAGATGTCAGTGATGTACAAGCAGCATATGGACTCTAGAA AGCAGACTCTCAATGAAGCATTGGGAAATTCCAGAGGCATTA
TATTCTAAACTGAAGAGGTTTAAGTCATATTGTTTTCAGAAGATGAAAATAGCTATGGATAAATTTTAATTGGGTAAAGTTTCACTGAGTCTTTTCCCCAGCAAAACAATAAAAAAGCCATTGGC TAA
CTCTGGTGGTCTCCCTTTATGCTATTAGCCATATTAGTCTTGCTTGTTACTGACTGTGTGTAAAGATGTAACTGGTTTGAGAACACTTGTA CCTTAATTGTGCTCCTGGAAACATATTTTGTGATATT
AACATACCCTTTTTCTATTTTCCACCTTCCCTTCTCCACATTGAAGGTTGCTAGTCCTCCTGTCATTACTCAGGAATGATGATACAGGGCAAGGAAGGAAGCAAACACAGA cCAAAGTCATTCTGTCC
ATTGTTTCTAAAAGCCCAAGCTTCAGAGGAAATGATTGGTTGTGGAGTATGCTGAGGAGGCAAAAAGAAGGAAATTCTATAGCATGAACAA ATGGAAAATTATTTCCATTAGGGTGAAAATCACCCTG
CCCCTTTTGAGCTAGTGGGTTGAAAGAAAAGAAAGAGATTTGTGAGCTGTTAGAGTAGAAGAGATCAACATCCTGCTTCTCAGGGCACCACAGGAAATGTTCTGAGACCTCCAAGAGGACCTGGC TGA
GCAGTTCTTAGGCAGAAGAACACACAAGCAATCTCATCAAGAAAGGGTCTGTGC T

1067	18.7	9.3	4.4	TeRefSeq_4	2	281	(1950)	C	L1MCa	LINE/L1	(6215)	293	1	1
1797	18.4	0.0	1.3	TeRefSeq_4	410	713	(1518)	C	AluJr	SINE/Alu	(0)	312	13	2
773	33.7	7.8	2.2	TeRefSeq_4	725	999	(1232)	C	MamGypsy2-I	LTR/Gypsy	(1693)	2473	2194	3
2256	0.0	0.0	0.4	TeRefSeq_4	1000	1231	(1000)	+	AluYd8	SINE/Alu	1	231	(68)	4
773	30.3	13.7	1.2	TeRefSeq_4	1233	1642	(589)	C	MamGypsy2-I	LTR/Gypsy	(1973)	2193	1719	3
458	29.8	8.5	7.3	TeRefSeq_4	1778	2211	(20)	+	MamGypLTR1c	LTR/Gypsy	13	451	(352)	5

>hg19 chr1 170602452 170604449
TTTGTCCACACTGAGCTGCCAACAACTTGTCAATTACAGTTGAAATCTTCATATCTTGATACTGGTTCTTGCAGAAGATTTTTGCTTCTGGTCTGGTAATTGTGATTTTCTGTATTAACCTATTTC TC
TATTTTGAGTTTGCCCTGTGACCTCAGTTTTCTGAAGGATATAAGAAGAGTGTTTATTTTCAGTTTGTTCAGCTTTTTTTGTTGTTGCTTTGTTTTGAGGATGAGAATGACAATTTCCAAGTAGC TTA
CATACTGGAACAGAGACTAGAAGTCTAGTTTGTGATATAAAAATTTATAAATACATGCTATTGCATTTGGCTAATTGCTCATCCCCTAAGAAAAGAGTGAGCATTCATTTGTCTATCTGTGCTTAG AT
GACATTGATTTTAGTTTCTTTATTTTTTTCTTTCTTTCTTTCTTTTTTATTTTTTGAGA CGTGGTATGGCTCTTTCACCCAGGCTGGAGTACAGTAGTATTCTCATGGCTCACTGCAACCTCAAACTT
CTGGGCTCAAGTAATCCTCTTGCCTCAGCCTCTTGAGTAATTGGAACTACATGCATGTGCCACCATGCTCAGCTAATTTGTTTTTATTTTGATTGTAGAGATGGGGTTTTGCTGTGTTGCCCAGGC TG
GTCTCAAACTCCTGGCCTTAAGAGATCCTCCTATTTCAGCCTCCCAAAGTGTGGGGATTACAGGAATTAGCCATGCCATCTGCCTAGATGACACTGTTTCTGAGAAGTGAATAATAATATTCTGA TGT
TTTCTATAATTTTGTGCTTAATTTCAATTTCACTATGTCAGATGAGTATTGGCTTTTAGCAATACTGCTTATATTCAGTGTAATGTGAGAACTGTTAAGTAGTCAGTAGCTGCTTAACAATCTAAA AA
GAATTCTCATTCTGGTAAGCCAAATGCCATCAGATAACTTTTTCTGGTAGATGATAATGCCACTCTGAAATGTCTACTTTTTGTGGAAAAAAAGTATG AAATAAATTGAGTGGTCCTTAGAAAGCTGG
CATTTGTTTCTAAAAACAATTATTTTTGATTTGGCACATCTTACATGCGTGACTACAATGTAGACTAAGGCATGCAGGCCCTGGGAAGGCAACTGCAACTTCCAATTTACTTTTTCTCTTTTTACC AC

AAAATCTACTGCTTTGATAGAATGGAGAGTCTTTCATTGAGTCATCCTTTACTAATGCTATTAATGAGATGTCAGTGATGTACAAGCAGCATATGGACTCTAGAA AGCAGACTCTCAATGAAGCATTG
GGAAATTCCAGAGGCATTATATTCTAAACTGAAGAGGTTTAAGTCATATTGTTTTCAGAAGATGAAAATAGCTATGGATAAATTTTAATTGGGTAAAGTTTCACTGAGTCTTTTCCCCAGCAAAAC AA
TAAAAAAGCCATTGGCTAACTCTGGTGGTCTCCCTTTATGCTATTAGCCA TgatATTAGTCTTGCTTGTTACTGACTGTGTGTAAAGATGTAACTGGTTTGAGAACACTTGTACCTTAATTGTGCTCC
TGGAAACATATTTTGTGATATTAACATACCCTTTTTCTATTTTCCACCTTCCCTTCTCCACATTGAAGGTTGCTAGTCCTCCTGTCATTACTCAGGAATGATGATACAGGGCAAGGAAGGAAGCAA AC
ACAGAtCAAAGTCATTCTGTCCATTGTTTCTAAAAGCCCAAGCTTCAGAGGAAATGATTGGTTGTGGAGTATGCTGAGGAGGCAAAAAGAAGGAAATTCTATAGCATGAACAAATGGAAAATTATTTC
CATTAGGGTGAAAATCACCCTGCCCCTTTTGAGCTAGTGGGTTGAAAGAAAAGAAAGAGATTTGTGAGCTGTTAGAGTAGAAGAGATCAACATCCTGCTTCTCAGGGCACCACAGGAAATGTTCTG AG
ACCTCCAAGAGGACCTGGCTGAGCAGTTCTTAGGCAGAAGAACACACAAGCAATCTCATCAAGAAAGGGTCTGTGC T

>KSI_AluYd8_2

>Scaffold3702-1178759-1179083
ATATATGTAATTTTATCTTTTTATTGCTTAGGTAAACTATTCTTTTCATGGCAGAGAGGAATCTTGGCTATCACCCTGTTCTACTCCTAATTACAGAAGGGGAAATGTGTGCCAGAGGGGACATGA CT
GGCACGGATCCTTGGAGAGGGGCTGGAGTGAGCATGTGAGTTCAGACTCCTGAGGTCCCTGGACACCTGCTCCTGGTCCTGGTGTGTAGCTCATGGTAGATGTTTGCTTTAGTGGGCTCATGCAT GTG
GAGCCCCAGTTCTTGCACCAGGCACAGAAGAGCCTTTGCACACCAGCTCACTGGGTGTTCCAGTCCAGCTCTGGGAGGTGGGCTCTGCCCATCTAGAGCTGGGCTGGCAGCTGGTGTCACTGCTTA AC
CTGCTCTGTCGCCCAGGGCAGGCAGCTTCCGCTCTGTGCTGCAGTTTGCTGTTCGTGCAACGGAGTGAGAATGTGACTTTCTCCCTGGTCCCCATGGGAACAGGTGGAACAAGGCGTCTAAAGTA CAT
GGAGCTTCGTGGAAGGCACCGTGGCAGCCAATAACGGGGCTGCATGGAAGGAGCCTCTCCCAGCCAGCAGGGCATGTGTCTTCTGCAGGGGGAGGTGCAGCGGCCGGCAGGCTCCGGGCAGTGTGC CT
GGGCCCCGGTTCTGCAACCCAAGTTCTCCAGGTTCTTCTTGTGGGTCCAGCTCCGTGCTCAGAGGGGGAGGTTCCAGAAGCTGAGGCATTTTCCCTTGCTTCGTTTACAGGAGTGGGAAGGCCTTCGC
CAGAAGCCCCTGGGAGAATTCACTTACCTTTCTGTTGGTCAgAGTTGGGTCCTTACCCCATTCTGAGAGCCTCAGGCAAGGAGAATGAACGGGGTTGCCCTCGCGCTGCGGGTCTCGGGCACCCACTG
CTAGGGTCCTAGAAGACCCTAAAGGGCTCcTGTCAGTGTGGTTTGTATTCATCACGATTTACCCTATTAGAAATGGGAAACTGATGAGTATT TAAAATATTTAAGccgggcgcggtggctcacgcctg
taatcccagcactttgggaggccgaggcgggcggatcacgaggtcaggagatcgagaccacggtgaaaccccgtctctactaaaaatacaaaaaattagccgggcgcagtggcgggcgcctgtag tcc
cagctactcgggaggctgaggcaggagaatggcgtgaacccagaaggcggagcttgcagtgagcggagatcgcgccacagcactcccgcctgggcgacagaacga gactccgtctcaaaaaaaaaaa
caaaaaaaaaaaaacaaaaaacaaacaaacaaaaaaaaaaataaaataaaaTatttaagTCAGTTAAAAGCAATGGTAATGAACCAATTACATGTTAAAATGAACATAAGCTTAATAAAAAATAACTAT
GTTTCAAAACAACAAAATGAGAGGAGGTGCATTTTTTTTTGTGATTCTGTTATGCAGCCTTAATCAAGAGGCTGGTTACCCTTGCATGCAGTCTTCTGCAATGTT TGGTTTGAAGCGTCAAAGACAAT
CTAGCCCCATAAACATATCTACTTGGAAAAGGAGGCATTTTAATAGGCTTTTCCGGCCATTGTAGCTATTTCATTTTGATTCTA CACCAGAACTCAAGTGACAGTTGCTTAAAGTTCAGTTTCACTGT
GGAATCTGAAATTGTGTCAGTAGAGTTTTTCCTACGCTGTTTTATTAAAATCCACTGGCCTGTCTTGCACACCAAGTGCATCTTTTACCCGGTGTGGAACACCAC GCACTGGACATTTGGAAATTATT
GGTCCACTGACAATTTCGCAGAACTTCTAAATGTTGACATATTTGTTATACAATCTCAGGAAAATCCCCACATTTGTTAATAATATTGTTGATTTTAACAGAAATGTCCTAGGAGTTGTTAGGCC CTG
GTGATGGGTACAAGTCttTTTTTTTTTTTTTTTTTTTTAAAGACAGTCTTACTCTGTCACCCAGGGCACGATCTCGGCTCACTGCAACCTCTGCCTCCTGGGTTC AGGAGATTCTTGTGCCTCAGCCT
CCCAAGTAGCTGGGATTACAGACATGCATCATCATGCCCAGCTAATTTCTTGTATTTTTAGTAGAGATGGGGTTTCACTATGTTGGCCAGGCTAGTCTCAAACTCCTGACCTCAAGTGATCTGCC CGC
CTCAGCCTCCCAAAGTGTTGAGATTACAGGCGTGAGCCACCGCACCTGGCCTGGGTACTAGTCTTTTAAAATTCTAATTTTCACTTGAAATCTCAAATTTTGACA TTGGTTTATGATGACAGACTCAC
TTCATTTTTGAGAAAATGACA

191	30.2	7.0	0.0	UnnamedSequence	46	131	(2194)	C	MIR3	SINE/MIR	(21)	187	96	1
2123	21.6	10.8	7.2	UnnamedSequence	908	1000	(1325)	C	Charlie1a	DNA/hAT-Charlie	(55)	1400	1310	2
2790	1.0	0.0	0.0	UnnamedSequence	1001	1298	(1027)	+	AluYd8	SINE/Alu	2	299	(0)	3
2123	21.6	10.8	7.2	UnnamedSequence	1299	1936	(389)	C	Charlie1a	DNA/hAT-Charlie	(146)	1309	657	2
2136	10.7	5.2	0.3	UnnamedSequence	1937	2227	(98)	C	AluSz	SINE/Alu	(7)	305	1	4
2123	21.6	10.8	7.2	UnnamedSequence	2228	2322	(3)	C	Charlie1a	DNA/hAT-Charlie	(799)	656	546	2

>Hg19 chr7:47550092-47552077
ATATATGTAATTTTATCTTTTTATTGCTTAGGTAAACTATTCTTTTCATGGCAGAGAGGAATCTTGGCTATCACCCTGTTCTACTCCTAATTACAGAAGGGGAAATGTGTGCCAGAGGGGACATGA CT
GGCACGGATCCTTGGAGAGGGGCTGGAGTGAGCATGTGAGTTCAGACTCCTGAGGTCCCTGGACACCTGCTCCTGGTCCTGGTGTGTAGCTCATGGTAGATGTTTGCTTTAGTGGGCTCATGCAT GTG
GAGCCCCAGTTCTTGCACCAGGCACAGAAGAGCCTTTGCACACCAGCTCACTGGGTGTTCCAGTCCAGCTCTGGGAGGTGGGCTCTGCCCATCTAGAGCTGGGCTGGCAGCTGGTGTCACTGCTTA AC
CTGCTCTGTCGCCCAGGGCAGGCAGCTTCCGCTCTGTGCTGCAGTTTGCTGTTCGTGCAACGGAGTGAGAATGTGACTTTCTCCCTGGTCCCCATGGGAACAGGTGGAACAAGGCGTCTAAAGTA CAT
GGAGCTTCGTGGAAGGCACCGTGGCAGCCAATAACGGGGCTGCATGGAAGGAGCCTCTCCCAGCCAGCAGGGCATGTGTCTTCTGCAGGGGGAGGTGCAGCGGCCGGCAGGCTCCGGGCAGTGTGC CT
GGGCCCCGGTTCTGCAACCCAAGTTCTCCAGGTTCTTCTTGTGGGTCCAGCTCCGTGCTCAGAGGGGGAGGTTCCAGAAGCTGAGGCATTTTCCCTTGCTTCGTTTACAGGAGTGGGAAGGCCTTCGC
CAGAAGCCCCTGGGAGAATTCACTTACCTTTCTGTTGGTCAaAGTTGGGTCCTTACCCCATTCTGAGAGCCTCAGGCAAGGAGAATGAACGGGGTTGCCCTCGCGCTGCGGGTCTCGGGCACCCACTG
CTAGGGTCCTAGAAGACCCTAAAGGGCTCtTGTCAGTGTGGTTTGTATTCATCACGATTTACCCTATTAGAAATGGGAAACTGATGAGTATT TAAAATATTTAAGTCAGTTAAAAGCAATGGTAATGA
ACCAATTACATGTTAAAATGAACATAAGCTTAATAAAAAATAACTATGTTTCAAAACAACAAAATGAGAGGAGGTGCATTTTTTTTTGTGATTCTGTTATGCAGCCTTAATCAAGAGGCTGGTTAC CC
TTGCATGCAGTCTTCTGCAATGTTTGGTTTGAAGCGTCAAAGACAATCTAGCCCCATAAACATATCTACTTGGAAAAGGAGGCATTTTAATAGGCTTTTCCGGCCATTGTAGCTATTTCATTTTG ATT
CTACACCAGAACTCAAGTGACAGTTGCTTAAAGTTCAGTTTCACTGTGGAATCTGAAATTGTGTCAGTAGAGTTTTTCCTACGCTGTTTTATTAAAATCCACTGGCCTGTCTTGCACACCAAGTGCAT
CTTTTACCCGGTGTGGAACACCACGCACTGGACATTTGGAAATTATTGGTCCACTGACAATTTCGCAGAACTTCTAAATGTTGACATATTTGTTATACAATCTCAGGAAAATCCCCACATTTGTT AAT
AATATTGTTGATTTTAACAGAAATGTCCTAGGAGTTGTTAGGCCCTGGTGATGGGTACAAGT CTTTTTTTTTTTTTTTTTTTTAAAGACAGTCTTACTCTGTCACCCAGGGCACGATCTCGGCTCACT
GCAACCTCTGCCTCCTGGGTTCAGGAGATTCTTGTGCCTCAGCCTCCCAAGTAGCTGGGATTACAGACATGCATCATCATGCCCAGCTAATTTCTTGTATTTTTAGTAGAGATGGGGTTTCACTA TGT

TGGCCAGGCTAGTCTCAAACTCCTGACCTCAAGTGATCTGCCCGCCTCAGCCTCCCAAAGTGTTGAGATTACAGGCGTGAGCCACCGCACCTGGCCTGGGTACTAGTCTTTTAAAATTCTAATTTT CA
CTTGAAATCTCAAATTTTGACATTGGTTTATGATGACAGACTCACTTCATTTTTGAGAAAATGAC A

2490	21.6	11.7	2.9	UnnamedSequence	908	1599	(387)	C	Charlie1a	DNA/hAT-Charlie	(55)	1400	657	1
2090	10.7	5.2	0.3	UnnamedSequence	1600	1888	(98)	C	AluSz	SINE/Alu	(9)	303	1	2
2490	21.6	11.7	2.9	UnnamedSequence	1889	1983	(3)	C	Charlie1a	DNA/hAT-Charlie	(799)	656	546	1

>KSI_AluYd8_3

>Scaffold16936-3927859-3928082
CACCAGCAGTGTAGAAGTGTTCCCTGATTGCTGCATCCATGCCAACTTCTACTGTTTTTGATTCTTGGATTATGGCCATTGTTGCAAGAGT gAGGTGGTATCGCATTGTGGTTTTGATTTGCATTTC
CCTGATCAcTAGTGATGTTGAGCATTTTTTCATATGTGTGTTGGTCATTTGTACATCTTCTTTTGAGAGTTGTCTATTCATGTCCTTAGCCCACTTTTTGATGGAATTGATTGTTTTCTCCTTGCTGA
TTTGTTTGAGTTCATTGTAGATTCTGGATATTAGTCCTTTGTCAGATGTATAGATTGTGAATATTTTCTCCCACTCTGTGGGTTGTCTGTTTATTCTGCTGACTGTTCCTTTTGCTGTGCAAAAG CTC
TTTAGTTTAATTAGGTCCCAGCCATGTATCTTTGTTTTTATGCAATTGCTTTTACCCAAAAGCAATTTTTGGTCATGAAATCCTTGCCTAAGCCAATGTCTAGAAGGGTTTTTCTAATGTTATCTTCT
AGAATTTTTATAGTTTCAGGTCTTAGATTTAGGTCCTTAATCCATCTTGAGTTGATTTTTGTATAAGGTGAGAGATGAGGATCCAGTTTCATTTTCCTACATGTGGCTAGCCAATTACCCCAGCA CAC
TTTGTTGAAAAGGGTGTTTTTCCCCCACTTTATGTTTTTGTTTGCTTTGTCAAAGATCAGTTGGCT aTAAGTATTTGGGTTTATTTCTGGGTTCTCTATTCTGTCCCATTGTTCTG tGTGCCTATTTT
TATGCCAGTACCAtACTGTTTTGGTGACTATGCCCTTAGAGTATAGTTTGAAATCAGGTAGTGTGATGCTTCCAGATTTGTTCTTTTTGCTTAGTCTTGCTTTGGCTATGCGGGCTATTTTTTGGTTC
CaTATGAATTTTAGAATTGTTTTTCTAACTCTGTGAAGAATGATGGTGGTATTTTGATGGAGATTGCATTGAATTTGTAGATTGCTTTTGGCAGTGTGGCCATTCTCACAATATTGATTCTACCCAT
CCATGAGCATGGGATGTGTTTTCATTTGTTTGTCATCTATGATTTATTTCAGCAGTGTTTTGTAGTTTTCCTTGTAGAAGCCTTTTGACTCCTTTTTTAGGTATATTCCTCAGTATTTTTTTTTTT TTT
GCAGCTATTGTAAAAGGGTTGAGTTCTTGATTTGATTATCTGCTTGGTCACTGTTGGTGTATAAAAGAGCTACTGATTTGAATATATTAATCTTGTATCTG cAAACTTTGCTGAATTCTCTTATCTGT
TCTAGGAGCTTTCTAGAGGCGTCCTTAGGATTTTCAAGGTAAACAATC ATATCGTCAGCAGTCAGGGACAGTTTGACTTCCTCTTCACTgATTTGGATGCCCTTTATTTCTTTCTCTTGTCTGATTGC
TCTGGCCAAGACTTCCAGTACTATGATGAAGAAGGGTGGTGAGAGTGGGCATCCTTGTTTTGcTCCCGTTCTCAGGAAGAATGCTTTCAGCTTTTCCCCAT cCAGTATTAAGTTGGCTGTGGGTTTGT
CATAGGTGGCTTTTAATTCATTAAGGTGTGTCCCTTGTATGCCAATTTTGCTGAGCTGTGCTCTTTTTAGTAATCTTGATACCTAACAGATTATACATACTAACTCATTT gCTTTACTGAAAACCTTA
TAAcAGTATTAAGAAAATTTTCAATATTAATACAgATAATAAAACTACAAAATGTAGTTCAAATTTGTGTATGCAGTTTTTAAATTACATTTCACTAAAAATTTACAATCAAATTAATTTCTTCTTCT
AAATTAATTTCTTCTGAAGTCATTCATATGCCCTTCCTATGTATAACTTG gTAAACACAGATGTGGTAAAAAATTTGGCTCCATTGTGTCAGGTTAGTTTAAATTTGAGGGTGTTGGCATATTAACAG
CTAATCAGACTAAAAAGATGATGTGATACTTTCAGCTATTAGGAAAGAAAAGGGTTTACCTTTTTTATGAAGAAAGAAC Tgatctccgctcactgcaagctccgccttccgggttcacgccattctcc
tgcctcagcctcccgagtagctgggactacaggcgcccgccactgcgcccggctaatttttttgtatttttagtagagacggggtttcaccgtggtctcgatctcctgacctcgtgatccacccgc ctc
ggcctcccaaagtgctgggattacaggcgtgagccaccgcgcccggccGTCTCCAAATTTCTGTACAATGGTTTTGTATCAACACCATCTGATTCATATA CCTCGTTTCTGGTCAATTATCTCTTCAA
TTATTTAATTTCTAGTACTGTTAGAATCCAtATATTTTCAAATACTCAGTTTTTAAAACTATATTAACTATGTTAAATTGTATTCTCATCTAAGGGGTTTTGTTTTTGCTGT TTTTTTTTTTTTTATCT
TGCTCCCTTTTCTGAGCTCTGTATTATTATACCAAGGTTTGCATTAGATAAATC cGTAACCACcCAACTGGGTTCTTCTTGCCTGCTGCCTAGAAAAAGCCAATACACTGAGAACAGCAGGTGTTACA
gCAGAGAAAGTTTACTTATTGCAAGGCAGTGGAGCAGAAGGATGGGACATATTTCTCAAATCAGTCTCTCTGATAATCCAGAAGCTAGGATTTTTCAAGGATAGTTAGGCAGCAGGGGGTTGGGAAGT
GGTTAGGGAATGGGGAATGCTGATTGGTGGGTCAGGAAAGAAATGACATGGGAGTCAAAGCTGTCTTCTTGGACTGAGACAGTCCCTGGGAGAAGTTAAGTCTGTTTCTTGGCATGGGTCACAGG ATG
GGTGGCAGTAGTTGGTCCATGgGAATTCAAGGCCTGAAAAATACCTTAAACACCAGTCTTAGGTTTTATAATAGTGATGTTATATATAGGAGCAATTAGGGAG tTTACGAATCTTGTGACTTCAGTCC
TAAACCATAATTCCAACCTTGTGGTTAATTTGTTAGTCTTACAAAAGTTGTTTTGGTCCCCAGGAAAGGAGGGGATTAGTTTTGGAAAGGGACTGTTATCATCTTTGTTTTAAAATCAAACTATA TAC
TAATTTCCTCCCATAGTTATCTTGGCCTGTACTCAGGAATGAGCAAGGACAGTTA gTTTGTGAGGTTAGAAGCAAGATGGAGTCAGCTATGTCAGATTTCTCTCACTGTCATGA TTTTTGCAAAGGCA
GTCAAATCTACATTCTCACAGTATTACCTTGCCCCAGTACCAGCCATTTATTTTCCTGCAAGGAAATTAAGGAGAATTCCATATAATTAGAAACAAAACTATATAAAAATATAAATACAGGCTCT TGA
AGTTTTGCCTGAAATGCTGTTTTGACTCATGGGTCAAAATTTGGTTTTACTTCACATATTATTGGTAGAATCATTGTTTTAATTACAGATGAAACTAATGTAGAAGTCCTGTTT CTCAGAAGCCTTTG
GATAAACTCAAATTTCTATGATATAAACATTCAAGCTACCCCAAATCAATTAAGAATGTTATAAAATAAAAATATAACCAATGCCTAATCCAATTGAATCCTATTAAATTTTTAAATTAATATCT AGA
AAATAATTTAGTTTTTATACTTTACAGCCATTTTCAGAACAAGTAAAAACTGTATTTATAGAAACATTATTTCATATAAAGAGTGGTGGAAAAAAGAAAGTTTTCCTATTTAAA TGAGTTTAAGAAAT
GCTAGGTGCAAAGaAAGGTTAGCACCTACCGTAATGTGTTTTGAATATAAAATAAAAGGATCAGAGTCTCACAGTCATCAACTCTTCATCCTCCTTGTCACCACCCAACTTATTTCTTGGAGCATTT
TAAGGACTAATATTCCTGAAAAATGCTATCTAAATCCAAATAGGAAGAATTTCAATATTCAGGATATTTTCTTTGAGCATCCAAAGGGACTGTAAAAATTAAATATACAGAATG AAACCAATCAGTTT
AGCAATGCATTATTATTATGTCTATTTCTCTGCTCATTATTGCCCCTTTGTTGAATATCAAGATGCCCAGAAGTAAAAATAAATTGTATCATTGAGAGAAATAGATGCACTGAATGAGTGGTTTT TAA
TTATCATGGTCACTTTTTGGATTTGGGGTGATTTTGGCAGTACCCTCAGTACTGATTATGTTTTAAATTATTGCTTTTCCTTGAGGAATCTGCCCTATGACTCAAGAAAAAGAA ATCTTCCTAGGAAG

10460	8.2	0.3	0.6	TeRefseq	1	1591	(2633)	C	L1PB	LINE/L1	(608)	5543	3958	1
285	26.9	9.2	1.1	TeRefseq	1608	1780	(2444)	C	L1MC5a	LINE/L1	(1571)	6459	6273	2
2196	0.5	0.0	0.0	TeRefseq	2001	2224	(2000)	C	AluYd8	SINE/Alu	(75)	224	1	3
16	16.5	0.0	0.0	TeRefseq	2402	2428	(1796)	+	(T)n	Simple_repeat	1	27	(0)	4
1821	24.6	5.0	9.5	TeRefseq	2488	3202	(1022)	C	LTR8	LTR/ERV1	(1)	690	5	5
12	18.4	5.4	0.0	TeRefseq	3281	3317	(907)	+	(ATATAA)n	Simple_repeat	1	39	(0)	6

>Hg19 chr20:8669594-8671605
CACCAGCAGTGTAGAAGTGTTCCCTGATTGCTGCATCCATGCCAACTTCTACTGTTTTTGATTCTTGGATTATGGCCATTGTTGCAAGAGT aAGGTGGTATCGCATTGTGGTTTTGATTTGCATTTC

CCTGATCAtTAGTGATGTTGAGCATTTTTTCATATGTGTGTTGGTCATTTGTACATCTTCTTTTGAGAGTTGTCTATTCATGTCCTTAGCCCACTTTTTGATGGAATTGATTGTTTTCTCCTTGCTGA
TTTGTTTGAGTTCATTGTAGATTCTGGATATTAGTCCTTTGTCAGATGTATAGATTGTGAATATTTTCTCCCACTCTGTGGGTTGTCTGTTTATTCTGCTGACTGTTCCTTTTGCTGTGCAAAAGCTC
TTTAGTTTAATTAGGTCCCAGCCATGTATCTTTGTTTTTATGCAATTGCTTTTACCCAAAAGCAATTTTTGGTCATGAAATCCTTGCCTAAGCCAATGTCTAGAAGGGTTTTTCTAATGTTATCT TCT
AGAATTTTTATAGTTTCAGGTCTTAGATTTAGGTCCTTAATCCATCTTGAGTTGATTTTTGTATAAGGTGAGAGATGAGGATCCAGTTTCATTTTCCTACATGTGGCTAGCCAATTACCCCAGCACAC
TTTGTTGAAAAGGGTGTTTTTCCCCCACTTTATGTTTTTGTTTGCTTTGTCAAAGATCAGTTGGCT gTAAGTATTTGGGTTTATTTCTGGGTTCTCTATTCTGTCCCATTGTTCTGaGTGCCTATTTT
TATGCCAGTACCAcACTGTTTTGGTGACTATGCCCTTAGAGTATAGTTTGAAATCAGGTAGTGTGATGCTTCCAGATTTGTTCTTTTTGCTTAGTCTTGCTTTGGCTATGCGGGCTATTTTTTGGTTC
CgTATGAATTTTAGAATTGTTTTTCTAACTCTGTGAAGAATGATGGTGGTATTTTGATGGAGATTGCATTGAATTTGTAGATTGCTTTTGGCAGTGTGGCCATTCTCACAATATTGATTCTACCCA T
CCATGAGCATGGGATGTGTTTTCATTTGTTTGTCATCTATGATTTATTTCAGCAGTGTTTTGTAGTTTTCCTTGTAGAAGCCTTTTGACTCCTTTTTAGGTATATTCCTCAGTATTTTTTTTTTTT T
tGCAGCTATTGTAAAAGGGTTGAGTTCTTGATTTGATTATCTGCTTGGTCACTGTTGGTGTATAAAAGAGCTACTGATTTGAATATATTAATCTTGTATCTG aAAACTTTGCTGAATTCTCTTATCTG
TTCTAGGAGCTTTCTAGAGGCGTCCTTAGGATTTTCAAGGTAAACAATCATATCGTCAGCAGTC AGGGACAGTTTGACTTCCTCTTCACTtATTTGGATGCCCTTTATTTCTTTCTCTTGTCTGATTG
CTCTGGCCAAGACTTCCAGTACTATGATGAAGAAGGGTGGTGAGAGTGGGCATCCTTGTTTTGtTCCCGTTCTCAGGAAGAATGCTTTCAGCTTTTCCCCATtCAGTATTAAGTTGGCTGTGGGTTTG
TCATAGGTGGCTTTTAATTCATTAAGGTGTGTCCCTTGTATGCCAATTTTGCTGAGCTGTGCTCTTTTTAGTAATCTTGATACCTAACAGATTATACATACTAACTCATTT tCTTTACTGAAAACCTT
ATAAtAGTATTAAGAAAATTTTCAATATTAATACAaATAATAAAACTACAAAATGTAGTTCAAATTTGTGTATGCAGTTTTTAAATTACATTTCACTAAAAAT TTACAATCAAATTAATTTCTTCTTC
TAAATTAATTTCTTCTGAAGTCATTCATATGCCCTTCCTATGTATAACTTG tTAAACACAGATGTGGTAAAAAATTTGGCTCCATTGTGTCAGGTTAGTTTAAATTTGAGGGTGTTGGCATATTAACA
GCTAATCAGACTAAAAAGATGATGTGATACTTTCAGCTATTAGGAAAGAAAAGGGTTTACCTTTTTTATGAAGAAAGAAC TctaatagctGTCTCCAAATTTCTGTACAATGGTTTGTATCAACACC
ATCTGATTCATATACCTCGTTTCTGGTCAATTATCTCTTCAATTAT TTAATTTCTAGTACTGTTAGAATCCAcATATTTTCAAATACTCAGTTTTTAAAACTATATTAACTATGTTAAATTGTATTCT
CATCTAAGGGGTTTTGTTTTTGCTGTTgtTTTTTTTTTTTATCTTGCTCCCTTTTCTGAGCTCTGTATTATTATACCAAGGTTTGCATTAGATAAATC tGTAACCACtCAACTGGGTTCTTCTTGCCT
GCTGCCTAGAAAAAGCCAATACACTGAGAACAGCAGGTGTTACA aCAGAGAAAGTTTACTTATTGCAAGGCAGTGGAGCAGAAGGATGGGACATATTTCTCAAATCAGTCTCTCTGATAATCCAGAAG
CTAGGATTTTTCAAGGATAGTTAGGCAGCAGGGGGTTGGGAAGTGGTTAGGGAATGGGGAATGCTGATTGGTGGGTCAGGAAAGAAATGACATGGGAGTCAAA GCTGTCTTCTTGGACTGAGACAGTC
CCTGGGAGAAGTTAAGTCTGTTTCTTGGCATGGGTCACAGGATGGGTGGCAGTAGTTGGTCCATG aGAATTCAAGGCCTGAAAAATACCTTAAACACCAGTCTTAGGTTTTATAATAGTGATGTTATA
TATAGGAGCAATTAGGGAGgTTACGAATCTTGTGACTTCAGTCCTAAACCATAATTCCAACCTTGTGGTTAATTTGTTAGTCTTACAAAAGTTGTTTTGGTCC CCAGGAAAGGAGGGGATTAGTTTTG
GAAAGGGACTGTTATCATCTTTGTTTTAAAATCAAACTATATACTAATTTCCTCCCATAGTTATCTTGGCCTGTACTCAGGAATGAGCAAGGACAGTTA aTTTGTGAGGTTAGAAGCAAGATGGAGTC
AGCTATGTCAGATTTCTCTCACTGTCATGATTTTTGCAAAGGCAGTCAAATCTACATTCTCACAGTATTACCTTGCCCCAGTACCAGCCATTTATTTTCCTGC AAGGAAATTAAGGAGAATTCCATAT
AATTAGAAACAAAACTATATAAAAATATAAATACAGGCTCTTGAAGTTTTGCCTGAAATGCTGTTTTGACTCATGGGTCAAAATTTGGTTTTACTTCACATATTATTGGTAGAATCATTGTTTTA ATT
ACAGATGAAACTAATGTAGAAGTCCTGTTTCTCAGAAGCCTTTGGATAAACTCAAATTTCTATGATATAAACATTCAAGCTACCCCAAATCAATTAAGAATGT TATAAAATAAAAATATAACCAATGC
CTAATCCAATTGAATCCTATTAAATTTTTAAATTAATATCTAGAAAATAATTTAGTTTTTATACTTTACAGCCATTTTCAGAACAAGTAAAAACTGTATTTATAGAAACATTATTTCATATAAAG AGT
GGTGGAAAAAAGAAAGTTTTCCTATTTAAATGAGTTTAAGAAATGCTAGGTGCAAAG gAAGGTTAGCACCTACCGTAATGTGTTTTGAATATAAAATAAAAGG ATCAGAGTCTCACAGTCATCAACTC
TTCATCCTCCTTGTCACCACCCAACTTATTTTCTTGGAGCATTTTAAGGACTAATATTCCTGAAAAATGCTATCTAAATCCAAATAGGAAGAATTTCAATATTCAGGATATTTTCTTTGAGCATC CAA
AGGGACTGTAAAAATTAAATATACAGAATGAAACCAATCAGTTTAGCAATGCATTATTATTATGTCTATTTCTCTGCTCATTATTGCCCCTTTGTTGAATATC AAGATGCCCAGAAGTAAAAATAAAT
TGTATCATTGAGAGAAATAGATGCACTGAATGAGTGGTTTTTAATTATCATGGTCACTTTTTGGATTTGGGGTGATTTTGGCAGTACCCTCAGTACTGATTATGTTTTAAATTATTGCTTTTCCT TGA
GGAATCTGCCCTATGACTCAAGAAAAAGAAATCTTCCTAGGAAG

10402	8.1	0.2	0.5	Hg19seq	1	1692	(2520)	C	L1PB	LINE/L1	(508)	5643	3958	1
288	26.3	9.2	1.1	Hg19seq	1709	1881	(2331)	C	L1MC5a	LINE/L1	(1571)	6459	6273	2
15	19.7	0.0	0.0	Hg19seq	2288	2316	(1896)	+	(T)n	Simple_repeat	1	29	(0)	3
1845	24.1	5.0	9.5	Hg19seq	2375	3090	(1122)	C	LTR8	LTR/ERV1	(0)	691	5	4
12	18.4	5.4	0.0	Hg19seq	3169	3205	(1007)	+	(ATATAA)n	Simple_repeat	1	39	(0)	5

AluYg6

Instruction
Green box: Primer information
Red letter: Target site duplication
Black letter: Insertion sequences
Green letter: Deleted sequenced by insertion-mediated deletion

>KSI_AluYg6_1

>Scaffold577-15815-16111
ATTTATAATTTTTATTAACAATGGAATCATTTATGAACTTTTTTATTATGAACTTTTTTATTACATTCAATTTT CACCCAACTCATTAAAAACTGGGAAACCAATATaAATTaTGCATTTTGCAATGT
GTTTCACAAAAAGTATGtTTATTGTCTAATACATTTTAATCAGAAGGCTTTAcAGTTTGTAAAATAGTTTGTAAACAGAgAATCAGAGATTAGAAAGACCTTGGGGAATCCATTAAATTCAATCTCCT
CCATGTACAGAGGCCTAAGGAGAATAGATAGTTTATTCAAGGTCATAGAATTAGTAACTGGTTTTGTCTGAATTAAAATTCAGGCCTTCTGACTAGATCATTAGTTGCTCTGAAATCTGTTTTAAG AG
ATTGTATGCAAAATCATACTGATAATGATATCTCCATTATAAATTTTATTTCTAGAGATAAACTTGGCTACAAAGATTA gAAAAACATAGTTGTAcTATTTCATGCAAAAATAACCTAGTATCTTATG
CTATAATAATATCTTGGCATTTTCTaTACCTCGCTTCAAGTTGTCAGTTAgCAATACATTTATTAGACTTCAATGTGCACAAaatagggtAATCTTTTTAAAGTCTGAtTTTCTCCATCTATGAAATG
GAGATAACAGTGTTAGGTCATAGGGTTGC cAtGgGAAAAGTAAATAAAAATGAATGTtAAaAAAAATTGTTCTATGAAATCTAAATAAAATGTTTTTAAAGAGTCAAGAAAAGCAAGTCACTTAAAAA
AAAAGGTGGAAGTCACAGAAGGAGAGGGAGAAAGATCCTATTGCTTTGCATATATCAAGTTCCTTTTCTACTTTAAGGAATCCAAACTGGAAATTGCTGATGCTGTATTATGGGTATAGTTTATA AAA
GAAAGATAAAATCAAATTCCAAACACTAAACGATACTCAAAAGACCCTGC cCTAACTAATAAATGGCATTTAAATgaCTTAACTTTAAGATATAATATTTAAGGggctgggcgtggtggctcacgcct
gtaatcccagcactttgggaggccgagacgggcggatcacgaggtcaggagattgagaccatcctggctaacacggtgaaaccccgtctctactaaaaatacaaaaattagccgggcatggtggc gcg
tgcctgtagtcccagctacacgggaggctgaggcaggagaatggcgtgaacccgggaggcggagctt gcagtgagtcgagatcgcgccactgcactccagcctgggcgacagagcgaaactccaaaaa
aaaaaaaaaaaaaaaaagatataatatttaaggTATGTGCATATCgTTAATGAATCCTCATTTTAATTAATATTTTTtAAATTATACTTAAAGTTCTGGAGTCCATGTGCAGAAcgTGCAGGTTACAT
AGGcATACACATGCCATGGTGGTTTGCTGcGCCCACCAACCTGTTATcTACATTAGGGACTTCTAATGCCATCcCTCCCCTAGCACAaCCCCCCcAAACAGGCCCTGGTGTGTGATATTTCCCTCCCT
GTGTCCATGTGTTCTCATTGTTCAACTCCCACTTATGAGTGAGAACATGCAGTGTTTGGTTTCTGTTCTTGTGTTAGTTTG CTGAGAATGATGGTTTCCAGCTTCTTCCATGTCCCTGCAAAGGACAT
GAACTCATCCTTTTTAATGGCTGCATAGTATTCCATGGTGTATATGTGCCACATATTCTTTACCCAGTCTATCATTGGTGGGCATTTGGGTTGGTTCCAAGTCTTTGCTATTGTGAACAATGCCACAA
TAAACATACGTGTGCATGTGTCTTTAGAGTAGCATGATGTATAATCCTTTGGGTACAACCCAGTAATGGGATTGCTGGGTCAAATGATATTTCTGGTTCTAGATCCTTGAGGAATCGCCACACTG TCT
TCCACAATGGTTGAACTAATTTACACTCCCACCAACAGTGGAAAAGCATTCCTATTTCTCCACATCCTCTCCAGCATCTGTTGTTTCCTGACTTTTTAAGGATTATCATTCTAACTGGCATGAGATGG
TATCTCATTGCAGTTTTGATTTGCATTTCTCTGATGACCAGTGATGATGAGCATTTTTTCATGTTTGTTGGCCACATAAATGTCTTCTTTTGTGAAGTGTCTGTTCATATCCTTCGCCCACTTTT TGA
TGGGATTGTTTATTTCTTTTAAATTTGTTTAAGTTCTTTGTAGATTCTGGATATTAGCCCTTTGTCAGATGGATAGATTGCAAAAATTTTCTCCCATTCTGTAGGTTGCCTGTTCACTCTGATGATAG
TTTCTTTTGCTGTGCAAAAGCTCTTTAGTTTAATTAGATCTCATTTATCAATTTTGGCTTCTGTTGCCGTTGCTTTTGGTGTTTTAGTCATGAAGTCTTTGCCCATGCCTATGTCCTGATTGGTT TTG
CCTAGGTTTTCTTCTAGGGTTTTTATGGTTTTAGGTCTTACGTTTAAGTCTTTAATCCAACTTGAGTTAATTTTTGTATAAGTTGTAAGGAAGGGGTCCAGTTTCAGTTTTCTGCATATGGCTAGCCA
GTTTTCTCAAAACTATTTATTAAATAGAGAATCTTTTCCCCATTGATTGTTTTTGTCAGGTTTGTCAACAATCAGAAGGTTATAGATCTGTGGCATTATTTCTGGGGCCTCTGTTCTGTTCCATT

274	37.1	9.3	1.2	KS_1	201	350	(1947)	C	MIR3	SINE/MIR	(9)	199	38	1
222	30.2	3.0	3.0	KS_1	602	700	(1597)	+	MIRb	SINE/MIR	112	210	(58)	2
521	26.7	6.6	2.8	KS_1	808	1000	(1297)	+	Tigger13a	DNA/TcMar-Tigger	456	656	(115)	3
2700	1.4	2.0	0.0	KS_1	1001	1297	(1000)	+	AluYg6	SINE/Alu	1	303	(8)	4
521	26.7	6.6	2.8	KS_1	1298	1346	(951)	+	Tigger13a	DNA/TcMar-Tigger	657	707	(64)	3
2425	8.1	2.7	0.0	KS_1	1353	1685	(612)	C	L1PA7	LINE/L1	(4)	6150	5809	5

>hg19 chr6:57508919-57510291
ATTTATAATTTTTATTAACAATGGAATCATTTATGAACTTTTTTATTATGAACTTTTTTATTACATTCAATTTT CACCCAACTCATTAAAAACTGGGAAAtCCAATATAATTtTGCATTTTGCAATGT
GTTTCACAAAAAGTATGcTTATTGTCTAATACATTTTAATCAGAAGGCTTTAtAGTTTGTAAAATAGTTTGTAAACAGAaAATCAGAGATTAGAAAGACCTTGGGGAATCCATTAAATTCAATCTCCT
CCATGTACAGAGGCCTAAGGAGAATAGATAGTTTATTCAAGGTCATAGAATTAGTAACTGGTTTTGTCTGAATT AAAATTCAGGCCTTCTGACTAGATCATTAGTTGCTCTGAAATCTGTTTTAAGAG
ATTGTATGCAAAATCATACTGATAATGATATCTCCATTATAAATTTTATTTCTAGAGATAAACTTGGCTACAAAGATTA tAAAAACATAGTTGTAtTATTTCATGCAAAAATAACCTAGTATCTTATG
CTATAATAATATCTTGGCATTTTCTgTACCTCGCTTCAAGTTGTCAGTTAtCAATACATTTATTAGACTTCAATGTGCACAAAATCTTTTTAAAGTCTGAgTTTCTCCATCTATGAAATGGAGATAAC
AGTGTTAGGTCATAGGGTTGCtAcGaGAAAAGTAAATAAAAATGAATGTaAAtAAAAAgagTTGTTCTATGAAATCTAAATAAAATGTTTTTAAAGAGTCAAGAAAAGCAAGTCACTTAAAAAAAAAG

GTGGAAGTCACAGAAGGAGAGGGAGAAAGATCCTATTGCTTTGCATATATCAAGTTCCTTTTCTACTTTAAGGAATCCAAACTGGAAATTGCTGATGCTGTATTATGGGTATAGTTTATAAAAGA AAG
ATAAAATCAAATTCCAAACACTAAACGATACTCAAAAGACCCTGCtCTAACTAATAAATGGCATTTAAATatCTTAACTTTAAGATATAATATTTAAGGTATGTGCATATCATTAATGAATCCTCATT
TTAATTAATATTTTTAAAATTATACTTAAAGTTCTGGAGTCCATGTGCAGAAAATGCAGGTTACATAGGTATACACATGCCATGGTGGTTTGCTGTGCCCACCAACCTGTTATTTACATTAGGGACTT
CTACTAATGCCATCTCTCCCCTAGCACCCCACCCCCCCAAAACAGGCCCTGGTGTGTGATAT TTCCCTCCCTGTGTCCATGTGTTCTCATTGTTCAACTCCCACTTATGAGTGAGAACATGCAGTGTT
TGGTTTCTGTTCTTGTGTTAGTTTGCTGAGAATGATGGTTTCCAGCTTCTTCCATGTCCCTGCAAAGGACATGAACTCATCCTTTTTAATGGCTGCATAGTATTCCATGGTGTATATGTGCCACATAT
TCTTTACCCAGTCTATCATTGGTGGGCATTTGGGTTGGTTCCAAGTCTTTGCTATTGTGAAC AATGCCACAATAAACATACGTGTGCATGTGTCTTTAGAGTAGCATGATGTATAATCCTTTGGGTAC
AACCCAGTAATGGGATTGCTGGGTCAAATGATATTTCTGGTTCTAGATCCTTGAGGAATCGCCACACTGTCTTCCACAATGGTTGAACTAATTTACACTCCCACCAACAGTGGAAAAGCATTCCT ATT
TCTCCACATCCTCTCCAGCATCTGTTGTTTCCTGACTTTTTAAGGATTATCATTCTAACTGG CATGAGATGGTATCTCATTGCAGTTTTGATTTGCATTTCTCTGATGACCAGTGATGATGAGCATTT
TTTCATGTTTGTTGGCCACATAAATGTCTTCTTTTGTGAAGTGTCTGTTCATATCCTTCGCCCACTTTTTGATGGGATTGTTTATTTCTTTTAAATTTGTTTAAGTTCTTTGTAGATTCTGGATA TTA
GCCCTTTGTCAGATGGATAGATTGCAAAAATTTTCTCCCATTCTGTAGGTTGCCTGTTCACT CTGATGATAGTTTCTTTTGCTGTGCAAAAGCTCTTTAGTTTAATTAGATCTCATTTATCAATTTTG
GCTTCTGTTGCCGTTGCTTTTGGTGTTTTAGTCATGAAGTCTTTGCCCATGCCTATGTCCTGATTGGTTTTGCCTAGGTTTTCTTCTAGGGTTTTTATGGTTTTAGGTCTTACGTTTAAGTCTTT AAT
CCAACTTGAGTTAATTTTTGTATAAGTTGTAAGGAAGGGGTCCAGTTTCAGTTTTCTGCATA TGGCTAGCCAGTTTTCTCAAAACTATTTATTAAATAGAGAATCTTTTCCCCATTGATTGTTTTTGT
CAGGTTTGTCAACAATCAGAAGGTTATAGATCTGTGGCATTATTTCTGGGGCCTCTGTTCTGTTCCATT

287	38.3	8.1	1.3	hg19	203	350	(1023)	C	MIR3	SINE/MIR	(13)	195	38	6
273	28.4	3.1	3.1	hg19	595	692	(681)	+	MIRb	SINE/MIR	113	210	(58)	7
623	26.1	11.3	0.4	hg19	803	1040	(333)	+	Tigger13a	DNA/TcMar-Tigger	456	719	(52)	8
2467	8.2	1.5	0.9	hg19	1043	1373	(0)	C	L1PA7	LINE/L1	(13)	6141	5809	9

>KSI_AluYg6_2

>Scaffold4172-702091-702405

CCTGGGTGACAGAGTGAGACTCCATCTTGGAAAAAAAAAAATTACTCAGATGTCCTCAAGGATACCGGGACAGGAACAGTCTCATAAATTACTGGCGAGATTATAAATTGGAACTTTTTTGGAGAGC AA
TTTAGTAGTATGTATTACTAGTCTTAAAAATGTTTGTACCTTTTGACTCGGTTATAACACTTTTGGATATTTGTCCCAAGGAAATTAGAGATGAACACAAAGATTTGTGTACAAAAATGCCTATC ACA
GTGTTATGGTAGTGTCAAAAGACAAAATTACAACAAATTTAAAGAGATCAATTGGCTTTATTTGAGATTCTAGAATTGGGCAACACTTTATTCCATAGAATAAGC gTTCATTGAGCTGAGCACAGAAG
TTTGGCTTTAAAGACAGAGAAGAGCCAAAGAAAGTGGAAGCAAAGAACAGAGTATATTAATTACTTTTAGACAGAACAGTAGAAAAATAACTGATTAGTTAACATCAGTTTATTTCAGGCTACCTTTT
TTGTGTGTAAGGATTGAAGCATCGGGAACTTCATTTATCAAGCTGATTGAAGATTTTAAAATGGCCAATTTGGGAAATTCTGTTATTTCTTTCTCCTGATTTCTTAGAAG gTCAGACAACTTAGTTTA
GATGATGTGGAACTTTAGCATGGGTGACTCCATTTCGATTTTTAGTCTGGTCTGTTGGGGCCTTCTGCAGAAACTTAGTCTGAAGCAATGGCCTCCTATAATTTTTATTTGACAGTAGCAAAAATTTA
GAAACAACCTGTGTCCAACCATTGGGTATTGTTAAATAAAATATTA tCTATATTATGAAACACTAAGCAACTTAAAAATCATGTTTTCAAAACCATCTAAAGATAGAAGAAAGTACTCTTAATACAAT
GGTAAATAATTGGATATAaAACTGCATATAGAGTATTaatattaacttttttttttttttttttttttttttttgggaaaaaaaaactccatatagcgtcaaatc ttttttttttttttttttttttt
tttttttttgagacggagtttcgctctgtcgcccaggctggagtgcagtggcgcgatctcgactcactgcaagctccgcctcccgggttcacgccattctcctgcctcagcctcccgtgtagc tgg
gactacaggcactcgccaccatgcccggctaatttttgtatttttagtagagacggggtttcatcgtgttacccaggatggtctcgatctcctgacctcgtgatccgcccttctcggcctcccaaagt
gctgggattacaggcatgagccaccgcgcccggccAATATTAACTTTTAAAAATGTATATGTATATATATGTATGCATAGCAAAATGATTGAAATACACCAGAAATGTTAAGAATGAGTAGAGTATTT
CCGAGATCTGCAGTTTTAAGAGAACTTTAATTTTTAATAATTTTCTGTATTTTTCCAAATTATCTATAACGTATTATATAGTTAGGAAGCATATTTTATATATTACATATAATGCCAATTTTGGGTCG
AGGTCTTTGGGTTGAATTTTTAAAAAACGAACAAGAGGTGCTTAACCTA GGAGAGATTCTGGGTCACTAGCCTTAGTTCTTATCATCAGTTAGGCTCCAGTGGGTTAAATCTCTTCTTTTTGTTACCT
TGTCCTCTGCCATTTCTCAAAGCGTTCTGAGTTTTCTACTACAGAAGTCAAGTATAAGGTCAAATTAATTTTCAAGCTGAT tGCATTTTTTTACTAAATAGTTCTTATTCTCGTCCTTAGGAATAAAA
AGCCAGAGGCTCACTGTTTCACCTGGGAAATAATCTATTTACTGAGACTAGCTACATGGTTAGGAGACGATAAGGATTCCTAGGATTAAGAACATTGTGAAAAGCCAGCAC aGTGGCTCACACCTATA
ATCCCAGCTCTTTGGGAGGCGTAGGCAGGAGAATCATAGTGAGTTATGATCGTGTCATTGTACCCCACCACAGCCTGAGCGACTCAGCGAGACCCTGTCTCTCAAAAAAGAAAAAAAAATATTACTGA
GCCTGGAGAGTTTGAGGAATGAGTTAGACAAGAACAAAGGGACTTCATTTATTGAGTACCTTTTTTCGCTCCGTGCAGTGTACCAAGCACTTTTATG CAGTCTTTCCTTCAATCTTCACTAACTACAA
ACCTAAGAGGTAAATATTTCTCTTGCAAAGTGAGAAACTAAAGTTAGGAGGGATTAAGTAGTCTGCCCAAAGTCACAGAGCTAATAACTATAATTACGATAGAGCTAAACCAAGCAGAAATGTAAAAC
CAGTAGTGTTT

273	15.0	0.0	0.0	Scaffold4172-702091-702405	1	40	(2275)	+	Alu	SINE/Alu	254	293	(8)	1
1455	25.8	5.9	7.6	Scaffold4172-702091-702405	42	268	(2047)	+	L1ME4a	LINE/L1	5490	5722	(402)	2
1970	15.7	12.3	7.1	Scaffold4172-702091-702405	269	754	(1561)	C	LTR54	LTR/ERV1	(0)	510	1	3
1455	25.8	5.9	7.6	Scaffold4172-702091-702405	755	941	(1374)	+	L1ME4a	LINE/L1	5723	5918	(206)	2
31	0.0	0.0	0.0	Scaffold4172-702091-702405	942	970	(1345)	+	(T)n	Simple_repeat	1	29	(0)	4
1455	25.8	5.9	7.6	Scaffold4172-702091-702405	971	1000	(1315)	+	L1ME4a	LINE/L1	5919	5933	(191)	2
2541	2.1	0.4	0.0	Scaffold4172-702091-702405	1001	1315	(1000)	C	AluYg6	SINE/Alu	(0)	311	1	5
1455	25.8	5.9	7.6	Scaffold4172-702091-702405	1316	1479	(836)	+	L1ME4a	LINE/L1	5934	6090	(34)	2
13	23.2	0.0	2.4	Scaffold4172-702091-702405	1480	1521	(794)	+	(TATTATA)n	Simple_repeat	1	41	(0)	6
551	20.7	17.1	3.1	Scaffold4172-702091-702405	1900	2039	(276)	+	FRAM	SINE/Alu	6	164	(12)	7
486	26.2	5.9	3.5	Scaffold4172-702091-702405	2094	2263	(52)	C	MIRb	SINE/MIR	(14)	254	81	8

>hg19 chr4:77022877-77024808

CCTGGGTGACAGAGTGAGACTCCATCTTGGAAAAAAAAAAATTACTCAGATGTCCTCAAGGATACCGGGACAGGAACAGTCTCATAAATTACTGGCGAGATTATAAATTGGAACTTTTTTGGAGAGC AA
TTTAGTAGTATGTATTACTAGTCTTAAAAATGTTTGTACCTTTTGACTCGGTTATAACACTTTTGGATATTTGTCCCAAGGAAATTAGAGATGAACACAAAGAT TTGTGTACAAAAATGCCTATCACA
GTGTTATGGTAGTGTCAAAAGACAAAATTACAACAAATTTAAAGAGATCAATTGGCTTTATTTGAGATTCTAGAATTGGGCAACACTTTATTCCATAGAATAAGC aTTCATTGAGCTGAGCACAGAAG
TTTGGCTTTAAAGACAGAGAAGAGCCAAAGAAAGTGGAAGCAAAGAACAGAGTATATTAATTACTTTTAGACAGAACAGTAGAAAAATAACTGATTAGTTAACA TCAGTTTATTTCAGGCTACCTTTT
TTGTGTGTAAGGATTGAAGCATCGGGAACTTCATTTATCAAGCTGATTGAAGATTTTAAAATGGCCAATTTGGGAAATTCTGTTATTTCTTTCTCCTGATTTCTTAGAAG tTCAGACAACTTAGTTTA
GATGATGTGGAACTTTAGCATGGGTGACTCCATTTCGATTTTTAGTCTGGTCTGTTGGGGCCTTCTGCAGAAACTTAGTCTGAAGCAATGGCCTCCTATAATTT TTATTTGACAGTAGCAAAAATTTA
GAAACAACCTGTGTCCAACCATTGGGTATTGTTAAATAAAATATTA cCTATATTATGAAACACTAAGCAACTTAAAAATCATGTTTTCAAAACCATCTAAAGATAGAAGAAAGTACTCTTAATACAAT
GGTAAATAATTGGATATAgAACTGCATATAGAGTATTAATATTAACTTTTAAAAATGTATATGTATATATATGTATGCATAGCAAAATGATTGAAATACACCAGAAATGTTAAGAATGAGTAGAGTATT
TCCGAGATCTGCAGTTTTAAGAGAACTTTAATTTTTAATAATTTTCTGTATTTTTCCAAATTATCTATAACGTATTATATAGTTAGGAAGCATATTTTATATATTACATATAATGCCAATTTTGG GTC
GAGGTCTTTGGGTTGAATTTTTAAAAAACGAACAAGAGGTGCTTAACCTAGGAGAGATTCTGGGTCACTAGCCTTAGTTCTTATCATCAGTTAGGCTCCAGTGGGTTAAATCTCTTCTTTTTGTTACC

TTGTCCTCTGCCATTTCTCAAAGCGTTCTGAGTTTTCTACTACAGAAGTCAAGTATAAGGTCAAATTAATTTTCAAGCTGAT gGCATTTTTTTACTAAATAGTTCTTATTCTCGTCCTTAGGAATAAA
AAGCCAGAGGCTCACTGTTTCACCTGGGAAATAATCTATTTACTGAGACTAGCTACATGGTTAGGAGACGATAAGGATTCCTAGGATTAAGAACATTGTGAAAAGCCAGCAC gGTGGCTCACACCTAT
AATCCCAGCTCTTTGGGAGGCGTAGGCAGGAGAATCATAGTGAGTTATGATCGTGTCATTGTACCCCACCACAGCCTGAGCGACTCAGCGAGACCCTGTCTCTCAAAAAAGAAAAAAAAATATTA CTG
AGCCTGGAGAGTTTGAGGAATGAGTTAGACAAGAACAAAGGGACTTCAT TTATTGAGTACCTTTTTTCGCTCCGTGCAGTGTACCAAGCACTTTTATGCAGTCTTTCCTTCAATCTTCACTAACTACA
AACCTAAGAGGTAAATATTTCTCTTGCAAAGTGAGAAACTAAAGTTAGGAGGGATTAAGTAGTCTGCCCAAAGTCACAGAGCTAATAACTATAATTACGATAGAGCTAAACCAAGCAGAAATGTA AAA
CCAGTAGTGTTT

273	15.0	0.0	0.0	hg19	1	40	(1892)	+	Alu	SINE/Alu	254	293	(8)	9
1653	25.2	7.5	1.8	hg19	42	268	(1664)	+	L1ME4a	LINE/L1	5490	5722	(402)	10
1926	16.2	12.3	7.1	hg19	269	754	(1178)	C	LTR54	LTR/ERV1	(0)	510	1	11
1653	25.2	7.5	1.8	hg19	755	1096	(836)	+	L1ME4a	LINE/L1	5723	6091	(33)	10
13	23.2	0.0	2.4	hg19	1097	1138	(794)	+	(TATTATA)n	Simple_repeat	1	41	(0)	12
566	20.0	17.1	3.1	hg19	1517	1656	(276)	+	FRAM	SINE/Alu	6	164	(12)	13
486	26.2	5.9	3.5	hg19	1711	1880	(52)	C	MIRb	SINE/MIR	(14)	254	81	14

>KSI_AluYg6_3

>Scaffold11661-935338-935394
ACAAATTTGCTGTGACACCCTGGGCAAAACCTTTCCCTCTGAGCCTCACTTTCCCCATAGCAGCTCAAGGGAGCTGGTTTTCTCAGTCCCTTCCAGCTCTAACATGAATAGAAATCTGTGGTCTG GAG
CATTTTGCACGCaCTCAGCAAAACTGTGATGTCATGGAGCACACACCCACAGACTTTTCTGCTCACAGGAGGATTGTAGATGCTCTGTGG AGCACCTAAATCACAGCAGGGACAGTCTGTGAGGCGCC
AAGAGAGAGTCCTCCCCACAGCCTCCATGTTACTGATCATGGCATTCCAGCAATTAACAGGGTCAGGGGGCAGTCCACTAGCCACCAGCCCTGCCCTGAGAATGCGCAAACCTGAGACCAA GGCTCCT
GCCCCCAGCCCAGAGCAGGCAGACTTACATCTTCTCAAACTAGACAGTCTAGTATTGATTCTTCTCCCCGCTTAATCCATGGGAAGGAATTCCCAGACTTTCCCACCAGTTGCAGAGCAGCTTTACAA
TAATTTGATCCAAACTGATCATCTCCTGGTTTTATATCCTGTGGCATATCCCTTGATCCCAGGTTTTGCTGACACCACAGCAAGGGATTGGAGGGCGTGTGTGTACACGCGTGCGTGTGTGTGTG TAT
GCATGTACCTGTATGTGTGTGCACATGCATGTGTGTGCGTGTGTGTGTGCGCGCGTGTGTGTAGATACCCACATCTGGAGAAACAGAACCAGAGAAACTCTGTCCACAGCTGAGGGAAGAAGTCACAT
TTCCTGGCAAAGAATGTGTTCATGGAGGGTAAATTTCGAGGcgcagggctcagaactgcagaggagaaacaggctcgggaggaacaagcaacccttgctggcagcatcctttaaaaacattttttttct
ggccaggcgtggtggctcatgcctgtaatcccagcactttgggaggccatgaCAGGCTCAGCTCTGTCATGGACAGCCTGGGGGCTCTGGACAAGTGTGCAGCCTCCCAGGGCCTCGCTTTCTTCTGT
GGGGAGGCACTCACAAGGCGCTCCAGTGAGAGGGGCCATGGGTATCCCTCTCCCCTGACAGTCCTTCCCTCCGGAGCTGGATCCGAGCATTGGGTCCCAGGACCTCATCACTATCCTCTGTCTCC CCA
TAGATTCCTCTTCTCCTGTAAATGACCACAGAGGAAGACAATGAATGAGGTGAAAGAATCCCTTCGCAGCATCGAGCAGAAGTACAAGCTCTTCCAGCAGCAGCAGCTCACCTTCACCGCCGCTCTGG
AGCACTGCAGGGAGAACGCCCACGACAAGATCCGGCCCATCTCCAGCATTGGACAGGTGGGGCTCCCGACCCCCACCCCGGCCGAGGGGCAACAAGC CCAGTCCCCTCTCACAAAAGATGAAACCACT
GGGGCCCAGAGAGGGGAAGGACGTTTGCGCCAGAATTAGGACCCGGACCCAGAACCTAAATGATGTAATGAGTCCTTCATTCGTTTCTCTACCAAATATGTAAGTGAATAACAACAAGTGAATAA AGA
GGCAGGTTTAAAACTCAGCCGGCCAGGGCTTAAGTCCTAGCTCTACCATGTGCCACTGCTGTGATCTCAGGCCAATGACTTCACCTTTCTGTGCCTCAGTGTCCTCATCTGTGACGC AGGTTCAGTGG
TCACCCATAGAAAACCAGGGATTTACTGGTGACCAGAGGGATCCTGTCCTTCCCATCAGATTCCACTGGGGGTTGAGGAACTGTGGGATGCCAAGCACACCGCCAGGTGCTTTCGCAGATGTTTT TAT
TTAACCTGCATCTCCTCCAGGGTTAGCTGCTGTCCAGGTTTGCTGTGCAGGACTTGGAGGCCTCAAGACCAAATGATCCTTCCAGGATGTTAGGGGCTGGAGCTGCAGTGCAGTTAG GAACACACACA
GGCCGGCCCTTCACAGCTTGGAGGGAGTCAGCT

272	24.8	12.4	0.0	UnnamedSequence	1	105	(1848)	+	MIR3	SINE/MIR	73	190	(18)	1
33	33.1	0.0	0.0	UnnamedSequence	609	702	(1251)	+	(GT)n	Simple_repeat	1	94	(0)	2
452	10.5	0.0	0.0	UnnamedSequence	897	953	(1000)	+	AluYg6	SINE/Alu	1	57	(254)	3
413	27.6	2.4	0.8	UnnamedSequence	1527	1650	(303)	+	MIR3	SINE/MIR	14	139	(69)	4

>hg19 chr9:135758177-135760095
CACTGACGATGCTGGTTAGACGTAAGGAAGCCTAGGACCCAGGGTGTCTTGGGCACAAATTTGCTGTGACACCCTGGGCAAAACCTTTCCCTCTGAGCCTCACTTTCCCCATAGCAGCTCAAGGG AGC
TGGTTTTCTCAGTCCCTTCCAGCTCTAACATGAATAGAAATCTGTGGTCTGGAGCATTTTGCACGCgCTCAGCAAAACTGTGATGTCATGGAGCACACACCCACAGACTTTTCTGCTCACAGGAGGAT
TGTAGATGCTCTGTGGAGCACCTAAATCACAGCAGGGACAGTCTGTGAGGCGCCAAGAGAGAGTCCTCCCCACAGCCTCCATGTTACTGATCATGGCATTCCAGCAATTAACAGGGTCAGGGGGCAGT
CCACTAGCCACCAGCCCTGCCCTGAGAATGCGCAAACCTGAGACCAAGGCTCCTGCCCCCAGCCCAGAGCAGGCAGACTTACATCTTCTCAAACTAGACAGTCTAGTATTGATTCTTCTCCCCGCTTA
ATCCATGGGAAGGAATTCCCAGACTTTCCCACCAGTTGCAGAGCAGCTTTACAATAATTTGATCCAAACTGATCATCTCCTGGTTTTATATCCTGTGGCATATCCCTTGATCCCAGGTTTTGCTG ACA
CCACAGCAAGGGATTGGAGGGCGTGTGTGTACACGCGTGCGTGTGTGTGTGTATGCATGTACCTGTATGTGTGTGCACATGCATGTGTGTGCGTGTGTGTGTGCGCGCGTGTGTGTAGATACCCACAT
CTGGAGAAACAGAACCAGAGAAACTCTGTCCACAGCTGAGGGAAGAAGTCACATTTCCTGGCAAAGAATGTGTTCATGGAGGGTAAATTTCGAG GtCAGGCTCAGCTCTGTCATGGACAGCCTGGGGG
CTCTGGACAAGTGTGCAGCCTCCCAGGGCCTCGCTTTCTTCTGTGGGGAGGCACTCACAAGGCGCTCCAGTGAGAGGGGCCATGGGTATCCCTCTCCCCTGACAGTCCTTCCCTCCGGAGCTGGA TCC
GAGCATTGGGTCCCAGGACCTCATCACTATCCTCTGTCTCCCCATAGATTCCTCTTCTCCTGTAAATGACCACAGAGGAAGACAATGAATGAGGTGA AAGAATCCCTTCGCAGCATCGAGCAGAAGTA
CAAGCTCTTCCAGCAGCAGCAGCTCACCTTCACCGCCGCTCTGGAGCACTGCAGGGAGAACGCCCACGACAAGATCCGGCCCATCTCCAGCATTGGACAGGTGGGGCTCCCGACCCCCACCCCGG CCG
AGGGGCAACAAGCCCAGTCCCCTCTCACAAAAGATGAAACCACTGGGGCCCAGAGAGGGGAAGGACGTTTGCGCCAGAATTAGGACCCGGACCCAGA ACCTAAATGATGTAATGAGTCCTTCATTCGT
TTCTCTACCAAATATGTAAGTGAATAACAACAAGTGAATAAAGAGGCAGGTTTAAAACTCAGCCGGCCAGGGCTTAAGTCCTAGCTCTACCATGTGCCACTGCTGTGATCTCAGGCCAATGACTT CAC
CTTTCTGTGCCTCAGTGTCCTCATCTGTGACGCAGGTTCAGTGGTCACCCATAGAAAACCAGGGATTTACTGGTGACCAGAGGGATCCTGTCCTTCC CATCAGATTCCACTGGGGGTTGAGGAACTGT
GGGATGCCAAGCACACCGCCAGGTGCTTTCGCAGATGTTTTTATTTAACCTGCATCTCCTCCAGGGTTAGCTGCTGTCCAGGTTTGCTGTGCAGGACTTGGAGGCCTCAAGACCAAATGATCCTT CCA
GGATGTTAGGGGCTGGAGCTGCAGTGCAGTTAGGAACACACACAGGCCGGCCCTTCACAGCTTGGAGGGAGTCAGC T

277	24.7	10.8	0.3	hg19	12	159	(1710)	+	MIR3	SINE/MIR	30	190	(18)	1
33	33.1	0.0	0.0	hg19	663	756	(1113)	+	(GT)n	Simple_repeat	1	94	(0)	2
413	27.6	2.4	0.8	hg19	1443	1566	(303)	+	MIR3	SINE/MIR	14	139	(69)	3

>KSI_AluYg6_4

>Scaffold14554-625319-625621
AGTCTCCTCCTTCCTGCTTTATCTGCAAGGGAGCCACCCCTGCTAGAGCTGCTTCTCCCTAGGGTTAACCCTGGGGTGCCCTCACACCAAGGGGCCCAGGTGTGAATCTTGGATCTATTACTAATT CA
AGAGCAGAGACTTTCAGTTCTTTGGCAATTTTTCTCATTTCTAAAATAGGGCTCAAAATAATAGTTCCAAAAAATAAAATAATAGTCCTCCCTGTCTCACAGCCTTTTTTTTTTTTT TGGAGGTGAAA
ATAATAAGATTTCCAATTTCACTCTGGCCTATCCTACTCCCAACCTCCTACTCCCTCTCTCTTCCACAGTGTGTTCTGGAAGTTAACTCCCTGCATCCTCCTGCTCTGGGAGTCCTGTTCGTCATC TC
ATGCCCAACATAACACAGAGCCAGGAGGAGAGACGGGTGTTCTCCTAGCTCTGTGTTGCCACGTGGACCCTGTCATTCTTCCACACT cTGGCAATAGTCCACCTTTCTGGAAGCACCACTGCCTGATC
TCCCACTGTTTAATCCTTCTCATCACTGTCCGCTTCTCAATCGTTCTTTCAGTCTAAGGGTTTTGATGTTAGTCCTCCTCTTCATTCTGTCCTGCATCACTGTGGAAAATCTCCTTGCTCAGGAGC AT
GACCCATCAATGCCCTAACTTTACACAGCCTTGATGTCCCCGGCTCCAGCACCCTCCACCTGCACTCCAGCGATGCTCCCAGGGCTACACCTTTAATCTTCTCAGCACCCCAAGCCCCTCTGCGG CTA
ACATCTTCACTTCCAGGATTCCTGCTTTCTGATCCCAACATTCCACCCTTTGGGCTTTCTCACTCCATTAACTCAACACCAGCTTTTCTACTCCATTGAGGTCCCCAGCTCCTCAATTCTTGTCTTTT
TTTCTGGACCCATCAGCTCCTCCCGGTTTTCaTTACTTCCCTACAAAGCCTGGCCCCATGGTGGAGTATATCAACCCGGCTCTCagaaacctcatctattgtcggccgggcgcggtggctcacgcctg
taatcccagcactttgggaggccgagacgggcggatcacgaggtcaggagatcgagaccatcctggctgacacggtgaaaccccgtctctactaaaaatacaaaaattagccgggcatggtggcgc gc
gcctgtagtcccagctactcgggaggctgaggcaggagaatggcgtgaacccgggaggcggagcttgcagtgagtcgagatcgcgccactgcgctccagcctgggcgacagagcgaaactccgtc tca
aaaaaaaaaaaaaaaaaAGAAACCTCATCTATTGTCTTCCTACCTCACCTTCTCTAAAACTCAATACGGCACTCCAAATTAATCAAATTGTCTACTCTCCCTGCACTCATAGCC tGATTATGGTGCTG
CTGGAGAATAAAAAAAAAAAAaaTCACACTGCCATACAGATGAGGTTGCCACAGAGCCACAGTCCCAATCCCAGGAGGCTCAGCCCCAGATGGCAGCTCCACCCCACACCCTCACCATCCTTCCCAGC
CCCCtGACCTCCCCTGCCTCTGTCCTCCCGACAATGGACCTGCTCCTCCACAGAGAAATTTGAGGCTATTAGACATGCACTCCCTCAATGTCACACCCTTTTACCTAGAAAATGACTGTCCTTACACT
ATCTTTGCCTCTTCCCCTCCAATGTCAAAAGAAGAGGTGCTGCCCTCCTTCCAA GGCTAAACACTTCTAACCCATTGTGTCCTCTGAGACTTAGCTCCACCGATCATCTCCTCTCAAATATGTTTCTG
ATGCTTCCTCCTCCACTGATGTCTTCTCCTTAGCAGATGCACTCAAGTCTCTCCATCTGAAAATGTCTCTGTCTCTCTCTCATGCTCCCACACACATACCCAAATTCCCCCTTCAATACACCCAA TGT
CCACTGTGAGGTAATACCTTCCCTTACTTACTCTTCAGTCCTAGGCTCCTGGGA AGAGTCTCAACTTTCTCTCCCTCCAACACTCCTAAACCTCCTCTACCTATTTTCTGAAATGGCTGTTGCACTGT
TATGGAATTAAGGCCACTCACTCTAAAGCACTGGGCAGTCTTAACCTTTCTGACTTTCCTGAAACTCTTCTTTGGATTTCTACAAGACTATTGTTTCCCTTTCAATACCTCCATCTGTGGCCACT CCT
TCTCAATCTCCTCTACTTCCACTCTCCAAGTGTTGGTGGGCCGCAATCACACTT TTATTATTTTATTTTTATTTTCTCATTTTTACATCGTGCCTGTGTTACCTCATCCATACCCATTGCTTGAG C

320	34.6	7.2	6.8	UnnamedSequence	639	999	(1303)	+	L2	LINE/L2	808	1184	(2235)	1
2794	1.3	0.3	0.0	UnnamedSequence	1000	1302	(1000)	+	AluYg6	SINE/Alu	1	304	(7)	2
320	34.6	7.2	6.8	UnnamedSequence	1303	1439	(863)	+	L2	LINE/L2	1185	1308	(2111)	1

>hg19 chr1:204237474-204239458
AGTCTCCTCCTTCCTGCTTTATCTGCAAGGGAGCCACCCCTGCTAGAGCTGCTTCTCCCTAGGGTTAACCCTGGGGTGCCCTCACACCAAGGGGCCCAGGTGTGAATCTTGGATCTATTACTAATT CA
AGAGCAGAGACTTTCAGTTCTTTGGCAATTTTTCTCATTTCTAAAATAGGGCTCAAAATAATAGTTCCAAAAAATAAAATAATAGTCCTCCCTGTCTCACAGCCTTTTTTTTTTTTT TtGGAGGTGAA
AATAATAAGATTTCCAATTTCACTCTGGCCTATCCTACTCCCAACCTCCTACTCCCTCTCTCTTCCACAGTGTGTTCTGGAAGTTAACTCCCTGCATCCTCCTGCTCTGGGAGTCCTGTTCGTCATCT
CATGCCCAACATAACACAGAGCCAGGAGGAGAGACGGGTGTTCTCCTAGCTCTGTG TTGCCACGTGGACCCTGTCATTCTTCCACACTaTGGCAATAGTCCACCTTTCTGGAAGCACCACTGCCTGAT
CTCCCACTGTTTAATCCTTCTCATCACTGTCCGCTTCTCAATCGTTCTTTCAGTCTAAGGGTTTTGATGTTAGTCCTCCTCTTCATTCTGTCCTGCATCACTGTGGAAAATCTCCTTGCTCAGGA GCA
TGACCCATCAATGCCCTAACTTTACACAGCCTTGATGTCCCCGGCTCCAGCACCCTCCACCTGCACTCCAGCGATGCTCCCAGGGCTAC ACCTTTAATCTTCTCAGCACCCCAAGCCCCTCTGCGGCT
AACATCTTCACTTCCAGGATTCCTGCTTTCTGATCCCAACATTCCACCCTTTGGGCTTTCTCACTCCATTAACTCAACACCAGCTTTTCTACTCCATTGAGGTCCCCAGCTCCTCAATTCTTGTCTTT
TTTTCTGGACCCATCAGCTCCTCCCGGTTTTCgTTACTTCCCTACAAAGCCTGGCCCCATGGTGGAGTATATCAACCCGGCTCTCAGAAACCTCATCTATTGTCTTCCTACCTCACCTTCTCTAAAAC
TCAATACGGCACTCCAAATTAATCAAATTGTCTACTCTCCCTGCACTCATAGCC aGATTATGGTGCTGCTGGAGAATAAAAAAAAAAAATCACACTGCCATACAGATGAGGTTGCCACAGAGCCACAG
TCCCAATCCCAGGAGGCTCAGCCCCAGATGGCAGCTCCACCCCACACCCTCACCATCCTTCCCAGCCCCC cGACCTCCCCTGCCTCTGTCCTCCCGACAATGGACCTGCTCCTCCACAGAGAAATTTG
AGGCTATTAGACATGCACTCCCTCAATGTCACACCCTTTTACCTAGAAAATGACTGTCCTTACACTATCTTTGCCTCTTCCCCTCCAATGTCAAAAGAAGAGGTGCTGCCCTCCTTCCAAGGCTA AAC
ACTTCTAACCCATTGTGTCCTCTGAGACTTAGCTCCACCGATCATCTCCTCTCAAATATGTTTCTGATGCTTCCTCCTCCACTGATGTC TTCTCCTTAGCAGATGCACTCAAGTCTCTCCATCTGAAA
ATGTCTCTGTCTCTCTCTCATGCTCCCACACACATACCCAAATTCCCCCTTCAATACA CCCAATGTCCACTGTGAGGTAATACCTTCCCTTACTTACTCTTCAGTCCTAGGCTCCTGGGAAGAGTCTC
AACTTTCTCTCCCTCCAACACTCCTAAACCTCCTCTACCTATTTTCTGAAATGGCTGTTGCACTGTTATGGAATTAAGGCCACTCACTC TAAAGCACTGGGCAGTCTTAACCTTTCTGACTTTCCTGA
AACTCTTCTTTGGATTTCTACAAGACTATTGTTTCCCTTTCAATACCTCCATCTGTGGCCACTCCTTCTCAATCTCCTCTACTTCCACTCTCCAAGTGTTGGTGGGCCGCAATCACACTTTTATT ATT
TTATTTTTATTTTCTCATTTTTACATCGTGCCTGTGTTACCTCATCCATACCCATTGCTTGAG

183	35.7	8.3	4.2	UnnamedSequence	640	1121	(862)	+	L2	LINE/L2	808	1308	(2111)	1

A**l**u**Yh9**

Instruction
Green box: Primer information
Red letter: Target site duplication
Black letter: Insertion sequences
Green letter: Deleted sequenced by insertion-mediated deletion

>KSI_AluYh9_1

>Scaffold9875-2429-2475
TGCTATTTTCCTCCAGGGATCATGTAAGCTGATCATGCAAGAGTTTACCTAATCCATAGCTTCCTCCGTTGCTAAGAGCTCCAGCATTCTCCAGCTGTACTCCATAAAGTCCTTCCTATACATCCC AT
CCTTTACTTACAGGACATATTCTTCCTACTGATGCTCAAGACCAAACCATTTTAGAAA tATATGTCTCATAGCAGAATTGTGCAAGTTTACTAAAAATGCTCTCAGATGTTTTATATCATGTTTGGTG
CCCTGAAAGTAATTTATGAATGGTTGCTAATGGAAAACACCTTCATTTCCTTCTTCAGCCTTTTTTCAACTCAATCAGGATAACTTTTTGTTCCCAAAACAGTCAAGCTTTACACTTCAGTATCT TTC
TTGTTTGTCTGTCAACCACTCTCAAGGATTTTCTATTTTCTTGCATATTTGTGGATAGTTTTAAAATCATTTAAGAACAGTATAGAGATTTTACATTTCATGTTATATTTAAAACCATTAATATGATT
TTATTTTAATGTAAGCTTAAaTATTTACAACTCCTGTCATATAATTTAATATTTCTTTTTGGGTAATAAGAGTGCTCTCAGTTTTTCTGGTTATAGTGAAGTTGCAGAAAAATTGTTTGTAAATA ATA
TTTTAAAAAGTTATTTACGTAAATTTTGAGTAGTGGGAAGGAAAAGACAAAATATTGGT GCATTTTTTTAGGTTCTGGTTAAACCCTAGCATGTTGTTTATAAACCTATACATATCTGTTTATGCTGA
CTCAAACTATGTATATGTAAACTGTTTCTACCATAACTGTATAGGCTTAAATATTTAATCTTACTAATTTTATAATAAAACATTCTACTGTTTTTTACAA aagtatttacattttttttGAGACGGAG
TCTCGCTCTGTCGCCCAGGCTGGAGTGCAGTGGCGGGATCTCGGCTCACTGCAAGCTCCG cctcccgggttcacgccattctcctgcctcagcctcccaagtagctgAAGTATTTACATTTTTTGAAC
ACTTTCCATTTACTTGTTTACTATTTTGCTTTAACTGTATGAATTATTTATGCCTGTACCTTTCTACTGGAACATCACTGATTTTCTCAGTAAATTGAATCAGTTTTTAATATTATAAT TTTTTATTG
TACATGATTTGCATGGAATTTCCAATTAATTTTCTTCTTGAATTTGATGTTTATTGGGGCTACAAAT GCCCATTTTTGTATTCTCAAATATTTTAGTATTTCACCTTTATGAAGTAAGAAACCTGATA
CTTAAAAAAAGTATTTCTTATGTCTCAATAAATGTCAAATTCATTTTGCATAGATATTGTACTGGATATTACAAATCACACACGGTAGAAACTGATGTCTCAAATAGTGCTGCTCCTAT CTGAACATT
CTATATTGTTAACTCCTGACTTCCTGACTGTAATTCAGTCTCCACGCTCATGAAACCGTGGTCCGTCTGTCTATGCATCTCACTTGCTTAAAATCCATCCATGGCTCCCTGATCACTCTCTGAGT AAA
GTTCAGTCTcCTTGTCAGGTCTATAAAATCTTAAAATCTTTTCTGATTCCATACCTCACATCACTCATCACAGTGAATACCCTGAGTTTTAGTCATGCTAACTTTTTGAAACTTCCCAA TTATGTTTT
TTCTTTATCTTGTTGTTGGTCTTTCACATTCTGTTTCTCCTAAGTATTGATTTTCTCCGCATCTACCTACCAAAATTATATGCAAGTTTGAACCTCACATGAAATATCATCTCCTTTCAGATCTA TTT
CCTGGCCTCCAAGAGGGTGTTTATTTTTTGTCTAAATTCTTGTAGTGTCTTTCATCTTTTTCTATTAATGTGCTTATTATATTGTTATAGTTTTTATATGTGGGATTTTGTATCTGTTA TATCTATAA
GCTGCCTTTAACTCACTGCTGCTGACCTGTGTGATCAAGTTGCAGGAGAAAAAATTTCTTGTTACATAGGCTGATGCAAAAA G

32	0.0	0.0	0.0	UnnamedSequence	880	909	(1138)	+	(T)n	Simple_repeat	1	30	(0)	1
440	4.3	0.0	0.0	UnnamedSequence	1001	1047	(1000)	C	AluYh9	SINE/Alu	(99)	212	166	2
185	25.9	9.1	5.3	UnnamedSequence	1528	1637	(410)	+	L2	LINE/L2	2823	2936	(483)	3
12	28.8	0.0	0.0	UnnamedSequence	1706	1747	(300)	+	(TTTCTC)n	Simple_repeat	1	42	(0)	4

>Hg19 chr1:196779929-196781796
TGCTATTTTCCTCCAGGGATCATGTAAGCTGATCATGCAAGAGTTTACCTAATCCATAGCTTCCTCCGTTGCTAAGAGCTCCAGCATTCTCCAGCTGTACTCCATAAAGTC CTTCCTATACATCCCAT
CCTTTACTTACAGGACATATTCTTCCTACTGATGCTCAAGACCAAACCATTTTAGAAA aATATGTCTCATAGCAGAATTGTGCAAGTTTACTAAAAATGCTCTCAGATGTTTTATATCATGTTTGGTG
CCCTGAAAGTAATTTATGAATGGTTGCTAATGGAAAACACCTTCATTTCCTTCTTCAGCCTTTTTTCAACTCAATCAGGATAACTTTTTGTTCCCAAAACAGTCAAGCTTT ACACTTCAGTATCTTTC
TTGTTTGTCTGTCAACCACTCTCAAGGATTTTCTATTTTCTTGCATATTTGTGGATAGTTTTAAAATCATTTAAGAACAGTATAGAGATTTTACATTTCATGTTATATTTAAAACCATTAATATGATT
TTATTTTAATGTAAGCTTAAgTATTTACAACTCCTGTCATATAATTTAATATTTCTTTTTGGGTAATAAGAGTGCTCTCAGTTTTTCTGGTTATAGTGAAGTTGCAGAAAAATTGTTTGTAAATAATA
TTTTAAAAAGTTATTTACGTAAATTTTGAGTAGTGGGAAGGAAAAGACAAAATATTGGTGCATTTTTTTAGGTTCTGGTTAAACCCTAGCATGTTGTTTATAAACCTATACATATCTGTTTATGC TGA
CTCAAACTATGTATATGTAAACTGTTTCTACCATAACTGTATAGGCTTAAATATTTAATCTTACTAATTTTATAATAAAACATTCTACTGTTTTTTACA AAAGTATTTACATTTTTTGAACACTTTCC
ATTTACTTGTTTACTATTTTGCTTTAACTGTATGAATTATTTATGCCTGTACCTTTCTACTGGAACATCACTGATTTTCTCAGTAAATTGAATCAGTTTTTAATATTATAATTTTTTATTGTACA TGA
TTTGCATGGAATTTCCAATTAATTTTCTTCTTGAATTTGATGTTTATTGGGGCTACAAATGCCCATTTTTGTATTCTCAAATATTTTAGTATTTCACCTTTATGAAGTAAGAAACCTGATACTTAAAA
AAAGTATTTCTTATGTCTCAATAAATGTCAAATTCATTTTGCATAGATATTGTACTGGATATTACAAATCACACACGGTAGAAACTGATGTCTCAAATAGTGCTGCTCCTATCTGAACATTCTAT ATT
GTTAACTCCTGACTTCCTGACTGTAATTCAGTCTCCACGCTCATGAAACCGTGGTCCGTCTGTCTATGCATCTCACTTGCTTAAAATCCATCCATGGCTCCCTGATCACTCTCTGAGTAAAGTTCAGT
CTtCTTGTCAGGTCTATAAAATCTTAAAATCTTTTCTGATTCCATACCTCACATCACTCATCACAGTGAATACCCTGAGTTTTAGTCATGCTAACTTTTTGAAACTTCCCAATTATGTTTTTTCTTTA
TCTTGTTGTTGGTCTTTCACATTCTGTTTCTCCTAAGTATTGATTTTCTCCGCATCTACCTACCAAAATTATATGCAAGTTTGAACCTCACATGAAATATCATCTCCTTTCAGATCTATTTCCTGGCC

TCCAAGAGGGTGTTTATTTTTTGTCTAAATTCTTGTAGTGTCTTTCATCTTTTTCTATTAATGTGCTTATTATATTGTTATAGTTTTTATATGTGGGATTTTGTATCTGTTATATCTATAAGCTG CCT
TTAACTCACTGCTGCTGACCTGTGTGATCAAGTTGCAGGAGAAAAAATTTCTTGTTACATAGGCTGATGCAAAAA G

12 28.8 0.0 0.0 UnnamedSequence 1527 1568 (300) + (TTTCTC)n Simple_repeat 1 42 (0) 1

AluYk12

Instruction

Green box: Primer information

Red letter: Target site duplication

Black letter: Insertion sequences

Green letter: Deleted sequenced by insertion-mediated deletion

>KSI_AluYk12_1

>Scaffold10546

GATTATGGTGACAGATATTTTCATGTTTTAATGTTTTAAGATATCTGACTGTAGTAACCTTTGTTTTCATGGTGCAGCTTGTAAGTTAAAGATTACTATCTCAATG
AATGgGTCATTTGAATCCTAAAGAGACACCCATATAGTGTGAGGAATTTTGTTCCCTTTTAAATGGACTTTATTTTTTAGAGCAGTTTTGGGTTCACAGCAAAGTT
GACTAAAAAGTACAGAGTTGCCATATATCCCTTGTTCCCACACAAGCACAGCCTCCCCAGCTATCAATATCTAGCAACAAGaTGGTGCATTTGTTATAATTAATGA
ACCTACTTTTACACATTATTATCACCCAAAGTGAACTGTTTACGTTAGTGTTCATTTTTCTATATTCTGTGGGTTTTGACCAAcGTCCCCACAATTGTAGTATTCT
GTAGAATGGTGTCACTGTCCTAAAAACTGCCTGAGCTTTGTCTAGTTATTGATCTTTTTACTGCTGTGATAGTTTTGTGTTTTCTAGAATGTCATAGAGTTGGAAT
CATAATATATATCCTTTTCAAACTGGCTTCTTTCACTCAGTAATACACATTAAAGATTCCTCAATGTCCTTTCATTTCTTGACAGCTCATTTCTTCCTGGGCACTG
AATAATATTCCATGACGTACCACAACTTATTTACCCATTTACTTACTAAAGAAATTCTTGGTTTCTTCCAAGATTTGACAAATATAAATAAAGCTGCTATAAGCAT
TTGTGTGGAGATTTTTGCACGGATATGCATTTTCAACACATTTGGGTAAATAATACCAAGGAACGTGATTGCTGGATTGCATATCATAATAAGTCTaTGTTTTGCT
TTCTAAGCAACTAACCGTCTACTAAAGTGGCTGtACAATTTTGCATTCCCACCAACAATGAATGAAGAATTGTTGTTCCACATCCTCATCAGCTCTTGGTGTTtTC
AGTTGTCTGACTTTTGACCATTCTAATTTTATCTcattgttgtcttttttgtttgtttgttttttgttttttgagacggagtctcactctttcgcccaagct
ggactgcagtggcgctatcccggctcactgcaagctccgCATTGTTGTCTTAATTTACATTTCCCTGATGACATGTAATGTGGATCATCTTTCCATATATTTATGG
CCATACATATACCTTCTTTGCTGAGGTTATCTGTTTAGGTCTTTtCCTCTTTTTTATCCAAATGTTCATTTTCTTTTTGTTGAATTTTAAGAGTTCTTTGTATAAT
TTAGGTAAAAATCTTTTATTAGATTTGTTTTGTTTTGTACTTATTCTATGCCAGTCTATGGCTTGTTTTCCCATTTGCTTGACAGTGTCATTTACAGAGCAGAAGT
TTTTAATTTTAATTAAGTCTAGTGTATAATGTTTTTTTAAAATAGTGCCTTTGGTGGTACCTGTATAAAGTCATCTCCATGCCCAAGATCATCTAGATTTTCTATG
TTATCTTCTCAGAATTTTTGTTTTGCATTTTACATTGAGGTATATGATCCATTTTGATTTAATTTTTGTGAAGGTTGTAAGGTCTGTATCTAGATCATTTATTTGC
ACGTAAATGCCCACTTGTTCCACCACTGTTTCTTGAAAAGACAATATATGAAAGATTTTTTAAAATCACCTACAATTTAAAAATAATTTATTTATTTACAATATAT
TTCATTTTAGCCTTTACATTTAATACTTGATGTCCATTAATGCTACACATTACTGCATGTTTGAGGGACCATAACATATAAGATAGAATTTTGCCAAATGATAAAG
ATTACTATCGTTGGGCCTGAATTATCTATAAGACTGTGGAGTATGAACGTAAAACAATGAGCCTAGTAGGAGCCTTCTTAGCTGTGGTCTTTTTTTAAGGCCTGCA
TTATATACTAGTTTCCATTAATGATATTTATAGAAAGTTAAACTTAGTGTTTGCCTACCAAGCACCTATACCCAGTAGTAGATGGTTTGCATATAGTTAGTTTCAT
CCTGATATAAAAGCTGCAGGTAATACTGCTATTCCCAATAAAGATAATGAGGCTCAGAAATTTAAACTGCCCAGAATCACACACAT

2697	19.7	8.7	1.1	KS	163	1000	(1100)	C	L1MC1	LINE/L1	(1)	6332	5428	1
824	5.0	0.0	0.0	KS	1001	1100	(1000)	C	AluYk12	SINE/Alu	(0)	311	212	2
2300	18.1	2.3	0.7	KS	1101	1635	(465)	C	L1MC1	LINE/L1	(895)	5438	4895	1
191	24.7	14.7	0.9	KS	1996	2097	(3)	C	MIRb	SINE/MIR	(64)	204	89	3

>hg19 chr13:86360574-86362565

GATTATGGTGACAGATATTTTCATGTTTTAATGTTTTAAGATATCTGACTGTAGTAACCTTTGTTTTCATGGTGCAGCTTGTAAGTTAAAGATTACTATCTCAATG
AATGtGTCATTTGAATCCTAAAGAGACACCCATATAGTGTGAGGAATTTTGTTCCCTTTTAAATGGACTTTATTTTTTAGAGCAGTTTTGGGTTCACAGCAAAGTT

GACTAAAAAGTACAGAGTTGCCATATATCCCTTGTTCCCACACAAGCACAGCCTCCCCAGCTATCAATATCTAGCAACAAGcTGGTGCATTTGTTATAATTAATGA
ACCTACTTTTACACATTATTATCACCCAAAGTGAACTGTTTACGTTAGTGTTCATTTTTCTATATTCTGTGGGTTTTGACCAAtGTCCCCACAATTGTAGTATTCT
GTAGAATGGTGTCACTGTCCTAAAAACTGCCTGAGCTTTGTCTAGTTATTGATCTTTTTACTGCTGTGATAGTTTTGTGTTTTCTAGAATGTCATAGAGTTGGAAT
CATAATATATATCCTTTTCAAACTGGCTTCTTTCACTCAGTAATACACATTAAAGATTCCTCAATGTCCTTTCATTTCTTGACAGCTCATTTCTTCCTGGGCACTG
AATAATATTCCATGACGTACCACAACTTATTTACCCATTTACTTACTAAAGAAATTCTTGGTTTCTTCCAAGATTTGACAAATATAAATAAAGCTGCTATAAGCAt
TTTGTGTGGAGATTTTTGCACGGATATGCATTTTCAACACATTTGGGTAAATAATACCAAGGAACGTGATTGCTGGATTGCATATCATAATAAGTCTgTGTTTTGC
TTTCTAAGCAACTAACCGTCTACTAAAGTGGCTGcACAATTTTGCATTCCCACCAACAATGAATGAAGAATTGTTGTTCCACATCCTCATCAGCTCTTGGTGTTgT
CAGTTGTCTGACTTTTGACCATTCTAATTTTATCTCATTGTTGTCTTAATTTACATTTCCCTGATGACATGTAATGTGGATCATCTTTCCATATATTTATGGCCAT
ACATATACCTTCTTTGCTGAGGTTATCTGTTTAGGTCTTTcCCTCTTTTTTATCCAAATGTTCATTTTCTTTTTGTTGAATTTTAAGAGTTCTTTGTATAATTTAG
GTAAAAATCTTTTATTAGATTTGTTTTGTTTTGTACTTATTCTATGCCAGTCTATGGCTTGTTTTCCCATTTGCTTGACAGTGTCATTTACAGAGCAGAAGTTTTT
AATTTTAATTAAGTCTAGTGTATAATGTTTTTTTAAAATAGTGCCTTTGGTGGTACCTGTATAAAGTCATCTCCATGCCCAAGATCATCTAGATTTTCTATGTTAT
CTTCTCAGAATTTTTGTTTTGCATTTTACATTGAGGTATATGATCCATTTTGATTTAATTTTTGTGAAGGTTGTAAGGTCTGTATCTAGATCATTTATTTGCACGT
AAATGCCCACTTGTTCCACCACTGTTTCTTGAAAAGACAATATATGAAAGATTTTTTAAAATCACCTACAATTTAAAAATAATTTATTTATTTACAATATATTTCA
TTTTAGCCTTTACATTTAATACTTGATGTCCATTAATGCTACACATTACTGCATGTTTGAGGGACCATAACATATaaAAGATAGAATTTTGCCAAATGATAAAGAT
TACTATCGTTGGGCCTGAATTATCTATAAGACTGTGGAGTATGAACGTAAAACAATGAGCCTAGTAGGAGCCTTCTTAGCTGTGGTCTTTTTTTAAGGCCTGCATT
ATATACTAGTTTCCATTAATGATATTTATAGAAAGTTAAACTTAGTGTTTGCCTACCAAGCACCTATACCCAGTAGTAGATGGTTTGCATATAGTTAGTTTCATCC
TGATATAAAAGCTGCAGGTAATACTGCTATTCCCAATAAAGATAATGAGGCTCAGAAATTTAAACTGCCCAGAATCACACACAT

3484	19.0	5.7	1.1	hg19	163	1525	(467)	C	L1MC1	LINE/L1	(1)	6332	4895	4
191	24.7	14.7	0.9	hg19	1888	1989	(3)	C	MIRb	SINE/MIR	(64)	204	89	5

HERV-K

Instruction
Green box: Primer information
Emerald box: LTR sequences
Red box: LTR recombination site
Red letter: Target site duplication
Black letter: Insertion sequences
Green letter: Deleted sequenced by insertion-mediated deletion

>KSI_HERVK_1

>Scaffold6057-11637-11951
ATCCCAGATGGGATCCTGTGCCAAATAGTAGGGCACTATATCAACAACCCACGGCGATGGTGTTTGATCCTACAGTACCAGCTAGTGGACAAGATAGTGCACTGCATGAGACCGTTGATACAGCCA GA
AAACAGGGAGATCTTGAGGCATGGCAGTATCCGGTAATGTTACAACCGATACCGGCCGGGAAAGGGCCAGGAAAGGGAGTCAAGCAGGAGCGTCTGTCCGAACGGAGGCTAGGTAAGAATATTTC ACC
ATGAAAATGTTAAAAGACATAAAGGAAGGAGCTAAACAATATGGACCCAACTCTCCTTATATGAGAACGTTATTAGATTCCATTGCTCATGGAAATAGACTTATTCCTTATGATTGGGAAATTTTA CC
TAAATCTTCCCTTTCACCCTCTCAGTATCTACAGTTTAAAACCTGGTGGATTGATGGAGTACAAGAACAGGTACGGAAAAATCAGGCTACTTATCCTGTTGTTAATATAGATGCAGACCAATTGC TAG
GAACACGTCCAAATTGGAGCACTATTAACCAACAATCAGTAATGCAAAATGAGGCTATTGAACAACTAGGGGCTATTTGCCTCAGGGCCTGGGAAAAGATTCAGGACCCAGGAACCAGTTAGAGAC AG
TTTTCAGACTGTTATATCATTCATTATGTTGATGATATTTTGTGTGCTGCAGAAACAAGAGACAAATTAATTGACTTTTACATGTTTCTGCAGACAGAGGTTGCAAACACAGGCCTGACAATAGC ATC
TGATAAGATTCAGACCTCCACTCCTTTTAATTATTTGGGAATGCAGGTAGAGGAAAGAAAAATTAAACCACAAAAAATAGAAATAAGAAAAGACACATTAAGAACATTAAATGACTTTCAAAAATT GC
TAcGAGATATTAATcGGATTGGGCCAACTCTAGGCATTCCTACTTATGCCATGTCAAATTTGTTTTCTATCTTGAGAGGGGATCCAGACTTGAATGGTAAAAG gACAATAACTCCAGAGGCAACTAAA
GAAATTGAATTAGTTGAAGAAAAAATTAATTCAGCACAAGTAAATAGAATAGATCACTTAGCCCCACTCCAATTTTTGATTTTTGCTACTGCACATTCTCCAACAGGCATTATTGTTCAAAATACA GA
TCTTGTGGAGTGGTCATTCCTTCCTCACAGTACAGTTAAGACTTTTACATTGTACTTAGATCAAATGGCTACATTAATTGGTCAGACAAGACTACGAATAGTAAAATTGTGTGGAAGTGACCCAG ATA
AAATCATTGTTCCTTTAAACAAGGAACAGGTTAGACAAGCCTTTATCAATTCTGCTGCATGGCAGATTGGTCTAGCTGATTTTGTGGGAATTATTGATAATCATTACCCAGAAACAAAAATCTTCC AG
TTTTTAAAATTGACTACTTGGATTTTACCTAAAATTACCAGACAAAAACCTTTAGAAAATGCTCTGACAGTGTTTACTGATGGTTTCAGCAATGGAAAAGTGGCTTACACTGGGCCAAAAGAATG AGT
CATTGAAACTCAATATCACTCAGCTCAAAGAGCAGAATTGGTTGCTGTCACTTCAGTGTTACAAGATTTTAATCAGCCTATTAACATTGTTTCAGATTCTGC cTATATAGTACAGGCTACAAAGGCTA
CAAAGCTCTAATCAAATATAGTATGGATTATCAGTTAAATCATCTGTTTAAATTGTTACAACAAACTGTAAGAAAAAGAAATTTCCCAGTTTATATTACTCATATTC aAGCACATACTAATTTACCAG
GGCAATTAACTAAGGAAAATGAACAAGCTGACTTGCTAGTATCATCTGCCTTGATGGAAGCACAAGAACTTCATGTCCTGACTCATGTAAATGCAACAGGATTAAAAAATAAATTTGATATCACAT GG
AAACAGGCAAAAAATATTGTACAACATTGTGCTCAGTGTCAAGTCTTACACCTGCCCACTCAGGAGGCAGGAGTTAATCTCAGAGATTTATGTCCTAATGCATTATGGCAAATGGATGTCACACA TGT
ACCTTCATTTGGAAAATTGTCATTTGTCCGCGTGACAGTTGATACTTATTCACATTTCATATGGGCAACCTGCCAGACAGGAGAAAGTACTTCCCATGTTAAAATACATTTATTATCTTATTTTGC TG
TCATGGGAGTTCCAGAAAAAATTAAAACAGATAATGGGCCAGGATACTGTAGTAAAGCATTTCAAAAATTCTTAAATCAGTGGAAAATTACACATACAACAGGAATCCCTTATAATTCCCAAGGA CAG
GCCATAATTGAAAGAACTAATAGAACACTCAAAGCTCAATTGGTTAAACAAAAAAGGGAAAAAGACAGTAAGGAGTATAACACTTCCCAGATGAACTTAATCTAGCACCCTATACTTTAAATTTTT TA
AACATTTATAGAAATCAGACCACTACTTCTGCAGAACAACATTTTACTGGTAAAAAGAACAGCCCACATGAGGGAAAACTGATTTGGTGGAAAGACAACAAAAGTAAAAC aTGGGAAACAGGGAAGGT
GATAACATGGGGGAGAGGTTTTTCTTGTGTTTCACCAGGAGAAAATCAGCTTCCTGTTTGGATACCCACTAGACATTTGAAGTTCTACAATAAACCCATCCCATCAGAAATGCAAATAAAAGTGCC TC
CACAGAGACAAAAAACCTGCAGTTGAGCATCATCGACCCACCAGGTGAACAAAATGGTGATATCAGAAGAACAGATGAAGTTGCCATCCACCAAGAAAGCGGAGCCGCCAACCTGGGCCCAGCTA AAG
AAGCTGACACAGTTAGCTGAAAAAAGTCTGAAAAACACAAGGGTAACACAAACTCCAGAGAATATGCTGCTTGCAGCTTTAATGATTGTATCAATGGTGGTAAGTCTCCCTATGTCTGCAGGAGCC GC
TACAGCTAACTATACTTACTGGGCCTATGTGCCTTTCCGACCCTTAATTCGGGCAGTCACTTGGATAGATAATCCTATTGAAGTATATGTTAATAATAGTGCATGGGTACCAGGCCCCACAGATG ACC
GTGGCCCTGCCCAACCTGAAGAAGAAGGAATGATGATAAACATTTCCATTGGGTATCATTATCCTCCTATTTGCCTGGGAAAAGCACCGGGATGCTTAATGCCTACAAACCAAAATTGGTTGGTAG AA
GTACCTACTGTCAGTGCTACCAGTAAATTCACTTATCACAGGTAAGTGAAATGTCACTCGGGTCACAAATAAATAATTTACAGGATTCTTCCTATAAAAGATCATTAAAATTTAGGCTTAAGGGA AAA
TCTTGCCCCAAGGAAATTCCAAAAGAATAAAAAGACTCAGAAGTCTTAGTTTGGGAAGAATGTGTGGCTGATACTGCGGTGGTATTACAAAACAATAAATTTGGAACTATTATAAACTGGGCCGCT TG
AGGCCAATTATATTATGATAGTACAGGCCAGACCCACTCATGTTCACAGGCTCCATCAGTCTGGCCCACTAATCCGGCCTATGATAGTGATTTAACTAAAAGGCTAGACCAGGTTTATAGAAGGC TAG
AATCACCCTATCCATGGAAATGGGGTGAAAAGGGGATTTCATCACCCCGCCCAAAGTTAGTTAGTCCTGTTGTTGCTCCTGAACACCCAGAATTATGGAAGCTCACTGTGGCCTCATACCACATTA GA
ATTTGGTCTGGAAATCAAGTTATGGGAACAAGAAATCATAAACCATATTTTACTATTAACCTAAAATCCAATCCGACAATTCCTTTGCAAAGTTATGTAAAACCCCCTCATATGCTAGTTGTAGG AAA
CATAGTTATTAAACCAGATTCCCAAACTATAACCTGTGAAAATTGTAGATTGTTTACTTGCATTGATTCAACTTTTGATTGGCAGCATCGTATTCTGTTAGTGAGGGCAAGAGAGGGTGCGAGGAT CC
CTGTGTCCATGGACCGACCGTGGGAGGCTTCTCCATCCGTACATATCTTAACAGAAGTATTAAAAGGAGTTCTAACTAGATCTAAAAGATTCATTTTTACTTTAATTGCAGTGATTATGGGTCTT ATT
GCAGTCACAGCTGCTGCTAGGGCTGCTGTAATTGCTTTACCCTCCTCTGTTCACACTGCAGAATATGTGAATAATTGGCAAAAGAATTCCTCAAAATTGTGGAATTCTCAGACTCAAATAGATCAA AA

ATTGGCAAATCAAATTAATGATCTTAGACAAACTGTCATTTGGATGGGAGATAGGCTCATAAGTTTGGAATATCTTTTTCAGTTACTGTGTGACTACAATACATCAGATTTTGTATTACACCTC AAG
CCTATAATGAATCTGAACATCACTGGGACATGGTTAGATGCCATCTACAAGGAAGAGAAGATAATCTTACTTTAGATATTTCAAAATTGAAAGAACAAATTTTTAAAACATCCAAAGCCCAGTTAA AT
TTGGTGCCAGAAACTGAGGCAATGGAAAAAGCTGTTGATAGCCTCACGAATCTTAA cCCTGTCACTTGGGTTAAAACCATTGGAAATTCCACTATTGCAAATTTTGTATTAATCCTTGTATGTCTGTC
CTCTCTATTGTTAGTCTACAGGTGTATCCAGCAGCTCCGGAGAGACAGCGGCTAGCGAGAACGGACCATGATGATGATGGCGGTTTT GTCAAAAAGAAAAGGGGATATGTAGGAAAAGAGAGAGAG
ATCAGACTGTTACTGTGTCTATGTAGAAAGGGAAGACATAAGAGACTCCATTTGAAAAAGACCTGTACTTTGAACAATTGCTTTGCTCAGATGTTGT cAATTTGTAGTTTTGCCCCAGCCACTTTGA
CCCAACCTGGAGCTCATAAAAACATGTGTTGTATGAAGTCAAGGTTTAAGGGATCTAGGGCT GTGCAGGATGTGCCTTGTTAACAAAATGTTTACAAGCAGTATACTTGGCAAAAGTCATTGCCATTC
TCTAGTtgtgtgactacaatacatcagattttgtattacacctcaagcctataatgaatctgaacatcactgggacatggttagatgccatctacaag gaagagaagataatcttactttagatatt
tcaaaattgaaagaacaaatttttaaaacatccaaagcccagttaaatttggtgccagaaactgaggcaatggaaaaagctgttgatagcctcacgaatcttaaacctgtcacttgggttaaaac cat
tggaaattccactattgcaaattttgtattaatccttgtatgtctgtcctctctattgTtagtctac aggtgtatccagcagctccggagagacagcggctagcgagaacggaccatgatgatgatgg
cggttttgtcaaaaagaaaaggggatatgtagggaaaagagagagagatcagactgttactgtgtctatgtagaaagggaagacataagagactccatttgaaaaagacctgtactttgaacaatt
gctttgctcagatgttgttaatttgtagttttgccccagccactttgacccaacctggagctcacaaaaacatgtgttgtatgaaatcaaggtttaagg gacctaggtgcaggatgtgccttgttaac
aaaatgtttacaagcagtatacttggcaaaagtcattgccattctctagt GTCAACAAACCACAGGCATAATGCACTGCAGAAACCCACAGGGAACTCTGCCCTTGAAAGCTGGGTATCGTCCAAGGT
TTCTCCCCATGTGATAGTCGGAAATATGGCCTCATGAGATGAGAAAGACCTGACTGTCCCCCAGCCCGACACCCGTAAAGGGTCTGTGCTGAGGTGGAT TGGTAAAAGAGGAAAGCCTCTTGCAaTTG
AGATAGAGGAAGGCCnnnnnnnnnnnnnnnnnnnnnnnnnnnnnnnnnnnnnATAAAACCCGATTGTACATTTGTTCAATTCTGAGATAGGAGAAAAACCGCCCTATGGTGGGAGGTGAGACATGTT
GGCAGCAATGCTGCCTTGTTATTCTTTACTCCACTGAGATGTTTGGGTGGAGAGAAACATAAATCTGGCCTATGTGCACATCCAGGCATAGTACTTTCCCTTGAACTTAATTATGACATAGATTC CTT
TGCTCACATGCTTTTTGCTGACCTTCTCCTTATTATCACCCTGCTCTCCTACTA CATTCCTTTCTGCTGAAATCATGAAAATAATAATCAATAAAAACTGAGGGAACTCAGAGACCGGTGCCGGTGCA
GGTCCTTGGTGTGCtaAGTGCCGCTCTCCTGGGCCCACTGTTGTTTCTCTATACTTTGTCACTGTGTCTTATTTCTTTTCTCAGTCTCCCATCCCACCTGACTAGAAATACCCACAGGTGTGGAGGGG
CAGGCCACCCCTTCAGATTATCAAGAACTCCAATGAAGCCAGCTAAATCTATTT GCCAATTGACTGAATTAACAAAGGCTTTCCAAATCTGATTTTTATTCATTGGAACTATAATCTTATCTGGATCT
GAGCCACAAAGTTTAACAACTCTAAGGTGAGCTTGCCCAATCAAGATTGCCATCTGATCCAAGTATACTGTGAGTGTTCTAACAGTATTGTGAGGCAGAAAAGACCATTCAACTAAGTCCTCACT CTG
AACTGTAACCCCTGTAGAAGAATGGAGAGTAGGGAACACAATGAACTGTAATGG TAAATTTGAGTCTATTCTATTAACCTGGGCCTGTTGTATTTTTTCTTCAATCATTTGTAATTCTTTGGCAGTCT
CAGGAGTCAGTTCTCATTTACTATTGAGAGCAGGGTCACCTCTTAAAATAGAGAACAGATTAGACATAGCATAGGTAGGGATTCCCAAAGGAGGTCAAATCCAATTGATATCCCCTAATAATTTT TGA
AAATCATTTAAAGTTTTCAGAGAGTCTTTTTGAATTTGAACCTCTTGGGGTCTA ATTGCCCTTTCCTGATCCTGCATTCCCAAATATTGGAAAGGAGTGGATGTTTGAATTTTATCTGGTGCTATGAG
CAATCCAGCATTGGCTACCACCTCCTGTAAATATGAATAACACTGGATAAGTCAGTCTTAACTTTTGACCGTACCCAATATGTCATCCATAAAATGAATGATATATGAATCAGGAAACTGATCTC TCA
CAGGTGGAATAGCCTTCCCCACAAAAGTTTGACAAATAGTAGGACTATTCAACA TATCCTGCAGCAGGACTTTCCAATGATATCTGGCTGCTGGTTCCTTGTTGTTCATAGCAGGAATAGTAAAAGCA
AATTTCACAAAATCAGACTCTGCTAGAAGAATATTTAAAAAGCAATCCTTCAAATCTATAATTACTAATGGCCAGTCTCTAGGAATCATGGTGGGGGATAGAAGCCCCAGCTGCAGGGCCCCAAT CAG
CTGAATGAATGTGTTCACCACACGTAAGTCGGTTAGCATGCGCCATCTGCTGGA TTTTTTCTTTATTACAAACACCGGCAAATTCTATGGAGAAAATGTGGGCTCAATGCTTCCTTTGTTTAATTGCT
CTTTGACTAATTCCTGTAAGGCCCCCAATTTTTCCTGAGTAAGTGGCCACAGCTCAACCCAAACAGGATTTTGGGTCTTCCATTTTAAGGGAATAGGACTTGGAGGCTCAACAGTGACCACCCCT AAA
AATGGTAACCTACATGGGAGGTTCCAAGATGGCCAAATAGGAACAGTTCCAGTC TAGAGCTCCCAGCATGAGCGATGCAGAAGACAGGTGATTTCTGCATTTCCAACTGAAATACCAGGTTCATCTCA
CTGGAGCTTGTCAGACAGTGGGTGCAGGACAGTGGGTGCAGCCCACCGAGTGAGACCTGAAGCAGGGCAAGGCACCACCTCACCTGGGAAGTGCAAGGGGTCAGGGAATTCCCTTTCCTAGCCAA GGG
AAGCTGTGACAGATGGCACCTGGAAAATCGGGTCACTCCCACCCTAATACTGTG CTGTTCCAATGGTCTTAGCAAATGGCACACCAGGAGATTATTTCCTACACCTGGCTTGGAGGGTCCCACACCCA
TGGAGCCTTGCTCATTGCTAGCACAGCAGTCTGAGATCGAACTGCAAGGTGGCAGCAAGGCTGGGGGAGGGGCGCCCACCATTGCTGAGACTTCAGTAGGTAAACAAAGCGGCTGGGAAGCTTGA ACT
GGATGGAGCCCACTGCAGCTCAAGGAGGCCTACCAGCCTCTGCAGACTCCACCT CTGGGTGCAGGGCATAGCTGAACAAAAGGCAGCAGAATCTTCTGCAGACTTAAATGTCCCTGTCTGACAGCTTT
GAAGAGAGTAGTGGTTCTCCCAGCATGGAGTTTGAGATCTGAGAACAAACAGACTGCCTCCTCAAGTGGCTCCCTGACACCTGAGTACCCTAACTGGGAGGCACCTCCCAGTAGGGGCTGACTGA TAC
CTCACACGGCCGGGTACCCCTCTGAGATGAAGCTTCCAGAGGAATGATCAGGCA GCAACATTTGCTATTCTGCAATATTCGCTATTCTGTAGCCTCTACTGGTCATATCCAGGCAAACAGGATCTAGA
GTGGACCTCCAGCAAACTCCAACAGACCTGCAGCTGAGGGTTCTGACTATTAGAAGGAAAACTAACAAACAAAAAGTACATCCACACCAAAACCCCATCTGTACGTGACCATCATCAAAGACAAA AGG
TAGATAAAAACACAAAGATGGGGAGAAGCCAGAGCAGAAAAGCTGAAAATTCTA AAAATCAGAGCACCTCTTCTCCTCCAAAGGAATGCAGCTCCTCACCAGCAATAGATCAAACCTGGATGGAGAAT
GACTTTGATGAGTTGAGAGAAGAAAGCTTCAGATGATCAAACTTCTCTGAGCTAAAGGAGGATGTTTGAACCCATCACAAAGAAGCTAAAAACCTTGAAAAAAGATTAGACGAATGGCTAACTGA ATA
ACCAGGGTAGAGAAGACCTTAAATGACCTGATGGAGCTGAAAGCCATGGCACGA GAACTACATGTGCAAGCTTCAGTAGCTGATTCAATCAACTGGAAGAAAGGGTATCAGTGATTGAAGATCAAATG
AATGAAATGATGTGAGAAGAGAAGTTTAGAGAAAAAAGAGTAAAAAGAAATGGAACAAAGCCTCCAAGAAATATGGGACTATGTGAAAAGACCAAATCTACATCTGATTGGTGTACCTGAAAGTG ATG
GGGAGAATGGAACAAAGTTGGAAAACAGTTTTCAGGATATTATCCAGGAGAATT TCCTCAACCTACCAAGGCAGGCCAACATTCAAATTCAGGAAATACAGAGAATGCCACAAAGATACTCCTCAAGT
AGAGCAACTCCAAGACACATAATTGTCAGATTCACCCAAGTTGAAATGAAGGAAAAAAATGTTAAGGGCAGCCAGAGAGAAAGGTCGAGTTACCCACAAAGGGAAGCCCACCAGACTAACAGTGGA TCT
CTCAGCAGAATCTCTACAAGCCAGAAGAGAGTGGAGGCCAATATTCAACATTCT TAAAGCAAAGAATTTTCAACCCAGAGTTTCATATCCAGCCAAACTAAGCTTCATAAGTGAAGGAGAAATAAAA T
acttnnnnnnnnnnnnnnnnnnnnnnnnnnnnnnnnnnnnnnnnnnnnnnnnnnnnnnnnnnnnnnnnnnnnnnnnnnnnnnnnnnnnnnnnnnnnnnnnnnnnnnnnnnnnnnnnnnnnn nnn
nnnnnnnnnnnnnnnnnnnnnnnnnnnnnnnnnnnnnnnnnnnnnnnnnnn nnnnnnnnnnnnnnnnnnnnnnnnnnnnnnnnnnnnnnnnnnnnnnnnnnnnnnnnnnnnnnnnnnnnnnn
nnnnnnnnnnnnnnnnnnnnnnnnnnnnnnCAGACTGGCAAATTGGATAAAGAGTCAAGACCCATCAGTGTGCTGTATTCAGGAGACCCATCTCATGTGCAGAGACACACATAGGCTCAAAATAAAG
GGATGGAGGAAGATCTACCAAGCAAATGGAAAACAAAAAAAAGCAGGGGTTGCA ATCCTAGTCTCTGATAAAACAGGCTTTAAACAACAAAGATCAAAAGAGACAAAGA Aagccattacataatggta

```
aagggatcaactcatcaagaagagttaactatcctaaatatatatgcacccaatacaggagcaaccagattaataaggcaagtccttagagacccacaaagagacttagactcccacacaataat aat
gagagattttaacacccctctgtcaacattagacagatccacaggacagaaagt taacaaggatatccaggaatt
```

2874	22.4	1.0	2.8	HERVK-int-Scaffold6057-11637-11951	15	626	(9689)	+	HERVK-int	LTR/ERVK	846	1446	(6090)	1	
27229	12.9	0.5	0.6	HERVK-int-Scaffold6057-11637-11951	625	4627	(5688)	+	HERVK-int	LTR/ERVK	3452	7447	(89)	1	
1089	26.7	1.4	10.3	HERVK-int-Scaffold6057-11637-11951	4628	4716	(5599)	+	SVA_F	Retroposon/SVA	853	1193	(182)	2	
2194	7.1	5.3	0.0	HERVK-int-Scaffold6057-11637-11951	4717	4998	(5317)	+	LTR5A	LTR/ERVK	1	297	(736)	3	
2103	15.2	0.0	0.0	HERVK-int-Scaffold6057-11637-11951	5001	5315	(5000)	+	HERVK-int	LTR/ERVK	7133	7447	(89)	4	
261	20.8	0.0	22.5	HERVK-int-Scaffold6057-11637-11951	5316	5402	(4913)	+	SVA_F	Retroposon/SVA	853	923	(452)	2	
7663	11.0	2.2	0.0	HERVK-int-Scaffold6057-11637-11951	5405	6415	(3900)	+	LTR5A	LTR/ERVK	1	1033	(0)	5	
10847	4.9	0.0	0.0	HERVK-int-Scaffold6057-11637-11951	6416	7692	(2623)	C	HERVK14C-int	LTR/ERVK	(3323)	4111	2835	6	
14123	18.8	0.4	0.7	HERVK-int-Scaffold6057-11637-11951	7696	10315	(0)	+	L1P1	LINE/L1	3	2612	(3534)	7	
2874	22.4	1.0	2.8	hg19	15	626	(8765)	+	HERVK-int	LTR/ERVK	846	1446	(6090)	8	*
27270	12.9	0.5	0.6	hg19	625	4627	(4764)	+	HERVK-int	LTR/ERVK	3452	7447	(89)	8	
261	20.8	0.0	22.5	hg19	4628	4714	(4677)	+	SVA_F	Retroposon/SVA	853	923	(452)	9	
8134	7.5	1.7	0.0	hg19	4717	5732	(3659)	+	LTR5A	LTR/ERVK	1	1033	(0)	10	
10847	4.9	0.0	0.0	hg19	5733	7009	(2382)	C	HERVK14C-int	LTR/ERVK	(3323)	4111	2835	11	
16632	9.4	0.4	0.0	hg19	7013	9391	(0)	+	L1P1	LINE/L1	3	2391	(3755)	12	

```
>hg19 chr8:146242971-146252361
ATCCCAGATGGGATCCTGTGCCAAATAGTAGGGCACTATATCAACAACCCACGGCGATGGTGTTTGATCCTACAGTACCAGCTAGTGGACAAGATAGTGCACTGCATGAGACCGTTGATACAGCCA GA
AAACAGGGAGATCTTGAGGCATGGCAGTATCCGGTAATGTTACAACCGATACCGGCCGGGAAAGGGCCAGGAAAGGGAGTCAAGCAGGAGCGTCTGTCCGAACGGAGGCTAGGTAAGAATATTTC ACC
ATGAAAATGTTAAAAGACATAAAGGAAGGAGCTAAACAATATGGACCCAACTCTCCTTATATGAGAACGTTATTAGATTCCATTGCTCATGGAAATAGACTTATTCCTTATGATTGGGAAATTTTA CC
TAAATCTTCCCTTTCACCCTCTCAGTATCTACAGTTTAAAACCTGGTGGATTGATGGAGTACAAGAACAGGTACGGAAAAATCAGGCTACTTATCCTGTTGTTAATATAGATGCAGACCAATTGC TAG
GAACACGTCCAAATTGGAGCACTATTAACCAACAATCAGTAATGCAAAATGAGGCTATTGAACAACTAGGGGCTATTTGCCTCAGGGCCTGGGAAAAGATTCAGGACCCAGGAACCAGTTAGAGAC AG
TTTTCAGACTGTTATATCATTCATTATGTTGATGATATTTTGTGTGCTGCAGAAACAAGAGACAAATTAATTGACTTTTACATGTTTCTGCAGACAGAGGTTGCAAACACAGGCCTGACAATAGC ATC
TGATAAGATTCAGACCTCCACTCCTTTTAATTATTTGGGAATGCAGGTAGAGGAAAGAAAAATTAAACCACAAAAAATAGAAATAAGAAAAGACACATTAAGAACATTAAATGACTTTCAAAAATT GC
TAgGAGATATTAATtGGATTGGGCCAACTCTAGGCATTCCTACTTATGCCATGTCAAATTTGTTTTCTATCTTGAGAGGGGATCCAGACTTGAATGGTAAAAG aACAATAACTCCAGAGGCAACTAAA
GAAATTGAATTAGTTGAAGAAAAAATTAATTCAGCACAAGTAAATAGAATAGATCACTTAGCCCCACTCCAATTTTTGATTTTTGCTACTGCACATTCTCCAACAGGCATTATTGTTCAAAATACA GA
TCTTGTGGAGTGGTCATTCCTTCCTCACAGTACAGTTAAGACTTTTACATTGTACTTAGATCAAATGGCTACATTAATTGGTCAGACAAGACTACGAATAGTAAAATTGTGTGGAAGTGACCCAG ATA
AAATCATTGTTCCTTTAAACAAGGAACAGGTTAGACAAGCCTTTATCAATTCTGCTGCATGGCAGATTGGTCTAGCTGATTTTGTGGGAATTATTGATAATCATTACCCAGAAACAAAAATCTTCC AG
TTTTTAAAATTGACTACTTGGATTTTACCTAAAATTACCAGACAAAAACCTTTAGAAAATGCTCTGACAGTGTTTACTGATGGTTTCAGCAATGGAAAAGTGGCTTACACTGGGCCAAAAGAATG AGT
CATTGAAACTCAATATCACTCAGCTCAAAGAGCAGAATTGGTTGCTGTCACTTCAGTGTTACAAGATTTTAATCAGCCTATTAACATTGTTTCAGATTCTGC aTATATAGTACAGGCTACAAAGGCTA
CAAAGCTCTAATCAAATATAGTATGGATTATCAGTTAAATCATCTGTTTAAATTGTTACAACAAACTGTAAGAAAAAGAAATTTCCCAGTTTATATTACTCATATTC gAGCACATACTAATTTACCAG
GGCAATTAACTAAGGAAAATGAACAAGCTGACTTGCTAGTATCATCTGCCTTGATGGAAGCACAAGAACTTCATGTCCTGACTCATGTAAATGCAACAGGATTAAAAAATAAATTTGATATCACAT GG
AAACAGGCAAAAAATATTGTACAACATTGTGCTCAGTGTCAAGTCTTACACCTGCCCACTCAGGAGGCAGGAGTTAATCTCAGAGATTTATGTCCTAATGCATTATGGCAAATGGATGTCACACA TGT
ACCTTCATTTGGAAAATTGTCATTTGTCCGCGTGACAGTTGATACTTATTCACATTTCATATGGGCAACCTGCCAGACAGGAGAAAGTACTTCCCATGTTAAAATACATTTATTATCTTATTTTGC TG
TCATGGGAGTTCCAGAAAAAATTAAAACAGATAATGGGCCAGGATACTGTAGTAAAGCATTTCAAAAATTCTTAAATCAGTGGAAAATTACACATACAACAGGAATCCCTTATAATTCCCAAGGA CAG
GCCATAATTGAAAGAACTAATAGAACACTCAAAGCTCAATTGGTTAAACAAAAAAGGGAAAAAGACAGTAAGGAGTATAACACTTCCCAGATGAACTTAATCTAGCACCCTATACTTTAAATTTTT TA
AACATTTATAGAAATCAGACCACTACTTCTGCAGAACAACATTTTACTGGTAAAAAGAACAGCCCACATGAGGGAAAACTGATTTGGTGGAAAGACAACAAAAGTAAAAC tTGGGAAACAGGGAAGGT
GATAACATGGGGGAGAGGTTTTTCTTGTGTTTCACCAGGAGAAAATCAGCTTCCTGTTTGGATACCCACTAGACATTTGAAGTTCTACAATAAACCCATCCCATCAGAAATGCAAATAAAAGTGCC TC
CACAGAGACAAAAAACCTGCAGTTGAGCATCATCGACCCACCAGGTGAACAAAATGGTGATATCAGAAGAACAGATGAAGTTGCCATCCACCAAGAAAGCGGAGCCGCCAACCTGGGCCCAGCTA AAG
AAGCTGACACAGTTAGCTGAAAAAAGTCTGAAAAACACAAGGGTAACACAAACTCCAGAGAATATGCTGCTTGCAGCTTTAATGATTGTATCAATGGTGGTAAGTCTCCCTATGTCTGCAGGAGCC GC
TACAGCTAACTATACTTACTGGGCCTATGTGCCTTTCCGACCCTTAATTCGGGCAGTCACTTGGATAGATAATCCTATTGAAGTATATGTTAATAATAGTGCATGGGTACCAGGCCCCACAGATG ACC
GTGGCCCTGCCCAACCTGAAGAAGAAGGAATGATGATAAACATTTCCATTGGGTATCATTATCCTCCTATTTGCCTGGGAAAAGCACCGGGATGCTTAATGCCTACAAACCAAAATTGGTTGGTAG AA
GTACCTACTGTCAGTGCTACCAGTAAATTCACTTATCACAGGTAAGTGAAATGTCACTCGGGTCACAAATAAATAATTTACAGGATTCTTCCTATAAAAGATCATTAAAATTTAGGCTTAAGGGA AAA
```

TCTTGCCCCAAGGAAATTCCAAAAGAATAAAAAGACTCAGAAGTCTTAGTTTGGGAAGAATGTGTGGCTGATACTGCGGTGGTATTACAAAACAATAAATTTGGAACTATTATAAACTGGGCCGCT TG
AGGCCAATTATATTATGATAGTACAGGCCAGACCCACTCATGTTCACAGGCTCCATCAGTCTGGCCCACTAATCCGGCCTATGATAGTGATTTAACTAAAAGGCTAGACCAGGTTTATAGAAGGC TAG
AATCACCCTATCCATGGAAATGGGGTGAAAAGGGGATTTCATCACCCCGCCCAAAGTTAGTTAGTCCTGTTGTTGCTCCTGAACACCCAGAATTATGGAAGCTCACTGTGGCCTCATACCACATTA GA
ATTTGGTCTGGAAATCAAGTTATGGGAACAAGAAATCATAAACCATATTTTACTATTAACCTAAAATCCAATCCGACAATTCCTTTGCAAAGTTATGTAAAACCCCCTCATATGCTAGTTGTAGG AAA
CATAGTTATTAAACCAGATTCCCAAACTATAACCTGTGAAAATTGTAGATTGTTTACTTGCATTGATTCAACTTTTGATTGGCAGCATCGTATTCTGTTAGTGAGGGCAAGAGAGGGTGCGAGGAT CC
CTGTGTCCATGGACCGACCGTGGGAGGCTTCTCCATCCGTACATATCTTAACAGAAGTATTAAAAGGAGTTCTAACTAGATCTAAAAGATTCATTTTTACTTTAATTGCAGTGATTATGGGTCTT ATT
GCAGTCACAGCTGCTGCTAGGGCTGCTGTAATTGCTTTACCCTCCTCTGTTCACACTGCAGAATATGTGAATAATTGGCAAAAGAATTCCTCAAAATTGTGGAATTCTCAGACTCAAATAGATCAA AA
ATTGGCAAATCAAATTAATGATCTTAGACAAACTGTCATTTGGATGGGAGATAGGCTCATAAGTTTGGAATATCTTTTTCAGTTACTGTGTGACTACAATACATCAGATTTTTGTATTACACCTC AAG
CCTATAATGAATCTGAACATCACTGGGACATGGTTAGATGCCATCTACAAGGAAGAGAAGATAATCTTACTTTAGATATTTCAAAATTGAAAGAACAAATTTTTAAAACATCCAAAGCCCAGTTAA AT
TTGGTGCCAGAAACTGAGGCAATGGAAAAAGCTGTTGATAGCCTCACGAATCTTAA aCCTGTCACTTGGGTTAAAACCATTGGAAATTCCACTATTGCAAATTTTGTATTAATCCTTGTATGTCTGTC
CTCTCTATTGTTAGTCTACAGGTGTATCCAGCAGCTCCGGAGAGACAGCGGCTAGCGAGAACGGACCATGATGATGATGGCGGTTTT GTCAAAAAGAAAAGGGGATATGTAGGGAAAAGAGAGAGAG
ATCAGACTGTTACTGTGTCTATGTAGAAAGGGAAGACATAAGAGACTCCATTTGAAAAAGACCTGTACTTTGAACAATTGCTTTGCTCAGATGTTGT tAATTTGTAGTTTTGCCCCAGCCACTTTGA
CCCAACCTGGAGCTCATAAAAACATGTGTTGTATGAAGTCAAGGTTTAAGGGATCTAGGGCT GTGCAGGATGTGCCTTGTTAACAAAATGTTTACAAGCAGTATACTTGGCAAAAGTCATTGCCATTC
TCTAGTGTCAACAAACCACAGGCATAATGCACTGCAGAAACCCACAGGGAACTCTGCCCTTGAAAGCTGGGTATCGTCCAAGGTTTCTCCCCATGTGAT AGTCGGAAATATGGCCTCATGAGATGAGA
AAGACCTGACTGTCCCCCAGCCCGACACCCGTAAAGGGTCTGTGCTGAGGTGGATTGGTAAAAGAGGAAAGCCTCTTGCA gTTGAGATAGAGGAAGGCCactgtctcctgcctgcccctgggaactga
acgtcttggtATAAAACCCGATTGTACATTTGTTCAATTCTGAGATAGGAGAAAAACCGCCCTATGGTGGGAGGTGAGACATGTTGGCAGCAATGCTGC CTTGTTATTCTTTACTCCACTGAGATGTT
TGGGTGGAGAGAAACATAAATCTGGCCTATGTGCACATCCAGGCATAGTACTTTCCCTTGAACTTAATTATGACATAGATTCCTTTGCTCACATGCTTTTTGCTGACCTTCTCCTTATTATCACC CTG
CTCTCCTACTACATTCCTTTCTGCTGAAATCATGAAAATAATAATCAATAAAAACTGAGGGAACTCAGAGACCGGTGCCGGTGCAGGTCCTTGGTGTGC cgAGTGCCGCTCTCCTGGGCCCACTGTTG
TTTCTCTATACTTTGTCACTGTGTCTTATTTCTTTTCTCAGTCTCCCATCCCACCTGACTAGAAATACCCACAGGTGTGGAGGGGCAGGCCACCCCTTCAGATTATCAAGAACTCCAATGAAGCC AGC
TAAATCTATTTGCCAATTGACTGAATTAACAAAGGCTTTCCAAATCTGATTTTTATTCATTGGAACTATAATCTTATCTGGATCTGAGCCACAAAGTTT AACAACTCTAAGGTGAGCTTGCCCAATCA
AGATTGCCATCTGATCCAAGTATACTGTGAGTGTTCTAACAGTATTGTGAGGCAGAAAAGACCATTCAACTAAGTCCTCACTCTGAACTGTAACCCCTGTAGAAGAATGGAGAGTAGGGAACACA ATG
AACTGTAATGGTAAATTTGAGTCTATTCTATTAACCTGGGCCTGTTGTATTTTTTCTTCAATCATTTGTAATTCTTTGGCAGTCTCAGGAGTCAGTTCT CATTTACTATTGAGAGCAGGGTCACCTCT
TAAAATAGAGAACAGATTAGACATAGCATAGGTAGGGATTCCCAAAGGAGGTCAAATCCAATTGATATCCCCTAATAATTTTTGAAAATCATTTAAAGTTTTCAGAGAGTCTTTTTGAATTTGAA CCT
CTTGGGGTCTAATTGCCCTTTCCTGATCCTGCATTCCCAAATATTGGAAAGGAGTGGATGTTTGAATTTTATCTGGTGCTATGAGCAATCCAGCATTGG CTACCACCTCCTGTAAATATGAATAACAC
TGGATAAGTCAGTCTTAACTTTTGACCGTACCCAATATGTCATCCATAAAATGAATGATATATGAATCAGGAAACTGATCTCTCACAGGTGGAATAGCCTTCCCCACAAAAGTTTGACAAATAGT AGG
ACTATTCAACATATCCTGCAGCAGGACTTTCCAATGATATCTGGCTGCTGGTTCCTTGTTGTTCATAGCAGGAATAGTAAAAGCAAATTTCACAAAATC AGACTCTGCTAGAAGAATATTTAAAAAGC
AATCCTTCAAATCTATAATTACTAATGGCCAGTCTCTAGGAATCATGGTGGGGGATAGAAGCCCCAGCTGCAGGGCCCCAATCAGCTGAATGAATGTGTTCACCACACGTAAGTCGGTTAGCATG CGC
CATCTGCTGGATTTTTTCTTTATTACAAACACCGGCAAATTCTATGGAGAAAATGTGGGCTCAATGCTTCCTTTGTTTAATTGCTCTTTGACTAATTCC TGTAAGGCCCCCAATTTTTCCTGAGTAAG
TGGCCACAGCTCAACCCAAACAGGATTTTGGGTCTTCCATTTTAAGGGAATAGGACTTGGAGGCTCAACAGTGACCACCCCTAAAAATGGTAACCTACATGGGAGGTTCCAAGATGGCCAAATAG GAA
CAGTTCCAGTCTAGAGCTCCCAGCATGAGCGATGCAGAAGACAGGTGATTTCTGCATTTCCAACTGAAATACCAGGTTCATCTCACTGGAGCTTGTCAG ACAGTGGGTGCAGGACAGTGGGTGCAGCC
CACCGAGTGAGACCTGAAGCAGGGCAAGGCACCACCTCACCTGGGAAGTGCAAGGGGTCAGGGAATTCCCTTTCCTAGCCAAGGGAAGCTGTGACAGATGGCACCTGGAAAATCGGGTCACTCCC ACC
CTAATACTGTGCTGTTCCAATGGTCTTAGCAAATGGCACACCAGGAGATTATTTCCTACACCTGGCTTGGAGGGTCCCACACCCATGGAGCCTTGCTCA TTGCTAGCACAGCAGTCTGAGATCGAACT
GCAAGGTGGCAGCAAGGCTGGGGGAGGGGCGCCCACCATTGCTGAGACTTCAGTAGGTAAACAAAGCGGCTGGGAAGCTTGAACTGGATGGAGCCCACTGCAGCTCAAGGAGGCCTACCAGCCTC TGC
AGACTCCACCTCTGGGTGCAGGGCATAGCTGAACAAAAGGCAGCAGAATCTTCTGCAGACTTAAATGTCCCTGTCTGACAGCTTTGAAGAGAGTAGTGG TTCTCCCAGCATGGAGTTTGAGATCTGAG
AACAAACAGACTGCCTCCTCAAGTGGCTCCCTGACACCTGAGTACCCTAACTGGGAGGCACCTCCCAGTAGGGGCTGACTGATACCTCACACGGCCGGGTACCCCTCTGAGATGAAGCTTCCAGA GGA
ATGATCAGGCAGCAACATTTGCTATTCTGCAATATTCGCTATTCTGTAGCCTCTACTGGTCATATCCAGGCAAACAGGATCTAGAGTGGACCTCCAGCA AACTCCAACAGACCTGCAGCTGAGGGTTC
TGACTATTAGAAGGAAAACTAACAAACAAAAAGTACATCCACACCAAAACCCCATCTGTACGTGACCATCATCAAAGACAAAAGGTAGATAAAAACACAAAGATGGGGAGAAGCCAGAGCAGAAA AGC
TGAAAATTCTAAAAATCAGAGCACCTCTTCTCCTCCAAAGGAATGCAGCTCCTCACCAGCAATAGATCAAACCTGGATGGAGAATGACTTTGATGAGTT GAGAGAAGAAAGCTTCAGATGATCAAACT
TCTCTGAGCTAAAGGAGGATGTTTGAACCCATCACAAAGAAGCTAAAAACCTTGAAAAAAGATTAGACGAATGGCTAACTGAATAACCAGGGTAGAGAAGACCTTAAATGACCTGATGGAGCTGA AAG
CCATGGCACGAGAACTACATGTGCAAGCTTCAGTAGCTGATTCAATCAACTGGAAGAAAGGGTATCAGTGATTGAAGATCAAATGAATGAAATGATGTG AGAAGAGAAGTTTAGAGAAAAAAGAGTAA
AAAGAAATGGAACAAAGCCTCCAAGAAATATGGGACTATGTGAAAAGACCAAATCTACATCTGATTGGTGTACCTGAAAGTGATGGGGAGAATGGAACAAAGTTGGAAAACAGTTTTCAGGATAT TAT
CCAGGAGAATTTCCTCAACCTACCAAGGCAGGCCAACATTCAAATTCAGGAAATACAGAGAATGCCACAAAGATACTCCTCAAGTAGAGCAACTCCAAG ACACATAATTGTCAGATTCACCCAAGTTG
AAATGAAGGAAAAAATGTTAAGGGCAGCCAGAGAGAAAGGTCGAGTTACCCACAAAGGGAAGCCCACCAGACTAACAGTGGATCTCTCAGCAGAATCTCTACAAGCCAGAAGAGAGTGGAGGCCA ATA
TTCAACATTCTTAAAGCAAAGAATTTTCAACCCAGAGTTTCATATCCAGCCAAACTAAGCTTCATAAGTGAAGGAGAAATAAAA Tcctttacagacaagcaaatgctgagagattttgtcaccactgg
gcctgccttacaagagctcctgaaggaagcactaaacatggaaaggaaaaaccagtaccagccactgcaaaaacatgccaaattgtaaagaccatcgatgctaggaagaaactgcatcaactaat gag
caaaataaccagctaactcaaaatgacaggatcaaattcacacataacaatattaaccttaaatgtaaatgggctaaatgctccaattacaagaca CAGACTGGCAAATTGGATAAAGAGTCAAGACC

CATCAGTGTGCTGTATTCAGGAGACCCATCTCATGTGCAGAGACACACATAGGCTCAAAATAAAGGGATGGAGGAAGATCTACCAAGCAAATGGAAAACAAAAAAAAGCAGGGGTTGCAATCCTA GTC
TCTGATAAAACAGGCTTTAAACAACAAAGATCAAAAGAGACAAAGAA

>KSI_HERVK14C_2

>Scaffold13916
GATTATTGTTCATTTACTTCTGGGTGGGAGGATGAAGGCATGTCATGATGGCAATGGGGTGGCAAGCCCTGGGGTTCCAGCCAAAAGGCAAGGTATGGTGTGAATGCTGAATCAAAGAACCGACAA TA
CTAAAATACCATCAGCAGTGAGGATATAGAGCTTTGAGCAGAGCAAAACATTAGAAGTTAATTTGTTAGGCTTGAGCAGTTGTTCACTGGAAACCCAAAGTAAATATAAAGCTTTGGTGCAGAAC TTA
GTTCTCACTGCACTTCCTGATTATTCTCTTCCCCTCTACTCTGATCTTAAGTATTTTAAGAAAAAAATGAAATATGCTGGGAAATTTTGGGAAGAGGAGGGACTAAAAATTTTTATAGCTCTGTTG GA
TATAAGCAATTCATGATGGGCCCTGAGCATCCTTTCAACTTCTTGCTCGGTATCCCCAAAATGCAGTGTTTCCAAGTCACCATCTTACTTGATCTTGCAGATGTGGCCCAAAACATTGAGGACTG CAG
GGTAAAGCTGCAGCAGCATGTGTGGGCACAAAAAGGGTCCCTGGGTCTGGGCCCTTGCTGGTCTCCAGAGGTATATGGGGAAGAGACAGCACCAACTCTGGGTGAAGAAGCCAAAGGAGAAAAAAA TA
AAAGCCTTCCTGCAACTCAGCCTTACAAAAAGTATGTACAAAAATCTTGCAGTGTACATCATATCTAAGGGTGAAAAAGTGAATGATTTTTCCCTAAGATCGTGAACAAGGTGGGCTGCCTATTC CTA
CAATTTCTGTTCAACATAGTACTGGAAATCCTAGCCAGTGTGGTGGGGGAAATAAAGAAATAATGGGCATACTGATTGCAAAAGTAGAAATAAAATTGTTCTTATTCACGAATGACATAATCATCT AT
GTATAAGGTCTTGAGGAATCTACATAAAAAGCTATTTGAGCGAATAAGGTCACAAGATAAAATATTAATTGTATTTCTGTGTACTAAGCAATGAACAATTGGGAATTAAAATTTTAGAATACCAT TTA
CAGAAGCATACAAAATCATTAAACATGGATGGATAAATTAAATATGTGTAAAAGTACAATACTTTGTTGAGAGAAATAAAAGAAGATGTAAATAAATGAAAGATATACCATGGTCATGTATCGGAA GA
CTGAATATTTTACAGATATTGTGTTAGTTTTCTATTATTCTGTAATAAATTAACAAAAATTTCAGGACAACACAAATTTATTGTTGTATCGTTTCTGTGACTCTGGAGTTGAGGCTAA TTTGTTGTTG
TTGTTGTTGTTGTTTATTGAGATGGAGAGACTCGCTCTGTTGCCCAGGCTGGAGTGCAATGGCAAGATCTCGGCTCACCGCAACCTCCGCCTCCCAGGTTCAAGCGATTCTCCTGCCTCAGCCTC CCA
AGTAGTGGGGATTACAGGCAAGCGCCACAATGCCCAGCTAATTTTTGTATTTTTAGTAGAGACGAGGTTTCACCATGTTGGCCAGGCTGGTCTGGAACTCGTGACCTCAAGTGATCCGCC CACCTCGG
CCTCCCAAAGTGCTGGGATTACAGGCCTGAGCCACAGCGCCCGGCTGCAAATTGGGGGTTTTAAGGGTAATCTGAGAAGCCAATCACATCCAGCTTACTAAAGTAGGACTATGGTAGAAACTTTT GGT
TGTAAGATTAATAAGTTTTGGAGAGCTAATGTACAGCATGGTGACTATAGTTAATAATACTTTATTGTTTAGTTGAAAGTCAGTAAAAGAGCAGATTTTTGAGTGCCCTAACTGCACACA CACACATG
CACATATGTTACATATGGTAACTCTGTGTGGTGATGGATGTATTAATGAATTTGATTGTAGTAATCACTACGCAATGTATACCTATATCAACTCATCACATTGTACACCTTGAATATATGCAATT TGA
ATTTGTCAATTATACATCAAAATGCTGAGAAAAAGTAGGACCATGAAGTGGAAGCTCATTTAGAGGCCTATGGGAAGCTTTTGTCTCTGTGTATGGTATCACCTCTTTGGGACCTAGCTT TTGATTGT
GAATCTAGAGCTGCTTTTGGTCAGCAGGGTCAGCAGTTGGAAAGATGAGCTAAATGCTGAGCAGAGGAAGTGATGACAAATTGGAATCTGTCAGGGACCTTTGCATCTGTTCATCACCATATTTG TGG
TATATTTGTATTTTTCAAAGATGGATGCAATAATATATTCCATCTTACATGCTCTTCTGCAATATGACTTGCCACTCCCCCAACAAGAGGTAGAGTGTATTTCTCCATCCCATTGGGCCT GGAACTAA
CTGTGAATACTTTGACTAGCAGATGATGGTGTAAATGGTACAATACCAGTTCTGGGCACAGCACTTACCTGGCATGAAAATGGATTCCTGCCACTTAGAACACTCACTATTGGAAT GCTTCCTCAAAG
AATCCAGCTTCCATGCTGTAGAAGCCCTAGCCACATGATGAGGCCACATGTAAGTGCTCTAGTCTATAGCACCAGCTGAATTCCCAGCCAACATCCAACCATGTGCATGAACCATCTTGG AGGCCCAG
TCCAGCTGACTATTTAGAGAAATCCAGCCCCCAGACACCATCTGACAAACTGTAAGACCCACTCAGCTGAACCTAGTCAACTCACAGAACCATTAGAGATAATAATAAGTTT tTAAGCCACTAAATCT
TGGGACAATTTGCTACATAACAATGGATAATCAGCAGTtTCTGACCATGAAGACCCTCCAAGAATTATAGCCATTGTTTTACTTTTACCTCCTAATCACACAGTTTCTTCTAATATGAAA CCATACAA
ATAAAGGGATTTCAGTAAATGTAGTTTT

TGTTGGGAAAAGCTGAGTGTTGGGAGAGAAGCTGAGGCAGGGCTTGCATGTCTGACATAATGTAACAGAGTCTTGGAACATGTCCGGGGTCCAGGGTCTAAAACCCCTCGTGGCCTTTGGAACAC CAA
GCTCTGTGCTAAAGGGTGGAAGGCTACCCTGATGCACCATAATCTAAGCCCAGGGCATAAAACCCCTC aTGGCTTGGATAGAATCCAGGGCTCagggcacaaaacccccccaggggcctctggaatgtg
cctagacttgctggctccttgctccttgctctcccaggattgattgtatcttgagttaaaagaacctgctctccattatctcaagtagcagagcatatgctaaaccatcacagctgtaaatcttg tgc
ttaatgcaatgcgccctttcaaccccacattctcaccacctgtttctttgatcaccaataaatagtctgggcttccagagctcggggcctt cacagcctccatacttagcgatggccccctgaactca
ctttctctctcaaactgtcttttctcattcctttgactccaccagacttcatcgctcccatgacctggtgttgggtccgatcaccccaaca ttcctggctgcccaacgtgaggcaacaaagatcccgg
tgaaggaacactagagcgtgtgaaagcggaggatgcattgtcaaaggacacccgaggataactgaaagaagctcggtgggaaagcttagca ctcggaagaaccaggttaacaatgatgggacaaagtg
aaagcaaacattctgcttacttgaatttcttaaggcacttattatgaagagggtgaggcatggggtaagaccaggggaatctgaggcttgtttattcagagggccccatttagagaaacagaaga caa
agtgaggacttagaaggagcctgggcaagtaaagtcatcttgaagcttcagtttcatggttaacctgcctctgctagagaaaaacagattt aatgagcacagaggcatgatatgggctgaatgtaaag
aaatttggactcaaggccccaaggaatatttgtttgagttgtggaatatgcttgattttggtatgttagcaattttatcagcatcattcattgcaagattcagggcattctggcatgcttccaaa gcc
cagagcatcactgatgcaagtgatactttgaaggacttgacaaaagtaacattgggagataatgtgaaatactacagtttggccaggaggg agtgggacccctcggatcctcgtatggtgtctgaagg
tctttgtgcagttgctgtggttttgagtttctctgggatagcttgtattttgccagcaaatgaaagctttggacctctgcagatgtcgcttggaggagcagtcaaagacatcttcaggttcatgc tca
tattcattatggtgtttgtggcctttatgattggaatgttcaacctctactcctactacattgatgtaaaacaaaatgaagccttcacaac agttgaagagagttttaagacactgttctgggctaca
tttgtgctttctgcagtgaaatcagtggtcatcaactatagccacaaattcattgaaaacattggttatgttctttatggagtctatgatgttacaatggtcattgttttgctaaatatgttaat tgc
aatgataaacagttcactccaggaaattgaggatgacgctgatgtggagtggaaatttgcaagggccaaactctcattttcctgctttggg gaggggagagcacttcctgttcccttcaatctggtgc
cgggtccagggtccctgttttgtctcctgctgaagcttaaagggtggatttctgagctctacccagggtcataaaaaaggtttccaggaagaggtagagctaagtaagtagagctaagtaagtac agc
taagtagggaaagacgggaacttgcaggaactaacaggtaccatagagacaggcagggacagatagagataggtgaagactagcaatataa agtcagtgccctaaagaggtacaatagtaaagactag
tagtataaggtcagtgccctaaagaggttctggtcagtgccttaaagaggtacagaagtagagagtagtgagtagtgaagactagcagagatttgcagggacagacaggaacattctgaattatg gaa
attagctatggctcaaagtccaatcaaaatctgagagggcaaatataaaggaatagagaggaggaggcaaggaaggataaagatgctctt tcttttctctccgacaggaccttttggattcaaagtt
gcgctagcaataggaaatgttactcttgatataaatacacattttattacatgccccgaatgtcgcttgtttacctgcattaattcaacctttgataaaaatcaaattatcttgataattaaagc cag
ggaaggagtttggatccctgtgtctctaaatagaccatgggagacatcgccctctatttatattgcaactgagatccttaaaaactcttat cccattccaaaagatttacagttgaaggccattggag

gctctacgtttgtggatttgttctggtggttgtatgcttgtgctgtctccttttagtctgcagatgtggaagctgcctctgaagaggaagccgcctctgaagaggaagccacctccgaagagaa agc
tgccatcaagaacaagcaatgagagctgtggcggttttacaaaaaagaaaaggggggcatgttgggagaagctgagtgttgggagaaacagaggcagggcttgcatgtctgaaataatgtaaaagagt
cttggaacatgtccaaggtccagggtctaaaacctctcgtggcctttggaacaccaagctctgtgctaaagggtggaaggctaccctgatgcaccataatctaagcccagggcataaacccctc atg
gcCTCTGGAATGTGTCTAGACTTGCTGGCTCCTTGCTTCTAGCCCTCTCAGGCTCCTAGATTGATTGTGTCTTAGAATTGGCCATATCAATGCTAAACCATCACAGCTGTAAATCATGTGCTTAATGC
AACATATCCTTTCGACCCCCACATTTTCACCACCTGTTTCTTTGTCTGATCACTAATAAATCGTCTGGACTTCCAGAGCTCGGGGACTTTGCAGCCTCCATACTTAGCAATGGCCGCCTGGACCC ACT
TTCTCAAACTGTCTTTTCTCATTCCTTTGACTCCACTGGACTTCATCACCCCCACAACC TGGTGCTGGGTCCGATCACCCCAACA
GTTTTGAGCTCAACACTCTAGTATAAAGGTAGACAATACAACATTCTAGTATATTTGGCTTGGCTGTATAACTTATGTTGTATATTGGAATGTGAGTGGACGTGGTGCAAGCAGAGGCTTTCGATGTA
CTTGTTTGTTCTTGTACTTCTGTTATCTTCCATGAAAAGAATATGCCCTGGGTAGCCTTCGATGCGATCACACAGTAATAAGATGCACGGGGCAGACTTGAATTCAACCCTTATTCTGGAGCCAAGCC
TAGCCAAGCCCAGTAGAGCCCACAGAAACCACAAATGACTAAGAACAAGAAATAAATACTTGTAAGTCACTGAGATTTGGGGGGTTATTTGTTACATCCCATTATTTCAGAAATAACCATCCATT ATA
ATGGGTAAATAAAAAAGAATCAAACATTTATTCTGCCTTTCTTGACGAAAGTACTTCAGGGTAACCAAATAGTTGATATGGAAAAGTTCTTCTTTATAGAG GAATCCCAGTTTATAAATGCAGGAAGG
ATGACAGGATAGTACCACTTTGCAAACTATAATGAAATGACAGCTATAGGCAAAGATCATCAAAAACTGCTAAAATTATTAGGTGAAAAGTTAATTGGGAAGTTTATAATAGATAGATCAGGGTG ACA
ATACATGAACAAACTAATTAGTGTTAACCTAACAAAAAAAGAGAGACAACCAGACATATGTGCCTCCTGACGTGATGCAGTAGGAAGTATAAAGGACACCA CGTATGATTTATTCTTGCTAAAATTTG
CATTTGTATCTGATAAACCTCTACACCAACCTATAGGAAATACAGGGTCAGAGGAACTGGTGAGTGGTGATATCAGTTACTGAGTTAAGGAAGACACCTTGGGGAAGGAGCAATTAGCAAAATCC AGA
GTGTGGGAAATTTTACAGGACAAACAACTGGTTCTTCCACAAAGACATTAAAAGGAAGGAGAAGAGAAGTGAAACCTATAATTAAAAGATTAAAGAGATAT AATAATCGACTCCAATATATCCACCTT
CTTTGGATCCTGATTCAAGCAAACAAACTGTAAAAAATGTAGGAGACAACCAGAGAAATTTTGATAGTAAATGGATATTATATGATATTAAGGAATACTTACCCTTAGATACAGTATCTTTAGAT ATG
ATTAAGGAATAGTTATCTTTAGATGTGATATTGTGATTATGTTTAAAAATTAGTCCCTATCTTTTAGAGGTACTTGCTGAAATATTTATGGATGAAATGAT ACATCTTGGTCTTATTTCAAAATGTGG
GGTGGTAGGTGGTGGCTGTAGAGGGAGCACTATTGGCTAAAAACCTAGGTTTTTGGCTGGAGCAATTAGGTGCATGGTGATACCAGTTAGTGATATAGGAAAGCCAGAGAAAGAAACAGGTTTTT ATC
AGGCCAAGGGGAGTGTGGCAGATTACAAGTTCTGTTTTTGATACATTAGATTTGAAATGCCTACTAGACATAGAAGAATAGATACCAAGCAGATGGTTGGA CACATAAGTCAGGTATTCAGCTGAGAG
GTCAGATTGGAGATATGTTTTTGGATGTCACCAGATTCCATAGTTTACACACTCTCTATGTGATCTTATCTTTAATATGTCTTTAATATGTCATCTTTAATCTATTTCTTCAACACTTCCTAGAT TAG
GTAGATGCTATTCTGTTTTCTGCAGTTTCGCCCACTCCTAAAAGGAGTGTAAGTTTGTTCAGCTATTTTGGATGACAACTTAGCAATATGCCTTAAAATTA AAATGCACATCCCCAAGTGGCCAAGCA
ATTCCACTTCTCTGTGTCTACCTTAAGGTAATATTTGCACATGGGCAAAAACAGACATACACAAGGACTCTCCCACAGCCTTGTTTATAACAGCAAAATAATTGAAGCAACCTAAATGTCCATCA ACA
TGGGAATGGCTAAATTAAAAATATTTCCATACTATGAATATTATGCAACAGTTAAAAAGAATGTGGGATAGCTCTATGTACTATAACTAAAAGGTCTTCAA GATATATTGTTGAATAAGGAAAGCAAG
TTGCAGAGGAATACTTTTATTTCTATTTTATACCTTTAAAAAACCTGTATATGTGCACATGTGTATGCATATATTTGTATATATGTATAAAAATTTAAAAATACTTCAAATAATATAGAACAATC AGA
TAACATTGGCTATATTTGGGTAGGATACTGGGTTTGGAGGCTAATCAAGATAACTTTTTAGCTTTATAAGTAATGTTTTAATTTTATAATGAAAAGATATT TACATGTTACTTGTAAAAATCATCTTT
TTTTTCTAATTTAaAAGAGTGGGAGTAATTACATGTAACCATCTTGAACCAAGTTATGCCAAACTATAGAGTGAAGTTATTAGTAACACCGGCTAGGGAGCAGGACTTCCTGGGTTTGAATCCTGGCT
CTACAATTTAGTCGCTGTCTGTGCTTGAGCAAGATGCATACATTCTTTATCCTTCTGTTTCCTCATTTTTAAGATGAAGCTAACAATATTATTTACTTCAG GGGGTGTGGTAAAGTCATATATGCAAA
AGGTTTAAAAGATTACCTGGTACATGGTAAATTATTACTGTCTTAGCTGTTTTTATTGTTATTATTTCCATTTCTTGATAGGCTTCTAGTTTGGTATATTAAATGTATTTGGATACTATTCTTGT GAA
TAAGATAATGAATGTGGGGCTGAAAAGTATTTTTTTTTTTTTGAGACGGAGTTTCACTCTTGATGCCCGAGCTGGAGTGCAATGGCATGACCTCGGCTCACTGCAACCTCTTCCTCCTGGGTTCAAGC
GATTCTTCTGCTTCAGCCTCCGGAGTAGCTGGGATTACAGGTGCCCACCACCACGCCCAGCTAATTTTTTGTATTTTTAGTAGAGACGGGGTTTCACCATGTTGGCCAGGCTAGTCTCGAACTCA GGT
GATCCACCAGCCTCGGCCTCCCAAAGTGCTGAGATTACAGGCGTGAGCCACCGTGCCCAGCTGAATGGTATTTTTAAAGAAAGTCATAGCAGATTAATTCACTAAACACTTTGCATACTTATCATGTG
CAATTGTGTCCAAATTGTTTTCCTTCATAGATTGCTAAATGTCTGAAAGGCCATATTGTGTCTTTCAATGGTTTTGTTAATTATTTGAAAGAAAGGAAAAGAAAACTGCGATGACATAAATCTTC AAG
GGAATACAAATTCCATTGATGACATGTGGGGTTGAATTCAATAAAATGAAAGAGTATTTTTTCATAAAGTAAAAGAGTCTGAAATTAGATCTTAAAGTTTCTCTCATCCAACAGCTATTCAATGCTGT
AATAAGCTCACCAGCATCCCAAGTAAGAATCATTCTATATCTTCTTAAGTGTTGTTTGAATGGGAGCCCATTACTTAATGAGGCAGACTATTCAATTGTTGGACAACTAGAACTGTACCTTATGT TGA
TGTTAAATAATCAGCTTCACAACTTCAACTGGTTCCTTTTGCCACTAAAGATACACAAATCAATTGTCTCTCATCTTTTACATGGCAACTTTTTGAATATTTGTAGACAATGGTCCTGCTACCCTGGA
ATCATTTCTTTCTTCAGGCTAAAAACTCCTGCACCTATTTGATTTCCCATCCTATCACTTTCCTTATATTCTGGACCAATTCCAGTTTAGTGGTGTTCTACTTAAACTGTAGGGTTTAGTATTTT TGT
TGCTGGGAAAGTACATCTGCTATTGTGGCTCATCACTTTTGCACATACCAGAGAGAGAATCTGGAGCAGTTATTCTTCTAATGACCCCATATAATTAACCC tTGTGGTGTCTAATTAAAAATAAACAT
ATATCAGGGTCCCTATGTTTCCCTGTGCAACTGTTCTAATTCACCTCTGTCCTCTGGCTACCATTTTACATAAGATATCCATAGGGCAGTTAATTTTGGA A

KS4	56	386	HERVL_40		918	1339	ERV	d	0.6891	1.5000	737
KS4	678	1169	L1ME_ORF2		1896	2435	NonLTR/L1	d	0.7455	1.8983	1677
KS4	1172	1261	MLT1E	497	593	ERV/ERV3	c	0.7363	1.6429	360
KS4	1299	1581	AluYb10		2	288	NonLTR/SINE/SINE1	c	0.8763	2.0667	1667
KS4	1591	1645	LTR33C	384	437	ERV/ERV3	c	0.7455	1.4444	241
KS4	1649	1954	L1MA9	770	1059	NonLTR/L1	d	0.8197	2.0000	1408
KS4	1955	2170	LTR33C	170	390	ERV/ERV3	c	0.7202	1.3902	876
KS4	2172	2718	MLT1G1	1	587	ERV/ERV3	d	0.6837	1.6067	1097

KS4	2728	2842	LTR33C	40	170	ERV/ERV3	c	0.6695	1.2000	347
KS4	2849	3447	LTR14B	4	608	ERV/ERV2	d	0.9435	1.2174	4485
KS4	3448	3669	HERV-K14I	1	218	ERV/ERV2	d	0.9000	1.2500	1665
KS4	3693	3813	MER69A	1	150	DNA/hAT	c	0.7258	1.5263	317
KS4	4763	4843	TAR1	27	116	Simple/Sat	d	0.7412	1.5455	246
KS4	4846	4925	HERVK3I	150	221	ERV/ERV2	d	0.8400	1.3333	391
KS4	5198	5358	HERV-K14CI	6574	6734	ERV/ERV2	d	0.7453	1.7826	709
KS4	5392	5588	HERVK	7373	7533	ERV/ERV2	d	0.7195	1.5926	485
KS4	5595	6129	LTR14B	1	608	ERV/ERV2	d	0.9140	1.3448	3632
KS4	6186	6261	MLT1H2	89	164	ERV/ERV3	d	0.6974	1.3125	257
KS4	6428	6480	MLT1I	319	376	ERV/ERV3	d	0.8519	1.2000	300
KS4	6517	6719	L1MC5	1236	1446	NonLTR/L1	d	0.7633	1.7600	839
KS4	6817	7448	L1MC5	1451	2040	NonLTR/L1	d	0.7478	1.7692	1563
KS4	7453	7699	L2	2329	2560	NonLTR/CR1	c	0.6966	1.7000	756
KS4	7826	8329	L1ME3C_3end	277	795	NonLTR/L1	d	0.7081	1.6667	1293
KS4	8508	8719	MIR	13	236	NonLTR/SINE/SINE2	d	0.7009	1.8235	720
KS4	8859	9134	AluSq	2	284	NonLTR/SINE/SINE1	c	0.9134	1.2222	1928
KS4	9403	9742	L3	4112	4470	NonLTR/CR1	c	0.6466	1.5909	585

Hg19	156	486	HERVL_40	918	1339	ERV	d	0.6891	1.5000	737
Hg19	778	1269	L1ME_ORF2	1896	2435	NonLTR/L1	d	0.7455	1.8983	1677
Hg19	1272	1361	MLT1E	497	593	ERV/ERV3	c	0.7363	1.6429	360
Hg19	1402	1684	AluYb10	2	288	NonLTR/SINE/SINE1	c	0.8763	2.0667	1667
Hg19	1694	1748	LTR33C	384	437	ERV/ERV3	c	0.7455	1.4444	241
Hg19	1752	2057	L1MA9	770	1059	NonLTR/L1	d	0.8197	2.0000	1408
Hg19	2058	2273	LTR33C	170	390	ERV/ERV3	c	0.7202	1.3902	876
Hg19	2275	2821	MLT1G1	1	587	ERV/ERV3	d	0.6837	1.5955	1079
Hg19	2828	2945	LTR33C	40	173	ERV/ERV3	c	0.6694	1.1935	361
Hg19	2952	3511	LTR14B	4	608	ERV/ERV2	d	0.9265	1.3600	4004
Hg19	3568	3643	MLT1H2	89	164	ERV/ERV3	d	0.6974	1.3125	257
Hg19	3810	3862	MLT1I	319	376	ERV/ERV3	d	0.8519	1.2000	300
Hg19	3899	4101	L1MC5	1236	1446	NonLTR/L1	d	0.7633	1.7600	839
Hg19	4199	4830	L1MC5	1451	2040	NonLTR/L1	d	0.7478	1.7692	1563
Hg19	4835	5081	L2	2329	2560	NonLTR/CR1	c	0.6966	1.7000	756
Hg19	5208	5711	L1ME3C_3end	277	795	NonLTR/L1	d	0.7081	1.6667	1293
Hg19	5890	6101	MIR	13	236	NonLTR/SINE/SINE2	d	0.7009	1.8235	720
Hg19	6242	6517	AluSq	2	284	NonLTR/SINE/SINE1	c	0.9134	1.2222	1928
Hg19	6786	7125	L3	4112	4470	NonLTR/CR1	c	0.6466	1.5909	585

>Hg19 chrY:59131987-59139339
GATTATTGTTCATTTACTTCTGGGTGGGAGGATGAAGGCATGTCATGATGGCAATGGGGTGGCAAGCCCTGGGGTTCCAGCCAAAAGGCAAGGTATGGTGTGAATGCTGAATCAAAGAACCGACAA TA
CTAAAATACCATCAGCAGTGAGGATATAGAGCTTTGAGCAGAGCAAAACATTAGAAGTTAATTTGTTAGGCTTGAGCAGTTGTTCACTGGAAACCCAAAGTAAATATAAAGCTTTGGTGCAGAAC TTA
GTTCTCACTGCACTTCCTGATTATTCTCTTCCCCTCTACTCTGATCTTAAGTATTTTAAGAAAAAAATGAAATATGCTGGGAAATTTTGGGAAGAGGAGGGACTAAAAATTTTTATAGCTCTGTTG GA
TATAAGCAATTCATGATGGGCCCTGAGCATCCTTTCAACTTCTTGCTCGGTATCCCCAAAATGCAGTGTTTCCAAGTCACCATCTTACTTGATCTTGCAGATGTGGCCCAAAACATTGAGGACTG CAG
GGTAAAGCTGCAGCAGCATGTGTGGGCACAAAAAGGGTCCCTGGGTCTGGGCCCTTGCTGGTCTCCAGAGGTATATGGGGAAGAGACAGCACCAACTCTGGGTGAAGAAGCCAAAGGAGAAAAAAA TA
AAAGCCTTCCTGCAACTCAGCCTTACAAAAAGTATGTACAAAAATCTTGCAGTGTACATCATATCTAAGGGTGAAAAAGTGAATGATTTTTCCCTAAGATCGTGAACAAGGTGGGCTGCCTATTC CTA

CAATTTCTGTTCAACATAGTACTGGAAATCCTAGCCAGTGTGGTGGGGGAAATAAAGAAATAATGGGCATACTGATTGCAAAAGTAGAAATAAAATTGTTCTTATTCACGAATGACATAATCATCT AT
GTATAAGGTCTTGAGGAATCTACATAAAAAGCTATTTGAGCGAATAAGGTCACAAGATAAAATATTAATTGTATTTCTGTGTACTAAGCAATGAACAATTGGGAATTAAAATTTTAGAATACCAT TTA
CAGAAGCATACAAAATCATTAAACATGGATGGATAAATTAAATATGTGTAAAAGTACAATACTTTGTTGAGAGAAATAAAAGAAGATGTAAATAAATGAAAGATATACCATGGTCATGTATCGGAA GA
CTGAATATTTTACAGATATTGTGTTAGTTTTCTATTATTCTGTAATAAATTAACAAAAATTTCAGGACAACACAAATTTATTGTTGTATCGTTTCTGTGACTCTGGAGTTGAGGCTAA TttgTTGTTG
TTGTTGTTGTTGTTGTTTATTGAGATGGAGAGACTCGCTCTGTTGCCCAGGCTGGAGTGCAATGGCAAGATCTCGGCTCACCGCAACCTCCGCCTCCCAGGTTCAAGCGATTCTCCTGCCTCAGCC TC
CCAAGTAGTGGGGATTACAGGCAAGCGCCACAATGCCCAGCTAATTTTTGTATTTTTAGTAGAGACGAGGTTTCACCATGTTGGCCAGGCTGGTCTGGAACTCGTGACCTCAAGTGATCCGCCCA CCT
CGGCCTCCCAAAGTGCTGGGATTACAGGCCTGAGCCACAGCGCCCGGCTGCAAATTGGGGGTTTTAAGGGTAATCTGAGAAGCCAATCACATCCAGCTTACTAAAGTAGGACTATGGTAGAAACTT TT
GGTTGTAAGATTAATAAGTTTTGGAGAGCTAATGTACAGCATGGTGACTATAGTTAATAATACTTTATTGTTTAGTTGAAAGTCAGTAAAAGAGCAGATTTTTGAGTGCCCTAACTGCACACACA CAC
ATGCACATATGTTACATATGGTAACTCTGTGTGGTGATGGATGTATTAATGAATTTGATTGTAGTAATCACTACGCAATGTATACCTATATCAACTCATCACATTGTACACCTTGAATATATGCAA TT
TGAATTTGTCAATTATACATCAAAATGCTGAGAAAAAGTAGGACCATGAAGTGGAAGCTCATTTAGAGGCCTATGGGAAGCTTTTGTCTCTGTGTATGGTATCACCTCTTTGGGACCTAGCTTTT GAT
TGTGAATCTAGAGCTGCTTTTGGTCAGCAGGGTCAGCAGTTGGAAAGATGAGCTAAATGCTGAGCAGAGGAAGTGATGACAAATTGGAATCTGTCAGGGACCTTTGCATCTGTTCATCACCATATT TG
TGGTATATTTGTATTTTTCAAAGATGGATGCAATAATATATTCCATCTTACATGCTCTTCTGCAATATGACTTGCCACTCCCCCAACAAGAGGTAGAGTGTATTTCTCCATCCCATTGGGCCTGG AAC
TAACTGTGAATACTTTGACTAGCAGATGATGGTGTAAATGGTACAATACCAGTTCTGGGCACAGCACTTACCTGGCATGAAAATGGATTCCTGCCACTTAGAACACTCACTATTGGAAT GCTTCCTCA
AAGAATCCAGCTTCCATGCTGTAGAAGCCCTAGCCACATGATGAGGCCACATGTAAGTGCTCTAGTCTATAGCACCAGCTGAATTCCCAGCCAACATCCAACCATGTGCATGAACCATCTTGGAGGCC
CAGTCCAGCTGACTATTTAGAGAAATCCAGCCCCCAGACACCATCTGACAAACTGTAAGACCCACTCAGCTGAACCTAGTCAACTCACAGAACCATTAGAGATAATAATAAGTTT aTAAGCCACTAAA
TCTTGGGACAATTTGCTACATAACAATGGATAATCAGCAGTaTCTGACCATGAAGACCCTCCAAGAATTATAGCCATTGTTTTACTTTTACCTCCTAATCACACAGTTTCTTCTAATATGAAACCATA
CAAATAAAGGGATTTCAGTAAATGTAGTTTT
TGTTGGGAAAAGCTGAGTGTTGGGAGAGAAGCTGAGGCAGGGCTTGCATGTCTGACATAATGTAACAGAGTCTTGGAACATGTCCGGGGTCCAGGGTCTAAAACCCCTCGTGGCCTTTGGAACAC CAA
GCTCTGTGCTAAAGGGTGGAAGGCTACCCTGATGCACCATAATCTAAGCCCAGGGCATAAAACCCCTCgTGGCTTGGATAGAATCCAGGGCTCgtgg TCTGGAATGTGTCTAGACTTGCTGGCTCCT
TGCTTCTAGCCCTCTCAGGCTCCTAGATTGATTGTGTCTTAGAATTGGCCATATCAATGCTAAACCATCACAGCTGTAAATCATGTGCTTAATGCAACATATCCTTTCGACCCCCACATTTTCAC CAC
CTGTTTCTTTGTCTGATCACTAATAAATCGTCTGGACTTCCAGAGCTCGGGGACTTTGCAGCCTCCATACTTAGCAATGGCCGCCTGGACCCACTTT CTCAAACTGTCTTTTCTCATTCCTTTGACTC
CACTGGACTTCATCACCCCCACAACCTGGTGCTGGGTCCGATCACCCCAACA
GTTTTGAGCTCAACACTCTAGTATAAAGGTAGACAATACAACATTCTAGTATATTTGGCTTGGCTGTATAACTTATGTTGTATATTGGAATGTGAGTGGACGTGGTGCAAGCAGAGGCTTTCGATGTA
CTTGTTTGTTCTTGTACTTCTGTTATCTTCCATGAAAAGAATATGCCCTGGGTAGCCTTCGATGCGATCACACAGTAATAAGATGCACGGGGCAGACTTGAATTCAACCCTTATTCTGGAGCCAAGCC
TAGCCAAGCCCAGTAGAGCCCACAGAAACCACAAATGACTAAGAACAAGAAATAAATACTTGTAAGTCACTGAGATTTGGGGGGTTATTTGTTACATCCCATTATTTCAGAAATAACCATCCATT ATA
ATGGGTAAATAAAAAGAATCAAACATTTATTCTGCCTTTCTTGACGAAAGTACTTCAGGGTAACCAAATAGTTGATATGGAAAAGTTCTTCTTTATAGAGGAATCCCAGTTTATAAATGCAGGAAGG
ATGACAGGATAGTACCACTTTGCAAACTATAATGAAATGACAGCTATAGGCAAAGATCATCAAAAACTGCTAAAATTATTAGGTGAAAAGTTAATTGGGAAGTTTATAATAGATAGATCAGGGTG ACA
ATACATGAACAAACTAATTAGTGTTAACCTAACAAAAAAAGAGAGACAACCAGACATATGTGCCTCCTGACGTGATGCAGTAGGAAGTATAAAGGACACCACGTATGATTTATTCTTGCTAAAATTTG
CATTTGTATCTGATAAACCTCTACACCAACCTATAGGAAATACAGGGTCAGAGGAACTGGTGAGTGGTGATATCAGTTACTGAGTTAAGGAAGACACCTTGGGGAAGGAGCAATTAGCAAAATCC AGA
GTGTGGGAAATTTTACAGGACAAACAACTGGTTCTTCCACAAAGACATTAAAAGGAAGGAGAAGAGAAGTGAAACCTATAATTAAAAGATTAAAGAGATATAATAATCGACTCCAATATATCCACCTT
CTTTGGATCCTGATTCAAGCAAACAAACTGTAAAAAATGTAGGAGACAACCAGAGAAATTTTGATAGTAAATGGATATTATATGATATTAAGGAATACTTACCCTTAGATACAGTATCTTTAGAT ATG
ATTAAGGAATAGTTATCTTTAGATGTGATATTGTGATTATGTTTAAAAATTAGTCCCTATCTTTTAGAGGTACTTGCTGAAATATTTATGGATGAAATGATACATCTTGGTCTTATTTCAAAATGTGG
GGTGGTAGGTGGTGGCTGTAGAGGGAGCACTATTGGCTAAAAACCTAGGTTTTTGGCTGGAGCAATTAGGTGCATGGTGATACCAGTTAGTGATATAGGAAAGCCAGAGAAAGAAACAGGTTTTT ATC
AGGCCAAGGGGAGTGTGGCAGATTACAAGTTCTGTTTTTGATACATTAGATTTGAAATGCCTACTAGACATAGAAGAATAGATACCAAGCAGATGGTTGGACACATAAGTCAGGTATTCAGCTGAGAG
GTCAGATTGGAGATATGTTTTTGGATGTCACCAGATTCCATAGTTTACACACTCTCTATGTGATCTTATCTTTAATATGTCTTTAATATGTCATCTTTAATCTATTTCTTCAACACTTCCTAGAT TAG
GTAGATGCTATTCTGTTTTCTGCAGTTTCGCCCACTCCTAAAAGGAGTGTAAGTTTGTTCAGCTATTTTGGATGACAACTTAGCAATATGCCTTAAAATTAAAATGCACATCCCCAAGTGGCCAAGCA
ATTCCACTTCTCTGTGTCTACCTTAAGGTAATATTTGCACATGGGCAAAAACAGACATACACAAGGACTCTCCCACAGCCTTGTTTATAACAGCAAAATAATTGAAGCAACCTAAATGTCCATCA ACA
TGGGAATGGCTAAATTAAAAATATTTCCATACTATGAATATTATGCAACAGTTAAAAAGAATGTGGGATAGCTCTATGTACTATAACTAAAAGGTCTTCAAGATATATTGTTGAATAAGGAAAGCAAG
TTGCAGAGGAATACTTTTATTTCTATTTTATACCTTTAAAAAACCTGTATATGTGCACATGTGTATGCATATATTTGTATATATGTATAAAAATTTAAAAATACTTCAAATAATATAGAACAATC AGA
TAACATTGGCTATATTTGGGTAGGATACTGGGTTTGGAGGCTAATCAAGATAACTTTTTAGCTTTATAAGTAATGTTTTAATTTTATAATGAAAAGATATTTACATGTTACTTGTAAAAATCATCTTT
TTTTTCTAATTTAgAAGAGTGGGAGTAATTACATGTAACCATCTTGAACCAAGTTATGCCAAACTATAGAGTGAAGTTATTAGTAACACCGGCTAGGGAGCAGGACTTCCTGGGTTTGAATCCTGGCT
CTACAATTTAGTCGCTGTCTGTGCTTGAGCAAGATGCATACATTCTTTATCCTTCTGTTTCCTCATTTTTAAGATGAAGCTAACAATATTATTTACTTCAGGGGGTGTGGTAAAGTCATATATGCAAA
AGGTTTAAAAGATTACCTGGTACATGGTAAATTATTACTGTCTTAGCTGTTTTTATTGTTATTATTTCCATTTCTTGATAGGCTTCTAGTTTGGTATATTAAATGTATTTGGATACTATTCTTGT GAA
TAAGATAATGAATGTGGGGCTGAAAAGTAtTTTTTTTTTTTTGAGACGGAGTTTCACTCTTGATGCCCGAGCTGGAGTGCAATGGCATGACCTCGGCTCACTGCAACCTCTTCCTCCTGGGTTCAAG
CGATTCTTCTGCTTCAGCCTCCGGAGTAGCTGGGATTACAGGTGCCCACCACCACGCCCAGCTAATTTTTTGTATTTTTAGTAGAGACGGGGTTTCACCATGTTGGCCAGGCTAGTCTCGAACTC AGG
TGATCCACCAGCCTCGGCCTCCCAAAGTGCTGAGATTACAGGCGTGAGCCACCGTGCCCAGCTGAATGGTATTTTTAAAGAAAGTCATAGCAGATTAATTCACTAAACACTTTGCATACTTATCATGT

GCAATTGTGTCCAAATTGTTTTCCTTCATAGATTGCTAAATGTCTGAAAGGCCATATTGTGTCTTTCAATGGTTTTGTTAATTATTTGAAAGAAAGGAAAAGAAAACTGCGATGACATAAATCTT CAA
GGGAATACAAATTCCATTGATGACATGTGGGGTTGAATTCAATAAAATGAAAGAGTATTTTTTCATAAAGTAAAAGAGTCTGAAATTAGATCTTAAAGTTTCTCTCATCCAACAGCTATTCAATGCTG
TAATAAGCTCACCAGCATCCCAAGTAAGAATCATTCTATATCTTCTTAAGTGTTGTTTGAATGGGAGCCCATTACTTAATGAGGCAGACTATTCAATTGTTGGACAACTAGAACTGTACCTTATG TTG
ATGTTAAATAATCAGCTTCACAACTTCAACTGGTTCCTTTTGCCACTAAAGATACACAAATCAATTGTCTCTCATCTTTTACATGGCAACTTTTTGAATATTTGTAGACAATGGTCCTGCTACCCTGG
AATCATTTCTTTCTTCAGGCTAAAAACTCCTGCACCTATTTGATTTCCCATCCTATCACTTTCCTTATATTCTGGACCAATTCCAGTTTAGTGGTGTTCTACTTAAACTGTAGGGTTTAGTATTT TTG
TTGCTGGGAAAGTACATCTGCTATTGTGGCTCATCACTTTTGCACATACCAGAGAGAGAATCTGGAGCAGTTATTCTTCTAATGACCCCATATAATTAACCC gTGTGGTGTCTAATTAAAAATAAACA
TATATCAGGGTCCCTATGTTTCCCTGTGCAACTGTTCTAATTCACCTCTGTCCTCTGGCTACCATTTTACATAAGATATCCATAGGGCAGTTAATTTTGGA A

L1HS

Instruction

Green box: Primer information

Red letter: Target site duplication

Black letter: Insertion sequences

Green letter: Deleted sequenced by insertion-mediated deletion

>KSI_L1HS_1

>Scaffold8776-3149934-3150755

AACCATCACCAATAATTTTAGAACATTCTCATCACCCAACAGAAAACCCCTGTACCATTTGCAGTCACGTTCCATCCCCCAGTCTGAAGCAACCAATCAGGCATCCTCTGTCTTTACAAATTTTCC TA
TTCTGGACATTTCATACAAATAGAATCAAATAATATGTCGTCTTTGGTATCTGGACTTTTTATTTATCATGATGTTTTCAAAGTTCATCCATGTCGTAGCATTTATCAGCATTTCATTATTTTCA AAT
GGCCAAATAATCCTCTGTCATATGAATATACCGTTTTGTTTATCCAGTCATCAGTTGATAAACATTTGGGTTGTTTCTACTTTTTGGTTATTATGAATAGTGCTGTTATAAATATTCCTGTACAAG TT
TTTGTGTGGACATATGTTTTCATTACTCTTTTTTTAATTTTTATTTTATTATTATACTTTAAGTTTTAGGGTACATATGCACA AcATGCAGGTTTGTTACATATGTATACATGTGCCATGTTGGTGTG
CTGCACCCATTAACTCGTCATTTAGCATTAGGTATATCTCCCAGTGCTCTTGATTATATACCTAGAAGTGAAATTGCTGGGTCATAGAGGAACTCTATATCTAACATTTTGAGAAACTGCCAAGCT GT
TTTCTAAAGTGACCACATCATTTTATAGTCCTACTAGCAATGTATATGAGTTTCAATTTCTTCCCATAT TCATCACTTTTTAATTTTGGCTCTTTTTGATTATAGCCATCCTAATGGGTATAAAGTAG
AGTCTCCCGTTTGTCAATCATGGCTATGATGCTAAAATTCTCATCTTCCTTTTTTTAAAGTAAGTTTTCTAATACAGAGTTCCTCCTAAAGAAAGCCCTGTTCCCCTTGGCATGTTTACAGTGACT CT
CACATAAAGGGAAAATTAATACAAACATACTGGACATTTTCCATGGGATGGGACCTACATTAAGCGTATATTATCTCACTAGATCTTCTCAACTGCTGTTTTGGATGGACAAGAACATACGACCA TGG
TAGTCTATTATTCTAAAAAGTTATTGAACAAAACATACCTATTTAAAACCCCAGTTGTAAAACCTCAGTGTCCCCAACCGTCTTGATTTCCCCAAATAGGTTGTTCCATTTCTCTCATGAGTGCCC TT
TAAGTATTTCATATTACAAAGGGAATTATTTTTCCAGTGACCTTGGCACACTGAAAACACCCCTGGCAAGCTCATTTAAGCTCACAGAACACCAGAGGAAGTAGTTAGAGAGAAAGCAGGTGGCT TCA
ATATACTACTTTGGCTTTCAGCTAGTAAGTGCTCTCCACATAACTCCTAATCAATGAGTTTTTCAGCAAAAGGTAGGTTGAGGGGAAGAGTGTTAGTGAGGAATGAGAGAGACTGATAAGTATTTG AG
GATTACATCACCCAAAATGAAAGACAAACTCATGAATTGAGTAGACAGAAGATTTGACTCACTCAGTTGCTCTATTGATGTAGCCAGATTATTTTCTTTCCTGAAAGTTGCACATAAGAGAATAA TCA
TCTAAAAGATATTCTTATTGTGTACTTTTATTCTGTAAATGTTCAAAAGCCAATATGTGCAAAACCATTTAGCAAAATGCCACCATGGGGTTCATAGTTTGTAATTTTAGAAAAGCAACCAAAGGC TG
CTGTACCTCATAAATTATTCAGTAAGGAGGTTTGAGTAAGATTGAAAAGTAGAAGTAAAACCTACACTCCCAGCAGGCGAAATATTGTTTGACATTTTTAATCATAATGAAACATACGACCTAGC GGG
GCAAAACTAAAACACATTTGCATGTCAAAGAGTAGGTTCCATTTAATTTGTTTAAGTTTCTTTGTTAGCATTTCTGTTTCTTATGAAATATCGTCAAGGTTATGAAGAATATAATAAGTATCTAAA TG
GTTGAGAGATATGAACACAGATAAAAACAGCTGATTTTCTGGCCAGTCTTTGGAAAGTGAGAAATTTGTATGCAAGAAGCCATTTCTCCCCTTTGCTTCTTTAGTGCAGTTTTATTGCATTTTCA GAA
GGCAAGAAAAGTAACCAAAGTATCGGCCAACTTACATTTATACAATGTTTTGTCTCCTGACTGTGCATACAATTAAGGACTTATACTCTTTTTCACATTTTTTGCCCTTACCTCAGGAAAAATGTC AT
GACAGCAAACCCTTAAGTTTAGGGGTTTACTTAATCATTTTATCCCTGTCTTAAAGGGAATGGAAAGGAGTTGGGCCTGTGATTTAGAGGAAAATTGAATTTCACAAGTTCAGATTTAGAGTTGA GGC
AACTTGAAACCTAAATTTTTCTTATCCTGAAAAGCTTTAAGATTATTGAAGAAAACAAAATGAACAGACAAAAAAAAACTCTTTCCACATATATAATCAATTTTTT aAATTTTGTGATAGGTTTTTAT
TTCTCATTGAATAAGCTGAAAAGTAACTTTAATGGCTAGTGAATTGATGTAAAGGAACACACATGTTTTCCATTGATTTTCCTCTTTTTAATTAATGCCATAATTTCTAGTTGAGAGAAGACTGA GAG
AAACTGATTGATGCTTAAAAAGATATTCTAACTTACCAGGAAATCATCCTGAGAACCTGTGGTTAAATGATTCAGTTGGCTTAACAATGTGTGGAAGAGAGAGTTTTGCAAGTGTTTACACTTCAC AA
AGACTTTCCAAGAGTTGGATTTTGTTGAGCTGACCTTTAATGCACAGAAGAGATGTTAGGATTGGAGACACGTGGGTATGGCTCTGTGTTTGTCTGGGTCTATTCTCTTGACTACTGCTTCTAGT AAG
GTTGGATGTTCCTCCAGGTCTGGGACAGAGCATGACACCTTTGGTTCTATAGCCCCAGTGTCTGACATTTACTGGAAAATCAAATATTTGAAAGAGTAAATGTGTAACTGCACAAATGAGTGAATG AA
ATGAAATTATGGCTAAATCTGGTCTCTTGGAAGGAACAGTGTTTCTTTGCATGTGATGGCATTTAAACGGATTTATGCAGTTGAATACTCACAGATAGCCCAGTCTAGGGTATGAGCCACAGGCC GTT
TACATAATTTGAATGAACTCATAAATGAGTTTATTTTAAAGGGGGACCTAAATATTAGTTGTTTTGCTGCAAAATTAGGTGAAATCACTCTTAATTTCCCAAATCTGTAATTTCTCCTACCAACCT CC
TTCTCTCACGAGCCTCTCTTACAGCAATTTTCCCAATTCTAGTGTCCTCAGTTTAGCTGGATCTCCAAACCCAATCCTGTCTCTGTCTTCTTCTTGCTATTTTGTTTTGGGTGA aTTACCTAATCTCT
CCTAACATAAATTGTTCATTTGTAAGAGGAGGTGCAAACACACTTCTCAGTGTTATGAGGTTTTAATGCAATCATAGATGTCTTTTACTGAGTACATAGAATCTTGTACATAGATTTGATCTTTTC AC
ATAGTCCCATATTTCTTGAGGCTTCGTTCATTTCTTTTCATTCTTTTTTCTCTAATTTTGTCGTCCCACTTTATTTCATTAATTTGATCTTCCATCACCGATATCCTTTCTTCCACTTGATCAAA TCA
GCTATTGAAGCTGGTGTGTGCTTCATGAAGTTCTCGTACTGTGGTTTTCAGCTCCATCAGATCATTTAAGCTCTTCTCTACACTGGTTATTCTAGTTAGCCATTTATCTAACCTTTTTTCAACATT TT
TAGCTTCCTTGCGATAGGTTAGAACATGCTCCTTTAGCTCAGAAAAGTTTGTTATTACTGACCTTCTGAAGCCTACTTCTGTCAACTTGTCAAACTCATTCTCTGTCCAGTTTTGTTCCCTTGCT GGC
AAGGAGTTGTGTTCCTTTGGAGGAGAAGAGGTGTTCTGGTTTTTGGAATTTTCAGCTTTTCTGCTCTGGTTTGTCCCCATCTTTGCGGTTTTATCTACCTTTGGTCTTTTATGTTTGTGACCTACA GT
TGGAGTTTTGGTGTGGATGCCCTTTTTGTGGATGTTGATGCGATTCCTTTCTGTTTGTTAGTTTTCCTTCCAACAGACAGTCCCCCTCACCTGCAGGTCTGTTGGAGTTTTCTGGAGGTCCTATC CAG
AACCTGTTTGCTTGGGTATCACCAGTGGAGGTTGCAGAACAGCAAATATTGCTGCCTGATCCTTCCTCTGGAAGCTTTGTCCCAGAGGGG cACCCACATGTATGAGGTGTCTGTCAGCCCCTCCTGAG
AGGTGTCTCCTAGTCAGGCTACATGGGGGTCAGGGACCCACTTGAGGAGGCAGTCTGTCCATTATCAGAGCTCAAACACTGTTCTGGGAAACCACTGATCTCTTTAGAGCTGTCAGGCAGGGATG TTT
AAGTCTGCAGAAGCTGTCTGCTGCCTTTTGTTCAGGTATGCCCTGCCCCCA cAGGTGGAATCTAGAGAGGCACTAGGCCTTGCTGAGCTGCAGTGGGCTCCACCCAGTTTAAGCTTCCCTGCCACTTT
GTTTATTATACTCTATGAGCATAGTACTGCCTACTCAAGCCTCAGCAATAGTGGACAGCCCTCCCCCTGCAAAGCTCCAGCATCGCAGGTCGAACTCAGACTGCTGTCCTAGCAGCAAGAAAGGC TCT

GTGAGCATGGGACCCACCAAGACAGGCACAGGATGGAATCTCTTGGTCTGCCGGTTGTCAAGACCATGGGAAAAGTGCAGTATTTGGGTAGGAGTATACCATTCATCCAGGTACAGCCACTCATGG CT
TCCCTTGGTTAGGAAAGGGAAATCTCCCAACcCCTTGCACCTCCCAGGTGATGCGATGCCCTGCCCTGCTTCTGCTTGCCCTCCGTAGGCTGCAATCACTGTCCAACCAGTCCCAGTGAAATGAACTA
GGTACCTCAGTTGCAAAAGCAGAAATTACCCGTCTTCTGCATCAGTCTTGCTGGGAGCTGTAGACCAGAGCTGTTTCTATA tAGCCATCTTGGAAGTGATTCCCTCCTTTAGTTCTTTGAGTATCTTT
ttttttttttttttttttttattatactctaagttttagggtacatgtgcacattgtgcaggttagttacatatgtatacatgtgccatgctggtgcgctgcacccactaatgtgtcatctagc att
aggtatatctcccaatgctatccctccccctcccccgaccccaccacagtccccagagtgtgatattccccttcctgtgtccatgtgatctcattgttcaattcccacctatgagtgagaatatg cg
gtgtttggtttttgttcttgcgatagtttactgagaatgatggtttccaatttcatccatgtccctacaaaggatatgaactcatcattttttatggctgcatagtattccatggtgtatatgt gcc
acattttcttaatccagtctatcattgttggacatttgggttggttccaagtctttgctattgtgaatagtgccgcaataaacatacgtgtgcatgtgtctttatagcagcatgatttatactcat tt
gggtatatacccagtaatgggatggctgggtcaaatggtatttctagttctagatccctgaggaatcgccacactgacttccacaatggttgaactagtttacagtcccaccaacagtgtaaaag tgt
tcctatttctccgcatcctcttcagcacctgttgtttcctgaatttttaatgattgccattctaactggtgtgagatgatatctcatagtggttttgatttgcatttctctgatggccagtgatga tg
agcatttcttcatgtgtttttggctgcataaatgtcttcttttgagaagtgtctgttcatggaga ctttAAGAGAGCTGTTTTAAAGTCTTTTTTTATTAGGTAgATCTGCTATGAAGTCTTTCTCA
AGGATAGTTCCTCTTTTGTGTTTGTGTTTTTTGTTTCCCTGTAAATGGGCCACACTTTCCAATTTAATTGTATGCCCCGTAGTTTTGTTGTTGTTGTTGAAAACTGGACATCTGAACTAATAATG TGG
CAGCTCTGAAAATCAGATTCTCCTCTTTCTCCAGGACTTGTTGTTTTCAATTTCTGTAGGCTGTCTCTGTGCTGATGATCAACCTGAGGTGTAAACTTAAATTCTC TTCAGGTGTTTTCTGAGCATGA
GCCTTTGCCTGGGTATGCACAGTAATTTTTAAAATGTTCCAAAGTATGCAGTTGCTTTTCAATGTCCTAGTGTAGTTTGGCTCCACAAAGAAGAAAAAGAGAAAAAGGAAAGTGATAAAAAAAAA AAA
GGCAGTGGACATTTCAATCTCCTCAACGTCATTCAACGGAAGGGAATTGGGTTTGTTTTAATGGGGGGGTGTGAAACAATAATAGCTGCTCACCTCTGGCTTAGGC CTCAGTGATTAGAAACAGCAAT
CAGAGTTCAGAACACAGATCCCCAATATTTAGAAGACAGGTCTTTATTGCTGTCCCACACCCCAGTTCCTACGATATGCATGGAAGCTGCTTCAGGAGCACATGCACCGGTGCCTGCCATGGGGC TGT
GCATGGGGAAGTAGTAGCTGCTGTTGAGCTAAAAGCTGAAATTGACCAAAGTTAACCACAACTGAGTGTTGAAGCCTTCTCTTGGAAGCTTCAAGGCTTTAATAGA CTCCATAGATCCAAAACAGTCA
TATAAGGCAGATTCTGCAAGCTTAATTGTTATGTAAGGGGGAGATAGATTCCTGGTGCTTCCTACCCAGTCATCTTCCTAGACTCCTCCTCTTGGCCTTCAGTTCTTAGTCTTCTTATCCATGTA GTT
TAATAATGGTTTAACAATTGCTGTGAGAATATGTGCACATATTTATATTTACTTATGCTCTTTATGATACTTCTGAGACCTATGAGCTAGAAATTGACTTATGTAA AGTGGCTTACGTATTTCCTTTG
GAGAAAAGAATGTTTTGGCTCATTTCAAAGTGTTGCATGCTCTCTAAGGAAAAATGCAATGTTTCTATACATATTTTAAACGAGTAAGAACCACAAAATTGTCTTACCTGTTCATGGAAATAATT GAG
TTACTAGTTATTGGAGTACTCTGTCCCATCACCTTTTTTTCTTCTGAATTGTGAGAGGGCTTGATTTAATGGGCCTGACTTTAGACATCGGAGAATTCTAATTCTA TTTCTCCTCTTACTAGTTTGGC
AATTTGGGAGAACTATTAACATCTCTATATCTCAAGTCTGTATTTGCTAAAATGGGAATAATAAGAACTAACTCAAAGGCATATTGTATACATGGTCAGCAAACATACAAAATGAGATGAAGTAT AAA
TATGCCTAGTCCCTAACTGGGCTTCAGCAAATGCCAGTTTATTTCCCTCCTTGAAGCTGAACTGGGCAGCTTGCTCTGCAGTACAGAGTTGCCTAAAATAATGCAA TGATGAGACTTGAATACCAGGT
CAGTAGCCTGCCAAGCTCTTTTTTCTAATCTCTCCTACTGATTCTTAAATATGGCCCAGATCCAAACTTCCCTTCAGGAGCTTGTTTTTATCCACAGCCCAATCTAGAATGTTTGGGTCCTTGAC TTG
ACTTAGTCTAGTTCCTGGAGAGGCTTACCTTGTCTGGCCTTTGTCTTTCTCTTTTAATCAATAGGAGTTATGCTTTTAATAAAGAAAAACACAGCATATTTCAGAA CAATGTATTATATGATAATTCC
AATCACAAAGAGCAAAGTTGACTCTCTCTGTTTTATCAGTATATTACTATTTGAATGTTTGTCCTCTTCAAAGCTAATCTTGAAATTTAATTGCCATTGTAACTGTATTAAGAGCAAGAAACTTT AAA
AGGTGATTGGGCCATGAAGGCTCCACCTTCAGGGATGGGATTAATGCAATTATAAGAGGTCAAATTTGCTCCCTTTTGCCTTTCTCAGCCCTTCCACCTTCTGCTA TGTGAGGATCCAGCCTACCTCC
CTCCAGAGGATGCAGAGTACAAaGCACATCCTTCTTGGAGGAGGAGAATGGCCTCACCAGACACCAAAACTGCCAATGCCTTGACCTTAGACTTCCAGCCTCCAGACCATGAGCCAATAAATTTCTGT
TAATTAAAAATTATGCAGTCACAGGTATTCTGTTATAGTAGCACAAATGAATTAAGGCAAGTTATCTCTTTTATAAGACTCTAGACCAATCGTTTACAACCAAATG TAGATTTGTATCTGATGGATAT
CTGGAGATATTTTTGGTTGTCATACCTGCAGGGGGTGCTGCTGGCATCTAGAGGGTAGTACAAGCCAAGGATACTGCTAAACATTTTACAATTTGCAGGACAGGCCTCATGACAAACAAAATTAT CTG
GTCCCCAATGTCAATAATAACAAGATTAAGAAGTCCTGATCTAGACTGAGCTGGGTATCCAGCTAGCCCCCTCAAACTCCCTCCCAATAAGCAAAGAGATATTAAT CTCCCTGACAGTAAGAGAAGGA
TGGGCAGCATTCCATTGCCATGGTCATCATACACTTATTAGGTACATGCCAGGGGTTCATCCCAACATGAACCTCTAAGACAAGATGTAAAGTAACTATAGTATATTAGATAACATACCACAGCT TGG
AGTTAGACAGATTTGTATCTGGTTTTTGCTCTGTCACTTAAAGCTATAGTTCTATGAGAAAGTAACTTGTGTTTCTGGAGCTTCAGCAACACTCAACAAAATGGTA GTGGTGGTATGCAGAAATTATG
TTTaTAATATCTTTAGAATAATGGTTGGAACATGGGAAACAAACAATAAATTGTAGATGTTACAGTTTTGACATCTTCATTACCATCATCGTCGTCGTCATCGTCATC GtcaTCATCATCATCATCAT
CATCATGTCTCATCTTAGCCTGACTTATAGCTATCTCCATGTTACAACTTAAGCTGTATGTGCCCACAATTCTGCATTTAAAAAAAACCATCTGTGTCAATTAACC TATGCATGACACAGTCAGGGAG
TCAATTCGAGGAGGGTGAAAGGAAATCAAGCAGTCAGAGAAAATGACATTTCATCAACTTGGCCCCACTTTCTATTAGCTTTTAGATTGATTTTCATGGAAATATATGATATGGGCTGGCATTAG AGG
AAAATGTCAACTTGCAAATGTGCTGGTTGTTGACAAGTACCCTATATTGCAGAATATTTTTATTCTTCTTTTCCTTTTTTTCCAAGATAATTTTTACATTTTTTTC TGGCTGTTTCTGAAACACTCAG
GCCTTCTCCAGTTTCATGCATTTGTTCACTCTATCACTTTGATCCTTTAAATTAGAAGAAATGTTACTGGTCTAAATTATAGAAAAGTCCAGAATTTCCTAGCTTTAAAGGGATCAGTCTAAGGC TCA
AGTTGTATCCTCAGTACCCAGTGTCTTTCCTTATTATCCCCTAAATCTGATCTCAACTGTTTTAGTCTCAGAGTCCCCCAGGTGTCAAGTCACTTATCAGTAGCTA TGGATTTATGTCTTTTCAGATT
GAATCCAGTGTCATGGGAGAATGTCTGTTTGTCTAATCCCTTAAGATCTTTAAGGGCAAAAAGCATATCTCAGTAATCTTTTAAACTCTTACAGTCTAGCATAGAATCCTACAAGTGTAGGAGTC TAC
TCACCAAATACATAGAAAAGACTGGTTGAATTAGATTTTCCAAGAATTCATATAGTTAAATATAGATAAGATACTCAAAAGGAAAAAAGAAAGAAAGTTGACTCCT AGAGAGAAAAGGCACTTAAGTT
GTACAACTGGTTAGTAGCAGAACATGAGAAACATTATTGATTGCTTGTAAGACAAGGTCATCCAATTTCCATTTCCAATAAATGTTGTGATGTGATTGCCGATGAAATGTTAGGAGATA cCTGCCAGT
GGGACTTCAGAGAGATGGTATTTTTGGCTTGTTTTTAACCTTCAAAATTGACCCATGAAAAGACACAGCCTCATTATTTCGCATAATGCTGCTTAGCAGTATATGC CAGATGGAACTCCAATAGACAT
TATTTGGCCATGGAAGGGGTGAACACAGAGAGGCTGGCAGAAAAGAAAGTTAGGAGGAGAAACCTAAGCCATGATGATGTCATTAAGCTGTTTAATGAACCAGGAATATCCTACTTCTACAGTTC TTC
TTCTTATGCAAGACAATAAATGTGTTTACCATTTAGGTCAATTATGTTAGGATTTCTGTAACTTGCAGCCAAAAATATACTCCTGGGTACCCAGAGAATGAGGGCT TTTCCCAAGAGATCAATTCACT
TATGCAGAAAGAAGACCTTTGGAATGAAAGCAGTTTTGTTTGGCCTAAACGTATGAAGAACATTGTCACCTGGTCAATAATTTCACAGATTCAAACTTTGTAGACAGTCTACTGGCCCCTTTTGA TGC
CCTTGCAGTCAGCATAATATTGCAAATATAGCTTCTTCCTTCAAGGAGTTTGGTCCCCCCAGAGACTTGAGAACTGATCTG cCCAGCCTGATAAGATATGTCATGCCAGCTTACTCTGTTTTTATCT

CAGCGCTACCAGGTATGTTGTAGGTATACTCAACATAAAGGTTAATTAGCAGATCCAATGACCTAGGTAATATGTATGTAGGAGATGAACATCTCTAGAAATAGATGAAAGGAAAAAGAAATGTG GGA
GGGAAGAAAAAAATATGAATCACCTTCATTTATCTTATTAATACATTACCTTAAATTCTGCTTTTAATTACTGGTAGGTCTGGTCTCAGGAAATGGCAGTAGAGTTG CACTTAGAATTCTGAGTTTTGG
AAAAAAATGAATGAGAAATATCTCTTCAATTAAAAAGAAGGAGCTGTTCAATGTGACTTCTTCCAAAATA T

3134	19.4	3.0	1.9	L1HS-Scaffold8776-3149934-3150755	1	421	(10401)	C	L1MB7	LINE/L1	(103)	6081	5655	13
1150	4.3	0.0	0.0	L1HS-Scaffold8776-3149934-3150755	422	560	(10262)	C	L1PA4	LINE/L1	(0)	6155	6017	14
3134	19.4	3.0	1.9	L1HS-Scaffold8776-3149934-3150755	561	774	(10048)	C	L1MB7	LINE/L1	(530)	5654	5439	13
279	32.9	5.1	0.0	L1HS-Scaffold8776-3149934-3150755	3273	3409	(7413)	+	MIRb	SINE/MIR	64	207	(61)	15
10721	11.1	1.2	1.6	L1HS-Scaffold8776-3149934-3150755	3436	4959	(5863)	C	L1P2	LINE/L1	(4662)	1522	5	16
7556	0.2	0.0	0.0	L1HS-Scaffold8776-3149934-3150755	5001	5822	(5000)	C	L1HS	LINE/L1	(0)	6155	5334	17
4164	21.4	3.5	3.1	L1HS-Scaffold8776-3149934-3150755	5827	6748	(4074)	C	L1M4c	LINE/L1	(5444)	940	16	18
262	29.5	5.7	0.8	L1HS-Scaffold8776-3149934-3150755	7133	7255	(3567)	+	MIR	SINE/MIR	50	178	(84)	19
1625	22.1	3.8	2.2	L1HS-Scaffold8776-3149934-3150755	7730	8123	(2699)	+	MLT1A1	LTR/ERVL-MaLR	9	408	(0)	20
850	20.0	2.9	2.9	L1HS-Scaffold8776-3149934-3150755	8189	8358	(2464)	+	MER20	DNA/hAT-Charlie	50	219	(0)	21
193	29.2	4.4	2.1	L1HS-Scaffold8776-3149934-3150755	8572	8662	(2160)	+	MIRc	SINE/MIR	35	127	(141)	22
31	10.9	0.0	0.0	L1HS-Scaffold8776-3149934-3150755	8788	8838	(1984)	+	(CAT)n	Simple_repeat	1	51	(0)	23
253	31.1	0.9	0.0	L1HS-Scaffold8776-3149934-3150755	8963	9068	(1754)	C	UCON105	DNA/hAT?	(46)	294	188	24
536	28.4	4.6	3.5	L1HS-Scaffold8776-3149934-3150755	9967	10193	(629)	+	MLT1L	LTR/ERVL-MaLR	278	600	(10)	25

3147	19.0	2.8	2.2	Hg19	1	421	(9562)	C	L1MB7	LINE/L1	(103)	6081	5655	1	
1106	4.3	0.7	0.0	Hg19	422	559	(9424)	C	L1PA4	LINE/L1	(0)	6155	6017	2	
3147	19.0	2.8	2.2	Hg19	560	776	(9207)	C	L1MB7	LINE/L1	(530)	5654	5439	1	
290	32.1	5.1	0.0	Hg19	3275	3411	(6572)	+	MIRb	SINE/MIR	64	207	(61)	3	
10726	11.1	1.2	1.6	Hg19	3438	4961	(5022)	C	L1P2	LINE/L1	(4662)	1522	5	4	
4320	21.2	3.4	3.0	Hg19	4957	5912	(4071)	C	L1M4c	LINE/L1	(1396)	1107	16	5	*
262	29.5	5.7	0.8	Hg19	6297	6419	(3564)	+	MIR	SINE/MIR	50	178	(84)	6	
1640	21.8	3.8	2.2	Hg19	6894	7287	(2696)	+	MLT1A1	LTR/ERVL-MaLR	9	408	(0)	7	
850	20.0	2.9	2.9	Hg19	7353	7522	(2461)	+	MER20	DNA/hAT-Charlie	50	219	(0)	8	
193	29.2	4.4	2.1	Hg19	7736	7826	(2157)	+	MIRc	SINE/MIR	35	127	(141)	9	
27	11.7	0.0	0.0	Hg19	7952	7999	(1984)	+	(CAT)n	Simple_repeat	1	48	(0)	10	
253	31.1	0.9	0.0	Hg19	8124	8229	(1754)	C	UCON105	DNA/hAT?	(46)	294	188	11	
536	28.4	4.6	3.5	Hg19	9128	9354	(629)	+	MLT1L	LTR/ERVL-MaLR	278	600	(10)	12	

>Hg19 chr10:110448478-110458460
AACCATCACCAATAATTTTAGAACATTCTCATCACCCAACAGAAAACCCCTGTACCATTTGCAGTCACGTTCCATCCCCCAGTCTGAAGCAACCAATCAGGCATCCTCTGTCTTTACAAATTTTCC TA
TTCTGGACATTTCATACAAATAGAATCAAATAATATGTCGTCTTTGGTATCTGGACTTTTTATTTATCATGATGTTTTCAAAGTTCATCCATGTCGTAGCATTTATCAGCATTTCATTATTTTCA AAT
GGCCAAATAATCCTCTGTCATATGAATATACCGTTTTGTTTATCCAGTCATCAGTTGATAAACATTTGGGTTGTTTCTACTTTTTGGTTATTATGAATAGTGCTGTTATAAATATTCCTGTACAAG TT
TTTGTGTGGACATATGTTTTCATTACTCTTTTTTTAATTTTTATTTTATTATTATACTTTAAGTTTTAGGGTACATATGCACA AATGCAGGTTTGTTACATATGTATACATGTGCCATGTTGGTGTGC
TGCACCCATTAACTCGTCATTTAGCATTAGGTATATCTCCCAGTGCTCTTGATTATATACCTAGAAGTGAAATTGCTGGGTCATAGAGGAACTCTATATCTAACATTTTGAGAAACTGCCAAGCTG TT
TTCTAAAGTGACCACATCATTTTATAGTCCTACTAGCAATGTATATGAGTTTCAATTTCTTCCCATAT TcagCATCACTTTTTAATTTTGGCTCTTTTTGATTATAGCCATCCTAATGGGTATAAAGT
AGAGTCTCCCGTTTGTCAATCATGGCTATGATGCTAAAATTCTCATCTTCCTTTTTTTAAAGTAAGTTTTCTAATACAGAGTTCCTCCTAAAGAAAGCCCTGTTCCCCTTGGCATGTTTACAGTGA CT
CTCACATAAAGGGAAAATTAATACAAACATACTGGACATTTTCCATGGGATGGGACCTACATTAAGCGTATATTATCTCACTAGATCTTCTCAACTGCTGTTTTGGATGGACAAGAACATACGAC CAT
GGTAGTCTATTATTCTAAAAAGTTATTGAACAAAACATACCTATTTAAAACCCCAGTTGTAAAACCTCAGTGTCCCCAACCGTCTTGATTTCCCCAAATAGGTTGTTCCATTTCTCTCATGAGTGC CC
TTTAAGTATTTCATATTACAAAGGGAATTATTTTTCCAGTGACCTTGGCACACTGAAAACACCCCTGGCAAGCTCATTTAAGCTCACAGAACACCAGAGGAAGTAGTTAGAGAGAAAGCAGGTGG CTT
CAATATACTACTTTGGCTTTCAGCTAGTAAGTGCTCTCCACATAACTCCTAATCAATGAGTTTTTCAGCAAAAGGTAGGTTGAGGGGAAGAGTGTTAGTGAGGAATGAGAGAGACTGATAAGTATT TG

AGGATTACATCACCCAAAATGAAAGACAAACTCATGAATTGAGTAGACAGAAGATTTGACTCACTCAGTTGCTCTATTGATGTAGCCAGATTATTTTCTTTCCTGAAAGTTGCACATAAGAGAAT AAT
CATCTAAAAGATATTCTTATTGTGTACTTTTATTCTGTAAATGTTCAAAAGCCAATATGTGCAAAACCATTTAGCAAAATGCCACCATGGGGTTCATAGTTTGTAATTTTAGAAAAGCAACCAAAG GC
TGCTGTACCTCATAAATTATTCAGTAAGGAGGTTTGAGTAAGATTGAAAAGTAGAAGTAAAACCTACACTCCCAGCAGGCGAAATATTGTTTGACATTTTTAATCATAATGAAACATACGACCTA GCG
GGGCAAAACTAAAACACATTTGCATGTCAAAGAGTAGGTTCCATTTAATTTGTTTAAGTTTCTTTGTTAGCATTTCTGTTTCTTATGAAATATCGTCAAGGTTATGAAGAATATAATAAGTATCTA AA
TGGTTGAGAGATATGAACACAGATAAAAACAGCTGATTTTCTGGCCAGTCTTTGGAAAGTGAGAAATTTGTATGCAAGAAGCCATTTCTCCCCTTTGCTTCTTTAGTGCAGTTTTATTGCATTTT CAG
AAGGCAAGAAAAGTAACCAAAGTATCGGCCAACTTACATTTATACAATGTTTTGTCTCCTGACTGTGCATACAATTAAGGACTTATACTCTTTTTCACATTTTTTGCCCTTACCTCAGGAAAAATG TC
ATGACAGCAAACCCTTAAGTTTAGGGGTTTACTTAATCATTTTATCCCTGTCTTAAAGGGAATGGAAAGGAGTTGGGCCTGTGATTTAGAGGAAAATTGAATTTCACAAGTTCAGATTTAGAGTT GAG
GCAACTTGAAACCTAAATTTTTCTTATCCTGAAAAGCTTTAAGATTATTGAAGAAAACAAAATGAACAGACAAAAAAAAACTCTTTCCACATATATAATCAATTTTTT tAATTTTGTGATAGGTTTTT
ATTTCTCATTGAATAAGCTGAAAAGTAACTTTAATGGCTAGTGAATTGATGTAAAGGAACACACATGTTTTCCATTGATTTTCCTCTTTTTAATTAATGCCATAATTTCTAGTTGAGAGAAGACT GAG
AGAAACTGATTGATGCTTAAAAAGATATTCTAACTTACCAGGAAATCATCCTGAGAACCTGTGGTTAAATGATTCAGTTGGCTTAACAATGTGTGGAAGAGAGAGTTTTGCAAGTGTTTACACTTC AC
AAAGACTTTCCAAGAGTTGGATTTTGTTGAGCTGACCTTTAATGCACAGAAGAGATGTTAGGATTGGAGACACGTGGGTATGGCTCTGTGTTTGTCTGGGTCTATTCTCTTGACTACTGCTTCTA GTA
AGGTTGGATGTTCCTCCAGGTCTGGGACAGAGCATGACACCTTTGGTTCTATAGCCCCAGTGTCTGACATTTACTGGAAAATCAAATATTTGAAAGAGTAAATGTGTAACTGCACAAATGAGTGAA TG
AAATGAAATTATGGCTAAATCTGGTCTCTTGGAAGGAACAGTGTTTCTTTGCATGTGATGGCATTTAAACGGATTTATGCAGTTGAATACTCACAGATAGCCCAGTCTAGGGTATGAGCCACAGG CCG
TTTACATAATTTGAATGAACTCATAAATGAGTTTATTTTAAAGGGGGACCTAAATATTAGTTGTTTTGCTGCAAAATTAGGTGAAATCACTCTTAATTTCCCAAATCTGTAATTTCTCCTACCAAC CT
CCTTCTCTCACGAGCCTCTCTTACAGCAATTTTCCCAATTCTAGTGTCCTCAGTTTAGCTGGATCTCCAAACCCAATCCTGTCTCTGTCTTCTTCTTGCTATTTTGTTTTGGGTGA gTTACCTAATCT
CTCCTAACATAAATTGTTCATTTGTAAGAGGAGGTGCAAACACACTTCTCAGTGTTATGAGGTTTTAATGCAATCATAGATGTCTTTTACTGAGTACATAGAATCTTGTACATAGATTTGATCTTT TC
ACATAGTCCCATATTTCTTGAGGCTTCGTTCATTTCTTTTCATTCTTTTTTCTCTAATTTTGTCGTCCCACTTTATTTCATTAATTTGATCTTCCATCACCGATATCCTTTCTTCCACTTGATCA AAT
CAGCTATTGAAGCTGGTGTGTGCTTCATGAAGTTCTCGTACTGTGGTTTTCAGCTCCATCAGATCATTTAAGCTCTTCTCTACACTGGTTATTCTAGTTAGCCATTTATCTAACCTTTTTCAACA TT
TTTAGCTTCCTTGCGATAGGTTAGAACATGCTCCTTTAGCTCAGAAAAGTTTGTTATTACTGACCTTCTGAAGCCTACTTCTGTCAACTTGTCAAACTCATTCTCTGTCCAGTTTGTTCCCTTG CTG
GCAAGGAGTTGTGTTCCTTTGGAGGAGAAGAGGTGTTCTGGTTTTTGGAATTTTCAGCTTTTCTGCTCTGGTTTGTCCCCATCTTTGCGGTTTTATCTACCTTTGGTCTTTTATGTTTGTGACCTA CA
GTTGGAGTTTTGGTGTGGATGCCCTTTTTGTGGATGTTGATGCGATTCCTTTCTGTTTGTTAGTTTTCCTTCCAACAGACAGTCCCCCTCACCTGCAGGTCTGTTGGAGTTTTCTGGAGGTCCTA TCC
AGAACCTGTTTGCTTGGGTATCACCAGTGGAGGTTGCAGAACAGCAAATATTGCTGCCTGATCCTTCCTCTGGAAGCTTTGTCCCAGAGGGG aACCCACATGTATGAGGTGTCTGTCAGCCCCTCCTG
AGAGGTGTCTCCTAGTCAGGCTACATGGGGGTCAGGGACCCACTTGAGGAGGCAGTCTGTCCATTATCAGAGCTCAAACACTGTTCTGGGAAACCACTGATCTCTTTAGAGCTGTCAGGCAGGGA TGT
TTAAGTCTGCAGAAGCTGTCTGCTGCCTTTTGTTCAGGTATGCCCTGCCCCCA gAGGTGGAATCTAGAGAGGCACTAGGCCTTGCTGAGCTGCAGTGGGCTCCACCCAGTTTAAGCTTCCCTGCCACT
TTGTTTATTATACTCTATGAGCATAGTACTGCCTACTCAAGCCTCAGCAATAGTGGACAGCCCTCCCCCTGCAAAGCTCCAGCATCGCAGGTCGAACTCAGACTGCTGTCCTAGCAGCAAGAAAG GCT
CTGTGAGCATGGGACCCACCAAGACAGGCACAGGATGGAATCTCTTGGTCTGCCGGTTGTCAAGACCATGGGAAAAGTGCAGTATTTGGGTAGGAGTATACCATTCATCCAGGTACAGCCACTCAT GG
CTTCCCTTGGTTAGGAAAGGGAAATCTCCCAAC tCCTTGCACCTCCCAGGTGATGCGATGCCCTGCCCTGCTTCTGCTTGCCCTCCGTAGGCTGCAATCACTGTCCAACCAGTCCCAGTGAAATGAAC
TAGGTACCTCAGTTGCAAAAGCAGAAATTACCCGTCTTCTGCATCAGTCTTGCTGGGAGCTGTAGACCAGAGCTGTTTCTATA cAGCCATCTTGGAAGTGATTCCCTCCTTTAGTTCTTTGAGTATCT
TTAAGAGAGCTGTTTAAAGTCTTTTTTTATTAGGTA aATCTGCTATGAAGTCTTTCTCAAGGATAGTTCCTCTTTTGTGTTTGTGTTTTTTGTTTCCCTGTAAATGGGCCACACTTTCCAATTTAAT
TGTATGCCCCGTAGTTTTGTTGTTGTTGTTGAAAACTGGACATCTGAACTAATAATGTGGCAGCTCTGAAAAT CAGATTCTCCTCTTTCTCCAGGACTTGTTGTTTTCAATTTCTGTAGGCTGTCTCT
GTGCTGATGATCAACCTGAGGTGTAAACTTAAATTCTC TTCAGGTGTTTTCTGAGCATGAGCCTTTGCCTGGGTATGCACAGTAATTTTTAAAATGTTCCAAAGTATGCAGTTGCTTTTCAATGTCCT
AGTGTAGTTTGGCTCCACAAAGAAGAAAAAGAGAAAAAGGAAAGTGATAAAAAAAAAAAAAGGCAGTGGACATTTCAATCTCCTCAACGTCATTCAACGGAAGGGAATTGGGTTTGTTTTAATGGG GGG
GTGTGAAACAATAATAGCTGCTCACCTCTGGCTTAGGC CTCAGTGATTAGAAACAGCAATCAGAGTTCAGAACACAGATCCCCAATATTTAGAAGACAGGTCTTTATTGCTGTCCCACACCCCAGTTC
CTACGATATGCATGGAAGCTGCTTCAGGAGCACATGCACCGGTGCCTGCCATGGGGCTGTGCATGGGGAAGTAGTAGCTGCTGTTGAGCTAAAAGCTGAAATTGACCAAAGTTAACCACAACTGA GTG
TTGAAGCCTTCTCTTGGAAGCTTCAAGGCTTTAATAGA CTCCATAGATCCAAAACAGTCATATAAGGCAGATTCTGCAAGCTTAATTGTTATGTAAGGGGGAGATAGATTCCTGGTGCTTCCTACCCA
GTCATCTTCCTAGACTCCTCCTCTTGGCCTTCAGTTCTTAGTCTTCTTATCCATGTAGTTTAATAATGGTTTAACAATTGCTGTGAGAATATGTGCACATATTTATATTTACTTATGCTCTTTAT GAT
ACTTCTGAGACCTATGAGCTAGAAATTGACTTATGTAAAGTGGCTTACGTATTTCCTTTGGAGAAAAGAATGTTTTGGCTCATTTCAAAGTGTTGCATGCTCTCTAAGGAAAAATGCAATGTTTCTAT
ACATATTTTAAACGAGTAAGAACCACAAAATTGTCTTACCTGTTCATGGAAATAATTGAGTTACTAGTTATTGGAGTACTCTGTCCCATCACCTTTTTTTCTTCTGAATTGTGAGAGGGCTTGAT TTA
ATGGGCCTGACTTTAGACATCGGAGAATTCTAATTCTA TTTCTCCTCTTACTAGTTTGGCAATTTGGGAGAACTATTAACATCTCTATATCTCAAGTCTGTATTTGCTAAAATGGGAATAATAAGAAC
TAACTCAAAGGCATATTGTATACATGGTCAGCAAACATACAAAATGAGATGAAGTATAAATATGCCTAGTCCCTAACTGGGCTTCAGCAAATGCCAGTTTATTTCCCTCCTTGAAGCTGAACTGG GCA
GCTTGCTCTGCAGTACAGAGTTGCCTAAAATAATGCAA TGATGAGACTTGAATACCAGGTCAGTAGCCTGCCAAGCTCTTTTTTCTAATCTCTCCTACTGATTCTTAAATATGGCCCAGATCCAAACT
TCCCTTCAGGAGCTTGTTTTTATCCACAGCCCAATCTAGAATGTTTGGGTCCTTGACTTGACTTAGTCTAGTTCCTGGAGAGGCTTACCTTGTCTGGCCTTTGTCTTTCTCTTTTAATCAATAGG AGT

TATGCTTTTAATAAAGAAAAACACAGCATATTTCAGAA CAATGTATTATATGATAATTCCAATCACAAAGAGCAAAGTTGACTCTCTCTGTTTTATCAGTATATTACTATTTGAATGTTTGTCCTCTT
CAAAGCTAATCTTGAAATTTAATTGCCATTGTAACTGTATTAAGAGCAAGAAACTTTAAAAGGTGATTGGGCCATGAAGGCTCCACCTTCAGGGATGGGATTAATGCAATTATAAGAGGTCAAAT TTG
CTCCCTTTTGCCTTTCTCAGCCCTTCCACCTTCTGCTA TGTGAGGATCCAGCCTACCTCCCTCCAGAGGATGCAGAGTACAA gGCACATCCTTCTTGGAGGAGGAGAATGGCCTCACCAGACACCAAA
ACTGCCAATGCCTTGACCTTAGACTTCCAGCCTCCAGACCATGAGCCAATAAATTTCTGTTAATTAAAAATTATGCAGTCACAGGTATTCTGTTATAGTAGCACAAATGAATTAAGGCAAGTTAT CTC
TTTTATAAGACTCTAGACCAATCGTTTACAACCAAATG TAGATTTGTATCTGATGGATATCTGGAGATATTTTTGGTTGTCATACCTGCAGGGGGTGCTGCTGGCATCTAGAGGGTAGTACAAGCCAA
GGATACTGCTAAACATTTTACAATTTGCAGGACAGGCCTCATGACAAACAAAATTATCTGGTCCCCAATGTCAATAATAACAAGATTAAGAAGTCCTGATCTAGACTGAGCTGGGTATCCAGCTA GCC
CCCTCAAACTCCCTCCCAATAAGCAAAGAGATATTAAT CTCCCTGACAGTAAGAGAAGGATGGGCAGCATTCCATTGCCATGGTCATCATACACTTATTAGGTACATGCCAGGGGTTCATCCCAACAT
GAACCTCTAAGACAAGATGTAAAGTAACTATAGTATATTAGATAACATACCACAGCTTGGAGTTAGACAGATTTGTATCTGGTTTTTGCTCTGTCACTTAAAGCTATAGTTCTATGAGAAAGTAA CTT
GTGTTTCTGGAGCTTCAGCAACACTCAACAAAATGGTA GTGGTGGTATGCAGAAATTATGTTT cTAATATCTTTAGAATAATGGTTGGAACATGGGAAACAAACAATAAATTGTAGATGTTACAGTTT
TGACATCTTCATTACCATCATCGTCGTCGTCATCGTCATC GTCATCATCATCATCATCATCATGTCTCATCTTAGCCTGACTTATAGCTATCTCCATGTTACAACTTAAGCTGTATGTGCCCACAATT
CTGCATTTAAAAAAAACCATCTGTGTCAATTAACCTAT GCATGACACAGTCAGGGAGTCAATTCGAGGAGGGTGAAAGGAAATCAAGCAGTCAGAGAAAATGACATTTCATCAACTTGGCCCCACTTT
CTATTAGCTTTTAGATTGATTTTCATGGAAATATATGATATGGGCTGGCATTAGAGGAAAATGTCAACTTGCAAATGTGCTGGTTGTTGACAAGTACCCTATATTGCAGAATATTTTTATTCTTC TTT
TCCTTTTTTTCCAAGATAATTTTTACATTTTTTTCTGGCTGTTTCTGAAACACTCAGGCCTTCTCCAGTTTCATGCATTTGTTCACTCTATCACTTTGATCCTTTAAATTAGAAGAAATGTTACTGGT
CTAAATTATAGAAAAGTCCAGAATTTCCTAGCTTTAAAGGGATCAGTCTAAGGCTCAAGTTGTATCCTCAGTACCCAGTGTCTTTCCTTATTATCCCCTAAATCTGATCTCAACTGTTTTAGTCT CAG
AGTCCCCCAGGTGTCAAGTCACTTATCAGTAGCTATGGATTTATGTCTTTTCAGATTGAATCCAGTGTCATGGGAGAATGTCTGTTTGTCTAATCCCTTAAGATCTTTAAGGGCAAAAAGCATATCTC
AGTAATCTTTTAAACTCTTACAGTCTAGCATAGAATCCTACAAGTGTAGGAGTCTACTCACCAAATACATAGAAAAGACTGGTTGAATTAGATTTTCCAAGAATTCATATAGTTAAATATAGATA AGA
TACTCAAAAGGAAAAAAGAAAGAAAGTTGACTCCTAGA GAGAAAAGGCACTTAAGTTGTACAACTGGTTAGTAGCAGAACATGAGAAACATTATTGATTGCTTGTAAGACAAGGTCATCCAATTTCCA
TTTCCAATAAATGTTGTGATGTGATTGCCGATGAAATGTTAGGAGATA tCTGCCAGTGGGACTTCAGAGAGATGGTATTTTTGGCTTGTTTTTAACCTTCAAAATTGACCCATGAAAAGACACAGCCT
CATTATTTCGCATAATGCTGCTTAGCAGTATATGCCAGATGGAACTCCAATAGACATTATTTGGCCATGGAAGGGGTGAACACAGAGAGGCTGGCAGAAAAGAAAGTTAGGAGGAGAAACCTAAGCCA
TGATGATGTCATTAAGCTGTTTAATGAACCAGGAATATCCTACTTCTACAGTTCTTCTTCTTATGCAAGACAATAAATGTGTTTACCATTTAGGTCAATTATGTTAGGATTTCTGTAACTTGCAG CCA
AAAATATACTCCTGGGTACCCAGAGAATGAGGGCTTTT CCCAAGAGATCAATTCACTTATGCAGAAAGAAGACCTTTGGAATGAAAGCAGTTTTGTTTGGCCTAAACGTATGAAGAACATTGTCACCT
GGTCAATAATTTCACAGATTCAAACTTTGTAGACAGTCTACTGGCCCCTTTTGATGCCCTTGCAGTCAGCATAATATTGCAAATATAGCTTCTTCCTTCAAGGAGTTTTGGTCCCCCCAGAGACT TGA
GAACTGATCTGtCCAGCCTGATAAGATATGTCATGCCAGCTTACTCTGTTTTTATCTCAGCGCTACCAGGTATGTTGTAGGTATACTCAACATAAAGGTTAATTAGCAGATCCAATGACCTAGGTAAT
ATGTATGTAGGAGATGAACATCTCTAGAAATAGATGAAAGGAAAAAGAAATGTGGGAGGGAAGAAAAAAATATGAATCACCTTCATTTATCTTATTAATACATTACCTTAAATTCTGCTTTTAATT ACT
GGTAGGTCTGGTCTCAGGAAATGGCAGTAGAGTTGCAC TTAGAATTCTGAGTTTTGGAAAAAAATGAATGAGAAATATCTCTTCAATTAAAAAGAAGGAGCTGTTCAATGTGACTTCTTCCAAAATA T

>KSI_L1HS_2

>Scaffold10609-167777-169006
GATGCTTAAATACAATTTTCTCAGCACCTGGTGAATAAATTTCATCAGATTATTTCCTATTAGAGATCCTCCTTCAAATTTGCCACATCTGTGTTATTTTTTTCCCTTTAGGAAAGAGCAGAAGAA TG
TGTTGAGAATTAACCCTGTCGTTTCAGTTTGTACATTCCTGTTATACCTCGAGATATTACTTTTTAATTTTAATTTTTATTTCCATGGTTTTGTGGGGTAAGGTGGTTTTTGGTTACATGGATAA GTT
ATTTAGTGGTGATTTCTGAGATTTTGGTGCACCTGTTACCCAAGCAGTGTACACTGTACCCAATGTGCAGTCTTTTATCCCTCACCCCCCTCCCACTCTTGTTCCCTGAGTCTGCAAAGACCATTA CA
TCATTCTTATGCTTCTCCATCAGTGTCTGTCTCAACATCACGGTGTGCACGTCAAGACAGTACGAAAATGACTTTTTTATTTTATAACTTTTTAATGCTATCTTTCCCGCACTTGATGATGTTGA GAC
ACTGATGGAGATGCCTGGGGCATTACAGGGAAATGTACCTCCAGTAATGCCACGTTTTCTTTTCGAGGTTACTTCTGAACAGCTTTAAAGAGGGCGCTGCCTGCCTCATAGTCTGCAGCCTCGTGG TG
ACCAGTTGGTCACCAGGGGGCAGGCTTGGATCACAAAAGTGTGTCACCGCTCATTTTTCTTCAAGAGAAAGAAGCGTATTGCTCTCGCTATAAAACTGTGGTGCAATTCATTTTTTACAAACCGG ATG
GTCGATAAAAATAAGTCATTTTGTATTATAACCATGAACTCAAGATATATCATTCATTTAAAAACTACGTTCTATATTTTTGTAAAATAAAAAAATACATTTTATCAATTCCAATTTTGTTTAATT TT
ACTCATTAAACATTGATACAGGTGAAGAAAATTTTTTTACAGATAGTTTTTGCCCTCTAATATTAACTGTACAAAGCAAAGTATTGTTGTAACAGTAGAAGAATAAACAGAAACTACTGACTAGC ATA
TGGCAAAATTTGGGATCTCAGATCTTTTAAAAATTATTTTATCATTACAAAAGTAATATCTACTTTATTGTTGAAAACAGAAATTACTGAGCTACAAAAAGGAAAAAAATACTAAGCAGGCTTCAT TC
CAATATTCGAAGGGAACTATTCTAAGATGTTGGTGCATTTTGTTAAGATGTATTTTAGATCAAGATGCCTCTATTTATCAGTTTGTCTTTCTCAAACTTAGAATCAGGCATTGTGTAACCTGCTT CTA
CTTTTCACTTAATAGCTTATTGTTGACAAGTTTCCTGTCATTTAATACAATTCTACATTATAACCTGTAGCAGCTTTGCATGATATTGTATCAATGTGCATTATTTACATATTCAATACCTTATTT TA
AGCTATTTAAAAAGTTATTTCTAATGTTTTGAGTAGCTCCTTTTAAGTGAAAATGAAACAACTTTTCCAAGACTTATGAAACATAATGCATACAGAGTTAACATTTTTCTTTTTAGACGGAGTCT CGC
TCTGTCGCCAGGCTGGAGTTCGATGGCATGATCTCGGCTCACTGCAACCTCTGCCTCCTGGGTTCAAGTGATTCTCCTGCCTCAACCTCCCAAGTAGCTGGGACTGCAGGCACATGCCACCACGCC CA
GCTTATTTTTGTATTTTTAGTAGAGACGGGGTTTCCCCATGTTGGCCAGGAAACTCTCCAACTCTTGACCTCTTGATCCACCCGCCTCGGCCTCCCAAAGTGCTGGGATTACAGGTGTAAGCCAC CGT
GCCTGGCCTAGAGTTAACATTTTTCATATTTAGAACCCTTTTTATACATTAAGGAGAACAATGCAGTTTACCTTCAAAGTAAAGATTAATATTATTTTGGTTACATGGATAAGTTATTTAGTGGTG AT
TTCTGAGATTTTGGTGCACCTGTTACCCAAGCAGCTGTTATCCAATCTTCAAAGTAAAGATTAATATTAGATAAGGATTCCACACTAATTTAATTTATAGCCTTTTGAAGAAAGATGAATTACAA TTT
AGAAATCATTTTCACACTTATTTTTGCTATTAATCCTATAACTCTCTTTCTTATGTCATTATGTAAAACCGCATTAAATAATCACTTACCAAAAGAAGTCATTTGAAATATGGTTCCTCACTATGA CT
AAGAATATAATACATTTATCTTATTCCCATTATTTAAGTCATCCTTATTTAATAGCACTTAGCAATACCTTAGTAGAATGCTTTTCCACATTTTTCACTTGCGTGGGCCAATTAAAACCATAATT CTT
AGAATAACTCCTAAACACAAACAATTAAAGCTATTGAGGACTATTCAGGTCTTATAAACTGCAACCCTGCTTTCAAGGTGATGTTTGTGCTGATTTGCATTGTACAAATGATTGTAGAATTAACCA GT
GCTTACAATCAGTGTGACATTGGGCAAATTGTTCAAAACATCTTAAACCCCATATTCCTTTTCTATAAAGCAGGATTAGACATATCTATCTTTGCAAAGTTACTGTAAATATTTGAAGTGTTTAT TTA
TTACAAACCTGTGTTGCCTGGCCCGCATTAATGGGCTTTGAAAGTAGGGTAGGCATCTGCTGTGTTACAATGGGCTGCTCTGAAGTCTCATCCTCATTCATAGGAGACAAGATGAGCTCTTGCACT TG
ATATCAGCCCTCTTCCTCCCTGTAAGGATCAAAAGTAAACTCAGGCATTGCGGTGAAGATCATGCCTAGGTTGTCAATTTCAGTGATCATGAGGGGAGCCACAATATGAGTTAAGACATGGCTTT CTG
GGCATTTTTGCCTTCATTAGCAGGGGTCAGGGAGGAGTGAGTGCTGGACCTTCATACATTGCTGGTGGGAATGTAAAATGAGTGGTCACATTGGAGTGCAGATTGGCAGTTCTGCAGAATGTTTAA CA
CAGAGTTACCACATGACCCAGCAATTCCAGTCCTAGATATATGCCCCCTCACACACAAAATTAAAAACTTAAATCCACAAGAAAATCTGTGCACAAATGTCCATTTCACCATGGTTCATAATAGT GCA
AAGGGCTATTCAGGAAACAACTTTGACCTATCAATGGAAAATGAAAGTATATTACATCCATAAAA tTGATTATTATTTGGTAATTAGAAAATGAACTTCTGATACATGCTACAAACCTTGAAAA Catg
ATGCTAAGTGAATGAAACCAATACAAATTACTAATGCTGTGTGATTCCATTTAATAAGATGTGTGCAGAAAGGGCAAATCTATAGGAACAAAAGATAGATTAGGGTTGGAAATGGGGAGATGGGGA AG
GGCAATGGGAAGTGACTGTAAATAGGTTGAGGGTTTCTTTTTTGGGTGATTAAAATGTTCAAAAATTAGGTTGTGGTAATGGTTGCACAATTTTGTGAATATACAAAAACCCATTGAATTGTACAC TT
TAAATGGATGAATTGTACCATATATAAATTATACCTTGGTAAAGCTGTTTAAGAAAAAGAAGTGTGAGTTGGCCATGCCTTCCTATGCCCAACCCCACACTGAGGGTTTCACCAAAACCCTCCGG TAC
TGTTTTCAAGACTCAATCTTGTGTTTTCCTTAGGCCTCCCAATTCCCAAGCCACCCTTTACCTTCACACTGTAAGCATTATAACGCTCTGCATGTGTCCTGTTCCCTTCTCTTCCCTCACTCCTCT CC
TGCTAATAATCTCTTTACCTTTTGAGGTGCAGGAAGCCTGTTCCCACCTTAGCATTTTCCCTACAGCCCTCAACTTGTGGCTTCTTCCTCTTCCACAGACCTAGTCGGTCTGGAGTTGATGTAGG TGT
TCTCTGCTCCTCAATTCTATGTCCAGACCTCTCCCTTTTTGTTCCCTAAAGACTCTCAACCTTGACTCTTATGTCACCAGATGACATGAGTTCAGTACCTCTTCCGTCATTGTCATCCATCAAGGT TG
TTCTTTTGGGTTTCTCAGTGATTTAGTTTGTGGCTTGTCTCTCTCC cTCACTCTTAATTATTAGTGATCTGAGTAGCTACAAAAATGATCATTTCAACAAGTTTGCCTTCAAATTCTGTTTGTTTTTT
TCTTGCAGTAACAACAACAAGCTCAACCATCCATTTTCATGATCATATCCAGACCTCACTGTTCAGTATATTAGCCATTTTTAGCTACATTTGCCTATTGTTCACTTTAAATGTGGCAATCAGAAC TG
AGACGTGCTGTAAGTATAAAATACACACTGGCTTCCAAAATAATAATGAATAATCATAATGATAATAATAATAATAGGAAATACCTTATTAAGTTTATTCTGATTGCAAATTGTAGTAGTAATAT TTT
ATCTATCCTGAATTAAGTAAAATATGTTAATAAACAAAATTTAGTGTTTCTTTTTATTTTTTTTTAAAGATAGATACTAAAACAGTTTGAATACATGTGTGTCTGACATTATATTTCTATTGGACG GT
GCTGCCCCAGGCCTTGGCACCAAGATACCAATAACTACAACTTCATCATTATCCTAGTTTTATGTATCTGGCTCTCTTCTGTATTTTTCAGACATTCCCTCTCATGACTCAACTTTAAGAGTTTT TCA
ACCCTACTATTATCTGTGATCTATTGATTCTACAATCTTTTCACCTTCACTTATTGCCTCAGTATCCCTTTTTTTCTCTTTGCTTCCAGCCTAAACTCCATGGACAATTGTTATAATAAATTCTTTG TG
TTATCCTTGTTCTTCTCTCCCATTATCGAACTTACTTGCAAAGTCACAACATTCATTTAACTCAGCTCTTATTTACTATAAGATGTCTAAGTACAGCTGAATATGACTGGATACAACGAATAACCATG
TTGATGTCCTCATGCTATTCCTGGAACGTGCAAGGAACACCATGGTCACTCTGCCAGGAATATACTTCTAGATGTCTCCCTGCCTAACTCCTTCATCTTCTTCATGCTTTTGTCCA GATTTACATTTC
TTtttttttttttttttttttttatactctaagttttagggtacatgtgcacattgtgcaggttagttacatatgtatacatgtgccatgctggtgcgctgcacccactaatgtgtcatctagcat t
aggtatatctcccactgctatccctccccctcccccgaccccaccacagtccccagagtgtgatattccccttcctgtgtccatgtgatctcattgttcaattcccacctatgagtgagaatatg cg
gtgtttggtttttgttcttgcgatagtttactgagaatgatggtttccaatttcatccatgtccctacaaaggatatgaactcatcatttttatggctgcatagtattccatggtgtatatgt gcc
acattttcttaatccagtctatcattgttggacatttgggttggttccaagtctttgctattgtgaatagtgccgcaataaacatacgtgtgcatgtgtctttatagcagcatgatttatactcat tt

gggtatatacccagtaatgggatggctgggtcaaatggtatttctagttctagatccctgaggaatcgccacactgacttccacaatggttgaactagtttacagtcccaccaacagtgtaaaag tgt
tcctatttctccgcatcctctccagcacctgttgtttcctgacttttaatgattgccattctaactggcgtgagatgatatctcatagtggttttgatttgcatttctctgatggccagtgatga tg
agcatttcttcatgtgtttttggctgcataaatgtcttcttttgagaagtgtctgttcatgtccttcgcccacttttgatggggttgtttgtttttttcttgtaaatttgtttgagttcattg tag
attctggatattagccctttgtcagatgagtaggttgcaaaaatttctcccatgttgtaggttgcctgttcactctgatggtagtttcttttgctgtgcagaagctctttagtttaattagatcc ca
tttgtcaattttgtcttttgttgccattgcttttggtgttttggacatgaagtccttgcccacgcctatgtcctgaatggtaatgcctaggttttcttctagggtttttatggttttaggtctaa cgt
ttaaatctttaatccatcttgaattgatttttgtataaggtgtaaggaagggatccagttccagctttctacatatggctagccaggtagtgtgatgcctccagctttgttcttttggcttaggat tg
acttggcgatgcgggctctttttggttccatatgaactttaaagtagtttttccaattctgtgaagaaagtcattggtagcttgatggggatggcattgaatctgtaaattaccttgggcagt atg
gccattttcacgatattgattcttcctacccatgagcatggaatgttcttccatttgtttgtgtcctcttttatttccttgagcagtggtttgtagttctccttgaagagatccttcacatccctt tt
aagttggattcctaggtattttattctctttgaagcaattgtgaatgggagttcactcatgatttggctctttgtttgtctgttattggtgtataagaatgcttgtgattttgaatattgattt tgt
atcctgagactttgctgaagttgcttatcagcttaaggagattttcagctgagacaatggggtttctagatatataatcatgtcgtctgcaaacagggacaatttgacttcctcttttcctaatt ga
ataccctttatttccttttcctgcctaattgccctggccagaacttccaacactatgttgaataggagtggtgagagagggcatccctgtcttgtgccagttttcaaagggaatgcttccagttt ttg
cccattcaaatagacacaataaaaaatgataaaggggatatcatcactgatcccacagaaatacaaactaccatcagagaatactgcaaacacctctatgcaaataaactagaaaatctagaagac at
ggatacattcctcgacacatacactctcccaagactaaaccaggaagaagttgaatctctgaatagaccaataacaggctctgaaattgtggcaataatcaatagtttaccaaccaaaaagagtc cag
gaccagatggattcacagccgaattctaccagaggtacatggaggaactggtaccattccttctgaaactattccaatcaatagaaaaagagggaatcctccctaactcattttatgaggccagca tc
attctgataccaaagccgggcagagacacaaccaaaaaagagaattttagaccaatatccttgatgaacattgatgcaaaaatcctcaataaaatactggcaaaccgaatccagcagcacatcaa aaa
gcttatccaccatgatcaagtgggcttcatccctgggatgcaaggctggttcaatatacgcaaatcaataaatgtaatccagcatataaacagagccaaagacaaaaaccacatgattatctcaat ag
atgcagaaaaagcctttgacaaaattcaacaacccttcatgctaaaaactctcaataaattaggtattgatgggacgtatttcaaaataataagagctatctatgacaaacccacagccaatatc ata
ctgaatgggcaaaaactggaagcattccctttgaaaaccggcacagatttacatttcttAGTGAAACTTACTCTGATGGCTCTATTTAAAGTTATAATCTTCAATAGCAAAGGCATGGAATCAACCTA
ACTGCCCATCAATGATAGACTAGATAAAGAAAATGTTGtACATATACACCATGGAATACTATGCAGCCATAAGAAAGAATGAGATTATGTCCTTTGCAGGAACATGGATGGAGCTGGAAACCATTATC
CTCAGCAAACTAATGCAGAAACAGAAAACCAAATACTGCATGTTCTCACTTATAAGTGACAGCTGAATGATGAGAACACATGGACATGTAAGAGTCAACAACACACACTGGGCCTATCAGAGGGT CAG
GGTGAGTGGAAGAAGAGTATCAGGAAAAATAGTTAATGGATGCTGGGCTTAACATCTAGGTGATGGGATAATCTGCACGGCAAAGCCATGGCACATGTCCACCTATGTAACACACCTGCACATGTACC
CCAAACTTAAAATAAAAGTTGGAAATAAAAAATAAATTCATTAAAATTAAAATTAAAAATCAATAAGAAGTTGTGTTCCTCACTTCTCTACAATCTCCTCAATTCTCCTTAACCTTCTCTGATTT CTT
TTCTCTCCTCTCCTCTGCCTTGCTTTACAATATTTTCCCTTCAGCAAGGCAAAATATACTCATTTATAACTGTCTTAAATGTAACTTTCTGCTTTAATGTAATCTCCAGAAGGGCATGAATCTTGTTT
TGTTCACTTAAGTAACAAAATTTCTGGAACTACCGGAGTATAGGCTCTTCAGAAATATTAATAGAATAAATGAGTTAATTAATAAAATTTTTTAATGTAAAATTAATGTTCATTTCATCAAACTTCAT
ATATATTTAAAATAATATATACCTATTTCAACACAGTTTCTCCACTTTTTAACTATTTGCATTAGGATGATAAGCAAAAAATTGTACACTAAAAAAATTCTTCTTGAACTCACAGGTAAGCACATTGG
CTGGGGGATTTGGGAACACTGGCAAAACCACACATAAACAAAAACAAAATTACACACCACACATGAATTATAAAGTATTTCTATATGGTTAAGCAATAATAGGCCAAAGATATATTTTCAATGTG TTA
ATATATATTCAATAAATTGATTAGAAAGAAAAATGCTTTATGGCTTTCAAAACTAAGTAGCTAAAAAAAATACAAGATTTTCAGTTGGAAATTTTAAGGTGTCTACAATCACAATTGTAACTTTGGGG
AAGAAGAGAAAGAGAGAGAAAGCAAAGAAAGAAAGAACAGGAAAGGAAGAGCGAGAGAAGGAGAGGAGAAGGAGAGAAAGAGAAGAAAGGAAAAAGAAAGGAAAGAAAGAAAAGAAAGAAAGAA AGA
GGGAGAGAGGAAGGCAGGCAAGAAAGAAAGACAAGAAAGCAAGAAGGAAAGAAGGAAGGAAAAGAGAAAGAAAGAAAGAAAAAAAAGAGAGAGGAAGGGAGGGAGGGAGAGAGGAAGGGAGGGAGGGA
GGAAGGAAGGAAGGAAGGAGAGAAAGACAAGAAAGCAAGAAAGCAAGAAGGAAGGAAGGAAGGAAAAGAGAAAGAAAGAAAAGGAAAGGAAAGAGAGAGGAAGGAAGGGAGGGAGGGAGGAAGGA AGG
AAGGAAGGAAGGACGGACGAGAGCTTCATAAAAATTTTCTGGTTTTAAAGTTATTTTATTTTTCTTAAAAATTTCTTTAAATAAATAGTCTGTTAGAGGTATTAATGTGTGTCACTCTTGCTTGGAAT
GAGGTTTATAAAGGGCACATGTTATTGTGTTTCTCTTCTGGGTAAAATTCTCTGATGATTCTCAGTTTCTGCAGGATAGAATCTTTGCCTGACACCGGGGTCCTTAGGTCGGATCCCTGCCTAGG TCT
ACAGCCTCATCTCCATTCACATTCCACTCCTCTTAGGCAGTGCATGTTTTATCTCTAAGTTCTTCCCAGCATCATCCTGGGAACTTTGAGTTTCACCAgTTTTCCCTGGTGGAGAAACACCGTAAATG
GGAATGCCGTGCAGACCTGAGATTATGTGACTCTACCATCAAGACTCAAAGGAGCTTTATTTGAAATAAAGGAGCTAGTGGTTGTGTGGGTGTAAATTAGATGATaTAATCTGTCAGCTCAGTTATTT
TTATTCACCACTCTCTTAACACATTTCAGTTTTAAGGGGGGGAGTGGGTTCAACTATGTAAAGTCATTGCTTTTATTTTAGCCAATTCAGAATAGTAGACTTCTAAATCGGGAAAGATCTCAGAAGAC
TTTTTTTCTTTATAATTGAGATATTTGCTATTTGTGTTCTTTGGGTGGAAAAAATGATTTAATGACGCATTTTTATTTAAAAAAGTGCCATTTTATCTGTATAAGAGGAAGATGTGATTGAAGTG TTA
TTTTAACACTCCTTCTATTAAAATTATAAAAACATTATATAATATGCTTTTAAAAAGTAGTGAATAAATGTGGTTGTTATATGAACACAAATATGTAGGCAATCAGCATGTCATGAGAATTATTAAAT
AAATTAAAATATTTGCAATATAATTTACATAATTCAGCAGTTGTATGTTAATCACaTAGTTGTCAGAAATAACTTCATTTTTTAACTTTTGTTTGATGTTTAAGCTTTTGTTAGTCTAATTATTTTCA
CACTTTTCTTTCAATATTTCTAACTTCATAAATATTGATAAGTAAATTAAAATCAAATCCTCACATCTAGAAACTGCTGATAATTCAAACAAAATTATTTTTTTCACAAAAAAGTGTTTTAAAATGTC
ATTAACTCAGTTAAAAAATAGAACTACCAAGTTAGTTTATAAAATATGAATTAATATAATTATAAAAGTATAGTCTATGTCTTTATTTTTATTTTAGAGACTAATACCTTTTGTGTTATTAAATA TAA
TTTCTGTTTTTTGCAATATCTCTGAACTCTCATGTCCCCCAGCAGCATACCCATCCCCtAACACCTACAAACAATGACAATAACCCTTTCCTGTACTCCAGTGAATTCGATGCCTTCGTAACACTAAC
TGATATCAAAGGAACAGGATTCCTCAAGTTGATCATGGTAGAAAAAAATTAGTTGAAAATTTAGGCACAAAAAAATTCAAAACAGTTTCATCAGAATGTTTTTGTTTTCACTTCTTCGGGTACATA AGT
GTCCCCTGTTCATTTTCAGTAATATCCACCAAACCAACGGCATCTCCTCTAAGTGAGGTCACCGCAGAAATCAAAAAACACACTACTAGCTTGTGAGGACTTGTCTGGATGGTTTATTAGCAGGATG
TGTCACTACCAACCCTTGGTCTGATGTGAAATTGAGTTTCGAAATGACACATATTTCCTGGATTCTCCAGTTAACCAGAGATTTCTTTCGGGCTTTCCCTTTTTTTTTCTCCCTTAGGCATCTGT AGA
AGAAAGAAAAAAGCAAAGTACTCCAAAAGACTTAAAGAAGCAGAATCAATTGTCTAATTTTATTCGAAAGGTTTTCTCTCTGCTGTTCTCACTA

1377	10.8	0.5	0.5	L1HS-Scaffold10609-167777-169006	202	405	(10825)	C	L1PB2	LINE/L1	(2)	6149	5946	12	
375	30.6	7.3	3.3	L1HS-Scaffold10609-167777-169006	1058	1439	(9791)	C	L1ME4a	LINE/L1	(2)	6122	5726	13	
2220	10.4	0.0	0.0	L1HS-Scaffold10609-167777-169006	1512	1800	(9430)	C	AluSc	SINE/Alu	(20)	289	1	14	
531	7.3	0.0	0.0	L1HS-Scaffold10609-167777-169006	1887	1954	(9276)	C	L1PB2	LINE/L1	(37)	6114	6047	15	
254	33.0	0.9	2.7	L1HS-Scaffold10609-167777-169006	2434	2545	(8685)	+	MIRb	SINE/MIR	80	189	(79)	16	
2657	20.7	4.5	3.1	L1HS-Scaffold10609-167777-169006	2862	3505	(7725)	+	L1MB7	LINE/L1	5522	6174	(10)	17	
1449	32.1	9.4	2.7	L1HS-Scaffold10609-167777-169006	3699	4152	(7078)	+	L2a	LINE/L2	416	906	(2513)	18	
910	23.1	2.7	6.2	L1HS-Scaffold10609-167777-169006	4153	4484	(6746)	+	MER33	DNA/hAT-Charlie	4	324	(0)	19	
1449	32.1	9.4	2.7	L1HS-Scaffold10609-167777-169006	4485	4868	(6362)	+	L2a	LINE/L2	907	1309	(2110)	18	
210	31.2	3.9	0.0	L1HS-Scaffold10609-167777-169006	4907	4983	(6247)	+	L2a	LINE/L2	3053	3132	(294)	18	
8219	0.4	0.0	0.0	L1HS-Scaffold10609-167777-169006	5001	6230	(5000)	C	L1HS	LINE/L1	(0)	6155	4926	20	
6114	2.3	0.0	0.0	L1HS-Scaffold10609-167777-169006	6225	6920	(4310)	C	L1HS	LINE/L1	(1414)	4732	4037	20	*
7188	0.9	0.0	0.0	L1HS-Scaffold10609-167777-169006	6918	7725	(3505)	+	L1HS	LINE/L1	3273	4080	(2066)	20	
3035	10.8	3.2	0.0	L1HS-Scaffold10609-167777-169006	7782	8224	(3006)	+	L1PA11	LINE/L1	5717	6173	(1)	21	
12	27.0	3.6	1.8	L1HS-Scaffold10609-167777-169006	8286	8340	(2890)	+	(TCTCC)n	Simple_repeat	1	56	(0)	22	
191	28.7	11.1	0.0	L1HS-Scaffold10609-167777-169006	8416	8523	(2707)	+	L2a	LINE/L2	3307	3426	(0)	18	
132	23.7	2.5	3.5	L1HS-Scaffold10609-167777-169006	8959	9361	(1869)	+	GA-rich	Low_complexity	1	399	(0)	23	

1377	10.8	0.5	0.5	Hg19	302	505	(8077)	C	L1PB2	LINE/L1	(2)	6149	5946	1
375	30.6	7.3	3.3	Hg19	1158	1539	(7043)	C	L1ME4a	LINE/L1	(2)	6122	5726	2
2220	10.4	0.0	0.0	Hg19	1612	1900	(6682)	C	AluSc	SINE/Alu	(20)	289	1	3
531	7.3	0.0	0.0	Hg19	1987	2054	(6528)	C	L1PB2	LINE/L1	(37)	6114	6047	4
254	33.0	0.9	2.7	Hg19	2534	2645	(5937)	+	MIRb	SINE/MIR	80	189	(79)	5
2602	20.8	5.0	3.1	Hg19	2962	3602	(4980)	+	L1MB7	LINE/L1	5522	6174	(10)	6
1473	32.0	9.4	2.7	Hg19	3796	4249	(4333)	+	L2a	LINE/L2	416	906	(2513)	7
910	23.1	2.7	6.2	Hg19	4250	4581	(4001)	+	MER33	DNA/hAT-Charlie	4	324	(0)	8
1473	32.0	9.4	2.7	Hg19	4582	4965	(3617)	+	L2a	LINE/L2	907	1309	(2110)	7
340	32.2	3.2	0.8	Hg19	5004	5128	(3454)	+	L2a	LINE/L2	3053	3180	(246)	7
3016	11.1	3.2	0.0	Hg19	5134	5576	(3006)	+	L1PA11	LINE/L1	5717	6173	(1)	9
12	27.0	3.6	1.8	Hg19	5638	5692	(2890)	+	(TCTCC)n	Simple_repeat	1	56	(0)	10
191	28.7	11.1	0.0	Hg19	5768	5875	(2707)	+	L2a	LINE/L2	3307	3426	(0)	7
132	23.7	2.5	3.5	Hg19	6311	6713	(1869)	+	GA-rich	Low_complexity	1	399	(0)	11

>Hg19 chr21:29065680-29074161
GATGCTTAAATACAATTTTCTCAGCACCTGGTGAATAAATTTCATCAGATTATTTCCTATTAGAGATCCTCCTTCAAATTTGCCACATCTGTGTTATTTTTTTCCCTTTAGGAAAGAGCAGAAGAA TG
TGTTGAGAATTAACCCTGTCGTTTCAGTTTGTACATTCCTGTTATACCTCGAGATATTACTTTTTAATTTTAATTTTTATTTCCATGGTTTTGTGGGGTAAGGTGGTTTTTGGTTACATGGATAA GTT
ATTTAGTGGTGATTTCTGAGATTTTGGTGCACCTGTTACCCAAGCAGTGTACACTGTACCCAATGTGCAGTCTTTTATCCCTCACCCCCCTCCCACTCTTGTTCCCTGAGTCTGCAAAGACCATTA CA
TCATTCTTATGCTTCTCCATCAGTGTCTGTCTCAACATCACGGTGTGCACGTCAAGACAGTACGAAAATGACTTTTTTATTTTATAACTTTTTAATGCTATCTTTCCCGCACTTGATGATGTTGA GAC
ACTGATGGAGATGCCTGGGGCATTACAGGGAAATGTACCTCCAGTAATGCCACGTTTTCTTTTCGAGGTTACTTCTGAACAGCTTTAAAGAGGGCGCTGCCTGCCTCATAGTCTGCAGCCTCGTGG TG
ACCAGTTGGTCACCAGGGGGCAGGCTTGGATCACAAAAGTGTGTCACCGCTCATTTTTCTTCAAGAGAAAGAAGCGTATTGCTCTCGCTATAAAACTGTGGTGCAATTCATTTTTTACAAACCGG ATG
GTCGATAAAAATAAGTCATTTTGTATTATAACCATGAACTCAAGATATATCATTCATTTAAAAACTACGTTCTATATTTTTGTAAAATAAAAAAATACATTTTATCAATTCCAATTTTGTTTAATT TT
ACTCATTAAACATTGATACAGGTGAAGAAAATTTTTTTACAGATAGTTTTTGCCCTCTAATATTAACTGTACAAAGCAAAGTATTGTTGTAACAGTAGAAGAATAAACAGAAACTACTGACTAGC ATA
TGGCAAAATTTGGGATCTCAGATCTTTTAAAAATTATTTTATCATTACAAAAGTAATATCTACTTTATTGTTGAAAACAGAAATTACTGAGCTACAAAAAGGAAAAAAATACTAAGCAGGCTTCAT TC

CAATATTCGAAGGGAACTATTCTAAGATGTTGGTGCATTTTGTTAAGATGTATTTTAGATCAAGATGCCTCTATTTATCAGTTTGTCTTTCTCAAACTTAGAATCAGGCATTGTGTAACCTGCTT CTA
CTTTTCACTTAATAGCTTATTGTTGACAAGTTTCCTGTCATTTAATACAATTCTACATTATAACCTGTAGCAGCTTTGCATGATATTGTATCAATGTGCATTATTTACATATTCAATACCTTATTT TA
AGCTATTTAAAAAGTTATTTCTAATGTTTTGAGTAGCTCCTTTTAAGTGAAAATGAAACAACTTTTCCAAGACTTATGAAACATAATGCATACAGAGTTAACATTTTTCTTTTTAGACGGAGTCT CGC
TCTGTCGCCAGGCTGGAGTTCGATGGCATGATCTCGGCTCACTGCAACCTCTGCCTCCTGGGTTCAAGTGATTCTCCTGCCTCAACCTCCCAAGTAGCTGGGACTGCAGGCACATGCCACCACGCC CA
GCTTATTTTTGTATTTTTAGTAGAGACGGGGTTTCCCCATGTTGGCCAGGAAACTCTCCAACTCTTGACCTCTTGATCCACCCGCCTCGGCCTCCCAAAGTGCTGGGATTACAGGTGTAAGCCAC CGT
GCCTGGCCTAGAGTTAACATTTTTCATATTTAGAACCCTTTTTATACATTAAGGAGAACAATGCAGTTTACCTTCAAAGTAAAGATTAATATTATTTTGGTTACATGGATAAGTTATTTAGTGGTG AT
TTCTGAGATTTTGGTGCACCTGTTACCCAAGCAGCTGTTATCCAATCTTCAAAGTAAAGATTAATATTAGATAAGGATTCCACACTAATTTAATTTATAGCCTTTTGAAGAAAGATGAATTACAA TTT
AGAAATCATTTTCACACTTATTTTTGCTATTAATCCTATAACTCTCTTTCTTATGTCATTATGTAAAACCGCATTAAATAATCACTTACCAAAAGAAGTCATTTGAAATATGGTTCCTCACTATGA CT
AAGAATATAATACATTTATCTTATTCCCATTATTTAAGTCATCCTTATTTAATAGCACTTAGCAATACCTTAGTAGAATGCTTTTCCACATTTTTCACTTGCGTGGGCCAATTAAAACCATAATT CTT
AGAATAACTCCTAAACACAAACAATTAAAGCTATTGAGGACTATTCAGGTCTTATAAACTGCAACCCTGCTTTCAAGGTGATGTTTGTGCTGATTTGCATTGTACAAATGATTGTAGAATTAACCA GT
GCTTACAATCAGTGTGACATTGGGCAAATTGTTCAAAACATCTTAAACCCCATATTCCTTTTCTATAAAGCAGGATTAGACATATCTATCTTTGCAAAGTTACTGTAAATATTTGAAGTGTTTAT TTA
TTACAAACCTGTGTTGCCTGGCCCGCATTAATGGGCTTTGAAAGTAGGGTAGGCATCTGCTGTGTTACAATGGGCTGCTCTGAAGTCTCATCCTCATTCATAGGAGACAAGATGAGCTCTTGCACT TG
ATATCAGCCCTCTTCCTCCCTGTAAGGATCAAAAGTAAACTCAGGCATTGCGGTGAAGATCATGCCTAGGTTGTCAATTTCAGTGATCATGAGGGGAGCCACAATATGAGTTAAGACATGGCTTT CTG
GGCATTTTTGCCTTCATTAGCAGGGGTCAGGGAGGAGTGAGTGCTGGACCTTCATACATTGCTGGTGGGAATGTAAAATGAGTGGTCACATTGGAGTGCAGATTGGCAGTTCTGCAGAATGTTTAA CA
CAGAGTTACCACATGACCCAGCAATTCCAGTCCTAGATATATGCCCCCTCACACACAAAATTAAAAACTTAAATCCACAAGAAAATCTGTGCACAAATGTCCATTTCACCATGGTTCATAATAGT GCA
AAGGGCTATTCAGGAAACAACTTTGACCTATCAATGGAAAATGAAAGTATATTACATCCATAAAA cTGATTATTATTTGGTAATTAGAAAATGAACTTCTGATACATGCTACAAACCTTGAAAA CATG
CTAAGTGAATGAAACCAATACAAATTACTAATGCTGTGTGATTCCATTTAATAAGATGTGTGCAGAAAGGGCAAATCTATAGGAACAAAAGATAGATTAGGGTTGGAAATGGGGAGATGGGGAAG GGC
AATGGGAAGTGACTGTAAATAGGTTGAGGGTTTCTTTTTTGGGTGATTAAAATGTTCAAAAATTAGGTTGTGGTAATGGTTGCACAATTTTGTGAATATACAAAAACCCATTGAATTGTACACTTT AA
ATGGATGAATTGTACCATATATAAATTATACCTTGGTAAAGCTGTTTAAGAAAAAGAAGTGTGAGTTGGCCATGCCTTCCTATGCCCAACCCCACACTGAGGGTTTCACCAAAACCCTCCGGTAC TGT
TTTCAAGACTCAATCTTGTGTTTTCCTTAGGCCTCCCAATTCCCAAGCCACCCTTTACCTTCACACTGTAAGCATTATAACGCTCTGCATGTGTCCTGTTCCCTTCTCTTCCCTCACTCCTCTCCT GC
TAATAATCTCTTTACCTTTTGAGGTGCAGGAAGCCTGTTCCCACCTTAGCATTTTCCCTACAGCCCTCAACTTGTGGCTTCTTCCTCTTCCACAGACCTAGTCGGTCTGGAGTTGATGTAGGTGT TCT
CTGCTCCTCAATTCTATGTCCAGACCTCTCCCTTTTTGTTCCCTAAAGACTCTCAACCTTGACTCTTATGTCACCAGATGACATGAGTTCAGTACCTCTTCCGTCATTGTCATCCATCAAGGTTGT TC
TTTTGGGTTTCTCAGTGATTTAGTTTGTGGCTTGTCTCTCTCC aTCACTCTTAATTATTAGTGATCTGAGTAGCTACAAAAATGATCATTTCAACAAGTTTGCCTTCAAATTCTGTTTGTTTTTTTCT
TGCAGTAACAACAACAAGCTCAACCATCCATTTTCATGATCATATCCAGACCTCACTGTTCAGTATATTAGCCATTTTTAGCTACATTTGCCTATTGTTCACTTTAAATGTGGCAATCAGAACTGA GA
CGTGCTGTAAGTATAAAATACACACTGGCTTCCAAAATAATAATGAATAATCATAATGATAATAATAATAATAGGAAATACCTTATTAAGTTTATTCTGATTGCAAATTGTAGTAGTAATATTTT ATC
TATCCTGAATTAAGTAAAATATGTTAATAAACAAAATTTAGTGTTTCTTTTTATTTTTTTTTAAAGATAGATACTAAAACAGTTTGAATACATGTGTGTCTGACATTATATTTCTATTGGACGGTG CT
GCCCCAGGCCTTGGCACCAAGATACCAATAACTACAACTTCATCATTATCCTAGTTTTATGTATCTGGCTCTCTTCTGTATTTTTCAGACATTCCCTCTCATGACTCAACTTTAAGAGTTTTTCA ACC
CTACTATTATCTGTGATCTATTGATTCTACAATCTTTTCACCTTCACTTATTGCCTCAGTATCCCTTTTTTCTCTTTGCTTCCAGCCTAAACTCCATGGACAATTGTTATAATAAATTCTTTGTGT TA
TCCTTGTTCTTCTCTCCCATTATCGAACTTACTTGCAAAGTCACAACATTCATTTAACTCAGCTCTTATTTACTATAAGATGTCTAAGTACAGCTGAATATGACTGGATACAACGAATAACCATGTTG
ATGTCCTCATGCTATTCCTGGAACGTGCAAGGAACACCATGGTCACTCTGCCAGGAATATACTTCTAGATGTCTCCCTGCCTAACTCCTTCATCTTCTTCATGCTTTTGTCCA GATTTACATTTCTTA
GTGAAACTTACTCTGATGGCTCTATTTAAAGTTATAATCTTCAATAGCAAAGGCATGGAATCAACCTAACTGCCCATCAATGATAGACTAGATAAAGAAAATGTTG cACATATACACCATGGAATACT
ATGCAGCCATAAGAAGAATGAGATTATGTCCTTTGCAGGAACATGGATGGAGCTGGAAACCATTATCCTCAGCAAACTAATGCAGAAACAGAAAACCAAATACTGCATGTTCTCACTTATAAGT GAC
AGCTGAATGATGAGAACACATGGACATGTAAGAGTCAACAACACACACTGGGCCTATCAGAGGGTCAGGGTGAGTGGAAGAAGAGTATCAGGAAAAATAGTTAATGG ATGCTGGGCTTAACATCTAGG
TGATGGGATAATCTGCACGGCAAAGCCATGGCACATGTCCACCTATGTAACACACCTGCACATGTACCCCAAACTTAAAATAAAAGTTGGAAATAAAAAATAAATTCATTAAAATTAAAATTAAA AAT
CAATAAGAAGTTGTGTTCCTCACTTCTCTACAATCTCCTCAATTCTCCTTAACCTTCTCTGATTTCTTTTCTCTCCTCTCCTCTGCCTTGCTTTACAATATTTTCCC TTCAGCAAGGCAAAATATACT
CATTTATAACTGTCTTAAATGTAACTTTCTGCTTTAATGTAATCTCCAGAAGGGCATGAATCTTGTTTTGTTCACTTAAGTAACAAAATTTCTGGAACTAC CGGAGTATAGGCTCTTCAGAAATATTA
ATAGAATAAATGAGTTAATTAATAAAATTTTTTAATGTAAAATTAATGTTCATTTCATCAAACTTCATATATATTTAAAATAATATATACCTATTTCAACACAGTTT CTCCACTTTTTAACTATTTGC
ATTAGGATGATAAGCAAAAAATTGTACACTAAAAAAATTCTTCTTGAACTCACAGGTAAGCACATTGGCTGGGGGATTTGGGAACACTGGCAAAACCACACATAAACAAAAACAAAATTACACAC CAC
ACATGAATTATAAAGTATTTCTATATGGTTAAGCAATAATAGGCCAAAGATATATTTTCAATGTGTTAATATATATTCAATAAATTGATTAGAAAGAAAAATGCTTT ATGGCTTTCAAAACTAAGTAG
CTAAAAAAAATACAAGATTTTCAGTTGGAAATTTTAAGGTGTCTACAATCACAATTGTAACTTTGGGGAAGAAGAGAAAGAGAGAGAAAGCAAAGAAAGAAAGAACAGGAAAGGAAGAGCGAGAG AAG
GAGAGGAGAAGGAGAGAAAGAGAAGAAAGGAAAAAGAAAGGGAAAGAAAGAAAAGAAAGAAAGAAAGAGGGAGAGAGGAAGGCAGGCAAGAAAGAAAGACAAGAAAG CAAGAAGGAAAGAAGGAAGGA
AAAGAGAAAGAAAGAAAGAAAAAAAAGAGAGAGGAAGGGAGGGAGGGAGAGAGGAAGGGAGGGAGGGAGGAAGGAAGGAAGGAAGGAGAGAAAGACAAGAAAGCAAGAAAGCAAGAAGGAAGGAA GGA

AGGAAAAGAGAAAGAAAGAAAAGGAAAGGAAAGAGAGAGGAAGGAAGGGAGGGAGGGAGGAAGGAAGGAAGGAAGGAAGGACGGACGAGAGCTTCATAAAAATTTTC TGGTTTTAAAGTTATTTTATT
TTTCTTAAAAATTTCTTTAAATAAATAGTCTGTTAGAGGTATTAATGTGTGTCACTCTTGCTTGGAATGAGGTTTATAAAGGGCACATGTTATTGTGTTTCTCTTCTGGGTAAAATTCTCTGATG ATT
CTCAGTTTCTGCAGGATAGAATCTTTGCCTGACACCGGGGTCCTTAGGTCGGATCCCTGCCTAGGTCTACAGCCTCATCTCCATTCACATTCCACTCCTCTTAGGCA GTGCATGTTTTATCTCTAAGT
TCTTCCCAGCATCATCCTGGGAACTTTGAGTTTCACCAaTTTTCCCTGGTGGAGAAACACCGTAAATGGGAATGCCGTGCAGACCTGAGATTATGTGACTCTACCATCAAGACTCAAAGGAGCTTTAT
TTGAAATAAAGGAGCTAGTGGTTGTGTGGGTGTAAATTAGATGATgTAATCTGTCAGCTCAGTTATTTTTATTCACCACTCTCTTAACACATTTCAGTTTTAAGGGG GGGAGTGGGTTCAACTATGTA
AAGTCATTGCTTTTATTTTAGCCAATTCAGAATAGTAGACTTCTAAATCGGGAAAGATCTCAGAAGACTTTTTTTCTTTATAATTGAGATATTTGCTATTTGTGTTCTTTGGGTGGAAAAAATGA TTT
AATGACGCATTTTTATTTAAAAAAGTGCCATTTTATCTGTATAAGAGGAAGATGTGATTGAAGTGTTATTTTAACACTCCTTCTATTAAAATTATAAAAACATTATA TAATATGCTTTTAAAAAGTAG
TGAATAAATGTGGTTGTTATATGAACACAAATATGTAGGCAATCAGCATGTCATGAGAATTATTAAATAAATTAAAATATTTGCAATATAATTTACATAATTCAGCAGTTGTATGTTAATCAC gTAGT
TGTCAGAAATAACTTCATTTTTTAACTTTTGTTTGATGTTTAAGCTTTTGTTAGTCTAATTATTTTCACACTTTTCTTTCAATATTTCTAACTTCATAAATATTGAT AAGTAAATTAAAATCAAATCC
TCACATCTAGAAACTGCTGATAATTCAAACAAAATTATTTTTTTCACAAAAAAGTGTTTTAAAATGTCATTAACTCAGTTAAAAAAATAGAACTACCAAGTTAGTTTATAAAATATGAATTAATAT AAT
TATAAAAGTATAGTCTATGTCTTTATTTTTATTTTAGAGACTAATACCTTTTGTGTTATTAAATATAATTTCTGTTTTTTGCAATATCTCTGAACTCTCATGTCCCC CAGCAGCATACCCATCCCCcA
ACACCTACAAACAATGACAATAACCCTTTCCTGTACTCCAGTGAATTCGATGCCTTCGTAACACTAACTGATATCAAAGGAACAGGATTCCTCAAGTTGATCATGGTAGAAAAAAATTAGTTGAA AAT
TTAGGCACAAAAAATTCAAAACAGTTTCATCAGAATGTTTTTGTTTTCACTTCTTCGGGTACATAAGTGTCCCCTGTTCATTTTCAGTAATATCCACCAAACCAACG GCATCTCCTCTAAGTGAGGTC
ACCGCAGAAATCAAAAAACACACTACTAGCTTGTGAGGACTTGTCTGGATGGTTTTATTAGCAGGATGTGTCACTACCAACCCTTGGTCTGATGTGAAATTGAGTTTCGAAATGACACATATTTC CTG
GATTCTCCAGTTAACCAGAGATTTCTTTCGGGCTTTCCCTTTTTTTTTCTCCCTTAGGCATCTGTAGAAGAAAGAAAAAAGCAAAGTACTCCAAAAGACTTAAAGAA GCAGAATCAATTGTCTAATTT
TATTCGAAAGGTTTTCTCTCTGCTGTTCTCACTA

>KSI_L1HS_3

>Scaffold12895-1876184-1877460
AGGCTAAACAAATTTGACAAACCTTCAGCTAGACTAAGAAAAACAGAGAAAAGACCCAAATGGATACAATCAGAAATGAAAAATGAAACATTACAATTGATATTGCAGAAATTCAAAAGTTCATTA GT
GGCTACTATGAGCAGCTACATACCAATAAATTGGCCTGGAATAAATTGACAATTTCTAAGACACATACAACCTACCAAGATTGAATCAGGAAGAAATCCAAACCCTGAACAGACCTGTAACAAAT AAT
AAGATCAAAACTGTCAAAAAGGCTCCCAGTAAAGAAAAGCCCAAGACCCGATGTCTTTACTGCTGAATTCTACCAAACATTTAAAGAATTAATACCAATCCTACTCAAACTATTATGAAACACAGA GA
AGGAAAGAATGCGTTGAAACTCATTTTATAAGGCCAATATTACCCTGATACCAAAACCAGACAAAGACACTTCAAAAAAAGAAAATTACAGGACAGTATCTCTGATGAATATGGATGCAAATATC CTG
AACAAAACACTAGCAAAACAAATTTAGCAATACATTAAAGAGATCATTAATCATGACCAAGTGGGATTTATCCCTGGGATGCAAGGATGCTTCAACATATGCAAATCAATCAATGTGATACACTAT AT
CAAGAGAAGGAAGGGCAAAAGCCATATGATCATTTCAATTGATGCTGAAAAATCATTTGATAAAATTCAAAATCCCTTCATAATAAAAACCAAAAAAAAAACTGAGTATAGAACGAATATGCCTC AAC
ACAATAAAAGCTATATATGACAGACCCACAGCTAGTATCATACCGAACAGGGACAAACTGAAAGTCTTTCCCCTGAGATCTGGAACACGACAAGAATGCTGGCTTTCAACACTGTTACTCAACACA GT
ACTGAACGTCCTAGCTAGAATAACCAGACAAGAGAAAAAAATAAAGGGCATTCGAAGTGGAAAGTAAGAAACCAAATTATTCTTGTTTTCGGATGATATAATCTTCTATTTGGAAAAACCTAAAA ACT
CTACAAAAAAGCTATTATAACTGATAAACAAATTTAGTAAAGGTGCAGGATACAAAATGAACATACAAAAATCAGTAGCATTTCTATATGCCAACAGTGAGCAATCTGAGAAAGACATTTAAAGAG TA
ATCCCATTTGCAATAATCACACATAAAATAAAATACCTAGGAATTAACCAAAGAAGTGAAAGTTTCTATAATAAACATGATAAAACACCGATGAAAAACATTGAAGAGACACAAAAAAAAAATGA AAA
AAAAATACTCCGTGTTCACAGATTGGAACAATCAATATTGTTATAATGTCTGTTCTACCTAGAGCAATCTACAGATTCAGTGCAAGGCCCTATAAAAATACCAATGACATTCTTCACAGAAATAGA AA
AAAAATCCTAAAATTTATATGGAACCACAAAAGACAAAGAATATCCAGAGCTATCCTGAGGAAAAAGAACTAAACTGTAGAAATCACATTATCTGACCTCAAATTATACTACAGAGCTATAGTAA ACA
AAACAGTGTGGTACTAGCACAAAAACAGACACATAGACCAATGGAACAGAATAAAGAACCCAGAAACAAATCCACACACTTACAGTGAACTCATTTTCGATGAAAGTGCCAAGGACATACACTGAG GA
AAAAATGGTCTCTTCAATAAACGATGCTGGGGAAATTGGATATCCACATACAGATGAATGAAAATAGACCCCTATCTCTCACCATATACAAAAATCAAATTAAAATTGATTAAAGACTTAAATCT AAG
ACCCTAAAGTATAAAACTACTACAAGAAAAC cTTGGGGAAATTCTCTAGGACATTGCCCTGGGCAAAAAATTTTTGAGTGATACCCCACAAGCACAGACAATCAAAGCCAAAACAGACAAATGGGATT
ACATCAAGTTAAAAAGCTTCTGAACAGCAAAGGAAACAGTTAACAAAGTGAAGAGACAACCCATGGAATGGGAGAAAATATTTGCAAACTACCCCTCTGACAAGGGATTAATAATCAGAATATGT AAG
AAGCTCAAACAACTCTAAAGGAAGAAAATTATAATCCAATCTAAAACTGGGCAAAAAATTTGAACAGGCATTTCTCAAAAGAAGACAGACAAATGATAAACAGTCATGTGAAAAGTGCTTGACATCTT
CGATAATCAAAGAAATGCAAATCAAAGCTACAATGAAATATCATCTCACCCCAGTTAAAATGGCTCATATCCAAAGACAGGCAATAACAAATGCTGACAAGCATATGGAGAAAAGGAACCCTCG TAC
ACTGTTGGTGGGAATGTAAGTTAGTACAACCTCTGTAGAGAACAGTTTGGAGTTTCCTCAAAAAACTAAAATTAGAGCTACCATAAGATCCAGCATTTCACTGATGGGCATATATCCCAAAGAGTG AA
AATCAGTATATCAAAAAGATATCTGCACTCCTATGTTTGTTGCATCACTGCTCGCAATAGTTAAGATTTGGAAGCACCCTAAGTGACCATCAACAGATGAATGGATAAAGAAAATGTGGTACATA TAT
ACAATGTACTACTATTCGGCCATGAAAAAGAATGAAATTCTGTCATTTGCAAAAACATAAATAGAACTAGAGATCATTATGTTAAGTGAAATAAGACAGGCACAGAAAAACAGACCACATATTCTC AC
TTATTTATGGGATCTAAAAATCAAAACAAGTGAACTTATGGATATAGAGAGCAGAAGGATGGAAACCAGAAGCTAAGTAGGGTAGTGGGGGTAGAGGGAGGTGGGGATAGTTAATGGGTGTGAAA AGG
TACTTAGAGAGGGGTGGCTAGCAAGATGGCCAAATAGGAACAGCTCTGGTCTGCAGCTCCCAGCAAGATCAAAGCACAAGGCAGGTGATTTCTGCATTTCCAACTGAGATACCCAGCTCATCTCTT TT
GGGTCTGGTTAGACAGTGGGTGCCGCCCACAGAGGGTGAGCTGAAACAGGGTGGTGCGTTGCCTCACCTGGGAAGCACAAGGGGTCAGGGTACTCCCTCCCTTAGGCAAGGGAAGCCATGAGGGA CTG
TGCCGTGAGGAACAGTGCATTCTGGCCCAGATACTATGCTTTTCCCATGGTCTTCACAACCCACAGACCAACAGATTCCCTTGGGTGCCTACACCACCAGGGCCCTGGGTTTCAAGTACAAAACTG GG
CGGCTATTTGGGCAGACACTGAGCTAGCTGCAGGAGATTTTTTTCATACCCCAATGGCAGCTGGAATGCCAGTGAGACAGAACCCTTCACTCCCCTGGAAAAGGGGCTGAAGCCAGGAATTCAA GTG
GTCTAGCTCAGTGGATCCTATCCCCATGGAGCCCAGCAAGCTAAGATTGACAGGCTTGAAATTCTCGCTGCCAGCACAGCAGTCTGAAGTCGACCTGAGACACATGAGCTTGGTGGGAGGGGCACC TG
CCATTACTGAGGCTTTTATAGGTGGTTTTCCCCTCGCAGCATAAAGAAAGCTGCCAGAAAGTTC cAACTGGGCGCAGCCCACCACAGCTCCACAAAGCTGCTGTAGCCAGACTGCCTCTCTAGATTTC
TCCTCTCCAGGCAGGGTATCTCTGAAAGAAAGGCAATAGCCCCAGTCAGGGGCCTATAGATAAAACTCCCGTCTCCCTGGGACAGAGGACCTGAGGGAAGGGGCAGCTGTGGGCGCAGCTTCAGCA GA
CTTAAACATTCCTGCCTGCCAGCTCTGAAGAGAGCACTCCCAGCTCCCAGCGCAGCGCTTGAGCTCTGCTAAGGGACAGACTACCTCCTCAAGTGGGTCCCTGACCCCCCTGCCTCCTGAATGGG AGA
CACCTCCCAGCAGGGGTCGAGAGACACCTCATACAGAAGAGCACTGGCTGGCATCTGGTGGGTGCCCCTCTGGGGATGAAGCTTCCAGAGGAAGGAACAGGTAGCAATCTTTGCTGTTCTGCAGCT TC
CACTGGTGATTCCCAGGCAAACAGCGTCTGGAGTGGACCTCCAATAAACTCCAGCAGACCTGCAGCAGAGGGGCCTGACTGTTAGAAGAAAAACTAACAAACAGAAAGAATAGCATCCACATCAA CAA
AAAGGATGTCCACACAGAAACCCCATCCAAAGTTGACCAACATCAAAGACCAAAGGTAGTTAAATCCACAGAGATGAGGAAAAACCAG cGCAAAAAGGCTGAAAATTCTAAAACCAGAATGCCTCTT
CTTCTCCAAAGGACCGCAACTCCTCGCCAGCAAGGGAACAAAACTGGACAGAGAATGAGTTTGACGAATTGACAGAAGTAGGCTTCAGAAGGTGGTAACAAACTCCTCTGAGCCAAAGGAGCATG TTC
TAACCAAATGCAAGGAAGCTAAGAACCTTGAAAAAAGGTTAGAGGGATTGCTAACTAGAATAACCAGTTTAGAGTAGAATATAAATGACCTAATGGAGCTGAAAAGCACAGCATGAGAACTTTGTG AA
GCACACACAAGTATCAATAGCCGAATCAAGCAACCAGAAGAAAGGATATCAGAGATTGAAGATCAACTTAATGAAATAAAGCATGTAGACAAGATTAGAGAAAAAAGAATGAAAAG gAATGAACAAAG
CCTCCAAGAAATATGGGACTATGTGAAAAGACCAAACCTACGTCTGATTGGTGTACCTGAAAGTGA cGGGGAGAATGGAACCAAGTTGGAAAACACTCCTCAGGATATTATCCAGGAGAACTTCCCTA
AACTAGCAAGACAGGCCAACATTCAAATTCAGGAAATACAGAGAACACCACAAAGATACTCCTCGTGAAGAGCAACCCCAAGACACGTAATTGTCAGATTCACCAAGGTTAAAAATGAAGGAAAA AAT
GTTAAGGGCAGCCAGAGAGAAAGGTCAGGTTTCCCACAAAGGCAAGCCCATCAGACTAAAAGCAGATCTCTCTGCAGAAACCCTACAAGCCAGAAGAAAGTGGGGGCCAA Tattcaacattctttttt
tttttttttttccaatttcatccatgtccctacaaaggatatgaactcatcatttttttatggctgcatagtattccatggtgtatatgtgccacattttcttaatccagtctatcattgttggacatt
tgggttggttccaagtctttgctattgtgaatagtgccgcaataaacatacgtgtgcatgtgtctttatagcagcatgatttatagtcctttgggtatatacccagtaatgggatggctgggtcaa at
ggtatttctagttctagatccctgaggaatcgccacactgacttccacaatggttgaactagtttacagtcccaccaacagtgtaaaagtgttcctatttctccacatcctctccagcacctgtt gtt
tcctgacttttttaatgattgccattctaactggtgtgagatgatatctcatagtggttttgatttgcatttctctgatggccagtgatgatgagcatttcttcatgtgtttttttggctgcataaat gt

cttcttttgagaagtgtctgttcatgtcctttgcccacttttgatggggttgtttgttttttcttgtaaatttgtttgagttcattgtagattctggatattagccctttgtcagatgagtag gtt
gcgaaaattttctcccatgttgtaggttgcctgttcactctgatggtagtttcttttgctgtgcagaagctctttagtttaattagatcccatttgtcaattttggcttttgttgccattgctttt gg
tgttttggacatgaagtccttgcccacgcctatgtcctgaatggtaatgcctaggttttcttctagggtttttatggttttaggtttaacgtttaaatctttaatccatcttgaattgatttttg tat
aaggtgtaaggaagggatccagtttcagctttctacatatggctagccagttttcccagcaccatttattaaatagggaatcctttccccattgcttgtttttctcaggtttgtcaaagatcagat ag
ttgtagatatgcggcattatttctgagggctctgttctgttccattgatctatatctctgttttggtaccagtaccatgctgttttggttactgtagccttgtagtatagtttgaagtcaggtag tgt
gatgcctccagctttgttcttttggcttaggattgacttggcaatgcgggctctttttttggttccatatgaactttaaagtagtttttttccaattctgtgaagaaagtcattggtagcttgatggg ca
aggtaatttacagATTCAACATTCTTAAATAAAAGAATTTTAAGCCTAGAATTTTATATCCAGCCAAACTAAGCTTCATAAGTGAAAGAAAAATAAAATCCTTTACAAACAAGCAAATGTTGAGATAC
TGTGTCACCACCAGGCCTGCCTTACAAGAACTTGTGAAGGAAGCACTAAATATGGAAAGGAAAAACCGGTATTAACCACTGGAAAAACAT GTAGAGACCAGAGACACTATGAAGAAACTGCATTAAAT
AAAATTTCAGGCCAATATCCCTGATGAACACCAATGTGAAAATCCTCAATAAAATACTGGCAAACCGAATCCAGCAGCACTTATCCACCATGATGAAGTCAGCTTCATCCCTGGGATGCAAGGCTGGT
TCAACATACACAAATCAATAAACGTAATCCATCACATAAACAGAACCAATAACAAAAACCACATGATTATCTCAATAGACACAGAAAAGGCCTTCAATAAATTCAACAACCCTTCATTCTAAAAT CTC
TCAATAAACTAGGTATTGATGGAATGTATCTCAAAATAATAAGAGCAATTAATGATGAACCCACAGCTAATATACTAAATGGGCAAAAGCTGGAAGCATTCCCTTTGAAAACCAGCACAAGACAAGGA
TGCCCTCTCTCACCACTCCTATTCAACATAGTATTGGAAGTTCTGGCCAGGGCAATCAGGCAAGAGAAAGAAATAAGGAGTATTCAAATAGGAAGAGGAGAAGTCACATTGTCTTTGTTTGCAGA TGA
CATGATTATATATTTAGAAAACCCCATCGTCTCAGCCCAAAATCTCCTTAAGCTGGTAAGCAACTTCAGCAAAGTCTCAAGATACAAAACCAATGTGCAAAAATCACAAGCATTCCTACACACCAACA
ATAGACAGAGAGCCAAATCATGAGTGAACTTCCATTCACAACTGCTACAAAGAGAATAAAATATCTAGAAATAAAACTTACAAGGGTTGTGCAGGACCTCTTCAAGAAGAACTACAAACCACTGC TCG
AGGAAATAAGTGAGGACATAAACAAATGGAAAAACATTCCATGCTAATGATAGGAAGAATCAATATCGCAAAAGTGGCCATACTGCCCAAAGTAATTTATAGATTCAATGCTATCCCCATCAAGCTAC
CATTGACTTTCTTCACAGAATTAGAAAAAAGTACTTTAAATTTCATATGGAACCAAAAAAGAGCTCATGTAGCCAAGACAATCCTAAGCAAAAAGAACAAAGCTGGAGGCATCACATTACCTGAC TTC
AAACTATACTATAAGGCTACATTAACCAAAACAGCACAGTACTGGTACCAAAATAGATATATAGACCAATGGAACAGAACAGAAGTCTCAGAAATAATGCCACATATCTATGTGATCTTTGACAAACC
TGACAAAAACAAGCAATGGGAAAAGGATTCCCTATTTAATAAATGGTATTGAGAAAATTCGCCATATGGAAAAAACTGAAACTGGACCCCTTTCTTACACCTTATACAAAAATTAACTCGAGATG GAT
TAAAGACTTAAACATAAGACTTAAAACCATAAAAATCCTAGAAGAAAACCTAGGCAATACCATTCAGGATGTAAGCATGGGCAAAGACTTCATGACTAAAACACAAAAAGCAATGACAACAAAAGCCA
AAATTGACAAATAAGATCTAATTAAACTAAGGAGCTTCTGCACAGCAAAAGAAACTATCATCAGAGTGAACAGGCAATCTACAGAATGGGAGAAAATTTTTGCAATCTATCCTTTTGACAAAGGG CTA
ATATCCAGAATCTATAAGGAACTTAAATAAATGTACAAAAAAAAAAACCAACGCCATTAAAAAGTGGGCAAAGGATATGAACAGACACTTCTCAAAAGAAGATATTTATGCAGCCAATAAACATATGA
AAAAAAGCTCATCATCACTGGTCATTAGAGAAATGCAAATCAAAACCACAATGAGACACTATCTCATGCCAGTTAGAATGGTGATTGCTAAAAAGTCAGGGAACAGCAGATTCTGGAGAGGATGT GGA
GAAATAGGAGCaCTTTTACACTCTTGGTGGGAGTGTAAATTAGTTCAACCATTGTGGAAGACAGTGTGGTGATTCCTCAGTGATCTAGAACCAGAAATACCTTTTGACCCAGCAATCCCATTACTGGG
TATATGCCCTAGAGATTACAAATCATTCTACTATAAAGACACATGCACACGTATGTTTATTGCAGTACTATTCACAATAGCAAAGACTTGAAACCAACCCAAATGCCCATCAATGATAGACTGGA TAA
AGAAAATGTGGCATATATACACCATGGAATACTGTGCAGCCATAAAAAGGATAAGTTCATGTCCTTTGCTGGGACATGGATGAAGCAGGAAACCATCATTCTCAGCAAACTAACACAGGAATAGAAAA
CCAAACACCGCATATTCTCACTCATAAGTGGGAGTTGAACAATGAGACACATTGATACAGGGAGGGGAACATCACACACTGGGGTCGAGGTGGGTAGATCACAAGGTCAAGAAATTGAGACCATC CTG
GCGAACATAGTGGAACCCTGTTGTGGGGCTAGGGGAGGGATAGCATTAGGAGAAGTACCTAATGTAGATGATGGTTTGATGGGTGCAGCAAACCACCATGGCACGGGTATACCTATGTAACAAACCTG
CACATTCTGTACATGTATcCCAAAACTTAAAGTATAATAATAAAGAAAAAGAAAAAATAGTTGGAAAGAATGAACAAGACCTACTATTTGATAGCACAACTAGTCAATAATAACTTGA cTGTATATTA
TAAAATAACTAAAAGAGTGTAATTGGATTGTTTGTAACATGAAGGATAAATGCTTAAGAGGATGGATACCTCATTCTCCATGATGTGATTATTTCACATAGCATGCCTGTATTAAAACATTTTGGATT
GGTCCCATGGTGTAATGGTTAGCACTCTGGGCTTTGAATCCAGCAATCCGAGTTCGAATCTTGGTGGGACCTTTCAAAGGAGTACATTTTGGCCAGGCGCAGTGGCTCACGCCTGTAATCCCAGC ACT
TTGAGAGGCCGAAGCAGGTGGATCACCTGAGTTCAGGAGTTCGAGACCAGCCTGACCAACATGGAGAAACTTTGTCTCTACTAAAAATACAAAATTAGCCAGCTGTGACAGTGCATGCCTGTAATCCC
AGCTAGTTGGGAGGCTGAGTCAGGAGAATCTCTTGAACCCAGGAGGTGGAGGTTGCAGTGAGCCAAGATCGTGCCATTGCACTCCAGCCTGGGCAACAAGAGTGAAACCCCATTAAAAAAAAAAA TTT
CGGGCCGGGTGCGGTGGCTCACACCTGTAATCCCAGCCCTTTTGGAGGCCAAGGCGGGTGGATCACGAGGTCAAAAGATCAAGACCATCCTGGCCAACATGGTGAAACCCTGTCTCTACTAAAAATAA
AAAAATTAGCCTGGCATGGTGGTGGGCGCCTGTAGTCTCAGTTACTTGGGAGGCTGAGGCAGGAGAATCACTTGAACCCAGGAATCGGAGATTGCAGTGAGCCGAGATTGCACCACTGCACTCCA GCC
TGGTGACAGAGTGAGACTCCATCTCAAAAAAATAATTAAAAAAAAGTTTCATGTACTCCATAAATGCATACACCTACTATATGCCCATAAAAATAAAAATAATAAAAAACTGGGAAAGTAAAACTGAA
CACAAATTTAGTAGAAGAAAAGAAATAATAAAGATAAGAGCATAAATACATGAAATTGAAATGAAAAAATACATAAAATATCAACAAAACAGAAGTTGGCTTTTTGAAAAGATAAACAAAATCCA CAA
ACCTTCAGCCAGCCAGATTAAGAAAAAAGAAAGACATCCCAAGTAATAAAAATCACAGATGAAAAAGGAAACAACAACTGATATCACAGAAATTCAAGGAATCATTAGAGACTAACATGAGCAATATG
CCAACAAATCGAAACACTTAGAAGAAATGGATATATCCTAGACACATACAACCTACAAAGATTGAACTACGGGAAAATCCAAAATCTGCATAGACCAATGACCGGTACTGAAATTGAAGCTGTAA TAA
AAATGCTTCCAGCAAACAAAAGCCTAAGACTCGATGGCTTTACTGTTGAATTCTATCAAACATTTAAAGAAGAACTAATACCAATCCAAATCAAACTATTCCAAAAAATAAAGGAGGGAATACTTCCA
AACTCATTCTACAAGGCCAGAATTACCCTTATATTAAAACCAGACAAAAACACATAAACAAACAAACAAACAAACAAACTACAGGCCAAACATTGACGCAAAAACATTCAACAAAATACTAGCAA ACT
GAATTTAACTACACACTCAAAAAATCGTTCATCATGACCAAGTGGGATTCATCCCAGATATGCAAAGATAGTTCAACAAACACTAATCAATCCATGTGATACATCATATCAAGATAATTTAGGAGAAA
AATCATATGATCATTTCAATTGATGCTGAAAATGCGTTTGGTAAAATTTGACATCCCTTCATGAAAAAACCTTAAAAAATGGGTATAGATGGAACATATCTCAACTCAATAAAAGCCATATGTGA CAG
ACAGACAGTATCATACTGAACAGGGAAAAACTGAAAGCCTTTGTTTTAAGATGTAGAACAGGACAAGGATGCCCACTTTTACCACTGTTATTCAACATAGTACTGGAAATCCTAGCTAGAGCAACCAG
ACAAGTGAAAGATATAAAAGGCATCTAAATTGGAAAGGAGTAAGTCCAATTATTCTGTTTGCAGATGATATAATCTTATATTTAGAGAAACTTAAAGACTCCACCAAAAAACTATTTGAACTGAT AAA
ACAATTCAGTAAAGTTGCAGGATATAAAATAAACATACAAAATAAGTAGCATTTCTGTATGCCAACAACAAACAATCTGAAAAAGAAACTAAAAAGTAATTCCATTTACAATAGCTACAAATAAAATT
AAGTACCTAGGCA

13987	11.9	1.1	0.3	L1HS-Scaffold12895-1876184-1877460	1	2827	(8450)	+	L1MA2	LINE/L1	3214	6062	(242)	9	
13243	8.5	0.6	0.2	L1HS-Scaffold12895-1876184-1877460	2829	4548	(6729)	+	L1PA7	LINE/L1	1	1727	(4734)	10	
8656	10.1	1.9	2.0	L1HS-Scaffold12895-1876184-1877460	3700	4988	(6289)	+	L1P2	LINE/L1	572	1858	(4326)	11	*
7222	0.7	0.0	0.0	L1HS-Scaffold12895-1876184-1877460	5001	6277	(5000)	C	L1HS	LINE/L1	(292)	5863	4587	12	
1620	13.9	2.8	0.4	L1HS-Scaffold12895-1876184-1877460	6246	6530	(4747)	+	L1PA7	LINE/L1	2145	2397	(4086)	10	*
12168	8.0	1.4	0.0	L1HS-Scaffold12895-1876184-1877460	6525	8783	(2494)	+	L1PA7	LINE/L1	4003	6294	(175)	10	
416	20.3	0.0	0.0	L1HS-Scaffold12895-1876184-1877460	8785	8853	(2424)	+	Alu	SINE/Alu	43	111	(200)	13	
1287	5.7	0.0	0.0	L1HS-Scaffold12895-1876184-1877460	8854	9010	(2267)	+	L1PA7	LINE/L1	5997	6153	(1)	14	
1430	11.0	3.5	13.9	L1HS-Scaffold12895-1876184-1877460	9012	9216	(2061)	+	L1MA2	LINE/L1	6046	6254	(50)	15	
637	2.8	0.0	0.0	L1HS-Scaffold12895-1876184-1877460	9217	9288	(1989)	+	tRNA-Gln-CAA	tRNA	1	72	(3)	16	
1430	11.0	3.5	13.9	L1HS-Scaffold12895-1876184-1877460	9289	9306	(1971)	+	L1MA2	LINE/L1	6255	6255	(49)	15	
2174	11.3	1.0	0.0	L1HS-Scaffold12895-1876184-1877460	9307	9597	(1680)	+	AluSp	SINE/Alu	1	294	(19)	17	
2316	9.4	0.0	0.7	L1HS-Scaffold12895-1876184-1877460	9603	9901	(1376)	+	AluSc	SINE/Alu	1	297	(12)	18	
1430	11.0	3.5	13.9	L1HS-Scaffold12895-1876184-1877460	9902	9955	(1322)	+	L1MA2	LINE/L1	6255	6304	(0)	15	
7294	14.2	3.0	0.7	L1HS-Scaffold12895-1876184-1877460	9959	11275	(2)	+	L1M1	LINE/L1	3072	4418	(1728)	19	

14041	11.9	1.0	0.3	Hg19	1	2828	(7140)	+	L1MA2	LINE/L1	3214	6062	(242)	1	
13289	8.9	1.0	0.6	Hg19	2830	5221	(4747)	+	L1PA7	LINE/L1	1	2397	(4086)	2	
12168	8.0	1.4	0.0	Hg19	5216	7474	(2494)	+	L1PA7	LINE/ L1	4003	6294	(175)	2	*
416	20.3	0.0	0.0	Hg19	7476	7544	(2424)	+	Alu	SINE/Alu	43	111	(200)	3	
1271	6.4	0.0	0.0	Hg19	7545	7701	(2267)	+	L1PA7	LINE/L1	5997	6153	(1)	4	
1444	10.6	3.5	13.9	Hg19	7703	7907	(2061)	+	L1MA2	LINE/L1	6046	6254	(50)	1	
637	2.8	0.0	0.0	Hg19	7908	7979	(1989)	+	tRNA-Gln-CAA	tRNA	1	72	(3)	5	
1444	10.6	3.5	13.9	Hg19	7980	7997	(1971)	+	L1MA2	LINE/L1	6255	6255	(49)	1	
2174	11.3	1.0	0.0	Hg19	7998	8288	(1680)	+	AluSp	SINE/Alu	1	294	(19)	6	
2316	9.4	0.0	0.7	Hg19	8294	8592	(1376)	+	AluSc	SINE/Alu	1	297	(12)	7	
1444	10.6	3.5	13.9	Hg19	8593	8646	(1322)	+	L1MA2	LINE/L1	6255	6304	(0)	1	
7294	14.2	3.0	0.7	Hg19	8650	9966	(2)	+	L1M1	LINE/L1	3072	4418	(1728)	8	

```
>Hg19 chr6:145495952-145505919
AGGCTAAACAAATTTGACAAACCTTCAGCTAGACTAAGAAAAACAGAGAAAAGACCCAAATGGATACAATCAGAAATGAAAAATGAAACATTACAATTGATATTGCAGAAATTCAAAAGTTCATTA GT
GGCTACTATGAGCAGCTACATACCAATAAATTGGCCTGGAATAAATTGACAATTTCTAAGACACATACAACCTACCAAGATTGAATCAGGAAGAAATCCAAACCCTGAACAGACCTGTAACAAAT AAT
AAGATCAAAACTGTCAAAAAGGCTCCCAGTAAAGAAAAGCCCAAGACCCGATGTCTTTACTGCTGAATTCTACCAAACATTTAAAGAATTAATACCAATCCTACTCAAACTATTATGAAACACAGA GA
AGGAAAGAATGCGTTGAAACTCATTTTATAAGGCCAATATTACCCTGATACCAAAACCAGACAAAGACACTTCAAAAAAAGAAAATTACAGGACAGTATCTCTGATGAATATGGATGCAAATATC CTG
AACAAAACACTAGCAAAACAAATTTAGCAATACATTAAAGAGATCATTAATCATGACCAAGTGGGATTTATCCCTGGGATGCAAGGATGCTTCAACATATGCAAATCAATCAATGTGATACACTAT AT
CAAGAGAAGGAAGGGCAAAAGCCATATGATCATTTCAATTGATGCTGAAAAATCATTTGATAAAATTCAAAATCCCTTCATAATAAAAACCAAAAAAAAAACTGAGTATAGAACGAATATGCCTC AAC
ACAATAAAAGCTATATATGACAGACCCACAGCTAGTATCATACCGAACAGGGACAAACTGAAAGTCTTTCCCCTGAGATCTGGAACACGACAAGAATGCTGGCTTTCAACACTGTTACTCAACACA GT
ACTGAACGTCCTAGCTAGAATAACCAGACAAGAGAAAAAAATAAAGGGCATTCGAAGTGGAAAGTAAGAAACCAAATTATTCTTGTTTTCGGATGATATAATCTTCTATTTGGAAAAACCTAAAA ACT
CTACAAAAAAGCTATTATAACTGATAAACAAATTTAGTAAAGGTGCAGGATACAAAATGAACATACAAAAATCAGTAGCATTTCTATATGCCAACAGTGAGCAATCTGAGAAAGACATTTAAAGAG TA
ATCCCATTTGCAATAATCACACATAAAATAAAATACCTAGGAATTAACCAAAGAAGTGAAAGTTTCTATAATAAACATGATAAAACACCGATGAAAAACATTGAAGAGACACAAAAAAAAAATGA AAA
AAAAATACTCCGTGTTCACAGATTGGAACAATCAATATTGTTATAATGTCTGTTCTACCTAGAGCAATCTACAGATTCAGTGCAAGGCCCTATAAAAATACCAATGACATTCTTCACAGAAATAGA AA
AAAAATCCTAAAATTTATATGGAACCACAAAAGACAAAGAATATCCAGAGCTATCCTGAGGAAAAAGAACTAAACTGTAGAAATCACATTATCTGACCTCAAATTATACTACAGAGCTATAGTAA ACA
AAACAGTGTGGTACTAGCACAAAAACAGACACATAGACCAATGGAACAGAATAAAGAACCCAGAAACAAATCCACACACTTACAGTGAACTCATTTTCGATGAAAGTGCCAAGGACATACACTGAG GA
AAAAATGGTCTCTTCAATAAACGATGCTGGGGAATTGGATATCCACATACAGATGAATGAAAATAGACCCCTATCTCTCACCATATACAAAAATCAAATTAAAATTGATTAAAGACTTAAATCT AAG
```

ACCCTAAAGTATAAAACTACTACAAGAAAACaTTGGGGAAATTCTCTAGGACATTGCCCTGGGCAAAAAATTTTTGAGTGATACCCCACAAGCACAGACAATCAAAGCCAAAACAGACAAATGGGATT
ACATCAAGTTAAAAAGCTTCTGAACAGCAAAGGAAACAGTTAACAAAGTGAAGAGACAACCCATGGAATGGGAGAAAATATTTGCAAACTACCCCTCTGACAAGGGATTAATAATCAGAATATGT AAG
AAGCTCAAACAACTCTAAAGGAAGAAAATTAaTAATCCAATCTAAAACTGGGCAAAAAATTTGAACAGGCATTTCTCAAAAGAAGACAGACAAATGATAAACAGTCATGTGAAAAGTGCTTGACATCT
TCGATAATCAAAGAAATGCAAATCAAAGCTACAATGAAATATCATCTCACCCCAGTTAAAATGGCTCATATCCAAAGACAGGCAATAACAAATGCTGACAAGCATATGGAGAAAAGGGAACCCTC GTA
CACTGTTGGTGGGAATGTAAGTTAGTACAACCTCTGTAGAGAACAGTTTGGAGTTTCCTCAAAAAACTAAAATTAGAGCTACCATAAGATCCAGCATTTCACTGATGGGCATATATCCCAAAGAGT GA
AAATCAGTATATCAAAAAGATATCTGCACTCCTATGTTTGTTGCATCACTGCTCGCAATAGTTAAGATTTGGAAGCACCCTAAGTGACCATCAACAGATGAATGGATAAAGAAAATGTGGTACAT ATA
TACAATGTACTACTATTCGGCCATGAAAAAGAATGAAATTCTGTCATTTGCAAAAACATAAATAGAACTAGAGATCATTATGTTAAGTGAAATAAGACAGGCACAGAAAAACAGACCACATATTCT CA
CTTATTTATGGGATCTAAAAATCAAAACAAGTGAACTTATGGATATAGAGAGCAGAAGGATGGAAACCAGAAGCTAAGTAGGGTAGTGGGGGTAGAGGGAGGTGGGGATAGTTAATGGGTGTGAA AAG
GTACTTAGAGAGGGGTGGCTAGCAAGATGGCCAAATAGGAACAGCTCTGGTCTGCAGCTCCCAGCAAGATCAAAGCACAAGGCAGGTGATTTCTGCATTTCCAACTGAGATACCCAGCTCATCTCT TT
TGGGTCTGGTTAGACAGTGGGTGCCGCCCACAGAGGGTGAGCTGAAACAGGGTGGTGCGTTGCCTCACCTGGGAAGCACAAGGGGTCAGGGTACTCCCTCCCTTAGGCAAGGGAAGCCATGAGGG ACT
GTGCCGTGAGGAACAGTGCATTCTGGCCCAGATACTATGCTTTTCCCATGGTCTTCACAACCCACAGACCAACAGATTCCCTTGGGTGCCTACACCACCAGGGCCCTGGGTTTCAAGTACAAAACT GG
GCGGCTATTTGGGCAGACACTGAGCTAGCTGCAGGAGATTTTTTTTCATACCCCAATGGCAGCTGGAATGCCAGTGAGACAGAACCCTTCACTCCCCTGGAAAAGGGGCTGAAGCCAGGAATTCA AGT
GGTCTAGCTCAGTGGATCCTATCCCCATGGAGCCCAGCAAGCTAAGATTGACAGGCTTGAAATTCTCGCTGCCAGCACAGCAGTCTGAAGTCGACCTGAGACACATGAGCTTGGTGGGAGGGGCAC CT
GCCATTACTGAGGCTTTTATAGGTGGTTTTCCCCTCGCAGCATAAAGAAAGCTGCCAGAAAGTTCgAACTGGGCGCAGCCCACCACAGCTCCACAAAGCTGCTGTAGCCAGACTGCCTCTCTAGATTT
CTCCTCTCCAGGCAGGGTATCTCTGAAAGAAAGGCAATAGCCCCAGTCAGGGGCCTATAGATAAAACTCCCGTCTCCCTGGGACAGAGGACCTGAGGGAAGGGGCAGCTGTGGGCGCAGCTTCAGC AG
ACTTAAACATTCCTGCCTGCCAGCTCTGAAGAGAGCACTCCCAGCTCCCAGCGCAGCGCTTGAGCTCTGCTAAGGGACAGACTACCTCCTCAAGTGGGTCCCTGACCCCCCTGCCTCCTGAATGG GAG
ACACCTCCCAGCAGGGGTCGAGAGACACCTCATACAGAAGAGCACTGGCTGGCATCTGGTGGGTGCCCCTCTGGGGATGAAGCTTCCAGAGGAAGGAACAGGTAGCAATCTTTGCTGTTCTGCAGC TT
CCACTGGTGATTCCCAGGCAAACAGCGTCTGGAGTGGACCTCCAATAAACTCCAGCAGACCTGCAGCAGAGGGGCCTGACTGTTAGAAGAAAAACTAACAAACAGAAAGAATAGCATCCACATCA ACA
AAAAGGATGTCCACACAGAAACCCCATCCAAAGTTGACCAACATCAAAGACCAAAGGTAGTTAAATCCACAGAGATGAGGAAAAACCAGtGCAAAAAGGCTGAAAATTCTAAAAACCAGAATGCCTCT
TCTTCTCCAAAGGACCGCAACTCCTCGCCAGCAAGGGAACAAAACTGGACAGAGAATGAGTTTGACGAATTGACAGAAGTAGGCTTCAGAAGGTGGTAACAAACTCCTCTGAGCCAAAGGAGCAT GTT
CTAACCAAATGCAAGGAAGCTAAGAACCTTGAAAAAAGGTTAGAGGGATTGCTAACTAGAATAACCAGTTTAGAGTAGAATATAAATGACCTAATGGAGCTGAAAAGCACAGCATGAGAACTTTGT GA
AGCACACACAAGTATCAATAGCCGAATCAAGCAACCAGAAGAAAGGATATCAGAGATTGAAGATCAACTTAATGAAATAAAGCATGTAGACAAGATTAGAGAAAAAAGAATGAAAAGaAATGAACAAA
GCCTCCAAGAAATATGGGACTATGTGAAAAGACCAAACCTACGTCTGATTGGTGTACCTGAAAGTGAtGGGGAGAATGGAACCAAGTTGGAAAACACTCCTCAGGATATTATCCAGGAGAACTTCCCT
AAACTAGCAAGACAGGCCAACATTCAAATTCAGGAAATACAGAGAACACCACAAAGATACTCCTCGTGAAGAGCAACCCCAAGACACGTAATTGTCAGATTCACCAAGGTTAAAATGAAGGAAA AAA
TGTTAAGGGCAGCCAGAGAGAAAGGTCAGGTTTCCCACAAAGGCAAGCCCATCAGACTAAAAGCAGATCTCTCTGCAGAAACCCTACAAGCCAGAAGAAAGTGGGGGCCAA TATTCAACATTCTTAAA
TAAAAGAATTTTAAGCCTAGAATTTTATATCCAGCCAAACTAAGCTTCATAAGTGAAAGAAAAATAAAATCCTTTACAAACAAGCAAATGTTGAGATACTGTGTCACCACCAGGCCTGCCTTACA AGA
ACTTGTGAAGGAAGCACTAAATATGGAAAGGAAAAACCGGTATTAACCACTGGAAAAACATGTAGAGACCAGAGACACTATGAAGAAACTGCATTAAATAAAATTTCAGGCCAATATCCCTGATGAAC
ACCAATGTGAAAATCCTCAATAAAATACTGGCAAACCGAATCCAGCAGCACTTATCCACCATGATGAAGTCAGCTTCATCCCTGGGATGCAAGGCTGGTTCAACATACACAAATCAATAAACGTA ATC
CATCACATAAACAGAACCAATAACAAAAACCACATGATTATCTCAATAGACACAGAAAAGGCCTTCAATAAATTCAACAACCCTTCATTCTAAAATCTCTCAATAAACTAGGTATTGATGGAATGT AT
CTCAAAATAATAAGAGCAATTAATGATGAACCCACAGCTAATATACTAAATGGGCAAAAGCTGGAAGCATTCCCTTTGAAAACCAGCACAAGACAAGGATGCCCTCTCTCACCACTCCTATTCAA CAT
AGTATTGGAAGTTCTGGCCAGGGCAATCAGGCAAGAGAAAGAAATAAGGAGTATTCAAATAGGAAGAGGAGAAGTCACATTGTCTTTGTTTGCAGATGACATGATTATATATTTAGAAAACCCCAT CG
TCTCAGCCCAAAATCTCCTTAAGCTGGTAAGCAACTTCAGCAAAGTCTCAAGATACAAAACCAATGTGCAAAAATCACAAGCATTCCTACACACCAACAATAGACAGAGAGCCAAATCATGAGTG AAC
TTCCATTCACAACTGCTACAAAGAGAATAAAATATCTAGAAATAAAACTTACAAGGGTTGTGCAGGACCTCTTCAAGAAGAACTACAAACCACTGCTCGAGGAAATAAGTGAGGACATAAACAAAT GG
AAAAACATTCCATGCTAATGATAGGAAGAATCAATATCGCAAAAGTGGCCATACTGCCCAAAGTAATTTATAGATTCAATGCTATCCCCATCAAGCTACCATTGACTTTCTTCACAGAATTAGAA AAA
AGTACTTTAAATTTCATATGGAACCAAAAAAGAGCTCATGTAGCCAAGACAATCCTAAGCAAAAAGAACAAAGCTGGAGGCATCACATTACCTGACTTCAAACTATACTATAAGGCTACATTAACC AA
AACAGCACAGTACTGGTACCAAAATAGATATATAGACCAATGGAACAGAACAGAAGTCTCAGAAATAATGCCACATATCTATGTGATCTTTGACAAACCTGACAAAAACAAGCAATGGGAAAAGG ATT
CCCTATTTAATAAATGGTATTGAGAAAATTCGCCATATGGAAAAAACTGAAACTGGACCCCTTTCTTACACCTTATACAAAAATTAACTCGAGATGGATTAAAGACTTAAACATAAGACTTAAAAC CA
TAAAAATCCTAGAAGAAAACCTAGGCAATACCATTCAGGATGTAAGCATGGGCAAAGACTTCATGACTAAAACACAAAAAGCAATGACAACAAAAGCCAAAATTGACAAATAAGATCTAATTAAA CTA
AGGAGCTTCTGCACAGCAAAAGAAACTATCATCAGAGTGAACAGGCAATCTACAGAATGGGAGAAAATTTTTGCAATCTATCCTTTGACAAAGGGCTAATATCCAGAATCTATAAGGAACTTAAA TA
AATGTACAAAAAAAAAAACCAACGCCATTAAAAAGTGGGCAAAGGATATGAACAGACACTTCTCAAAAGAAGATATTTATGCAGCCAATAAACATATGAAAAAAAGCTCATCATCACTGGTCATT AGA
GAAATGCAAATCAAAACCACAATGAGACACTATCTCATGCCAGTTAGAATGGTGATTGCTAAAAAGTCAGGGAACAGCAGATTCTGGAGAGGATGTGGAGAAATAGGAGCgCTTTTACACTCTTGGTG
GGAGTGTAAATTAGTTCAACCATTGTGGAAGACAGTGTGGTGATTCCTCAGTGATCTAGAACCAGAAATACCTTTTGACCCAGCAATCCCATTACTGGGTATATGCCCTAGAGATTACAAATCAT TCT

ACTATAAAGACACATGCACACGTATGTTTATTGCAGTACTATTCACAATAGCAAAGACTTGAAACCAACCCAAATGCCCATCAATGATAGACTGGATAAAGAAAATGTGGCATATATACACCATGG AA
TACTGTGCAGCCATAAAAAGGATAAGTTCATGTCCTTTGCTGGGACATGGATGAAGCAGGAAACCATCATTCTCAGCAAACTAACACAGGAATAGAAAACCAAACACCGCATATTCTCACTCATA AGT
GGGAGTTGAACAATGAGACACATTGATACAGGGAGGGGAACATCACACACTGGGGTCGAGGTGGGTAGATCACAAGGTCAAGAAATTGAGACCATCCTGGCGAACATAGTGGAACCCTGTTGTGGG GC
TAGGGGAGGGATAGCATTAGGAGAAGTACCTAATGTAGATGATGGTTTGATGGGTGCAGCAAACCACCATGGCACGGGTATACCTATGTAACAAACCTGCACATTCTGTACATGTAT tCCAAAACTTA
AAGTATAATAATAAAGAAAAAGAAAAAATAGTTGGAAAGAATGAACAAGACCTACTATTTGATAGCACAACTAGTCAATAATAACTTGA tTGTATATTATAAAATAACTAAAAGAGTGTAATTGGATT
GTTTGTAACATGAAGGATAAATGCTTAAGAGGATGGATACCTCATTCTCCATGATGTGATTATTTCACATAGCATGCCTGTATTAAAACATTTTGGATTGGTCCCATGGTGTAATGGTTAGCACT CTG
GGCTTTGAATCCAGCAATCCGAGTTCGAATCTTGGTGGGACCTTTCAAAGGAGTACATTTTGGCCAGGCGCAGTGGCTCACGCCTGTAATCCCAGCACTTTGAGAGGCCGAAGCAGGTGGATCACC TG
AGTTCAGGAGTTCGAGACCAGCCTGACCAACATGGAGAAACTTTGTCTCTACTAAAAATACAAAATTAGCCAGCTGTGACAGTGCATGCCTGTAATCCCAGCTAGTTGGGAGGCTGAGTCAGGAG AAT
CTCTTGAACCCAGGAGGTGGAGGTTGCAGTGAGCCAAGATCGTGCCATTGCACTCCAGCCTGGGCAACAAGAGTGAAACCCCATTAAAAAAAAAAAATTTCGGGCCGGGTGCGGTGGCTCACACCTG TA
ATCCCAGCCCTTTTGGAGGCCAAGGCGGGTGGATCACGAGGTCAAAAGATCAAGACCATCCTGGCCAACATGGTGAAACCCTGTCTCTACTAAAAATAAAAAAATTAGCCTGGCATGGTGGTGGG CGC
CTGTAGTCTCAGTTACTTGGGAGGCTGAGGCAGGAGAATCACTTGAACCCAGGAATCGGAGATTGCAGTGAGCCGAGATTGCACCACTGCACTCCAGCCTGGTGACAGAGTGAGACTCCATCTCAA AA
AAATAATTAAAAAAAAGTTTCATGTACTCCATAAATGCATACACCTACTATATGCCCATAAAAATAAAAATAATAAAAAACTGGGAAAGTAAAACTGAACACAAATTTAGTAGAAGAAAAGAAAT AAT
AAAGATAAGAGCATAAATACATGAAATTGAAATGAAAAAATACATAAAATATCAACAAAACAGAAGTTGGCTTTTTGAAAAGATAAACAAAATCCACAAACCTTCAGCCAGCCAGATTAAGAAAAA AG
AAAGACATCCCAAGTAATAAAAATCACAGATGAAAAAGGAAACAACAACTGATATCACAGAAATTCAAGGAATCATTAGAGACTAACATGAGCAATATGCCAACAAATCGAAACACTTAGAAGAA ATG
GATATATCCTAGACACATACAACCTACAAAGATTGAACTACGGGAAAATCCAAAATCTGCATAGACCAATGACCGGTACTGAAATTGAAGCTGTAATAAAAATGCTTCCAGCAAACAAAAGCCTAA GA
CTCGATGGCTTTACTGTTGAATTCTATCAAACATTTAAAGAAGAACTAATACCAATCCAAATCAAACTATTCCAAAAAATAAAGGAGGGAATACTTCCAAACTCATTCTACAAGGCCAGAATTAC CCT
TATATTAAAACCAGACAAAAACACATAAACAAACAAACAAACAAACAAACTACAGGCCAAACATTGACGCAAAAACATTCAACAAAATACTAGCAAACTGAATTTAACTACACACTCAAAAAATCG TT
CATCATGACCAAGTGGGATTCATCCCAGATATGCAAAGATAGTTCAACAAACACTAATCAATCCATGTGATACATCATATCAAGATAATTTAGGAGAAAAATCATATGATCATTTCAATTGATGC TGA
AAATGCGTTTGGTAAAATTTGACATCCCTTCATGAAAAAACCTTAAAAAATGGGTATAGATGGAACATATCTCAACTCAATAAAAGCCATATGTGACAGACAGACAGTATCATACTGAACAGGGAA AA
ACTGAAAGCCTTTGTTTTAAGATGTAGAACAGGACAAGGATGCCCACTTTTACCACTGTTATTCAACATAGTACTGGAAATCCTAGCTAGAGCAACCAGACAAGTGAAAGATATAAAAGGCATCT AAA
TTGGAAAGGAGTAAGTCCAATTATTCTGTTTGCAGATGATATAATCTTATATTTAGAGAAACTTAAAGACTCCACCAAAAAACTATTTGAACTGATAAAACAATTCAGTAAAGTTGCAGGATATAA AA
TAAACATACAAAATAAGTAGCATTTCTGTATGCCAACAACAAACAATCTGAAAAAGAAACTAAAAAGTAATTCCATTTACAATAGCTACAAATAAAATTAAGTACCTAGGC A

>KSI_L1HS_4

>Scaffold13660-1094413-1095879
AATCCCAGCACTTTGGGAAGAAGAGGCAGGCAGATCACTTGAAGTCAGCAGTTTGAGACCAGCCTGGTCAGCATGGTGAAACCCTGTCTCTACTAAAAATAAAAAAATTAGCCAGGTGTGGTGCCA CA
TGCCTGTAATTCCAGCTACTTGGGAGGCTGAGGCATGAGAATCACTTGAACCCGGGAGGTGGAGGTTGCACTCCAGCCTGGACGACAGAGTGAGACTC TATGTCAAAATAAATAAATAAAAATAAATC
AAAGCTGAGAAAATTTATTACACTAAAATAGAAAAATTTTAAAAGAAAAACTACTATGAAAATAATGGAATAAGAAGTTACAACACTGGAAAAGAAATTTGTGAGCTTTGTGAGGACAGGGACTG TGT
CCATTTTATTCATCATTATATCTCACCATGTTCTGTAGTGCCTAAAATCAAAGCTGCAGCACCCAAGATACTTTAAGTACTTACAACCATTCCCAAAT GATGGATACTTACCTACAGAGAAGACTCCA
CATCATAAACCACAAACATTATTCTATTGCTTTCTTCTGTTGCTGTTCATTCCTTAGAGAAAAAAGAAGAGATATATA gAAGTGGTGACTTAACCAGACAATTGTTTAAAAATATGCATTTAGTGTAG
AAACTATGTGTTCAGGATTGTGCAGGTTGCTGAGAAGAATATATAGAAATATAAAACAAGAGTCTCTTCCCAAAGGAGTTTACAGCCTACTTGGGAGA TTGTGACAAATATGAAAAAATACTTGACAA
ATCTGGACCATTTAAATAATGCTATTTATGCTATAGCCATACATAGGACAGAGATAACACTTCCAGATGTGGTAATTGGGAAATATATCATGCAGGAGGAAAGATTTGATCTATGTATTGAAGGA GGG
TAAGTTTATGGGCAGGAAGAGAGCATGGAAGCAAGCATCCCACGGGAGGGATGCTTTGTAGGAAATAAAAATTTAAGGTGCTTTGGGGAAAGGGCAAT CTCATCAGAGTGGAAGGAGACAGATTAGAA
GCATAGATTCTTTAAGAATAAATATGAATGAGTAGCTGGAAATTTTCTGAGAAAGAATAGTAGTGATGTGAACATAAAAGCCTAACTAGTTATGATAAATGTATCCGTAAGTAAAGTAATTAAGC CAG
TTTGGGGTTGGCAGAGGAATTGTGCCAGACATCTGTGGATTTTGCTACCCAGCAGCATTCGCTCTTCTCCTGGTTGTGGGGCCCCAGCCCTGTTGCTA TTACCTGGAACTAAAGGTTAAGATGATGGT
TCAAAGATGAAGCCACCATGGAAGAGAGCATAGCGGACAGATGGAGAGAAACTGCATCCAGGTGACCCCATTTGTACTAAACCTGGTTACCTGGTTTTTCTTTAGTACATATGCCAGTTTGAATT GGT
TTTGTCATTTGAAATGAAGAgTGAAAACTGACAGAAGAATAGACAGACAGAAAGATCAGGAGAGCAGAGTAAAGAATTCAGAAACAGACTGATATACA CGTGAGAATTTCCCCTCACTGGACATTCCT
CAGTCTTTGCTCTGAATCTCTTCCCCACTTCTGTAGTATCCCAGGTCTCAGGCCCTAGACTTCTTATCTATTCTTAGATGAATGAATTCAGTCTTGTGGTATGATGTATCTGTTTGCACATGACT CCT
AAGTTTATATTTCCAGTTTTTGTTATTCTTCTGAGATTCAGACCCATATATTCACTTGTCCACCAAGATTCTCCACTTGAGTGTATAAACGGATCTGA AGTGTAACATGTCCTATACCAAACTCCTGA
CTCCCAACCCCCCCTTACCCTAAATCTGCTCCTTTCCTAAGAATCTCAGGCTCATAAAATGGCAGCATTGTCCACTCAGTTTCCCAGGCCAAAAACCTAG gAGTTACCCTTGAGTCTTTTCTTTCCTA
TAACTCTCACATTTAGGCCATCAGCAAGTCTTGTTGATTCTACCTTCAAAACACAGCCTGGATCTGAGCACTTCTTATCTCCTTATAGGAGGTAAGTA GATATGTAGGTTCCCAACATAGTCCAAGCC
AGCACCATCTCTTGCTTGGATTATTGTAATAGCTTCTAAGTGGCTTCTCAACTTCCTTTT cTCCCCCTTATGGTCTATTCTACACACgTTCGTTAGAACAGTCTTTTCAAACACAAGTCAGATCATGT
TACTTGCTTGTTCAAAGCCAGTGTTTTCCCATGACATTTTTAATATCCCAAATCATTACCATGGTCTCCAGGACCCTGCAAAATCTGACTTCTGCCTA GATTTCTCAATCATCTCTGCTTCTCTCTCA
CTCACTCTGCTACAGTCACACTGGCTTCTTCATTCTTCCTACAACATGCCAAATACATGTCTACCTTAGGGCCTTTACACTTCTTGCTTTGGATCTTTTGATGTCTCATTCCCTCACCTTATTCA GGT
TCTCCTCAAAGTGGCCTTTCCTTTCCACCTCCCCAGACCCTAACCTTTCTTGGTTCTTTAGAACACTTATTACTATTTTGTTTATTGTCTGTTCTTCT CATTAAATGAAAACTCCATGAAGCAGGAAT
GTTGTCCTCTTTAATAGTATATTCCTAACATTGGAATAATGCCTGGTGTATAGTTTGAGCCAATACATATTTTTGAATGACTTATTAAATGAAATTTTTATATGATAGAGGTAGCACAGCATATC ATT
TTGTAAGAGATTTATTATCCAATGACTGGTTTTGAGAAAACTAGTTAGACATTTTAGGAGAAGATGTATGCTTAATAAGAGGATATAATGAACAGTTT TgTGCCAACATACTTGACAATTTAGATGAA
ATAAACAAATTCCAAAAAACCTGATATACCAAAGCTAACACAAGTAAAAATAGAAAACCTGAATAGAGCTATATCTAGTAAAGAGTTTAATTCATTATTAAAGACCTTCGCATCAAACAGCAGAC AAA
CAAATAAATTTTCTGGGGCAGATGGCTTTTCCGGTAAAGTCTTCCAAACACTTAAAATAAATAATAACATCAATCTTACATACTCTTTCAGAGAATAG AAAAAAAAGTAACATTTCCCAGCTCATTCT
CAAGAAAAAACATTATATCAAAAGCTGACACAGACATTACAAGAAAAGAAAATTAAAGACCAAACTCTCCTGGACATAGATACAGAAATACTAAGCAAAATATAATCAAAATGAATTTAGTGATA CAT
AAAAAAGATAATACATTATAACCAAGTGGGATAAACTTTAGGAATTCAAGAGTAGTTTAACATTTGAAAATAAATCAAGATAATACCTTGTAGTAACA GAATAAAGGAGTAAAAATATACATACAATC
TACATAGATGTAGAAAAGGATTTGATAAAATTCAGTGTTGATTTATGATTTTTAAAAAGCCTAAGCAAAGGAAATTTGTTAATCTGATAATGAGTGCCTAAAAATATCTTAAACATCATTCTTGA TGG
TGAAATGTTGAAAGTCTTCTCCTTGAGATATCTGCTAGCATCACTTTAATTTAGCATTGCATTAGAGATCCTAGCCATTGTAGCAAGGCAAGAGAAAG AAATAGAAAGTATATGGTTTGGAAAGTAAG
AAATAAAACTGTCATTATCAATAGGCAACATGACTGTATATATAGAAAACCCAAAAGAATCTATAGATAAACTATTAGAATTAATAAGTAAATTTAAGCAGGGGT gCTGGATACCAGGTCAATATATA
AAAACCAATTATACTTACATATACTAGCAATaAAAAATAGAAAATGAAATTTAAAAAACAATACCATTTACAATAGCCTCAGAAAACACCGAATTCAT AGGAATAAATGTGACAGAGATGTAAGATTT
CTACATTGAAAACAAGAAGACATACTGAGAGAAATTGAAGAAAACTTAAGTAGATGGAAAGATATACCATGTTCATAGAATGGATGACTCAATATTGTGAAGATGTCAACTTTCCTTAAATTAAT TTG
TATATTATACAAGCCATTCAAAATTCTAGCAGGCTTTTTAAATGGATATCAACAAACTGAATATAAAATCTGCATGGAAAAGAAAAGGGCCGAGTTAA GCCATCACAATAATTAAGAATATGAAGGAG
GCTGAGATAACTTATGGCACCAAATATTTAGAATTATTATAAAGCTATTGTAATTAAGTCAATGTGGCATCTGTGCTAAAATAGACAAATGGCCCTATTGGGGAGGAAATATAATTTTTCCTCAA CCT
CTATGAGTTCTTAGTTGAGACAGATTCCTGTAACAAAAGACAAGATTAACAAGATAAAAACAGACAAGCTAATTAACATGTGTAGCACACATCATGAG GGACAAACTTTAATGAAAAGTATCTCAAAG
CAATGGCTTAGAACTCAGGCTTATATAGCATCTTCAACAAACAGCAATACATTTGTAGACAAATGAAAGGACAAATGAAAAGATTTTAGGTTTCCAAGGGTGGGAAATGATGGGAAGGTAAATAT ATG
GGAGGAAACTAATGGAGTGAGGTTTATTTTCAGGTTCCTCTGATGCTGTCTATGGGCTGATAAAGAGTCTAGAGTTTTCTCTAGTCAAGGAGAACTTA TATCCTGCCTGTAGGCAGAAGGTAGGATAG
GGTGAGCTTTTTCTCTGTTTACTGCTTCTTAATTGCCTTTTGCTAAAAAGT gATTTTTATGTCAGAGGCATATTTTGGGGTGACATATTCCTGTTTCCTTAAGCCCAATGGAACAGATTAGAGAATTT
AGAAAGAGATCTTTATATGATCACTTGATTTATGACAAAGTTGACACTAAAATACAGTAGAGAAAGGATGTCTTTTCAATAAATAGTGCTGACTCAATTTAGTATCCATACGGGAAAAAAAGAAT CTT
CCTCAAGCCATTCACAAATATCAATTCTAGCTGCATTGTAGGTCTATATATA TTAGGTAAGACAATAAAGCTCTTAAAAGAAAACATAAGAGAATATCTGTATATCCTTAGAATAGCCA AACATTTCT
Tttttttttttttttttttttattatactctaagttttagggtacatgtgcacattgtgcaggttagttacatatgtatacatgtgccatgctggtgcgctgcacccactaatgtgtcatctagcatt
aggtatatctcccaatgctatccctccccctcccccgaccccaccacagtc cccagagtgtgatattccccttcctgtgtccatgtgatctcattgttcaattcccacctatgagtgagaatatgcg
gtgtttggtttttgttcttgcgatagtttactgagaatgatggtttccaatttcatccatgtccctacaaaggatatgaactcatcatttttatggctgcatagtattccatggtgtatatgt gcc
acattttcttaatccagtctatcattgttggacatttgggttggttccaagt ctttgctattgtgaatagtgccgcaataaacatacgtgtgcatgtgtctttatagcagcatgatttatagtccttt

gggtatatacccagtaatgggatggctgggtcaaatggtatttctagttctagatccctgaggaatcgccacactgacttccacaatggttgaactagtttacagtcccaccaacagtgtaaaag tgt
tcctatttctccacatcctctccagcacctgttgtttcctgacttttaatgattgccattctaactggtgtgagatgatatctcatagtggttttgatttgcatttctctgatggccagtgatgatg
agcatttcttcatgtgtttttggctgcataaatgtcttcttttgagaagtgtctgttcatgtccttcgcccacttttgatggggttgtttgtttttttcttgtaaatttgtttgagttcattg tag
attctggatattagccctttgtcagatgagtaggttgcaaaaatttctcccatttgtaggttgcctgttcactctgatggtagtttcttttgctgtgcagaagctctttagtttaattagatccca
tttgtcaattttggcttttgttgccattgcttttggtgttttggacatgaagtccttgcccacgcctatgtcctgaatggtaatgcctaggttttcttctagggtttttatggttttaggtttaa cgt
ttaaatctttaatccatcttgaattgattttgtataaggtgtaaggaagggatccagtttcagctttctacatatggctagccagttttcccagcaccatttattaaatagggaatcctttccccat
tgcttgtttttctcaggtttgtcaaagatcagatagttgtagatatgcggcattatttctgagggctctgttctgttccattgatctatatctctgttttggtaccagtaccatgctgttttggt tac
tgtagccttgtagtatagtttgaagtcaggtagtgtgatgcctccagctttgttcttttggcttaggttagtccttctgtgtaatgtttaacatttcttAAACATTACACAGAAGGACTAACTATAAA
GGAAAATATTGATAAACTAGATTAAACTAAACTCAAGAAATTCTGATCATCAAAAGATACTATTAAGGGAATGAAAGAG CAAGCCCAGAGTAGGAGAAGATACTATAAATAACTACAAATCAATATGA
GAAAGGCAAGTAACCCAAAAGAAAAGTGGACAAAAAGCTTGAAAAAACACTT TACACAGAGAATGTTCAAATGGCTTACAAATATGTTAAAAGTTATATCAATTGCATTCATTGTCAGAGAAATGCAA
ATTAAATCTACAAAAAGGTATGTCTACATATGCTTCAGAATGGCTAAATTTAAAAAGTCGGACAATACCAAATGTTGACAAGAACATGGAACAACTAGACTCTCATATATTATTGGTGGGAATGT AAA
CTGATACAGCTATTATGGAAGACCGTTGGGCAGTATCTACTGGGCAGTATCT ACTAAAGCTGAACATACACTCTGTACCCGCAATTCCCCACTCCTAGGTATATGACAAACAAATGTAGTATATATAT
TTGCCAGAAGACATATATAAGAAGGTACATAGCATTCATAATATTCATAATAGCCTCAAACTGGAAGCAACCCAGATATCCAAAGACAGTAGAATAGATAAATAAATCAATATATGGAATATATA CAG
AAATGAGAATATGTAATCTATAATCACATGCAAAAACATGGGCGTATGTCAC AAACATAATGCTGAGAAAAAGAAGCCAAACACTCAAACAGGCAGCACTAATCTCTGGTAATAGAAATCAGGATAGT
GGTTACCTGGGGGTGGTAATGACTAGAAGCGGGAATTAAGGGGACTTTTAGGGTGTGAGTCATATTTGGTTTCTTGGTCTGGGTGTATGGTATGGGATGGGGTTATTTTGTGAAAATTCATCAAG CTG
TACAATGATGCCTTGTGCTCTTTTCTGTATGTATCTTATACTTCATTAAATT TTTTATTTAAAAAAACCTGGATCCTTACTTCAATTCTTAAACCAAAGTAAGTTACAGCTAGATTACATATTTAAAT
ATAAAATGAAAGGTCCACAATTAAACATGGATGAACTTTTTTTTATAATCTCAGAAACATGAATTGGAAAAAATTTCTTTGAAAAGAAAATTAAAATGGTAATAAATAAATATCAACATGAGGA AAA
TATTTATAAAATAATAATGTATTATGTGTTGCCAGTCAGACCATCAGACATT AAAAGATACAAAATACTCAGTGTTGCTAAGGTTTGGTGAAAGGGTACTCTCATGTTTGATTATTAGAATAATTTGT
TTCTACAGCATTTTTGGAGGACTTTAAAATGTGCATACTATTTGACCTAACAGTACATTTCTAGGAATTTTATCTTAGGAAATATTTGCCTACATAAATATATCAGGATAGTAATTAAAGCATTT TTA
TAATATCGAAACTGGAAATACTTTAAATGTCTATGAATAGAGTACTGGTTAT ATAAGGTACACCCTGTTTAATAAAGTATACTATGTCTGGTAGAAAGAATGAGGTTGATCTGTATCAGTGGATGAAG
TCATATGGCCAGGAGATACTGATTGCTAAATGAAAAGAGTAAGTTGATGAACATTTCTGTAGTGTGATCTCATTTAAAGAAATATATATTTCATTGGAAAATTCTGGGAAAATATACACCAAACT ATT
TACATAAAATGATCACATATAGTCAAAAATATTTATGTGCATGTATTAAAAT CAGTAGGAACTATACCAAAATGTCAACACTGGCTCTCTCGGGGGATTACTGAGGCATTTAAATTTATTTTTGTATA
CCTTTGTATTCCAAATTCCCTACAATGAGTGTATGTTGATTTAATAACCAGAAGATGCTAAATAGGATTATTAAATTTTGTCTGTAT tTTACCAGTTTCATGATAAAGATCAAACTCCTAATGAAAGC
ATTTCTTTCCTGTCCTTACTGCCTGTCTGTCCACCTTTATCTGGCACTGACC TTCCATATGCACCCTGCATTTCGTTTGTATACAGAACACAGATACTTTCTTATTGGAAATAATTTAAACAATGAGG
AGCACATAGAGAATAAAACGTGGAAACTCGACCTTTTTCTGTACGTTTGCTCACATATGTATGTGTATATACAAATATAGTCTTTTTAAAAGCATAAATGGGATGCTTGCCTCCTCCATCTTAAC CAC
ATTCCATTACTTGCTGTTCCTTGAACCATTCATGCTGTTTCAGCCCCAaGTAACTAGCCAAAGGCCCCTTTTACCCTTTCTTCCTGTCCCTGTGCAACCAGCAATTACAGTGTCTAATTATTTGATGT
TTTTCCTAAAGACTTTTGATGCAAGAGGTTCAAAAGGCACAAAGCATGAAAGGAAAGCAGACGTAGCCATTGCCATGTAGGCAGCTGAAGGATGATGGAGAATTGGCAGGATACTGTTTCCAGTT ATT
CACTGAGATTTTTATCCCTCTGGTTTCTCTTAGTGATGGCACATCCCCTGCC TAGGCAGCTAGGGTGTTCCCTACTCATTGCCTCAGGGGACAAGTGACATAGTGGATTTCAGAAATCGATGCTTAGC
TTTTGATTTGCTACAGTCTGGTTCTTGTGTGGCACCAAAGGACTCAATGCATAGACAAAACATTTAGAAAGATGCCCATGAGTAAGAAAAGGCTTCTTTAGTACCTTTAAAGCCCCTTCCTAATG GGG
TGATATTGTCCTTGGTGAGAAGAGAGGAGGAGAGCTGGAGGGAAGGAGGAAA GGCAGATGTCCGTGGTTCCTTAGGAGGCAGGTCTTGCTCTGTGTCTACGCTGAATTCTGAAGGGCAGTGACCATGC
AAACCCCTCACAGGTCTGGCATGCCAGAGAGCAGAGGAAACCCATGCCCCTGTATTTAGGCAAGGCTCAGAGGCTTTGGCACTCACTGAGTAAGGCCCTGGAATGTAGACTTAATTCTGGGGAGA TGG
GGTTGGAGGAGAGACCTTGAATTGCATGGAATTAGTCTCTAACAGAGGGTTG CATTATAAATTAAGATGATTAGAGAACAGGTAAGTTGCATTCTTTACATACCTGAGCTTGAAAACTGAGAATTGTC
CTCATAACAAATAGAAGAATTCTGATTTTATTTCTTTGCTTTGGTGGTGGTGCTGGTTGGATTTTGATGTTGGCCATGAGCCCCGGGGATTTCCTTAAGCATGATCCTCGGAAGCTGTCTCACTT TCT
CCCTGGCTTTGCCTCTCCACAGGGCTGCTGACCACCAGCAGCACGGACCTCT GGAGGCCAAAACAGGCCTGACAGTCACAGATGTGTTTGCCATCTCATCTTTATGTGAGTGGAGTAAGGAAAGATGA
AAGCCACAGAATGAGACCTTTCTGGACATGACTGTACAGGAGCCCAGCCTGGAATGCACTCAGAGTTGATGCTGAATAAACTAAGAGATGCAAATAAAAACCAACAAGGAACCCTTCTTTAACGT ATT
AAATTTGAGGGAAAATTCTTGTCATTGACTTGCATTCATTCAAGGGTTGGTT AAGTAATTTTCAGACCTAAATTTGTTATTGATgATAGCATTTAGAGTGAATTTTGGATATCACTCAGTAACTTCAG
CTTCATTGGTGCCTAGAAAGTTCACCTaAGCAGATGTCACAACACAATAAGCTGATGGAAATGAGAGGACAGATCGGCATCACTGGACCTACAATAGAGGAGGTTATTTCTTTGTGGCCATTCTAGGT
ACTCTGTGAAGCTCAGGATGTGGATATTGTTGGCGCAGCTTTATTTGTGCTC TGTTGTCCCGCCATCATTTCAGATTTGCTGATCTTTGCCACAAGTCCGAATCAGCTCATCCATTTTCTCACCTTTC
TCTGCCTTTTCCCCACCTCCTGCTTGTGCTTTGCACCCCCTCCTTCCTTGCTGTGTGCTGGAGCCGCGTGACTTTCTCCTTTCCCCGAACTCTCACCCTGCTTGCCTTTGGTCCCTTCTTTCTCT GTG
ATGTGAACATCCTTGCTTAAATGCAGCTGTATTGTTCATTTTGTGGCATGCC ATTTCCTTTCCACCTGGCAGACTCTCCACCAGCTGGGAAGGGACATGTATCTGCTCTTCAGGAAATGCTGTCCTTT
GGGTGGAAAAGGACTGGCTTCCTGGCAGTGCCAAAATGAAGATCTTTCTCTGCTGAAAACAAAACAAAACCCCTTTATCTGTGAAACTTCCTGTTTCCTCCACTGAAAGCCGTGCTTCTCCAACA TGG
GGCATTATGCCAGGGGCACATGAAATCATAGGACAGACTGGGTGCATCTTCC CGGATGCTGCTTGAAACTTTGGGGGGAAATAGTTTGACTTTTTCTTCCTCGTAAAAGCTGTTGCCATACATGGGT
AGAGGAATATTATGGAGGGCAATGTAAAATTCATGTGGCATTTAAGGATTAAACAGGAGATGTAAAAATTTCTTGTTTCTGGTAAGCTGCTTTGGGTTATGTCTTCAAAATTCCTGGGGTGTCTT TCT
GCTTCTCTGCTGTTGGGAAGGACTAAAATGGAGACTTCTGAGCAGCACCTAA AGTATTtCTTGTAAGTGGAAGACTGCTATGGGTTTGACCCAGCACATTTGGCAGCTTGGGCTGATTTAATGAAGTT
GTACAAAATTTTGAAATATTAATAGAGATGTCTTCCTTCCCCAGCCCTTAAAATTTCCCCATGGCCCTCCCCTTATTCTGTTATTAATTCATTGCTGGAAGCATTTGGACTGTTATAATAGGAAT GTA

AAGATAAATAAACAGGTAGGGCTCCTGTTGAGATTTTAAATATCTGTATTTTCAAATTCATAGCAGAGACAAAGCAAGACTTTTCATATGGAAGAGTTTGGGCTTGAACATCAAAAGCAAATCTTTGA
AGAAACTGAGGTGTTATGGGTTGAATTGTGTCTCCCCAAAAATCAGATGTTGAAATCTTAACTTCTAGTATCTCA

1651	12.6	9.4	0.0	L1HS-Scaffold13660-1094413-1095879	1	254	(11213)	+	AluSz	SINE/Alu	27	304	(8)	14
265	28.3	0.0	0.0	L1HS-Scaffold13660-1094413-1095879	356	408	(11059)	+	L2b	LINE/L2	3259	3311	(64)	15
409	30.4	7.6	3.0	L1HS-Scaffold13660-1094413-1095879	622	949	(10518)	C	L2d	LINE/L2	(15)	3449	3053	16
271	24.7	0.0	3.9	L1HS-Scaffold13660-1094413-1095879	1287	1366	(10101)	+	MLT1K	LTR/ERVL-MaLR	387	463	(132)	17
2046	29.9	10.0	2.3	L1HS-Scaffold13660-1094413-1095879	1520	2640	(8827)	+	L2a	LINE/L2	2159	3420	(6)	18
5665	21.9	4.0	1.7	L1HS-Scaffold13660-1094413-1095879	2761	4194	(7273)	+	L1ME3	LINE/L1	3338	4804	(1342)	19
1471	22.6	11.7	2.6	L1HS-Scaffold13660-1094413-1095879	4196	4708	(6759)	C	MER34B	LTR/ERV1	(4)	561	4	20
1327	21.8	4.6	0.0	L1HS-Scaffold13660-1094413-1095879	4710	4993	(6474)	+	L1ME3	LINE/L1	4797	5093	(1053)	19
8227	0.4	0.0	0.0	L1HS-Scaffold13660-1094413-1095879	5001	6467	(5000)	C	L1HS	LINE/L1	(0)	6155	4689	21
367	28.8	1.3	1.3	L1HS-Scaffold13660-1094413-1095879	6495	6649	(4818)	+	L1MD	LINE/L1	5089	5243	(903)	22
2941	21.9	11.9	2.6	L1HS-Scaffold13660-1094413-1095879	6650	7469	(3998)	+	L1ME3	LINE/L1	5089	6153	(9)	19
701	27.6	12.3	5.1	L1HS-Scaffold13660-1094413-1095879	7671	8375	(3092)	+	L1ME3G	LINE/L1	5406	6119	(5)	23
228	30.6	12.9	6.3	L1HS-Scaffold13660-1094413-1095879	9417	9594	(1873)	+	MamGypLTR1c	LTR/Gypsy	600	788	(15)	24
574	28.8	6.1	7.6	L1HS-Scaffold13660-1094413-1095879	10764	11056	(411)	+	Charlie16a	DNA/hAT-Charlie	52	340	(2)	25
333	31.8	8.7	3.3	L1HS-Scaffold13660-1094413-1095879	11139	11369	(98)	+	MamGypLTR4	LTR/Gypsy	10	252	(686)	26
445	14.1	0.0	0.0	L1HS-Scaffold13660-1094413-1095879	11404	11467	(0)	+	MLT1C	LTR/ERVL-MaLR	1	64	(403)	27

1846	12.9	8.6	0.0	Hg19	75	354	(9705)	+	AluSz	SINE/Alu	1	304	(8)	1
265	28.3	0.0	0.0	Hg19	456	508	(9551)	+	L2b	LINE/L2	3259	3311	(64)	2
409	30.4	7.6	3.0	Hg19	722	1049	(9010)	C	L2d	LINE/L2	(15)	3449	3053	3
271	24.7	0.0	3.9	Hg19	1387	1466	(8593)	+	MLT1K	LTR/ERVL-MaLR	387	463	(132)	4
12	21.5	4.4	2.2	Hg19	1519	1591	(8468)	+	A-rich	Low_complexity	1	74	(0)	5
2060	29.8	10.0	2.3	Hg19	1620	2740	(7319)	+	L2a	LINE/L2	2159	3420	(6)	6
5640	21.8	4.2	1.7	Hg19	2861	4292	(5767)	+	L1ME3	LINE/L1	3338	4804	(1342)	7
1471	22.4	11.7	2.6	Hg19	4294	4806	(5253)	C	MER34B	LTR/ERV1	(4)	561	4	8
2941	22.0	10.4	2.0	Hg19	4808	6061	(3998)	+	L1ME3	LINE/L1	4797	6153	(9)	7
701	27.6	12.3	5.1	Hg19	6263	6967	(3092)	+	L1ME3G	LINE/L1	5406	6119	(5)	9
228	30.6	12.9	6.3	Hg19	8009	8186	(1873)	+	MamGypLTR1c	LTR/Gypsy	600	788	(15)	10
574	28.8	6.1	7.6	Hg19	9356	9648	(411)	+	Charlie16a	DNA/hAT-Charlie	52	340	(2)	11
333	31.8	8.7	3.3	Hg19	9731	9961	(98)	+	MamGypLTR4	LTR/Gypsy	10	252	(686)	12
445	14.1	0.0	0.0	Hg19	9996	10059	(0)	+	MLT1C	LTR/ERVL-MaLR	1	64	(403)	13

>Hg19 chr1:42478993-42488951
AATCCCAGCACTTTGGGAAGAAGAGGCAGGCAGATCACTTGAAGTCAGCAGTTTGAGACCAGCCTGGTCAGCATGGTGAAACCCTGTCTCTACTAAAAATAAAAAAATTAGCCAGGTGTGGTGCCA CA
TGCCTGTAATTCCAGCTACTTGGGAGGCTGAGGCATGAGAATCACTTGAACCCGGGAGGTGGAGGTTGCACTCCAGCCTGGACGACAGAGTGAGACTCTATGTCAAAATAAATAAATAAAAATAA ATC
AAAGCTGAGAAAATTTATTACACTAAAATAGAAAAATTTTAAAAGAAAAACTACTATGAAAATAATGGAATAAGAAGTTACAACACTGGAAAAGAAATTTGTGAGCTTTGTGAGGACAGGGACTGT GT
CCATTTTATTCATCATTATATCTCACCATGTTCTGTAGTGCCTAAAATCAAAGCTGCAGCACCCAAGATACTTTAAGTACTTACAACCATTCCCAAATGATGGATACTTACCTACAGAGAAGACT CCA
CATCATAAACCACAAACATTATTCTATTGCTTTCTTCTGTTGCTGTTCATTCCTTAGAGAAAAAAGAAGAGATATATA aAAGTGGTGACTTAACCAGACAATTGTTTAAAAATATGCATTTAGTGTAG
AAACTATGTGTTCAGGATTGTGCAGGTTGCTGAGAAGAATATATAGAAATATAAAACAAGAGTCTCTTCCCAAAGGAGTTTACAGCCTACTTGGGAGATTGTGACAAATATGAAAAAATACTTGA CAA
ATCTGGACCATTTAAATAATGCTATTTATGCTATAGCCATACATAGGACAGAGATAACACTTCCAGATGTGGTAATTGGGAAATATATCATGCAGGAGGAAAGATTTGATCTATGTATTGAAGGAG GG
TAAGTTTATGGGCAGGAAGAGAGCATGGAAGCAAGCATCCCACGGGAGGGATGCTTTGTAGGAAATAAAAATTTAAGGTGCTTTGGGGAAAGGGCAATCTCATCAGAGTGGAAGGAGACAGATTA GAA

GCATAGATTCTTTAAGAATAAATATGAATGAGTAGCTGGAAATTTTCTGAGAAAGAATAGTAGTGATGTGAACATAAAAGCCTAACTAGTTATGATAAATGTATCCGTAAGTAAAGTAATTAAGCC AG
TTTGGGGTTGGCAGAGGAATTGTGCCAGACATCTGTGGATTTTGCTACCCAGCAGCATTCGCTCTTCTCCTGGTTGTGGGGCCCCAGCCCTGTTGCTATTACCTGGAACTAAAGGTTAAGATGAT GGT
TCAAAGATGAAGCCACCATGGAAGAGAGCATAGCGGACAGATGGAGAGAAACTGCATCCAGGTGACCCCATTTGTACTAAACCTGGTTACCTGGTTTTTCTTTAGTACATATGCCAGTTTGAATTG GT
TTTGTCATTTGAAATGAAGAaTGAAAACTGACAGAAGAATAGACAGACAGAAAGATCAGGAGAGCAGAGTAAAGAATTCAGAAACAGACTGATATACACGTGAGAATTTCCCCTCACTGGACATTCCT
CAGTCTTTGCTCTGAATCTCTTCCCCACTTCTGTAGTATCCCAGGTCTCAGGCCCTAGACTTCTTATCTATTCTTAGATGAATGAATTCAGTCTTGTGGTATGATGTATCTGTTTGCACATGACTC CT
AAGTTTATATTTCCAGTTTTTGTTATTCTTCTGAGATTCAGACCCATATATTCACTTGTCCACCAAGATTCTCCACTTGAGTGTATAAACGGATCTGAAGTGTAACATGTCCTATACCAAACTCC TGA
CTCCCAACCCCCCCTTACCCTAAATCTGCTCCTTTCCTAAGAATCTCAGGCTCATAAAATGGCAGCATTGTCCACTCAGTTTCCCAGGCCAAAAACCTAG aAGTTACCCTTGAGTCTTTTCTTTCCTA
TAACTCTCACATTTAGGCCATCAGCAAGTCTTGTTGATTCTACCTTCAAAACACAGCCTGGATCTGAGCACTTCTTATCTCCTTATAGGAGGTAAGTAGATATGTAGGTTCCCAACATAGTCCAA GCC
AGCACCATCTCTTGCTTGGATTATTGTAATAGCTTCTAAGTGGCTTCTCAACTTCCTTTT tTCCCCCTTATGGTCTATTCTACACACaTTCGTTAGAACAGTCTTTTCAAACACAAGTCAGATCATGT
TACTTGCTTGTTCAAAGCCAGTGTTTTCCCATGACATTTTTAATATCCCAAATCATTACCATGGTCTCCAGGACCCTGCAAAATCTGACTTCTGCCTAGATTTCTCAATCATCTCTGCTTCTCTC TCA
CTCACTCTGCTACAGTCACACTGGCTTCTTCATTCTTCCTACAACATGCCAAATACATGTCTACCTTAGGGCCTTTACACTTCTTGCTTTGGATCTTTTGATGTCTCATTCCCTCACCTTATTCAG GT
TCTCCTCAAAGTGGCCTTTCCTTTCCACCTCCCCAGACCCTAACCTTTCTTGGTTCTTTAGAACACTTATTACTATTTTGTTTATTGTCTGTTCTTCTCATTAAATGAAAACTCCATGAAGCAGG AAT
GTTGTCCTCTTTAATAGTATATTCCTAACATTGGAATAATGCCTGGTGTATAGTTTGAGCCAATACATATTTTTGAATGACTTATTAAATGAAATTTTTATATGATAGAGGTAGCACAGCATATCA TT
TTGTAAGAGATTTATTATCCAATGACTGGTTTTGAGAAAACTAGTTAGACATTTTAGGAGAAGATGTATGCTTAATAAGAGGATATAATGAACAGTTT TTGCCAACATACTTGACAATTTAGATGAAA
TAAACAATTCCAAAAAACCTGATATACCAAAGCTAACACAAGTAAAAATAGAAAACCTGAATAGAGCTATATCTAGTAAAGAGTTTAATTCATTATTAAAGACCTTCGCATCAAACAGCAGACAA AC
AAATAAATTTTCTGGGGCAGATGGCTTTTCCGGTAAAGTCTTCCAAACACTTAAAATAAATAATAACATCAATCTTACATACTCTTTCAGAGAATAGAAAAAAAAGTAACATTTCCCAGCTCATT CTC
AAGAAAAAACATTATATCAAAAGCTGACACAGACATTACAAGAAAAGAAAATTAAAGACCAAACTCTCCTGGACATAGATACAGAAATACTAAGCAAAATATAATCAAAATGAATTTAGTGATACA TA
AAAAAGATAATACATTATAACCAAGTGGGATAAACTTTAGGAATTCAAGAGTAGTTTAACATTTGAAAATAAATCAAGATAATACCTTGTAGTAACAGAATAAAGGAGTAAAAATATACATACAA TCT
ACATAGATGTAGAAAAGGATTTGATAAAATTCAGTGTTGATTTATGATTTTTAAAAAGCCTAAGCAAAGGAAATTTGTTAATCTGATAATGAGTGCCTAAAAATATCTTAAACATCATTCTTGATG GT
GAAATGTTGAAAGTCTTCTCCTTGAGATATCTGCTAGCATCACTTTAATTTAGCATTGCATTAGAGATCCTAGCCATTGTAGCAAGGCAAGAGAAAGAAATAGAAAGTATATGGTTTGGAAAGTA AGA
AATAAAACTGTCATTATCAATAGGCAACATGACTGTATATATAGAAAACCCAAAAGAATCTATAGATAAACTATTAGAATTAATAAGTAAATTTAAGCAGGGGT aCTGGATACCAGGTCAATATATAA
AAACCAATTATACTTACATATACTAGCAATAAAAATAGAAAATGAAATTTAAAAAACAATACCATTTACAATAGCCTCAGAAAACACCGAATTCATAGGAATAAATGTGACAGAGATGTAAGATTTCT
ACATTGAAAACAAGAAGACATACTGAGAGAAATTGAAGAAAACTTAAGTAGATGGAAAGATATACCATGTTCATAGAATGGATGACTCAATATTGTGAAGATGTCAACTTTCCTTAAATTAATTT GTA
TATTATACAAGCCATTCAAAATTCTAGCAGGCTTTTTAAATGGATATCAACAAACTGAATATAAAATCTGCATGGAAAAGAAAAGGGCCGAGTTAAGCCATCACAATAATTAAGAATATGAAGGAGGC
TGAGATAACTTATGGCACCAAATATTTAGAATTATTATAAAGCTATTGTAATTAAGTCAATGTGGCATCTGTGCTAAAATAGACAAATGGCCCTATTGGGGAGGAAATATAATTTTTCCTCAACC TCT
ATGAGTTCTTAGTTGAGACAGATTCCTGTAACAAAAGACAAGATTAACAAGATAAAAACAGACAAGCTAATTAACATGTGTAGCACACATCATGAGGGACAAACTTTAATGAAAAGTATCTCAAAGCA
ATGGCTTAGAACTCAGGCTTATATAGCATCTTCAACAAACAGCAATACATTTGTAGACAAATGAAAGGACAAATGAAAAGATTTTAGGTTTCCAAGGGTGGGAAATGATGGGAAGGTAAATATAT GGG
AGGAAACTAATGGAGTGAGGTTTATTTTCAGGTTCCTCTGATGCTGTCTATGGGCTGATAAAGAGTCTAGAGTTTTCTCTAGTCAAGGAGAACTTATATCCTGCCTGTAGGCA GAAGGTAGGATAGGG
TGAGCTTTTTCTCTGTTTACTGCTTCTTAATTGCCTTTTGCTAAAAAGTaATTTTTATGTCAGAGGCATATTTTGGGGTGACATATTCCTGTTTCCTTAAGCCCAATGGAACAGATTAGAGAATTTAG
AAAGAGATCTTTATATGATCACTTGATTTATGACAAAGTTGACACTAAAATACAGTAGAGAAAGGATGTCTTTTCAATAAATAGTGCTGACTCAATTTAGTATCCATACGGGAAAAAAAGAATCTTCC
TCAAGCCATTCACAAATATCAATTCTAGCTGCATTGTAGGTCTATATATATTAGGTAAGACAATAAAGCTCTTAAAAGAAAACATAAGAGAATATCTGTATATCCTTAGAATAGCCA AACATTTCTTA
AACATTACACAGAAGGACTAACTATAAAGGAAAATATTGATAAACTAGATTAAACTAAACTCAAGAAATTCTGATCATCAAAAGATACTATTAAGGGAATGAAAGAG CAAGCCCAGAGTAGGAGAAGA
TACTATAAATAACTACAAATCAATATGAGAAAGGCAAGTAACCCAAAAGAAAAGTGGACAAAAAGCTTGAAAAAACACTTTACACAGAGAATGTTCAAATGGCTTACAAATATGTTAAAAGTTAT ATC
AATTGCATTCATTGTCAGAGAAATGCAAATTAAATCTACAAAAAGGTATGTCTACATATGCTTCAGAATGGCTAAATTTAAAAAGTCGGACAATACCAAATGTTGACAAGAACATGGAACAACTAGAC
TCTCATATATTATTGGTGGGAATGTAAACTGATACAGCTATTATGGAAGACCGTTGGGCAGTATCTACTGGGCAGTATCTACTAAAGCTGAACATACACTCTGTACCCGCAATTCCCCACTCCTA GGT
ATATGACAAACAAATGTAGTATATATATTTGCCAGAAGACATATATAAGAAGGTACATAGCATTCATAATATTCATAATAGCCTCAAACTGGAAGCAACCCAGATATCCAAAGACAGTAGAATAGATA
AATAAATCAATATATGGAATATATACAGAAATGAGAATATGTAATCTATAATCACATGCAAAAACATGGGCGTATGTCACAAACATAATGCTGAGAAAAAGAAGCCAAACACTCAAACAGGCAGC ACT
AATCTCTGGTAATAGAAATCAGGATAGTGGTTACCTGGGGGTGGTAATGACTAGAAGCGGGAATTAAGGGGACTTTTAGGGTGTGAGTCATATTTGGTTTCTTGGTCTGGGTGTATGGTATGGGATGG
GGTTATTTTGTGAAAATTCATCAAGCTGTACAATGATGCCTTGTGCTCTTTTCTGTATGTATCTTATACTTCATTAAATTTTTTATTTAAAAAAACCTGGATCCTTACTTCAATTCTTAAACCAA AGT
AAGTTACAGCTAGATTACATATTTAAATATAAAATGAAAGGTCCACAATTAAACATGGATGAACTTTTTTTTTATAATCTCAGAAACATGAATTGGAAAAAATTTCTTTGAAAAGAAAATTAAAATGG
TAATAAATAAATATCAACATGAGGAAAATATTTATAAAATAATAATGTATTATGTGTTGCCAGTCAGACCATCAGACATTAAAAGATACAAAATACTCAGTGTTGCTAAGGTTTGGTGAAAGGGT ACT
CTCATGTTTGATTATTAGAATAATTTGTTTCTACAGCATTTTTGGAGGACTTTAAAATGTGCATACTATTTGACCTAACAGTACATTTCTAGGAATTTTATCTTAGGAAATATTTGCCTACATAAATA

TATCAGGATAGTAATTAAAGCATTTTTATAATATCGAAACTGGAAATACTTTAAATGTCTATGAATAGAGTACTGGTTATATAAGGTACACCCTGTTTAATAAAGTATACTATGTCTGGTAGAAA GAA
TGAGGTTGATCTGTATCAGTGGATGAAGTCA TATGGCCAGGAGATACTGATTGCTAAATGAAAAGAGTAAGTTGATGAACATTTCTGTAGTGTGATCTCATTTAAAGAAATATATATTTCATTGGAAA
ATTCTGGGAAAATATACACCAAACTATTTACATAAAATGATCACATATAGTCAAAAATATTTATGTGCATGTATTAAAATCAGTAGGAACTATACCAAAATGTCAACACTGGCTCTCTCGGGGGA TTA
CTGAGGCATTTAAATTTATTTTTGTATACCT TTGTATTCCAAATTCCCTACAATGAGTGTATGTTGATTTAATAACCAGAAGATGCTAAATAGGATTATTAAATTTTGTCTGTAT aTTACCAGTTTCA
TGATAAAGATCAAACTCCTAATGAAAGCATTTCTTTCCTGTCCTTACTGCCTGTCTGTCCACCTTTATCTGGCACTGACCTTCCATATGCACCCTGCATTTCGTTTGTATACAGAACACAGATAC TTT
CTTATTGGAAATAATTTAAACAATGAGGAGCACATAGAGAATAAAACGTGGAAACTCGACCTTTTTCTGTACGTTTGCTCACATATGTATGTGTATATACAAATATAGTCTTTTTAAAAGCATAAATG
GGATGCTTGCCTCCTCCATCTTAACCACATTCCATTACTTGCTGTTCCTTGAACCATTCATGCTGTTTCAGCCCCA cGTAACTAGCCAAAGGCCCCTTTTACCCTTTCTTCCTGTCCCTGTGCAACCA
GCAATTACAGTGTCTAATTATTTGATGTTTT TCCTAAAGACTTTTGATGCAAGAGGTTCAAAAGGCACAAAGCATGAAAGGAAAGCAGACGTAGCCATTGCCATGTAGGCAGCTGAAGGATGATGGAG
AATTGGCAGGATACTGTTTCCAGTTATTCACTGAGATTTTTATCCCTCTGGTTTCTCTTAGTGATGGCACATCCCCTGCCTAGGCAGCTAGGGTGTTCCCTACTCATTGCCTCAGGGGACAAGTG ACA
TAGTGGATTTCAGAAATCGATGCTTAGCTTT TGATTTGCTACAGTCTGGTTCTTGTGTGGCACCAAAGGACTCAATGCATAGACAAAACATTTAGAAAGATGCCCATGAGTAAGAAAAGGCTTCTTTA
GTACCTTTAAAGCCCCTTCCTAATGGGGTGATATTGTCCTTGGTGAGAAGAGAGGAGGAGAGCTGGAGGGAAGGAGGAAAGGCAGATGTCCGTGGTTCCTTAGGAGGCAGGTCTTGCTCTGTGTC TAC
GCTGAATTCTGAAGGGCAGTGACCATGCAAA CCCCTCACAGGTCTGGCATGCCAGAGAGCAGAGGAAACCCATGCCCCTGTATTTAGGCAAGGCTCAGAGGCTTTGGCACTCACTGAGTAAGGCCCTG
GAATGTAGACTTAATTCTGGGGAGATGGGGTTGGAGGAGAGACCTTGAATTGCATGGAATTAGTCTCTAACAGAGGGTTGCATTATAAATTAAGATGATTAGAGAACAGGTAAGTTGCATTCTTT ACA
TACCTGAGCTTGAAAACTGAGAATTGTCCTC ATAACAAATAGAAGAATTCTGATTTTATTTCTTTGCTTTGGTGGTGGTGCTGGTTGGATTTTGATGTTGGCCATGAGCCCCGGGGATTTCCTTAAGC
ATGATCCTCGGAAGCTGTCTCACTTTCTCCCTGGCTTTGCCTCTCCACAGGGCTGCTGACCACCAGCAGCACGGACCTCTGGAGGCCAAAACAGGCCTGACAGTCACAGATGTGTTTGCCATCTC ATC
TTTATGTGAGTGGAGTAAGGAAAGATGAAAG CCACAGAATGAGACCTTTCTGGACATGACTGTACAGGAGCCCAGCCTGGAATGCACTCAGAGTTGATGCTGAATAAACTAAGAGATGCAAATAAAAA
CCAACAAGGAACCCTTCTTTAACGTATTAAATTTGAGGGAAAATTCTTGTCATTGACTTGCATTCATTCAAGGGTTGGTTAAGTAATTTTCAGACCTAAATTTGTTATTGAT cATAGCATTTAGAGTG
AATTTTGGATATCACTCAGTAACTTCAGCTT CATTGGTGCCTAGAAAGTTCACCTgAGCAGATGTCACAACACAATAAGCTGATGGAAATGAGAGGACAGATCGGCATCACTGGACCTACAATAGAGG
AGGTTATTTCTTTGTGGCCATTCTAGGTACTCTGTGAAGCTCAGGATGTGGATATTGTTGGCGCAGCTTTATTTGTGCTCTGTTGTCCCGCCATCATTTCAGATTTGCTGATCTTTGCCACAAGT CCG
AATCAGCTCATCCATTTTCTCACCTTTCTCT GCCTTTTCCCCACCTCCTGCTTGTGCTTTGCACCCCCTCCTTCCTTGCTGTGTGCTGGAGCCGCGTGACTTTCTCCTTTCCCCGAACTCTCACCCTG
CTTGCCTTTGGTCCCTTCTTTCTCTGTGATGTGAACATCCTTGCTTAAATGCAGCTGTATTGTTCATTTTGTGGCATGCCATTTCCTTTCCACCTGGCAGACTCTCCACCAGCTGGGAAGGGACA TGT
ATCTGCTCTTCAGGAAATGCTGTCCTTTGGG TGGAAAAGGACTGGCTTCCTGGCAGTGCCAAAATGAAGATCTTTCTCTGCTGAAAACAAAACAAAACCCCTTTATCTGTGAAACTTCCTGTTTCCTC
CACTGAAAGCCGTGCTTCTCCAACATGGGGCATTATGCCAGGGGCACATGAAATCATAGGACAGACTGGGTGCATCTTCCCGGATGCTGCTTGAAACTTTGGGGGGAAATAGTTTTGACTTTTTC TTC
CTCGTAAAAGCTGTTGCCATACATGGGTAGA GGAATATTATGGAGGGCAATGTAAAATTCATGTGGCATTTAAGGATTAAACAGGAGATGTAAAAATTTCTTGTTTCTGGTAAGCTGCTTTGGGTTAT
GTCTTCAAAATTCCTGGGGTGTCTTTCTGCTTCTCTGCTGTTGGGAAGGACTAAAATGGAGACTTCTGAGCAGCACCTAAAGTATT cCTTGTAAGTGGAAGACTGCTATGGGTTTGACCCAGCACATT
TGGCAGCTTGGGCTGATTTAATGAAGTTGTA CAAAATTTTGAAATATTAATAGAGATGTCTTCCTTCCCCAGCCCTTAAAATTTCCCCATGGCCCTCCCCTTATTCTGTTATTAATTCATTGCTGGAA
GCATTTGGACTGTTATAATAGGAATGTAAAGATAAATAAACAGGTAGGGCTCCTGTTGAGATTTTAAATATCTGTATTTTCAAATTCATAGCAGAGACAAAGCAAGACTTTTCATATGGAAGAGT TTG
GGCTTGAACATCAAAAGCAAATCTTTGAAGA AACTGAGGTGTTATGGGTTGAATTGTGTCTCCCCAAAAATCAGATGTTGAAATCTTAACTTCTAGTATCTC A

LTR5HS

Instruction
Green box: Primer information
Red letter: Target site duplication
Black letter: Insertion sequences
Green letter: Deleted sequenced by insertion-mediated deletion

>KSI_LTR5HS_1

>Scaffold1239-174695-175663
AAAACCCGAAAGTGTACACAGAGAATATGCAACAGTGAACCCAACGAAAAGATGACAGTGAACCCAGAGAAAA GACGACAGTGAACCCAAAGATAAATTGACAGTGAACCTAAAGAAAAGATGACAGT
GAACTTAGAGCATAGAGAATGGTACACCCAGAAAATACGACAGCAGACCCAGAGTTTCAGCAATAAAGAACCCAG AGGAAAGACAATAGGGCACCCAGAGGAGAAGACAGTGAATCCAGATAATTGAC
AATGGTGAATTGAGAGAAAATACGACAGTGAACACAGAGAAAAGACTACAGTAA ACCCAGATGAAAGGTGACCGTGCATCAAGAGAAAATACAGCAGTGAACTCAGAGAAAAATGACGGTGAACCCAG
AGAAAAGACAACAGTAAACCCAGAGAAACAAAAACAGTGCACCCAGAGAAAAGATGCCAGTGAATCTAAGAAAAT ACAGCAGTAAGCCCAGAGAGAAGATGACAGTGAACCCAGATAAAAGACGATGG
TGCACCCAGAAAAAAGACGACCATGAAGCCAGAGAGAAAATGACAGTGAACCCACAGAAAATAAACAGTGAACCTAGAAAAAACACAGCAGTGAACCCAGAGGAAAGATGACATTGCTCCCAGAG AAA
AGACGACAGTGAACCCAAGAAAGGATGACAGTGAACCTGGAGAAAATACAACAGTGAACCCAGAGAAAAGACAAC GGTAAACCCAGAAGAAAGATGAAAATGAACCCAGAGAAAACGTGACAGTGAAC
CCAGAGAAAATACAACATTGAACCCAGAGAAAAGTCGACAGTGAAACCAGAGAAAAGACGATGGTGCACCCAGAAAAAAGATGACGGTGAACCTAGAGAAAAGACGACGATGAATCCAGAGAAAA TGT
GACAGTGAACCCAGAGAAATAACAAGAGTGAACACACAGAAAATATGACAGTGAACACAGAGAAAAGATGATGGT ACACCCAGAGAAAAGATGATGGTACGCCTGTGGGAAAAGCAAGAGAGATCAG
ATTGTTACTGTGTCTGTGTAGAAAGAAGTAGACATAGGAGACTCCATTTTGTTCTGTACTAAGAAAAATTCTTCTGCCTTGAGATTCTGTTAATCTATAACCTTACCCCCAACCCCGTGCTCTCTGAA
ACATGTGCTGTGTCAAACTCAGAGTTAAATGGATTAAGGGCGGTGCAAGATGTGCTTTGTTAAACAGATGCTTGAAGGCAGCATGCTCCTTAAGAGTCATCACCACTCCCTAATCTCAAGTACCCAGG
GACACAAAAACTGCGGAAGGCCGCAGGGACCTCTGCCTAGGAAAGCCAGGTATTGTCCAAGGTTTCTCCCCATGTGATAGTCTGAAATATGGCCTCCTGGGAAGGGAAAGACCTGACCGTCCCCCAGC
CCGACATCCGTAAAGGGTCTGTGCTGAGGAGGATTAGTGAAAGAGGAAGGAATGCCTCTTGCAGTTGAGACAAGAGGAAGTCATCTGTCTCCTGCCCGTCCCTGGGCAATGGAATGTCTCGGTATAAA
ACCCGATTGTATGCTCCATCTACTGAGATAGGGAAAAGCCACCTTAGGGCTGGAGGTGGGACCTGCGGGCAGCAATACTGCTTTGTAAAGCATTGAGATGTTTATGTGTATGCATATCTAAAAGCACA
GCACTTAATCCTTTACATTGTCTATGATGCAAAGACCTTTGTTCACGTGTTTGTCTGCTGACCCTCTCCCCACAATTGTCTTGTGACCCTGACACATCCCCCTCTTCGAGAAACACCCACAGATGATC
AATAAATACTAAGGGAACTCAGAGGTTGGCGGGATCCTCCATATGCTGAACGCTGGTTCCCCCGGTCCCCTTATTTCTTTCTCTATACTTTGTCTCTGTGTCTTTTTCTTTTCCAAATCTCTCGTCCC
ACCTTACGAGAAACACCCACAGGTGTGGAGGGGCAACCCACCCCTACATACGCCCAGAGAAAAGACGACATTGCACCCAGAGAAAAGATGACAGTGAACCTAGAGAACAGATAGCCCAGAGAAATGAC
GACAATGAACCCAGAGAAAAGACGACAGTGAACCCAGAGAAAATACAACAGTGAACCCAGAGAA AAGCTGACAGTGACCACAGAGAAAAGACAATCGTGCACCCAGAGAAAACACGACAGTGCACCCA
GAGAAAATACAACAGTGAACCCAGAGGAAAGACGACAGTGAACCCAGAGAAAATACAACAGTGAACCCAGAGGAAAGACGATAGTGAACGC AGAGAAAAGACAACAGTGAACCCAGAGAAAATATGAA
TGTGATCCCAGAGAAAAGACAACAGTGCACTCAGAGAAAAGATGATAGTGAACCCAGAGTAAAGACGACAGTGAACCTAGAAAAGATGACAGTGCAACCAGAAAAAAATACAACAGTGAGACCAAG GAA
AGGCGAGAGTTTACCCAGAGAAAAGACAATGGTGCACCCAGAGAAAAGATAATGGTGCACCCAGAGAA AAGACAATGGTGAAACCAGAAAAAAAGACAGTGGACCCAGAGAACATAGGATAGTGAACC
AAGAGAAAAGACGACAGTGAACCCAGAGAAAGGACGAAGGTGCACCCAGAGAAAAGATGACAGTGAACCTGGAGGAAAGACGACAGTGAACCCAGAGAAAAGTTGACAATGAATCCAGAGAAAAT ACA
ACAGTGAATCCAGAGAAAAGATGAAAGTGAACCCAGAGAAGAGACGATGGTGCAACCAGAGAAAAGATGACGGTGAACCAGAGAAAAGAAG ACAGTGAAACCAGAGAAAAGATGATGGTGAACCCAGA
GAAAAGATGACAGTGAATCCAGAGAAAATGCAACAGTGACCACAGAAAAAAGACGACAAGGCATCCAGAAAAAACAAGACAGTGTACCCAGAAAATATACCACAGTGAACCCAGTGAAAAGACAA CAG
TGCACCCAGAGAAACGACAACAGT

8843 1.4 0.0 0.1 UnnamedSequence 1000 1968 (1000) + LTR5_Hs LTR/ERVK 1 968 (0) 1

>hg19 chr6:161265881-161275519
AAAACCCAAAAGTGTACACAAAGAATATGCAACAGTGAACCCAACGAAAAGATGACAGTGAACCCAGAAAAAAGATCACAGTGAACCCAAAGATAAATTGACAGTGAACCTAAAGAAAAGATGAC AGT
GAACTTAGAGCATAGAGAATGGTACACCCAGAAAATACGACAGCAGACCCAGAGTTTCAACAATAAAGAACCCAGAGGAAAGACAATAGTGCACCCAGAGGAGAAGACAGTGAATCCAGATAATT GAC
AATGGTGAATTGAGAGAAAATACGACAGTGAACACAGAGAAAAGACTACAGTAA ACCCAGATGAAAGGTGACCGTGCATCAAGAGAAAATACAGCAGTGAACTCAGAGAAAAATGACGGTGAACCCAG
AGAAAAGACAAAGGTGCACCCAGAGAAAAGACAACAGTAAACCCAGAGAAACAAAAACAGTGCACCCAGAGAAAAGATGCCAGTGAATCTAAGAAAATACAGCAGTAAGCCCAGAGAGAAGATGA CAG
TGAACCCAGATAAAAGACGATGGTGCACCCAGAAAAAAGACGACCATGAAGCCAGAGAGAAAATGACAGTGAA CCCACAGAAAATAAACAGTGAACCTAGAAAAAACACAACAGTGAACCCAGAGGAA
AGATGACATTGCTCCCAGAGAAAAGACGACAGTGAACCCAAGAAAGGATGACAGTGAACCTGGAGAAAATACAACAGTGAACCCAGAGAAAAGACAGCGGTAAACCCAGAAGAAAGATGAAAATGAAC
CCAGAGAAAACGTGACAGTGAACCCAGAGAAAATACAACATTGAACCCAGAGAAAAGTCGACAGTGAAACCAG AGAAAAGACGATGGTGCACCCAGAAAAAAGATGACGGTGAACCTAGAGAAAAGAC
GACGATGAATCCAGAGAAAATGTGACAGTGAACCCAGAGAAATAACAACAGTGAACACACAGAAAATATGACAGTGAACACAGAGAACAGACGATGGTACA CCCAGAGAAAAGATGATGGTACGCCCA

GAGAAAAGACGACATTGCACCCAGAGAAAAGATGACAGTGAACCTAGAGAACAGATAGCCCAGAGAAATGACG ACAATGAACCCAGAGAAAAGACGACAGTGAACCCAGAGAAAATACAACAGTGAAC
CCAGAGAAAAGCTGACAGTGACCACAGAGAAAAGACAATCGTGCACCCAGAGAAAACACGACAGTGCACCCAGAGAAAATACAACAGTGAACCCAGAGGAAAGACGACAGTGAACCCAGAGAAAATAC
AACAGTGAACCCAGAGGAAAGACGATAGTGAACGCAGAGAAAAGACAACAGTGAACCCAGAGAAAATATGAAT GTGATCCCAGAGAAAAGACAACAGTGCACTCAGAGAAAAGATGATAGTGAACCCA
GAGTAAAGACGACAGTGAACGTAGAAAAGATGACAGTGCAACCAGAAAAAATACAACAGTGAGACCAAGGAAAGGCGACAGTTTACCCAGAGAAAAGACAATGGTGCACCCAGAGAA AAGACAATGGT
GAAACCAGAAAAAAAGACAGTGGACCCAGAGAACATAGGATAGTGAACCAAGAGAAAAGACGACAGTGAACCC AGAGAAAGGACGAAGGTGCACCCAGAGAAAAGATGACAGTGAACCCGGAGGAAAG
ACGACAGTGAACCCAGAGAAAAGTTGACAATGAATCCAGAGAAAATACAACAGTGAATCCAGAGAAAAGATGAAAGTGAACCCAGAGAAGAGACGACGGTGCAACCAGAGAAAAGATGATGGTGA ACC
AGAGAAAAGAAGACAGTGAAACCAGAGAAAATATGATGGTGAACCCAGAGAAAAGATGACAGTGAATCCAGAG AAAATGCAACAGTGACCACAGAAAAAAGATGACAATGCATCCAGAAAAAACAAGA
CAGTGTACCCGAAAATATACCACAGTGAACCCAGTGAAAAGACAACAGTGCACCCAGAGAAACGACAACAGT

>KSI_LTR5HS_2

>Scaffold1807-1181863-1182787
TTATAGGACAATACAGACAAAGTATAATAGACGTCTTGCCCTCAAGGAGTCTATGGAAGCAACTGAATATGTGCACAAACG CACTAAAAAATAATAAAACAGGATATGATTACATGGCAAATAGACAG
TAAATGATACAAGACTaCATGGAAACAGATGTCATGCCCCCTTCCTGGTAGATTTCTAACCATCTAAAACCAGTAGAAAAGGCTCTCCTGGGCATGCTCTATAATTTTCTCAGGGGTACTCCAGCCAC
AGGCAAATCACCACTCACCCCGGCCTCTCAAATCTTAGAAACAAGAGCTCTGCTAGTTTAAAAATGCTTTGGTTTTACTACTTACTCTGGCTACGAAATTTTTTGTTCATAATTGTAGCTTTTTT CTA
CCAAATTCTCATTTTAAAATCCTCCCCTCTTACCCACCTCTCATTCCTTGCTGTAGGCCATGTAGTTGTGTTCTGTTTGTTTGTTTGTTTGTTTGTTTGTTTTTAAACTTTTGGTCTTACCATACCTT
TGCTTTAATCCTAGAGCATCTGTTAAGCTTTAAAGGAAAGAGCATAAATGAATGCACACATATACTCTTAGAGCCTATAAAATCAGCTTCCTACTCCACCAGGGAGGTCTGAAGGAGCCTTTGGT TTC
TGGTAAAGCAAATGGATGAGGATCGCCGCTAACATTCACTTCTCCCACGAGAAGCCTCCCCAGGCTGATTGCTATAGTCGCTAGTGCAGAC tGCGACCTGATCTGCTCAGGCTGCCAGACTTCAGAAG
CCTCAGCCAGGTTCAGAGCCTCCCTAATCCTAATGGGCTAGGAGCCCTGGGTGTCTCCAGATAACCCATTTTCCAGCCTTCCAAGAAAGAGGAGTAGTGGGGCAGTGGGCGAGAGAGAATTGGGT TTC
AGTGCTGTTTTAATGTAAACTGCTGGGGGCTGGGAGCTCCCCTTTTGCTCTCCTTGTTTTGGTTCCTACCCCAGAAACATAAAGTATTACTGATAAACATGGATAATTAAATTTGGATAAAAACTACT
TGAACCTGAAGAGTTCATTCCAATTTGTATAACAGCAATTGATGTGGGTTGTGTATTTAAAGGAATATTTAAATTTCATGCAGCAATTTGCAAGTTCAAATGAAAAGCTATTTGGCACATTTGGA TAC
TGGGTTGCAAGGGAAATAAGCAAAATGTACACATGAGGTACATGCCATCATATAATTGTGTACATTTTTTATTTGTCACAAAGCATTATAAAAACAATAGAGAAAAACTATGAAGGAAAAAAAGGTC
ACCACTGGGAAAAAAAAAAACTTATTTCCATATGTTTTGCCTGTGTGTTTATGGAAGGCTTTTTATTCAAAATTTTGGTTTTTATTCAAAAAACTTTGCATAAACATCTTCCTGTGTTGCTTCA CCT
AGTCTTCTGGTTAATGTGCCATAATTTGTTCTCCTCTTTTTGATCACTGGGTTTTTCTAATTTTTTGTTCTCACAACTAGGACTGATGGGAGATTTTTTTGTAATACAGATATTTTCTGACATGTAAG
AAATGAAAACATACACTTAAAAAAAACTATTTCATTTAAGCACAGACATACCCTAAATATAGTGTTAAGTTGTTTCTTAACTATGTGGTGATTCCTGCTCCGGCCTGGACTCCAGCCTGGAGAGC CCT
TTTTTGATTTCCAGGCCTCAGGTTCTTAAAAGAGAAGCAAAACAACTTTTCCCAAACTGGTGTGGTAAAGCCTTTAAGAAATCTAGTTCCGGCCGGGTGCAGTGGCTCACGCCTGTAATCCCAGCACT
TTGGGAGGCCAAGGCAGGTGGATCACCTGAGGTTGGCCTGTCGGCCTGACCAACATGGAGAAGCCCCTTCTCTACTAAAAATACAAAATTAGCCGGGCGTGGTGGCACATGCCTGTAATCCCAGC TAA
TCAGGAGGCTGAGTCAGGAGAATCGCTTGAACCTGGGAGGCAGAGGTTGCAGTGAGCCGAGATCGAGACACTGCACTCCAGCCTGGGCAACAAGAGCGAAACTCCACCTCAAAAAAAAAAAAAAGAAGA
AAGGAAAAGAAATCTAGTTCCACACGTTATCCCTTTCCCGCTCCCTCATCTGTGCTGCTCCCCCAACAAAAACACACAAACTTGAGCTTTAAGACATGCCTTCACCGCAGCCACCCGGCTCTTTC TTG
TACCCCTTGTCTAGACCTGAGTTGATAAAAGTGAATGATGGGTGTGTGGGCTTAGAGGCACTGCCTCGGGCCTCAGTTCTGGGAAGACCCTTAATTACTGGATGATGAACAGTGACAGAAGAAGGAAG
CAGCCATATAGAAAACTTAACTGCCCTGCTAAAGAGGCAGCCTAGATAGTATGTACAATCCCAGCCTTCGAAATTAGACCAGAATTTAACAGCCACCTCTGCCATGTACTGGTTGTGTGACTTAA AGT
AAGCTGCTCAACTTCTCTGCAATTATTTCCCCATCTGCAAAATGGGGATAATAATAATAGTACCCAACATCATAACGTTCAGGGCATGAATAACTAAGATACTGCATGAAAAGTTTTAGTATGGTGCC
TGGCATAGCAAACTCTCAGTGATAGACAACTTCTATTGCCCTGTTCTTTAAATAGTCTTAGATCCCGGCAATCAGCCCGTAGAAGTCACGCTCCCCAGGATTCAAAGACAAGGCCGCCCCTCACC TCT
TCCCTACTCACCTATCAGGCTGCCTTAGTGTGCTGTCATTTTCTCCTTCAAGGCTGCCTCTGATGTTCATTTCACCAGGATGATTCATCGGGATGGGTCAGGGCCAGTGGCGAGTTAGCCTGATAGCC
CAGCTGGATCTTCCCCTGAAATCTGATGGAAGCACACTTCACCGCCCGTCAGGACTAAAGTCCGGCATTGGTGAGCAGTAAGAACAGCCCCTCTCCAGTGTGGGAGGAGGGGCCTCGAGTAGACC CCA
ATGACTGACCCTCATCAGCTCTCCAGGAAGTGCCTGCCCAGTGGCATCCAGAAGAGGTGCCCAGGTCCTGTGTCTCCCCAGGGCACACTGGGGTTTGCCACTGGGGTTAGGTTTCCTGGGGACTTCTC
AATGACACTGTGAAAACCTAGCTCACCTCCTTGGTGATCGTACTCTCAGCCACTAAAAGGTTTCCTAGGGCACTTAGAAGTTGAGTTCAGACAATAATAATATCCTGGTCGTCTGGTGAAATCGT TAG
CATCTGAGGGGCTGCTTTGCAGTACCCTCTGTTGGGCTTGCGGCTCCTGCCTTTGTATGTTTAACCTTTAAACAAGGG GGTTGGCAGAAGGAAGCAAGCTGTTCTGTCACAGCTGGTCCTGGCGATGC
CAGAGGAGGCCCAAGCTGGAGCTTCCTAGTTAGCTTTGGCTTTTGCCTAAAGAGGATTTCAGGAATAGGGCAGGACACAGGGACCCAGCCAACACCAGGAATAAAAGCCAAGCGTCTACTCCATG CTT
TTGGTTCTGGCTGGCCAGGGCATCTGACACCAGGGAACATCTTTCCTTTCACACTCCATTGTCCTGGGGCTTCAATTACATTCTTGACTGAGAGCCCAGGAATGAGGATAGCGGGCACTCCAGAAATT
AACCTTAGAGAAGTGACTACGTGAGTTTTTAGCCTACTCGCCTCCCTAACAATGGTGCACACCATTGCTGCTCCAGGTGGAGGAGTCACTGCCACCTCCACTAGCTGAGCAAACATCCCTTGGCC TCA
GATCTGGGATTAAAAGGAAgCAGGAGGTCAGCTCCCCACTCATTAGAGCTGGATTATCCTACGAGGGTTTGTACTGCGTGCATAGACAAAAGCATAGCACCCAAAAGAATGAGACACAGCGACGGAAT
TTTGTTATTTTATATTTCAAAAAAAGGCATTGGCTAAGTATAGTGGCTCATGCCTGTAATCCCAGCACTTAGGGAGGCCGAGGCAGGAGGATCACTTGAGCCAAGGAGTTTAACATCAGCCTGGG CAA
CATGGCAAAACCCCATCTCTACAAAAAAAATATAAAAAATTAGCTGGGTGTGGTGGCACACGCCTGTGGTCCCAGCTACTTTGGAGGCTGAAATGAGCCTAGGATCAATTGAACCTAGGAAGTCGAGG
CTGCGGTGAGCCGTGATCACACCGCTGCACTCCAACCTGGGCGGCAACAGAGCGAGACCCTGTCTCAAAAAAGAAAAGGCATTGACAAAGTCATTATTGGAAAAATCTGAATGTATGTAGTCTT ATT
TCCATTTGTTTCATAGTTTAGTGATGTTTTATCTTGAGTTACAAATCCCTTTGGTGCAAAGGATGATGTCTTATGCTAGCTGCACATCTCGTCGGTCTTATAGCTCACCACCACAGAGAG cTGAGCCC
TCCACAAGCCCCTGGTCTGTGTCCTTGACTCTCCCAAGGCCACCACCCCACCTTTCCCCACACTCAGAGCCATCAGCATCACCACCACCATTGATCGTATGAAGACATGCAGACCACTTTTATAC TGC
ATGTGCTTTGTTGCAGGTACTTTTGTGAATCTGCAACACTGAGAAAAACtCgAGTTCAATCCTTAGTTCTTCCCAGTTAACAACTGGGGGATTTGAAGCAAGTTACTCAGCCTTTCTGAATTGCAGTT
TACTCAGCCTCTCTGAATGTCAGTTTCCTCCTCTGTAAGAGCCTGCGCCT cATAGTTGTGTTAAAGAACAAAAGCTATCCCAGcTACTTGGAAGGCCGACATGGGAGGAGTGCTTGAGGCCAGGAGTT
CAAGACCAGCCTGGGCCATAAGCAAGACCCTGTCTCTACAAAAAAAAAAAAAAAAAAAAGTTGTTTTAATTAGCCAGGTGTGGTTGTACATCCCTGTAGTCCTAGCTACTTGGAGCCTGAGGTGGAAA
GATCACTTGAGCCCAGGAGTTGGAGACTACAATGAGCTATGATCTCGCCACTGCATTCCAG CCTGGGTGACAGTTGCTCTGATCCCCAGAGCAACTCTCTAAAAAAAAAAAAAAaAAAAAaaaaaaa
aaaaaaaaaaaggtgggacgagagatttggaaaagaaaaagacacagagacaaagtatagagaaagaaataaggggacccggggaaccagcgttcagcatatggaggatcccgccagcctctgagttcc
cttagtatttattgattattcgtgggtgtttctccgagaggggatgtgtcagggtcacaagacaattgtggggagagggtcagcagacaaacacgtgaacaaaggtctttgcatcatagacaat gta
aaggattaagtgctgtgcttttagatatgcatacacataaacatctcaatgctttacaaagcagtattgctgcccgcaggtcccacctccagccctaaggcggtttttccctatctcagtagatggag
catacaatcgggtttataccgagacattccattgcccagggacaggcaggagacagatgccttcctcttgtctcaactgcaagaggcattccttcctcttttactaatcctcctcagcacagac cct

ttacgggtgtcgggctgggggacggtcaggtctttcccttcccacgaggccatatttcagactatcacatggggagaaaccttggacaatacctggctttcctaggcagaggtccctgcggccttccg
cagtttttgtgtccctgggtacttgagattagggagtggtgatgactcttaaggagcatgctgccttcaagcatctgtttaacaaagcacatcttgcaccgccctcaatccattcaactctgagt tga
cacagcacatgtttcagagagcacggggttgggggtaaggttatagattaacagaatctcaaggcagaagaattttcttagcacataacaaaatggagtctcctatgtctacttctttctacacaga
cacagtaacaatttgatctctcttgcttttccccacAGAAAAAAGGCCAGGTGTGATGACTCACATCTATAATCCCAGCACAGGGAGATCGAGGCAGGAAGATCACTGAACCCAGGAGTT cAAGACAA
GCCTGGGCAACGTAGTGAGACCTCATCTCTACAAAAATAAATAAATAACCAGGCATGGTGGCATGTACCTGTAGTGCCAGCTACTTGGGAAGCTGAGGCGGGAGGATTGCCTGAGCCCAGGAGGT CAA
GGGTGCAGTGAGaCACGATTGCACCACTGCATTCCAGCCTGGGTGATGGAATGAGACCCTGTCTCTATTTAAATAAGTAAATAAACAAACAAATAAATAAAAAAGAATAAAAGGTAAAACAT GTCTGA
GGCCTGCTACAAGGCAGATTACCTATAAATATTAGAGTTATTCATGCCCTTTCTTCACTCCTGATCCGCCTAAAAAGAAGATAGAGTAGGTCCCTTCTCTAGGGAAAAGGAA CCACAACCACCACCAT
CTCAGCTTGAGGGACTTGTAGCGAAGCTTTACCCTATGAACCAACCTCGGGAGTCTCCGGCCTCCATTGCCCCACACCTTGGAAGGGGAGGGGCTTCTCTGGAGAGAGGTTTGTTTAAATGT CATTAT
TTTCTGAAATCATTGCCCAATTTTTCCTATTTTACAGTCAGCCATCCACTATGCATCAGGTGAGTGCGTCTGAGAAGCCTATAGCTGAGGAGTCCTCGGGGAATGAGGTCATCTGGAAACGCAAG CCT
CCCTCGGTTTCAGCAACTCATGCACGTGAAGACAGGCCCCTTCCCCAGGCCCCTGTTGCTGCGAAACGGTGTGGGAGGGTGAGGAAAGGTGTAGAGAGGCACTCCCCACACTTTAATGAGCG CAGGAG
CCCGTCAGTGATCTTGTCCAGATGCAGATTCTGTTGTGGTGGGGGCTGGCTCCAGCCTCTGCGTTTCTTGCCAGCTCTGGGTGATGCCGGTGCTGCTGGCCTGTGGGCCACACTTTGAGTAGCAG GGC
TCTGAGATATGATGTGGTGTGTAACATGGGCATTATTCAGAGGGACTGTAGGTGTCACGGTGGTTGGTGGCAAGAAAAAGAAACCAGTTCATTCAGCTCAGGATTCCCAGCCCTGGAAGGGA GCACCA
ATGGCCAGGCACCGGGCTTCACTCCAGCCTCAGCCCTGGGACGGTAAGGCCCCGGGAGAAGTTCCATAAGACTGTGTACAGTGAAAAAGAGGTCATTCCTCAATTCTGCATTCGAAAATCGGGGC ATC
TTAGGAAGGGGACATGAGCACTAGGTGTCCAAAACCCACAAATGCCCACTCCAGCAGGGATTCCTTGGAGCCAGGTGAGTGGGCGGAGAGTGAGATCTGGAGGCCCTGATGAGTCTGAGACA GCGCCT
CCTATTTGAGGTCATCTCTCTCTCTGGCTGAAAGAAGGAGGGGTGTGCATAGCAGGGCTGCTGGGGAGGGAGAGAGGAGGGAGGAAGGAGTCTGTGGGCTCTACAATGAAACTGGAGGCCCAAGT CCT
CCCGGGACCACAGCGTTCTTAGCGGGAGCCAACGCACAGGCCGGACTAGGAGGTGACAAGTGGGGCATGCTAGGGTACAAAATTTAAGGAGGTGCTTACTCTCAAGTCATAAAAGTACTTGC ATATGA
ATGCCTCCTTAAAAtttTTTTTTTTTTTTTTTTTTGAGACGGAATCTTGCTATGTTGCCCAGGCTGGAGTGCGGTGGTGCGATCTTCGCTCACTGCAAGCTCCGCCTTCCGGGTTCACGCCATTCTCCT
GCCTCAGCCTCCTGAGTAGCTGGGACTACAGGCACCTGCCACCATGCCCGGCTAATTTTTTGTATTTTTAGTAGAGACGGGGTTTCACCGTGTTAG gCACGGGGTCTCGATCTCCTGACCTCGTGAT
CTGCCTGCCTTGGCCTCCCAAAGTGCTGGGATTACAGGCGTGAGCCACCATGCCCAGCCAAATTTTTTTATTTTTTATTTTACTTTTAAGAGATGGGATCTTGCTCTGTCACCCAGGGGACAGT GCA
GTAATATGATTGTAGCTCACTGCAGCCTCCAACTCCTAGACCCAAGCAATCCTCCCT tCtGCCTCAGCCTCCTGAGTAGCTGGGACTACAGGCAGGTGCCACCACACCCAGCTAATTTTAAT TTATTT
TTCGTAGAAACAGGGTCTCACTATGTTGCCCAGGCTGGTCTCAAACTCCTGGCCTCAAGTGATCTTTCTGCCTCAGCCTCCCAAACTGCTAAGATTACAGGTGTTGGCCACCGCACCCAGCCCCT TCT
TAAATTTTGCAAGCTAGTCACTTCACCTGACTCCAGCCCTGCTAAGGAAGAGTAGTCACACTTTGGTCTCCTCTTTACTCTTCCACCTGGGCAAGACACCTCTGGGCCTCTTCTTTTCCCTC AAGCAA
AAAAGAACAAGGTAATAGTGAGGAAGGACGATGCTTACCATAAACATCTTCAGAACTTATTTACCTTGAAATGGGTAGCACCAGACCCAGGGCCTGGCATGCTCAATCAATGTTTGTTCAATGAA TTA
ATCAATCCCACCACTAAATGGAAAGGATCTTTCATTGTTTGACAACAGTTGTCCCTCTCAGAGGGCAGAGGCTATTTTTGCATCATCTTTGCACTTAGTGTGTTCCTGGAATATAACAGAGG TCAATA
GAAGTCTACTGAACTTGATGGAGGTGAAACTCATTTCACCTGAAGGTAAAGTTAGTAGTTGT aTAcAAGGTGTCAGATGAGGGACCATCTCCAAAATATTTTCAACTCTTTTGAAATTCCTCTATTAA
TAACTTATTCCTTTGACTTTCATTTTCCTTACCCACTTGTCTTATTTTAAAATGCCAGTGCCAGTAGGGAAAGGTTACATTAACCAAAAAACAAACTTTAACTTCTTTGCGTTTTTGCCCTG GGAAAC
CTCTAAATCGGAATTTGGCAAACTATGGTCTATGGGCAAAATTCAGCCCATCACCTGTTTTTGTACAACCTGAGAGCTAAGAATGGCTTTTTAATTTTTTTAGTGGTTGAAAAAAAAAACTCAAA AGA
GGAATAATCGTTTATGATATGTGAAGATTATATGAAATTCAAATTTCAGTGTTTACAAATAAAGTTTTATTGGAACATAGCCGCATTTATTCACTTAAGCAGTGTCTGTGGCTGCTTTGGTG CTATGA
TAACAGAGTTAAGAAGTTGTGACAGAGACCTCATGGCCCACAAAGTTTAAGATATTTGCTATCTGGCCCTTTACCGAAAAAGTTTGTTGACTCTGAGGGGGGAGCTATCTGTACTCCTGTATTTAT CCC
ATCAACTTGTTTTGGTAATTTTTTTACAAAATCACTAGATATAATCTCATTTTAATAGAAACCATCGCTTAGAGAGATAAATAGATTCCCAAAGAGTTTATGTATAAATAGAAGTCTTATTA ATAAAA
TCCTATTTTTAGTATTTCTGTTTAAAATATTTTAGGTTAGACAAATGAAAATGTTGTCTCTTCTGGTTTTGGAGGACAGGTTTGTCAGAAGCAGGGACTGTGTTACCCTAGAGAAATCTCCTTTT TAT
CTTGaCTTTTGATTTTGTTTTTGTTTTTTGTTTTGTTTCGTTTTGTTTTGAGACAGGCTCTTGCTCTGTTGCCCAGGCTGGAGTGCAGTGGTGCCATCATAACTCACTGCAACCTGGACCTC CTGGGC
TTAAGTGATTCCCCCACCTCAGCCTCCTGAGTAGCTGGGACTACAGGTGCATGCCACCATGCCTAGATAAATTTTTGTATTTTTTGTAGAAACAGGGTTTCTCCATGTTGCCCAGGCTAGTCTTG AAC
TCCTGATCTCAAGTGATCCTCCTGCCTTGGCCTCCCAAAGTGCTGGGATTACAGGCATCAGCCACAGTGCCTGGCCTAGTTTGTCTTTTACTATGGGTCGTTCTTTGGGCTCAGGCCTATCG GCACTG
GTGCATGCCATACTTAATCTCAGAGACTCAGCTTGCTGGAGGCAGACTGAAAGCAAGTATCTCGAAGATGTAGTTTTGAGTTATGACCCAAGCAAGGGAGAGACTTTTCCCAGGGGTAGAGAAGG ACA
TGATTTACTGGAATAGTCCCAAGAATATGCCAGGAAATCATTTGTCTGCAGCTAAATGGTTTCCATACTGCAATGAGTAGGAGGAGTAAACAGGCAAGGATGGGAATTCCAGAAGTGATTTT TCTCCC
ATTCTCCCCAACCCCCAAGTTTATTGGTAAATGTGTGATTGTCATTTTGCCCCCTGCACCTCATATCCCCACATGTACTCTCTCGCCAAACTTACGGTTTTGTAGCTGATCAACTTCAAGCAAAG TAC
ACTTGGGCTGGGTTGCCAGCCTAAGAATTTTCAAGTAGAGAAAGCTGGTGAAATCTGATCCAATGTGATTCCCCTGGTTCTATGGAATTCTAGATTTTCCCGGATTTGCCAGAGAGATTTTG TTGGTC
TCACCAATATGAGCCTTGAGTGTCGCAGCAGGGTAATGCCTAAGAATTCCCGGGTCAATAGCAGCATGTGAGCCTTCTAGCTGCACTCTGCATTGCTGAGAGCAATTTTAAAGTCCATTCAATGG ACA
GGATGTGAAATGAGGGGAGTTCATGGAAGGCGACAGGATTTTACTCTGCCTGCCCACCTCAGTCGATAGTGAAGCTTTCAAACTCCCTGTTTTCATAGCTTAATGGAGTGTATGTTATGAAA TACCTT
TAAACAAAGAATCCCTAATCTTTTCCTGATGTGAAGAAACTTTGAATTTTCCCAGAGTCATAAAATCAGAGATGCTAGAATGGAAAGGGAGACCTCATTTTCCAGATGTAGAACTGAAGCCCAGA CTG
CACTTGGTGAAGCAGGTTACCCGAGGTCTGCAGCTGAGGGGCAGCAGGGTTCACGTTACACGCCAGGGTCCCTGACACCACACCCAGAGCATTTCCCCTTATGCTACAGTCTCCCCATGGCT GATAGG
GGTGGGAAGTGGGGGCAGAACTACAAAGACAGATTCTTGGTAGAT

31	0.0	0.0	3.0	KS_5	453	486	(10439)	+	(TGTT)n	Simple_repeat	1	33	(0)	1
2261	10.1	2.3	0.0	KS_5	1755	2060	(8865)	+	AluSp	SINE/Alu	1	313	(0)	2

586	31.6	3.7	1.6	KS_5	2342	2582	(8343)	+	MIR	SINE/MIR	2	247	(15)	3	
259	24.5	11.3	4.5	KS_5	2629	2752	(8173)	+	X26_DNA	DNA/TcMar-Tigger	27	158	(733)	4	
271	19.8	1.4	16.5	KS_5	3841	3882	(7043)	+	MamTip1	DNA/hAT-Tip100	85	120	(147)	5	
1943	12.3	0.0	3.5	KS_5	3883	4174	(6751)	+	AluJb	SINE/Alu	12	293	(19)	6	
271	19.8	1.4	16.5	KS_5	4175	4270	(6655)	+	MamTip1	DNA/hAT-Tip100	121	205	(62)	5	
12	22.0	3.9	3.9	KS_5	4392	4442	(6483)	+	(CCA)n	Simple_repeat	1	51	(0)	7	
294	34.8	0.0	1.1	KS_5	4522	4614	(6311)	+	MIRc	SINE/MIR	44	135	(133)	8	
222	21.7	0.0	0.0	KS_5	4603	4648	(6277)	+	MIRb	SINE/MIR	102	147	(121)	9	*
1558	12.9	1.0	9.5	KS_5	4685	4993	(5932)	+	AluJr	SINE/Alu	28	312	(0)	10	
8515	1.5	0.0	0.0	KS_5	5001	5925	(5000)	C	LTR5_Hs	LTR/ERVK	(43)	925	1	11	
1706	18.1	1.8	0.0	KS_5	5933	6255	(4670)	+	AluJb	SINE/Alu	1	312	(0)	12	
453	24.8	7.8	3.1	KS_5	6758	6911	(4014)	C	MER5A1	DNA/hAT-Charlie	(5)	161	1	13	
13	8.7	4.0	0.0	KS_5	7360	7384	(3541)	+	(GGGA)n	Simple_repeat	1	26	(0)	14	
454	24.8	8.4	1.2	KS_5	7463	7565	(3360)	+	MER96	DNA/hAT-Tip100	4	111	(64)	15	
2449	7.3	0.3	0.3	KS_5	7566	7867	(3058)	C	AluY	SINE/Alu	(9)	302	1	16	
1945	15.8	0.0	2.6	KS_5	7871	8186	(2739)	C	AluJr	SINE/Alu	(4)	308	1	17	
454	24.8	8.4	1.2	KS_5	8187	8233	(2692)	+	MER96	DNA/hAT-Tip100	115	167	(8)	15	
235	19.4	12.0	1.2	KS_5	8376	8450	(2475)	+	L2b	LINE/L2	3313	3387	(0)	18	
1954	19.0	0.0	0.9	KS_5	8847	9181	(1744)	+	MER58B	DNA/hAT-Charlie	7	338	(3)	19	
2148	13.7	0.0	1.0	KS_5	9490	9804	(1121)	C	AluJb	SINE/Alu	(0)	312	1	20	
203	32.8	1.9	5.3	KS_5	10717	10860	(65)	C	MIR3	SINE/MIR	(119)	149	9	21	

31	0.0	0.0	3.0	hg19	453	486	(9507)	+	(TGTT)n	Simple_repeat	1	33	(0)	22	
2261	10.1	2.3	0.0	hg19	1755	2060	(7933)	+	AluSp	SINE/Alu	1	313	(0)	23	
586	31.6	3.7	1.6	hg19	2342	2582	(7411)	+	MIR	SINE/MIR	2	247	(15)	24	
259	24.5	11.3	4.5	hg19	2629	2752	(7241)	+	X26_DNA	DNA/TcMar-Tigger	27	158	(733)	25	
271	19.8	1.4	16.5	hg19	3841	3882	(6111)	+	MamTip1	DNA/hAT-Tip100	85	120	(147)	26	
1943	12.3	0.0	3.5	hg19	3883	4174	(5819)	+	AluJb	SINE/Alu	12	293	(19)	27	
271	19.8	1.4	16.5	hg19	4175	4270	(5723)	+	MamTip1	DNA/hAT-Tip100	121	205	(62)	26	
12	22.0	3.9	3.9	hg19	4392	4442	(5551)	+	(CCA)n	Simple_repeat	1	51	(0)	28	
293	34.8	0.0	1.1	hg19	4522	4614	(5379)	+	MIRc	SINE/MIR	44	135	(133)	29	
222	21.7	0.0	0.0	hg19	4603	4648	(5345)	+	MIRb	SINE/MIR	102	147	(121)	30	*
1474	14.1	1.0	10.5	hg19	4685	4996	(4997)	+	AluJr	SINE/Alu	28	312	(0)	31	
1706	18.1	1.8	0.0	hg19	5004	5326	(4667)	+	AluJb	SINE/Alu	1	312	(0)	32	
453	24.8	7.8	3.1	hg19	5829	5982	(4011)	C	MER5A1	DNA/hAT-Charlie	(5)	161	1	33	
13	8.7	4.0	0.0	hg19	6431	6455	(3538)	+	(GGGA)n	Simple_repeat	1	26	(0)	34	
454	24.8	8.4	1.2	hg19	6534	6636	(3357)	+	MER96	DNA/hAT-Tip100	4	111	(64)	35	
2448	7.0	0.3	0.3	hg19	6637	6935	(3058)	C	AluY	SINE/Alu	(12)	299	1	36	
1945	15.8	0.0	2.6	hg19	6939	7254	(2739)	C	AluJr	SINE/Alu	(4)	308	1	37	
454	24.8	8.4	1.2	hg19	7255	7301	(2692)	+	MER96	DNA/hAT-Tip100	115	167	(8)	35	
235	19.4	12.0	1.2	hg19	7444	7518	(2475)	+	L2b	LINE/L2	3313	3387	(0)	38	
1954	19.0	0.0	0.9	hg19	7915	8249	(1744)	+	MER58B	DNA/hAT-Charlie	7	338	(3)	39	
2148	13.7	0.0	1.0	hg19	8558	8872	(1121)	C	AluJb	SINE/Alu	(0)	312	1	40	
203	32.8	1.9	5.3	hg19	9785	9928	(65)	C	MIR3	SINE/MIR	(119)	149	9	41	

>hg19 chr15:63369598-63379590
TTATAGGACAATACAGACAAAGTATAATAGACGTCTTGCCCTCAAGGAGTCTATGGAAGCAACTGAATATGTGCACAAACGCACTAAAAAATAATAAAACAGGATATGATTACATGGCAAATAGAC AG
TAAATGATACAAGACTgCATGGAAACAGATGTCATGCCCCCTTCCTGGTAGATTTCTAACCATCTAAAACCAGTAGAAAAGGCTCTCCTGGGCATGCTCTA TAATTTTCTCAGGGGTACTCCAGCCAC
AGGCAAATCACCACTCACCCCGGCCTCTCAAATCTTAGAAACAAGAGCTCTGCTAGTTTAAAAATGCTTTGGTTTTACTACTTACTCTGGCTACGAAATTTTTTGTTCATAATTGTAGCTTTTTT CTA
CCAAATTCTCATTTTAAAATCCTCCCCTCTTACCCACCTCTCATTCCTTGCTGTAGGCCATGTAGTTGTGTTCTGTTTGTTTGTTTGTTTGTTTGTTTGTT TTTAAACTTTTGGTCTTACCATACCTT
TGCTTTAATCCTAGAGCATCTGTTAAGCTTTAAAGGAAAGAGCATAAATGAATGCACACATATACTCTTAGAGCCTATAAAATCAGCTTCCTACTCCACCAGGGAGGTCTGAAGGAGCCTTTGGT TTC
TGGTAAAGCAAATGGATGAGGATCGCCGCTAACATTCACTTCTCCCACGAGAAGCCTCCCCAGGCTGATTGCTATAGTCGCTAGTGCAGAC aGCGACCTGATCTGCTCAGGCTGCCAGACTTCAGAAG
CCTCAGCCAGGTTCAGAGCCTCCCTAATCCTAATGGGCTAGGAGCCCTGGGTGTCTCCAGATAACCCATTTTCCAGCCTTCCAAGAAAGAGGAGTAGTGGGGCAGTGGGCGAGAGAGAATTGGGT TTC
AGTGCTGTTTTAATGTAAACTGCTGGGGGCTGGGAGCTCCCCTTTTGCTCTCCTTGTTTTGGTTCCTACCCCAGAAACATAAAGTATTACTGATAAACATG GATAATTAAATTTGGATAAAACTACT
TGAACCTGAAGAGTTCATTCCAATTTGTATAACAGCAATTGATGTGGGTTGTGTATTTAAAGGAATATTTAAATTTCATGCAGCAATTTGCAAGTTCAAATGAAAAGCTATTTGGCACATTTGGA TAC
TGGGTTGCAAGGGAAATAAGCAAAATGTACACATGAGGTACATGCCATCATATAATTGTGTACATTTTTTATTTGTCACAAAGCATTATAAAAACAATAGA GAAAAACTATGAAGGAAAAAAAGGTC
ACCACTGGGAAAAAAAAAAACTTATTTTCCATATGTTTTGCCTGTGTGTTTATGGAAGGCTTTTTATTCAAAATTTTGGTTTTTATTCAAAAAACTTTGCATAAACATCTTCCTGTGTTGCTTCA CCT
AGTCTTCTGGTTAATGTGCCATAATTTGTTCTCCTCTTTTTGATCACTGGGTTTTTCTAATTTTTTGTTCTCACAACTAGGACTGATGGGAGATTTTTTTG TAATACAGATATTTTCTGACATGTAAG
AAATGAAAACATACACTTAAAAAAAACTATTTCATTTAAGCACAGACATACCCTAAATATAGTGTTAAGTTGTTTCTTAACTATGTGGTGATTCCTGCTCCGGCCTGGACTCCAGCCTGGAGAGC CCT
TTTTTGATTTCCAGGCCTCAGGTTCTTAAAAGAGAAGCAAAACAACTTTTCCCAAACTGGTGTGGTAAAGCCTTTAAGAAATCTAGTTCCGGCCGGGTGCA GTGGCTCACGCCTGTAATCCCAGCACT
TTGGGAGGCCAAGGCAGGTGGATCACCTGAGGTTGGCCTGTCGGCCTGACCAACATGGAGAAGCCCCTTCTCTACTAAAAATACAAAATTAGCCGGGCGTGGTGGCACATGCCTGTAATCCCAGC TAA
TCAGGAGGCTGAGTCAGGAGAATCGCTTGAACCTGGGAGGCAGAGGTTGCAGTGAGCCGAGATCGAGACACTGCACTCCAGCCTGGGCAACAAGAGCGAAA CTCCACCTCAAAAAAAAAAAAAGAAGA
AAGGAAAAGAAATCTAGTTCCACACGTTATCCCTTTCCCGCTCCCTCATCTGTGCTGCTCCCCCAACAAAAACACACAAACTTGAGCTTTAAGACATGCCTTCACCGCAGCCACCCGGCTCTTTC TTG
TACCCCTTGTCTAGACCTGAGTTGATAAAAGTGAATGATGGGTGTGTGGGCTTAGAGGCACTGCCTCGGGCCTCAGTTCTGGGAAGACCCTTAATTACTGG ATGATGAACAGTGACAGAAGAAGGAAG
CAGCCATATAGAAAACTTAACTGCCCTGCTAAAGAGGCAGCCTAGATAGTATGTACAATCCCAGCCTTCGAAATTAGACCAGAATTTAACAGCCACCTCTGCCATGTACTGGTTGTGTGACTTAA AGT
AAGCTGCTCAACTTCTCTGCAATTATTTCCCCATCTGCAAAATGGGGATAATAATAATAGTACCCAACATCATAACGTTCAGGGCATGAATAACTAAGATA CTGCATGAAAAGTTTTAGTATGGTGCC
TGGCATAGCAAACTCTCAGTGATAGACAACTTCTATTGCCCTGTTCTTTAAATAGTCTTAGATCCCGGCAATCAGCCCGTAGAAGTCACGCTCCCCAGGATTCAAAGACAAGGCCGCCCCTCACC TCT
TCCCTACTCACCTATCAGGCTGCCTTAGTGTGCTGTCATTTTCTCCTTCAAGGCTGCCTCTGATGTTCATTTCACCAGGATGATTCATCGGGATGGGTCAG GGCCAGTGGCGAGTTAGCCTGATAGCC
CAGCTGGATCTTCCCCTGAAATCTGATGGAAGCACACTTCACCGCCCGTCAGGACTAAAGTCCGGCATTGGTGAGCAGTAAGAACAGCCCCTCTCCAGTGTGGGAGGAGGGGCCTCGAGTAGACC CCA
ATGACTGACCCTCATCAGCTCTCCAGGAAGTGCCTGCCCAGTGGCATCCAGAAGAGGTGCCCAGGTCCTGTGTCTCCCCAGGGCACACTGGGGTTTGCCAC TGGGGTTAGGTTTCCTGGGGACTTCTC
AATGACACTGTGAAAACCTAGCTCACCTCCTTGGTGATCGTACTCTCAGCCACTAAAAGGTTTCCTAGGGCACTTAGAAGTTGAGTTCAGACAATAATAATATCCT GGTCGTCTGGTGAAATCGTTAG
CATCTGAGGGGCTGCTTTGCAGTACCCTCTGTTGGGCTTGCGGCTCCTGCCTTTGTATGTTTAACCTTTAAACAAGGGGGTTGGCAGAAGGAAGCAAGCT GTTCTGTCACAGCTGGTCCTGGCGATGC
CAGAGGAGGCCCAAGCTGGAGCTTCCTAGTTAGCTTTGGCTTTTGCCTAAAGAGGATTTCAGGAATAGGGCAGGACACAGGGACCCAGCCAACACCAGGAATAAAAGCCAAGCGTCTACTCCATG CTT
TTGGTTCTGGCTGGCCAGGGCATCTGACACCAGGGAACATCTTTCCTTTCACACTCCATTGTCCTGGGGCTTCAATTACATTCTTGACTGAGAGCCCAGGA ATGAGGATAGCGGGCACTCCAGAAATT
AACCTTAGAGAAGTGACTACGTGAGTTTTTAGCCTACTCGCCTCCCTAACAATGGTGCACACCATTGCTGCTCCAGGTGGAGGAGTCACTGCCACCTCCACTAGCTGAGCAAACATCCCTTGGCC TCA
GATCTGGGATTAAAAGGAAtCAGGAGGTCAGCTCCCCACTCATTAGAGCTGGATTATCCTACGAGGGTTTGTACTGCGTGCATAGACAAAAGCATAGCACC CAAAAGAATGAGACACAGCGACGGAAT
TTTGTTATTTTATATTTCAAAAAAAGGCATTGGCTAAGTATAGTGGCTCATGCCTGTAATCCCAGCACTTAGGGAGGCCGAGGCAGGAGGATCACTTGAGCCAAGGAGTTTAACATCAGCCTGGGCAA
CATGGCAAAACCCCATCTCTACAAAAAAAAATATAAAAAATTAGCTGGGTGTGGTGGCACACGCCTGTGGTCCCAGCTACTTTGGAGGCTGAAATGAGCCTA GGATCAATTGAACCTAGGAAGTCGAGG
CTGCGGTGAGCCGTGATCACACCGCTGCACTCCAACCTGGGCGGGCAACAGAGCGAGACCCTGTCTCAAAAAAGAAAAGGCATTGACAAAGTCATTATTGGAAAAATCTGAATGTATGTAGTCTT ATT
TCCATTTGTTTCATAGTTTAGTGATGTTTTATCTTGAGTTACAAATCCCTTTGGTGCAAAGGATGATGTCTTATGCTAGCTGCACATCTCGTCGGTCTTAT AGCTCACCACCACAGAGAGgTGAGCCC
TCCACAAGCCCCTGGTCTGTGTCCTTGACTCTCCCAAGGCCACCACCCCACCTTTCCCCACACTCAGAGCCATCAGCATCACCACCACCATTGATCGTATGAAGACATGCAGACCACTTTTATAC TGC
ATGTGCTTTGTTGCAGGTACTTTTGTGAATCTGCAACACTGAGAAAAAC cCaAGTTCAATCCTTAGTTCTTCCCAGTTAACAACTGGGGGATTTGAAGCAA GTTACTCAGCCTTTCTGAATTGCAGTT
TACTCAGCCTCTCTGAATGTCAGTTTCCTCCTCTGTAAGAGCCTGCGCCT tATAGTTGTGTTAAAGAACAAAAGCTATCCCAGaTACTTGGAAGGCCGACATGGGAGGAGTGCTTGAGGCCAGGAGTT
CAAGACCAGCCTGGGCCATAAGCAAGACCCTGTCTCTACAAAAAAAAAAAAAAAAAAAA AaaaGTTGTTTTAATTAGCCAGGTGTGGTTGTACATCCCTGTAGTCCTAGCTACTTGGAGCCTGAGGTGG
AAAGATCACTTGAGCCCAGGAGTTGGAGACTACAATGAGCTATGATCTCGCCACTGCATTCCAG CCTGGGTGACAGTTGCTCTGATCCCCAGAGCAACTCTCTAAAAAAAAAAAAAAAgAAAAAgaaa
gaaAGAAAAAAGGCCAGGTGTGATGACTCACATCTATAATCCCAGCACAGGGAGATCGAGGCA GGAAGATCACTGAACCCAGGAGTTgAAGACAAGCCTGGGCAACGTAGTGAGACCTCATCTCTACA
AAAATAAATAAATAACCAGGCATGGTGGCATGTACCTGTAGTGCCAGCTACTTGGGAAGCTGAGGCGGGAGGATTGCCTGAGCCCAGGAGGTCAAGGGTGCAGTGAG cCACGATTGCACCACTGCATT
CCAGCCTGGGTGATGGAATGAGACCCTGTCTCTATTTAAATAAGTAAATAAACAAACAAATAA ATAAAAAAGAATAAAAGGTAAAACATGTCTGAGGCCTGCTACAAGGCAGATTACCTATAAATATT
AGAGTTATTCATGCCCTTTCTTCACTCCTGATCCGCCTAAAAAGAAGATAGAGTAGGTCCCTTCTCTAGGGAAAAGGAA CCACAACCACCACCATCTCAGCTTGAGGGACTTGTAGCGAAGCTTTACC
CTATGAACCAACCTCGGGAGTCTCCGGCCTCCATTGCCCCACACCTTGGAAGGGGAGGGGCTT CTCTGGAGAGAGGTTTGTTTAAATGTCATTATTTTCTGAAATCATTGCCCAATTTTTCCTATTTT
ACAGTCAGCCATCCACTATGCATCAGGTGAGTGCGTCTGAGAAGCCTATAGCTGAGGAGTCCTCGGGGAATGAGGTCATCTGGAAACGCAAGCCTCCCTCGGTTTCAGCAACTCATGCACGTGAA GAC

AGGCCCCTTCCCCAGGCCCCTGTTGCTGCGAAACGGTGTGGGAGGGTGAGGAAAGGTGTAGAG AGGCACTCCCCACACTTTAATGAGCGCAGGAGCCCGTCAGTGATCTTGTCCAGATGCAGATTCTG
TTGTGGTGGGGGCTGGCTCCAGCCTCTGCGTTTCTTGCCAGCTCTGGGTGATGCCGGTGCTGCTGGCCTGTGGGCCACACTTTGAGTAGCAGGGCTCTGAGATATGATGTGGTGTGTAACATGGG CAT
TATTCAGAGGGACTGTAGGTGTCACGGTGGTTGGTGGCAAGAAAAAGAAACCAGTTCATTCAG CTCAGGATTCCCAGCCCTGGAAGGGAGCACCAATGGCCAGGCACCGGGCTTCACTCCAGCCTCAG
CCCTGGGACGGTAAGGCCCCGGGAGAAGTTCCATAAGACTGTGTACAGTGAAAAAGAGGTCATTCCTCAATTCTGCATTCGAAAATCGGGGCATCTTAGGAAGGGGACATGAGCACTAGGTGTCC AAA
ACCCACAAATGCCCACTCCAGCAGGGATTCCTTGGAGCCAGGTGAGTGGGCGGAGAGTGAGAT CTGGAGGCCCTGATGAGTCTGAGACAGCGCCTCCTATTTGAGGTCATCTCTCTCTCTGGCTGAAA
GAAGGAGGGGTGTGCATAGCAGGGCTGCTGGGGAGGGAGAGAGGAGGGAGGAAGGAGTCTGTGGGCTCTACAATGAAACTGGAGGCCCAAGTCCTCCCGGGACCACAGCGTTCTTAGCGGGAGCC AAC
GCACAGGCCGGACTAGGAGGTGACAAGTGGGGCATGCTAGGGTACAAAATTTAAGGAGGTGCT TACTCTCAAGTCATAAAAGTACTTGCATATGAATGCCTCCTTAAATTTTTTTTTTTTTTTTTGA
GACGGAATCTTGCTATGTTGCCCAGGCTGGAGTGCGGTGGTGCGATCTTCGCTCACTGCAAGCTCCGCCTTCCGGGTTCACGCCATTCTCCTGCCTCAGCCTCCTGAGTAGCTGGGACTACAGGC ACC
TGCCACCATGCCCGGCTAATTTTTTTGTATTTTTAGTAGAGACGGGGTTTCACCGTGTTAG cCACGGGGTCTCGATCTCCTGACCTCGTGATCTGCCTGCCTTGGCCTCCCAAAGTGCTGGGATTACA
GGCGTGAGCCACCATGCCCAGCCAAATTTTTTTATTTTTTATTTTACTTTTTAAGAGATGGGATCTTGCTCTGTCACCCAGGGGACAGTGCAGTAATATGATTGTAGCTCACTGCAGCCTCCAAC TCC
TAGACCCAAGCAATCCTCCCTcCcGCCTCAGCCTCCTGAGTAGCTGGGACTACAGGCAGGTGC CACCACACCCAGCTAATTTTAATTTATTTTTCGTAGAAACAGGGTCTCACTATGTTGCCCAGGCT
GGTCTCAAACTCCTGGCCTCAAGTGATCTTTCTGCCTCAGCCTCCCAAACTGCTAAGATTACAGGTGTTGGCCACCGCACCCAGCCCCTTCTTAAATTTTGCAAGCTAGTCACTTCACCTGACTC CAG
CCCTGCTAAGGAAGAGTAGTCACACTTTGGTCTCCTCTTTACTCTTCCACCTGGGCAAGACAC CTCTGGGCCTCTTCTTTTCCCTCAAGCAAAAAAGAACAAGGTAATAGTGAGGAAGGACGATGCTT
ACCATAAACATCTTCAGAACTTATTTACCTTGAAATGGGTAGCACCAGACCCAGGGCCTGGCATGCTCAATCAATGTTTGTTCAATGAATTAATCAATCCCACCACTAAATGGAAAGGATCTTTC ATT
GTTTGACAACAGTTGTCCCTCTCAGAGGGCAGAGGCTATTTTTGCATCATCTTTGCACTTAGT GTGTTCCTGGAATATAACAGAGGTCAATAGAAGTCTACTGAACTTGATGGAGGTGAAACTCATTT
CACCTGAAGGTAAAGTTAGTAGTTGTtTAgAAGGTGTCAGATGAGGGACCATCTCCAAAATATTTTCAACTCTTTTGAAATTCCTCTATTAATAACTTATTCCTTTGACTTTCATTTTCCTTACCCAC
TTGTCTTATTTTAAAATGCCAGTGCCAGTAGGGAAAGGTTACATTAACCAAAAAACAAACTTT AACTTCTTTGCGTTTTTGCCCTGGGAAACCTCTAAATCGGAATTTGGCAAACTATGGTCTATGGG
CAAAATTCAGCCCATCACCTGTTTTTGTACAACCTGAGAGCTAAGAATGGCTTTTTAATTTTTTTAGTGGTTGAAAAAAAAAACTCAAAAGAGGAATAATCGTTTATGATATGTGAAGATTATAT GAA
ATTCAAATTTCAGTGTTTACAAATAAAGTTTTATTGGAACATAGCCGCATTTATTCACTTAAG CAGTGTCTGTGGCTGCTTTGGTGCTATGATAACAGAGTTAAGAAGTTGTGACAGAGACCTCATGG
CCCACAAAGTTTAAGATATTTGCTATCTGGCCCTTTACCGAAAAAGTTTGTTGACTCTGAGGGGGAGCTATCTGTACTCCTGTATTTATCCCATCAACTTGTTTTGGTAATTTTTTTACAAAATC ACT
AGATATAATCTCATTTTAATAGAAACCATCGCTTAGAGAGATAAATAGATTCCCAAAGAGTTT ATGTATAAATAGAAGTCTTATTAATAAAATCCTATTTTTAGTATTTCTGTTTAAAATATTTTAGG
TTAGACAAATGAAAATGTTGTCTCTTCTGGTTTTGGAGGACAGGTTTGTCAGAAGCAGGGACTGTGTTACCCTAGAGAAATCTCCTTTTTATCTTG tCTTTTGATTTTGTTTTTGTTTTTTGTTTTGT
TTCGTTTTGTTTTGAGACAGGCTCTTGCTCTGTTGCCCAGGCTGGAGTGCAGTGGTGCCATCA TAACTCACTGCAACCTGGACCTCCTGGGCTTAAGTGATTCCCCCACCTCAGCCTCCTGAGTAGCT
GGGACTACAGGTGCATGCCACCATGCCTAGATAAATTTTTGTATTTTTTGTAGAAACAGGGTTTCTCCATGTTGCCCAGGCTAGTCTTGAACTCCTGATCTCAAGTGATCCTCCTGCCTTGGCCT CCC
AAAGTGCTGGGATTACAGGCATCAGCCACAGTGCCTGGCCTAGTTTGTCTTTTACTATGGGTC GTTCTTTGGGCTCAGGCCTATCGGCACTGGTGCATGCCATACTTAATCTCAGAGACTCAGCTTGC
TGGAGGCAGACTGAAAGCAAGTATCTCGAAGATGTAGTTTTGAGTTATGACCCAAGCAAGGGAGAGACTTTTCCCAGGGGTAGAGAAGGACATGATTTACTGGAATAGTCCCAAGAATATGCCAG GAA
ATCATTTGTCTGCAGCTAAATGGTTTCCATACTGCAATGAGTAGGAGGAGTAAACAGGCAAGG ATGGGAATTCCAGAAGTGATTTTTCTCCCATTCTCCCCAACCCCCAAGTTTATTGGTAAATGTGT
GATTGTCATTTTGCCCCCTGCACCTCATATCCCCACATGTACTCTCTCGCCAAACTTACGGTTTTGTAGCTGATCAACTTCAAGCAAAGTACACTTGGGCTGGGTTGCCAGCCTAAGAATTTTCA AGT
AGAGAAAGCTGGTGAAATCTGATCCAATGTGATTCCCCTGGTTCTATGGAATTCTAGATTTTC CCGGATTTGCCAGAGAGATTTTGTTGGTCTCACCAATATGAGCCTTGAGTGTCGCAGCAGGGTAA
TGCCTAAGAATTCCCGGGTCAATAGCAGCATGTGAGCCTTCTAGCTGCACTCTGCATTGCTGAGAGCAATTTTAAAGTCCATTCAATGGACAGGATGTGAAATGAGGGGAGTTCATGGAAGGCGA CAG
GATTTTACTCTGCCTGCCCACCTCAGTCGATAGTGAAGCTTTCAAACTCCCTGTTTTCATAGC TTAATGGAGTGTATGTTATGAAATACCTTTAAACAAAGAATCCCTAATCTTTTCCTGATGTGAAG
AAACTTTGAATTTTCCCAGAGTCATAAAATCAGAGATGCTAGAATGGAAAGGGAGACCTCATTTTCCAGATGTAGAACTGAAGCCCAGACTGCACTTGGTGAAGCAGGTTACCCGAGGTCTGCAG CTG
AGGGGCAGCAGGGTTCACGTTACACGCCAGGGTCCCTGACACCACACCCAGAGCATTTCCCCT TATGCTACAGTCTCCCCATGGCTGATAGGGGTGGGAAGTGGGGGCAGAACTACAAAGACAGATTC
TTGGTAGAT

>KSI_LTR5HS_3

>Scaffold7830-16787-17000
TAAGGAGATTAAATCAGTAATAAAGAGTCTCCCATTTGAGAAAAAGCCCAGGGCCTTATGACTTCATGGATGAATTCTACCAAATATTTTAAAAAA AGAGCTAACATCAATCTTTTTCAAATGCTACC
CCCCAACcGAAATGGAGAAGAGGGAATATTTCTCAACTCTTTTTTATAAGGCCAGCATAACCCTGACACTAAATCCAGACGAGGACATTAC cAGGAAAGTACAGGCCAATATTCTTGATGAACATAGAT
GCAAAAATCcTCAACAAAATACTAGCAAATCAAATTCAACAGCACATTAAAATGGTCACTCACCATGATCAAGTGGGATTTATCCCTTGGTTACAAAGATGGTTCAATACATGCAAACCTATAAATGT
AATACACCATATTAACAGAATGAAGGACAAAAACCATATGATCATCTCAATAGATGCATAAAAAGAATTTGACAAAATTCAGCATTCTTTCATGATTAAAAACTGTCAAGAAATTACGTATAGAGGTA
ATGTGCCTCAGCACAATAAAGGCTATATATGACAAGCCCACAGCTAACATTATACTCAATGTTAAAAAGTTGAAAACATTTCCTCTAAGATCTGGAACAAGAGAATTATGCCCACTTTTACTACT TCA
ACTCGACTGAGTATTAGAAGTTCCCATCAGAACAATTGAGCAAGAGAAAGACGTAAAAGGCATTCAAATAGGAAAGGACAGAGTAAAATTGTCACTATTTGCTGGTGACATTATCTTATATATAGAAA
ACCCTAAAGACCCCACCAAAAACCTTTTAGAACTAATAAaCAAATACAGTAAACTTACAGGATACAAATTCAACATTAAAAAATCAATAGCATTGGCCAGGTGCAGTGGCTCACACCTGTAATCCCAG
CACTCTGGGAGGCCcAGGTtGGCCGATTGAGGTCAGGAGATCGAGACAATCCTGCCTAACATGGTGAAACACCATCTCTACTAAAAATACAAAAATTAGCCAGGAGTGGTGGTGCATGCCTGTAGTCC
CAGCTACTCAGGAGGCTGAGGCAGGAGAATCACTTGAACCCAGGAAGTGGAGGTGGCAGTGAGCCAAGATCATGCCACTGCACTCTGGCCTGGGCTACAGAGGGAGACTCCGTCTCAAAAAAAAA AAA
TATTAATAGCATTTCAGCCAGGTGGGGTGGCTCACACCTGTAATCCCAGGACTTTGGGAGGCAGATTGCTTGAGATCAGGAGTTTGAGACCAGCCTGGGCAACATAGTGAGACCCCCATCTCTACTAA
AAATACAAAAATTAGCCGGGTGTGGTAGTGTTTGCCTGAAGTCCCAGCTACTCAGGAGGCTGAGGCACAAGCATCACTTGAACCTGACAGGTGGAGGTTGCAGTGAGCTGAGATTGCACCACTGC ACT
CCAGCCTGGGCAAGAGAGTGAACTCGGTCTCAAAAAAAAAAAAAAAAAAAAAGAAAGAAAAGAAAAGAAAAAAAAAGAAAAGGAAAAAAAAAAGAAAGAAAAGAATAAGAAATCAGTAGCATTTCTATACA
CTAAcAATGAACTATCCAAAAAAAGAGAAATCCCATTTACAATAACTACACACACACACACACACACACACACGTAATACTTAGAAATAATGTTAACTAAATAGGTCAAAGACCTTTACACTGAGAAC
AAAACATTTATAAAACAAATTAAATAATATACAAATAAATAGAAAGCTATTCAGTGTTCATAGCTTGAAAGAATTAATACCATAAAAATGTCTATTATACCAAAAGTGATCTACAAATTCATGCAATC
CTATCAAAATTCCAATGTCATTTTTCACAGAATAGAAAAAAAATCCTAAAATTTTGTATGAAACAACAGAAAAACCCAAATAGCCAAAGCAATCATCAGCAAATAGAACAAAGCTAGAGGCATTA CAC
TACGTGATTTTAAATTATACTACAAAGCTATAGTAACTAAAACAGCAGGGTACTGGCATAAAAATAAAGACATTGACCAATGGA tCAAAATAGCCCAGAAATAAATCCATACATGTACAGTTAATTAA
TTTTTGGCAAAAGTGCCAAGAATACACCATGGGAAAAAGATAGTCTCTTCAGAAAATAGTGCTAAGAAAAGTGTAAGTCTACATGCAAAAGAATTAAATTGGACTCTTTTCTGACACCATATATG CAA
ATCAACTAAAAATGGTCTGAACACTTAAATGTACACTGAAAACTGTAAAACTGTAAAAAGAAAATACAGGGGAAAAACTGCATAACATTAGTCTTGGTGACGATTTTTTAGATTTGGCCCAAAAAGCA
AAAATAGACAAATAAGATTACAACAAACTTAAAAATTTCTGtATAGTGAAGGAAACAATTAACGATGTGAAGAGACAGTCTGCAAATTGGGAGAAAATATTTGCAATCCATACACTCAATAAGGAGCT
AATATTCAAAGTGTGTAAGGAATTCAAGCAACTCAGTAACAAGAAAACAAATAATTCAATTTAAAAATGAGCTGGGTGCGGTGGCTCACGCCTGTAATCCCAGCACTTTGGGAGAACAAGGTGGGCAG
ATCATGATGTCAAGAGATTGAGACCATCCTGGCCAACATGGTGAAACCTCGTCCCTACTAAAAATACAAAAATTATCTGGGCATGGTGGCACGCGCCTATAAAAATTAGCTAAGCATGGTGGCGC GCA
CCTATAGTCCCAGCTACTCAGGGGGCTGAGGCAGAAGAATCACTTGAACCAGGGAGGCGGAGGTTACAGTAAGCCCAGATCACACCACTGCCCTCCAGCCTGGCAACAGAGCTAGCGAGACTCCGTCT
CAAAAAAAAAAAAAAAAAAAaTGAGCAAGGGGACTAAACACACATTTCTCAAAAGATATACAAACGGTCAAAAGGTATTTGAAAAAATATTCAACTTAAGTAATCATCAGGAAAATGCAATTTAAAAC
TACAATGAGCTATCATCTCACACCAGTCAGAATGGCTATTGTCAAAAAGACAAGCAATAAGAAGTGTTGGTGAAGATGTAGAGAAAAGTAAACCCTTGTACACTGTTGGTGGGAATGTAAATTAG CAT
AGTCTTTATGGAAAACTGTATGATGTTCCTTTAAAAATTACTAATAAAGCTGCCATATGATCCAGCAATCCCACTTATGGGTATTTACCCAAAAGATTTGAAATCAGTTTATTGAAGAGACATCTGCA
CTCTCATTGTTATTGCAGCACTATTCACAATAGCCAAGATATGGAATCATCCCAAGTGTCCATCAATGGATGAATGTGTAAAGAAAATGTGGTGCACTTATACAATGAAATATTATTCATCCATA AAA
AAGAATAAAATTCTATCATTTGCAGCAACATTGCAGGACCTGGAGGGTATATGCTAAGTGAAAGAAGCCAGACATGGAAAAACAAATACTACCTGATCTCACTTACATGTGGAATCTAAAATGTTAAA
GAAGCAGAGAATGGAATGGTGGTTACCAGGGCCTGGGGTAGGAGTAGGGGAATGGAAAGACTTTGGTCATAGGATACAAAATTCCAAGTAGACAGGAGGAATACATTCAGGAGATCTATTGTACA GCA
AGGTAATTATAGTTATATAATTATGGAGTATAATTATATACTCCATAATTGCTAAGCGAATAAGTTGATTTTAAATTTTCTCACCACAAAAAAATAAGTATGGGAAGTAATGAACATGTTAATTAGCT
TGATTTAGTCATTCCATAATGGATACATATCTCTAAACATCAATTGAAAAATAAACACGTAAATAGTAAAATAGAAATAAGTTAATCCACACATG CACACCACATTTCACCTGGCAGTTCTACTTAAT
CTATTATATAACGTGCATCATCTAATATCTTAAACAATGTGCCACATCTAATATGTTATACAATGTGCTTCTTTATCTCACTGTGCTTTTCCCACTAAAATGTAAGCTTGACAAAGGCAAGGATTTTT
ACCTTTTTTTCCCTGTTGTGTCTCTGGCCACTCTTACAGTTGGGCACTAAATAAATATTGAATGAATAAGTGGATGGAGAGAGGGAAGGAAG gGAGGAAGGAGGGAGGAAAAGAAGGGAGGGAGGACG
AGAGAAAGAAAGGAGAAGGAAGAAAGATAAAGAAAGGAGGGATAGAGGAAAGGAGATGAGAGTGTTATGTGCTCCTGAGTATGAGGATGACGATACGACATCTCGTGTTTGGGTACTGAGTTGGATTT
TAAAATCGCATGGTGCAGTAGCACATAGCAAACATTCAAAGACTGTGTGCTGCCTTAATGAACAAGCTTCCTTAACTTGCGTCTTCCACTTCCCTTAATTACTGCAACCTCCTCTTTTTCCTCCA ACC
AGAGAAGGTCTGTAATGGAATTCTTGTGCCTTCAGGGGGCCACAGGACAAAATCTGCAGCTGGAGGATCACTTCCTATCTCTACTTCCTCCTGGCAGAAGGCAGAGCAAGCCCCCTGAGCATTCCAAG
CCCCTGCTCCGATGTACCTTGTCAGGGTTAAAAGCACAGCAGGTTGGTGTGTGCGGAGACCGGTAAATAAGAAACAATGGCTTTGGTGTTTGGAATGTAGAAAAGCTGCTCAAATCGCTCAGGTG GGT
GCTCTATGAAAGTAACTTCCATTGGTTCCAGTTTTATTTCATCTTGCCTGTAAGTGCGAAGATTGTTTAC AGAGGCATGCCCACCGTCCTAGGCCCATAGTAGGACCAAAATGTTGATGCTGAACAT
TTTTGTTTTCTATGTTTGCTTTAACTGGGGAGTCACAAGTTCAAACAGCCACAGGAACCAATCCTGGGGTGAAGAGTAGGCTGTGTTGGGGACT Gtggtaaactagacggctcatgctgacacccagt
tccactgagggtggccacggatgtagggttgccagagttgtcacatctccatctctctctctctctttctctctctctcattctctctttctctctcattctcattcttcctctatcattctctct
cattctctcatctgtttaacaaagcacatcttgcaccgcccttaatccatttaactctgagttgacacagcacatgtttcagagagcacggggttgggggtaaggtcacagaatctcaaggcagaaga
atttttcttagtacagaacaaaatggagtctcctatgtctacttctttctacacagacacagtaacaatctgatctctcttgcttttccccacaTGGTAAACTAGACGGCTCATGCTGACACCCAGTT
CCACTGAGGGTGGCCACGGATGTAGGGTTGCCAGAGTTGTCACATCTCCATCTCTCTCTCTCTCTCTTTCTCTCTCTCTCATTCTCTCTTTCTCTCTCATTCTCATTCTTCCTCTATCATTCTCT CTC
ATTCTCTGTCTCATTCTCTTTCTCTCATTCTCTCTCTCATTCTCTGTCTCATTCTCTTTCTCTCATTCTCTCTCTCATTCTCTGTCTTTCTCATTCTTTCTTTCTCATTCTCTCTCAGTCTCTGTCTC

ATTCTTTTCTCCCATTCTTTCTCTCTCCCATTCTCTCCTTCTCTCTCTCATTCTCATTTTCTCCTTTTCTCTCTCTCATTCTCTCTCTCTCGCATTCACTCTTTCTCTCAGTCTCAGTCTCATTC TCA
CTCTCATTCTCCCTTTCTCTCCCTCATTCTCTCTTTTTTCTCATTCTCTCTCATTCTCTCTCACTGTCATTCTgcggaTATCATTCTTTCATTCACTCTCCTCTCTCATTCTCTCTCTCAGTCTCTGT
CTCATTCTCTCTCATTCTCTCTTCCTCTCTCTCATTCTTTCTCCCCAAGATAAGCTGAATGTCTGAATTTTCTGTGAAATGTAATTTTTAAATGCTAGAAAATGACCTGATTTGCTTTGAAACACCTA
TATTTAGATTCTGTATTGTTCATTTATTTATGTATACAGTACAGAGTTGTGCAAGCATCACCAGAATCTAATTTTGAGTACATTTATATCATTTCAGTAAGAAATAGTGTGCCCCTTAGTGGTCACCC
CTTCATTTTTCTCCTGTCCCCCACCAAGCCTAGACAATATTTTCTGTCTGTATAGATTTGCCTATTCTGGACGTTTCATATAAATGAAATAATACCAAACATGGTCTTTTGTGACTGGCTTCTTT CAC
TTGGTGTAGTGTTTCGGGAGTTATCTTTGTTTTGGCCTATATCAGAACTCTATTTCTTTTTGTTGGTGGTGGTAAATAATATTCTATTGTATGGCTACATGACATTCTGTCTGTTTATTCATTAGCCA
TCAAACAAGCATATCACAGGCTGGTCCCAGCCCTGGGGGACCCATTTTTCACCATGCAACAGCCTCCTGGCTGGTGTCCCCATCTCCTGTTCAATCCCCC cGCCCTCTGCTGACACAGGGTGTGCATG
CAACCAGCCAGACAGCCCAGGGTAGAAATATTGGGACAATTTTTCTAAACCAAGGCTTTGAGCATGTCACTCTCCTCCATCCCCACCTTTATGCTGAAATCACCCTCCTCTCTTGGCTTCAGAGTGGA
GCTCCAGCCCCTTAGTCCAAAACAAAAACTTTTGCAAGAGCTGGCCCCACCACCCTCCACACCCTTCCCTGCTCACAAACCCTGTGCTCCAACACACCATGTCACCCTCTCCTCTCAAAGCCTGA GCA
TGTCCAGTCCTCTGTCCCCTTGCTCATGCTGTGCCACTGGTGTGTAATTCACCAATTGTGGGTCATTCTCCTCCACTGAGTGGGGAACTGTTCTGGAAGGAGAAGGGGTATTCTCTGTCCTCAGGACT
TGGGACAAAGCAGGTACATAATGAATGTCTGACGGTGGAATAGACACAGGCCATTCCTCCATCTAAAATGCCCTTCCCTACATTGTCCATCTCACATTTGACAGCAGCTCTCCTTGCTCACCCAC ACC
TGATCCAAGCACTGATTATCTAGTTCGTCTCTGTGCTCATGCAGCACCTAGCTCTGTGTGTGCGTGCATGTACGTATGTGTGTGCGAGCGTTTGCGTGACTTCATCAGATTTCAGTCCTTGGGGACAA
GCTTTTCCCCATATTGTTAAAAATTCAGTCTGTGCAATAATTTGTTGAGGCCTAATTTGCATGCACTGAGATGCAACCATTTAAGTGTATAGTTTGATGTGTTTCAAGAAATGTTCACCTGTGGA TTA
TACTCATGTAGCCACAGCTGCCATCAAAATATAGAACATCCCCATCACCCCAGAAAGCTCTTCTGTGCCCCTTCCAGGTCAATGCCCTCACGCCCCATCCAAGGCAACCACTGATCTCTCCTGACACC
ACAGCTCAGCGTTTGCGGCTGTGGGGCTTCATAGGCACGGACTGAGGCAGCAGACAATCTTGCGTGTCTGTCTTCATTTGCGCGGCGCAGCATCTGTGAGATTCAGCCATGCAGTCGGGTCTGTC GTG
AATTCACTCTTTTTGCCCCATTCCCAGCACAGTGCCTGAGTGTCAAATTGAATAGACTTCAATTGCTGTGAGGTGATTAATAGCAAATTGTGTGTTATTCATGAATAAAGAAAAAAGATGCAACGTGT
TCTGGGAGGTGGAAAAGAGAAAGAAACCCACCCCGACCCCGCCTTTCTGTTCTCACCTAACGAAGTCTGGAGTTCAGCAAGAAAAGGTGTGAGGGGGTTTTCAGGAAACAGATAGGCCACCAGG GTC
CAAATCAGAGGTAGAGTGGCACATCCAGCCCAAATGGAAACAAACGATGCAAGCCTATGGCTTAAATCAGAAATTAATCTCTACTGGTAGTTTCCATGAACTCTTCTCCAGGTAAACATCACATGTAA
AAGTAAGTTCCTGTCATTATAGAATAATTAGATTATAAGAACTAAATcTATAGTGTATACTACGCATTACTGCAGAATTGTAGAAATTATGCAACCCAGTCCTTAACAGAATTAAATGGGAATCTATA
TTTTTTCTCAGTAAGTCTTTATTGAATGAATGGAGTCATATTCTGTTTGTGTTTTTCTGTGCTTTTGCAATTTCTCAAATTAAAGATAAATAAACGGTCTCTTCTTGGCCTCGTGGCTAAGATCA AGT
GTAAAGACAAGTAAATGGTAGTTGAATGAAAGAAACCAGGAAAAGTTATTTTTGGAGTGTGTAGGGTGTGCCTCGCCTCCTTGGACCCACCTCGTTTCTCCCAAGAGCAGACCCAGATACACTAACTC
GATCTGCCTCCCGCCTCCTCCTTCTGCTTTAATCAACTGTGGGTCCTGCTCTCTTCAATGCCTGCGTGTCAGCTAAGTTTGCAATCACCCCGTTTACAGCTTGAAAATGAAGCCCTGTCTTTTCT GCA
AGTAAGAATCTTCCTGAACCATGCATCCAAATGTCACATTTCCAAACATGTTGTGAGCTGAGGACTCCAAGTCATAAACTTCAAAGCAAGTACTTGTAATGGGAATTCTGACTGACAGTCCCTTAATT
ACAGTCTTTAAATATGCACAGTCCCTGACaGAGCCAGGCTTTCTGAGGAAAATTCCTGCCTAACTGGTTGAAAATGTGAACAGTGGGGTATAATTCCCTGAGCTGCAAGGAGCCACAGATCAGAGAAA
ATGCAAGAGAAACTCTAAAGACCCTTGTAGGATGAATTTTGTCTTTTGCAGGCGGTATGAAAACCGAAAGAGGGGAGACAACTGGGACAAGGAGAAACAAATATTTCCTGTGCTTGAAGCTGACACAC
ATGGTTTATTTGCCGGTGTTTGGAGAGGTTAGAAAGCAAAGATAGCAGAGGAAGAATAGGTTTATCCTTGAGGACAGAACATGCATGCTGGTGAGTGGGAAGGGACTAAAAACAAGGTGGCTGGG CCG
GGCGCAGTGGCTCATGCCTATAATCCCATCAcTTTGGGAGGCCTcGGAGGGAGGATCACTTGAGGTCAAGAGTTAGAGACCAGCTTGGTGAAACCCCGTCTCTATTAAAAATAAAAAATTAGCTGGGT
GTGGTGGCTCGTGCCTATAATCCCAGCTACTACTCAGGAGGCTGAGGCAGGAGAATTGCTTGAACCTGGGAGGCGGAGGTTGCAGTGAGCCTAGATGGTACCACTGCACTCCGGCCTGGGCAAAA GAG
CAAGATTTCGTCTCAAACAACAAACAAACAAAAAATGACAGGGTGGCCCAAAACAAGGCAGTCCCACTGATCACGGACTCCAGTGACATTAACAGAGACCAGGGGGACATGGTTGCCCCTGTGTGCCC
TGGGTCACATCACAGCCACTCCTTATTGGCCCTAGCCACAGGATGTaCTATGTCTCAGGCAGTGGCGTGTGTGCAGGTAACTcCTCCATCAGAGCCCCAGAAGGCAGCATGGCATCAGGTTTGGCAGT
CTTATGCCACGTTGAGATCAGAGACCTTGTTTTATTAGCACAGGGTGCTCAGCCAACAAGAATGTCAAGCTGGTGTTTTTGCTACCACAGCCCCAGTTTAATGACAGAGAGTAACCTTGATATGTAGT
TTGGTGACCAGTTAGGCAGGAAATTTATGGCAAAACACAGAAATGACCACACTGGAGTGGATTTAAGTCCTGATGCTGCCAATTAACAGCTATGTAGCTTTGCCCTTGGGCCAGAGTTACCACGT AAA
ACACAAATATCCAGCTAAATCTGAATTTCAGATATATAACAGATAATTTTCCACATATAACACATACTAAAACATTATCCATTGTTTATCTAAAATGCAAAGGTAACTGAGTGCTCTGATTTTAATTT
GCTAAATCTGGCCAACGACTTTCTCTTGCTGAACCTCATTTTCCTACTGTTTCCAAGTGGACAGCAGTGCATGTCTCAAGGGGGTTTGAGAC tAGCTGAGATGCTGCTGGCCCAGCACAGGGTTCCTA
CACTATGAGGGTGATCAACTGAGCTCTCAGTGCTTCTTCTCCCTCAGGATCTTTGTCTGCCTTTTAGCTCTTTCCAAATGTTAAACTTTCCATAAAGGCCAATGTTATGTGTCCCCCATGGCAGAACA
TTTCTTTTCTCACCATAGAAATCAATTTATTGTATATTTCAAAATAACTAAAAGAATGAAATTGGAGTGTCCCTAACACAAAGAAATACTCAGTGCTTGAGGTGATGGATACTCCAGTTGCTCTG ATC
TAATCACAACACATCATATGCTTGTATCAAAATATCATCTGTACCCCATAAACATGTGAAACTATTATGCATCCATGATTAAATATAAAAGTGAATAAAAAA

4975	28.4	2.2	4.2	KS_3	1	862	(9352)	+	L1MA8	LINE/L1	3474	4331	(1815)	1
2095	11.7	1.0	0.0	KS_3	863	1152	(9062)	+	AluSc8	SINE/Alu	1	293	(18)	2
4975	28.4	2.2	4.2	KS_3	1153	1168	(9046)	+	L1MA8	LINE/L1	4332	4344	(1802)	1
1994	13.3	3.7	0.3	KS_3	1169	1468	(8746)	+	AluSz	SINE/Alu	2	311	(1)	3
4975	28.4	2.2	4.2	KS_3	1469	1583	(8631)	+	L1MA8	LINE/L1	4345	4408	(1738)	1
30	0.0	0.0	0.0	KS_3	1584	1609	(8605)	+	(AC)n	Simple_repeat	1	26	(0)	4
5407	27.5	2.1	4.0	KS_3	1610	2502	(7712)	+	L1MA8	LINE/L1	4409	5325	(966)	1
2083	8.7	0.0	12.4	KS_3	2503	2837	(7377)	+	AluSc	SINE/Alu	2	299	(10)	5

5407	16.1	1.6	1.9	KS_3	2838	3754	(6460)	+	L1MA8	LINE/L1	5326	6240	(51)	1
394	28.9	13.2	0.0	KS_3	3885	4043	(6171)	+	L2a	LINE/L2	3247	3426	(0)	6
25	19.7	8.7	3.3	KS_3	4044	4158	(6056)	+	GA-rich	Low_complexity	1	121	(0)	7
41	21.8	0.0	0.0	KS_3	4910	5000	(5214)	+	(TCTCTC)n	Simple_repeat	1	91	(0)	8
1878	1.9	3.7	0.0	KS_3	5001	5214	(5000)	C	LTR5_Hs	LTR/ERVK	(746)	222	1	9
182	28.1	0.6	0.4	KS_3	5293	5803	(4411)	+	(TCTCTC)n	Simple_repeat	1	512	(0)	10
1351	21.8	2.9	4.5	KS_3	5929	6269	(3945)	C	L1MB8	LINE/L1	(87)	6091	5756	11
181	34.7	9.4	1.6	KS_3	6699	6815	(3399)	+	L2c	LINE/L2	3248	3373	(14)	12
1038	27.7	1.1	4.3	KS_3	7081	7438	(2776)	C	L1ME2	LINE/L1	(3)	6161	5815	13
233	11.8	0.0	0.0	KS_3	8034	8067	(2147)	+	U2	snRNA	2	35	(153)	14
194	29.1	3.0	6.2	KS_3	8387	8452	(1762)	C	MER131	DNA?	(126)	89	26	15
2002	11.5	3.4	1.0	KS_3	8828	9123	(1091)	+	AluSz	SINE/Alu	1	303	(9)	16
199	28.9	0.0	0.0	KS_3	9530	9574	(640)	+	MIRc	SINE/MIR	53	97	(171)	17
571	19.2	18.6	0.0	KS_3	9585	9740	(474)	C	MER53	DNA/hAT	(1)	192	8	18
939	20.8	0.0	0.0	KS_3	10008	10190	(24)	+	L1MA4	LINE/L1	6109	6291	(9)	19

4983	28.4	2.2	4.3	hg19	1	863	(8975)	+	L1MA8	LINE/L1	3474	4331	(1815)	20
2132	11.4	1.0	0.0	hg19	864	1153	(8685)	+	AluSc8	SINE/Alu	1	293	(18)	21
4983	28.4	2.2	4.3	hg19	1154	1169	(8669)	+	L1MA8	LINE/L1	4332	4344	(1802)	20
2003	13.3	3.6	0.3	hg19	1170	1470	(8368)	+	AluSz	SINE/Alu	2	312	(0)	22
4983	28.4	2.2	4.3	hg19	1471	1585	(8253)	+	L1MA8	LINE/L1	4345	4408	(1738)	20
37	0.0	0.0	0.0	hg19	1586	1617	(8221)	+	(AC)n	Simple_repeat	1	32	(0)	23
5407	27.4	2.1	4.1	hg19	1618	2510	(7328)	+	L1MA8	LINE/L1	4409	5325	(966)	20
2074	8.7	0.0	12.5	hg19	2511	2844	(6994)	+	AluSc	SINE/Alu	2	298	(11)	24
5407	16.1	1.6	1.9	hg19	2845	3761	(6077)	+	L1MA8	LINE/L1	5326	6240	(51)	20
394	28.9	13.2	0.0	hg19	3892	4050	(5788)	+	L2a	LINE/L2	3247	3426	(0)	25
25	19.7	8.7	3.3	hg19	4051	4165	(5673)	+	GA-rich	Low_complexity	1	121	(0)	26
191	27.6	0.6	0.0	hg19	4917	5427	(4411)	+	(TCTCTC)n	Simple_repeat	1	514	(0)	27
1351	21.8	2.9	4.5	hg19	5553	5893	(3945)	C	L1MB8	LINE/L1	(87)	6091	5756	28
181	34.7	9.4	1.6	hg19	6323	6439	(3399)	+	L2c	LINE/L2	3248	3373	(14)	29
1038	27.7	1.1	4.3	hg19	6705	7062	(2776)	C	L1ME2	LINE/L1	(3)	6161	5815	30
233	11.8	0.0	0.0	hg19	7658	7691	(2147)	+	U2	snRNA	2	35	(153)	31
194	29.1	3.0	6.2	hg19	8011	8076	(1762)	C	MER131	DNA?	(126)	89	26	32
1984	11.8	3.4	1.0	hg19	8452	8747	(1091)	+	AluSz	SINE/Alu	1	303	(9)	33
199	28.9	0.0	0.0	hg19	9154	9198	(640)	+	MIRc	SINE/MIR	53	97	(171)	34
571	19.2	18.6	0.0	hg19	9209	9364	(474)	C	MER53	DNA/hAT	(1)	192	8	35
939	20.8	0.0	0.0	hg19	9632	9814	(24)	+	L1MA4	LINE/L1	6109	6291	(9)	36

>hg19 chr19:29850943-29860780

TAAGGAGATTAAATCAGTAATAAAGAGTCTCCCATTTGAGAAAAAGCCCAGGGCCTTATGACTTCATGGATGAATTCTACCAAATATTTTAAAAAA AaGAGCTAACATCAATCTTTTTCAAATGCTAC
CCCCCAAaGAAATGGAGAAGAGGGAATATTTCTCAACTCTTTTTTATAAGGCCAGCATAACCCTGACACTAAATCCAGACGAGGACATTAC aAGGAAAGTACAGGCCAATATTCTTGATGAACATAGA
TGCAAAAATCaTCAACAAAATACTAGCAAATCAAATTCAACAGCACATTAAAATGGTCACTCACCATGATCAAGTGGGATTTATCCCTTGGTTACAAAGATGGTTCAATACATGCAAACCTATAAATG
TAATACACCATATTAACAGAATGAAGGACAAAAACCATATGATCATCTCAATAGATGCATAAAAAGAATTTGACAAAATTCAGCATTCTTTCATGATTAAAAACTGTCAAGAAATTACGTATAGA GGT
AATGTGCCTCAGCACAATAAAGGCTATATATGACAAGCCCACAGCTAACATTATACTCAATGTTAAAAAGTTGAAAACATTTCCTCTAAGATCTGGAACAAGAGAATTATGCCCACTTTTACTACTTC
AACTCGACTGAGTATTAGAAGTTCCCATCAGAACAATTGAGCAAGAGAAAGACGTAAAAGGCATTCAAATAGGAAAGGACAGAGTAAAATTGTCACTATTTGCTGGTGACATTATCTTATATATA GAA
AACCCTAAAGACCCCACCAAAAACCTTTTAGAACTAATAAgCAAATACAGTAAACTTACAGGATACAAATTCAACATTAAAAAATCAATAGCATTGGCCAGGTGCAGTGGCTCACACCTGTAATCCCA

GCACTCTGGGAGGCCaAGGTgGGCCGATTGAGGTCAGGAGATCGAGACAATCCTGCCTAACATGGTGAAACACCATCTCTACTAAAAATACAAAAATTAGCCAGGAGTGGTGGTGCATGCCTGTAGTC
CCAGCTACTCAGGAGGCTGAGGCAGGAGAATCACTTGAACCCAGGAAGTGGAGGTGGCAGTGAGCCAAGATCATGCCACTGCACTCTGGCCTGGGCTACAGAGGGAGACTCCGTCTCAAAAAAAAAAA
ATATTAATAGCATTTCAGCCAGGTGGGGTGGCTCACACCTGTAATCCCAGGACTTTGGGAGGCAGATTGCTTGAGATCAGGAGTTTGAGACCAGCCTGGGCAACATAGTGAGACCCCCATCTCTA CTA
AAAATACAAAAATTAGCCGGGTGTGGTAGTGTTTGCCTGAAGTCCCAGCTACTCAGGAGGCTGAGGCACAAGCATCACTTGAACCTGACAGGTGGAGGTTGCAGTGAGCTGAGATTGCACCACTGCAC
TCCAGCCTGGGCAAGAGAGTGAACTCGGTCTCAAAAAAAAAAAAAAAAAAAA AaGAAAGAAAAGAAAAGAAAAAAAAGAAAAGGAAAAAAAAAGAAAGAAAAGAATAAGAAATCAGTAGCATTTCTATA
CACTAAaAATGAACTATCCAAAAAAAGAGAAATCCCATTTACAATAAC TacacacACACACACACACACACACACACACGTAATACTTAGAAATAATGTTAACTAAATAGGTCAAAGACCTTTACA
CTGAGAACAAAACATTTATAAAACAAATTAAATAATATACAAATAAATAGAAAGCTATTCAGTGTTCATAGCTTGAAAGAATTAATACCATAAAAATGTCTATTATACCAAAAGTGATCTACAAA TTC
ATGCAATCCTATCAAAATTCCAATGTCATTTTTCACAGAATAGAAAAAAAATCCTAAAATTTTGTATGAAACAACAGAAAAACCCAAATAGCCAAAGCAATCATCAGCAAATAGAACAAAGCTAGAGG
CATTACACTACGTGATTTTAAATTATACTACAAAGCTATAGTAACTAAAACAGCAGGGTACTGGCATAAAAATAAAGACATTGACCAATGGA aCAAAATAGCCCAGAAATAAATCCATACATGTACAG
TTAATTAATTTTTGGCAAAAGTGCCAAGAATACACCATGGGAAAAAGATAGTCTCTTCAGAAAATAGTGCTAAGAAAAGTGTAAGTCTACATGCAAAAGAATTAAATTGGACTCTTTTCTGACACCAT
ATATGCAAATCAACTAAAAATGGTCTGAACACTTAAATGTACACTGAAAACTGTAAAACTGTAAAAAGAAAATACAGGGGAAAAACTGCATAACATTAGTCTTGGTGACGATTTTTTAGATTTGG CCC
AAAAAGCAAAAATAGACAAATAAGATTACAACAAACTTAAAAATTTCTG cATAGTGAAGGAAACAATTAACGATGTGAAGAGACAGTCTGCAAATTGGGAGAAAATATTTGCAATCCATACACTCAAT
AAGGAGCTAATATTCAAAGTGTGTAAGGAATTCAAGCAACTCAGTAACAAGAAAACAAATAATTCAATTTAAAAATGAGCTGGGTGCGGTGGCTCACGCCTGTAATCCCAGCACTTTGGGAGAAC AAG
GTGGGCAGATCATGATGTCAAGAGATTGAGACCATCCTGGCCAACATGGTGAAACCTCGTCCCTACTAAAAATACAAAAATTATCTGGGCATGGTGGCACGCGCCTATAAAAATTAGCTAAGCATGGT
GGCGCGCACCTATAGTCCCAGCTACTCAGGGGGCTGAGGCAGAAGAATCACTTGAACCAGGGAGGCGGAGGTTACAGTAAGCCCAGATCACACCACTGCCCTCCAGCCTGGCAACAGAGCTAGCG AGA
CTCCGTCTCAAAAAAAAAAAAAAAAAATGAGCAAGGGGACTAAACACACATTTCTCAAAAGATATACAAACGGTCAAAAGGTATTTGAAAAAATATTCAACTTAAGTAATCATCAGGAAAATGCAAT
TTAAAACTACAATGAGCTATCATCTCACACCAGTCAGAATGGCTATTGTCAAAAAGACAAGCAATAAGAAGTGTTGGTGAAGATGTAGAGAAAAGTAAACCCTTGTACACTGTTGGTGGGAATGT AAA
TTAGCATAGTCTTTATGGAAAACTGTATGATGTTCCTTTAAAAATTACTAATAAAGCTGCCATATGATCCAGCAATCCCACTTATGGGTATTTACCCAAAAGATTTGAAATCAGTTTATTGAAGAGAC
ATCTGCACTCTCATTGTTATTGCAGCACTATTCACAATAGCCAAGATATGGAATCATCCCAAGTGTCCATCAATGGATGAATGTGTAAAGAAAATGTGGTGCACTTATACAATGAAATATTATTC ATC
CATAAAAAAGAATAAAATTCTATCATTTGCAGCAACATTGCAGGACCTGGAGGGTATATGCTAAGTGAAAGAAGCCAGACATGGAAAAACAAATACTACCTGATCTCACTTACATGTGGAATCTAAAA
TGTTAAAGAAGCAGAGAATGGAATGGTGGTTACCAGGGCCTGGGGTAGGAGTAGGGGAATGGAAAGACTTTGGTCATAGGATACAAAATTCCAAGTAGACAGGAGGAATACATTCAGGAGATCTA TTG
TACAGCAAGGTAATTATAGTTATATAATTATGGAGTATAATTATATACTCCATAATTGCTAAGCGAATAAGTTGATTTTAAATTTTCTCACCACAAAAAAATAAGTATGGGAAGTAATGAACATGTTA
ATTAGCTTGATTTAGTCATTCCATAATGGATACATATCTCTAAACATCAATTGAAAAATAAACACGTAAATAGTAAAATAGAAATAAGTTAATCCACACATG CACACCACATTTCACCTGGCAGTTCT
ACTTAATCTATTATATAACGTGCATCATCTAATATCTTAAACAATGTGCCACATCTAATATGTTATACAATGTGCTTCTTTATCTCACTGTGCTTTTCCCACTAAAATGTAAGCTTGACAAAGGCAAG
GATTTTTACCTTTTTTTCCCTGTTGTGTCTCTGGCCACTCTTACAGTTGGGCACTAAATAAATATTGAATGAATAAGTGGATGGAGAGAGGGAAGGAAG aGAGGAAGGAGGGAGGAAAAGAAGGGAGG
GAGGACGAGAGAAAGAAAGGAGAAGGAAGAAAGATAAAGAAAGGAGGGATAGAGGAAAGGAGATGAGAGTGTTATGTGCTCCTGAGTATGAGGATGACGATACGACATCTCGTGTTTGGGTACTGAGT
TGGATTTTAAAATCGCATGGTGCAGTAGCACATAGCAAACATTCAAAGACTGTGTGCTGCCTTAATGAACAAGCTTCCTTAACTTGCGTCTTCCACTTCCCTTAATTACTGCAACCTCCTCTTTT TCC
TCCAACCAGAGAAGGTCTGTAATGGAATTCTTGTGCCTTCAGGGGGCCACAGGACAAAATCTGCAGCTGGAGGATCACTTCCTATCTCTACTTCCTCCTGGCAGAAGGCAGAGCAAGCCCCCTGAGCA
TTCCAAGCCCCTGCTCCGATGTACCTTGTCAGGGTTAAAAGCACAGCAGGTTGGTGTGTGCGGAGACCGGTAAATAAGAAACAATGGCTTTGGTGTTTGGAATGTAGAAAAGCTGCTCAAATCGC TCA
GGTGGGTGCTCTATGAAAGTAAACTTCCATTGGTTCCAGTTTTATTTCATCTTGCCTGTAAGTGCGAAGATTGTTTACAGAGGCATGCCCACCGTCCTAGGCCCATAGTAGGACCAAAATGTTGATGC
TGAACATTTTTGTTTTCTATGTTTGCTTTAACTGGGGAGTCACAAGTTCAAACAGCCACAGGAACCAATCCTGGGGTGAAGAGTAGGCTGTGTTGGGGACT GTGGTAAACTAGACGGCTCATGCTGAC
ACCCAGTTCCACTGAGGGTGGCCACGGATGTAGGGTTGCCAGAGTTGTCACATCTCCATCTCTCTCTCTCTCTCTTTCTCTCTCTCTCATTCTCTCTTTCTCTCTCATTCTCATTCTTCCTCTATCAT
TCTCTCTCATTCTCTGTCTCATTCTCTTTCTCTCATTCTCTCTCTCATTCTCTGTCTCATTCTCTTTCTCTCATTCTCTCTCTCATTCTCTGTCTTTCTCATTCTTTCTTTCTCATTCTCTCTCAGTC
TCTGTCTCATTCTTTTCTCCCATTCTTTCTCTCTCCCATTCTCTCCTTCTCTCTCTCATTCTCATTTTCTCCTTTTCTCTCTCTCATTCTCTCTCTCTCGCATTCACTCTTTCTCTCAGTCTCAGTCT
CATTCTCACTCTCATTCTCCCTTTCTCTCCCTCATTCTCTCTTTTTTCTCATTCTCTCTCATTCTCTCTCACTGTCATTCT ctcttTATCATTCTTTCATTCACTCTCCTCTCTCATTCTCTCTCTCA
GTCTCTGTCTCATTCTCTCTCATTCTCTCTTCCTCTCTCTCATTCTTTCTC CCCAAGATAAGCTGAATGTCTGAATTTTCTGTGAAATGTAATTTTTAAATGCTAGAAAATGACCTGATTTGCTTTGA
AACACCTATATTTAGATTCTGTATTGTTCATTTATTTATGTATACAGTACAGAGTTGTGCAAGCATCACCAGAATCTAATTTTGAGTACATTTATATCATTTCAGTAAGAAATAGTGTGCCCCTT AGT
GGTCACCCCTTCATTTTTCTCCTGTCCCCCACCAAGCCTAGACAATATTTTCTGTCTGTATAGATTTGCCTATTCTGGACGTTTCATATAAATGAAATAATACCAAACATGGTCTTTTGTGACTGGCT
TCTTTCACTTGGTGTAGTGTTTCGGGAGTTATCTTTGTTTTGGCCTATATCAGAACTCTATTTCTTTTTGTTGGTGGTGGTAAATAATATTCTATTGTATGGCTACATGACATTCTGTCTGTTTA TTC
ATTAGCCATCAAACAAGCATATCACAGGCTGGTCCCAGCCCTGGGGGACCCATTTTTCACCATGCAACAGCCTCCTGGCTGGTGTCCCCATCTCCTGTTCAATCCCCC tGCCCTCTGCTGACACAGGG
TGTGCATGCAACCAGCCAGACAGCCCAGGGTAGAAATATTGGGACAATTTTTCTAAACCAAGGCTTTGAGCATGTCACTCTCCTCCATCCCCACCTTTATGCTGAAATCACCCTCCTCTCTTGGC TTC
AGAGTGGAGCTCCAGCCCCTTAGTCCAAAACAAAAACTTTTGCAAGAGCTGGCCCCACCACCCTCCACACCCTTCCCTGCTCACAAACCCTGTGCTCCAACACACCATGTCACCCTCTCCTCTCAAAG
CCTGAGCATGTCCAGTCCTCTGTCCCCTTGCTCATGCTGTGCCACTGGTGTGTAATTCACCAATTGTGGGTCATTCTCCTCCACTGAGTGGGGAACTGTTCTGGAAGGAGAAGGGGTATTCTCTG TCC
TCAGGACTTGGGACAAAGCAGGTACATAATGAATGTCTGACGGTGGAATAGACACAGGCCATTCCTCCATCTAAAATGCCCTTCCCTACATTGTCCATCTCACATTTGACAGCAGCTCTCCTTGCTCA
CCCACACCTGATCCAAGCACTGATTATCTAGTTCGTCTCTGTGCTCATGCAGCACCTAGCTCTGTGTGTGCGTGCATGTACGTATGTGTGTGCGAGCGTTTGCGTGACTTCATCAGATTTCAGTC CTT
GGGGACAAGCTTTTCCCCATATTGTTAAAAATTCAGTCTGTGCAATAATTTGTTGAGGCCTAATTTGCATGCACTGAGATGCAACCATTTAAGTGTATAGTTTGATGTGTTTCAAGAAATGTTCACCT

GTGGATTATACTCATGTAGCCACAGCTGCCATCAAAATATAGAACATCCCCATCACCCCAGAAAGCTCTTCTGTGCCCCTTCCAGGTCAATGCCCTCACGCCCCATCCAAGGCAACCACTGATCT CTC
CTGACACCACAGCTCAGCGTTTGCGGCTGTGGGGCTTCATAGGCACGGACTGAGGCAGCAGACAATCTTGCGTGTCTGTCTTCATTTGCGCGGCGCAGCATCTGTGAGATTCAGCCATGCAGTCGGGT
CTGTCGTGAATTCACTCTTTTTGCCCCATTCCCAGCACAGTGCCTGAGTGTCAAATTGAATAGACTTCAATTGCTGTGAGGTGATTAATAGCAAATTGTGTGTTATTCATGAATAAAGAAAAAAG ATG
CAACGTGTTCTGGGAGGTGGAAAAGAGAAAGAAACCCACCCCGACCCCGCCTTTCTGTTCTCACCTAACGAAGTCTGGAGTTCAGCAAGAAAAGGTGTGAGGGGGTTTTTCAGGAAACAGATAGGCCA
CCAGGGTCCAAATCAGAGGTAGAGTGGCACATCCAGCCCAAATGGAAACAAACGATGCAAGCCTATGGCTTAAATCAGAAATTAATCTCTACTGGTAGTTTCCATGAACTCTTCTCCAGGTAAAC ATC
ACATGTAAAAGTAAGTTCCTGTCATTATAGAATAATTAGATTATAAGAACTAAATtTATAGTGTATACTACGCATTACTGCAGAATTGTAGAAATTATGCAACCCAGTCCTTAACAGAATTAAATGGG
AATCTATATTTTTTCTCAGTAAGTCTTTATTGAATGAATGGAGTCATATTCTGTTTGTGTTTTTCTGTGCTTTTGCAATTTCTCAAATTAAAGATAAATAAACGGTCTCTTCTTGGCCTCGTGGC TAA
GATCAAGTGTAAAGACAAGTAAATGGTAGTTGAATGAAAGAAACCAGGAAAAGTTATTTTTGGAGTGTGTAGGGTGTGCCTCGCCTCCTTGGACCCACCTCGTTTCTCCCAAGAGCAGACCCAGATAC
ACTAACTCGATCTGCCTCCCGCCTCCTCCTTCTGCTTTAATCAACTGTGGGTCCTGCTCTCTTCAATGCCTGCGTGTCAGCTAAGTTTGCAATCACCCCGTTTACAGCTTGAAAATGAAGCCCTG TCT
TTTCTGCAAGTAAGAATCTTCCTGAACCATGCATCCAAATGTCACATTTCCAAACATGTTGTGAGCTGAGGACTCCAAGTCATAAACTTCAAAGCAAGTACTTGTAATGGGAATTCTGACTGACAGTC
CCTTAATTACAGTCTTTAAATATGCACAGTCCCTGACgGAGCCAGGCTTTCTGAGGAAAATTCCTGCCTAACTGGTTGAAAATGTGAACAGTGGGGTATAATTCCCTGAGCTGCAAGGAGCCACAGAT
CAGAGAAAATGCAAGAGAAACTCTAAAGACCCTTGTAGGATGAATTTTGTCTTTTGCAGGCGGTATGAAAACCGAAAGAGGGGAGACAACTGGGACAAGGAGAAACAAATATTTCCTGTGCTTGAAGC
TGACACACATGGTTTATTTGCCGGTGTTTGGAGAGGTTAGAAAGCAAAGATAGCAGAGGAAGAATAGGTTTATCCTTGAGGACAGAACATGCATGCTGGTGAGTGGGAAGGGACTAAAAACAAGG TGG
CTGGGCCGGGCGCAGTGGCTCATGCCTATAATCCCATCAtTTTGGGAGGCCTtGGAGGGAGGATCACTTGAGGTCAAGAGTTAGAGACCAGCTTGGTGAAACCCCGTCTCTATTAAAAATAAAAAATT
AGCTGGGTGTGGTGGCTCGTGCCTATAATCCCAGCTACTACTCAGGAGGCTGAGGCAGGAGAATTGCTTGAACCTGGGAGGCGGAGGTTGCAGTGAGCCTAGATGGTACCACTGCACTCCGGCCT GGG
CAAAAGAGCAAGATTTCGTCTCAAACAACAAACAAACAAAAAATGACAGGGTGGCCCAAAACAAGGCAGTCCCACTGATCACGGACTCCAGTGACATTAACAGAGACCAGGGGGACATGGTTGCCCCT
GTGTGCCCTGGGTCACATCACAGCCACTCCTTATTGGCCCTAGCCACAGGATGTgCTATGTCTCAGGCAGTGGCGTGTGTGCAGGTAACTtCTCCATCAGAGCCCCAGAAGGCAGCATGGCATCAGGT
TTGGCAGTCTTATGCCACGTTGAGATCAGAGACCTTGTTTTATTAGCACAGGGTGCTCAGCCAACAAGAATGTCAAGCTGGTGTTTTTGCTACCACAGCCCCAGTTTAATGACAGAGAGTAACCTTGA
TATGTAGTTTGGTGACCAGTTAGGCAGGAAATTTATGGCAAAACACAGAAATGACCACACTGGAGTGGATTTAAGTCCTGATGCTGCCAATTAACAGCTATGTAGCTTTGCCCTTGGGCCAGAGT TAC
CACGTAAAACACAAATATCCAGCTAAATCTGAATTTCAGATATATAACAGATAATTTTCCACATATAACACATACTAAAACATTATCCATTGTTTATCTAAAATGCAAAGGTAACTGAGTGCTCTGAT
TTTAATTTGCTAAATCTGGCCAACGACTTTCTCTTGCTGAACCTCATTTTCCTACTGTTTCCAAGTGGACAGCAGTGCATGTCTCAAGGGGGTTTGAGAC cAGCTGAGATGCTGCTGGCCCAGCACAG
GGTTCCTACACTATGAGGGTGATCAACTGAGCTCTCAGTGCTTCTTCTCCCTCAGGATCTTTGTCTGCCTTTTAGCTCTTTCCAAATGTTAAACTTTCCATAAAGGCCAATGTTATGTGTCCCCCATG
GCAGAACATTTCTTTTCTCACCATAGAAATCAATTTATTGTATATTTCAAAATAACTAAAAGAATGAAATTGGAGTGTCCCTAACACAAAGAAATACTCAGTGCTTGAGGTGATGGATACTCCAG TTG
CTCTGATCTAATCACAACACATCATATGCTTGTATCAAAATATCATCTGTACCCCATAAACATGTGAAACTATTATGCATCCATGATTAAATATAAAAGTGAATAAAAA A

>KSI_LTR5HS_4

>Scaffold13397-1849507-1849652
AAAAAACAAAGAAGTCCCAGACCAGAAAGATTAACAGCCGAATTCTACCAGACATACAAAGAACAGCTGGTACCAATTCTAGTGAAACTATTCCAAAAAATCGAGGAGGAGGGACTCCTCCCTAAT TC
TATGGAGCCAGCATCACCTGATACCAAAACTTGGCAAAGACACAACCGAAAAAGACTACAGGACAATATCCCTAATGAACATAGATGCAAAAATCCTCAACAAAATACTAGCAAACCGAATCCAG CAG
CTAATTCAATAAGCTAATTCACCATGATCACATAGGCTTATAGGCTTGATTTCTAGGATGAAAGTTTGGTTCAACATAAGCAAATCAATATATGTGATTCACCACACAAGCAGAATTAAAAACAAT TG
GCTAGGCATGGTGGTTCACATCTGTAATTCCAGCACTTTAAGCAGCCAAGGCAGGAGGATCACATGAGACGAAGAGTTTGAGACCAGCCTGGGAAACATAGTGAGATACTCTCTCTACCAAAAAA AAA
AAAAAAAAAAAAAAAAAaTTAGCTAGGTATGGTGGCACGTGCCTGTAGTCGCAGCTACTCAGTGGGCTGAGGTAGGAGAACTGCTTAAGCCCAGTAGGCCAAGGCTGCAGTTGGCCATGATCATAC C
actccagcctgggaaacagagtgagactttgtcttgaaacacatacatacatacatacaaacaaaaaacatatgatcatctcaatagatgcagaaaaagcttttgataaaatcttacatcccctc atg
atagaaacccccaagaaactagacatcaaaggaacatacctcaaaataatgagagctatctgtgataaacccacagccaacatcatactgaatgggcaaaactggaaacattccccttgagaactg ga
acaagataaggatgcctattctcagcactcattcttcatagtactagaaagaagtgttacccagagcaatcatccaagagaaagaaataaaagtcatccaaataggggaaaaaaaagaagtcaaa cta
tctctctttatggatgatatgattctattcatagaaaaccctaaagactccaccaaaaggctcctagaatggataaatgactttggcaatgtttcaggatacaaaatcaatgtacaaaaatcagta gt
atttttatacaccaataatgttcaatctaagagccaaatcaagaacacaatccctttacaatagccacaaaaaaaatgaaataccggccaggcgcagtggctcacgcctgtaatcccagcactttg gga
ggctgaggtgggtagatcatgaagtcaggagatcgagaccatcctggctaacacggtgaaaccccatctctactaaaaaatacaaaaaattagctgggcgtggtggcgggcgcctgtagtcccagc ta
ctcgagaggctgaggcaggagaatggcatgaacctgggaggcagagcttgcagtgagccaagatagcaccactgt ACTCCAGCCTGGGCGACAGAGCGAGACTCCGTCTCAAAAAAAAAAAAAAAT
GAAATACCTAGAAACATATCTGACCAAGGAGGTGAAAGATCTCTACAAGGAGAACTACAAAACACTGCTAAAAGCAATCACAGATGACACAAACAAATAGAAAAACATTCCATGTTCATGGATTGG AA
GAATCAATATTATAAAAATGGCCATATTGCCCAAAGCAATCTATAAATTCAATGCTATTCCTATCAAACTACCAATGTCATTTTTCACAGAACTAGAAAAAAACTATACTAAAATTCATATGGAA CCT
AAAAACACCTGAAAAGCCAAGGCAATCCTAAGGAAAAAGAACAAAGCCAGTGGTATCACATTAACTAACTTCAAACCATACTAATAAGGCTATGGCAACCAAAACTGTACCACTCATAGTACTGGT AT
AAAAACACAGACCAATGGAACAGAATAGAGAACCCAGAAACAAAGCTGCACACCTACAGCCATCTGATCTTTGACAAAGTTAACAACAACAAAAAAAGCAATGGGGAAAGGATTCCTTATTCAAT AAA
TGGTGCTGGCATAGCTGGCTAGTCATAAGCAGAAGAATGAAACTGGACCCCTACCTTTCACCATATGAAAAAATTAATTCAATTTATAAGAATCCTAGAAGAAAACCTAGGAAACACCATTCTGGA CA
TCAACCTTTGGAAATAATTTATACTTAAGCCCTGAAAAGCAACTACAACAAAAACAAAAATTGACAAGTGGGATCTAAGTAAATTAAATGGCTTCTGCACAGCAAAAGAAACTATCAACAGAGTA AAC
AGATGACCTAGAGAATGGGAGAAAATATTCACAAACTACTCACCCAACAAAGGCTTAATATCCAGAATCTATAAGGAACTTAAACAACTGAACAAGCAAAAACTAAATAACCTCATTAAAAAAATG GG
AAAAAGACATGAACAGACACTTCTCAAAAGAAGATATACAGGCAGCCAACAAGCATTTAAGAGTGTTCCACATAACTAATCATCAGAGAAATGCAAATAAAACCACAATGAGTTGCCATCTCACA GCA
GTCAGAATAGCTATTATTTAAAAAAGGAATGCCTGTACACTGTTGGTGAGAATGTAAATTAGCTCAGCCACTATGGAAAGCTATTTGGAGATTTCTCAAATAACTTAGAACTACCATTCAATCCAG CA
ATCTCGTTACTGGGTATACATCCAGAGAAAACAAATCATTCTACCAAAAAGACATATGCACTCGCATGTTCATGGCAGCACAATTCACAACAGCAAACACATGGAATCAACCTAGGTGCTCATTA ACA
GTAGACTGGATAAAGAAAATGTGGTACAGATACACCTCGGAATACAATGCAGCCATAAAGAAAACAAAATCACATCCATTGAAACAACATAGATGCAGCTGTGAATTAACACAGGAACAGATAACC AA
ATAATGCATGTTCTCACTTCTAAGTGGGAGATAAACATTGGGTACTCATGGACAGAAAGATGACAACAATAGACACTGGGGACTACTAGAGGAGTGAGGGAGAGAAGGAAGGTTGAAAAACTAAC TAT
TGGTTACTATTCTCAGTACCTGAGTGATGGGATCATTTGTACCCCAAACCTCAGCATCGTGCAATATAACCAAGTAATAAATTTATACATGTACCTCCCAAATCTAAAAGTTGAAAAAAAATTTTA AA
AAGAATAAATGTTATTCACCATTATTAATGTGATTAGGTGTAATTTAATCTTGACTGAAAATAGCTAATTGTTTAACTAAAAGTTTAGTTTTTCCTATAGCATATCTAATATAAGTTGAAAGTGT CTT
TCTTAAAATGTATGAGATCCTTACAATGGAATACTATCTGACAATTATAAGAAATGGAGTACTAATATAAACTACAGTGTGGATGAGCTTTGAAAACATTATGCTAAGTGAAAGATGTCAGTCACA AA
AGACCACATAATACAAAATTTGTTTATATGAAATATCCAGAGCAGGCAAACATATAGAAACAAAAAATAAATAAGTGGTTGTCAGGAATTGGGAAGAAGTTAATGAGGAATGACTGATAACAGGT AAA
GAGTTTCTTCTGAAGGTGATGAAAATGTTTAAAAATCCAATTATGGAGATGACCATAAAAATCCATGAATATACTACAAAACACTGAATTTTATATATTAAATCAGTAAATTTTGTGCTATATATA TT
TTATCTCAATAAGGCTCTTAAAAATATATAAGGAAAATTTCTTTCAGCCTAGTTGAACACATTGAAAACTAATGATCCCAAAGGACAGACATTAGAAAAGTTACAGCAATATTTAGACGTCTAT CAA
ATCACTAAAAGGGTTTAATGTGTTTCTTATATTTGTTTATTTTAAATTTGTGTATGAAAACGTGATTACTGGTGAATTCTAACAAACATTTAAGGGAGAAATAGTATTAGTTTTATACAAACTCTT CG
AGGAAGTAGGAGAGGAGATACCTGTCCCCAATTTATTCTATGATGCCAGCATGGCTTTGATACCAAAACCAGAAAAAGATATTAGAAAAAAATAAAACTACTGACAAATAAGGATACTACATCAC AAC
AAAGTTGGGCTTATCCAGGAAaATAAGGTTGTTTTAACATTCAAAAGCATAAATATAACTCAACATACTAACAAATTAGTACACACACATGCACGCATGCACACA TACACACACACACACACACAC
ACGACTACAATAGATGCAGTAAAGTCATTTGATAAAATCCAAAATCCATTCCTCATAAAAACTCTCAACAAAATGAGAATTAAAGGAAACTTTGTTCATTTAATAAAGAGCAACTATGAAAAATA TAT
AGGTAGCATCATATTTAATGGCAAAAGACTGAATGATTTCACCCTGCTATGGTTTGAACATGCCTCCACCTTCACAAAAGCATATGTTGGAAACTTAATCCCCAGTTGCATTAGTCCATTTTCACA CC
AGTATAAAGAACTGCCTGAGACTGGGTAAGTTATAAAGAAAACAGGTTTCACTGACTCACAGTTCCACAGGCTGTGAAGGAGTCATGGCTGGGGAGGCCTCAGGAAACTTACAATCATGGCAGAAGGC
GAAGCAGAGGCCAGCACATCTTCTCATGACTGGCAGGAAAGAGAGACAGTGAAGGGGGAAGTGCTACACACTTTTAAACAACCAGATCTCATGATAACTCAGCATCGTGAGAACAGCAAGGGGGAG AT
CTGCCCCCATGATCCAATCACCTCCCAACAGGCCCTTCCTCCACCACTGAAGATCAT AACTCAACATGAGATTTGTGTGGGGACACAGAGCCAAACCATATTACCAATACAATAGTGTTGGGATGTGC
AGCCTAATGGGAGGTATTTAGGTCATGCAGGCTCCACCCTCATGAATGGATTAATGCCATTTATAAAAAGGCTTGAGCCTGCAAGTTTGATCTCTTGCTCTTTCTTGCCCTCTCTTTGCCCTTCT GCC
ATGAGAtgtgtaggggtgggttgcccctacacacctgtgggtgtttctcgtaaggtgggacgagagatttggaaaagaaaaagacacagagacaaagtatagagaaagaaataaggggacccggggaa
ccagcgttcagcatatggaggatccccttatttctttctctatactttgtctctgtgtctttttcttttccaaatctctcgtcccaccttacgagaaacacccacagg TGTGTAGGGGCAACCCACCC
CTACAAGATGACTCACCAAACAGGTCCTTATGATATGCCAGCCCTTCAATCTTAGAC TTCCCAGCGTCTCGAACCTTGAGCCAACACACTTCTGTTCATTATAAATTACCCAGTCTGATATTCCGTTA
GCACAAAATGGACTATGACACTAAGACCTGCAATAACACAAGAATGTCCACTGTTACCACTTTTATTCAATATTCTACTAGAAGTTCT GGACAGTGCAATCAGCAAGAAAAATAAATAAAAAGCATTT

ATATTTGACAGGAAAAGGTAAAACTATCTTTATTCTCAAACAACATGAATGTCTTTA TAGAAAATCTGATGGACACTACTAAGAAGCTACTAGAACTAATACATGAATTTATCAAGATTGTAGGTTAT
GGCTGGGCTTGGTGGCTCACGCCTGTAATCCCAGCACTTTGGGAGGCTGAGGCGAGCAGATCACGAGGTCAAGAGATCAAGACCATCCTGGCCAACATGGTGATACCCTGTCTGTTGTGGGAAGT CAG
GGACCCCAAATCAAGGGATTGGCTGAAGCCATGGCAGAATAACGTGGattgtgaagatttcatggacatttattagttccccaaatcaatatttttataatttcttacgcctgtctttactgcaatct
ctgaacataaATTGTGAAGATTTCATGGACATTTATTAGTTCCCCAAATCAATATTTTTATAATTTCTTACGCCTGTCTTTACTGCAATCTCTGAACATAAATTGTGAAGATTTCATGGACATTTATC
ACTTCCCCAATCAATACCCTTGTGATTTCCTACGCCTGTCTTTAATCTCTTAATCCT GTCATCTTCATAAGCTAAGGAGGATGTATGTCGCCTCAGGACACTGTGATGATTGCGTTAACTGCACAAAT
TGTTTGTAGAGCATGTGTGTTTGAACAATATGAAATCTGGGCACCTTGAAAAAAGAACAGGATAACAGCAATGTTCAGGGAACAAGAGAGATAACCTTAAACTCTGACCGCTGGTGAGCCGGGCA GAA
CAGAGCCATATTTCTCTTCTTTCAAAAGCAAATGGGAGAAATATCACTGAATTCTTT TTCTCAGCAAGGAACATCCCTGAGAAAGAGAATGCATCCCTGAGGGTAGGCCTCTAAAATGGCCGCTTCGG
GGGGCAGCTGTCTTTTATGGTCGAAGCTGTAGGGATGAAATAAGCCCCAGTCTCCGGTAGCACTCCCAGGCTTATTAGGACGAGGAAATCCCCGCCTAATAAATTTGGTCAGACCGGTTGTCTG CTC
TCAAACCCTGTCTCCTGATAAGATGTTATCAATGACAATGCGTGCCCGAAACTTCAT TAGCAATTTTAATTTAGCCCTGGTCCTGTGGTCCTGTGATCTTGCCCTGCCTCCATTTGCCTTGTGATATT
CTATTACCTTGTGAAGTATGTGATCTCTGTGACCCACACCCTATTCATACACTCCCTCCCCTTTTGAAATCACTAATAAAAACTTGCTGGTTTTGCGGCTTGT GGGGCATCACGGAACCTGCCGACAT
GTGATGTTGCCCCTGGATACCCAGCTTTAAAATTTCTCTATTTTGTACTCTGTCCTT TTATTTCTCAGACCAGCCGACACTTAGGGAAAATAGAAAAGAACCTACGTGACTATCGGGGACAGGTTCTC
CCGATACCTGTCTCTACTAAAAATACAAAAATTAGCTGGG gGTGGgGGCGTGTGACTGTAGTCCCAGCTACTTGGGAGGCTGAGGCAGGAGAATCACTTGAACCCAGAAGG tGGAGGTTGCAGTGAGC
TGAGATCATGCCACTGCACTCCAGCCTGGTGACTGGAGCCAGACTGCATCTCAAAAA AAAAAAGATTGTGGGTTATAAGATCAATATACAAAAGTTAATTGTATTTTTATGTATTAGCGACAAATAAT
AAAGAATTGAAATTTCATAAACAATGTCATTTATAATAGAACTCAGGAATTTTAAAACACCTGGGAAATACTTAGAGATAAATACTTAGAATTAAAGCCAACAAAAGATGTGCAAGACCTGAAAA CTA
TAAAACATTGAGAGATAAATGAAAGAAAATCTAACTAAATGGAGTAATATATATATA TATATATACTTTGACCCTATGTTGAAATACTCAATCTTGTTAAGAGGTCAATTCTCCCAAAATAGTTCTGT
AGATTCAACACAATCTCAATTAAAATCCCAGTTTGTTCTTAAGGGAAAATGACAAGTTGGTTCTAAAATGCAGATGAAAATACAAAGGACCTAGAACAGGCAAAACCAGTCTGAAAAAGGACAAA TTT
GGGGCCAACGTTGCCTGATTTCAAATCTTATTATAAAGCTACAGTAATCAAGGCAAT GTATTATTGGCATCAAGACAAATAGATCAGTAGAGCAGAATAGAGTCCAAGAATGGACCCACATACATGGA
CAATTGATTTTTGACAAAGATGCAAAGGTAGTTCAATAAAGAAAGGGTTGCCTTTTTAACAAATGCTGCTGAAACAAGTGGCTATCCATATGTAAAACAAACAAAACTTAGCACCATACATACAA ATA
ATTAACTCAGAATGGGTCACAGACCTAACTATAAACCTAAAACTATAGAGCTTTTTG AAGAAAACATAGAAGTTCTTTATGACTTTTGGTTAAGGAATATTCTCTTAGATGCAACACAATAAGCAAAA
CACATGAAAGAAAAAATGTAATAAAAGACTTTGTGAAAAATAAAACATTTGCTCTTCAAAAGACACTGTTAGGCACTTTGGGAGGCTGATGTGGGTGGATCACCTGAGGTCAGGAGTTCAAGACC AGC
CCGACCAACATGGCAAAACCCCATCTCTACTAAAAATGCAAAAATTAACCAGGCATG ATGGCACATGCCTGTAATCCCAGCTACTTGGGAGGCTGAGGCGGGAGAATTGCTTGAACCTGGGAGGTGGA
GGTTGCAGTGAGtCAAGATTGCTCCATTGCACTCCAGCCTGGGCAACAAGAGCAAAACCCCCCCCACCAAAAAAAAAAAAA AGACACTGTTAGGAGGCTGAAAAGACAAGCTACTGACTGGGAAAAAA
ATATTTGCAAAGCACATATACCATAAAAGACTTGTATACAGGATACATAAAGAACAT TCCAAAAGTAATAAGAAACAAGCAACCTAATAATAAAGTGGGCAAAATATTTAAACAGATACTTTGCCAAA
GAAGATACATGAATCACAAAAAATTGCAtGAAAAGATGCTCAACATCATTAGTCATTAAGAGAGTTGCAAATTAAAGCCACAATGAAATGTGGGAAGAAAGGAATATGCAATACCAAGATGTCATGAA
TAAAACAATATTCCAACATCAAAGACAACAGAAATGGACTAGGTAAACACTCTAAGG CAGGGGTCCCCAACTCCCAGGCCACAGACTGGTATCAGTATGGGTCTGTGGCCTGTTAGGAAGGGGGACCA
CACAGCAGGAGGTGAGCAACAGGTGAGCAAGCATTACCACCTGAGCTCTGCCTCTGTCAGATCAGAGGTGGCATTAGATTCTCATAGGACCCTAAACCCTATTGTGAACTGTG aATGTGCAGGATCTA
GGCTGTGTACTCCTTATGAGAATCTAATGCCTCATGATCTGAGGTGGTACAGTTTCA ACCCGAAATCATCCCCACCCTCAACCCTTCCCCCGTAGAAAAACTGTCTTCCACTAACCTGGTTCCTGGTG
CCAAAAAGGTAGGGGACTGCTGCTCTAGGAATTGCTTATTCACCATCAGTAGCAGAAACCCAGAATTTCAAGATTTTTGTTACTGATGGTAAGTAAACAATTCTAAAATATGGAATAGATCAATC AGA
AATATTGTTAAAGTTAAAGCCCACAGATGTGCCTATGCATCTTTGTGAGAATAATGA TAGATGAAACAGCAATCTCAAATATAGCAACAGCCATGAGCTCCTACAAAAAAAAAAAAACCCTGTTGCTG
AAATATACAGCCTTGTTGCCCACACAAAGCCTGTTGGTGGTCTCTTCACACAGATGCGCGTGACATTTGGTGCCAAAACCCGGGATAGGAGGACTCTTTCGGGAGACTGGTCCCCTGTCCTCGCC CTC
GATTCATGAGGAGATCCACCTACAACATCAGGTCCTCAGACCACCAGCCCAAGGAAC ATCTCACCAATTTCAAATCAGGTAAGCAGTCTTTTCACTCTTCTCCAGCCTTTCTCACTACCCTTCAATCT
TCCTCTCTCACTACCCTTCAATCTCCCTGCCCTTCCAATTCCAGTTGTTTTTCCTCTCTAGTAGAGACAAGAGGACACATTTTATCCGTGGACCCAAAACTCTGGCGCAGTCCCGGACTCGGGAA GAC
AGTCTTCCCTTGGTGTTTAATCACTGTGGGGATGCCTGCCTTGGTCATTCACCCACA TTCCCTTGGTGGCAAGTCAACTgTGGGGACgCCTGGTTTGGCTGCTCACCCACATTGCAGCCCAGGGCTGC
TCACCATCCCCTTCTCCGTGTCTCTGCCTTTCTCTTTAAACTTACCTCCTTCACTATGGGCAACCTTGCGACCTCCATTCCTCCTTCTTCTCCCTTAGCCTATGTTCTCAAGAACTTAAAACCTC TTC
AACTCACACCTGACCTAAAACTGAAACGTCTTATTTTCTTCTGCAATACCACTTGGC TCCAGTACAAACTCGACAGTGGTTCCAAATAGCCAGAAAATGGCACTTTTAATTTCTCCATTTTACAAGAG
CTGGATGATTTTTGTCAAAAAATGGTCAAATGGGTCTGAGGTGCGCCTGCTGTCCAGGCATTCTTTTACACATTGGTCCCTCCCTAGTCTCTGCTCCCAATGCAATTCATCCCACATCTTTCTTC TTT
CTCTCTTGTCTGTTCCTTCAGTCTCCACCCAAAG

10525	11.5	3.8	4.7	KS_4	8	383	(9763)	+	L1PA16	LINE/L1	3512	3884	(2262)	1
1456	20.6	1.7	5.2	KS_4	384	679	(9467)	+	AluJr	SINE/Alu	1	286	(26)	2
10525	11.5	3.8	4.7	KS_4	680	1236	(8910)	+	L1PA16	LINE/L1	3885	4407	(1739)	1
2521	6.7	0.0	0.3	KS_4	1237	1535	(8611)	+	AluYk3	SINE/Alu	1	298	(13)	3
10525	11.6	7.8	2.4	KS_4	1536	3193	(6953)	+	L1PA16	LINE/L1	4408	6162	(4)	1
1673	24.1	0.7	0.5	KS_4	3332	3736	(6410)	+	L1MB8	LINE/L1	5772	6177	(1)	4
930	25.1	1.1	0.7	KS_4	3906	4195	(5951)	+	L1M4	LINE/L1	3536	3899	(2247)	5
31	3.3	0.0	0.0	KS_4	4196	4226	(5920)	+	(CA)n	Simple_repeat	1	31	(0)	6

930	25.1	2.7	1.8	KS_4	4227	4392	(5754)	+	L1M4	LINE/L1	3900	4062	(2084)	5	
764	15.6	0.7	9.5	KS_4	4397	4458	(5688)	+	MSTD	LTR/ERVL-MaLR	1	54	(342)	7	
1950	16.9	13.1	0.7	KS_4	4459	4839	(5307)	C	MSTA	LTR/ERVL-MaLR	(0)	428	1	8	
764	17.2	1.2	10.1	KS_4	4840	4998	(5148)	+	MSTD	LTR/ERVL-MaLR	55	204	(204)	7	
1321	0.0	0.0	0.0	KS_4	5001	5146	(5000)	C	LTR5_Hs	LTR/ERVK	(0)	968	823	9	
1010	0.9	0.0	0.0	KS_4	5140	5253	(4893)	+	LTR5_Hs	LTR/ERVK	855	968	(0)	10	*
678	21.9	5.3	0.0	KS_4	5254	5396	(4750)	+	MSTD	LTR/ERVL-MaLR	246	396	(0)	7	
1032	22.0	1.7	1.3	KS_4	5397	5631	(4515)	+	L1M4	LINE/L1	4066	4301	(1845)	5	
2191	9.4	0.0	2.8	KS_4	5633	5744	(4402)	+	AluSc	SINE/Alu	1	112	(197)	11	
7601	7.1	12.3	6.7	KS_4	5745	6918	(3228)	+	MER11B	LTR/ERVK	1	1236	(0)	12	
2191	9.4	0.0	2.8	KS_4	6919	7103	(3043)	+	AluSc	SINE/Alu	113	290	(19)	11	
3526	22.9	3.9	5.2	KS_4	7104	8008	(2138)	+	L1M4	LINE/L1	4290	5188	(958)	5	
1944	12.8	0.4	0.0	KS_4	8009	8274	(1872)	+	AluSp	SINE/Alu	34	300	(13)	13	
3526	18.1	2.6	1.9	KS_4	8275	8537	(1609)	+	L1M4	LINE/L1	5189	5443	(736)	5	
2111	14.5	0.3	3.2	KS_4	8634	8982	(1164)	+	MER1B	DNA/hAT-Charlie	1	339	(0)	14	
531	6.0	1.5	0.0	KS_4	9215	9281	(865)	+	LTR7B	LTR/ERV1	397	464	(0)	15	
2195	20.4	5.2	2.5	KS_4	9282	10146	(0)	+	HERVH-int	LTR/ERV1	1	1210	(6503)	15	
10525	11.5	3.8	4.7	hg19	8	383	(9453)	+	L1PA16	LINE/L1	3512	3884	(2262)	16	
1462	20.7	1.7	4.9	hg19	384	678	(9158)	+	AluJr	SINE/Alu	1	286	(26)	17	
10525	11.5	3.8	4.7	hg19	679	1235	(8601)	+	L1PA16	LINE/L1	3885	4407	(1739)	16	
2548	6.6	0.0	0.3	hg19	1236	1537	(8299)	+	AluYk3	SINE/Alu	1	301	(10)	18	
10525	11.6	7.8	2.4	hg19	1538	3195	(6641)	+	L1PA16	LINE/L1	4408	6162	(4)	16	
1673	24.1	0.7	0.5	hg19	3334	3738	(6098)	+	L1MB8	LINE/L1	5772	6177	(1)	19	
961	24.9	1.6	1.0	hg19	3908	4197	(5639)	+	L1M4	LINE/L1	3536	3899	(2247)	20	
40	2.6	0.0	0.0	hg19	4198	4236	(5600)	+	(CA)n	Simple_repeat	1	39	(0)	21	
961	24.8	2.7	1.8	hg19	4237	4402	(5434)	+	L1M4	LINE/L1	3900	4062	(2084)	20	
1294	20.8	10.5	8.8	hg19	4407	4468	(5368)	+	MSTD	LTR/ERVL-MaLR	1	54	(342)	22	
1950	16.9	13.1	0.7	hg19	4469	4849	(4987)	C	MSTA	LTR/ERVL-MaLR	(0)	428	1	23	
1294	20.8	10.5	8.8	hg19	4850	5176	(4660)	+	MSTD	LTR/ERVL-MaLR	55	396	(0)	22	
1032	22.0	1.7	1.3	hg19	5177	5411	(4425)	+	L1M4	LINE/L1	4066	4301	(1845)	20	
2256	8.4	0.0	2.8	hg19	5413	5524	(4312)	+	AluSc	SINE/Alu	1	112	(197)	24	
7862	8.8	0.8	2.0	hg19	5525	6607	(3229)	+	MER11C	LTR/ERVK	1	1071	(0)	25	
2256	8.4	0.0	2.8	hg19	6608	6792	(3044)	+	AluSc	SINE/Alu	113	290	(19)	24	
3512	23.0	3.9	5.2	hg19	6793	7697	(2139)	+	L1M4	LINE/L1	4290	5188	(958)	20	
1968	12.4	0.4	0.0	hg19	7698	7964	(1872)	+	AluSp	SINE/Alu	34	301	(12)	26	
3512	18.5	2.6	1.9	hg19	7965	8227	(1609)	+	L1M4	LINE/L1	5189	5443	(736)	20	
2134	14.2	0.3	3.2	hg19	8324	8672	(1164)	+	MER1B	DNA/hAT-Charlie	1	339	(0)	27	
531	6.0	1.5	0.0	hg19	8905	8971	(865)	+	LTR7B	LTR/ERV1	397	464	(0)	28	
2195	20.4	5.2	2.5	hg19	8972	9836	(0)	+	HERVH-int	LTR/ERV1	1	1210	(6503)	28	

>hg19 chr10:101011117-101020952
AAAAAACAAAGAAGTCCCAGACCAGAAAGATTAACAGCCGAATTCTACCAGACATACAAAGAACAGCTGGTACCAATTCTAGTGAAACTATTCCAAAAAATCGAGGAGGAGGGACTCCTCCCTAAT TC
TATGGAGCCAGCATCACCTGATACCAAAACTTGGCAAAGACACAACCGAAAAAGACTACAGGACAATATCCCTAATGAACATAGATGCAAAAATCCTCAACAAAATACTAGCAAACCGAATCCAGCAG
CTAATTCAATAAGCTAATTCACCATGATCACATAGGCTTATAGGCTTGATTTCTAGGATGAAAGTTTGGTTCAACATAAGCAAATCAATATATGTGATTCACCACACAAGCAGAATTAAAAACAA TTG

GCTAGGCATGGTGGTTCACATCTGTAATTCCAGCACTTTAAGCAGCCAAGGCAGGAGGATCACATGAGACGAAGAGTTTGAGACCAGCCTGGGAAACATAGTGAGATACTCTCTCTACCAAAAAAAAA
AAAAAAAAAAAAAAAAATTAGCTAGGTATGGTGGCACGTGCCTGTAGTCGCAGCTACTCAGTGGGCTGAGGTAGGAGAACTGCTTAAGCCCAGTAGGCCAAGGCTGCAGTTGGCCATGATCATAC CA
CTCCAGCctgggaaacagagtgagactttgtcttgaaacacatacatacatacatacaaacaaaaaacatatgatcatctcaatagatgcagaaaaagcttttgataaaatcttacatcccctcatga
tagaaacccccaagaaactagacatcaaaggaacatacctcaaaataatgagagctatctgtgataaacccacagccaacatcatactgaatgggcaaaactggaaacattccccttgagaactg gaa
caagataaggatgcctattctcagcactcattcttcatagtactagaaagaagtgttacccagagcaatcatccaagagaaagaaataaaagtcatccaaataggggaaaaaaaagaagtcaaactat
ctctctttatggatgatatgattctattcatagaaaaccctaaagactccaccaaaaggctcctagaatggataaatgactttggcaatgtttcaggatacaaaatcaatgtacaaaaatcagta gta
ttttatacaccaataatgttcaatctaagagccaaatcaagaacacaatccctttacaatagccacaaaaaaatgaaataccggccaggcgcagtggctcacgcctgtaatcccagcactttgggag
gctgaggtgggtagatcatgaagtcaggagatcgagaccatcctggctaacacggtgaaaccccatctctactaaaaatacaaaaaattagctgggcgtggtggcgggcgcctgtagtcccagc tac
tcgagaggctgaggcaggagaatggcatgaacctgggaggcagagcttgcagtgagccaagatagcaccactgtactccagcCTGGGCGACAGAGCGAGACTCCGTCTCaaaAAAAAAAAAAAAAAAA
ATGAAATACCTAGAAACATATCTGACCAAGGAGGTGAAAGATCTCTACAAGGAGAACTACAAAACACTGCTAAAAGCAATCACAGATGACACAAACAAATAGAAAAACATTCCATGTTCATGGAT TGG
AAGAATCAATATTATAAAAATGGCCATATTGCCCAAAGCAATCTATAAATTCAATGCTATTCCTATCAAACTACCAATGTCATTTTTCACAGAACTAGAAAAAAACTATACTAAAATTCATATGGAAC
CTAAAAACACCTGAAAAGCCAAGGCAATCCTAAGGAAAAAGAACAAAGCCAGTGGTATCACATTAACTAACTTCAAACCATACTAATAAGGCTATGGCAACCAAAACTGTACCACTCATAGTACT GGT
ATAAAAACACAGACCAATGGAACAGAATAGAGAACCCAGAAACAAAGCTGCACACCTACAGCCATCTGATCTTTGACAAAGTTAACAACAACAAAAAAAGCAATGGGGAAAGGATTCCTTATTCAATA
AATGGTGCTGGCATAGCTGGCTAGTCATAAGCAGAAGAATGAAACTGGACCCCTACCTTTCACCATATGAAAAAATTAATTCAATTTATAAGAATCCTAGAAGAAAACCTAGGAAACACCATTCT GGA
CATCAACCTTTGGAAATAATTTATACTTAAGCCCTGAAAAGCAACTACAACAAAAACAAAAATTGACAAGTGGGATCTAAGTAAATTAAATGGCTTCTGCACAGCAAAAGAAACTATCAACAGAGTAA
ACAGATGACCTAGAGAATGGGAGAAAATATTCACAAACTACTCACCCAACAAAGGCTTAATATCCAGAATCTATAAGGAACTTAAACAACTGAACAAGCAAAAACTAAATAACCTCATTAAAAAA ATG
GGAAAAAGACATGAACAGACACTTCTCAAAAGAAGATATACAGGCAGCCAACAAGCATTTAAGAGTGTTCCACATAACTAATCATCAGAGAAATGCAAATAAAACCACAATGAGTTGCCATCTCACAG
CAGTCAGAATAGCTATTATTTAAAAAAGGAATGCCTGTACACTGTTGGTGAGAATGTAAATTAGCTCAGCCACTATGGAAAGCTATTTGGAGATTTCTCAAATAACTTAGAACTACCATTCAATC CAG
CAATCTCGTTACTGGGTATACATCCAGAGAAAACAAATCATTCTACCAAAAAGACATATGCACTCGCATGTTCATGGCAGCACAATTCACAACAGCAAACACATGGAATCAACCTAGGTGCTCATTAA
CAGTAGACTGGATAAAGAAAATGTGGTACAGATACACCTCGGAATACAATGCAGCCATAAAGAAAACAAAATCACATCCATTGAAACAACATAGATGCAGCTGTGAATTAACACAGGAACAGATA ACC
AAATAATGCATGTTCTCACTTCTAAGTGGGAGATAAACATTGGGTACTCATGGACAGAAAGATGACAACAATAGACACTGGGGACTACTAGAGGAGTGAGGGAGAGAAGGAAGGTTGAAAAACTAACT
ATTGGTTACTATTCTCAGTACCTGAGTGATGGGATCATTTGTACCCCAAACCTCAGCATCGTGCAATATAACCAAGTAATAAATTTATACATGTACCTCCCAAATCTAAAAGTTGAAAAAAAATT TTA
AAAAGAATAAATGTTATTCACCATTATTAATGTGATTAGGTGTAATTTAATCTTGACTGAAAATAGCTAATTGTTTAACTAAAAGTTTAGTTTTTCCTATAGCATATCTAATATAAGTTGAAAGTGTC
TTTCTTAAAATGTATGAGATCCTTACAATGGAATACTATCTGACAATTATAAGAAATGGAGTACTAATATAAACTACAGTGTGGATGAGCTTTGAAAACATTATGCTAAGTGAAAGATGTCAGTC ACA
AAAGACCACATAATACAAAATTTGTTTATATGAAATATCCAGAGCAGGCAAACATATAGAAACAAAAAATAAATAAGTGGTTGTCAGGAATTGGGAAGAAGTTAATGAGGAATGACTGATAACAGGTA
AAGAGTTTCTTCTGAAGGTGATGAAAATGTTTAAAAATCCAATTATGGAGATGACCATAAAAATCCATGAATATACTACAAAACACTGAATTTTATATATTAAATCAGTAAATTTTGTGCTATAT ATA
TTTTATCTCAATAAGGCTCTTAAAAATATATAAGGAAAATTTTCTTTCAGCCTAGTTGAACACATTGAAAACTAATGATCCCAAAGGACAGACATTAGAAAAGTTACAGCAATATTTAGACGTCTATC
AAATCACTAAAAGGGTTTAATGTGTTTCTTATATTTGTTTATTTTAAATTTGTGTATGAAAACGTGATTACTGGTGAATTCTAACAAACATTTAAGGGAGAAATAGTATTAGTTTTATACAAACT CTT
CGAGGAAGTAGGAGAGGAGATACCTGTCCCCAATTTATTCTATGATGCCAGCATGGCTTTGATACCAAAACCAGAAAAAGATATTAGAAAAAAATAAAACTACTGACAAATAAGGATACTACATCACA
ACAAAGTTGGGCTTATCCAGGAAtATAAGGTTGTTTTAACATTCAAAAGCATAAATATAACTCAACATACTAACAAATTAGTACACACACATGCACGCATGCACACA TacacacacACACACACACAC
ACACACACACACGACTACAATAGATGCAGTAAAGTCATTTGATAAAATCCAAAATCCATTCCTCATAAAAACTCTCAACAAAATGAGAATTAAAGGAAACTTTGTTCATTTAATAAAGAGCAACTATG
AAAAATATATAGGTAGCATCATATTTAATGGCAAAAGACTGAATGATTTCACCCTGCTATGGTTTGAACATGCCTCCACCTTCACAAAAGCATATGTTGGAAACTTAATCCCCAGTTGCATTAGT CCA
TTTTCACACCAGTATAAAGAACTGCCTGAGACTGGGTAAGTTATAAAGAAAACAGGTTTCACTGACTCACAGTTCCACAGGCTGTGAAGGAGTCATGGCTGGGGAGGCCTCAGGAAACTTACAATCAT
GGCAGAAGGCGAAGCAGAGGCCAGCACATCTTCTCATGACT GGCAGGAAAGAGAGACAGTGAAGGGGGAAGTGCTACACACTTTTAAACAACCAGATCTCATGATAACTCAGCATCGTGAGAACAGCA
AGGGGGAGATCTGCCCCCATGATCCAATCACCTCCCAACAGGCCCTTCCTCCACCACTGAAGATCATAACTCAACATGAGATTTGTGTGGGGACACAGAGCCAAACCATATTACCAATACAATAG TGT
TGGGATGTGCAGCCTAATGGGAGGTATTTAGGTCATGCAGGCTCCACCCTCATGAATGGATTAATGCCA TTTATAAAAAGGCTTGAGCCTGCAAGTTTGATCTCTTGCTCTTTCTTGCCCTCTCTTTG
CCCTTCTGCCATGAGATGTGTAGGGGCAACCCACCCCTACAAGATGACTCACCAAACAGGTCCTTATGATATGCCAGCCCTTCAATCTTAGACTTCCCAGCGTCTCGAACCTTGAGCCAACACACTTC
TGTTCATTATAAATTACCCAGTCTGATATTCCGTTAGCACAAAATGGACTATGACACTAAGACCTGCAA TAACACAAGAATGTCCACTGTTACCACTTTTATTCAATATTCTACTAGAAGTTCT GGAC
AGTGCAATCAGCAAGAAAAATAAATAAAAAGCATTTATATTTGACAGGAAAAGGTAAAACTATCTTTATTCTCAAACAACATGAATGTCTTTATAGAAAATCTGATGGACACTACTAAGAAGCTACTA
GAACTAATACATGAATTTATCAAGATTGTAGGTTATGGCTGGGCTTGGTGGCTCACGCCTGTAATCCCA GCACTTTGGGAGGCTGAGGCGAGCAGATCACGAGGTCAAGAGATCAAGACCATCCTGGC
CAACATGGTGATACCCTGTCTGTTGTGGGAAGTCAGGGACCCCAAATCAAGGGATTGGCTGAAGCCATGGCAGAATAACGTG GATTGTGAAGATTTCATGGACATTTATTAGTTCCCCAAATCAATAT
TTTTATAATTTCTTACGCCTGTCTTTACTGCAATCTCTGAACATAAATTGTGAAGATTTCATGGACATT TATCACTTCCCCAATCAATACCCTTGTGATTTCCTACGCCTGTCTTTAATCTCTTAATC
CTGTCATCTTCATAAGCTAAGGAGGATGTATGTCGCCTCAGGACACTGTGATGATTGCGTTAACTGCACAAATTGTTTGTAGAGCATGTGTGTTTGAACAATATGAAATCTGGGCACCTTGAAAA AAG
AACAGGATAACAGCAATGTTCAGGGAACAAGAGAGATAACCTTAAACTCTGACCGCTGGTGAGCCGGGC AGAACAGAGCCATATTTCTCTTCTTTCAAAAGCAAATGGGAGAAATATCACTGAATTCT
TTTTCTCAGCAAGGAACATCCCTGAGAAAGAGAATGCATCCCTGAGGGTAGGCCTCTAAAATGGCCGCTTCGGGGGGCAGCTGTCTTTTATGGTCGAAGCTGTAGGGATGAAATAAGCCCCAGTC TCC
GGTAGCACTCCCAGGCTTATTAGGACGAGGAAATCCCCGCCTAATAAATTTGGTCAGACCGGTTGTCT GCTCTCAAACCCTGTCTCCTGATAAGATGTTATCAATGACAATGCGTGCCCGAAACTTC

ATTAGCAATTTTAATTTAGCCCTGGTCCTGTGGTCCTGTGATCTTGCCCTGCCTCCATTTGCCTTGTGATATTCTATTACCTTGTGAAGTATGTGATCTCTGTGACCCACACCCTATTCATACAC TCC
CTCCCCTTTTGAAATCACTAATAAAACTTGCTGGTTTTGCGGCTTGTGGGGCATCACGGAACCTGCCG ACATGTGATGTTGCCCCTGGATACCCAGCTTTAAAATTTCTCTATTTTGTACTCTGTCC
TTTTATTTCTCAGACCAGCCGACACTTAGGGAAAATAGAAAAGAACCTACG TGACTATCGGGGACAGGTTCTCCCGATACCTGTCTCTACTAAAAATACAAAAATTAGCTGGGcGTGGtGGCGTGTGA
CTGTAGTCCCAGCTACTTGGGAGGCTGAGGCAGGAGAATCACTTGAACCCAGAAGGcGGAGGTTGCAGTGAGCTGAGATCATGCCACTGCACTCCAGCCTGGTGACTGGAGCCAGACTGCATCTCAAA
AAAAAAAAGATTGTGGGTTATAAGATCAATATACAAAAGTTAATTGTATTTTTATGTATTAGCGACAAATAATAAAGAATTGAAATTTCATAAACAATGTCATTTATAATAGAACTCAGGAATTT TAA
AACACCTGGGAAATACTTAGAGATAAATACTTAGAATTAAAGCCAACAAAAGATGTGCAAGACCTGAAA ACTATAAAACATTGAGAGATAAATGAAAGAAAATCTAACTAAATGGAGTAATATATATA
TATATATATACTTTGACCCTATGTTGAAATACTCAATCTTGTTAAGAGGTCAATTCTCCCAAAATAGTTCTGTAGATTCAACACAATCTCAATTAAAATCCCAGTTTGTTCTTAAGGGAAAATGA CAA
GTTGGTTCTAAAATGCAGATGAAAATACAAAGGACCTAGAACAGGCAAAACCAGTCTGAAAAAGGACAA ATTTGGGGCCAACGTTGCCTGATTTCAAATCTTATTATAAAGCTACAGTAATCAAGGCA
ATGTATTATTGGCATCAAGACAAATAGATCAGTAGAGCAGAATAGAGTCCAAGAATGGACCCACATACATGGACAATTGATTTTTGACAAAGATGCAAAGGTAGTTCAATAAAGAAAGGGTTGCC TTT
TTAACAAATGCTGCTGAAACAAGTGGCTATCCATATGTAAAACAAACAAAACTTAGCACCATACATACA AATAATTAACTCAGAATGGGTCACAGACCTAACTATAAACCTAAAACTATAGAGCTTTT
TGAAGAAAACATAGAAGTTCTTTATGACTTTTGGTTAAGGAATATTCTCTTAGATGCAACACAATAAGCAAAACACATGAAAGAAAAAATGTAATAAAAGACTTTGTGAAAAATAAAACATTTGC TCT
TCAAAAGACACTGTTAGGCACTTTGGGAGGCTGATGTGGGTGGATCACCTGAGGTCAGGAGTTCAAGAC CAGCCCGACCAACATGGCAAAACCCCATCTCTACTAAAAATGCAAAAATTAACCAGGCA
TGATGGCACATGCCTGTAATCCCAGCTACTTGGGAGGCTGAGGCGGGAGAATTGCTTGAACCTGGGAGGTGGAGGTTGCAGTGAG cCAAGATTGCTCCATTGCACTCCAGCCTGGGCAACAAGAGCAA
AACCCCCCCCACCAAAAAAAAAAAAAAAaGACACTGTTAGGAGGCTGAAAAGACAAGCTACTGACTGGGA AAAAAATATTTGCAAAGCACATATACCATAAAAGACTTGTATACAGGATACATAAAGAA
CATTCCAAAAGTAATAAGAAACAAGCAACCTAATAATAAAGTGGGCAAAATATTTAAACAGATACTTTGCCAAAGAAGATACATGAATCACAAAAAATTGCA cGAAAAGATGCTCAACATCATTAGTC
ATTAAGAGAGTTGCAAATTAAAGCCACAATGAAATGTGGGAAGAAAGGAATATGCAATACCAAGATGTC ATGAATAAAACAATATTCCAACATCAAAGACAACAGAAATGGACTAGGTAAACACTCTA
AGGCAGGGGTCCCCAACTCCCAGGCCACAGACTGGTATCAGTATGGGTCTGTGGCCTGTTAGGAAGGGGGACCACACAGCAGGAGGTGAGCAACAGGTGAGCAAGCATTACCACCTGAGCTCTGC CTC
TGTCAGATCAGAGGTGGCATTAGATTCTCATAGGACCCTAAACCCTATTGTGAACTGTGcATGTGCAGGATCTAGGCTGTGTACTCCTTATGAGAATCTAATGCCTCATGATCTGAGGTGGTACAGTT
TCAACCCGAAATCATCCCCACCCTCAACCCTTCCCCCGTAGAAAAACTGTCTTCCACTAACCTGGTTCCTGGTGCCAAAAAGGTAGGGGACTGCTGCTCTAGGAATTGCTTATTCACCATCAGTA GCA
GAAACCCAGAATTTCAAGATTTTTGTTACTGATGGTAAGTAAACAATTCTAAAATATGGAATAGATCAA TCAGAAATATTGTTAAAGTTAAAGCCCACAGATGTGCCTATGCATCTTTGTGAGAATAA
TGATAGATGAAACAGCAATCTCAAATATAGCAACAGCCATGAGCTCCTACAAAAAAAAAAAAAACCCTGTTGCTGAAATATACAGCCTTGTTGCCCACACAAAGCCTGTTGGTGGTCTCTTCACAC AGA
TGCGCGTGACATTTGGTGCCAAAACCCGGGATAGGAGGACTCTTTCGGGAGACTGGTCCCCTGTCCTCG CCCTCGATTCATGAGGAGATCCACCTACAACATCAGGTCCTCAGACCACCAGCCCAAGG
AACATCTCACCAATTTCAAATCAGGTAAGCAGTCTTTTCACTCTTCTCCAGCCTTTCTCACTACCCTTCAATCTTCCTCTCTCACTACCCTTCAATCTCCCTGCCCTTCCAATTCCAGTTGTTTT TCC
TCTCTAGTAGAGACAAGAGGACACATTTTATCCGTGGACCCAAAACTCTGGCGCAGTCCCGGACTCGGG AAGACAGTCTTCCCTTGGTGTTTAATCACTGTGGGGATGCCTGCCTTGGTCATTCACCC
ACATTCCCTTGGTGGCAAGTCAACTtTGGGGACaCCTGGTTTGGCTGCTCACCCACATTGCAGCCCAGGGCTGCTCACCATCCCCTTCTCCGTGTCTCTGCCTTTCTCTTTAAACTTACCTCCTTCAC
TATGGGCAACCTTGCGACCTCCATTCCTCCTTCTTCTCCCTTAGCCTATGTTCTCAAGAACTTAAAACC TCTTCAACTCACACCTGACCTAAAACTGAAACGTCTTATTTTCTTCTGCAATACCACTT
GGCTCCAGTACAAACTCGACAGTGGTTCCAAATAGCCAGAAAATGGCACTTTTAATTTCTCCATTTTACAAGAGCTGGATGATTTTTGTCAAAAAATGGTCAAATGGGTCTGAGGTGCGCCTGCT GTC
CAGGCATTCTTTTACACATTGGTCCCTCCCTAGTCTCTGCTCCCAATGCAATTCATCCCACATCTTTCT TCTTTCTCTCTTGTCTGTTCCTTCAGTCTCCACCCAAAG

SVA

Instruction

Green box: Primer information

Red letter: Target site duplication

Black letter: Insertion sequences

Green letter: Deleted sequenced by insertion-mediated deletion

>KSI_SVA_F_1 (We have tried to verify that there is insertion in this region, but we couldn’t confirmed the exact sequences by high GC-content concentrated in this insertion region.)

>SVA_F-Scaffold8597-396226-396311
GATATATCAAGACAGTGGTGCTAAGCATGTTCAGGATGTTTAAGATACAAGTTGGAGAAAAATACTTAAGAAAAGCTTCTCTTTCAGAAAGCAATTTGGCCATCTGTATTAAGAACTATAAAAATG TG
TAAACCATATCTGAACCCATAATCCCACATCTGAAAATCTAGCCTGGGAAAATGATCCTAAATGTGGAACAAACCAACACAGAATAAACTTCAGACTCCAAGATGTTCATTATGGTATTACTTAC ACT
CACGGAAAATTGGACATTACCTAAaGGACTTCTAAGAGAGAAAAGACAAGTTAATTATGATGGAATTCTACGATCAACTACTATGAAGGTATGGAAAGTGATGATGATGAAGACTATGAAATAACATG
GGAAGATGCTGATGATAAAGAATTTAAGGAAAAAGCAGGATACAAAAGTTTATACACACACAGCATGAGCTCAACTGTGGTTTAATTTAAAAGAAGTTAAACTCTCAGACCCTGTATGACGGGCT GGC
TTCCAGCAACCTCAGCATTTCCGAGAGAGCCCACAGAGGCCCTGGTCTTGCCTAGGAGACCCCAGGGCCTGAGGATGGCACACTCATGGGATTATTTACATAGCCTCCCCCTGACCAGCAGGGCCC TG
GCTCTGGGTTGATTTGAGGACATGTGAACATTACCAGGGTGGTGGTGGCAGCGGTGGTGGCAGCAGCAGTGGCAGC aGTAGTTAGGTCGGCATTCCTCTCTCTCTCTCTCTCTCTAGGAGGCGGAAAG
TACAGAAGAGAAGGTAGGTGGAGAATAAGAAGCAGATCAGAGAGGAACTCCTCCTTCCACTTGCTTTGCTACAAACAGGATTAACAAGTAAAGGTAAAAGGCCCCTGCTAGAAGGAAAACAAAAAA GA
AGAAAAAGAGAAAAGGGTGGATGCAAGGGAGAGAGGTGAGGAAAAGAGAGGCAAATGTCAAAGCTGCAAGGGAAAAGGGGCCCATTCTGATGACCACTGTTCCAATGCAAAAACAAATTCCTAAT AAG
CTGTCATTCCTTGTCACAGAGTCATGGGTGAGCAGAAAAAATGGC gTTTCCCAGAACAATGTCCATGGAAGCAATTCACCAGCAAATTGAGTCCCCAGAGAACAGAGACACACATAGACTAGGTGGGG
CACACAGCAAAGGGGCAACACTCAAACAGTGATGGTGCAGAGACACTCCCTGGCCTTCCCACTCCCATTCCCCACCCCGGCAAGCTCCAAAGGGTCCAAGTGCCCGAAGGAAGGGGAGGCTACAG GAC
AGGACCAGCCCTACCCCACTCCTCCCAAGCTCCGTTCCCACACACATCAATCTGTGCACTGACAGCTGAGGTAGAAAATGGAAGGCCGGGATGGGGGGATGGTAGAGATGATCAAGATAGAAACCA AA
CCAATGAGGCCATAAGCCCAGGCAGCTGTCCTCACTAATGGGATAGACCCAGGAGTCTGCTTCTCCCCAGTTGATCTTGACCTTGTGTAGGAACCAGCTGCTTGGACCAGTGACAGAGACAACTG CTG
AGGTAGGGTGACAGATACTCTTGCTCACTGAGGGTACAAAGGACTGTCTCCAAATCCATCCTCAACCAGCCTGGAACTATCACCCCATCATCCTAAAACAGTGCACAGAAGAAAAGGAAACAGTAG AA
AACTTCAAAGCCAGACAGAGTCCTCAGGGCAGCCCTCCCTCCATTTCTCCACAGCCTTTCAGGCAGGCTCCAGCTCTGCTTCCTGGCCATCGTGTGCTTACCTGTGGAGCCTTCCCTC cGCAGGAACA
ATCAGAAGACCATTTGCCTTACTCTGGGGGATGGGGGGAGGTGGGAGGTTCAAAATTCAAAGTGAGACCATAAAGGTGCTGAGTCCAAAGGACCCTAAAGGAGTTTTGTGACCATTAACAATCACA AG
GCTAAGGTATTAATAGGTTTTCTGCATTTTTCCTGATAAGGGAGGTCACCCCAAGGACTGTCACAGGCAGCCTGTGCTCTGGTCAGCATTGAGCTGGAAGCTTCGGGGAGAGGCCCAAGGGAGGG ATC
CTGCAAGTTCCCAAGGGTCAGGACCTGGCCCCACTGGGAACGGAGGCTTCACTTCCAAGAGGCACAGATTTAGGCAGAAGACCAGGAGTAGGGAGTCCTGAGACTAGGGATAAACAAAGGGGCAGG AA
TCCTGGAGAGAAGCCCCGTGCTCCACTCACTGACTGCGTCTGTACTGACTATGTACCAGCTGTGTGGTCAG gGAGAAAGAAGGGAGGCAGACATGAGGGACTCAGAGTCCGGGGGCTGGGGTGAGGCT
GGAACTCAGATAATCATCCCTTTCCAGAAAGTTCTGAGCAGCCTGGGAGCAGCAGGGAAGGAGAAGTCAGCTCTATTCCAGGGGCTCCACAGAACTAATGAGGTTGGGGAGGGAAGCTTAAAAGATAG
CCAGAGACACACTAGGAACACAGGGGACAGCATAAGCAAAGGCACAC gGAGGtGAGAACAGAGGTGTCTGGGTGGAGATGGGGAGTCCAGTGAAGTGGAGAGGAGGAGGAAGTGGCTGGAGGTAGGTC
AGGGTCTCGGATGCTAAGCTTGGGCCTTCCGACCATTTAGACCGTTGCCTGTAGGCAGTCGTGAAATGCTAACAGGTTTAAAAGAGGAAGTAACATGGTCAGAACCAGAAAAATCCTGGGCAGCT GAG
GGAGAGGAACAGAGGTGCCAGAAGATATGCAATCGGAATTGGAGAGACCAGTT CTGTGTACTGGCTGTGGGGCAAAGGGCTGCCATGGTTGTGACAAACCCACCTGGCCAGCTGTGCCTGCATTCCTT
ACTCATTTTGTAATTTTTATGATGGGCAATCCCACAAACATTCTGCATTCCTTAATTTAGGGC TAGTGCTACCAGGTGGTGACCTGCCCTCCCTTCACCTTGAGTGAAGTCTCGTTTCCGGGTTTGAG
TATTAGGAATGCAGCATGCATCCCTGTTTGCTATGATGTCTTCTCCTTCTGT TGCTTCTTTTCTTGCTGCCGTTGAAGTTGGAATGCTATATCTTGAAATCCACGTCTCAGAGTACAAGGAGTCAAA
TAGTATTCTCCCATGCTGGTGAGCTAAGGTCAGGGGTGGAGGAGAGACCTTAAGGAAAAAGAGCAACTCTTGTTCTTCTATATCTCAACCAGGAAAGCCTCTCCCGGCTCATTCC Ttcaatatcctt
TTTTTTTTTTTTTTTTTTTTTTAAATTTATTTTTTTATTGATAATTCTTGGG TGTTTCTCACAGAAGGGGATTTGGCAGGGTCATGGGCAATAGTGGAGGGAAGGTCAGCAAAAAAACAAGTGAACA
AAGGTCTCTGGTTTTCCTAGGCAGAGGACCCTGCGGCCTTCCGCAGTGTTTGTGTCCCTGGGTACTTGAGATTAGGGAGTGGTGATGACTCTTAAGGAGCATGCTGCCTTCAAGCATCTGTTTAA CAA
AGCACATCTTGCACCGCCCTTAATCCATTTAACCCTGAGTGGACACAGCACAT GTTTCAGAGAGCACTGGGTTGGGGGTAAGGTCACAGATCAACAGGATCCCAAGGCAGAAGACTTTTTCTTAGTGC
AGAACAAAATGAAAAGTCTCCCATGTCTACTTCTTTCTACACAGACACGGCAACCATCCGATTTCTCAATCTTTTCCCCACCTTTCCCGCCTTTCTATTCCACAAAGCCGCCATTGTCATCCTGG CCC
GTTCTCAATGAGCTGTTGGGCACACCTCCCAGACGGGGTGGTGGCCGGGCAGA GGGGCTCCTCACTTCCCAGTAGGGGCGGCCGGGCAGAGGCGCCCCTCACCTCCCGGACGGGGCGGCTGGCCGGGC
AGGGGGGCTGACCCCCCCCACCTCCCTCCCGGACGGGGCGGCTGGCCGGGCGGGGGGCTGACCCCCCCACCTCCCTCCCGGATGGGGCGGCTGGCCGGGTGGGGTGCCGACCCCCCCACCTCCCT CCC
GGACGGGGCGGCTGGCCAGGCGGGGGGCCGACCCCCCCACCTCCCTCCCGGAC GGGGCGGCTGGCCGGGCGGGGGGCCGACCCCCCCACCTCCCTCCCGGATGGGGCGGCTGGCCGGGCAGAGGGGCT
CCTCACTTCCCAGTAGGGGCGGCCGGGCAGAGGCGCCCCTCACCTCCCGGACGGGGCGGCTGGCCGGGCGGGGGGGCTGACCCCCCCACCTCCCTCCCGGACGGGGTGGCTGGCCGGGCTGAGGG GCT
CCTCACTTCCCAGTAGGGGCGGCCGGGCAGAGGCGCCCCTCACCTCCCGGACG GGGCGGC(GC) richGACCCCCCACCTCCCTCCCGGATGGGGTGGCTGCCGGGCGGAGACGCTCCTCACTTCCCA

GATGGGGTGGCTGCCGGGCGGAGAGGCTCCTCACTTCTCAGACGGGGCGGTTGCCAGGCAGAGGGTCTCCTCACTTCTCAGATGGGGCGGCCGGGCAGAGACGCTCCTCACCTCCCAGACGGGGT CTC
GGCCGGGCAGAGGCGCTCCTCACGTCCCAGAGGGGGCGGCGGGGCAGAGGCGC TCCCCACATCTCAGACGATGGGCGGCCGGGCAGAGACGCTCCTCACTTCCTAGATGGGATGGCGGCCGGGAAGAG
GCGCTCCTCACTCCTAGATGGGATGGCGCCGCGAGACGTccaggccaacacagcgaacccccgtctccaccaaaaccagtcaggcgtggcggcgcgtgcctgcaatcgcaggcactcggcaggct tcc
ctgaacccgcgagctggaagccgagaccagctggtcgctgccgggcccgctag tagcccacggagaagccgcccacagccggacaccagagccttgagggccgccatcttcaaaaacctaccctcgta
attttttcTCAATATCCTTATTTTAAGATATAAGTTAGTACAATAATTACAGAGGCATAAGCCAAATGGGAGTGGTTAACAGCTTACTATAGACAGAGTATGAGTCATAACAACTTGTGAAAGTTCAC
TCCTGTGTGTAAAGCTTGATACGTGGCCCTATTTTGAAGAGGCTTATAGGCCCGGATGGCCTCTGAGATTCTGCCACCTGGCTCACTCCACTATCCATTACCCTCTGACTCCAAGGAGTTCAAA CCA
ATAGGAGGAAGGGTCCTGATGCGGGCCCTGAGTGGGGGTGCAGCAGGAGGCGGTGGGAGCCATGAAGAA TTCACCACCACgGAAGCAGGCAGAGGAAGCAGGCAGAGGAAACAGGCAGGATGAATGGA
GAGTGGAGAGGCAGGAGTGGACCCTCCTAGTAACGGCTGGTATTTACTGAAGGTTTACCATGAGAAGGATGCCTCATAACCCTCCCCACCAAGGAGGAAACTGAGGCCTGCAGAGCTTAAGTAAC CTG
CCTGAGGTCACTGACTGTAAAGTGGCAGTGCCAGGACTGAACCCAGGCAGTCTGAGCACACAGCACACT CTCAGCCCTGGATACCAGACTGTGAGCAACACTGGGGGCACCCTGGCCATGAGGAGTGT
CACTGATGCCCTGAGAAGGCACTAAACCTGGAGTCAGACGCTGGGGTTTGAGCCGCAGCCCTACTGTTGTCTATGTGACTTTCGGAGCCTGTCCCTCTCTGAGCCTGTTTCCTCACCTGTGACAT GGA
GCAGAAGTTTCTACATTGTCTGGCCTCAGGGGTCTAGGCAAATCCTAGCAGTGACTGAATGGGAAGCCA AGGAAGTGGAAATGGGACATGGCCCCTACTTCAAGTAGAATGAAACCCTTGTGACCTGC
TGTAAGCGGAAGGTTGTGTAGCATTTCAATGGGGCAGAAAGAAATCTGATACTGAAAAGCCTGTTTGGGAACCCTTGGACTAAATGTCTCAGAAGCCACTCCATTTTGAGCATTACTTCTGTCTA ATC
TTAAATGGTATGTGTGTGTGTGTACATGTGTGTGCATGTGTATGTGTGTGTGGGAATGAAAAGTCAAAA TGACAGCAGGGTGTCTGaGCCAGGGATAAGCTGAGAGGCTATAAAGAGGGCTAACAGCC
CATAAGGTTTATTTGCTCAACAAACAGTGCAGAAAGGTCAAAACCAGGTGCTACAAAATCCCTTTCTTCTTCTAGAATATAACCATAAAACAGATAACACAGGCAGTTCCTAATTTGTAGACAGG ATG
TGCTCCAAATGTTTCTCCTTCCACCTCTTTTTTTTCAGGGTTCAGGTTTCCCATTACCATTTTATTATT TATTAGGTAATATTCTTTACTGGAAAGGTATGGCAGAGTTTAAGCATTTCTCAACTTAG
TTATCTGGACTCTGACCATATTTTCCCAAAATGATCAATTAGGGTGAGATTGGAGCAGATGATAAGAGAGTAGCTGGGGAAGTGGGAGAAAAAGC gGAAAAGATTCTCCTAAGGTGGAAAGTTAACCC
TTGGAGAAGGAAAGGATGAAGAGAAAAAAAAGTATGAGGAAAAGCAAAGAAAAAAAAAAGATGATATTG ACAAGATAAGAAAAAAGGAGGTATATATGTTTGAAAAACCAAAACTAAAATATTCCTGA
TGCTAAGTTGTGgAAGTGGGAAGAGGAGGGAGGGAGGGAGGACGGGCAGTTCCTAGTGTTCCAGGGAAAGCAGGGAGGCAGCTGGGAGGAGATGCTAACTTAGGCCCGCTTTCCCCAAAGTGTGACTC
ACAAAACACTCATCACTTGAGACGGTTCTTGGAAAAGTTGTCTCGTCAGGCAAGCTCAGGAAACACTTC TGGGGTTACAGTGTACACCGGGGTACCAAAGGCTCTGCGGGAATGAGACCTGCTTTACA
TGGTTCAACCCAACATTCCTCAAGCGTATTTCGCTCCATTTGGGGAAGCACACTTTTGGAGAAGCACTCTCTTAGGTGGTGCCCAGTGTTAGCCAGCACAGCTGTGCCACCGGTCTGAGCTGGCT TGG
TTAGTTGTTCCATCTACTCGGATTGAGGTGGGCAGGAGCATGTAGCTGGCAGAGGACATTCAGAAATCT ACcGTGTTGAGCCTGGGGAGTATGTAAATTGTATCATAGGAATTTAAGCTGATGGCTAG
ATTTTACTGGTCCAATATTTGACTTCAAAACAGGGAAGTCTCACATTCACCTAAACTGCAACATATCTAGACGTAGCATCCCACCCTCCAGTCTTCCTTGAAAACAGCCACCTCAGTTCAAAAAT TCA
GCATCATCGCTAACTCTTTTATCCTCTTTACCCAAGTATATATTTTATTTATTTATTTATTTTAT TTTTTTGAGACAGAGTCTCACTCTGTTGCCCAGGCTGGAGTGCAGTGGCATGATCTCGG
CTCACTGCAACCTCTGCCTCCTGGGTTCAAGAGATTCTCCTGCCTCAGCCTCCCGAGTAGCTGGGATTACAGCCGCCCACCACTATGTCCAGCTAATTTTTGTATTTTTAGTAGAGACGGGGTTT CGC
CATGTTGGCCAGGCTGGTCTTGAACTCCTGACTTCAAGTGATCCGCCTGCCTTGGCCTCCCAAAGTGCT GGGATTACAGGCATGAGCCACTGCGCCTAGGCCCAAATATATATTTTAACCTACCTATA
TGGCACCCAGACCACAGCTGTCTGTCTTCTACTCAGGCAGATTTGTGGGATGCTATCAGATGCCCTGCTGAATAAGATATGACAGCATTGTATCCACAGTACATCCCTCATCTACTGGCATAGCA ACT
GTCAAGAGAGAAACGAGATGCAGCTGGCACGGCTTATTAGTCATCCCTGCTGGCTCCTGGTGATGGTGG CCGCCTCTTCCAGGTGCTCGCTGCCACTGCTTTAATAACAGGCTCCTGAATCTTGCCGT
CATTGGTATGTGCTAACTGGCATCAGACAAATGTTTCTCAATCACAGAGCTTTTCTTAAATGAATATGTGTTGGGTTAAAGTGACCCGCATTTTGCAGGACATTTACCTGTTCTCTCTTGCATTC TCC
AATATTCTTCCAAGGTGACCCACAACTGCGTGGCTGTCTTGCTTGCAGGTGTTCATAGGGCCCTGGTCT GTACCTCACTTGGCCGAAGAATTCAAACAGCCTCAAGGGGTTTGCCCACCACCTGCTCT
CCTGCTTCAGTTTCCTTGTAACAGGGCTTGTTCTGCTCTCTCCTGCCTCACAGGCTTAACTCTGACACCTGG cCCCAGAGCTTCTGGTGCTTTCCTACCCCAGTGGGCATCTCAATTTCCAGGAACAT
GCAAGGCTCCTTTCTGCCTTAAGGTCCCTGCTCACTGGCCCCAGCTGAGAAGCCCATCTCCCTGCAGAC TTTAGAACCTAGTTTATATCCTGGTTCTTCCAAGCCATTGCTCTCTCTCCTTCCTCTGA
GCTTCCAAAGCATTTGGCAGCCACCATCCAATCACAGGATATTTGGGTCTGTTAACAAATTGTTCCCTGGGAAGGTCCCACTTGCCAGAAGTGGGTTGGAGGCTTCGGGACAGGGCAGGACTGAG ACC
TTACACTTCTTCAGAATCTCCCTGCAGCAAGTGATGCAGCACAATGCCACAGCAGTCCTCGAGTGCTTC CCCTGGGGTCATAGGGAAGGTGCACAGAGTTCCTTGACCTTGATGGAAGCCGTAAACTG
CCAACAATCCTCCTACCTAAAGCAGAGTTTATCTCGAAATGAAATGGACATTGGGCCTTGTAGAAATGGGCGTACTCAGACTCTTCAAATGAATTTCAATGAAACTGGGTGCTTAGATAAGTTAG ATG
TGTCATTGCACCTAGGAGGTTATAGTCTAAAAACATGAATTTAAGTGTTAGGGGTAATCTTGAAATATC AAAGTTATACTCTTGGGAAAATAATGCTCCTCAAATAAACTGAAAAATTTATTTCAATA
AAGATGCCTCTTAGATCAATCTGATTCTCCATGCCATGGTGATGAAAGTTGACCTATCCTTAGCATTACTTTCCTAAGCAGGGCTAAAGCCCAACACATATGGATCAGACGAGCAGTGAGAAACT TTT
TCTCAGACTCGGCCCAGTCCACATAACTCTCAGATGGCCCACGAATCAGACTTCTCCAGATATTTCACC AGGGGCTGGCATGTAGGCATGTGACCTGAACTAAGGGCACTGTCTCCAGGTGCCCCAGG
AGACACaCTCTGACTGACTGGATAAACTTTGAAGTTACCTAAAAGTCTGACATCTAAATGGTGGTTTTATTTTTAAAAGGAAAATACACAGCTACACAAAGCCAGGGAATAATCAGTGTGTACTTTGG
AATAATTAGGGTTGTCTTTTTTCCCAATCTTGCTGGAAGGCTACTTCCAGAAAACAAAAACAGAACAAC GAAAACACTCCAGCATTTCCCTTGGCTATAGAGTTGACAGCAATAGGCAAGCACACTGT
TTGGCGTTTACCTCATCTGCACTGGTGGTGGCTGGGGGGCTGGTTCCTCGCTGTAAGTGCCAGGT aTCTGTGAAATCACTCCCCTTCCCCTGCCCTCATTACACACACATCTGATACACATCTGCATG
CACACACACCCTCTCTCATTCCACCTTCCCAGGCCTCACCTGGTTGATAACATAGATGCCATAGTCCAG CTGCTGGCGCTGCAGGACTGGGTGCAAATAATATAGCCAGTACTTGAGGTGCTCCTGCC
GGTTGCGGAATGGAATGATGATGGCCACCTTGTGAGGAGAGACGCAGTCCCTGGGGGCATAGCGGCCGCCCATCTTCACATTTGGGTTCTGCTTTGCCACGAGCTCCAGGTCCACAGGCATGTTA AAC
TCAAT

SW perc perc perc query position in query matching repeat position in repeat

score	div.	del.	ins.	sequence	begin	end	(left)		repeat	class/family	begin	end	(left)	ID
868	29.2	1.0	6.6	UnnamedSequence	79	463	(9262)	+	L1ME4c	LINE/L1	5563	5927	(156)	1
228	16.7	0.0	0.0	UnnamedSequence	739	774	(8951)	+	LTR40A1	LTR/ERVL	258	293	(291)	2
267	28.2	16.4	4.7	UnnamedSequence	2709	2879	(6846)	+	LTR89	LTR/ERVL?	20	209	(670)	3
26	0.0	0.0	0.0	UnnamedSequence	3199	3223	(6502)	+	(T)n	Simple_repeat	1	25	(0)	4
6265	8.8	6.4	3.3	UnnamedSequence	3227	4725	(5000)	C	SVA_F	Retroposon/SVA	(8)	1367	146	5
226	36.9	11.7	1.4	UnnamedSequence	4873	5136	(4589)	+	LTR89	LTR/ERVL?	584	874	(5)	6
443	29.2	4.9	1.2	UnnamedSequence	5298	5460	(4265)	C	MIR	SINE/MIR	(93)	169	1	7
375	29.3	9.5	0.6	UnnamedSequence	5500	5657	(4068)	+	MIRc	SINE/MIR	11	182	(86)	8
25	18.6	0.0	0.0	UnnamedSequence	5888	5932	(3793)	+	(GT)n	Simple_repeat	1	45	(0)	9
423	31.1	17.8	0.0	UnnamedSequence	6628	6807	(2918)	C	MER103C	DNA/hAT-Charlie	(2)	295	84	10
2440	8.8	0.0	0.0	UnnamedSequence	7208	7512	(2213)	C	AluSx1	SINE/Alu	(2)	310	6	11
394	30.9	6.9	4.2	UnnamedSequence	7588	7820	(1905)	C	CR1-3_Croc	LINE/CR1	(1633)	1972	1734	12
328	36.8	8.4	0.0	UnnamedSequence	7915	8104	(1621)	C	L3	LINE/CR1	(1428)	2671	2466	13
219	36.2	8.4	5.0	UnnamedSequence	8273	8487	(1238)	+	L2d	LINE/L2	3156	3377	(48)	14
874	28.1	1.6	6.3	hg	79	443	(7606)	+	L1ME4c	LINE/L1	5563	5911	(172)	15
235	16.7	0.0	0.0	hg	739	774	(7275)	+	LTR40A1	LTR/ERVL	258	293	(291)	16
250	27.0	16.4	4.7	hg	2709	2879	(5170)	+	LTR89	LTR/ERVL?	20	209	(670)	17
411	24.0	3.9	1.5	hg	3659	3785	(4264)	C	MIR	SINE/MIR	(132)	130	1	18
321	27.0	10.3	0.0	hg	3825	3950	(4099)	+	MIRc	SINE/MIR	11	149	(119)	19
25	18.6	0.0	0.0	hg	4213	4257	(3792)	+	(GT)n	Simple_repeat	1	45	(0)	20
249	31.3	17.7	0.0	hg	4952	5131	(2918)	C	MER103C	DNA/hAT-Charlie	(2)	295	84	21
2434	9.0	0.0	0.3	hg	5532	5842	(2207)	C	AluSx1	SINE/Alu	(2)	310	1	22
346	26.4	8.6	6.7	hg	5912	6086	(1963)	C	CR1-3_Croc	LINE/CR1	(1633)	1972	1795	23
230	36.8	8.4	0.0	hg	6239	6428	(1621)	C	L3	LINE/CR1	(1428)	2671	2466	24

>chr9:33127360-33135408
GATATATCAAGACAGTGGTGCTAAGCATGTTCAGGATGTTTAAGATACAAGTTGGAGAAAAATACTTAAGAAAAGCTTCTCTTTCAGAAAGCAATTTGGCCATCTGTATTAAGAACTATAAAAATG TG
TAAACCATATCTGAACCCATAATCCCACATCTGAAAATCTAGCCTGGGAAAATGATCCTAAATGTGGAACAAACCAACACAGAATAAACTTCAGACTCCAAGATGTTCATTATGGTATTACTTAC ACT
CACGGAAAATTGGACATTACCTAAgGGACTTCTAAGAGAGAAAAGACAAGTTAATTATGATGGAATTCTACGATCAACTACTATGAAGGTATGGAAAGTGATGATGATGAAGACTATGAAATAACATG
GGAAGATGCTGATGATAAAGAATTTAAGGAAAAAGCAGGATACAAAAGTTTATACACACACAGCATGAGCTCAACTGTGGTTTAATTTAAAAGAAGTTAAACTCTCAGACCCTGTATGACGGGCT GGC
TTCCAGCAACCTCAGCATTTCCGAGAGAGCCCACAGAGGCCCTGGTCTTGCCTAGGAGACCCCAGGGCCTGAGGATGGCACACTCATGGGATTATTTACATAGCCTCCCCCTGACCAGCAGGGCCC TG
GCTCTGGGTTGATTTGAGGACATGTGAACATTACCAGGGTGGTGGTGGCAGCGGTGGTGGCAGCAGCAGTGGCAGC gGTAGTTAGGTCGGCATTCCTCTCTCTCTCTCTCTCTCTAGGAGGCGGAAAG
TACAGAAGAGAAGGTAGGTGGAGAATAAGAAGCAGATCAGAGAGGAACTCCTCCTTCCACTTGCTTTGCTACAAACAGGATTAACAAGTAAAGGTAAAAGGCCCCTGCTAGAAGGAAAACAAAAAA GA
AGAAAAAGAGAAAAGGGTGGATGCAAGGGAGAGAGGTGAGGAAAAGAGAGGCAAATGTCAAAGCTGCAAGGGAAAAGGGGCCCATTCTGATGACCACTGTTCCAATGCAAAAACAAATTCCTAAT AAG
CTGTCATTCCTTGTCACAGAGTCATGGGTGAGCAGAAAAAATGGC aTTTCCCAGAACAATGTCCATGGAAGCAATTCACCAGCAAATTGAGTCCCCAGAGAACAGAGACACACATAGACTAGGTGGGG
CACACAGCAAAGGGGCAACACTCAAACAGTGATGGTGCAGAGACACTCCCTGGCCTTCCCACTCCCATTCCCCACCCCGGCAAGCTCCAAAGGGTCCAAGTGCCCGAAGGAAGGGGAGGCTACAG GAC
AGGACCAGCCCTACCCCACTCCTCCCAAGCTCCGTTCCCACACACATCAATCTGTGCACTGACAGCTGAGGTAGAAAATGGAAGGCCGGGATGGGGGGATGGTAGAGATGATCAAGATAGAAACCA AA
CCAATGAGGCCATAAGCCCAGGCAGCTGTCCTCACTAATGGGATAGACCCAGGAGTCTGCTTCTCCCCAGTTGATCTTGACCTTGTGTAGGAACCAGCTGCTTGGACCAGTGACAGAGACAACTG CTG
AGGTAGGGTGACAGATACTCTTGCTCACTGAGGGTACAAAGGACTGTCTCCAAATCCATCCTCAACCAGCCTGGAACTATCACCCCATCATCCTAAAACAGTGCACAGAAGAAAAGGAAACAGTAG AA
AACTTCAAAGCCAGACAGAGTCCTCAGGGCAGCCCTCCCTCCATTTCTCCACAGCCTTTCAGGCAGGCTCCAGCTCTGCTTCCTGGCCATCGTGTGCTTACCTGTGGAGCCTTCCCTC tGCAGGAACA
ATCAGAAGACCATTTGCCTTACTCTGGGGGATGGGGGGAGGTGGGAGGTTCAAAATTCAAAGTGAGACCATAAAGGTGCTGAGTCCAAAGGACCCTAAAGGAGTTTTGTGACCATTAACAATCACA AG

GCTAAGGTATTAATAGGTTTTCTGCATTTTTCCTGATAAGGGAGGTCACCCCAAGGACTGTCACAGGCAGCCTGTGCTCTGGTCAGCATTGAGCTGGAAGCTTCGGGGAGAGGCCCAAGGGAGGG ATC
CTGCAAGTTCCCAAGGGTCAGGACCTGGCCCCACTGGGAACGGAGGCTTCACTTCCAAGAGGCACAGATTTAGGCAGAAGACCAGGAGTAGGGAGTCCTGAGACTAGGGATAAACAAAGGGGCAGG AA
TCCTGGAGAGAAGCCCCGTGCTCCACTCACTGACTGCGTCTGTACTGACTATGTACCAGCTGTGTGGTCAG aGAGAAAGAAGGGAGGCAGACATGAGGGACTCAGAGTCCGGGGGCTGGGGTGAGGCT
GGAACTCAGATAATCATCCCTTTCCAGAAAGTTCTGAGCAGCCTGGGAGCAGCAGGGAAGGAGAAGTCAGCTCTATTCCAGGGGCTCCACAGAACTAATGAGGTTGGGGAGGGAAGCTTAAAAGAT AG
CCAGAGACACACTAGGAACACAGGGGACAGCATAAGCAAAGGCACAC aGAGGgGAGAACAGAGGTGTCTGGGTGGAGATGGGGAGTCCAGTGAAGTGGAGAGGAGGAGGAAGTGGCTGGAGGTAGGTC
AGGGTCTCGGATGCTAAGCTTGGGCCTTCCGACCATTTAGACCGTTGCCTGTAGGCAGTCGTGAAATGCTAACAGGTTTAAAAGAGGAAGTAACATGGTCAGAACCAGAAAAATCCTGGGCAGCTG AG
GGAGAGGAACAGAGGTGCCAGAAGATATGCAATCGGAATTGGAGAGACCAGTTCTGTGTACTGGCTGTGGGGCAAAGGGCTGCCATGGTTGTGACAAACCCACCTGGCCAGCTGTGCCTGCATTC CTT
ACTCATTTTGTAATTTTTATGATGGGCAATCCCACAAACATTCTGCATTCCTTAATTTAGGGCTAGTGCTACCAGGTGGTGACCTGCCCTCCCTTCACCTTGAGTGAAGTCTCGTTTCCGGGTTTG AG
TATTAGGAAATGCAGCATGCATCCCTGTTTGCTATGATGTCTTCTCCTTCTGTTGCTTCTTTTCTTGCT GCCGTTGAAGTTGGAATGC TATATCTTGAAATCCACGTCTCAGAGTACAAGGAGTCAAA
TAGTATTCTCCCATGCTGGTGAGCTAAGGTCAGGGGTGGGAGGAGAGACCTTAAGGAAAAAGAGCAACTCTTGTTCTTCTATATCTCAACCAGGAAAGCCTCTCCCGGCTCATTCC TTCAATATCCTT
ATTTTAAGATATAAGTTAGTACAATAATTA CAGAGGCATAAGCCAAATGG GAGTGGTTAACAGCTTACTATAGACAGAGTATGAGTCATAACAACTTGTGAAAGTTCACTCCTGTGTGTAAAGCTTGA
TACGTGGCCCTATTTTGAAGAGGCTTATAGGCCCGGGATGGCCTCTGAGATTCTGCCACCTGGCTCACTCCACTATCCATTACCCTCTGACTCCAAGGAGTTCAAACCAATAGGAGGAAGGGTCC TGA
TGCGGGCCCTGAGTGGGGGTGCAGCAGGAGGCGGTGGGAGCCATGAAGAA TTCACCACCACaGAAGCAGGCAGAGGAAGCAGGCAGAGGAAACAGGCAGGATGAATGGAGAGTGGAGAGGCAGGAGTG
GACCCTCCTAGTAACGGCTGGTATTTACTGAAGGTTTACCATGAGAAGGATGCCTCATAACCCTCCCCACCAAGGAGGAAACTGAGGCCTGCAGAGCTTAAGTAACCTGCCTGAGGTCACTGACT GTA
AAGTGGCAGTGCCAGGACTGAACCCAGGCAGTCTGAGCACACAGCACACT CTCAGCCCTGGATACCAGACTGTGAGCAACACTGGGGGCACCCTGGCCATGAGGAGTGTCACTGATGCCCTGAGAAGG
CACTAAACCTGGAGTCAGACGCTGGGGTTTGAGCCGCAGCCCTACTGTTGTCTATGTGACTTTCGGAGCCTGTCCCTCTCTGAGCCTGTTTCCTCACCTGTGACATGGAGCAGAAGTTTCTACAT TGT
CTGGCCTCAGGGGTCTAGGCAAATCCTAGCAGTGACTGAATGGGAAGCCA AGGAAGTGGAAATGGGACATGGCCCCTACTTCAAGTAGAATGAAACCCTTGTGACCTGCTGTAAGCGGAAGGTTGTGT
AGCATTTCAATGGGGCAGAAAGAAATCTGATACTGAAAAGCCTGTTTGGGAACCCTTGGACTAAATGTCTCAGAAGCCACTCCATTTTGAGCATTACTTCTGTCTAATCTTAAATGGTATGTGTG TGT
GTGTACATGTGTGTGCATGTGTATGTGTGTGTGGGAATGAAAAGTCAAAA TGACAGCAGGGTGTCTGgGCCAGGGATAAGCTGAGAGGCTATAAAGAGGGCTAACAGCCCATAAGGTTTATTTGCTCA
ACAAACAGTGCAGAAAGGTCAAAACCAGGTGCTACAAAATCCCTTTCTTCTTCTAGAATATAACCATAAAACAGATAACACAGGCAGTTCCTAATTTGTAGACAGGATGTGCTCCAAATGTTTCT CCT
TCCACCTCTTTTTTTTCAGGGTTCAGGTTTCCCATTACCATTTTATTATT TATTAGGTAATATTCTTTACTGGAAAGGTATGGCAGAGTTTAAGCATTTCTCAACTTAGTTATCTGGACTCTGACCAT
ATTTTCCCAAAATGATCAATTAGGGTGAGATTGGAGCAGATGATAAGAGAGTAGCTGGGGAAGTGGGAGAAAAAGC aGAAAAGATTCTCCTAAGGTGGAAAGTTAACCCTTGGAGAAGGAAAGGATGA
AGAGAAAAAAAAGTATGAGGAAAAGCAAAGAAAAAAAAAAAGATGATATTG ACAAGATAAGAAAAAAGGAGGTATATATGTTTGAAAAACCAAAACTAAAATATTCCTGATGCTAAGTTGT GAAGTGGG
AAGAGGAGGGAGGGAGGGAGGACGGCAGTTCCTAGTGTTCCAGGGAAAGCAGGGAGGCAGCTGGGAGGAGATGCTAACTTAGGCCCGCTTTCCCCAAAGTGTGACTCACAAAACACTCATCACT TGA
GACGGTTCTTGGAAAAGTTGTCTCGTCAGGCAAGCTCAGGAAACACTTCT GGGGTTACAGTGTACACCGGGGTACCAAAGGCTCTGCGGGAATGAGACCTGCTTTACATGGTTCAACCCAACATTCCT
CAAGCGTATTTCGCTCCATTTGGGGAAGCACACTTTTGGAGAAGCACTCTCTTAGGTGGTGCCCAGTGTTAGCCAGCACAGCTGTGCCACCGGTCTGAGCTGGCTTGGTTAGTTGTTCCATCTAC TCG
GATTGAGGTGGGCAGGAGCATGTAGCTGGCAGAGGACATTCAGAAATCTA CtGTGTTGAGCCTGGGGAGTATGTAAATTGTATCATAGGAATTTAAGCTGATGGCTAGATTTTACTGGTCCAATATTT
GACTTCAAAACAGGGAAGTCTCACATTCACCTAAACTGCAACATATCTAGACGTAGCATCCCACCCTCCAGTCTTCCTTGAAAACAGCCACCTCAGTTCAAAAATTCAGCATCATCGCTAACTCT TTT
ATCCTCTTTACCCAAGTATATATTTTATTTATTTATTTATTTATTTTATT TTTTTGAGACAGAGTCTCACTCTGTTGCCCAGGCTGGAGTGCAGTGGCATGATCTCGGCTCACTGCAACCTCTGCCTC
CTGGGTTCAAGAGATTCTCCTGCCTCAGCCTCCCGAGTAGCTGGGATTACAGCCGCCCACCACTATGTCCAGCTAATTTTTGTATTTTTAGTAGAGACGGGGTTTCGCCATGTTGGCCAGGCTGG TCT
TGAACTCCTGACTTCAAGTGATCCGCCTGCCTTGGCCTCCCAAAGTGCTG GGATTACAGGCATGAGCCACTGCGCCTAGGCCCAAATATATATTTTAACCTACCTATATGGCACCCAGACCACAGCTG
TCTGTCTTCTACTCAGGCAGATTTGTGGGATGCTATCAGATGCCCTGCTGAATAAGATATGACAGCATTGTATCCACAGTACATCCCTCATCTACTGGCATAGCAACTGTCAAGAGAGAAACGAG ATG
CAGCTGGCACGGCTTATTAGTCATCCCTGCTGGCTCCTGGTGATGGTGGC CGCCTCTTCCAGGTGCTCGCTGCCACTGCTTTAATAACAGGCTCCTGAATCTTGCCGTCATTGGTATGTGCTAACTGG
CATCAGACAAATGTTTCTCAATCACAGAGCTTTTCTTAAATGAATATGTGTTGGGTTAAAGTGACCCGCATTTTGCAGGACATTTACCTGTTCTCTCTTGCATTCTCCAATATTCTTCCAAGGTG ACC
CACAACTGCGTGGCTGTCTTGCTTGCAGGTGTTCATAGGGCCCTGGTCTG TACCTCACTTGGCCGAAGAATTCAAACAGCCTCAAGGGGTTTGCCCACCACCTGCTCTCCTGCTTCAGTTTCCTTGTA
ACAGGGCTTGTTCTGCTCTCTCCTGCCTCACAGGCTTAACTCTGACACCTGG tCCCAGAGCTTCTGGTGCTTTCCTACCCCAGTGGGCATCTCAATTTCCAGGAACATGCAAGGCTCCTTTCTGCCTT
AAGGTCCCTGCTCACTGGCCCCAGCTGAGAAGCCCATCTCCCTGCAGACT TTAGAACCTAGTTTATATCCTGGTTCTTCCAAGCCATTGCTCTCTCTCCTTCCTCTGAGCTTCCAAAGCATTTGGCAG
CCACCATCCAATCACAGGATATTTGGGTCTGTTAACAAATTGTTCCCTGGGAAGGTCCCACTTGCCAGAAGTGGGTTGGAGGCTTCGGGACAGGGCAGGACTGAGACCTTACACTTCTTCAGAAT CTC
CCTGCAGCAAGTGATGCAGCACAATGCCACAGCAGTCCTCGAGTGCTTCC CCTGGGGTCATAGGGAAGGTGCACAGAGTTCCTTGACCTTGATGGAAGCCGTAAACTGCCAACAATCCTCCTACCTAA
AGCAGAGTTTATCTCGAAATGAAATGGACATTGGGCCTTGTAGAAATGGGCGTACTCAGACTCTTCAAATGAATTTCAATGAAACTGGGTGCTTAGATAAGTTAGATGTGTCATTGCACCTAGGA GGT
TATAGTCTAAAAACATGAATTTAAGTGTTAGGGGTAATCTTGAAATATCA AAGTTATACTCTTGGGAAAATAATGCTCCTCAAATAAACTGAAAAATTTATTTCAATAAAGATGCCTCTTAGATCAAT
CTGATTCTCCATGCCATGGTGATGAAAGTTGACCTATCCTTAGCATTACTTTCCTAAGCAGGGCTAAAGCCCAACACATATGGATCAGACGAGCAGTGAGAAACTTTTTCTCAGACTCGGCCCAG TCC
ACATAACTCTCAGATGGCCCACGAATCAGACTTCTCCAGATATTTCACCA GGGGCTGGCATGTAGGCATGTGACCTGAACTAAGGGCACTGTCTCCAGGTGCCCCAGGAGACAC gCTCTGACTGACTG
GATAAACTTTGAAGTTACCTAAAAGTCTGACATCTAAATGGTGGTTTTATTTTTAAAAGGAAAATACACAGCTACACAAAGCCAGGGAATAATCAGTGTGTACTTTGGAATAATTAGGGTTGTCT TTT
TTCCCAATCTTGCTGGAAGGCTACTTCCAGAAAACAAAAACAGAACAACG AAAACACTCCAGCATTTCCCTTGGCTATAGAGTTGACAGCAATAGGCAAGCACACTGTTTGGCGTTTACCTCATCTGC
ACTGGTGGTGGCTGGGGGGCTGGTTCCTCGCTGTAAGTGCCAGGT gTCTGTGAAATCACTCCCCTTCCCCTGCCCTCATTACACACACATCTGATACACATCTGCATGCACACACACCCTCTCTCATT

CCACCTTCCCAGGCCTCACCTGGTTGATAACATAGATGCCATAGTCCAGC TGCTGGCGCTGCAGGACTGGGTGCAAATAATATAGCCAGTACTTGAGGTGCTCCTGCCGGTTGCGGAATGGAATGATG
ATGGCCACCTTGTGAGGAGAGACGCAGTCCCTGGGGGCATAGCGGCCGCCCATCTTCACATTTGGGTTCTGCTTTGCCACGAGCTCCAGGTCCACAGGCATGTTAAACTCAA T

>KSI_SVA_F_2 (We have tried to verify that there is insertion in this region, but we couldn’t confirmed the exact sequences by high GC-content concentrated in this insertion region.)

>SVA_F-Scaffold15806-1280400-1280864
GAGTCATTTCTTCAAAACAAATGACTGAGGTGGAAAATTACTTACCTTGAATAAATTAATTGGAAAATCAGAGAACACTGGGTTTATTTAGGATGAGGTTGTTTGGTATGTGTATGGGAGGGTAGAAT
TCCTAATTGCTCATCTGACTGGGTTCAAAATGTAATACTAGATATTTGTGTTGCAATTCAGTTGGTACTTTTGGTATAGGGCTAACTTATCTTGCGTGTAATTTTTTTTTTTTTTTTT Ttttttttt
tttttattATGAAATCTGGTGCTGTTGCCCAGGCTGGAGTGCAGTGGT GTGATCTTGGCTCACTACAACCTCCGTCTCCCAGGTTCAAGGGATTCTCATGCCTCAGCCTCCCGAGTAGCTGGGATTA
CAGGCGCCGGCCACCTTGCCTGGCTAATTTTTGTATTTTTAGTAGAGACGAGGTTTCACCATGTTGGCCAGGCTGGTCTTGAACTCCTGACCTCAAGTGATCCACCTGCCTCGGCTTCCCAAAGT GCT
GGCATTACAGGCTCGCTCAGGCATCTTGCCTTGTAATTCTCATGATAGT AATGGCTATTTTTTTCTTGCCTTAGAGTTGTAAGTAAAAATTCCTTAATTACACATTAAGGTTTGATCTTTAATTTTAC
AATGTTTGAGTCATTTTGTTACTTCTTTTCTCCCAGAATGACTTGCGTAGCTCTAAATGATTTTAGTTAATTTCACATCTGTTTGCCTTTCTTCTAAAATGACCCCTAGAATCTCAGCTTAACTA AGG
AAAATGTCAAGTGGGTGTTGTTTCTTTGTTAGTGGTTTTGGCCTAGACT ATCTAAAGTTTGGCAAATTACTCACAAAGTATGTTAATTGGCATCACATTCCAATCAGTGTACATAGCATTTTTTGAGG
AACACTTGACACACGGTTTTATTTTTAGACCAGATTCTAAGGGGTTTTACTGGGTGGGGCTTAACAATCCTAAAGCTAGTTTACGGTTTTAAAATCTTTATGATTTAGAGGTTGTTTACATTTTTT TGT
TAATAAATGGGAAGCAGCAGGCAGTGGCAGTCAATTTTGTTTGTTTCTT TTTTTGTTTTTTTTGAGACGGAGTTTCGTTCTTGTTGCCCAGGCTGGAGTGCAGTGGCATGATCTTTCCTCACCACAGC
CTCTGCCTCCTGGGTTCAAGCGATTCTCCTGCCTCAGCCTCCTGAGTAGCTGGGATTACAGGCATGCGCCACCACACCTGGCTAATTTTGTATTTTTAGTAGAGACAGGGTTTCACTGTGTTGGT CAT
GCTGGTCTTGAACTCCCTAACTCAGGTGATCTGCCTGCCTCAGCCTCCC AAAGTGCTGGGATTACAGGCGTGAGCCACCACGCCCAGCCCTCACATAACTTTTATGATATTATGTTCTTATAATTGTT
CCATTATTAATTATAATTAATCTCTtACTGTGCCTAATTTATATGTTAAACTTGATCATGGGTATGTATGTACAGGAAAAAACATAGTGTATACAGTATAGTATACTGTTCTTGCTTTCAGGCATTCA
TTGGTAGTCTTGGAACATATTCCAAGTGGATATGGAAGCACTACTATGT GATGGAATGTTACTCAGTAATAAAAAGAAAGGATGTACTGGTGTATACTACAACATTGGAAACATATTAAGTAAAAGAA
ACCATGCAGGAAAGACCACATATTGAATTATTCCATTTATATGTAATGTCCAGAATAGGAAAATCCTTAGTGACAGAAAGTAGATCAGGGGCTGAGGGATGTAGGGAATGGTCAGTGACTGTGAT AGG
GTTTTTTTTGTTTTGTTTTGTTTTTTTTTTGGGTGATAAAAGTGTTCTA CAATTATGGAGATGGATCCACAACTGTGAATATAATAAAAGCCATTTTATTGTGTACTTTTTTTTTTTTTGAGATGTTTT
GCTCTTGTTGCCCAGGCTGGAGTGCAGTGGTGCACAATCTCAGCTCACCGCAACCTCTGCCTTGGGTTCAAGCGATTCTCCTGCCTCAGCCTCCTGAGTAGCTGGGGTTACAGGCATGCACCACC ATG
CCCGACTAATTTTGTATTTTTAGTAGAGATGGGGTTTCTCCATGATAGT GAGACTGGTCTCGAACTCCCGACCTCAGGTAATCTGCCCACCTCGGCCTCCCAAAGTGCTGGGATTTACAGGTGTGAGC
CACTGCGCCTGGCCCCTGCCTGGAAATTCTTAAGAATTAAATATTATTTTCTTTGAGTAAGTAATAATGTCAGATGTCACAAAATTTAAAGGATATAGAAAGTTATGTTGTGCATAGTTATTCTA CGT
TCTCCTTTGTTCTCTAGCTACCCAGCTTCCTTTCACAGAGGGAAATGTG AATTCTTATGTATCCTATTTTGTGACGACACAAAAATATTTAAGGGTAGATGTGCTGGCCTTACTTTCTTGCTCAAATC
TTCCAGGCTCTCCCTGGCTACCAGACTAAGTATATACAATTCTTGCTTCTGTGTCTTGCTCACATGGCTTTCTCATAGTAATAGTATGTTTAGTGGAAATCAAAAGCCAGTATTTTTAGGCCCCT GTA
GGTGCAGGCCAGTGCACTTGGTGCAGCATAAGGCAAGTTCCTACCTTCA AGGACCTTATCTAATTGAGAGAGGCTGACATTTCCTTACTTTTATCTATTCCTCTTTTTTTTTTTTTT tCCCGAGATGG
AGTTTCACTCTTATCACAAAGGCTGGCGTACAGTGGCGTGATGTTGGTTCACTGCAACCTCTGTCTCCCGGGTTCAAGTGATTCTCCTGTCTCAGCTTCCCAAGTAGCAGGGATTACAGGCATCT GCC
ACCACGCCCGGCTAAATTTTGTATTTTTAGTAGAGATGGGGTTTCATGA TGTTGGCCAGACTGGTCTTGAACTCCTGACTGCAGATGATCCACCTGTCTCAGCCTCCCAGAATGCTGGGATTACAGGT
GTGAGCCACCGCAGTGGCCAAGTTGAATTTCTTGTATAGATGTCTTATGTCTCAGTTTAAAAATGAGGCATGTCTTACTCTTGCCTATATATTTCCAGTGGGGCATGTCTTATCCTTTTTTATAT ATC
TCCAGTTCTCAAAACCTTGTCTTACACAGCAAACATTAATCAGAGGTTA ATCACTTATGGATTTAGTTTCACTCTACTGCCATCTCTCCAAAATGCTTACAGTAAAGAGGCTACACTTACTAAATTCA
AACTTAGAACTTTGTAACTTAGGACCTGTCTGTTGGAGGAGGTAAGTTGCTTAAGCTTCTCCCTCTATTGGGCATTATATGTAGAGTGCTCTAGGTTCTGCCTGCTAGCTCCTTGATGACATTTG CCT
GCTTTAGCAGGACACTGACTCTTCAGCTCAAGCAGCTGTTACCATGCTG GCTATTTCCTTTTGCATTCATAGGTTTTAGATATGGAAGAAAGTTATACCCAGAGAGAATGGGGGGATAAATGATAATC
TTCTACCATTTTTGACCTTAGTCTGGTCCAGCATTATAGTGTGATTTTGAGAGGAGAGATTGTGATTTCCCTGGCTAGTATTATCTAGTATTGCCTGACAATGCAAAATGGACCCCTAGGCTGTC TTA
TGTAAGTTGGAAGGGGGCGGATTGGGGGTGGGGTGTGTGTGTGTGTATC TACAATCCAGTGACATTTAACAACATTCACAATGTCTTAAAACCAT tGCCACTACTCGTTTCCAGAACCTTTTTGTTAC
CTCAAACAGAAACAGTACTCATTAAGCAAGAACTCCTCATTCCTCCCTATGCCCAGTTCCTCGTAACCCCTATTCTACTTTCTCTCTATGAATTTGCCTATTCCAGGTACCTCATGTAAGGGGAG TCA
TACACTATCTGGCCTTTTTTGTCTGGCTTATTTTACTTAGCACATTTTTGAGGTTTATCCATGTGGTGGTGGCATGTATCTGTATTTTATTCCTTT TTATGGCTGAATAATATTTCATTGTATGAATA
TATCATTTTGTTTATCTCCTCATCAGTGATGGACACTTGGATTATTTCCACCTTTTGGTTGTTGTAAATAATGCTGCAGTGAACATTGGTGTACAAATATCTGTTGGAGCCCCTGCTTTCAGTGC CTT
AGGGTAATGCACAGGAGTAGAATTACTGGATCATATGGTAATTCTGTGTTTACTTTTTGAGGAACTGCCAAACTGTTTTGCACAGTGACTGCACCA TTTTACATTTCCCACCAGCAAAGCACAAGGGT
TCCAGTTTTGCCACATCTTTTCCAACACTTGTTATTTTTTCTTTTTTAATTTTTAAAGTAACAATCTTGGGCTGGGCACGGTGGCTCATGCCTGTAATCCCAGCACTTTGGGAGGCCGAGGCGGG CGG
ATCACCTGAGGTCAGGAGTTCGAGACCAGCCTGGCCAACATGGTGAAACCCCGTCTCTACTAAAAGTACAAAAAAAATTCGCTGGGCTTGTTGGCG CATGCCTGTAATCCCAGCTACTTGGGAGGTTG
AGGCAGGAGAATCGCTTGAcCTCGGGAGGCAGAGGTTGCAGTGAGCTGAGATTGCGCCATTGCACTCCACAAGAGTGAGACTTCGTCTCAAAAAATAAATAAATAAAATAACAACC CTAATGACTGTG
AAGTGGTTGTAGGCTTTTTGCATTTTAAAAAATTATTATTTTTACTATTTTAGAGATGGCGGGGgaGGGGGGTCTCACTATGTTTACCAGGTTGGTCTCGAACTCCTGGCCTCCATTGATCCTCCCAT
TTTAGCCTCCTAAAGTACTGGGATTACAGGCTTGAGCCACTGCGCCCAGCCCTCCTTTTCTTCTTATTGCTACTTAGATGTAATGGGCAAACCAGAACCTTTGAATTTGGCTATATTTGGATCTGATG
GGTTTTTTCCTCCCTCTTAAACTTGTGTATAATGTTGTTCAAAGGATACATTATGTACACAATTAATGACATCTTTTTAAAA AGAACACTTTCTTTCtttttttttttttttttttttttttttt
ttttttaatttatttttttattgataattcttgggtgtttctcacagaggggatttggcagggtcatgggacaatagtggagggaaggtcagcagataaacaagtgaacaaaggtctctggttt tcc
taggcagaggaccctgcggccttccgcagtgtttgtgtccctgattacttgagattagggattggtgatgactcttaacgagcatgctgccttcaa gcatctgtttaacaaagcacatcttgcaccgc
ccttaatccatttaaccctgagtggacacagcacatgtttcagagagcacagggttgggggtaaggtcacagatcaacaggatcccaaggcagaagaatttttcttagtacagaacaaaatgaaa agc

ctcccatgcctacttctttctacacagccacggcaaccatccgatttccccatctctcccccccctttcccgcctttctattccacaaa (GC)richagggagagggagagggagagggagagggaga
gggagagggagagggagaggctaattggcaactattatttcaccgtgtgtcagaacactttctttcATCCTTGTGAGGTGTAAGAAGAAAAATAAGCaAAAAAAAAAAATGAATAAAGTTTTTCTATA
AGTTGATACACACAGTTGTAGTTACTTTATACTCATACCCTTTTTTTGTGTTGTGAGTGACACAGTCTCACTCCATTGCCCAGGCTGGAGTGCAGTGGTGCG ATCTCCACTCACCGTAGCCTTGACCT
CCCAGGCACAAGCCATCTTCCCTCCTGAACCTGCTGAGTAGCTGGGACTACAGGTGTGTGCCACCACACTCAGCTAATTTTTGTATTTTTTGTAGAGAT GGGGTTTTGCCATGTTGCCCAGGCTGATG
TCGAAGTGCTGAGCTCAAGTGATTCTCCCGCCTTGGCCTCCCAAAATGCTGGGATTATAGGCATGAGCCACTGTGCCTGGCCTCTCATACTTTTTTATTCTATAA TGTATTTAGGGCCAGCCTCTGTG
GCTCATGCCTATAATCCCAGCACTTTGGGAGGCCAAGGAGGGAGGATTGCTTGAGGCCAGGAGTTCAAAACCAGCCTGGGCATCATAGTGAG AcCCCCATCTCTAAAACCAAAAAAACTAGTCAGGCA
TGTGGTATGCGCCTGTAGTCCCAGCTACTTGGTAGGCTGAGGAGAGAGGATTGTTTGAGTCCAGGAATTGGAGGCTGCAATAAGCTATGATTGTGCCATGGTGCTCTAGCCTGGGCAACAGAACAAGA
TGCTGTCTCTTGAAAAAAAAAAAAAAGAAAAAGAAAAGATGAATAGTCTGTCTGAAATGGCCGGCAGTCGCAGCTGCTGACCTGACCTGCATCTACTGT ATAAGTCAAGTAGTTCAACTTTTTATTAT
AATGTCATGGCTTTTCTTTGCTTCTTGGGAGCGCTTCCAGCATCACTAGTGGTACTTCATATGGGACCCATGCTGTTATTCAAAGTTTATGGTATTGTACTGTACATGATGAAAAATACAGAAGA CCC
ATGAGAGATcACTTTTTTTTTTTTGAGACATAGTCTTGCTCTGTCGCCCAGGCTGGAGTGCAATGGCATGATCTCAGCTCTCCTCTGCCTCCTGGGTTC AAGTGATTCTCCTGCCTCAGCCTCCCTAG
TAGCTGGGATTGCAGGCACTCGCTACCACAACCGGCTAATGTTTATATTTTTAGTAGAGACAGGGTTTCATCATGTTGGCCAGGCTGGTCTCGAACTCCTGACCTCAAGTAATCCACCAGCTCGG CCT
CCCAAAGTGCTAAGATGACAGGAATGAGCCAATGTGCCCAGCCAAGAGATATCACTTTTTACTGCAATAGGAAATGTACTGGAGAGACCAACTGCTCAG AGGGACACAAGGCCTTTTAAGTGGACACA
TGCAACTTGTAACACTTGAGTTCACTGCAATAGAAAGAAGAGGTGGCTATGAAATTACTGTAGTAATATAGTTTGTACAACAGGTTTGTTTTCTTTTGAGGCAAAGTCTCACTCTGTCACCCAGG CTG
GAGTGCAGTAGCAAGATCTCAGCTCACTGCAACCTCTGCCTCTCAGGTTCAAGCGATTCTCCTGCCTCAGTCTCCCAAGTAGCTAGGATTACAGGCCTG TGCCACTATGCCCAGCTAATTTTTGTATT
TTTAGTAGAGATGGGGTTTCACCCTGTTGCCCAGGGTGGTCTCAAACTCCTGGCCTCAAGCGATCTACCCGCATCATCCTCCCAAAGTGCTGGGCTTATAGGCATGAGCTACCGCACCCAGCCAA CAT
CATTTAATTTTATGCAGTTATTTATTTATTTAATTAAAAAAAaaaTTTTTTTTTGAGATGGAGTCTCACTGTCTTGCCCAGGCTGGAGTGCAGTGGCT TAATCTTGGCTCACTGTACTCACTGCAAC
CTCTGTCTCCCAGGTTCAAGTGATTCTCCTGCCTCAGTCTCCTGAGTAGCTGTATAACACCTGGCTAATTTTTGTATTTTTAGTAGAGACAGGGTTTCACCATGTTGGACAGGCTGGTCTCAAC CTC
CTGACCTCAAGTGATCCACCCGCCTCTGCCTCCCAAGTGCTGGGATTACAGATGTGAGCCACTGCACCTGGCTTCAGTTATTTAATATGCTGCAGTTA TTTAATATACTGCATCTTAACATTTGTTT
ACATTTCTCTCAAGTGTGAATGTGTGTCTGTGCAAATGGTGTTATGTATGGTCTGTAAGTATATGCATAAGTTTTGATTTTTTTTTTCTTTGAGACCGAGTCTAGCTTTGTCGCC tAGGCTGGAGTGC
AGTGGTGCGAACTTGGCTCACTGCAGCCCCTGCCTCCTGGGTTCAAGTGATTCTC Ttgcctcagcctcccgagtagctgggattacaggcaccggccac catgcccagttaattttgtatttttagt
agagacggggcttcactgtgttggccaggctggtctcaaactcctgaccttatgatccgcccatctcggcctcccaaagttctgggattacaggtgtgagtcactacgcctggcctatttttatt tat
tattgtttttttgagatggagtcttgctctgtctcccaggttggagtgcagtggcatgatgttggcttactgcagactctgcctcctgggttcaagcga ttctccTGCCTCAGCCTCCCGAGTAGCTG
GGATTACAGGTACTCATCACCCTGCCCAGCTAATTTTTATATCATTTATTTATTTATTTATTTATTTATTTATTTA TTTTTAGAGATGGAGTTTCGCTCTTGTTGCCCAGGCTAGAGTGCAGTGGCGC
GATCTCGGCTCACCAC

SW score	perc div.	perc del.	perc ins.	query sequence	position in query begin	end	(left)		matching repeat	repeat class/family	position in repeat begin	end	(left)	ID
35	0.0	0.0	0.0	KSI_SVA_F_2	230	262	(8066)	+	(T)n	Simple_repeat	1	33	(0)	1
2024	10.0	0.0	0.0	KSI_SVA_F_2	266	524	(7804)	C	AluSx1	SINE/Alu	(33)	279	21	2
2348	10.6	0.3	0.0	KSI_SVA_F_2	1059	1369	(6959)	C	AluSp	SINE/Alu	(1)	312	1	3
1032	19.0	3.8	0.0	KSI_SVA_F_2	1371	1581	(6747)	+	MER44A	DNA/TcMar-Tigger	120	338	(1)	4
1169	19.0	9.3	4.0	KSI_SVA_F_2	1587	1898	(6430)	+	L1MB8	LINE/L1	5794	6121	(57)	5
2182	8.9	2.0	1.0	KSI_SVA_F_2	1899	2190	(6138)	C	AluSp	SINE/Alu	(18)	295	1	6
355	28.7	6.7	2.6	KSI_SVA_F_2	2220	2369	(5959)	C	L1ME4b	LINE/L1	(1)	6144	5989	7
212	24.7	23.6	0.8	KSI_SVA_F_2	2529	2634	(5694)	C	L2d2	LINE/L2	(2)	3462	3333	8
2079	14.3	0.0	0.0	KSI_SVA_F_2	2664	2956	(5372)	C	AluSq2	SINE/Alu	(12)	301	9	9
3626	16.3	1.7	0.8	KSI_SVA_F_2	3634	4271	(4057)	C	L1MB4	LINE/L1	(60)	6120	5477	10
2217	8.1	3.0	1.0	KSI_SVA_F_2	4294	4588	(3740)	+	AluSx	SINE/Alu	1	301	(11)	11
717	17.0	0.0	8.5	KSI_SVA_F_2	4647	4787	(3541)	C	FLAM_C	SINE/Alu	(3)	130	1	12
39	0.0	0.0	0.0	KSI_SVA_F_2	4963	4998	(3330)	+	(T)n	Simple_repeat	1	36	(0)	13
4167	2.1	0.2	0.0	KSI_SVA_F_2	5001	5465	(2863)	C	SVA_F	Retroposon/SVA	(8)	1367	902	14
57	0.0	0.0	0.0	KSI_SVA_F_2	5466	5516	(2812)	+	(AGGGAG)n	Simple_repeat	1	51	(0)	15
1930	16.1	0.0	0.7	KSI_SVA_F_2	5665	5962	(2366)	C	AluJb	SINE/Alu	(16)	296	1	16
1884	16.0	1.3	1.3	KSI_SVA_F_2	5995	6301	(2027)	+	AluJr	SINE/Alu	1	307	(5)	17
2147	15.6	2.8	8.6	KSI_SVA_F_2	6302	6532	(1796)	C	Tigger2b_Pri	DNA/TcMar-Tigger	(309)	759	534	18
2050	11.5	2.4	0.0	KSI_SVA_F_2	6533	6819	(1509)	C	AluSz	SINE/Alu	(18)	294	1	19

2147	15.6	2.8	8.6	KSI_SVA_F_2	6820	6988	(1340)	C	Tigger2b_Pri	DNA/TcMar-Tigger	(535)	533	373	18
2116	13.9	0.0	0.0	KSI_SVA_F_2	6989	7283	(1045)	C	AluSz	SINE/Alu	(17)	295	1	20
2147	15.6	2.8	8.6	KSI_SVA_F_2	7284	7309	(1019)	C	Tigger2b_Pri	DNA/TcMar-Tigger	(696)	372	355	18
1847	10.6	6.0	3.4	KSI_SVA_F_2	7334	7617	(711)	C	AluSz	SINE/Alu	(20)	292	2	21
470	13.7	2.4	16.7	KSI_SVA_F_2	7627	7749	(579)	C	Tigger2b_Pri	DNA/TcMar-Tigger	(696)	372	265	18
2236	11.6	0.0	0.0	KSI_SVA_F_2	7750	8043	(285)	C	AluSg	SINE/Alu	(16)	294	1	22
1329	15.5	1.8	0.5	KSI_SVA_F_2	8046	8228	(100)	C	AluSx3	SINE/Alu	(7)	304	122	23
39	0.0	0.0	0.0	KSI_SVA_F_2	8229	8264	(64)	+	(ATTT)n	Simple_repeat	1	36	(0)	24
1329	15.5	1.8	0.5	KSI_SVA_F_2	8265	8299	(29)	C	AluSx3	SINE/Alu	(191)	120	83	23
229	0.0	0.0	0.0	KSI_SVA_F_2	8300	8325	(3)	C	Alu	SINE/Alu	(52)	249	224	25

2215	9.3	0.0	0.0	hg	232	511	(7218)	C	AluSx1	SINE/Alu	(12)	300	21	26
2348	10.6	0.3	0.0	hg	1046	1356	(6373)	C	AluSp	SINE/Alu	(1)	312	1	27
1021	19.4	3.8	0.0	hg	1358	1568	(6161)	+	MER44A	DNA/TcMar-Tigger	120	338	(1)	28
1156	17.2	15.1	0.0	hg	1574	1779	(5950)	+	L1MB8	LINE/L1	5794	6029	(149)	29
33	2.6	2.6	0.0	hg	1780	1818	(5911)	+	(GTTTT)n	Simple_repeat	1	40	(0)	30
1156	17.2	15.1	0.0	hg	1819	1896	(5833)	+	L1MB8	LINE/L1	6030	6120	(58)	29
2182	8.9	2.0	1.0	hg	1897	2188	(5541)	C	AluSp	SINE/Alu	(18)	295	1	31
355	28.7	6.7	2.6	hg	2218	2367	(5362)	C	L1ME4b	LINE/L1	(1)	6144	5989	32
212	24.7	23.6	0.8	hg	2527	2632	(5097)	C	L2d2	LINE/L2	(2)	3462	3333	33
2062	14.7	0.0	0.0	hg	2662	2954	(4775)	C	AluSq2	SINE/Alu	(12)	301	9	34
3642	16.1	1.7	0.8	hg	3632	4269	(3460)	C	L1MB4	LINE/L1	(60)	6120	5477	35
2241	7.8	3.0	1.0	hg	4292	4586	(3143)	+	AluSx	SINE/Alu	1	301	(11)	36
750	15.8	0.0	6.9	hg	4645	4783	(2946)	C	FLAM_C	SINE/Alu	(13)	130	1	37
1930	16.1	0.0	0.7	hg	5060	5357	(2372)	C	AluJb	SINE/Alu	(16)	296	1	38
1914	16.0	1.3	1.0	hg	5390	5695	(2034)	+	AluJr	SINE/Alu	1	307	(5)	39
2147	15.6	2.8	8.6	hg	5696	5926	(1803)	C	Tigger2b_Pri	DNA/TcMar-Tigger	(309)	759	537	40
2050	11.5	2.4	0.0	hg	5927	6213	(1516)	C	AluSz	SINE/Alu	(18)	294	1	41
2147	15.6	2.8	8.6	hg	6214	6382	(1347)	C	Tigger2b_Pri	DNA/TcMar-Tigger	(532)	536	373	40
2116	13.9	0.0	0.0	hg	6383	6677	(1052)	C	AluSz	SINE/Alu	(17)	295	1	42
2147	15.6	2.8	8.6	hg	6678	6703	(1026)	C	Tigger2b_Pri	DNA/TcMar-Tigger	(696)	372	355	40
1865	10.5	5.9	3.4	hg	6725	7010	(719)	C	AluSz	SINE/Alu	(18)	294	2	43
470	13.7	2.4	16.7	hg	7020	7142	(587)	C	Tigger2b_Pri	DNA/TcMar-Tigger	(696)	372	265	40
2251	11.2	0.0	0.0	hg	7143	7436	(293)	C	AluSg	SINE/Alu	(16)	294	1	44
1329	15.5	1.8	0.5	hg	7439	7621	(108)	C	AluSx3	SINE/Alu	(7)	304	122	45
48	0.0	0.0	0.0	hg	7622	7665	(64)	+	(ATTT)n	Simple_repeat	1	44	(0)	46
1329	15.5	1.8	0.5	hg	7666	7700	(29)	C	AluSx3	SINE/Alu	(191)	120	83	45
229	0.0	0.0	0.0	hg	7701	7726	(3)	C	Alu	SINE/Alu	(52)	249	224	47

>hg19 chr3:12693447-12701175
GAGTCATTTCTTCAAAACAAATGACtaTGAGGTGGAAAATTACTTACCTTGAATAAATTAATTGGAAAATCAGAGAACACTGGGTTTATTTAGGATGAGGTTGTTTGGTATGTGTATGGGAGGGTAGA
ATTCCTAATTGCTCATCTGACTGGGTTCAAAATGTAATACTAGATATTTGTGTTGCAATTCAGTTGGTACTTTTGGTATAGGGCTAACTTATCTTGCGTGTAATTTTTTTTTTTTTTTTT TgagATGA
AATCTGGTGCTGTTGCCCAGGCTGGAGTGCAGTGGTGTGATCTTGGCTCACTACAACCTCCGTCTCCCAGGTTCAAGGATTCTCATGCCTCAGCCTCCCGAGTAGCTGGGATTACAGGCGCCGGCCA
CCTTGCCTGGCTAATTTTTGTATTTTTAGTAGAGACGAGGTTTCACCATGTTGGCCAGGCTGGTCTTGAACTCCTGACCTCAAGTGATCCACCTGCCTCGGCTTCCCAAAGTGCTGGCATTACAG GCT

CGCTCAGGCATCTTGCCTTGTAATTCTCATGATAGTAATGGCTATTTTTTTCTTGCCTTAGAGTTGTAAGTAAAAATTCCTTAATTACACATTAAGGTTTGATCTTTAATTTTACAATGTTTGAGTCA
TTTTGTTACTTCTTTTCTCCCAGAATGACTTGCGTAGCTCTAAATGATTTTAGTTAATTTCACATCTGTTTGCCTTTCTTCTAAAATGACCCCTAGAATCTCAGCTTAACTAAGGAAAATGTCAA GTG
GGTGTTGTTTCTTTGTTAGTGGTTTTGGCCTAGACTATCTAAAGTTTGGCAAATTACTCACAAAGTATGTTAATTGGCATCACATTCCAATCAGTGTACATAGCATTTTTTGAGGAACACTTGACACA
CGGTTTTATTTTTAGACCAGATTCTAAGGGGTTTTACTGGGTGGGGCTTAACAATCCTAAAGCTAGTTTACGGTTTTAAAATCTTTATGATTTAGAGGTTGTTTACATTTTTTGTTAATAAATGG GAA
GCAGCAGGCAGTGGCAGTCAATTTTGTTTGTTTCTTTTTTTGTTTTTTTTGAGACGGAGTTTCGTTCTTGTTGCCCAGGCTGGAGTGCAGTGGCATGATCTTTCCTCACCACAGCCTCTGCCTCCTGG
GTTCAAGCGATTCTCCTGCCTCAGCCTCCTGAGTAGCTGGGATTACAGGCATGCGCCACCACACCTGGCTAATTTTGTATTTTTAGTAGAGACAGGGTTTCACTGTGTTGGTCATGCTGGTCTTG AAC
TCCCTAACTCAGGTGATCTGCCTGCCTCAGCCTCCCAAAGTGCTGGGATTACAGGCGTGAGCCACCACGCCCAGCCCTCACATAACTTTTATGATATTATGTTCTTATAATTGTTCCATTATTAATTA
TAATTAATCTCTcACTGTGCCTAATTTATATGTTAAACTTGATCATGGGTATGTATGTACAGGAAAAAACATAGTGTATACAGTATAGTATACTGTTCTTGCTTTCAGGCATTCATTGGTAGTCTTGG
AACATATTCCAAGTGGATATGGAAGCACTACTATGTGATGGAATGTTACTCAGTAATAAAAAGAAAGGATGTACTGGTGTATACTACAACATTGGAAACATATTAAGTAAAAGAAACCATGCAGGAAA
GACCACATATTGAATTATTCCATTTATATGTAATGTCCAGAATAGGAAAATCCTTAGTGACAGAAAGTAGATCAGGGGCTGAGGGATGTAGGGAATGGTCAGTGACTGTGATAGGGTTT Tttttgtt
ttgTTTTGTTTTGTTTTGTTTTTTTTTTGGGTGATAAAAGTGTTCTACAATTATGGAGATGGATCCACAACTGTGAATATAATAAAAGCCATTTTATTGTGTACTTTTTTTTTTTTTGAGATGTTTTGC
TCTTGTTGCCCAGGCTGGAGTGCAGTGGTGCACAATCTCAGCTCACCGCAACCTCTGCCTTGGGTTCAAGCGATTCTCCTGCCTCAGCCTCCTGAGTAGCTGGGGTTACAGGCATGCACCACCAT GCC
CGACTAATTTTGTATTTTTAGTAGAGATGGGGTTTCTCCATGATAGTGAGACTGGTCTCGAACTCCCGACCTCAGGTAATCTGCCCACCTCGGCCTCCCAAAGTGCTGGGATTTACAGGTGTGAGCCA
CTGCGCCTGGCCCCTGCCTGGAAATTCTTAAGAATTAAATATTATTTTCTTTGAGTAAGTAATAATGTCAGATGTCACAAAATTTAAAGGATATAGAAAGTTATGTTGTGCATAGTTATTCTACG TTC
TCCTTTGTTCTCTAGCTACCCAGCTTCCTTTCACAGAGGGAAATGTGAATTCTTATGTATCCTATTTTGTGACGACACAAAAATATTTAAGGGTAGATGTGCTGGCCTTACTTTCTTGCTCAAATCTT
CCAGGCTCTCCCTGGCTACCAGACTAAGTATATACAATTCTTGCTTCTGTGTCTTGCTCACATGGCTTTCTCATAGTAATAGTATGTTTAGTGGAAATCAAAAGCCAGTATTTTTAGGCCCCTGT AGG
TGCAGGCCAGTGCACTTGGTGCAGCATAAGGCAAGTTCCTACCTTCAAGGACCTTATCTAATTGAGAGAGGCTGACATTTCCTTACTTTTATCTATTCCTCTTTTTTTTTTTTTT cCCCGAGATGGAG
TTTCACTCTTATCACAAAGGCTGGCGTACAGTGGCGTGATGTTGGTTCACTGCAACCTCTGTCTCCCGGGTTCAAGTGATTCTCCTGTCTCAGCTTCCCAAGTAGCAGGGATTACAGGCATCTGC CAC
CACGCCCGGCTAAATTTTGTATTTTTAGTAGAGATGGGGTTTCATGATGTTGGCCAGACTGGTCTTGAACTCCTGACTGCAGATGATCCACCTGTCTCAGCCTCCCAGAATGCTGGGATTACAGGTGT
GAGCCACCGCAGTGGCCAAGTTGAATTTCTTGTATAGATGTCTTATGTCTCAGTTTAAAAATGAGGCATGTCTTACTCTTGCCTATATATTTCCAGTGGGGCATGTCTTATCCTTTTTTATATAT CTC
CAGTTCTCAAAACCTTGTCTTACACAGCAAACATTAATCAGAGGTTAATCACTTATGGATTTAGTTTCACTCTACTGCCATCTCTCCAAAATGCTTACAGTAAAGAGGCTACACTTACTAAATTCAAA
CTTAGAACTTTGTAACTTAGGACCTGTCTGTTGGAGGAGGTAAGTTGCTTAAGCTTCTCCCTCTATTGGGCATTATATGTAGAGTGCTCTAGGTTCTGCCTGCTAGCTCCTTGATGACATTTGCC TGC
TTTAGCAGGACACTGACTCTTCAGCTCAAGCAGCTGTTACCATGCTGGCTATTTCCTTTTGCATTCATAGGTTTTAGATATGGAAGAAAGTTATACCCAGAGAGAATGGGGGGATAAATGATAATCTT
CTACCATTTTTGACCTTAGTCTGGTCCAGCATTATAGTGTGATTTTGAGGAGGAGATTGTGATTTCCCTGGCTAGTATTATCTAGTATTGCCTGACAATGCAAAATGGACCCCTAGGCTGTCTT ATG
TAAGTTGGAAGGGGGCGGATTGGGGGTGGGGTGTGTGTGTGTGTATCTACAATCCAGTGACATTTAACAACATTCACAATGTCTTAAAACCAT cGCCACTACTCGTTTCCAGAACCTTTTTGTTACCT
CAAACAGAAACAGTACTCATTAAGCAAGAACTCCTCATTCCTCCCTATGCCCAGTTCCTCGTAACCCCTATTCTACTTTCTCTCTATGAATTTGCCTATTCCAGGTACCTCATGTAAGGGGAGTC ATA
CACTATCTGGCCTTTTTTGTCTGGCTTATTTTACTTAGCACATTTTTGAGGTTTATCCATGTGGTGGTGGCATGTATCTGTATTTTATTCCTTTTTATGGCTGAATAATATTTCATTGTATGAATATA
TCATTTTGTTTATCTCCTCATCAGTGATGGACACTTGGATTATTTCCACCTTTTGGTTGTTGTAAATAATGCTGCAGTGAACATTGGTGTACAAATATCTGTTGGAGCCCCTGCTTTCAGTGCCT TAG
GGTAATGCACAGGAGTAGAATTACTGGATCATATGGTAATTCTGTGTTTACTTTTTGAGGAACTGCCAAACTGTTTTGCACAGTGACTGCACCATTTTACATTTCCCACCAGCAAAGCACAAGGGTTC
CAGTTTTGCCACATCTTTTCCAACACTTGTTATTTTTTCTTTTTTAATTTTTAAAGTAACAATCTTGGGCTGGGCACGGTGGCTCATGCCTGTAATCCCAGCACTTTGGGAGGCCGAGGCGGGCG GAT
CACCTGAGGTCAGGAGTTCGAGACCAGCCTGGCCAACATGGTGAAACCCCGTCTCTACTAAAAGTACAAAAAAAATTCGCTGGGCTTGTTGGCGCATGCCTGTAATCCCAGCTACTTGGGAGGTTGAG
GCAGGAGAATCGCTTGAaCTCGGGAGGCAGAGGTTGCAGTGAGCTGAGATTGCGCCATTGCACTCCACAAGAGTGAGACTTCGTCTCAAAAAAATAAATAAATAAAATAACAACC CTAATGACTGTGAA
GTGGTTGTAGGCTTTTTGCATTTTAAAAAATTATTATTTTTACTATTTTAGAGATGGCGGGGGGGGGGTCTCACTATGTTTACCAGGTTGGTCTCGAACTCCTGGCCTCCATTGATCCTCCCATTTTA
GCCTCCTAAAGTACTGGGATTACAGGCTTGAGCCACTGCGCCCAGCCCTCCTTTTCTTCTTATTGCTACTTAGAT GTAATGGGCAAACCAGAACCTTTGAATTTGGCTATATTTGGATCTGATGGGTT
TTTTCCTCCCTCTTAAACTTGTGTATAATGTTGTTCAAAGGATACATTATGTACACAATTAATGACATCTTTTTAAAA AGAACACTTTCTTTCATCCTTGTGAGGTGTAAGAAGAAAAATAAGCAAA
AAAAAAAATGAATAAAGTTTTTCTATAAGTTGATACACACAGTTGTAGTTACTTTATACTCATACCCTTTTTTTGTGTTGTGAGTGACACAGTCTCACTCCATTGCCCAGGCTGGAGTGCAGTGG TGC
GATCTCCACTCACCGTAGCCTTGACCTCCCAGGCACAAGCCATCTTCCCTCCTGAACCTGCTGAGTAGCTGGGACTACAGGTGTGTGCCACCACACTCAGCTAATTTTTGTATTTTTGTAGAGATGG
GGTTTTGCCATGTTGCCCAGGCTGATGTCGAAGTGCTGAGCTCAAGTGATTCTCCCGCCTTGGCCTCCCAAAATGCTGGGATTATAGGCATGAGCCAC TGTGCCTGGCCTCTCATACTTTTTTATTCT
ATAATGTATTTAGGGCCAGCCTCTGTGGCTCATGCCTATAATCCCAGCACTTTGGGAGGCCAAGGAGGGAGGATTGCTTGAGGCCAGGAGTTCAAAACCAGCCTGGGCATCATAGTGAG ACCCCATCT
CTAAAACCAAAAAAACTAGTCAGGCATGTGGTATGCGCCTGTAGTCCCAGCTACTTGGTAGGCTGAGGAGAGAGGATTGTTTGAGTCCAGGAATTGGAGGCTGCAATAAGCTATGATTGTGCCATGGT
GCTCTAGCCTGGGCAACAGAACAAGATGCTGTCTCTTGAAAAAAAAAAAAAAGAAAAAGAAAAGATGAATAGTCTGTCTGAAATGGCCGGCAGTCGCAGCTGCTGACCTGACCTGCATCTACTGT ATA
AGTCAAGTAGTTCAACTTTTTATTATAATGTCATGGCTTTTCTTTGCTTCTTGGGAGCGCTTCCAGCATCACTAGTGGTACTTCATATGGGACCCATGCTGTTATTCAAAGTTTATGGTAT TGTACTG
TACATGATGAAAAATACAGAAGACCCATGAGAGATgACTTTTTTTTTTTTGAGACATAGTCTTGCTCTGTCGCCCAGGCTGGAGTGCAATGGCATGATCTCAGCTCTCCTCTGCCTCCTGGGTTCAAG
TGATTCTCCTGCCTCAGCCTCCCTAGTAGCTGGGATTGCAGGCACTCGCTACCACAACCGGCTAATGTTTATATTTTTAGTAGAGACAGGGTTTCATCATGTTGGCCAGGCTGGTCTCGAA CTCCTGA
CCTCAAGTAATCCACCAGCTCGGCCTCCCAAAGTGCTAAGATGACAGGAATGAGCCAATGTGCCCAGCCAAGAGATATCACTTTTTACTGCAATAGGAAATGTACTGGAGAGACCAACTGCTCAG AGG
GACACAAGGCCTTTTAAGTGGACACATGCAACTTGTAACACTTGAGTTCACTGCAATAGAAAGAAGAGGTGGCTATGAAATTACTGTAGTAATATAGTTTGTACAACAGGTTTGTTTTCTT TTGAGGC

AAAGTCTCACTCTGTCACCCAGGCTGGAGTGCAGTAGCAAGATCTCAGCTCACTGCAACCTCTGCCTCTCAGGTTCAAGCGATTCTCCTGCCTCAGTCTCCCAAGTAGCTAGGATTACAGGCCTG TGC
CACTATGCCCAGCTAATTTTTGTATTTTTAGTAGAGATGGGGTTTCACCCTGTTGCCCAGGGTGGTCTCAAACTCCTGGCCTCAAGCGATCTACCCGCATCATCCTCCCAAAGTGCTGGGC TTATAGG
CATGAGCTACCGCACCCAGCCAACATCATTTAATTTTATGCAGTTATTTATTTATTTAATTAAAAAA AttTTTTTTTTTTGAGATGGAGTCTCACTGTCTTGCCCAGGCTGGAGTGCAGTGGCTTAAT
CTTGGCTCACTGTACTCACTGCAACCTCTGTCTCCCAGGTTCAAGTGATTCTCCTGCCTCAGTCTCCTGAGTAGCTGTATAACACCTGGCTAATTTTTTGTATTTTTAGTAGAGACAGGGT TTCACCA
TGTTGGACAGGCTGGTCTCAACCTCCTGACCTCAAGTGATCCACCCGCCTCTGCCTCCCCAAGTGCTGGGATTACAGATGTGAGCCACTGCACCTGGCTTCAGTTATTTAATATGCTGCAGTTAT TTA
ATATACTGCATCTTAACATTTGTTTACATTTCTCTCAAGTGTGAATGTGTGTCTGTGCAAATGGTGTTATGTATGGTCTGTAAGTATATGCATAAGTTTTGATTTTTTTTTTCTTTGAGAC CGAGTCT
AGCTTTGTCGCCcAGGCTGGAGTGCAGTGGTGCGAACTTGGCTCACTGCAGCCCCTGCCTCCTGGGTTCAAGTGATTCTC TTGCCTCAGCCTCCCGAGTAGCTGGGATTACAGgcaccggccaccatg
cccagttaattttgtatttttagtagagacggggcttcactgtgttggccaggctggtctcaaactcctgaccttatgatccgcccatctcggcctcccaaagttctgggattacaggtg tgagtca
ctacgcctggcctatttttatttattattgtttttttgagatggagtcttgctctgtctcccaggttggagtgcagtggcatgatgttggcttactgcagactctgcctcctgggttcaagcgat tct
cctgcctcagcctcccgagtagctgggattacagGTACTCATCACCCTGCCCAGCTAATTTTTTATATCATTTATTTATTTATTTATTTATTTATTTA TttatttatTTTAGAGATGGAGTTTC
GCTCTTGTTGCCCAGGCTAGAGTGCAGTGGCGCGATCTCGGCTCACCA C

Data Note S2. Sequence information of the manually inspected TASV deletion regions

NAHR-ARMDs

Alu-Alu recombination mediated deletion

>NAHR_ 1_Scaffold809 654223-656521
KSD_NAHR_1

>Scaffold809 654223 656521_NAHR
ACATGTCTGGTTGGCCACAACCCAGTATTCAGAATTAGTCACATGGACACTCCTAACTTCGAGGGAGTCAGGAAGAGTAGCATCACCACGTAGCTGGAGGGGAGAGAACAGGAAAAGCTGATGAGT TC
ACTAATGTCTGTCACAGAAGGTATATTGTCTCTGGAAAGCTTCCATTCCTTTTTAAAAATGGTATTTGTACCCTAGGTAATGCC TCAAAGTGTCATTTGTCTCTACCCTGTTGAGCCCTTACAAATAA
TAATAAATTGTATAATTGGATACAAATTATAAGATTATAAAAAGAACGATAAGTATGAAACATACAAAGATTATTAAAAAATGAAGAACTGAATTTACAATATATGCTTCAG CAAATCTATACTGGAG
TCTGTTGGGGACAGTTGTGTGAGTTGATCAACTGAAACACCCTGCCCTTTTTTCTTTTCCTAATTATTTTTCTTTTTACTAACC TGTTCGATATCTTGCCTGATTTGATGGAATCAAATGACTGATTT
GCTGTCATCCAAGCAAAACCACCTACATGACATGGACAAGAAACCTATGAAGATGATTTCTCTTCAGTTTGCTAAATTAGAAAATGTATGCAAATTAACTCAGCGAGGCCTGTTAAC CACTCTTAATT
CAATCTCGAGAGTCCTCCTTTTAATATGTCAAGTGTTCCACTCACATTGCAATTTTCTGCAGTTAGACTTTATATCCTATTATA TCTATGTCTATTTAAGTGTATGTACACAAATACACACATATATT
TAAGAGAATTAAGAGGAATGGGCATTGTTTTATTTTAGAGCCCTAGGAAACTCCAAAACATTTTAGGATGACCTACTTTTAAATTTTTCCTATCTTCTTACTCTTTGCCCCTACCAGTCAAAGGA ATT
TAAGATATGTTTTGAAGAATCACAAGCCATGAAAGAAATCTGC CTGGGTGCAGTGGCTCACACCTGTAATCCCAGCACTTTGGG AGGCCGAGGCAGGCAGATCACAAGGTCAGGAGTTCGAGACCAGC
CTGACTAATATGGTGAAACCCCATCTCTACTGAAAATACAAAAATTAGCTGGGCGT GGTAGCAGGCGCCTGTAATCCCAGCTACTTGGGAGGCTAAGGGAGGAGAATCGCTTGAACCTGGAAGGCGGA
GGTTGCAGTGAGCTGAGATCACACCACTGCACCCCAGCCTGGGCAACAGAGTAAGGCTTTGTCTCAAAAAAAAAAAAAAAAAAAAA TCTTCAAGTCTAGTTAAGCCTTGATTAATCATGATGCTTAGG
AAAGACAGTTTTCTAGTTATTTTAATGAGTTTAAGAAGAAACCTATTTTAACCTGGTT GCTGGGAACTTTCCAAAAATAGGTAAGTGTACTTTTCTGCACACCTCATCCTTTAATAAATACA GTATCG
TGAATGTAACTGAAGCTAGTGAATCAGGGTTTCATCTTGCTGAAAAGCTAGTATCATTGTATATATTGTCCTGTAGATGATAAAGTTTTTTCTTTGGTGTAATGAACTTAGATGACTCATTTCTA CAG
ACTTAAAATACACTTAGCTTATTTTAGCAGAAATAGTTTTATGTTCAAAGTATGAACT AATTTGGCAAATACAAATTGGAGAAGAAAGTGTATTCAAATGGATTTCAAAATAATAACACCTGTT ATTC
GGGGCACTTACTATATGTCAGACACTTTGATGAACAACCTTAAATACATTACTTACCGCTGTTATATTTTTCCTCCGCTTATTTACTGAATCACCTCCCCAGTGTTCAAAATCATGCTCTACTCA CTG
AAGGATGTGCAGTTCTGTAACAAATTATATAATACATTAATAAGATGTTTTTTAACTT AGTCACAGGAAATCTTTTCAAATTACTGATTAGCATTCAATAGGATTGCTATTTTCATTTCCTATCCTGT
ATGTTAAAGATTAATGATTAAAATAAATCCTGAAAATGCTAATAACATTCTAAGTAGAACATCATTAAAGAAAAATAATTGGAGTTATATCGCATTACTAAACCATTATTAGGCATAGGCATTTA ATT
AGATATTTCATCAAAATGCAATTTTGGAGTACCAGGGgTTTTTTTTTGTTTTTTTTTTTTTTGGCCTGAAAGACTAGGAATCTGGAATCTAATGAGAACTATTTTTCTTTGCCGTTTGGGCAACATGAT
GGGACCTGGACCCAAGGAAGTTTTACAGACCCTGGTCGGGCAGTCTGATGCCACTGAGTGGTGACTTCCAGGGAGTCAGTCAGTTTTGCCCTGTTCCATACAAACCACCTTCAACCTCAGC C

353	24.2	9.0	6.6	S	12	144	(2154)	C	MLT1I	LTR/ERVL-MaLR	(308)	136	1	1
2373	10.4	0.0	0.0	S	940	1238	(1060)	+	AluSg	SINE/Alu	3	301	(9)	2
189	26.5	4.2	4.2	S	1645	1715	(583)	C	MIRb	SINE/MIR	(0)	268	198	3
19	4.5	0.0	0.0	S	2087	2109	(189)	+	(T)n	Simple_repeat	1	23	(0)	4
226	20.0	12.0	0.0	S	2249	2298	(0)	+	MamSINE1	SINE/tRNA-RTE	75	130	(160)	5

353	24.2	9.0	6.6	hg19	12	144	(2483)	C	MLT1I	LTR/ERVL-MaLR	(308)	136	1	6
2427	9.5	0.0	0.3	hg19	940	1243	(1384)	+	AluSg	SINE/Alu	3	305	(5)	7
2362	10.9	0.0	0.0	hg19	1264	1566	(1061)	+	AluSx1	SINE/Alu	1	303	(9)	8
189	26.5	4.2	4.2	hg19	1973	2043	(584)	C	MIRb	SINE/MIR	(0)	268	198	9
21	4.1	0.0	0.0	hg19	2414	2438	(189)	+	(T)n	Simple_repeat	1	25	(0)	10
226	20.0	12.0	0.0	hg19	2578	2627	(0)	+	MamSINE1	SINE/tRNA-RTE	75	130	(160)	11

>hg19 Chr8:99463925-99466551
ACATGTCTGGTTGGCCACAACCCAGTATTCAGAATTAGTCACATGGACACTCCTAACTTCGAGGGAGTCAGGAAGAGTAGCATCACCACGTAGCTGGAGGGGAGAGAACAGGAAAAGCTGATGAGT TC
ACTAATGTCTGTCACAGAAGGTATATTGTCTCTGGAAAGCTTCCATTCCTTTTTAAAAATGGTATTTGTACCCTAGGTAATGCCTCAAAGTGTCATTTGTCTCTACCCTGTTGAGCCCTTACAAA TAA
TAATAAATTGTATAATTGGATACAAATTATAAGATTATAAAAAGAACGATAAGTATGAAACATACAAAGATTATTAAAAAATGAAGAACTGAATTTACAATATATGCTTCAGCAAATCTATACTGG AG
TCTGTTGGGGACAGTTGTGTGAGTTGATCAACTGAAACACCCTGCCCTTTTTTCTTTTCCTAATTATTTTTCTTTTTACTAACCTGTTCGATATCTTGCCTGATTTGATGGAATCAAATGACTGA TTT
GCTGTCATCCAAGCAAAACCACCTACATGACATGGACAAGAAACCTATGAAGATGATTTCTCTTCAGTTTGCTAAATTAGAAAATGTATGCAAATTAACTCAGCGAGGCCTGTTAACCACTCTTAA TT
CAATCTCGAGAGTCCTCCTTTTAATATGTCAAGTGTTCCACTCACATTGCAATTTTCTGCAGTTAGACTTTATATCCTATTATATCTATGTCTATTTAAGTGTATGTACACAAATACACACATAT ATT

TAAGAGAATTAAGAGGAATGGGCATTGTTTTATTTTAGAGCCCTAGGAAACTCCAAAACATTTTAGGATGACCTACTTTTAAATTTTTCCTATCTTCTTACTCTTTGCCCCTACCAGTCAAAGGAA TT
TAAGATATGTTTTGAAGAATCACAAGCCATGAAAGAAATCTGC CTGGGTGCAGTGGCTCACACCTGTAATCCCAGCACTTTGGGAGGCCGAGGCAGGCAGATCACAAGGTCAGGAGTTCGAGACCAGC
CTGACTAATATGGTGAAACCCCATCTCTACTGAAAATACAAAAATTAGCTGGGCGTGgtggcacatgcccgtaatcccagctactcaggaggctgaggcaggagaatcacttgaacccaggaggcaga
aggttgcagtgggccaagatcacaccactgcgctccagcctgggtgacagagcgagactctgtctcgaaaaaaaaaaaaaaaaaaaaaaaacaagaaagaaa tcaagtctaggccaggcttggtggct
catgcctgtaatcccagcactttgggaggctgaggccagcagatcacttgaggtcaagagttcaagaccagcctgaccaacatggtgaaaccctgtctctactaaaaatacaaaaattagctgat cgt
gGTAGCAGGCGCCTGTAATCCCAGCTACTTGGGAGGCTAAGGGAGGAGAATCGCTTGAACCTGGAAGGCGGAGGTTGCAGTGAGCTGAGATCACACCACTGCACCCCAGCCTGGGCAACAGAGTAAGG
CTTTGTCTCAAAAAAAAAAAAAAAAAAAAATCTTCAAGTCTAGTTAAGCCTTGATTAATCATGATGCTTAGGAAAGACAGTTTTCTAGTTATTTTAATGAGTTTAAGAAGAAACCTATTTTAACCTGG
TTGCTGGGAACTTTCCAAAAATAGGTAAGTGTACTTTTCTGCACACCTCATCCTTTAATAAATACAGTATCGTGAATGTAACTGAAGCTAGTGAATCAGGGTTTCATCTTGCTGAAAAGCTAGTA TCA
TTGTATATATTGTCCTGTAGATGATAAAGTTTTTTCTTTGGT GTAATGAACTTAGATGACTCATTTCTACAGACTTAAAATACACTTAGCTTATTTTAGCAGAAATAGTTTTATGTTCAAAGTATGAA
CTAATTTGGCAAATACAAATTGGAGAAGAAAGTGTATTCAAATGGATTTCAAAATAATAACACCTGTTATTCGGGGCACTTACTATATGTCAGACACTTTGATGAACAACCTTAAATACATTACT TAC
CGCTGTTATATTTTTCCTCCGCTTATTTACTGAATCACCTCC CCAGTGTTCAAAATCATGCTCTACTCACTGAAGGATGTGCAGTTCTGTAACAAATTATATAATACATTAATAAGATGTTTTTTAAC
TTAGTCACAGGAAATCTTTTCAAATTACTGATTAGCATTCAATAGGATTGCTATTTTCATTTCCTATCCTGTATGTTAAAGATTAATGATTAAAATAAATCCTGAAAATGCTAATAACATTCTAA GTA
GAACATCATTAAAGAAAAATAATTGGAGTTATATCGCATTAC TAAACCATTATTAGGCATAGGCATTTAATTAGATATTTCATCAAAATGCAATTTTGGAGTACCAGG GttTTTTTTTTTGTTTTTTT
TTTTTTGGCCTGAAAGACTAGGAATCTGGAATCTAATGAGAACTATTTTTCTTTGCCGTTTGGGCAACATGATGGGACCTGGACCCAAGGAAGTTTTACAGACCCTGGTCGGGCAGTCTGATGCC ACT
GAGTGGTGACTTCCAGGGAGTCAGTCAGTTTTGCCCTGTTCC ATACAAACCACCTTCAACCTCAGCC

>Chimp
ACATGTCTGGTTGGCCACAACCCAGTATTgAGAATTAGTCACATGGcCACTCCTAACTTCGAGGGAGTCAGGAAGAGTAGCATCACCA tGTAGCTGGAGGGGAGAGAACAGGAAAAGCTGATGAGTTC
ACTAATGTCTGTCACAGAAGGTATATTGTCTCTGGAAAGCTTCCATTCCTTTTTAAAAATGGTATTTGTACCCTAGGTAATGCCTCAAAGTGTC ATTTGTCTCTACCCTGTTGAGCCCTTACAAATAA
TAAATTGTATAATTGGATACAAATTATAAGATTATAAAAAGAACGATAAGTATGAAACATACAAAGATTATTAAAAAATGAAGAACTGAATTTACAATATATGCTTCAGCAAATCTATACTGGAG TCT
GTTGGGGACAGTTGTGTGAGTTGATCAACTGAAACACCCTGCCCTTTTTTCTTTTCCTAATTATTTTTCTTTTTACTAACCTGTTCGATATCTT GCCTGATTTGtTGGAATCAAATGACTGATTTGCT
GTCATCCAAGCAAAACCACCTACATGACATGGACAAGAAACCTATGAAGATGATTTCTCTTCAGTTTGCTAAATTAGAAAATGTATGCAAATTAA tTCAGCGAGGCCTGTTAACCACTCAATTCAATC
TCGAGAGTCCTCCTTTTAATATGTCAAGTGTTCCACTCACATTGCAATTTTCTGC cGTTAGACTTTATATCCTATTATATCTATGTCTATTTAAGTGTATGTACACAAATACATATATTTAAGAGAAT
TAAGAGGAATGGGCATTGTTTTATTTTAGAGCCCTAGGAAACTCCAAAACATTTTAGGATGACCTACTTTTAAATTTTTCCTATCTTCTTACTCTTTGCCCCTACCAGTCAAAGGAATTTAAGAT ATG
TTTTGAAGAATCACAAGCCATGAAAGAAATCTGCCTGGGTGCAGTGGCTCACACCTGTAATCCCAGCACTTTGGGAGGCCGAGGCAGGCAGATC ACAAGGTCAGGAGTTCGAGACCAGCCTGACTAAT
ATGGTGAAACCCCATCTCTAtTaAAAATACAAAAATTAGCTGGGCGTGgtggcacatgcccgtaatcccagctactcaggaggctgaggcaggagaatcacttgaacccaggaggcagaaggttgcag
tgggccaagatcacaccactgcgctccagcctgggcgacagagcaagactctgtctcggaaaaaaaaaaaaaaaaaacaagaaagaaatctagt ctaggccaggcttggtggctcatgcctgtaatcc
cagcactttgggaggctgaggccagcagatcacttgaggtcaagagttcaagaccagcctgaccaacatggtgaaaccctgtctctactaaaaatacaaaaattagctgattgtg GTAGCAGGCGCCT
GTAATCCCAGCTACTTGGGAGGCTAAGGGAGGAGAATCGCTTGAACCTGGAAGGCGGAGGTTGCAGTGAGCTGAGATCACACCACTGCACCCCA GCCTGGGCAACAGAGTAAGGCTTTGTCTCAAAAA
AAAAAAAAAAAAATCTTCAAGTCTAGTTAAGCCTTGATTAATCATGA cGCTTAGGAAAGACAGTTTTCTAGTTATTTTAATGAGTTTAAGAAGAAACCTATTTTAACCTGGTTGCTGGGAACTTTCCA
AAAATAGGTAAGTGTACTTTTCTGCACACC cCATCCTTTAATAAATACAGTATCGTGAATGTAACTGAAGCTAGTGAATCAGGGTTTCATCTTG CTGAAAAGCTAGcATCATTGTATATATTGTCCTG
TAGATGATAAAGTTTTTTCTTTGGTGTAATGAACTTAGATGACTCATTTCTACAGACTTA cAATACACTTAGCTTATTTTAGCAGAAATAGTTTTATGTTCAAAGTATGA gCTAATTTGGCAAATACA
AATTGGAGAAGAAAGTGTATTCAAATGGATTTCAAAATAATAACACCTGTTATTCGGGGCACTTACTATATGTCAGAC gCTTTGATGAACAACCTTAAATACATTACTTACCGCTGTTATATTTTTCC
TCCGCTTATTTACTGAATCACCTCCCCAGTGTTCAAAATCATGCT tTACTCACTGAAGGATGTGCAGTTCTGTAACAAATTATATAATACATTAATAAGATGTTTTTTAACTTAGTCACAGGAAATCT
TTTCAAATTACTGATTAGCATTCAATAGGATTGCTATTTTCATTTCCTATCCTGTATGTTAAAGATTAATGATTAAAATAAATCCTGAAAATGC TAATAACATTCTAAGTAGAAtATCATTAAAGAAA
AATAATTGGAGTTATATCaCATTACTAAACCATTATTAGGCATAGGCATTTAATTAGATATTTCATCAAAATGCAATTTTGGAGTACCAGG GttTTTTTTTTTGgtttttttTTTTTTTTTTTTTGG
CCTGAAAGACTAGGAATCTGGAATCTAATGAGAACTATTTTTCTTTGCCGTTTGGGCAACATGATGGGACCTGGACCCAAGGAAGTTTTACAGACCCTGGT tGGGCAGTCTGATGCCACTGAGTGGTG
ACTTCCAGGGAGTCAGTCAGTTTTGCCCTaTTCCATACAAACCACCTTCAACCTCAGCC

Homologous Sequences
CGTGGT

>NAHR_ 2_ Scaffold3189 441568 443877
KSD_NAHR_2
>Scaffold3189 441568 443877 SINE/Alu AluY NAHR 2309

CTATTGGAGCCATTTTCCCAACAGCATGCGCTTACTTTATGTCTCTATCACATTTTGACAATTCTCACGATATTTCAAACTTTTTCATTAATTATTATAACTGTTACGGTGATCTGTGATCAGTGA AC
TTTGATCACTATTATAATTGTTTAGGGGCACCACAAACTGCACCCATGTAA GATGGTAAACTTAATAAACATGTGTGCTCCTACTGATCCACTGACCAGTTGTTTCTAGTCTCTCTGCCTCTCCTCAG
ACCTCTCTATTCCCTGAGACACAACAATATTGGAATTAGGCCAATTAATAACCCTAGAATGGCCTCCAAGTGATCAAGTGAAAGGAAAAGTCACATGTCTCTCACTTTAAATCAAATCTAGAATT GAT
TAAGCTTAGTGAGAAAGACAGATCAAAAGCTGAGATAAGCTGAAAGCTATG CTTCTTGTGACAAAACAGCCTTAGATTGGAGAGAGATACATTTAGGACTTTAATAGCTAGAGAGAAGAAGGCAGTGG
CTGGTTTCAAAAGACAGGCTGACTTGTTTGTTAGGGGCTAAAGCAGCTGGTGACTTTAAGTGGAAACCAATGTTCATTTACCATTCTGAAAATCCGAGAGCCCTTAACAATTATGCTCAATCAAT TCT
GCCTGTGCTCTATAAATGAAACATCACAGCCTGAAGAAGAAAGCACATCTG TTTACAGCATAGTTTACTGAATATTTTAAGTGCACTATTGAGACCTACTGCTCAAAAAAGATTCCTTTTGAAGTATT
ACTGCTCTCCAACAGTGCAACTGGTCACCCAAGAGCTCTGATGAAGATGTACAAGATTAATGTTGTTTCCATGCCTGCTAACACAACGTCTATTCCGCTGTCCATGGATCACAGAGCTATTTCTA CTT
TCAGGTCTTACTTAAGAAATACATTTCAGCCAGGTGTGGTGGCTCACACCTGTAATCCCAGGACTTTGGGAGGCTGAGGCGGGTGGATCACGAGGTCAGGAGTTCGAGGACCATCCTGACTAACACAG
TGAAACCCCGTCTCTACTAAAAATCCAAAAAAATTAGCTGGGTGTGTTGGCGGGTGCCTGTAGTCCCAGCTACTCGGGAGGCTGAGGCA GGCGGATCACGAGGTCAGGAGATCgAGACCAtCCTGGCT
AACAcaGTGAAACCCCGTCTCTACTAAAAATcCAAAAAAATTAGCTGGGtGTGGTGGCGGGCGCCtGTAcTCCCAGcTACTCaGGAGGCTGAGGCAGGgGAATgGCgTGAACCCGGGAGGCAGAGGTT
GCAGTGAGCCAAGATCACGCCACTGCACTCCAGCCTGGCGACAGAGTCAGACTCCGTCTCAAAAAAAAAAAAAAGAAAAGAAAAAAACAAAA CTGCCTCTGCAACTGGCACATTTCCCAAAAAAGCATAG
CTAAGAGATGAAGCTCAAGACAGTGAAAGGGCCCAGATTGCACAGCGGTG TGAAAGCCAtGCTGAGGGTTTGGGACTTTATCCGGGGAGCAATAGGAAGCCATTGTTGGCTGACATCACTGCTATGCA
TTGCAACTCTGGCGACCATGTAGAGAATGAGGAAAAGGGAGAGTGCATTTGGGAAACCACGTGAGTGGTATTATAATTCTCCAGGCACAAGCTAGTATCATCTTATTTTAGGACGGTGGCAGTGG AGA
GGGACACATTCAACAGGACTTGTTGATGGATTGGAATTGAGATACAGTAG TAAAGTAGCAAAGTCACCCTGCTTCACTGGGTGAATGGTAAAATGTGAACACTAGAAGAGGTATAAATTTTAGGGTAA
GAGAGTGAGATTAATTTTTTATATATGTAGTTGTATAATCTAATCTAATAAGGAGGCAAGGTCGGGTAGAAAGGATTCATGCCTTAATGCTAATGCTTTTGGACTAAATAACTAAGTTTCCATTC AGT
TATAATGGTCTTCCACAAGGAAGTGCCATCTTTTGCAGAATTTATTCCCC CAAAGAGCTCGTGGCTGAAATATAACTCCTGGAATTTGCCTGGAATTTGAATTCCTCAGAATCATCCACACCAAGTTA
ATAATTAAAGGTTCCTCATACTGTCTACATTTGTTTTTTTCACCTGTGTACAGGATTCTGATTTCACAAAATCCTCCCAGCAGTATTATTTAACCTAGTGTCAAAGAAAGGTGTGTATATTCCAG CAA
CCCAGAGGCAAGTCTCTACCAGCTCAAGAAGGAAAAAGGTGGGAGGCTGC AGAAGCCACAGAAACTGACCCTGCCCAGAGCCAAAGCAGACAAGAGGGCTCCTGCAAAGTTAATCTTCTTTGAGATTT
TCTCA

2832	14.7	2.9	0.2	S	1	449	(1860)	+	Tigger1	DNA/TcMar-Tigger	100	560	(1858)	1	*
3088	12.8	3.8	0.4	S	444	923	(1386)	+	Tigger1	DNA/TcMar-Tigger	983	1478	(940)	2	
1013	9.1	0.0	0.8	S	925	1056	(1253)	+	AluYj4	SINE/Alu	2	132	(179)	3	
2314	9.0	0.7	0.7	S	1061	1370	(939)	+	AluY	SINE/Alu	2	311	(0)	4	
268	33.1	17.9	0.7	S	1410	1730	(579)	C	L2	LINE/L2	(466)	2953	2570	5	

2832	14.7	2.9	0.2	hg19	1	449	(2038)	+	Tigger1	DNA/TcMar-Tigger	100	560	(1858)	6	*
3088	12.8	3.8	0.4	hg19	444	923	(1564)	+	Tigger1	DNA/TcMar-Tigger	983	1478	(940)	7	
2322	6.9	0.3	0.7	hg19	925	1214	(1273)	+	AluY	SINE/Alu	2	290	(21)	8	
2558	6.1	0.3	0.3	hg19	1239	1548	(939)	+	AluSc8	SINE/Alu	2	311	(0)	9	
279	32.9	17.9	0.7	hg19	1588	1908	(579)	C	L2	LINE/L2	(466)	2953	2570	10	

>hg19 Chr11:130188112-130190598

CTATTGGAGCCATTTTCCCAACAGCATGCGCTTACTTTATGTCTCTATCACATTTTGACAATTCTCACGATATTTCAAACTTTTTCATTAATTATTATAACTGTTACGGTGATCTGTGATCAGTGAAC
TTTGATCACTATTATAATTGTTTAGGGGCACCACAAACTGCACCCATGTAAGATGGTAAACTTAATAAACATGTGTGCTCCTACTGATCCACTGACCAGTTGTTTCTAGTCTCTCTGCCTCTCCT CAG
ACCTCTCTATTCCCTGAGACACAACAATATTGGAATTAGGCCAATTAAT AACCCTAGAATGGCCTCCAAGTGATCAAGTGAAAGGAAAAGTCACATGTCTCTCACTTTAAATCAAATCTAGAATTGAT
TAAGCTTAGTGAGAAAGACAGATCAAAAGCTGAGATAAGCTGAAAGCTATGCTTCTTGTGACAAAACAGCCTTAGATTGGAGAGAGATACATTTAGGACTTTAATAGCTAGAGAGAAGAAGGCAG TGG
CTGGTTTCAAAAGACAGGCTGACTTGTTTGTTAGGGGCTAAAGCAGCTG GTGACTTTAAGTGGAAACCAATGTTCATTTACCATTCTGAAAATCCGAGAGCCCTTAACAATTATGCTCAATCAATTCT
GCCTGTGCTCTATAAATGAAACATCACAGCCTGAAGAAGAAAGCACATCTGTTTACAGCATAGTTTACTGAATATTTTAAGTGCACTATTGAGACCTACTGCTCAAAAAAGATTCCTTTTGAAGT ATT
ACTGCTCTCCAACAGTGCAACTGGTCACCCAAGAGCTCTGATGAAGATG TACAAGATTAATGTTGTTTCCATGCCTGCTAACACAACGTCTATTCCGCTGTCCATGGATCACAGA GCTATTTCTACTT
TCAGGTCTTACTTAAGAAATACATTTCAGCCAGGTGTGGTGGCTCACACCTGTAATCCCAGGACTTTGGGAGGCTGAGGCGGGTGGATCACGAGGTCAGGAGTTCGAGGACCATCCTGACTAACACAG
TGAAACCCCGTCTCTACTAAAAATCCAAAAAAATTAGCTGGGTGTGTTG GCGGGTGCCTGTAGTCCCAGCTACTCGGGAGGCTGAGGCAGgagaatggcgtgaacccgggaggcagagctttcagtga
gctgagatcgcccaccgcactccagcctgggtgacagcgagactccgtctcaaaaaaaaa ggggggggggaggggaataagaagccaggcgcggtggctcacgcctgtaatcccagcactttagg

```
aggctgaggcggGCGGATCACGAGGTCAGGAGATCaAGACCAcCCTGGCTAACAtgGTGAAACCCCGTCTCTACTAAAAATaCAAAAAAATTAGCTGGGcGTGGTGGCGGGCGCCcGTAgTCCCAGgT
ACTCgGGAGGCTGAGGCAGGaGAATcGCtTGAACCCGGGAGGCAGAGGTTGCAGTGAGCCAAGATCACGCCACTGCACTCCAGCCTGGCGACAGAGTCAGACTCCGTCTCAAAAAAAAAAAAAGAAAA
GAAAAAACAAAA]CTGCCTCTGCAACTGGCACATTTCCCAAAAAAGCATAGCTAAGAGATGAAGCTCAAGACAGTGAAAGGGCCCAGATTGCACAGCGGTGTGAAAGCCAcGCTGAGGGTTTGGGACT
TTATCCGGGGAGCAATAGGAAGCCATTGTTGGCTGACATCACTGCTATGCATTGCAACTCTGGCGACCATGTAGAGAATGAGGAAAAGGGAGAGTGCATTTGGGAAACCACGTGAGTGGTATTAT AAT
TCTCCAGGCACAAGCTAGTATCATCTTATTTTAGGACGGTGGCAGTGGAGAGGGACACATTCA ACAGGACTTGTTGATGGATTGGAATTGAGATACAGTAGTAAAGTAGCAAAGTCACCCTGCTTCAC
TGGGTGAATGGTAAAATGTGAACACTAGAAGAGGTATAAATTTTAGGGTAAGAGAGTGAGATTAATTTTTTATATA TGTAGTTGTATAATCTAATCTAATAAGGAGGCAAGGTCGGGTAGAAAGGATT
CATGCCTTAATGCTAATGCTTTTGGACTAAATAACTAAGTTTCCATTCAGTTATAATGGTCTT CCACAAGGAAGTGCCATCTTTTGCAGAATTTATTCCCCCAAAGAGCTCGTGGCTGAAATATAACT
CCTGGAATTTGCCTGGAATTTGAATTCCTCAGAATCATCCACACCAAGTTAATAATTAAAGGTTCCTCATACTGTCTACATTTGTTTTTTTCACCTGTGTACAGGATTCTGATTTCACAAAATCC TCC
CAGCAGTATTATTTAACCTAGTGTCAAAGAAAGGTGTGTATATTCCAGCAACCCAGAGGCAAG TCTCTACCAGCTCAAGAAGGAAAAAGGTGGGAGGCTGCAGAAGCCACAGAAACTGACCCTGCCCA
GAGCCAAAGCAGACAAGAGGGCTCCTGCAAAGTTAATCTTCTTTGAGATTTTCTCA
```

2783	14.4	4.0	0.2	Chimp	1	444	(6382)	+	Tigger1	DNA/TcMar-Tigger	100	560	(1858)	1	*
8985	11.6	3.7	4.2	Chimp	439	919	(5907)	+	Tigger1	DNA/TcMar-Tigger	983	1464	(954)	2	
2412	8.2	0.3	0.7	Chimp	920	1212	(5614)	+	AluY	SINE/Alu	2	293	(18)	3	
8985	11.6	3.7	4.2	Chimp	1213	1231	(5595)	+	Tigger1	DNA/TcMar-Tigger	1465	1469	(949)	2	
2553	8.1	0.0	0.3	Chimp	1232	1527	(5299)	+	AluYk3	SINE/Alu	2	296	(15)	4	
8985	11.6	3.7	4.2	Chimp	1528	1877	(4949)	+	Tigger1	DNA/TcMar-Tigger	1470	1829	(589)	2	
1865	18.8	1.0	1.0	Chimp	1878	2170	(4656)	C	AluJr4	SINE/Alu	(19)	293	1	5	
8985	11.6	3.7	4.2	Chimp	2171	2642	(4184)	+	Tigger1	DNA/TcMar-Tigger	1830	2291	(127)	2	
1594	12.2	3.6	0.4	Chimp	2643	2865	(3961)	C	MER30	DNA/hAT-Charlie	(0)	230	1	6	
8985	11.6	3.7	4.2	Chimp	2866	2981	(3845)	+	Tigger1	DNA/TcMar-Tigger	2292	2416	(2)	2	
2538	9.3	0.0	0.0	Chimp	3097	3396	(3430)	C	AluSx1	SINE/Alu	(11)	301	2	7	
826	32.4	8.2	3.8	Chimp	3431	3601	(3225)	C	L2c	LINE/L2	(9)	3378	3216	8	
1889	15.7	10.3	2.7	Chimp	3602	3862	(2964)	+	MER47A	DNA/TcMar-Tigger	1	289	(77)	9	
2310	13.0	0.0	0.0	Chimp	3863	4163	(2663)	+	AluSz	SINE/Alu	1	301	(11)	10	
1889	15.7	10.3	2.7	Chimp	4164	4242	(2584)	+	MER47A	DNA/TcMar-Tigger	290	366	(0)	9	
826	32.4	8.2	3.8	Chimp	4243	4520	(2306)	C	L2c	LINE/L2	(172)	3215	2910	11	
1824	24.3	6.2	1.1	Chimp	4639	5137	(1689)	+	L1MC5	LINE/L1	7017	7540	(407)	12	
1122	18.2	1.3	2.6	Chimp	5180	5410	(1416)	+	L1MB8	LINE/L1	5942	6169	(9)	13	
17	30.4	0.0	0.0	Chimp	5411	5454	(1372)	+	(AT)n	Simple_repeat	1	44	(0)	14	
2666	7.5	0.3	0.0	Chimp	5584	5891	(935)	+	AluSx4	SINE/Alu	2	310	(0)	15	
352	33.1	17.9	0.7	Chimp	5932	6252	(574)	C	L2c	LINE/L2	(466)	2953	2570	8	

2832	14.7	2.9	0.2	S	1	449	(1860)	+	Tigger1	DNA/TcMar-Tigger	100	560	(1858)	1	*
3088	12.8	3.8	0.4	S	444	923	(1386)	+	Tigger1	DNA/TcMar-Tigger	983	1478	(940)	2	
1013	9.1	0.0	0.8	S	925	1056	(1253)	+	AluYj4	SINE/Alu	2	132	(179)	3	
2314	9.0	0.7	0.7	S	1061	1370	(939)	+	AluY	SINE/Alu	2	311	(0)	4	
268	33.1	17.9	0.7	S	1410	1730	(579)	C	L2	LINE/L2	(466)	2953	2570	5	

2832	14.7	2.9	0.2	hg19	1	449	(2038)	+	Tigger1	DNA/TcMar-Tigger	100	560	(1858)	6	*
3088	12.8	3.8	0.4	hg19	444	923	(1564)	+	Tigger1	DNA/TcMar-Tigger	983	1478	(940)	7	
2322	6.9	0.3	0.7	hg19	925	1214	(1273)	+	AluY	SINE/Alu	2	290	(21)	8	
2558	6.1	0.3	0.3	hg19	1239	1548	(939)	+	AluSc8	SINE/Alu	2	311	(0)	9	
279	32.9	17.9	0.7	hg19	1588	1908	(579)	C	L2	LINE/L2	(466)	2953	2570	10	

>Chimp

CTATTGGAGCCATTTTCCCAACAGCATGCaCTTACTTTATGTCTCTATCACATTTTGACAATTCTCACaATATTTCAAACTTTTTCATTAATTATaATAACTGTTAtGGTGATCTGTGATCAGTGAAC
TTTGATCACTATTgTAATTGTTTAGGGGCACCACAAACcGCACCCATGTAAGATGGTAAACTTAATAAACATGTGTGCTCgTACTGATCCACTGACCAGTTGTTTCTAGTCTgTCTGCCTCTCCTCAG
ACCTCTCTATTCCCTGAGACACAACAATATTGGAATTAGGCCAATTAATAACCCTAGAATGGCCTCCAA GTGATCAAGTGAAAGGAAAAGTCACAcGTCTCTCtCTTTAAATCTAGAATTGATTAAGC
TTAGTGAGAAAGACAGATCAAAAGCTGAGATAAGCTGAAAGCTATGCTTCTTGTGACAAAACAGCCTTAGATTGGA aAGAGATACATTTAGGACTTTcATAGCTAGAGAGAAGgcGGCAGTGGCTGGT
TTCAAAAGACAGGCTGACTcaTTTGTTAGGGGCTAAAGCAGCcGGTGACTTTAAGTGGAAACCAATGTTCATTTACCATTCTGAAAATCCGAGAGCCCTTAACAATTATGCTCAATC tATTCTGCCTG
TGCTCTATAAATGAAACATCACAGCCTGAAGgAGAAAGCACATCTGTTTACAGCATAGTTTACTGAATATTTTAAGcGCACTATTGAGACCTACTGCTCAAAAAAGATTCCTTTcGAAGTATTACTGC
TCTCCAACAGTGCAACTGGTCACCCAAGAGCTCTGATGAAGATGTACAAGATTAATGTTGTTTCCATGC CTGCTAACACAACGTCTATTCtGCTaTCCATGGATCACAGAGCTATTTCTACTTTCAGG
TCTTACTTAAGAAATACATTTCAGCCAGtTGTGGTGGCTCACACCTGTAATCCCAGcACTTTGGGAGGCTGAGGCGGGTGGATCACGAGGTCAGGAGTTCGAGGACCATCCTG gCTAACACAGaGAAA
CCCCGTCTCTACTAAAAATCCAAAAAAATTAGCTGGGTGTGTTGGCGGGTGCCTGTAGTCCCAGCTACT CaGGAGGCTGAGGCAGgagaatggcgtgaacccgggaggcggagctttcagcgagctga
gattgcccaccgcactccagcctgggtgacagagagatactccgtctcaaaaaaaaaaaaagcgggggggggaataagaagccaggtgtggtggttcacgcctgtaatcccagcactttaggaggc tga
ggcgggcggatcacgaggtcaggagatcaagaccatcctggctaacatggtgaaaccccgtctctacta aaaatacaaaaaaattagctgggcgtggtggcgggcgcccgtagtcccaggtactcggg
aggctgaggcaggagaacggcatgaatctgagaggcagaggttgcagtgagccgcgatagcaccactgcactccagcctggacgacagagcgagactccgtctcaaaaaaaaaaaaaaaatacatt tca
aaaggccttagctaccatgtatagtgattcctctgatggatctaggcaaagtgaaaaccttctggaaag gattcaccattctagatgtcattaagaacatccatgatttgtgggagaaggtcaaaata
tcaacattaacaggactttggaagaagttgattccacccctcatgactttgaagggttcaagactccagtagaggaagtaactacagatatgctagaaatagcaagagaactagaatttgaagtg gaa
cctgaagatatgattgaattgttgcaatctcataatgaaacttgaacaataaggagttgcttctggtgg agggccaaaggaagtgttttgtttttgaggcagggtcttgctctgtcacccaggctgg
agtgcagtggtgcaatcatacctcactgtaacctcaaactcctcggctcaaacaatattcctacctcagcctcccaattagctgggactacaggtgcatgccaccacgtctggctaattaaaaaa att
gttttgtagagatacggtcttgctatgttgcctaggctggtcttgaacttctggtcttaagtgagtcc cttaccttggcttctcaaaatactgagattataggcatataccacacccagccaaaaat
agtttcttgagatggaatctactcatgataaacataaaggtgaagatgctgggaactttgttgaaatgacaacaaagaacttagaatattacataaacgtagttgataaagcagcagcggggttt gag
aggactgactccaatttgcaagacattctactgcaggtaaaatgctaccaaacagcattgcatgctac agagaaatcttttgtgaaataaagagtcaattgatgcatcaaacttcattgttgtctca
tttttaaagaaattgctgtagccaccccaaccttcagcaaccaccattttgctcagtcagcagccatcaacagtgaggcaagaccctccaccagcaaaaagactatgactcgctgaaggttcaga tga
tcattagcacttttaagcaatacagtatttttaatttttatttttttagatataatgctcttgtacact taattgactatagcaggggtgtccaatcttctggcttccttgggccacattggaagaat
aagaattgtgttgggccacacagaaaatatactgacactagcaatagctgatgtgctagaaaaaattgcaaaaaaaatctcataatcctttaggaaagtttacaaatttgtgtttggccacattc aaa
gctgtcctgggttgcatgtagcctgcgggccatgggttggacaatcttggaccatagtatagtgtaaac ataactttatatgtactgagaagccataaagtgtgtgtgacccactttattgtgatgg
tctttaactgaacccacgatatctctgaggtatgtctatactgggatttcaacctgatatcttaggttctggggccagattacttagatttgaatatcagaagaactcagtaaatcttccatctt cac
tctttacaaattgggataaagtactttctttttttttttttttgagatggagtctcgctccgtcgccca ggctggagtgcagtggtatgatcttggctcactgcaagctctgcctcccgggttcaagc
aattctcctgtcacagcctcctgagtagctgggactaccggtgcctgccaccatgcccagcttatttttgcatttttagtagagacggggtttcaccctgttggtcaggctggtctcaaactcct gac
ctcaggtgatccacctgcctcggcctcccaaagttctgggattacaggcgtgagccaccgcacccggct aagataaagtattttctttttcccaaatatgacttcaacatttatttttgagcatata
ctatattccaatcattaagttaatcttagaagaaaaatagtagatatgattgctgccttcatggaacttatggtttagtgaggaggcagacattatacaaatgatcagacatatgcttatcacaa ata
agcataaatatgaactgcagacagtccccaacttgagatttttttgactttacaacagtgtaaagtgata aatatacaaaagagactgtacttcacatgtctatacaaccattctatttctactttca
caataaagtaaatgagatatttaatactttagtgtaaaataggctttgtgttggatgatcttgcccaactgtaggcaaatgtaaatgttctcagcacatttaaggtaggccaggtatgtttggta ggt
tagatgtattaaatgtatttttggctgggtgtggtggctgatgcctttaatcccagcactttgggaagc cgaggtgggcggataacttgaagtcagaagtttgagaccagcccgggccatgtggtgaa
accccatctctactaaaaacacaaaaattaggtggacatggtgatgcatgcctgtaatcccagctactagggaggctgaggcaggagaatcgcttgaacccgggaggcagaggttgcagtgagct gag
attgcaccactgcactccagcctgggagacagagtgagactccatctcaaataaataaataaataaatg catttttgacttatgatatttttaacttacaatgggtttattgcaatataaccccatca
taagttgaggagcatctgtataagaagtgccatgaaggtcaaacagagggtggtacgaggaggatataatcagaagaccaacctagtcttccttaagaaaacaatgattgaaaggtaagtaagagt tag
tggaagttggagaaagctttccaggtgagggaatatgggcaaaggccctgaaatggaaaaaactctggt gacttaggaaatgaaaataggtcagttggcttagatatagcgagagaggaggaaagtgg
cctgaaaggaggctgcagagatggggaccaaatcatgcaggaccttacaaaaggagtaggatcccaaagattcaaggtcttgggggtcaggttaaggactttggatcaggatggtagtggctgaa aag
gagagaagtggcaacaattaagagattatttggtaaaagggaaataatcaagcatttatcatgctttct tatataaacaatatcactaggtaatcgaataatcgaaaaggggcaatttccctttgtag
aaacattgtacaatagctagtaaatgaataatgatacaattatatcaccacttaaaaactcctaatgaatgaattgatctgggcatttctcattaatggctgccaacatcacaaaaagaaggaca agc
atacactgtgtatctctcagtggaaacgtacattgccacctataaagtaatattgccaaaaattaaact tgaatctgataaaacatttagatcctactgccaaattacacaaaatgcagctaagggaa
cataaatacaccacagggatgcagttgacaaaatccagattgtggcaaacactatagggcaaacaacctagtcccttttttagaaatgctggggaaaaaagagaaagggaaaacaatgcagaggac tta
accgttataccagccaaacatggcacagctctcactgaaactgcaaaaaagaaatatatggcagatctg tacagagagaaagtaactaagttagtggttcctagggccgaggagttgggggaaaatgg
gagtaactgctaacaggtatgtgtttctttctgagtaatgagaatgttataaaattgattatggtgatggttgcaaagctctgtgaatatgccaaaagccatttaattacatatcttaaatgggt gaa
ttgtatggtatgtgaactatatctcaagaaatctgacatatgtatatgtgtgtgtctatatatgtgtat ctatatgtaaaagaagagatctgaggattgaaaggaaatcagctaaggaacggagtaca
gtatttctacacagaagaaataatatgcgtaatggagaaacagcatagcattttagaagattaaaaaaaaaaaaaaaactgccagtcatgatggctcacgcctgtaatcccagcactttgggaggc cga
ggcagGCGGATCACaAGGTCAGGAGtTCGAGACCAgCCaGGCcAAtAtgcTGAAACCCCGTCTCTACTAAAAATAcAAAAATTAGCTGGGTGTGGTGGCGtGCaCCTaTAgTCCCAGCTACTCAGGAG
GCTGAGGCAGGaGAATcGCtTGAACCCGGGAGGCAGAGGTTGCAGTGAGCCgAGATCACGCCACTGCACTCCAGCCTGGCGACAGAGTCAGACTCCGTCTCaAAAAAAAAAAAAAGAAAAGAAAAAAC

AAAACTGCCTCTGCAACTGGCACATTTCCtgAAAAAGCATAGCTAAGAGATGAAGCTCAAGACAGTGAAAGGGCCCAGA cTGCACAGCGGTGTGAAAGCCATGCTGAGGGTTTGGGACTTTATCCtGG
GAGCAATAGGAAGCCATTGTTGGCTGACATCACTGCTATGCATTGCAACTCTGGCGACCATGTAGAGAATGAGGAAAAGGGAGAGTGCATTTGGGAAACCAC aTGAGTGGTATTATAATTCTCCAGGC
ACAAGCTAGTATCATCTTATTTTAGGACGGT GGCAGTGGAGAGGGACAgATTCAACAGGACTTGTTGATGGATTGGAATTGAGATACAGTAGTAAAGTAG gAAAGTCACCCTGCTTCACTGGGTGAAT
GGTAAAATGTGAACACTAGAAGAGGTATAAATTTTAGGGTAAGAGAGTGAGATTAATTTTTTATATATGTAGTTGTATAATCTAATCTAATAAGGAGGCAAGGTCGGGTAGAAAGGATTCATGCC TTA
ATGCTAATGCTTTTGGACTAAATAACTAAGT TTCCATTCAGTTATAATGGTCTTtCACAAGGAAGTGCCATCTTTTGCAGAATTTATTCCCCCAAAGAGCTCGTGaCTGAAATATAACTCCTGGAATT
TGCCTGGAATTTGAATTCCTCAGAATCATCCACACCAAGTTAATAATTAAAGGTTCCTCATACTGTCTACATTTGTTTTTTTCACCTGTGTACAGGATTCTGATTTCACAAAATCCTCCCAGCAG TAT
TATTTAACCTAGTaTCAAAGAAAGGTGTGTATATTCCAGCAACCCAGAGGCAAGTCTCTACCAGCTCAAGAAGGAAAAAGGTGGGAGGCTGCAGAAGCCACAGAAACTGACCCTGCCCAGAGCCAAA a
CAGACAAGAGGGCTCCTGCAAAGTTAATCTTCTTTGAATTTT

Homologous Sequences =
GGAGGCTGAGGCAGG

>NAHR_3_ Scaffold16047 983136 985437
KSD_NAHR_3

>Scaffold16047 983136 985437_NAHR
TATTGGAAGGTGGAGAATAGAGGAAGAGGCTGGAAATGGAGGCTTGGCAGGGAGCCCTTGACTATCTGGGAAGGA aCTGAAAATGGATCTGAAGTTAGCACAAATGACACAAGAAAAAATAAAAACTC
GAAGAGTTCTGTAACCTTGAAAGAGATTGAACCAGAAGTTGCAAATCCTTTCATAAAGAAAACATGAGGCCCAGATGGCTTTCTGAGCAGGGTCAAGCCATCGCAGAAGGAACACATAATTCTAACCC
GAACAATGTCTTCTGGAGAAAAAGAGAGAACACTCCCCAGCTTCTTGGGAGACTGGCAAGAGCTTGACTCCGGTTGGAAAAGAATGGCCTCAGAAAGGAAAATTACAGACAAATCTCACTCATGA CCT
TGGATGCAAAAATCCTAAACACAATATTAGCAAATGGAATTTGGCAACATATTAAAAAGATTACTACATCATGACAAGTCGGGTTTATCTTAGGAAGCTAAGACTGATCTAACAGAAACATCAATTAA
TACAATTCATTAGCAAAGAAAAGCCATGTAATTTTTGCAAGTGATACAAAAAAGGACTTGAGGAAATTAATTATCCGCTTGCAATTAAATAAAAATCTCTTGCACACTAGGAAAAGAATAAAATA CTT
TAACCTGATAAGCTGCATGTCAAAAAAAAAAAAAAAAAaACTAGAGTCAACAGAAATTTTGAAAGCTTTTTTTTTTTTTAAAGATCAAGAACAAAGCAGGTATTCCCACTATTCCCACTTCTATTCAGCA
TTTTATTGCAGGTCCTAGCTAGCGGAGAAAAAAAGAGAAAGGAAGAAAAAAGATAATCAGTCGATAAAAGATGAGAATTAATAAGAGAATTCAGCAAAGTGGCTGGATAAAATAAAAATCAATAT ACC
AAAGCCAGCCATATTTCTACATTTCAGCAAGAATCAGCAAATGCAATTCTGAAAAATATATCATGAAGTGACATCAGTGAAACTACCTAG GAATAAATCTAAAAAAGGTTTTGCAGAAGCTCTTTGTG
GAGATACTTAAAAAACCAGTTTGAAAGAGAAATGTAGGCAGGGCGCAGTGGCTCACgTCTGTAATCCCAGCACTTTGGGAGGCCGAGGCGGGtGGATCATGAGGTCAGGAGtTCGAGACCAgCtTGGt
cAACAtaGTGAAACCCGaCTCTACTAAAAACACAAAAAAATTGGCTGGGTGTGGTGGCGGGCACCTGTATTTCCAGCTACTCAGGAGGCTAAGGCAGGAGAATTGTTTGAACCTGGGAGGAAGAGGTT
GCAGTGAGTTGAGATTGTGCCATTGCACTCCAGCCCGGGTTACAGTGTGAGATTCTGAAAGAAGAAAAGAAAAGAAAGAAGAGGGA GAAGGAAGGAAGGAAGGAAGGAAGGAGAGAACGAAAGAAAGA
AATAGAAAGAAAGAAAGAAAGAAAGAAAAAGAAAGAAAGAAAGAGAAAGAAAGAAAGAAAGAAAGAAAGAAAGAAAGAAAGAAAGAAAGAAAGAAAATGTATCATGTTCTTGAACAGGAAGACA
ACATTGTGAAGGGGTTAATTTTTCCCAAATTGGTCTATTCTGGGTCAATGCAATTCTAACCAAAATCCCAATAGGGTTTGTTGTTGCTGTTTTGTTTTGGTGTAACTTGATAAGCTGATTGGAAAATA
CATATGGAGCAGGCACGGTGGCTCACACCTGTAATCCCAGCCATTTGAGAGGCTGAGGCGGGTGGATCACTTGAGGTCAGGAGTTTGAGACCAACCTGAACAACATGGCGAAACCCTGTCTCTACTAA
GAACACAAAAATTAGCCGGGCGCGGTGGTGCAAACCTGTAATCCCAGCTACTCAGGAGGCTGAGGCATGAGAATTGCTTGAACCTGGGAGGCGGAGGTGTCAGTGAGCCGAGATTGCGCCACTGC ACT
CCAGCCTGGGTGACAGAGCAGGACTCTGTCTCAAAAAAAAAAAAAAAAAAAAAGAAAAGAAAAAGAAAAAGAAAAAAAGAAGAAAAAGAAAGAAAATGCATATGGAAATGTAAAGAGCCAACGATTGCTC
AAATAATCCTGAAGAGATAAAAGGTGGGTGGGTTGGGGGTGAAGGGGAGAAATATTCAGAGGTAATGAAGGTGGTGCTCAGATAGACAAATGGACCAACGGACAGCCACACCACAGACCTGCATC CAT
GTGGAAGTTTGGTTTATGACGCCTCCAGCACGGAAGATCTTGGAGAAAAGTGTTTTATTCCACCAATGCTGCTGGAAGGATGGGGTGTCCTCAGGGGAAAAAGTGAAATTAGATCCATGCCTCA C

1837	27.6	5.4	2.3	S	92	1057	(1244)	+	L1M5	LINE/L1	3418	4484	(1662)	1
2048	13.7	1.7	0.3	S	1061	1360	(941)	+	AluSx3	SINE/Alu	1	304	(7)	2
27	0.0	0.0	0.0	S	1367	1392	(909)	+	(GAAG)n	Simple_repeat	1	26	(0)	3
52	1.3	5.1	2.5	S	1394	1472	(829)	+	(AGAA)n	Simple_repeat	1	81	(0)	4
511	24.7	1.0	11.0	S	1473	1672	(629)	+	L1M5	LINE/L1	4476	4657	(1489)	1
2369	11.0	0.0	0.0	S	1674	1982	(319)	+	AluSz6	SINE/Alu	4	312	(0)	5
316	32.3	13.4	0.9	S	2011	2301	(0)	+	L1M5	LINE/L1	4644	4970	(1176)	1

1842	27.6	5.4	2.3	hg19	95	1060	(1445)	+	L1M5	LINE/L1	3418	4484	(1662)	6
2837	2.6	0.0	0.0	hg19	1064	1373	(1132)	+	AluYe5	SINE/Alu	1	310	(0)	7
1082	17.9	2.3	0.0	hg19	1376	1548	(957)	+	AluSg4	SINE/Alu	127	303	(7)	8
27	0.0	0.0	0.0	hg19	1555	1580	(925)	+	(GAAG)n	Simple_repeat	1	26	(0)	9
66	2.1	6.3	0.0	hg19	1582	1676	(829)	+	(AGAA)n	Simple_repeat	1	101	(0)	10
511	24.7	1.0	11.0	hg19	1677	1876	(629)	+	L1M5	LINE/L1	4476	4657	(1489)	6
2369	11.0	0.0	0.0	hg19	1878	2186	(319)	+	AluSz6	SINE/Alu	4	312	(0)	11
316	32.3	13.4	0.9	hg19	2215	2505	(0)	+	L1M5	LINE/L1	4644	4970	(1176)	6

>hg19 Chr11:70444368-70446872
TATTGGAAGGTGGagaAGAATAGAGGAAGAGGCTGGAAATGGAGGCTTGGCAGGGAGCCCTTGACTATCTGGGAAGGA gCTGAAAATGGATCTGAAGTTAGCACAAATGACACAAGAAAAAATAAAAA
CTCGAAGAGTTCTGTAACCTTGAAAGAGATTGAACCAGAAGTTGCAAATCCTTTCATAAAGAAAACATGAGGCCCAGATGGCTTTCTGAGCAGGGTCAAGCCATCGCAGAAGGAACACATAATTC TAA
CCCGAACAATGTCTTCTGGAGAAAAAGAGAGAACACTCCCCAGCTTCTTGGGAGACTGGCAAGAGCTTGACTCCGGTTG GAAAAGAATGGCCTCAGAAAGGAAAATTACAGACAAATCTCACTCATGA
CCTTGGATGCAAAAATCCTAAACACAATATTAGCAAATGGAATTTGGCAACATATTAAAAAGATTACTACATCATGACAAGTCGGGTTTATCTTAGGAAGCTAAGACTGATCTAACAGAAACATC AAT
TAATACAATTCATTAGCAAAGAAAAGCCATGTAATTTTTGCAAGTGATACAAAAAAGGACTTGAGGAAATTAATTATCC GCTTGCAATTAAATAAAAATCTCTTGCACACTAGGAAAAGAATAAAATA

CTTTAACCTGATAAGCTGCATGTCAAAAAAAAAAAAAAAAgACTAGAGTCAACAGAAATTTTGAAAGCTTTTTTTTTTTTAAAGATCAAGAACAAAGCAGGTATTCCCACTATTCCCACTTCTATTCA
GCATTTTATTGCAGGTCCTAGCTAGCGGAGAAAAAAAAGAGAAAGGAAGAAAAAAAGATAATCAGTCGATAAAAGATGAGA ATTAATAAGAGAATTCAGCAAAGTGGCTGGATAAAATAAAAATCAATAT
ACCAAAGCCAGCCATATTTCTACATTTCAGCAAGAATCAGCAAATGCAATTCTGAAAAATATATCATGAAGTGACATCAGTGAAACTACCTAGGAATAAATCTAAAAAAGGTTTTGCAGAAGCTC TTT
GTGGAGATACTTAAAAAACCAGTTTGAAAGAGAAATGTAGGCAGGGCGCAGTGGCTCACaTCTGTAATCCCAGCACTTTGGGAGGCCGAGGCGGGcGGATCATGAGGTCAGGAGaTCGAGACCAtCcT
GGctAACAcgGTGAAAcCCCGtCTCTACTAAAAAtacagaaaattagccgggcgaggtggcgggcgcctgtagtcccagctactcgggaggctgaggcaggagaatggcgtgaaccccaggggcgga
gcctgcagtgagccgagattgcgcCACtgcactccagcctgggcgacagcgagactccgt ctcaaaaaaaaaaaaaaaaaaaaaaaaaaAAAAAAATTGGCTGGGTGTGGTGGCGGGCACCTGT
ATTTCCAGCTACTCAGGAGGCTAAGGCAGGAGAATTGTTTGAACCTGGGAGGAAGAGGTTGCAGTGAGTTGAGATTGTGCCATTGCACTCCAGCCCGGGTTACAGTGTGAGATTCTGAAAGAAGA AAA
GAAAAGAAAGAAGAGGGAGAAGGAAGGAAGGAAGGAAGGAAGGAGAGAACGAAAGAAAGA AATaaaaAGAAAGAAAGAAAGAAAGAAAGAAAAAGAAAGAAAGAAAGAgaaagaaagaaaGAAAGAAA
GAAAGAAAGAAAGAAAGAAAGAAAGAAAGAAAGAAAGAAAGAAAGAAAATGTATCATGTTCTTGAACAGGAAGACAACATTGTGAAGGGGTTAATTTTTCCCAAATTGGTCTATTCTGGGTCAAT GCA
ATTCTAACCAAAATCCCAATAGGGTTTGTTGTTGCTGTTTTGTTTTGGTGTAACTTGATA AGCTGATTGGAAAATACATATGGAGCAGGCACGGTGGCTCACACCTGTAATCCCAGCCATTTGAGAGG
CTGAGGCGGGTGGATCACTTGAGGTCAGGAGTTTGAGACCAACCTGAACAACATGGCGAAACCCTGTCTCTACTAAGAACACAAAAATTAGCCGGGCGCGGTGGTGCAAACCTGTAATCCCAGCT ACT
CAGGAGGCTGAGGCATGAGAATTGCTTGAACCTGGGAGGCGGAGGTGTCAGTGAGCCGAG ATTGCGCCACTGCACTCCAGCCTGGGTGACAGAGCAGGACTCTGTCTCAAAAAAAAAAAAAAAAAAAG
AAAAGAAAAAGAAAAAGAAAAAAAGAAGAAAAAGAAAGAAAATGCATATGGAAATGTAAAGAGCCAACGATTGCTCAAATAATCCTGAAGAGATAAAAGGTGGGTGGGTTGGGGGTGAAGGGGAG AAA
TATTCAGAGGTAATGAAGGTGGTGCTCAGATAGACAAATGGACCAACGGACAGCCACACC ACAGACCTGCATCCATGTGGAAGTTTGGTTTATGACGCCTCCAGCACGGAAGATCTTGGAGAAAAGTG
TTTTATTCCACCAATGCTGCTGGAAGGATGGGGTGTCCTCAGGGGAAAAAGTGAAATTAGATCCATGCCTCA C

>NAHR_4_ Scaffold15799 363849 364156

KSD NAHR 4

>Scaffold15799 363849-364156_NAHR

GGGGTTGGGGAGCCTGGGATAATAGCCTTGGTGGGCTGGCTCCGGAGTTAGAACCGGGTGGAGAGAAGTGGGTGGAGGCCTCCTGGTTTGAAGGCAACTGTGATAAGACCTACAGATAGAATGGGT GT
GGGGACTGAGGGATAGGGAGCAATCGAGGAAGACTCCTTACCCAGGTAGGAACACAGACACTGAACTCTAGACCAGAGCTTCTTGAGGCATGTTCCCCAGAACACCCAATATGCAGTTTGTGTTT GGG
CAGGCAGGGGCAATGGGTAGGGAGGGATGAGTAGGGTCTAAAAATTTTGGAATCCTTATTTATTGCATCACTTCTTGAGAGTACTATATGTACATCAGCATATTAAAAAAATCTGAGATGCCCTTC AT
AAAGAAAGCTGGTTAATCTTTAAATCTGTTTTCCTTAAGCTTATTTAGCCATAGGATCTTTTTTTCTGTAATAAATACATAAAGAAGACTAACAATGGGAAAGGTTTCTCTAGATTTAAAAGTTG GAC
ATCCAAAGTTATATAGGATCCTCCCCACTCTTGTGAAAATAAAATGACTGTGTACCCTAAAGGCAGAAATGCACCTTGCTTTTATGTGTAAAATAGGTTTAAGGTTAGAAGGCAAGAGTTCATATC AT
GTATTTGTATGGCGTATTTTTTAGACTTCATTTTGGGAAAATGAAGATCAAGTAGGAATTTATGAATTCCTTGCTTTGTATTTTATTCATCTTTTTTTTTTTTAAGTTAAAAAATACA GGCCAGGCAT
GGCGGCTCATGCCTATAATCCCAGCACTTTGGGAGGCTGAGGTGGGAGGATCACAAGGTCAGGAGTTCGAGACCAGCCTGGCCAATATGGAGAAATCCTGTCTCTACTAAAAATACAAAATTTAG CCG
gGTGTGGTGGTGCATGTCTGTAGTCCCAGCCACTCAGGAGGCTGAGGCAGAATACTTGCTTGAACCTGGGAGGCAGGGGTTGCAGTGAGCCGAGATCACGCCACTGCATTCCAACCTG GGTGACAGGG
CAAGACTCCATCAAAAAAAAAAAAAACCAGTAAAACAGGCCAGGCGCGGTGGCTCACACCTGTAATCCCAGCAATTTGGGAGGCCGAGAAGGGTGGATCACGAGGTCAGGAGATTGAGACCATCCTGGC
TAACACGGTGAAACCCCGTCTCTACTAAAAATACAAAAAAATTAGCCGGGCGTGGTGGCGGGCACCTGTAGTCCCAGCTACTCAGGAGGCTGAGACAGGAAAATGGCGTGAACCC gGGAGGTGGAGCT
TGCAGTGAGCCGAGATCGTGCCACTGCACTCCAGCCTGGGCGACAGAGCGGGACTCCGTCTCAAAAAAAAACCAAAAAAACAACAAAAATGCTTTTCATTTTTGAGTGGATGGGGAGTCATCAGAA AGG
GAAGAAACCCTTTCTTTTTTTTTTTTTTCCTGGCCTTCAGGTATCTAGTAATAGCTTTGGAAGAGCTTTAGAGACATAGCTTCTTGACCTGTTAACAGACATAGCTGTTCTTGAGCCT GTCTTCTCTT
CCCCAGGAGAAGATGGGCTCCTGCAGGTTCCATGGAAGCTGATCAGCTGCCAGAATCAGTAGGTAAGAAGCATTTTTTTCTAAAACTACATGTGGCCCTTTCAGGCACACAACCTCCCAAGCTGC CCC
ATGCTGAAGAGATTTGCAcGGATGATCGAACAGAGAGCTGTGGACACATCCTTGTACATACTGCCCAAGGAAGACAGGTGAGGGGCTCTCACTGCTGGCATGAAGTCACGCTTGGTCA GACATCCAAG
TGTATGGGGAAGAGGAGTTCATCAGGCCCACTCACACCTGACAGCTGATGTCTCCTCGTGTAAGGAGGCTGAACCCCATTCTGCCCACCTACCAGTAGGCCCTAGGTCCTCGCACTTCTACAGCT GGG
ACTGGTGGCCTAGTGCTTCTGTTCCCTGTTATGCAGAACCAGGAGCCACCACCCTCATGACTTTATCACTTGCCTTTTCCTTTTGCTTTAAGGTTTAAAGGATTTGGGTGGGGGGAGC TGAAGAAAGA
ATATTCATTTCTGTCTTTAGTCATCATGTTACAATTTGCAAACCATTTTCACATTCTCACTGTGAGGTGAGAGAGATACCTTTCTCCCTGGCTTTATAGATAAAGAAACTGAGGCTGGGAGATAT TGC
CTACCTGGTCTAGGCCCAGCTAGCAAGTGATGGAGCCAGAACTTGGACCCAGATCCACTGACTGCCTATTCTGTGTTTTCGCTATATTACTAGCAGTTAGGGTTCTGTTTCCTGACTA CCAGAATGGA
AAG

231	27.6	5.5	3.6	100433	57	165	(2142)	C	L2	LINE/L2	(750)	2669	2559	1
361	27.9	4.8	1.2	100433	291	457	(1850)	C	MER103C	DNA/hAT-Charlie	(69)	228	56	2
298	25.8	2.2	0.0	100433	648	736	(1571)	C	hAT-1_Mam	DNA/hAT-Tag1	(3222)	250	160	3
2185	11.7	0.7	0.0	100433	759	1049	(1258)	+	AluSx4	SINE/Alu	1	293	(17)	4
2581	5.8	0.0	0.3	100433	1061	1368	(939)	+	AluY	SINE/Alu	1	307	(4)	5
423	31.2	6.9	0.5	100433	2093	2266	(41)	C	MIRc	SINE/MIR	(74)	194	10	6

231	27.6	5.5	3.6	Hg19	57	165	(2449)	C	L2	LINE/L2	(750)	2669	2559	7
361	27.9	4.8	1.2	Hg19	291	457	(2157)	C	MER103C	DNA/hAT-Charlie	(69)	228	56	8
298	25.8	2.2	0.0	Hg19	648	736	(1878)	C	hAT-1_Mam	DNA/hAT-Tag1	(3222)	250	160	9
2196	12.0	0.0	0.0	Hg19	759	1049	(1565)	+	AluSx4	SINE/Alu	1	291	(19)	10
2448	6.8	0.0	0.3	Hg19	1061	1355	(1259)	+	AluY	SINE/Alu	1	294	(17)	11
2566	6.2	0.0	0.3	Hg19	1367	1674	(940)	+	AluY	SINE/Alu	1	307	(4)	12
15	5.6	0.0	0.0	Hg19	1725	1743	(871)	+	(T)n	Simple_repeat	1	19	(0)	13
423	31.2	6.9	0.5	Hg19	2400	2573	(41)	C	MIRc	SINE/MIR	(74)	194	10	14

>Hg19 chr1:147123686-147126299

GGGGTTGGGGAGCCTGGGATAATAGCCTTGGTGGGCTGGCTCCGGAGTTAGAACCGGGTGGAGAGAAGTGGGTGGAGGCCTCCTGGTTTGAAGGCAACTGTGATAAGACCTACAGATAGAATGGGT GT
GGGGACTGAGGGATAGGGAGCAATCGAGGAAGACTCCTTACCCAGGTAGGAACACAGACACTGAACTCTAGACCAGAGCTTCTTGAGGCATGTTCCCCAGAACACCCAATATGCAGTTTGTGTTT GGG
CAGGCAGGGGCAATGGGTAGGGAGGGATGAGTAGGGTCTAAAAATTTTGGAATCCTTATTTATTGCATCACTTCTTGAGAGTACTATATGTACATCAGCATATTAAAAAAATCTGAGATGCCCTT CAT
AAAGAAAGCTGGTTAATCTTTAAATCTGTTTTCCTTAAGCTTATTTAGCCATAGGATCTTTTTTTCTGTAATAAATACATAAAGAAGACTAACAATGGGAAAGGTTTCTCTAGATTTAAAAGTTG GAC

ATCCAAAGTTATATAGGATCCTCCCCACTCTTGTGAAAATAAAATGACTGTGTACCCTAAAGGCAGAAATGCACCTTGCTTTTATGTGTAAAATAGGTTTAAGGTTAGAAGGCAAGAGTTCATATCA T
GTATTTGTATGGCGTATTTTTTAGACTTCATTTTGGGAAAATGAAGATCAAGTAGGAATTTATGAATTCCTTGCTTTGTATTTTATTCATCTTTTTTTTTTTTTAAGTTAAAAAATACA GGCCAGGCAT
GGCGGCTCATGCCTATAATCCCAGCACTTTGGGAGGCTGAGGTGGGAGGATCACAAGGTCAGGAGTTCGAGACCAGCCTGGCCAATATGGAGAAATCCTGTCTCTACTAAAAATACAAAATTTAG CCG
tGTGTGGTGGTGCATGTCTGTAGTCCCAGCCACTCAGGAGGCTGAGGCAGAATACTTGCTTGAACCTGGGAGGCAGGGGTTGCAGTGAGCCGAGATCACGCCACTGCATTCCAACCTGGGTGACAG GG
CAAGACTCCATTctcaaaaaaaaaaaaccagtaaaacaggccgggcgcggtggctcacacctgtaatcccagcaatttgggaggccgagaagggtggatcacgaggtcaggagattgagaccatcctggc
taacacggtgaaaccccgtctctactaaaaatacaaaaaaattagccgggcgtggtggcgggcacctgtagtcccagctactcaggaggctgagacaggaaaatggcgtgaaagcgggaggtgga gct
tgcagtgagccgagatcacgccactgcattccaacctgggtgacagggcaagactccatctCAAAAAAAAAAAAACCAGTAAAACAGGCCAGGCGCGGTGGCTCACACCTGTAATCCCAGCAATTTGG
GAGGCCGAGAAGGGTGGATCACGAGGTCAGGAGATTGAGACCATCCTGGCTAACACGGTGAAACCCCGTCTCTACTAAAAATACAAAAAAATTAGCCGGGCGTGGTGGCGGGCACCTGTAGTCCC AGC
TACTCAGGAGGCTGAGACAGGAAAATGGCGTGAACCCaGGAGGTGGAGCTTGCAGTGAGCCGAGATCGTGCCACTGCACTCCAGCCTGGGCGACAGAGCGGGACTCCGTCTCAAAAAAAACCAAAAAA
ACAACAAAAATGCTTTTCATTTTTGAGTGGATGGGGAGTCATCAGAAAGGGAAGAAACCCTTTCtTTTTTTTTTTTTTTCCTGGCCTTCAGGTATCTAGTAATAGCTTTGGAAGAGCTTTAGAGACAT
AGCTTCTTGACCTGTTAACAGACATAGCTGTTCTTGAGCCTGTCTTCTCTTCCCCAGGAGAAGATGGGCTCCTGCAGGTTCCATGGAAGCTGATCAGCTGCCAGAATCAGTAGGTAAGAAGCATTTT T
TTCTAAAACTACATGTGGCCCTTTCAGGCACACAACCTCCCAAGCTGCCCCATGCTGAAGAGATTTGCA aGGATGATCGAACAGAGAGCTGTGGACACATCCTTGTACATACTGCCCAAGGAAGACAG
GTGAGGGGCTCTCACTGCTGGCATGAAGTCACGCTTGGTCAGACATCCAAGTGTATGGGGAAGAGGAGTTCATCAGGCCCACTCACACCTGACAGCTGATGTCTCCTCGTGTAAGGAGGCTGAACCC C
ATTCTGCCCACCTACCAGTAGGCCCTAGGTCCTCGCACTTCTACAGCTGGGACTGGTGGCCTAGTGCTTCTGTTCCCTGTTATGCAGAACCAGGAGCCACCACCCTCATGACTTTATCACTTGCC TTT
TCCTTTTGCTTTAAGGTTTAAAGGATTTGGGTGGGGGGAGCTGAAGAAAGAATATTCATTTCTGTCTTTAGTCATCATGTTACAATTTGCAAACCATTTTCACATTCTCACTGTGAGGTGAGAGAGA T
ACCTTTCTCCCTGGCTTTATAGATAAAGAAACTGAGGCTGGGAGATATTGCCTACCTGGTCTAGGCCCAGCTAGCAAGTGATGGAGCCAGAACTTGGACCCAGATCCACTGACTGCCTATTCTGT GTT
TTCGCTATATTACTAGCAGTTAGGGTTCTGTTTCCTGACTACCAGAATGGAAAG

Homologous Sequences =

TTGCAGTGAGCCGAGATCACGCCACTGCATTCCAACCTGGGTGACAGGGCAAGACTCCATCTCAAAAAAAAAAA

>NAHR_5_ Scaffold1019 1951464 1953753
KSD_NAHR_5
>Scaffold1019 1951464 1953753 NAHR
TTTGGGCAAAGCCACTGTACACAGTACCCAAATAATATCTGGAATCAGATCGCTCATTGTACTTATAGAAAATTAGAGTGTTCTTTGGTCTATTCATATTGTTGGAGTTTCCCCTTTGTCTTTCCT GG
GTGGGGAAGTTAAAGAGGAATCTCACAGTTATTTAAGTTGGTGGGATAGAGATGTAGCTCATGTTGTTCTCCTTCTGAGACGCACCCACCTGCAGGAAGGGGAATGATTAACCCACCACATGAGG GGG
TTTGGCTGAGGTGCAGGAACCAGCATAGAATGAGATAGGAGAACCCAAGTAACTCACCCATCTTCCCACCTGCTGTGATGATAACTGGGGTCATTTATCCACAATGCTTAAAGTGACGGAGACAAC CA
TAGGCATTTGCAAATTCCTTGTCTGCCTGCCTATTTACCTAGGACAGACTTACATTTTGACTTGCCCTTTCCTCCCCAGTTGGACTGGAATTTGAGATGTTCCATCAGTCTGTTTAATCCTTTAG GTC
TGACCATGATCTGAGATGAAGTTTGTTCCTTGGTTTTGCTTTTGCTGGTTTGTGATCTTGGTTCTCGACTTAGACTTGGAAGGCTGGACAGAGTCAGAGAGATTGGCAGTGATAATCTTTACACGT GC
TTTCTCCTTATGTCATTTTACATTTGGAGCAATCCCTTGAACTCATAGGATGAGTATTGTTAGTTCCATTTTACAGACAAGAAAAGAAGGCTCAGAGTAGTGAAATGACTTGCCCTTTGAGTAGT TGA
TGTCTAACTCCAAATATTTATTCCACTATTTTCTCTACTAATCATGGTATTTCTCATCACCATTGCTATGCCTGTGAGTAGAATTTTTATCCCTGTTTTCAGAGGGAAAACACTAGGATTTTAAGA AA
CTTTTCCCCCTTGATAATCCATCGATTCAGTAGTTGTGCCAGATACCAACCAGAGACCTCTTTGTAGGTCACACTCAATCCCTAACAAGTCTAAAAGAAACAG GGCCAGGCATGGTGGCTCATGCCTG
TAATCCTAGCACTTTGGGAGGCTGAGGTGGGTGGATTGCCTGAGCTCAGGAATTTGAGACCAGCCTGGGCAACACAGTGAAACCCCATCTCTACTAAAATACAAAAAAATTAGCTGGGTGTAGCAG CG
TGCACCTGTAATCCCGGCTACTTGGAAGGCTAAGGCAGGAGAATCGCTTGAGCCCAGGAGGCGGA GGTTGCAGTGAGCCGAGATTGCTCCATTGCACTCTAGCCTGGGCAACAGAGCAAGACTCCGTC
TCaaaaaaacccatttccccttagcccagggaaaaaagacaaaaaccctttcaaaAAAAAAAAAAAAAAAAAAAAAAAAAAAAAAAGAAAGAAACAGCAGGGCTCTTAATATACCCCTTAGTGGAGATAA
ATCCTATAAAATTACACTTCAGCAGCAAAATAGACAAGCACAGTAATTTTGGTGTGAAATGTGCAAAGTCTTATTTACAGAACATCTCATGATGCTGTTCCCAGATGTCTTTTTAAAGAAATTTT TAG
TTACATGCAAAGGAACTGAAAACCAGGAATATAATTGGTAAGAAACGTAAATCTGT CCAAAAAAAACAATTGCAACAACTTGATGAGATATATATTTTCTCAGCATCAGCATAAAAGAAAGAACATTC
TGATCCAGAAATGGTATGTAAGTAAATAAACTTTAATTGGTTTGACAATGCCCCTTTTCCTGGCAGTGCCAAGAATCCTAGCATTATAATTACACAGTTCTGGGCAGACATGTGGTTTCCATTAA GAA
AGACTTGTGATTGACTGAGTTCATGAGGCATGTCAGAAAAAACACATCAATGTCTT TATAAGAAGGGAAGAGAAATGGATCTAAATGTATTTATAACTTCACAAGATACACATTCATAGTTTCAAAAG
GGCATACATTTCTTTAATTCTGATGAAAAAAAAAAATCATTAGGGAAAGCCATTGAGAGCATTTTGGCCTCCACTGCTACATGAGCACAGTACAGTGACTTGCATTTAGGTAAAAGGAATACTTT TTT
TTTTTTTTTCCCTTACAAGGATTTATTTAAGGATTCTGGAAGGCAAACGTTGAGGA AAGTCTTAGTTGAATAAGCTGTTGCTTCATGGTTTAAACGTTCCCCAACCTCCTTTTTTCCTTTAAATGGCT
TTACCATTAACAAAAAGATTTTTCAGATCCGTTCTCTCTTAGCCCCGTGTGGTGAGCCAAGCAGGATTTAGTAGTCTATCTCCCC T

346	29.4	5.6	0.0	S	630	755	(1534)	C	MIRc	SINE/MIR	(39)	229	97	1
2123	13.1	0.0	0.3	S	1000	1289	(1000)	+	AluSx	SINE/Alu	1	289	(23)	2
32	5.3	0.0	0.0	S	1360	1399	(890)	+	(A)n	Simple_repeat	1	40	(0)	3

346	29.4	5.6	0.0	hg19	630	755	(1572)	C	MIRc	SINE/MIR	(39)	229	97	4
940	13.6	0.8	0.0	hg19	1000	1131	(1196)	+	AluJo	SINE/Alu	1	133	(179)	5
1795	15.0	6.5	0.7	hg19	1137	1429	(898)	+	AluSz	SINE/Alu	2	311	(1)	6

>hg19 Chr5:158419028-158421354
TTTGGGCAAAGCCACTGTACACAGTACCCAAATAATATCTGGAATCAGATCGCTCATTGTACTTATAGAAAATTAGAGTGTTCTTTGGTCTATTCATATTGTTGGAGTTTCCCCTTTGTCT TTCCTGG
GTGGGGAAGTTAAAGAGGAATCTCACAGTTATTTAAGTTGGTGGGATAGAGATGTAGCTCATGTTGTTCTCCTTCTGAGACGCACCCACCTGCAGGAAGGGGAATGATTAACCCACCACATGAGG GGG
TTTGGCTGAGGTGCAGGAACCAGCATAGAATGAGATAGGAGAACCCAAGTAACTCACCCATCTTCCCACCTGCTGTGATGATAACTGGGGTCATTTATCCACAATGCTTAAAGTGACGGAG ACAACCA
TAGGCATTTGCAAATTCCTTGTCTGCCTGCCTATTTACCTAGGACAGACTTACATTTTGACTTGCCCTTTCCTCCCCAGTTGGACTGGAATTTGAGATGTTCCATCAGTCTGTTTAATCCTTTAG GTC
TGACCATGATCTGAGATGAAGTTTGTTCCTTGGTTTTGCTTTTGCTGGTTTGTGATCTTGGTTCTCGACTTAGACTTGGAAGGCTGGACAGAGTCAGAGAGATTGGCAGTGATAATCTTTA CACGTGC
TTTCTCCTTATGTCATTTTACATTTGGAGCAATCCCTTGAACTCATAGGATGAGTATTGTTAGTTCCATTTTACAGACAAGAAAAGAAGGCTCAGAGTAGTGAAATGACTTGCCCTTTGAGTAGT TGA
TGTCTAACTCCAAATATTTATTCCACTATTTTCTCTACTAATCATGGTATTTCTCATCACCATTGCTATGCCTGTGAGTAGAATTTTTATCCCTGTTTTCAGAGGGAAAACACTAGGATTT TAAGAAA
CTTTTCCCCCTTGATAATCCATCGATTCAGTAGTTGT GCCAGATACCAACCAGAGACCTCTTTGTAGGTCACACTCAATCCCTAACAAGTCTAAAAGAAACAG GGCCAGGCATGGTGGCTCATGCCTG
TAATCCTAGCACTTTGGGAGGCTGAGGTGGGTGGATTGCCTGAGCTCAGGAATTTGAGACCAGCCTGGGCAACACAGTGAAACCCCATCTCTACTAAAATACAAAAA AATTAGCTGGGTGTAGCAGCG
TGCACCTGTAATCCCGGCTACTTGGAAGGCTAAGGCAGGAGAATCGCTTGAGCCCAGGAGGCGGA Ggtgaaaccccatctctactaaaatacaaaaaaattagctgggtgtagcagcgtgcacctgta
atcccggctacttggaaggctaaggcaggagaatcgcttgagcccaggaggcggag GTTGCAGTGAGCCGAGATTGCTCCATTGCACTCTAGCCTGGGCAACAGAGCAAGAC TCCGTCTCAAAAAAAA
AAAAAAAAAAAAAAAAAAAAGAAAGAAACAGCAGGGCTCTTAATATACCCCTTAGTGGAGATAAATCCTATAAAATTACACTTCAGCAGCAAAATAGACAAGCACAGTAATTTTGGTGTGAAATGTG
CAAAGTCTTATTTACAGAACATCTCATGATGCTGTTCCCAGATGTCTTTTTAAAGAAATTTTTAGTTACATGCAAAGGAACTGAAAACCAGGAATATAATTGGTAAGAAACG TAAATCTGTCCAAAAA
AAACAATTGCAACAACTTGATGAGATATATATTTTCTCAGCATCAGCATAAAAGAAAGAACATTCTGATCCAGAAATGGTATGTAAGTAAATAAACTTTAATTGGTTTGACAATGCCCCTTTTCC TGG
CAGTGCCAAGAATCCTAGCATTATAATTACACAGTTCTGGGCAGACATGTGGTTTCCATTAAGAAAGACTTGTGATTGACTGAGTTCATGAGGCATGTCAGAAAAAACACAT CAATGTCTTTATAAGA

AGGGAAGAGAAATGGATCTAAATGTATTTATAACTTCACAAGATACACATTCATAGTTTCAAAAGGGCATACATTTCTTTAATTCTGATGAAAAAAAAAATCATTAGGGAAAGCCATTGAGAGC ATT
TTGGCCTCCACTGCTACATGAGCACAGTACAGTGACTTGCATTTAGGTAAAAGGAATACTTTTTTTTTTTTTTTCCCTTACAAGGATTTATTTAAGGATTCTGGAAGGCAAA CGTTGAGGAAAGTCTT
AGTTGAATAAGCTGTTGCTTCATGGTTTAAACGTTCCCCAACCTCCTTTTTTCCTTTAAATGGCTTTACCATTAACAAAAAGATTTTTCAGATCCGTTCTCTCTTAGCCCCGTGTGGTGAGCCAA GCA
GGATTTAGTAGTCTATCTCCCCT

Homologous Sequences =
GTGAAACCCCATCTCTACTAAAATACAAAAAAATTA

>NAHR_6_ Scaffold10740 3485204 3487498
KSD_NAHR_6

>Scaffold10740 3485204 3487498 SINE/Alu AluY NAHR 2294
GAAGAATATTTATGTTTTGGGTGGGAAACGACTTTCTAAATAGGACTGAAACTGAAACCACAAGGAAAGACTCACAGATTTAACTACATAAAATTAAAACTTGTATACAATAATAGAAATTACAAA GA
AACTTAAAAATCAACCATAGACATGGAAGAATATTTGCAATACATGAAAGAGAAGATTAGTATTTATAAGGCATAAAGCACACCTATACATCAGTAAGAATAATTCAGAGATCCAATAGAAACACAGA
TAAAGGAAAAGACAGCTATTTCACTGAAGAAATACAAATTGCCAATACGCTTGTGAAACGATGCTCAACACTAAACATAGAGAAATGCAAAGTGAAGCAAATGTTACATTCCAGGCCATGGCAGA AAT
ACAAAACATCCAACATCGGCAAGGATGCATAATGATGGACACCTTCACACAACTGTGGTGAAATGTAAATATGTGTAGCTCTTTGGGTGGACAATTTGGCAGTATTCATCTGAATTTAAAATGTACGT
GGTCTTGACCCAGAAAATTCATTTGTAAGAGTTTATCATGCAGCAATACTTGAACAGGTGCACTACAATGTACACAACAACTGCAATATTGTTTTTCATAGCAAAAAATGAAAATACCCTAAGAT TCC
ATGAATAGGTTAATACAATGGAACACCACACAtCCAGTAAAAAACAATGAAGCATGTGTTTATATATATAATATCTTAAAGAGATAATGAGTTTAAAAATTAAGCTATAAATAAAATGTGTTTCCAGT
AGTTTTGTTAATTATATACAAATGTGTATAAAAAGTGAAGAGAATGGAATAGGCAGAGAAAGTTGAAGTGGTTCCAGGAGCCTTATACTTTTTGTATTGCTTTAATTTTCACAAGTATTTTATAA TCC
TGAAATAAATGTAAGTGAGGAGAAATACATTCATTTCCATCACATTCATTGTGGTGTAATTCTTCCTAGTATTGGCCCAAATCAGACATACTTCAAATAGTTGC GGCCGGGCACGGTGTGTCATGCCT
GTAATCCCAGCACTTTAGGAGCCCCAGGTGGGCGGATCACGAGGTCAGGAGATCAAGACCATCCTGGCTAACACGGTGAAACCCTGTCTCTACTAAAAATACAAAAAATTAGCCGGGCATGGTGG CGG
GCGCCTGTAGTCCCAGCTACCTGGGAGGCTGAGGCAGGAGAATGGTGTGAACCTGGGAGGTGGAGCTTGCAGTGAGCTGA GATTGTGCCACTGCAGTCCAGCCTGGGCGACAGAGCAAGACTCCGTCT
CAAAAAAAAAACAAACCTACACATACTTATATACTGGCAATcTCTTTTTCTTGGATTATGATTCAGATGTCTCCTAGGAAATTTCTAAATCCTAGTTGAAGGCTCAGCTCTTTGCTGCACTTTTGAGA
ACTACACTATTTCATTGTCATTATTATTACACTGTGATGTCAGGCATCTTTTCATTGACCTGTATCTCCACCTCAGTTCTAGGAAGGAGGGAGGTGGAGGAAGGAAAAGAGACTGGCTTTAGCAC CTA
ACACATGCCATCTTTGTGCAAGGTACCTGGCACAGCTTACATCACCTCATCTTTACAAGCTCCCTAGGAGGTGAGAtTGTACCCGTTTAACAGACGAGGAGGCTCAGGTGCGTCTCTAAAATCAGCAA
AGTCAGGGTGTCACTTCTTCAGCATGATTTGAGTGTTTTGACCTATAAAACTGGGAATGTCATTCAGTTCAACCTAAAAATAGCATACACAAAGAAATGTGCACTGTTCATAATTATACAGCTGC CCA
GAAAACTCCCCAGTCTGCCAAGCCCCCTTCCACCACCCTGACTTCTAACATCACTGAACTCTAGAGAAATGGAGCCGTAAAGCGTGTGCTCTTTCAtGTGACTTTTTTTGTTGAATAtGTTTTTGAGA
TTTATCTCTGTTGTTGTGGGTAGCAGCAGTTGGTTCATTCTCGTGTGCTCCAATTTATTTATTCATTTTACCCATGGATATATGGGATATTTATCACTTTTGGCTATTAAGTGCAATGCTGCTAT TGG
TTTTTGGTGAACCTATTTGTAATTTCTTGTACATATATACGTAGGGGTGCAACTGCTGGGTTTTTCATCAGAAGTGGGCCATGCTGCCCATCTGCCTGCAGAATGGGACCCATTTGCCAGGCCCTGTC
ACTTGCCAGGCCCATTACATGCTTTCTACAAGGTAGCTGCTCAGCAGATGGAGATTACAGCATCATACAGGCTAAATATTGCTTTTAGAGGTATAGCTGGACAGAGAGGCCAAATCC T

1009	27.2	9.8	1.7	S	1	812	(1482)	+	L1ME3G	LINE/L1	5053	5943	(202)	1
2437	7.5	0.0	0.0	S	1001	1295	(999)	+	AluY	SINE/Alu	1	295	(16)	2
210	37.0	4.2	0.0	S	1526	1644	(650)	C	MIRb	SINE/MIR	(20)	248	125	3
240	16.7	0.0	0.0	S	1700	1741	(553)	+	MADE2	DNA/TcMar-Mariner	39	80	(0)	4
622	23.2	24.1	0.3	S	1822	2109	(185)	C	L1ME3A	LINE/L1	(194)	5979	5621	5

1009	27.2	9.8	1.7	hg19	1	812	(3633)	+	L1ME3G	LINE/L1	5053	5943	(202)	6
545	29.4	6.3	6.5	hg19	941	1000	(3445)	C	L1ME4c	LINE/L1	(102)	5981	5919	7
2482	7.4	0.0	0.0	hg19	1001	1299	(3146)	+	AluY	SINE/Alu	1	299	(12)	8
545	29.4	6.3	6.5	hg19	1300	1685	(2760)	C	L1ME4c	LINE/L1	(165)	5918	5537	7
254	28.0	13.2	4.3	hg19	1911	2212	(2233)	C	L1ME4a	LINE/L1	(4)	6120	5793	9
3404	10.4	1.4	0.8	hg19	2302	2795	(1650)	+	L1PA13	LINE/L1	5666	6162	(1)	10
252	24.7	0.0	1.2	hg19	2828	2913	(1532)	C	L1ME4a	LINE/L1	(768)	5356	5272	9
2349	6.2	0.0	4.1	hg19	3138	3444	(1001)	+	AluY	SINE/Alu	1	295	(16)	11
240	16.7	0.0	0.0	hg19	3851	3892	(553)	+	MADE2	DNA/TcMar-Mariner	39	80	(0)	12
596	24.1	24.1	0.3	hg19	3973	4260	(185)	C	L1ME3A	LINE/L1	(194)	5979	5621	13

>hg19 Chr10:122225594-122230038
GAAGAATATTTATGTTTTGGGTGGGAAACGACTTTCTAAATAGGACTGAAACTGAAACCACAAGGAAAGACTCACAGATTTAACTACATAAAATTAAAACTTGTATACAATAATAGAAATTACAAA GA
AACTTAAAAATCAACCATAGACATGGAAGAATATTTGCAATACATGAAAGAGAAGATTAGTATTTATAAGGCATAAAGCACACCTATACATCAGTAAGAATAATTCAGAGATCCAATAGAAACAC AGA
TAAAGGAAAAGACAGCTATTTCACTGAAGAAATACAAATTGCCAATACGCTTGTGAAACGATGCTCAACACTAAACATAGAGAAATGCAAAGTGAAGCAAATGTTACATTCCAGGCCATGGCAGAA AT
ACAAAACATCCAACATCGGCAAGGATGCATAATGATGGACACCTTCACACAACTGTGGTGAAATGTAAATATGTGTAGCTCTTTGGGTGGACAATTTGGCAGTATTCATCTGAATTTAAAATGTA CGT
GGTCTTGACCCAGAAAATTCATTTGTAAGAGTTTATCATGCAGCAATACTTGAACAGGTGCACTACAATGTACACAACAACTGCAATATTGTTTTTCATAGCAAAAAATGAAAATACCCTAAGATTCC

ATGAATAGGTTAATACAATGGAACACCACACAcCCAGTAAAAAACAATGAAGCATGTGTTTATATATATAATATCTTAAAGAGATAATGAGTTTAAAAATTAAGCTATAAATAAAATGTGTTTCCAGT
AGTTTTGTTAATTATATACAAATGTGTATAAAAAGTGAAGAGAATGGAATAGGCAGAGAAAGTTGAAGTGGTTCCAGGAGCCTTATACTTTTTGTATTGCTTTAATTTTCACAAGTATTTTATAAT CC
TGAAATAAATGTAAGTGAGGAGAAATACATTCATTTCCATCACATTCATTGTGGTGTAATTCTTCCTAGTATTGGCCCAAATCAGACATACTTCAAAT **AGTTGC**GGCCGGGCACGGTGTGTCATGCCT
GTAATCCCAGCACTTTAGGAGCCCCAGGTGGGCGGATCACGAGGTCAGGAGATCAAGACCATCCTGGCTAACACGGTGAAACCCTGTCTCTACTAAAAATACAAAAAATTAGCCGGGCATGGTGGC GG
GCGCCTGTAGTCCCAGCTACCTGGGAGGCTGAGGCAGGAGAATGGTGTGAACCTGGGAGGTGGAGCTTGCAGTGAGCTGA Gatggtgccactgcactccagcctgggtgacagagcaagactctgtct
caaaaaaaaaaaaaaaaa**agttgc**agttccgaggtatatttttgtatagtgcttttccttaactattacatcataaaatttcaccacgtttcttcatgattttcaaaatgattctttcaataactaga
taatattctgtgtgttcacaatctaccaaatcatcttcaaatgttgaccatttgagttttaataatttttatttgacaaatatatttactttgttttgtatggaacacagcaaaaaaatctttgt atg
tgaggattttggtgtctcttccattagtgcctccatgtatttttttaccaagaattctaagtcaaagggcaggaataattttatggctgtttatgtactgcaatattgctcttcaaaacagttgtaccc
atttactattccacctgagcagccgatcacatttgaaaacacaaacttatacattctggatagtaatctttactctctgatttttcttaagtattttagttgataaaaaatgtagaaaggttgat att
ttgacacattgtcactttaaatatttctaaagcaaacacaaaaaaatcattcagtgccctatcctggaatatttcattggcctctcctttgttctgtaacagaaaacatctatattgattttttgat
tgtgcatgaaaataataaatgcttactataaaataatttagacataaacatttaaaaagcaaatattacataatcccatcagcacaaaataaccaccgttaaaactgtattatgtatctttatag aga
tataggcacaattgattttattacattaccacattaaaatagcatcacgcacacttttctaatctgcttttatcacttaacaattgttataaatgtcttctcaagtaaacaaataactacctactata
gaactaacttcaatagctgcacattctttcattgtagcacataaaaagcttctactaatgtatactcattgttattttgtcgttttcagatccatatcttgcattgctatggaatcacatattta cat
tctataataaagacacgtacacactatgttcattgcagcactattcacaatagcaaagacatggaatcaacctaaatgcccgtcagtggtagaccggataaagaaaatgtggtacatacacaccatga
actactatgcggccacaacaaagaatgagatcatgtcctttgcaggaacatggatggggctggaggtcattatccttagcaagataatgcaggaacagaaaaccaagtaccacatgttctcactt ata
agtggaaagataagaacacatgaacacatagaggagaacaacagacactgaggcctactagaagcgggtgagtgggtgggaggaggaaaaggatcaggaaaagtgactaacaggtactagtcttgata
cctgggtgacaaaataatcagtacaacaaactcccataatatgagtttacctctgtaacaaacctgcatatgtacccctgaacttaaaataaaagttaacataaaaatatcacggatttaaagtg tgt
cagtttatgaactttgttagttttctgctcgtatctgttgcctatttttctgttgatgagctcttctcttttctttttaattggtaagagctctctgaatgagggagcattaacccttaactctgta
aatgaggacaacgctacctcctgagaggtgtcataattaaagcctatgactgtgacaagtgcctagtacaaaggtggcacatggtaggcattgaagtgttcttccttagtagctagtaggtgtct cct
tctccacctccctgccttcctataaccaaggtgatgatagatgtcaattaaaatat**acctacaca**ggccgggcgcggtggctcacgcctgtaatcccagcactttgggaggctgaggcgggcagatca
cgaggtaagaggattgagaccatgctggctaacatggtgaaaccccatctctacttaaaaaaaaataaaattacaaaaaaattagccaggcatggtggcgggtgcctgtagtcccaggtactcgg gag
gctgaggcaggagaatggcgtgaacctgggaggcggagcttgcagtgagccgag ATTGTGCCACTGCAGTCCAGCCTGGGCGACAGAGCAAGACTCCGTCTCAAAAAAAAAAACAA**ACCTACACA**TACT
TATATACTGGCAATtTCTTTTTCTTGGATTATGATTCAGATGTCTCCTAGGAAATTTCTAAATCCTAGTTGAAGGCTCAGCTCTTTGCTGCACTTTTGAGAACTACACTATTTCATTGTCATTATTAT
TACACTGTGATGTCAGGCATCTTTTCATTGACCTGTATCTCCACCTCAGTTCTAGGAAGGAGGGAGGTGGAGGAAGGAAAAGAGACTGGCTTTAG CACCTAACACATGCCATCTTTGTGCAAGGTACC
TGGCACAGCTTACATCACCTCATCTTTACAAGCTCCCTAGGAGGTGAGAgagTGTACCCGTTTAACAGACGAGGAGGCTCAGGTGCGTCTCTAAAATCAGCAAAGTCAGGGTGTCACTTCTTCAGCAT
GATTTGAGTGTTTTGACCTATAAAACTGGGAATGTCATTCAGTTCAACCTAAAAATAGCATACACAAAGAAATGTGCACTGTTCATAATTATACAGCTGCCCAGAAAACTCCCCAGTCTGCCAAG CCC
CCTTCCACCACCCTGACTTCTAACATCACTGAACTCTAGAGAAATGGAGCCGTAAAGCGTGTGCTCTTTCA cGTGACTTTTTTTGTTGAATAcGTTTTTGAGATTTATCTCTGTTGTTGTGGGTAGCA
GCAGTTGGTTCATTCTCGTGTGCTCCAATTTATTTATTCATTTTACCCATGGATATATGGGATATTTATCACTTTTGGCTATTAAGTGCAATGCTGCTATTGGTTTTTGGTGAACCTATTTGTAA TTT
CTTGTACATATATACGTAGGGGTGCAACTGCTGGGTTTTTCATCAGAAGTGGGCCATGCTGCCCATCTGCCTGCAGAATGGGACCCATTTGCCAGGCCCTGTCACTTGCCAGGCCCATTACATGCTTT
CTACAAGGTAGCTGCTCAGCAGATGGAGATTACAGCATCATACAGGCTAAATATTGCTTTTAGAGGTATAGCTGGACAGAGAGGCCAAATCC T

Homologous Sequences =
GAGAT

>NAHR_7_ Scaffold1016 4856679 4858976
KSD_NAHR_7

>Scaffold1016 4856679 4858976 SINE/Alu AluSx1 NAHR 2297

TTCCTTGACATCCGTGGAAAAGGGTTACATTTCCAGGGGCAACTGTTCCTTAACTTCCAGCTTCTAGAAGGTCATCCACTGTGGTGATGGGATGTGAAGGAACAGCATCCCAGCCAGTGTACGGGG AA
AGCAGCTGGACTGGGTGGGGAGGTCGGCAGGGCAGGCCCCAGAGGTGTGGATTTTATTTCACATCCATGGAAGCCTCTGGGGGCCTCACAGCATCTGCTTGACGTTTTACAGCTTGGCTCTGGCTTCT
GTGTGAGTAGTGAGGACGTGGCTGCAGAGCGAGTGAGGAGACGACTGGGGGCAACAGTGGCCTGGAGAAAGGAGAGGCAAGGGAGTGAGGTGGGATTAGGCTGCATTTTAAAAGTAGAGCTGGCA CGA
TTGATGATGAGAGATGGGCATGCGAAGAGGAAAAAGCAGGCTGCATCCTGGGGTTTTCTGGGGCCACAGGGTGAGCAGTTCTTCTGAGATGGGAGTACTGGGGAGCTGAGATTAGTTTAGGGTTGGAG
GCAGCAGAGATGCCCAGCGACCTCTGGGCTGGATGGAAACGAGTTGTCGTTAGTGTTCGGTGTGTTCGTGGGGCTGCATGCTATTTGCTGGATCCCTGTTAATGCGAAGCTTAGTGCAGTGACCT GCG
TCCAAAGCAGTGCCTGAGTGCCTGTTAGACATACAGATTCCCAGGCCACTCCCGAGACTGCATCAGGGTGTCTTGGAGATGGATCCAGGAATCTGAGTTTTAACAAGCTCTCAAGGTGACTCTGTGTG
TGTTGAACTTTGGTGAGCACTGACGTAGGGGAAGAACAGTGTTCAGAAGAAGGCTGAAGACTCTACCCCAAGGGCATTGAGGAGGCAGAAAGGAGCACTAGATCCAGAAACTGAGGTGGAGCAGT CCA
GGAGGCAGGTGGAGAACCAGGAGTGGGCGGAGAGAGGAAGTAACCAGCCATGCACCATATTGACGGAAGGTGATGCAAGATGATGACCAAGAATTGTTAACT GGGCCGGGCGTGGTGGCTCACACCTG
TAATCCCAGCACTTTGGGAAGCCCAGGCGGACAGATCACCTAAGGCCAGGAGCTCAAGACCAGCCTGGCCAACGTGGTGAAACCCCATCTCTACTAAAAATACAAAAATTAGCTAGACGTGGTGG CAG
GTACCTGTAATCCCAGCCACCTGGGGAGCTGAGGCAAGAGAATCGCTTGAACCTGGGAGGTGGAGGCTGCAGTGAGCCGAGATCGCGCCATTGTACTCCAG CCTGGGCAACAGAGCAAGACTCTGTCT
CAAAAAAAAAAAAAAAATGTTGTTTATTGGATGTGGAAAGGCAGAGGCCGTTAGCACCCTTGGCACATCGTTCCTCCGTCAGCGTAGTGGGAGTGAAGGCCTTGTTGCCACAAGTTTGAGAGCCGGTA
AGAGGAGAGGAGCACAGCCGGGACAGAGATGCTCTTGACAGTGAGCAGCACTGAGGAAACACCTCAAGCCTGTGGCCCTGACTCCAAGGAGGAACAGCTGGCATGGCTGTGCTTTTCTCTAGCCA CAT
TCACTCCTCAGTACAAGGGTAGAAAAGTGGATTATCCAGATTCGGGATTTCCCAGACAAGTGCAAGGAGGGAGAGAAGGGTGTGTATAAGAGTGTGGTTATTGTGCTGGACGTGGGATCAAAGCTGGG
GACAGAGGAGGGTGAGGGGCAATGCCACTGGATTGGAGGTTCTCGTGAGGACA cGTTTTGGGATGCAGGTATTTGAAAAAGGGAGCCGGAAAAGTAGGCGATGTGAGCAGAGTGGGATGTGTGAGGTG
GAGACTCGGGAGGCAGTGAGTGATGCCATCGGTAAACATCATTGCCGTCATTACATTATCATTGCAAGATCATTACATTAACATTAGCATGATCACTGTTGATAAGCATGTTTAGTGTGATTTGTGGA
ACAAGGAGATGAGCTGATGGGTGAGAAGGTTCAGGAAGGGAGAGGGCAGGCAGCGGTCGTCCCATCTCTGTGGATCTTAAAAGCAACAAGAATGATAACAAGAATAGAAGTGGAGACAAAAACAG CAA
GCCAGGTGCTAAGTTTTTCATGAAGGAAGGGGAAACTTAATAGATGATGTGAGCCCCAGAGAACAGGTTTTTAAAGAAGAGACCAGAGGGGACATCCATCCTCTTAGATGTTTGAGCCAGAGCAAGAA
CAAGTGGAGAGCACTCAGGGGAGATGGAGATATAAGGACTTGTGCTGATGGCGGACTGTGAGCTTTGCAGGGTGCAGTAGGAGGTTGAGGTGGTTGGGAATGATGTGCACTTGAGACAGG T

472	28.3	3.5	0.0	S	646	790	(1507)	+	MER5A	DNA/hAT-Charlie	40	189	(0)	1
213	36.6	1.3	8.8	S	892	999	(1298)	C	L2	LINE/L2	(1287)	2132	2031	2
2355	10.7	0.0	0.0	S	1000	1297	(1000)	+	AluSx1	SINE/Alu	1	298	(14)	3
213	36.6	1.3	8.8	S	1298	1419	(878)	C	L2	LINE/L2	(1389)	2030	1918	2
621	29.6	11.6	7.5	S	1451	2081	(216)	C	L2	LINE/L2	(2054)	1365	711	2
274	28.7	5.7	1.6	S	2158	2281	(16)	C	L2	LINE/L2	(2953)	466	338	2

472	28.3	3.5	0.0	hg19	646	790	(1641)	+	MER5A	DNA/hAT-Charlie	40	189	(0)	4
2407	10.5	0.0	0.0	hg19	1000	1304	(1127)	+	AluSx1	SINE/Alu	1	305	(7)	5
995	9.6	0.0	0.0	hg19	1307	1431	(1000)	+	AluSx3	SINE/Alu	175	299	(12)	6
193	40.3	0.0	0.8	hg19	1434	1553	(878)	C	L2	LINE/L2	(1383)	2036	1918	7
627	29.7	11.7	7.6	hg19	1585	2215	(216)	C	L2	LINE/L2	(2054)	1365	711	7
274	28.7	5.7	1.6	hg19	2292	2415	(16)	C	L2	LINE/L2	(2953)	466	338	7

>hg19_Chr9:140972914-140975344

TTCCTTGACATCCGTGGAAAAGGGTTACATTTCCAGGGGCAACTGTTCCTTAACTTCCAGCTTCTAGAAGGTCATCCACTGTGGTGATGGGATGTGAAGGAACAGCATCCCAGCCAGTGTACGGGG AA
AGCAGCTGGACTGGGTGGGGAGGTCGGCAGGGCAGGCCCCAGAGGTGTGGATTTTATTTCACATCCATGGAAGCCTCTGGGGGCCTCACAGCATCTGCTTGACGTTTTACAGCTTGG CTCTGGCTTCT
GTGTGAGTAGTGAGGACGTGGCTGCAGAGCGAGTGAGGAGACGACTGGGGGCAACAGTGGCCTGGAGAAAGGAGAGGCAAGGGAGTGAGGTGGGATTAGGCTGCATTTTAAAAGTAGAGCTGGCA CGA
TTGATGATGAGAGATGGGCATGCGAAGAGGAAAAAGCAGGCTGCATCCTGGGGTTTTCTGGGGCCACAGGGTGAGCAGTTCTTCTGAGATGGGAGTACTGGGGAGCTGAGATTAGTT TAGGGTTGGAG
GCAGCAGAGATGCCCAGCGACCTCTGGGCTGGATGGAAACGAGTTGTCGTTAGTGTTCGGTGTGTTCGTGGGGCTGCATGCTATTTGCTGGATCCCTGTTAATGCGAAGCTTAGTGCAGTGACCT GCG
TCCAAAGCAGTGCCTGAGTGCCTGTTAGACATACAGATTCCCAGGCCACTCCCGAGACTGCATCAGGGTGTCTTGGAGATGGATCCAGGAATCTGAGTTTTAACAAGCTCTCAAGGT GACTCTGTGTG
TGTTGAACTTTGGTGAGCACTGACGTAGGGGAAGAACAGTGTTCAGAAGAAGGCTGAAGACTCTACCC CAAGGGCATTGAGGAGGCAGAAAGGAGCACTAGATCCAGAAACTGAGGTGGAGCAGTCCA
GGAGGCAGGTGGAGAACCAGGAGTGGGCGGAGAGAGGAAGTAACCAGCCATGCACCATATTGACGGAAGGTGATGCAAGATGATGACCAAGAATTGTTAACTG GGCCGGGCGTGGTGGCTCACACCTG

TAATCCCAGCACTTTGGGAAGCCCAGGCGGACAGATCACCTAAGGCCAGGAGCTCAAGACCAGCCTGGCCAACGTGGTGAAACCCCATCTCTACTAAAAATACAAAAATTAGCTAGACGTGGTGG CAG
GTACCTGTAATCCCAGCCACCTGGGGAGCTGAGGCAAGAGAATCGCTTGAACCTGGGAGGTGGAGGCTGCAGTGAGCCGAGATCGCGCCATTGTACTCCAG Cctgggcaacagagccagactccatct
caaaaaaaaaaaaaaaaaaaaaaaagagggaggctgagacaggagactcgctagaacccgggaggtggaggttgcagtgagccaaaattgcgccactatactccagc CTGGGCAACAGAGCAAGACTCT
GTCTCAAAAAAAAAAAAAAAAAaaTGTTGTTTATTGGATGTGGAAAGGCAGAGGCCGTTAGCACCCTTGGCACATCGTTCCTCCGTCAGCGTAGTGGGAGTGAAGGCCTTGTTGCCACAAGTTTGAGAG
CCGGTAAGAGGAGAGGAGCACAGCCGGGACAGAGATGCTCTTGACAGTGAGCAGCACTGAGGAAACACCTCAAG CCTGTGGCCCTGACTCCAAGGAGGAACAGCTGGCATGGCTGTGCTTTTCTCTAG
CCACATTCACTCCTCAGTACAAGGGTAGAAAAGTGGATTATCCAGATTCGGGATTTCCCAGACAAGTGCAAGGAGGGAGAGAAGGGTGTGTATAAGAGTGTGGTTATTGTGCTGGACGTGGGATCAAA
GCTGGGGACAGAGGAGGGTGAGGGGCAATGCCACTGGATTGGAGGTTCTCGTGAGGACA tGTTTTGGGATGCAGGTATTTGAAAAAGGGAGCCGGAAAAGTAGGCGATGTGAGCAGAGTGGGATGTGT
GAGGTGGAGACTCGGGAGGCAGTGAGTGATGCCATCGGTAAACATCATTGCCGTCATTACATTATCATTGCAAGATCATTACATTAACATTAGCATGATCACTGTTGATAAGCATGTTTAGTGTGATT
TGTGGAACAAGGAGATGAGCTGATGGGTGAGAAGGTTCAGGAAGGGAGAGGGCAGGCAGCGGTCGTCCCATCTCTGTGGATCTTAAAAGCAACAAGAATGATAACAAGAATAGAAGTGGAGACAA AAA
CAGCAAGCCAGGTGCTAAGTTTTTCATGAAGGAAGGGGAAACTTAATAGATGATGTGAGCCCCAGAGAACAGGTTTTTAAAGAAGAGACCAGAGGGGACATCCATCCTCTTAGATGTTTGAGCCAGAG
CAAGAACAAGTGGAGAGCACTCAGGGGAGATGGAGATATAAGGACTTGTGCTGATGGCGGACTGTGAGCTTTGCAGGGTGCAGTAGGAGGTTGAGGTGGTTGGGAATGATGTGCACTTGAGACAG GT

Homologous Sequences

TACTCCAGCCTGGGCAACAGAGC

>Chimp

TTCCTTGACATCCGTGGAAAAGGGTTACATTTCCAGGGGCAACTGTTCCTTAACTTCCAGCTTCTAGAAGGTCATCCACTGTGGTGATGGGATGTGAAGGAACAGCATCCCAGCCAGTGTACGGGG AA
AGCAGCTGGACTGGGTGGGGAGGTCGGCAGGGC tGGCCCCAGAGGTGTGGATTTTATTTCACATCCATGGAAGCCTCTGGGGGCCTCACAG gATCTGCTTGACGTTTTACAGCTTGGCTCTGGCTTCT
GTGTGAGTAGTGAGGACGTGGCTGCAGAGCGAGTGAGGAGACGACTGGGGGCAACAGTGGCCTGGAGAAAGGAGAGGCAAGGGAGTGAGGTGGGATT gGGCTGCATTTTAAAAGTAGAGCTGGCACGA
TTGATGATGAGAGATGGGCATGCGAAGAGGAAAAAGCAGGCTGCATCCTGGGGTTTTCTGG aGCCACAGGGTGAGCAGTTCTTCTGAGATGGGAGTAC TGGGGAGCTGAGATTAGgTcAGGGTTGGAG
GCAGCAGAGATGCCCAGCGACCTCTGGGCTGGATGGAAA tGAGTTGTCGTTAGTGTT tGGTGTGTTCGTGGGGCTGCATGCTATTTGCTGGATCCCTGTTAATGCGAAGCTTAGTGCAGTGACCTGCG
TCCAAAGCAGTGCCTGAGTGCCTGTTAGACATACAGATTCCCAGGCCACTCCCGAGACTGCATCAGGGTGTCTTGGAGATGGATCCAGGAATCTGAGT TTTAACAAGCTCTCAAGGTGACTCTGTGTG
TGTTGAACTTTGGTGAGCACTGACGTAGGGGAAGAACAGTGTTCAGAAGAAGGCTGAAGACTCTACCCCAAGGGCATTGAGGAGGCAGAAAGGAGCACTAGATCCAGAAACTGAGGTGGAGCAGT CCA
GGAGGCAGGTGGAGAACCAGGAGTGGGCGGAGAGAGGAAGTAACCAGCCATGCACCATATTGACGGAAGGTGATGCAAGATGATGACCAAGAATTGTT AACTGGGCCGGGCaTGGTGGCTCACACCTG
TAATCCCAGCACTTTGGGAAGCCCAGGCGGACAGATCACCTAAGGCCAGGAGCTCAAGACCAGCCTGGCCAAC aTGGTGAAACCCCATCTCTACTAAAAATACAAAAATTAGC cAGACGTGGTGGCAG
GTACCTGTAATCCCAGCCACCTGGGGAGCTGAGGCAAGAGAATCGCTTGAACCTGGGAGGTGGAGGCTGCAGTGAGCCGAGATCG tGCCATTGTACTCCAGCctgggcaacagagccagactccgtct
caaaaaaaaaaaaaaaaaaaagagggaggctgagacaggagactcgctagaacccgggaggtggaggttgcagtgagccaaaattccgccactatactccagc CTGGGCAACAGAGCAAGACTCTGTC
TCAAAAAAAAAAAAAAAAATGGTTTATTGGATGTGGAAAGGCAGAGGCCGTTAGCgCCCTTGGCACATtGTTCCTCCaTCAGCGTAGTGGGAGTGAAGGCCTTGTTGCCgCAAGTTTGAGAGCCGGTAA
GAGGAGAGGAGCACAGCCGGGACAGAGATGCTCTTGACAGTGAGCAGCACTGAGGAAACACCTCAAGCCTGTGGCCCTGACTCCAAGGAGGAACAGCTGGCATGG tTGTGCTTTTCTCTAGCCACATT
CACTCCTCAGTACAAGGGTAGAAAAGTGGATTATCCAGATTCGGGATTTCCCAGACAAGTGCAAGGAGGGAGAGAAG GGTGTGTATAAGAGTGTGGTTATTGTGCTGGAC aTGGGATCAAAGCTGGGG
ACAGAGGAGGGTGAGGGGCAATGCCACTGGgTTGGAGGTTCTCGTGAGGACACGTTTTGGGATGCAGGTATTTGAAAAAGGGAGCCGGAAAAGTAGGCGATGTGAGCAGAGTGGGATGTGTGAGGTGG
AGACTCGGGAGGCAGTGAGTGATGCCATCGGgAAtCATCATTGCCGTCATTACATTATCATTGCAAGATCATTACAT TAACATTAGCATGATCACTGTTGATAAGCATGTTTAGTGTGATTTGT aGAA
CAAGGAGATGAGCTGATGGGTGAGAAGGTTCAGGAA tGGAGAGGGCAGGCAGCGGTCGTCCCATCTCTGTGGATCTTAAAAGCAACAAGAATGATAACAAGAATAGAAGTGGAGACAAAAACAGCAAG
CCAGGTcCTAAGTTTTTCATGAAGGAAGGGGAAACTTAATAGATGATGTGAGCCCCAGAGAACAGGTTTTTAAAGAA GAGACCAGAGGGGACATCCATCCTCTTAGATGTTTGAGCCAGAGCAAGAAC
AAGTGGAGAGCACTCAGGGGAGATGGAGATATAAGGAC gTGTGCTGATGGCGGAC cGTGAGCTTTGCAGGGTGCAGTAGGAGGTTGAGGTGGTTGGGAATGATGTGCACTTGAGACAGG T

>NAHR_8_ Scaffold4058 474463 476587
KSD_NAHR_8

>Scaffold4058 474463 476587 SINE/Alu AluYc NAHR 2124
AACACTTCTCATTAGGGCAGGACTGTGAAGCTTCTTCTGTAGGAGAAACAGAGACTTGAGTTAGGGAGAGTTTTAAGAGACTTCTGCTGCTGGCTTTGTTGACTGGTGGGAGGGTAACAGTGGCCT CA
TGTTTAACAACAGTAATTCTAAACACTTTTAATTTTTACATTTTTCAGTAAATTATTAATTATTCCTGAACTCTCT GATTTTGTATTACTGGAATGCAATTTCAGGTGATTATGACATGACTCAAAGA
AGAAGACAAAATAGGTTAATTACCAGTATCATTTCATACATGGACAAACTGAGGTTCAGTTTAAAAGTTTAAATAATTTTTAAAGAATATCTGGCAAGTTAGTGCTAAACACAGACAGAAGCAAA GTC
TCTAGACATTCTGAATAGTACCTTTTCCACAATATCACAATGTTTCCTTAGTAGGTATGCCCGGAGTAGAGCCCCG TTTTGTGGTGCCTGAAGCGTATACAATTTTGGAATCACTCTAAAAAAAAAAA
ACTCCATTGCTAGGGCTTGTCCCACAGCCTTGGAAGGGATCTGTATGACAGGCCCTGAAAATTAAGCTTCATTCGGTTCATAGTAAATCGGCCTCTGAATCTACCTGATGAAAACAACAAGATTT TAC
TTAGGAGTTCATGACCTCTTTTTGACTGATATTCTTAGGGCCACGATTTCCTTACATGATATGACCTATTTCTTAA CAGTGACAGGCCACATTTGGGGTCCCTCCGAGGAGTGATGTGGCTTTCAGAG
CAAATCACATATGCTGATTCCCCCACCACTGTAGGCAAAGTCTATCACTTAAATTCAAATTCATTAATTATTATGAAATTCTCTGAAAAAATTAATTCTTAATTTAAATAGCGTTGCCATTTAAA ATG
TTCAAGGTTTTTCTGTATAGTGGCTACATAAAAGTGGGACTGTGGTTCTTTCTCAAGTTGGGAGAAAATATGAGGG AAATAGTGCAACTAAAAAAAAAATCCCGCTCGGGAGGCTGAGGCAGGAAGATG
GCGTTAACCCGGGAGGCGGAGCTTGCAGTGAGTCGAGATCGTGCCACTGCACTCCAGTCTGGGCAACAGAGCAAGACTCCGTCTCTAAAAAAAAACAAAAATCCTGCTTACAGATTTAAAAAAAGCAGT
AAAATAAAAAATGAACACTGAAAATTTATATATGACAGTAACTGTTAGGAAAAGGAGAAATGCTACAAAGTAATTT CTATGTATTTGAAAAGTGGTAGTCAGGTATAAGACTTGGTTTTCAGTTTCAT
CTTTAGATCTGTATTTATATTAATCCAATAACATGCTTTAACTGAATAATAACTATGTTATGTTTGTACTATGTTCAAGAAGGAGTGGATAATAACACTAAATAATCAGTAACTTTTAAGGAGTA ATT
TTTTTAAGGAGTGGGTGATAACAAATATGAGTGAGGTCTTATTCCTTTCTTTAGGAGCTTATAATACTTTTGAGTG TCAAGGATAGCAATGGTATATAAATGACATGATTAAAATGATAAAATGAATA
AAAATGTGAATTTACAAAACAGAGTAGTCTCTTTGGAGTAATCAGGGAAGACTTAATGCAAAAAGCAGCCTTGCAGGATATATCAGATTGATTCCAAAAAGGAGAAGTAATGTAGACGTGTATGC ATT
CTAACCATGTGGACAGCCTTAATCAAACACTAATGACAGCTTAAGATCTCAAACTCCCTCTGCTCCCATTCAACCA TTTAATGAGGACCAACTATATGCCAGGTACTCCAGTAGTGCCTTCTGTTCTG
CTGTTCCCAGGGCAAGGGGGGCTAAGTGGTTAGTGGACATTCAGGCTATGTTTCCTTCGGCCTGAAATGGTAAACTAGAACCTCAAGCAAAATTGAAAATAGCCACAACATAGCTCATCTACTGA CCA
CTCAACTGTAATCACTATCATTCATTATTTGCCTAATTAAACATAAGATTTATCTCTACTAATGCAGAGGCTTTTA TTTATATTTTCAGGTATAATATGCCCTGGCTTATTATCACAGATAGGAAAGT
ATTAAGATTGCAGGAAATAGAAAAATACTCTATGCAGAGAGTACTTCTTCTAATCAACAGTGGAATGTTGACTGG T

12	12.9	5.6	2.7	S	157	192	(1932)	+	(TTAATTA)n	Simple_repeat	1	37	(0)	1
255	37.0	3.5	3.0	S	227	434	(1690)	C	MIR1_Amn	SINE/MIR	(64)	204	1	2
522	26.4	7.4	0.0	S	447	609	(1515)	C	Arthur1A	DNA/hAT-Tip100	(2)	175	1	3
1015	8.0	0.0	0.0	S	1000	1124	(1000)	+	AluYc	SINE/Alu	160	284	(15)	4
203	14.6	7.3	0.0	S	1731	1771	(353)	C	L2c	LINE/L2	(7)	3380	3337	5

12	12.9	5.6	2.7	hg19	157	192	(2048)	+	(TTAATTA)n	Simple_repeat	1	37	(0)	6
255	37.0	3.5	3.0	hg19	227	434	(1806)	C	MIR1_Amn	SINE/MIR	(64)	204	1	7
522	26.4	7.4	0.0	hg19	447	609	(1631)	C	Arthur1A	DNA/hAT-Tip100	(2)	175	1	8
911	4.5	6.3	0.0	hg19	1000	1110	(1130)	+	AluYa8	SINE/Alu	171	288	(22)	9
1041	7.2	0.0	0.0	hg19	1116	1240	(1000)	+	AluYc	SINE/Alu	160	284	(15)	10
203	14.6	7.3	0.0	hg19	1847	1887	(353)	C	L2c	LINE/L2	(7)	3380	3337	11

>hg19 Chr8:92399362-92401601
AACACTTCTCATTAGGGCAGGACTGTGAAGCTTCTTCTGTAGGAGAAACAGAGACTTGAGTTAGGGAGAGTTTTAAGAGACTTCTGCTGCTGGCTTTGTTGACTGGTGGGAGGGTAACAGTGGCCT CA
TGTTTAACAACAGTAATTCTAAACACTTTTAATTTTTACATTTTTCAGTAAATTATTAATTATTCCTGAACTCTCTGATTTTGTATTACTGGAATGCAATTTCAGGTGATTATGACATGACTCAA AGA
AGAAGACAAAATAGGTTAATTACCAGTATCATTTCATACATGGACAAACTGAGGTTCAGTTTAAAAGTTTAAATAATTTTTAAAGAATATCTGGCAAGTTAGTGCTAAACACAGACAGAAGCAAAG TC
TCTAGACATTCTGAATAGTACCTTTTCCACAATATCACAATGTTTCCTTAGTAGGTATGCCCGGAGTAGAGCCCCGTTTTGTGGTGCCTGAAGCGTATACAATTTTGGAATCACTCTAAAAAAAAA AAA
ACTCCATTGCTAGGGCTTGTCCCACAGCCTTGGAAGGGATCTGTATGACAGGCCCTGAAAATTAAGCTTCATTCGGTTCATAGTAAATCGGCCTCTGAATCTACCTGATGAAAACAACAAGATTTT AC
TTAGGAGTTCATGACCTCTTTTTGACTGATATTCTTAGGGCCACGATTTCCTTACATGATATGACCTATTTCTTAACAGTGACAGGCCACATTTGGGGTCCCTCCGAG GAGTGATGTGGCTTTCAGAG
CAAATCACATATGCTGATTCCCCCACCACTGTAGGCAAAGTCTATCACTTAAATTCAAATTCATTAATTATTATGAAATTCTCTGAAAAAATTAATTCTTAATTTAAATAGCGTTGCCATTTAAAA TG
TTCAAGGTTTTTCTGTATAGTGGCTACATAAAAGTGGGACTGTGGTTCTTTCTCAAGTTGGGAGAAAATATGAGGGAAATAGTGCAACTAAAAAAAAAATCCCG CTCGGGAGGCTGAGGCAGGAAGATG
GCGTTAACCCGGGAGGCGGAGcttgcagtgagccgagatcccgccactgcactccagcctgggcaacagagcgagactaaaaaaaa tcccgctcgggaggctgaggcaggagaacggcgtgaacccgg
gaggtggag TTGCAGTGAGTCGAGATCGTGCCACTGCACTCCAGTCTGGGCAACAGAGCAAGACTCCGTCTCTAAAAAAAAACAAAAATCCTGCTTACAGATTTAAAAAAAGCAGTAAAATAAAAAA T

GAACACTGAAAATTTATATATGACAGTAACTGTTAGGAAAAGGAGAAATGCTACAAAGTAATTTCTATGTATTTGAAAAGTGGTAGTCAGGTATAAGACTTGGTTTTCAGTTTCATCTTTAGATC TGT
ATTTATATTAATCCAATAACATGCTTTAACTGAATAATAACTATGTTATGTTTGTACTATGTTCAAGAAGGAGTGGATAATAACACTAAATAATCAGTAACTTTTAAGGAGTAATTTTTTTAAGG AGT
GGGTGATAACAAATATGAGTGAGGTCTTATTCCTTTCTTTAGGAGCTTATAATACTTTT GAGTGTCAAGGATAGCAATGG TATATAAATGACATGATTAAAATGATAAAATGAATAAAAATGTGAATT
TACAAAACAGAGTAGTCTCTTTGGAGTAATCAGGGAAGACTTAATGCAAAAAGCAGCCTTGCAGGATATATCAGATTGATTCCAAAAAGGAGAAGTAATGTAGACGTGTATGCATTCTAACCATG TGG
ACAGCCTTAATCAAACACTAATGACAGCTTAAGATCTCAAACTCCCTCTGCTCCCATTCAACCATTTAATGAGGACCAACTATATGCCAGGTACTCCAGTAGTGCCTTCTGTTCTGCTGTTCCCA GGG
CAAGGGGGGCTAAGTGGTTAGTGGACATTCAGGCTATGTTTCCTTCGGCCTGAAATGGTAAACTAGAACCTCAAGCAAAATTGAAAATAGCCACAACATAGCTCATCTACTGACCACTCAACTGT AAT
CACTATCATTCATTATTTGCCTAATTAAACATAAGATTTATCTCTACTAATGCAGAGGCTTTTATTTATATTTTCAGGTATAATATGCCCTGGCTTATTATCACAGATAGGAAAGTATTAAGATT GCA
GGAAATAGAAAAATACTCTATGCAGAGAGTACTTCTTCTAATCAACAGTGGAATGTTGACTGG T

Homologous Sequences
AACCCGGGAGGCGGAGCTTGCAGTGAG

>Chimp
AACACTTCTCATTAGGGCAGGACTGTGAAGCTTCTTCTGTAGGAGAAACAGAGAC cTGAGTTAGGGAGAaTTTTAAGAGAtTTCTGCTGCTGGCTTTGTTGACTGGTGGGAGGGTAACAGTGGCCTCA
aGTTTAACAACAGTAATTCTAAACACTTTTAATTTTTACATTTTTCAGTAAATTATTAATTATTCCTGAACTCTCTGATTTTGTATTACTGGAATGCAATTTCAGGTGATTATGACATGACTCAAA GA
AGAAGACAAAATAGGTTAATTACCAGTATCATTTCATACATGGACAAACTGAGGTTCAGTTTAAAAGTTTAAATAATTTTTAAAGAATATCTGGCAAGTTAGTGCTAAACACAGACAGAAGCAAA GTC
TCTAGACATTCTGAATAGTACCTTTTCCACAATATCACAATGTTTCCTTAGTAGGTATGCCCGGAGTAGAGCCCCGTTTTGTGGTGCCTGAAGCGTATACAATTTTGGAATCAC aCTaAAAAAAAAAA
AACTCCATTGCTAGGGCTTGTCCCACAGCCTTGGAAGGGATCTGTATGACAGGCCCTGAAAATTAAGCTTCATTCGGTTCATAGTAAATCGGCCTCTGAATCTACCTGATGAAAACAACAAGATT TTA
CTTAGGAGTTCATGACCTCTTTTTGAtTGATATTCTTAGGGCCACGATTTCCTTACATGATATGACCTATTTCTTAACAGTGACAGGCCACATTTGGGGTCCCTCCGAGGAGTGA cGTGGCTTTCAGA
GCAAATCACATATGCTGATTCCCCCACCACTGTAGGCAAAGTCTATCACTTAAATTCAAATTCATTAATTATTATGAAATTCTCTGAAAAAATTAATTCTTAATTTAAATAGCGTTGCCATTTAA AAT
GTTCAAGGTTTTTCTGTATAGTGGCTACATAAAAGTGGGACTGTGGTTCTTTCTCAAGTTGGGAGAAAATATGAGGGAAATAGTGCAACTAAAAAAAAATCCCGCTCGGGAGGCTGAGGCAGGAA aAT
GGCGTgAACCCGGGAGGCGGAGcttgcagtgagccgagatacgccactgcactccagcctgggcaacagagcgagactaaaaaaaaatcccgctcgggaggctgaggcaggagaatggcgtgaactcg
gaaggtggagCTTGCAGTGAGTCGAGATCGTaCCACTGCACTCCAGcCTGGGCAACAGAGCgAGACTCCGTCTCTAAAAAAAAAaaaAAAAATCCTGCTTACAGATTTAAAAAAAGCAGTAAAATAAAA
AATGAACACTGAAAATTTATATATGACAGTAACTGTTAGGAAAAGGAGAAATGCTACAAAGTAATTTCTATGTATTTGAAAAGTGGTAGTCAGGTATAAGACTTGGTTTTCAGTTTCATCTTTAG ATC
cGTATTTATATTAATCCAATAACATGCTaTTAACTGAATAATAACTATGTTATGTTTGTACTATGTTCAAGAAGGAGTGGATAATAACACTAAATAATCAGTAACTTTTAAGGAGTAATTTTTTTAAG
GAGTGGGTGATAACAAATATGAGTGAGGTCTTATTCCTTTCTTTAGGAGCTTATAATACTTTTGAGTGTCAAGGATAGCAATGGTATATAAATGACATGATTAAAATGATAAAATGAATAAAAAT GTG
AATTTACAAAACAGAGTAGTCTCTTTGGAGTAATCAGGGAAGACAATGCAAAAAGCAGCCTTGCAGGATATATCAGATTCCAAAAAGGAGAAGTAATGTAGACaTGTATGCATTCTAACCATGTGGAC
AGCCTTAATCAAACACTAATGACAGCTTAAGATCTCAAACTCCCTCTGCTCCCATTCAACCATTTAATGAGGACCAACTATATGCCAGGTACTCCAGTAGTGCCTTCTGTTCTGCTG TttcTCCCAGG
GCAAGGGGGGCTAAGTGGTTAGTGGACATTCAGGCTATGTTTCCTTaGGCCTGAAATGGTAAACTAGAACCTCAAGCAAAATTGAAAATAGCCACAACATAGCTCATCTACTGACCACTCAACTGTAA
TCAtTATCATTCATTATTTGCCTgATTAAACATAAGATTTAcCTCTACTAATGCAGAGGCTTTTATTTATATTTTCAGGTATAATATGCCCTGGCTT cTTATCAgAGATAGGAAAGTATTAAGAcTGC
AGGAAATAGAAAAATACTCTAcGCAGAGAGTACcTgTTCTAATCAACAGTGGAATGTTGACTGGT

12	12.9	5.6	2.7	Chimp	157	192	(2049)	+	(TTAATTA)n	Simple_repeat	1	37	(0)	1
335	37.0	3.2	2.8	Chimp	227	434	(1807)	C	MIR1_Amn	SINE/MIR	(64)	204	1	2
530	26.2	6.7	0.0	Chimp	447	610	(1631)	C	Arthur1A	DNA/hAT-Tip100	(2)	175	1	3
942	2.9	1.0	0.0	Chimp	1001	1102	(1139)	+	AluYc	SINE/Alu	160	262	(37)	4
1100	6.3	0.0	0.0	Chimp	1117	1243	(998)	+	AluYc	SINE/Alu	160	286	(13)	5
220	14.6	7.3	0.0	Chimp	1845	1885	(356)	C	L2c	LINE/L2	(7)	3380	3337	6

>NAHR_9_ Scaffold14588 5465171 5467475
KSD_NAHR_9

>Scaffold14588 5465171 5467475 SINE/Alu AluY NAHR 2304
CTGGGGTTGCCCAAAGCAGAGGCTGACTGACATGCACAGAATGAGACCCCTGCCCCTGTGGGGTTTTAGAGCTGCCACTTCAATGGTGACTTCTCACTGGGCACTGCCATAAGACATGAAAGTCTA CA
GGCTTTGTACCCACTCACACATGTCTTCCTTGGACGAACTTGTACCTGATCTTCCAATCTTTCTCCTTCTAAGC CCCTGATGGAGCACCCGTTCATTTCTCACTGCCAACGGTCCATATATATTCATA
TCTCAACCCACTTCCACTTCTCTGTCAATGGAACAGGAACAGGTGAGTGTGTGGCTGGAAGTTCTATGGAGTTAGGACATTTTCCTTCATCGCCGTCTTTTGGGGCTATGCTGGAGCTGAAATGC AGC
AATAACCCAATTTTGGCTAGCACCAGTATATCATGCTGACCAGTCTTTGAACCATAAACCAGTCCCAAGTCATT CGGTTGATCATAAATCACCCCAACAACCAACAGTACCGCCACCACCACTGCCAC
CACCACCGACACCACCATGATGCCATAATTGTGAGCTCAAGGACGGGTTTCACTACTTCCAAAGTCGATGACAGGGTAGACTGGGCCTCCTGTTCCTGTGCTTATTTATGTCTTCCAGATTGACT GAG
GCCTGAGTCAAATTAGGTGCCTCCAGAATGACATCCTGAGGGTATGTTTTCAAGAGCCTGATACCAATGCTCTC AGCCTAAGTCCCTCAGGAAATGGAGCCTGGAACTTAAAAAATAATAAATAAAAT
ATATATGTTACTACTGTATTAGAGAGTCCATTAGAAGAGAAGCAGGGTGAGGGAAAGGGTGTGACCAGAGAAAGAGAGACTTTGCAGAGGGTGTTGGTAAACAGACCAATGCTTGGTATGCAGTG GGG
TTCATTGCTCAGTCTCACGGGATTATCTCCCAAGGGGCCATATGAATCACTTCATCTTCAGACAACCCAACCGT GGGAAGTAAGCGGTAAAAATATATTTGCTGGCTGGGCACAGTGGCTCACGCCTG
TAATCCCAGCACTTTGGGAGGCCGAGGCGGGTGGATCACAAGGgCAGGAGATGGAGACCATCCTGGCTAACACGGTGAAACCCCGTCTCTACTAAAAATACAAAAAATTAGCCAGGCGTGGTGACGGG
CGCCTGTAGTCCCAGCTGCTCAGGAGGCTGAGGCAGGAGAATGGCGTGAcCCCGGGAGGCGGAGCTTGCCATGAGCTGAGACCACGCCACTGCACTCCATCCTGGGCAACAGAGCAAGACTCCGTCTC
AAAAAAAAAAAAAAAAACCAAAAAACTTGCCAGCCCCTGCATCCAATTGGTGAAACATTTGCCCCATGAGCTGGTAACTCACCTACCTTTCTGAGTTGCATAGACGTGGGTCCTTGAATGTATCTAAAT
AGCATCTCAGCATCACAAGCAAAGCCCTGCATGGGATTAGTGGTAATGTG GCAGGGACACAGGGCAAAGCGCAACGTGTTCGCAGGTTACAGCCCCATGAGTTTGGTTGGAGCCATGCAGAGTTGGTT
TCTAAAGTAGCTACTGGGCAGAAACAAGTATCCCAAGGCCCTGGAAAGAGCTAAGGTCAAGAAGATCTGAAGAAGTTGAATGTATGAAACAGGTTTTACACCTCTGTCACTTTCCTTCTCCAGAT CCC
ACTTTTTCTTTCTTTCTTTTTCTTTTTCTTTTTTTTTTTTTTAAGATGGA GTGTCATTCTGTTGCCCATTCTGGAGTGCAGTGACACGATCTCAGCTCACTGCAACTTCTGCCTGCTGGGTTCAAGCG
ATTCTCCTGCCTCAGGTTCCCAAGTACCGGGGTGTACAGGTACCCAA tACCACCCCTGGCTAATTTTTGTATTTTTTGTAGATACAGGGTTTCACCATGTTGGTAAGGCTGGTGTCAAACTCCTGACC
TCAAGTGATCCACCCACCTCGGCCTCCCAAAGTGCTGGGATTACGGGCGT GAGCCACTGCGCCTGACCCTCTTTCTTAATTTTTATTGTTCTACTAGAGTATGTGTCCCTGCTAGTAGCTGTCATTTG
CAGTACTGGTAATGCATGCTCCCTCTGTCCTTCCTTCTACCTGCCAAATATATTACCTACTAAAAACAAAATTGTAAAATAAGCAAAGAGAGTTTTTGTCCTCTTCTCATTTCCTATTTGTTTTT CCT
CTCTTCATCCTTTTGAGATTGTCTCCCCAAGATATCCTTCAGCTTAGTAC AACAGATATTTATTATATGCTTTTTATGAGCTTGGTATTGTGCTACAGAATAAGAATGCAGAGATTAAAGCTGTGGC C

745	34.4	6.6	2.2	S	2	438	(1866)	C	ERV3-16A3_I-int	LTR/ERVL	(1333)	3893	3438	1
24	22.9	0.0	0.0	S	474	529	(1775)	+	(CAC)n	Simple_repeat	1	56	(0)	2
334	36.0	6.1	9.1	S	807	999	(1305)	C	LTR16E2	LTR/ERVL	(82)	486	299	3
2522	7.2	0.0	0.0	S	1000	1304	(1000)	+	AluY	SINE/Alu	1	305	(6)	4
334	36.0	6.1	9.1	S	1305	1553	(751)	C	LTR16E2	LTR/ERVL	(270)	298	56	3
2125	14.7	0.0	0.0	S	1677	1988	(316)	C	AluSx1	SINE/Alu	(0)	312	1	5

671	32.9	7.2	2.7	hg19	18	458	(2016)	C	ERV3-16A3_I-int	LTR/ERVL	(1655)	4012	3438	1
24	22.9	0.0	0.0	hg19	474	529	(1945)	+	(CAC)n	Simple_repeat	1	56	(0)	2
2800	2.0	0.0	0.0	hg19	1000	1301	(1173)	+	AluY	SINE/Alu	1	302	(9)	3
1401	8.7	0.0	0.0	hg19	1302	1474	(1000)	+	AluYc	SINE/Alu	121	293	(6)	4
249	25.4	0.0	1.5	hg19	1483	1550	(924)	C	LTR16D	LTR/ERVL	(327)	324	258	5
2168	14.4	0.0	0.0	hg19	1847	2158	(316)	C	AluSx1	SINE/Alu	(0)	312	1	6

>hg19 Chr10:10808679-10811152
CTGGGGTTGCCCAAAGCAGAGGCTGACTGACATGCACAGAATGAGACCCCTGCCCCTGTGGGGTTTTAGAGCTGCCACTTCAATGGTGACTTCTCACTGGGCACTGCCATAAGACATGAAAGTCTA CA
GGCTTTGTACCCACTCACACATGTCTTCCTTGGACGAACTTGTACCTGATCTTCCAATCTTTCTCCTTCTAAGCCCCTGATGGAGCACCCGTTCATTTCTCACTGCCAACGGTCCATATATATTC ATA
TCTCAACCCACTTCCACTTCTCTGTCAATGGAACAGGAACAGGTGAGTGTGTGGCTGGAAGTTCTATGGAGTTAGGACATTTTCCTTCATCGCCGTCTTTTGGGGCTATGCTGGAGCTGAAATGCA GC
AATAACCCAATTTTGGCTAGCACCAGTATATCATGCTGACCAGTCTTTGAACCATAAACCAGTCCCAAGTCATTCGGTTGATCATAAATCACCCCAACAACCAACAGTACCGCCACCACCACTGC CAC
CACCACCGACACCACCATGATGCCATAATTGTGAGCTCAAGGACGGGTTTCACTACTTCCAAAGTCGATGACAGGGTAGACTGGGCCTCCTGTTCCTGTGCTTATTTATGTCTTCCAGATTGACTG AG
GCCTGAGTCAAATTAGGTGCCTCCAGAATGACATCCTGAGGGTATGTTTTCAAGAGCCTGATACCAATGCTCTCAGCCTAAGTCCCTCAGGAAATGGAGCCTGGAACTTAAAAAATAATAAATAA AAT
ATATATGTTACTACTGTATTAGAGAGTCCATTAGAAGAGAAGCAGGGTGAGGGAAAGGGTGTGACCAGAGAAAGAGAGACTTTGCAGAGGGTGTTGGTAAACAGACCAATGCTTGGTATGCAGTGG GG
TTCATTGCTCAGTCTCACGGGATTATCTCCCAAGGGGCCATATGAATCACTTCATCTTCAGACAACCCAACCGTGGGAAGTAAGCGGTAAAAATATATTTGCTGGCTGGGCACAGTGGCTCACGCCTG

TAATCCCAGCACTTTGGGAGGCCGAGGCGGGTGGATCACAAGG tCAGGAGATGGAGACCATCCTGGCTAACACGGTGAAACCCCGTCTCTACTAAAAATACAAAA Aattagccgggcgtggtggcggg
cgcctgtagtcccagctactcgggaggctgaggcaggagaatggcgtgaacccgggaggcggagcttgcagtgagccgagatcgcgccactgcactccagcctgggcgacagagcgagactccgt ctc
aaaaaaaaaaaaaaaaaaaaATTAGCCAGGCGTGGTGACGGGCGCCTGTAGTCCCAGCTGCTCAGGAGGCTGAGGCAGGAGAATGGCGTGA aCCCGGGAGGCGGAGCTTGCCATGAGCTGAGACCACG
CCACTGCACTCCATCCTGGGCAACAGAGCAAGACTCCGTCTCAAAAAAAAAAAAAAAAACCAAAAAA CTTGCCAGCCCCTGCATCCAATTGGTGAAACATTTGCCCCATGAGCTGGTAACTCACCTACC
TTTCTGAGTTGCATAGACGTGGGTCCTTGAATGTATCTAAATA GCATCTCAGCATCACAAGCAAAGCCCTGCATGGGATTAGTGGTAATGTGGCAGGGACACAGGGCAAAGCGCAACGTGTTCGCAGG
TTACAGCCCCATGAGTTTGGTTGGAGCCATGCAGAGTTGGTTTCTAAAGTAGCTACTGGGCAGAAACAAGTATCCCAAGGCCCTGGAAAGAGCTAAGGTCAAGAAGATCTGAAGAAGTTGAATGT ATG
AAACAGGTTTTACACCTCTGTCACTTTCCTTCTCCAGATCCCACTTTTTCTTTCTTTCTTTTTCTTTTTCT TTTTTTTTTTTTTAAGATGGAGTGTCATTCTGTTGCCCATTCTGGAGTGCAGTGACA
CGATCTCAGCTCACTGCAACTTCTGCCTGCTGGGTTCAAGCGATTCTCCTGCCTCAGGTTCCCAAGTACCGGGGTGTACAGGTACCCAA cACCACCCCTGGCTAATTTTTGTATTTTTTGTAGATACA
GGGTTTCACCATGTTGGTAAGGCTGGTGTCAAACTCCTGACCTCAAGTGATCCACCCACCTCGGCCTCCCA AAGTGCTGGGATTACGGGCGTGAGCCACTGCGCCTGACCCTCTTTCTTAATTTTTAT
TGTTCTACTAGAGTATGTGTCCCTGCTAGTAGCTGTCATTTGCAGTACTGGTAATGCATGCTCCCTCTGTCCTTCCTTCTACCTGCCAAATATATTACCTACTAAAAACAAAATTGTAAAATAAG CAA
AGAGAGTTTTTGTCCTCTTCTCATTTCCTATTTGTTTTTCCTCTCTTCATCCTTTTGAGATTGTCTCCCCA AGATATCCTTCAGCTTAGTACAACAGATATTTATTATATGCTTTTTATGAGCTTGGT
ATTGTGCTACAGAATAAGAATGCAGAGATTAAAGCTGTGGCC

Homologous sequence

TTAGCC

>NAHR_10_ Scaffold528 3222812 3223115
KSD_NAHR_10

>Scaffold528 3222812-3223115_NAHR

CCACCCTCTCCTTATAAACCTTTTACTTAATCCCCCAAATCCCCGACAGTCCTCCAGTGTTCCTTCAACTAGCTAACTCCCTTCCACTCCTTCAGCTACCTCTAACACCCTCACTGACTCTCTCTC CC
TGAATGACTATCCCAATACCTTGTCACTGATCATTTTCCCATACATATACACATGCACCCCCACTAACATATTTATCTGCCACACCAGGGGGAAGACAGCATGAGAAAGTTGGAACCAAAGTCTG TAT
TTTGCAGGGAAAATTTAGCCCTTGGAGCAGATGAACGTGTGCCTTCAAAAGAGGCAGTGTTTATATAGACTAGGAGAATCCTTGACTCTTTCCTTTCACTTTCTCCCTGTCTCTCCACCATCTACA CA
CCTAGTTACCTGTGCTGGTTTCTTTCCATAATGAAAAAAATAGCTAATACACTTTCTGCCAAGCACTAAGCCCTTTATACAGATTAAATTACTGTAAAATTATCAAATCACTCTAAAATGTCATT GTT
CCATTCCATTCCCACCAGCAGCATCCTAGTTCAGGACTGAATAACAAACTCTCAGTTCTCTGGCTTCACATTCCCATTTCCATATGTATCCAGATGAATCCTCTTAACCTCTGCTGCGACTTCCGC TG
CATGCAATCCTTTCACAGATCTCCAAAAATAGTCAAGATCTCCCATACCTGGACCTCATCCGCTGTTGATGACAACCGTAACTCCTCTTTCACTCCCCAGACCCAATCCAAGTCAGCAAATTCCA TAG
GTTCTTCATTCAAAATAGATCCAGAATTTGACCACATCTAACCATCTTCATGACTACTACTATGGTCTAAATCACCACAATCTATCACCTGGATTTCTACAATAGCCTCCTAATTGGTTACCCTGC TT
CTACTTTTCCCCTTCTACAGTCTCTTCTTAATATAAAAGATAGAGCCTAAATAGAAGCCAAATCATTATCACGTCTGTGCTTAAGATTGTCTAGTGGTGTGGGT GGGCATGGTGGCTCACACCTGTAA
TCCCAGCACTTTGGGAGGCCGAGGAGAGTGGATCACGAGGTCAAGAGATAGAGACCATCCTGGCTAAAATGGTGAAACCCCATCTCTACTAAAAATACAAAAAATTAGCCGGGCATGGTGGCGGGCGC
CTGTAGTACCAGCTGCTCGGGAAGCTGAGGCAGGAGAATGGTGTGAACCCAGGAGGCAGAGCTTGCAGTGAGCGAAGATTGTGCCACTGCACTCCAGCCTGGGCAACAGAGCGAGACTCCATCTC AAA
AAAAAATAAAATAAAATACAAAAATATAAAAATTAGCCAGGCATGATGGCGGGTGCAGTGAGCCGAGATCGTACCACTGCATTCCAGCCTGGGCAACAGAGCGAGACTCTGTCTCAAAAAAAAAAAAA
AAAGATTGTCTAGTGGTTTCCCATTTTACCATGAATAAAAGGCCAGAGTCTACATTGGCTTTCAAGATGTGACATGATCTAATATCCTGCTGGTGTCAATACTACTCTCTCTTCCCTAGCTCACA CAT
TTTAGCTACACTAACTTCCTAGCTAGTCTTCAAACACACCAGGCAGCAGCACATTTGTTCTCCACCTACCTGGAACATTTCCCCTCCCTACCCCAACTATCTGCGTGGCACACTCCATACTTTCCT TC
AGATCTCTGCTCAAATGTCATATTTATCAAAAGGTTCTTTCCTTATCAGCTCCCACTCTGCACTGTCACTTCCCTTTCCCCT cACTCTGCTCTATATTTTTCTATAGTACTCATCACTACCTGTCATA
TACTTACTGGTTTCTATGTTTATTGTCTGTCTCCTCAAGCTGGACTATAACCTCCATGAGGGCAAGAACTTTGTTTAGAATAGTGCCTGGCACTTAATAAGTTCTTAGTAAGCATTTATTGAATGA AT
AAATTGTTCCCTGCATAAACCTTTTACTCTAAACATGCTTGGTCTTCTCACTGTTCCAATATAAATAATTGTGGTGGCTAATATTTAAGTGCATTCAATGTGCCAGGAATTTGACATGTTTTTTT TTA
TCCTATTCAATCTTACAAAACTGACAACTCCATGAGATAAAAGCTATTTTTATCCACGTTTAACAGTTGAGAAAACTGAGGCTCAAAGAGGTTAAGTAACTTGCCCAAGATCAGACAGCTAATTAG TG
GCAAAAATAGGACTCAAACTGGGTTAGTCTGACACCAAAACACCCATTCTCCTAATCTTTATATTATCCTGCCTCCCTATAAGGCTTTTTCCTTTCTGTGAACTATTTGTGTAAGTAGCTCTTTG CA

1058	25.2	4.9	1.0	155746	706	993	(1310)	+	L2a	LINE/L2	2557	2855	(564)	1
2392	9.2	0.0	0.0	155746	1001	1304	(999)	+	AluYj4	SINE/Alu	5	308	(3)	2
637	9.3	0.0	0.0	155746	1328	1413	(890)	+	Alu	SINE/Alu	215	300	(12)	3
1057	26.7	16.4	2.2	155746	1417	1924	(379)	+	L2a	LINE/L2	2848	3426	(0)	1
736	26.9	4.4	3.2	155746	1998	2247	(56)	C	MIR	SINE/MIR	(8)	254	2	4

1339	26.6	9.1	4.9	hg19	706	1000	(1436)	+	L2a	LINE/L2	2557	2848	(571)	5
1400	7.1	27.7	3.0	hg19	1001	1129	(1307)	+	AluSx3	SINE/Alu	5	131	(180)	6
2420	9.1	0.0	0.0	hg19	1130	1437	(999)	+	AluYj4	SINE/Alu	1	308	(3)	7
1400	7.1	27.7	3.0	hg19	1438	1546	(890)	+	AluSx3	SINE/Alu	132	299	(12)	6
1339	26.3	15.2	2.8	hg19	1547	2057	(379)	+	L2a	LINE/L2	2849	3426	(0)	5
736	26.9	4.4	3.2	hg19	2131	2380	(56)	C	MIR	SINE/MIR	(8)	254	2	8

>hg19 Chr20:33475918-33478353

CCACCCTCTCCTTATAAACCTTTTACTTAATCCCCCAAATCCCCGACAGTCCTCCAGTGTTCCTTCAACTAGCTAACTCCCTTCCACTCCTTCAGCTACCTCTAACACCCTCACTGACTCTCTCTC CC
TGAATGACTATCCCAATACCTTGTCACTGATCATTTTCCCATACATATACACATGCACCCCCACTAACATATTTATCTGCCACACCAGGGGGAAGACAGCATGAGAAAGTTGGAACCAAAGTCTG TAT
TTTGCAGGGAAAATTTAGCCCTTGGAGCAGATGAACGTGTGCCTTCAAAAGAGGCAGTGTTTATATAGACTAGGAGAATCCTTGACTCTTTCCTTTCACTTTCTCCCTGTCTCTCCACCATCTACA CA
CCTAGTTACCTGTGCTGGTTTCTTTCCATAATGAAAAAAATAGCTAATACACTTTCTGCCAAGCACTAAGCCCTTTATACAGATTAAATTACTGTAAAATTATCAAATCACTCTAAAATGTCATT GTT
CCATTCCATTCCCACCAGCAGCATCCTAGTTCAGGACTGAATAACAAACTCTCAGTTCTCTGGCTTCACATTCCCATTTCCATATGTATCCAGATGAATCCTCTTAACCTCTGCTGCGACTTCCGC TG
CATGCAATCCTTTCACAGATCTCCAAAAATAGTCAAGATCTCCCATACCTGGACCTCATCCGCTGTTGATGACAACCGTAACTCCTCTTTCACTCCCCAGACCCAATCCAAGTCAGCAAATTCCATAG
GTTCTTCATTCAAAATAGATCCAGAATTTGACCACATCTAACCATCTTCATGACTACTACTATGGTCTAAATCACCACAATCTATCACCTGGATTTCTACAATAGCCTCCTAATTGGTTACCCTGC TT
CTACTTTTCCCCTTCTACAGTCTCTTCTTAATATAAAAGATAGAGCCTAAATAGAAGCCAAATCATTATCACGTCTGTGCTTAAGATTGTCTAGTGGTGTGGGT GGGCATGGTGGCTCACACCTGTAA
TCCCAGCACTTTGGGAGGCCGAGgcgggcgggtcacaaggtcaggagtttgagaccagcctggccaacatggtgaaaacctgtctctactaaaaatac aaaaattggccaggcgcagtggcacacgcc

tgtaatcccagcactttgggaggccgagGAGAGTGGATCACGAGGTCAAGAGATAGAGACCATCCTGGCTAAAATGGTGAAACCCCATCTCTACTAAAAATACAAAAAATTAGCCGGGCATGGTGGCG
GGCGCCTGTAGTACCAGCTGCTCGGGAAGCTGAGGCAGGAGAATGGTGTGAACCCAGGAGGCAGAGCTTGCAGTGAGCGAAGATTGTGCCACTGCACTCCAGCCTGGGCAACAGAGCGAGACTCC ATC
TCAAAAAAAAATAAAATAAAATACAAAAATATAAAAATTAGCCAGGCATGATGGCGGGTGCAGTGAGCCGAGATCGTACCACTGCATTCCAGCCTGGGCAACAGAGCGAGACTCTGTCTCAAAAAAAA
AAAAAAAAGATTGTCTAGTGGTTTCCCATTTTACCATGAATAAAAGGCCAGAGTCTACATTGGCTTTCAAGATGTGACATGATCTAATATCCTGCTGGTGTCAATACTACTCTCTCTTCCCTAGCTCA
CACATTTTAGCTACACTAACTTCCTAGCTAGTCTTCAAACACACCAGGCAGCAGCACATTTGTTCTCCACCTACCTGGAACATTTCCCCTCCCTACC CCAACTATCTGCGTGGCACACTCCATACTTT
CCTTCAGATCTCTGCTCAAATGTCATATTTATCAAAAGGTTCTTTCCTTATCAGCTCCCACTCTGCACTGTCACTTCCCTTTCCCCT gACTCTGCTCTATATTTTTCTATAGTACTCATCACTACCTG
TCATATACTTACTGGTTTCTATGTTTATTGTCTGTCTCCTCAAGCTGGACTATAACCTCCATGAGGGCAAGAACTTTGTTTAGAATAGTGCCTGGCACTTAATAAGTTCTTAGTAAGCATTTATT GAA
TGAATAAATTGTTCCCTGCATAAACCTTTTACTCTAAACATGCTTGGTCTTCTCACTGTTCCAATATAAATAATTGTGGTGGCTAATA TTTAAGTGCATTCAATGTGCCAGGAATTTGACATGTTTTT
TTTTATCCTATTCAATCTTACAAAACTGACAACTCCATGAGATAAAAGCTATTTTTATCCACGTTTAACAGTTGAGAAAACTGAGGCTCAAAGAGGTTAAGTAACTTGCCCAAGATCAGACAGCT AAT
TAGTGGCAAAAATAGGACTCAAACTGGGTTAGTCTGACACCAAAACACCCATTCTCCTAATCTTTATATTATCCTGCCTCCCTATAAG GCTTTTTCCTTTCTGTGAACTATTTGTGTAAGTAGCTCTT
TGCA

Homologous Sequences =

CCTGTAATCCCAGCACTTTGGGAGGCCGAGG

>NAHR_11_ Scaffold16040 198931 199238
KSD_NAHR_11

>Scaffold16040 198931-199238_NAHR
CTGCCACAGGAAGGCAGCCAGTACCTGGGACCTGCCAAGTTCACATGGCAGAAGCCACAGCTTCCTTTGGGCCCTTCACCCTGGTCACAGGCACAGAGAGAAACCCAGTGGCCTTGCCTGCTTGCT CC
CAGGACCCCCACCCCCCTGCTCCCAGGCTGTGATGGGCGAGACCAGCTCCACCTCCAGAGCAGCTGGGGGGCATGTAAGGGCTAGTTCCTGGAAGACCCGCTGTGGCTGCAGGCTTTGGTGGAGA GGC
CTGTACAAGGATGAGATTGCAGCCAGGGCCAGGGGAAGGAGGTTTCTGGAATCACTGATGGCTGTTTCGTCCAGGCTTGCAAGCTCTTCCACGTGCAGCCCTTGCTCCTCTCCCCTCCTCTCCCTG CC
TGTGGTTCCCCTACTTCCCACCTGCAGTGGCTGCCTGACTCCCCGTGCTGAAGATTGGCTGAGCATCCACCGAGACACCCTCATCCTCACTCCGATTGTGTGTGGCAGATTGGGGCCCACCAGCA TCA
CAGATGAGGGCACTGAGGGGCTCCGGGAGGAAAAGATGCACGTCCAGGATCTCACAGCTGGTCAAAGGCACAGCCAGGCTCTAATGGTGTTCCCCTGACTCCAGGTTCATTGTTCTTTCCTGGATT CG
GTCCAGATTAACCCAGCACTGGCTTGTCAGAAATCCTCAAGGGCTTCTAGTTGCCTGGAGCTGAAACCTGCAAACTTTTCTGAGCTTGCATCCCTATGAGTGAAACTTTCTGCACACACTCTCCA TTA
TGTGTCCATTTAAATTATTTCTAAAGTATTTGCATGTGTTAATGTACTCTCACAGTCTGTGCATTAGAAAACATTCAAATGCCGTGTAAAGGTGAGATAAAGCCTGCGTAGTGGCACGCTCCTGTA GT
CTGAGCTACCCAAGAGGCTGAGGCAGGAGGATCGCTTGAGCCCAGGAGTTCAAGTCCAGCCTGAGCAACACAGCAAGACCCACCTCTTAATAAAACAAATAAGA GGCCGGGTGTGGTGGCTTACGCCC
ATAATCCCAGCACTTTGGGAGGCCGAGGCGGGCAGATCACGAGGATCGGGAGATTGAGACTATCTTAGCCAACATGGTGAAACCCCTTGTCTACTAAAATACAAAAAATTAGCGGGCGAGGTGGCA TG
TGCCTGTAATCCCAGCTACTTGGGAGGCTGAGGCAGGGGAATCACTTGAACCCGGGAGGCGGAGGTTGCAGTGAGCTGAGATCGTGCCACTGCACTCCAGCCTGGTGACAGAGCAAGACTCTG TCTCA
AAAAAAAAAAAAAAAAAAAAAGAAAGAGATAAATATATTACAAACACAAATAAATAGAAATAATAGCAATAGAAATAGGGTCGTTAATATTTTCTCCCTGCCTCTGGGGATTAGCCTGTGAGTACCGAT
GGAGTACACGTACATCCTTGCCTTGGAGACAACAGGCCTGGAGAATAAGCAACTCCTGGATGTGGTACAGGGTGTTCTATCATCTAAGCCTTCAGCAGCTTT cTTCCTGCTGCCCACAGAGCACTGTT
AAGGTGCCAGAAAGGCCACAACCATATTCAACTTTACATCTGTGCCCACACTCCCTGTCATCCTTTCCTCTCTCCCCCTTGTCAAATCCCCCATGTTGATGAACTCCAAACCAAAACCCAGATCC TCT
GCAAAGCCCTCCTCGCCTACCGTGACCCGGGCATTTGTACCAGCCATTCTGCCTCTGGATCTCAGCCTGCTTTGTATCTCCACTAAACTATCCTGCTTGGCATGCCACATCTCATTGAGCACTTA ATG
CAGGTACCGTTATTATTTTTTTATTTTTGAAGAGACGGAGTCTCGAACTATTGCCCAGGCTGGTCTCAAACTACTGGCCTCAAGCAGTTATCCTGCCTCAGCCTCCCAAAATGCAGGGTTTACAG GCA
TGAGCTACTGCAACCAACAATTACTTCCATTTTATAGCTGGGGAAACTGAGGCTCAGAGAGGTTAAGTAATTTTCCAGAAATCACACAGTAAATGGCAAATGGATTTGTACCCAGGCCTGACTTC CGA
GTCTAACTGTAAGCACTAAGCTACATGAAACAAGGTTGGCCTCTTTCATTCCTGTTGATTCTTCCCAGCACCTAGCATAGAAATAAATATATATATAAAAAATAAATTGCTGAATAAATGATTGA ATG
AATTAATTACCCAGGCCAACACCAAGAAGCCTGGGGAAAAGAGCCCACTTCATTCCTGATACAAAATGTATTACCTCCTGTACCTCAAGAGTCCTGAGCAAGACTTTAAACTTCAGATGTGGCTG AAT
CTG

374	23.5	3.2	3.2	766	510	632	(1675)	C	MIRc	SINE/MIR	(126)	142	20	1
401	25.0	2.2	1.4	766	708	845	(1462)	C	Charlie7a	DNA/hAT-Charlie	(7)	265	127	2
769	16.4	0.8	0.0	766	874	995	(1312)	+	FLAM_A	SINE/Alu	6	128	(14)	3
2317	9.2	1.0	0.3	766	1001	1304	(1003)	+	AluSc8	SINE/Alu	1	306	(5)	4
231	27.8	11.6	5.1	766	1313	1441	(866)	C	Charlie7a	DNA/hAT-Charlie	(133)	139	3	5
732	20.9	2.9	0.3	766	1802	1953	(354)	C	FLAM_C	SINE/Alu	(1)	301	2	6
448	20.8	10.0	0.0	766	1954	2073	(234)	C	MIR	SINE/MIR	(126)	136	5	7
20	23.3	1.8	0.0	766	2129	2183	(124)	+	A-rich	Low_complexity	1	56	(0)	8

374	23.5	3.2	3.2	hg19	510	632	(1903)	C	MIRc	SINE/MIR	(126)	142	20	9
401	25.0	2.2	1.4	hg19	708	845	(1690)	C	Charlie7a	DNA/hAT-Charlie	(7)	265	127	10
769	16.4	0.8	0.0	hg19	874	995	(1540)	+	FLAM_A	SINE/Alu	6	128	(14)	11
2334	8.9	1.0	0.3	hg19	1001	1304	(1231)	+	AluSc8	SINE/Alu	1	306	(5)	12
1946	6.6	0.0	0.0	hg19	1305	1532	(1003)	+	AluSc	SINE/Alu	77	304	(5)	13
231	27.8	11.6	5.1	hg19	1541	1669	(866)	C	Charlie7a	DNA/hAT-Charlie	(133)	139	3	14
732	20.9	2.9	0.3	hg19	2030	2181	(354)	C	FLAM_C	SINE/Alu	(1)	301	2	15
448	20.8	10.0	0.0	hg19	2182	2301	(234)	C	MIR	SINE/MIR	(126)	136	5	16
20	23.3	1.8	0.0	hg19	2357	2411	(124)	+	A-rich	Low_complexity	1	56	(0)	17

>hg19 chr16:74761672-74764206
CTGCCACAGGAAGGCAGCCAGTACCTGGGACCTGCCAAGTTCACATGGCAGAAGCCACAGCTTCCTTTGGGCCCTTCACCCTGGTCACAGGCACAGAGAGAAACCCAGTGGCCTTGCCTGCTTGCT CC
CAGGACCCCCACCCCCCTGCTCCCAGGCTGTGATGGGCGAGACCAGCTCCACCTCCAGAGCAGCTGGGGGGCATGTAAGGGCTAGTTCCTGGAAGACCCGCTGTGGCTGCAGGCTTTGGTGGAGA GGC
CTGTACAAGGATGAGATTGCAGCCAGGGCCAGGGGAAGGAGGTTTCTGGAATCACTGATGGCTGTTTCGTCCAGGCTTGCAAGCTCTTCCACGTGCAGCCCTTGCTCCTCTCCCCTCCTCTCCCTG CC
TGTGGTTCCCCTACTTCCCACCTGCAGTGGCTGCCTGACTCCCCGTGCTGAAGATTGGCTGAGCATCCACCGAGACACCCTCATC CTCACTCCGATTGTGTGTGGCAGATTGGGGCCCACCAGCATCA

CAGATGAGGGCACTGAGGGGCTCCGGGAGGAAAAGATGCACGTCCAGGATCTCACAGCTGGTCAAAGGCACAGCCAGGCTCTAATGGTGTTCCCCTGACTCCAGGTTCATTGTTCTTTCCTGGATT CG
GTCCAGATTAACCCAGCACTGGCTTGTCAGAAATCCTCAAGGGCTTCTAGTTGCCTGGAGCTGAAACCTGCAAACTTTTCTGAGCTTGCATCCCTATGAGTGAAACTTTCTGCACACACTCTCCA TTA
TGTGTCCATTTAAATTATTTCTAAAGTATTTGCATGTGTTAATGTACTCTCACAGTCTGTGCATTAGAAAACATTCAAATGCCGTGTAAAGGTGAGATAAAGCCTGCGTAGTGGCACGCTCCTGTA GT
CTGAGCTACCCAAGAGGCTGAGGCAGGAGGATCGCTTGAGCCCAGGAGTTCAAGTCCAGCCTGAGCAACACAGCAAGACCCACCTCTTAATAAAAC AAATAAGAGGCCGGGTGTGGTGGCTTACGCCC
ATAATCCCAGCACTTTGGGAGGCCGAGGCGGGCAGATCACGAGGATCGGGAGATTGAGACTATCTTAGCCAACATGGTGAAACCCCTTGTCTACTAAAATACAAAAAATTAGCGGGCGAGGTGGCA TG
TGCCTGTAATCCCAGCTACTTGGGAGGCTGAGGCAGGGGAATCACTTGAACCCGGGAGGCGGAGGTTGCAGTGAGCTGAGATCGTGCCACTGCACTCCAGCCTGGTGACAGAGCAAGACTCTG Tctca
aaaaaaaaaaaaaaaaaaaaaaaatcaagaccatcctggccaacatgatgaaaccccgtctctactaaaaatacaaaaatcagctgggcgtggtggtgcgtgcctgtagtcccagctactcaggaggc
tgaggcaggagaatcacttgaacccaggaggcggaggttgcagtgagctgagatcatgccactgcactccagcctggcgacagaacgagactctgt CTCAAAAAAAAAAAAAAAAAAAAAGAAAGAGA
TAAATATATTACAAACACAAATAAATAGAAATAATAGCAATAGAAATAGGGTCGTTAATATTTTCTCCCTGCCTCTGGGGATTAGCCTGTGAGTACCGATGGAGTACACGTACATCCTTGCC TTGGAG
ACAACAGGCCTGGAGAATAAGCAACTCCTGGATGTGGTACAGGGTGTTCTATCATCTAAGCCTTCAGCAGCTTT tTTCCTGCTGCCCACAGAGCACTGTTAAGGTGCCAGAAAGGCCACAACCATATT
CAACTTTACATCTGTGCCCACACTCCCTGTCATCCTTTCCTCTCTCCCCCTTGTCAAATCCCCCATGTTGATGAACTCCAAACCAAAACCCAGATCCTCTGCAAAGC CCTCCTCGCCTACCGTGACCC
GGGCATTTGTACCAGCCATTCTGCCTCTGGATCTCAGCCTGCTTTGTATCTCCACTAAACTATCCTGCTTGGCATGCCACATCTCATTGAGCACTTAATGCAGGTACCGTTATTATTTTTTTATT TTT
GAAGAGACGGAGTCTCGAACTATTGCCCAGGCTGGTCTCAAACTACTGGCCTCAAGCAGTTATCCTGCCTCAGCCTCCCAAAATGCAGGGTTTACAGGCATGAGCTACTGCAACCAACAATTACT TCC
ATTTTATAGCTGGGGAAACTGAGGCTCAGAGAGGTTAAGTAATTTTCCAGAAATCACACAGTAAATGGCAAATGGATTTGTACCCAGGCCTGACTTCCGAGTCTAACTGTAAGCACTAAGCTACA TGA
AACAAGGTTGGCCTCTTTCATTCCTGTTGATTCTTCCCAGCACCTAGCATAGAAATAAATATATATATAAAAAATAAATTGCTGAATAAATGATTGAATGAATTAATTACCCAGGCCAACACCAA GAA
GCCTGGGGAAAAGAGCCCACTTCATTCCTGATACAAAATGTATTACCTCCTGTACCTCAAGAGTCCTGAGCAAGACTTTAAACTTCAGATGTGGCTGAATCT G

406	25.7	3.0	3.0	Chimp	466	631	(1887)	C	MIRc	SINE/MIR	(80)	188	20	1
397	25.7	2.2	1.4	Chimp	707	844	(1674)	C	Charlie7a	DNA/hAT-Charlie	(7)	265	127	2
716	17.6	0.8	0.0	Chimp	873	997	(1521)	+	FLAM_A	SINE/Alu	6	131	(11)	3
2172	8.2	0.7	0.3	Chimp	1000	1290	(1228)	+	AluSc8	SINE/Alu	1	292	(19)	4
1831	7.7	0.0	0.0	Chimp	1291	1523	(995)	+	AluSc	SINE/Alu	77	309	(0)	5
12	10.7	12.5	0.0	Chimp	1529	1568	(950)	+	A-rich	Low_complexity	1	45	(0)	6
682	22.4	2.9	0.3	Chimp	2013	2164	(354)	C	FLAM_C	SINE/Alu	(1)	301	2	7
474	20.0	10.0	0.0	Chimp	2165	2284	(234)	C	MIR	SINE/MIR	(126)	136	5	8
17	25.8	1.8	0.0	Chimp	2336	2394	(124)	+	A-rich	Low_complexity	1	60	(0)	9

>Chimp
CTGCCACAGGAAGGCAGCCAGTACCTGGGACCTGCCAAGTTCACATGGCAGAAGCCACAGCTTCCTTTGGGCCCTTCACCCTGGTCACAGGCACAGAGAGAAACCCAGTGGCCTTGCCTGCTTGCT CC
CAGGACCCCCACaCCCCTGCTCCCAGGCTGTGATGGGCGAGACtAGCTCCACCTCCAGgGgAGCTGGGGGCATGTAAGGGCTAGTTCCTGGAAGACCCGCTGTGGCTGCAGGCTTTGGTGGAGAGGCC
TGTACAAGGATGAGATTGCAGCCAGGGCCAGGGGAAGGAGGTTTCTGGAATCACTGATGGCTGTTTC aTCCAGGCTTGCAAGCTCTTCCACGTGCAGCCCTTGCTCCTCTCCCCTCCTCTCCCTGCCT
GTGGTTCCCCTACTTCCCACCTGCAGTGGCTGCCTGACTCCCCGTGCTGAAGATTGGCTGAGCATCCACCGAGACACCCTCATCCTCACTCCGATTGTGTGTGGCAGATTGGGGCCCACCAGCAT CAC
AGATGAGGGCACTGAGGGGCTCCGGGAGGAAAAGATGCACGTCCAGGATCTCACAGCTGGTCAAAGGCACAGCCAGGCTCTAATGGTGTTCCCCTGACTCCAGGTTCATTGTTCTTTCCTGGATTC GG
TCCAGATTAACCCAGCACTGGCTTGTCAGAAATCCTCAAGGGCTTCTAGTTGCCTGGAGCTGAAACCTGCAAACTTTTCTGAGCTTGCATCCCTATGAGTGAAACTTTCTGCACACACTCTCCAT TAT
GTGTCCATTTAAATTATTTCTAAAGTATTTGCATGTGTTAATGTACTCTCACAGTCTGTGCATTAGAAAACATTCAAATGCCGTGTAAAGGTGAGATAAAGCCTGCGTAGTGGCACGCTCCTGTAG TC
TGAGCTACCCAAGAGGCTGAGGtAGGAGGATCGCTTGAGCCCAGGAGTTCAAGTCCAGCCTGAGCAACACAGCAAGACCCACCTCTTAATAAAACAAATAAGAGGCCGG aTGTGGTGGCTcACGCCCg
TAATCCCAGCACTTTGGGAGGCCGAGGCGGGCgGATCACGAGGATCaGGAGATTGAGACTATCTTAGCCAACATGGTGAAACCCCgTGTCTACTAAAATACAAAAAATTAGCtGGGCGAGGTGGCAcG
TGCCTGTAATCCtAGCTACTTGGGAGGCTGAGGCAGGGGAATCACTTGAACCCGGGAGGCGGAGGTTGCAGTGAGCTGAGATCGTGCCACTGCACTCCAGCCTGGTGACAGAGCAAGACTCTGTCTCA
AAAAAAAAAATCgAGACCATCCTGGCCAACATGATGAAACCCCGTCTCTACTAAAAATACAAAAATCAGCTGGGCGTGGTGGTGCGTGCCTGTAGTCCCAGCTACTCAGGAGGCTGAGGCAGGAGAAT
CACTTGAACCCAGGAGGCGGAGGTTGCAGTGAGCTGAGATCATGCCACTGCACTCCAGCCTGGCGACAGAACGAGACTCTGTCTCAAAAAAAAAAAAAA gAAAAAgAGAGATAAATATATTACAAACA
CAAATAAATAGAAATAATAGCAATAGAAATAGGGTCGTTAATATTTTCTCCCTGCCTCTGGGGATTAGCCTGTGAGTACCGATGGAGTACACGTACATCCTTGCCTTGGAGACAACAGGCCTGGAG AA
TAAGCAACTCCTGGATGTGGTACAGGGTGTTCTATCATCTAAGCCTTCAGCAGCTTT ccTCCTGCTGCCCACAGAGCACTGTTAAGGTGCCAGAAAGGCCACAACCA cATTCAACTTTACATCTGTGC
CCACACTCCCTGTCATCCcTTCCTCTCTCCCCCTTGTCAAATCCCCCATGTTGATGAACTCCAAACCAAAACCCAGATCCTCTGCAAAGCCCTCCTCGCCTACCGTGACCCGGGCATTTGTACCAGCC
ATTCTGCCTCTGGATCTCAGCCTGCTTTGTATCTCCACTAAACTATCCTGCTTGGCATGCCACATCTCATTGAGCACTTAATGCAGGTACCGTTATTATTTTTTTATTTTTGAAGAGACGGAGTC TtG
AACTATTGCCCAGGCTGGTCTCAAACTACTGGCC cCAAGCAGTTATCCTGCCTCAGCCTCCCAAAATGCAGGGTTTACAGGCATGAGCTACTGCAACCAACAATTACTTCCATTTTATAGCTGGGGAA
ACTGAGGCTCAGAGAGGTTAAGTAATTTTCCAGAA gTCACACAGTAAATGGCAAATGGATTTGTACCCAGGCCTGACTTCCGAGTCTAACTGTAAGCACTAAGCTACATGAAACAAGGTTGGCCTCTT

TCATTCCTGTTGATTCTTCCCAGCAtCTAGCATAGAAATAAatATATATATATAAAATAAATTGCTGAATAAATGATTGAATGAATTAATTACCCAGGCCAACACCAAGAAGCCTGGGGAAAAGAGCC
CACTTCATTCCTGATACAAAATGTATTACCTCCTGTACCTCAAGAGTCCTGAGCAAGACTTTAAACTTCAGATGTGGCTGAATCT G

Homologous Sequences

AGACTCTGTCTCAAAAAAAAAAAAAAAAAAAAA

>NAHR_12_ Scaffold16040 1171017 1171314
KSD_NAHR_12

>Scaffold16040 1171017 1171314_NAHR

TTCCTTGTTGACTGTGTCTTTAACTATGGCTGTCTTAAGATTTTGTCACCTATTATTGTTTTCTTTTGCTTTGATTCTTCTCCAAAAAATGACTTATAACTACAGTCCAGGGCTTGCTTCTTTGAC TC
CTGAGTCTGAAAAAGCCACCACCCCCTGCTAAATCTTAAGCATTAACACCAGTTGAAGTCTTATCTTCAGACTCAGTAGAAGATGACAATCAAAATGAATTGTTTTCATGAGACACGGGTCTTTG GGA
GAGTTCATAAAAAAAAAAAATCTTTTTTTTTTTTTTTTTTTTTTGAGACAGATTTTCTCTTTGTTACCCAGGTTGGAATGCAGTGGCACAAGCTTGGCTCACTGCAACCTCTGTCTCCTAGGTTCAGA
TGATTCTCTTGCCACAGCCACATGAGTGGCTGGGATTACAGGTGTGTGTCACCATGACTGACTAATTTTTATATTTTTGTTAGAAACAGGGTTTGCCATGTTGGCTGGGCTAGTCTGGAACTCCT GAC
CTCAAGTGATCTGCCCACCTCGGCTTCCCAAAGTGCTGAGATTATAGGTGTGAGCCACCGTTCCCAGCCAAAAAGGACTCTTAAATGCAGATTTCTGATAACTTTGGAGATTGTGCCATTGGATGA GA
GAGAAAATTTCCAGGGCATGAATGGAAAGGCTGACATGTTCATAAATATTGCTGACCTATTTTGAAGCACAGCAGGGAGTTGATTGCATGGATTGGACTAATGGAGGACTGAAATAAATTTGTAT TGC
TTTTTGTTGATGTTGTTTATTACAAACATTGCTGATTCTTCAGAGTCTGGAGAGCTTTTTTTTTTGAGCTATTTATACCCTTTAACAATTGAGTAGAGTATACTCTTGTAAACAGAATTTGAGGCT TA
TTTCTCTCTCTGCCTAATTGCTCAAGAAcTTGTAAATTAGTTGTGAATGTTCTTCATTCCTGGCAACATGTTTGCATAAGTGCAATAAGAATCTGTTTTCTGT CCGGGTGTAGTGGCTCACGCCGTAA
TCCCAGCACTTTGGGAGCCTGAGGTGGGTGGATCACGAGGTCAGGAGATTGAGACCATCCTGGCTAACAT GGTGAAACCCCGTCTCTACTAAAAATACAAAAAATTAGCCGGGCGTGGTAGCGGGCGC
CTGTAGTCCCAGCTACTCGGGAGGCTGAGGCAGGAGAATGGCGTGAACCCGGGAGGCGGAGCTTGCAGTGAGCCGAGATCGCGCCACTGCACTCCAGCCTGGGCGACAGAGCGAGACTCCGTCTC AAA
AAAAAAAAAAAAAAGAATCGTTTTCTTTTGTAATGGGACACAGTTTGAGGAACTGGTTATTTTCCCAGGGCTTTGACCGAAATGCCTTTGTGGGAGGCTCTAGCAAGGCCATTTTAGGAGAGGCTATG
TGGACAATGATTCTTGCTGCACTTTGTGTGGGTAATCAGGCCAAGTATATGGGACTGAAGCTTATTTTGCAGGTAGGTTGGTTCTGCTGTGATTTGTCTTTGGCGGAAATTGGGGACTGAAGAGA GAA
AGACTGTGTTTCTGAAGAAAACTACAGTATTAGATTAACCTTTGATTCCTGGGTGGCCACGTGGTCGCCCATAGCATGGAGCTGCCGACAACGTTCTTCCTCAGCATGAAGGAGCCAGTAAGGCTG GC
GATCAGGTTCCTCATGATTGAGGAATCAATAAATAGAAAAAGCGGACTGGACTGAAATTGACCCAATAGTCTTATAGACAGTTTTTTTTGAATAAACATAGAAATCAACCCTTCTGGTCTTAAAG GAT
GAAACTTAACATTTATTTTATCTGAGTTCCTTCCGCAGGAAAGAAGCCCCCAGGCCTCTCAAAAAGTATCAAAGAACTGAGAGTCACCAGATAGTCACATCCAAACGATTGAATGCCAGGCCCTTC AT
TCATCATGAATGATTCCTTACCCTTTCCAAGTTCCTGTTGTCCCATGCGTAGTTACGTTTCTTCCCTGCTATGTAACTCCCTAATTTAATCAGTAAGAGTGATGGATTTGAGACTGATCTCCTGT CTC
CTCAGCTGTAGTGCCCGATTAAAGCCTTCTTCTTTGGCAATAATTATTGTTTCGTGATTGGCTTTCCATGTGGCCAGCAGCAGGACCTAGACTGAAGCCCTGGTATTTTGGTAACATGAACATAGA CT
GGTCCTGGCTAGTTTTGGAGGCAGATAATGCAGGTAAAAAGAGTCTTCAGCAAGGCTTCCCTTCTAACAAAAAGCAGCCCCCAAATCATTTCTTTTCTAACAAAAAGGCTTTTCTATGCC T

741	9.1	4.0	2.4	14955	1	124	(2173)	+	LOR1-int	LTR/ERV1	4901	5026	(3094)	1	*
870	16.4	0.0	0.0	14955	120	247	(2050)	+	LOR1-int	LTR/ERV1	3821	3948	(4172)	2	
2848	10.3	4.5	7.6	14955	248	277	(2020)	+	LOR1-int	LTR/ERV1	5021	5042	(3078)	2	
1967	17.4	0.3	0.0	14955	278	581	(1716)	C	AluSz	SINE/Alu	(7)	305	1	3	
2848	10.3	4.5	7.6	14955	582	999	(1298)	+	LOR1-int	LTR/ERV1	5043	5464	(2656)	2	
2650	3.7	0.3	0.0	14955	1000	1297	(1000)	+	AluY	SINE/Alu	3	301	(10)	4	
2848	11.2	0.6	2.0	14955	1298	1621	(676)	+	LOR1-int	LTR/ERV1	5392	5650	(1108)	2	
539	19.8	0.0	0.0	14955	1621	1711	(586)	+	LOR1-int	LTR/ERV1	8030	8120	(0)	2	*
2289	20.4	3.4	0.4	14955	1717	2164	(133)	+	LOR1b	LTR/ERV1	1	461	(0)	2	
595	11.4	1.1	1.1	14955	2195	2283	(14)	+	LTR9A1	LTR/ERV1	29	117	(611)	5	

741	9.1	4.0	2.4	hg19	1	124	(2484)	+	LOR1-int	LTR/ERV1	4901	5026	(3094)	6	*
870	16.4	0.0	0.0	hg19	120	247	(2361)	+	LOR1-int	LTR/ERV1	3821	3948	(4172)	7	
2848	10.1	4.4	8.0	hg19	248	277	(2331)	+	LOR1-int	LTR/ERV1	5021	5042	(3078)	7	
1760	18.9	0.4	0.0	hg19	278	584	(2024)	C	AluSz	SINE/Alu	(0)	312	1	8	
2848	10.1	4.4	8.0	hg19	585	1002	(1606)	+	LOR1-int	LTR/ERV1	5043	5464	(2656)	7	
2528	7.2	0.3	0.0	hg19	1003	1307	(1301)	+	AluY	SINE/Alu	3	308	(3)	9	
2840	1.0	0.0	0.0	hg19	1308	1608	(1000)	+	AluY	SINE/Alu	1	301	(10)	10	
2848	11.1	0.6	2.1	hg19	1609	1932	(676)	+	LOR1-int	LTR/ERV1	5392	5649	(1109)	7	
539	19.8	0.0	0.0	hg19	1932	2022	(586)	+	LOR1-int	LTR/ERV1	8030	8120	(0)	7	*
2289	20.4	3.4	0.4	hg19	2028	2475	(133)	+	LOR1b	LTR/ERV1	1	461	(0)	7	
595	11.4	1.1	1.1	hg19	2506	2594	(14)	+	LTR9A1	LTR/ERV1	29	117	(611)	11	

>hg19 chr16:75722411-75725018

TTCCTTGTTGACTGTGTCTTTAACTATGGCTGTCTTAAGATTTTGTCACCTATTATTGTTTTCTTTTGCTTTGATTCT TCTCCAAAAAATGACTTATAACTACAGTCCAGGGCTTGCTTCTTTGACTC
CTGAGTCTGAAAAAGCCACCACCCCCTGCTAAATCTTAAGCATTAACACCAGTTGAAGTCTTATCTTCAGACTCAGTAGAAGATGACAATCAAAATGAATTGTTTTCATGAGACACGGGTCTTTG GGA
GAGTTCATAAAAAAAAAAAATCTTTTTTTTTTTTTTTTTTTTTTTtttGAGACAGATTTTCTCTTTGTTACCCAGGTTGGAATGCAGTGGCACAAGCTTGGCTCACTGCAACCTCTGTCTCCTAGGTTC
AGATGATTCTCTTGCCACAGCCACATGAGTGGCTGGGATTACAGGTGTGTGTCACCATGACTGACTAATTTTTATATTTTTGTTAGAAACAGGGTTTGCCATGTTGGCTGGGCTAGTCTGGAACT CCT
GACCTCAAGTGATCTGCCCACCTCGGCTTCCCAAAGTGCTGAGATTAT AGGTGTGAGCCACCGTTCCCAGCCAAAAAGGACTCTTAAATGCAGATTTCTGATAACTTTGGAGATTGTGCCATTGGATG
AGAGAGAAAATTTCCAGGGCATGAATGGAAAGGCTGACATGTTCATAAATATTGCTGACCTATTTTGAAGC ACAGCAGGGAGTTGATTGCATGGATTGGACTAATGGAGGACTGAAATAAATTTGTAT
TGCTTTTTGTTGATGTTGTTTATTACAAACATTGCTGATTCTTCAGAG TCTGGAGAGCTTTTTTTTTTGAGCTATTTATACCCTTTAACAATTGAGTAGAGTATACTCTTGTAAACAGAATTTGAGGC
TTATTTCTCTCTCTGCCTAATTGCTCAAGAAtTTGTAAATTAGTTGTGAATGTTCTTCATTCCTGGCAACATGTTTGCATAAGTGCAATAAGAATCTGTTTTCTGT CCGGGTGTAGTGGCTCACGCCG
TAATCCCAGCACTTTGGGAGCCTGAGGTGGGTGGATCACGAGGTCAGG AGATTGAGACCATCCTGGCTAACATGgtgaaaccccgtctctactaaaaatacaaaaaattagctgggcatggtggcggg
cgcctgtagtcctagctactcgggaggctgaggcaggagaatggcgtgaacctgggaggcagagcttgaagtgagctgagatcgtgccactgcactctagcctgggagacagagcaaaactctgt ctc
aaaaaaaaaaaaaaaaaaaaaaaaaaggccgggcgcggtggctcacgcctgtaatcccagcactttgggaggccgaggcgggcggatcacgaggtcaggagatcgagactatcctggctaacacgGT
GAAACCCCGTCTCTACTAAAAATACAAAAAATTAGCCGGGCGTGGTAGCGGGCGCCTGTAGTCCCAGCTACTCGGGAGGCTGAGGCAGGAGAATGGCGTGAACCCGGGAGGCGGAGCTTGCAGTG AGC
CGAGATCGCGCCACTGCACTCCAGCCTGGGCGACAGAGCGAGACTCCGTCTCAAAAAAAAAAAAAAAAAAAGAA TCGTTTTCTTTTGTAATGGGACACAGTTTGAGGAACTGGTTATTTTCCCAGGGCT T
TGACCGAAATGCCTTTGTGGGAGGCTCTAGCAAGGCCATTTTAGGAGAGGCTATGTGGACAATGATTCTTGCTGCACTTTGTGTGGGTAATCAGGCCAAGTATATGGGACTGAAGCTTATTTT GCAGG
TAGGTTGGTTCTGCTGTGATTTGTCTTTGGCGGAAATTGGGGACTGAAGAGAGAAAGACTGTGTTTCTGAAGAAAACTACAGTATTAGATTAACCTTTGATTCCTGGGTGGCCACGTGGTCGCCCAT A
GCATGGAGCTGCCGACAACGTTCTTCCTCAGCATGAAGGAGCCAGTAAGGCTGGCGATCAGGTTCCTCATGATTGAGGAATCAATAAATAGAAAAAGCGGACTGGACTGAAATTGACCCAATAGT CTT
ATAGACAGTTTTTTTTGAATAAACATAGAAATCAACCCTTCTGGTCTTAAAGGATGAAACTTAACATTTATTTTATCTGAGTTCCTTCCGCAGGAAAGAAGCCCCCAGGCCTCTCAAAAAGTATC AAA
GAACTGAGAGTCACCAGATAGTCACATCCAAACGATTGAATGCCAGGCCCTTCATTCATCATGAATGATTCCTTACCCTTTCCAAGTTCCTGTTGTCCCATGCGTAGTTACGTTTCTTCCCTGCT ATG
TAACTCCCTAATTTAATCAGTAAGAGTGATGGATTTGAGACTGATCTCCTGTCTCCTCAGCTGTAGTGCCCGATTAAAGCCTTCTTCTTTGGCAATAATTATTGTTTCGTGATTGGCTTTCCATG TGG
CCAGCAGCAGGACCTAGACTGAAGCCCTGGTATTTTGGTAACATGAACATAGACTGGTCCTGGCTAGTTTTGGAGGCAGATAATGCAGGTAAAAAGAGTCTTCAGCAAGGCTTCCCTTCTAACAA AAA
GCAGCCCCCAAATCATTTCTTTTCTAACAAAAAGGCTTTTCTATGCC T

Homologous Sequences
GGTGAAACCCCGTCTCTACTAAAAATACAAAAAATTAGC

>NAHR_13_ Scaffold4044 579296 579606
>KSD_NAHR_13
>Scaffold4044 579296 579606_NAHR

GATTGAGGGAGGCTCACACAGATGTATGAAGATCAACCATGTCTTTATAACTGCAGAAGCTTAGTTATGAGTGTATATGGAAGCTCATTATACTAATTTTTATTTTTATGACTATTTGAAATTTTC CA
TAATAAAAAATTAGAAACAACTATGGAAGGGAATGAAAAATTTACCTAAACTTTTAGGACCTAATTTAAAGCCTGTTTACCCTACACTGTTTGCAGAAGAGAATGAAACTGACATCCAGAAAGAG GCA
GAGACAGAGACCCCAGAAGCACAAACCAGCCCTCATTCTCACTATTCCCAGCCCGGCCCCTCCCCTCCCAATTCCTTTTTTGTGTACGTTAAAGTTGGGACTTGTTCACTTAGAACCCAAAGGCAC TG
ACCAATGGAAAACTATAAATATTTGCATTCATCTTCTGGGAGTATACAGTATTATCTTGGGAATGCACCAAAACAGAAAAGAAGTTAACACTAAATTTGTTAACTTTTGACCATTTTCAAAAAAT CAA
GTCTACTTTTCAAATGTTGAATATGTCTGCAaCATAGTCTCTTAGTACCGTTTCCCATGAAGAGGAACCAGGACTCCTTGGGAAATTGCTGATTCCTGGTCTGGGCATTAAATGCCCAAGATGAGCCT
GGAGTATCTTTGGATTCCAGAAATCAGAGACTGCAAAGACTAGCAGGGTCAGGTCAAAAAAACATGGGCTGCCTGCCAGCCAGCATGACCAATTTAAGCACTGAAGTTTGAAACACATCAAATGT TTT
AAATCCATGAGTTAATAATGACACTTAAGAAAATGAAAAGAAAAAGAAACCTCTTTGGTCACCTTTGGAGGGTGATAGTGAAGCAACTCATTTATTCTGAAAACAAGAAAATAAGGAGAAAGAAAC AA
AGTATTTTATTCTAACTTTCCTATACAAATTGTACCTCCACATATAAAATAACTGATGAGCAAAAACAAATTTTTCAGAATTCCAGCAATAAATGCAGAAGGGA GGCTGGGTGCGGTGGCTCACACCT
GTAATCCCAGCACTTTGAGAGGCTGTGGTGGGTGGATCACTTGAGCCCAGGAGTTTGAGACTAGcATGGGCAACATAGTGAAACCTCATCTCTACAACAAATACAAAAAAATTATCTGGGCATGGTGA
TGTGCACCTGTGGTGATGTGCACCTGTGATCCCAGCTACTTAGGAGGCTGAGGTGGGAGGATCACTTGAGCCCAGGAGATGGAAGTTGCAGTGAGCTGAGATCATGCCACTGCATTCCAGCCTGG GTG
ACAGAGTGAGACTCTGTCTCAAAAAAAATAAGTACAGAAGGAATAATATCACCATTAcACAGCTCTTAAACAACGGATTTGAGGAATAATCATCAATACTACCCCAAAACCATTAGGTGAAAAGCTGA
CAGGAAACTTTATAATGGAAAGTTCCGACTAGCAACATAGCACATGTACAGAAGAAGAGAGGATATCCTACCTGCTTGAAAATTCTTTTCTTTACAGGGAGGAAAGGTCAATTAGAAATACAAAG GGA
AGAAGAAATTCACCAGTTTACACCATAACTACTCTTTTACATTAACCACTGTTGTAGATGAAAGTCTGCAGTCTCAAGGGCATGGC AGGTGTTCTGTATAGTCTGGGCCATCTTCCTTAAAGCTGGTG
TTCACCAAGTACAAAAGCGCCTACCAGAAATATTTGTCAGCGTCCAACAGACATGGCAAGGCTATTCCATATTCTTTTCTTTTCAGGAAAGCTCTGCCCTTCTCATGATATCCCATAGCTAACAT AAG
GGCCTAGGAAAAAATACAAATTTAAAAAACATCACTTTAAAATATAATAAGGCAAATTATATTAAAAGCATATTACTTTTTAAAAAACAAAGTAAAAAAAATGAAAACAAGATCATAACTCAATCT AA
AAGCCAAAATTAAGAAAAATATAATTAATTAAAGCTTTATTTCATTTAAATACTGAAGCCAAACACTTCATTTATTATAGCTGATACAATCCTAACCAGCACTTGGCCCTTTGCTTTCATTAGTT TAT
TTTCGTATCCACTGATCTGGATTCTACTTCTAATATTCTATTCAGACTAAACATATTAAAAAGCAGCCAGTAATGCTTGTATCATTTTTATTTTATTTATTTTATTTTTTTGAGACGGAGTCTCAT TC
TGTCGTCCAGGCTGGAGGGCAGTGGTGCGATCTCGGGTCACTGCAACCTCTGCCTCCTGGGTTCAAGCGATTCTCCTGCCTCAGCCTGCCGAGCAGCTAGGACTACAGGCATGAGCCACCATGCC CGG
CTAATT

425	25.6	1.5	1.5	963	18	148	(2162)	+	L1MC5	LINE/L1	7816	7946	(1)	1
12	26.4	2.2	0.0	963	223	267	(2043)	+	(AGACAG)n	Simple_repeat	1	46	(0)	2
1792	21.4	9.8	6.2	963	547	1000	(1310)	+	L1MC5a	LINE/L1	6665	7138	(892)	3
1937	13.3	0.0	6.2	963	1001	1308	(1002)	+	AluJb	SINE/Alu	1	290	(22)	4
1792	21.4	9.8	6.2	963	1309	1445	(865)	+	L1MC5a	LINE/L1	7139	7276	(754)	3
1325	10.7	0.0	0.0	963	2133	2310	(0)	C	AluSx3	SINE/Alu	(3)	308	131	5

425	25.6	1.5	1.5	hg19	18	148	(2307)	+	L1MC5	LINE/L1	7816	7946	(1)	6
12	26.4	2.2	0.0	hg19	223	267	(2188)	+	(AGACAG)n	Simple_repeat	1	46	(0)	7
1747	21.5	9.6	8.0	hg19	543	1000	(1455)	+	L1MC5a	LINE/L1	6661	7138	(892)	8
987	11.5	0.0	1.5	hg19	1001	1139	(1316)	+	FLAM_C	SINE/Alu	1	137	(6)	9
1799	15.2	0.0	6.3	hg19	1150	1452	(1003)	+	AluJb	SINE/Alu	6	290	(22)	10
1747	21.5	9.6	8.0	hg19	1453	1589	(866)	+	L1MC5a	LINE/L1	7139	7276	(754)	8
1325	10.7	0.0	0.0	hg19	2278	2455	(0)	C	AluSx3	SINE/Alu	(3)	308	131	11

>hg19 chr7:151056712-151059166

GATTGAGGGAGGCTCACACAGATGTATGAAGATCAACCATGTCTTTATAACTGCAGAAGCTTAGTTATGAGTGTATATGGAAGCTCATTATACTAATTTTTATTTTTATGACTATTTGAAATTTTC CA
TAATAAAAAATTAGAAACAACTATGGAAGGGAATGAAAAATTTACCTAAACTTTTAGGACCTAATTTAAAGCCTGTTTACCCTACACTGTTTGCAGAAGAGAATGAAACTGACATCCAGAAAGAG GCA
GAGACAGAGACCCCAGAAGCACAAACCAGCCCTCATTCTCACTATTCCCAGCCCGGCCCCTCCCCTCCCAATTCCTTTTTTGTGTACGTTAAAGTTGGGACTTGTTCACTTAGAACCCAAAGGCACTG
ACCAATGGAAAACTATAAATATTTGCATTCATCTTCTGGGAGTATACAGTATTATCTTGGGAATGCACCAAAACAGAAAAGAAGTTAACACTAAATTTGTTAACTTTTGACCATTTTCAAAAAATCAA
GTCTACTTTTCAAATGTTGAATATGTCTGCAtCATAGTCTCTTAGTACCGTTTCCCATGAAGAGGAACCAGGACTCCTTGGGAAATTGCTGATTCCTGGTCTGGGCATTAAATGCCCAAGATGAGCCT
GGAGTATCTTTGGATTCCAGAAATCAGAGACTGCAAAGACTAGCAGGGTCAGGTCAAAAAAACATGGGCTGCCTGCCAGCCAGCATGACCAATTTAAGCACTGAAGTTTGAAACACATCAAATGT TTT
AAATCCATGAGTTAATAATGACACTTAAGAAAATGAAAAGAAAAAGAAACCTCTTTGGTCACCTTTGGAGGGTGATAGTGAAGCAACTCATTTATTCTGAAAACAAGAAAATAAGGAGAAAGAAAC AA
AGTATTTTATTCTAACTTTCCTATACAAATTGTACCTCCACATATAAAATAACTGATGAGCAAAAACAAATTTTTCAGAATTCCAGCAATAAATGCAGAAGGGA GGCTGGGTGCGGTGGCTCACACCT

GTAATCCCAGCACtttgggaggccaaggcaggaggatcacatgagtccaggagttcaagatcagcctgagcaacacagggagaccccatctgtataattaaaaaaaaaaaaaaaaaaaaaaa gccgac
acagcgactcacacctgtaataccagcacTTTGAGAGGCTGTGGTGGGTGGATCACTTGAGCCCAGGAGTTTGAGACTAG tATGGGCAACATAGTGAAACCTCATCTCTACAACAAATACAAAAAAAT
TATCTGGGCATGGTGATGTGCACCTGTGGTGATGTGCACCTGTGATCCCAGCTACTTAGGAGGCTGAGGTGGGAGGATCACTTGAGCCCAGGAGATGGAAGTTGCAGTGAGCTGAGATCATGCCA CTG
CATTCCAGCCTGGGTGACAGAGTGAGACTCTGTCTCAAAAAAAAATAAGTACAGAAGGAATAATATCACCATTAtACAGCTCTTAAACAACGGATTTGAGGAATAATCATCAATACTACCCCAAAACCA
TTAGGTGAAAAGCTGACAGGAAACTTTATAATGGAAAGTTCCGACTAGCAACA TAGCACATGTACAGAAGAAGAGAGGATATCCTACCTGCTTGAAAATTCTTTTCTTTACAGGGAGGAAAGGTCAAT
TAGAAATACAAAGGGAAGAAGAAATTCACCAGTTTACACCATAACTACTCTTTTACATTAACCACTGTTGTAGATGAAAGTCTGCAGTCTCAAGGGCATGGC AgGGTGTTCTGTATAGTCTGGGCCAT
CTTCCTTAAAGCTGGTGTTCACCAAGTACAAAAGCGCCTACCAGAAATATTTGTCAGCGTCCAACAGACATGGCAAGGC TATTCCATATTCTTTTCTTTTCAGGAAAGCTCTGCCCTTCTCATGATAT
CCCATAGCTAACATAAGGGCCTAGGAAAAAATACAAATTTAAAAAACATCACTTTAAAATATAATAAGGCAAATTATATTAAAAGCATATTACTTTTTAAAAAACAAAGTAAAAAAAATGAAAAC AAG
ATCATAACTCAATCTAAAAGCCAAAATTAAGAAAAATATAATTAATTAAAGCTTTATTTCATTTAAATACTGAAGCCAA ACACTTCATTTATTATAGCTGATACAATCCTAACCAGCACTTGGCCCTT
TGCTTTCATTAGTTTATTTTCGTATCCACTGATCTGGATTCTACTTCTAATATTCTATTCAGACTAAACATATTAAAAAGCAGCCAGTAATGCTTGTATCATTTTTATTTTATTTATTTTATTTT TTT
GAGACGGAGTCTCATTCTGTCGTCCAGGCTGGAGGGCAGTGGTGCGATCTCGGGTCACTGCAACCTCTGCCTCCTGGGT TCAAGCGATTCTCCTGCCTCAGCCTGCCGAGCAGCTAGGACTACAGGCA
TGAGCCACCATGCCCGGCTAATT
>Chimp
GATTGAGaGAGGCTCACACAGATGTATGAAGATCAACCATGTCTTTATAACTGCAGAAGCT cAGTTATGAGTGTATATGGAAGCTCATTATACTAATTTTTATTTTTATGtCTATTTGAAATTTTCCA
TAATAAAAAATTAGAAACAACTATGGAAGGGAATGAAAAATTTACCTA AACTTTTAGGACCTAATTTAAAGCCTGTTTACCCTACACTGTTTGCAGAAGAGAATGAAACTGACATCCAGAAAGAGGCA
GAGACAGAGACCCCAGAAGCACAAACCAGCCCTCATTCTCACTATTCCCAGCCCGGCCCCTCCCCTCCCAATTCCTTTTTTGTGTACGTTAAAGTTGGGACTTGTTCACTTAGAACCCAAAGGCA CTG
ACCAATGGAAAACTATAAATATTTGCATTCATCTTCTGGGAGTATACA GTATTATCTTGGGAATGCACCAAAACAGAAAAGAAGTTAACACTAAATTTGTTAACTTTTGACCATTTTCAAAAAATCAA
GTCTACTTTTCAAATGTTGAATATGTCTGCATCA cAGTCTCTTAGTACCaTTTCCCATGAAGAGGAACCAGGACTCCTTGGGAAATTGCTGATTCCTGGTCTGGGCATTAAATGCCCAAGATGAGCCT
GGAGTATCTTTGGATTCCAGAAATCAGAGACTGCAAAGACTAGCAGGG TCAGGTCAAAAAAACATGGGCTGCCTGCCAGCCAGCATGACCAATTTAAGCACTGAAGTTTGAAACACATCAAATGTTTT
AAATCCATGAGTTAATAATGACACTTAAGAAAATGAAAAGAAAAAGAAACCTCTTTGGTCACCTTTGGAGGGTGATAGTGAAGCAACTCATTTATTCTGAAAACAAGAAAATAAGGAGAAAGAAA CAA
AGTATTTTATTCTAACTTTCCTATACAAATTGTACCTCCACATATAAA ATAACTGATGAGCAAAAACAAATTTTTCAGAATTCCAGCAATA cATGCAGAAGGGAGGCTGGGTGCGGTGGCTCACACCT
GTAATCCCAGCACTTTGGGAGGCCAAGGCAGGAGGATCACATGAGTCCAGGAGTTCAAGATCAGCCTGAGCAACACAGGGAGACCCCATCTGTATAATTAAAAAAAAAAAAAAA AGCCGACACAGCGgC
TCACACCTGTAATACCAGCACTTTGAGAGGCTGTGGTGGGTGGATCAC TTGAGCCCAGGAGTTTGAGACTAGcATGGGCAACATAGTGAAACCTCATCTCTACAACAAATACAAAAAAATTATCTGGG
CgTGGTGATGTGCACCTGTGGTGATGTGCACCTGTGATCCCAGCTACTT gGGAGGCTGAGaTGGGAGGATCACTTGAGCCCAGGAGATGGAAGTTGCAGTGAGCTGAGATCATGCCACTGCATTCCAG
CCTGGGTGACAGAGTGAGACTCTGTCTCAAAAAAAAATAAtaataaGTACAGAAGGAATAATATCACCATTATACAGCTCTTAAACAACGGATTTGAGGAATAATCATCAATACTACCCCAAAACCATT
AGGTGAAAAGCTGACAGGAAACTTTATAATGGAAAGTTCCGACTAGCAACATAGCACATGTACAGAAGAAGAGAGGATATCCTACCTGCTTGAAAATTCTTTTCTTTACAGGGAGGAAAGGTCAA TTA
GAAATACAAAGGGAAGAAGAAATTCACCAGTTTACACCATAACTAC TCTTTTACATTAACCACTGTcGTAGATGAAAGTCTGCAGTCTCAAGGGCATGGCAGGGTGTTCTGTATAGTCTGGGCCATCT
TCCTTAAAGCTGGTGTTCACCAAGTACAAAAGCGCCTACCAGAAATATTTGTCAGCGTCCAACAGACATGGCAAGGCTATTCCATATTCTTTTCTTTTCAGGAAAGCTCTGCCCTTCTCATGATA TCC
CATAGCTAACATAAGGGCCTAGGAAAAAATACAAATTTAAAAAACA TCACTTTAAAATATAATAAGGCAAATTATATTAAAAGCATATTACTTTTTAAAAAACAAAGTAAAAAAAATGAAAACAAGAT
CATAACTCgATCTAAAAGCCAAAATTAAGAAAAATATAATTAATTAAAGCTTTATTTCATTTAAATACTGAAGCCAAACACTTCATTTATTATAGCTGATACAATCCTAACCAGCACTTGGCCCTTTG
CTTTCATTAGTTTATTTTCaTATCCACTGATCTGGATTCTACTTCTAATATTCTATTCAGACTAAACATATTAAAAAGCAGCCAGTAATGCTTGTATCATTTTTATTT cATTTATTTTATTTTTTTGA
GACGGAGTCTCATTCTGTCGTCCAGGCTGGAGGGCAGTGGTGCGATCTCGGGTCACTGCAACCTCTGCCTCCTGGGTTCAAGCGATTCTCCTGCCTCAGCCTGCCGAGCAGCTAGGACTACAGGC ATG
AGCCACCgTGCCCGGCTAATT

Homologous Sequences
CCAGCACTTTG

>NAHR_14_ Scaffold10901 250430 250739
KSD_NAHR_14

>Scaffold10901 250430 250739_NAHR

CAGAAGGTTTTCAAtTTACGTTGCTCAGATAACCAAGAAAATCACTATTAGTGGCCACTATAGCCTTATGAAATGTATTTCTTTTTTTTTATTTTATTTTTTAAGTTTTTATTTTTTTAGAGACTGAG
CCTCATTATGTTGCCCAGGCTGCAGTGCAGTGGCTATACAAAGGTGCACTACAGTCTTGAACTCCTGGGCTCAAGGGACCCTCTCGCCTCAGCCTCCCAAGTAGCTGGTACTCTACAGGTGCATG CCA
CCTCTGCTGTCTGAAATATATTTCTTTTCTTTTTCTTTTTTTTTGTTTTTTGTTTTTGAGACAGGGTCTTGCTCTGTCGCCCAGGCTGTAGTGCAGTGGCACAATCTCCGCTCACTGCAACCTCCGCC
TCCTGGGTTCAAGCGATTCTCCTTCCTCAGGCTCCTGAGTAGCTGGGACTACAGGCGTGTGCCACCACACCTGGCTAATTTTTGTATTTTTAGTAGAGACGGGGTTTCACCCTGTTGATCAGGCT GGT
CTCGAACTCCTGACCTAATGATGCACCTGCCTCGGCCTCCCAAAGTGCTGGGATTACAGGCATGAGCCGCAGCGCCAGGCCTGAAATGTATTTCTTTTGTTGTTGTTGTTGTTGTTGTTGTT GAG
ATGGAGTATCGCTCTTGTTGCCGCGGTTGGACCGCAATGACAGGATCTAGGCTCACTGCAACCTCCGCCTCCCGGGTTCAAGTGATTCTCCTGTCTCAGCCTCCCAAGTAGCTAGGATTACAGGC ATG
TACCACCATGCCTGGCTAATTTTGTATTTTTAGTAGAGACAGGGTTTCTCCGTGTTGGTAAGGCTGGTCTCGGAACTCCTGATCTCAGGTGATCCGCCCACCGTGGCCTCCCAAAGTGCTGGGATTAC
AGGCATGAGCCACCgTGCCCAGCCTATGAAATGTGTTTCTTAAATAGTAAGACTTGAAAGTCAAAATCACTCCTTGATCCATGGGGATTAAGAATGGATGCA aAGGCCgGGcacaGTGGCTCATGCCT
GTAATCCCAGCACaTTGGGAGGCCaAGGTGGgCAGATCACgAGGTCAgGAGtTtGAGACCAgCCTGaCCAACAtGGTGAAACCCCGcCTgTACTAAAAATACAAAAATTAGCTaGaCGTGGTGGTGcG
TGCCTGTAaTCCCAGCTACTcaGGAGGCTGAGGtgGGAGAATcGCTcaAACCtGGGAGGggGAGGTTGCAGTGAGaTGAGATCCTGCCATTGCACTCCGGCCTGGGCGACAGAGCTAGATTCCATCTC
AAAAAaaaaagAAAAAAGAAAAAAGAAAAAAAGAAAAAAAAGAATGGATGCAGAATGGATGTTGTGTTAGCAGGCATGAAAACAACATTACTCTCCTCCTTGTACATCTCTATCAGAGCTCTTGGGTG
AACAGGTGCATTCTCAATGAGAAGTAATATTTTGAAAGGCATCTTTTTTTCTGAGCAGTAGGTCTCAACAGTGGACTTATACTACTTAATAAACTGTGCTATAAACAGATGTGATGTCATCCAGG CTT
TGTTGTTCCATTTATAGAGCACAGGCAGTAGACTTAGCATAATTCTTAAGGTCTCTAGGATTTTCTGAGCATTGGTTTCAACTTAAGGTCACCAGCTGCATTAGCCTCTAA tAAGAGGGTCAGCCTTT
CCTTAGCTTAGAAGCTTTGAAGCCAGTCATTGACTTCTCTCTAGCTATGAAAGTCCTAGATGGCATCTTCTTCCAATAAAAGGCTTTTCATCTGCATTGAAAATCTGTTGTTTAGTGTAGCTACC TTC
ATCAATGATCTTAGCTAGATCTTCTGAATAACTTGCTGCGGCTTCTCCATCAGCACTTGCTACTTCATCTTGCACTTTTATGTTACGGAAACAGCTTCTTTCTTTAAACCCC ATGAACCAACCTCTGC
TAGCTTTCAATTTTTATTCTACAGCTTCTTTATCTCTCTCAGCCTTCACAGAATTGAAAAGAGTTAGGGTCTTGCTCTGGATTAGGCTTTGGCTTAAGGGAATGTTGTGGCTGGTTTGATCTTCT ATC
CAGACCACTCAGACTTTTTCCATATCAGCAATAAGGCTGTTTTGCTTTTTTATTGTTTGTGTGTTGAATGGAGTAGCACTTTCAATTTCCTTTAAGAAGTTTTCTTTTGCAT TCACAACTGTTGGGGG
CAAGAGGCCTAGTTTTGGCCTATCTCTCCTTTTGATATGCCTTCCTCACTAAGCTTAATCATTTCTAGCTTCTGATTTAAAGAGAGAGGACTGTGAGTCTTCCTTTCATTGAATACTTAGAAGCC ACT
GTAGG

488	16.9	1.2	0.0	823	1	83	(2226)	C	Tigger1	DNA/TcMar-Tigger	(870)	1548	1465	1
924	13.8	6.6	2.9	823	92	258	(2051)	C	FAM	SINE/Alu	(3)	182	10	2
2361	10.1	0.0	0.0	823	286	593	(1716)	C	AluSg	SINE/Alu	(2)	308	1	3
32	0.0	0.0	0.0	823	609	638	(1671)	+	(TTG)n	Simple_repeat	1	30	(0)	4
2072	12.2	0.3	0.3	823	639	920	(1389)	C	AluSp	SINE/Alu	(31)	282	1	5
7361	8.5	2.6	4.2	823	921	1000	(1309)	C	Tigger1	DNA/TcMar-Tigger	(937)	1481	1420	1
2361	11.3	0.0	0.0	823	1001	1310	(999)	+	AluSg	SINE/Alu	1	310	(0)	6
7361	8.5	2.6	4.2	823	1311	2309	(0)	C	Tigger1	DNA/TcMar-Tigger	(999)	1419	419	1

473	18.1	1.2	0.0	hg19	1	83	(2508)	C	Tigger1	DNA/TcMar-Tigger	(870)	1548	1465	7
924	13.8	6.6	2.9	hg19	92	258	(2333)	C	FAM	SINE/Alu	(3)	182	10	8
2361	10.1	0.0	0.0	hg19	286	593	(1998)	C	AluSg	SINE/Alu	(2)	308	1	9
43	0.0	0.0	0.0	hg19	609	647	(1944)	+	(TTG)n	Simple_repeat	1	39	(0)	10
2057	12.5	0.3	0.3	hg19	648	929	(1662)	C	AluSp	SINE/Alu	(31)	282	1	11
498	9.5	1.3	2.7	hg19	930	1005	(1586)	C	Tigger1	DNA/TcMar-Tigger	(937)	1481	1407	7
2069	8.4	0.0	0.0	hg19	1010	1260	(1331)	+	AluSc	SINE/Alu	1	251	(58)	12
2348	11.6	0.0	0.0	hg19	1289	1598	(993)	+	AluSg	SINE/Alu	1	310	(0)	13
7154	8.5	2.9	0.8	hg19	1608	2591	(0)	C	Tigger1	DNA/TcMar-Tigger	(996)	1422	419	7

>hg19 chrX:44436368-44438958

CAGAAGGTTTTCAAcTTACGTTGCTCAGATAACCAAGAAAATCACTATTAGTGGCCACTATAGCCTTATGAAATGTATTTCTTTTTTTTTATTTTATTTTTTAAGTTTTTATTTTTTTAGAGACTGAG
CCTCATTATGTTGCCCAGGCTGCAGTGCAGTGGCTATACAAAGGTGCACTACA GTCTTGAACTCCTGGGCTCAAGGGACCCTCTCGCCTCAGCCTCCCAAGTAGCTGGTACTCTACAGGTGCATGCCA
CCTCTGCTGTCTGAAATATATTTCTTTTCTTTTTCTTTTTTTTTGTTTTTTGTTTTTGAGACAGGGTCTTGCTCTGTCGCCCAGGCTGTAGTGCAGTGGCACAATCTCCGCTCACTGCAACCTCC GCC

TCCTGGGTTCAAGCGATTCTCCTTCCTCAGGCTCCTGAGTAGCTGGGACTACA GGCGTGTGCCACCACACCTGGCTAATTTTTGTATTTTTAGTAGAGACGGGGTTTCACCCTGTTGATCAGGCTGGT
CTCGAACTCCTGACCTAATGATGCACCTGCCTCGGCCTCCCAAAGTGCTGGGATTACAGGCATGAGCCGCAGCGCCAGGCCTGAAATGTATTTCTTTTGTTGTTGTTGTTGTTGTTGTTGTT Gtt
gttgttgAGATGGAGTATCGCTCTTGTTGCCGCGGTTGGACCGCAATGACAGGATCTAGGCTCACTGCAACCTCCGCCTCCCGGGTTCAAGTGATTCTCCTGTCTCAGCCTCCCAAGTAGCTAGGATT
ACAGGCATGTACCACCATGCCTGGCTAATTTTGTATTTTTAGTAGAGACAGGGTTTCTCCGTGTTGGTA AGGCTGGTCTCGGAACTCCTGATCTCAGGTGATCCGCCCACCGTGGCCTCCCAAAGTGC
TGGGATTACAGGCATGAGCCACCaTGCCCAGCCTATGAAATGTGTTTCTTAAATAGTAAGACTTGAAAGTCAAAATCACTCCTTGATCCATGGGGATTAAGAATGGATGCA gAGGCCaGGtgtgGTGG
CTCATGCCTGTAATCCCAGCACtTTGGGAGGCCgAGGTGGaCAGATCACaAGGTCAaGAGaTcGAGACCAtCCTGgCCAACAgGGTGAAACCCCGtCTcTACTAAAAATACAAAAATTAGCTcGgCGT
GGTGGTGtGTGCCTGTAgTCCCAGCTACTtgGGAGGCTGAGGcaGGAGAATtGCTtgAACCcGGGAGGcaGAGGTTGCAGTGAGcTGAGattgccccactgcattccaaaaaaaaaaaaaaaagaatg
gatgcaaaggccgggcacagtggctcatgcctgtaatcccagcacattgggaggccaaggtgggcagatcacgaggtcaggagtttgagaccagcctgatcaacatggtgaaaccccgtctgtactaa
aaatacaaaaattagctagacgtggtggtgcgtgcctgtaatcccagctactcaggaggctgaggtgggagaatcgctcaaacctgggagggggaggttgtagtgagatgag ATCCTGCCATTGCACT
CCGGCCTGGGCGACAGAGCTAGATTCCATCTCAAAAAAAAAAAGAAAAAAGAAAAAAAGAAAAAAAAAGAATGGATGCAGAATGGATGTTGTGTTAGCAGGCATGAAAACAACATTACTCTCCTCCTTG
TACATCTCTATCAGAGCTCTTGGGTGAACAGGTGCATTCTCAATGAGAAGTAATATTTTGAAAGGCATCTTTTTTTCTGA GCAGTAGGTCTCAACAGTGGACTTATACTACTTAATAAACTGTGCTAT
AAACAGATGTGATGTCATCCAGGCTTTGTTGTTCCATTTATAGAGCACAGGCAGTAGACTTAGCATAATT CTTAAGGTCTCTAGGATTTTCTGAGCATTGGTTTCAACTTAAGGTCACCAGCTGCATT
AGCCTCTAAcAAGAGGGTCAGCCTTTCCTTAGCTTAGAAGCTTTGAAGCCAGTCATTGACTTCTCTCTAGCTATGAAAGTCCTAGATGGCATCTTCTTCCAATAAAAGGCTTTTCATCTGCATTGAAA
ATCTGTTGTTTAGTGTAGCTACCTTCATCAATGATCTTAGCTAGATCTTCTGAATAACTTGCTGCGGCTT CTCCATCAGCACTTGCTACTTCATCTTGCACTTTTATGTTACGGAAACAGCTTCTTTC
TTTAAACCCCATGAACCAACCTCTGCTAGCTTTCAATTTTTATTCTACAGCTTCTTTATCTCTCTCAGCCTTCACAGAATTGAAAAGAGTTAGGGTCTTGCTCTGGATTAGGCTTTGGCTTAAGG GAA
TGTTGTGGCTGGTTTGATCTTCTATCCAGACCACTCAGACTTTTTCCATATCAGCAATAAGGCTGTTTTG CTTTTTTATTGTTTGTGTGTTGAATGGAGTAGCACTTTCAATTTCCTTTAAGAAGTTT
TCTTTTGCATTCACAACTGTTGGGGGCAAGAGGCCTAGTTTTGGCCTATCTCTCCTTTTGATATGCCTTCCTCACTAAGCTTAATCATTTCTAGCTTCTGATTTAAAGAGAGAGGACTGTGAGTC TTC
CTTTCATTGAATACTTAGAAGCCACTGTAGG

Homologous Sequences
GGCC

>NAHR_15_ Scaffold2051 3593 3886
>KSD_NAHR_15
>Scaffold2051 3593 3886_NHAR

GGGAATAAAAAGGCTCTTGGCACTTCAAATATTTTAATGTTCTTAATTCCACAGTAAAATTAAAATTACAAAATTTACCTGCTTTGAATGTTTCTCTGTCCTTCAATTCTAAGGCTTTATTTGGAA GA
GAAATTTTCATTCCACAGGTAGGCTAAATGGGTTTGGAAACACAATAATTACTATATAATGTATACTTCATAAAATAAGTAGTTAATATTCAAGATAAAAATATAAAAGTTATTACCTTCAGAAG ACC
ATCTTTATCAGGAGAgTCTAAAAGAAAAGGGACATATATAATTGATTATACGCAAGCCTGTCAAAGTCTACCAAACATTCATGCAGTGTGAATATGAAGATGAATCCTCATGCTTGGATTGAAAAGAG
ATTACACTAGGTTTTGGGGTCTTTTGGGTTATTGTGTTTGTTAACATGCCAGAGTGACAGAAATATACCTAAGAAAATTTAAAAAATAAATTTCCTCAAACATGCTGTGAAGATTTCAACAGTCA AAA
TATATTTCAAGTGACATTAACTAAAGCAGAAAATCCTAATACCAGTAAGAGTAAGCTG tCATATAAGGAAAGAGGTTGAATTTAGTAAGATGACCAAGACATGACCCCTCCACCAAAAATAAATAAAT
AAATGTCCAAGCTAATGGTAGAATGCTATGACATATTATTAAAGAAACACATCAGAATCACTTATGTCAATATACTACAAGTAGCTATTATGTTCTTCAATTTGTCCTGTAATTTAAGAATTGCA AAG
CTCCGATGAGAATTCAAAGGTACAATTTACTTATCATTGCAGAGAATGTTCTAAATT tAtCAGCATAAATATCTACTTATAGAATaAAAGTTTATAAATATATTTATTTTTGAACCTCTGTGAAATAC
GAGTATTTTTAcATTTCTGGGGTTCTcTAGTTTAGTCTTCTTAAAATTTCgTGATCCACTTATACAGAAAAGTCAAAGCATACCCATCAACAAACTAATACAGT CCAGGTGCCATGGCTCATGCCTGT
AACTCCAGAACCTTGGAAGCCTGAAGAGGGCGGATCTCTCGAG gCCAGGAGTTCCAAAGCAGCCTGGCCAAAATAGCGAAACCCTGTCTCCACAAAAAATACAAAAATCATCCAGGTATGGTGGCACA
TGCCTGTAACCCCAGCTACTTGGGAGCCGGAGCTGTGAGAATCACTTGAACCCAGGA GGCCCAGGTGGCAGTGAGTCAAGATCATGCCCCTGCACTCCAGCCTGGATGACAGAGCGAGACTCTGTCTC
AACAAAAGAAAAAACACCTGATATGAAAGTATCAAAAAcTAGATGTCAATTTCCAGAGACTTTTTACATATCATTAACCTGCATATTTCAATGTTAGAATAATGGTTATTGAAAATAACAGTTTTATT
GTTCAAGGAATGAATTCTACAATTGCTTTGCTTTGCTAAAACTAATTCTATTTGTTATACCATGCGAGTTGTTAAACCACTGTTATGAAAAAGCTTTTATAGAAAGCTTAGCTTCAAGAAAAAAA CAG
ACATTTCTTTGACATAGAAATATATCTCATTTCCTCAAATAATATGAATACAACATGCTTAATCTCTCATTTTTCTCTACTTTGTTATTTTTCAGTAAGACACACTTTAATATATTTAGACATGTC AA
GTATATATGTTGTTTCCTTTGGAGTTGATTGATCAGTAATGAAGACACTTCAGGGGACATAAACTCTCTAAAACACACA cAATTTATGTcCAAGCgaTTTAAGGCTAAATATGCATGACCGTGTTGCA
GGTATGTGTTTTAGAAAAAAAGTGCTTAAAATTGTATTCAATACACTTTAAATAAAAACCCGCTTTAATTACGATGAGAAGGTCGTTCTCGATTCATTAATATCTTTAAACTTTACTTCTGAAGCA CT
GAAGAAACCTCAGAAATTCGTATATAGGAAATGATACCATTAATTCATCTGCTTGTTTTAGGTAAAAATATGATAAATGTACATCCTCACTAAGTGCAAAGAAAGCAGAGTGTCATGAGATAGGA AAT
GAGTATGTAACCAAAATATTTTCCTTTATGAAGAAAATAGCTGGCTTTGACCTTTCTAAGACAAGATAAAAAAGCAAAACACGCAGAGATGAAACATTCAGAGCTATCTCATCATCAACGAACCGT TT
ATCACTTGGCATTTAGGAATCCTATTTATGGTTGAAAACTCTGGTTCTAAAATGTGATCTGATTATCCCTGAAGTTCAAAATCCATGCAGAGGAAGCAAGCAGGGAAATACTACCT T

1786	18.7	0.0	0.0	60010	1001	1294	(999)	+	AluSz6	SINE/Alu	3	296	(16)	1

1819	18.4	0.0	0.0	hg19	1001	1294	(14925)	+	AluSz6	SINE/Alu	3	296	(16)	2
19971	2.6	0.0	0.0	hg19	1295	4395	(11824)	+	L1PA2	LINE/L1	3054	6155	(0)	3
15	10.7	3.3	0.0	hg19	5363	5392	(10827)	+	(TTTTTA)n	Simple_repeat	1	31	(0)	4
3094	6.0	11.1	0.0	hg19	5677	6090	(10129)	C	L1PA4	LINE/L1	(0)	6155	5696	5
2095	11.7	1.3	0.3	hg19	6093	6392	(9827)	C	AluY	SINE/Alu	(8)	303	1	6
501	22.3	14.3	0.6	hg19	7285	7424	(8795)	+	MER5A1	DNA/hAT-Charlie	2	160	(6)	7
2058	10.3	0.0	6.7	hg19	11246	11565	(4654)	+	AluY	SINE/Alu	1	300	(11)	8
1733	18.5	1.7	0.3	hg19	11633	11924	(4295)	C	AluSx1	SINE/Alu	(16)	296	1	9
16	5.2	0.0	0.0	hg19	12110	12129	(4090)	+	(CA)n	Simple_repeat	1	20	(0)	10
6852	7.4	2.9	0.1	hg19	12935	13804	(2415)	+	MER11D	LTR/ERVK	1	894	(3)	11
13	23.0	1.8	5.6	hg19	13955	14010	(2209)	+	A-rich	Low_complexity	1	54	(0)	12
1718	19.7	0.0	0.0	hg19	14927	15220	(999)	+	AluSz6	SINE/Alu	3	296	(16)	13

>hg19 chr18:14806536-14822754

GGGAATAAAAAGGCTCTTGGCACTTCAAATATTTTAATGTTCTTAATTCCACAGTAAAATTAAAATTACAAAATTTACCTGCTTTGAATGTTTCTCTGTCCTTCAATTCTAAGGCTTTATTTGGAAGA
GAAATTTTCATTCCACAGGTAGGCTAAATGGGTTTGGAAACACAATAATTACTATATAATGTATACTTCATAAAATAAGTAGTTAATATTCAAGATAAAAATATAAAAGTTATTACCTTCAGAAG ACC
ATCTTTATCAGGAGAcTCTAAAAGAAAAGGGACATATATAATTGATTATACGCAAGCCTGTCAAAGTCTACCAAACATTCATGCAGTGTGAATATGAAGATGAATCCTCATGCTTGGATTGAAAAGAG
ATTACACTAGGTTTTGGGGTCTTTTGGGTTATTGTGTTTGTTAACATGCCAGAGTGACAGAAATATACCTAAGAAAATTTAAAAAATAAATTTCCTCAAACATGCTGTGAAGATTTCAACAGTCA AAA
TATATTTCAAGTGACATTAACTAAAGCAGAAAATCCTAATACCAGTAAGAGTAAGCTGcCATATAAGGAAAGAGGTTGAATTTAGTAAGATGACCAAGACATGACCCCTCCACCAAAAATAAATAAAT
AAATGTCCAAGCTAATGGTAGAATGCTATGACATATTATTAAAGAAACACATCAGAATCACTTATGTCAATATACTACAAGTAGCTATTATGTTCTTCAATTTGTCCTGTAATTTAAGAATTGCAAAG
CTCCGATGAGAATTCAAAGGTACAATTTACTTATCATTGCAGAGAATGTTCTAAATTcAaCAGCATAAATATCTACTTATAGAATgAAAGTTTATAAATATATTTATTTTTGAACCTCTGTGAAATAC
GAGTATTTTTAtATTTCTGGGGTTCTgTAGTTTAGTCTTCTTAAAATTTCcTGATCCACTTATACAGAAAAGTCAAAGCATACCCATCAACAAACTAATACAGTCCAGGTGCCATGGCTCATGCCTGT

AACTCCAGAACCTTGGAAGCCTGAAGAGGGCGGATCTCTCGAGtCCAGGAGTTCCAAAGCAGCCTGGCCAAAATAGCGAAACCCTGTCTCCACAAAAAATACAAAAATCATCCAGGTATGGTGGCACA
TGCCTGTAACCCCAGCTACTTGGGAGCCGGAGCTGTGAGAATCACTTGAACCCAGGAGgcggaggtggcagtgagtcaagatcgtgccactgcgctccagcctggatgacagagtgagactctgtctc
aacaaaagaaaaaacgcaataaaaaatgataaaggggatatcaccaccgatcccacagaaatacaaactaccatcagagaatactacaaacacctctacgcaaataaactagaaaatctagaataaat
agataaattcctcgatacacacactctcccaagactaaaccaggaagaagttgaatctctgaatagaacaataacaggatctgaaattgtggcaataatcaatagtttaccaaccaaaaagagtc cag
gaccagatggattcacagccgaattctaccagtggtacaaggaggaactggtaccattccttctgaaactattccaatcaatagaaaaagagggaatcctccctaactcattttatgaggccagcatc
attctgataccaaagccgggcagagacacaacagaaaaagagaattttagaccaatatccttgatgaacattgatgcaaaaatcctcaataaaatactggcaaaatgaatccagcagcacatcaa aaa
gcttatccaccatgatcaagtgggcttcatccctgggatgcaaggctggttcaatatatgcaaatcaataaatgtaatccagcatataaacagagccaaagacaaaaaacacatgattatctcaatag
atgcagaaaaagcctttgacaaaattcaacaacccttcatgctaaaaacactcaataaattaggtattgatgggatgtatctcaaaataataagagctatctatgacaaacccacagccaatatc ata
ctgaatgggcaaaaactggaagcattccctttgaaaactggcacaatacagggatgccctctctcaccactcctgttcaacatagtgttggaagttctggccagggcaatgaggcaggagaaggaaat
aaagggtatttaattaggaaaagaggaagtcaaattgtccctctttgcaaacgacatgattgtatatctagaaaaccccattgtctcaacccaaaatctccttaagctgataagcaaattcagca aag
tctcaggatacaaaatcaatgtacaaaaatcacaagcattcttatacaccaataacagacaaacagagagccaaatcatgagtgaactcccattcacaattgcttcaaagagaataaaataaatagga
atccaacttacaagggatgtgaaggacctcttcaaggagaactacaaaccactgctcaatgaaataaaagaggataaaacaaatggaagaacattccatgctcatgggtaggaagaatcaatatc gtg
aaaatggccatactgcccaaggttatttacagattcaatgccatccccatcaagctaccaatgactttcttcacagaattggaaaaaactactttaaagttcatatggaaccaaaaatgagcccgtgt
cgccaagtcaatcctaagccaaaagaacaaagctggaggcatcacagtacctgacttcaaactatactacaaggctacagtaaccaaaacagcatggtactggtaccgaaacagagatatagatc aat
ggaacagaacagagccctcagaaataacgccacatatctacaactatctgatctttgacaaacctgagaaaaacaagcaatggggaaaggattccctatttaataaatggtgctgggaaaactggcga
gccatatgtagaaagctgaaactggatcccttccttacaccttatacaaaaagtaattcaagatggattaaagacttaaacgttagacctaaaaccataaaaaccctagaagaaaacctaggcat tac
cattcaggacataggcatgggcaaggacttcatgtctaaaacaccaaaagcaatggcaacaaaagacaaaattgacaaatgggatctaattaaactaaagagcttctgcacagcaaaggaaactacca
tcagagtgaacaggcaacctacagaatgggagaaaatttcgcaacctactcatctgacaaagggctaatatccagaatctacaatgaactcaaacaaatttacaagaaaaaaacaaacaacccc atc
aaaaagtgggcgaaggacatgaacagacacttctcaaaagaagacatttatgcagccaaaaaacacatgaaaaaatgctcatcatcactggccatcagagaaatgcaaatcaaaaccactatgagata
ccatctcacaccagttagaatggcaatcattaaaaagtcagaaaaccacaggtgctggagaggatgtggagaaataggaacactttgacactgttggtgggactgtaaactagttcaaccattgt gga
agtcagtgtggcgattcctcagggatctagaactggaaataccatttgacccagccatcccattactgggtatatacccaaaggactataaatcatgctgctataacgacacatgcacacgtatgttt
attgcagcattattcacaatagcaaagacttggaaccaacccaaatatccaacaatgatagactggattaagaaaatgtggcacatatacaccatggaattctatgcagccataaaaaatgatga gtt
catgtcctttgtagggacatggatgaaactggaaatcatcattctcagtaaactatcacaagaacaagaaaccaaacaccgcatattctcactaataggtgggaaatgaacaatgagaacacatggac
acaggaaggggaacatcacactctggggactgttgtagggtgggggaggggggagggatagcattaggagatatacctaatggtagatgacgagttggtgggtgcagcgcaccagcatggcaca tgt
atacatatgtaactaacctgcacaatgtgcacatgtaccctaaaacttaaagtataattaaaaaaaaagaaaaaacacctaatatgaaagtatcaaaaattagaagtcaatttccagagaattttata
tatcattaacctgcatatttcaatgttagaataatggttactgaaaataacagttttattatttgttgctaaaactaattctgtttgttatacaatgcgagttgctaaaacactgttatgaaaa agc
tcttataaaaaacttagcttcaagaaaaaacagacatttctttgacatagaaatatatctcctttcatcaaataatatgaataaaacatgcttaatctctcatttttctctacttttatttctcagt
acaacatactttaatatatttagacatatcaagtatatatgctgtttcccttcagagatgattgatcaataatgaagacacttcagggatataaactctctaaaacacaaaaaatttatgtccaa gca
ttttaaggctaaatacatgaccatattgcaggtaggtgttttataaaaaaagtgcttaaaattgtattcaacacactttaaataaaaacctgctttaattatgatgagaaggtcattctcgattcatt
aatatctttaaactttacttctgaagcactgaagaaacctcagaatttcatatataatgataccattaatacatctgtttgttttaggtaaaaatatgataaatgtacatcctcactaagtgcaa aga
aagcagagtgtcatgagataggaaatgaatatgtaaccaaaatattttcctttatgaagaaaatagctggctttgacctttctaagacaagataaaaaagcaaaacatgcagagatgaaacattcaga
gctatctcatcatcaacgaaccgtttatcacttggcatttaggaatcctatgtatggttgaaaactctggttctaaattgtgatctgattaccccctgaagttcaaaatccatgcagaggaaggaa gca
ggggaatactgcctttgttatttttttattactttattttataaaggtgtacactggagtttttagagtcaggacaggtcacatggcatggctctaaagattagtgagccacatttgtaatggct
attgaaatgtatggctgtgaatccttgcattttaattacttcacttttaatttctaatatagcatgaagtaagacatacacttaaacaaaaccttgttgacatctgtaattatatttatgttgct ttt
gagtcatcatgaagagagtctaattaaacgtagtgcaaagtaaaaatgcacagtcagaaatgtttctttatttttttattttaatattatactttaagttttagggtacatgtgcacaatgtgcaggt
tcccattaactcgtcatttagcattaggtatatctcctaatgctatccctcctccaaccccacaacagtccccggagtgtgatgttccccttcctgtgtccatgtgttctcattgttcaattcac acc
tatgagtgagaacacgcagtgtttggtttttttgtccttgcgagagtttgctgagaatgatggtttccagtttcattcatgtccctacaaaggacatgaacttatcatttttttatggctgcgtagtgtt
ccatggtgtatatgtgccatactttcttaatccagtctagcgttgttggacatttggtttggttccaagtctttgctgttgtgaatagtgccacaataaataaattcttttttttttttttttt cac
agagtctggccctgtcacccaggctggagtgtaggtgcatgatcttggctcactgcaatctctgcctcatgggttctcacattctcctgcctcagcctcccgagtagctgggactagaggcacccacc
accatgcttggctaatttttttgtatttttttcatagagacggggtttcaccatgttagccaggatggtctcgatctgctgacctcgtgatccgcatgcctctgcctcccaaagggctgggattaca ggc
gtgaaccacagtgcccggcctgaaatcttgaaataaatgaaaaacgagctaatcgctgaacaaaaaaaataaaaaatcactgtggaatgacaagaacattctacaaacatttatatgtgatttttgcaa
aaagtgttaatgccaatgtgtatatgctgagtgatgaggagataagtgatcctgaatcagaggagcaaatcgtgacactgagaaaataaatgcaaaagctgaacgtagaatgctataccatgtgt ctt
taatgcaacacattataataatttatatcattacattacaaatattcaccatgttctttaatatgtcctgtcactgagaaggtaaaaagtttggatgagagttcagctgaatgtagaactgacatctc
agcagtgaaagtgttatgaattgatcagcttggatatatacttagagaataatactaaatataaatatccactaattttcacacccatatggcataacggtatgccaacattttttgtactttgta gct
tagccatctcaatattccttgatccactcatggaggaagatgtacaaaactcatcaggaatagcaaatataataagctttcaatattgacatatttgttttaaatttagttgcaacagacttattaaa
tattaataacattttctatgttaaaatcactataatacctatgaataagaacaatttaggtattcaagcaataaattcagaattttctttcttcttctctggctaaaaacatactcaaaagtaag agc

aacataacgctttctcctaaccttttctgtcttgaccctttatccaattactcttcttccacatttctcccatgaaaggcctctcctttttgaccctctatccaattactcttcttccacatttttccc
atgaaaggcctctcctcttgctactcagtctgaggtccaaggaccaccagcatcagcatcgcctgacaacttactaaaaatgcagaatccctagtcggctgaatcacagtgtgaattgttaacaa ggt
tttcagacggttttctaaagtttgaaaacttacggtctatacggagtgtatatgacaaattatatacatgtgtggccgtgcatatatgcttattctcaactattgcaaatagaacacagctctccatg
gtcagctgcttccatccctaatggcttcccaccagtgagaaagacaggaagagtcagaatattttttgctgtaattctctggtatgttttggcactggaaagtcactttatattcttcccaaattt cta
accatacccgatcttttcctaaagaatcatttcattttaccatcctccattatataaacctgcctcttgttccttcccgtcctctctgtactctctgtctttctcgatcttcatttcctcttttacta
ctttctcctttcttatttctcattttcctcagctcttggaataccttgaattcaaactaataattcagttcctttccagcactctctatgtagtctgctgccaacacatgataagacgtataac tta
tcaccatttcacttctcttttttttccatgctttgcattcaggaggtaatttcttcagctattaggggatatccctcctcatcagttcagagaaacatttggtcaaatgacttttaaataaaatatg
gttcttaccttctaattttccacttaaggtatcgaattctttttgatgtgtagccttgggtaaacacccatcattctgtaagagagcctcaaggaaactctgttaaaagtaatatcaataaataa ttt
caatggagaaatcccaataatgaagagtatcaaaagttcctaattaaaatcttttctaaaaacacagtttcctactttataaggaattcgacttaagagcagacatgtgttgaaacccaaaacattct
gatagaaaacctcacataacactctccacatcaacttacctttacgttcatgaggctattaacttggttactgagcaggaaaaacaaatcacactcacgaaagaattcactcgtgcatatttcag aaa
gtaactctcttaaaaatatctaacttgtgttgtagtcctcattaaaagtaaatacgccatttcaaacaaaatacaatacacttgctactagccataacacgtactgcattaaacatttaaaatattca
tgatcatagattgtttagtgttaaaaatgatgcatgccgtgaagagaacttttagagtctgtcatataatgcatgcacggaatgtttaaaaagttgctttattttggtaaacaggggcttaaaac aag
caacatgtttttgacaaaatttatgctgatagaaaaagtgcttgaagagaatgtaaatgcagtaagaaaagaaattatgtcatgtctaaatgaaagccaacaaatattaaagtctttttgtctgacga
caaatcagataaaatctacttgtctactgacttccaccctttgctcttaccaccaggcagcagctgcttgtttctaagaggtcagatattttctatgtctttccacagttgtgaccttaaaatac atc
actattatttcacactcatgctgctgcagcttgactgcctgccacaaagggactcgaggagttctacttgtgaaaataatttatatttttcaaaagattactcaaccaatactcttgttactgtagtc
ccacaaggtttcctagtcctcatgctcctctttctcctgaaatgttccctcagatctatactgaacaatacagaaatgtcaatacattcaagtcccaggcattaacttttcaggtcccaacttttt aca
atactgcagtttaaactgtctctctacgaatccagttagaactttttgcacatctagatacaccacagaatcataaatttgcccacagctggactcagtgtaattcttttgcttttagacaaacttgct
tcagatcccatgtgaatttctagtgacttctaagccttatttttttacactataccacaccggcttcttaaattaagcctgaaaattagcagaatttcactacgaagcagtcatctacttagcaca tag
atcctctattaagcgtattctgaaaaatatcaaaaataatttgggagtgacatatgcagaggtgagaaaataaagtctaagcctctgcttttatgtttttgtatcatgcaatactagtattaaagaaa
attaataatacacagtacctcaaaatcccaagaattttcttcatcatcccttttgtttggattctgatgggaacatctgagctataaaaggttaatcacagatacattcatgagaacatttctcta cta
taaactttaaaaacatatataaatctactttcattcacgaatctgcggtttttcctctgtagcccgtatattctttttgttgattacaatcactacttctatagtcaatccgttagaaatgaaatcta
aaaaaacggaaaattaacattatatcattcgttagaatgcagaaaaatagaaatttgactaactcgtagagttatttcttcacaaagcagttacatcctcttctcccgtgaaacacagtgtgtcc tat
acttgctgttcattcagaaaactttaattacctatcatctctcacttctttgtaaatttttatttctagtattacttagtttgattgactctgcaatctctattacacagttcttcaaaaaagatga
tttatcaacgaacaagatttttttagggatattaactttctgatgttagtaaactatatgtccaacacttttacatttcaatatgccatgacagtatattgggtgtgttttgggtgtctatatgtt tta
ggaagataagcttaaaaaactttattgcatataaattaagaactgctttaattacaatgacatagttctcccctaactcaacaatgtcttcagagtcagcttgtgacaactaggagaaacatcagaa
atgtataggaaaaaataattgccctaagtaatctcacatccccacatgttataggataaaatcaaggcccacacaagacttgaggagctctcaggccaatgagacagaaaatgaatttataccaa aaa
taacgttcagctttgtttatcaaccaaaagtacacattacaactctttgaatcaggttaaagaaaaaagcaaacatgcacgactaaagcctaaaagactgtatcatcgttaaaggtacgtttatcatt
tgacatccatcaactgtgtatctggtttttaaaagtaaatttaacatgtaatctcagcatccctaaagttcacatttcatgcagagggaccaagaaactaaaatcatactttggtatgccaccat ttt
cactcatgtttagaagtgtaccctggatcgtttcagagatctaatgtgttgcagtagcatagctctaataatctgtgggtcgcagttggaattgttagtggaatgtatgattgcacaaggaaatactt
taaatattttagtttttaatttctcacaactaaaaagtcagtagatataactaacttaaagactctgaaattttcatgttttcatttttatttatcagcataataattgtgtacttaatggaattt tat
aaacaatctgacatttaaaaagacagttaattgtgtcctttatggacgtctctaaaaattataatgctgatcaaaacagaatgttgtcaaaaattacactgtacaaccacctatgcattagaagttga
aaaaaatttttatgtattaaatatgaccacgtacacatgttggaaaatgtttaaacatgcataagaatacttttaaaaagtagtttagaatattggttacttctaggcaataaatggtactagtgt aaa
aagactctgggccgggcgcggtggctcacgcctctaatcccagcactttaggaggccgaggtgggtggattacaaggtcagcaggtcaagaccatcctggctaacatggtgaaaccccgtttctacta
aaaatacaaagaatgagccaggcgttgtggtgggcgcctggattcccagctacttgggaggcccaggcaggagaatggcgtgaagccgggaggtggagcttgcagtgcgccaagatcacaccact gct
ctccagcctgggcgagacaacgagatgggcgagacagcgagacgccatatcaaaatttaaaaaaaaaagaaaatttaagacgtcaaaacaaattctacacatagtcagaaccacacacatatgttgtg
aatttatggttgtttgtttgctttttgagatggagtctcactctgtcactcaggctaaagtggcacaagcttggctcactgcaacttctgcctcatgggttcaagcaattctcctgcctcagcct cca
gaatagctgagattacgggcacccaccaccacaaccggctattgtttgttatttttagtagcgatggggtttcactgtgttgaccaggctcgtctgcgactcctgaccttaagtaatctgcccacctt
ggtctcctaaattgccaggattacaggcatgtgccaccatgcacagcctgttgtaacatgatttaaacatgcatgggaatgacaacaaaaattaattcaggctgttttgtacctctaggcaatta att
acagtagcataaaaagacaccaaggccaaaagctttagagtcacacttctaaaacagtgtgtctagcaaagagtcatcaagtagtgatttttaaaaattatatgtcacacacacacacacactcagtc
acctaatcagaaaaacaagaaactgatttctttagattctcagaatgacaacactcacaactgtcacgtcagcaagtgaaactttcctcaaagtaaagatcagaattccagtcagcatttgaac cac
tgaaaatatttcacagtttcaaaattcacctgccgtaaaactgagcacgtacagaaattgcaaattggaaactttacactatcgggatattaaagaagaaccacatgacgtaaagcagaattttttttc
taaatccctaattcttatgcttctaaatggcattttctcaaacataaatactaacatttgcattatttgtatcaaattttcccatcaaattttcaaaagaaaacaactttgggaataaaaggctc tca
gcatttcaaatagtttaatattcttaattccagagtaaaatttaaattacaaagtttacctgcttttaatgtttctctgtcctttaattctaaggctttatttggaagagaaactttccttacacagg
taggctaaatgggtttggaaacacaaaaattaatagataatgtatacttcataaaatatgtaggtaatattcaagataaaaatataaaagttattaccttcagaagaccatctttatcaggagac tct
aaaagaaaagggacatatataattgattatatgcaagcctgacaaagcctaccaaatgttcatagagtgtgaatatgaagatgaatcctcatgcttggattgaaaagggattacactaggttttgggg
tcttttgggttattgtatttgttaacatgtcaactgtaggaagactccctgaaactattgctatggaatgaaagatgcaatgctcctgattattgtaaaacaaaattgcatgcaggattgtgtaa aga

caatgccaggttggactgccagaatgagccaacagcgcgtgatgtgtttccccctgcagagagcctgtgaatggacgtgcagtcagggaggttttgcatcaccaagattcctatcccagaaaggcata
tgttcatagctctgggaatggaatgctacccttgtggagagcctataaacagatgcatggggggtgcctgtccatatggataagatagtgctatgaacgccctcatctcgccatggctcttctag gcc
tctttagggttaaggcatactcccttctgagaatttctggtctaaccagttgtctagcttcacattgtgtttctatggattgtttctaaccagcttttgctgcaactgttactgctgattaatatctt
gctaatcataggttatggaaagactgtgtttctttttaaggctctgttagaaattaccgatgcacacactatattgtaaattcttatccctgtgtactgtatttctgcatacagatgttagtta aag
aattacttcatccccatgtgaccatctcacctcaaaatcaaatgaccctaaatccctcattaacccacccccaccctcactaaacttaatagtaaatgcgggtatatctagtgaattgttggcaccac
gggaacacaaggctgtgattcccctggacccagctttcactatcttgtctgtgtctattctttctcaacctgccgatccacctgggaacaaagacaaggccccattgcattgcaggctgctggac aga
tatccacatgaggcaaagctgaatttttttatctctaaatcttatgcttctaaatgacattttctcaaacataaatactaacatttgcattatttgtatcaaattttcccaccaaattttctaaaga
aaacaacattgggaataaaaggctcttggcatttcaaatagtttaatattcttaattccagattaaaatttaaattacaaaatttacctgctttgagtgtttctctgtcctttaattctaaggct tta
tttggaagagaaactttccttccacaggtaggctaaatgggtttggaaacacaaaaattaatatataatgtatacttcaaaaatatgtagttaatattcaacatagaaatataaaagtaattaccttc
agaagaccatctttatcaggagactctaaaagaaaagggacatatataattgattatatgcaagcctgacaaagcctaccaagcattcgtacaatgtgaatatgaagatgagtcctcatgcttgg att
gaaaaggattacactaggttttggggtcttttgggtttttgtatttgttaacatgccaatgggacagaaatatacttaagaaaatttaaaaaataaatttcatcaaacgtgctatgaagatttcaac
agtcaaaatatatttcaactgacaataactaaagtagaaaaatcctaataccagtaagattaatctgccgtatagggaaagaagttgaatttattaagatgaccaagtcatgacccccccgccaaa aaa
aaaataagtcctaggtaatggtagaatgctatgacatattgttaaggaaacacatcagaatctcttatgtcaatatactgcaagtagctattatgttcttcaatttgtcctgtaatttaagaattgca
aagctccgatgagaattcaaaggtacaatttacttatcattgcagaggatgttctaaatttatcagcataaatatctacttatagaataaaagttatgaatatatttattttgatactctgtga tat
aggagtatttttacatttatggggttctctggtttagtcctctcaaaatttcctgatccacttatacagaaaagtcaaagcacacccatccacaaactaatacagt ccaggtgccgtggctcatgcct
gtatctccagaaccttggaaacccgaagagggtggatctctcaaggccaggagttccaaagcagcctggccaaaatagcgaaaccctgtctccacaaaaaatacaaaaatcatccaggtatggtg gca
catgcctgtaaccgcagctacttgggagccggagctgtgagaatcacttgaacccaggagGCCCAGGTGGCAGTGAGTCAAGATCATGCCCCTGCACTCCAGCCTGGATGACAGAGCGAGACTCTGTC
TCAACAAAAGAAAAAACACCTGATATGAAAGTATCAAAAAtTAGATGTCAATTTCCAGAGACTTTTTACATATCATTAACCTGCATATTTCAATGTTAGAATAATGGTTATTGAAAATAACAGTTTTA
TTGTTCAAGGAATGAATTCTACAATTGCTTTGCTTTGCTAAAACTAATTCTATTTGTTATACCATGCGAGTTGTTAAACCACTGTTATGAAAAAGCTTTTATAGAAAGCTTAGCTTCAAGAAAAA AAC
AGACATTTCTTTGACATAGAAATATATCTCATTTCCTCAAATAATATGAATACAACATGCTTAATCTCTCATTTTTCTCTACTTTGTTATTTTTCAGTAAGACACACTTTAATATATTTAGACATGTC
AAGTATATATGTTGTTTCCTTTGGAGTTGATTGATCAGTAATGAAGACACTTCAGGGGACATAAACTCTCTAAAACACACA aAATTTATGTgCAAGCatTTTAAGGCTAAATATGCATGACCGTGTTG
CAGGTATGTGTTTTAGAAAAAAAGTGCTTAAAATTGTATTCAATACACTTTAAATAAAAACCCGCTTTAATTACGATGAGAAGGTCGTTCTCGATTCATTAATATCTTTAAACTTTACTTCTGAAGCA
CTGAAGAAACCTCAGAAATTCGTATATAGGAAATGATACCATTAATTCATCTGCTTGTTTTAGGTAAAAATATGATAAATGTACATCCTCACTAAGTGCAAAGAAAGCAGAGTGTCATGAGATAG GAA
ATGAGTATGTAACCAAAATATTTTCCTTTATGAAGAAAATAGCTGGCTTTGACCTTTCTAAGACAAGATAAAAAAGCAAAACACGCAGAGATGAAACATTCAGAGCTATCTCATCATCAACGAACCGT
TTATCACTTGGCATTTAGGAATCCTATTTATGGTTGAAAACTCTGGTTCTAAAATGTGATCTGATTATCCCTGAAGTTCAAAATCCATGCAGAGGAAGCAAGCAGGGAAATACTACCT T

>Chimp
GGGAATAAAAAGGCTCTTGGCAtTTCAAATATTTTAATGTTCTTAATTCCAgAtTAAAATTAAAATTACAAAATTTACCaGCTTTGAATGTTTCTCTGTCCTTCAATTCTAAGGCTTTATTTGGAAGA
GAAATTTTCATTCCACAGGTAGGCTAAATGGGTTTGGAAACACAATAATTACTATATAATGTATACTTCATAAAATAAGTAGTTAATATTCAAGATAAAAATATAAAAGTTATTACCTTCAGAAG ACC
ATCTTTATCAGGAGAcTCTAAAAGAAAAGGGACATATATAATTGATTATAtGCAAGCCTGTCAAAGTCTACCAAACATTCATGCAGTGTGAATATGAAGATGAATCCTCATGCTTGGATTGAAAAGAG
ATTACACTAGGTTTTGGGGTCTTTTGGGTTATTGTGTTTGTTAACATGCCAG cGTGACAGAAATATACCTAAGAAAATTTAAAAAATAAATTTCCTCAAACATGCTGTGAAGATTTC cACAGTCAAAA
TATATTTCAAGTGACATTAACTAAAGCAGAAAATCCTAATACCAGTAAGAGTAAGCTG cCATATAAGGAAAGAGGTTGAATTTAGTAAGATGACCAAGACATGACCCCTCCACCAAAAATAAATAAAT
AAATGTCCAAGCTAATGGTAGAATGCTATGACATATTgTTAAAGAAACACATCAGAATCACTTATGTCAATATACTACAAGTAGCTATTATGTTCTTCAATTTGTCCTGTAATTTAAG AATTGCAAAG
CTCCGATGAGAATTCAAAGGTACAATTTACTTATCATTGCAGAGAATGTTCTAAATT cATCAGCATAAATATCTACTTATAGAATgAAAGTTTATAAATATgTTTATTTTTGAACCTCTGTGAAATAC
GAGTATTTTTACATTTCTGGGGTTCTCTAGTTTAGTCTTCTTAAAATTTC cTGATCCACTTATACAGAAAAGTCAAAGCATACCCATCAACAAACTAATACAGTCCAGGTGCCATGGC TCATGCCTGT
AACTCCAGAACCTTGGAAGCCTGAAGAGGGCGGATCTCTCGAGGCCAGGAGTTCCAAAGCAGCCTGGCCAAAATAGCGAAAC Cctgtctccacaaaaaatacaaaaatcatccgggtatggtggcaca
tgcctgtaaccccagctacttgggagccggagctgtgagaatcacttgaacccaggaggcccaggtggcagtgagtcaagatcatgccactgcactccagcctggataacacagtgag actctgtctc
aacaaaagaaaaaacaccctaacatcacaattaaaagaactagaaaagcaagaacaaacacattcaaaagctagcagaaggcaagaaataactaaaatcagagcagaactgaaggaaatagagac aca
aaaaaacccttcaaaaaattaatgaatccaggagctggtttttgaaaggatcaacaaaattgatagaccgctagcaagactaataaagaaaaaaagggagaagaatcaaatagatgca ataaaaaatg
ataaaggggatatcaccaccgatcccacagaaatacaaactaccatcagagaatactacaaacacctctacgcaaataaactagaaaatctagaagaaatggatacattcctcgacacatacact ctc
ccaagactaaacaaggaagaagttgaatctctgaatagaccaataacaggagctgaaattgtggcaataatcaatagcttaccaatcaaaaagagtccaggaccagatggattcacag ccgaattcta
ccagaggtacaaggaggaactggtaccattccttctcaaactattcgaatcaatagaaaaagagggaatcctccataactcattttatgaggccagcatcattctgaaaccaaagccaggcagag ata
caacagaaaaagagaattttagaccaatatccttgatgaacattgatgcaaaaatcctcaataaaatactggcaaaatgaatccagcagcacatcaaaaagcttatccaccatgatca agtgggcttc
atccctgggatgcaaggctggctcaatatacgcaaatcaataaatgtaatccagcatataaacagagccaaagacaaaaaacacatgattatctcaatagatgcagaaaaagcctttgacaaaat tca
acaacccttcatgctaaaaactctcaataaattaggtattgatgggatgtatctcaaaataataagagctatctatgacaaacccacagccaatatcatactgaatgggcaaaaactg gaagcattcc

ctttgaaaactggcacaagacagggatgccctctctcaccactcctgttcaacatagtgttggaagttctggccagggcaatgaggcaggagaaggaaataaagggtatttaattaggaaaagag gaa
gtcaaattgtccctctttgcagacaacatgattgtatatctagaaaaccccattgtctcaacccaaaatctccttaagctgataagcaacttcagcaaagtctcagaatacaaaatca atgtacaaaa
atcacaagcattcttatacaccaataacagacaaacagagagccaaatcatgagtgaactcccattcacaattgcttcaaaagaataaannnnnnnnnnaataaaataaataggaatccaactac aag
ggagtggaaggacctcttcaggagaactacaaaccactgctcaatgaaataaaagaggataaaacaaatggaagaacattccatgctcatgggtaggaagaatcaatatcgtgaaaat ggccatactg
cccaaggttatttacagattcaatgccatccccatcaagctaccaatgactttcttcacagaattggaaaaaactactttaaagttcatatggaaccaaaaaagagcccgtgtcgccaagtcaat cct
aagccaaaagaacaaagctggaggcatcacagtacctgacttcaaactatactacaaggctacagtaaccaaaacagcatggtactggtaccgaaacagagatatagatcaatggaac agaacacagc
cctcagaaataacgccacatatctacaactatctgatctttgacaaacctgagaaaaacaagcaatggggaaaggattccctatttaataaatggtgctgggaaaactggcgagccatatgtaga aag
ctgaaactggatcccttccttacaccttatacaaaaagtaattcaagatggattaaagacttaaacgttagacctaaaaccataaaaaccctagaagaaaacctaggcattaccattc aggacatagg
catgggcaaggacttcatgtctaaaacaccaaaagcaatggcaacaaaagacaaaattgacaaatgggatctcattaaactacagagcttctgcacagcaaaagaaactaccatcagagtgaaca ggc
aacctacagaatgggagaaaattttcgcaacctactcatctgacaaagggctaatatccagaatctacaatgaactccaacaaatttacaagaaaaaaacaaccccatcaaaaagtgg gtgaaggaca
tgaacagacacttctcaaaagaagacatttatgcagccaaaaaacacatgaaaaaatgctcatcatcactggccatcagagaaatgcaaatcaaaaccacaatgagataccatctcacaccagtt aga
atggcaatcattaaaaagtcaggaaacaacaggtgctggagaggatgtggagaaataggaacacttttacactgttggtgggactgtaaactagttcaaccattgtggaagtcagtgt ggcgattcct
cagggatctagaactagaaataccatttgacccagccatcccattactgggtatatacccaaaggactataaatcttgctgctataaagacacatgcacacgtacgtttattgcggcattattca caa
tagcaaagacttggaaccaacccaaatgtccnnnnnnnnnnaaattttcgcaacctactcatctgacaaagggctaatatccagaatctacaatgaactccaacaaatttacaagaaa aaaacaaccc
catcaaaaagtgggtgaaggacatgaacagacacttctcaaaagaagacatttatgcagccaaaaaacacatgaaaaaatgctcatcatcactggccatcagagaaatgcaaatcaaaaccacaa tga
gataccatctcacaccagttagaatggcaatcattaaaaagtcaggaaacaacaggtgctggagaggatgtggagaaataggaacacttttacactgttggtgggactgtaaactagt tcaaccattg
tggaagtcagtgtggtgattcctcagggatctagaactggaaataccatttgacccagccatcccattactgggtatatacccaaaggactaaaaatcatgctgctataaagacacatgcacacg tat
gtttattgcagcattattcacaatagcaaagacttggaaccaacccaaatgtccaacaatgatagactggattaagaaaatgtggcacatatacaccatggaattctatgcagccata aaaaatgatg
agttcatgtccttcgtagggacatggatgaaactggaaatcatcattctcagtgaactatcgcaagaacaagaaaccaaacaccgcatattctcactaataggtgggaattgaacaatgagaaca cat
ggacacaggaaggggaacatcacactctggggaccgttgtagggtgggggtagggggggagggatagcattaggatatatacctaatggtagatgacgagttggtgggtgcagcgcacc agcatggcac
atgtatacatatgtaactaacctgcacaatgtgcccatgtaccctaaaacttaaagtataataaaaaaaaagaaaaaacaagaaaaaaccccctaatatgaaagtatcaaaaattagaagtcaattt cca
gagaattttatatatcattaacctgcatatttcaatgttagaataatggttactgaaaataacagttttattattttgttgctaaaactaattctgtttgttataccatgtgagttg ttaaaacact
gttatgaaaaagctcttataaaaaacttaacttcaagaaaaaacagacatttctttgacatagaaatatatctcctttcatcaaataatatgaataaaacatgcttgatctctcatttttctcta ctt
tttgtttttcagtacaacatactttaatatatttagacatatcaagtatatatgctgtttcccttgagagatgattgatcaataatgaagacacttcaggatataaactccctaaaa cacaaaaaat
ttatgtccaagcattttaaggctaaatacatgaccgtattgcaggtaggtgttatataaaaaaagtgcttaaaattgtattcaacacactttaaagaaaaacctgctttaattatgatgagaagg tca
ttctcgattcattaatatctttaaactttacttctgaagcactgaagaaacctcagaatttcatatataagaaatgataccattaatacatctgtttgttttaggtaaaaatatgata aatgtacatc
ctcactaagtgcaaagaaagcagagtgtcatgagatatgaaatgtaatgtaaccaaaatattttcctttatgaagaaaatagctggctttgacctttctaagacaagataaaaaagcaaaacatg cag
agatgaaacattcagagctatctcatcatcaacgaaccgtttatcacttggcatttaggaatcctatgtatggttgaaaactctggttctaaattgtgatctgattacctctgaagtt caaaatccat
gcagaggaaggaagcaggggaatactacctttgttatttttttattactttttattttttataaaggtgtacactggagtttttttagagtcaggacacgtcacatggcatggctctaaagattagtg agc
cacatttgtaatgggtattgaaatgtatggctgtgaatccttgcattttaagtacttcacttttaatttctaatatagcgtgaagtaagacgtacacttaaacaaaggcttgttgaca tctttaatta
tatttatgttgcttttgagtcatcatgaagagagtctaattaaacgtagtgcaaaataaaaatgcacagtcagaaatgttttctttatttttttattttaatattattatactttaagttttaggg tac
atgtgcacaatgtgcaggtttgttacatatgtatacatgcgccatgttggtgtgctgcacccattaactcgtcatttagcattaggtatatctcctaatgctatccctcctccaaccc cacaacagtc
cccggagtgtgatgttccccttcctgtgtccatgtgttctcattgttcaattcacacctatgagtgagaacacgcagtgtttggtttttttgttcttgcgagagtttgctgagaatgatggtttcc agt
ttcatccatgtccctacaaaggacatgaacttatcatttttttatggctgcgtagtgttccatggtgtatatctgccatactttcttaatccagtctagcgttgttggacatttggttt ggttccaagt
ctttgctattgtgaatagtgccacaataaataaattctttttttttttttcacagagtctggccctgtcacccaggctggagtgtaggtgcacgatcttggctcactgcaatctctgcctcatggg ttc
tcacattctcctgcctcagcctcccgagtagctgggactagaggcacccaccaccatgcttggctaatttttttgtatttttttcatagagacggggtttcaccatgttagccaggatgg tctcgatctg
ctgacctcgtgatccacatgcctctgcctcccaaagggctgggattacaggcgtgaaccacagtgcccagcctgaaatcttgaaataaatgaaaaacgagctaatcgctgaacaaaaaaataaaa aat
cactgtggaatgacaagaacattctacaaacatttatatgtgatttttgcaaaaagtgttaatcccaatgtgtatatgctgagtgatgaggagataagtgatcttgaatcagaggagc aaatcgtgac
actgagaaaataaatgcaaaagctgaacgtagaatgctacaccatgtatctttaatgcaacacattataataatttatatcattacattacaaatattcatcatgttctttaatatgtcctgtca cag
agaaggtaaaaagtttggatgagagttcagctgaatgtagaactgacatctcagcagtgaaagtgttatgaattgatcagtttggatatatacttagagaataatactaaatttaata accactaatt
ttcatacccatatggtataacggtatgccaacatttttgtaatttgtagcttagccatctcaatattccttgatccactcatggaggaagatgtacaaaactcatcaggaatagcaaatataata agc
tttcaatattgacatatttgttttaaatttagttgcaacagacattaaatattaataacattttctatgttaaaatcaatacaatacgtatgaataagaacaatttaggtattcaagc aataaattca
gaattttctttcttcttctctggctaaaaacatactcaaaagtaagagcaacataaggctttctcctaacctttttctgtcttgaccctttatccaactactcttcttccatatttctcccatgaa agg
cctctcctcttgctactcggtctgaggtccaaggaccaccagcatcagcatcgcctgacaacttactaaaaatgcagaatccctagtcggctgaatcacagtgtgaattgttaacaag gttttcagat
gatttttctaaagtttgaaaacttatggtctatacggagtgtatatgacgaattatatacatgtgtggccgtgcatatatgcttattctcaactattgcaaatagaacacagctctccatggtcag ctg
cttccatccctaatggcttcccaccagtgagaaacacaggaagagtcagaatatttttgctgtaattctctggtatgttttggcactagaaagtcactttatattcttcccaaatttc taaccatacc

cgatcttttcctaaagaatcatttcattttaccatcctccattatataaacctgcctcttgttccttcccatcctctctgtactctctgtctttctcgatcttcatttcctcttttactactttc tcc
tttcttatttctcattttcctcagctcttggaataccttgaattcaaactaataattcagttcctttccagcactctcaatgtagtctgctgccaatacatgataagacgtataact tatcaccatt
tcacttctcttttttccccatgctttgcattcaggaggtaattttcttcagctattagggatatccctcctcatcagttcagagaaacatttggtcaaatgacttttaaataaaatatggtt ctt
accttctaattttccacttaaggtatcgaattctttttgatgtgtagccttgggtaaacacacatcattctgtaagagagtctcaaggaaactctgttaaaagtaatatcaataaata atttcaatgg
agaaatcccaataatgaagagtatcaaaagttcctaattaaaatcttttctgaaaacacagtttcctactttacaagcaattcgtcttaagagcagacgtgttgaaacccaaaacattctgatag aaa
acctcacataacactctccacatcaacttacctttacgttcatgaggctattaacttggttactgagcaggaaaaacaaatcacactcacgaaagaattcactcgtgcatatttcaga aagtaactct
cttaaaaatatctaacttgtgttgtagtcctcattaaaagtaaatatgccatttcaaacaaaatacaatgcacttgctactagccataacacgtactgcattaaacatttaaaatattcatgatc ata
gaattgtttagtgttaaaaatgatgcatgccgtgaagagaacttttagagtctgccatataatgcatgcacggaatgtttaaaaagctgctttattttggtaaacaggggcttaaaac aagcaacatg
tttttgacaaaatttatgctgatagaaaaagtgcttgaagagaatgtaaatgcagtaagaaaagaaattacgtcatgtctaaatgaaagccaacaaatattaaagtctttttgtctgacgacaaa tca
gataaaatctacttgtttactgacttccaccctttgctcttaccaccaggcagcagctgcttgtttctaagaggtcagatattttctatgtctttccacagttgtgaccttaaaatac atcactatta
tttcacgctcatgctgctgcagcttgactgcctgccacaaagggactcgaggagttctacttgtgaaaataatttatatttttcaaaagattactcaaccaatactcttgttactctagtcccac aag
gtttcctagtcctcatgctcctctttctcctgaaatgttccctcagatctatactgaacaatacagaaatgtcaatacattcaagtcccaggcattaacttttcaggtcccaactttt acaatactgc
agtttaaactgtctctctatgaatccagttagaacttttgcacatctagatacaccacagaatcataaatttgcccacagctggactcagtgtaattcttttgcttttaggcaaacttgcttcag atc
ccatgtgaacttctagtgacttctaagccttatttttacactataccacaccggcttcttaaattaagcttacagattagcagaatttcactatgaagcagtcatctacttagcaca tagatcctct
attaagcgtattctgaaaaatatcgaaaataatttgggagtgacatatgcagaggtgagaaaataaagtctaagcctctgcttttatgtttttgtatcatgcaatactagtattaaagaaaatta ata
atacacagtacctcaaaatcccaagaatgttcttcatcgtcctttgtttggattctgatgggaacatctgagctataaaaggttaatcacagatacattcatgagaacatttctcta ctataaactt
taaaaacatatataaatctactttcattcacgaatctgcggtttttcctctgtagcccgtatattctttttgttgattacaatcactacttctatagtcaatccgttagaattgaaatctaaaaa aac
cggaaaattaacattatatcattcgttagaatgcagaaaaatagaaatttgactaactcgtagaattatttcttcacaaagcagtcatatcctcttctcccgtgaaacacagtgtgtc ctatacttgc
tgttcattcagaaaactttaattacctgtcatctctcacttctttgtagatttttatttctactattacttagtttgattgactctgcaatctctattacacagttcttcaaaaaagatgattt atc
aacgaacaagatttttagggatattaactttctgatgttagtaaactatatgtccaacacttttacatttcaatatgccatgacagtatattgggtgtgttttgggtgtctatatgt tttaggaagg
taagcttaaaaactttttattgcatataaattaagaactgctttaattacaatgacatagttcttccctaactcaacaatgtcttcagagtcagcttgtgacaactaggagaaacatcagaaatg tat
aggaaaaaataattgccctaagtaatctcacatccccacatgttataggataaaatcaaggcccacacaagacttgaggagctctcaggccaatgagacagaaaatgaatttatacca aaaataacgt
tcagctttctttatcaaccaaaagtacacattacaactatttgaatcaggttaaagaaaaaagcaaacatgcacaactaaagcttaaaagactgtatcatcgttaaaggtacgtttatcatttga cat
ccatcaactgtgtatctggtttttaaaagtaaatttaacgtgtgatctcagcatccctaaagttcacatttcatgcagagggaccaagaaactaaaatcatactttggtatgccacca ttttcactca
tgtttagaagtctaccctggatcgtttcagagatctaatgtgttgcagtaccatagctctaataatctgtgggtcgcagttcgaattgttagtggaatgtatgattgcacaaggaaatactttaa aga
ttttagttttaatttctcacaactaaaaagtcagtagatataactaacttaaagactctgaaattttcatgttttcatttttattcattatttatcagcataataattgtgtacttaa tggaatttca
taaacaatctgatatttaaaaagacagttaattgtgtcctttatggacgtctctaaaaattataatgctgatcaaaacagaatgttgccaaaaattacactgtacaaccacgtatgcattagaag ttg
aaaaaaatttacatattaaatatgaccacgtacacatgttggaaaatgtttaaacatgcataagaatacttttaaaaagtagtttagagtattggttacttctaggcaagaaatggt actagtgtaa
aaagactctgggccgggcgtggtggctcacgcctctaatctcagcactttaggaggccgaggtgggtggattacaaggtcagcaggtcgagaccatcctggctaacatggtgaaaccccgtttct act
aaaaatacaaagaatgagccaggcgttgtagtgggtgcctggattcccagctacttgggaggcccaggcaggagaatggcatgaacccgggaggtggagcttgcagtgcgccaagatc acaccactgc
tctccagcctgggcaagacaacgagatgggcgagagagcgagatgccatatgaaaatttttaaaaaaaaaaaaagaaaatttaagacgtgaaaacaaattctacacatagtcagaaccacacacata tgt
tgtgaatttatggttgtttgcttgctttttgagatggagtctcactctgtcactcaggctaaagtggcacaagcttggctccctgcaacttctgcctcatgggttcaagggattctcc tgcctcagcc
tccagtgtagctgagattacgggcacccaccaccacaaccggctattgtttgttatttttagtagcgatggggtttcactgtgttgaccaggctcgtctgcgactcctgaccttaagttatctgc cca
ccttggtctcctaaattgccaggattacaggcatgtgccgccatgcacagcctgttgtaacatgatttaaacatgcatgggaatgacaacaaaaattaattcaggctgttttgtacct ctaggcaatt
aattacagtagcataaaaagacaccaaggccaaacgctttagagtcacacttctaaaacagtgtgtctagcaaagagtcatcaagtaatgattttaaaaattatatgtcacacacacacacaca cac
tcagtcacctaatcagaaaagcaagaaactgatttactttagattctcagagtgacaacactcacaactgtcacgtcagcaagtgaaactttactcaaagtaaagatcagaattccaa tcagcatttg
aaccactgcaaatatttcacagtttcaaaattcacctgccttaaaaccgagcacgtacagaaattgcaaattggaaactttatactatcgagatattaaagaagatccacatgaagtaaagcaga att
tttttctaaattcctaattcttatgcttgtaaatggcatttctcaaacatgaatactaacgttgccattattttatcaaatttcccatcgaattttgaaaagaaaacaacattggg aataaaaagg
tgctcagcatttcaaatagtttaatattcttaattccacagtaaaattaaaattgcaaaatttacctgctttgaatgtttctctgtccttcaattctaaagctttatttggaagagaaaatttcc ttc
cacaggtaggctaaatcggtttggaaacacaaaaataacatataatgtatacttcataaaatatgtaggtaatattcaagacaaaaatataaaagttattaccttcagaagaccatca ttatcaggag
actctaaaagaaaagggacatacataattgattatatgcaagcctgaaaaagcctaccaaatgttcatacagtgtgaatatgaagatgaatcctcatgcttggattgaaaagggattacactagg ttt
tggggtcttttgggttattgtatttgttaacatgccaactgtagggagacccccctgaaactattgctatggaaagaaagatgcaatgctcctgattattgtaaatacaaaactgcatg caggattgtg
taaagacaatgccaggttggactgccagaatgagccaacagcgcgtgatgtgcttccccctgcagagagcctgtgaatggatgtgcagtcagggaggtttcgcatcaccaagattcctatcccag aaa
ggcatatgttcattgctcttgtggagagcctataaacggacgcactgggggcgcctgtccatatggataagatagtgctatgaacgccctcatcttgccacggctcttctaggcctc tttagggtta
ctgcatactcccttctgagaatttctggtctaaccggttgtctagcttcacttcctgtttctatggattgtttggaaccagcttttgctgcaactgttactactgattaatatcttgctaatcat ggg
ttatggaaagactgtgtttctttttaaggctctgttagaaattaccgatgcacacagtacattgtaaattcttatccctgtatactgtatttctgcatacagatgttagttacagaa ttacttcatc

cccatgtaaccatctcacctcataatcaaatgatcctaaatccctcattaacccacccccaccctcactaaacttaataataaacgcgggtatatccagtgaattgttggcaccacgggaacaca agg
cagtgattcccctggacccagctttcactatcttgtctgtgtctattctttctcaacctgccgatccacctgggaacaaagacaaggccccattgcattgcaggctgctggacagata tccacatgag
gcaaagctgaattttttgttaatctctaaatcttatgcttgtaaatggcattttctcaaacataaatgctaacattggcattatttgtatcaaattttcccatcaaattttcaaaataaaacaa cat
tgggaataaaaggctctcggcatttcaaatagtttaatattcttaattccagattaaaagttaaattacaaaatttacctgctttgaatgtttctctgtcctttaattctaaggcttt atttggaaga
gaaactttccttccacaggtaggctaaatgggtttggaaacacaaaaattaatacataatatatgcttcctaaaatctgtagttagtattcaagatagctatataaaagttattaccttcagaag acc
atctttaacaggagactctaaaagtaaagggacatatataattgattatatgcaagcctgacaaagcctaccaagcatttgtacaatgtgaatatgaagatgaatcctcatgcttgga ttgaaaaggg
attacactaggttttggggtcttttgggcttttgtatttgttaacatgccaatgggacagaaatatacttaagaaaatttaaaaaataaatttcatcaaacttgctatgaagatttcaaaagtca aaa
tatatttcaactgacaataactaaattagaaaaatcctaataccagtaagattaatctgccgtatagggaaagaagttgaatttagtaagatgaccaagttatgacccccctgccaaaa aaaaaaaaag
tcctagctaatggaagaatgctatggcatattgttaaggaaacacatcagaatctcttatgtcaatatactgcaagtagctattatgttcttcaattttcctgtaatttaagaattgccaagct gcg
atgagaattcaaaggtacaatttacttatcattgcagaggatattctaaatttatcagcataaatatctacttatagaataaaagtttatgaatgtatttatttctgatcctctgtga tataggagta
tttttacatttatggggttctccggtttagtcctctcaaaatttcctgatccacttatacagaaaagtcaaagcacacccatcaacaaactaatacagtccaggtgccatggctcatgcctgtaa ctc
cagaaccttggaagcccgaagagggcagatctctcaaggcctggagttccaaagcagcctggccaaaatagcgaaacc
CTGTCTCCACAAAAAATACAAAAATCATCCAGGTATGGT GGCACATGCCTGTAACCCCAGCTACTTGGGAGCCGGAGCTGTGAGAATCACTTGAACCCAGGAGGCCCAGGTGGCAGTGAGTCAAGATC
ATGCCgCTGCACTCCAGCCTGGATGACAGAGtGAGACTCTGTCTCAACAAAAGAAAAAACACCTGAT gTGAAAGTATCAAAAACTAGATGTCAATTTCCAGAGACTTTTTACATATCATTAACCTGCA
TATTTCAATaTTAGAgTAATGGTTATTGAAAATAACAGTTTTATTGTTCAgGGAAcGAATTCTACAATTGCTTTGTTGCTAAAACTAATTCTATTTGTTATACCATGtGAGTTGTTAAgCCACTGTTA
TaAAAAAGgTTTTATAGAAAGCTTAGCTTCAAGAAAAAACAGACATTTCTTTGACATAGAAATATATCTC cTTTCaTCAAATAATATGAATAaAACATGCTTAATCTCTCATTTTTCTCTACTTTGTT
ATTTTTCAGTAAGACACACTcTAATATATTTAGACATaTCAAGTATATATGTTGTTTCCTTTGaGAGTTaATTGATCAaTAATGAAGACACTTCAGGGAtATAAACTgTCTAAAACACACAaAATTTA
TGTgCAAGCatTTTAAGGCTAAATATGCATGACCGTGTTGCAGGTATGcGTTTTAGAAAAAAAGTGCTTAAAATTGTATTCAATACACTTTAAATAAAAACCCGCTTTAATTACGATGAGAAG aTCaT
TCTCGATTCATTAATATCTTTAAACTTTACTTCTGAAGCACTGAAGAAACCTCAGAAATTC aTATATAGGAAATGATACCATTAATTCATCTGCTTGTTTTAGGTAAAAATATGATAAATGTACATCC
TCACTAAGTGCAAAGAAAGCAGAGTGTCATGAGATAGGAAATGA aTATGTAACCAAAATATTTTCCTTTATGAAGAAAATAGCTGGCTTTGACCTTTCTAAGACAAGATAAAAAAGCAAAACACGCA G
AGATGAAACATTCAGAGCTATCTCATCATCAACGAACCGTTTATCACTTGGCATTTAGGAATCCTAT gTATGGTTGAAAACTCTGGTTCTAAA cTGTGATCTGATTATCCCTGAAGTTCAAAATCCAT
GCAGAGGAAGCAAGCAGGGTACTAC

Homologous Sequences

CAGCTACTTGGGAGCCGGAGCTGTGAGAATCACTTGAACCCAGGAGGC

>NAHR_16_ Scaffold8735 415612 415919
KSD_NAHR_16

>**Scaffold8735 415612 415919_**NAHR

GCAGGCAGCCTGAGATTTTAAAAAGGAGGCAATTCTCTGAAATAAAATGTGAGCCACAAGTTGGGCGCTGTACCTCACGCCTGTAATCCCAGTACTTCGGGAGGCCGAAGAGGGTGGATCATCTGA GG
TTCAGAGTTCGAGATCAGTCTGGCCAACATGGTGAAACCCCGTCTCTAAAACAATACAAAAAAATTAGCTGGGCATGGTGGCAGGCGCCTGTAATCCCAGCTACTTGGGAGGCTGGGGCAGGAGA ATC
GCTTGATCCTGGGCGGTGGAGGTTGCAGTGAGACGAGATCACGCCACTGCACTCCAGTATGGACAACAAGAGTGAAACTCTGTCTCAAAAAAAATAAATAAATAAAATAAAATCATAAAATGTGAG CC
ACATGTAAATTTTAAATTTTCTAGTAGCCAAACTTTATTTTATATTTTTATTTTTTTAGACGGAGTCTCACATTGTTGCCTGGGCTGGAGTGCAATGGCGCGATTTCTGCTCACTGCAACCTCTG CCT
CCCGGGTTCAAGCGATTCTCCTGCCTCAGCCTCCCAAGTAGCTGAGATAAAAGGCGCTGGCCACCATGCCCGGCTAATTTGTAGCCAAACTTTAAAAAGAGTTTAGCTCTATTTTTAAAAAGAAAC AA
AGAACAGGTGAAATTGATTGTAACAATTTAACCCAATATATCGAAAATATTGTCATTTTAATATGTGAGAAATATGTAATTATTAATGAAGTGTGTCTATATATATGGAACTAAACCTTTGAAAC TAA
CTCGTATTTTACCTTTCTAGCACATCGCAGTTCAGACCAGGCGCATTCCAGGCAGCCAGTAGCAACACAGGGCCAATAGCTGCTACATTGAAGTGCAGCTCTGACATCAGGAGGGTGAACGACCTG AA
CATCCCTTTTCTGCCGGAGGTGAGGAGAGAGCCTCTCCCTACCAACATCTCTCTTCGGTTCTGAGGGTAGAACAGACAGTGGTCATAGACAGAGCAGAGGGCA GGCCGGGCGGAGTGGCTCACGCCTG
TAATCCCAGCACTTTGGGAGGCCGAGGCGGGCGGATCACGAGGTCAGGAGATCGAGACCATCCCGGCTAAAACGGTGAAACCCCGTCTCTACTAAAAATACAAAAAATTAGCCGGGCGTAGTGGCG GG
CGCCTGTAGTCCCAGCTACTTGGGAGGCTGAGGCAGGAGAATGGCGTGAACCCGGGAGGCGGAGCTTGCAGTGAGCCGAGATCCCGCCACTGCACTCCAGCCTGGGCGACAGAGCGAGACTC aGTCTC
AAAAAAAAAAAAAAAAAAAAAAAAAAA GAGCAGAGGGCAACAGGAATAAAATAACCATAGGTAAACCACTGCTGACCACCTATTGCCCTCCTTCCTTCTAGAGATCAGACAGTGAACAAAGGATGATG
GGGCCAAAGGAGAAGAGAACCCTATGTCTCTTCAGGCCC aTCCACACTCTTGACCTCCAATTTTTAGATATGAAAAAAAAATAGATTAAAGGCAAGCTTATCTTGCTATTTGGCCTTGGCCCTAATGG
TCAGGCCGTGGTTGTCTATTTTCTCCTGTGGTATGAGGGACTGTGTGGGTATAGGCACCAATCACATGCATACAATGTCTACGTGTATTTCTGCATTACTCAATATTACCTTACAAGACATCCAA CTC
TAAATCAGAGGAAAAAATAGTACCTTTAGAGTGTCCACTG TGTGAAGGTAAGTAGTACTGAATCTGTCATTAAATCCTGGCATCCTGTCTGCACTGGGTGCATGTATTAGTTAGCTATTGCAGCATAA
CAAACCATCCAAAACTTATTAGCTCATAATTGAAATGGTCAGCCATTTGGGCTGGGCTCAGTGGGGCCATTTCTCTAGTCTCAGCTGAGCATTTTTAGACATGTATCATCAGCTGCTTGTTGACT AAG
CAGCTGTGTTTCTGGGAGTGAGCTTCTGCTTCTGGAGCTG TCAACAGGGGCAACTTTTTTTCTCCTTTCCATGGTATCTTATTCTCTGACTGGCCAACGTGGGTTTTTCCCATGGAGATGGCAGCATT
CTGAAATAAAAACAGAAGCATTCAAGACCATTTGAGCCTGGGCCCCAAACTAGCCCACTGTCATGTCCACAGGTTTCTACTGCCCTGAGCAAGTACGACCCGCCAGATCTAGTGTTCAGAAAACA GAT
TCTGGATCTCAATGGAACAACTGTAAAAGCACCTGGCAAT GGGCAAGAATACAGGAGGATGAAAAATTGCTACCATTTTGGTAATCATTATTATTCTGCCCTTTTTAATGTATGTGGTTTATAGAACT
CTT

2149	11.1	0.7	1.0	a	63	368	(1939)	+	AluSq	SINE/Alu	5	309	(4)	1
289	9.1	2.3	0.0	a	371	414	(1893)	C	MER33	DNA/hAT-Charlie	(18)	306	262	2
1212	13.1	0.0	0.0	a	418	592	(1715)	C	AluSg4	SINE/Alu	(7)	303	129	3
728	27.4	7.6	0.4	a	629	866	(1441)	C	MER33	DNA/hAT-Charlie	(69)	255	1	2
2881	1.0	0.0	0.0	a	1000	1307	(1000)	+	AluYa5	SINE/Alu	1	308	(3)	4
1281	30.1	8.8	0.4	a	1767	2263	(44)	C	MLT1H	LTR/ERVL-MaLR	(0)	549	11	5
2149	11.1	0.7	1.0	hg19	63	368	(2120)	+	AluSq	SINE/Alu	5	309	(4)	6
289	9.1	2.3	0.0	hg19	371	414	(2074)	C	MER33	DNA/hAT-Charlie	(18)	306	262	7
1212	13.1	0.0	0.0	hg19	418	592	(1896)	C	AluSg4	SINE/Alu	(7)	303	129	8
728	27.4	7.6	0.4	hg19	629	866	(1622)	C	MER33	DNA/hAT-Charlie	(69)	255	1	7
2934	0.6	0.0	0.0	hg19	1000	1310	(1178)	+	AluYa5	SINE/Alu	1	311	(0)	9
1354	8.0	4.0	0.0	hg19	1313	1488	(1000)	+	AluSx	SINE/Alu	129	311	(1)	10
1281	30.1	8.8	0.4	hg19	1948	2444	(44)	C	MLT1H	LTR/ERVL-MaLR	(0)	549	11	11

>hg19 chr19:21512841-21515328

GCAGGCAGCCTGAGATTTTAAAAAGGAGGCAATTCTCTGAAATAAAATGTGAGCCACAAGTTGGGCGCTGTACCTCACGCCTGTAATCCCAGTACTTCGGGAGGCCGAAGAGGGTGGATCATCTGA GG
TTCAGAGTTCGAGATCAGTCTGGCCAACATGGTGAAACCCCGTCTCTAAAACAATACAAAAAAATTAGCTGGGCATGGTGGCAGGCGCCTGTAATCCCAGCTACTTGGGAGGCTGGGGCAGGAGA ATC
GCTTGATCCTGGGCGGTGGAGGTTGCAGTGAGACGAGATCACGCCACTGCACTCCAGTATGGACAACAAGAGTGAAACTCTGTCTCAAAAAAAATAAATAAATAAAATAAAATCATAAAATGTGAG CC
ACATGTAAATTTTAAATTTTCTAGTAGCCAAACTTTATTTTATATTTTTATTTTTTTAGACGGAGTCTCACATTGTTGCCTGGGCTGGAGTGCAATGGCGCGATTTCTGCTCACTGCAACCTCTG CCT
CCCGGGTTCAAGCGATTCTCCTGCCTCAGCCTCCCAAGTAGCTGAGATAAAAGGCGCTGGCCACCATGCCCGGCTAATTTGTAGCCAAACTTTAAAAAGAGTTTAGCTCTATTTTTAAAAAGAAAC AA
AGAACAGGTGAAATTGATTGTAACAATTTAACCCAATATATCGAAAATATTGTCATTTTAATATGTGAGAAATATGTAATTATTAATGAAGTGTGTCTATATATATGGAACTAAACCTTTGAAAC TAA

CTCGTATTTTACCTTTCTAGCACATCGCAGTTCAGACCAGGCGCATTCCAGGCAGCCAGTAGCAACACAGGGCCAATAGCTGCTACATTGAAGTGCAG CTCTGACATCAGGAGGGTGAACGACCTGAA
CATCCCTTTTCTGCCGGAGGTGAGGAGAGAGCCTCTCCCTACCAA CATCTCTCTTCGGTTCTGAGGGTAGAACAGACAGTGGTCATAGACAGAGCAGAGGGCAGGCCGGGCGGAGTGGCTCACGCCTG
TAATCCCAGCACTTTGGGAGGCCGAGGCGGGCGGATCACGAGGTCAGGAGATCGAGACCATCCCGGCTAAAACGGTGAAACCCCGTCTCTACTAAAAATACAAAAAATTAGCCGGGCGTAGTGGCG GG
CGCCTGTAGTCCCAGCTACTTGGGAGGCTGAGGCAGGAGAATGGCGTGAACCCGGGAGGCGGAGCTTGCAGTGAGCCGAGATCCCGCCACTGCACTCCAGCCTGGGCGACAGAGCGAGACTC cGTCTC
Aaaaaaaaaaaaaaaaaaaaaaaaaaaaaaaaaaaaattagctgggctggtggcgcatgcctgtaatcccagctactcgggaggcagaagaatcgcttgaacctggaaggcagaggttgcagtgagcc
aagatcgggtcactgcactccagcctgggtggcagagcgagactccgcctcaac AAAAAAAAAAAAAAAAAAAAAAAAAAGAGCAGAGGGCAACAGGAATAAAATAACCATAGGTAAACCACTGCTGA
CCACCTATTGCCCTCCTTCCTTCTAGAGATCAGACAGTGAACAAAGGATGATGGGGCCAAAGGAGAAGAGAACCCTATGTCTCTTCAGGCCC gTCCACACTCTTGACCTCCAATTTTTAGATATGAAA
AAAAAATAGATTAAAGGCAAGCTTATCTTGCTATTTGGCCTTGGCCCTAATGGTCAGGCCGTGGTTGTCTATTTTCTCCTGTGGTATGAGGGACTGTGTGGGTATAGGCACCAATCACATGCATA CAA
TGTCTACGTGTATTTCTGCATTACTCAATATTACCTTACAAGACATCCAACTCTAAATCAGAGGAAAAAATAGTACCTTTAGAGTGTCCACTGTGTGAAGGTAAGTAGTACTGAATCTGTCATTA AAT
CCTGGCATCCTGTCTGCACTGGGTGCATGTATTAGTTAGCTATTGCAGCATAACAAACCATCCAAAACTTATTAGCTCATAATTGAAATGGTCAGCCATTTGGGCTGGGCTCAGTGGGGCCATTTCTC
TAGTCTCAGCTGAGCATTTTTAGACATGTATCATCAGCTGCTTGTTGACTAAGCAGCTGTGTTTCTGGGAGTGAGCTTCTGCTTCTGGAGCTGTCAACAGGGGCAACTTTTTTTCTCCTTTCCAT GGT
ATCTTATTCTCTGACTGGCCAACGTGGGTTTTTCCCATGGAGATGGCAGCATTCTGAAATAAAAACAGAAGCATTCAAGACCATTTGAGCCTGGGCCCCAAACTAGCCCACTGTCATGTCCACAG GTT
TCTACTGCCCTGAGCAAGTACGACCCGCCAGATCTAGTGTTCAGAAAACAGATTCTGGATCTCAATGGAACAACTGTAAAAGCACCTGGCAATGGGCAAGAATACAGGAGGATGAAAAATTGCTA CCA
TTTTGGTAATCATTATTATTCTGCCCTTTTTAATGTATGTGGTTTATAGAACTCT T

Homologous Sequences = undefined

>NAHR_17_ Scaffold12434 3240591 3240887
KSD_NAHR_17

>Scaffold12434 3240591 3240887_NAHR
AAAAACTTGAAACTGCTGCTAGGGCTGAGCATACAAGATTTGAATATTTGGGAAGATCCAGTATTAAAAAGTTGAACCTGGTTTCCCCCCGAGTCTATGAATTTAATCTAGCCTTATTTAAAACAC TA
AAAGGCTCTTTTAAAGTTAGGCAGGTTGATTCTGAGGTTCAAAAGAAAAAAATGTGAAAAGATGCAGAACATTTCTGAAAATGTAAGAGGAAATCAGCCCTTCTGCATTTGAAAACTGATCATAA AAG
TATAGTTTTCAAACAGTTTGGGACTGGCCCATAAAAGGACAGACAACTGGTTAAAAGGGACAGATTAGAGGGGCCAGAAATACATATGGGAATTTACAT CtTTTTTTTTTTTTTTTTTTATCTGAGATGG
AGTCTCGGTTTGTCACCCAGGCTGGAGTGCAGTGGCGCGATCTTGGGTCAC gGAAACCTCTGCCTCCCGGATTCAAGCTATTCTCCTGCCTCAGCCTC aCGAGTAGCTGGGATTACAGGCACACGCCA
CCACACCTGGCTAATTTTATGTATTTTTAGTAGAGATGGGGTTTCACTGTGTTAGCCAGGGTGGTCTCGATCTCCTGACCTCGTGATCCGCCCACCTTGGCCTCCCAAAGTGCTGGGATTACAGGC AT
GAGCCACCATACCCAGCTGGGAATTTACATCTTAACAGTGGAATTTCATATCATTGAAAACGAGATGGACTTTTCTCCATAAAACTAAGACACTCAATAGCCATCTGGAAAGGTATAAAGTTAGA GGT
AGATTGGAGATTTATTATTTATTTATTTATTTATTTTTTAATTTTTTTTGAGAGAGAGTCTTACTCTGTCACTTAGGCTGGAGTGCAGTGGCATCATCTCGGCTCACTGCAACCTCCACCTCCTGG GT
GGAGGCGATTCTCCTGCCTCAGCCTCCCAAGTAGCTGGGATTACAGGCGTGCGCCACCATCCCCAGCCTAGATTGCAGATTTAAATGTCAAAAACGACACCTCAGGCAGGGCGTGGTGGCTCACG CCT
GTAATCCCAGCACTTTGAGAGGCCGAGGCAAGTGTATCACCTGAGGTCAGGAGTTCGTGACCAGCCTGGGCAACATGTTGAGACCCCCATCTCTACTAAAAATACAAAAATTAGCTGGGTGTGGTG GT
GTGCTCCTGTAATCCCAGCTACTTGGGAGGCTGAGGCAGGAGAATGGCTTGAACCCGGGAGGCAGAGATTGCAGTGAGCCAGGATGACGCCACTGCACTCCAGCCTGGGTGACAGAGAGAGACCC TGT
CTCAAAAAGAAAAAAAAAGTTCATAACTCATGTAAGTTATGAGTGGCTAAATTCCTGGCATTACATTCTATTTGTATTAATAGCTATTGGGCTCACTGTTAACACAAGGTTTGGAACAGAGCCAGAG TT
AAATGTATAAATATATACATTTGCTGTGTGAAGGATTAATTGTTCCTTTTCAACTCTCAGAAACCTACATCCTCCTACTCCAACATCATTCCTGACTGGCAAACTCCTATAACGCCCTCAGTTAC CTC
ATCAAACCTCCCCACCTTGGGTGTTCCATCTCACCCAGAGAAAAGTCCCAAGTCCCCACCAGGAGCTTTGAGGTCTTGTAAGATCTGGCTGTCACCTCCTCTCTTGCGCGGCCTGCTTTTTCTTGG GC
TTTCTTCTCAACACTCCCTTGCTCCCTCTGCTGCCAGGCACCACGGGTTTCTGCTGTTCTTCAAGTACACCAGGCAGGCTCCAGCCTCAGGGCCTTTGCAC gGATTTTTtTTTTTTcctgttgTTTTG
AGAcGAAGTCTCGCTCTGTCACCCAGGCTGGAGTGCAGTGGCGGGATCTCGGCTCACTGCAAGCTCCGCCTCCCAGGTTCAAGCCATTCTCCTGCCTCAGCCTCCCGAGTAGCTGGGACTACAAGCGC
CCGCCACCACGCCCGGCTAATTTTTTGTATTTTAGTAGAGACAGGATTTCACCATGTTGCCCAGCTAGTCTTGAACTCCTGAGCTCAGGCAATCCACCCGCCTTGGCCTCCCAAAGTGCTGGGAT TAC
AGGCGTGAGCCGCCGTGCCCGGCCGCACTGACTGTTCCTTCTGCTTGGAACACAGTTCCCTCACATGTTCACAAGGTGGCTCTCTCAC tTCCTTCAGGTCTTTGCTCCAAATGTCACCTCCATGAAAC
CTTTACTTGGCACACTGTAAATTATAACATCCTCTCCCAATACACACTCTCTATGCCTGTCTCTCTCGCTCTGTCTCTCTCTTTTTTTTTTTTTTTTTT TGACAGAGTCTtaCTCTGTcGC

347	30.7	5.4	3.8	a	51	354	(1942)	+	L1M5	LINE/L1	4531	4843	(1300)	1
2325	10.3	0.0	0.0	a	357	657	(1639)	C	AluSc8	SINE/Alu	(9)	302	2	2
1184	13.5	0.0	1.1	a	786	963	(1333)	C	AluSx	SINE/Alu	(0)	312	137	3
2254	10.1	0.0	0.3	a	1001	1297	(999)	+	AluSx	SINE/Alu	1	296	(16)	4
811	22.6	3.8	8.1	a	1538	1768	(528)	+	L2a	LINE/L2	2839	3048	(371)	5
2371	7.9	0.7	0.3	a	1769	2072	(224)	C	AluSx1	SINE/Alu	(7)	305	1	6
811	22.6	3.6	5.9	a	2073	2257	(39)	+	L2a	LINE/L2	3049	3231	(195)	5
16	0.0	0.0	0.0	a	2258	2275	(21)	+	(T)n	Simple_repeat	1	18	(0)	7

347	30.7	5.4	3.8	hg19	51	354	(3358)	+	L1M5	LINE/L1	4531	4843	(1300)	8
2366	9.7	0.0	0.0	hg19	357	656	(3056)	C	AluSc8	SINE/Alu	(10)	301	2	9
1184	13.5	0.0	1.1	hg19	785	962	(2750)	C	AluSx	SINE/Alu	(0)	312	137	10
2254	10.1	0.0	0.3	hg19	1000	1296	(2416)	+	AluSx	SINE/Alu	1	296	(16)	11
446	25.8	1.8	10.4	hg19	1537	1765	(1947)	+	L2	LINE/L2	2839	3049	(370)	12
2348	8.3	0.7	0.3	hg19	1769	2070	(1642)	C	AluSx1	SINE/Alu	(8)	304	2	13
1223	12.9	0.0	1.1	hg19	2199	2376	(1336)	C	AluSx	SINE/Alu	(0)	312	137	14
2254	10.1	0.0	0.3	hg19	2414	2710	(1002)	+	AluSx	SINE/Alu	1	296	(16)	15
819	22.4	3.6	8.3	hg19	2951	3182	(530)	+	L2a	LINE/L2	2839	3049	(370)	16
2401	7.3	0.7	0.3	hg19	3183	3485	(227)	C	AluSx1	SINE/Alu	(8)	304	1	17
819	22.2	3.2	5.9	hg19	3486	3670	(42)	+	L2a	LINE/L2	3050	3231	(195)	16
286	4.8	0.0	0.0	hg19	3671	3712	(0)	C	Alu	SINE/Alu	(1)	300	259	18

>hg19 chr19:46941528-46945239
AAAAACTTGAAACTGCTGCTAGGGCTGAGCATACAAGATTTGAATATTTGGGAAGATCCAGTATTAAAAAGTTGAACCTGGTTTCCCCCCGAGTCTATGAATTTAATCTAGCCTTATTTAAAACAC TA

AAAGGCTCTTTTAAAGTTAGGCAGGTTGATTCTGAGGTTCAAAAGAAAAAAATGTGAAAAGATGCAGAACATTTCTGAAAATGTAAGAGGAAATCAGCCCTTCTGCATTTGAAAACTGATCATAA AAG
TATAGTTTTCAAACAGTTTGGGACTGGCCCATAAAAGGACAGACAACTGGTTAAAAGGGACAGATTAGAGGGGCCAGAAATACATATGGGAATTTACAT CTTTTTTTTTTTTTTTTTATCTGAGATGGA
GTCTCGGTTTGTCACCCAGGCTGGAGTGCAGTGGCGCGATCTTGGGTCAC tGAAACCTCTGCCTCCCGGATTCAAGCTATTCTCCTGCCTCAGCCTC cCGAGTAGCTGGGATTACAGGCACACGCCAC
CACACCTGGCTAATTTTATGTATTTTTAGTAGAGATGGGGTTTCACTGTGTTAGCCAGGGTGGTCTCGATCTCCTGACCTCGTGATCCGCCCACCTTGGCCTCCCAAAGTGCTGGGATTACAGGCA TG
AGCCACCATACCCAGCTGGGAATTTACATCTTAACAGTGGAATTTCATATCATTGAAAACGAGATGGACTTTTCTCCATAAAACTAAGACACTCAATAGCCATCTGGAAAGGTATAAAGTTAGAG GTA
GATTGGAGATTTATTATTTATTTATTTATTTATTTTTTAATTTTTTTTGAGAGAGAGTCTTACTCTGTCACTTAGGCTGGAGTGCAGTGGCATCATCTCGGCTCACTGCAACCTCCACCTCCTGGG TG
GAGGCGATTCTCCTGCCTCAGCCTCCCAAGTAGCTGGGATTACAGGCGTGCGCCACCATCCCCAGCCTAGATTGCAGATTTAAATGTCAAAAACGACACCTCAGGCAGGGCGTGGTGGCTCACGC CTG
TAATCCCAGCACTTTGAGAGGCCGAGGCAAGTGTATCACCTGAGGTCAGGAGTTCGTGACCAGCCTGGGCAACATGTTGAGACCCCCATCTCTACTAAAAATACAAAAATTAGCTGGGTGTGGTGG TG
TGCTCCTGTAATCCCAGCTACTTGGGAGGCTGAGGCAGGAGAATGGCTTGAACCCGGGAGGCAGAGATTGCAGTGAGCCAGGATGACGCCACTGCACTCCAGCCTGGGTGACAGAGAGAGACCCT GTC
TCAAAAAGAAAAAAAAGTTCATAACTCATGTAAGTTATGAGTGGCTAAATTCCTGGCATTACATTCTATTTGTATTAATAGCTATTGGGCTCACTGTTAACACAAGGTTTGGAACAGAGCCAGAGTTA
AATGTATAAATATATACATTTGCTGTGTGAAGGATTAATTGTTCCTTTTCAACTCTCAGAAACCTACATCCTCCTACTCCAACATCATTCCTGACTGGCAAACTCCTATAACGCCCTCAGTTACC TCA
TCAAACCTCCCCACCTTGGGTGTTCCATCTCACCCAGAGAAAAGTCCCAAGTCCCCACCAGGAGCTTTGAGGTCTTGTAAGATCTG GCTGTCACCTCCTCTCTTGCGCGGCCTGCTTTTTCTTGGGCT
TTCTTCTCAACACTCCCTTGCTCCCTCTGCTGCCAGGCACCACGGGTTTCTGCTGTTCTTCAAGTACACCAGGCAGGCTCCAGCCTCAGGGCCTTTGCAC tGActtttttttttcttttttttttga
gaagaagtctcgctctgtcacccaggctggagtgcagtggcgggatctcggctcactgcaagctccgcctcccaggttcaagccattctcctgcctcagcctcccgagtagctgggactacaagcg cc
cgccaccacgcccggctaatttttgtatttagtagagacaggatttcaccatgttgcccagctagtcttgaactcctgagctcaggcaatccacccgccttggcctcccaaagtgctgggatt aca
ggcatgagccaccatacccagctgggaatttacattttaacagtggaatttcatatcattgaaaacgagatggacttttctccataaaactaagacactcaatagccatctggaaaggtataaagtta
gaggtagattggagatttattatttatttatttttttttttttttaattttttttgagagagagtcttactctgtcacttaggctggagtgcagtggcatcatctcggctcactgcaacctccacc tcc
tgggtggaggcgattctcctgcctcagcctcccaagtagctgggattacaggcgtgcgccaccatcctcagcctagattgcagatttaaatgtcaaaaacgacacctcaggcagggcgtggtggct ca
cgcctgtaatcccagcactttgagaggccgaggcaagtgtatcacctgaggtcaggagttcgtgaccagcctgggcaacatgttgagacccccatctctactaaaaatacaaaaattagctgggt gtg
gtggtgtgctcctgtaatcccagctacttgggaggctgaggcaggagaatggcttgaacccgggaggcagagattgcagtgagccaggatgacgccactgcactccagcctgggtgacagagagag ac
cctgtctcaaaaagaaaaaaaaagttcataactcatgtaagttatgagtggctaaattcctggcattacattctatttgtattaatagctattgggctcactgttaacacaaggtttggaacagag cca
gagttaaatgtataaatatatacatttgctgtgtgaaggattaattgttccttttcaactctcagaaacctacatcctcctactccaacatcattcctgactggcaaactcctataacgccctcag tt
acctcatcaaacctccccaccttgggtgttccatctcacccagagaaaagtcccaagtccccaccaggagctttgaggtcttgtaagatctggctgtcacctcctctcttgcgcggcctgctttt tct
tgggctttcttctcaacactcccttgctccctctgctgccaggcaccacgggtttctgctgttcttcaagtacaccaggcaggctccagcctcagggcctttgcactgac ttttttTTTTTcTTTTTT
TTTTGAGAaGAAGTCTCGCTCTGTCACCCAGGCTGGAGTGCAGTGGCGGGATCTCGGCTCACTGCAAGCTCCGCCTCCCAGGTTCAAGCCATTCTCCTGCCTCAGCCTCCCGAGTAGCTGGGACTACA
AGCGCCCGCCACCACGCCCGGCTAATTTTTGTATTTAGTAGAGACAGGATTTCACCATGTTGCCCAGCTAGTCTTGAACTCCTGAGCTCAGGCAATCCACCCGCCTTGGCCTCCCAAAGTGCT GGG
ATTACAGGCGTGAGCCGCCGTGCCCGGCCGCACTGACTGTTCCTTCTGCTTGGAACACAGTTCCCTCACATGTTCACAAGGTGGCTCTCTCAC cTCCTTCAGGTCTTTGCTCCAAATGTCACCTCCAT
GAAACCTTTACTTGGCACACTGTAAATTATAACATCCTCTCCCAATACACACTCTCTATGCCTGTCTCTCTCGCTCTGTCTCTCTCTTTTTTTTTTTTTTTTT TtgaGACAGAGTCTcgCTCTGTtGC

327	31.2	4.7	3.4	Chimp	51	355	(1719)	+	L1M5	LINE/L1	4531	4843	(1300)	1
2299	9.8	0.3	0.0	Chimp	358	653	(1421)	C	AluSc8	SINE/Alu	(13)	298	2	2
34	0.0	5.1	0.0	Chimp	774	812	(1262)	+	(ATTT)n	Simple_repeat	1	41	(0)	3
1130	13.0	0.0	0.0	Chimp	814	967	(1107)	C	AluSx	SINE/Alu	(22)	290	137	4
2270	9.7	0.0	0.3	Chimp	1005	1302	(772)	+	AluSx	SINE/Alu	1	297	(15)	5
535	24.8	1.7	11.3	Chimp	1539	1770	(304)	+	L2	LINE/L2	2838	3049	(370)	6
2603	5.3	0.0	0.0	Chimp	1774	2074	(0)	C	AluY	SINE/Alu	(10)	301	1	7

>Chimp
AAAAACTTGAAACTGCTGCTAGGGCTGAGCATACAAGATTTGAATATTTGGGAAGATCCAGTATTAAAAAGTTGAACCTGGTTTCCCCC tGAGTCTATGAATTTAAgCTAGCCTTATTTAAAACACTA
AAAGGCTCTTTTAAAGTTAGGCAGGTTGATTCTGAGGTTCAAAAGAgAAAAAATGTGAAAAGATaCAGAACATTTCTGAAAATGTAAGAGGAAATCAGCCCTTCTGCATTTGAAAACTGATCATAAAA
GTATAGTTTTCAAACAGTTTGGGACTGGCCCATAAAAGGACAGACAACTGGTTAAAAGGGACAGATTAGAGGGGCCAGAAATACATATGGGAATTTACATCTTTTTTTTTTTT TATCTGAGATGGAGT
CTCGGTTTGTCACCCAGGCTGGAGTGCAGTGGCGcgatttgggtcactgaaacctctGCCTCCCgGaTTCAAGCtATTCTCCTGCCTCAGCCTCCCGAGTAGCTGGGAtTACAgGCaCaCGCCACCAC
aCCtGGCTAATTTTaTGTAtTTTTAGTAGAGACggggtttcactgtgttagccagggtggtctcgatctcctgacctcgtgatccgtcca CCTTGGCCTCCCAAAGTGCTGGGATTACAGGCATGAGC
CACCATACCCAGCTaGGAATTTACATcTTAACAGTGGAATTTCATATCATTGAAAACGAGATGGACTTTTCTCCATAAAACTAAGACACTCAATAGCCATCTGGAAAGGTATAAAGTTAGAGGTAGAT
TGGAGATTTATTATTTATTTATTTatttatttatttaTTTTTTAATTTTTTTTGAGAGAGAGTCTTACTCTGTCACTTAGGCTGGAGTGCAGTGGCATCATCTCGGCTCACTGCAACCTCCACCTCCT
GGGTGGAGGCGATTCTCCTGCCTCAGCCTCCCAAGTAGCTGGGATTACAGGCGTGCGCCACCATCC cCAGCCTAGATTGCAGATTTAAATGTCAAAAACGACACCTCAGGCAGGGCGTGGTGGCTCAC

GCCTGTAATCCCAGCACTTTGAGAGGCCGAGGCAAGTGTATCACCTGAGGTCAGGAGTTCGTGACCAGCCTGGGCAACATGTTGAGACCCCC gTCTCTACTAAAAATACAAAAATTAGCTGGGTGTGG
TGGTGTGCTCCTGTAATCCCAGCTACTTGGGAGGCTGAGGCAGGAGAATGGCTTGAACCCGGGAGGCAGAGATTGC tGTGAGCCAaGATGACGCCACTGCACTCCAGCCTGGGTGACAGAGA aAGACC
CTGTCTCAAAAAaaAAAAAAAAGTTCATAACTCATGTAAGTTATGAGTGGCTAAATTCCTGGCATTACATTCTATTTGTATTAATA tCTATTGGGCTCACTGTTAACACAAGGTTTGGAACAGAGCCA
GAGTTAAATGTATAAATATATACATTTGCTGTGTGAAGGATTAATTGTTCCTTTTCAACTCTCAGAAACCTACATCCTCCTACTCCAACATC gTTCCTGACTGGCAAACTCCTATAACGCCCTCAGTT
ACCTCATCAAACCTCCCACCTTGGGTGTTCCATCTCACCCAGAGAAAAGTCCCAAGTCCCCACCAGGAGCTTTGAGGTCTTGTAAGATCTGGCTGTCACCTCCTCTCTTGCG tGGCCTGCTTTTTCTT
GGGCTTTCTTCcCAACACTCCCTTGCTCCgTCTGCTGCCAGGCACCACtGGTTTCTGCTGTTCTTCAAGTACACCAGGCAGGCTCCgGCCTCAGGGCCTTTGCACTGACTTTTTTTTTTTTTTTTTTTT
TGAaAAGAAGTCTCGCTCTGTCgCCCAGGCTGGAGTGCAGTGGCGGGATCTCGGCTCACTGCAAGCTCCGCCTCCCAGGTTCAAGCCATTCTCCTGCCTCAGCCTCCCGAGTAGCTGGGACTACAAGC
GCCCGCCACCACGCCCGGCTAATTTTTTGTAtTTTTAGTAGAGACgGGATTTCACtATGTTagccaggatggtctccatctcttgatctcgtgatccgcctGCCTcGGCCTCCCAAAGTGCTGGGATT
ACAGGCGTGAGCCaCCaTGCCCGGCC

Homologous Sequences =

TTTTTTTTTTTCTTTTTTTTTTGAGAAGAAGTCTCGCTCTGTCACCCAGGCTGGAGTGCAGTGGCGGGATCTCGGCTCACTGCAAGCTCCGCCTCCCAGGTTCAAGCCATTCTCCTGCCTCAGCC TCC
CGAGTAGCTGGGACTACAAGCGCCCGCCACCACGCCCGGCTAATTTTTTGTATTTTAGTAGAGACAGGATTTCACCATGTTGCCCAGCTAGTCTTGAACTCCTGAGCTCAGGCAATCCACCCGCC TTG
GCCTCCCAAAGTGCTGGGATTACAGGC

>NAHR_18_ Scaffold6455 96896 97177
KSD_NAHR_18

>Scaffold6455 96896 97177_NAHR
CAAGTAGCTGGGATTAGAGGCACGCGCCACCtCaCCTGGCTAATTTTGTATTTTTAGTAGAGTTAGGGTTTCTCCATGTTGGTCAGGCTGGTCTCAAACTCCCGACCTCAGGTGATCCACCCGCCTCG
GCCTCCCAAAGTGCTGGATTACAGGTGTGAGCCACCGCACCCGGCCTAAATCTTTGATTAAATGAAATAAATGCTGAAAAATTCATAAAACTGTAAGCAAACAAAAATTGCCCCCAAACACAGAG ATT
CTTATAAGAAGCTTCTATTTACCTATTTCCATTAGCATCAAAACTGATAAATTAACAAGGGTGGTTTTCATGTAATTGCTGGAGGCAGGAACATTTATCGATGCTTCTAGCTTTACACACAGGC gCaC
ACATGCATCCTTGCATTTACATCAAGGTCAATTAATCAGCTAAATCTAAGAAATTTTTTTCTACTCTGTTTAATTCTCAGTTGGTACTTACTCTGCTCTAAGATGCTGCATTTACAAAGTGAAT GTG
GGAAAGCCAGTTCCTTCTAAGGACATCCTAGTTATGTTTCTTTTTCCATAGGAGAATCCATTTACTGGAGCCAGAAACCTACTCCATCATCGAACGCCAGCCCTTGGTCTGAGCCTGCGGCTGTAGAT
GTGGAACTCACAGCATATGCATTGTTGGCCCAGCTTACCAAGCCCAGCCTGACTCAAAAGGAGATAGCGAAGGCCACTAGCATAGTGGCTTGGTTGGCCAAGCAAC gCAATGCATATGGGGGCTTCTC
TTCTACTCAGGTAAACAGCCTGTTCTCCCACTGCCACTTATCAGGTAGAATTAGATCTTGTGTGGAGATAGAGGAAAACATATTGTCCTTGCCTTCTCGGTCCAGGGGCTTAGAGAGAGGAAAAGCCT
GTGAACAATATATGGAAGTATTCATAAAATGGGTTATAAGAGCACATTAATAGTACCAGATGTATTTAATTTTCTTTTTT TgTTGTTGAATGTGAAACATTGGGGCCGGGCATGGTGGCTCATGCCTG
TAATaCCAGTGCTTTGGGAGGCCGAGGCAGGCGGATCACCTGAGGTCGGGAGTTCGAGACCAGCCTGACCAACATGGAGAAACCCCATCTCTACTAAAAATACAAAATTAGCCAGGCGTGGTGGCGGG
gGCCTGTAATCCCAGCTACCTGGGAGGCTGAGGCAGGAGAATCGCTTGAACCCGGGAGGCAGAGGTTGCGGTGAGCCGAGATGGCGCCATTGCACTCCAGCCTGGGCAACAAGAGTTAAAACTCCG TC
TCAACAACAACAACACAACAACAACAACAAaAATTTTAACTTTGTTTCAGAAAGATTAATCAGCCACTTGGTGTGTGAGATAATTTAGAGAGGAGAAACATTGGACTTGAAGAGATCAGGAGACTGTT
TCCTTTATCTAGGTTAGGGGTAAGGGGAAATTGGGAAATTGAATGAGTGGTGTGGCAGTATGGAAAAAACAGAGGGGTAGAATTGTATGATAAACTCAAATCATAAAAAATTATTTTAGATTATA ATG
AAGAGAGTAGTCATTAATCTATGTATTCTGAAAATATTAAATATCTACCATGTCCAAATCACTTTGATGAGGCTACAGAAATAAC aAAGAAAATgTATTTCTTGTTCTCATGAAGATTATATTCTGAT
TTGTTGAAATTAGCCAATTTCACTGAAGATATAACATGTAAATCAGACCTGAAAGAATGAACATCTTTTAATAGGTTGCATGTTTGGTAAAAATGTTAAAAGCACCAAGTTTAGGCCAGG cGTGGTGG
CTCACGCCTGTAATCCCAGCACTTTGGGAGGCCGAGGCAGGTGGATCACGAGGTCAGGAGATCGAGACCACGGTGAAACCCCATCTCTACTAAAAATACAAAAAATTAGCCGGGCACAGTGGTGGGCG
CCTGTAGTCCCAGCTACTCGGGAGGCTGAGGCAGGAGAATGGCGTGAACCCAGGAGGC gGAGCTTGCAGTGAGCCAAGATCGCGCCACTGCACTCCAGCCTGGGAGACAGAGCAAGACTCCATCTCAA
AAAAAAAAAAAAAAAAAAAAAAGCACCAAGTTTAAATATGGTAATGACTTAATTGAAACTTAAAAAAATTATATGGTACTATG tCTCCCTGTTTCAGGGAAATTCTTGGCCAAAGATCACAAAATACT
CCTTCTTGCTCGGTCCTGAATAATTATAATATATAATAATATTTTTGGAAAGCAGTGGGTGAAATGTTTACAACTCTCAGTGACAGGTATATGTCAGAATCCTT T

1383	7.5	1.1	0.0	a	1	174	(2107)	C	AluSp	SINE/Alu	(137)	176	1	1
380	28.1	1.5	3.0	a	207	342	(1939)	C	Helitron1Na_Mam	RC/Helitron	(840)	222	89	2
2341	6.0	0.3	0.3	a	1000	1281	(1000)	+	AluSp	SINE/Alu	1	282	(31)	3
27	0.0	3.5	0.0	a	1282	1310	(971)	+	(CAA)n	Simple_repeat	1	30	(0)	4
2604	4.4	0.0	0.0	a	1778	2070	(211)	+	AluYc	SINE/Alu	1	293	(6)	5
468	23.9	4.3	5.9	a	2138	2275	(6)	C	LTR83	LTR/ERVL	(0)	780	645	6

1425	6.3	1.1	0.0	hg19	1	174	(3138)	C	AluSp	SINE/Alu	(137)	176	1	7
380	28.1	1.5	3.0	hg19	207	342	(2970)	C	Helitron1Na_Mam	RC/Helitron	(840)	222	89	8
2455	9.5	0.0	0.0	hg19	1001	1305	(2007)	+	AluSq2	SINE/Alu	1	305	(8)	9
1912	12.6	0.0	12.5	hg19	1654	2003	(1309)	+	AluSz	SINE/Alu	2	312	(0)	10
2316	7.1	0.3	0.3	hg19	2028	2309	(1003)	+	AluSp	SINE/Alu	1	282	(31)	11
31	0.0	3.1	0.0	hg19	2310	2341	(971)	+	(CAA)n	Simple_repeat	1	33	(0)	12
2601	5.1	0.0	0.0	hg19	2806	3101	(211)	+	AluYc	SINE/Alu	1	2 96	(3)	13
468	23.9	4.3	5.9	hg19	3169	3306	(6)	C	LTR83	LTR/ERVL	(0)	780	645	14

>hg19 chr12:9015827-9019138
CAAGTAGCTGGGATTAGAGGCACGCGCCACCaCgCCTGGCTAATTTTGTATTTTTAGTAGAGTTAGGGTTTCTCCATGTTGGTCAGGCTGGTCTCAAACTC CCGACCTCAGGTGATCCACCCGCCTCG
GCCTCCCAAAGTGCTGGATTACAGGTGTGAGCCACCGCACCCGGCCTAAATCTTTGATTAAATGAAATAAATGCTGAAAAATTCATAAAACTGTAAGCAAACAAAAATTGCCCCCAAACACAGAG ATT
CTTATAAGAAGCTTCTATTTACCTATTTCCATTAGCATCAAAACTGATAAATTAACAAGGGTGGTTTTCATGTAATTGCTGGAGGCAGGAACATTTATCGA TGCTTCTAGCTTTACACACAGGCaCgC
ACATGCATCCTTGCATTTACATCAAGGTCAATTAATCAGCTAAATCTAAGAAATTTTTTTCTACTCTGTTTAATTCTCAGTTGGTACTTACTCTGCTCTAAGATGCTGCATTTACAAAGTGAAT GTG
GGAAAGCCAGTTCCTTCTAAGGACATCCTAGTTATGTTTCTTTTTCCATAGGAGAATCCATTTACTGGAGCCAGAAACCTACTCCATCATCGAACGCCAGC CCTTGGTCTGAGCCTGCGGCTGTAGAT
GTGGAACTCACAGCATATGCATTGTTGGCCCAGCTTACCAAGCCCAGCCTGACTCAAAAGGAGATAGCGAAGGCCACTAGCATAGTGGCTTGGTTGGCCAAGCAAC aCAATGCATATGGGGGCTTCTC

TTCTACTCAGGTAAACAGCCTGTTCTCCCACTGCCACTTATCAGGTAGAATTAGATCTTGTGTGGAGATAGAGGAAAACATATTGTCCTTGCCTTCTCGGTCCAGGGGCTTAGAGAGAGGAAAAGCCT
GTGAACAATATATGGAAGTATTCATAAAATGGGTTATAAGAGCACATTAATAGTACCAGATGTATTTAATTTTCTTTTTT TttTTGTTGAATGTGAAACATTGGGGCCGGGCATGGTGGctcatgcct
gtcatcgcagcactttgagaggccgaggcaggcggatcacctgaggtcaggagttcgagaccagtctggccaacatgatgaaaccccatctctactaaaaatacaaaaattagccaggtgcgatggca
ggtgcctgtaatcccagctacttaggaggctgagacaggagaatcgcttgaacccaggaggcagaggttgcagtgagctgagatcatgccattgcactccagtctgggtgacaagagtgaaactc tgt
ctccaaaaaaaaaaaaaaaaaaaaagcaacatcatgaggtccatgttttacatgcaagtaggatatatataattatggctaaatgtacatattaataaactaggaagaccattatggggtctgtaaag
ataacaccaagattcatgggcacatatccttctatccttcttctgctattgcaggtattagaaagctaataagatatagtctgaactctaaaaagtttacagtctagaagaaaagcgaggacaaa gtc
tatgtattacaaagaggatctggtttataatataagaaacatgcaaaaggtacaacatatcgaatttcaaaatgggagagaaagctcatatcagcttgagggataagaaaagtcttagccgggcacag
tgactcacgcctataatcccagcactttggcagcctgaggcggttggatcactggaggtcaggagttcaagatcagcctggtcaacatggtgaaaccccatctctactaaaatacaaaaattagc tgg
atgtggccccatctctactaaagatacaaaaattagctggatgtggtgacatgttcctgtagtcccagctactcgggaggctgaggcagaagaattgcttgaacccggaaggcagaggttgcagtgag
ctgagatcgtgccactgcactccagcctgagtgacaaagcagcactctgtgtcaacaacaacaataaacaaaaacaaacaaaaacaacaacaaacaaaaattttttta ggctggacacagcagCTCATG
CCTGTAATcCCAGTGCTTTGGGAGGCCGAGGCAGGCGGATCACCTGAGGTCGGGAGTTCGAGACCAGCCTGACCAACATGGAGAAACCCCATCTCTACTAAAAATACAAAATTAGCCAGGCGTGGTGG
CGGGtGCCTGTAATCCCAGCTACCTGGGAGGCTGAGGCAGGAGAATCGCTTGAACCCGGGAGGCAGAGGTTGCGGTGAGCCGAGATGGCGCCATTGCACTCCAGCCTGGGCAACAAGAGTTAAAACTC
CGTCTCAACAACAACAACACAACAACAACAACAAcAATTTTAACTTTGTTTCAGAAAGATTAATCAGCCACTTGGTGTGTGAGATAATTTAGAGAGGAGAAACATTGGACTTGAAGAGATCAGGAGAC
TGTTTCCTTTATCTAGGTTAGGGGTAAGGGGAAATTGGGAAATTGAATGAGTGGTGTGGCAGTATGGAAAAAACAGAGGGGTAGAATTGTATGATAAACTCAAATCATAAAAAATTATTTTAGAT TAT
AATGAAGAGAGTAGTCATTAATCTATGTATTCTGAAAATATTAAATATCTACCATGTCCAAATCACTTTGATGAGGCTACAGAAATAAC tAAGAAAATaTATTTCTTGTTCTCATGAAGATTATATTC
TGATTTGTTGAAATTAGCCAATTTCACTGAAGATATAACATGTAAATCAGACCTGAAAGAATGAACATCTTTTAATAGGTTGCATGTTTGGTAAAAATGTTAAAAGCACCAAGTTTAGGCCAGG tGTG
GTGGCTCACGCCTGTAATCCCAGCACTTTGGGAGGCCGAGGCAGGTGGATCACGAGGTCAGGAGATCGAGACCACGGTGAAACCCCATCTCTACTAAAAATACAAAAAATTAGCCGGGCACAGTGGTG
GGCGCCTGTAGTCCCAGCTACTCGGGAGGCTGAGGCAGGAGAATGGCGTGAACCCAGGAGGC aGAGCTTGCAGTGAGCCAAGATCGCGCCACTGCACTCCAGCCTGGGAGACAGAGCAAGACTCCATC
TCAAAAAAAAAAAAAAAAAAAAAAAAaaaGCACCAAGTTTAAATATGGTAATGACTTAATTGAAACTTAAAAAAATTATATGGTACTATG cCTCCCTGTTTCAGGGAAATTCTTGGCCAAAGATCACA
AAATACTCCTTCTTGCTCGGTCCTGAATAATTATAATATATAATAATATTTTTGGAAAGCAGTGGGTGAAATGTTTACAACTCTCAGTGACAGGTATATGTCAGAATCCTT T

>Chimp
CAAGTAGCTGGGATTAcAGGCACaCGCCACCAtGCCTGGCTAATTTTGTgTTTTTAGTAGAGTTgGGGTTTCTCCATGTTGGTCAGGCTGGTCTCgAACTCCCGACCTCAGGTGATCCACCCGCCTCG
GCCTCCCAAAGTGCTGGATTACAGGTGTGAGCCACCGCACCCGGCCTAAATCTTTGATTAAATGAAATAAATGCTGAAAAATTCATAAAACTGTAAGCAAACAAAAATTGCCCCCAAACACAGAG ATT
CTTATAAGAAGCTTCTATTTACCTATTTCCATTAGCATCAAAACTGATAAgTTAACAAGGGTGGTTTTCATGTAATTGCTGGAGGCAGGAACATTTATCGATGCTTCTAGCTTTACAC gCAGGCgCaC
ACATGCATCCTTGCATTTACATCAAGGTCAATTAATCAGCTAAATCTAAGAAATTTTTTTCTACTCTGTTTAATTCTCAGTTGGTACTTACTCTGC cCTAAGATGCTGCATTTACAAAGTGAATGTG
GGAAAGCCAGTTCCTTCTAAGGACATCCTAGTTATGTTTCTTTTTCCATAGGAGAATCCATT cACTGGAGCCAGAAACCTACTCCATCATCGAACGCCAGCCCTTGGTCTGAGCCTGCGGCTGTAGAT
GTGGAACTCACAGCATATGCATTGTTGGCCCAGCTTACCAAGCCCAGC tTGACTCAAAAGGAGATAGCGAAGGCCACTAGCA cAGTGGCTTGGTTGGCCAAGCAACgCAATGCATATGGGGGCTTCTC
TTCTACTCAGGTAAACAGCCTGTTCTCCCACTGCCACTTATCAGGTAGAATTAGATCTTGTGTGGAGATAGAGGAAAACATATTGTCCTTGCCTTCTCGGTCCAGGGGCTTAGAGAGAGGAAAAGCCT
GTGAACgATATATGGAAGTATTCATAAAATGGGTTATAgGAGCACATTAATAGTACCAGATGTATTTAATTTTCTTTTTTTgTTGTTGAATGTGAAACAcTGGGGCCGGGCgTGGTGGCTCATGCCTG
TCATCGCAGCACTTTGgGAGGCCGAGGCAGGCGGATCACCTGAGGTCAGGAGTTCGAGACCAGTCT cGCCAACATGATGAAACCCCgTCTCTACTAAAAATACAAAAATTAGCCAGGTGtGATGGCAG
GTGCCTGTAATCCCAGCTACTTAGGAGGCTGAGACAGGAGAATCGCTTGAACCCAGGAGGCAGAGGTTGCAGTGAGCTGAGAT CtggcctTGCCATcCCAGTCTGGGGACAAGAGTGAAACTCTGTtT
CCAAAAAAAAAAAGCAACATCATGAGGTCCATGTTTTACATGCAAGTAGGATATATATAATTATGGCTAAATGTACATATTAATAAACTAGGAAGACCATTATGGGGTCTGTAAAGATAACACCAAG
ATTCATGGGCACATATCCTTCTTCTGCTATTGCAGGTATTAGAAAGCTAATAAGATATAGTCTGAACTCTAAAAAGTTTACAGTCTAGAAGAAAAGCGAGGACAAAGTCTATGTATTACAAAGAGGAT
CTGGTTTATAATATAAGAAACATGCAAAAGGTACAACATATCGAATTTCAAAATGGGAGAGAAAGCTCATATCAGCTTGAGGGATAAGAAAAGTCTTAGCCGGGCACAGTGACTCACGCCTA cAATCC
CAGtACTTTGGCAGCCTGAGGCGGaTGGATCACTGGAGGTCAGGAGTTCAAGATCAGCCTGGTCAACATGGTGAAACCCCATCTCTACTAAAATACAAAAATTAGCTGGATGTGGCCCCATCTCTACT
AAAGATACAAAAATTAGCTGGATGTGGTGACATGTTCCTGTAGTCCCAGCTACTCGGGAGGCTGAGGCAGAAGAATTGCTTGAACC tGGAAGGCAGAGGTTGCAGTGAGCTGAGATCGcGCCACTGCA
CTCCAGCCTGAGTGACAAAGCAGCACTCTGTGTCAACAACAACAATAAACAAAAACAAACAAAAACAACAACAAACAAAAATTTTTTAGGCTGGACACAG tgGCTCATGCCTGTAATCCCAGTGCTTT
GGGAGGCCGAGGCAGGCGGATCAtCTGAGGTCGGGAGTTCGAGACCAGCCTGAtCAACATGGAGAAACCCCATCTCTACTAAAAATACAAAATTAGCCAGGCGTGGTGGC aGGTGCCTGTAATCCCAG
CTACCTGGGAGGCTGAGGCAGGAGAATgGCTTGAACCCGGGAGatgGAGGTTGCGGTGAGCCGAGATGGCGCCATTGCACTCCAGCCTGGGCAACAAGAGTT AAAACTCtGTCTCAACAACAACAACA
CAACAACAACAACAAaAATTTTAACTTTGTTTCAGAAAGATTAATCAGCCACTTGGTGTGTGAGATAATTTAGAGAGGAGAAACATTGGACTTGAAGAGATCAGGAGACTGTTTCCTTTATCTAGGTT
AGGGGTAAGGGGAAATTGGGAAATTGAATGAGTGGTGTGGCAGTA gGGAAAAAACAGAGGGGTAGAATTGTATGATAAACTCAAATCATAAAAAATTATTTT AGATTATAATGAAGAGAGTAGTCATT
AATCTATGTATTCTGAAAATATTAAATATCTACCATGTCCAAATCACTTTGATGAGGCTACAGAAATAACTAAGAAAAT gTATTTCTTGTTCTCATGAAGATTATATTCTGATTTGTTGAAATTAGCC
AATTTCACTGAAGATATAACATGTAAATCAGACCTGAAAGAATGAACATCTTTTAATAGGTTGCATGTTTGGTAAAAATGTTAAAAGCACCAAGTTTAGGCC AGGcGTGGTGGCTCACGtCTGTAATC
CCAGCACTTaGGGAGGCCGAGGCAGGTGGATCACGAGGTCAGGAGATCGAGACCAC aGTGAAACCCCgTCTCTACTAAAAATACAAAAAATTcGCCGGGCACAGTGGTGGGCGCCTGTAGTCCCAGCT
ACTCGGGAGGCTGAGGCAGGAGAATGGCGTGAACtCAGGAGGCgGAGCTTGCAGTGAGCCAAGATCGCGCCACTGCACTCCAGCCTGGGAGACAGAGCAAGA CTCCATCTCAAAAAAAAAAAAAAAA

AAAAAAAAAAaCACaAAGTTTAAATATGGTAATGACTTAATTGAAACTTAAAAAAATTATATGGTACTATGCCTCCCTGTTTCAGGGAAATTCTTGGCCAAAGATCACAAAATACTCCTTCTTGCTCG
GTCCTGAATAATTATAATATATAATAATATTTTTGGAAAGCAGTGGGTGAAATGTTTACAACTCTCAGTGACAGGTATATGTCAGAATCCTT T

Homologous Sequences

GCTCATGCCTGT

>NAHR_19_ Scaffold1060 496749 497073
>KSD_NAHR_19
>Scaffold1060 496749 497073_NAHR

CATTCCAGCTTGAGCAACAGAGTGAGACTCCATCTCAAATAAATAAATAAATAAACAAACAAATCAAGATCTGAATTTGAGAACTGACTTTAGGATTCATGCAACCTTTAGCACATTCCTAACCTT CT
TGAATGTCAGTATCCATCCCTGCCAAGCAGGAACAACAATTCTCATTTCACAAAACAACACCTTCACCTCATGTGGGGCTCATGTGTTGCAGAATATTTTGTCATAAAATGTTCTATTTTTACCT TTC
ATTTCTGAGCCTCACATTAAAAGATGGATTATCAtTTACACACTTTCTTAAAATTCCAGAGGTTTATAAGTAACATTAGCAGGATCTCTGTGTTCCCTCAGCCTTGTTTGTCTTCCCAACTATGCTCA
TTTCTAAAAACTTCAATGCTCTTTTGAATTGCGAGTAAGTACTCTTCTGCAGACCAAGGGTATGAAAAAAAGGGGAAGGACAAATTAATGGGTATACATAACTTGGAGGCACTAAAAGTCATCA AAT
GATTATTATGTTTTCTTTAAATGGTTGTTATGAATaGCAATTATTATACTACACCATCAGTTCTTCATAAATGCATCTCAAAGAAAAAAAAAAACTCACCCAGGACAGAGGAATGGCAGATTTATTGT
ATTTAGATAAATTTCTGTCTAGCATCCTGTGAAcAGGAGCTTGAGATGATATTGTAAATACATGGCTTCTCTTAGGACATACCGTGGCGTTCCACGCCACCAATAGGGTGAAGTGGATTGTGCATTTC
CTGCCAAGCAGCCAATGAGAGTCAAAACTGGGTCTAAAATACTGTGCTGTAAAAAGAACTTAAGAGCTAGTTGTCAGTTGTGTTTATTATGCTATAAACTTTTAAACATCACTTTCCTGATCGCCC TT
AGATTGCTAGTTTAGAAGTAGATTGATGATTCAGAGTTCTTATCATCCTTCCTGAGATATTCACCATGAATTGCCAAGTTATTTTTAAGAAATCAATGTGTGG CAGGTGCGGTGGCTCATGCCTGTAT
TCCCAGCACTTTGGGGCACTTTGGGAGGCCAATGCAGGCAAATCACTTGAGGCCAGAAGTTCAAGACTATCCTGGCCAACATGGTGAAACCCCGTCTCAACTAAAAATACAAAA ATTAGCTGGGCATG
GTGGTATGTGCCTTTAATCCCAACTACTCAGGAGGCTGAGACATGAGAATCGCTTGAACCTGGAAAGCGGAGGTTGCAGTGAGCTGAGATCACGCCACTGCACTCCAGCCTGTGCAATAGAGCAA GAT
TCTGTCCCCCCCTCAAAAAAAAAAAAAGAAGAAGAAAAGAAAAAAATCAATGGATCTTTCTTTCCCTACCTCACTTCCCCTCATCAAAGAAAACAAATTTTAACTATT tTAGTGTTGTAAAACTATT
CTAAGTAATTCAGCTAGTTCCTCTCTCTACACCCACATACTGCTTTTGTCTGTAAAATACAACAAGTGTTCTGGGTCAGTTTCCAATGTTCTATGCAGTGCCTAAAATGGTGGGCACTAGAAGGA GAA
ACTGAGTGATATGACTGACTAGAAGACCCTAGCATTCATCCCTTCCACAAAGAGAGACAAAACAACAAATAAACAACTACATTTTAAGAAAAATAATCAAAGGAGCGTGCTGGAATATGTCAAAAC AG
CAAGAGAAACCTTGGAGAGCATAGAAGCTCATGATATCCACATAGAGAATGGAAGGATGCACTGGACCCCACCACCCCATTCTCCAGCCAGGATCAGCCAGGAATCAGGAGGAACTTCTCCCTAA AGC
AAAAAGGGAGGCAAGAGGATCCCAGTAGCTCTCACCAATATCTTGGATACCTACAGTTTTCCTTGTTGGGATCCCCTACAGCCCTCACAGGCCCAGCTGAGGGAGCTGCCTAGAGGCCACATAGCG GC
ACTCCCCCAGAAAAGGAGCCAAcACTGTGCCCCACCCCCTGTGGCCTGCACAGCTACTGCACTATGCCATCTTGAAACTAGAAATACTGCTGGAGTGTGTCTTGCTCCAGAGTTGAGTAGCCGTGGCT
CCTTTTCATCCTTGAGTCTAAGGAGCCACTGAATCACCTCAGCCTGGTGGCCTGACATCCCAGAGCCAAGCTGTGAGCAACTGCTATACCCTTCCTCATGGGGCCATGCAGTGACAGAACTGTTTC AT
TATCCCTCTGCCTACCCTCTGGACTGAGCTGAATCTGTAtAGTCCCTCCTGAAGAAATGGTACTTTGGCAGAACAgCTCCATCTTCTGAATTCCAGAACCTGAGCTGCTGGGGAAGGGGACGTGCCTC
AAAGACATAGATCCTGGTGC

299	18.2	0.0	0.0	a	1	55	(2269)	+	Alu	SINE/Alu	246	300	(1)	1
188	39.1	7.7	0.0	a	66	180	(2144)	+	MIRc	SINE/MIR	47	172	(96)	2
2118	12.6	0.0	5.2	a	1000	1324	(1000)	+	AluSz6	SINE/Alu	4	312	(0)	3
3896	21.3	3.6	0.5	a	1541	2286	(38)	+	L1M2a	LINE/L1	13	927	(6241)	4

299	18.2	0.0	0.0	hg	1	55	(2457)	+	Alu	SINE/Alu	246	300	(1)	5
188	39.1	7.7	0.0	hg	66	180	(2332)	+	MIRc	SINE/MIR	47	172	(96)	6
2539	5.8	0.0	3.8	hg	1000	1326	(1186)	+	AluYb8	SINE/Alu	4	318	(0)	7
1244	13.5	0.0	3.4	hg	1327	1511	(1001)	+	AluSz6	SINE/Alu	134	312	(0)	8
3873	21.0	3.5	0.0	hg	1729	2433	(79)	+	L1M2a	LINE/L1	13	742	(6426)	9

>hg19 chr9:77523017-77525528

CATTCCAGCTTGAGCAACAGAGTGAGACTCCATCTCAAATAAATAAATAAATAAAC AAACAAATCAAGATCTGAATTTGAGAACTGACTTTAGGATTCATGCAACCTTTAGCACATTCCTAACCTTCT
TGAATGTCAGTATCCATCCCTGCCAAGCAGGAACAACAATTCTCATTTCACAAAACAACACCTTCACCTCATGTGGGGCTCATGTGTTGCAGAATATTTTGTCATAAAATGTTCTATTTTTACCT TTC
ATTTCTGAGCCTCACATTAAAAGATGGATTATCAcTTACACACTTTCTTAAAATTCCAGAGGTTTATAAGTAACATTAGCAGGATCTCTGTGTTCCCTCAGCCTTGTTTGTCTTCCCAACTATGCTCA
TTTCTAAAAACTTCAATGCTCTTTTGAATTGCGAGTAAGTACTCTTCTGCAGACCAAGGGTATGAAAAAAAGGGGAAGGACAAATTAATGGGTATACATAACTTGGAGGCACTAAAAGTCATCA AAT
GATTATTATGTTTTCTTTAAATGGTTGTTATGAATgGCAATTATTATACTACACCATCAGTTCTTCATAAATGCATCTCAAAGAAAAAAAAAAACTCACCCAGGACAGAGGAATGGCAGATTTATTGT
ATTTAGATAAATTTCTGTCTAGCATCCTGTGAAtAGGAGCTTGAGATGATATTGTAAATACATGGCTTCTCTTAGGACATACCGTGGCGTTCCACGCCACCAATAGGGTGAAGTGGATTGTGCATTTC
CTGCCAAGCAGCCAATGAGAGTCAAAACTGGGTCTAAAATACTGTGCTGTAAAAAGAACTTAAGAGCTAGTTGTCAGTTGTGTTTATTATGCTATAAACTTTTAAACATCACTTTCCTGATCGCC CTT
AGATTGCTAGTTTAGAAGTAGATTGATGATTCAGAGTTCTTATCATCCTTCCTGAGATATTCACCATGAATTGCCAAGTTATTTTTAAG AAATCAATGTGTGGCAGGTGCGGTGGCTCATGCCTGTAT
TCCCAGCACTTTGGGGCACTTTGGGAGGCCAATGCAGGCAAATCACTTGAGGCCAGAAGTTCAAGACTATCCTGGCCAACATGGTGAAACCCCGTCTCAACTAAAAATACAAAA Aattagccgggcgc
ggtggcgggcgcctgtagtcccagctactcgggaggctgaggcaggagaatggcgtgaacccgggaagcggagcttgcagtgagccgag attgcgccactgcagtccgcagtccggcctgggcgacag
agcgagactccgtctcaaaaaaaaaaaaaaaaaaaaaaaaaaaa TTAGCTGGGCATGGTGGTATGTGCCTTTAATCCCAACTACTCAGGAGGCTGAGACATGAGAATCGCTTGAACCTGGAAAGCG

GAGGTTGCAGTGAGCTGAGATCACGCCACTGCACTCCAGCCTGTGCAATAGAGCAAGATTCTGTCCCCCCCTCAAAAAAAAAAAAA AaGAAGAAGAAAAAGAAAAAAATCAATGGATCTTTCTTTCCCT
ACCTCACTTCCCCTCATCAAAGAAAACAAATTTTAACTATTcTAGTGTTGTAAAACTATTCTAAGTAATTCAGCTAGTTCCTCTCTCTACACCCACATACTGCTTTTGTCTGTAAAATACAACAAGTG
TTCTGGGTCAGTTTCCAATGTTCTATGCAGTGCCTAAAATGGTGGGCACTAGAAGGAGAAACTGAGTGATATGACTGACTAGAAGACCCTAGCATTCATCCCTTCCACAAAGAGAGACAAAACAA CAA
ATAAACAACTACATTTTAAGAAAAATAATCAAAGGAGCGTGCTGGAATATGTCAAAACAGCAAGAGAAACCTTGGAGAGCATAGAAGCTCATGATAT CCACATAGAGAATGGAAGGATGCACTGGACC
CCACCACCCCATTCTCCAGCCAGGATCAGCCAGGAATCAGGAGGAACTTCTCCCTAAAGCAAAAAGGGAGGCAAGAGGATCCCAGTAGCTCTCACCAATATCTTGGATACCTACAGTTTTCCTTGTTG
GGATCCCCTACAGCCCTCACAGGCCCAGCTGAGGGAGCTGCCTAGAGGCCACATAGCGGCACTCCCCCAGAAAAGGAGCCAA tACTGTGCCCCACCCCCTGTGGCCTGCACAGCTACTGCACTATGCC
ATCTTGAAACTAGAAATACTGCTGGAGTGTGTCTTGCTCCAGAGTTGAGTAGCCGTGGCTCCTTTTCATCCTTGAGTCTAAGGAGCCACTGAATCACCTCAGCCTGGTGGCCTGACATCCCAGAG CCA
AGCTGTGAGCAACTGCTATACCCTTCCTCATGGGGCCATGCAGTGACAGAACTGTTTCATTATCCCTCTGCCTACCCTCTGGACTGAGCTGAATCTG TAgAGTCCCTCCTGAAGAAATGGTACTTTGG
CAGAACAaCTCCATCTTCTGAATTCCAGAACCTGAGCTGCTGGGGAAGGGGACGTGCCTCAAAGACATAGATCCTGGTG C

Homologous Sequences
TTAGC

>NAHR_20_ Scaffold14588 879917 880191
KSD_NAHR_20
>**Scaffold14588 879917 880191**_NAHR

TCAGACTGGACAGAGGCCCTGATGTCCGAGCTGAGCAGGCTTATGCTGCGGGAGCCACCACCCCTCAGACGGGCCGCAGCTCAGGAAAGAAGCCCCTCTGTAAGCCGGAGCCCCCAGCAGGGCTCC AA
ACTGAAGTCAATGTCCAGGATCTGCCGCTAGGCCTGGAGGTGGGGGCAGGGAAGCAGGCATGGCATGCCGGGCCTTTGTCCCCTCCATGCTATTTGGAGACCATTAGTGGCCTTCTCAGCAGGCA GAG
TAAGGAGACAGACAGGCTATCAGCAGATGAGGAGACGCTAAAAGCCCTGGGCTGCCATGCTCTTGATGTCCAGTGACCTAGAACAAACAGTTCCTCGAAAACAGATGCTTTTCACTGCCCAGGGTG AG
CCGGGCATCCTGAGCACATGAGTGGAACAAACAAGCACAAAGCACCAAGCACCCAGGGGCTTTCTTGTGTCCCCAGGGGCACACGGAGAGATGGTGGCCTCCCCGAAAGCCAGTGAGGCACACCC CAC
CGTGCCCAGGGTCATTCTGGCAGCTGTAGCTGCCATATCAGGAAAGTAGCAGCAACTCCTTGGAAACAATACCCATCCACTGACCCTTCCCCTAGGCCAAGGGTCCCTCCTGGTCTTACCAGGTGT CC
CCACcGCACCGCTGCTCCTAAGAGCACCCAGATATGCAGAGACAGACGCACACACATACTCTCAGCTGTGCACATACGCAGAATATTTCTGTCGGAACCACGCCAAGGAAGCAGAGGCTGATTGATTT
ATCCAACAGGGGATTATGTCTACCCACTAATCATAGGAACAAATGTCCTCTGGCAGTAGACATTTAGGGAGGGAAAATAACAA TAACAATAAAGACAGCAGAGTAAATACACAGGGCAAAAGGTCAGA
CTGGATTTCAAACCCAAATAAACTGCCAGTCCCTTCTTGGCAAAGCAGAGCCATTCTCCCTATGCACTGCTCTGTCGCATAGTAAGATTCAAAGAGAGGGGCGCTATAACTGGGGATATAGCTCA GGG
GTAGAGCATTTGACTGCAGAACAAGAGAGGGGtGCACCGGGCACaGTGGCTCAgGCCTGTAATACTTTGGGAGGCCGAGGCGGGCGGATCACgAGGTCAGGAGATCGAGACCACAGCGAAACCCCATC
TCTATTAAAAATACAAAACATTAGCCAGGCGTGATGGCAGGCGCCTGTAGTCCCAGCTACTCAGGAGGCTAAGCCAGGAGAATGGCGTGAACCTGGGAGGCGCAGCTTGCAGTGAGCCAAGGTCA CGC
CACTGCACTCCAGaggggggacccccgcctaaaAAAAAAAAAAAAAAAAAAGAAAAGGTAAGTATTGGCTGGGCATGGTCTCCCAGCACTTTGGGCAGCAGAGGAGGGCAGACCACCTGAGGTCAGGAG
TTCGAAACCAGCCTGGTCAACATGGCAAAACCCCGTCTATACTAAAAATACAAAAATTAGCCAGGCGTGGTAGCAGGCACCTGTAATCCCATCTGCTGAGGTAGGAGAATCGCTTGAACCTGGGA GGC
AGAGGTTGCAGTGAGCCGAGATTGTACCAATGCACTCCAGCCTGGGAAACAGAGCAAAACTCTGTCTCAAAAAAACAAAAAAAAAATTATTGAGTTTAACACAAGATTTTCCAAAGAGTATTTGTG TC
TCATTTCATTTTATGTAAATGTTCAATATTTTAACTTCTTTTTCACACACCTAAAGTTTTTGAAAGTTGTGAAACTTAAATGTGTTTAGATGTGGACTTTAGAGGTCAACTGAAACATGCCTGGG GGA
ACACAGATGTCACCGGGTTACAGGGGAAGGAACAACTGTGTAAAGACCCTGGGTAACAGACTAAACACCCGAATTGAATCTGGGGGACTCGAGCTCAAAGTGTTGTCATGGTTACAACCATGGCCA CG
TGGGTAAGTCCTCCCAGAAAGTCACCAAGTCCCAGGCACGGAACTTCAATTCTGCAAGGTTGCTACGTTCATGACACGCTCGCTCACTGGGCACATGCACCAGGTACATGCACTGGGTACCCGTG AGA
CAGAGGCTACTTGGGGGATACAGGGTCAATGTGGCTGAGTAGTGCCTTTGGGCTAAGCCCTGGCTGTCTACAAGCCCGCAGTACCATTAGTGTACAACGGAGACTGCCTACTGGGGCCCGTTCCCT GC
AGGCAAACATACACAGGAAGCCAAAAGAAAACTGAAGGCTGGCTGGTGAACACTCAGCCGTGGGATACAGTGACACAAGGAGCCAGTTATGGAAAGC A

350	4.5	0.0	0.0	114227	1008	1051	(1223)	+	tRNA-Cys-TGY	tRNA	1	44	(31)	1
1968	9.8	5.8	0.0	114227	1061	1335	(939)	+	AluYc	SINE/Alu	3	293	(6)	2
1917	11.0	3.4	0.0	114227	1360	1622	(652)	+	AluSx1	SINE/Alu	29	300	(12)	3

350	4.5	0.0	0.0	Hg19	1011	1054	(2193)	+	tRNA-Cys-TGY	tRNA	1	44	(31)	4	
971	8.2	1.6	0.0	Hg19	1064	1185	(2062)	+	AluY	SINE/Alu	3	126	(185)	5	
322	17.9	3.0	3.0	Hg19	1187	1253	(1994)	+	AluYh7	SINE/Alu	76	142	(169)	6	
322	17.9	3.0	3.0	Hg19	1237	1303	(1944)	+	AluYh7	SINE/Alu	76	142	(169)	7	
322	17.9	3.0	3.0	Hg19	1287	1353	(1894)	+	AluYh7	SINE/Alu	76	142	(169)	8	
322	17.9	3.0	3.0	Hg19	1337	1403	(1844)	+	AluYh7	SINE/Alu	76	142	(169)	9	
322	17.9	3.0	3.0	Hg19	1387	1453	(1794)	+	AluYh7	SINE/Alu	76	142	(169)	10	
320	14.3	4.1	0.0	Hg19	1437	1485	(1762)	+	AluYh9	SINE/Alu	76	126	(185)	11	*
1886	7.3	0.8	0.0	Hg19	1487	1720	(1527)	+	AluYk3	SINE/Alu	76	311	(0)	12	
2383	8.2	0.0	0.0	Hg19	2018	2308	(939)	+	AluYc	SINE/Alu	1	291	(8)	13	
1917	11.0	3.4	0.0	Hg19	2333	2595	(652)	+	AluSx1	SINE/Alu	29	300	(12)	14	

>Hg19 chr10:6250785-6254031

TCAGACTGGACAGAGGCCCTGATGTCCGAGCTGAGCAGGCTTATGCTGCGGGAGCCACCACCCCTCAGACGGGCCGCAGCTCAGGAAAGAAGCCCCTCTGTAAGCCGGAGCCCCCAGCAGGGCTCC AA
ACTGAAGTCAATGTCCAGGATCTGCCGCTAGGCCTGGAGGTGGGGGCAGGGAAGCAGGCATGGCATGCCGGGCCTTTGTCCCCTCCATGCTATTTGGAGACCA TTAGTGGCCTTCTCAGCAGGCAGAG
TAAGGAGACAGACAGGCTATCAGCAGATGAGGAGACGCTAAAAGCCCTGGGCTGCCATGCTCTTGATGTCCAGTGACCTAGAACAAACAGTTCCTCGAAAACAGATGCTTTTCACTGCCCAGGGT GAG
CCGGGCATCCTGAGCACATGAGTGGAACAAACAAGCACAAAGCACCAAGCACCCAGGGGCTTTCTTGTGTCCCCAGGGGCACACGGAGAGATGGTGGCCTCCC CGAAAGCCAGTGAGGCACACCCCAC
CGTGCCCAGGGTCATTCTGGCAGCTGTAGCTGCCATATCAGGAAAGTAGCAGCAACTCCTTGGAAACAATACCCATCCACTGACCCTTCCCCTAGGCCAAGGGTCCCTCCTGGTCTTACCAGGTG TCC

CCACtGCACCGCTGCTCCTAAGAGCACCCAGATATGCAGAGACAGACGCACACACATACTCTCAGCTGTGCACATACGCAGAATATTTCTGTCGGAACCACGC CAAGGAAGCAGAGGCTGATTGATTT
ATCCAACAGGGGATTATGTCTACCCACTAATCATAGGAACAAATGTCCTCTGGCAGTAGACATTTAGGGAGGGAAAATAACAA TaacAACAATAAAGACAGCAGAGTAAATACACAGGGCAAAAGGTC
AGACTGGATTTCAAACCCAAATAAACTGCCAGTCCCTTCTTGGCAAAGCAGAGCCATTCTCCCTATGCACTGCTCTGTCGCATAGTAAGATTCAAAGAGAGGGGCGCTATAACTGGGGATATAGCTCA
GGGGTAGAGCATTTGACTGCAGAACAAGAGAGGGGcGCACCGGGCACgGTGGCTCAcGCCTGTAATcccagccctctgggaggccgaggcgggtggatcatgaggtcaggagatcaagactatcctgg
ctaacacggcgaaaccccgtctattaaaaacactatcaagactatcctggctaacatggcgaaaccccgtctattaaaaacactatcaagactatcctggctaacatggcgaaaccccgtctattaaa
aacactatcaagactatcctggctaacatggcgaaaccccgtctattaaaaacactatcaagactatcctggctaacatggcgaaaccccgtctattaaaaacactatcaagactatcctggcta aca
tggcgaaaccccgtctattaaaaacactatcaagactatcctggctaacatggcgaaaccccgtctat taaaaacactatcaagactatcctggctaacatggcgaaaccccgtctattaaaaacaca
aaaaattagccggccatgttggcgggcgcctgtagtcccagctactcgggaggctgaggcaggagaatggcgtgaacccgggaggcggagcttgcagtgagccgagatagcgccactgcactcca gcc
tgggtgacagagcgaaacttcgtctcaaaaaaaaaaaaaacaaaaaaacaaaaaaaaacaaaaaaaaa caaagagaggggcgcctgaggtgtttgcacctgtccctcccttcagacacccaccactgt
cttggacagatggggtaatcacaaatccttactaaatctttttgtcttattctccaacctcagccccttttaaaaactcacagcacatctcatgtttctaattctgtgaccctcagtttcttctt ctc
ttctcaatgggccttttaccaccaaaaaagagacaccccccacccgcctcatcaccctccctggcaa ggcacagtgattagaaaagataagtattaggccgggcacggtggctcacgcctgtaatcc
cagcACTTTGGGAGGCCGAGGCGGGCGGATCACaAGGTCAGGAGATCGAGACCACAGCGAAACCCCATCTCTATTAAAAATACAAAACATTAGCCAGGCGTGATGGCAGGCGCCTGTAGTCCCAGCTA
CTCAGGAGGCTAAGCCAGGAGAATGGCGTGAACCTGGGAGGCGCAGCTTGCAGTGAGCCAAGGTCACGCCACTGCACTCCA GtctgggtgacagagtgagacaccgtctcAAAAAAAAAAAAAAAAG
AAAAGGTAAGTATTGGCTGGGCATGGTCTCCCAGCACTTTGGGCAGCAGAGGAGGGCAGACCACCTGAGGTCAGGAGTTCGAAACCAGCCTGGTCAACATGGCAAAACCCCGTCTATACTAAAAATAC
AAAAATTAGCCAGGCGTGGTAGCAGGCACCTGTAATCCCATCTGCTGAGGTAGGAGAATCGCTTGAACCTGGGAGGCAGAGGTTGCAGTGAGCCGAGATTGTACCAATGCACTCCAGCCTGGGAA ACA
GAGCAAAACTCTGTCTCAAAAAAACAAAAAAAAAATTATTGAGTTTAACACAAGATTTTCCAAAGAGTATTTGTGTCTCATTTCATTTTATGTAAATGTTCAATATTTTAACTTCTTTTTCACACACC
TAAAGTTTTTGAAAGTTGTGAAACTTAAATGTGTTTAGATGTGGACTTTAGAGGTCAACTGAAACATGCCTGGGGGAACACAGATGTCACCGGGTTACAGGGGAAGGAACAACTGTGTAAAGACCCTG
GGTAACAGACTAAACACCCGAATTGAATCTGGGGGACTCGAGCTCAAAGTGTTGTCATGGTTACAACCATGGCCACGTGGGTAAGTCCTCCCAGAAAGTCACCAAGTCCCAGGCACGGAACTTCAATT
CTGCAAGGTTGCTACGTTCATGACACGCTCGCTCACTGGGCACATGCACCAGGTACATGCACTGGGTACCCGTGAGACAGAGGCTACTTGGGGGATACAGGGTCAATGTGGCTGAGTAGTGCCTT TGG
GCTAAGCCCTGGCTGTCTACAAGCCCGCAGTACCATTAGTGTACAACGGAGACTGCCTACTGGGGCCCGTTCCCTGCAGGCAAACATACACAGGAAGCCAAAAGAAAACTGAAGGCTGGCTGGTGAAC
ACTCAGCCGTGGGATACAGTGACACAAGGAGCCAGTTATGGAAAGCA

Homologous Sequences = GCCTGTAATCCCAGC

>NAHR_21_ Scaffold12649 2345765 2346044
KSD_NAHR_21

>Scaffold12649 2345765 2346044_NAHR

AGCAACTCTGCAGCTCCAACCTCTTAGGTTCATACCTGCCTTTTTGCCAAATTCTCCTTCCAGCTCGGCCTGTGCCCCAGTCAGGGGGAGGTCCACCATCTCTAGGCACACCACTTCTGCCAGGGA CT
CCTCACGGCTCCACAGCACCACCTTCCCTGCTACAGGAAACATTTTTACAAAGCTAAGCCTGGGGCTGGGCACAGTGGCTCATGCCTGTAATCCCAGCACTTTGGGA GGCCAAGGTGGGTGAATCTCT
TGAGCCCAGGAATTTGAGACCAGCCTGGACAACAGGGCAAAACCTTATCTCAACAAAAAATAAATATCAGGGCTGGGCACAGTGGCTCACGCCTGTAATCCCAGCACTTTGGGAGGCCAAGGCGG GCA
GATCACGAGGTCAGGAGATCGAGACCATCCTGGCTAACATGGTGAAACCCCTCTCTACTAAAAATACAAAAAATTAGCCAGGCATGATGGTGGGCGCCTGTAGTTCC AGCTACTCGGGAGGCTGAGGC
AGGAGAATGGCGTGAACCCGGGAGGTGGAGGTTGCAGTGAGCCGAGAT cGTGCCATTGCACTCCAGCCTGGGCGACACAGCGAGACTCCGTCTTGAAATAAAATAAAATAAAATAAAAATAAATAAAT
ATCAAAAAATTAGCCAAGCATGGTGGCGAGCGCCTGTAGTCACAGCTACTTAGGAGGCTGAGATAGGAAGATCACTTGAGCCCGGGAGATTGAGGCTGCAGTGAGCT ATGATCACGCCACTGTACTCC
AGACTGGGCAACAGTGAGACCTTGTCTCAAAAAATAAATAAATAAATAAACAAAAGCTAAGCCTGTCTCCCAGATTTTCTAGAGAGACTCTTCTCGCTGACTCAGGGATTACTACAAGCTCTTGC ATA
AAAGGAGAAATGGCAGAGCAAGGGGCTGGAATCAATAGGTCAAAGGAGGAAAGTTCTGTATCCCACCGAGAATGCCAAGTTATTCTTCAAAAGCAGGTAACGGGGCC GGGCATAGTGGCTCATGCCTG
TAATCCCAGCACTTTGGGAGGCCAAGGCGGGCAGATCACCTGAGGTTAGGAGCTTGAGACCAGCCTGGGCCAACATGGTGAAACCCTGTCTCTTACTAAAAATACAAAATTAGCGCCTATAATCC CAG
CTACTCAGGAGGCTGAGGCAGGAGAATCGCTTGAACCAGGAGGCAGAAGTTGCAAAGAGCTGAGATTACACCATTCCACTCCAGCCTGGGCAACAAGAGCAAAACTC TGTCTCAAAAAAGAAAAAAAG
AGCCAGGCACGGTGGCTCACACCTGTAATCCCAGCACTTTGGGAGGCCGAGGCAGGCGGATCACTTGAGGTCAGGAATTCGAGACCAGCCTGGCCAACATGGGGAAACCCTGTCTCTACTGAAAATAC
AAAAATTAGCTGGTCGTGGTGGCACACGCCTGTAATCCCAGCTACTCAGGAGGCTGAGGCAGGAGAATCGCTTGAATCCGAAAGGTGGAGATTGCAGTGGGCCGAGA TCATGCCACTGCACTCCAGCC
TGGGCAAGAGAGCAAGACTCTATCTCAAAAAAAAAAAAAAAAAAAAAAAAAaGCAGGTGACATTTGGCTATGAACGAAACACTCAGGGCACACTCCACTTGAATCTCTTCCAGAAACCTGCAGCCTCCC
AAAAAACAGTCTGATTGGAACACATTGGAAGGCCTGGGGAAATAAAAAAATGGGTTGAATGAGGTCTACTTACCCAACTGCTGCAGGAAAAGTAGCAGATGATCCTC TGTCTGCACCAAAGCCCGGTA
GCCCACTGAGTCATCCTTCTTCAAGAACACCTGGATATACAGCTATAAGCCACAGTCAAGACCGGTGTAGTCAGTGAGGATCCAAATGCCTCTGAGAGCACCAGACCCCAGGAGTCAAAGCCTGG ACT
TAACAGAGGTACAGGCCATGGCATTATTTGATGTCTAAACAATGTAGCTATTATCTGGATGCCAGTAAGTGATACATATTCTTAGGAGCCAACATAATGATGCTGGG GTCTCCCAGACCTTTCCATGG
AAGAACCTGAGTACTCCACGGGTACCACGCCATTCATCCATGCTCTAATCACACCCCACAAAGTGAGCAGGAGGCCGGGATTAGACTTTCCGCTTTAGAAACGAAGAAGCCACAGCCCAAGCAGC TGA
GGAACACAACCAGATGCACACAGAGGCAATACTGGGTAGTGATGAGGAGTCCAGGCTGTGGGGTTAGACCTGAATCTGAACCGCAGTGACACCTCTTACTGG C

1950	13.9	0.6	5.2	a	192	326	(1953)	+	AluJb	SINE/Alu	1	127	(185)	1
2499	7.4	0.3	0.0	a	327	635	(1644)	+	AluY	SINE/Alu	1	310	(1)	2
1950	13.9	0.6	5.2	a	636	823	(1456)	+	AluJb	SINE/Alu	128	309	(3)	1
1999	9.3	6.8	0.7	a	1000	1279	(1000)	+	AluSq2	SINE/Alu	1	297	(16)	3
2505	8.5	0.0	0.0	a	1282	1587	(692)	+	AluSz	SINE/Alu	2	307	(5)	4

1950	13.9	0.6	5.2	hg	192	326	(2263)	+	AluJb	SINE/Alu	1	127	(185)	5
2484	7.8	0.3	0.0	hg	327	635	(1954)	+	AluY	SINE/Alu	1	310	(1)	6
1950	13.9	0.6	5.2	hg	636	823	(1766)	+	AluJb	SINE/Alu	128	309	(3)	5
1999	9.3	6.8	0.7	hg	1000	1279	(1310)	+	AluSq2	SINE/Alu	1	297	(16)	7
2346	9.0	0.3	1.0	hg	1282	1581	(1008)	+	AluSp	SINE/Alu	2	299	(14)	8
2506	8.5	0.0	0.0	hg	1592	1897	(692)	+	AluSz	SINE/Alu	1	306	(6)	9

>hg19 chr1:19561589-19564177

AGCAACTCTGCAGCTCCAACCTCTTAGGTTCATACCTGCCTTTTTGCCAAATTCTCCTTCCAGCTCGGCCTGTGCCCCAGTCAGGGGGAGGTCCACCATCTCTAGGCACACCACTTCTGCCAGGGA CT
CCTCACGGCTCCACAGCACCACCTTCCCTGCTACAGGAAACATTTTTACAAAGCTAAGCCTGGGGCTGGGCACAGTGGCTCATGCCTGTAATCCCAGCACTTTGGGAGGCCAAGGTGGGTGAATC TCT
TGAGCCCAGGAATTTGAGACCAGCCTGGACAACAGGGCAAAACCTTATCTCAACAAAAAATAAATATCAGGGCTGGGCACAGTGGCTCACGCCTGTAATCCCAGCACTTTGGGAGGCCAAGGCGG GCA
GATCACGAGGTCAGGAGATCGAGACCATCCTGGCTAACATGGTGAAACCCCTCTCTACTAAAAATACAAAAAATTAGCCAGGCATGATGGTGGGCGCCTGTAGTTCCAGCTACTCGGGAGGCTGA GGC
AGGAGAATGGCGTGAACCCGGGAGGTGGAGGTTGCAGTGAGCCGAGAT tGTGCCATTGCACTCCAGCCTGGGCGACACAGCGAGACTCCGTCTTGAAATAAAATAAAATAAAATAAAAATAAATAAAT
ATCAAAAAATTAGCCAAGCATGGTGGCGAGCGCCTGTAGTCACAGCTACTTAGGAGGCTGAGATAGGAAGATCACTTGAGCCCGGGAGATTGAGGCTGCAGTGAGCTATGATCACGCCACTGTAC TCC
AGACTGGGCAACAGTGAGACCTTGTCTCAAAAAATAAATAAATAAATAA ACAAAAGCTAAGCCTGTCTCCCAGATTTTCTAGAGAGACTCTTCTCGCTGACTCAGGGATTACTACAAGCTCTTGCATA
AAAGGAGAAATGGCAGAGCAAGGGGCTGGAATCAATAGGTCAAAGGAGGAAAGTTCTGTATCCCACCGAGAATGCCAAGTTATTCTTCAAAAGCAGGTAACGGGGCCGGGCATAGTGGCTCATGCCTG
TAATCCCAGCACTTTGGGAGGCCAAGGCGGGCAGATCACCTGAGGTTAG GAGCTTGAGACCAGCCTGGGCCAACATGGTGAAACCCTGTCTCTTACTAAAAATACAAAATTAGCGCCTATAATCCCAG
CTACTCAGGAGGCTGAGGCAGGAGAATCGCTTGAACCAGGAGGCAGAAGTTGCAAAGAGCTGAGATTACACCATTCCACTCCAGCCTGGGCAACAAGAGCAAAACTCTGTCTCAAAAAAGAAAAA AAG

AGCCAGGCACGGTGGCTCACACctgtaatcccagcactttgggaggccgaggcaggtggatcacctgaggtcaggagtttgagaccagcctgacaaacatggtgaaaccccatctctactaaaatac
aaaattagccaggcgtggtggtgcatgcctgtaatcccagctacttgggaggctgaggcaggagaatcacttaaacctgggaggcagaggttgcagtgagctgagatcatgccactgtactccag cag
tctgagcaacaagagcaaaactccgtctcaaaaaaaaaaaaaaaagcaggtgacaggccaggcacggtggctcacacCTGTAATCCCAGCACTTTGGGAGGCCGAGGCAGGCGGATCACTTGAGGTCA
GGAATTCGAGACCAGCCTGGCCAACATGGGGAAACCCTGTCTCTACTGAAAATACAAAAATTAGCTGGTCGTGGTGGCACACGCCTGTAATCCCAGCTACTCAGGAGGCTGAGGCAGGAGAATCG CTT
GAATCCGAAAGGTGGAGATTGCAGTGGGCCGAGATCATGCCACTGCACT CCAGCCTGGGCAAGAGAGCAAGACTCTATCTCAAAAAAAAAAAAAAAAAAAAAAA AGCAGGTGACATTTGGCTATGAAC
GAAACACTCAGGGCACACTCCACTTGAATCTCTTCCAGAAACCTGCAGCCTCCCAAAAAACAGTCTGATTGGAACACATTGGAAGGCCTGGGGAAATAAAAAAATGGGTTGAATGAGGTCTACTT ACC
CAACTGCTGCAGGAAAAGTAGCAGATGATCCTCTGTCTGCACCAAAGCC CGGTAGCCCACTGAGTCATCCTTCTTCAAGAACACCTGGATATACAGCTATAAGCCACAGTCAAGACCGGTGTAGTCAG
TGAGGATCCAAATGCCTCTGAGAGCACCAGACCCCAGGAGTCAAAGCCTGGACTTAACAGAGGTACAGGCCATGGCATTATTTGATGTCTAAACAATGTAGCTATTATCTGGATGCCAGTAAGTG ATA
CATATTCTTAGGAGCCAACATAATGATGCTGGGGTCTCCCAGACCTTTC CATGGAAGAACCTGAGTACTCCACGGGTACCACGCCATTCATCCATGCTCTAATCACACCCCACAAAGTGAGCAGGAGG
CCGGGATTAGACTTTCCGCTTTAGAAACGAAGAAGCCACAGCCCAAGCAGCTGAGGAACACAACCAGATGCACACAGAGGCAATACTGGGTAGTGATGAGGAGTCCAGGCTGTGGGGTTAGACCT GAA
TCTGAACCGCAGTGACACCTCTTACTGGC

>Chimp
AGCAACTCTGCAGCTCCAACCTCTTAGGTTCATACCTGCCTTTTTGCCAAATTCTCCTTCCAGCTCGGCCTGTGCCCCAGTCAGGGGGAGGTCCACCATCTCTAGGCACACCACTTCTGCCAGGGA CT
CCTCgCGGCTCCACAGCACCACCTTCCCTGCTACAGGAAACATTTTTACAAAGCTAAGCCTGGGGCTGGGCACAGTGGCTCATGCCTGTAATCCCAGCACTTTGGGAGGCCAAGGTGGGTGAATCTCT
TGAGCCCAGGAATTTGAGACCAGCCTGGACAACAGGGCAAAACCTTATCTCAACAAAAAATAAATATCAGGGCTGGGCAC gGTGGCTCACGCCTGTAATCCCAGCACTTTGGGAGGCCAAGGCGGGCA
GATCACaAGGTCAGGAGATCGAGACCATCCTGGCTAACATGGTGAAACCCCTCTCTACTAAAAATACAAAAAATTAGCCAGGC gTGATGGTGGGCGCCTGTAGTTCCAGCTACTCGGGAGGCTGAGGC
AaGAGAATGGCGTGAACCCGGGAGGTGGAGGTTGCAGTGAGCCaAGATcGTGCCATTGCACTCCAGCCTGGGCGACACAGCGAGACTCCGTCT cGAAATAAAATAAAATAAAATAAAAATAAATAAAT
ATCAAAAAATTAGCCAAGCATGGTGGCGAGCGCCTGTAGTCACAGCTACTTAGGAGGCTGAGATAGGAAGATCACTTGAGCCC aGGAGATTGAGGCTGCAGTGAGCTATGATCAtGCCACTGTACTCC
AGACTGGGCAACAGTGAGACCTTGTCTCAAAAAATAAATAAATAAATAAACAAAAGCTAAGCCTGTCTCCCAGATTTTCTAGAGAGACTCTTCTCGCTGACTCAGGGATTACTACAAGCTCTTGC ATA
AAAGGAGAAATGGCAGAGCAAGGGGCTGGAATCAATAGGTCAAgGGAGGAAAGTTCTGTATCCCACCGAGAAgGCCAAGTTATTCTTCAAAAGCAGGTAACGGGGCCGGGCATAGTGGCTCATGCCTG
TAATCCCAGCACTTTGGGAGGCCAAGGCGGGCAGATCACCTGAGGTTAGGAGCTTGAGACCAGCCTGGGCCAACATGGTGAAACCCTGTCTCTTACTAAAAATACAAAATTAGCGCCT gTAATCCCAG
CTACTCAGGAGGCTGAGGCAGGAGAATCGCTTGAACCAGGAGGCAGAAGTTGCAAAGAGCTGAGATTACACCATTCCACTCCAGCCTGGGCAACAAGAGCAAAACTCTGTCTCAA AAAAGAAAAAAAG
AGCCAGGCACGGTGGCTCACACCTGTAATCCCAGCACTTTGGGAGGCCGAGGCAGGTGGATCACCTGAGGTCAGGAGTTTGAGACCAGCCTGACAAACAT GTGAAACCCCATCTCTACTAAAAATACA
AAATTAGCCAGGCGTGGTGGTGCATGCCTGTAATCCCAGCTACTTGGGAGGCTGAGGCAGGAGAATCACTTAAACCTGGGAGGCAGAGGTTGCAGTGAGCTGAGATCATGCCACT GTACTCCAGCAGT
CTGAGCAACAAGAGCAAAACTCCGTCTCAAAAAAAAAAAAAAAAGCAGGTGACAGGC tAGGCACGGTGGCTCACACCTGTAATCCCAGCACTTTGGGAGGCCGAGGCAGGCGGATCACTTGAGGTCAG
GAATTCGAGACCAGCCTGGCCAACATGGGGAAACCCTGTCTCTACTGAAAATACAAAAATTAGCTGGTCGTGGTGGC gCAtGCCTGTAATCCCAGCTACTCAGGAGGCTGAGGCA GGAGAATCGCTTG
AATCCGAAAGGTGGAGATTGCAGTGGGCCGAGATCATGCCACTGCACTCCAGCCTGGGCAAGAGAGCAAGACTCTATCTCAA AtAAAAAAAAAAAAAAAGCAGGTGACATTTGGCTATGAAtGAAACA
CTCAGGGCACACTCCACTTGAATCTCTTCCAGAAACCTGCAGCCTCCCAAAAAACAGTCTGATTGGAACACATTGGAAGGCCTGGGGAAATAAAAAAATGGGTTGAATGAGGTCT ACTTACCCAACTG
CTGCAGGAAAAGTAGCAGATGATCCTCcGTCTGCACCAAAGCCCGGTAGCCCACTGAGTCATCCTTCTTCAAGAACACCTGGATATACAGCTATAAGCCACAGTCAAGACCGGTGTAGTCAGTGAG Ga
tccaaatgcctctgagagcaccagaccccaagATCCAAATGCCTCTGAGAGCACCAGACCCCAaGAGTCAAAGCCTGGACTTAACAGAGGTACAGGCCATaGCATTATTTGATGTCTAAACAAcGTAG
CTATTATgTGGATGCCAGTAAGTGATACATATTCTTAGGAGCCAACATAATGATGCTGGGGTCTCCCAGACCTTTCCATGGAAGAAC tTGAGTACTCCACGGGTACCACGCCATTCATCCATGCTCTA
ATCACACCCCACAAAGTGAGCAGGAGGCCGGGATTAGACTTTCtGCTTTAGAAACGAAGAAGCCACAGCCCAAGCAGCTGAGGAACACAACCAGATGCACACAGAGGCAATACTG GGTAGTGATGAGG
AGTCCAGGCTGTGGGGTTAGACCTGAATCTGAACCGCAGTGACACCTCTTACTGGC

Homologous Sequences
GCCAGGCACGGTGGCTCACACCTGTAATCCCAGCACTTTGGGAGGCCGAGGCAGG

>NAHR_22_Scaffold8597 478573 478869
KSD_NAHR_22

>Scaffold8597 478573 478869_NAHR
GGCACAGCTTGGAGCCTGTTCCCCTGGGGAGGGTGGCACTGAGTGATGCAAGCCTGGGTCCCAAGAAACCCTGGCCCTGATGCTGCTGCCCCCTCTCTGGGAGCAGGAAGTCTCCAGGGGAAAGGA CT
ATTTCTCTAGAGCCCTGGAAATCCCAGATTAACCCTCCTACTAGGTAGATCTACAGTGTTCCCTCTGAGCATACCAGCATCTCAAGTTTCCCACTAAAGCAGGGGGCTGGGCCAGGCTTGGCTCC AGA
GCTGCGAACAGCCTGGACAGAGCTGACATCAGCAGTTAGGCCAGGTTGGGGAAGGGTAGGGTTAGCCCCCAGCAGCTGGGCTGTCTCACCCATAGGGGAGGAGAGAAGAAGAGGGAGCAGCAGAAG AA
GGGAGGAGGAAAAGCAGGAAAAGGAAAGTGAGCCCAGGGAAGTTTGCTCAGGACAGGCTTTTGCTGTAGGGTGGAGGAATTTCTCTAGCGGTAGTGTACCACTACACTTCAGCCTGGGAGCAGGA TTC
CTCCAGCTGTAAAGTGGAGTAATCCGATGCATGTTATCTCCCCGCTTAACAACTGACGTCCAAAACCCCTGCCTATGTCTTCATCTTTATTTCATCTCAGACCATTTCCCGCCATGCATGGACCTT CC
CATAGAATCACTTATAGATCCCTCCAAAGGCCATGCTAATCCATGACTACATGCCTTTGGGTATACAGTTCTCTGTTTGAATTGCCTTTCCATGATTTATCTAGCTGGAAATTTCCTTAAAATAT CAC
CTTCTCCATGAAGTATTCCCCAATTCTGATAAAATTATTCTTGCCTCTGTGTTCAAATCTAGGTGTCTAAGATACATTAGAATGACCATTTATTAATCTAGTAAGTATTCTCAAACTTTAGTGTGC AT
CAGAATGACCTGGGAGTCTTGTTAAAACACAGATTACTGGGATCCAACCCCAGATTTCTGATTTCAGTTCTGACCTGTAGTGGGGTCAAGAATGTTCACTTCT GGCTGGGCGCAGTGGCTCACACCTG
TAAATCCCAGCACTTTGGGAGGCTGAGGCGGGTGGATCACTTGAGGTCAGGAGTTCGAGACCAGTCTGGCCAACATAGTGAAACCCCACCTCTACTAAAAATGCAAAAATTAGCCGGATGTGGTGG CG
GGTGCCTGTAAGCCCAGCTACTCAGGAGGCTGAGGCAGGAGAATC aCTTGAACCCAGGAGGCgGAGGTTaCAGTGAGCtGAGATAGCGCCAtTGCACTCcAGCaTGGGCgACAGAGcaAGACTCtgTC
TCAAAAAaAAAAAAAAGTACATAATAACAAACTGTGATAATTGATCTGAAAGAAAAGAATATTACAAGAAAGAATAATATCAAGAACCTATTTCAGATT gTGTGGTCAGGTAAGACCTTTTTGGGAAG
GGGACGTTTATACAGGCAGACATCCAGTACTGTAAACAAACTTCTATAAGGTGTGTTTATTGCACTGTATTGTTACCTAGGTTTAAGTTGTCCAGCTAACCACCAGACTGTGTACTTCTTGAAAT GTA
GAATCTTGTCAATATTTGTGCCCTCAGCATGCTGCACAGAGTAGGGCCTTGGTAGCTAGTGAAAGAATGAATGGAAAGATGGAGGGATCATGGGAAGAATGAGCAAATAAATAAAGGAAACATCAG GT
GGCTGTATGAGTGCATACTTGCATGAATGGATGATGGATAGGTAACGGAGGGATGGGTGACTGTCTCCAGCAGCAACACTGACAACCAGGATTCCTGGTGGTACCAGTGAGACAAGAGTAGAAAC TGA
TAGGAACTTAGCTGATTCCTTTACTATATTTCCAAACCATGTCCATCATTTAAGGTGACCACCAATGGCCTCTCCTCCCCAAAAAGCCCCTTGATTACCATTGCTAAGCCCTACTCCACACGTGAA TG
AATTGCTGTCACCTCCTGTACTCCCACACATGCCACACCACACTTACGTCGACAAACGTAATTGCCTTGCGCTTGCCATCTACTGATCACCTGGGGTACAGGTTTCTCAGGGCAAGCCATGCTCT CTG
TGTCAGTCTTAGAAGGGATAAAGGGATAGAGAGAGGAAGAAAAGAAAGTTTCTTTCCTACATCTGAGCCAAAGAAGGTACAGAGCTCAGTGGGCAGTGAGGAGATTCTTGCTCTGATAATTCATCA CA
TGACTTCAAAAAAGCACTTTAAATGACACATTTTTTCTAGATATGTGTCTTAGGCCTCCTAAGAGGAGTTTATTTAGAGACCCAGTTTGAGTCACAATAAAGAGTTAAAAAAAACACCC A

14	25.2	4.2	0.0	197298	360	407	(1889)	+	GA-rich	Low_complexity	1	50	(0)	1
181	35.2	13.5	1.5	197298	578	822	(1474)	+	L2c	LINE/L2	2923	3196	(191)	2
647	16.5	1.6	2.4	197298	876	999	(1297)	C	MER5A	DNA/hAT-Charlie	(6)	183	61	3
2402	8.4	0.0	0.3	197298	1000	1296	(1000)	+	AluSx1	SINE/Alu	1	296	(16)	4
180	26.0	0.0	0.0	197298	1368	1417	(879)	C	L2a	LINE/L2	(248)	3178	3129	5
245	29.0	9.9	0.9	197298	1508	1608	(688)	+	L2c	LINE/L2	3278	3387	(0)	2

14	25.2	4.2	0.0	hg	360	407	(2749)	+	GA-rich	Low_complexity	1	50	(0)	6
181	35.2	13.5	1.5	hg	578	822	(2334)	+	L2c	LINE/L2	2923	3196	(191)	7
981	14.5	1.5	8.7	hg	876	999	(2157)	C	MER5A	DNA/hAT-Charlie	(6)	183	71	8
2417	8.4	0.0	0.3	hg	1000	1298	(1858)	+	AluSx1	SINE/Alu	1	298	(14)	9
981	14.5	1.5	8.7	hg	1299	1369	(1787)	C	MER5A	DNA/hAT-Charlie	(119)	70	1	8
342	28.7	3.3	11.1	hg	1417	1600	(1556)	C	L2a	LINE/L2	(0)	3426	3256	10
1681	17.4	3.2	0.0	hg	1604	1885	(1271)	+	AluJr	SINE/Alu	1	291	(21)	11
2115	8.4	0.0	0.4	hg	1895	2156	(1000)	+	AluSx3	SINE/Alu	36	296	(15)	12
245	29.0	9.9	0.9	hg	2368	2468	(688)	+	L2c	LINE/L2	3278	3387	(0)	7

>hg19 chr9:33211889-33215044
GGCACAGCTTGGAGCCTGTTCCCCTGGGGAGGGTGGCACTGAGTGATGCAAGCCTGGGTCCCAAGAAACCCT GGCCCTGATGCTGCTGCCCCCTCTCTGGGAGCAGGAAGTCTCCAGGGGAAAGGACT
ATTTCTCTAGAGCCCTGGAAATCCCAGATTAACCCTCCTACTAGGTAGATCTACAGTGTTCCCTCTGAGCATACCAGCATCTCAAGTTTCCCACTAAAGCAGGGGGCTGGGCCAGGCTTGGCTCC AGA
GCTGCGAACAGCCTGGACAGAGCTGACATCAGCAGTTAGGCCAGGTTGGGGAAGGGTAGGGTTAGCCCCCAG CAGCTGGGCTGTCTCACCCATAGGGGAGGAGAGAAGAAGAGGGAGCAGCAGAAGAA
GGGAGGAGGAAAAGCAGGAAAAGGAAAGTGAGCCCAGGGAAGTTTGCTCAGGACAGGCTTTTGCTGTAGGGTGGAGGAATTTCTCTAGCGGTAGTGTACCACTACACTTCAGCCTGGGAGCAGGA TTC
CTCCAGCTGTAAAGTGGAGTAATCCGATGCATGTTATCTCCCCGCTTAACAACTGACGTCCAAAACCCCTGC CTATGTCTTCATCTTTATTTCATCTCAGACCATTTCCCGCCATGCATGGACCTTCC

CATAGAATCACTTATAGATCCCTCCAAAGGCCATGCTAATCCATGACTACATGCCTTTGGGTATACAGTTCTCTGTTTGAATTGCCTTTCCATGATTTATCTAGCTGGAAATTTCCTTAAAATAT CAC
CTTCTCCATGAAGTATTCCCCAATTCTGATAAAATTATTCTTGCCTCTGTGTTCAAATCTAGGTGTCTAAGA TACATTAGAATGACCATTTATTAATCTAGTAAGTATTCTCAAACTTTAGTGTGCAT
CAGAATGACCTGGGAGTCTTGTTAAAACACAGATTACTGGGATCCAACCCCAGATTTCTGATTTCAGTTCTGACCTGTAGTGGGGTC **AAGAATGTTCACTTCT**GGCTGGGCGCAGTGGCTCACACCTG
TAAATCCCAGCACTTTGGGAGGCTGAGGCGGGTGGATCACTTGAGGTCAGGAGTTCGAGACCAGTCTGGCCAACATAGTGAAACCCCACCTCTACTAAAAATGCAAAAATTAGCCGGATGTGGTG GCG
GGTGCCTGTAAGCCCAGCTACTCAGGAGGCTGAGGCAGGAGAATC gCTTGAACCCAGGAGGCaGAGGTTgCAGTGAGCcGAGATAGCGCCAcTGCACTCtAGCcTGGGCaACAGAGtgAGACTCCaTC
TCAAAAAataaaa**aagaatgttcacttct**aacaagttcccatgtattgttgatcctgctggtccaaggatcacactttgagaaccactgatttaccacaagaaattctcaaccctcagtatggtcagt
gcttacttattaatttattcagtgaatgtctactgagggcctgctatgtgccagacatggaccaggtgttgagtgttcaatggtaagcaaagcccacattgaa cttgcccacataggacttgcctcta
tggagcttccagtcaactatggaagacatattaagcaaataaaaataccattaagtatataataacaggccaggagtggtggctcatgcctgtaatcccagcactttgggaggtagagacagagg ctt
gcttgagcccaggagtttgagaccagcctgggcaatatagtgacacctcgtctctacaaaaaatttaaaaattagccaagcatggtagcacacacttggagtc cttgctacttaggaggctgaggtgg
gaggatagcttgagctcaggaggcagaggtgtactgagacagcacttacactccagcctgggtgacagagtaagactctatctcaaaaaagaagtacataat actttgggaggccgaggcgggcggat
cacgaggtcaggagttcgaaaccagcctgaccaacatggtgaaaccccgtctctactagaaatacaaaaaaattagctgggtgtggtggcatgcacttgtaat cccagctgctcaggaggttgaggca
ggagaatcacttgaacccaggaggcggaggttacagtgagctgagatagcgccattgcactccagcatgggcgacagagcaagactctgtctcaaaaaaa AAAAAAAGTACATAATAACAAACTGTG
ATAATTGATCTGAAAGAAAAGAATATTACAAGAAAGAATAATATCAAGAACCTATTTCAGATT aTGTGGTCAGGTAAGACCTTTTTGGGAAGGGGACGTTTATACAGGCAGACATCCAGTACTGTAAA
CAAACTTCTATAAGGTGTGTTTATTGCACTGTATTGTTACCTAGGTTTAAGTTGTCCAGCTAACCACCAGACTGTGTACTTCTTGAAATGTAGAATCTTGTCAATATTTGTGCCCTCAGCATGCT GCA
CAGAGTAGGGCCTTGGTAGCTAGTGAAAGAATGAATGGAAAGATGGAGGGATCATGGGAAGAATGAGCAAATAAATAAAGGAAACATCAGGTGGCTGTATGAGTGCATACTTGCATGAATGGATGATG
GATAGGTAACGGAGGGATGGGTGACTGTCTCCAGCAGCAACACTGACAACCAGGATTCCTGGTGGTACCAGTGAGACAAGAGTAGAAACTGATAGGAACTTAGCTGATTCCTTTACTATATTTCCAAA
CCATGTCCATCATTTAAGGTGACCACCAATGGCCTCTCCTCCCCAAAAAGCCCCTTGATTACCA TTGCTAAGCCCTACTCCACACGTGAATGAATTGCTGTCACCTCCTGTACTCCCACACATGCCAC
ACCACACTTACGTCGACAAACGTAATTGCCTTGCGCTTGCCATCTACTGATCACCTGGGGTACAGGTTTCTCAGGGCAAGCCATGCTCTCTGTGTCAGTCTTAGAAGGGATAAAGGGATAGAGAG AGG
AAGAAAAGAAAGTTTCTTTCCTACATCTGAGCCAAAGAAGGTACAGAGCTCAGTGGGCAGTGAG GAGATTCTTGCTCTGATAATTCATCACATGACTTCAAAAAAGCACTTTAAATGACACATTTTTT
CTAGATATGTGTCTTAGGCCTCCTAAGAGGAGTTTATTTAGAGACCCAGTTTGAGTCACAATAAAGAGTTAAAAAAAACACCC A

Homologous Sequences

TGAGGCAGGAGAATC

>NAHR_23_ Scaffold10228 4171017 4171314
KSD_NAHR_23
>**Scaffold10228 4171017 4171314**_NAHR

GGTTGAGCAAGCTCTTTTCTATTTCTGAATGTTGAATATTTTTATTATGAAAGGGTTTGGGATTGTACCAAATGCTTTTTCTGCATCAATTGAGATCATGTGGGTTTTGCTCTTTATTCTATAATA TA
ATGTATTACATTAATTAATTTTAATATGTTGAAGTAAACCTGCATTCTTGGGATGTCCACTTTATTATAGTACATTACCAGCCTGGCCAACATGGCGAAACCCCATCTCTACTAAAAATACAAAA ATT
AGCTGGGCGTGGTGGTAGGTGCCTGTAATCACTGCTACTCAGGAGGCTGAGGCAGGAGAATTGCTTGAACCTAGGAGGCAGAGGTTGCTGTGAGCCAAGATTGCGCCACTGCACTCCAGCCTGGGC AA
CGAGTGAAACTCCATCTCAAAAACAAACAAACAAAAAAAAAAACCAAATTGCCACCTTTGTCATAAACCAAGTATCAAATATTCATTTGTCTCTGTTCTATTGGTGAGTTTTATTCTTGAACCAAG AAT
ATACTATCTTAATTTCTGTAGTTTTTATAATAAGCTTTGACAACAAATAAGGCAGTTGTCTTCTTCGATTCTTTTTTAAAAAAAA aTTTTAAGATACGGGGTCTCACTATGTTTGTCAGACCGATCTC
TAACTCCTGGGATCAAGCAATCATTTCATCTCAGCCTCCCAAGTAGACAGGACTACAGGCACGCACCACTACACCCAGCTCAAATCTTGGCTATAAAAAAGTATCAGCTTGTCAACTCCCACACA AAA
AGAAGAAAAGCAAGGAGGAGGAGAGAAACTGTCGGTATTTTTATTAGAATTGCACTGAATCTATGTATCAATTTGGAGGATAACATATTTTACAATATTGAGTTTCACATCATGAATAAGAGATAT CT
TCCAAATCATTTGGACCTTCTTACATAAAATTTTCTCTATAGAGGCCGGGTGTGGTGGTTCACATCTGTAATCCCAGCACTTCGTGAGGCTAAGGCGGGCGGATCACCTGAGGTCAGGAGTTCAAGAC
CAGCCTGACCAACGTGGTGAAATCCCATCTCTACTAAAAATACAAAATTAGCTGGGCATGGTGGCACATGCCTGTAATCCCAGCTATTTGGGAGGCTGAGGCAGGAGAATCACTTGAACCTGGGAG GC
GAGGGTTGCAGTGAGCCAAGATCACACCATTGCACTCCAGCCTGGGTGACAGAGCGAGTCTCGGTCTCAAAAATAAATAAATAAATAACTTATTAATTCAAATAGTATAACTTTTGGATTTTCTACAT
AACAAATAATATAAAAAGAAAGTTTTATTCCTCCCTTTCCACCTTTCTTTCATTCCTTTTCTTGTTTTAGTACATAGCTAAAGACCTAGAGTAAAATGCTGAATCGTAATAGAATAGCAGACATCC TT
ATCTAAAAATCTCAGAAGAATTCTAACTTAGTTTCAGTTTTCCCAGAAGCAGACCCGGAGACAAGGATCTGTGTACAGGAATTTACTGGGAGGTGAAGGAAAACATTGGTTGGGGAGTGGGAAAG GGA
GACAAGGAAGGGAAGGTGGCCAATAGAGGCATGTGTTATCAGGCTTATCTCTGGAGCAGAATTCTGCTGGGGGGACTCTGGGAGCCAGTGTAAACTACATGCCTCAGAGTTCTCTCACCCCAGATG GG
AGGGAGCTGTGGTATTCAGACACAGAGTCTTGCCAGTTATTGTTTAGAGCTGCTGTGGGATGGAGCTTGAATTCCCCAGCACTTCCAACTGCCATGGGAGTGGAAAACTGTAGTCAAGCAGCAAA GAA
AGCCCTCAGGCAAGAAGTGCAGATGCTGGCAGCTGAAAACGGTAGTGTGTACACTGAAATGGTAAAAGTGACGGATACAGGTAAGTTGCCAACAGTGTCTTCTACAAACCCCTTCAACATTTTACT GC
ATTAACAAGTAACATTTGCAGTCCCCATAGGATAACGGCAGCTGCATAAGTCCAGCCTTTCTAGACACGCTCCTAAAATCATTTTGTCAAT aAAGAGAGAAAGAAGGGGAAGAATACACATAATTGCT
GAGCATGGTGGCTCACACCTGTAATACCAGGCCAAGGTGGGGGGAATTGGTTGAGTCCAGGAGTTCAAGACCAGTCTGGGCAACATAGTGAGACCGTGTCTCTACTAAAAATTAAAAAAAATTAGC TG
GGTGTGGGGGCGCATGCCTGTAGTCCCAGCTGCTCAGGGGGACTGAGGCAGGAGGATCACTTGAGCCTGGGAAGTTGAGGC tGCAGTGAGCCAAGATTGCATCACTGCACTCCAGCCTGGG

917	20.7	3.9	0.0	6857	1	203	(2094)	C	L1M4	LINE/L1	(2152)	3994	3784	1
1702	9.5	0.9	0.0	6857	205	426	(1871)	+	AluSq	SINE/Alu	84	307	(6)	2
656	22.7	14.8	10.5	6857	431	598	(1699)	C	L1M5	LINE/L1	(1271)	4875	4688	3
671	22.3	0.0	0.0	6857	599	719	(1578)	C	FLAM_A	SINE/Alu	(21)	121	1	4
656	22.7	14.8	10.5	6857	720	934	(1363)	C	L1M5	LINE/L1	(1459)	4687	4477	3
2229	11.7	0.3	0.0	6857	940	1237	(1060)	+	AluSx	SINE/Alu	1	299	(13)	5
473	22.1	13.4	4.5	6857	1240	1434	(863)	C	L1M5	LINE/L1	(1868)	4278	4061	3
1697	22.9	5.8	1.0	6857	1435	1898	(399)	C	LTR16C	LTR/ERVL	(3)	486	1	6
473	22.1	13.4	4.5	6857	1899	1925	(372)	C	L1M5	LINE/L1	(2086)	4060	4037	3
1548	15.1	4.4	1.5	6857	2046	2297	(0)	+	AluJb	SINE/Alu	2	260	(52)	7

917	20.7	3.9	0.0	hg	1	203	(2599)	C	L1M4	LINE/L1	(2152)	3994	3784	8
1702	9.5	0.9	0.0	hg	205	426	(2376)	+	AluSq	SINE/Alu	84	307	(6)	9
1313	24.3	13.9	7.6	hg	431	597	(2205)	C	L1M5	LINE/L1	(1271)	4875	4688	10
680	22.1	0.0	0.0	hg	598	719	(2083)	C	FLAM_A	SINE/Alu	(20)	122	1	11
1313	24.3	13.9	7.6	hg	720	939	(1863)	C	L1M5	LINE/L1	(1459)	4687	4463	10
2225	11.2	0.3	0.0	hg	940	1225	(1577)	+	AluSx	SINE/Alu	1	287	(25)	12
50	0.0	0.0	0.0	hg	1226	1268	(1534)	+	(AC)n	Simple_repeat	1	43	(0)	13
1313	24.3	13.9	7.6	hg	1269	1445	(1357)	C	L1M5	LINE/L1	(1684)	4462	4279	10
2151	11.8	0.0	0.3	hg	1446	1742	(1060)	+	AluSc	SINE/Alu	1	296	(13)	14
1313	24.3	13.9	7.6	hg	1743	1939	(863)	C	L1M5	LINE/L1	(1868)	4278	4061	10
1697	22.9	5.8	1.0	hg	1940	2403	(399)	C	LTR16C	LTR/ERVL	(3)	486	1	15
1313	24.3	13.9	7.6	hg	2404	2430	(372)	C	L1M5	LINE/L1	(2086)	4060	4038	10
1531	15.5	4.4	1.5	hg	2551	2802	(0)	+	AluJb	SINE/Alu	2	260	(52)	16

>hg19 chr2:220231493-220234294
GGTTGAGCAAGCTCTTTTCTATTTCTGAATGTTGAATATTTTTATTATGAAAGGGTTTGGGATTGTACCAAATGCTTTTTCTGCATCAATTGAGATCATGTGGGTTTTGCTCTTTATTCTATAATA TA
ATGTATTACATTAATTAATTTTAATATGTTGAAGTAAACCTGCATTCTTGGGATGTCCACTTTATTATAGTACATTACCAGCCTGGCCAACATGGCGAAACCCCATCTCTACTAAAAATACAAAA ATT
AGCTGGGCGTGGTGGTAGGTGCCTGTAATCACTGCTACTCAGGAGGCTGAGGCAGGAGAATTGCTTGAACCTAGGAGGCAGAGGTTGCTGTGAGCCAAGATTGCGCCACTGCACTCCAGCCTGGGC AA
CGAGTGAAACTCCATCTCAAAAACAAACAAACAAAAAAAAAAACCAAATTGCCACCTTTGTCATAAACCAAGTATCAAATATTCATTTGTCTCTGTTCTATTGGTGAGTTTTATTCTTGAACCAAG AAT
ATACTATCTTAATTTCTGTAGTTTTTATAATAAGCTTTGACAACAAATAAGGCAGTTGTCTTCTTCGATTCTTTTTTAAAAAAAA tTTTTAAGATACGGGGTCTCACTATGTTTGTCAGACCGATCTC
TAACTCCTGGGATCAAGCAATCATTTCATCTCAGCCTCCCAAGTAGACAGGACTACAGGCACGCACCACTACACCCAGCTCAAATCTTGGCTATAAAAAA GTATCAGCTTGTCAACTCCCACACAAAA
AGAAGAAAAGCAAGGAGGAGGAGAGAAACTGTCGGTATTTTTATTAGAATTGCACTGAATCTATGTATCAATTTGGAGGATAACATATTTTACAATATTGAGTTTCACATCATGAATAAGAGATAT CT
TCCAAATCATTTGGACCTTCTTACATAAAATTTTCTCTATAGAGGCCGGGTGTGGTGGTTCACATCTGTAATCCCAGCACTTCGTGAGGCTAAGGCGGGCGGATCACCTGAGGTCAGGAGTTCAAGAC
CAGCCTGACCAACGTGGTGAAATCCCATCTCTACTAAAAATACAAAATTAGCTGGGCATGGTGGCACATGCCTGTAATCCCAGCTATTTGGGAGGCTGAGGCAGGAGAATCACTTGAACCTGGGAG GC
GAGGGTTGCAGTGAGCCAAGATCACACCATTGCACTCCAGCctgggtgacagagcaagactctgtctcaaaaaacacacacacacacacacacacacacacacacacacacaaattttctctgt
agagatcttgcataactttattgacagatacttgattttatgcttgtagaagtaaaccttttaaaaatttcgttttctacccatttttttattggtatgtagaaataaaattgatatttgtatgt tga
tttttcagccagctgtattgataaactctctcattacggccgggtgcagtggcttacacctgtaatcccagcactttggaggccgaggcaggccgatcacgaggccaggagatcaagaccatcctgg
ccagcatggtgaaaccctgtctctactaaaagtacaaaaattacctgggtgtggtggtgtgcacctgtagtcccagctactcgggagactgaggcaggagaatggcttgaaccttggaggcagag gtt
gaagtgaaccaagatcacaccactgcactccagcCTGGGTGACAGAGCGAGTCTCGGTCTCAAAAATAAATAAA TAAATAACTTATTAATTCAAATAGTATAACTTTTGGATTTTCTACATAACAAAT
AATATAAAAAGAAAGTTTTATTCCTCCCTTTCCACCTTTCTTTCATTCCTTTTCTTGTTTTAGTACATAGCTAAAGACCTAGAGTAAAATGCTGAATCGTAATAGAATAGCAGACATCCTTATCT AAA
AATCTCAGAAGAATTCTAACTTAGTTTCAGTTTTCCCAGAAGCAGACCCGGAGACAAGGATCTGTGTACAGGAATTTACTGGGAGGTGAAGGAAAACATTGGTTGGGGAGTGGGAAAGGGAGACAAGG
AAGGGAAGGTGGCCAATAGAGGCATGTGTTATCAGGCTTATCTCTGGAGCAGAATTCTGCTGGGGGGACTCTGGGAGCCAGTGTAAACTACATGCCTCAGAGTTCTCTCACCCCAGATGGGAGGG AGC
TGTGGTATTCAGACACAGAGTCTTGCCAGTTATTGTTTAGAGCTGCTGTGGGATGGAGCTTGAATTCCCCAGCA CTTCCAACTGCCATGGGAGTGGAAAACTGTAGTCAAGCAGCAAAGAAAGCCCTC
AGGCAAGAAGTGCAGATGCTGGCAGCTGAAAACGGTAGTGTGTACACTGAAATGGTAAAAGTGACGGATACAGGTAAGTTGCCAACAGTGTCTTCTACAAACCCCTTCAACATTTTACTGCATTA ACA
AGTAACATTTGCAGTCCCCATAGGATAACGGCAGCTGCATAAGTCCAGCCTTTCTAGACACGCTCCTAAAATCA TTTTGTCAATgAAGAGAGAAAGAAGGGGAAGAATACACATAATTGCTGAGCATG
GTGGCTCACACCTGTAATACCAGGCCAAGGTGGGGGGAATTGGTTGAGTCCAGGAGTTCAAGACCAGTCTGGGCAACATAGTGAGACCGTGTCTCTACTAAAAATTAAAAAAAATTAGCTGGGTG TGG
GGGCGCATGCCTGTAGTCCCAGCTGCTCAGGGGGACTGAGGCAGGAGGATCACTTGAGCCTGGGAAGTTGAGGC cGCAGTGAGCCAAGATTGCATCACTGCACTCCAGCCTGGG

Homologous Sequences
TGCACTCCAGCCTGGGTGACAGAGC

>NAHR_24_ Scaffold5430 3029171 3029452
KSD_NAHR_24

>Scaffold5430 3029171 3029452_NAHR

TTTTAACATGCTGAATTGATTGTTTTTTTTTTTTTAGTGTCCTATAGATATACCTGGCCATAATAATTAGTAAGTAGCCATTTAGGTTGCTTTTAATATCATATTTTTGAAATTGTATTGTTCAATA CA
AATTAATTGAAAGTACTGAGTGTAAACCTCAGGACAAAACTACTGAGAAATATTAAAGGTTTTAATTTTATACCCAACTATTTAGTGATACTGGTTTTGCCTACATGTGGTGGATCTTTAGAAGA TGA
AAGAGATGTGTGGAATAATGATAATCCTGATGATGGTAATAATAATGATAGCAATGTTTATTATGCACAGGGCACTCTGTAAATTATTCCATTTATTTCCTTTAATTCTTGCTAAAAAAGCCATCT GA
AACAAGTATGTCTATTTATTTTACTTCTAAGGAAAATGAGAATATGAGAATTATAAAGAGGATAAGTAACACTGTTTTTTTTTTTTTTTTGAAACAGAGTCTTGCTCTGTTGCCCAGGCCACAGT GCA
GTGATATGATCTCGGCTCACTGCAATCTTCACCTCCCAGAGTCAAATGATTCTCCTGCCTCAGCCTCCCGAGTACCTGGGATTACAGGTGCGCACTACCATGCCCGGTTTGTTTTTGTATTTTTAG TA
GAGACGGGGTTTCACTATGTTAGCCATGCTGGTGTCATACTCCTGACCTCAAGTAGTCCACCCACCTTGGCCTTCCAAAGTGCCAGGATTACAGGTATGAGCCACTGCACCCGGCCCTGAGATGT ATT
TTAAAGGCAGAGACAGCAGGACATATGGGGTGGATATTGATGATAAGAACAAAGGAAAAATTAAAGATGACACCTTGGTTTTTGGTTTGAGCTCAAATAGCTGATGGTGACATTTACTATGAGAAA GA
CTAGAAAAGGAACAGGCTTCGGGGACAAAAGTAGGAGGCTCTATTAGGTGTACTAAGTTTGAGTTGCCTAGTTACATCTAAGAGAAGGTGTCAAATAGGCCACTGGATATGTGAGTCTGGAGTAG CAA
ACAGAGCAGGGCTAGAGATAGAAGTGTGCAATCGTTGGCTGGGTGCGGTGGCTCACGCCTGTAATCCCAGCACTTTGGGAGGCCGAGGTGGGCAGATCACCTGAGGTCAGGAGTTTGAGAATAGCCTG
GCCAACAgaGaGAAACCCTGTCTCTACTGAAAATACAAAATTAGCCGGGTGTGGTGGTACATGCTTGTAATCCCAGCTACTCGGGAGGCTGAGGCAGGAGAATCGCTTGAATCCTGGAGGCAGAGGTT
GCGGTGAGCCAAGATCACGCCATTGCACTCCAGCCTGGGCAACAAGAGCGAAACTCCATCTCAAACAAAAACAAAAACAAAAACAAAA GAAGCGTGCAATTGTCGGGTGGTGTTTACAGCCCTAGAAC
TAGGTGGGACCATTGGGTAGAGTATATGCTTTGAGAAGGGAAAGGACTCATGATAGAGCCCTGAGGCATCCTAATATTTAGAGATTCATAGAGAGAATGAGAAGGGCTAGCCAGTGACAGTGGCC AAG
GAGAAACATTAAGGCATATTTCTTTCATTAATAAAACTAAATTTTAAAAAATCAAAGTTATACACATTCATAGTCTCACTGTGTTACC CAGACTGAAGTGCAGTGGCATGATCTGGGCTCACTGCAAC
CTCTACCTCCTGGGCTCAAGTGATGCTCCCACCTCAGCCTCCCAAGTGGCTGGGACTACAGGCACACCACCATGTCTGGCTAATTTTTGTATTTTTTTTGGTACAGATGGAGTTCCACCATGTTG CCC
AGGCTGGTTTCAAACTCCTGAGCTCAAGTGATCCACCTGCCTCGGCCTCTTAGAGTGCTGGGATTACAGGCATGAGCCACCATGCCAG GCCCCATATTCTTAAAAATAAAATTGTTCTACAGGGCTTT
TGCAATGAAAAGAACAGTTATCTGCTTCATCCTTCTCCATTACTCAATCCCGATGCCCAGAGGCAACTACTTTAAACTCTTTCAGTTGCTTCTTCTGTGTTTTCATTTGTTATTTCTTTTCTTCT TTT
GCCATCTCTATAGAGATATCATGTCTCTATAGAGACATCCTCTTTAAGTAAAGACTTACTCTTTACTCCTATCACTCATCCTCACACA CACTTGCACATATTCACGCAAACACAGTTTCTCACTATCA
TTTCATTATAATTACATCACATATTTTGTTAAATCAATATATGGTATTAATTACTCTGACTTTGTAAATGTTATTGATAACTGAGCCGTATAGTATACTATGAT T

13	26.6	0.0	0.0	a	270	306	(1975)	+	(AAT)n	Simple_repeat	1	37	(0)	1
2099	15.1	0.0	0.0	a	459	756	(1525)	C	AluSz	SINE/Alu	(14)	298	1	2
303	32.4	6.6	1.7	a	827	1052	(1229)	C	L2-1_AMi	LINE/L2	(611)	624	388	3
2274	8.9	0.3	0.0	a	1061	1342	(939)	+	AluSp	SINE/Alu	1	283	(30)	4
27	0.0	0.0	0.0	a	1343	1368	(913)	+	(AAACAA)n	Simple_repeat	1	26	(0)	5
277	35.4	1.9	5.2	a	1386	1543	(738)	C	L2	LINE/L2	(1097)	2322	2170	6
657	23.9	6.7	9.5	a	1587	1608	(673)	C	HAL1	LINE/L1	(10)	2497	2480	7
1806	15.6	0.7	1.1	a	1609	1883	(398)	C	AluSz	SINE/Alu	(38)	274	1	8
657	23.9	6.7	9.5	a	1884	2281	(0)	C	HAL1	LINE/L1	(28)	2479	2088	7

13	26.6	0.0	0.0	hg	270	306	(2312)	+	(AAT)n	Simple_repeat	1	37	(0)	9
2099	15.1	0.0	0.0	hg	459	756	(1862)	C	AluSz	SINE/Alu	(14)	298	1	10
303	32.4	6.6	1.7	hg	827	1052	(1566)	C	L2-1_AMi	LINE/L2	(611)	624	388	11
2242	10.4	0.3	0.3	hg	1061	1359	(1259)	+	AluSq2	SINE/Alu	1	299	(14)	12
23	5.6	0.0	5.1	hg	1378	1418	(1200)	+	(TTTGT)n	Simple_repeat	1	39	(0)	13
2010	10.4	0.4	0.0	hg	1421	1679	(939)	+	AluSp	SINE/Alu	24	283	(30)	14
27	0.0	0.0	0.0	hg	1680	1705	(913)	+	(AAACAA)n	Simple_repeat	1	26	(0)	15
277	35.4	1.9	5.2	hg	1723	1880	(738)	C	L2	LINE/L2	(1097)	2322	2170	16
657	23.9	6.7	9.5	hg	1924	1945	(673)	C	HAL1	LINE/L1	(10)	2497	2480	17
1806	15.6	0.7	1.1	hg	1946	2220	(398)	C	AluSz	SINE/Alu	(38)	274	1	18
657	23.9	6.7	9.5	hg	2221	2618	(0)	C	HAL1	LINE/L1	(28)	2479	2088	17

>hg19 chr13:46004261-46006878

TTTTAACATGCTGAATTGATTGTTTTTTTTTTTTTAGTGTCCTATAGATATACCTGGCCATAATAATTAGTAAGTAGCCATTTAGGTTGCTTTTAATATCATATTTTTGAAATTGTATTGTTCAATACA

AATTAATTGAAAGTACTGAGTGTAAACCTCAGGACAAAACTACTGAGAAATATTAAAGGTTTTAATTTTATACCCAACTATTTAGTGATACTGGTTTTGCCTACATGTGGTGGATCTTTAGAAGA TGA
AAGAGATGTGTGGAATAATGATAATCCTGATGATGGTAATAATAATGATAGCAATGTTTATTATGCACAGGGCACTCTGTAAATTATTC CATTTATTTCCTTTAATTCTTGCTAAAAAAGCCATCTGA
AACAAGTATGTCTATTTATTTTACTTCTAAGGAAAATGAGAATATGAGAATTATAAAGAGGATAAGTAACACTGTTTTTTTTTTTTTTTTGAAACAGAGTCTTGCTCTGTTGCCCAGGCCACAGT GCA
GTGATATGATCTCGGCTCACTGCAATCTTCACCTCCCAGAGTCAAATGATTCTCCTGCCTCAGCCTCCCGAGTACCTGGGATTACAGGT GCGCACTACCATGCCCGGTTTGTTTTTGTATTTTTAGTA
GAGACGGGGTTTCACTATGTTAGCCATGCTGGTGTCATACTCCTGACCTCAAGTAGTCCACCCACCTTGGCCTTCCAAAGTGCCAGGATTACAGGTATGAGCCACTGCACCCGGC CCTGAGATGTATT
TTAAAGGCAGAGACAGCAGGACATATGGGGTGGATATTGATGATAAGAACAAAGGAAA AATTAAAGATGACACCTTGGTTTTTGGTTTGAGCTCAAATAGCTGATGGTGACATTTACTATGAGAAAGA
CTAGAAAAGGAACAGGCTTCGGGGACAAAAGTAGGAGGCTCTATTAGGTGTACTAAGTTTGAGTTG CCTAGTTACATCTAAGAGAAGGTGTCAAATAGGCCACTGGATATGTGAGTCTGGAGTAGCAA
ACAGAGCAGGGCTAGAGATAGAAGTGTGCAATCGTTGGCTGGGTGCGGTGGCTCACGCCTGTAATCCCAGCACTTTGGGAGGCCGAGGT GGGCAGATCACCTGAGGTCAGGAGTTTGAGAATAGCCTG
GCCAAgatggtgaaacccatctctaataaaaatacaaaaaactagccaggcatggtggcacgtggctgtaatcccagatactcgggaggctgaggcaggagaattgcttgaacctgtgaagcagaggt
tgcagtgagccaagatcacaccattgcactccaacctgggcaacaagagtgaaaatccatctcaaaaaaaaaaaaaaaaa ttcaatcaaaccatactctttgttttttttttctttgtgtttttgttttg
ttttgttttgagtgcaatcccagcactttgggaagccaaggcaggcagatcacttgaggctaggagttcgagaccagcctaggcaa CAtgGtGAAACCCTGTCTCTACTGAAAATACAAAATTAGCCG
GGTGTGGTGGTACATGCTTGTAATCCCAGCTACTCGGGAGGCTGAGGCAGGAGAATCGCTTGAATCCTGGAGGCAGAGG TTGCGGTGAGCCAAGATCACGCCATTGCACTCCAGCCTGGGCAACAAGA
GCGAAACTCCATCTCAAACAAAAACAAAAACAAAAACAAAAGAAGCGTGCAATTGTCGGGTGGTGTTTACAGCCCTAGAACTAGGTGGGACCATTGGGTAGAGTATATGCTTTGAGAAGGGAAAGGAC
TCATGATAGAGCCCTGAGGCATCCTAATATTTAGAGATTCATAGAGAGAATGAGAAGGGCTAGCCAGTGACAGTGGCCA AGGAGAAACATTAAGGCATATTTCTTTCATTAATAAAACTAAATTTTAA
AAAATCAAAGTTATACACATTCATAGTCTCACTGTGTTACCCAGACTGAAGTGCAGTGGCATGATCTGG GCTCACTGCAACCTCTACCTCCTGGGCTCAAGTGATGCTCCCACCTCAGCCTCCCAAGT
GGCTGGGACTACAGGCACACCACCATGTCTGGCTAATTTTTGTATTTTTTTTGGTACAGATGGAGTTCCACCATGTTGCCCAGGCTGGTTTCAAACTCCTGAGCTCAAGTGATCCACCTGCCTCGGCC
TCTTAGAGTGCTGGGATTACAGGCATGAGCCACCATGCCAGGCCCCATATTCTTAAAAATAAAATTGTTCTACAGGGCTTTTGCAATGAAAAGAACAGTTATCTGCTTCATCCTTCTCCATTACT CAA
TCCCGATGCCCAGAGGCAACTACTTTAAACTC TTTCAGTTGCTTCTTCTGTGTTTTCATTTGTTATTTCTTTTCTTCTTTTGCCATCTCTATAGAGATATCATGTCTCTATAGAGACATCCTCTTTAA
GTAAAGACTTACTCTTTACTCCTATCACTCATCCTCACACACACTTGCACATATTCACGCAAACACAGTTTCTCACTATCATTTCATTATAATTACATCACATATTTTGTTAAATCAATATATGG TAT
TAATTACTCTGACTTTGTAAATGTTATTGATAACTGAGCCGTATAGTATACTATGATT

Homologous Sequences
CAACATGGTGAAACCC

>NAHR_25_ Scaffold5954 2043283 2043584
KSD_NAHR_25

>Scaffold5954 2043283 2043584_NAHR
CTCATATATGGCAGGGACATTGGAATTATCAGACTGGGAATTTAAAACAAGTATGATTAACAGGTGAAGTGCTCTAATGGAAAAAGTAGACAACATGCAAAAACAGGCAATACAGGCTGAGAAGTG GA
AATCCTAAGAATGAAAAAGCCATGGGAGAGATTTGAAAAGCACTGTAACAGAAATGGAGAATGACTTTAATGGACAC tTGAGTAGACTGGGTGTGGCTGAGGAAAGACTCTGAGCCTGAAGACATGTA
ATGAAAACTTCTAGGTTGGGTGCGGTGGCTCATGCCTGAAATGCCAGCATTTTGGGAGGCTGGGGCGGGAGGATTGCTTGAGGCCAGGAGTTCAAGACCAGTCTGGGCAACATAGTGACACCCTGT CT
TTACAAAGTTTTTTTTGTTGTCGTTTTTTTTGTTTTTGAGACAGACTCTCCCTCTGTCGCCCAGGCTGGAGTGCGATGACACAATCTCAGTTCACTGCAACCTCTGCCTCTCGGGTTCAGGAGATTCT
CCCGCATCAGCCTCCCGAGCAGCTGGGATTACAGGCACCCGCCATCATGCCCAGCTAATTTTTTTGGTATTTTTGTAGAGACAGGGTTTCACCATGTTGGCCAGGCTGGTCTCAAACTCCTGACCT CA
GGTGATCCACCCACCTCGGCCTCCCAGAGTGCTGGGATTACAGGCATGAGCCACCGTGCCCGGCCACAAAAAGTTTTTAAAAATAAGCTGGGCTTGGAGGCATATACCCTGTGGTCCTAGCTA tTTGG
GAAGCTGAGGTAACAGGATGGCTTGAGCCCAGGAGATTAAGGCTGCAGTGAGCCATGATTGTACCACTGCACTCCAACCTGGCTGATAGAGCAAGACCCTGCCAAATAAATAAATAAATATCTCTA GG
GCTATCACTAAAAAAAGGAAAGAGAAGCATACCTGACTTGCTAAGAAAGGAGAAAAAATGGAATCACACAAAACATGCAATCAAAACCAAAAAAGGCAGAAAAAGAGTGGAAGACAAAATAAGGA ACA
ACGAACCAGGGCAACAAAAACAGAAAACAGTAACCAGGCCGGGCgCGGcGGCTCACGCCCGTCATCCCAGCACTTCCGGAGGCCGAGGCGGGCGGATCACGA gGTCAAGACTATCCTGGCTAACATGG
TGAAACCCCGTCTCTACTAAAAATgCAAAAAATTAGCCGGGCGcGGTGgCGGCGCCTGTAGTCCCAGCTACTCGGGAGGCTGAGGCAGGAGAATGGCGTGAACCCAGGAGGC gGAGCTTGCAGTGAG
CCGAGATCGCGCCACTGCACTCCAGCCTGGGCGACAGAGCGAGACTCTGTCT CaaAAAAAAAAAAAAAAAAACAAAAATAAATAAAATAAAATCTATTATAAGGAAAAAAAGTTAACATAGTATTATC
AAATGGAAATTCCACTCCTAAATATAcACTGAAGAAATTTGAAAACATTTAAAGAAACATTTGTACCCAAATGTTCACAGCAGCACTATTCACAATAGCCAAAAGGTGGAAACAACTCAAAGTTGATC
AAgTGATAAACAAAATGTGGTACAGCCCACACAGTAGATATGACTCAGCCATGTAAGGAATG aAGCTCTAACAGGCTAAAACATGAATGACCCTGAAAACATGCCCAGTGAGAGAAGCCGTCAGAAAA
GAACACATATTAGGATTCCATTCATATGAAATGTCAAAAGAACAAAAGAATAAAAAG GaAAAAAAAGAGAAACAACATACGAAATGTCAAGAACAGCCAAATCTCTAGAGACAGAGGCAGATGAGTCA
TTTCTTAGGGTGGTGGAGGGGCgCCGGGGCAATAGGAGAGTGATAACTGAAGGGTCTGGGGGTTTCTTCGTGAGGTGATGGAAATGTCCTCAAGTTGACTGTGGTCTTGGTTGCAGGATTCTGTGACT
GTAAAAACCAATGGCCGGGCGCAGTGGCTCACACCTGTCATCCCAGCACTTTGGGAGGCC GAGGAGGGCGGATCACGAGGTCAGGAGGTCGAGATCATCCTAGCTATCTCTACTAAAAATGCAAAAAA
TTAGCCGGGCGTGGTGACGGGTGCCTGTAGTCCCAGCTACTGGGGAGGCGGAGCTTCGAGTG tGCGCTACTGCACTCCAGCCTGGGCGACAGAGCAAGACTCCGTCT CaaaAAAAAAAAAAAAAAAAA
CCAAAAAAATAACAACAACAATGAAATGTGCACGTTAAATGGCTGTACGTTCTATATGTC AGTAAAGCTGGTTTTTAAAAAACATAAAGAATGTGAAGTTCACTGTCTTCCTCAAAAACATGAT A

1363	17.2	4.1	0.7	45673	1	269	(2032)	+	L1MCa	LINE/L1	864	1141	(5367)	1	
1720	17.1	0.7	3.0	45673	270	392	(1909)	+	AluJr	SINE/Alu	1	122	(190)	2	
2309	10.5	0.3	1.0	45673	393	705	(1596)	C	AluSx1	SINE/Alu	(1)	311	1	3	
1720	17.1	0.7	3.0	45673	706	886	(1415)	+	AluJr	SINE/Alu	123	297	(15)	2	
792	13.2	5.3	1.1	45673	890	1058	(1243)	+	L1MCa	LINE/L1	2105	2280	(4228)	1	
2573	4.3	2.6	0.0	45673	1061	1362	(939)	+	AluY	SINE/Alu	1	310	(1)	4	
1521	19.0	6.4	11.8	45673	1387	1932	(369)	+	L1MB7	LINE/L1	5593	6110	(74)	5	
1575	9.0	18.4	0.0	45673	1933	2176	(125)	+	AluYc	SINE/Alu	1	289	(10)	6	
600	22.4	4.7	12.5	45673	2177	2258	(43)	+	L1MB7	LINE/L1	6099	6178	(0)	5	

1384	16.9	4.1	0.7	hg19	1	269	(3349)	+	L1MCa	LINE/L1	864	1141	(5367)	7	
1735	16.8	0.7	3.0	hg19	270	392	(3226)	+	AluJr	SINE/Alu	1	122	(190)	8	
2318	10.5	0.3	1.0	hg19	393	706	(2912)	C	AluSx1	SINE/Alu	(0)	312	1	9	
1735	16.8	0.7	3.0	hg19	707	887	(2731)	+	AluJr	SINE/Alu	123	297	(15)	8	
792	13.2	5.3	1.1	hg19	891	1059	(2559)	+	L1MCa	LINE/L1	2105	2280	(4228)	7	
1023	8.4	0.0	0.0	hg19	1062	1192	(2426)	+	AluY	SINE/Alu	1	131	(180)	10	
2429	9.7	0.0	0.0	hg19	1196	1506	(2112)	+	AluY	SINE/Alu	1	311	(0)	11	
1021	18.2	9.3	0.0	hg19	1511	1735	(1883)	+	L1MCa	LINE/L1	2745	2990	(3331)	7	*
2115	17.8	3.1	0.7	hg19	1715	2127	(1491)	+	L1MCa	LINE/L1	4550	4972	(1349)	7	
895	18.0	0.7	0.0	hg19	2128	2277	(1341)	+	AluJo	SINE/Alu	1	151	(161)	12	
574	9.2	7.5	0.0	hg19	2279	2383	(1235)	+	L1MC	LINE/L1	5329	5446	(708)	13	
2495	5.3	2.7	0.0	hg19	2384	2683	(935)	+	AluY	SINE/Alu	1	308	(3)	14	
1546	19.2	6.4	11.6	hg19	2708	3252	(366)	+	L1MB7	LINE/L1	5593	6110	(74)	15	

```
1563    8.7 18.7  0.0  hg19       3253  3493  (125) + AluYc    SINE/Alu        1    286   (13)  16
 605   22.9  4.7 12.5  hg19       3494  3575   (43) + L1MB7    LINE/L1      6099   6178    (0)  15
```

>hg19 chr19:2908335-2911952

CTCATATATGGCAGGGACATTGGAATTATCAGACTGGGAATTTAAAACAAGTATGATTAACAGGTGAAGTGCTCTAATGGAAAAAGTAGACAACATGCAAAAACAGG CAATACAGGCTGAGAAGTGGA
AATCCTAAGAATGAAAAAGCCATGGGAGAGATTTGAAAAGCACTGTAACAGAAATGGAGAATGACTTTAATGGACAC aTGAGTAGACTGGGTGTGGCTGAGGAAAGACTCTGAGCCTGAAGACATGTA
ATGAAAACTTCTAGGTTGGGTGCGGTGGCTCATGCCTGAAATGCCAGCATTTTGGGAGGCTGGGGCGGGAGGATTGCTTGAGGCCAGGAGTTCAAGACCAGTCTGGGCAACATAGTGACACCCTG TCT
TTACAAAGTTTTTTTTGTTGTCGtTTTTTTTTGTTTTTGAGACAGACTCTCCCTCTGTCGCCCAGGCTGGAGTGCGATGACACAATCTCAGTTCACTGCAACCTCTGCCTCTCGGGTTCAGGAGATTC
TCCCGCATCAGCCTCCCGAGCAGCTGGGATTACAGGCACCCGCCATCATGCCCAGCTAATTTTTTTGGTATTTTTGTAGAGACAGGGTTTCACCATGTTGGCCAGGCTGGTCTCAAACTCCTGAC CTC
AGGTGATCCACCCACCTCGGCCTCCCAGAGTGCTGGGATTACAGGCATGAGCCACCGTGCCCGGCCACAAAAAGTTTTTAAAAATAAGCTGGGCTTGGAGGCATATACCCTGTGGTCCTAGCTA cTTG
GGAAGCTGAGGTAACAGGATGGCTTGAGCCCAGGAGATTAAGGCTGCAGTGAGCCATGATTGTACCACTGCACTCCAACCTGGCTGATAGAGCAAGACCCTGCCAAATAAATAAATAAATATCTC TAG
GGCTATCACTAAAAAAAGGAAAGAGAAGCATACCTGACTTGCTAAGAAAGGAGAAAAAATGGAATCACACAAAACATGCAATCAAAACCAAAAAAGGCAGAAAAAGAGTGGAAGACAAAATAAGG AAC
AACGAACCAGGGCAACAAAAACAGAAAACAGTAACCAGGCCGGGCcCGGtGGCTCACGCCCGTCATCCCAGCACTTCCGGAGGCCGAGGCGGGCGGATCACGA cgtcaggagatggagaccatcctgg
ccaacacagggaaaccccgtctctactaaaaatataaaaattaggccaggcgcattggctcacgcctgtaaccccaacacttcgggaggccgaggcgggcagatcacgatgtcaggagattgagacca
tcgtggctaacatggtgaaaccctgtctctactaaaaatacaaaaaattagctgggcatggcggggagtacctgtagtcccagctgctggggaggctgaggca ggagaatggcgtgaacccaggaggc
ggcgcttgcagtgagcagagatcgcgccactgcactccagcctgggaaacagagtgagactctgtctcaaaaaaaaaaaaaaaaaaaaaaagaaaagaaaagaaacagatcaagcacgtggaacatc aat
aagcacagagttgaattcaacagcacccccaatgatgaatgtctgtagacaacttcaacaacagcagaaaacacattcttgtcaagctcacgtggaacactcc tcaacctagactgtattttggaca
taaaacaccttaacaagtttaaaagaatatcgtacatcatatgctcttagacaatactggaattaaatcagcattgccctaaaataaaacatacaggtataaatctaacaaaatgtgcacaagat ctt
aggaaaattgtaaaactctgacagaagaaatcaaagaactaagtgaatagttattccatgtccacgtatagaagacccaatattgtcaagacattggttcttc ccaacttgatctatagattcaacac
aatcccaatcaaaatcccagcaagttatttggtggatattgacaaactgattgtaaagtttatatggagaggtaaaagactcaaaataccaacacagtattgaaggggaaggttcaagttggaag act
gacactatgcattttccaggctttctggagagctgtagtcatcaggagagtgcgatgtagagtaaagcatgtacaaataggccaggtgtggtggcacatgcct gtaatcccagcatgttgggaggcca
aggtgggaggattgcttgagctcaggagtttcacatcaccctgggcaacacagtgagaccccatttctaaaattaagaaaaaattagccaggtgtggaggctaaagaccttaagacaactcatct gtg
aagatatagatggcagatgagtatataaaaaatgctccacatcatgtcatcagggaaatgcaaattaaaacaagatacc ggccaggcacagaggctcatgcctgtaatcctagcactttgggaggccg
aggcgtgcggatcacgacGTCAAGACTATCCTGGCTAACATGGTGAAACCCCGTCTCTACTAAAAAT aCAAAAAATTAGCCGGGCGtGGTGtCGGCGCCTGTAGTCCCAGCTACTCGGGAGGCTGAG
GCAGGAGAATGGCGTGAACCCAGGAGGC aGAGCTTGCAGTGAGCCGAGATCGCGCCACTGCACTCCAGCCTGGGCGACAGAGCGAGACTCTGTCT CAAAAAAAAAAAAAAAAAACAAAAATAAATAAAA
TAAAATCTATTATAAGGAAAAAAAGTTAACATAGTATTATCAAATGGAAATTCCACTCCTAAATATA tACTGAAGAAATTTGAAAACATTTAAAGAAACATTTGTACCCAAATGTTCACAGCAGCACT
ATTCACAATAGCCAAAAGGTGGAAACAACTCAAAGTTGATCAA cTGATAAACAAAATGTGGTACAGCCCACACAGTAGATATGACTCAGCCATGTAAGGAATGgAGCTCTAACAGGCTAAAACATGAA
TGACCCTGAAAACATGCCCAGTGAGAGAAGCCGTCAGAAAAGAACACATATTAGGATTCCATTCATATGAAATGTCAAAAGAACAAAAGAATAAAAAG GAAAAAAAGAGAAACAACATACGAAATGTC
AAGAACAGCCAAATCTCTAGAGACAGAGGCAGATGAGTCATTTCTTAGGGTGGTGGAGGGGC tCCGGGGCAATAGGAGAGTGATAACTGAAGGGTCTGGGGGTTTCTTCGTGAGGTGATGGAAATGTC
CTCAAGTTGACTGTGGTCTTGGTTGCAGGATTCTGTGACTGTAAAAACCAATGGCCGGGCGCAGTGGCTCACACCTGTCATCCCAGCACTTTGGGAGGCCGAGGAGGGCGGATCACGAGGTCAGG AGG
TCGAGATCATCCTAGCTATCTCTACTAAAAATGCAAAAAATTAGCCGGGCGTGGTGACGGGTGCCTGTAGTCCCAGCTACTGGGGAGGCGGAGCTTCGAGTG cGCGCTACTGCACTCCAGCCTGGGCG
ACAGAGCAAGACTCCGTCTCAAAAAAAAAAAAAAAAAACCAAAAAAATAACAACAACAATGAAATGTGCACGTTAAATGGCTGTACGTTCTATATGTCAGTAAAGCTGGTTTTTAAAAAACATAAAGAA
TGTGAAGTTCACTGTCTTCCTCAAAAACATGATA

Homologous Sequences

GCGGATCACGACGTCA

>NAHR_26_ Scaffold5954 2874436 2874742
>KSD_NAHR_26

>Scaffold5954 2874436 2874742_ NAHR
ATCTGTAAAACGGGGATCCCGGTTTTGACCCTGAGCAGTATTTCAGGGAAGGCTCAAAGGTTTAAGCATCTGCCACTAAGGATCCCCTGGGCTGCCTCTCCATGCTTCTAACTCCACTCGGCCCC CA
GTGGGAAAGCCAAAGGTGAATTACAGTGTCCCAGGGCTAGAGGGGGCCCCCTGCCCCATGGAAACCATCTACGCTAAGGTGCTGTCATTCTGGGCACGGAATGACATTTTTGAGATGGAGTCTTG CTC
TGTCTCTGGGGCTGGAGTGCAGTGGTGCGATCCCGGCTCACTGCAACCTCTGCCTCCCAGGTTCAAGCAATTCTCCTGCCTCAGCCTCCCAAGTAGCTGGAATTATAGGCACACGCCAACACACCT GG
CTAATTTTTGTATTTTTAGTAGAGACAGAGTTTCACCACGTTTGCCAGGCTGGTCTCAAACTCCTGGGCTCAAGTGACCCACCTGCCTTGGCCTCCCAAAATGCTGGGATTACAGGCGTGAGCCA CCG
TGTCTGGTCCAGAGGAGAACTGAAAGAACACACACACACAGGCAATCAAGAATGCTGAGGCCGGGTCGGTGGCTCTCGCTTGAAATCCCAGCACTTTGAGTGGCTGAGGC gGGCAGATCACTTGAaCC
CAGAAGCTCGAGACCAGGCCGGCCAACACAGCAAAACCCCATTTCTATTTTCAATTTTTAAAAAAAAGAAAGAAAGGAAACAAATTAATTCCCCTCTGTATAGGCTATAAAGTCTTACCGGTTCC CAT
ATTAATTAATGAATTAAGtAGACACATAAAGCACCtttctTTTTTTTTTTTTTTTTGTACTTTTAGTAGGACAGGGTTTCACCATGTTAGCCAGGCTGGTCTTGAACTCCTGgCCTCGTaATCCgCCCG
TCTCGGCCTCCCAAAGTGCTGGGATTACAGGCGTGAGCCACCGCATCCGGCCTCATTTTGTATTTGTATGAGAGTAATGATTTTACTATTTTGGGAAAAAATATCCTTTAATATCATAAAGCCAA ATG
TCTTCAGGCCAGCATTTAAAGCTCTCCCTTCATTTTGGCCGGACGCGGTGGCTCACGCCTGTAATCCCAaCACTTTGGGAGGtCGAGGCGAGCGAATCACCTGAGATCAGGAGTTCGAGACCAGCCTG
ACCAATAtGGcGAAACCCTGTCTCTACTAAAAATACAAAAATTAGCTGGGCGTGGT GGCGGGCGCCTGTAGTCCCAGCTACTCGGGAGGCTGAGGCAGGAGTATGGCGTGAACCCGGGAGGCGGAGCT
TGCAGTGAGCCGAGATCGCGCCACCGCACTCCAGCCTGGGCGACAGAGCGAGACTCCGTTTCAAAAAAATATAAAAAGTAAAATAAA GTTACAAAGCGCCTCTCACGCATGTTACCCTCTGTGCAGCC
ATTGGCTCCATTACTGCGCTCTTGTTTCATGTCTGTCTTTCCCCACCAAAATATGCGCCCCACGAGAGCAGGAAGCTTGGCCTGCCTTGCTCACTGCTGAATCTCCAAGGCCTGGTGACCCCACA CTC
GCGCATACAGTAAGTGCACAATAAATGCCCGTTCGCTCCCTGGGCGACCCCTCCCCCCAGCCTAGGCTTCCGCTAACCTCAATCCATTCCAAAGGGTGTCAGGTTCCACCCTGAGCAAACACAGCT CC
CCTGGCCCCTGGCCAGCCCTAAAGAGGAAGAACAAGTACCCATACTCCCTGCCCTGGACTTTCCCC aGGACTCAGGGCCACGAGGAAGCACCAAGCCAGGGGGCAGGACTTTCTCGAGATGCTGAGCC
TGAGGCATCAGCTGGGGGCAGGGCCAGCCTCCAGAGCACCACCCCCGCCACCAGCTCCCTGTCCT tTGCGTCTTGACTCTTGGCCGCCTGACATTTCTACTTGGCTGTGCGGCCCGGGGTAAG tGGCT
GTCCTTCTCTGAGCCAGGGTTCATCCTCTACAAACCAGGAAGCTGATGAGACCAGAggaCTgCGATTCGGACCGGGATAGCTCGTCTTGACCCCAGAATGACCGAGAATCTGTTACCCGCTTTATTAC
ATCCGGCACTCAACAGCCCTGgGgCTTGGTGACTCCATATCCCCCCcCCCCTTTTTTTTTTTTTTTTTGAGACAGAGTCTCACTCTGTTGTCCAGGCTGGAGTGCAGTGGCACAATCTCGGCTCACTG
CAACCTCCACCTCCTGGGTTCAAGCAATTCTCCTGCCTCAGCCTCCGGAGTAGCTGGGATTACAAGTGCT cGCCACCATATTCGGCTCATTTTTTGTATTTTTAGTAGAGACAGGGTTCCACCATGTT
GG

2170	12.2	0.0	0.0	a	235	521	(1785)	C	AluSz	SINE/Alu	(25)	287	1	1
723	16.0	0.7	7.4	a	571	714	(1592)	+	FLAM_C	SINE/Alu	1	135	(8)	2
312	23.4	0.0	0.0	a	727	790	(1516)	+	LTR37A	LTR/ERV1	289	352	(74)	3
1079	6.9	0.8	0.0	a	819	948	(1358)	C	AluSx3	SINE/Alu	(180)	131	1	4
288	13.6	0.0	3.4	a	949	1009	(1297)	+	LTR37A	LTR/ERV1	368	426	(0)	3
2497	7.5	0.0	0.0	a	1061	1367	(939)	+	AluSx1	SINE/Alu	1	307	(5)	5
428	28.7	0.0	2.9	a	1430	1569	(737)	+	L2a	LINE/L2	3279	3414	(12)	6
1630	10.1	0.0	0.0	a	2100	2306	(0)	C	AluSx3	SINE/Alu	(13)	298	92	7

2170	12.2	0.0	0.0	hg	235	521	(2075)	C	AluSz	SINE/Alu	(25)	287	1	8
712	16.0	0.7	7.4	hg	571	714	(1882)	+	FLAM_C	SINE/Alu	1	135	(8)	9
297	22.0	0.0	0.0	hg	727	785	(1811)	+	LTR37A	LTR/ERV1	289	347	(79)	10
1135	6.1	0.0	0.0	hg	814	944	(1652)	C	AluSx3	SINE/Alu	(180)	131	1	11
288	13.6	0.0	3.4	hg	945	1005	(1591)	+	LTR37A	LTR/ERV1	368	426	(0)	10
2459	7.1	0.0	0.3	hg	1057	1353	(1243)	+	AluSx1	SINE/Alu	1	296	(16)	12
2507	5.6	1.3	0.0	hg	1357	1658	(938)	+	AluY	SINE/Alu	1	306	(5)	13
428	28.7	0.0	2.9	hg	1721	1860	(736)	+	L2a	LINE/L2	3279	3414	(12)	14
1633	10.5	0.0	0.0	hg	2388	2596	(0)	C	AluSx3	SINE/Alu	(11)	300	92	15

>hg19 chr19:3709957-3712552
ATCTGTAAAACGGGGATCCCGGTTTTGACCCTGAGCAGTATTTCAGGGAAGGCTCAAAGGTTTAAGCATCTGCCACTAAGGATCCCCTGGGCTGCCTCTCCATGCTTCTAACTCCACTCGGCCCC CA
GTGGGAAAGCCAAAGGTGAATTACAGTGTCCCAGGGCTAGAGGGGGCCCCCTGCCCCATGGAA ACCATCTACGCTAAGGTGCTGTCATTCTGGGCACGGAATGACATTTTTGAGATGGAGTCTTGCTC
TGTCTCTGGGGCTGGAGTGCAGTGGTGCGATCCCGGCTCACTGCAACCTCTGCCTCCCAGGTTCAAGCAATTCTCCTGCCTCAGCCTCCCAAGTAGCTGGAATTATAGGCACACGCCAACACACCT GG

CTAATTTTTGTATTTTTAGTAGAGACAGAGTTTCACCACGTTTGCCAGGCTGGTCTCAAACTCCTGGGCTCAAGTGACCCACCTGCCTTGGCCTCCCAAAATGCTGGGATTACAGGCGTGAGCCA CCG
TGTCTGGTCCAGAGGAGAACTGAAAGAACACACACACACAGGCAATCAAGAATGCTGAGGCCGGGTCGGTGGCTCTCGCTTGAAATCCCAGCACTTTGAGTGGCTGAGGC tGGCAGATCACTTGAgCC
CAGAAGCTCGAGACCAGGCCGGCCAACACAGCAAAACCCCATTTCTATTTTCAATTTTTAAAAAAAAGAAAGAAAGGAAACAAATTAATTCCCCTCTGTATAGGCTATAAAGTCTTACCGGTTCC CAT
ATTAATTAATGAATTAAGcAGACACATAAAGCACCTTTTTTTTTTTTTTTGTACTTTTAGTAGaGACAGGGTTTCACCATGTTAGCCAGGCTGGTCTTGAACTCCTGaCCTCGTgATCCaCCCGTCTC
GGCCTCCCAAAGTGCTGGGATTACAGGCGTGAGCCACCGCATCCGGCCTCATTTTGTATTTGTATGAGAGTAATGATTTTACTATTTTGGGAAAAAATATCCTTTAATATCATAAAGCCAAATGT CTT
CAGGCCAGCATTTAAAGCTCTCCCTTCATTTTGGCCGGACGCGGTGGCTCACGCCTGTAATCCCAgCACTTTGGGAGGcCGAGGCGAGCGAATCACCTGAGATCAGGAGTTCGAGACCAGCCTGACCA
ATAcGGtGAAACCCTGTCTCTACTAAAAATACAAAAATTAGCTGGGCGTGGTGgtgggcgcctgtaatcccagctacttgggaggctgagacaggagaatcgctttgaacccaggaggtggaggttgc
agcgagcggagatcgttccactgcactccagcctgggcaacagagcgagactccatctcaaaaaaaaataaaataaggccgggcatggtggctcacgcctgtaatcccagcactttgggaggctgagg
tgggcggatcacaaggtcaggagagaccatcttggctaccacggtgaaaccccgtctgtactaaaaatacaaaaaattagccgggtgtggtgGCGGGCGCCTGTAGTCCCAGCTACTCGGGAGGCTGA
GGCAGGAGTATGGCGTGAACCCGGGAGGCGGAGCTTGCAGTGAGCCGAGATCGCGCCACCGCACTCCAGCCTGGGCGACAGAGCGAGACTCCGTTTCAAAAAAATATAAAAAGTAAAATAAA GTTACA
AAGCGCCTCTCACGCATGTTACCCTCTGTGCAGCCATTGGCTCCATTACTGCG CTCTTGTTTCATGTCTGTCTTTCCCCACCAAAATATGCGCCCCACGAGAGCAGGAAGCTTGGCCTGCCTTGCTCA
CTGCTGAATCTCCAAGGCCTGGTGACCCCACACTCGCGCATACAGTAAGTGCACAATAAATGCCCGTTCGCTCCCTGGGCGACCCCTCCCCCCAGCCTAGGCTTCCGCTAACCTCAATCCATTCC AAA
GGGTGTCAGGTTCCACCCTGAGCAAACACAGCTCCCCTGGCCCCTGGCCAGCC CTAAAGAGGAAGAACAAGTACCCATACTCCCTGCCCTGGACTTTCCCCgGGACTCAGGGCCACGAGGAAGCACCA
AGCCAGGGGGCAGGACTTTCTCGAGATGCTGAGCCTGAGGCATCAGCTGGGGGCAGGGCCAGCCTCCAGAGCACCACCCCCGCCACCAGCTCCCTGTCCT cTGCGTCTTGACTCTTGGCCGCCTGACA
TTTCTACTTGGCTGTGCGGCCCGGGGTAAGcGGCTGTCCTTCTCTGAGCCAGGGTTCATCCTCTACAAACCAGGAAGCTGATGAGACCAG ACTcCGATTCGGACCGGGATAGCTCGTCTTGACCCCAG
AATGACCGAGAATCTGTTACCCGCTTTATTACATCCGGCACTCAACAGCCCTG tGcCTTGGTGACTCCATATCCCCCCaCCCCttTTTTTTTTTTTTTTTTTTGAGACAGAGTCTCACTCTGTTGTCCA
GGCTGGAGTGCAGTGGCACAATCTCGGCTCACTGCAACCTCCACCTCCTGGGTTCAAGCAATTCTCCTGCCTCAGCCTCCGGAGTAGCTGGGATTACAAGTGCT tGCCACCATATTCGGCTCATTTTT
TGTATTTTTAGTAGAGACAGGGTTCCACCATGTTGG

Homologous Sequences

GTGGTGG

>NAHR_27_Scaffold5954 4053811 4054119
KSD_NAHR_27

>Scaffold5954 4053811 4054119_NAHR

TCCCAAAGTGCTGGGCAACAGATCGAGATCCTGTCTGCAAGAAAACAGTACAACACAAAAAAACTAAGTGTCTTGGCTGGGTGCGGTGGCTCACACCTGTAATCCCAGCACTCTGGGAGGCCGAGG CC
TGCAGACCATGTGAGGTCGGGAGTTTGAGACCAGCCTGACCAACATGGAGAAACCCCGTTTCTACTAAAAATACAAAATTAGCCTGGTGTGGTGGTGCATGCCTGTAATCCCAGCTACTCGCAAG GCT
GAGGCAGGAGAATTGCTTGAACCCAGGAGGCAGAGGTTCTGGTGAGCCGAGATCATGCCATTGCACTCCAACCTGGGCAACAAGAGTGAAACTCCTTCTCAAAAACAAAAACAAAAAAGACTAGGC GT
GGTGGCTCATGCCTGTAATCCCAACACTTTGGGAGGCTAAGGCAGGTGGATCACAAGGTCAGGAGATTGGGACCATCCTGGCCAACATGGTGAAACCCTGTCTCTACTGAAAATACAAAAATTAG CTG
GGTGTGGTGGCAGGTGCCTGTGATCCCAACTACTAGGGAGGCTGAGGTAGGAGAATCGCTTGAACCCGGGAGGCGGAGGTTGCAGTGAG cCGAGATTTTACTACTGCACTCCAGCCTGGCAACACAGC
AAGACTCTGTCTTAAATAAATAAATCAATAAAGTGTCTTAGTTGGTCCCACCCTATGGAGAGGCTGGGT cTTCCaGAATCTGCTGGAATGTGGCAGGGGtCACTAAATGCAAATGTGTCATGGCTGCA
GGGTTGACAGACACCCTGCAGCAAGAGGGAGGGAGGTACCAGTATGTTGCAGTTCGAAGGTGGCTGGAGAAGTATTTCAGGGCCATTCCCCCAAGAAGACAAACCCTGGACCAGTCTTTTTAAAAT TT
AATTTAATTTAATTTTAGTATTATAAAGAAAAATTAAAAAACGGGCCGGGCcTGGTGGCTCAcGCCTGTAATCCCAGCACTTTGGGAGGtCGAGGCGGGtGGATCACGAGGTCGGGAGATCGAGACtA
TCCTGGtTAACAtGGTGAAACCCCGTCTCTACTAAAAATACAAAAATTAGC gaGaCATGGTGGCActCGCCTaTAATCCCAGCTACTCaGGAGGCTGAGaCAGGAGAATtGCTTGAACCtGGGAGGCG
GAGGTTGCAGTGAGCCGAGATCCCGCCACTGCACTCCAGCCTGTACGACAGAGCAAGACTCCGTCTCAAAAAAAAAAAGACAAAGAAAAA TTAAAAACTAGACACAGGGTCTCCCTATGTTGGCCAGGC
TGGTCTCAAACTCCTAACCTCAAAGACATCTGCCCACCTTGGCCTCCCAAAGTGCTGGGATTACAGACACGAGCCACAGCGCCCAGCCTCAGCCGCTGTTTCATTTCAGCCATGCAGGGCCTTGC AGG
CAAAGCCAGGCCTTTTGGATTTTACTTCAAATGCTGTAGGGACCTGATGGAGATTTTCAGTGAGGGAACAGGATGGCCTTTTT gATGCTGAGAGTTCTTTTGTTGCTCCAGGAATACAATTGACAGGT
AAAATTCAGGATGCCCAATTAAATCAGAATTGCAGATAAACAATACATTTTTTTGAGACAGAGTTTTGTGCACTGTCTCTCTCTCTCTCTCTCTCTC TaTATATATATATATATTTTTTTTTTT
TTTGAGAAGCTAATTTTTGTATTTTTAGTAGAGACAGGGTTTCGCCATGTTGGCCAGGCTGGTCTCACACTCCTGACCTCAAGTGATCC GCCCACCTCGGCCTCCCAAAGTGCTGGGCGGCAGATCGA
GATCCTGTTTCTAAGAAAACTAAAACAGTACAACACA

2282	10.3	0.3	0.0	a	75	374	(1934)	+	AluSp	SINE/Alu	1	301	(12)	1
2139	13.1	0.0	0.0	a	375	664	(1644)	+	AluSc	SINE/Alu	1	290	(19)	2
14	22.9	2.1	2.1	a	888	935	(1373)	+	A-rich	Low_complexity	1	48	(0)	3
2413	8.4	0.3	0.7	a	940	1248	(1060)	+	AluSc8	SINE/Alu	1	308	(3)	4
795	14.3	0.0	0.8	a	1250	1368	(940)	C	AluSz	SINE/Alu	(194)	118	1	5
185	35.4	2.1	1.0	a	1391	1487	(821)	C	L2	LINE/L2	(503)	2916	2819	6
311	17.5	3.2	0.0	a	1528	1590	(718)	C	MER53	DNA/hAT	(5)	188	124	7
29	0.0	0.0	0.0	a	1613	1637	(671)	+	(TC)n	Simple_repeat	1	25	(0)	8
954	4.6	0.0	0.0	a	2152	2260	(48)	C	AluSz	SINE/Alu	(174)	138	30	9

2282	10.3	0.3	0.0	hg	75	374	(2469)	+	AluSp	SINE/Alu	1	301	(12)	10
2124	13.4	0.0	0.0	hg	375	664	(2179)	+	AluSc	SINE/Alu	1	290	(19)	11
14	22.9	2.1	2.1	hg	888	935	(1908)	+	A-rich	Low_complexity	1	48	(0)	12
2407	7.2	0.0	0.0	hg	940	1229	(1614)	+	AluSc	SINE/Alu	1	290	(19)	13
2528	6.4	0.0	1.3	hg	1501	1812	(1031)	+	AluY	SINE/Alu	1	308	(3)	14
795	14.3	0.0	0.8	hg	1814	1932	(911)	C	AluSz	SINE/Alu	(194)	118	1	15
185	35.4	2.1	1.0	hg	1955	2051	(792)	C	L2	LINE/L2	(503)	2916	2819	16
311	17.5	3.2	0.0	hg	2092	2154	(689)	C	MER53	DNA/hAT	(5)	188	124	17
46	0.0	0.0	0.0	hg	2177	2215	(628)	+	(TC)n	Simple_repeat	1	39	(0)	18
2354	8.7	0.0	0.3	hg	2229	2526	(317)	C	AluSq2	SINE/Alu	(15)	298	2	19
2242	7.8	0.0	0.0	hg	2528	2795	(48)	C	AluSz	SINE/Alu	(15)	297	30	20

>hg19 chr19:4884495-4887337

TCCCAAAGTGCTGGGCAACAGATCGAGATCCTGTCTGCAAGAAAACAGTACAACACAAAAAAACTAAGTGTCTTGGCTGGGTGCGGTGGCTCACACCTGTAATCCCAGCACTCTGGGAGGCCGAGG CC
TGCAGACCATGTGAGGTCGGGAGTTTGAGACCAGCCTGACCAACATGGAGAAACCCCGTTTCTACTAAAAATACAAAATTAGCCTGGTGTGGTGGTGCATGCCTGTAATCCCAGCTACTCGCAAG GCT
GAGGCAGGAGAATTGCTTGAACCCAGGAGGCAGAGGTTCTGGTGAGCCGAGATCATGCCATTGCACTCCAACCTGGGCAACAAGAGTGAAACTCCTTCTCAAAAACAAAAACAAAAAAGACTAGGC GT

GGTGGCTCATGCCTGTAATCCCAACACTTTGGGAGGCTAAGGCAGGTGGATCACAAGGTCAGGAGATTGGGACCATCCTGGCCAACATGGTGAAACCCTGTCTCTACTGAAAATACAAAAATTAG CTG
GGTGTGGTGGCAGGTGCCTGTGATCCCAACTACTAGGGAGGCTGAGGTAGGAGAATCGCTTGAACCCGGGAGGCGGAGGTTGCAGTGAG tCGAGATTTTACTACTGCACTCCAGCCTGGCAACACAGC
AAGACTCTGTCTTAAATAAATAAATCAATAAAGTGTCTTA GTTGGTCCCACCCTATGGAGAGGCTGGGTtTTCCgGAATCTGCTGGAATGTGGCAGGGGcCACTAAATGCAAATGTGTCATGGCTGCA
GGGTTGACAGACACCCTGCAGCAAGAGGGAGGGAGGTACCAGTATGTTGCAGTTCGAAGGTGGCTGGAGAAGTATTTCAGGGCCATTCCCCCAAGAAGACAAACCCTGGACCAGTCTTTTTAAAAT TT
AATTTAATTTAATTTTAGTATTATAAAGAAAAATTAAAAAACG GGCCGGGCgTGGTGGCTCAtGCCTGTAATCCCAGCACTTTGGGAGGcCGAGGCGGGcGGATCACGAGGTCGGGAGATCGAGACcA
TCCTGGcTAACAcGGTGAAACCCCGTCTCTACTAAAAATACAAAAATTAGC cgGgCATGGTGGCAggCGCCTgTAATCCCAGCTACTCgGGAGGCTGAGgCAGGAGAATcGCTTGAACCcGGGAGGCG
GAGGttgcagtgagccgacattgcactactgcactccagcctggcaacacagcaagactctgtcttaaataaataaa tcaataaagtgtcttagttggtcccaccctatggagaggctgggttttccg
gaatctgctggaatgtggcaggggtcactaaatgcaaatgtgtcatggctgcagggttgacagacaccctgcagcaagagggagggaggtaccagtatgttgcagttcgaaggtggctggagaag tat
ttcagggccattcccccaagaagacaaaccctggatcagtctttttaaaatttaatttaattttagtattataaagaaaaatttaaaaaaca ggccgggcgtggtggctcatgcctgtaatcccagca
ctttgggaggccgaggcgggcggatcacgaggtcgggagatcgagaccatcctggctaacatggtgaaaccccgtctctactaaaaatacaaaaaaaattagccaggcgtggcggcggacaccta tag
tcccagctattcgggagactgaggcaggagaatggtgtgaaaccgggaggcggagc TTGCAGTGAGCCGAGATCCCGCCACTGCACTCCAGCCTGTACGACAGAGCAAGACTCCGTCTCAAAAAAAA A
AGACAAAGAAAAATTAAAAACTAGACACAGGGTCTCCCTATGTTGGCCAGGCTGGTCTCAAACTCCTAA CCTCAAAGACATCTGCCCACCTTGGCCTCCCAAAGTGCTGGGATTACAGACACGAGCCA
CAGCGCCCAGCCTCAGCCGCTGTTTCATTTCAGCCATGCAGGGCCTTGCAGGCAAAGCCAGGCCTTTTGGATTTTACTTCAAATGCTGTAGGGACCTGATGGAGATTTTCAGTGAGGGAACAGGA TGG
CCTTTTTcATGCTGAGAGTTCTTTTGTTGCTCCAGGAATACAATTGACAGGTAAAATTCAGGATGCCCAATTAAATCAGAATTGCAGAT AAACAATACATTTTTTTTGAGACAGAGTTTTGTGCACTG
TCTCTCTCTCTCTCTCTCTCTCTCT

Homologous Sequences = GGGAGGCGGAGGTTGCAGTGAGCCGA

>NAHR_28_Scaffold1016 4524114 4524424
KSD_NAHR_28

>**Scaffold1016 4524114 4524424_**NAHR
TGGGCAAGATGACAAAACCCTGTCTCTACTAAAAATACAAAAATTAGCCAGGTGTGGTGGCGCAAGTGTATAGTCCCAGCTATTTGTGGGGCCAAGGCAGGAGAATTGCTTGAGCCCAGGAGGTTG AG
GCTGCACTGTGTCGTGTTCATGCCACTGAACTCCAGCCTGGGTGACAAAGTAAGACCCTGTCTCAAAAAAATGGAAAAGGAAAAGGAAAAGGAAAGAAAAAAAGTGATAGCTGGCTAAGAATTTCT TAA
ACATAAATAGAGATCAAGTCAAAGATCCAAGAAGCTCAGCGAAACCTAAACAAAATAAATACCAAAAAAGCAAAACAAAACAACAAAACCCACCACACTCAGGCACATCATTTTCAAACTGCTGAA AA
CCAAAGCAAATATTGGTGGCAGACAGAGAAAAATGACATATAGTATGCAAAGGAACAGAGGCAAGTTCAGCCAATGTCTCATTAGAAACCATGCAAGCCACACTCGAGTAACCTCTTTAAGGGCT GGG
GGGTGGGGAACTGCCAACCCAGAATTCTATGTCCCTGGAAGTAAGTATCTTTGAAAAATTAAGACAAAACAACTTTTTCAAACAAACAAAAGCTGAGAGAATTCATTATCAGCAGCCTTGTGCTAC CA
AAAAAAGAGTTATTGGAAAGTCTTTAGATACAATATAATAATACCACAAAGACAGAAATTTGGATACAAAGGAATAAAGATCTCTGGATATGTTTAAGATGAAGGTTAAACTATTCTTATGTTTA ATC
AAACTAAAAAAAAAATACTTAAAGCAAAAATAATAGCCAATATACTGTAGAGTTTTAGCATATATAAAATTAAAACGCAGAAACAAGATACAAGACAGGACCTACTGTAAGATTCTTACACTGTATG TT
ATGTGGTATAATGATTTGTTGGAGGTAGACTGTGATAAATTAAAGATGTGTATTGTACACTCCAGGGAAACCATTCAAAACATTAGAAAAAAGTTCAAATAATAAAGCCAATATTGAAATAAATT GGA
ATCATAAAAAAATTCAAATTCAAAAGAATGCAGAAAAGGCTGGGTGTGGTAGCTCACGACTGTAATCTCAGCACTTTGAGAGGCTGAGGCGGGCAGATCACCTGAGCTCAGGAGTTCGAGACCAGCCTG
GCCAACATGGGGAAACCCCGTCTCTACTAAAAATACAAAAAATTAGCCGGGCGTAGTGGCGGGCGCCTGTAGTCCCAGCTACTTGGGAGGCTGAGGCaGGAGAATGGCGTGAACCCGGGAGGCGGAGC
TTGCAGTGAGCCGAGATCCCGCCACTGCACTCCAGCCTGGGCGACAGAGCAAGACTCCGTCTCAAAAAAAAAAAAAAAAAAAAAAAAAAAA ATACAAAAAATTAGCCGGGCGTGGTGGCGCACATCTGT
AATCCCAACTACTTGGGAGGCTGAGGCAAAAGAATCGCTTGAACCTGGGAGGCAGCGATTGCAGTGAGCCGAGATCCTGCCACTGCACTCCAACCCGGGCAACAAGAGTGAAACTCTGTCTCAGA AAA
AAAAAAAAAAAAAAAAAaaaaCACACAGAAAATGAAGAAAACAGGAAAACAGATGGTATAAGTGGAAAAACTATTACGATGTTAGCCTGCAATCCAGCTATATTAATACTTACATTAAATGGAGATAA
TCTAAATATATCAACTTAACAAAGGGACCGTGATGCAGTCATGTCTCACGTAACAACACGGTGTGTTCTGGGAAATGTGTCACTGGGTGATTTTGCTGGTCTGAACATCACAGGCTGTACTTGCA TGA
GCCTAAATGGTGTCGCCCACTCCACACTCAGGTTATGGTACCCAATGTTGCTCCTCGGCTACAAAGTTGTACAAACATGACTGTACTGAATACTGTAGGCAGCTGTAATACAACAGGAAGTATATG TA
CATCTAAACATATCTGATCACAAAAAAATTTGCACTAAAAATACAAAACAGGTTGAACACCCCAAATCCAAAAATCTGAAATGCAAAATGTTCCAAAATCCAAAATTTTCTGAGGGCCAATATGAC GTT
CAACAGAAATGTTCACTGAGGCATTTTTAATTTTTTTTTAATTTGAGATGGAGTCTTGCTCTGTCGCCCAGGCTGGAGTGCAGTAGTGAGATCTTGGCACGCTGCAATCTCCACCTCCCGGGTTCA AG
AGATTCTCCTGTCTCAGCCTCCCAAGTAGCTCGGATTACAAGCTCCCGCCACCACGCCCATCTAATTTTTGTATTTTTGGTAGAGACTGGATTTCCCCATGTTGGCCGGGCTGGTTTTGAACTCC TGA
CCTCAA

1336	19.4	0.0	0.0	a	1	222	(2088)	+	AluJb	SINE/Alu	91	312	(0)	1
1551	28.0	8.9	4.3	a	239	1060	(1250)	+	L1MEf	LINE/L1	1157	2014	(4186)	2
2588	5.8	0.0	0.7	a	1061	1371	(939)	+	AluYa5	SINE/Alu	1	309	(2)	3
1409	11.0	0.0	0.0	a	1376	1557	(753)	+	AluSp	SINE/Alu	129	310	(3)	4
319	24.1	6.6	1.6	a	1562	1683	(627)	+	L1MEf	LINE/L1	2008	2135	(4065)	2
1180	22.2	3.4	1.1	a	1700	1963	(347)	C	Tigger3a	DNA/TcMar-Tigger	(0)	348	79	5
606	20.2	0.0	0.0	a	1968	2071	(239)	+	Tigger4a	DNA/TcMar-Tigger	1	104	(132)	6
1697	10.5	0.0	1.7	a	2072	2309	(1)	C	AluSx1	SINE/Alu	(13)	299	66	7

1338	18.2	0.0	1.4	hg	1	225	(2227)	+	AluJb	SINE/Alu	91	312	(0)	8
1787	28.1	7.9	4.6	hg	239	1060	(1392)	+	L1MEf	LINE/L1	1157	2014	(4186)	9
2233	10.1	0.0	6.9	hg	1061	1197	(1255)	+	AluSp	SINE/Alu	1	133	(180)	10
2898	1.0	0.0	0.3	hg	1198	1509	(943)	+	AluYa5	SINE/Alu	1	311	(0)	11
2233	10.1	0.0	6.9	hg	1510	1699	(753)	+	AluSp	SINE/Alu	134	306	(7)	10
1787	28.1	7.9	4.6	hg	1700	1825	(627)	+	L1MEf	LINE/L1	2015	2135	(4065)	9
1180	22.2	3.4	1.1	hg	1842	2105	(347)	C	Tigger3a	DNA/TcMar-Tigger	(0)	348	79	12
606	20.2	0.0	0.0	hg	2110	2213	(239)	+	Tigger4a	DNA/TcMar-Tigger	1	104	(132)	13
1697	10.5	0.0	1.7	hg	2214	2451	(1)	C	AluSx1	SINE/Alu	(13)	299	66	14

>hg19 chr9:140662047-140664498
TGGGCAAGATGACAAAACCCTGTCTCTACTAAAAATACAAAAATTAGCCAGGTGTGGTGGCGCAAGTGTATAGTCCCAGCTATTTGTGGGGCCAAGGCA GGAGAATTGCTTGAGCCCAGGAGGTTGAG
GCTGCACTGTGTCGTGTTCATGCCACTGAACTCCAGCCTGGGTGACAAAGTAAGACCCTGTCTCAAAAAAATGGAAAAGGAAAAGGAAAAGGAAAGAAAAAAAGTGATAGCTGGCTAAGAATTTCT TAA

ACATAAATAGAGATCAAGTCAAAGATCCAAGAAGCTCAGCGAAACCTAAACAAAATAAATACCAAAAAAGCAAAACAAAACAACAAAACCCACCACACT CAGGCACATCATTTTCAAACTGCTGAAAA
CCAAAGCAAATATTGGTGGCAGACAGAGAAAAATGACATATAGTATGCAAAGGAACAGAGGCAAGTTCAGCCAATGTCTCATTAGAAACCATGCAAGCCACACTCGAGTAACCTCTTTAAGGGCT GGG
GGGTGGGGAACTGCCAACCCAGAATTCTATGTCCCTGGAAGTAAGTATCTTTGAAAAATTAAGACAAAACAACTTTTTCAAACAAACAAAAGCTGAGAG AATTCATTATCAGCAGCCTTGTGCTACCA
AAAAAAGAGTTATTGGAAAGTCTTTAGATACAATATAATAATACCACAAAGACAGAAATTTGGATACAAAGGAATAAAGATCTCTGGATATGTTTAAGATGAAGGTTAAACTATTCTTATGTTTA ATC
AAACTAAAAAAAAATACTTAAAGCAAAAATAATAGCCAATATACTGTAGAGTTTTAGCATATATAAAATTAAAACGCAGAAA CAAGATACAAGACAGGACCTACTGTAAGATTCTTACACTGTATGTT
ATGTGGTATAATGATTTGTTGGAGGTAGACTGTGATAAATTAAAGATGTGTATTGTACACTCCAGGGAAACCATTCAAAACATTAGAAAAAAGTTCAAATAATAAAGCCAATATTGAAATAAATT GGA
ATCATAAAAAATTCAAATTCAAAAGAATGCAGAAAAGGCTGGGTGTGGTAGCTCACGACTGTAATCTCAGCACTTTGAGAGGCTGAGGCGGGCAGATCA CCTGAGCTCAGGAGTTCGAGACCAGCCTG
GCCAACATGGGGAAACCCCGTCTCTACTAAAAATACAAAAAATTAGgctgggcgcggtggctcacgcctgtaatcccagcactttgggaggccgaggcgggcggatcacgaggtcaggagatcgagac
catcccggctaaaacggtgaaaccccgtctctactaaaaatacaaaaaattagCCGGGCGTAGTGGCGGGCGCCTGTAGTCCCAGCTACTTGGGAGGCTGAGGC gGGAGAATGGCGTGAACCCGGGA
GGCGGAGCTTGCAGTGAGCCGAGATCCCGCCACTGCACTCCAGCCTGGGCGACAGAGCAAGACTCCGTCTCAAAAAAAAAAAAAAAAAAAAAAAAAAAA AaaaaaaaaaaaTACAAAAAATTAGCCGGGC
GTGGTGGCGCACATCTGTAATCCCAACTACTTGGGAGGCTGAGGCAAAAGAATCG CTTGAACCTGGGAGGCAGCGATTGCAGTGAGCCGAGATCCTGCCACTGCACTCCAACCCGGGCAACAAGAGTG
AAACTCTGTCTCAGAAAAAAAAAAAAAAAAAAAAAACACACAGAAAATGAAGAAAACAGGAAAACAGATGGTATAAGTGGAAAAACTATTACGATGTTAGCCTGCAATCCAGCTATATTAATACTTACA
TTAAATGGAGATAATCTAAATATATCAACTTAACAAAGGGACCGTGATGCAGTCA TGTCTCACGTAACAACACGGTGTGTTCTGGGAAATGTGTCACTGGGTGATTTT GCTGGTCTGAACATCACAGG
CTGTACTTGCATGAGCCTAAATGGTGTCGCCCACTCCACACTCAGGTTATGGTACCCAATGTTGCTCCTCGGCTACAAAGTTGTACAAACATGACTGTACTGAATACTGTAGGCAGCTGTAATAC AAC
AGGAAGTATATGTACATCTAAACATATCTGATCACAAAAAATTTGCACTAAAAATACAAAACAGGTTGAACACCCCAAATCCAAAAATCTGAAATGCAAAATGTTCCAAAATCCAAAATTTTCTG AGG
GCCAATATGACGTTCAACAGAAATGTTCACTGAGGCATTTTTAATTTTTTTTTAATTTGAGATGGAGTCTTGCTCTGTCGCCCAGGCTGGAGTGCAGTAGTGAGATCTTGGCACGCTGCAATCTC CAC
CTCCCGGGTTCAAGAGATTCTCCTGTCTCAGCCTCCCAAGTAGCTCGGATTACAAGCTCCCGCCACCACGCCCATCTAATTTTTGTATTTTTGGTAGAGACTGGATTTCCCCATGTTGGCCGGGC TGG
TTTTGAACTCCTGACCTCAA

Homologous Sequences

GAAACCCCGTCTCTACTAAAAATACAAAAAATTA

>NAHR_29_Scaffold9291 345963 346276
KSD_NAHR_29

>**Scaffold9291 345963 346276_**NAHR
CATGGGCAGCTGCCTCGCCCTCATCCCCAGCTTGGTGCTGGCACTTCTCTGGGGTATAGACATCTAATGACTGAGTCCCTGCCGTTGAGAGGCTACAGTCTGGTGGGGGAGGCGGCAGGTTGGCAA AG
CTTACAGAAATGAAATGGAGATTGCAAAAGCTCTCTGAAGAGACGAGGCTGGGCTGGGGTGATCAGGGAGGCAGGAGCCAGTGCAACTGAAGAAAGATGGAGTTTGGAGGTGCTGAGTTCTGCAG ATG
AGTGTTCCAGGCACAGACGGCCCCAAAAGCTGCTTTAGACTGGAAATGTCTGGGCTCCAGACCCCTCCCCAGGGCCCCAAACAGCAGTAAGCCTATCTCTAAAGAGCTGAGGTCAGAAAAGGCATG TG
GAGGCCCCACTGGGGAACATGACTTGAGTTAACCTCTCATGAAAGTACTTGCCTGGCTTCTGACTCTTCAAGAAAGGCAAATAGAATAAAGAGGCTTCCCACAGAATTTCAAGATGACAGAGAAT CTG
TGGATGAATTCTGCTAGTGAAAGACAGTGCATATCACAACCTGGATGGAACTGGAGACTATTATTCTAAGTGAAGTAACTCAGGAATGGAAAACCAAACATTGTATGTTCTCACTCATAAGTTGGA GC
TAAGCTATGAGGATGCAAAGGCATAAGAATGATACAACGGACATCAGGGACTCTGGGGAAAGGGTGGGAGGGGGGTGAGGGATAAAAGACTACAAATTGGGTTCAGTCTATACTGCTTAGGTGAT GGG
TGCACCAAAATCTCACAGATCACCACTAAAGAACTTACTCATATAACCAAATACCACCTGTTCTCCCAAAAACCTATGGAAATAAAAAAAAAAAAAAAAAAAAA AaGAAAGACAGTGCATATCCCTTCT
AGGACTCATAGAGCAAAAGAGGCTCGCATAAAGCTATCCTGCT GGCCTGGTGCGGTGGCTCACGCCTGTAATCCCAGCACTTTGGGAGGCTGAGGCGGGCGGATCACATGAAGTCAGGAGgTTGAGAC
CAGCCTGGCCAACATGGCGAAACCCCGTCTCTACTAAAAATATAAAAAATTATCCCAGGTGTGGTGGTGCAAGCCTGTAATCCCAGCTACTCGAGAGGCTGAGGCAGGAAAATCGCTTGAACCCA GGA
GGAGGTGGAGATTGCAATGATCCGAGATTGCGCCATTGCACCCCAGCCTGGGCAACAAGAGTTTTGAAACTCCATCTTAAAAAAAAAAAAAAAAAAAAAAA AGCTATTCTGCTGCCTGGAATTCTGATT
CCTCATGCGCTGGTGTTCCACCTTTGAGGAGATGGGCAGGGGGAATAGATGTTTTAGAGAACAGCACTGAGGACTAAGTCACAGGATGTTCAAGTTAACAAAATTGCACATATTTGCTGACTTTT CCT
TCAAGGGCCTCTAGGTTAAAAATGAGGTTGTCCAGCCAGGCGTGGTAGCTCACGCCTGTAATCCCAGCACTTTGAGAGAATCGCTTGAGCCCAGGAGTTCAAGACCAGCCTCGGTTGGGCA ACAGAGC
GAGAACCCACCTTGACAAAATTTTAAAAAAGGGGGGCTGTTCTTCTTGAAGACTCAAGTACATAAGCTAATAAAGGCTCTGATAAGTCCTGCAATTTACAAACCAGTTTAACTATATCTCAGATT TCT
CCAAATTTGACATGAAGTCTATTCACTAGGTAACATCCAATAACAATAACAACAACACTTTAGGAAGATTGAGCTAGTGCAACCTCTTATTGTAAATGGTGTCTTAGGAATTAATACCGTG GGTCCTA
GAATCTGGTCATCCTTATTAGAAAGGGGTCCTAGAGGTCTCATATCAGGACTCCAGGTTCCCATCTGGACAGTATAGGCAGGAAGAGTTTGGGGAAGGGGCTGGCTTTTCCCTCTCACCTGAGCT AGA
GCCCCAGAAGGCTTGCCACTCCCAGGCTGGCTCTCCTGCCAGGTGGCAGGACTCCCTTCACCTGCCTCAGCAAGTCATTGAAGTGACCTCCCTGGCCAGAAGCTCTGGCCTTGCTTCTTCC CAGGGCT
TTTTCCTTACAAATAGTAGAAAAGGAAAATGGTAGCTGCACTCTGTATTTTAATGTGATACGTTCAGGTTGAGCTTTTATATTTCTAACCTACTCCCACGCCTACTCTTTCAGATCCAGGTGTGA GTT
TAAATGCAAACCATACCAATTTCTTTCTCAGAAGAGCATCTTACTACTAACAGCTCAATACCATGAGTTAGCACTGGCTGAAATGTTTTAGAGAACCCAAACTTATTTGGAGAGCAAGACA TTCAGAA
CAAAAACGT

2410	6.8	0.0	0.3	a	549	877	(1436)	+	L1PB1	LINE/L1	5843	6151	(0)	1
2259	9.6	0.0	2.6	a	940	1253	(1060)	+	AluSp	SINE/Alu	1	306	(7)	2
669	12.2	11.4	3.8	a	1443	1565	(748)	+	AluJr4	SINE/Alu	2	133	(179)	3
347	25.0	9.8	0.0	a	1578	1709	(604)	C	MER103C	DNA/hAT-Charlie	(107)	190	46	4

2410	6.8	0.0	0.3	hg	549	858	(1592)	+	L1PB1	LINE/L1	5843	6151	(0)	5
1133	6.0	0.0	0.0	hg	939	1071	(1379)	+	AluSz6	SINE/Alu	1	133	(179)	6
2177	10.9	0.0	2.6	hg	1078	1390	(1060)	+	AluSp	SINE/Alu	3	307	(6)	7
669	12.2	11.4	3.8	hg	1580	1702	(748)	+	AluJr4	SINE/Alu	2	133	(179)	8
347	25.0	9.8	0.0	hg	1715	1846	(604)	C	MER103C	DNA/hAT-Charlie	(107)	190	46	9

>hg19 chr18:48214409-48216858
CATGGGCAGCTGCCTCGCCCTCATCCCCAGCTTGGTGCTGGCACTTCTCTGGGGTATAGACATCTAATGACTGAGTCCCTGCCGTTGAGAGGCTACAGTCTGGTGGGGGAGGCGGCAGGTTGGCAA AG
CTTACAGAAATGAAATGGAGATTGCAAAAGCTCTCTGAAGAGACGAGGCTGGGCTGGGGTGATCAGGGAGGCAGGAGCCAGTGCAACTGAAGAAAGATGGAGTTTGGAGGTGCTGAGTTCTGCAG ATG
AGTGTTCCAGGCACAGACGGCCCCAAAAGCTGCTTTAGACTGGAAATGTCTGGGCTCCAGACCCCTCCCCAGGGCCCCAAACAGCAGTAAGCCTATCTCTAAAGAGCTGAGGTCAGAAAAGGCATG TG
GAGGCCCCACTGGGGAACATGACTTGAGTTAACCTCTCATGAAAGTACTTGCCTGGCTTCTGACTCTTCAAGAAAGGCAAATAGAATAAAGAGGCTTCCCACAGAATTTCAAGATGACAGAGAAT CTG
TGGATGAATTCTGCTAGTGAAAGACAGTGCATATCACAACCTGGATGGAACTGGAGACTATTATTCTAAGTGAAGTAACTCAGGAATGGAAAACCAAACATTGTATGTTCTCACTCATAAGTTGGA GC
TAAGCTATGAGGATGCAAAGGCATAAGAATGATACAACGGACATCAGGGACTCTGGGGAAAGGGTGGGAGGGGGGTGAGGGATAAAAGACTACAAATTGGGTTCAGTCTATACTGCTTAGGTGATGGG
TGCACCAAAATCTCACAGATCACCACTAAAGAACTTACTCATATAACCAAATACCACCTGTTCTCCCAAAAACCTATGGAAATAAAAAAAAAAAAAAAAAAAAA AGAAAGACAGTGCATATCCCTTCTA
GGACTCATAGAGCAAAAGAGGCTCGCATAAAGCTATCCTGCTGGCCTGGTGCGGTGGCTCACGCCTgtaatcccagcactttgggaggctgaggcgggcggatcacatgaagtcaggagtttgagacc
agcctggccaacatggcaaaccccgtctctactaaaaatataaaaaattatcccaggtgtggtggtgcaagcatGTAATCCCAGCACTTTGGGAGGCTGAGGCGGGCGGATCACATGAAGTCAGGAG
tTTGAGACCAGCCTGGCCAACATGGCGAAACCCCGTCTCTACTAAAAATATAAAAAATTATCCCAGGTGTGGTGGTGCAAGCCTGTAATCCCAGCTACTCGAGAGGCTGAGGCAGGAAAATCGCTT GA

ACCCAGGAGGAGGTGGAGATTGCAATGATCCGAGATTGCGCCATTGCACCCCAGCCTGGGCAACAAGAGTTTTGAAACTCCATCTTAAAAAAAAAAAAAAAAAAAAAA AaGCTATTCTGCTGCCTGGA
ATTCTGATTCCTCATGCGCTGGTGTTCCACCTTTGAGGAGATGGGCAGGGGGAATAGATGTTTTAGAGAACAGCACTGAGGACTAAGTCACAGGATGTTCAAGTTAACAAAATTGCACATATTTGC TG
ACTTTTCCTTCAAGGGCCTCTAGGTTAAAAATGAGGTTGTCCAGCCAGGCGTGGTAGCTCACGCCTGTAATCCCAGCACTTTGAGAGAATCGCTTGAGCCCAGGAGTTCAAGACCAGCCTCGGTTGGG
CAACAGAGCGAGAACCCACCTTGACAAAATTTTAAAAAAGGGGGGCTGTTCTTCTTGAAGACTCAAGTACATAAGCTAATAAAGGCTCTGATAAGTCCTGCAATTTACAAACCAGTTTAACTATAT CT
CAGATTTCTCCAAATTTGACATGAAGTCTATTCACTAGGTAACATCCAATAACAATAACAACAACACTTTAGGAAGATTGAGCTAGTGCAACCTCTTATTGTAAATGGTGTCTTAGGAATTAATA CCG
TGGGTCCTAGAATCTGGTCATCCTTATTAGAAAGGGGTCCTAGAGGTCTCATATCAGGACTCCAGGTTCCCATCTGGACAGTATAGGCAGGAAGAGTTTGGGGAAGGGGCTGGCTTTTCCCTCTCA CC
TGAGCTAGAGCCCCAGAAGGCTTGCCACTCCCAGGCTGGCTCTCCTGCCAGGTGGCAGGACTCCCTTCACCTGCCTCAGCAAGTCATTGAAGTGACCTCCCTGGCCAGAAGCTCTGGCCTTGCTT CTT
CCCAGGGCTTTTTCCTTACAAATAGTAGAAAAGGAAAATGGTAGCTGCACTCTGTATTTTAATGTGATACGTTCAGGTTGAGCTTTTATATTTCTAACCTACTCCCACGCCTACTCTTTCAGATCC AG
GTGTGAGTTTAAATGCAAACCATACCAATTTCTTTCTCAGAAGAGCATCTTACTACTAACAGCTCAATACCATGAGTTAGCACTGGCTGAAATGTTTTAGAGAACCCAAACTTATTTGGAGAGCA AGA
CATTCAGAACAAAAACGT

Homologous Sequences

TGTAATCCCAGCACTTTGGGAGGCTGAGGCGGGCGGATCACATGAAGTCAGGAGTTTGAGACCAGCCTGGCCAACATGGC

>NAHR_30_ Scaffold4319 2114943 2115248
KSD_NAHR_30

>**Scaffold4319 2114943 2115248_**NAHR
TTTCACACACCCATCCTTTTTGTTCAGAACACCAATTGATTTTTAAAGTGTGTTATCCTTTATTTACTTTAGAGACAGAAAACACAGAGAGAGTGACTTCTCCGGGTTGCAAATTGTCAGTGATGG AG
CCAGGATTAAGAACTCGGGCTGGCCAGCCCTCTGTGGATCGTATGATGTTTATGTATACGATGCCC gTTCACTCTTCTGCCCTGCCAGCAAACAGAGCTGATGAATATGAGGAGAATGGCAGCGAAGA
GCTTGTTTCCATTCTAAAAAGGCAAGAAGCCTAGGAAGGCTCATTAGGAGAAAATATTGAATGGGCAGCTTTCGAGTTTCACCTGTGATCTGAGAGGGAAAGTGTATTTTCACCGAAGCCATTATG TA
ACTGGTTCCGGGGGGCAGAGAGGAGTGACCAGAGAAGAGAATGAGGGCAAGAGACCAGGAGAGCAAAGACAGGGTCACCATCTAGAGGGCAGGGGCTACATCTGACATCCCCTGTAGACCCTCTG GAT
GCAGCCAAGGTTTAGAGGTTAGAGCACTGCTTTCATATCAGACGAGTGGAATTAGGATTGCAGCGGTGTGTCCTTGGACAAAGCACTGATCCTCTCTGGCCCTCAATTTTCTTGTTTATGAAATGG GA
ATAATACCACTAACCTTACCAGTTTTTGTGGGTGGATGGAGATATATAAAACCTACTTTTAGAAGGCATGATATATAGTAAACGTTCAGTGCTTTATAGGTGATTTCCAAATCATCTGTAGTAAC TAT
GGCTTTTTTACAAGCATGGAAATAATGTGTGTTTATTTAAAAAAAAAAGAAACTATTACTAAAAAGCATAAAGAAGAAAGTAGAAAATATTCAAATCCTACCACTCAGAGATAGCCACCCATCACA TC
CAGGCATGTGCACCTCCTCCCTTTCTCCTCCACATGTACAAATATGTACATATTTTGAAGCCAAAAACAAAATGTGATAATGCCATATATGAAATTTGGAACTTATCTCTTTTCATTTAACAATA TCT
GGTGGGTATTATTTCAGGTCAGAAAATATGATTTTAGGCCAGGTGCAGTGGCTCACGACTGTAATCTCAACATTTTGGGAGGCTGAGGTGGGCGGATCACTTGAGGTCAGGAGTTCGAGACCAGCCTG
GCCAACATGGTGAAACTCCATCTCTACTAAAAATATAAAAATTAGCTAGGCATGATGGTGGGCAGCTGTAATCCCA GCTACTTGGGAAGCTGAGGCAGGAGAATTGCTTGAACCTGGGAGGTGGAGGT
TGCAGTGAGCCGGAATTACGCCACTGCAGTCCAGCCTGGGTGACAGGGTAAGACTCCATCTCAAAAAATAAAATAAATAAAATAAA CTAATAAAATAAATAAGAAGAGGCATCAGAAGCCCAGCCAGA
GCTTGGGAGTTACCTGATGTGCAGGGGGAGGCCGCTGAAGGGTGTTGGTTGAGGGAGGGGGTGATGTAATTTTGGAGTTGGAGTTAAGGGATTGGGTGTCCTCACGGTTTCCCTCATTCATGCCT TCA
CTCATGAGACAGTTACAGAGCACCAAGGCTCAGCGAGGTGCAGGACTGGCCTGGGGCGGGGGGTGAGGGGAACCTGTGCTGTCTGACCCCCCAGCAGCCTCTCCAGGGCCTCCTCGGCTGGGCTGA TA
GACCTGTGTTTTAGCTTGAACCTCTGTCCACCACCCTTCAGGGAGGACCTCACACACGCCTTGGCCGGCGTGTGATGGGCACTCC cAGGAGGCATGTGGTGTGAGCCAGAGTCGGATTGGAACTCAGG
ATGGGAACAACGCCTGCACAAGGAGAACGTCCATAAACAGCTGCAGAAGACCAAAAGGGAGGGGTGGAGGA Gggaggcgggcttcact GGAGTCCTCCCTCCACCATTGCTGTTGTTATGACCCAGCC
ACTCCCTGGGGGCTCACTCCCTCTGGAAAATGGTAGGTTAGAAAAGACCAGTGATTCTCTCTCTTTCTCTTTTGCTTTTTTTTTTTAAGAGACCAAGTCTCGCTATATTGCCCAGGCTGATCTTG AGC
GCCTGACCTCAAGCAGTCCTCCTGCCTCAGCCTCCCGAGTAGCTGAGATCACAGGTGCATGCCACTGCGCCCAGCTTGACATTGATTCTATGATAACAGCAGCCCGTGGAATGGTGGAAGCAGCCA GA
CACACTTGGGAATTACCTTGGGTGAGTCTATTGGAACCTCAGCTTCCCCATCTGTAAAATGGGATGGTTGTTACTCCCTTGCTGGGCCACTGTGAAGGTGAGACGTGCCGTAGGAGCACGCCGAG CAC
A

428	29.2	6.4	1.8	a	574	730	(1575)	+	MIRb	SINE/MIR	86	249	(19)	1
398	32.6	5.0	4.2	a	772	1053	(1252)	C	L1ME4a	LINE/L1	(3)	6121	5838	2
2262	12.8	0.0	0.0	a	1061	1366	(939)	+	AluSx1	SINE/Alu	1	306	(6)	3
853	17.2	0.0	0.0	a	1996	2123	(182)	C	FLAM_A	SINE/Alu	(14)	128	1	4
251	25.9	10.6	0.0	a	2191	2275	(30)	+	MIRb	SINE/MIR	95	188	(80)	5

428	29.2	6.4	1.8	hg	574	730	(6285)	+	MIRb	SINE/MIR	86	249	(19)	6
398	32.6	5.0	4.2	hg	772	1053	(5962)	C	L1ME4a	LINE/L1	(3)	6121	5838	7
1776	11.1	0.4	0.0	hg	1061	1294	(5721)	+	AluSx1	SINE/Alu	1	235	(77)	8
286	28.2	5.9	1.6	hg	2079	2197	(4818)	C	L1MC5a	LINE/L1	(0)	8030	7907	9
554	23.8	0.0	0.0	hg	2279	2400	(4615)	C	L1MC5a	LINE/L1	(248)	7782	7661	9
571	21.1	2.5	4.5	hg	2485	2643	(4372)	C	L1ME4a	LINE/L1	(427)	5697	5542	7
21	27.8	2.8	1.4	hg	2696	2767	(4248)	+	GA-rich	Low_complexity	1	73	(0)	10
31	18.4	0.0	0.0	hg	3264	3319	(3696)	+	(T)n	Simple_repeat	1	56	(0)	11
2423	9.2	0.0	0.0	hg	3582	3884	(3131)	C	AluSq2	SINE/Alu	(10)	303	1	12
336	5.3	0.0	3.5	hg	4037	4095	(2920)	+	L1MA4	LINE/L1	6240	6296	(4)	13
341	22.0	1.1	1.1	hg	4796	4887	(2128)	+	MIRb	SINE/MIR	35	126	(142)	14
206	33.1	4.2	7.5	hg	5376	5727	(1288)	C	L2a	LINE/L2	(21)	3405	3013	15
2249	12.8	0.0	0.0	hg	5788	6092	(923)	+	AluSx1	SINE/Alu	2	306	(6)	16
853	17.2	0.0	0.0	hg	6706	6833	(182)	C	FLAM_A	SINE/Alu	(14)	128	1	17
251	25.9	10.6	0.0	hg	6901	6985	(30)	+	MIRb	SINE/MIR	95	188	(80)	14

>hg19 chr22:37142115-37149129

TTTCACACACCCATCCTTTTTGTTCAGAACACCAATTGATTTTTAAAGTGTGTTATCCTTTATTTACTTTAGAGACAGAAAACAC AGAGAGAGTGACTTCTCCGGGTTGCAAATTGTCAGTGATGGAG
CCAGGATTAAGAACTCGGGCTGGCCAGCCCTCTGTGGATCGTATGATGTTTATGTATACGATGCCC aTTCACTCTTCTGCCCTGCCAGCAAACAGAGCTGATGAATATGAGGAGAATGGCAGCGAAGA
GCTTGTTTCCATTCTAAAAAGGCAAGAAGCCTAGGAAGGCTCATTAGGAGAAAATATTGAATGGGCAGCTTTCGAGTTTCACCTG TGATCTGAGAGGGAAAGTGTATTTTCACCGAAGCCATTATGTA
ACTGGTTCCGGGGGGCAGAGAGGAGTGACCAGAGAAGAGAATGAGGGCAAGAGACCAGGAGAGCAAAGACAGGGTCACCATCTAGAGGGCAGGGGCTACATCTGACATCCCCTGTAGACCCTCTGGAT
GCAGCCAAGGTTTAGAGGTTAGAGCACTGCTTTCATATCAGACGAGTGGAATTAGGATTGCAGCGGTGTGTCCTTGGACAAAGCACTGATCCTCTCTGGCCCTCAATTTTCTTGTTTATGAAATG GGA
ATAATACCACTAACCTTACCAGTTTTTGTGGGTGGATGGAGATATATAAAACCTACTTT TAGAAGGCATGATATATAGTAAACGTTCAGTGCTTTATAGGTGATTTCCAAATCATCTGTAGTAACTAT
GGCTTTTTTACAAGCATGGAAATAATGTGTGTTTATTTAAAAAAAAAAGAAACTATTACTAAAAAGCATAAAGAAGAAAGTAGAAAATATTCAAATCCTACCACTCAGAGATAGCCACCCATCAC ATC
CAGGCATGTGCACCTCCTCCCTTTCTCCTCCACATGTACAAATATGTACATATTTTGAA GCCAAAAACAAAATGTGATAATGCCATATATGAAATTTGGAACTTATCTCTTTTCATTTAACAATATCT
GGTGGGTATTATTTCAGGTCAGAAAATATGATTTTAGGCCAGGTGCAGTGGCTCACGACTGTAATCTCAACATTTTGGGAGGCTGAGGTGGGCGGATCACTTGAGGTCAGGAGTTCGAGACCAGCCTG
GCCAACATGGTGAAACTCCATCTCTACTAAAAATATAAAAATTAGCTAGGCATGATGGT GGGCAGCTGTAATCCCAGctactcgggaggctgaggcaagagaatctcttgaacccaggatggggaggt
tacagtgagcgagactctgtctcaaatatatgtatcatatatatcacatatatatgatatatatatcatatatatgatatgtgatatatatgatatatatcatatatatgtgatatatgtgatatata
tgatatatatcatatatgtgatatatatatgatatatatgatatatatatgatatatat gatatatatgacatatatgatatatatgatatatatgatatatatgtgacatatatgatatatatatga
tatatatgtgatatacatcatatatatatcacatatatatcatatcatatatcacatatatatcatatcatatatcacatatatatcatatcatatatatcatatataacatatatatcatatat atc
atatataacatatatatcatatgtatcatatatatcatatatatcatatgtatcatata tatcatatatatcatatatatctcatatatctcatatatatcatatatatcatatataaatcatatata
tcatatatatgatatataaatcatatatgtatcatatatatcatatatgtatcatatatacatatatatgatacatatatgtatcatatatatgatgcatatatgtatcatgtatatctcatata tat
catatatatctcatatatatcatatctcatatatctcatatatctcatatatatcatat atatgatcatatatgatcatatatgatatatatgatcatatatgatatatatatcccatatatatcata
tatatgatatatatgtgatttacatggtctttttatttctttttaaatcgtgagaattgtaaaacctacacaaaaacagaacagcatgatgaacgctcatttacctgacagccagattcggaac ttc
caagatcctgccgcacttgctctgtcctgctcccctgccatgatggtggtggttacgat cgtttaaaccaaatgccagccctccagtcactgcctctaaaccaaccacaaagccattatcacaccaca
taaaattaatagtaattctttaggagcatctactgttcagtccacagtcaaatttccctgatttactcccaaaggttcttttgcagttggtttattacgtgacccacgcataatgatttccagtt gaa
ggacaccaggtttccaggtggtcactcaggaaaacagccaaggacagtgtccccttgca catatctgtggacccctgtccaattatttctgcaggacatacattcctagaaagggacattccaagtca
aagtctacgtactttttaaaaaagcttttaatagcatttgacaaattgccctccagataaattgtaccagtttacactcctaggagtggggactggcaccctggacagctctgggtcgttaatg aaa
agaaattagagagagagaaggcaggtggagagaaaaagagagaaggaagagagggctgg agtgagagggagcagagtgacactgcagagctctggtgccccctgggtgccagcctgcccgagccagag
aggagctcagagaaccaagggtatcaaccaaccagcggctgcaccagattaagccctaaagcaacgattcagtcgagaccgagaacaaagctgaagcaaagactcttcgatccattctaattagg agc
tgcctttccagagaaatcaagagcaggctgccacggctgaaagcaaaaaaaagaaggaa aagaaaaaaactgctgcaacccagctgtgcgcgaagcttgtctgttttcttaaaatcccaagaaagagc
tggttctactgtgctcacagtccaggggaccctgttgttctgagagttgtaggatcccactaggagcaaagatctctaccaccaaaggtcagggtgcgttcagcactgggacctcgctaaggac agg
gagagggcgggaaggagacccatgtgtgggagcaatgaccttgctgtggcggctgctgc tgcattgtttgtttttttttgtttgtttttatttttgttttgttttttatttttttttgtttttgctatagct
aaaaactaaaatttatttcaggtttgagaaaagcaaataaaattaagatacatttgaagcatggaactacacacaaaaatggagaacaggctggttctggccctacagcctgtgctgttggaggt tcc
ctcttgggtggacgccatgatgcattgcttaccttcaatgtaccctctccggttcacac tagtatatttatatgttgattacaagtacccaaaaaagaactagtccgtggataaaaatgacctaattt
tctttttcttttttttttgagatgaagtctcattcttgttgcccaggctggagtgcaatggcacaatctcggctcactgcaacctccacttcctgggttcaagtgattagcctgcctcagcctccc gag
tagctgggattacaggtgcctgccactacacccggataatttttgtatttttagtagag ttggggtttcaccatattggccaggctggtctcgaactcctgacctcaggtcatccgcccgccttggcc
tcccaaagtgctgggattacaggcgtgagccactgcacccggcccaaatgacctaattttcaattgaccaagccattccaacagtaagcatcaaatactctgagataaggactgtactgagttca tag
tgccaagtccagaaatcccctggtcaccatatcatttatcagatggcatttaaattaaa agcagacagtcaaaatatcacatatacccccccaaaatatgtacaactatgatatatcaattaaaaaat
tgtttaaggcatgtaccctctagattatggtggattcttgcagtatgaaaggaagtttttaagcaaagaagaaagttgcatgaacaagtatcctattattttcaaaaaaatcagcagctcataca ttg
tatcttgattttatttcacatggcaactctttaaagtggctgtttgtgtcccactgcgc aaaagaagaaaatgcaccccagacagatgacatgacacttggctgcttcttgtgacctcaggctccttc
tcgacatctgtgagtctttttttgcctcctggctcattgcctcattgtagattccaacagcagcaaccttgcccaggggcaagctgaatgcaaggtacaagcaggggcttgaacccctgtgagcca cac
tgtgctcccagaatctcccccaaagcctcttccagcgtgtttcctctcactcaaccttc acagccaccccaggaagccaaggcaactgttttacgttacggctgaggacgcaatgggcccatgacctg
cgttccccagtgcaggctcctagcgatgtcacagctaccgtcagactctgtgaatctcaggaaacctccagaggaaggtcaggcccttcccaaccacctccctgcaggtgcttgctgatctcagg ggc
agaaggtggtgacggtcgagaagcaatgtggggcggtttcacctgcacgcggcacccaa ggctttggaatcacacagcctctgggtgcgagtctttgctcagctctacctcactgagtggccttggac
aagtcacttcgcctctctgggccctaacatcacctacttcagatgtttgctgagggttcacagcctggcgatagctgcagacccccccagcccctgctgagtgggacactcgccgctcaccctatt gtt
tcggttgttgccgctgctgttgtgaccctatcctggataatcccaggggtggagtctgc tgccctccacccttcgtccaaaggagctctgggactaggaggcctgggtccccggtgagtgacccctgg
gaagagtggcttgccatggccccagagaggactggagagaggcccagcaggggctaggaggcctgagcccagtcggtctcttgccctggctgagagcgcctttgccttggggcctctggcatgac act
agtgggttagcaatccctggagtcctcgcgtctctcacttctttgggtttccacgcaca gcgtgcaggcaagaagcgtttaaggagcagcctcgacacagagcccctgtgtctcaacaggccctggct
ttttgaccaccagccaccagccaggccccgctgtaggtgcaggggcttaagagctggacagggcagatgagcctcctactccttgctccatggagcacacaagctggtgagggagagagacaata agt
cagtttgcaatcccgtagaaaaccacaggttgtaaaaatcactggaaagaaaagaacag aaccctctatacaagactccaaaggagtcttagcagggaggaggtgtccagcagcagcctctctgtgga
gacatgaaggagcaggagtcaggcagtgaacggggagttgtacattagccagaggaaatagcatgtttgagggtcctgcggcagaggaggttggcccacaaaaggccagaactcctgggcactga gtg

aagggagcacggggaaaagagtttccagccgagcgcagtggctcacacctgtaatcccagcactttgggaggctgaggcgggtggatcactggaggtcaagagttcaagaccaacttggccaacatgg
tgaaactccaactctactaaaaacacaaaaattagttgggcgcggtggcgggcgtctgtaatcctag CTACTTGGGAAGCTGAGGCAGGAGAATTGCTTGAACCTGGGAGGTGGAGGTTGCAGTGAGC
CGGAATTACGCCACTGCAGTCCAGCCTGGGTGACAGGGTAAGACTCCATCTCAAAAAAT AAAATAAATAAAATAAACTAATAAAATAAATAAGAAGAGGCATCAGAAGCCCAGCCAGAGCTTGGGAGT
TACCTGATGTGCAGGGGGAGGCCGCTGAAGGGTGTTGGTTGAGGGAGGGGGTGATGTAATTTTGGAGTTGGAGTTAAGGGATTGGGTGTCCTCACGGTTTCCCTCATTCATGCCTTCACTCATGA GAC
AGTTACAGAGCACCAAGGCTCAGCGAGGTGCAGGACTGGCCTGGGGCGGGGGGTGAGG GGAACCTGTGCTGTCTGACCCCCCAGCAGCCTCTCCAGGGCCTCCTCGGCTGGGCTGATAGACCTGTGTT
TTAGCTTGAACCTCTGTCCACCACCCTTCAGGGAGGACCTCACACACGCCTTGGCCGGCGTGTGATGGGCACTCC tAGGAGGCATGTGGTGTGAGCCAGAGTCGGATTGGAACTCAGGATGGGAACAA
CGCCTGCACAAGGAGAACGTCCATAAACAGCTGCAGAAGACCAAAAGGGAGGGGTGGAG GAGGGAGTCCTCCCTCCACCATTGCTGTTGTTATGACCCAGCCACTCCCTGGGGGCTCACTCCCTCTGG
AAAATGGTAGGTTAGAAAAGACCAGTGATTCTCTCTCTTTCTCTTTTGCTTTTTTTTTTTAAGAGACCAAGTCTCGCTATATTGCCCAGGCTGATCTTGAGCGCCTGACCTCAAGCAGTCCTCCT GCC
TCAGCCTCCCGAGTAGCTGAGATCACAGGTGCATGCCACTGCGCCCAGCTTGACATTGATTCT ATGATAACAGCAGCCCGTGGAATGGTGGAAGCAGCCAGACACACTTGGGAATTACCTTGGGTGAG
TCTATTGGAACCTCAGCTTCCCCATCTGTAAAATGGATGGTTGTTACTCCCTTGCTGGGCCACTGTGAAGGTGAGACGTGCCGTAGGAGCACGCCGAGCAC A

Homologous Sequences
AGCTACT

>NAHR_31_ Scaffold7862 241399 241694
KSD_NAHR_31

>Scaffold7862 241399 241694_NAHR
TAGAAAAGGGAAAGCAATAAGAACAGGAACCAGATCAGTTACTGCCCAGGGCTAGACTGTTAGAAAGAAAGCGACCATCACAGCATATTCTCACTCGCAGGTGGGAACTGAACAATGAGAACACAT GG
ACACAGGAAGGGGAACATCACATTCCGGGGGGAGGGGGAAGGGATAGCTTTAGGAGATATATCTAATGCTAAATGACAAGTTAATGGGTGCAGCAC ACCAGCATGGCACATGTAAACATATGTAACTA
ACCTGCACATTGTGCACATGTACCCTAAAACTTAAAGTATAATAATAATAAAATAAAATAAAGTGATCAAAACAAAACAAAAACAAACAAAAATAACACACACACAAAAAACCCCACAATAAACA AAA
ATCCAACCAAACAAAAAGGCACCAAGAAACTTCAAGAATTGATCTAACTGTTCTATATCGTGATTGTAGTGGTTTCACACACTTTATGTGTTTGTC AAAACTCACTGAATTGTATTAAAAACAGCAAA
TATCATTGTTGGAAAATGCTTCAATAGACCTGAACTAATAGATAAATAGAAGGAAATAAAAGAAAACATCATAGGGCACCAAGTAAAAACAAGGTTCATATATATAATATAGATGGTGGTCTTGG TTG
TAAAATGGTGAAACATCTTCATAGTCAGAATAAATAGGCTTGAAAACTTTCTTGTGTCAACTGGTTCATCACAAATATCATTTAAAAGTATCTTTT ATGGCTGGGTGCAGTGGCTCAAACCTAAAATC
CCACTTTGGGAGGCTAAGGCAAAAGACCAGCCTGGGCAATGCAGTGAGACATTGTCTCTACAAACAACAACAGAAGGAGAAGGAGAAGTAGAAGAGAAAAGAAAAGAAAAGAAAAAAATAGCCAG GCA
TGGTGGCACATGCCTGTAGTCTCAGCTACTCAGGAGGCTAGGGGAGAAGATCTCTTGAACCTAGGAGGTTGAGGCTGCAGTGAGACCTAATAGCAC CACTGCACTCCAGTCTGAGTGACAGACCAAGA
CCCTGTATCAAAGAAATAAAATAAAAATCAAATGAAGGCAGGGCGCAGTGGCTCACGCCTGTAATCCCAACACTTTGGGAGACTGAGGTGGGGGCAGATCATGA GGTCAGGAGTTCGAGACCAGCCTG
GCCAACACGGTGAAAGCCCATCTCTACTAAAAATACAAAAATTAGCTGGGCATGGTGGCACGTGCCAGTAATCCCAGCTACTGGGGAGGCTGAGGCAAGAGAATTGCTTGAACTCAGGAAGCAGA GGT
TGCAATGCGCCGAGATCAAACCACTGCACTCAGCCTGGGCGACAGAGCAGGACTCTGTCTCAAAAAAAAAAAAAAAAATAATAA TTGTAATAAAAATACTCTTTACCAAATAAATATAAAACAAAATATA
TTTCAATAAATCATTATTTCTGTTTTTGCTCCAAATACTATACCAACTTGCATTTTTAGACAAGAGATACACATAAACACATTCATTTACATATAATTCTTCTGTTGCCAAAGGCATGCATGGAA AAA
TATCCTATTTCAACATGTTTTTCTGAGTATTATCTGCAAGTCCATGAACCAGAAATTATCCCATGGAAAAAAAAATACAAAAAAATTAAAAAATAAAGATGTAGTCT TAGTTATATAACCTTTTAAATT
ATAGCAATGCTTTGCATTTTGTCATATAATGACCTAAATGCATTCACAGTTTTTTCTATAAGTGTATATAATTTTTATATTGTTGTTTATATATATGGGAATGGTCAGAAATATTTTGGTTTACT TAT
TTCATGTTTATTGGCTTCATTTTTAGAAAAACTTTTTTTCTGCATAAATTCTGGATAAATGAAGCTATCTCATCTTTGATAAGCTATGTCAGCCATTTCTGCTGAA ACCATCTGCATCTAATTTCTAA
AGGAAAAACTGCATAGAAGCAATAAAATCAAACAAGGTGATAACCCAAAAATAATTCTTTCAGAATGATTAGAGATTTCCCCTCTTTTGCCATTTCCATTTTAACTTGATCATATGACCTTCTCT TTT
GGTAATATAATAATTCAGAATTAATCTCTACAAGCTTTCATGAATAATTTAGTTAGTGATGGTAGAAAGGAATGCAATTAATTTCTGGGGTAAATTCCAGATCATC ATAAAAACCTGGAAGGGGACCA
TTTGTATATTTAATTAATAAATGGTAACATGTACACAATTTTTTTCTACTCAAATTAGCACTGTGATACTGATAATTGCTCAATATGAAATTCTCACCAAGTGCAATTACAGGGAAAT C

1767	6.5	7.4	0.0	101044	79	309	(1986)	+	L1PA3	LINE/L1	5905	6152	(3)	1
496	26.5	5.3	0.0	101044	395	545	(1750)	+	L1ME1	LINE/L1	6011	6169	(10)	2
1093	16.9	9.3	14.0	101044	739	1051	(1244)	+	AluJb	SINE/Alu	1	300	(12)	3
2222	10.5	0.3	0.7	101044	1061	1356	(939)	+	AluSg	SINE/Alu	1	295	(15)	4
16	19.2	0.0	0.0	101044	1603	1632	(663)	+	A-rich	Low_complexity	1	30	(0)	5

1767	6.5	7.4	0.0	hg	79	309	(2294)	+	L1PA3	LINE/L1	5905	6152	(3)	6
496	26.5	5.3	0.0	hg	395	545	(2058)	+	L1ME1	LINE/L1	6011	6169	(10)	7
1093	16.9	9.3	14.0	hg	739	1051	(1552)	+	AluJb	SINE/Alu	1	300	(12)	8
1950	15.1	0.3	0.7	hg	1061	1359	(1244)	+	AluSx4	SINE/Alu	1	298	(12)	9
2222	10.5	0.3	0.7	hg	1369	1664	(939)	+	AluSg	SINE/Alu	1	295	(15)	10
16	19.2	0.0	0.0	hg	1911	1940	(663)	+	A-rich	Low_complexity	1	30	(0)	11

>hg19 chr7:145500290-145502892
TAGAAAAGGGAAAGCAATAAGAACAGGAACCAGATCAGTTACTGCCCAGGGCTAGACTGTTAGAAAGAAAGCGACCATCACAGCATATTCTCACTCGCAGGTGGGAACTGAACAATGAGAACACAT GG
ACACAGGAAGGGGAACATCACATTCCGGGGGGAGGGGGAAGGGATAGCTTTAGGAGATATATCTAATGCTAAATGACAAGTTAATGGGTGCAGCACACCAGCATGGCACATGTAAACATATGTAA CTA
ACCTGCACATTGTGCACATGTACCCTAAAACTTAAAGTATAATAATAATAAAATAAAATAAAGTGATCAAAACAAAACAAAAACAAACAAAAATAACACACACACAAAAAACCCCACAATAAACAA AA
ATCCAACCAAACAAAAAGGCACCAAGAAACTTCAAGAATTGATCTAACTGTTCTATATCGTGATTGTAGTGGTTTCACACACTTTATGTGTTTGTCAAAACTCACTGAATTGTATTAAAAACAGC AAA
TATCATTGTTGGAAAATGCTTCAATAGACCTGAACTAATAGATAAATAGAAGGAAATAAAAGAAAACATCATAGGGCACCAAGTAAAAACAAGGTTCATATATATAATATAGATGGTGGTCTTGGT TG
TAAAATGGTGAAACATCTTCATAGTCAGAATAAATAGGCTTGAAAACTTTCTTGTGTCAACTGGTTCATCACAAATATCATTTAAAAGTATCTTTTATGGCTGGGTGCAGTGGCTCAAACCTAAAATC
CCACTTTGGGAGGCTAAGGCAAAAGACCAGCCTGGGCAATGCAGTGAGACATTGTCTCTACAAACAACAACAGAAGGAGAAGGAGAAGTAGAAGAGAAAAGAAAAGAAAAGAAAAAAATAGCCAGG CA
TGGTGGCACATGCCTGTAGTCTCAGCTACTCAGGAGGCTAGGGGAGAAGATCTCTTGAACCTAGGAGGTTGAGGCTGCAGTGAGACCTAATAGCACCACTGCACTCCAGTCTGAGTGACAGACCA AGA
CCCTGTATCAAAGAAATAAAATAAAAATCAAATGAAGGCAGGGCGCAGTGGCTCACGCCTGTAATCCCAACACTTTGGGAGACTGAGGTGGGGGCAGATCATGA G gtcaggagttcgagaccagcctg

gccaacacggtgaaagcccatctctactaaaaatacaaaaattagctgggcatggtggcacatgcctgtagtctcagctactcaggaggctaggggagaagatctcttgaacctaggaggttgag gct
gcagtgagacctaatagcaccactgcactccagtctgagtgacagaccaagaccctgtatcaaagaaataaaataaaaa tcaaatgaaggcagggcgcagtggctcacgcctgtaatcccaacacttt
gggagactgaggtgggggcagatcatgagGTCAGGAGTTCGAGACCAGCCTGGCCAACACGGTGAAAGCCCATCTCTACTAAAAATACAAAAATTAGCTGGGCATGGTGGCACGTGCCAGTAATCCCA
GCTACTGGGGAGGCTGAGGCAAGAGAATTGCTTGAACTCAGGAAGCAGAGGTTGCAATGCGCCGAGATCAAACCACTGCACTCAGCCTGGGCGACAGAGCAGGACTCTGTCTCAAAAAAAAAAAA AAA
TAATAATTGTAATAAAAATACTCTTTACCAAATAAATATAAAACAAAATATATTTCAATAAATCATTATTTCTGTTTTTGCTCCAAATACTATACCAACTTGCATTTTTAGACAAGAGATACACATAA
ACACATTCATTTACATATAATTCTTCTGTTGCCAAAGGCATGCATGGAAAAATATCCTATTTCAACATGTTTTTCTGAGTATTATCT GCAAGTCCATGAACCAGAAATTATCCCATGGAAAAAAAATA
CAAAAAAATTAAAAAATAAAGATGTAGTCT TAGTTATATAACCTTTTAAATTATAGCAATGCTTTGCATTTTGTCATATAATGACCTAAATGCATTCACAGTTTTTTCTATAAGTGTATATAATTTTT
ATATTGTTGTTTATATATATGGGAATGGTCAGAAATATTTTGGTTTACTTATTTCATGTTTATTGGCTTCATTTTTAGAAAAACTTTTTTTCTGCATAAATTCTGGATAAATGAAGCTATCTCAT CTT
TGATAAGCTATGTCAGCCATTTCTGCTGAAACCATCTGCATCTAATTTCTAAAGGAAAAACTGCATAGAAGCAATAAAATCAAACAAGGTGATAACCCAAAAATAATTCTTTCAGAATGATTAGAGAT
TTCCCCTCTTTTGCCATTTCCATTTTAACTTGATCATATGACCTTCTCTTTTGGTAATATAATAATTCAGAATTAATCTCTACAAGCTTTCATGAATAATTTAGTTAGTGATGGTAGAAAGGAAT GCA
ATTAATTTCTGGGGTAAATTCCAGATCATCATAAAAACCTGGAAGGGGACCATTTGTATATTTAATTAATAAATGGTAACATGTACACAATTTTTTTCTACTCAAATTAGCACTGTGATACTGATAAT
TGCTCAATATGAAATTCTCACCAAGTGCAATTACAGGGAAATC

Homologous Sequences

GGCAGGGCGCAGTGGCTCACGCCTGTAATCCCAACACTTTGGGAGACTGAGGTGGGGGCAGATCATGAGGTCAGGAGTTCGAGACCAGCCTGGCCAACACGGTGAAAGCCCATCTCTACTAAAAA TAC AAAAATTAGCTGGGCATGGTGGCAC

>NAHR_32_ Scaffold1058 790080 790212
KSD_NAHR_32

>Scaffold1058 790080 790212_NAHR
CCTGGGCAACAAGGGCTAGACTCCATCTCAAAAAAAAAAAAAAAGACCACCCCAAACTGAGGCTTAGAATTACAAACATAATGAAAAATAAAACAAAATTTTGACACATTAAAAAAAAGGAATTACC AA
CATAGAAAGGTGTTTGAGAAATACTGTTAATTAAACGAGACGGGTGCATCTGCGTGAACGTAGGAAGGAAGCGTAGCCGGATTATGAAATGCACGAACACCCTGGGAGGGGTGAGCTTTCCCACA CTT
TGCTCTTTGGGCTGTGGGGTTGTAGTGATTTTCTTCTTTTTGCCAATCTGTAGGTTTCCAATCTGTGTTTGCTCCCTGGGTATGCATTTGGGAA aTTCAAAAAAATTCTGTGGCTGTTGAGTCAACCA
ACCTCCTCCTCCCTGGTTCTTGAGGCTTTGCAACAGTTGGGCTCCCACCAATCCGCCCTACCACTAACTGATC cGCCACCGATCCGTGGCACGAACAGTGACTGTGGCCTAAGCACGGGTTGTCGCGT
TGCTCTCCAGCCTTTCCAACCTTCCTGTCAAGCCTCTCTTCCTCTCACAAAGCCTCCCAGGTGCACCTGGGAGCAGGTACCTCACCTCCAGCCGCCTTCCCCCTGGCTTGAAAGTGCCTTCCTTAT CA
CCTACACCATCCTTTCACTTATCCAGGGTGTGGCTTCTCTCTCCTAGCAGGTTGTAAATTTCCTGGGCCTGCAGACTGAGCCTCAGCCCAGGGTTTACTCACTCCCACCCTG tCTGGAACACAGTGAG
AGTTCATTgAGGAAATGACTACAAGGCACAGGGGCTCTGGTCTGGCTGGAACTGATGGTGTAAATGGGGAGAAAAGGGTGATGTATCAATGAGACAGGGACTGAAAAAGACAA aGCTGTGCCCAGAAC
TAAGTTCTATGTTGTATGCTGTGGAGTAAAAGGGCAGGGTTC aCCGGGCTTGGTGGCTCACACCTGTAATCCCAGCACTTTGGGAGGCCGAGGCAGGTGGATCACCTGAGGTCAGGAGTTCAAGACCA
GCCTGGTCAACAAGGGGAAACCCTGTCTCTACTAAAAGTACAAACATT GGCCAAGTGCGGTGGCTTACGCCTGTAATCCCAGCACTTTGGGAGCCTGAGGTGGGC gGATCATGAGGTCAGGAGTTTGA
GACCAGCCTGGCCAACATGGTGAAACCCCATCTCTTCTAAAAATAAAAAAAAATAATAAAAAATAAAAAACTAGCCAGGCACGATGGCAGGCGTCTGTAATCCCAGCTACTCAGGAGGCTGAGGCACG
AGAATCGCTTGAACCCAGGAGACGGAGATTGCAGTGAGCCAAGATCTTGCCACTGCACTCCAACCTGGGCTAGCGAGCAAGACTCTGTCTCAA TATATATATAGTTGTTTATTGTCTATTTCTCCACC
CCAACTAGAATGTAAGCTCCAAAAAAAACAGGAAACTTGGAATTTATTTATTTATTTAGAGATGGGGTCTTGCTCTGCCACCCAGGCTGGATGGAGTGCAGTGGTGTGATCACAACTCATTGCAG CCA
CGGTCTCCTGGGCTCAAGCAATCCTCCCACCTCAGCCTCCCAAATAGCTGGGACTACAGGCACACACCA cCATGCCTGGCTAATTAAAAAACAAACAAACAAACAAACAAACAAAGCACTTTGTAGAG
ATGAAGTCTCACTATGTTTCCCAGACTGGTCTTGAAATCCTGGGTTCGAGTGATCCTCCCACCTCGGCCTCCCTAAGTGTTGGGATCACAGGTGTGAGCCACCATGCTC aGTGCCTTGGATTTTATTC
ATCGCTTCACCCTCAGTGCCAAACCAGCAGTAGGCAGGCAGTTCATCGTTGTTAAATAAACGTTTGTCGGC tGGGCGCGGTAGCTCACACCTGTAATCCCAGCACTTTGGGAGGCCAAGGCAGGCGGA
TCACCTGAGGTCTGGAGTTTGAGACCAGCCTGACCAACATGGAGAAACCCCGTCTCTACTAAAAATACAAAATTGGCCGGGCGTGATGGCGCATGCCTGTAATCCCAGCT ACTCGGGAGGCTGAGGCA
GGAGAATGGCTTGAACCcGGGAGGCGGAGGTTGCTGTGAGCCAAGATCGCGCCATTGCACTCCAGCCTGGGCAACAAGAGTGA A

316	13.3	0.0	0.0	114481	1	45	(2087)	+	AluSq2	SINE/Alu	255	299	(14)	1
1051	9.0	0.0	0.0	114481	940	1072	(1060)	+	AluSx	SINE/Alu	3	135	(177)	2
1977	11.6	0.0	6.4	114481	1073	1373	(759)	+	AluSx3	SINE/Alu	1	283	(28)	3
346	27.8	0.0	11.3	114481	1378	1450	(682)	+	L2a	LINE/L2	3272	3335	(91)	4
1798	17.4	0.7	1.4	114481	1451	1624	(508)	C	AluJo	SINE/Alu	(14)	298	129	5
28	0.0	0.0	0.0	114481	1625	1651	(481)	+	(AAAC)n	Simple_repeat	1	27	(0)	6
1798	17.4	0.7	1.4	114481	1652	1775	(357)	C	AluJo	SINE/Alu	(184)	128	3	5
346	27.8	0.0	11.3	114481	1776	1859	(273)	+	L2a	LINE/L2	3336	3413	(13)	4
2319	5.5	0.4	0.0	114481	1861	2131	(1)	+	AluSp	SINE/Alu	1	272	(41)	7

316	13.3	0.0	0.0	hg	1	45	(2956)	+	AluSq2	SINE/Alu	255	299	(14)	8
1061	9.0	0.0	0.0	hg	939	1072	(1929)	+	AluSx	SINE/Alu	2	135	(177)	9
2373	9.2	0.3	0.0	hg	1073	1367	(1634)	+	AluSg	SINE/Alu	1	296	(14)	10
778	28.2	3.3	10.6	hg	1675	1935	(1066)	+	L2a	LINE/L2	3018	3267	(159)	11
1967	11.2	0.0	7.1	hg	1936	2238	(763)	+	AluSx1	SINE/Alu	2	284	(28)	12
778	28.2	3.3	10.6	hg	2239	2315	(686)	+	L2a	LINE/L2	3268	3335	(91)	11
1798	17.4	0.7	1.4	hg	2316	2489	(512)	C	AluJo	SINE/Alu	(14)	298	129	13
33	0.0	0.0	0.0	hg	2490	2520	(481)	+	(AAAC)n	Simple_repeat	1	31	(0)	14
1798	17.4	0.7	1.4	hg	2521	2644	(357)	C	AluJo	SINE/Alu	(184)	128	3	13
778	28.2	3.3	10.6	hg	2645	2728	(273)	+	L2a	LINE/L2	3336	3413	(13)	11
2319	5.5	0.4	0.0	hg	2730	3000	(1)	+	AluSp	SINE/Alu	1	272	(41)	15

>hg19 chr17:1911535-1914535
CCTGGGCAACAAGGGCTAGACTCCATCTCAAAAAAAAAAAAAAAGACCACCCCAAACTGAGGCTTAGAATTACAAACATAATGAAAAATAAAACAAAATTTTGACACATTAAAAAAAAGGAATTACCAA

CATAGAAAGGTGTTTGAGAAATACTGTTAATTAAACGAGACGGGTGCATCTGCGTGAACGTAGGAAGGAAGCGTAGCCGGATTATGAAATGCACGAACACCCTGGGAGGGGTGAGCTTTCCCACA CTT
TGCTCTTTGGGCTGTGGGGTTGTAGTGATTTTCTTCTTTTTGCCAATCTGTAGGTTTCCAATCTGTGTTTGCTCCCTGGGTATGCATTTGGGAA gTTCAAAAAAATTCTGTGGCTGTTGAGTCAACCA
ACCTCCTCCTCCCTGGTTCTTGAGGCTTTGCAACAGTTGGGCTCCCACCAATCCGCCCTACCACTAACTGATC tGCCACCGATCCGTGGCACGAACAGTGACTGTGGCCTAAGCACGGGTTGTCGCGT
TGCTCTCCAGCCTTTCCAACCTTCCTGTCAAGCCTCTCTTCCTCTCACAAAGCCTCCCAGGTGCACCTGGGAGCAGGTACCTCACCTCCAGCCGCCTTCCCCCTGGCTTGAAAGTGCCTTCCTTA TCA
CCTACACCATCCTTTCACTTATCCAGGGTGTGGCTTCTCTCTCCTAGCAGGTTGTAAATTTCCTGGGCCTGCAGACTGAGCCTCAGCCCAGGGTTTACTCACTCCCACCCTGcCTGGAACACAGTGAG
AGTTCATTcAGGAAATGACTACAAGGCACAGGGGCTCTGGTCTGGCTGGAACTGATGGTGTAAATGGGGAGAAAAGGGTGATGTATCAATGAGACAGGGACTGAAAAAGACAA gGCTGTGCCCAGAAC
TAAGTTCTATGTTGTATGCTGTGGAGTAAAAGGGCAGGGTTC gCCGGGCTTGGTGGCTCACACCTGTAATCCCAGCACTTTGGGAGGCCGAGGCAGGTGGATCACCTGAGGTCAGGAGTTCAAGACCA
GCCTGGTCAACAAGGGGAAACCCTGTCTCTACTAAAAGTACAAACATT GGCCAAGTGCGGTGGCTTACGCCTGTAATCCCAGCACTTTGGGAGCCTGAGGTGGGC aGATCATGAGGTCAGGAGTTTGA
GACCAGCCTGGCCAACatggtgaatcccatctctactaaaaacacaaaaattagctgggcgtggtggtgtgtgc ctgtaatctcagctactcgagaggctgaggtaggagaattgcttgaagccagga
ggtggaggttgcaatgagccgagatcacgccactgcactccagcctgggcgacagagcaagactccgtctcaaaaaaaaaaaaaaaa ggcaggattcatgggaatcaatctctgaccttatactcaaa
ctgcagcctgggccacataaacagctgagaaagagaggcatctgaccccgatgtaaaggggatccccagatcg gccacttctcctcctgcccgctgccatagctgttgccttggtccctgaacccct
ggccaagtggctgatggacttgggactgaccgcaagttctggtactctccaaaggaatctcaaaccactccacaggccacaacacagaatgccggccctgtccacagccccagccacggagacag cca
ggtactgtagcaagcacatgcctgacgcagggcctttgcacctgctattgttccttctgccaggagagccctcc ccagggagaagcagctctaatgtctgcagcgccctccctcactccctcacttcg
cttaggcctctgttcgaatgtccgtctatcagaaagacttctctgccaatctcctaaaataaccgctctctggtcaccctctgttcttcaactgttaattttcttcaaagcacttaccccacctg ata
gaatatagttgtttagccaggcgcggtagctcacgcctataatcccagcactttgggaggccgatgtgggtaga tcacttgaggccaggagttcaagaccagcctggcctacATGGTGAAACCCCATC
TCTTCTAAAAATAAAAAAAtAATAATAAAAAATAAAAAACTAGCCAGGCACGATGGCAGGCGTCTGTAATCCCAGCTACTCAGGAGGCTGAGGCACGAGAATCGCTTGAACCCAGGAGACGGAGATTG
CAGTGAGCCAAGATCTTGCCACTGCACTCCAACCTGGGCTAGCGAGCAAGACTCTGTCTCAA TATATATATAGTTGTTTATTGTCTATTTCTCCACCCCAACTAGAATGTAAGCTCCAAAAAAAACAG
GAAACTTGGAATTTATTTATTTATTTAGAGATGGGGTCTTGCTCTGCCACCCAGGCTGGATGGAGTGCAGTGGTGTGATCACAACTCATTGCAGCCACGGTCTCCTGGGCTCAAGCAATCCTCCC ACC
TCAGCCTCCCAAATAGCTGGGACTACAGGCACACACCAtCATGCCTGGCTAATTAAAaaacAAACAAACAAACAAACAAACAAACAAAGCACTTTGTAGAGATGAAGTCTCACTATGTTTCCCAGACT
GGTCTTGAAATCCTGGGTTCGAGTGATCCTCCCACCTCGGCCTCCCTAAGTGTTGGGATCACAGGTGTGAGCCACCATGCTC gGTGCCTTGGATTTTATTCATCGCTTCACCCTCAGTGCCAAACCAG
CAGTAGGCAGGCAGTTCATCGTTGTTAAATAAACGTTTGTCGGCcGGGCGCGGTAGCTCACACCTGTAATCCCAGCACTTTGGGAGGCCAAGGCAGGCGGATCACCTGAGGTCTGGAGTTTGAGACCA
GCCTGACCAACATGGAGAAACCCCGTCTCTACTAAAAATACAAAATTGGCCGGGCGTGATGGCGCATGCCTGTAATCCCAGCTACTCGGGAGGCTGAGGCAGGAGAATGGCTTGAACC tGGGAGGCGG
AGGTTGCTGTGAGCCAAGATCGCGCCATTGCACTCCAGCCTGGGCAACAAGAGTGA A

Homologous Sequences

ACATGGTGAA

>NAHR_33_ Scaffold10990 1044469 1044767
KSD_NAHR_33

>Scaffold10990 1044469 1044767_NAHR

CTGATGCTGTGGCCAAAAGTCTCAGAGAATCGCAGGTATCTTATGAAGGAGAAAGAAAATAACACATGTAGAACAAAACAAGATGGCATAATACCTGCCTCAAAGCTTGCAGTGGCAGCTTTGGCA TT
AGATTCTGCCCAGCTCAGTCCCCAGAATATGGATTCCCCTCCATGGAAGCATCTCATTGCTAAAAACCCTTACAAGATGTGTGAGAAACTTCTTCCCCCTTAAATGGCTGACTGAACAAATTTAA CGG
AGGCTGTTATCCACTGAGTTTAGTATCAAAGCTCTGAGATCCAGTTCTCTTTGAGTTTTTGCTTTCATCTGGAATCTTGCGCCCCTGCCCTCTTTGCTGCCGTCACTGCACAATGTCAAACAGCTG GA
GTCTGGGCAGCACCAGAATCCAACTGTCTCAGCCTAACAAGTGAAGGGATGGGGGAAGAACAGATGGCGGGAGATGAGGAAGGTGGGGGTGTCATGGAGGACGAAAGGGGGCTGTGCTTGCTTTA GAG
AATCTGATATCTTGGAAAAGTGGTCTCACGTGGAATTCAGTGTCAATAGATTCTATTTGTCTAGGGCTTTGCAAAATCAAGCACACAGAAGTGGCCTTGGACTATTCTATTTTTCATTTCTCTAAC CC
AGCTTCACCTCTCACTCAAACTTTTACTAATTTTCCCTCCATTTATTGAGTGCCTACCAAGGGTTTATATATACATATATACACATATATATATGTGTATGT aTATATATATGTGTATACATATATAT
GTGTATATATAGAGAGAGAAATAGTACATATATATTATTTCCAATATTCACAGAAACTAGGGTTTAGGGATGTAATTAATTTGTCTAAGCTCATGCAGCTTATTTAAGACTGGATTCAAACCCAGG TT
TGTCTTGATGCCACAATATTCTTTTTTTAAAAATCTCATCCTCGGCCAAGCACAATGGCTCACGCCTGTAGTCCTAGCACTTTCGGAGGACGAGGCAGGTGGACCAGTTGAGCCCAGGAGTTCGAGAC
CAGCCAGGCCAACTTGGAGAAACACTGTCTCTACTAAAAATACAAAAAATTAGCCGGCCTGGTAGCAGGCACCTGTAGTCCCAGCTACTCCAGAGGCTGAGGCAGGAGAATGG tGTGAAcCCGGGAGG
tGGAGCTTGCAGTGAGCCAAGATCACACCACTGCACTCCAGCCTGGAAGACAGAGTGAGACCCTGTCTCTAAATAAATAAA aAAAATATCATTCTCCCCCTGGGGTACACTTGTCCATAGGGAGCCTT
GTGGGGATCTGGTGATTAATTCCTAAAAGTCAACGGAACATATAGCCAGTAGGAATGCCTGAGTTGACAGCCTGGGGCTAGAGAAGTCAGTCCCAGCCAGTTCAACTTGTATAAGGAATAGATGG CAT
CATACCAGGCTCACCTGTAGTTCTGCCTGAACCATGCAATTTAGGGTTTAATTATACGCTGTCTTGCATTGTTTCTACTAATTGTT GCATAGGTCAATCTTATACTTTCATTCATCATTCTTCCTTCA
GTCAACAAATATTTTAGAGCAACTGGGCATATTTCTTCTCCAGCAGCCCTTGCAATACCCAGCATAGAA aGGTAAGCCAACTCAGATAACTAGTTAATGGTCCAGTCAAGAGCAAATGAGGGCTAATT
TTCCTAATTATAAATTAATGGATTATGTTATGCATCTTGAAATTGTACTGAGATATTGCCTTAATAAATAGACACTTTATACACTC AACAGCTTGAATTTTAAAGCAGAAAGTTGGGTTCATGAATAT
CTCATTTCCAGTATCATTTATCAAGATAAAATGTCTTTATCCAAATATAAGCTTTTGAGAATAGTTATGTGTACAAAAGCCAATTTACTAATATTGTTAAAACTGTGAATTTTGGTATCTCGTTT ATT
TGTTTTTTTTTGTTTTTGAGATGGAGTCTCcCTCTATTGCCCAGGCTGGAGTGCAGTGGCACGATCTCAACCCACTGCAACCTCCC GCCTCCCGGGTTCAAGCGACTCTTCTGCCTCAGCCTCCTGAG
TAGCAGGGATTATAGGCATGCGCCACCACGCCTGGCTAATTTTTGCATTTTTAGTAGAGATGGGGTTTCACCATGTTGGTCAGGCTGGTCTCAAAACTCCTGACCTCGTGATCCGCCCCCCTCAG CCT
CCCAAAGTGCTGGGGTTACAGGCGTGAGTCACCGCACCTGGCCGGTATGTCATTTTTAATCTTAACCCATACATCAGAACTCCATT CTCTTCCTGTATATAATATCCTTAGACTCTTACATT

42	17.6	0.0	0.0	a	706	779	(1519)	+	(TA)n	Simple_repeat	1	74	(0)	1
266	29.8	9.7	1.4	a	782	920	(1378)	C	MIRb	SINE/MIR	(93)	175	22	2
1959	14.4	0.3	0.7	a	940	1238	(1060)	+	AluY	SINE/Alu	1	298	(13)	3
311	17.7	2.2	8.1	a	1420	1511	(787)	+	L2d	LINE/L2	3206	3292	(133)	4
2337	9.5	0.0	0.7	a	1915	2219	(79)	C	AluSg	SINE/Alu	(7)	303	1	5

.

40	19.6	0.0	0.0	hg	706	779	(1656)	+	(TA)n	Simple_repeat	1	74	(0)	6
266	29.8	9.7	1.4	hg	782	920	(1515)	C	MIRb	SINE/MIR	(93)	175	22	7
1971	12.5	1.4	0.7	hg	940	1234	(1201)	+	AluY	SINE/Alu	1	297	(14)	8
787	14.3	18.6	0.0	hg	1235	1374	(1061)	+	AluSx	SINE/Alu	134	299	(13)	9
311	17.7	2.2	8.1	hg	1556	1647	(788)	+	L2d	LINE/L2	3206	3292	(133)	10
2372	9.2	0.0	0.7	hg	2051	2356	(79)	C	AluSg	SINE/Alu	(6)	304	1	11

>hg19 chr3:112923035-112925469

CTGATGCTGTGGCCAAAAGTCTCAGAGAATCGCAGGTATCTTATGAAGGAGAAAGAAAATAACACATGTAGAACAAAACAAGATGGCATAATACCTGCCTCAAAGCTTGCAGTGGCAGCTTTGGCA TT
AGATTCTGCCCAGCTCAGTCCCCAGAATATGGATTCCCCTCCATGGAAGCATCTCATTGCTAAAAACCCTTACAAGATGTGTGAGAAACTTCTTCCCCCTTAAATGGCTGACTGAACAAATTTAA CGG
AGGCTGTTATCCACTGAGTTTAGTATCAAAGCTCTGAGATCCAGTTCTCTTTGAGTTTTTGCTTTCATCTGGAATCTTGCGCCCCTGCCCTCTTTGCTGCCGTCACTGCACAATGTCAAACAGCTG GA
GTCTGGGCAGCACCAGAATCCAACTGTCTCAGCCTAACAAGTGAAGGGATGGGGGAAGAACAGATGGCGGGAGATGAGGAAGGTGGGGGTGTCATGGAGGACGAAAGGGGGCTGTGCTTGCTTTAGAG
AATCTGATATCTTGGAAAAGTGGTCTCACGTGGAATTCAGTGTCAATAGATTCTATTTGTCTAGGGCTTTGCAAAATCAAGCACACAGAAGTGGCCTTGGACTATTCTATTTTTCATTTCTCTAAC CC
AGCTTCACCTCTCACTCAAACTTTTACTAATTTTCCCTCCATTTATTGAGTGCCTACCAAGGGTTTATATATACATATATACACATATATATATGTGTATGT gTATATATATGTGTATACATATATAT
GTGTATATATAGAGAGAGAAATAGTACATATATATTATTTCCAATATTCACAGAAACTAGGGTTTAGGGATGTAATTAATTTGTCTAAGCTCATGCAGCTTATTTAAGACTGGATTCAAACCCAGG TT
TGTCTTGATGCCACAATATTCTTTTTTTAAAAATCTCATCCTCGGCCAAGCACAATGGCTCACGCCTGTAGTCCTAGCACTTTCGGAGGACGAGGCAGGTGGACCAGTTGAGCCCAGGAGTTCGAGAC

CAGCCAGGCCAACTTGGAGAAACACTGTCTCTACTAAAAATACAAAAAATTAGCCGGCCTGGTAGCAGGCACCTGTAGTCCCAGCTACTCCAGAGGCTGAGGCAGGAGAATGG cGTGAACCGGGAGGc
GGAGCttgcagtgagccgagatcacacaactgcactccagcctgggtgacagagcaagacactgtcaaaaaaaaaaaaaaaa tcaactgggcatggtggtgtgcgcctgtaatcccagctactcaggt
ggctgaggcagaggTTGCAGTGAGCCAAGATCACACCACTGCACTCCAGCCTGGAAGACAGAGTGAGACCCTGTCTCTAAATAAATAAA tAAAATATCATTCTCCCCCTGGGGTACACTTGTCCATAG
GGAGCCTTGTGGGGATCTGGTGATTAATTCCTAAAAGTCAACGGAACATATAG CCAGTAGGAATGCCTGAGTTGACAGCCTGGGGCTAGAGAAGTCAGTCCCAGCCAGTTCAACTTGTATAAGGAATA
GATGGCATCATACCAGGCTCACCTGTAGTTCTGCCTGAACCATGCAATTTAGGGTTTAATTATACGCTGTCTTGCATTGTTTCTACTAATTGTTGCATAGGTCAATCTTATACTTTCATTCATCA TTC
TTCCTTCAGTCAACAAATATTTTAGAGCAACTGGGCATATTTCTTCTCCAGCAGCCCTTGCAATACCCAGCATA GAAcGGTAAGCCAACTCAGATAACTAGTTAATGGTCCAGTCAAGAGCAAATGAG
GGCTAATTTTCCTAATTATAAATTAATGGATTATGTTATGCATCTTGAAATTGTACTGAGATATTGCCTTAATAAATAGACACTTTATACACTCAACAGCTTGAATTTTAAAGCAGAAAGTTGGG TTC
ATGAATATCTCATTTCCAGTATCATTTATCAAGATAAAATGTCTTTATCCAAATATAAGCTTTTGAGAATAGTT ATGTGTACAAAAGCCAATTTACTAATATTGTTAAAACTGTGAATTTTGGTATCT
CGTTTATTTGTTTTTTTTTtGTTTTTGAGATGGAGTCTCgCTCTATTGCCCAGGCTGGAGTGCAGTGGCACGATCTCAACCCACTGCAACCTCCCGCCTCCCGGGTTCAAGCGACTCTTCTGCCTCAG
CCTCCTGAGTAGCAGGGATTATAGGCATGCGCCACCACGCCTGGCTAATTTTTGCATTTTTAGTAGAGATGGGG TTTCACCATGTTGGTCAGGCTGGTCTCAAAACTCCTGACCTCGTGATCCGCCCC
CCTCAGCCTCCCAAAGTGCTGGGGTTACAGGCGTGAGTCACCGCACCTGGCCGGTATGTCATTTTTAATCTTAACCCATACATCAGAACTCCATTCTCTTCCTGTATATAATATCCTTAGACTCT TAC
ATT

Homologous Sequences
TTGCAGTGAGCC

>NAHR_34_Scaffold15785 392507 392805
KSD_NAHR_34

```
>Scaffold15785 392507 392805_NAHR
gcgtgaaccactgcgcccggccctgtttgtctgtttgtttaaggcagcgtatggctctgtcgttcag GGTGGAGTGCAGTGGCGCAATCATGACTCACTGCAGCCTTGAACTCCTGAATGCCGCCTTG
AATTCCTGGATTCCAGCGACTCTCCCACCTCAACCTCCTGAGTAGCTGAGACCAAAGGCACGTGCCACACTCCCGGCTAATTTAT TtttaTTTATTTATTTATTTATTTATTTATTTATTTATTTAGA
GACATAGTTTTGCTCTTGTTGCCCCAGGCTGGAGTGCAATGGCGCGATCTTGGTTCACCACAACCTCC ATCTCCTGGGTTCAAGCGATTCTCCTGCCTCAGCCTCCTGAGTAGCTGGGATTACAGGCA
TGTGTCACCATGCCCAGCTAATTTTGTATTTTTAGTAGAGATGGGGTTTCTCCATAAATTTTCCTATTTTTTGTAGAGACCGGAGTCTCACTCTGTTGCCCAGGCCGGTCTTGAACTCCTGGCCT CAA
ACCATCCTCCCGCCGCAGCCTCCCAAAGTGCTGGTATTACAGGCGTGAGCCACTACACCTGACCGAAA TTTTCATTTTATAAGGAGGGACGCTTCAGGCCTGGAGGAATTAGCTAGCCTAGAGACCCA
GGCTGTCAATGGCAGGTGTGGGACTCATATCCCGTTCTGTGAGACTTGCAAGCATGGGACAACATTGCTTCCATCTGTGCCCCTTGCTGTAAGGAGCCCGTGTTAGGAACCCCTCGAGTTCAGGG TCT
GAAACCACCCTGCAGCTGGCGGGGTGGAGTCTGAGCGCTTTTCAGTACCCCCACTGCCCCACCCTGGT CTGAGCCACCTTCATCTCCTGCCTGGGCTCATCACCCCACTGTGCTTTCTCATTCTGCTG
TGCCCTCTTCAGCAGCCAGGGTGACTTTGTGAACCTTCAGATAAAGCCTGCACCCTGCCTGGGACTTTGCAAGGCTGCACTTTGTGCATAAAATTACATTGCA GGCCAGGCGCGGTGCCTCACGCCTG
TAATCCCAACACTTTGGGAGGCTAAGGTGGGCGAATCACGAGGTCAGGAGGAGATCGAGACCATCTTA GCCAACATGGTGAAACCTCGTCTCTACTAAAATT aAAAAAAAAAGTTAGCCAAGTGTGGT
GGCACGCACCTGTAGTCCTGGCTACTTGGGAGGCTGAGGCAGGGGAATCGCTTGAACCTGGGAGGCAGAGGTTGCAGTGAGCCAAGATCGCGCCCTGCACTCTAGCCTGGCAACAGAGCAAGACT CCG
TCTCAAAAAAAAAAAAAATTACcCTCCAGCCCCTGGCAAAGGCCTTTCCCTGGTAGTCACACATCAAA AGTTTGTAAGGGCTCATTTATCTGTCTCCCTGGTTACTATTTGCCTCTATCACCCACCCC
ACTGCCTGCCGCCTTTTCCCCCTCTGAAAAGACATCAGGGTCATTTTTGCTCCAGGGGTTCTA aATTCTGTGTTTCCTCTGCCAGGAAtAGcCCcCCTGCAGGTTTTCTGTTACTGGATGGAAAGTCT
TGGCTGTGAGTTGGTCAGGGGCTTGCGCGTTGAACCGAGAATTGCAACACAAGGCCGGGCACGGTGAC TCACCCCTATAATCTCAGCACTTTGGGGAGGCCGAGGCGGGTGGATCACCTGAGGTCAGG
GGTTCAAGACCAGCCTGGCCAACATGGCGAAACCCTGTCTCTACTGAAAATACAAAAACTAGGCCAGGCACGGTGGCTCATGCCTGTAATCCCAACACTTTGGGAGGCTGAGGCAGGAGGATCAC TTG
AAGTCAGGAGTTCAAGACCAGCCTGGCCAACATGGCGAAACCCCGTCTCTCCTAAAAATTAGCTGGAT GTGGTGGCACATGCCTGTAATCCCAGCTACTCGGGAGGCTGAGGCAGGAGAATTGCTTGA
ACCCGGGAGATGGAGGTTGCAGTGACCCAAAATCACACCACTGCACTCCAGCCTGGGCAACAAGAGCAAAACTCCATCTCAAAAATAATAAATAAATAAAAATTGCAACAAAAACCCCACAAAGC AAC
AACGACAACAACAAAAAACAAATCAACAAAAGACAAACAAATGGCGTAAAAAGCACAGATTGATTGAC ACGAAGTTCAAGAGACCACACAGCTCAAGAGACCCCTCAATTAGGGTCTTCATTAAGCAG
AAAGAATGTGGTTAACACTCATGGCTGAAGTGGAAACTTCTGTCTTGTTATCACAGAAGTGCAGATGTGGCCTCTATGCTGCCTTATCTTGCCCAGAACTGGCTGCACCTGCTGATCTTTT G
```

856	16.1	0.5	12.0	A	26	211	(2087)	C	AluJr	SINE/Alu	(15)	297	131	1
1784	11.2	0.3	14.1	A	215	576	(1722)	C	AluSp	SINE/Alu	(0)	313	1	2
303	19.1	1.5	0.0	A	795	862	(1436)	+	L2	LINE/L2	2647	2715	(704)	3
2223	9.4	0.3	2.4	A	1000	1298	(1000)	+	AluSc	SINE/Alu	1	293	(16)	4
252	26.8	1.4	1.4	A	1517	1588	(710)	C	MER51E	LTR/ERV1	(0)	640	569	5
1074	8.8	0.0	0.7	A	1589	1725	(573)	+	AluSx	SINE/Alu	1	136	(176)	6
2162	11.1	2.7	0.3	A	1726	2022	(276)	+	AluSz	SINE/Alu	1	304	(8)	7
357	16.2	23.5	5.5	A	2027	2196	(102)	C	MER51E	LTR/ERV1	(34)	448	250	5
777	8.0	0.0	0.0	A	2199	2298	(0)	C	MER51E	LTR/ERV1	(334)	148	49	5

695	12.7	0.7	16.3	hg	3	144	(2800)	C	AluJr	SINE/Alu	(59)	253	131	8
1784	11.2	0.3	14.1	hg	148	505	(2439)	C	AluSp	SINE/Alu	(0)	313	1	9
303	19.1	1.5	0.0	hg	724	791	(2153)	+	L2	LINE/L2	2647	2715	(704)	10
2224	9.6	0.3	2.4	hg	929	1229	(1715)	+	AluSc	SINE/Alu	1	295	(14)	11
266	25.4	1.4	1.4	hg	1448	1519	(1425)	C	MER51C	LTR/ERV1	(0)	640	569	12
1098	8.0	0.0	0.7	hg	1520	1656	(1288)	+	AluSx	SINE/Alu	1	136	(176)	13
2226	8.7	3.5	0.0	hg	1657	1944	(1000)	+	AluSx	SINE/Alu	1	298	(14)	14
252	26.8	1.4	1.4	hg	2163	2234	(710)	C	MER51E	LTR/ERV1	(0)	640	569	15
1074	8.8	0.0	0.7	hg	2235	2371	(573)	+	AluSx	SINE/Alu	1	136	(176)	16
2162	11.1	2.7	0.3	hg	2372	2668	(276)	+	AluSz	SINE/Alu	1	304	(8)	17
357	16.2	23.5	5.5	hg	2673	2842	(102)	C	MER51E	LTR/ERV1	(34)	448	250	15
777	8.0	0.0	0.0	hg	2845	2944	(0)	C	MER51E	LTR/ERV1	(334)	148	49	15

>hg19 chr7:6488184-6491127

GGTGGAGTGCAGTGGCGCAATCATGACTCACTGCAGCCTTGAACTCCTGAATGCCGCCTTGAATTCCTGGATTCCAGCGACTCTCCCACCTCAACCTCCTGAGTAGCTGAGACCAAAGGCACGTGC CA
CACTCCCGGCTAATTTATTTTTATTTATTTATTTATTTATTTATTTATTTAGAGACATAGTTTTGCTCTTGTTGCCCCAGGCTGGAGTGCAATGGCGCGATCTTGGTTCACCACAACCTCCATC
TCCTGGGTTCAAGCGATTCTCCTGCCTCAGCCTCCTGAGTAGCTGGGATTACAGGCATGTGTCACCATGCCCAGCTAATTTTGTATTTTTAGTAGAGATGGGGTTTCTCCATAAATTTTCCTATT TTT
TGTAGAGACCGGAGTCTCACTCTGTTGCCCAGGCCGGTCTTGAACTCCTGGCCTCAAACCATCCTCCCGCCGCAGCCTCCCAAAGTGCTGGTATTACAGGCGTGAGCCACTACACCTGACCGAAATTT
TCATTTTATAAGGAGGGACGCTTCAGGCCTGGAGGAATTAGCTAGCCTAGAGACCCAGGCTGTCAATGGCAGGTGTGGGACTCATATCCCGTTCTGTGAGACTTGCAAGCATGGGACAACATTGC TTC
CATCTGTGCCCCTTGCTGTAAGGAGCCCGTGTTAGGAACCCCTCGAGTTCAGGGTCTGAAACCACCCTGCAGCTGGCGGGGTGGAGTCTGAGCGCTTTTCAGTACCCCCACTGCCCCACCCTGGTCTG
AGCCACCTTCATCTCCTGCCTGGGCTCATCACCCCACTGTGCTTTCTCATTCTGCTGTGCCCTCTTCAGCAGCCAGGGTGACTTTGTGAACCTTCAGATAAAGCCTGCACCCTGCCTGGGACTTTGCA
AGGCTGCACTTTGTGCATAAAATTACATTGCAGGCCAGGCGCGGTGCCTCACGCCTGTAATCCCAACACTTTGGGAGGCTAAGGTGGGCGAATCACGAGGTCAGGAGGAGATCGAGACCATCTTAGCC
AACATGGTGAAACCTCGTCTCTACTAAAATTtAAAAAAAAAGTTAGCCAAGTGTGGTGGCACGCACCTGTAGTCCTGGCTACTTGGGAGGCTGAGGCAGGGGAATCGCTTGAACCTGGGAGGCAGAGG
TTGCAGTGAGCCAAGATCGCGCCCTGCACTCTAGCCTGGCAACAGAGCAAGACTCCGTCT Caaaaaaaaaaaaaaaattacactccagcccctggcaaaggcctttccctggtagtcacacatcaaaa
gtttgtaagggctcatttatctgtctccctggttactatttgcctctatcacccaccccactgcctgccgccttttcccccctctgaaaagacatcagggtcatttttgctccaggggttctacattct
gtgtttcctctgccaggaatagccccccctgcaggttttctgttactggatggaaagtcttggctgtgagttggtcaggggcttgcgtgttgaaccgagaattgcaacacaaggccgggcacggtg act
cacgcctgtaatctcagcactttggggaggccgaggcgggtggatcacctgaggtcaggggttcaagaccagcctggccaacacggcgaaaccctgtctctactgaaaatacaaaaacta ggccaggc
acggtggctcatgcctgtaatcccaacactttgggaggctgaggcaggaggatcacctgaagtcaggagttcaagaccagcctggccaacatggcgaaaccccgtctctcctaaaaattagctgg atg
tggtggcacatgcctgtaattccagctactccggaggctgaggcaggggaatcgcttgaacctgggaggcggaggttgcagtgagccaagatcgcgccctgcactctagcctggcaacagagcaagac
tccgtctcaaAAAAAAAAAAAAAATTACaCTCCAGCCCCTGGCAAAGGCCTTTCCCTGGTAGTCACACATCAAAAGTTTGTAAGGGCTCATTTATCTGTCTCCCTGGTTACTATTTGCCTCTATCACC
CACCCCACTGCCTGCCGCCTTTTCCCCCTCTGAAAAGACATCAGGGTCATTTTTGCTCCAGGGGTTCTA cATTCTGTGTTTCCTCTGCCAGGAAgAGtCCtCCTGCAGGTTTTCTGTTACTGGATGGA
AAGTCTTGGCTGTGAGTTGGTCAGGGGCTTGCGCGTTGAACCGAGAATTGCAACACAAGGCCGGGCACGGTGACTCACCCCTATAATCTCAGCACTTTGGGGAGGCCGAGGCGGGTGGATCACCTGAG
GTCAGGGGTTCAAGACCAGCCTGGCCAACATGGCGAAACCCTGTCTCTACTGAAAATACAAAAACTAGGCCAGGCACGGTGGCTCATGCCTGTAATCCCAACACTTTGGGAGGCTGAGGCAGGAG GAT
CACTTGAAGTCAGGAGTTCAAGACCAGCCTGGCCAACATGGCGAAACCCCGTCTCTCCTAAAAATTAGCTGGATGTGGTGGCACATGCCTGTAATCCCAGCTACTCGGGAGGCTGAGGCAGGAGAATT
GCTTGAACCCGGGAGATGGAGGTTGCAGTGACCCAAAATCACACCACTGCACTCCAGCCTGGGCAACAAGAGCAAAACTCCATCTCAAAAATAATAAATAAATAAAAATTGCAACAAAAACCCCA CAA
AGCAACAACGACAACAACAAAAAACAAATCAACAAAAGACAAACAAATGGCGTAAAAAGCACAGATTGATTGACACGAAGTTCAAGAGACCACACAGCTCAAGAGACCCCTCAATTAGGGTCTTCATT
AAGCAGAAAGAATGTGGTTAACACTCATGGCTGAAGTGGAAACTTCTGTCTTGTTATCACAGAAGTGCAGATGTGGCCTCTATGCTGCCTTATCTTGCCCAGAACTGGCTGCACCTGCTGATCTT TTG

Break point =

GAGGTTGCAGTGAGCCAAGATCGCGCCCTGCACTCTAGCCTGGCAACAGAGCAAGACTCCGTCTCAAAAAAAAAAAAAA

>NAHR_35_ Scaffold259 1176504 1176805
KSD_NAHR_35

>Scaffold259 1176504 1176805_NAHR
GTTGTAATAAAAACACTTAAAATGAGATCTGCTTGTTACCAGTGGAGGGCGTTCAGGTTCTTCGCATTTTGAACAAAGAACTGGACAGAACGCACAAACAAAACAAGGAGCAAACAAATTTTCATT TA
TTGAAAATGAAAGTACACTCCACAGGGTGGGAGCAGGCCAAGCATAGGGGCTCAAGAGCCCGGATTCAGAATCTTTTGGGGTCCAAATACCTCCTAGAAGTTTGCCATTGGCCACTTGGTGTTCA CCC
CATGCAAATGAATAGTGGTCCACAATCAGAGGCTGAAGTGAAGTTACAAAGTTACACTTCTATGCAAATATCTGATTGGCTGCAGAAAGCAACCAATCAGAGGAACTGTCCATTTTCCATCTGCAG AG
CAGAAAAGCAGGGGGTTTGCAAAGGGAGTAGCTTCTGGTCCTTTTGTTACTTAGGCATGGAAAGTTGGGATTTTCCTTTCGATTTAGCTCTAGAAATTCAGTGTGAATCAGCCTTAGGTTCCCTG CCT
CCAGACCCTATTCTCCTGCCtCATACTCTCTTAACAAATTTTTAAGTAAACAGGTACACAGTATTGTTAACTATGAGCACGGTGTTGTATAGCAGATATCTAGAATATGTTTATCTTGTATAACTGAA
ACTTTATACCTCTTGAATAGCAACTCTCCATTTTGTTACCGAAACACCAGGGATTCAGTCTGAGTCCTGCTGTTTGCTGCACAGATAGCCAACGGCTGAGATGATTACTGCCAAGGAAGAAGGCT TTA
ATAGAGTGCTGCAGTAGGGGAGATGAGAGATCAGTCTCAAATCCATCTCCCTGACTGGCTAAAATTAGGGGTTTATATAGCAGAGAAAAAAATGTAACAATGTATGGGAAAACAGGAATTCTGAAG GG
CTAAGGAAGCAATCATGATGAATGAGGGGCCTGGCATCTCATTGTCTGGATGTGATGATCTGGTGAGTTTCCGTTCTTTGATACCTTCTGAGAGGCCTGGTATTTAATTGTCTGGATCTGGTGAG TTT
CAAGCTTTAAGTTTATTTTTTAAAAACTGTCTATGTCCAGGCGCGGTGGCTCACGCCTGTAATCCCAGCACTTTGGGAGGCCGA GGCGGGTGGcTCACGAGGTCAGGAGATCGAGACCATCCTGGCTA
ACACGGTGAAACCCCGTCTCTAAAAATACAAAAAATTAGCTGGGCGTGGTGGCGGGCGCCTGTAGTCCCAGCTACTCGGGAGGCTGAGGCAGGAGAATGGCGTGAACCCGGGAGGCG tAGCTTGCAGT
GATCCGAtGTGGCGCCACTtCACTCCAGCCTGGGCGACAGAGCGAGACTCCGTCTCAAAAAAAAAAAAAAGACAAAGAAAAAGCAAAGAAAAGAAAGCAAGTGCACAAAGAGAGAATGAGGTCAAGTG
GGGGAGGCAATGAGTTCAATCTGGCAGGAATGCACAGCAGGTTATAAAGATGGCCCCAGAACACGGGAGCTGGGCCATGGATGCATGCAGGGGTTTGACCTGGGGTTGGGGGGACAAG CAGGAATCTC
CAGGTAGCAATCTTGGTGGTGGTGGGGTTCTGGCAGCTCTGCCTGTGGTGCAGCATCAGGGGTTAAGGACGTCCTCCAGAGGAGCGGCGAGGGAAACTAGGGGTGTCAGTAGACGATCTGGGATT CCA
GGCCGCCTCCAGCCTGGCTGAGAACTGCCGGATCAAACAGAAAGCAGAGaTGATGCTTTCTCATTCAGACGGGGCTGGGTGGGAAGGAAAGGCTGTGGCTTTATGGCACACACAAAGT GTGTGGGGTC
AGGACATACCGCAGATCCCCAGCTCCCTGAACCCTCCGCCTGCCTCTCCCGCGCTGGCCCAGCCCCGCAGGCAGAGGCACGAGCCATTGATGCAGTATTTGAAGGTGGCTGAACCCAAGCAGGAG GTG
CAGCCTCTACCCGAGAGGGGGTGCAGGCAGAAGAGTGGGAGAGAAGCCTGGAGGTCTCCTGTGGCCAACGGCCCCATGCCTGCCCCTCAGCAGCATGGGATGACTGGCGAACCCCAG CCGACCCCAC
ATTTACTAGACTCCACGGCTACCGGTAGGGTTACAAGTTTAAGTGACTCTGAGGCCTAAACCTCCGCCCGATTTCTGGGGAGAGTCCAGCAGCGCGCCCTGGTGGCCAACCGTGCACCTCCCAGG TAG
GGAAAGGCCCGCGGGGTCCCTCCTGCCTGCGGGATCCTCAGGCCAGTCCCGCGGGCGCTGCTCTGCTCTCAGCGCTACCCACCTGCAAGGGGCACGGCTCTGACCACAGTAGAGGGAC AGCCCGT

1921	14.2	9.4	0.9	144623	34	535	(1766)	C	MER51A	LTR/ERV1	(0)	634	1	1	
846	14.1	0.0	2.2	144623	536	673	(1628)	C	L1MB1	LINE/L1	(34)	6134	6000	2	
2100	17.0	0.9	0.3	144623	674	1010	(1291)	C	LOR1b	LTR/ERV1	(0)	461	123	3	
2577	4.3	1.0	0.0	144623	1061	1362	(939)	+	AluY	SINE/Alu	3	307	(4)	4	

1921	14.4	9.4	0.9	Hg19	34	535	(5999)	C	MER51A	LTR/ERV1	(0)	634	1	5	
846	14.1	0.0	2.2	Hg19	536	673	(5861)	C	L1MB1	LINE/L1	(34)	6134	6000	6	
2100	17.0	0.9	0.3	Hg19	674	1010	(5524)	C	LOR1b	LTR/ERV1	(0)	461	123	7	
2523	3.2	0.0	0.0	Hg19	1061	1368	(5166)	+	AluY	SINE/Alu	3	311	(0)	8	
4089	13.1	1.9	2.6	Hg19	1398	1929	(4605)	C	L1MB1	LINE/L1	(165)	6003	5476	6	
32	0.0	0.0	0.0	Hg19	1930	1959	(4575)	+	(T)n	Simple_repeat	1	30	(0)	9	
4089	13.1	1.9	2.6	Hg19	1960	2109	(4425)	C	L1MB1	LINE/L1	(693)	5475	5326	6	*
8926	12.3	1.2	3.0	Hg19	2107	2925	(3609)	+	L1M3	LINE/L1	3812	4599	(1541)	10	
2422	9.1	0.0	0.0	Hg19	2926	3223	(3311)	C	AluSx1	SINE/Alu	(14)	298	1	11	
8926	12.3	1.2	3.0	Hg19	3224	3913	(2621)	+	L1M3	LINE/L1	4600	5294	(846)	10	
19	0.0	0.0	0.0	Hg19	4136	4156	(2378)	+	(A)n	Simple_repeat	1	21	(0)	12	
574	26.7	8.9	1.1	Hg19	4625	4865	(1669)	C	MIRb	SINE/MIR	(0)	262	7	13	
2678	3.6	1.0	0.0	Hg19	5291	5595	(939)	+	AluY	SINE/Alu	1	308	(3)	14	

>Hg19 chr1:110185976-110192509
GTTGTAATAAAAACACTTAAAATGAGATCTGCTTGTTACCAGTGGAGGGCGTTCAGGTTCTTCGCATTTTGAACAAAGAACTGGACAGAACGCACAAACAAAACAAGGAGCAAACAAATTTTCATT TA
TTGAAAATGAAAGTACACTCCACAGGGTGGGAGCAGGCCAAGCATAGGGGCTCAAGAGCCCGGATTCAGAATCTTTTGGGGTCCAAATACCTCCTAGAAGTTTGCCATTGGCCACTTGGTGTTCA CCC

CATGCAAATGAATAGTGGTCCACAATCAGAGGCTGAAGTGAAGTTACAAAGTTACACTTCTATGCAAATATCTGATTGGCTGCAGAAAGCAACCAATCAGAGGAACTGTCCATTTTCCATCTGCAG AG
CAGAAAAGCAGGGGGTTTGCAAAGGGAGTAGCTTCTGGTCCTTTTGTTACTTAGGCATGGAAAGTTGGGATTTTCCTTTCGATTTAGCTCTAGAAATTCAGTGTGAATCAGCCTTAGGTTCCCTG CCT
CCAGACCCTATTCTCCTGCCgCATACTCTCTTAACAAATTTTTAAGTAAACAGGTACACAGTATTGTTAACTATGAGCACGGTGTTGTATAGCAGATATCTAGAATATGTTTATCTTGTATAACTGAA
ACTTTATACCTCTTGAATAGCAACTCTCCATTTTGTTACCGAAACACCAGGGATTCAGTCTGAGTCCTGCTGTTTGCTGCACAGATAGCCAACGGCTGAGATGATTACTGCCAAGGAAGAAGGCTTTA
ATAGAGTGCTGCAGTAGGGGAGATGAGAGATCAGTCTCAAATCCATCTCCCTGACTGGCTAAAATTAGGGGTTTATATAGCAGAGAAAAAAATGTAACAATGTATGGGAAAACAGGAATTCTGAAG GG
CTAAGGAAGCAATCATGATGAATGAGGGGCCTGGCATCTCATTGTCTGGATGTGATGATCTGGTGAGTTTCCGTTCTTTGATACCTTCTGAGAGGCCTGGTATTTAATTGTCTGGATCTGGTGAG TTT
CAAGCTTTAAGTTTATTTTTTAAAAACTGTCTATGTCCAGGCGCGGTGGCTCACGCCTGTAATCCCAGCACTTTGGGAGGCCGA Ggcgggcggatcacgagatcaggagatcgagaccatcctggcta
acacggtgaaaccccgtctctactaaaagcacgaaaaattagccgggcgtggtggcgggcgcctgtagtcccagctacttgggaggctggggcaggagaatggcgtgaacccggaggcggagct tgc
agtgagccgagatcgcgccactgcactccagcgtgagcgacagagcgagactccgtctcaaaacaaaacaaaacaaaacaaaaca aaaactgtctctggacagttggattggtttaatttcctcctc
tcccgatcccctgaaaaccgtcattctactttgtttctatgagttggcctattttggataccccatgtcagtggaatcatgcagtatttgttcttctgtgacgagctaatttcacttagcataat gtc
ctccaggttcatccatagtgttatatattgcagaatttccttctttttaagactgattaatatttcactgaatgtacataccaaa ttttctttattcattcatctgttgatgaatagttaggtcattt
tcatgtcttggatattgtgaataatgctacaatgaacaaaggactgctaatatctctttgggatccttcagttcttttgaatatatacccagaagtgggattgctggatcatttggtagttctat ttt
tcattcttttatgaacctctatattgttttccacagtggctgcacaattttactttcccaccaaccgtgtacaaaagttccaatt tctcctcaacagcacttgttaccagttgggagcttgtctcact
ttctttaccttttttttttttttttttttttttttttgacaatagccactctaacaggtgtgaagtgatatttcattgtggttttgatttgcatttctcttatgattagtgatgttgagcatc ttt
tcatatacctgttggccatttgtatgctttcttttgagaaatgtctattcaagtcctttgcaaggatggctcatcatatgtaaaa caattaatgtgacacaccacgttaacaaaatgaaggataaaca
tcaaatgatcatctcaatagatgcagaaaaagcatttgacaaaattcaacatcttttcatagtaaaaactctcaacaaactaggtataaaaggaatttccttgacaaaataaagaccatatatg aca
agctcacagctaacattatactcaacaatgaaaaactgaaagctttgtctgtaagatcaggaacaaggcaaagaatacccgctct tgccacttctagtcaacatagtactggaagtcctaacggagca
attaggcaaggaaaagaaatttaaaagtcatacaaattggaaacgattaagtaacactatctctgtttgcagatgacataatcttatatgtagaaaccctaaagactccacacacaaaaaaaaa ctt
agaatgaataaacaaattccataaagttgcaggatacaagatcaacatacaaaagttagttatgttttggtacactaacaacaaa gtatccaaaagaaaattacaaaaaacaatcccatttgcaatag
caccaagaagaataaaatacttaggaataaactcaaccaaggagatgaaagacttgtatgtaacactgatgaaagaaatcaaagaaaactacataatattgatgaaagaaatcaaagaagacaca agt
aaatggaaaaacatcccatgtttatagattgaaagatctaataatgtttaaatgttcacgctactttgatctacagattcaatgt aatccctatcaaaatctaatggcatttttttttttttttgag
acaaagtctcactctcttgcccaggctggagtgcagtggtgcgatctcagctcactgcaacctctgcctcctgggttcaagcagttctcctgcctcagcctcccgagtagctgggattacaggca ccc
gccaccatgcctgcctaattttttatatttttagtagagacggggttttaccatgttggccaggctggtctcgaactcctgacctc aggtgatccacccatcttggcttcccaaagtgctgggatgaca
ggcatgagccaccacacccagcctctaatggcatttttttacagaaataggaaaaaataatcctaaaattcatatggaaccacaaaagatcccaaataaccaacacaatcttgagaaagaagaac aaa
gctggaggcatcaacttcctgattccaaaatatattacaaagctatagtaattaaaacagtatggtactgtcatataaacagaca caaggaccaatggaacagaacagacagcccagaaataacctaa
gtgtatataatcaactgatcttcaacagggtgccaagaatacacaatggacaaacgatggcctctttaacaaatggttttgggaaaactgaatatggacatacaaaagaataaaattggacccct gtc
ttacaccacactcaaaaatgagctcacaatggattaaagatttaaatataacacctaaacctataagactcctagaagaaaacat aggagaaagtcttcatgacattggtctaagcgatgatttcttt
tatatatcaaaagcacaggcaacaaaagcaaaaatagacgaatggaaccacatcaaactaaaaaccttctgcacagcaaaagaaacaacagtgaaaaggcagtctatggaattggagaaaatatt tgc
aaaccacatatctgagaagaggttagctaacatccaaaatacatatagaactcccacaactcaatagcaaaaattactttaaagc ttttaactatagccttaaaatataattgaataaaatatgtgta
tattaaatgtattttgaataaactgtagtaacttttctaaataaacagaaaatcataccttattctgtacacatacctgtatctctctgcattagtgtatccagtacaattaaccatctactctc att
attatatttaattagagtaaccaatcttcttgtttctcaaaaaaaaaaaaaaaaaaaaaagccccgagaaaagaaagtttaaaac tgtccagctaagactggccacggtgccaccatgcccagcccag
tactgatattttctgagatttgatctcacacttggctgcggggcttttacctaattcctttgagaacaatcctgcctctgctacctttggccagctattggaaggggcatccagctctctgccag cca
cctcaccctactcagacaggggggcccaactccacctcaaaccaggagaagtggaaaagagtctttttttcctgctgttcgtctgaa ggtgcaggtcatccctaagtgcacgtggcagtccaggtctccc
ctgtgccctggaacatccttctccctggcctctgctcctgggtgtcttgagaaacataacttcttgatcaccaatttaagtatgacagtggggtttagaaaaaagctatgttccctttaaagaag ggg
cttatttgcttttgctaataatcccagccaatacttactgagtgtctatgacttgtctactatatatacctcctcaccaaattct caaaacatctctttgaattatttctttcttccctcattttatg
gatgagaaaactgaggctcaggaaaattaagtaacttgcccaaggacacacagctagcaagtcaccaagttgtatctgaccagtccagtcaaccccaagacctgggtctacccaggacaccacac tgc
ctacaaaacgctgcagggtgaaacccacaggatccaaagagccaacaacaatccaagactttccagacaccgtgggggctgatagt agacaaacttggccctaggccaagaggccctcacgtgccaagc
actgtaggcatttcttagaattctttgtgtcttttgttttcattatcagcaagaaatgctacttcacaaggctggaatatttcgtgtaatgtgatcctataaacacacgctaagcggggagtaaa caa
ggcagcagacatgccggagagagacctgagcccgaatgcgtgggaaagcttcagagcagtgcaccggggaacacaaacatcattg gctgcaaaaaaggtccctgtaatgacctttgtcatttggggtt
tactgttgctgtttccctaaatacaaagaaagtgcacaaagaggccaggcgcagtggctcacgcctgtaattccagcactttgggaggccgag GCGGGTGGaTCACGAGGTCAGGAGATCGAGACCAT
CCTGGCTAACACGGTGAAACCCCGTCTCTAAAAATACAAAAAATTAGCTGGGCGTGGTGGCGGGCGCCTGTAGTCCCAGCTACTC GGGAGGCTGAGGCAGGAGAATGGCGTGAACCCGGGAGGCGgAG
CTTGCAGTGATCCGAgGTGGCGCCACTgCACTCCAGCCTGGGCGACAGAGCGAGACTCCGTCT CaAAAAAAAAAAAAAAGACAAAGAAAAAGCAAAGAAAAGAAAGCAAGTGCACAAAGAGAGAATGA
GGTCAAGTGGGGGAGGCAATGAGTTCAATCTGGCAGGAATGCACAGCAGGTTATAAAGATGGCCCCAGAACACGGGAGCTGGGCC ATGGATGCATGCAGGGGTTTGACCTGGGGTTGGGGGGACAAGC
AGGAATCTCCAGGTAGCAATCTTGGTGGTGGTGGGGTTCTGGCAGCTCTGCCTGTGGTGCAGCATCAGGGGTTAAGGACGTCCTCCAGAGGAGCGGCGAGGGAAACTAGGGGTGTCAGTAGACGA TCT
GGGATTCCAGGCCGCCTCCAGCCTGGCTGAGAACTGCCGGATCAAACAGAAAGCAGAG gTGATGCTTTCTCATTCAGACGGGGCTGGGTGGGAAGGAAAGGCTGTGGCTTTATGGCACACACAAAGTG
TGTGGGGTCAGGACATACCGCAGATCCCCAGCTCCCTGAACCCTCCGCCTGCCTCTCCCGCGCTGGCCCAGCCCCGCAGGCAGAGGCACGAGCCATTGATGCAGTATTTGAAGGTGGCTGAACCC AAG

CAGGAGGTGCAGCCTCTACCCGAGAGGGGGTGCAGGCAGAAGAGTGGGAGAGAAGCCTGGAGGTCTCCTGTGGCCAACGGGCCCC ATGCCTGCCCCTCAGCAGCATGGGATGACTGGCGAACCCCAGC
CGACCCCACATTTACTAGACTCCACGGCTACCGGTAGGGTTACAAGTTTAAGTGACTCTGAGGCCTAAACCTCCGCCCGATTTCTGGGGAGAGTCCAGCAGCGCGCCCTGGTGGCCAACCGTGCA CCT
CCCAGGTAGGGAAAGGCCCGCGGGGTCCCTCCTGCCTGCGGGATCCTCAGGCCAGTCCCGCGGGCGCTGCTCTGCTCTCAGCGCT ACCCACCTGCAAGGGGCACGGCTCTGACCACAGTAGAGGGACA
GCCCGT

Homologous Sequences =

TCAGGAGATCGAGACCATCCTGGCTAACACGGTGAAACCCCGTCTCTA

>NAHR_36_ Scaffold13926 246583 246875
KSD_NAHR_36

>**Scaffold13926 246583 246875_**NAHR

TCCCCTAATTTTTGTGGGAGAAAGCTGAAGGCAAAATAATTGAATGTGGTATTTCA tTCCTAGATGAATCCTAGAGGCACCCATGGCATCTTACTTCCCTTAAATAGAAAAGAACCCTTCTCTTCCCT
TCGTCTTTGCATAAGCCTTTGGAGTTATCCTCTGTCAGCTCAGACATTTGGTTTCTCCATAATCCACACATATGAAAATCACAATCATTATTATTTCATGACTTTTTTTTAATACAGTCATGCGC CAC
ATAATGTTTGGTCAACAACAGAGCACATCTATGACAGTGGTGCCATAAGATTATAAAACTATAATTTTACTGTACCTTTTCTATGTTTAAATATGTTTACATACACACAAATACTTACCATTGTGT TA
CAGTTGGGTACAGCACTTGGTACAGTAGCATGCTGTGCAGGTTTGTAGCCTAGGAGCAATAGGCTGTACTATATAGCCTAGATGTATAGTAGGCTATACCATCCAGGTTTGTGTAAGTACACTCT ATG
CTATTCACACGACGATGAACATTGCCAAATGATGCTTTTCTCAAAACATATCCCTATCATTAATCGATGTGACTGTATTAAAATCTCTAGATATAATGGGACCTTTGAATACTGAGTCTATGTTAG TC
ATTGTACCAAAATGTATATCTGGTTTTTACTCTTTTTTACAAAATGTTTTGTTGCTTGAACATCTCCTAAGTAGGATGTAGCAGAAACTAGAAGGGTGTCCAGTCTTTTGGCTTCCCTGGGCCAC ACT
GGAAGAGGAATTGTCTTAGGCCCCACGTAAAATACACTAACACTAATGATAGCTGATAAGCTAAAATAATAATAATAATAATAATAATAATAATA AGCCAGGTGTGGTGGCTCATGCCTGTAATCCCA
GCACTTTGGGAGGATGAGGCAGGCAGATCACCTGAGGTCAGGAGTTTGAGACCAGCCTGGCCAACCTGGTGAAACCCCATCTCTATTAAAAATACAGACATTA GGCCAGGCGCGGTGGCTCATGCCTG
TAATCCCACCACTTTGGGAGGCCGAGGCGGGTGGATCACAAGGTCAGGAGATCGAGACCATCCTGGCTAACATGGTGAAACCCTGTCTCTACTAAAAATACAAAAAATTAGCCGGGCTTGGTGGCG GG
CGCCTGTAGTCCCAGCTACTCGGGAGGCTGAGGCAGGAGAATCGCTTGAACCCTGGAGGCAGAAGTTGCAGTGAGCTGAGATTGCACCATTGCACTCTAACCTGGGTGATAGAGCGCGACTCCATCTC
AAAAAAAAAAAAATTGTAAAAAATATAATGTTTTAAGAAAGTTTATGAATTTGTGTTGGGCTGCATTTAAAGCCATACTTGGCCTCATGGCTCGTGGGCCAGACAAGCTTGAACTAGAATTTTCAAA AC
CTACTTTCTCTAAGCCTCTGTCTACCTTGGACAAAATTGTGGGAGTAGAGGAGGAATTGACAGGAATAGGGTAGACAGTGTGGGTTCATATTAAGACATTAAGAAAACTAATGGAGTTGGATGTA GAA
TAtTCTTTTCCTGTAATCTTTCTGCTTCTACCTAAAGACCACCCCATTTTTGGTAGACGATTAGTCATTTTAAAGTCCAAGAAACGTGGAGGAATCTGAAGCATATTAGCTTTCTCAGAACTCAATTT
TATCTGAAACTCAGGAATTCCTTTTAGTAACTTTTTCAATATCATTTGGTTCTAGCTGTGCTCCTTTCATCATTTCATCTTAATCATAATCCTCAAGTATTTATTGACTATGAAGGATCCCTGCT ATA
TACCAGGGAAACGGTCCTGGAGATAGAAGGTGCTACATGCCTTGGGTCAGAAGCAAAACAGGAAATCTATGATGATTCTTCAGGTCCCAATTAAATCTGGGCTATCGCCAGCAAGAACAGCCTCATTC
CCTCTCTGTTCCTTTCCACTTTTGTTTGGGAAAAGGTGAAGAAGACACAGTAGGGTACCACCACCTCTGATAACCAGGCTGCTGTTAGTTCTGTTCGAGGAGTCAATCTTCTTCCAAATAACCAT ACC
TTACTCTGTTTTCTGATATGATTTGCTAGCCTCGGAAAATATAAGAATGAAAGAAGGATGAGGAAGGAATAGAAGCAACTAGATGTGGATACTTGGTTCAGTTGGGAAAGTGGGATTATATGTAGAAT
ATACAGAGAAGAAAGGAGAAGAATATAATGTAAACATATGGAAAAGGCCTGAACCAGTTCAAATTAATTTATATAGAAAGGGGTATGGAGTCAAATGCGCCTATTTGCCTCCCAA T

2199	13.7	1.7	0.9	39320	243	587	(1705)	+	Tigger3a	DNA/TcMar-Tigger	1	348	(0)	1
799	8.1	0.0	0.0	39320	734	832	(1460)	C	MER30	DNA/hAT-Charlie	(4)	226	126	2
35	0.0	0.0	0.0	39320	833	864	(1428)	+	(AAT)n	Simple_repeat	1	32	(0)	3
1052	10.4	0.0	0.0	39320	865	999	(1293)	+	AluSx	SINE/Alu	2	136	(176)	4
2413	7.8	0.0	0.0	39320	1000	1292	(1000)	+	AluSc8	SINE/Alu	1	293	(18)	5
573	10.6	12.8	0.0	39320	1297	1390	(902)	C	MER30	DNA/hAT-Charlie	(124)	106	1	2

2199	13.7	1.7	0.9	hg	243	587	(1886)	+	Tigger3a	DNA/TcMar-Tigger	1	348	(0)	6
1199	11.1	14.9	0.9	hg	732	832	(1641)	C	MER30	DNA/hAT-Charlie	(1)	229	112	7
38	0.0	0.0	0.0	hg	833	867	(1606)	+	(AAT)n	Simple_repeat	1	35	(0)	8
2163	10.8	0.0	4.1	hg	868	1002	(1471)	+	AluSx1	SINE/Alu	2	129	(183)	9
2567	6.3	0.0	0.0	hg	1003	1304	(1169)	+	AluY	SINE/Alu	1	302	(9)	10
2163	10.8	0.0	4.1	hg	1305	1473	(1000)	+	AluSx1	SINE/Alu	130	294	(18)	9
1199	11.1	14.9	0.9	hg	1474	1571	(902)	C	MER30	DNA/hAT-Charlie	(119)	111	2	7

>hg19 chr12:66709516-66711988

TCCCCTAATTTTTGTGGGAGAAAGCTGAAGGCAAAATAATTGAATGTGGTATTTCA cTCCTAGATGAATCCTAGAGGCACCCATGGCATCTTACTTCCCTTAAATAGAAAAGAA CCCTTCTCTTCCCT
TCGTCTTTGCATAAGCCTTTGGAGTTATCCTCTGTCAGCTCAGACATTTGGTTTCTCCATAATCCACACATATGAAAATCACAATCATTATTATTTCATGACTTTTTTTTAATACAGTCATGCGCCAC
ATAATGTTTGGTCAACAACAGAGCACATCTATGACAGTGGTGCCATAAGATTATAAAACTATAATTTTACTGTACCTTTTCTATGTTTAAATATGTTTACATACACACAAATACTTACCATTGTG TTA
CAGTTGGGTACAGCACTTGGTACAGTAGCATGCTGTGCA GGTTTGTAGCCTAGGAGCAATAGGCTGTACTATATAGCCTAGATGTATAGTAGGCTATACCATCCAGGTTTGTGTAAGTACACTCTATG
CTATTCACACGACGATGAACATTGCCAAATGATGCTTTTCTCAAAACATATCCCTATCATTAATCGATGTGACTGTATTAAAATCTCTAGATATAATGGGAC CTTTGAATACTGAGTCTATGTTAGTC
ATTGTACCAAAATGTATATCTGGTTTTTACTCTTTTTTA CAAAATGTTTTGTTGCTTGAACATCTCCTAAGTAGGATGTAGCAGAAACTAGAAGGGTGTCCAGTCTTTTGGCTTCCCTGGGCCACACT
GGAAGAGGAATTGTCTTAGGCCCCACGTAAAATACACTAACACTAATGATAGCTGATAAGCTAAAATAATAATAATAATAATAATAATAATAATA AtaaGCCAGGTGTGGTGGCTCATGCCTGTAATC
CCAGCACTTTGGGAGGATGAGGCAGGCAGATCACCTGAG GTCAGGAGTTTGAGACCAGCCTGGCCAACCTGGTGAAACCCCATCTCTATTAAAAATACAGACATTA GGCCAGGCGCGGTGGCTCATGC

CTGTAATCCCACCACTTTGGGAGGCCGAGGCGGGTGGATCACAAGGTCAGGAGATCGAGACCATCCTGGCTAACATGGTGAAACCCTGTCTCTACTAAAAATACAAAAAATTAGCCGGGCTTGGT GGC
GGGCGCCTGTAGTCCCAGCTACTCGGGAGGCTGAGGCAGgagaatggcatgaacctggaaggcggagattgcagtgagccgagatcatgccactgcactccagcctgggtgatacagcaagactccat
ctcaaaaaaaaaaaaaaaaaaaaatacaaacattagctgagtgtggtggtgggcacctgtaatcccagctagtcgggaggctgagacagGAGAATCGCTTGAACCCTGGAGGCAGAAGTTGCAGTGAG
CTGAGATTGCACCATTGCACTCTAACCTGGGTGATAGAGCGCGACTCCATCTCAAAAAAAAAAAAATTGTAAAAAATATAATGTTTTAAGAAAGTTTATGAATTTGTGTTGGGCTGCATTTAAAGCCAT
ACTTGGCCTCATGGCTCGTGGGCCAGACAAGCTTGAACTAGAATTTTCAAAACCTACTTTCTCTAAGCCTCTGTCTACCTTGGACAAAAT TGTGGGAGTAGAGGAGGAATTGACAGGAATAGGGTAGA
CAGTGTGGGTTCATATTAAGACATTAAGAAAACTAATGGAGTTGGATGTAGAATAaTCTTTTCCTGTAATCTTTCTGCTTCTACCTAAAGACCACCCCATTTTTGGTAGACGATTAGTCATTTTAAAG
TCCAAGAAACGTGGAGGAATCTGAAGCATATTAGCTTTCTCAGAACTCAATTTTATCTGAAACTCAGGAATTCCTTTTAGTAACTTTTTC AATATCATTTGGTTCTAGCTGTGCTCCTTTCATCATTT
CATCTTAATCATAATCCTCAAGTATTTATTGACTATGAAGGATCCCTGCTATATACCAGGGAAACGGT CCTGGAGATAGAAGGTGCTACATGCCTTGGGTCAGAAGCAAAACAGGAAATCTATGATGA
TTCTTCAGGTCCCAATTAAATCTGGGCTATCGCCAGCAAGAACAGCCTCATTCCCTCTCTGTTCCTTTCCACTTTTGTTTGGGAAAAGGT GAAGAAGACACAGTAGGGTACCACCACCTCTGATAACC
AGGCTGCTGTTAGTTCTGTTCGAGGAGTCAATCTTCTTCCAAATAACCATACCTTACTCTGTTTTCTGATATGATTTGCTAGCCTCGGAAAATATAAGAATGAAAGAAGGATGAGGAAGGAATAG AAG
CAACTAGATGTGGATACTTGGTTCAGTTGGGAAAGTGGGATTATATGTAGAATATACAGAGAAGAAAGGAGAAGAATATAATGTAAACAT ATGGAAAAGGCCTGAACCAGTTCAAATTAATTTATATA
GAAAGGGGTATGGAGTCAAATGCGCCTATTTGCCTCCCAAT

Break point =
CAGGAGAAT

>NAHR_37_ Scaffold9881 149226 149541
KSD_NAHR_37

>**Scaffold9881 149226 149541_**NAHR
CTGAGGAAATTAACAACCCGGGCGTTTCAGCCCCCTGTGAACTTGATTGGAGCATGGTCACAATTTGTAACGAAATGTGCTTGCATGCCCAGTGCTAAAGATTTCTCTCTGTCTACCCTCTAGGCT GT
GGAAAGGTCTGTTCTAACCATTAGCTTGTATCTGGAGCTCAGATGGTGCCGGACACTCCGTAGGAGCTCAGATCTGTTGATAGAAGCCACTAGAGAATGGGACAAGCAACAGGAGGTGTTTTGCT GCC
TAGTAAACTTTTTTTGAGATGAAGTTTCACTTTTGTTGCCCAAGCTGGAGTGCAGTGGTGCCTTCTCGGCTCACCGCAACCTCTGCCTCCTGGCTTCAAGCGATTCTCCTGCCTCAGCCTCCCAAG CA
GCTGGGATGACAGGCACCCGCCACCATGCCCGGCTAATTTTTTTTGTATATTTAGTAGAAATGAGGTTTTGCCATGTTGGCCAGGCTGGTCTCGAGTTCCTGACCTCAAGTGCTCTGCCCGC gTCGGC
CTCCCAAAGTGCTGGGATTACAGGCGTGAGCCACTGCGCCCGGCTCTACCCATCTTTCTTACATGATGATGTTCAGCAGCCAGCCTGATAGGGGAGCATTGTTTGTCCTTTTTTTCCTTATTGCAT TT
TCCCGCATAGGTGCCAGGGCTCAGTAGCATAAATCCAGGTAGAACTGGGAAGTTCCAGGTATCTGGGCTGTTTGCTCTGGTGGACAAAGAGCAGCTGCAAGGTGGG gCTTGATTAGCCAGTGAAGTCT
GAGGATCCAGAGGGAAACTTATTCCTTTCTTTTGTTGAAATCAACATGAGCAGGAAATGGATCTTTCCCCCAATCAGAGAACTTCACATGTTTTTCTAATAGAATAGCAGTGTCTGAGGTATATAC At
GGGGAGTGGCCCCCCGGGAAAACGAGGCCATGAGGCTCCTGGCATGGAGATGACACACGAAGACTCTCGTAACTT cTAGAGGGGAAAATAAAGCAGTCATTCCAGGCTGGGCATGGTGGCTCACGTCT
GTAATCCCAGCACTTTGGGAGGCCGAGGCAGGTGGATCACCTGAGGTCAGGAGTTCGAGACCAGCCTGGCCAACATGGTGAAACCCCAACTCTACTAAAAATAAAAAATTAAAAAAAAA AcATTAGTT
GGGCATGGTGCTGGGCACCaGTAATCCCAGCTACTCAGGAGGCTGAGGCAGGAGAATTGCTTGAACCCGGGAGGCAGA GATTGCAGTGAGCCAAGATCACACCACTGCACTCCAGCCTGGGTGACAAG
AGTGAAACTCCATCTGAAATAAAATAAAATATAAAAGTTAAAGCAGTCATCCTGGCTTTTAGGAACCCGCAGAATCAGGA aGTAAaCTaAAAAGTCGTCTCCCTTTTGTCACGATGGAATGTGAAATA
GGTGCATTTTCCTTCTGACTTATGATTCTGTTTCACGGTGCCTCTGCAGAAATTCAGTACGTCCCCATGGGCACCTGTGT CCTTCAGGAGTTGAGGAAATTTTCTCCATTCCTTGTCCCTGTCCCTAG
ACTCTGCCAGCTCGTGCATGTAGAAGCTCCCGGGAAAACTGTCAACCTGTGCACATTCAAGACCGCCATATACTTGTTATTGAAAAGCCATCAGTCATGTGAGCAGAACTTTTTTATGGTTGAGA TTG
TCCCCAGGGAAACCCGCCTTGCAATTCAGACCTTCCACGGAGATCGATTCCAAACTCTCATCCCGTTCCCACTTCCTCGT TCCCATGGAGGCTGCGGTTCTCTCTCTCTGTGCCGCTTACAGATTGTC
ATACCTATTTCCAAAAATGACACAGAAAACTTCAACGTGTGGCTAAATTTGAACAGAGAAAAATAAGCAAAGAATTCAAGGCGCGAGGCGTTTTCAGGGAGAGAGGGAGAGCTTTTGCATGGTTT GAA
GGGATGATCTTTAATGAGTTGAGGGTCACCTAGAATAGAATTAAACTGTAGAGAGCAAATTGTTGGAAATGCAAGGAGAA ACTTCGGAGAAGACTCCAACACAGCCTGGATCTGGGAGATGAGACTCA
GGTAACAAAAAGTTTGAATAACATGGTTAATAAGATTGTGGTAACAGATAGGACTGAACTTTGCACTCTGCAAACAATATACATTTTAAAGTGCTCTTGGAATAGTTAGAAAAATCAATCATCTG TTA
AATCTGAAAAAAAAcAAAAAAAcaaaaaAACAAaaAAAAaCCCACCCCAAACCAATACATTCCAAAAAAGCAGACATTGTACGGGTTCTAATCTCTGGCTGCAAGTAAGCATAACTGGAATTAATTTT
TAAAAGCTTAA

2201	10.3	0.0	1.0	41503	265	556	(1759)	C	AluSq	SINE/Alu	(23)	290	2	1
2214	10.1	0.0	4.3	41503	1001	1316	(999)	+	AluSq2	SINE/Alu	1	303	(10)	2
231	34.1	6.5	0.0	41503	2053	2175	(140)	+	L1M5	LINE/L1	2602	2732	(3414)	3
24	9.5	0.0	0.0	41503	2183	2216	(99)	+	(A)n	Simple_repeat	1	34	(0)	4

2227	9.9	0.0	1.0	hg	265	556	(1911)	C	AluSq	SINE/Alu	(23)	290	2	5
1829	8.1	0.0	5.1	hg	1002	1248	(1219)	+	AluSx1	SINE/Alu	1	235	(77)	6
679	7.5	0.0	0.0	hg	1249	1328	(1139)	+	AluSp	SINE/Alu	156	235	(78)	7
1070	12.2	0.0	0.0	hg	1329	1476	(991)	+	AluSq2	SINE/Alu	156	303	(10)	8
231	34.1	6.5	0.0	hg	2212	2334	(133)	+	L1M5	LINE/L1	2602	2732	(3414)	9
19	8.2	0.0	0.0	hg	2342	2367	(100)	+	(A)n	Simple_repeat	1	26	(0)	10

>hg19 chr19:7202900-7205366
CTGAGGAAATTAACAACCCGGGCGTTTCAGCCCCCTGTGAACTTGATTGGAGCATGGTCACAATTTGTAACGAAATGTGCTTGCATGCCCAGTGCTAAAGATTTCTCTCTGTCTACCCTCTAGGCT GT
GGAAAGGTCTGTTCTAACCATTAGCTTGTATCTGGAGCTCAGATGGTGCCGGACACTCCGTAGGAGCTCAGATCTGTTGATAGAAGCCACTAGAGAATGGGACAAGCAACAGGAGGTGTTTTGCT GCC
TAGTAAACTTTTTTTGAGATGAAGTTTCACTTTTGTTGCCCAAGCTGGAGTGCAGTGGTGCCTTCTCGGCTCACCGCAACCTCTGCCTCCTGGCTTCAAGCGATTCTCCTGCCTCAGCCTCCCAAG CA
GCTGGGATGACAGGCACCCGCCACCATGCCCGGCTAATTTTTTTTGTATATTTAGTAGAAATGAGGTTTTGCCATGTTGGCCAGGCTGGTCTCGAGTTCCTGACCTCAAGTGCTCTGCCCGC cTCGGC
CTCCCAAAGTGCTGGGATTACAGGCGTGAGCCACTGCGCCCGGCTCTACCCATCTTTCTTACATGATGATGTT CAGCAGCCAGCCTGATAGGGGAGCATTGTTTGTCCTTTTTTTCCTTATTGCATTT
TcCCCGCATAGGTGCCAGGGCTCAGTAGCATAAATCCAGGTAGAACTGGGAAGTTCCAGGTATCTGGGCTGTTTGCTCTGGTGGACAAAGAGCAGCTGCAAGGTGGG tCTTGATTAGCCAGTGAAGTC
TGAGGATCCAGAGGGAAACTTATTCCTTTCTTTTGTTGAAATCAACATGAGCAGGAAATGGATCTTTCCCCCAATCAGAGAACTTCACATGTTTTTCTAATAGAATAGCAGTGTCTGAGGTATATA CA
cGGGGAGTGGCCCCCCGGGAAAACGAGGCCATGAGGCTCCTGGCATGGAGATGACACACGAAGACTCTCGTAACTT aTAGAGGGGAAAATAAAGCAGTCATTCCAGGCTGGGCATGGTGGCTCACGTC
TGTAATCCCAGCACTTTGGGAGGCCGAGGCAGGTGGATCACCTGAGGTCAGGAGTTCGAGACCAGCCTGGCCAACATGGTGAAACCCCAACTCTACTAAAAATAAAAAATTAAAAAAAAA AATTAGTT

GGGCATGGTGCTGGGCACCtGTAATCCCAGCTACTCAGGAGGCTGAGGCAGGAGAATTGCTTGAACCCGGGAGGCAGA Gattgcagtgagccgggagcctgtaatcccagctactcaggaggctgagg
caggagaattgcttgaacccgggaggcagagattgcagtgagccgggagcctgtaatcccagctactcaggaggctgaggcaggagaattccttgaacctgggaggcagag ATTGCAGTGAGCCAAGA
TCACACCACTGCACTCCAGCCTGGGTGACAAGAGTGAAACTCCATCTGAAATAAAATAAAATATAAAA GTTAAAGCAGTCATCCTGGCTTTTAGGAACCCGCAGAATCAGGAgGTAAgCTAAAAGTCG
TCTCCCTTTTGTCACGATGGAATGTGAAATAGGTGCATTTTCCTTCTGACTTATGATTCTGTTTCACGGTGCCTCTGCAGAAATTCAGTACGTCCCCATGGGCACCTGTGTCCTTCAGGAGTTGA GGA
AATTTTCTCCATTCCTTGTCCCTGTCCCTAGACTCTGCCAGCTCGTGCATGTAGAAGCTCCCGGGAAAACTGTCAACCTGT GCACATTCAAGACCGCCATATACTTGTTATTGAAAAGCCATCAGTCA
TGTGAGCAGAACTTTTTTATGGTTGAGATTGTCCCCAGGGAAACCCGCCTTGCAATTCAGACCTTCCACGGAGATCGATTCCAAACTCTCATCCCGTTCCCACTTCCTCGTTCCCATGGAGGCTG CGG
TTCTCTCTCTCTGTGCCGCTTACAGATTGTCATACCTATTTCCAAAAATGACACAGAAAACTTCAACGTGTGGCTAAATTTGAACAGAGAAAAATAAGCA AAGAATTCAAGGCGCGAGGCGTTTTCAG
GGAGAGAGGGAGAGCTTTTGCATGGTTTGAAGGGATGATCTTTAATGAGTTGAGGGTCACCTAGAATAGAATTAAACTGTAGAGAGCAAATTGTTGGAAATGCAAGGAGAAACTTCGGAGAAGAC TCC
AACACAGCCTGGATCTGGGAGATGAGACTCAGGTAACAAAAAGTTTGAATAACATGGTTAATAAGATTGTGGTAACAGATAGGACTGAACTTTGCACTCT GCAAACAATATACATTTTAAAGTGCTCT
TGGAATAGTTAGAAAAATCAATCATCTGTTAAATCTGAAAAAAAAA aAAAAAAAAACAAcAAAAcCCCACCCCAAACCAATACATTCCAAAAAAGCAGACATTGTACGGGTTCTAATCTCTGGCTGCAA
GTAAGCATAACTGGAATTAATTTTTAAAAGCTTAA

Break point =

GGGAGGCAGAGATTGCAGTGAGCC

>NAHR_38_ Scaffold13294 428732 429020
>KSD_NAHR_38

>**Scaffold13294 428732 429020_**NAHR
CTAGACAATGTAATAGAGCCTGCTATGGCACAATGATCTAGTTTTGTTTTTCATTTAATTATGTTCCTGGTTATTCTTGTGAGTATTGACTATCTAGAAAACTTATTCACCCATATGCCATATTTT AG
GATTGAAAGGAATTTTGGACCTCTAAACTTTTCAGACAAGGCAACTGAGGCCCAGAATGATTAGATCTTTGGCCCAGGGGAAGAAAATAGGTTGGTGTCAGCTGGCTTTAACAGTCTTGGCTCCC TGA
CTTCCAGAACAGTTCTATCCCCAGTCAGATTGCTTCTGGTTCGTAAAGTGGGGTAGCAAAGTGGTGACCCGATGACTTCTTTTAAGCTAATAGTCCATATTTAAAAATTGACAGATTA tACATAGGAA
TCTGGACTTCTGGTTTCTCTTCAAAGACTTGGATCCACACTTCTGAAAAACAGATGGTTGGCGTTCATAGTGGCTTCTGCTTCTATACAGGTTATGTGCTTTTCAAATTACCAGAGTCGCCACGC TTT
CCTATTGCCTCCCAGATACTGAGGTTGACTGTCAGCTGCCACTTGTATTTATTTTTACTTTTGAAATTCCTCCATGTTGCACTATTATTTTTCTAATAGTAGAATTAAGACGAAAGTGAACTATTT CC
TTTACCAATGTCTTTTTTTTTTTTTTTtAAGATGGAGTCTCACTCTGTTGCCTAGGCTGGTGTGCGGTGGCGCGATCTCGGTTCACTGCAACCTTCGCCTCCCGGGTTCAAGTGATTCTCCTGCCTCA
GCCTCCCAAGTAGCTGGGATTATAGCGCGTACCACCATGCCCGGCTAATTTTTGTATTTTTAGTAGGGACAGGGTTTCACCATGTTGGGCAGGCTGGTCTCAAACTCCTGACCTCAAGTGATCCACCC
ACTTCAGCCTTACAAAGTGCTGGGATTATAGGTGTGAGCCACTGTGCCTAGCAAAGTGGGCTGTATTTCAAGAAGAAATGGATATTTTAAAAAGTAAAGTGTT GGCCGGGCACGGTGGCTCACACCTG
TAATCCCAGCACTTTGGGAGGCCAAGGTGGGCGGATCACAAGGTCAGGAGATTGAGACCATCCTGGCTAACACGGTGAAA CCCCGTCTCTACTAAAAATACAAAATTAGCCGGTTTTGGTGGCGCATG
CCTGTAATCCCAGCTACTCGGGAAGGCTGAGGCAGGAGAATCACTTGAACCCAGGAGGCAGAGGTTGCAGTGAGCTGAGATCACGCCATTGCACTCCAGCCTGGGCAACAGAAAGAGACTCCATC TCA
AAAAAAAATTATATGTATTATATACACATATAATATATATATTGTGTGTATATATATTTTTATTTTAGAGAGACAGTCTGTCTCTGTCATCCAGGCTGGAGTGCAGTGGTGTGATCATGGCTCACTGC
AGCCTCGACTTCCTGGGCTCAACCCATCTTCCAGTCCTTCCAGCTTCGCCCTCCTGAGTAGCTGGGTCCACAGACATGAGCCACCATGCCTGGCCATTTTTTCTTTTTTCTTTTTTTTTGTAGAA ACG
GTTTCTCACAGTGTTGCCCAGGCTGGTCTTGAACTCCTAGGCTCAAGTGATCCTCCCACTTTGGCCTCCCAAAGTGTTGGGGTTACAGACGTGAGCCACTCTACCTGGCTTCATGATACTATGAAA CA
AATCTATTTGGCACCTGTAGTCACTGGAAGTTGCAACTCTTATGCTAAGTTCTAACACCATAATCAGAAGAACTTTGGATATTTCACTGTGAGGTTGTTGCATGCCTTTGACAAGGTGGGATTTT TTT
TTTTTTTtAATTTTTAGAGATGGGTTCTTGTTATATTGCCCAGACTGGAATGCAGAGTGCAGGCTATTCATAGGCATGATCAAAGTGTACTACAGCCTCAAAATTGTGGGATCAAGCCATCCTTCTGC
CTCAGCCTCCTTCATGGCTGGGACTACAGGTGCCCTGCTACCTTTGACAAGTTTTGAAAGATTTTCATAGCATTGAGGGGAGAAGAAAAGTGAAACAAAGAGAAGAAATGGGACCCTTCTTTATC CTC
CCTCCCCTCAACAGAACTATTAAAACCCGAGATCAGTTTGTTTAGACACTAGTCATATTGTCCAAAAGATTGCGCATTAAGAATCTCTGTCTTAACTTTAGGCAGAATTATCTTGAACTGTTCTAG TA
GGATGGTAATCATGACAGCACAGCTACTGGGGAAATTAGAACTGAGGTTTCCTCTCTTCCGTTTGCTGAAATAACAGCAAAACTGCTTGAAGAAAACGCACTATATTATTC T

243	36.4	2.2	0.8	A	147	292	(1996)	C	MIR3	SINE/MIR	(58)	150	5	1
884	27.5	6.6	7.0	A	336	652	(1636)	+	Charlie8	DNA/hAT-Charlie	82	397	(2060)	2
2306	10.5	0.3	0.0	A	653	948	(1340)	C	AluSz	SINE/Alu	(14)	298	2	3
2322	8.0	0.7	0.3	A	1000	1288	(1000)	+	AluSc8	SINE/Alu	1	290	(21)	4
22	14.7	6.4	0.0	A	1290	1336	(952)	+	(TA)n	Simple_repeat	1	50	(0)	5
1645	16.2	0.0	5.8	A	1337	1645	(643)	C	AluJb	SINE/Alu	(19)	293	2	6
892	17.5	0.0	4.7	A	1786	1962	(326)	C	FAM	SINE/Alu	(7)	178	10	7

243	36.4	2.2	0.8	hg	147	292	(2336)	C	MIR3	SINE/MIR	(58)	150	5	8
900	27.1	6.6	7.0	hg	336	652	(1976)	+	Charlie8	DNA/hAT-Charlie	82	397	(2060)	9
2337	10.5	0.0	0.0	hg	653	948	(1680)	C	AluSz	SINE/Alu	(15)	297	2	10
2611	5.6	0.0	0.3	hg	1000	1302	(1326)	+	AluY	SINE/Alu	1	302	(9)	11
2243	9.3	0.7	0.3	hg	1339	1629	(999)	+	AluSp	SINE/Alu	1	292	(21)	12
22	14.7	6.4	0.0	hg	1631	1677	(951)	+	(TA)n	Simple_repeat	1	50	(0)	13
1645	16.2	0.0	5.8	hg	1678	1986	(642)	C	AluJb	SINE/Alu	(19)	293	2	14
883	17.6	0.0	4.8	hg	2127	2302	(326)	C	FAM	SINE/Alu	(8)	177	10	15

>hg19 chr1:224639203-224641830
CTAGACAATGTAATAGAGCCTGCTATGGCACAATGATCTAGTTTTGTTTTTCATTTAATTATGTTCCTGGTTATTCTTGTGAGTATTGACTATCTAGAAAACTTATTCACCCATATGCCATATTTT AG
GATTGAAAGGAATTTTGGACCTCTAAACTTTTCAGACAAGGCAACTGAGGCCCAGAATGATTAGATCTTTGGCCCAGGGGAAGAAAATAGGTTGGTGTCAGCTGGCTTTAACAGTCTTGGCTCCC TGA
CTTCCAGAACAGTTCTATCCCCAGTCAGATTGCTTCTGGTTCGTAAAGTGGGGTAGCAAAGTGGTGACCCGATGACTTCTTTTAAGCTAATAGTCCATATTTAAAAATTGACAGATTA cACATAGGAA

TCTGGACTTCTGGTTTCTCTTCAAAGACTTGGATCCACACTTCTGAAAAACAG ATGGTTGGCGTTCATAGTGGC TTCTGCTTCTATACAGGTTATGTGCTTTTCAAATTACCAGAGTCGCCACGCTTT
CCTATTGCCTCCCAGATACTGAGGTTGACTGTCAGCTGCCACTTGTATTTATTTTTACTTTTGAAATTCCTCCATGTTGCACTATTATTTTTCTAATAGTAGAATTAAGACGAAA GTGAACTATTTCC
TTTACCAATGTCTTTTTTTTTTTTTTTAAGATGGAGTCTCACTCTGTTGCCTAGGCTGGTGTGCGGTGGCGCGATCTCGGTTCACTGCAACCTTCGCCTCCCGGGTTCAAGTGATTCTCCTGCCTCAG
CCTCCCAAGTAGCTGGGATTATAGgCGCGTACCACCATGCCCGGCTAATTTTTGTATTTTTAGTAGGGACAGGGTTTCACCATGTTGGGCAGGCTGGTCTCAAACTCCTGACCTCAAGTGATCCAC CC
ACTTCAGCCTTACAAAGTGCTGGGATTATAGGTGTGAGCCACTGTGCCTAGCAA AGTGGGCTGTATTTCAAGAAGAAATGGATATTTTAAAAAGTAAAGTGTTGGCCGGGCACGGTGGCTCACACCTG
TAATCCCAGCACTTTGGGAGGCCAAGGTGGGCGGATCACAAGGTCAGGAGATTGAGACCATCCTGGCTAACACGGTGAAA Ccccgtctctactaaaaatacaaaaacattagccaggtgtggtgatgg
gcacctgtagtcccagctactcgggaggctgaggcaggagaatggcatgaacctgggaggcagagcttgcagtgagccgagatcgcgccactgcactccagcctgggcaacagagcgaaactctg tct
caaaaaaaaaaaaaaaaaaaagggtttcagggccaaaagaagatagcttaagattatggccaggtgcggtggctcgtgcctgtaatcccagcactttgggaggctgaggcgggcagatgatctgagg
tcagaagttcaagaccagcctgaccaatatggagaaac CCCGTCTCTACTAAAAATACAAAATTAGCCGGTTTTGGTGGCGCATGCCTGTAATCCCAGCTACTCGGGAAGGCTGAGGCAGGAGAATCA
CTTGAACCCAGGAGGCAGAGGTTGCAGTGAGCTGAGATCACGCCATTGCACTCCAGCCTGGGCAACAGAAAGAGACTCCATCTCAAAAAAAAA TTATATGTATTATATACACATATAATATATATATT
GTGTGTATATATATTTTTATTTTAGAGAGACAGTCTGTC TCTGTCATCCAGGCTGGAGTGCAGTGGTGTGATCATGGCTCACTGCAGCCTCGACTTCCTGGGCTCAACCCATCTTCCAGTCCTT CCAG
CTTCGCCCTCCTGAGTAGCTGGGTCCACAGACATGAGCCACCATGCCTGGCCATTTTTTCTTTTTTCTTTTTTTTTGTAGAAACGGTTTCTCACAGTGTTGCCCAGGCTGGTCTTGAACTCCTAGGCT
CAAGTGATCCTCCCACTTTGGCCTCCCAAAGTGTTGGGG TTACAGACGTGAGCCACTCTACCTGGCTTCATGATACTATGAAACAAATCTATTTGGCACCTGTAGTCACTGGAAGTTGCAACTCTTAT
GCTAAGTTCTAACACCATAATCAGAAGAACTTTGGATATTTCACTGTGAGGTTGTTGCATGCCTTTGACAAGGTGGGATTTTTTTTTTTTTTAATTTTTAGAGATGGGTTCTTGTTATATTGCCC AGA
CTGGAATGCAGAGTGCAGGCTATTCATAGGCATGATCAA AGTGTACTACAGCCTCAAAATTGTGGGATCAAGCCATCCTTCTGCCTCAGCCT CCTTCATGGCTGGGACTACAGGTGCCCTGCTACCTT
TGACAAGTTTTGAAAGATTTTCATAGCATTGAGGGGAGAAGAAAAGTGAAACAAAGAGAAGAAATGGGACCCTTCTTTATCCTCCCTCCCCTCAACAGAACTATTAAAACCCGAGATCAGTTTGT TTA
GACACTAGTCATATTGTCCAAAAGATTGCGCATTAAGAA TCTCTGTCTTAACTTTAGGCAGAATTATCTTGAACTGTTCTAGTAGGATGGTAATCATGACAGCACAGCTACTGGGGAAATTAGAACTG
AGGTTTCCTCTCTTCCGTTTGCTGAAATAACAGCAAAACTGCTTGAAGAAAACGCACTATATTATTC T

Break point =

GAAACCCCGTCTCTACTAAAAATACAAAA

>NAHR_39_ Scaffold5081 840701 841027
KSD_NAHR_39

>**Scaffold5081 840701 841027_**NAHR
CAGTTTGGGTTATCTGTGTGACTGTCATTAGAGACTTCATAAACATGCATCTTCTCCACCCTCTTCCCAGCAGATGACAGGGCTGAACTTTTTCTCCCCTTTTAAAAGTGGATGTGGCACGTAACT TG
CTGTGGCCATTGATATGTGAATGGACAAGGAATATTTTCCTATAGGTTACAAGCTTTCAGAACCAATGCATGACTTGCCCCATTTCCTCTTACAC ACTGTGGGAACTGTGAAAACATGCACTGTGTTG
AAGCTTTCATCAGTGTGAACCTTGAAAAACTATGAATGGCCGAGGATCCCTGCCATCCCACCTTGGAAAAGTAGTATGAGTTAGAAATTAACTTAGTTATCTTTAGCCACTGAGATTTTTAGGAT TAT
TCATTACTTTAGTAAAACAGTGAATTCTGACTGATATCTTATAACCCAACTTCTGAAACTGAAAATATTTAGTGCTGACTGAGAGGGATTTGGGA TAATATATGATGAGAATTATGATTATGAGAGGG
TAAtTATTTTATTTATTAAAAATTAAATAGGTTTTTAATGTGTGGCAACTATAAAAATGTATTTATGACTTCACTTGGTCCCTGAGATTTACAGATTTCAGTGTGTTCCTAAATTTTCTGACTCTGAG
TCTGAGAACTGACAATATTACCTTATTTTGGAAATATGTCTCTAAAATGACAGCAGACAAAGCTGTAGTGAGAGCTAATTTTGCAACTCAGTGGT CAGTGAATCAGACTTCAAGGAGAGACTGCcAGG
AGGAGAAGGTTCACAGAAATATAATAAAGTCAATTTTCACCTTTGGGATTATTTTATTAGGTTTAGCGTTCTATTCTTGAGATTTGTTATTAGCCAATGCTTATGGCAATGAATGAGTTAGAT gACAT
TCAATGAGAAAATATGCAAAAAATTACGTGAAGAATTAAAATATCAAGATTATTTAGCACAGCATTTTGCTAATGGAAATACATGACTAATAAAA ATAATTATCGGCCGGGCGCAGTGGCTCACGCCT
GTAATCCCAGCACTTTGGGAGGCCGAGGCGGGTGGATCACGAGGTCAGGAGATCGAGACCATCCTGGCTAACAGGGTGAAACCCCGTCTCTACTAAAAAATACAAAAGCATTAGCTGGGCATGGT CGC
GGGCGCCTGTAGTCCCAGCTACTCGGGAGGCTGAGGCGGGAGAATGGCCTGAACCCGGGAG cCAGAGCTTGCAGTGAGCCAAGATAGCGCCACTGCACTCCAGCCTGGGCGACAGAGCGAGACTCCGT
CTCAAAAAAAAAAAAAAAAAcaaAAAACAAAAAACAAACAAAAAAAAATTATCACATAACAGAGAGATCTATCTAAGACTCTAAATTTAAGCCAAGAGATATAACATATCCACCAAAGAGACAGAAATA
ATCTAAGAGAAAAAAGTGAGACACTGTTTCTAAATAGTTTCTGAACAGTTTCTGAAATTGTAGGAATCTAACTGGGAAGAAACTTTAACTTTTTGTAAATGGAGAATCAAAGCCTCCCTAAGCAT GTC
TCCTGGCTACTGTCATCCTGGCCTGACTTGAATCAAAATCACAAGTGTCCTGTGTCTAGATAAGTCTTTCGATCAAAGCTCTACCTAGTGTTCTTGTCATTTCTGGTATGATTTCTTATATTTTTGCC
CCTGGTTCATGCGTTATAAAGCAGGATATTGAAACGCTAATAAGTCTTTGGCTGAACTTTAGAATCACAGCAGTGACAGCAGCAAACATACCCTCCTTTGTTCTCTTTCTTGTGCTGAAGATACT GAg
ATATTCTTATCTGCCAGAGCCAAATCAGAGCCCTAACTTgCCTGATAGCTGGAACTGCATCTTTCtTTTTTTTTATTATTCTTAACAGTGTGTACTTGTATTGAAGaATTACATGATAAAATAGATGGA
AAAACACTCTTTGATATTCTTGAGAAAGATTTAGGAGCAAAAGTAATAGTCACTGAGGATGTAAAGTTTAAAGCAATAGTGAACAAGATAGGAAACATGAACACAATTAGGAGATATTGGTAGGA TCT
TGAAGGCTGAAGTTCAAATACAGTTTCTTGCACAAGGACAGCTGGATGTAGAGTCTCAATCCTTTAGTGGAAGTCTGACATAACCACTATCGGgTTGTTGTTCAATCATTAATATCTTGGCGAAAACC
CAAGTGGAACGTCTGCAGAAAAGTCTGGTCAAGGCTATTTCACACATTTGTTACAGATGCCCAGaCATGTCTTCATTTTGGTATGTCGTTCTTCTGTCCATACAAGCCACATGTTGAAAATAtGTTGG
GTCTTGATGCTTATCCAGACCT

678	33.6	4.2	2.8	A	63	420	(1906)	+	MLT1I	LTR/ERVL-MaLR	47	409	(2)	1
2587	5.4	0.0	0.6	A	1001	1313	(1013)	+	AluYk3	SINE/Alu	1	311	(0)	2

678	33.6	4.2	2.8	hg	63	420	(2206)	+	MLT1I	LTR/ERVL-MaLR	47	409	(2)	3
2395	6.5	0.0	0.7	hg	1001	1293	(1333)	+	AluYk3	SINE/Alu	1	291	(20)	4
2920	0.7	0.0	0.0	hg	1296	1641	(985)	+	AluY	SINE/Alu	1	311	(0)	5

>hg19 chr5:8025990-8028615
CAGTTTGGGTTATCTGTGTGACTGTCATTAGAGACTTCATAAACATGCATCTTCTCCACCCTCTTCCCAGCAGATGACAGGGCTGAACTTTTTCTCCCCTTTTAAAAGTGGATGTGGCACGTAACT TG
CTGTGGCCATTGATATGTGAATGGACAAGGAATATTTTCCTATAGGTTACAAGCTTTCAGAACCAATGCATGACTTGCCCCATTTCCTCTTACACAC TGTGGGAACTGTGAAAACATGCACTGTGTTG
AAGCTTTCATCAGTGTGAACCTTGAAAAACTATGAATGGCCGAGGATCCCTGCCATCCCACCTTGGAAAAGTAGTATGAGTTAGAAATTAACTTAGTTATCTTTAGCCACTGAGATTTTTAGGAT TAT
TCATTACTTTAGTAAAACAGTGAATTCTGACTGATATCTTATAACCCAACTTCTGAAACTGAAAATATTTAGTGCTGACTGAGAGGGATTTGGGATAATATATGATGAGAATTATGATTATGAGAGGG
TAAaTATTTTATTTATTAAAAATTAAATAGGTTTTTAATGTGTGGCAACTATAAAAATGTATTTATGACTTCACTTGGTCCCTGAGATTTACAGATTTCAGTGTGTTCCTAAATTTTCTGACTCTGAG
TCTGAGAACTGACAATATTACCTTATTTTGGAAATATGTCTCTAAAATGACAGCAGACAAAGCTGTAGTGAGAGCTAATTTTGCAACTCAGTGGTCA GTGAATCAGACTTCAAGGAGAGACTGCaAGG
AGGAGAAGGTTCACAGAAATATAATAAAGTCAATTTTCACCTTTGGGATTATTTTATTAGGTTTAGCGTTCTATTCTTGAGATTTGTTATTAGCCAATGCTTATGGCAATGAATGAGTTAGAT aACAT
TCAATGAGAAAATATGCAAAAAATTACGTGAAGAATTAAAATATCAAGATTATTTAGCACAGCATTTT GCTAATGGAAATACATGACTAATAAAAATAATTATCGGCCGGGCGCAGTGGCTCACGCCT
GTAATCCCAGCACTTTGGGAGGCCGAGGCGGGTGGATCACGAGGTCAGGAGATCGAGACCATCCTGGCTAACAGGGTGAAACCCCGTCTCTACTAAAAAATACAAAAGCATTAGCTGGGCATGGT CGC
GGGCGCCTGTAGTCCCAGCTACTCGGGAGGCTGAGGCGGGAGAATGGCCTGAACCCGGGAG gCAGAGCTTGCAGTGAGCCAAGATAGCGCCACTGCACTCCAGCctgggcaacagagagagactctat
cttaaaaaaaaaacaggccgggcgcggtggctcacgcctgtaatcccagcactttgggaggccgaggcgggcggatcacgaggtcaggagatcgagatcatcctggctaacacggtgaaaccccgtct
ctactaaaaatacaaaaaattagccgggcgtggtagcgggcgcctgtagtcccagctactcgggaggctgaggcaggagaatggcgt gaacccgggaggcggagcttgcagtgagccgagatcgcgcc
actgcactccagcCTGGGCGACAGAGCGAGACTCCGTCTCAAAAAAAAAAAAAAAAAaaaaaaaaaacAAAACAAAAAACAAACAAAAAAAAATTATCACATAACAGAGAGATCTATCTAAGACTCTAA
ATTTAAGCCAAGAGATATAACATATCCACCAAAGAGACAGAAATAATCTAAGAGAAAAAAGTGAGACACTGTTTCTAAATAGTTTCTGAACAGTTTCTGAAATTGTAGGAATCTAACTGGGAAGA AAC
TTTAACTTTTTGTAAATGGAGAATCAAAGCCTCCCTAAGCATGTCTCCTGGCTACTGTCATCCTGGCCTGACTTGAATCAAAATCACAAGTGTCCTGTGTCTAGA TAAGTCTTTCGATCAAAGCTCTA

CCTAGTGTTCTTGTCATTTCTGGTATGATTTCTTATATTTTTGCCCCTGGTTCATGCGTTATAAAGCAGGATATTGAAACGCTAATAAGTCTTTGGCTGAACTTTAGAATCACAGCAGTGACAGCAGC
AAACATACCCTCCTTTGTTCTCTTTCTTGTGCTGAAGATACTGAaATATTCTTATCTGCCAGAGCCAAATCAGAGCCCTAACTTaCCTGATAGCTGGAACTGCATCTTTCTTTTTTTATTATTCTTAA
CAGTGTGTACTTGTATTGAAGcATTACATGATAAAATAGATGGAAAAACACTCTTTGATATTCTTGAGAAAGATTTAGGAGCAAAAGTAATAGTCACTGAGGATGTAAAGTTTAAAGCAATAGTGAAC
AAGATAGGAAACATGAACACAATTAGGAGATATTGGTAGGATCTTGAAGGCTGAAGTTCAAATACAGTTTCTTGCACAAGGACAGCTGGATGTAGAGTCTCAATCCTTTAGTGGAAGTCTGACAT AAC
CACTATCGGaTTGTTGTTCAATCATTAATATCTTGGCGAAAACCCAAGTGGAACGTCTGCAGAAAAGTCTGGTCAAGGCTATTTCACACATTTGTTACAGATGCCCAGgCATGTCTTCATTTTGGTAT
GTCGTTCTTCTGTCCATACAAGCCACATGTTGAAAATAcGTTGGGTCTTGATGCTTATCCAGACCT

Homologous Sequences =
GCGCCACTGCACTCCAGCCTGGGC

>NAHR_40_ Scaffold4023 421910 422195
>KSD_NAHR_40

>**Scaffold4023 421910 422195_**NAHR

CTGAGCTTTAAAAATTTTTAATGTTTGTATTGTACTGGTGGGGAGTCTTAGGAGCCTGGCCAAAAACTTGGTGTTTCAGGAACATTTCAGTTGACAGACTTTGTTGATTTTCTTCTATTTCTTCAT CT
TTTAATTGGGTATTTTATATACATATGCAACTTGTAATTGGGTATTGTACATTTTGAGTATAAACTTTTTGTAAAAAAGAAACCTAACAATTTAAATTCATGGCATTTTGTTTTTATTACTTATA TCA
GAAAAGTTGCCAAAATAGGACCCTGGCTTTTTCTTCAAATCATAGGAATCTCTAATAAAGCTTTGACAAACCCAGCCACTCTGATGGAAATCATCGCATACTACGCACTGCCACCAGGAATCACAT CA
GAAGTCTTCTTTGCTGTGTAAAAGTAGCTCAGGGGCACCAAAATCTTCTGCAGAGCTTTAGTGTGTGTCTGGGAAACTTGAGATAGAGTTGTGGATTCCCATGCATCATCTCCTCCCTGCTCATG AGT
GGGCACGCTGGGTGTTGGCGCCCTTTCCTGATGAACACTGATGCATCCTCAGGGTTCTTCTCAACCAGTCCTCCAGGGACTCTCTCCAGGGAGTTAATCAGAGTTGGCATTCCAGATAAATTTTAA AT
TCGATCCCTCTCTTTGATAGAAGGGGGGCCTAAGATATTCTCAAAATTACCTGTCTTGAAATGAGATTTATCCAAGGTGAGGCTGTACAGTAAGAGTGCGGGCTTTGTCATCACATGACTAGATA CTG
AGTCCTGGCTCTATCCCTTAATAGTTGAATGGTAGTGAACTGGTCACTTCGTTTCTTCAAGTTTTGGTTTTTTGTTTTGTTTTTTAATCTGTGAAATGGAAAGAGGTCATACCTTTTTCAGAATAC TG
AGGATTAAATACAACAATTTTTGTAAAGTGCTTAGTAGACTATCTAGCCCAATAGTTCTCAGATTGTTAGTATTAGAACCCATTTATACTGTTAAAGATTATT GGCCAGGCGCAATGGCTCACACCTG
TAATCCCAGCACTTTGGGAGGCcGAGGtGGGTGGATCACGAGGTCAGGAGATtGAaACCATCCTGGCTAACAtgGTGAAACcCCGTCTCTACTAAAAATACAAAAAATTAGCCAGGTcCAGTGGCGGG
CGCCTGTAGCCGCAGCTACTCGGGAGGCTGAGGCAGGAGAATGGCaTGAACCTGGGAGGCAGAGCTTGCAGTGAGCCGAGATAGTGCCACTGAACTCCAGCCT GGACGACAGAGCGAGACTCCGTCTC
AAAAATAATAATAATAATTAGCCGGGCACAGTGGCAGGCGCCTGTAATCCCAGCTACTCGGGAGGCTGAGGCAGGAGAAT gGCaTGAACCtgGGaGGCAGAGcTTGCAGTGAGCCGAGATTGCGCCAC
TGCACTCCAGCCTGGGCGACAGAGTGAGACTGCATCTCAAAAAAAGAAAAAAAAAAAAAAAGATTATTGAGGACCCCATAGCATTTTTGTTTATTAAAAATTAA AACTGAAAAATTTTATGAATATGTA
TTACTTTTAGAAATTATAATAAAACCCCACTGTATATGAACATAGATAGCATATTTTATGAAAAATAATTGTATTTTCCAAAGACAAGTTAGTGAGTAGCTTATGTGCTTCTGCATTCAACCTGT TGC
AATATCACACATCCTGCAGATTCTGGAAAATTCCATGTACACATATGAGTGAATCAGAGTCAAAAAGACAAATAATATCCTAGTAGTAGTTTGATGATGTGCC CAGGAATGGACAGCTGACCCATTTC
TAGCCAGAGATTCAAGGAAAAGTTTGCTGGGAAGAGGCATTCCGTTCCAAATAAAGAGACCAAGGTTTCTAAAAAGAAGGTCTTTTGCCCTTTTGATTATTTGGAATGTAGACATGATGCTTTGG TTT
GTCACAGCTGTTTGGGGACAAGATCGAGAGTAAAAGTCAACACATTCAGGGTGATGGAGCAGAATGACAGGAAGAGCTCTGTTCTTGAGAGCACTGTTGAACA GCTGAACCAGCTCCTACAAGCCTTA
CCTACCAGCTTTGAACAAAAAAAAAATTTTGTTTAAATAGTTTCAGATAGTTTTGGCTGAGTGTGGTGGCTCATGCCTATAATCCCAGCACTTTGGGAAGCCCAGGCAGGTGGATCACTTGAGGTC AGG
AGTTTGAGACCAGCCTGGCCAACATGGGGAAACTGCGTCTCTACTAAAAATACGAAAATTAGCCTGGTATGGTGCTGTGTGCCTGTAGTCCCAGCTACTTGGG AGGCTG

326	29.8	4.5	3.2	129992	494	649	(1636)	C	MER117	DNA/hAT-Charlie	(38)	159	2	1
407	33.5	4.5	6.4	129992	725	947	(1338)	+	MIRb	SINE/MIR	16	234	(34)	2
2363	8.0	0.0	0.0	129992	1000	1285	(1000)	+	AluYk3	SINE/Alu	1	286	(25)	3
1466	6.9	0.0	0.0	129992	1296	1468	(817)	+	AluYc	SINE/Alu	119	291	(8)	4
652	16.9	29.6	1.1	129992	1499	1758	(527)	C	Charlie1b	DNA/hAT-Charlie	(108)	415	82	5
527	28.9	13.8	3.2	129992	1769	2051	(234)	+	MLT1L	LTR/ERVL-MaLR	197	508	(102)	6
1352	13.0	0.0	0.0	129992	2102	2285	(0)	+	AluSz	SINE/Alu	1	184	(128)	7

326	29.8	4.5	3.2	hg	494	649	(1778)	C	MER117	DNA/hAT-Charlie	(38)	159	2	8
399	33.5	4.5	6.5	hg	725	944	(1483)	+	MIRb	SINE/MIR	16	231	(37)	9
655	17.4	18.0	4.4	hg	946	999	(1428)	C	Charlie1b	DNA/hAT-Charlie	(0)	523	469	10
2367	6.1	0.0	8.0	hg	1000	1142	(1285)	+	AluSg4	SINE/Alu	1	129	(181)	11
2401	6.7	0.0	0.0	hg	1143	1427	(1000)	+	AluYk3	SINE/Alu	2	286	(25)	12
2367	6.1	0.0	8.0	hg	1428	1610	(817)	+	AluSg4	SINE/Alu	130	302	(8)	11
655	18.1	33.8	2.5	hg	1611	1900	(527)	C	Charlie1b	DNA/hAT-Charlie	(55)	468	82	10
527	28.9	13.8	3.2	hg	1911	2193	(234)	+	MLT1L	LTR/ERVL-MaLR	197	508	(102)	13
1352	13.0	0.0	0.0	hg	2244	2427	(0)	+	AluSz	SINE/Alu	1	184	(128)	14

>hg19 chr18:18959781-18962207

CTGAGCTTTAAAAATTTTTAATGTTTGTATTGTACTGGTGGGGAGTCTTAGGAGCCTGGCCAAAAACTTGGTGTTTCAGGAACATTTCAGTTGACAGACTTTGTTGATTTTCTTCTATTTCTTCATCT
TTTAATTGGGTATTTTATATACATATGCAACTTGTAATTGGGTATTGTACATTTTGAGTATAAACTTTTTGTAAAAAAGAAACCTAACAATTTAAATTCATGGCATTTTGTTTTTATTACTTATA TCA
GAAAAGTTGCCAAAATAGGACCCTGGCTTTTTCTTCAAATCATAGGAATCTCTAATAAAG CTTTGACAAACCCAGCCACTCTGATGGAAATCATCGCATACTACGCACTGCCACCAGGAATCACATCA
GAAGTCTTCTTTGCTGTGTAAAAGTAGCTCAGGGGCACCAAAATCTTCTGCAGAGCTTTAGTGTGTGTCTGGGAAACTTGAGATAGAGTTGTGGATTCCCATGCATCATCTCCTCCCTGCTCATG AGT
GGGCACGCTGGGTGTTGGCGCCCTTTCCTGATGAACACTGATGCATCCTCAGGGTTCTTC TCAACCAGTCCTCCAGGGACTCTCTCCAGGGAGTTAATCAGAGTTGGCATTCCAGATAAATTTTAAAT

TCGATCCCTCTCTTTGATAGAAGGGGGGCCTAAGATATTCTCAAAATTACCTGTCTTGAAATGAGATTTATCCAAGGTGAGGCTGTACAGTAAGAGTGCGGGCTTTGTCATCACATGACTAGATA CTG
AGTCCTGGCTCTATCCCTTAATAGTTGAATGGTAGTGAACTGGTCACTTCGTTTCTTCAA GTTTTGGTTTTTTGTTTTGTTTTTTAATCTGTGAAATGGAAAGAGGTCATACCTTTTTCAGAATACTG
AGGATTAAATACAACAATTTTTGTAAAGTGCTTAGTAGACTATCTAGCCCAATAGTTCTCAGATTGTTAGTATTAGAACCCATTTATACTGTTAAAGATTATT GGCCAGGCGCAATGGCTCACACCTG
TAATCCCAGCACTTTGGGAGGCtGAGGcGGGTGGATCACGAGGTCAGGAgttcaagacgagcctggccaagatggtgaaaccccgtctctactaaaaatacaataattagccaggcacgccgggcgcg
gtggctcacacctgtaatcccagcactttggaaggccgaggtgggcggatcacaaggtcaggt GATcGAgACCATCCTGGCTAACAcaGTGAAACaCCGTCTCTACTAAAAATACAAAAAATTAGCCA
GGTgCAGTGGCGGGCGCCTGTAGCCGCAGCTACTCGGGAGGCTGAGGCA GGAGAATGGCgTGAACCTGGGAGGCAGAGCTTGCAGTGAGCCGAGATAGTGCCACTGAACTCCAGCCTGGACGACAGAG
CGAGACTCCGTCTCAAAAATAATAATAATAATTAGCCGGGCACAGTGGCAGGCGCCTGTAATCCCAGCTACTCGGGAGGCTGAGGCAGGAGAAT tGCtTGAACCcaGGtGGCAGAGaTTGCAGTGAGC
CGAGATTGCGCCACTGCACTCCAGCCTGGGCGACAGAGTGAGACTGCAT CTCAAAAAAAGAAAAAAAAAAAAAAAGATTATTGAGGACCCCATAGCATTTTTGTTTATTAAAAATTAAAACTGAAAAAT
TTTATGAATATGTATTACTTTTAGAAATTATAATAAAACCCCACTGTATATGAACATAGATAGCATATTTTATGAAAAATAATTGTATTTTCCAAAGACAAGTTAGTGAGTAGCTTATGTGCTTC TGC
ATTCAACCTGTTGCAATATCACACATCCTGCAGATTCTGGAAAATTCCA TGTACACATATGAGTGAATCAGAGTCAAAAAGACAAATAATATCCTAGTAGTAGTTTGATGATGTGCCCAGGAATGGAC
AGCTGACCCATTTCTAGCCAGAGATTCAAGGAAAAGTTTGCTGGGAAGAGGCATTCCGTTCCAAATAAAGAGACCAAGGTTTCTAAAAAGAAGGTCTTTTGCCCTTTTGATTATTTGGAATGTAG ACA
TGATGCTTTGGTTTGTCACAGCTGTTTGGGGACAAGATCGAGAGTAAAA GTCAACACATTCAGGGTGATGGAGCAGAATGACAGGAAGAGCTCTGTTCTTGAGAGCACTGTTGAACAGCTGAACCAGC
TCCTACAAGCCTTACCTACCAGCTTTGAACAAAAAAAAATTTTGTTTAAATAGTTTCAGATAGTTTTGGCTGAGTGTGGTGGCTCATGCCTATAATCCCAGCACTTTGGGAAGCCCAGGCAGGTG GAT
CACTTGAGGTCAGGAGTTTGAGACCAGCCTGGCCAACATGGGGAAACTG CGTCTCTACTAAAAATACGAAAATTAGCCTGGTATGGTGCTGTGTGCCTGTAGTCCCAGCTACTTGGGAGGCT G

Homologous Sequences = **undefined**

>NAHR_41_ Scaffold4023 5375949 5376243
KSD_NAHR_41

>Scaffold4023 5375949 5376243_NAHR
CTCAGCTCACTGCAACCTCCACCTCCCAGGTTCAAGCCATTCTCCTGCCTCAGCCTCCCAAGTAGCTGGGACAACAGGTGTGTGCCACCACACCTGGCTAATTTTTTATTTTTAGTAGAGATGGGG CT
TCACAATATTGGCCAGGCTGGTCTCAAACTCCTGACCTCAGGTGATCTGCTGGCCTTGGCCTCCCAAAGTGTTGGCATTACAGGCGTGAGCCACCGCACCTGGCCTAATCTCATTCTTTTTTTTT TCT
TTTTTGTTTTCTGAGATATAGTTTCACTCTTGTTGCCCAGGTTGGAGTGCAGTGGCATGATTTCGCCTCACTGCAACCCTCTCCCCCGCCACTGACCCCACCTGGGTTCAAGCGATTCTCCTGCCT CA
GCCTCCCGAGTAGCTGGGATTACAAGCATGTGCCACCATGCCTGGTTAATTTTATATTTTTAGTAGAAATGGGGTTTCTCCATGTTGGTCAGGCTGGTCTCGAACTCCCGATCTTAGGTGATCCA CCC
GCCTCGGCCTCCCAAATTGCTGGGATTACAGGCGTGAGCCACCATGCCTGTCCTAATCTCATTCTTAATGTCAGCTATATTGTCTCTAAAATACGGTTAATACCTTCTGAAAAATATCATAAGGGT TA
AATAGAATAAGTATACAGAGTCTGCATATCACAGAATTCTGGGGAACACCATTTATGTGGAATACAATGTTGCACAGCAGAAGTGTCATGAATATTTAAGGTACTTATGCAGCATTTGTTGATAG GTA
CTCTGTGGTTGCTATTTAGCTAACATGTATGAGGACCCTCCAAATTCTCAGCCGTCTGAAAGTATGGGTCTTTAAGTTATCTCAGTGCCTACCTGTCCCTATTTTTTAAATTAAAAAGATGATGCT TA
CTGTAGAAAATTTGGAAAACAGAAAAATTACACAAAAATATTACCCTCCCCCCAAAGTTTTGAGTATATTTAAAAAATCTTCTTTTAAAAAAAAATTGACACAGGGTTGGGCATGGTGACTCACAT CTT
GTAATCCCAGCACTTTGGGAGGCCGAGGCAGGCAGATCACTTGTGGGCATGAGTTCGAGACCAGCCTCGCCAACATAGCAAAATCCTGTCCCTACTGAAAGCACAAAAACTAGCCAAGCGTTGTGG TG
CATACCTCTAATCCCAGGTACTTGGGAGGCTGAGGCACGAGAATCACTTGAGCCCAGATGTATGCAGTGGGCCAAGATCGTGCCACTGCACTCCAGCCTGGGCAACAGAACAAGACTCTGTCTCA AAA
AAAAGTAAAAAAAATTGAGACAGGGTCTTGCTGTCACCCAGGCTGGAATGCATTAGTACAATCAGGGCTCACTTCAGACTGA CCTCCCTAGCATAAGCAGTTCTCCTACGTCAAACCTACTGAGTGAC
TGGGACTACAGGTGTGCCTCACTATGCCCAGCTAATTTTTTTTTTTTTTGGTACATTTGGGATCTCACTGTTTCCCAGGCTGGCCTTGAACTCCTGGGCTCAAGTGATCTTCCTGCCTTAGCCT CCC
AAAGTGCTGGGATTACAGGCGTGAGCGACTGCACCTGGCC CCTAATCTCCTTTTTAAATGTCATCTTTATTGAGGTAAAATTTT TGTTATTAAAAAAGGGGAACCTAGGCTGGGCGCAGTGGCTCACG
CCTGTAATCCCAGCACTTTGGGAGGCTGAGGTGGGTGGATCATGACGTCAGGAGATCAAGACCATCCTGGCCAACATGGTGAAACACCATCTCTACTAAAAATACAAAAATTAGCTGGGCGTGGT GGT
ACGTGCCTGTAATCCCAGCTACTCTGGAGGCTGAGGCAGGAGGATCACTTGAACCAGGGAGCTGGAGGTTACAGTGAACCGAGA TTGTGCCACTGCACTCCAATCTGGTGACAGAACGAGACTCCATA
TCAAAAAAAAAAAAAAAAAGAGGAACCTGGCTGGGTGCGGTGGCTCATGCCTGT tATCCCACCACTTTGGGAGGCCAAGGCAGGTGGATTACTTGAGGCCAGGAGTTCGAGACCAGCCAGGCCAACAT
GGCAAAACTCGGTCTCTACTAAAAATACACAAAAATTAGCCAGGGTATTGTGGTACATGCCTGTAGTGTGAGCTACTTGGGAGG AGAGGCTTGAGAATCGCTTGAACCCAGGAGCAGAGGTTGCAGTG
ATCCAAGATTGCACTCCAGCCTGGGCAACAGAGCAAAACTCTCTCTCTCTCAAAAAAAAAAAAAGATCATTTACGGTGAATTGAGTGCTGAAACATTTAATAAAGGTGTAGCACCA A

1777	10.7	0.4	0.0	132805	1	233	(2061)	C	AluSx	SINE/Alu	(78)	234	1	1
2177	10.8	0.3	4.8	132805	242	565	(1729)	C	AluSp	SINE/Alu	(3)	310	1	2
1927	14.9	2.0	0.3	132805	1000	1294	(1000)	+	AluSz6	SINE/Alu	1	300	(12)	3
1568	18.1	1.8	1.1	132805	1295	1576	(718)	C	AluJb	SINE/Alu	(28)	284	1	4
2382	9.1	0.0	0.0	132805	1644	1939	(355)	+	AluSc5	SINE/Alu	1	296	(13)	5
1816	11.9	3.4	3.1	132805	1949	2241	(53)	+	AluSz6	SINE/Alu	1	294	(18)	6

1777	10.7	0.4	0.0	hg	1	233	(2710)	C	AluSx	SINE/Alu	(78)	234	1	7
2177	10.8	0.3	4.8	hg	242	565	(2378)	C	AluSp	SINE/Alu	(3)	310	1	8
1927	14.9	2.0	0.3	hg	1000	1294	(1649)	+	AluSz6	SINE/Alu	1	300	(12)	9
1568	18.1	1.8	1.1	hg	1295	1576	(1367)	C	AluJb	SINE/Alu	(28)	284	1	10
1734	17.7	1.0	1.0	hg	1644	1943	(1000)	+	AluJb	SINE/Alu	1	300	(12)	11
1568	18.1	1.8	1.1	hg	1944	2225	(718)	C	AluJb	SINE/Alu	(28)	284	1	12
2382	9.1	0.0	0.0	hg	2293	2588	(355)	+	AluSc5	SINE/Alu	1	296	(13)	13
1843	11.6	3.4	3.1	hg	2598	2890	(53)	+	AluSz6	SINE/Alu	1	294	(18)	14

>hg19 chr18:23826564-23829506
CTCAGCTCACTGCAACCTCCACCTCCCAGGTTCAAGCCATTCTCCTGCCTCAGCCTCCCAAGTAGCTGGGACAACAGGTGTGTGCCACCACACCTGGCTAATTTTTTATTTTTAGTAGAGATGGGG CT
TCACAATATTGGCCAGGCTGGTCTCAAACTCCTGACCTCAGGTGATCTGCTGGCCTTGGCCTCCCAAAGTGTTGGCATTACAGGCGTGAGCCACCGCACCTGGCCTAATCTCATTCTTTTTTTTT TCT
TTTTTGTTTTCTGAGATATAGTTTCACTCTTGTTGCCCAGGTTGGAGTGCAGTGGCATGATTTCGCCTCACTGCAACCCTCTCCCCCGCCACTGACCCCACCTGGGTTCAAGCGATTCTCCTGCCT CA
GCCTCCCGAGTAGCTGGGATTACAAGCATGTGCCACCATGCCTGGTTAATTTTATATTTTTAGTAGAAATGGGGTTTCTCCATGTTGGTCAGGCTGGTCTCGAACTCCCGATCTTAGGTGATCCA CCC
GCCTCGGCCTCCCAAATTGCTGGGATTACAGGCGTGAGCCACCATGCCTGTCCTAATCTCATTCTTAATGTCAGCTATATTGTCTCTAAAATACGGTTAATACCTTCTGAAAAATATCATAAGGGT TA
AATAGAATAAGTATACAGAGTCTGCATATCACAGAATTCTGGGGAACACCATTTATGTGGAATACAATGTTGCACAGCAGAAGTGTCATGAATATTTAAGGTACTTATGCAGCATTTGTTGATAG GTA
CTCTGTGGTTGCTATTTAGCTAACATGTATGAGGACCCTCCAAATTCTCAGCCGTCTGAAAGTATGGGTCTTTAAGTT ATCTCAGTGCCTACCTGTCCCTATTTTTTAAATTAAAAAGATGATGCTTA
CTGTAGAAAATTTGGAAAACAGAAAAATTACACAAAAATATTACCCTCCCCCCAAAGTTTTGAGTATATTTAAAAAATCTTCTTTTAAAAAAAAATTGACACAGGGTTGGGCATGGTGACTCACAT CTT

GTAATCCCAGCACTTTGGGAGGCCGAGGCAGGCAGATCACTTGTGGGCATGAGTTCGAGACCAGCCTCGCCAACATAGCAAAATCCTGTCCCTACTGAAAGCACAAAAACTAGCCAAGCGTTGTGG TG
CATACCTCTAATCCCAGGTACTTGGGAGGCTGAGGCACGAGAATCACTTGAGCCCAGATGTATGCAGTGGGCCAAGATCGTGCCACTGCACTCCAGCCTGGGCAACAGAACAAGACTCTGTCTCA AAA
AAAAGTAAAAAAAATTGAGACAGGGTCTTGCTGTCACCCAGGCTGGAATGCATTAGTACAATCAGGGCTCACTTCAGACTGA Cctccctagcataagcagttctcctacgtcaaacctactgagtgac
tgggactacaggtgtgcctcactatgcccagctaattttttttttttttttggtacatttgggatctcactgtttcccaggctggccttgaactcctgggctcaagtgatcttcctgccttagcct ccc
aaagtgctgggattacaggcgtgagcgactgcacctggcccctaatctcctttttaaatgtcatctttattgaggtaaaatttttgttattaaaaaaggggaacctaggctgggcgcagtggctcacg
cctgtaatcccagcactttgggaggctaaggcaggaagatcacttgagcccaggagttcaaggccagcctgggaaacagtgagatcccaaatgtaccaaaaaaaaaaaaaaattagctgggcata gtg
aggcacacctgtagtcccagtcactcagtaggtttgacgtaggagaactgcttatgctagggaggtcagtctgaagtgagccctgattgtactaatgcattccagcctgggcaacagaacaagact ct
gtctcaaaaaaaagtaaaaaaaattgagacagggtcttgctgtcacccaggctggaatgcattagtacaatcagggctcacttcagactgac CTCCCTAGCATAAGCAGTTCTCCTACGTCAAACCTA
CTGAGTGACTGGGACTACAGGTGTGCCTCACTATGCCCAGCTAATTTTTTTTTTTTTTTGGTACATTTGGGATCTCACTGTTTCCCAGGCTGGCCTTGAACTCCTGGGCTCAAGTGATCTTCCTG CCT
TAGCCTCCCAAAGTGCTGGGATTACAGGCGTGAGCGACTGCACCTGGCCCCTAATCTCCTTTTTAAATGTCATCTTTATTGAGGTAAAATTTT TGTTATTAAAAAAGGGGAACCTAGGCTGGGCGCAG
TGGCTCACGCCTGTAATCCCAGCACTTTGGGAGGCTGAGGTGGGTGGATCATGACGTCAGGAGATCAAGACCATCCTGGCCAACATGGTGAAACACCATCTCTACTAAAAATACAAAAATTAGCT GGG
CGTGGTGGTACGTGCCTGTAATCCCAGCTACTCTGGAGGCTGAGGCAGGAGGATCACTTGAACCAGGGAGCTGGAGGTTACAGTGAACCGAGA TTGTGCCACTGCACTCCAATCTGGTGACAGAACGA
GACTCCATATCAAAAAAAAAAAAAAAAAGAGGAACCTGGCTGGGTGCGGTGGCTCATGCCTGT aATCCCACCACTTTGGGAGGCCAAGGCAGGTGGATTACTTGAGGCCAGGAGTTCGAGACCAGCCA
GGCCAACATGGCAAAACTCGGTCTCTACTAAAAATACACAAAAATTAGCCAGGGTATTGTGGTACATGCCTGTAGTGTGAGCTACTTGGGAGG AGAGGCTTGAGAATCGCTTGAACCCAGGAGCAGAG
GTTGCAGTGATCCAAGATTGCACTCCAGCCTGGGCAACAGAGCAAAACTCTCTCTCTCTCTCAAAAAAAAAAAAGATCATTTA CGGTGAATTGAGTGCTGAAACATTTAATAAAGGTGTAGCACCAA

Break point =

TTGAGACAGGGTCTTGCTGTCACCCAGGCTGGAATGCATTAGTACAATCAGGGCTCACTTCAGACTGACCTCCCTAG CATAAGCAGTTCTCCTACGTCAAACCTACTGAGTGACTGGGACTACAGGTG
TGCCTCACTATGCCCAGCTAATTTTTTTTTTTTTTTGGTACATTTGGGATCTCACTGTTTCCCAGGCTGGCCTTGAACTCCTGGGCTCAAGTGATCTTCCTGCCTTAGCCTCCCAAAGTGCTGGG ATT
ACAGGCGTGAGCGACTGCACCTGGCC

>NAHR_42_ Scaffold9447 740344 740637
KSD_NAHR_42

>Scaffold9447 740344 740637_NAHR
ACCCAGGTCCTGGCACTGTTCTAGTTTCCAGAATTCAGGGAATTTATTAGCATTTGACCAAAATAAATCGGTTGTGAGCCTGCTTTACTGATGGCTCCAATATTAAATATTTCTTACTCTGTGTCA GG
GAGTTTCTGTCTTGTGACTACTATGGTGACTGGGTCTGTGTTTTTCCAGGTCTCTGGCCCATTGCTCCCTGTCTGACTGAGCCCTCCTATGCTTTCTGGGGATTTGGGCTGAGCCTCAGTTTCTC TTC
TGCATGGGTGTTAGATGATTTTTTTTTTGTTTTTCTTTTTTCTTTTTTTT TGAGACAGAGTCTCTCCCTGTCACCCAGGCTGGAGTGCAGTGGCGTGATCTCGGCTCACGGCAACCTCCGCCTCCCGG
GTTCAAGCAATTCTCCTGCCTCAGCCTCCCGAGTAGCTGGGACTACAGGCGCCTGCCACCAC aCCTGGCTAACTTTTGTATTTTTAGTAGAGACGGGGTTTCACCATATTGGTCAGGCTGATCTCGAA
CTCCTGACCTTGTGATCCGCCCACCTTGGCCTCCCAAAGTGCTGGGATTACAGGTGTGAGCCACTGTGCCTGGCCTAGATGACTATTTTATAGGCGGAGATGATAACCACAGAATGTTGGCTGAAT AT
TGAGGGCACTTTGATCCAGATACAAGAAAGAAGCTTGTGGAACTTACTGAGTCATCTCCTGCTAGAATAGTTCAGAAGTTTCAGCAATCCCATATATTATACAGTGTATAACAAAGACAAGGCTT AAA
ATAAAGGATTCACGTTAGAATTCTTCTTAACCAAAAACAACTCCACACAATTGCAACAATTATAGCTGGAAAAATAACTCCCAATGTTCACTATCCTAACACATCAAATTTCTTCATTTCTCTATT CT
CTTTTTTTAGTTTATACCCATACAAATATATCATTTTCACTTAGCTATAACCAAAGCAGAGATTCAAATTAGTATACCAGATCTACTCTAAAGAGAAAATACA GCCGGGCGCaGTGGCTCcCGCCTGT
CATCCCAGCACTTTGGGAGGCCGAGGCGGGCGGATCACGA GGTCAGGAGATCGAGACCATCCCGGCTAAAATGGTGAAACCCCGTCTCTACTAAAAATACAAAAAATTAGCCGGG tGTGGTGGCGGGc
GCCTGTAGTCCCAGCTACTCGGGAGGCTGAGGCAGGAGAATGG cGtGAACCCGGGAGGCGGAGCTTGCAGgGAGCcGAGATCGCGCCaCTGCACTCCAGCCTGGGCGACAGAGCGAGACTCCGTCTCA
AAACAAAAAAAAATTACAATTTTTTAAAGTGAACTGAACTTCCTCTGTGAGAAAAAGATTATCTCACCACCGCTGGAAGATCCTTGAAGAGGGTGAGACACTTTGCTGTATGAATTGCGAACATATTC
TCAATGTTGCCTGCTGCTTTGATCCCAGACGTGACCCAGACCCATGTTAAAACCAACTCCT cACAGGTTTATTCATCACCCCACTTTCCTTCCAGTTACGGCTTTGCGAGTCTAGGAAACAGGCT tTC
AGTAAAGGAAACACAGCGGCCATCAACGCAGTCTAGCTGTGCGGAAGGAGCGCTCAGCCCCGGCGAGAGGTGTGGCTGCTGCCGCCCCTTTTCGGGGGACTCCTCCATGACGTTGCGTTATTTGAA TT
TGATGTTCAGATAACACAAGTTTTAAAGATACATGTAAAAATTGTTCTACAATATGGAAGAAACGTGATTTTGGTGGTAGAAGATGACAGCCTGGGGGATATGCGGTGGGACTAGTTGGAGGCAA AGC
TGCACATTTGGCAAAACTTGTCTTAGAATCACCTTGCCCCTTCTTCTCCTTCTCATCCTCACAGACACTCTGTATTCCCCAACTAGACCGAGAGCTCTTGATGAGTTAATTAACGCCGAGGTTTTA GT
GGCTCCATTCCCAGCCCTTCGGGAAGGGTATAAATTTCAGAAGGAAAACCTCAGGGTACAATATTTAACCCACGTCAGTGGTTCTCTAAGAGCGGAATTTCAAGTGCCGCATTCCTGGAAGGGGA GGA
ATTTGCTGGATTAAAGGCTTCCTGGCCTGCAATT aTaTTATATGAGAGCATACCTACTCAATACGTATAAAGACACCTTGAAAAAATATTCAAGATGTACGAAAAGGAAATTGGGGAGATAACTGCAA
GACTTAGAGAAGAATGAGGGACAGGAAATTGCATATCAGCTTTATTGAAAGAAAGGGTATTTGTTGTGTCCGCTGTGGGAATGCTCTATGCCTATCGCCTCTTCTCAAGCACGACA C

2541	8.1	0.0	0.0	168643	278	587	(1706)	C	AluSx4	SINE/Alu	(0)	310	1	1
259	31.8	1.5	3.0	168643	836	971	(1322)	C	L1ME4c	LINE/L1	(58)	6025	5892	2
2639	3.1	0.0	0.0	168643	1000	1293	(1000)	+	AluY	SINE/Alu	2	295	(16)	3
189	39.8	7.6	0.0	168643	2003	2120	(173)	C	MamTip2	DNA/hAT-Tip100	(169)	136	10	4

2556	7.7	0.0	0.0	hg	279	588	(1839)	C	AluSx4	SINE/Alu	(0)	310	1	5
259	31.8	1.5	3.0	hg	837	972	(1455)	C	L1ME4c	LINE/L1	(58)	6025	5892	6
1192	2.3	0.0	0.0	hg	1001	1131	(1296)	+	AluY	SINE/Alu	2	132	(179)	7
2507	4.1	0.7	0.0	hg	1136	1427	(1000)	+	AluYm1	SINE/Alu	2	295	(16)	8

>hg19 chr4:187849685-187852111
ACCCAGGTCCTGGCACTGTTCTAGTTTCCAGAATTCAGGGAATTTATTAGCATTTGACCAAAATAAATCGGTTGTGAGCCTGCTTTACTGATGGCTCCAATATTAAATATTTCTTACTCTGTGTCA GG
GAGTTTCTGTCTTGTGACTACTATGGTGACTGGGTCTGTGTTTTTCCAGGTCTCTGGCCCATTGCTCCCTGTCTGACTGAGCCCTCCTATGCTTTCTGGGGATTTGGGCTGAGCCTCAGTTTCTCTTC
TGCATGGGTGTTAGATGATTTTTTTTTTGTTTTTCTTTTTTCTTTTTTTT TtGAGACAGAGTCTCTCCCTGTCACCCAGGCTGGAGTGCAGTGGCGTGATCTCGGCTCACGGCAACCTCCGCCTCCCG
GGTTCAAGCAATTCTCCTGCCTCAGCCTCCCGAGTAGCTGGGACTACAGGCGCCTGCCACCACgCCTGGCTAACTTTTGTATTTTTAGTAGAGACGGGGTTTCACCATATTGGTCAGGCTGATCTCGA
ACTCCTGACCTTGTGATCCGCCCACCTTGGCCTCCCAAAGTGCTGGGATTACAGGTGTGAGCCACTGTGCCTGGCCTAGATGACTATTTTATAGGCGGAGATGATAACCACAGAATGTTGGCTGA ATA
TTGAGGGCACTTTGATCCAGATACAAGAAAGAAGCTTGTGGAACTTACTGAGTCATCTCCTGCTAGAATAGTTCAGAAGTTTCAGCAATCCCATATATTATACAGTGTATAACAAAGACAAGGCTTAA
AATAAAGGATTCACGTTAGAATTCTTCTTAACCAAAAACAACTCCACACAATTGCAACAATTATAGCTGGAAAAATAACTCCCAATGTTCACTATCCTAACACATCAAATTTCTTCATTTCTCTA TTC
TCTTTTTTTAGTTTATACCCATACAAATATATCATTTTCACTTAGCTATAACCAAAGCAGAGATTCAAATTAGTATACCAGATCTACTCTAAAGAGAAAATACA GCCGGGCGCgGTGGCTCaCGCCTG
TCATCCCAGCACTTTGGGAGGCCGAGGCGGGCGGATCACGA Ggtcaggagatcgagacccttctggctaacacggtgaaaccccgtctctactaaaaatacaaaaaa attagccgggcgcggtggcgg
ggcctgtagtcccagcactttgggaggccgaggcgggcggatcacgag GTCAGGAGATCGAGACCATCCCGGCTAAAATGGTGAAACCCCGTCTCTACTAAAAATACAAAAAATTAGCCGGG cGTGGT
GGCGGGCCTGTAGTCCCAGCTACTCGGGAGGCTGAGGCAGGAGAATGG tGgGAACCCGGGAGGCGGAGCTTGCAGtGAGCtGAGATCGCGCCcCTGCACTCCAGCCTGGGCGACAGAGCGAGACTCC
GTCTCAAAACAAAAAAAAATTACAATTTTTTAAAGTGAACTGAACTTCCTCTGTGAGAAAAAGATTATCTCACCACCGCTGGAAGATCCTTGAAGAGGGTGAGACACTTTGCTGTATGAATTGCGAAC

ATATTCTCAATGTTGCCTGCTGCTTTGATCCCAGACGTGACCCAGACCCATGTTAAAACCAACTCCT tACAGGTTTATTCATCACCCCACTTTCCTTCCAGTTACGGCTTTGCGAGTCTAGGAAACAG
GCTcTCAGTAAAGGAAACACAGCGGCCATCAACGCAGTCTAGCTGTGCGGAAGGAGCGCTCAGCCCCGGCGAGAGGTGTGGCTGCTGCCGCCCCTTTTCGGGGGACTCCTCCATGACGTTGCGTTATT
TGAATTTGATGTTCAGATAACACAAGTTTTAAAGATACATGTAAAAATTGTTCTACAATATGGAAGAAACGTGATTTTGGTGGTAGAAGATGACAGCCTGGGGGATATGCGGTGGGACTAGTTGG AGG
CAAAGCTGCACATTTGGCAAAACTTGTCTTAGAATCACCTTGCCCCTTCTTCTCCTTCTCATCCTCACAGACACTCTGTATTCCCCAACTAGACCGAGAGCTCTTGATGAGTTAATTAACGCCGAGGT
TTTAGTGGCTCCATTCCCAGCCCTTCGGGAAGGGTATAAATTTCAGAAGGAAAACCTCAGGGTACAATATTTAACCCACGTCAGTGGTTCTCTAAGAGCGGAATTTCAAGTGCCGCATTCCTGGA AGG
GGAGGAATTTGCTGGATTAAAGGCTTCCTGGCCTGCAATTgTgTTATATGAGAGCATACCTACTCAATACGTATAAAGACACCTTGAAAAAATATTCAAGATGTACGAAAAGGAAATTGGGGAGATAA
CTGCAAGACTTAGAGAAGAATGAGGGACAGGAAATTGCATATCAGCTTTATTGAAAGAAAGGGTATTTGTTGTGTCCGCTGTGGGAATGCTCTATGCCTATCGCCTCTTCTCAAGCACGACA C

Break point =

TCCCAGCACTTTGGGAGGCCGAGGCGGGCGGATCACGAGGTCAGGAGATCGAGACC

>NAHR_43_ Scaffold16034 9256984 9257264
KSD_NAHR_43

>Scaffold16034 9256984 9257264_NAHR
CCACATTCACATAACTTTTATTACAGTATATTGTTATAATTGTTCTATTTTATTATGAGTTGTTAATTTCTTACTGTGCCAATTTTATAAATTAAATTTTATTATAGGTATGTGTAGGAAAAAAGT AT
ATACTGTATAGGGCTAAATACTCTCTGCAGTTTCAGACACCCACCGGGGGTCTTGAAACATAAGGGAGGACTACTATGTTTGATTATGTGAGTATGAAGAAAAATAGAAGATACACGTCAACACC AGT
AACTTTAAAAGAGGGTGGGGGGCCAGGCACAGTAGCTCACACCTGTAATCCCAACACTTCAGGAGGCTGAGGCAGGAGGATTGTTTGAGCTCAGGAGTTTCAGACCAGCCTGGGGCTACATAGTGA GA
CAGTCTTTTAAAACACACACACAGACACACACACACACCCCCCAAAAAAAAACTCCGGCACGTATAATAGAGTCCCCCCGCTTTTATTTTTTTTTTCAGACGGAGTCTCTCTTTGTCAGGCTGGA ATA
CAGTGGTGCGATCTCAGCTCACTGCAACCTCCAACTCCCTGGTTCAAGCAATTCTCCTGCCTCAGCCCCCCAAGTAGATGGGACTACAGGCATGTGCCCATGCCCGGCTAATTCTTGTATTTTTAG TA
GAGACAGGAGTTCACCATGTTGGCCAGGTTGGTCTCGATCTCCTGACCTCGTGATTCACCTGCCTCGGCCTCCCAAAGTGCCAGGATTACAGGTGTGAGCCACCACGCCCGCCAATAGAGTCCCT GTT
ATGTCAAATGTATGCCTTTGTATAAATACATGAAATTAAAAGATGAATTTAGACATTGTAGCAAGCTGAATAATGTCCCCCAAAATATCCAGGTCCTAATCCCTAGAACCTGTGAATGTTGCCTTG AG
TGGCAAAATGGACTTTGCAGATGTGATTAAGTTACACACCGTGAGATGGAGAGATTATCCTGTGGGCCATAATGTAATCACAGTGTCATAGGAGGGAAACAGA GGCCAGGCATGGAGGCTCACACCTG
TAATCCCAGCACTTTGGAAGGCTGAAGCGGGTGGATCACGAGGTCAGGAGTTCGAGACCAGTTTTGCCACCATGGCGAAACCCCCATCTCTACTAAAAATACAAAAATTATCCAGGCATGGTGGTG CA
CGCCTGTAATCCCAGCTACTCAGGAGGCTGAGGCAGGAGAATCACTTGAATGCAGGAGGCAGA GGTTGCAGTGAGTTGAGATCGTGCCATTGCACTCCAGCCTGGGTGACATAGCAAGACTGTGTCCC
CACCACCACCACCACCAAAAAAAAGGAGGGAAACAGAAAGAAAAGAAAGTGATATAATGTAAGCAGAGGTTGAAGTGATGCGCTTTGAAGACAGAGAAAGGCTAACACAAGCCAAGGAATACATGC AGC
CACCAGAAGCTCAGAGGCTCCAGAAGAAAGTAGCCCTGACACCTTTAGGCTTGACAACCGGCCAGTAAAACTGATTTCAGACTTCTGACCTCCAGAACTGTAAGAGAATAAATCTACGTTGTTTT AAG
CCACTAAATTGGTGATTTATTACAGCGGCCATAGGAAACAAATGCAGATGTGTATACACTAATTTATAGAAATCAGGGTACACTGTCAAGTGAAAAAAGCAAATTTTTAGGGTAACTGTATAATA CAG
TTACATTTTAATAAAAGCTACCAACTAAATATCTTTATATATACATATATTAATACATTGATAGACATATATTTATATTTGCTGTGTTTATTATATAAGCAAGAAAAAGGATTTGGAAGAATACA TAA
CAGGTTATTGATAATGGTTAAATCATGTGAGTGGTTGGGGATGGGGCGGGGAATGAATAAGGGGGTGAGAGATTGCCATATATATCTTCATTTTGTGCTCTTGCTATGAGCATATACTGCCTTTG CAA
TTTAAAAAATGTACAATTAAGACATTTTTCAATAGGTCACATTAATCTTAATTTAAAACATATTAAGCTCAGCTGGGCGCAGTGGCTCATGCCTGTAATCCCAGCACTTTGAGAGGCCAAGGCAG GTG
GATCACCTGAGGTCAGGAGTTCAAGACTAGCCCCACCAACATGGTGAAACCCCATCTCTACTAAATACAAAAAAAGTAGCCTGGCATGGTGGCACATGACTCTAATTCCAGCTACTTGGGCAGTA GCT
GAGGCAGGAGAATTCCTTGAACCCAGGAGACAGAGGTTGCAGTGAGCCGAGAGTGTGCCATTGCACTCCAGCCTGGGCAACAAGAGCAAAACTCCGTCTCAAA C

1164	10.4	11.8	0.9	199827	1	203	(2077)	+	MER44A	DNA/TcMar-Tigger	114	338	(1)	1
742	16.5	1.6	0.8	199827	277	397	(1883)	+	FLAM_C	SINE/Alu	1	122	(21)	2
20	4.1	0.0	0.0	199827	398	422	(1858)	+	(CA)n	Simple_repeat	1	25	(0)	3
2134	10.5	1.8	0.0	199827	466	751	(1529)	C	AluSc5	SINE/Alu	(15)	294	4	4
1729	18.0	13.0	6.9	199827	825	999	(1281)	+	MLT1D	LTR/ERVL-MaLR	1	192	(313)	5
2047	12.8	0.0	0.4	199827	1000	1280	(1000)	+	AluSg	SINE/Alu	1	280	(30)	6
1729	18.0	13.0	6.9	199827	1281	1582	(698)	+	MLT1D	LTR/ERVL-MaLR	193	505	(0)	5
21	29.3	1.4	1.4	199827	1690	1761	(519)	+	(TA)n	Simple_repeat	1	72	(0)	7
2007	12.8	0.0	1.1	199827	1992	2279	(1)	+	AluSp	SINE/Alu	2	286	(27)	8

1164	10.4	11.8	0.9	hg	1	203	(2273)	+	MER44A	DNA/TcMar-Tigger	114	338	(1)	9
742	16.5	1.6	0.8	hg	277	397	(2079)	+	FLAM_C	SINE/Alu	1	122	(21)	10
20	4.1	0.0	0.0	hg	398	422	(2054)	+	(CA)n	Simple_repeat	1	25	(0)	11
2134	10.5	1.8	0.0	hg	466	751	(1725)	C	AluSc5	SINE/Alu	(15)	294	4	12
874	19.4	7.5	10.9	hg	825	999	(1477)	+	MLT1D	LTR/ERVL-MaLR	1	192	(313)	13
2047	12.8	0.0	0.4	hg	1000	1280	(1196)	+	AluSg	SINE/Alu	1	280	(30)	14
874	19.4	7.5	10.9	hg	1281	1359	(1117)	+	MLT1D	LTR/ERVL-MaLR	193	247	(258)	15
836	12.5	0.0	0.0	hg	1365	1476	(1000)	+	AluSx3	SINE/Alu	170	281	(30)	16
13	0.0	0.0	0.0	hg	1477	1493	(983)	+	(CAC)n	Simple_repeat	1	17	(0)	17
1020	19.8	15.4	2.5	hg	1499	1778	(698)	+	MLT1D	LTR/ERVL-MaLR	191	505	(0)	13
21	29.3	1.4	1.4	hg	1886	1957	(519)	+	(TA)n	Simple_repeat	1	72	(0)	18
2007	12.8	0.0	1.1	hg	2188	2475	(1)	+	AluSp	SINE/Alu	2	286	(27)	19

>hg19 chr11:107731852-107734327

CCACATTCACATAACTTTTATTACAGTATATTGTTATAATTGTTCTATTTTATTATGAGTTGTTAATTTCTTACTGTGCCAATTTTATAAATTAAATTTTATTATAGGTATGTGTAGGAAAAAAGTAT
ATACTGTATAGGGCTAAATACTCTCTGCAGTTTCAGACACCCACCGGGGGTCTTGAAACATAAGGGAGGACTACTATGTTTGATTATGTGAGTATGAAGAAAAAATAGAAGATACACGTCAACACC AGT
AACTTTAAAAGAGGGTGGGGGGCCAGGCACAGTAGCTCACACCTGTAATCCCAACACTTCAGGAGGCTGAGGCAGGAGGATTGTTTGAGCTCAGGAGTTTCAGACCAGCCTGGGGCTACATAGTGAGA
CAGTCTTTTAAAACACACACACAGACACACACACACACCCCCCAAAAAAAAACTCCGGCACGTATAATAGAGTCCCCCCGCTTTTATTTTTTTTTTCAGACGGAGTCTCTCTTTGTCAGGCTGGA ATA
CAGTGGTGCGATCTCAGCTCACTGCAACCTCCAACTCCCTGGTTCAAGCAATTCTCCTGCCTCAGCCCCCCAAGTAGATGGGACTACAGGCATGTGCCCATGCCCGGCTAATTCTTGTATTTTTAGTA
GAGACAGGAGTTCACCATGTTGGCCAGGTTGGTCTCGATCTCCTGACCTCGTGATTCACCTGCCTCGGCCTCCCAAAGTGCCAGGATTACAGGTGTGAGCCACCACGCCCGCCAATAGAGTCCCT GTT
ATGTCAAATGTATGCCTTTGTATAAATACATGAAATTAAAAGATGAATTTAGACATTGTAGCAAGCTGAATAATGTCCCCCAAAATATCCAGGTCCTAATCCCTAGAACCTGTGAATGTTGCCTTGAG
TGGCAAAATGGACTTTGCAGATGTGATTAAGTTACACACCGTGAGAT GGAGAGATTATCCTGTGGGC CATAATGTAATCACAGTGTCATAGGAGGGAAACAGA GGCCAGGCATGGAGGCTCACACCTG
TAATCCCAGCACTTTGGAAGGCTGAAGCGGGTGGATCACGAGGTCAGGAGTTCGAGACCAGTTTTGCCACCATGGCGAAACCCCCATCTCTACTAAAAATACAAAAATTATCCAGGCATGGTGGT GCA
CGCCTGTAATCCCAGCTACTCAGGAGGCTGAGGCAGGAGAATCACTTGAATGCAGGAGGCAGA Ggttgcagtgagttgagatcgtgccattgcactccagcct gggtgacatagcaagactgtgtccc
caccaccaccaccaccaaaaaaaaggagggaaacagaaagaaaagaaagtgatataatgtaagcagaggttgaagtgatgcgctt tactcaggaggctgaggcaggagaatcacttgaacgcgggaggc
agagGTTGCAGTGAGTTGAGATCGTGCCATTGCACTCCAGCCTGGGTGACATAGCAAGACTGTGTCCC CACCACCACCACCACCAAAAAAAAGGAGGGAAACAG AAAGAAAAGAAAGTGATATAATGTA
AGCAGAGGTTGAAGTGATGCGCTTTGAAGACAGAGAAAGGCTAACACAAGCCAAGGAATACATGCAGCCA CCAGAAGCTCAGAGGCTCC AGAAGAAAGTAGCCCTGACACCTTTAGGCTTGACAACCG
GCCAGTAAAACTGATTTCAGACTTCTGACCTCCAGAACTGTAAGAGAATAAATCTACGTTGTTTTAAGCCACTAAATTGGTGATTTATTACAGCGGCCATAGG AAACAAATGCAGATGTGTATACACT
AATTTATAGAAATCAGGGTACACTGTCAAGTGAAAAAAGCAAATTTTTAGGGTAACTGTATAATACAGTTACATTTTAATAAAAGCTACCAACTAAATATCTTTATATATACATATATTAATACA TTG
ATAGACATATATTTATATTTGCTGTGTTTATTATATAAGCAAGAAAAAGGATTTGGAAGAATACATAACAGGTTATTGATAATGGTTAAATCATGTGAGTGGT TGGGGATGGGGCGGGGAATGAATAA
GGGGGTGAGAGATTGCCATATATATCTTCATTTTGTGCTCTTGCTATGAGCATATACTGCCTTTGCAATTTAAAAAATGTACAATTAAGACATTTTTCAATAGGTCACATTAATCTTAATTTAAA ACA
TATTAAGCTCAGCTGGGCGCAGTGGCTCATGCCTGTAATCCCAGCACTTTGAGAGGCCAAGGCAGGTGGATCACCTGAGGTCAGGAGTTCAAGACTAGCCCCA CCAACATGGTGAAACCCCATCTCTA
CTAAATACAAAAAAAGTAGCCTGGCATGGTGGCACATGACTCTAATTCCAGCTACTTGGGCAGTAGCTGAGGCAGGAGAATTCCTTGAACCCAGGAGACAGAGGTTGCAGTGAGCCGAGAGTGTG CCA
TTGCACTCCAGCCTGGGCAACAAGAGCAAAACTCCGTCTCAAAC

Homologous Sequences =

GGAGGCAGAGGTTGCAGTGAGTTGAGATCGTGCCATTGCACTCCAGCCTGGGTGACATAGCAAGACTGTGTCCC

>NAHR_44_Scaffold16034 2009358 2009671
>KSD_NAHR_44

>**Scaffold16034 2009358 2009671**_NAHR

CTGAGGCAGGAGAATAGCGTGAACCCAGGAGGCAGAGCTTGCAGTGAGCTGAGATCACGCCACTGCCCTCCAGCCTGGGTGACAGAGCGAGACTCCATCTCAAAAAAAAAAAAAAAATGATTGTGAT AC
TAAAAAATTTGGATAGAGTTCTCAAGTGTATGAGTCAGGAAAAACCATGTGTTTGTACCGTGGATAGCTCCCAATAATGCTTTTTTTT TGCCTCCTGGGTTCAAGCAGTTCTCCTGCCTCAGCCTCCT
GAATAGCTGGGACTACAGGCATGCGCCATCACGCCTGGTGAATTTTTGTATTTTTAGTAGAGATGGGGTTTTACCATGTCAGCCAGGCTGGTCTTGAACTCCTGATCTTGATTGATTCACCCACCT CA
GCCTCCCAAAGTGCTGGGATTATACGCATGAGCCACAGCGCCTGGCCAGCTCCCAGTAATTCTGTTCTCTCCTTGAGCCTCTGTCATGAGGTAGCCTCCACTAACTCTGTGTTTCCCCATGCCCA GAT
TGGAGACCaTGTCATGGCATTTGTCAATTACAATGCCTGGGCAGAGGTGGTCTGCACACCAGTGGAGTTTGTCTACAAGATCCCGGATGACATGAGCTTCTCCGAGGCTGCTGCATTCCCCATGAACT
TCGTCACAGCCTATGTGATGCTGTTTGAAGTTGCCAACCTCCGGGAAGGGATGTCTGTGCTCGTGCACTCAGCTGGTGGGGGCGTGGTAAGTCAGCTGTTTGTAACTTCTCTTCTTTAAGGTCAT GAT
GGTGGAAGTGGAGTGACACCCCCCTGAGGTCTTATGTGAGCTATGTCCTTTAGCAGGAATCATCACTATGGTTCTGGTAGAGTTCACATGGACAGCAAAGCCCATATAGATGATTGCATTGGGGTA GG
ATAAGCCTGAGAGATGGTTTCTCTGGAGTTTTGGAAGGTCACCAAAATGGCCTGTGAGGACACAATGGCTTTCAGAGATAGTTCCTGTTGAGAAGCATTCTCA GGCCGGGCGCCGTGACTCACGCCTG
TAATCCCAGCACTTTGGGAGGCAGAGGCGGGCAGATCACGAAGTCAGGAGATCAAG cCCATCCTGGCTAACACGGTGAAACCCCGTCTCTACTAAAAATAAAAAAAAAAAA AAAATTAGCCGGGCGTA
GTGGCaGGCGCCTGTAGTCCCAGCTACT cGGGAGGCTGAGGCAGaAGAATGGCGTGAACCCGGGAGGCAGAGCTTGCAGTGAGCCGAGAT tgCGCCACTGCACTCCAGCCTGGGCGACAGAGCGAGAC
TCCGTCTCAAAAAAAAAAAAAAAGAAAAAGAGAAGCATTCTCAACAGTCTTGGTATCATGATGAAAAGTTGTGTCAATCATTGTAAGA tGTCTCACAAGTCATCTACTACTGCTATTATTTGAAATGAC
AAAATGGTCATTTTGTCAGTGATTTAGTTTTTCAATAAATTGCAGTGGACTTGAGATGGTTCCCATATTAATGACATTCTTATTAATTTGT ATTCTGCTATATTTTCTTGAGTGGGCAGAGATTGTA
CCAGCACACTAGAAATCATACATAAACCTAAGTACATTCATGAAAGTGGTTGCACATGTCAGCGTCATCTGTCAGGTGTGTCTCTGCCAAGGCGGAGAATGGTGGAAATCAAAATTTCTGCTCTA GAT
TTCAGGGATGGCAGAAACTTCTCTCATGGTTTGTCTGTGTCTCATTGCCAGGCATTCAGTGTATATTGCATGCC cGCTACATGGCAAGCACTATGTCTGTCATTGGTCTACTGGGGTCTGGGTGGAAC
GTTAGTGTTTCTGAGAGTGGAAGGTACTACCTACTAGTTCAAAACATATTAATTAAACACATAACATGTTCTAGACTCTGTGGATATAGAGATTAATCCCCTATCTTAGAGG cTTTGAGAGGTTCTCA
GAGTCACTGATTCTGTAGTTTGATGAACTCAGAAATAAACTCTTAGGATTTGGTGTATCTCAGAGATATTTCACTGGAGGTTTTGCTAGCAT ACCGTTGATTTTAAAAAAGAAAAACTTGGCTTTCTG
TAACTATTAAAACTCTATATAGTGTAGAGCACATTTCCGTTGTTT tTATCTAAGAAAACAAGCTCACATCCAGAACCATGAAGGCAAAGATAGGAAACTTTAGGAATGCCTTAAGAAAAATATCTTCA
ATCATTTTAGCAAATTTGCTGATACCTTTCGACCATGAATAAAGACATATAAAAGCTAAGCATCCCGTAGAAAAGAAATGGCTTTCAGTTCC AGTGAATCTGTTAACAGCAAGCAAGCCATTTAGAAT
GCTGGCATT

982	6.9	0.0	0.0	A	1	116	(2197)	+	AluYc	SINE/Alu	169	284	(15)	1
1547	13.1	0.0	0.9	A	218	431	(1882)	C	AluSg	SINE/Alu	(98)	212	1	2
2572	5.1	0.0	2.6	A	1000	1313	(1000)	+	AluY	SINE/Alu	1	306	(5)	3
821	29.5	5.3	5.3	A	1317	1635	(678)	+	Chap1_Mam	DNA/hAT-Charlie	7	325	(17)	4

982	6.9	0.0	0.0	hg	1	116	(2462)	+	AluYc	SINE/Alu	169	284	(15)	5
2241	12.5	0.0	0.7	hg	209	512	(2066)	C	AluSg	SINE/Alu	(8)	302	1	6
2623	5.0	0.0	2.2	hg	1081	1398	(1180)	+	AluY	SINE/Alu	1	311	(0)	7
1635	2.2	0.0	0.0	hg	1401	1578	(1000)	+	AluYa5	SINE/Alu	130	307	(4)	8
808	29.8	5.3	5.3	hg	1582	1900	(678)	+	Chap1_Mam	DNA/hAT-Charlie	7	325	(17)	9

>hg19 chr16:77858552-77861129

CTGAGGCAGGAGAATAGCGTGAACCCAGGAGGCAGAGCTTGCAGTGAGCTGAGATCACGCCACTGCCCTCCAGCCTGGGTGACAGAGCGAGACTCCATCTCAAAAAAAAAAAAAAAATGATTGTGAT AC
TAAAAAATTTGGATAGAGTTCTCAAGTGTATGAGTCAGGAAAAACCATGTGTTTGTACCGTGGATAGCTCCCAATAATGCTTTTTTTT Ttttttttttttggaggcagagtcttgctttgttgccca
ggctggagtgcaatggcatgatctcagctcactgcaacctccGCCTCCTGGGTTCAAGCAGTTCTCCTGCCTCAGCCTCCTGAATAGCTGGGACTACAGGCATGCGCCATCACGCCTGGTGAATTTTT
GTATTTTTAGTAGAGATGGGGTTTTACCATGTCAGCCAGGCTGGTCTTGAACTCCTGATCTTGATTGATTCACCCACCTCAGCCTCCCAAAGTGCTGGGAT TATACGCATGAGCCACAGCGCCTGGCC
AGCTCCCAGTAATTCTGTTCTCTCCTTGAGCCTCTGTCATGAGGTAGCCTCCACTAACTCTGTGTTTCCCCATGCCCAGATTGGAGACC gTGTCATGGCATTTGTCAATTACAATGCCTGGGCAGAGG
TGGTCTGCACACCAGTGGAGTTTGTCTACAAGATCCCGGATGACATGAGCTTCTCCGAGGCTGCTGCATTCCCCATGAACTTCGTCACAGCCTATGTGATG CTGTTTGAAGTTGCCAACCTCCGGGAA
GGGATGTCTGTGCTCGTGCACTCAGCTGGTGGGGGCGTGGTAAGTCAGCTGTTTGTAACTTCTCTTCTTTAAGGTCATGATGGTGGAAGTGGAGTGACACCCCCCTGAGGTCTTATGTGAGCTAT GTC
CTTTAGCAGGAATCATCACTATGGTTCTGGTAGAGTTCACATGGACAGCAAAGCCCATATAGATGATTGCATTGGGGTAGGATAAGCCTGAGAGATGGTTT CTCTGGAGTTTTGGAAGGTCACCAAAA
TGGCCTGTGAGGACACAATGGCTTTCAGAGATAGTTCCTGTTGAGAAGCATTCTCA GGCCGGGCGCCGTGACTCACGCCTGTAATCCCAGCACTTTGGGAGGCAGAGGCGGGCAGATCACGAAGTCAG

GAGATCAAGaCCATCCTGGCTAACACGGTGAAACCCCGTCTCTACTAAAAATAAAAAAAAAAAA Aaattagccgggcgtagtggcaggcgcctgtagtcccagctactcgggaggctgaggcagaaga
atggcgtgaacccgggaggcagagcttgcagtgagccgagattgcgccactgcactccagcctgggcgacagagccagactccatctcaaaaaaaaagaaaaaaaaaaaaaaaaagaa agAAATTAGC
CGGGCGTAGTGGCgGGCGCCTGTAGTCCCAGCTACTtGGGAGGCTGAGGCAGgAGAATGGCGTGAACCCGGGAGGCAGAGCTTGCAGTGAGCCGAGATccCGCCACTGCACTCCAGCCTGGGCGACAG
AGCGAGACTCCGTCTCAAAAAAAAAAAAAAaGAAAAAGAGAAGCATTCTCAACAGTCTTGGTATCATGATGAAAAGTTGTGTCAATCATTGTAAGA cGTCTCACAAGTCATCTACTACTGCTATTATT
TGAAATGACAAAATGGTCATTTTGTCAGTGATTTAGTTTTTTCAATAAATTGCAGTGGACTTGAG ATGGTTCCCATATTAATGACATTCTTATTAATTTGTATTCTGCTATATTTTCTTGAGTGGGCA
GAGATTGTACCAGCACACTAGAAATCATACATAAACCTAAGTACATTCATGAAAGTGGTTGCACATGTCA GCGTCATCTGTCAGGTGTGTCTCTGCCAAGGCGGAGAATGGTGGAAATCAAAATTTCT
GCTCTAGATTTCAGGGATGGCAGAAACTTCTCTCATGGTTTGTCTGTGTCTCATTGCCAGGCATT CAGTGTATATTGCATGCCtGCTACATGGCAAGCACTATGTCTGTCATTGGTCTACTGGGGTCT
GGGTGGAACGTTAGTGTTTCTGAGAGTGGAAGGTACTACCTACTAGTTCAAAACATATTAATTAAACACATAACATGTTCTAGACTCTGTGGATATAGAGATTAATCCCCTATCTTAGAGG tTTTGAG
AGGTTCTCAGAGTCACTGATTCTGTAGTTTGATGAACTCAGAAATAAACTCTTAGGATTTGGTGT ATCTCAGAGATATTTCACTGGAGGTTTTGCTAGCATACCGTTGATTTTAAAAAAGAAAAACTT
GGCTTTCTGTAACTATTAAAACTCTATATAGTGTAGAGCACATTTCCGTTGTTTgTATCTAAGAAAACAAGCTCACATCCAGAACCATGAAGGCAAAGATAGGAAACTTTAGGAATGCCTTAAGAAAA
ATATCTTCAATCATTTTAGCAAATTTGCTGATACCTTTCGACCATGAATAAAGACATATAAAAGC TAAGCATCCCGTAGAAAAGAAATGGCTTTCAGTTCCAGTGAATCTGTTAACAGCAAGCAAGCC
ATTTAGAATGCTGGCATT

Homologous Sequences =

CGCCACTGCACTCCAGCCTGGGCGACAGAGC

>NAHR_45_ Scaffold7842 100452 100732
KSD_NAHR_45

>**Scaffold7842 100452 100732**_NAHR

GTGCCCAACCGCCAAACTCGATCTCTATCTCTCTCTCTCTCAGAGTCTCTCTCTGTCACACACACACAAACACACACACACGCACATACACACAGAGATACCCATGATATACACACAACCCTCATT TT
TCACCATTTGGAAGTTCGATGTCCAATTGAGAGCAGTGACTCCCCTTGCCAATTCCAGGAAATAAAACTAAGCACAGATTGCCAGCTCACTGCGTGTCCGTGGCCTCATGTCTTTTTGGGAAGTG GCC
TCTGTCTCTGGAGCAGGGAGAGCATACAAAGTGTTTCACTTCACTGGGCATTGTGGCTCATGCCTGTAATCCCAGCACTTTGGGAGGCCAAGGTGGGCAGATCACGAGGTCCGGAGATCGAGACCA TC
CTGGCTAACACGGTGAAACCCCGTTTCTACTAAAAATACAAAAAGCTGGGCGTGGAGGCGGGCGCCTGTAGTCCCAGCTACTCGGGAGGCTGAGGCAGGAGAATGGCGTGAACCCAGGAGGCAGA GCT
TCCAGTGAGCCGAGATCTTGCCACTGCACTCCAGCCTGGGCAACACAGCAAGACTCTGTCTCAAAAAAAAAAAAAAAAATCAGCCAGGTGTTGTGGTACGTGTCTGCAGTCCCAGCTGCTTGGGAGG CT
GAGGTGGGAGAATCGCTTGAACCTGGGAGGCAGAGGTTGCAGTGAGCTGAGATTGCACCACTGTACTCCAGCCTGGGCAACA gAGCAAGACTCTGTCTCAAAAAAATAAATACATAAATAAAAATGCT
TAAACATTTGAGAAGAATCAAGCCCGGAAGGGACCATAAGGACTCCCCACACAGGAGGACGCGTGCCTCAGGGCACTCGGGAATCAGTACTGTAGTGACATCTAAATCTGTACTCAGGAAGCAAGT CA
GTCCTAACACCAGAGCAGCTTCAGATTAAAAAATACAAATCAACTGGACGCAGTGGCTCACATTTGTAATCCCAGCACTTTGGGAGGAAGAGGCAAGAGGGTTGCTTGAACCCAGGAGTTTAACA CCA
GCCTGGGCATCATAGTGAGCGTTCATCTCTCCAAAAAAAAAAAAAAAGAGCCAAGTGCGGTCCCAGTCACTCAGGGGGCTGAAGTGGGAGGGTCACTTGTGCCCAGAAGTTGGAGACTGTAGAGAGC TA
TGATTGCGCCACTACACTCCAGCCTGGGCGACCGAGCAAGACCCTGTCTCTAAAAAAATAAGAAAGAATAGT CTGGGTGCAGTGGCTCACACCTGTAACCCCAGCATTTTGGGAGGCCAAGGTGGGCA
GATCACAAGGTCAGGAGTTCGAGACCAGCCTGGCCAATATGGTGAAACCCCGTCTCTACTAAAAATATAAAAAAATTAGCCAGGCGTGGTGTCGGGCACCTGTAGTCCCAGCTACTCAGGAGGCTG AG
GCAGGAGAATCGCTGGAACCCGGGAGGCAGAAGTTGCAGTGAACTGAAATCGCGCCACTGCACTCCAGCCTGGGTGACAGAGTGAGACTCCATCTCTAAATAAATA AAAAGGAATGAAAGGAGGGAAG
GAGGGAAGGAGGGAGGGAGGGAGGGAGGCAGGCATCCTCTTGTGGACTGTTTTTTACTTTGTTGGTTTTCTCCACCGGAATACTACATTGTGAGTTGCCACAGTTACTCACCTTTGGCTCTGCC TCA
GTGGCGACCCATGGAGTGATGTTCCAAATGCCTACTCTGCAAACAGTCCTGATTGTGCAGTGAGCAGCACTACAACTACATCAATGTAGTTGATTGATGGATGTCATTTA AAGACAGAAATGTGTGCA
GAGGTGAGTGCAGAAGGGTGAAGGGGACTTCCAGGCACAAAACCTTGAGATCCATCCTAATCCTTCACCAACGACTCGAATTTTTTTTTTTTTTATTTTTGTGGGTACATAGTAGGTGTATATATT TAT
GGGGCACATAAGACATTTTGATACAGGCATGCAATGTATAATAATCACATCGGGGGGCCGGGCGCAGTGGCTCACACCTGTAATCCCAGCACTTTGGGAGGCCGAGGCGG GTGGGTCAGGAAGTCAGG
AGTTCGAGACCAGCCTGACCAACATGGTGAAACCCCGTCTCTACTAAAAATACAAAAATAAGCCGGGTGTGGTGGCCCATGCCTGTAATCCCAGCTACTCGGGAGGCTGAGGCATGAGAATTGCT TGA
ACCCGGGAGGCAGAAGTTGCAGTGAGCTGAGATAGTGACACTGCACTCCAGCCTGGCCAACAGAGCAAGACTCCGTCTCAAAAAAAAATCATATCAGGATAAAT G

19	19.2	0.0	0.0	4877	22	57	(2223)	+	(TC)n	Simple_repeat	1	36	(0)	1	*
30	19.3	0.0	1.7	4877	57	117	(2163)	+	(CA)n	Simple_repeat	1	60	(0)	2	
2367	7.2	1.4	0.0	4877	300	590	(1690)	+	AluY	SINE/Alu	3	297	(14)	3	
1184	15.1	0.0	0.0	4877	593	764	(1516)	+	AluSx3	SINE/Alu	135	306	(5)	4	
1533	19.2	6.0	0.0	4877	940	1220	(1060)	+	AluJr	SINE/Alu	3	300	(12)	5	
2279	9.9	0.0	0.3	4877	1225	1518	(762)	+	AluSx3	SINE/Alu	3	295	(16)	6	
28	7.8	0.0	0.0	4877	1528	1568	(712)	+	(GGAG)n	Simple_repeat	1	41	(0)	7	
721	8.3	0.0	0.0	4877	1880	1975	(305)	C	L1MA3	LINE/L1	(0)	6305	6210	8	
2344	8.0	0.0	0.0	4877	1976	2263	(17)	+	AluSg	SINE/Alu	1	288	(22)	9	

19	19.2	0.0	0.0	Hg19	22	57	(4024)	+	(TC)n	Simple_repeat	1	36	(0)	10	*
30	19.3	0.0	1.7	Hg19	57	117	(3964)	+	(CA)n	Simple_repeat	1	60	(0)	11	
2367	7.2	1.4	0.0	Hg19	300	590	(3491)	+	AluY	SINE/Alu	3	297	(14)	12	
1158	15.7	0.0	0.0	Hg19	593	764	(3317)	+	AluSx3	SINE/Alu	135	306	(5)	13	
1533	19.2	6.0	0.0	Hg19	940	1220	(2861)	+	AluJr	SINE/Alu	3	300	(12)	14	
2234	9.3	0.0	0.4	Hg19	1225	1548	(2533)	+	AluSx3	SINE/Alu	3	311	(0)	15	
184	27.7	0.0	0.0	Hg19	1549	1595	(2486)	C	MIRb	SINE/MIR	(138)	130	84	16	
2365	9.0	0.0	0.7	Hg19	1780	2078	(2003)	+	AluSz	SINE/Alu	1	297	(15)	17	
230	30.7	4.3	0.8	Hg19	2108	2222	(1859)	C	L1ME4a	LINE/L1	(57)	6067	5949	18	
1070	15.8	0.6	0.0	Hg19	2434	2597	(1484)	C	AluJb	SINE/Alu	(11)	301	137	19	
21	0.0	0.0	0.0	Hg19	2721	2743	(1338)	+	(CA)n	Simple_repeat	1	23	(0)	20	
96	22.3	3.8	3.2	Hg19	2760	3021	(1060)	+	GA-rich	Low_complexity	1	264	(0)	21	

2593	5.4	0.0	0.0	Hg19	3022	3319	(762)	+	AluY	SINE/Alu	2	299	(12)	22
96	22.3	3.8	3.2	Hg19	3320	3369	(712)	+	GA-rich	Low_complexity	265	314	(0)	23
721	8.3	0.0	0.0	Hg19	3681	3776	(305)	C	L1MA3	LINE/L1	(0)	6305	6210	24
2344	8.0	0.0	0.0	Hg19	3777	4064	(17)	+	AluSg	SINE/Alu	1	288	(22)	25

>Hg19 chr7:97654283-97658363
GTGCCCAACCGCCAAACTCGATCTCTATCTCTCTCTCTCTCAGAGTCTCTCTCTGTCACACACACACAAACACACACACACGCACATACACACAGAGATACCCATGATATACACACAACCCTCATTTT
TCACCATTTGGAAGTTCGATGTCCAATTGAGAGCAGTGACTCCCCTTGCCAATTCCAGGAAATAAAACTAAGCACAGATTGCCAGCTCACTGCGTGTCCGTGGCCTCATGTCTTTTTGGGAAGTG GCC
TCTGTCTCTGGAGCAGGGAGAGCATACAAAGTGTTTCACTTCACTGGGCATTGTGGCTCATGCCTGTAATCCCAGCACTTTGGGAGGCCAAGGTGGGCAGATCACGAGGTCCGGAGATCGAGACCATC
CTGGCTAACACGGTGAAACCCCGTTTCTACTAAAAATACAAAAAGCTGGGCGTGGAGGCGGGCGCCTGTAGTCCCAGCTACTCGGGAGGCTGAGGCAGGAGAATGGCGTGAACCCAGGAGGCAGA GCT
TCCAGTGAGCCGAGATCTTGCCACTGCACTCCAGCCTGGGCAACACAGCAAGACTCTGTCTCAAAAAAAAAAAAAAAAATCAGCCAGGTGTTGTGGTACGTGTCTGCAGTCCCAGCTGCTTGGGAGGCT
GAGGTGGGAGAATCGCTTGAACCTGGGAGGCAGAGGTTGCAGTGAGCTGAGATTGCACCACTGTACTCCAGCCTGGGCAACA cAGCAAGACTCTGTCTCAAAAAAATAAATACATAAATAAAAATGCT
TAAACATTTGAGAAGAATCAAGCCCGGAAGGGACCATAAGGACTCCCCACACAGGAGGACGCGTGCCTCAGGGCACTCGGGAATCAGTACTGTAGTGACATCTAAATCTGTACTCAGGAAGCAAGTCA
GTCCTAACACCAGAGCAGCTTCAGATTAAAAAATACAAATCAACTGGACGCAGTGGCTCACATTTGTAATCCCAGCACTTTGGGAGGAAGAGGCAAGAGGGTTGCTTGAACCCAGGAGTTTAACA CCA
GCCTGGGCATCATAGTGAGCGTTCATCTCTCCAAAAAAAAAAAAAAAGAGCCAAGTGCGGTCCCAGTCACTCAGGGGGCTGAAGTGGGAGGGTCACTTGTGCCCAGAAGTTGGAGACTGTAGAGAGCTA
TGATTGCGCCACTACACTCCAGCCTGGGCGACCGAGCAAGACCCTGTCTCTAAAAAAATAAGAAAGAATAGT CTGGGTGCAGTGGCTCACACCTGTAACCCCAGCATTTTGGGAGGCCAAGGTGGGCA
GATCACAAGGTCAGGAGTTCGAGACCAGCCTGGCCAATATGGTGAAACCCCGTCTCTACTAAAAATATAAAAAAATTAGCCAGGCGTGGTGTCGGGCACCTGTAGTCCCAGCTACTCAGGAGGCTGAG
GCAGGAGAATCGCTGGAACCCGGGAGGCAGAAGTTGCAGTGAACTGAAATCGCGCCACTGCACTCCAGCCTGGGTGACAGAGTGAGACTCCATCTCTAAATAAATA Aataaataaataaataaataaa
taaataaataaactgagtctcagagggattagattgcctgccctgggccacagaactagctgtaatcatggtcccgagtctttgtgttgggagaaatattagtttggaagctcagtttcctcctctgc
cccccaggtggacctgacctgactcatgtgtgcaaatgaaggcatctccctgcgtgcccacatttgtcgagccctttctttccttccccttccttttaaagatgtcttgtcttgggctgggcgc agt
ggctcacgcctgtaatctcagcactttgggaggctgaggcggcggatcacttgaggtcaggagttcgagaccagcctggccaaaagggtgaaaccccgtttctactaacaatacaaaaattagccgg
gcatatggtggtgcatgcctgtaatttcagctactcaggacgctgagacggaagaatcgcttgaacccgggaggtggagattgcagtgagctgagatcacgccactgcaccccagcctgggcaac aga
gcaagactctgtctcaaaaaaaaaaaaaaacatttttgaggaaaggcagaagcattggaagaaattataagaaaacataaaaataattatccataattccttcaactaggtaaaaccactataaatgt
ttgcctagcgccttccagtgtttttctgtacacacacaaatatgcacagaccaaaaactatatacttttaaaaatgtaaagactttatttgccaggtagggttttagaaagttgaaactaattca ttc
tctaaccgtgtatgagagtatgttcctcctgcaccttcatcaaatattcgtgttcctttttgttaaattatgcatatgttttcctagtgcagtttccctttttcttgcattaagtggcagttctctaata
cttttttttttttttttagagacagggtcttgctatgtcacccaggctgaagtgcagtggtgcgatcatagccattgcagccttgaactcccaggctcaagggatcctctagcggcagtgtcc caa
gtagctgggactacaagtgcgtgtcaccatgcctggcttcctgatactttttctgctttcctgccccagatttgcaatccaggcagaaagaaaatctccactggatcccacggacaaagacgccccat
cttggggagctggagtcatgaatgtcagtgtgcacacacacacacacacacacacggaaagagagagagagagaaagcaagaaagagagaagtaaggaagaaggagagagaaagaaagaagagaa agg
aaagagaaagagacaaaagaaaaggaaagagaaaggaagaaagaaaagaaaaaaaggaaaggggcagaaggaaggaagaaggggaaagaagaaagaaaggaaaagaaagaaagaaaagagaaagaga
gaaaggaagaaaggaaggaaagaaggaaggaaggaaggaaagaaggaaggcaggcaggcaggcaggcatgcagtcca gccaggcgcggtggctcacgcctgtaatcccagcattttgggaggcagagg
cgggtggatcacaaggtcaggagatcgagaccatcctggctaacacggtgaaaccccgtctctactaaaaatacaaaaaattagccaggtgcggtggcgggcacctgtagtcccagctactagggagg
ctgaggcaggagaatggtgtgaacccggaggcagagcttgcggtgagccgagatcgcaccactgcactccagcctgggtgacagagcaagactccgtctcaaaaaaaaaaaaaa AAAGGAATGAAA
GGAGGGAAGGAGGGAAGGAGGGAGGGAGGGAGGGAGGCAGGCATCCTCTTGTGGACTGTTTTTTTACTTTGTTGGTTTTCTCCACCGGAATACTACATTGTGAGTTGCCACAGTTACTCACCTTTGGC
TCTGCCTCAGTGGCGACCCATGGAGTGATGTTCCAAATGCCTACTCTGCAAACAGTCCTGATTGTGCAGTGAGCAGCACTACAACTACATCAATGTAGTTGATTGATGGATGTCATTTAAAGACA GAA
ATGTGTGCAGAGGTGAGTGCAGAAGGGTGAAGGGGACTTCCAGGCACAAAACCTTGAGATCCATCCTAATCCTTCACCAACGACTCGAATTTTTTTTTTTTTTATTTTTGTGGGTACATAGTAGGTGTA
TATATTTATGGGGCACATAAGACATTTTGATACAGGCATGCAATGTATAATAATCACATCGGGGGGCCGGGCGCAGTGGCTCACACCTGTAATCCCAGCACTTTGGGAGGCCGAGGCGGGTGGGT CAG
GAAGTCAGGAGTTCGAGACCAGCCTGACCAACATGGTGAAACCCCGTCTCTACTAAAAATACAAAAATAAGCCGGGTGTGGTGGCCCATGCCTGTAATCCCAGCTACTCGGGAGGCTGAGGCATGAGA
ATTGCTTGAACCCGGGAGGCAGAAGTTGCAGTGAGCTGAGATAGTGACACTGCACTCCAGCCTGGCCAACAGAGCAAGACTCCGTCTCAAAAAAAAATCATATCAGGATAAAT G

Homologous Sequences =

AAA

>NAHR_46_ Scaffold5150 9152828 9153128
KSD_NAHR_46

>**Scaffold5150 9152828 9153128_**NAHR
AGACTATTAAGGGCAAAGACACTTCTCTTATAATCAACTTCAGCTATTAAAGCCATCCAGAATTTTTTCTCATATTGAAAAAAATTTATCCTCTACCCCCTGATGGCTACAAGAAAGGGTTTGGGC CA
TTTGTTCCAAATCTCATATATAACTCTATTATAGAGCTATTATGCATGTTAAGAACATCTAAATCCAGTCTAGTCTGATGTACCTATTATCAGAATTTGTGAAAATGCCACTTATTTAAAAATTA TTT
CTTAAGACTGCTCATGTATAGAAAGTTTCTGAATATAGTCAGACTTCCTTTGCAGTAAGAAAAAGGATGATAAAAGGGTATGAAACACTTTGAATAAAAATTAAATATTACAAATGTATTACAATA CT
GATTATAATAACTCTAAGTAATTTTTTGTTTGTTTAAAAAAATAATGTATCCCTATTGATTGGCAAGTATGCTACCAAATCCTGAGGATTCATTCCTGATATTCCATGCAATGAATGGACACAAT ATC
TTAAGTGTTTACCAGGACATGTTCTTGCAATTTAAATATATAATAATGGATTAATAATATAGTTTTTACCATTCTATAAGTGTCAGTTAAAGTTATATAATAGCCAAAAATCTAACCGCAGTCACA CA
CACACTGCCTAACATTTCAGTCAATGGTGGACCACATATATAATGGTAGTCTCATAAGGTTATAATGGAGCTGAAAAATTTCTATTGCTTAGTAATGTCATAGCCATCATAATATCACAGTGCAA CAC
ATTATTTATATGTTTGTGGTGATGCTGGTGTAAACAAACTAACTGTGCTGCCAGTCATACAAAAGTACAGGACACTGGGAGGCTGAGGCAGCGGATTACGAGGTCAGGAGTTTGAGAGCAGCCTGT GC
AATATGGTGAAACCACGTCTCTACTAAAAAATACAAAAAAAAT GGCCGGGCGCGGTGGCTCACGCTTGTAATCCCAGCACTTTGGGAGGCCGAGGCGGG gGGATCACGAGGTCAGGAGATCGAGACCA
CGGTGAAACCCCGTCTCTACTAAAAATAAAAAAAAAATTAGCCGGGTGTGGTGGCGGGCGCCTGTAGTCCCAGCTACTCGGAGAGGCTGAGGCA GGAGAATCGCTTGAACCGGGGAGGCAGAGGTTGC
AGTGAGCTGAGATCGCCCCACTGCACTCCAGCCAGGGTGACAGAGAGAGACTCCATCTCA AATTAAATAAATAATAAATAAATAAAAATATATAAAAAAT AGGACATACAATTTTGCACAGTATGTAA
TATTTAGTAAGTATAGTAAATGATTATGTTACTTGTTTATGTATTTACCAGACTATACTTTTTATCATTATTTTAGTGTGTACTACTTTTACTTATGAAAAATGTTAACTGTTAAACAGCCTCAG TCA
GTCCTTAAGGAGGTATTCCAGAAGAAGGCATTATCATCATAGGCAATGACAGCTCCATGCGTGTTATTGCCCCTGAAGTTCTTCCAGTGAGACAAGATGT GGAAGTGGAGGACAGTGATGTTGATGAT
CGTGACCCTTGTTTGTCCTGGACTAATAATGTGTGTGTTTGTGTTTTAGATTTTAACAGAAAAGTTTAAGAAGTAAAATAAATAAATAAATTGTTAAAATAAGAATATAAAGTAAAAAAATATAT TTT
ACAGCTGTTCAGCATGTTTGTGTTTTAAGCTAAGTGTTATAAAAAAGAATCAAAAAGTTAATTTAAAAAAGATTTTTAAGCATAGCATTTCAATATCCTT TGACCATTGTGGTATGAATAAAGTTGC
TTGAATCAAGAAAGCAACACAAATTATAGCATCAATACTATCTATGTATCTAGAAATACATAAAATGAAATGAAAATGATTAGTGAAGGAATTCATCTTAAAAGTCAATTAACTTACTGAAATTT AAG
GATAAATGTTATGAACCAATTGTAAATAGGGGTGTTTTCCTAATTGTGATAAAAAGAGGGGTTGCCATATCTGTCAGTCAATATGTTGTCTTTCTTCGGT GTCAAGATGTATGTGTCCTATGAAACAT
TTTCATATTTTTTATTTTGAATGCTCAAACTGATCATTTTTACATCCATGCTTTCTCATTAAATTGATTTCTTCACCAGATACTGCTTCAAGCTGTCTACCTCTAATACCACTCTTAAATTCTG TAT
ATTTTTGGAGATATTTCTCAAGGTCTGTTCCCATCAGAAGATGAAGTTGATAAGTAATATGGAATTTGAATTACATATGTCCCACTTTAGGTAAAGAAGT TAAGCTCTCCATCTTACAAGGCTG

1219	18.5	0.0	0.0	20016	652	835	(1465)	+	Tigger3b	DNA/TcMar-Tigger	21	204	(1027)	1
650	11.0	1.0	1.0	20016	836	935	(1365)	+	AluSx4	SINE/Alu	32	131	(179)	2
2313	6.6	0.0	1.4	20016	940	1240	(1060)	+	AluYc3	SINE/Alu	1	297	(3)	3
2693	12.3	6.1	0.8	20016	1243	1732	(568)	+	Tigger3b	DNA/TcMar-Tigger	195	710	(521)	4

3802	14.1	4.3	2.3	Hg19	652	835	(1634)	+	Tigger3b	DNA/TcMar-Tigger	21	204	(1027)	5
1820	11.7	0.3	2.2	Hg19	836	939	(1530)	+	AluSx3	SINE/Alu	32	132	(179)	6
2596	3.1	0.0	0.7	Hg19	940	1231	(1238)	+	AluYc3	SINE/Alu	1	290	(10)	7
1820	11.7	0.3	2.2	Hg19	1232	1409	(1060)	+	AluSx3	SINE/Alu	133	308	(3)	6
3802	14.1	4.3	2.3	Hg19	1410	1901	(568)	+	Tigger3b	DNA/TcMar-Tigger	205	710	(521)	5

>Hg19 chr8:112405211-112407679
AGACTATTAAGGGCAAAGACACTTCTCTTATAATCAACTTCAGCTATTAAAGCCATCCAGAATTTTTTCTCATATTGAAAAAAATTTATCCTCTACCCCCTGATGGCTACAAGAAAGGGTTTGGGC CA
TTTGTTCCAAATCTCATATATAACTCTATTATAGAGCTATTATGCATGTTAAGAACATCTAAATCCAGTCTAGTCTGATGTACCTATTATCAGAATTTGTGAAAATGCCACTTATTTAAAAATTA TTT
CTTAAGACTGCTCATGTATAGAAAGTTTCTGAATATAGTCAGACTTCCTTTGCAGTAAGAAAAAGGATGATAAAAGGGTATGAAACACTTTGAATAAAAATTAAATATTACAAATGTATTACAATA CT
GATTATAATAACTCTAAGTAATTTTTTGTTTGTTTAAAAAAATAATGTATCCCTATTGATTGGCAAGTATGCTACCAAATCCTGAGGATTCATTCCTGATATTCCATGCAATGAATGGACACAAT ATC
TTAAGTGTTTACCAGGACATGTTCTTGCAATTTAAATATATAATAATGGATTAATAATATAGTTTTTACCATTCTATAAGTGTCAGTTAAAGTTATATAATAGCCAAAAAT CTAACCGCAGTCACACA
CACACTGCCTAACATTTCAGTCAATGGTGGACCACATATATAATGGTAGTCTCATAAGGTTATAATGGAGCTGAAAAATTTCTATTGCTTAGTAATGTCATAGCCATCATAATATCACAGTGCAACAC
ATTATTTATATGTTTGTGGTGATGCTGGTGTAAACAAACTAACTGTGCTGCCAGTCATACAAAAGTACAGGACACTGGGAGGCTGAGGCAGCGGATTACGAGGTCAGGAGTTTGAGAGCAGCCTGT GC
AATATGGTGAAACCACGTCTCTACTAAAAAATACAAAAAAAAT GGCCGGGCGCGGTGGCTCACGCTTGTAATCCCAGCACTTTGGGAGGCCGAGGCGGG tGGATCACGAGGTCAGGAGATCGAGACCA
CGGTGAAACCCCGTCTCTACTAAAAATAAAAAAAAAATTAGCCGGGTGTGGTGGCGGGCGCCTGTAGTCCCAGCTACTCGGAGAGGCTGAGGCA Ggagaatggcgtgaacccgggaggcggagcttgc
agtgagccgagactgcgccgctgtactccagcctgggtgacagagcgagactccgtctcaaaaaaa aaaaaaaaaaaaattagccaggtgtggtggcatgcgcctgtagtaccagctatttgggaggc

tgagacagGAGAATCGCTTGAACCGGGGAGGCAGAGGTTGCAGTGAGCTGAGATCGCCCCACTGCACTCCAGCCAGGGTGACAGAGAGAGACTCCATCTCA AATTAAATAAATAATAAATAAATAAAA
ATATATAAAAAATAGGACATACAATTTTGCACAGTATGTAATATTTAGTAAGTATAGTAAATGATTATGTTACTTGTTTATGTATTTACCAGACTATACTTTTTATCATTATTTTAGTGTGTACT ACT
TTTACTTATGAAAAATGTTAACTGTTAAACAGCCTCAGTCAGTCCTTAAGGAGGTATTCCAGAAGAAGGCATTATCATCATAGGCAATGACAGCTCCATGC GTGTTATTGCCCCTGAAGTTCTTCCAG
TGAGACAAGATGTGGAAGTGGAGGACAGTGATGTTGATGATCGTGACCCTTGTTTGTCCTGGACTAATAATGTGTGTGTTTGTGTTTTAGATTTTAACAGAAAAGTTTAAGAAGTAAAATAAATA AAT
AAATTGTTAAAATAAGAATATAAAGTAAAAAAATATATTTTACAGCTGTTCAGCATGTTTGTGTTTTAAGCTAAGTGTTATAAAAAAGAATCAAAAAGTTA ATTTAAAAAAGATTTTTAAGCATAGCA
TTTCAATATCCTTTGACCATTGTGGTATGAATAAAAGTTGCTTGAATCAAGAAAGCAACACAAATTATAGCATCAATACTATCTATGTATCTAGAAATACATAAAATGAAATGAAAATGATTAGT GAA
GGAATTCATCTTAAAAGTCAATTAACTTACTGAAATTTAAGGATAAATGTTATGAACCAATTGTAAATAGGGGTGTTTTCCTAATTGTGATAAAAAGAG GGGTTGCCATATCTGTCAGTCAATATGTT
GTCTTTCTTCGGTGTCAAGATGTATGTGTCCTATGAAACATTTTCATATTTTTTATTTTGAATGCTCAAACTGATCATTTTTACATCCATGCTTTCTCATTAAATTGATTTTCTTCACCAGATAC TGC
TTCAAGCTGTCTACCTCTAATACCACTCTTAAATTCTGTATATTTTTGGAGATATTTCTCAAGGTCTGTTCCCATCAGAAGATGAAGTTGATAAGTAATAT GGAATTTGAATTACATATGTCCCACTT
TAGGTAAAGAAGTTAAGCTCTCCATCTTACAAGGCTG

Homologous Sequences =

CAGGAGAAT

>NAHR_47_ Scaffold2632 4512664 4512809
KSD_NAHR_47

>Scaffold2632 4512664 4512809_NAHR
CCAGCTATGGATGAAGTAAACCCTGGTGCTAATTAGACTGCCTCTCTGGATGGTGCAAGCTGTGAGCCTTAGCATTGTCTGTGTGGTGCTAATTTTGCAGGTATTCAGAATGCAAGAGCTGTGAAG AT
ATGATTGCCTCCACCTAGATTTCCAAGTATGTCTCCCACATCCTCTGGAATTAGGCAGAAATGTGCCACTGGGTGGAGCCTCCACAGAGAGCCTTCACTAGAGCAATGTCCAGCAGAATTAGAAC TGT
GAGGTCAGGCAATTGTACAGAGTCCTTCCTAGGTCAGTGTCTAATGGCCACCCCAAGCCTTCAGTACTGTAGAGACACCGGCATGCAGCACCAGCCTGTGAGAGCTCCAGGTATTTATTTCAATAT GT
GATAGCTGAAGCATGGGCTGCACTCAACAAAGCCAcGAAGGAAAAATTGCCCAAGGCCATGGGGATTCCACCCCTACCCCTGTTTTTCTGGAAATTGGAACATGAATTCAGGGATTATTGTCAAGCCT
TAAGATTTAATGTTGTTTGACCTGTTGGGTTTTAGACTTACTTGGGATATCTTACTTCACTTGTCTTTTTTCCTATGTCTCTCTTTTGAAATGAGAATGTTTATCCTCTGTCTGTTCCACCATTGT AT
TTTAGGAGCACATAACTTGTTTGACTTCACAAGTTCACAGTTGGAGAAAAATTTGATGCTGGAACAAGTTAAGACTCCTGGGCTACTGGAATGGAGTGAATGTATTTTGTTTGTGAGAAGGAGAT GAA
TGTGGGAATCAAGGGGGAGTGTAGTTCTGTGGGGTAAATGTCACCCAAAATTTATGTATTGGAAACAATTCCTATTACAAGTGTTGAGAGGTGAGGCCTGCTGGAAGGTGCTTATGTCATAAGGGC TT
CATTCTCATGAATAGCTTAATGTCACTATAACAAGGAATTATGGGTTGGGTGCGGTGGCTCATGCCTATAATCCCAGCACTTTGAGAGGCCGAGGCAGGTGGGTCATCTGAGTTCAGGAGTTCGA GAC
CAGCCTGGCCAACAGGGTGAAACCCAGTCTCTACTAAAAATACGGAAAATTAGCTGGCCATGCCAGGCACGGTGGCTCACACCTGTAATCCCAGCACTTTGGGAGGCCAAGGTGGGTGGATCACTT GA
GGTCAGGAGTcAGAGACCAGCCTGGCCAAAAAAATTAGCTGGACATGGTGGTGGGCACCCATAAcCCCAGCTACTCTGGAGGCTGAGGCAGGAAAATCACTTGAACCCGGGAGGCTGAGGTTGCAGTGT
GTCAAGATCACACCATTGTACTCCAGCCTGGGCAACAAGAGCCAAACTCCATCTTAAAAAAAAAAAAAAAAAAAAaGAAAAGTAACCAGGCATGGTAGCATGTGCCTGTAGTCCCAGCTACTCAGGAGG
CGGAGGCAGGAGAATGGCTTGAACTGGGAGGCATAGGTTGCAATGAGTGAGCCGAGATTGTGCCACTGCACTCCAGCCTGGCGACAGAGCAAGACTTGGTCTCAAACAAACAAAGGAACTGTGGA AGG
AGGTTCACTCCCATTCCATATTTTGCCATGTGAGAACACAGAAAGAAGGCCTTCACCAGATGCTGGCCCATTAATCTTGAACTTCTCGGTCTCAGGAAATGTAAGAAATAAATATCTGTTCTTTAT AA
ATTACATAATTTTATATATCCTGTGAATCAGCATTAAATTAACTAAGACACTAAAGCATTTATTAAAGTAGTAAATATTTAAATAATCATATACATAAAAGTAAAAATAAATAAAATTTAATGAT CTC
AGACAAACAAAAATAGCTTTCAATAACAGCAAAGTAACTATATTTAAGGTTGCCATTTAAGGGACAATTGTCAAACTTAATTGTTCAATAACTAAAAGAATCTTTAAAAAAAACACTTGATAATTA TA
CTGCTAAATTTATTTAGCTTTATTAAGTATTTTTTCTTCTAAAAGAACATATTTTTCAAATATTAAGGGAAGTAGCCACAATTCCAATAACTGCCCTTTTTGCTTATATTTTAGTAAACATACAA TTT
AAGTAGAACAGAACACAACTTCATTGAGATAGGCCTAAATACATGTAAATTAAATACAACTGGGTTGATAGTTATAATAGCAGAATTTTTTATGTA A

2757	26.7	8.4	7.4	30986	3	783	(1362)	+	MSTD-int	LTR/ERVL-MaLR	858	1646	(5)	1
709	26.6	12.5	6.5	30986	812	939	(1206)	+	MSTD	LTR/ERVL-MaLR	27	151	(214)	1
1954	11.0	0.7	5.8	30986	940	1085	(1060)	+	AluSx	SINE/Alu	1	141	(171)	2
1877	11.6	11.6	0.0	30986	1086	1360	(785)	+	AluSq2	SINE/Alu	2	308	(5)	3
1954	11.0	0.7	5.8	30986	1361	1522	(623)	+	AluSx	SINE/Alu	142	293	(19)	2
710	24.9	5.3	0.6	30986	1523	1714	(431)	+	MSTD	LTR/ERVL-MaLR	152	396	(0)	1
15	23.5	0.0	0.0	30986	1736	1775	(370)	+	A-rich	Low_complexity	1	40	(0)	4

2773	26.6	4.2	3.9	Hg19	3	783	(1506)	+	MSTD-int	LTR/ERVL-MaLR	858	1646	(5)	5
605	21.9	3.4	0.0	Hg19	807	925	(1364)	+	MSTD	LTR/ERVL-MaLR	17	139	(257)	5
1306	15.8	0.0	4.7	Hg19	940	1161	(1128)	+	AluSx	SINE/Alu	1	212	(100)	6
1374	10.5	0.9	7.9	Hg19	1165	1230	(1059)	+	AluSx3	SINE/Alu	79	140	(171)	7
1913	10.9	11.7	0.0	Hg19	1231	1504	(785)	+	AluSq2	SINE/Alu	2	307	(6)	8
1374	10.5	0.9	7.9	Hg19	1505	1666	(623)	+	AluSx3	SINE/Alu	141	292	(19)	7
710	24.7	4.6	0.0	Hg19	1685	1858	(431)	+	MSTD	LTR/ERVL-MaLR	215	396	(0)	5
15	23.5	0.0	0.0	Hg19	1880	1919	(370)	+	A-rich	Low_complexity	1	40	(0)	9

>Hg19 chr13:57667964-57670252
CCAGCTATGGATGAAGTAAACCCTGGTGCTAATTAGACTGCCTCTCTGGATGGTGCAAGCTGTGAGCCTTAGCATTGTCTGTGTGGTGCTAATTTTGCAGGTATTCAGAATGCAAGAGCTGTGAAG AT
ATGATTGCCTCCACCTAGATTTCCAAGTATGTCTCCCACATCCTCTGGAATTAGGCAGAAATGTGCCACTGGGTGGAGCCTCCACAGAGAGCCTTCACTAGAGCAATGTCCAGCAGAATTAGAAC TGT
GAGGTCAGGCAATTGTACAGAGTCCTTCCTAGGTCAGTGTCTAATGGCCACCCCAAGCCTTCAGTACTGTAGAGACACCGGCATGCAGCACCAGCCTGTGAGAGCTCCAGGTATTTATTTCAATAT GT
GATAGCTGAAGCATGGGCTGCACTCAACAAAGCCAtGAAGGAAAAATTGCCCAAGGCCATGGGGATTCCACCCCTACCCCTGTTTTTCTGGAAATTGGAACATGAATTCAGGGATTATTGTCAAGCCT
TAAGATTTAATGTTGTTTGACCTGTTGGGTTTTAGACTTACTTGGGATATCTTACTTCACTTGTCTTTTTTCCTATGTCTCTCTTTTGAAATGAGAATGTTTATCCTCTGTCTGTTCCACCATTGT AT

TTTAGGAGCACATAACTTGTTTGACTTCACAAGTTCACAGTTGGAGAAAAATTTGATGCTGGAACAAGTTAAGACTCCTGGGCTACTGGAATGGAGTGAATGTATTTTGTTTGTGAGAAGGAGAT GAA
TGTGGGAATCAAGGGGGAGTGTAGTTCTGTGGGGTAAATGTCACCCAAAATTTATGTATTGGAAACAATTCCTATTACAAGTGTTGAGAGGTGAGGCCTGCTGGAAGGTGCTTATGTCATAAGGGC TT
CATTCTCATGAATAGCTTAATGTCACTATAACAAGGAATTATGGGTTGGGTGCGGTGGCTCATGCCTATAATCCCAGCACTTTGAGAGGCCGAGGCAGGTGGGTCATCTGAGTTCAGGAGTTCGAGAC
CAGCCTGGCCAACAGGGTGAAACCCAGTCTCTACTAAAAATACGGAAAATTAGCTGGCCATGCCAGGCACGGTGGCTCACACCTGTAATCCCAGCACTTTGGGAGGCCAAGGTGGGTGGATCACTT GA
GGTCAGGAGTtAGAGACCAGCCTGgccaacagggtgaaacccagtctctactaaaaatacgggaaattagctggccatgccaggcacggtggctcacacctgtaatcccagcactttgggaggccaag
gtgggtggatcacttgaggtcaggagttcgagaccagcttgGCCAAAAAATTAGCTGGACATGGTGGTGGGCACCCATAAtCCCAGCTACTCTGGAGGCTGAGGCAGGAAAATCACTTGAACCCGGGA
GGCTGAGGTTGCAGTGTGTCAAGATCACACCATTGTACTCCAGCCTGGGCAACAAGAGCCAAACTCCATCTTAAAAAAAAAAAAAAAAAAA AGAAAAGTAACCAGGCATGGTAGCATGTGCCTGTAGTC
CCAGCTACTCAGGAGGCGGAGGCAGGAGAATGGCTTGAACTGGGAGGCATAGGTTGCAATGAGTGAGCCGAGATTGTGCCACTGCACTCCAGCCTGGCGACAGAGCAAGACTTGGTCTCAAACAA ACA
AAGGAACTGTGGAAGGAGGTTCACTCCCATTCCATATTTTGCCATGTGAGAACACAGAAAGAAGGCCTTCACCAGATGCTGGCCCATTAATCTTGA ACTTCTCGGTCTCAGGAAATGTAAGAAATAAA
TATCTGTTCTTTATAAATTACATAATTTTATATATCCTGTGAATCAGCATTAAATTAACTAAGACACTAAAGCATTTATTAAAGTAGTAAATATTTAAATAATCATATACATAAAAGTAAAAATA AAT
AAAATTTAATGATCTCAGACAAACAAAAATAGCTTTCAATAACAGCAAAGTAACTATATTTAAGGTTGCCATTTAAGGGACAATTGTCAAACTTAA TTGTTCAATAACTAAAAGAATCTTTAAAAAAA
ACACTTGATAATTATACTGCTAAATTTATTTAGCTTTATTAAGTATTTTTTCTTCTAAAAGAACATATTTTTCAAATATTAAGGGAAGTAGCCACAATTCCAATAACTGCCCTTTTTGCTTATAT TTT
AGTAAACATACAATTTAAGTAGAACAGAACACAACTTCATTGAGATAGGCCTAAATACATGTAAATTAAATACAACTGGGTTGATAGTTATAATAG CAGAATTTTTTATGTAA

Homologous Sequences =

TGGCCAA

>NAHR_48_Scaffold490 141155 141455
KSD_NAHR_48

>Scaffold490 141155 141455_NAHR
GTAGTCTTGATCCATACAGGTGCTGAATTTAAAATAATgCATTTCATCCAACCACAATAATTTCAGAATAACAAGTATTTTACTTGTGTTTTCAAAAATTCCATGAAGTTGACACATATTCCTTTAAG
AATAATTACTTATTTTTCAACCCAATCCATATTTTTCACAGATTACAGCTAATCTCCTTAAACATCCCTATAAAACTCATCCAGTTTTTATCTTGTACCTCTTGTTTCCCCCCACACCACCTCCC CAC
TGCCCCCCACCCACCACTTTTTTCTCAACCCTCCATCAC AAAATACCAAAAATTATTCAAGCAAATCAGGACTATAGTTGCAGAGTGGGGAAAGAAATAGCCAGAAGTCAGCCAAGCTTGAGTCCTTT
TTAGTGTTAGGGACAAAAATCTCATTCTATATGCATTATTTCTAAATAATGTCCCAGCACTATTAAGCATTCAATGTGGTTCATAGAAAAGTGCTTTCCAAAAGAGATCCAGCTTCCTGTCCACC AAC
AGGCCTCACAGGTGATACAATTCTAAATTTGGCCTTTAC CTTGTCAATGTATCTCCATTGTCATCTGTCCTGAGAAGCCTGTCTCAAACT cACAGGAGACTGAAGTTTAACAATCCAATTAGACTGCT
TCTCCTCCCACCCATCTTGAGAGATACTCCTTCCAGGGTATTGAATAATGATGTTTCCTCATTCAGGGTACCACCTTCTCATTAGCAGAACAGTTCATAATAAAAATGCTACTGACTGGCCGGGT GCA
GTGGCTCATGCCTATAATCTCAGCACATTGGGGGGCCAA AGAGAGAGGATTGCTTGAGGCCTGGAGTTTTTGACCAGCCTGGGCAACATAGCAAAATCCTGTCTCTACAAAATATTTTTAAAATGAAT
AAGCCAGGTATGATGGCATACACCTATAGTCCTAGGTATTCGGGAGGCTGAGGTGGAAGGATTGCCTGACCCGGGCATTTGAGGCTGCAGTGAGCTATGATCATGCTGCTGTACTCCAGCCCAGG TGA
CAaAGTGAGACCTTTTTTAAAAAAATGCTATTTGGCAGCCGGGCGCGGTGGCTCACGCCTGTAATCCTAGCACTTTGGAAGGCCGAGGAGGGCGGATCACGAGGTCAGGAGATGGAGACCATCCAAGCT
AACACAGTGAAACCCCCTCTCTACTAAAAATACAAAAAATTAGCC GTGCATGGTGGCGGGTGCCTGTAGTCCCAGCtACTTGGAGCCTGAGGCAGGAGAATGGCGTGAACCCGGGAGGCGGAGCTTGC
CAAGATCGCGCCACTGCACTCCAGCCGAGGTGACAGAGC GACACTCCGTCTCaaAAAAAAAAAAAAAAAAAAGAACAGAAAAGAAAAAAATGCTATTTGGCAAGCAGAGAATAAAAACAAATGGAAATA
CATATGATCTTAAGTAAAATTTCATATTTATGCTACAGGTGGTGCTGTAGGTGGAGGATCACTGCCCCACAATACCCTGAAGCGAGTGGAGTCTTTGTGTGCCCTACTTCCCAGTCTCCAGGCTG CTC
AATTGTAGTTCCCTCTTCTGTTTATCCAGACTGACTGTC TTCTGTGTCCTCAACTTTTTCTCCATGTAAAGTACAATACAATTGGGTTCCAATTACTAAATTAAAATCACATTCTGGAACTGCCTGCT
TGAAATTACACCCAGGTGACCCTTACTGGAGTTTTGTATGTTCCAAAACACTTCAGCCACATATTTACAGTCTCCAGACTTACTCCCACAGATGGCGAACTTTATTTCCGCCATTTCCAATGTAA GAG
GAAACATCTCACTGCTGTCCCCATTGCATTGCTGCCATT CAACAGAAGAACAAAAATCTATTAATATCTCTTAGGCATGTTGAATTAATCTCCCCACTCTCATTTTGTCTCAAATCTACACTTCTCGT
TACCCTAAAGTCGTGGAAATGTAAAAATAAAGATATTCATTTGTATGTATCAGGGTCACCAGTCAAAGCAGATACTAGTTAAAATGTTTGGAGAGGGGCAAAGTCTGATATGGGTATTAGTTGGA GCT
CTCTACTAGACGCTTTCATAGATTTTCCCCTTATTTTGC AAATATTACTTTGATACAGCACAAATCGAATTTATGTTTCACAATTCACACAGCTGACTGACTTGTTTCATGTTCTATAATAATTCCAG
CTGCTATTTTACCTTTTATCCCCTCTCTCACAGTGTTAGCCTATCACTTGAGCATTTAGGAGGGGTGGGACTGTGTTAGAACACATCTGGCATGTAGTCAACCTGATGATTCAATATGGCGGG A

321	34.6	5.7	5.9	36106	19	390	(1910)	C	LTR103_Mam	LTR/ERV1?	(45)	430	60	1
1648	19.2	0.7	1.7	36106	758	1048	(1252)	+	AluJr	SINE/Alu	1	288	(24)	2
2370	7.0	2.7	0.0	36106	1061	1361	(939)	+	AluY	SINE/Alu	2	310	(1)	3

332	34.3	5.7	5.9	Hg19	19	390	(2138)	C	LTR103_Mam	LTR/ERV1?	(45)	430	60	4
1663	18.9	0.7	1.7	Hg19	758	1048	(1480)	+	AluJr	SINE/Alu	1	288	(24)	5
2608	5.0	0.0	0.0	Hg19	1061	1363	(1165)	+	AluY	SINE/Alu	2	304	(7)	6
1684	9.6	3.5	0.0	Hg19	1364	1592	(936)	+	AluY	SINE/Alu	75	311	(0)	7

>Hg19 chr1:170223074-170225601
GTAGTCTTGATCCATACAGGTGCTGAATTTAAAATAATaCATTTCATCCAACCACAATAATTTCAGAATAACAAGTATTTTACTTGTGTTTTCAAAAATTCCATGAAGTTGACACATATTCCTTTAAG
AATAATTACTTATTTTTCAACCCAATCCATATTTTTCACAGATTACAGCTAATCTCCTTAAACATCCCTATAAAACTCATCCAGTTTTTATCTTGTACCTCTTGTTTCCCCCCACACCACCTCCC CAC
TGCCCCCCACCCACCACTTTTTTCTCAACCCTCCATCAC AAAATACCAAAAATTATTCAAGCAAATCAGGACTATAGTTGCAGAGTGGGGAAAGAAATAGCCAGAAGTCAGCCAAGCTTGAGTCCTTT
TTAGTGTTAGGGACAAAAATCTCATTCTATATGCATTATTTCTAAATAATGTCCCAGCACTATTAAGCATTCAATGTGGTTCATAGAAAAGTGCTTTCCAAAAGAGATCCAGCTTCCTGTCCACC AAC
AGGCCTCACAGGTGATACAATTCTAAATTTGGCCTTTAC CTTGTCAATGTATCTCCATTGTCATCTGTCCTGAGAAGCCTGTCTCAAACT aACAGGAGACTGAAGTTTAACAATCCAATTAGACTGCT
TCTCCTCCCACCCATCTTGAGAGATACTCCTTCCAGGGTATTGAATAATGATGTTTCCTCATTCAGGGTACCACCTTCTCATTAGCAGAACAGTTCATAATAAAAATGCTACTGACTGGCCGGGT GCA
GTGGCTCATGCCTATAATCTCAGCACATTGGGGGGCCAA AGAGAGAGGATTGCTTGAGGCCTGGAGTTTTTGACCAGCCTGGGCAACATAGCAAAATCCTGTCTCTACAAAATATTTTTAAAATGAAT
AAGCCAGGTATGATGGCATACACCTATAGTCCTAGGTATTCGGGAGGCTGA GGTGGAAGGATTGCCTGACCCGGGCATTTGAGGCTGCAGTGAGCTATGATCATGCTGCTGTACTCCAGCCCAGGTGA
CAgAGTGAGACCTTTTTTAAAAAAATGCTATTTGGCAGCCGGGCGCGGTGGCTCACGCCTGTAATCCTAGCACTTTGGAAGGCCGAGGAGGGCGGATCACGAGGTCAGGAGATGGAGACCATCCAAGCT
AACACAGTGAAACCCCCTCTCTACTAAAAATACAAAAAATTAGCC Gggcatggtggcgggcgcctgtagtcccagctactcgggaggatgaggcaggagaatggcgtgaacccgggaggcggagactg
cagtgagccgagattgcaccactgcactccagcctgggc gacagagcgagactccgcctcaaaaaaaaaaaaaaaaaaaaaaagatcaaggccatcttggccaacacgatgaaaccctgtctctacta
aaaataccaaaaattagccg TGCATGGTGGCGGGTGCCTGTAGTCCCAGC cACTTGGAGCCTGAGGCAGGAGAATGGCGTGAACCCGGGAGGCGGAGCTTGCCAAGATCGCGCCACTGCACTCCAGCC

GAGGTGACAGAGCGACACTCCGTCTCAAAAAAAAAAAAAAAAAGAACAGAAAAGAAAAAAATGCTATTTGGCAAGCAGAGAATAAAAACAAATGGAAATACATATGATCTTAAGTAAAATTTCATATT
TATGCTACAGGTGGTGCTGTAGGTGGAGGATCACTGCCCCACAATACCCTGAAGCGAGTGGAGTCTTTGTGTGCCCTACTTCCCAGTCTCCAGGCTGCTCAATTGTAGTTCCCTCTTCTGTTTATCCA
GACTGACTGTCTTCTGTGTCCTCAACTTTTTCTCCATGTAAAGTACAATACAATTGGGTTCCAATTACTAAATTAAAATCACATTCTGGAACTGCCTGCTTGAAATTACACCCAGGTGACCCTTACTG
GAGTTTTGTATGTTCCAAAACACTTCAGCCACATATTTACAGTCTCCAGACTTACTCCCACAGATGGCGAACTTTATTTCCGCCATTTCCAATGTAAGAGGAAACATCTCACTGCTGTCCCCATT GCA
TTGCTGCCATTCAACAGAAGAACAAAAATCTATTAATATCTCTTAGGCATGTTGAATTAATCTCCCCACTCTCATTTTGTCTCAAATCTACACTTCTCGTTACCCTAAAGTCGTGGAAATGTAAAAAT
AAAGATATTCATTTGTATGTATCAGGGTCACCAGTCAAAGCAGATACTAGTTAAAATGTTTGGAGAGGGGCAAAGTCTGATATGGGTATTAGTTGGAGCTCTCTACTAGACGCTTTCATAGATTT TCC
CCTTATTTTGCAAATATTACTTTGATACAGCACAAATCGAATTTATGTTTCACAATTCACACAGCTGACTGACTTGTTTCATGTTCTATAATAATTCCAGCTGCTATTTTACCTTTTATCCCCTCTCT
CACAGTGTTAGCCTATCACTTGAGCATTTAGGAGGGGTGGGACTGTGTTAGAACACATCTGGCATGTAGTCAACCTGATGATTCAATATGGCGGG A

Homologous Sequences =

AAAAATTAGCCG

>NAHR_49_ Scaffold11012 428385 428560
KSD_NAHR_49

>Scaffold11012 428385 428560_NAHR
GTTGTGTTTTCTGGAGCACCTGGAGTCCCCTCTGTCCCTGGCCATCCCTCCTTCCCCACACCTCACCCCTGTACCTCACCTTCCCCACCCCCCTGCCTCACCCCTGCACCTCACCCTCCCCA tGCTTC
CCGTATCAGGGAGCTTCTTAAACACAGAATGGGGTGTTAAGATGATTGGAGGGCAGCAGCCTATTGGAATCCCGATGAACGTGGAAAGGTGTCAGGGAGGGGTAGCTGTGGTGTGGAGGAGCCCC ACA
TTTTCGTCCCTGTCTTGTCACAAAGAAGGAGCCCATCGCTGTGATTAGAAGGACTTAGGCCGGGCATGGTGGCTCACACCTGTAATCCCAGCACTTTGGCAGGCTGAGGCAG tTtGATTcCCTGAGGT
CAGGAGTTCAAGACCAGCTGGCCAACATGGAGAAACCCCGTCTCTACTAAAAATACAAAAAATTAGCCAGGCATGGTGGTGTGTGCCTATAATCCTAGCTCCTTGGGAGGCTGAGGCAGGAGAAT TGT
TTGAACCAGGGAAGCGGAGGTTACAGTGACCCGAGATCACACCATTGCACTCCAGTCTGGGCGACAGAGCAAGACTCTCTCTCAAAAACAAACAAAGGAAAAAAACAAACAAAAAGGCCGGGG CTCGG
TGGCTCATGCCTGTAATCCCAGCACTTTGGGAGGCCGAGGCGGTGGATTGCCTGAGGTCAGGAGTTGGAGACCAGCCTGGCTAAGATAGTGAAACCCCATCTTTACTAAAAATACAAAAATTAGC TGG
GCGTGATGGCAGGCGCCTGTAATCCCAGCTACTCGGGAGGCTGATGAGGGAGAATCATTTGAACCTCGGGAGGGCAGATGTTGCAGTGAGTTGAGATCGCGCCACTGTACTCCAGCCTGGGCG ACAAG
AGTGAAACTCCTTAAAAAAATAAAAAGGAAACAAACAAACAAACAAAAAATCAGCTGGGCATAGTGGTGGGGGCCTGTAGTCCCAGGTACTTGGGAGGCTGAGGCAGGAGAATTGCTTGAACCTG GGT
GGCTGATATTGCAGTGAGGCAAGATCATGCCACTGCACTCCAGCCTGGGTGATAGAGTGAGACTCCATCTTAAAAAAGGAAACAAAAAAAAGCCACAAAGAAGGACTTAGAATTGGGTGCTAA GCAGG
CCTCTTCCTACTGATAGGTGGACACATCTATTTCTTTATTATTATTATTATTATTA TTTTTGAGACAGAGTCTTGCTCTGTTGCCCAGGCTGGAGTGCGGTGGCATGATCTCGGCTCACTGCAAGCTC
CGCCTCCCCTGTTCACGCCATTCTCCTGCCTCAGCATCCCGAGTAGTTGGGACTACAGGCGCCCGCCACCACGCCCGGCTAATTTTTTGTATTTTTAGTAGAGATGGGGTTTCACCATGTTAG CCAGt
ATGGTCTCGATCTCCTGACCTCGTGATCTGCCCGCCTCGGCCTCCCAAAGTGCTGGGATTACAGGCGTGAGCCACCGCGCCTGGCC TATTTCTTTATTTTTAATTTTTTTTTATTTTTTATTTTTGA
GACGGAGTCTCGCTCTGTTGCCCAGGCTAGAGTGCAGTGAGCGATCTTGGCTCACTGCAAGCTCCACCTCCCGGGTTCAAGTGATTCTCGTGCCTCAGCCTTCTGAGTCGCTGGGACTACAGG TGCCC
GCCACCACGCCTGGCTAAATTTTTGTATTTTTAGTAGAGACAGGGTTTCTCCGTGTTAGCCAGGATGGTCTCAATCTCCTGACTTCGTGATCCACTCGCCTTGGCCTCCCAAAGTGTTGGGATTA CAG
GcGTGAGCCACCATGCCCGGCCTGGCCACATCTATTTCTGATGGATAAAAGTGGATTCCTGAATATGTGATATGCCTCTCTGGGGACCTGTGGAATGACATTTTGCATGTCAGACCAGCACAG TGCTT
TTATTCTAGACTTTTCTAAACCTTCCCCTCTAAACCAGGGAAAGCGAGTCGGTTCCCCGGTTCCTCATCATGGGTAATCAGAACAGATCTCTGATTTAGGAAGTTGGTTTTTTTTTTTTTTTTTT TTT
GAAGCAGAGTTTTGCTCTTGTTGCCCAGGCTGGAGTGCAGTGGCGCGATCTTGGCTCACTGCAACCTCTGCCCCCTGGGTTCAAGCGATTCTTCTGCCTCAGCCTCCCCAGTAGCTGGGATTA CAGG

2165	13.1	0.3	0.3	43499	314	625	(1550)	+	AluSx	SINE/Alu	1	312	(0)	1
2182	10.6	1.3	1.0	43499	628	939	(1236)	+	AluSq2	SINE/Alu	1	313	(0)	2
1162	16.1	0.0	0.0	43499	942	1115	(1060)	+	AluSg4	SINE/Alu	127	300	(10)	3
19	0.0	0.0	0.0	43499	1188	1208	(967)	+	(TTA)n	Simple_repeat	1	21	(0)	4
2481	5.2	0.0	0.0	43499	1209	1494	(681)	C	AluY	SINE/Alu	(25)	286	1	5
2388	8.7	0.3	0.7	43499	1505	1814	(361)	C	AluY	SINE/Alu	(2)	309	1	6
1246	6.8	0.0	0.0	43499	2028	2175	(0)	C	AluSq	SINE/Alu	(9)	304	157	7

2241	12.2	0.3	0.3	Hg19	314	625	(1870)	+	AluSx	SINE/Alu	1	312	(0)	8
2182	10.6	1.3	1.0	Hg19	628	939	(1556)	+	AluSq2	SINE/Alu	1	313	(0)	9
1162	16.1	0.0	0.0	Hg19	942	1115	(1380)	+	AluSg4	SINE/Alu	127	300	(10)	10
20	6.0	2.7	0.0	Hg19	1188	1208	(1287)	+	(TTA)n	Simple_repeat	1	21	(0)	11
2507	4.9	0.0	0.0	Hg19	1209	1494	(1001)	C	AluY	SINE/Alu	(25)	286	1	12
20	6.0	2.7	0.0	Hg19	1495	1504	(991)	+	(TTA)n	Simple_repeat	1	11	(0)	13
2560	5.5	0.3	0.7	Hg19	1505	1814	(681)	C	AluY	SINE/Alu	(2)	309	1	14
2373	9.0	0.3	0.7	Hg19	1825	2134	(361)	C	AluY	SINE/Alu	(2)	309	1	15
1246	6.8	0.0	0.0	Hg19	2348	2495	(0)	C	AluSq	SINE/Alu	(9)	304	157	16

>Hg19 chr16:501240-503734
GTTGTGTTTTCTGGAGCACCTGGAGTCCCCTCTGTCCCTGGCCATCCCTCCTTCCCCACACCTCACCCCTGTACCTCACCTTCCCCACCCCCCTGCCTCACCCCTGCACCTCACCCTCCCCA cGCTTC
CCGTATCAGGGAGCTTCTTAAACACAGAATGGGGTGTTAAGATGATTGGAGGGCAGCAGCCTATTGGAATCCCGATGAACGTGGAAAGGTGTCAGGGAGGGGTAGCTGTGGTGTGGAGGAGCCCC ACA
TTTTCGTCCCTGTCTTGTCACAAAGAAGGAGCCCATCGCTGTGATTAGAAGGACTTAGGCCGGGCATGGTGGCTCACACCTGTAATCCCAGCACTTTGGCAGGCTGAGGCAG gTgGATTaCCTGAGGT

CAGGAGTTCAAGACCAGCTGGCCAACATGGAGAAACCCCGTCTCTACTAAAAATACAAAAAATTAGCCAGGCATGGTGGTGTGTGCCTATAATCCTAGCTCCTTGGGAGGCTGAGGCAGGAGAAT TGT
TTGAACCAGGGAAGCGGAGGTTACAGTGACCCGAGATCACACCATTGCACTCCAGTCTGGGCGACAGAGCAAGACTCTCTCTCAAAAACAAACAAAGGAAAAAAACAAACAAAAAGGCCGGGGCTCGG
TGGCTCATGCCTGTAATCCCAGCACTTTGGGAGGCCGAGGCGGTGGATTGCCTGAGGTCAGGAGTTGGAGACCAGCCTGGCTAAGATAGTGAAACCCCATCTTTACTAAAAATACAAAAATTAGC TGG
GCGTGATGGCAGGCGCCTGTAATCCCAGCTACTCGGGAGGCTGATGAGGGAGAATCATTTGAACCTCGGGAGGGCAGATGTTGCAGTGAGTTGAGATCGCGCCACTGTACTCCAGCCTGGGCGACAAG
AGTGAAACTCCTTAAAAAAATAAAAAGGAAACAAACAAACAAACAAAAAATCAGCTGGGCATAGTGGTGGGGGCCTGTAGTCCCAGGTACTTGGGAGGCTGAGGCAGGAGAATTGCTTGAACCTG GGT
GGCTGATATTGCAGTGAGGCAAGATCATGCCACTGCACTCCAGCCTGGGTGATAGAGTGAGACTCCATCTTAAAAAAGGAAACAAAAAAAAAGCCACAAA GAAGGACTTAGAATTGGGTGCTAAGCAGG
CCTCTTCCTACTGATAGGTGGACACATCTATTTCTTTATTATTATTATTATTATTA TTTTTGAGACAGAGTCTTGCTCTGTTGCCCAGGCTGGAGTGCGGTGGCATGATCTCGGCTCACTGCAAGCTC
CGCCTCCCCTGTTCACGCCATTCTCCTGCctcagcatcccgagtagttgggactacaggcgcccgccaccacgcccggctaatttttttgtatttttagtagagatggggtttcaccatgttagccagg
atggtctcgatctcctgacctcgtgatctgcccgcctcggcctcccaaagtgctgggattacaggcgtgagccaccgcgcctggcc tatttcttta ttttttaatttttttttttatttttttatttttga
gacggagtctcgctctgttgcccaggctagagtgcagtgagcgatcttggctcactgcaagctccacctcccgggttcaagtgattctcgtgc CTCAGCATCCCGAGTAGTTGGGACTACAGGCGCCC
GCCACCACGCCCGGCTAATTTTTTGTATTTTTAGTAGAGATGGGGTTTCACCATGTTAGCCAG gATGGTCTCGATCTCCTGACCTCGTGATCTGCC CGCCTCGGCCTCCCAAAGTGCTGGGATTACAG
GCGTGAGCCACCGCGCCTGGCCTATTTCTTTATTTTTTAATTTTTTTTTTATTTTTTATTTTTGAGACGGAGTCTCGCTCTGTTGCCCAGGCTAGAGTGCAGTGAGCGATCTTGGCTCACTGCAAGCTC
CACCTCCCGGGTTCAAGTGATTCTCGTGCCTCAGCCTTCTGAGTCGCTGGGACTACAGGTGCCCGCCACCACGCCTGGCTAAATTTTTGTATTTTT AGTAGAGACAGGGTTTCTCCGTGTTAGCCAGG
ATGGTCTCAATCTCCTGACTTCGTGATCCACTCGCCTTGGCCTCCCAAAGTGTTGGGATTACAGG tGTGAGCCACCATGCCCGGCCTGGCCACATCTATTTCTGATGGATAAAAGTGGATTCCTGAAT
ATGTGATATGCCTCTCTGGGGACCTGTGGAATGACATTTTGCATGTCAGACCAGCACAGTGCTTTTATTCTAGACTTTTCTAAA CCTTCCCCTCTAAACCAGGGAAAGCGAGTCGGTTCCCCGGTTCC
TCATCATGGGTAATCAGAACAGATCTCTGATTTAGGAAGTTGGTTTTTTTTTTTTTTTTTTTTGAAGCAGAGTTTTGCTCTTGTTGCCCAGGCTGGAGTGCAGTGGCGCGATCTTGGCTCACTG CAA
CCTCTGCCCCCTGGGTTCAAGCGATTCTTCTGCCTCAGCCTCCCCAGTAGCTGGGATTACAG G

Homologous Sequences =

TGCCTCAGCATCCCGAGTAGTTGGGACTACAGGCGCCCGCCACCACGCCCGGCTAATTTTTTGTATTTTTAGTAGAGATGGGGTTTCACCATGTTAGCCAGGATGGTCTCGATCTCCTGACCTCG TGA
TCTGCCCGCCTCGGCCTCCCAAAGTGCTGGGATTACAGGCGTGAGCCACCGCGCCTGGCC

>NAHR_50_ Scaffold12159 1028338 1028449
KSD_NAHR_50

>Scaffold12159 1028338 1028449_NAHR
AAGTTTTCAAGGCATGAATTTCTTACCAGTCCGTTATTATGATAACGAAAAGGAATGGATCCCCAGGAACATCTTTTCTGACTGGTTTCACAAACGTCTGTGAGCAGCTTGTGCTCATTGCAGGGA AG
CAGGATGGAATGACCAGTGCAATATTTTGTTATTCCTTGGCAACTGTTCTGCTCATCTTTCAGCTGAAATTCTCGTCAAAAATATGTTTATGC TATGCAATATCCCCCAAATGTGACTTCAAAAATTC
AGCCATTTGACTAGGGTATCCTTAGATTAGTGAGGAATAAATATAAAAATACTTTCTTGAACAGCATGCTAGCAGCAGTGAACAGAGGCATGGGTGAGGAAGGTTTTCAAAAAGGATCACATAAA CAT
AAAACAGCCGAGATAGTTCTGAATCAAGTTGATTGTGATAATAGTGATGATGAAGATACCATTGTTAACACTGCAGAAAAAGTGACTATAGAC GACATGCCAGAAATATGTGATTGGCTTATTGAAGG
ACTAGAGCAGTGTGATGAAATCATATCCATTTATAAAATTGAAGAGAGAATTCCAAGACAAAATGTTAATGAGGCTGCCCATCATTAATGAGGCAGATGACTCAAAAAACATTTTAAAGAGCCAC CCA
GCAGAATTCCTCCTTATCTTAGAGGACCTGCTTCCTGGTCTCTCAATGGCCTCCGATGTTTCTTCTCACCTAAAAAAAAAAAATACAGTGTACA GGCCGGGCGTGGTGGCTCACGCCTGTAATCCCAGC
ACTTTGGGAGGCTGAGGCGGGCGGATCACAAGGTCAGGAGATCGAGACCATCCTGGCTAACACAGTGAAACCCCGTCTTTACTAAAAATACAAAAAATTAGCTGGGCGTGTCGGCGTGCGCCTGT AGT
TCCAGTTGCTGGGGAGGCTGCGGCAGGAGAATGGCGTGAACCCGGGAGGCGGAGCTTGCAGTGAGCCGAGATCGTGCCACTGCACTCCAGCCT AGGCAACAGAGCGATACTGCATCGCAAACAACAAC
AACAACAAAAAAAAAAAcAACAAAAAAAATACAGTATGGGGAGGCTGAGGCAGGAGAATGGCGTGAACTCGGGAGGCGGAGCTTGCTGTGAGCCGAGATCACTCCAG CCTGGGCGACAGAGCGAGACTCC
ATCTCAAAAAAAAAAAAAAATACAGTGTACATAACCTTTTCATAAAACACAGCATCATAGCTGGAGACTGAAAGCCTGCTGTTGTGTGTTTTT GCTGAAGTTTAACAGCTGATCCAAGTATTCTGGTG
ATGTTACTGTGCTGCTTAGTTACCATGAATGCATGATCTTTTCACTGTATCAATGGTATGTCATCCTTTTTCCTGTTAAATACTTATGTGTGAATAAGTATAAGGAAATAGTTGCTTAGCAGTAT TAT
ATATTTTCAGAGTCACAAATGATGGTGTTGCCAAACAATCACAGGCTGGCTACATGGATGACTGAGATAGTGAACACTTTTGCTTTCTGAGGT TCAGTGTACACAATTAGTTTCATGCACAGAATTAA
TTAAAAATTGTATTAAGTTACTTTCAGGTTATATGTATAAGATATATGTGAAACATAAGTGCATTTCATGTTGAGACTTGGGTCCCATCCCAAGATAGCTTATTATATATATGCAAATATTCCAA AAT
CCAAAAAATCTGAAATCTGAAGCATTTCTGGTCCAAAGCGTTTCAGATAAAGGATGTTTAACCTGTATAATGCTGATAAATTTTCACCAGTTG AACAATTAGCTAAAATAATATGCTAATCCTATCAG
GGCCAAGAAAGTGACTCAGTCAAAAAATTTCTTATTCCTCTGTAAACCAGAATGTGTGCCCAATAAAAAGGAACATAGAGACCAGTCATGGTACAGAAGTCAAGAGGGAAGCTTGAGAAAAGATC TCC
CCAAGGAGCAAAGGAGTGTGGTAGCATGCAGAGCCTGAGAGTCAGCATACCAGGGAGAAGCAGGGGAGATGGTCAGAGGCAAAGTGGTTCAGG GTTAATACCAAGACTGCTACCCACATTAAAGTGTT
CTGTGCTACTTTTGGTAAGGTGCATGTGAAGACTGGCTGAGCTGAAATGACCAGAGGTAGGG T

2570	11.4	1.1	0.0	52725	3	370	(1741)	+	Tigger4	DNA/TcMar-Tigger	1154	1525	(1206)	1
2049	12.5	5.4	3.9	52725	384	733	(1378)	+	Tigger4	DNA/TcMar-Tigger	1824	2178	(553)	2
2433	8.5	0.0	0.0	52725	734	1040	(1071)	+	AluYj4	SINE/Alu	1	307	(4)	3
931	2.7	8.9	0.0	52725	1061	1172	(939)	+	AluYc	SINE/Alu	163	284	(15)	4
3555	12.9	1.4	0.2	52725	1173	1729	(382)	+	Tigger4	DNA/TcMar-Tigger	2168	2731	(0)	5

2570	11.4	1.1	0.0	Hg19	3	370	(1879)	+	Tigger4	DNA/TcMar-Tigger	1154	1525	(1206)	6
2049	12.5	5.4	3.9	Hg19	384	733	(1516)	+	Tigger4	DNA/TcMar-Tigger	1824	2178	(553)	7
2460	8.4	0.0	0.0	Hg19	734	1043	(1206)	+	AluYj4	SINE/Alu	1	310	(1)	8
1033	3.2	8.0	0.0	Hg19	1061	1185	(1064)	+	AluYc	SINE/Alu	163	297	(2)	9
931	2.7	8.9	0.0	Hg19	1199	1310	(939)	+	AluYc	SINE/Alu	163	284	(15)	10
3555	12.9	1.4	0.2	Hg19	1311	1867	(382)	+	Tigger4	DNA/TcMar-Tigger	2168	2731	(0)	11

>Hg19 chr5:85987520-85989768
AAGTTTTCAAGGCATGAATTTCTTACCAGTCCGTTATTATGATAACGAAAAGGAATGGATCCCCAGGAACATCTTTTCTGACTGGTTTCACAAACGTCTGTGAGCAGCTTGTGCTCATTGCAGGGA AG
CAGGATGGAATGACCAGTGCAATATTTTGTTATTCCTTGGCAACTGTTCTGCTCATCTTTCAGCTGAAATTCTCGTCAAAAATATGTTTATGCTATGCAATATCCCCCAAATGTGACTTCAAAAA TTC
AGCCATTTGACTAGGGTATCCTTAGATTAGTGAGGAATAAATATAAAAATACTTTCTTGAACAGCATGCTAGCAGCAGTGAACAGAGGCATGGGTGAGGAAGGTTTTCAAAAAGGATCACATAAAC AT
AAAACAGCCGAGATAGTTCTGAATCAAGTTGATTGTGATAATAGTGATGATGAAGATACCATTGTTAACACTGCAGAAAAAGTGACTATAGACGACATGCCAGAAATATGTGATTGGCTTATTGA AGG
ACTAGAGCAGTGTGATGAAATCATATCCATTTATAAAATTGAAGAGAGAATTCCAAGACAAAATGTTAATGAGGCTGCCCATCATTAATGAGGCAGATGACTCAAAAAACATTTTAAAGAGCCACC CA
GCAGAATTCCTCCTTATCTTAGAGGACCTGCTTCCTGGTCTCTCAATGGCCTCCGATGTTTCTTCTCACCTAAAAAAAAAAAATACAGTGTACAGGCCGGGCGTGGTGGCTCACGCCTGTAATCCC AGC
ACTTTGGGAGGCTGAGGCGGGCGGATCACAAGGTCAGGAGATCGAGACCATCCTGGCTAACACAGTGAAACCCCGTCTTTACTAAAAATACAAAAAATTAGCTGGGCGTGTCGGCGTGCGCCTGTA GT
TCCAGTTGCTGGGGAGGCTGCGGCAGGAGAATGGCGTGAACCCGGGAGGCGGAGCTTGCAGTGAGCCGAGATCGTGCCACTGCACTCCAGCCTA GGCAACAGAGCGATACTGCATCGCAAACAACAAC
AACAACAAAAAAAAAAAaAACAAAAAAAATACAGTATGGGGAGGCTGAGGCAGGAGAATGGCGTGAACTCGGGAGGCGGAGCTTGCTGTGAGCCGAGATCACTCCAG Cctgggcgatagagcgagactcc

atctcaaaaaaaaaaaaaaaaaaaaaaaaaaaatacagtgtacaaggggaggctgaggcaggagaatggcgtgaactcgggaggcggagcttgctgtgagccgagatcactccagc CTGGGCGACAGA
GCGAGACTCCATCTCAAAAAAAAAAAAAAATACAGTGTACATAACCTTTTCATAAAACACAGCATCATAGCTGGAGACTGAAAGCCTGCTGTTGTGTGTTTTTGCTGAAGTTTAACAGCTGATCCAAG
TATTCTGGTGATGTTACTGTGCTGCTTAGTTACCATGAATGCATGATCTTTTCACTGTATCAATGGTATGTCATCCTTTTTCCTGTTAAATACTTATGTGTGAATAAGTATAAGGAA ATAGTTGCTTA
GCAGTATTATATATTTTCAGAGTCACAAATGATGGTGTTGCCAAACAATCACA GGCTGGCTACATGGATGACTGAGATAGTGAACACTTTTGCTTTCTGAGGTTCAGTGTACACAATTAGTTTCATGC
ACAGAATTAATTAAAAATTGTATTAAGTTACTTTCAGGTTATATGTATAAGATATATGTGAAACATAAGTGCATTTCATGTTGAGACTTGGGTCCCATCCCAAGATAGCTTATTATA TATATGCAAAT
ATTCCAAAATCCAAAAAATCTGAAATCTGAAGCATTTCTGGTCCAAAGCGTTTCAGATAAAGGATGTTTAACCTGTATAATGCTGATAAATTTTCACCAGTTGAACAATTAGCTAAAATAATATG CTA
ATCCTATCAGGGCCAAGAAAGTGACTCAGTCAAAAAATTTCTTATTCCTCTGTAAACCAGAATGTGTGCCCAATAAAAAGGAACATAGAGACCAGTCATGGTACAGAAGTCAAGAGG GAAGCTTGAGA
AAAGATCTCCCCAAGGAGCAAAGGAGTGTGGTAGCATGCAGAGCCTGAGAGTCAGCATACCAGGGAGAAGCAGGGGAGATGGTCAGAGGCAAAGTGGTTCAGGGTTAATACCAAGACTGCTACCC ACA
TTAAAGTGTTCTGTGCTACTTTTGGTAAGGTGCATGTGAAGACTGGCTGAGCTGAAATGACCAGAGGTAGGG T

Homologous Sequences =

GGGAGGCTGAGGCAGGAGAATGGCGTGAACTCGGGAGGCGGAGCTTGCTGTGAGCCGAGATCACTCCAGCCTGGGCGA

>NAHR_51_ Scaffold1763 2657355 2657665
KSD_NAHR_51

>Scaffold1763 2657355 2657665_NAHR
GTCACTACCTCCAGGGACCAGGTGTGCTGTCAAGGCAGAACAGTGTGGTGCAGAGGTTATCTGCATGGTCCTGGAGTTGGCCTTCTTGGGTTCAAATCCCAGCCCTGCTACTGCCTGGTTCTGTGA TC
TAGGGAGAGTTACTTGACATTTCTAAACATTTCTGATTTCAGCCTATAAAATGAGGCCAATAATAAGAGTATCTGCCCCGCCTAGACAGGATTAAATGAGGTAGTGCTTAGCATAGCTCAGTGAG CAC
TGCTGGTGTCTCTGTGTTGGAGCCTTCCCACCCCTGCTCTCCTGCAGGCAGTGTTAGTGGCCCCCATCCTGTTCCCTCATGGCCCCTGAGCTCATGATTCTCCCCCTGCTAGATGGTGTGCATTTT GA
AAGTGGGGACAACTTTCCTCCATTTATGCAAGGCCTCACTCAGAGAAGCCCCTTAACAAAGGGCTACTGGAATAAACGAAGGAATGGATGAAGAAACAAAGGCCAGGCAGAGTAAAACCAAGAAG ACG
TTGCATAATTGAAGGCAGCAATTGGGCATTTTCACCTCAACAATGCTAGCACCTATTACATCAGCTGGACTGGACAGTGCTTTCACACTTGCAAAATGCATCCAAAAGGACAATGAAGTCCCAGTT AC
TTGGGAGGCTGAGGTGGGAGGATCGCTTGAGTCCAGGAGTTGGAGGCCACAGTGAACTGTGATCACACTGCTACAGTCCAGCCTGGGCAACAGAGTGAGACCCCGTCTTTAATCAACAACAACAA CAA
AAAGGATGATCAGGCCAGACGCGGTGGCTCATTTGAGGTCAGGAGTTTGAGACCGGCCTGGCCAACATGGTGAAACCCCATCTCTACCAAAAATACAAAAAAATTAGCCAGGC cTGATGGTGGGCAAC
TGTACTCCCAGCTACTCAGGAGGCTGAGGCAGGAGAATTGCTTGAACCTGGGAGGCACTGGTTGCAGTGAGCTGAGATCGCACCACTGCACTCACTCTAGCCTGGGCAACAGAGCAAGACTCTGT CTC
AAAAAAAAAACAAAAAAAAAACAAAAAAAAAAAACCGCCGGGTGCGGTGGCTCATGCCTGTAATCCCGGCACTTTGGGAGGCCGAGG gAGGTGGATCACCTGAGGTCAGGAGTTCGTGACCAGCCTGG
CCAACATGGTGAAACCCCATCTCTACTAAAAATACAAAAATTAGCTGGGCGTGGTGGCAGGCGCCTGTAATCCCAGCTACTCGGGAGGCTGAGGCA GGAGAATCGCTTGAACCCAGAAGGTGGAGGTg
GCAGTGAGCCAAGATGGCACCATTGTACTCCAGCCTGGGCGACAGAGTGAGACTCTGTCTCAAAACAACACAACAACAACAACTAAAAAAA CAAAAAAGGATGATCAAGGATATAGGATGATAGTGCT
CTCCTTCCAATGACTTCACATTTGAACAAGTGATCAGACATGCAAACTGGCTACTATGATTCACTATCATAAAATGCCATGCTGTGGGAACACAAGAAAGGGTGCACTTCATTCTTTTTTTTTTT TTT
TGAGACTGAGTCTCACTCTGTTGCCCAGGCTGGAGTGCAGTGGCTTGATCTCAGCTCACTGCAACCTCCACCTCCCAGGTTCAAGTGATTCTCCTGCCTCAGCCTCCTGAGTAGCTGGGATTACAG GT
GCATGCCACCACACTGGCTAATTTTTGTATTTTTATTAGAGACAGAGTTTTGCCATGTTGGCCAGGCTGGTCTTGAACTTCTGATCTCAGGTGATTCGCCTGCCTCGGCCTCCCAAAGTGCTGGG ATT
ACAGGCGTGAGCCACTGTGCCCGGCCAGGTGCACTTAATTCTTCCTGGCTGTGAGGGTGAAGGAAAGCTACAAAAGTTAAGTGCCCTGGAGCCAATCTTTGAAGTAAAGAGACTGGCGGGTAGAAA GG
CATAGGATAGAGTCATAGAGTCACTCAGCAGAAGCAAAGGCACAGAGGGAACTGCCTGATGGATCCTAAGAGTGCGAAGGAATTCCTCACTGGCTAGAGTGTGGGTGTCATGTAGTTGGATAGAG TAA
TGGCCCCCCAAAGATATCCATGTCCTCACCCTGGAGCCTGTGAATAATCCTCATATAGCAGAAGAGACTCTGCAGGTGTGATGAAACGAATGATCTTAGGATGGGGAGGTTACCCTGCATTATCCA CA
TGGGCCCTAAATGCAATCACATGTCCTCCTCCTAAGAGGGAGGCGGGAGAACAGGTTCAGTGGAAGGTGGTGTGACTAAGCAAGTAGAGGGAGAAAAGGTGACGTGATGTGGATCGCGGGCCAAG GAT
TGCAGG

497	27.6	5.6	4.0	179820	40	235	(2075)	+	MIR	SINE/MIR	1	199	(63)	1
895	17.8	0.0	0.0	179820	626	771	(1539)	+	FRAM	SINE/Alu	23	168	(8)	2
1880	10.9	0.4	2.6	179820	780	1054	(1256)	+	AluSx1	SINE/Alu	44	312	(0)	3
2421	9.3	0.0	0.0	179820	1061	1371	(939)	+	AluSx1	SINE/Alu	2	312	(0)	4
2381	9.4	0.3	0.0	179820	1520	1818	(492)	C	AluSx	SINE/Alu	(12)	300	1	5
999	22.8	2.0	2.0	179820	2031	2281	(29)	+	MLT1D	LTR/ERVL-MaLR	1	251	(254)	6

497	27.6	5.6	4.0	Hg19	40	235	(2214)	+	MIR	SINE/MIR	1	199	(63)	7
910	13.8	0.0	8.4	Hg19	626	779	(1670)	+	FRAM	SINE/Alu	23	163	(13)	8
1891	10.9	0.4	2.6	Hg19	780	1054	(1395)	+	AluSx1	SINE/Alu	44	312	(0)	9
910	13.8	0.0	8.4	Hg19	1055	1067	(1382)	+	FRAM	SINE/Alu	164	176	(0)	8
2419	6.8	2.0	0.0	Hg19	1072	1366	(1083)	+	AluSq10	SINE/Alu	2	302	(11)	10
988	13.2	0.0	0.0	Hg19	1367	1510	(939)	+	AluSx3	SINE/Alu	168	311	(0)	11
2381	9.4	0.3	0.0	Hg19	1659	1957	(492)	C	AluSx	SINE/Alu	(12)	300	1	12
999	22.8	2.0	2.0	Hg19	2170	2420	(29)	+	MLT1D	LTR/ERVL-MaLR	1	251	(254)	13

>Hg19 chr12:112062487-112064935
GTCACTACCTCCAGGGACCAGGTGTGCTGTCAAGGCAGAACAGTGTGGTGCAGAGGTTATCTGCATGGTCCTGGAGTTGGCCTTCTTGGGTTCAAATCCCAGCCCTGCTACTGCCTGGTTCTGTGA TC
TAGGGAGAGTTACTTGACATTTCTAAACATTTCTGATTTCAGCCTATAAAATGAGGCCAATAATAAGAGTATCTGCCCCGCCTAGACAGGATTAAATGAGGTAGTGCTTAGCATAGCTCAGTGAGCAC
TGCTGGTGTCTCTGTGTTGGAGCCTTCCCACCCCTGCTCTCCTGCAGGCAGTGTTAGTGGCCCCCATCCTGTTCCCTCATGGCCCCTGAGCTCATGATTCTCCCCCTGCTAGATGGTGTGCATTT TGA
AAGTGGGGACAACTTTCCTCCATTTATGCAAGGCCTCACTCAGAGAAGCCCCTTAACAAAGGGCTACTGGAATAAACGAAGGAATGGATGAAGAAACAAAGGCCAGGCAGAGTAAAACCAAGAAGACG

TTGCATAATTGAAGGCAGCAATTGGGCATTTTCACCTCAACAATGCTAGCACCTATTACATCAGCTGGACTGGACAGTGCTTTCACACTTGCAAAATGCATCCAAAAGGACAATGAAGTCCCAGT TAC
TTGGGAGGCTGAGGTGGGAGGATCGCTTGAGTCCAGGAGTTGGAGGCCACAGTGAACTGTGATCACACTGCTACAGTCCAGCCTGGG CAACAGAGTGAGACCCCGTCTTTAATCAACAACAACAACAA
AAAGGATGATCAGGCCAGACGCGGTGGCTCATTTGAGGTCAGGAGTTTGAGACCGGCCTGGCCAACATGGTGAAACCCCATCTCTACCAAAAATACAAAAAAATTAGCCAGGCaTGATGGTGGGC AAC
TGTACTCCCAGCTACTCAGGAGGCTGAGGCAGGAGAATTGCTTGAACCTGGGAGGCACTGGTTGCAGTGAGCTGAGATCGCACCACTGCACTCACTCTAGCCTGGGCAACAGAGCAAGACTCTGTCTC
AAAAAAAAAACAAAAAAAAAACAAAAAAAAAAcaaaaaaaaaaAACCGCCGGGTGCGGTGGCTCATGCCTGTAATCCCGGCACTTTGGGAGGCCGAGG tAGGTGGATCACCTGAGGTCAGGAGTTCGT
GACCAGCCTGGCCAACATGGTGAAACCCCATCTCTACTAAAAATACAAAAATTAGCTGGGCGTGGTGGCAGGCGCCTGTAATCCCAGCTACTCGGGAGGCTGAGGCA Ggagaatccctcaaacccggg
aggcagaggtgagccgagatcgcaccatcgcactccagcctgggagacataagcgagactttgtctcaaaaaaaaaaaaaaaaaaa gcaactcgggaggctgaggcagGAGAATCGCTTGAACCCAGA
AGGTGGAGGTtGCAGTGAGCCAAGATGGCACCATTGTACTCCAGCCTGGGCGACAGAGTGAGACTCTGTCTCAAAACAACACAACAACAACAACTAAAAAAA CAAAAAAGGATGATCAAGGATATAGG
ATGATAGTGCTCTCCTTCCAATGACTTCACATTTGAACAAGTGATCAGACATGCAAACTGGCTACTATGATTCACTATCATAAAATGCCATGCTGTGGG AACACAAGAAAGGGTGCACTTCATTCTTT
TTTTTTTTTTTTGAGACTGAGTCTCACTCTGTTGCCCAGGCTGGAGTGCAGTGGCTTGATCTCAGCTCACTGCAACCTCCACCTCCCAGGTTCAAGTGATTCTCCTGCCTCAGCCTCCTGAGTAG CTG
GGATTACAGGTGCATGCCACCACACTGGCTAATTTTTGTATTTTTATTAGAGACAGAGTTTTGCCATGTTGGCCAGGCTGGTCTTGAACTTCTGATCTC AGGTGATTCGCCTGCCTCGGCCTCCCAAA
GTGCTGGGATTACAGGCGTGAGCCACTGTGCCCGGCCAGGTGCACTTAATTCTTCCTGGCTGTGAGGGTGAAGGAAAGCTACAAAAGTTAAGTGCCCTGGAGCCAATCTTTGAAGTAAAGAGACT GGC
GGGTAGAAAGGCATAGGATAGAGTCATAGAGTCACTCAGCAGAAGCAAAGGCACAGAGGGAACTGCCTGATGGATCCTAAGAGTGCGAAGGAATTCCTC ACTGGCTAGAGTGTGGGTGTCATGTAGTT
GGATAGAGTAATGGCCCCCCAAAGATATCCATGTCCTCACCCTGGAGCCTGTGAATAATCCTCATATAGCAGAAGAGACTCTGCAGGTGTGATGAAACGAATGATCTTAGGATGGGGAGGTTACC CTG
CATTATCCACATGGGCCCTAAATGCAATCACATGTCCTCCTCCTAAGAGGGAGGCGGGAGAACAGGTTCAGTGGAAGGTGGTGTGACTAAGCAAGTAGA GGGAGAAAAGGTGACGTGATGTGGATCGC
GGGCCAAGGATTGCAGG

Homologous Sequences =

ACTCGGGAGGCTGAGGCAGGAGAATC

NHEJ-ARMDs

NHEJ-ARMDs
Alu-Alu recombination mediated deletion

>NHEJ_1

KSD_NEHJ_1

>Scaffold15803 87485 89866 NHEJ
TTTATAGGTTCTCTCTCCCAGTATTCACAGACTTTTAATGAGAGCTGTAGCTCACTTGAGTTGAGGGAATAAAAGACAAGGGAG tGCcAGGATTCCTCCCTTTGCTCTTCACATTGTGACTGGTGAAC
AAGGCCGACTTCATTCCACAGCTGGATTGATATGTAAATTTACTCCGACTAATTGGCTTCCCCAGCTGTCTCCAGAGGGCGGATGTATTCTTTATAAAGTGCTGTCTAGGGCTGCATTGAGAACA TTA
GAACTACTTAATTGAATATCAATGAATCTTTATAAACTAATAATGATGGGGAGCCTGGGAAGCCTGATGTAGATTTGGCATATTGAGAAGAGGAGTGCCCTGATTTGAATTTGTGGGAAGTGAAAT TT
TCTCTGAGTTTTACAAAACCCAATTTAGGCTTCTTTAATCTAAAATCCTTGAAAGCCCCACTGGTGCCTTCTTGAACATGTCATTTGGTGAATGTTGGACGTGAGCAAGTGTGTCAGTCTGTGTC AAG
AGATTACCTCCCTCCTTTTCCTAATACAGTAAAACTCAATGATTGTTGTGGTTAATTCTTGCTCATGCAGGAAGAGTCACTACTCCTTTCCACAAGTCACTCCTCCTTTCCCTTAGGGACTGGGGG AG
TGCATCCAAACTGAGTATACCTTGTTGCTCCTTGCACAGGGCTCCTTGCAGAGCTGATATTCAGAAACTGAGTGAGCATAGATCTTTGCCTTTAAGTCCTACCAGGAAGTTCACTGAATCAAACC AAA
CCCAACCAAAGCAAAGCACCAAACCAAACAAGAATAACAAACAACAAACATAACTCTGTGTAGTTCAAAGTAGTGGGGTTGATATTGCTGGATCACTGGGCCATTCTGAGGTCCACTGAGATGTGC CT
AGGATGAGGATTTCCTTTTTCTTGTCTTCTCTCCCTCCCTCTACTACCTTGTGCTATCAGACATTCAAAGGCCTAGGGGGGCAGGTCACCTGAGGTTGGGAGTTCGAGACCAGCCTGACCAACAT GGA
GAAACCCTGTTTCTACTAAAAAAATACAAAATTTAAGGCcGGGCGTGGTGGCTCAAGCCTGTAATCCCAGCACTTTGGGAGGCCAAGGtGAGTGAATCACGAGGTCAGGAGGTCAAGACCATCCTGGC
TAACACGGTGAAACCCCGTCTCTACTAAAAATACAAAAAAATTAGTCAAGCGTGGTGGCGGGCGCCTGTAGTCCCAGATACTCGGGAGGCTGAGGCAGGAGAATGGCGTGAATCTGGGAGACGGA GCG
GCGCATGCCTaTAATCCCAGCTACTCAGGAGGCCGAGGCAGGAGAATTGCTTGAACCTGGGAGGCGGAGGTTGCGGTGAGCCGAGATGGTGCCATTGCACTCCAGCCTGGGCAACAAGAGTGAAACTC
CATCTCAAGTAAATAAATAAATAAAAATAAAAAATAAAACTGTACGGTATCACTGTCAAAAATAGATGTAGATTAATGGAACAAAATAAAAAATCTAGAAACAGGCCCCAGTATATAAAAATTTATCA
TGCATGAAAGGTGTTGTTTCTGACCTGGAAAAATAATTATTTATCCCATAAAAGGTACTGAGATAATTGGATAGCTATTTGGTGTAAAAAGTTATTAATAAAAGTAGACCTCACAGGATGCCCCTA AA
CAAATTctgattGtTTAGAATTTGATTACAAAAAGAACTGCAAGTCAAGAAAGTCATCATAACTAAAATTAAAAGGCAAATGACAGACTCCGAACAATATTTACAATACATATATAGCAAGGATTCTG
ACAAGTCGATAAGAAAAAGATGAAATCACTCAATAGAAATGTGGACAAAGGACATGAGCAGATAATTCATGATGAAGAAATGCAAATGGCCAATGAGCATGAAATCCTTATTAATGCTAAAAACCA CT
AACTTAACAGTACCCTAATTACTGTTTATTAAATGGACAGTTTTTCTTTAAGCACTAATGCCCAATTGTGGCAGAGGTGTGCAGAGGAGGACATTCACCTACCCGCTAGAAGGCAATTGGTAATT TGC
ATCAAGAGCCTTAAAATGGTTCTTAGACTTTGGCCCAGAAATTCTACTTATTGGCACACACCTTGAACTGCTTCAGAGATATGTTCAAAGATTTATGTATATAGGTGTTACTCATAATTAGCTCAA AT
TGGAAATAACCTAAATGCCCACCAAGAACTATTTAGTTAAATATATTAGTTAATCATATTATTAGTTACATACTTGTGCTATCATTATATGAATGCTACACATTTTAAAATAATATTTAAAAGAC TGT
TGGTGATATTGCTGCTTGTGTTACACTATATAAAGTGAAAACAAACAAGATATGAAGTAGAAATCAAGGTTACTCT C

20	10.5	4.9	0.0	S	757	797	(1584)	+	(AACCA)n	Simple_repeat	1	43	(0)	1
17	19.8	3.0	6.2	S	798	822	(1559)	+	A-rich	Low_complexity	1	64	(0)	2
1823	10.0	4.2	0.8	S	965	1060	(1321)	+	AluSp	SINE/Alu	44	137	(176)	3
1830	6.8	0.0	0.5	S	1061	1279	(1102)	+	AluYm1	SINE/Alu	1	218	(93)	4
1823	10.0	4.2	0.8	S	1280	1442	(939)	+	AluSp	SINE/Alu	149	311	(2)	3
339	32.0	3.9	5.9	S	1443	1648	(733)	+	L1ME4a	LINE/L1	4761	4962	(1184)	5
774	26.1	9.2	4.8	S	1683	2360	(21)	+	L1ME4a	LINE/L1	5138	5899	(225)	5

20	10.5	4.9	0.0	hg19	757	797	(1677)	+	(AACCA)n	Simple_repeat	1	43	(0)	6
17	19.8	3.0	6.2	hg19	798	822	(1652)	+	A-rich	Low_complexity	1	64	(0)	7
2000	9.1	0.0	2.2	hg19	965	1060	(1414)	+	AluSp	SINE/Alu	44	133	(180)	8
2466	6.9	1.3	0.3	hg19	1061	1363	(1111)	+	AluYm1	SINE/Alu	1	306	(5)	9
2000	9.1	0.0	2.2	hg19	1364	1541	(933)	+	AluSp	SINE/Alu	134	311	(2)	8
339	32.0	3.9	5.9	hg19	1542	1747	(727)	+	L1ME4a	LINE/L1	4761	4962	(1184)	10
774	26.3	11.3	4.1	hg19	1762	2453	(21)	+	L1ME4a	LINE/L1	5126	5899	(225)	10

>hg19 ChrX:150408277-150410750

TTTATAGGTTCTCTCTCCCAGTATTCACAGACTTTTAATGAGAGCTGTAGCTCACTTGAGTTGAGGGAATAAAAGACAAGGGAGcGCtAGGATTCCTCCCTTTGCTCTTCACATTGTGACTGGTGAAC
AAGGCCGACTTCATTCCACAGCTGGATTGATATGTAAATTTACTCCGACTAATTGGCTTCCCCAGCTGTCTCCAGAGGGCGGATGTATTCTTTATAAAGTGCTGTCTAGGGCTGCATTGAGAACA TTA
GAACTACTTAATTGAATATCAATGAATCTTTATAAACTAATAATGATGGGGAGCCTGGGAAGCCTGATGTAGATTTGGCATATTGAGAAGAGGAGTGCCCTGATTTGAATTTGTGGGAAGTGAAATTT
TCTCTGAGTTTTACAAAACCCAATTTAGGCTTCTTTAATCTAAAATCCTTGAAAGCCCCACTGGTGCCTTCTTGAACATGTCATTTGGTGAATGTTGGACGTGAGCAAGTGTGTCAGTCTGTGTC AAG
AGATTACCTCCCTCCTTTTCCTAATACAGTAAAACTCAATGATTGTTGTGGTTAATTCTTGCTCATGCAGGAAGAGTCACTACTCCTTTCCACAAGTCACTCCTCCTTTCCCTTAGGGACTGGGGGAG
TGCATCCAAACTGAGTATACCTTGTTGCTCCTTGCACAGGGCTCCTTGCAGAGCTGATATTCAGAAACTGAGTGAGCATAGATCTTTGCCTTTAAGTCCTACCAGGAAGTTCACTGAATCAAACC AAA
CCCAACCAAAGCAAAGCACCAAACCAAACAAGAATAACAAACAACAAACATAACTCTGTGTAGTTCAAAGTAGTGGGGTTGATATTGCTGGATCACTGGGCCATTCTGAGGTCCACTGAGATGTGCCT
AGGATGAGGA[TTTCCTTTTTCTTGTCTTCTCTCCCTCCCTCTACTACCTTGTGCTATCAGACATTCAAAGGCCTAGGGGGGCAGGTCACCTGAGGTTGGGAGTTCGAGACCAGCCTGACCAACA TGG
AGAAACCCTGTTTCTACTAAAAAAATACAAAATTTAAGGCtGGGCGTGGTGGCTCAAGCCTGTAATCCCAGCACTTTGGGAGGCCAAGGcGAGTGAATCACGAGGTCAGGAGGTCAAGACCATCCTGG
CTAACACGGTGAAACCCCGTCTCTACTAAAAATACAAAAAAATTAGTCAAGCGTGGTGGCGGGCGCCTGTAGTCCCAGATACTCGGGAGGCTGAGGCAGGAGAATGGCGTGAATCTGGGAGACGG AGC
ttgcagtgagctgagattgcgccactgcactccagcctgggaacagagactccatctccaaaaaaaaaaaaaaaaaaaaaaaattagccaggtgtggtGGCGCATGCCTgTAATCCCAGCTACTCAG
GAGGCCGAGGCAGGAGAATTGCTTGAACCTGGGAGGCGGAGGTTGCGGTGAGCCGAGATGGTGCCATTGCACTCCAGCCTGGGCAACAAGAGTGAAACTCCATCTCAAGTAAATAAATAAATAAA AAT
AAAAAATAAAA]CTGTACGGTATCACTGTCAAAAATAGATGTAGATTAATGGAACAAAATAAAAAATCTAGAAACAGGCCCCAGTATATAAAAATTTATCATGCATGAAAGGTGTTGTTTCTGACCTG
GAAAAATAATTATTTATCCCATAAAAGGTACTGAGATAATTGGATAGCTATTTGGTGTAAAAAGTTATTAATAAAAGTAGACCTCACAGGATGCCCCTAAACAAAT TGaTTAGAATTTGATTACAAAA
AGAACTGCAAGTCAAGAAAGTCATCATAACTAAAATTAAAAGGCAAATGACAGACTCCGAACAATATTTACAATACATATATA GCAAGGATTCTGACAAGTCGATAAGAAAAAGATGAAATCACTCAA
TAGAAATGTGGACAAAGGACATGAGCAGATAATTCATGATGAAGAAATGCAAATGGCCAATGAGCATGAAATCCTTATTAATGCTAAAAACCACTAACTTAACAGTACCCTAATTACTGTTTATTAAA
TGGACAGTTTTTCTTTAAGCACTAATGCCCAATTGTGGCAGAGGTGTGCAGAGGAGGACATTCACCTACCCGCTAGAAGGCAATTGGTAATTTGCATCAAGAGCCTTAAAATGGTTCTTAGACTTTGG
CCCAGAAATTCTACTTATTGGCACACACCTTGAACTGCTTCAGAGATATGTTCAAAGATTTATGTATATAGGTGTTACTCATAATTAGCTCAAATTGGAAATAACCTAAATGCCCACCAAGAACT ATT
TAGTTAAATATATTAGTTAATCATATTATTAGTTACATACTTGTGCTATCATTATATGAATGCTACACATTTTAAAATAATATTTAAAAGACTGTTGGTGATATTGCTGCTTGTGTTACACTATATAA
AGTGAAAACAAACAAGATATGAAGTAGAAATCAAGGTTACTCTC

Microhomology = undefined

>NHEJ_2

KSD_NEHJ_2

>Scaffold16357 164440 166710 NHEJ
TGGTTACCTTGTTTACAAATGGGGAAACTGAGGTCAGAAAGGGGATGTAACTTGAGCAAATCCTCACACAAGACTTAGTTTCCTTGAGACTGACTGAAGTGGGGATTAAGGACCTTCCTCTTAACC TG
GTTTTGAGACTAAGAGAACTGGTGTGTGCACTTCCCCACCCAGCCCACAAGGTTTTTGCAAATTGTAGCAAGATGTGAGATACAGAGAGACAGATGTTATCTGGGTCAAACTTGAGATCCAAGTT GCA
TGGGCTGGTAGTTAAGGAAATAGGCTTTCGATCCAGACCAATCTTGACTCCCACTTATTTCCAGTTGTTTGACTCTGGGTGAGTTGTTTAACCTTGGTCATCTTCCGTTTCCTCATTATAGCAATG GG
GAAAATGCAGAAGATTACAGCAGGAGTTAAATGAGACAATGGACAAAAAGCCCTTAGCAGTGTGCCTGGCATTATGACGTGGTCAATGAAATGGTAGCTATGTCATCATTATTTAACTTTGGGTT GAA
AATGTTTGTCATCTAGGGCCTGCTGTGTCCTTATAACGCACAGGTCAATCAGTTCTCAAGCACCCGACCTTGTCCCTAAGGGCTAGACTTAGAGGGTTGGTGGAGGTTAGGATGAAGAGCAATGTT GG
GGAGGACCTTGGAGTCCCTTGCTGTTTTCCTTGCTGTTGAGAGACTGACTTGGAGAATGAGGTCTGCTCCTTTAATGCCCACAGCGACTCCTGCTCCAGTCCCTTTTCAGGCCAGGCCTGAAAGC CAG
CTGAGCTGGCCACTCCTGTCAGTGGCAGCTCTCCATCTCTCACTCCCCTCCTCTTCACCACCCTTCTCCACCTCTGACTCAGCAGGAGGGGGCCTGGAGCCCAGTCCCACATCTGGGCCTCTCCAC CC
TTTTCTGTTGCTGTTAGTAAGCAAGGGCCACACCCATGGGGTGACTACTCCAATTTCAGCAGGGACCGACTCCGATTATTGAGTCTTTTAAAAAAATTTTTGA GGCCGGGCGTGGTGGCTCACGCCTG
TAATCCCAGCACTTTGGGAAGCCGAGGCGGGCGGATCACGAGGTCACGAGACCATCCTGGATAACACGGTGAAACCCCGTCTCTACTAAAAATACAAAAAAATAGCCGGGCATGGTGGCAGGCGCC TG
TAGTCCCAGTTACTTGGGAGGCTGAGGCAGGAGAATGGCGTGAACCCAGGAGGCAGAGTTTGCAGTGAGCCGAGATCGAGCCACTGCAGTCCGGCCTGGGCAACAGAGCGAGACT CTGCAGTCATAAC
TCACTACAACCTCCAACTGGGCTCAAGCTATCCTCCCACCTCAGCCTCCCAAGTAGCTAGGACTACAGCTGTGCACCACCACGTGTGGCC AATGATGAAGTCTGAAGTGGCAAAGGTCTCAGAACTCT
GGGTTCATACACTGGAAGGGGACACCAAGGAATAGTTCAGAGGCTGAGAGGAATGGGGGTGACTGTGGGTACCTGGATGGGAAGGGATGACAAGATAGAGCAGGATGGCCAAGTATGCCCCGGCC TGT
GTCTGGAGAAGCAAGGCACCTCCTGTGTACAATGGGGACCCTAATTCCCCACTCAACTTGTGGGGGTGAAGAGCACAGGCTTTGGAATCAGATCAACCTGAGTTTAAATCCCAGCTGGGCTGTTTT CC
AGCTGTGTGACTTACGGCAAGTCTTTGAGCCCTTTTCCCCCTCTTTGAGCCTGCTTCCTCAGCTGTCAGAGATAAGGACCTTCCTTGCAGTGCTGTTGTGATGATTAAATGAAATCACAGATGTA AAG
AGTGCTTGGTACAACAGGAGGCACAGGGTAAATGCTTGGGTAATGGAACTACTGTCATTATCACCCATCTGGCTGCATGTTACAAGGTCAATGAGATAATGGATACAAAATGTAAACTAGATGAAC AT
AAAGGCTTATTATATAGACCAGTGTGTCCTGAAGAAAACTGGTGTCCTAAAGTGTGTCCTGCAGAAAACTAGTTCCCTGGAATGTTAATAGCTGTTTCTAAATAAAGGTTTTGCAGGCAATATGT TTT
GAGAATGCAGAGTTAAATGAGAATACAATAAGTTTCTTTATTGTTAAAGTATTTAATCCCTAATAGTGATATACACTGTGAATCTTCAAGAGGACAGAGTAAGCAAACCTAACTGACCAGGAGTGG CT
AGAGTATTGTCAGTTCCACAGAACAAATGTGAGAGAAAGATCTGGATTTGGTGTTAGGGTGCCTTTAACATCTAACACTGGTTAACTCTCCTT T

231	21.2	2.9	2.9	S	12	79	(2191)	C	MIR3	SINE/MIR	(68)	140	73	1
515	28.3	8.0	2.4	S	262	490	(1780)	+	MIRb	SINE/MIR	12	267	(1)	2
2265	5.9	2.2	0.0	S	1000	1270	(1000)	+	AluY	SINE/Alu	1	277	(34)	3
637	16.0	3.0	0.0	S	1274	1373	(897)	C	AluJr	SINE/Alu	(77)	235	133	4
727	26.2	4.0	5.8	S	1605	1851	(419)	+	MIRb	SINE/MIR	26	268	(0)	5
582	25.2	7.8	3.0	S	1962	2153	(117)	+	MER103C	DNA/hAT-Charlie	5	205	(92)	6
268	16.6	6.5	3.1	S	2209	2270	(0)	+	MER113	DNA/hAT-Charlie	152	215	(306)	7

231	21.2	2.9	2.9	hg19	12	79	(2270)	C	MIR3	SINE/MIR	(68)	140	73	8
515	28.3	8.0	2.4	hg19	262	490	(1859)	+	MIRb	SINE/MIR	12	267	(1)	9
2561	5.0	2.0	0.0	hg19	1000	1301	(1048)	+	AluY	SINE/Alu	1	308	(3)	10
1001	16.2	1.9	0.0	hg19	1302	1448	(901)	C	FRAM	SINE/Alu	(26)	150	1	11
727	26.2	4.0	5.8	hg19	1684	1930	(419)	+	MIRb	SINE/MIR	26	268	(0)	12
582	25.2	7.8	3.0	hg19	2041	2232	(117)	+	MER103C	DNA/hAT-Charlie	5	205	(92)	13
268	16.6	6.5	3.1	hg19	2288	2349	(0)	+	MER113	DNA/hAT-Charlie	152	215	(306)	14

>hg19 Chr20:44463856-44466204
TGGTTACCTTGTTTACAAATGGGGAAACTGAGGTCAGAAAGGGGATGTAACTTGAGCAAATCCTCACACAAGACTTAGTTTCCTTGAGACTGACTGAAGTGGGGATTAAGGACCTTCCTCTTAACCTG
GTTTTGAGACTAAGAGAACTGGTGTGTGCACTTCCCCACCCAGCCCACAAGGTTTTTGCAAATTGTAGCAAGATGTGAGATACAGAGAGACAGATGTTATCTGGGTCAAACTTGAGATCCAAGTT GCA
TGGGCTGGTAGTTAAGGAAATAGGCTTTCGATCCAGACCAATCTTGACTCCCACTTATTTCCAGTTGTTTGACTCTGGGTGAGTTGTTTAACCTTGGTCATCTTCCGTTTCCTCATTATAGCAATGGG
GAAAATGCAGAAGATTACAGCAGGAGTTAAATGAGACAATGGACAAAAAGCCCTTAGCAGTGTGCCTGGCATTATGACGTGGTCAATGAAATGGTAGCTATGTCATCATTATTTAACTTTGGGTT GAA
AATGTTTGTCATCTAGGGCCTGCTGTGTCCTTATAACGCACAGGTCAATCAGTTCTCAAGCACCCGACCTTGTCCCTAAGGGCTAGACTTAGAGGGTTGGTGGAGGTTAGGATGAAGAGCAATGTTGG

GGAGGACCTTGGAGTCCCTTGCTGTTTTCCTTGCTGTTGAGAGACTGACTTGGAGAATGAGGTCTGCTCCTTTAATGCCCACAGCGACTCCTGCTCCAGTCCCTTTTCAGGCCAGGCCTGAAAGC CAG
CTGAGCTGGCCACTCCTGTCAGTGGCAGCTC TCCATCTCTCACTCCCCTCCTCTTCACCACCCTTCTCCACCTCTGACTCAGCAGGAGGGGGCCTGGAGCCCAGTCCCACATCTGGGCCTCTCCACCC
TTTTCTGTTGCTGTTAGTAAGCAAGGGCCACACCCATGGGGTGACTACTCCAATTTCAGCAGGGACCGACTCCGATTATTGAGTCTTTTAAAAAAATTTTTGAGGCCGGGCGTGGTGGCTCACGCCTG
TAATCCCAGCACTTTGGGAAGCCGAGGCGGGCGGATCACGAGGTCACGAGACCATCCTGGATAACACGGTGAAACCCCGTCTCTACTAAAAATACAAAAAAATAGCCGGGCATGGTGGCAGGCGCCTG
TAGTCCCAGTTACTTGGGAGGCTGAGGCAGGAGAATGGCGTGAACCCAGGAGGCAGAGTTTGCAGTGAGCCGAGATCGAGCCACTGCAGTCCGGCCTGGGCAACAGAGCGAGACT Ccgtctcaaaaaa
aaaaaaaaaaaaaaaaaaaaattttgagacagggtctcgttctgtcactcaggctggagtgcagtgg TGCAGTCATAACTCACTACAACCTCCAACTGGGCTCAAGCTATCCTCCCACCTCAGCCTCC
CAAGTAGCTAGGACTACAGCTGTGCACCACCACGTGTGGCCAATGATGAAGTCTGAAGTGGCAAAGGTCTCAGAACTCTGGGTTCATACACTGGAAGGGGACACCAAGGAATAGTTCAGAGGCTGAGA
GGAATGGGGGTGACTGTGGGTACCTGGATGGGAAGGGATGACAAGATAGAGCAGGATGGCCAAGTATGCCCCGGCCTGTGTCTGGAGAAGCAAGGCACCTCCTGTGTACAATGGGGACCCTAATT CCC
CACTCAACTTGTGGGGGTGAAGAGCACAGGCTTTGGAATCAGATCAACCTGAGTTTAAATCCCAGCTGGGCTGTTTTCCAGCTGTGTGACTTACGGC AAGTCTTTGAGCCCTTTTCCCCCTCTTTGAG
CCTGCTTCCTCAGCTGTCAGAGATAAGGACCTTCCTTGCAGTGCTGTTGTGATGATTAAATGAAATCACAGATGTAAAGAGTGCTTGGTACAACAGGAGGCACAGGGTAAATGCTTGGGTAATGG AAC
TACTGTCATTATCACCCATCTGGCTGCATGTTACAAGGTCAATGAGATAATGGATACAAAATGTAAACTAGATGAACATAAAGGCTTATTATATAGA CCAGTGTGTCCTGAAGAAAACTGGTGTCCTA
AAGTGTGTCCTGCAGAAAACTAGTTCCCTGGAATGTTAATAGCTGTTTCTAAATAAAGGTTTTGCAGGCAATATGTTTTGAGAATGCAGAGTTAAATGAGAATACAATAAGTTTCTTTATTGTTA AAG
TATTTAATCCCTAATAGTGATATACACTGTGAATCTTCAAGAGGACAGAGTAAGCAAACCTAACTGACCAGGAGTGGCTAGAGTATTGTCAGTTCCA CAGAACAAATGTGAGAGAAAGATCTGGATTT
GGTGTTAGGGTGCCTTTAACATCTAACACTGGTTAACTCTCCTTT

Microhomology = undefined

>NHEJ_3

KSD_NEHJ_3

>Scaffold11358 1342234 1342528_NHEJ

AAAATTAGCCAGGTGTGGTGGTGCATGCCTGTAGTCCCAGCTGCTTGGGAGGCTGAGGCAGGAGAATCTCTTGAACCTGGGAGGTGAAGGTTGCAGTGAGCTGAGATTGAGGCAGTGAGCTGAGAT TG
AGCCACTGCACTCCAGCCTGGGTGACAGCGAGACTCTGTCTCAAAAAAAAAAAAAAATCAGTATGCTTCTACCACATTTGCCTTAAATGTTTTGGTCAAATGGAATGAATCTTGCTTTGAAAACAC CTT
ATAAACCTCCTGAATGCTACAGAAATTGAGCTGGTTGGTAACAGGTACTCCACCTTCCCCACCCCCTACAACACTGACAGCATTTCAAGGACCTTGGAAAGGGGAGAAATTCCTAATAATAGAGCA CC
ACGGAATTACCTGGATGGAAGTGCAAACACTGGCCACAGAGTCGGGCCCGTGGCTCTGGATGTATTGTGTTGGGTCAGAAGCTCAGAGGGAACAAAGGAGTGGGGACAGTACCTGTGAGTTTGTT TTA
AGAAGGGCAGGAAAGCCGATAGGTTGGCTGAAAGGGCCTGGAAGAGAGGATATCACTTTCACCCAGGTCTTATTTAAACCATTCTACGTAGGTGCAAATTTAGTCTCTTTTTAAAGATTACCAGGA AA
GGGGATTCTACTGTCTCTCTTAGTAGCTTATCCTTTTATTAGAAGTCAATTTTATGCCTAGTCTTTGATTATTGTTCATTTTTTTCTATTTTATTTGCCTCTTGTTGCATACTCATAAGAAATCA TTT
TGCACTGATGAAAACAACTCTTTCCCTGTTGCTCCAGAGCAGAGTGAATTGTACAAAACAAATTCTTGAATCTAAAGTTCATTACAAGGTGGTTGTTTGGATTGAACACAGGCTGCCCCTGAGGAA TG
GCAGGTTCTGCAGGAAAATAGCCAAGTGCAAAAGCCTATTTGACCCCATGTACATCTACACCTGATAAATTCTCATCAGCCCTTTAAAAAAGTTTCTGACCAG GGCCGGGCGCGGTGGCTCACGCCTG
TAATCCCAGCACTTTGGGAGGCCGAGGCGGGTGGATCATGAGGTCAGGAGATCGAGACCATCCTGGCTAACAAGGTGAAACCCCGTCTCTACTAAAAATACAAAAAATTAGCCGGGCGCGGTGGTG GG
CGCCTGTAGTCCCAGCTACTCGGGAGGCTGAGGCAGGAGAATGGCGTGAACCCGGGAAGCGGAGCTTGCAGTGAGCCGAGATTGCACCACTGCAGTCCGCAGTCCGGCCTGGGCGACAGAGCGAG ACT
CCGTCTCAAAAAAAGACCAGCCTGACCAATATGGAGAAACCCCGTCTCTGCTAAAAATACAAAATTAGCTGGGCATGGTAGTGCATGCCTGTAATCCCAGCTACTCGAGAGGCTGAGGCAGGAGAATC
ACTTGAACCCGGGAGGCGGAGGTTGCGGTGAGCCGAGATTGCACCATTGCACTCCAGCCTGGGCAACAAGAGTGAAACTCCATCTCAAAAAAAAAA GTTTCTGTGAGGAATGCTTGCTTTACTAAAAA
AAGAAAACGGGCAGAATAGACTTTGAGGACGTACTTCTAAGGCACATTAGATTTTGTGATAATGCGCATAAATGTGTGTGT GTGTGTATGTGTATGCATATATATTTGTAAGTACCTGGATCTGGATC
CCCTTAAAATCCACATCATTAGGCTCACATTTGCCTCAGGCAGCAAGGCCACCATAACTTGGCAGATGGGGTGTTGCGACTAGCAGGTGGCCAGAGTCAAGGAGGAGGCCACCTTTCTGGCTGGC ACC
TCTGTTTTTAAGGACCACACCATTTATGCACTCGGATAAACTATCTCTATCTTTAGGGCTTCTTTGCTTTCTTGTCCTTTCTGACATACAGACTCAGAAGCCATTGAATTGAGCCTTCTCCTGTAG GA
ACCCACTCTACAACCTCCGTGAGAAATGGTCCTCAGCATTGGAGGTTGGACCGGATTCTCTAAGGCCTCTGCCAGCATCATGACACAATGCATTATGCCCAGAAATCTGGGTGGTGTAACTATCT TCA
GGATGCCTACAGTCTAGTTGGGGGAAACATTGTTGTATGAGAGCAATGATTACATGTCCTCTAGTACTAGATAGGACAGGCACAAGGGAGGCTTTAGACAGAGCATTTAAATTCGTCTTAAAGAAT CA
GAGAAGTCTTCAGCCTCAATTCAAAGATGTTTGTCTTGAGTACTGGTTGGGAGTAACATTTGTTCCCAGAGCTCTTATGGGACCTTATTATATACTAGCCAGTCAACCCTTAAAAGA C

1148	8.2	1.1	11.4	a	1	184	(2110)	+	AluSx3	SINE/Alu	129	295	(16)	1
260	34.7	5.3	0.0	a	587	681	(1613)	C	Plat_L3	LINE/CR1	(83)	3503	3404	2
2787	1.0	0.0	0.0	a	1000	1294	(1000)	+	AluYb8	SINE/Alu	1	295	(23)	3
1767	6.7	0.5	0.0	a	1295	1504	(790)	+	AluSp	SINE/Alu	83	293	(20)	4
17	9.1	0.0	0.0	a	1609	1632	(662)	+	(TG)n	Simple_repeat	1	24	(0)	5
14	23.0	0.0	0.0	a	1633	1645	(649)	+	(TGTATG)n	Simple_repeat	1	37	(0)	6
219	27.1	5.7	0.0	a	1885	1954	(340)	C	L3	LINE/CR1	(34)	4065	3992	7
180	13.9	4.4	4.4	a	1961	2005	(289)	+	MIR3	SINE/MIR	145	189	(19)	8

1148	8.2	1.1	11.4	hg19	1	184	(2215)	+	AluSx3	SINE/Alu	129	295	(16)	9
260	34.7	5.3	0.0	hg19	587	681	(1718)	C	Plat_L3	LINE/CR1	(83)	3503	3404	10
2931	1.0	0.0	0.0	hg19	1000	1310	(1089)	+	AluYb8	SINE/Alu	1	311	(7)	11
2405	7.9	0.3	0.0	hg19	1317	1608	(791)	+	AluSp	SINE/Alu	1	293	(20)	12
16	8.7	4.0	0.0	hg19	1713	1737	(662)	+	(TG)n	Simple_repeat	1	26	(0)	13
14	21.6	2.6	0.0	hg19	1738	1750	(649)	+	(ATGTGT)n	Simple_repeat	2	40	(0)	14
219	27.1	5.7	0.0	hg19	1990	2059	(340)	C	L3	LINE/CR1	(34)	4065	3992	15
180	13.9	4.4	4.4	hg19	2066	2110	(289)	+	MIR3	SINE/MIR	145	189	(19)	16

>hg19 chr4:38882653-38885051

AAAATTAGCCAGGTGTGGTGGTGCATGCCTGTAGTCCCAGCTGCTTGGGAGGCTGAGGCAGGAGAATCTCTTGAACCTGGGAGGTGAAGGTTGCAGTGAGCTGAGATTGAGGCAGTGAGCTGAGAT TG
AGCCACTGCACTCCAGCCTGGGTGACAGCGAGACTCTGTCTCAAAAAAAAAAAAAAATCAGTATGCTTCTACCACATTTGCCTTAAATGTTTTGGTCAAATGGAATGAATCTTGCTTTGAAAACACCTT
ATAAACCTCCTGAATGCTACAGAAATTGAGCTGGTTGGTAACAGGTACTCCACCTTCCCCACCCCCTACAACACTGACAGCATTTCAAGGACCTTGGAAAGGGGAGAAATTCCTAATAATAGAGCACC

ACGGAATTACCTGGATGGAAGTGCAAACACTGGCCACAGAGTCGGGCCCGTGGCTC TGGATGTATTGTGTTGGGTCAGAAGCTCAGAGGGAACAAAGGAGTGGGGACAGTACCTGTGAGTTTGTTTTA
AGAAGGGCAGGAAAGCCGATAGGTTGGCTGAAAGGGCCTGGAAGAGAGGATATCACTTTCACCCAGGTCTTATTTAAACCATTCTACGTAGGTGCAAATTTAGTCTCTTTTTAAAGATTACCAGG AAA
GGGGATTCTACTGTCTCTCTTAGTAGCTTATCCTTTTATTAGAAGTCAATTTTATG CCTAGTCTTTGATTATTGTTCATTTTTTTCTATTTTATTTGCCTCTTGTTGCATACTCATAAGAAATCATTT
TGCACTGATGAAAACAACTCTTTCCCTGTTGCTCCAGAGCAGAGTGAATTGTACAAAACAAATTCTTGAATCTAAAGTTCATTACAAGGTGGTTGTTTGGATTGAACACAGGCTGCCCCTGAGGA ATG
GCAGGTTCTGCAGGAAAATAGCCAAGTGCAAAAGCCTATTTGACCCCATGTACATC TACACCTGATAAATTCTCATCAGCCCTTTAAAAAAGTTTCTGACCAG GGCCGGGCGCGGTGGCTCACGCCTG
TAATCCCAGCACTTTGGGAGGCCGAGGCGGGTGGATCATGAGGTCAGGAGATCGAGACCATCCTGGCTAACAAGGTGAAACCCCGTCTCTACTAAAAATACAAAAAATTAGCCGGGCGCGGTGGT GGG
CGCCTGTAGTCCCAGCTACTCGGGAGGCTGAGGCAGGAGAATGGCGTGAACCCGGG AAGCGGAGCTTGCAGTGAGCCGAGATTGCACCACTGCAGTCCGCAGTCCGGCCTGGGCGACAGAGCGAGACT
CCGTCTCAAAAAAaaaaaaaaaaaaaaaaaagtttctgaccaggtgcagtggctcacacctgtaatcccagcactttgggaggccgaggggaccggatcacctgaggtcaggagttcg AGACCAGCCTG
ACCAATATGGAGAAACCCCGTCTCTGCTAAAAATACAAAATTAGCTGGGCATGGTA GTGCATGCCTGTAATCCCAGCTACTCGAGAGGCTGAGGCAGGAGAATCACTTGAACCCGGGAGGCGGAGGTT
GCGGTGAGCCGAGATTGCACCATTGCACTCCAGCCTGGGCAACAAGAGTGAAACTCCATCTCAAAAAAAAAA GTTTCTGTGAGGAATGCTTGCTTTACTAAAAAAAGAAAACG GGCAGAATAGACTTT
GAGGACGTACTTCTAAGGCACATTAGATTTTGTGATAATGCGCATAAATGTGTGTG TGgTGTGTATGTGTATGCATATATATTTGTAAGTACCTGGATCTGGATCCCCTTAAAATCCACATCATTAGG
CTCACATTTGCCTCAGGCAGCAAGGCCACCATAACTTGGCAGATGGGGTGTTGCGACTAGCAGGTGGCCAGAGTCAAGGAGGAGGCCACCTTTCTGGCTGGCACCTCTGTTTTTAAGGACCACAC CAT
TTATGCACTCGGATAAACTATCTCTATCTTTAGGGCTTCTTTGCTTTCTTGTCCTTTCTG ACATACAGACTCAGAAGCCATTGAATTGAGCCTTCTCCTGTAGGAACCCACTCTACAACCTCCGTGAG
AAATGGTCCTCAGCATTGGAGGTTGGACCGGATTCTCTAAGGCCTCTGCCAGCATCATGACACAATGCATTATGCCCAGAAATCTGGGTGGTGTAACTATCTTCAGGATGCCTACAGTCTAGTTG GGG
GAAACATTGTTGTATGAGAGCAATGATTACATGTCCTCTAGTACTAGATAGGACAGGCAC AAGGGAGGCTTTAGACAGAGCATTTAAATTCGTCTTAAAGAATCAGAGAAGTCTTCAGCCTCAATTCA
AAGATGTTTGTCTTGAGTACTGGTTGGGAGTAACATTTGTTCCCAGAGCTCTTATGGGACCTTATTATATACTAGCCAGTCAACCCTTAAAAGA C

1124	8.7	1.1	11.4	Chimp	1	183	(1886)	+	AluSx3	SINE/Alu	129	294	(17)	1
255	34.7	5.3	0.0	Chimp	585	679	(1390)	C	Plat_L3	LINE/CR1	(83)	3503	3404	2
2349	8.9	0.3	0.0	Chimp	991	1281	(788)	+	AluSp	SINE/Alu	1	292	(21)	3
31	0.0	0.0	0.0	Chimp	1386	1412	(657)	+	(TG)n	Simple_repeat	1	27	(0)	4
236	28.6	5.7	0.0	Chimp	1660	1729	(340)	C	L3	LINE/CR1	(34)	4065	3992	5
200	13.9	4.4	4.4	Chimp	1736	1780	(289)	+	MIR3	SINE/MIR	145	189	(19)	6
219	34.6	1.4	10.3	Chimp	1815	1962	(107)	C	L2b	LINE/L2	(97)	3278	3143	7

>Chimp
AAAATTAGCCAGGTGTGGTGGTGCATGCCTGTAGTCCCAGCTGCTTGGGAGGCTGAGGCAGGAGAATCTCTTGAACCTGGGAGGTGAAGGTTGCAGTGAGCTGAGA
TTGAGGCAGTGAGCTGAGATTGAGCCACTGCACTCCAGCCTGGGTGACAGCaAGACTCTGTCTCAAAAAAAAAAAAAATCAGTATGCTTCTACCACATTTGCCTTAA
ATGTTTGGTCAAATGGAATGAATCTTGCTTTGAAAACACCTTATAAACCTCCTGAATGCTACAGAAATTGAGCTGGTTGGTAACAGGTACTCCACCTTCCCCACCC
CCTACAACACTGACAGCATTTCAAGGACCTTGGAAAGGGGAGAAATTCCTAATAATAGAGCACCACGGAATTACCTGGATGGAAGTGCAAACACTGGCCACAGAGT
tGGGCCCGTGGCTCTGGATGTATTGTGTTGGGTCAGAAGCTCAGAGGGAACAAAGGAGTGGGGACAGTACCTGTGAGTTTGTTTTAAGAAGGGCAGGAAAGCCGAT
AGGTTGGCTGAAAGGGCCTGGAgGAGAGGATATCACTTTCACCCAGGTCTTATTTAAACCATTCTACGTAGGTGCAAATTTAGTCTCTTTTTAAAGATTACCAGGA
AAGGGGATTCTACTGTCTCTCTTAGTAGCTTATCCTTTTATTAGAAGTCAATTTTATGCCTAGTCTTTGATTATTGTTCATTTTTTTCTATTTTATTTGCCTCTTG
TTGCATACTCATAAGAAATCATTTTGCACTGATGAAAACAACTCTTTCCCTGTTGCTCCAGAGCAGAGTGAATTGTACAAAACAAATTCTTGAATCTAAAGTTCAT
TACgAGGTGGTTGTTTGGATTGAACACAGGCTGCCCCTGAGGAATGGCAGGTTCTGCAGGAAAATAGCCAAGTGCAAAAGCCTATTTGACCCCATGTACATCTACA
CCTGATAAATTCTCATCAGCCCTTTAAAAAGTTTCTGACCAGGTGCgGTGGCTCACACCTGTAATCCCAGCACTTTGGGAGGCCGAGGGGACCGGATCgCCTGAGG
TCAGGAGTTCGAGACCAGCCTGACCAATATGGAGAAACCCCGTCTCTGCTAAAAATACAAAATTAGCTGGGCATGGTAGTGCATGCCTGTAATCCCAGCTACTCaA
GAGGCTGAGGCAGGAGAATCACTTGAACCCGGGAGGCGGAGGTTGCaGTGAGCCGAGATTGCACCATTGCACTCtAGCCTGGGCAACAAGAGTGAAACTCCATCTC
AAAAAAAAAGTTTCTGTGAGGAATGCTTGCTTTACTAAAgAAAGAAAACtGGCAGAATAGACTTTGAGGACGTACTTCTAAGGCACATTAGATTTTGTGATAATGC
GCATAAATGTGTGTGTGtGTGTGTgTGTGTgTGTATATTTGTAAGTACCTGGATCTGGgTCCCCTTAAAATCCACATCATTAGGCTCACATTTGCCTCAGGCAGCA
AGGCCACCATAACTTGGCAGgTGGGGTGTTGCGACTAGCAGGTGGCCAGAGTCAAGGAGGAGGCCACCTTTCTGGCTGGCACCTCTGTTTTTAAGGACCACACCAT

TTATGCACTCGGATAAACTATCTCTATCTTTAGGGCTTCTTTGCTTTCTTGTCCTTTCTGACATACAGACTCAGAAGCCATTGAATTGAGCCTTCTCCTGTAGGAA
CCCACTCTACAACCTCCGTGgGAAATGGTCCTCAGCATTGGAGGTTGGACCGGATTCTCTAAGGCCTCTGCCAGCATCATGACACAATGCATTATGCCCAGAAATC
TGGGTGGTGTAACTATCTTCAGGATGCCTACAGTCTAGTTGGGGGAAACATTGTTGTATGAGAGCAATGATTACATGTCCTCTAGTACTAGATAGGACAGGCACAA
GGGAGGCTTTAGACgGAGCATTTAAATTCGTCTTAAAGAgTCAGAGAAGTCTTCAGCCTCAATTCAAAGATGTTTGTCTTGAGTACTGGTTGGGAGTgACATTTGT
TCCCAGAGCTCTTgTGGGACCTTATTATATACTAGCCAGTCAACCCTTAAAAGAC
Microhomology: A

>NHEJ_4

KSD_NEHJ_4

>Scaffold16618 102066 102379_NHEJ
GAAAAGGAAAAGAAAAGAAGAGAAAAGACTGGAGAAAATCTTCAGGACCTAGAACTAGGAGAAGAGTTCCTAGACATGACATCAAAAGCACAATTAAGAAAAGAAGGACATTGATACATTGACTGT CT
TAGTTCATTTTGTATTACCATAACAGAATACCTGAGACTGAGTAATTTATAAAGAAAAGGCTTATTTAGCTCAGTTCTGTAGGCTGGGAAGTTCTAGGGCATGTCCCTGGCTTCTGGTGAGGACT TTC
TCACTGTCATAACATGGTTGAGAACATAAAAGGGAAAGCAGATGCATGCAAAGAGGCAAAATCCACAGAGGCACCCTGGCTTTTAACAACACACTGTCATGGGAACTAACCCATTCCTGTGAAAAC CA
ATACAGTCTGGCCAGAGCAAAGACTCCACTCACTACAGCAAGAACTGCACCAAGCCATGCATGAGTGATCTG tCCCCATGACCCAAACACCTCCCACTAGGCCCCACCTCCCAACACCACCTCACTGA
AGATCAAATTTCAACATGAGTTTTGGTGGCGACAAGCTCAAACCATAGCATCAGCCTCAACAAAACTAAAACTTTCGCTATGTGCAAGACTGTTAAGATGCTGAAAAGACAAGCCACAGGCTGAAA AA
AAAATTTGCAAACCATATATCCAACAAGACATGCAAAAAACTCAAAACTCAACATTAAAAACAAAAACAATCTTATTAGAAATGGGCAAAAGACATGAAGGAAATTTCACTGAAGAGGATATATG ATG
GCAAATAAACACACAAAAAGATGTTCAACATCATTAGCCATAAGGGAAATGCAAATTAAAACTACAATGAGTTATCAACACATACCTATCAGAATGGCTAAAGAATAGTGACAACACGAAATGCTG AT
GGCTGCAAAGAAACTGAATCACTCATACATTGCTAGTGGAAATACATAACGGCACAGGCACTCTGGAAGACAGCTTGTGGTTTCCTTAAAAACTAAACATGCG GGCCGGGCGCGGTGGCTCACACCTG
TAATCCCAGCACTCTGGGAGGCCGAGGCGGGCAGATCACAAGGTCAGGAGATAGAGACCATCCTGGCTAACACAGTGAAACCCCATCTCTACTAAAAATACAAAAAATTAGCCGGGTGTGGTGGTG GG
CGCCTGTAGTCCCAGCTACT cGGGAGACTGAGGCAGGAGAATGGCATGAACCCAGGAGGCAGAGGTTGCAGCGAGCCAAGATCACACCACTGCACTCCAGCCTGGGCGACAGAGCAAGACTCCGTCTC
AAAAAAAAAAAAAACTATTAAAAATACAAAAAATTAGCCAGGCATGGTGACACGTGCCTGTAATCCCAGCTACTCAGGAGGCTGAGGCAGGAGAATCGCTTGAACCCAGGAGGCAGAGGTTGCAGTGA
GCCGAGATTGCACCACTGCACTCCAGACTGGGCAACAGAGCAAGTCTCCATCTCAAACAAAACAAAACAAAACAAAAAAA CTAAACATGCAACTACTATACAACCCATTAATTCCACTCCTGGGCTTT
TTTACAGAGAAATGAAGACTTATGGTTATACAAAAGCATGTACGTGAATGTTCACAGCAGGTTTATTTAAATTAGCCAAAAACTAGAAACAACCTAAATGTTCTTCATAAGAATGGTTAACTTACA CA
CATTTATTATCTCACAGTTTTCATGGATCAGGAATCTGGGCACAGCTTAACCAGGTCCTCTGCTCAGGTCTCATAAGGCTGCATGCAATCAGGTGCCGGCAGGACTACAGTGTCATCAGCTGCTT GAC
TGGGGAAACTCTGCTTCCAGGTTCCCACACAATTGTCGACAGAATTGATCTTCTTACATCTATAGGGCTGAGGTCCCCATTTTTTCTCATCATTTACAGATTGCCTTCCATTCCCTGCCACATGGC CC
TCTCCATAGACATTCATACATAATTTTTTGGTTCTTTAAGACTAGCAGGCCCTCCCTCCAAAAGTAACTACGTGAGATGATAGATAAATTAGTCTGCTTCACTATAGTAACCACTTTACTATCTA TGT
GTATCCCATAACATGTCTTAAACCTCAAATGTACACAATAAAATTTATTTAA tAAAACAAAGACTAGGGGGAAAATCTTTCTCTTCAGTCTAAGACAGAGTGCTATATAAAGAAACACAACCATGGGA
GTGACATGCCACCATCTTTGCCATATCCTATTGCTTAGAGGCAAGTCACAGTTTCCACTCATACTCAAGGGAAGGGGATGGTACAAAAATGTGGATCACTGGGGTTTGCCTGACACACCAAGTCA AAC
AGCGGGTAA

447	21.0	0.0	1.0	a	21	121	(2192)	+	L1MD	LINE/L1	5043	5142	(1004)	1
2727	14.1	2.3	0.7	a	124	559	(1754)	C	MSTB2	LTR/ERVL-MaLR	(0)	457	15	2
1900	13.1	5.4	0.0	a	567	998	(1315)	+	L1MD	LINE/L1	5145	5608	(634)	1
2543	6.7	0.0	1.6	a	1000	1313	(1000)	+	AluY	SINE/Alu	1	309	(2)	3
1330	10.9	0.0	0.0	a	1314	1488	(825)	+	AluSx	SINE/Alu	134	308	(4)	4
908	17.9	2.4	0.0	a	1489	1656	(657)	+	L1MD	LINE/L1	5599	5770	(472)	1
1078	24.2	10.0	4.0	a	1660	1969	(344)	C	MLT1E1A	LTR/ERVL-MaLR	(57)	623	296	5
362	25.9	2.6	2.6	a	1984	2098	(215)	+	L1MA9	LINE/L1	6168	6282	(30)	6
470	25.3	0.1	30.0	a	2149	2272	(41)	C	MLT1E1A	LTR/ERVL-MaLR	(399)	161	50	5

447	21.0	0.0	1.0	hg	21	121	(2329)	+	L1MD	LINE/L1	5043	5142	(1004)	7
2737	13.8	2.3	0.7	hg	124	559	(1891)	C	MSTB2	LTR/ERVL-MaLR	(0)	457	15	8
2568	14.0	7.1	2.2	hg	567	999	(1451)	+	L1MD	LINE/L1	5145	5608	(634)	7
2615	6.5	0.0	0.0	hg	1000	1306	(1144)	+	AluY	SINE/Alu	1	307	(4)	9
2568	15.0	3.3	4.8	hg	1307	1317	(1133)	+	L1MD	LINE/L1	5609	5609	(633)	7
2311	10.7	0.0	0.3	hg	1318	1626	(824)	+	AluSx	SINE/Alu	1	308	(4)	10
2568	15.0	3.3	4.8	hg	1627	1794	(656)	+	L1MD	LINE/L1	5609	5770	(472)	7
1078	24.2	10.0	4.0	hg	1798	2107	(343)	C	MLT1E1A	LTR/ERVL-MaLR	(57)	623	296	11
362	25.9	2.6	2.6	hg	2122	2236	(214)	+	L1MA9	LINE/L1	6168	6282	(30)	12
470	25.3	0.1	30.0	hg	2287	2410	(40)	C	MLT1E1A	LTR/ERVL-MaLR	(399)	161	50	11

>hg19 chr18:45293797-45296247
GAAAAGGAAAAGAAAAGAAGAGAAAAGACTGGAGAAAATCTTCAGGACCTAGAACTAGGAGAAGAGTTCCTAGACATGACATCAAAAGCACAATTAAGAAAAGAAGGACATTGATACATTGACTGT CT
TAGTTCATTTTGTATTACCATAACAGAATACCTGAGACTGAGTAATTTATAAAGAAAAGGCTTATTTAGCTCAGTTCTGTAGGCTGGGAAGTTCTAGGGCATGTCCCTGGCTTCTGGTGAGGACT TTC
TCACTGTCATAACATGGTTGAGAACATAAAAGGGAAAGCAGATGCATGCAAAGAGGCAAAATCCACAGAGGCACCCTGGCTTTTAACAACACACTGTCATGGGAACTAACCCATTCCTGTGAAAAC CA
ATACAGTCTGGCCAGAGCAAAGACTCCACTCACTACAGCAAGAACTGCACCAAGCCATGCATGAGTGATCTG cCCCCATGACCCAAACACCTCCCACTAGGCCCCACCTCCCAACACCACCTCACTGA
AGATCAAATTTCAACATGAGTTTTGGTGGCGACAAGCTCAAACCATAGCATCAGCCTCAACAAAACTAAAACTTTCGCTATGTGCAAGACTGTTAAGATGCTGAAAAGACAAGCCACAGGCTGAAA AA
AAAATTTGCAAACCATATATCCAACAAGACATGCAAAAAACTCAAAACTCAACATTAAAAACAAAAACAATCTTATTAGAAATGGGCAAAAGACATGAAGGAAATTTCACTGAAGAGGATATATG ATG
GCAAATAAACACACAAAAAGATGTTCAACATCATTAGCCATAAGGGAAATGCAAATTAAAACTACAATGAGTTATCAA CACATACCTATCAGAATGGCTAAAGAATAGTGACAACACGAAATGCTGAT
GGCTGCAAAGAAACTGAATCACTCATACATTGCTAGTGGAAATACATAACGGCACAGGCACTCTGGAAGACAGCTTGTGGTTTCCTTAAAAACTAAACATGCG GGCCGGGCGCGGTGGCTCACACCTG
TAATCCCAGCACTCTGGGAGGCCGAGGCGGGCAGATCACAAGGTCAGGAGATAGAGACCATCCTGGCTAACACAGTGAAACCCCATCTCTACTAAAAATACAAAAAATTAGCCGGGTGTGGTGGTG GG
CGCCTGTAGTCCCAGCTACTtGGGAGACTGAGGCAGGAGAATGGCATGAACCCAGGAGGCAGAGGTTGCAGCGAGCCAAGATCACACCACTGCACTCCAGCCTGGGCGACAGAGCAAGACTCCGTCTC
AAAAAAAAAAAAAAaaaaaaaaaaaacctaaacatgtggcagagcacagtggctcacacctgtaatcctagcacttttggaggccaagttgggcggatcacctgaggtcacgagttcgagaccatcct
ggccaacatggtgaaaccctgtctCTATTAAAAATACAAAAAATTAGCCAGGCATGGTGACACGTGCCTGTAATCCCAGCTACTCAGGAGGCTGAGGCAGGAGAATCGCTTGAACCCAGGAGGCAGAG
GTTGCAGTGAGCCGAGATTGCACCACTGCACTCCAGACTGGGCAACAGAGCAAGTCTCCATCTCAAACAAAACAAAACAAAACAAAAAAA CTAAACATGCAACTACTATACAACCCATTAATTCCACT
CCTGGGCTTTTTTACAGAGAAATGAAGACTTATGGTTATACAAAAGCATGTACGTGAATGTTCACAGCAGGTTTATTTAAATTAGCCAAAAACTAGAAACAACCTAAATGTTCTTCATAAGAATGGTT
AACTTACACACATTTATTATCTCACAGTTTTCATGGATCAGGAATCTGGGCACAGCTTAACCAGGT CCTCTGCTCAGGTCTCATAAGGCTGCATGCAATCAGGTGCCGGCAGGACTACAGTGTCATCA
GCTGCTTGACTGGGGAAACTCTGCTTCCAGGTTCCCACACAATTGTCGACAGAATTGATCTTCTTACATCTATAGGGCTGAGGTCCCCATTTTTTCTCATCATTTACAGATTGCCTTCCATTCCCTGC
CACATGGCCCTCTCCATAGACATTCATACATAATTTTTTGGTTCTTTAAGACTAGCAGGCCCTCCCTCCAAAAGTAACTACGTGAGATGATAGATAAATTAGTCTGCTTCACTATAGTAACCACT TTA
CTATCTATGTGTATCCCATAACATGTCTTAAACCTCAAATGTACACAATAAAATTTATTTAAaAAAACAAAGACTAGGGGGAAAATCTTTCTCTTCAGTCTAAGACAGAGTGCTATATAAAGAAACAC
AACCATGGGAGTGACATGCCACCATCTTTGCCATATCCTATTGCTTAGAGGCAAGTCACAGTTTCCACTCATACTCAAGGGAAGGGGATGGTACAAAAATGTGGATCACTGGGGTTTGCCTGACA CAC
CAAGTCAAACAGCGGGTA
>Chimp
GAAAAGGAAAAGAAAAGAAGAgaaaaGAAAAGACTGGAGAAAATCTTCAGGACCTAGAACTAGGAGAAGAGTTCCTAGACATGACATCAAAAGCACAATTAAGAAA
AGAAGGACATTGATcCATTGACTGTCTTAGTTCATTTTGTATTACCATAACAGAATACCTGAGACTGAGTAATTTATAAAGAAAAGGCTTATTTAGCTCAGTTCTG
TAGGCTGGGAAGTTCTAGGGCATGgCCCTGGCTTCTGGTGAGGACTTTCTCACTGTCATAACATGGTTGAGAACATAAAAGGGAAAGCAGATGCATGCAAAGAGGC
AAAAcCCACAGAGGCACCCTGGCTTTTAACAACACACTGTCATGGGAACTAACCCATTCCTGTGAAAACCAATAgAGTCTGGCCAGAGCAAAaACTCCACTCACTA
CAGCAAGAACTGCACCAAGCCATGCATGAGTGATCTGCCCCCATGACCCAAACACCTCCCACTAGGCCCCACCTCCCAACACCACCTCACTGAAGATCAAATTTCA
AtATGAGTTTTGGTGGCGACAAGCTCAAACCATAGCATCAGCCTCAACAAAACTAAAACTTTCGCTATGTGCAAGACTGTTAAGATGCTGAAAAGACAAGCCACAG
GCTGAAAAAAAAATTTGCAAACCAcATATCCAACAAGACATGCAAAAAACTCAAAACTCAACATTAAAAACAAAAACAATCTTATTAGAAATGGGCAAAAGACATG
AAGGAAATTTCACTGAAGAGGATATATGATGGCAAATAAACACACAAAAAGATGTTCAACATCATTAGCCATAAGGGAAATGCAAATTAAAACTACAATGAGTTAT
CAACACATACCTATCAGAATGGCTAAAGAATAGTGACAACACcAAATGCTGATGGCTGCAgAGAAACTGAATCACTCATACATTGCTAGTGGAAATACATAAtGGC
ACAGGCACTCTGGAAGACAGCTTGTGGTTTCCTTAAAAACTAAACATGCGGGCCGGGCaCGGTGGCTCACACCTGTAATCCCAGCACTCTGGGAGGCCGAGGCGGG
CAGATCACAAGGTCAGGAGATcGAGACCATCCTGGCTAACACAGTGAAACCCCATCTCTACTAAAAATACAAAAAATTAGCCGGaTGTcGTGGTGGGCGCCTGTAG
TCCCAGCTACTcGGGAGACTGAGGCAGGAGAATGGCATGAACCCAGGAGGCAGAGGTTGCAGCGAGCCAAGATCACACCACTGCACTCCAGCCTGGGCGACAGAGC
AAGACTCCGTCTCAAAAAAAAAAAAAAACCTAAACATGTGGCAGAGCACAGTGGCTCACACCTGTAATCCTAGCACTTTTGGAGGCCAAGTTGGGCGGATCACCTG
AGGTCAgGAGTTCGAGACCATCCTGGCCAACATGGTGAAACCCTGTCTCTATTAAAAATACAAAAAATTAGCCAGGCATGGTGACACGTGCCTGTAATCCCAGCTA
CTCAGGAGGCTGAGGCAGGAGAATCGCTTGAACCCAGGAGGCAGAGGTTGCAGTGAGCCGAGATTGCACCACTGCACTCCAGACTGGGCgACAGAGCAAGTCTCCA
TCTCAAACAAAACAAAACAAAACAAAAAAACTAAACATGCAACTACTATACAACCCATTAATTCCACTCCTGGGCTTTTTTACAGAGAAATGAAGACTTATGGTTA
TACAAAAGCATGTACGTGAATGTTCACAGCAGGTTTATTTAAATTAGCCAAAAACTAGAAACAACCTAAATGTTCTTCATAAGAATGGTTAACTTACACACATTTA
TTATCcCACAGTTTTCATGGATCAGGAATCTGGGCACAGCTTAACCAGGTCCTCTGCTCAGGTCTCATAAGGCTGCATGCAATCAGGTGCCaGCAGGACTACAGTG
TCATCAGCTGCTTGACTGGGGAAACTCTGCTTCCAGGTTCCCACACAATTGTCaACAGAATTGATCTTCTTACATCTATAGGGCTGAGGTCCCCATTTTTTCTCAT
CATTTACAGATTGCCTTCCATTCCCTGCCACATGGCCCTCTCCATAGACATTCATACATAATTTTTTGGTTCTTTAAGACTAGCAGGCCCTCCCTCCAAAAGTAAC

TACGTGAGATGATAGATAAATTAaTCTGCTTCACTATAGTAACCACTTTACTATCTATGTGTATCCCATAACATGTCTTAAACCTCAAATGTACACAATAAAATTT
ATTTAAAAAAACAAAGACTAGGGGGAAAATCTTTCTCTTCAGTCTAAGACAGAGTGCTATATAAAGAAACACAACCATGGGAGTGACATGCCACCATCTTTGCCAT
ATCCTATTGCTTAGAGGCAAGTCACAGTTTCCACTCATACTCAAGGGAAGGGGATGGTACAAAAATGTGGATCACTGGGGTTTGCCTGACACACCAAGTCAAACAG
CGGGTA

Microhomology = undefined

>NHEJ_5

KSD_NEHJ_5

>Scaffold1016 4101021 4101251_NHEJ
TTTATAAAAATGACTTTTCTATTGAGATGAACACATGGTTTTTCTTTATTAATCAGTTAGTATGATGCATTTGCACCATTTAATGTTTCTATGTTAAACCAACCTTACATTTTTGGGATGGACTGC AC
TTGACCATTATGTATTCCTTTGGGGTAACTTTTATTCCATTTGCTTAAATTTTGTTAGGGATGTTTGCATCCATGTCCATGAGGGATCTTGGTCTGTCATTGTCTTTTCTTGTAATATATTTTTA CTT
TTGGTATCAGAAAAATGCTTGCCTGATAGAATGAGCTTGGAATTACTCCCTTGCCTTTCCATTTTCTGGGAAAACTGTGTGTAGTTGGTATTACCTCTAACCTCTAACCGAAAACCATTTTCCTTA AA
ATAGGGCTATTCGGATTGCCTATGTCTTTTTGAATGAGCTTTGGTTGTTTTTGTCTTTCAGGGAATTTGTCCATTTTATCTAAGTTGTCAAACTTATTGGCTTAAAGTTGTTCATAATAGTCTCC TGT
TATATGTTTATTATCTGTAGAGTCTGTAGTGATGTCAATGCTCCCATTCCCAGTGCTGGTGATTTGTGTCTTTTTGTCCTGCAGTAATTCTATGATCTATGTCATTTCTAGGTCAGTTTCTACTCA TC
ACATTTGGCCTCATATTTCCTCTTCTCTGCATGCCTGGTAATTTTTTTTTTTTTTTTTAAGACAGAGTCTCGCCCTGTCGCCCAGGCTAGAGTGCAGTGGCGtGATCTCAGCTCACTGCAACCTCCG
CCTCCCGGGTTCAAGTAATTCTCCTGTCTCACCCTCCAGAGTAGCTGGGATTATAGGCTCCTGCCACCACACCTGGCTAATTTTTGTATTTTTAGTAGAGACGGGGTTTCACCATGTTAGCCAGGA TG
GTCTCGATCTCCTGACCTCGTgATCCgtccgcctcggccttGGAGAATGGCATGAACCCAGGAGGCGGAGCTTGCAGTGAGCCGAGATGGCACCACTGCACTCCAGCCTGGGCGACAGAGCAAGATTC
TGCCTCAAAAAAAAAAAGTAGGCCTTGCCAGGTGCAGTGGGAGGATCGCTTAAGGCCAGGAGTTTGAAACCAGCCTGgGCAACGTGACAAAACCCCGTCTCTACAAAAAATACAAAAATTAGCCAGCC
GTGGTGGGGCATGCCTATAGTCCTGCTACTCAGGGCTGAGGTGGGAGGGTCACTTGAGGCCAGGAGGTTGAGGCTGCAGTGAGCCTTGATCACACCACTGCAGTCCAGCCTGGGGGACAGAGTGA GAT
CCTGTGTCAAATATATACATTTAATTTTAAAAAAGTTTTAAGTAGGAGTTACAGTTTAAAGTGGCAGTTAAACACAAGCATCTATTGCCTCTTTGCTTTAAAATGAAGACAAAAATTTCAAGAGAA TA
AGAGGGGAGACGGGAGCTGGAACCCGGAAGACAGATGTCCAGGCAGCGACTGTCCTCGGAGGCCTGAGAGTGCTGAACCTGCAAATGCAAGGAGCCAGTTGGCTTCGGAGCCCCCGGGAGGCACA GGA
AGTGGAGGCCCAGATTCCTCCAAGAAGCTGGGCTGGAAACAGGGTCTCCgGGGGGTCTTTGTGAGCGGCACGGTGTTCTGGCCACAGCTGAGGACAGGGCTGGGGCTCCGTGCTGAAACCAGGAGGAC
ACAGGTGAGACCCTTGCTGGAGGTGCACAGACGCTTCTCAGCCCACAGCACCTCTGGCCTTCCAGGGCTACCAGGCCACGCCGTGCACACAGGGAGGTGGTTCCTCTGCAGGGCAGTGTTGGGCA GGA
GCAGTTAGGGCTGTGGGGCCTCTCGCCAGCCACACGCATGCTAACACTAACACACTGGGATGGTTAACACTGAGTGTGAACTGGATTGGATGGACAGATGCAAAGTATTGATCCTGGGTGTGTCTG TG
AGGGTGTTGCCAAAGGAGATTCACCTCCGACTGAGTGGACTGGGAGAGGCAGACGCACCCTTAATCTCAGTGGGCACCGTCTCATCAGCTGCCAGCAAGGCTGGAATATAAAGCAGGCAGAAAAA CAT
GAGAAGGCTAGATGGGCCTACCCTCCCAGCCTACATCTTTCTCCCATGCTGGACGTCTCCTGCCCTCGAACATCAGACTCTAAGTTCTTCAGCTTTGAGACTCGGGCTGGCTCTCTGTGCTCCTCA GC
TTGCACATGGCCTATTGTGGGACCCTGTGATCGTGTGAGTTAATACTTAATAAA

1089	25.1	3.3	1.8	a	18	354	(1876)	C	L1MEc	LINE/L1	(2238)	4083	3742	1	
851	23.7	1.9	0.0	a	372	586	(1644)	C	L1MEc	LINE/L1	(2662)	3659	3441	1	
230	15.9	23.3	2.4	a	579	681	(1549)	C	L1MEc	LINE/L1	(5450)	950	827	1	*
2080	7.5	0.4	0.0	a	682	936	(1294)	C	AluSc8	SINE/Alu	(12)	299	44	2	
865	7.7	0.0	0.0	a	938	1041	(1189)	+	AluYc	SINE/Alu	177	280	(19)	3	
1562	14.3	1.3	0.0	a	1061	1291	(939)	+	AluJr	SINE/Alu	52	285	(27)	4	
2596	12.6	0.0	0.5	a	1848	2230	(0)	+	MLT2A1	LTR/ERVL	1	381	(63)	5	

1089	25.1	3.3	1.8	hg	18	354	(4327)	C	L1MEc	LINE/L1	(2238)	4083	3742	6	
851	23.7	1.9	0.0	hg	372	586	(4095)	C	L1MEc	LINE/L1	(2662)	3659	3441	6	
230	15.9	23.3	2.4	hg	579	681	(4000)	C	L1MEc	LINE/L1	(5450)	950	827	6	*
2420	8.0	0.3	0.0	hg	682	980	(3701)	C	AluSc8	SINE/Alu	(11)	300	1	7	
638	27.7	7.2	1.1	hg	981	1413	(3268)	C	L1MEc	LINE/L1	(5758)	642	121	6	*
880	19.9	5.9	2.5	hg	1412	1648	(3033)	+	L1ME3	LINE/L1	4106	4350	(1796)	8	
433	28.3	3.1	5.5	hg	1451	1674	(3007)	+	L1MC	LINE/L1	4151	4369	(1777)	9	*
2262	13.0	0.0	0.0	hg	1686	1993	(2688)	+	AluSz	SINE/Alu	1	308	(4)	10	
2067	20.9	9.2	4.1	hg	2018	2895	(1786)	+	L1ME3	LINE/L1	5119	6054	(108)	8	
1019	16.7	0.0	0.0	hg	2899	3054	(1627)	C	AluJb	SINE/Alu	(20)	292	137	11	
2354	5.4	0.0	7.9	hg	3178	3492	(1189)	+	AluY	SINE/Alu	1	292	(19)	12	
1547	14.7	1.3	0.0	hg	3512	3742	(939)	+	AluJr	SINE/Alu	52	285	(27)	13	
2596	12.6	0.0	0.5	hg	4299	4681	(0)	+	MLT2A1	LTR/ERVL	1	381	(63)	14	

>hg19 chr9:140252217-140256897

TTTATAAAATGACTTTTCTATTGAGATGAACACATGGTTTTTCTTTATTAATCAGTTAGTATGATGCATTTGCACCATTTAATGTTTCTATGTTAAACCAACCTTACATTTTTGGGATGGACTGC AC
TTGACCATTATGTATTCCTTTGGGGTAACTTTTATTCCATTTGCTTAAATTTT GTTAGGGATGTTTGCATCCATGTCCATGAGGGATCTTGGTCTGTCATTGTCTTTTCTTGTAATATATTTTTACTT
TTGGTATCAGAAAAATGCTTGCCTGATAGAATGAGCTTGGAATTACTCCCTTGCCTTTCCATTTTCTGGGAAAACTGTGTGTAGTTGGTATTACCTCTAACCTCTAACCGAAAACCATTTTCCTT AAA
ATAGGGCTATTCGGATTGCCTATGTCTTTTTGAATGAGCTTTGGTTGTTTTTG TCTTTCAGGGAATTTGTCCATTTTATCTAAGTTGTCAAACTTATTGGCTTAAAGTTGTTCATAATAGTCTCCTGT
TATATGTTTATTATCTGTAGAGTCTGTAGTGATGTCAATGCTCCCATTCCCAGTGCTGGTGATTTGTGTCTTTTTGTCCTGCAGTAATTCTATGATCTATGTCATTTCTAGGTCAGTTTCTACTCATC
ACATTTGGCCTCATATTTCCTCTTCTCTGCATGCCTGGTAAtTTTTTTTTTTTTTTTTTTAAGACAGAGTCTCGCCCTGTCGCCCAGGCTAGAGTGCAGTGGCG cGATCTCAGCTCACTGCAACCTCC
GCCTCCCGGGTTCAAGTAATTCTCCTGTCTCACCCTCCAGAGTAGCTGGGATTATAGGCTCCTGCCACCACACCTGGCTAATTTTTGTATTTTTAGTAGAGACGGGGTTTCACCATGTTAGCCAG GAT
GGTCTCGATCTCCTGACCTCGTaATCCacccaccttggcctcccaaagtgctgggattacaggcatgagccacagtgcccggcctaactttcccctatttctgaggcaagagccttcttagaactata
gtcaatgccccgccaattaggaagcttttaaaactctgggtcttgggagcagatcctgttcccagctctttgtaaggcttgaaaacttttccctgtaatgtgttcaggtggcccttcctctgctt tgt
gtgagttcctcacacgcagggtgctctttacttgggcgaattctcaagggcgctccctgcagagctccacagctctttttctgggcatctcttctctgcgaactcagggaagcacccagtgtctcctg
ggttccccttccctcgcatgttgctggaagtttctccaggagggaagatggagcgattggcagcctcgcttcccatcactcgggcatcactgccctgagccacctgttgcctaatgtctgagaac tgc
tgtttttattcaatactgtaccaaggacccatgccaggcgggaaggcaggaaaataagtgcaaagaccgggtacaaagaaacaactgtcattttctcagaagatatgtttttatatgtagggaaggaa
aaccaaaaaatctacagataaattattagaattaataagtaaatttagcaagatcactggttacaaggtaagtttataaaagccaattgtatttctttatacttgcaataaagctttctcaaata gga
tataaaatcactaagaataaaggctgggtgcggtggctcacacctgtattcccagcactttgggaggttgaggcaggcggatcacttgaggtcaggagttccagaccagcctagcaaacatggtgaaa
tctcgtctctactaaaaatacaaaaattagctgggcatggtagcagatgcctgtagttccagctacttaggaggctgaggcaggagaatcacttgaatctgggaggcagagattgcagtaaaccg ata
tcgtgccattgcacaccagcctgggtgacacagcgagactccatctcaaaaaaaaaaaaaaaagaagaagaaaaggaaggaaggatgaaagaaagagaataacagaagaagattgacaaatttgactta
ataccttctggacgtcaaaggaaatcattgagagagtgaaaaggcaacttccagaccaagagaagattatttacagtacatattttacaaaagactgtatccagagtatttttaagcaaaaaacc tac
aaagcaaataaacaataaaagacaaaacaaaccaagtttttaaatgagcaaaagccttgaataagcacatcacaaaagaggatatccagatagccaatgtaggaaaagttgctaagcaccattaatat
cagggaaatgcaaattaagccacaaagaaataccaccacctcccatcaggaaggctaaaatttaaaagtcgaacataccaagtggtgacaaggacatggagcaaccagaaccctcacgcactgct ggt
gggatgtgcactggtgcagacacttcagaaaattatttagcagtttctactaaagcaaaaaaagtatgccaaccctatgacatatgttgtccgagtccacttagcatatgcccgagagaaatgagctc
attatccacaaaagatcgcagtcatggcagcttaatcatcatctgtgaaacacgggagcagccctagtccccagagcagcagtggatgcagtgatcacggttcacaaactcagctgtgagagact gaa
cctcgtcacactcagcagcgtggacagagactgcatggaagaagccagatccaaaagtggcaaagtgtccctattctatcattccatttgcatcaagttcaaaatcgggcaaataatctctggccata
gaagtcagagaagtgtgggatggggatacacagagatggacacgggagaaactttggggtgctggaatgtcctttatttacattttactttagagacagggtctcactctgttacccaggctgga gtg
cagtgacacaaccacagctcactgcagactcaacctcctcggctcgagcgatcctcctgcctcagcctcccaaggtgatgggattacaggcgtgagccaccgcacctggcctggaacgttctatgttg
agatttgggcagtgggtttaccggtgcatttgcacccatgattggcataccttaccgtgtgtcacttatacctcagtaaatagtttatcttaaaaagta ggccttggccgggcacggtggctcacgcc
tgtaatcccagcactttgggaggccgaggcaggcagatcatgaggtcaggagatcgagactaacctggctaacacagtgaaaccatcctggctaacacagtgaaaccccatctctactaaaaatacaa
aaaattacccaggcgtggtggcgggcgcctgtagtcccagctactctggaggctgaggca GGAGAATGGCATGAACCCAGGAGGCGGAGCTTGCAGTGAGCCGAGATGGCACCACTGCACTCCAGCCT
GGGCGACAGAGCAAGATTCTGCCTCAAAAAAAAAAAGTAGGCCTTGCCAGGTGCAGTGGAGGATCGCTTAAGGCCAGGAGTTTGAAACCAGCCTG aGCAACGTGACAAAACCCCGTCTCTACAAAAA
ATACAAAAATTAGCCAGCCGTGGTGGGGCATGCCTATAGTCCTGCTACTCAGGGCTGAGGTGGGAGGGTCACTTGAGGCCAGGAGGTTGAGGCTGCAGTGAGCCTTGATCACACCACTGCAGTCC AGC
CTGGGGGACAGAGTGAGATCCTGTGTCAAATATATACATTTAATTTTAAAAAAGTTTTAAGTAGGAGTTACAGTTTAAAGTGGCAGTTAAACACAA GCATCTATTGCCTCTTTGCTTTAAAATGAAGA
CAAAAATTTCAAGAGAATAAGAGGGGAGACGGGAGCTGGAACCCGGAAGACAGATGTCCAGGCAGCGACTGTCCTCGGAGGCCTGAGAGTGCTGAACCTGCAAATGCAAGGAGCCAGTTGGCTTC GGA
GCCCCCGGGAGGCACAGGAAGTGGAGGCCCAGATTCCTCCAAGAAGCTGGGCTGGAAACAGGGTCTCC aGGGGGTCTTTGTGAGCGGCACGGTGTTCTGGCCACAGCTGAGGACA GGGCTGGGGCTCC
GTGCTGAAACCAGGAGGACACAGGTGAGACCCTTGCTGGAGGTGCACAGACGCTTCTCAGCCCACAGCACCTCTGGCCTTCCAGGGCTACCAGGCCACGCCGTGCACACAGGGAGGTGGTTCCTC TGC
AGGGCAGTGTTGGGCAGGAGCAGTTAGGGCTGTGGGGCCTCTCGCCAGCCACACGCATGCTAACACTAACACACTGGGATGGTTAACACTGAGTGTGAACTGGATTGGATGGACA GATGCAAAGTATT
GATCCTGGGTGTGTCTGTGAGGGTGTTGCCAAAGGAGATTCACCTCCGACTGAGTGGACTGGGAGAGGCAGACGCACCCTTAATCTCAGTGGGCACCGTCTCATCAGCTGCCAGCAAGGCTGGAA TAT
AAAGCAGGCAGAAAAACATGAGAAGGCTAGATGGGCCTACCCTCCCAGCCTACATCTTTCTCCCATGCTGGACGTCTCCTGCCCTCGAACATCAGACTCTAAGTTCTTCAGCTTT GAGACTCGGGCTG
GCTCTCTGTGCTCCTCAGCTTGCACATGGCCTATTGTGGGACCCTGTGATCGTGTGAGTTAATACTTAATAA A

Microhomology = undefined

>NHEJ_6

KSD_NEHJ_6

> Scaffold1058 516096 516304_NHEJ

AATCTCTACCTCCCGGGTTCAAGGGATTCTCCTGCCTCAGCCTCCCGAGTAGCTTGGACTACAGGCGCCCGCCACCATGCCCAGCTAATTTTTGTATTTTTAGTAGAGACGGGGTTTCACCATATT GG
CCAGGATGGTCTCAATCTCCTGACCTCGTGATCCGCCTGCCTCAGCCTCCCAACGTGCTGGGATTACAGGTGTGAGCCACCGTGCCCGGCCAATTTTTGTATTTTTTTTTTTTTAGTAGAGACAGG TTT
CACCATGTTGGCCAGGCTGATCTCAAACTCCTGACCTCAAGTGATCCGCCCGCCTTGGCCTCCCAAAGTGCTGGGATTATAGGCAGGAGCCACTGCGCCCAGCTCTGATTGGATAAAATTCCGTGC CA
GGCTGTCTCAGGTGTGGCCTCTCTTGCTTCTAGCCTAAATGTGGTCCCCTTGGCCTGGGTATTGATCCCCGGGCCAGACAGCTGTGGCTGAGATGGCTGTATTTGTTAGCTTCCCAAGGCAAGTG TGA
CACAGAGCACAGCCACTTCTGCAGGGAGGAGCCAGAAGCCACTTGGGCAGCCCTC gTGGGAGGGGGCTGTGGGTGAGGCTGGCCCGGGAACGCGTCCTGGCTTCCCTGCAGCTCATTCTGAAAGGCCA
GTCTGATCCACCTCCCCGAGGCTCCCCTTTCCTGCTTCCCCTTTCCTCCAGGCTGTGTCCTGCGTACTCTTCCTTCATCCTTCGTCTCCTTTCCTTAGGCCCCGTCAGCCTTTCTTGTCCTCAGG CCC
TCTGCAGACAGGCCCAAACAGCCAGAACCCGGGGCTTATCCTCCCACGGGCAGGGGTTCTCCACACGGGTACTCTGCCTGGAACCCAAGGAGTCACAGCTGCAGGAGGCCCTCGAGCCCTGTGGTG TC
CACACTGCTTTTGCTGCAGAATCCCTTCAAATCAAATCTTAATGGCTGGGCACGGTGGCTCACGCCTGTAATCCCAGCACTGTGGGAGGCTGAGGCAGGTGGATCACCTGAGGTCTGGAGTTTGAGAC
CAGCCTGGCCCACATGGTGAAACCCCGTCTCTACTAAAAATACATCAATTGGCCAGGTGAGATGGTGAGCGCCTGTAGTCCCAGCTACTCGGGAGGCTGAGGCAGGAGAATCACTTGAACCCG Gctca
aGTGGCATGATCTTGGCTCActGCAACCTCTGCCTCCCGGGTTCAAGCAATTCTCCTGCCTCAGCCTCCCGAGTTGCTGCGATTACAAGC tCCCGTCACCACAGAAGCCCAGCTCATTTTTGTATTTT
TAGTAGAGATGAGGTTTTGCCATGTTGGTCAGGCTGGTCTTGAACTCCTGACCTCAGGTGATCTGCCTGCCTCGGCCTCCCAAAGTGCTAGGATTACAGGCGTGAGCCCCCATGCCCGGCC ACACTGC
TAGAAACTTTAAAAGCATCTCATTCGGTTACGTGTCCGCCTGCAGTGGGACAGGTAGTATGACTCCACCCCACTGTGAGGAAGTGGAGGAGCAGTGGGGTCAGTCACACGCCTGGCAGGATGGCC AGG
AGCCCCATTGTCTGCCTTAGGAGCACCTGCTGGCCCCACCCCCACTTAGCTTCGGGGCTTTCTGTCCTCATGCTCTTCCCTTCCCTTTTCTGTAGGTGGCTCATTTCCCCTTTAAGAACAACATGA GC
TTTGTGGTCCTTGTACCCACCCACTTTGAATGGAACGTGTCCCAGGTACTGGCCAACCTGAGTTGGGACACCCTGCACCCACCTCTGGTGTGGGAGAGGCCCACCAAGGTCCGGCTGCCTAAGCT GTA
TCTGAAACACCAAATGGACCTGGTGGCCACCCTCAGCCAGCTGGGTAAGGAGGAGGGTGCGGGCGAGCCCCCGAGGTCAGGCTGGGCAGGGCGGGTAAGGAGGAAGCGTCTGGCTGGCAGTGGAGC TG
AGTCAGAGCTGTCCTGTGTCCTGCTCTTTTCCAGGGCTTCGGGAGC aGGGAGAGGGTTGAATATGAGCCCCCAGACCCTCTGTCCTAGAGGAAGTTGGGGTGGCCCGGGGAATGGGGCTCTGCTTTGC
TGCCCAAGACCTCGCATTGCAAGGAGAGTCTGGGTAGAGAGCCTGGGATGGCATGTGAGCCTCGCTGGCTTCCCTGGAAGGGGAAGGAGGAGAAGCTGGGGTGTCACACTGTTAAAGAAATGCTGG GT
GGAGTGGCTCATGCCTGTAATCCCAGCACGTT

1809	6.9	0.0	0.0	a	4	219	(1989)	C	AluSc	SINE/Alu	(93)	216	1	1
1033	6.4	0.7	5.2	a	220	359	(1849)	C	AluSz	SINE/Alu	(177)	135	2	2
14	30.9	2.7	1.4	a	663	735	(1473)	+	(TCCTCCT)n	Simple_repeat	1	74	(0)	3
1643	9.6	0.0	0.0	a	940	1148	(1060)	+	AluSx1	SINE/Alu	1	209	(103)	4
1896	9.6	0.0	1.6	a	1153	1401	(807)	C	AluSx1	SINE/Alu	(67)	245	1	5
250	15.4	0.0	0.0	a	2170	2208	(0)	+	Alu	SINE/Alu	2	40	(93)	6

1809	6.9	0.0	0.0	hg	4	219	(2716)	C	AluSc	SINE/Alu	(93)	216	1	7
1033	6.4	0.7	5.2	hg	220	359	(2576)	C	AluSz	SINE/Alu	(177)	135	2	8
14	30.9	2.7	1.4	hg	663	735	(2200)	+	(TCCTCCT)n	Simple_repeat	1	74	(0)	9
2321	10.4	0.0	0.0	hg	940	1238	(1697)	+	AluSq	SINE/Alu	1	299	(14)	10
2404	8.4	0.0	1.6	hg	1241	1549	(1386)	+	AluY	SINE/Alu	1	304	(7)	11
2248	10.6	0.0	1.3	hg	1826	2128	(807)	C	AluSq2	SINE/Alu	(14)	299	1	12
250	15.4	0.0	0.0	hg	2897	2935	(0)	+	Alu	SINE/Alu	2	40	(93)	13

>hg19 chr17:1653522-1656456

AATCTCTACCTCCCGGGTTCAAGGGATTCTCCTGCCTCAGCCTCCCGAGTAGCTTGGACTACAGGCGCCCGCCACCATGCCCAGCTAATTTTTGTATTTTTAGTAGAGACGGGGTTTCACCATATT GG
CCAGGATGGTCTCAATCTCCTGACCTCGTGATCCGCCTGCCTCAGCCTCCCAACGTGCTGGGATTACAGGTGTGAGCCACCGTGCCCGGCCAATTTTTGTATTTTTTTTTTTTTAGTAGAGACAGG TTT
CACCATGTTGGCCAGGCTGATCTCAAACTCCTGACCTCAAGTGATCCGCCCGCCTTGGCCTCCCAAAGTGCTGGGATTATAGGCAGGAGCCACTGCGCCCAGCTCTGATTGGATAAAATTCCGTGC CA
GGCTGTCTCAGGTGTGGCCTCTCTTGCTTCTAGCCTAAATGTGGTCCCCTTGGCCTGGGTATTGATCCCCGGGCCAGACAGCTGTGGCTGAGATGGCTGTATTTGTTAGCTTCCCAAGGCAAGTG TGA
CACAGAGCACAGCCACTTCTGCAGGGAGGAGCCAGAAGCCACTTGGGCAGCCCTC cTGGGAGGGGGCTGTGGGTGAGGCTGGCCCGGGAACGCGTCCTGGCTTCCCTGCAGCTCATTCTGAAAGGCCA
GTCTGATCCACCTCCCCGAGGCTCCCCTTTCCTGCTTCCCCTTTCCTCCAGGCTGTGTCCT GCGTACTCTTCCTTCATCC TTCGTCTCCTTTCCTTAGGCCCCGTCAGCCTTTCTTGTCCTCAGGCCC

TCTGCAGACAGGCCCAAACAGCCAGAACCCGGGGCTTATCCTCCCACGGGCAGGGGTTCTCCACACGGGTACTCTGCCTGGAACCCAAGGAGTCACAGCTGCAGGAGGCCCTCGAGCCCTGTGGTG TC
CACACTGCTTTTGCTGCAGAATCCCTTCAAATCAAATCTTAAT GGCTGGGCACGGTGGCTCACGCCTGTAATCCCAGCACTGTGGGAGGCTGAGGCAGGTGGATCACCTGAGGTCTGGAGTTTGAGAC
CAGCCTGGCCCACATGGTGAAACCCCGTCTCTACTAAAAATACATCAATTGGCCAGGTGAGATGGTGAGCGCCTGTAGTCCCAGCTACTCGGGAGGCTGAGGCAGGAGAATCACTTGAACCCG GGTGG
CAgaggttgcagtgagccaagatcatgccactgcactccagcctggacaataagagtgaaactctgtcttgaaaaaaaaaaaaaaa gaggctgggtgtggtttacaggctcatgcctgtaatcccagc
actttgggaggccaaggcaggtggatcacgaggtcaggagattgagaccatcctggctaacacggtgaaaccccatctcttctaaaaatacaaaaaattagccgggcgtagtggcgggcacctgta at
cccagctactcgggaggctgaggcagaagaatggcgtgaatccacgaggcggaggttgcggtgagccgagattgcgccactgcactccagcctgggcgacagtgcgagactccaactcaaaagaa aag
aaaaaaaaagaaatcttaagccaaatcacaatatatttacacagctggcaaagtcacaagagggaggtgtgggagggtggggcgggcagcactcggcagtggctcacccacagaacccagctgtgg ta
tgacagccagtgaccctgtctgtggtacatctggagaagctgagacaaagacaggcgattccttacgccgggtcacacggccagctggtagccggtgaagagagaaaaccaccaggtattattga gca
ccgtctgtgacagcggacactgctacaatgttgttttttttcttttttttgagacggattttttactcctgtcccccaggctggagtgcagtggct TGATCTTGGCTCAtgGCAACCTCTGCCTCCCGGG
TTCAAGCAATTCTCCTGCCTCAGCCTCCCGAGTTGCTGCGATTACAAGC gCCCGTCACCACAGAAGCCCAGCTCATTTTTGTATTTTTAGTAGAGATGAGGTTTTGCCATGTTGGTCAGGCTGGTCTT
GAACTCCTGACCTCAGGTGATCTGCCTGCCTCGGCCTCCCAAAGTGCTAGGATTACAGGCGTGAGCCCCCATGCCCGGCC ACACTGCTAGAAACTTTAAAAGCATCTCATTCGGTTACGTGTCCGCCT
GCAGTGGGACAGGTAGTATGACTCCACCCCACTGTGA GGAAGTGGAGGAGCAGTGGGGTCAGTCACACGCCTGGCAGGATGGCCAGGAGCCCCATTGTCTGCCTTAGGAGCACCTGCTGGCCCCACCC
CCACTTAGCTTCGGGGCTTTCTGTCCTCATGCTCTTCCCTTCCCTTTTCTGTAGGTGGCTCATTTCCCCTTTAAGAACAACATGAGCTTTGTGGTCCTTGTACCCACCCACTTTGAATGGAACGT GTC
CCAGGTACTGGCCAACCTGAGTTGGGACACCCTGCACCCACCTCTGGTGT GGGAGAGGCCCACCAAGGTCCGGCTGCCTAAGCTGTATCTGAAACACCAAATGGACCTGGTGGCCACCCTCAGCCAGC
TGGGTAAGGAGGAGGGTGCGGGCGAGCCCCCGAGGTCAGGCTGGGCAGGGCGGGTAAGGAGGAAGCGTCTGGCTGGCAGTGGAGCTGAGTCAGAGCTGTCCTGTGTCCTGCTCTTTTCCAGGGCT TCG
GGAGCgGGGAGAGGGTTGAATATGAGCCCCCAGACCCTCTGTCCTAGAGG AAGTTGGGGTGGCCCGGGGAATGGGGCTCTGCTTTGCTGCCCAAGACCTCGCATTGCAAGGAGAGTCTGGGTAGAGAG
CCTGGGATGGCATGTGAGCCTCGCTGGCTTCCCTGGAAGGGGAAGGAGGAGAAGCTGGGGTGTCACACTGTTAAAGAAATGCTGGGTGGAGTGGCTCATGCCTGTAATCCCAGCACGT T

Microhomology = GT

>NHEJ_7

KSD_NEHJ_7

>Scaffold10752 157469 157754_NHEJ
ATTTTTCTTTTTTTTTAAGCTATATAAATATTTGATCCACCTGAAATTTTGCCAGGTATATAGTGAGAACTATGGCTCCCCATTGTACTAATACCATACCTCACTGTTTGAAATGCCACCTTTATTT AC
TTCATTTCTAAATGCATTTGAATCCTTTTCTGGTTCTCTCTTTCAGCGATTCATCTGTCTGTATATTTGCTCGTACAATTCTATTTTAATTATTGTACCTTTATAATAT TTTTAAAATATCTGGTATG
GCTAGTGTCTCCATATTGCTCTTTCTTTTTCACACATTTCCCAGCTGTTAATTTTAGAATTTTTTCTAGTTCTGAAGCTATTAATTTATTATTTTATTGAGACTTTTTTTTTTTTTTTCTGATAT GGA
ATCTCGCTCTGTTGCCCAGGCTGGAGTGTAGTAGCGTGATCTTGGCTCACTGCAACCTCCGCCTCCTAGGTTCAAGCAATTTTGAACCTGCTTCAGCCTCCCAAGTAGC TGAGATTACAGGCGCCTGC
CACCATGCCTCCCTAATTTTTGTATTTTTAGTGGAGATGGGGTTTCAAACATGTTGGCCAGGCTGATCTTGAACTTCTGACCTTAAGTGATCCCCCCGCCTCGGCCTCCCAAAGTGCTGGCTGGG ATT
ACAGGCATGAGCCACTGTGCTTGGCCGGGACTACATTCTTTTTATAAATTAACCCAAGGATAATTGATATCCATATGTTAACTCTTGCTGTCCAGGAACAGGGTGCACT GTACATTGACTTAAGTCTC
TGTGTGTGTTCTTCAAATAGTATTTACATTTTTTTTTTTTTTAGAGAGGCAATACATGCTTATGGTTATTCTTAGGTATTTTATCTTTTAGTTTGTTTATTATTCTCATTCATTTCTTAATTTAA AGG
AATAATGTGTTTTAGCTACACGCACATGGAAAAAGTTATCATT GGCCAGGCGCGGTGGCTCATGCCTATAATCCCAGCACTTTGGGAGGCCGAGGCAGGCGGATCATGA GGTTATGAGATCGAGACCA
TTCTGGCTAACACGGTAAAACCCCATCTCTACTAAAAATACAAAAAATTAGCTGGGCATGGTGGCATGTGCCTATAGTCCCAGCTACTCAGGAGGCTGAGGCAGGAGAATGGCTTGAACCTGGGA GGC
AGAGGTTGCAGTGAGCCGAGATTGTCCCACTGCACTCCAGCCTGGGTGACAGAG cGAGACTCCATCTCAAAAAAATCTCGCTCTGTGGCCCAGGTGGGAGTGCAGTGGC GCAATCTCGGCTCACTGCA
AGCTCCGCCTCCCAGGTTCACGCCATTCTCCTGCCTCAGCCTCCCGAGTAGCTGGGACTACAGGCGCCCGCCACCACGCCCGGCTAATTTTTTTGTATTTTTAGTAGAGACGGGGTTTCACTGT GTT
AGCCAGGATGGTCTCGATCTCCTGACCTCGTGATCCGCCCGCCTCGGCCTCCCAAAGTGCTGGGATTACAAGCGTGAGCCACCGCGCCCGGCC GACAGACTTTCATTTTTAATTGTTAGTCTGTTTTC
TGCTACTGACCGCCTTTTTGTCACTTTACATATCTTCTCTGAGCCTTAGTTTCCTCATCTGTAAATTGAAGAATCTAAAACAGATATCTAAAGCCTGTCTTCCCGCTCTGCCCACATTGATTCTG ATG
TGATTAACTTCCTAGAGGCCTTATATCTGCTCAGGGAAGCCCTCTTGATGCCAGGACCTATGTGACTCCCAGTGGTTGAGGACCATCTGAGCTCTGGATTTCTAGTCCC TTGTGGCCAGGCTGGTGGG
CCACCATCAAGGTCATGTCAGGAAGCTCACTGTCCACCTTCTAGGGCTAATTTGTTTGGTTGGGTAGTTAAAGGTTTAGGGTCCTGGTTTGGAATTGCTTGAAGTGCCCTAGTATCTCCTCGGGA GTT
CGCCTTTCCTTTTCATTCCCTCCTCTGGACCCTTAGCTCTCAGACCTACTATCCTCAGGCTGTCCACATGTCTCCCAGTGCAACCCTCATTGTCAACTCCCTCCTTTCC CTCTCTGCCTTCTGGCCCT
CTCCCCCTGTCCCCTCAATACTCAGGGAAGGAAGTGGAGCCTTGGGTTGAAAGAACTGGTTTAGCAACGAGTCAGGTTATGTTCTTATCCACCTTTGCCTTTCAATAGCTATGTGATCTTTGGTA AGG
TTTAGAGAGAATAAATGCAAAGTACTAACCCATGATTACTCCAGCAGGGGTCTTAGTGAATTGGTTTGCTCATTAGTGGTTAATAGTATTAATAGTAGCTTATTACAT A

255	31.2	12.1	3.9	135629	98	359	(1926)	C	L1M5	LINE/L1	(1257)	4889	4595	1
2165	11.4	0.0	2.7	135629	360	666	(1619)	C	AluSx1	SINE/Alu	(13)	299	1	2
255	31.2	12.1	3.9	135629	667	857	(1428)	C	L1M5	LINE/L1	(1552)	4594	4400	1
2357	8.4	0.0	0.0	135629	940	1225	(1060)	+	AluSc8	SINE/Alu	1	286	(25)	3
2570	0.7	0.0	0.7	135629	1226	1501	(784)	C	AluYi6_4d	SINE/Alu	(37)	274	1	4
318	20.1	6.7	9.8	135629	1556	1660	(625)	+	MIR3	SINE/MIR	100	201	(7)	5

255	31.2	12.1	3.9	hg	98	359	(2062)	C	L1M5	LINE/L1	(1257)	4889	4595	6
2165	11.4	0.0	2.7	hg	360	666	(1755)	C	AluSx1	SINE/Alu	(13)	299	1	7
255	31.2	12.1	3.9	hg	667	857	(1564)	C	L1M5	LINE/L1	(1552)	4594	4400	6
2477	8.3	0.0	0.0	hg	940	1240	(1181)	+	AluSc8	SINE/Alu	1	301	(10)	8
2653	0.3	0.0	0.7	hg	1330	1637	(784)	C	AluYi6_4d	SINE/Alu	(0)	311	1	9
318	20.1	6.7	9.8	hg	1692	1796	(625)	+	MIR3	SINE/MIR	100	201	(7)	10

>hg19 chr20:34512824-34515244
ATTTTTCTTTTTTTTTAAGCTATATAAATATTTGATCCACCTGAAATTTTGCCAGGTATATAGTGAGAACTATGGCTCCCCATTGTACTAATACCATACCTCACTGTTTGAAATGCCACCTTTATTT AC
TTCATTTCTAAATGCATTTGAATCCTTTTCTGGTTCTCTCTTTCAGCGATTCATCTGTCTGTATATTTGCTCGTACAATTCTATTTTAATTATTGTACCTTTATAATATTTTTAAAATATCTGGT ATG
GCTAGTGTCTCCATATTGCTCTTTCTTTTTCACACATTTCCCAGCTGTTAATTTTAGAATTTTTTCTAGTTCTGAAGCTATTAATTTATTATTTTATTGAGACTTTTTTTTTTTTTTTCTGATATG GA
ATCTCGCTCTGTTGCCCAGGCTGGAGTGTAGTAGCGTGATCTTGGCTCACTGCAACCTCCGCCTCCTAGGTTCAAGCAATTTTGAACCTGCTTCAGCCTCCCAAGTAGCTGAGATTACAGGCGCC TGC
CACCATGCCTCCCTAATTTTTGTATTTTTAGTGGAGATGGGGTTTCAAACATGTTGGCCAGGCTGATCTTGAACTTCTGACCTTAAGTGATCCCCCCGCCTCGGCCTCCCAAAGTGCTGGCTGGGA TT
ACAGGCATGAGCCACTGTGCTTGGCCGGGACTACATTCTTTTTATAAATTAACCCAAGGATAATTGATATCCATATGTTAACTCTTGCTGTCCAGGAACAGGGTGCACTGTACATTGACTTAAGTCTC
TGTGTGTGTTCTTCAAATAGTATTTACATTTTTTTTTTTTTTAGAGAGGCAATACATGCTTATGGTTATTCTTAGGTATTTTATCTTTTAGTTTGTTTATTATTCTCATTCATTTCTTAATTTAAAGG

AATAATGTGTTTTAGCTACACGCACATGGAAAAAGTTATCATT GGCCAGGCGCGGTGGCTCATGCCTATAATCCCAGCACTTTGGGAGGCCGAGGCAGGCGGATCATGAGGTTATGAGATCGAGACCA
TTCTGGCTAACACGGTAAAACCCCATCTCTACTAAAAATACAAAAAATTAGCTGGGCATGGTGGCATGTGCCTATAGTCCCAGCTACTCAGGAGGCTGAGGCAGGAGAATGGCTTGAACCTGGGAG GC
AGAGGTTGCAGTGAGCCGAGATTGTCCCACTGCACTCCAGCCTGGGTGACAGAG tGAGACTCCATCTCAAAAAAAaaaaaaaaaaaaagttaccattttgaaactgaactgtatttgattcattttg
gtgaatcctggttttcaaaacatgaagtcattggtgagacagactttcatttttttttttttttttttttttgagacggagTCTCGCTCTGTGGCCCAGGTGGGAGTGCAGTGGCGCAATCTCGGC
TCACTGCAAGCTCCGCCTCCCAGGTTCACGCCATTCTCCTGCCTCAGCCTCCCGAGTAGCTGGGACTACAGGCGCCCGCCACCACGCC CGGCTAATTTTTTTGTATTTTTAGTAGAGACGGGGTTTC
ACTGTGTTAGCCAGGATGGTCTCGATCTCCTGACCTCGTGATCCGCCCGCCTCGGCCTCCCAAAGTGCTGGGATTACAAGCGTGAGCCACCGCGCCCGGCC GACAGACTTTCATTTTTAATTGTTAGT
CTGTTTTCTGCTACTGACCGCCTTTTTGTCACTTTACATATCTTCTCTGAGCCTTAGTTTCCTCATCTGTAAATTGAAGAATCTAAAA CAGATATCTAAAGCCTGTCTTCCCGCTCTGCCCACATTGA
TTCTGATGTGATTAACTTCCTAGAGGCCTTATATCTGCTCAGGGAAGCCCTCTTGATGCCA GGACCTATGTGACTCCCAGTGGTTGAGGACCATCTGAGCTCTGGATTTCTAGTCCCTTGTGGCCAGG
CTGGTGGGCCACCATCAAGGTCATGTCAGGAAGCTCACTGTCCACCTTCTAGGGCTAATTTGTTTGGTTGGGTAGTTAAAGGTTTAGG GTCCTGGTTTGGAATTGCTTGAAGTGCCCTAGTATCTCCT
CGGGAGTTCGCCTTTCCTTTTCATTCCCTCCTCTGGACCCTTAGCTCTCAGACCTACTATCCTCAGGCTGTCCACATGTCTCCCAGTGCAACCCTCATTGTCAACTCCCTCCTTTCCCTCTCTGC CTT
CTGGCCCTCTCCCCCTGTCCCCTCAATACTCAGGGAAGGAAGTGGAGCCTTGGGTTGAAAGAACTGGTTTAGCAACGAGTCAGGTTAT GTTCTTATCCACCTTTGCCTTTCAATAGCTATGTGATCTT
TGGTAAGGTTTAGAGAGAATAAATGCAAAGTACTAACCCATGATTACTCCAGCAGGGGTCTTAGTGAATTGGTTTGCTCATTAGTGGTTAATAGTATTAATAGTAGCTTATTACAT A

Microhomology = undefined

>NHEJ_8

KSD_NEHJ_8

>Scaffold9444 5609992 5610293_NHEJ
ATTTTTCCCTGTATCCCTAGGGTGTGGCACATAGTAGGTGCTTAGTAAACTTATTTTGCATTCAATAATGCCAAGTAGAATAATTCATAGATTATGGCAAAATAAAAGAATTTGGCAAAATTGCAA GA
TTGAAGATTGCTTTCAGAAAAAATGTTCCAACCCCTGTTCAAAAGTACATAGCTTGCTGTGTTGTTGAAGTGCCAGCACGAGGAGGACCATGCCAACACATCCATTTATTGCCCATTCTGGGTGT GTT
GAGCGTTTTAAGTTTGCTATGCTTGTAAAAATCAAGGAATGGCAATGGTATCAACTTTCATGAACACAGTCGTTCAGCTGAACCAAGTATCAGTGATAACTTCTGTTTTGAATTTTAGAAATATAT CT
CTGAGGTTAGAATGTTGATTCATGCATATTCCAGCTGGCTTTCCCTATGAGTATAAATAGGGAAAACAAGGCTATTGGCCCAAATGTAAGTTGTATTCCAAGGTTTGGTCCAAAGACCAGAGAAT CCC
TAAGTAGGAAGACAGTTTTGTTTTCCCCTCTTTTACTGGTTAACTTTGAGCAGGTCACTTATATTTGGGGGCACAGAAGATGAAGATGACCCTGAGCTTGTCATGTTCACTGTTAATTGTTCTACT AA
CACCCTAGACTTCATCATCTTGTTATAGCACTTATCACAGTTGCTTTTAAATCATCATGCAGTTATATGTTTATTGTCTGCCTTTTCCACTAGAATAAAGCCTCTGCTGACATAAGGTCTATGTT TGT
CTTGTTAATTGCTCTCCCTACCACCTAGCACCAATGCCTGGCATTTGGTTGGCTCTCAGTATTTGTGAAATGAATGAATGAGATGATCTTGTCCAAAGAAGACAGGGAAAGATTCTTAGAGGTTAA AA
AAGAAAAATTCGGCCGGGTGTGGTGGCTCACACCTGTAATCCCAGCACTTTGGGAGGCCGAGGCGGGCAGATTACCCGATGTCAGGAGTTCAAGACCAGCCTGACCAACATGGTGAAACCCCATCTCT
ACTAAAAATACAAAAAGTAGCAAGGCGTGTGGAGGGCACCTGTAATCCCAGCTACTTGGGAGGCTGAGACAGGAGAATCGCTTGAACCCAGGAGGCGGAG GTTGAAGACCAGCCTGGTCAACATAGTG
AAACCCTGTCTCTACAAAAAAATACACACACACAAAATTAGCCAGGCTTGGTAGC aGGTGCCTGTAATCCCAGCTACTCGGGAGGCTGAGGTGGGAGAATCACTTGAACCTGGGAGGTGGAGGTTGCA
GTGAGCCGAGATCGTGCCACTGCACTCCAGCCTGGGCAACAGAGCAAGACTCAGTCTAAAAAAAAAAAAAAAGAAAAGAAAAA TCCACATACTGTGGTTGTGACTAGTTTTGCAAACCATTCCTATTGA
TAACATTGCAATGCCTCTAGATAcTACCAACTAGAAGCCACGCTAAAATGAAACTGTATCAATGAGGAGAGTCTAGGTTATGCTACAGTAACAAGCCTTAAAACCTCAGTAGCTTACAACAATACAGG
TTATTTCTCACTGGATACATGAACATGGTGTTGCCTCCACTACGTGTCCTGTTTACTCTTCAGCCAAGGCTGATAGCAGCCTCTGTCTGGAACATTAAACTTTATCAGGGCAGAGGAAGAAGCGA GTG
ACAAAGCACGAACTGGTTTTTAAAGCTTCTTCCTCAAATGCTAGGCACTACTTTTG TTCATGTTTTATTGGTTGAAGAATGTTTGCCTGTTGTCAATAAGATGCAGAAGTATGAAGCATACATTGGCT
TTTAAAGCTTTTGCCTAGAAGTTGGTGGCATATGGCACTTCTGCTCACATTTAATTGGCCAAAGCAAGCCGTGGCCAAGCACTGATCAAGTGGCAGAGAAATATAGTCCATCTCCAGGGAGAAAC AGC
AAATATGTTTGAATAGTAACATGATTTTTCAAATACATCAAGAGAAAAAAATAAGC ATGGAGAATGTTGTACAGTAGATTTTAAAGGATATAACATGGGTGGTTTCCAGAATTCGTTAACTACCCTAA
AGACCCTCAGCATCTGGTCATTTGTAATTTGGGGAAGCTTTGCCTTGCCATCCCAAGATCTGCGTTGTTTATATCTCTGAGGCAGAAATAGCTTATAGCGATAAATCGTGAGTGAGAGAATTCTC CAT
TTAGAGATTTTCACAGGTCCCCAGAGCAGACCTTTTCCAATGTCAAGTGTCCACTG GAATTTCAAGTGCAGACTAAGGAGCATGTCAGCATGAAATCTGAAAATTTAAAAATAGAATTATTAAG A

230	24.5	0.0	0.0	a	1	49	(2252)	+	L2c	LINE/L2	3319	3367	(20)	1
428	26.0	6.8	2.8	a	642	847	(1454)	+	L2a	LINE/L2	3213	3426	(0)	2
1174	8.9	0.0	0.0	a	908	1053	(1248)	+	AluSx	SINE/Alu	1	146	(166)	3
1916	11.3	0.3	5.6	a	1061	1362	(939)	+	AluSx1	SINE/Alu	20	306	(6)	4
843	29.0	7.2	0.0	a	1463	1741	(560)	C	MLT1I	LTR/ERVL-MaLR	(0)	411	113	5
529	22.4	4.1	4.0	a	1777	1923	(378)	C	MLT1I	LTR/ERVL-MaLR	(329)	220	64	6
258	28.8	0.0	0.0	a	2006	2078	(223)	+	Tigger12A	DNA/TcMar-Tigger	22	94	(725)	7

230	24.5	0.0	0.0	hg	1	49	(2378)	+	L2c	LINE/L2	3319	3367	(20)	8
428	26.0	6.8	2.8	hg	642	847	(1580)	+	L2a	LINE/L2	3213	3426	(0)	9
2348	9.6	0.3	0.0	hg	908	1208	(1219)	+	AluSq2	SINE/Alu	1	302	(11)	10
1924	8.8	0.0	3.1	hg	1227	1488	(939)	+	AluSx1	SINE/Alu	53	306	(6)	11
843	29.0	7.2	0.0	hg	1589	1867	(560)	C	MLT1I	LTR/ERVL-MaLR	(0)	411	113	12
529	22.4	4.1	4.0	hg	1903	2049	(378)	C	MLT1I	LTR/ERVL-MaLR	(329)	220	64	13
258	28.8	0.0	0.0	hg	2132	2204	(223)	+	Tigger12A	DNA/TcMar-Tigger	22	94	(725)	14

>hg19 chr9:133451065-133453491
ATTTTTCCCTGTATCCCTAGGGTGTGGCACATAGTAGGTGCTTAGTAAACTTATTTTGCATTCAATAATGCCAAGTAGAATAATTCATAGATTATGGCAAAATAAAAGAATTTGGCAAAATTGCAA GA
TTGAAGATTGCTTTCAGAAAAAATGTTCCAACCCCTGTTCAAAAGTACATAGCTTGCTGTGTTGTTGAAGTGCCAGCACGAGGAGGACCATGCCAACACATCCATTTATTGCCCATTCTGGGTGTGTT
GAGCGTTTTAAGTTTGCTATGCTTGTAAAAATCAAGGAATGGCAATGGTATCAACTTTCATGAACACAGTCGTTCAGCTGAACCAAGTATCAGTGATAACTTCTGTTTTGAATTTTAGAAATATAT CT
CTGAGGTTAGAATGTTGATTCATGCATATTCCAGCTGGCTTTCCCTATGAGTATAAATAGGGAAAACAAGGCTATTGGCCCAAATGTAAGTTGTATTCCAAGGTTTGGTCCAAAGACCAGAGAAT CCC
TAAGTAGGAAGACAGTTTTGTTTTCCCCTCTTTTACTGGTTAACTTTGAGCAGGTCACTTATATTTGGGGGCACAGAAGATGAAGATGACCCTGAGCTTGTCATGTTCACTGTTAATTGTTCTACT AA

CACCCTAGACTTCATCATCTTGTTATAGCACTTATCACAGTTGCTTTTAAATCATCATGCAGTTATATGTTTATTGTCTGCCTTTTCCACTAGAATAAAGCCTCTGCTGACATAAGGTCTATGTT TGT
CTTGTTAATTGCTCTCCCTACCACCTAGCACCAATGCCTGGCATTTGGTTGGCTCTCAGTATTTGTGAAATGAATGAATGAGATGATCTTGTCCAAAGAAGACAGGGAAAGATTCTTAGAGGTTAA AA
AAGAAAAATTCGGCCGGGTGTGGTGGCTCACACCTGTAATCCCAGCACTTTGGGAGGCCGAGGCGGGCAGATTACCCGATGTCAGGAGTTCAAGACCAGCCTGACCAACATGGTGAAACCCCATCTCT
ACTAAAAATACAAAAAGTAGCAAGGCGTGTGGAGGGCACCTGTAATCCCAGCTACTTGGGAGGCTGAGACAGGAGAATCGCTTGAACCCAGGAGGCGGAG Gttgcagtgagctgagaatgtgccattg
cactccagcctgggcaagaagagcaaaaccttgtctcgaaaaaaaaaaaaaaaaaagccgggtgcagtggctcacaggcggatcacctgaggtcaggagTTGAAGACCAGCCTGGTCAACATAGTGAA
ACCCTGTCTCTACAAAAAAATACACACACACAAAATTAGCCAGGCTTGGTAGC gGGTGCCTGTAATCCCAGCTACTCGGGAGGCTGAGGTGGGAGAATCACTTGAACCTGGGAGGTGGAGGTTGCAGT
GAGCCGAGATCGTGCCACTGCACTCCAGCCTGGGCAACAGAGCAAGACTCAGTCTAAAAAAAAAAAAAAAGAAAAGAAAAA TCCACATACTGTGGTTGTGACTAGTTTTGCAAACCATTCCTATTGATA
ACATTGCAATGCCTCTAGATAtTACCAACTAGAAGCCACGCTAAAATGAAACTGTATCAATGAGGAGAGTCTAGGTTATGCTACAGTAACAAGCCTTAAAACCTCAGTAGCTTACAACAATACAGGTT
ATTTCTCACTGGATACATGAACATGGTGTTGCCTCCACTACGTGTCCTGTTTACTCTTCAGCCAAGGCTGATAGCAGCCT CTGTCTGGAACATTAAACTTTATCAGGGCAGAGGAAGAAGCGAGTGAC
AAAGCACGAACTGGTTTTTAAAGCTTCTTCCTCAAATGCTAGGCACTACTTTTGTTCATGTTTTATTGGTTGAAGAATGTTTGCCTGTTGTCAATAAGATGCAGAAGTATGAAGCATACATTGGC TTT
TAAAGCTTTTGCCTAGAAGTTGGTGGCATATGGCACTTCTGCTCACATTTAATTGGCCAAAGCAAGCCGTGGCCAAGCAC TGATCAAGTGGCAGAGAAATATAGTCCATCTCCAGGGAGAAACAGCAA
ATATGTTTGAATAGTAACATGATTTTTCAAATACATCAAGAGAAAAAAATAAGCATGGAGAATGTTGTACAGTAGATTTTAAAGGATATAACATGGGTGGTTTCCAGAATTCGTTAACTACCCTA AAG
ACCCTCAGCATCTGGTCATTTGTAATTTGGGGAAGCTTTGCCTTGCCATCCCAAGATCTGCGTTGTTTATATCTCTGAGG CAGAAATAGCTTATAGCGATAAATCGTGAGTGAGAGAATTCTCCATTT
AGAGATTTTCACAGGTCCCCAGAGCAGACCTTTTCCAATGTCAAGTGTCCACTGGAATTTCAAGTGCAGACTAAGGAGCATGTCAGCATGAAATCTGAAAATTTAAAAATAGAATTATTAAG A

Microhomology: Gttg

>NHEJ_9

KSD_NEHJ_9

>Scaffold14085 161832 162050_NHEJ
CAGGAAAAGGTGTGAAAAAGCTCTTGAGGTGATGGGAAGGGGTGTGCTGTTTTGAAATGGGAAGATAAAAACAGAGCCATGGGGGTTCTGCACCATCGCCTTGGAACAAATGGTGTCTGCCTCCAC AT
CTGCAATGACTCAGATCTCTGCTCTGGGCATACTTGCAGTCAAATCAGGGCTGCCCAGACATCCTGTGTTTGTGAAATGCTGAGAAAGACTAACTGGCTTCCAGCAGTTGCCTACTCATCCACCC TCT
TCCAAACACCCAGGCTGGGGAGTAACATTTTGGGGATGAGTCCTTGTAATGCTGCTTCTCTCTGGAGAGCAATGTGTGAAAATTGACCCAAGGAGTTGTCTCATGGGATGAGGCATAATTGGCTGT CT
TTTTTCGGAACCTCCTTGAGGCTCATCTACCCCCGTGCCCTGAGCTCACTGGGGCCTGCCTGGCCAAACAGAATATACCCATTAAGGGAATAAATTTCCAGGCCACAATAACAAGCATCACCACT TTG
AAAAGAAAAAGCCATGAACTGAAATTCTTCAgAATAAGAATTTTTAATTAGCCCAATAAACACATTAAAAGATATAAGGAAGATACTAGAACTATGAAACAAATGAACACAAAAACACCAAAAAGAAC
CGACAAGAAATACTAAGTTGAAAACATAACAGTTGAGGCcGGGCACGGTGGCTCAAGCCTGTAATCCCAGCACTTTGGGAGGCCAAGGAGGGTGAATCAACTGAGGTCAGGAGTTCGAGACCAGCCTG
ACCAATATGGTGAAACCCCGTCTCTACTAAAAATACAAAAACTAGCCAGGCGTGGTGGTGCATGCCTGTAATCCCAGCTACTCAGGAGACTGAGGCAGGAGAATTGCTTGAACCTGGGAGGCAGAG GT
TGCAGTGAGCCGAGGTCGTGCCACAGCACTACAGCCTGGGTGACAGAGCAAGACTCCATCTCAAAAAAAAAAAAAAAGAAAGAAAAGAAAATATAACAGTTAAT GCCAGGTGCAGCAGCTAACTCCTGT
AATCACAGCACTTTGGGAGGCCGAGGCGGGCAGATCACCTGAGGTCAGAAGTTTGAGACCAGCCTGACCAACATGGAGAAACCCCGTCTCTACTAAAAATACAAAATTAACCAGGTGTGGTGGCAT TT
GCCTGTAATCCCAGCTACTCAGGAGGCTGAGGCAGGAGAATTGCTTGAATCCAGGAGGTGGAAGT TAGCTCTTTGGTGTAGGGAAAAGAAAGAGAGATCAGACTGTCACTGTGTCTATGTAGAAAGGA
AAGGCATAAGAAATTCCATTTTGACCTATACCTTGAACTATTGCTTTGCTGAGATGTTGTTAATTTGTAACTTTGCCCTAGCCACTTCGCCCCAGCCACTTCGCCCCAGCCACTTTGCCCCAACTT TG
AGCTCACAAAAACATGTGTTGTATGGAATCAAGGTTTAAGGGTTCTAGGGTTGTGCAGGACGTGCCTTGTTAACAAAATGTTTACAAGCAGTATGCTTGGTAAAAGTCATTGCCATTCTCTAGTC TCA
ATAAACCAGGGGCACAATGCACTGTGAAAAGCTGCAGGGACCTCTGCGCTGAAAAGCCGGTATTGTCCAAGGTTTCTCCCCATGTGATAGTCTGAAATATGGCCTCGTGAGATGAGAAAGACCTGA CT
GTCCCCCAGCCTGACACCCGTAAAGGGTCTATGCTGAGGTGGATTAGTAAAAGAGGAAAGCCTCTTGCAGTTGAGATAGAGGAAGGCCACTGTCTCCTGCCTGAGATAGGAGAAAAACCACCCTA TGG
CAGGAGGTGAGACATGTTGGCAGCTATGCTGCCTTGTTATTCTTTACTCCACTGAGATGTTTGGGCaGAGAGAAACATAAATCTGGCCTACGTGCACATCCAGGCATAGTACCTCCCCTTGAACTTAA
TTATGACATAGATTCTTTTGCTCACATTTTTTTTTTTTTTTTTGCTGACCTTCTCCTTATTATCACCCTGCTCTCCTACTGCATTCCTCTTGCTAAGATAATGAAAATAATAATCAATACAAACTGA
GGGAACTCAGAGACCAGTGCCAGTGCATGTCCTTGGTATGCTGAGTGCCGGTCTCCTGGACCCACTGTTGTTTCTCTATACTTTGTCTCTGTGTCTTATTTCTTTTCTCAGTCTCTCATCCCACCT GG
CAAGATATCCCACAGGTGTGGAGGGGCAGGTCACCCCTTTAC

483	31.8	12.1	2.6	a	94	439	(1779)	+	L1MEh	LINE/L1	278	655	(1580)	1
361	21.8	2.2	1.5	a	542	676	(1542)	+	L1MEh	LINE/L1	844	979	(1256)	1
2467	9.3	0.0	0.0	a	677	986	(1232)	+	AluSx	SINE/Alu	1	310	(2)	2
1656	11.4	0.5	0.0	a	1000	1218	(1000)	+	AluSp	SINE/Alu	2	221	(92)	3
7184	8.1	6.8	2.2	a	1229	2217	(1)	+	LTR5A	LTR/ERVK	1	1033	(0)	4

483	31.8	12.1	2.6	hg19	94	439	(1902)	+	L1MEh	LINE/L1	278	655	(1580)	5
359	20.5	2.3	1.5	hg19	548	676	(1665)	+	L1MEh	LINE/L1	850	979	(1256)	5
2452	9.7	0.0	0.0	hg19	677	986	(1355)	+	AluSx	SINE/Alu	1	310	(2)	6
2251	11.0	0.7	0.0	hg19	1000	1299	(1042)	+	AluSp	SINE/Alu	2	303	(10)	7
231	14.3	0.0	0.0	hg19	1308	1349	(992)	+	Alu	SINE/Alu	1	42	(91)	8
7188	8.0	6.8	2.4	hg19	1350	2340	(1)	+	LTR5A	LTR/ERVK	1	1033	(0)	9

>hg19_chr17:21754887-21757227
CAGGAAAAGGTGTGAAAAAGCTCTTGAGGTGATGGGAAGGGGTGTGCTGTTTTGAAATGGGAAGATAAAAACAGAGCCATGGGGGTTCTGCACCATCGCCTTGGAACAAATGGTGTCTGCCTCCAC AT
CTGCAATGACTCAGATCTCTGCTCTGGGCATACTTGCAGTCAAATCAGGGCTGCCCAGACATCCTGTGTTTGTGAAATGCTGAGAAAGACTAACTGGCTTCCAGCAGTTGCCTACTCATCCACCC TCT
TCCAAACACCCAGGCTGGGGAGTAACATTTTGGGGATGAGTCCTTGTAATGCTGCTTCTCTCTGGAGAGCAATGTGTGAAAATTGACCCAAGGAGTTGTCTCATGGGATGAGGCATAATTGGCTGT CT
TTTTTCGGAACCTCCTTGAGGCTCATCTACCCCCGTGCCCTGAGCTCACTGGGGCCTGCCTGGCCAAACAGAATATACCCATTAAGGGAATAAATTTCCAGGCCACAATAACAAGCATCACCACT TTG
AAAAGAAAAAGCCATGAACTGAAATTCTTCAaAATAAGAATTTTTAATTAGCCCAATAAACACATTAAAAGATATAAGGAAGATACTAGAACTATGAAACAAATGAACACAAAAACACCAAAAAGAAC
CGACAAGAAATACTAAGTTGAAAACATAACAGTTGAGGCtGGGCACGGTGGCTCAAGCCTGTAATCCCAGCACTTTGGGAGGCCAAGGAGGGTGAATCAACTGAGGTCAGGAGTTCGAGACCAGCCTG
ACCAATATGGTGAAACCCCGTCTCTACTAAAAATACAAAAACTAGCCAGGCGTGGTGGTGCATGCCTGTAATCCCAGCTACTCAGGAGACTGAGGCAGGAGAATTGCTTGAACCTGGGAGGCAGAG GT
TGCAGTGAGCCGAGGTCGTGCCACAGCACTACAGCCTGGGTGACAGAGCAAGACTCCATCTCAAAAAAAAAAAAAAAGAAAGAAAAGAAAATATAACAGTTAAT GCCAGGTGCAGCAGCTAACTCCTGT
AATCACAGCACTTTGGGAGGCCGAGGCGGGCAGATCACCTGAGGTCAGAAGTTTGAGACCAGCCTGACCAACATGGAGAAACCCCGTCTCTACTAAAAATACAAAATTAACCAGGTGTGGTGGCAT TT

GCCTGTAATCCCAGCTACTCAGGAGGCTGAGGCAGGAGAATTGCTTGAATCCAGGAGGTGGAAGT Tgcggtgagccaagatcacagcattgcactccagcctgggcaatgagagcaaactctggctca
aaaaaaaaaaaaaaaaaaaggtgggggggcggggctcagtggctcaggcctgtaaccccAGCTCTTTGGTGTAGGGAAAAGAAAGAGAGATCAGACTGTCACTGTGTCTATGTAGAAAGGAAAGGCAT
AAGAAATTCCATTTTGACCTATACCTTGAACTATTGCTTTGCTGAGATGTTGTTAATTTGTAACTTTGCCCTAGCCACTTCGCCCCAGCCACTTCGCCCCAGCCACTTTGCCCCAACTTTGAGCT CAC
AAAAACATGTGTTGTATGGAATCAAGGTTTAAGGGTTCTAGGGTTGTGCAGGACGTGCCTTGTTAACAAAATGTTTACAAGCAGTATGCTTGGTAAAAGTCATTGCCATTCTCTAGTCTCAATAAA CC
AGGGGCACAATGCACTGTGAAAAGCTGCAGGGACCTCTGCGCTGAAAAGCCGGTATTGTCCAAGGTTTCTCCCCATGTGATAGTCTGAAATATGGCCTCGTGAGATGAGAAAGACCTGACTGTCC CCC
AGCCTGACACCCGTAAAGGGTCTATGCTGAGGTGGATTAGTAAAAGAGGAAAGCCTCTTGCAGTTGAGATAGAGGAAGG CCACTGTCTCCTGCCTGAGATAGGAGAAAAACCACCCTATGGCAGGAGG
TGAGACATGTTGGCAGCTATGCTGCCTTGTTATTCTTTACTCCACTGAGATGTTTGGGC gGAGAGAAACATAAATCTGGCCTACGTGCACATCCAGGCATAGTACCTCCCCTTGAACTTAATTATGAC
ATAGATTCTTTTGCTCACATTTTTTTTTTTTTTTTTTttGCTGACCTTCTCCTTATTATCACCCTGCTCTCCTACTGCATTCCTCTTGCTAAGATAATGAAAATAATAATCAATACAAACTGAGGGAA
CTCAGAGACCAGTGCCAGTGCATGTCCTTGGTATGCTGAGTGCCGGTCTCCTGGACCCACTGTTGTTTCTCTATACTTTGTCTCTGTGTCTTATTTCTTTTCTCAGTCTCTCATCCCACCTGGCA AGA
TATCCCACAGGTGTGGAGGGGCAGGTCACCCCTTTAC
Microhomlogy= undefined

>NHEJ_10

KSD_NEHJ_10

>Scaffold752 343330 343639_NHEJ
GAGGCTGTCGGGGGAGGATTGCTTACACCCAGGAGGTAGAGGCTGTTGTGAGCCATGATCATGCCACTGCACTCCAGCCTAGGCAACAGAGCAACACCCTGTCT CAAAAAAAAAAAAAAAAAAAGCCTA
CAAGTAAAGCAATAAATGATGAATAAATCAATTTTTTAAATGGGTGAAAGAT TTGAATAGACATTTCTCCAAAGAACATACGCAAATGTCAGCCGGGCACTGTGGCTCACACCTGTAATACCAGCACT
TTCGGAGGCCGAGGAGGGCAGATCACGAGGTCAGGAGTTCGAGACCAGCCTGACCAACATGGTGAAACCCTGTCTCTACTAAAAATACAAAAATTAGCCGGGCATGGCGCGTGCCTGTAATCCCA GCT
ACTCAGGAGGCTGAGGCAGGAGAATCACTTGAACCCAGGAAGGGGAGATTGC AGTGAGCTGAGATCGCACCACTGCAGTTCAGCCTGGGTGGCAGAGCGAGACTCTGTCTCAAAATAAAATAAAATAA
AATAAAAAAGAACATATACAAATGTCCAATAAGCACATGAAAAAATGATCATCAGGGAAATGCAAATCATAACAACAATGAGAAACCAGTTAACACCCATTCAGTTGCTATAATCAAAAAGACAA GGG
ATCGGGTGGGAGATTTAAAAAGAAGACATCCAGCAAGTGTTGAAAAGGATAT GGAGAAATCGGAACCCTCATACATTGCTGATGAGAACATCAAATGAAGACACATGTCCACACAAAAACATGTTCAC
AAATGTTCATGTAGAATTATTCATAATAGGCCGGGCACGGTGGCTTATGCCTGTAATCCCAGCACTTTGGGAGGCCGAGGTGGGTGGATCACCTGAGGTCAGGAGTTTGAGACCAGCCTGGCCAACAT
GGTGAAACCCCGTCTCTACTAAAAATACAGAATTAGCCAGGCATGGTGGCGC ATGCCTGTAATCTCAGCTACTTGGGACGCTGAGGCAGGAGAATCACTTGAACCCGGGAGGCAGA GGCTCACGCCTG
TAATCCCAGCACTTTGGGAGGCCAAGGCGGGCGGATCACGAGGCCAGGAGATCGAGACCATCCTGGCTAACACGGTCAAACCCCGTCTCTACTAAAAATACAAAAAATTAGCCGGGCGTGGTAGC GGG
CGCCTGTAGTCCCAGCTATTCGGGAGGCTGAGGCAGGAGAATGGCGTGAACCTGGGAGGTGGAGCTTGCAGTGAGCCGAGATCGTGCCACTGCACTCCAGCCTGGGTGACAGAGCGAG AGTCCATCTC
AAAATAAATAAATAAATAAAAATAAATAAAGACTGAGTCCTCCTTCAAAAAAGAAGATATAAAAAGGAGAAGATCAGTAACTGTGACTGTATCTATGTCCCAAAATACATAAAATTAAAAAGACTAAA
CGGGGAAATATTTGCAACAGTTAGGACACTGTTAATATTCACAATATATAAAGACTTAGATATCAGAATAGCTATCTAAGATAGCACAGATGGGTAAATGACAGTCTCTTCAGACTCA TTTGCTTAGA
AGAAACAGAAGCCATTCTAGCTCTCTCGCTTTTCCTCTCTTTTTTTATTTTCTGGTAGAGATAGGGTCTTGCTACTCCTGGCCTCCGCCTCACAAAGTGTTGGGATTATAGGCATGAACCATCAT GCC
CAGCCCACTCTAGCTATCTTACTGTAAAGGTATAACAGGTAGTCTCCTAGTCATCCAAGGACAAGAAAAGTAGTATACACAGGGAATGGTCCTGGAAAGGCAGGAACAGAGACTGCCC ACTCTCTCAT
AGGCCTGCATAGGTAGCTCTTCTTTCTGCATGTGTTGTATAAAGTCGCTGCATCACTTAGCTCAAATTCTGAAGACTCCGATTGACAAGCCATGAATTGTTTCACTTGGATCAGGTATCCATTCC TTG
TCCAAATAGCTGTCATTGTTATAAAAATGGGGCTTTACATTATCTGAGAAGGGGCATTATCTGAGAAGGGGGTAAATAAGTGCTTGGGAAATATATGCAACCTCATCCTTGGGAATCA AAGAAATTTA
AATAATGAGAGACTTATTTTCTGCCCATTAAATCATCAGTGATTAACGGTGACAATCAATGCTGACACACTGCTGGGATAGGATGTAAATTGGGTCTGGTCTGGAAAACAATTTTGCTGTATGTA TTA
AAGACCTTCAGAATGAATATAACCTACGACCCAATAATTCCACTTCTAGGAA aCTATGCTAAGGGAATGAATAATCAGAGATATAGGCAAGATTTAATATGTTAATATCTTTGTAGTA TTATTTATAA
TTTTT

814	15.4	0.0	0.0	138544	1	123	(2186)	+	AluJb	SINE/Alu	178	300	(12)	1
934	15.5	5.1	18.1	138544	125	219	(2090)	+	L1M4	LINE/L1	5280	5359	(815)	2
2324	9.5	1.0	0.0	138544	220	524	(1785)	+	AluSg	SINE/Alu	2	309	(1)	3
934	13.5	43.0	14.5	138544	525	796	(1513)	+	L1M4	LINE/L1	5360	5721	(463)	4
1693	7.3	0.5	0.0	138544	797	1000	(1309)	+	AluSx	SINE/Alu	1	205	(107)	5
2508	6.5	0.0	0.3	138544	1001	1310	(999)	+	AluY	SINE/Alu	3	311	(0)	6
337	18.9	0.0	0.0	138544	1617	1669	(640)	C	Alu	SINE/Alu	(248)	53	1	7
252	23.7	3.4	0.0	138544	1875	1933	(376)	C	MLT1M	LTR/ERVL-MaLR	(473)	199	139	8
723	23.9	17.1	3.2	138544	2007	2305	(4)	+	L1M4	LINE/L1	5385	5723	(401)	2

823	15.3	0.0	0.0	hg	1	124	(2298)	+	AluJb	SINE/Alu	178	301	(11)	9
934	15.5	5.1	18.1	hg	126	220	(2202)	+	L1M4	LINE/L1	5280	5359	(815)	10
2324	9.5	1.0	0.0	hg	221	525	(1897)	+	AluSg	SINE/Alu	2	309	(1)	11
934	13.5	43.0	14.5	hg	526	797	(1625)	+	L1M4	LINE/L1	5360	5721	(463)	12
2474	8.7	0.3	0.0	hg	798	1109	(1313)	+	AluSq	SINE/Alu	1	313	(0)	13
2596	6.1	0.0	0.0	hg	1113	1423	(999)	+	AluY	SINE/Alu	1	311	(0)	14
337	18.9	0.0	0.0	hg	1730	1782	(640)	C	Alu	SINE/Alu	(248)	53	1	15
252	23.7	3.4	0.0	hg	1988	2046	(376)	C	MLT1M	LTR/ERVL-MaLR	(473)	199	139	16
750	23.5	17.1	3.2	hg	2120	2418	(4)	+	L1M4	LINE/L1	5385	5723	(401)	10

>hg19 chrX:54123149-54125570

GAGGCTGTCGGGGGAGGATTGCTTACACCCAGGAGGTAGAGGCTGTTGTGAGCCATGATCATGCCACTGCACTCCAGCCTAGGCAACAGAGCAACACCCTGTCTCa
AAAAAAAAAAAAAAAAAAAGCCTACAAGTAAAGCAATAAATGATGAATAAATCAATTTTTTAAATGGGTGAAAGATTTGAATAGACATTTCTCCAAAGAACATACGC
AAATGTCAGCCGGGCACTGTGGCTCACACCTGTAATACCAGCACTTTCGGAGGCCGAGGAGGGCAGATCACGAGGTCAGGAGTTCGAGACCAGCCTGACCAACATG
GTGAAACCCTGTCTCTACTAAAAATACAAAAATTAGCCGGGCATGGCGCGTGCCTGTAATCCCAGCTACTCAGGAGGCTGAGGCAGGAGAATCACTTGAACCCAGG
AAGGGGAGATTGCAGTGAGCTGAGATCGCACCACTGCAGTTCAGCCTGGGTGGCAGAGCGAGACTCTGTCTCAAAATAAAATAAAATAAAATAAAAAAGAACATAT
ACAAATGTCCAATAAGCACATGAAAAAATGATCATCAGGGAAATGCAAATCATAACAACAATGAGAAACCAGTTAACACCCATTCAGTTGCTATAATCAAAAAGAC
AAGGGATCGGGTGGGAGATTTAAAAAGAAGACATCCAGCAAGTGTTGAAAAGGATATGGAGAAATCGGAACCCTCATACATTGCTGATGAGAACATCAAATGAAGA
CACATGTCCACACAAAAACATGTTCACAAATGTTCATGTAGAATTATTCATAATAGGCCGGGCACGGTGGCTTATGCCTGTAATCCCAGCACTTTGGGAGGCCGAG
GTGGGTGGATCACCTGAGGTCAGGAGTTTGAGACCAGCCTGGCCAACATGGTGAAACCCCGTCTCTACTAAAAATACAGAATTAGCCAGGCATGGTGGCGCATGCC
TGTAATCTCAGCTACTTGGGACGCTGAGGCAGGAGAATCACTTGAACCCGGGAGGCAGAGgttgcagtgagccgagaccgtgtcactgcactccatcctgggcaac
aagaacgaaactccatctcaaaaaaaaaaaaaaaaaagtaatataaaaaaataggccgggcacagtgGCTCACGCCTGTAATCCCAGCACTTTGGGAGGCCAAGGCG
GGCGGATCACGAGGCCAGGAGATCGAGACCATCCTGGCTAACACGGTCAAACCCCGTCTCTACTAAAAATACAAAAAATTAGCCGGGCGTGGTAGCGGGCGCCTGT
AGTCCCAGCTATTCGGGAGGCTGAGGCAGGAGAATGGCGTGAACCTGGGAGGTGGAGCTTGCAGTGAGCCGAGATCGTGCCACTGCACTCCAGCCTGGGTGACAGA
GCGAGAGTCCATCTCAAAATAAATAAATAAATAAAAATAAATAAAGACTGAGTCCTCCTTCAAAAAAGAAGATATAAAAAGGAGAAGATCAGTAACTGTGACTGTA
TCTATGTCCCAAAATACATAAAATTAAAAAGACTAAACGGGGAAATATTTGCAACAGTTAGGACACTGTTAATATTCACAATATATAAAGACTTAGATATCAGAAT
AGCTATCTAAGATAGCACAGATGGGTAAATGACAGTCTCTTCAGACTCATTTGCTTAGAAGAAACAGAAGCCATTCTAGCTCTCTCGCTTTTCCTCTCTTTTTTTA
TTTTCTGGTAGAGATAGGGTCTTGCTACTCCTGGCCTCCGCCTCACAAAGTGTTGGGATTATAGGCATGAACCATCATGCCCAGCCCACTCTAGCTATCTTACTGT
AAAGGTATAACAGGTAGTCTCCTAGTCATCCAAGGACAAGAAAAGTAGTATACACAGGGAATGGTCCTGGAAAGGCAGGAACAGAGACTGCCCACTCTCTCATAGG
CCTGCATAGGTAGCTCTTCTTTCTGCATGTGTTGTATAAAGTCGCTGCATCACTTAGCTCAAATTCTGAAGACTCCGATTGACAAGCCATGAATTGTTTCACTTGG
ATCAGGTATCCATTCCTTGTCCAAATAGCTGTCATTGTTATAAAAATGGGGCTTTACATTATCTGAGAAGGGGCATTATCTGAGAAGGGGGTAAATAAGTGCTTGG
GAAATATATGCAACCTCATCCTTGGGAATCAAAGAAATTTAAATAATGAGAGACTTATTTTCTGCCCATTAAATCATCAGTGATTAACGGTGACAATCAATGCTGA
CACACTGCTGGGATAGGATGTAAATTGGGTCTGGTCTGGAAAACAATTTTGCTGTATGTATTAAAGACCTTCAGAATGAATATAACCTACGACCCAATAATTCCAC
TTCTAGGAAtCTATGCTAAGGGAATGAATAATCAGAGATATAGGCAAGATTTAATATGTTAATATCTTTGTAGTATTATTTATAATTTTT

Micrhomology = GG

>NHEJ_11

KSD_NEHJ_11

> Scaffold9667 6902633 6902936_NHEJ

TAATGTATACTTCTCACTTCACTAACAGATCTGTGCTTTTACATACATACATACATGTAGTTCCTTTTGTATGTTTTTCAAAAACTCACCCACAGAACCTGAACAATACTAAATTCTTCACTCCCA TT
GGCTACTTCAAGAAATATTATGCTGTCTTGCTTATCCTGAGTTCTAGAAATGTTAAGTTTGTAATATACTATTAACCTCCCACTCTCCTTCAGAAATCAGTGTCTATGAACCACATCAGTAACAC TAG
AAGATAATTCCATGTCACATAAATTTGCTGTAACTAAAGGTGAACTAAATTATCGACACACTCATACACAAAGTTCATCAAATACATATTTAAATTTATCTTAGTACAGGGTACTGTACTTTACCC AA
CTCAAAAAAATTTACTTTCTGAAAATATTTCACACTTTAGCCAATTAAAATTTTTTTAATCATATGCATAGCTTTTTCCTTTCACAAAAATTATTTCTTCAAATATCCTCTAACACTTTGTATCC TAT
TTCTCCGGATGGTCAAAATAAGCTTTTAAAAACCTTCCAGGTTGGGGCTGGGCGTGGTGGCTCATGCCTGTAATCCCAGCACTTTGGGAGGCCGAGGCAGGAAGATCACAAGGTTAGGAGATCCAG AC
CATCCTGGCTAACACGGTGAAACCCGGCCTCTACTTAAAATACAAAAAATTAGCCGGGCGTGGTGGCAGTCCCAGCTACTTGGGAGGCTGAGGCAGGAGAATGGCATGAACCTAGGAGGCGGAGC TTG
CAGTGAGCCGAGACTACGCCACTGCACTCCAGTCTGGGGGACAGAGTGAGACTCCATCTCCAAAAAAAAAAAAAAAAAAAAA AgatgcccagggggggatagggtctcgTGTGATCCCAACACTTTGG
GAAGCCAAGGCAGGAGGACTGCTTAAGCCCAAGAGTTCAAGACCAGCCTCGGCAACATAGGGAGATGAAGTCTCTACAAAAAATTAAAAAAAAAAAAAAAATT GGCCGGGTGCAGTGTCTCATGCCTG
TAATCCCAACATTTTGGGAGGCCCAGGTGGGCAGACCACAAGGTCAAGAGATCGAGACCATCCTGGCCAACACAGTGAAACCCTATCTCTACTAAAAATACAAAAAATTAGCTGGGTGTGGTGGCG GG
CACCTGCAGTCCCAGCTATCAGGAGGCTGTGGCGGAAGAATCGCTTGAACCCGGGAGGTGGAGGTAGCAGCGAGCCGAGATCGAGCCACTGCACTGCACTC CAAGTCAGGAGaTgGAGgCCAGCCTGG
CCAACAGGGCGAAATCTCGTCTCTACTAAAAATACAAAATTAGCCAGGCGTGGTGGCATGCACCTGTAATCCCAGCTACTCGGGAGGCTGTGGGCAGGAGAGTTTCTTGAACTCGGGAGGCAGAGG TT
ATAGTGAGCCAAAATTGTGCCATTGCACTCCAGCCTGGGCAAAAAGAGTGAAACTCTGTATTTAAAAAAAAAAAATAAAAATAATAAAAAA TAAATATAAAAAATAAATAAATTAGCCAGGCATGGTAA
CATGCAACTGTAGTCCCAGCTACTTAGGGGGCTGAGGTGTAAGGATCCCTTTTACCTGGCAGGTTGAGGCTGCAATAAGCTGTGATCACACCACTACACCCCAGCCTAGGTAACAGAGTGAGAAAT TA
GACCCCTGTCTCAAAGAAACACACACACACAAAAAAACTTTCCTAATCACCACCTGTGAAAAGAAAGGGGACCTTGGGGGAACTACCTAATAAGCCAGACCCATACAGAAACCACTATCCACTTT AAT
CTGGTTTATGTTTTATTATAAATTGTGCTTATTCACTGGAATTATTAATTAACATACTGTCTATCCATCCTGTACAATAATTTCTCATCTATCCTCTAATATTTACCAAGTCTCTGTACGTAACAG GC
ACTGTTCTAGGGCACTGGAAAAAAATAAGTGAAATGTGTGCCTTCAAAGAGTTTACGGTTATTGGAGATTGAGGATGATCAAGAGAAAATTCCTTCCTCAGTTATCTTGTAGTCTACAAAGAAAT GCA
GGCAACTCAATGGACAACTATTAAAGCATGAAATAAATCCCTGTGAAGGAATATGTTGTGGGTACTATGGAAATAGTGGGAAGAAAAGTCTAACCTTATAATAGCATAACAAGGAAATCAAGTAGA GG
AAATGCAGTTGAAAACTGGGTTTATGGGACAAATATAAGTTAATCTGAGGAATATGAGACTTCTAGATATACAGGGAATGGCAGGTAAAGCGAAGAGGTAAGAAAATTCATATAGAAGTGAGGGA AT

2294	8.2	3.4	0.0	A	558	850	(1453)	+	AluY	SINE/Alu	1	303	(8)	1
698	16.4	0.0	0.0	A	878	987	(1316)	+	AluJo	SINE/Alu	24	133	(179)	2
1828	12.3	0.4	0.0	A	1000	1250	(1053)	+	AluSc8	SINE/Alu	1	252	(59)	3
1585	14.8	0.4	0.4	A	1255	1498	(805)	+	AluSp	SINE/Alu	68	311	(2)	4
822	21.1	0.0	5.2	A	1523	1683	(620)	+	AluJb	SINE/Alu	137	289	(23)	5
204	24.9	17.2	2.6	A	1890	1988	(315)	C	L2a	LINE/L2	(16)	3448	3336	6

2312	8.1	3.4	0.0	hg	558	852	(1580)	+	AluY	SINE/Alu	1	305	(6)	7
1396	17.7	0.0	15.6	hg	860	1004	(1428)	+	AluJr	SINE/Alu	1	126	(186)	8
2259	10.7	0.3	1.6	hg	1005	1314	(1118)	+	AluSc8	SINE/Alu	1	306	(5)	9
2231	12.2	0.3	0.3	hg	1317	1627	(805)	+	AluSp	SINE/Alu	1	311	(2)	10
1396	17.7	0.0	15.6	hg	1628	1812	(620)	+	AluJr	SINE/Alu	127	289	(23)	8
204	24.9	17.2	2.6	hg	2019	2117	(315)	C	L2a	LINE/L2	(16)	3448	3336	11

>hg19 chr5:118219863-118222294

TAATGTATACTTCTCACTTCACTAACAGATCTGTGCTTTTACATACATACATACATGTAGTTCCTTTTGTATGTTTTTCAAAAACTCACCCACAGAACCTGAACAA
TACTAAATTCTTCACTCCCATTGGCTACTTCAAGAAATATTATGCTGTCTTGCTTATCCTGAGTTCTAGAAATGTTAAGTTTGTAATATACTATTAACCTCCCACT
CTCCTTCAGAAATCAGTGTCTATGAACCACATCAGTAACACTAGAAGATAATTCCATGTCACATAAATTTGCTGTAACTAAAGGTGAACTAAATTATCGACACACT
CATACACAAAGTTCATCAAATACATATTTAAATTTATCTTAGTACAGGGTACTGTACTTTACCCAACTCAAAAAAATTTACTTTCTGAAAATATTTCACACTTTAG
CCAATTAAAATTTTTTTAATCATATGCATAGCTTTTTCCTTTCACAAAAATTATTTCTTCAAATATCCTCTAACACTTTGTATCCTATTTCTCCGGATGGTCAAAA
TAAGCTTTTAAAAACCTTCCAGGTTGGGGCTGGGCGTGGTGGCTCATGCCTGTAATCCCAGCACTTTGGGAGGCCGAGGCAGGAAGATCACAAGGTTAGGAGATCC

AGACCATCCTGGCTAACACGGTGAAACCCGGCCTCTACTTAAAATACAAAAAATTAGCCGGGCGTGGTGGCAGTCCCAGCTACTTGGGAGGCTGAGGCAGGAGAAT
GGCATGAACCTAGGAGGCGGAGCTTGCAGTGAGCCGAGACTACGCCACTGCACTCCAGTCTGGGGGACAGAGTGAGACTCCATCTCCAAAAAAAAAAAAAAAAAAA
AAaatcgtccaggttgggtatagtggctcatgccTGTGATCCCAACACTTTGGGAAGCCAAGGCAGGAGGACTGCTTAAGCCCAAGAGTTCAAGACCAGCCTCGGC
AACATAGGGAGATGAAGTCTCTACAAAAAATTAAAAAAAAAAAAAAAAAATTGGCCGGGTGCAGTGTCTCATGCCTGTAATCCCAACATTTTGGGAGGCCCAGGTGGG
CAGACCACAAGGTCAAGAGATCGAGACCATCCTGGCCAACACAGTGAAACCCTATCTCTACTAAAAATACAAAAAATTAGCTGGGTGTGGTGGCGGGCACCTGCAG
TCCCAGCTATCAGGAGGCTGTGGCGGAAGAATCGCTTGAACCCGGGAGGTGGAGGTAGCAGCGAGCCGAGATCGAGCCACTGCACTGCACTCCagcctgggcgaca
gagcaagactccacctcaaaaaaaaaaaaaaaaaaaaaaattggccaggcgcagtggctcacacctgtaatcccagcactttgggaggccgaggtgggcggatc
acctgAAGTCAGGAGtTtGAGaCCAGCCTGGCCAACAGGGCGAAATCTCGTCTCTACTAAAAATACAAAATTAGCCAGGCGTGGTGGCATGCACCTGTAATCCCAG
CTACTCGGGAGGCTGTGGCAGGAGAGTTTCTTGAACTCGGGAGGCAGAGGTTATAGTGAGCCAAAATTGTGCCATTGCACTCCAGCCTGGGCAAAAGAGTGAAA
CTCTGTATTTAAAAAAAAAAATAAAAATAATAAAAAATAAATATAAAAAATAAATAAATTAGCCAGGCATGGTAACATGCAACTGTAGTCCCAGCTACTTAGGGGG
CTGAGGTGTAAGGATCCCTTTTACCTGGCAGGTTGAGGCTGCAATAAGCTGTGATCACACCACTACACCCCAGCCTAGGTAACAGAGTGAGAAATTAGACCCCTGT
CTCAAAGAAACACACACACACAAAAAAACTTTCCTAATCACCACCTGTGAAAAGAAAGGGGACCTTGGGGGAACTACCTAATAAGCCAGACCCATACAGAAACCAC
TATCCACTTTAATCTGGTTTATGTTTTATTATAAATTGTGCTTATTCACTGGAATTATTAATTAACATACTGTCTATCCATCCTGTACAATAATTTCTCATCTATC
CTCTAATATTTACCAAGTCTCTGTACGTAACAGGCACTGTTCTAGGGCACTGGAAAAAAATAAGTGAAATGTGTGCCTTCAAAGAGTTTACGGTTATTGGAGATTG
AGGATGATCAAGAGAAAATTCCTTCCTCAGTTATCTTGTAGTCTACAAAGAAATGCAGGCAACTCAATGGACAACTATTAAAGCATGAAATAAATCCCTGTGAAGG
AATATGTTGTGGGTACTATGGAAATAGTGGGAAGAAAAGTCTAACCTTATAATAGCATAACAAGGAAATCAAGTAGAGGAAATGCAGTTGAAAACTGGGTTTATGG
GACAAATATAAGTTAATCTGAGGAATATGAGACTTCTAGATATACAGGGAATGGCAGGTAAAGCGAAGAGGTAAGAAAATTCATATAGAAGTGAGGGAAT

Microhomology = A

>NHEJ_12

KSD_NEHJ_12

>Scaffold16538 3186318 3186584_NHEJ
AAAAAAGCACTAATTTAAGTAGTAGGAGGAATTGTGGTAGGCTGGATAATGAAGCCCCACTGCATATGTCTTTCTTCTAATCCTCAGAACCTGTGAATGTTCCCCCTCTAAGCAAAAGAGAATTTG CA
GGTGTGATTAGTTAAGGGTCTTGAAGTTGGGAGATTGTTCAGGATTATCTAGGTGGGCCTGTTGTAATAATAGGATCTTTAGAAGAGGAGTGAAGAAAGAGCCAGAGTCAGAGAAAGAGATGCAA TGA
AGGAAAGAGGCTGGAATGACGCTCTTTGAAGATTGAGAAAGAGCTGGGTGCGGTGGCTCACACCTGTAATCTCAACACTTTGGGAGGCCAAAGCAGATGATCACTTGAAATCAGGAGTTCGAGAAC AG
CCTGGCCAACACAGGGAAACCGCCTCCCTCTAAAATTACAAAAATTAGCTGGGAGTGGTGGCAGTGCCTGTAATCCCTGCTACTCAGGAGGCTGAGGCAGGAGAATCACTTGAACCCAGGAGGCA GAG
GTTGCAGTGAGCTGAGATTGCACCACTGCACTCCTGCCTGGGTGACAGAGCAAGACTCCATCTCAAAAAAAAAAAAAGAAAAAAAAGATTGAGAAAGAGGCCACAGGCTAAGGAATACAGGCAGCCAC TA
GCAGCTAAAAAAGGCAAGGACACAGATCCTTCCCCTCAGGGCTTCCAAAAGGAAACAGTCCTGCCAACATTTTGAATTTTGCCCAGTGAAAATGATTGCAGACTTCTGCCCTCCAAAATGTAAAA GAA
ATGTGTGTTGTTTTCTGCCAGGCACAGTGGCTCACGCCTGTAATCTCAGCACTTTTGGAGGCTGAGGCGGGCAGATCACATGAGGTCAGGAGTTCAAGACCAGCCTGGCCAACATGGTGAAACTATAT
CTCTACTAAAACACAAAAATTAGCTGGGCATGGTGGCACACACCTGTAATCCCAGCTACTCAGGAGGATGAGGCAGGAGAATTGCTTGAACCTGGGAGGCAGA GGCACTTTGGGAGGCTGAGGCAGGT
GGATCACGAGGTCAAGAGATGGAGACCATCCTGGCCAACATGGTAAAACCCCGTCTCTACTAAAAATACAAAAATTAGCTGGGCATGGGGGCACGCGCCTGTAATCCCAGCTACTCGGGAGGCTGA GG
CAGGAGAATTGCTTGAACCAGGGAGGCAGAGGTTGCAGTCAACCAAGATCGCACCACTGCACTGCAGCCCGGTGACAGAGCAAGACTCAGTCT CaAAAAAAAAAAAAAAAAAAAAGATTGTATTGTTT
TAAACCACTAAATGGGCTTTAAAAATCATCGCTTCTGTCCCCGATACATTGCAAAATTTGGAAGGGTCTTGAAATCATAAAGAAAATAAATATTTTGTATTTTGACTTTGTTATGTTCAATGTAGT CA
ATTTCTATCTTTTAATCTCTTAAAATATCATACTCATACTTGTCTTCATACAATTCCTTTTTAGAATAAACAAATACTATTTGGAATAGAGTGTGTGTGTGTTTATTTTAATAAATTGAATGTTA AAA
TTTACCTCTTAGACTTGAATTTATAAATTATATTATGCCAAGTAAAAATTCTTACTAGAATATTGGCATAAGGAAAAAAAAAAAGTAGATAAATTCTCAGAAAATAACATGTTTACAACTCTAAAAA TA
CTGAGAAATTCAGTAAATTAAATAGAAATATTCCTACTCTATATCAGTTCATATCAGCATATTCAATGTGGGTTGAAGGGAGAGGGCTGCCAGACATGAAGGTGAGTGTGTGAGGTCCTTGAAGG TGA
AAATGAGACACAGGGTAGGATAAGGTAGTGAAGTCTTAGAAAAATAGAAAAAAAATGCAAAGGGTTTAAAAAACACTTAAAAAGCAAGGTGTTCCAAGGACACTCGGTGAAAGGCCCATAGAGCTT AT
CTCTGATTAGCTATTATCCAGTCTCTTCTCTACACTATATCACCAGATAATAGCAAATAAAATTCAGGCCGGGCACGGTGGCTCACACCTATAATCCAAGCACTTTGGGAGGCCGAAGCAGGCAG ATC
ATTTGAGGCCAGGAGTTCAAGAGCAGCCTGGCCAACACGATGAAACCCCCCTCTCTCCTAAAAATACAAAAATTAGCTAGGCTTGGTGGTGGGCACCTGTAATCCAGCTACTCGTGAGGCTGAGGC AG
GAGAATCCCTTGAACCCAGGAGGCAGAGGTTGCAGTGAGCTGAGATCACACCACTGGCCCTCCAGCCTGGGCAACAAGAACAAAACTCC G

1872	22.4	2.4	2.4	A	33	298	(1968)	+	MLT1D	LTR/ERVL-MaLR	1	264	(241)	1
2116	12.4	1.0	0.0	A	299	596	(1670)	+	AluSz	SINE/Alu	2	302	(10)	2
1872	22.4	2.4	2.4	A	597	782	(1484)	+	MLT1D	LTR/ERVL-MaLR	265	453	(52)	1
1653	11.1	0.5	0.0	A	785	1001	(1265)	+	AluSx	SINE/Alu	2	219	(93)	3
2158	8.2	0.0	0.0	A	1002	1267	(999)	+	AluSc	SINE/Alu	34	300	(9)	4
12	24.3	1.9	3.9	A	1371	1422	(844)	+	(TATTTTG)n	Simple_repeat	1	51	(0)	5
2032	11.1	0.4	0.7	A	1987	2266	(0)	+	AluSq	SINE/Alu	1	279	(34)	6

1872	22.4	2.4	2.4	hg	33	298	(2086)	+	MLT1D	LTR/ERVL-MaLR	1	264	(241)	7
2116	12.4	1.0	0.0	hg	299	596	(1788)	+	AluSz	SINE/Alu	2	302	(10)	8
1872	22.4	2.4	2.4	hg	597	782	(1602)	+	MLT1D	LTR/ERVL-MaLR	265	453	(52)	7
2262	11.3	0.3	0.0	hg	785	1086	(1298)	+	AluSq	SINE/Alu	2	304	(9)	9
2440	8.0	0.0	0.0	hg	1087	1385	(999)	+	AluSc	SINE/Alu	1	299	(10)	10
12	24.3	1.9	3.9	hg	1489	1540	(844)	+	(TATTTTG)n	Simple_repeat	1	51	(0)	11
2032	11.1	0.4	0.7	hg	2105	2384	(0)	+	AluSq	SINE/Alu	1	279	(34)	12

>hg19 chr8:68309318-68311701
AAAAAAGCACTAATTTAAGTAGTAGGAGGAATTGTGGTAGGCTGGATAATGAAGCCCCACTGCATATGTCTTTCTTCTAATCCTCAGAACCTGTGAATGTTCCCCC
TCTAAGCAAAAGAGAATTTGCAGGTGTGATTAGTTAAGGGTCTTGAAGTTGGGAGATTGTTCAGGATTATCTAGGTGGGCCTGTTGTAATAATAGGATCTTTAGAA
GAGGAGTGAAGAAAGAGCCAGAGTCAGAGAAAGAGATGCAATGAAGGAAAGAGGCTGGAATGACGCTCTTTGAAGATTGAGAAAGAGCTGGGTGCGGTGGCTCACA
CCTGTAATCTCAACACTTTGGGAGGCCAAAGCAGATGATCACTTGAAATCAGGAGTTCGAGAACAGCCTGGCCAACACAGGGAAACCGCCTCCCTCTAAAATTACA

AAAATTAGCTGGGAGTGGTGGCAGTGCCTGTAATCCCTGCTACTCAGGAGGCTGAGGCAGGAGAATCACTTGAACCCAGGAGGCAGAGGTTGCAGTGAGCTGAGAT
TGCACCACTGCACTCCTGCCTGGGTGACAGAGCAAGACTCCATCTCAAAAAAAAAAAAAGAAAAAAAAGATTGAGAAAGAGGCCACAGGCTAAGGAATACAGGCAGCC
ACTAGCAGCTAAAAAAGGCAAGGACACAGATCCTTCCCCTCAGGGCTTCCAAAAGGAAACAGTCCTGCCAACATTTTGAATTTTGCCCAGTGAAAATGATTGCAGA
CTTCTGCCCTCCAAAATGTAAAAGAAATGTGTGTTGTTTTCTGCCAGGCACAGTGGCTCACGCCTGTAATCTCAGCACTTTTGGAGGCTGAGGCGGGCAGATCACA
TGAGGTCAGGAGTTCAAGACCAGCCTGGCCAACATGGTGAAACTATATCTCTACTAAAACACAAAAATTAGCTGGGCATGGTGGCACACACCTGTAATCCCAGCTA
CTCAGGAGGATGAGGCAGGAGAATTGCTTGAACCTGGGAGGCAGAGattgcagtgagccgagatggcgctactgcactccagcctgggctacaagcatgaaactct
gcctcaaaaaaaaaaaaaaaaaaaaggccaggcgtggtggctcacgcctgtaatcccaGCACTTTGGGAGGCTGAGGCAGGTGGATCACGAGGTCAAGAGATGGA
GACCATCCTGGCCAACATGGTAAAACCCCGTCTCTACTAAAAATACAAAAATTAGCTGGGCATGGGGGCACGCGCCTGTAATCCCAGCTACTCGGGAGGCTGAGGC
AGGAGAATTGCTTGAACCAGGGAGGCAGAGGTTGCAGTCAACCAAGATCGCACCACTGCACTGCAGCCCGGTGACAGAGCAAGACTCAGTCTCAAAAAAAAAAAAA
AAAAAAAGATTGTATTGTTTTAAACCACTAAATGGGCTTTAAAAATCATCGCTTCTGTCCCCGATACATTGCAAAATTTGGAAGGGTCTTGAAATCATAAAGAAAA
TAAATATTTTGTATTTTGACTTTGTTATGTTCAATGTAGTCAATTTCTATCTTTTAATCTCTTAAAATATCATACTCATACTTGTCTTCATACAATTCCTTTTTAG
AATAAACAAATACTATTTGGAATAGAGTGTGTGTGTGTTTATTTTAATAAATTGAATGTTAAAATTTACCTCTTAGACTTGAATTTATAAATTATATTATGCCAAG
TAAAAATTCTTACTAGAATATTGGCATAAGGAAAAAAAAAAAGTAGATAAATTCTCAGAAAATAACATGTTTACAACTCTAAAAATACTGAGAAATTCAGTAAATTA
AATAGAAATATTCCTACTCTATATCAGTTCATATCAGCATATTCAATGTGGGTTGAAGGGAGAGGGCTGCCAGACATGAAGGTGAGTGTGTGAGGTCCTTGAAGGT
GAAAATGAGACACAGGGTAGGATAAGGTAGTGAAGTCTTAGAAAAATAGAAAAAAAAATGCAAAGGGTTTAAAAAACACTTAAAAAGCAAGGTGTTCCAAGGACACT
CGGTGAAAGGCCCATAGAGCTTATCTCTGATTAGCTATTATCCAGTCTCTTCTCTACACTATATCACCAGATAATAGCAAATAAAATTCAGGCCGGGCACGGTGGC
TCACACCTATAATCCAAGCACTTTGGGAGGCCGAAGCAGGCAGATCATTTGAGGCCAGGAGTTCAAGAGCAGCCTGGCCAACACGATGAAACCCCCCTCTCTCCTA
AAAATACAAAAATTAGCTAGGCTTGGTGGTGGGCACCTGTAATCCAGCTACTCGTGAGGCTGAGGCAGGAGAATCCCTTGAACCCAGGAGGCAGAGGTTGCAGTGA
GCTGAGATCACACCACTGGCCCTCCAGCCTGGGCAACAAGAACAAAACTCCG

Microhomology = undefined

>NHEJ_13

KSD_NEHJ_13

>Scaffold4023 2512543 2512833_NHEJ
CAGTCTGTTTTCTCAGGGTATCCACTTCATCTATGGTCCAGTTTACCTAAAAATCCAATCCTTTCTTTGTTTCTCTTCAAGTAAAATCACCTCCTAGTCTTTCTTTCCTTTGTTGTCCTTCAATTT TT
AGGGTTTTGTATGTCTTTTGTATTAAATGATTTTTTTTCAAACTAAT aCTGCATTTTAGGATTTTGTATGTTGTTTGCATTAAATGATTTTTTCAAACCAATTCTGCATAGGAGTCATAGTTTTTGCT
GTGTTTTAGCATGTTACGGACTTTTTTTAAACCAACTCAGTTAAGGTCAATTTCTATTTCTCTTATGTATATTTATGTATATTAATTGAATTTACAGCACTTTATGAGTGAAGGAATTGTGTTTTA TC
CTTCTGGGTATCTTTTCCATTTTTTTCCTATTATTGACCAAGTAGAATTTACTTTTTTAAGTCTAAAACCATTAAACGGCTTTAGGCAAGGACTCTGCTGATCCTTCACATGCTCTAAAAAAATG GAG
TGGACTGGTGCTGGGCAAGTTTATATATCTACTTTTTTCCCAGGGATTAGTCCACACAGAATTCCCCTTGTCTGAGCTCCTAAATGCCACAATTATGGCTAACTGCTTATGCGTTGTTCTACACTT CC
TCTTCTTCCTTGAAATCTATTTACTACATAATTCAATATGCTCTTCCCTACTTTTCTGATATTTTTATTCTTATATGGAGAAAAGGAAAGGATTAACTGAGCATCTGAGTCACAGCCTGAAATGC CCT
TAACAAGCTTCATTATGACAATAAATATTTAGTTTACTGTGTGCTTAGCTTTGGCCTACATACTGGAGGAAATCAAAGATCCTGAAGCTCTTGCCTCACCTTGCTAAAGAACAGAGAAAAAAGAAA CG
CAATTCCTCACATCACTGAGAAGTGTTGCTCAGACAAAATGCTATTCTATTGAACAGCAAAGCCCTAGCAATGAGGTTCTTGAGGAAAGAGAAGGAGCCCCATG CGCGGTGGCTCACGCCTGTAATCC
CAACACTTTGGGAAGCCGAGGCGGGAGGATCACAAGGTCAGGAGTTCGAGACCAGCCTGACCAACATAGTGAAACCCCGTCTCTACTAAAAATACAAAAATTAGCCGGGCGTGGTGGTATGCGCCT GT
AACCCCAGCTACTCAGGAGGCTGCGGCAGGATAATCACTTGAACCCGGGAGGTGGAGGTTGCAGTGAGCCGAGA cTGAGCCACTGCACTCCAGCCTAGGAGACAGAGTGAGACTCCTTCTCAAAAAAA
AAAAAAAAAAGCAGAGGTTGCAGTGGGCCAAGATCATACCACTGCACTCCAGCCTTGGCAACAGAGCAAGACTCTGTCT CAAAAAAAAAAATTGGAGCCATTGAAAGGCATGCAAGCAAATTTCTTAT
GCTTATTATCCACTTGTAACTCTTTTCTTTTTTTTTTTGAGATGGAGTCTTGCTCTGTCACCCAGGCTGGAATGTAATGATGCAATCTTGGCTCACTGCAACCTTTGCCTCCCAGTTCAAGTGGTGTT
CCTGCCTCAGCCTCCCAAGTAGCCATGACTACAGGCACGCACCACCATGCCCAGCTAATTTTTGGCTACTCTCGAACTCCTGACCTCAGATGATCCACCTGTCTTGGTCTCTCAAAGTGCTGGGA TTA
CAGGCATGAGCCACTGTGCCTGGCCTTGTATCTCTTTTT TCAGTGAATTGTCTACTTCTGCTGTTTACCCATTTATTTTCACTGAGGTTTTAGTATTTATTAATTTAATAAATTCTTTGTATTTTACA
GATGCCAACCACTTGTCCAGAACAGAAATGAACTTTGCCTCTGAGCTGAAGGCTCAATTGCTATGAGGGGAAGAAGGACAGCTGGCTGCCTAGGGCCTGATTACAGCTGGAACCTCCCAAAGGAT GGA
GGAACACGTCATCAGCCAGGGCCAGCCCAGGCCACCAAG CCAGAGCTCAAGGGTGTGGTGCTTTCTCCTCCCTCCCTACCTAGGGCAGCTTCCTTTGACAGCACACTTGCTGCTTCAAACTTCTAACA
AATATCGACCATTCTTTCAAACAATAGAAGGGGGTCAGAATGAGCTATATAAGATCTTTTTCAAAAGAATTTTCTTTCTACGAATAAATAGGAGAACTGCTGAAGGAGAAAATTACACTAAAAAA AAT
GGAAATAAACTGAAATGGAGGCTTCAAAAAACTAGAAGA GAGCATCCTCTACTTTAGACAAAAAAAAAAaCTTCACTAATTCAAAAGAAAATTAATGTGGCAGAGAAATTACAT

2392	7.6	0.0	0.0	A	1000	1290	(1000)	+	AluSg	SINE/Alu	7	297	(13)	1
613	12.3	0.0	0.0	A	1291	1371	(919)	+	AluSx3	SINE/Alu	212	292	(19)	2
1570	15.4	15.1	0.0	A	1431	1689	(601)	C	AluSx	SINE/Alu	(14)	298	1	3
2439	8.0	0.0	0.0	hg	1000	1299	(1215)	+	AluSg	SINE/Alu	7	306	(4)	4
1833	13.8	4.4	0.0	hg	1321	1595	(919)	+	AluJb	SINE/Alu	19	305	(7)	5
1579	15.4	15.0	0.0	hg	1655	1914	(600)	C	AluSx	SINE/Alu	(13)	299	1	6

>hg19 chr18:21011372-21013885
CAGTCTGTTTTCTCAGGGTATCCACTTCATCTATGGTCCAGTTTACCTAAAAATCCAATCCTTTCTTTGTTTCTCTTCAAGTAAAATCACCTCCTAGTCTTTCTTT
CCTTTGTTGTCCTTCAATTTTTAGGGTTTTGTATGTCTTTTGTATTAAATGATTTTTTTTCAAACTAATtCTGCATTTTAGGATTTTGTATGTTGTTTGCATTAAA
TGATTTTTTCAAACCAATTCTGCATAGGAGTCATAGTTTTTGCTGTGTTTTAGCATGTTACGGACTTTTTTTAAACCAACTCAGTTAAGGTCAATTTCTATTTCTC
TTATGTATATTTATGTATATTAATTGAATTTACAGCACTTTATGAGTGAAGGAATTGTGTTTTATCCTTCTGGGTATCTTTTCCATTTTTTTCCTATTATTGACCA
AGTAGAATTTACTTTTTTAAGTCTAAAACCATTAAACGGCTTTAGGCAAGGACTCTGCTGATCCTTCACATGCTCTAAAAAAATGGAGTGGACTGGTGCTGGGCAA
GTTTATATATCTACTTTTTTCCCAGGGATTAGTCCACACAGAATTCCCCTTGTCTGAGCTCCTAAATGCCACAATTATGGCTAACTGCTTATGCGTTGTTCTACAC
TTCCTCTTCTTCCTTGAAATCTATTTACTACATAATTCAATATGCTCTTCCCTACTTTTCTGATATTTTTATTCTTATATGGAGAAAAGGAAAGGATTAACTGAGC
ATCTGAGTCACAGCCTGAAATGCCCTTAACAAGCTTCATTATGACAATAAATATTTAGTTTACTGTGTGCTTAGCTTTGGCCTACATACTGGAGGAAATCAAAGAT
CCTGAAGCTCTTGCCTCACCTTGCTAAAGAACAGAGAAAAAAGAAACGCAATTCCTCACATCACTGAGAAGTGTTGCTCAGACAAAATGCTATTCTATTGAACAGC
AAAGCCCTAGCAATGAGGTTCTTGAGGAAAGAGAAGGAGCCCCATGCGCGGTGGCTCACGCCTGTAATCCCAACACTTTGGGAAGCCGAGGCGGGAGGATCACAAG
GTCAGGAGTTCGAGACCAGCCTGACCAACATAGTGAAACCCCGTCTCTACTAAAAATACAAAAATTAGCCGGGCGTGGTGGTATGCGCCTGTAACCCCAGCTACTC

AGGAGGCTGCGGCAGGATAATCACTTGAACCCGGGAGGTGGAGGTTGCAGTGAGCCGAGAtTGAGCCACTGCACTCCAGCCTAGGAGACAGAGTGAGACTCCTTCT
CAAAAAAAAAAAAAAAAAgaaagagaaggagcaatttgggagccattgacgcctgtaattccagcactttgggaggccaaggcgggagcccaggagttgaagacca
gcctgggcgacatggtgagaccctatcttgacaaaaaatacaaaaattagccggacatggttgtgcaagctgtgctcccagctacttgggaggctgaggtgggagg
atcacttaagcatgggagGCAGAGGTTGCAGTGGGCCAAGATCATACCACTGCACTCCAGCCTTGGCAACAGAGCAAGACTCTGTCTCaaaaaaaaaaaaAAAAAA
AAAAATTGGAGCCATTGAAAGGCATGCAAGCAAATTTCTTATGCTTATTATCCACTTGTAACTCTTTTCTTTTTTTTTTTtGAGATGGAGTCTTGCTCTGTCACCC
AGGCTGGAATGTAATGATGCAATCTTGGCTCACTGCAACCTTTGCCTCCCAGTTCAAGTGGTGTTCCTGCCTCAGCCTCCCAAGTAGCCATGACTACAGGCACGCA
CCACCATGCCCAGCTAATTTTTGGCTACTCTCGAACTCCTGACCTCAGATGATCCACCTGTCTTGGTCTCTCAAAGTGCTGGGATTACAGGCATGAGCCACTGTGC
CTGGCCTTGTATCTCTTTTTTCAGTGAATTGTCTACTTCTGCTGTTTACCCATTTATTTTCACTGAGGTTTTAGTATTTATTAATTTAATAAATTCTTTGTATTTT
ACAGATGCCAACCACTTGTCCAGAACAGAAATGAACTTTGCCTCTGAGCTGAAGGCTCAATTGCTATGAGGGGAAGAAGGACAGCTGGCTGCCTAGGGCCTGATTA
CAGCTGGAACCTCCCAAAGGATGGAGGAACACGTCATCAGCCAGGGCCAGCCCAGGCCACCAAGCCAGAGCTCAAGGGTGTGGTGCTTTCTCCTCCCTCCCTACCT
AGGGCAGCTTCCTTTGACAGCACACTTGCTGCTTCAAACTTCTAACAAATATCGACCATTCTTTCAAACAATAGAAGGGGGTCAGAATGAGCTATATAAGATCTTT
TTCAAAAGAATTTTCTTTCTACGAATAAATAGGAGAACTGCTGAAGGAGAAAATTACACTAAAAAAAATGGAAATAAACTGAAATGGAGGCTTCAAAAAACTAGAA
GAGAGCATCCTCTACTTTAGACAAAAAAAAAACTTCACTAATTCAAAAGAAAATTAATGTGGCAGAGAAATTACAT
Microhomology =G

>NHEJ_14

KSD_NEHJ_14

>Scaffold16034 4091341 4091647_NHEJ
ACATTGTGGCTTCCCAGATGGGAGCAGATAGAAGAGACCACATGAAAGCTTTTAGCATCCTCTGTTCTTCAAAGCTTGCCGTTCATATCCCCAGACCAGGTGGTCAGCAGGGTCGAATGTGGTTCA TG
GGCCTGACTCTGAGGTCTCTTTTTTATGAGTTCAAACCCAACATTGACCACCAGGGAAGACTTAATCTATTTAATATATACCCTTCTCTGGGGTCCCAGATGGTGATGAGGCCAAAGCAAAGGTA TCA
AACCACATGTGGTCTTTAAATAGCCCTTGATGTTTGTCACAGGGGTTGAGCCCGGGGCCTGGACCCATTCCAGTTGGTAATTGATAATTCCAACTGGTAATTATGTGCTCTGGGTAGTTTCGGTTG GC
ATCTGCACATCCTGTCCTCAAATGGCGAGAGGTGTTCCCAGATTTCATGCCAGAGGTGGCTCCATTTCAAGCCCCATAAAGTTTCTAAATACCTTTGGAATGCTGCAGGATAAAAGATACTGTCC TCA
TTAAACACATGGGCTTCTCAAAAGAAAACCCATTACTCTCTGATGATGACAGTTTTTCCTGATCCTTATGTTCCTTGTTAAAGGAAAATGTCACTTAATAAAGAAGACACACATAACTCTTTCCAA GA
ATTAACTAATTATGCCAACCAACCTCCTAAGGAGAGAGACAAGTGATTTGGTTACAAATCATTATATCCTTCAGGATATAGAGTTGTTCTAGATTTTCTTGATGCTTTTTTGTCTTCCTTTTTAT TTT
TCACTTTTTGCTATATAATTAATGAAAACCCTTAGATTGAGGTCCGTTCATTTCTCTCCCTATAGCGTACCCGTCCATTTAAGAAGATGTAGGCA GCTGGGTGCGGTGGCTCAAGCCTGCAGTCCCAG
CACTTTGGGAGCCCGAGGCAGGTGGATCTCCTGAGGTCGGGAGTTCAAGACTAGCCTGACCAACATGGAGAAACCCTGTCTCTACTAAAAATACAAAAAATTAGCTGGGCGTGGTGGCGCATG CCTGT
AATCCCAGCACTTTGGAAGGCTGAGGCCGGCAGATCACAAGGTCAGGAGATCGAGATCATCCTGGCTAACATGGTGAAACCCCATCTCTACTTAAAAATAAATAAATAAATAAATACAAAAATTA GT
CAGGCGTGGCAGCATGTGCCTGTAGTCCCAGCTACTCGGGAGGCTGAGGCAGGAGGATGACGTGAACCCAGGAGGCAGAGCTTGCAGTGAGCCAAGATTACACTACTGCACTCCAGCCTGGATGA CCG
AGCAAGACTCTGTCTCAAAAAATAAATGGAATAGACGCTGGCCAGAGATAAAATTGTAGTCTTCCTCTTCCTGTTCTATCAAAGGAATTCCAAAGCCAGACTCAGCTAAATACCTAACATGTTGATCA
TGGAGATGACCACTTTCAAAACCCATGTGGAGGAGCCTTAGATTTTAATGGCCAACCTTACAGATTTCTCTATTTCCTGAGAGTGTATCACGCTTGTTGTAAACATGTCTGGAAAGAACACATAC TCC
AGCCATGACCTATGTGTTTAGTTCTTTATTACTGTGTAACAAATTACTACAAAACGAGGATCTGTAAAACAACAGCTTTTATTCTCAATTTGTTGGTTTAACTGGACTCAGCTGGGTGGTTATTAT GC
TGGTCTCACTTAGGGTCTCTCAAGCACTTGCAGCAAATGATGGCTGGGGCTGGAAACATCTGAAGACAGAGACACTGTGATGGCTCTCATTTTCTTCCTGCCATCTCAGGACTATTTCTTTCCAT GTG
GCCATTCCAGACGAGTAGATAAACTTCTTGTAGGGTGACTCAGGGCTCCCAAGAGTGAACATTTCATGAAGTAAGAAGCAAAAGTGACAGTCCTCTTAAAGGCTGGGCCTGGAATCAACATTATAT TA
CTTCCTCTGCATTCTATAGCCTAACGTGAGTGCCAGGCCAGCTCAGATTTCATTTGAAATTCACTTGGAAGTGATCACACAAGTGTGTGAAAACCAGGAGATGGTTCACTGGGGGCCACCCTTGT AGA
ACAGCCATTATTGTCTTTCTTGTCAACCCCCATCAAAAGCAATTCATCCCATATTTGAGCATAGATGCATCAAATCCTGCTCACTCCACTCAGATCTAGTGCTGCTTCTTCTCTGGCCTGTCCCCT CC
TCAATTTTCGTTCATATTTTAAGTTTGTGTTTTCTGCTTTCCCTTCACAGCATCAACATGGACCCATTTCCCCAGTTTTCAAATTCACTGAAAGCTCATAACGACTTCCACACTCTTAACTTGAA ATC
TG

356	27.4	20.7	1.8	197967	233	507	(1799)	+	AmnSINE1	SINE/5S-Deu-L2	225	550	(25)	1
1048	9.1	0.0	0.0	197967	864	995	(1311)	+	AluSp	SINE/Alu	2	133	(180)	2
2071	11.1	0.0	5.9	197967	1000	1306	(1000)	+	AluY	SINE/Alu	2	291	(20)	3
697	25.3	8.1	13.1	197967	1551	2054	(252)	C	MLT1H2	LTR/ERVL-MaLR	(0)	489	8	4

356	27.4	20.7	1.8	hg	233	507	(1998)	+	AmnSINE1	SINE/5S-Deu-L2	225	550	(25)	5
2257	9.7	1.7	0.3	hg	864	1163	(1342)	+	AluSp	SINE/Alu	2	305	(8)	6
2095	10.8	0.0	5.9	hg	1199	1505	(1000)	+	AluY	SINE/Alu	2	291	(20)	7
697	25.3	8.1	13.1	hg	1750	2253	(252)	C	MLT1H2	LTR/ERVL-MaLR	(0)	489	8	8

>hg19 chr16:79921796-79924300
ACATTGTGGCTTCCCAGATGGGAGCAGATAGAAGAGACCACATGAAAGCTTTTAGCATCCTCTGTTCTTCAAAGCTTGCCGTTCATATCCCCAGACCAGGTGGTCA
GCAGGGTCGAATGTGGTTCATGGCCTGACTCTGAGGTCTCTTTTTTATGAGTTCAAACCCAACATTGACCACCAGGGAAGACTTAATCTATTTAATATATACCCT
TCTCTGGGGTCCCAGATGGTGATGAGGCCAAAGCAAAGGTATCAAACCACATGTGGTCTTTAAATAGCCCTTGATGTTTGTCACAGGGGTTGAGCCCGGGGCCTGG
ACCCATTCCAGTTGGTAATTGATAATTCCAACTGGTAATTATGTGCTCTGGGTAGTTTCGGTTGGCATCTGCACATCCTGTCCTCAAATGGCGAGAGGTGTTCCCA
GATTTCATGCCAGAGGTGGCTCCATTTCAAGCCCCATAAAGTTTCTAAATACCTTTGGAATGCTGCAGGATAAAAGATACTGTCCTCATTAAACACATGGGCTTCT
CAAAAGAAAACCCATTACTCTCTGATGATGACAGTTTTTCCTGATCCTTATGTTCCTTGTTAAAGGAAAATGTCACTTAATAAAGAAGACACACATAACTCTTTCC
AAGAATTAACTAATTATGCCAACCAACCTCCTAAGGAGAGAGACAAGTGATTTGGTTACAAATCATTATATCCTTCAGGATATAGAGTTGTTCTAGATTTTCTTGA
TGCTTTTTTGTCTTCCTTTTTATTTTTCACTTTTTGCTATATAATTAATGAAAACCCTTAGATTGAGGTCCGTTCATTTCTCTCCCTATAGCGTACCCGTCCATTT
AAGAAGATGTAGGCAGCTGGGTGCGGTGGCTCAAGCCTGCAGTCCCAGCACTTTGGGAGCCCGAGGCAGGTGGATCTCCTGAGGTCGGGAGTTCAAGACTAGCCTG

ACCAACATGGAGAAACCCTGTCTCTACTAAAAATACAAAAAATTAGCTGGGCGTGGTGGCGCATGCctgtaatcccagctattggggaggctgaggcaggagaatt
gcttgaacctggaaggtggagattgcggtgtgccaagattgtgccattgcactccagcctggacaagagtgaaactccatcaaaaaaaaaaaaaaaaaaagaatta
tattctattcttgtttagaatagacgctggcagctgggcgtggtggctcatgcCTGTAATCCCAGCACTTTGGAAGGCTGAGGCCGGCAGATCACAAGGTCAGGAG
ATCGAGATCATCCTGGCTAACATGGTGAAACCCCATCTCTACTTAAAAATAAATAAATAAATAAATACAAAAAATTAGTCAGGCGTGGCAGCATGTGCCTGTAGTC
CCAGCTACTCGGGAGGCTGAGGCAGGAGGATGACGTGAACCCAGGAGGCAGAGCTTGCAGTGAGCCAAGATTACACTACTGCACTCCAGCCTGGATGACCGAGCAA
GACTCTGTCTCAAAAAATAAATGGAATAGACGCTGGCCAGAGATAAAATTGTAGTCTTCCTCTTCCTGTTCTATCAAAGGAATTCCAAAGCCAGACTCAGCTAAAT
ACCTAACATGTTGATCATGGAGATGACCACTTTCAAAACCCATGTGGAGGAGCCTTAGATTTTAATGGCCAACCTTACAGATTTCTCTATTTCCTGAGAGTGTATC
ACGCTTGTTGTAAACATGTCTGGAAAGAACACATACTCCAGCCATGACCTATGTGTTTAGTTCTTTATTACTGTGTAACAAATTACTACAAAACGAGGATCTGTAA
AACAACAGCTTTTATTCTCAATTTGTTGGTTTAACTGGACTCAGCTGGGTGGTTATTATGCTGGTCTCACTTAGGGTCTCTCAAGCACTTGCAGCAAATGATGGCT
GGGGCTGGAAACATCTGAAGACAGAGACACTGTGATGGCTCTCATTTTCTTCCTGCCATCTCAGGACTATTTCTTTCCATGTGGCCATTCCAGACGAGTAGATAAA
CTTCTTGTAGGGTGACTCAGGGCTCCCAAGAGTGAACATTTCATGAAGTAAGAAGCAAAAGTGACAGTCCTCTTAAAGGCTGGGCCTGGAATCAACATTATATTAC
TTCCTCTGCATTCTATAGCCTAACGTGAGTGCCAGGCCAGCTCAGATTTCATTTGAAATTCACTTGGAAGTGATCACACAAGTGTGTGAAAACCAGGAGATGGTTC
ACTGGGGGCCACCCTTGTAGAACAGCCATTATTGTCTTTCTTGTCAACCCCCATCAAAAGCAATTCATCCCATATTTGAGCATAGATGCATCAAATCCTGCTCACT
CCACTCAGATCTAGTGCTGCTTCTTCTCTGGCCTGTCCCCTCCTCAATTTTCGTTCATATTTTAAGTTTGTGTTTTCTGCTTTCCCTTCACAGCATCAACATGGAC
CCATTTCCCCAGTTTTCAAATTCACTGAAAGCTCATAACGACTTCCACACTCTTAACTTGAAATCTG

Microhomology = CATGCCTGTAATCCCAGC

>NHEJ_15

KSD_NEHJ_15

>Scaffold5150 9912351 9912634_NHEJ
CCACCATTCTACTCCCTATTTTTGATACCTAATATAATTGGGATCGTGCTCTATTTGTCTTGTTAAATAATTATTTCAATTTGCTTAATGTCTTCTAGGAGGCTCTGCCACATTCCTAGATACAGT AT
CTAGGAATACATATAACAAATTATGTATGCAAAAGTATACACTATCTAGGGGAGCATAGTGATGTTATGGTGAATTTGAGTTTTTCTGGTAAATTACAATTTTCTACAGATGCCCTTGAATTTTC TAT
CATAATGACACAGTTCATTTTTTTCCAATAATTACTTCTTTAATTTTTCTTTCTTATTGAACTGAGCAGAACTTCCAAAACAATGCTGAATAAAAAGAAAGATAATAATGAAGGATAACATTTGTT AA
TATGTTATGTGCAAGTTTCCATTCAAAGTACATGTGATAATTTATTTAAAACTGACAACATGCCAAATGAGGTAGATAATATAATTATTTCATTTTTTTTCAGATGAGAAAAATGAGATCGAAAG AAG
TTGAGTAAGTGGCTCAACCTTTTGCAGCTAATCATAACAGAGCCAGGATTCCACCTAGCAGCCTGCCGTATAACTCATTTCCTTAGCATGGTCTCTCAAGTACAAATAACAGTGGCAGGCCAGTTT GT
ATGTATCTGATTTTAAAGGACTACCTCTGATTATTTACTACCAAT cTTTGCTATCATTTGTAATAGTACTCTTTTTGTCTTAACAGCTTTATTGAAGTATAATTAATACACACAAAATTGCATGTATT
TAATACACATGGATGTGTTCAGTTGTATGCTTGCACCATAAAACAATCACCACAATCAAAGTAATAGATATCTCTATCATCCCCAAAAGTTTCCTCATGTGCCTTTGTGTGTGTGTGTGGTGGGAG AG
AGTAAAAACACTTAACAAAGGATCTACCTACTTAAAAAAATTTGGCTGAGCGTGGTGGCTCATGCCTGTAATCCCAGAACTTTGGGATGCTGAGGCAGGTGGATCACCTGAAGTCAGGAGATTGAGAC
CAGCCAGGCCAACATGGTGAAACCTCCTCTCTACTAAAAATACAAAACATAGCCAGGCGTGGTGGTGGGCACCTGTAATCCCAGCTACTCAGGAGGCTGAAGCAGGAGAATCGCTTGAACCCGGGA GG
TGGAGGTTGCAGTGAGCCCAGATCGTGCCACTGCACTCCAGCCTGGGTGACAGAGTGAGACACCATCTCA ACAACATGGTGAAACACCATATCTAATAAAAATACAAAAATTATCAGGGCCTGGTGGT
GCACAGCTGTAGTCCCAGCTACTTGGGAGGCTAAGGAAGGAAAATAGCTTGAGCCCGGGAGGTGGAGGTTGCAGTGCACCCAGATCATGCCACTGCACTCTAGCCTGGGCGATGGAGTGAGACCCT GT
CTCAAAAAAAGAAAAAACAACATTAAGTGCACAATACAGTATGGCTTACCATAGACATGTTGTATAGCAGATCTCTAGAATTCATTCAATTTGCATAACTTGAATTATACCCACTGAATAAAAACTAC
TCATTTGCCTCTTCCTACATCCCCTCACAACCACCGTTCTACTCCCTAGTTTAGATAACTAATATAAGTGGAATCATGCAGTATTTGTCTTGTTACATAATTATTTAAATTTGCATAATATCCTTT AG
GTTCTGCCACATTGTTGCATATGGCAGGATTTTACTCCTTTTTAAGGCTGAACAATATCCCATTGCATGTATACAACAAATTTTCTTTAACCATTCATCTGTTGATAGACATTTAAATTGTTTTC ATA
TCTTGTCTATTGTGAATAATGCTTCAATAAACACGGAAGTGCAGATATCTCTTCAAGATCCTAATTTCAATTCTTTCAGATAAATATTCAGAAATGAGATTGTTGGATCCTATGCTAGTTTCATTA TT
AATTTTTGAAGGAATCCCATAACTGTTTTCCTTAGTGGCTGTATCATTTCAGATTCCCACCAACAGAATACAAAGGTCTCAGCTTCTCCATAAACTTACCAGTATCTATCTTTTTTTAAAAAAGA AAT
AGACATGCTAACAAGTGTGAAATAATATCCCTTTGTGATCTATATTTGTATTGCCCTCAGCTTAGTGATGTTAAGCACTGTTCTTATCTTCGCCATTTATATGTCTTCCTTGGAGATATGTCTATT CA
AATCTTTAGTTCATTTTTTAATTAGAAGATTTGTATTTTTGCTGTTGAGTTCTAGGAGTTCCTTATACATCTTAGATATTAACCACTTATCAAACATATGGTTCCC A

285	20.7	6.2	0.0	20160	1	99	(2184)	C	L1MB1	LINE/L1	(316)	5975	5856	1
282	22.6	9.6	1.0	20160	258	351	(1932)	C	L1M5	LINE/L1	(1939)	4207	4106	2
510	28.2	5.4	1.4	20160	373	577	(1706)	C	MIR	SINE/MIR	(10)	252	40	3
889	18.8	2.6	5.9	20160	714	939	(1344)	C	L1MA10	LINE/L1	(0)	6334	6116	4
2198	10.2	0.0	0.0	20160	940	1223	(1060)	+	AluSx1	SINE/Alu	1	284	(28)	5
1337	15.3	0.0	0.0	20160	1224	1425	(858)	+	AluSx	SINE/Alu	95	296	(16)	6
4052	18.0	4.6	0.6	20160	1431	2279	(4)	C	L1MB1	LINE/L1	(52)	6116	5227	7

285	20.7	6.2	0.0	Hg19	1	99	(2308)	C	L1MB1	LINE/L1	(316)	5975	5856	8
282	22.6	9.6	1.0	Hg19	258	351	(2056)	C	L1M5	LINE/L1	(1939)	4207	4106	9
510	28.2	5.4	1.4	Hg19	373	577	(1830)	C	MIR	SINE/MIR	(10)	252	40	10
4739	19.1	4.4	3.8	Hg19	714	939	(1468)	C	L1MA10	LINE/L1	(0)	6334	6120	11
2414	9.4	0.0	0.0	Hg19	940	1247	(1160)	+	AluSx1	SINE/Alu	1	308	(4)	12
4739	19.1	4.4	3.8	Hg19	1248	1261	(1146)	C	L1MA10	LINE/L1	(215)	6119	6117	11
2014	13.5	0.0	0.0	Hg19	1262	1549	(858)	+	AluSz	SINE/Alu	9	296	(16)	13
4739	18.5	4.1	3.0	Hg19	1550	2403	(4)	C	L1MA10	LINE/L1	(218)	6116	5227	11

>Hg19 chr8:113165197-113167603
CCACCATTCTACTCCCTATTTTTGATACCTAATATAATTGGGATCGTGCTCTATTTGTCTTGTTAAATAATTATTTCAATTTGCTTAATGTCTTCTAGGAGGCTCTGCCACATTCCTAGATACAGT AT
CTAGGAATACATATAACAAATTATGTATGCAAAAGTATACACTATCTAGGGGAGCATAGTGATGTTATGGTGAATTTGAGTTTTTCTGGTAAATTACA ATTTTCTACAGATGCCCTTGAATTTTCTAT

CATAATGACACAGTTCATTTTTTTCCAATAATTACTTCTTTAATTTTTCTTTCTTATTGAACTGAGCAGAACTTCCAAAACAATGCTGAATAAAAAGAAAGATAATAATGAAGGATAACATTTGT TAA
TATGTTATGTGCAAGTTTCCATTCAAAGTACATGTGATAATTTATTTAAAACTGACAACATGCCAAATGAGGTAGATAATATAATTATTTCATTTTTT TTCAGATGAGAAAAATGAGATCGAAAGAAG
TTGAGTAAGTGGCTCAACCTTTTGCAGCTAATCATAACAGAGCCAGGATTCCACCTAGCAGCCTG CCGTATAACTCATTTCCTTAGCATGGTCTCTCAA GTACAAATAACAGTGGCAGGC CAGTTTGT
ATGTATCTGATTTTAAAGGACTACCTCTGATTATTTACTACCAAT aTTTGCTATCATTTGTAATAGTACTCTT TTTGTCTTAACAGCTTTATTGAAGTATAATTAATACACACAAAA TTGCATGTATT
TAATACACATG GATGTGTTCAGTTGTATGC TTGCACCATAAAACAATCACCACAATCAAAGTAATAGATATCTCTATCATCCCCAAAAGTTTCCTCATGTGCCTTTGTGTGTGTGTGTGGTGGGAGAG
AGTAAAAACACTTAACAAAGGATCTACCTACTTAAAAAAATTT GGCTGAGCGTGGTGGCTCATGCCTGTAATCCCAGAACTTTGGGATGCTGAGGCAG GTGGATCACCTGAAGTCAGGAGATTGAGAC
CAGCCAGGCCAACATGGTGAAACCTCCTCTCTACTAAAAATACAAAACATAGCCAGGCGTGGTGGTGGGCACCTGTAATCCCAGCTACTCAGGAGGCTGAAGCAGGAGAATCGCTTGAACCCGGG AGG
TGGAGGTTGCAGTGAGCCCAGATCGTGCCACTGCACTCCAGCCTGGGTGACAGAGTGAGACACCATCTCA Aaaaaaaaaaaaaaaaaaaaaaaaa tttaagcagccaga gctgtggctcatccctgta
atcccagcactttgggaggccgaggtgagagaatcacttgaggtcaggagttcaagaccagcctggc C AACATGGTGAAACACCATATCTAATAAAAATACAAAAATTATCAGGGCCTGGTGGTGCAC
AGCTGTAGTCCCAGCTACTTGGGAGGCTAAGGAAGGAAAATAGCTTGAGCCCGGGAGGTGGAGGTTGCAGTGCACCCAGATCATGCCACTGCACT CTAGCCTGGGCGATGGAGTGAGACCCTGTCTCA
AAAAAAGAAAAAACAACA TTAAGTGCACAATACAGTATGGCTTACCATAGACATGTTGTATAGCAGATCTCTAGAATTCATTCAATTTGCATAACTTGAATTATACCCACTGAATAAAAACTACTCAT
TT GCCTCTTCCTACATCCCC TCACAA CCACCGTTCTACTCCCTAGT TTAGATAACTAATATAAGTGGAATCATGCAGTATTTGTCTTGTTACATA ATTATTTAAATTTGCATAATATCCTTTAGGTTC
TGCCACATTGTTGCATATGGCAGGATTTTACTCCTTTTTAAGGCTGAACAATATCCCATTGCATGTATACAACAAATTTTCTTTAACCATTCATCTGTTGATAGACATTTAAATTGTTTTCATAT CTT
GTCTATTGTGAATAATGCTTCAATAAACACGGAAGTGCAGATATCTCTTCAAGATCCTAATTTCAATTCTTTCAGATAAATATTCAGAAATGAGA TTGTTGGATCCTATGCTAGTTTCATTATTAATT
TTTGAAGGAATCCCATAACTGTTTTCCTTAGTGGCTGTATCATTTCAGATTCCCACCAACAGAATACAAAGGTCTCAGCTTCTCCATAAACTTACCAGTATCTATCTTTTTTTAAAAAAGAAATA GAC
ATGCTAACAAGTGTGAAATAATATCCCTTTGTGATCTATATTTGTATTGCCCTCAGCTTAGTGATGTTAAGCACTGTTCTTATCTTCGCCATTTA TATGTCTTCCTTGGAGATATGTCTATTCAAATC
TTTAGTTCATTTTTTAATTAGAAGATTTGTATTTTTGCTGTTGAGTTCTAGGAGTTCCTTATACATCTTAGATATTAACCACTTATCAAACATATGGTTCCC A

Microhomology = undefined

>NHEJ_16

KSD_NEHJ_16

>Scaffold1772 874007 874289_NHEJ
GGTCATTTAAAAGTGTGTGGCACCTTCCTCTTCCCTCTCTCTTGCTGCTCAGGCCATGTGACATGCCGGCTCCCCCTTCACCTTCCACCATGATTGAAGTTTCCTGTGACCTCCCTAGTAGCCAAG GA
GATACCAGTGCTATGCTTCTTGTACAGCCTGCAGAACTGTGAGCCAATTAAACCTCTTTTCTTTATAAATTACCCAGTCTCAGGTATTTCTTTACAGCAATGCAGGAATGACCTAATACACCAGA TTA
GCCTGCTGGTCTTAGCGGGAGGATGAGAGACACATAGAACTGACCTATCCCTGCTAATGTACTCTAGCCAATCCTGGGTTAAAGCGCCTGTAGTCCCATCTACTCTAGAGGCTGAGGTAGGAGGAT CG
CTTGAGCCCAGGAGTTTAAGTCTAGCTTGTGCAATATAGTGGGATCTGTCTCAGAAAAAAAAAAAAAAGCAGAGCAACCAGCCATTTCACAGGCACATGTGTGAAACAGGCTTAAATGCAAGAACT CCA
ATAATGCATGTTTTGAGCCACTGAGTTTTGGCATAGTTTGTTACACAGCAATAGCTAACTGTTACAGGATACCAAGGAGTTCTACAAGAAAGTGACATTTGGGCTGAGACTTAAATGTTAAATAGG TA
TCAGATGAAGAATGCAGGGCAGAGTGTCTTGATGAAGGAAGAGGCATGTATGAAAGACTTCAGGTGAGAAAGAGCTTGGTGCACTCAAATAACTGGAAGAAACTGTGGTTTGGAGAAGGGAATGA GCT
GACGCCAGAGAGGAAGCAGGGCTGGATCATGTGTGGTTTCTCATATAGTCTTCTTAGAGATTTTAAGATTAAGTTTCAAAAATTGAACTCAGAAGAGAGCTATTTATGATCAAAAACATAAAATCA AA
GGATTGTTGTCTTCATTATTATTAATCTAGAACTGTGCTGTCT GGCCAGGCATGGTGGCTCATGCCTGTAATCCCAGCACTTTGGGAGGTTAAGGCGGGCAGATCATGAGGTCAGGAGATCGAGACCA
TCCTGGCTAACATAGTGAAACCCCGTCTCTACTAAAAATAAAAAAAATTAGCTGGGTGTGGTGGCACGTGCCTGTAGTCCCAGCTACTCGGGAGGCTGAGGCAAGAGAATCGCTTAAACCCGGGAG GC
AGAGATTGCAGTGAGCCAAGATCACGCCACTGTACTCCAGCCTGGGCGACAGAGCAAGACTTTGTCTC AAGTCTCACTCTGTTGCCCAGGCTGGAGTGTAGTGGCATCATCTCAGCTCACTGCAACCT
CCGCCTACCAGGTTCAAGTGATTCTCCTGGCTCAGCCTCCTGCGTAGCTGGGATTATAGGCACCCACCACCACGCCTGGCTAATTTTTGTATTTTTAGTAGAGATAGGGTTTCACCATATTGGCCA GG
CTGGTCTTGAACTCCTGACCTCAGGTGATCCACCCGCCTTGGCCTCCCAAAGTGCTGGGATTACAAGTGTGAGCCACCGTGCCCGGC AATTAAAATTAAATAAGATAAAAAATTCAGTTCCTCACTTA
CACTAGACTCATTTCAAATGCTCAGTAGCCCCATGTGGCTAGTGGCCACTATATTGGCAAAGCAAATATAGAACATTTTCAGTATCACAGAAAGTTTTATTGGAGAGGGCATTCGTTTCTTATTGC TG
CAGCATGAATTACTACAAAGTTAGCAGCTTAAAGCAACATAAATGTATTATCTCGTAGTTTCCATAGGTCAGGAATCTGGGTACGGATTAGCGAGGCCCTCTACTCAGGGTCTCACCAGGCTGAA ATC
AAGGTGTGAACTGGGATCTCCTCTGAGGCTCGGGGTCATCTTCCAAGCTCAGTAGTTGTTGGCAGAATTCATTTCCTTGCATCTTTGTGACCAAGGGACAGCTTAATGCTCCCAGAGACCA cGCTCAG
GTCCTTACCATGTGACCTCCTCTTCTtACAACATCAAAGCTTGCTTCTTTCTTCCAGAGGCCAGCAGCATATATTCTCTGACCTTCAGTTTCTTTTAAAGGGCTTGCCTGATTCGATCAGGCCCACCC
AGGATAGAGCTCCTTTTTGATTAACTCCAAGTCAACTGATGAGTAGCCTAGTCAAGGGAGTGACATCTCACCACATTCTCTGATCCTGCTACACTCAAGGGGAGGGGGTATATCCACCAGTGGGGC AG
GGAATCTTGGGGGCCATCTAACATTCTGCtTACCGcAGaCATCAGTGATCTAGAAAGTAGAGAAAGGTTAACTAAATGAATCAACGGGGAAGGCAAGTAGTGGGA G

1752	11.7	1.6	0.0	47724	1	248	(2034)	+	MSTA	LTR/ERVL-MaLR	177	428	(0)	1
700	16.2	0.9	0.0	47724	341	451	(1831)	+	FLAM_A	SINE/Alu	19	130	(12)	2
411	12.1	0.0	0.0	47724	521	578	(1704)	+	MLT1H	LTR/ERVL-MaLR	492	549	(0)	3
391	33.3	12.6	0.8	47724	587	801	(1481)	C	L2b	LINE/L2	(225)	3150	2911	4
2337	8.8	0.0	0.0	47724	940	1222	(1060)	+	AluSc8	SINE/Alu	1	283	(28)	5
2206	9.5	0.0	0.0	47724	1223	1495	(787)	C	AluSx1	SINE/Alu	(37)	275	2	6
904	15.5	0.7	0.0	47724	1496	1643	(639)	C	MER3	DNA/hAT-Charlie	(55)	154	6	7
2291	18.1	17.8	2.8	47724	1645	2213	(69)	C	MLT1E	LTR/ERVL-MaLR	(1)	652	1	8

1752	11.7	1.6	0.0	Hg19	1	248	(2150)	+	MSTA	LTR/ERVL-MaLR	177	428	(0)	9
700	16.2	0.9	0.0	Hg19	341	451	(1947)	+	FLAM_A	SINE/Alu	19	130	(12)	10
411	12.1	0.0	0.0	Hg19	521	578	(1820)	+	MLT1H	LTR/ERVL-MaLR	492	549	(0)	11
391	33.3	12.6	0.8	Hg19	587	801	(1597)	C	L2b	LINE/L2	(225)	3150	2911	12
2554	8.4	0.0	0.0	Hg19	940	1248	(1150)	+	AluSc8	SINE/Alu	1	309	(2)	13
1012	17.6	0.9	11.8	Hg19	1249	1325	(1073)	C	MER3	DNA/hAT-Charlie	(0)	209	154	14
2319	9.1	0.0	0.0	Hg19	1326	1611	(787)	C	AluSx1	SINE/Alu	(25)	287	2	15
1012	17.6	0.9	11.8	Hg19	1612	1759	(639)	C	MER3	DNA/hAT-Charlie	(56)	153	6	14
2290	18.2	17.8	2.8	Hg19	1761	2326	(72)	C	MLT1E	LTR/ERVL-MaLR	(1)	652	4	16

>Hg19 chr14:90123008-90125405
GGTCATTTAAAAGTGTGTGGCACCTTCCTCTTCCCTCTCTCTTGCTGCTCAGGCCATGTGACATGCCGGCTCCCCCTTCACCTTCCACCATGATTGAAGTTTCCTGTGACCTCCCTAGTAGCCAAG GA

GATACCAGTGCTATGCTTCTTGTACAGCCTGCAGAACTGTGAGCCAATTAAACCTCTTTTCTTTATAAATTACCCAGTCTCAGGTATTTCTTTACAGCAATGCAGGAATGACCTAATACACCAGATTA
GCCTGCTGGTCTTAGCGGGAGGATGAGAGACACATAGAACTGACCTATCCCTGCTAATGTACTCTAGCCAATCCTGGGTTAAAGCGCCTGTAGTCCCATCTACTCTAGAGGCTGAGGTAGGAGGA TCG
CTTGAGCCCAGGAGTTTAAGTCTAGCTTGTGCAATATAGTGGGATCTGTCTCAGAAAAAAAAAAAAAAAGCAGAGCAACCAGCCATTTCACAGGCACATGTGTGAAACAGGCTTAAATGCAAGAACTCCA
ATAATGCATGTTTTGAGCCACTGAGTTTTGGCATAGTTTGTTACACAGCAATAGCTAACTGTTACAGGATACCAAGGAGTTCTACAAGAAAGTGACATTTGGGCTGAGACTTAAATGTTAAATAG GTA
TCAGATGAAGAATGCAGGGCAGAGTGTCTTGATGAAGGAAGAGGCATGTATGAAAGACTTCAGGTGAGAAAGAGCTTGGTGCACTCAAATAACTGGAAGAAACTGTGGTTTGGAGAAGGGAATGAGCT
GACGCCAGAGAGGAAGCAGGGCTGGATCATGTGTGGTTTCTCATATAGTCTTCTTAGAGATTTTAAGATTAAGTTTCAAAAATTGAACTCAGAAGAGAGCTATTTATGATCAAAAACATAAAATCAAA
GGATTGTTGTCTTCATTATTATTAATCTAGAACTGTGCTGTCT GGCCAGGCATGGTGGCTCATGCCTGTAATCCCAGCACTTTGGGAGGTTAAGGCGGGCAGATCATGAGGTCAGGAGATCGAGACCA
TCCTGGCTAACATAGTGAAACCCCGTCTCTACTAAAAATAAAAAAAATTAGCTGGGTGTGGTGGCACGTGCCTGTAGTCCCAGCTACTCGGGAGGCTGAGGCAAGAGAATCGCTTAAACCCGGGA GGC
AGAGATTGCAGTGAGCCAAGATCACGCCACTGTACTCCAGCCTGGGCGACAGAGCAAGACTTTGTCTC Aaaaaaaaaaaaaaaaaaaaaaaaagaactgtgctgtccagtaggttagccactaggcgc
tatggctacccaagcttaaattagctaaaattattattattattatttttgagacggAGTCTCACTCTGTTGCCCAGGCTGGAGTGTAGTGGCATCATCTCAGCTCACTGCAACCTCCGCCTACCAGG
TTCAAGTGATTCTCCTGGCTCAGCCTCCTGCGTAGCTGGGATTATAGGCACCCACCACCACGCCTGGCTAATTTTTGTATTTTTAGTAGAGATAGGGTTTCACCATATTGGCCAGGCTGGTCTTG AAC
TCCTGACCTCAGGTGATCCACCCGCCTTGGCCTCCCAAAGTGCTGGGATTACAAGTGT GAGCCACCGTGCCCGGCAATTAAAATTAAATAAGATAAAAAATTCAGTTCCTCACTTACACTAGACTCAT
TTCAAATGCTCAGTAGCCCCATGTGGCTAGTGGCCACTATATTGGCAAAGCAAATATAGAACATTTTCAGTATCACAGAAAGTTTTATTGGAGAGGGCATTCGTTTCTTATTGCTGCAGCATGAA TTA
CTACAAAGTTAGCAGCTTAAAGCAACATAAATGTATTATCTCGTAGTTTCCATAGGTCAGGAATCTGGGTACGGATTAGCGAGGCCCTCTACTCAGGGTCTCACCAGGCTGAAATCAAGGTGTGAACT
GGGATCTCCTCTGAGGCTCGGGGTCATCTTCCAAGCTCAGTAGTTGTTGGCAGAATTCATTTCCTTGCATCTTTGTGACCAAGGGACAGCTTAATGCTCCCAGAGACCA tGCTCAGGTCCTTACCATG
TGACCTCCTCTTCTcACAACATCAAAGCTTGCTTCTTTCTTCCAGAGGCCAGCAGCAT ATATTCTCTGACCTTCAGTTTCTTTTAAAGGGCTTGCCTGATTCGATCAGGCCCACCCAGGATAGAGCTC
CTTTTTGATTAACTCCAAGTCAACTGATGAGTAGCCTAGTCAAGGGAGTGACATCTCACCACATTCTCTGATCCTGCTACACTCAAGGGGAGGGGGTATATCCACCAGTGGGGCAGGGAATCTTG GGG
GCCATCTAACATTCTGCcTACCGtAGgCATCAGTGATCTAGAAAGTAGAGAAAGGTTAACTAAATGAATCAACGGGGAAGGCAAGTAGTGGGAG

Microhomology = A

>NHEJ_17

KSD_NEHJ_17

>Scaffold7830 453697 453797_NHEJ
GGAGAATCGCTTGAACCCAGGAGGCGGAGGTTGCAGTGAGTCGAGATTGCACCATTGCACTCCAGCCTGGGCAACAAGAGTGAAACTCCATCTCGAAAAAAAAAAAAAAAGAAAGAAAAAGTACAT CC
CATCAAATCTGCCCCTTCTTAAAACCCTCTGACAGCATCCCACTGATTGTGATCACACCACATCTCCTGTAGGGAGCACGAGGCCCCATCTGGCTGGGCTGGTGCTAGTGCCGTCTCCTCCTGGG CCT
CCTTACCTGcGGCCCCTCTGTTCCTGGCTTGTTGGCTCACTCCCCTTTTCTCTGATACTCCATGCCTTTTGCTTCTTTGGTGCGTTTGCACCTGTTCCTACGCCTCCCACTCCCCGCCAATATTTCCC
CAGCTTTCTTAGTGAAGGCTCCTTCAGCATCGACGTCCCCTCCTGGAGGAGGCCTTTCCTGGCCACAATCTCTAAATTCCATTGTCTCCAGCTCAGTGCTGTTATTTTATGTTTCACCTCTCACC AAT
TTCCTCTCAGCATCTTCTTTTGCTTCTGTTGTTTTCATTGTAAAAATTTTCAAACATACACGCAAGTAAAGAGATGATAGTATAATGAGCCCTCATTGACTTATCCTACAGATTGAATAATTAAAA TG
CCGTCAGATCTGCTTCCTTGAGCCAGGGCTTTTTCTCTTGGCTGAAGAATCATAAGACAGATTCCAAACATGATGTCATTTTACTGTTACTCACTTGAGTATGCATTTTGAAAAAGGATGAGTGG TTT
CTTTTTTTTTTTTTTTGAAACAGAATCTCACTCTGTCACCAGGCTGGAGTGCAGTGGGGCAGTCTCGGCTCATTGCTGTCTCTGCCTCTCAGGTTCAAGTGATTCTCCTGCCTCAGCCTCCTCAG TA
GCTGGGACTATAGGCACATGCCACCATGTCCAGCTAATTTTTGTATTTTTAGTAGAGACAGGTTTCACCATGTTGGCCAGGATGGTCTCGATCTCGTGACTTCATGATCCACCTGCCTCGGCCTC CCA
AAGTGCTGGGATTACAGGTGTGAGCCACTGCGCCTGTAATCGCAGCACTTTGGGAGGCTGAAGCAGGCGGATCACTTGAGGTCAAGGGTTCGAGACCAGCCTGGCCAACATGGTGAAACCCTGTCACT
ACTAAAAATTAGCTGGAATATGAAACAATTTTCCTAGTTGTCCCAAATATGTCTTTTAATTGTTGGCCTGAGTCACAATCCCAACTGGATCCACGCATCACATCTGCTTATTGTATCTTTTTGTCTTT
TGTACCCTAACACAGCCCCCTCTTTTCCATGTTTGCTGCAGAAACCGGGTCAGTCTCCTGTGGGATCATTGCACATCCTGGATTTGTCTGTTGGCTTTTTCATGGAGTTGTTTAACTCGTTCCTCT CT
CCCCATATTCTTTGGAGATGGAAGCAAGCTCTAGAAGCCCAATTAGATTCGTGTCTAGAGGTGATGCTGTGTGTTCACCTGTCCTCAGCTCCAGGACCCGTGGTGTCTGGCTGTCCCACTCTCAT GAT
GCTGAGATTGGCCAGTGGGGTCAGGTGGTGGATGACAGCCTTATCGTTCCATGGTCAAGTTCCCCATCAGCCCCTGACCCAACAGTTTCATGCATCGATAATGTTTCCTGAATCAGTCATTCATTT GA
TTAGGGTTTGCAAAATGGGTGCCTTTTCTATTTCCTTCATTTCTTCCAAACTAGAGGCATACCTGGGGAGGGACAGGGTTTGAAAGCAGTCTACCCTGATGCAGCAATGGGAGTAGGAATTCATA GTC
TGTAGAGAACTTAAAAACAATCATGGCTGGGCGTGGTGGCTCACACCTGTAATCCCAACACTTTGGGAGACTGAGGCGGGCAGCTCACTTGAGGTCAGAGGTTCAAGACCAGCCTGGCCGGCATGG TG
AAACACCATCTCTACTAAAAATAAAAAAAATTAGCCGGGCATGATGGCGGGAGCCTGTAGTCTCAGCTACTTGGGAGGCTGAGGCAGGAGAATCGCTTGA aCCCGGGAGGTGGAGGTTGCAGTGAGCT
GAGATGTTGCCACTGCACTCCAGGCTGGACAACACAGCGAGACTCATCGCAA

983	8.3	0.0	0.0	49695	1	120	(1980)	+	AluSq2	SINE/Alu	190	309	(4)	1
536	31.1	2.7	1.8	49695	544	762	(1338)	C	L1MC5a	LINE/L1	(12)	8018	7798	2
2222	11.9	0.3	0.0	49695	766	1060	(1040)	C	AluSc	SINE/Alu	(10)	299	4	3
820	7.9	0.0	0.0	49695	1061	1161	(939)	+	AluSz	SINE/Alu	26	126	(186)	4
1352	23.6	10.7	2.8	49695	1179	1710	(390)	C	L1MC5a	LINE/L1	(324)	7706	7134	2
308	24.1	2.0	8.5	49695	1717	1816	(284)	+	MER97a	DNA/hAT-Tip100	2	95	(795)	5
2054	11.6	0.0	0.4	49695	1817	2093	(7)	+	AluSx1	SINE/Alu	1	276	(36)	6

983	8.3	0.0	0.0	hg19	1	120	(2086)	+	AluSq2	SINE/Alu	190	309	(4)	7
737	28.9	3.3	3.0	hg19	544	765	(1441)	C	L1MC5a	LINE/L1	(12)	8018	7798	8
2267	11.4	0.3	0.0	hg19	766	1063	(1143)	C	AluSc	SINE/Alu	(10)	299	1	9
737	28.9	3.3	3.0	hg19	1064	1140	(1066)	C	L1MC5a	LINE/L1	(233)	7797	7718	8
922	8.0	0.0	0.0	hg19	1155	1267	(939)	+	AluSz	SINE/Alu	14	126	(186)	10
1352	23.6	10.7	2.8	hg19	1285	1816	(390)	C	L1MC5a	LINE/L1	(324)	7706	7134	8
308	24.1	2.0	8.5	hg19	1823	1922	(284)	+	MER97a	DNA/hAT-Tip100	2	95	(795)	11
2037	11.9	0.0	0.4	hg19	1923	2199	(7)	+	AluSx1	SINE/Alu	1	276	(36)	12

>hg19 chr19:30288409-30290614
GGAGAATCGCTTGAACCCAGGAGGCGGAGGTTGCAGTGAGTCGAGATTGCACCATTGCACTCCAGCCTGGGCAACAAGAGTGAAACTCCATCTCGAAAAAAAAAAAAAAAGAAAGAAAAAGTACATCC
CATCAAATCTGCCCCTTCTTAAAACCCTCTGACAGCATCCCACTGATTGTGATCACACCACATCTCCTGTAGGGAGCACGAGGCCCCATCTGGCTGGGCTGGTGCTAGTGCCGTCTCCTCCTGGG CCT
CCTTACCTGtGGCCCCTCTGTTCCTGGCTTGTTGGCTCACTCCCCTTTTCTCTGATACTCCATGCCTTTTGCTTCTTTGGTGCGTTTGCACCTGTTCCTACGCCTCCCACTCCCCGCCAATATTTCCC

CAGCTTTCTTAGTGAAGGCTCCTTCAGCATCGACGTCCCCTCCTGGAGGAGGCCTTTCCTGGCCACAATCTCTAAATTCCATTGTCTCCAGCTCAGTGCTGTTATTTTATGTTTCACCTCTCACC AAT
TTCCTCTCAGCATCTTCTTTTGCTTCTGTTGTTTTCATTGTAAAAATTTTCAAACATACACGCAAGTAAAGAGATGATAGTATAAT GAGCCCTCATTGACTTATCCTACAGATTGAATAATTAAAATG
CCGTCAGATCTGCTTCCTTGAGCCAGGGCTTTTTCTCTTGGCTGAAGAATCATAAGACAGATTCCAAACATGATGTCATTTTACTGTTACTCACTTGAGTATGCATTTTGAAAAGGATGAGTGGTTT
CTTTTTTTTTTTTTTTTGAAACAGAATCTCACTCTGTCACCAGGCTGGAGTGCAGTGGGGCAGTCTCGGCTCATTGCTGTCTCTGCCTCTCAGGTTCAAGTGATTCTCCTGCCTCAGCCTCCTCAGTA
GCTGGGACTATAGGCACATGCCACCATGTCCAGCTAATTTTTGTATTTTTAGTAGAGACAGGTTTCACCATGTTGGCCAGGATGGTCTCGATCTCGTGACTTCATGATCCACCTGCCTCGGCCTC CCA
AAGTGCTGGGATTACAGGTGTGAGCCACTGCGCccggccgagtgttttcttatataaccacaccagtaacacacctaccaaaatgaacactaattcttggtatcagtttatattcacatgtattcata
caggctcacacCTGTAATCGCAGCACTTTGGGAGGCTGAAGCAGGCGGATCACTTGAGGTCAAGGGTTCGAGACCAGCCTGGCCAACATGGTGAAACCCTGTCACTACTAAAAAT TAGCTGGAATATG
AAACAATTTTCCTAGTTGTCCCAAATATGTCTTTTAATTGTTGGCCTGAGTCACAATCCCAACTGGATCCACGCATCACATCTGCTTATTGTATCTTTTTGTCTTTTGTACCCTAACACAGCCCCCTC
TTTTCCATGTTTGCTGCAGAAACCGGGTCAGTCTCCTGTGGGATCATTGCACATCCTGGATTTGTCTGTTGGCTTTTTCATGGAGTTGTTTAACTCGTTCCTCTCTCCCCATATTCTTTGGAGATGGA
AGCAAGCTCTAGAAGCCCAATTAGATTCGTGTCTAGAGGTGATGCTGTGTGTTCACCTGTCCTCAGCTCCAGGACCCGTGGTGTCTGGCTGTCCCACTCTCATGATGCTGAGATTGGCCAGTGGG GTC
AGGTGGTGGATGACAGCCTTATCGTTCCATGGTCAAGTTCCCCATCAGCCCCTGACCCAACAGTTTCATGCATC GATAATGTTTCCTGAATCAGTCATTCATTTGATTAGGGTTTGCAAAATGGGTGC
CTTTTCTATTTCCTTCATTTCTTCCAAACTAGAGGCATACCTGGGGAGGGACAGGGTTTGAAAGCAGTCTACCCTGATGCAGCAATGGGAGTAGGAATTCATAGTCTGTAGAGAACTTAAAAACA ATC
ATGGCTGGGCGTGGTGGCTCACACCTGTAATCCCAACACTTTGGGAGACTGAGGCGGGCAGCTCACTTGAGGTC AGAGGTTCAAGACCAGCCTGGCCGGCATGGTGAAACACCATCTCTACTAAAAAT
AAAAAAAATTAGCCGGGCATGATGGCGGGAGCCTGTAGTCTCAGCTACTTGGGAGGCTGAGGCAGGAGAATCGCTTGA gCCCGGGAGGTGGAGGTTGCAGTGAGCTGAGATGTTGCCACTGCACTCCA
GGCTGGACAACACAGCGAGACTCATCGCAA

Microhomology = CC

>NHEJ_18

KSD_NEHJ_18

>Scaffold6763 6877483 6877613_NHEJ
AATAGCCACTGCACTCCAGCCAGTGCAACATAGCAATAATTTGTATCTAGGAAAATAAAACAAAAAGGCCGGGCATGGTGGCTCACGCCTGTAATCCCAACACTTTGGGAGGCCGAGGCAGGCGGA Tg
AGCTGAGGTCAGGgGgTCAAGACCAGCCTGGCCAACGTGGTGAAACCCCGTCTCTACTAAAAATACAAAAGTTAGCCGGGCATGGTGGCAGGCG CCTGTAGACCCAGCTCAGGAGGCTGAGGCAGGAG
TATTGCTTGAACCTGGGAGGCGGAGGTTGCTGTGAGCCGAGATCACGCCACCGCACTCCAGCCTAGGTGACAGAGCAAGACTCCGTCTCAAAATAAATAAATACATAAACAAATAAGTAAAACAA ATA
ACCTAACATTTAAAAAAAATGTATTACTGGCTGGGCACAGTGGCTCATGCCTGTAATCCCAGCACTTTGGGAGGCCGAGGTGGGTGGATTGCCT CAGCTCAAGAGTTCAAGACCAGTCTGGTCAACAT
GGTGAAATTCAGTCTCTACTAAAAATACAAAAAAATTAGCTGGGCATGGCGGTGTGCGCCTGTAGTCCCAGCTATATTACTAAATAGCTTTTTTGGTGAGGATATGCCAAGAACCTCAAGTTTGA ACC
GCTTCTTATCCAAGGCTGCCATCTCAGTTATCTTTGATATGCTACGAAGCACCTGGCATAAGTTAAACAGAATTTATATGTGCCACGCGATATG AAGACGGCATACTAAGCATTTGTCACGTTAAAAA
TAGCTGTGCTGGCCGGGCGCGGTGGCTCACGCCTGTTATCCCAGCACTTTGGGAGGCCGAGGCGGGTGGATCACGAGGTCAGGAGATAGAGACCATCCTGGCTAACACGATGAAATCCCTTCTCTACT
AAAAATACAAAAAAAATTAGCCGGGCGTGGTGGCAGGCGCCTGTAGTCCCAGCTACGCAGGAGGCTGAGGCAGGAGAATGGCGTGAACCCGGCA GGCGAAGCTTGCGGTGAGCCGAGATCGCGCCACT
GCACTCCAGCCTGGGCGGCAGAGCGAGACTCCGTAGTCCCAGCTACTCGGGAGGCTGAGGCAGGAGAATCGCTTGAACCCGGGAGGTGGAGGTTGCCACTGCACTCCAGCTTGGGCAACAGAGCGAGA
CTCCCTCTCAAAAAACAAACAAAAACAAATAAAATAAAATAAAATAAATAAAAGTTTTGCTATGTTGCTCAGGCTGGTCTCTAACTAATGGGCTCAAGCAATCATCCCGCATCAGCATCTATAGTAGC
TGGGACTACAAGCCTGCACTACGACACCTGGCTGGCCCTTTTTGAATATCCTATTTAGTGGCATTAAGTACATTCACCTTAGGCAACCATCCATCTCTAGAATTTTTCCATCATGCCAATATGAA ATT
CTGTACATATTAAATGATAACTTCCCATTCCCTCCTCCC CCACAGAACCTGGTAACCACCTTCCTTCCTTCCTTCCTTCCTTCCTTCCTTCCTTCCTTCCTTCCTTC CtTTCCTTCCTCCCTtCCTCC
CTCCCTCCCTCCCTCCCTCCCTCCCTTCCTCCCTTCCTTCCTCCCTTCCTCCCTTCCCTTCCTCCCTTCCTTCCTTTTTGTGTGTGACAGAGTTTTGCTCTTGTTGCCCAGGCTGGAGTGCAAT GGC
GAGATCTCGGCTCACCACAACCTCCACCTCCTGGGTTCAAGCCATTCTCCTGCCTCAGCCTCCCGAGTAGATGGGATTACAGGCATGAGCCACCACACCTGGCTAATTTTGCATTTTTAGTGGAGACG
GGATTTCTCCATGTTGGTCAGGCTGGTCTCGAACTCCCGACCTCAGGTGACCCACCTGCCTCGACCTCCCAAAGTGCTAGGATTACAGGCGTAAGACCCTGTGCCCAGCTATTCTTTCTTTCTTT CTT
TATGAAGGTGACTATTTTAGGATCTCACATAAGAGGAAT CCACACAATATTTATTCTTTTGTGTCTGGGTTGTTCCATTTAGTATGAGGTTTTCAACGTTCCTCCATGTTGTAGCATGTATCAGAATA
TCACTCCTTTTTGTGAGTAAATAATATTCCATTGTATGTGTATACCACATTTTGTTTATCCATTCATCAGGTGATGAACAT T

317	21.2	0.0	0.0	82141	1	66	(2064)	+	7SLRNA	srpRNA	252	317	(3)	1
[truncated: 200,722 more chars]
